# Supplementary material for: Every which way? On predicting tumor evolution using cancer progression models
Source: PLoS Comput Biol. 2019 Aug 2;15(8):e1007246. doi: 10.1371/journal.pcbi.1007246 (PMC6693785; doi:10.1371/journal.pcbi.1007246)

Supporting Information for “Every which way? On predicting tumor evolution using cancer progression models”

Ramon Diaz-Uriarte[1,2*], Claudia Vasallo[1,2]

1 Dept. Biochemistry, Universidad Autónoma de Madrid, Madrid, Spain
2 Instituto de Investigaciones Biomédicas “Alberto Sols” (UAM-CSIC), Madrid, Spain

* ramon.diaz@iib.uam.es

S1 Figure. Plots of simulated fitness landscapes and fitness graphs.

Contents

1 Fitness landscapes: explanation of plots **1**

2 Seven genes **3**

3 Ten genes **634**

---

1. Fitness landscapes: explanation of plots

Here we show the 1260 fitness landscapes used. The first section, “Seven genes”, section 2, shows those for 7 genes, the second section, “Ten genes’, ’ section 3, those for 10 genes. In each section, the first 210 fitness landscapes are fully representable fitness landscapes, the next 210 are the “Local maxima” fitness landscapes. The next 210 correspond to Rough Mount Fuji (RMF) fitness landscapes. Landscapes are not ordered according to any criterion within type of fitness landscape.

In each page, the following figures are shown:

DAG of restrictions (top left) The true DAG of restrictions. This only applies properly to the representable fitness landscapes. In the local maxima fitness landscapes, not all paths between genotypes are available. (See below). For the RMF landscapes this, of course, is not available, since there is no underlying DAG of restrictions.

The true DAG of restrictions could be a tree. Among the representable fitness landscapes, 30% and 2%, of the landscapes with 7 and 10 genes, respectively, were trees (i.e., can be represented by OT and CAPRESE).

Fitness landscape (bottom left) As it says, the fitness landscape. Boxes surround the fitness maxima in the 7-genes landscapes. To minimize clutter, genotype labels are not shown in the 10-genes landscapes.

ID and landscape characteristics (center) The ID of the fitness landscape (a random string that matches the value of ID in the data tables), the number of accessible genotypes, the number of fitness maxima or peaks. Values for “removed edges” and “prop rem edges” denote, respectively, the number and proportion of edges in the fitness graph that were removed; this only applies to the “Local maxima” landscapes; thus, these values are 0 for “Representable” fitness landscapes, and NA for RMF fitness landscapes.

Fitness graph from DAG of restrictions (top right) The fitness graph implied by the DAG of restrictions. Not available for RMF (since there is no DAG of restrictions).

True fitness graph (bottom right) The actual fitness graph that corresponds to the fitness landscape. For "Representable" fitness landscapes this would be the same as the Fitness graph from DAG of restrictions, so we do not show it (to make the size of the files smaller).

Thus, a simple way to tell what kind of landscape you are looking at in any random page is this:

- If there is a fitness graph on the top right and no fitness graph in the bottom right corner: a representable fitness landscape.
- If there is a fitness graph on the top right and another fitness graph in the bottom right corner (with, necessarily, fewer paths than the graph in the upper right corner and at least two local maxima): a local maxima fitness landscape.
- If there is only a fitness graph on the bottom right: a RMF fitness landscape.

2. Seven genes

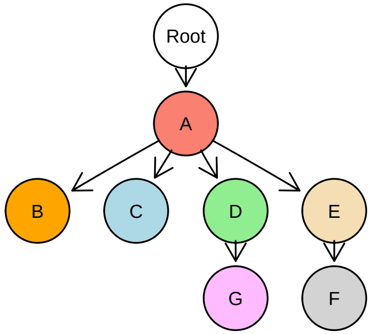

ID	1010kGD9gL10Sq9tJsP
acc genotypes	37
peaks no back	1
removed edges	0
prop rem edges	0

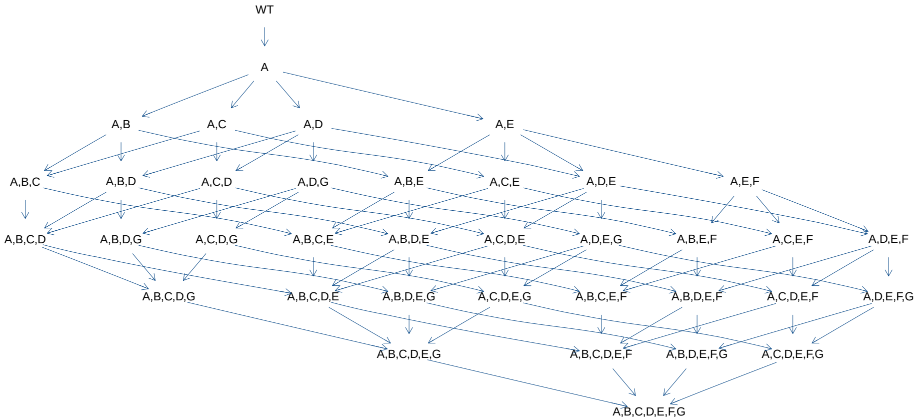

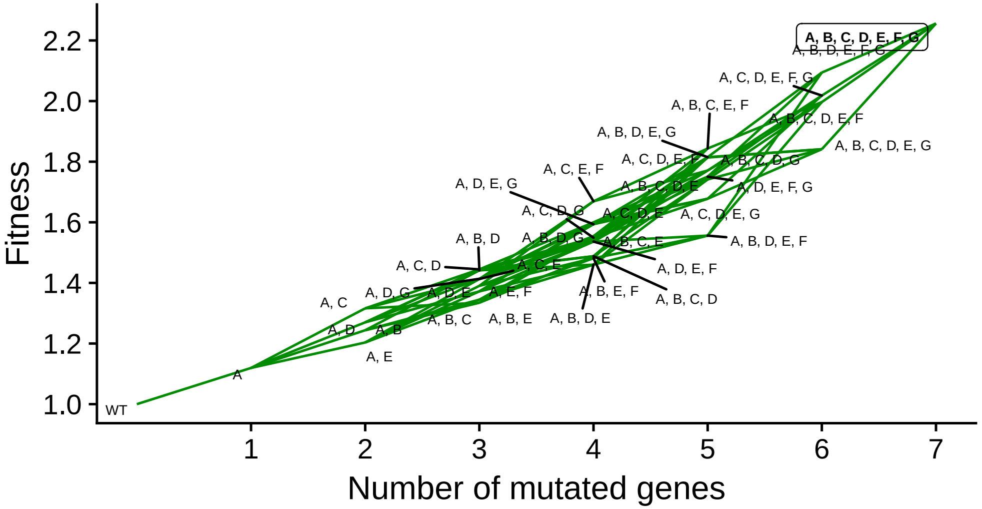

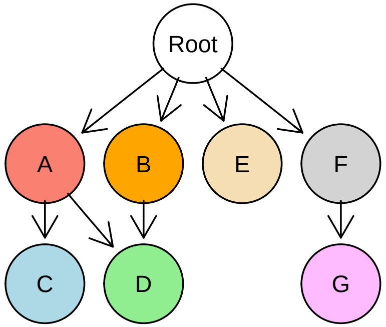

ID 10Dgx1l4YRhmToSuW
acc genotypes 48
peaks no back 1
removed edges 0
prop rem edges 0

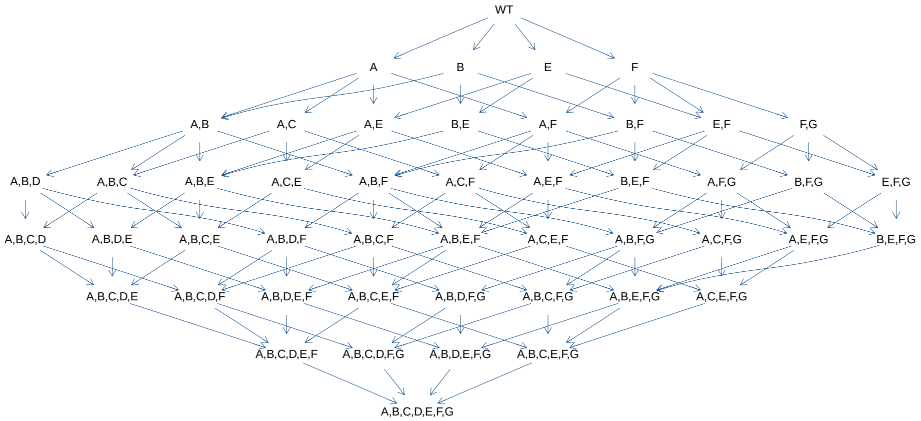

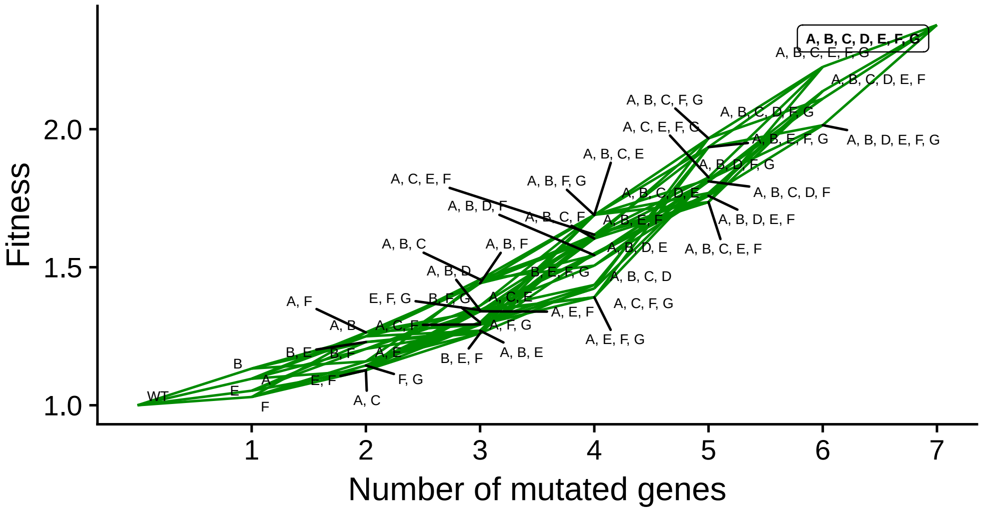

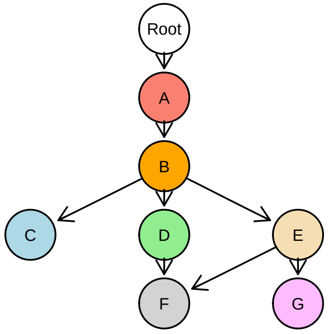

ID	1g3AE5zjGYmnuTbC
acc genotypes	18
peaks no back	1
removed edges	0
prop rem edges	0

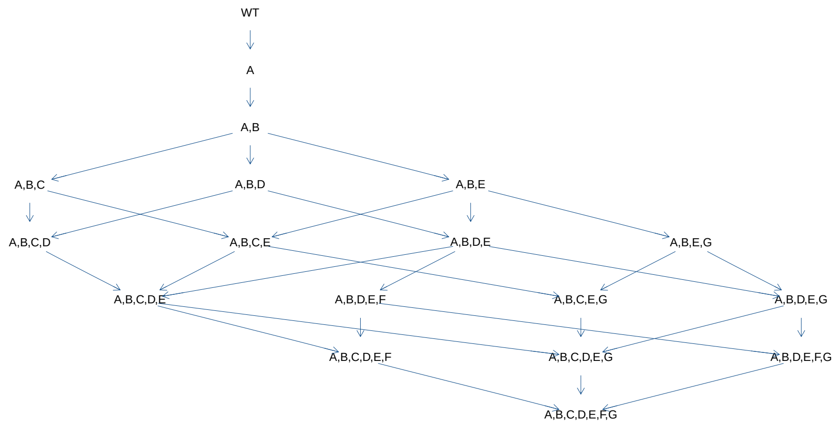

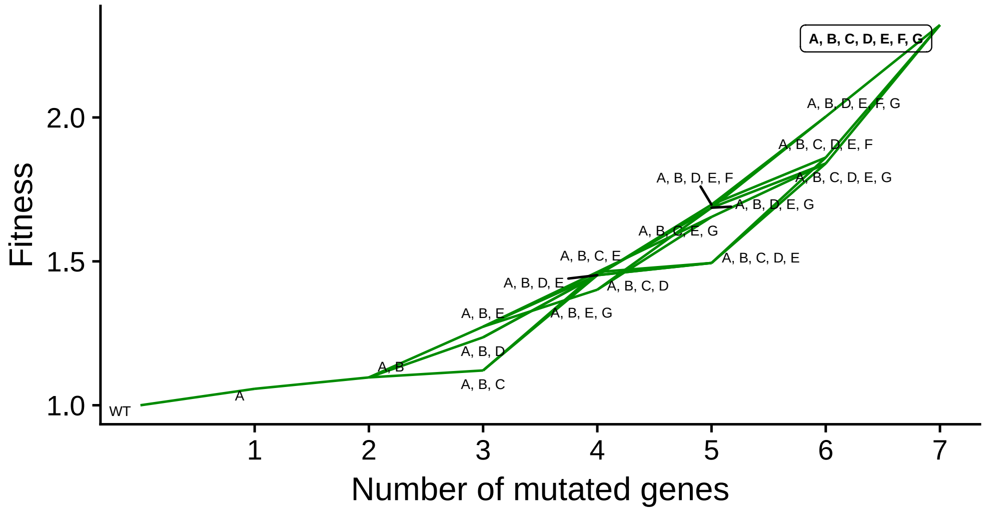

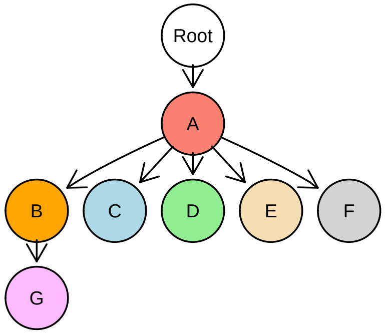

ID	1HutaalyylPzhYlD
acc genotypes	49
peaks no back	1
removed edges	0
prop rem edges	0

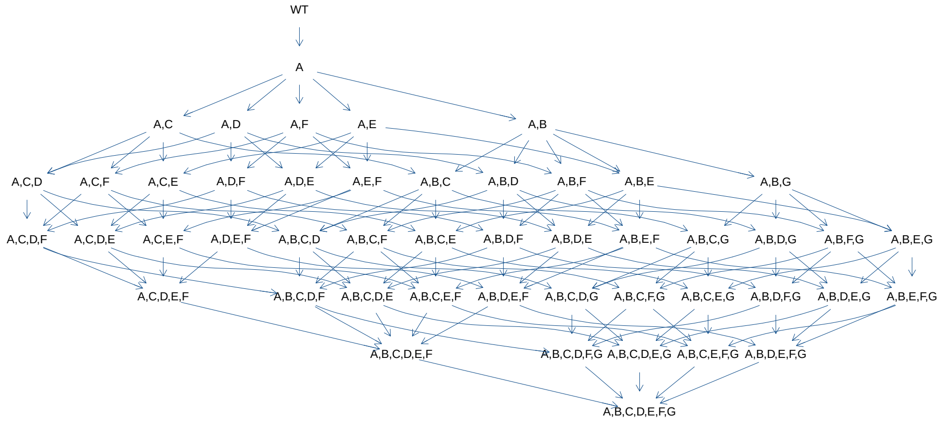

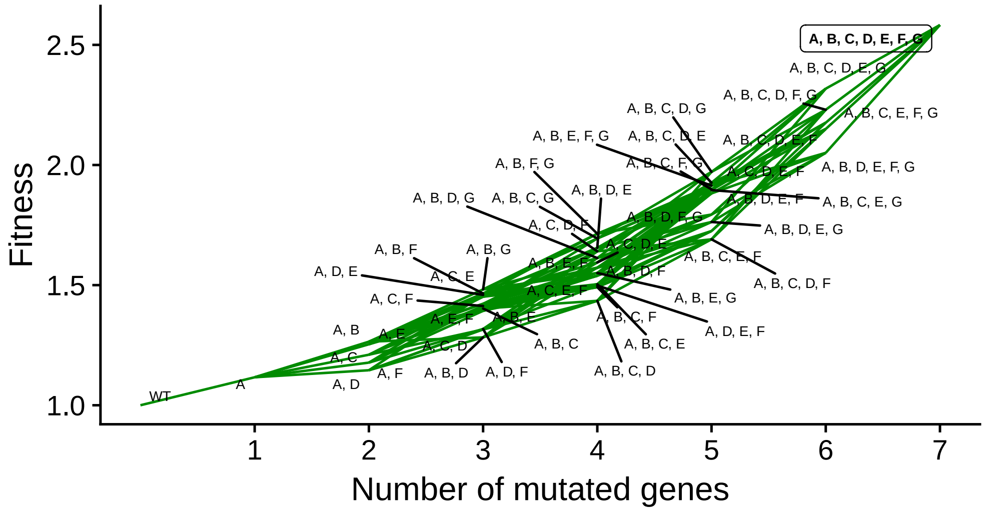

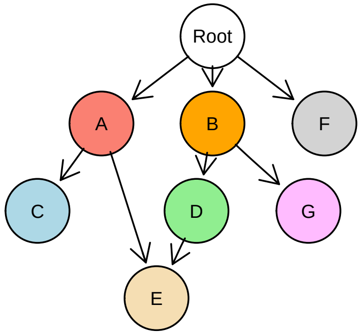

ID	1Mj1yzbmqyN510otm
acc genotypes	38
peaks no back	1
removed edges	0
prop rem edges	0

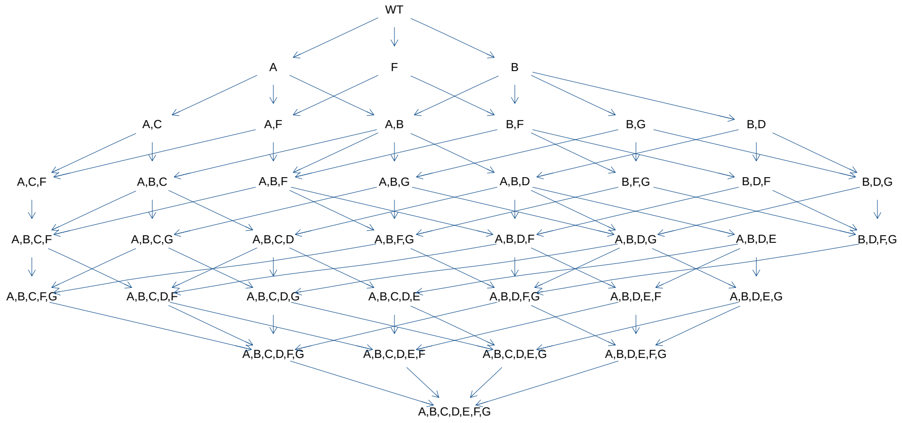

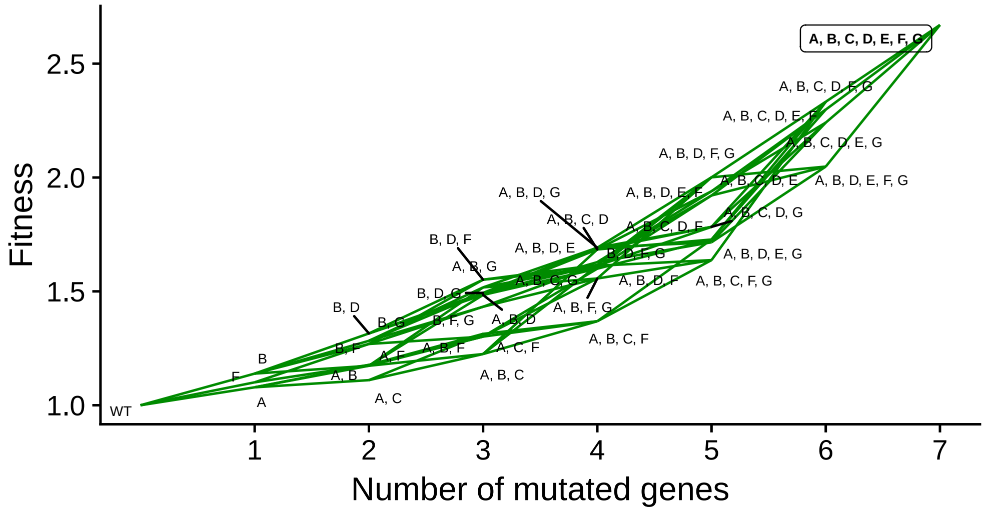

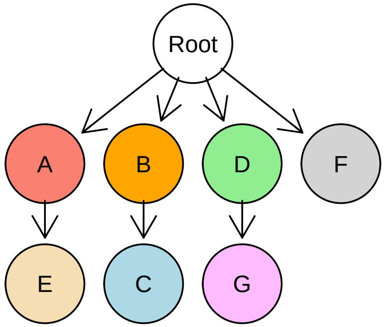

ID 1OIh71InKRui4v2e
acc genotypes 54
peaks no back 1
removed edges 0
prop rem edges 0

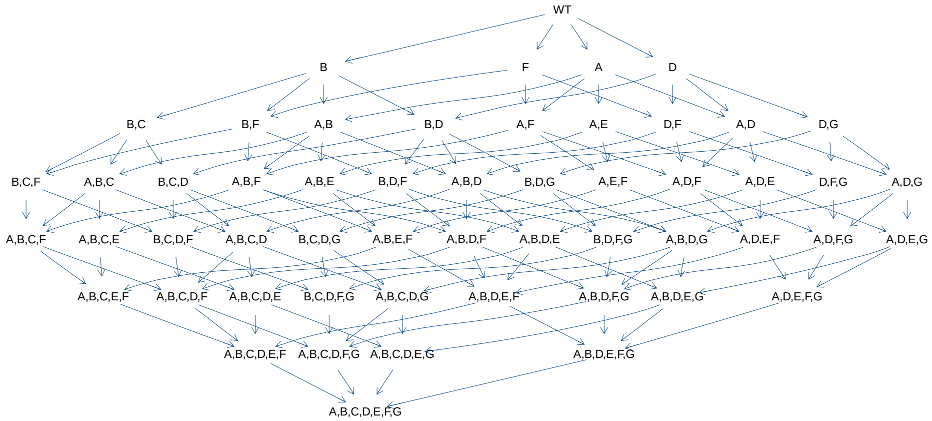

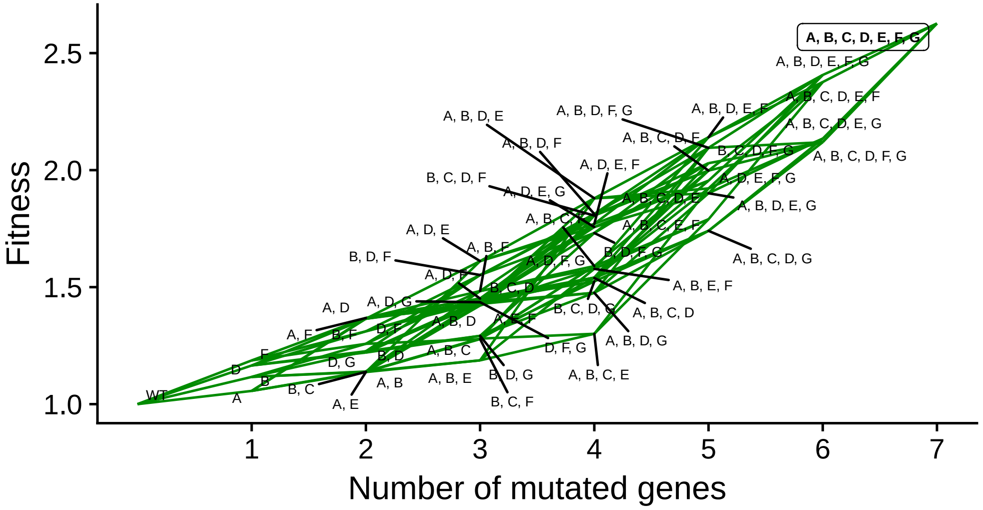

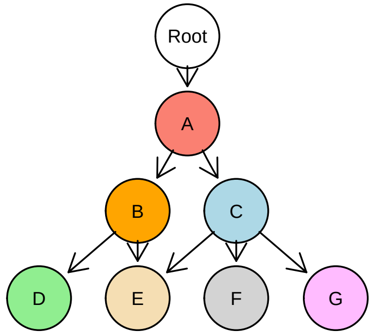

ID 1uosnPLLoevoyoqh
acc genotypes 24
peaks no back 1
removed edges 0
prop rem edges 0

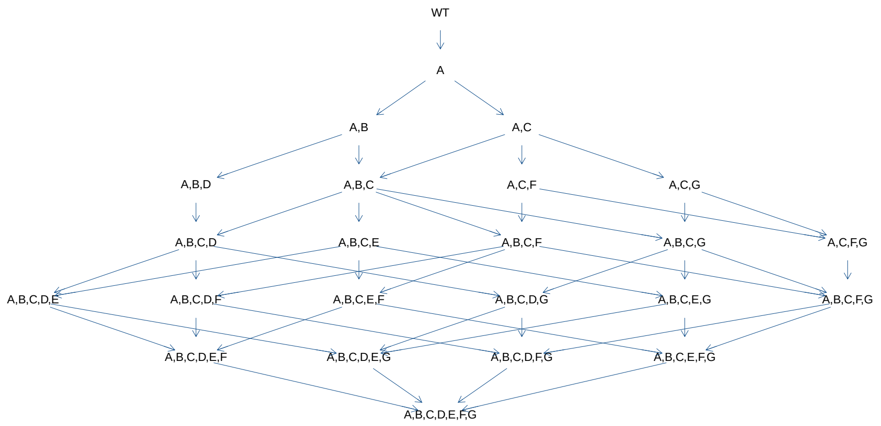

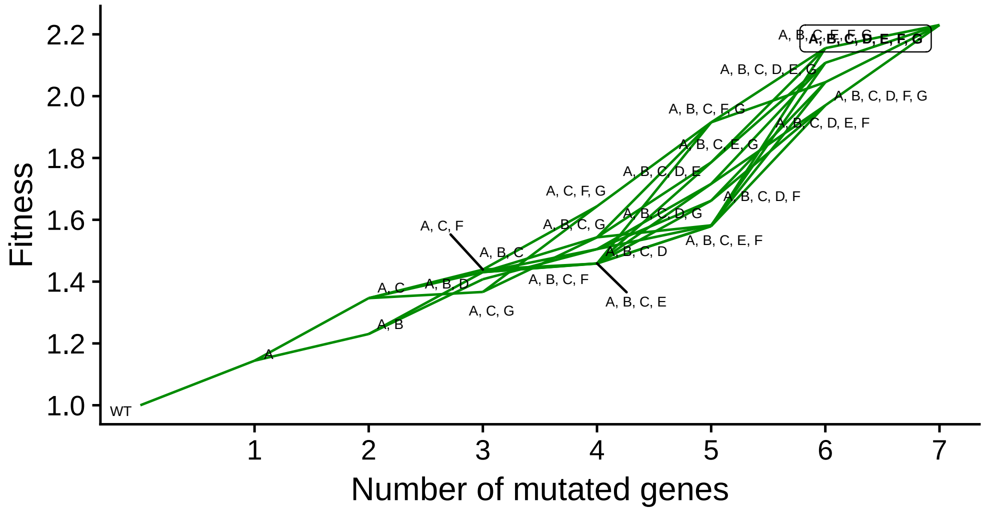

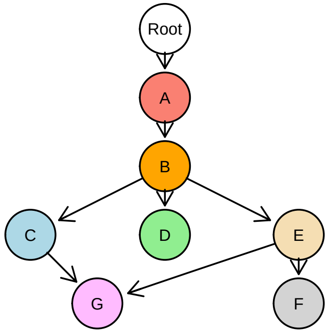

ID	29lH82hKYXw3zuT10
acc genotypes	18
peaks no back	1
removed edges	0
prop rem edges	0

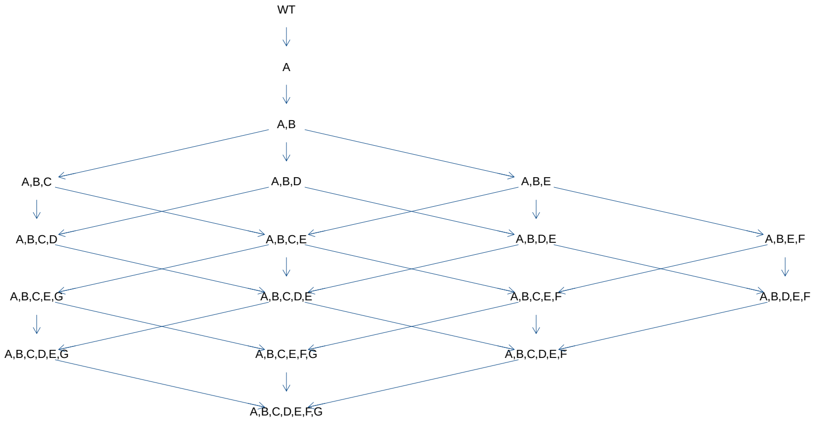

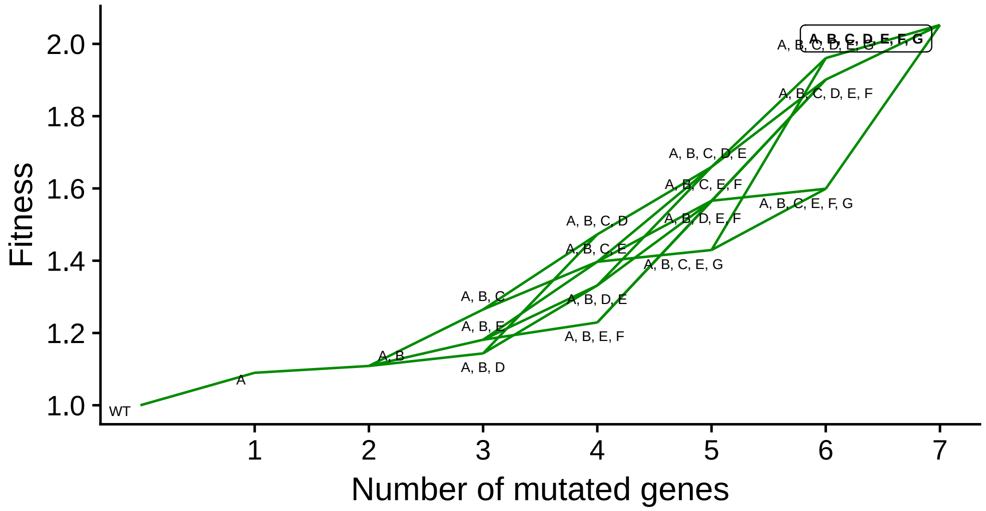

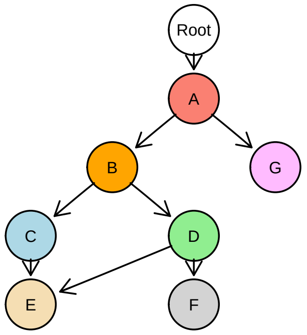

ID	2FyhSdCFo4Nq5dT10
acc genotypes	19
peaks no back	1
removed edges	0
prop rem edges	0

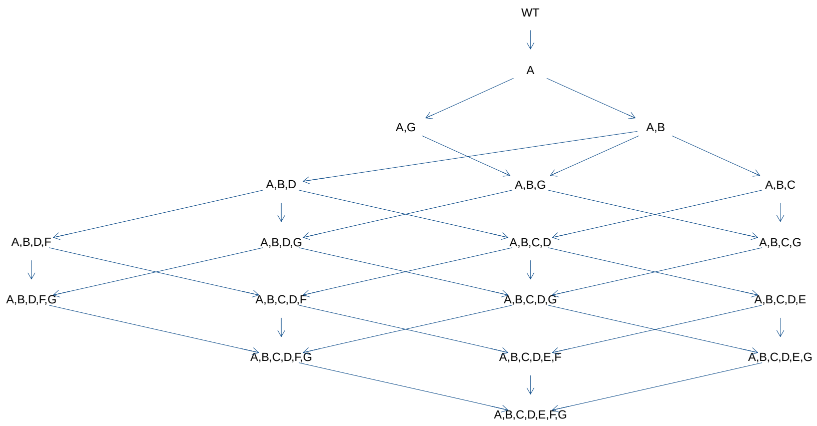

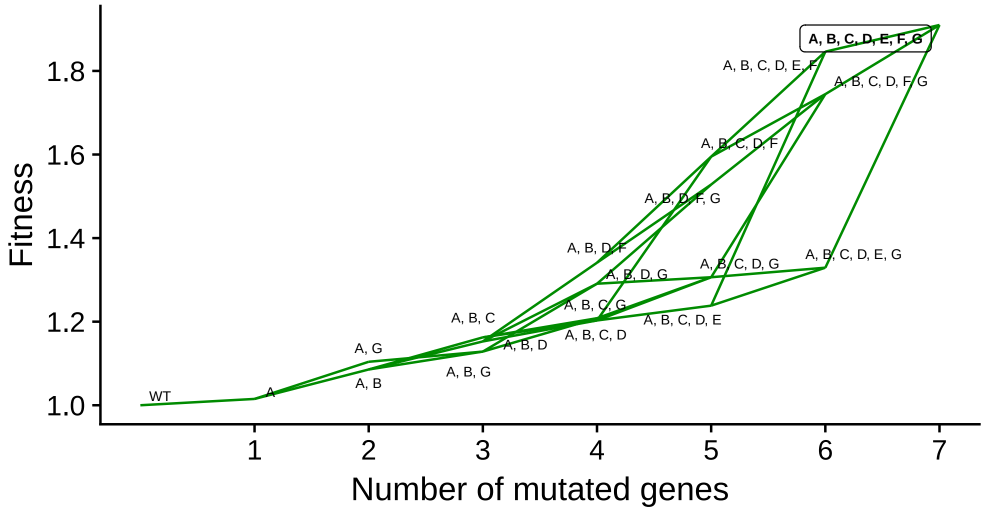

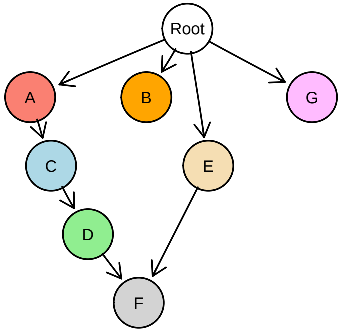

ID	2qPuA9kEjZvpHzWR
acc genotypes	36
peaks no back	1
removed edges	0
prop rem edges	0

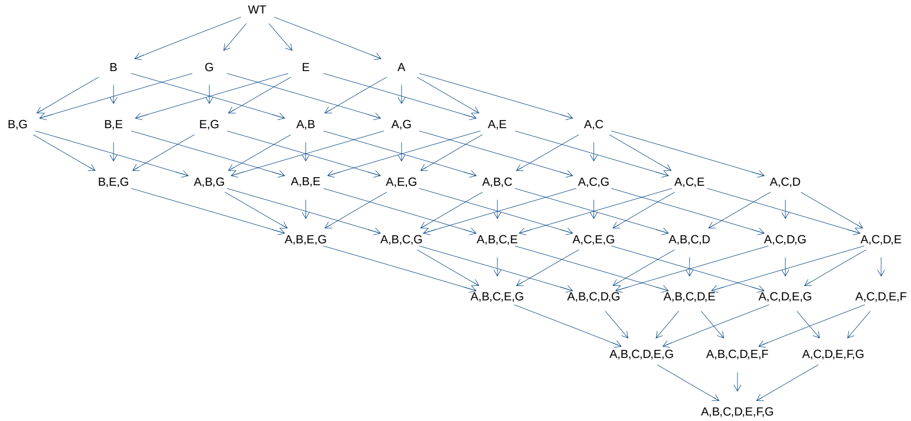

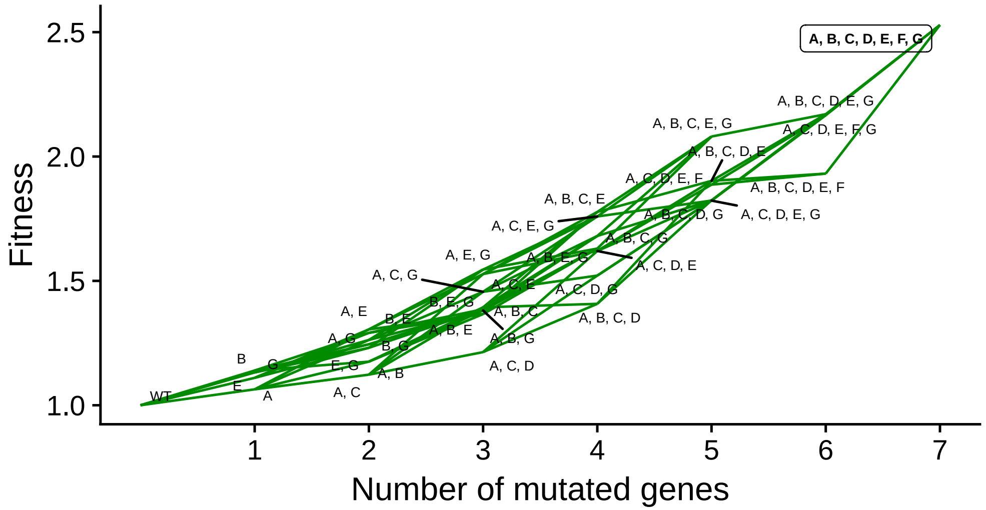

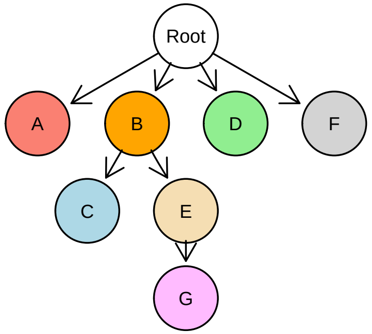

ID	2yfABs8hSv10jftX2
acc genotypes	56
peaks no back	1
removed edges	0
prop rem edges	0

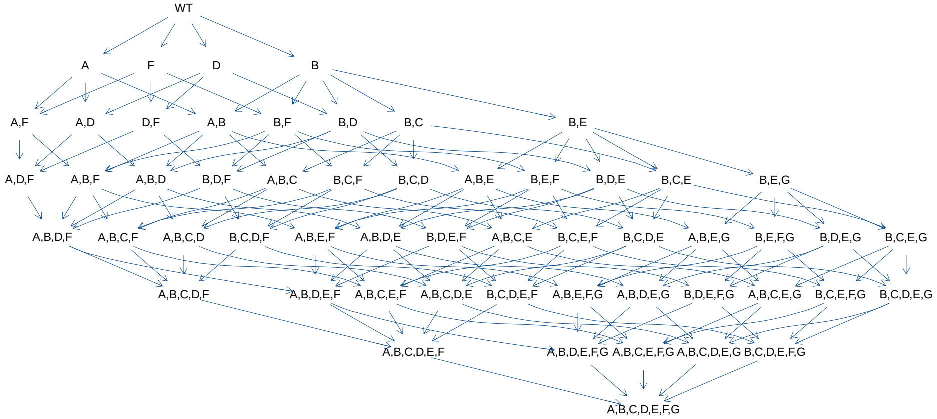

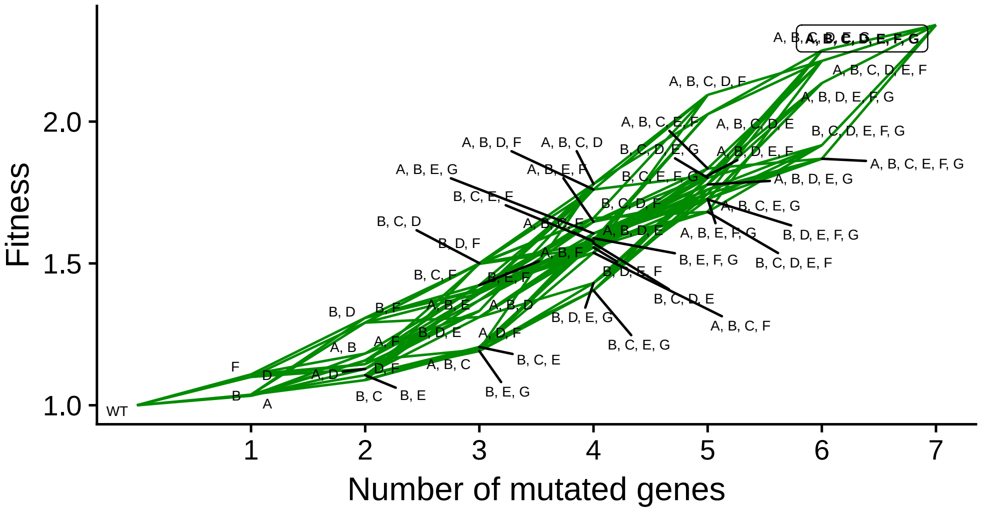

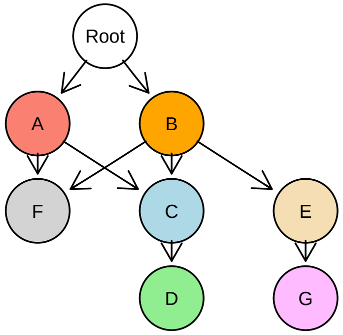

ID	3PMBKfaRwTHJEyd8
acc genotypes	23
peaks no back	1
removed edges	0
prop rem edges	0

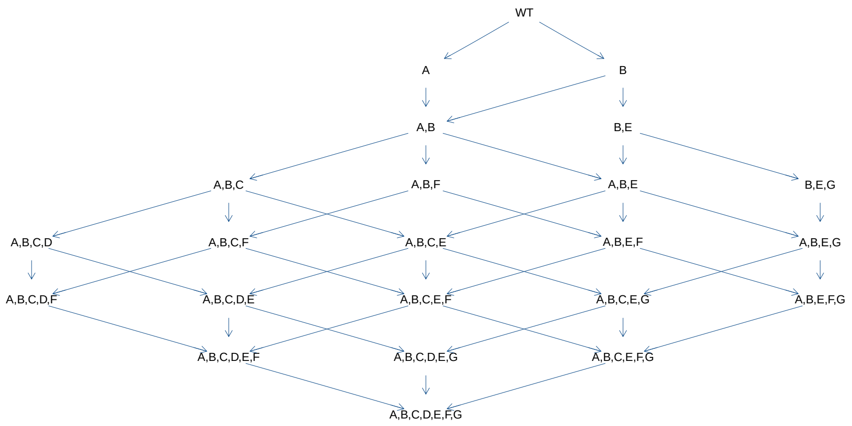

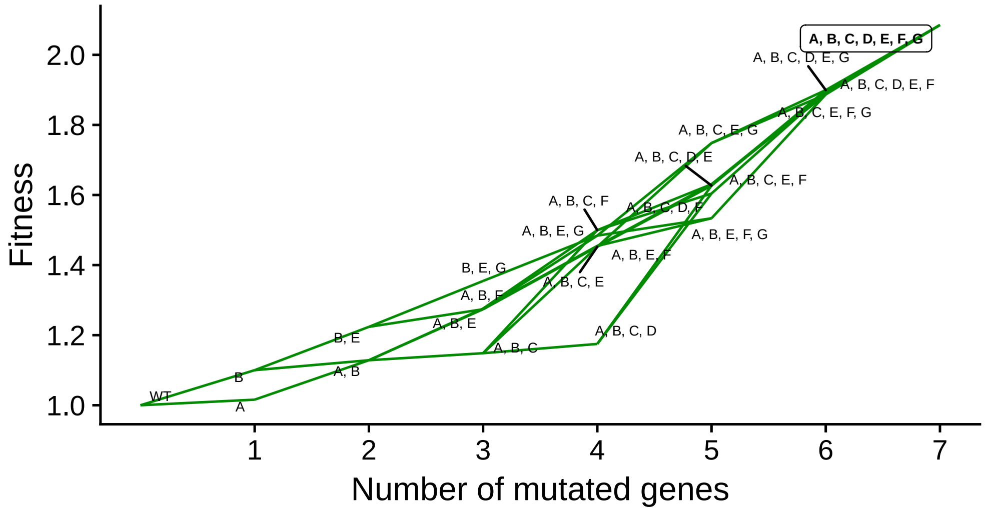

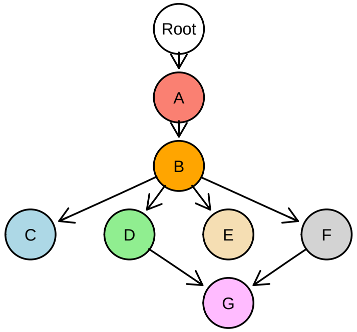

ID	3qyRJOswye1eG84H
acc genotypes	22
peaks no back	1
removed edges	0
prop rem edges	0

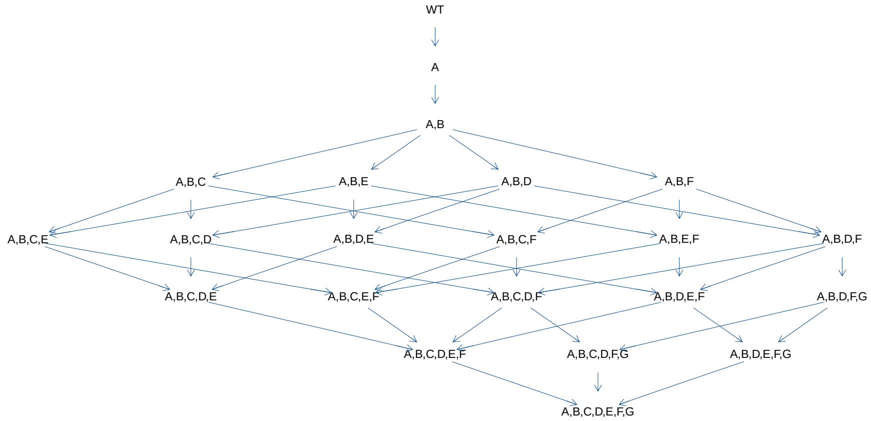

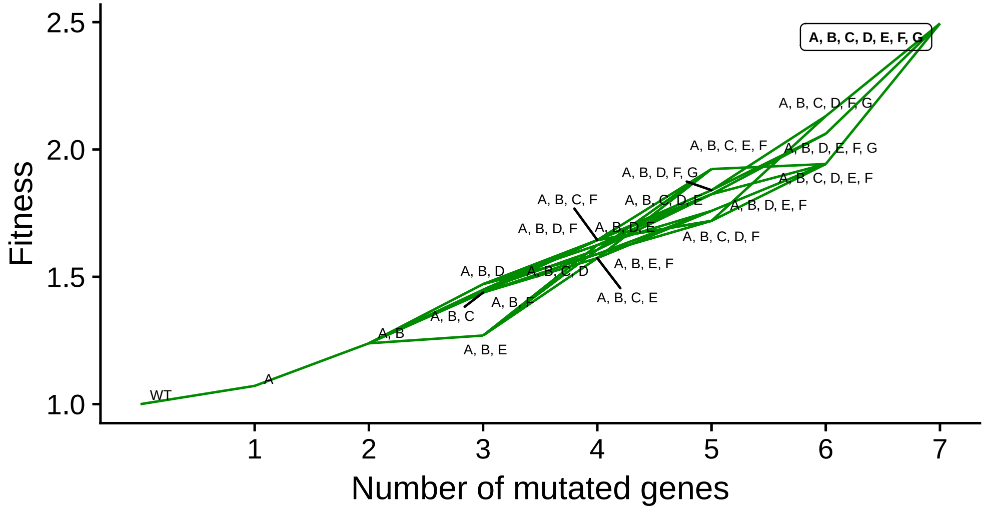

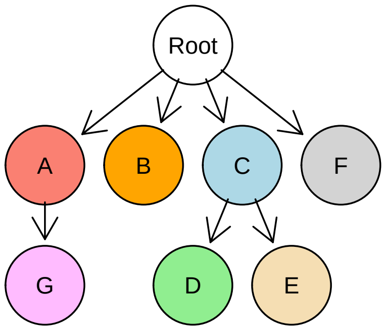

ID	3YPBDYDpA3ERbdMp
acc genotypes	60
peaks no back	1
removed edges	0
prop rem edges	0

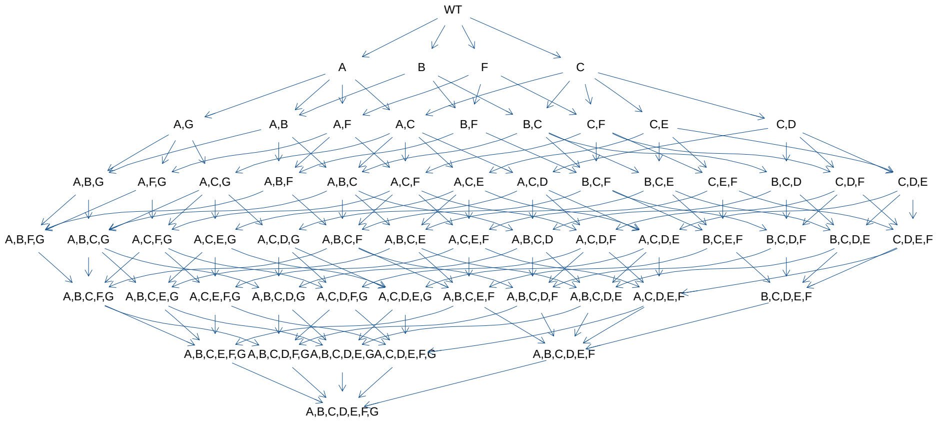

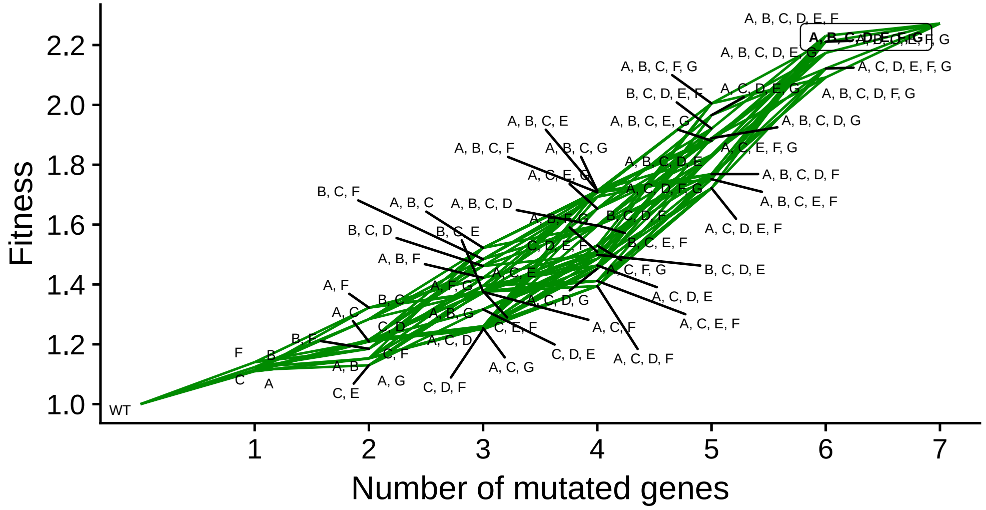

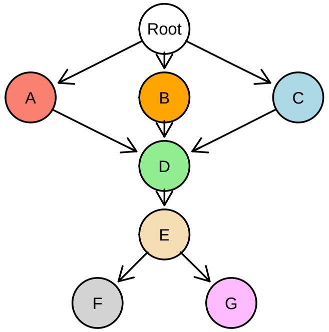

ID	417teASz4uBqi4qQ
acc genotypes	13
peaks no back	1
removed edges	0
prop rem edges	0

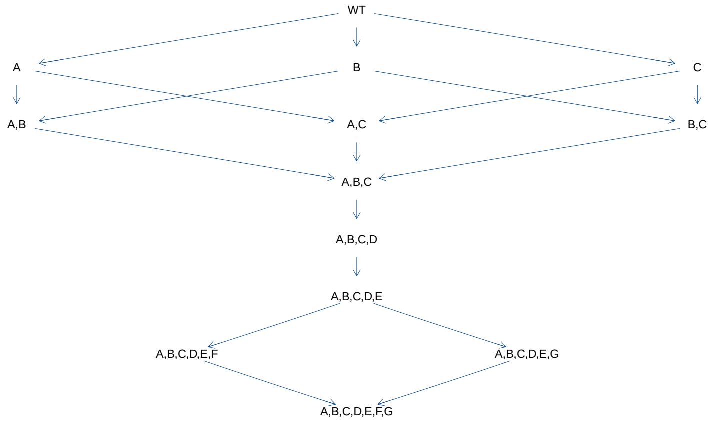

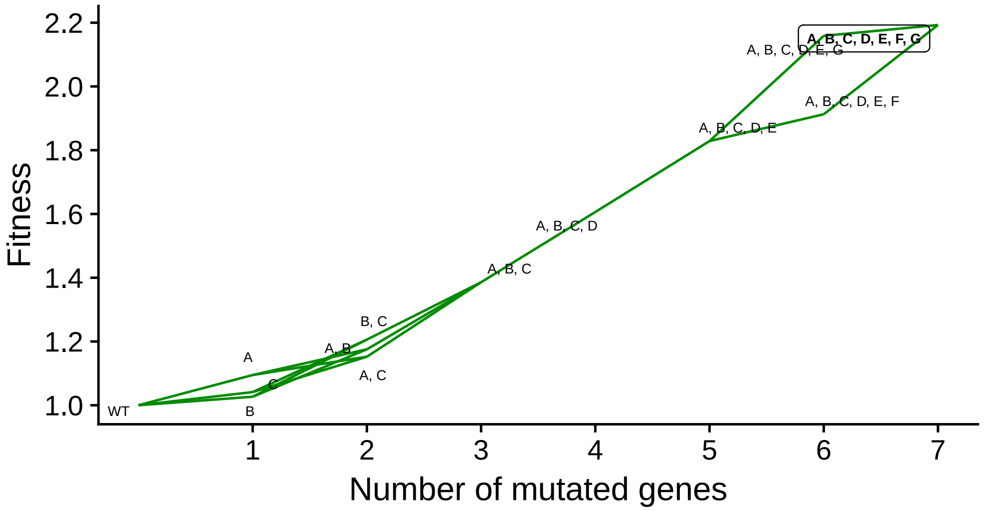

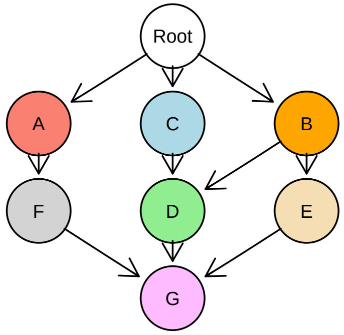

ID	46rFIBbA10BhomWGN
acc genotypes	25
peaks no back	1
removed edges	0
prop rem edges	0

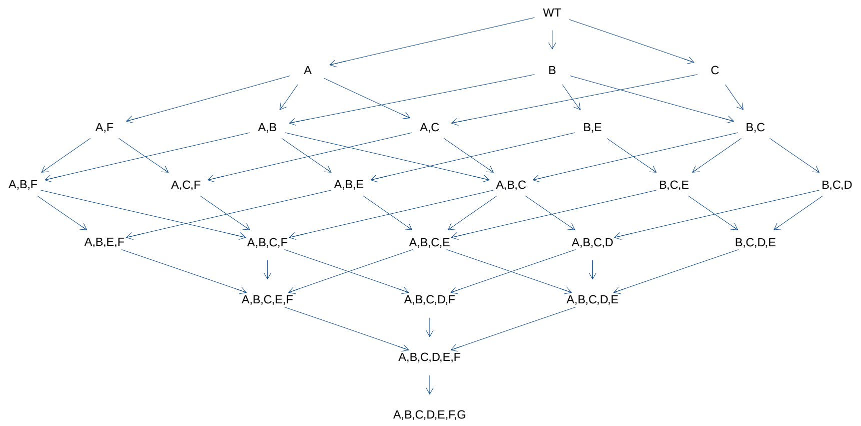

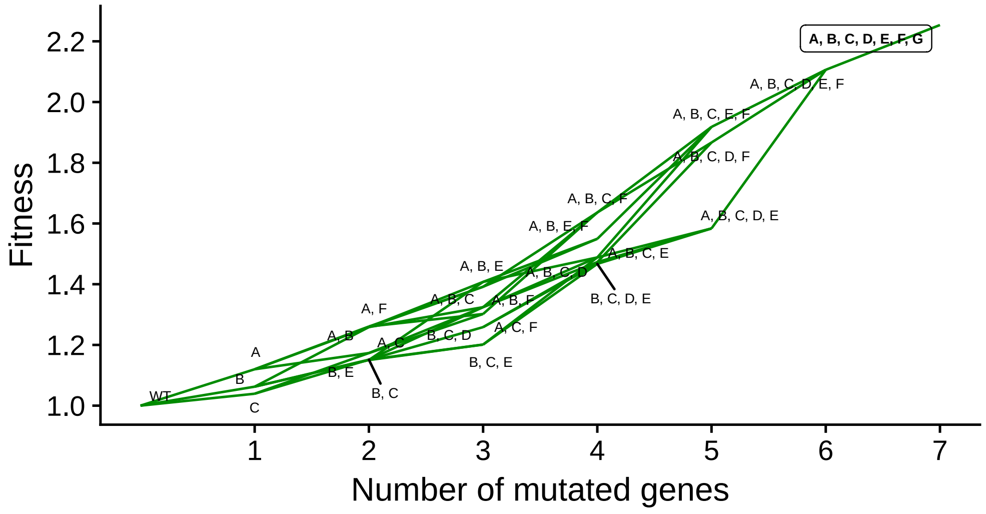

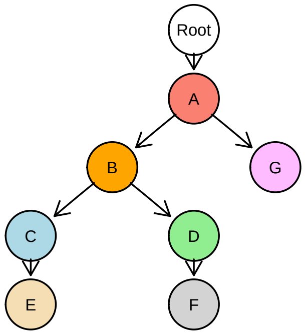

ID	4mouiGQselPBUfsX
acc genotypes	21
peaks no back	1
removed edges	0
prop rem edges	0

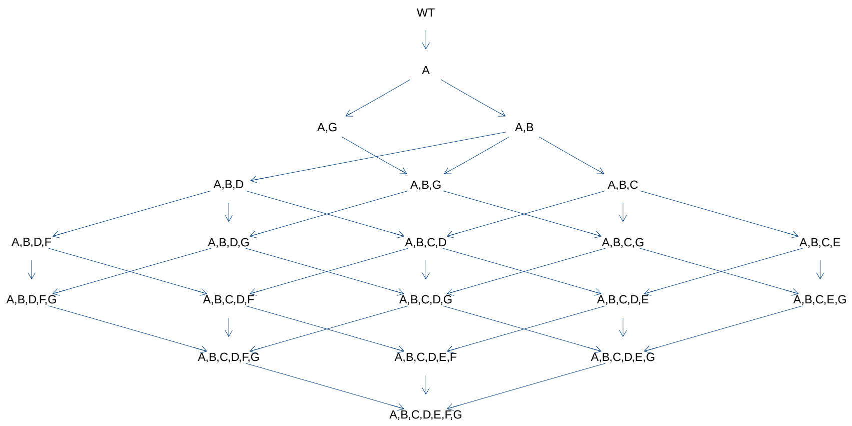

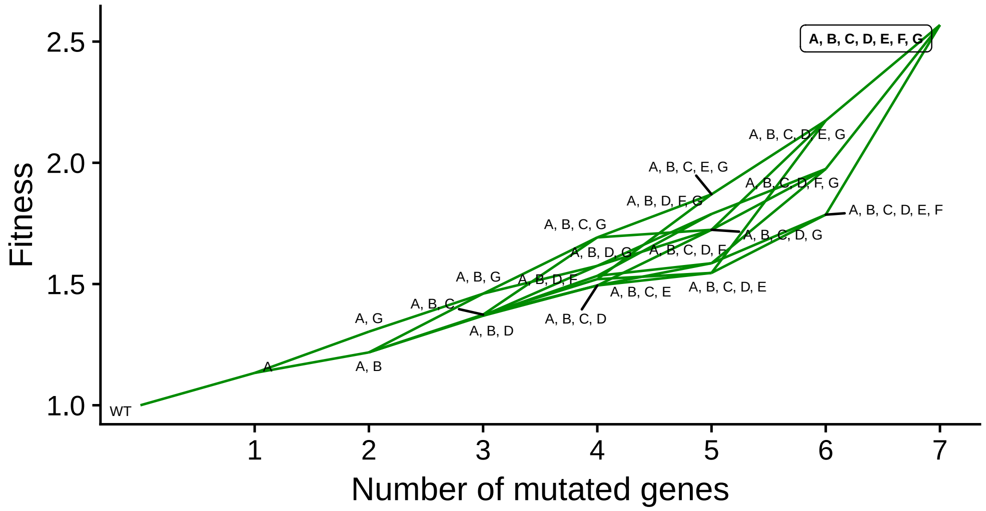

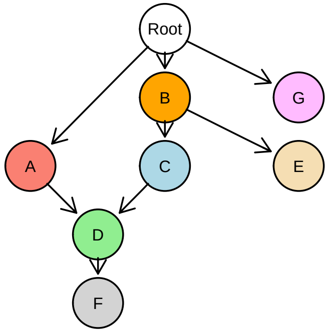

ID	4RoAEsprOB10Yv3U6
acc genotypes	28
peaks no back	1
removed edges	0
prop rem edges	0

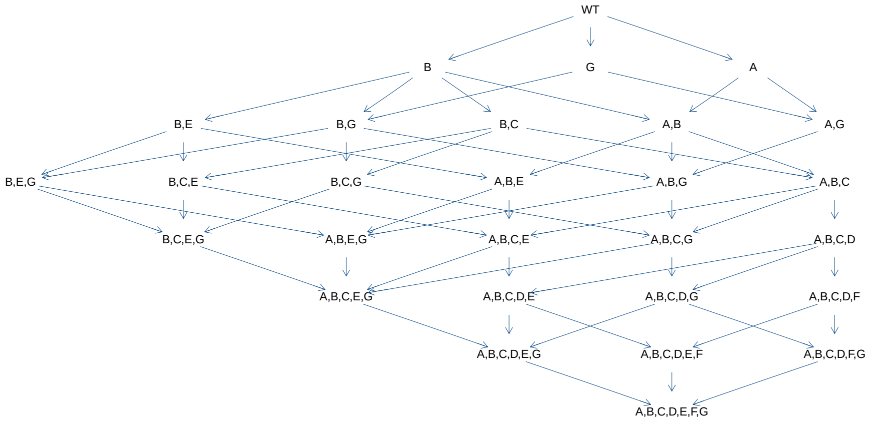

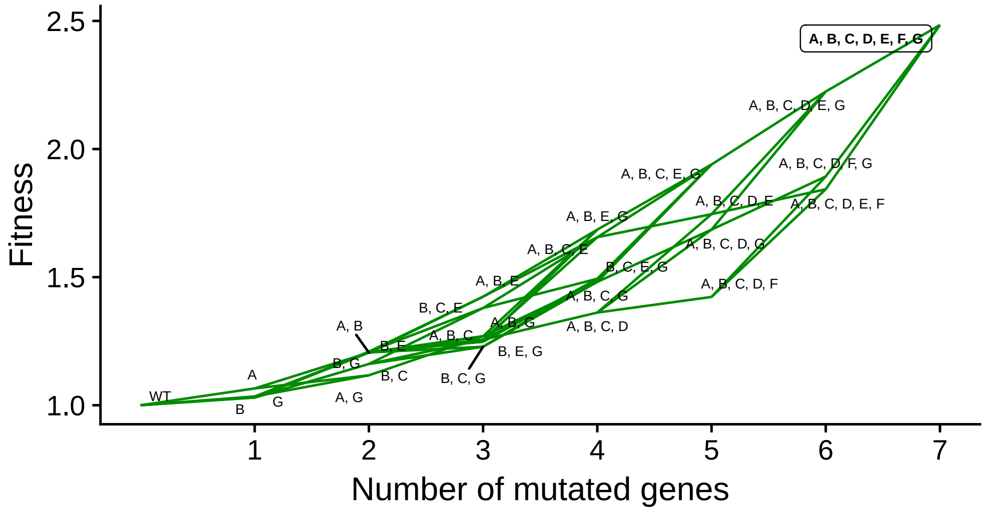

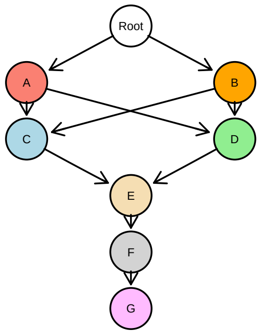

ID	4yUgjvZI1UgonSqo
acc genotypes	10
peaks no back	1
removed edges	0
prop rem edges	0

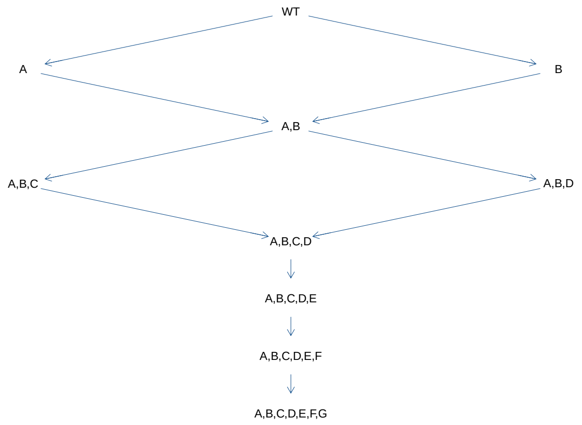

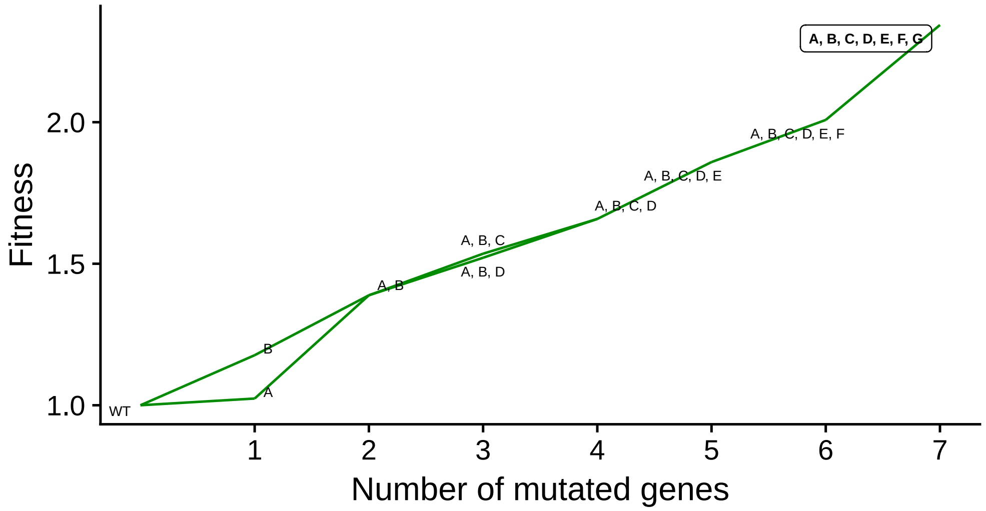

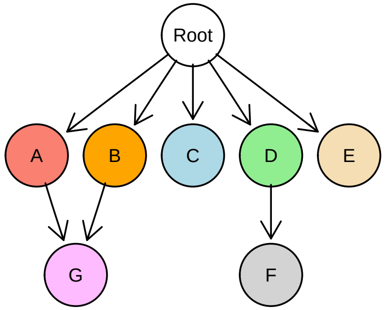

ID	5fz101Z59qIyO10Rxn
acc genotypes	60
peaks no back	1
removed edges	0
prop rem edges	0

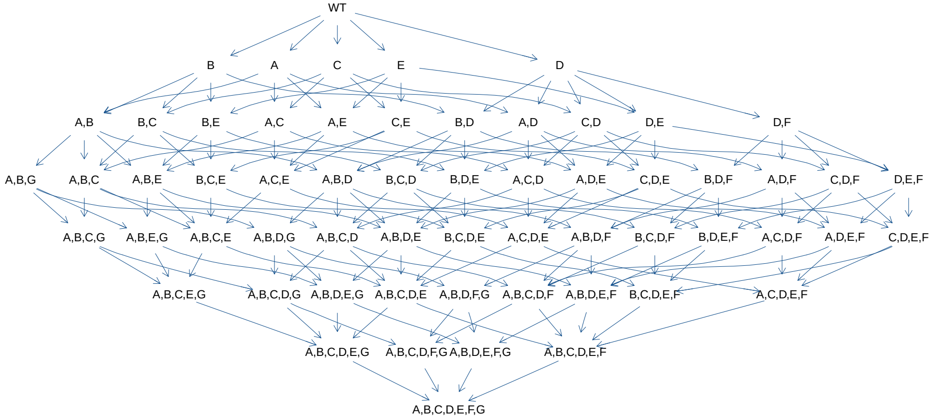

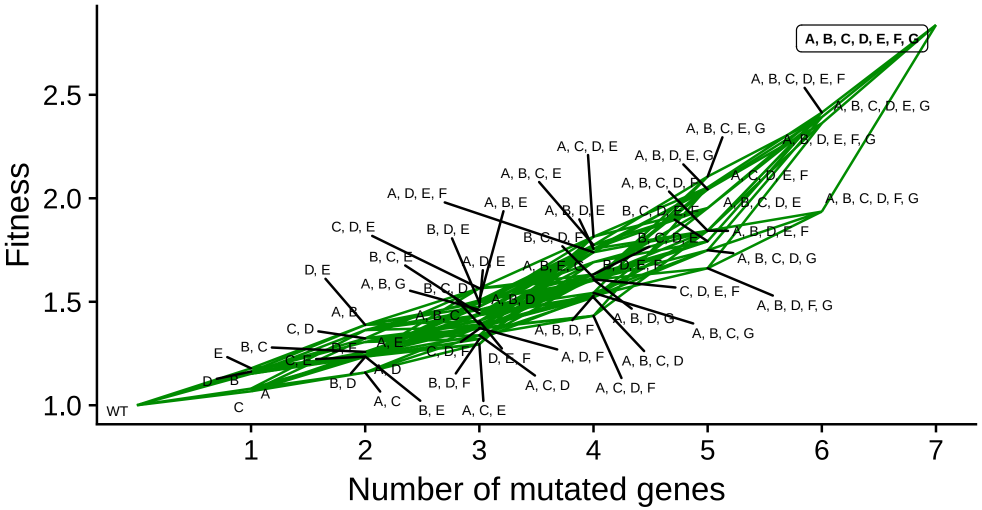

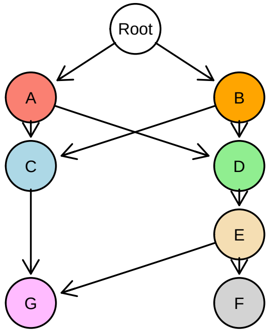

ID	6pjAHfBKJ10Ocigjg
acc genotypes	13
peaks no back	1
removed edges	0
prop rem edges	0

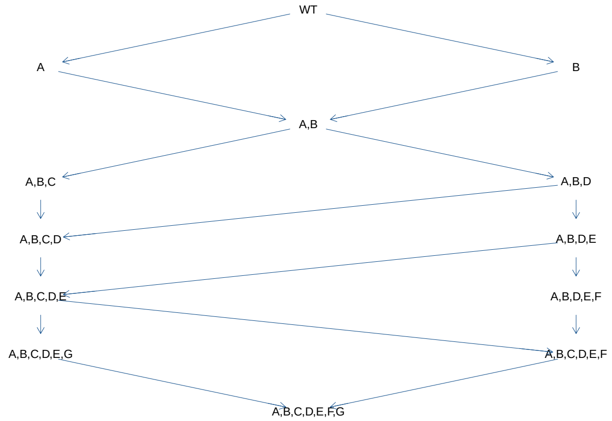

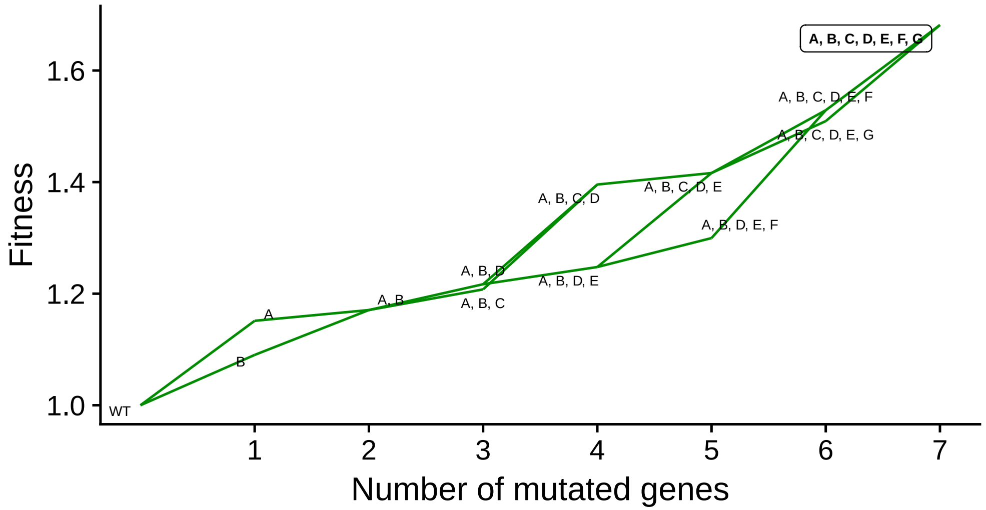

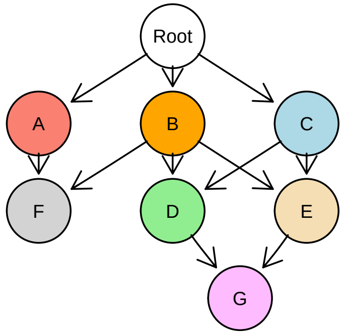

ID	718HviJL2vTZgZyJ
acc genotypes	22
peaks no back	1
removed edges	0
prop rem edges	0

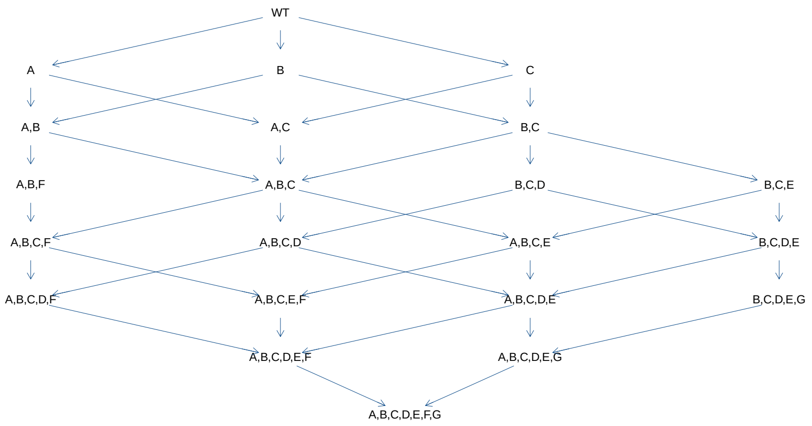

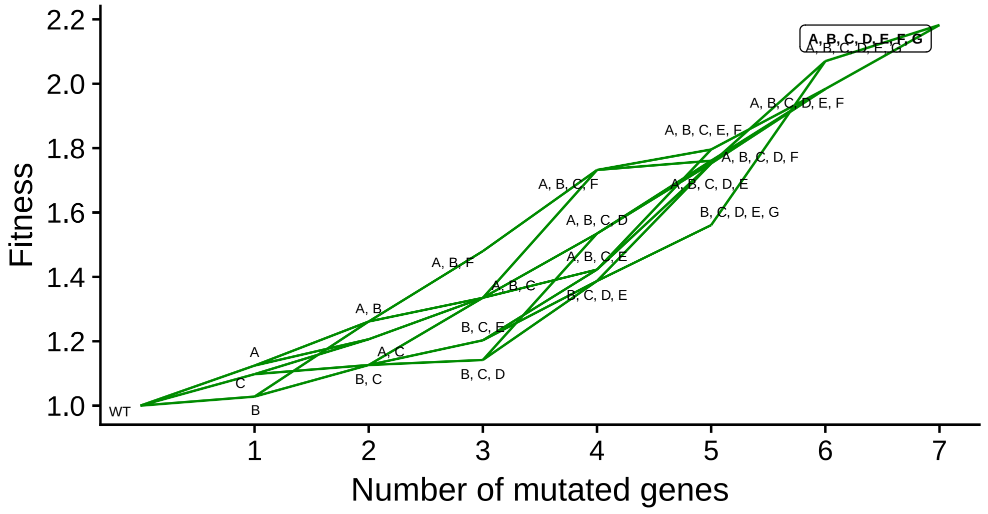

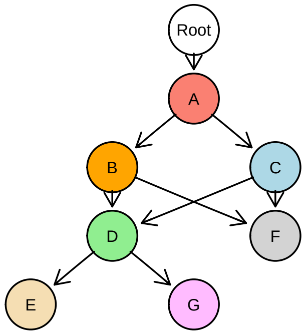

ID	7GFrRtiQ5Cyt3w8k
acc genotypes	14
peaks no back	1
removed edges	0
prop rem edges	0

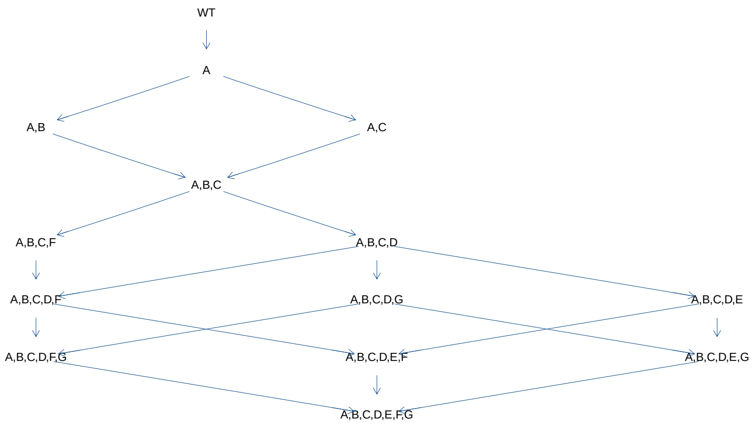

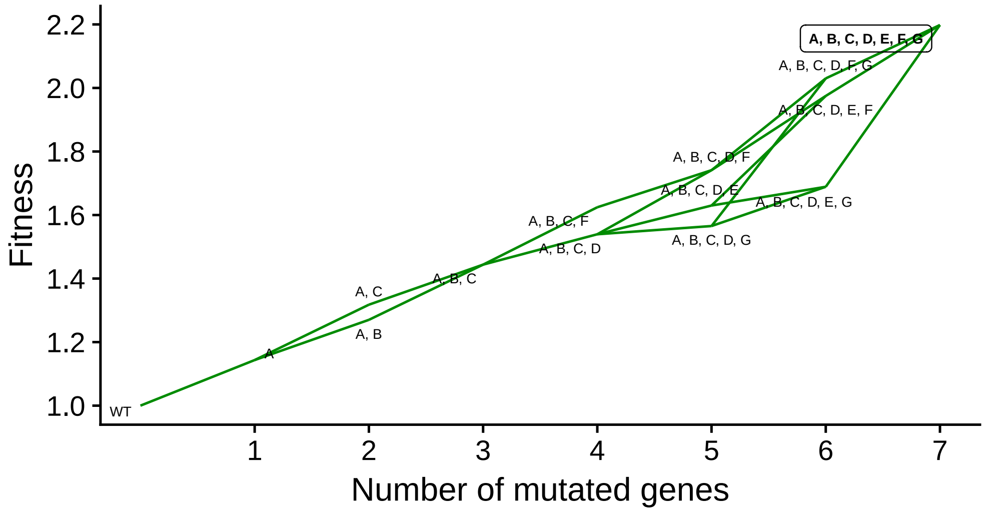

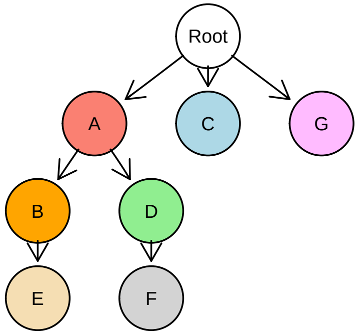

ID	7O6M3PSFd6vyICJi
acc genotypes	40
peaks no back	1
removed edges	0
prop rem edges	0

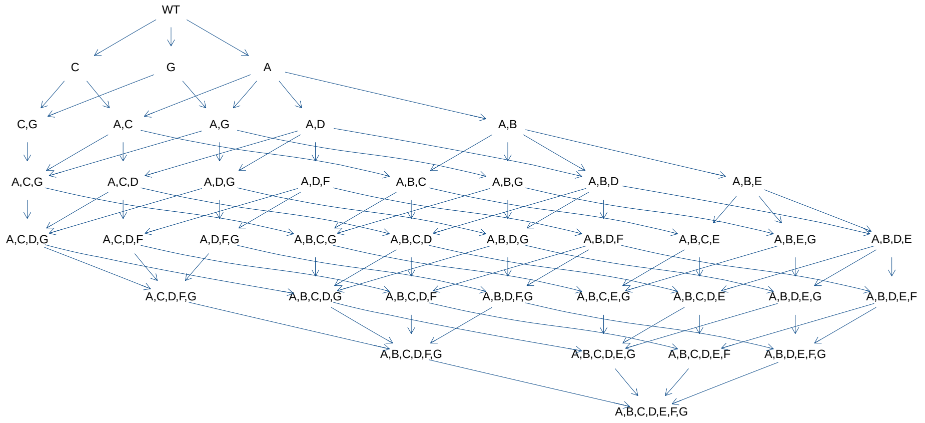

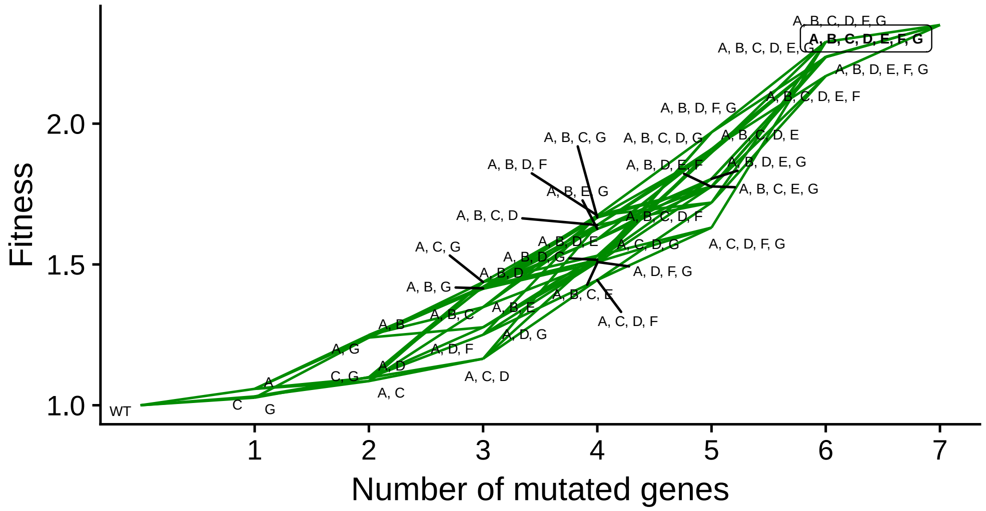

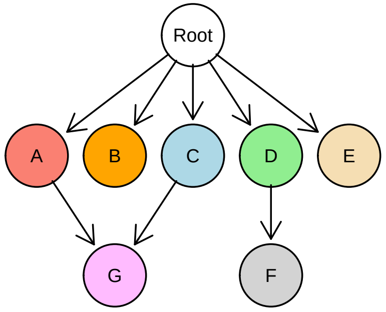

ID	8GEzhn1FLKorAfUm
acc genotypes	60
peaks no back	1
removed edges	0
prop rem edges	0

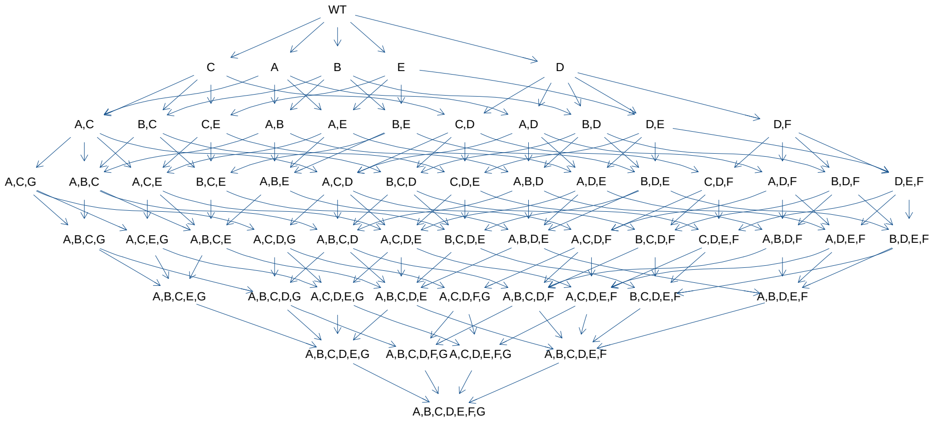

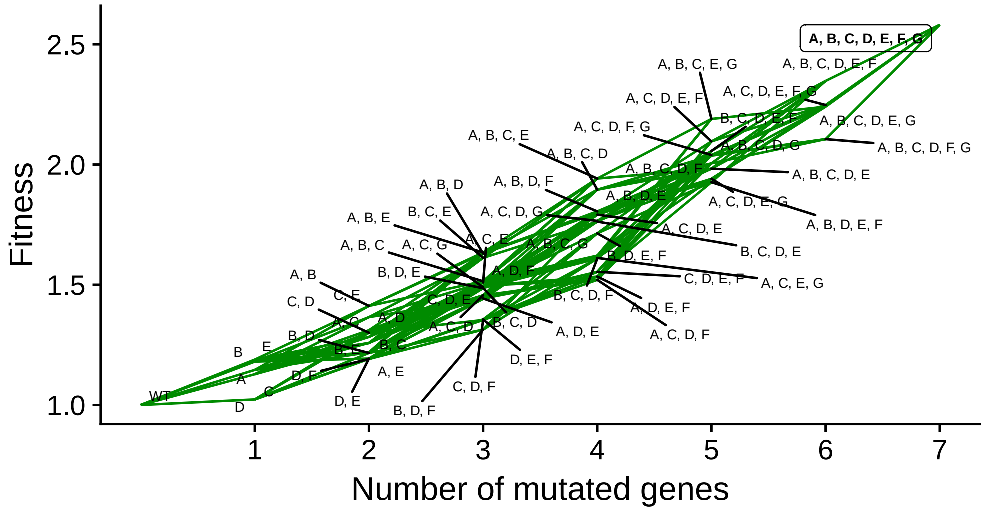

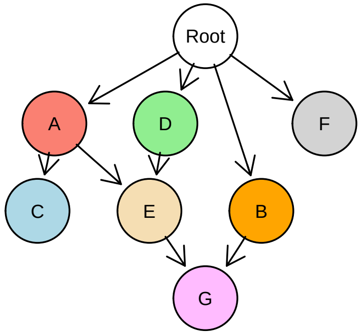

ID	8hAYcae857ZhZJr10
acc genotypes	36
peaks no back	1
removed edges	0
prop rem edges	0

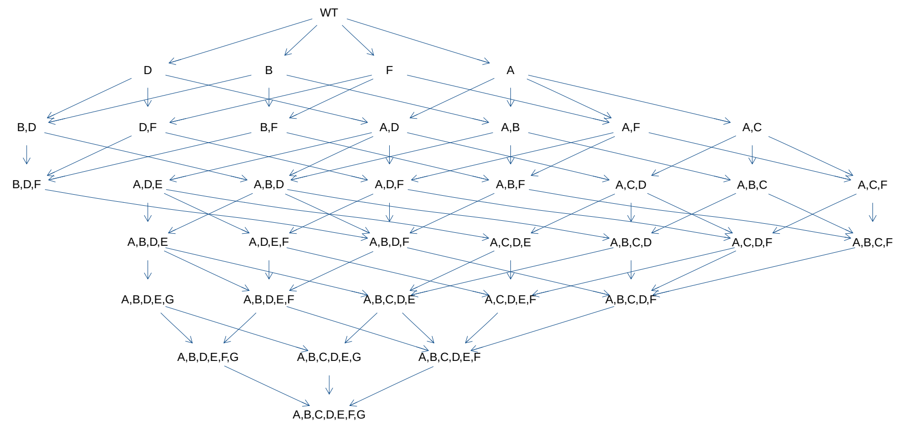

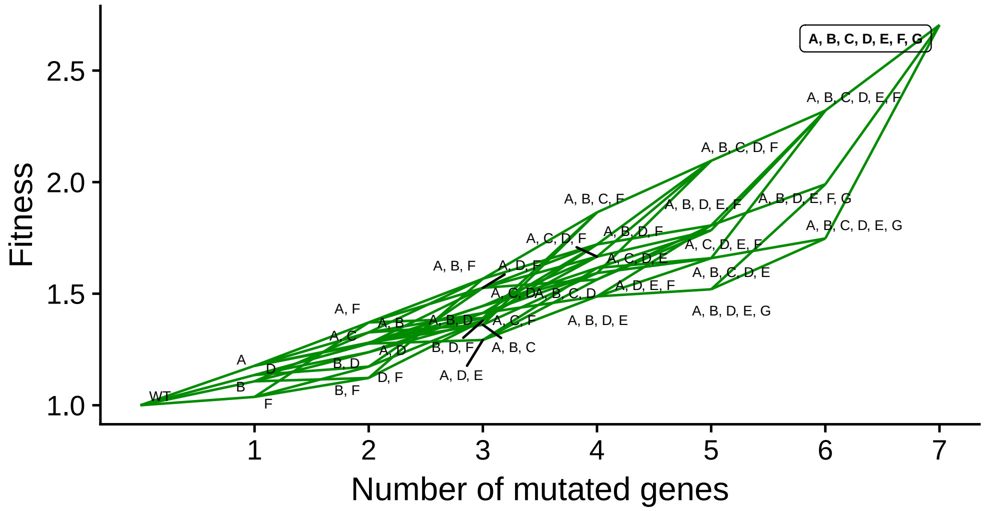

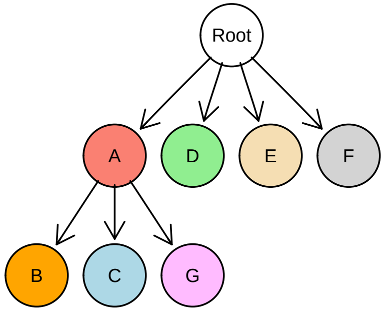

ID	8Zr6qLC9yn9cyhAY
acc genotypes	72
peaks no back	1
removed edges	0
prop rem edges	0

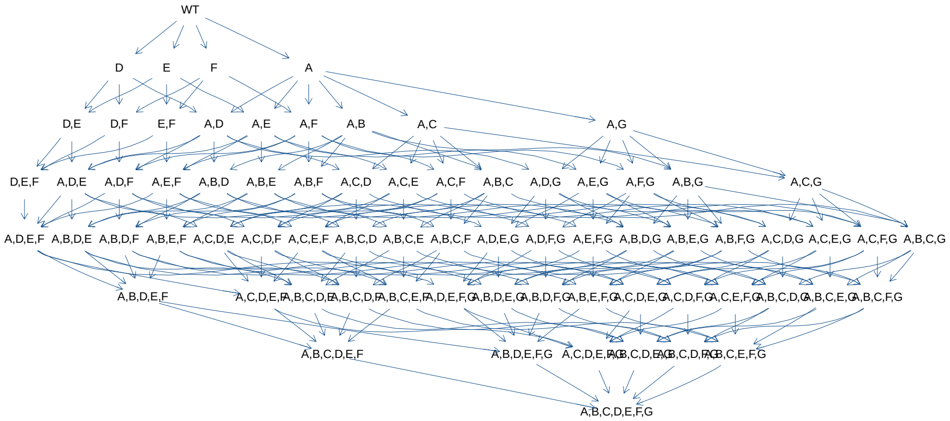

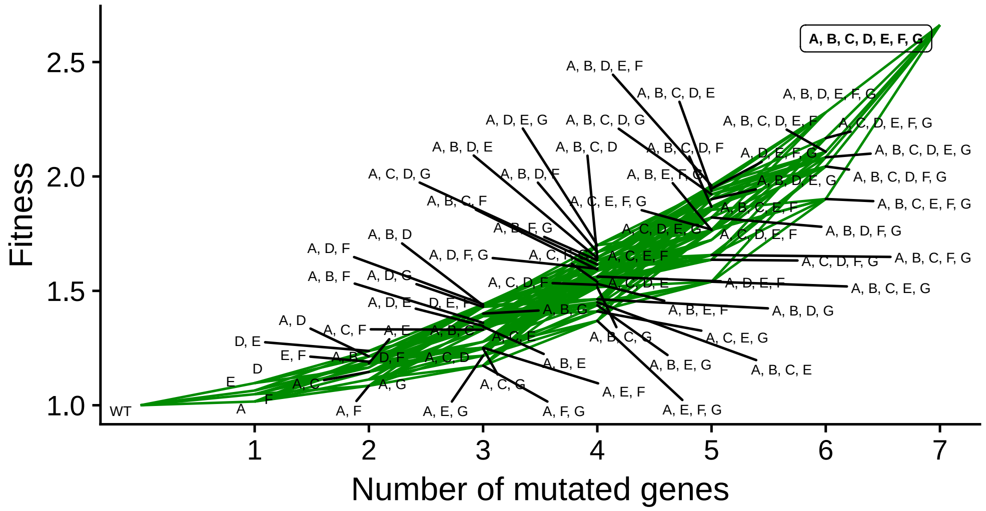

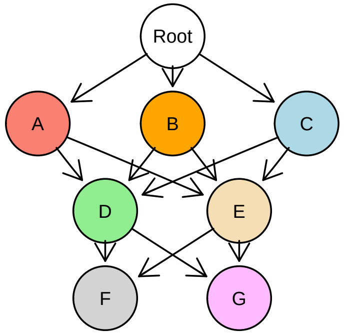

ID	9o10QUimqcWrPHbGH
acc genotypes	14
peaks no back	1
removed edges	0
prop rem edges	0

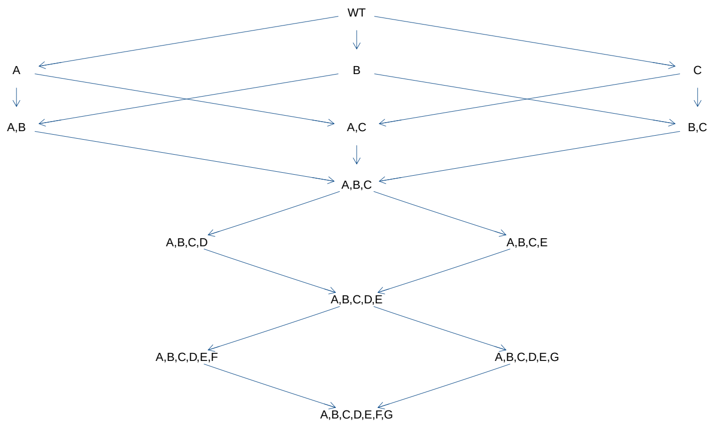

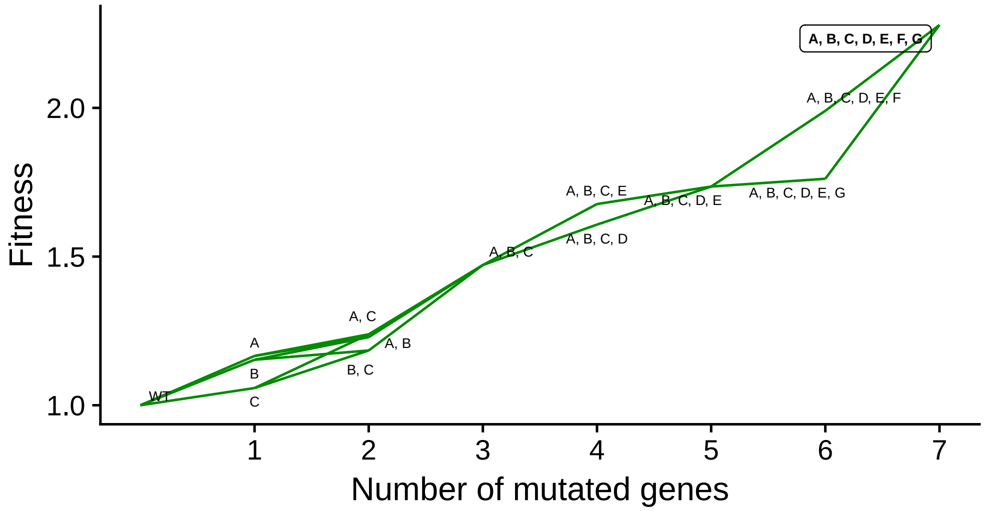

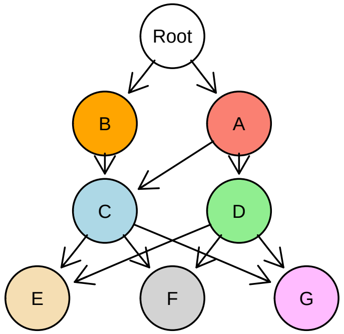

ID a2tStINqtyWzD10eQ
acc genotypes 15
peaks no back 1
removed edges 0
prop rem edges 0

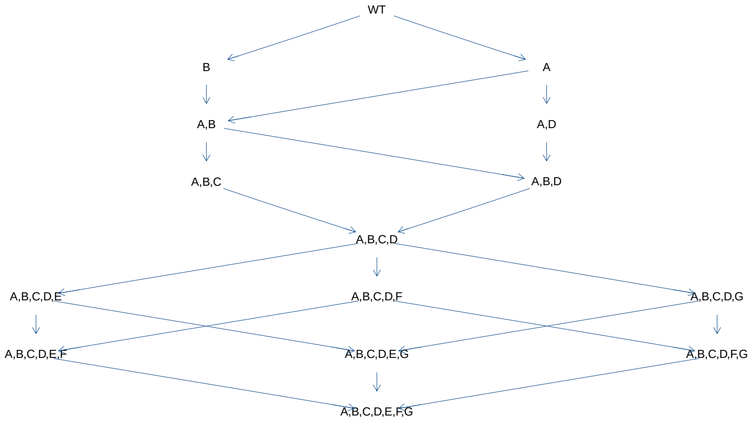

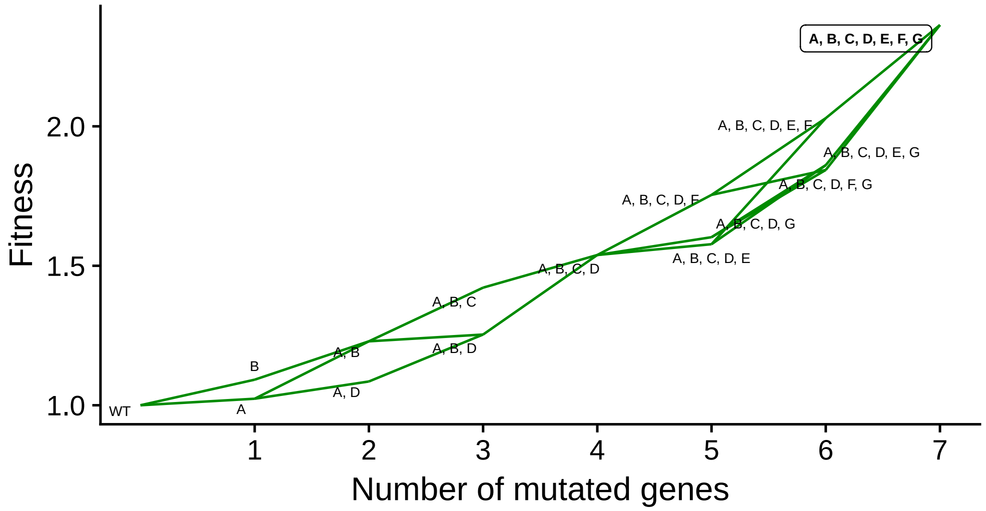

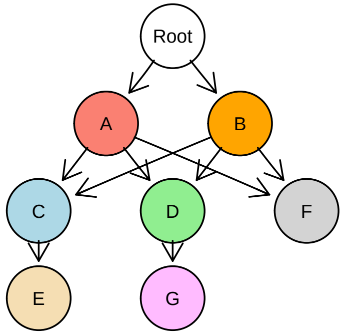

ID a7XZXptDjjWuSFhl
acc genotypes 21
peaks no back 1
removed edges 0
prop rem edges 0

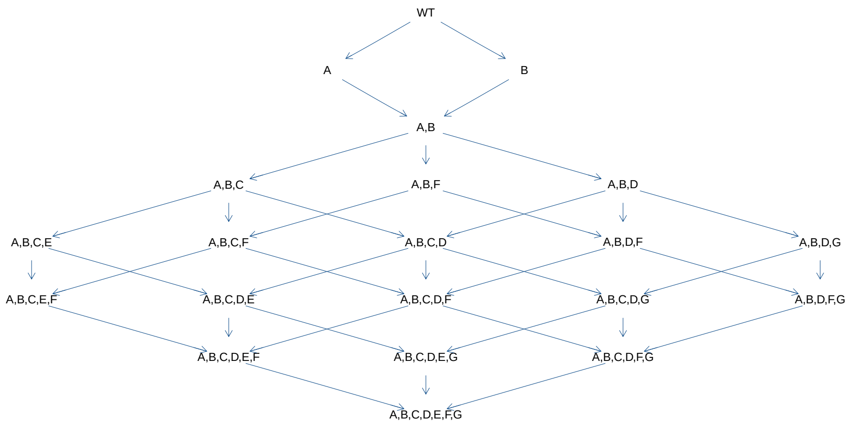

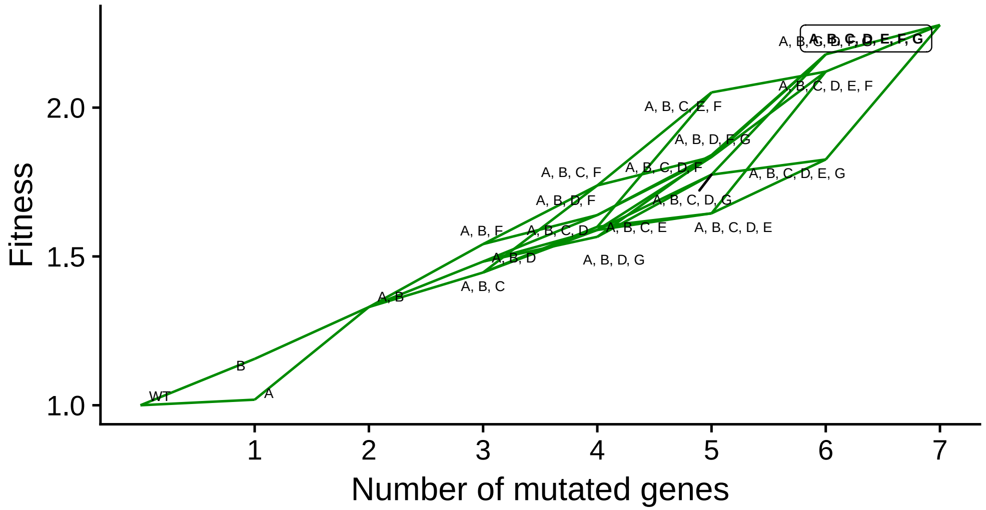

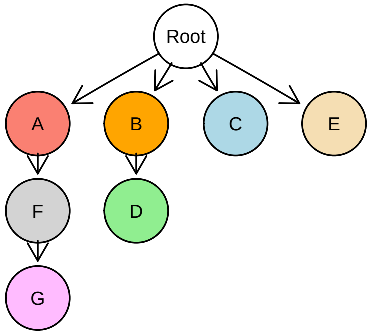

ID	aGBVZklQ4oOGxWTb
acc genotypes	48
peaks no back	1
removed edges	0
prop rem edges	0

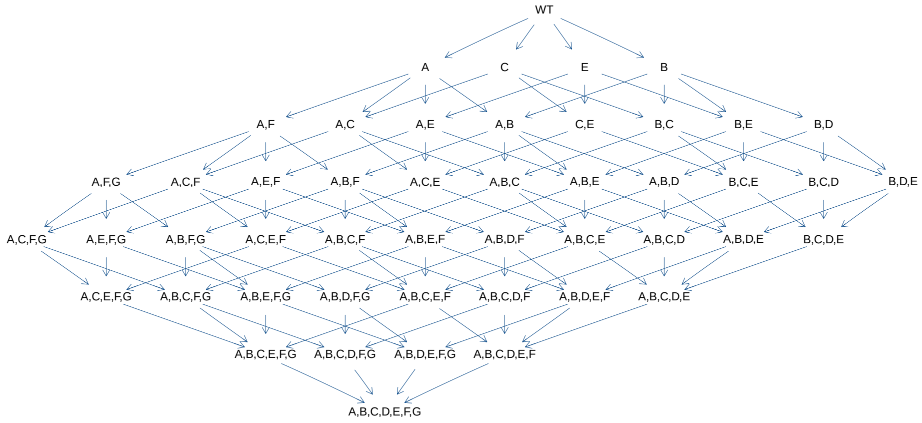

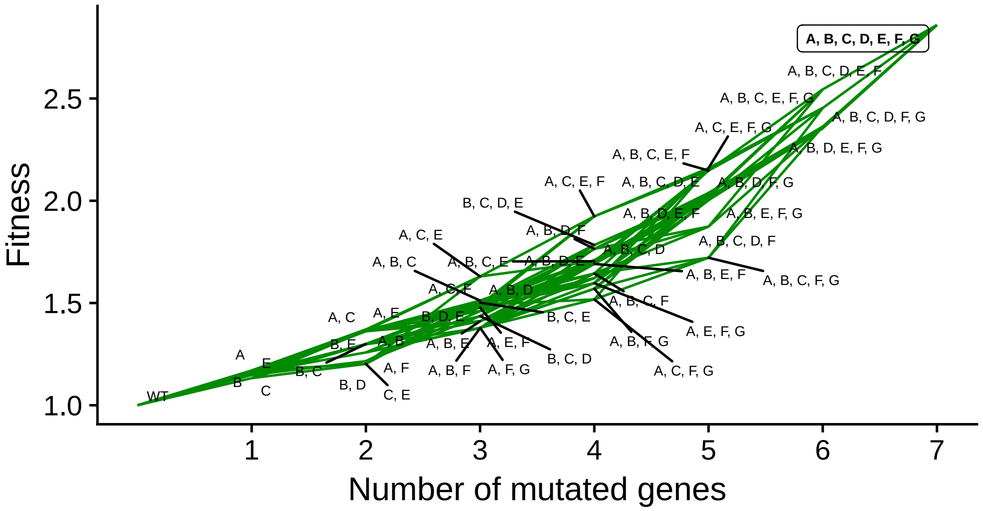

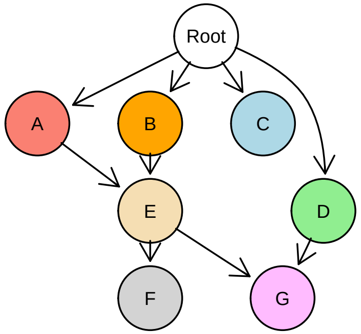

ID	AlBDGRuJ29dXk10wK
acc genotypes	28
peaks no back	1
removed edges	0
prop rem edges	0

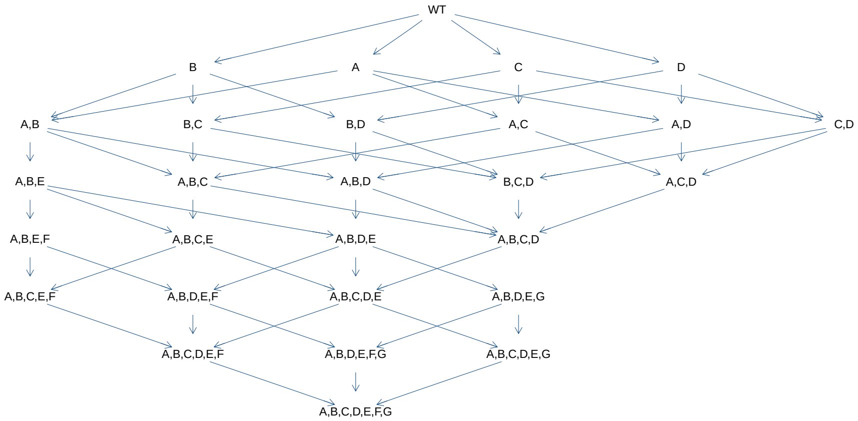

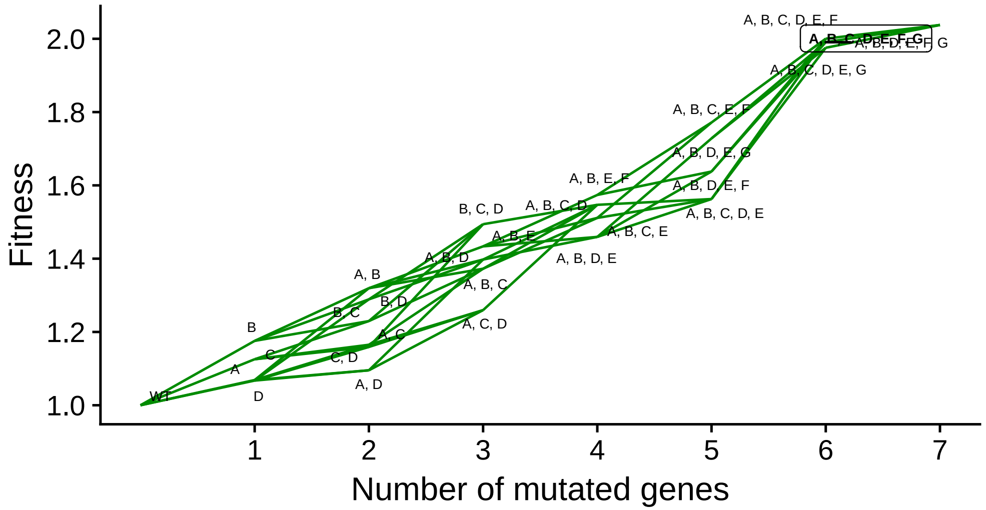

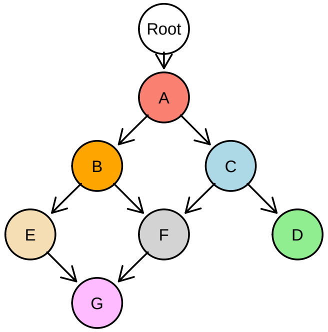

ID	ALtLgLoV6JFZgc9s
acc genotypes	16
peaks no back	1
removed edges	0
prop rem edges	0

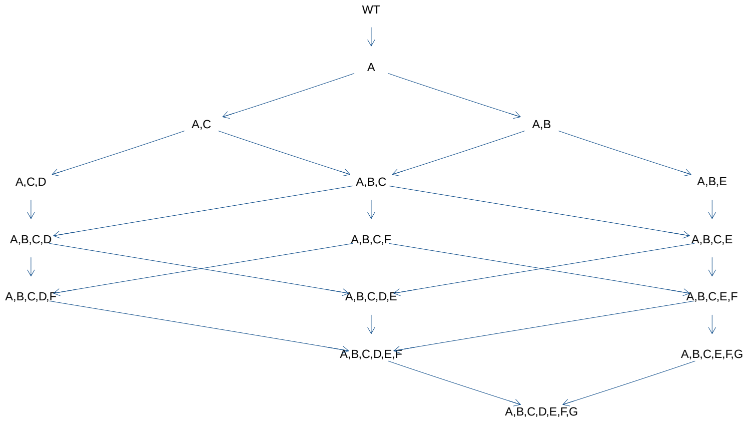

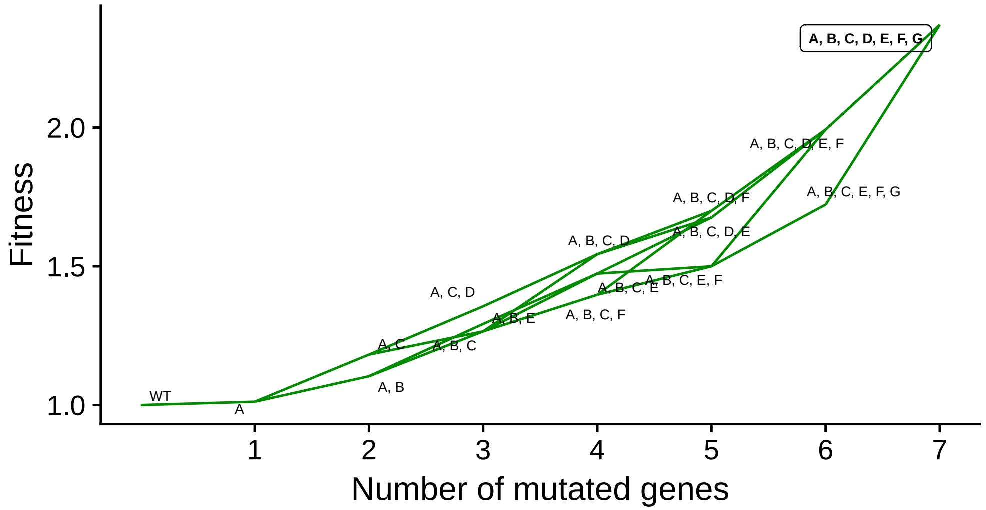

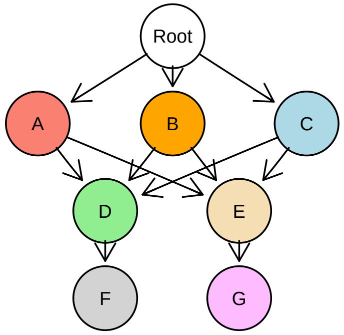

ID	AomkJD2bjXlfY6bL
acc genotypes	16
peaks no back	1
removed edges	0
prop rem edges	0

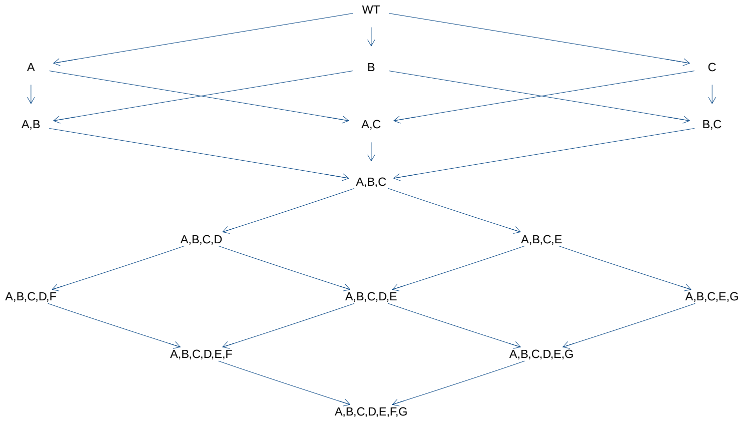

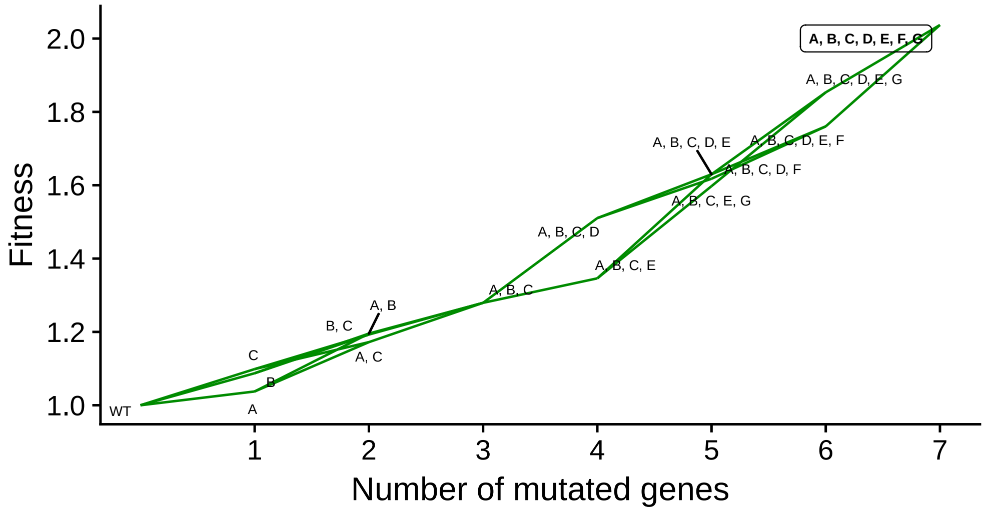

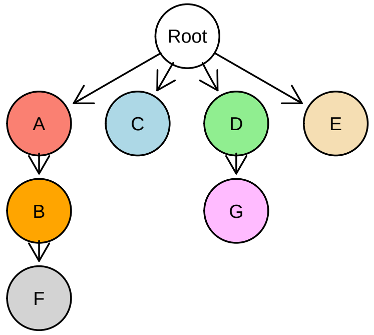

ID	AQcGRsclzSVWoU7Z
acc genotypes	48
peaks no back	1
removed edges	0
prop rem edges	0

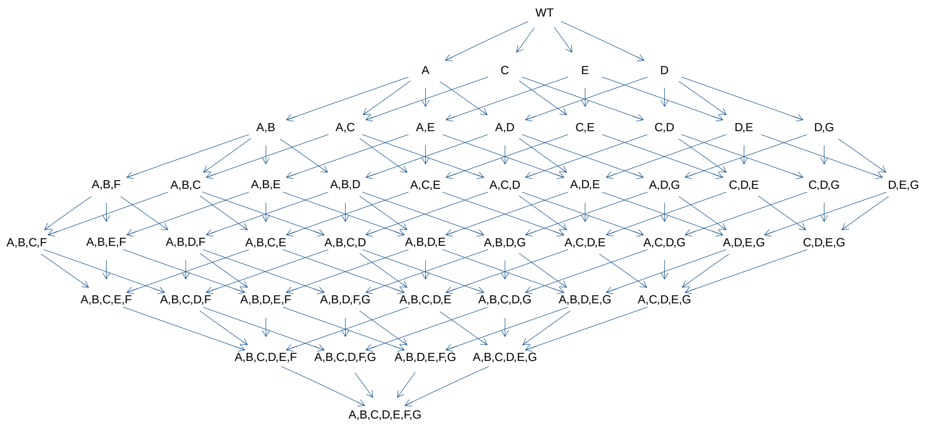

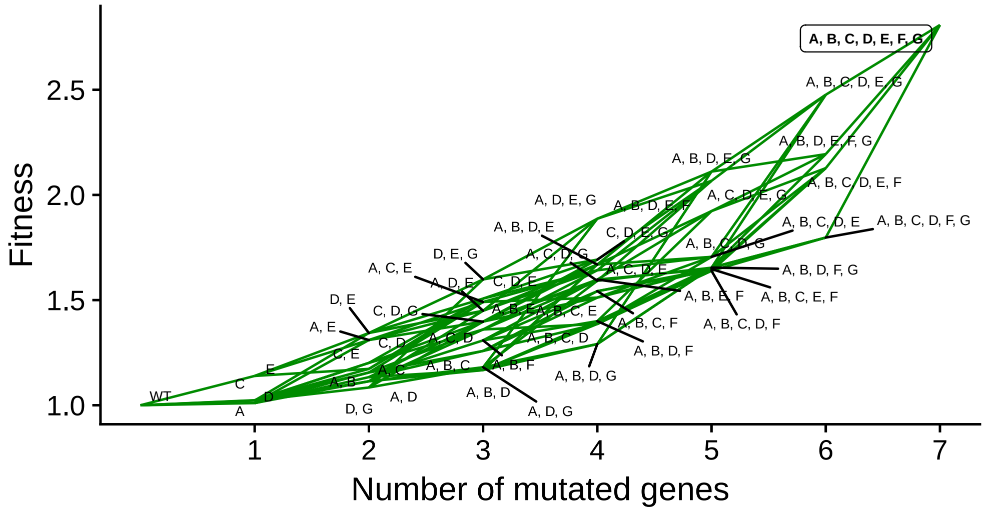

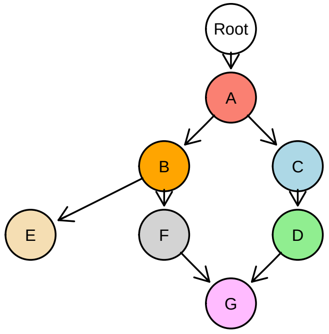

ID	Azj8j6q13euWbW53
acc genotypes	18
peaks no back	1
removed edges	0
prop rem edges	0

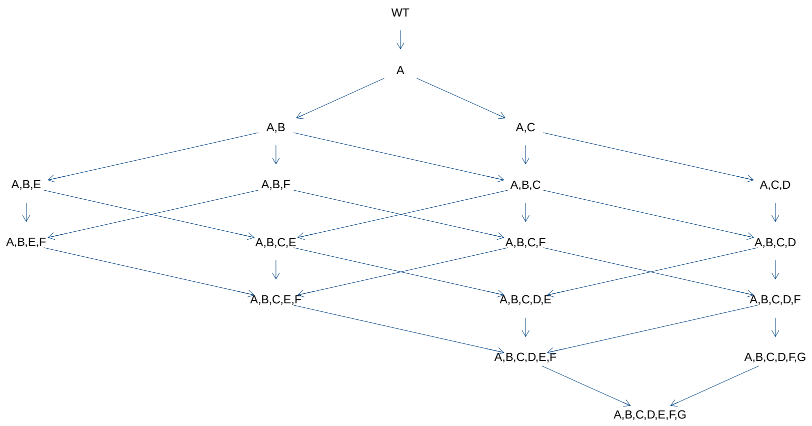

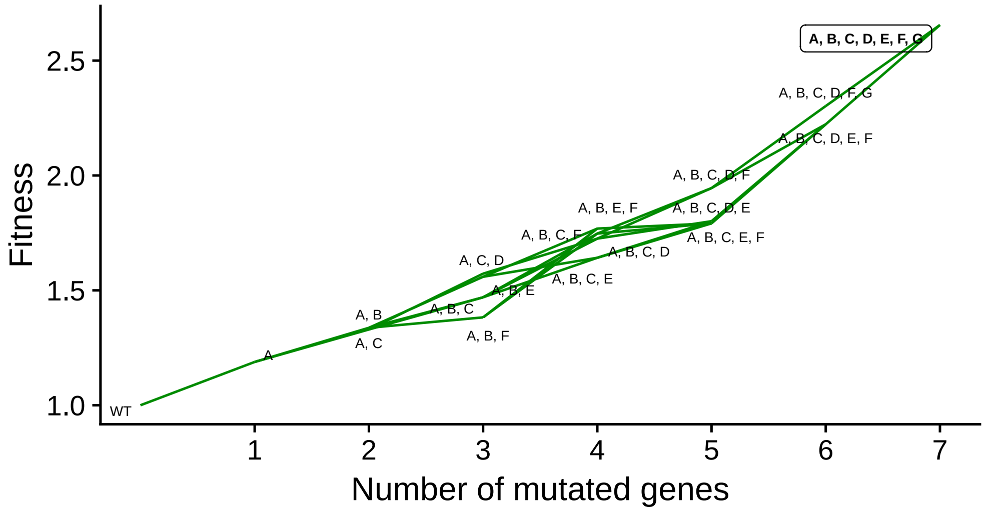

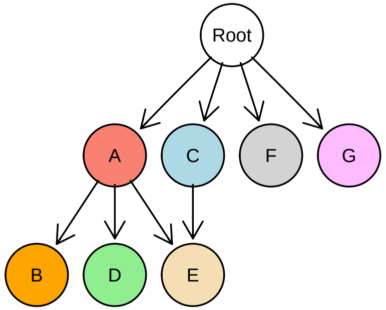

ID	B3l8D10NQwXKC9sLs
acc genotypes	56
peaks no back	1
removed edges	0
prop rem edges	0

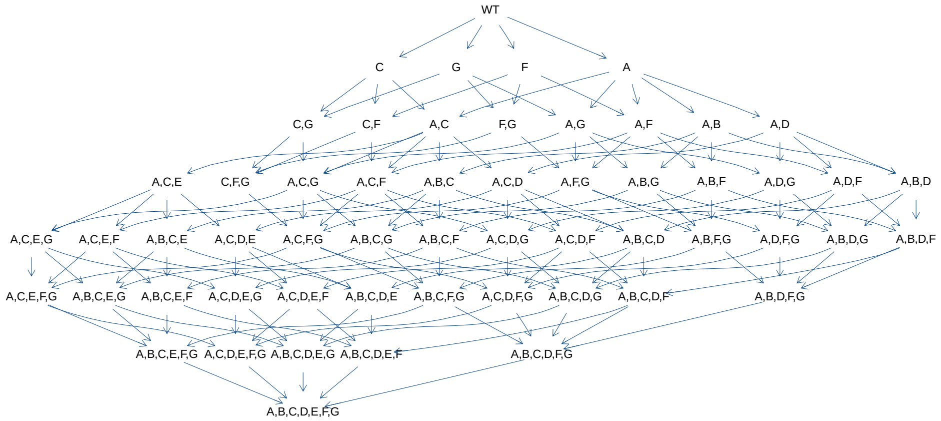

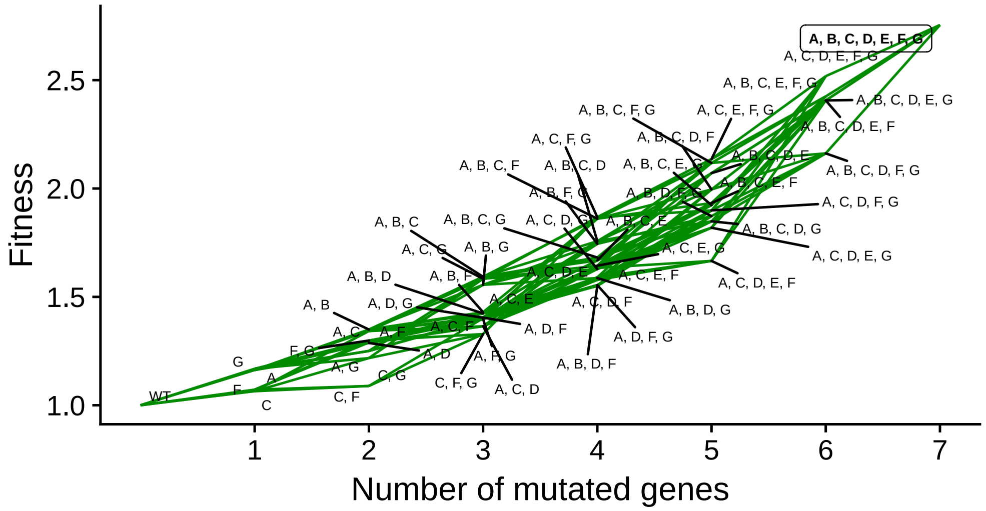

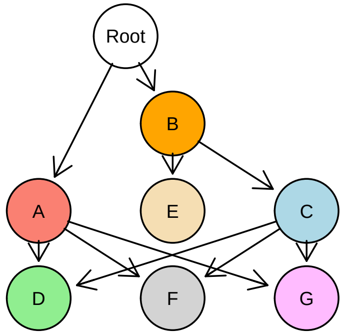

ID	BawvXOS7sG8kOijC
acc genotypes	24
peaks no back	1
removed edges	0
prop rem edges	0

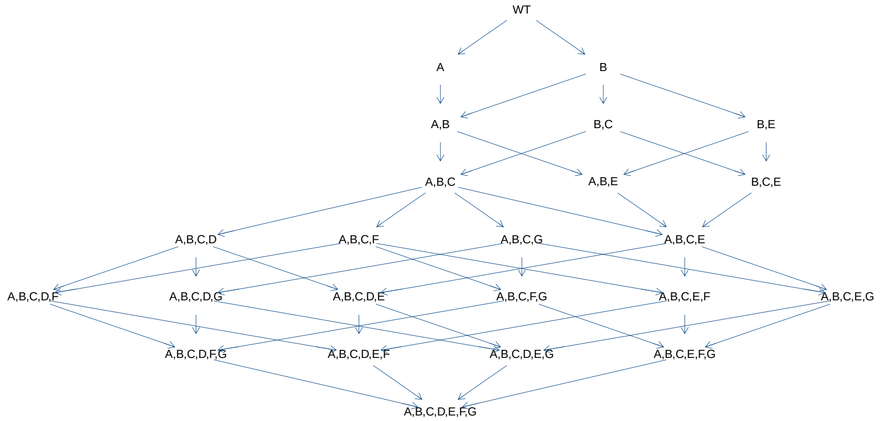

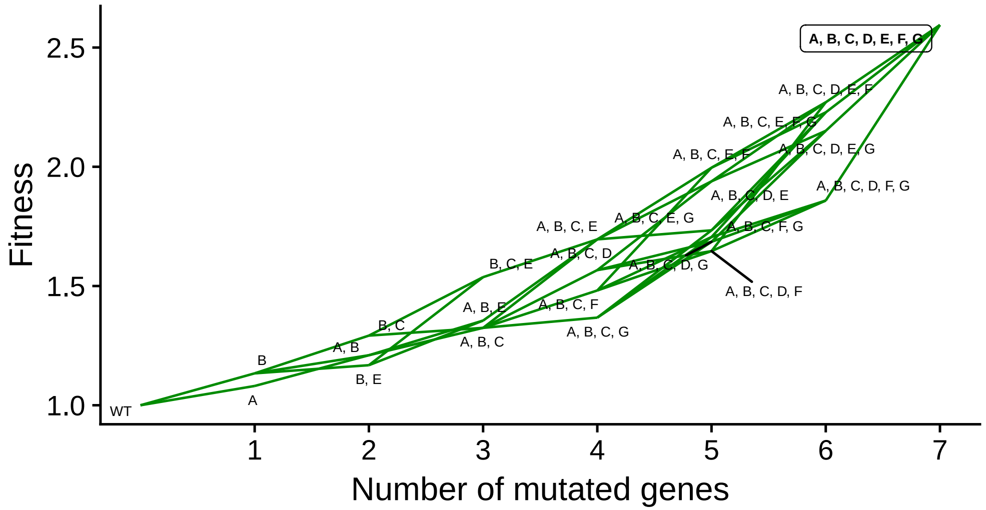

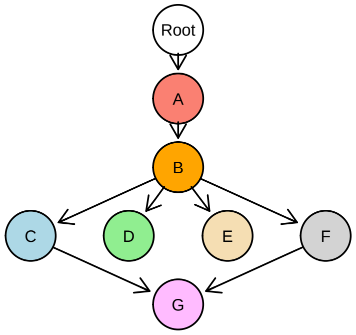

ID BFElrFle1os3fm18
acc genotypes 22
peaks no back 1
removed edges 0
prop rem edges 0

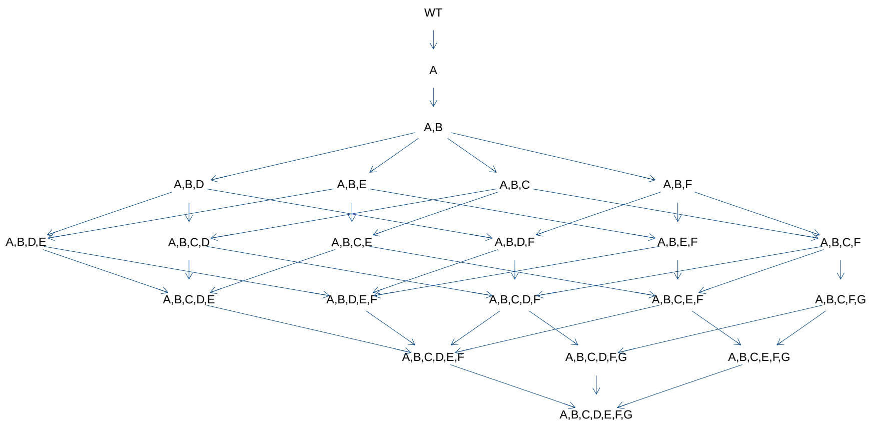

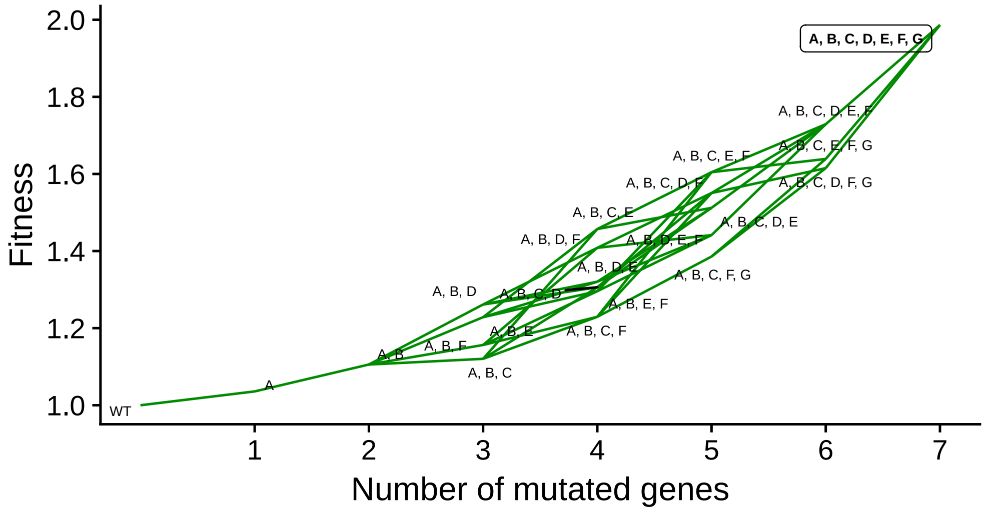

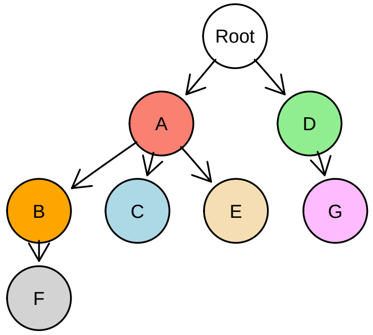

ID	bFmYC2KrBktypLw3
acc genotypes	39
peaks no back	1
removed edges	0
prop rem edges	0

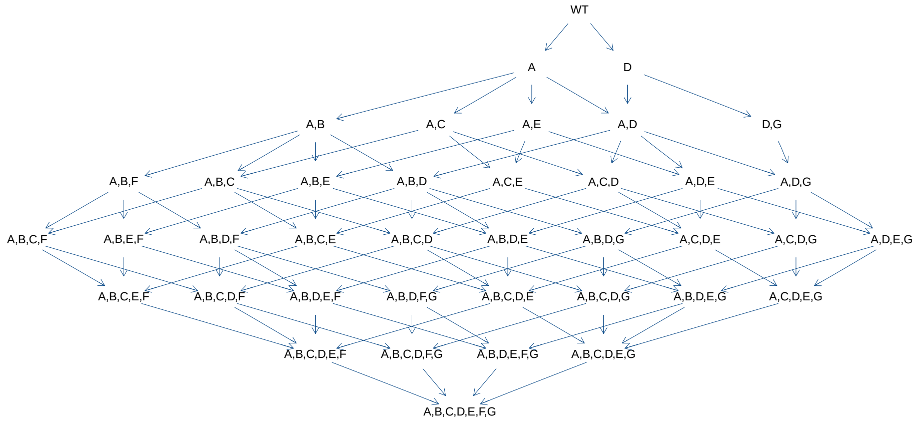

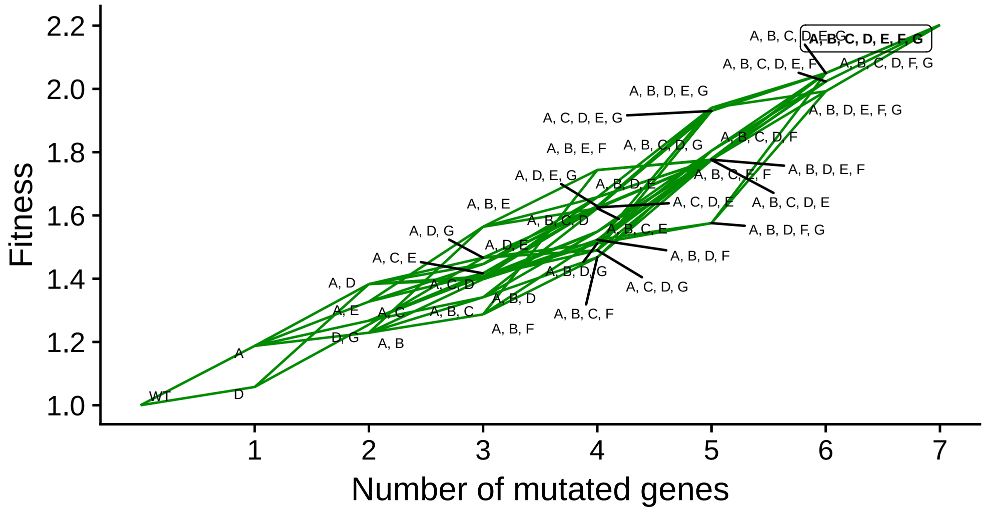

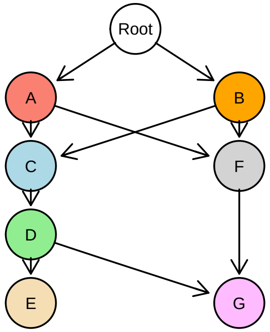

ID	BfsxwvogFr6VDw4S
acc genotypes	13
peaks no back	1
removed edges	0
prop rem edges	0

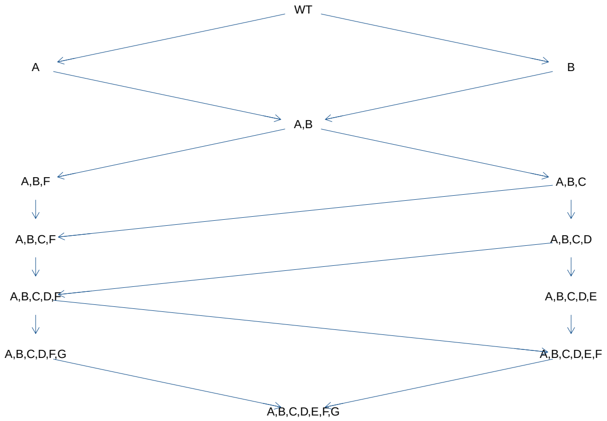

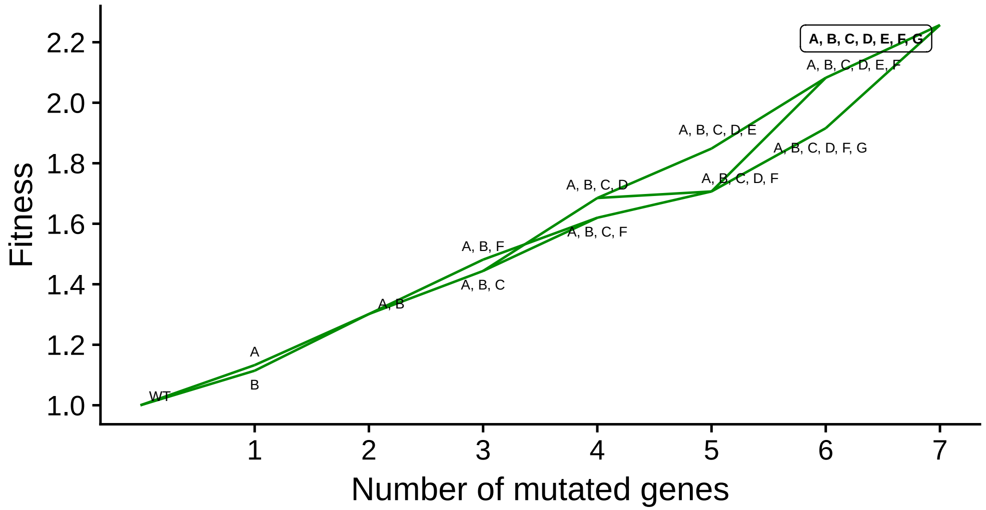

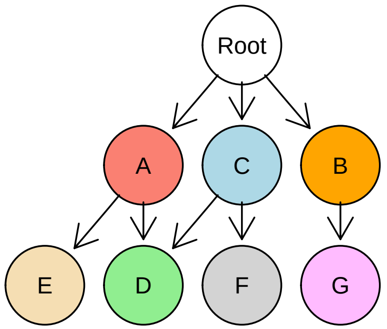

ID bJlgffVcPbF6GUJl
acc genotypes 39
peaks no back 1
removed edges 0
prop rem edges 0

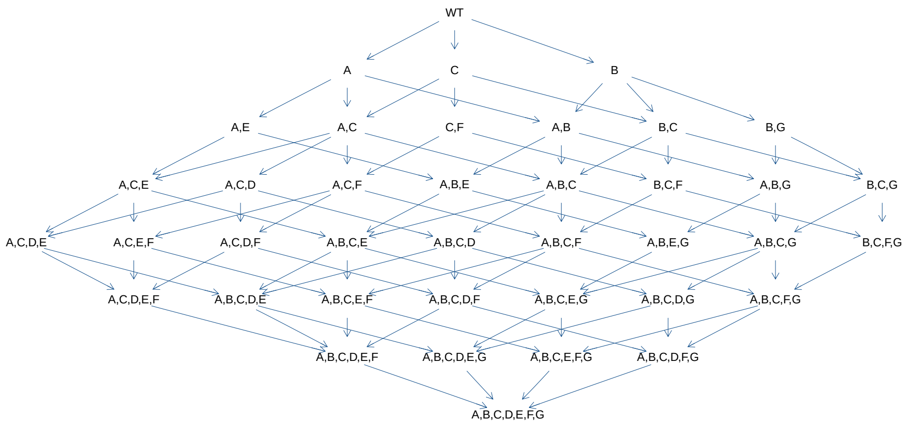

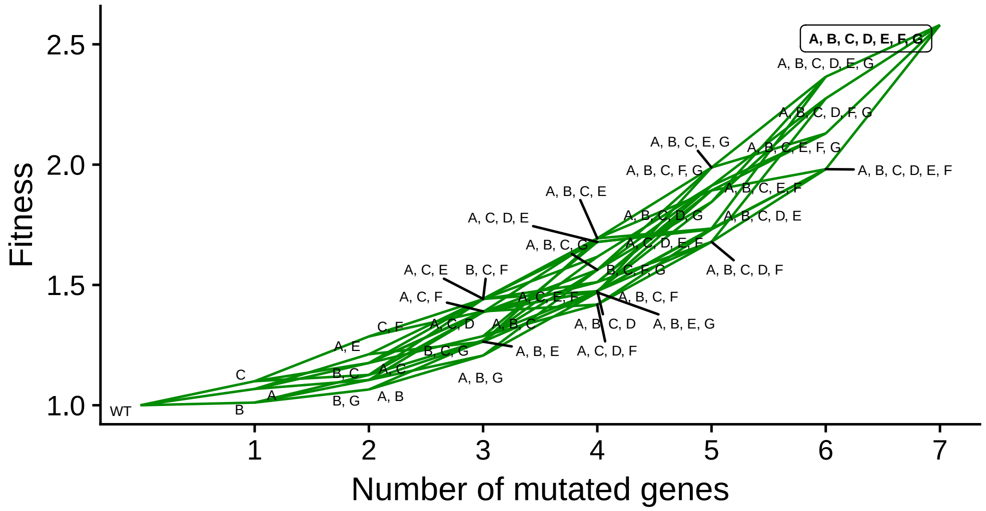

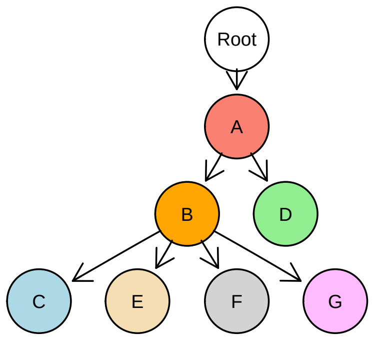

ID	bqjCnNng38uQA1OY
acc genotypes	35
peaks no back	1
removed edges	0
prop rem edges	0

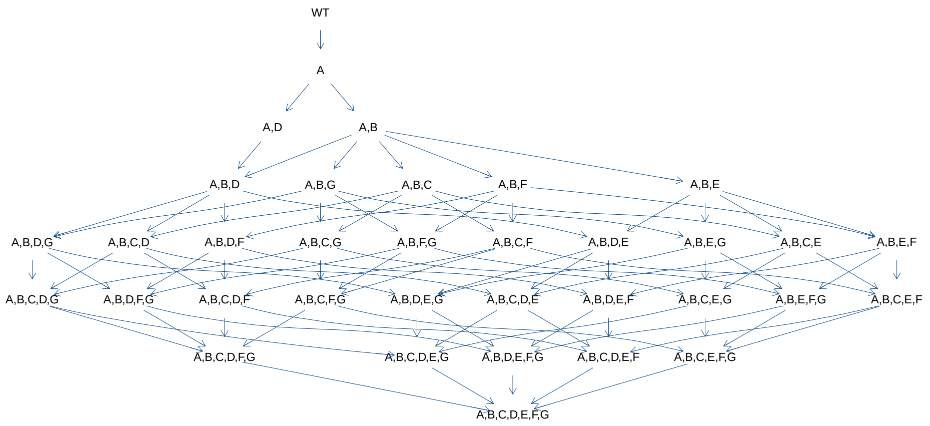

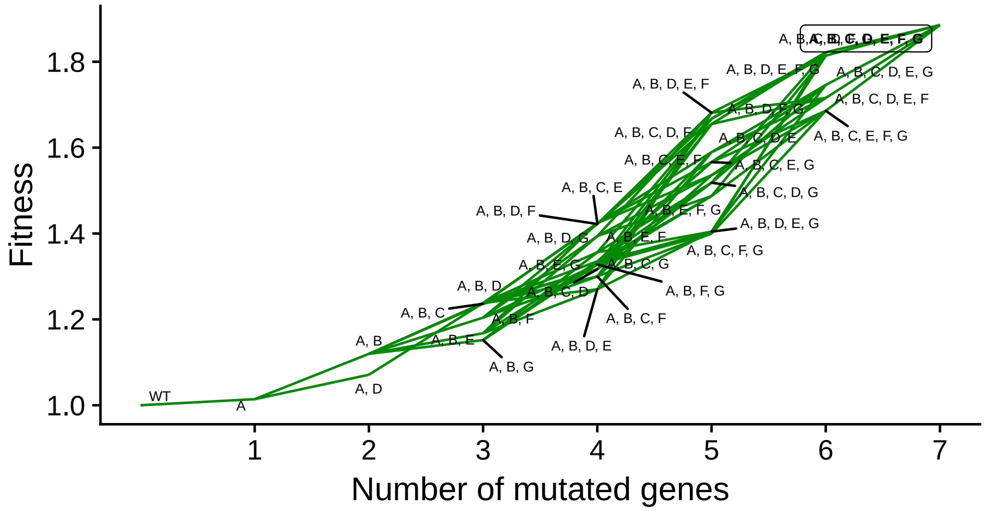

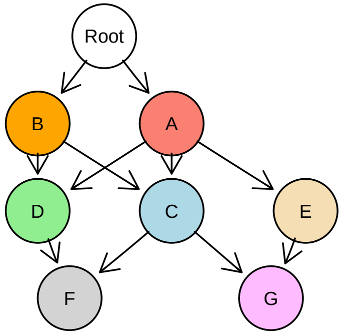

ID	btsq9bPlvHef8E4b
acc genotypes	17
peaks no back	1
removed edges	0
prop rem edges	0

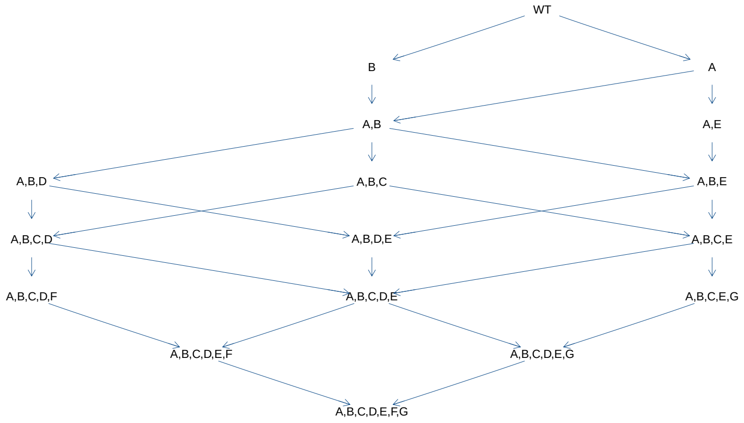

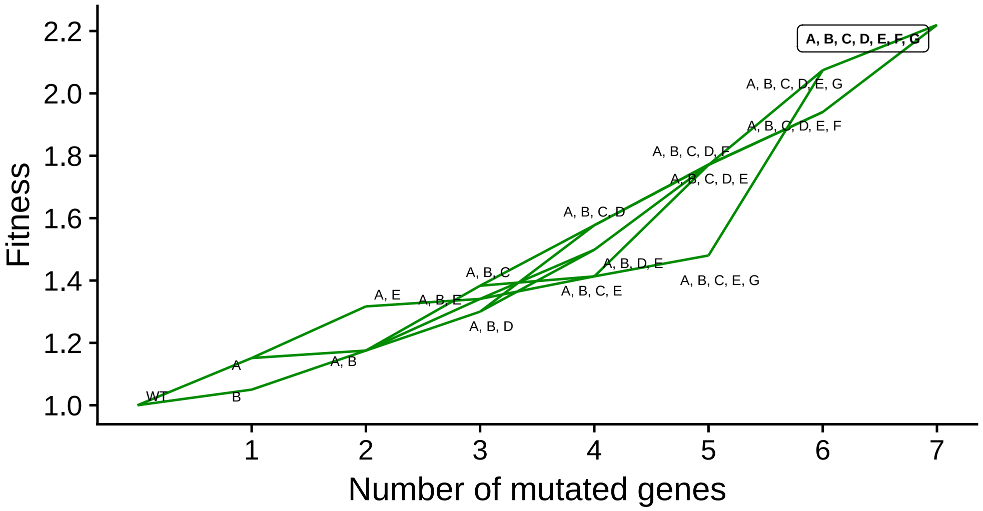

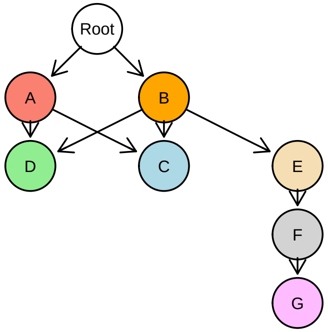

ID	BuXQklRXr18pe1ec
acc genotypes	22
peaks no back	1
removed edges	0
prop rem edges	0

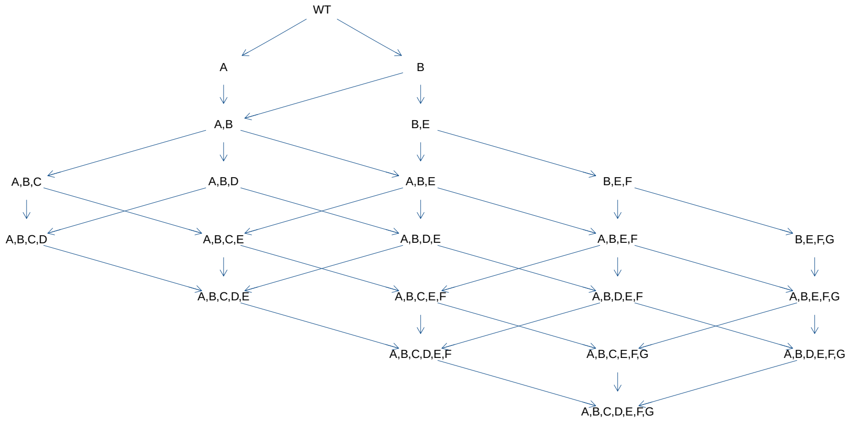

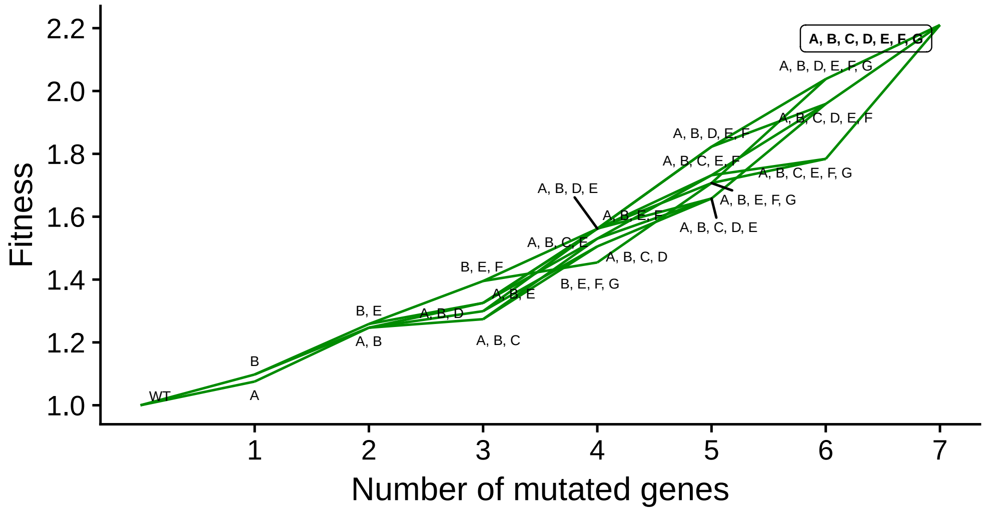

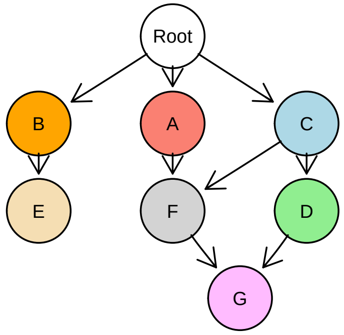

ID	cPY2aEYEnXRtcOVx
acc genotypes	27
peaks no back	1
removed edges	0
prop rem edges	0

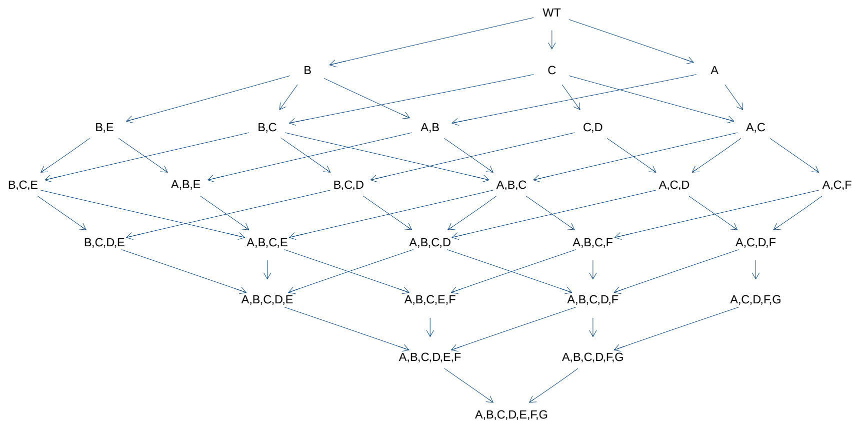

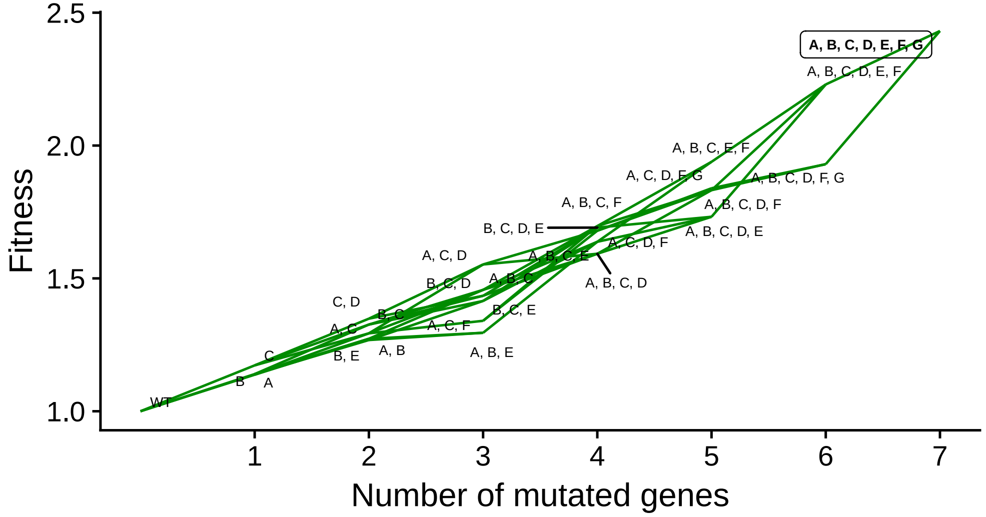

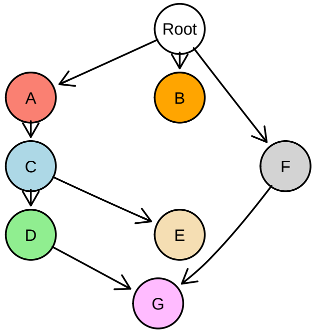

ID	cTShsHTaSM2pFU3Z
acc genotypes	28
peaks no back	1
removed edges	0
prop rem edges	0

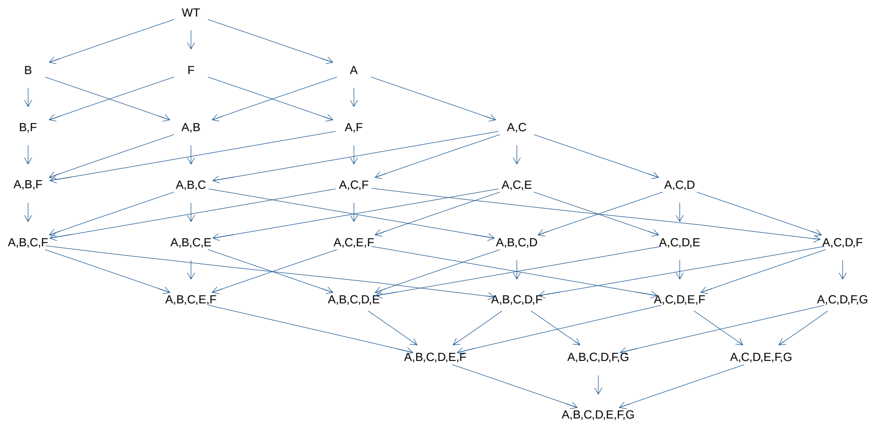

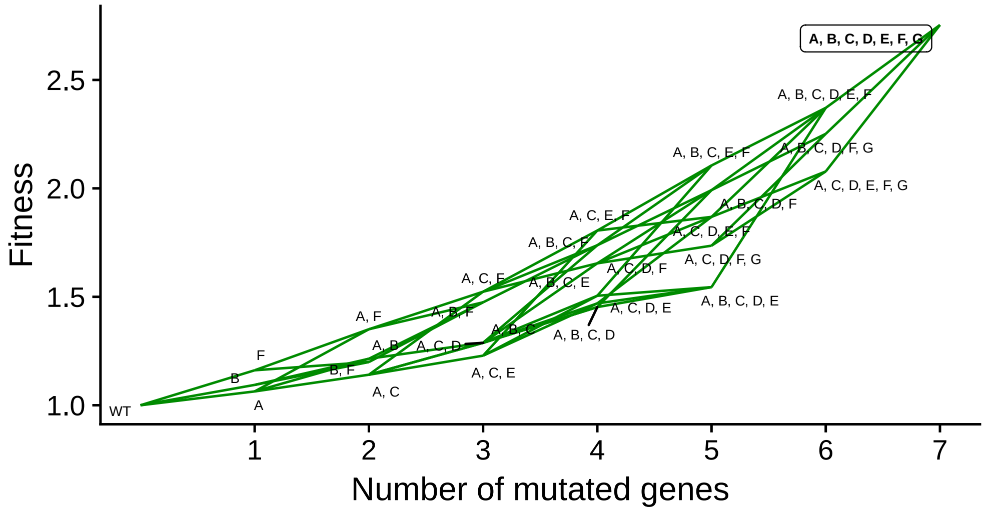

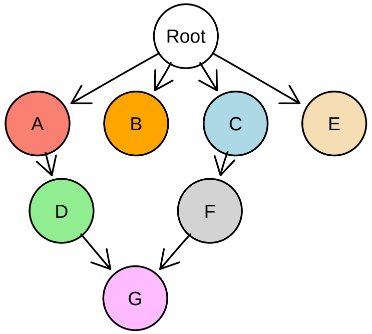

ID	cZ5tgt8lsWr92I8O
acc genotypes	40
peaks no back	1
removed edges	0
prop rem edges	0

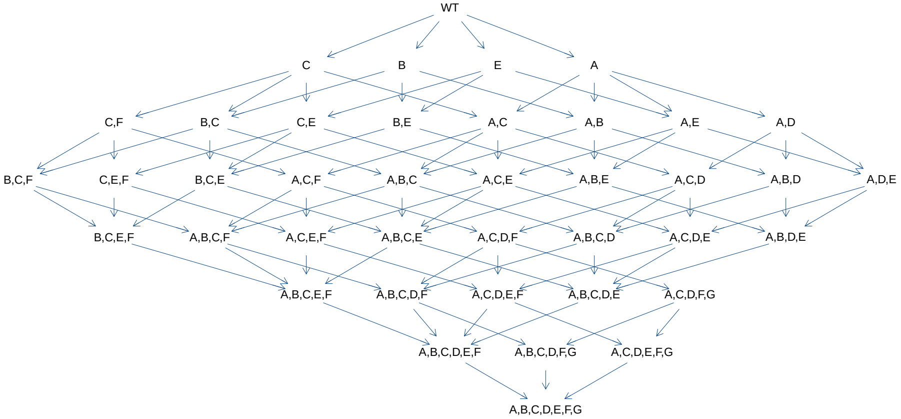

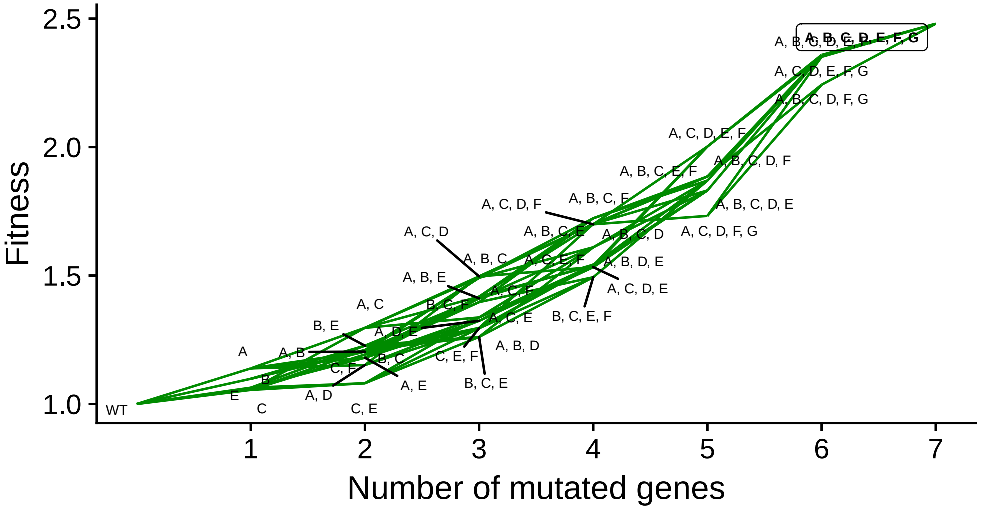

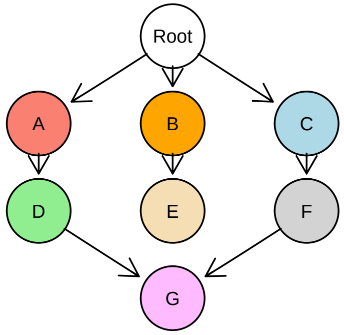

ID	D10upTTNDLyeed8b9
acc genotypes	30
peaks no back	1
removed edges	0
prop rem edges	0

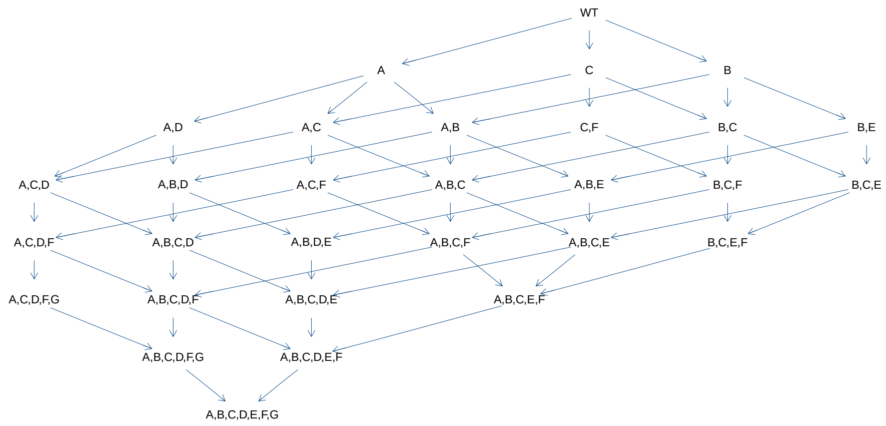

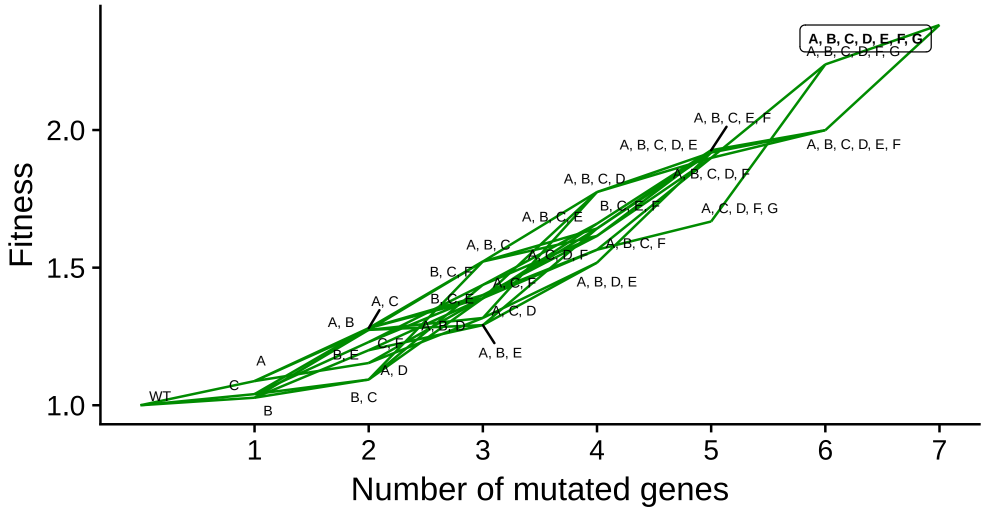

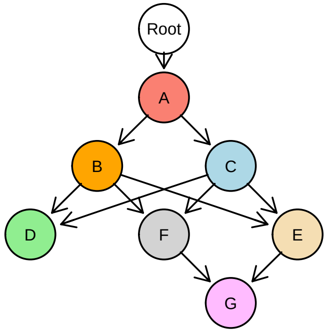

ID DaQ1lMqJ1ktP10uHB
acc genotypes 14
peaks no back 1
removed edges 0
prop rem edges 0

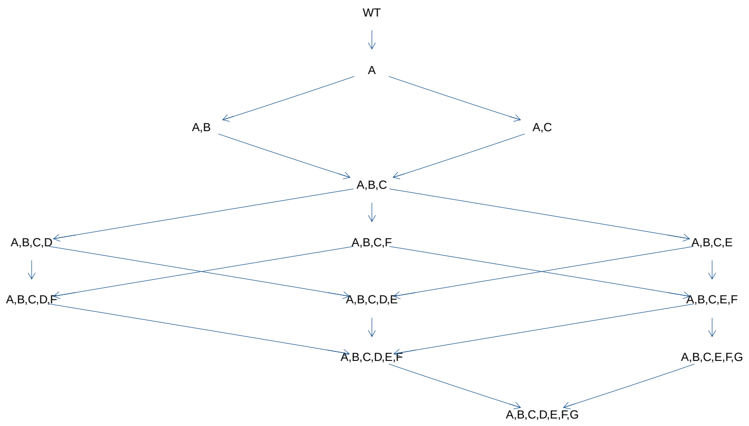

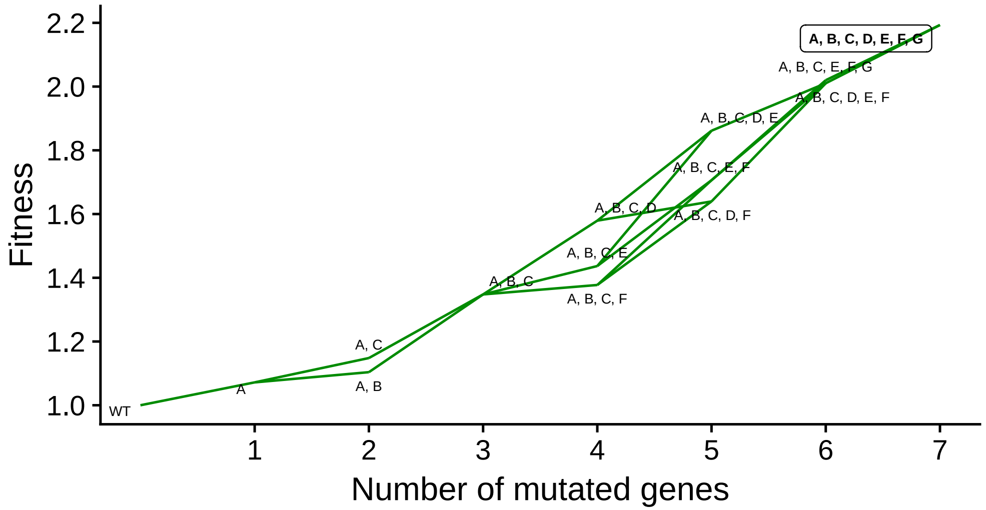

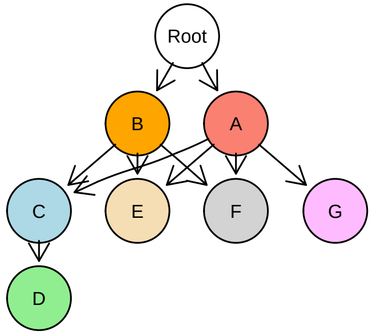

ID	dG5EKqdkRReFl4LP
acc genotypes	28
peaks no back	1
removed edges	0
prop rem edges	0

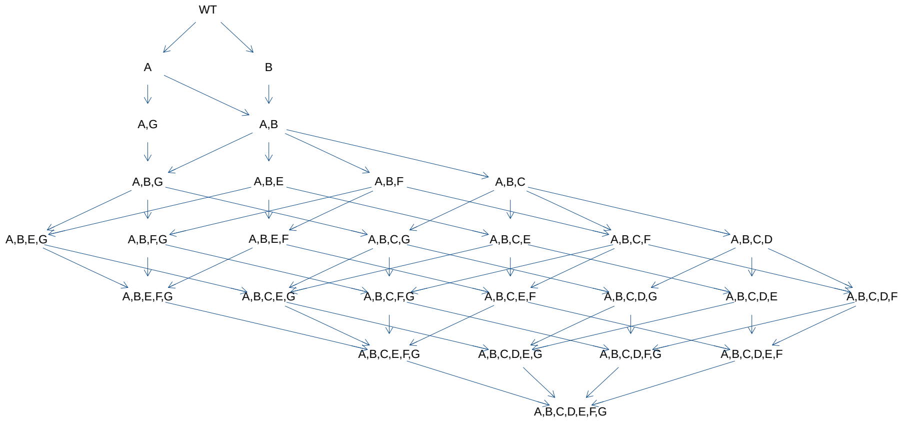

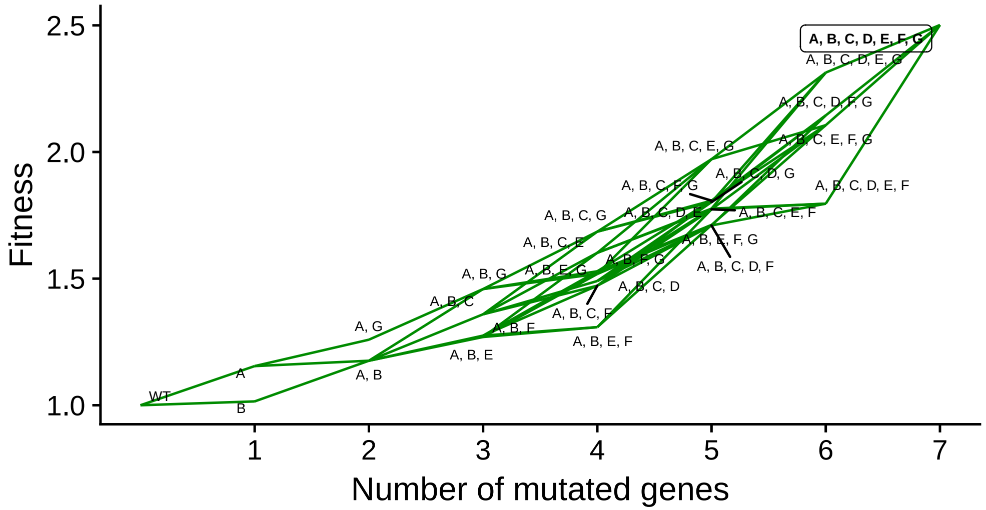

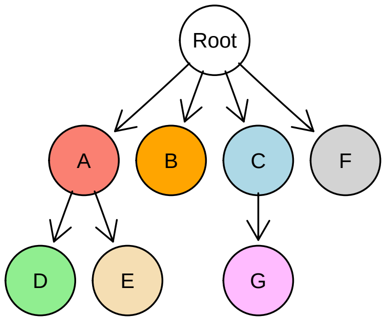

ID	DMErt7pbZBAcNnQg
acc genotypes	60
peaks no back	1
removed edges	0
prop rem edges	0

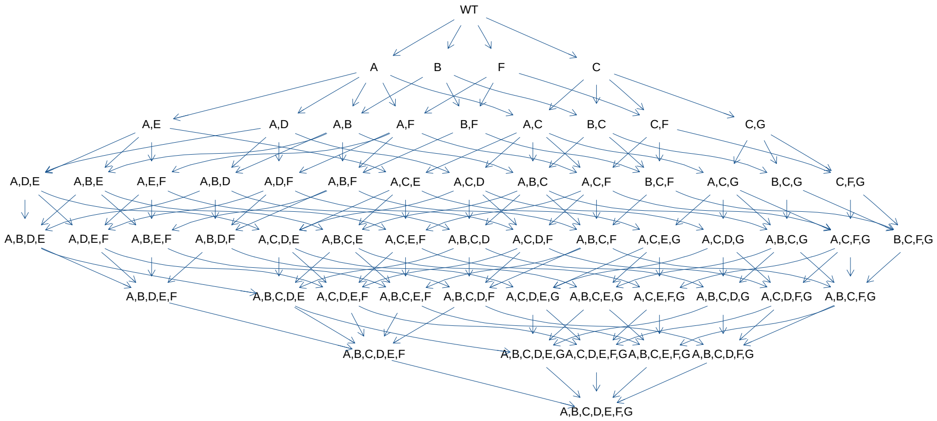

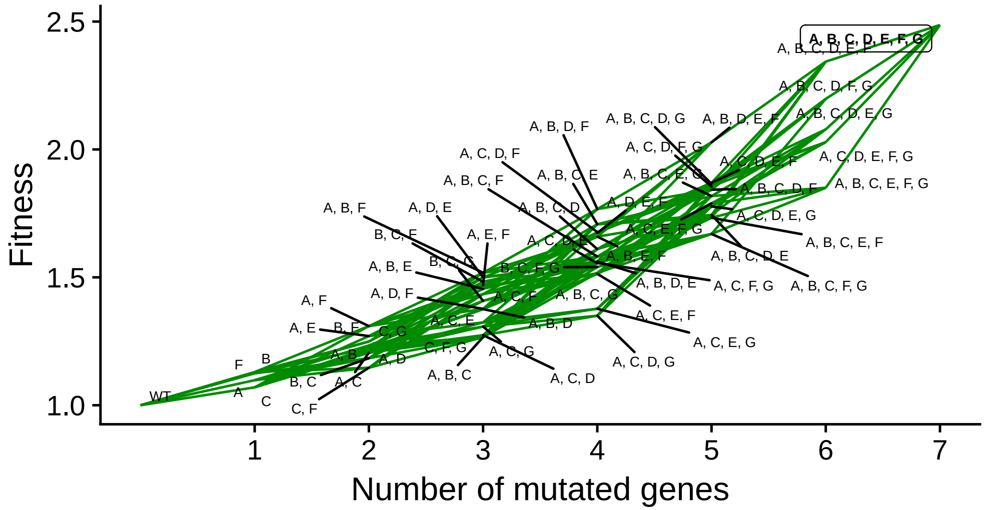

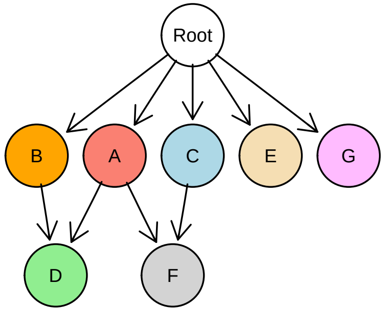

ID	dmZHV2wAZ3zFiHOq
acc genotypes	52
peaks no back	1
removed edges	0
prop rem edges	0

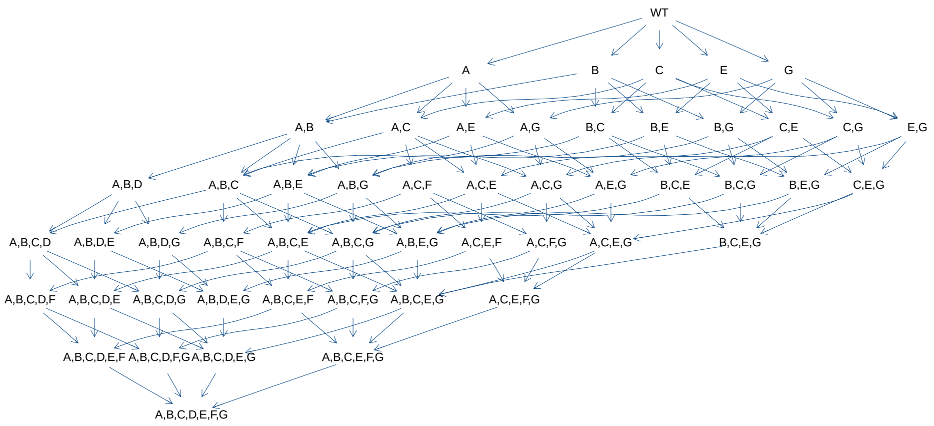

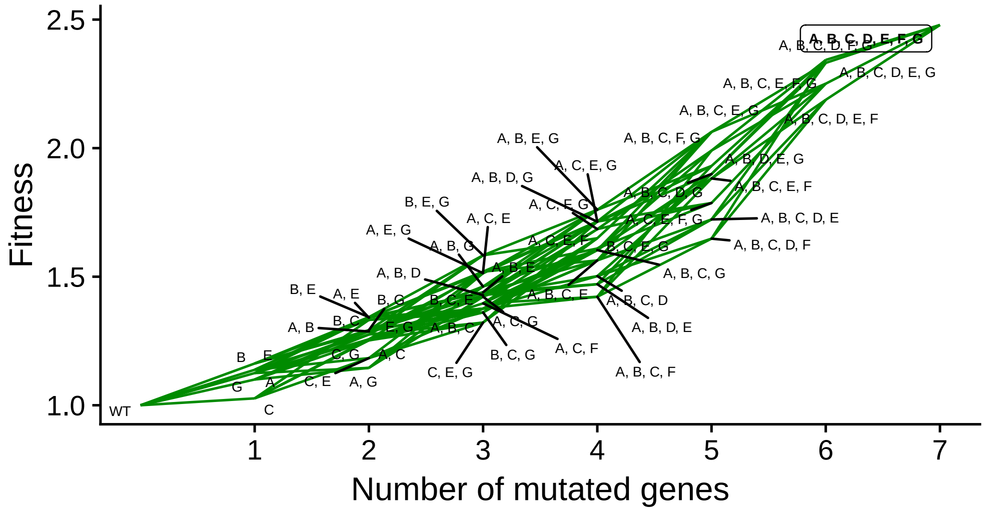

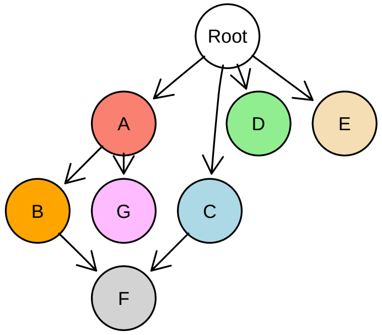

ID	DNdxF6nsuNX9Tqlx
acc genotypes	48
peaks no back	1
removed edges	0
prop rem edges	0

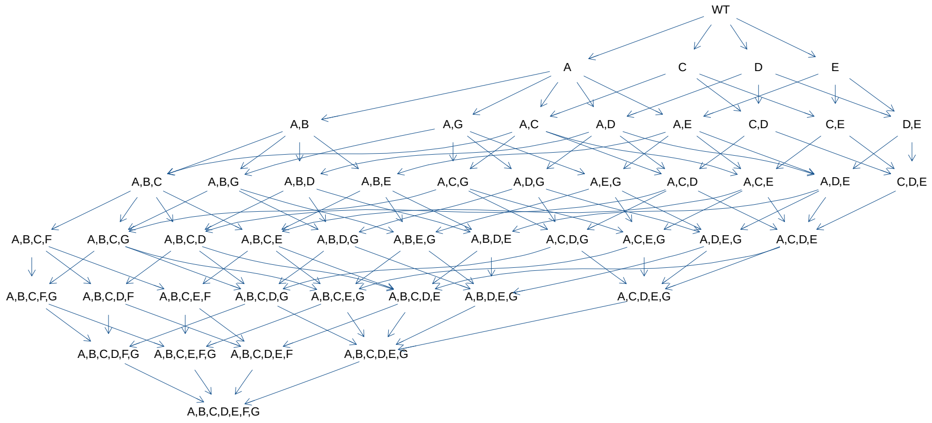

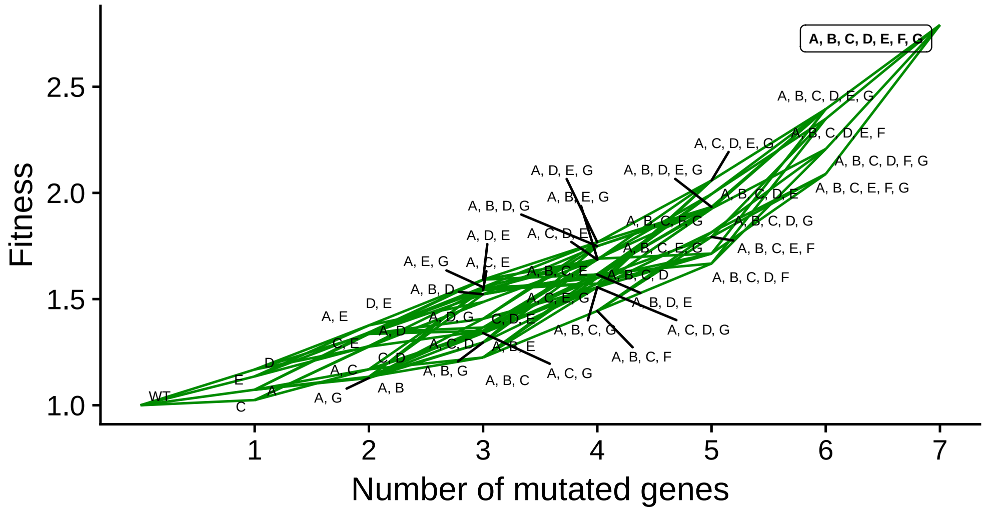

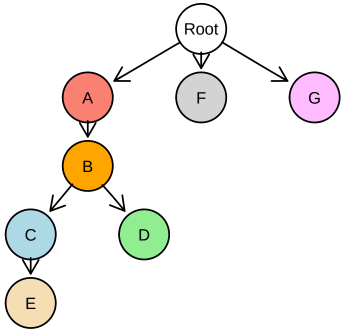

ID Dv8B7RHWDguRzH1W
acc genotypes 32
peaks no back 1
removed edges 0
prop rem edges 0

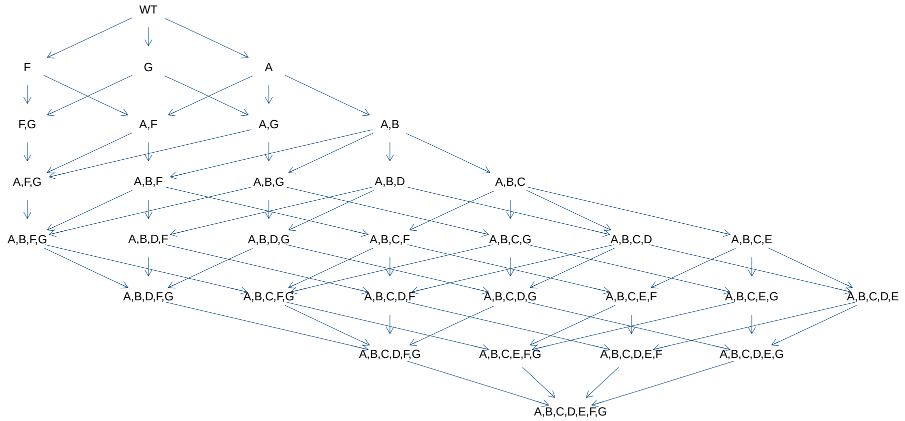

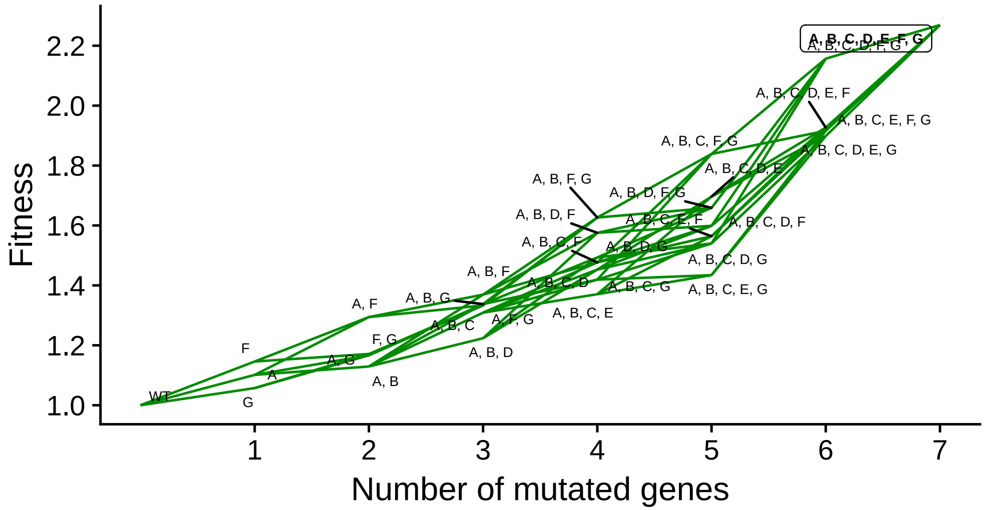

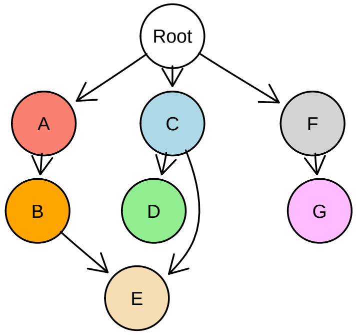

ID	e6d6sLGltLVqnRO4
acc genotypes	33
peaks no back	1
removed edges	0
prop rem edges	0

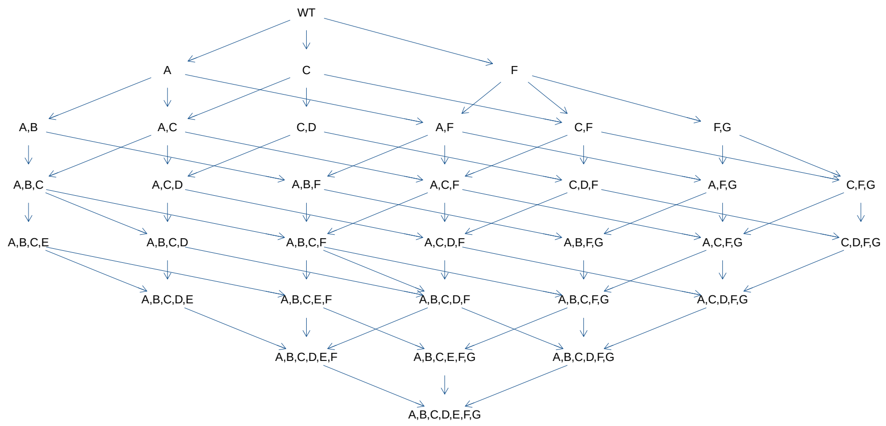

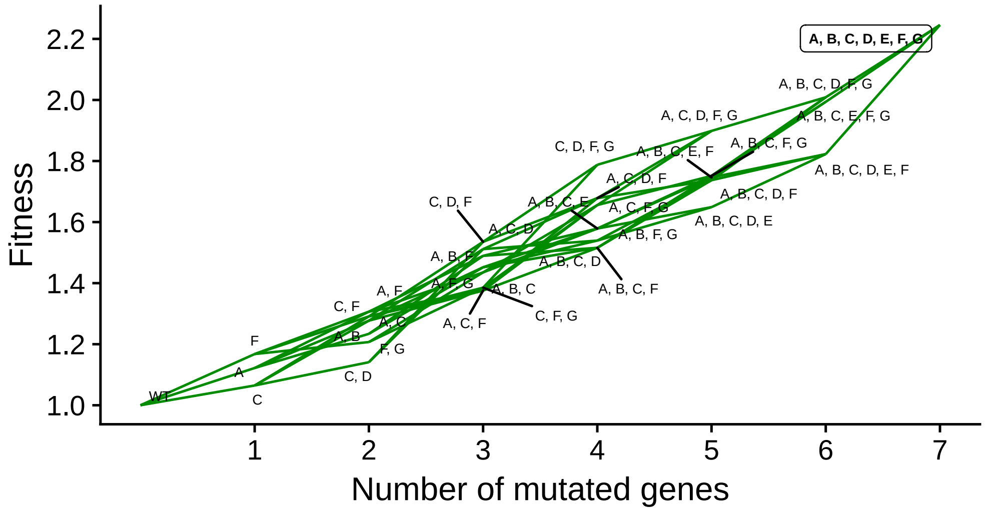

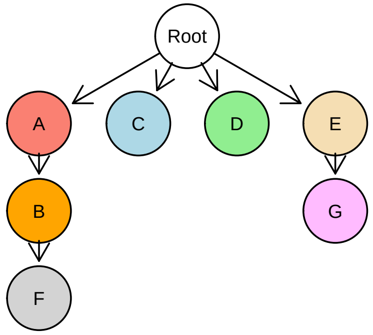

ID	E7PUOGD47fKBfgGx
acc genotypes	48
peaks no back	1
removed edges	0
prop rem edges	0

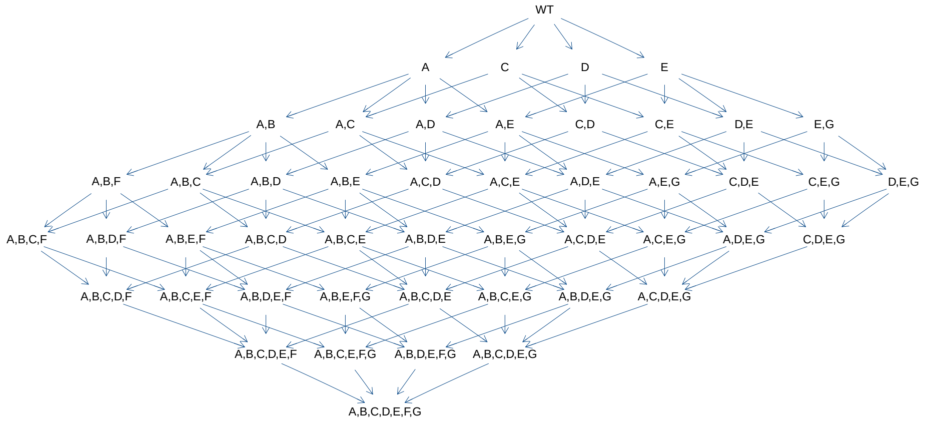

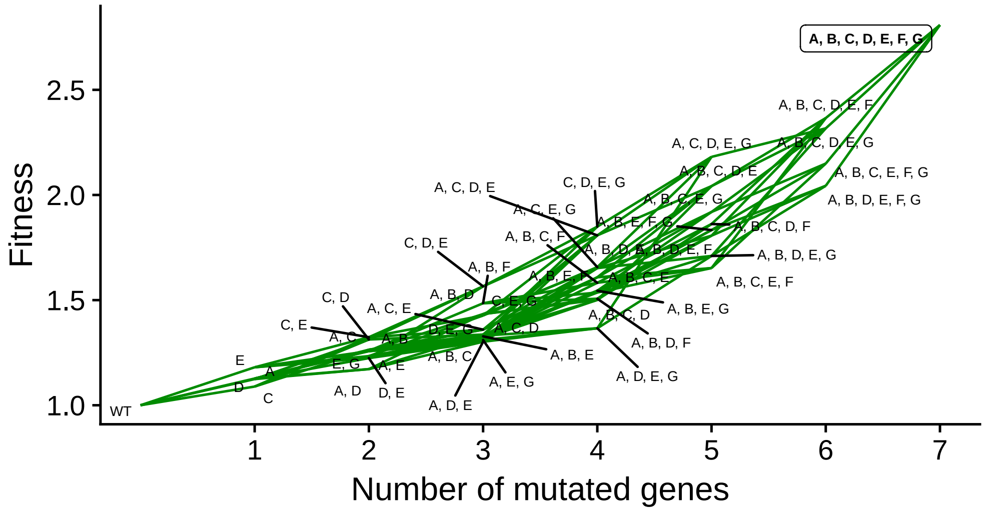

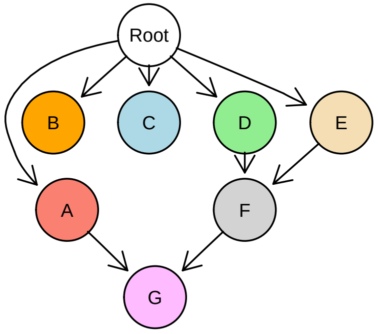

ID	eEiiU6IXTAitvbXD
acc genotypes	44
peaks no back	1
removed edges	0
prop rem edges	0

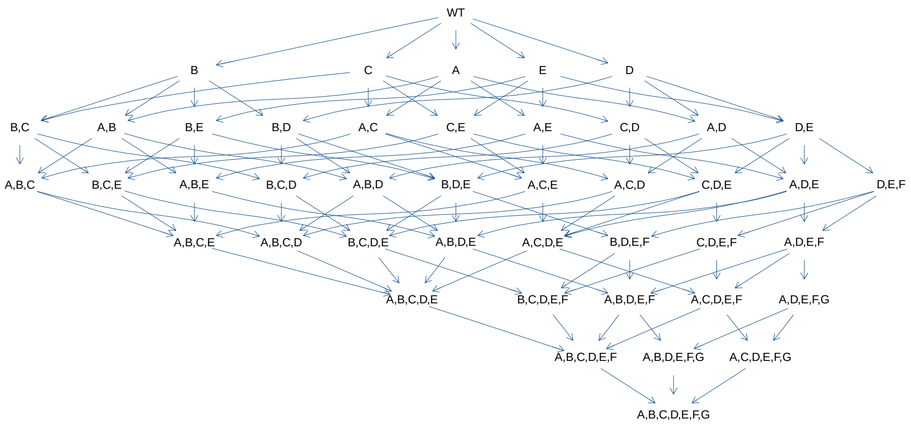

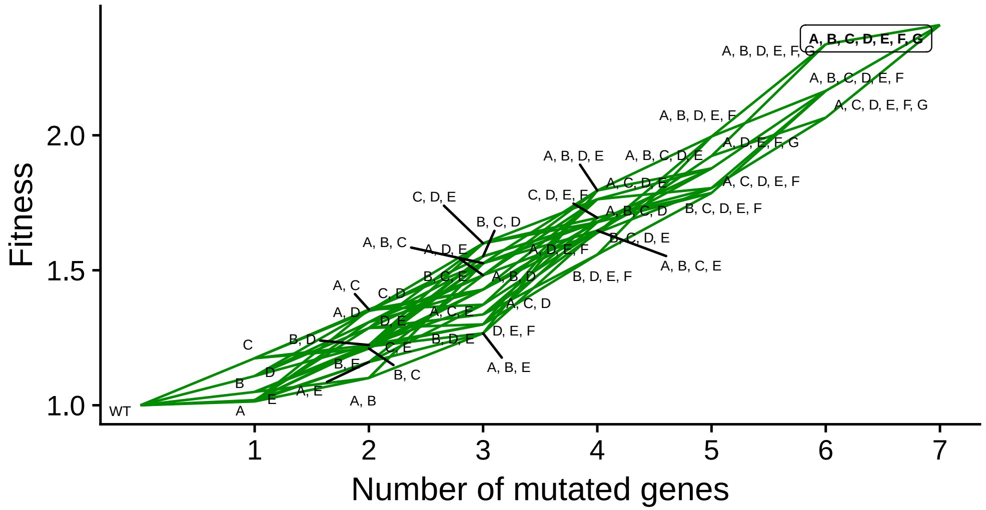

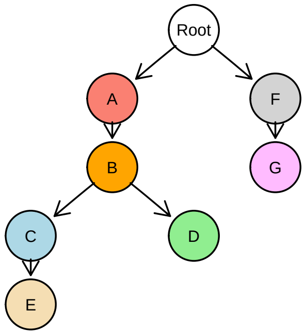

ID EF3dPOLYSD4SFyjp
acc genotypes 24
peaks no back 1
removed edges 0
prop rem edges 0

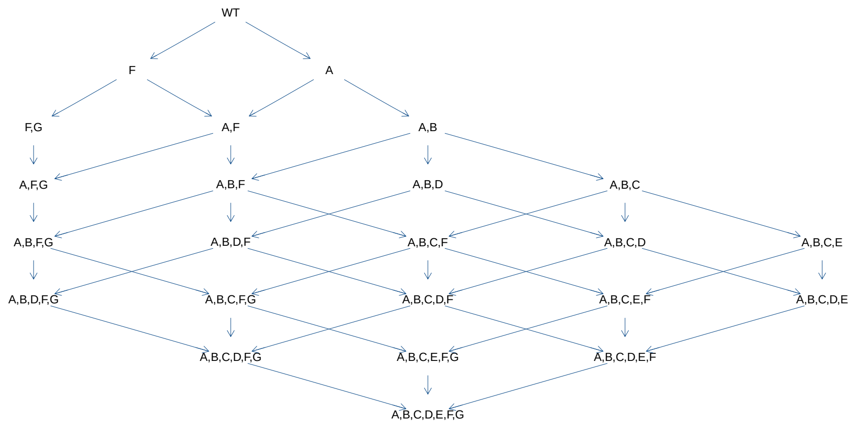

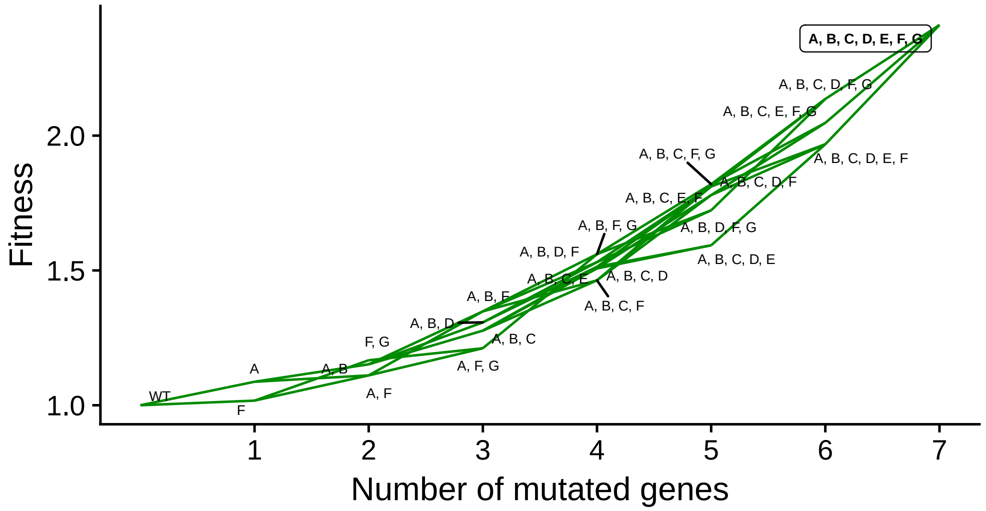

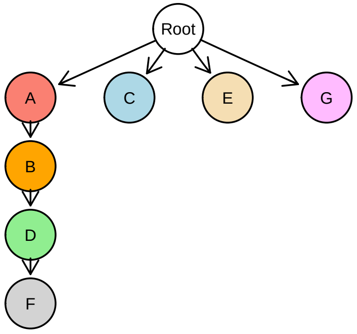

ID	EikpyAkVdLnWHzZF
acc genotypes	40
peaks no back	1
removed edges	0
prop rem edges	0

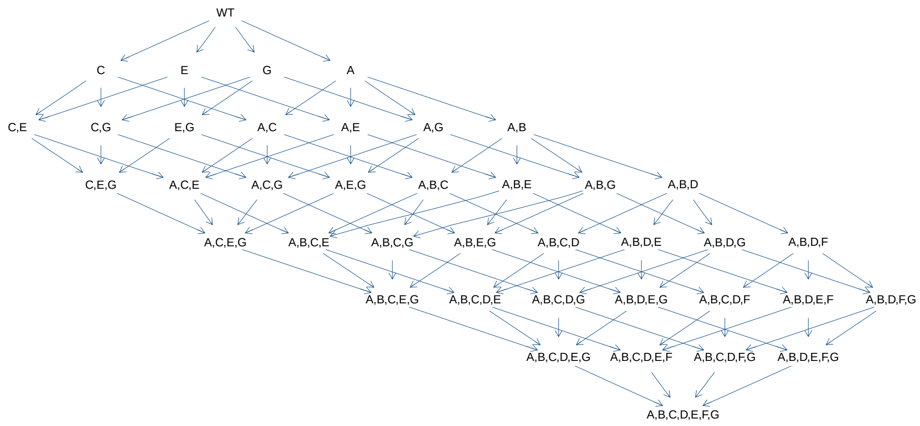

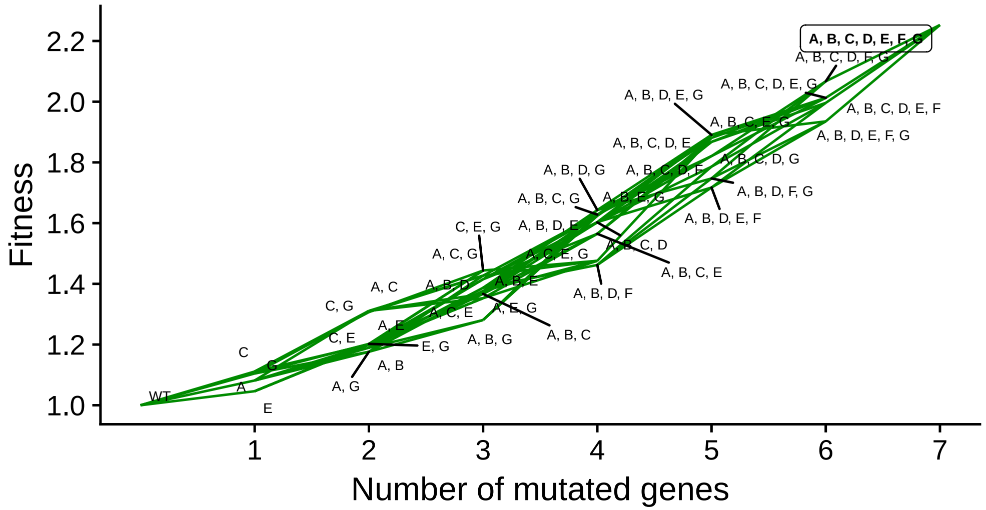

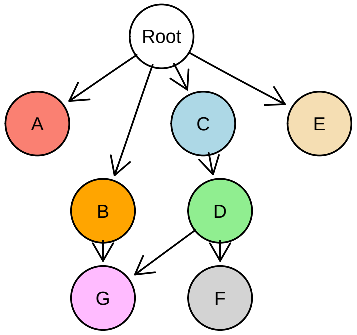

ID	EL1Qu5ixDsldHIqT
acc genotypes	40
peaks no back	1
removed edges	0
prop rem edges	0

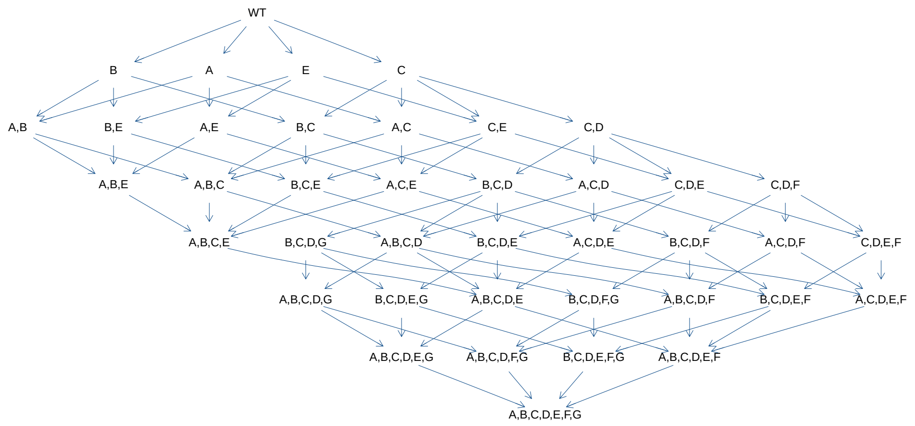

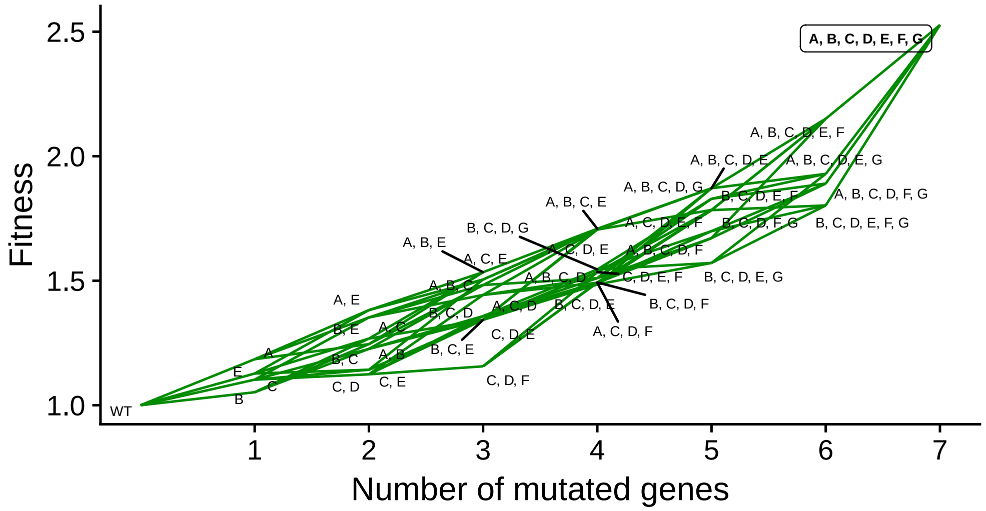

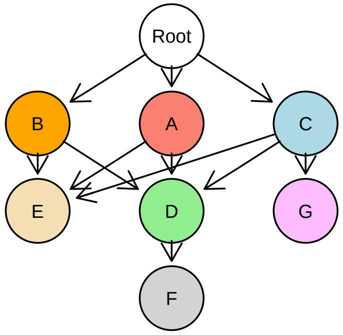

ID	eyXg2MFYlT9dyyLY
acc genotypes	22
peaks no back	1
removed edges	0
prop rem edges	0

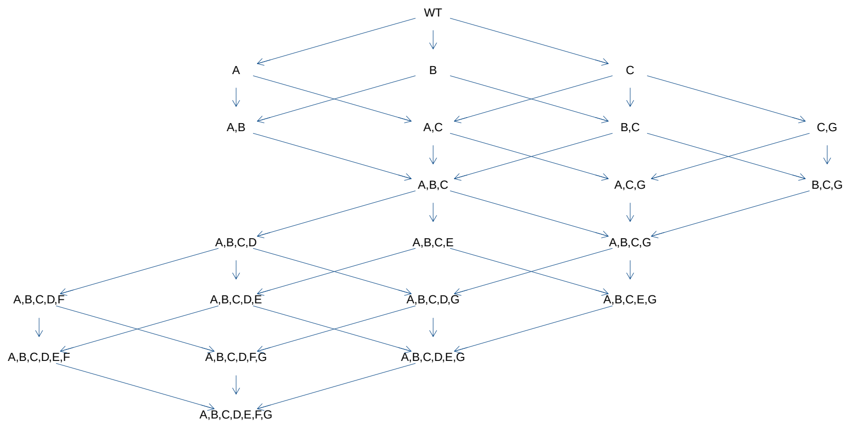

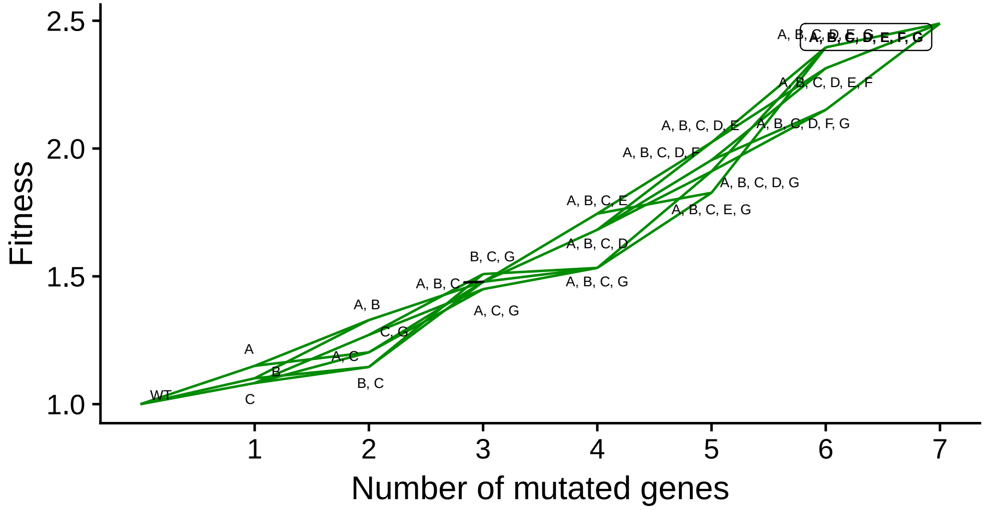

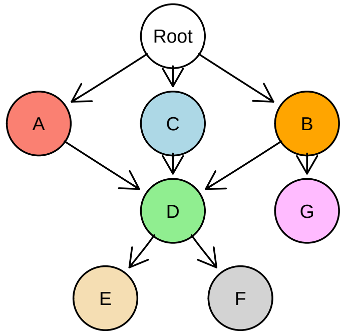

ID	FcKyPNlgfuFUAoV10
acc genotypes	20
peaks no back	1
removed edges	0
prop rem edges	0

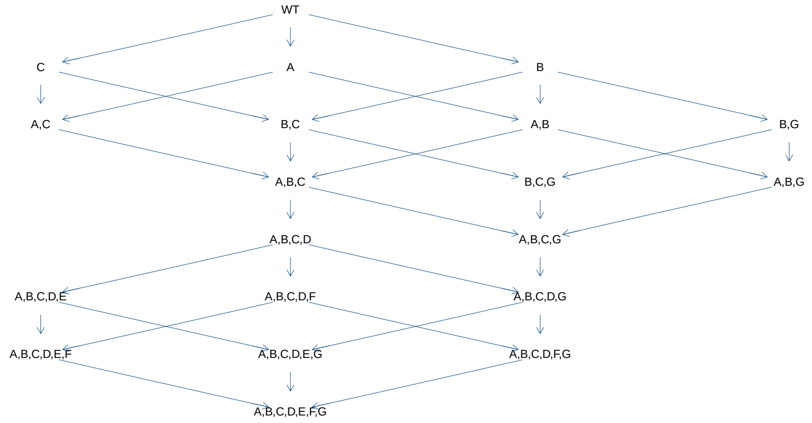

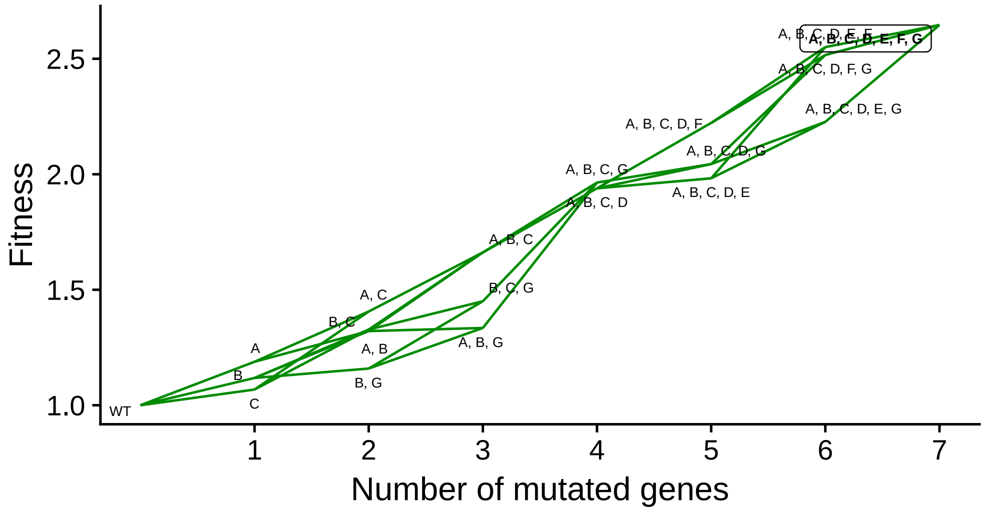

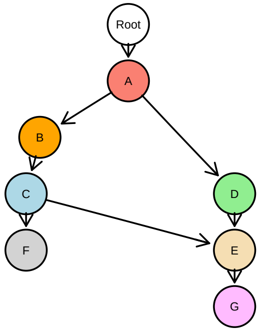

ID	FL34ukOM10yOTdr2W
acc genotypes	13
peaks no back	1
removed edges	0
prop rem edges	0

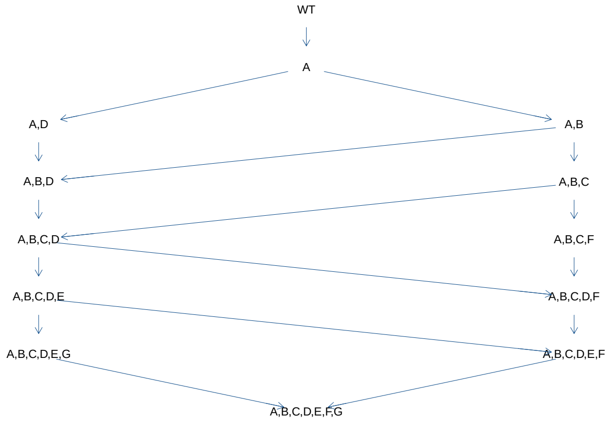

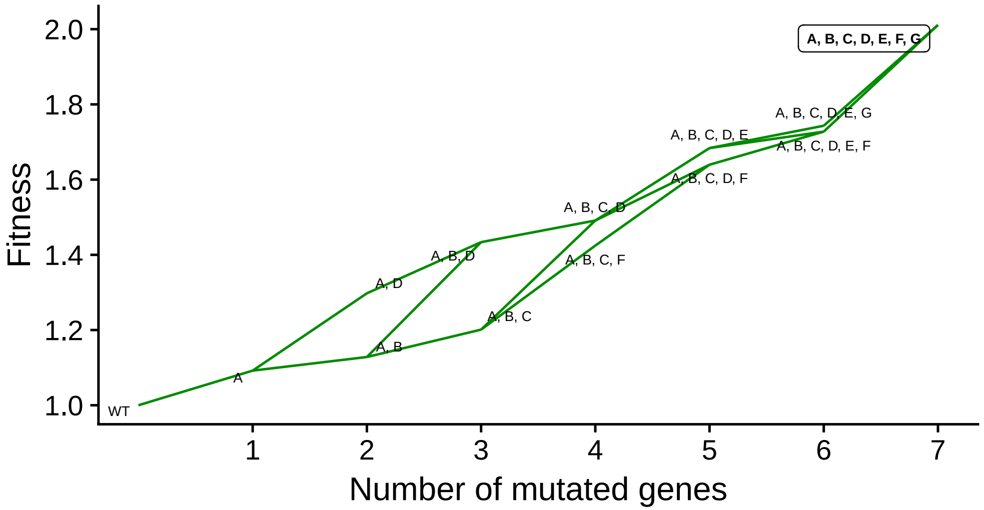

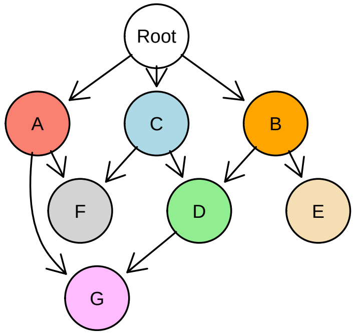

ID	fMD9ePnbEbDWW2mR
acc genotypes	25
peaks no back	1
removed edges	0
prop rem edges	0

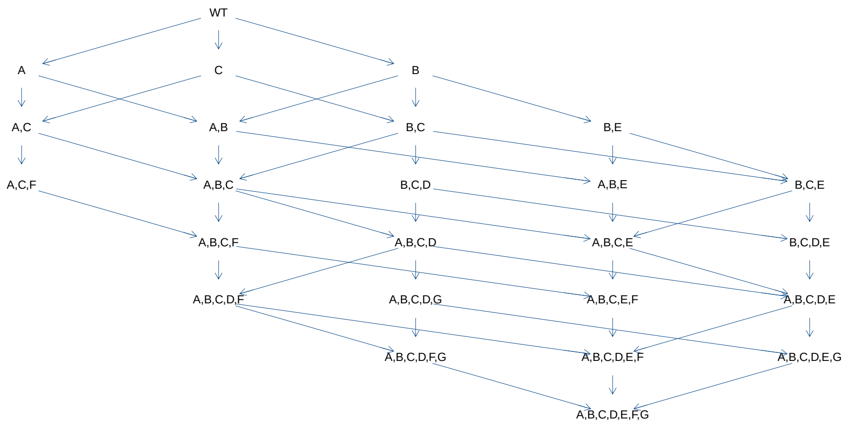

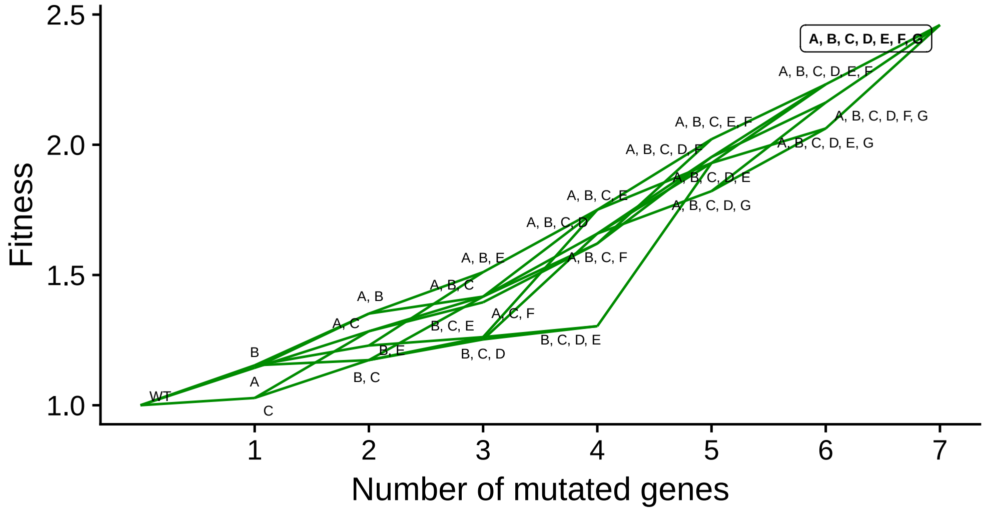

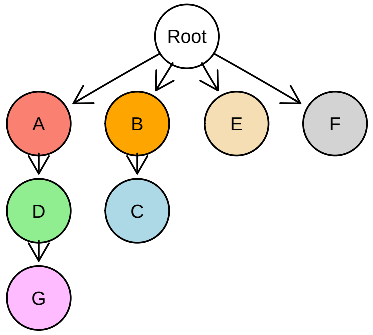

ID	fO3JEhzhobZEAWxH
acc genotypes	48
peaks no back	1
removed edges	0
prop rem edges	0

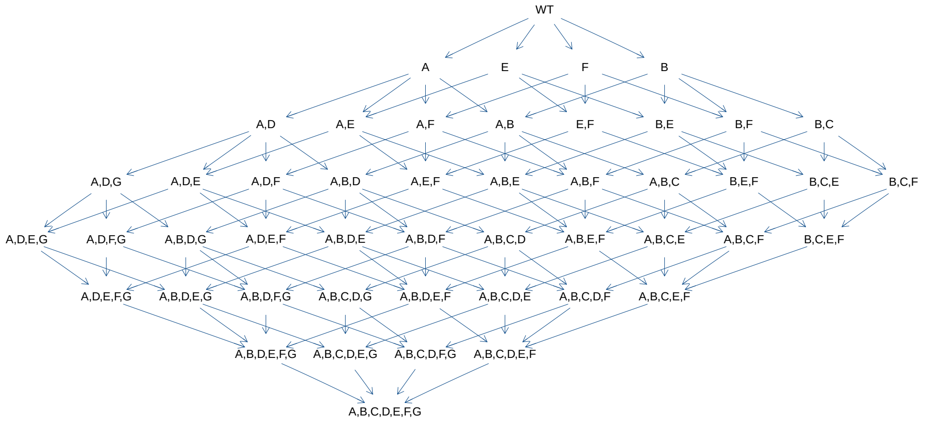

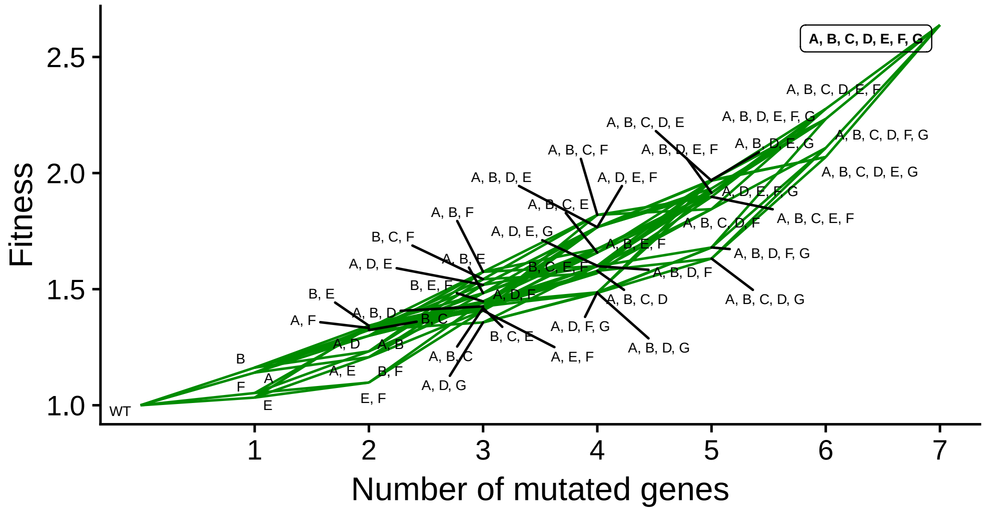

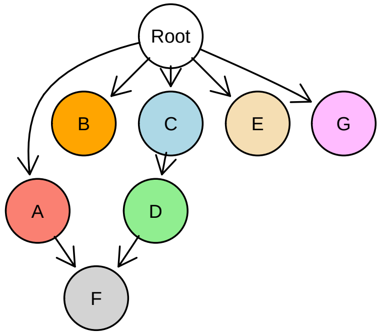

ID FoHYHTCWCEdt7JW8
acc genotypes 56
peaks no back 1
removed edges 0
prop rem edges 0

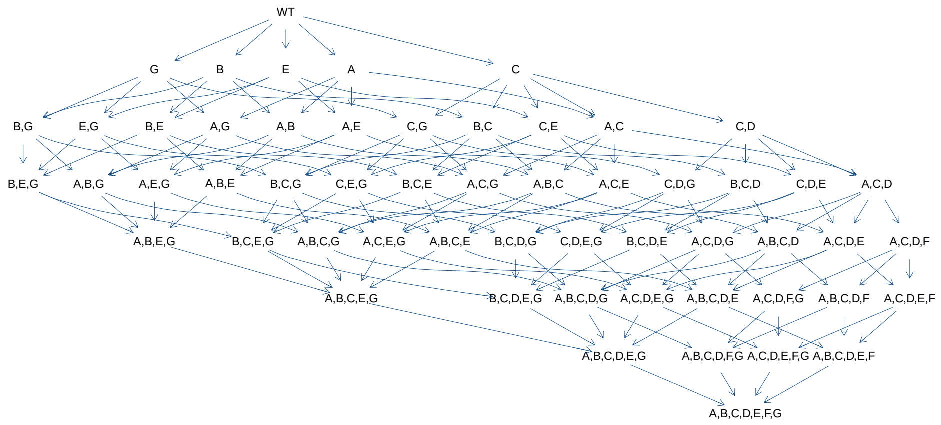

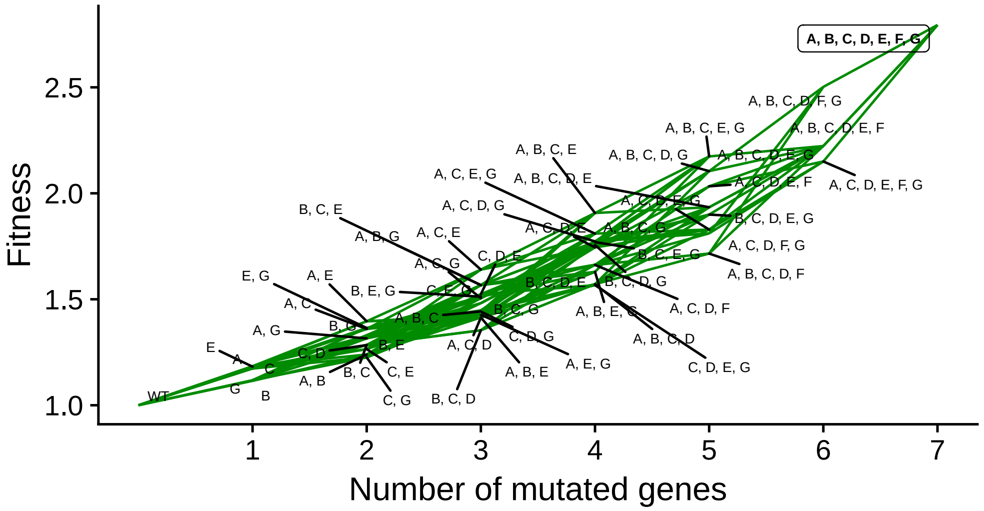

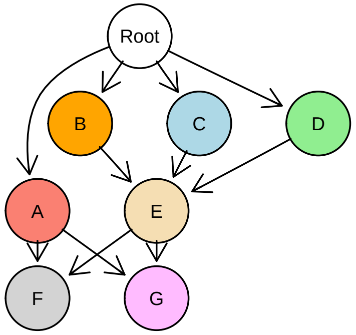

ID	FoocHDFetllLLSqj
acc genotypes	21
peaks no back	1
removed edges	0
prop rem edges	0

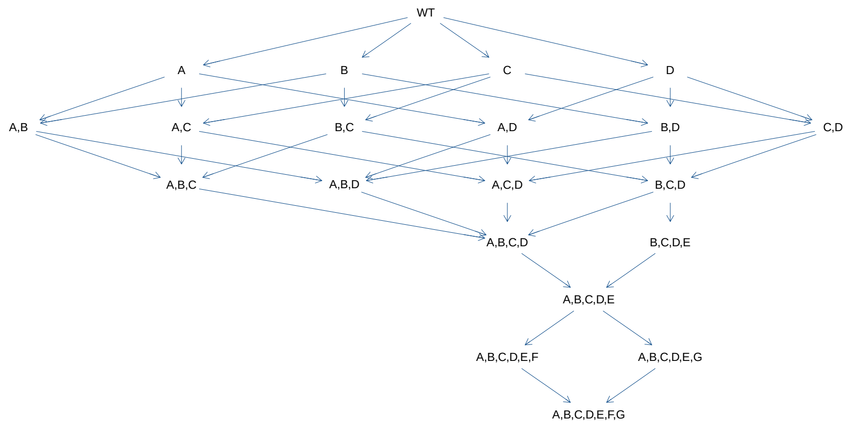

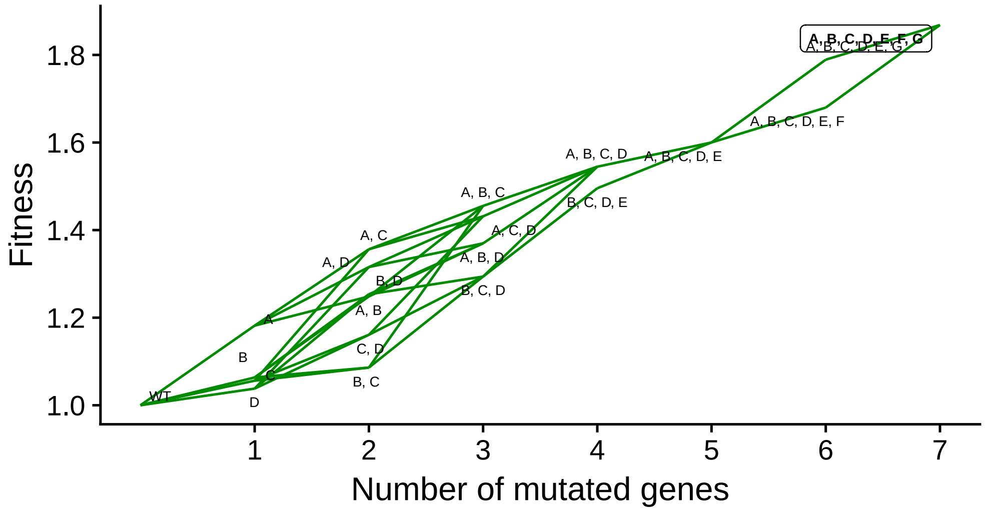

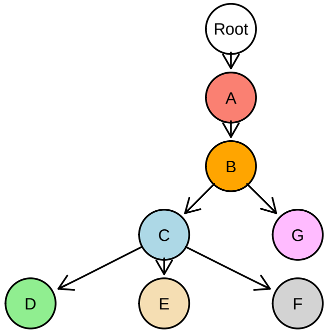

ID	Gb23niBOgzn9YAzw
acc genotypes	20
peaks no back	1
removed edges	0
prop rem edges	0

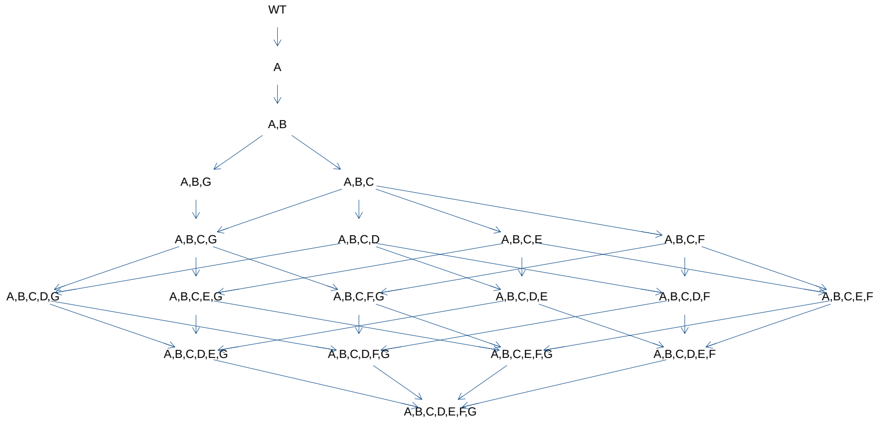

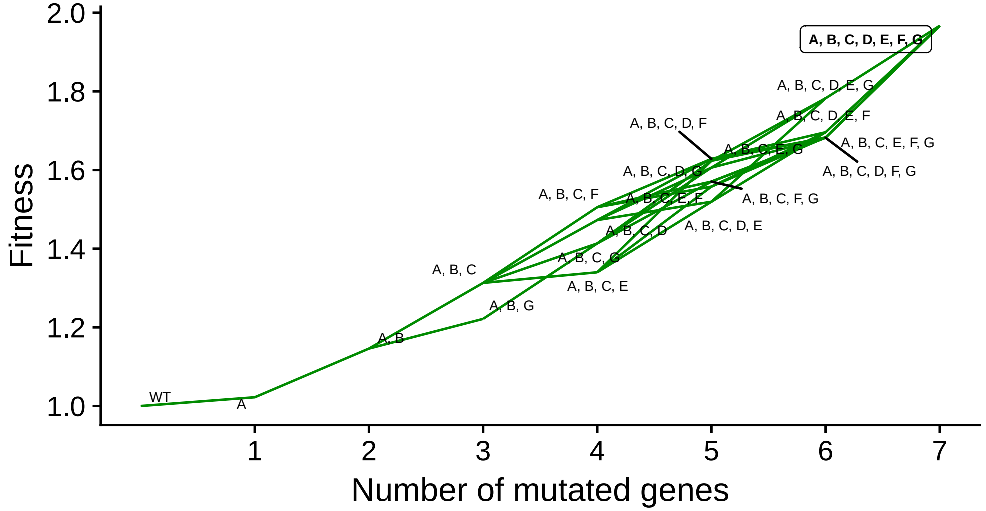

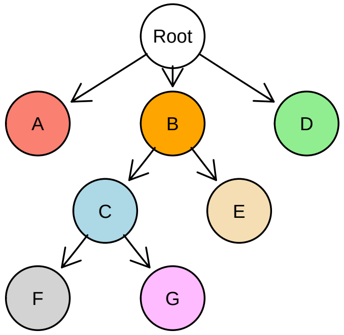

ID	GIWxSFRzKt1hZIMN
acc genotypes	44
peaks no back	1
removed edges	0
prop rem edges	0

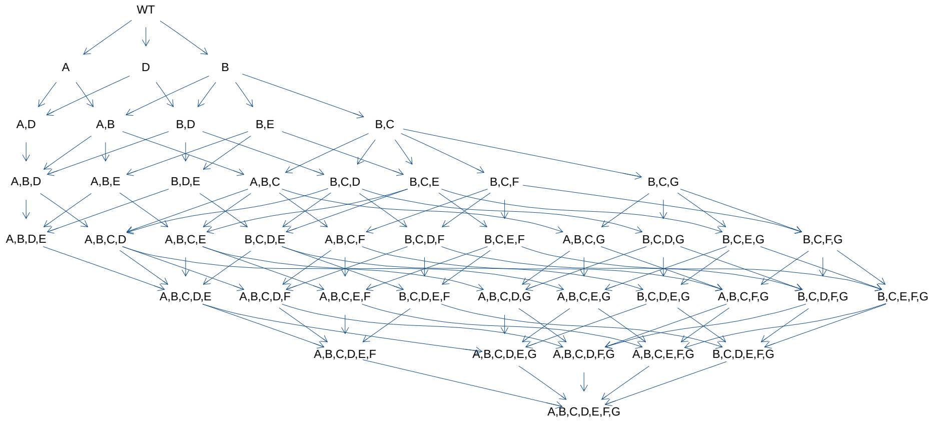

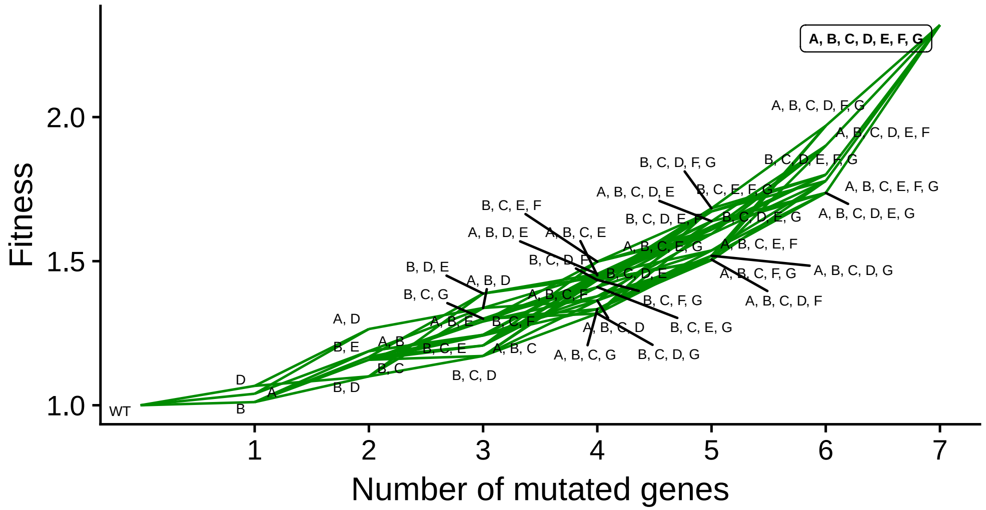

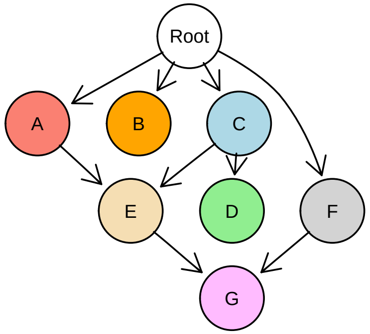

ID	gLwa1VlNXzVMwvea
acc genotypes	36
peaks no back	1
removed edges	0
prop rem edges	0

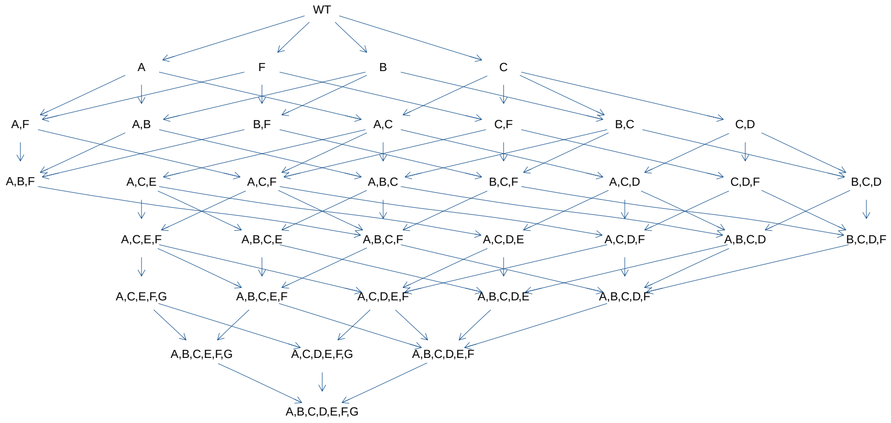

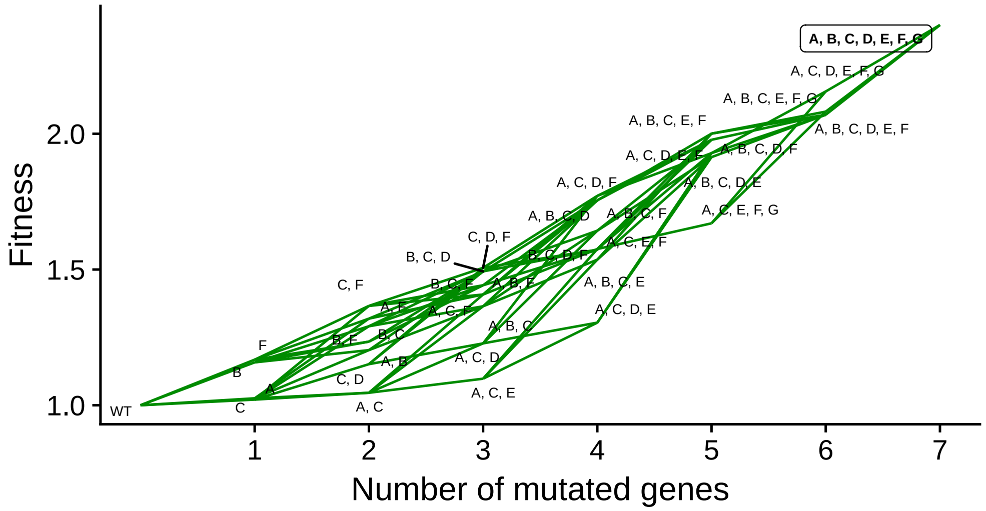

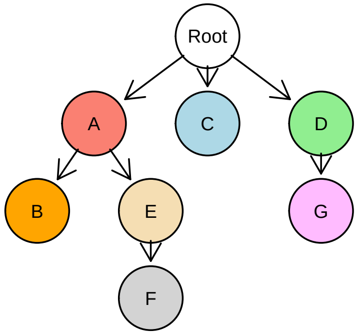

ID	Gn2HkZc73V1xJWfm
acc genotypes	42
peaks no back	1
removed edges	0
prop rem edges	0

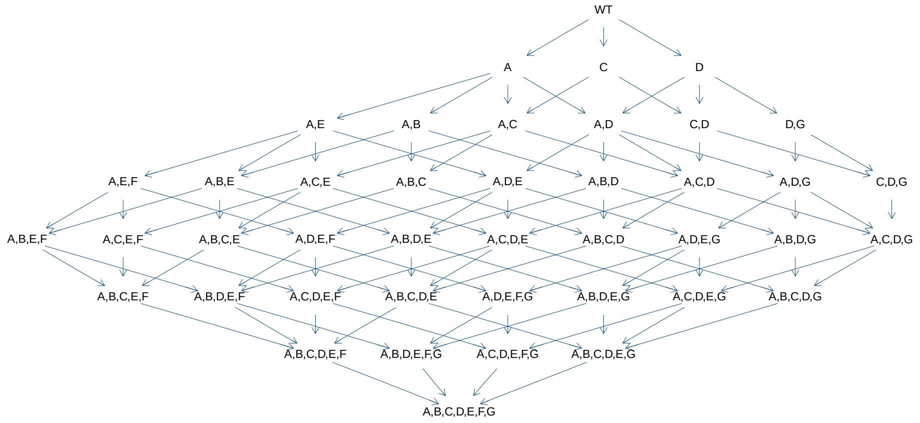

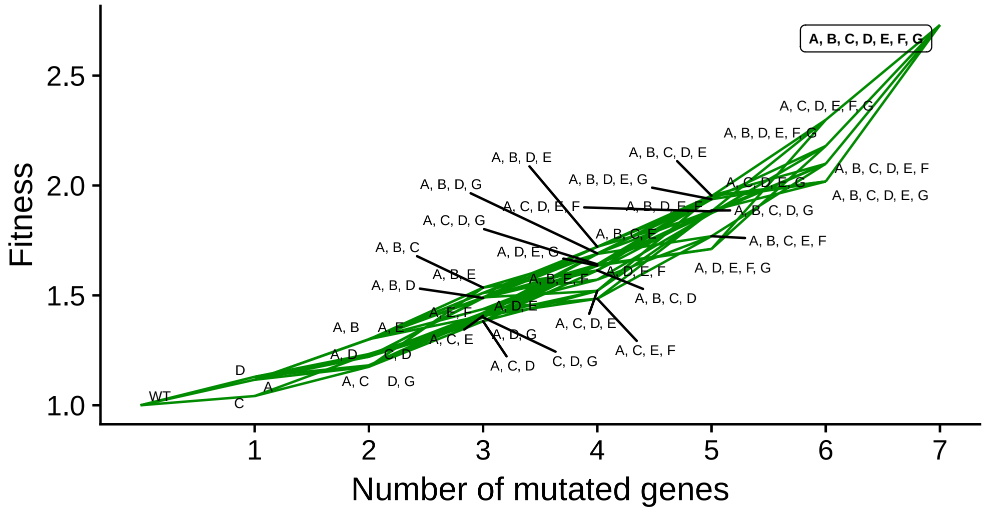

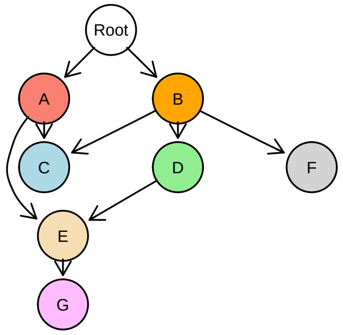

ID	GNq25Sjl71oPTH7O
acc genotypes	22
peaks no back	1
removed edges	0
prop rem edges	0

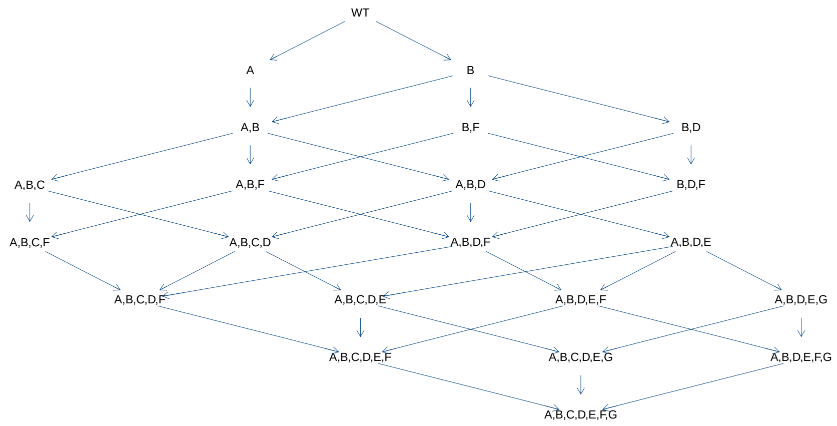

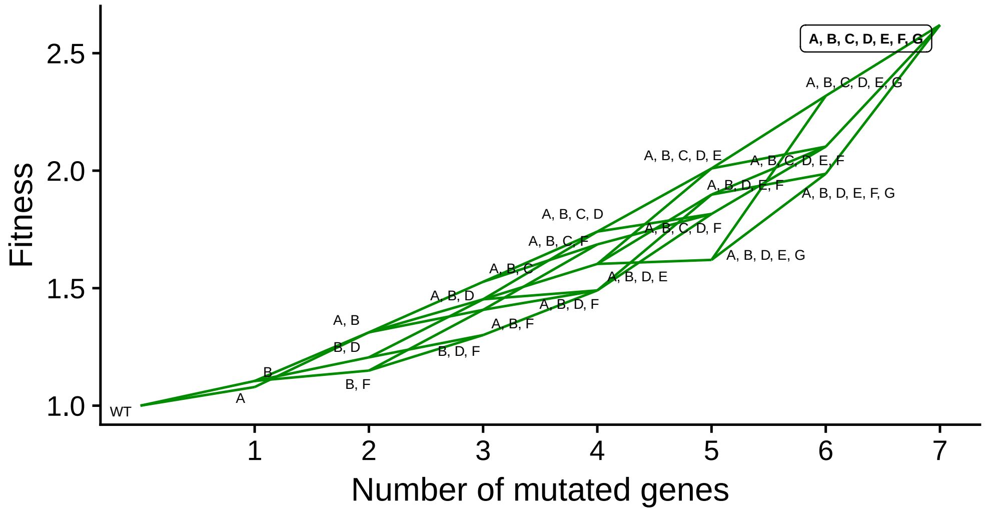

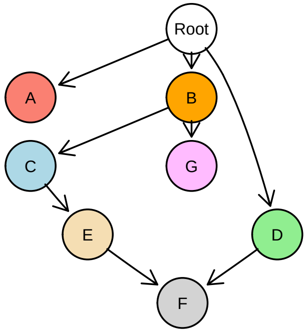

ID	gNsBkqVxNevMiAxL
acc genotypes	32
peaks no back	1
removed edges	0
prop rem edges	0

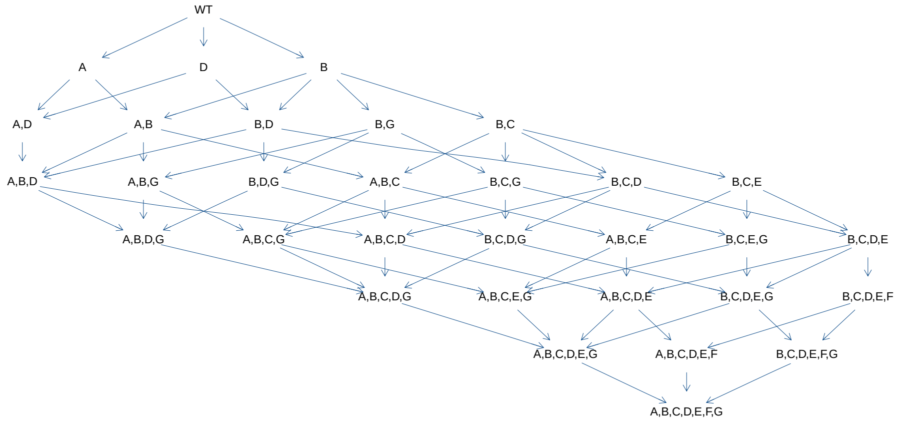

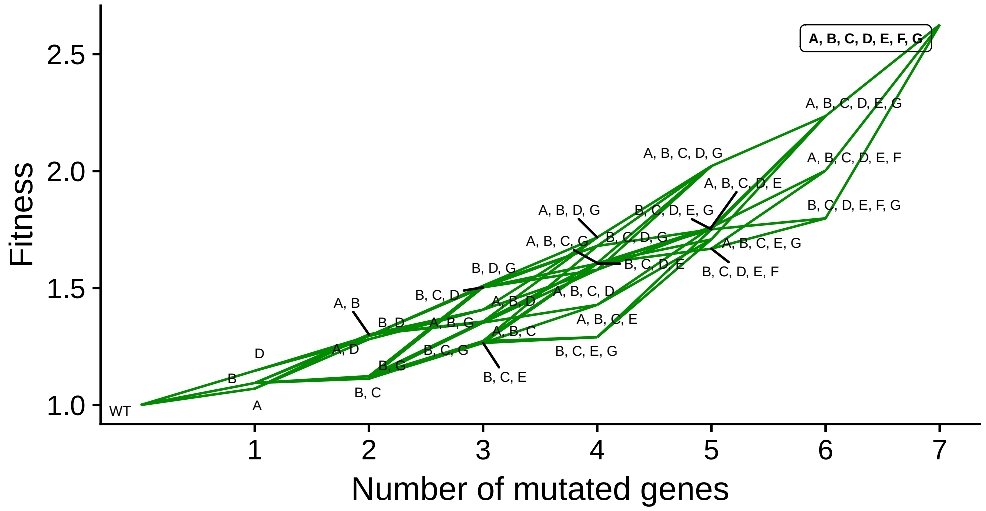

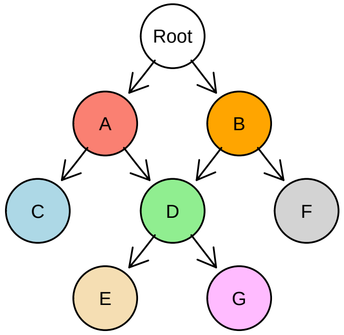

ID	Gp543eGHWygA1P8H
acc genotypes	25
peaks no back	1
removed edges	0
prop rem edges	0

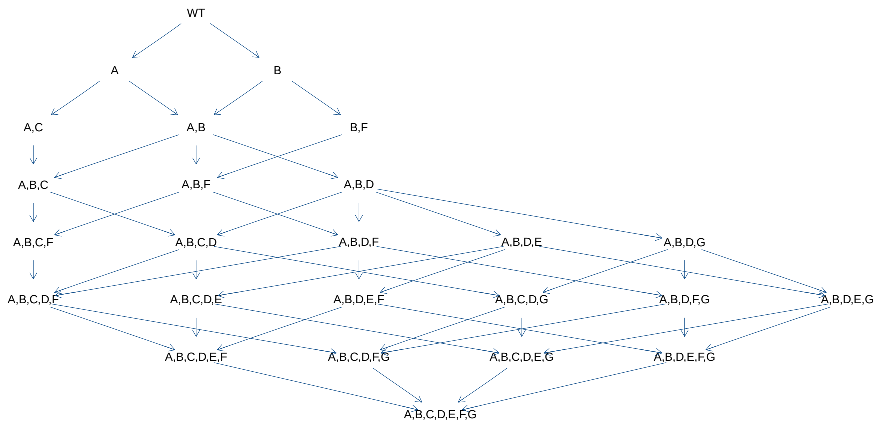

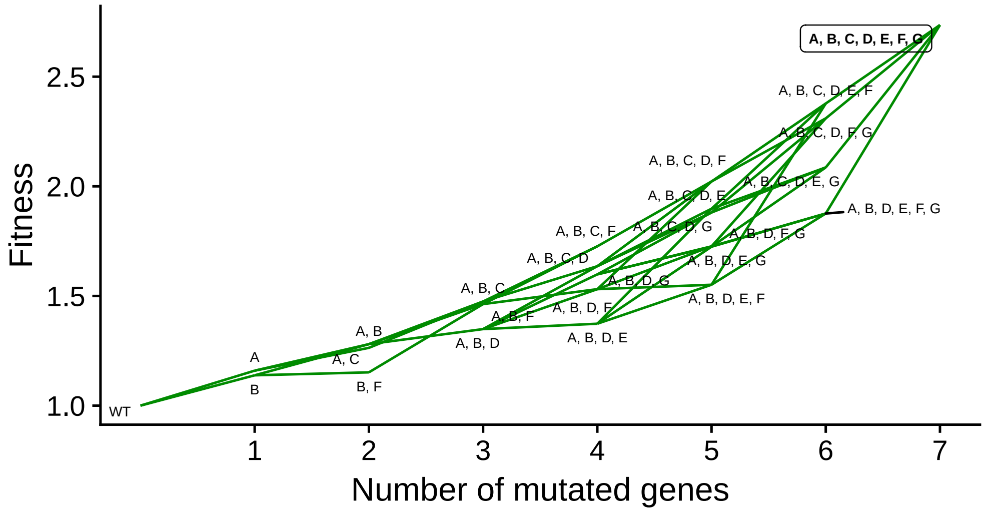

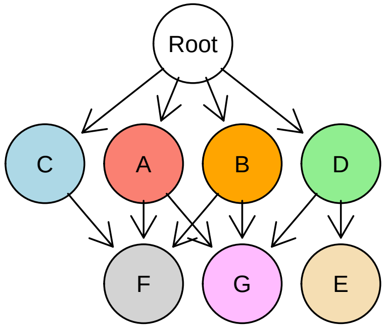

ID	gtuJ3lZP3MzeoW31
acc genotypes	33
peaks no back	1
removed edges	0
prop rem edges	0

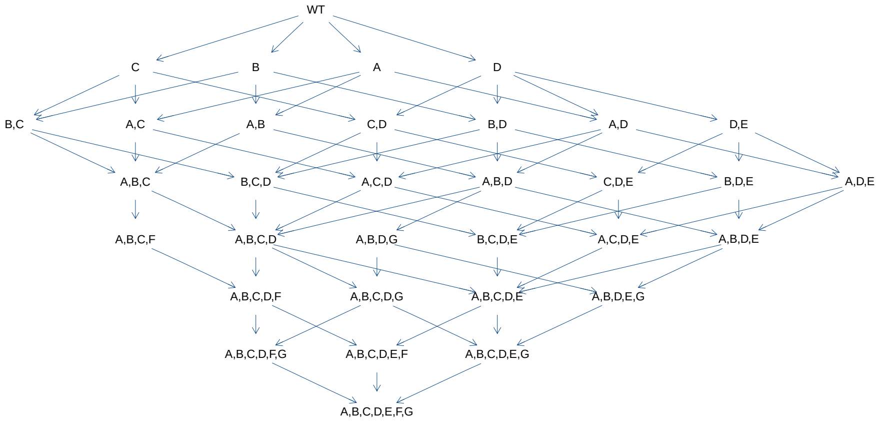

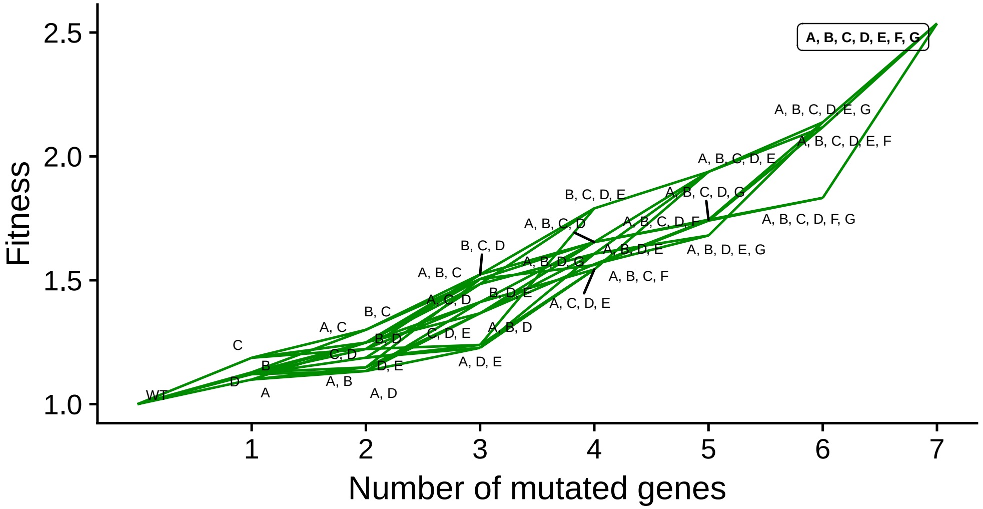

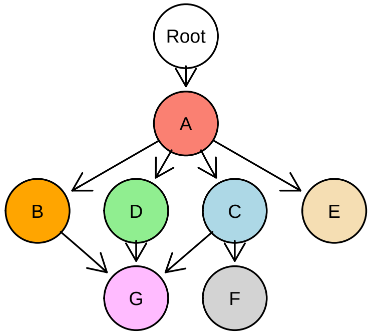

ID	GyBZiyDvewoQU6ri
acc genotypes	29
peaks no back	1
removed edges	0
prop rem edges	0

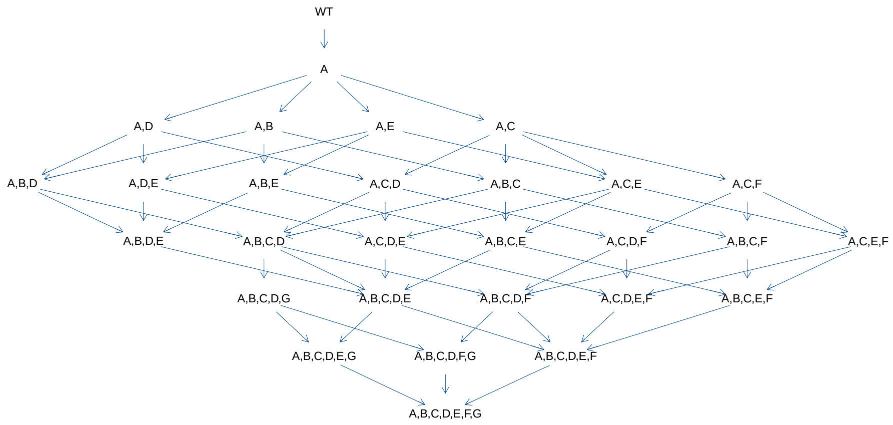

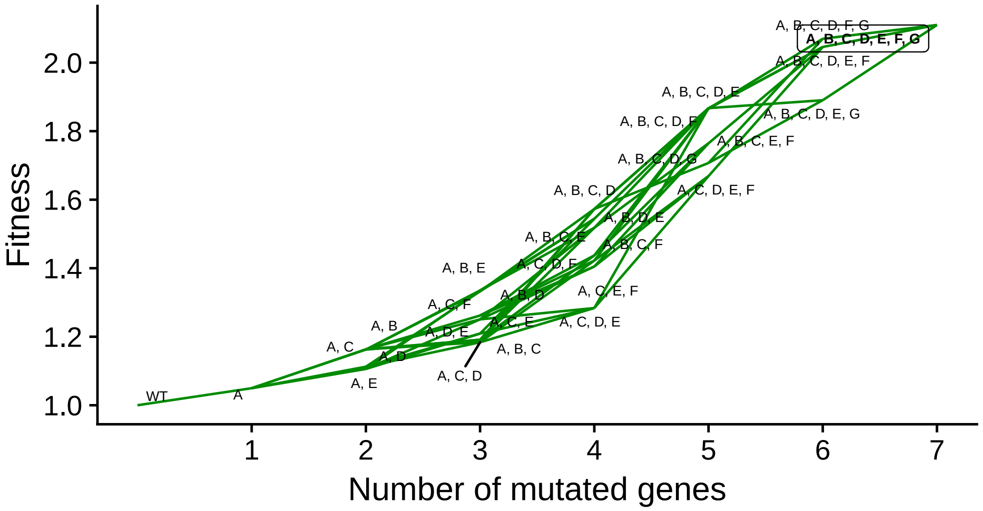

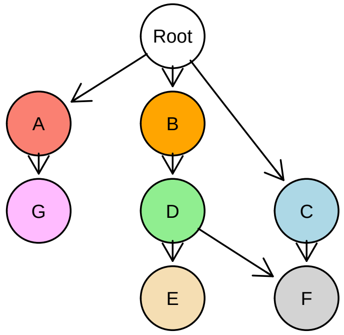

ID	h5XuDSnadWt672QY
acc genotypes	30
peaks no back	1
removed edges	0
prop rem edges	0

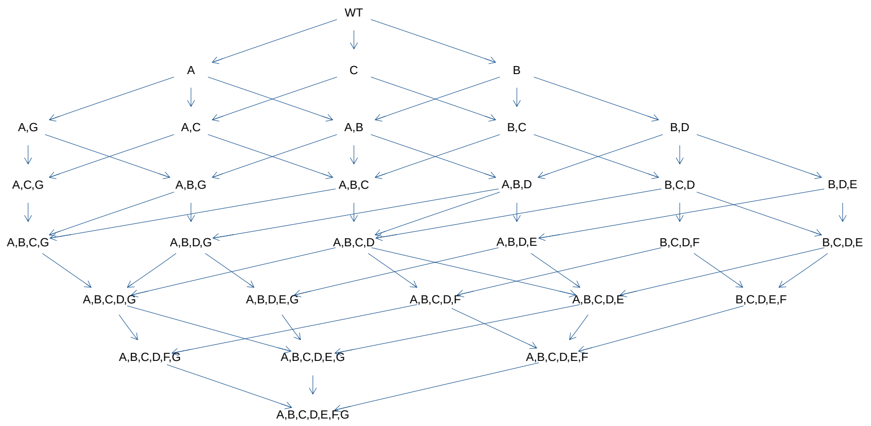

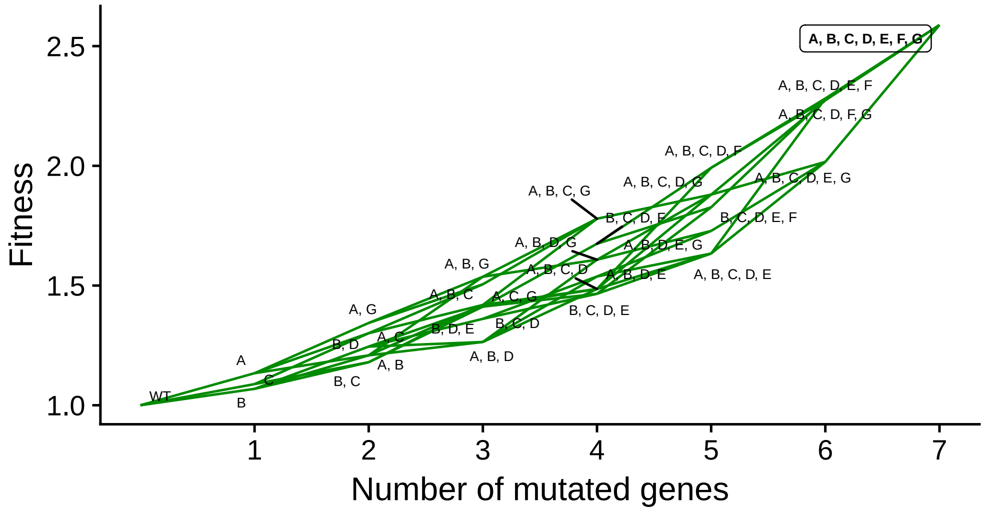

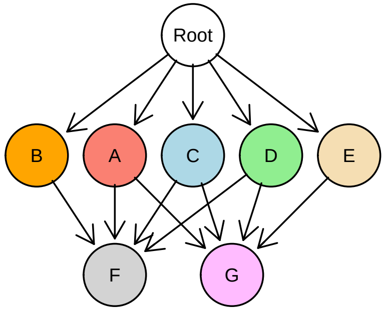

ID	h88O5DNbc34DtJRm
acc genotypes	37
peaks no back	1
removed edges	0
prop rem edges	0

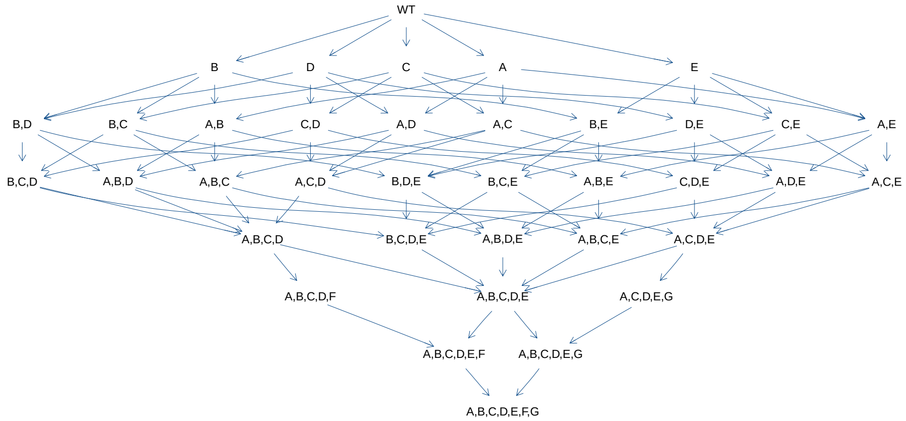

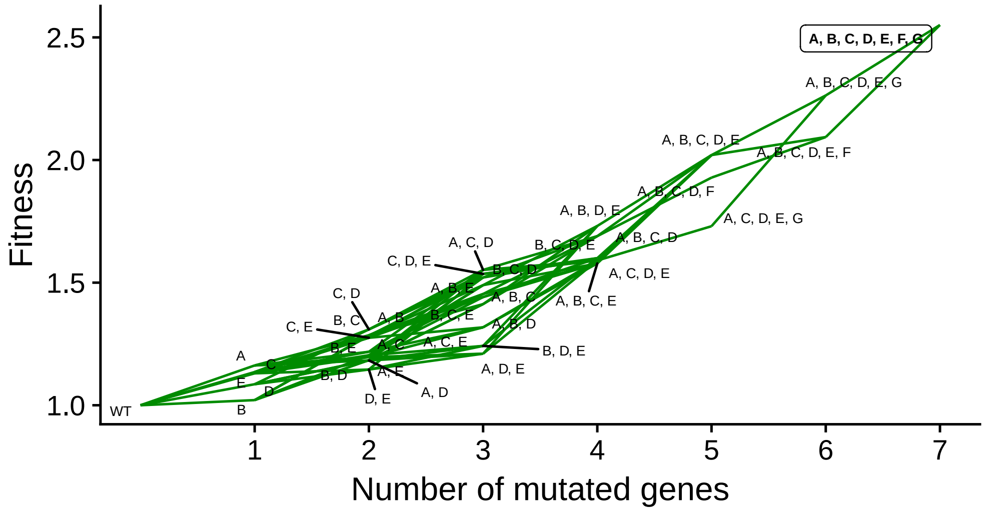

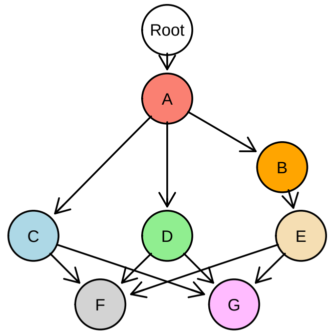

ID	Hbk7A4ZWDH110SIas
acc genotypes	16
peaks no back	1
removed edges	0
prop rem edges	0

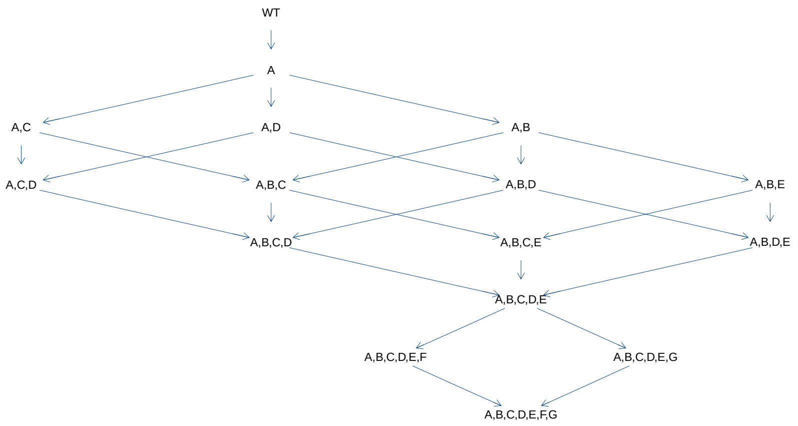

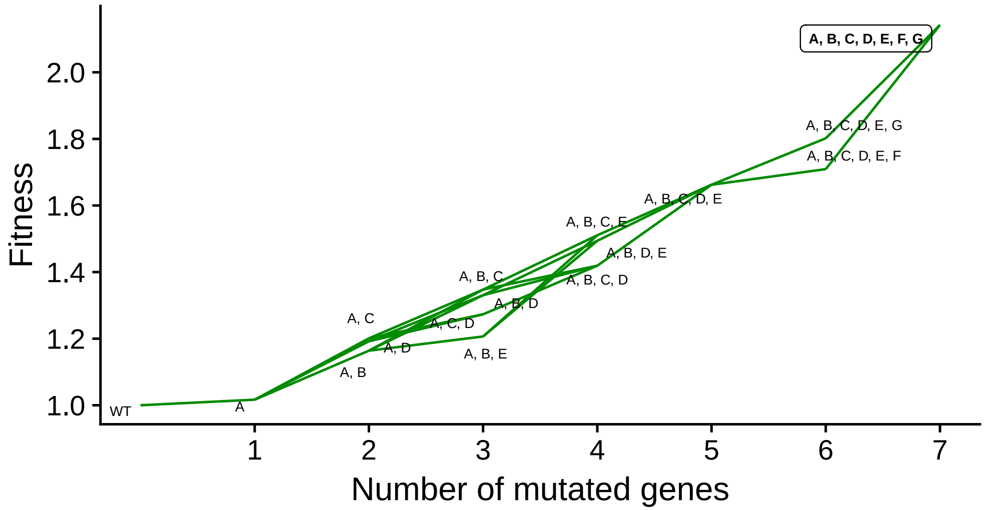

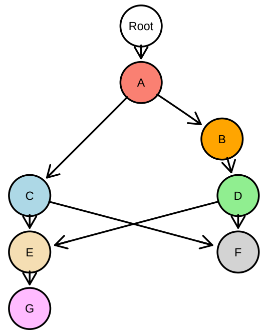

ID	HCfuosyRn2Mnwy2Y
acc genotypes	12
peaks no back	1
removed edges	0
prop rem edges	0

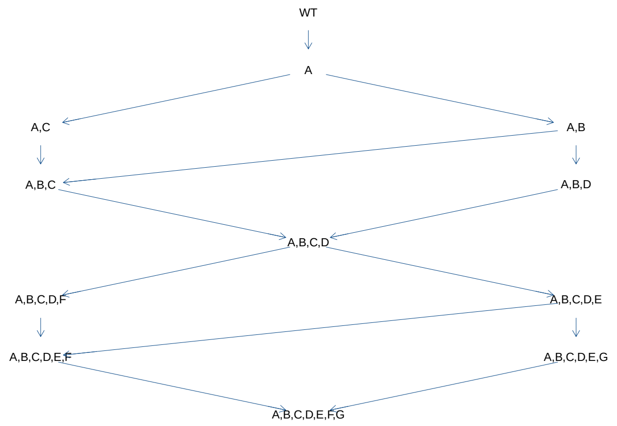

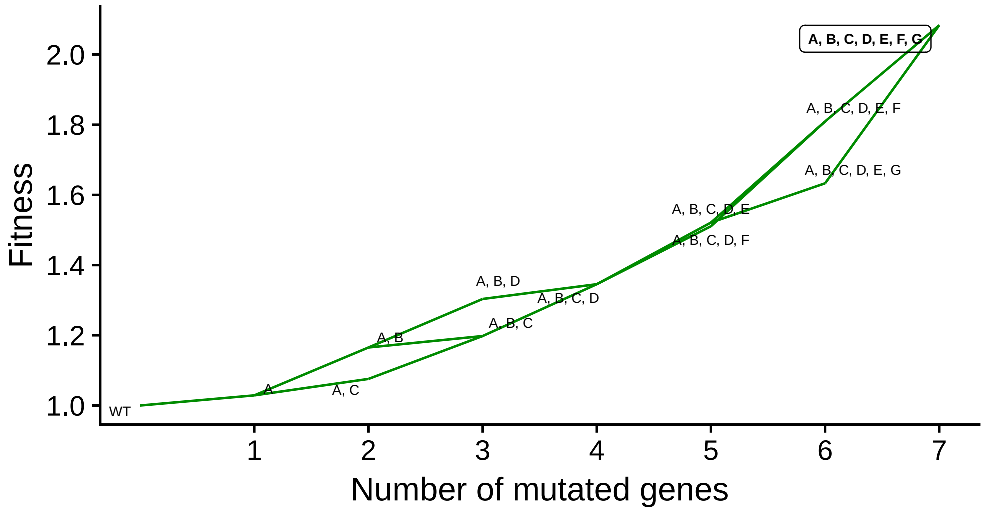

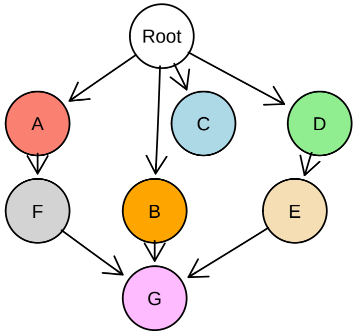

ID hEfBL8MoVyBlRoMA
acc genotypes 38
peaks no back 1
removed edges 0
prop rem edges 0

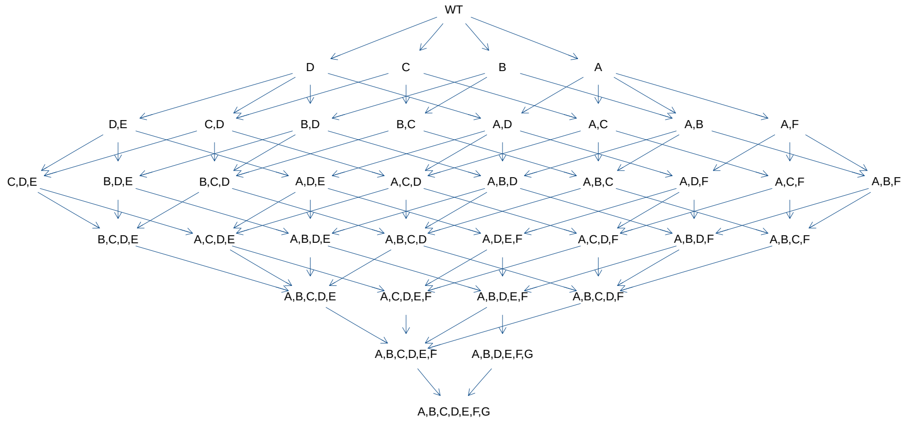

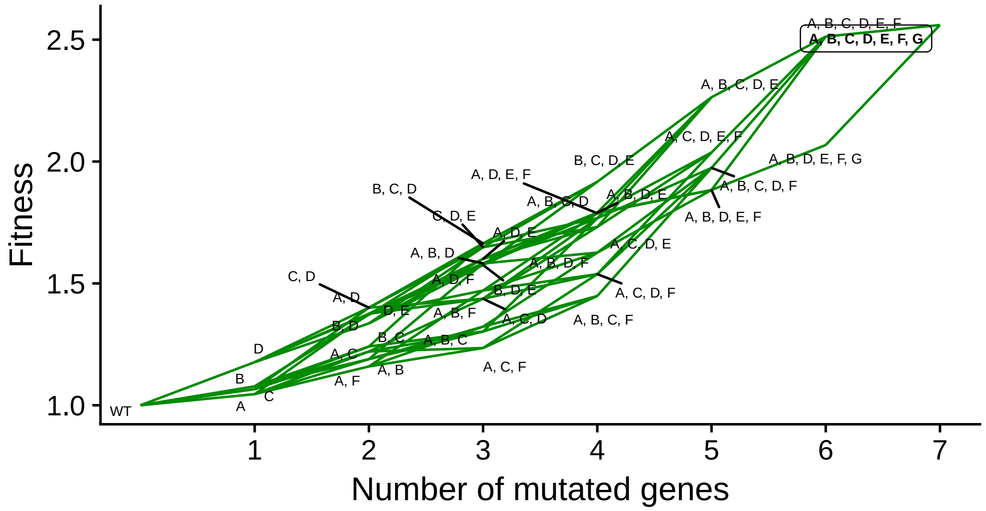

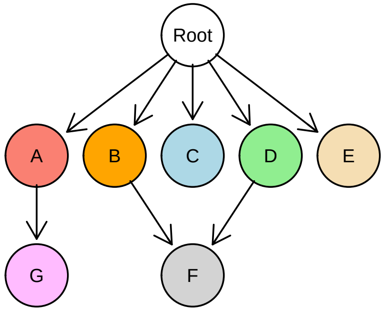

ID	HF510lGzHhUvaK1Wc
acc genotypes	60
peaks no back	1
removed edges	0
prop rem edges	0

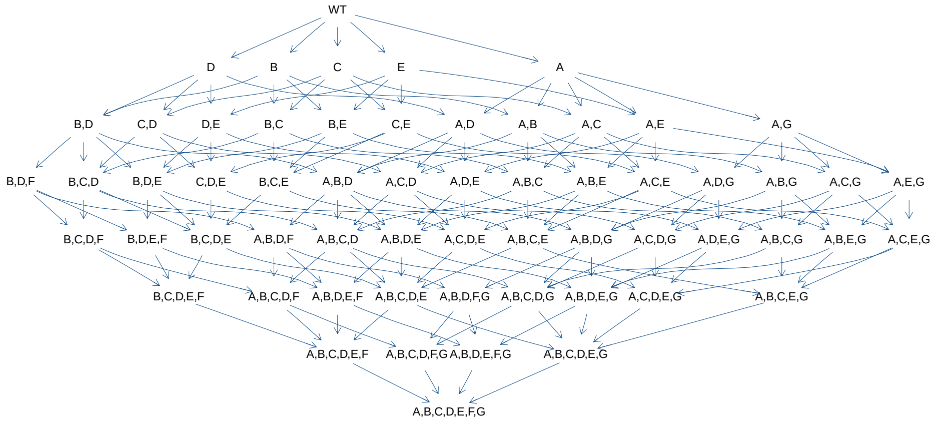

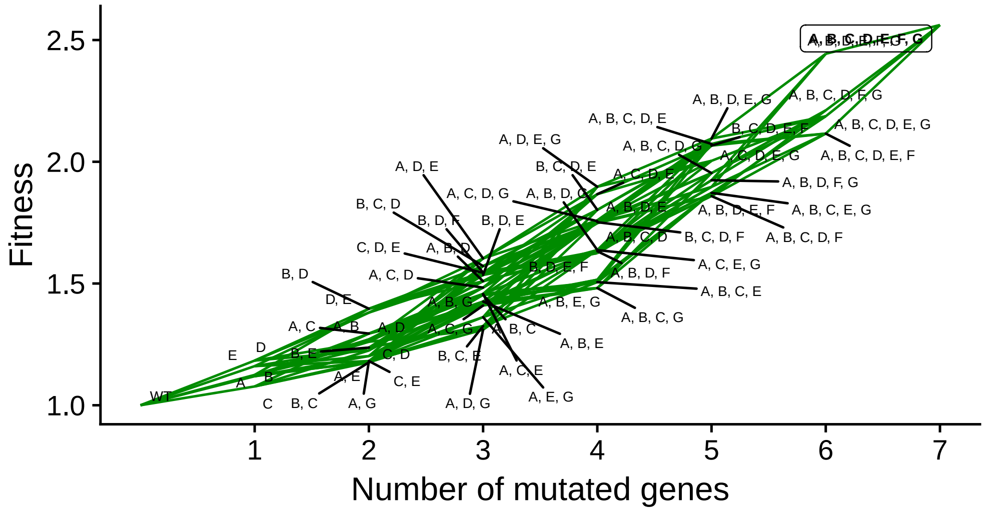

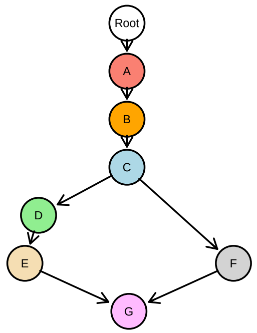

ID	HfNvtnFRbnBZJbsa
acc genotypes	10
peaks no back	1
removed edges	0
prop rem edges	0

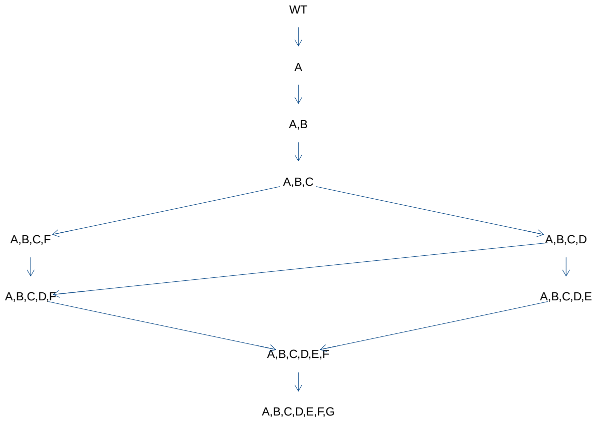

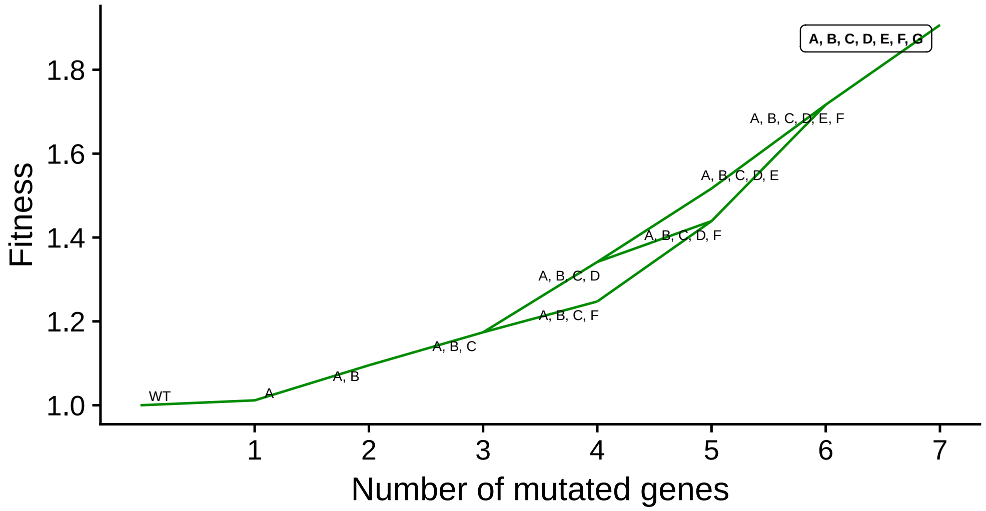

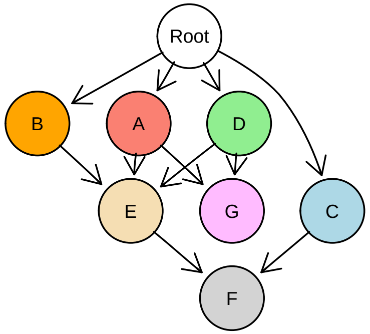

ID	HGZXE3cU1toI8NwB
acc genotypes	26
peaks no back	1
removed edges	0
prop rem edges	0

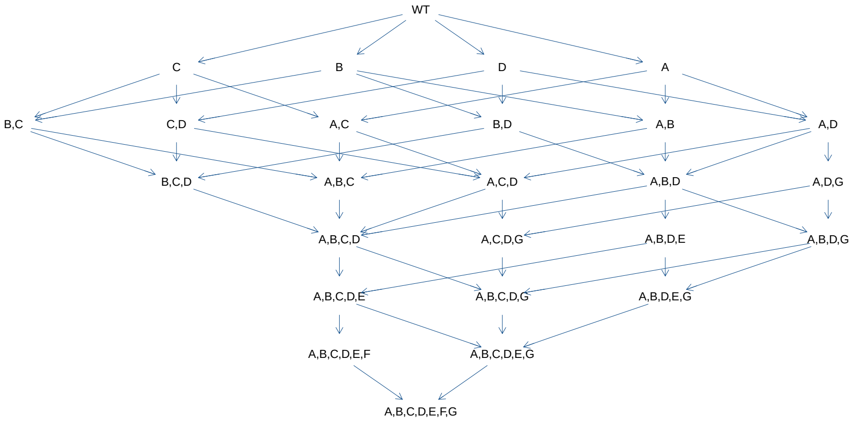

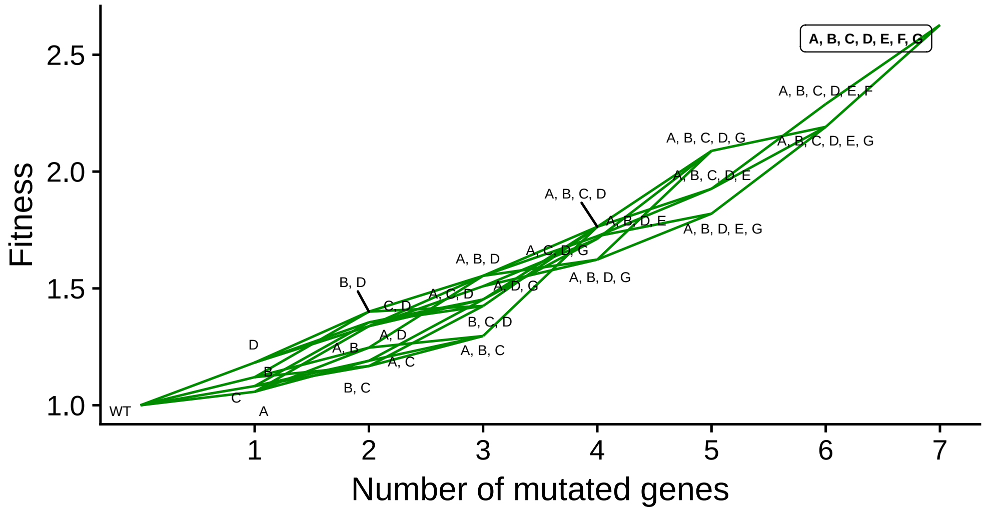

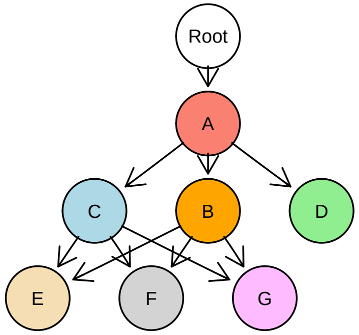

ID	HhTnaJyFFi4y9Ptx
acc genotypes	23
peaks no back	1
removed edges	0
prop rem edges	0

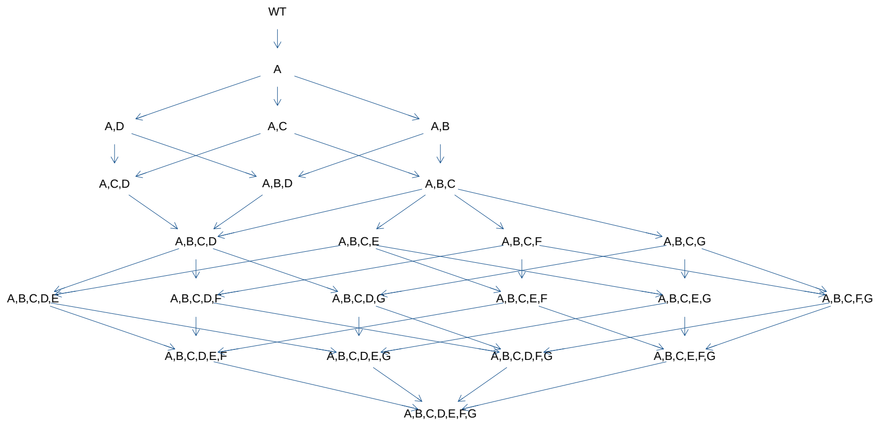

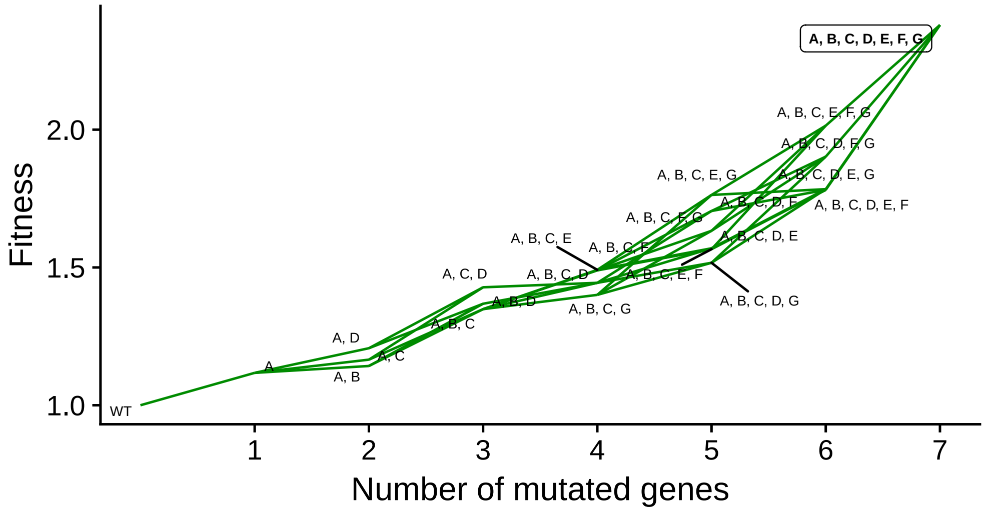

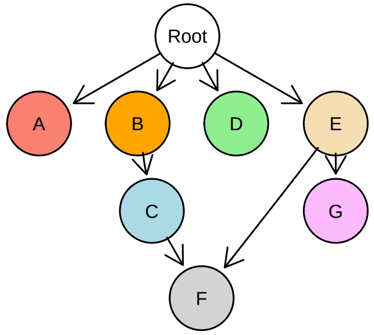

ID hiqO72oQ9p210Vxcp
acc genotypes 44
peaks no back 1
removed edges 0
prop rem edges 0

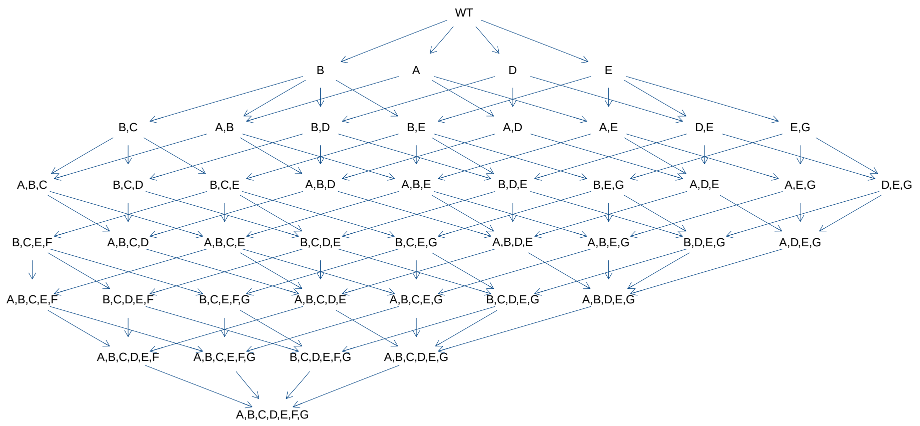

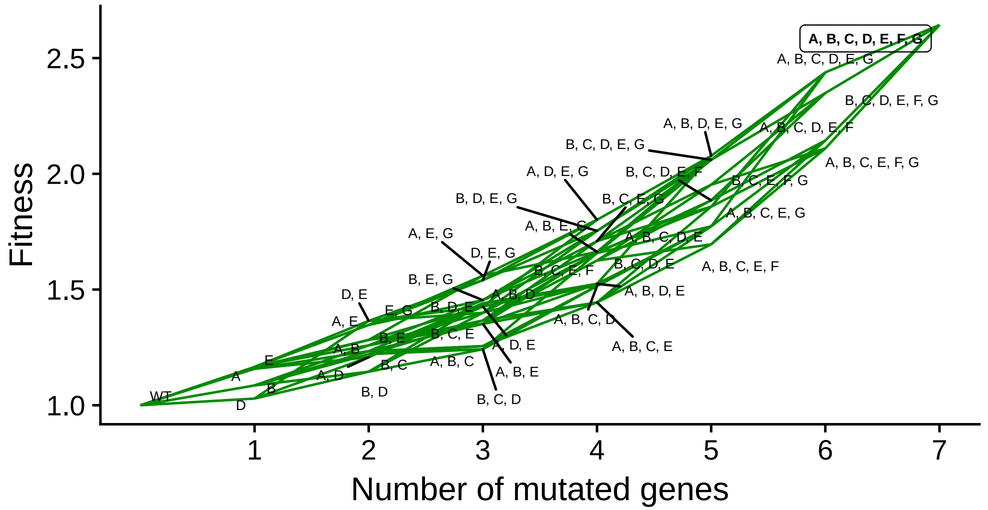

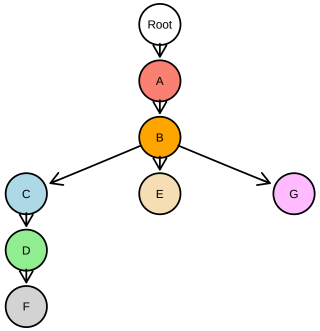

ID	hTuov2KGeLAIrrac
acc genotypes	18
peaks no back	1
removed edges	0
prop rem edges	0

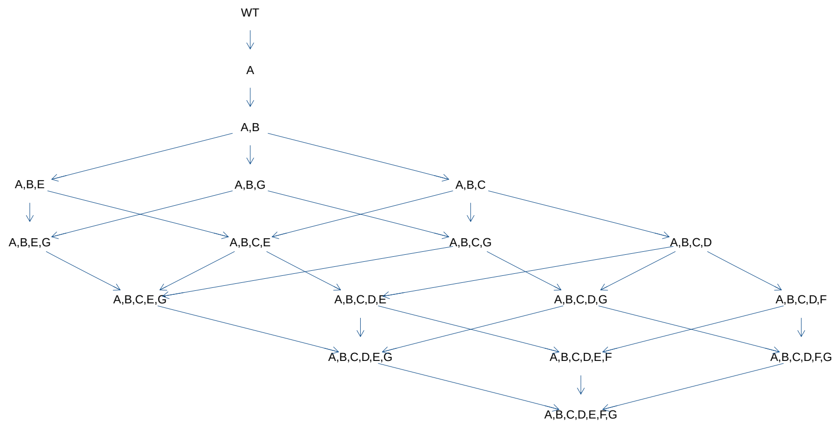

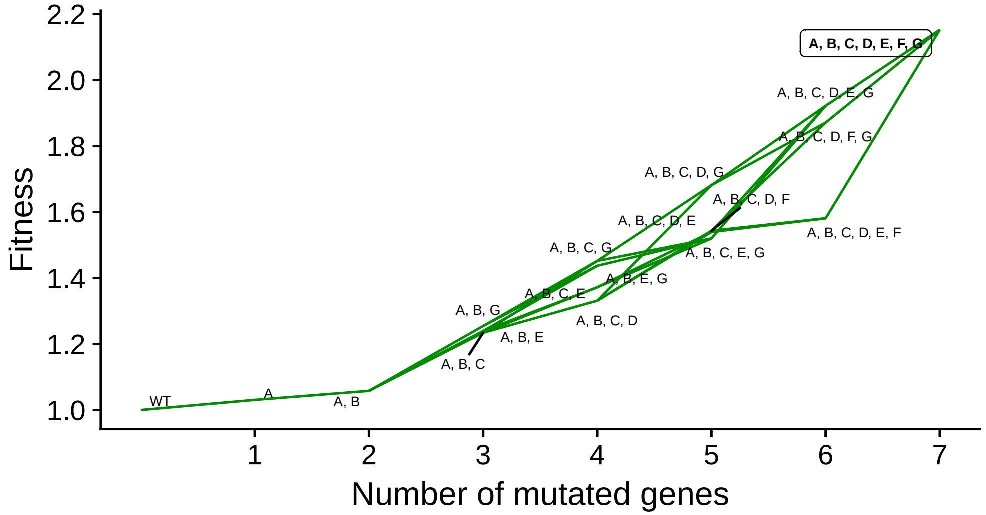

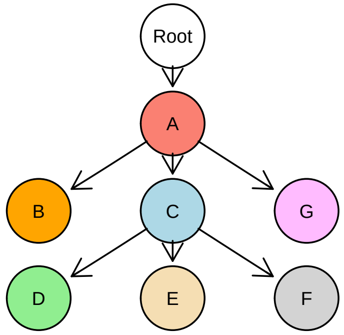

ID	hyGvt7bXxXmlBy4c
acc genotypes	37
peaks no back	1
removed edges	0
prop rem edges	0

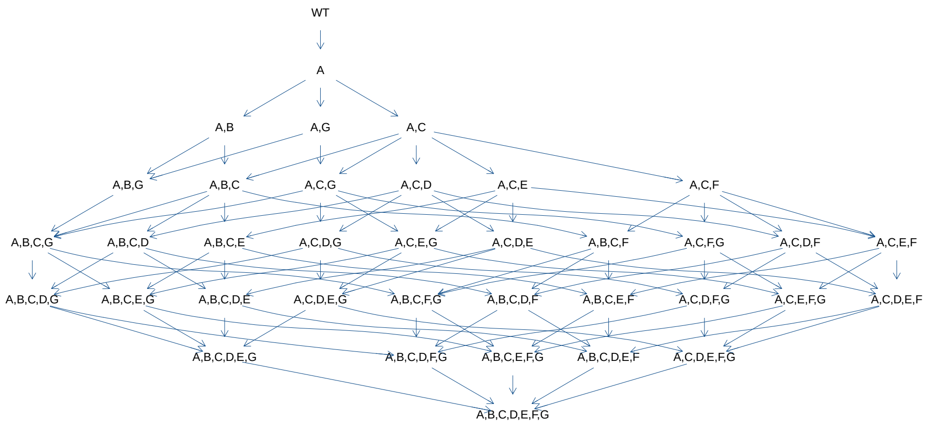

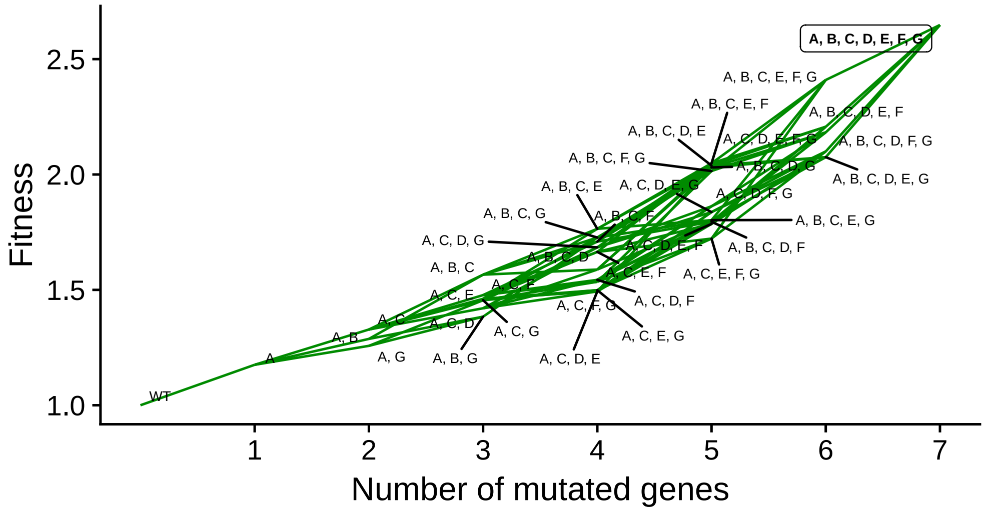

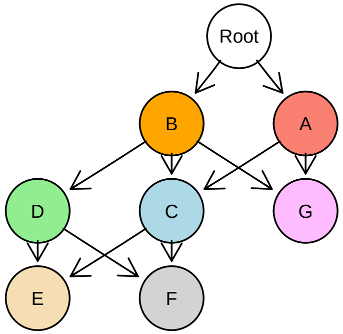

ID	HYyO10PwiQqDuWK2r
acc genotypes	18
peaks no back	1
removed edges	0
prop rem edges	0

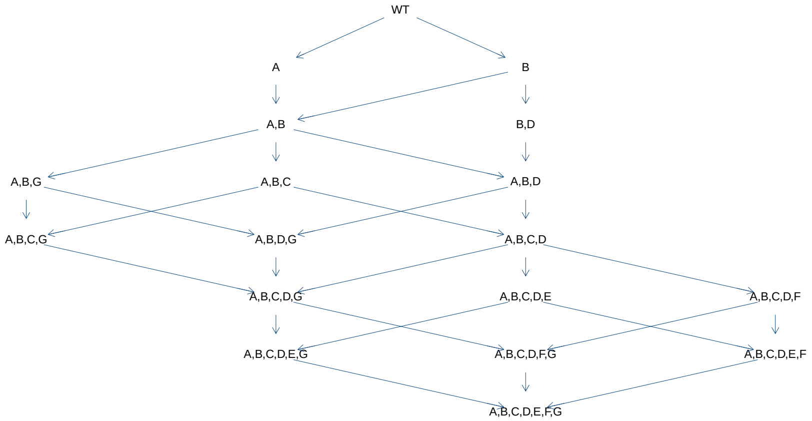

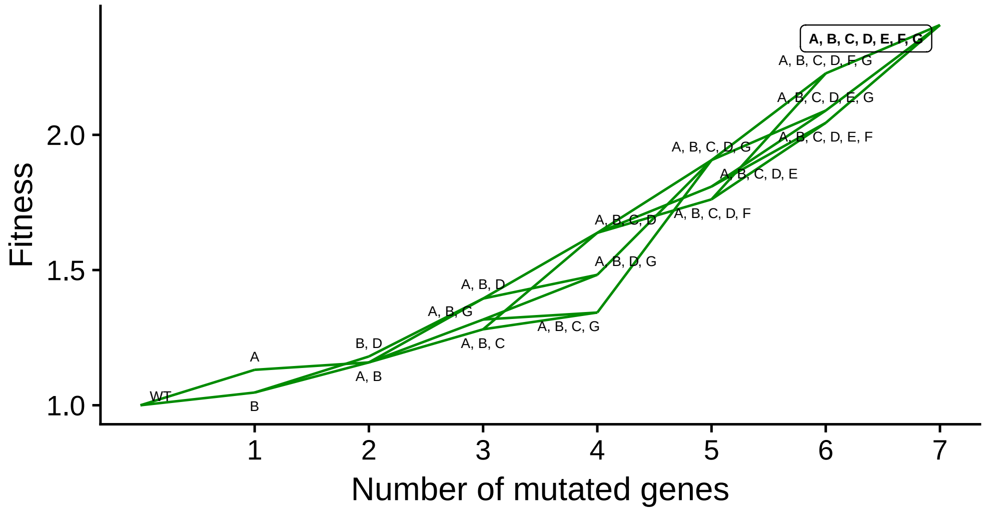

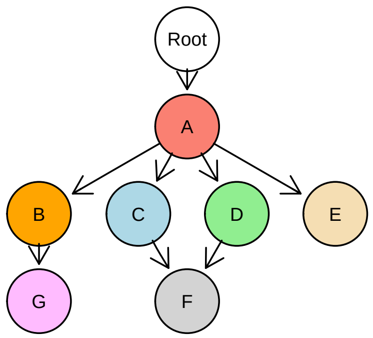

ID	IFOFBmVbzrLBfqR1
acc genotypes	31
peaks no back	1
removed edges	0
prop rem edges	0

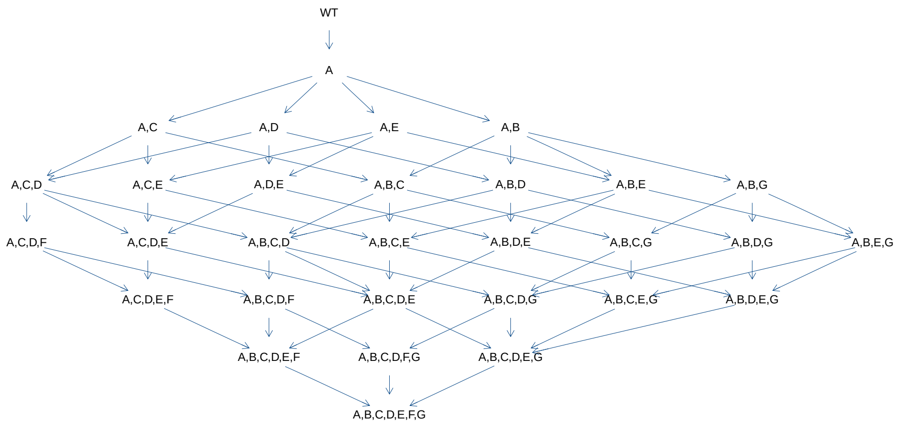

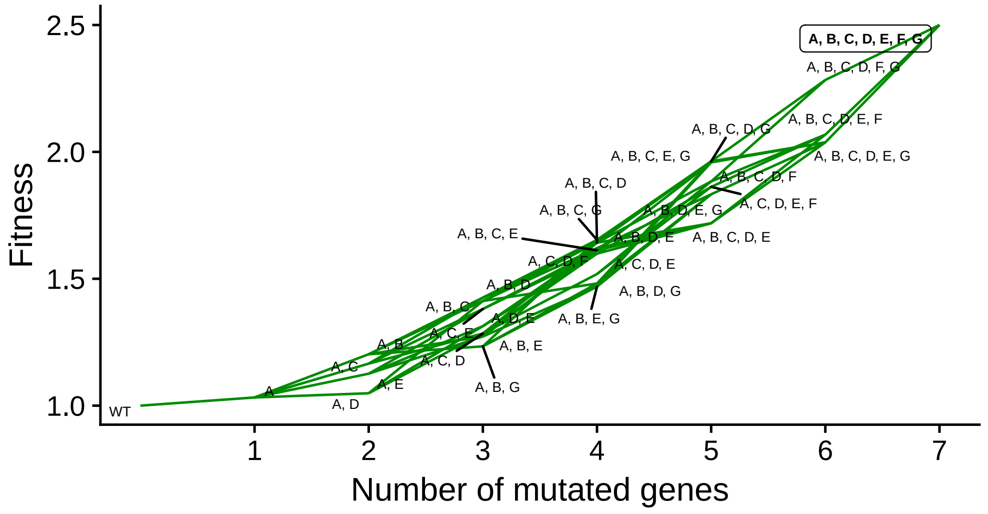

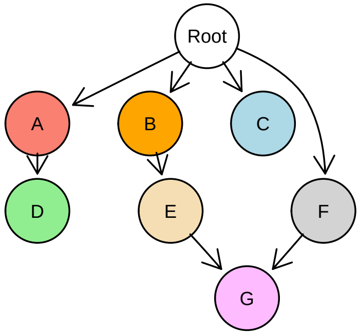

ID	IJ15PYr1XTbhonDL
acc genotypes	42
peaks no back	1
removed edges	0
prop rem edges	0

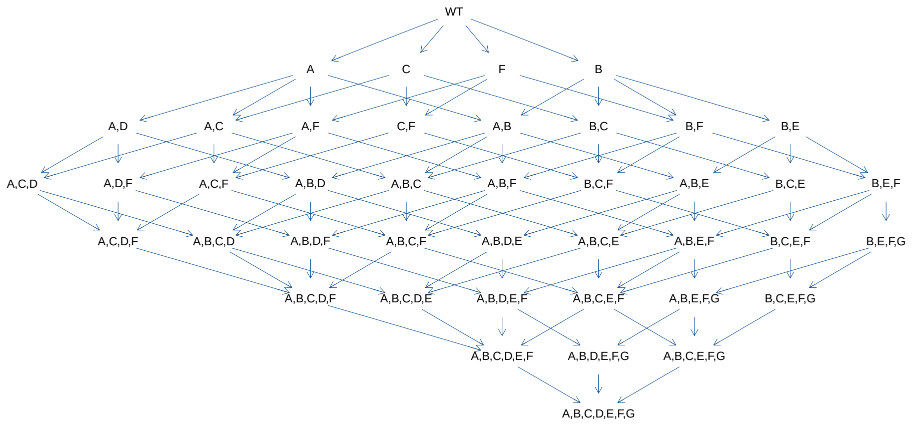

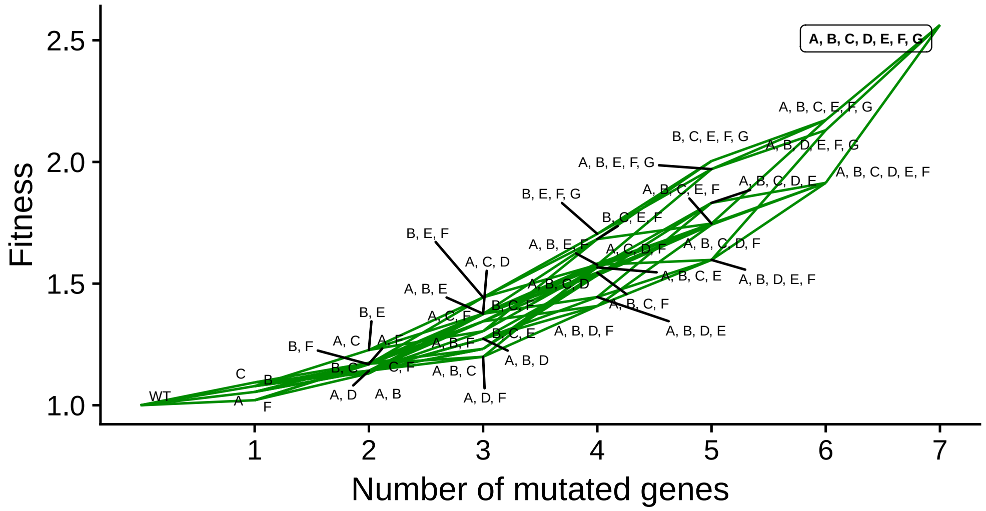

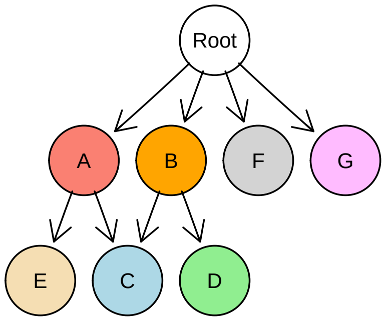

ID	iKHz2mPyuFYjaiPo
acc genotypes	52
peaks no back	1
removed edges	0
prop rem edges	0

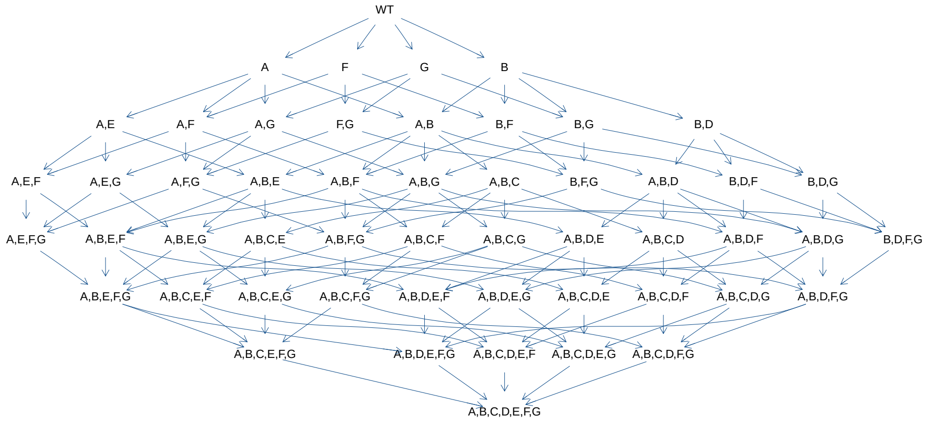

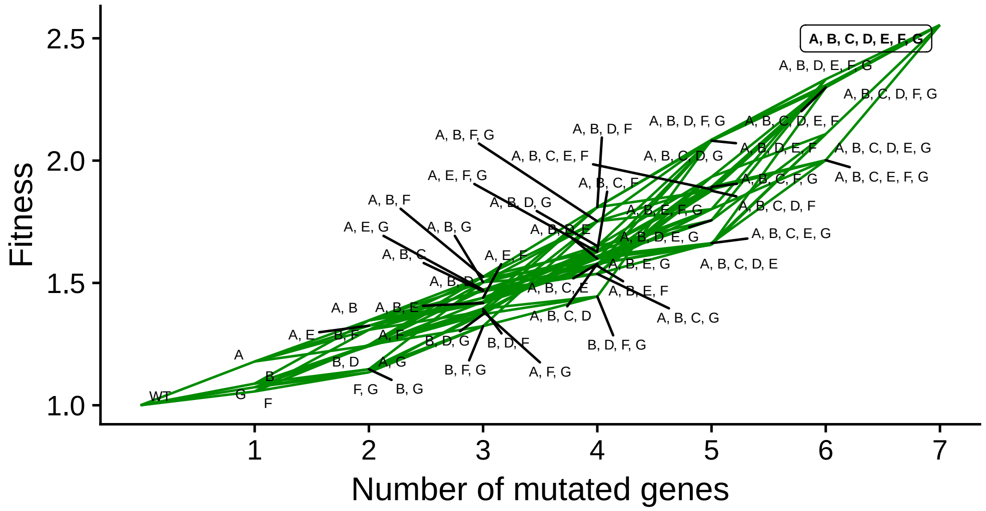

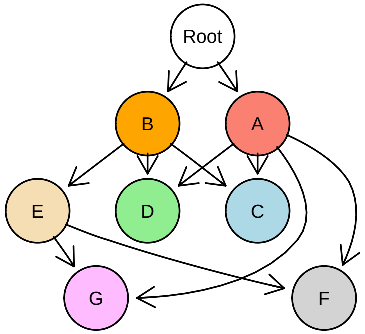

ID	imGyIoiov7jJGQPM
acc genotypes	24
peaks no back	1
removed edges	0
prop rem edges	0

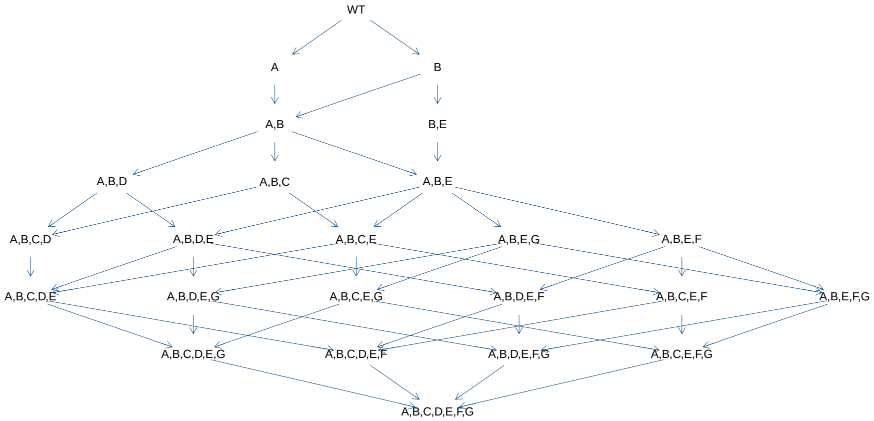

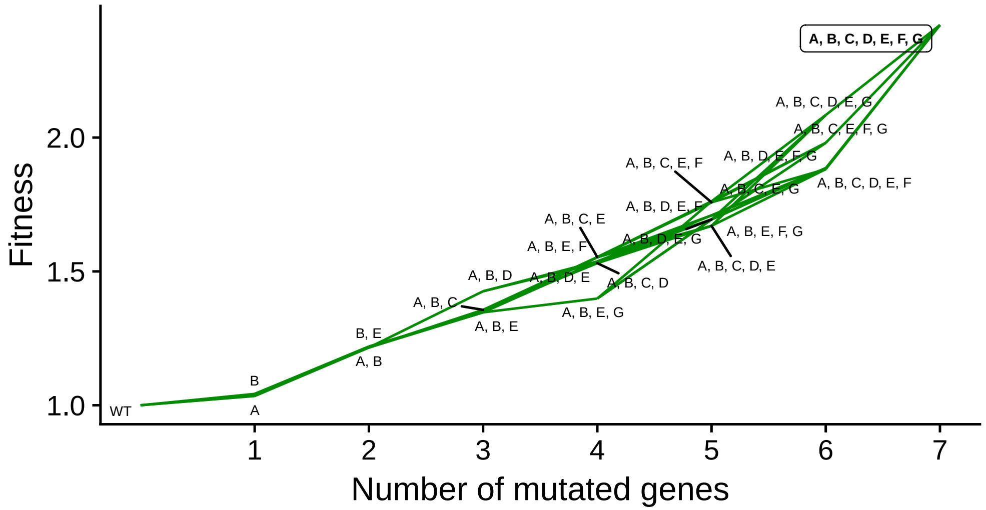

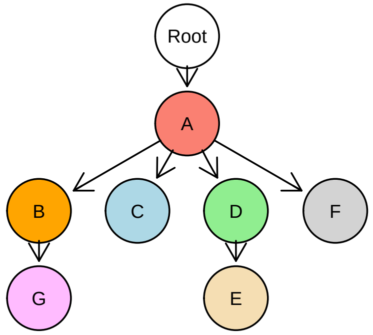

ID	Iq2ILDZ3MG3ndder
acc genotypes	37
peaks no back	1
removed edges	0
prop rem edges	0

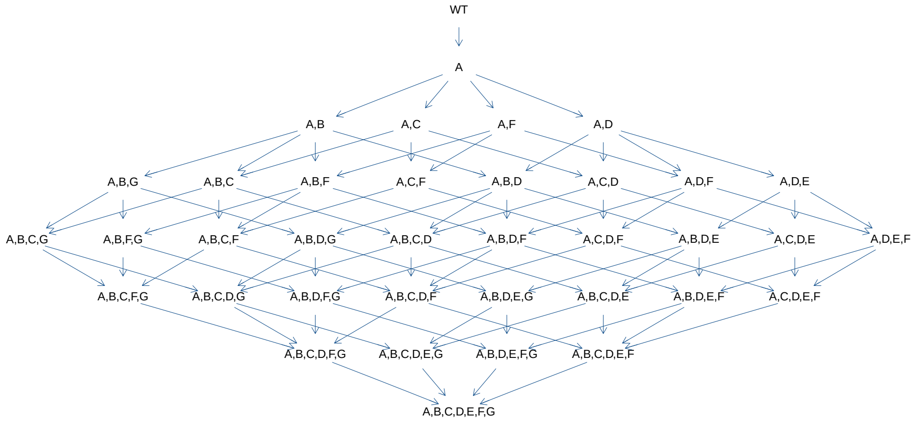

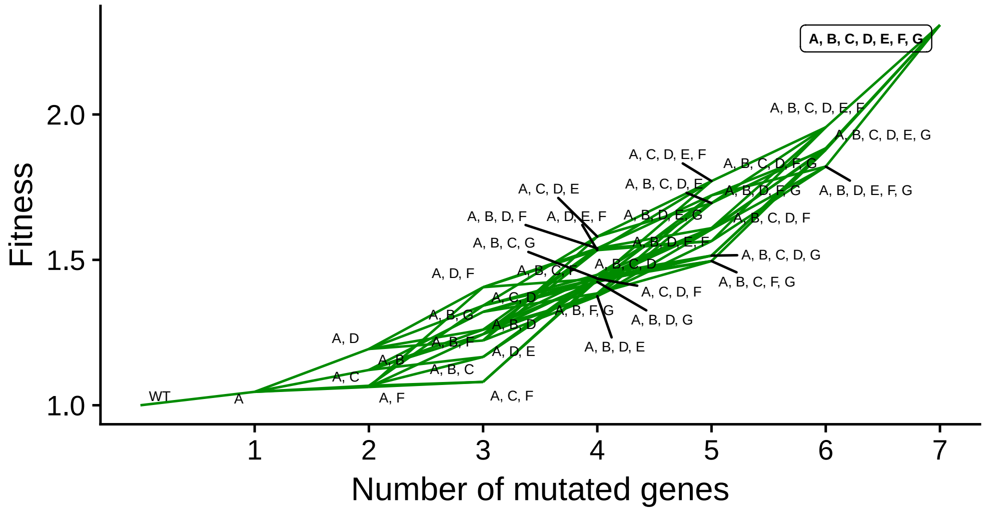

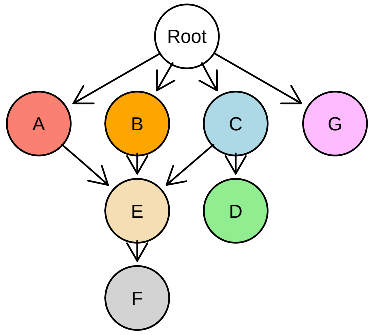

ID	IRpnRnTLcDvmGnyE
acc genotypes	32
peaks no back	1
removed edges	0
prop rem edges	0

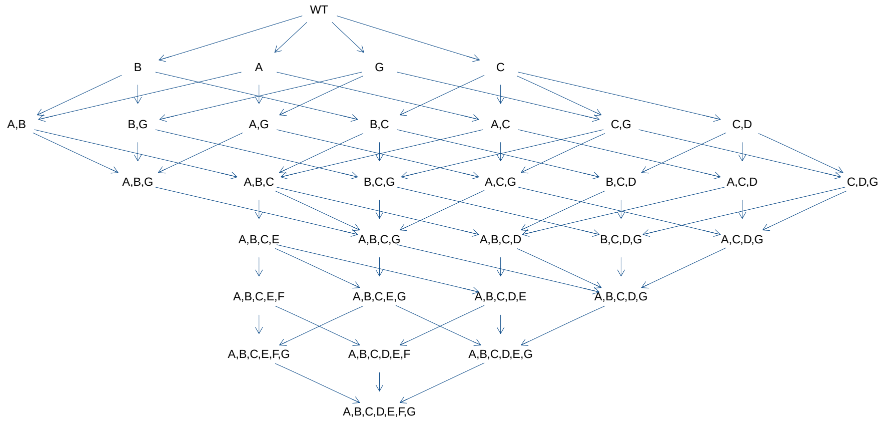

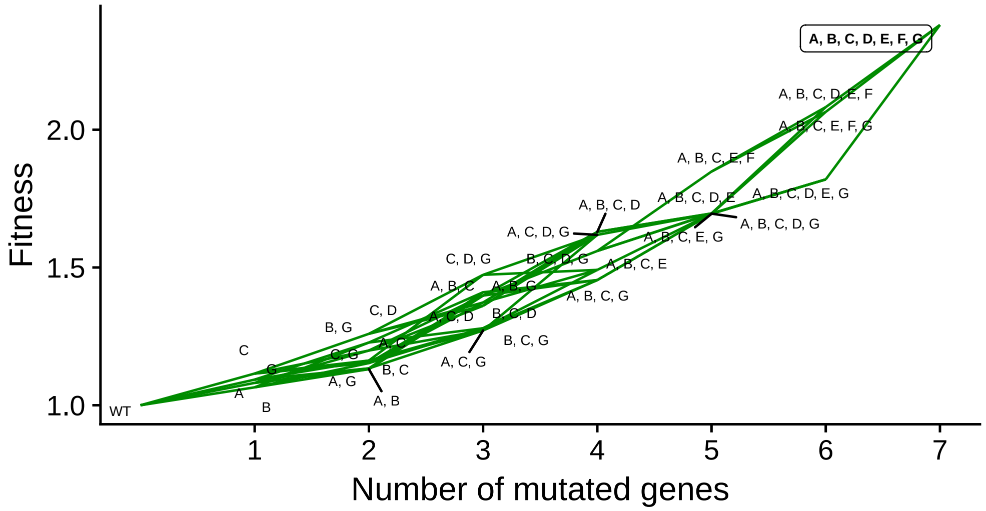

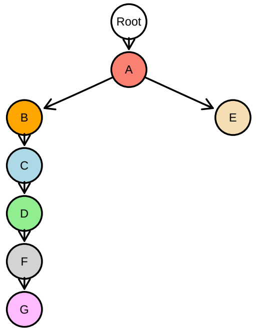

ID	iU2gBfqvhEyguOWr
acc genotypes	13
peaks no back	1
removed edges	0
prop rem edges	0

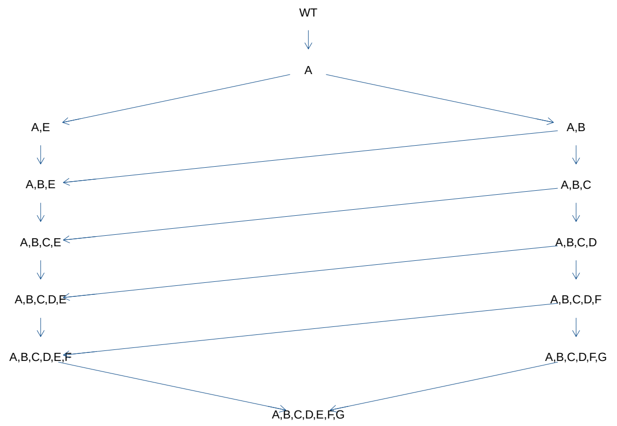

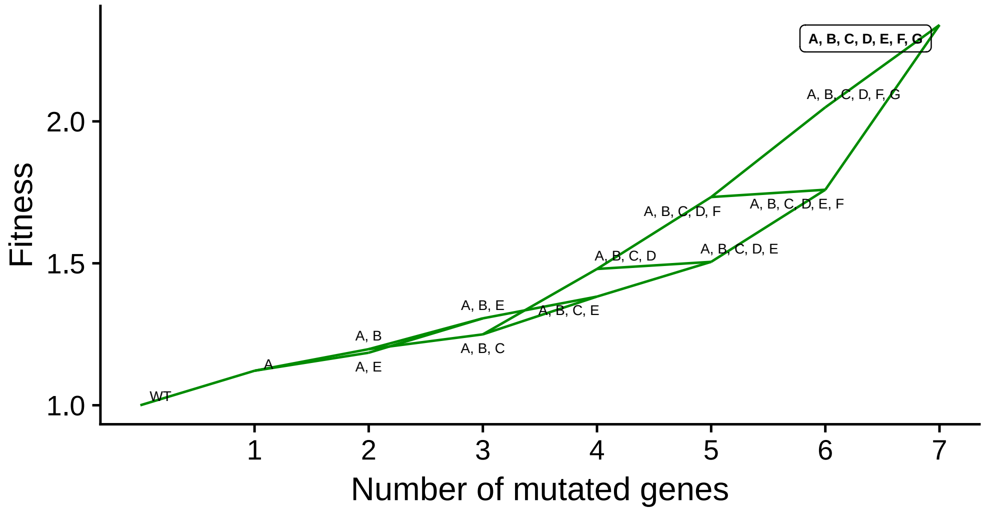

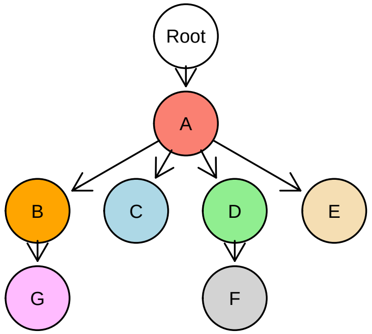

ID	IySPgbGJgYzQfV5k
acc genotypes	37
peaks no back	1
removed edges	0
prop rem edges	0

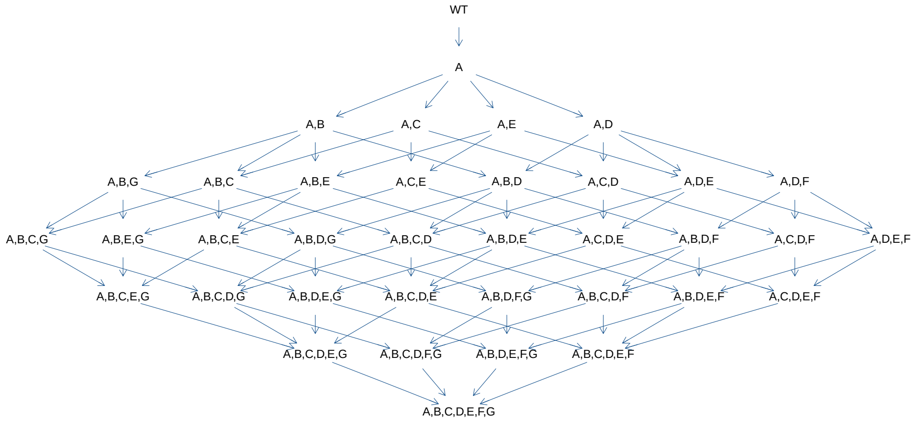

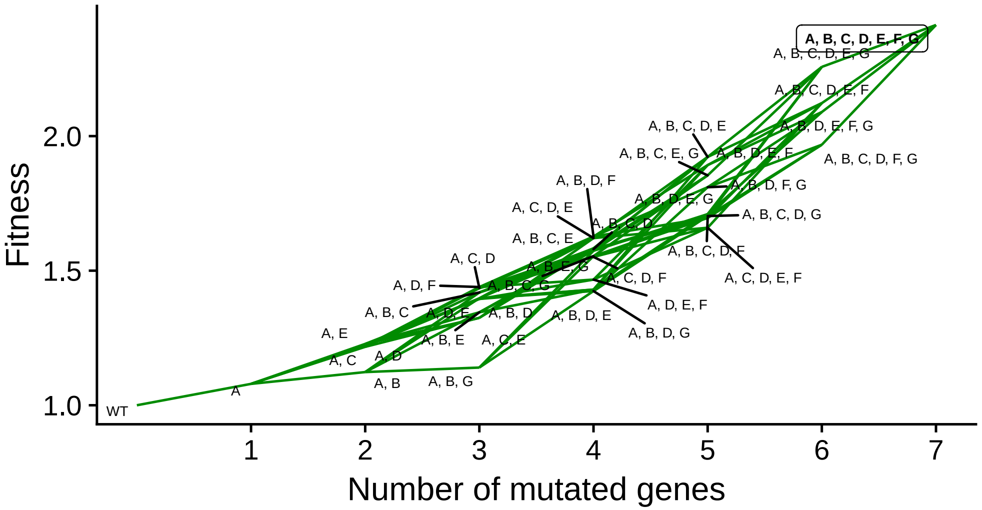

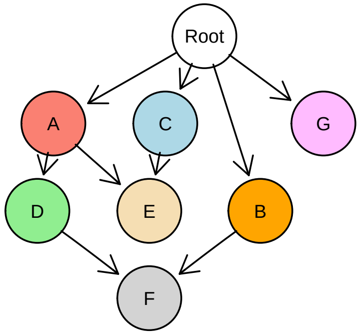

ID	J5q7C41n6m4aEUp10
acc genotypes	38
peaks no back	1
removed edges	0
prop rem edges	0

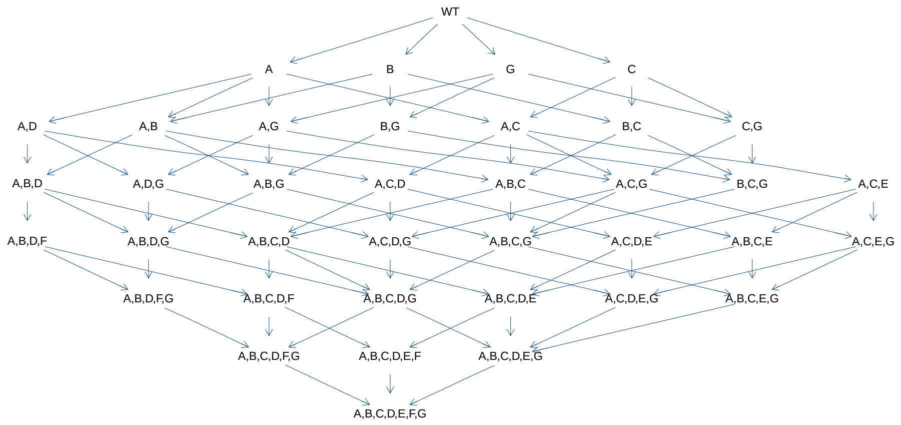

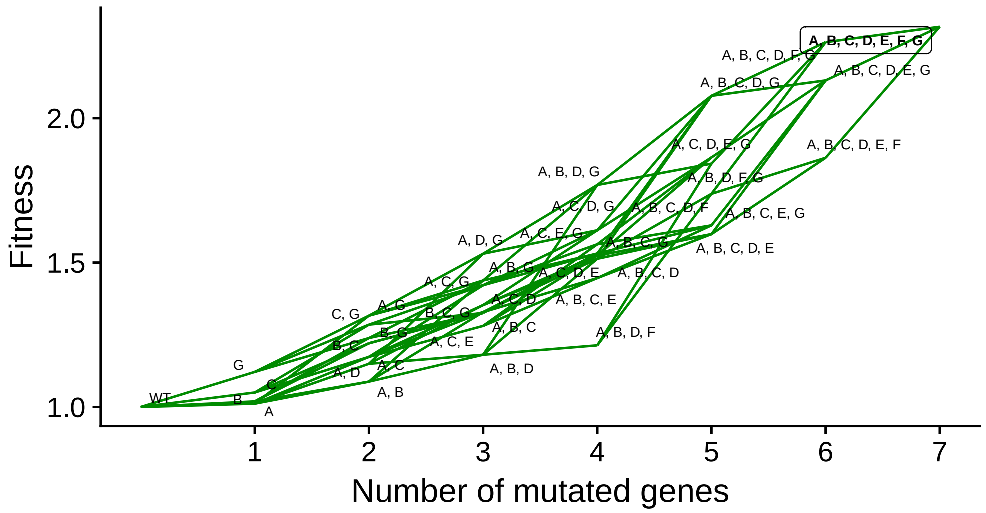

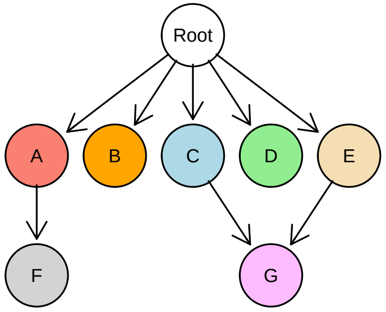

ID	j5YLGuLuDa6cvNb2
acc genotypes	60
peaks no back	1
removed edges	0
prop rem edges	0

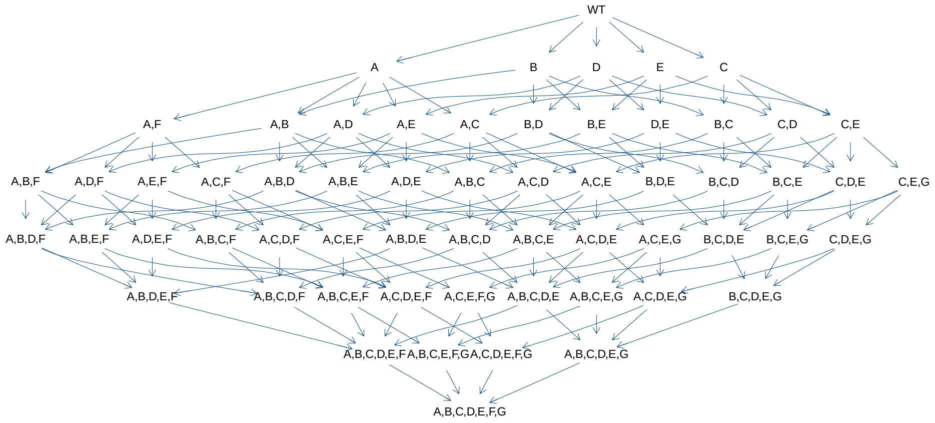

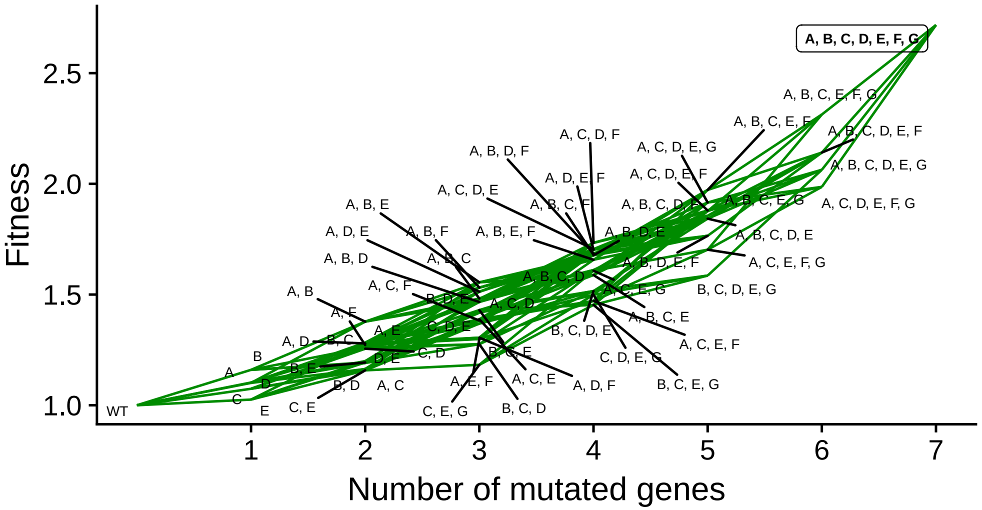

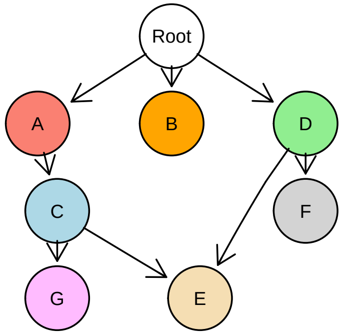

ID	J6Itoaqms6HIPc7B
acc genotypes	32
peaks no back	1
removed edges	0
prop rem edges	0

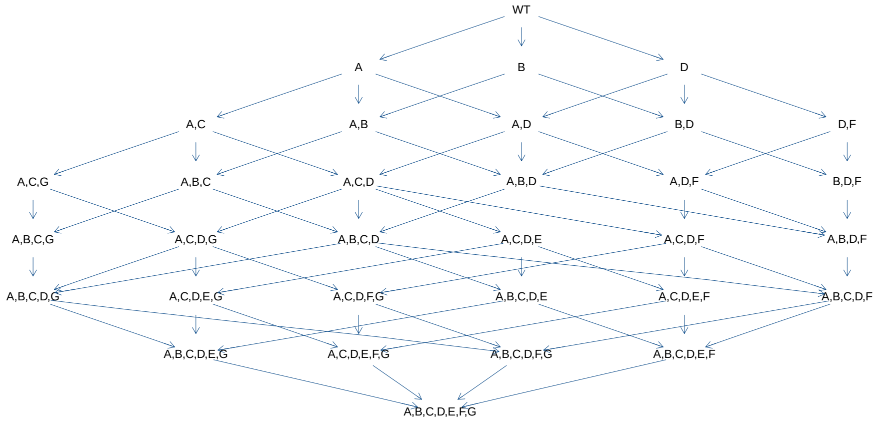

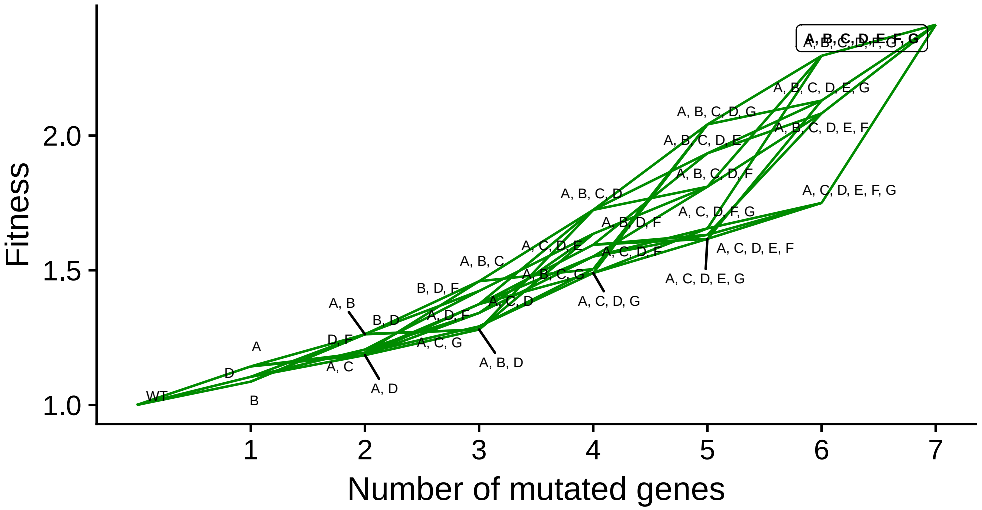

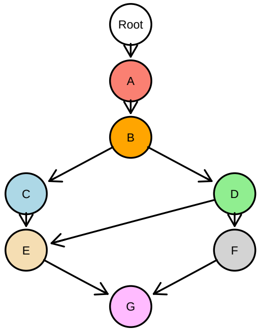

ID j71sbZK1fQ6ihd8O
acc genotypes 11
peaks no back 1
removed edges 0
prop rem edges 0

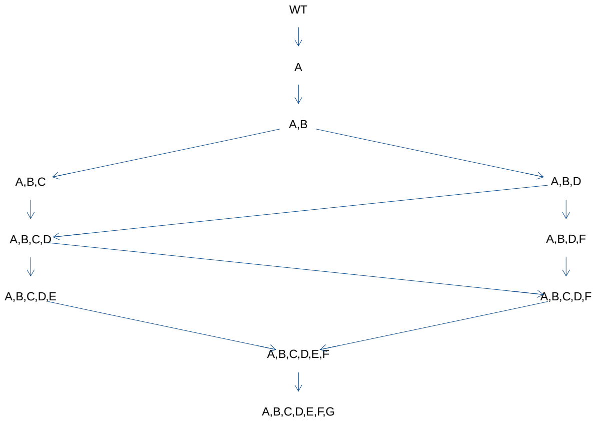

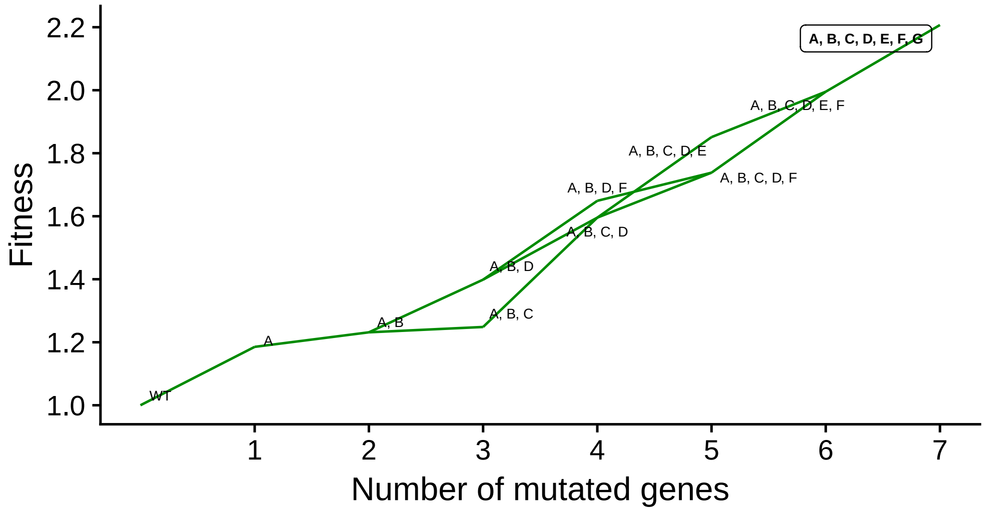

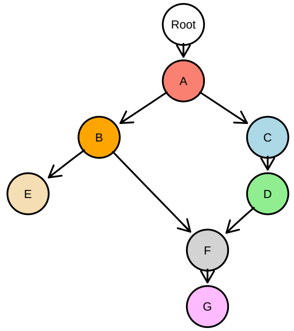

ID	JLERmKvl2w2lquvc
acc genotypes	14
peaks no back	1
removed edges	0
prop rem edges	0

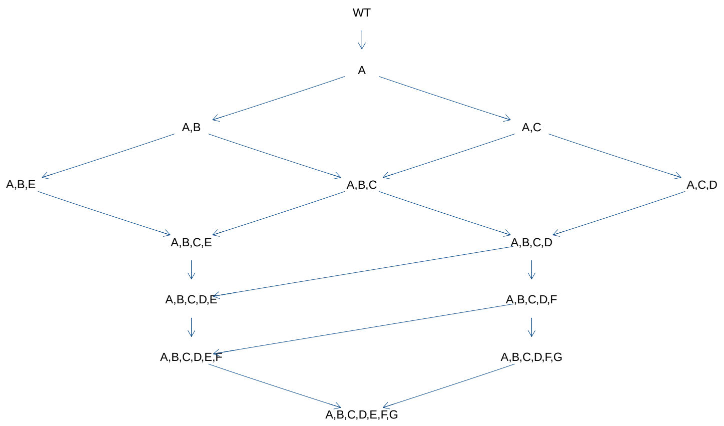

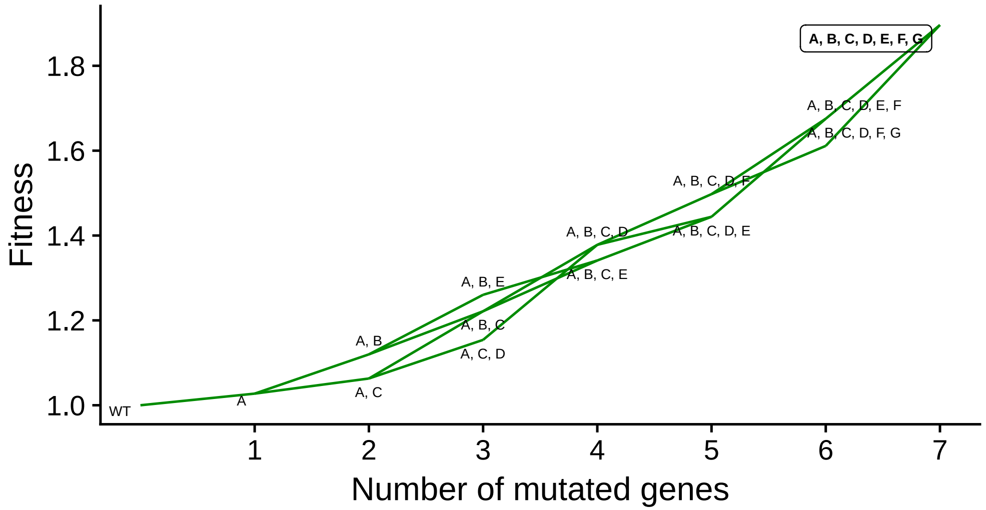

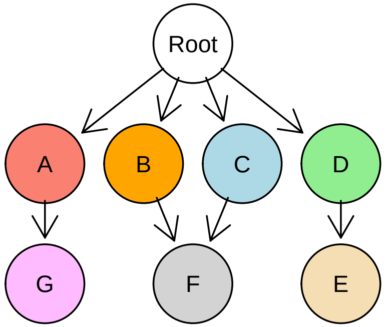

ID	JoTSpt4LngDDMASj
acc genotypes	45
peaks no back	1
removed edges	0
prop rem edges	0

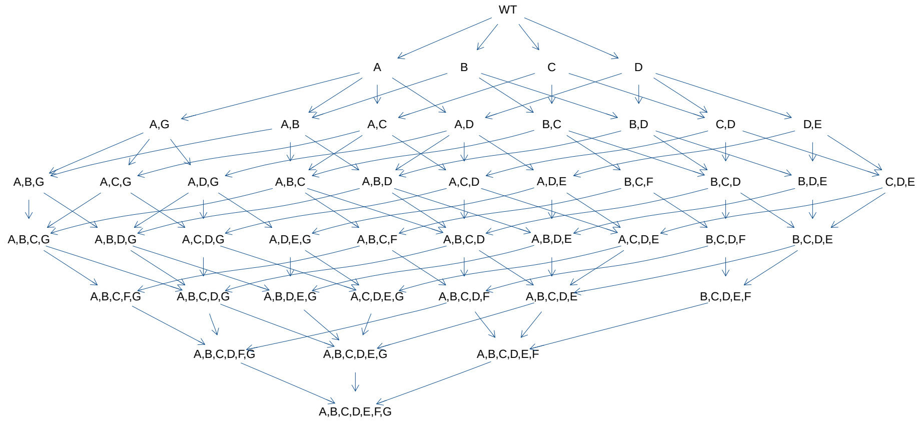

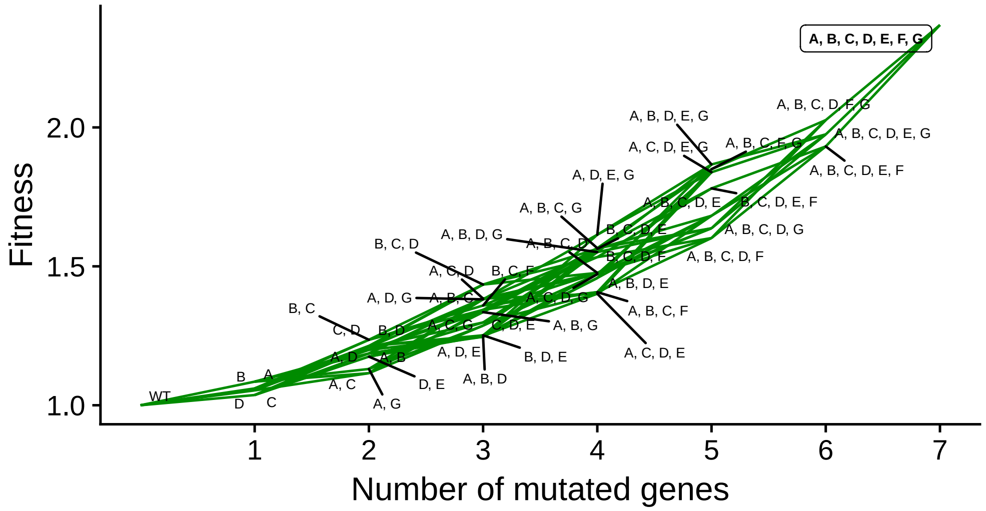

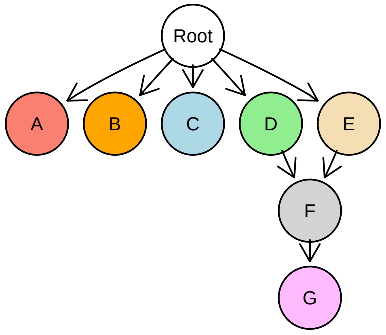

ID	jq7mqYXKFpls2uXm
acc genotypes	48
peaks no back	1
removed edges	0
prop rem edges	0

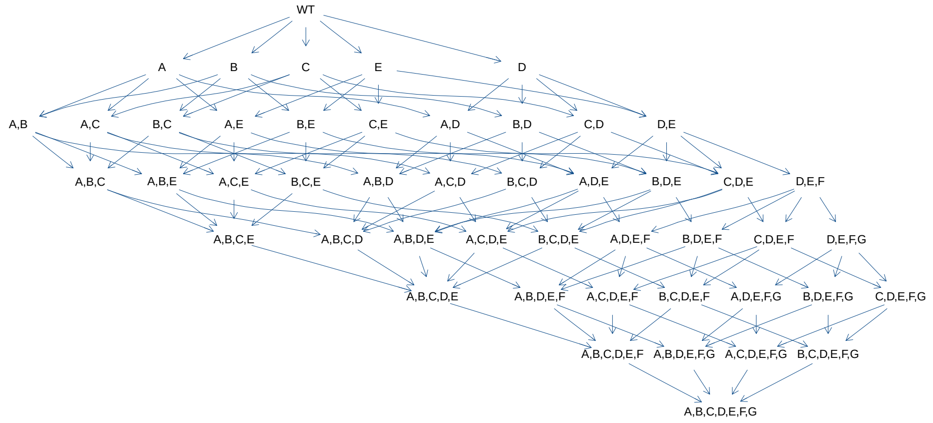

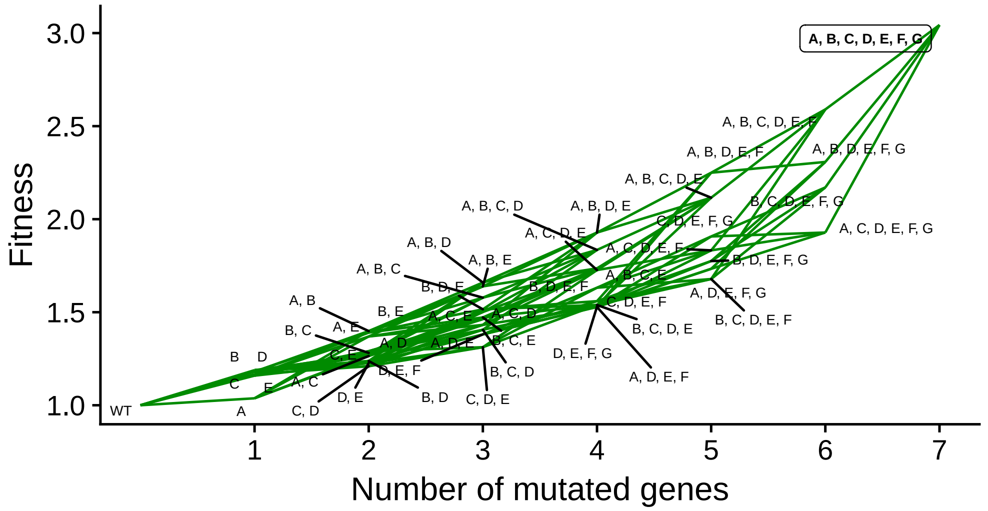

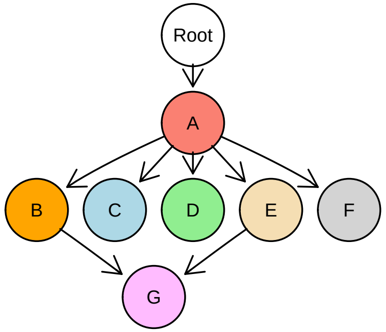

ID	JVEFSk5ORIOz9YE7
acc genotypes	41
peaks no back	1
removed edges	0
prop rem edges	0

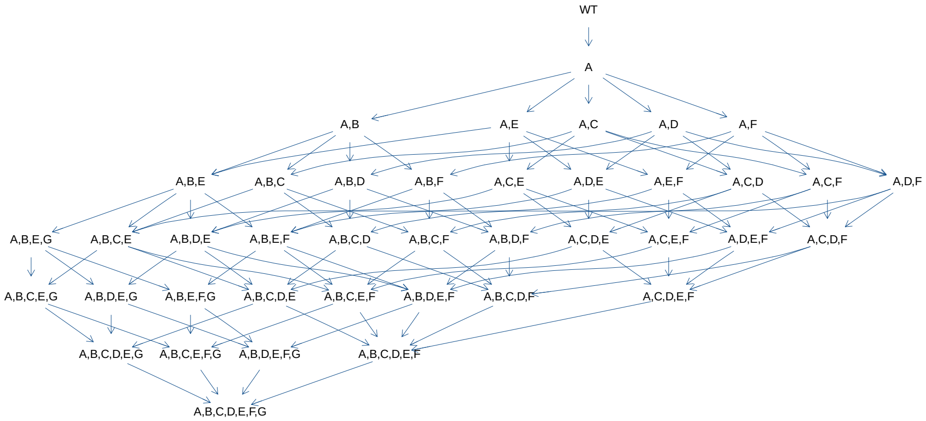

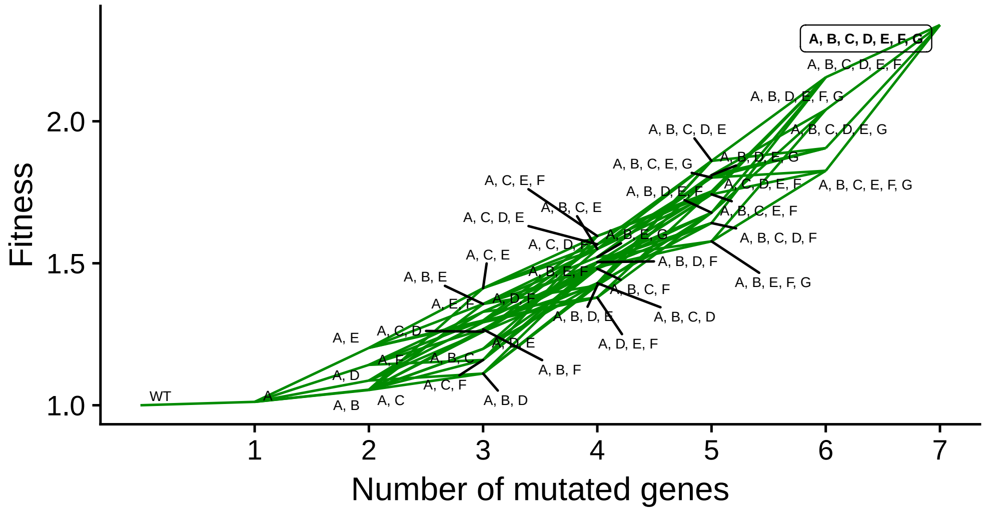

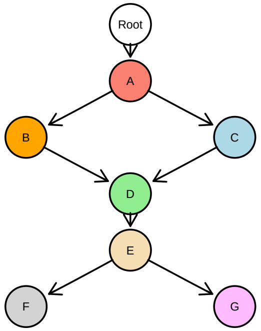

ID	K9x9g2eq1VT8fICv
acc genotypes	10
peaks no back	1
removed edges	0
prop rem edges	0

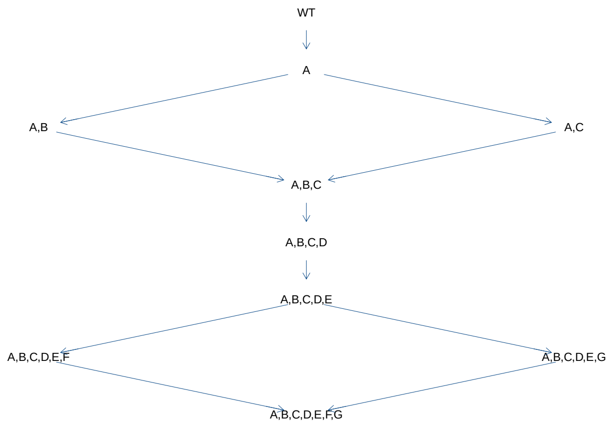

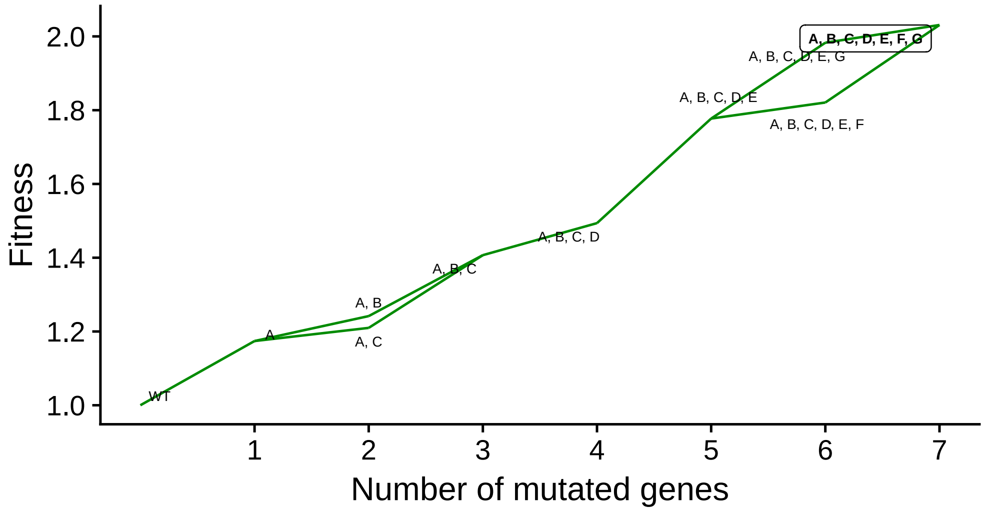

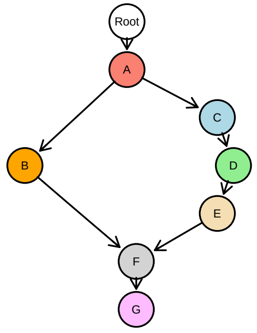

ID kBK5zr8jiuD8Etoo
acc genotypes 11
peaks no back 1
removed edges 0
prop rem edges 0

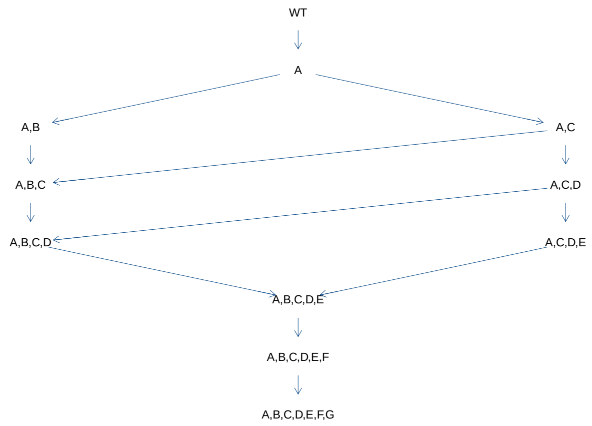

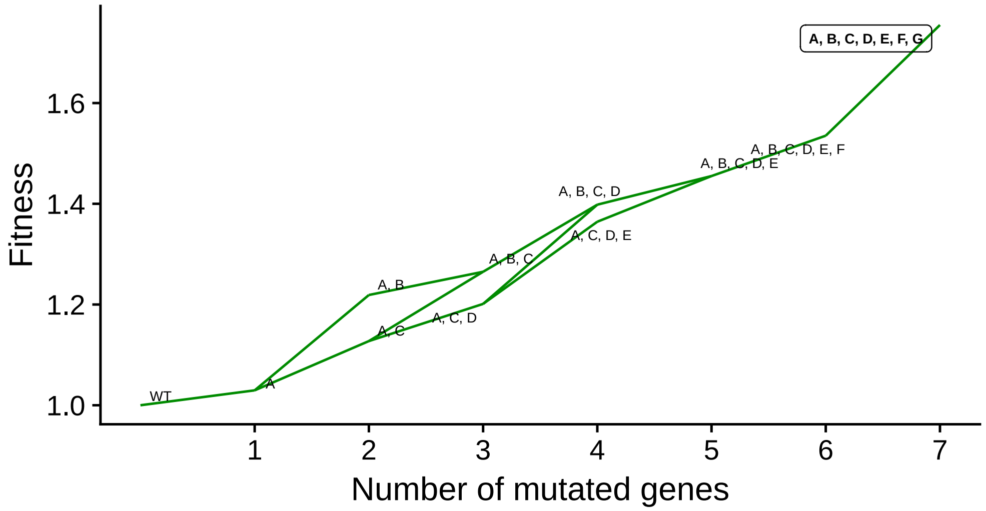

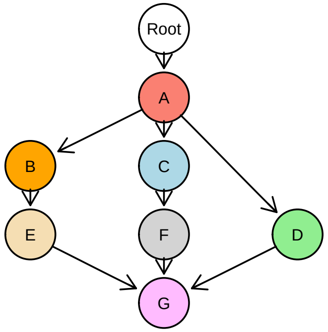

ID	KctNTtEFQr1oMvyT
acc genotypes	20
peaks no back	1
removed edges	0
prop rem edges	0

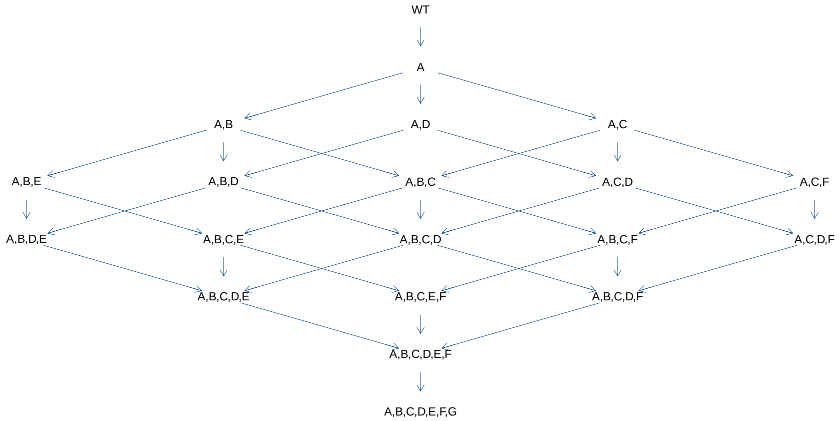

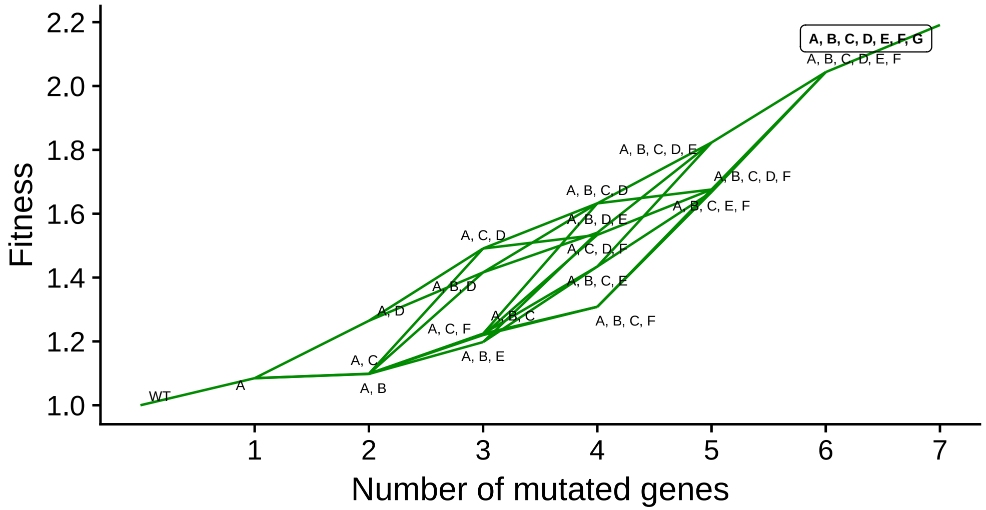

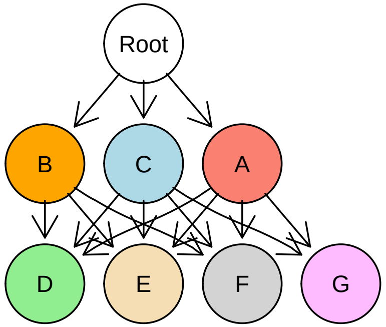

ID	kf9mgLp5ZKwjcVhz
acc genotypes	24
peaks no back	1
removed edges	0
prop rem edges	0

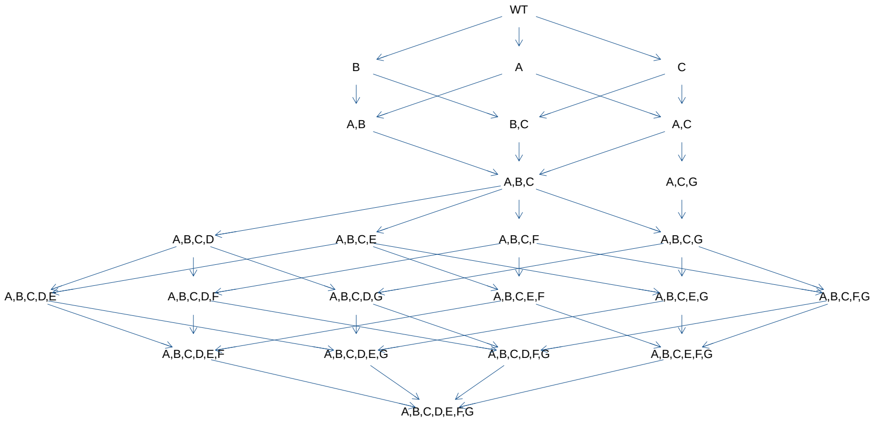

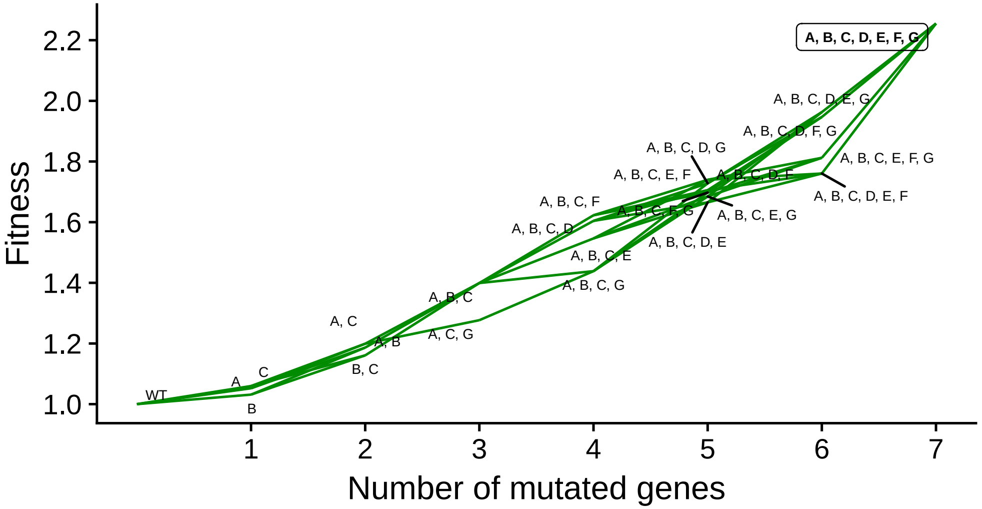

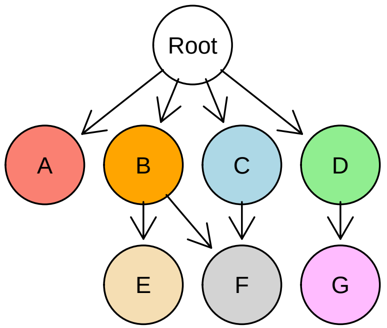

ID KtmVfIOzxH44NUrR
acc genotypes 48
peaks no back 1
removed edges 0
prop rem edges 0

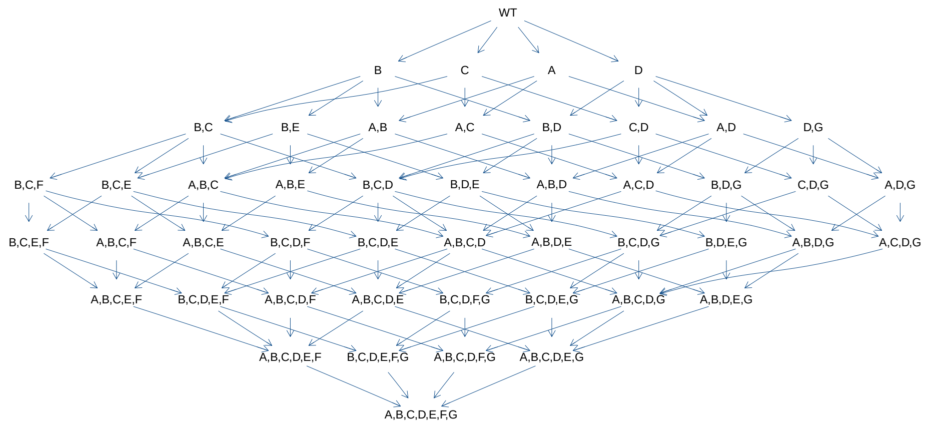

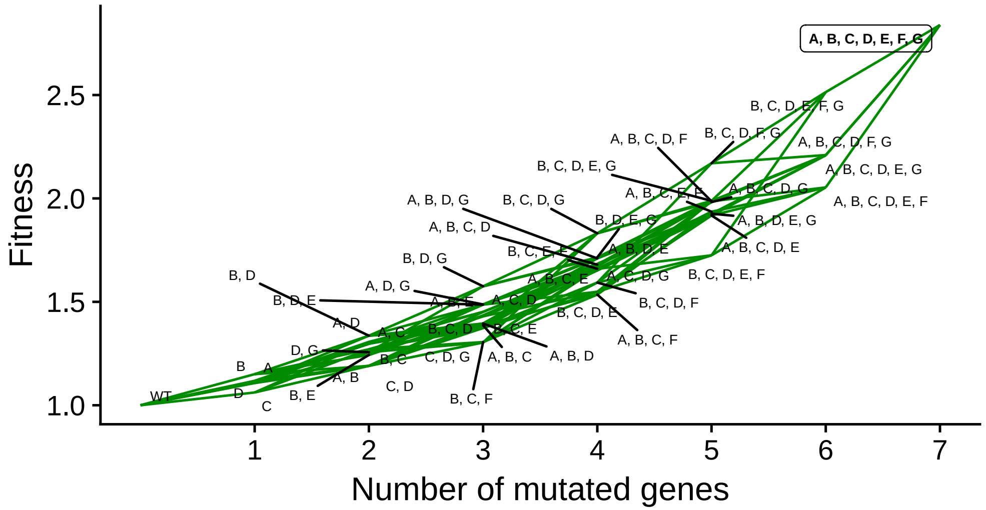

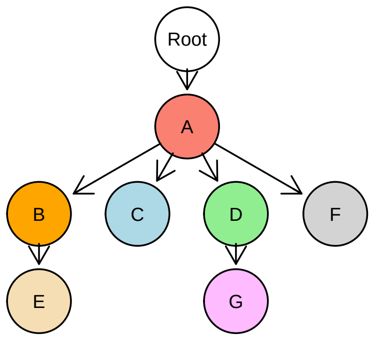

ID	KZkcV1r2fzi9JPSr
acc genotypes	37
peaks no back	1
removed edges	0
prop rem edges	0

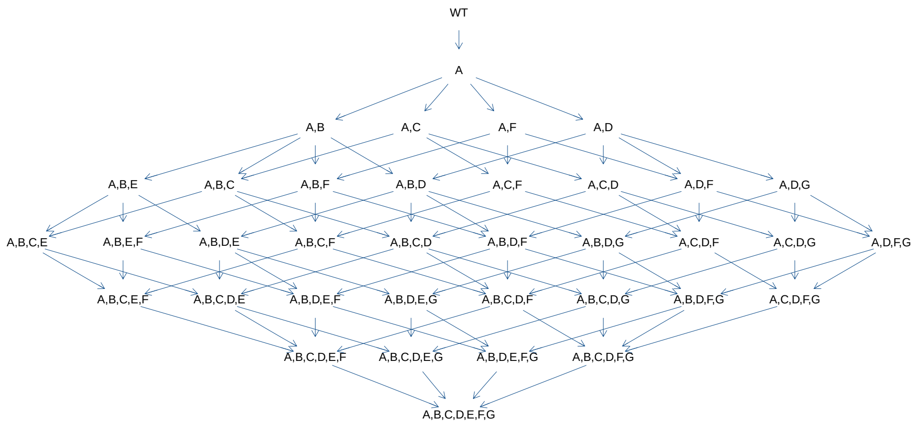

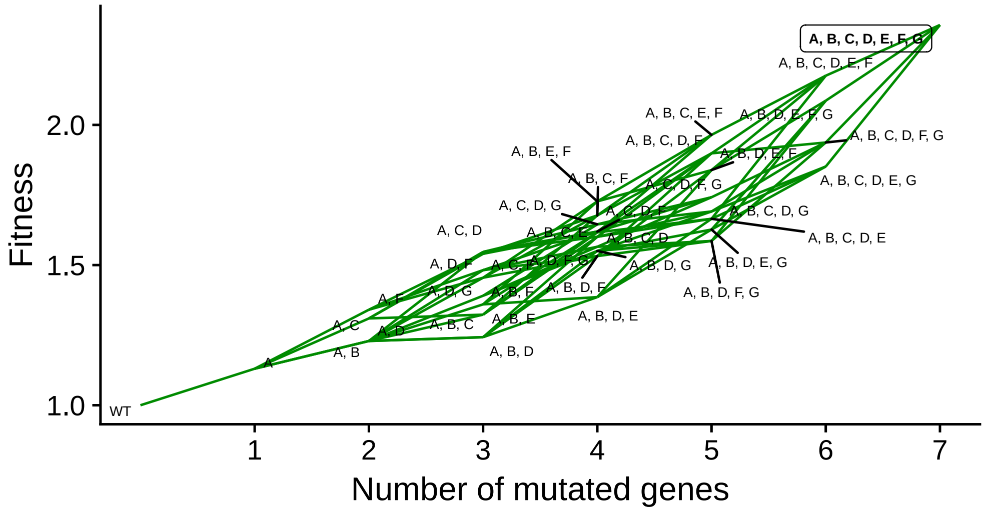

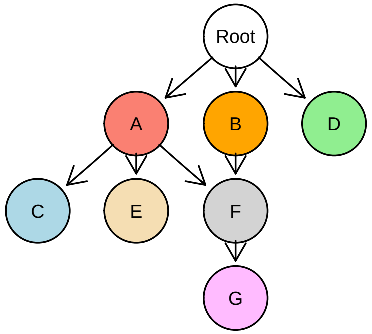

ID	Lke10loDf84oef4BC
acc genotypes	36
peaks no back	1
removed edges	0
prop rem edges	0

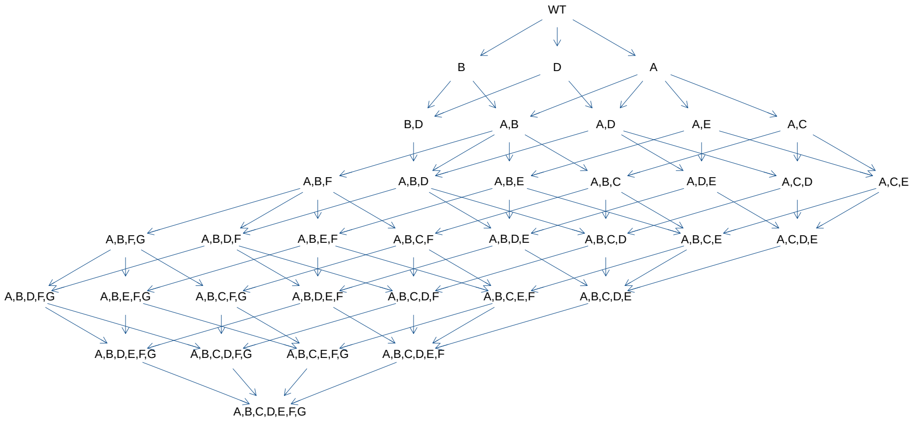

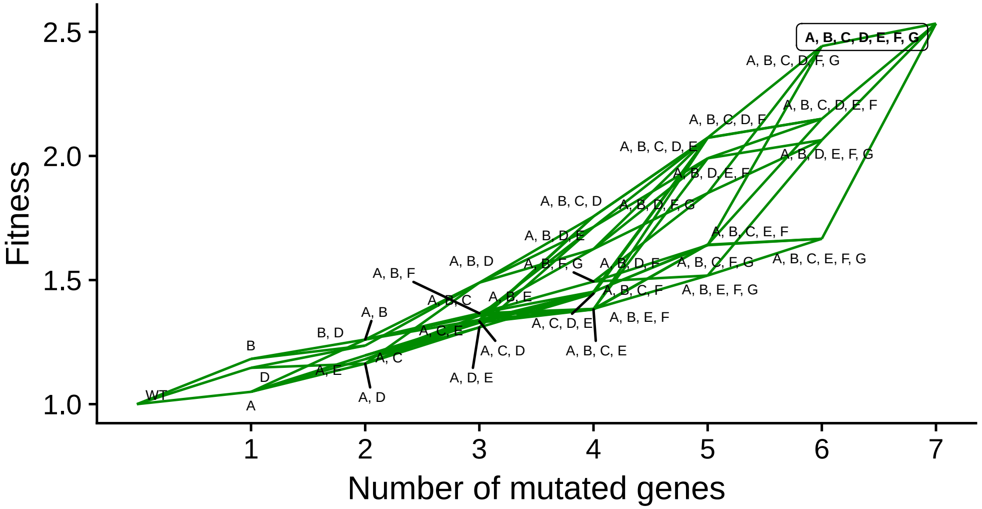

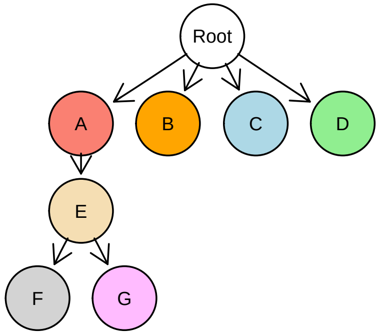

ID	LIVHZ106EHMqYUsuF
acc genotypes	48
peaks no back	1
removed edges	0
prop rem edges	0

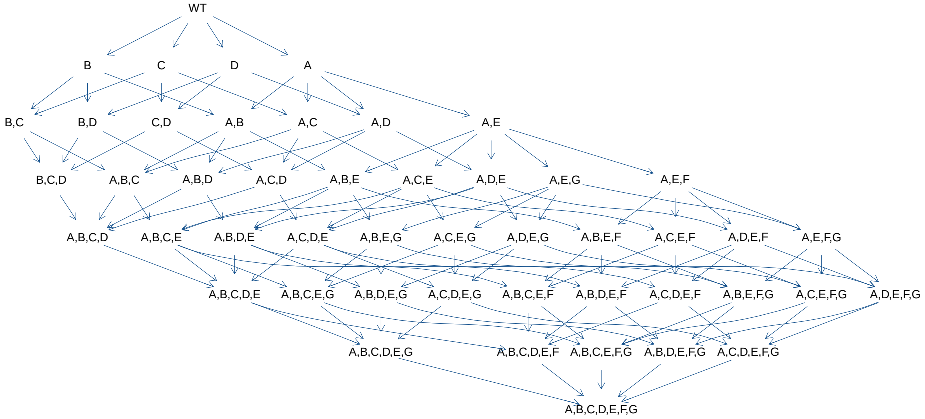

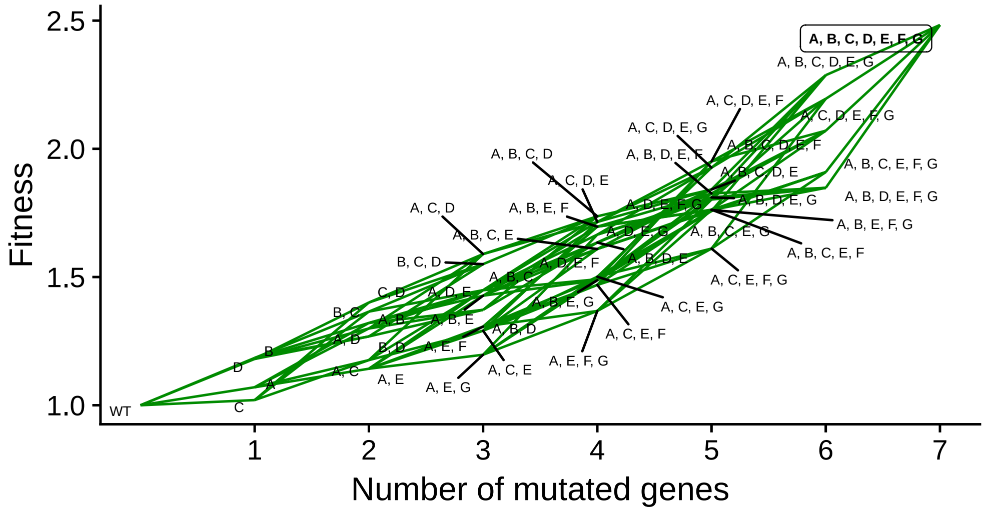

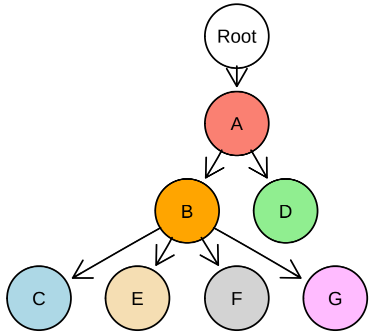

ID	LpANxOHMhj1cYLtN
acc genotypes	35
peaks no back	1
removed edges	0
prop rem edges	0

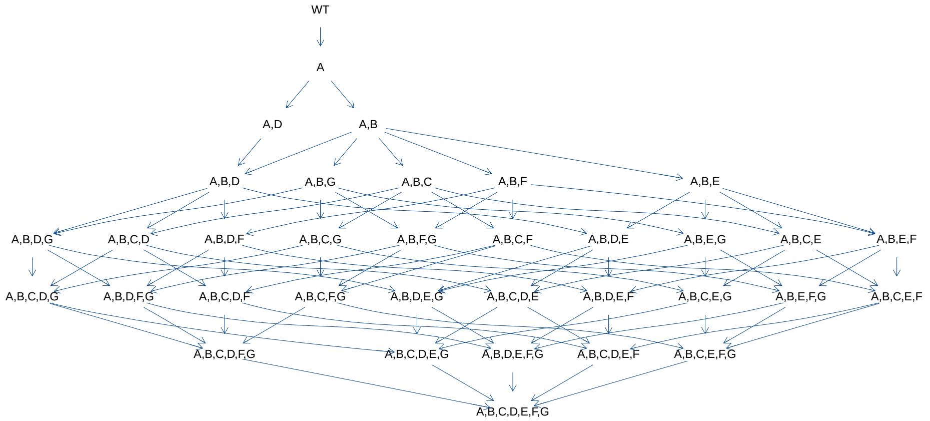

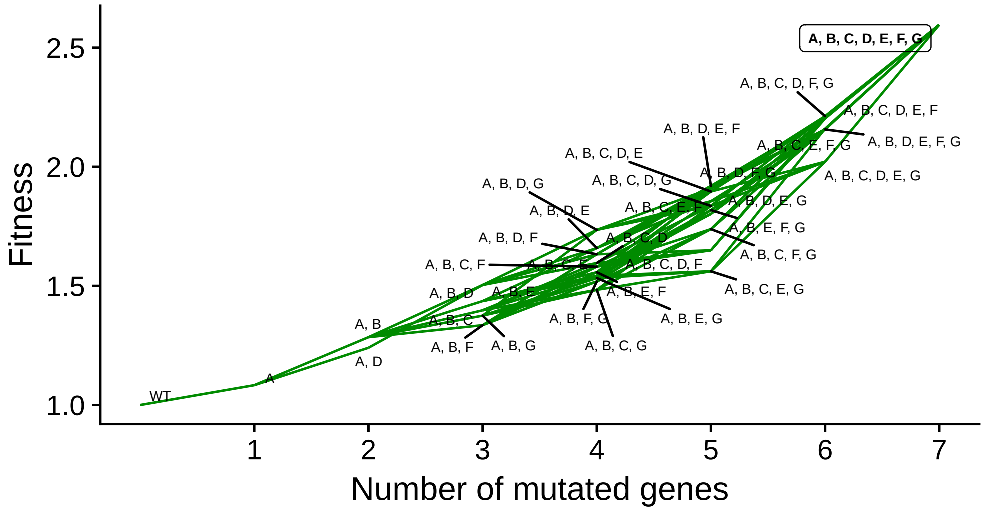

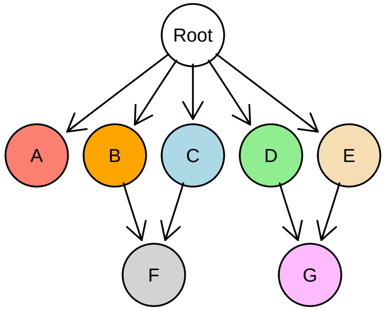

ID	LTgRqknZ6BY1cfLB
acc genotypes	50
peaks no back	1
removed edges	0
prop rem edges	0

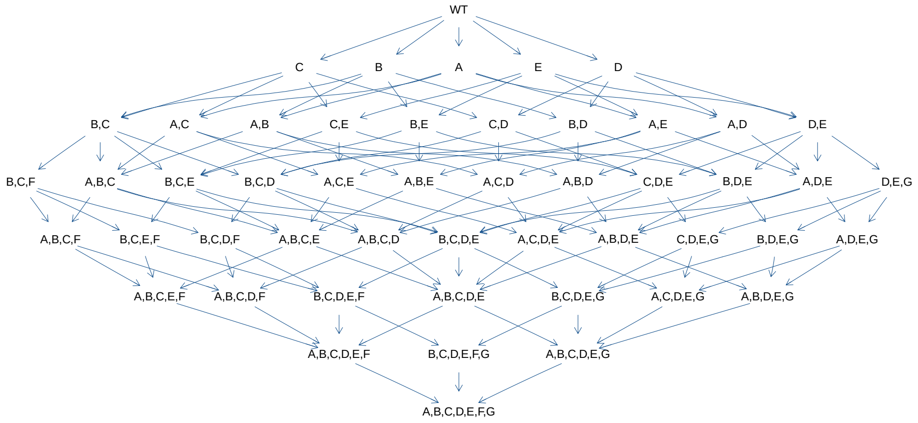

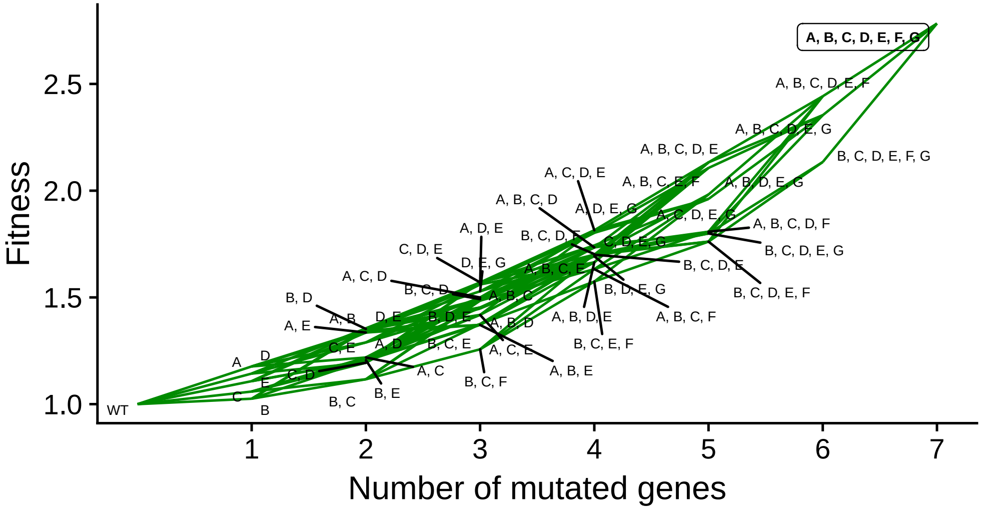

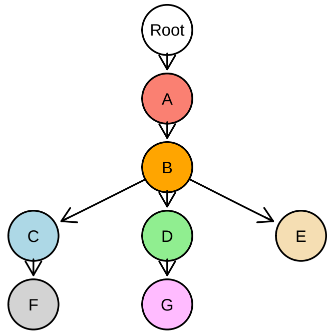

ID	Mdi5kYcCCepKSprh
acc genotypes	20
peaks no back	1
removed edges	0
prop rem edges	0

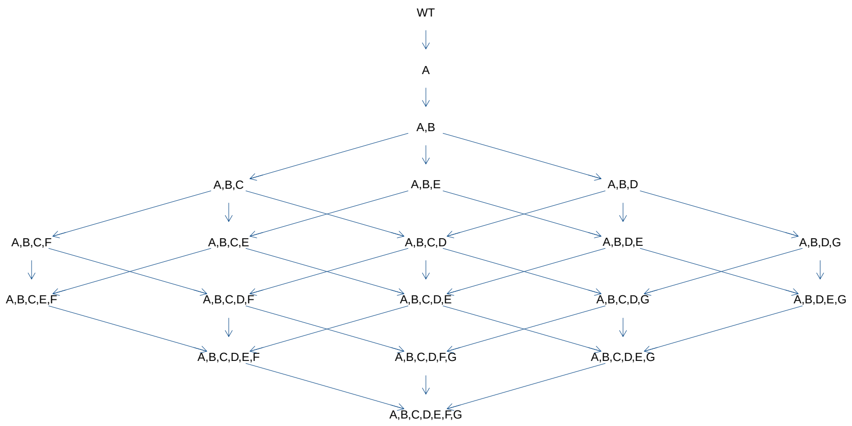

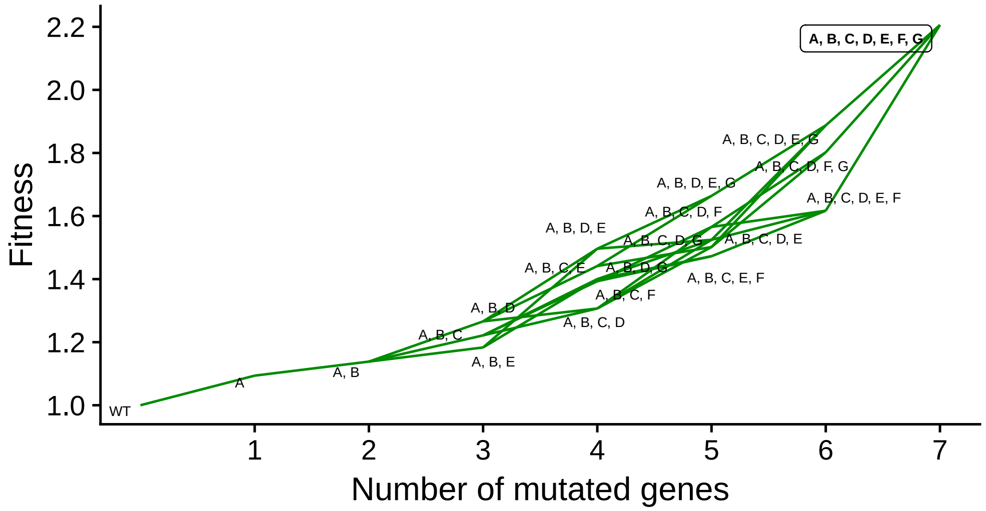

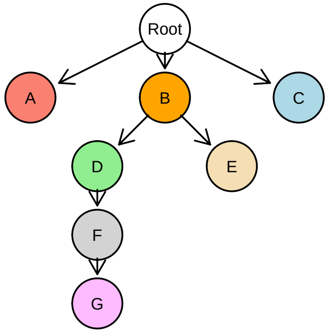

ID	mEX5RjVtCmtkvI6C
acc genotypes	36
peaks no back	1
removed edges	0
prop rem edges	0

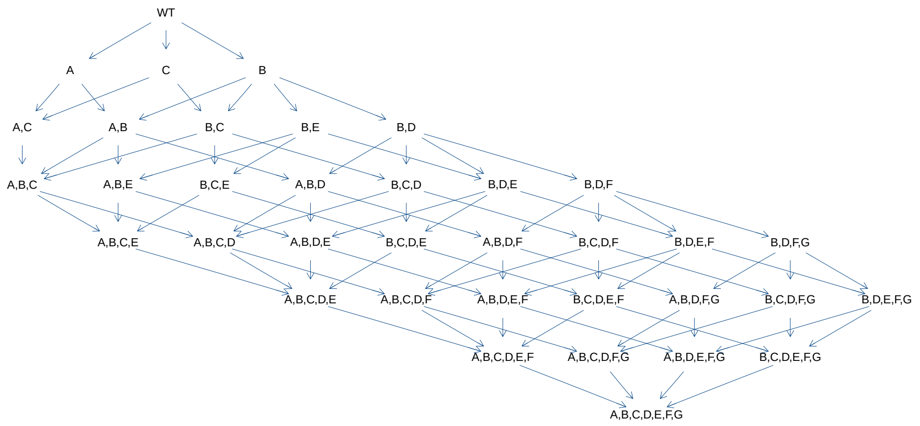

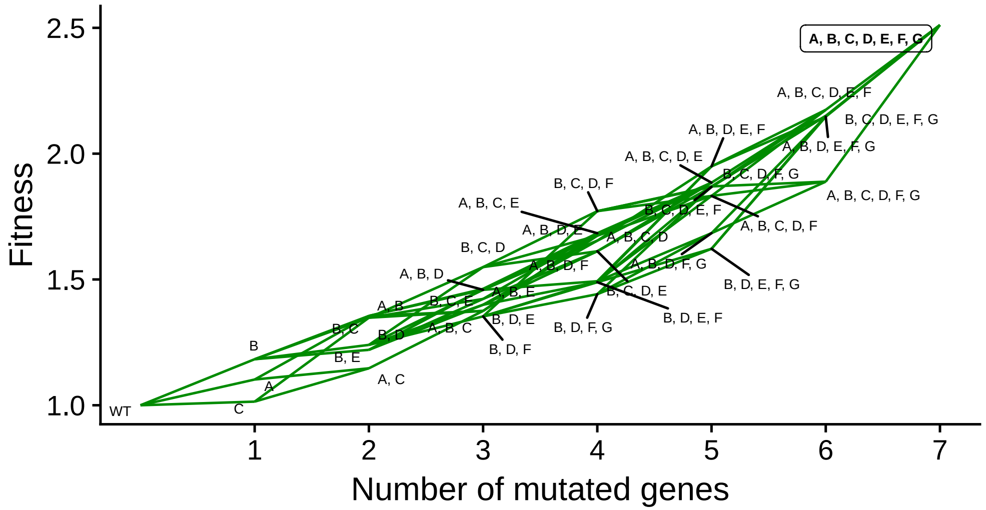

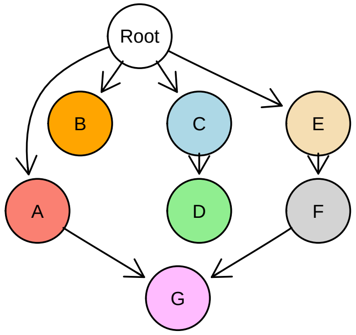

ID MHHdzPsMBi26wD8j
acc genotypes 42
peaks no back 1
removed edges 0
prop rem edges 0

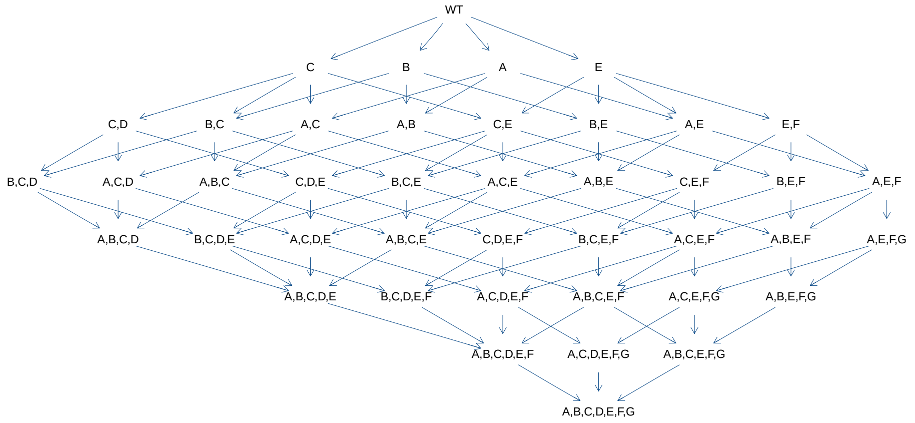

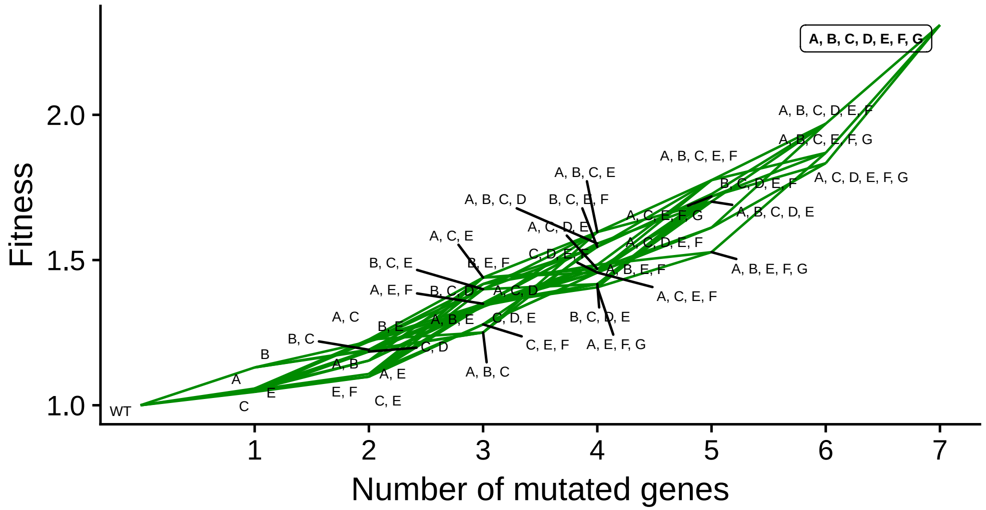

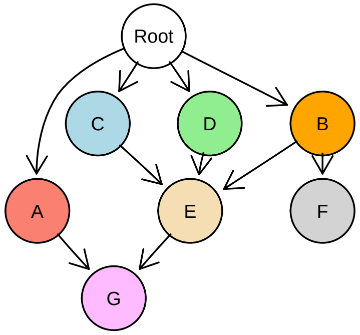

ID MiiXBqQH10YjcXVMI
acc genotypes 30
peaks no back 1
removed edges 0
prop rem edges 0

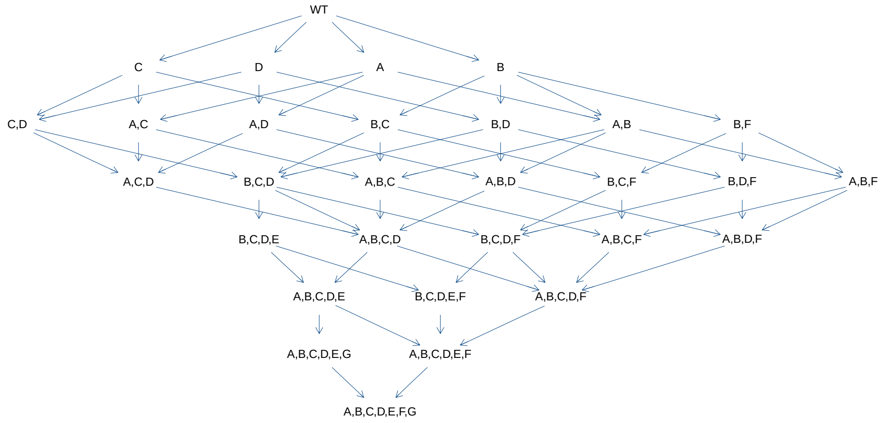

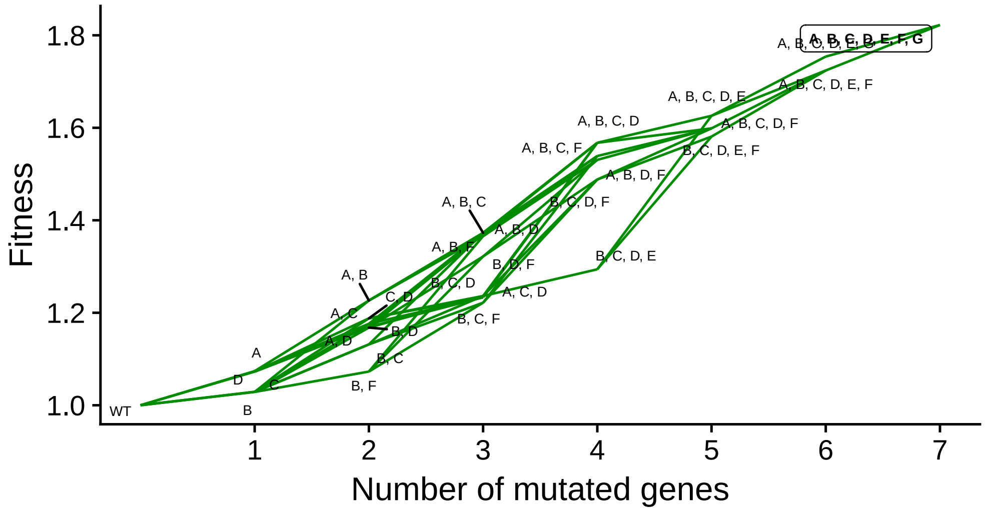

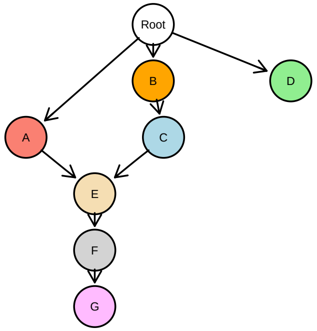

ID	MJEstF1cdVazElfA
acc genotypes	18
peaks no back	1
removed edges	0
prop rem edges	0

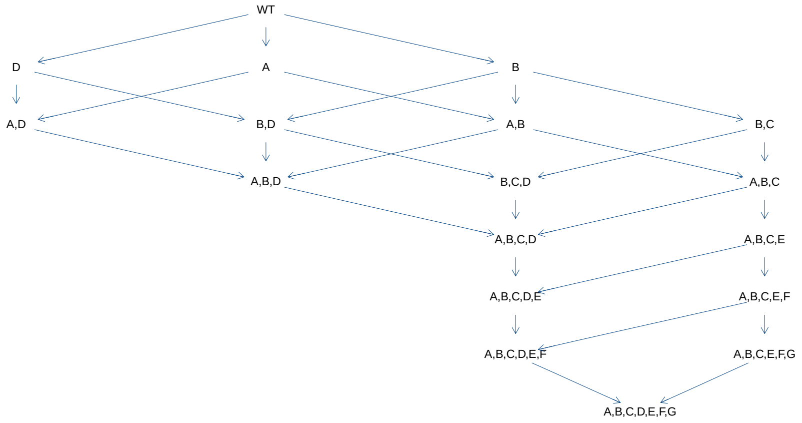

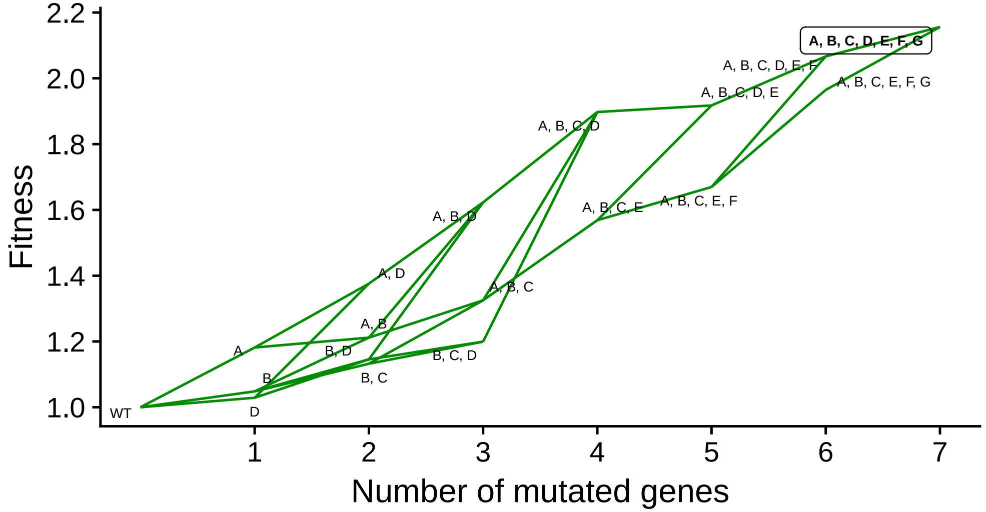

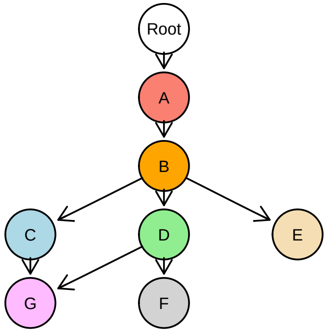

ID	MJMydL6WRmXU5Lio
acc genotypes	18
peaks no back	1
removed edges	0
prop rem edges	0

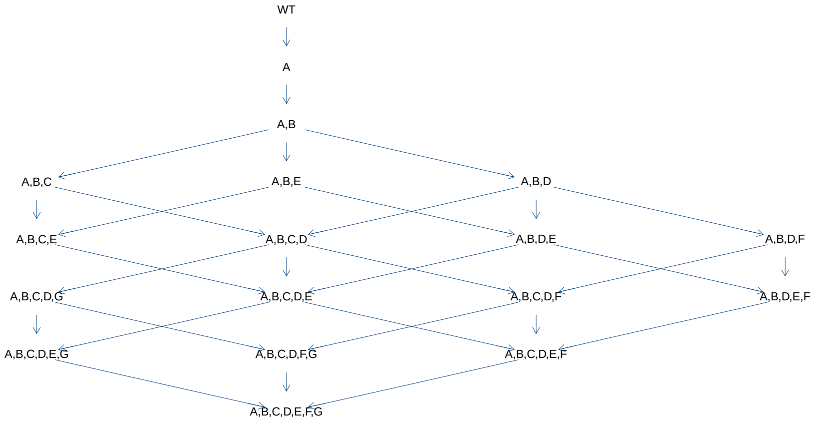

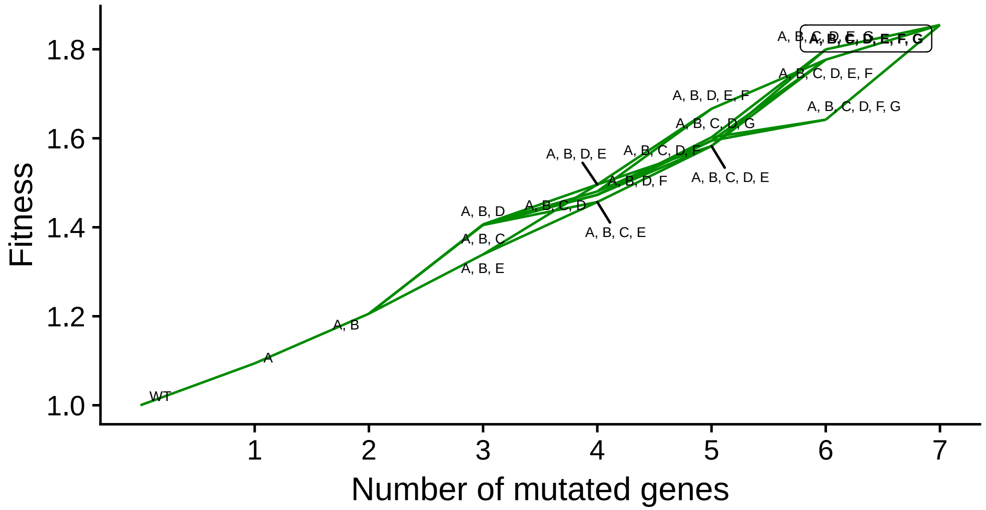

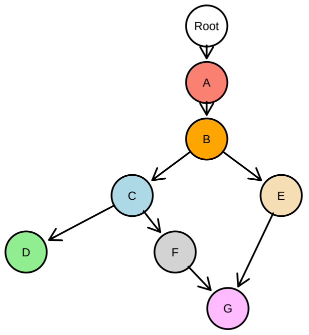

ID	mJyMPts2CCp9bFCw
acc genotypes	14
peaks no back	1
removed edges	0
prop rem edges	0

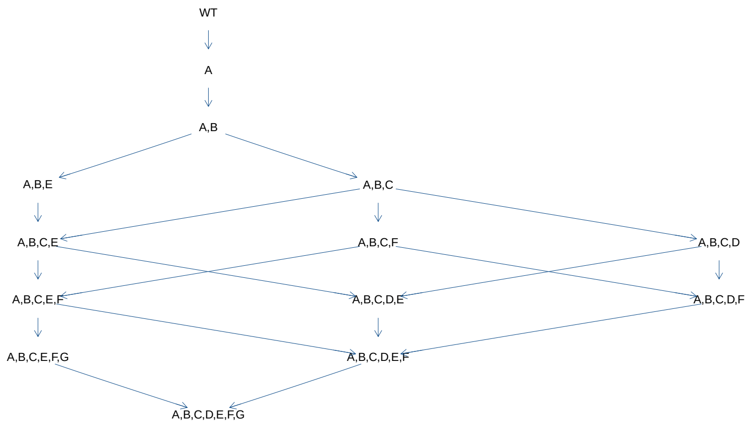

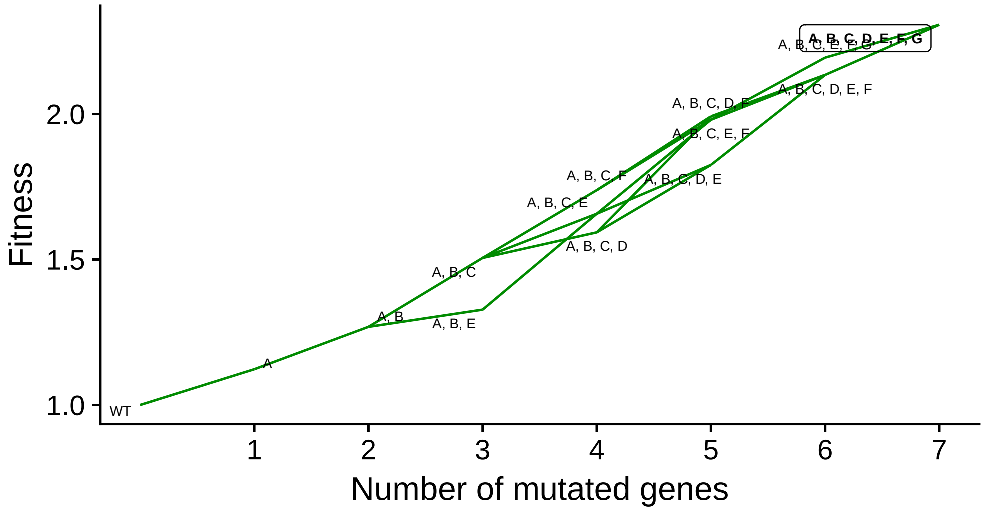

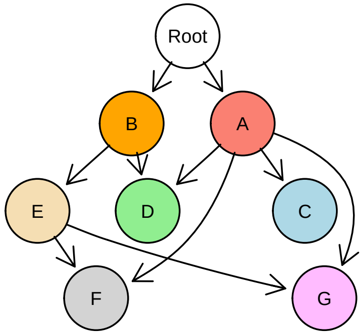

ID MmQTibreqvyLDuMK
acc genotypes 25
peaks no back 1
removed edges 0
prop rem edges 0

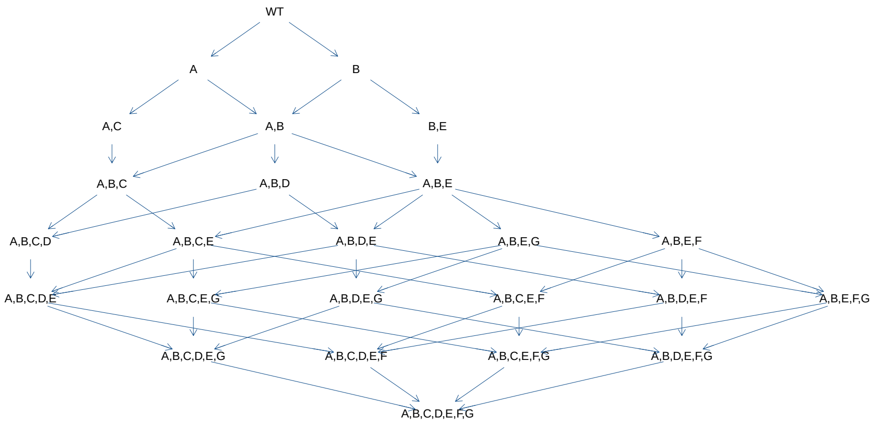

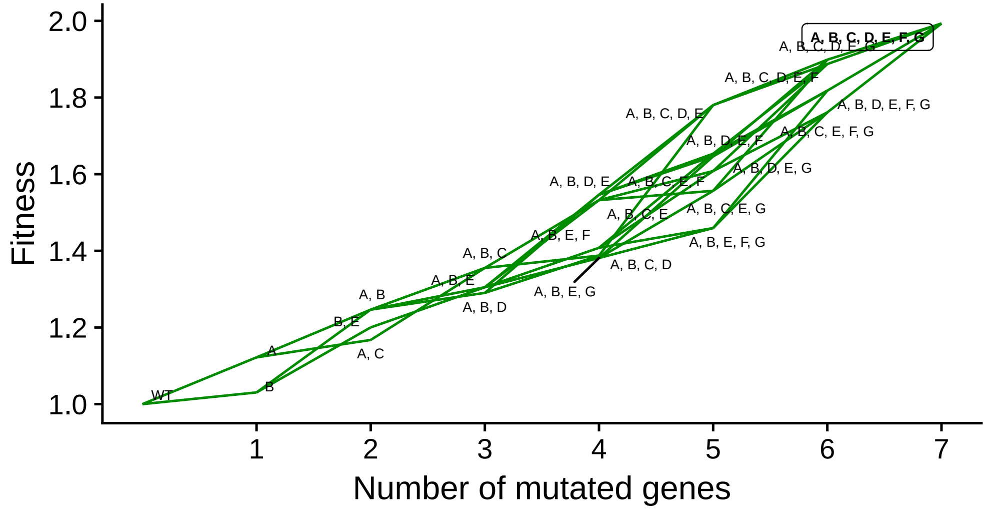

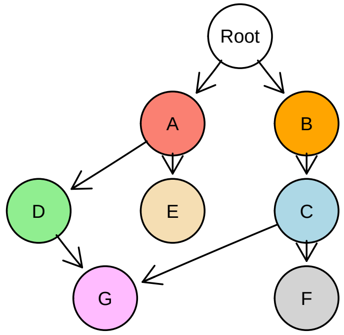

ID	msMIql4eo3nmvydj
acc genotypes	24
peaks no back	1
removed edges	0
prop rem edges	0

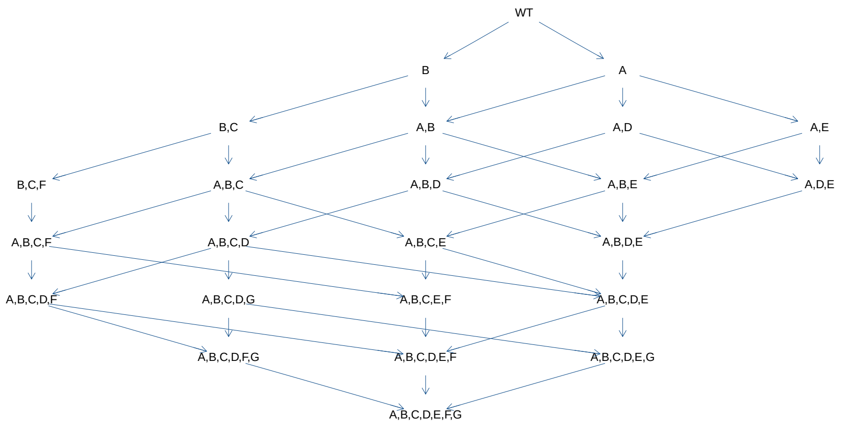

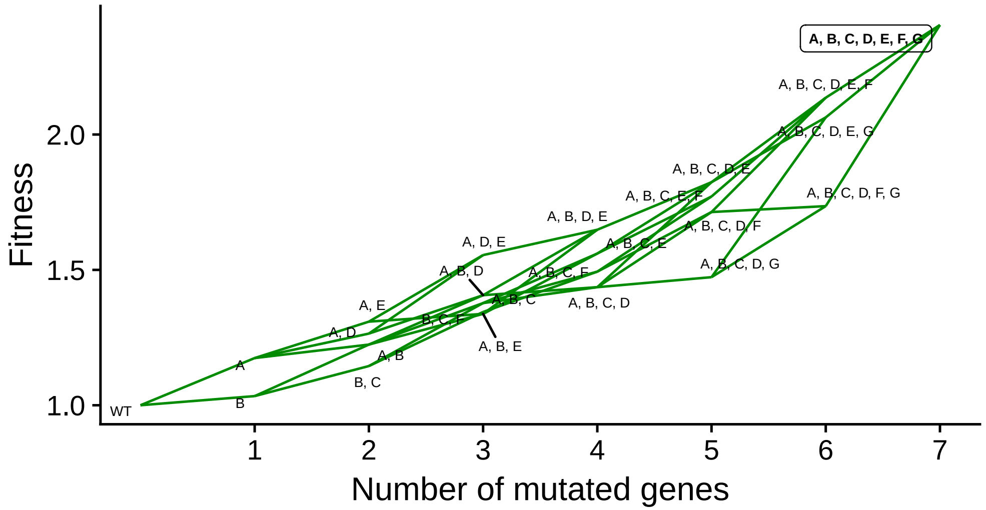

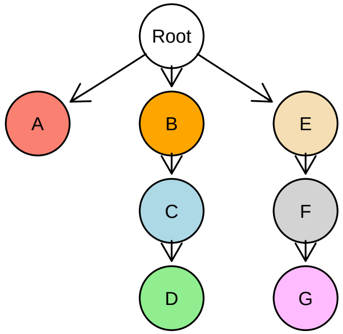

ID	mzgsSEJyju3Unz2A
acc genotypes	32
peaks no back	1
removed edges	0
prop rem edges	0

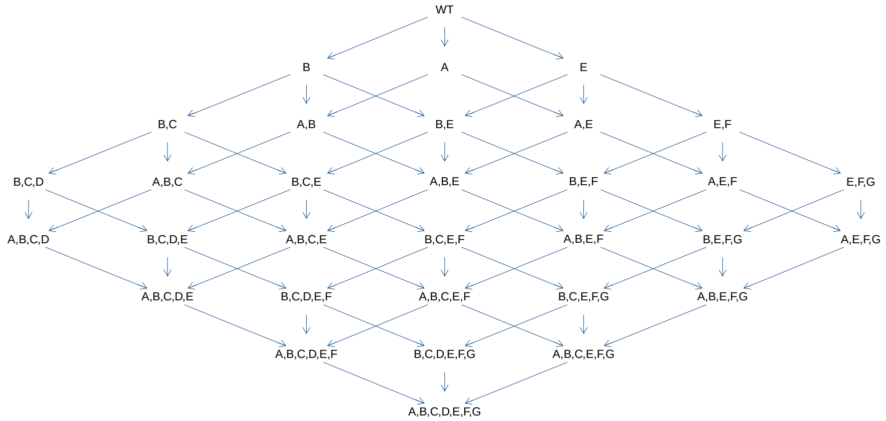

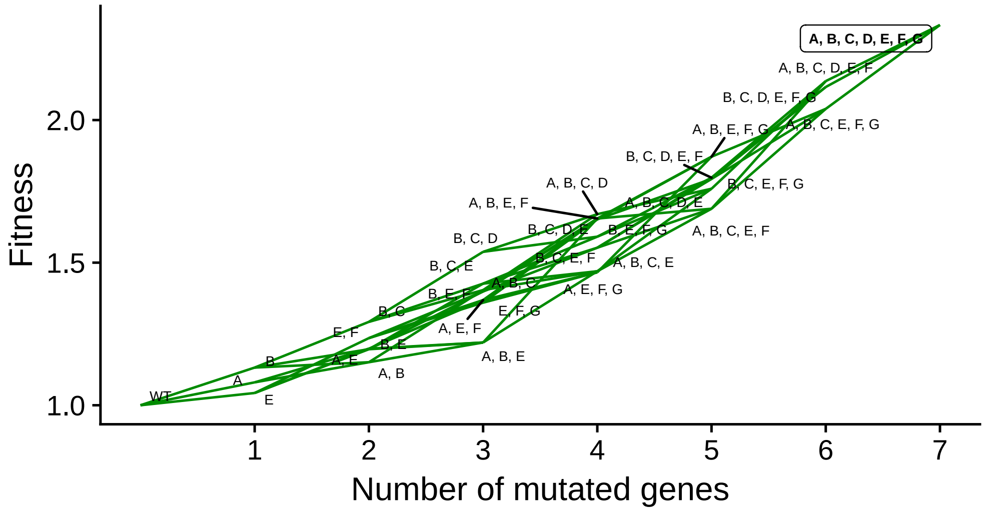

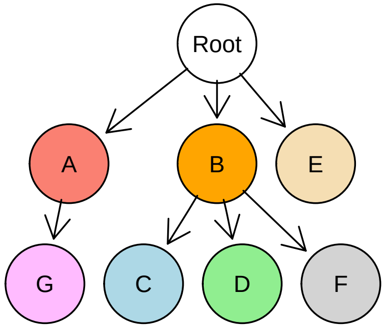

ID	N4iitsjj5WGvCNiO
acc genotypes	54
peaks no back	1
removed edges	0
prop rem edges	0

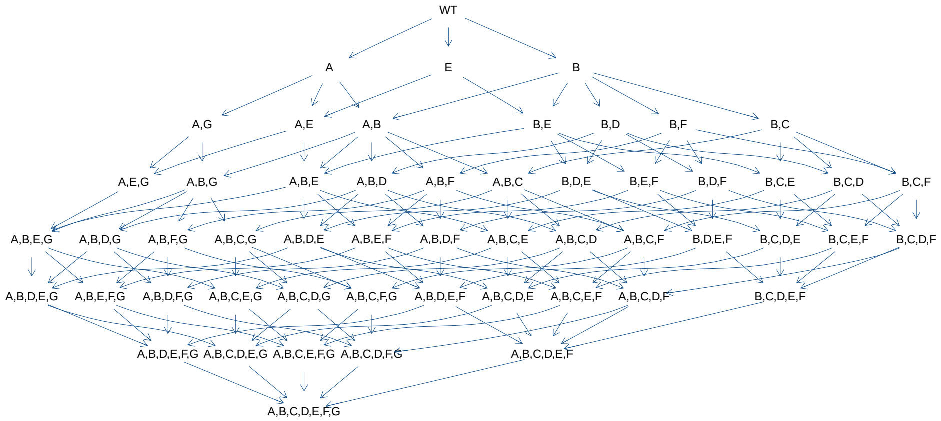

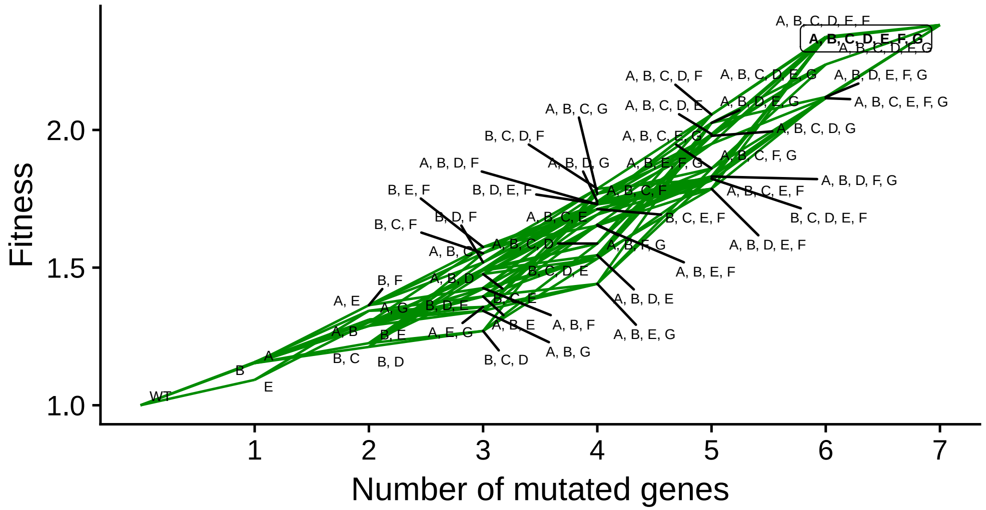

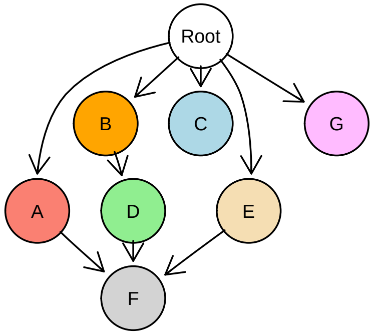

ID	N9wjEenhGOlChUML
acc genotypes	52
peaks no back	1
removed edges	0
prop rem edges	0

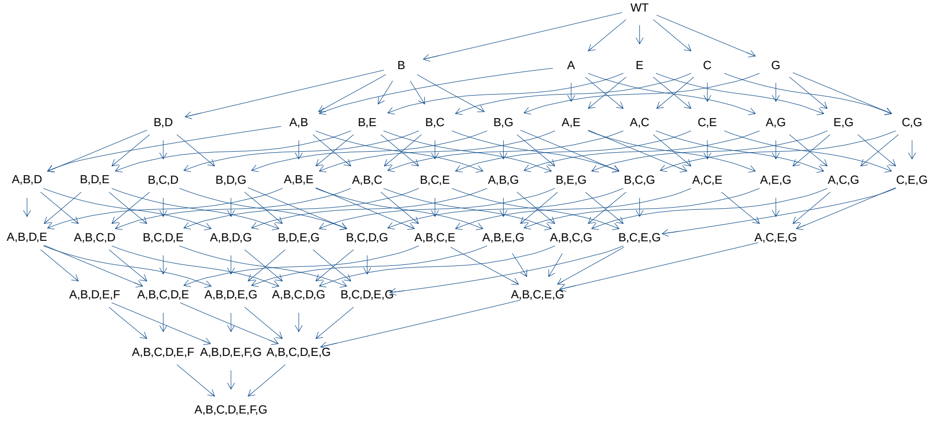

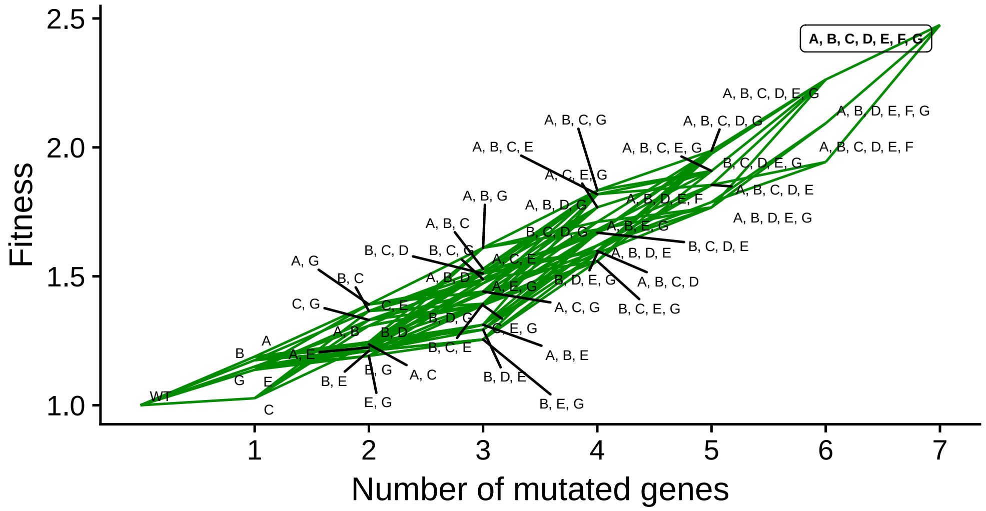

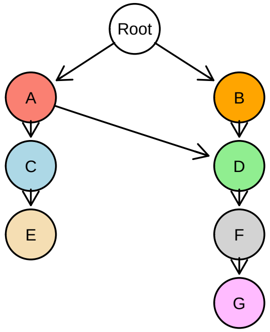

ID nbY47YBy9tCkfebb
acc genotypes 17
peaks no back 1
removed edges 0
prop rem edges 0

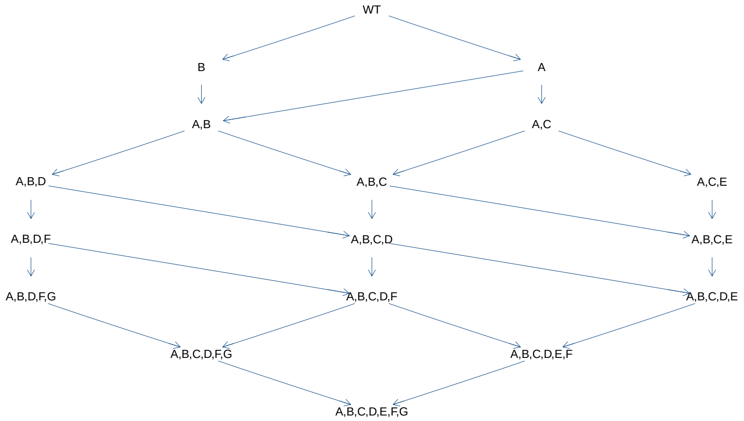

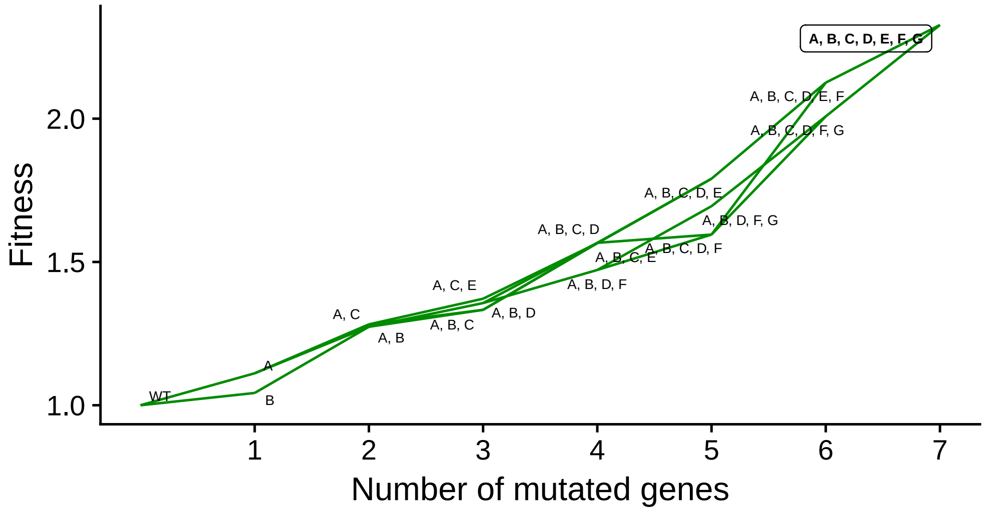

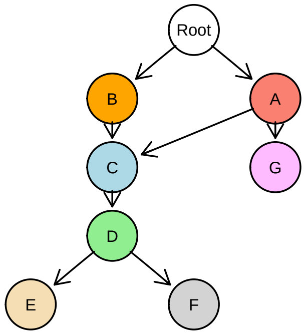

ID NEtTcxoCuFV4dbsx
acc genotypes 16
peaks no back 1
removed edges 0
prop rem edges 0

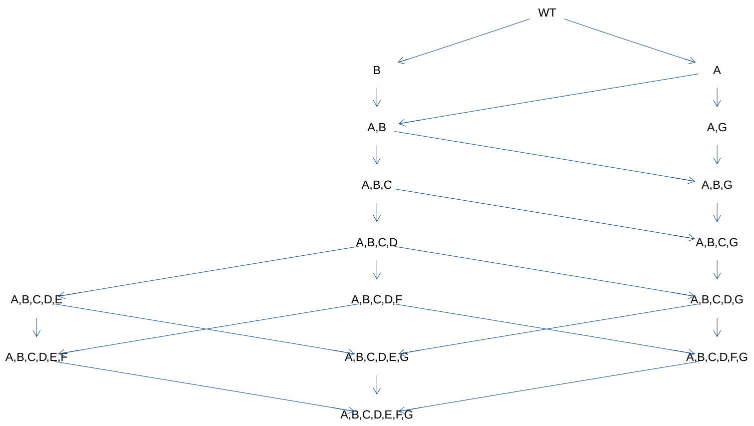

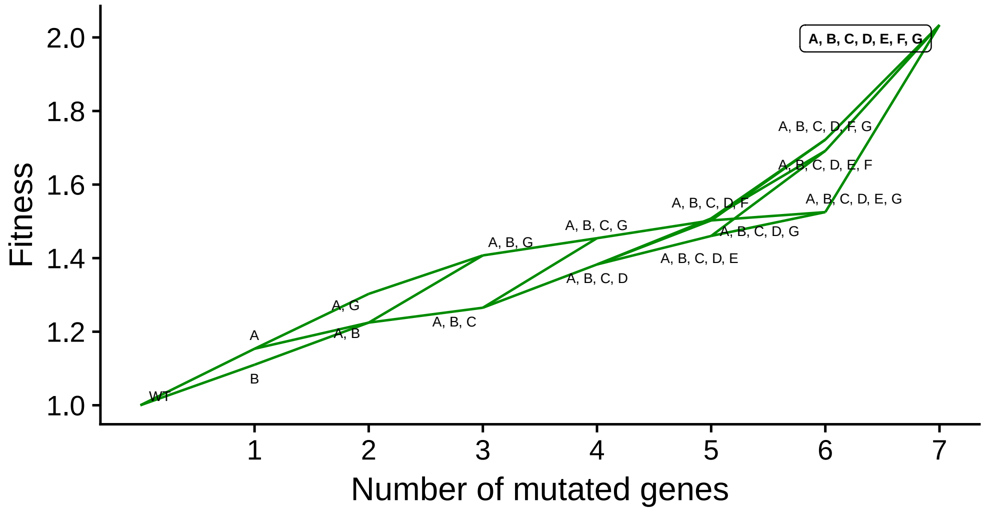

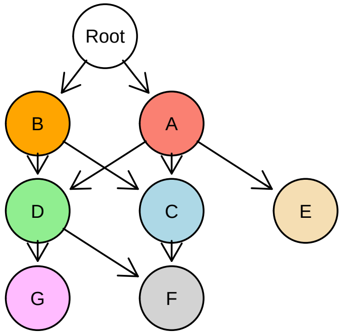

ID	NL2plam10LkyAR93o
acc genotypes	20
peaks no back	1
removed edges	0
prop rem edges	0

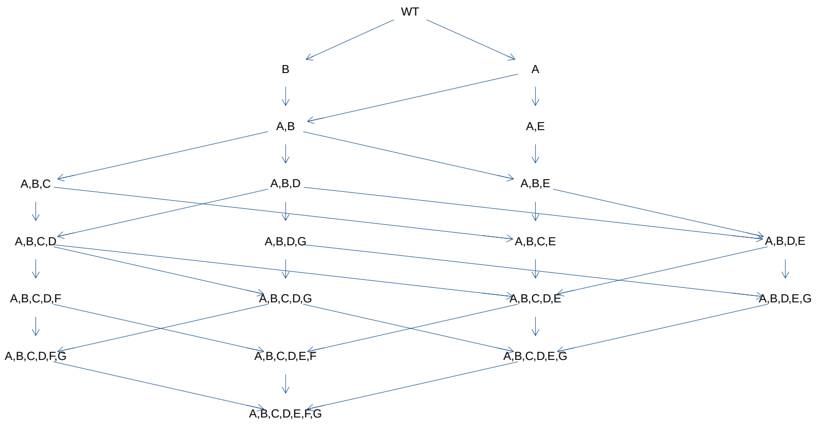

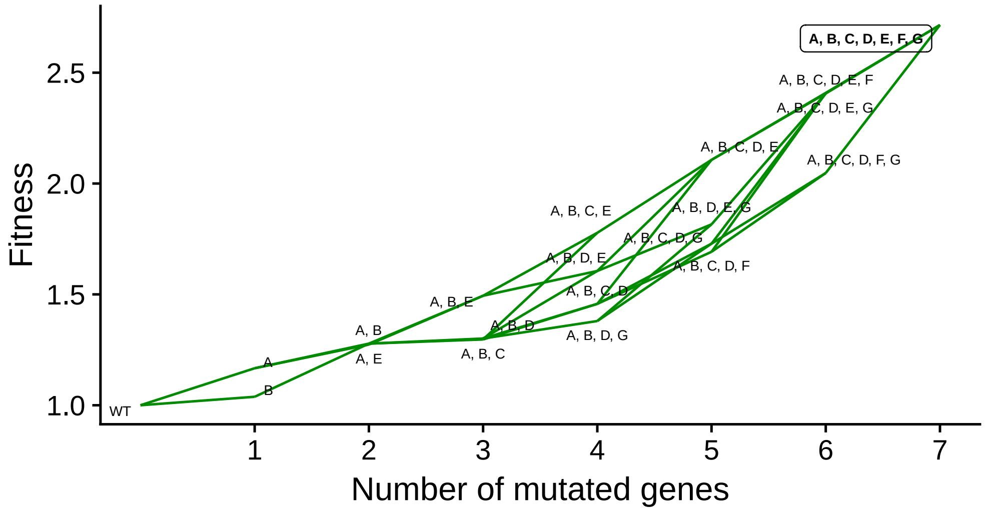

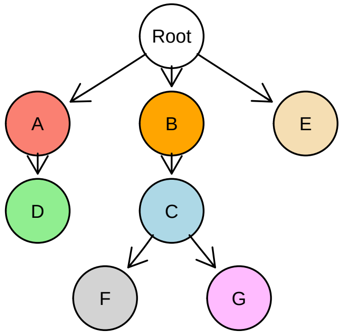

ID	nNXWsbAmror4SleI
acc genotypes	36
peaks no back	1
removed edges	0
prop rem edges	0

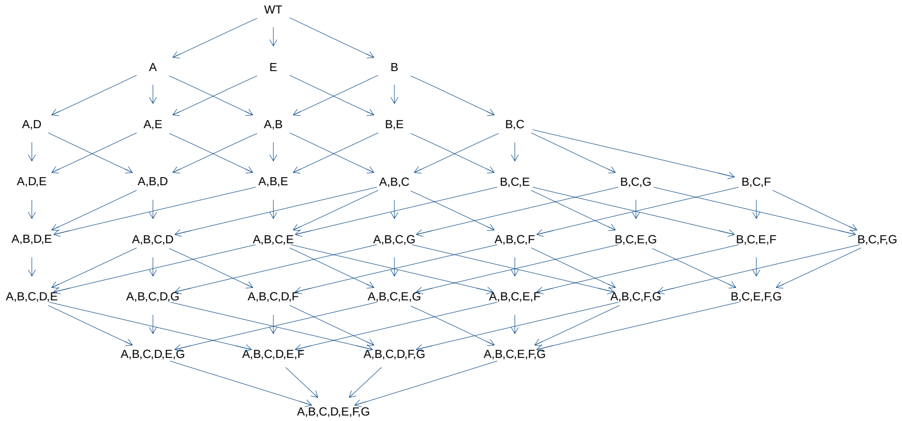

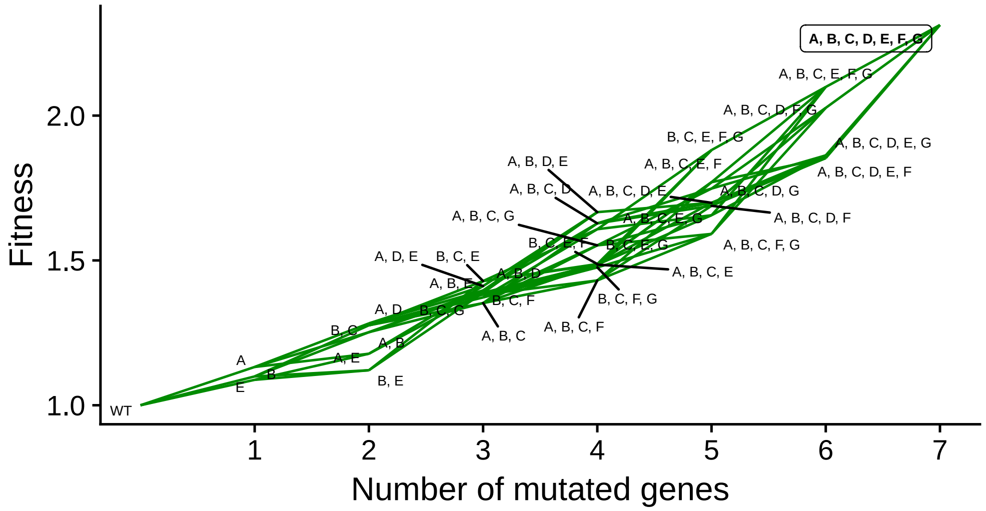

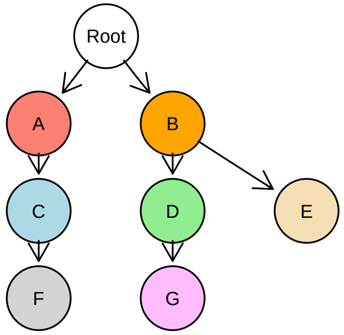

ID	NP6G6AnYJEoAMZzC
acc genotypes	28
peaks no back	1
removed edges	0
prop rem edges	0

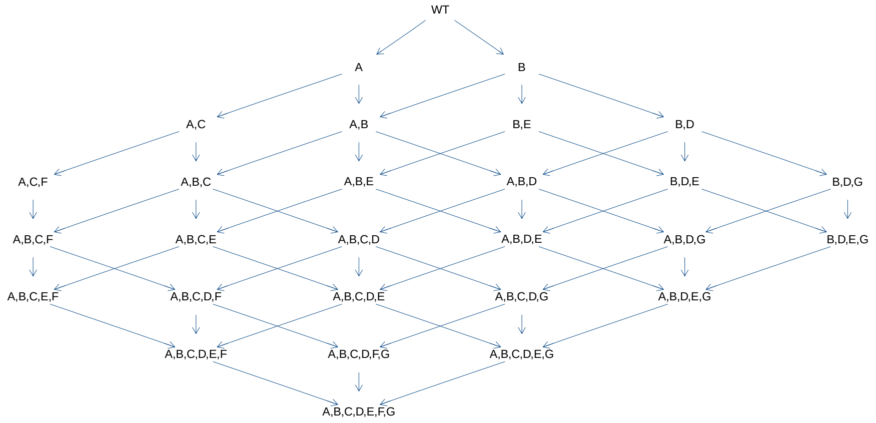

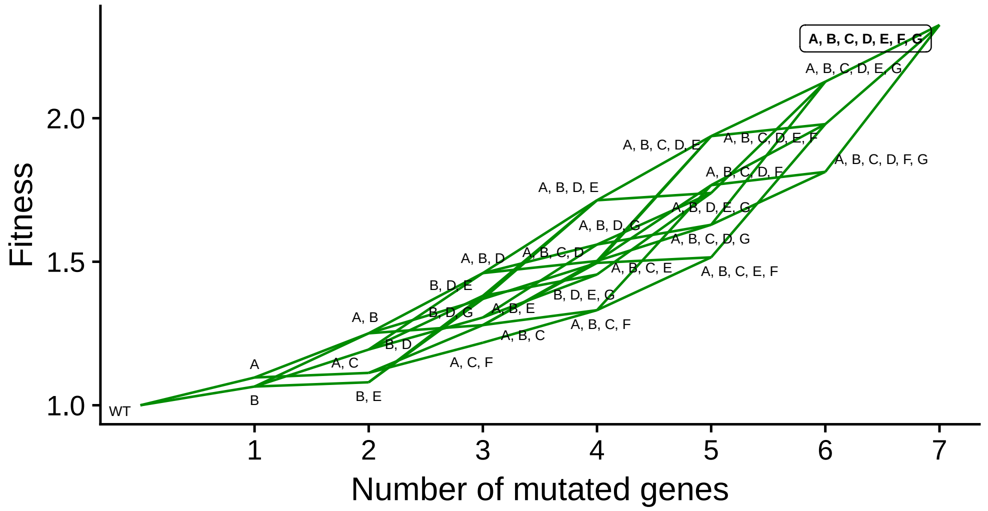

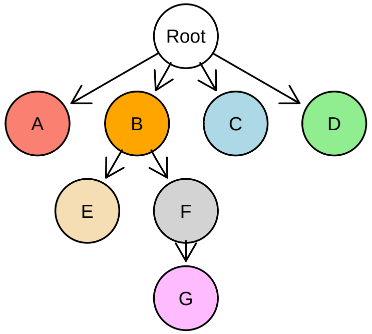

ID	NQkZsXiFwR5kJZQb
acc genotypes	56
peaks no back	1
removed edges	0
prop rem edges	0

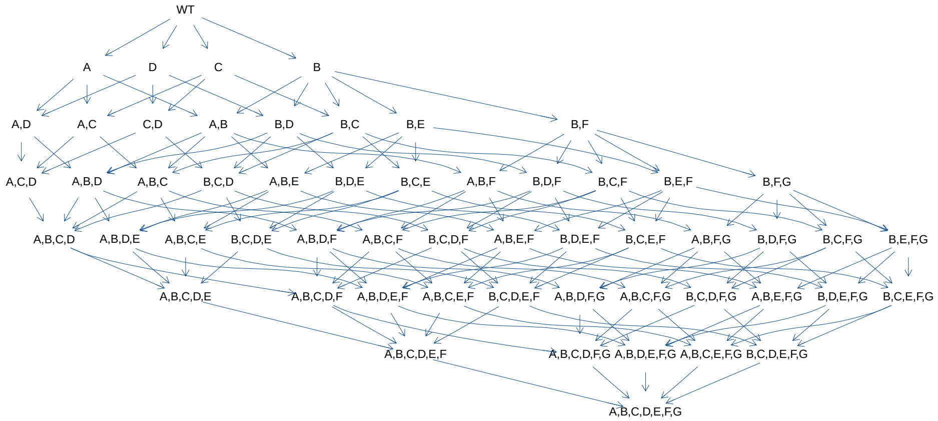

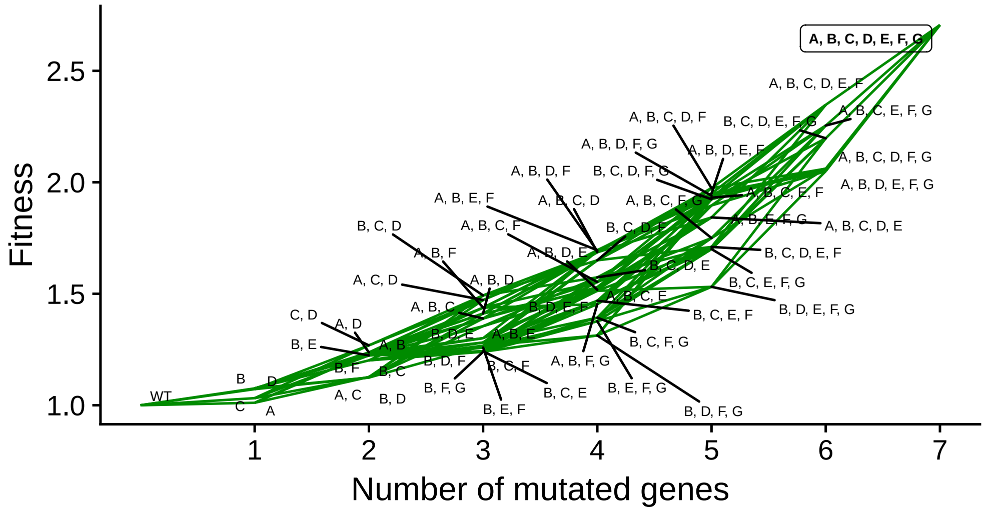

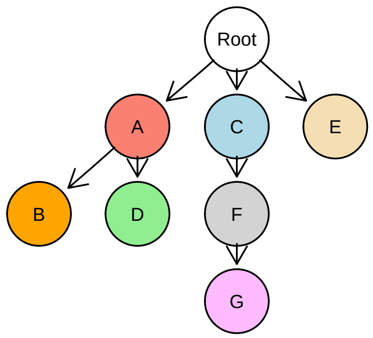

ID	O9dkG7dXRCSpbF7s
acc genotypes	40
peaks no back	1
removed edges	0
prop rem edges	0

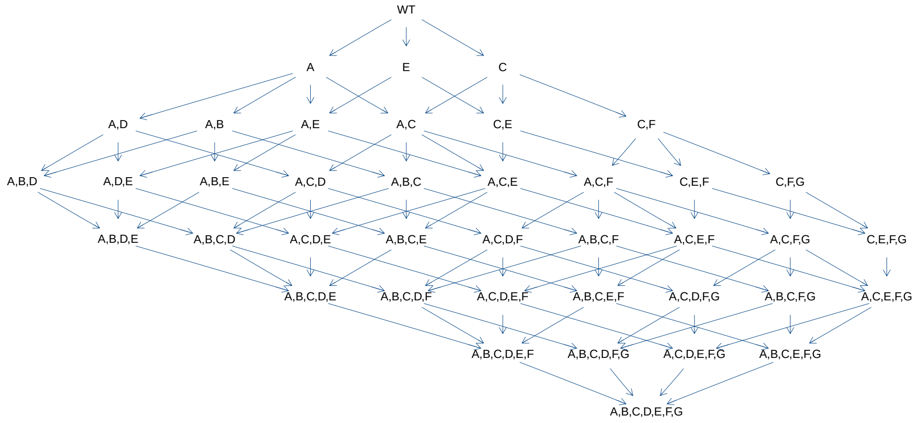

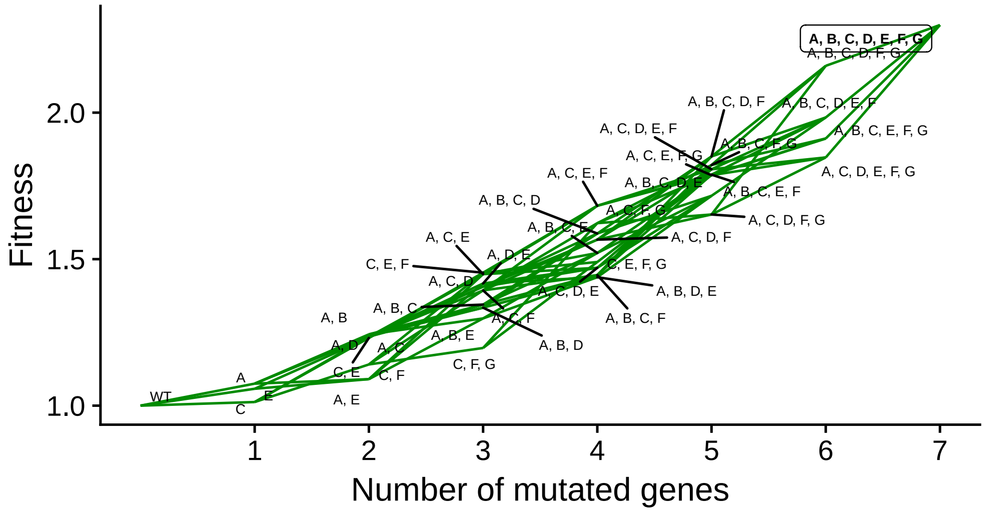

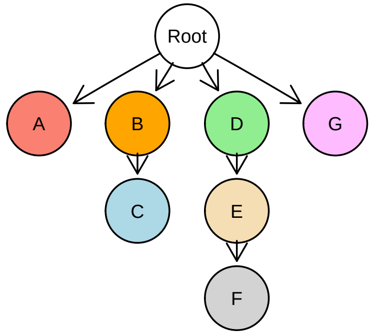

ID	o9zATJwfW4X1dLDs
acc genotypes	48
peaks no back	1
removed edges	0
prop rem edges	0

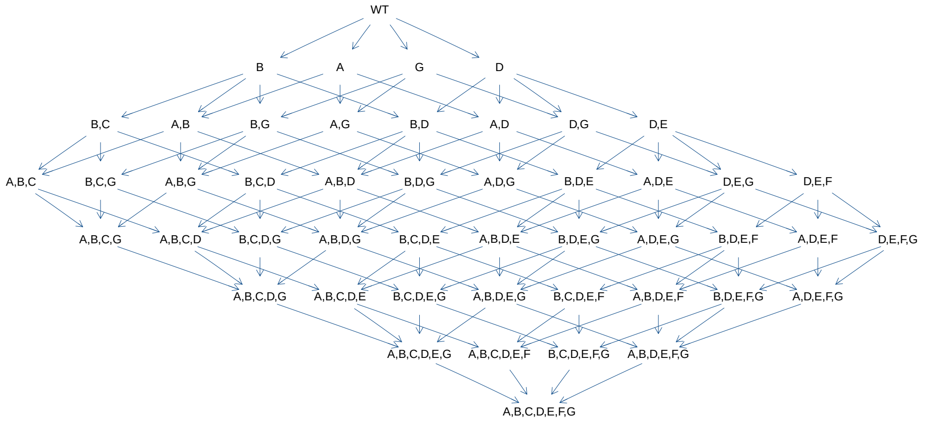

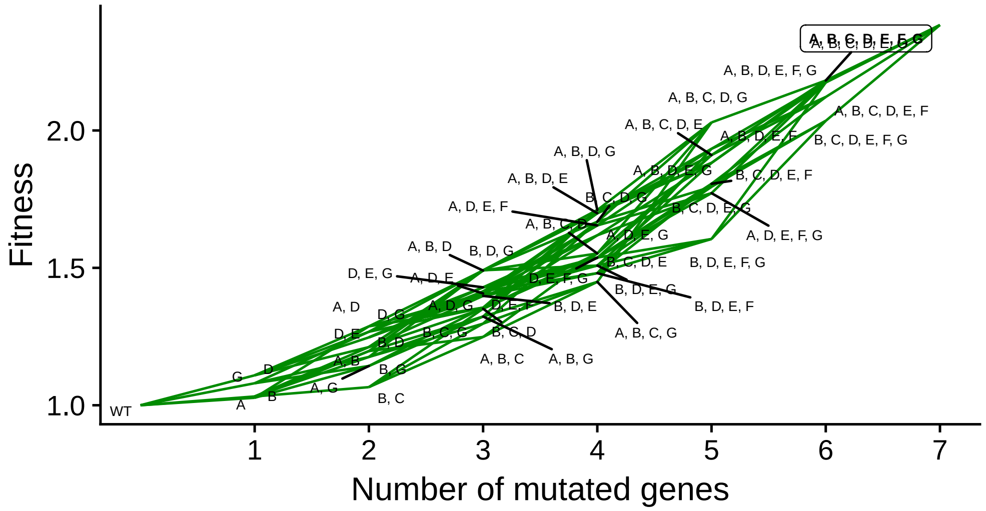

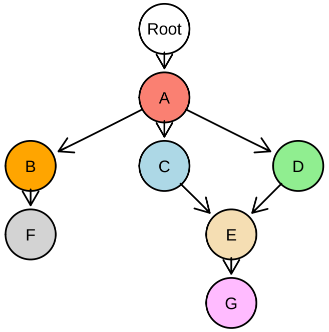

ID	oZm9q2fpYypg1gEQ
acc genotypes	19
peaks no back	1
removed edges	0
prop rem edges	0

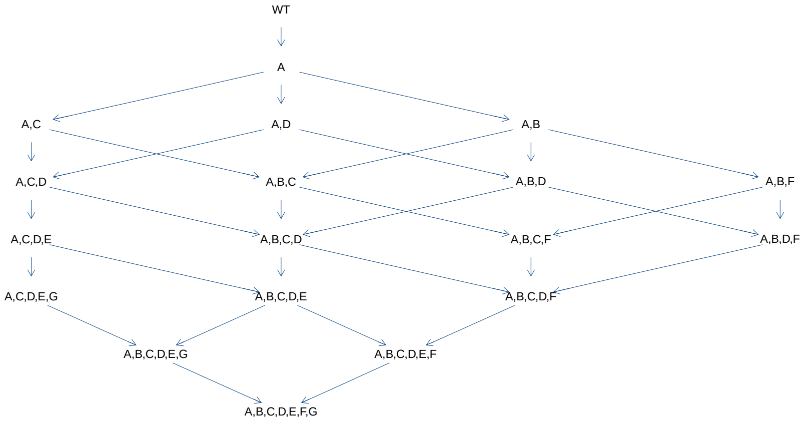

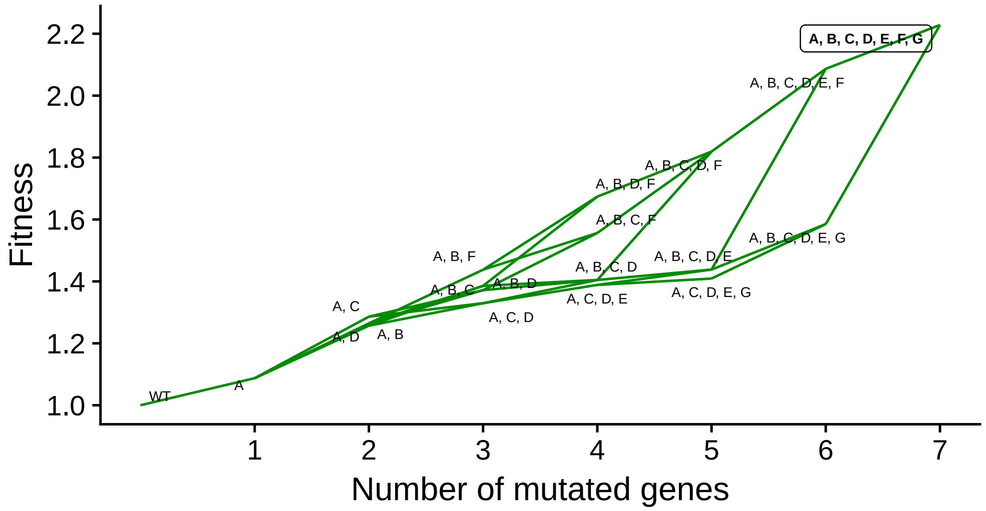

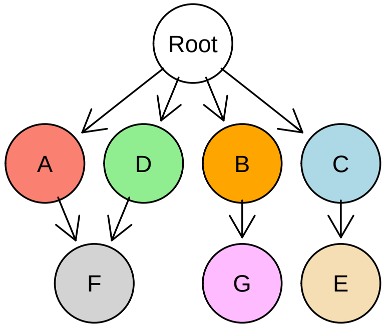

ID	P6fkPLOb9jfitZfv
acc genotypes	45
peaks no back	1
removed edges	0
prop rem edges	0

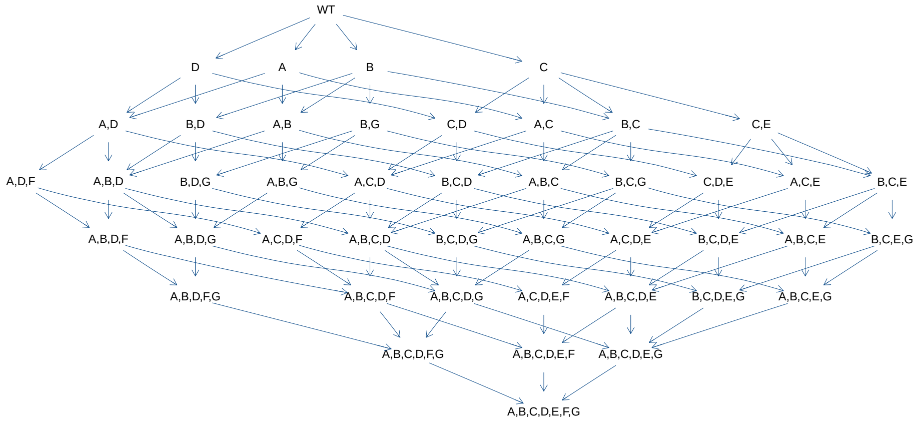

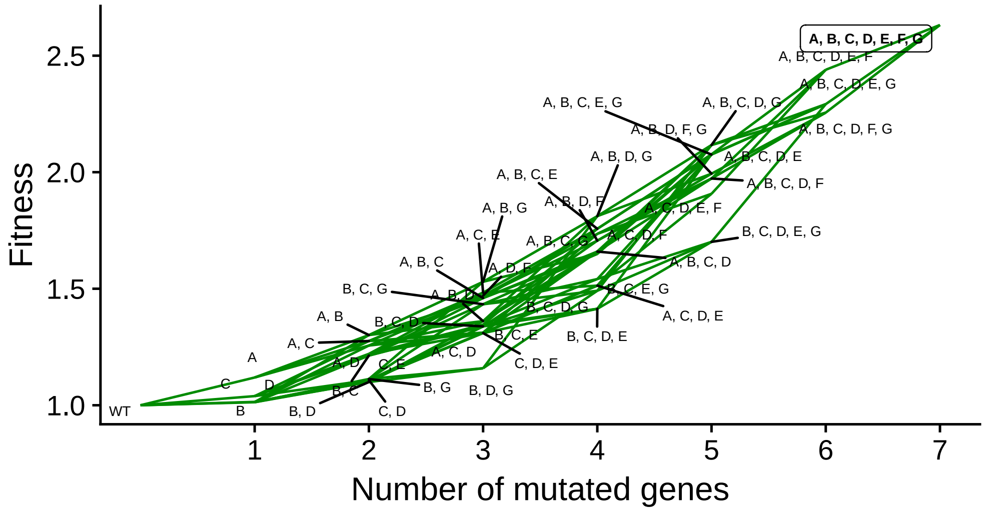

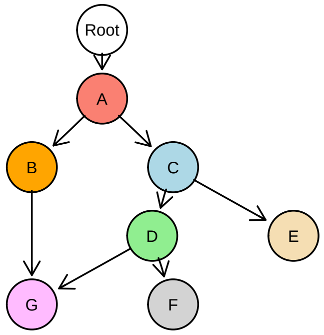

ID	pDRZFntEs1roodY8
acc genotypes	19
peaks no back	1
removed edges	0
prop rem edges	0

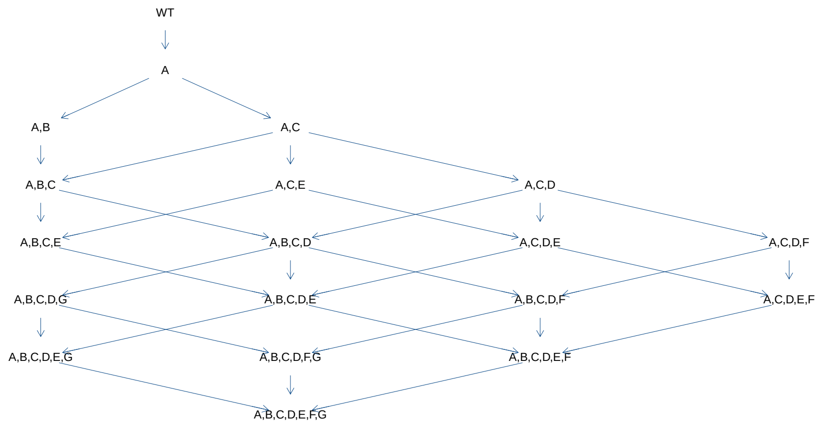

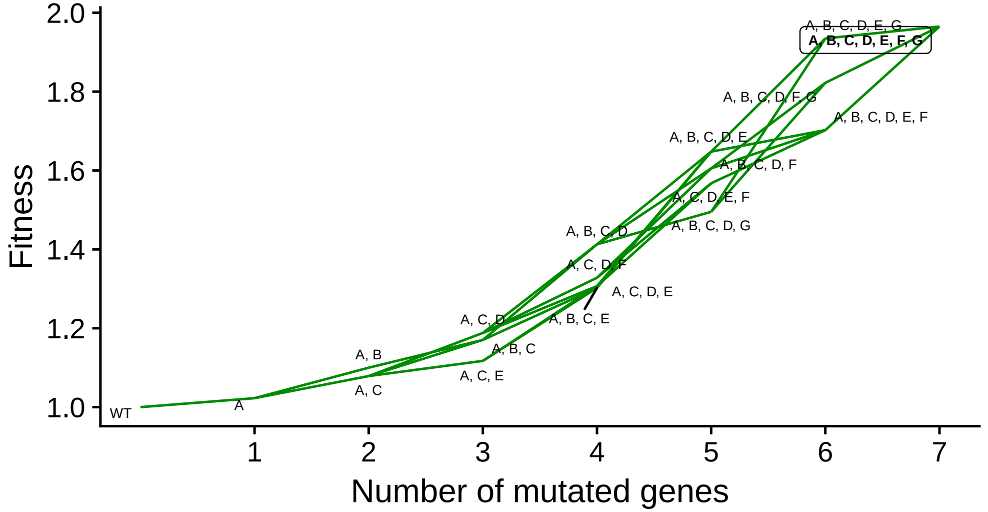

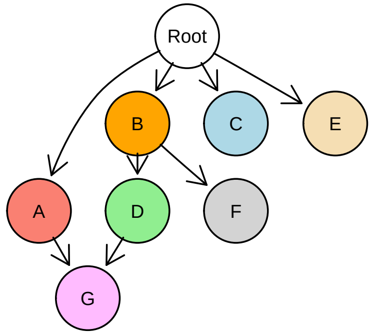

ID	pJAvDquRk5XGCjrp
acc genotypes	48
peaks no back	1
removed edges	0
prop rem edges	0

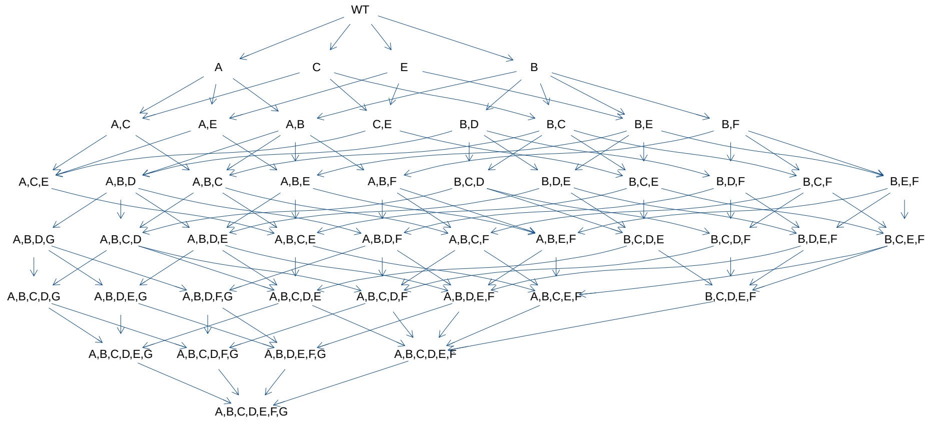

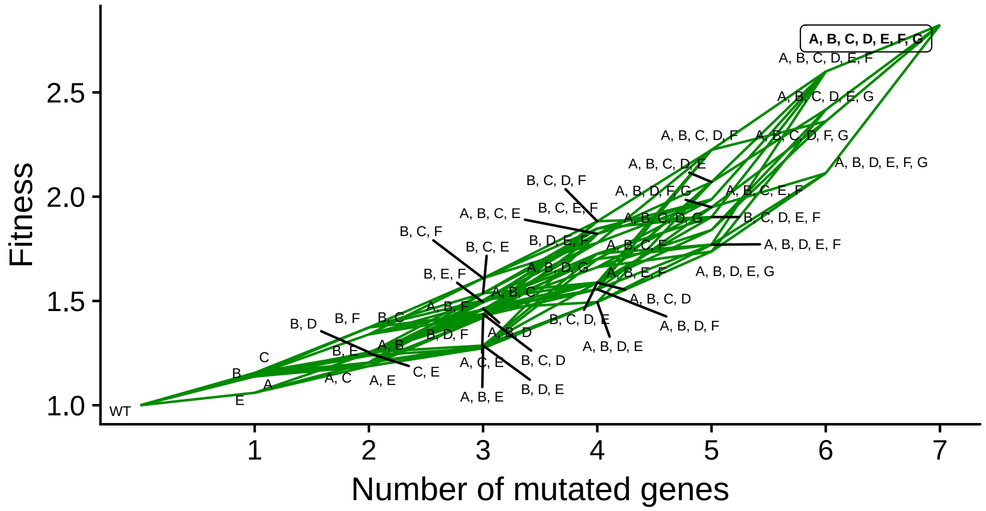

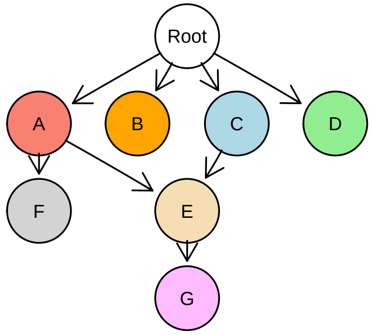

ID	PQUizfzfBmdE7gFY
acc genotypes	40
peaks no back	1
removed edges	0
prop rem edges	0

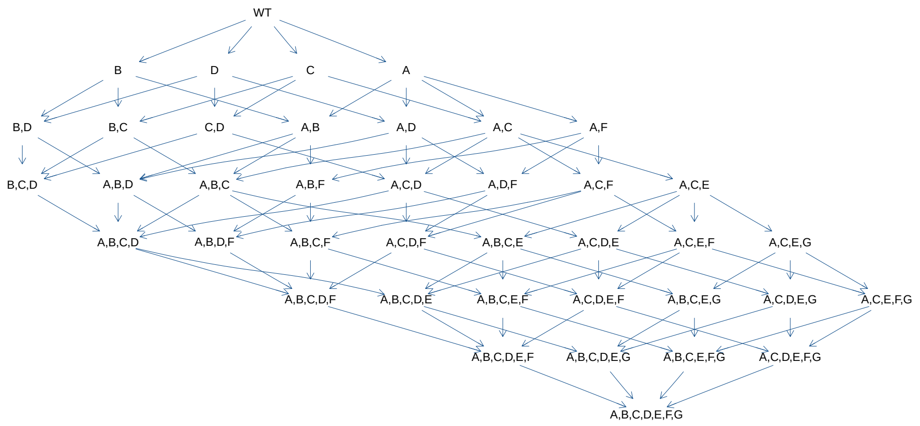

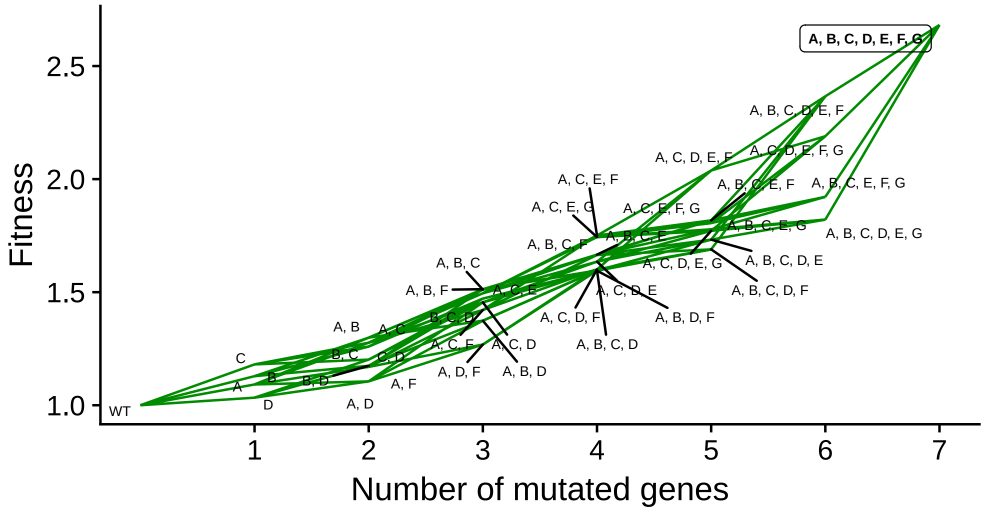

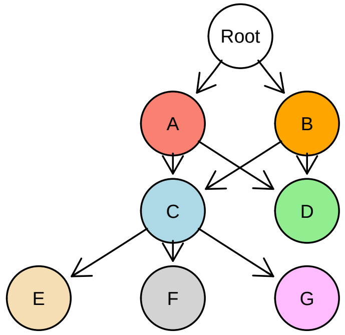

ID	q10sOtkvPmaBzPDOV
acc genotypes	21
peaks no back	1
removed edges	0
prop rem edges	0

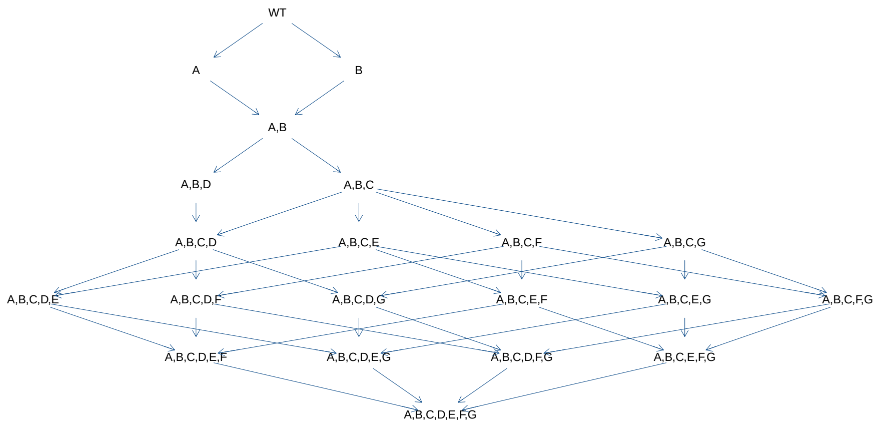

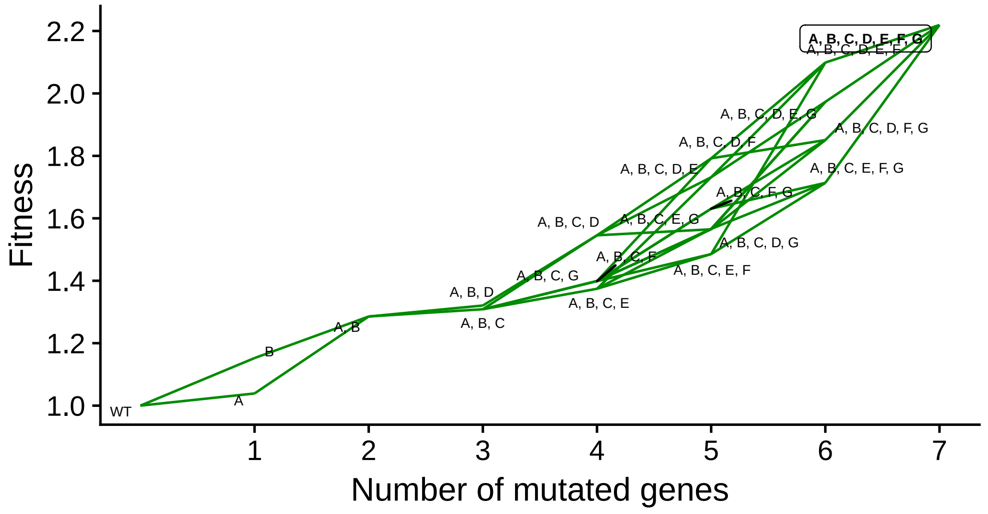

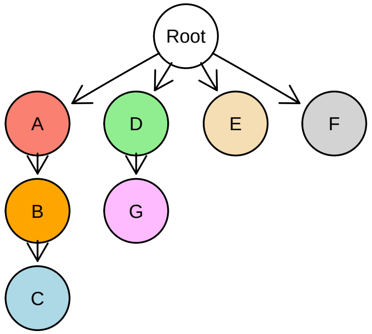

ID	q10upXvME3iKLaWcZ
acc genotypes	48
peaks no back	1
removed edges	0
prop rem edges	0

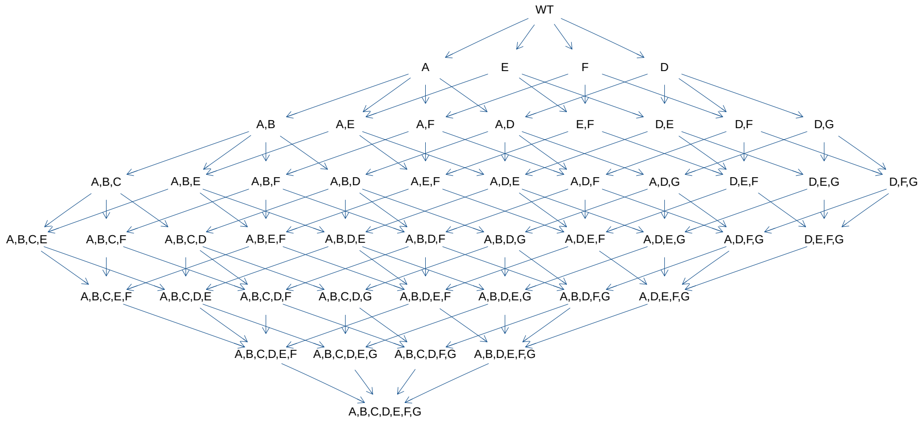

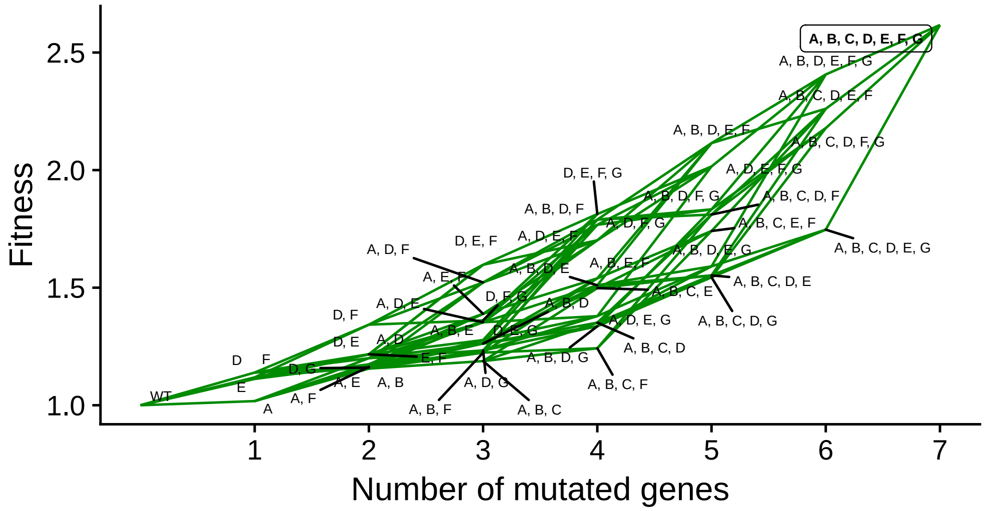

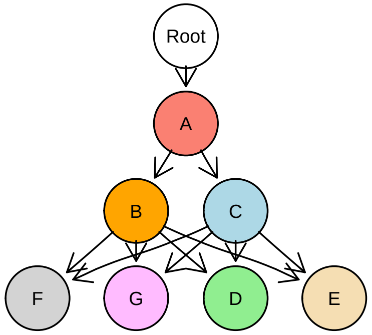

ID	q3bB6O4aHcWy2GNL
acc genotypes	20
peaks no back	1
removed edges	0
prop rem edges	0

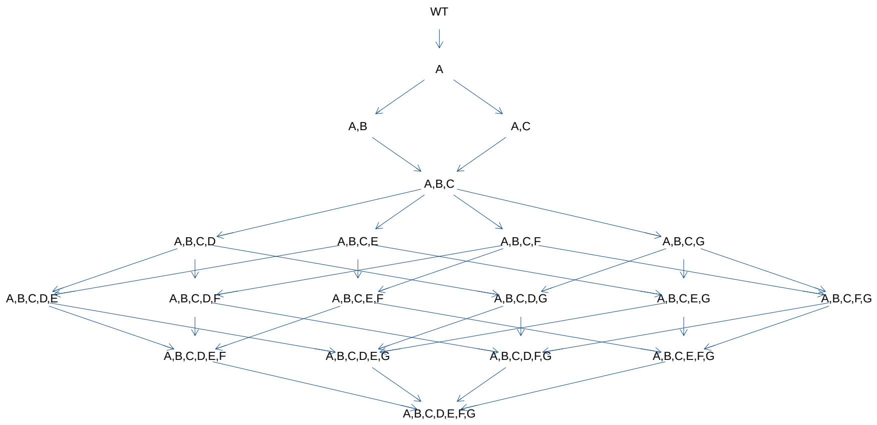

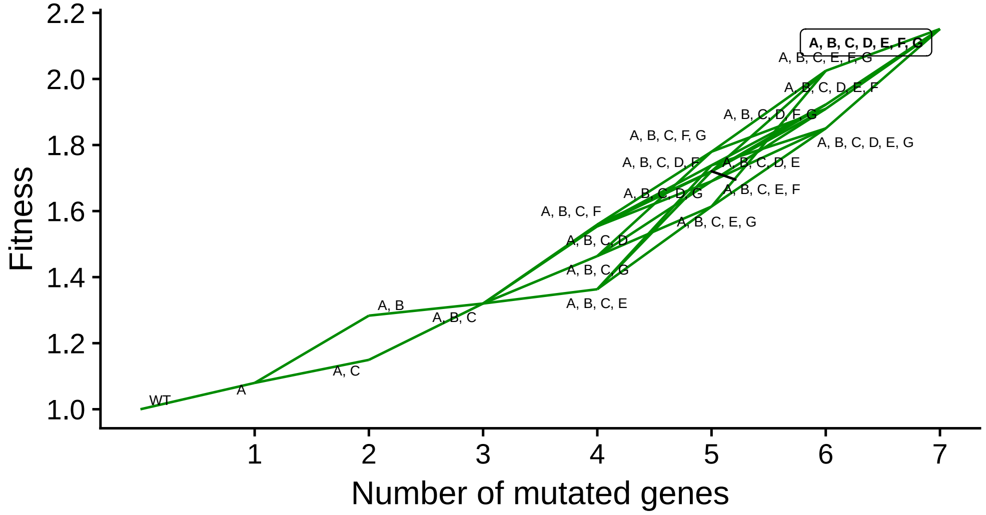

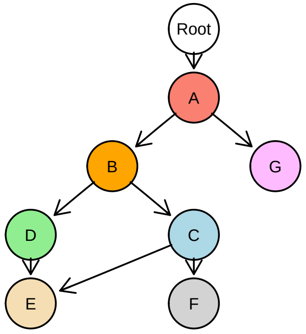

ID	qDTXWJ10Vx4dad6eV
acc genotypes	19
peaks no back	1
removed edges	0
prop rem edges	0

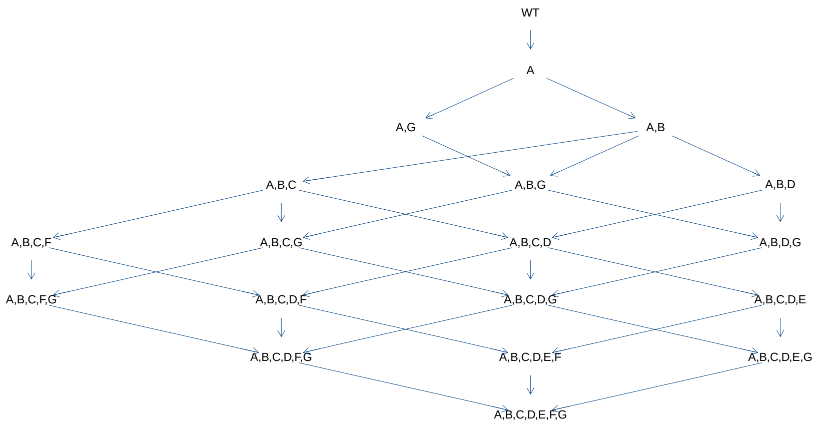

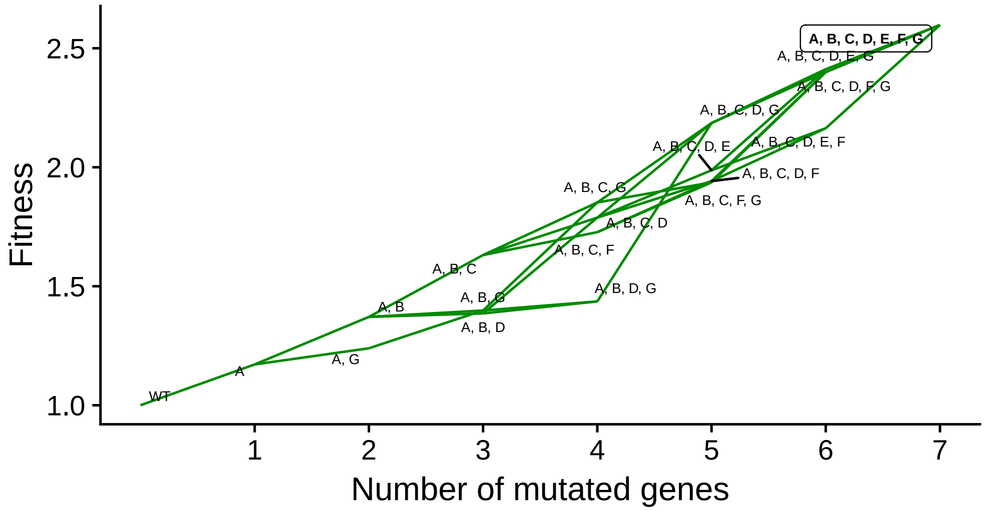

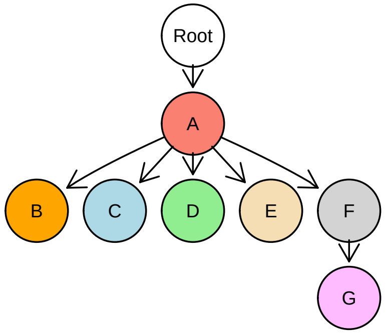

ID	qdVBLPcBGWHIqm6a
acc genotypes	49
peaks no back	1
removed edges	0
prop rem edges	0

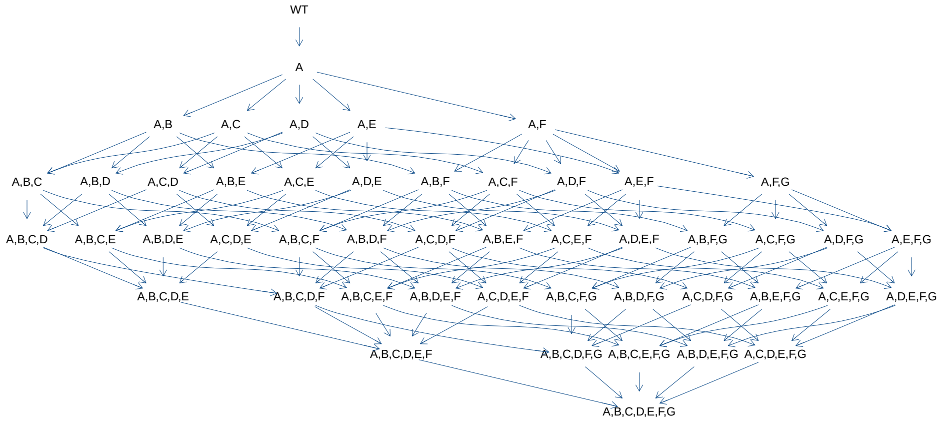

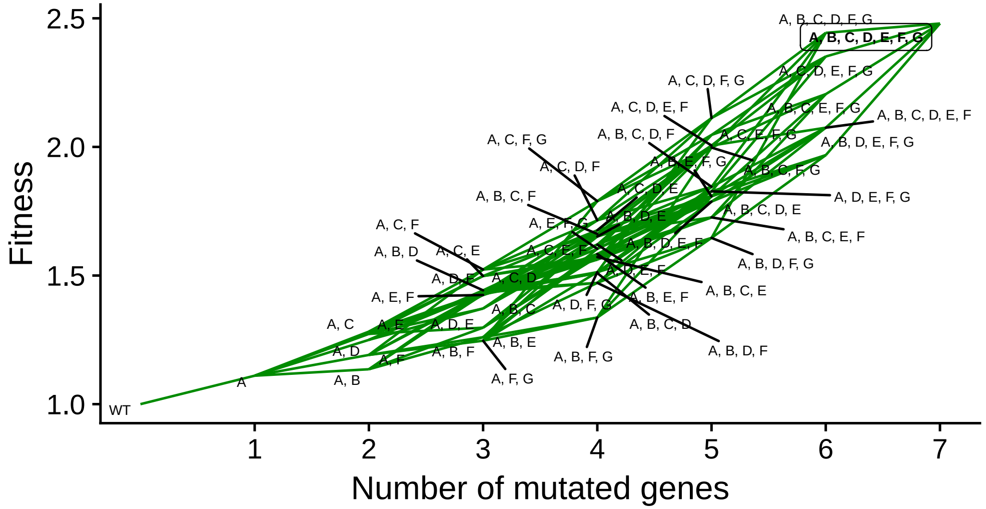

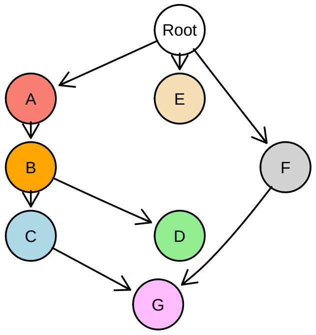

ID	QfKhJqFLfL10D8u110
acc genotypes	28
peaks no back	1
removed edges	0
prop rem edges	0

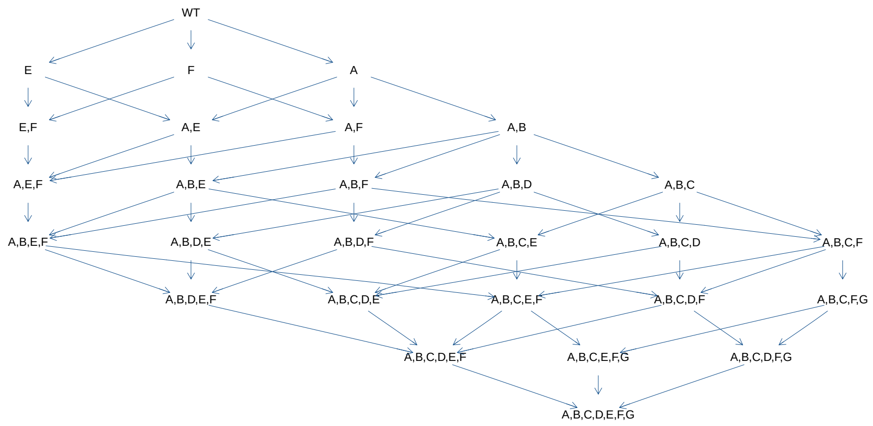

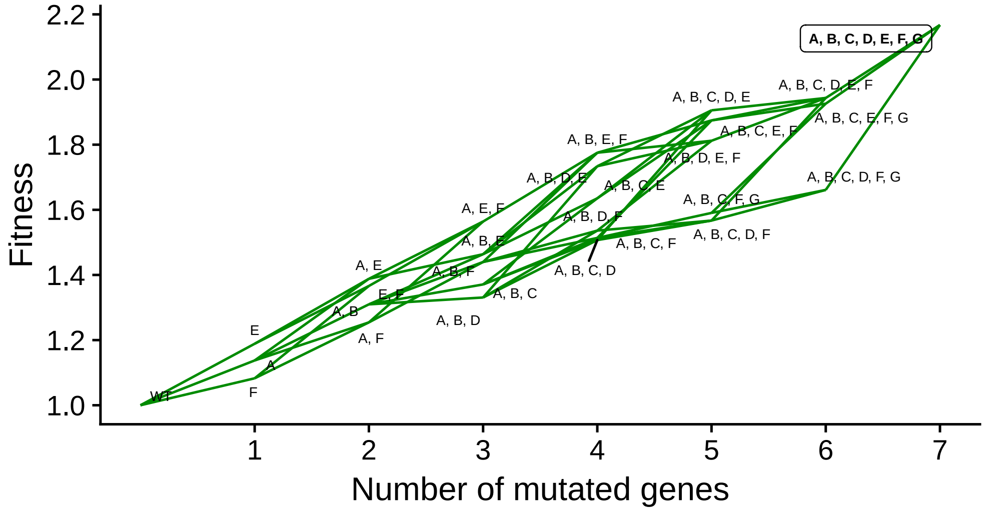

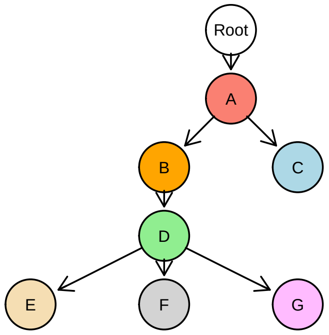

ID	qhzHLgirL8adNfAJ
acc genotypes	21
peaks no back	1
removed edges	0
prop rem edges	0

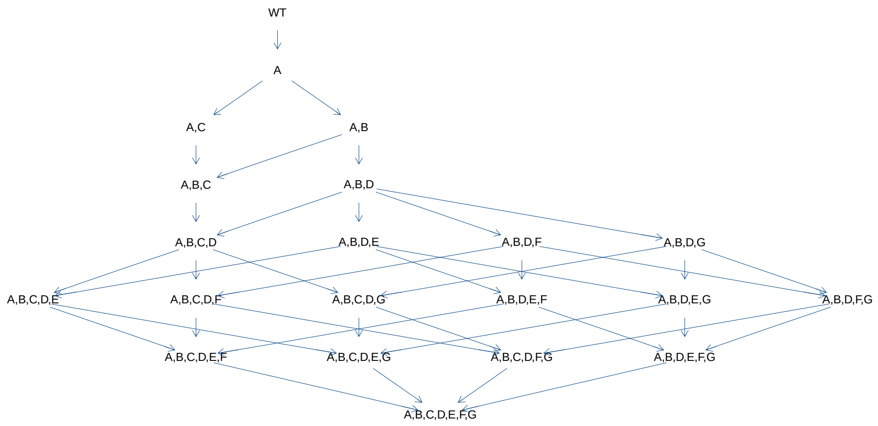

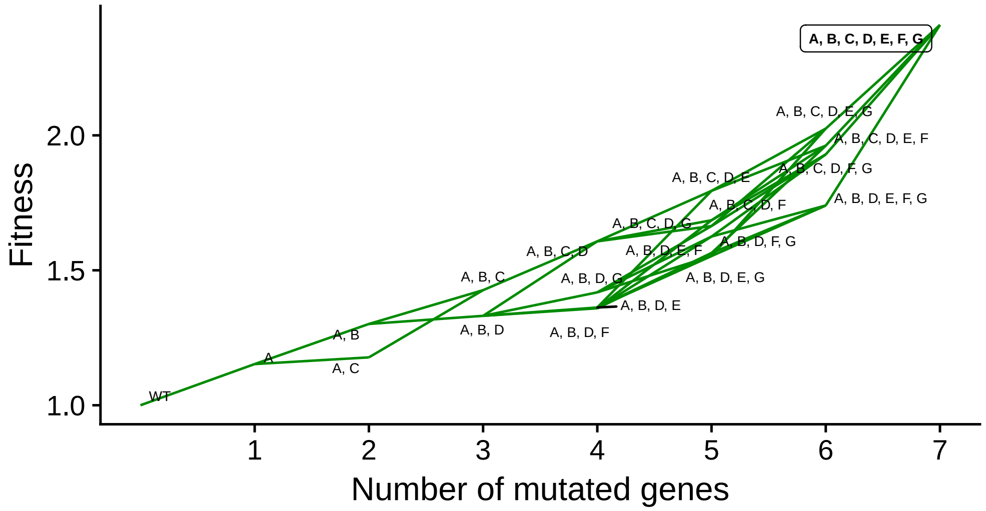

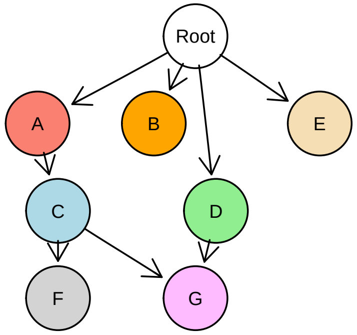

ID	qIEabZH111rt10AQ2
acc genotypes	40
peaks no back	1
removed edges	0
prop rem edges	0

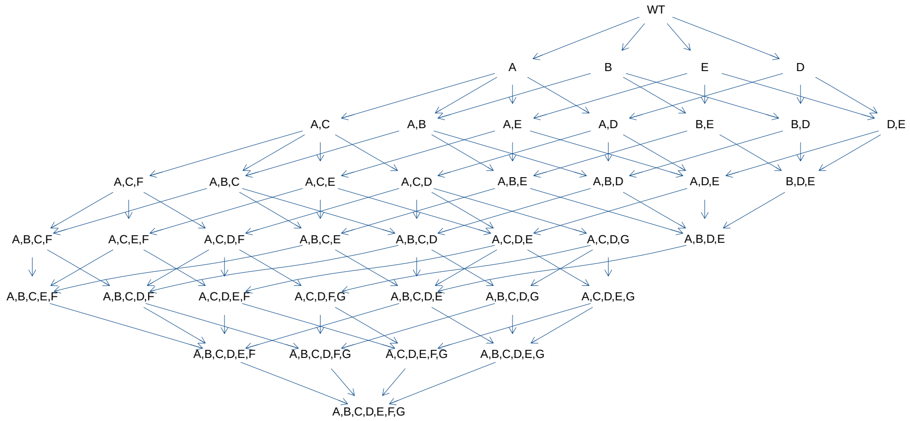

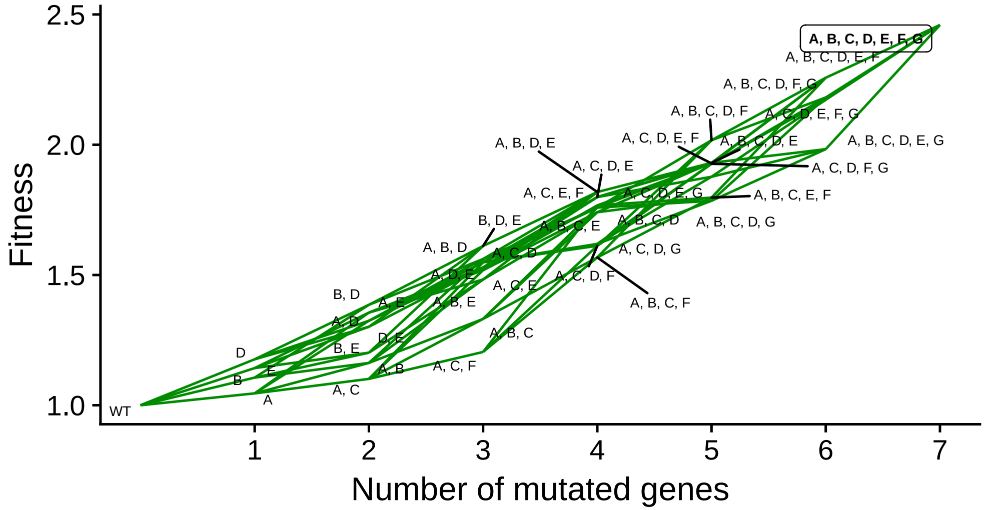

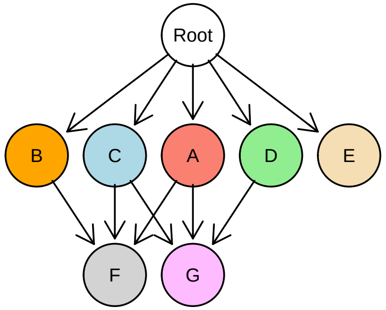

ID	qsBbMjGmsMVjLZVW
acc genotypes	42
peaks no back	1
removed edges	0
prop rem edges	0

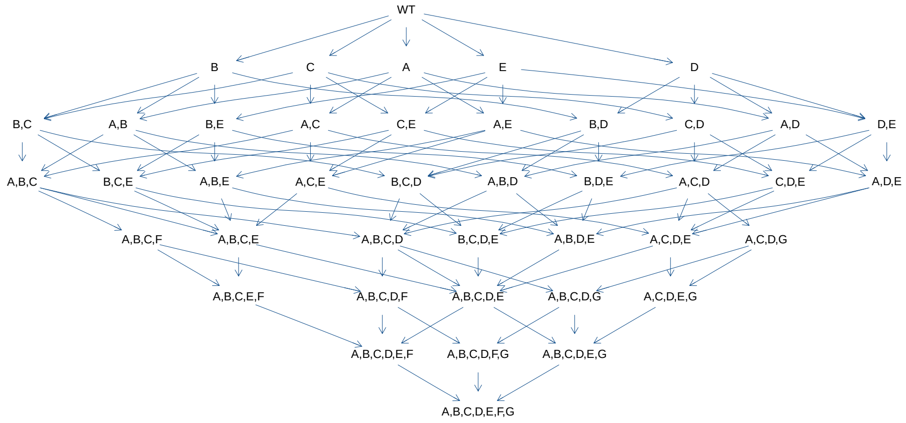

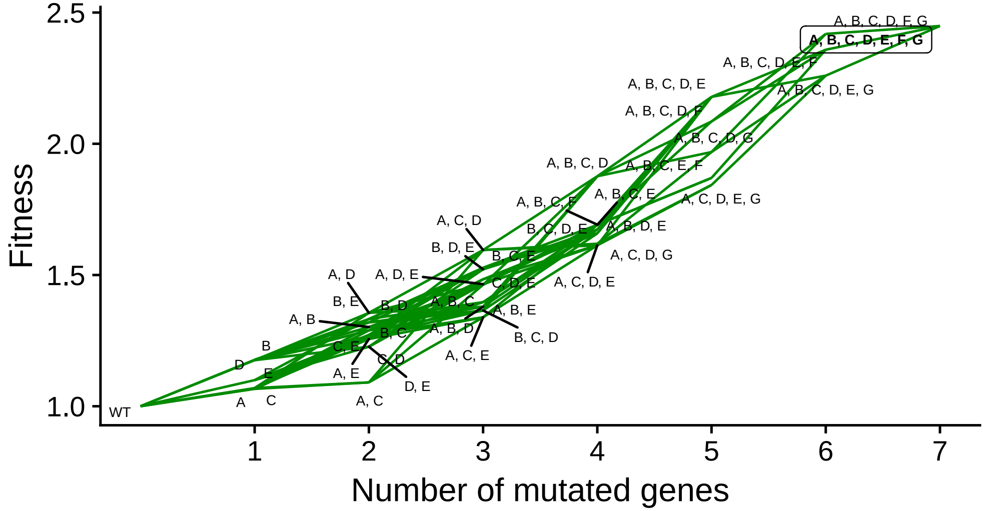

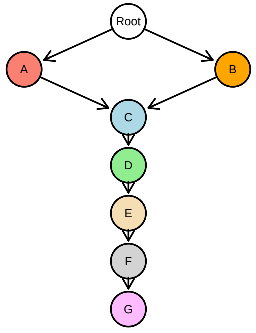

ID	QTVh104soQUgOBfKi
acc genotypes	9
peaks no back	1
removed edges	0
prop rem edges	0

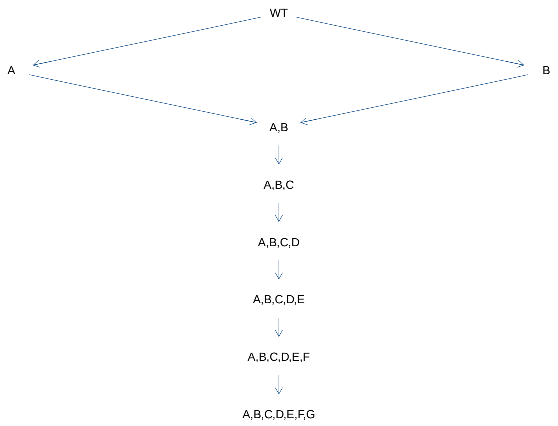

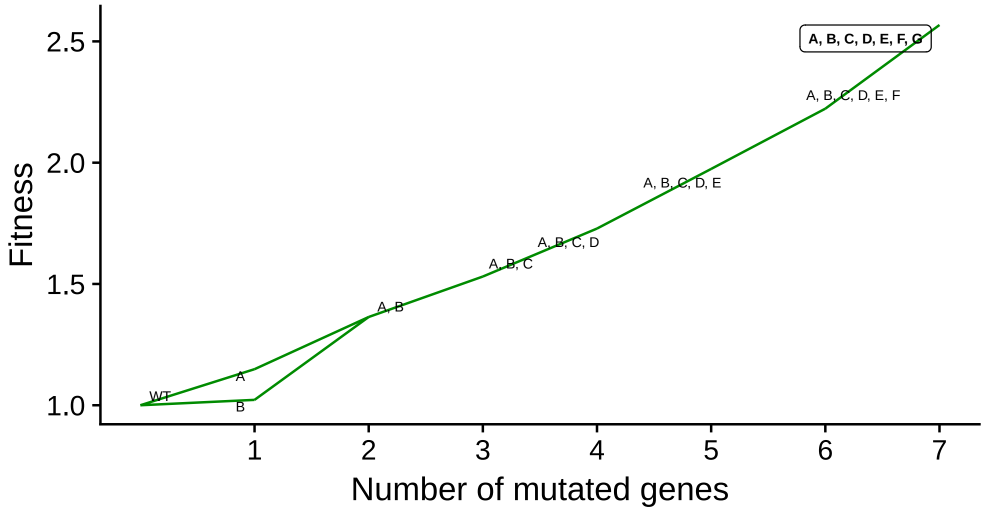

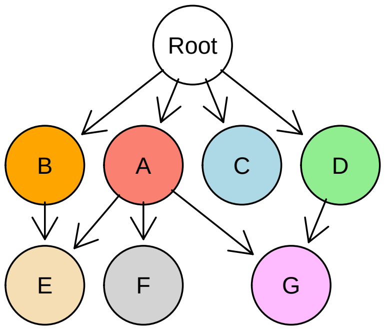

ID	qYgVxb5ntdQ1oibs
acc genotypes	44
peaks no back	1
removed edges	0
prop rem edges	0

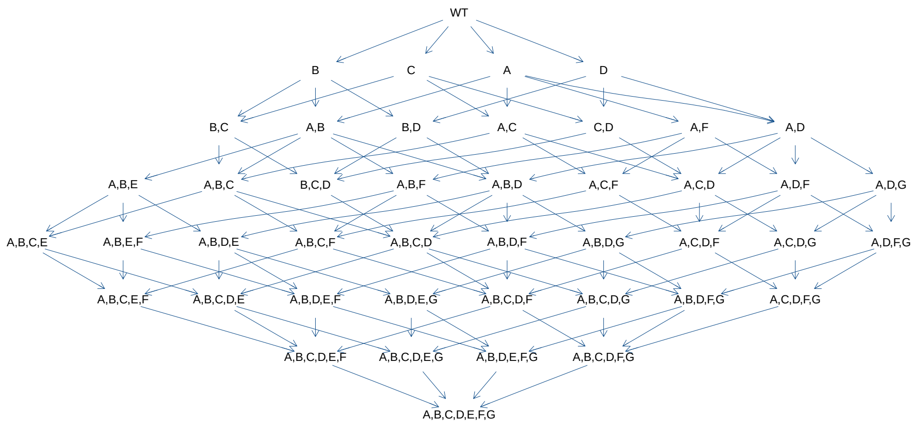

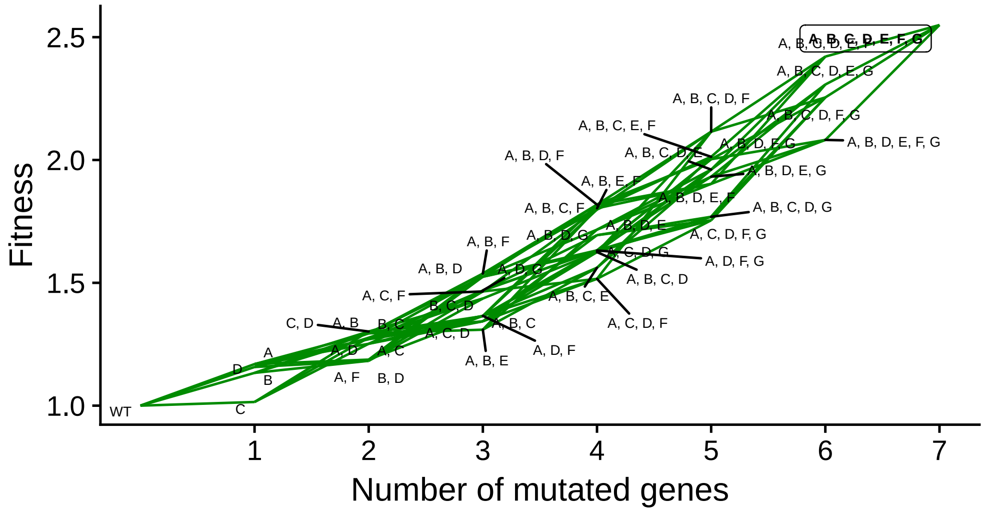

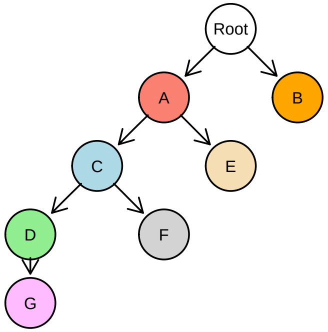

ID	r6gSASnFWWRSp10wF
acc genotypes	30
peaks no back	1
removed edges	0
prop rem edges	0

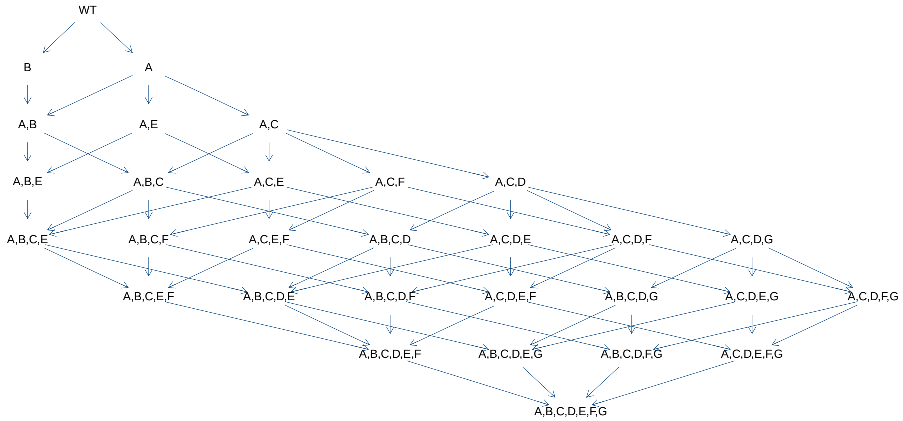

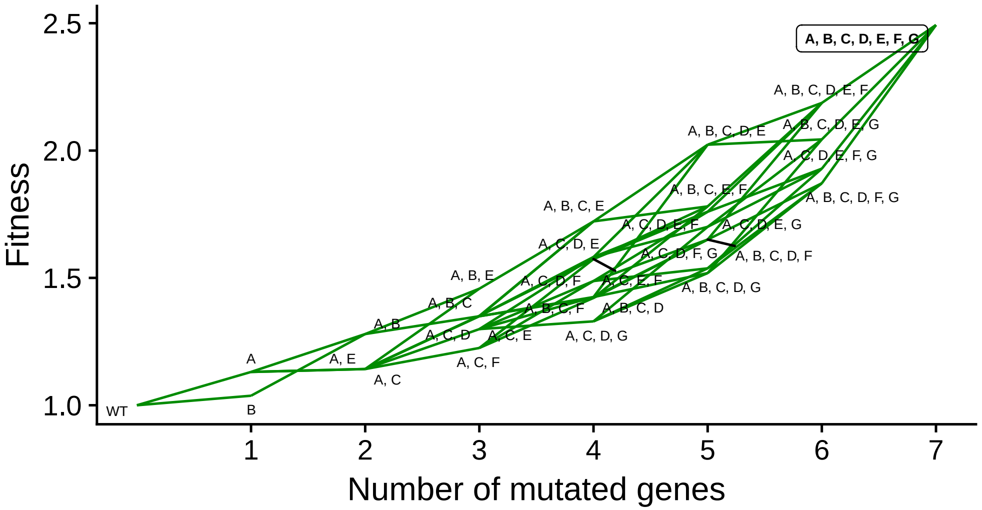

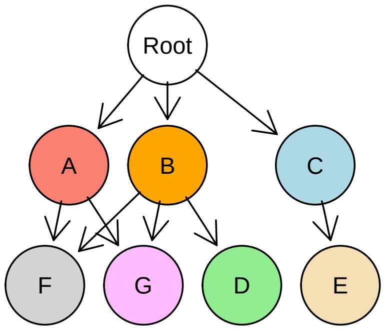

ID rcySfhqbP6f5Ys45
acc genotypes 36
peaks no back 1
removed edges 0
prop rem edges 0

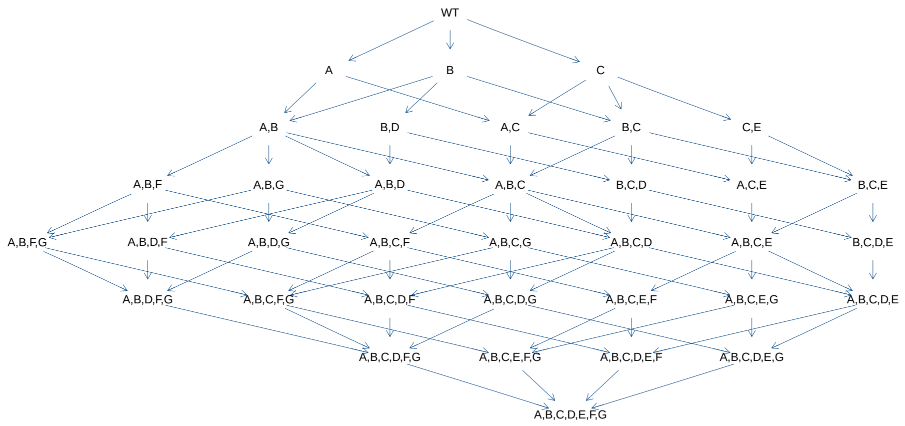

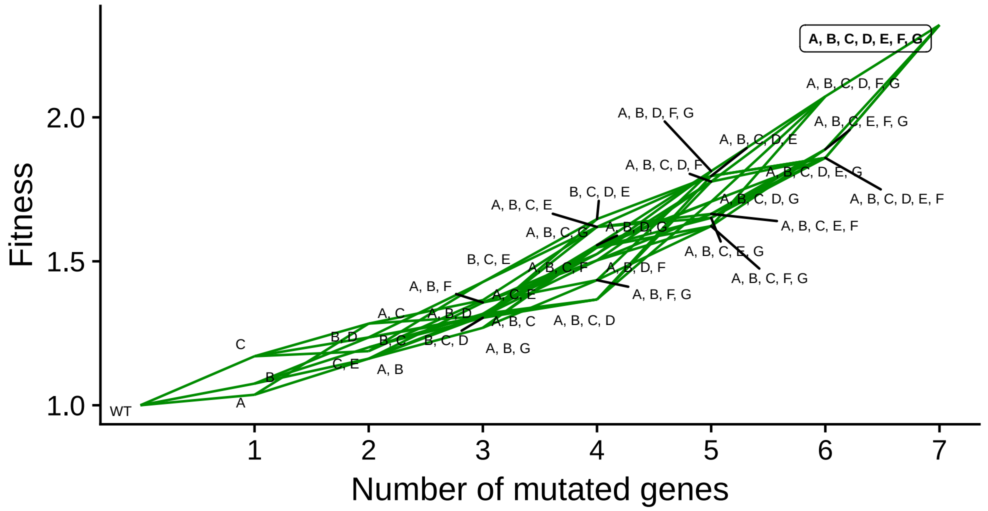

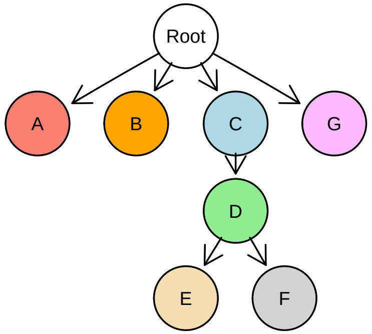

ID	RgRwHiCY10NBQ5ya4
acc genotypes	48
peaks no back	1
removed edges	0
prop rem edges	0

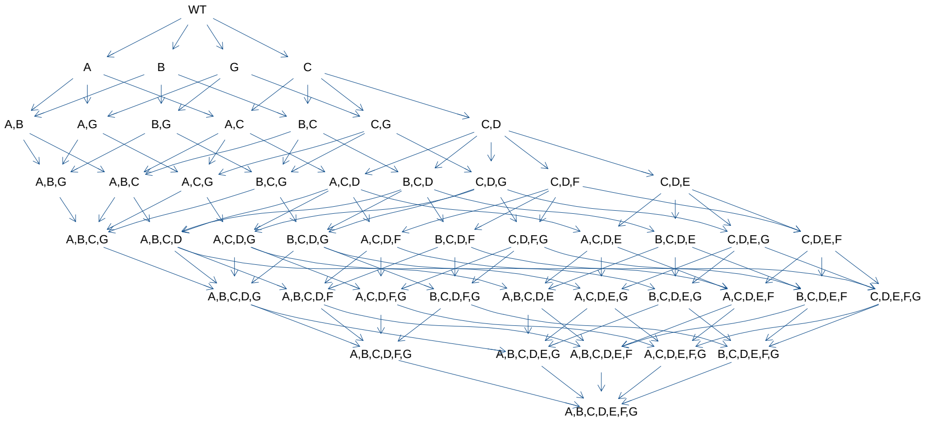

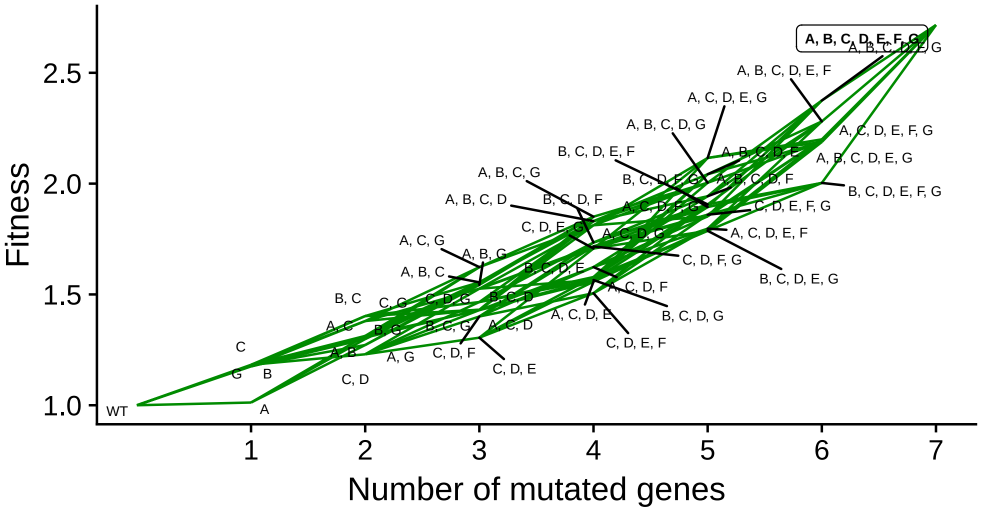

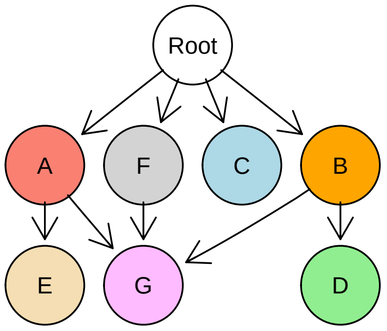

ID	RKaZWcfptCPVxJUF
acc genotypes	44
peaks no back	1
removed edges	0
prop rem edges	0

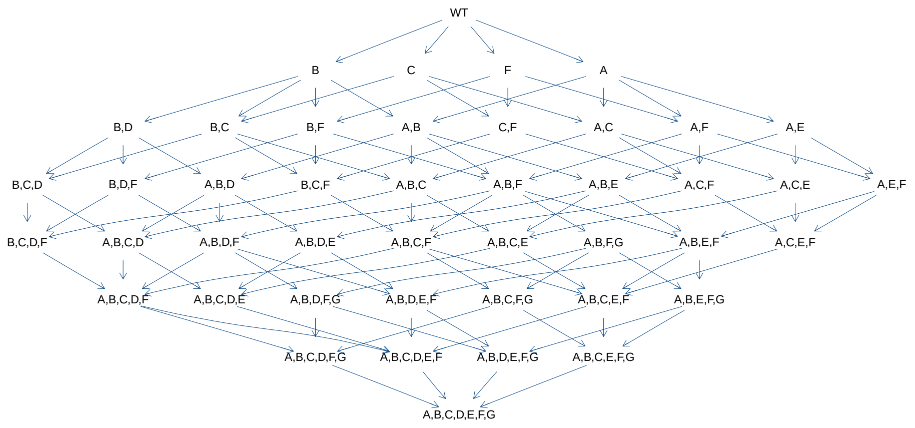

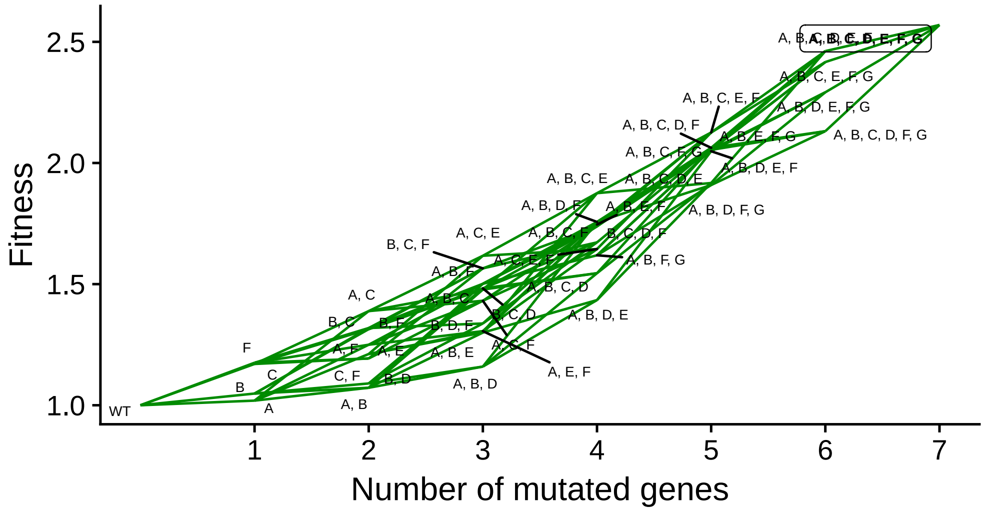

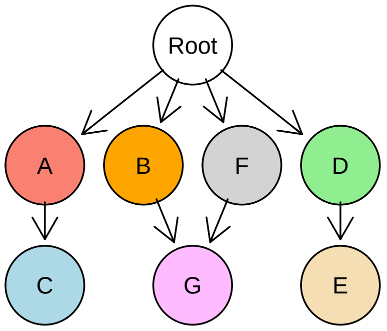

ID	rokOVrDugL5VXneF
acc genotypes	45
peaks no back	1
removed edges	0
prop rem edges	0

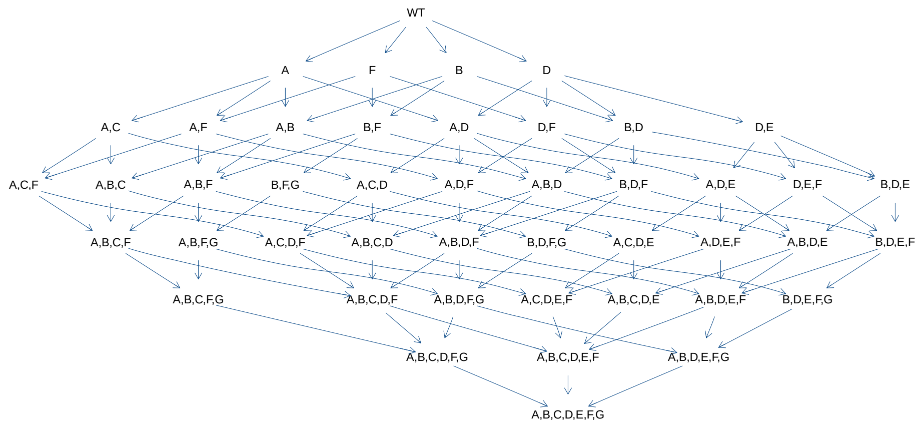

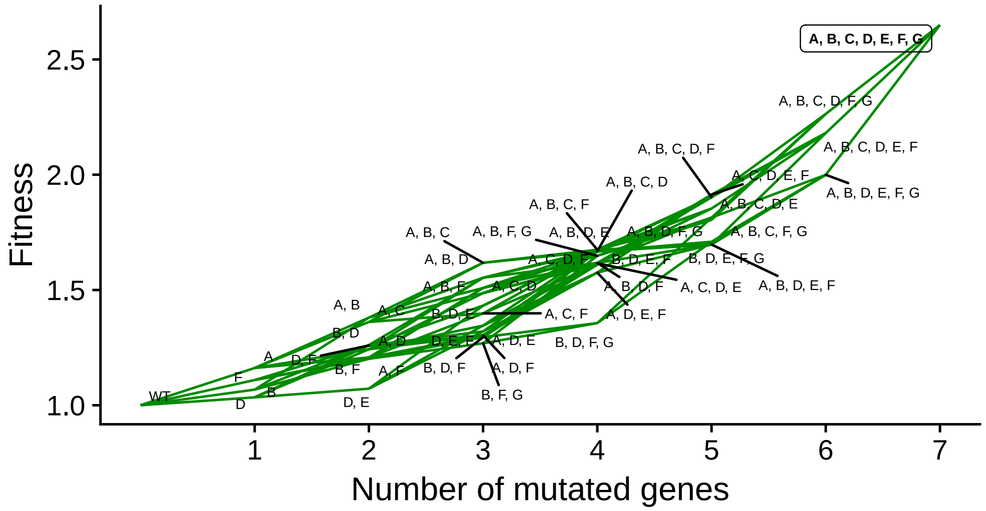

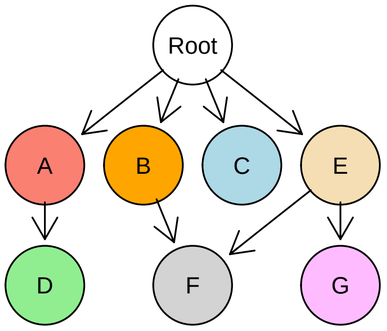

ID	ryICF10Z10RhI71ONm
acc genotypes	48
peaks no back	1
removed edges	0
prop rem edges	0

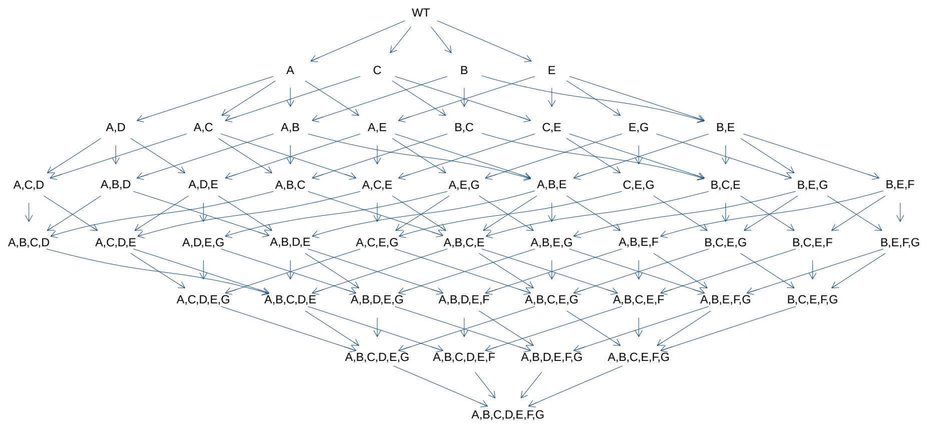

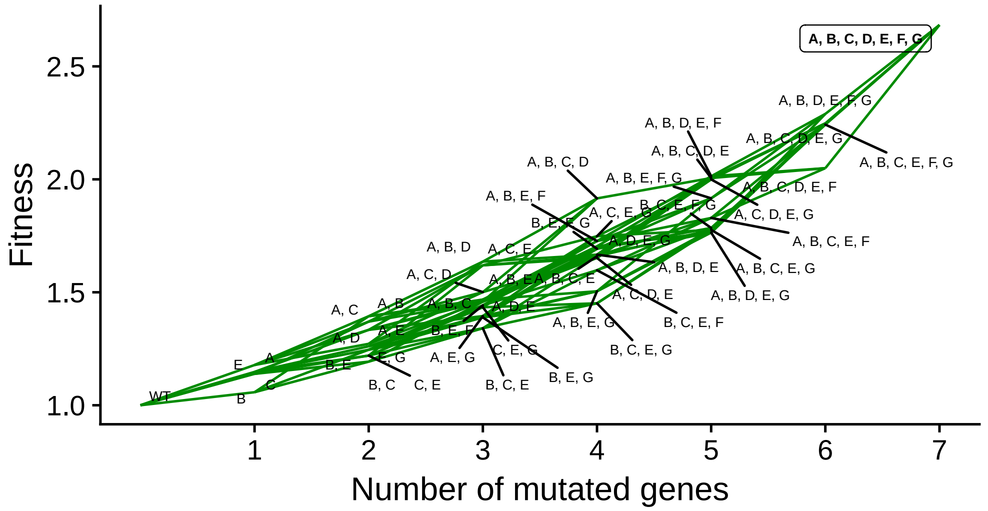

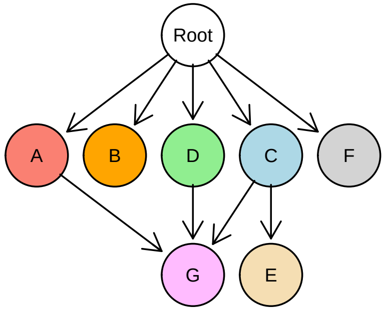

ID	s6QENCe4O6bJtquJ
acc genotypes	56
peaks no back	1
removed edges	0
prop rem edges	0

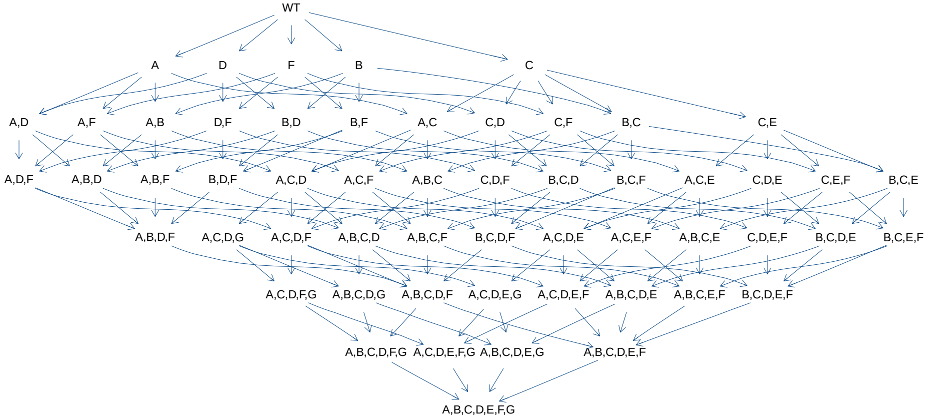

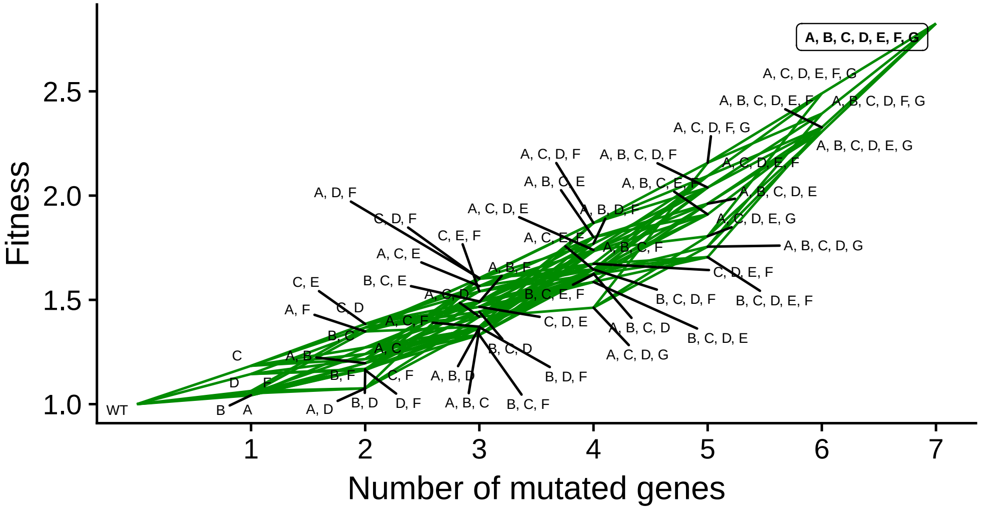

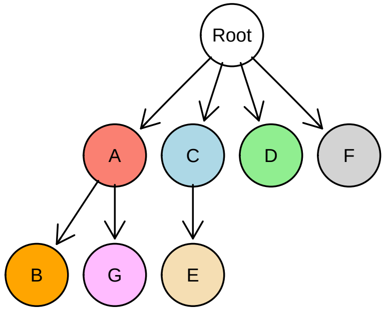

ID	SeULzsEELispb2JY
acc genotypes	60
peaks no back	1
removed edges	0
prop rem edges	0

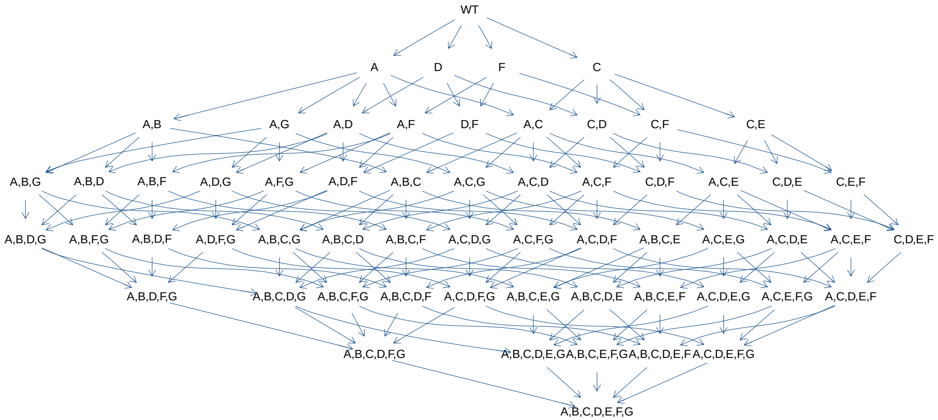

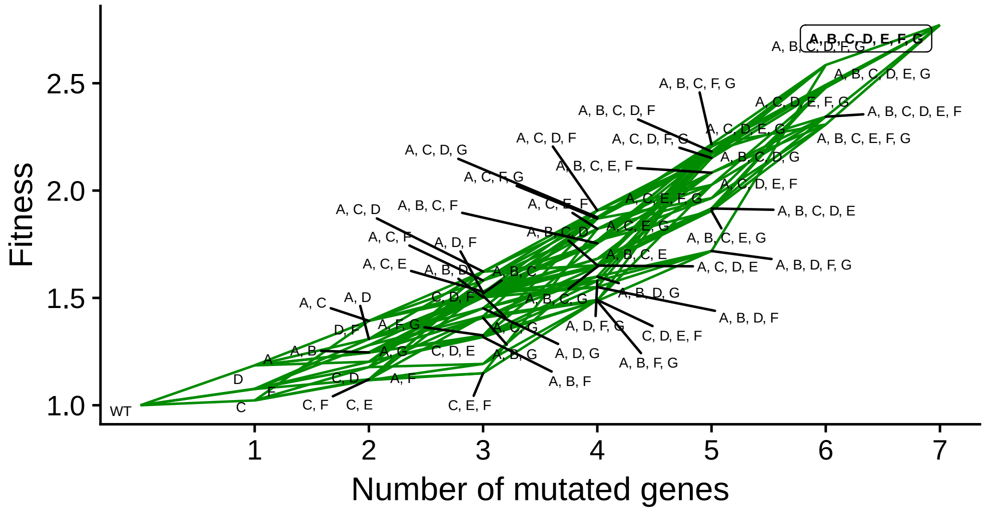

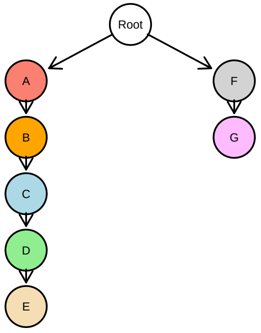

ID	sJxeTmNhXKbZaZnQ
acc genotypes	18
peaks no back	1
removed edges	0
prop rem edges	0

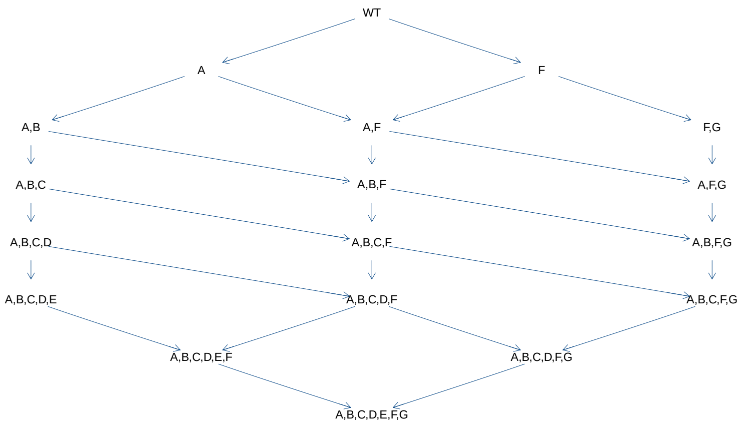

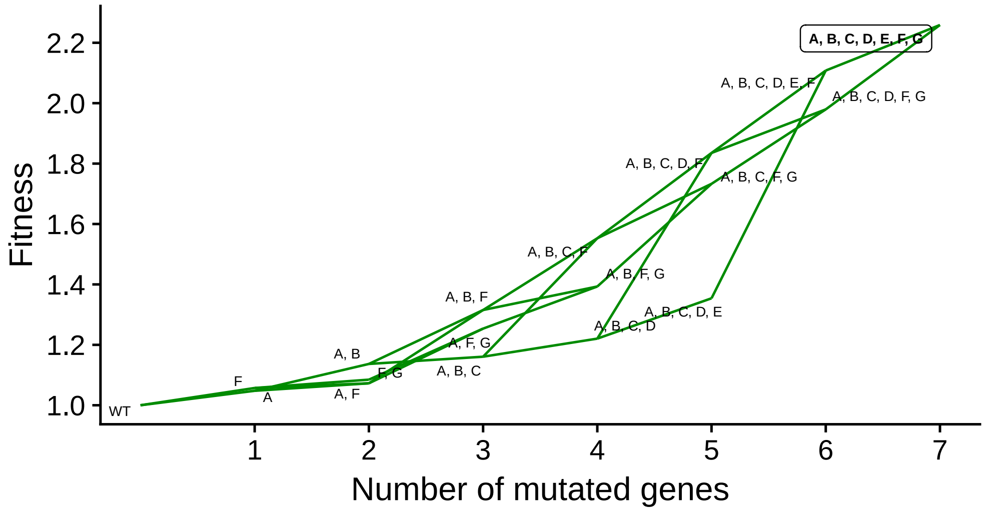

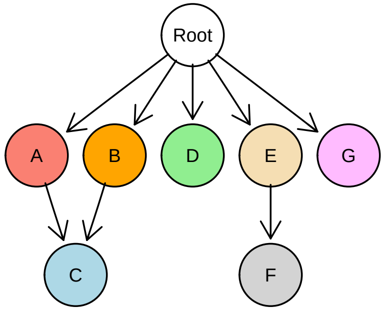

ID	SUTWyy1ERdbEqE8j
acc genotypes	60
peaks no back	1
removed edges	0
prop rem edges	0

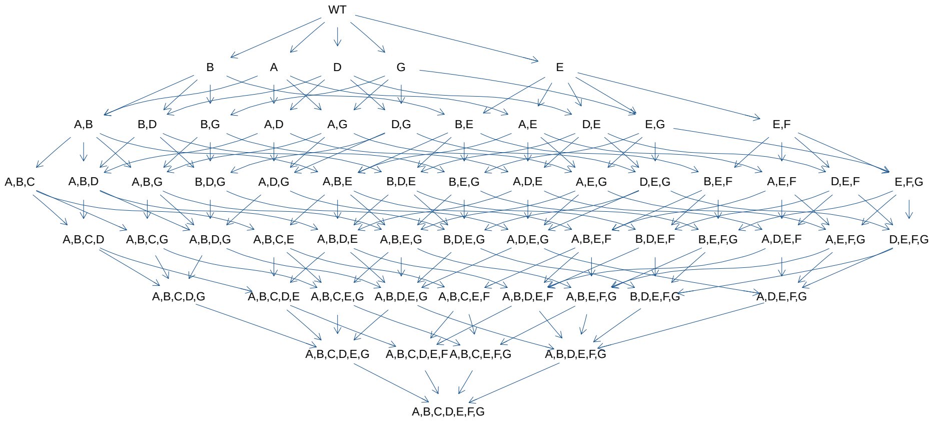

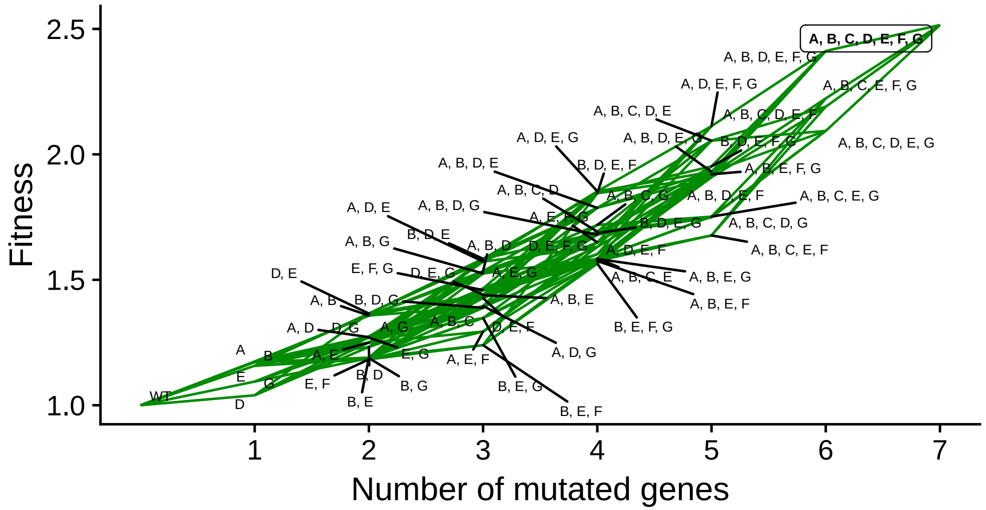

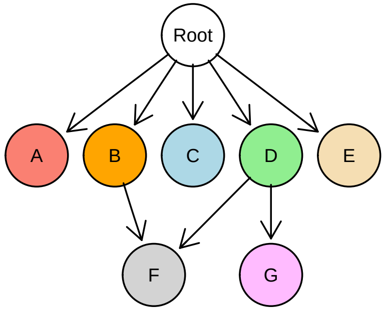

ID	TEOeR5kKW73StsGe
acc genotypes	64
peaks no back	1
removed edges	0
prop rem edges	0

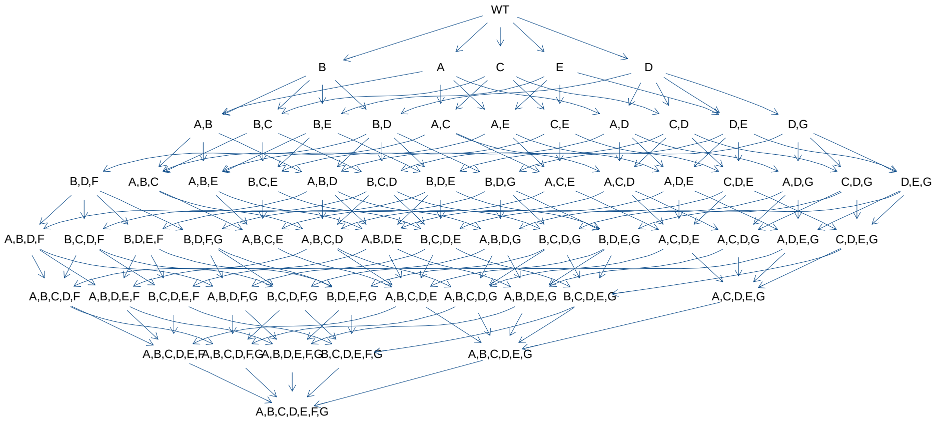

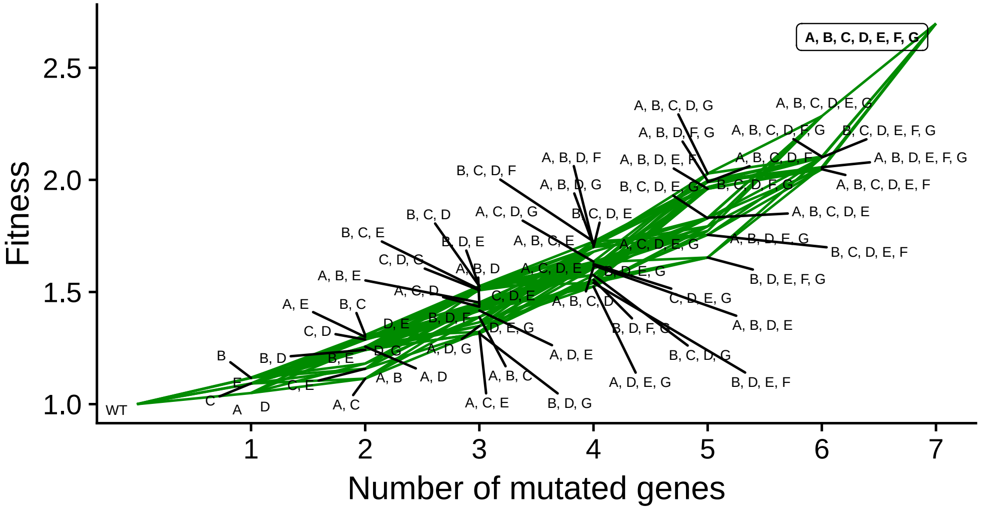

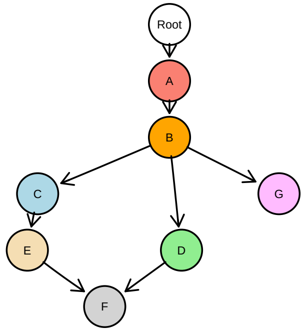

ID	TqrLOOQde8L7lZjK
acc genotypes	16
peaks no back	1
removed edges	0
prop rem edges	0

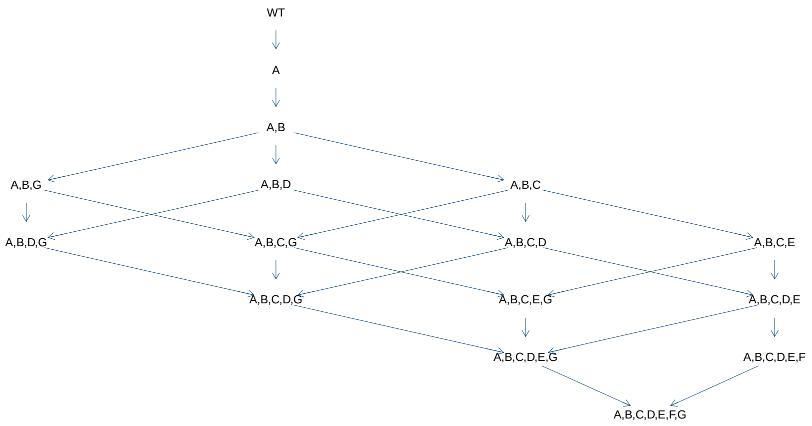

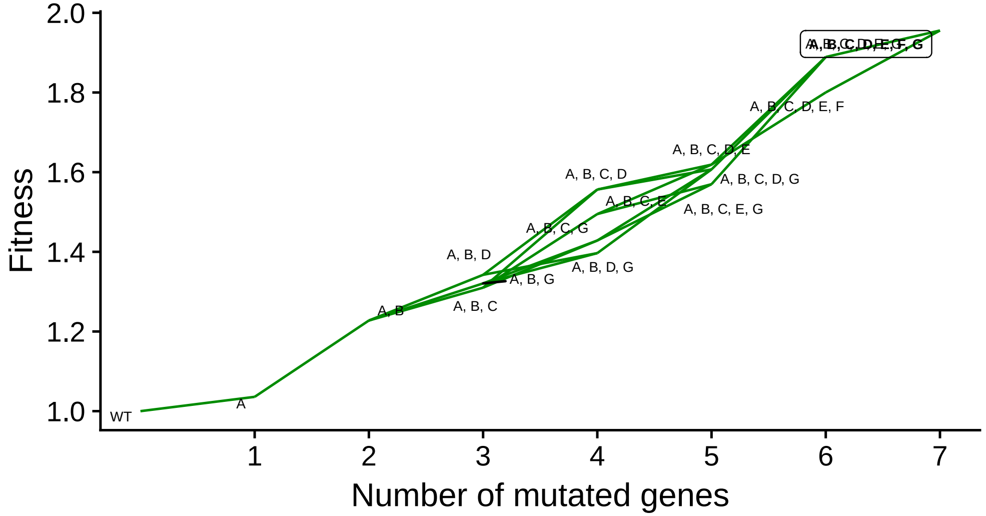

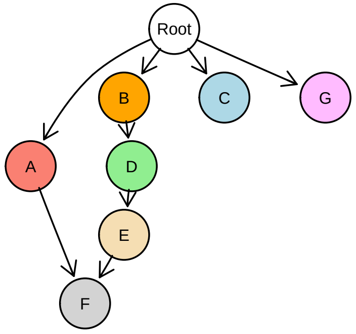

ID	tRehYFGQClcbWhJZ
acc genotypes	36
peaks no back	1
removed edges	0
prop rem edges	0

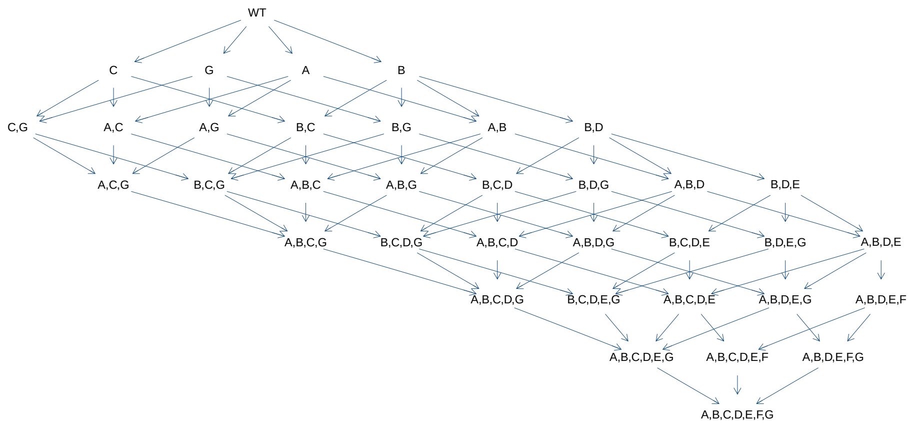

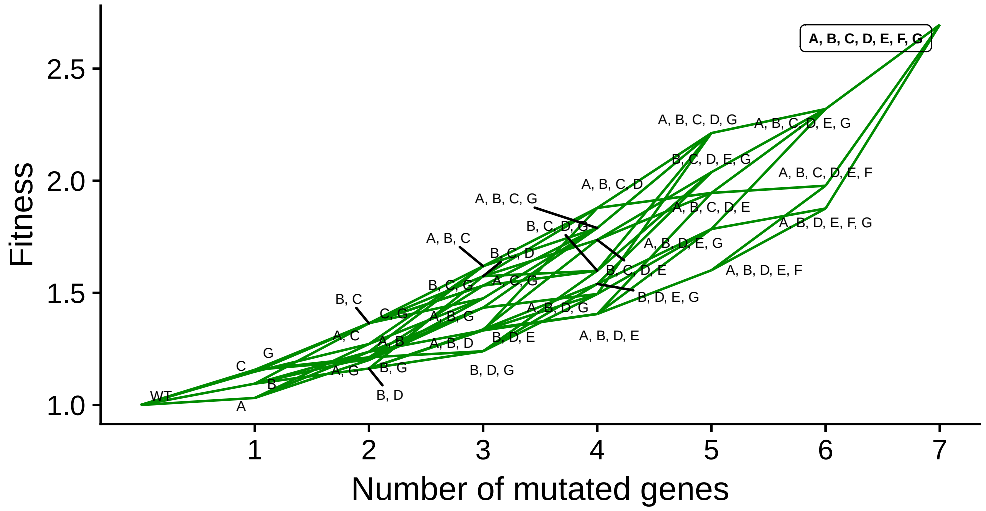

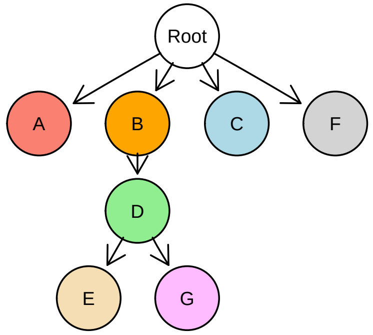

ID	TX10VAp1TpO185dpx
acc genotypes	48
peaks no back	1
removed edges	0
prop rem edges	0

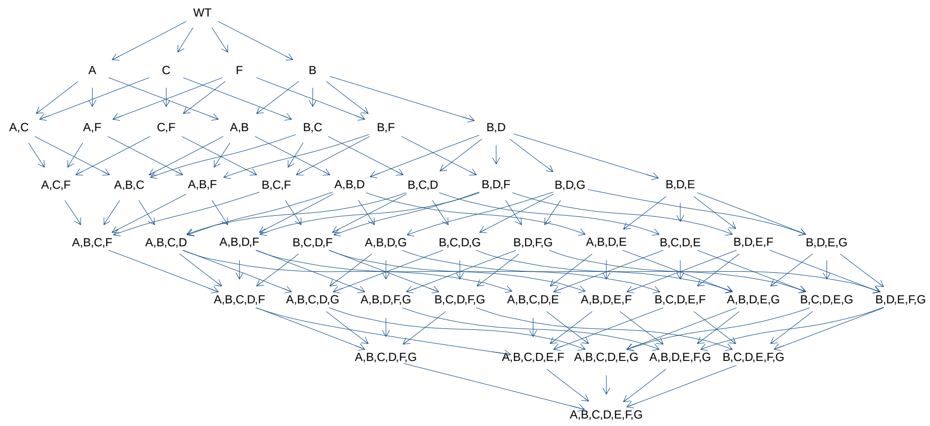

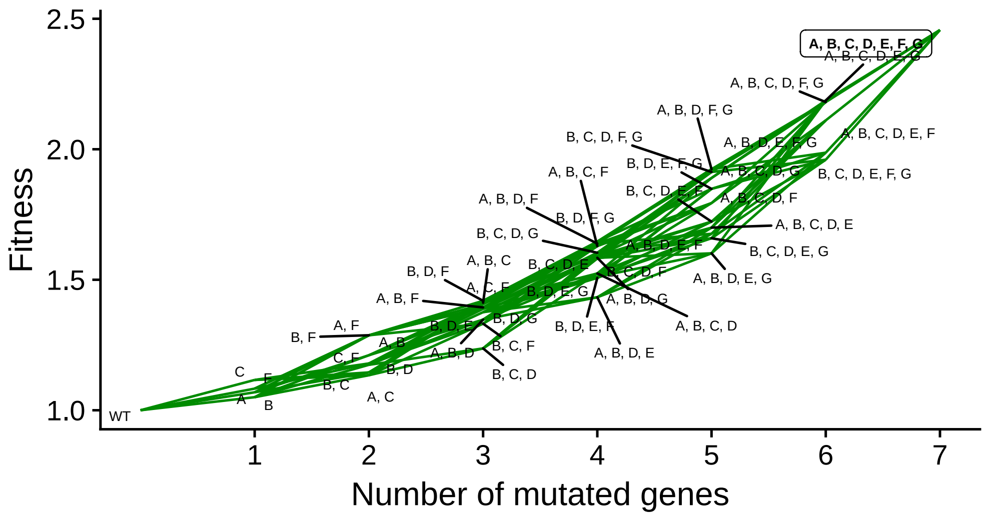

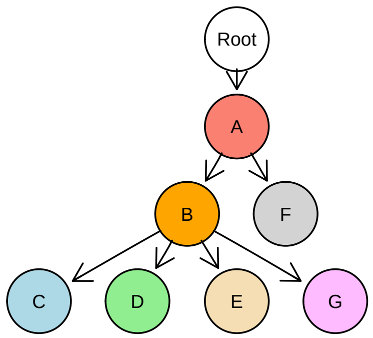

ID	tZE7JJadDSWCdZhf
acc genotypes	35
peaks no back	1
removed edges	0
prop rem edges	0

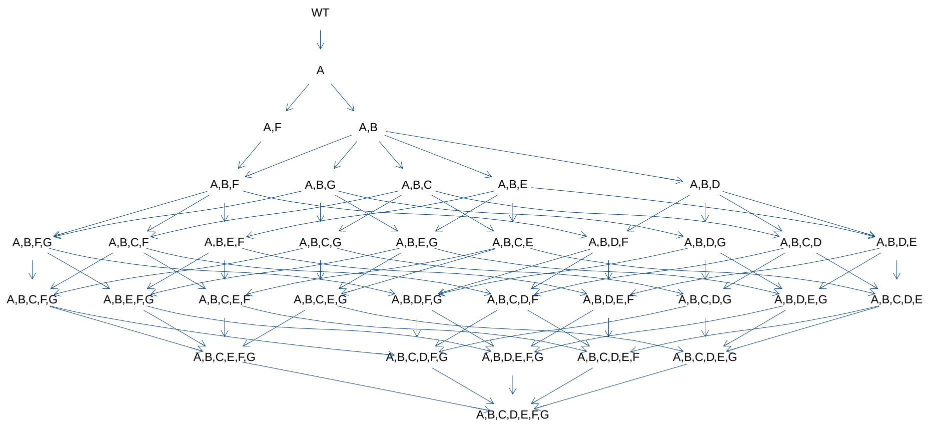

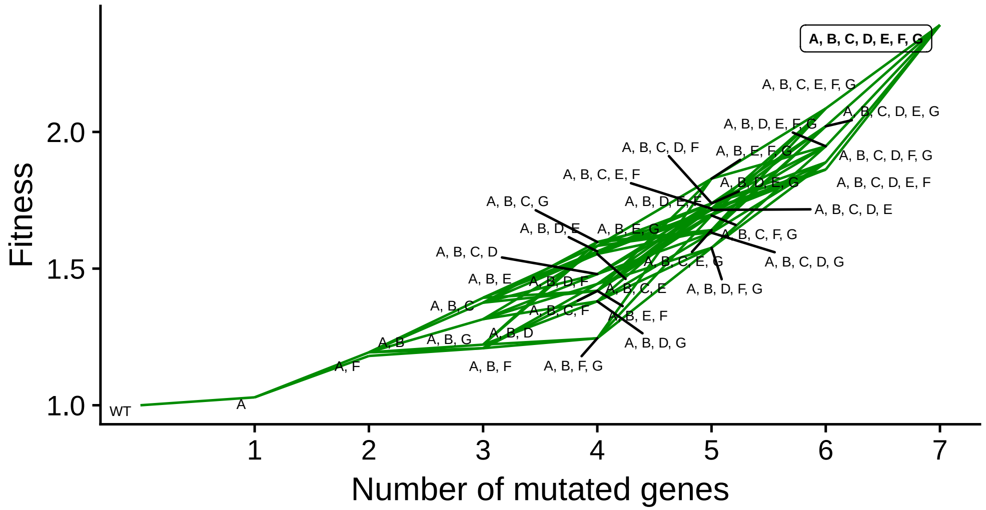

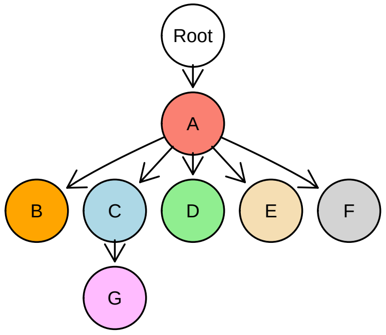

ID TZvpRpDnbfQvlt4G
acc genotypes 49
peaks no back 1
removed edges 0
prop rem edges 0

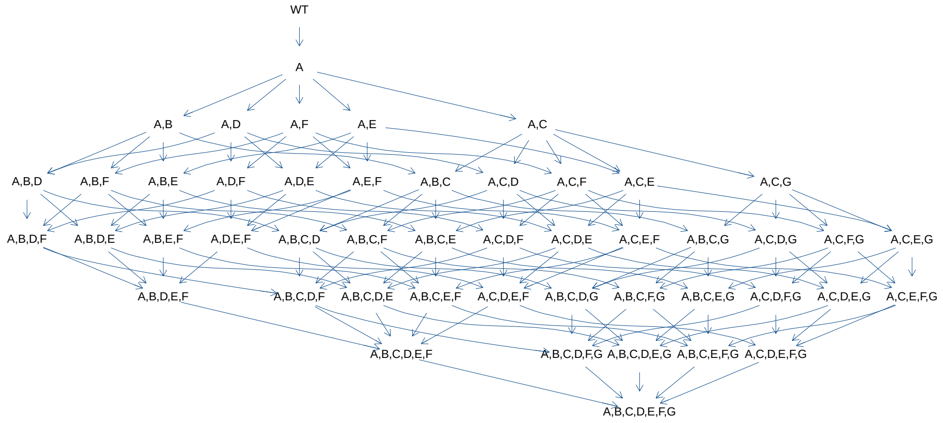

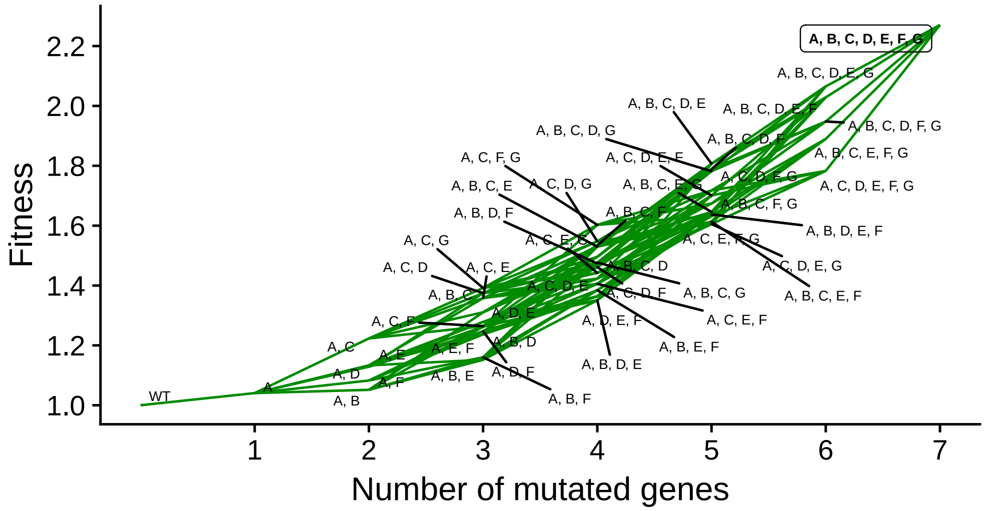

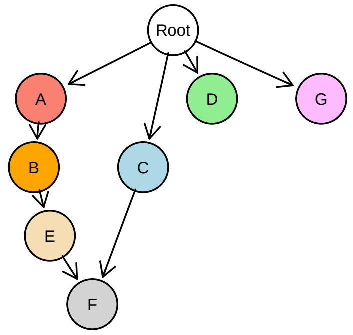

ID U9FVyqSn82tFhAtl
acc genotypes 36
peaks no back 1
removed edges 0
prop rem edges 0

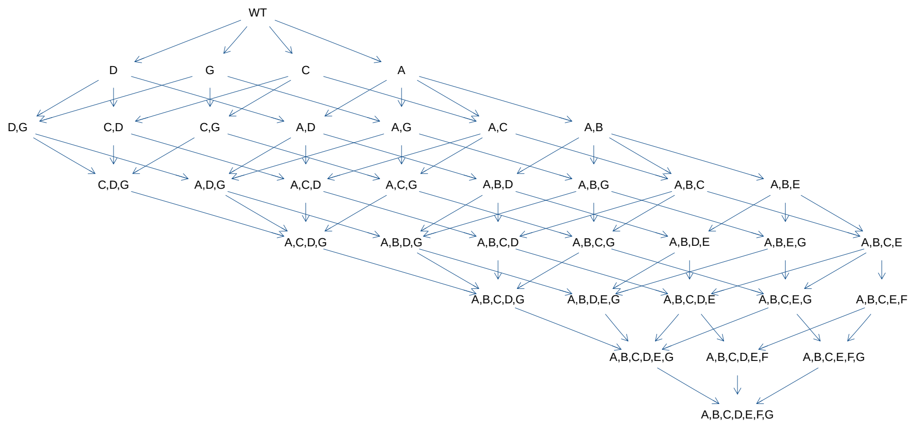

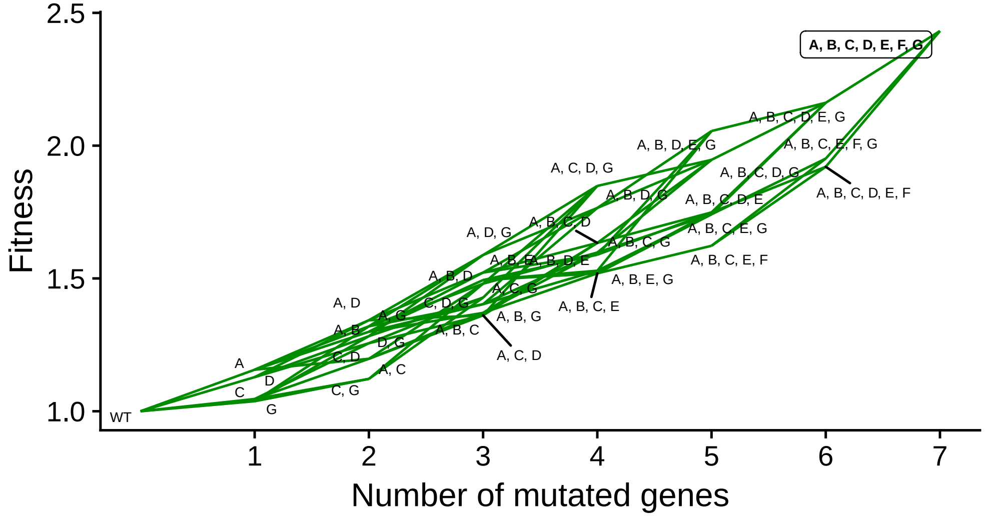

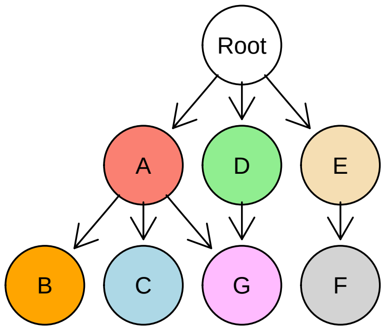

ID	U9tqKXHuH1LkO5mF
acc genotypes	42
peaks no back	1
removed edges	0
prop rem edges	0

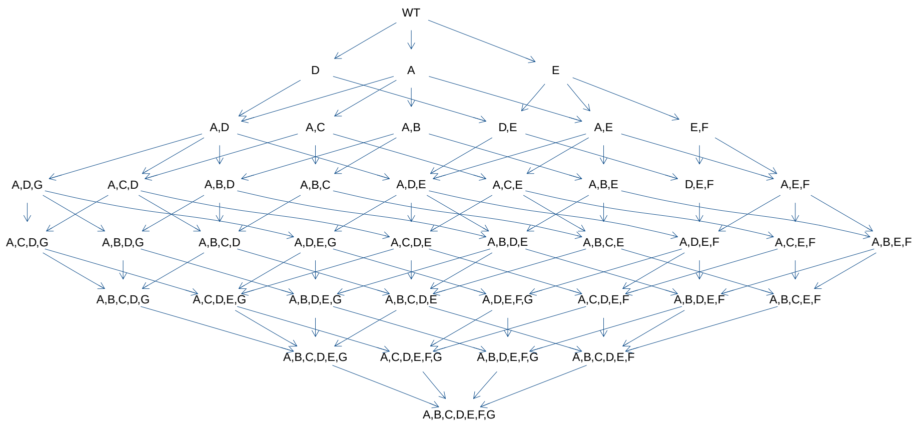

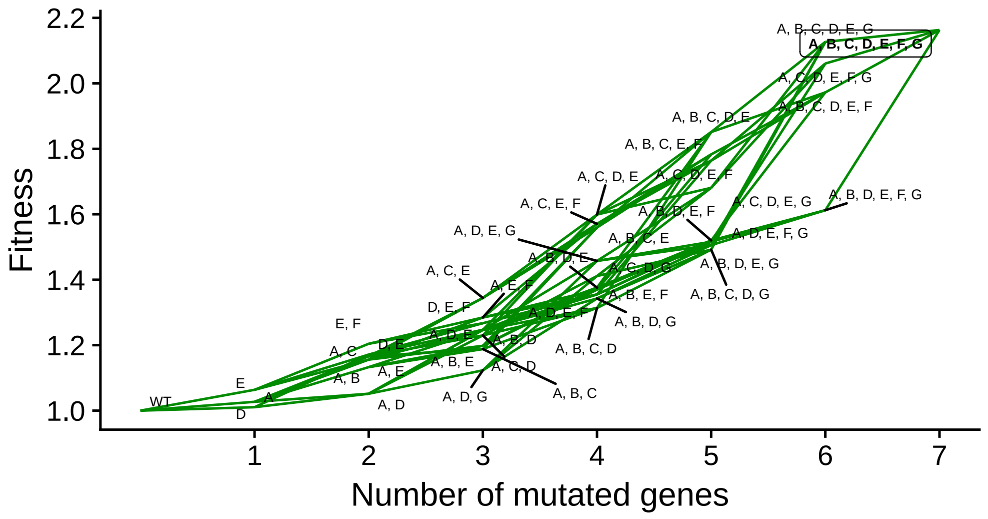

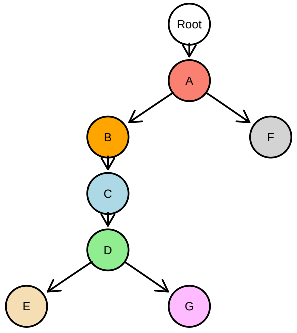

ID	Ua2QSlSPNSRgJaJY
acc genotypes	15
peaks no back	1
removed edges	0
prop rem edges	0

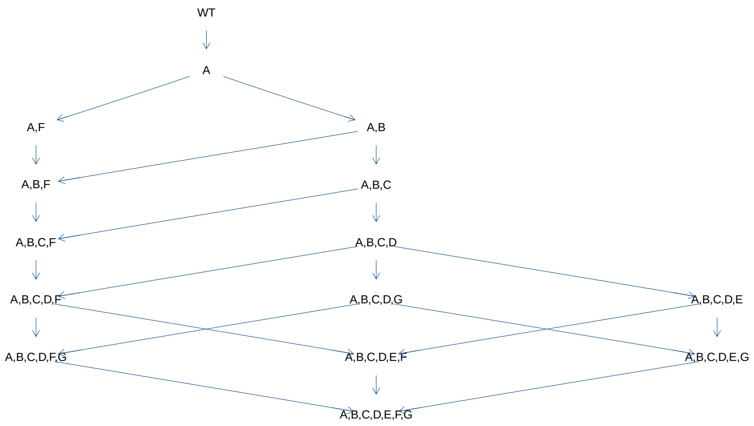

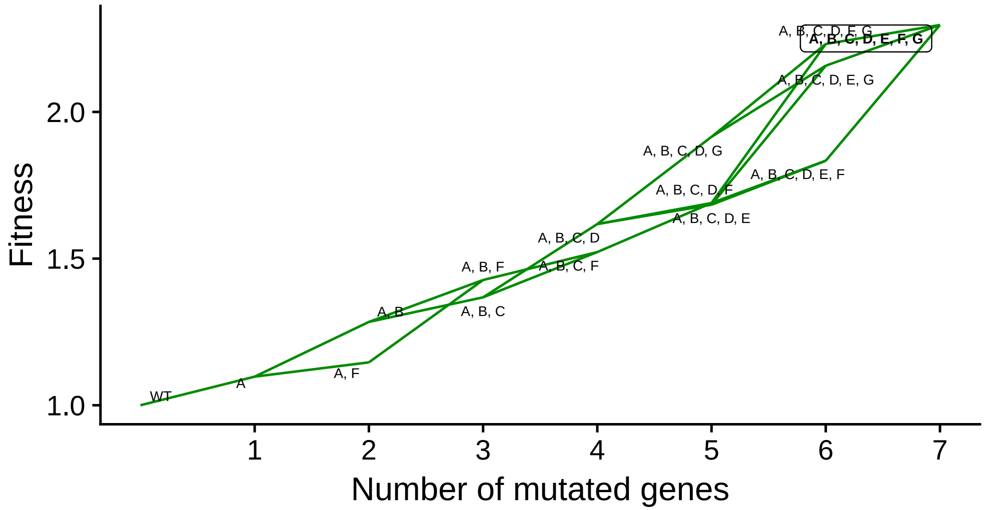

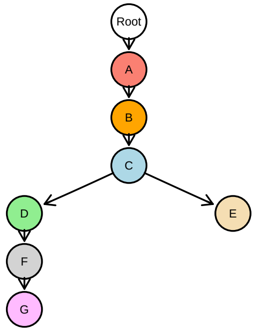

ID	UDkNV5Dxa4w10lh6h
acc genotypes	11
peaks no back	1
removed edges	0
prop rem edges	0

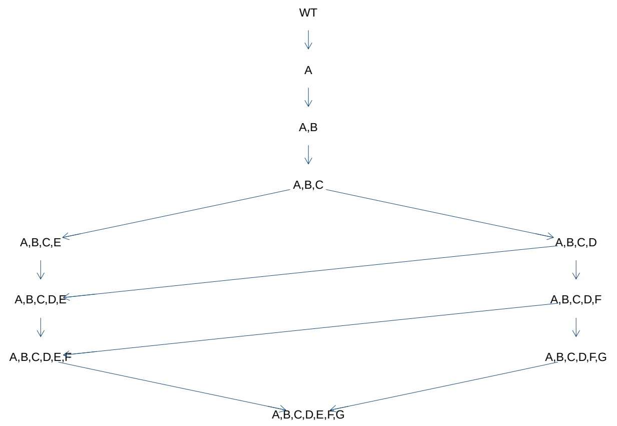

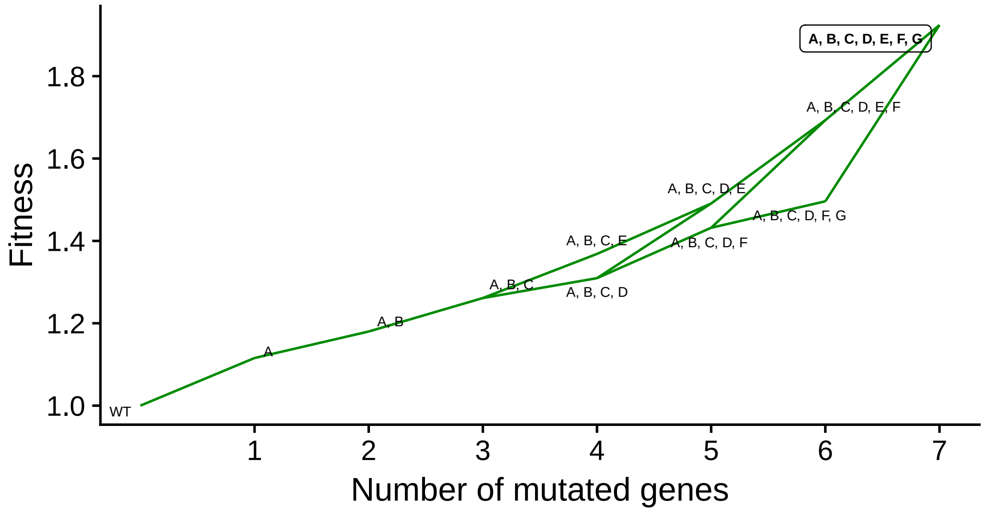

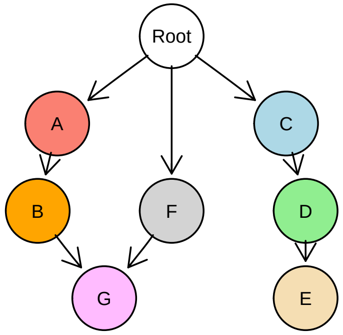

ID uggvrwXSAwaMkYSi
acc genotypes 28
peaks no back 1
removed edges 0
prop rem edges 0

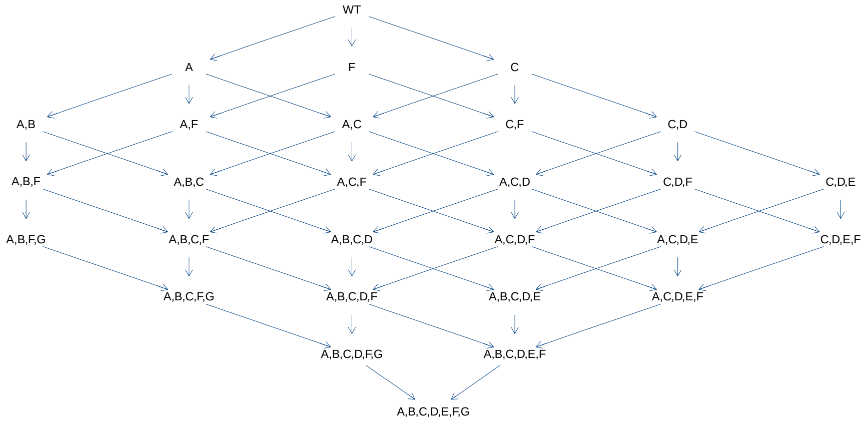

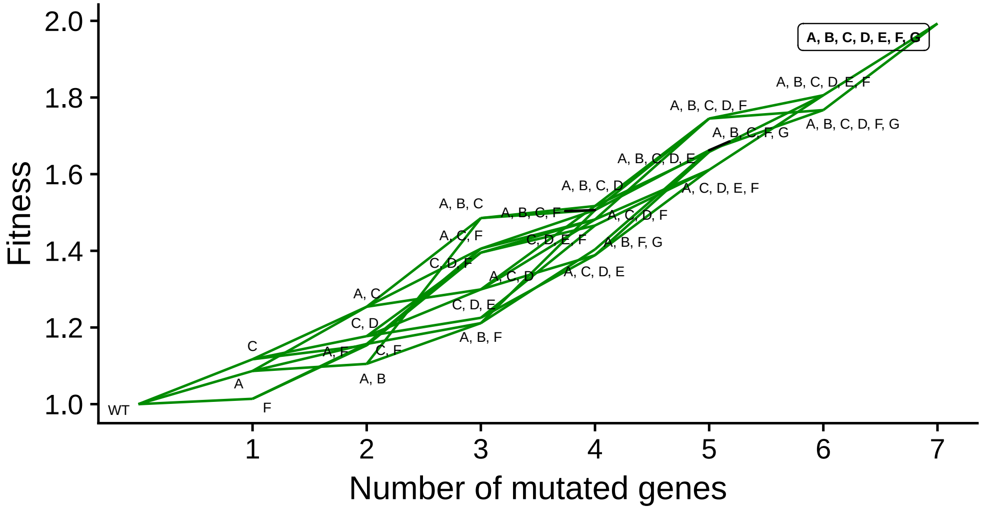

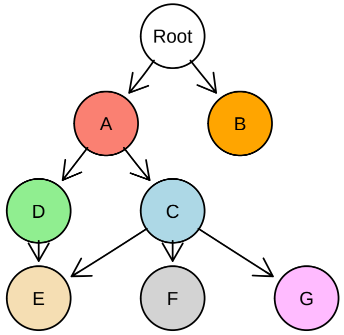

ID	UqGRanXLIlQe1z2o
acc genotypes	30
peaks no back	1
removed edges	0
prop rem edges	0

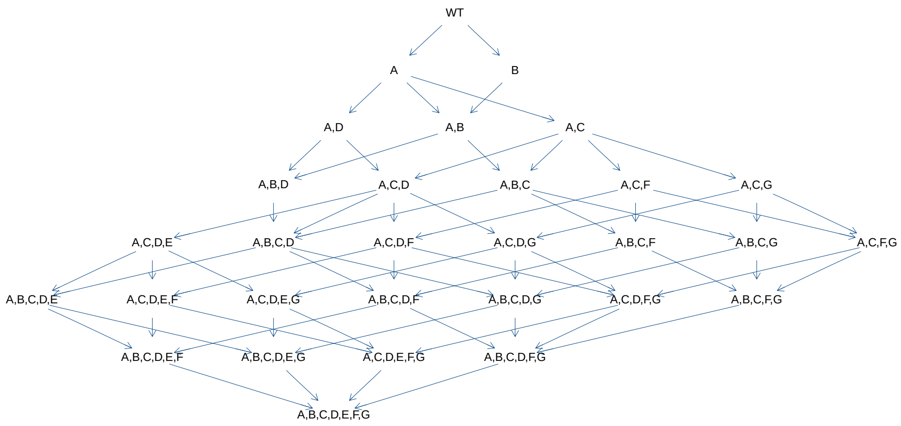

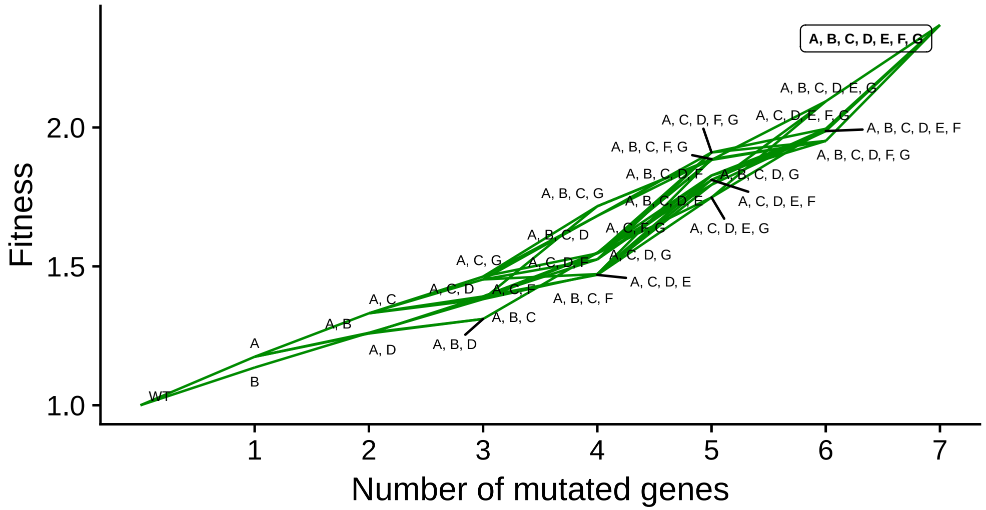

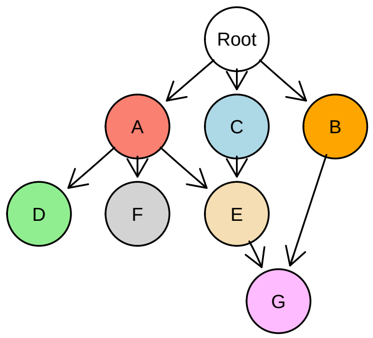

ID	uvOx4rGT8Tr6iHvm
acc genotypes	32
peaks no back	1
removed edges	0
prop rem edges	0

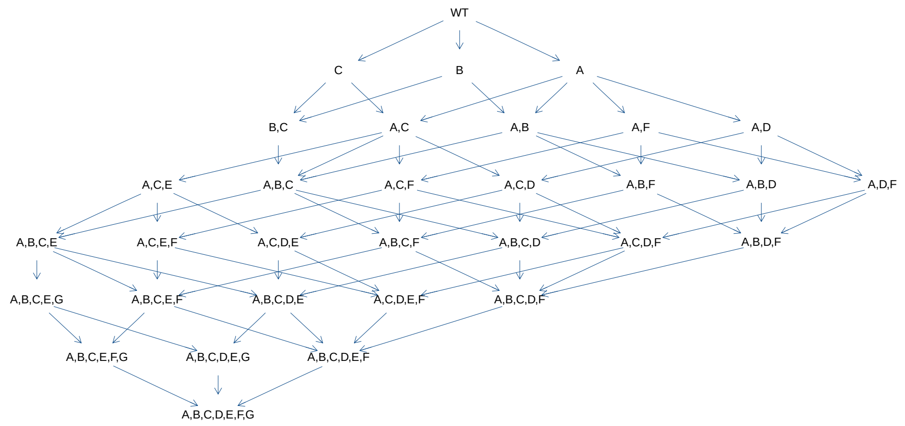

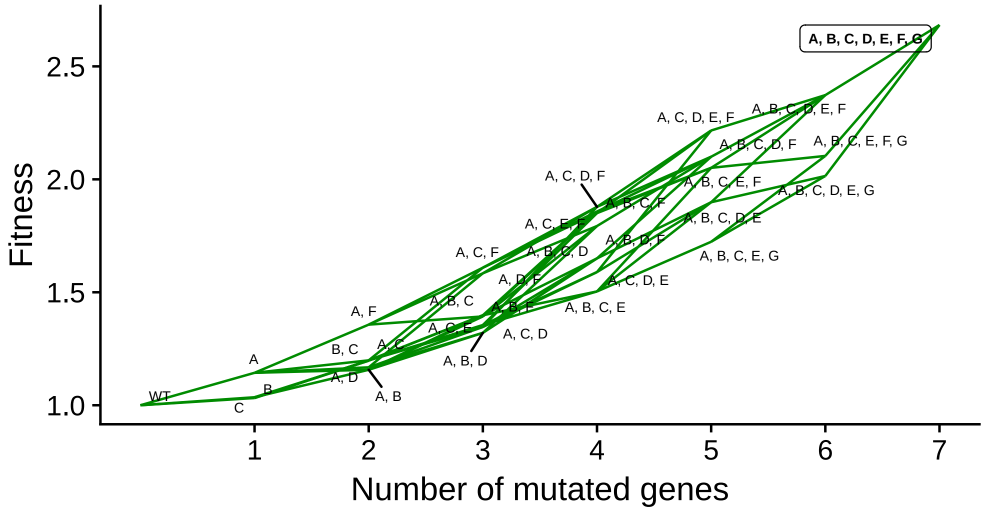

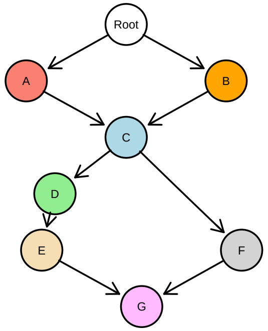

ID	uW1bRozHBgg4IHvp
acc genotypes	11
peaks no back	1
removed edges	0
prop rem edges	0

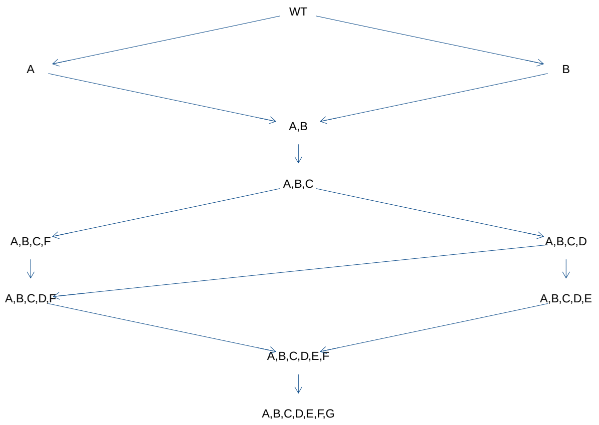

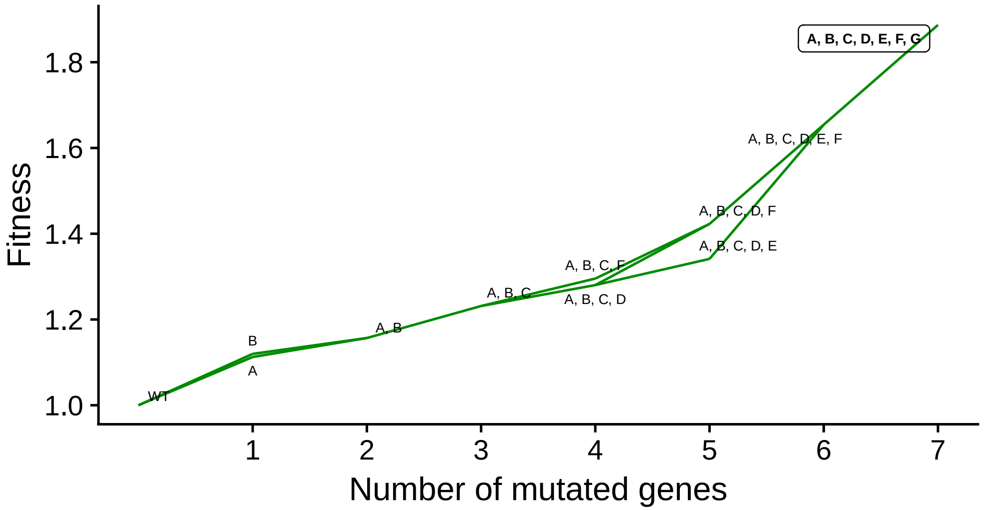

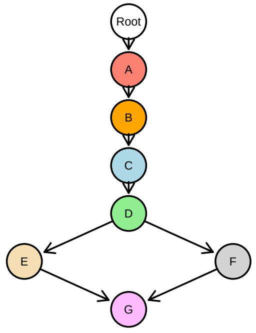

ID	uXxo7pBZAOc10w5w3
acc genotypes	9
peaks no back	1
removed edges	0
prop rem edges	0

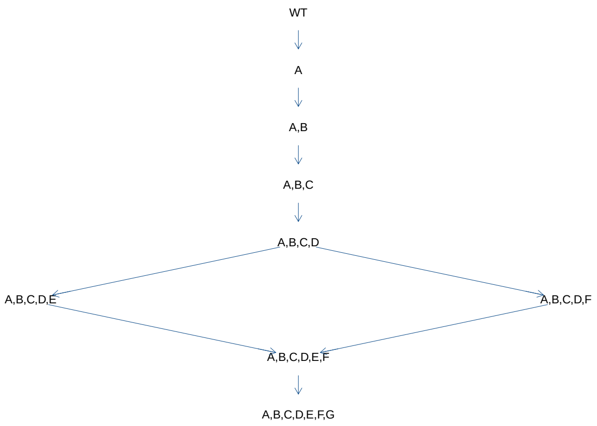

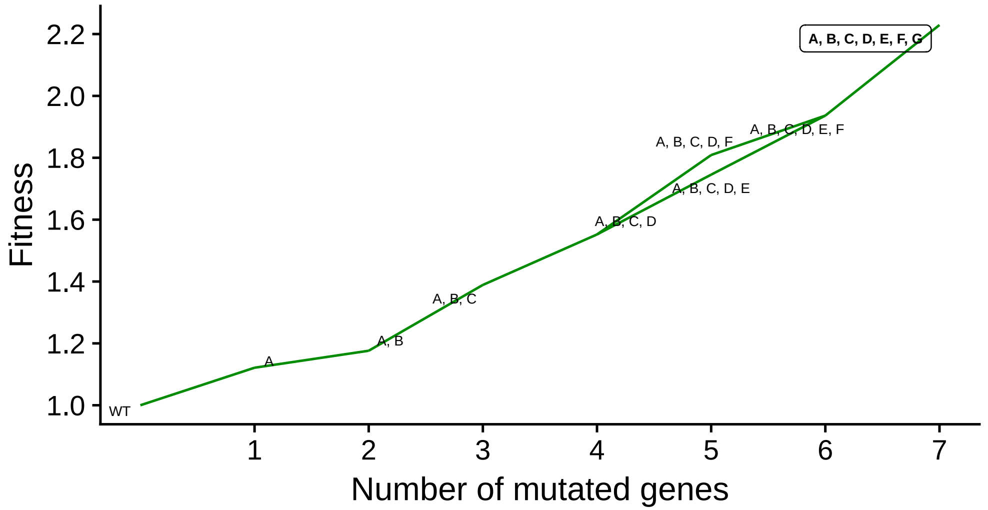

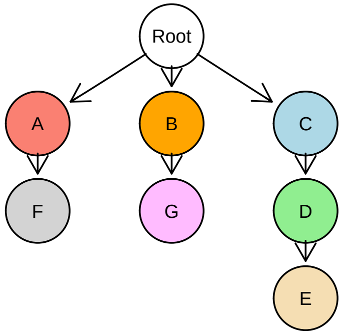

ID	vG10cQRZVOxdasUPn
acc genotypes	36
peaks no back	1
removed edges	0
prop rem edges	0

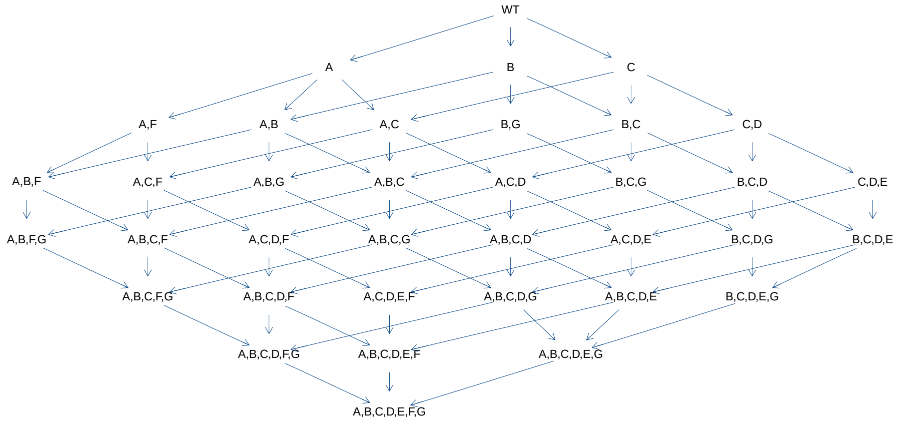

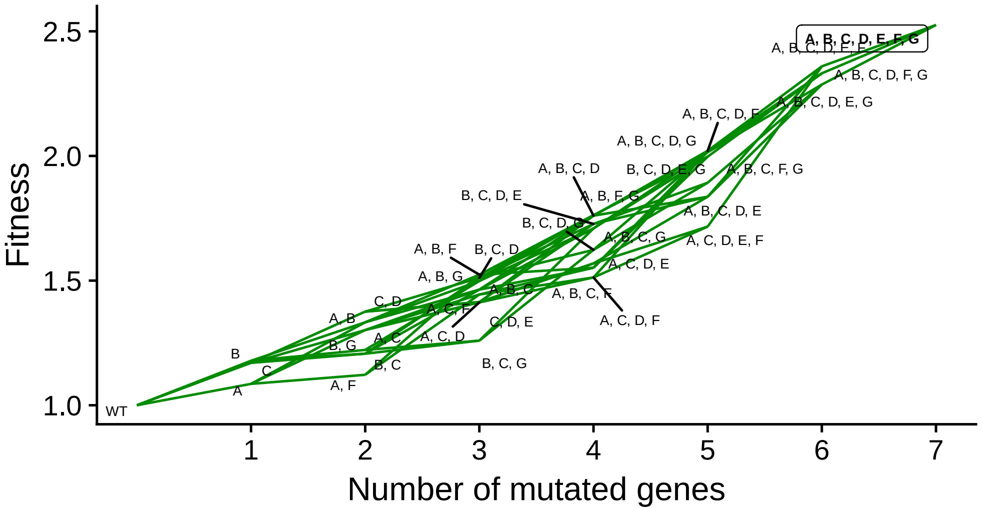

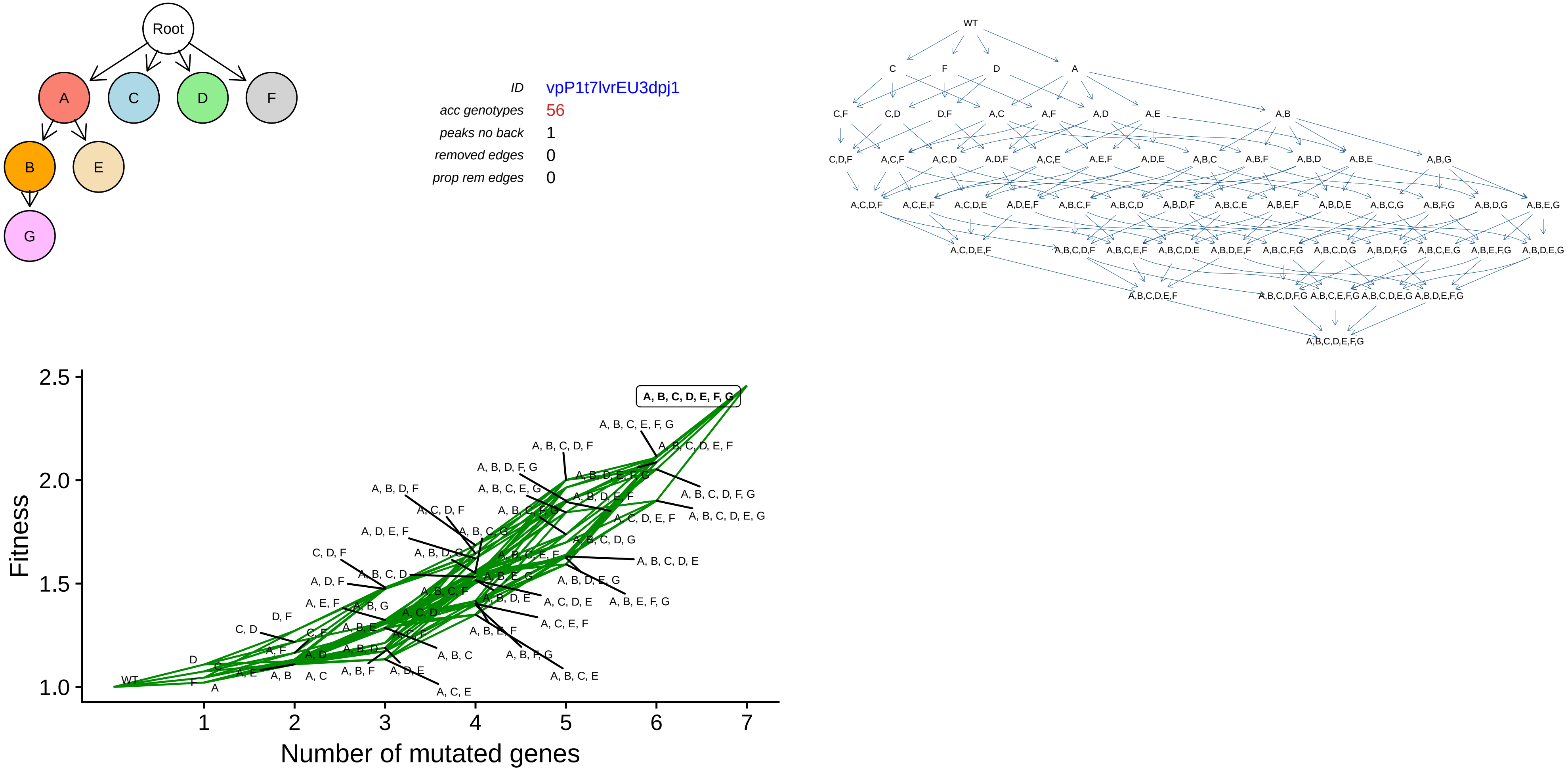

Root
A
C
D
F
B
E
G
ID vpP1t7lvrEU3dpj1
acc genotypes 56
peaks no back 1
removed edges 0
prop rem edges 0
WT
A,B,C,D,E,F,G
Fitness
Number of mutated genes
A, B, C, D, E, F, G

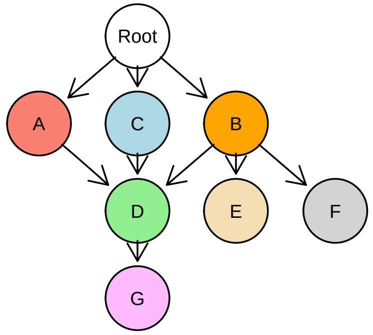

ID	vq10rX5oSabdGhDLE
acc genotypes	28
peaks no back	1
removed edges	0
prop rem edges	0

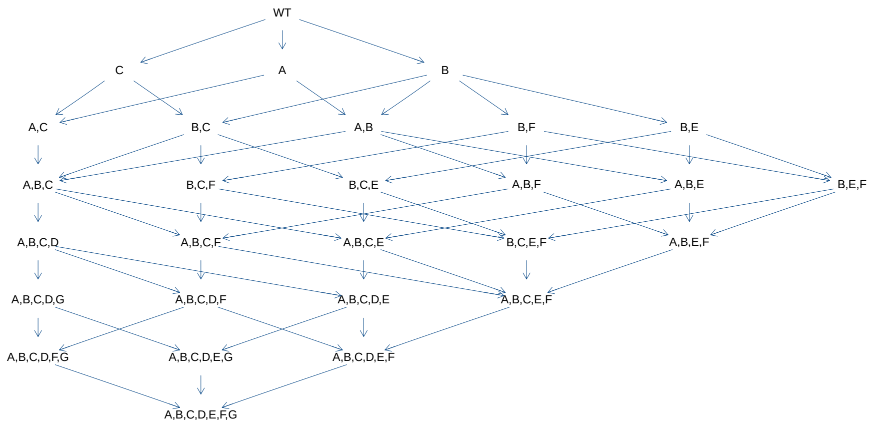

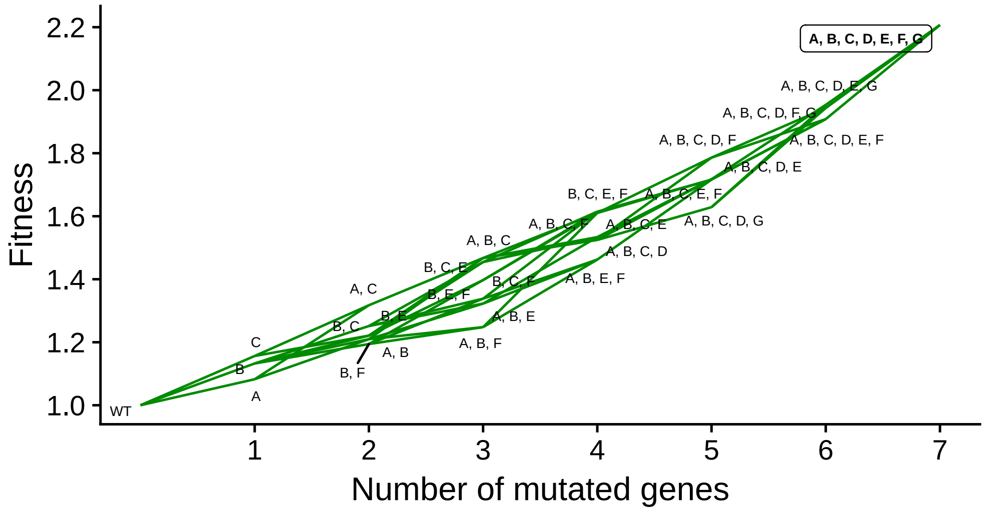

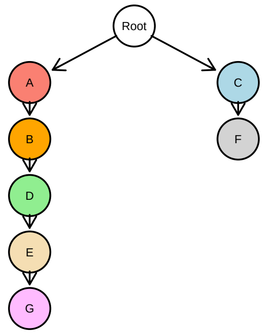

ID	vrlakeKZSaVC1Lzx
acc genotypes	18
peaks no back	1
removed edges	0
prop rem edges	0

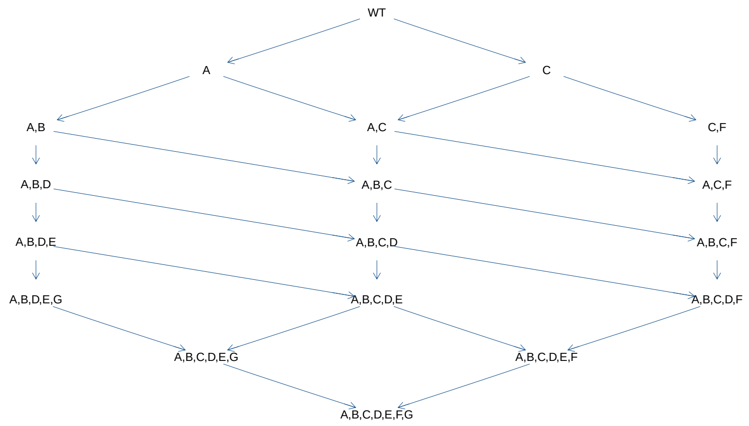

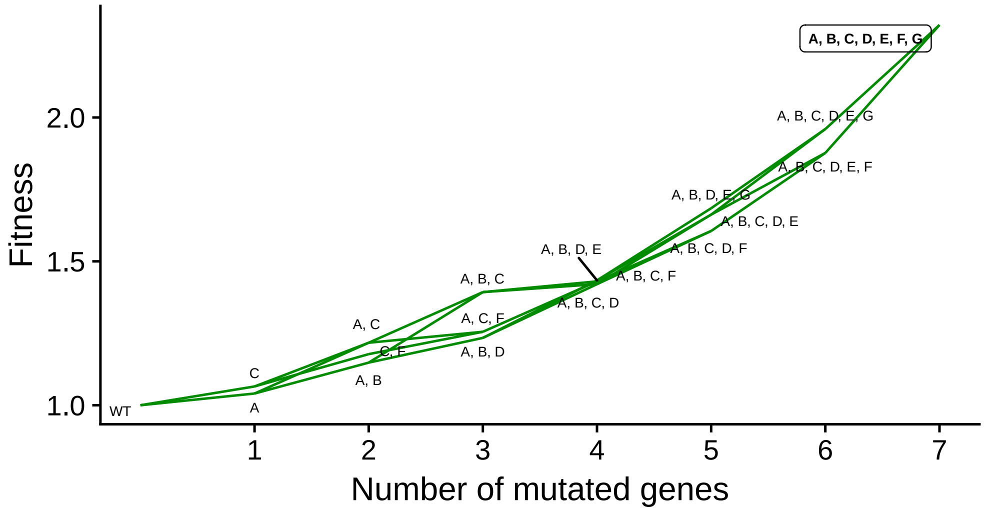

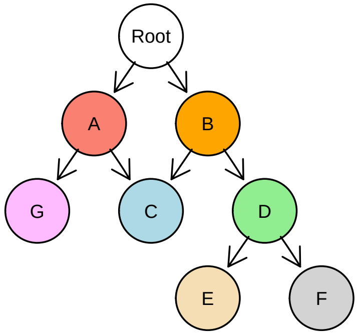

ID	VYbhtDvalyAvnLsQ
acc genotypes	28
peaks no back	1
removed edges	0
prop rem edges	0

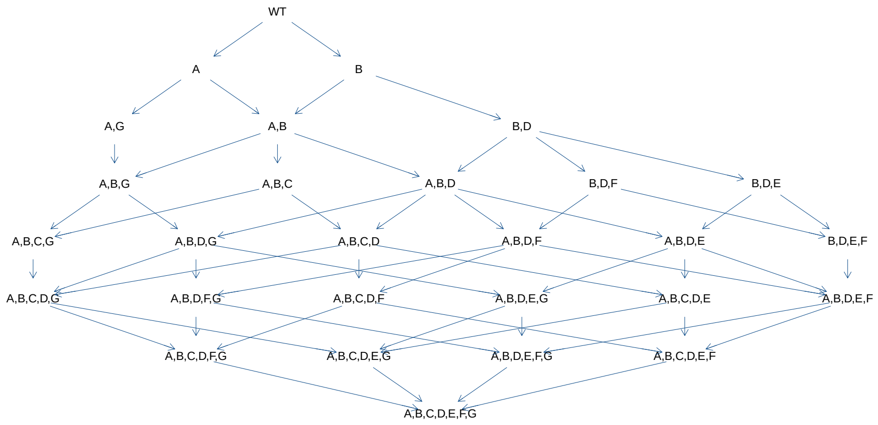

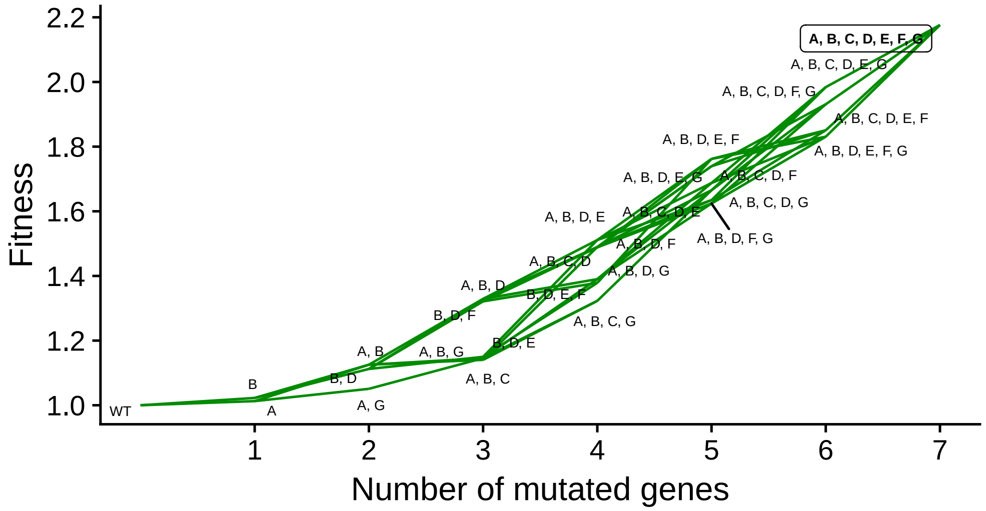

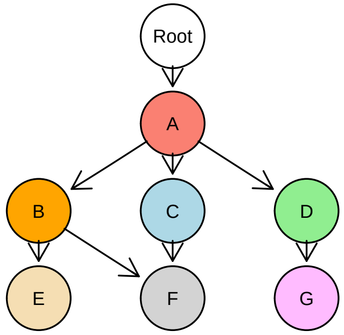

ID	Wc5FDMqc5Bb6oeDH
acc genotypes	25
peaks no back	1
removed edges	0
prop rem edges	0

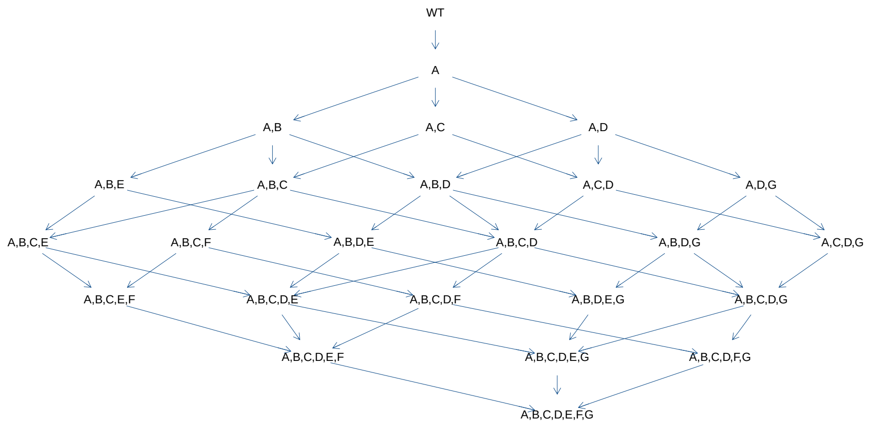

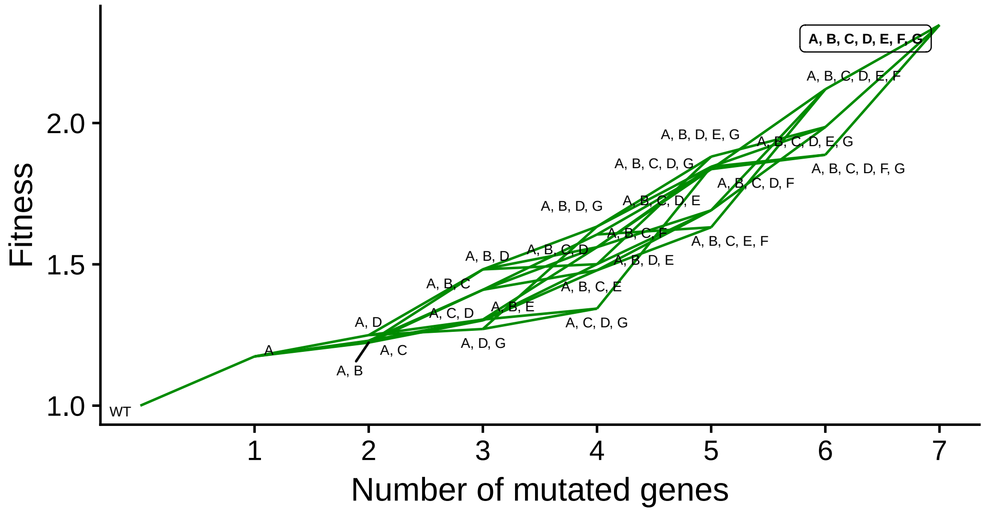

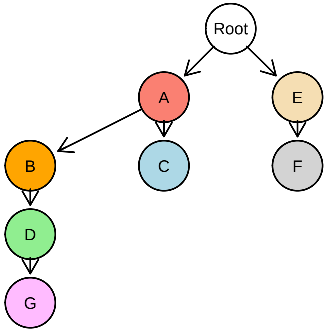

ID	Wh8iukI5tW3rKboV
acc genotypes	27
peaks no back	1
removed edges	0
prop rem edges	0

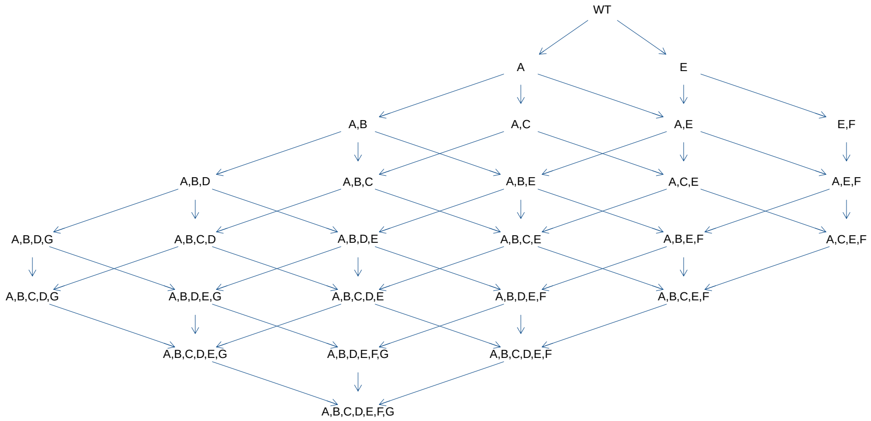

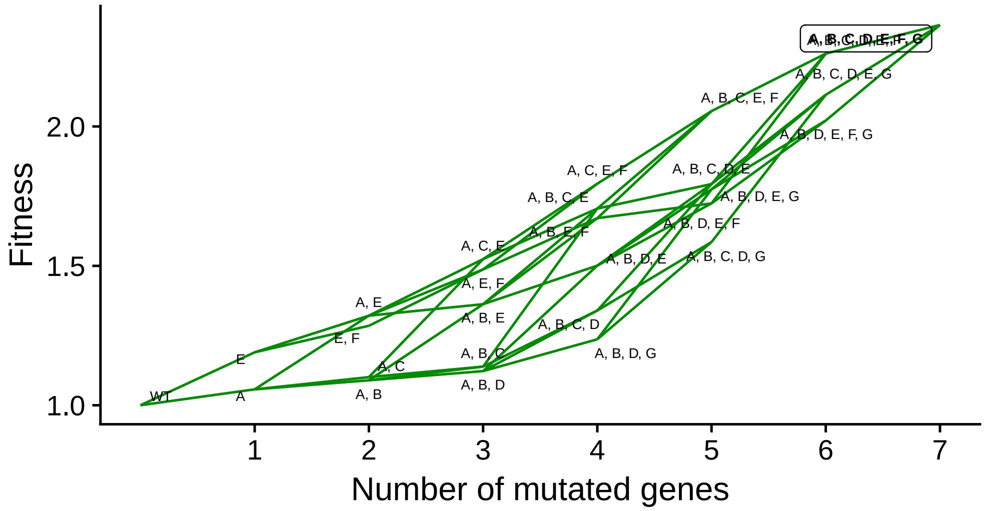

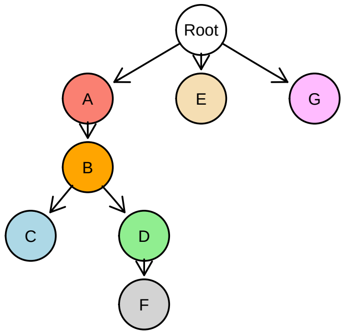

ID	wKElTkPsWorfdIcX
acc genotypes	32
peaks no back	1
removed edges	0
prop rem edges	0

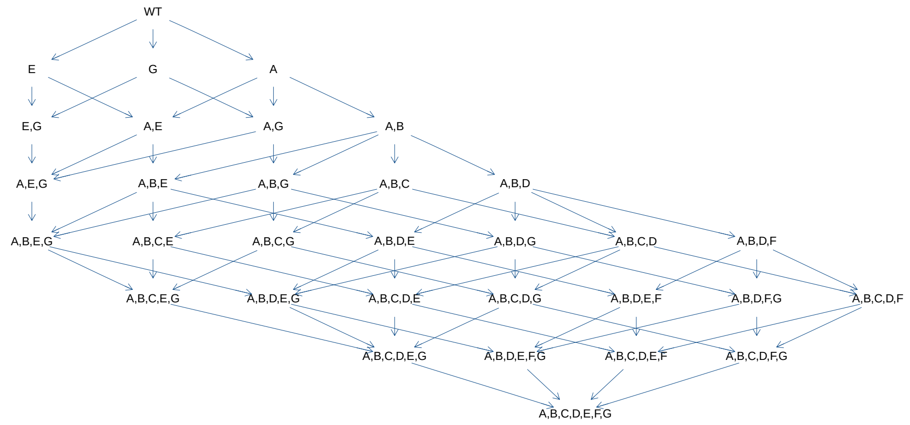

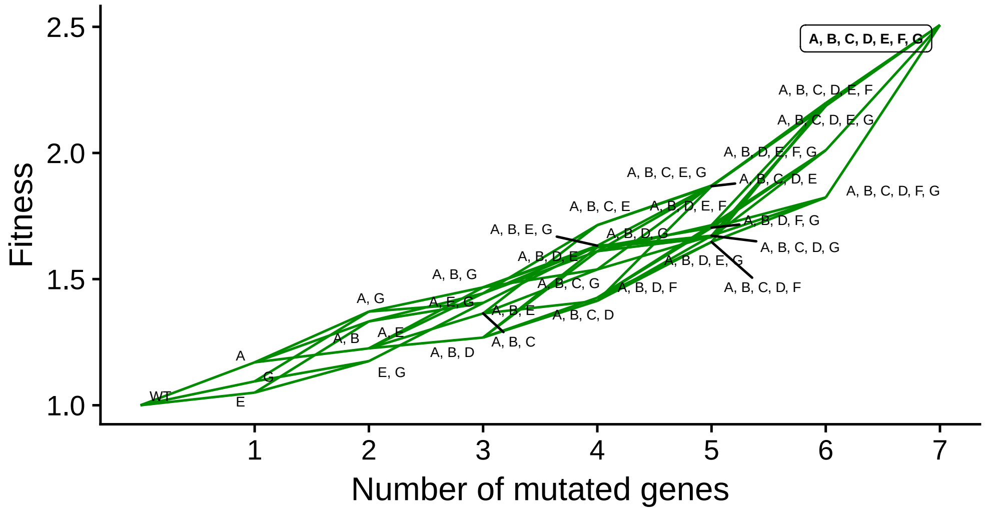

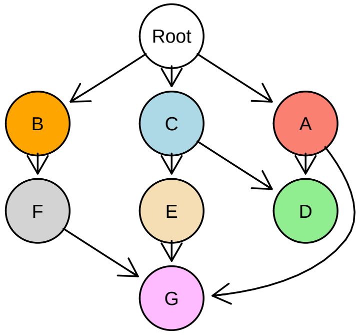

ID	wLlgb17DLATLHcdE
acc genotypes	26
peaks no back	1
removed edges	0
prop rem edges	0

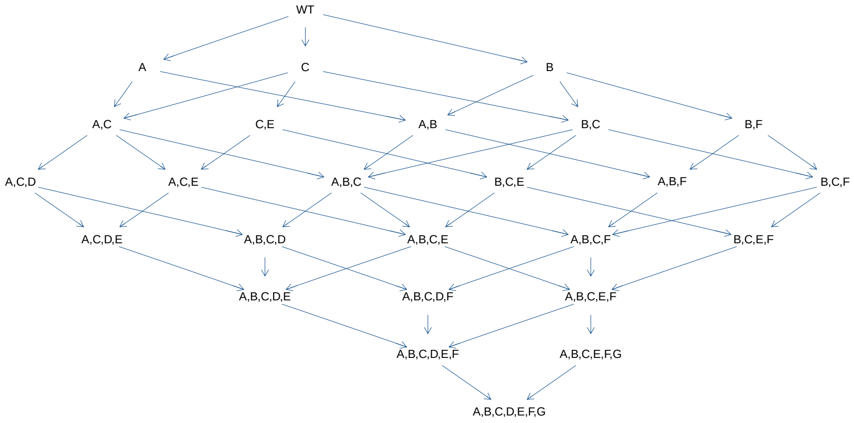

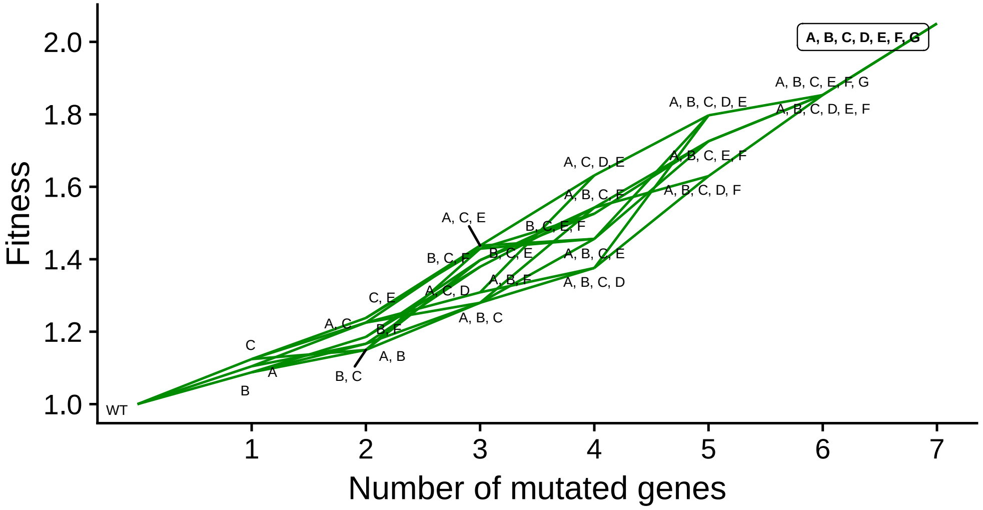

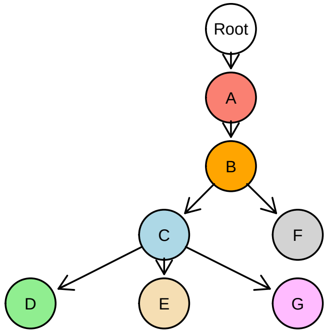

ID	wNAatqlHhqMbIvMb
acc genotypes	20
peaks no back	1
removed edges	0
prop rem edges	0

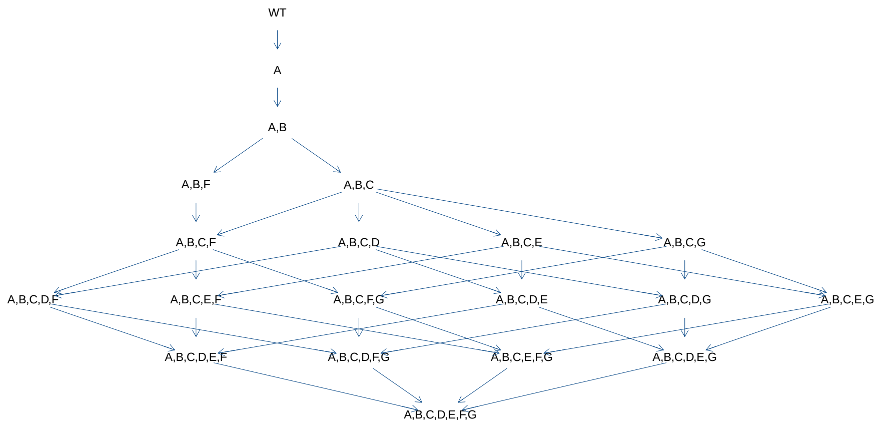

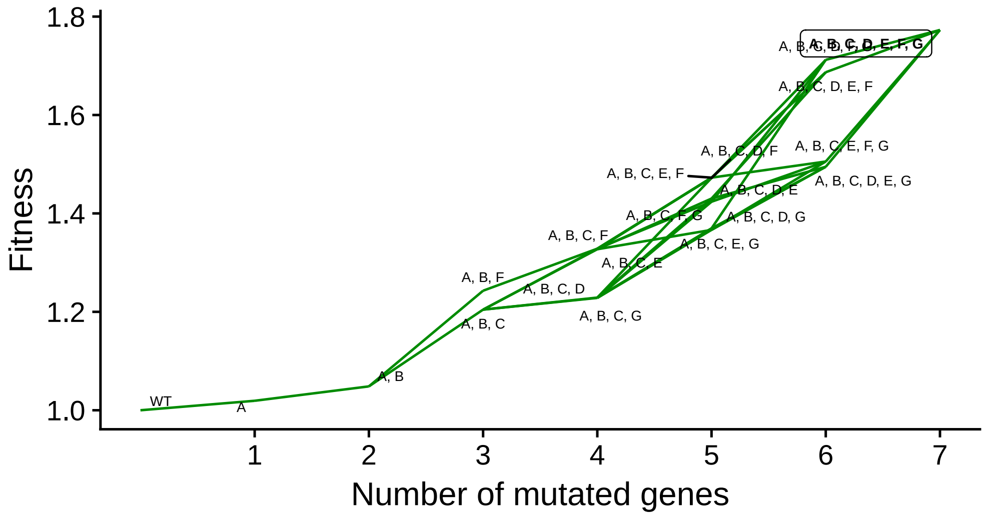

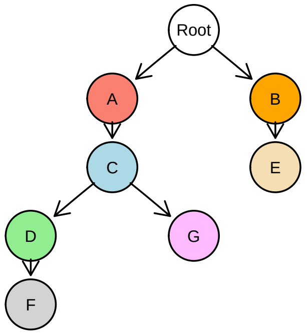

ID Wo7CkQUAYwfIkfX4
acc genotypes 24
peaks no back 1
removed edges 0
prop rem edges 0

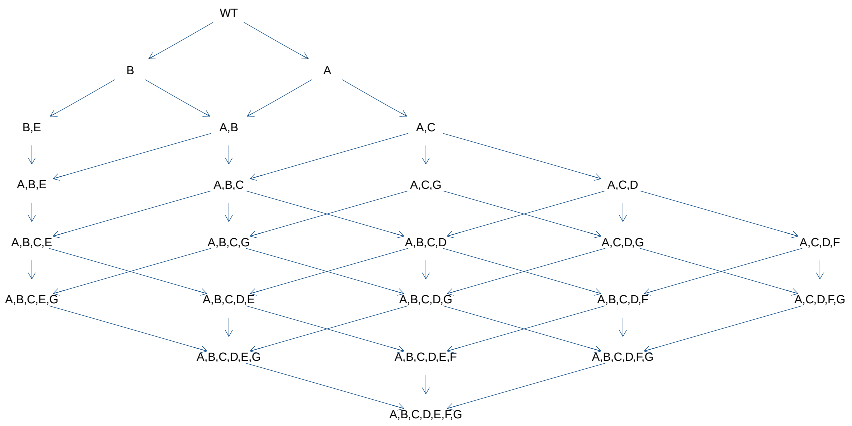

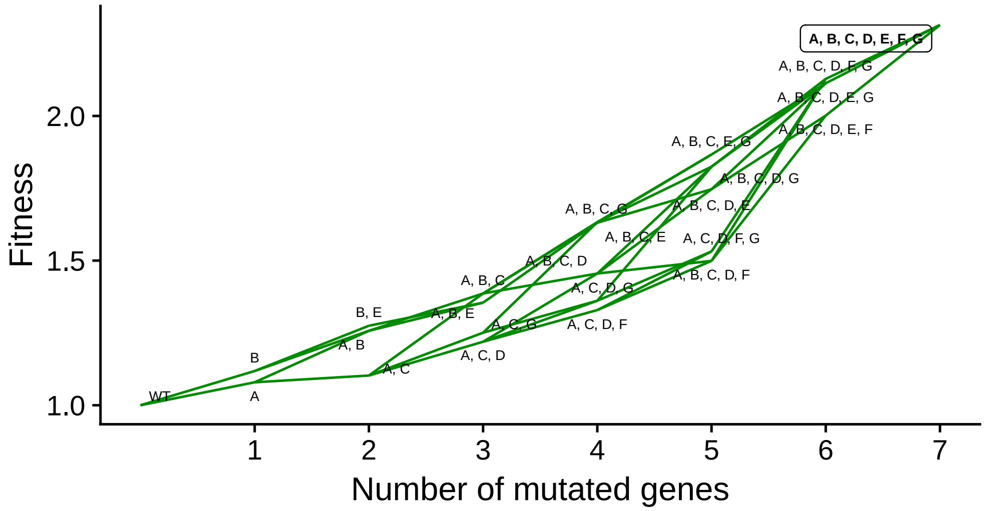

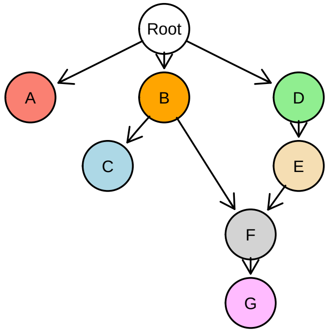

ID	WoqvN8oF47jbnoBK
acc genotypes	26
peaks no back	1
removed edges	0
prop rem edges	0

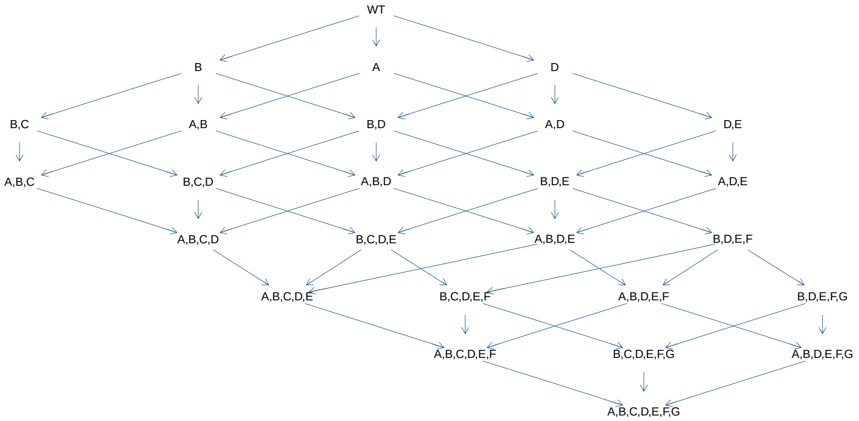

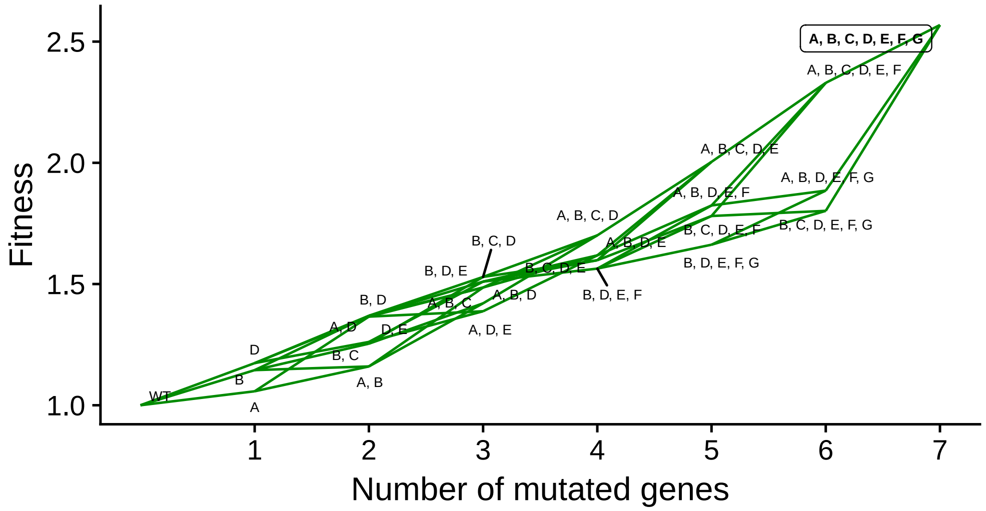

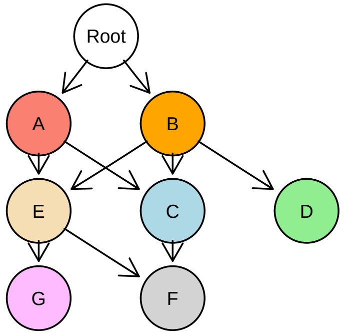

ID	WQ5ixaPdtGNgofTu
acc genotypes	20
peaks no back	1
removed edges	0
prop rem edges	0

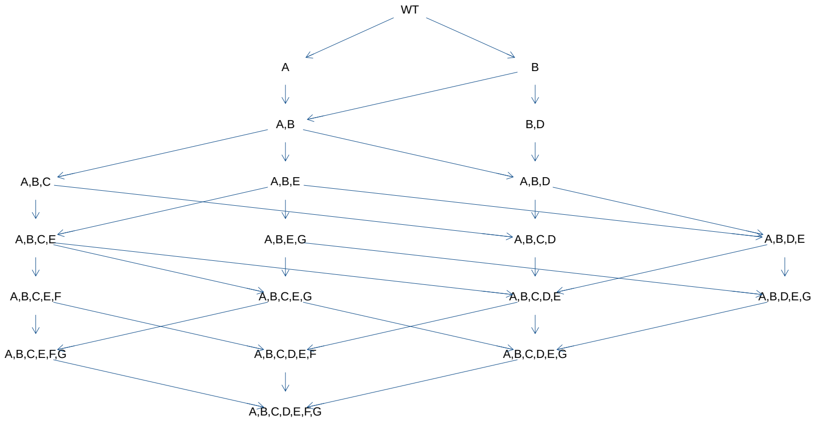

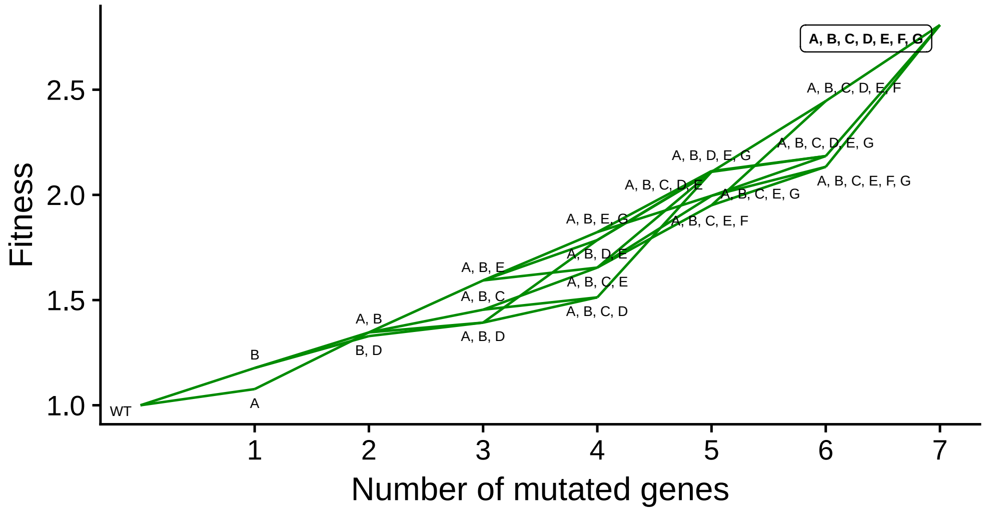

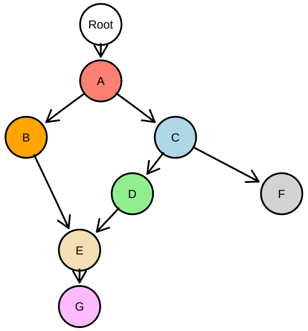

ID	wrcr1dFF7SQNSwts
acc genotypes	15
peaks no back	1
removed edges	0
prop rem edges	0

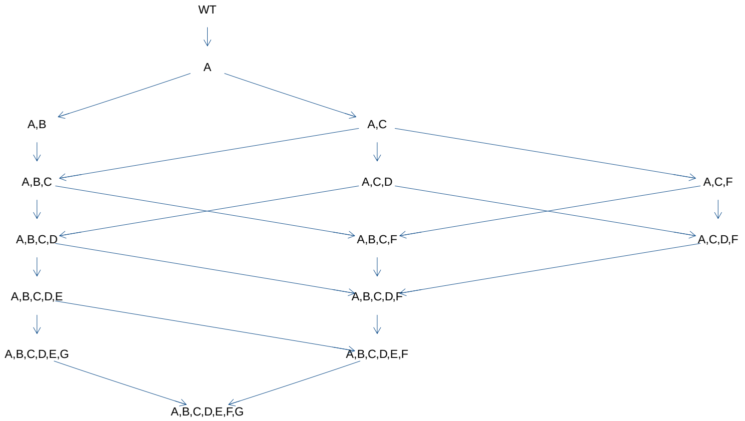

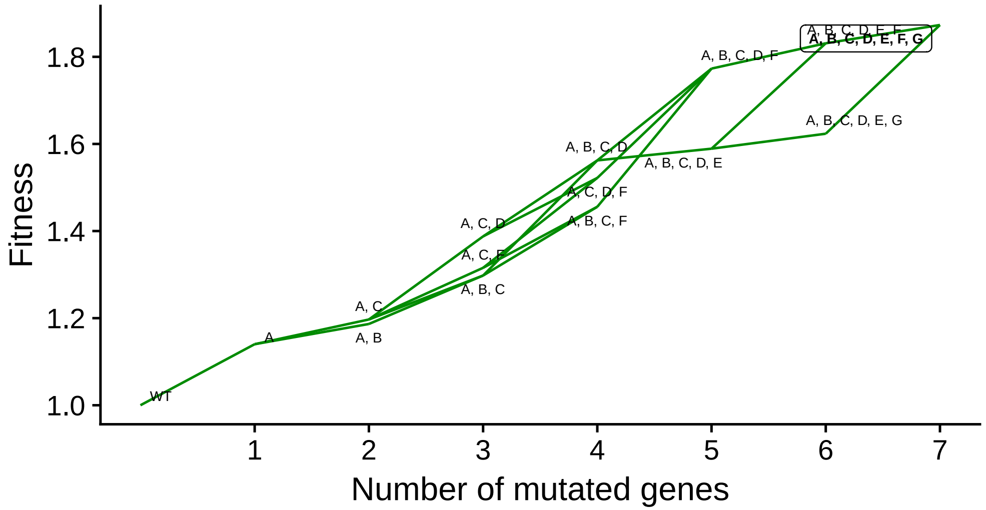

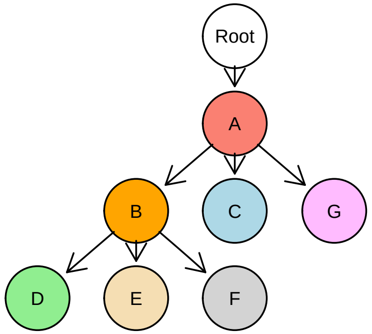

ID	wrRTzMwK5mQOC85K
acc genotypes	37
peaks no back	1
removed edges	0
prop rem edges	0

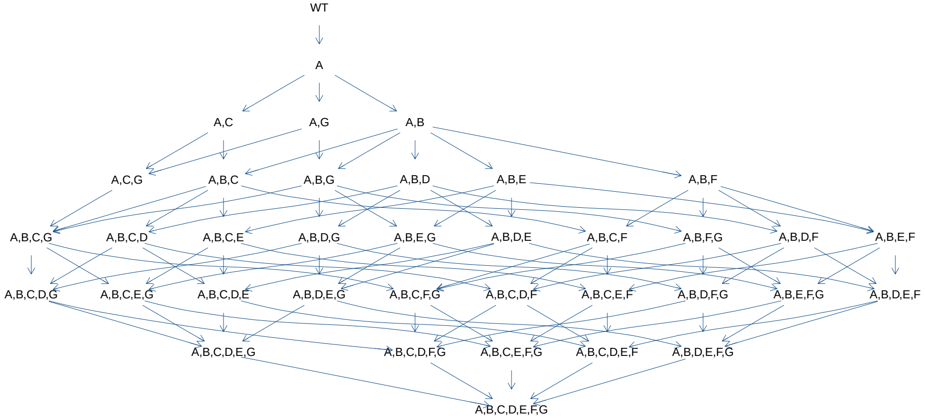

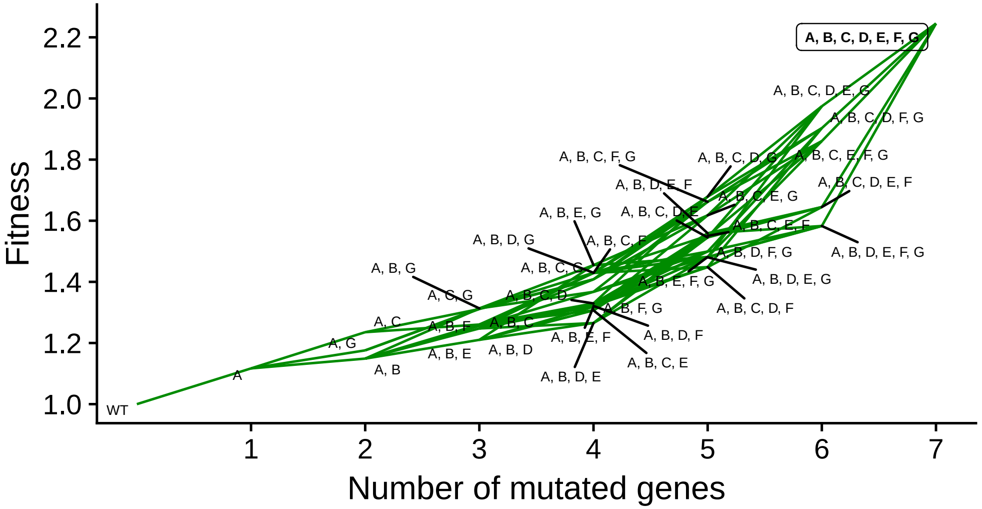

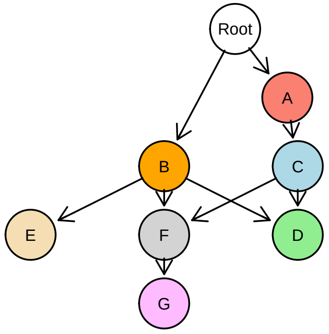

ID	WTwHv3VrTVwsxPNF
acc genotypes	19
peaks no back	1
removed edges	0
prop rem edges	0

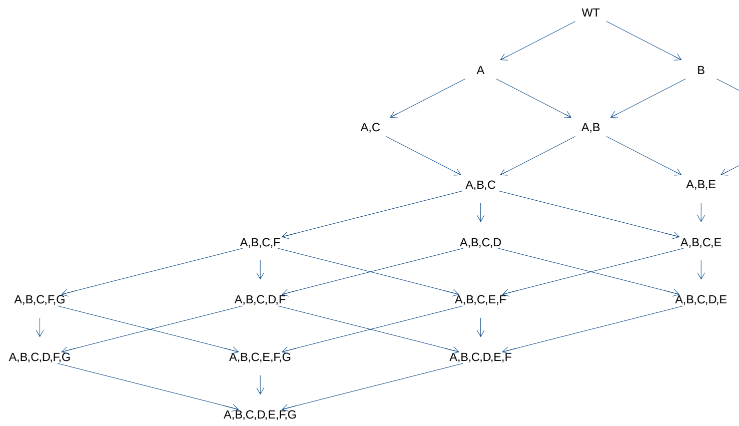

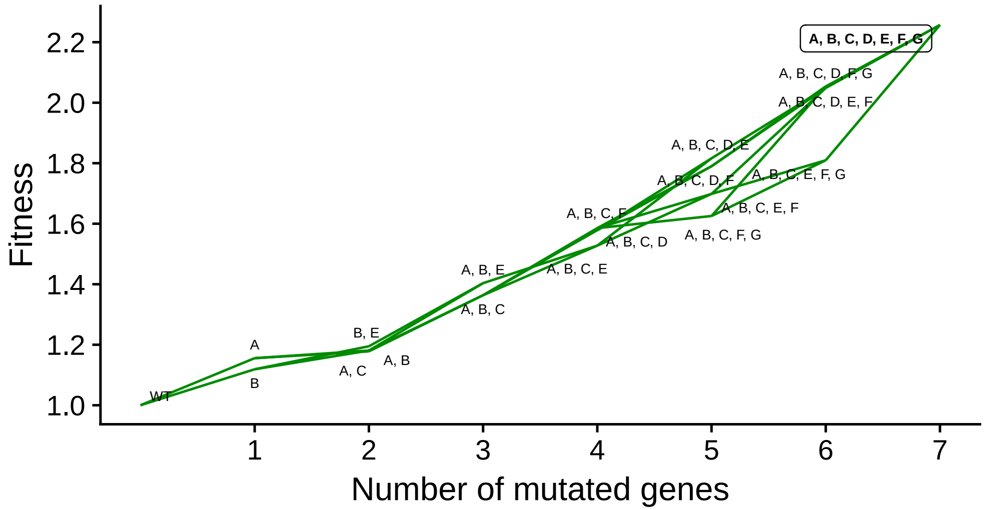

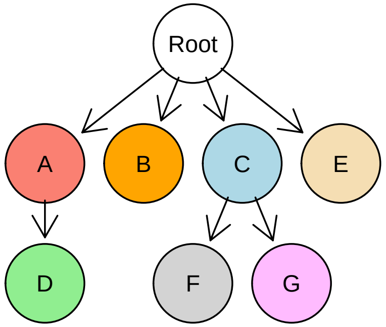

ID	wu54FEWu694xA1Ko
acc genotypes	60
peaks no back	1
removed edges	0
prop rem edges	0

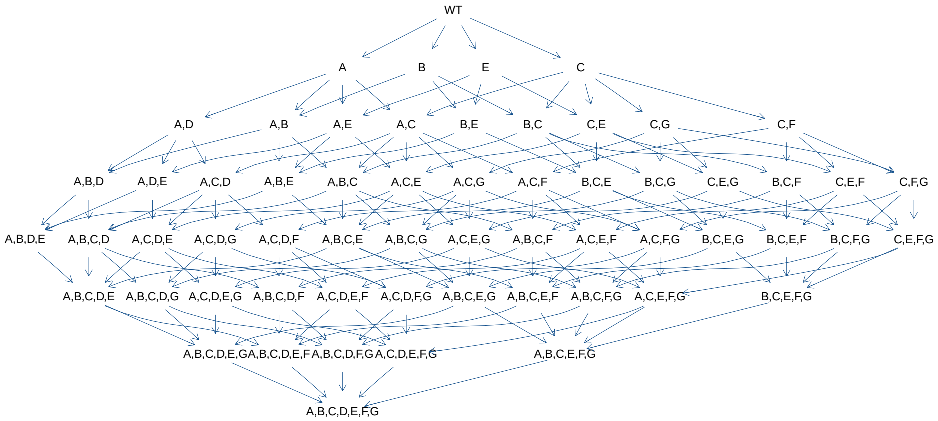

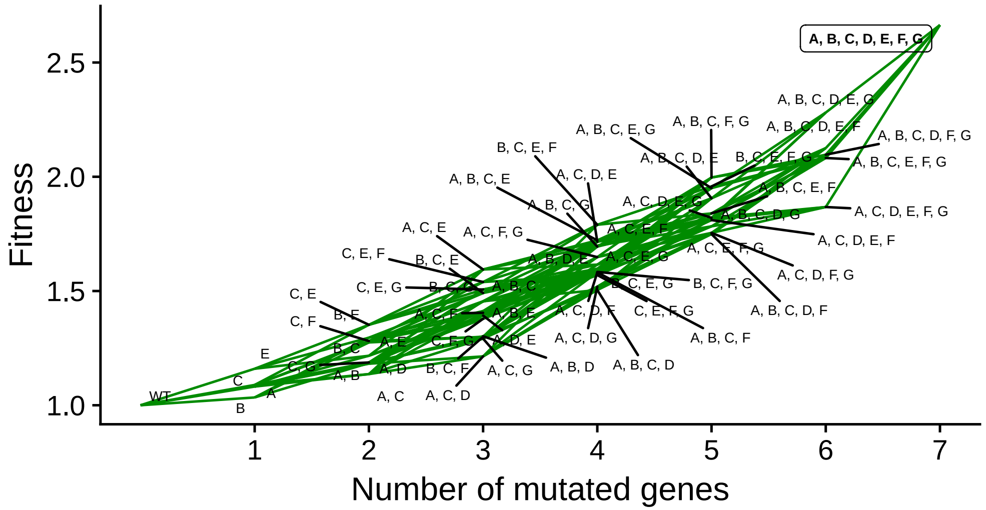

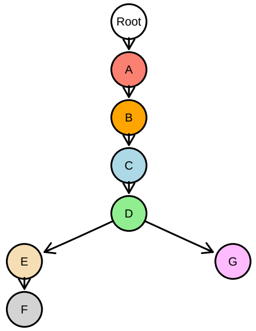

ID	wXqQ210QBkvlJzh1K
acc genotypes	10
peaks no back	1
removed edges	0
prop rem edges	0

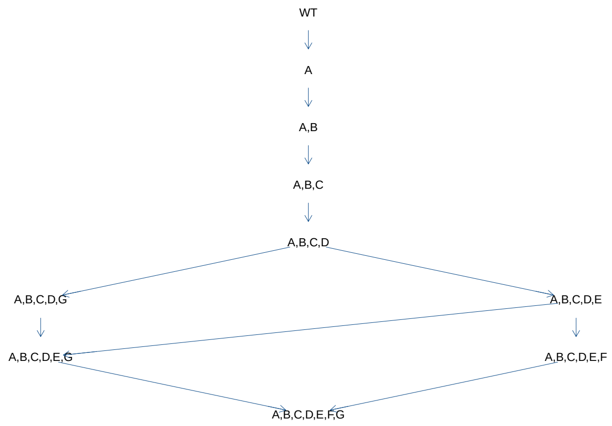

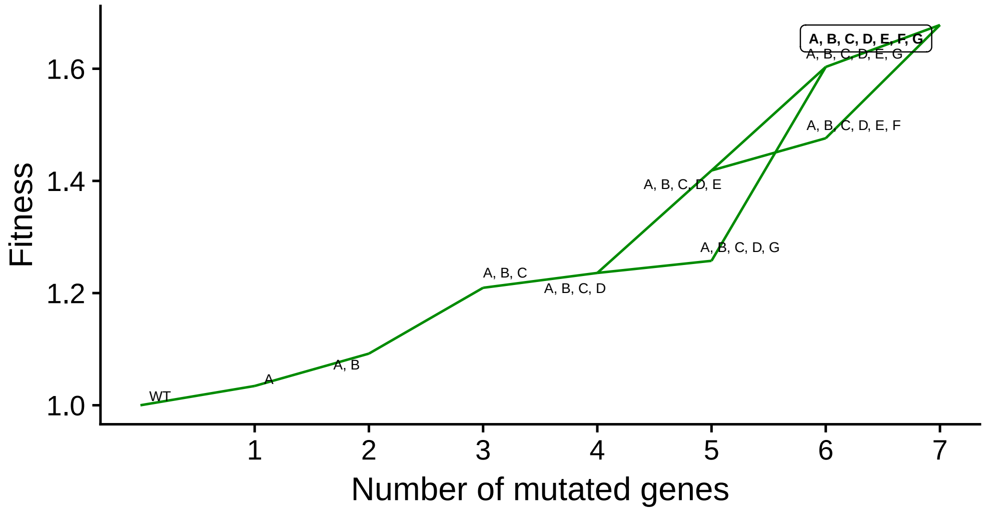

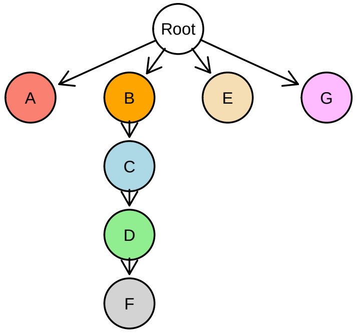

ID	WzFbSkNFewLZM2Lc
acc genotypes	40
peaks no back	1
removed edges	0
prop rem edges	0

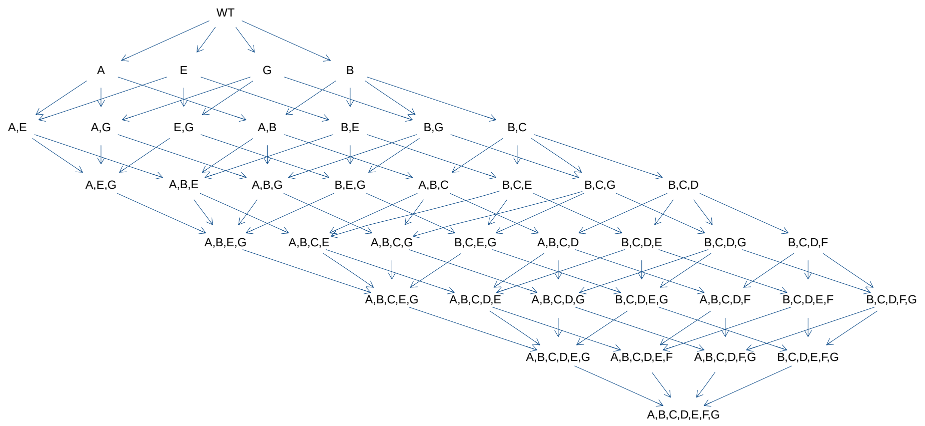

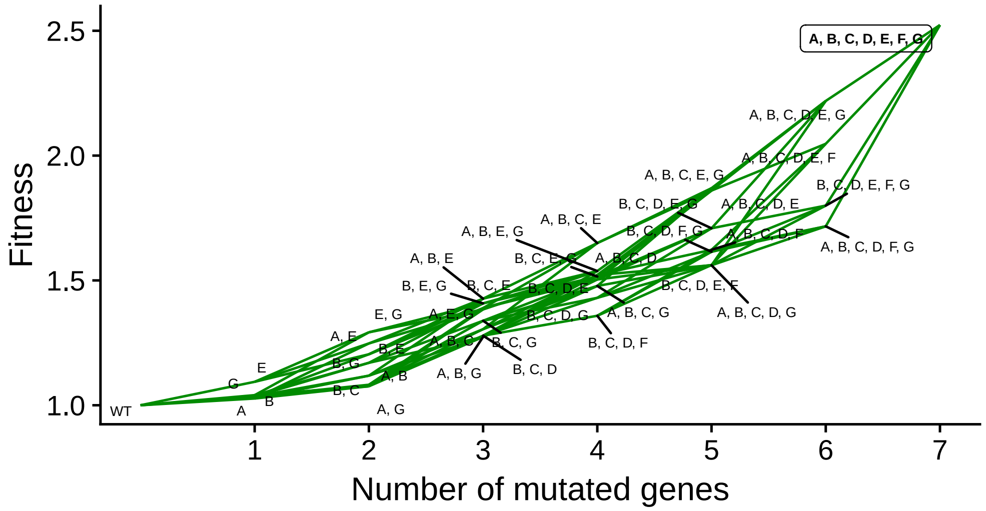

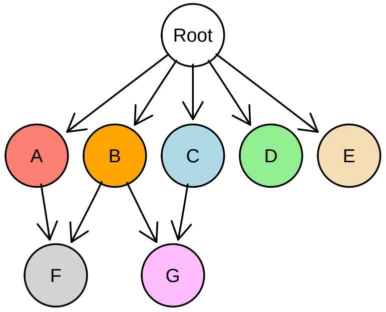

ID wZHRlWE1jjVBM1mQ
acc genotypes 52
peaks no back 1
removed edges 0
prop rem edges 0

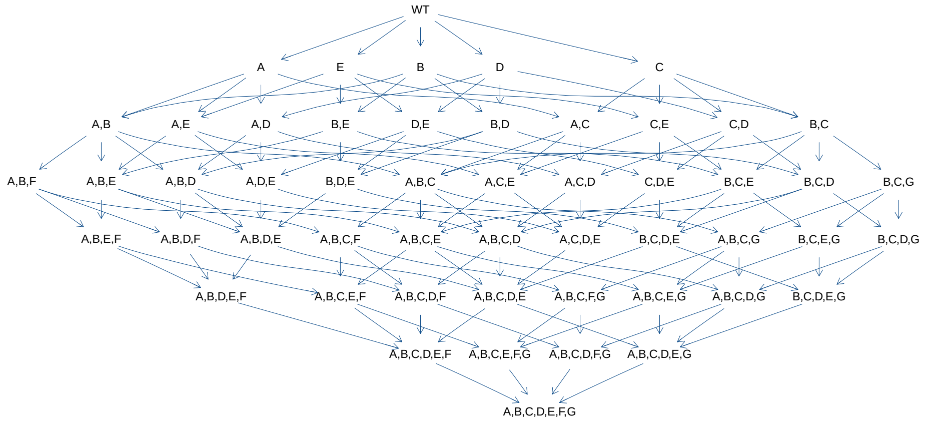

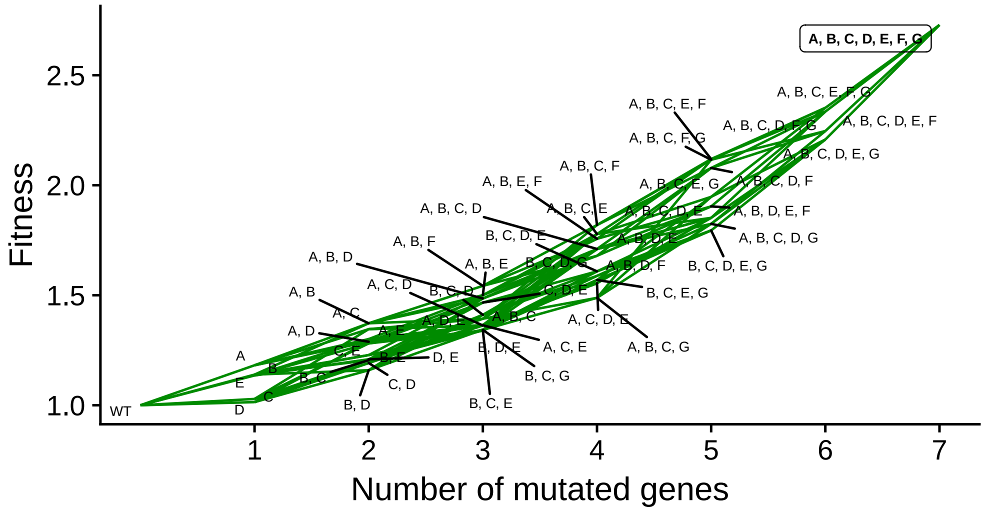

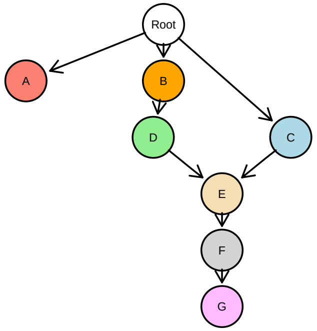

ID	xBMt9dOS3zBvChSy
acc genotypes	18
peaks no back	1
removed edges	0
prop rem edges	0

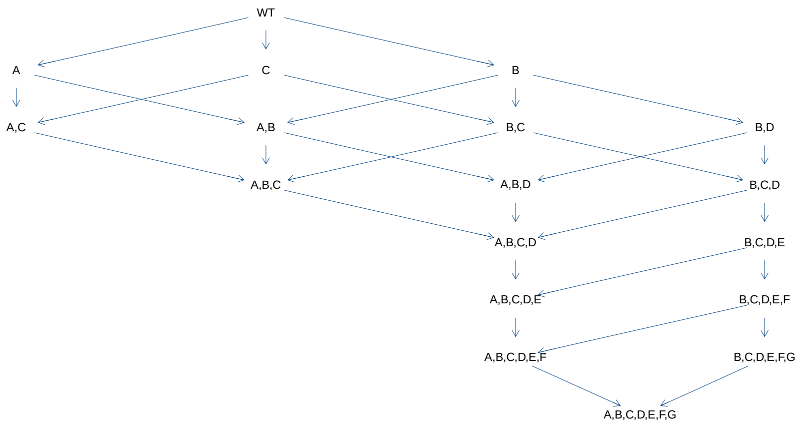

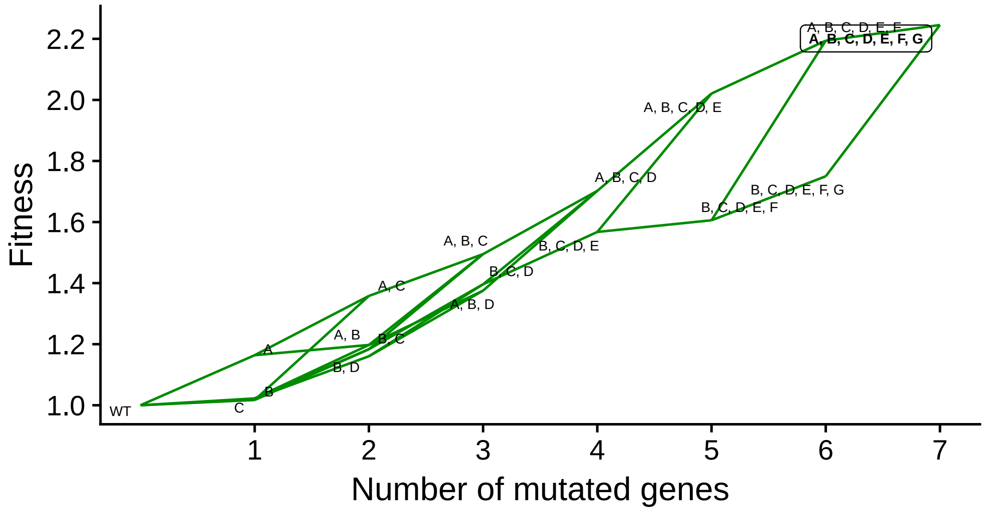

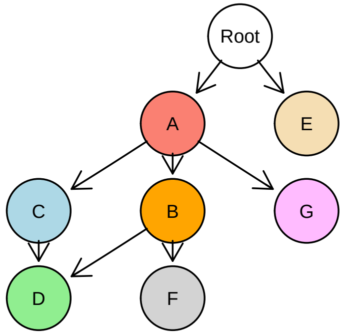

ID	xkgudeGhJGOlP7X7
acc genotypes	34
peaks no back	1
removed edges	0
prop rem edges	0

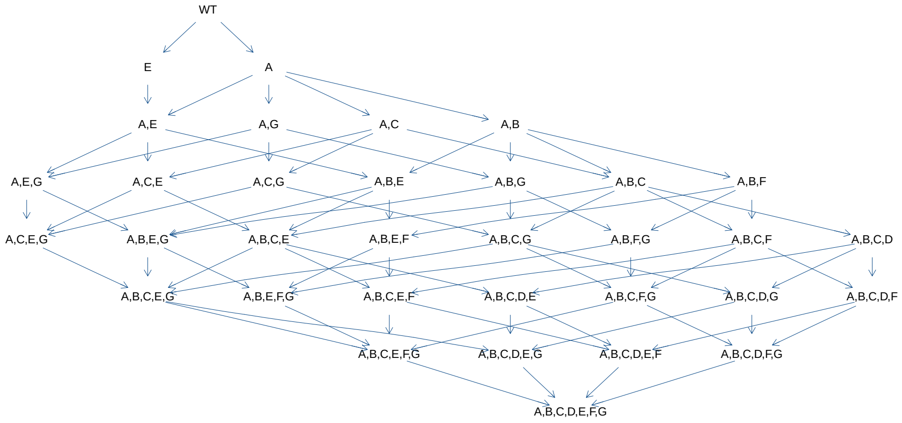

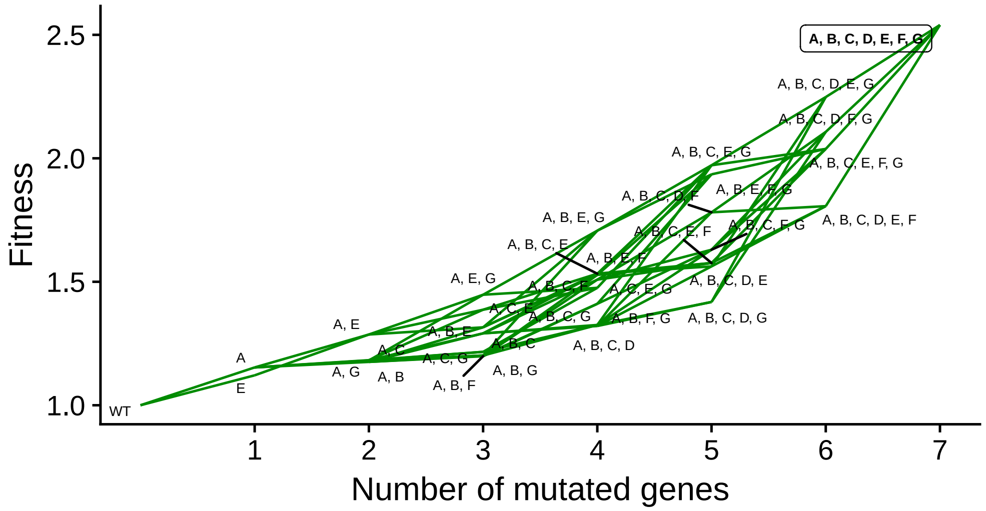

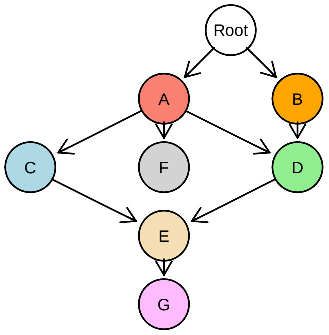

ID	XolBQBDPoWH3DwDQ
acc genotypes	18
peaks no back	1
removed edges	0
prop rem edges	0

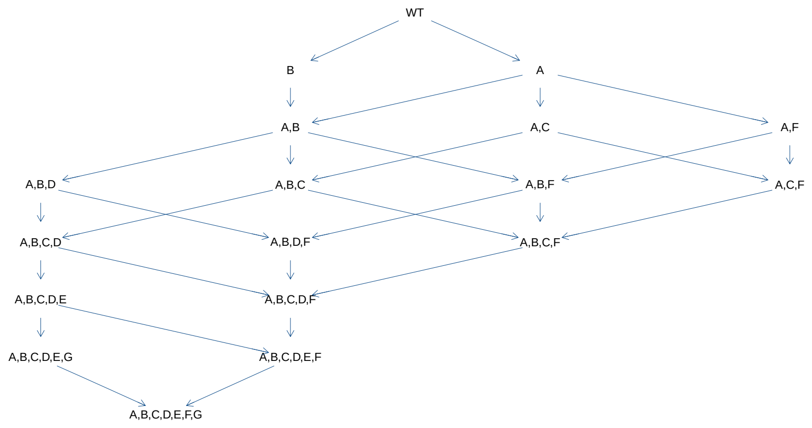

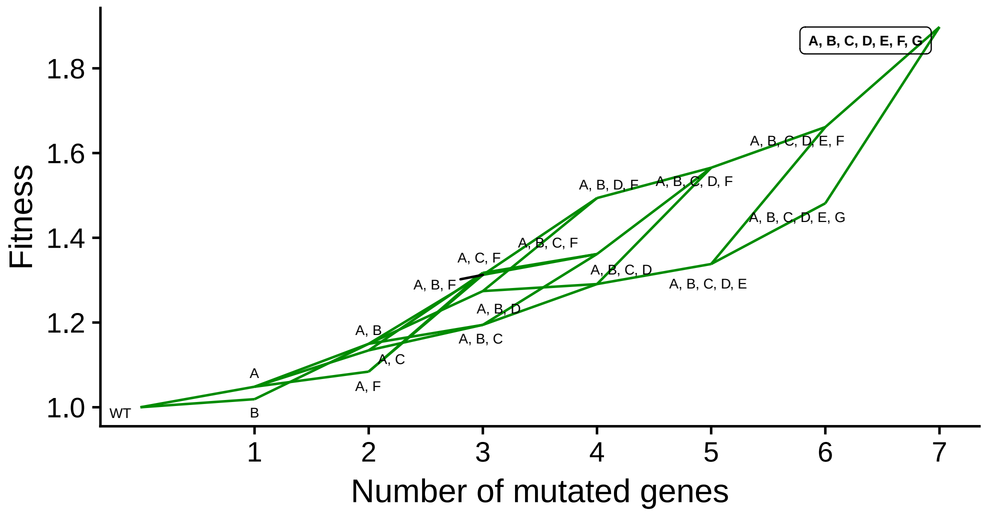

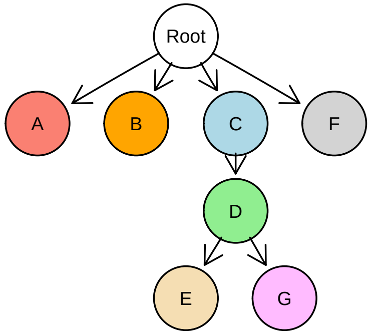

ID	XRovuAJWINYiVJHn
acc genotypes	48
peaks no back	1
removed edges	0
prop rem edges	0

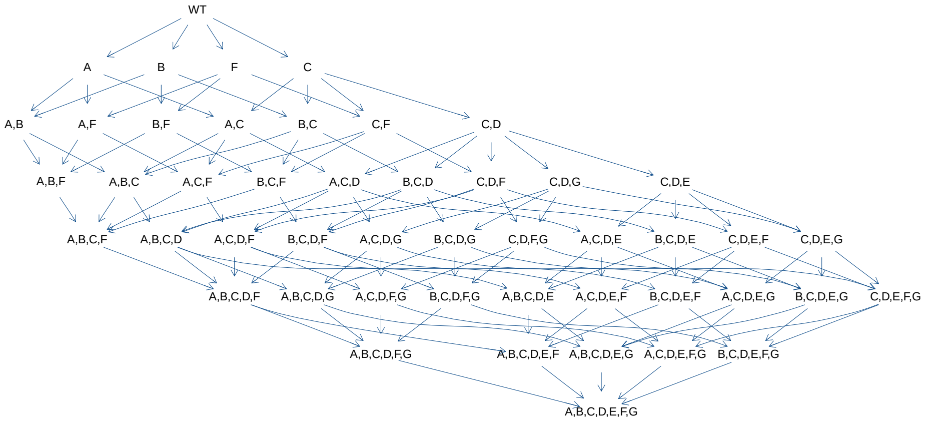

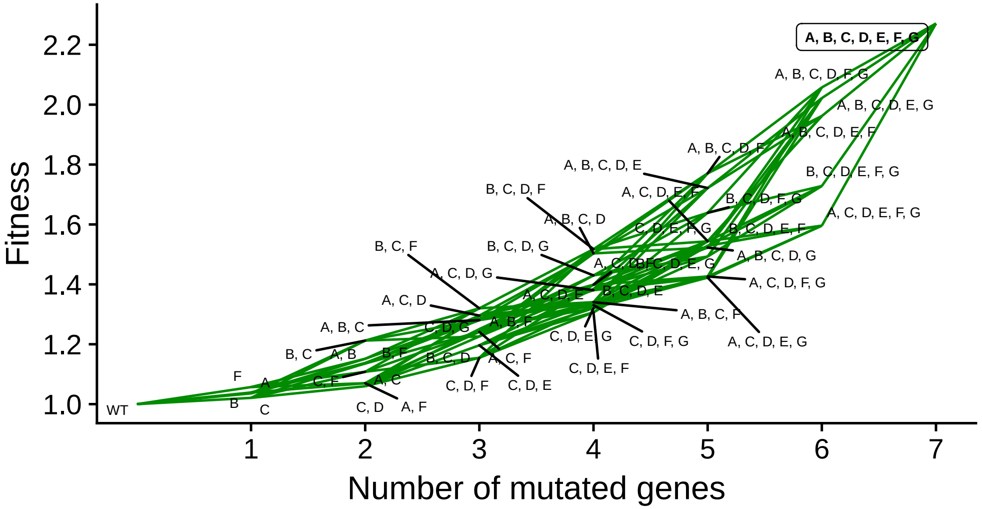

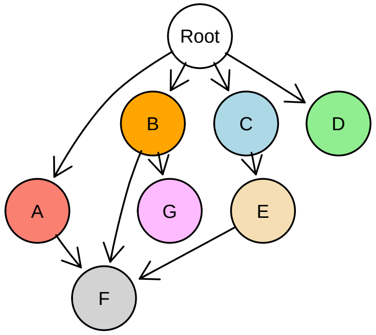

ID	xVDfqEWHyqTK7PeA
acc genotypes	40
peaks no back	1
removed edges	0
prop rem edges	0

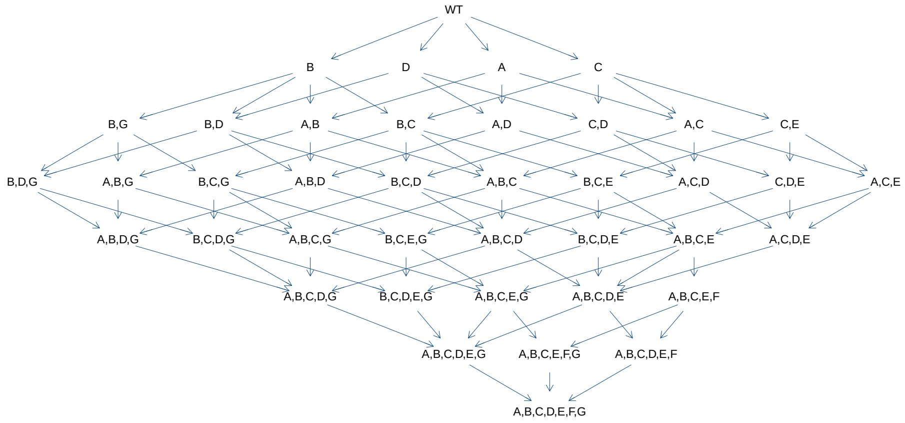

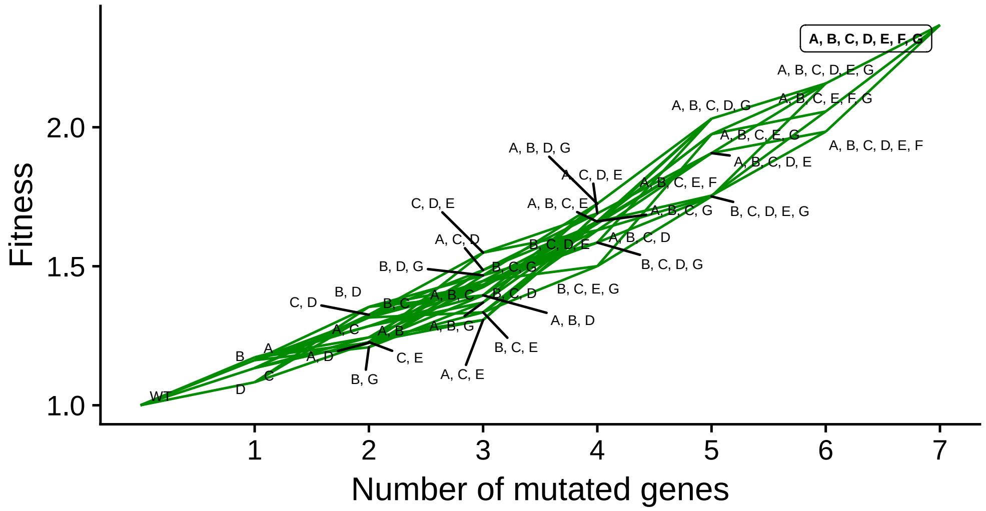

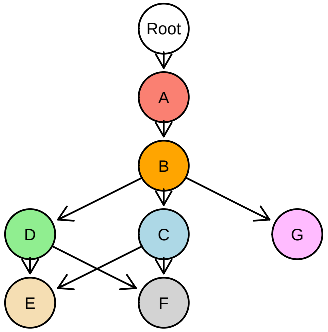

ID	y46oBk106Lnsh10yaQ
acc genotypes	16
peaks no back	1
removed edges	0
prop rem edges	0

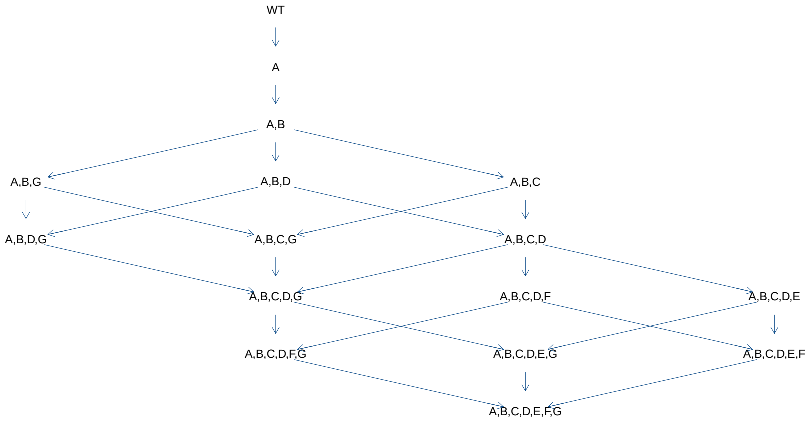

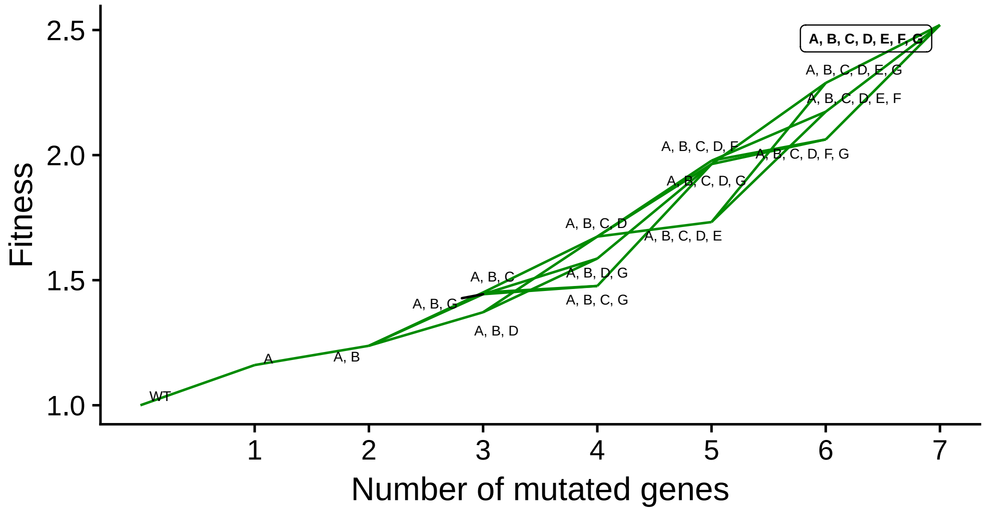

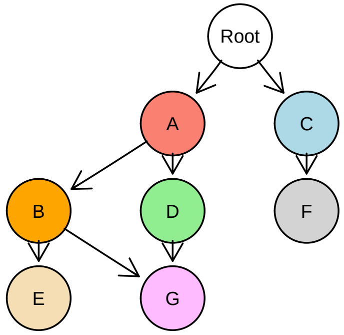

ID	YbCf9soBzZRhuiw10
acc genotypes	27
peaks no back	1
removed edges	0
prop rem edges	0

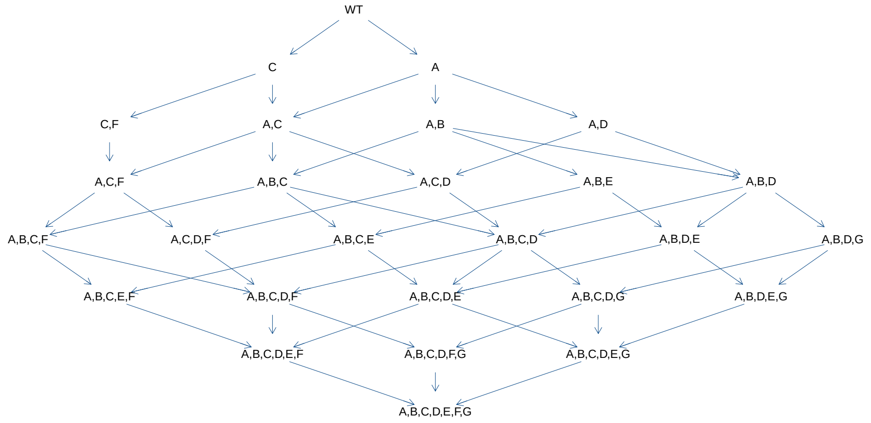

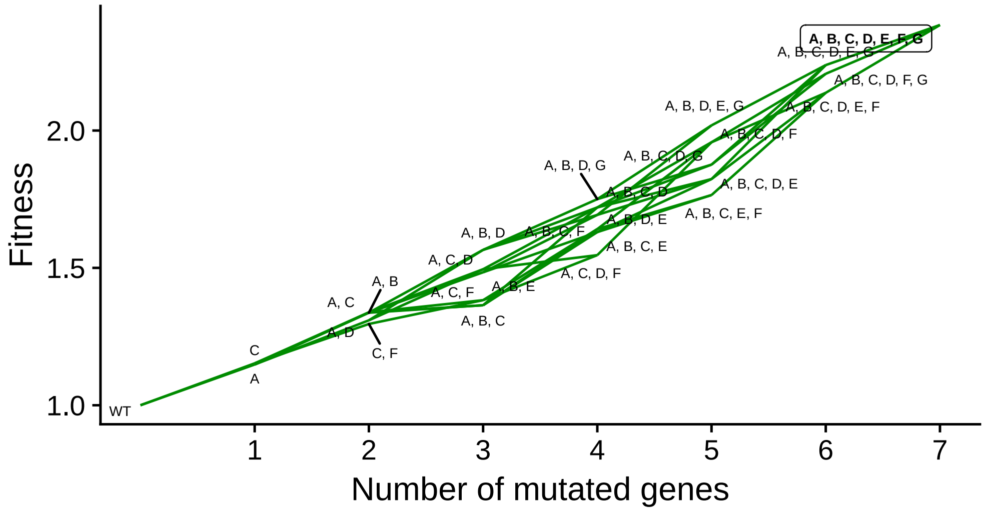

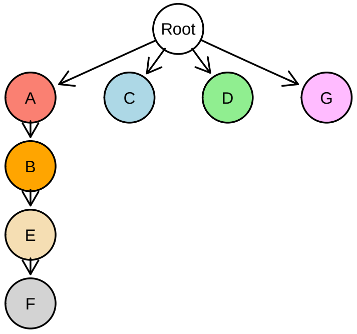

ID	YFmY1csTgroJgPCG
acc genotypes	40
peaks no back	1
removed edges	0
prop rem edges	0

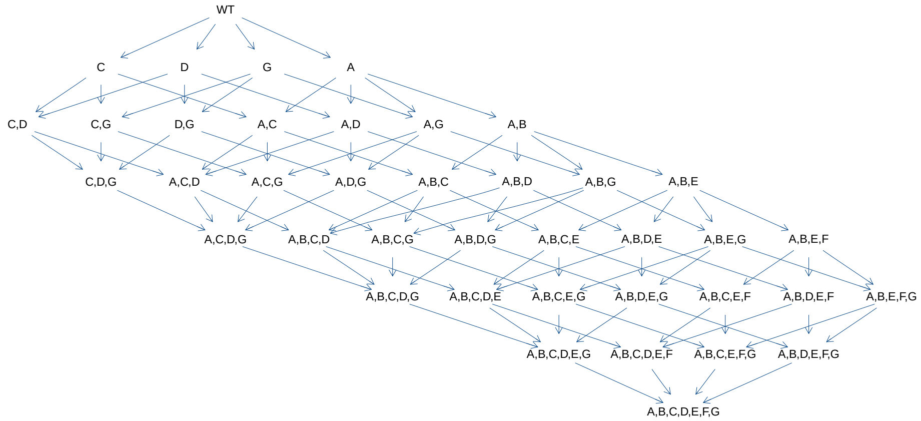

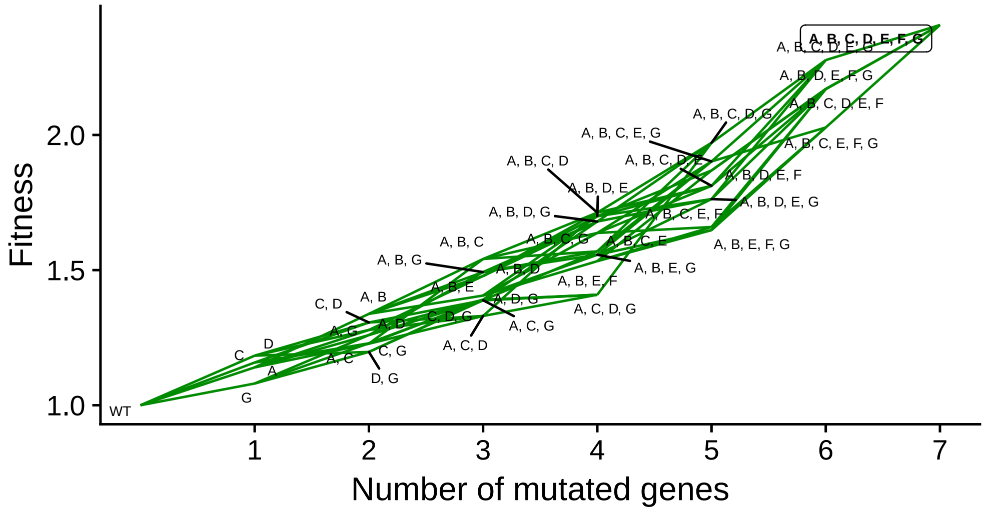

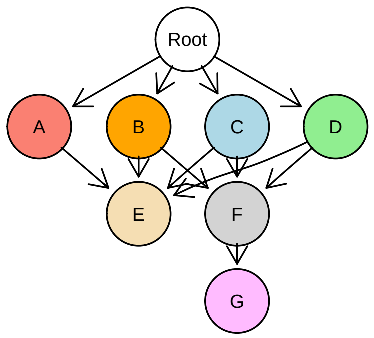

ID	yjwFeMSZVW68gVPI
acc genotypes	23
peaks no back	1
removed edges	0
prop rem edges	0

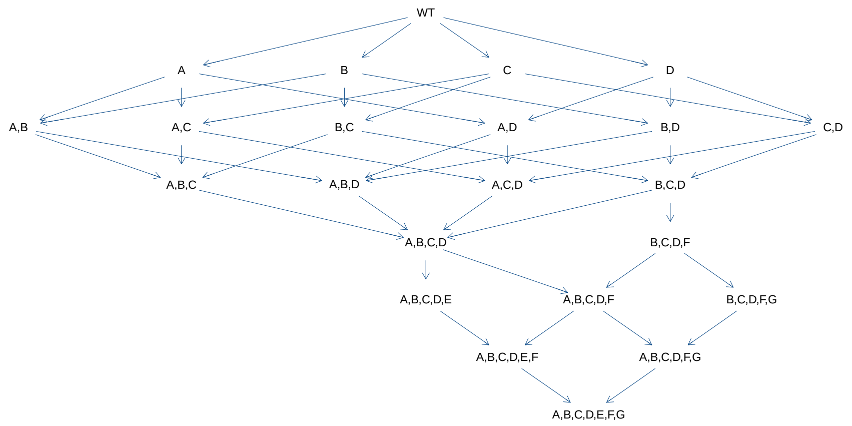

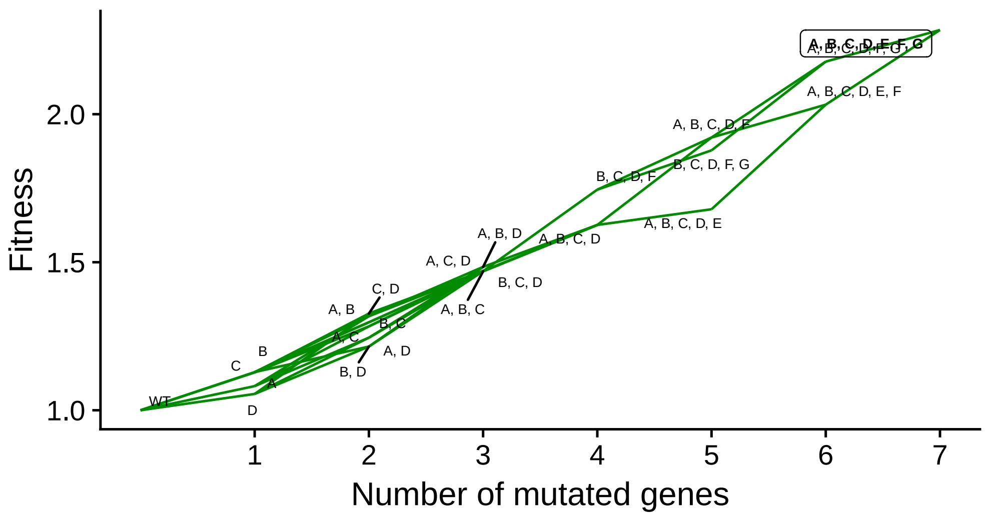

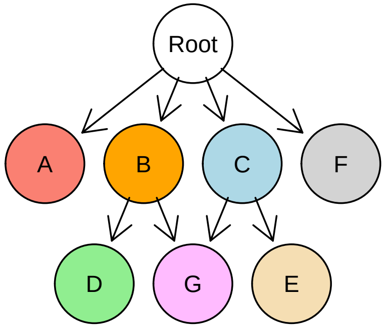

ID	yVlAr6nny7pIzy1a
acc genotypes	52
peaks no back	1
removed edges	0
prop rem edges	0

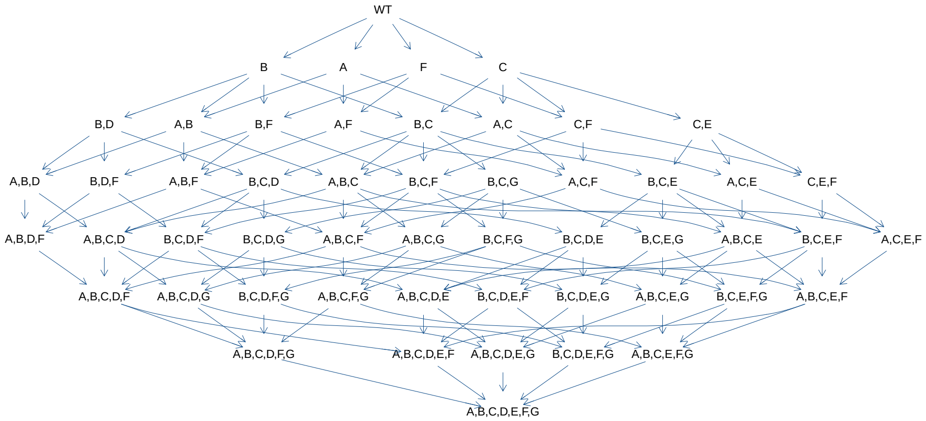

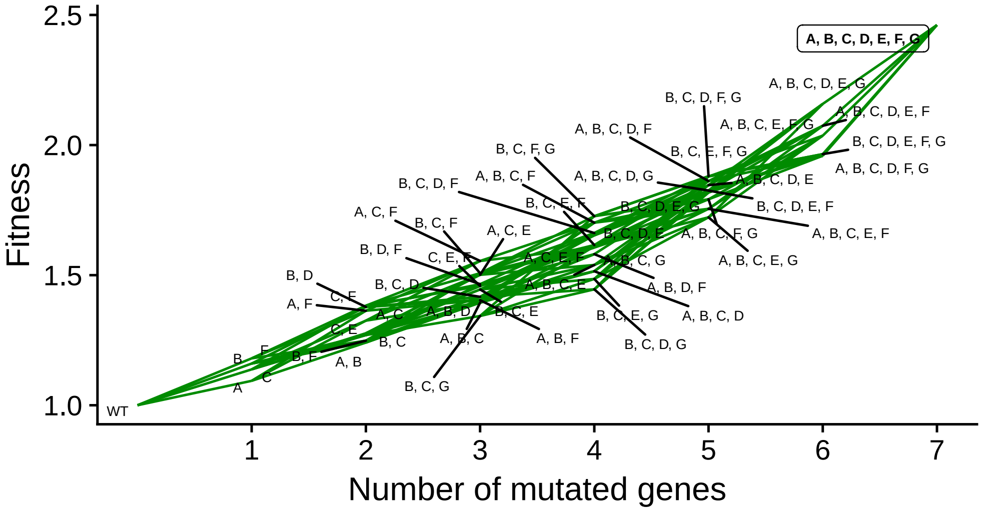

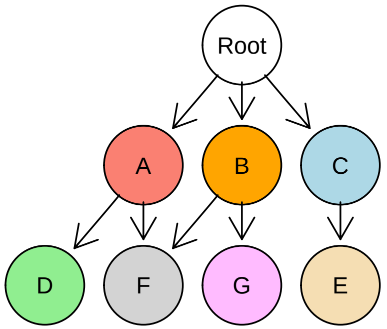

ID	YZtw9jhxSkZdMuV2
acc genotypes	39
peaks no back	1
removed edges	0
prop rem edges	0

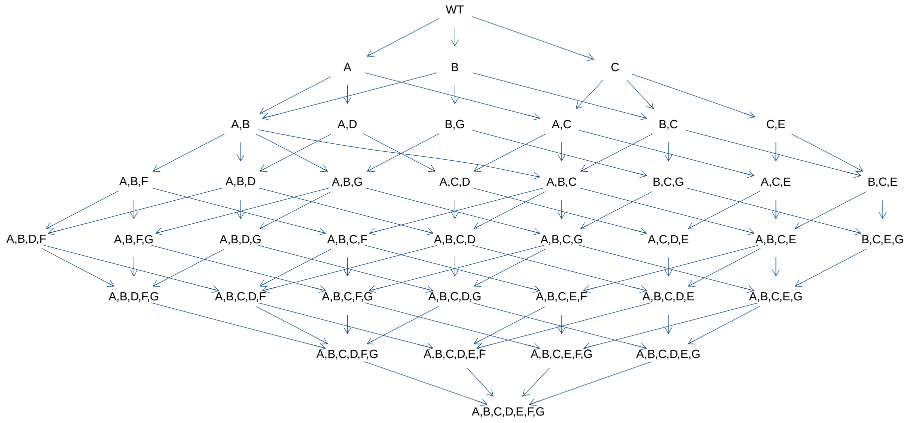

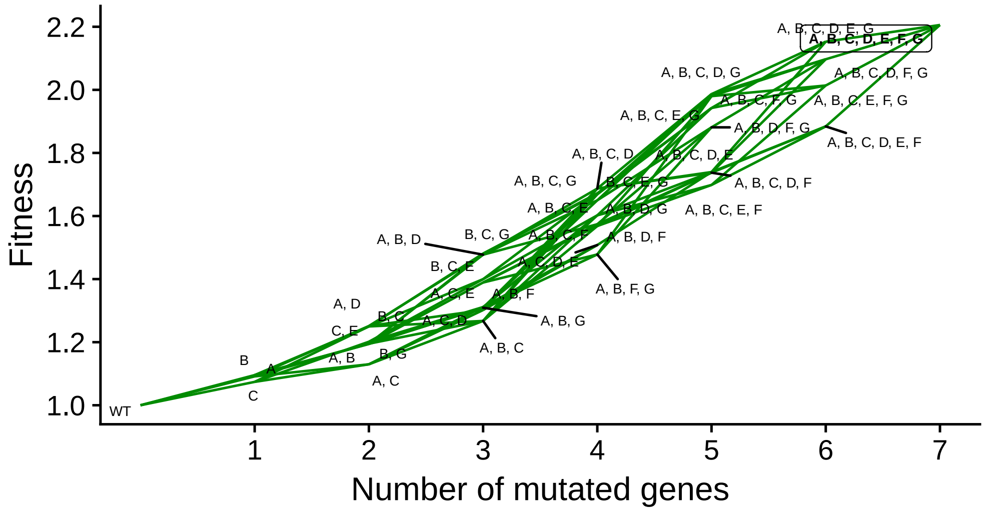

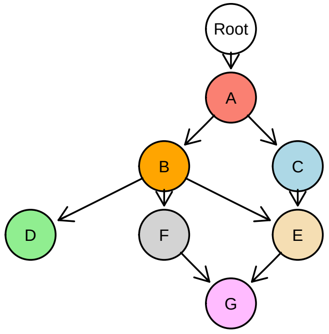

ID	Z5HeEO7UheuYVWra
acc genotypes	17
peaks no back	1
removed edges	0
prop rem edges	0

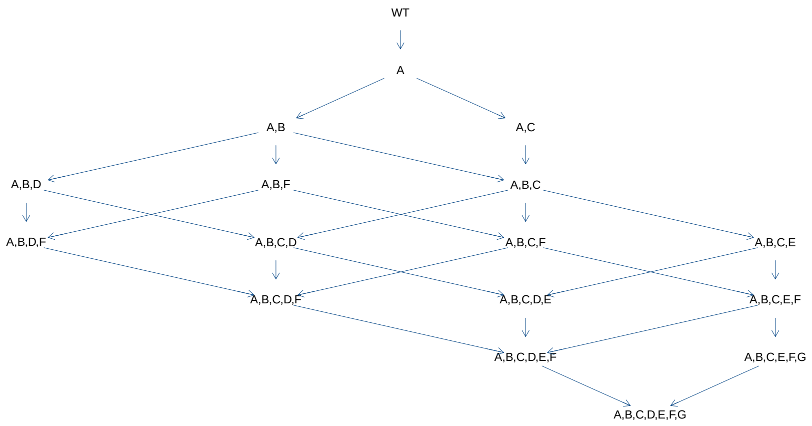

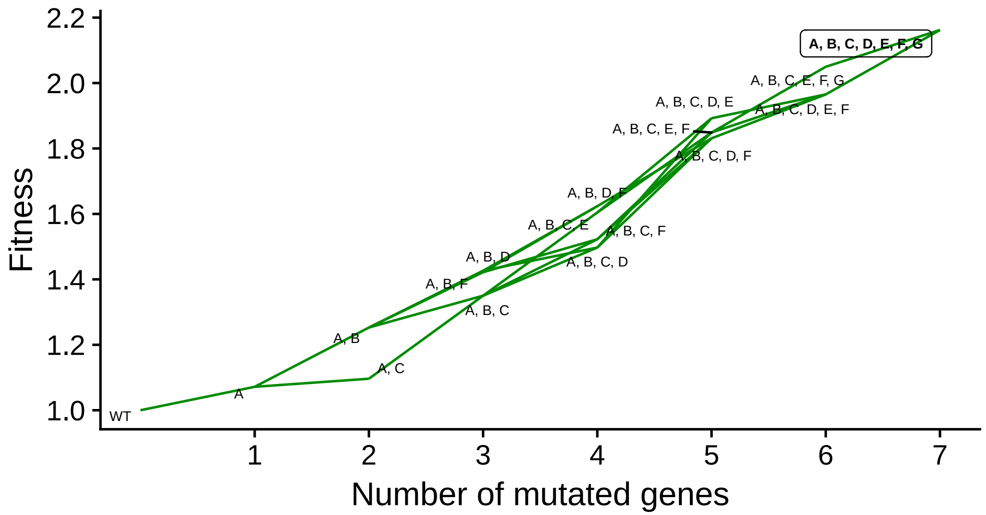

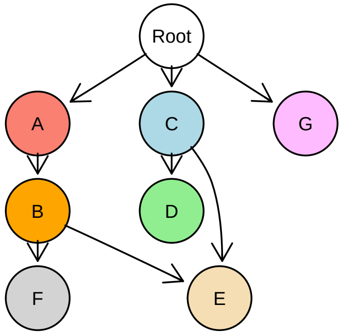

ID	zy1cmG2IWriY25OZ
acc genotypes	32
peaks no back	1
removed edges	0
prop rem edges	0

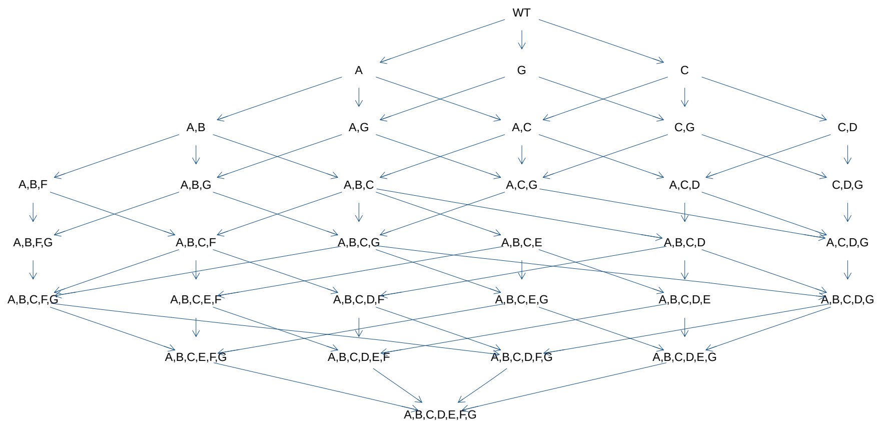

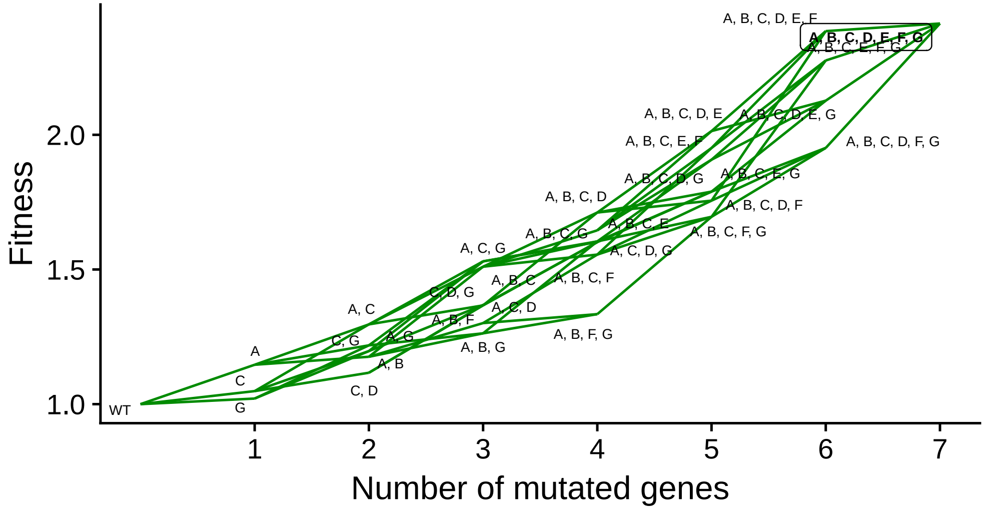

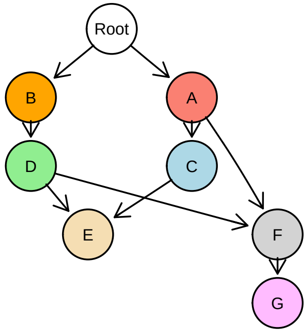

ID	10cxjmXXiW5qEyv73
acc genotypes	16
peaks no back	3
removed edges	2
prop rem edges	0.087

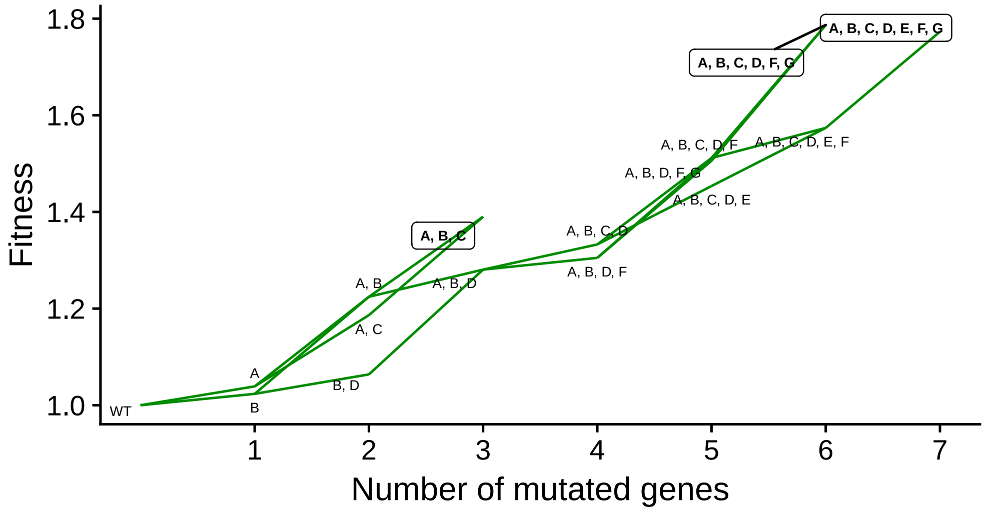

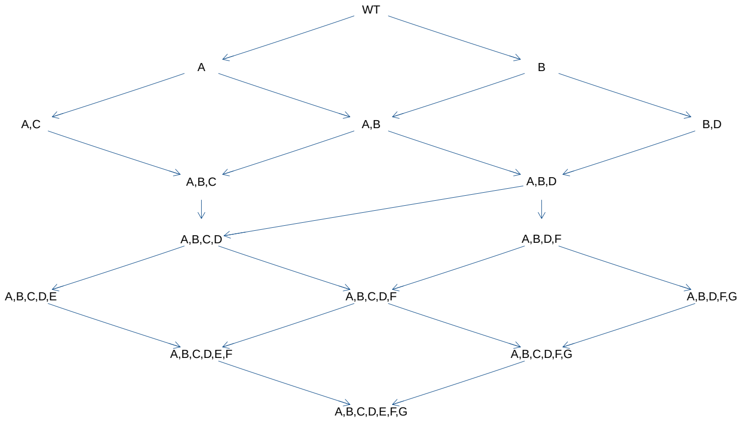

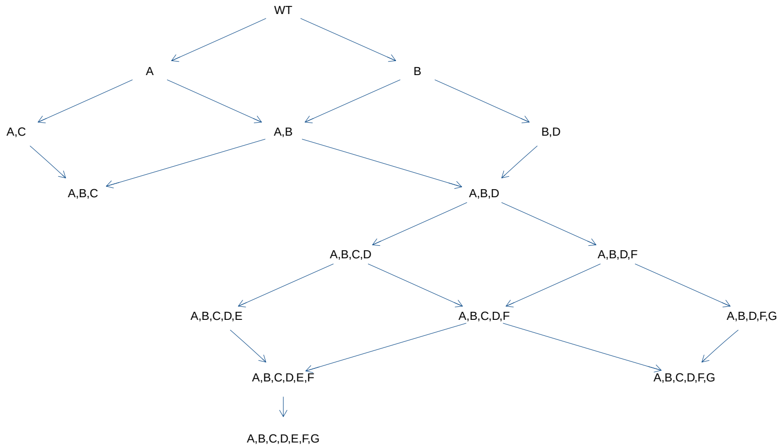

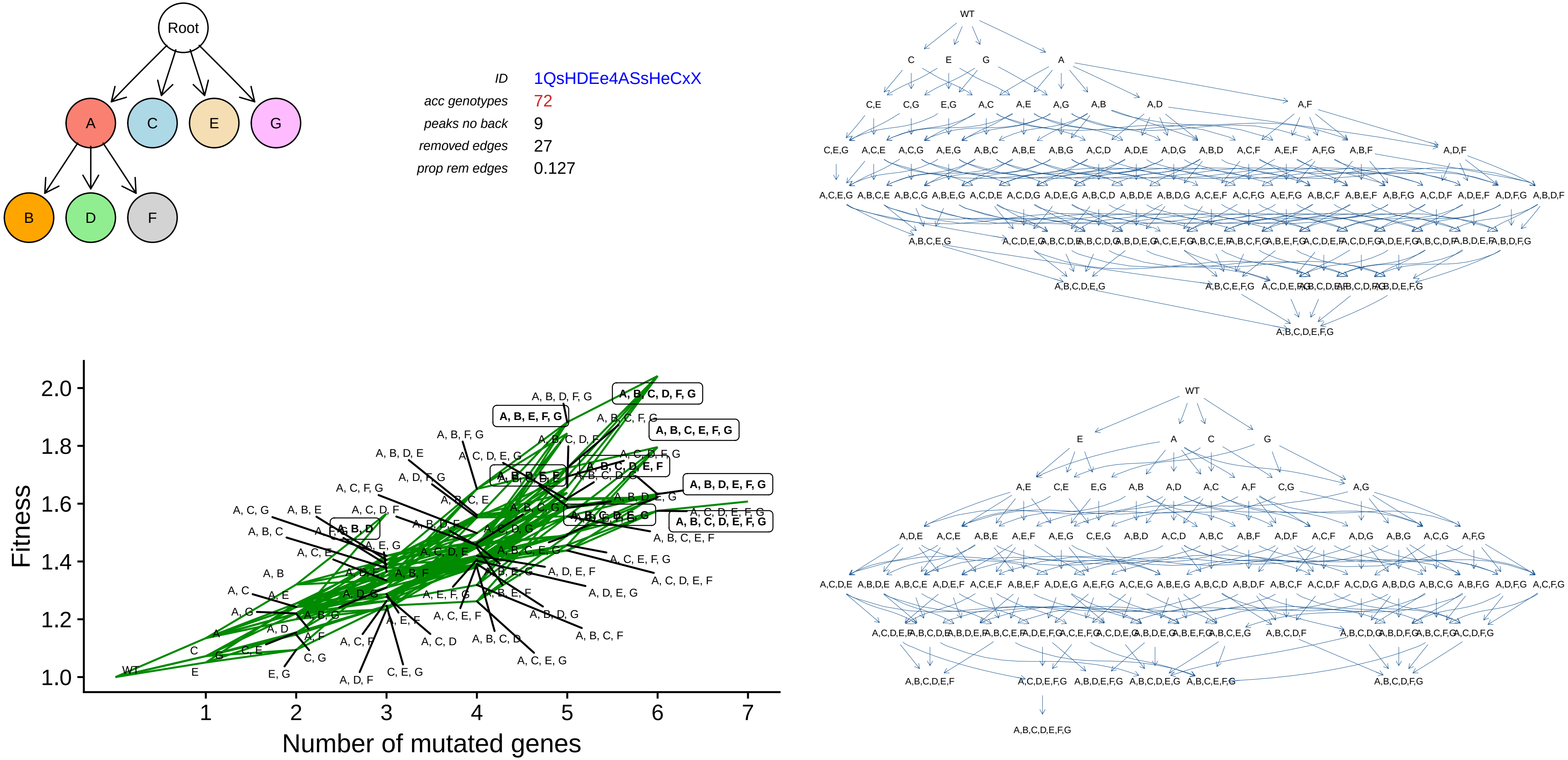

Root
A
C
E
G
B
D
F
ID 1QsHDEe4ASsHeCxX
acc genotypes 72
peaks no back 9
removed edges 27
prop rem edges 0.127
Fitness
Number of mutated genes
1.0
1.2
1.4
1.6
1.8
2.0
1
2
3
4
5
6
7
WT
A, B, C, D, F, G
A, B, E, F, G
A, B, C, E, F, G
A, B, D, E, F, G
A, B, C, D, E, F, G
A, B, D
A,B,C,D,E,F,G

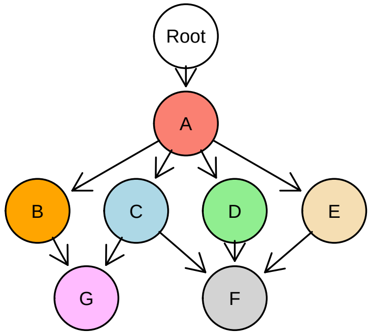

ID	2hKTyl9dwrqbrkDP
acc genotypes	24
peaks no back	4
removed edges	8
prop rem edges	0.174

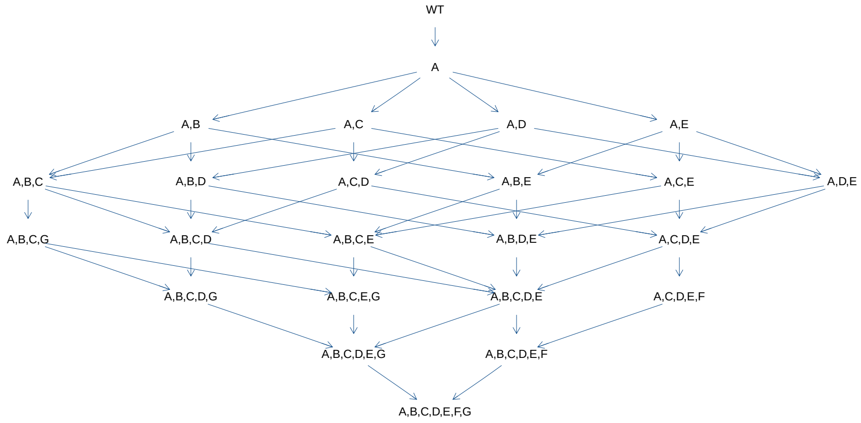

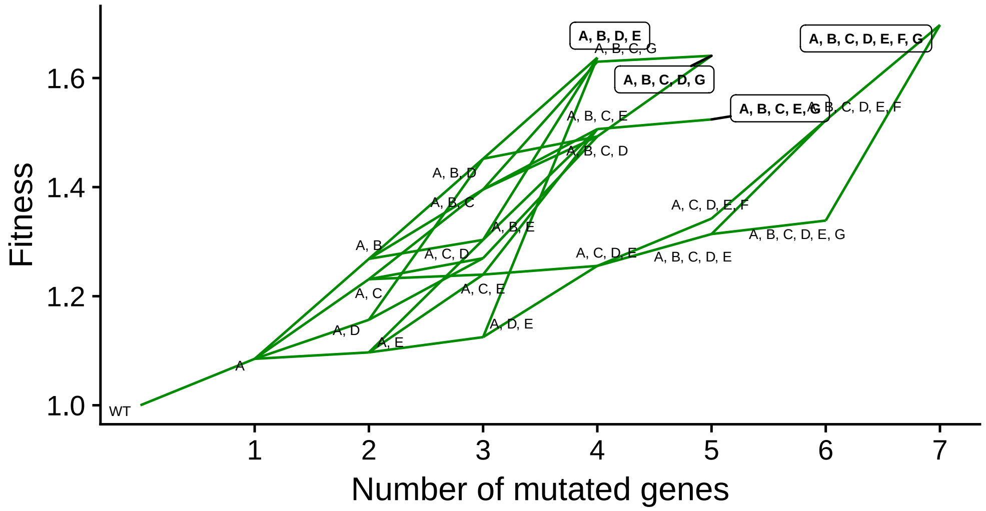

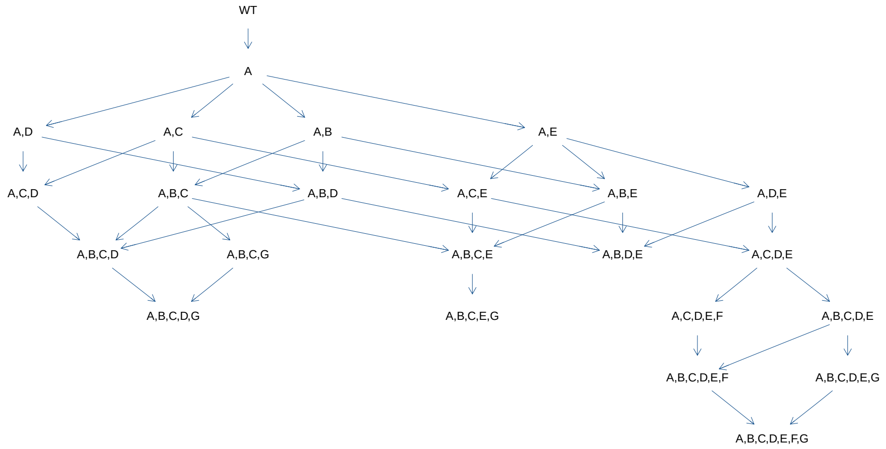

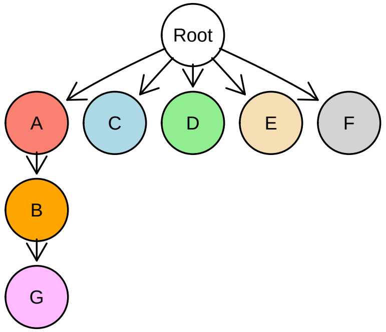

ID	3F1PDe5foEoGbGug
acc genotypes	64
peaks no back	9
removed edges	31
prop rem edges	0.176

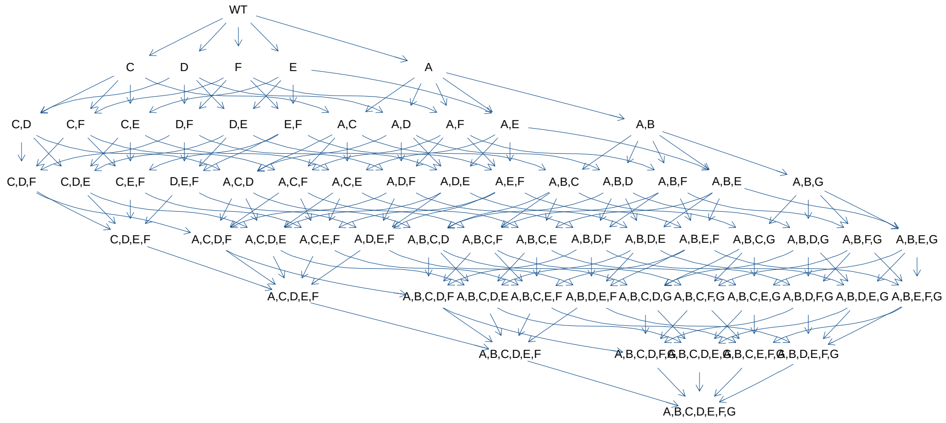

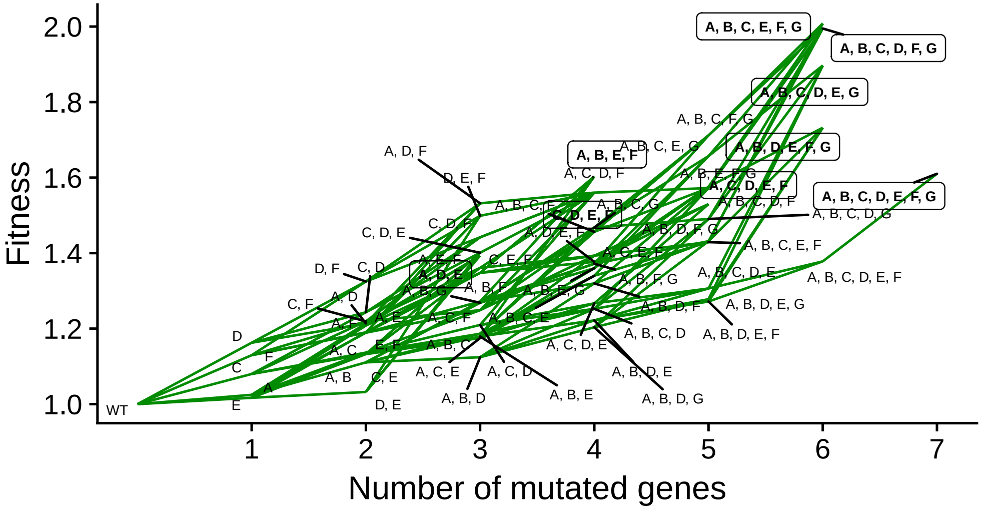

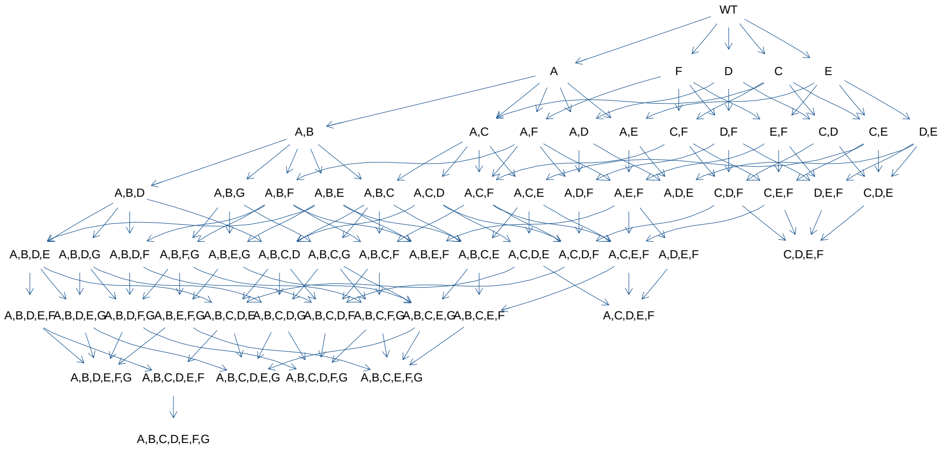

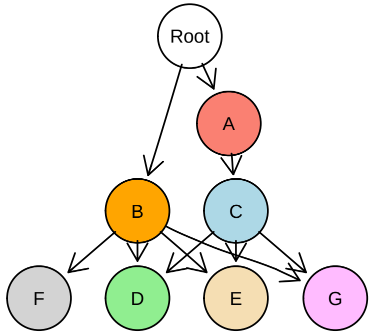

ID	3HeBeujvSFoM1FDd
acc genotypes	23
peaks no back	5
removed edges	7
prop rem edges	0.163

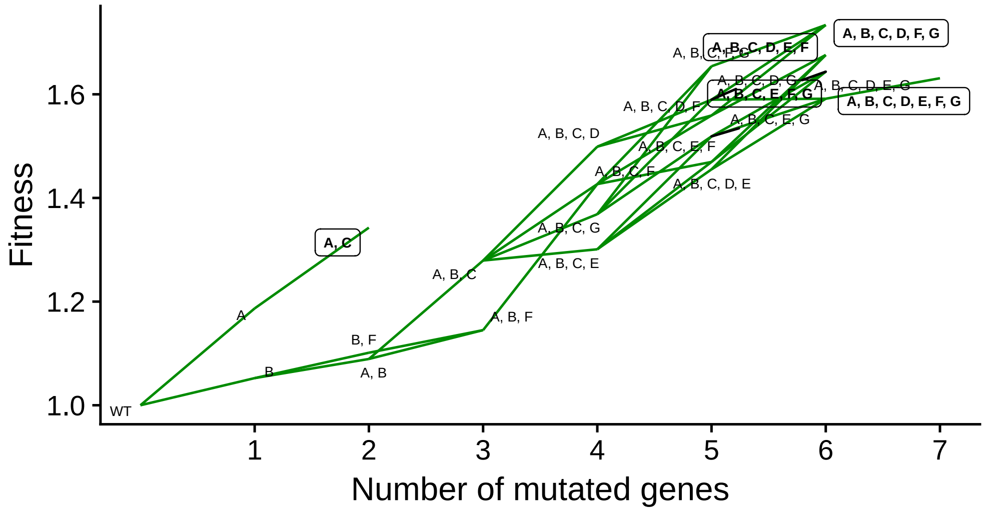

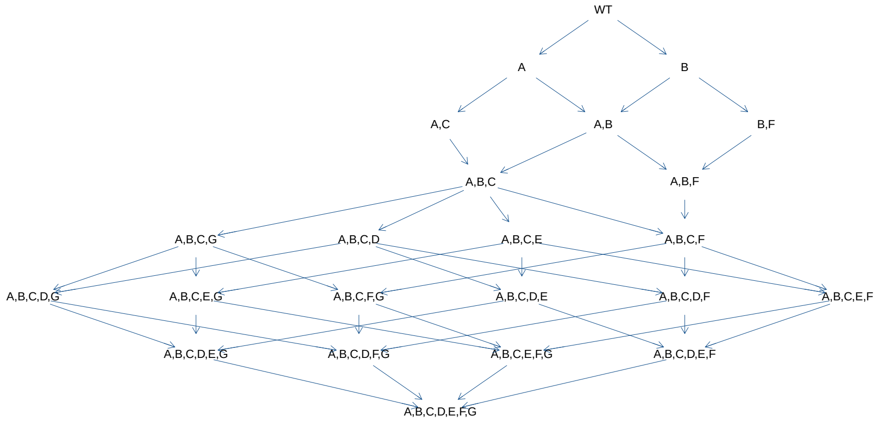

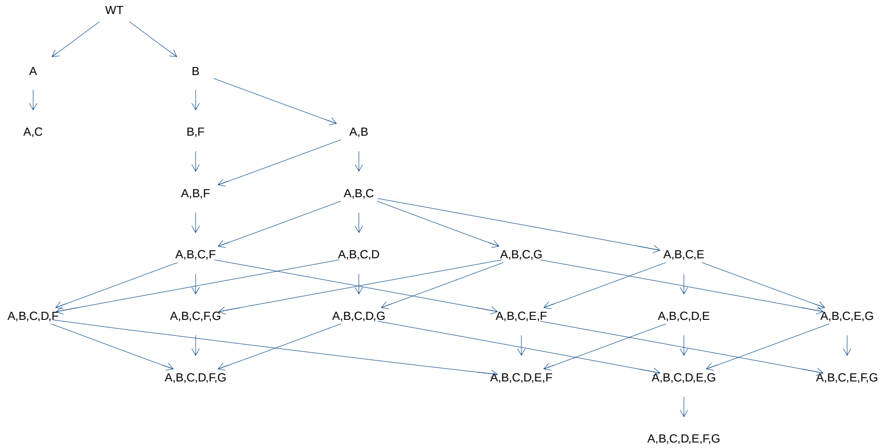

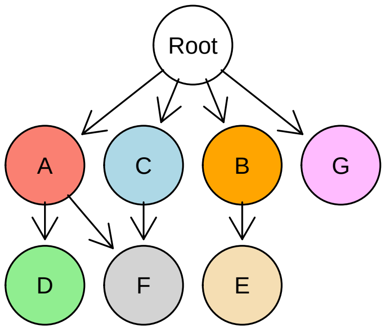

ID	3zLLGRM6bZ1renKV
acc genotypes	48
peaks no back	7
removed edges	25
prop rem edges	0.216

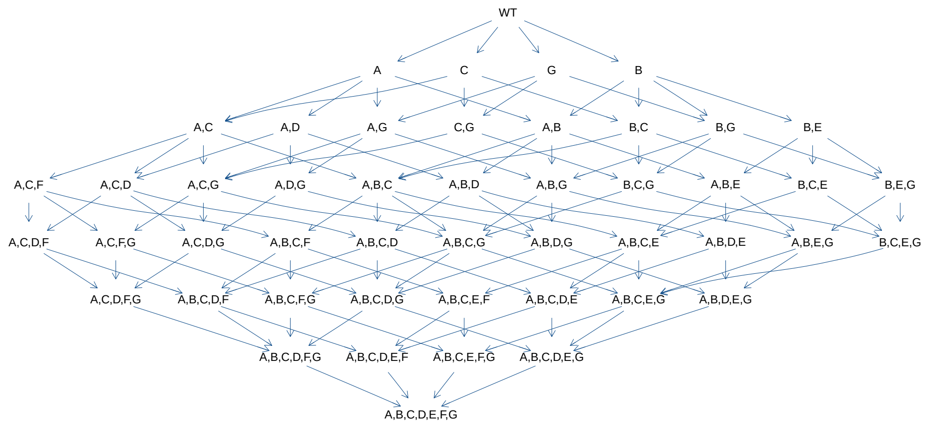

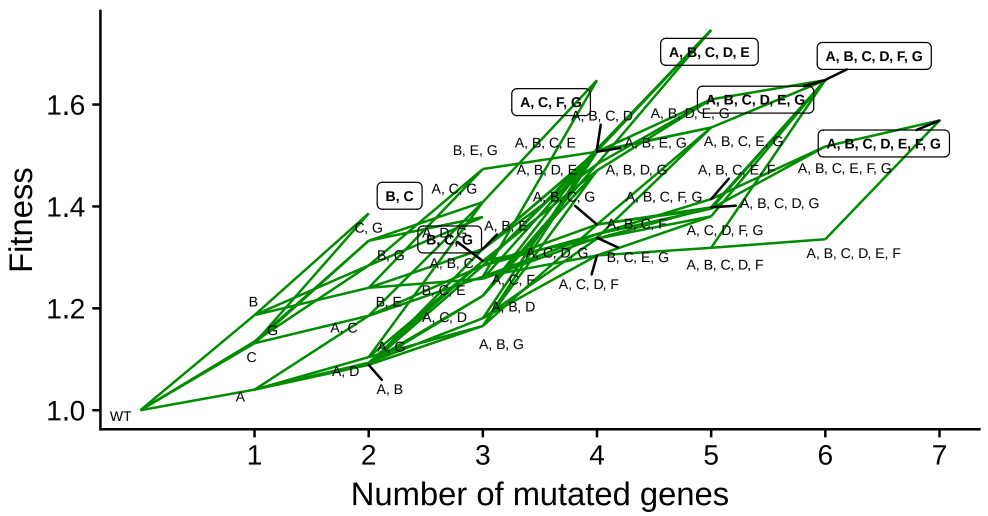

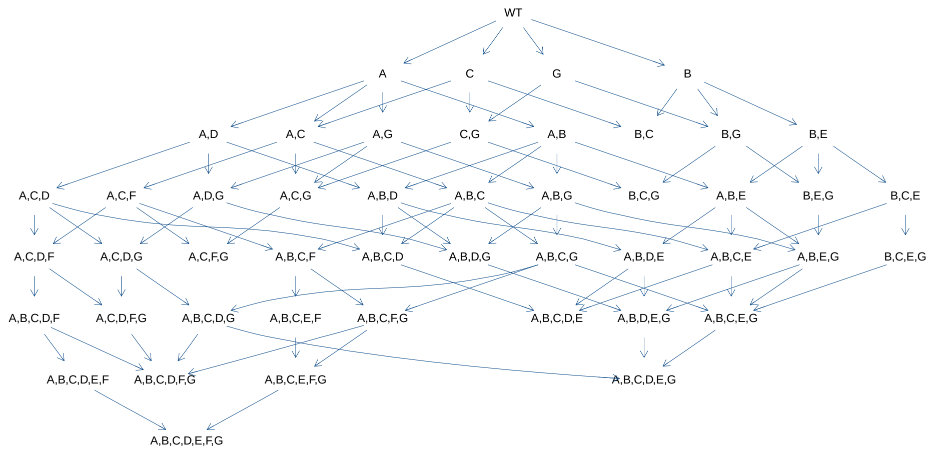

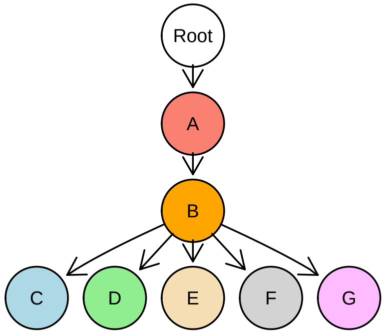

ID	410cr63nve1pNTOmC
acc genotypes	34
peaks no back	5
removed edges	5
prop rem edges	0.061

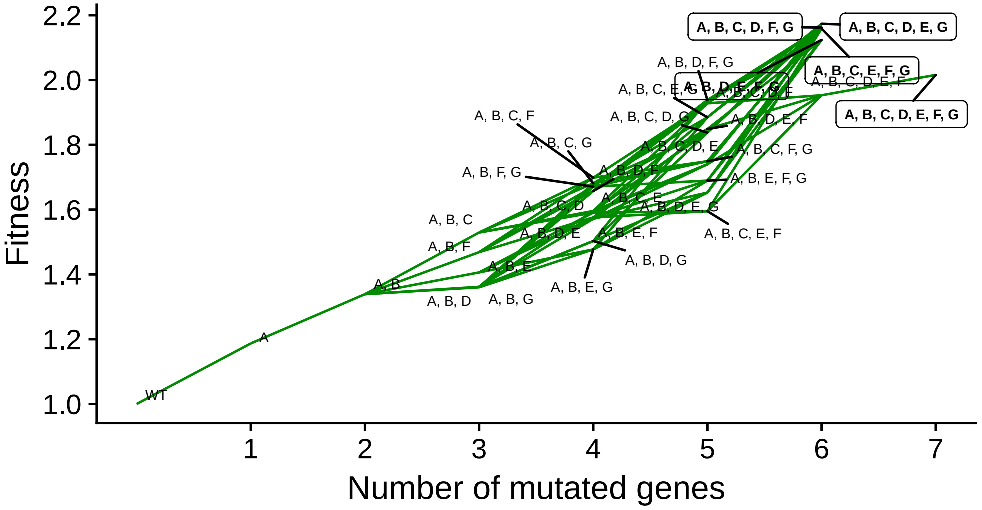

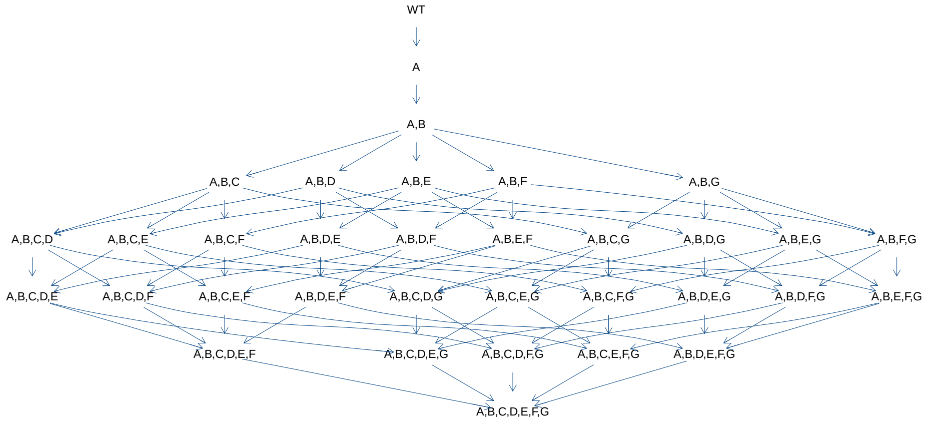

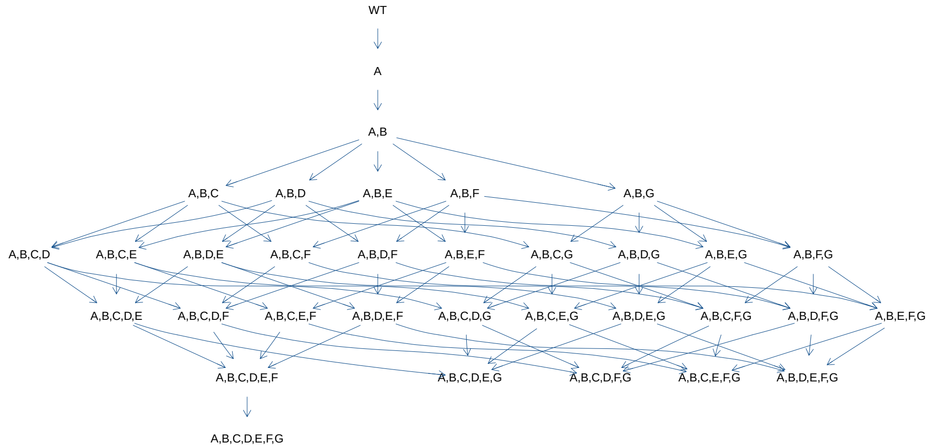

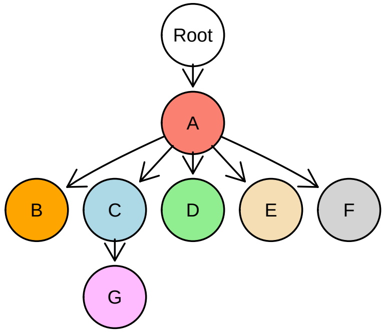

ID	4K18VlZE2xqKOhrH
acc genotypes	49
peaks no back	8
removed edges	22
prop rem edges	0.171

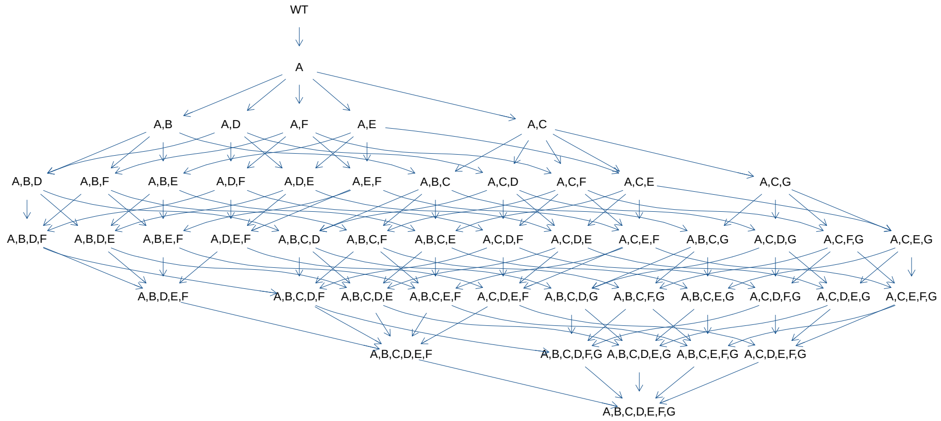

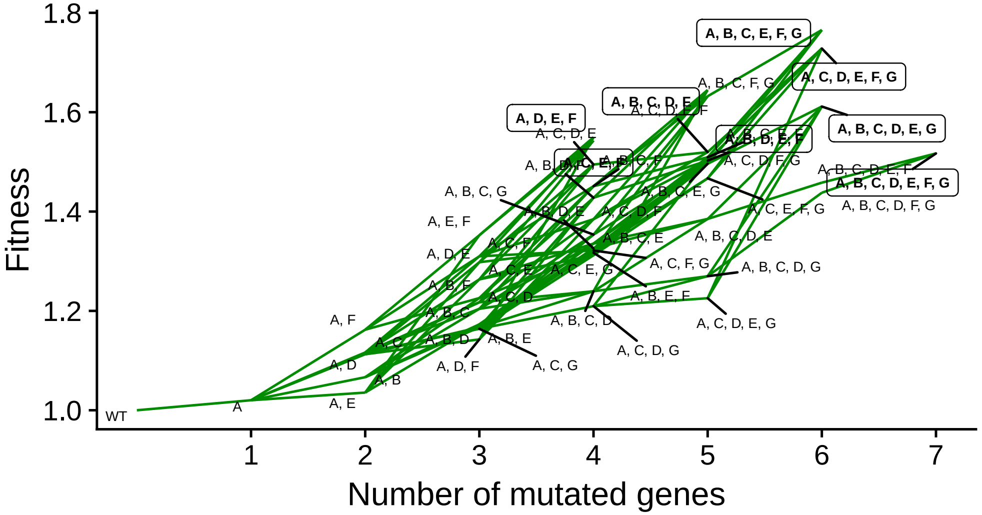

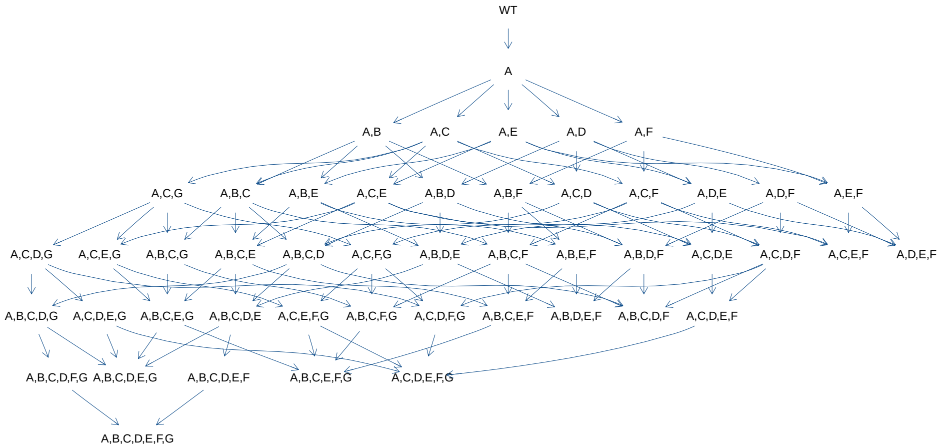

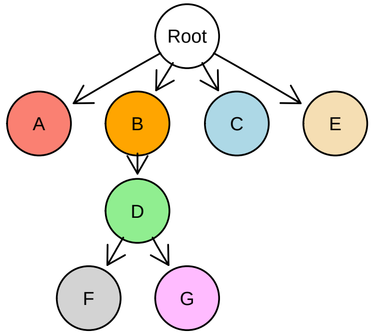

ID	4KzuP1MMEwOUSymI
acc genotypes	48
peaks no back	10
removed edges	26
prop rem edges	0.217

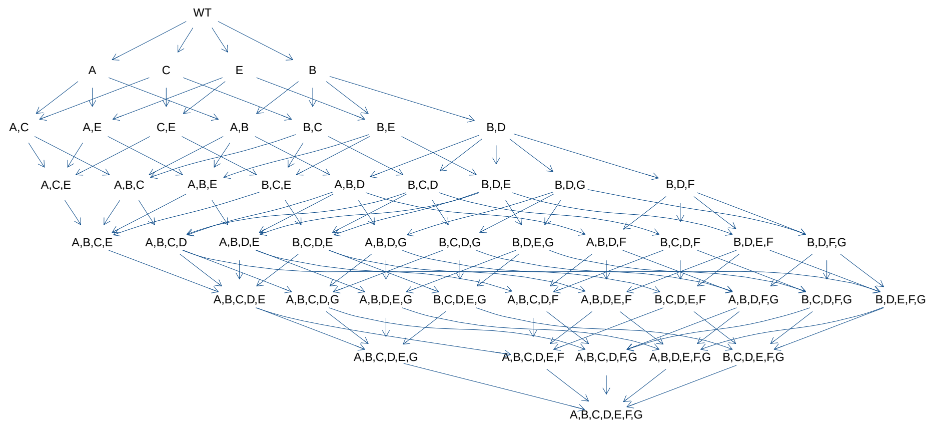

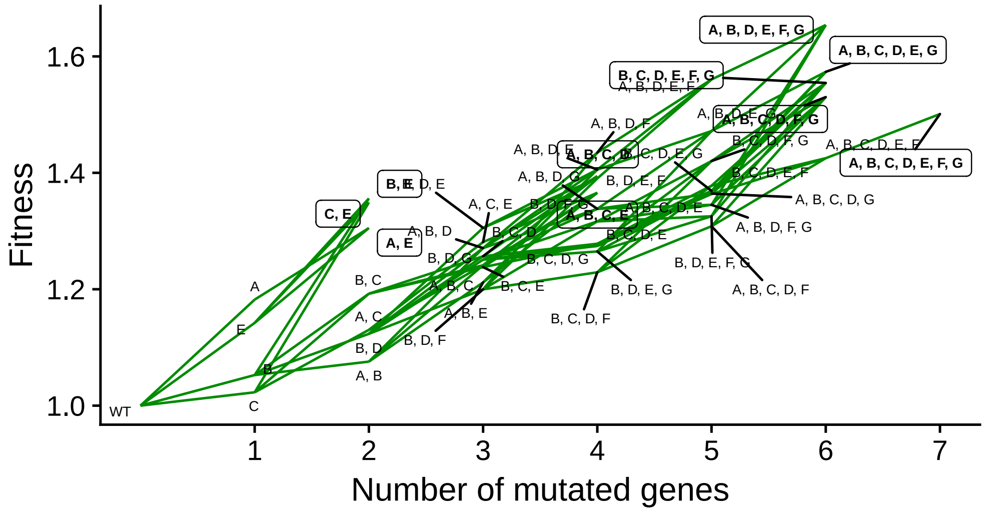

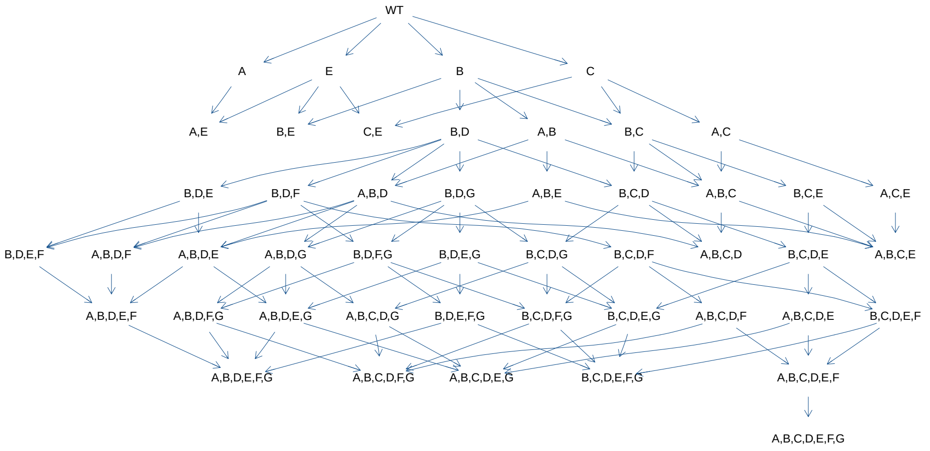

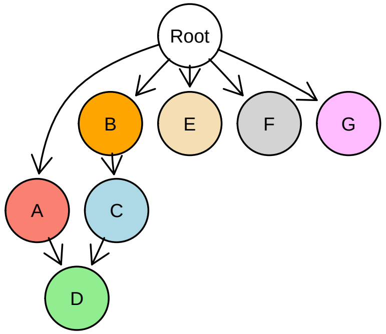

ID	4uRd77KBIQ1QZeh9
acc genotypes	56
peaks no back	9
removed edges	36
prop rem edges	0.243

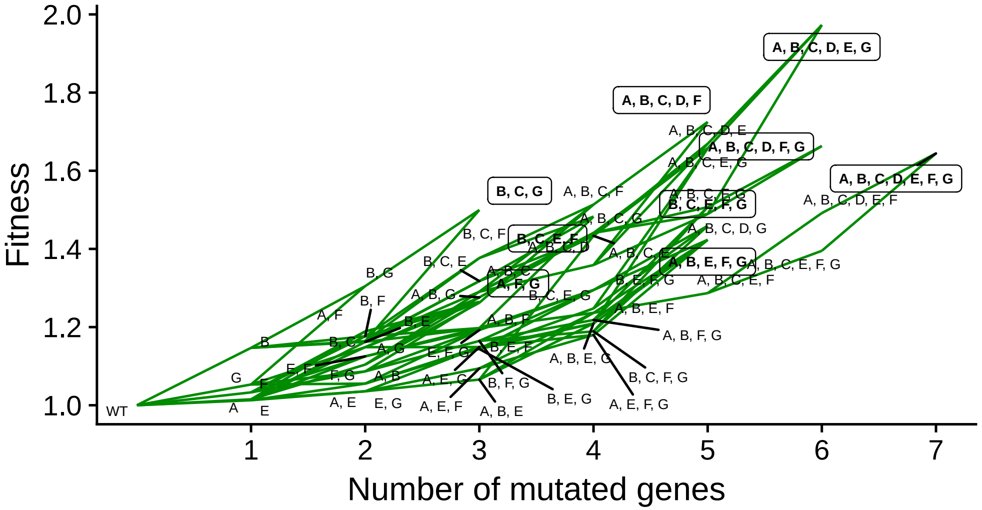

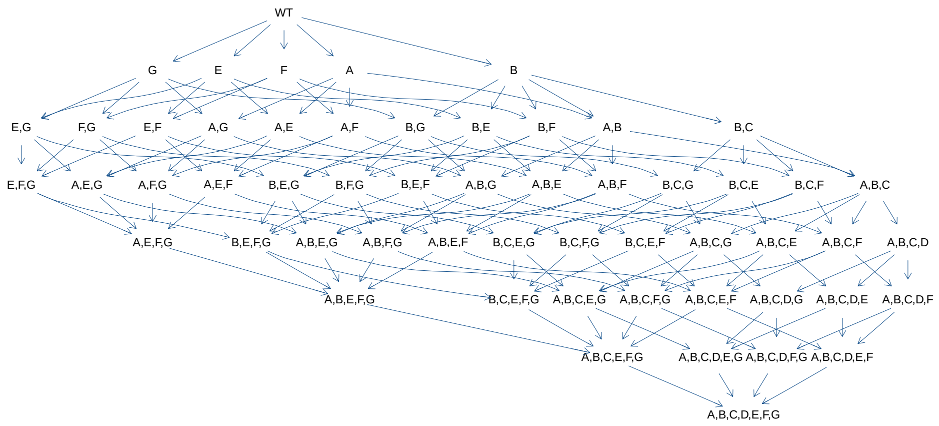

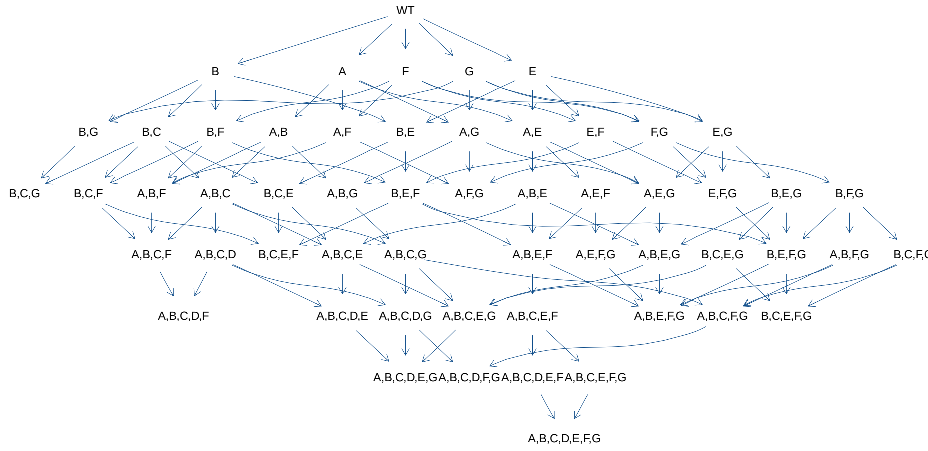

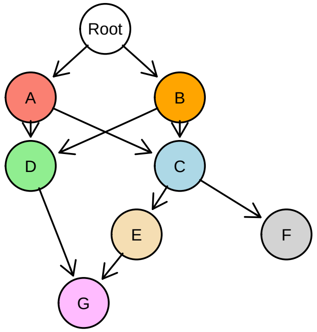

ID	4xM3Wofst2xw10jJ6
acc genotypes	15
peaks no back	2
removed edges	2
prop rem edges	0.091

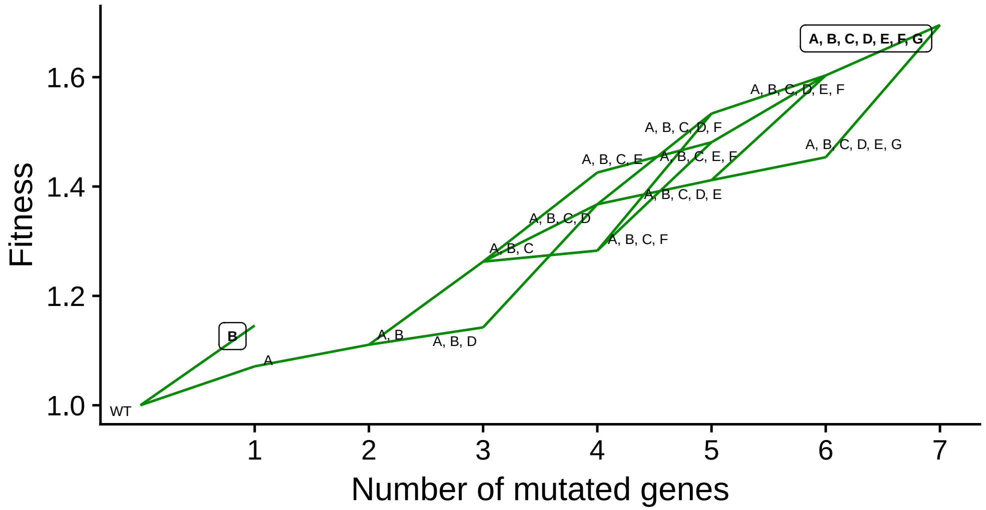

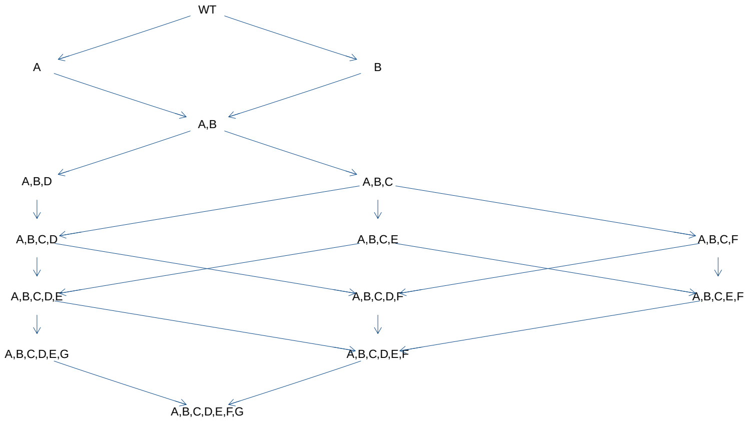

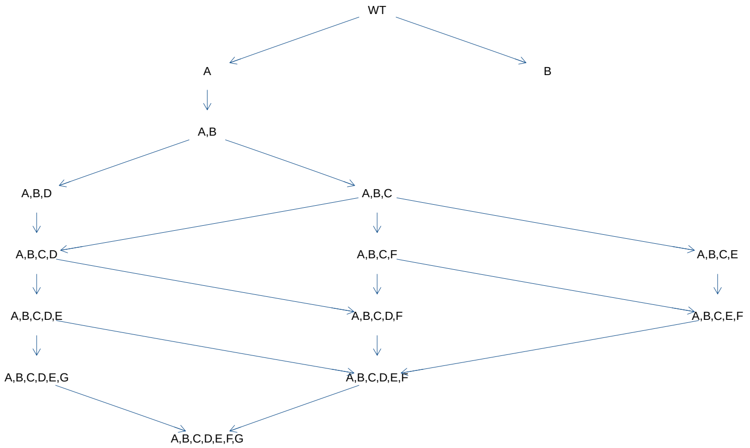

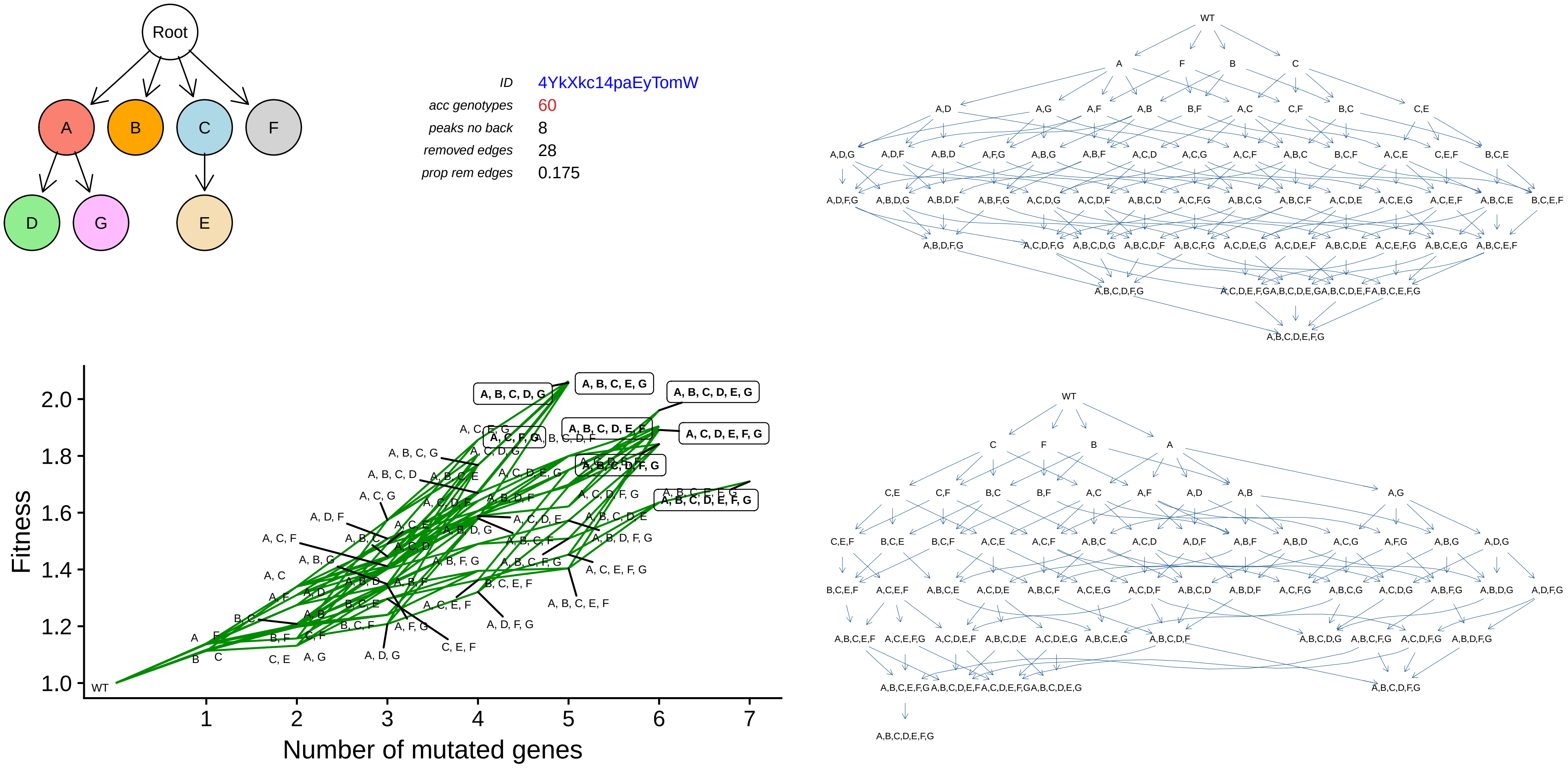
Root
A
B
C
F
D
G
E
ID 4YkXkc14paEyTomW
acc genotypes 60
peaks no back 8
removed edges 28
prop rem edges 0.175
Fitness
Number of mutated genes
A, B, C, D, G
A, B, C, E, G
A, B, C, D, E, G
A, C, D, E, F, G
A, B, C, D, E, F, G
WT

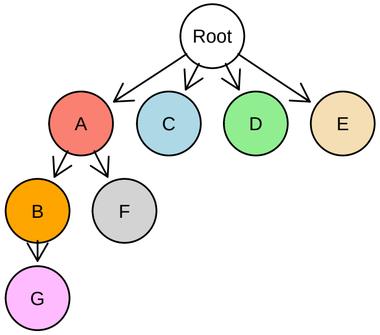

ID	5cLLbdWPenssPhaD
acc genotypes	56
peaks no back	7
removed edges	20
prop rem edges	0.135

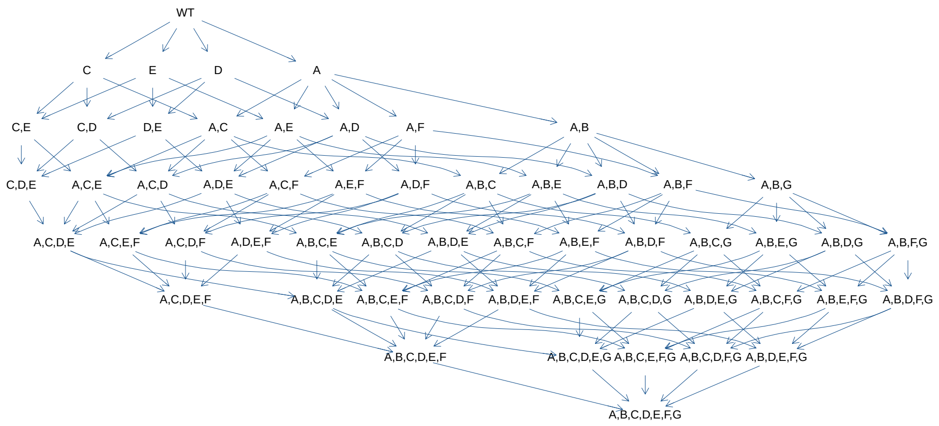

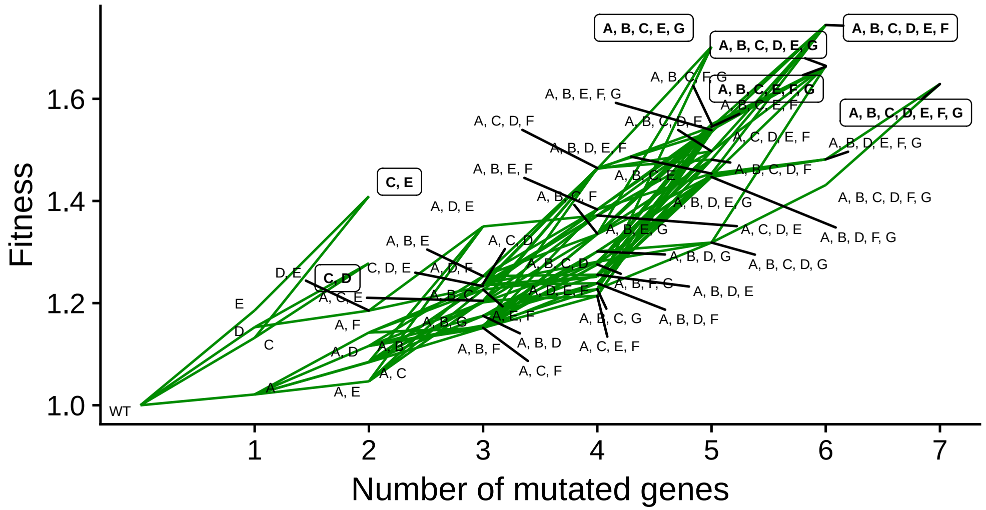

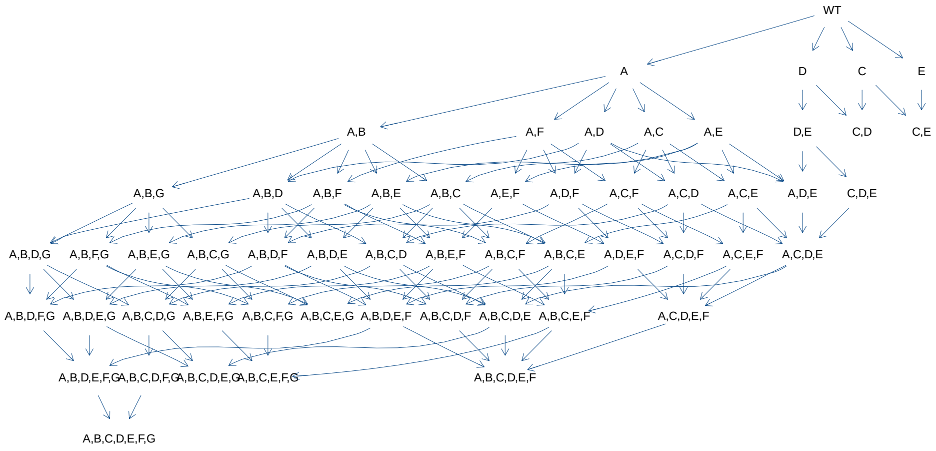

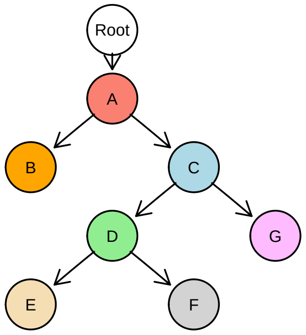

ID	5EI6S7TMXAPy65pq
acc genotypes	23
peaks no back	2
removed edges	3
prop rem edges	0.068

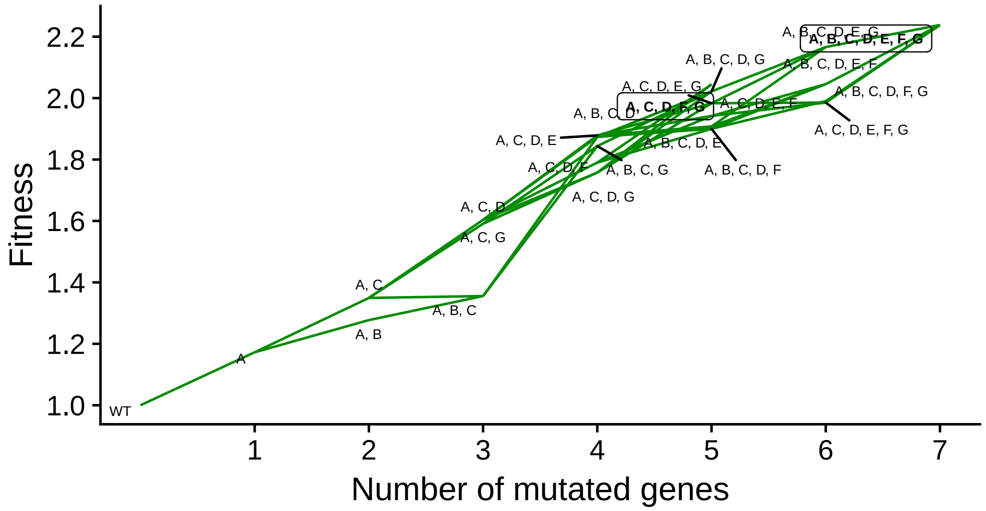

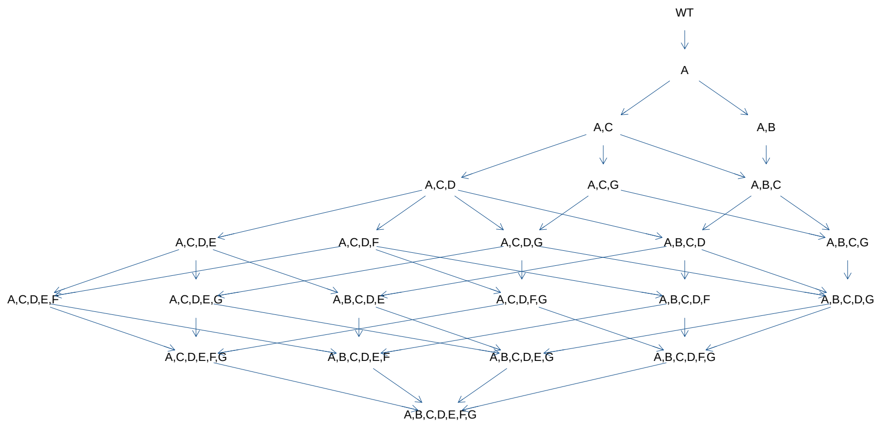

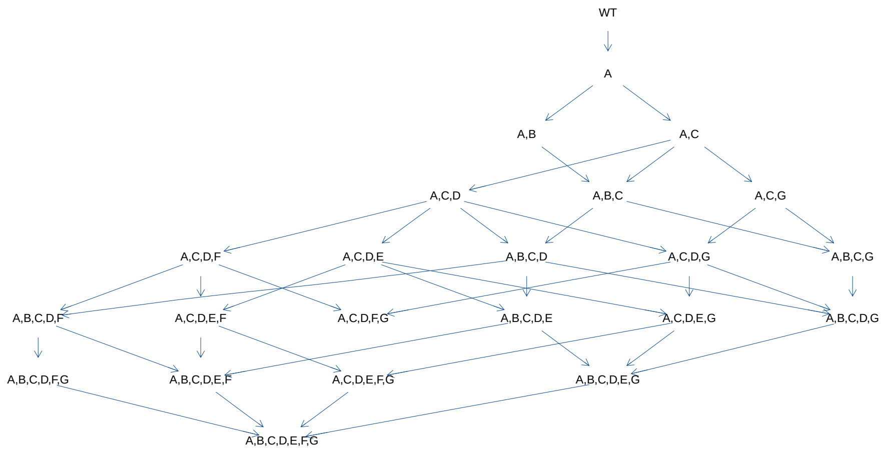

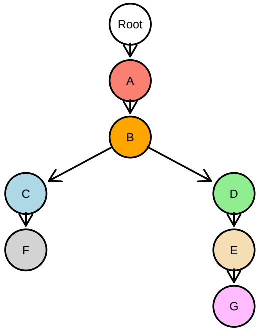

ID	5gEdOQwKmmjGCuwA
acc genotypes	14
peaks no back	3
removed edges	4
prop rem edges	0.211

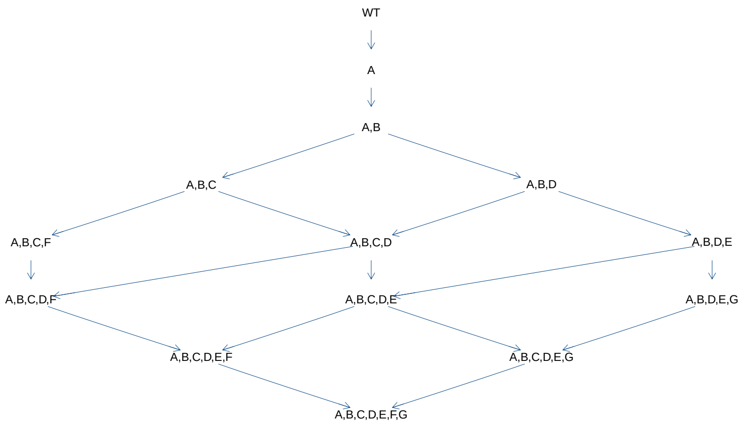

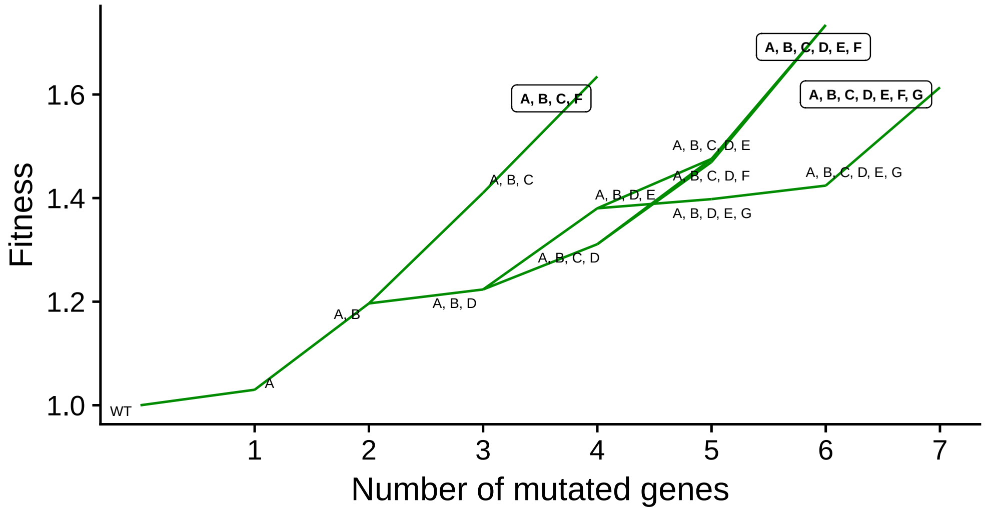

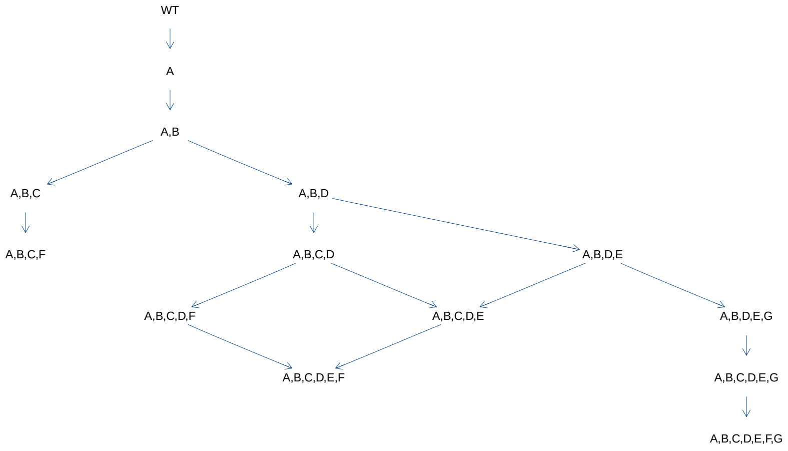

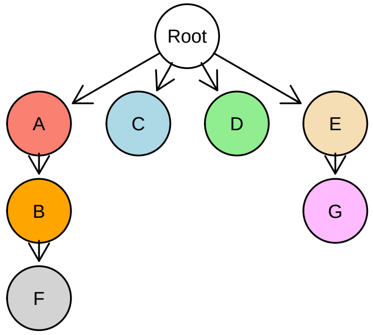

ID 5kZqKEOHzI10BWV78
acc genotypes 48
peaks no back 7
removed edges 16
prop rem edges 0.138

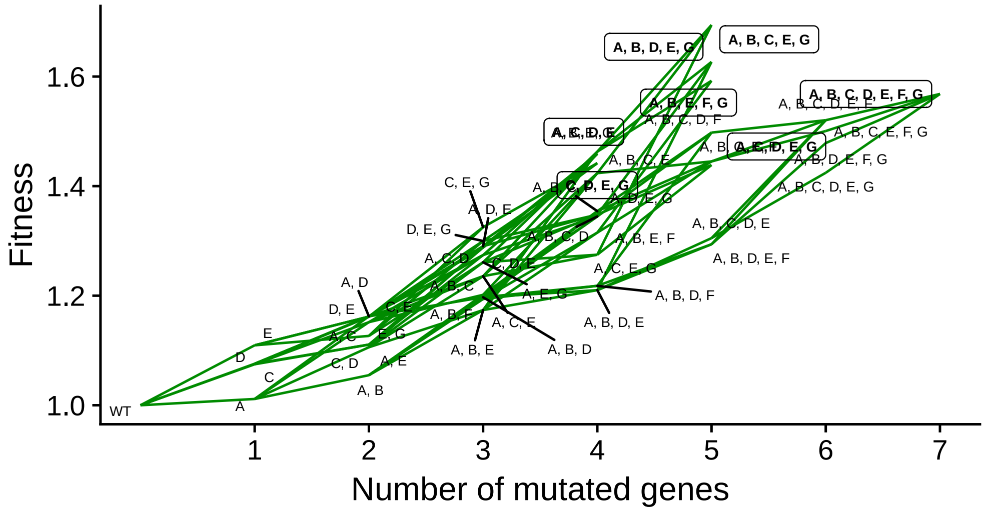

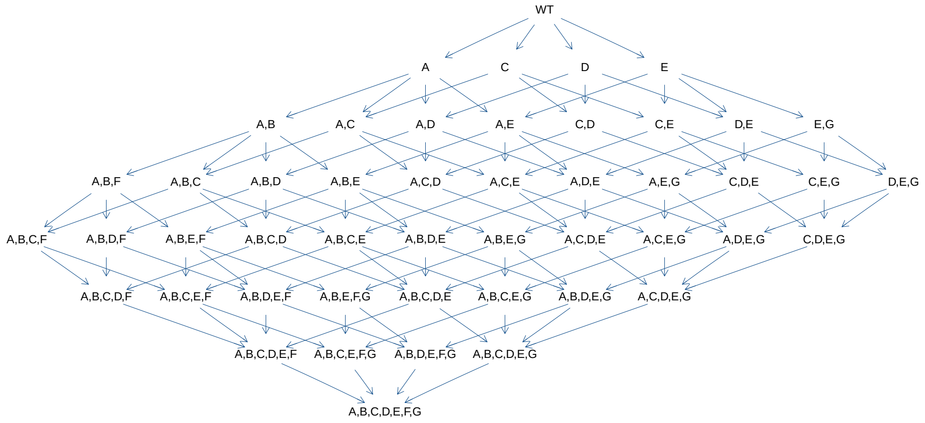

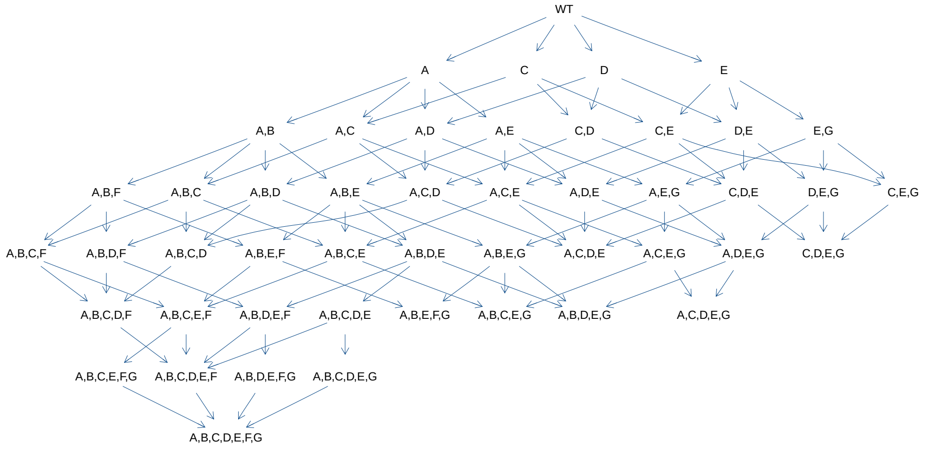

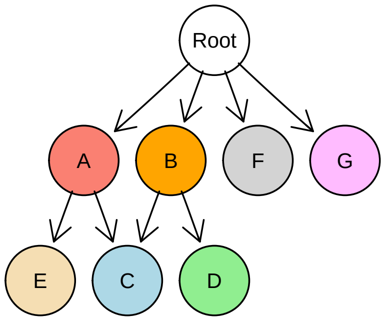

ID	6h4QpMCcKm22zn5f
acc genotypes	52
peaks no back	9
removed edges	31
prop rem edges	0.235

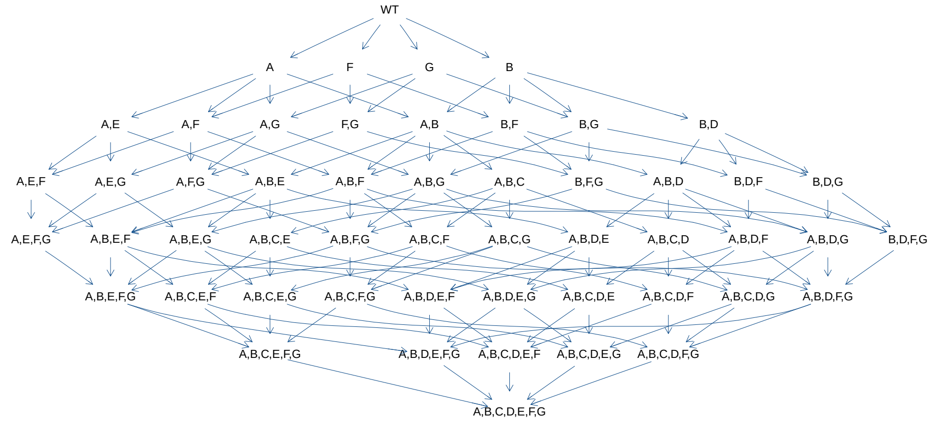

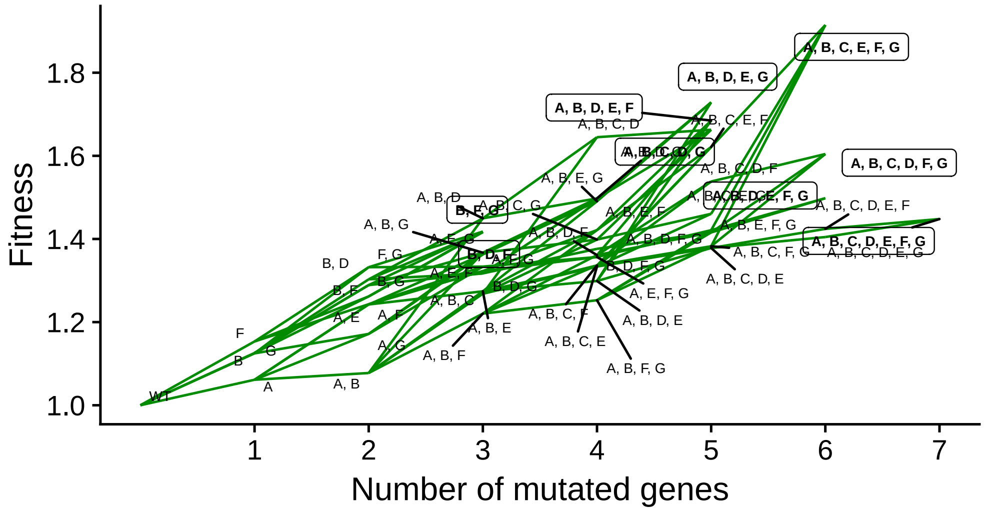

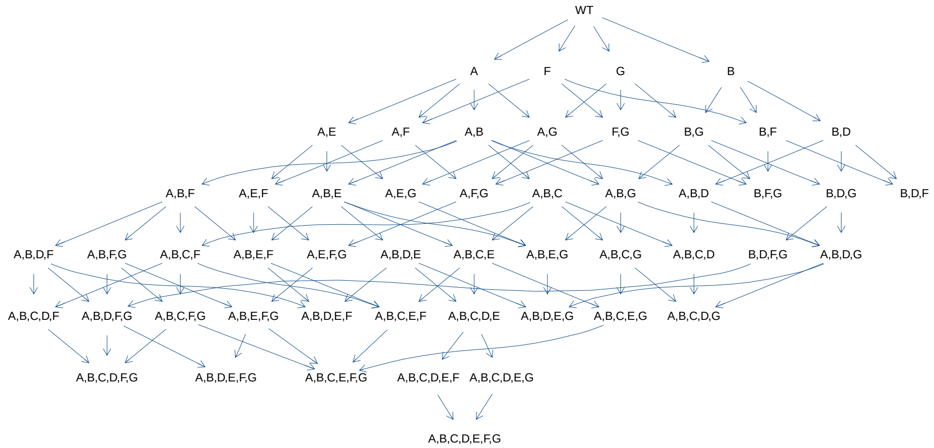

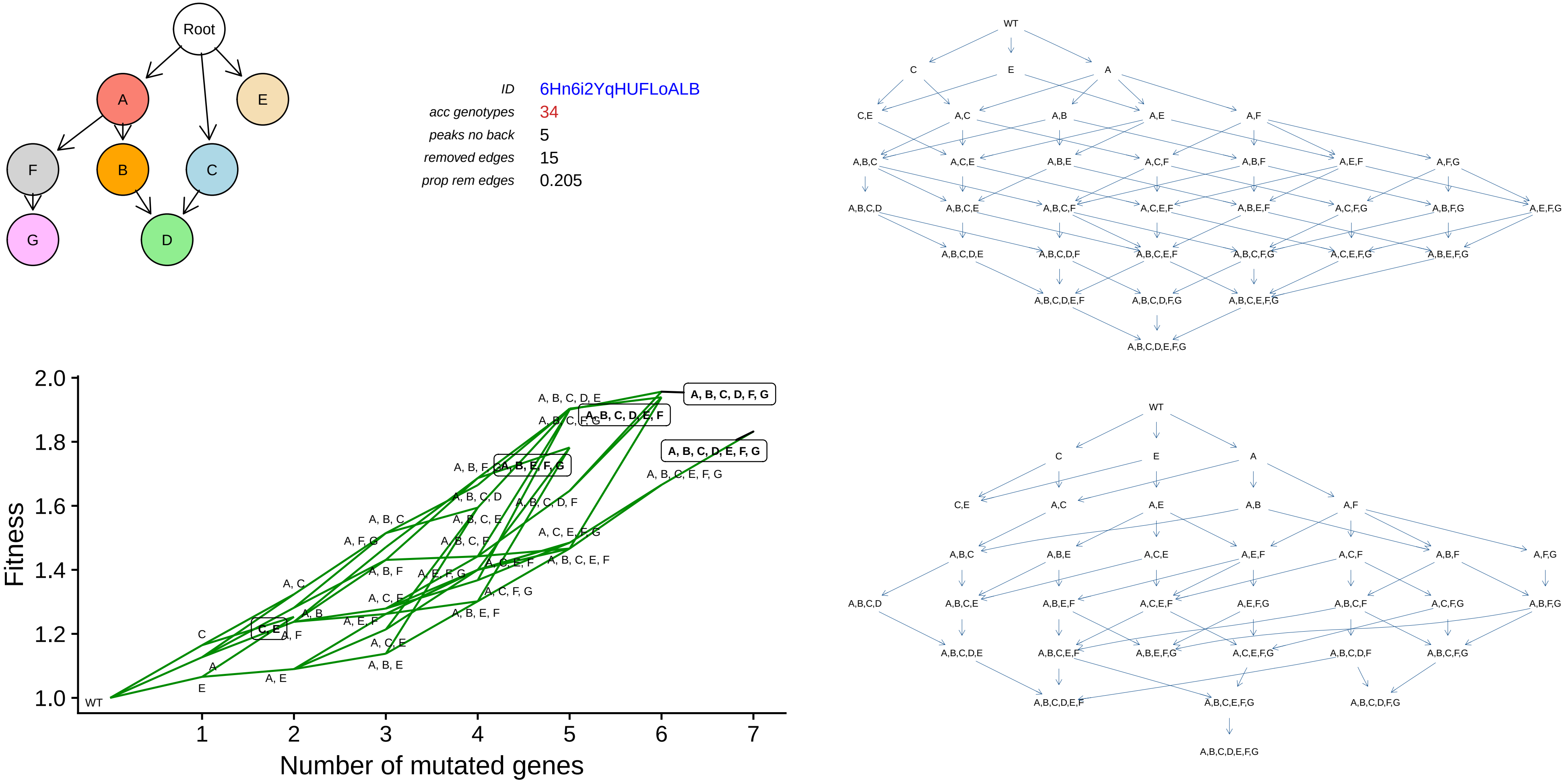
Root
A
B
C
D
E
F
G
ID 6Hn6i2YqHUFLoALB
acc genotypes 34
peaks no back 5
removed edges 15
prop rem edges 0.205
Fitness
Number of mutated genes
A, B, C, D, F, G
A, B, C, D, E, F
A, B, C, D, E, F, G
A, B, E, F, G
C, E
WT
A,B,C,D,E,F,G

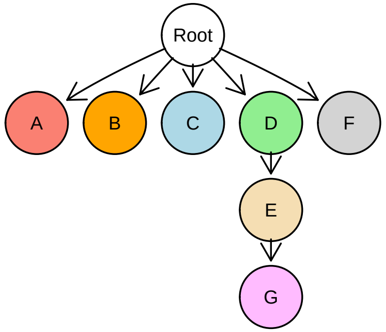

ID 6JONUdvaeCCGnS7Y
acc genotypes 64
peaks no back 8
removed edges 34
prop rem edges 0.193

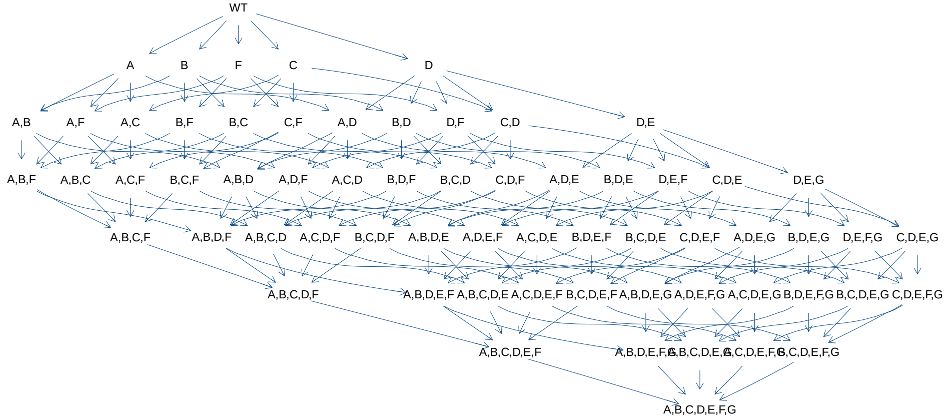

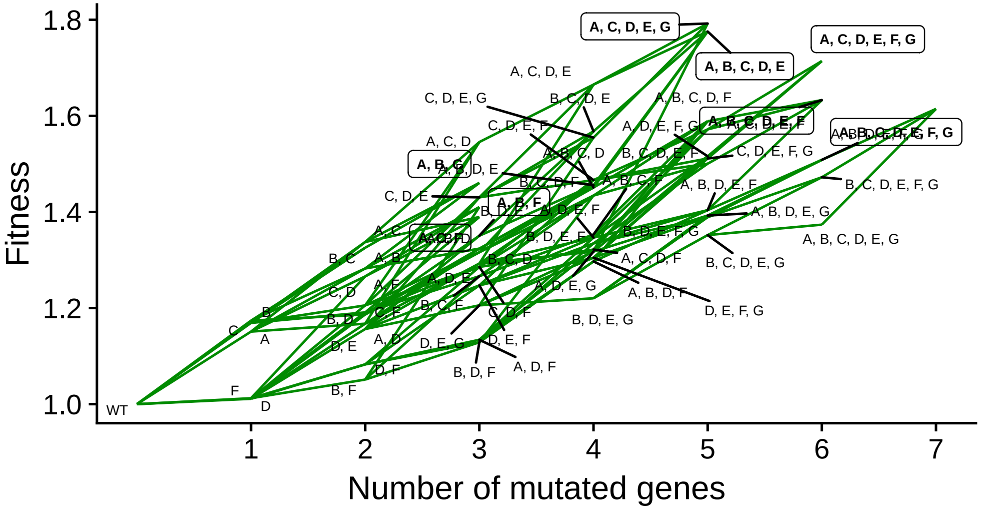

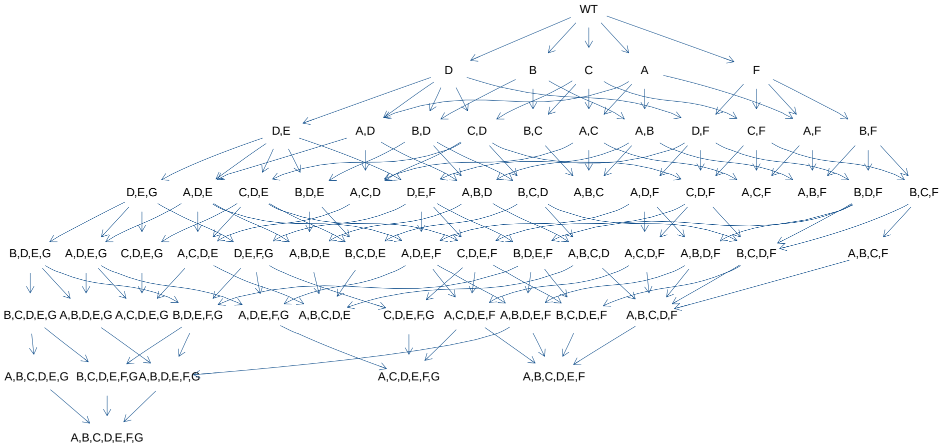

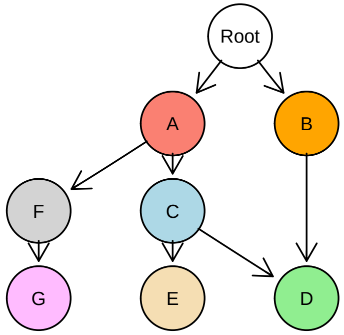

ID	6V3IXXX77zzMpI58
acc genotypes	26
peaks no back	2
removed edges	9
prop rem edges	0.184

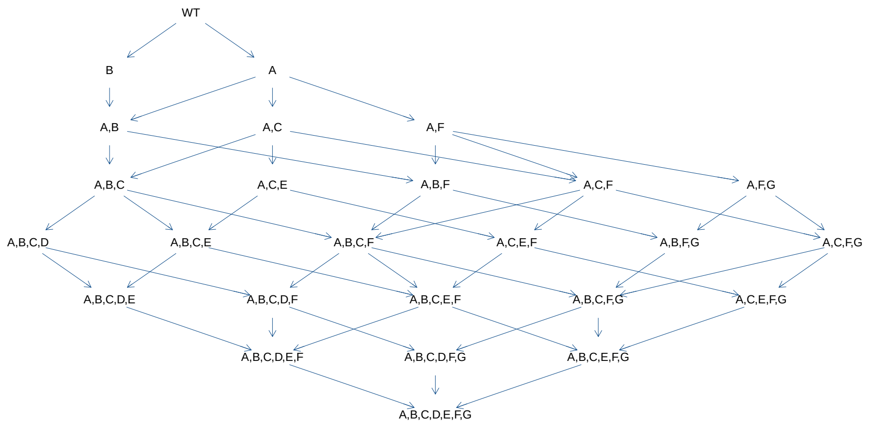

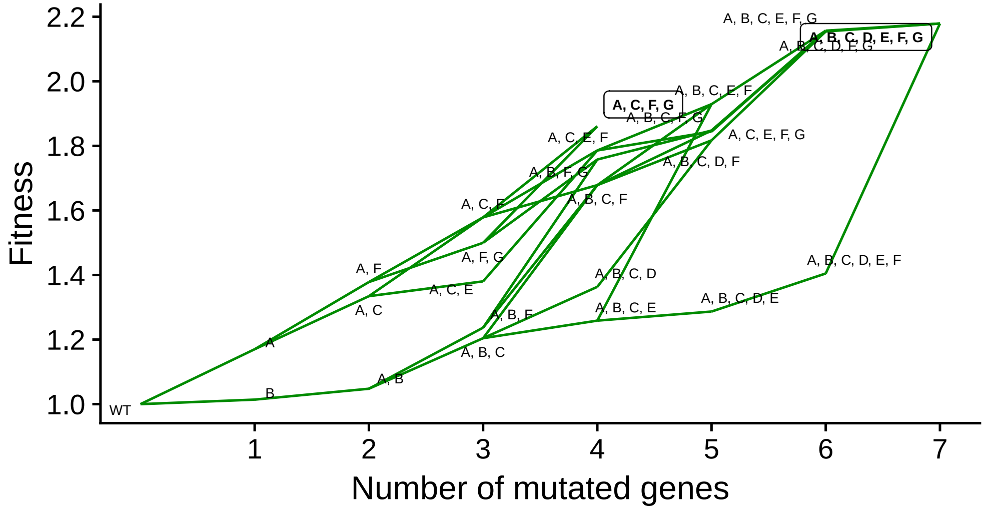

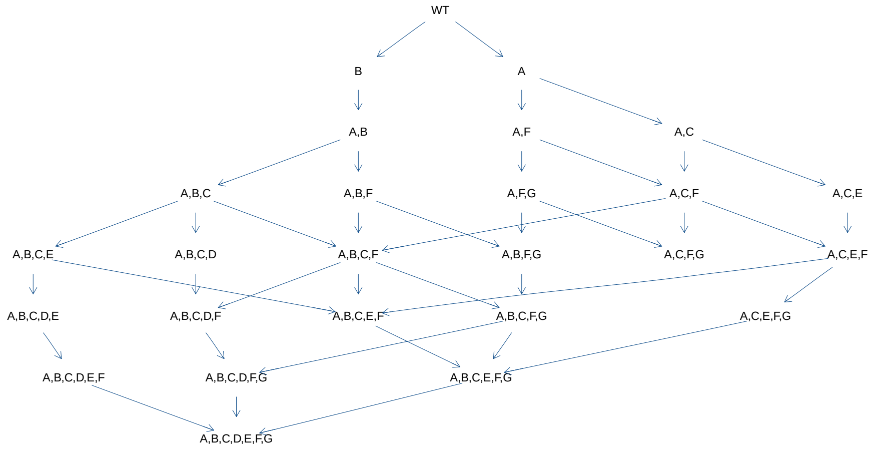

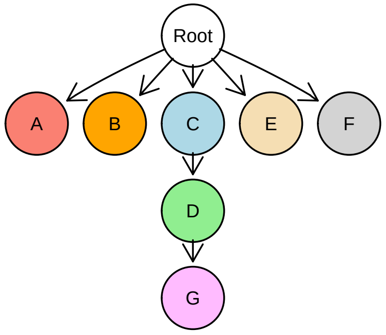

ID	738zqMpeBSiZXfez
acc genotypes	64
peaks no back	7
removed edges	26
prop rem edges	0.148

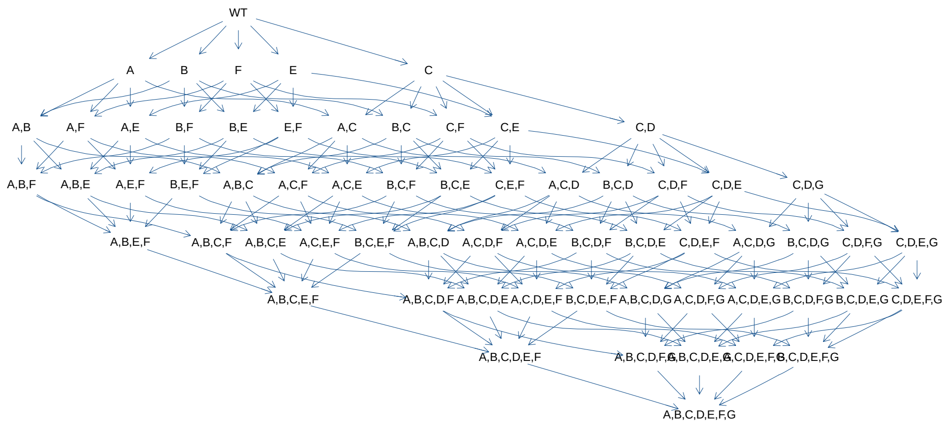

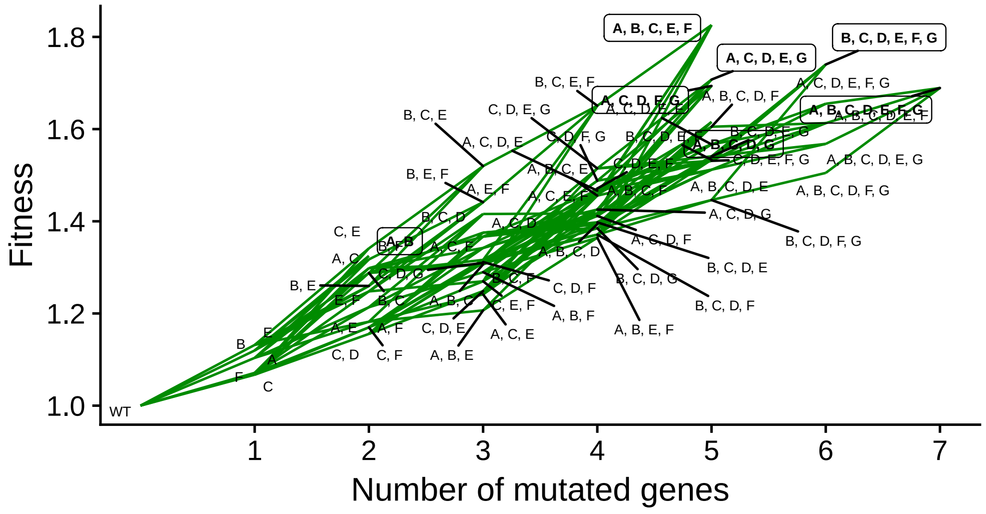

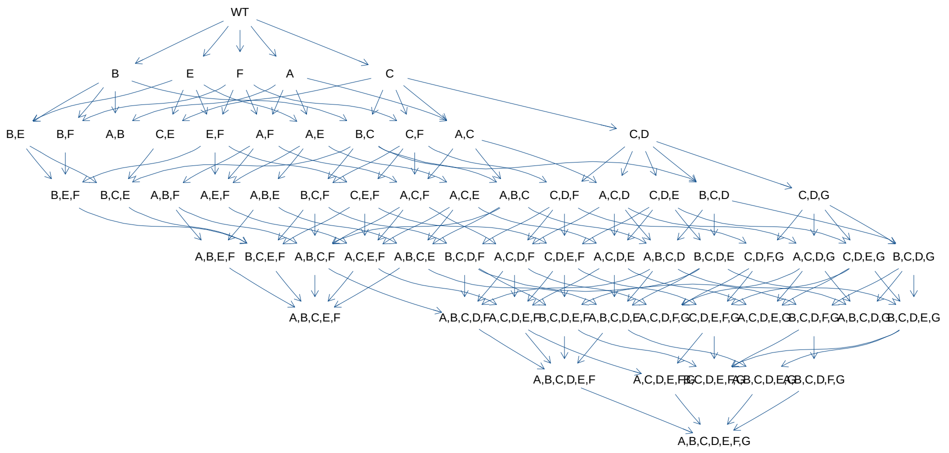

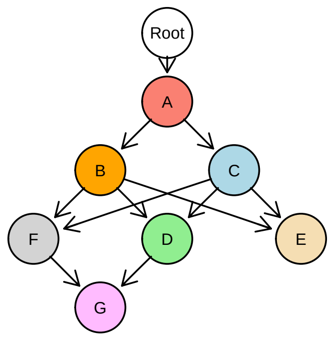

ID	7E10pIyu7UguIUl8I
acc genotypes	14
peaks no back	2
removed edges	1
prop rem edges	0.05

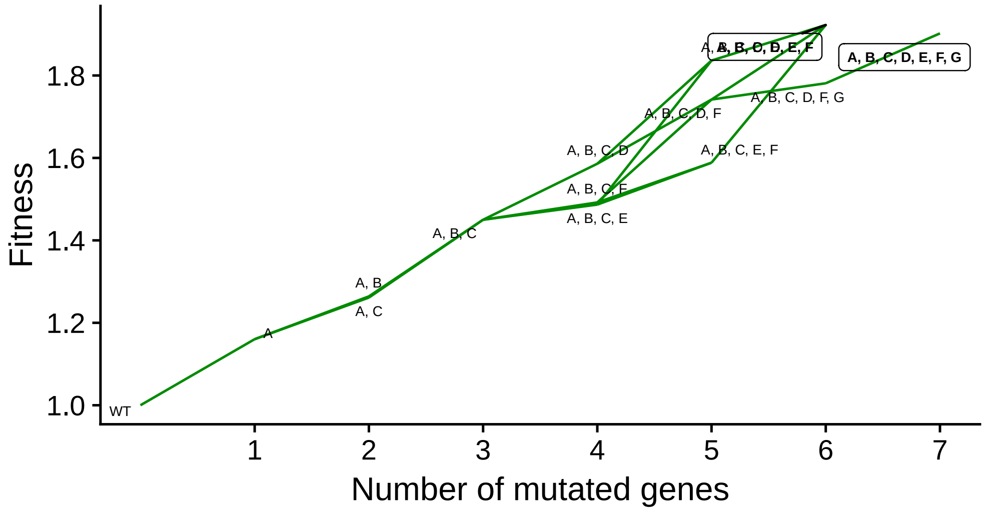

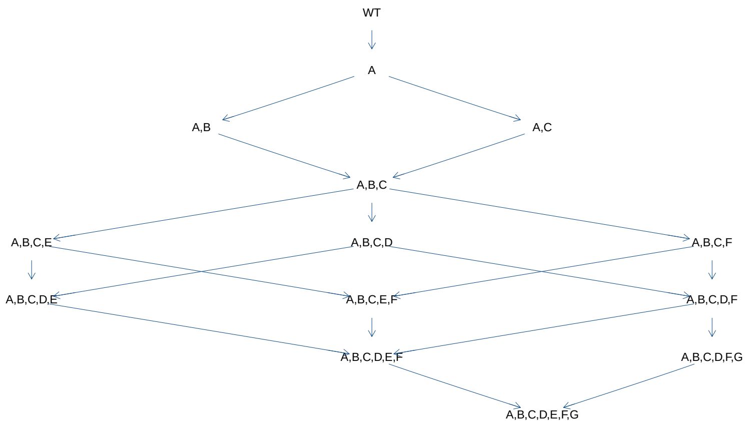

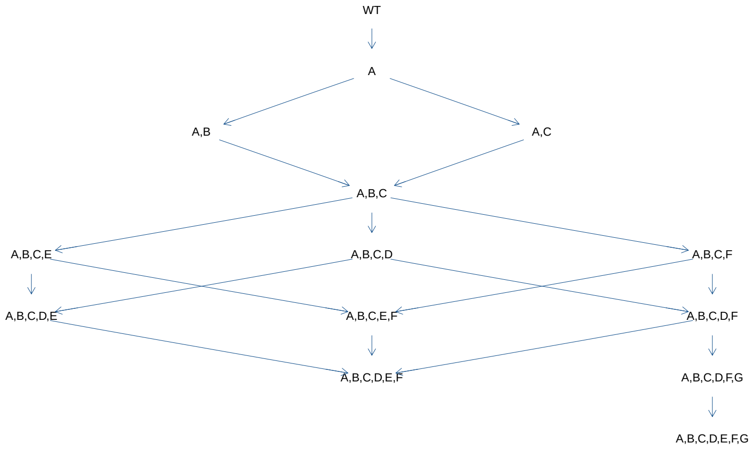

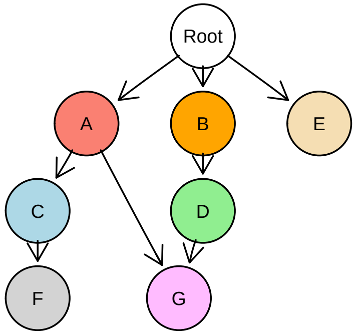

ID	7L5tLW7iVltNxTJ6
acc genotypes	30
peaks no back	6
removed edges	14
prop rem edges	0.237

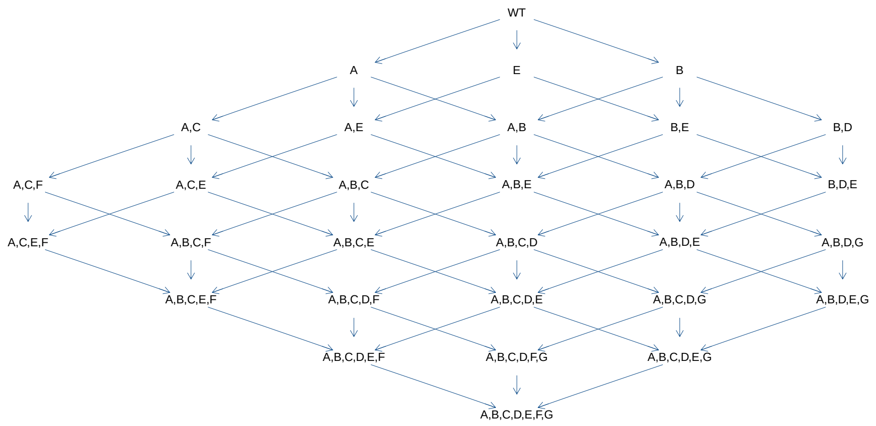

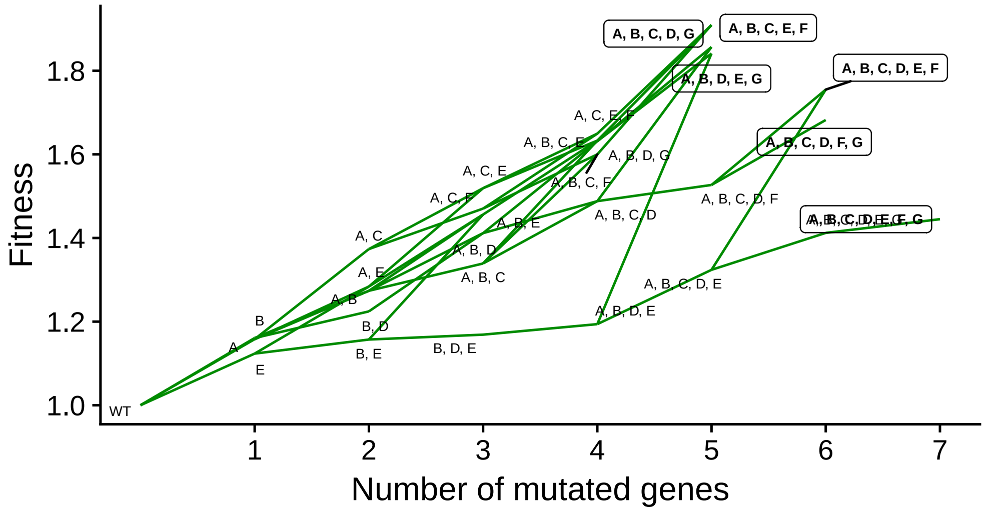

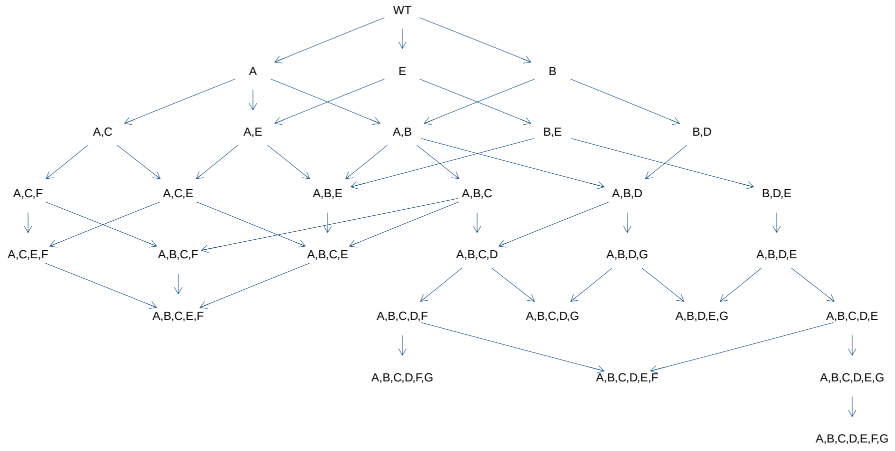

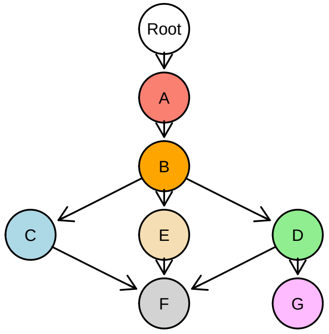

ID	7N2SgGN48X5QWxk9
acc genotypes	16
peaks no back	2
removed edges	5
prop rem edges	0.2

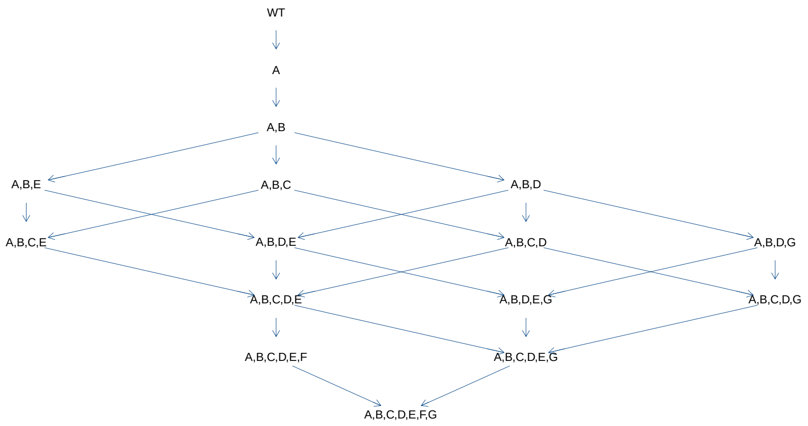

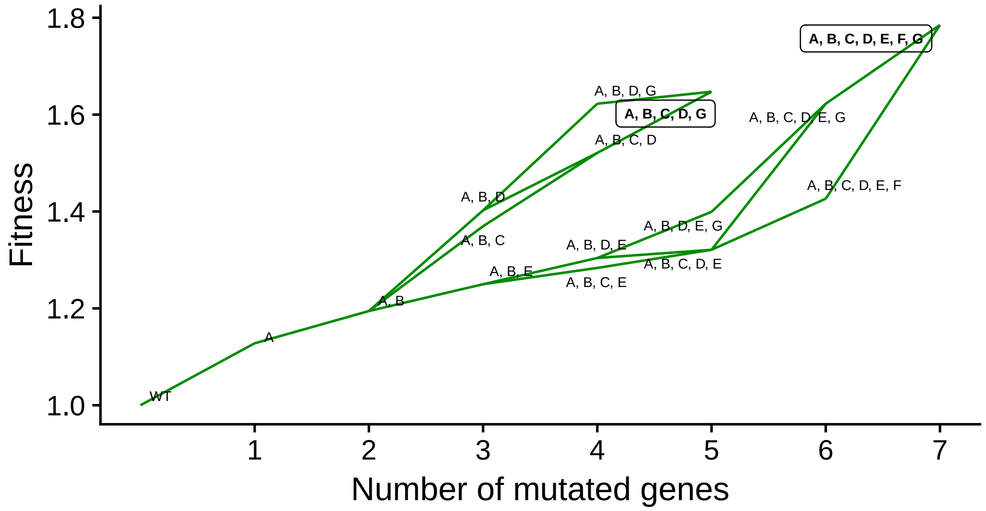

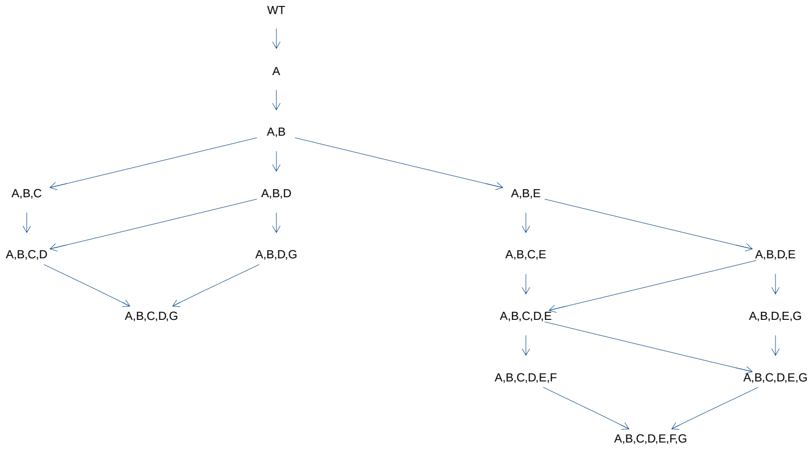

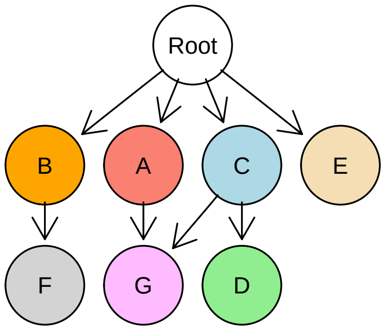

ID	7N9Bf18TzdbZ2zab
acc genotypes	48
peaks no back	6
removed edges	24
prop rem edges	0.207

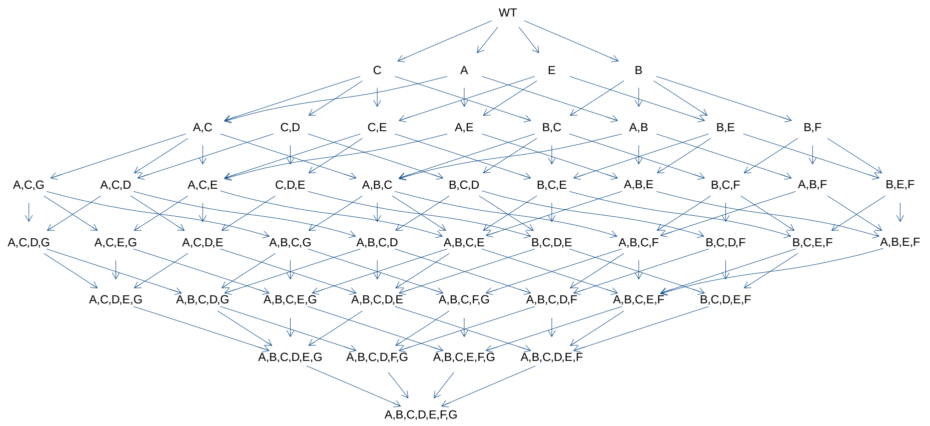

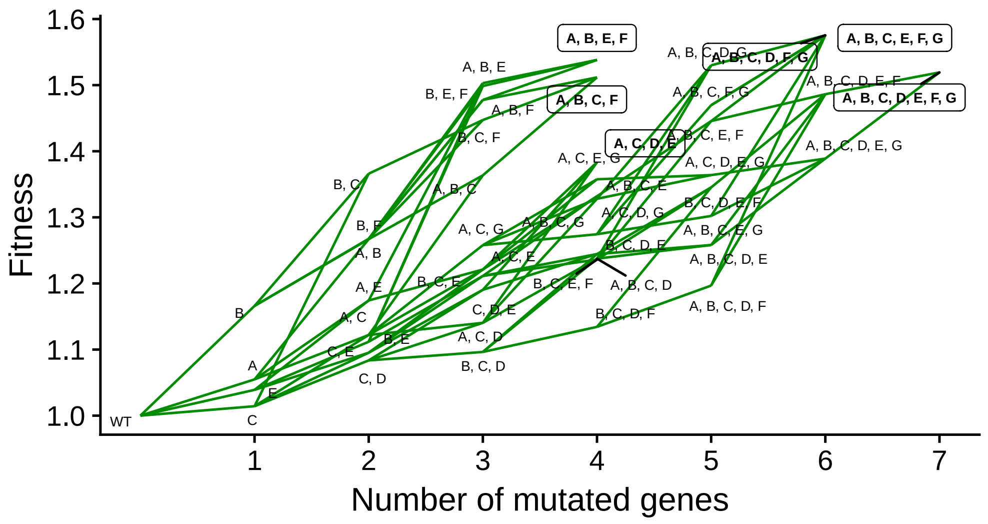

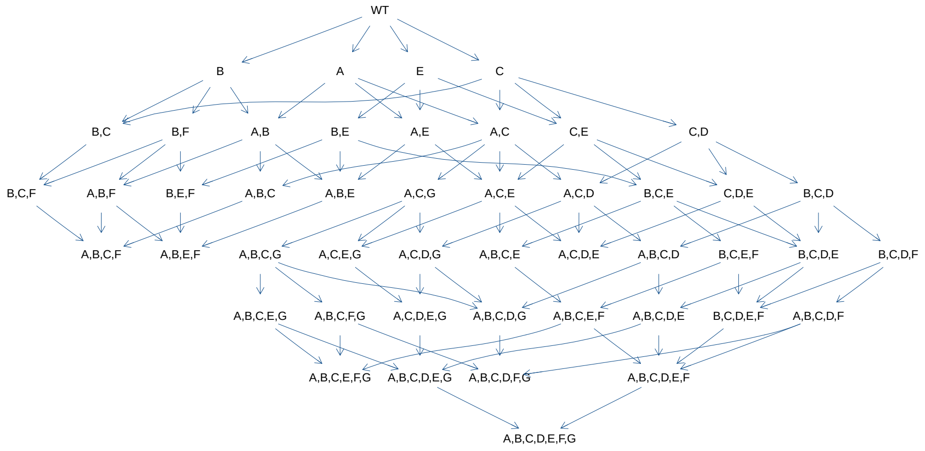

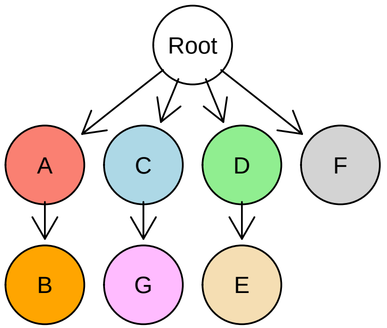

ID	7wKXobmcjD38LD6b
acc genotypes	54
peaks no back	8
removed edges	29
prop rem edges	0.215

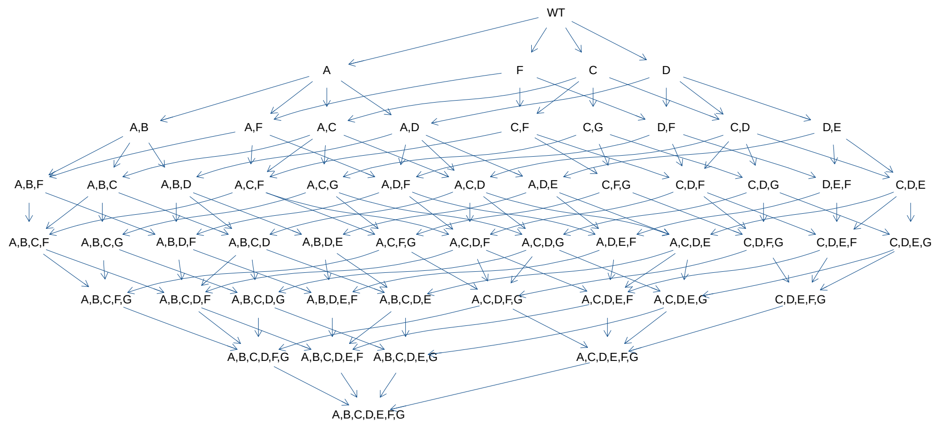

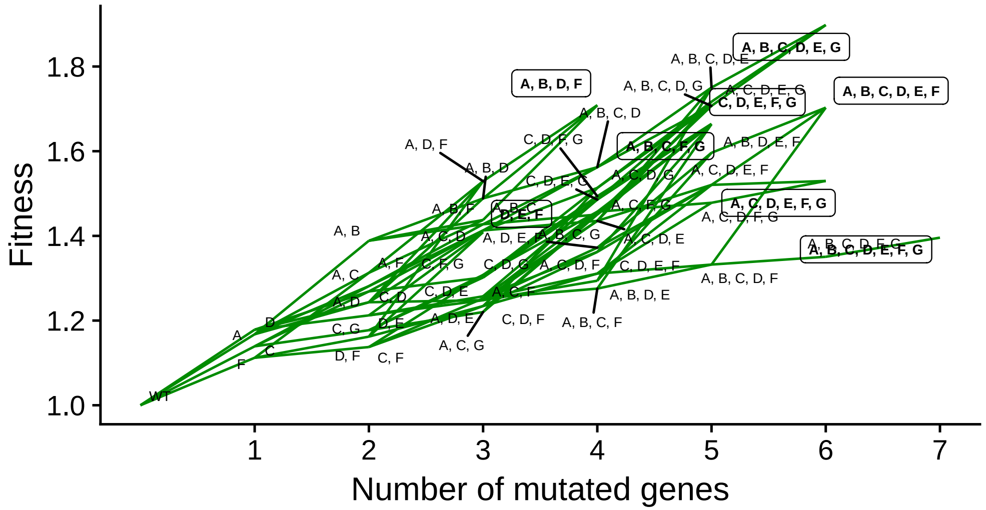

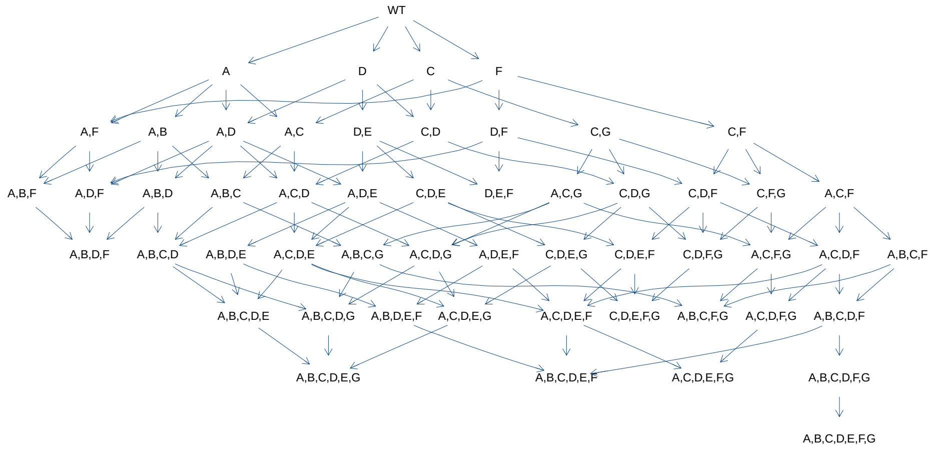

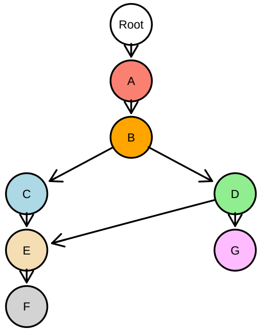

ID	8jUIKcUqRbPGoMif
acc genotypes	12
peaks no back	3
removed edges	2
prop rem edges	0.133

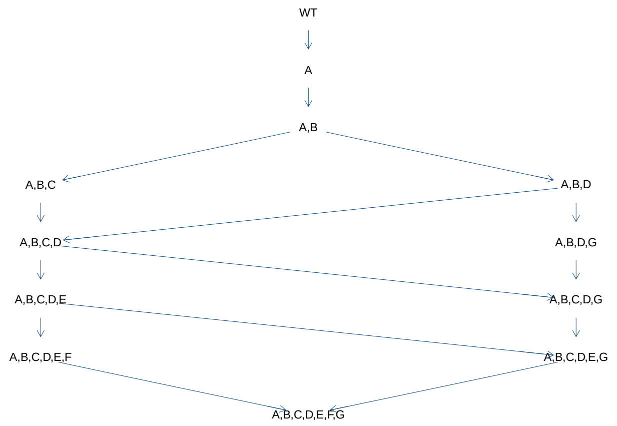

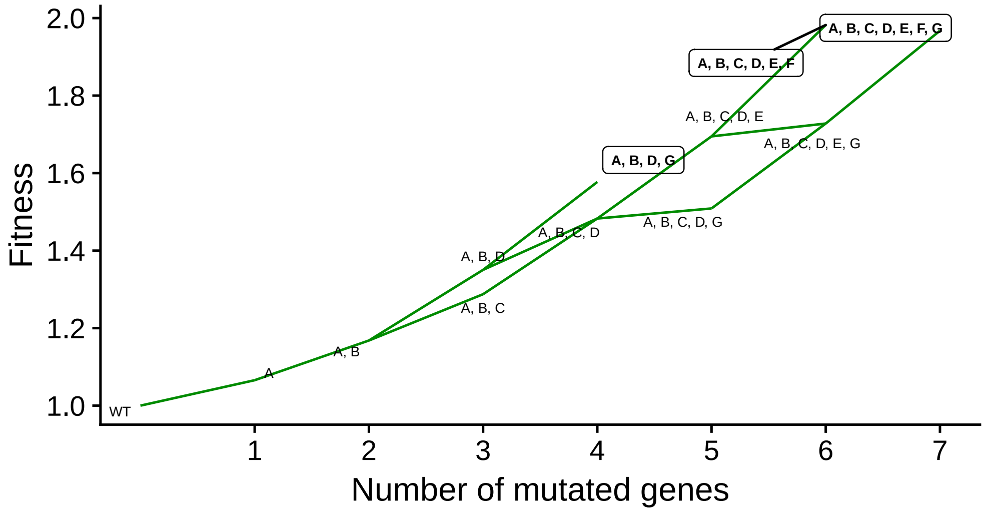

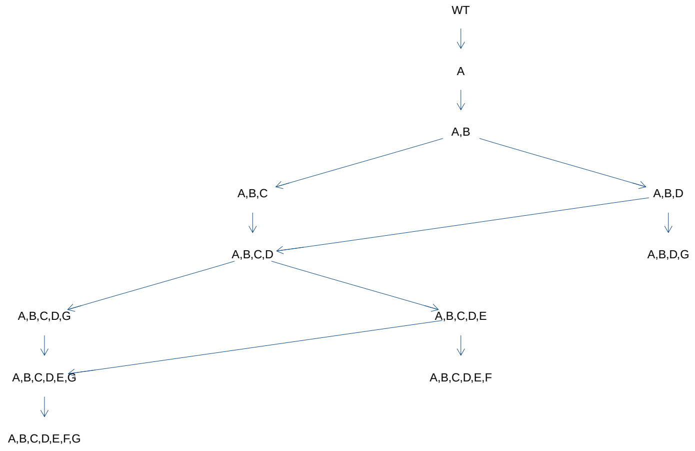

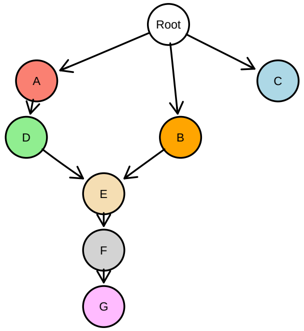

ID	8mSMlsAi8LuLuzZC
acc genotypes	18
peaks no back	4
removed edges	4
prop rem edges	0.138

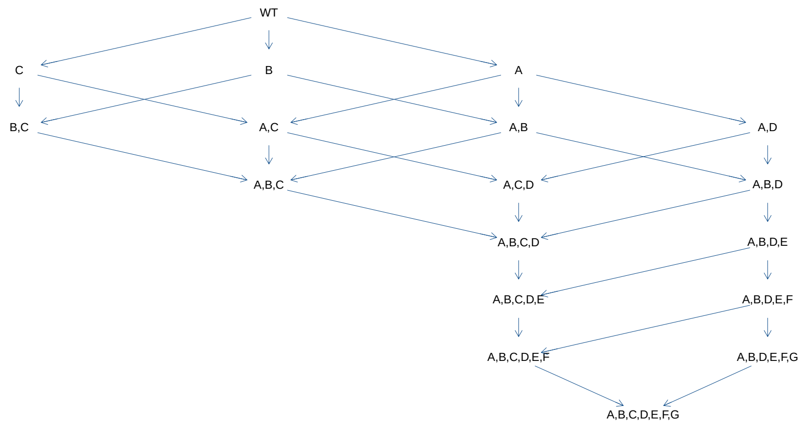

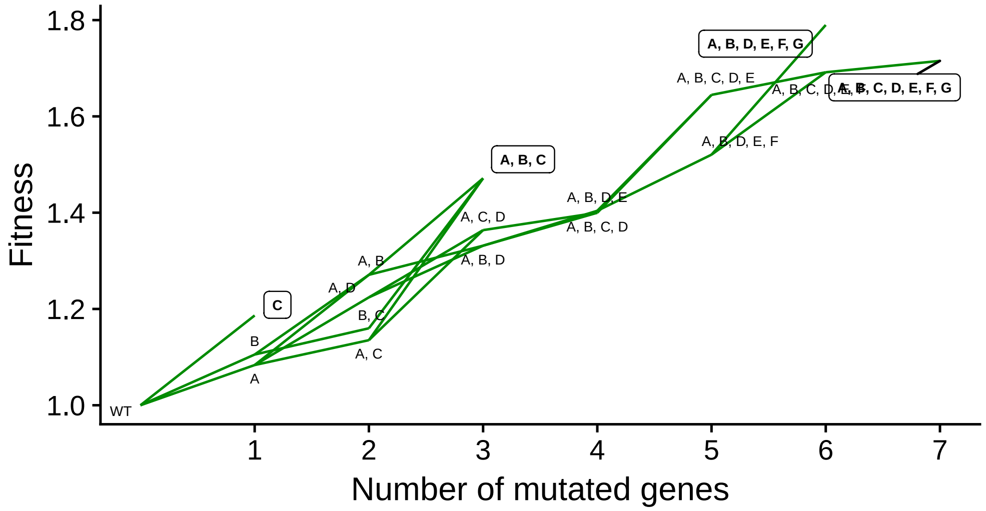

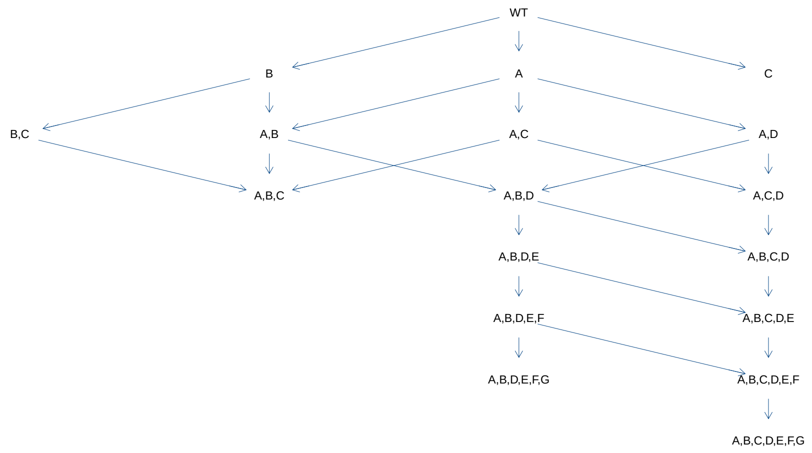

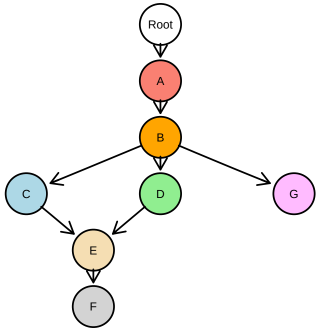

ID	8QlFQCUlUVfC10PZr
acc genotypes	14
peaks no back	2
removed edges	1
prop rem edges	0.05

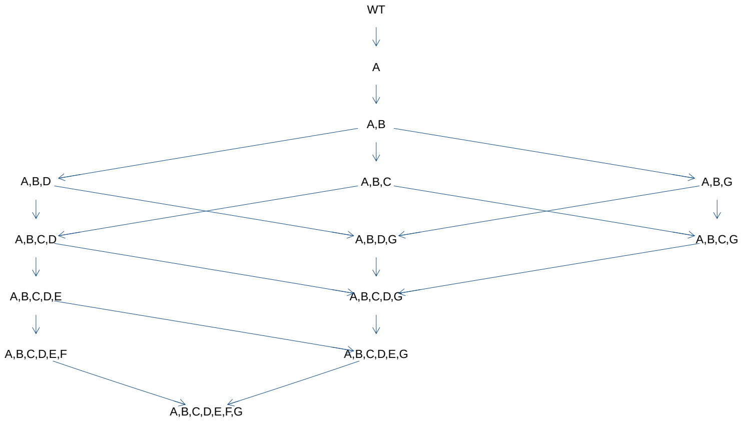

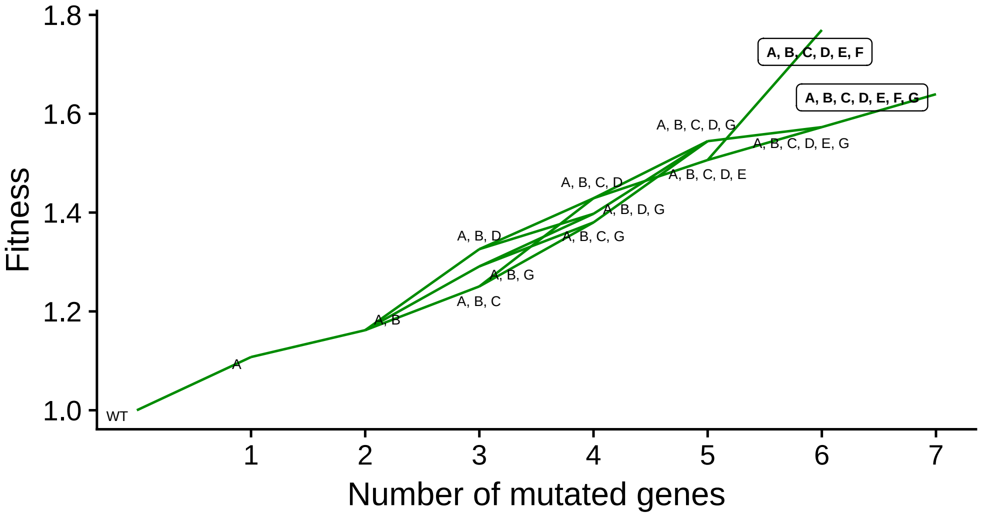

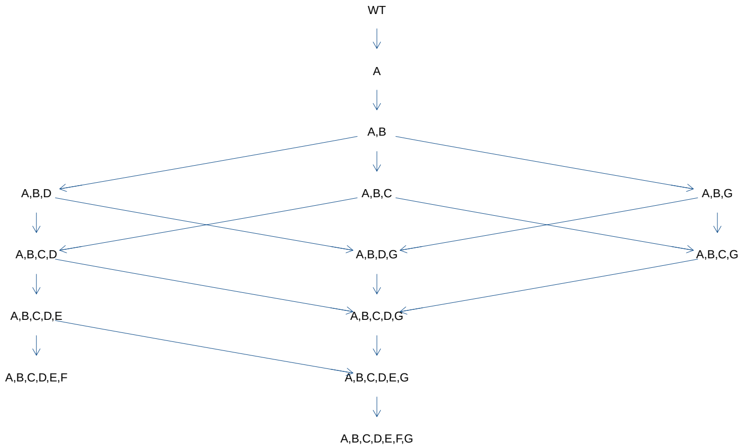

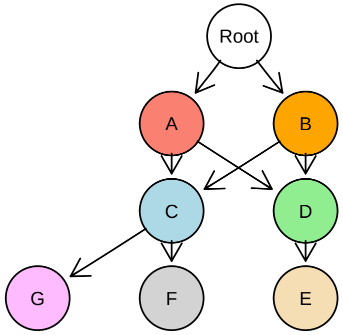

ID 8u1FzrFscU1z8xqB
acc genotypes 18
peaks no back 4
removed edges 3
prop rem edges 0.103

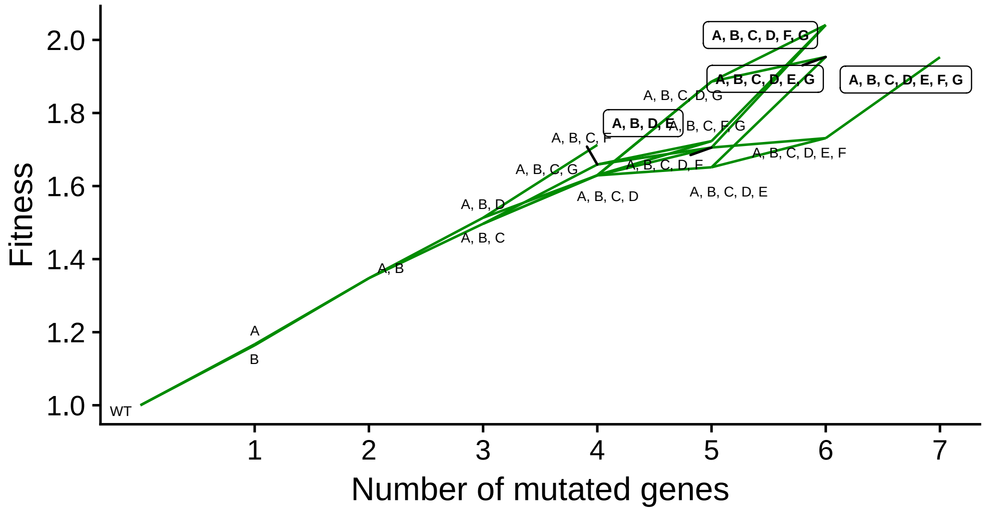

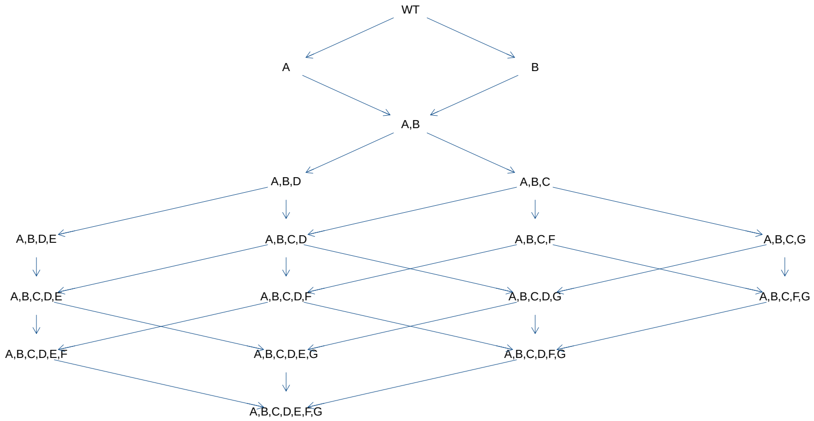

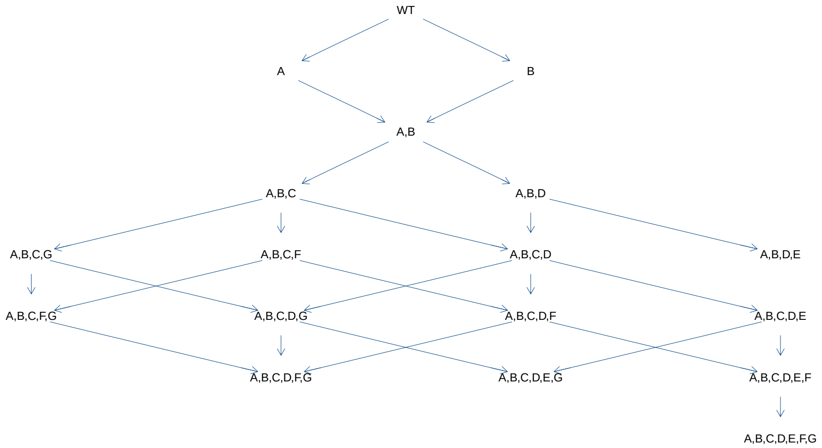

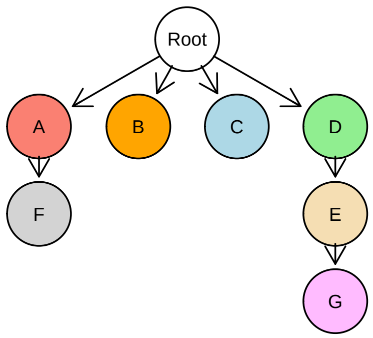

ID	9INXHxuzCY7gqqhs
acc genotypes	48
peaks no back	6
removed edges	26
prop rem edges	0.224

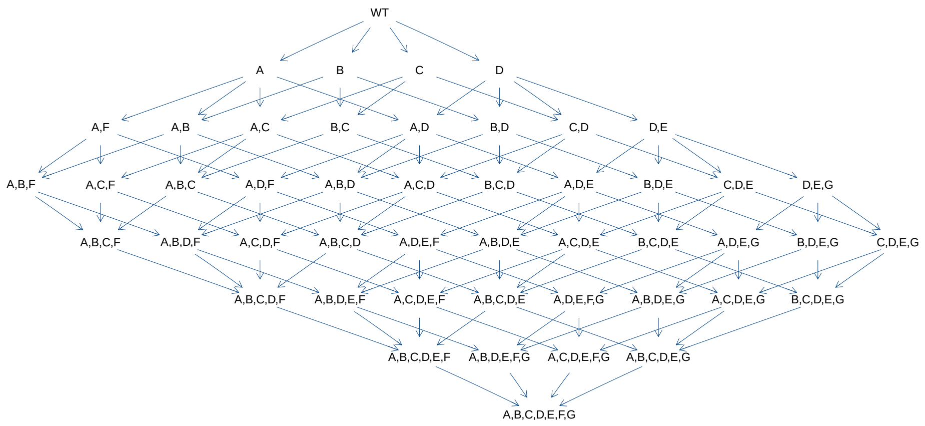

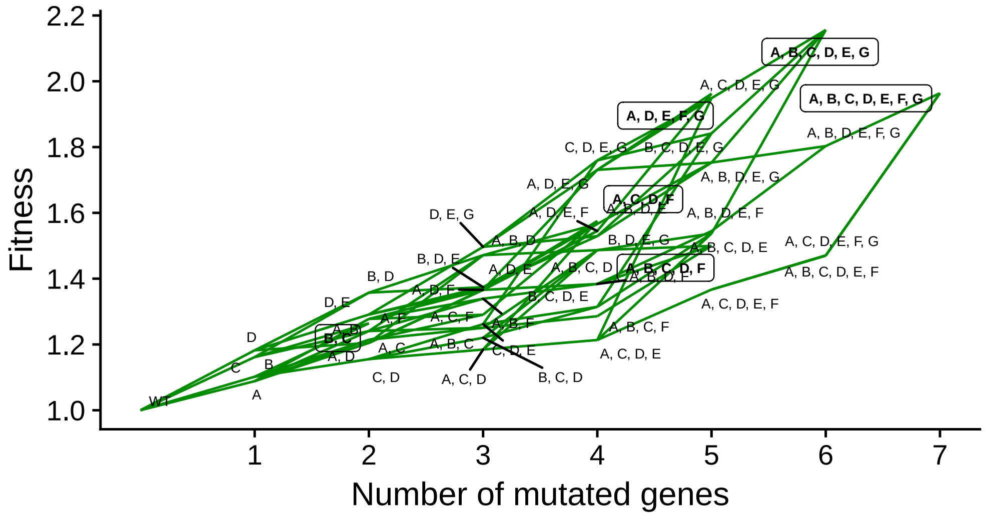

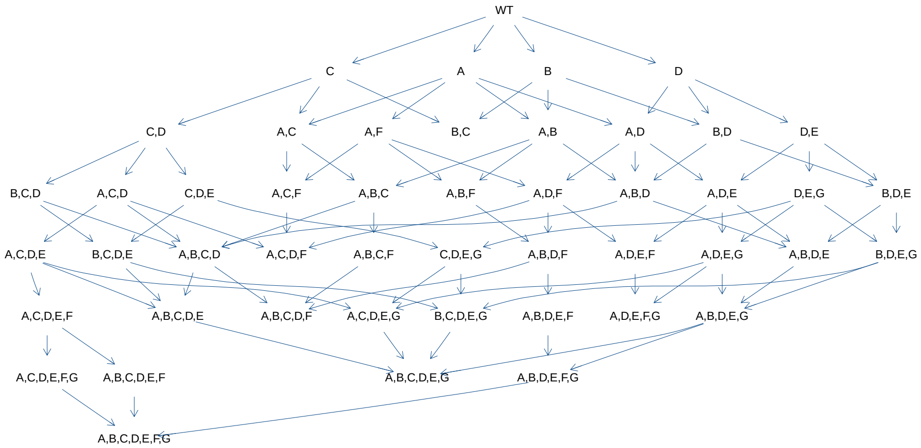

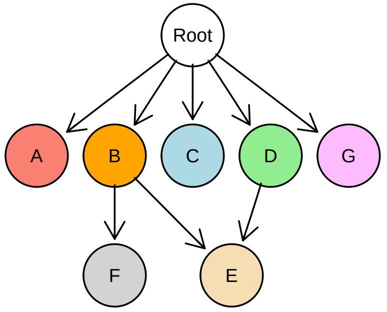

ID	9p9QxtT6PnU5iRpy
acc genotypes	64
peaks no back	9
removed edges	37
prop rem edges	0.21

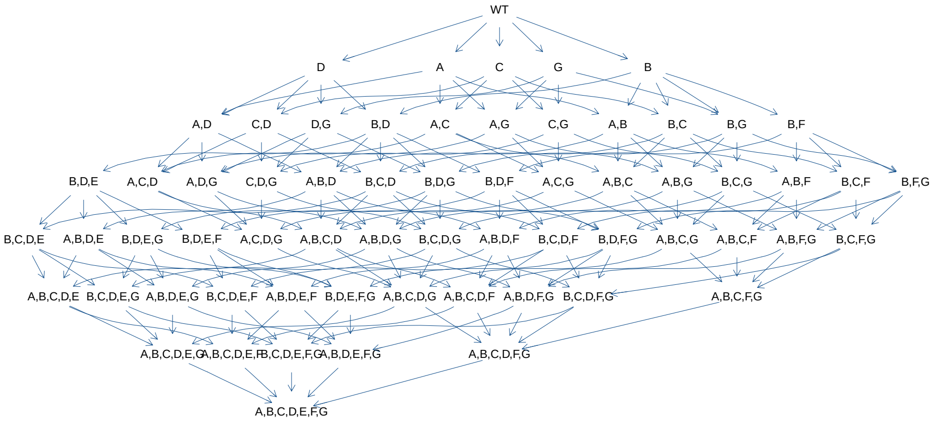

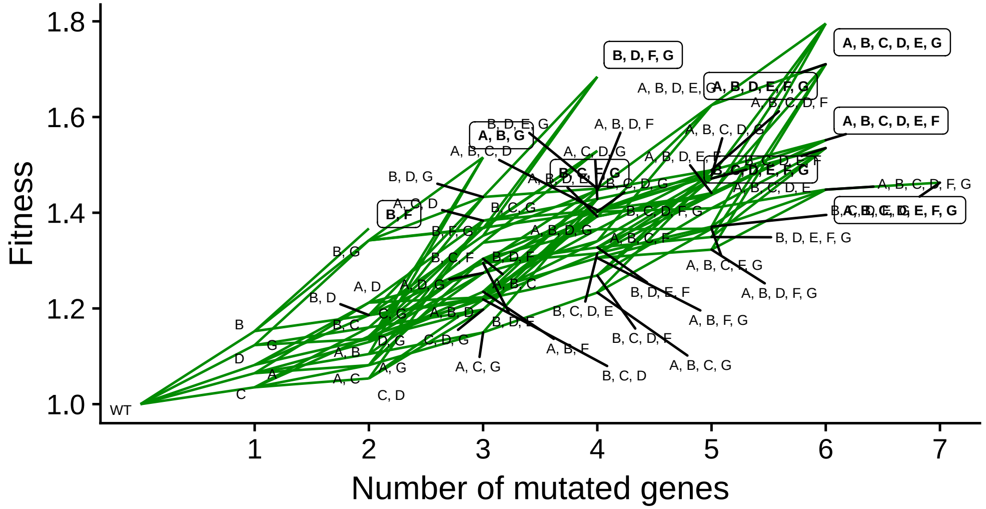

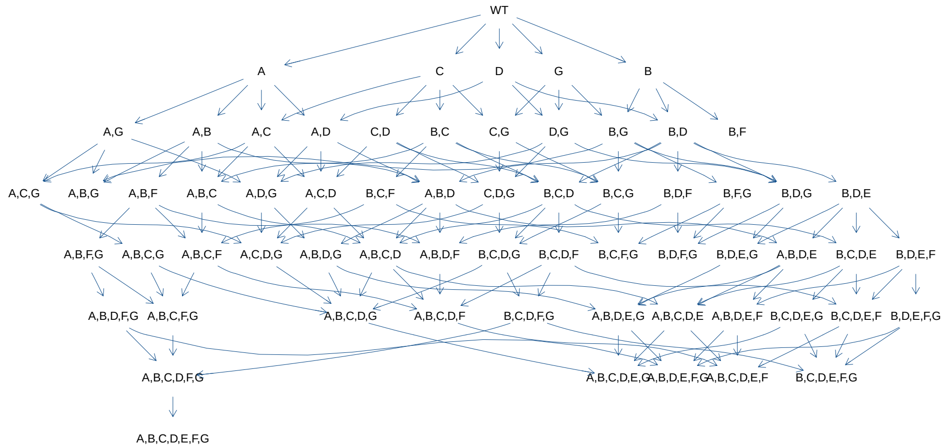

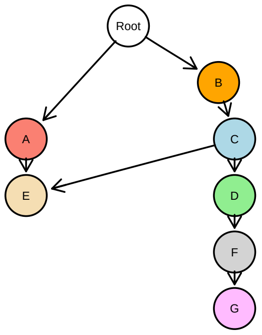

ID	9xXGITgcqHNk6QAO
acc genotypes	16
peaks no back	4
removed edges	4
prop rem edges	0.174

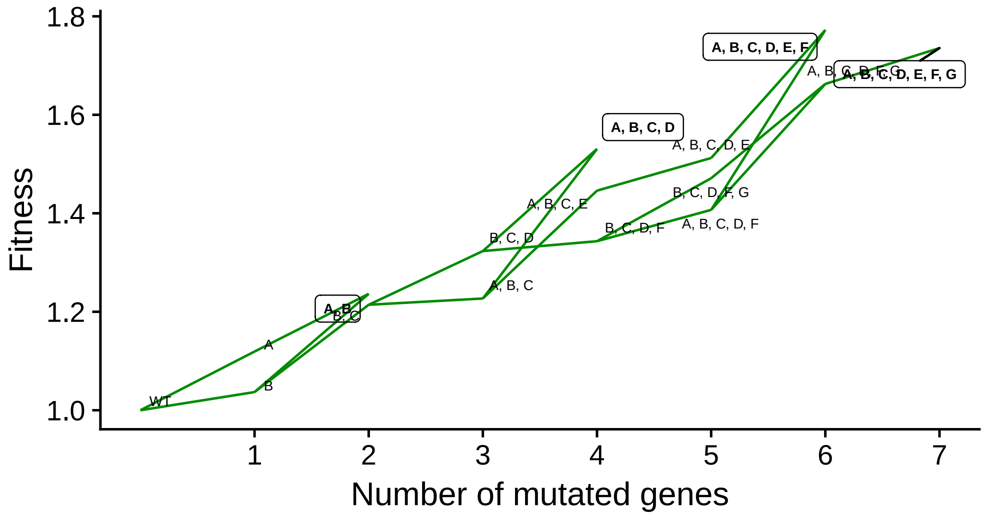

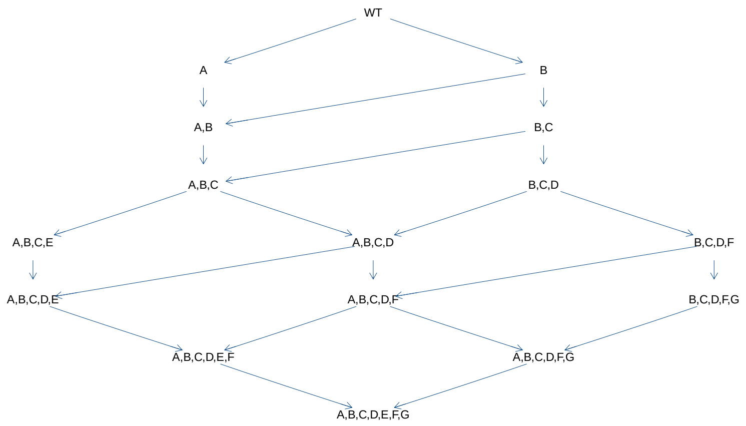

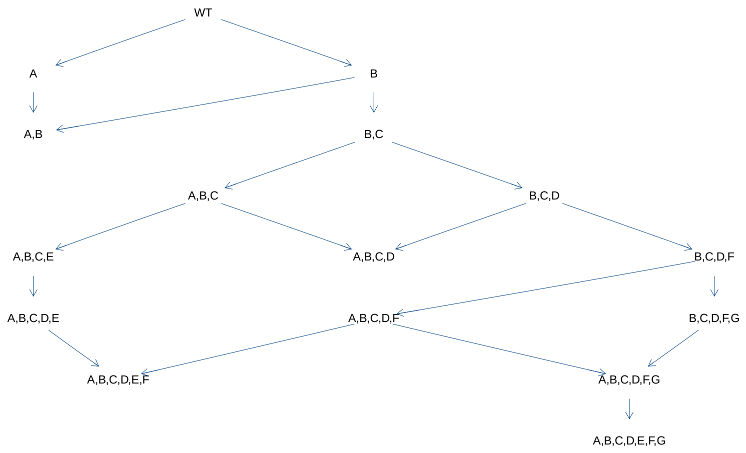

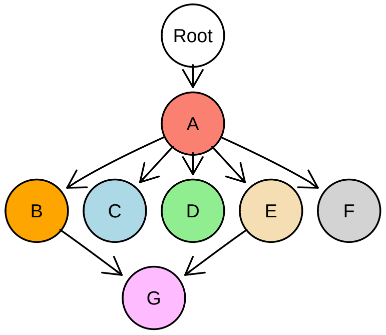

ID 9yJVB9KVObV7syFm
acc genotypes 41
peaks no back 6
removed edges 16
prop rem edges 0.158

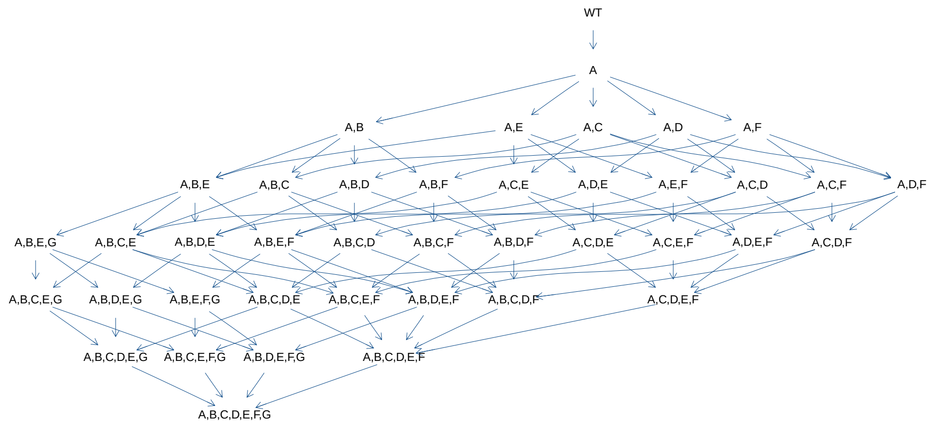

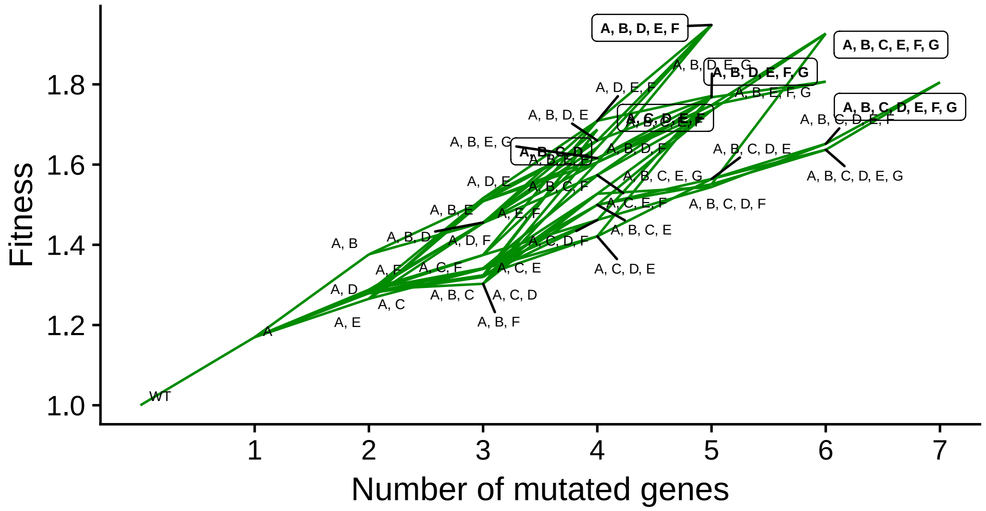

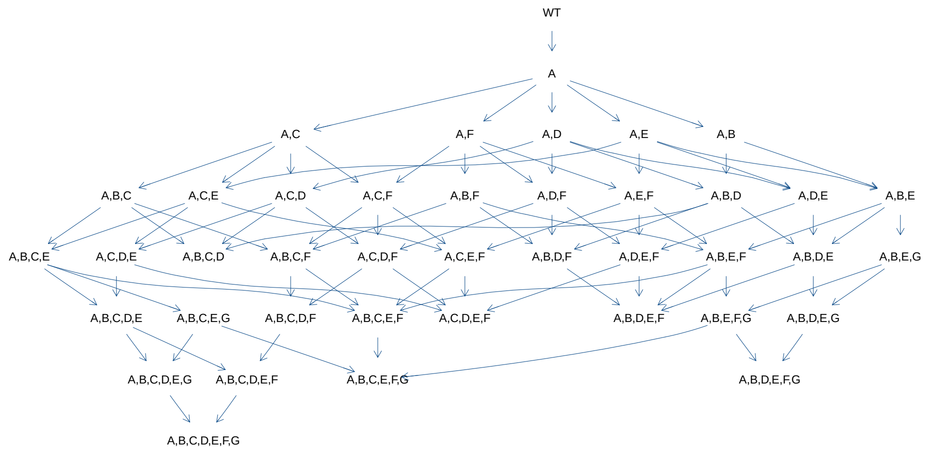

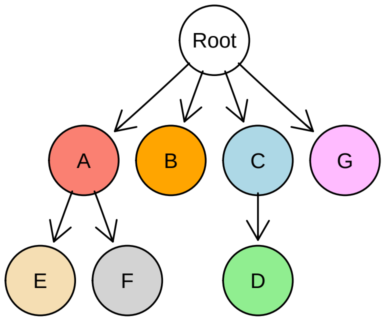

ID	a71ZlUjjTtaY98zg
acc genotypes	60
peaks no back	7
removed edges	21
prop rem edges	0.131

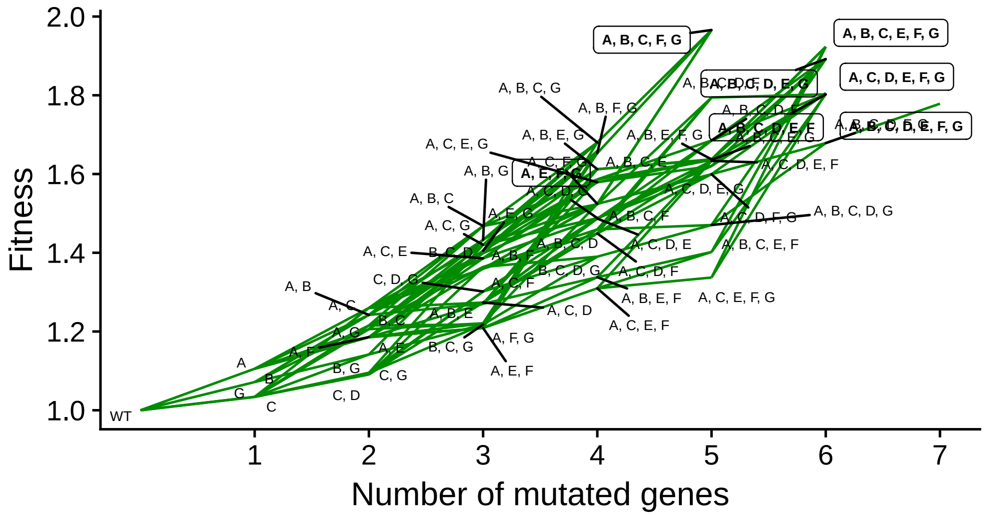

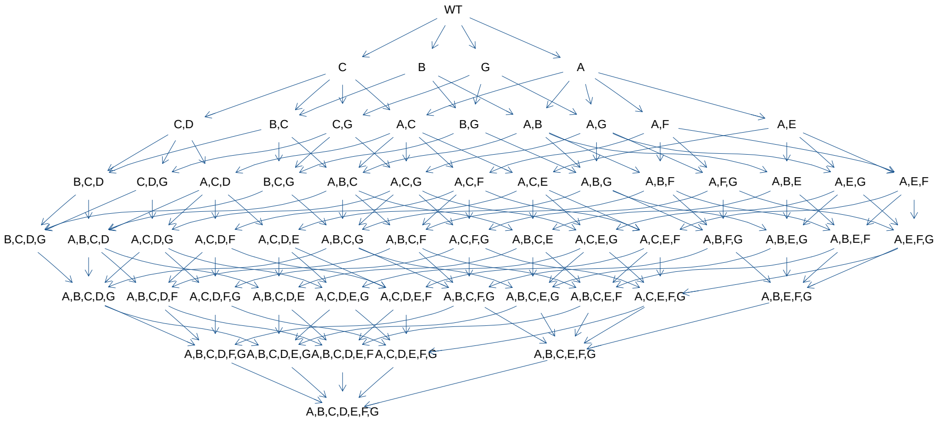

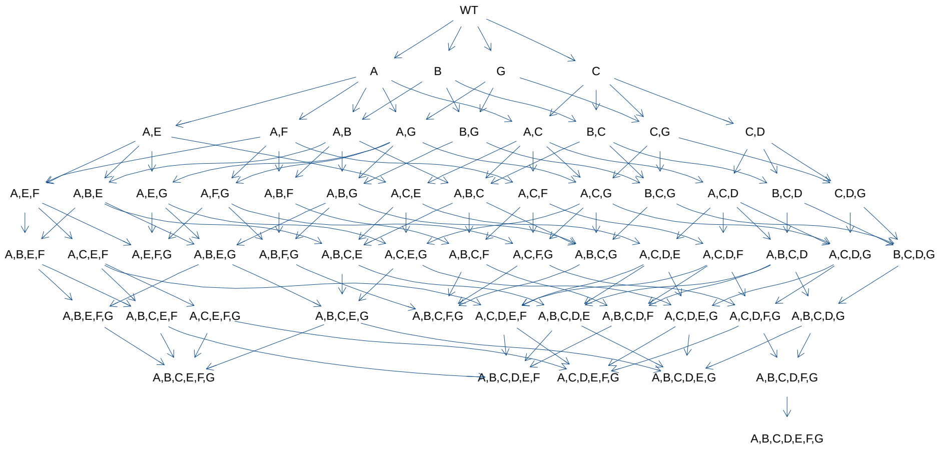

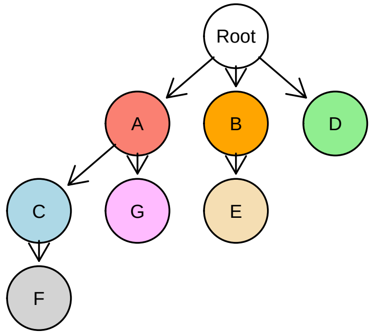

ID	aABp8Hfk3fLTX4co
acc genotypes	42
peaks no back	7
removed edges	17
prop rem edges	0.175

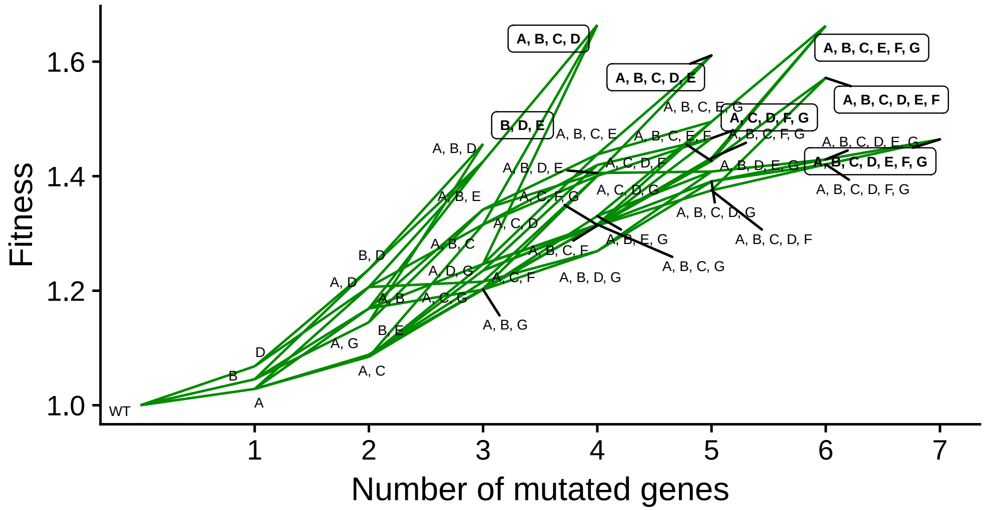

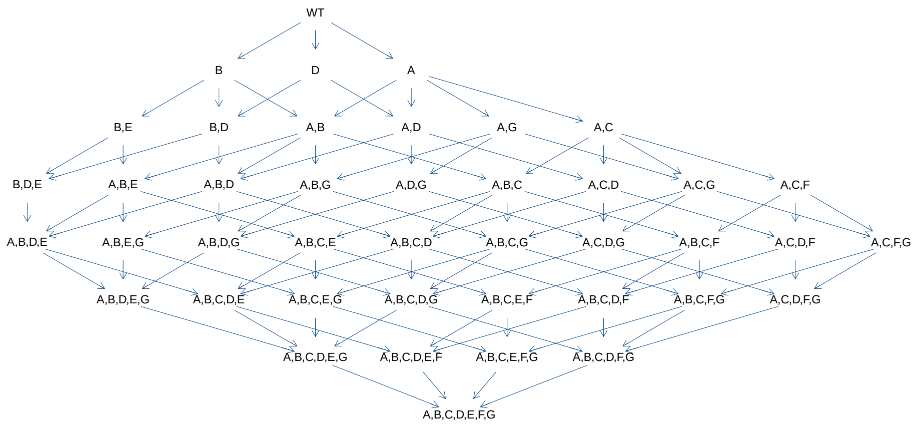

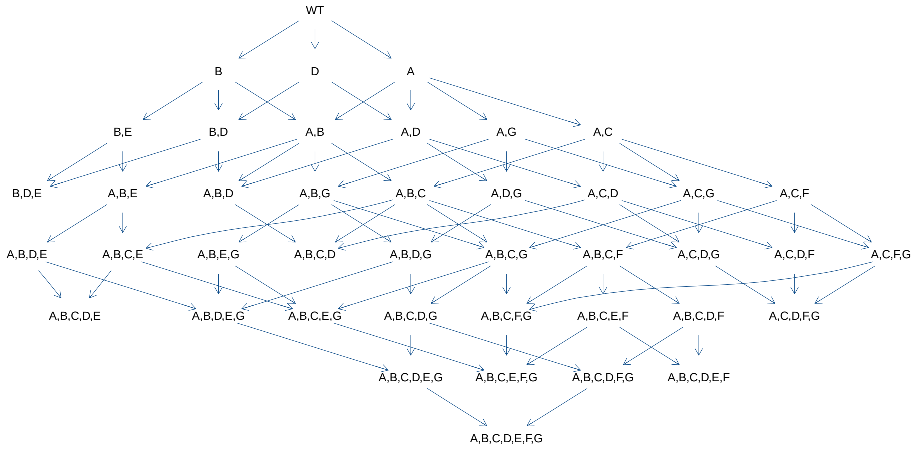

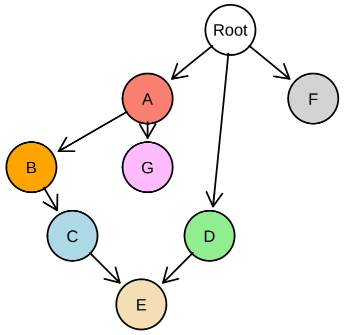

ID AeBhYdSJ32SIaHgW
acc genotypes 32
peaks no back 3
removed edges 8
prop rem edges 0.118

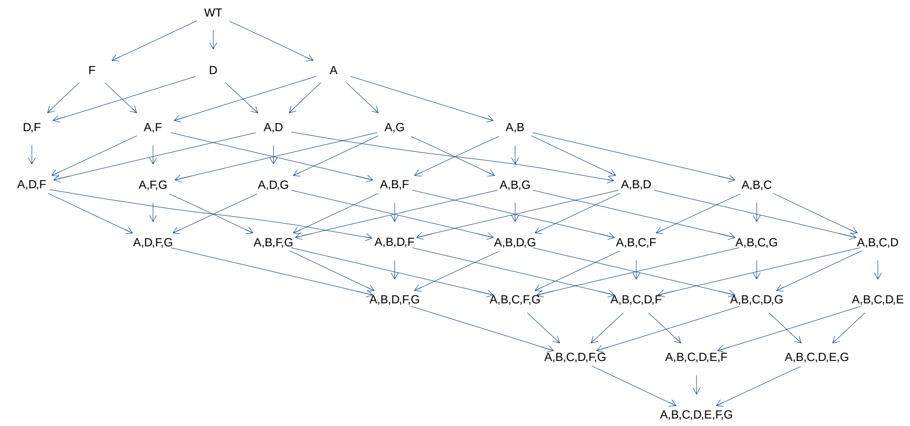

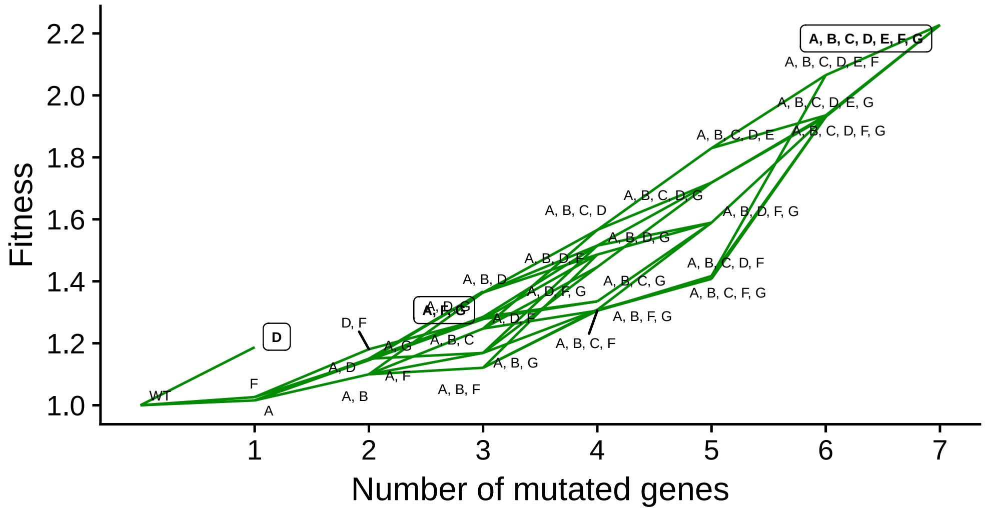

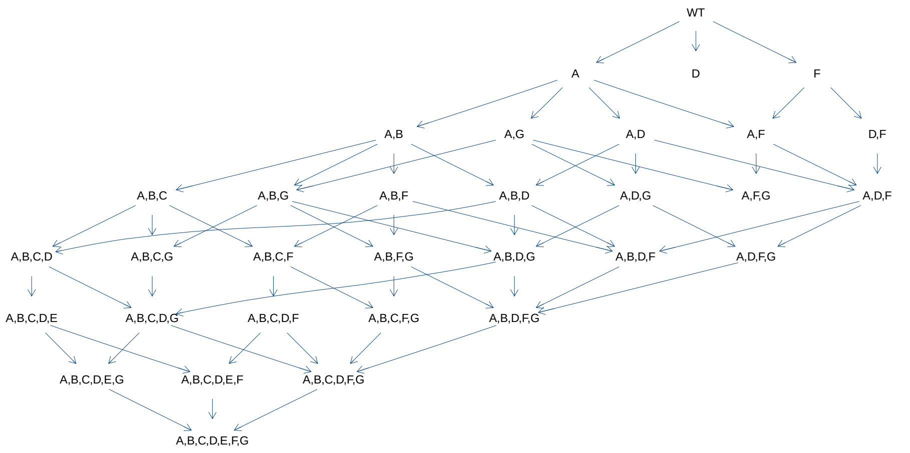

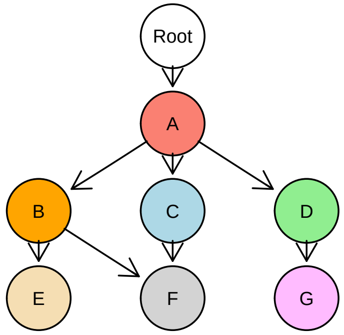

ID	ANTtoA35KKWUX10uQ
acc genotypes	25
peaks no back	3
removed edges	5
prop rem edges	0.106

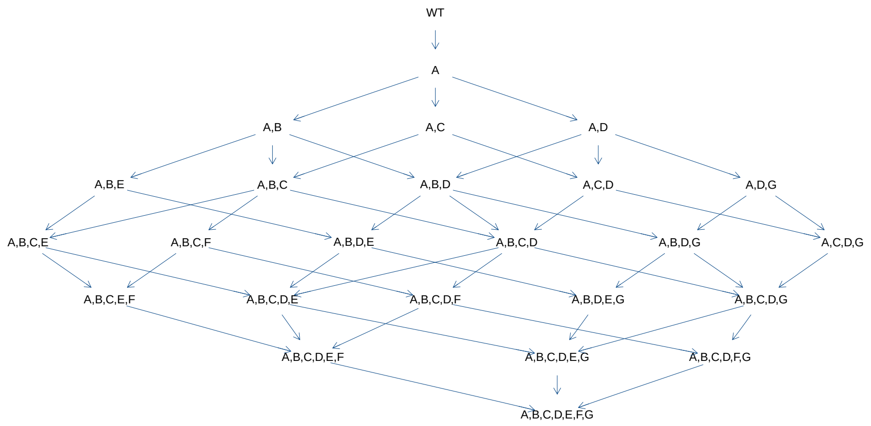

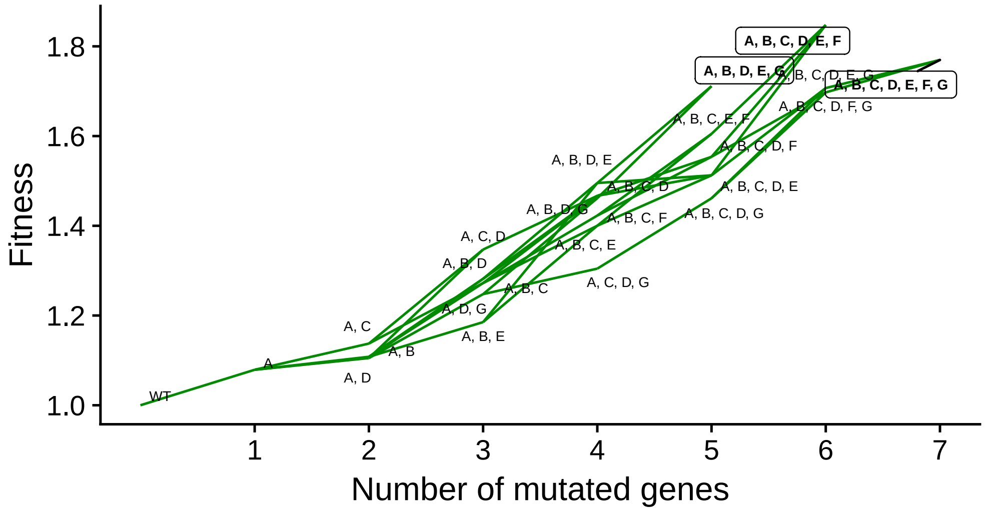

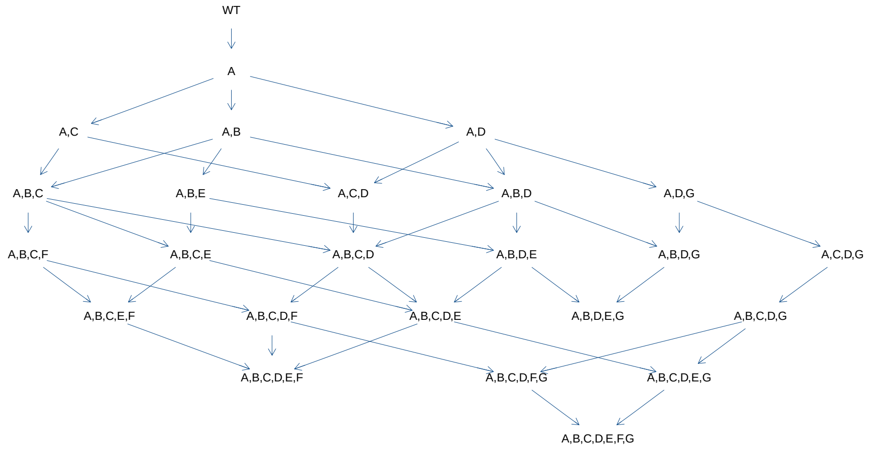

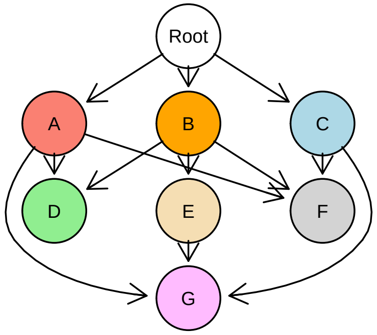

ID	Asdds4Nn2MDRrNBH
acc genotypes	24
peaks no back	5
removed edges	6
prop rem edges	0.136

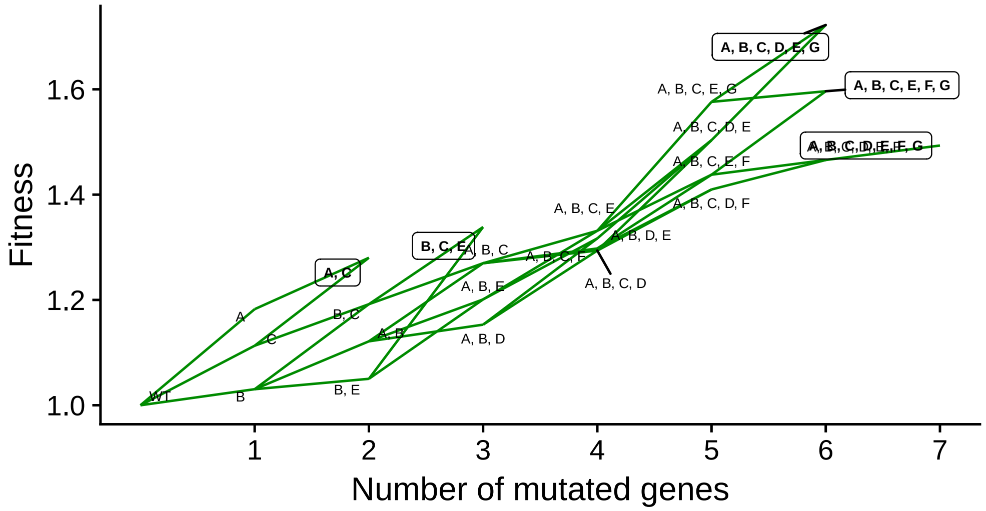

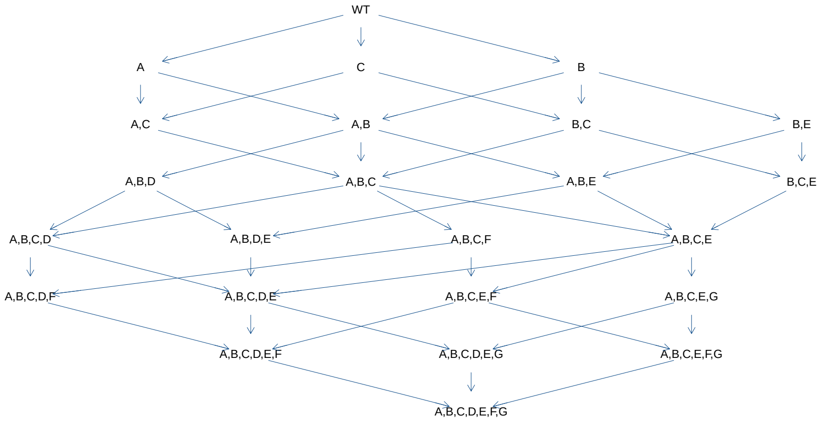

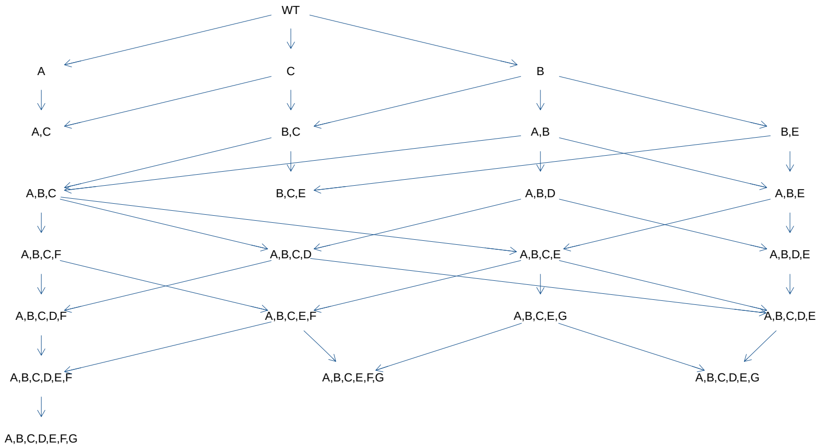

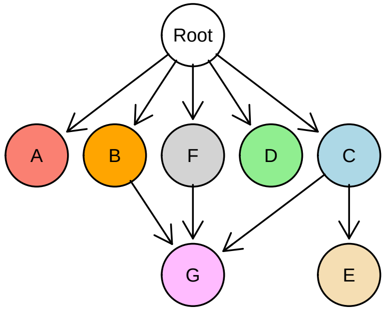

ID aW6TSVzKS8gXsaxO
acc genotypes 56
peaks no back 7
removed edges 28
prop rem edges 0.189

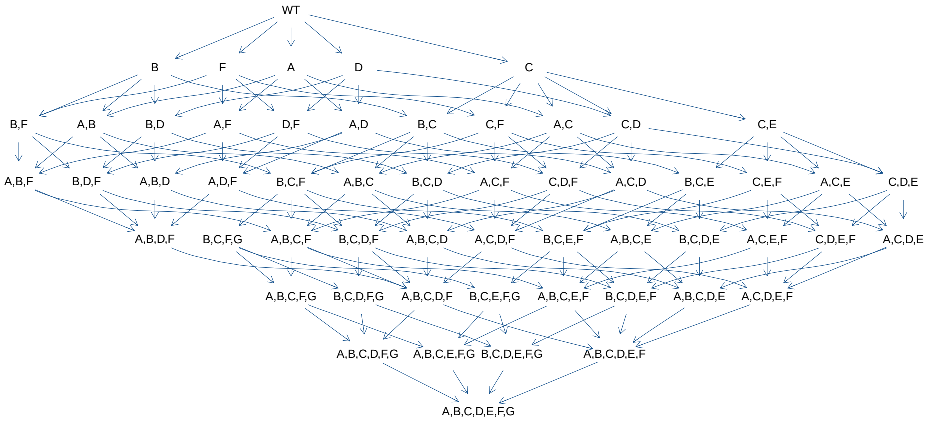

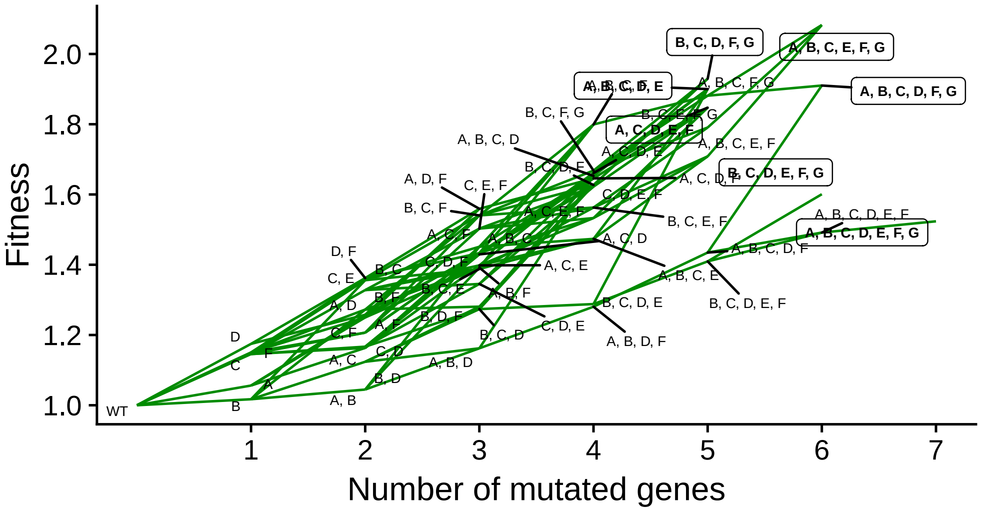

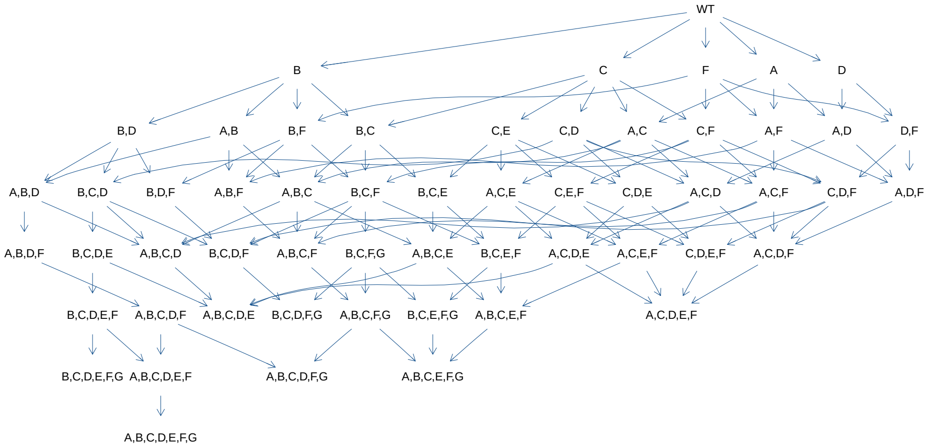

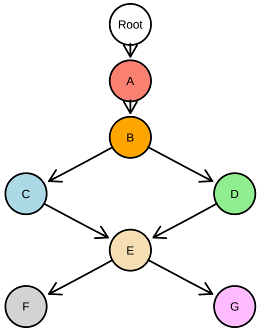

ID	AXnGmWZxzz5kfly4
acc genotypes	10
peaks no back	2
removed edges	1
prop rem edges	0.091

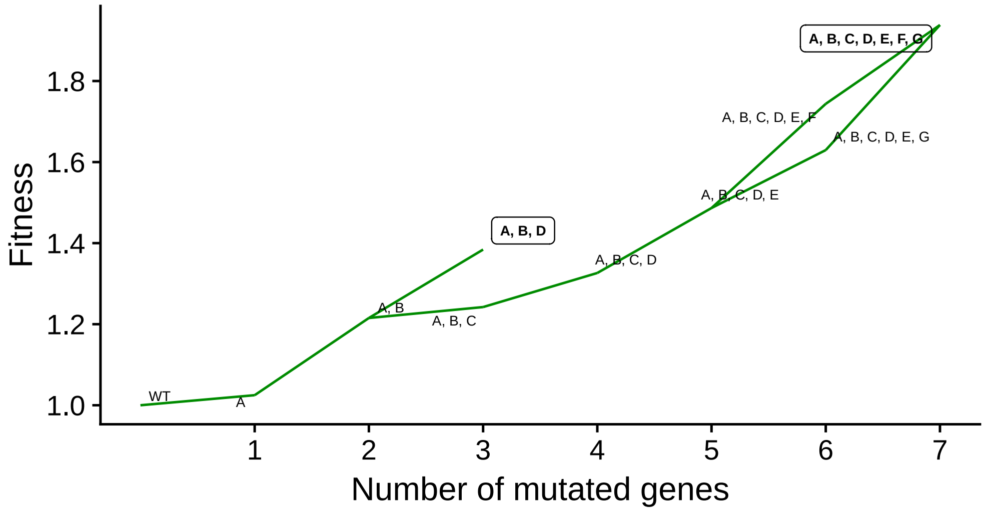

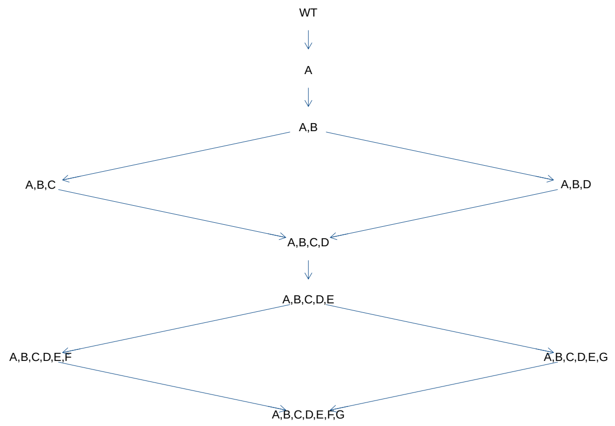

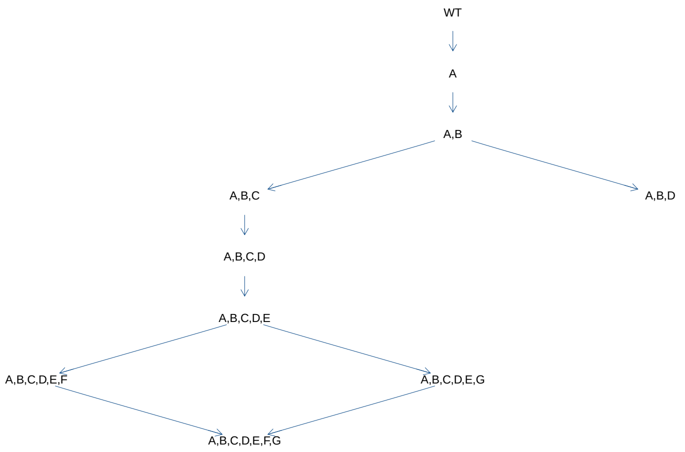

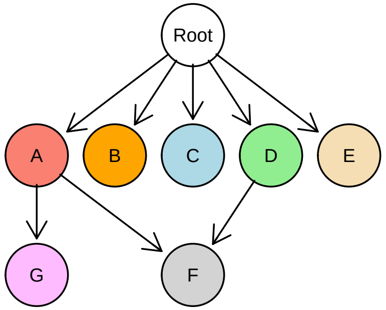

ID	AYfLkIxUEKLpymWH
acc genotypes	64
peaks no back	9
removed edges	33
prop rem edges	0.188

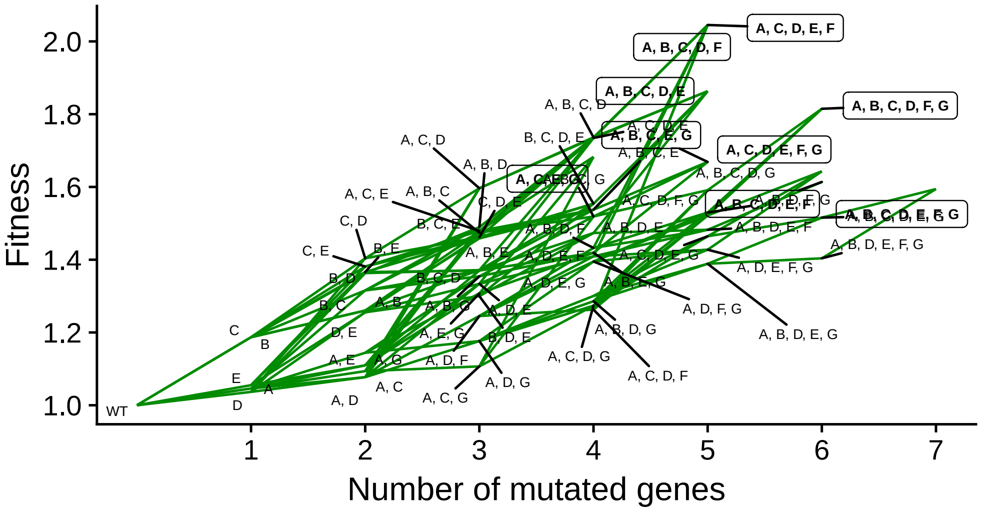

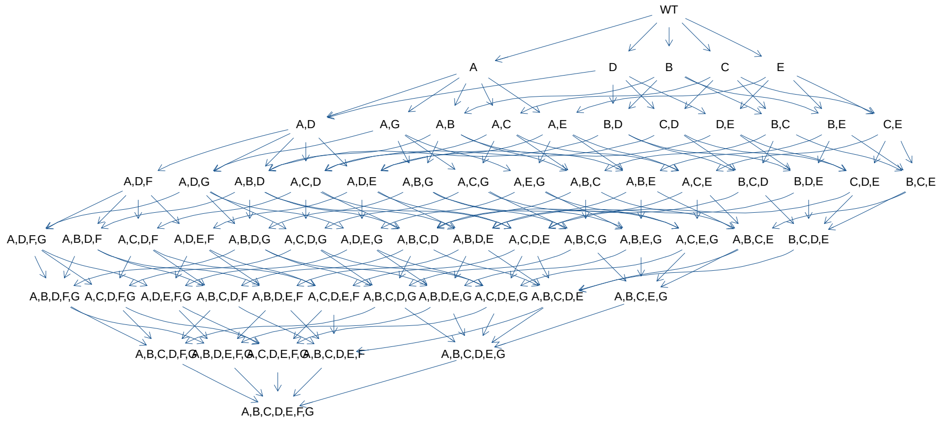

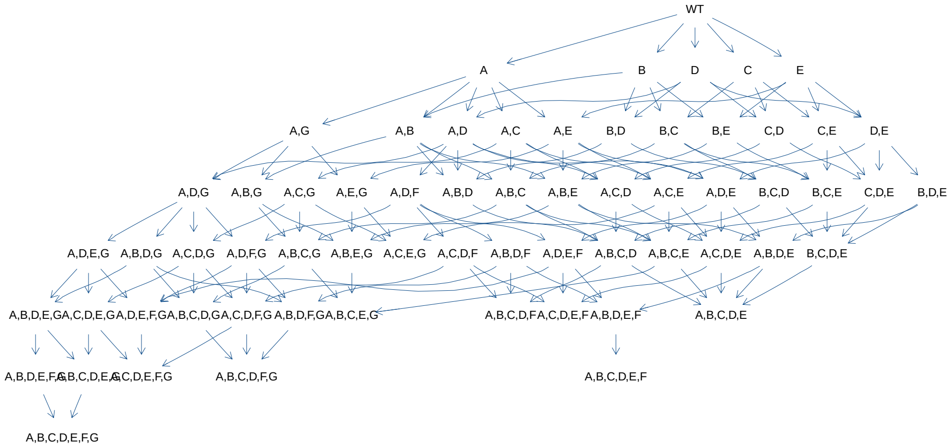

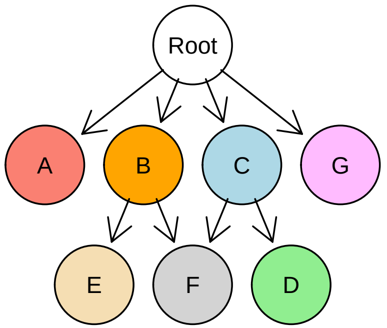

ID	b8dcfCJtzgN6gupz
acc genotypes	52
peaks no back	7
removed edges	20
prop rem edges	0.152

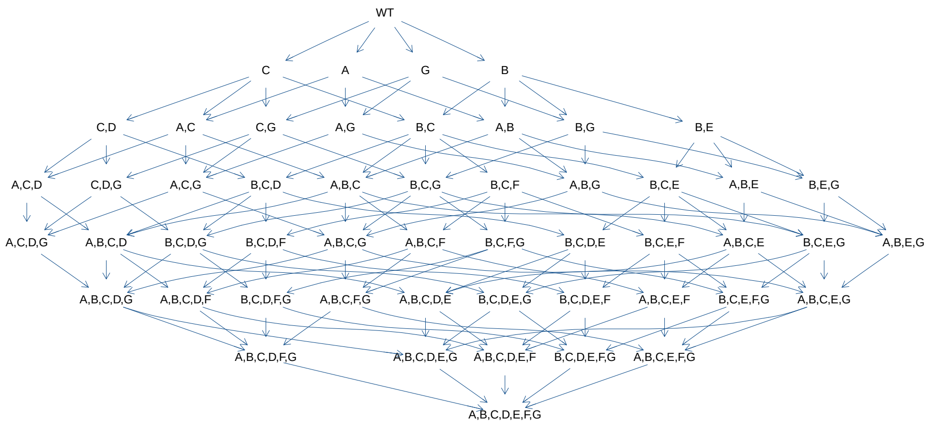

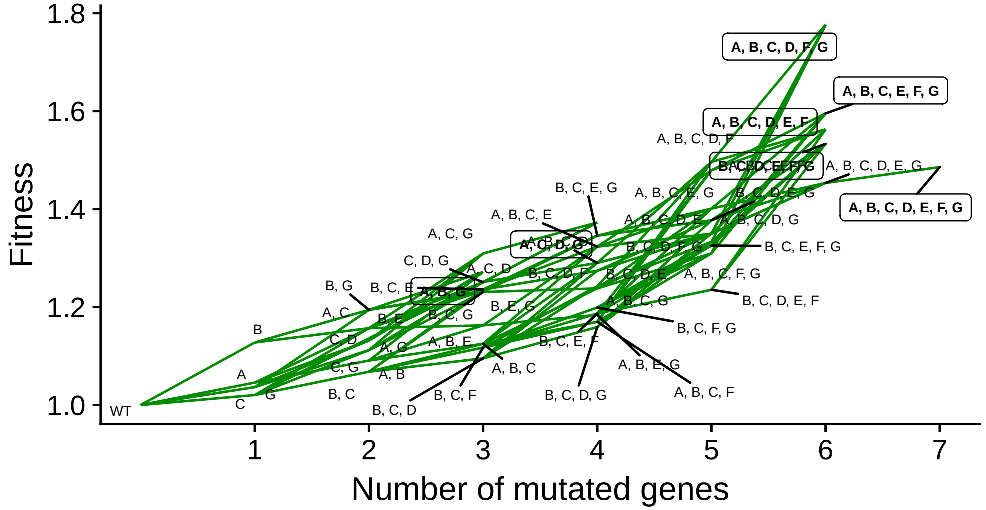

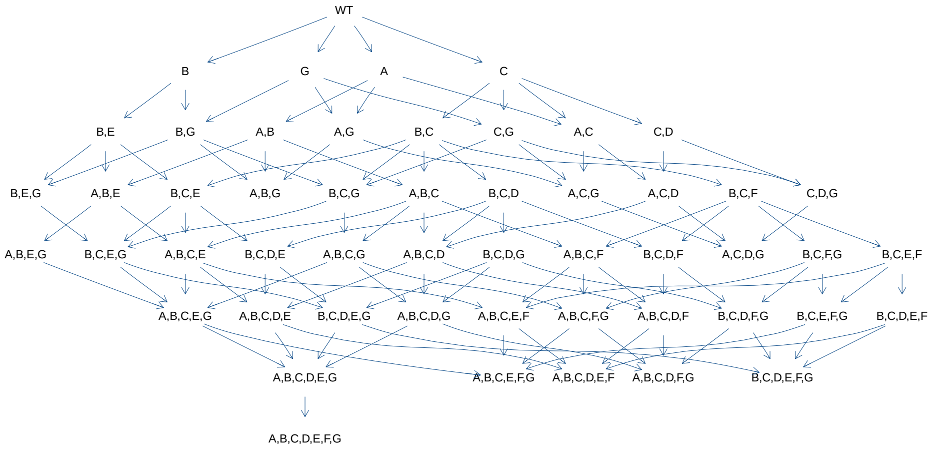

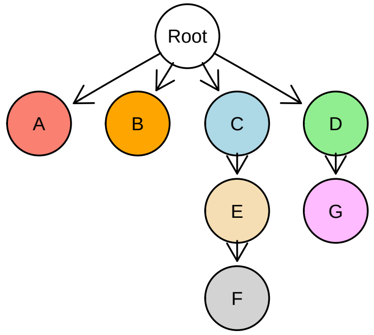

ID	b91s8fHjp10RnRLDw
acc genotypes	48
peaks no back	8
removed edges	25
prop rem edges	0.216

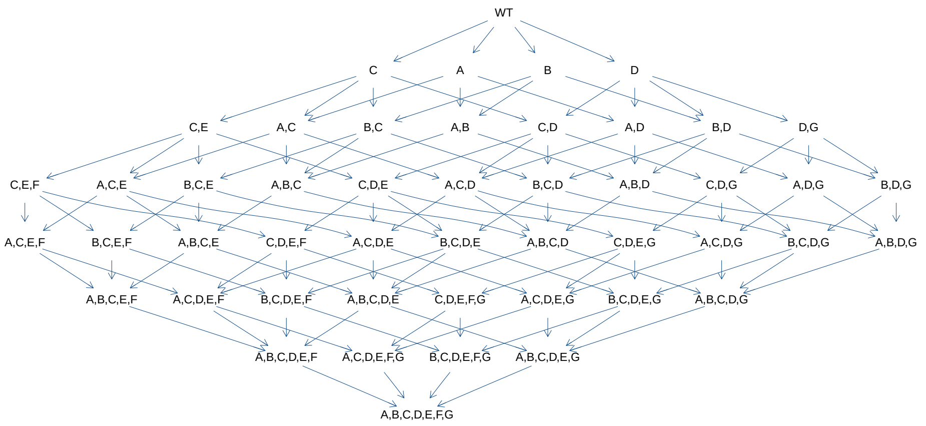

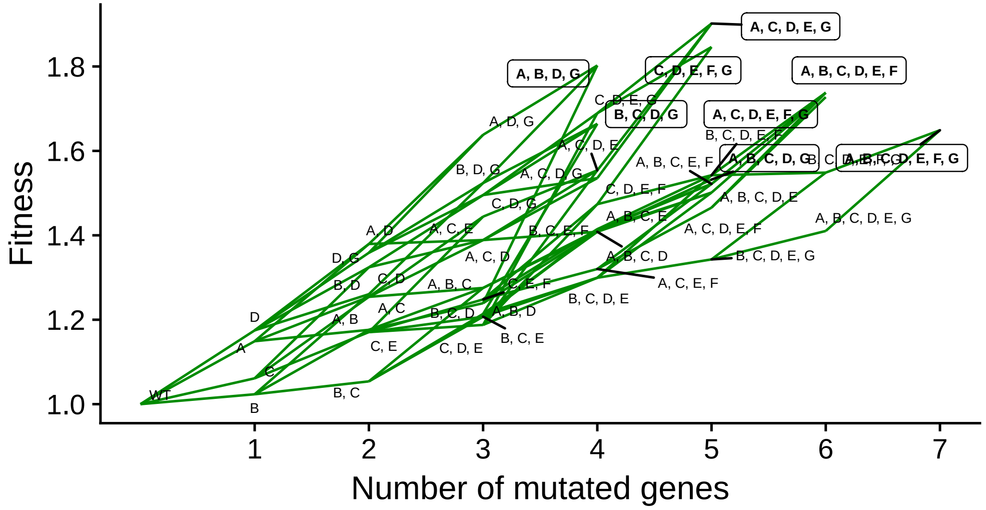

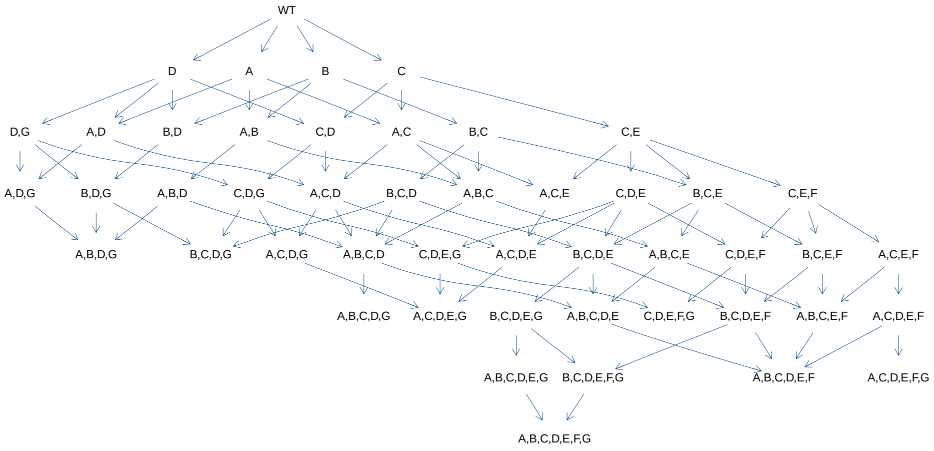

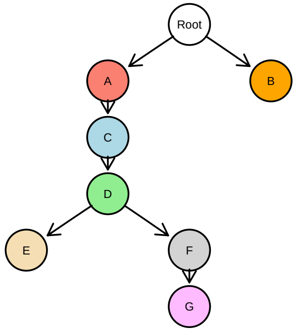

ID	bAM8Odp4yapUk10Fb
acc genotypes	18
peaks no back	4
removed edges	4
prop rem edges	0.138

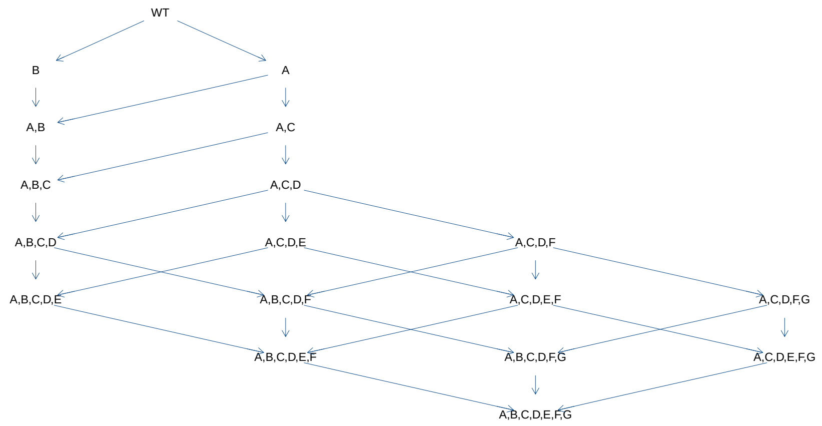

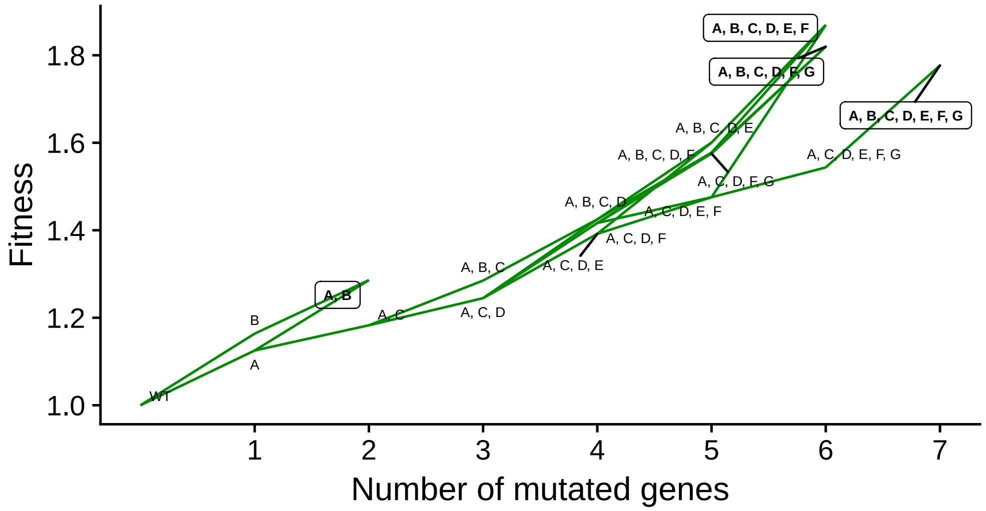

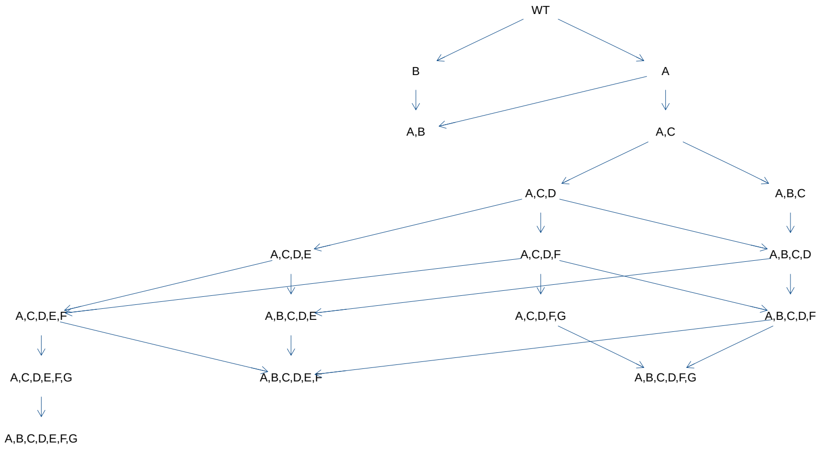

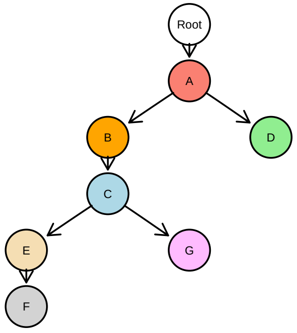

ID	bCsk2Qo5VMVm55fM
acc genotypes	17
peaks no back	2
removed edges	3
prop rem edges	0.111

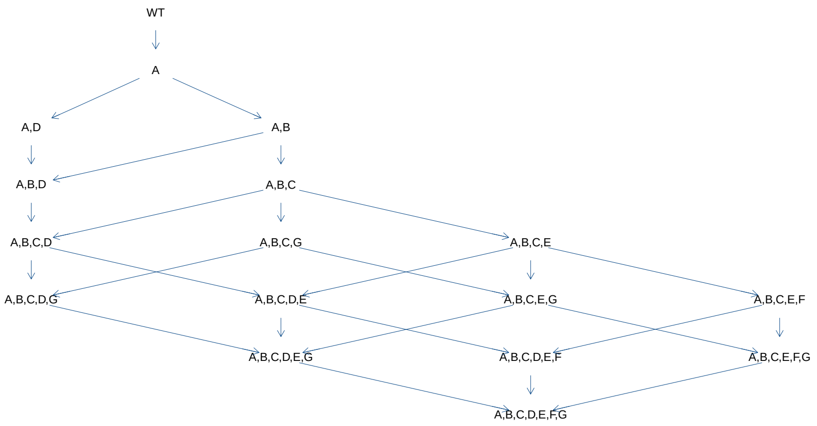

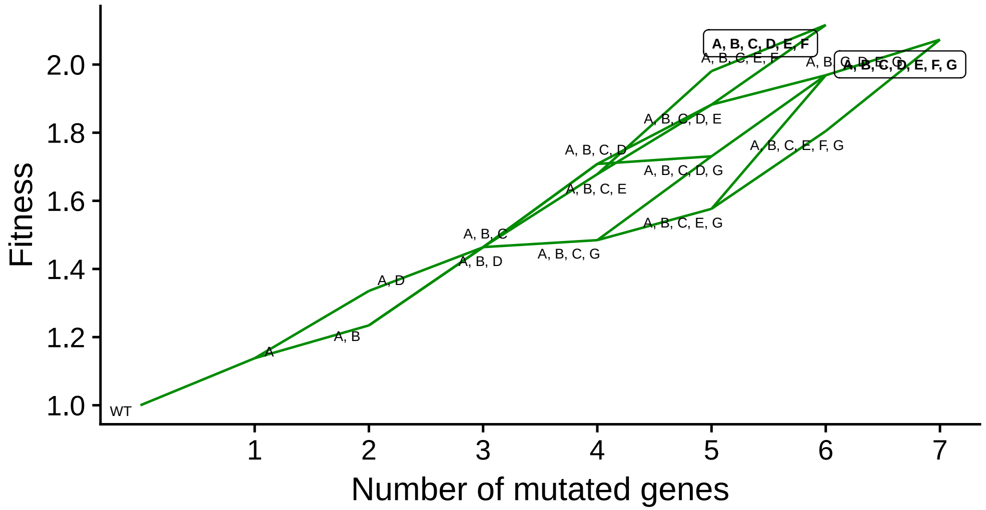

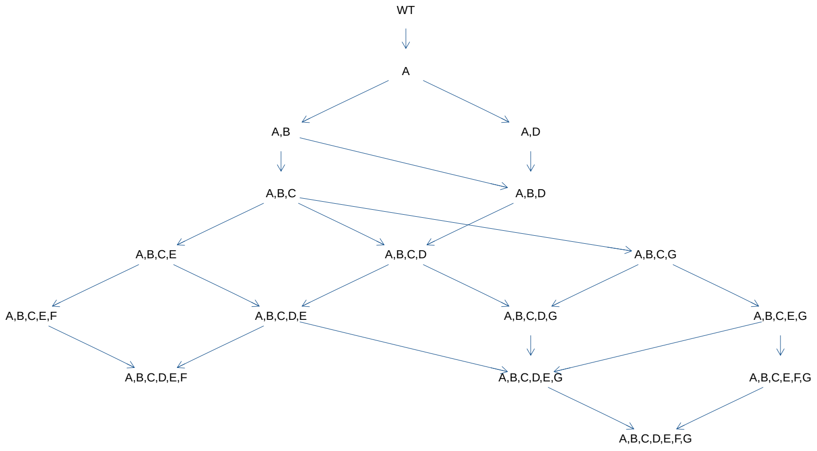

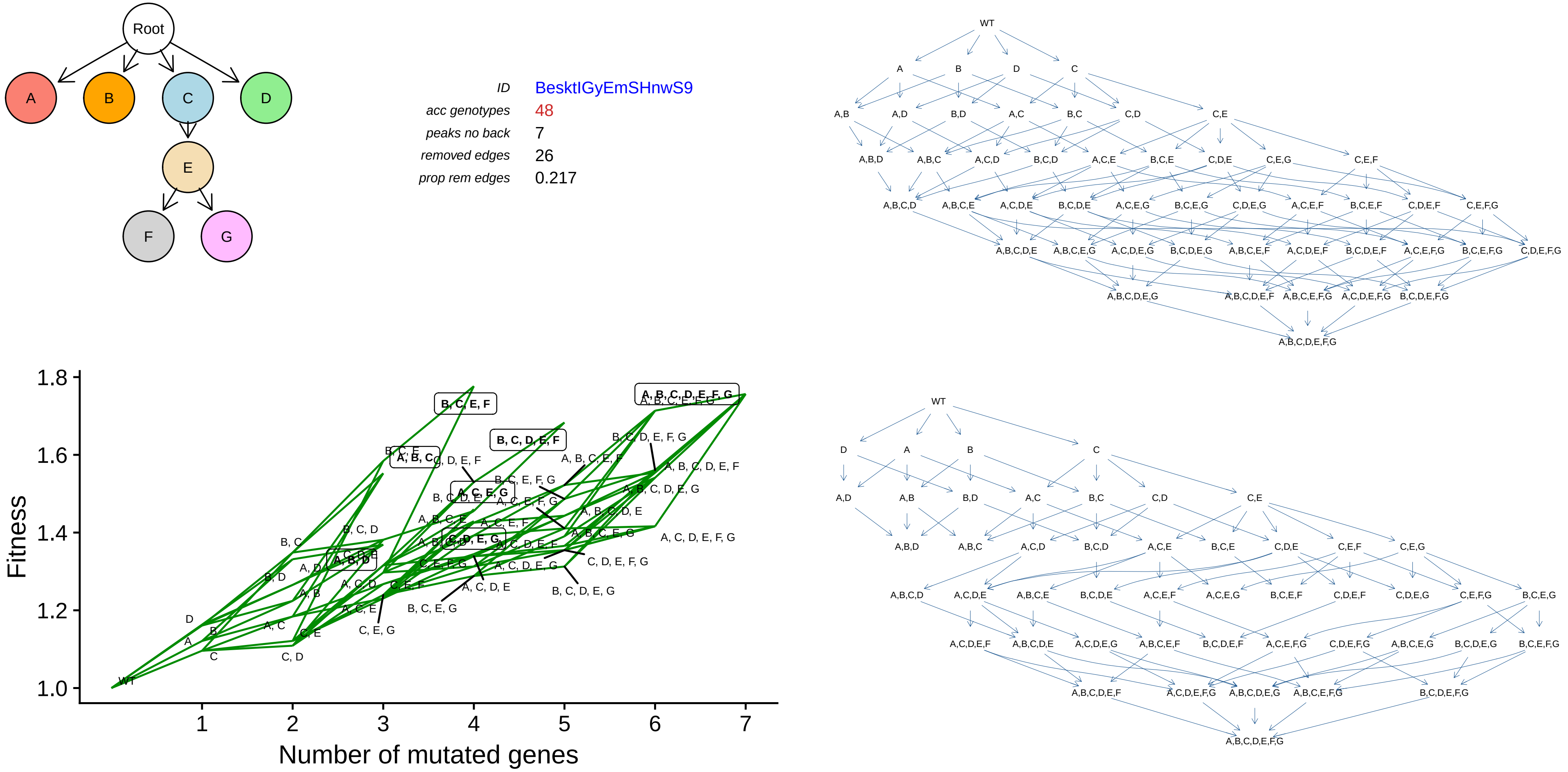
Root
A
B
C
D
E
F
G
ID BesktIGyEmSHnwS9
acc genotypes 48
peaks no back 7
removed edges 26
prop rem edges 0.217
Fitness
Number of mutated genes
WT

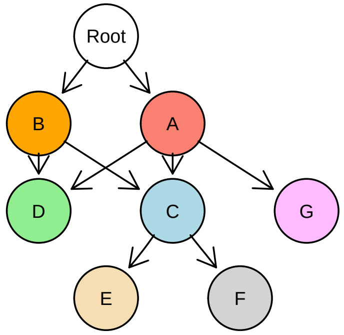

ID	Bm107M6KzXSHrdvsu
acc genotypes	24
peaks no back	2
removed edges	3
prop rem edges	0.065

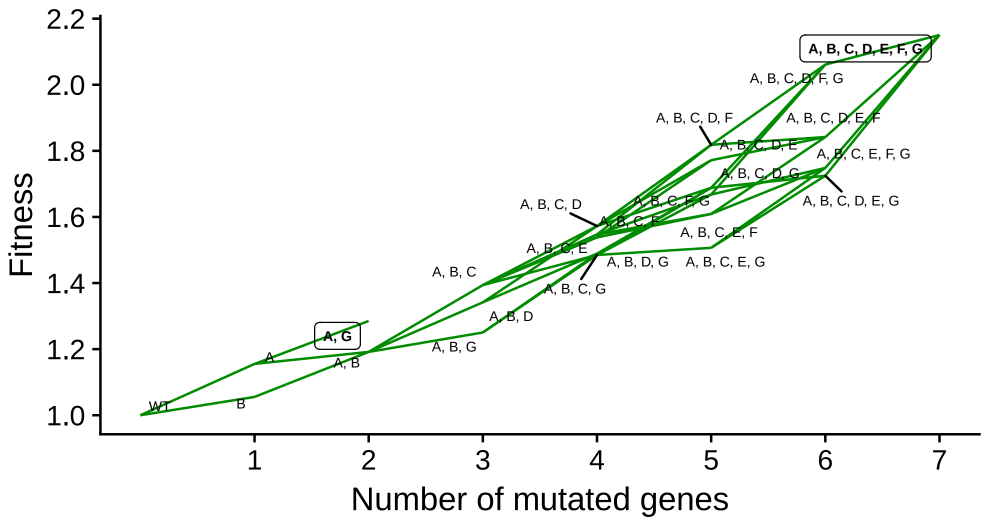

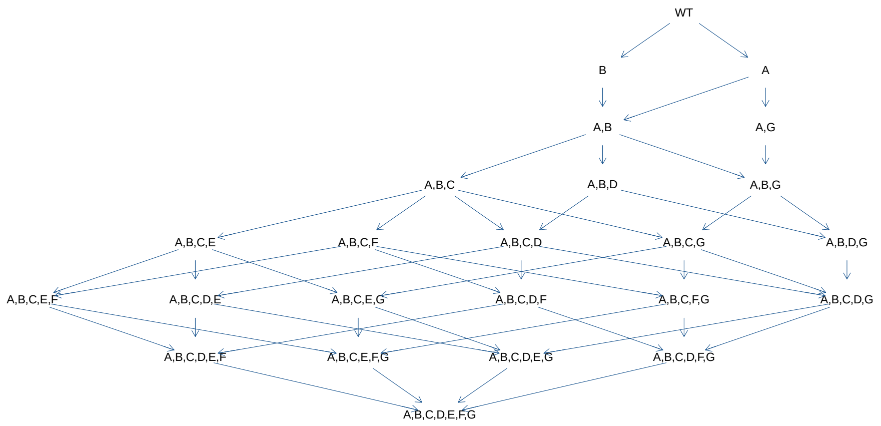

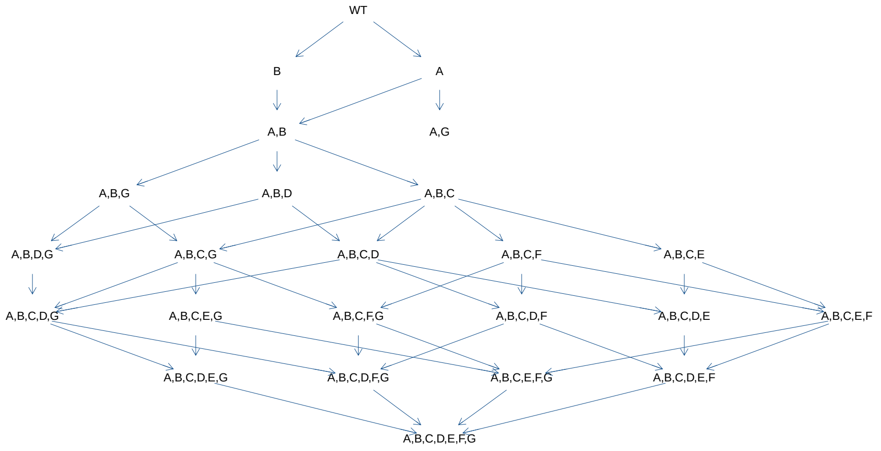

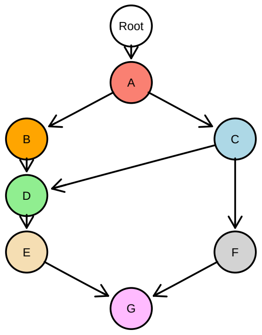

ID Bm9gSkay1l4nALeA
acc genotypes 12
peaks no back 2
removed edges 1
prop rem edges 0.067

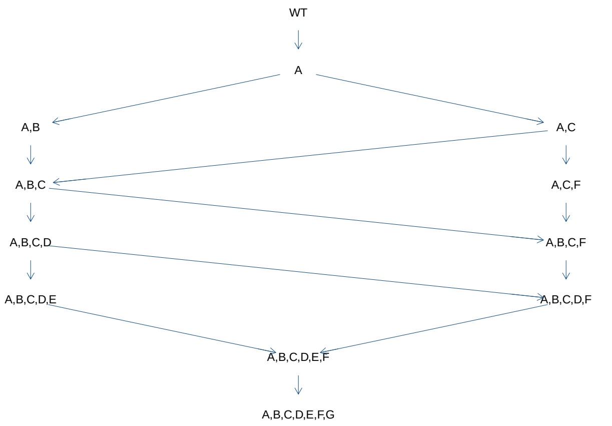

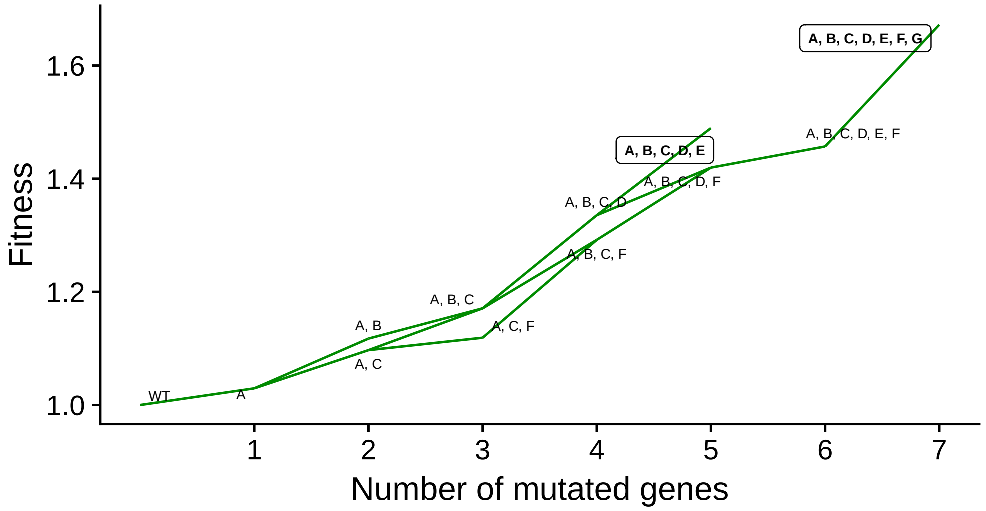

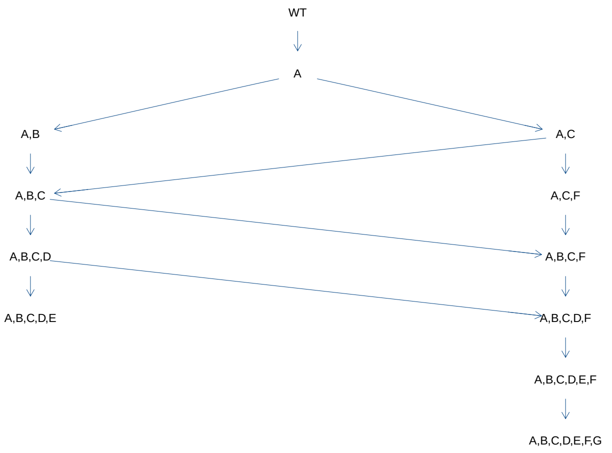

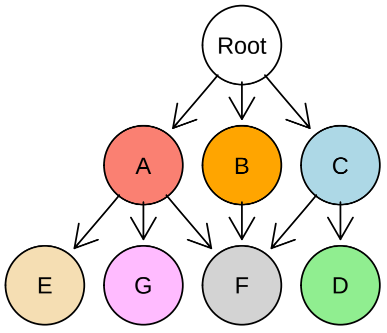

ID bpHispMQClsXVnvs
acc genotypes 38
peaks no back 6
removed edges 9
prop rem edges 0.106

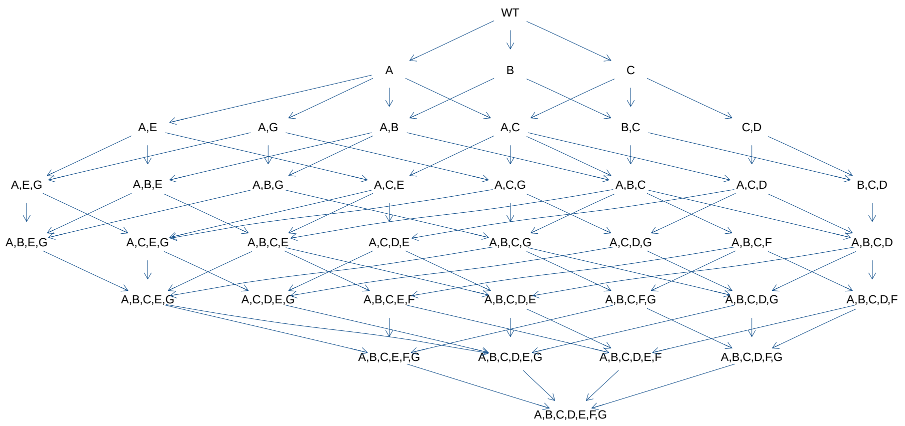

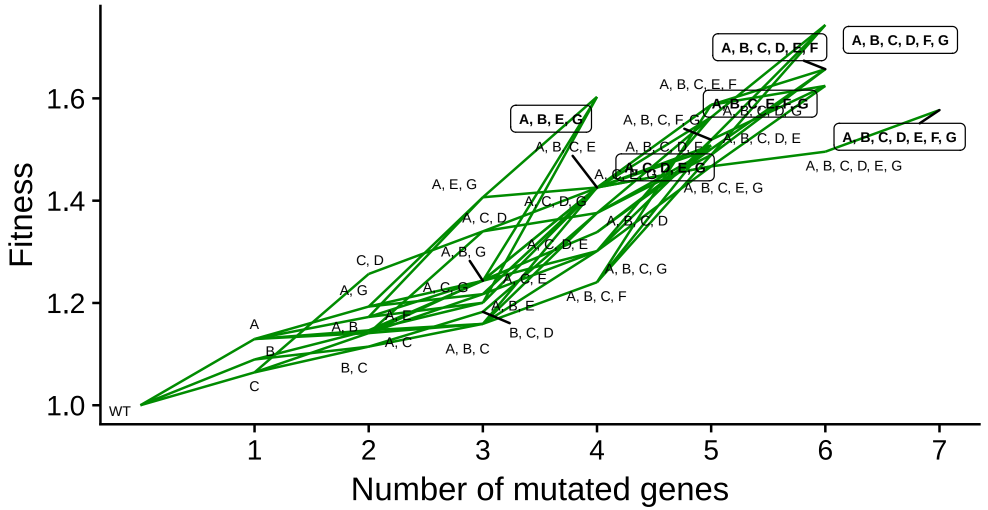

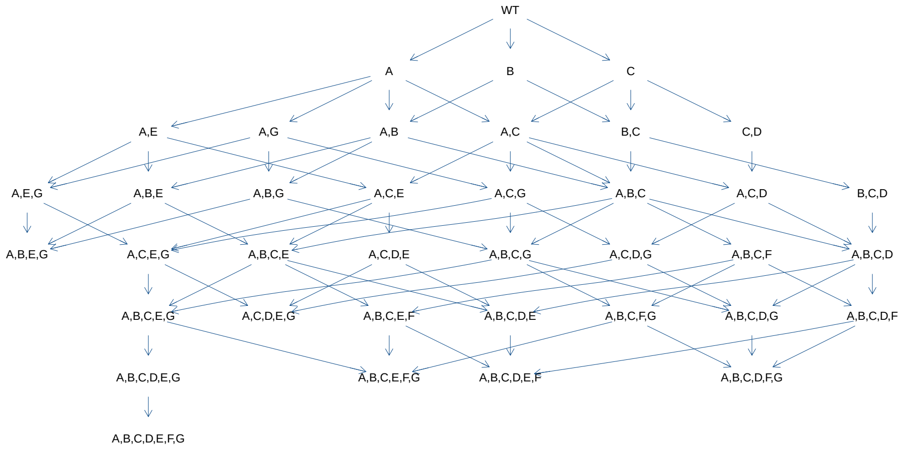

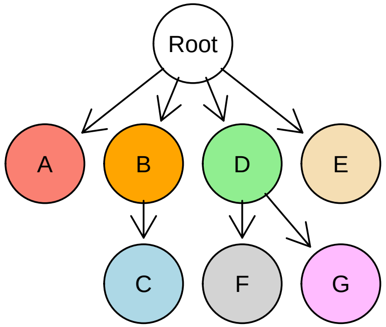

ID	BQW2TKpC1B6DJ7jN
acc genotypes	60
peaks no back	4
removed edges	27
prop rem edges	0.169

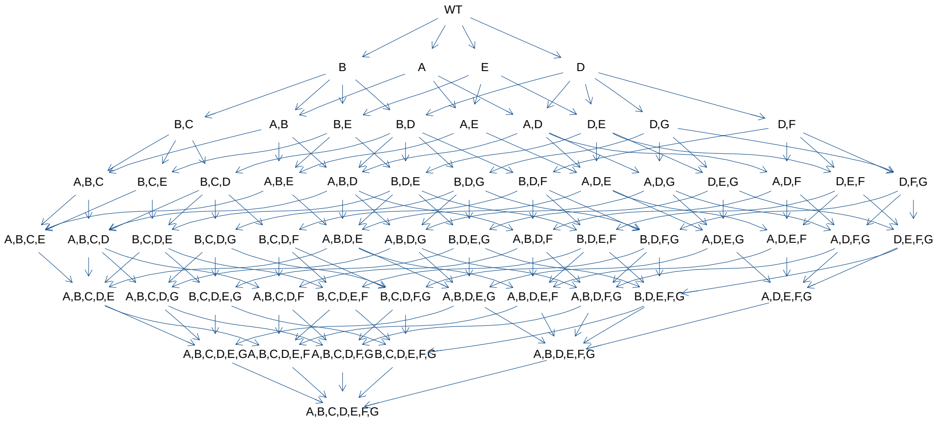

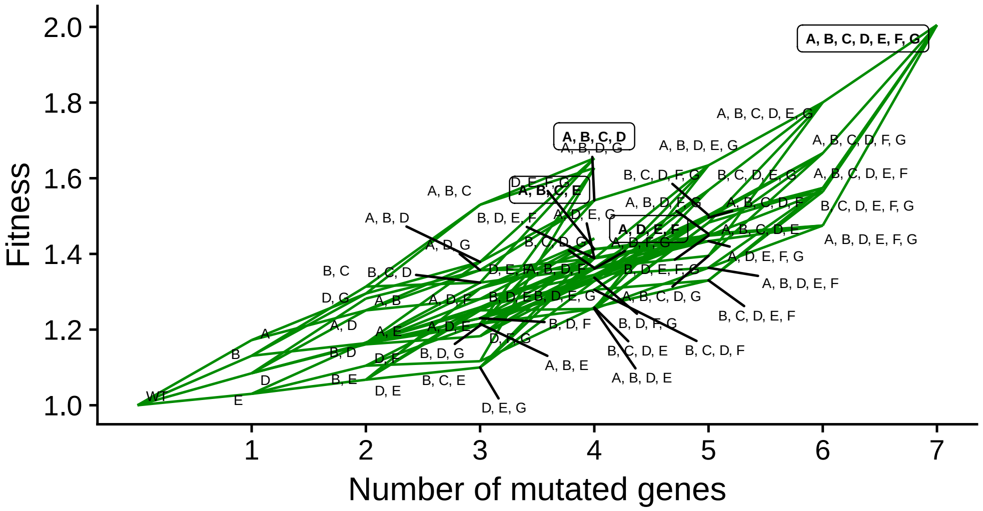

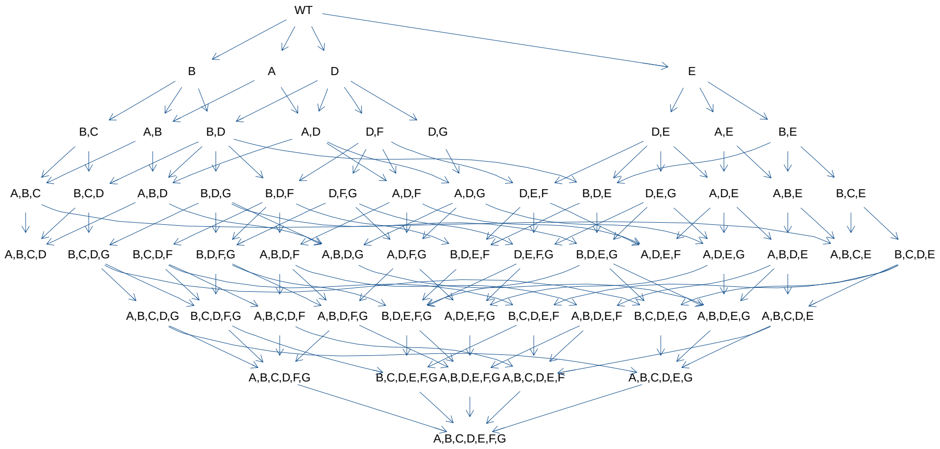

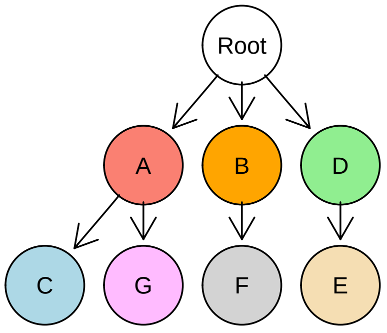

ID	BXlHohn6cCcfd6nR
acc genotypes	45
peaks no back	8
removed edges	20
prop rem edges	0.19

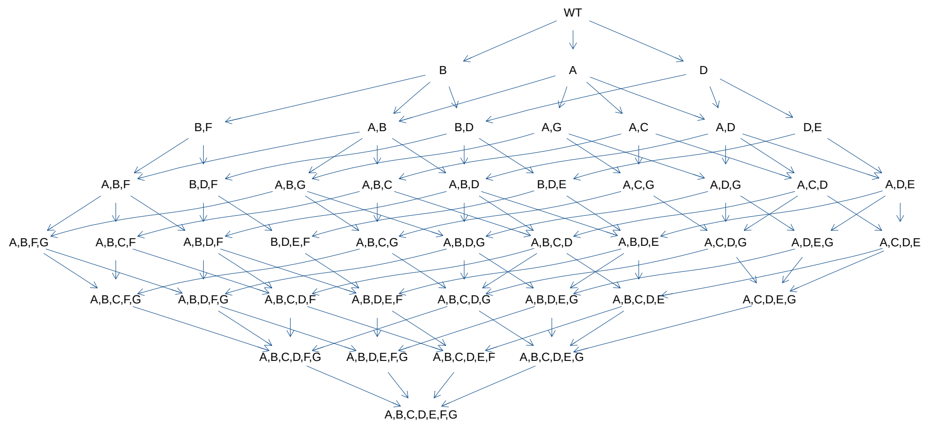

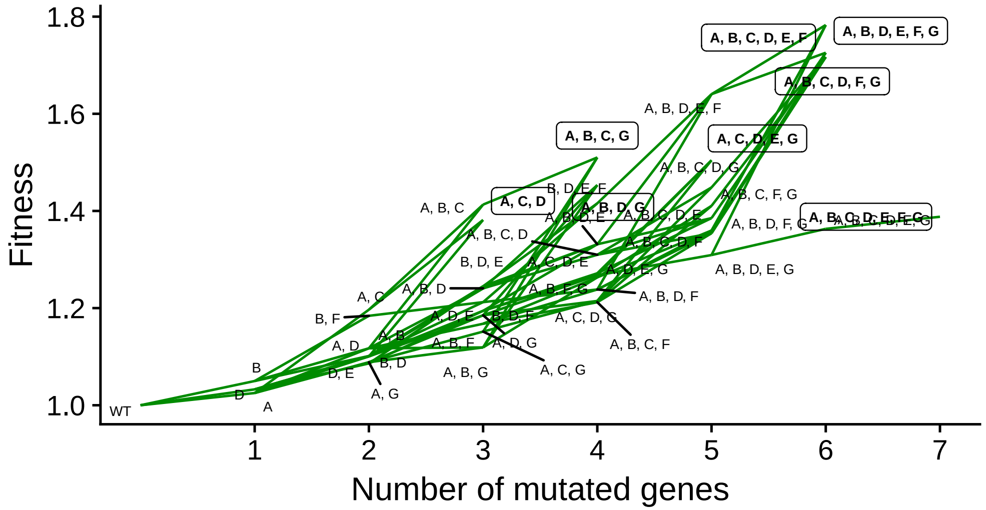

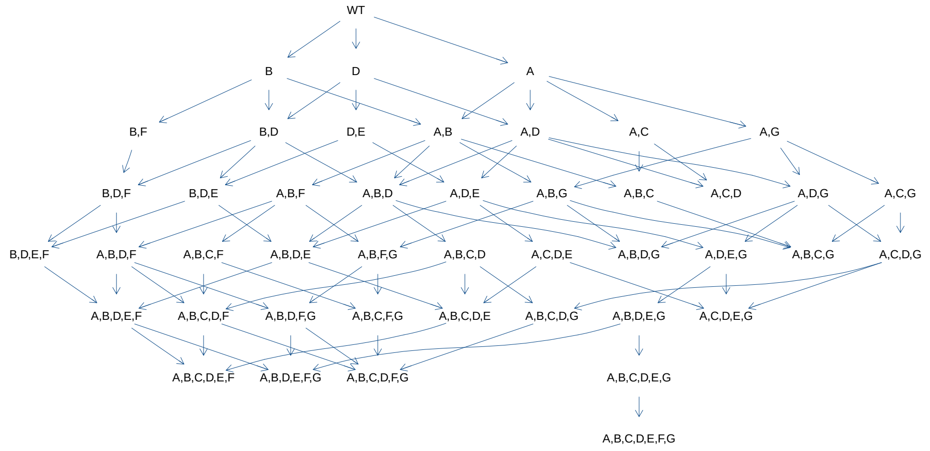

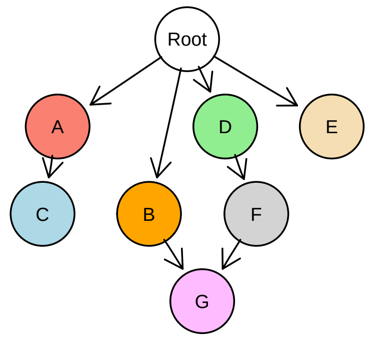

ID BzCzics10JIcdE102O
acc genotypes 42
peaks no back 6
removed edges 16
prop rem edges 0.165

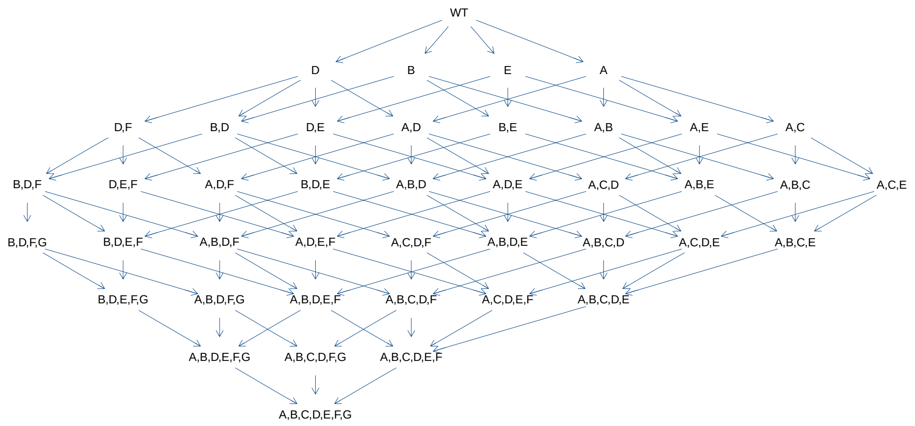

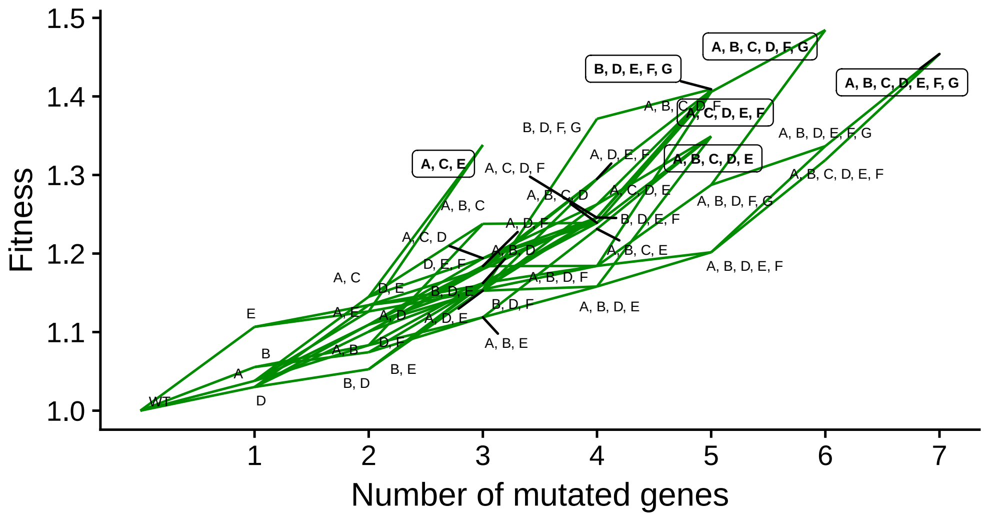

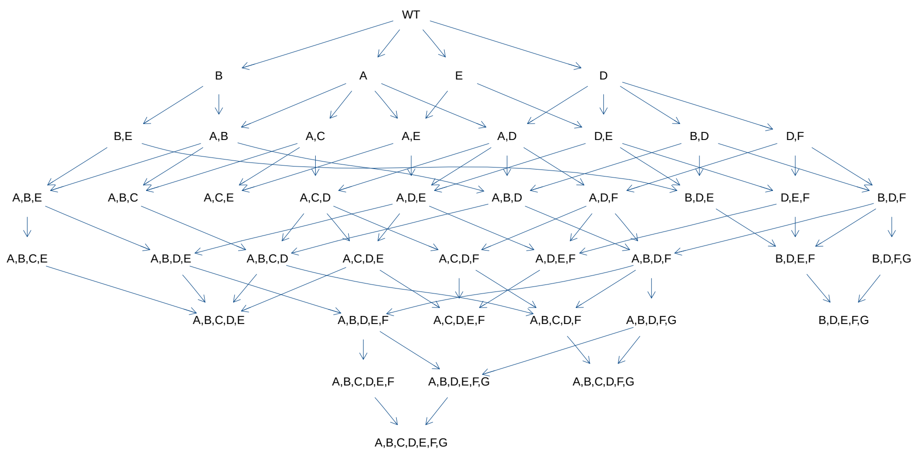

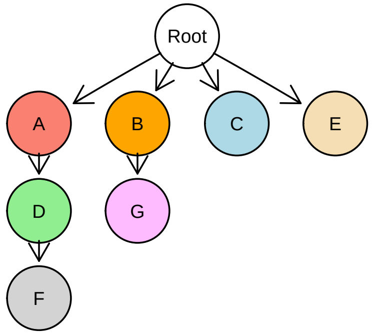

ID	c9GVdRVUtkHv3yR7
acc genotypes	48
peaks no back	4
removed edges	23
prop rem edges	0.198

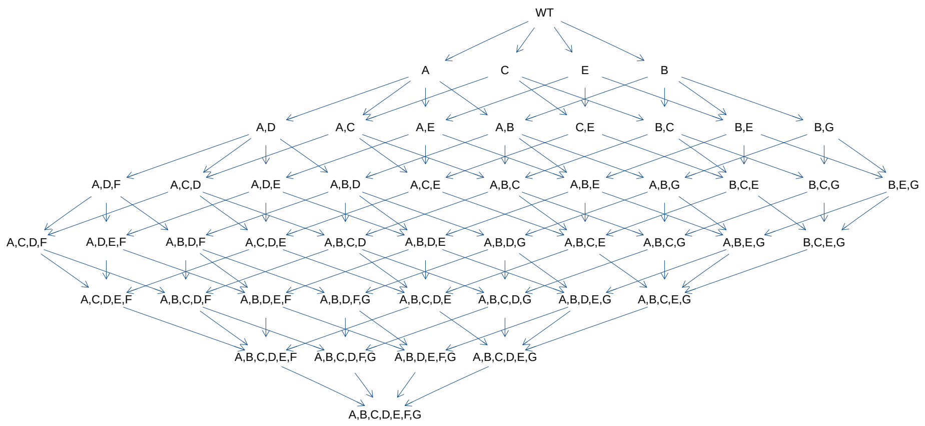

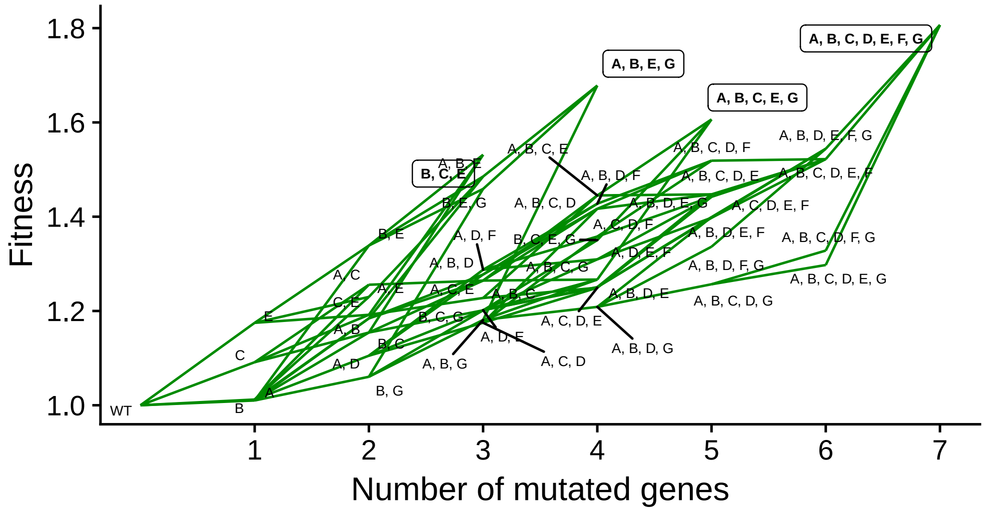

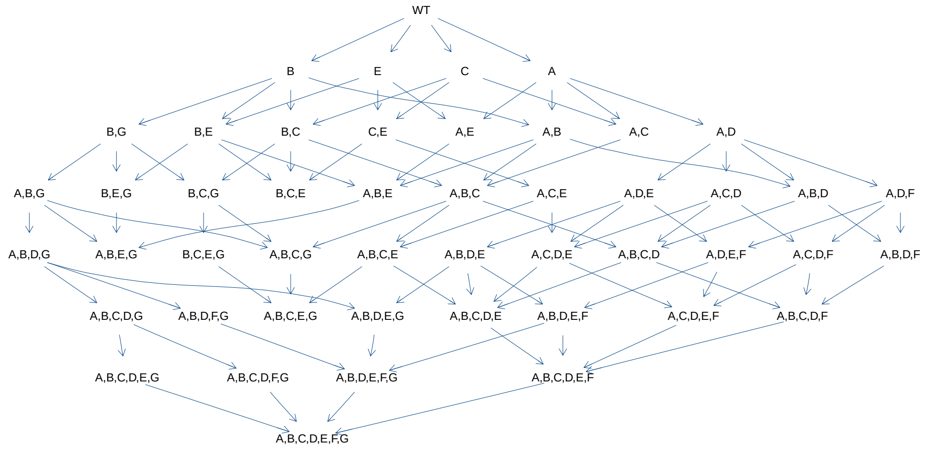

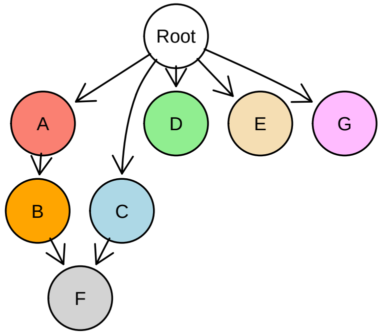

ID	C9MQvf10YWmVwuUpW
acc genotypes	56
peaks no back	9
removed edges	32
prop rem edges	0.216

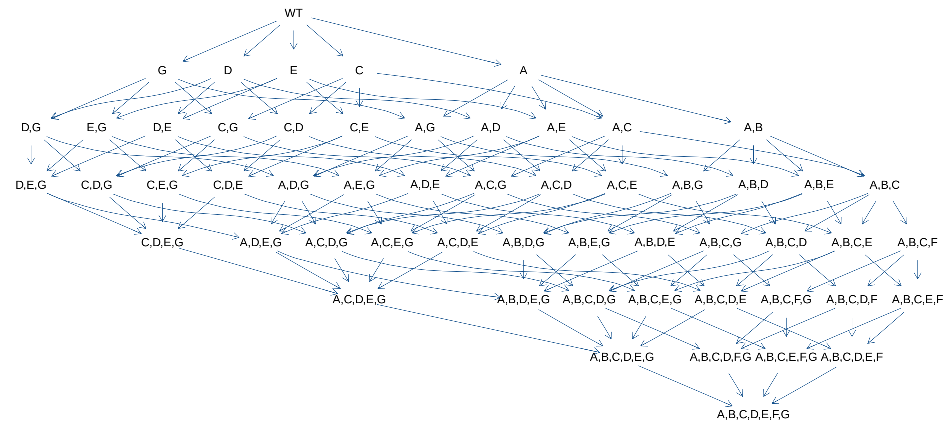

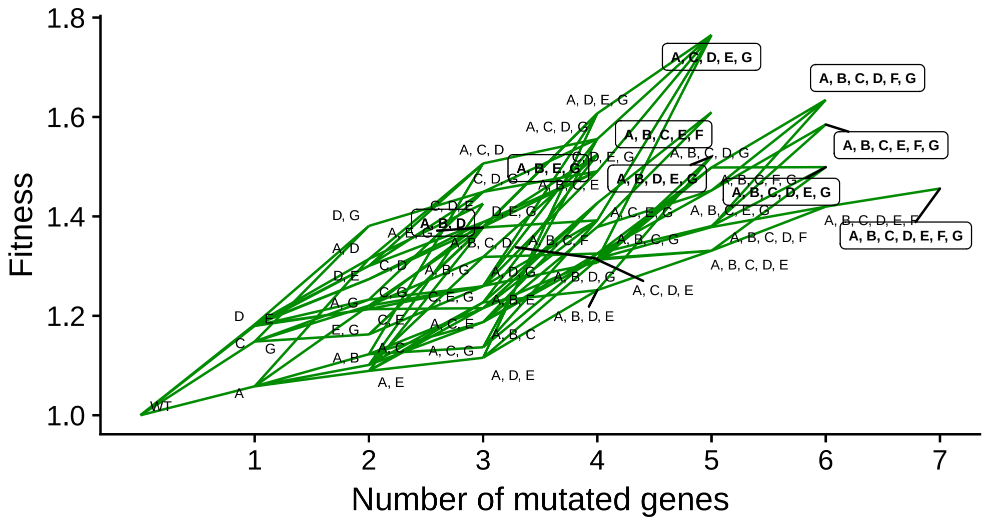

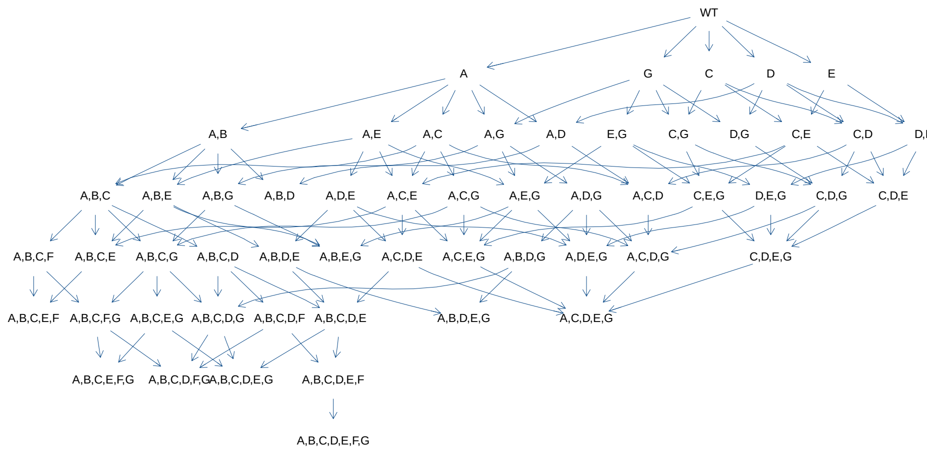

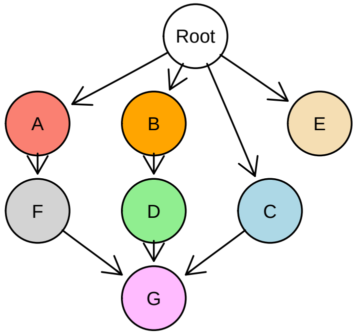

ID	ce4Pp9mhcgxMJhs9
acc genotypes	38
peaks no back	3
removed edges	9
prop rem edges	0.103

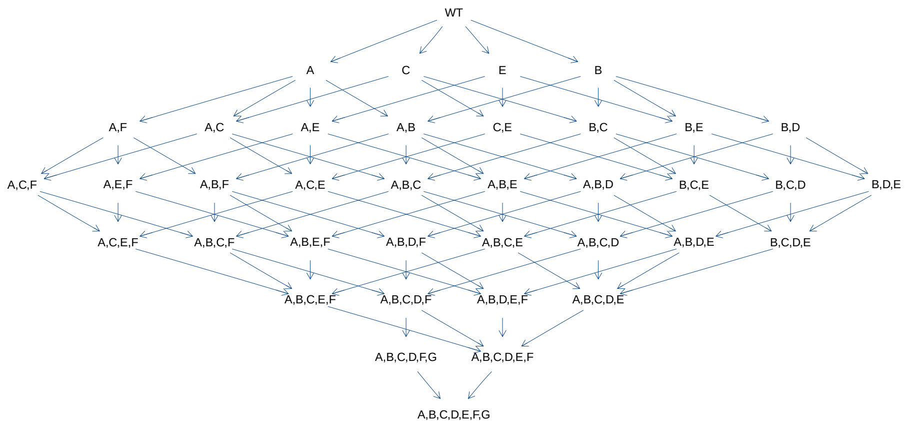

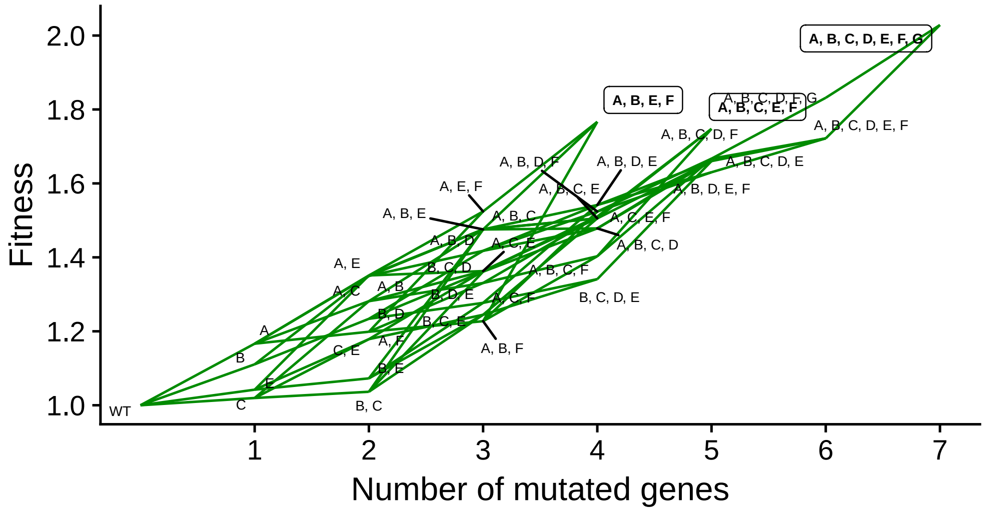

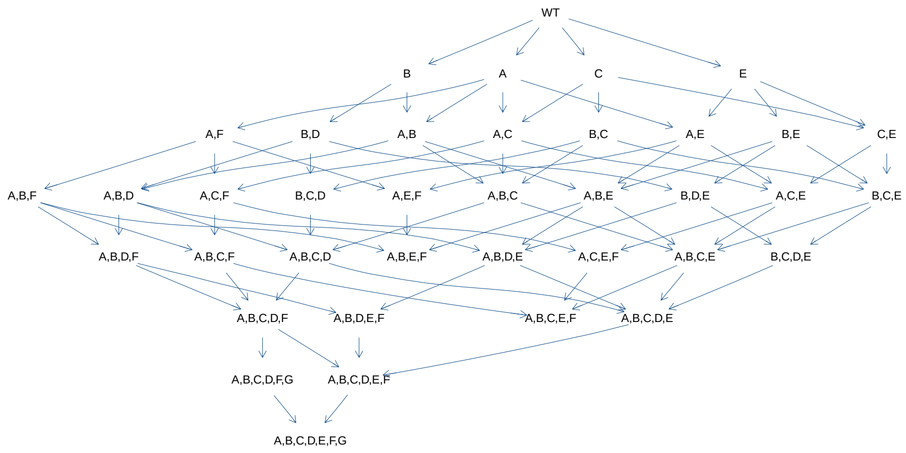

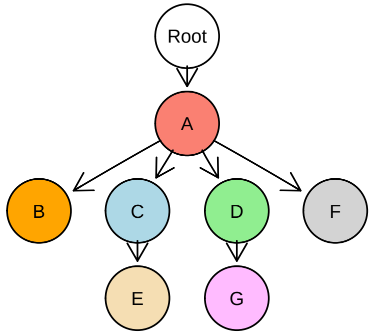

ID	cfODc4wi4uNS9zkM
acc genotypes	37
peaks no back	6
removed edges	16
prop rem edges	0.188

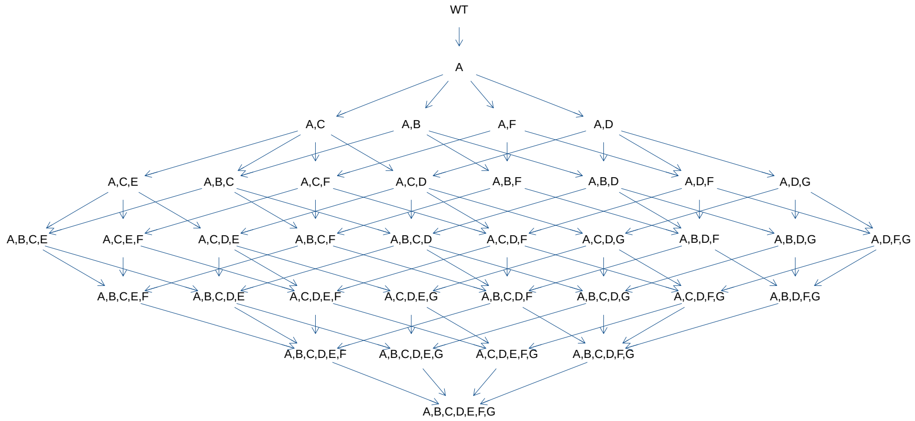

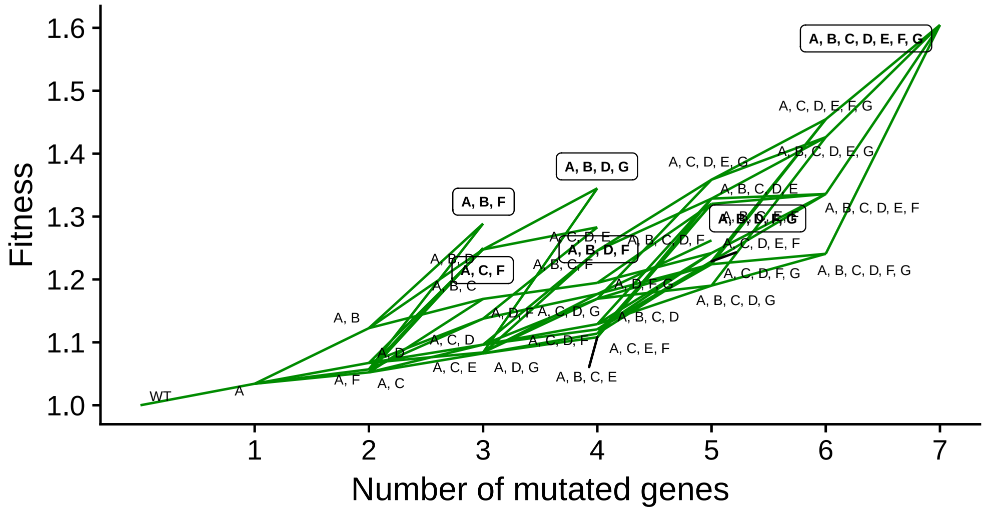

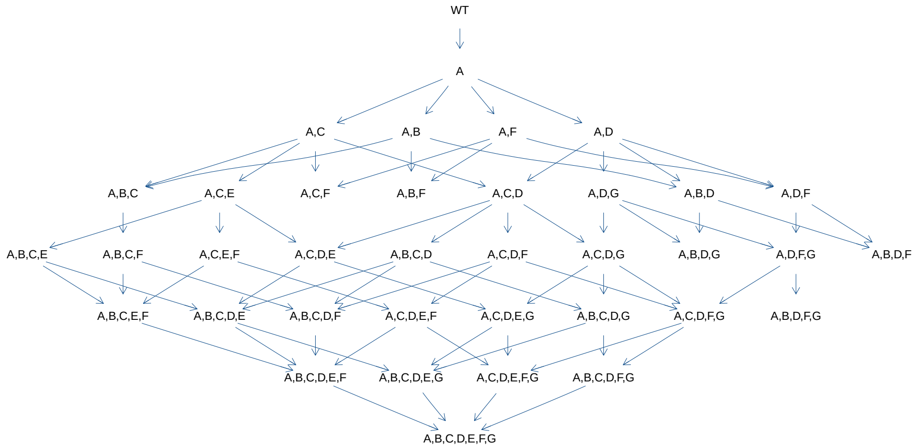

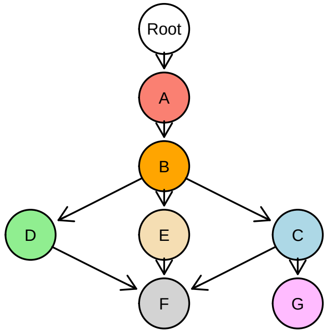

ID chTnx8SjPrqKECWb
acc genotypes 16
peaks no back 3
removed edges 5
prop rem edges 0.2

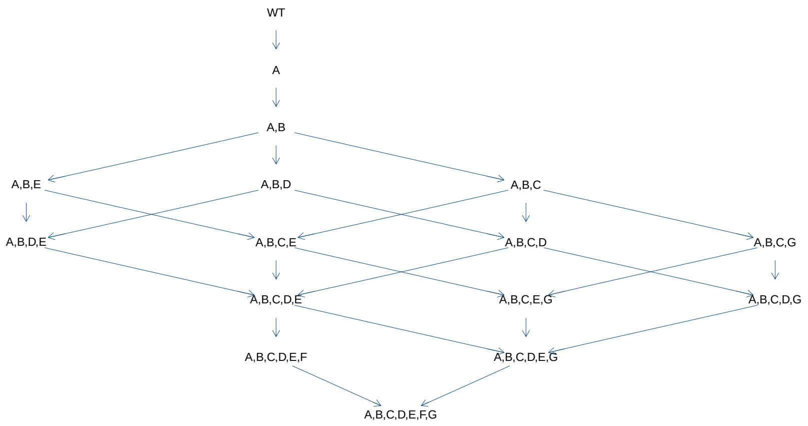

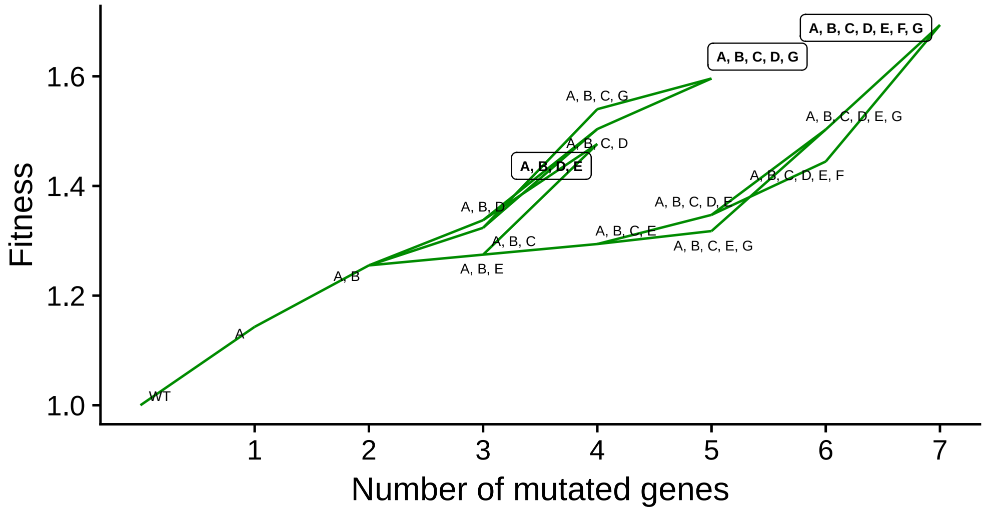

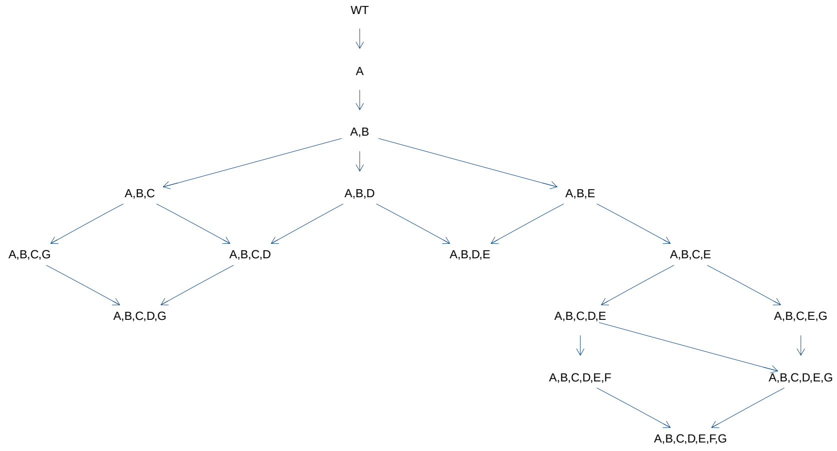

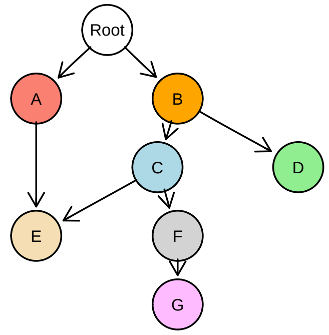

ID	ClxqJzXIXlPDwCzC
acc genotypes	24
peaks no back	5
removed edges	7
prop rem edges	0.159

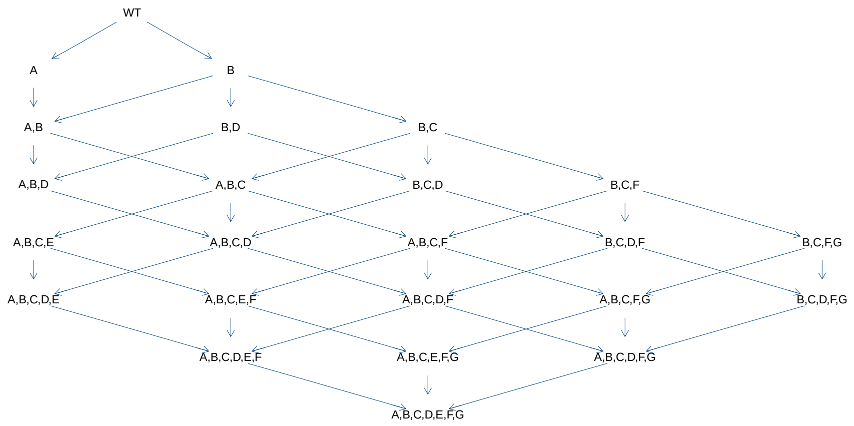

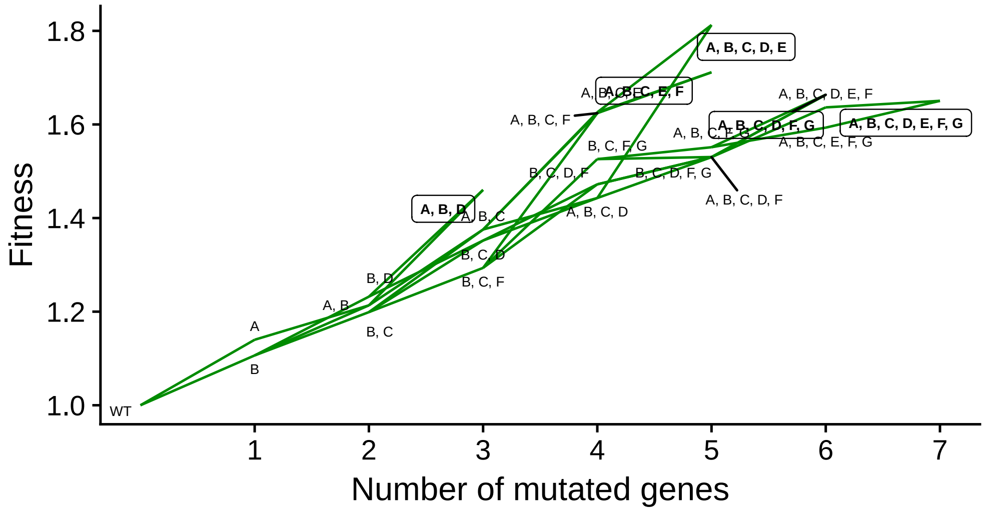

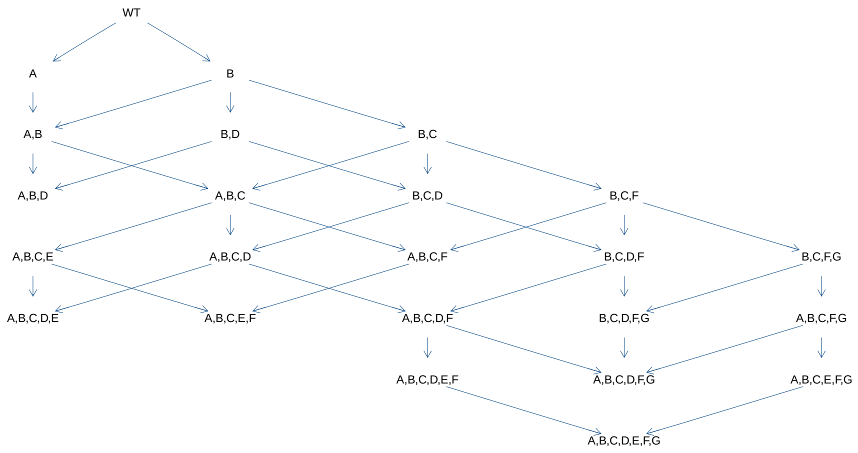

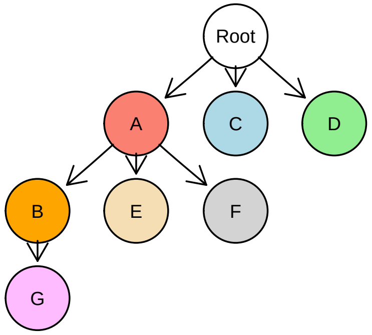

ID	cPTyfbkkNctnZY8i
acc genotypes	52
peaks no back	7
removed edges	30
prop rem edges	0.221

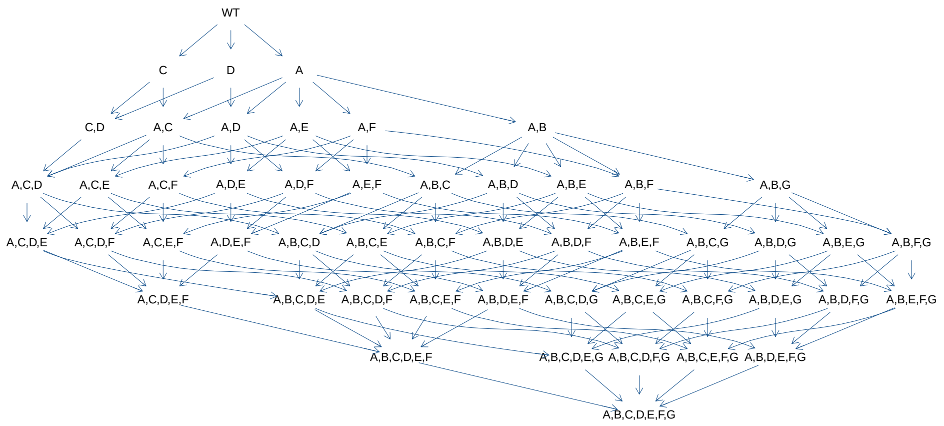

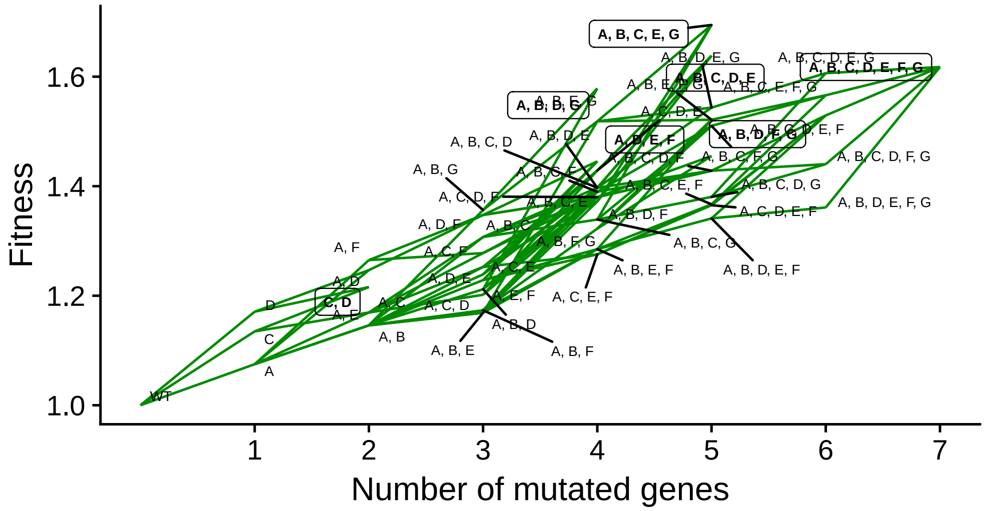

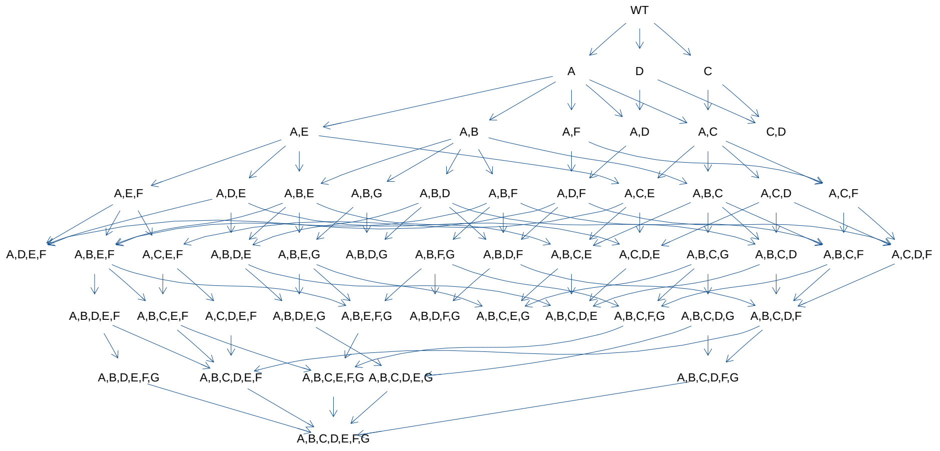

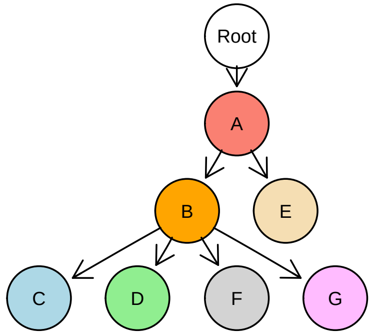

ID cPvlM3BD3obpDIZu
acc genotypes 35
peaks no back 4
removed edges 6
prop rem edges 0.071

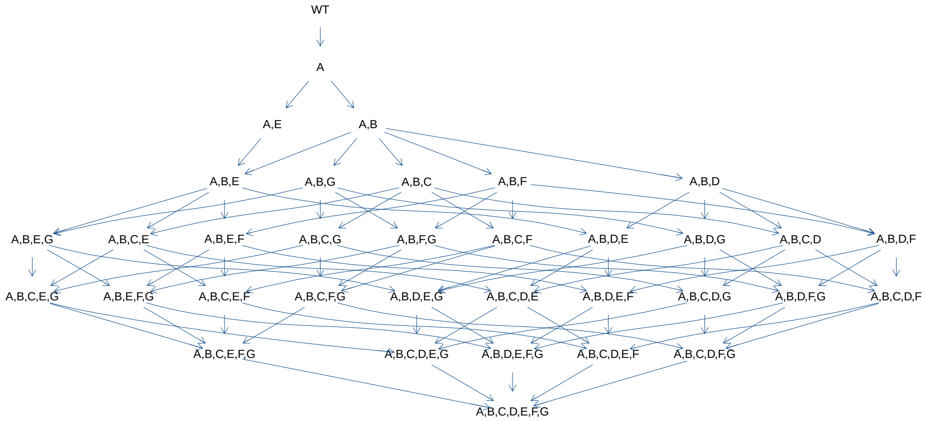

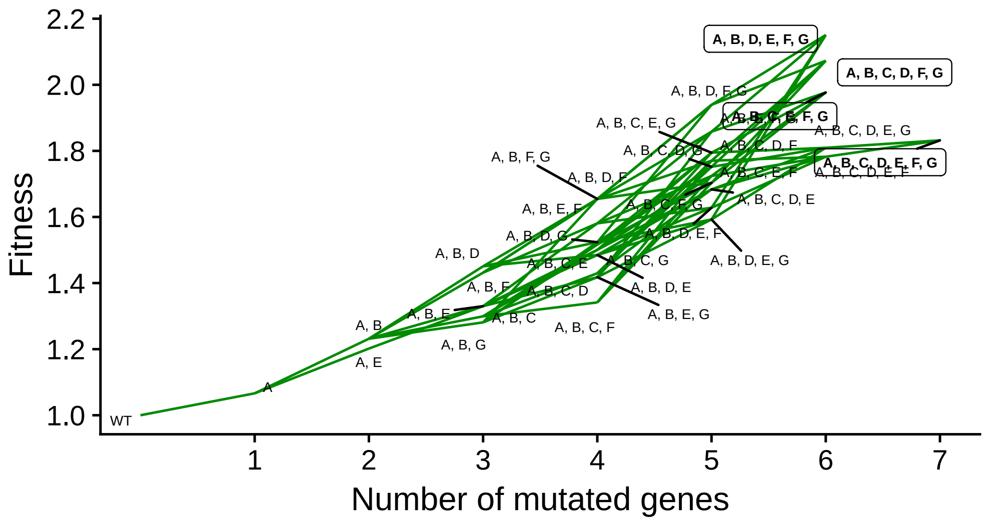

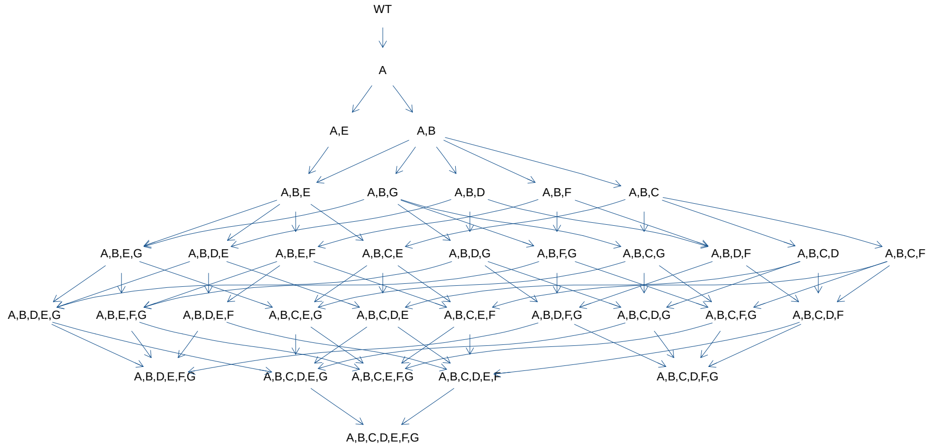

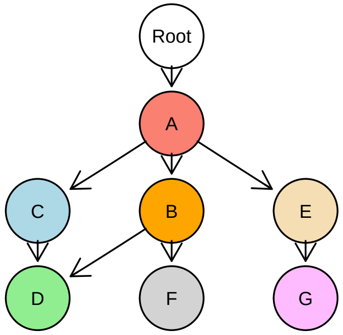

ID	CQaJm9lTvQh26obo
acc genotypes	25
peaks no back	4
removed edges	11
prop rem edges	0.234

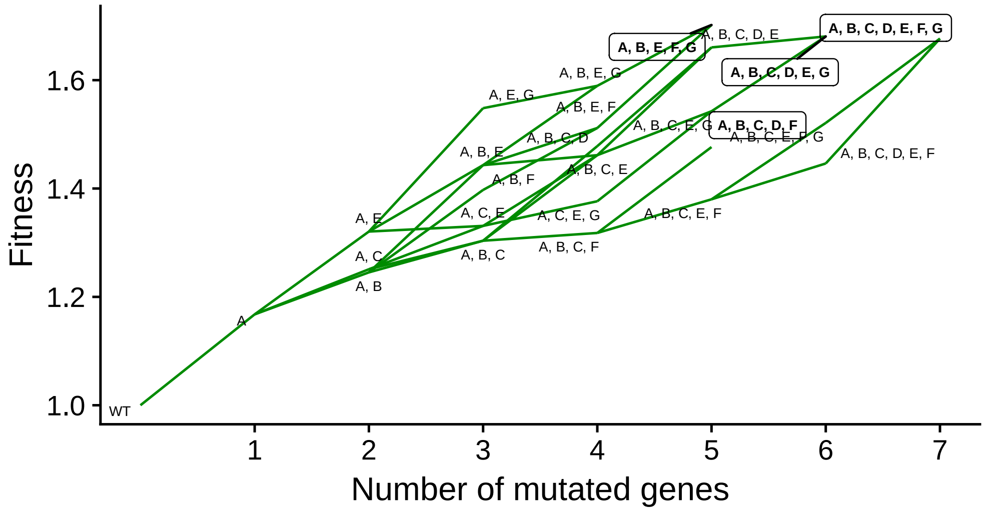

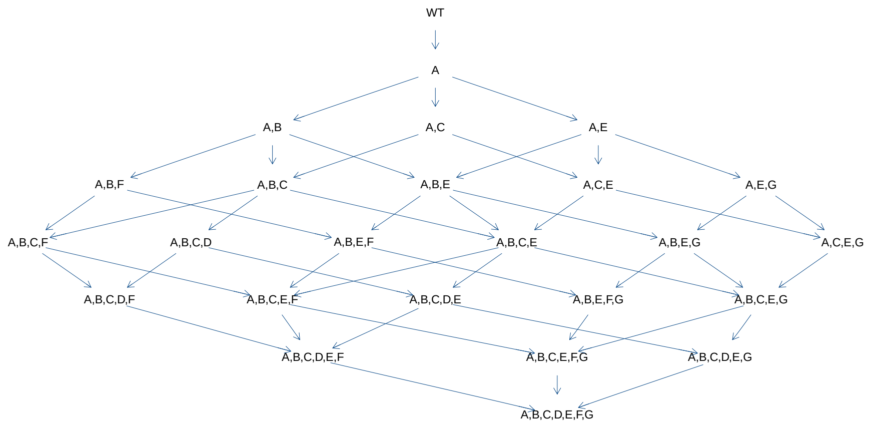

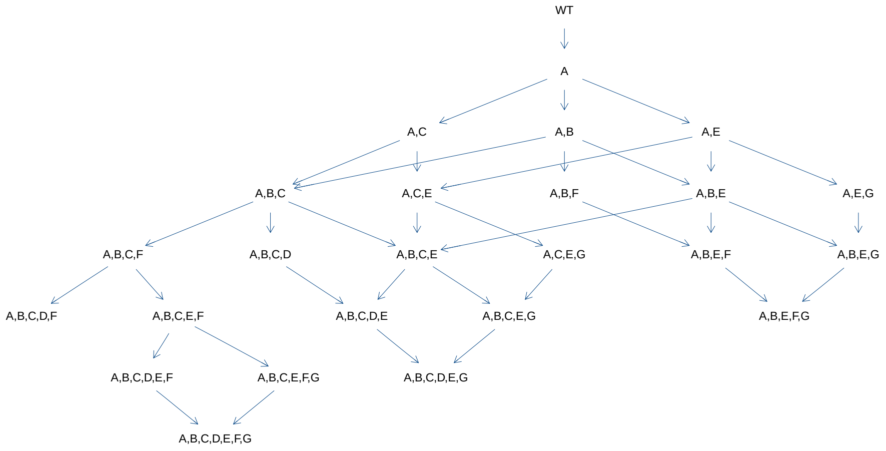

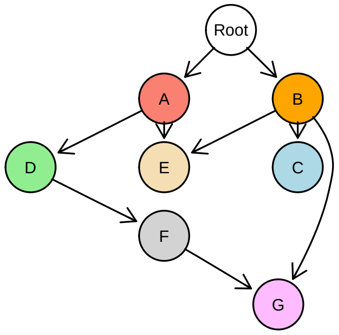

ID cQdnO9XMvDNKOW39
acc genotypes 22
peaks no back 4
removed edges 5
prop rem edges 0.132

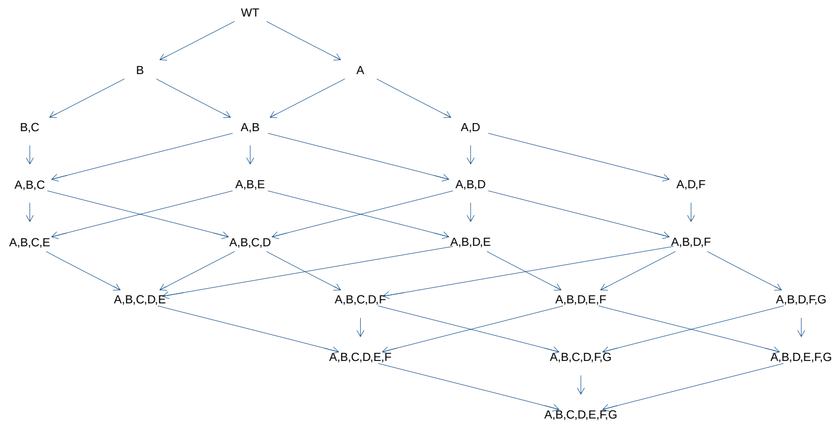

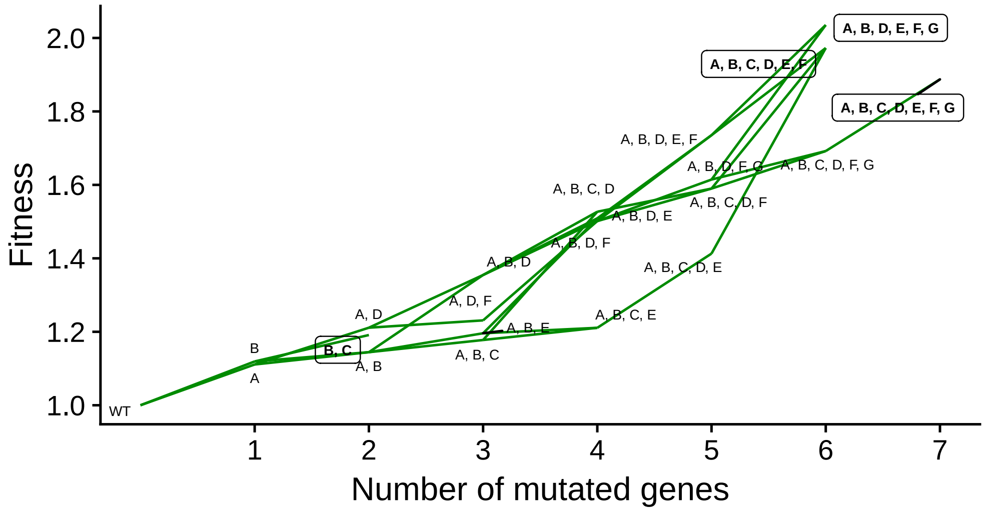

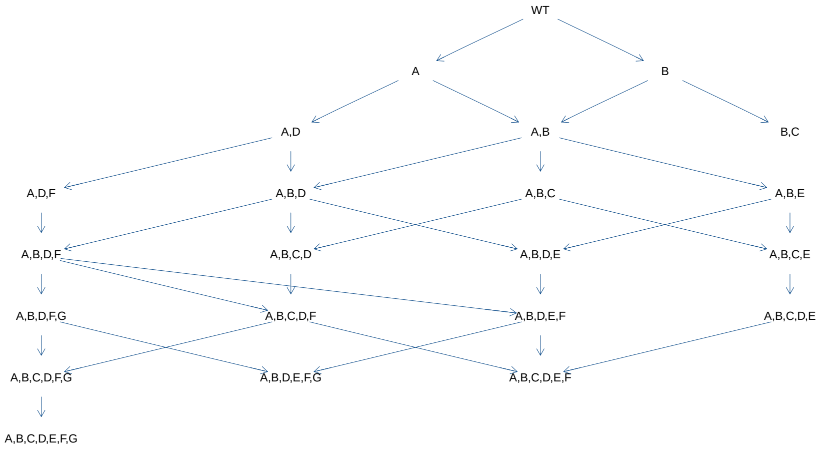

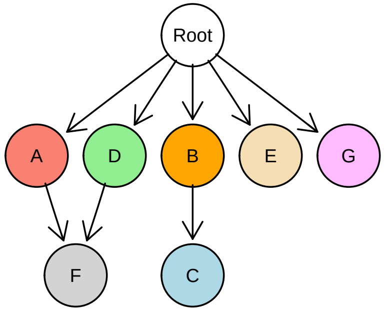

ID	cwFWkTsrBGFO2dfv
acc genotypes	60
peaks no back	7
removed edges	29
prop rem edges	0.181

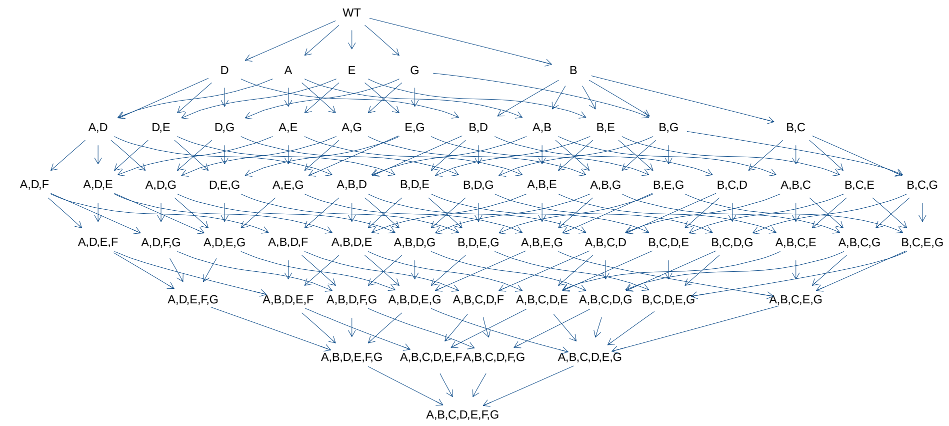

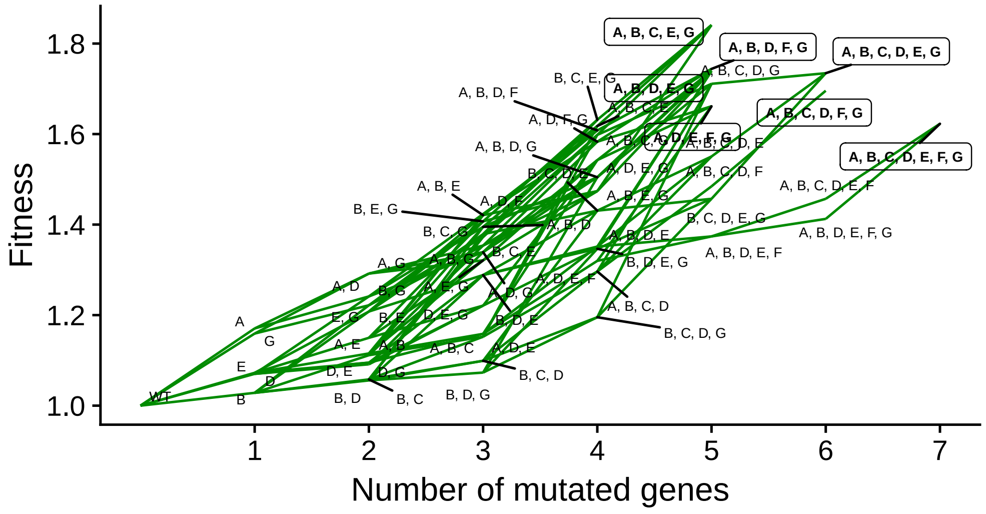

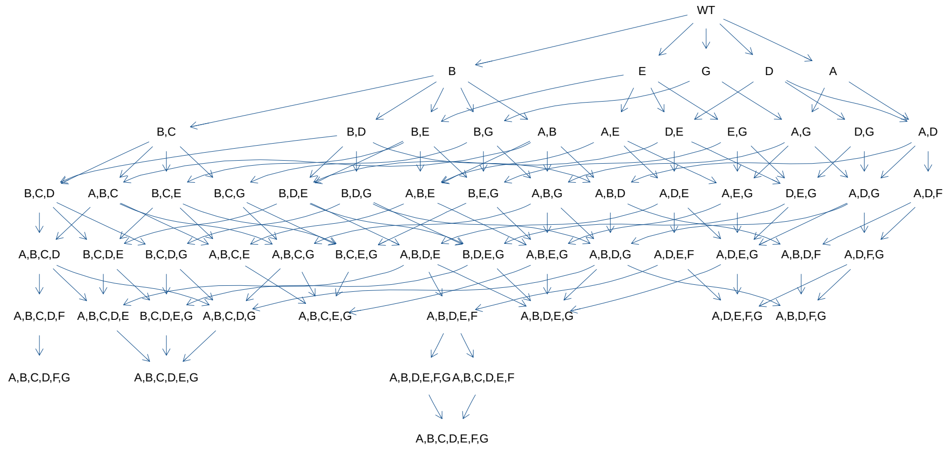

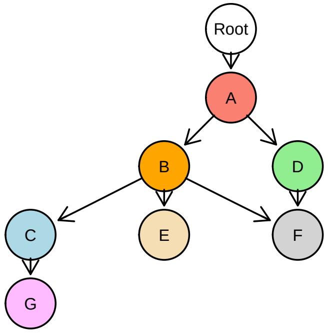

ID	D1wA9X4HhUWYpfHR
acc genotypes	21
peaks no back	5
removed edges	8
prop rem edges	0.216

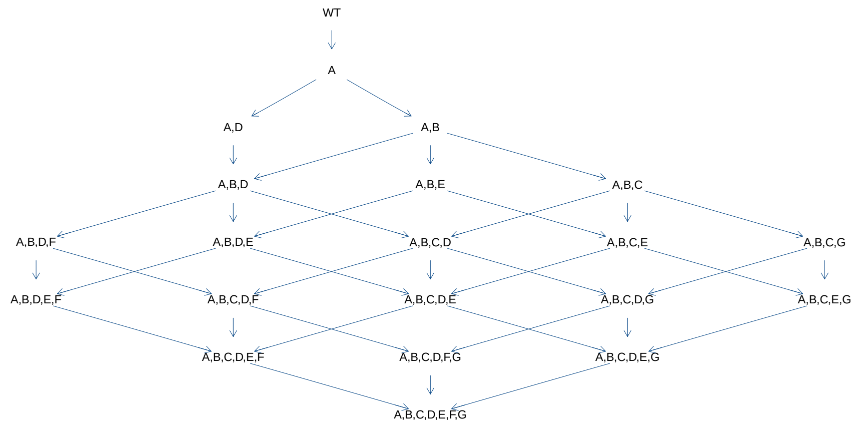

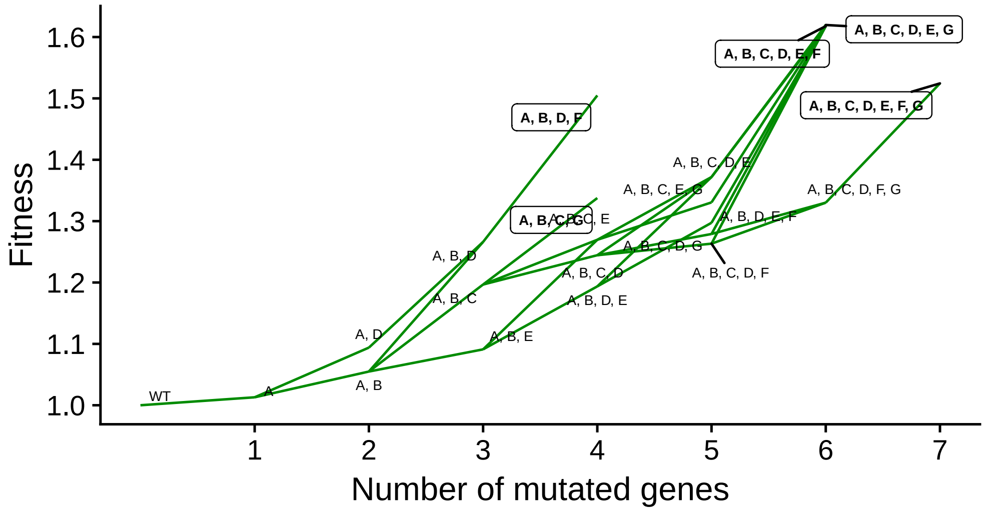

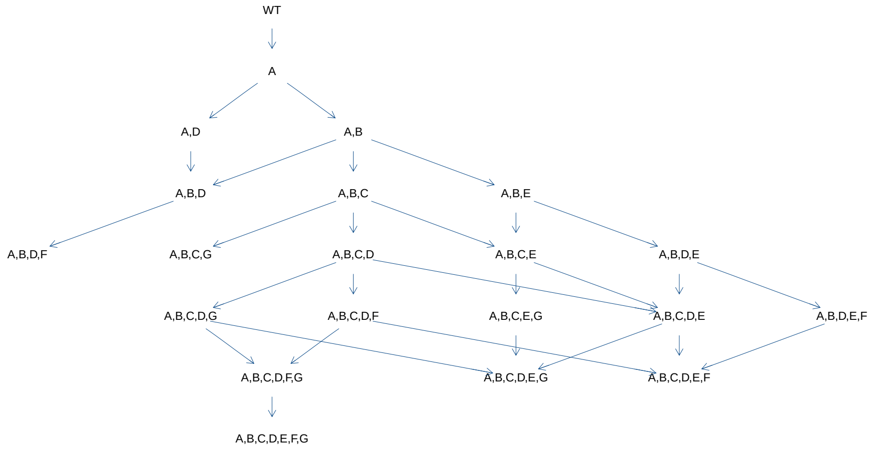

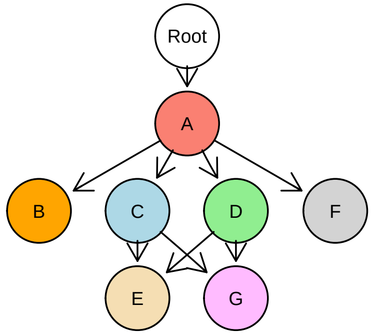

ID	DBuyPIOfmeKuioDc
acc genotypes	29
peaks no back	5
removed edges	9
prop rem edges	0.148

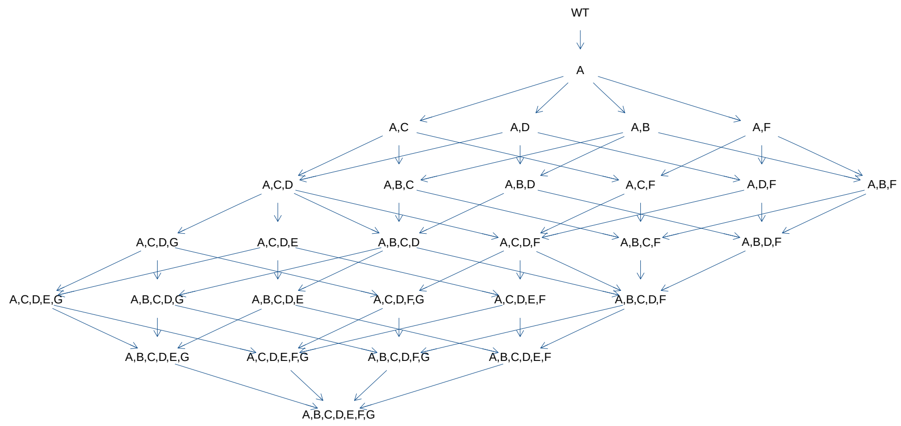

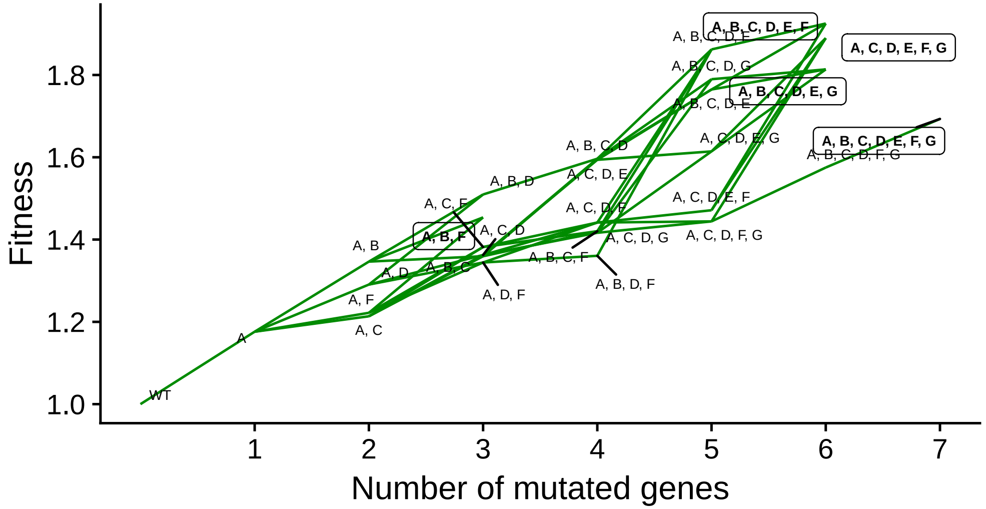

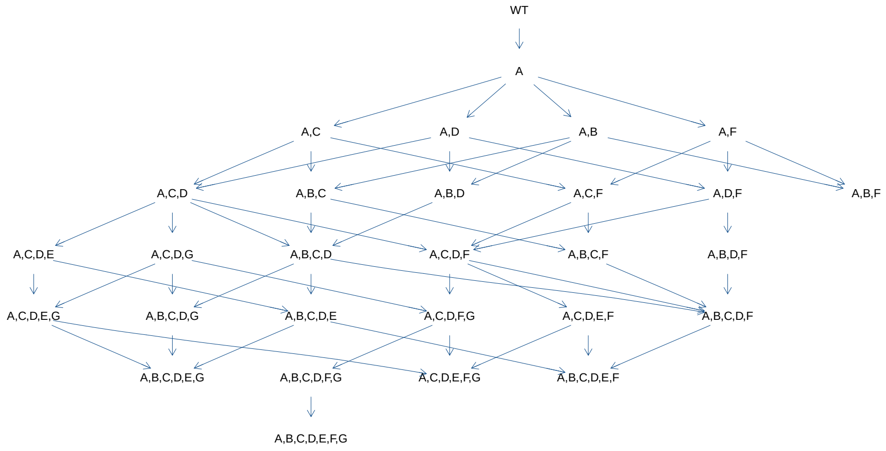

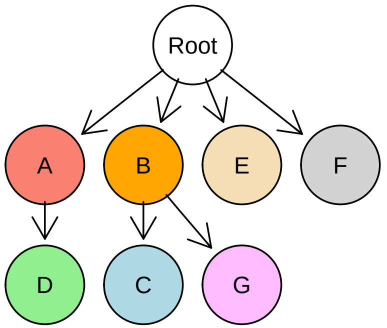

ID	DDAFd9YlDa3wcgyX
acc genotypes	60
peaks no back	8
removed edges	32
prop rem edges	0.2

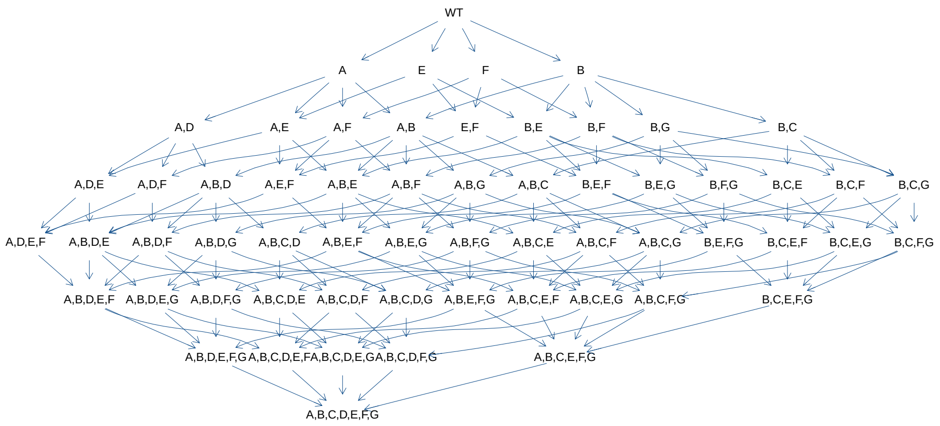

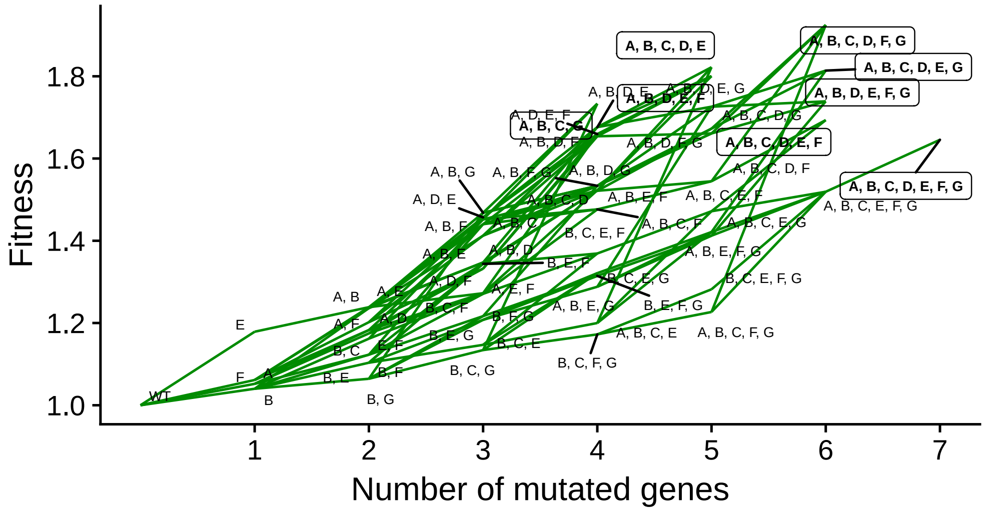

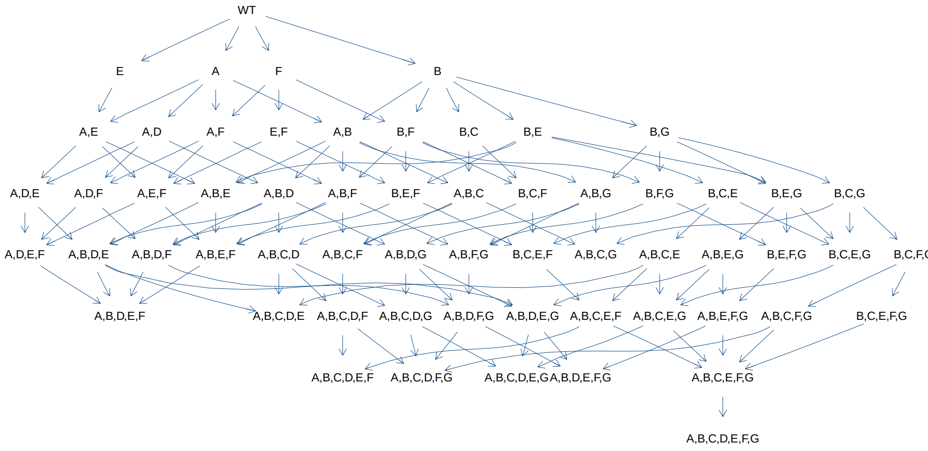

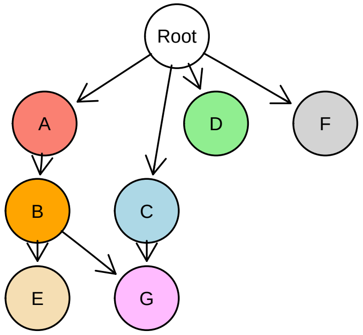

ID	DE10Mak4NoN6QJYUY
acc genotypes	40
peaks no back	4
removed edges	13
prop rem edges	0.141

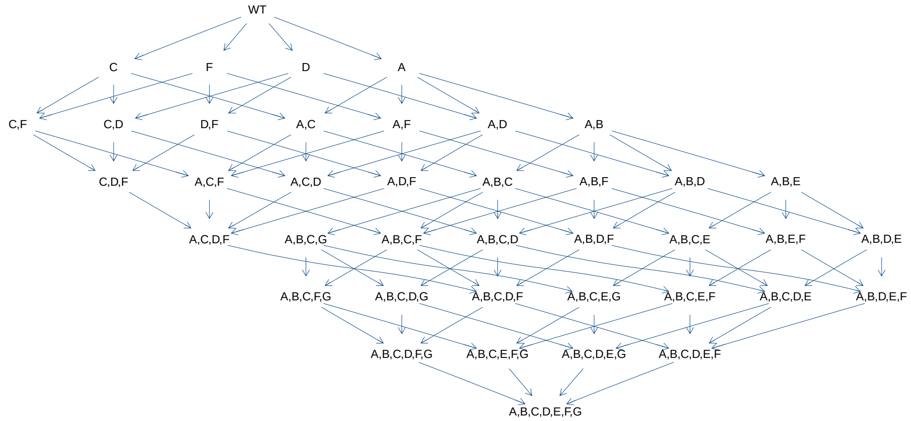

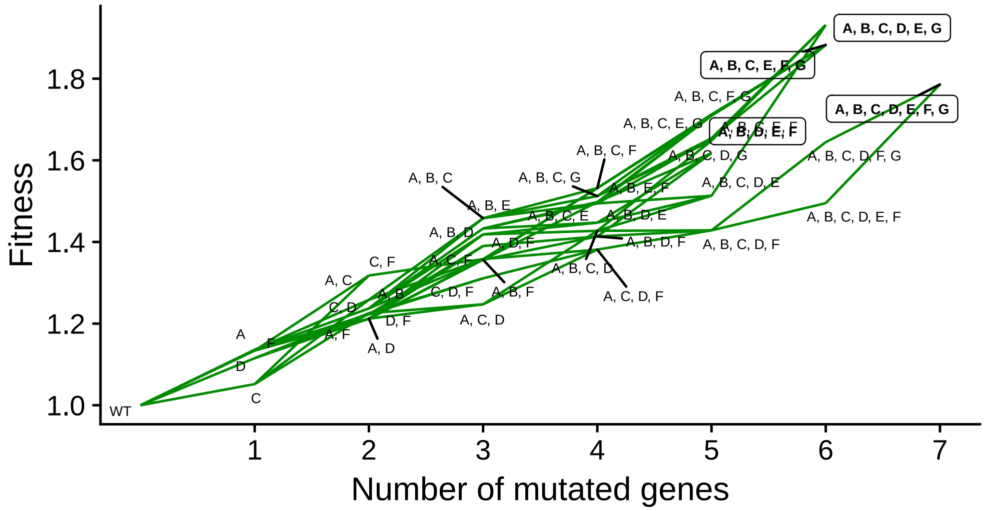

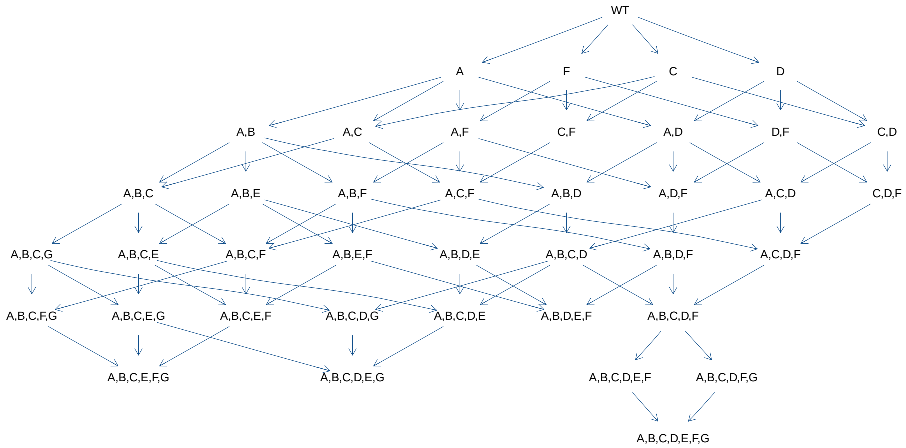

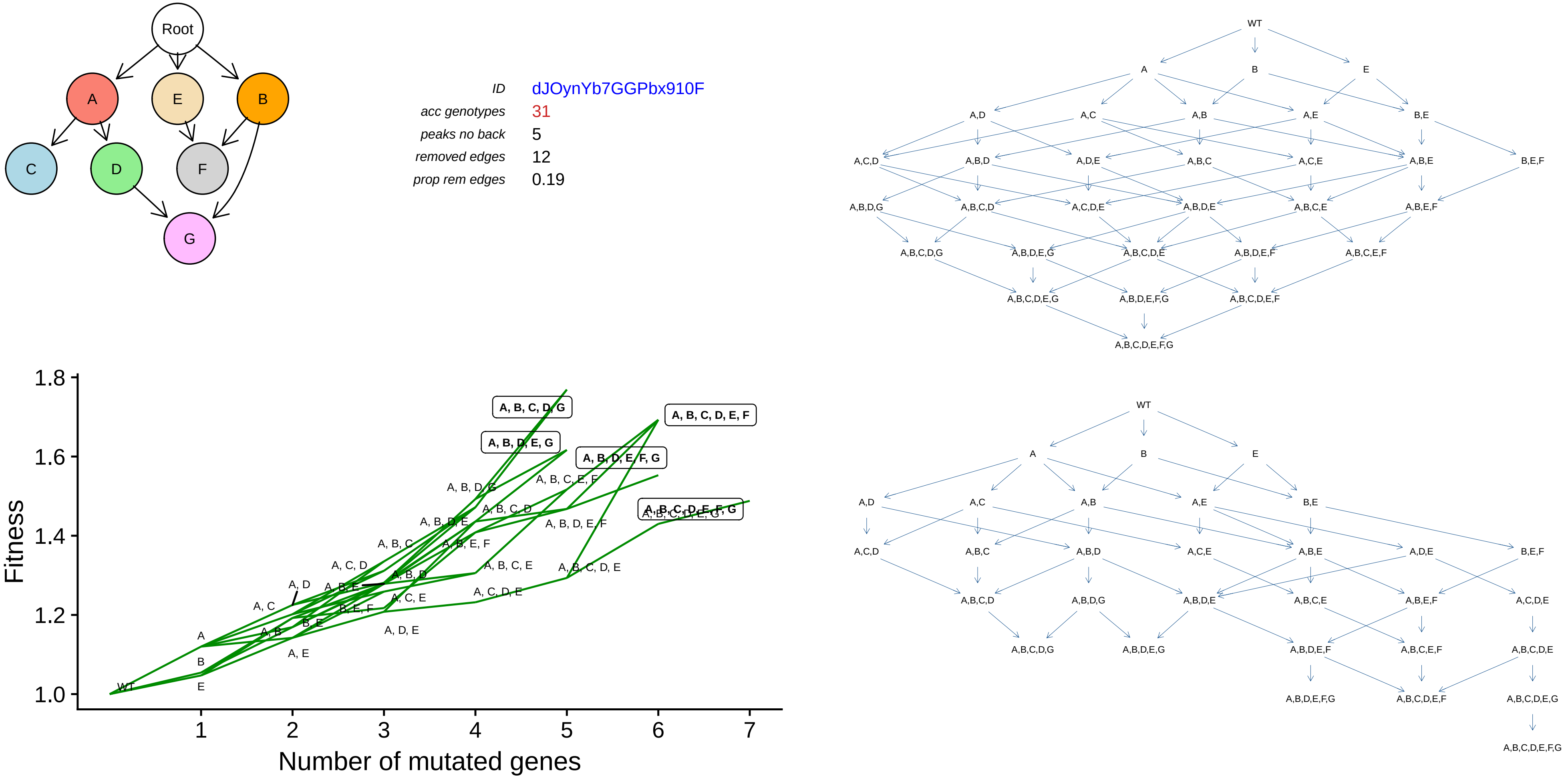
Root
A
E
B
C
D
F
G
ID dJOynYb7GGPbx910F
acc genotypes 31
peaks no back 5
removed edges 12
prop rem edges 0.19
Fitness
Number of mutated genes
A, B, C, D, G
A, B, C, D, E, F
A, B, D, E, G
A, B, D, E, F, G
A, B, C, D, E, F, G
WT
A,B,C,D,E,F,G

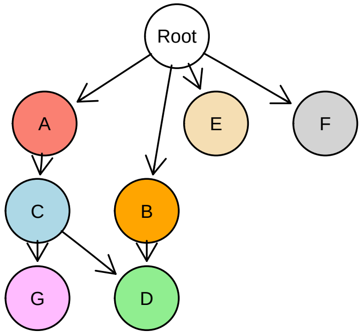

ID	DM6wSFi5wdVWUqiH
acc genotypes	40
peaks no back	6
removed edges	12
prop rem edges	0.13

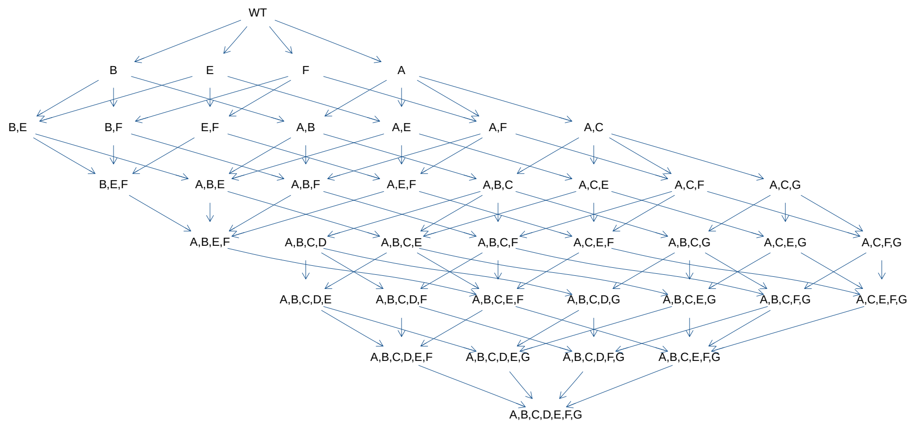

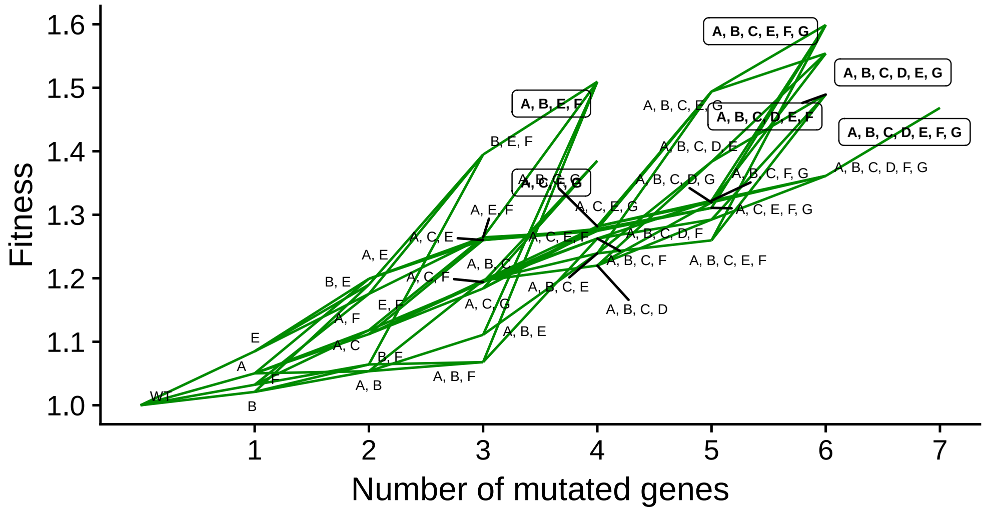

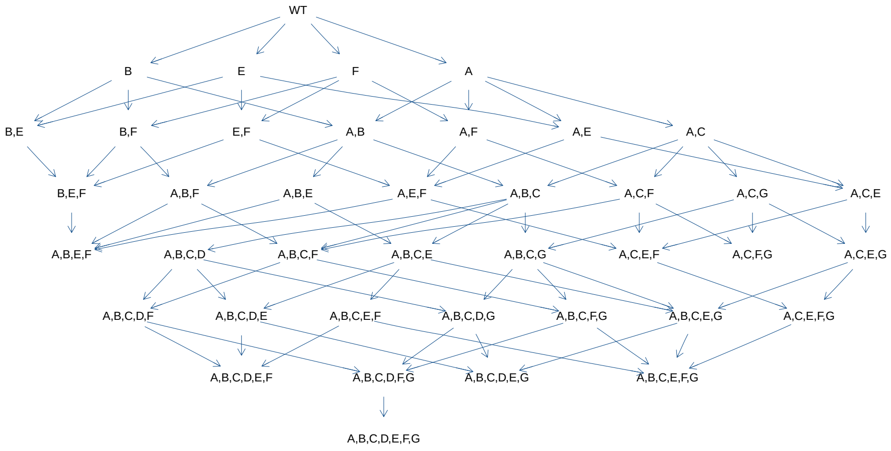

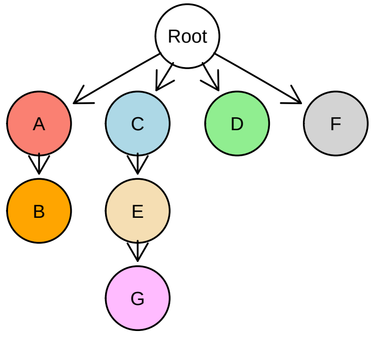

ID dO8v6amgefufggoE
acc genotypes 48
peaks no back 7
removed edges 24
prop rem edges 0.207

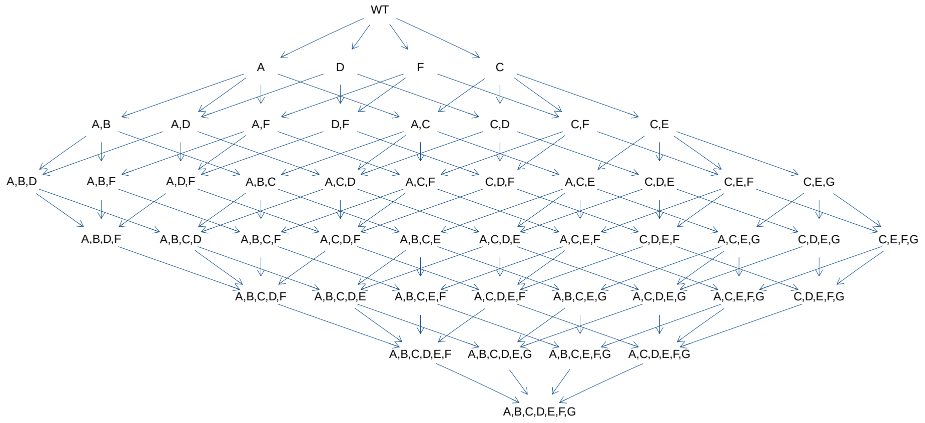

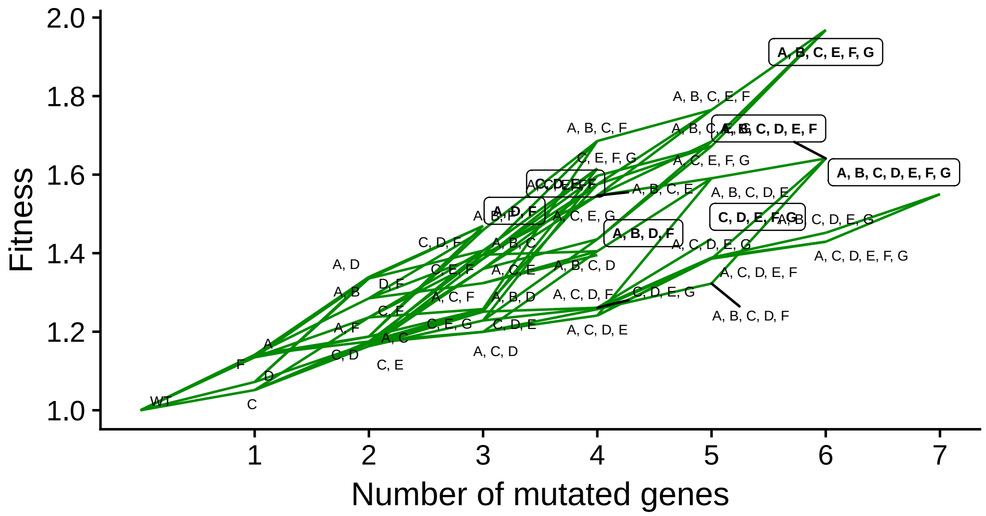

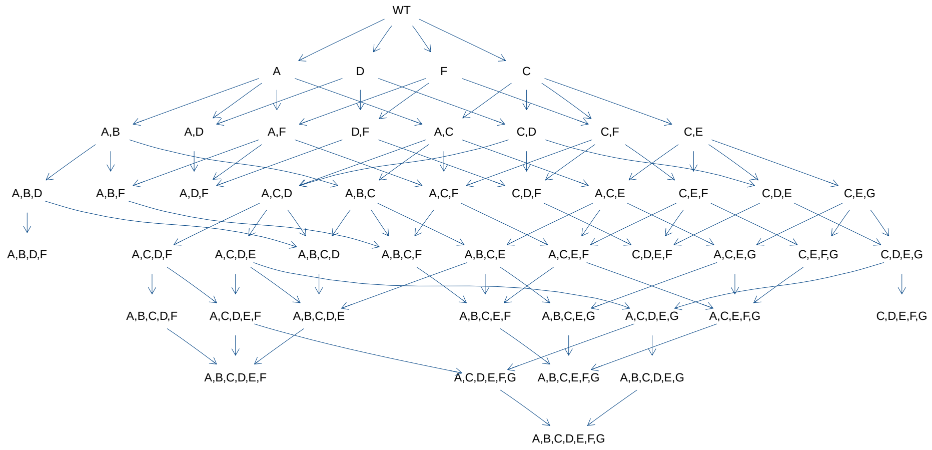

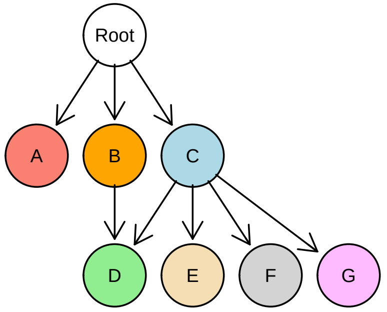

ID	dPDOh3Us3MOolJnT
acc genotypes	52
peaks no back	9
removed edges	26
prop rem edges	0.191

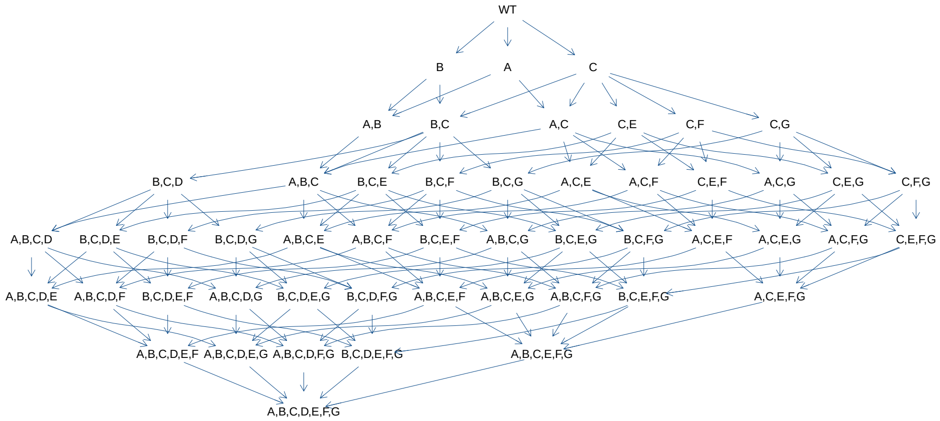

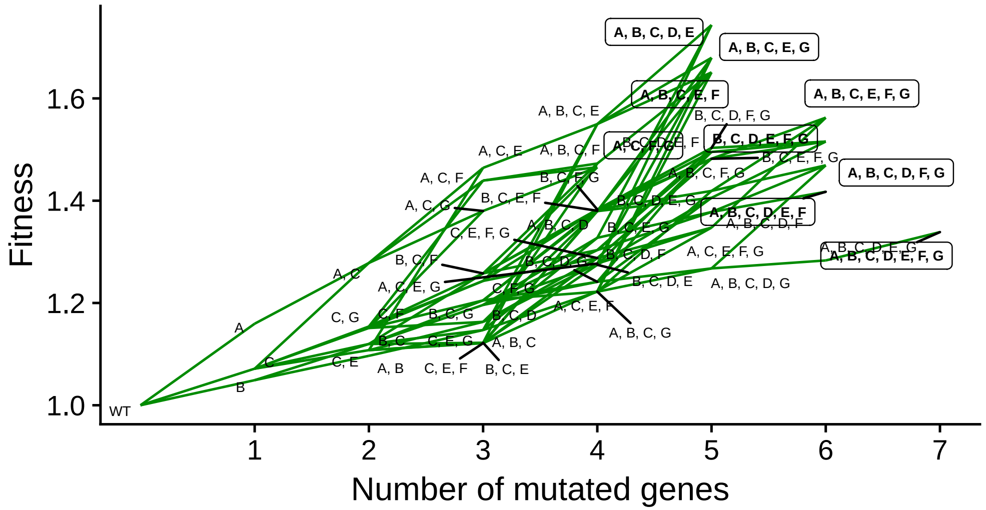

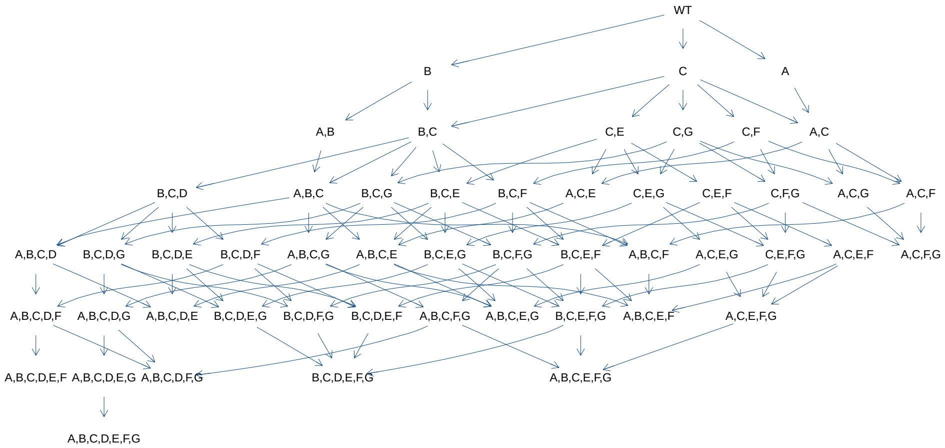

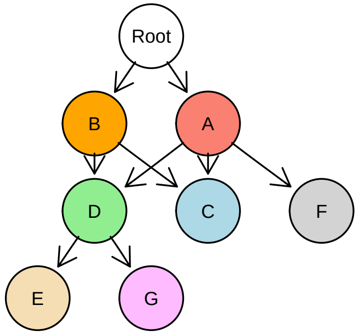

ID	E4Ngwmi2x89iIda9
acc genotypes	24
peaks no back	4
removed edges	4
prop rem edges	0.087

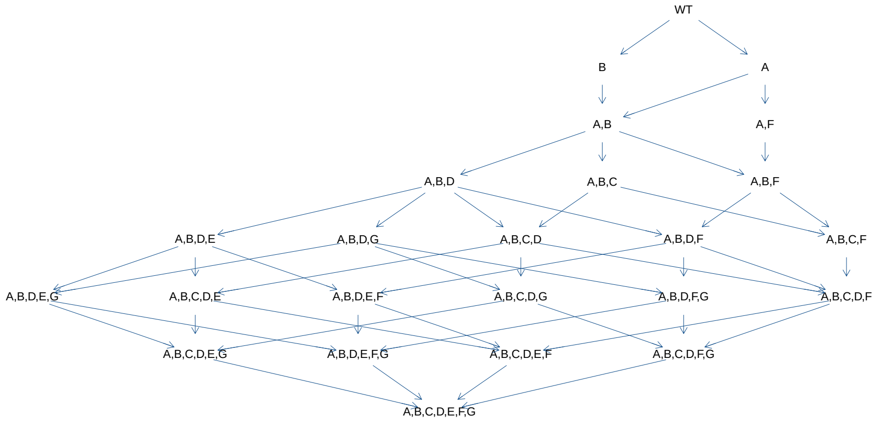

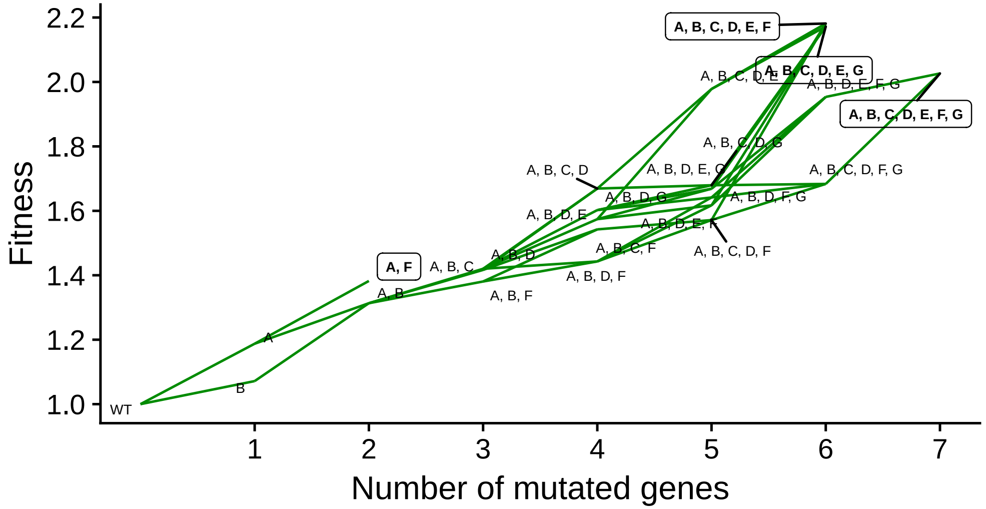

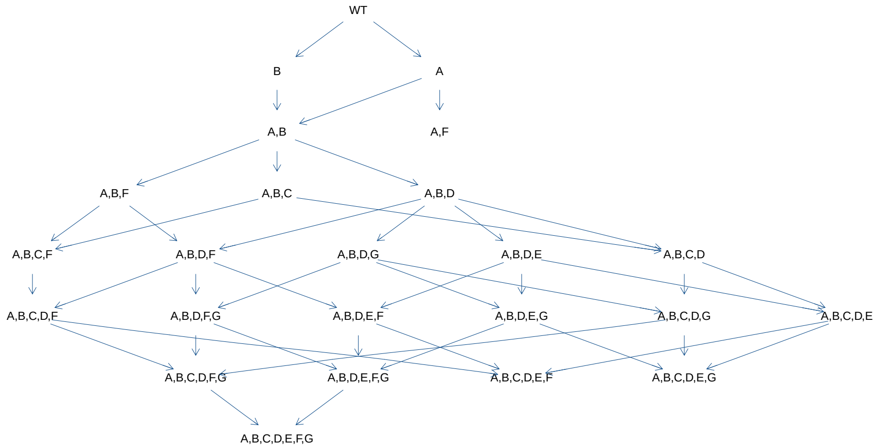

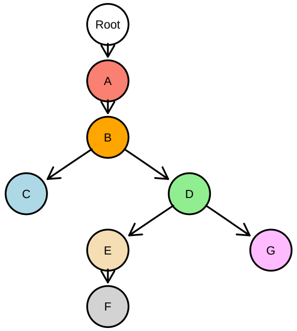

ID	ejQdvM3QTzcrMUgC
acc genotypes	16
peaks no back	4
removed edges	4
prop rem edges	0.16

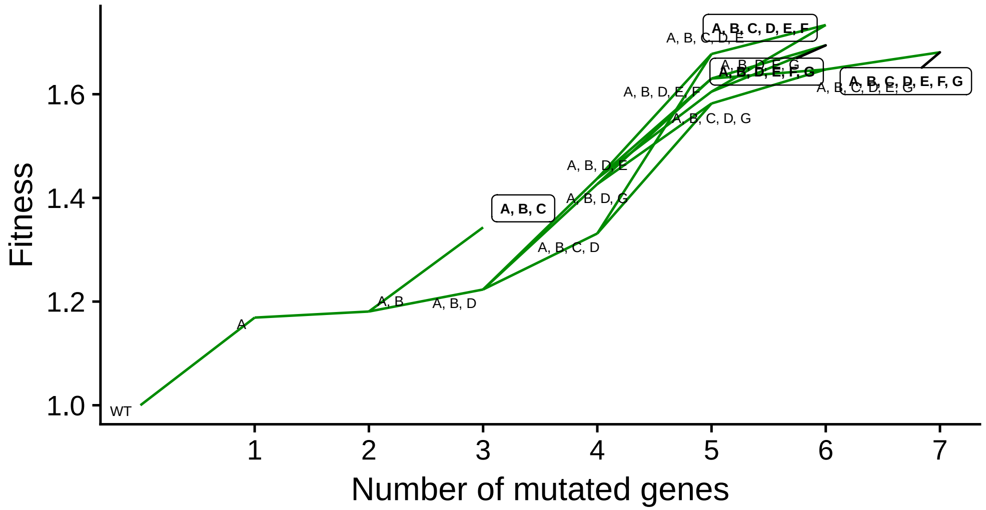

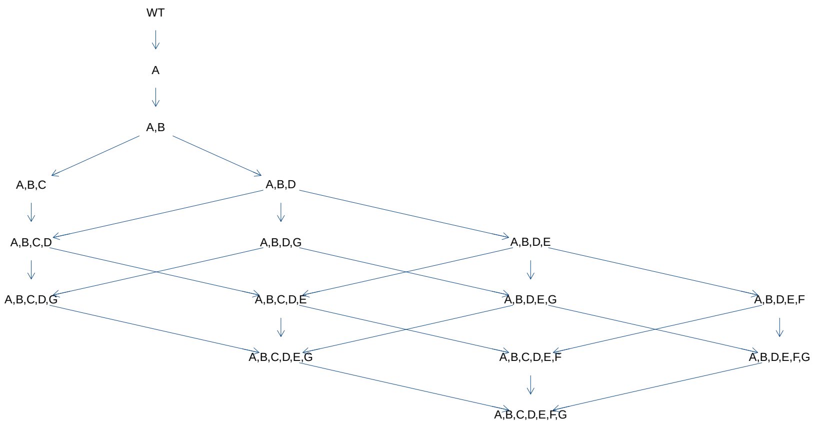

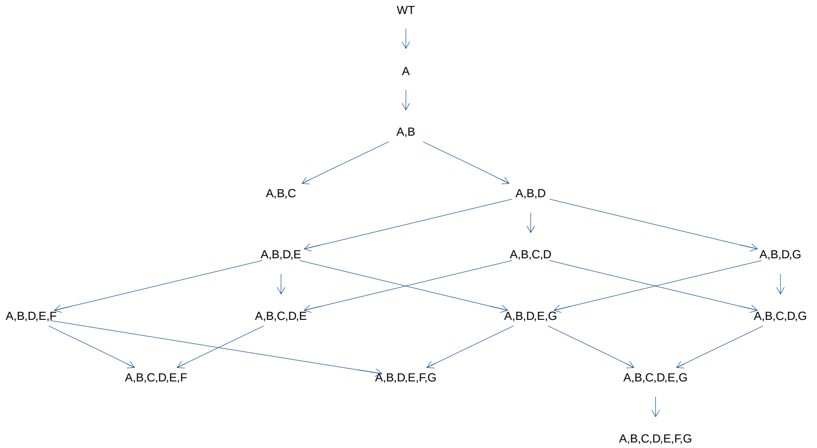

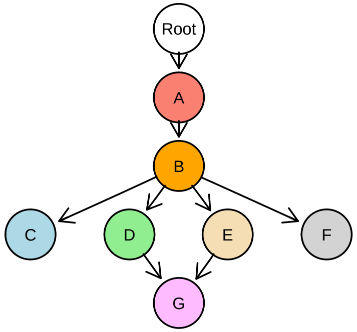

ID	eNAunG4FXTpwgDbz
acc genotypes	22
peaks no back	4
removed edges	9
prop rem edges	0.214

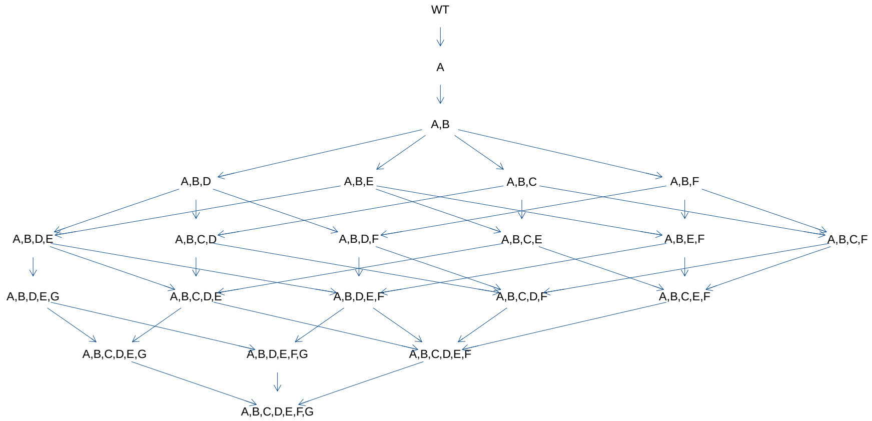

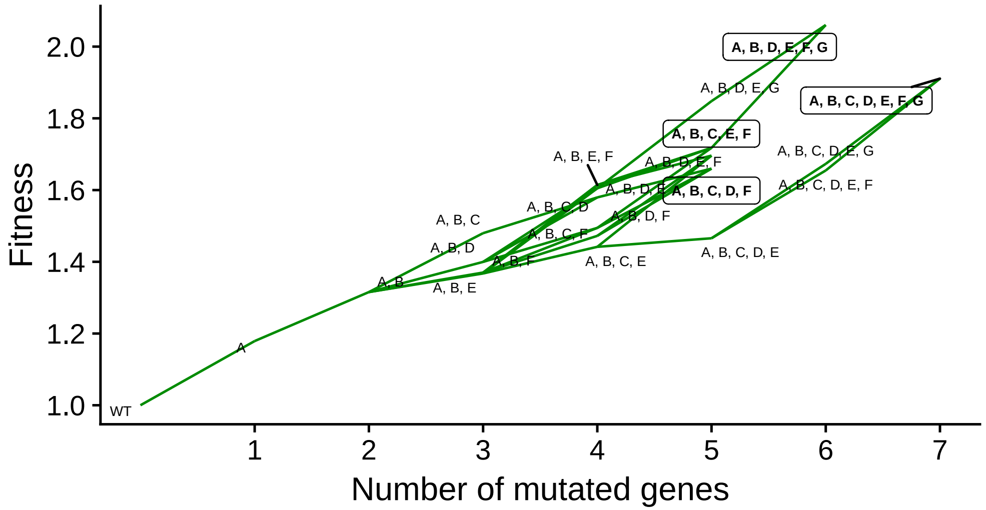

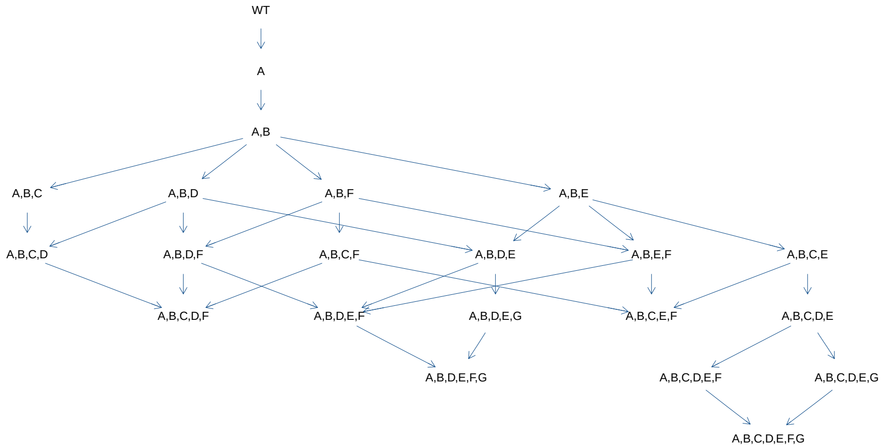

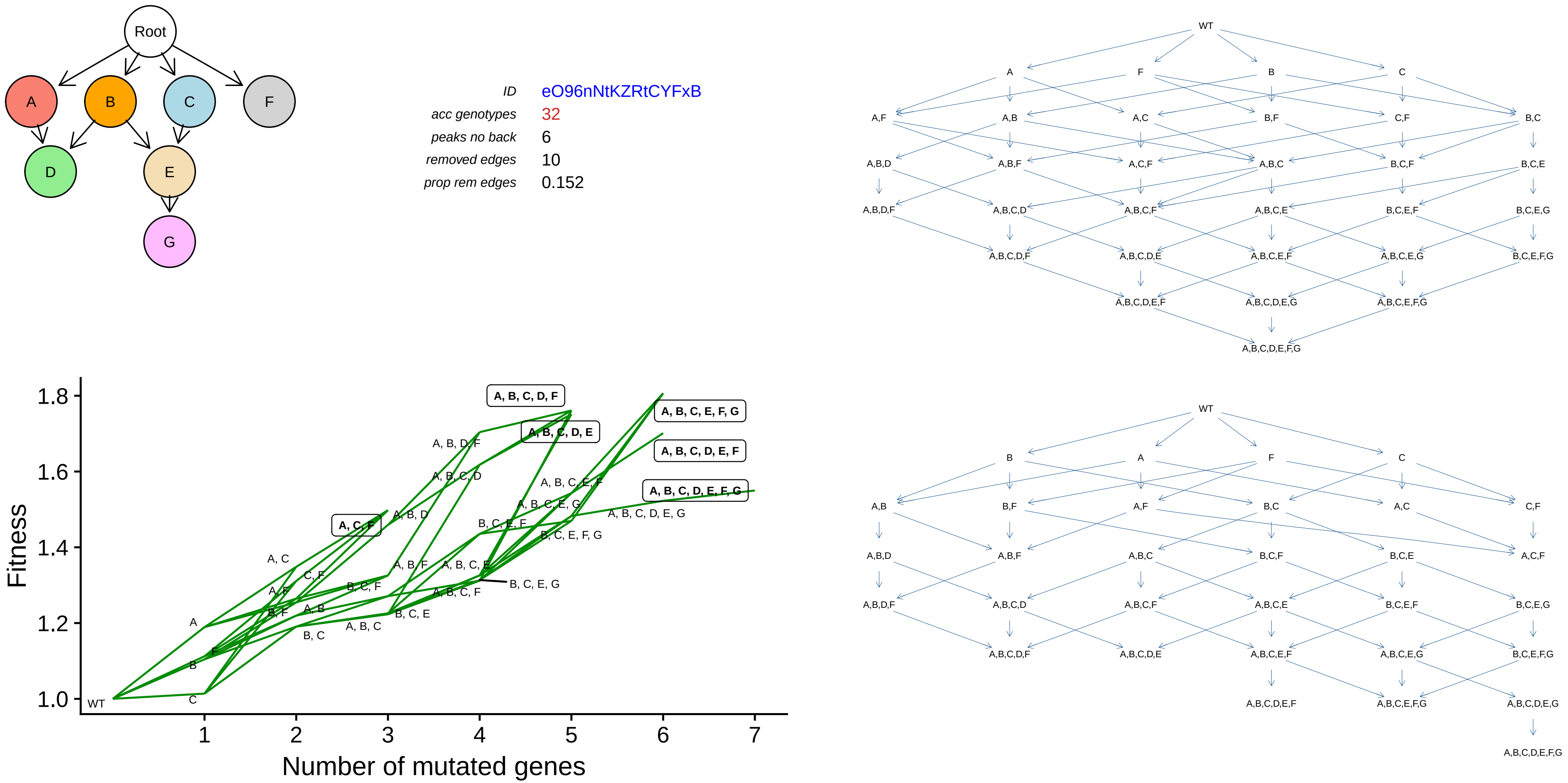
Root
A
B
C
F
D
E
G
ID eO96nNtKZRtCYFxB
acc genotypes 32
peaks no back 6
removed edges 10
prop rem edges 0.152
Fitness
Number of mutated genes
A, B, C, D, F
A, B, C, D, E
A, B, C, E, F, G
A, B, C, D, E, F
A, B, C, D, E, F, G
A, C, F
WT

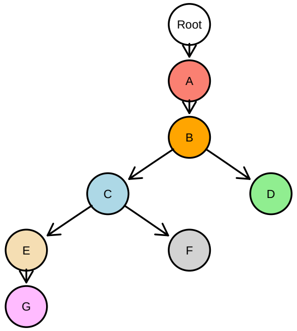

ID	EtcHaQHBlLbIjaZQ
acc genotypes	16
peaks no back	2
removed edges	2
prop rem edges	0.08

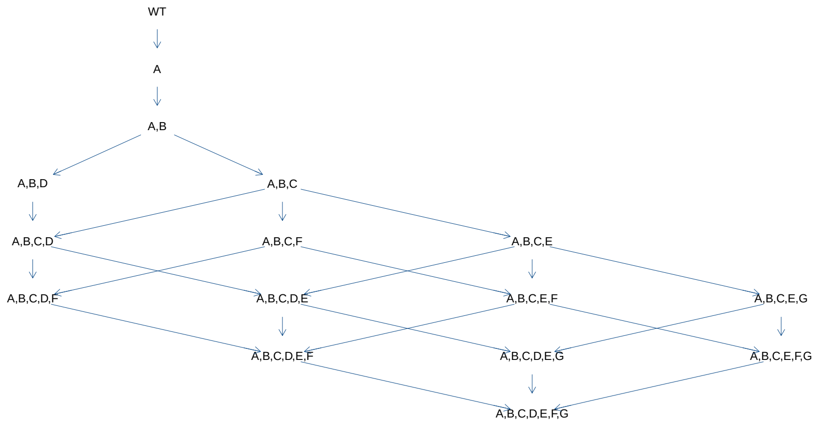

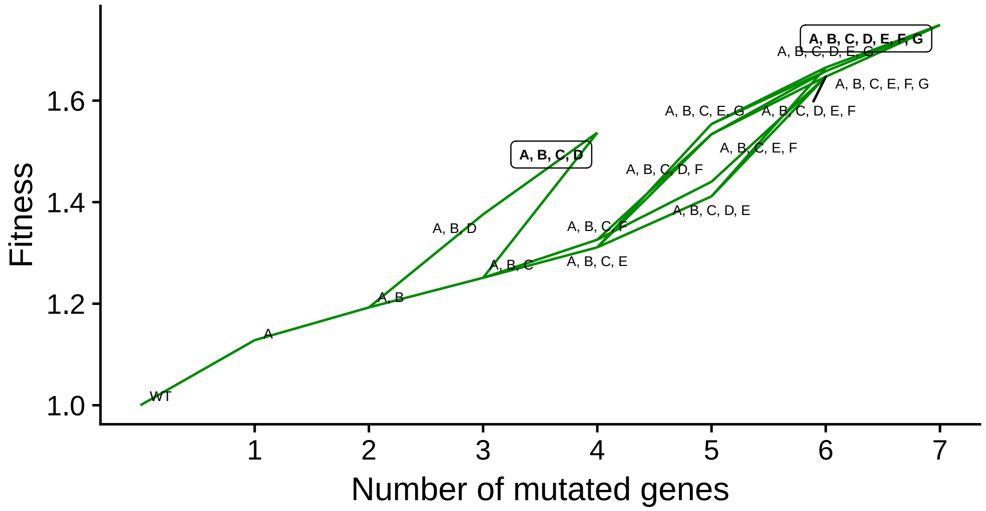

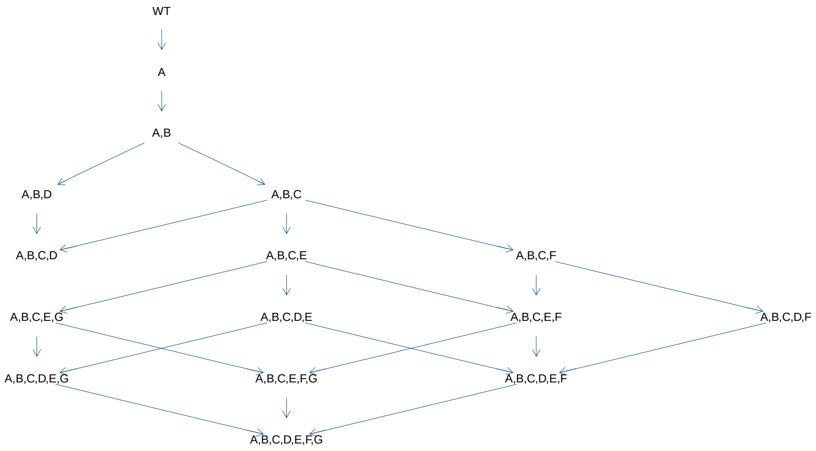

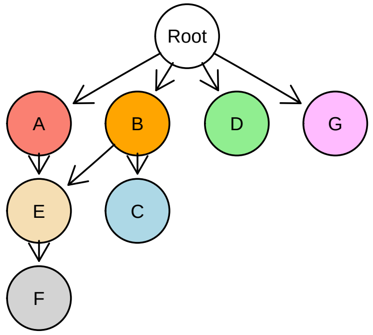

ID	f103lT84L6WhInZWQ
acc genotypes	40
peaks no back	5
removed edges	13
prop rem edges	0.141

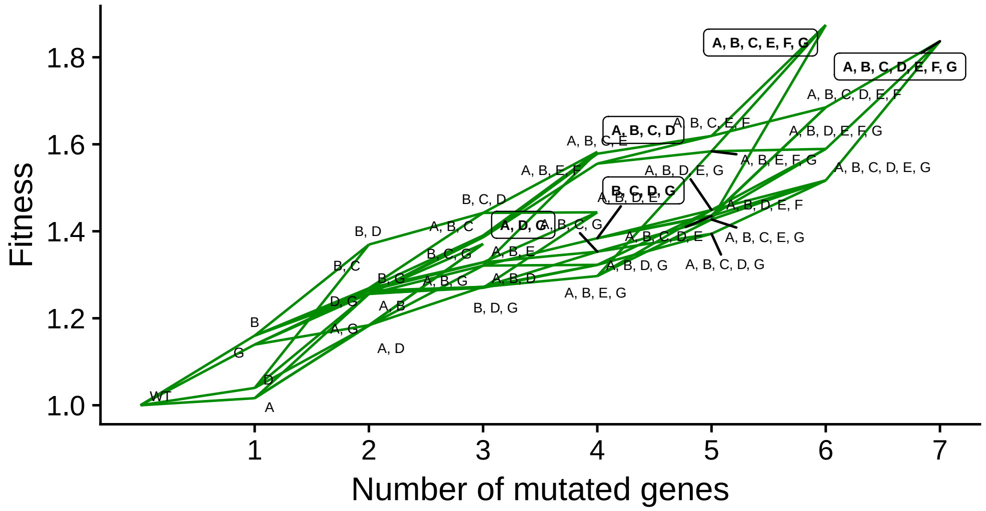

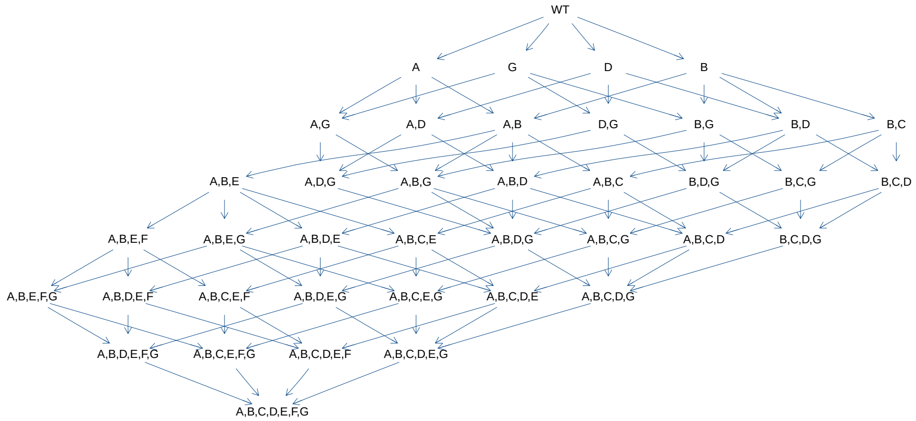

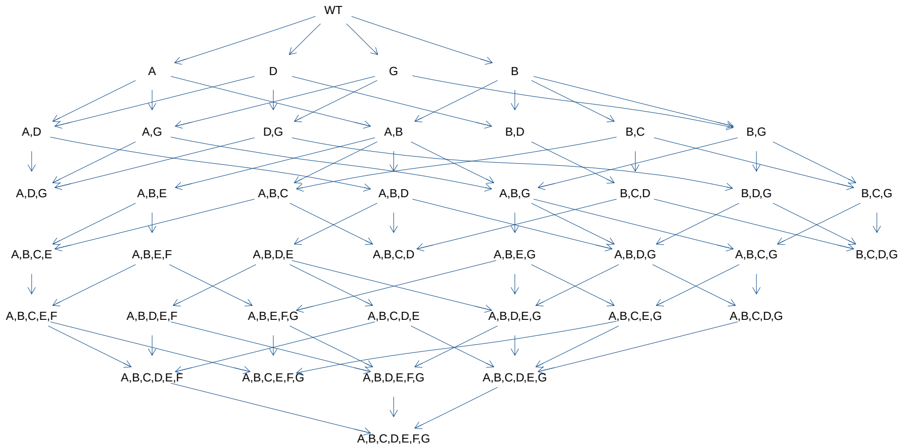

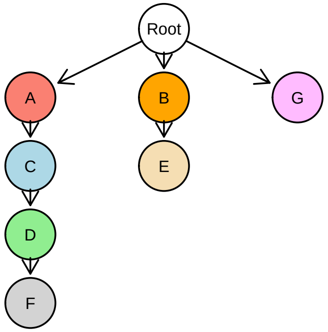

ID Fhypo3U5P7710LvXb
acc genotypes 30
peaks no back 6
removed edges 8
prop rem edges 0.136

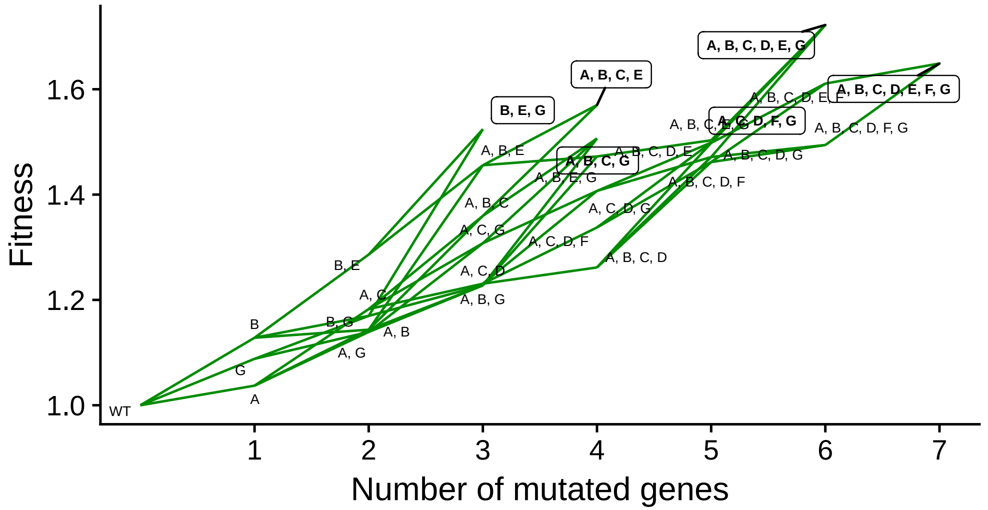

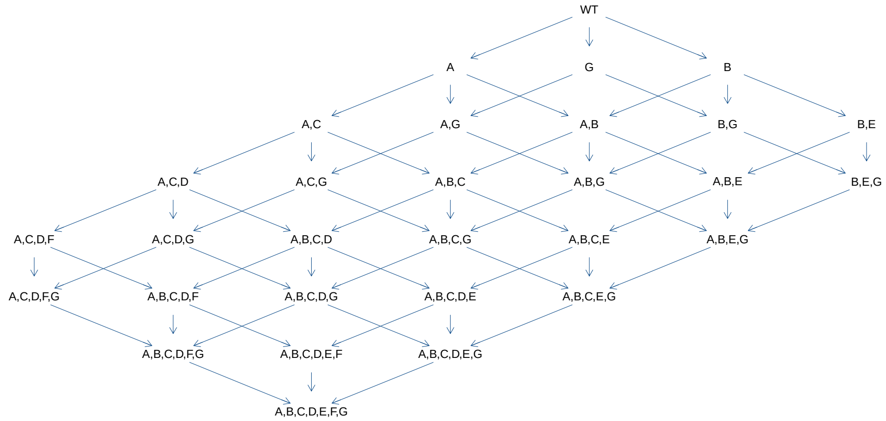

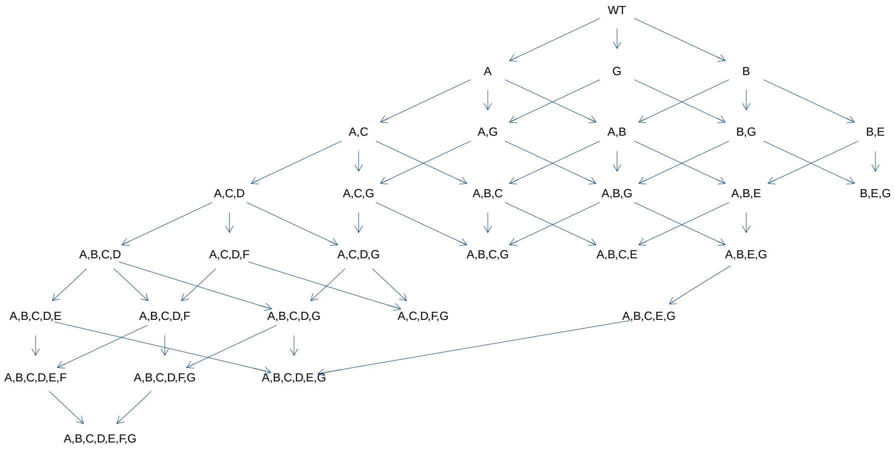

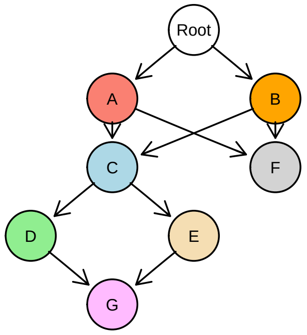

ID FJcdcp4ZT1UXlBCC
acc genotypes 15
peaks no back 3
removed edges 3
prop rem edges 0.136

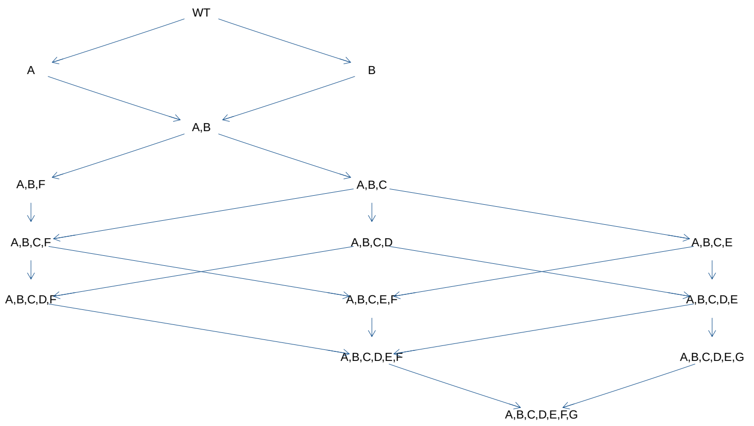

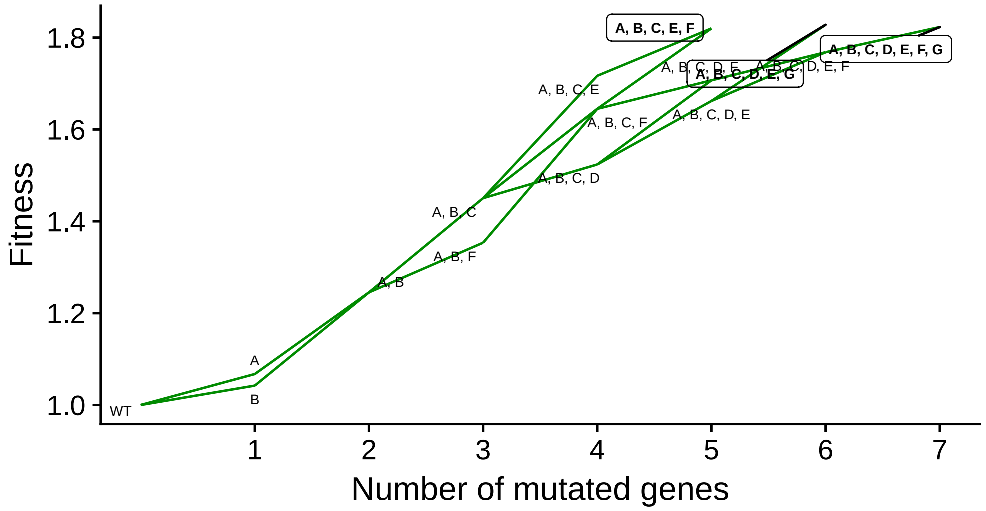

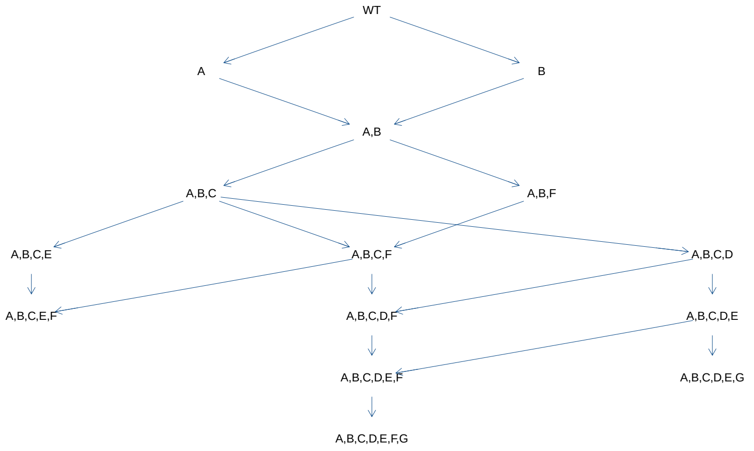

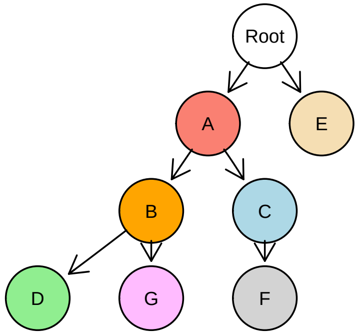

ID	FMNyhwmR9SOSPPYf
acc genotypes	32
peaks no back	6
removed edges	15
prop rem edges	0.221

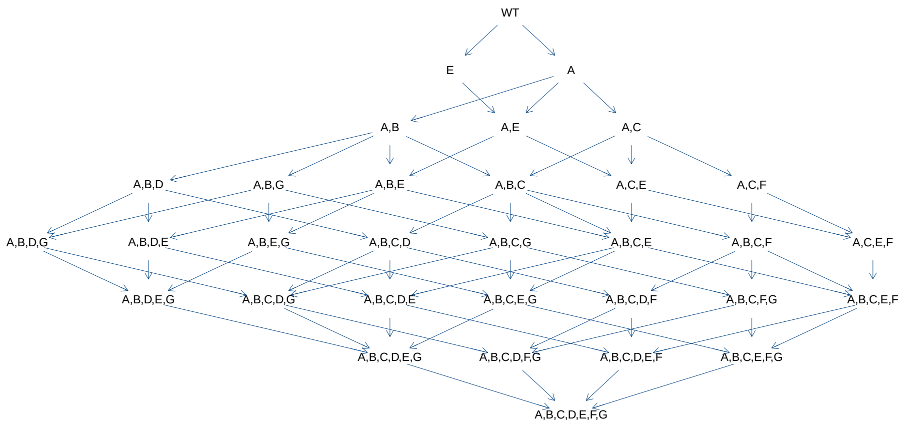

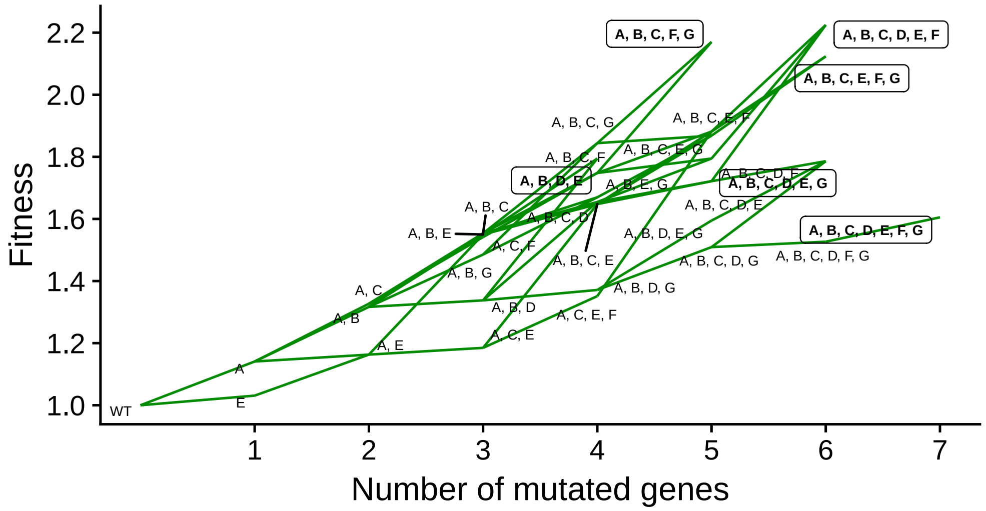

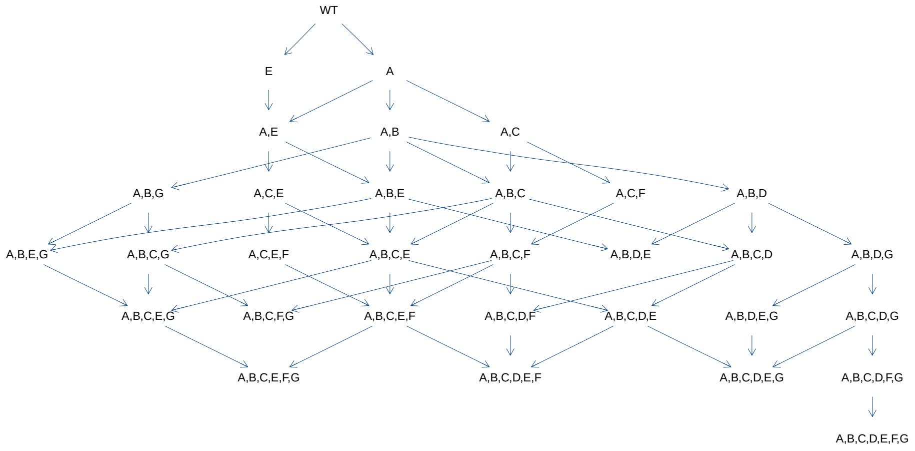

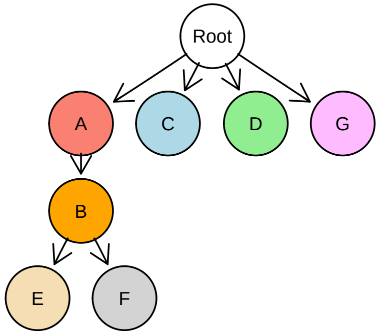

ID	FTNjYXrURUcCxqz9
acc genotypes	48
peaks no back	9
removed edges	27
prop rem edges	0.225

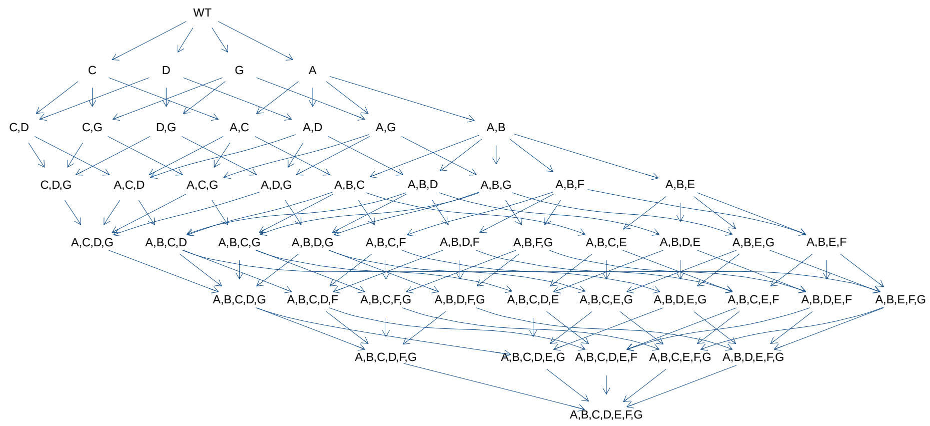

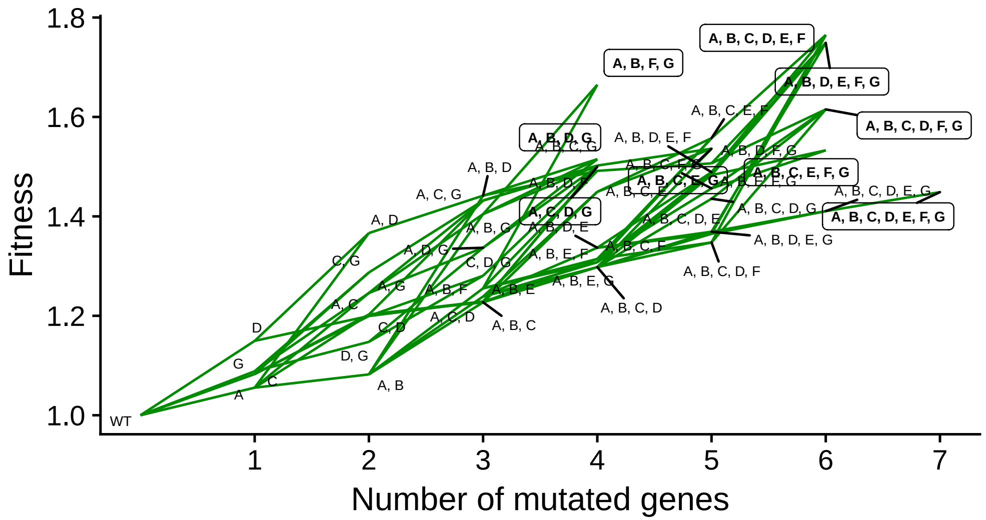

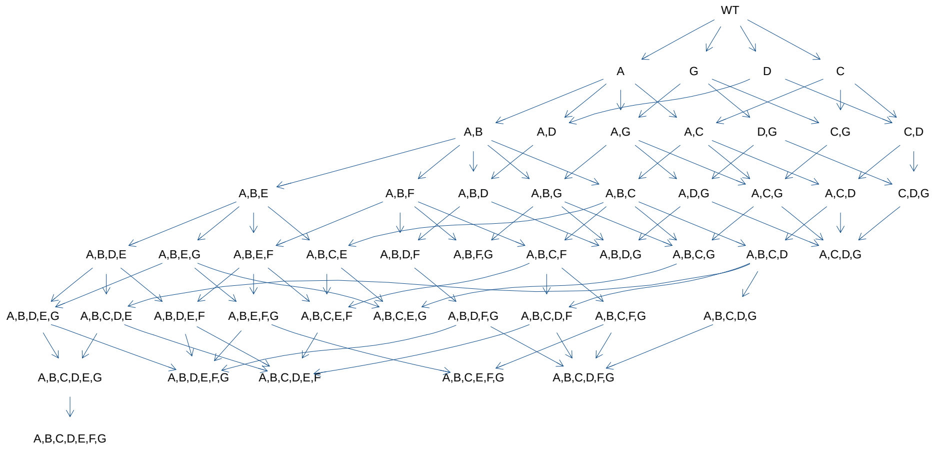

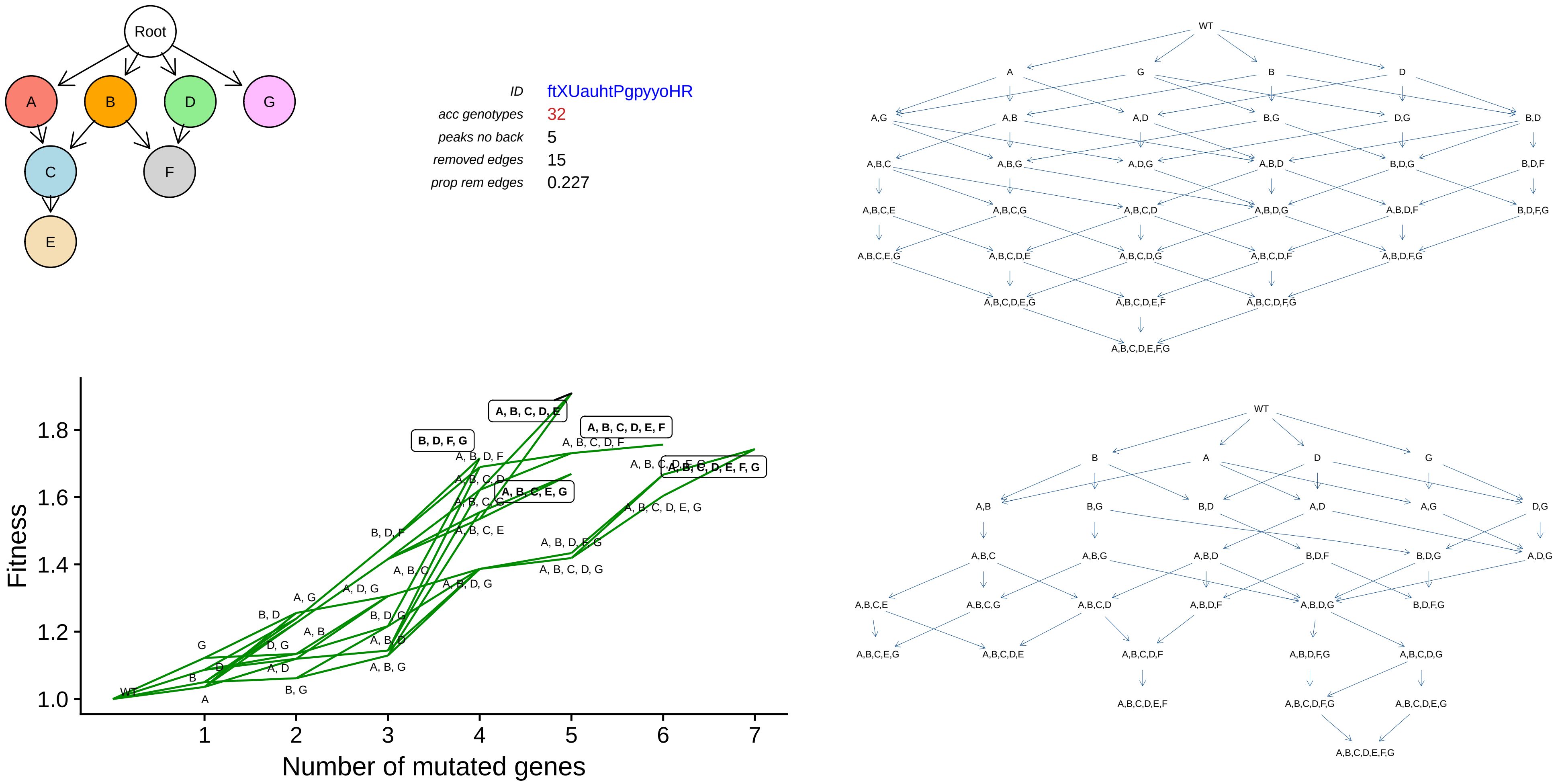

Root
A
B
D
G
C
F
E
ID ftXUauhtPgpyyoHR
acc genotypes 32
peaks no back 5
removed edges 15
prop rem edges 0.227
Fitness
Number of mutated genes
A, B, C, D, E
A, B, C, D, E, F
B, D, F, G
A, B, C, E, G
A, B, C, D, E, F, G
WT
A,B,C,D,E,F,G

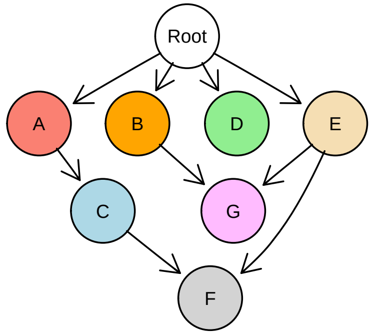

ID	fW757Yty4nJQzn3B
acc genotypes	36
peaks no back	5
removed edges	10
prop rem edges	0.128

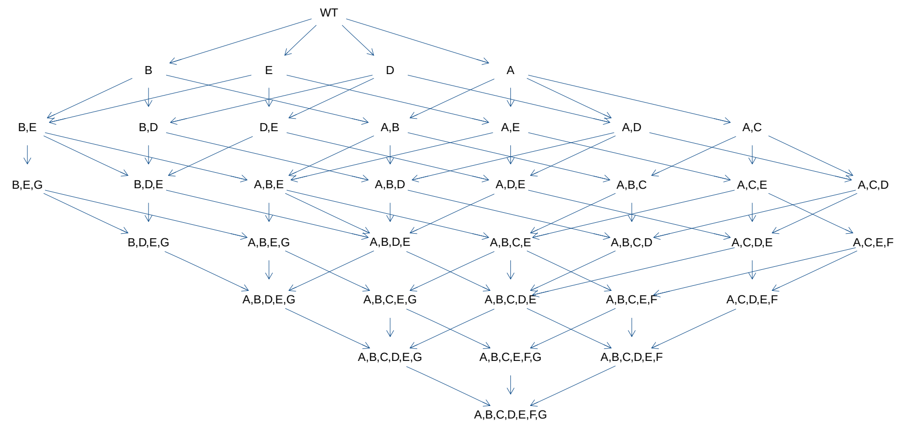

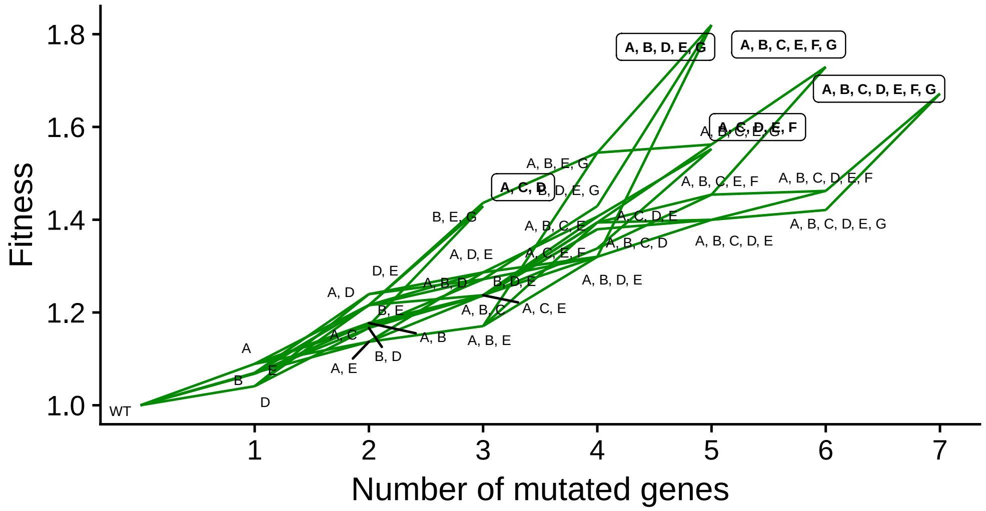

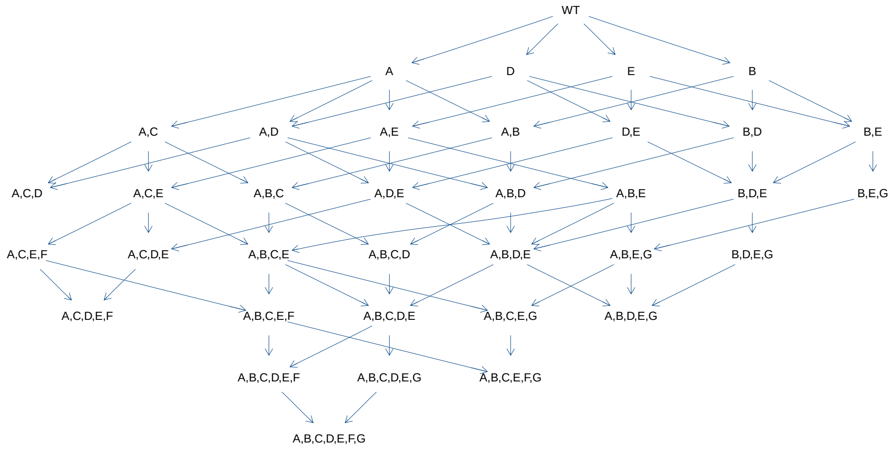

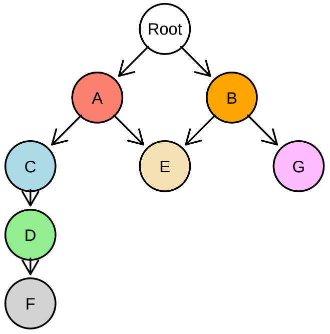

ID	g38nzkCLq72TPfvC
acc genotypes	23
peaks no back	5
removed edges	8
prop rem edges	0.2

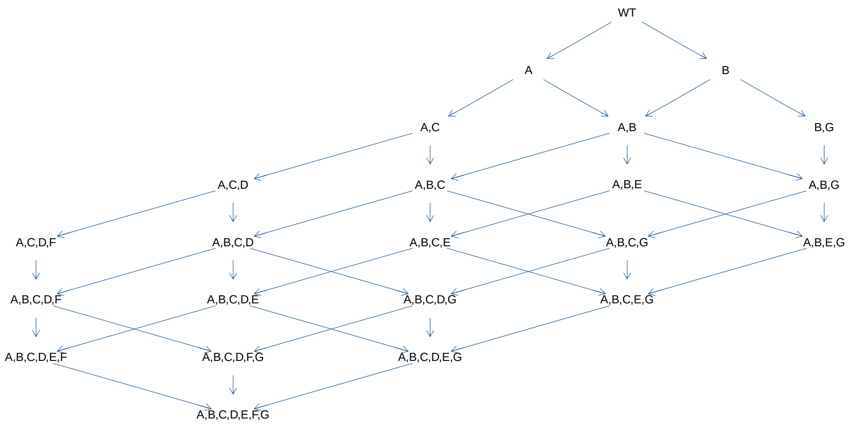

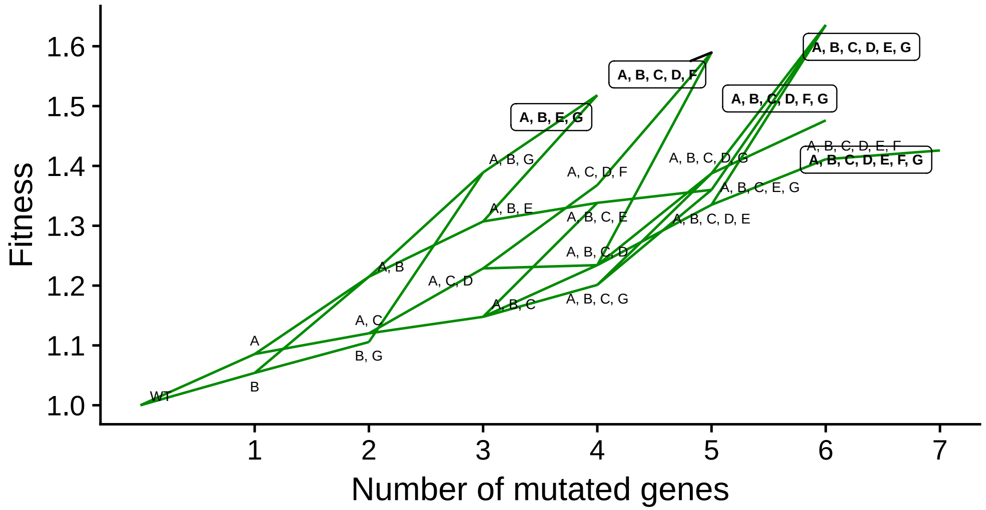

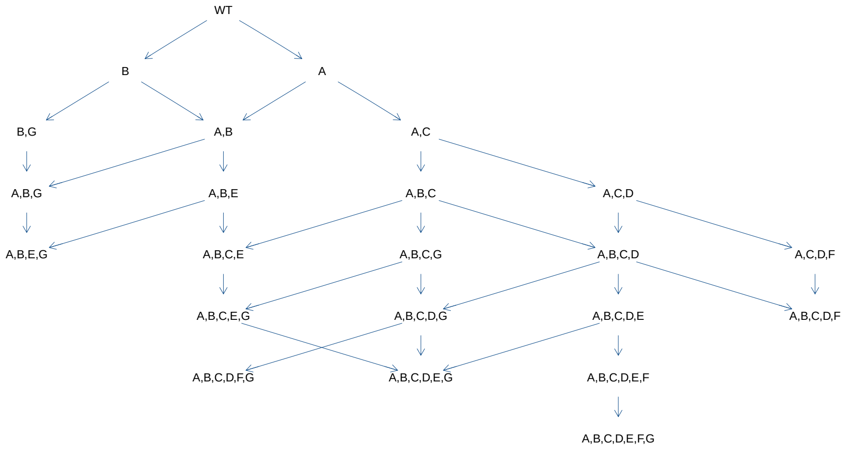

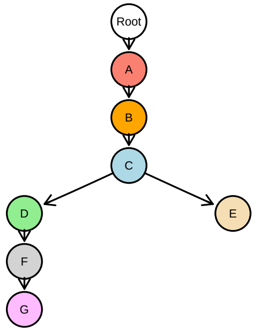

ID	GedZaWDeb1029Mf88
acc genotypes	11
peaks no back	2
removed edges	2
prop rem edges	0.154

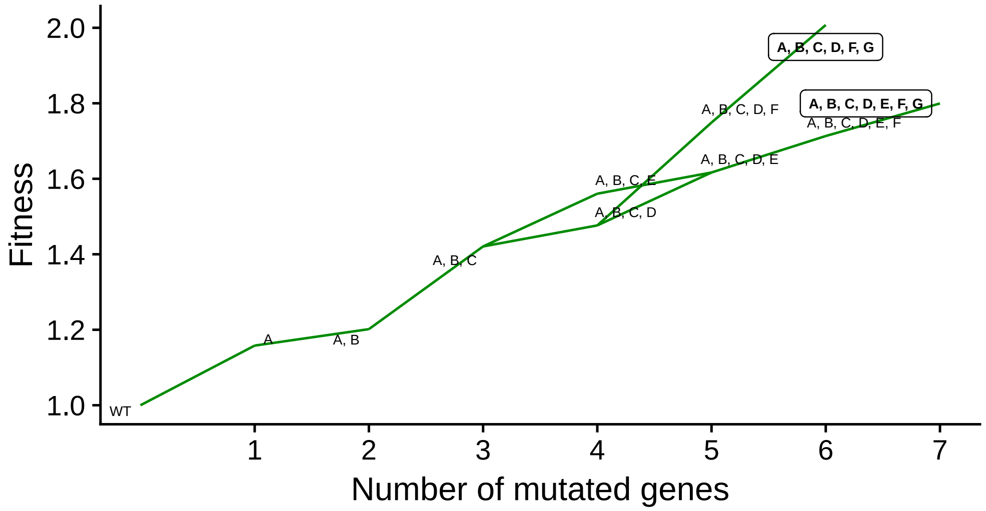

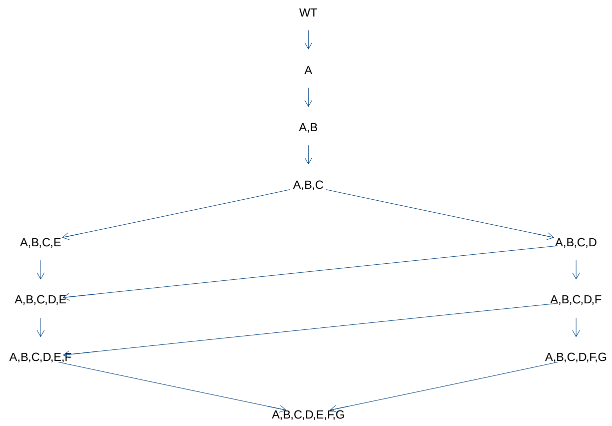

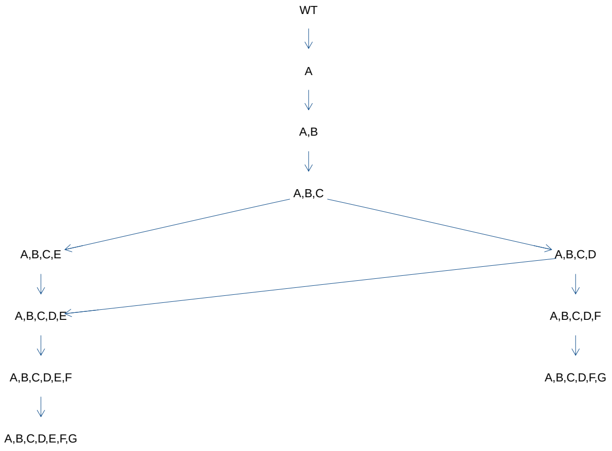

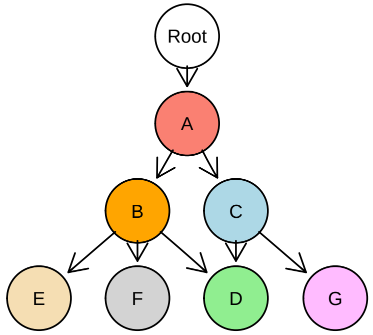

ID	gfkgOLnyr7gjNQkC
acc genotypes	24
peaks no back	5
removed edges	10
prop rem edges	0.217

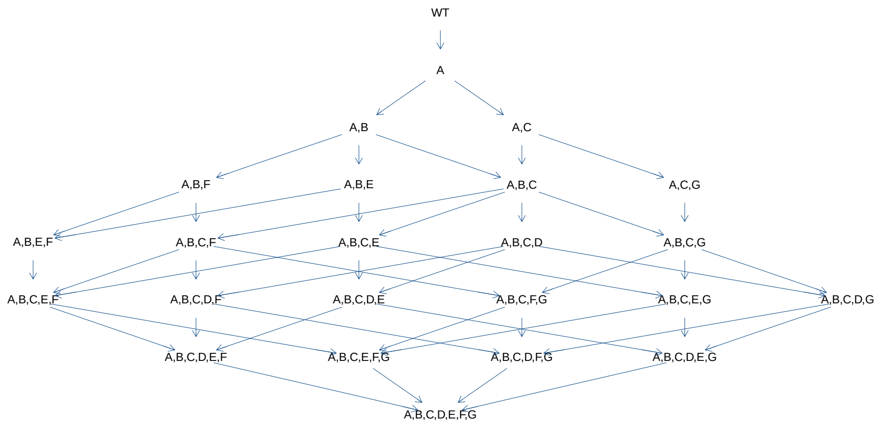

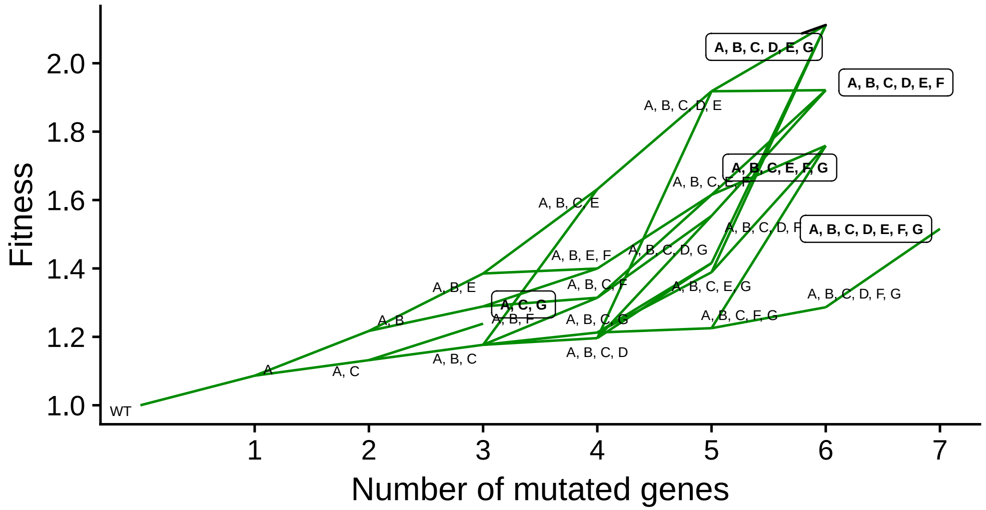

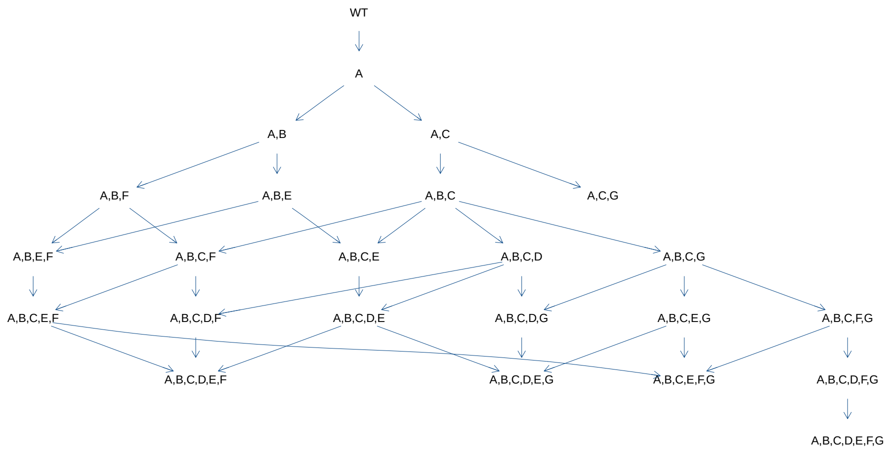

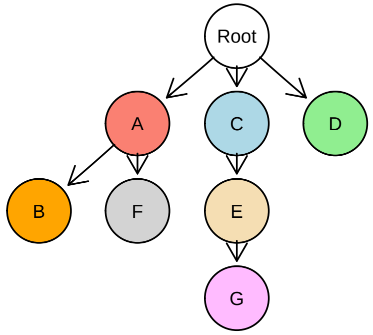

ID	gKfKrRLHCELf6w8t
acc genotypes	40
peaks no back	2
removed edges	8
prop rem edges	0.089

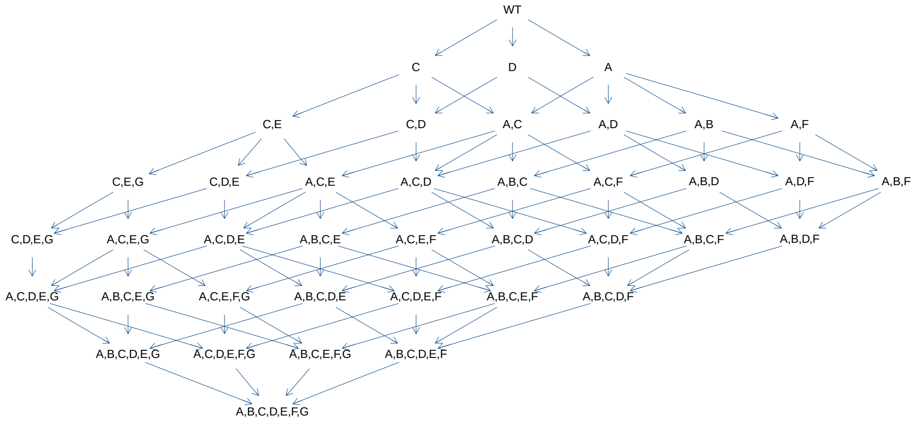

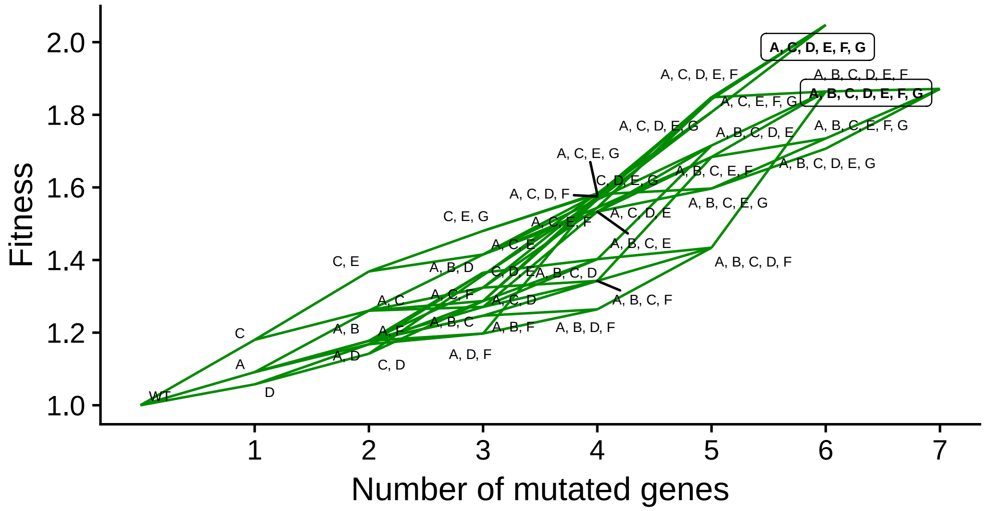

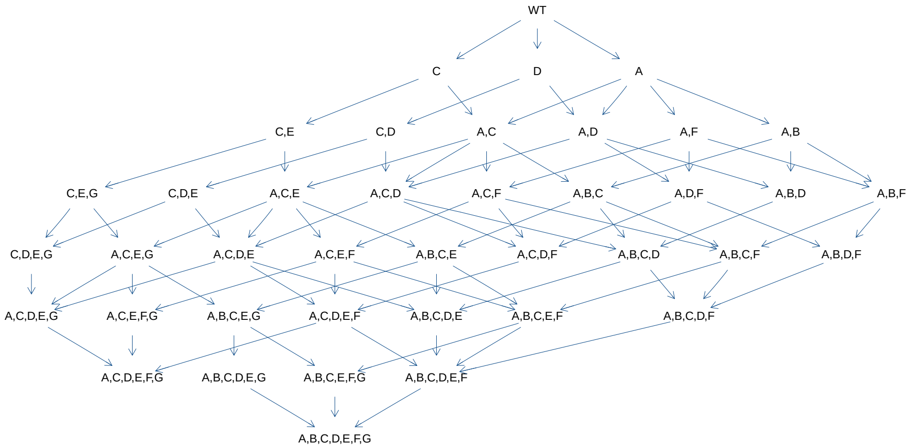

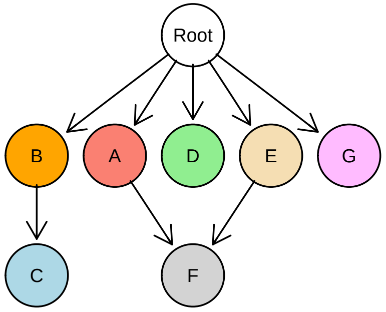

ID	GM3TL5IyxTmLPLFB
acc genotypes	60
peaks no back	9
removed edges	25
prop rem edges	0.156

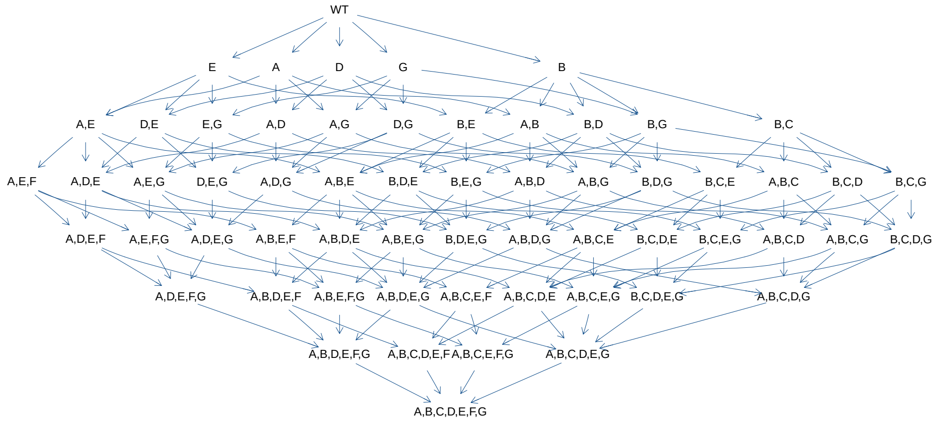

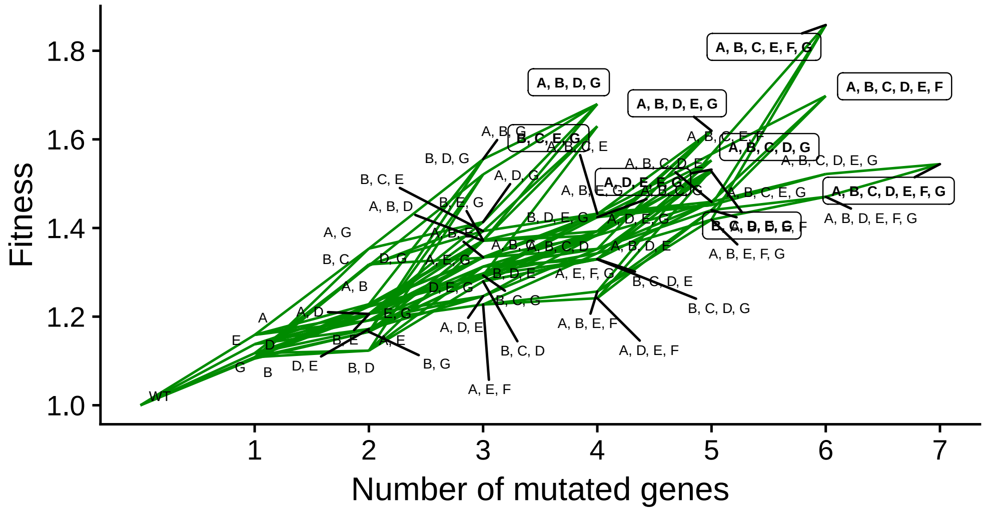

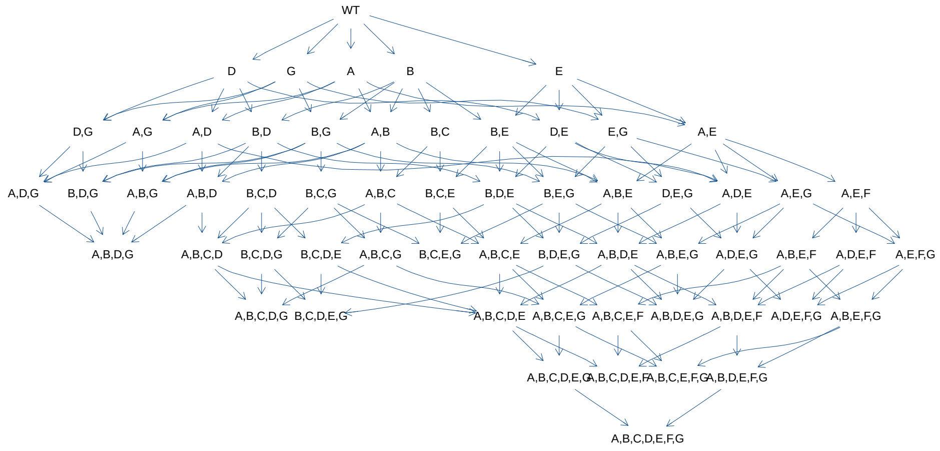

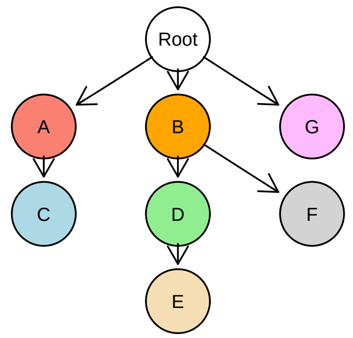

ID	gOGMou3PVNxUeDQ5
acc genotypes	42
peaks no back	6
removed edges	18
prop rem edges	0.186

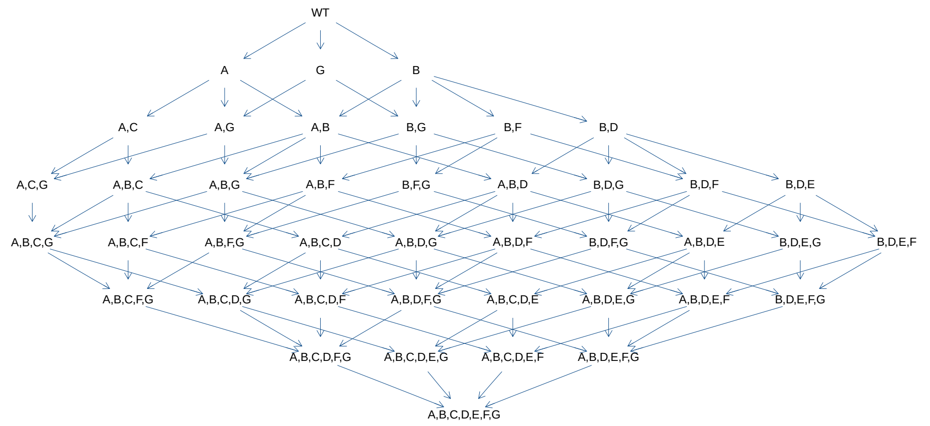

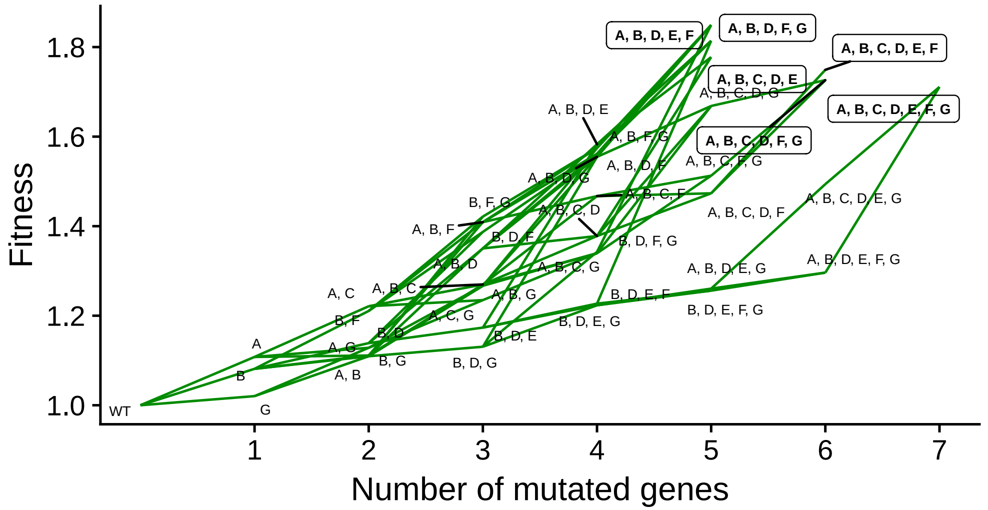

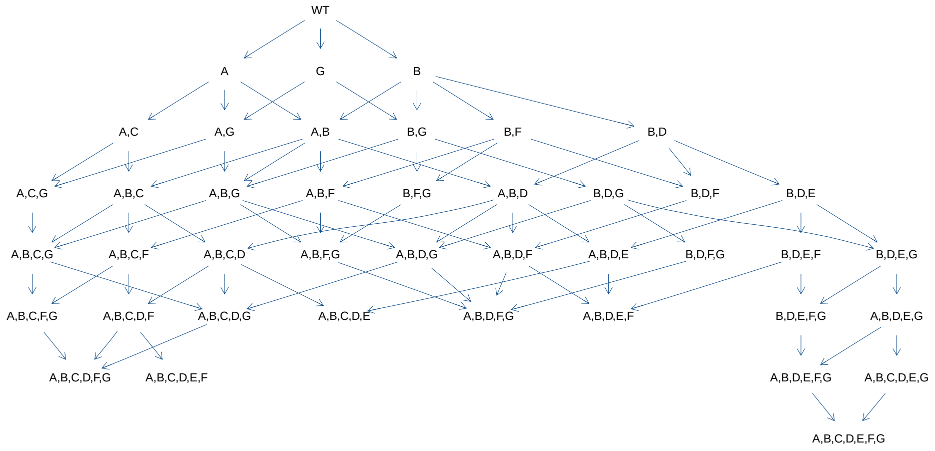

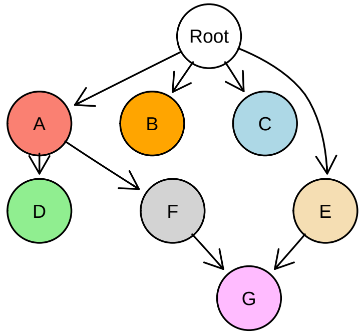

ID GwFwG10TqvAnPNr82
acc genotypes 48
peaks no back 7
removed edges 33
prop rem edges 0.275

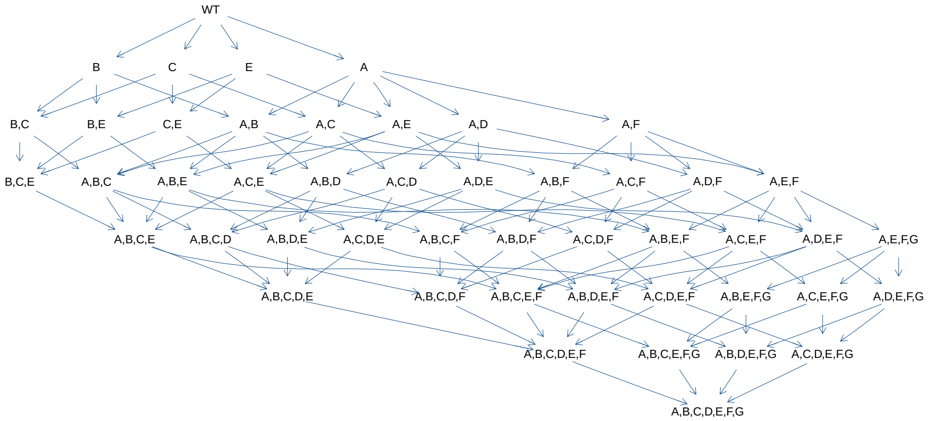

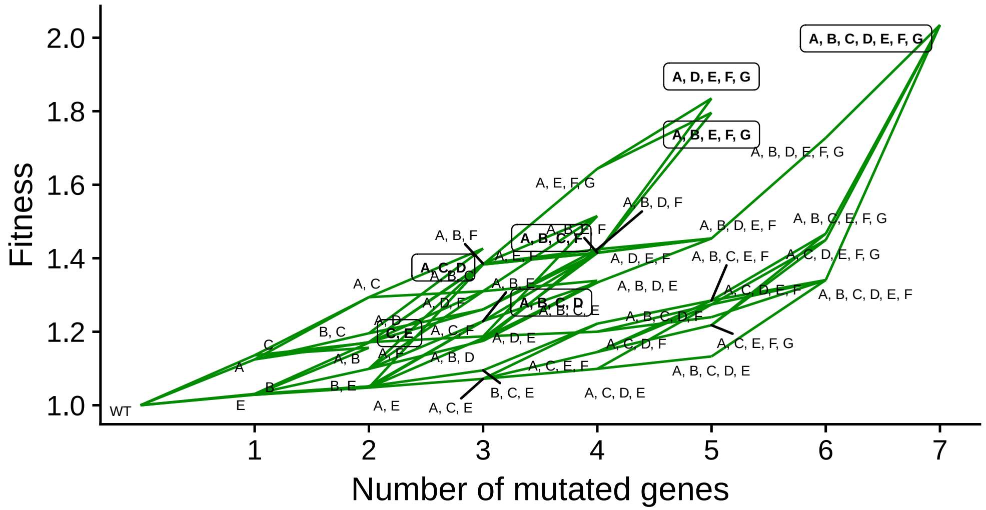

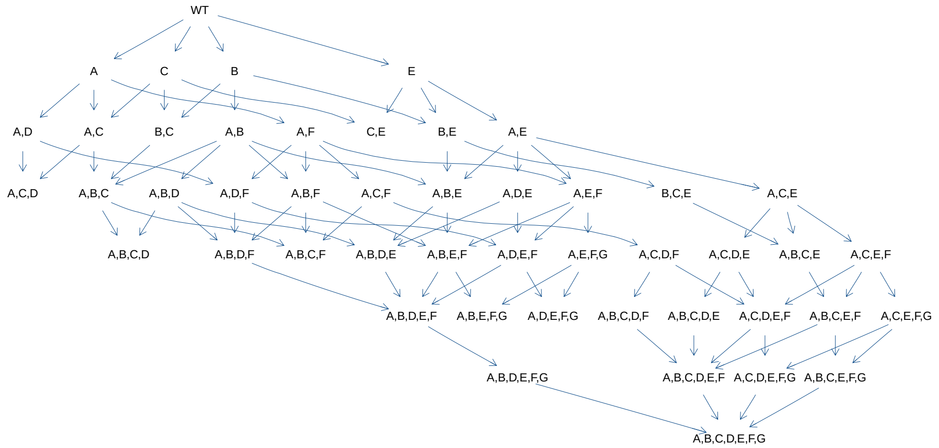

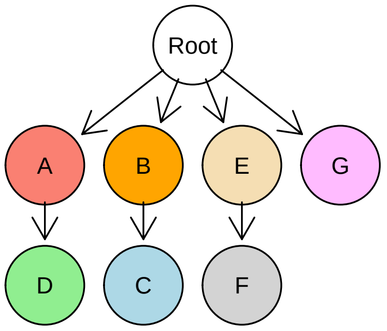

ID	haC6AnZXOHVkOVUX
acc genotypes	54
peaks no back	6
removed edges	21
prop rem edges	0.156

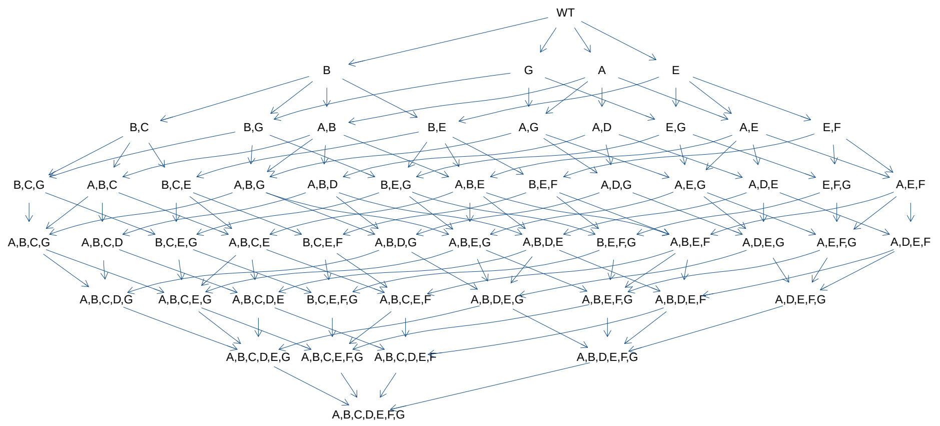

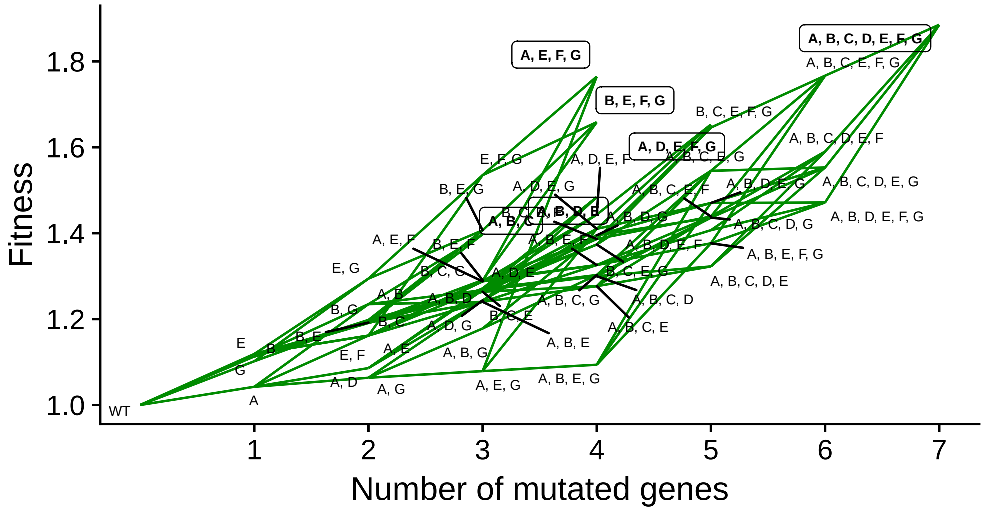

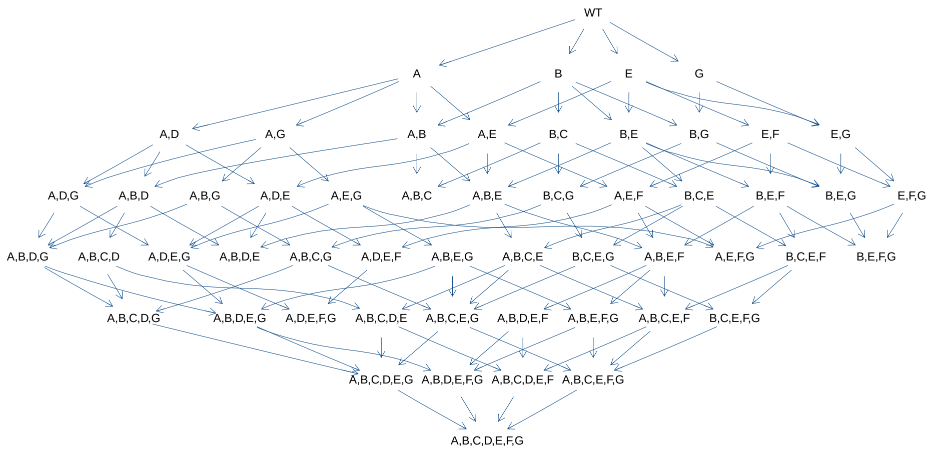

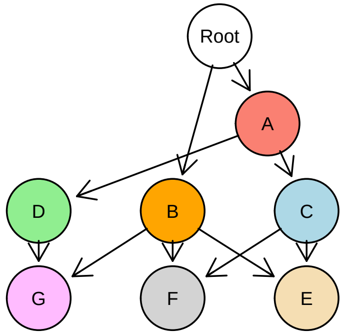

ID	Hc4dDsccE1OXyhxX
acc genotypes	21
peaks no back	5
removed edges	7
prop rem edges	0.194

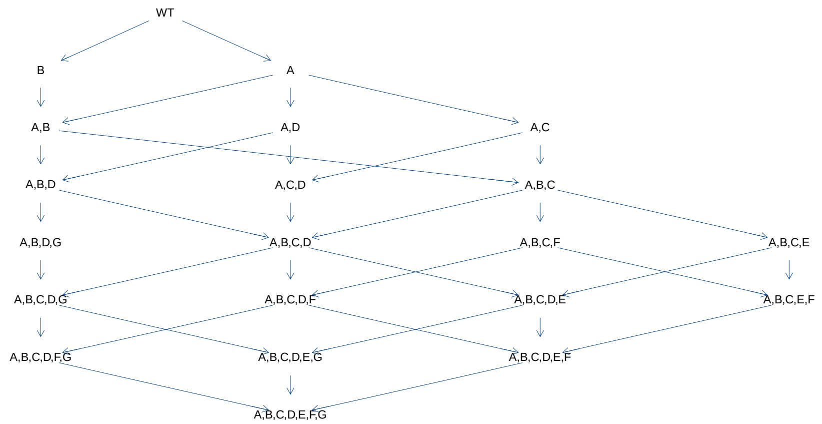

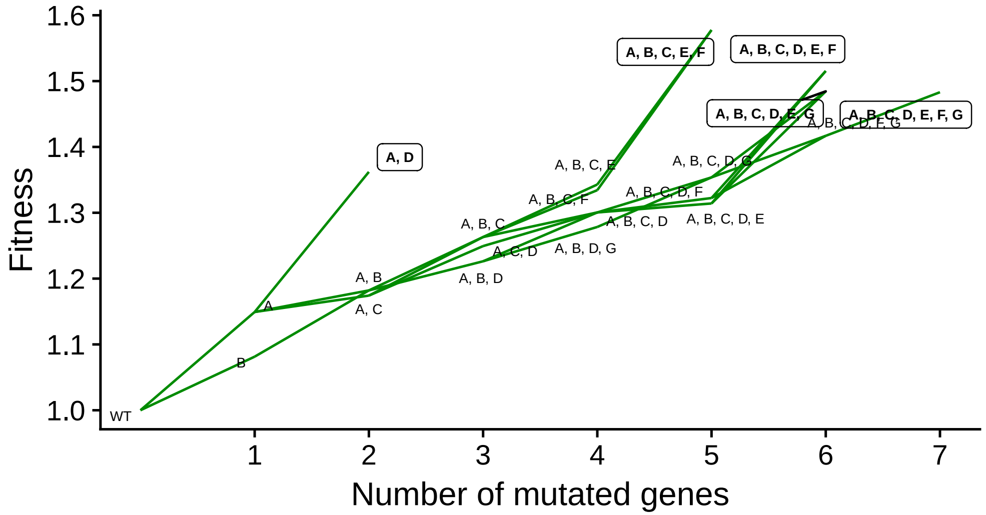

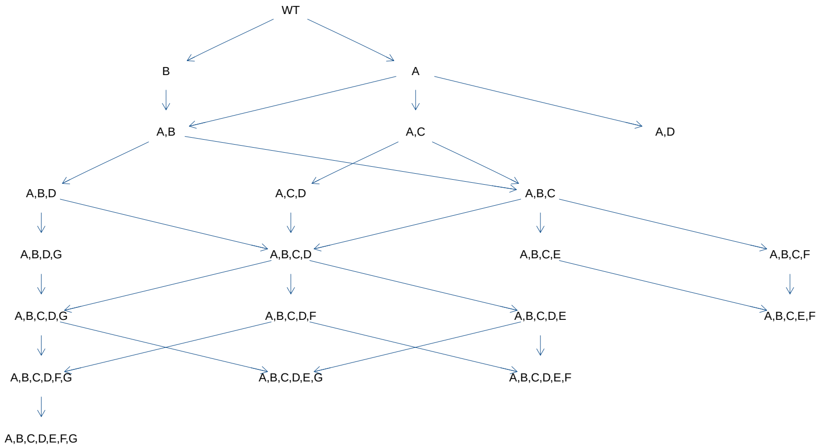

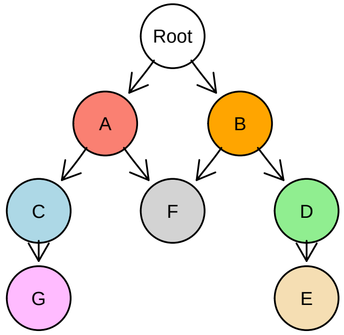

ID	HFe7qJ1sPtvbaUtG
acc genotypes	25
peaks no back	4
removed edges	5
prop rem edges	0.111

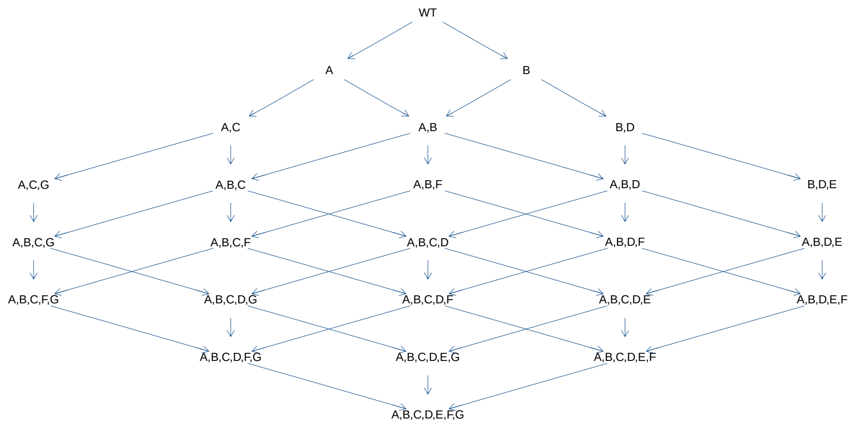

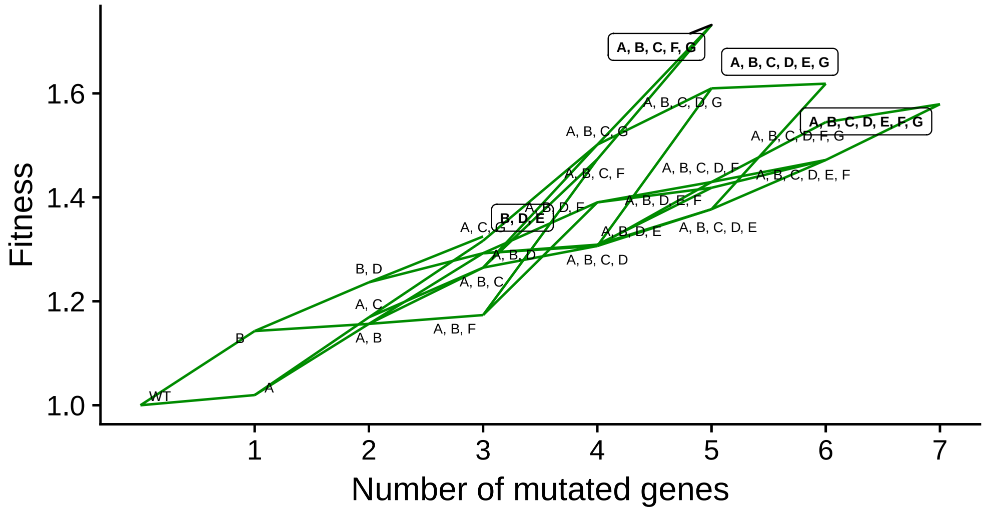

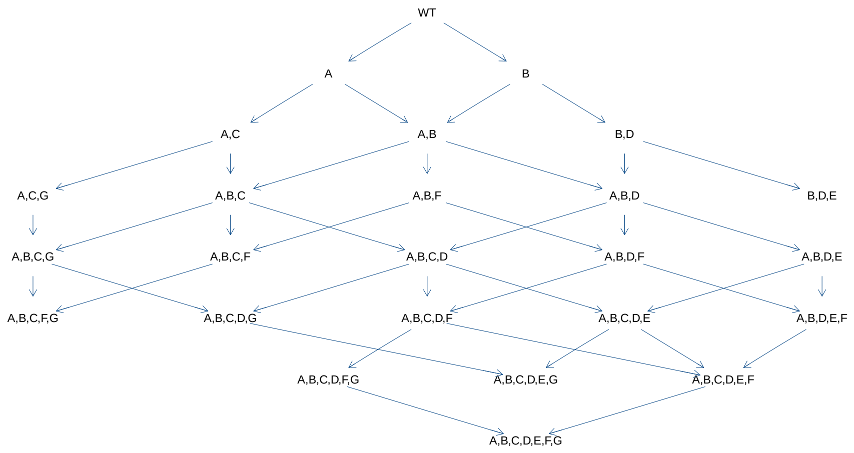

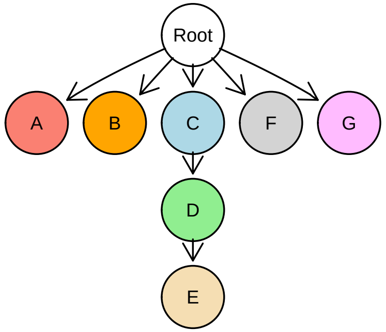

ID	hIoXRsRm4d7aXmN4
acc genotypes	64
peaks no back	2
removed edges	22
prop rem edges	0.125

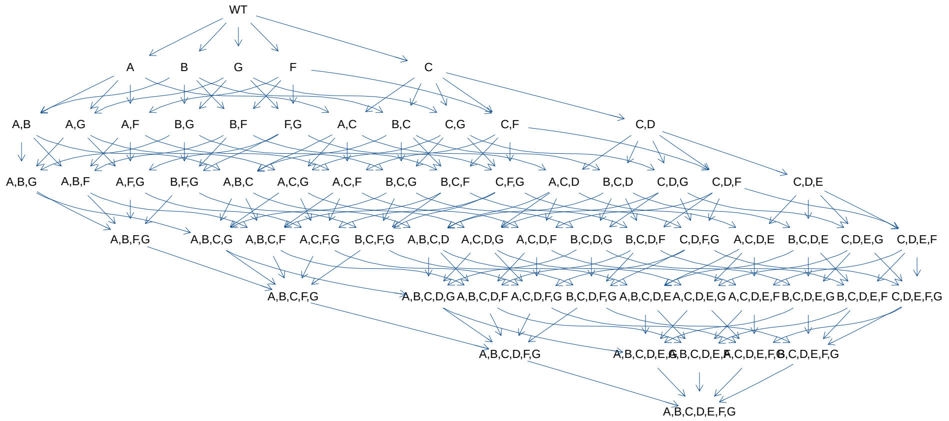

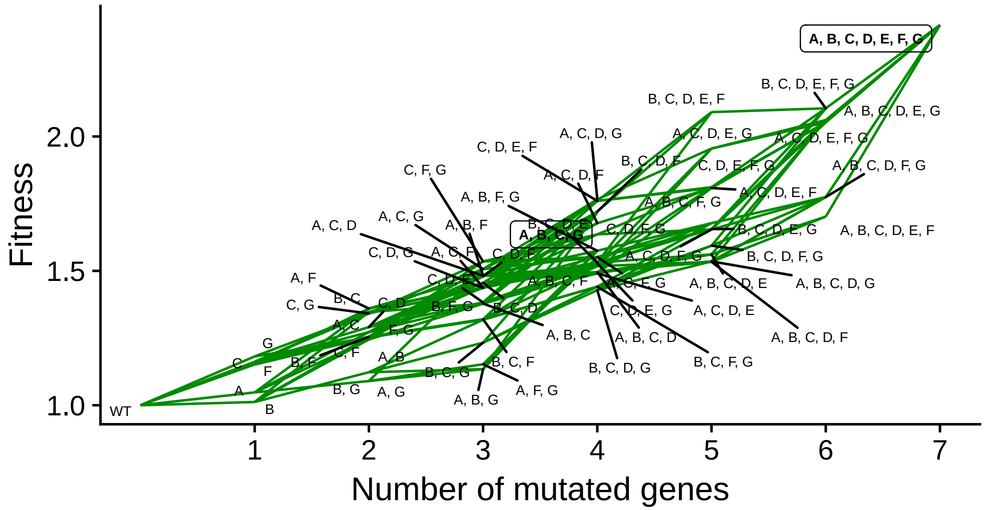

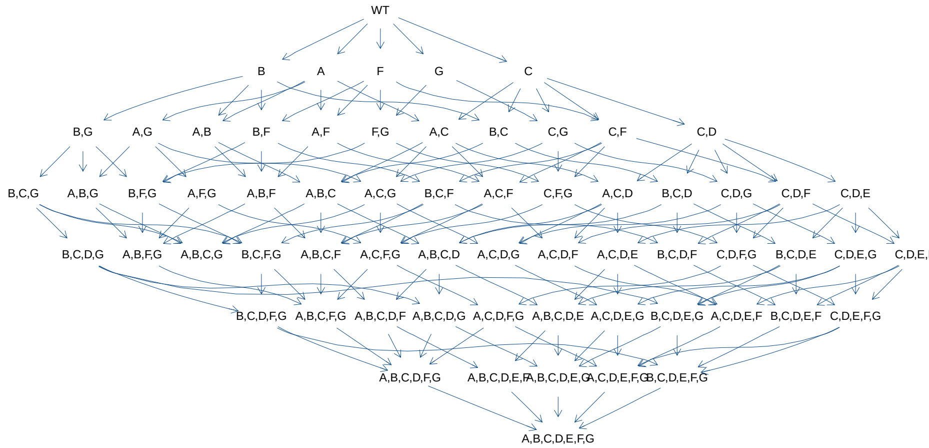

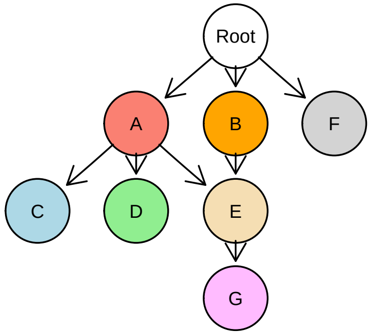

ID HizemNX910W6f2YLA
acc genotypes 36
peaks no back 5
removed edges 12
prop rem edges 0.15

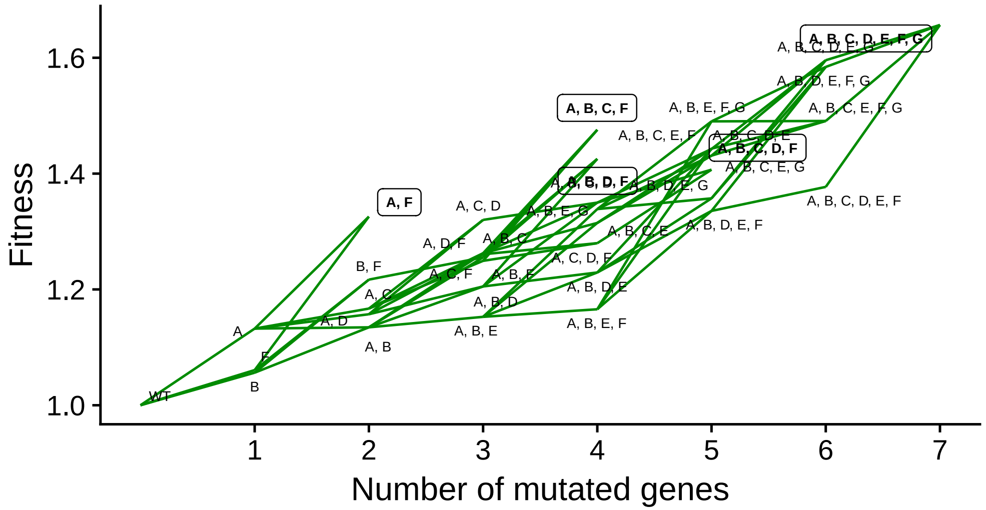

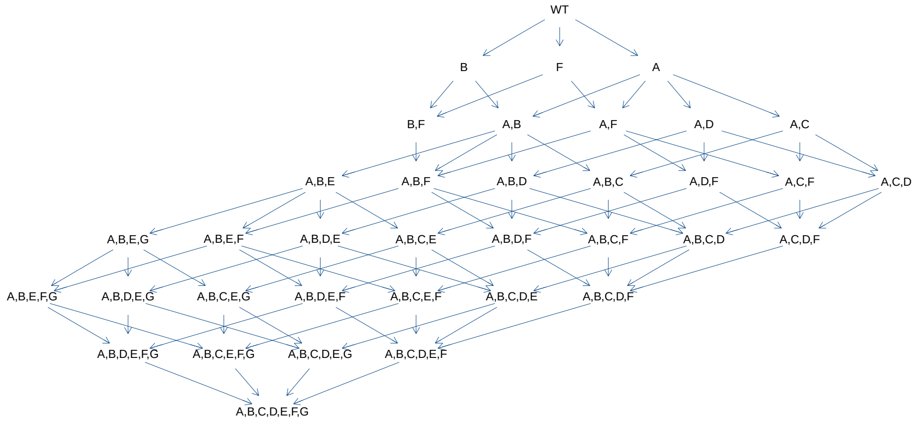

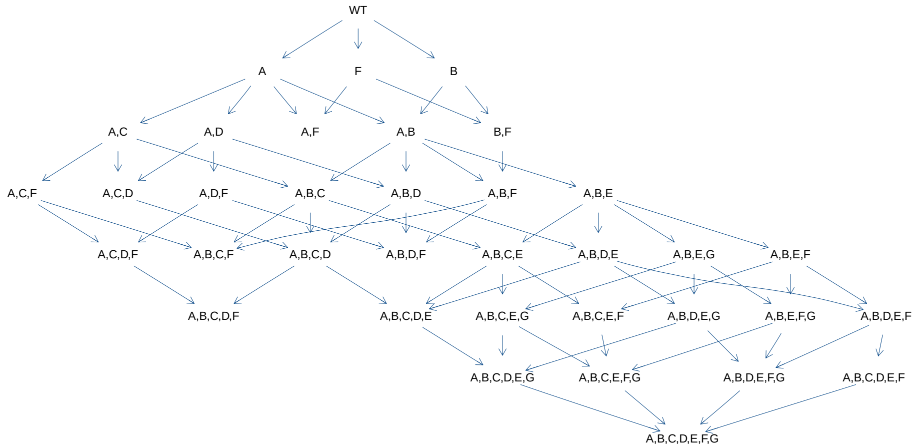

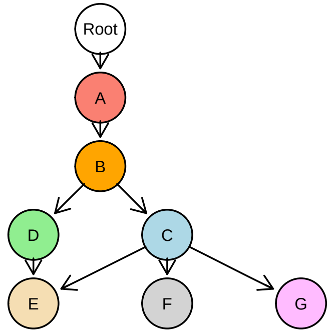

ID	hw8kQ4g44p4XAkDa
acc genotypes	16
peaks no back	2
removed edges	3
prop rem edges	0.12

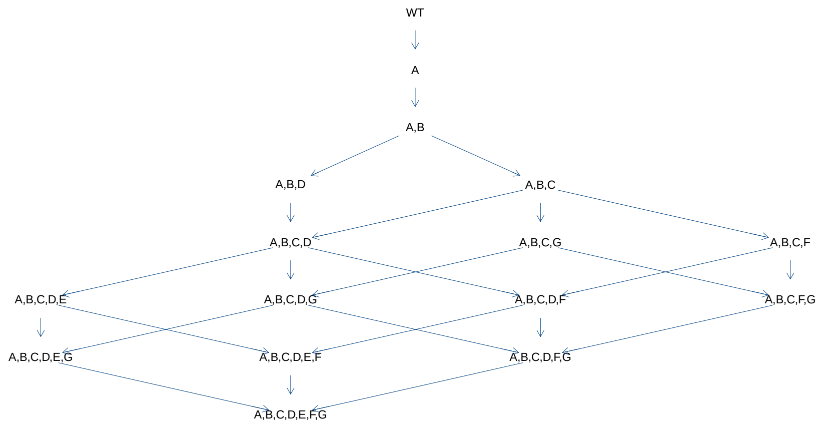

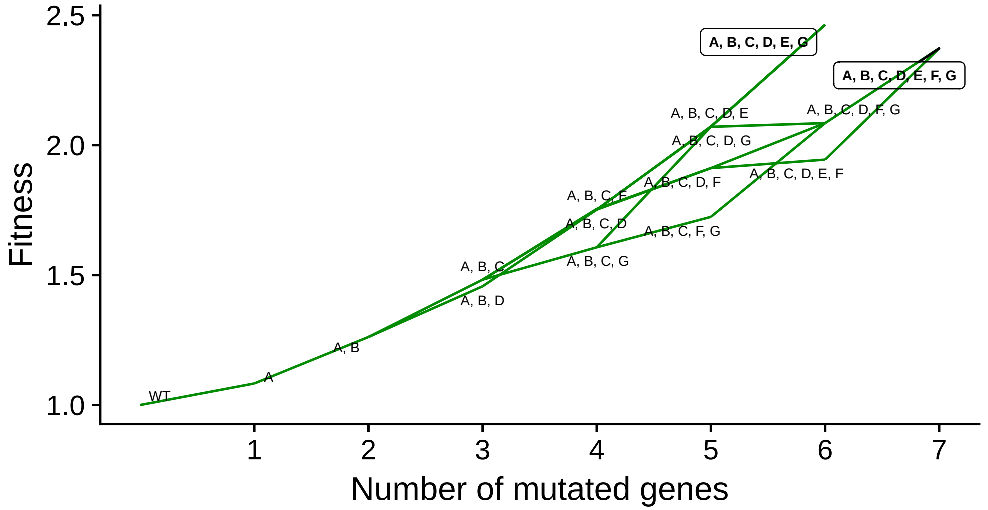

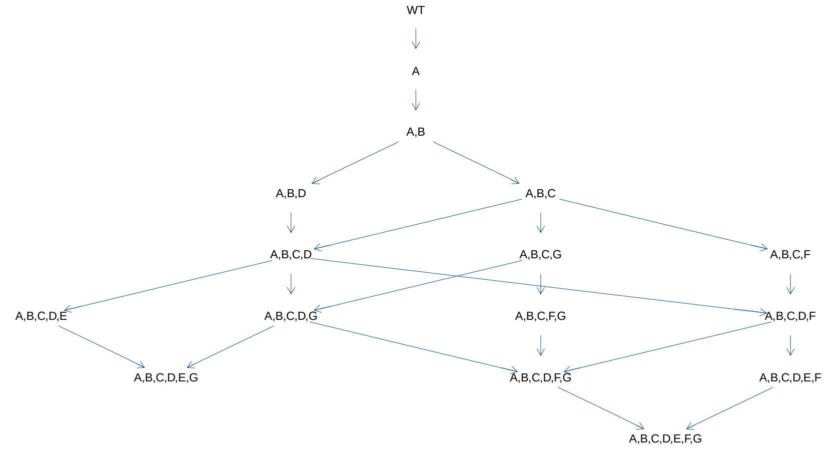

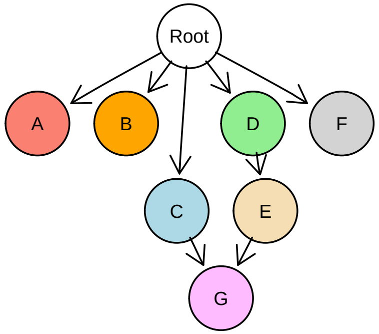

ID	Hy5Py5ryXo10HeUYG
acc genotypes	56
peaks no back	8
removed edges	35
prop rem edges	0.236

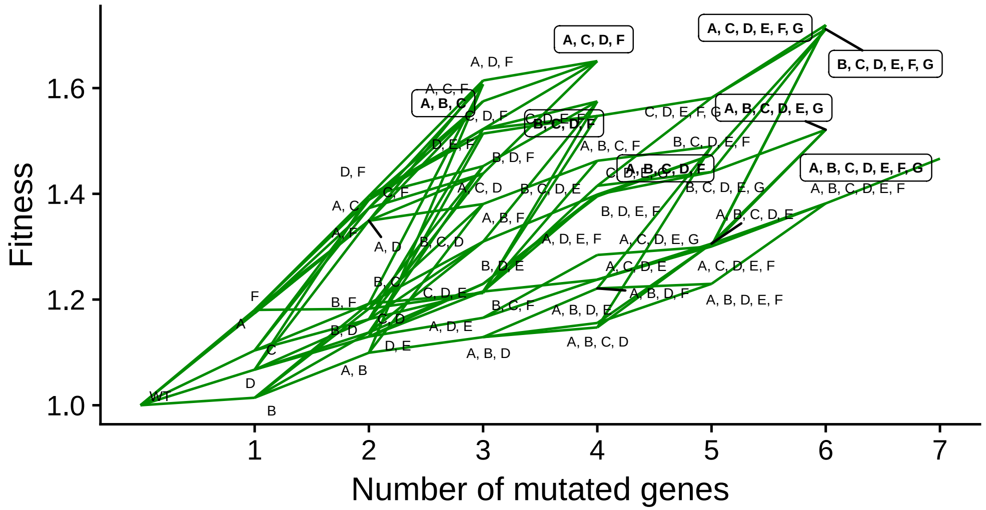

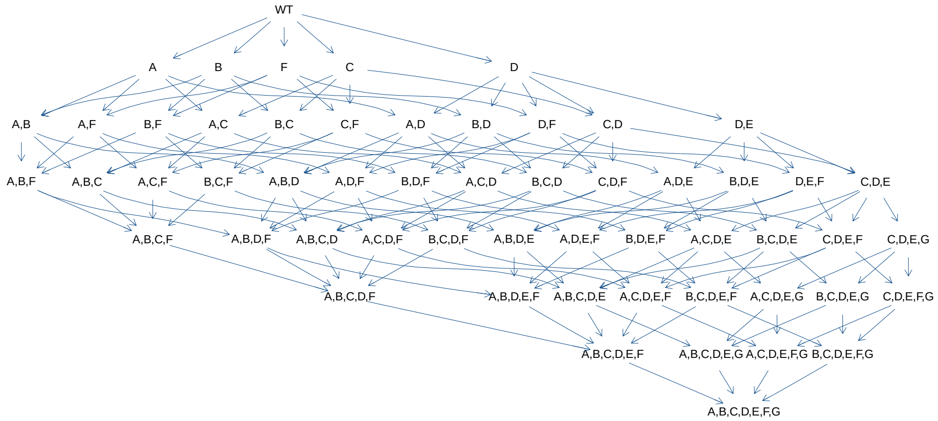

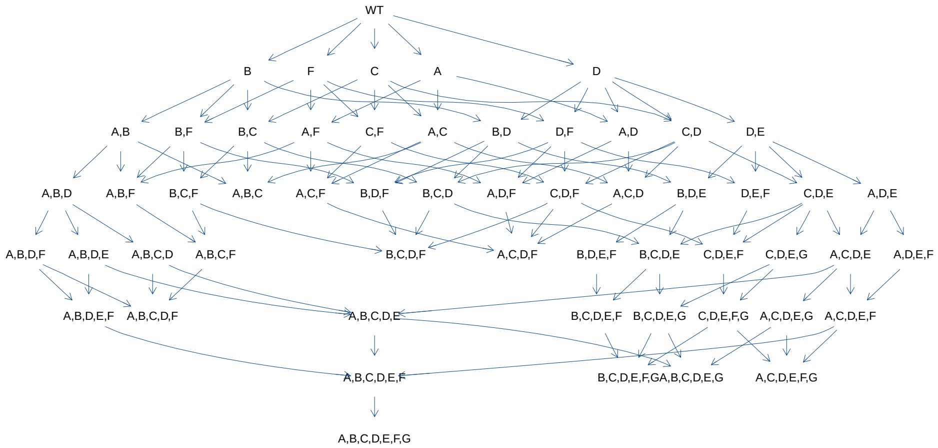

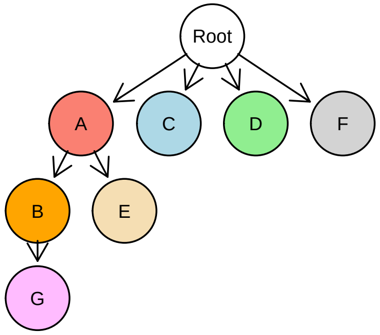

ID	Id85CzgL2g6AV3510
acc genotypes	56
peaks no back	9
removed edges	38
prop rem edges	0.257

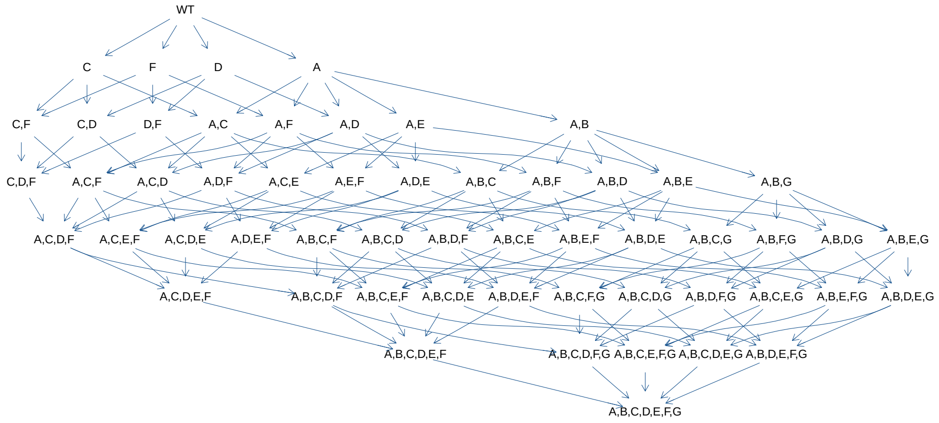

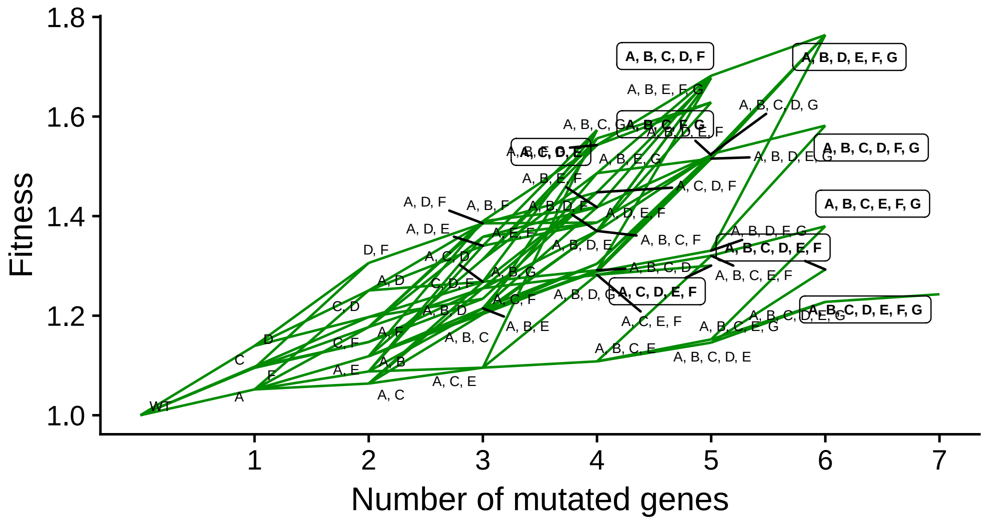

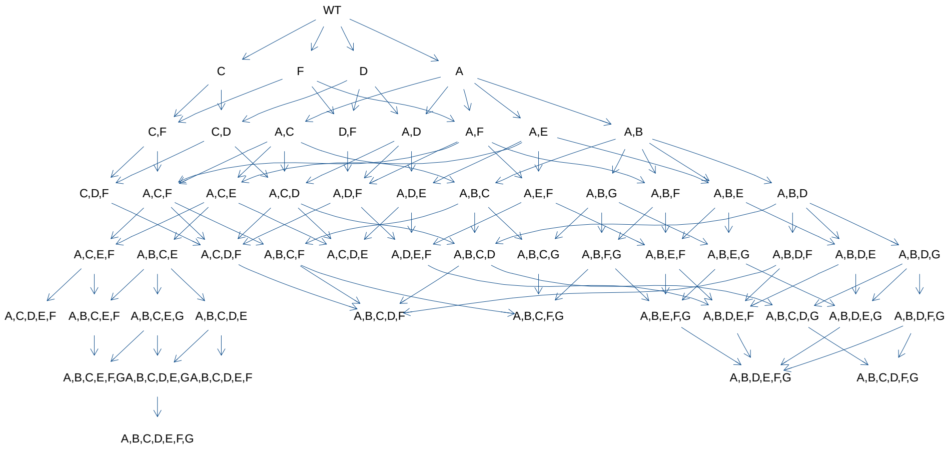

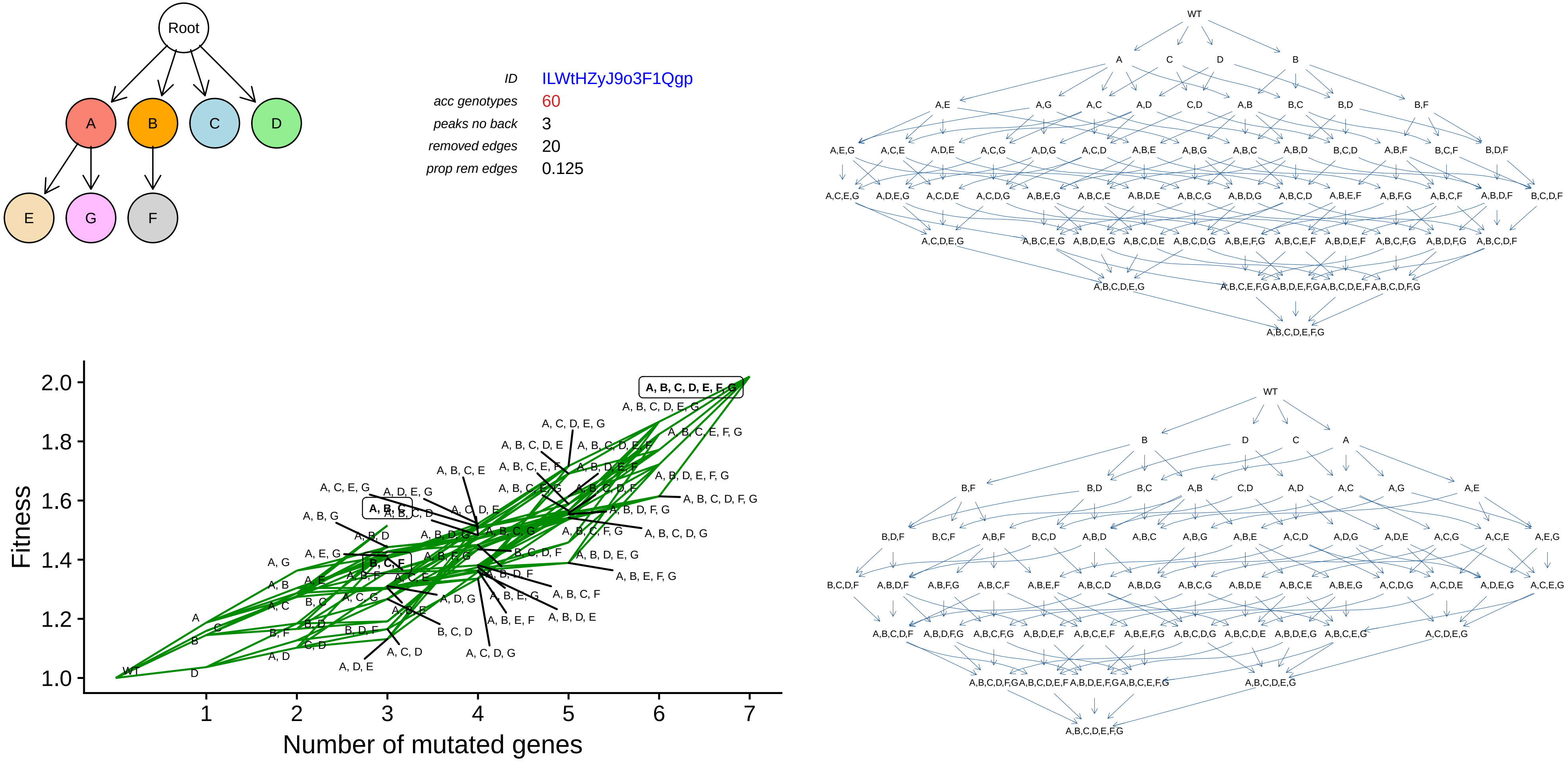
Root
A
B
C
D
E
G
F
ID
ILWtHZyJ9o3F1Qgp
acc genotypes
60
peaks no back
3
removed edges
20
prop rem edges
0.125
Fitness
Number of mutated genes
A, B, C, D, E, F, G
A, B, C
B, C, F
WT
A,B,C,D,E,F,G

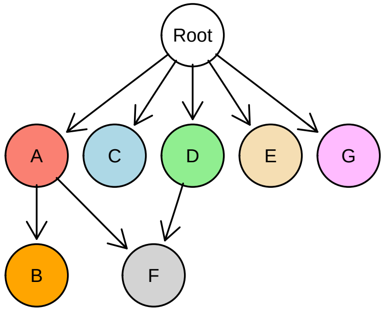

ID	JABP9YQJqeNrAKVt
acc genotypes	64
peaks no back	8
removed edges	35
prop rem edges	0.199

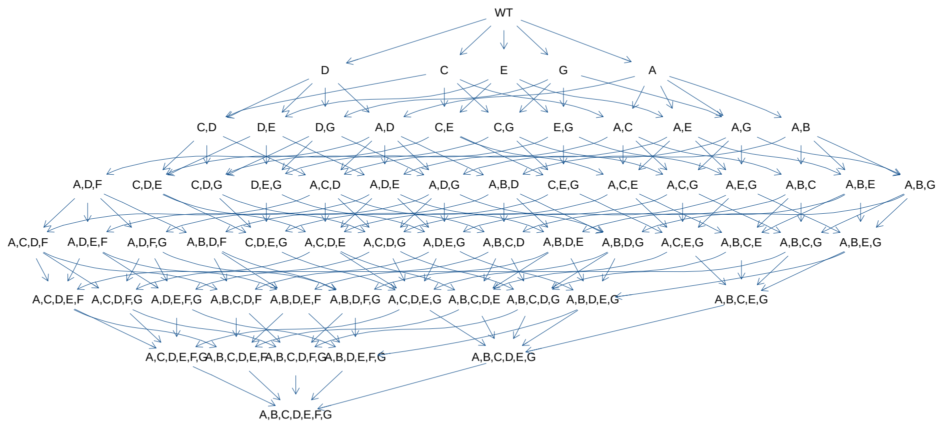

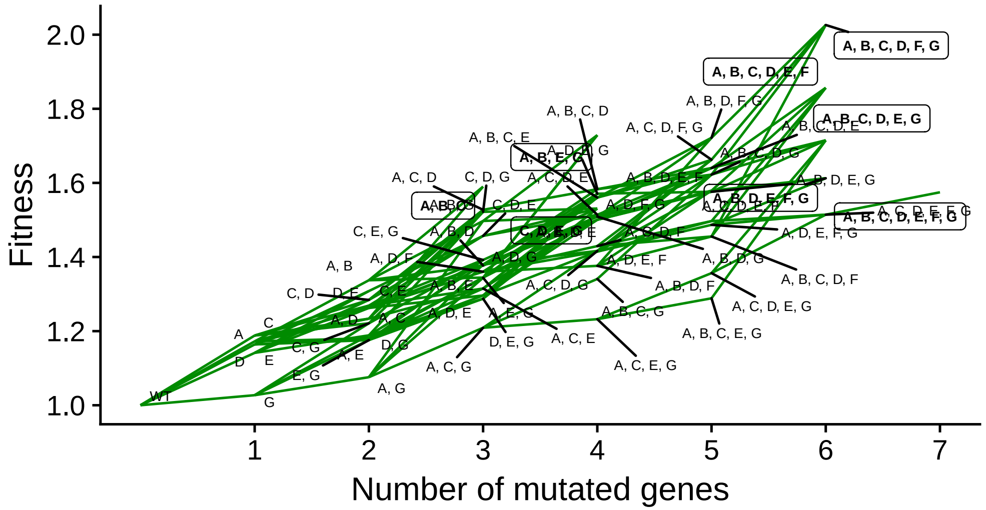

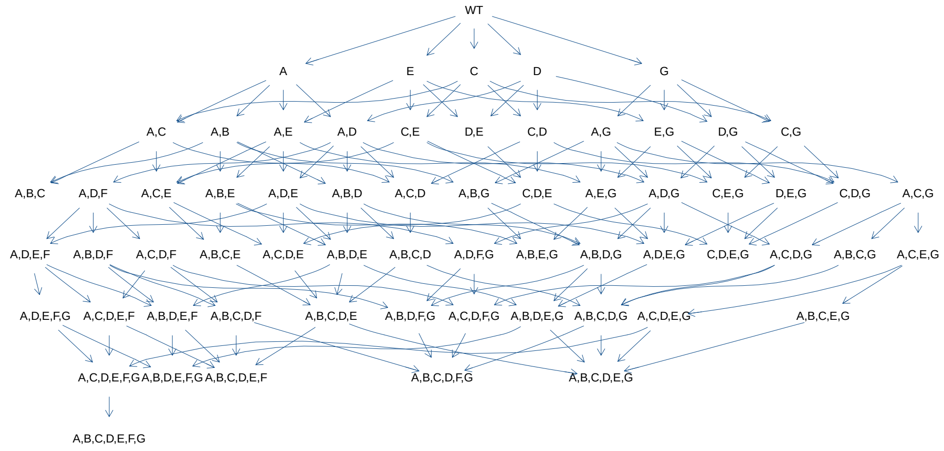

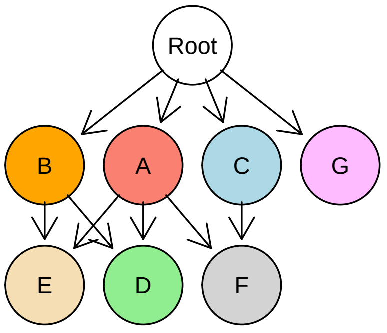

ID	jFHaUyfw10mPN2R1P
acc genotypes	38
peaks no back	4
removed edges	15
prop rem edges	0.176

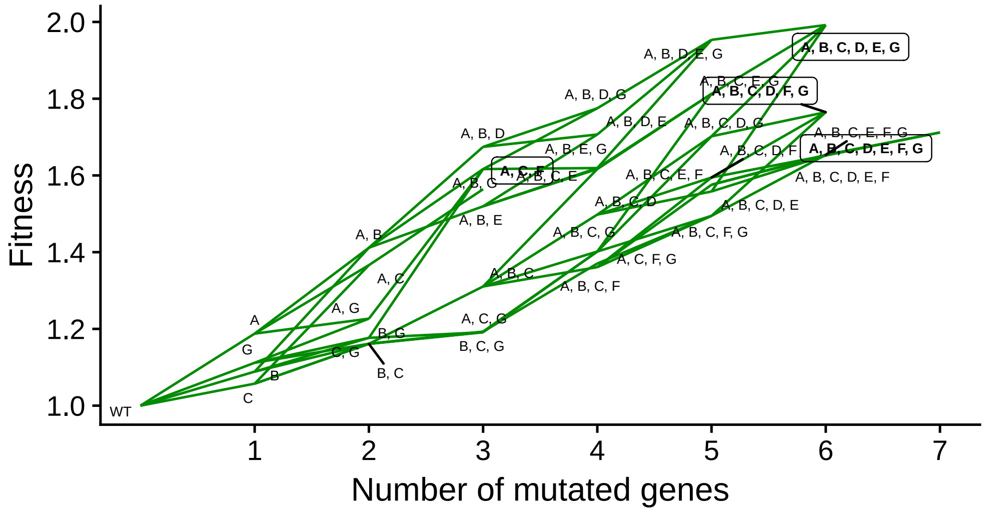

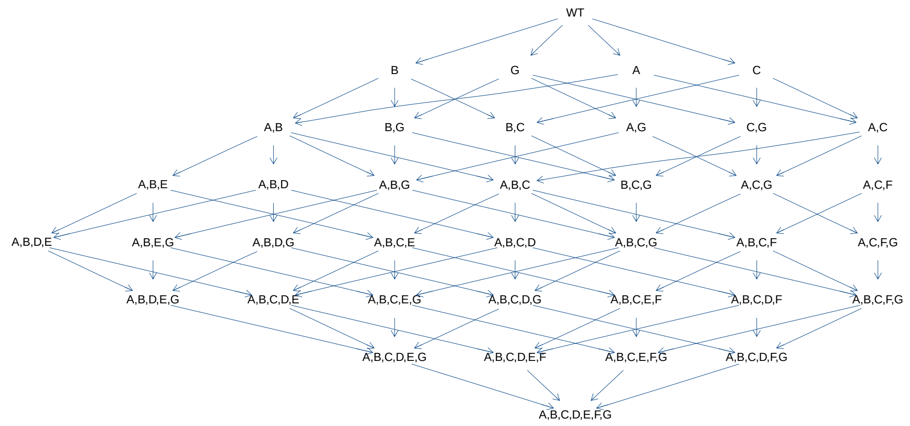

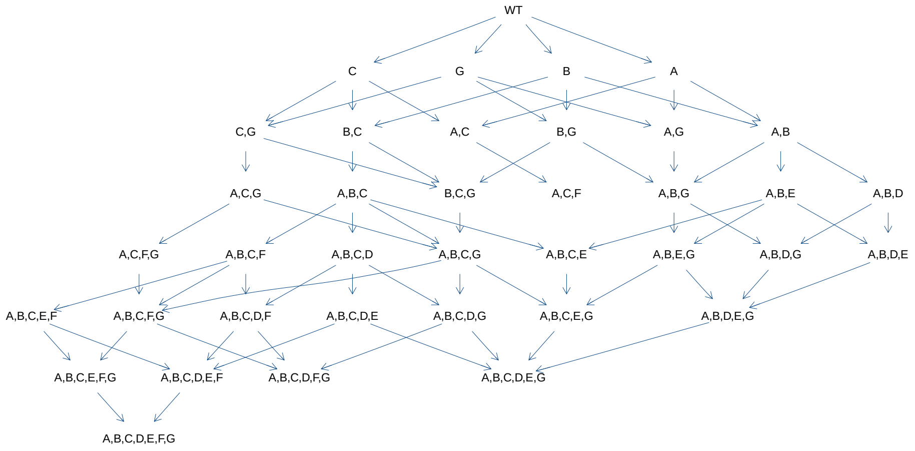

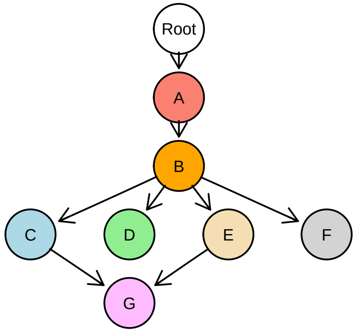

ID	jj397ue810e35pKQe
acc genotypes	22
peaks no back	3
removed edges	5
prop rem edges	0.119

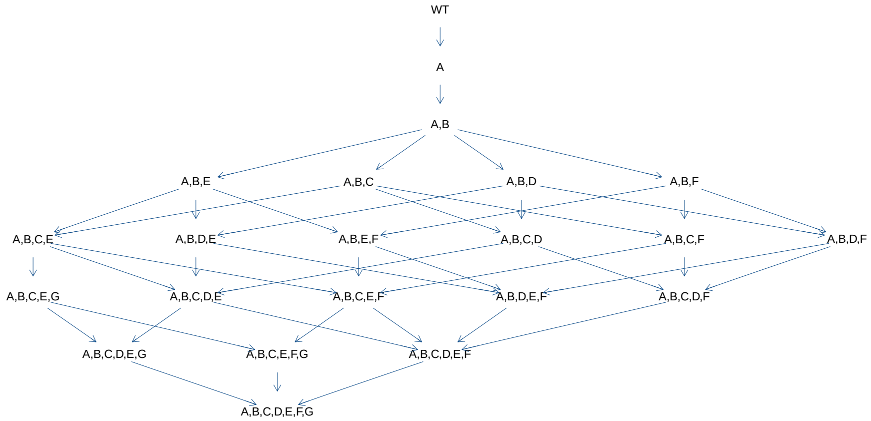

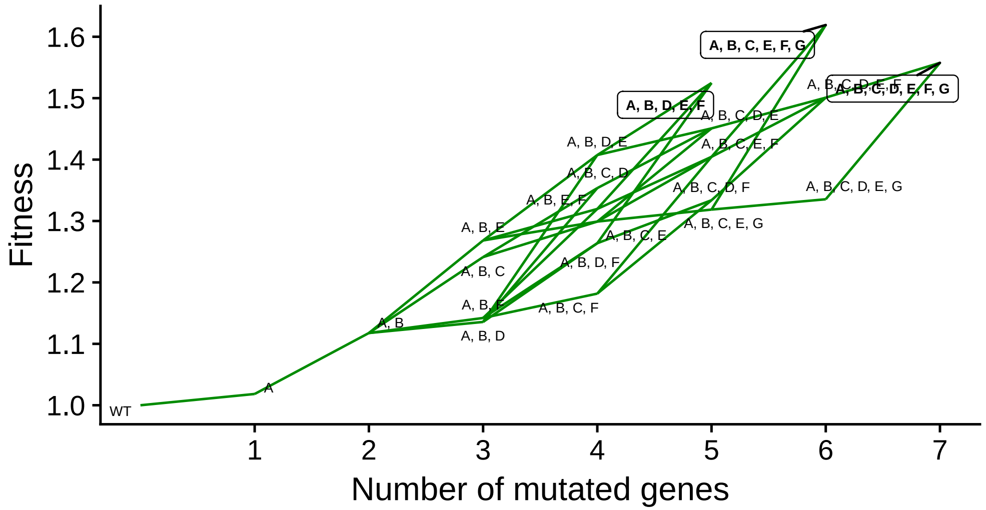

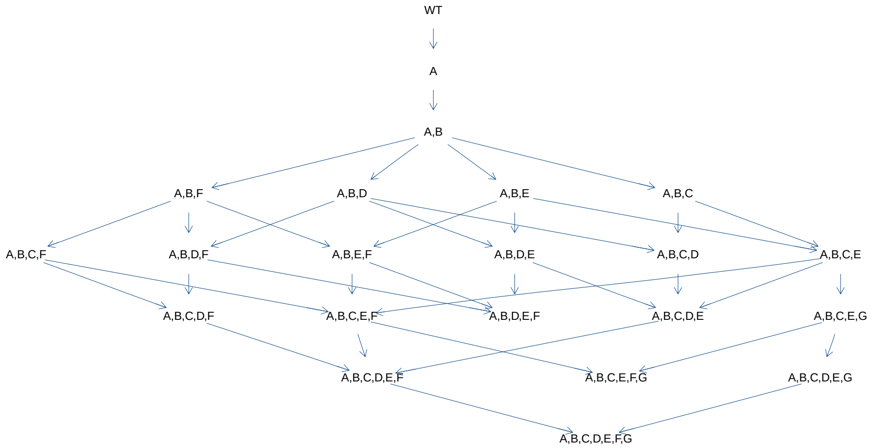

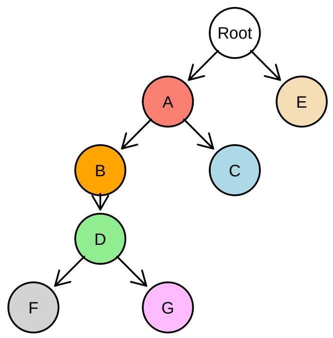

ID jle1t255I110i164X
acc genotypes 26
peaks no back 3
removed edges 4
prop rem edges 0.078

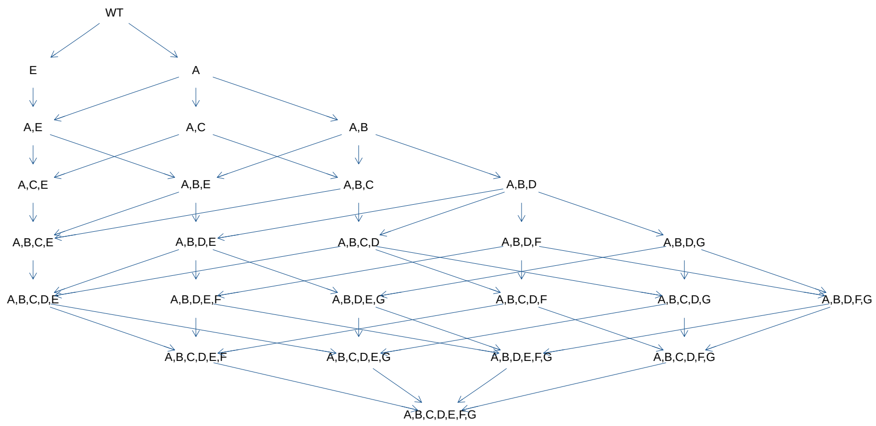

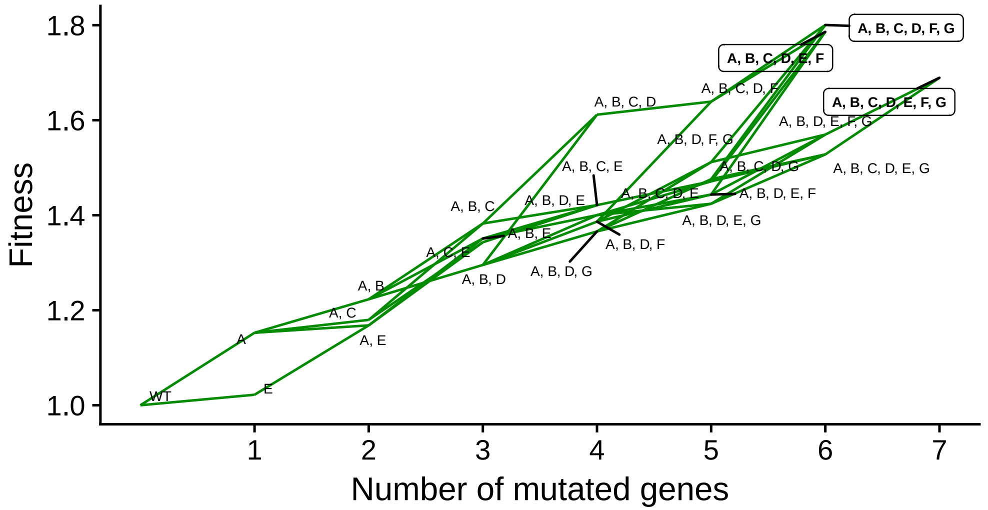

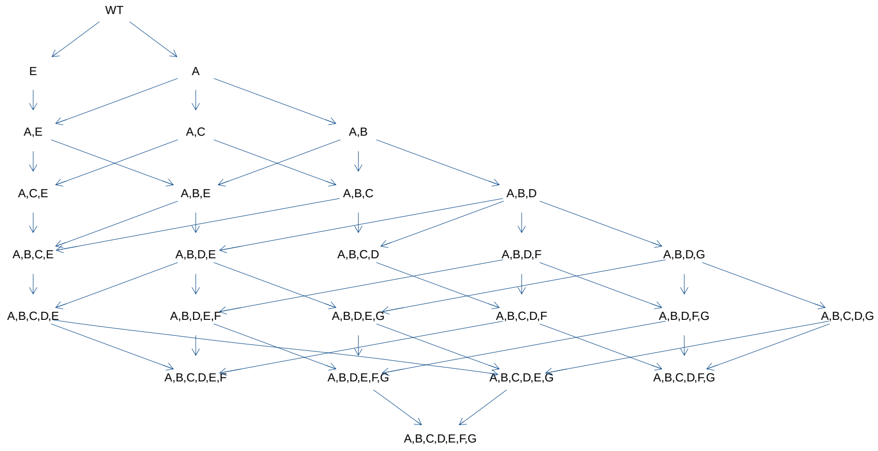

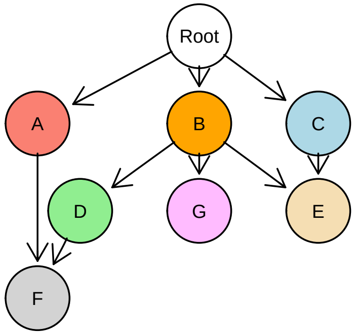

ID jXkcIl8ifcrBbL2U
acc genotypes 34
peaks no back 2
removed edges 4
prop rem edges 0.055

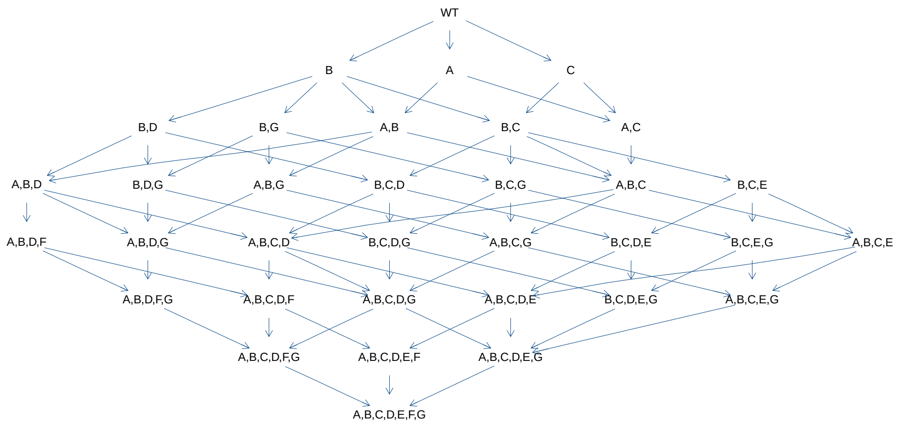

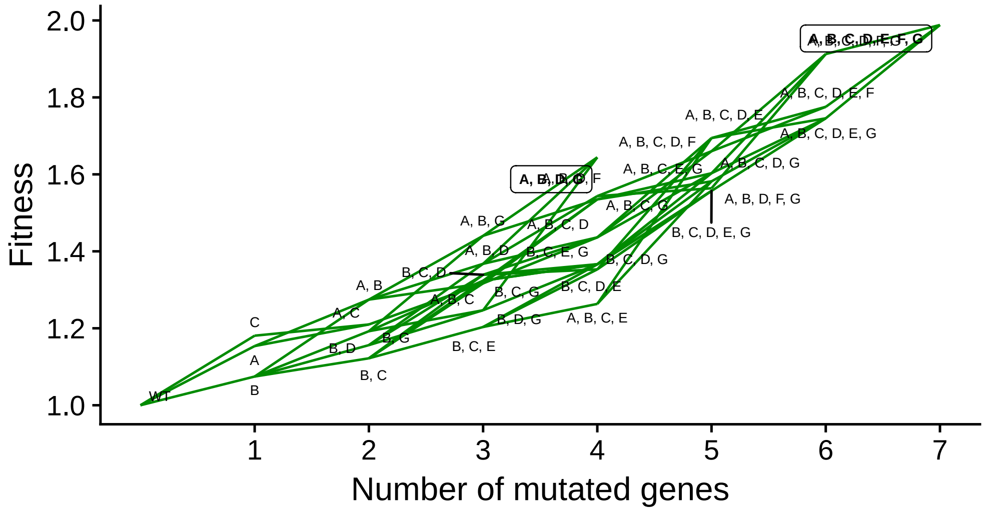

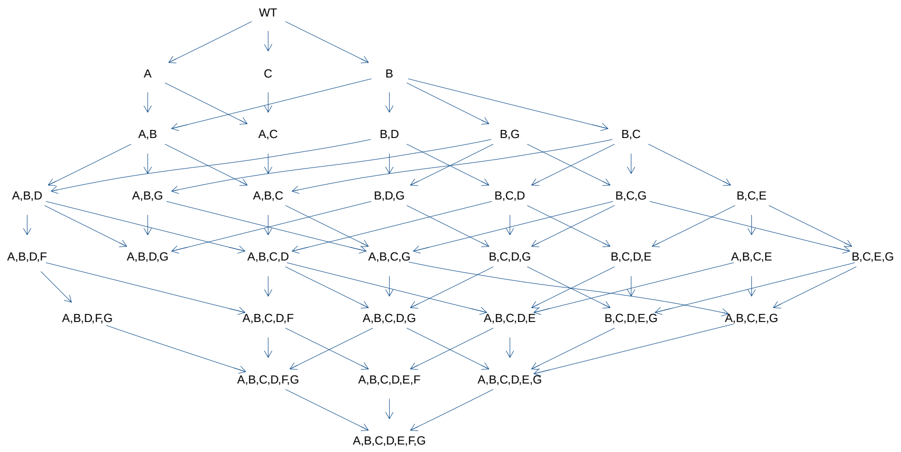

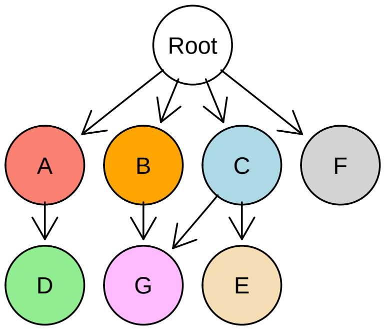

ID JXRG1xH8vZXQkcqA
acc genotypes 48
peaks no back 11
removed edges 27
prop rem edges 0.233

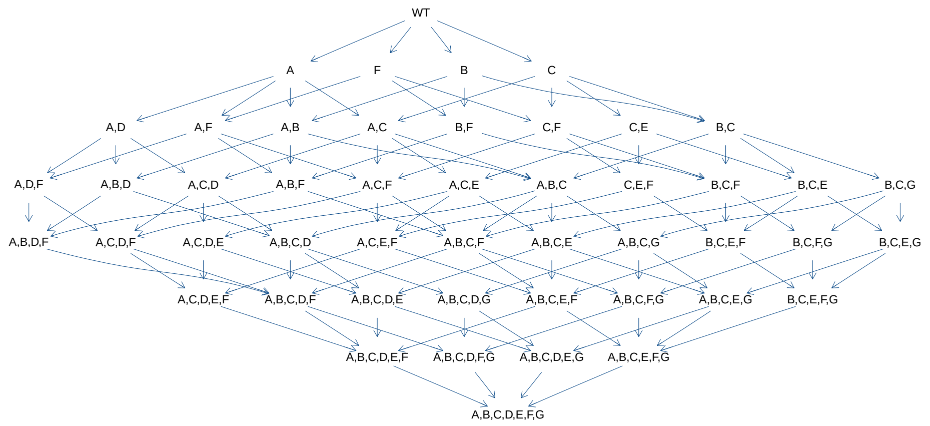

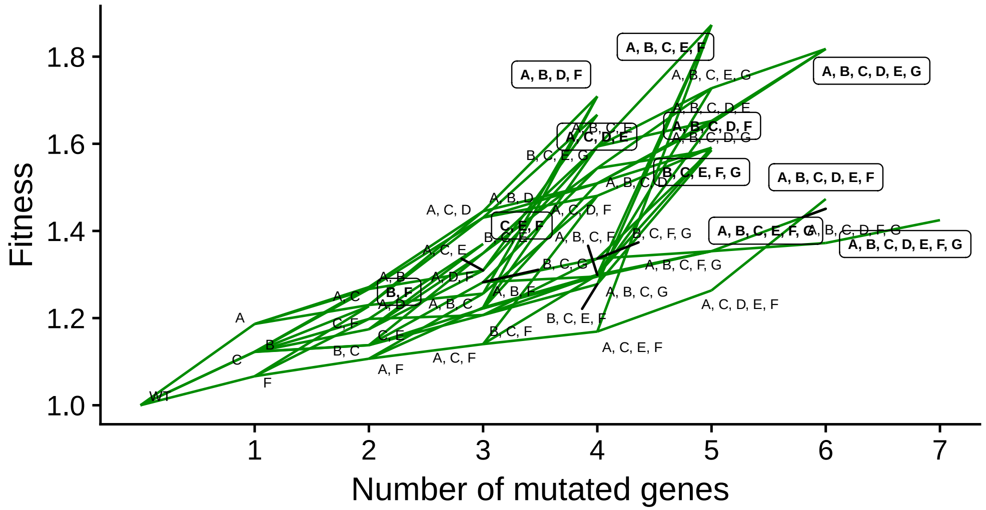

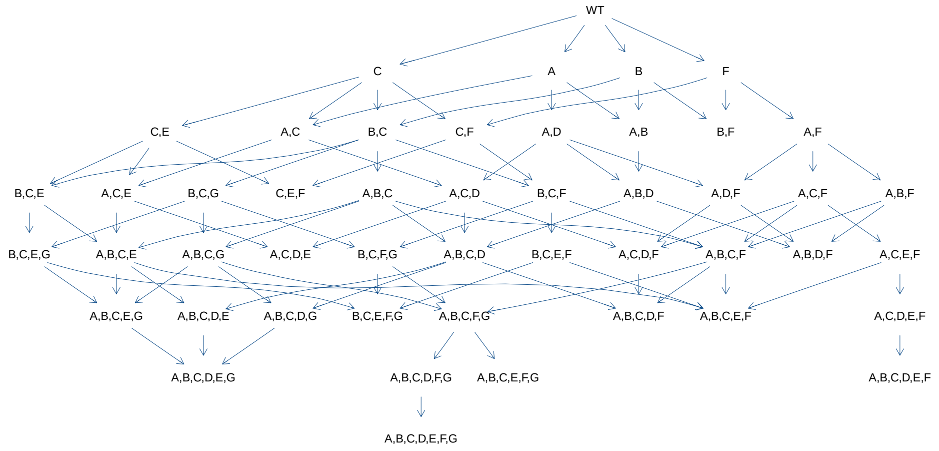

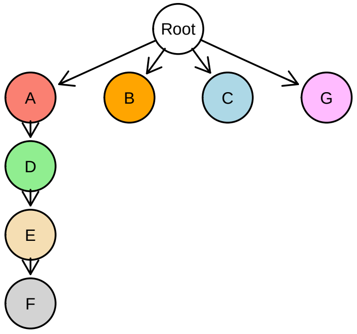

ID	k58HtWC5MFWCpF19
acc genotypes	40
peaks no back	7
removed edges	12
prop rem edges	0.13

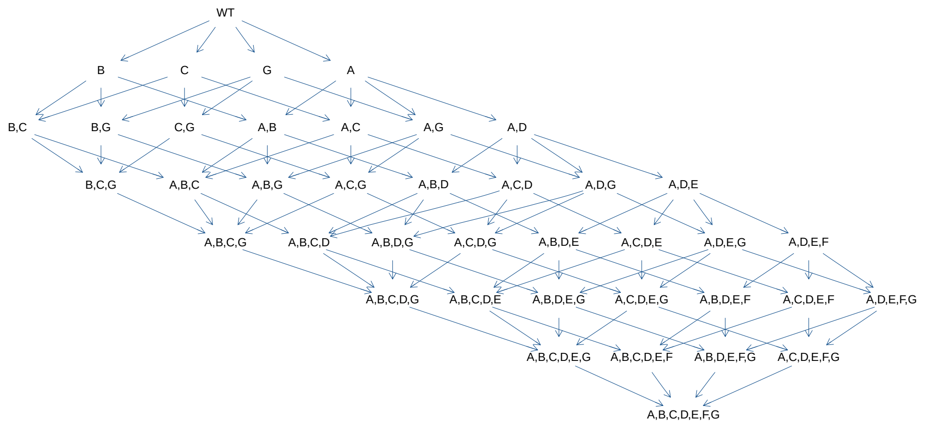

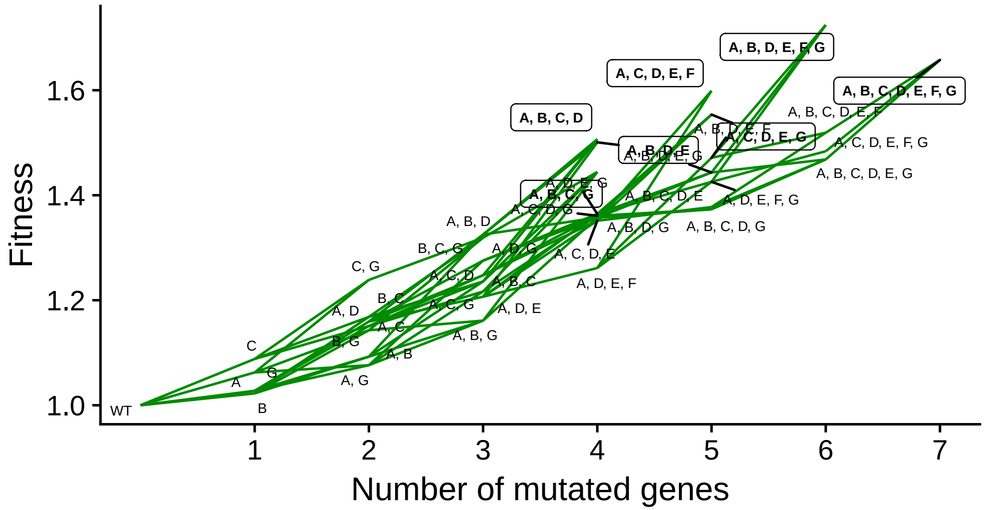

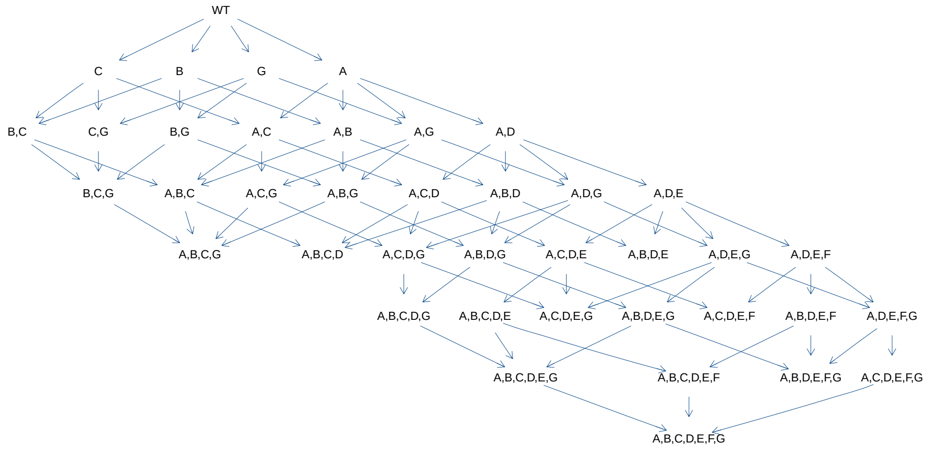

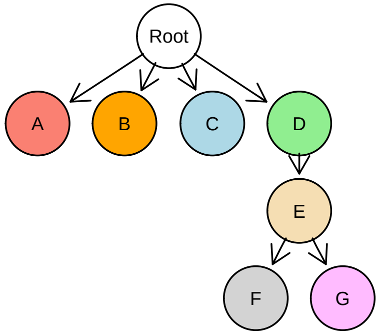

ID	kHBNd5rumamnFMto
acc genotypes	48
peaks no back	7
removed edges	25
prop rem edges	0.208

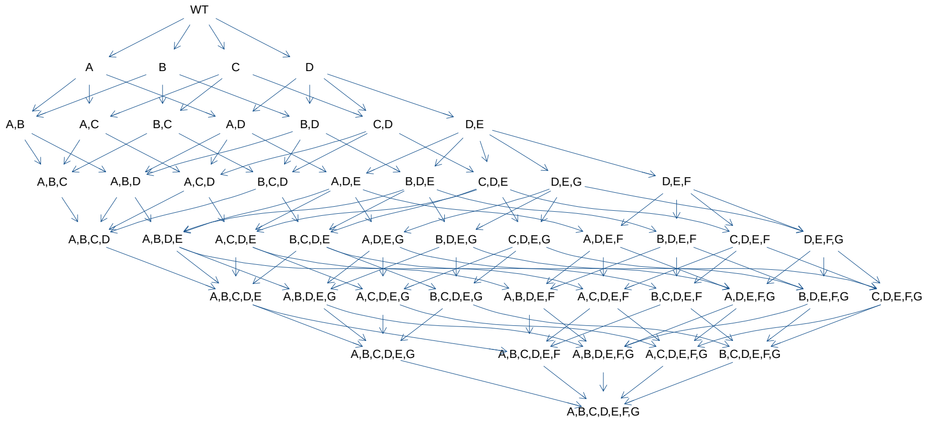

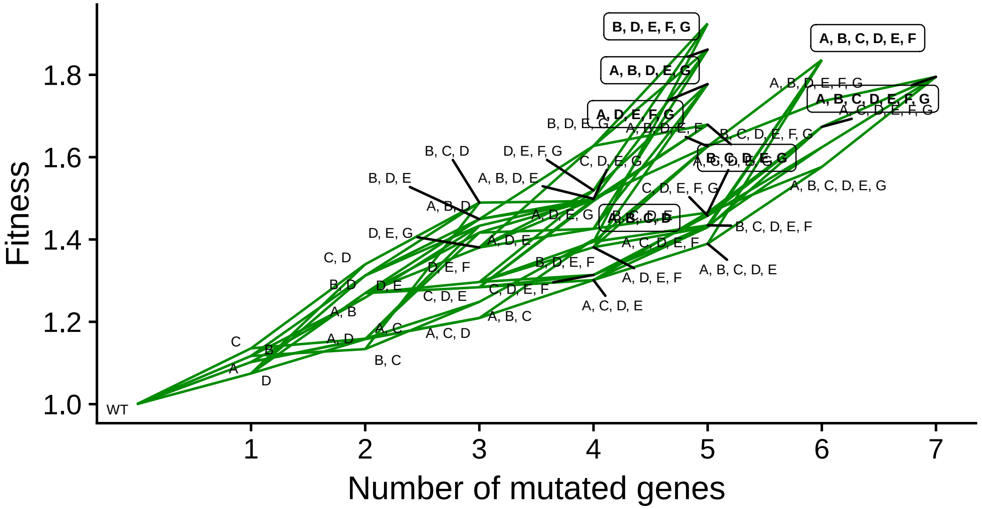

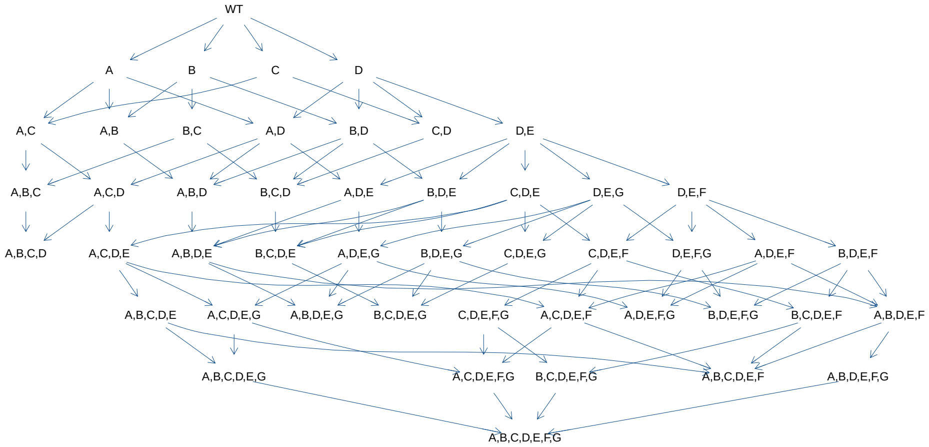

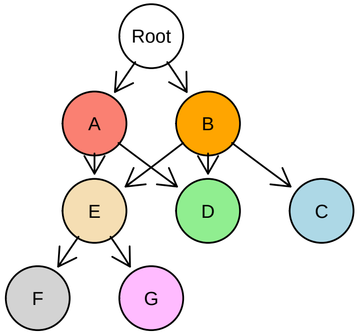

ID	kj6bITZoZLt3OYX2
acc genotypes	24
peaks no back	5
removed edges	8
prop rem edges	0.174

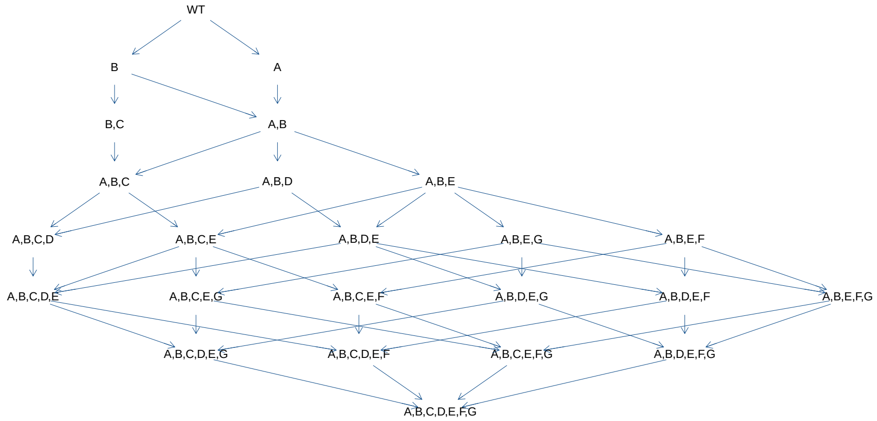

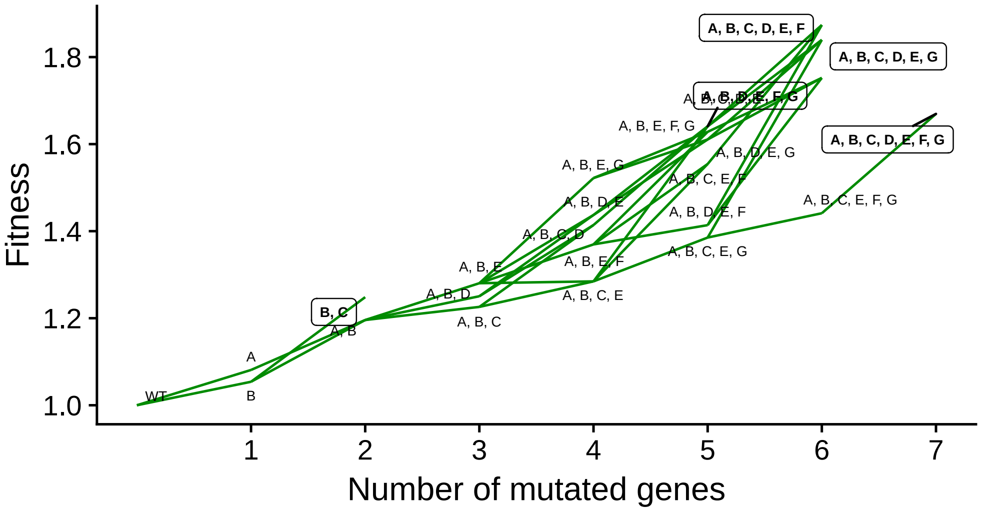

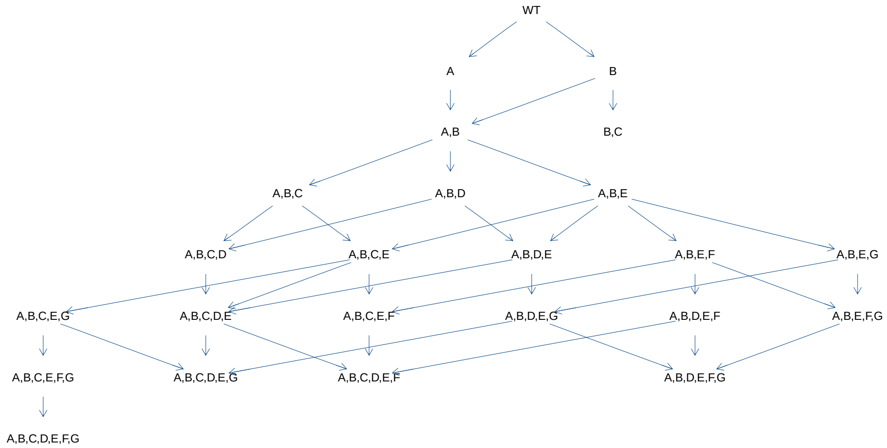

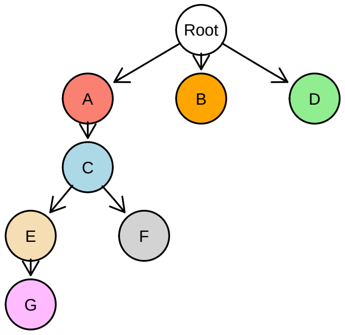

ID	L8C8OoieIFLvU98x
acc genotypes	32
peaks no back	7
removed edges	17
prop rem edges	0.25

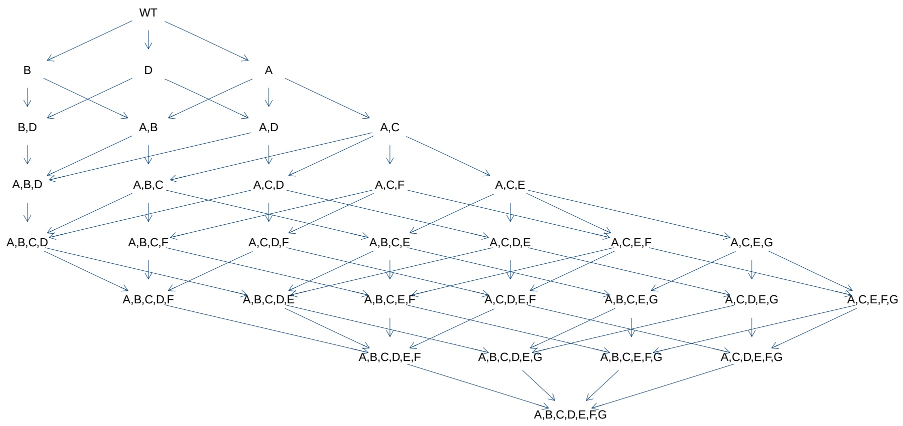

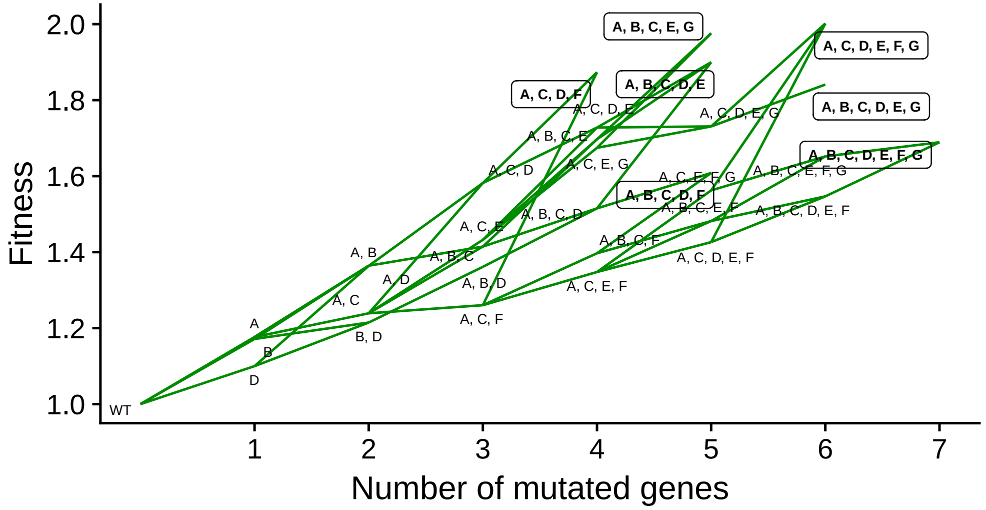

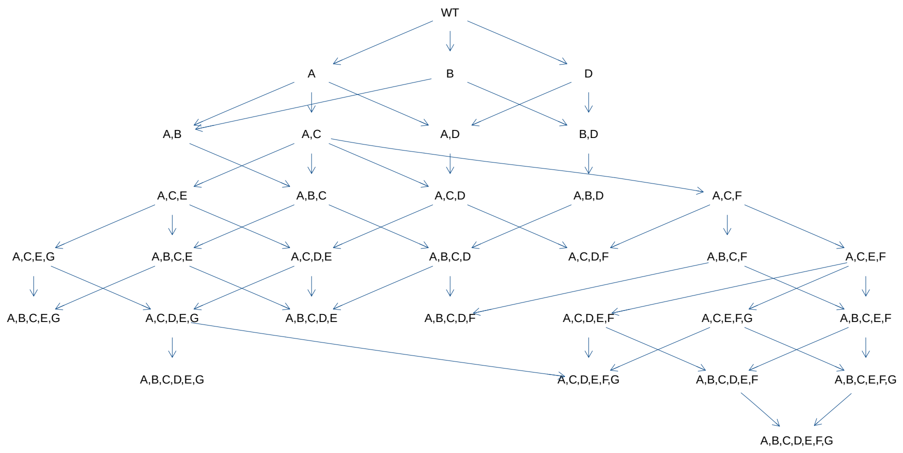

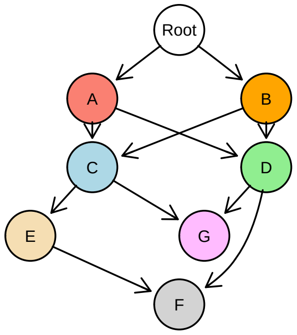

ID L9U6EXaxa4AgbWdQ
acc genotypes 13
peaks no back 3
removed edges 2
prop rem edges 0.118

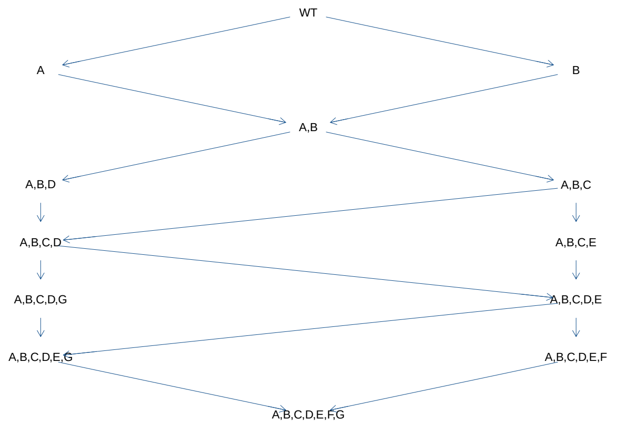

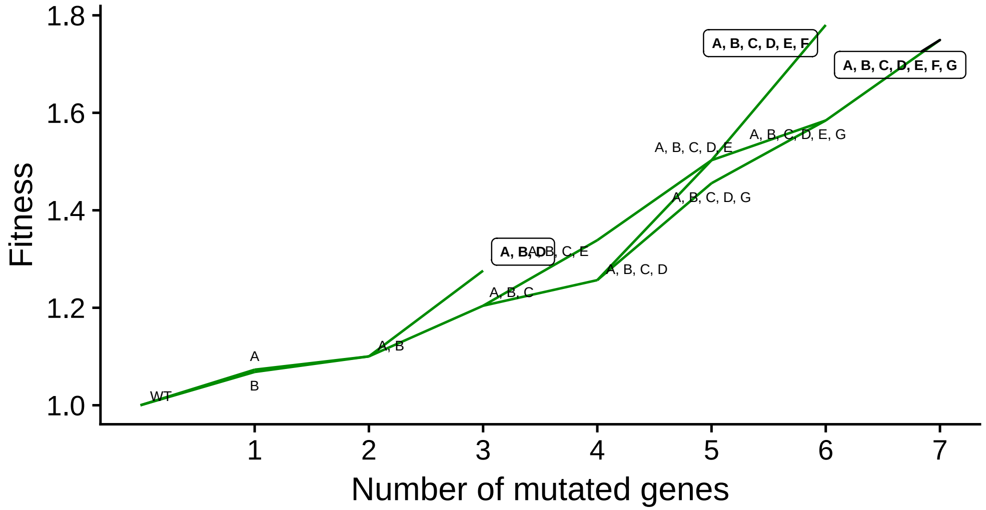

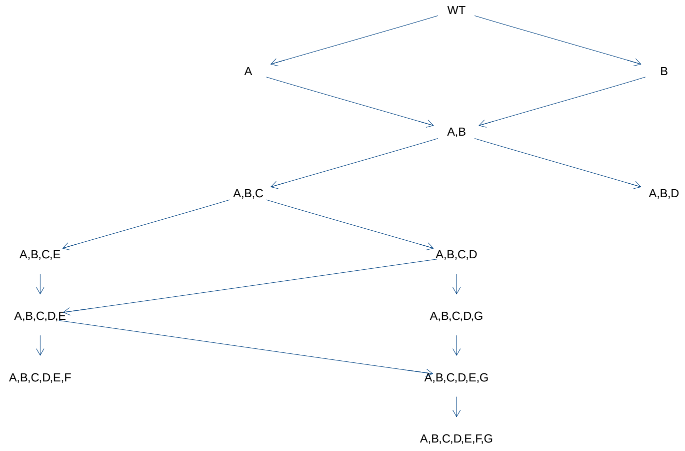

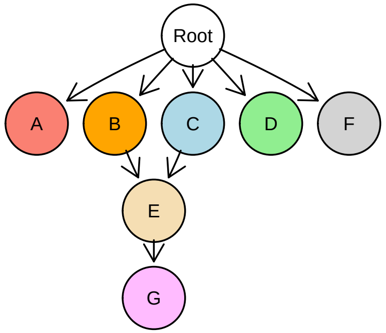

ID	IDsDxkW7aWMDoTYv
acc genotypes	48
peaks no back	8
removed edges	27
prop rem edges	0.225

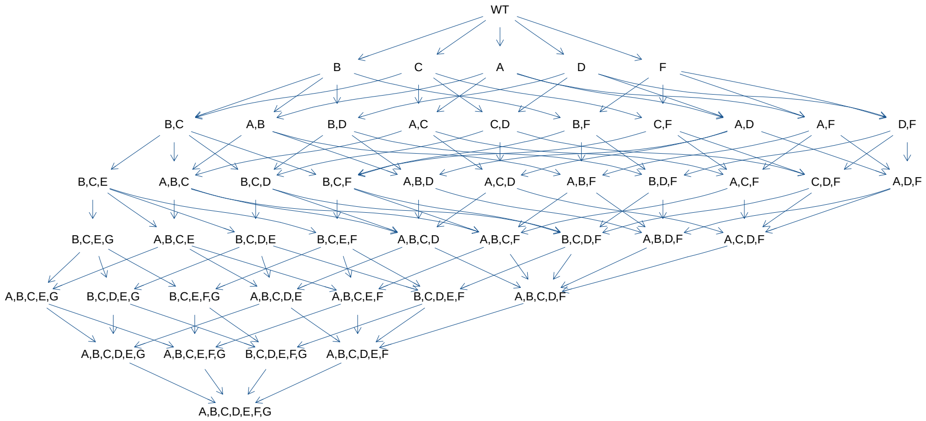

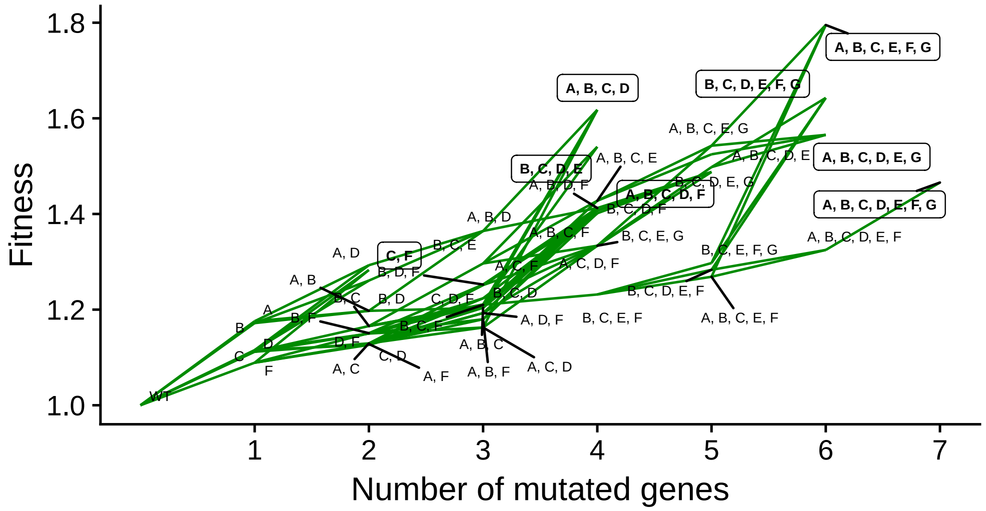

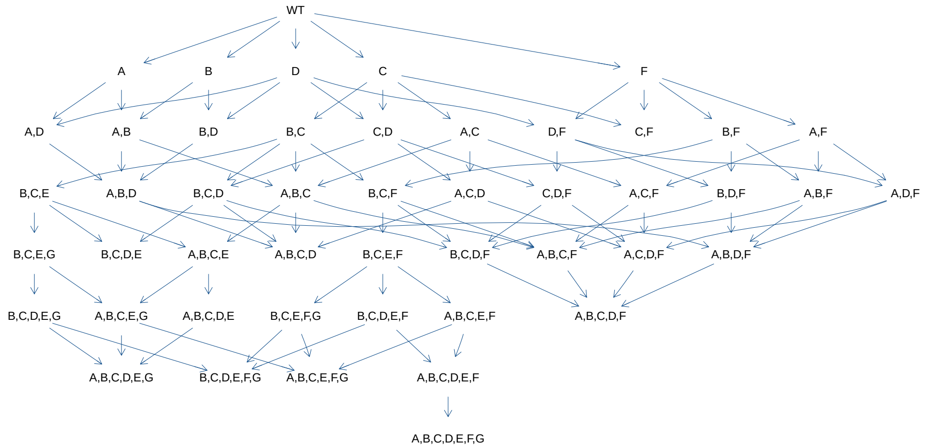

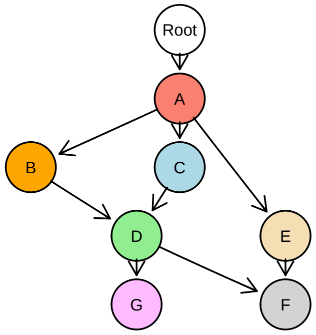

ID	Lh10CtFMPrzzV6ihq
acc genotypes	15
peaks no back	2
removed edges	1
prop rem edges	0.045

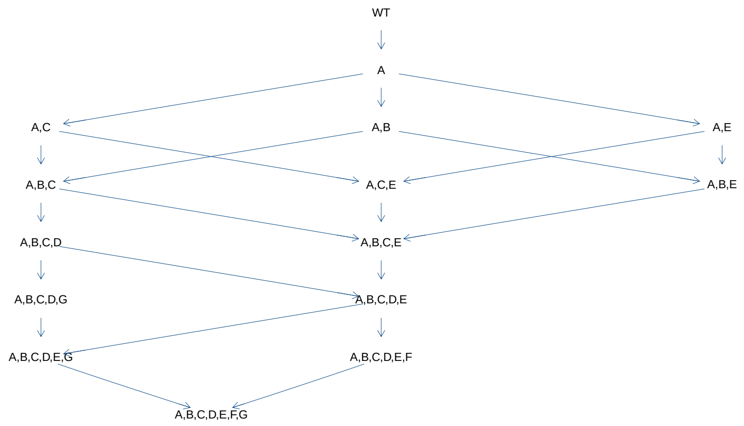

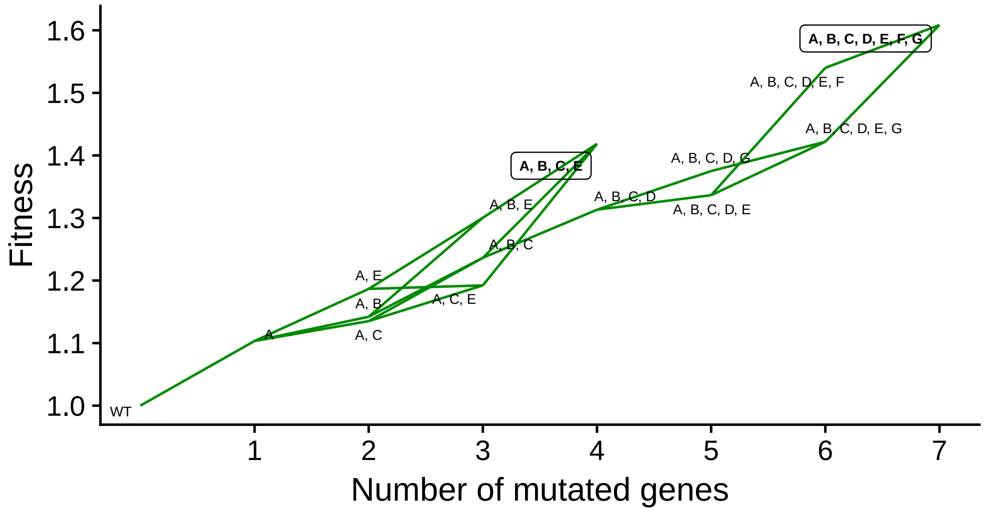

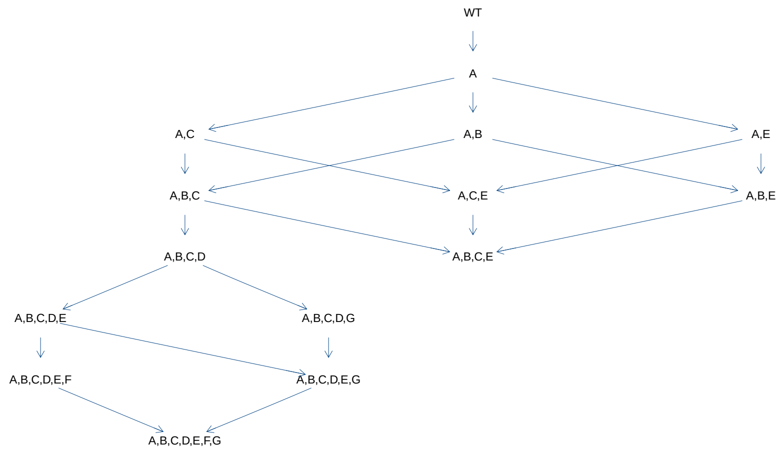

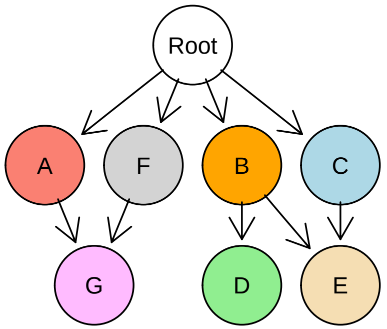

ID LqgKBG79CNzADijg
acc genotypes 40
peaks no back 6
removed edges 14
prop rem edges 0.156

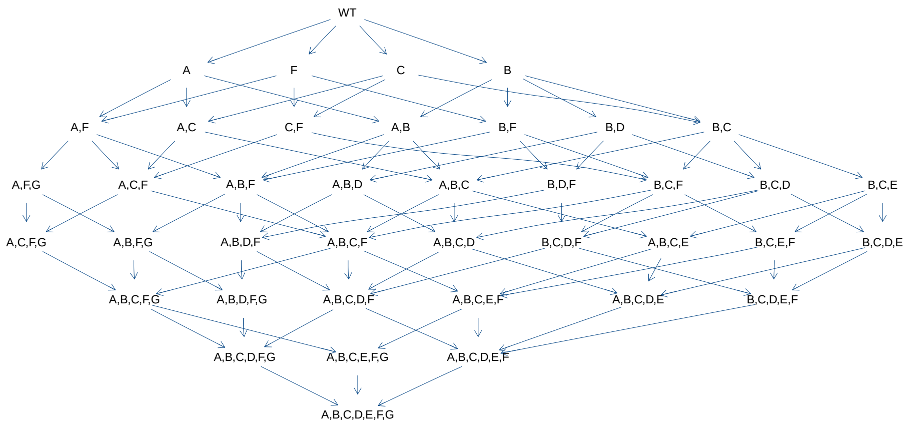

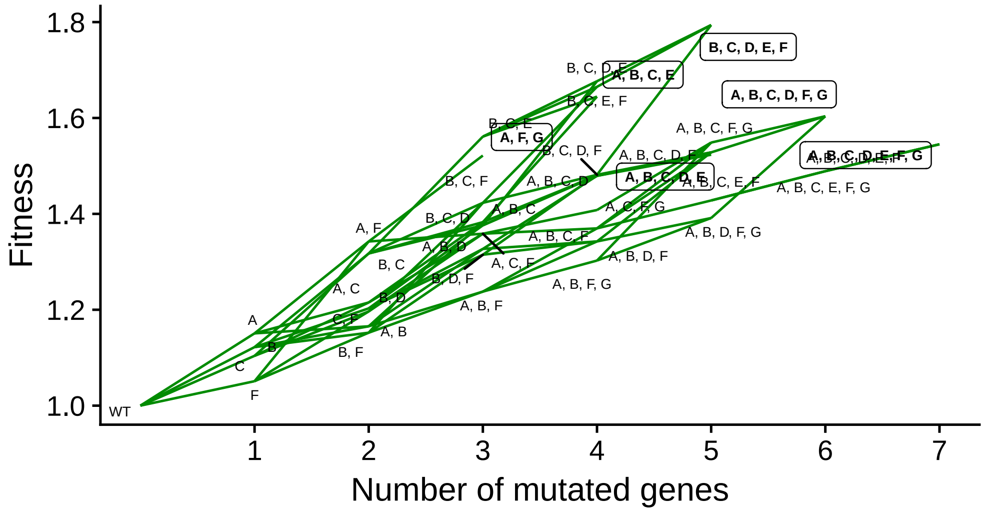

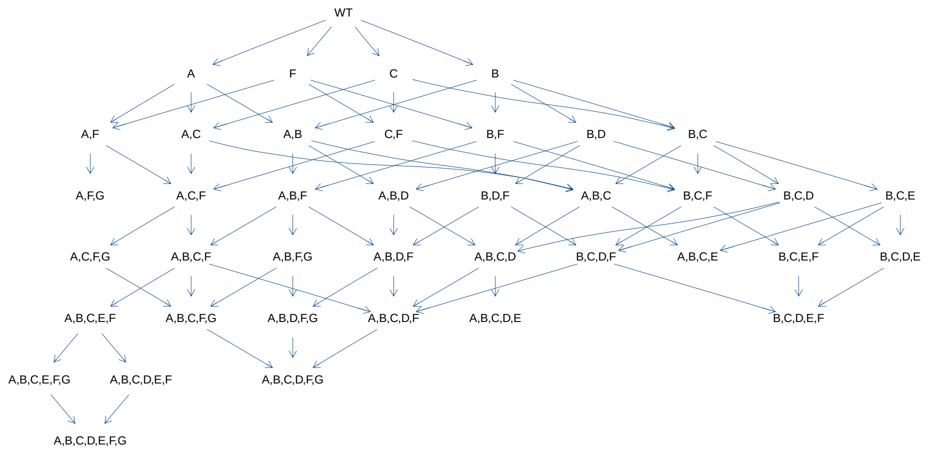

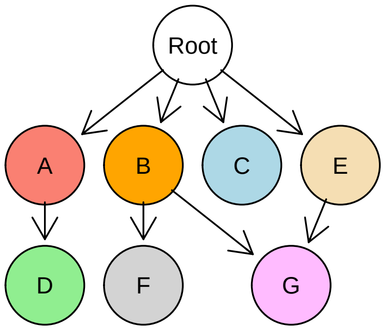

ID	lsG2NZmtk5W2NxDs
acc genotypes	48
peaks no back	7
removed edges	17
prop rem edges	0.147

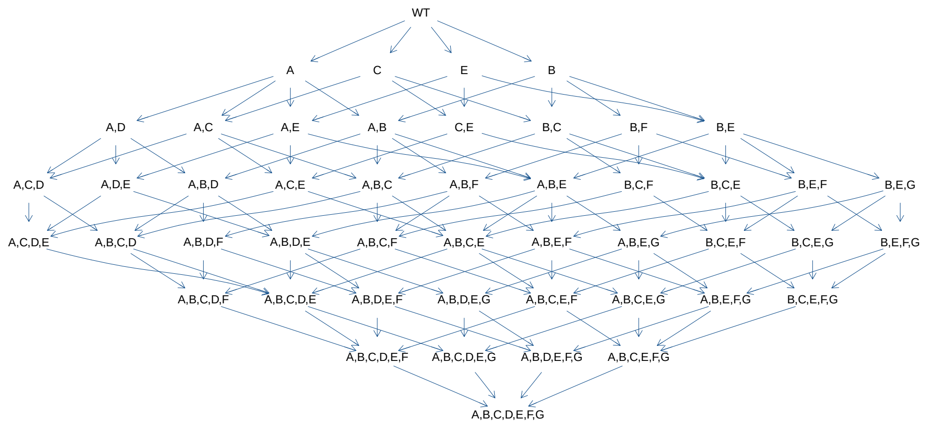

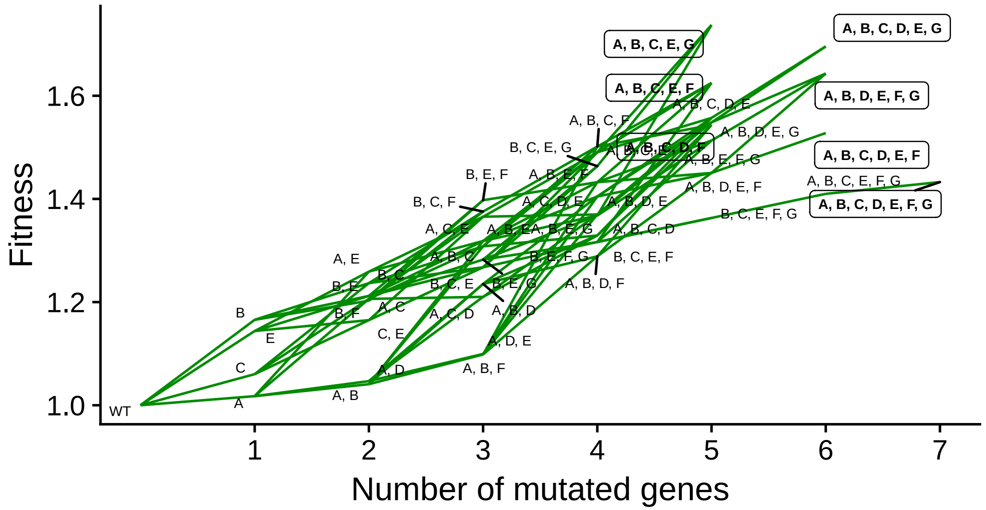

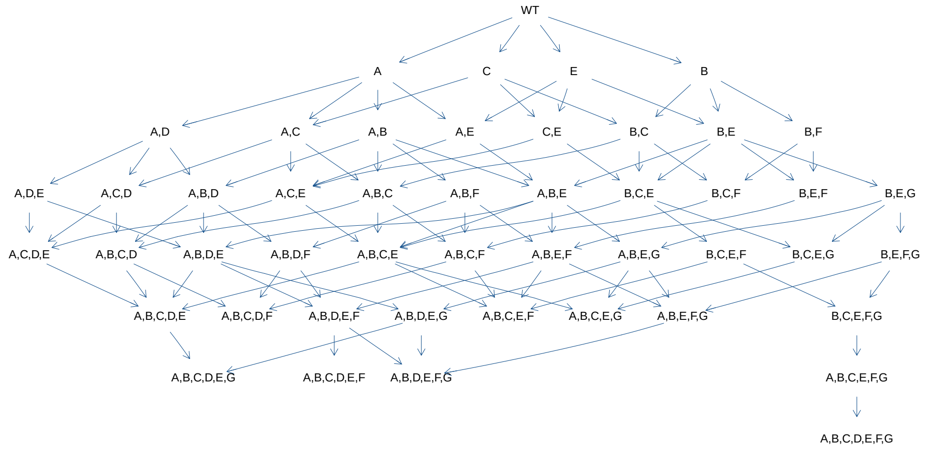

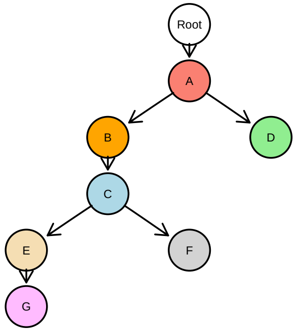

ID	lsjclB1mAC8mHR9Q
acc genotypes	17
peaks no back	3
removed edges	3
prop rem edges	0.111

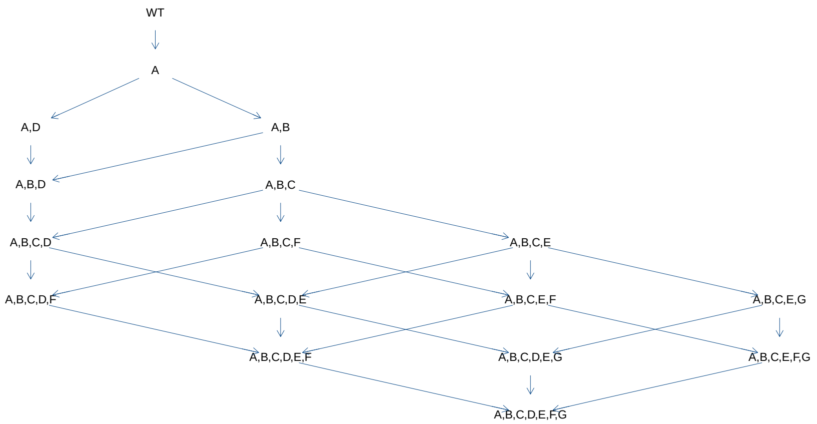

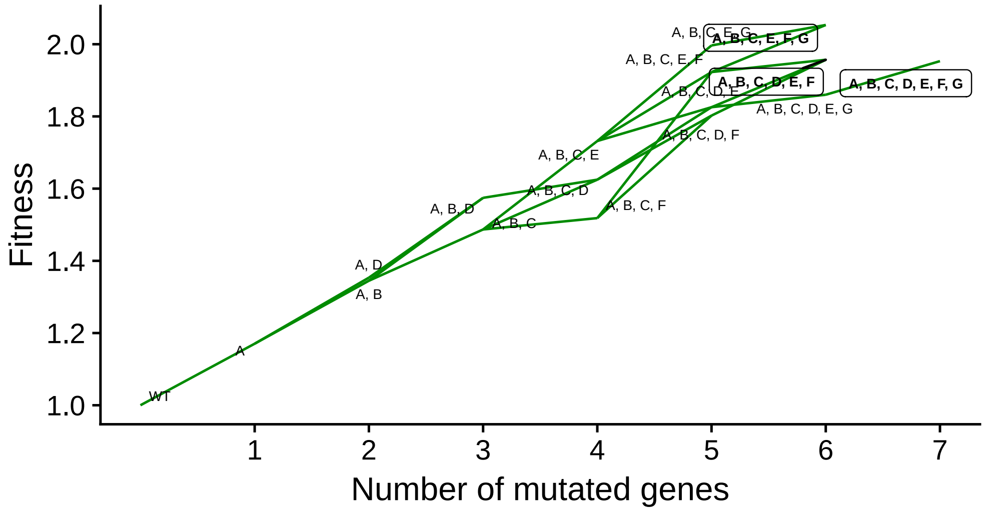

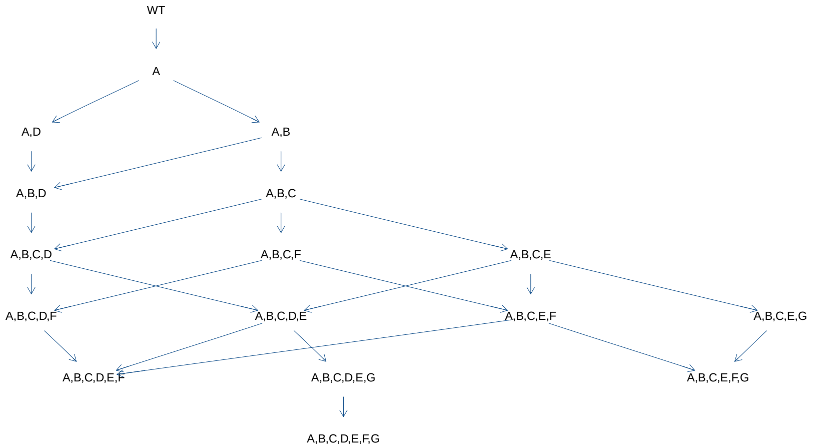

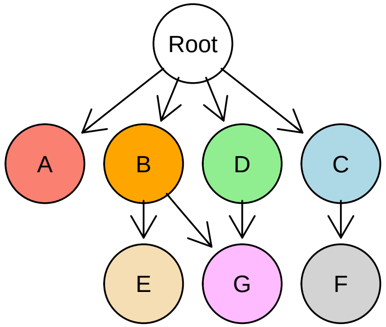

ID	LSnOGiz5m5AoyDGr
acc genotypes	48
peaks no back	7
removed edges	17
prop rem edges	0.147

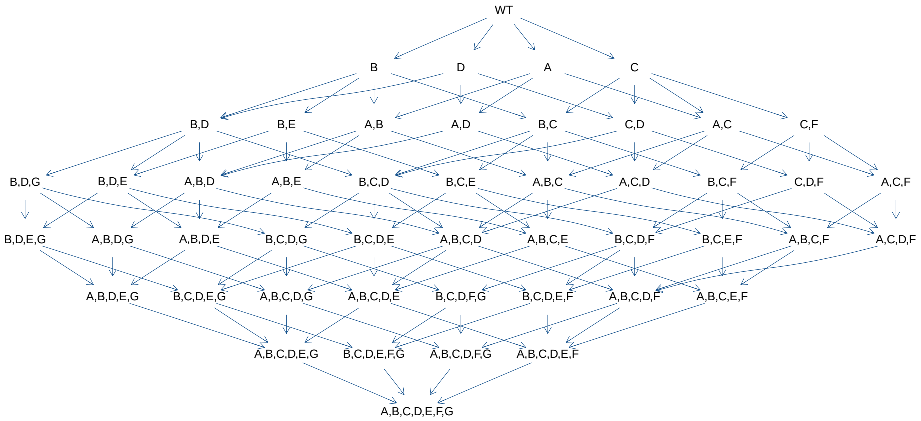

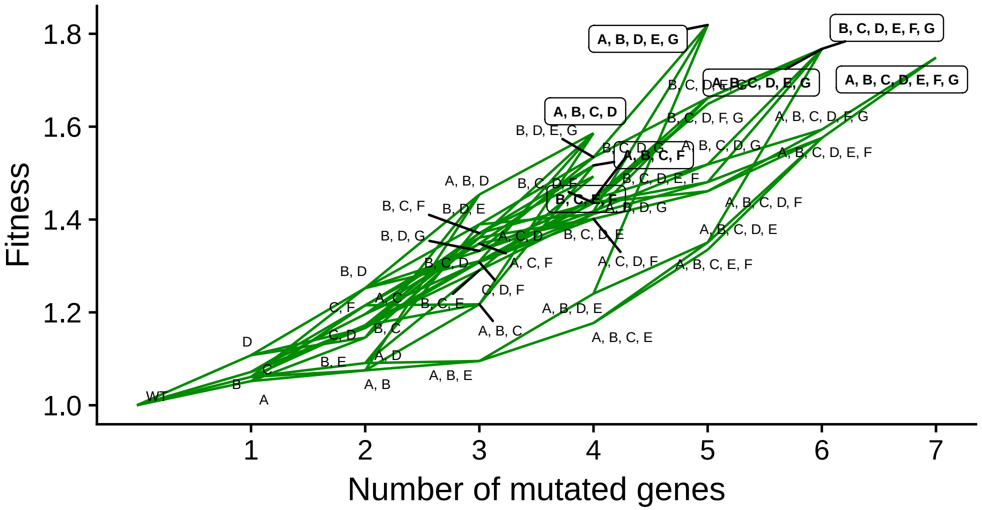

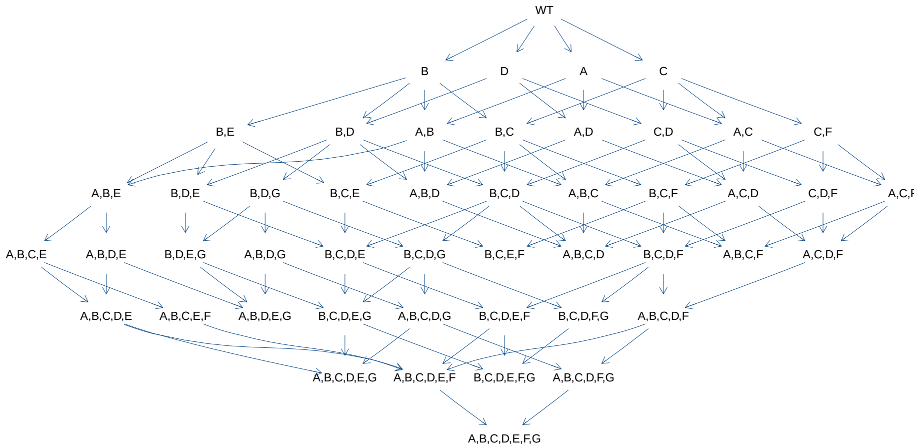

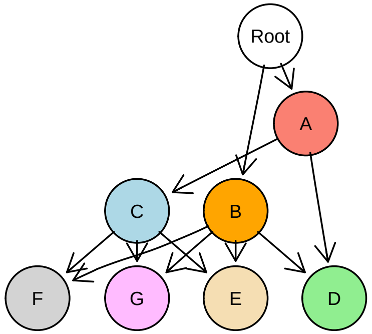

ID LSXwTnPZnOfXSdzA
acc genotypes 22
peaks no back 2
removed edges 2
prop rem edges 0.049

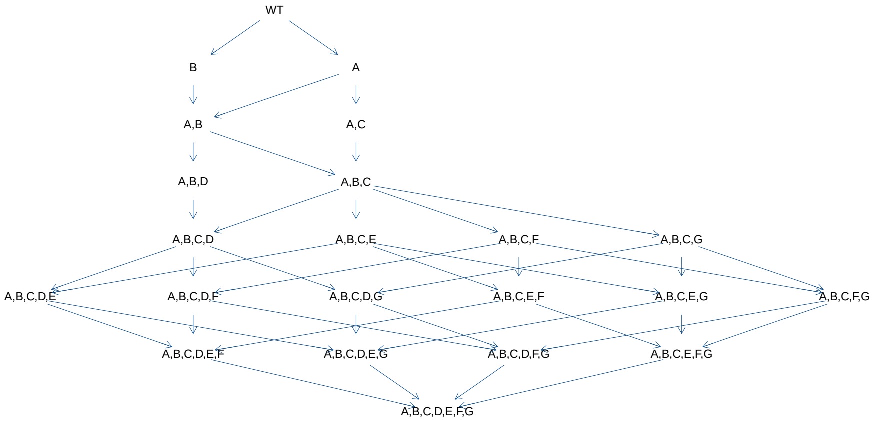

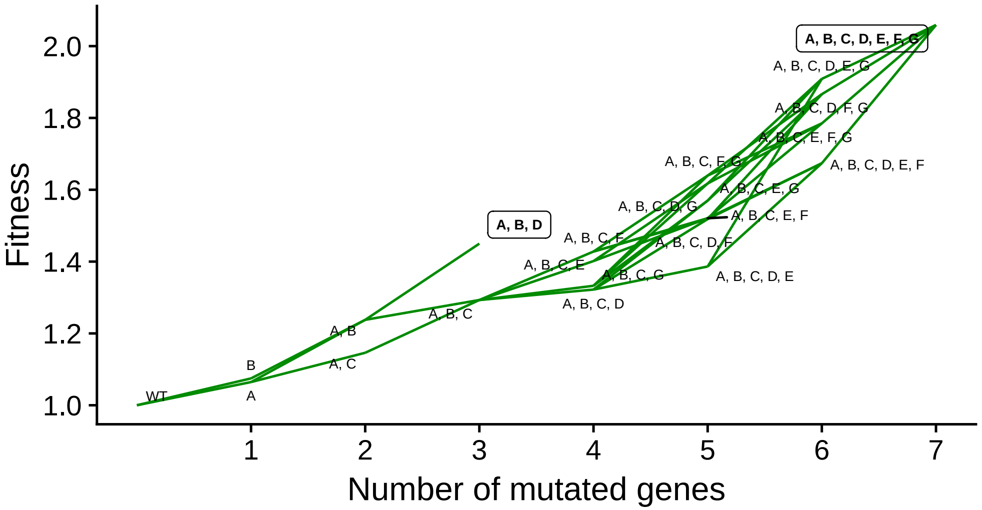

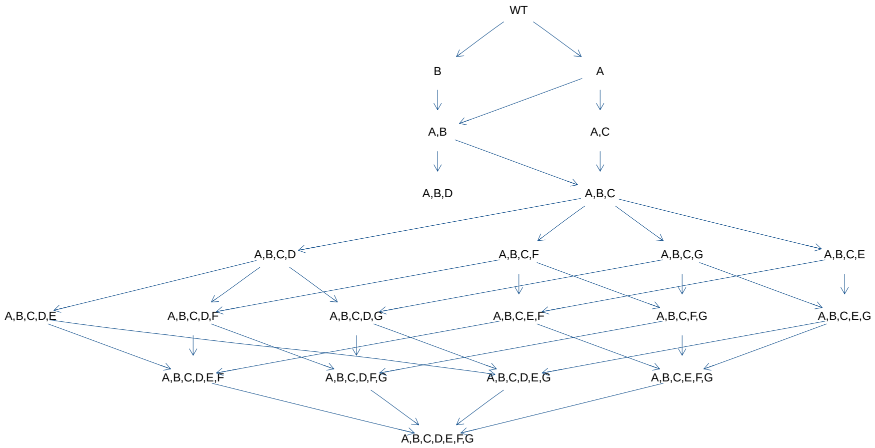

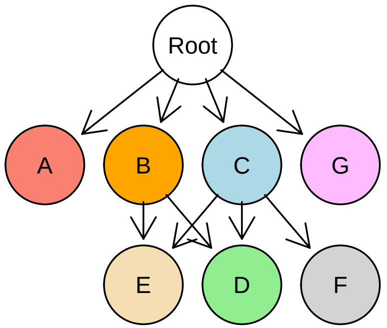

ID	lsZUuCCknMZno10B8
acc genotypes	48
peaks no back	8
removed edges	21
prop rem edges	0.175

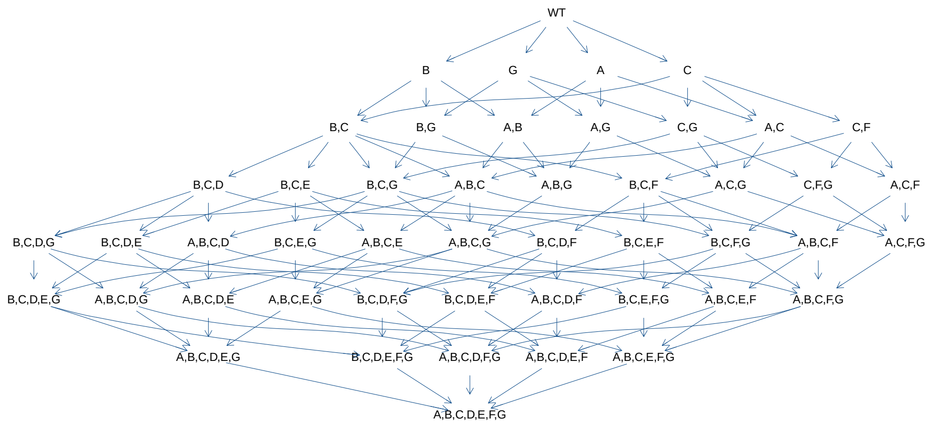

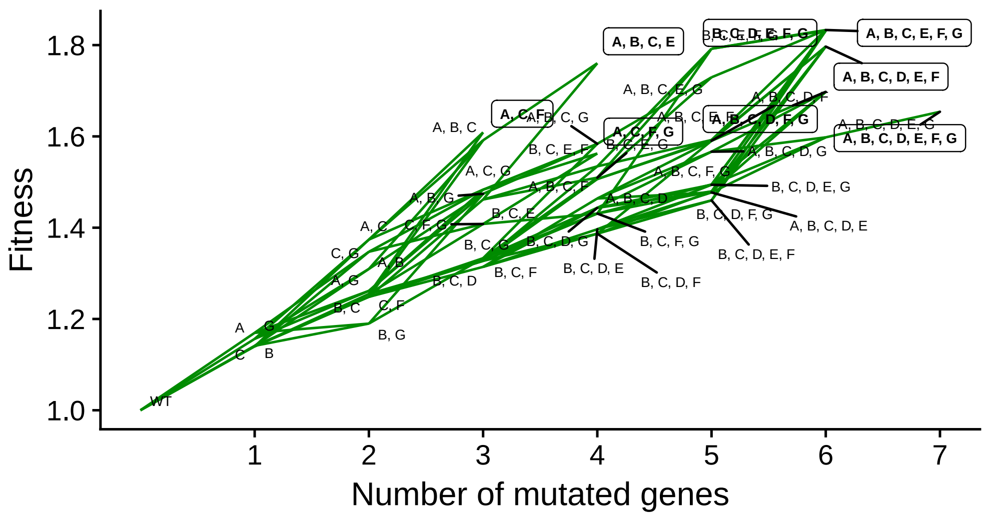

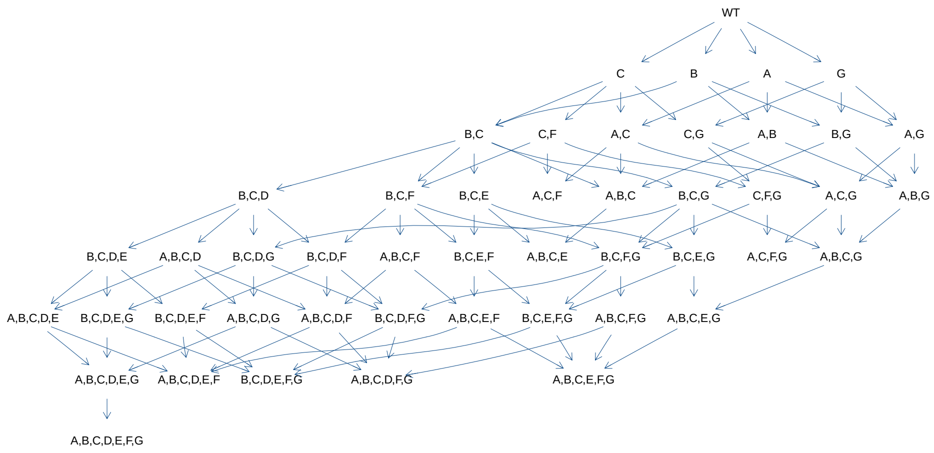

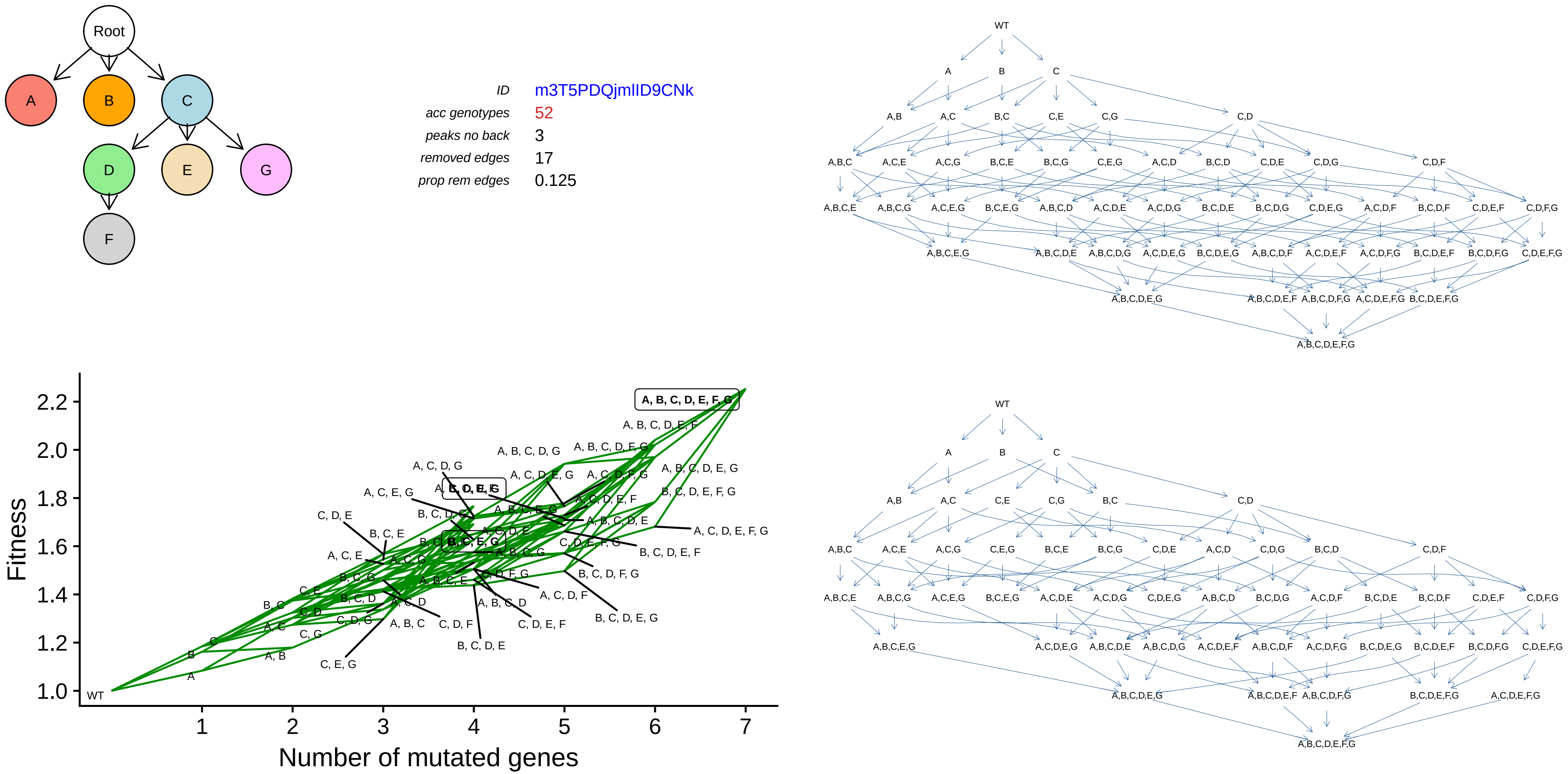
Root
A
B
C
D
E
G
F
ID m3T5PDQjmlID9CNk
acc genotypes 52
peaks no back 3
removed edges 17
prop rem edges 0.125
Fitness
Number of mutated genes
A, B, C, D, E, F, G
WT

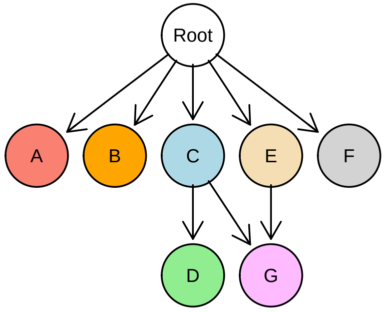

ID	m8kM7ayaSoitwqmo
acc genotypes	64
peaks no back	11
removed edges	36
prop rem edges	0.205

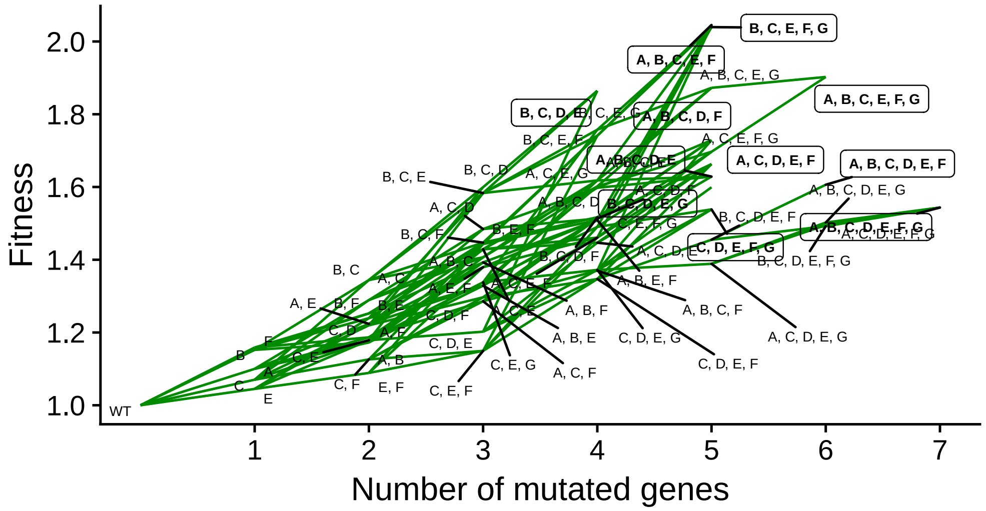

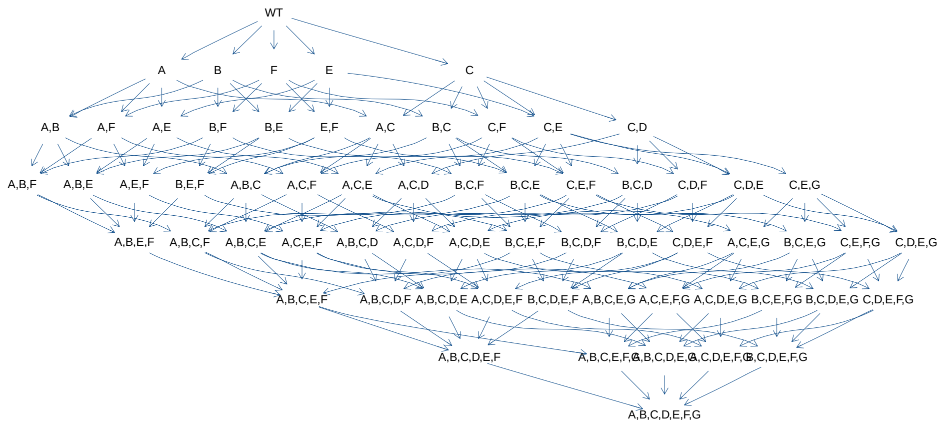

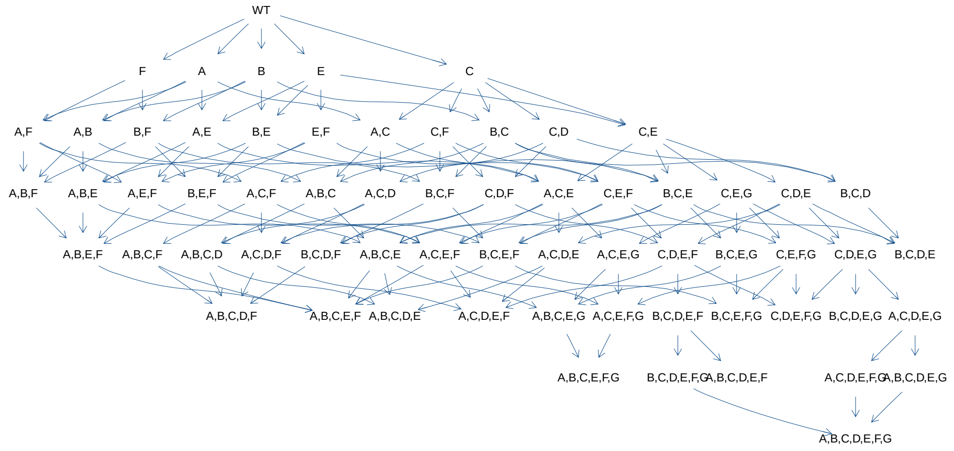

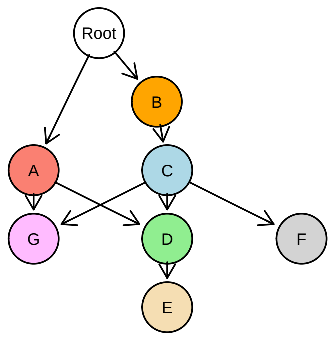

ID	MHkrL757u9iPj10kv
acc genotypes	18
peaks no back	4
removed edges	4
prop rem edges	0.138

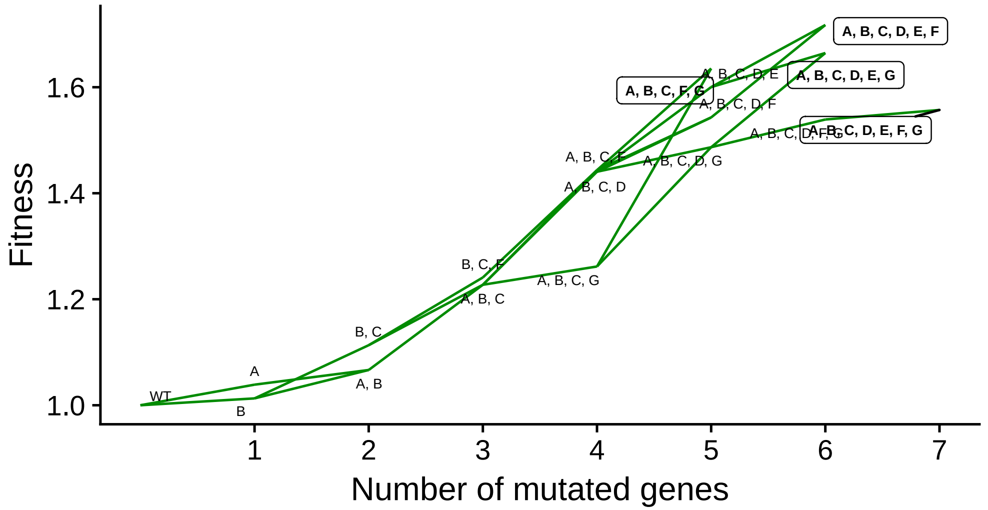

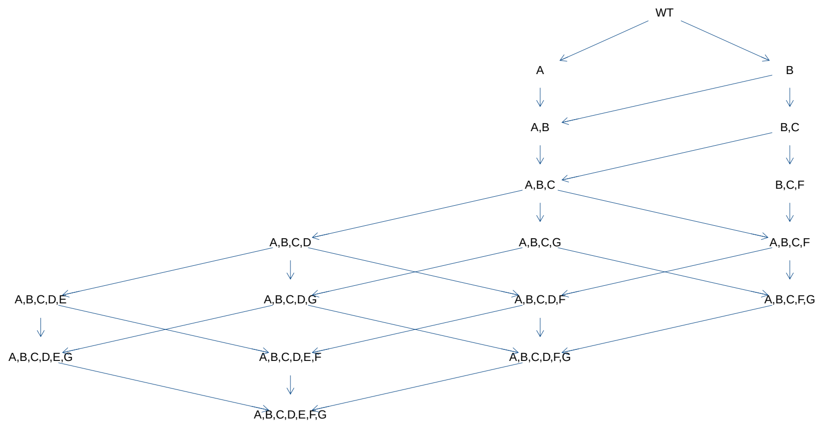

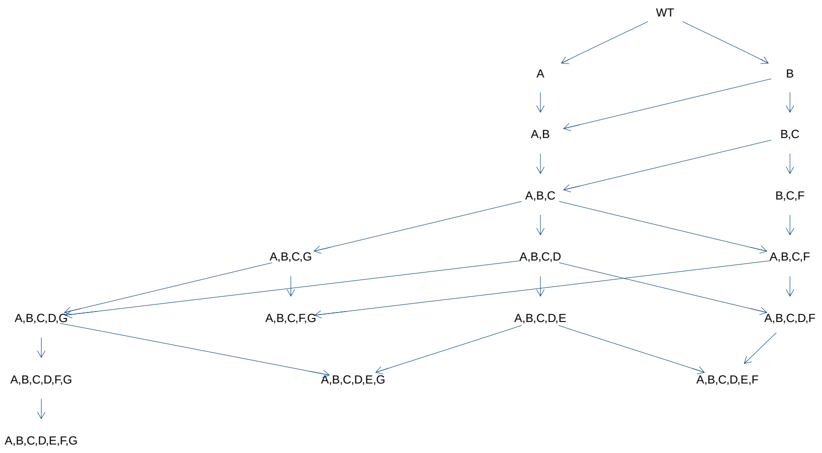

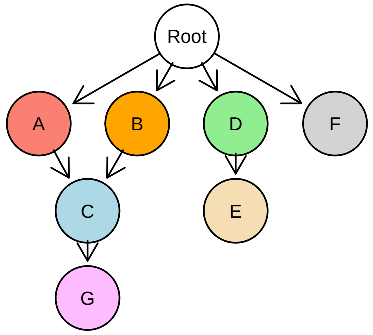

ID	MifrHKJzEqocbcMH
acc genotypes	36
peaks no back	3
removed edges	9
prop rem edges	0.115

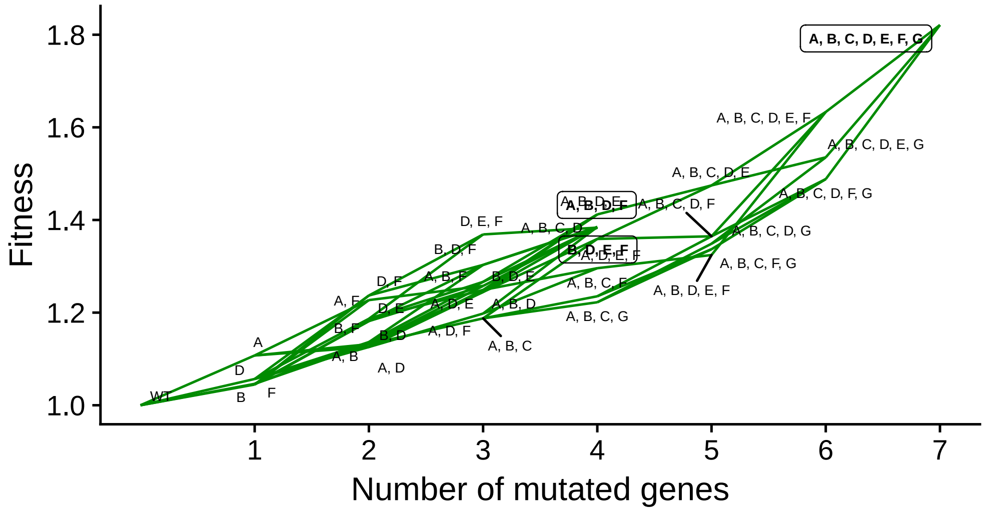

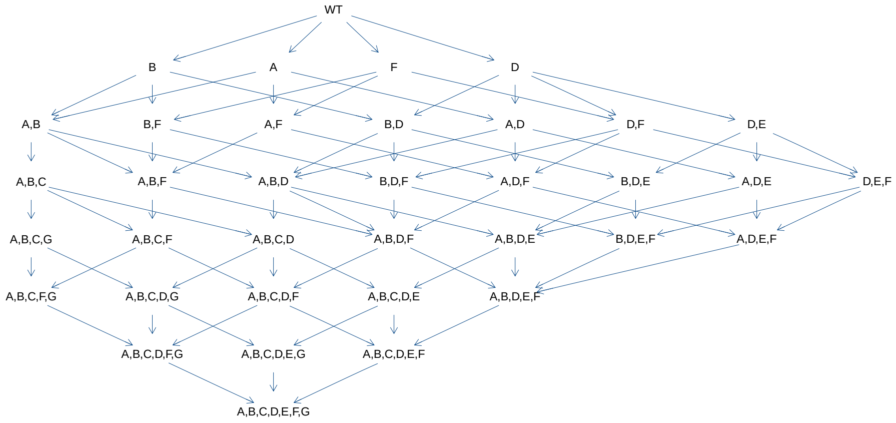

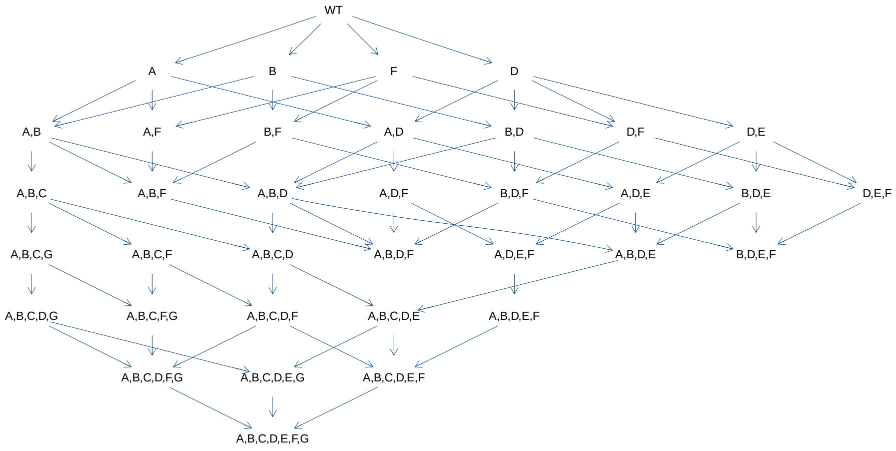

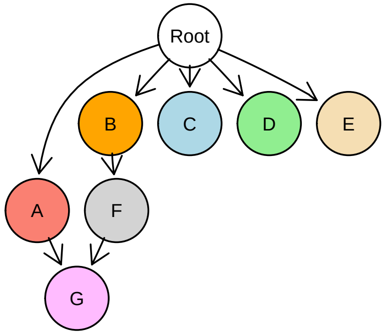

ID	mtZbHQ97NJy8xIsf
acc genotypes	56
peaks no back	10
removed edges	26
prop rem edges	0.176

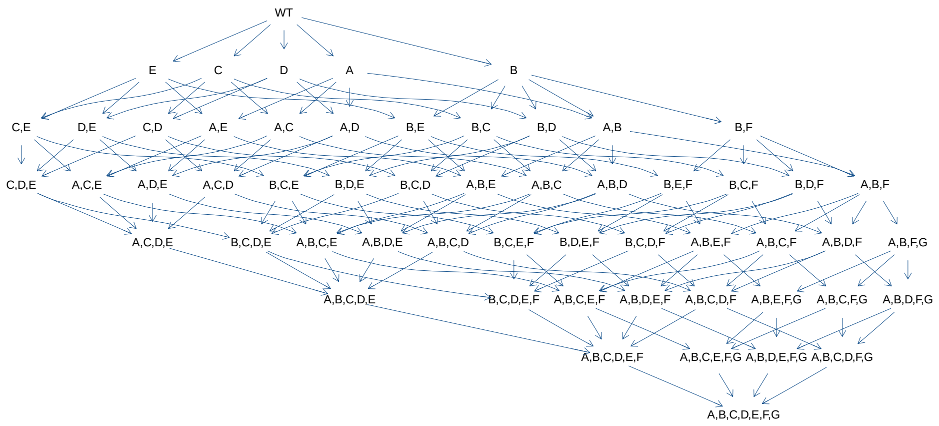

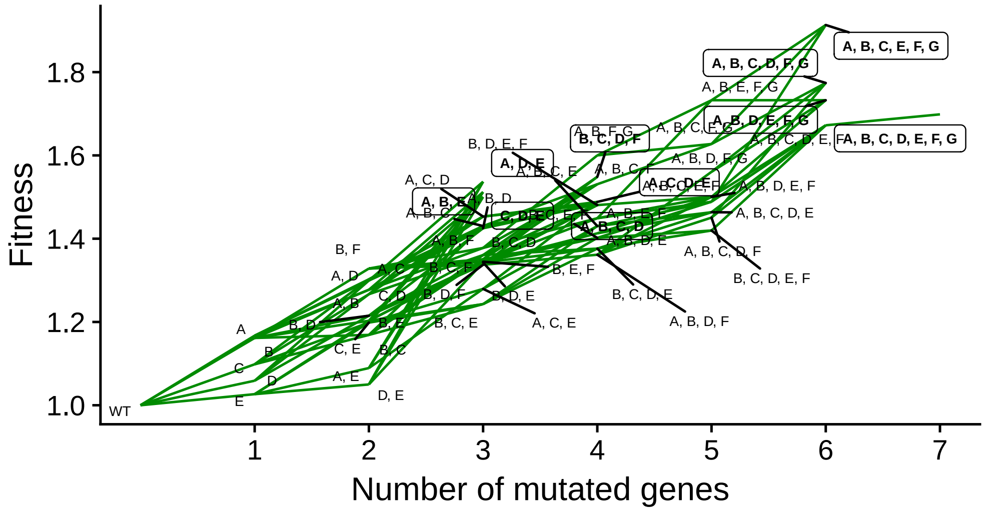

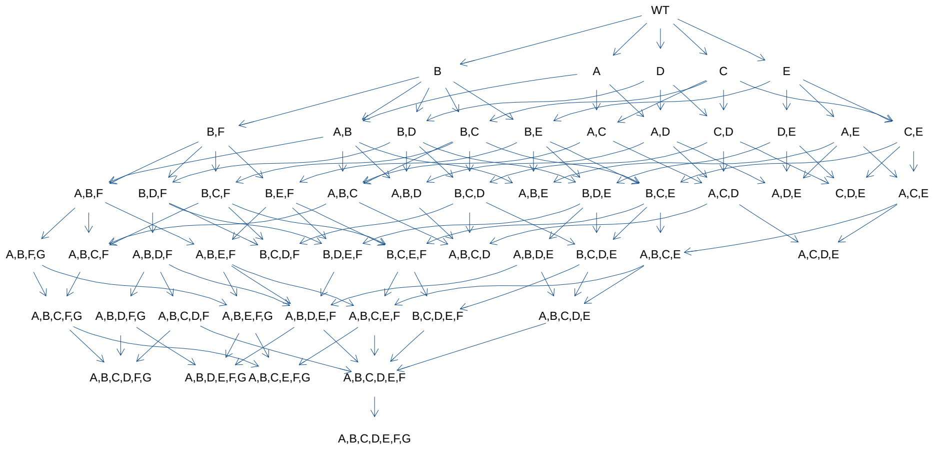

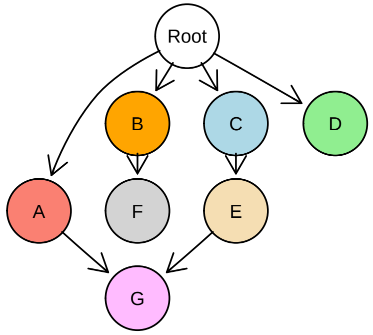

ID MuAVAUt10hjGNt5AD
acc genotypes 42
peaks no back 7
removed edges 15
prop rem edges 0.155

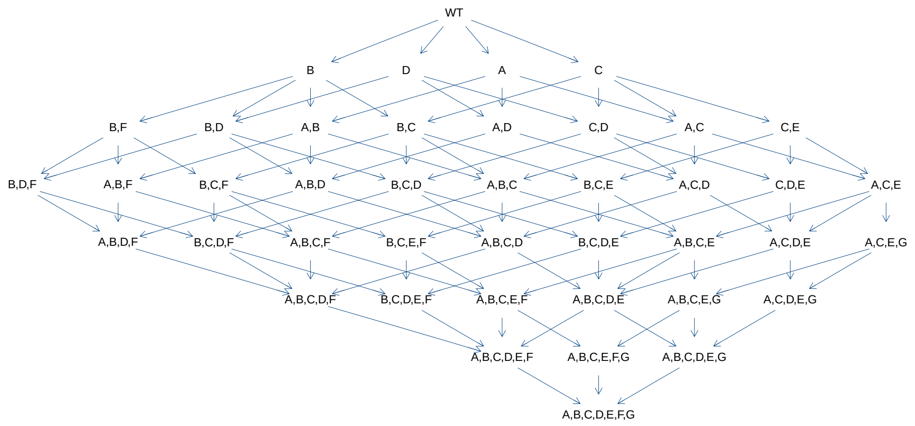

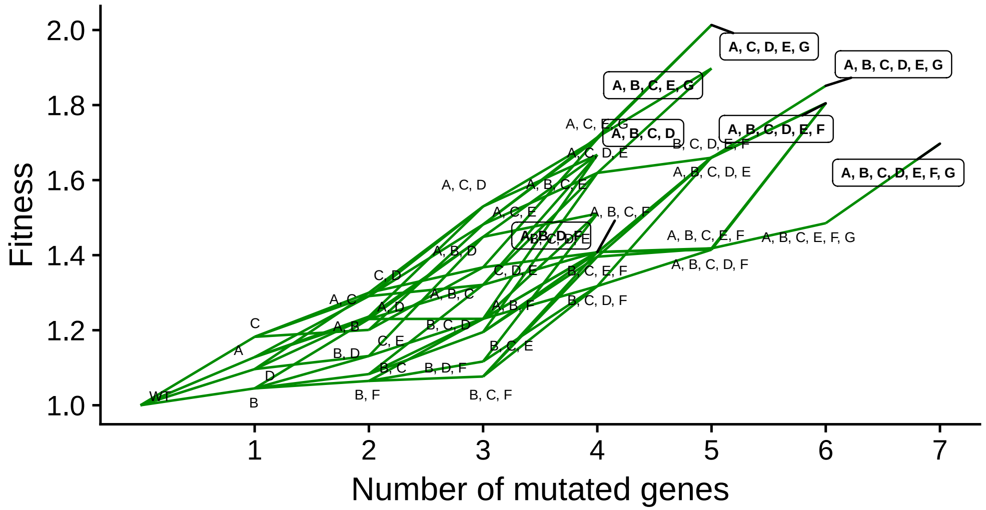

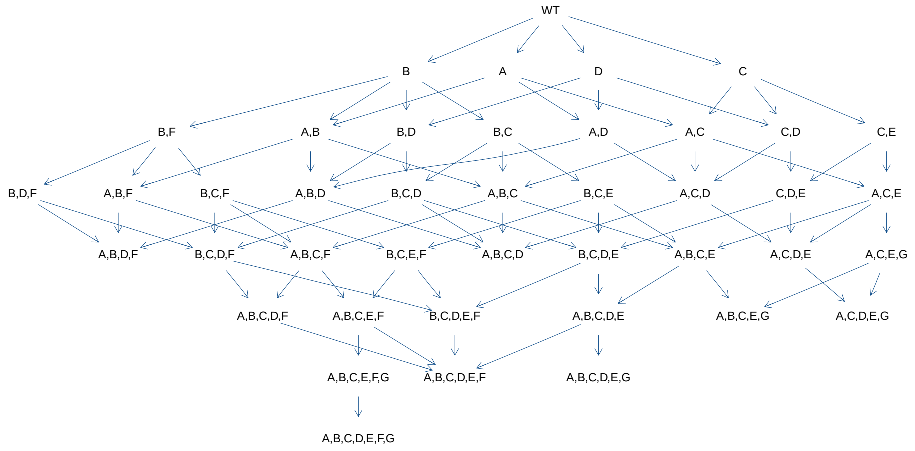

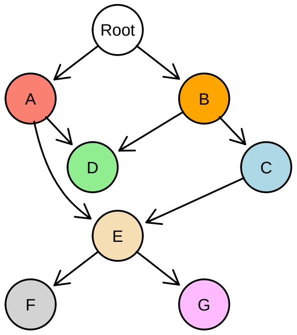

ID	mZBzm7cVIBm3NJil
acc genotypes	16
peaks no back	2
removed edges	2
prop rem edges	0.083

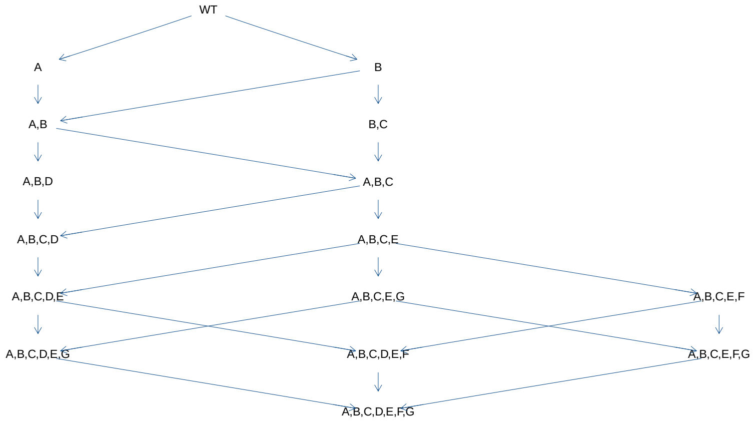

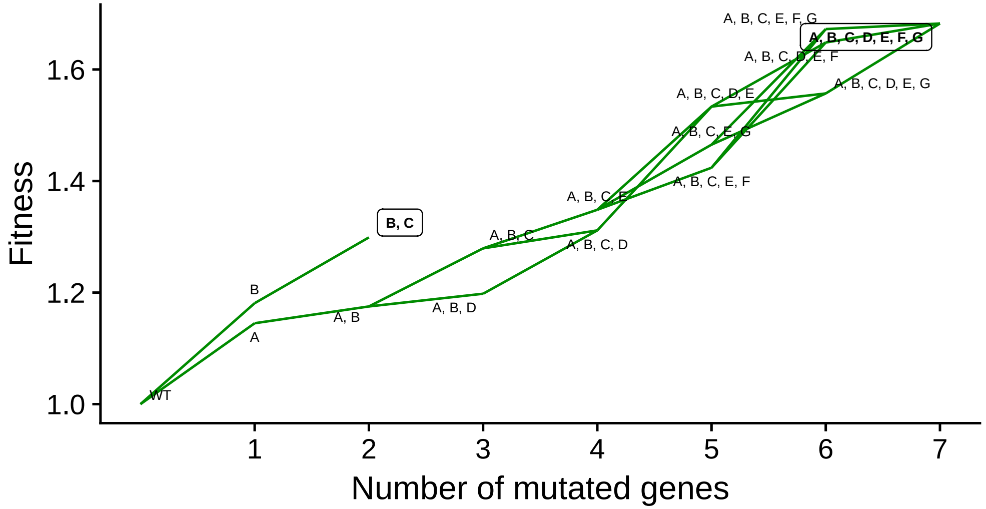

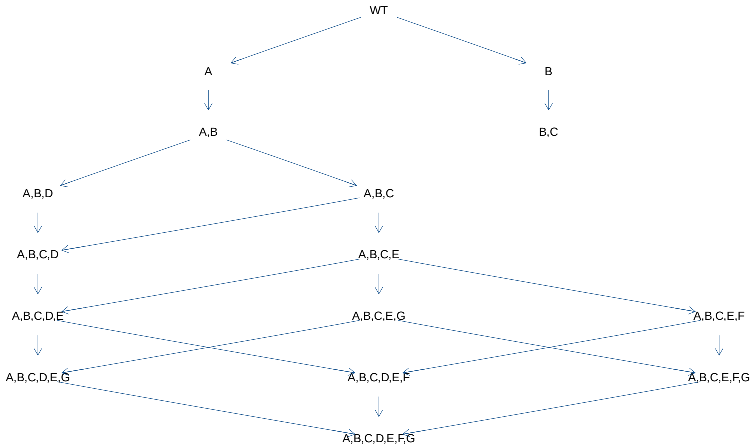

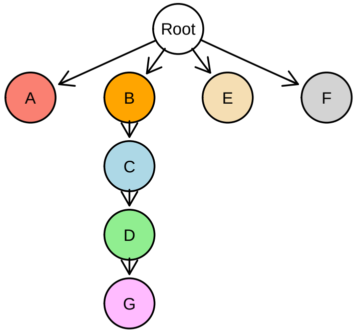

ID	N7LwhGSvS3wDt8P2
acc genotypes	40
peaks no back	7
removed edges	13
prop rem edges	0.141

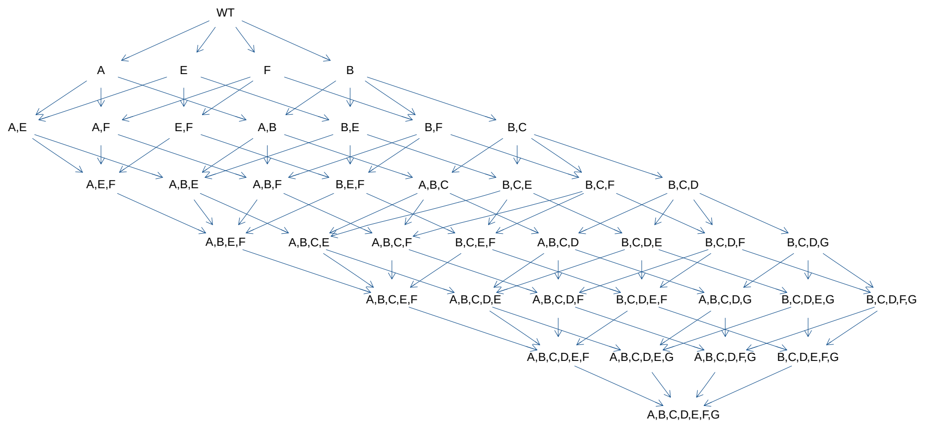

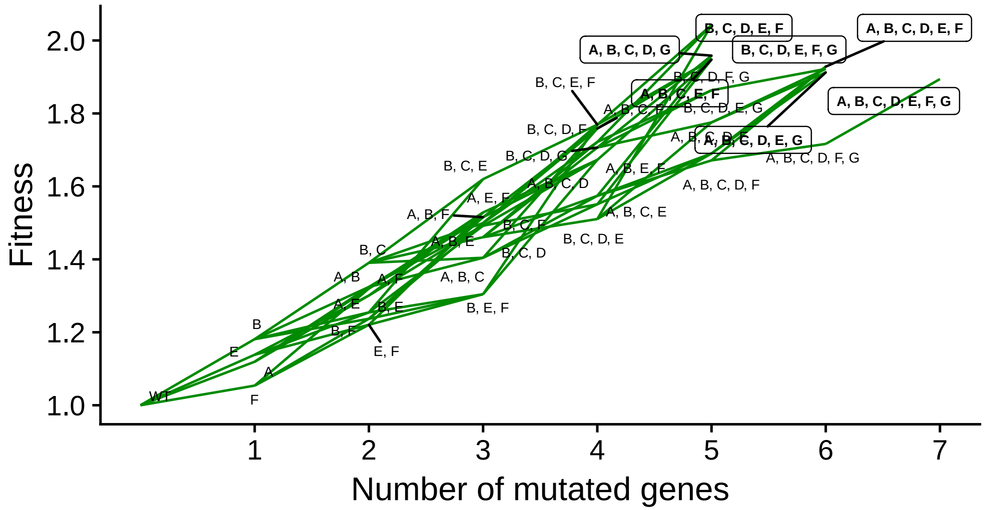

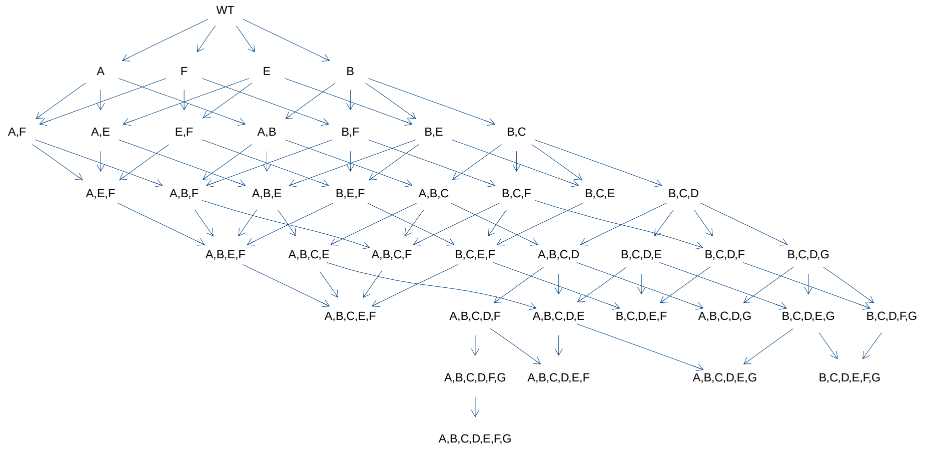

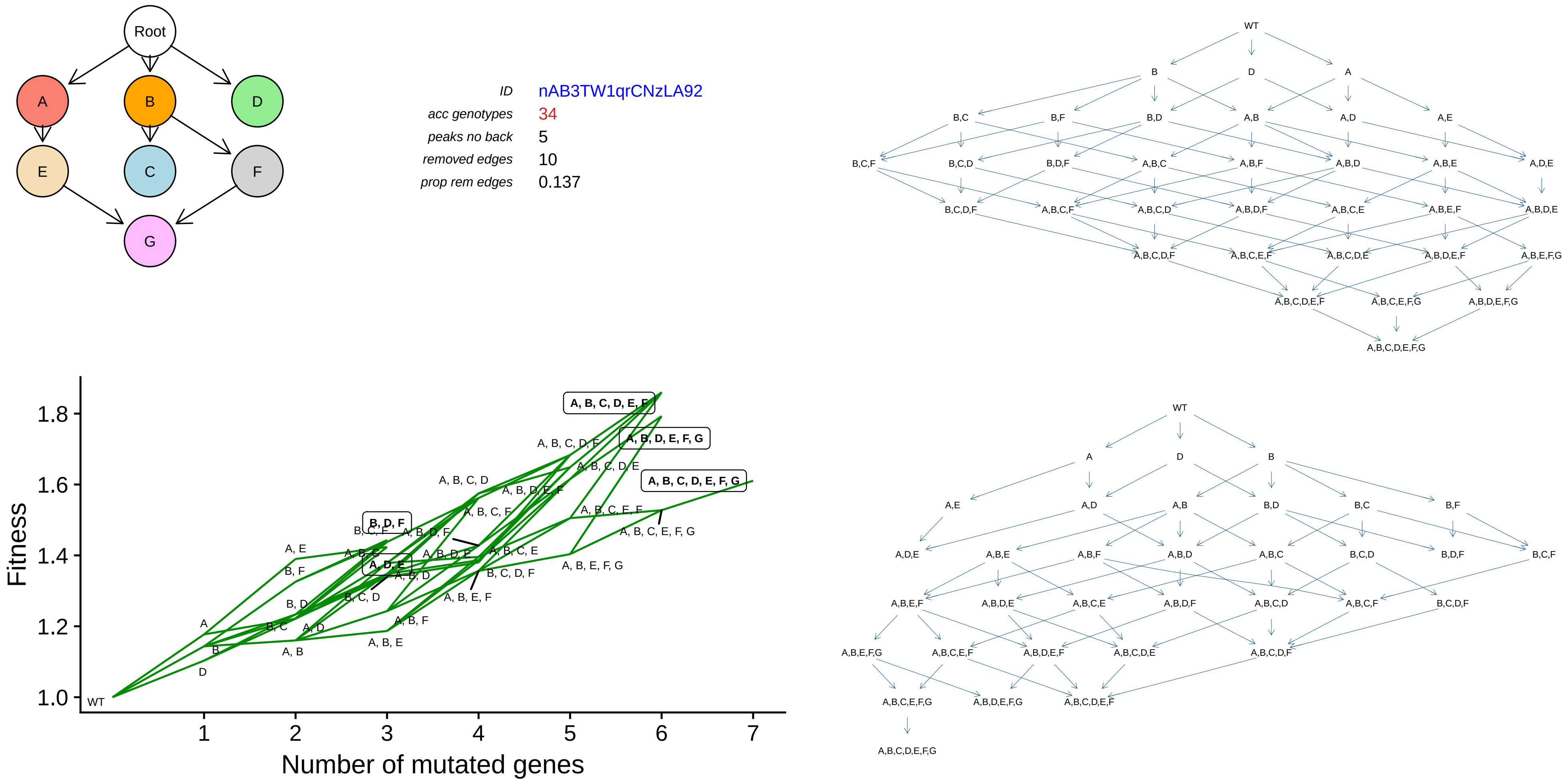

Root
A
B
D
E
C
F
G
ID nAB3TW1qrCNzLA92
acc genotypes 34
peaks no back 5
removed edges 10
prop rem edges 0.137
Fitness
Number of mutated genes

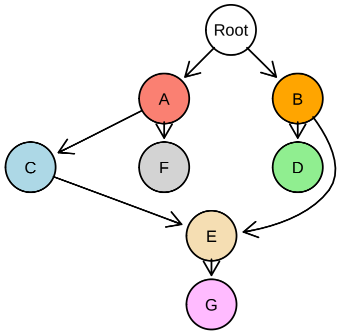

ID	Nc8hEZJTul6Jz10xm
acc genotypes	23
peaks no back	5
removed edges	9
prop rem edges	0.22

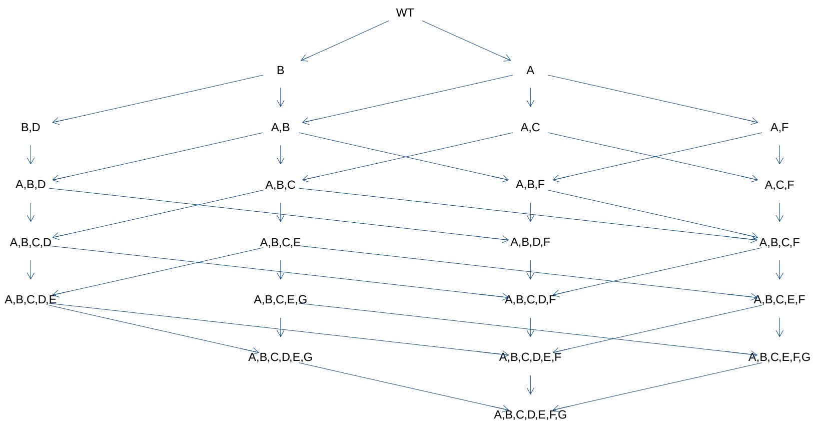

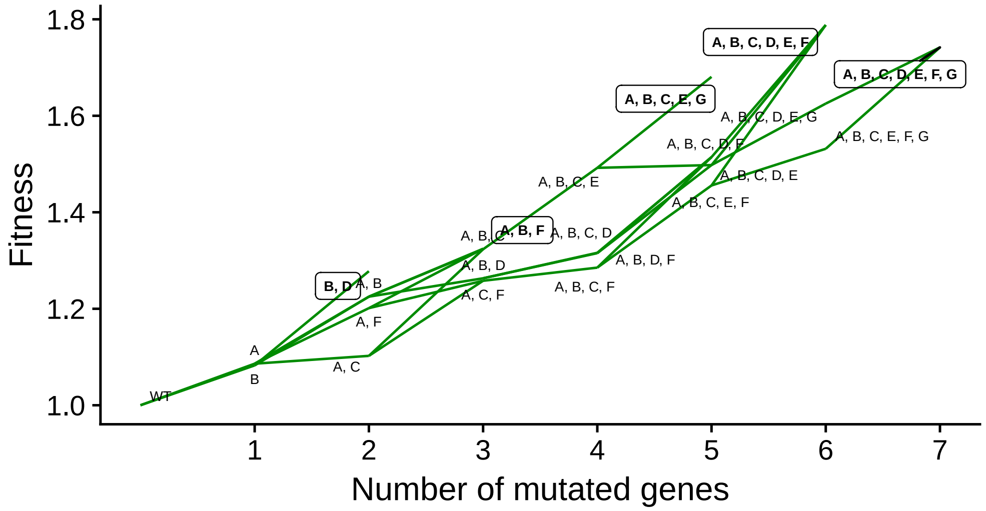

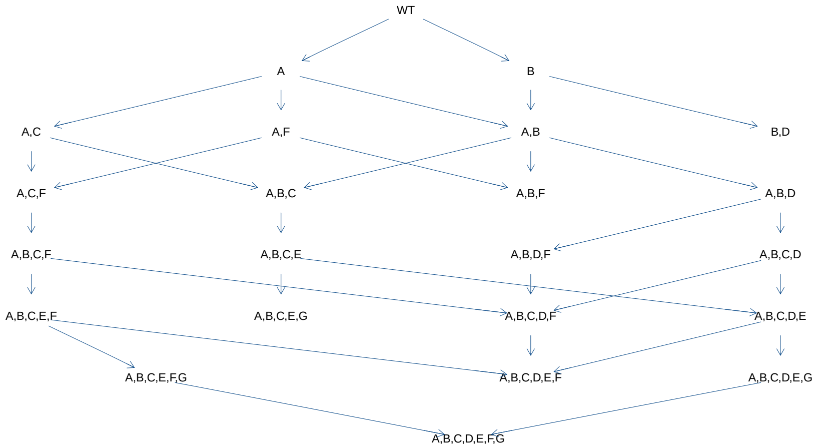

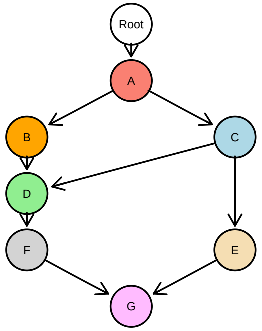

ID	nCI8mAj78ZzZKlBn
acc genotypes	12
peaks no back	2
removed edges	1
prop rem edges	0.067

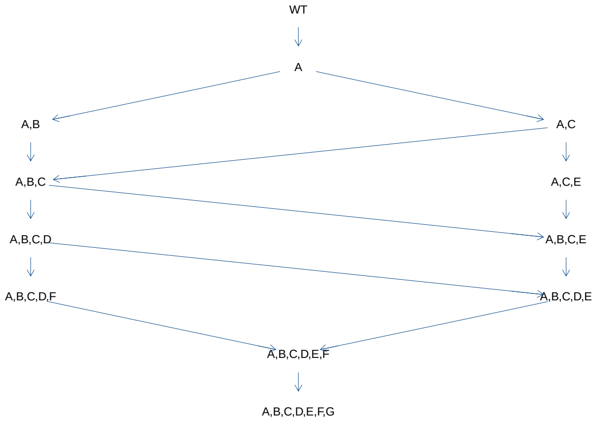

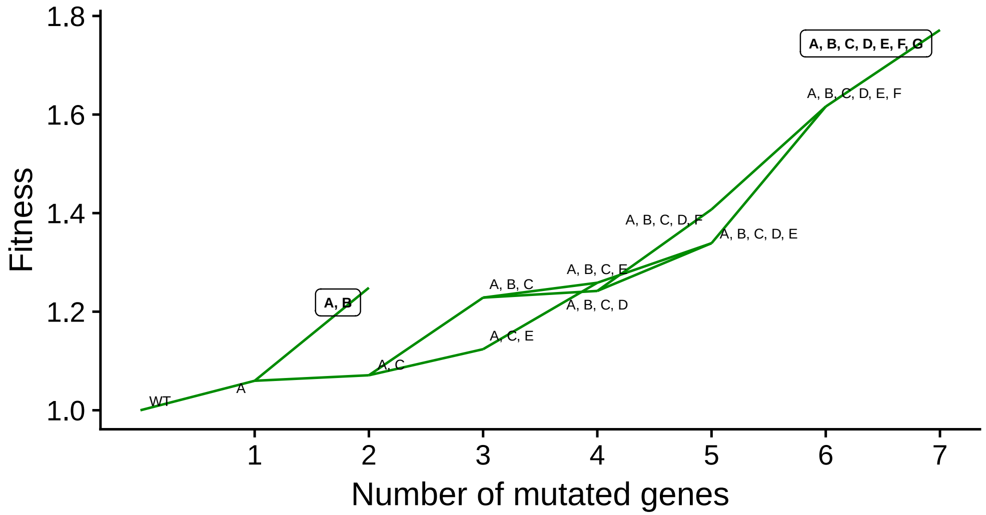

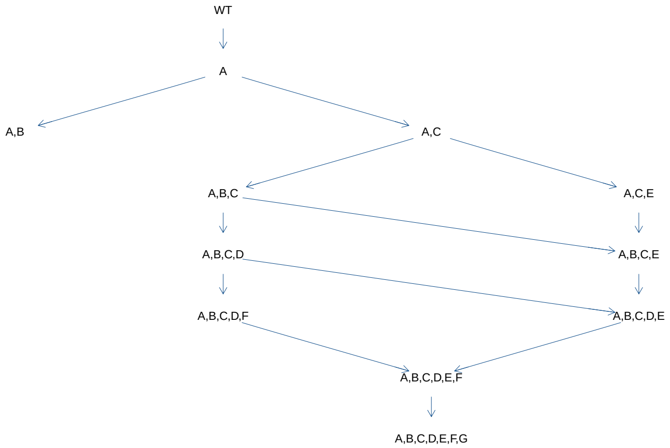

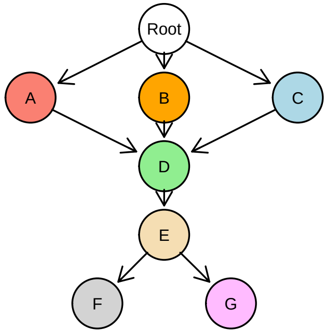

ID	nKSE4LBW1z9FriV10
acc genotypes	13
peaks no back	2
removed edges	2
prop rem edges	0.111

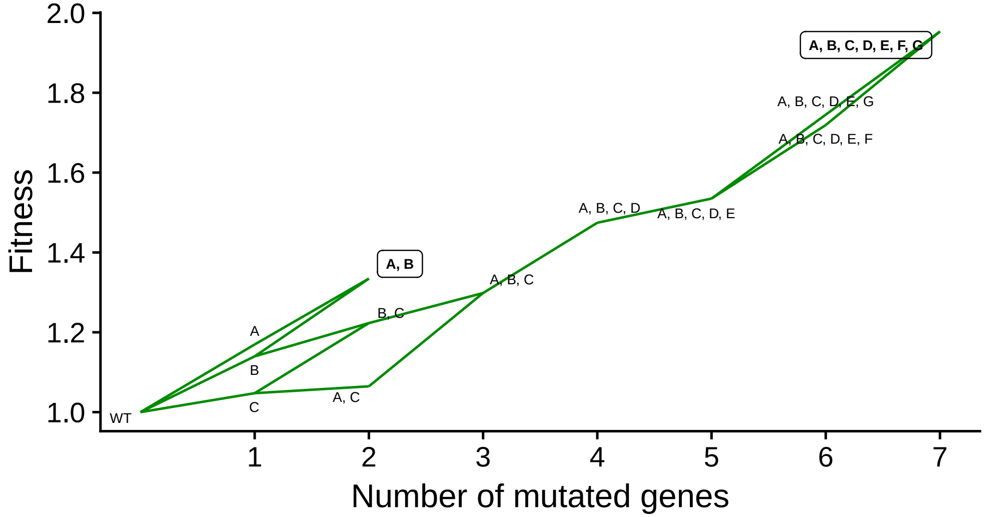

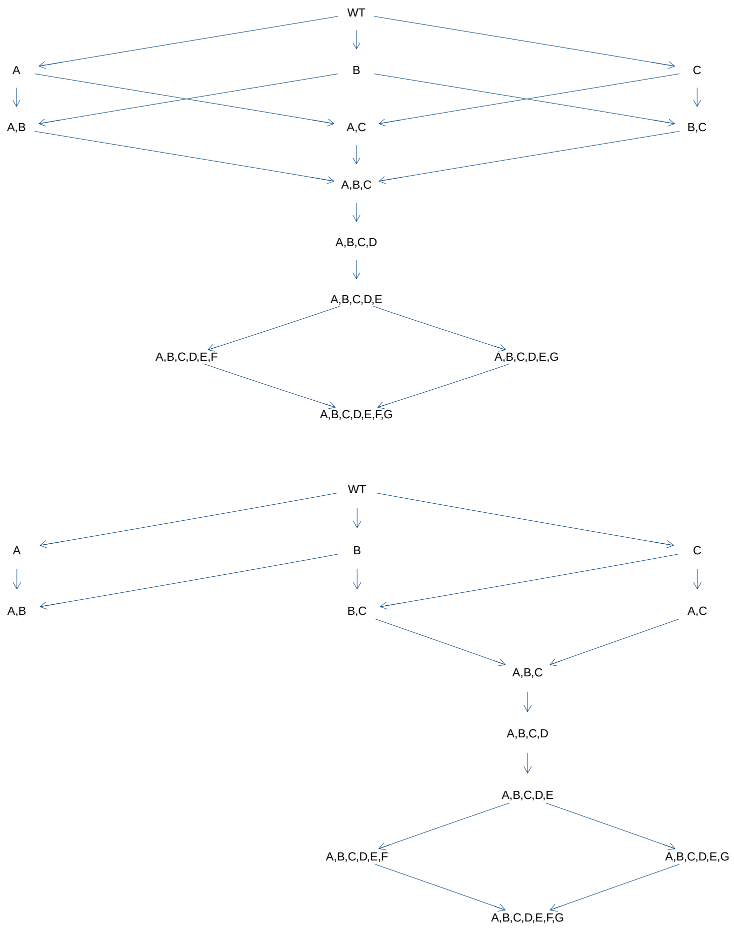

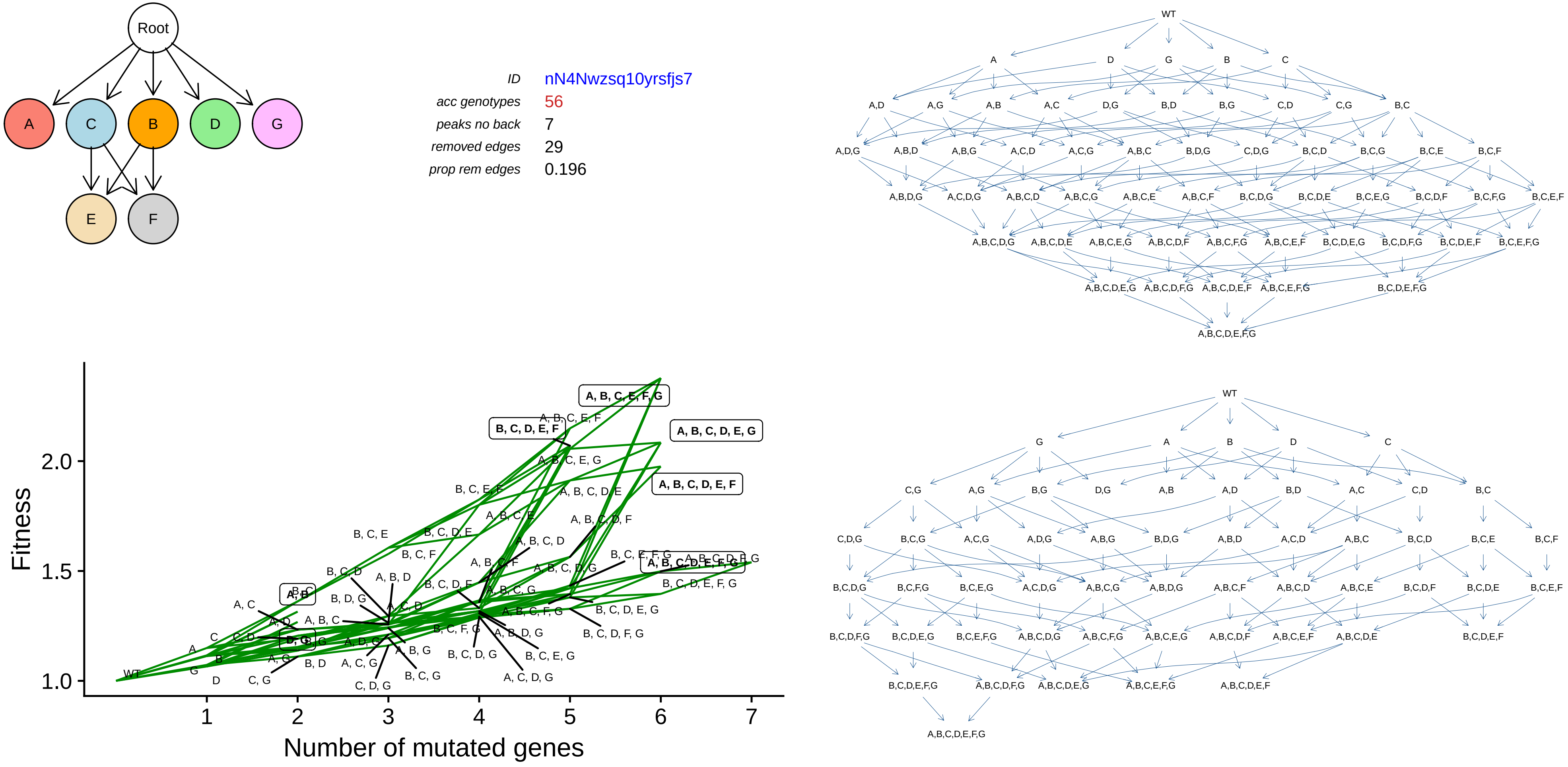
Root
A
C
B
D
G
E
F
ID nN4Nwzsq10yrsfjs7
acc genotypes 56
peaks no back 7
removed edges 29
prop rem edges 0.196
Fitness
Number of mutated genes
A, B, C, E, F, G
B, C, D, E, F
A, B, C, D, E, G
A, B, C, D, E, F
WT
A,B,C,D,E,F,G

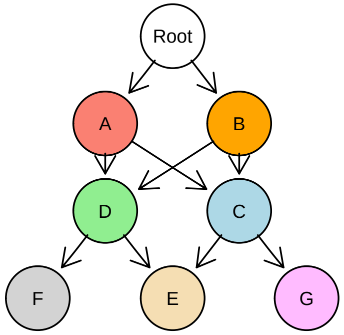

ID	NnQqInZk8XUxrSDx
acc genotypes	16
peaks no back	5
removed edges	5
prop rem edges	0.208

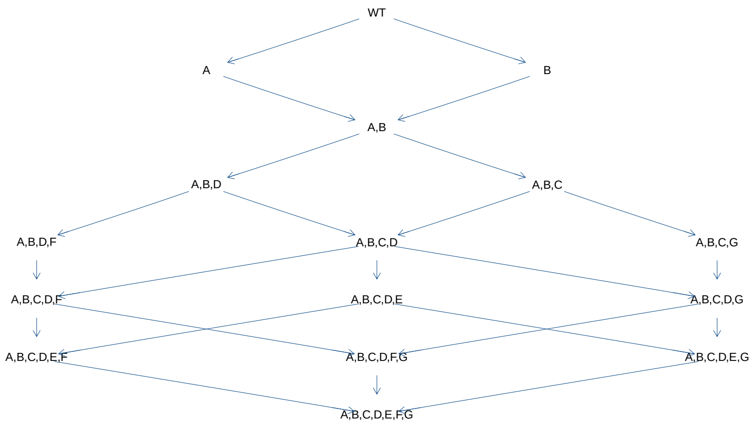

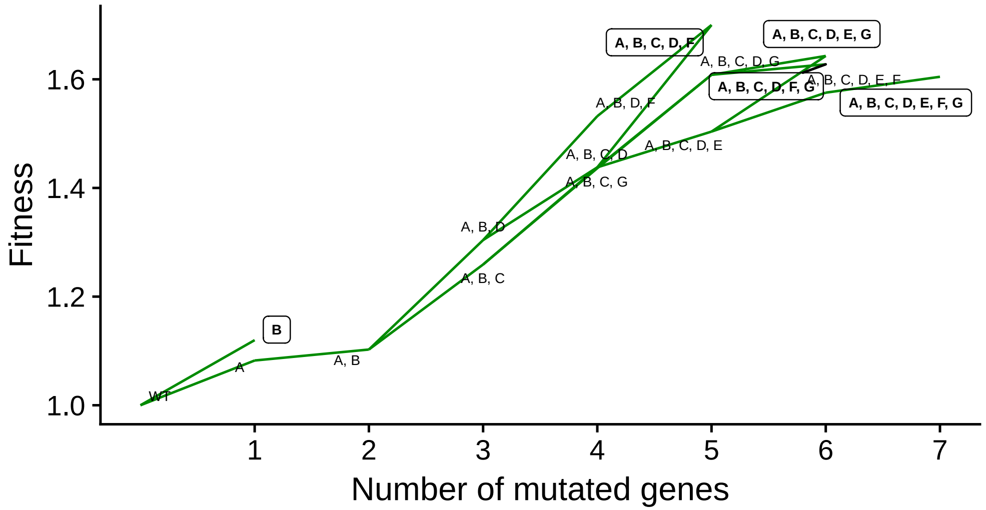

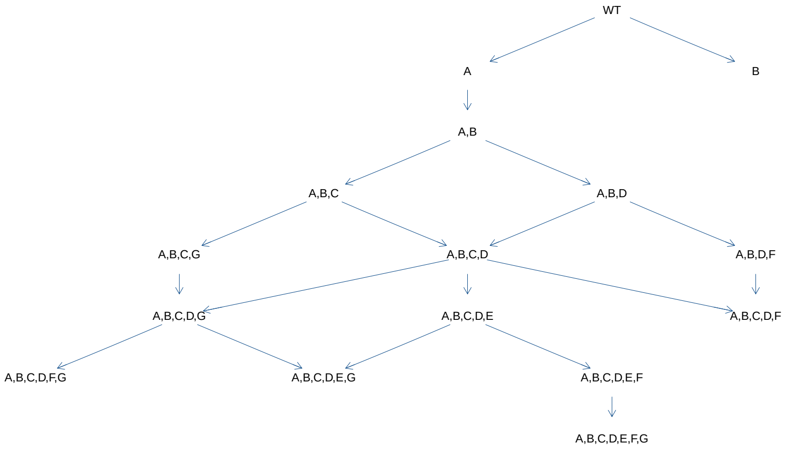

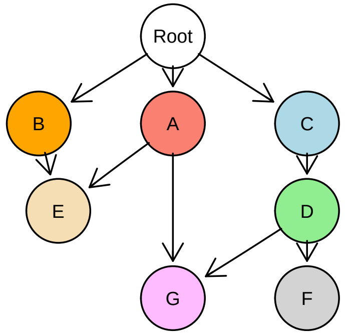

ID	nX7dlkO9zvyl4JCM
acc genotypes	26
peaks no back	2
removed edges	5
prop rem edges	0.104

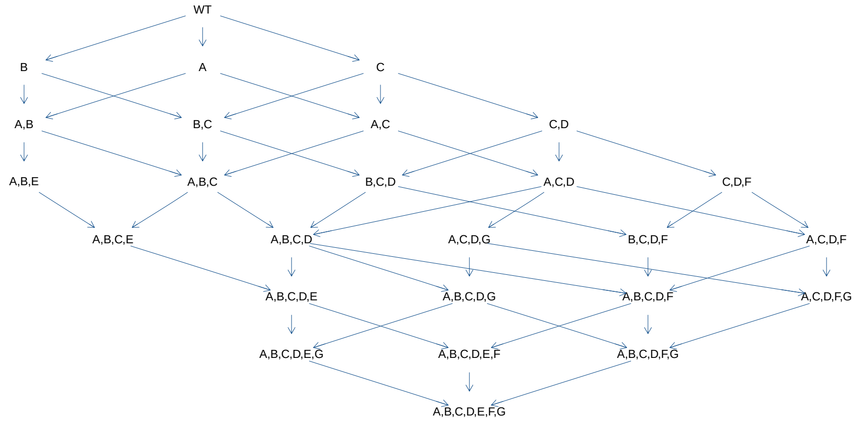

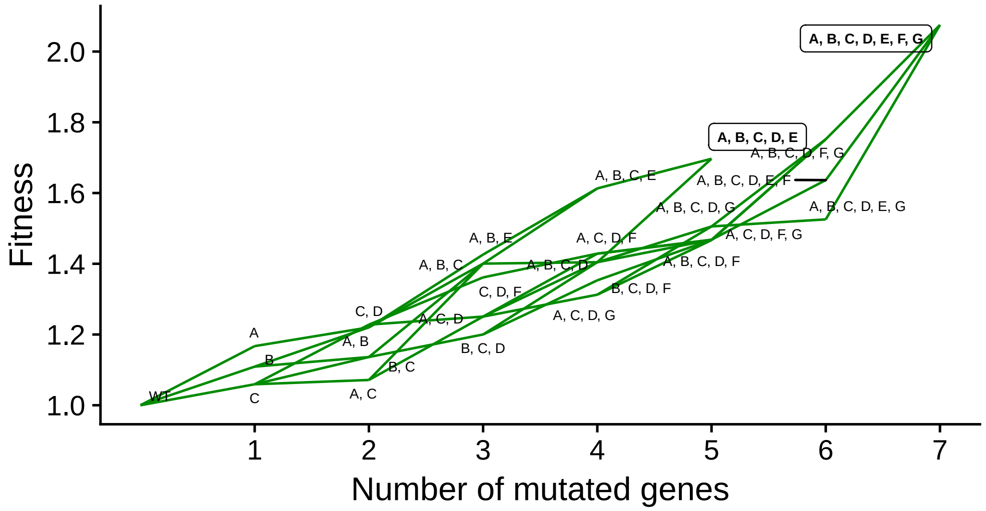

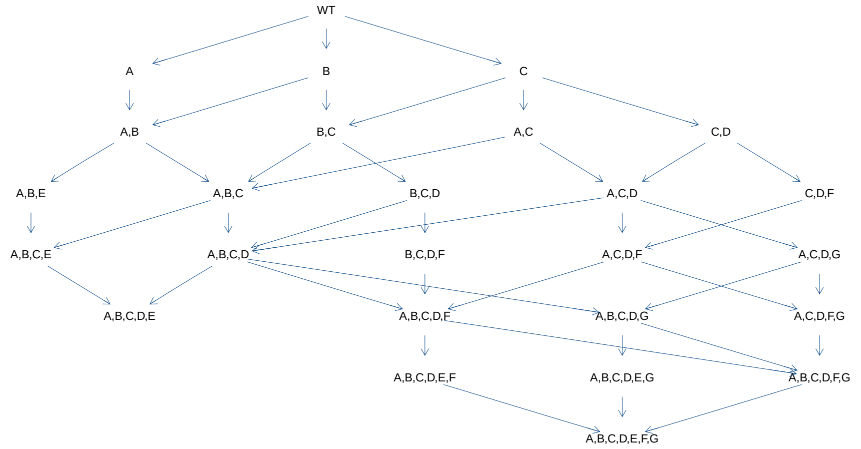

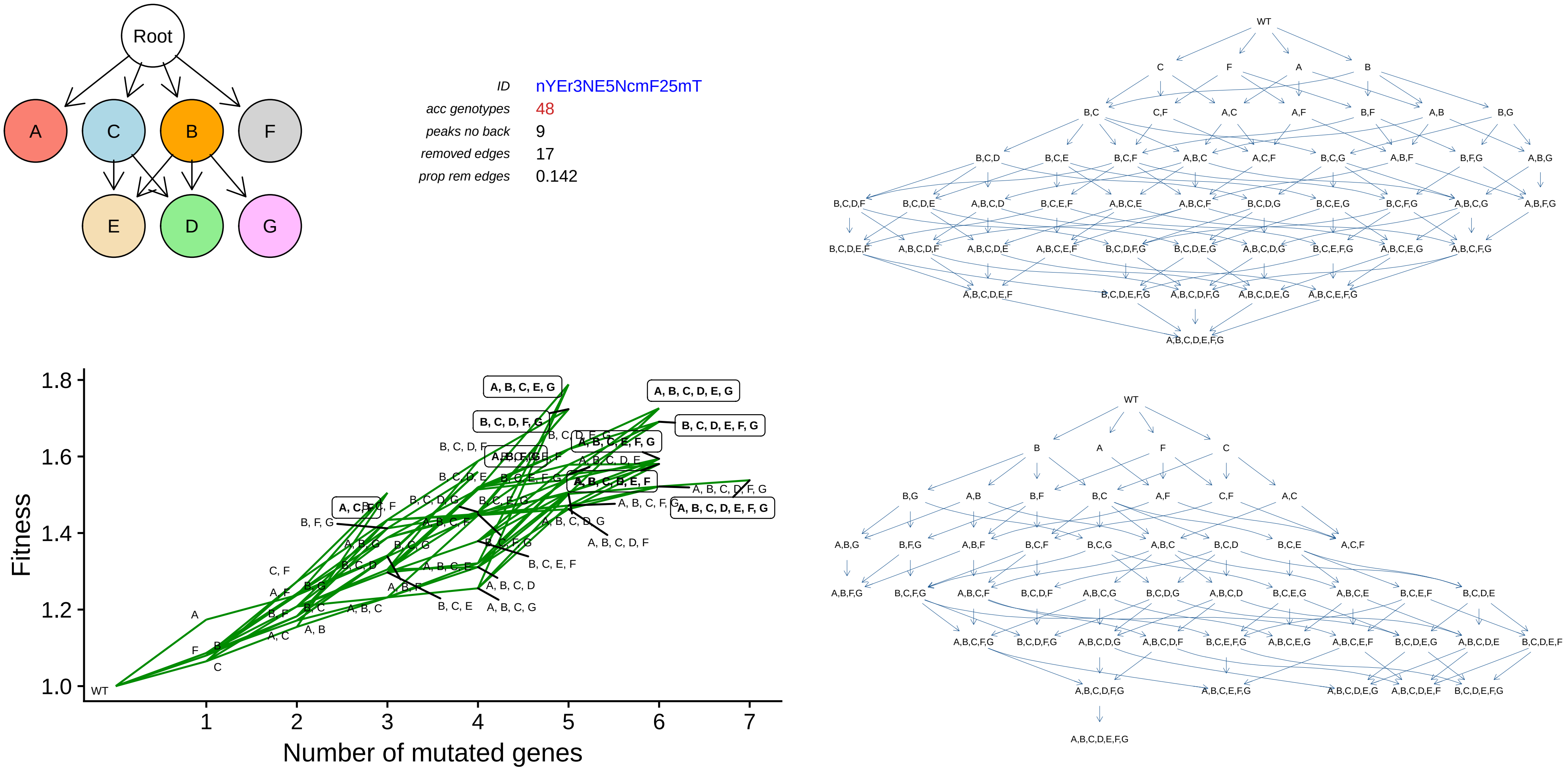
Root
A
C
B
F
E
D
G
ID nYEr3NE5NcmF25mT
acc genotypes 48
peaks no back 9
removed edges 17
prop rem edges 0.142
Fitness
Number of mutated genes
WT
A, B, C, E, G
A, B, C, D, E, G
B, C, D, E, F, G
B, C, D, F, G
A, B, C, E, F, G
A, B, C, D, E, F
A, B, C, D, E, F, G
A, B, C, D, F, G
A, B, C, D, F
A, B, C, F, G
A, B, C, D, G
B, C, E, F
A, B, C, D
A, B, C, G
B, C, E
A, B, C
A, B
A, C
C, F
B, F, G
A,B,C,D,E,F,G

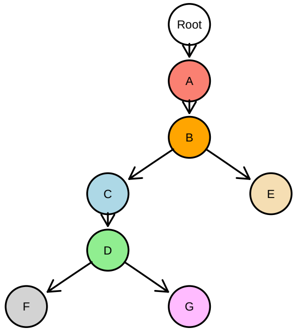

ID	o4Y3hPxIjQmaWRx1
acc genotypes	14
peaks no back	2
removed edges	1
prop rem edges	0.05

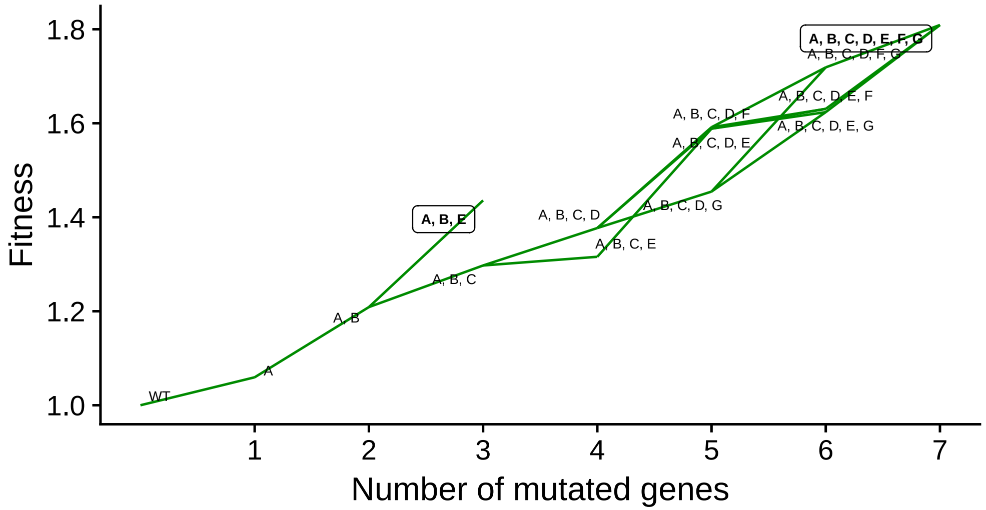

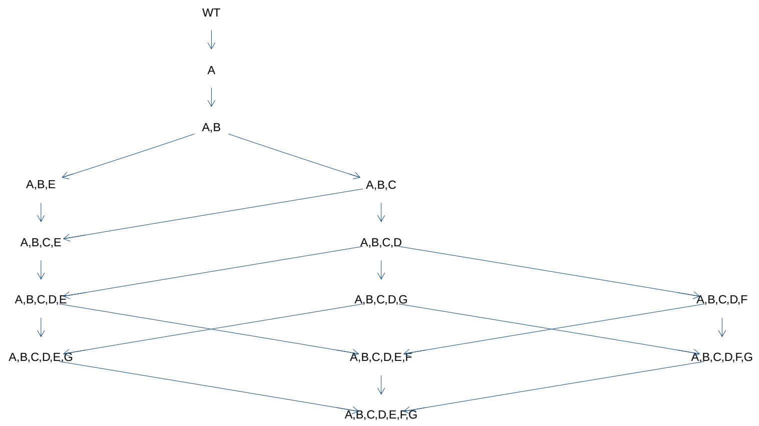

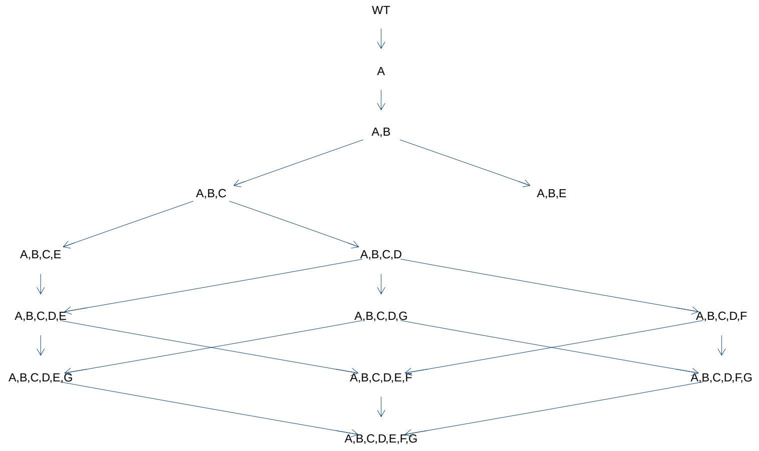

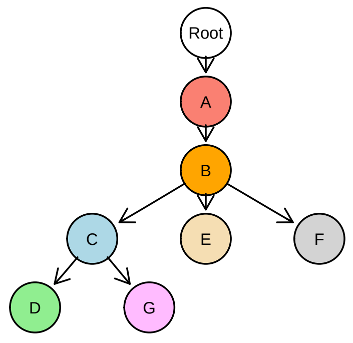

ID	OaMMWW2waVOxtGK3
acc genotypes	22
peaks no back	5
removed edges	5
prop rem edges	0.119

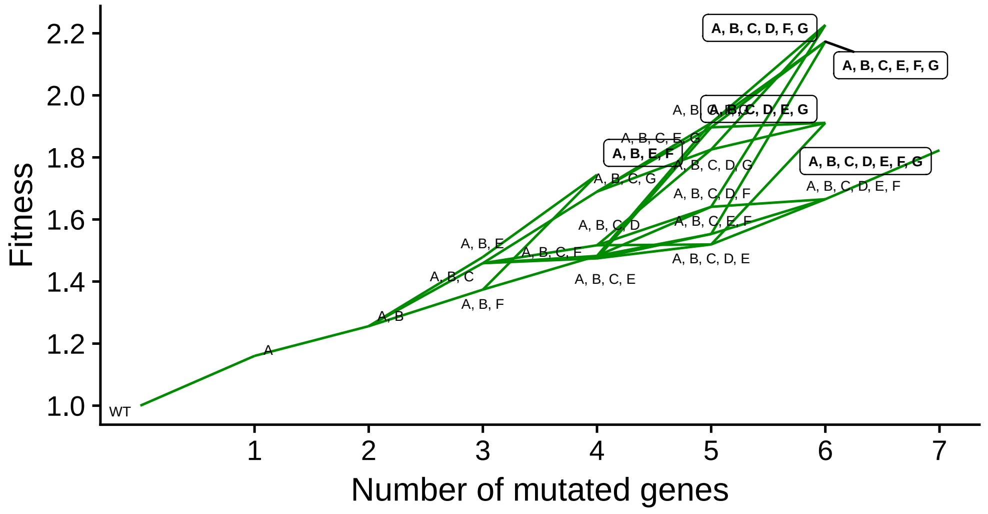

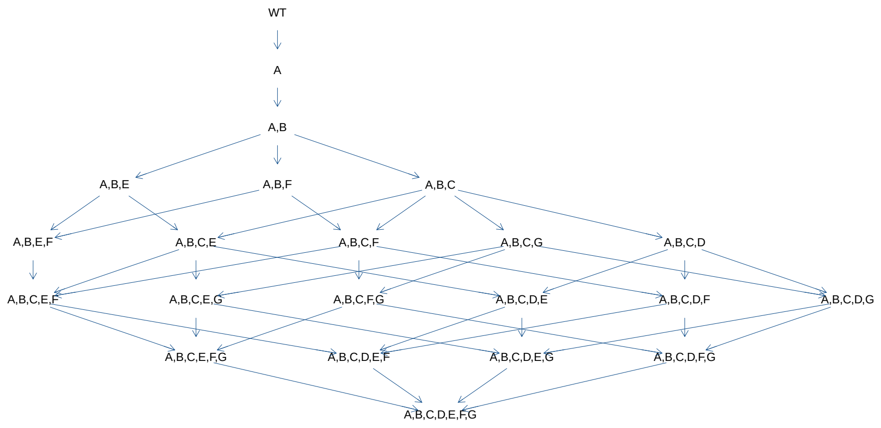

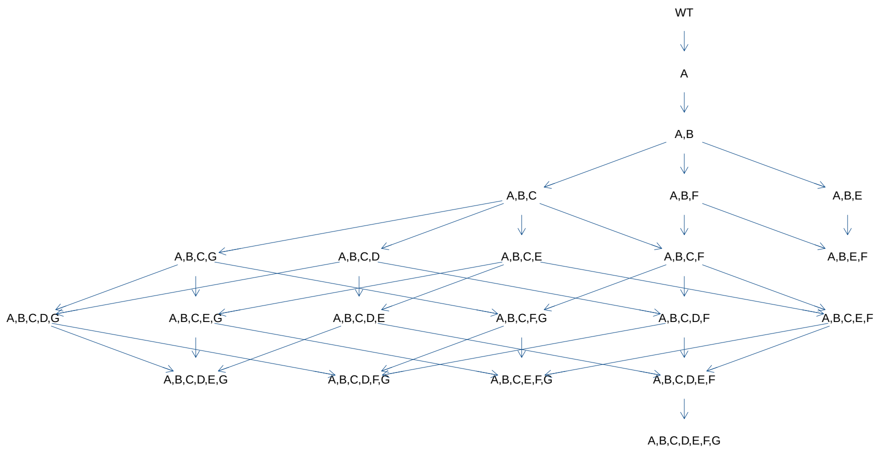

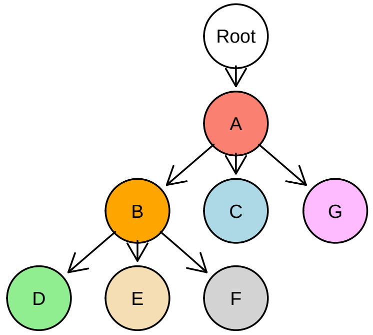

ID	ODbe43XDGuHvXueq
acc genotypes	37
peaks no back	3
removed edges	6
prop rem edges	0.067

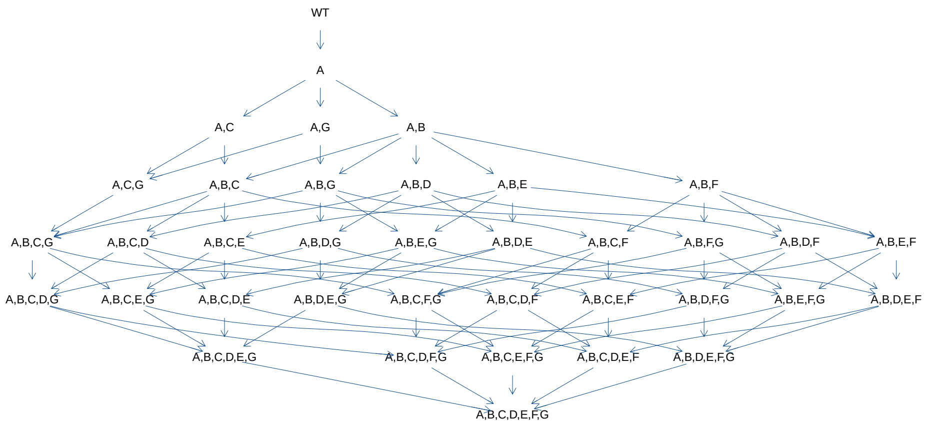

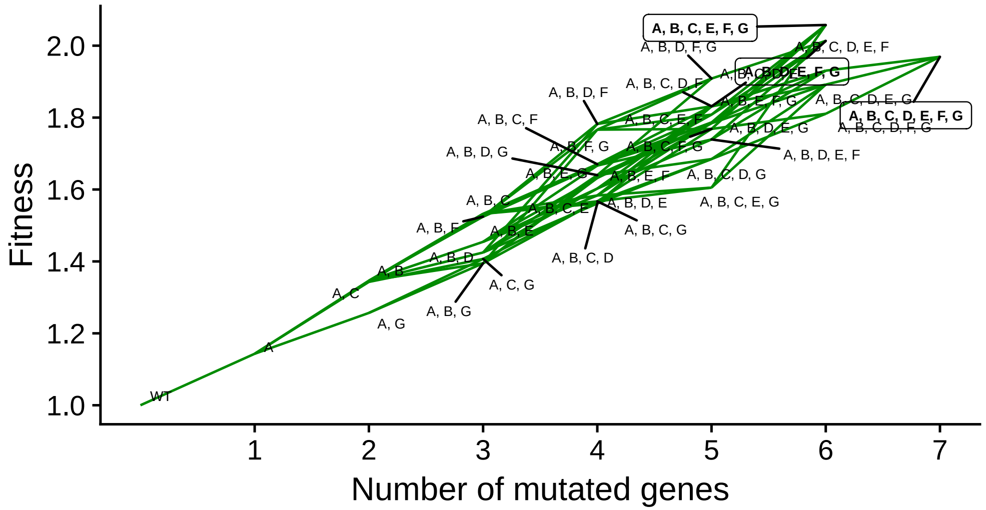

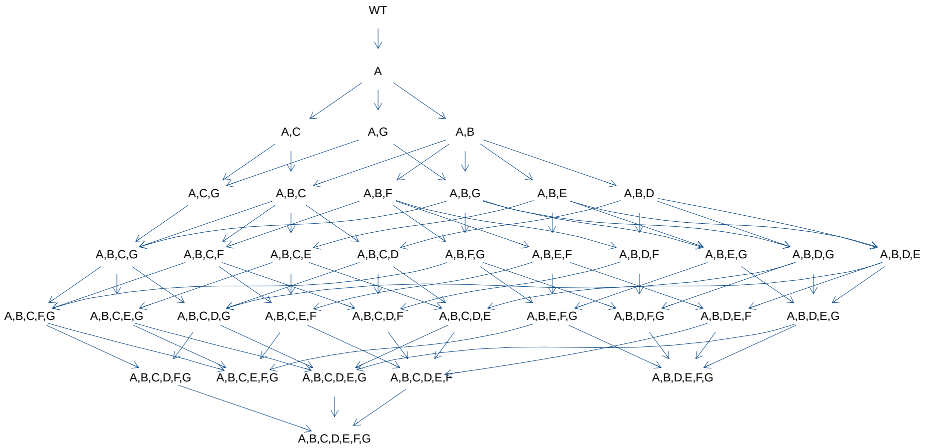

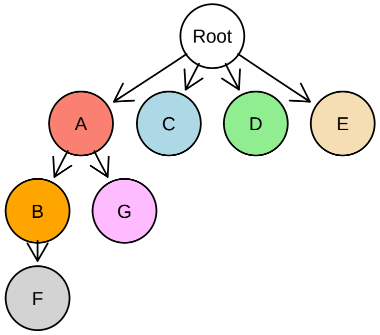

ID	OflKufVm9LrZy9VH
acc genotypes	56
peaks no back	8
removed edges	27
prop rem edges	0.182

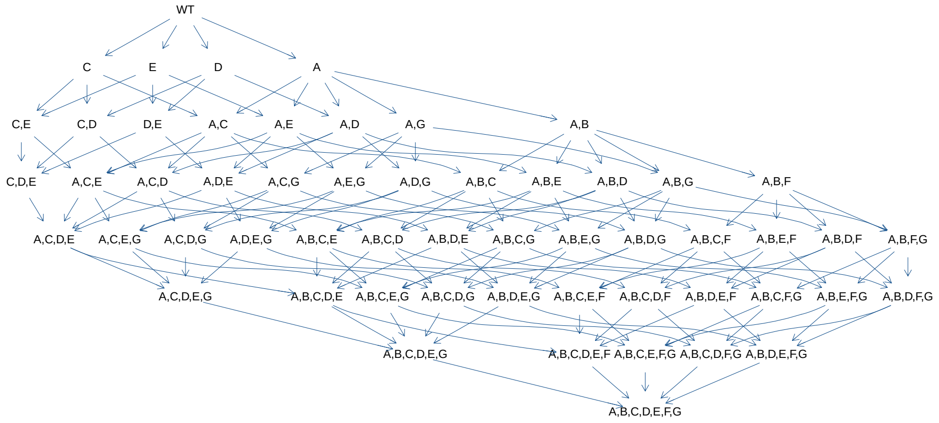

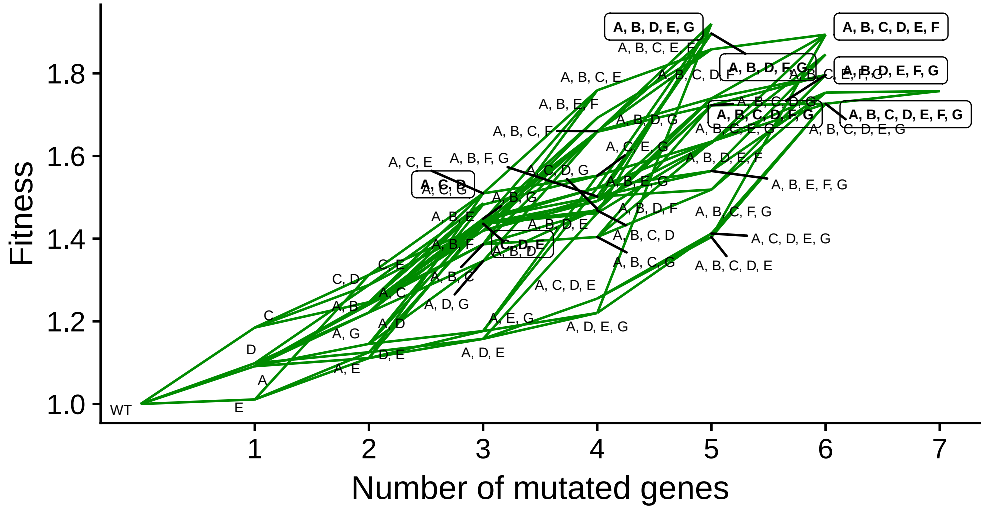

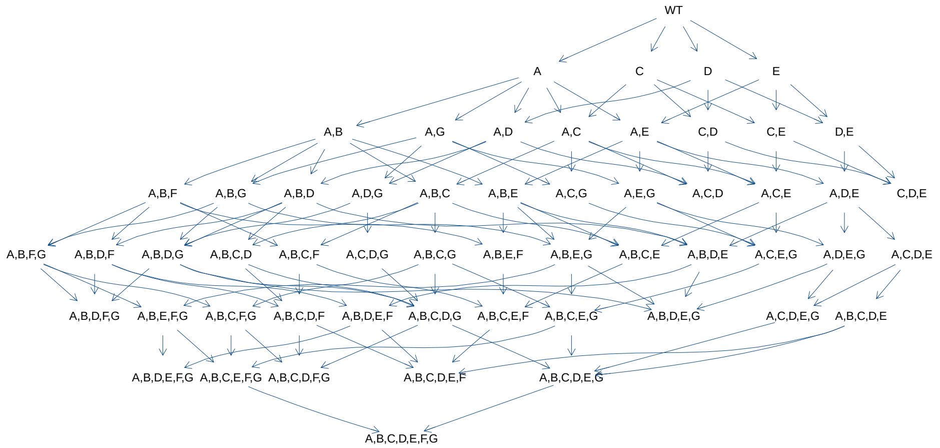

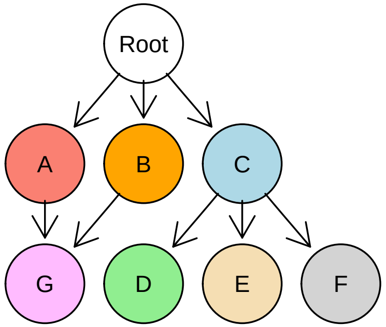

ID	OMDWvgSAoX1WSQy1
acc genotypes	45
peaks no back	3
removed edges	13
prop rem edges	0.118

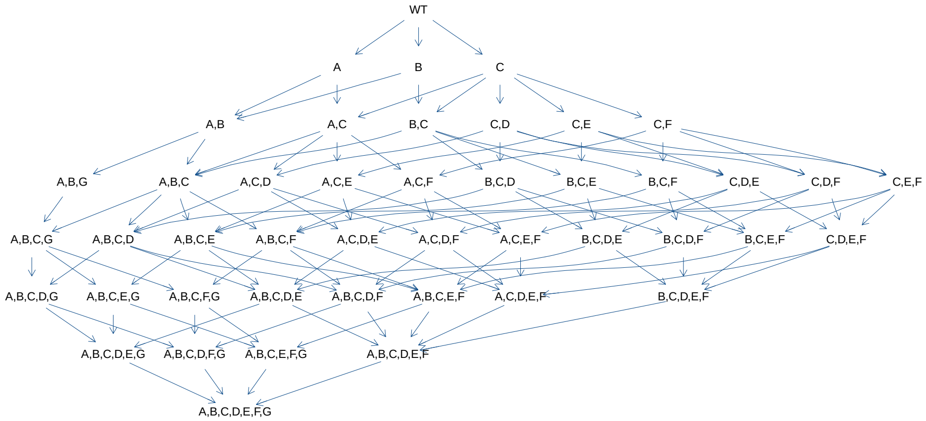

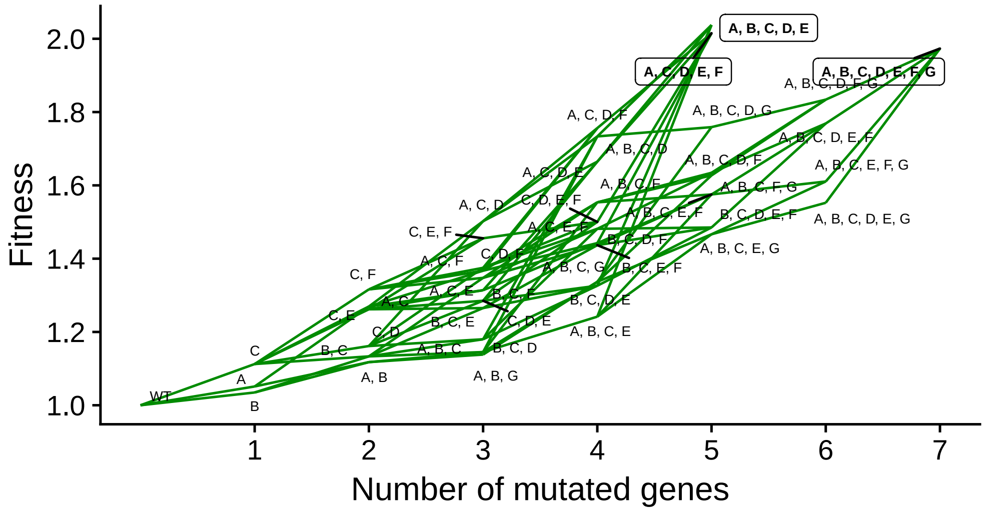

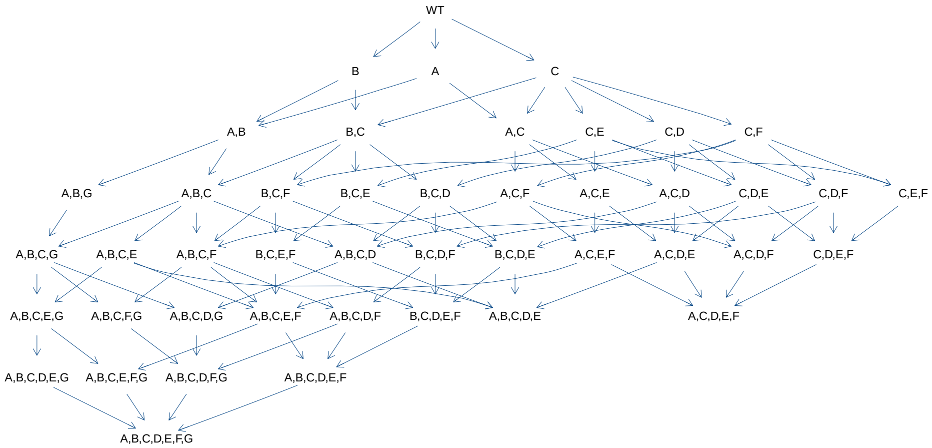

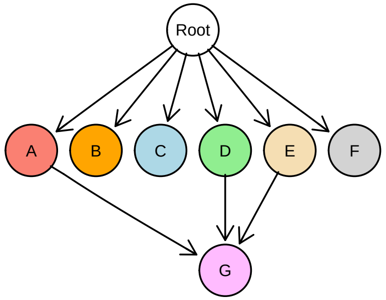

ID	OWPmZOSW53BSDQD7
acc genotypes	72
peaks no back	9
removed edges	44
prop rem edges	0.208

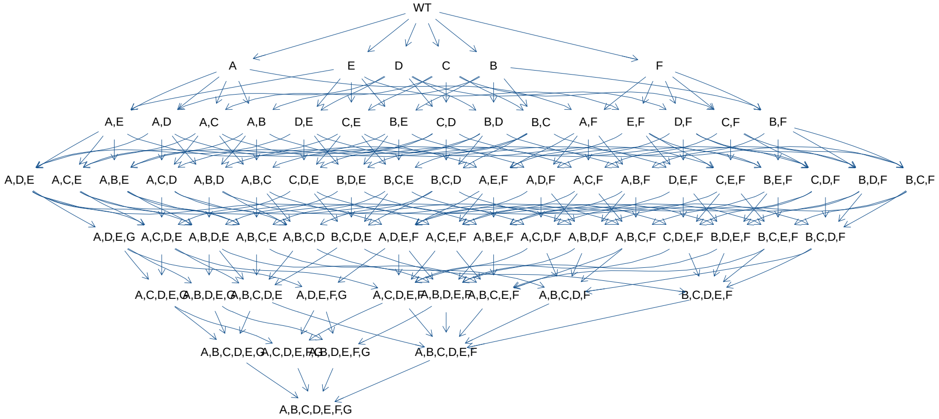

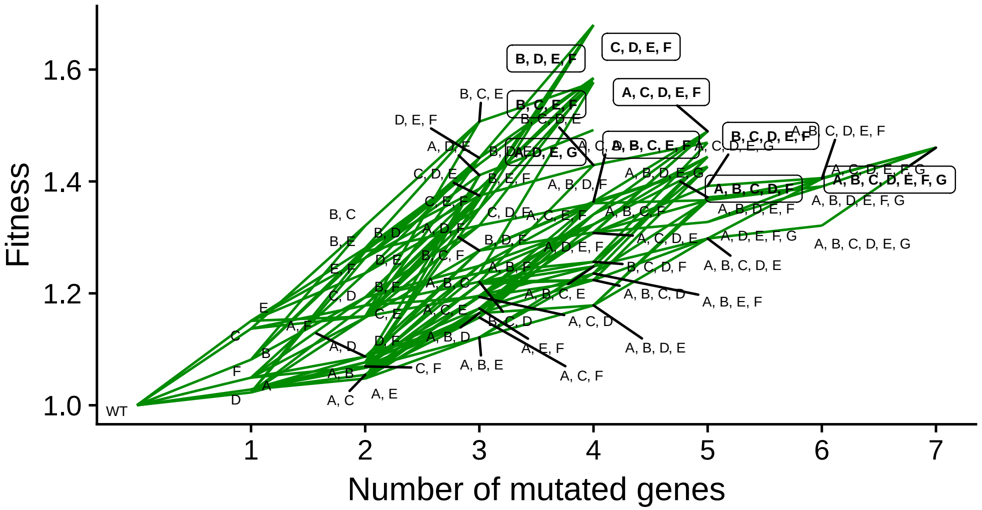

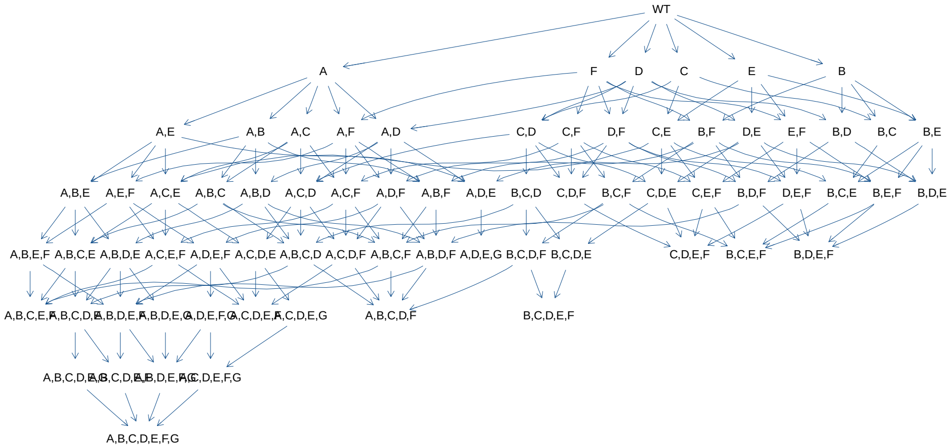

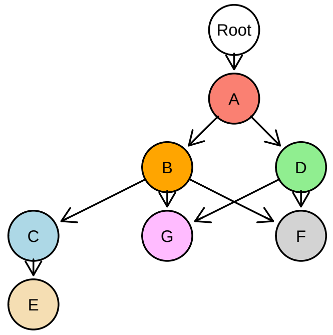

ID	OyMB3UqWrH3e9QQY
acc genotypes	18
peaks no back	4
removed edges	3
prop rem edges	0.103

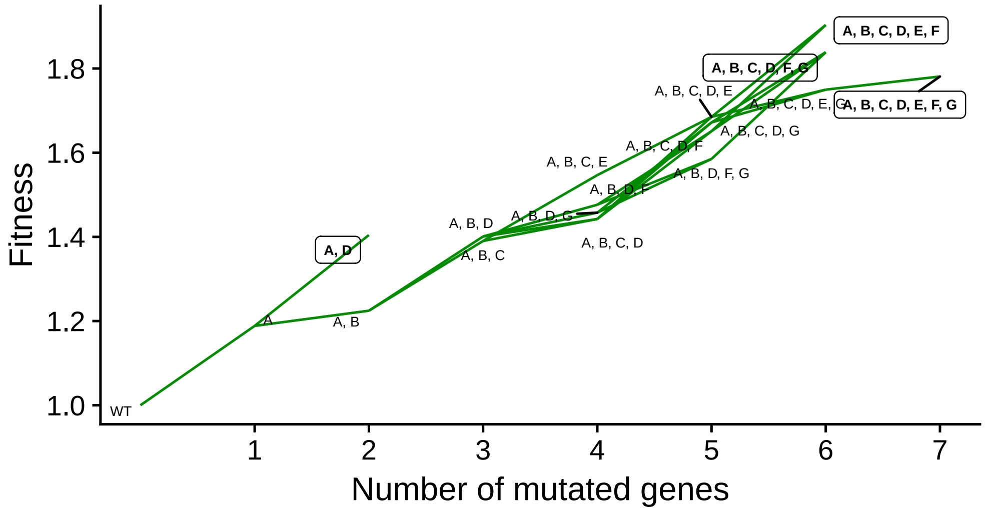

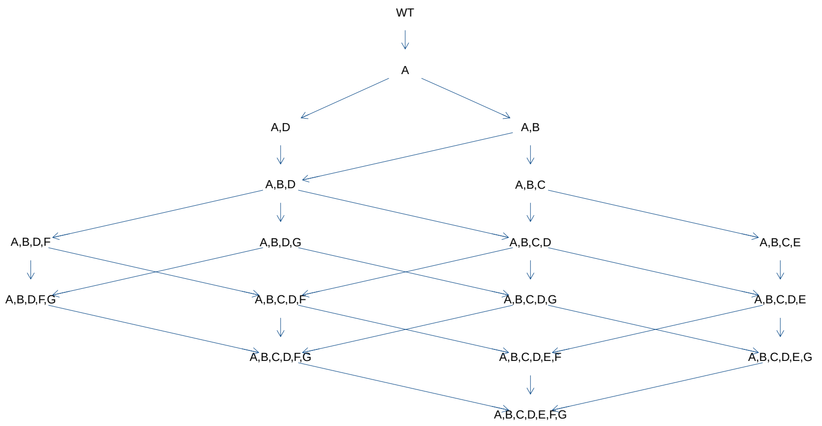

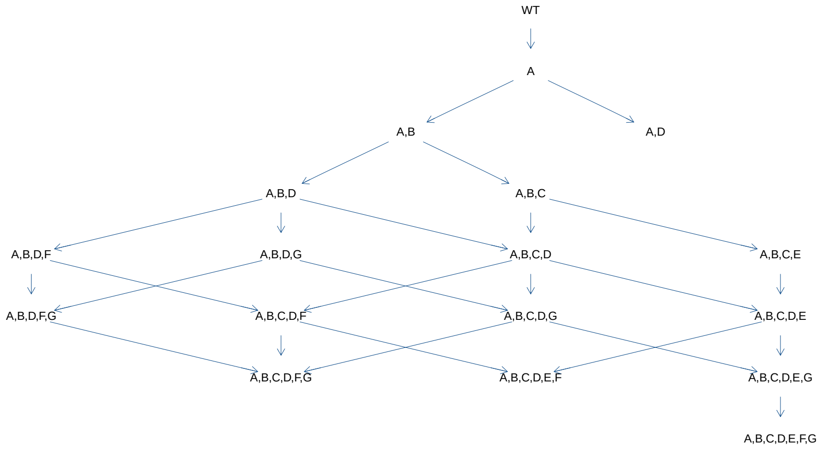

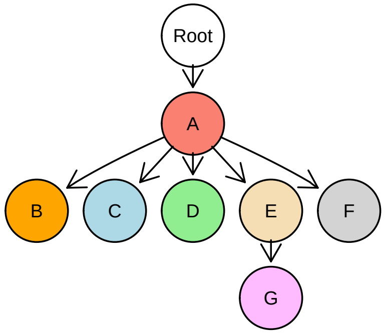

ID	PKsB3Byp3SIOQBQf
acc genotypes	49
peaks no back	7
removed edges	20
prop rem edges	0.155

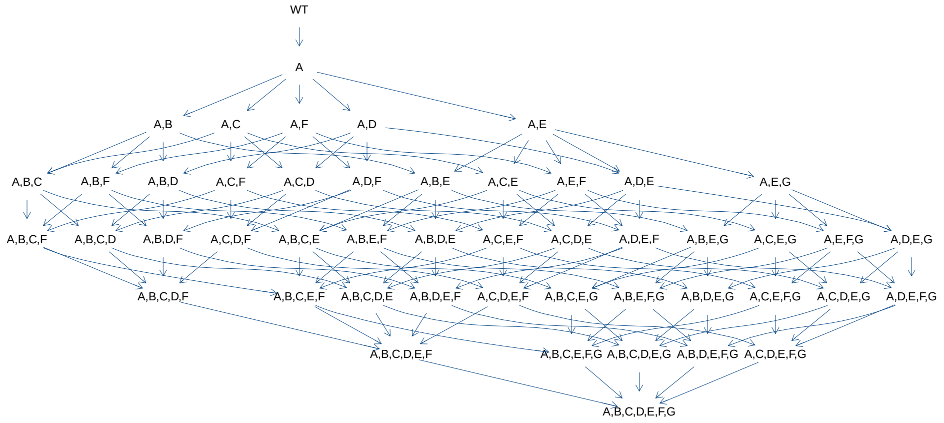

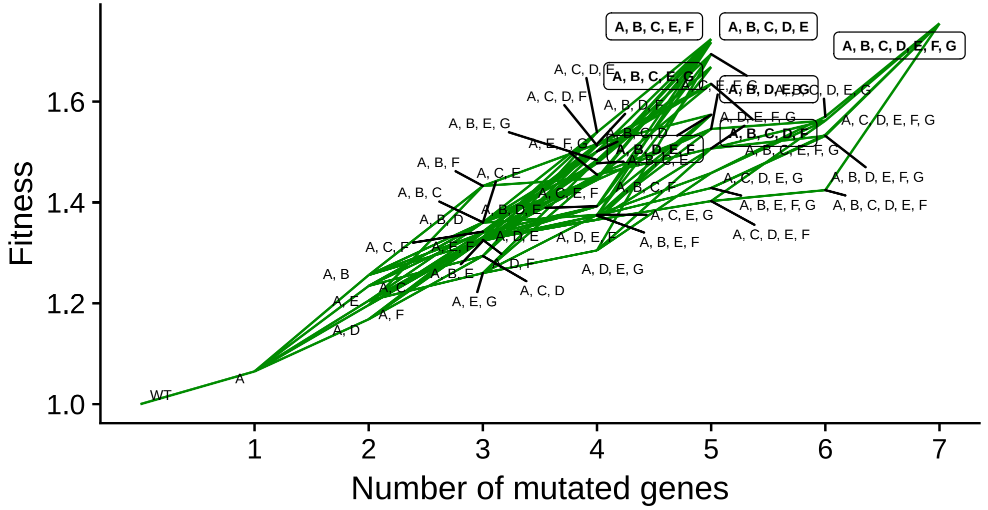

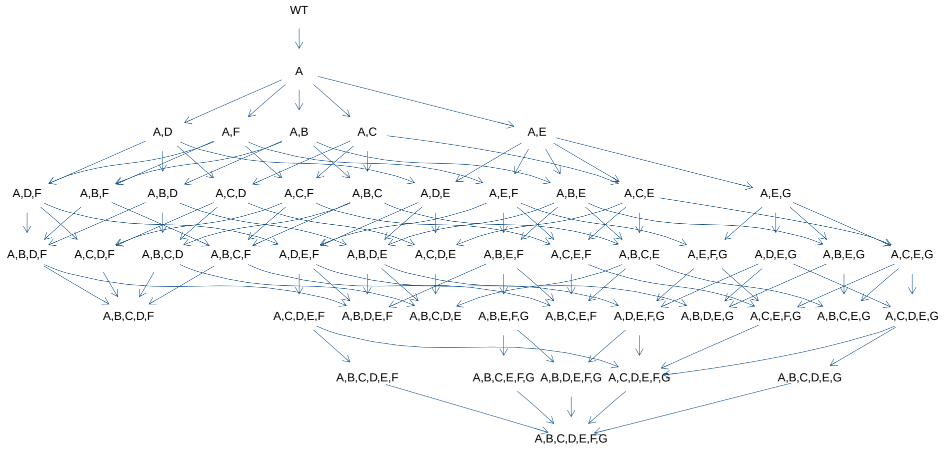

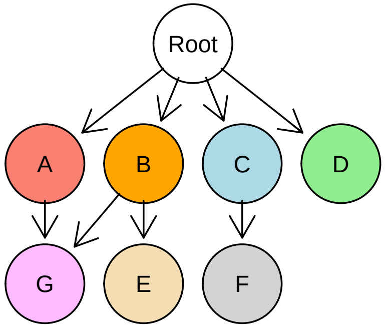

ID	pP2pcse7FrkoEWZp
acc genotypes	48
peaks no back	6
removed edges	20
prop rem edges	0.172

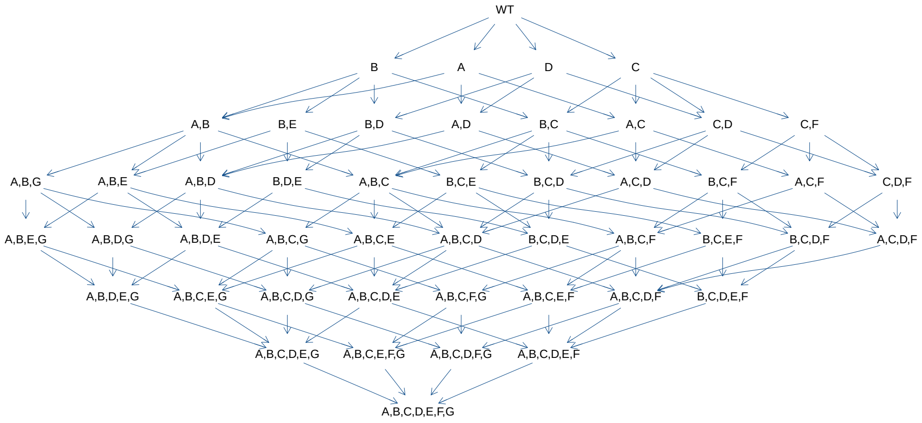

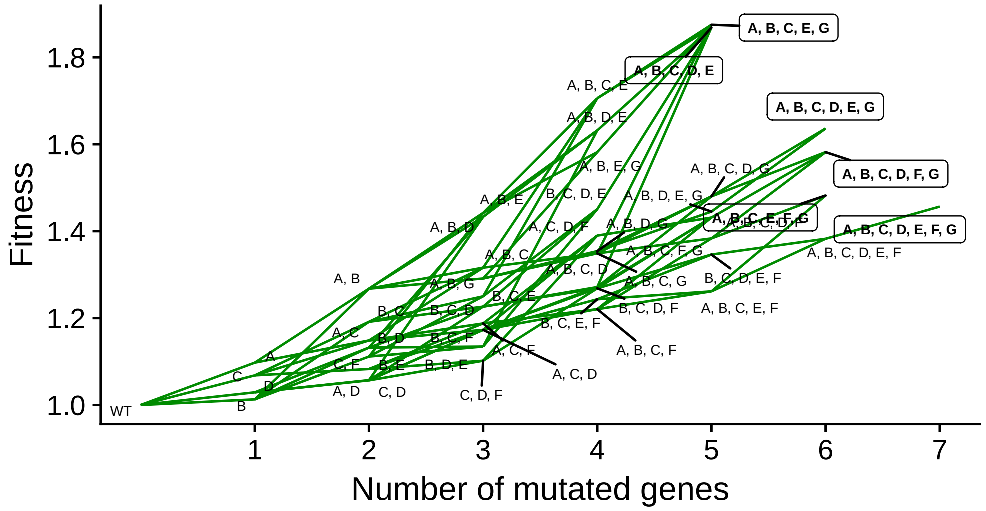

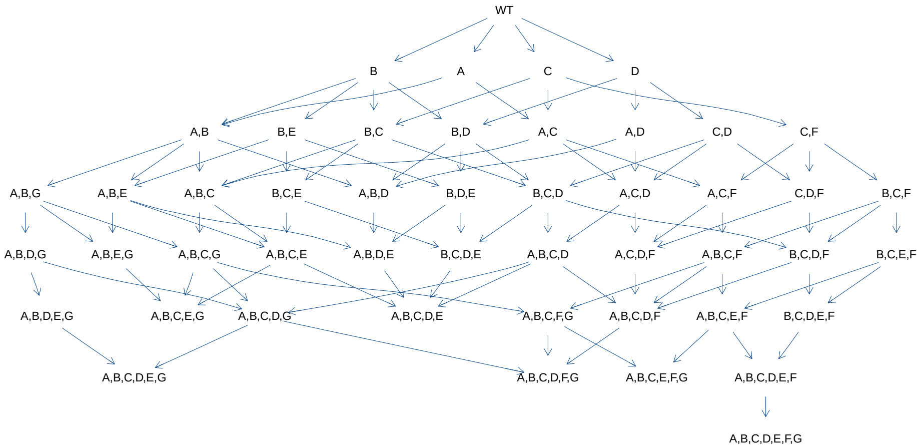

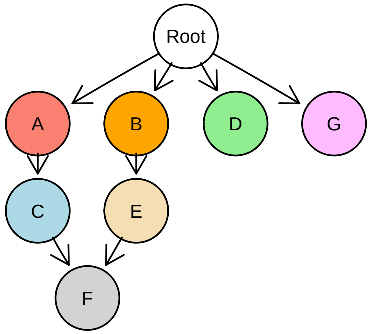

ID	PPvpOC9kPl3xygTj
acc genotypes	40
peaks no back	8
removed edges	19
prop rem edges	0.207

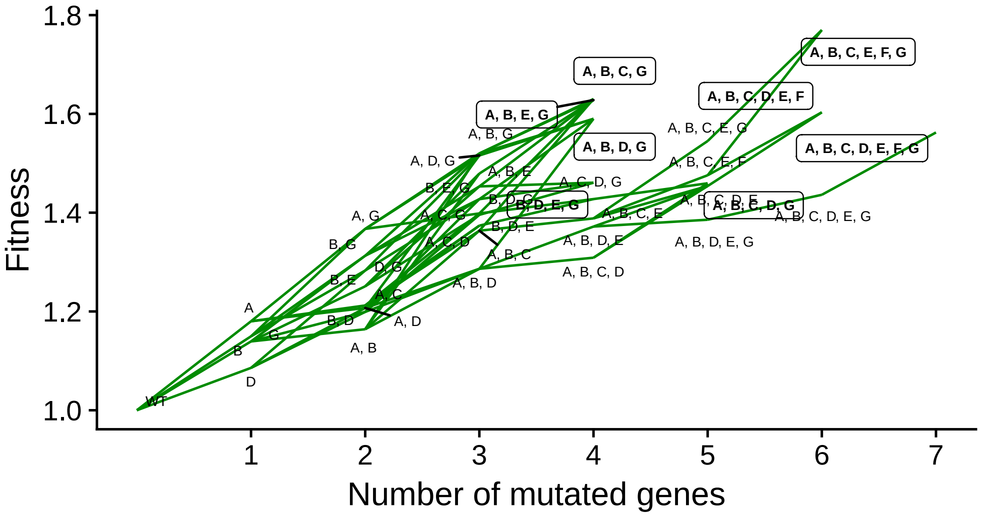

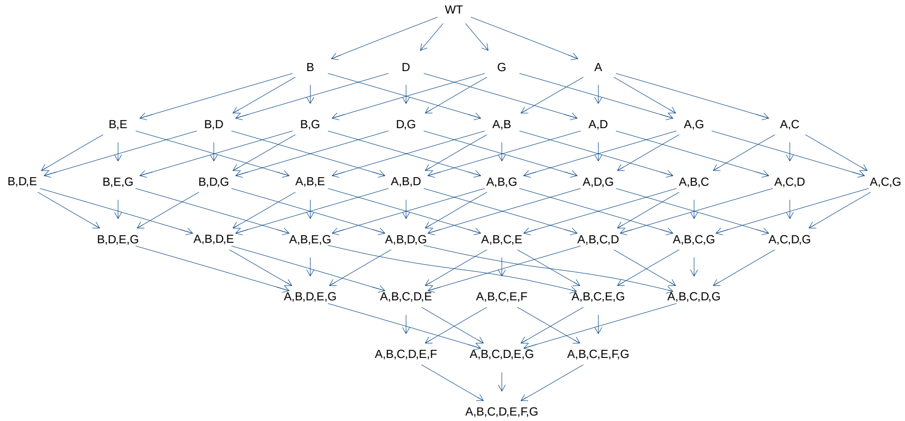

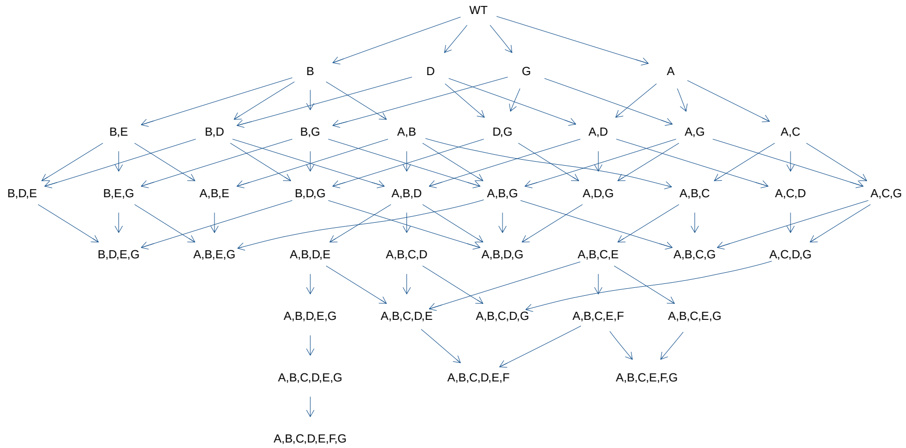

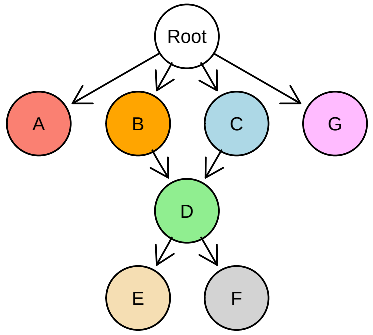

ID	pSBATEcePfnX7JWh
acc genotypes	32
peaks no back	2
removed edges	7
prop rem edges	0.103

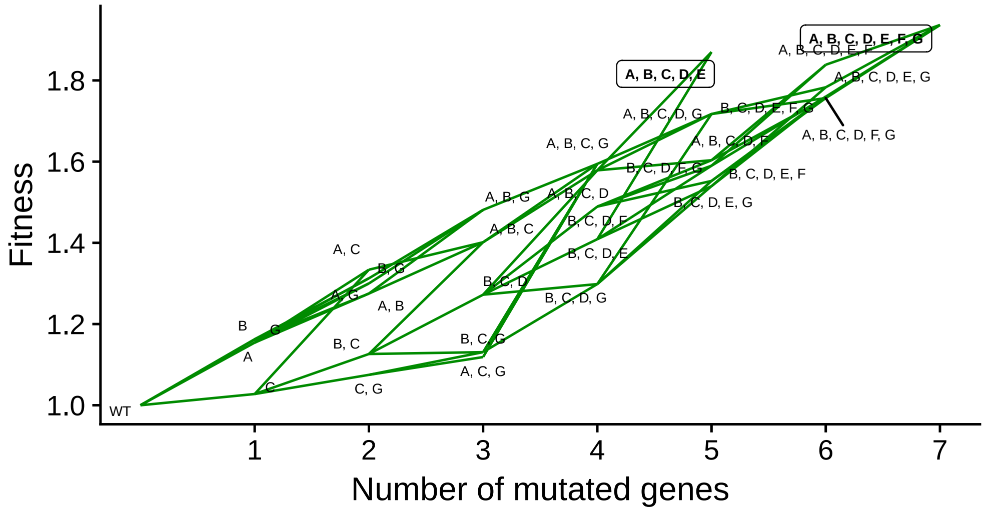

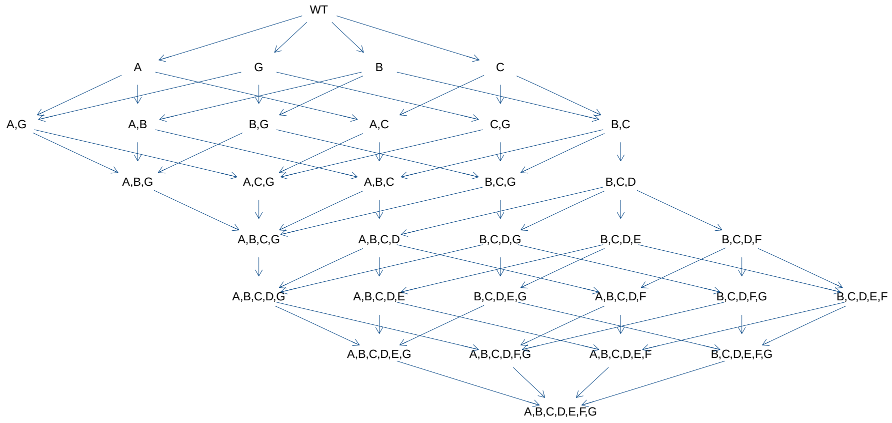

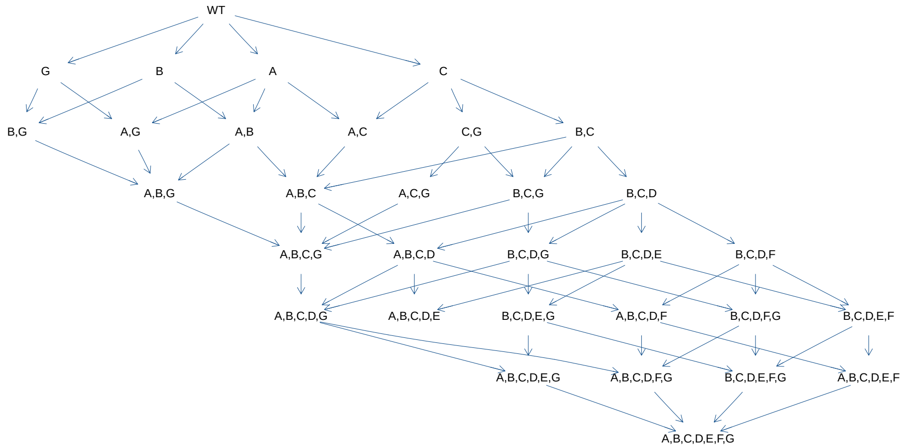

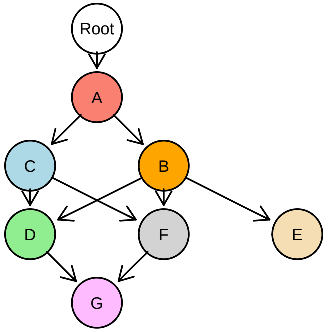

ID	pui6npZKf4iG9VmR
acc genotypes	15
peaks no back	3
removed edges	3
prop rem edges	0.136

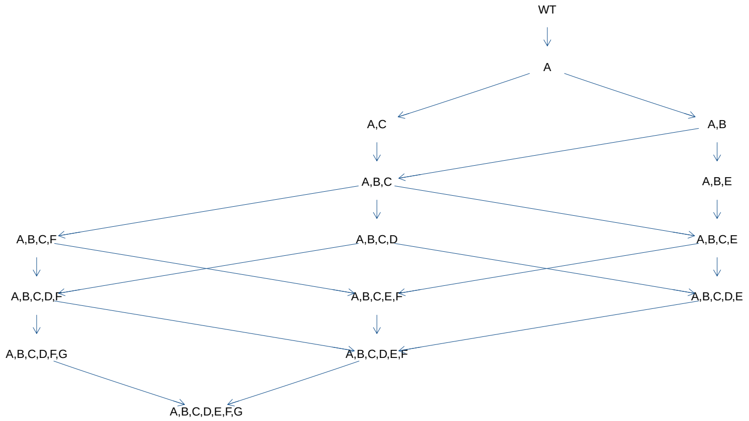

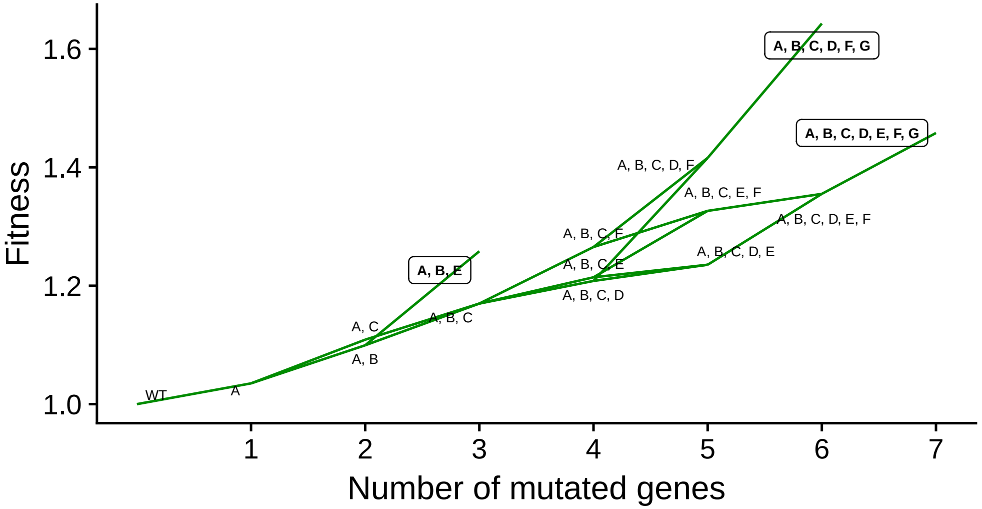

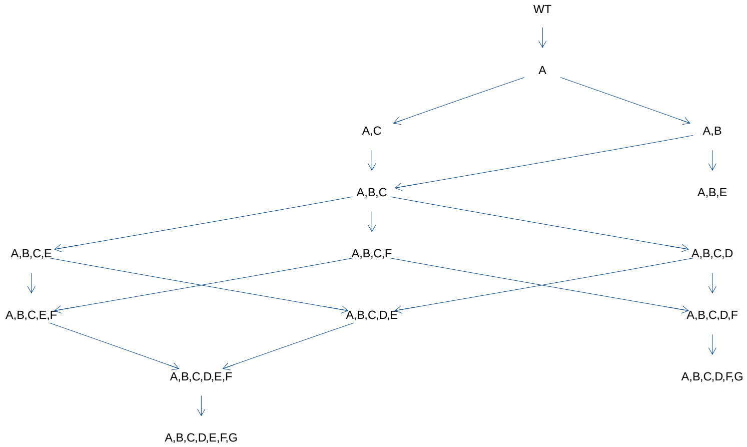

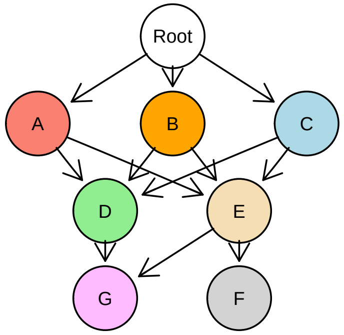

ID	Q7PLBKdNNwZTd7om
acc genotypes	15
peaks no back	2
removed edges	2
prop rem edges	0.091

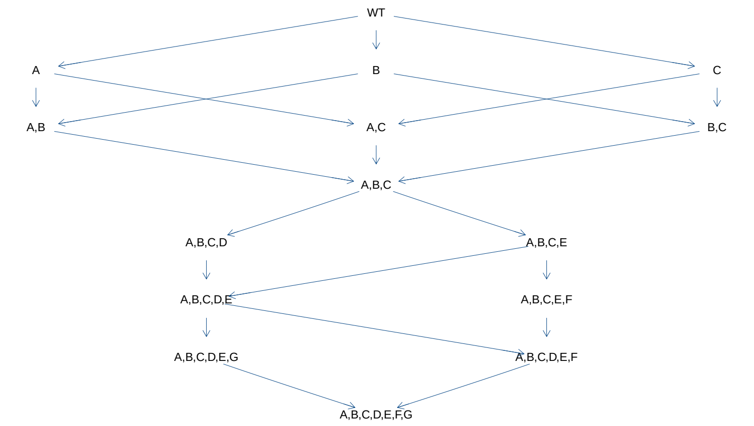

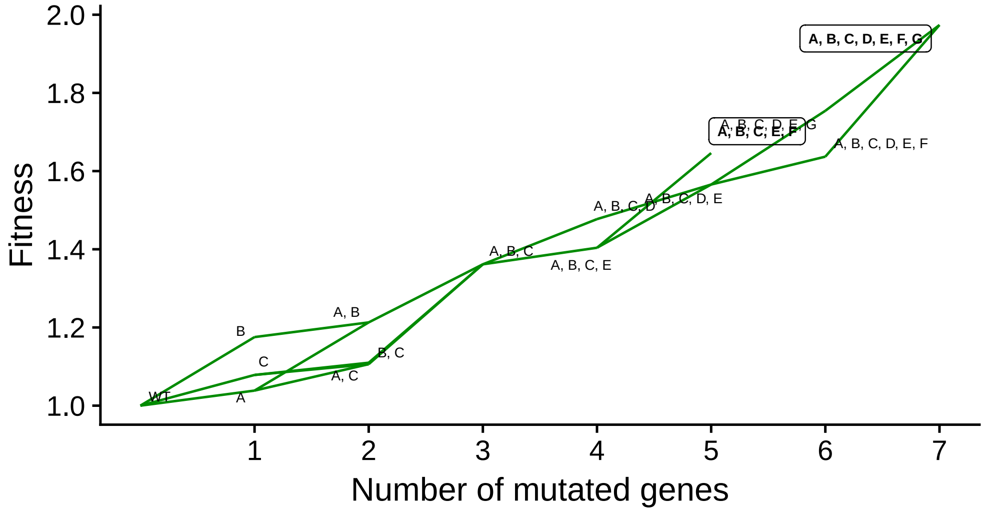

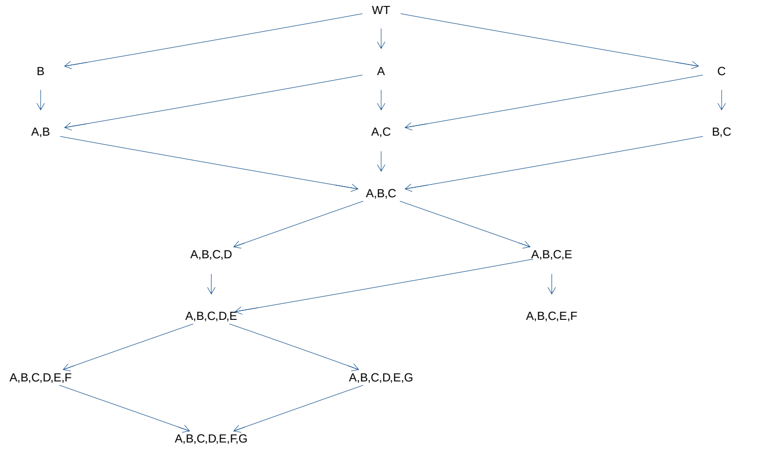

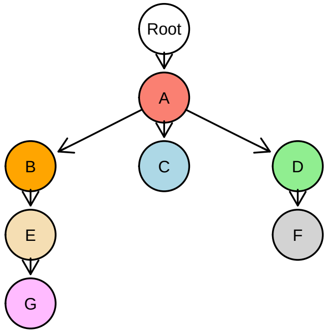

ID qEpRgGAzFDFL2rE5
acc genotypes 25
peaks no back 3
removed edges 10
prop rem edges 0.213

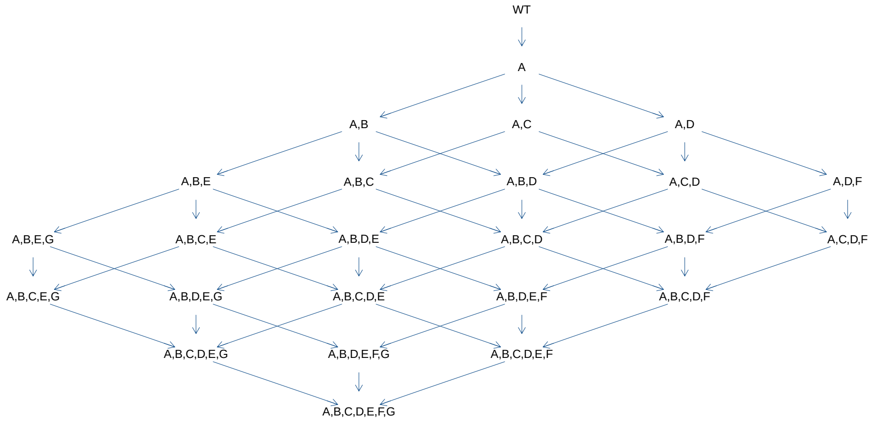

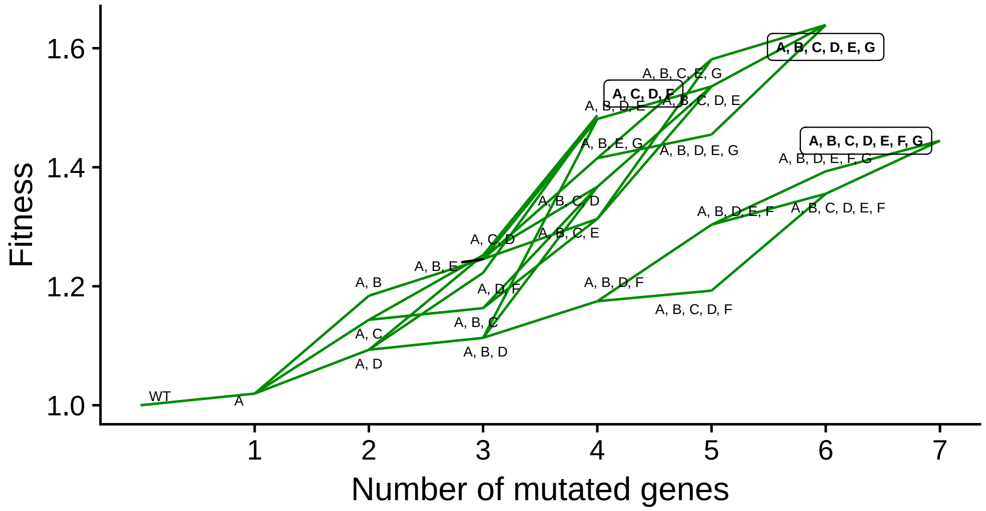

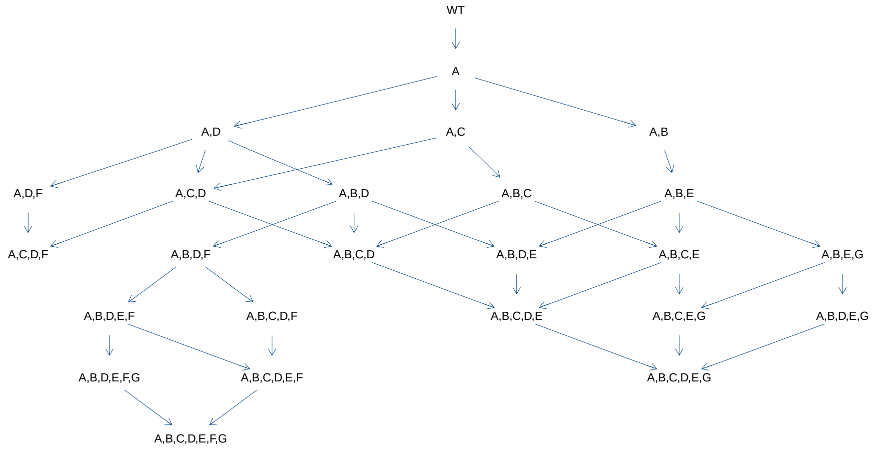

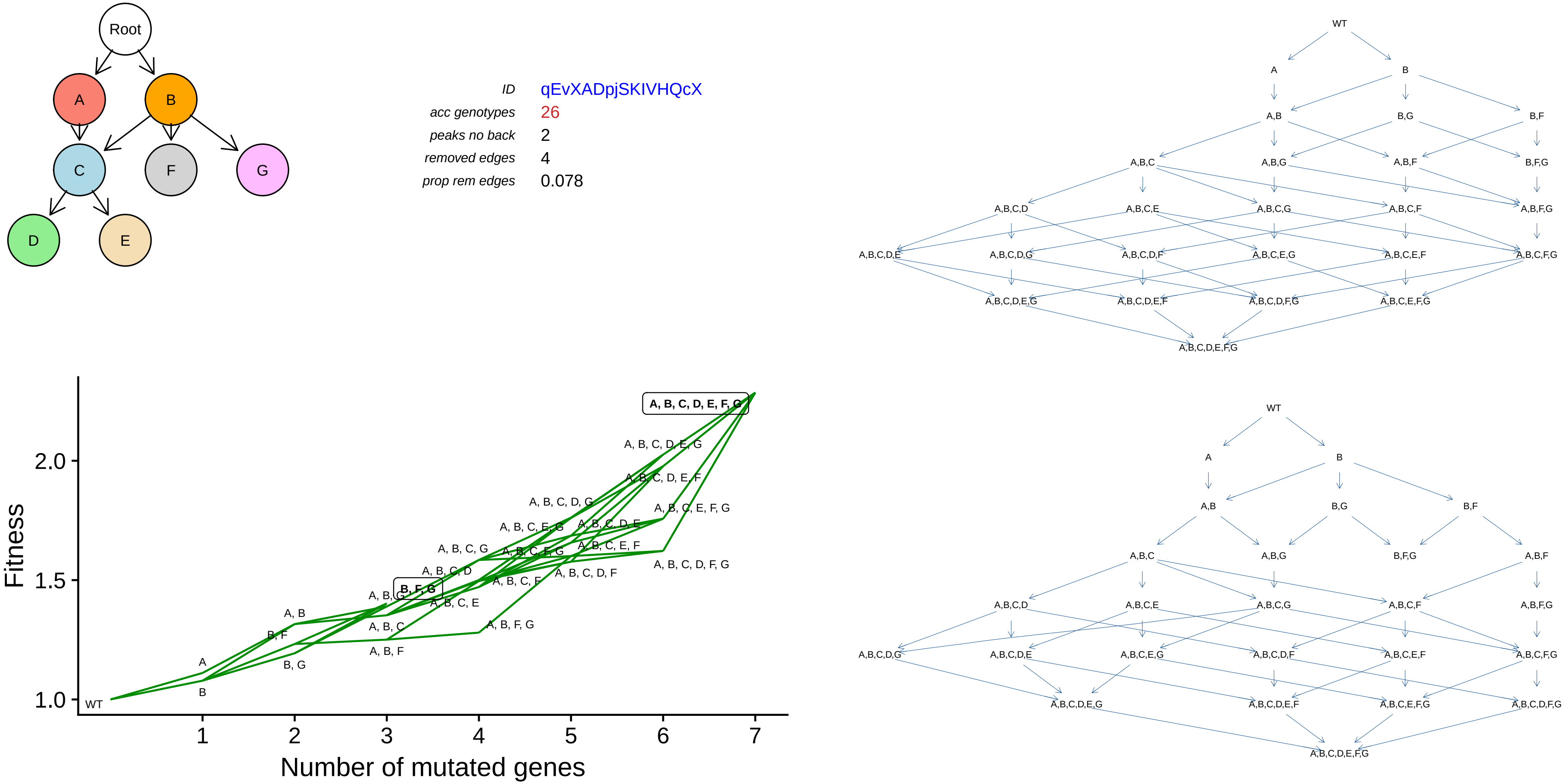

Root
A
B
C
F
G
D
E
ID qEvXADpjSKIVHQcX
acc genotypes 26
peaks no back 2
removed edges 4
prop rem edges 0.078
Fitness
Number of mutated genes
WT
A, B, C, D, E, F, G
B, F, G

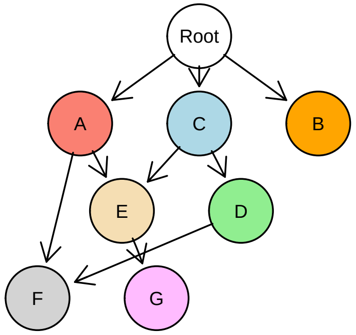

ID qp7i3jRVfJ15Ghst
acc genotypes 26
peaks no back 6
removed edges 12
prop rem edges 0.245

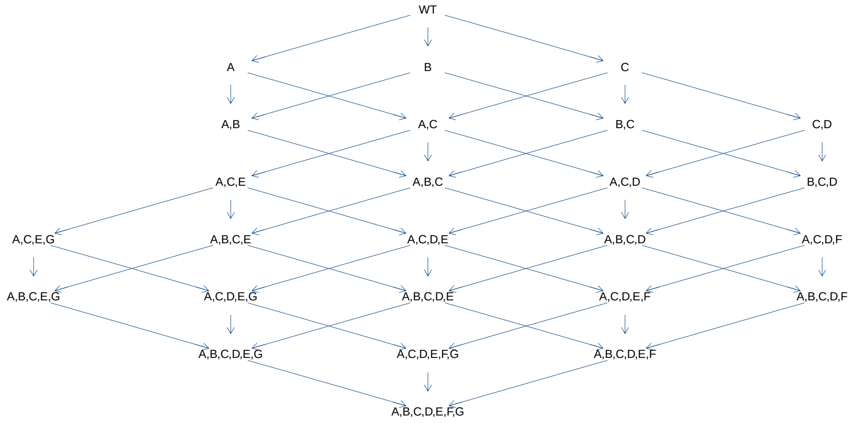

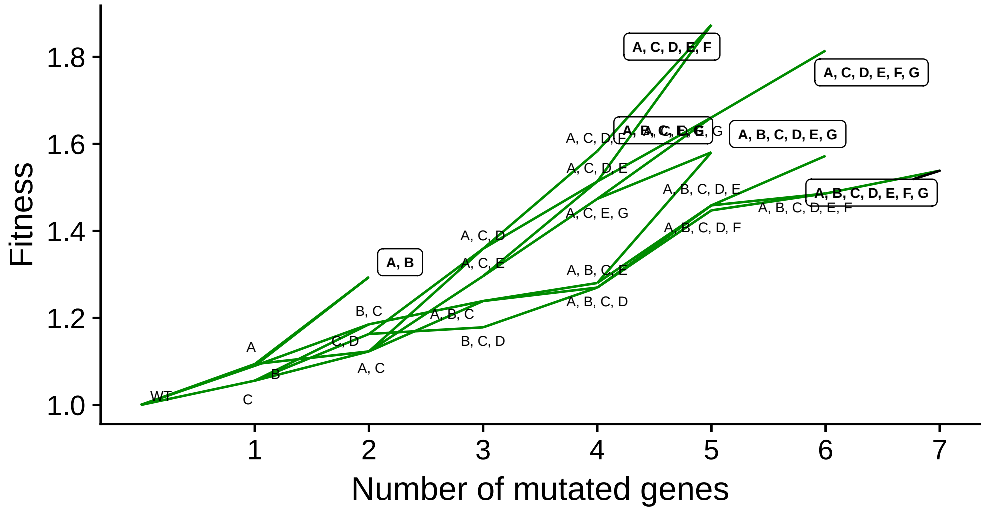

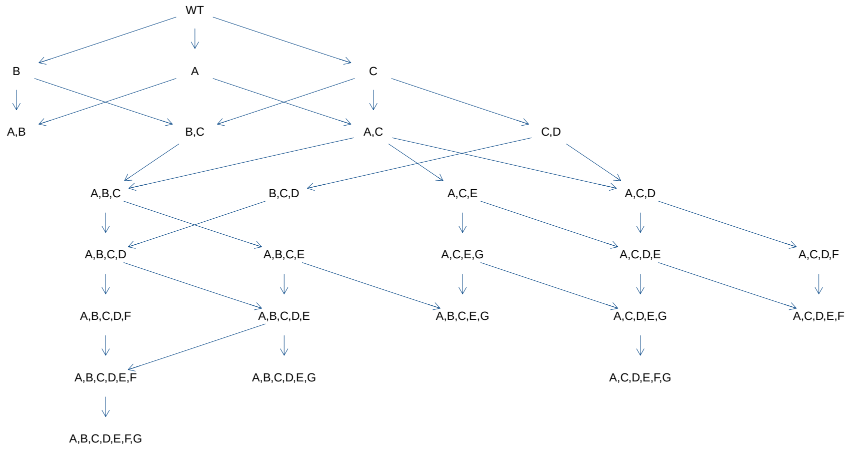

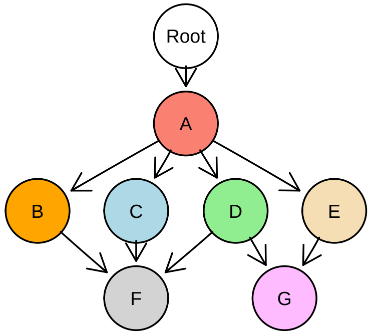

ID	QqSOOn7ihVdtBpuj
acc genotypes	24
peaks no back	4
removed edges	4
prop rem edges	0.087

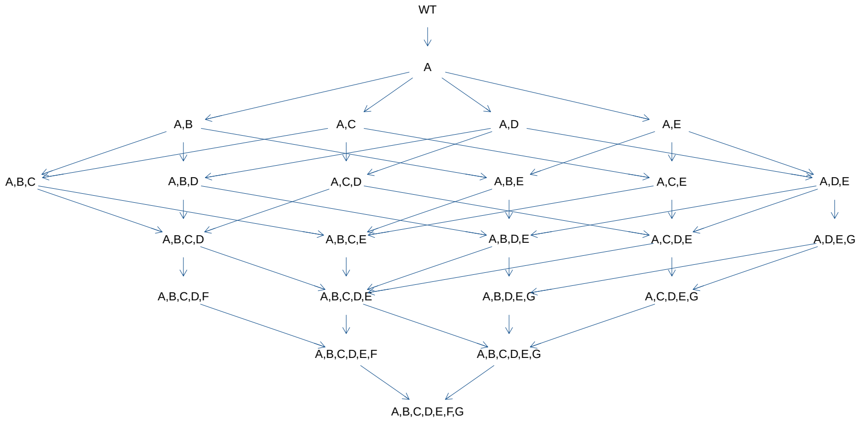

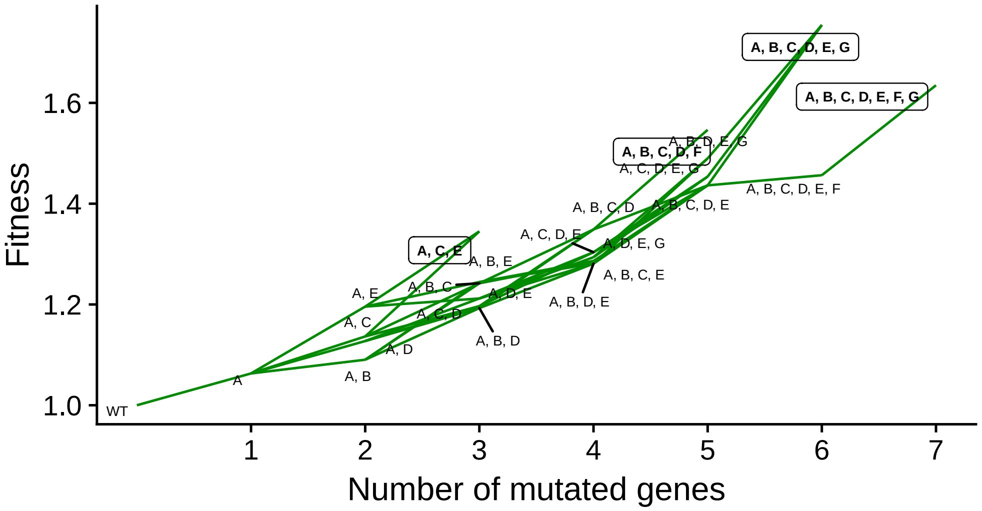

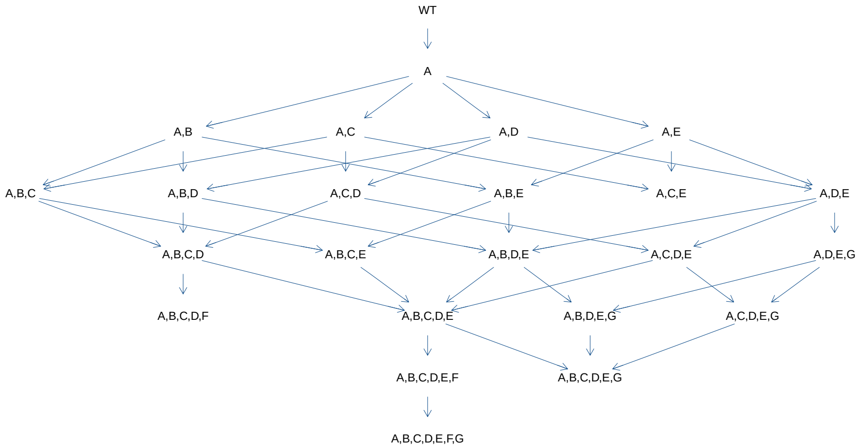

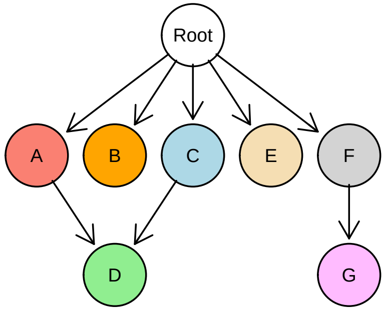

ID	qUS7LZwsUxmyH3in
acc genotypes	60
peaks no back	7
removed edges	32
prop rem edges	0.2

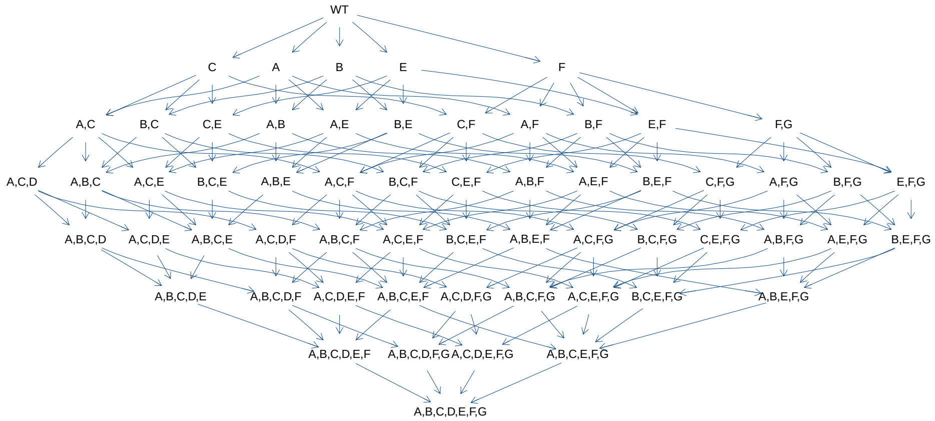

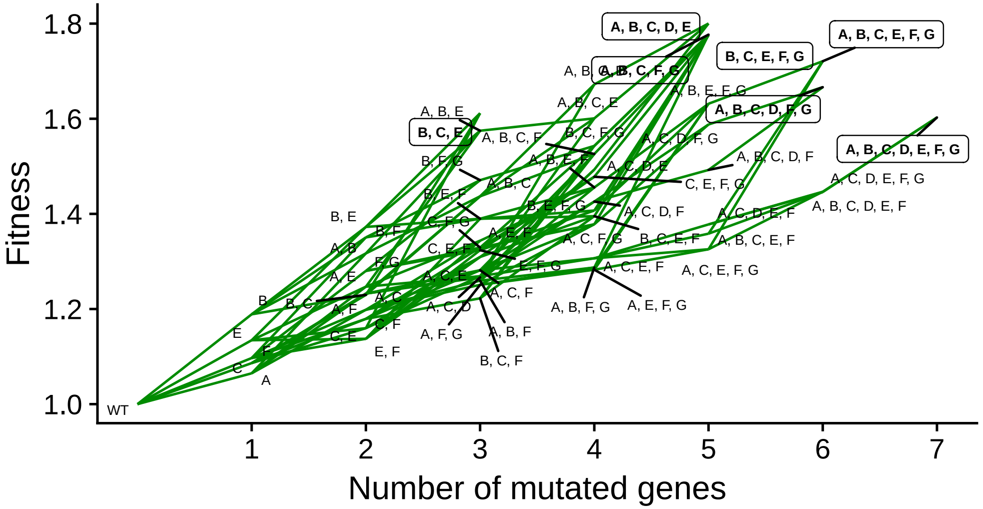

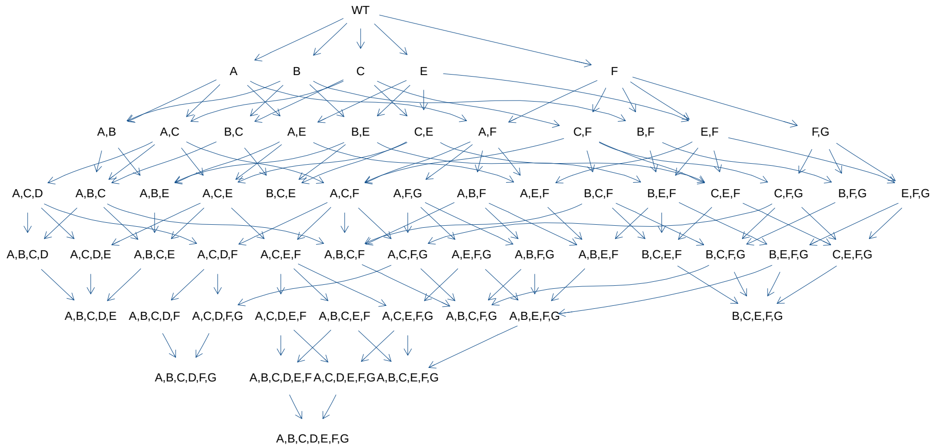

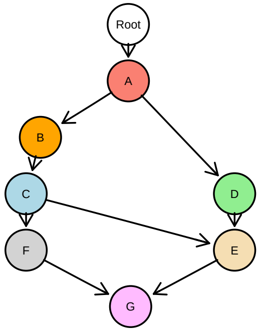

ID	R102qzAOOheDkzBdz
acc genotypes	12
peaks no back	2
removed edges	1
prop rem edges	0.067

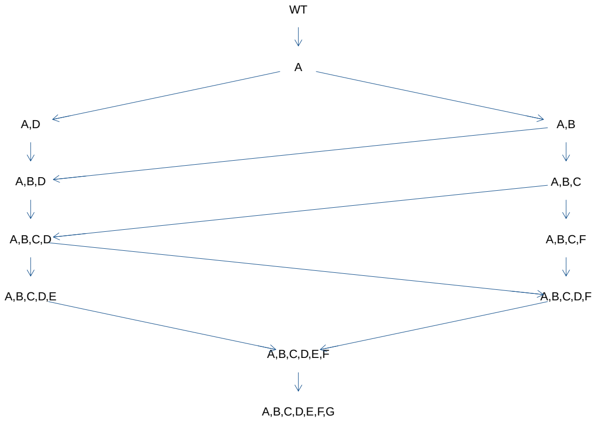

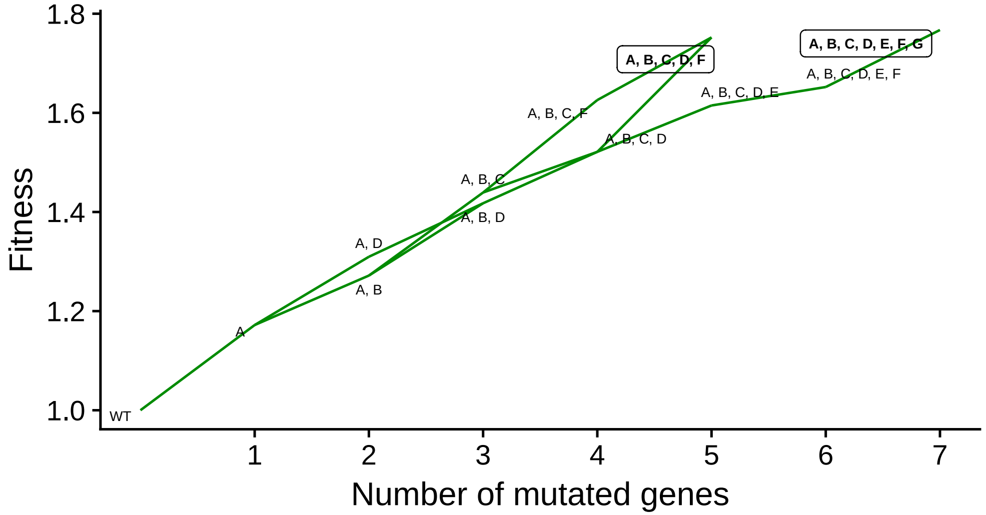

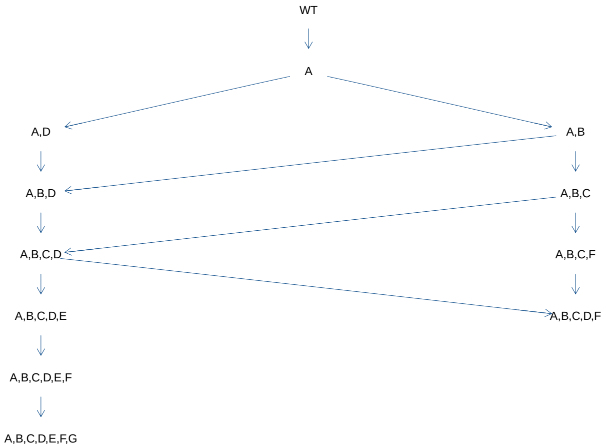

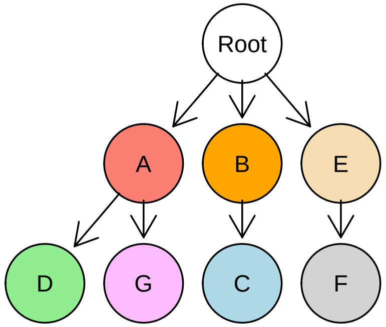

ID	RXEIHdhCdDnzsWrB
acc genotypes	45
peaks no back	6
removed edges	13
prop rem edges	0.124

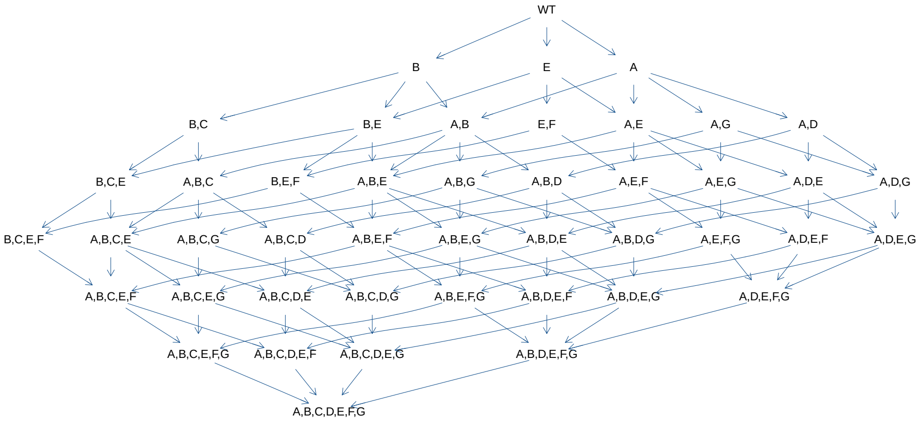

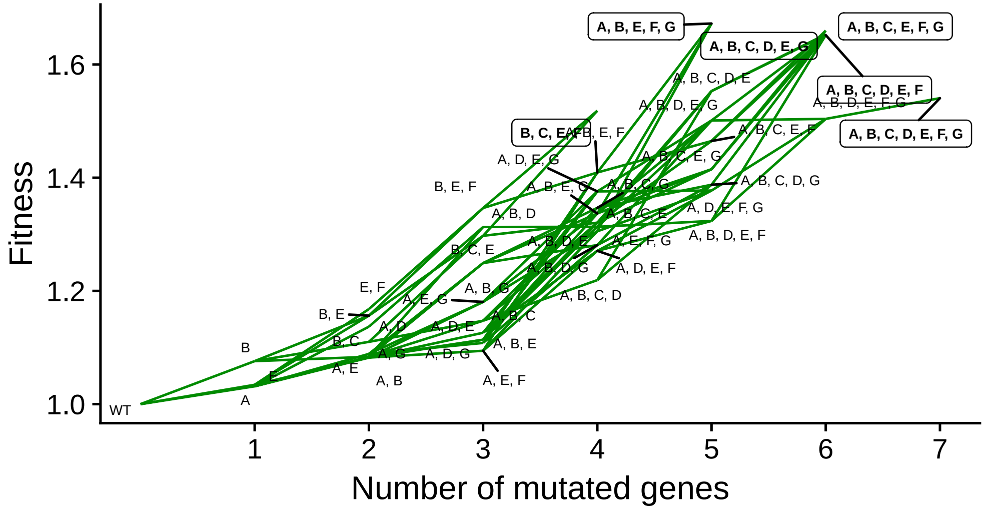

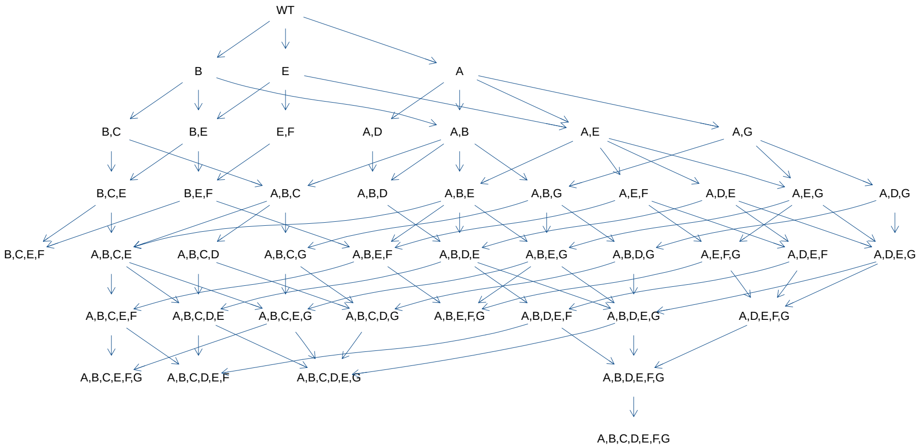

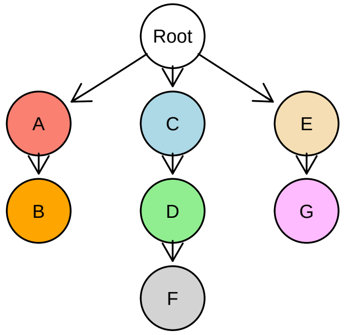

ID ScvT9EQPUJ5Ak10em
acc genotypes 36
peaks no back 2
removed edges 8
prop rem edges 0.107

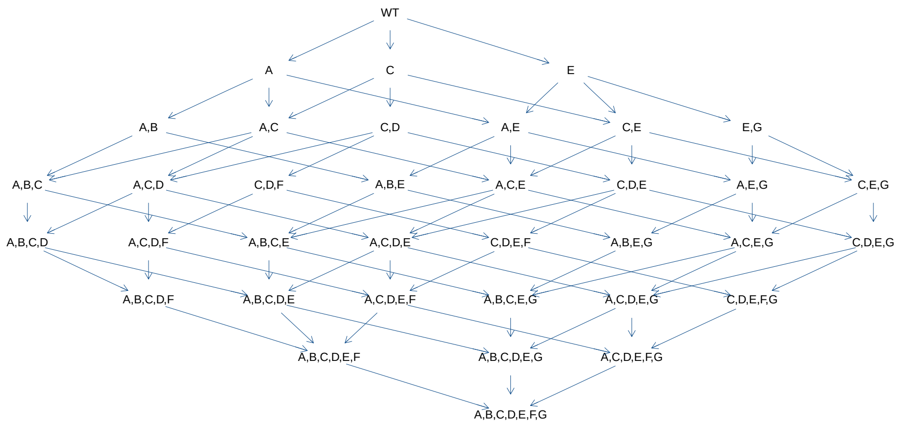

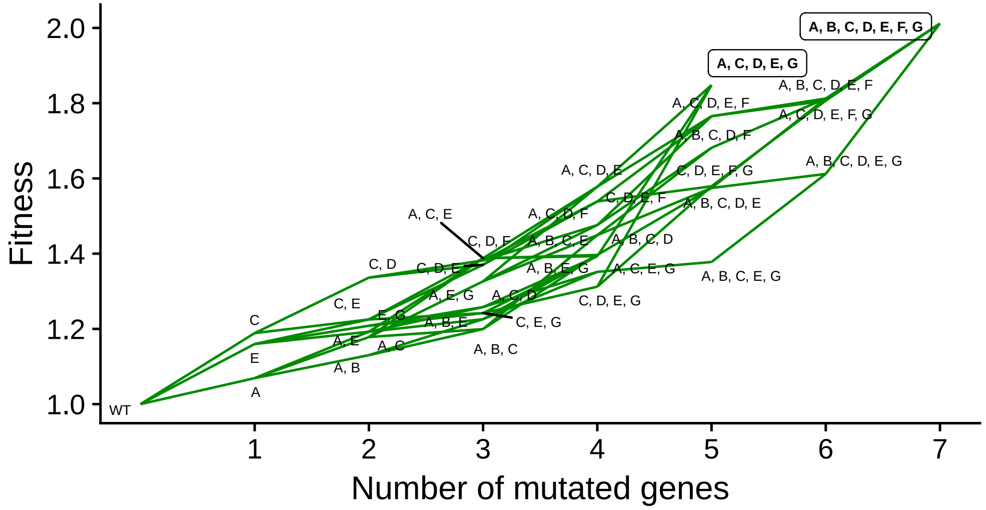

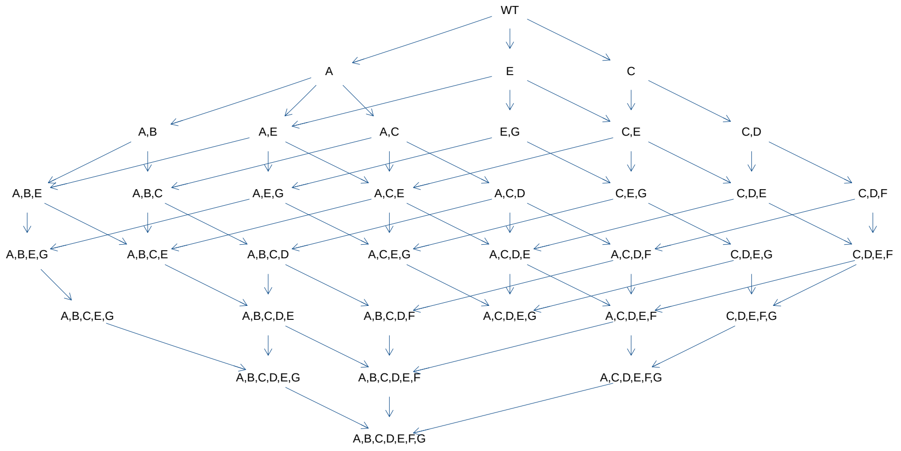

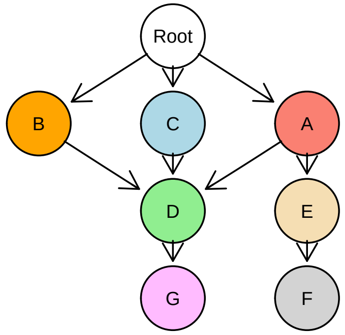

ID	sLsugLzPftgJ7O1e
acc genotypes	22
peaks no back	2
removed edges	3
prop rem edges	0.079

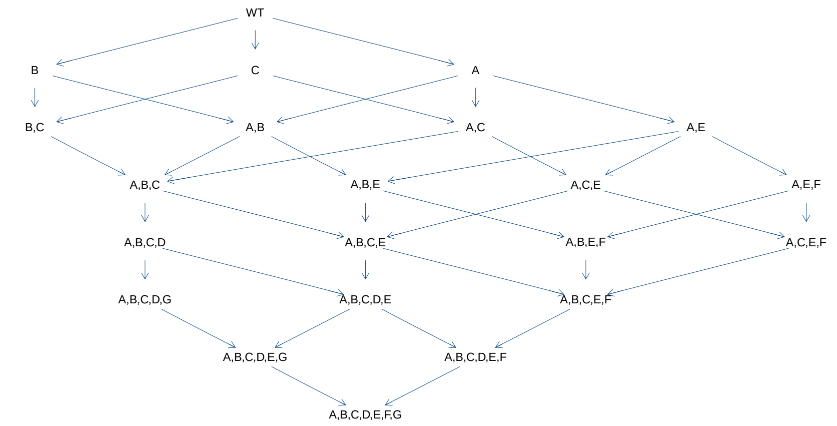

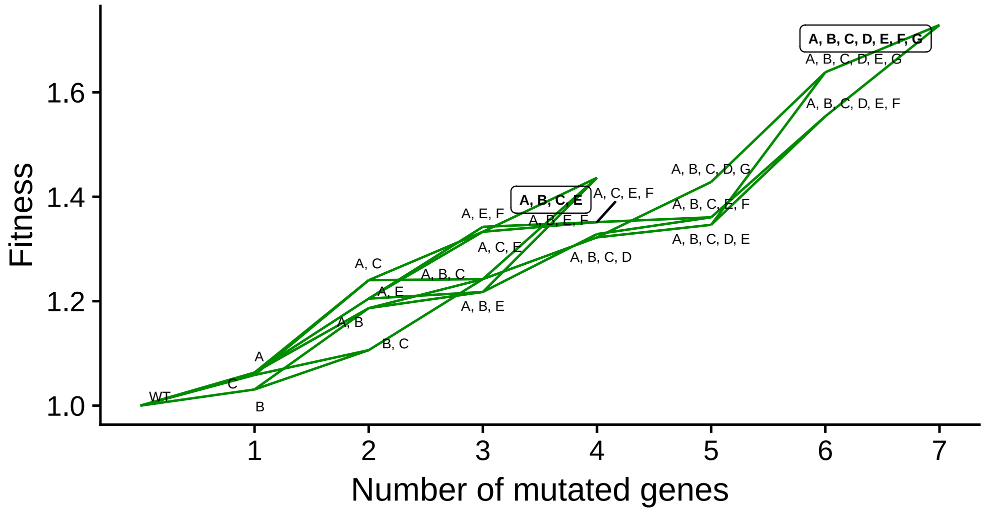

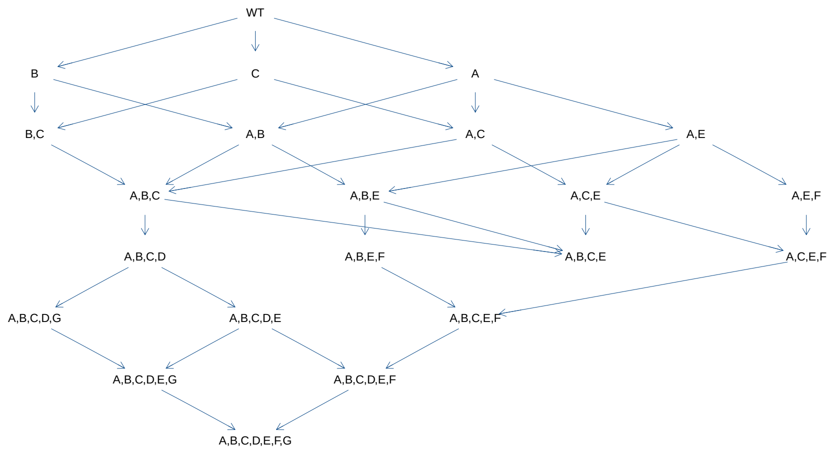

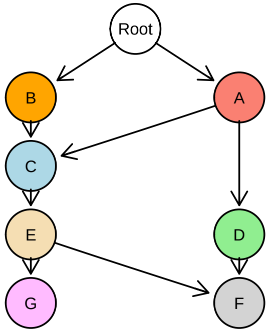

ID	SnRRjceljqckEUkX
acc genotypes	14
peaks no back	3
removed edges	2
prop rem edges	0.105

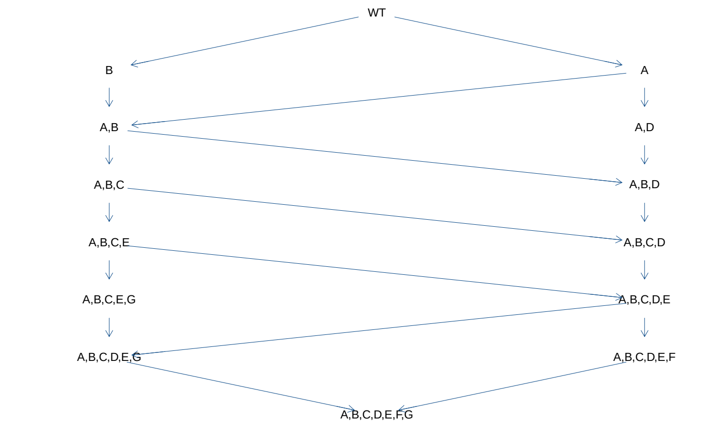

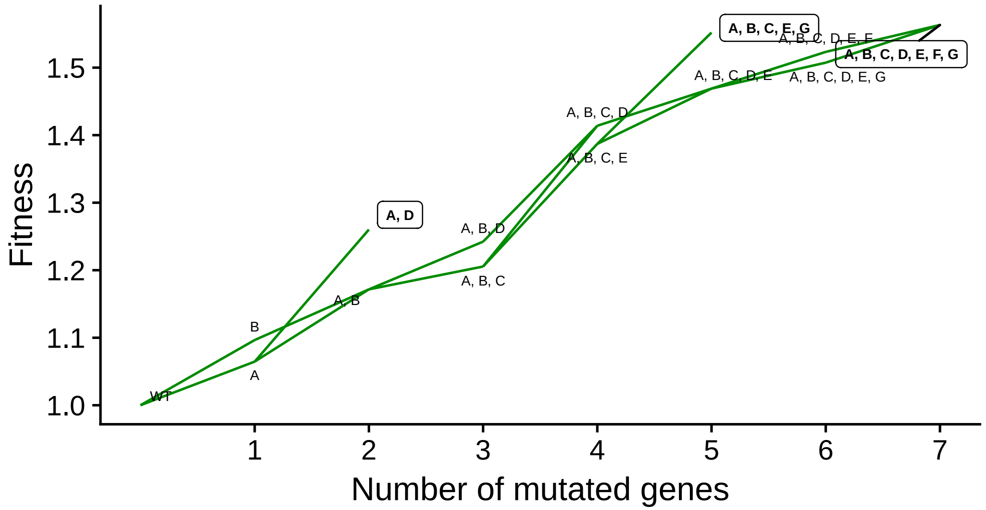

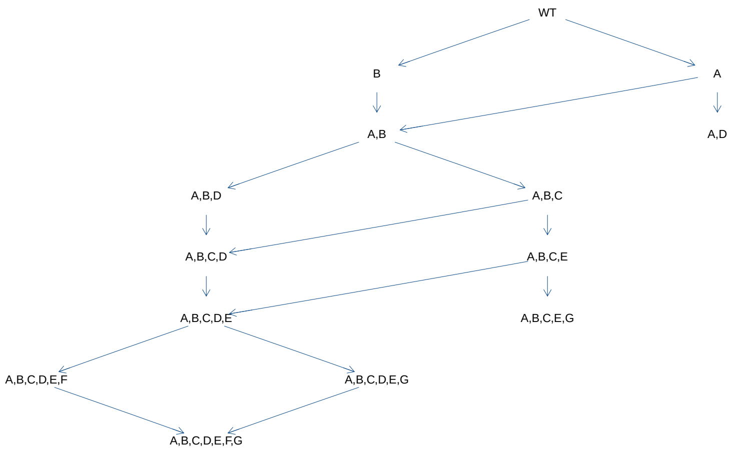

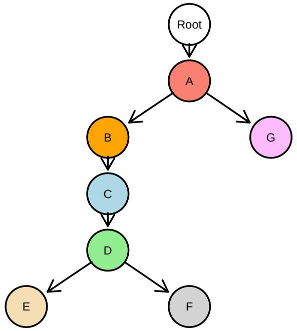

ID t2kQYfWf61wq10313
acc genotypes 15
peaks no back 2
removed edges 2
prop rem edges 0.091

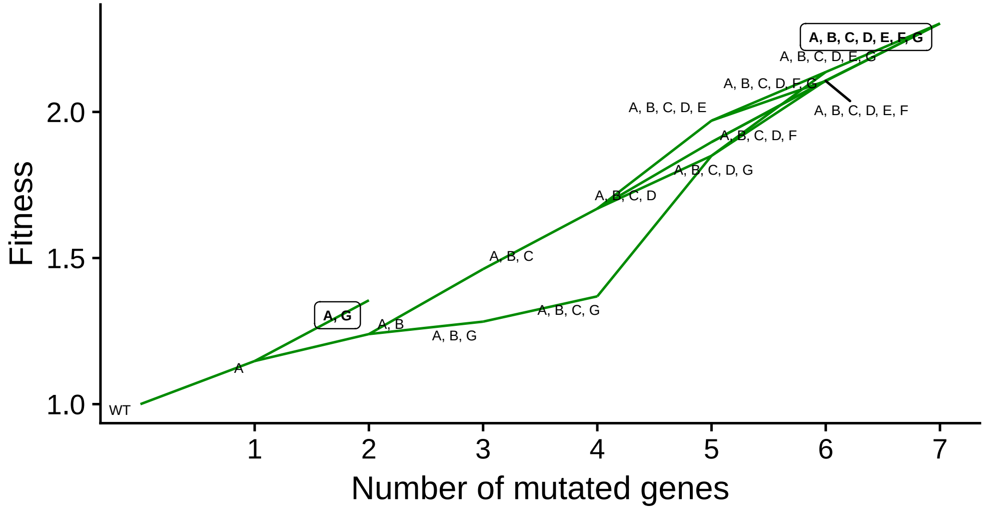

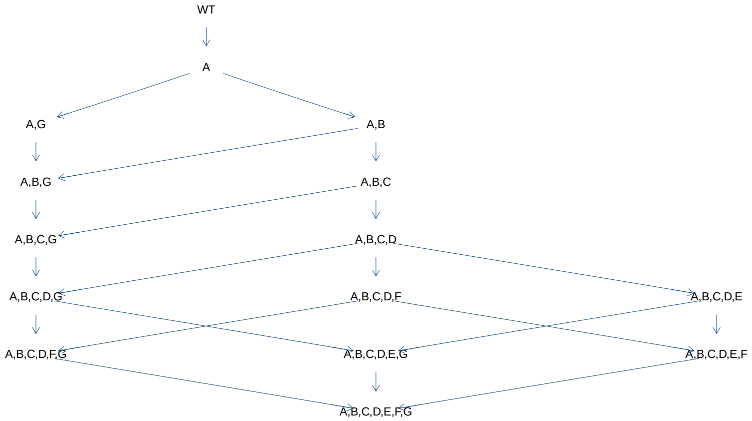

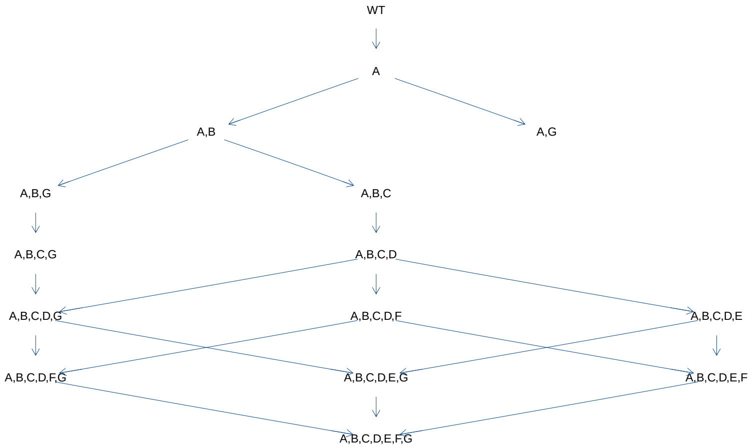

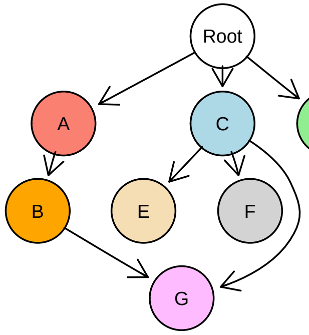

ID t4BmCLHxEiv103ES6
acc genotypes 38
peaks no back 8
removed edges 17
prop rem edges 0.2

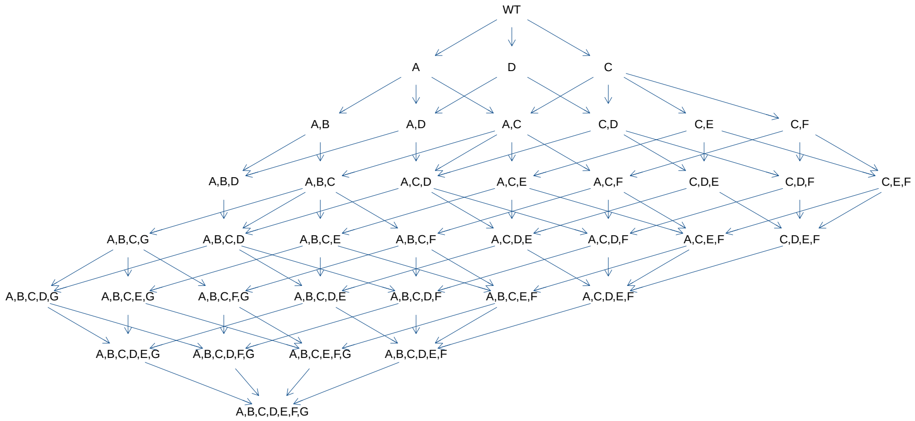

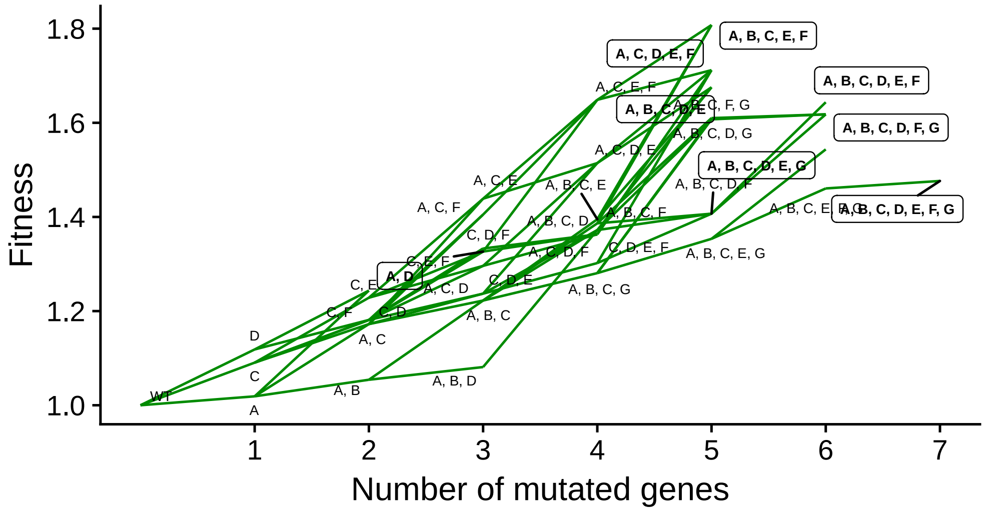

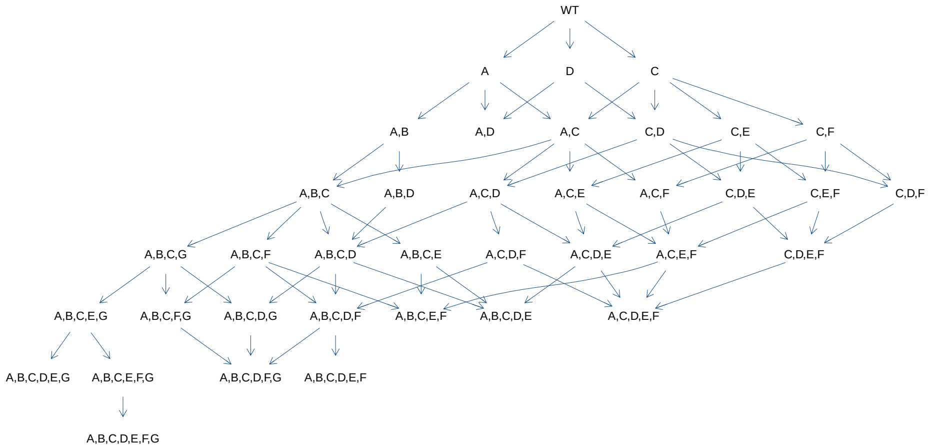

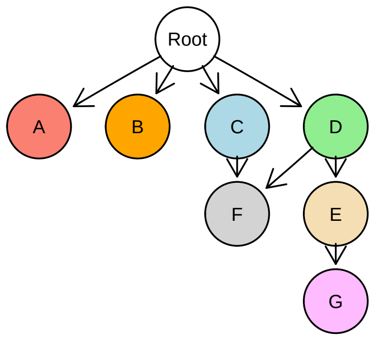

ID	T9n9yW1qk7hj10GjU
acc genotypes	44
peaks no back	7
removed edges	25
prop rem edges	0.24

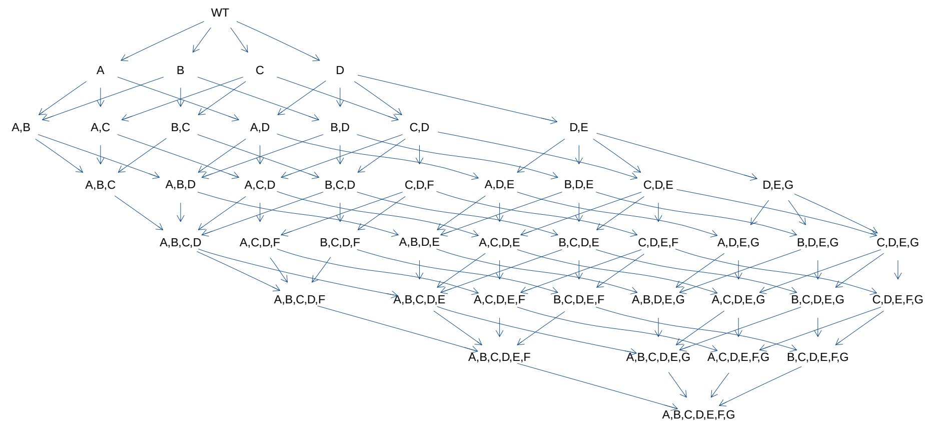

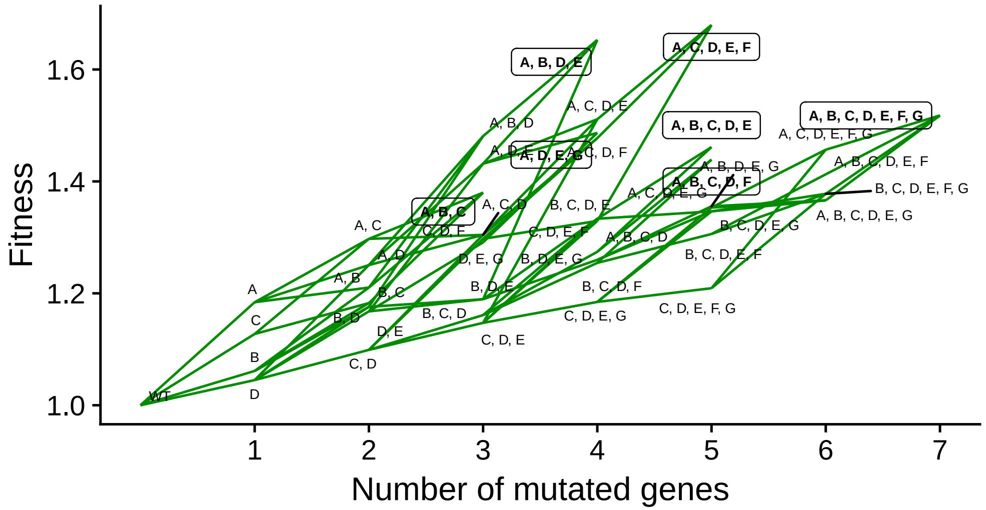

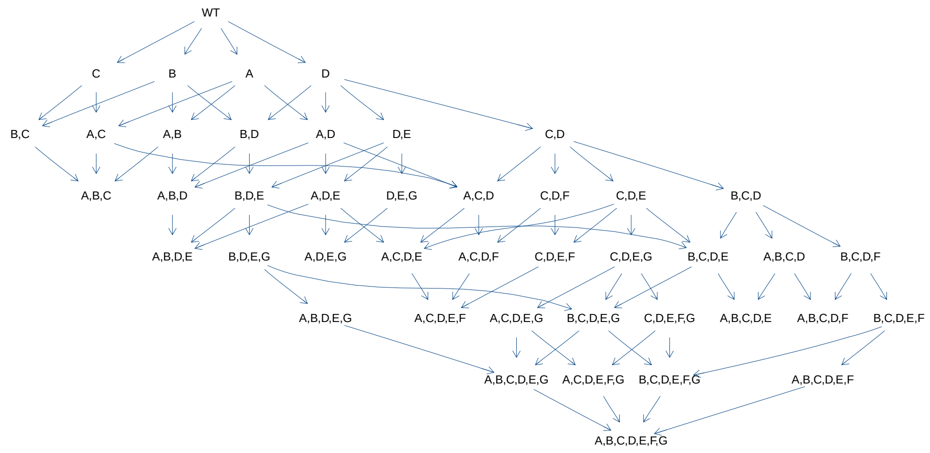

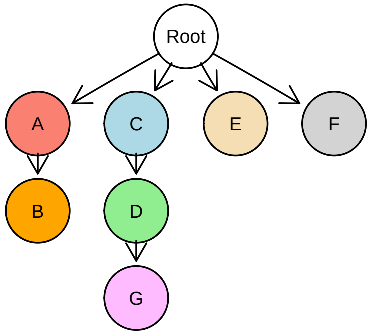

ID	TcQu1TxfmYIxeOT4
acc genotypes	48
peaks no back	6
removed edges	19
prop rem edges	0.164

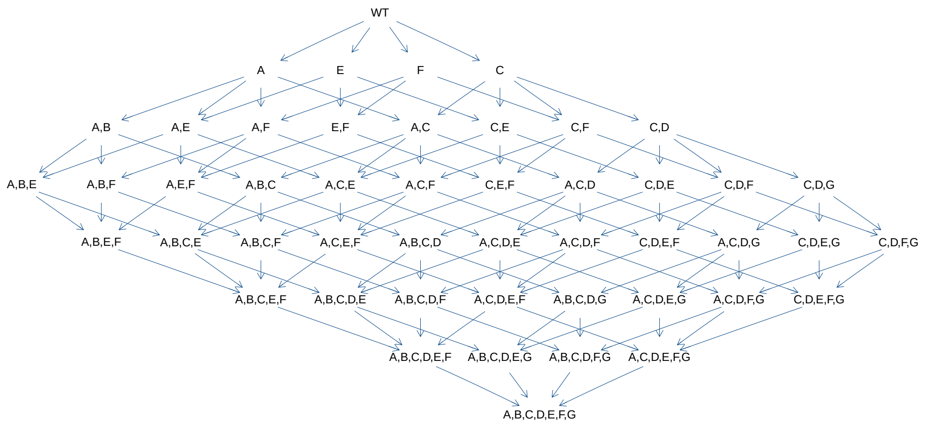

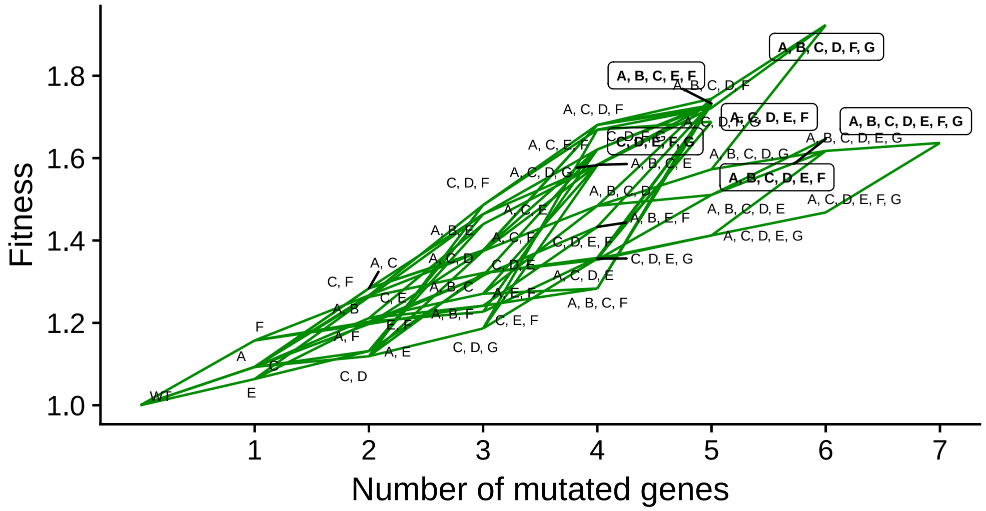

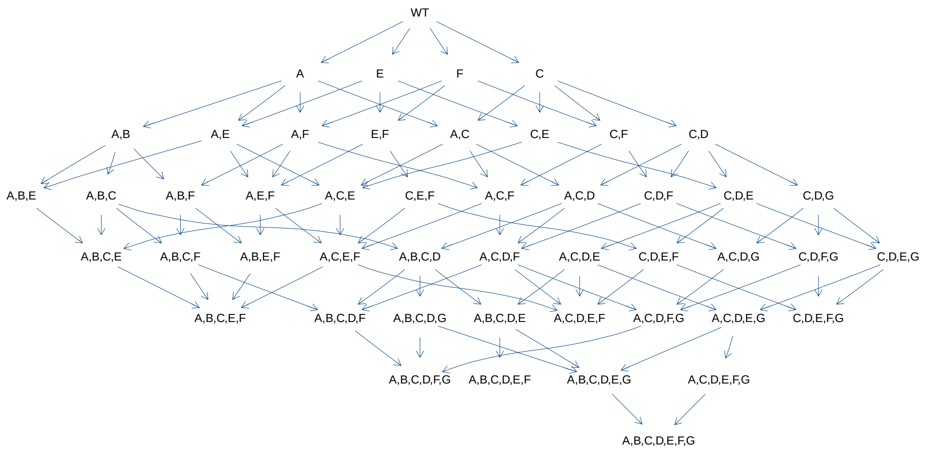

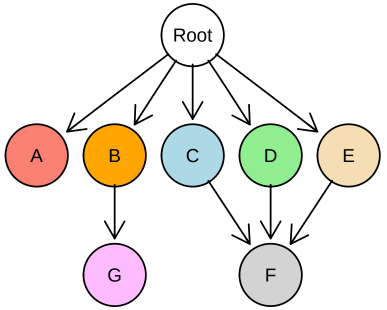

ID tdjsoypZIFJzlC5O
acc genotypes 54
peaks no back 7
removed edges 23
prop rem edges 0.163

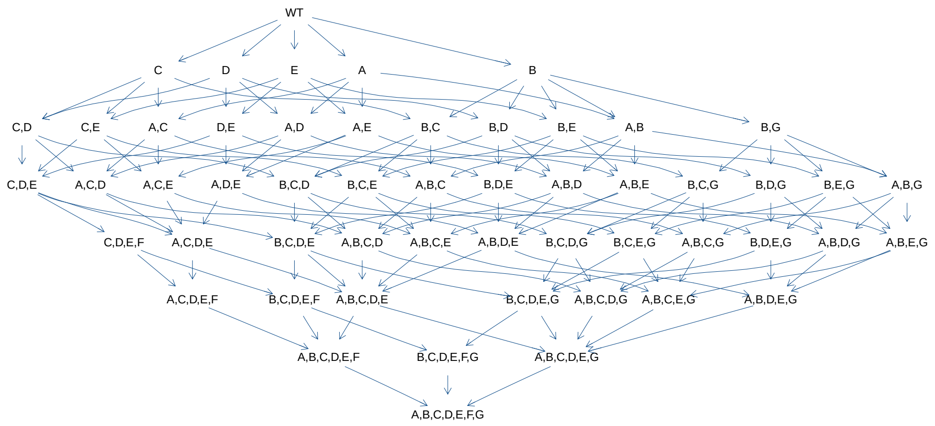

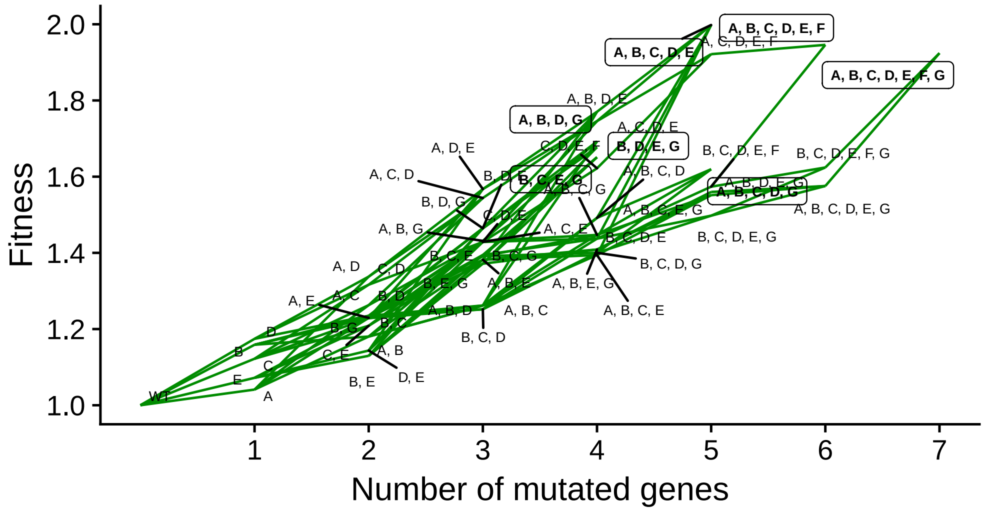

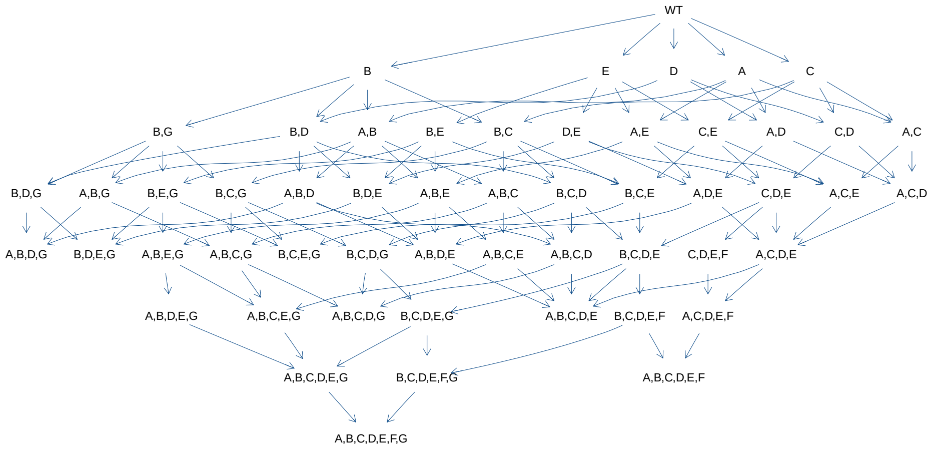

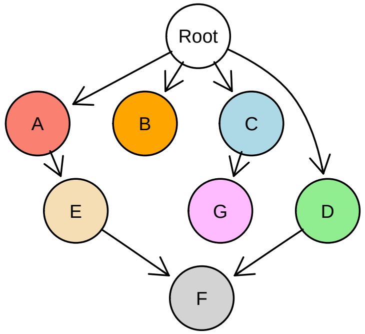

ID	THM3Re8DzitZ9K5X
acc genotypes	42
peaks no back	6
removed edges	12
prop rem edges	0.124

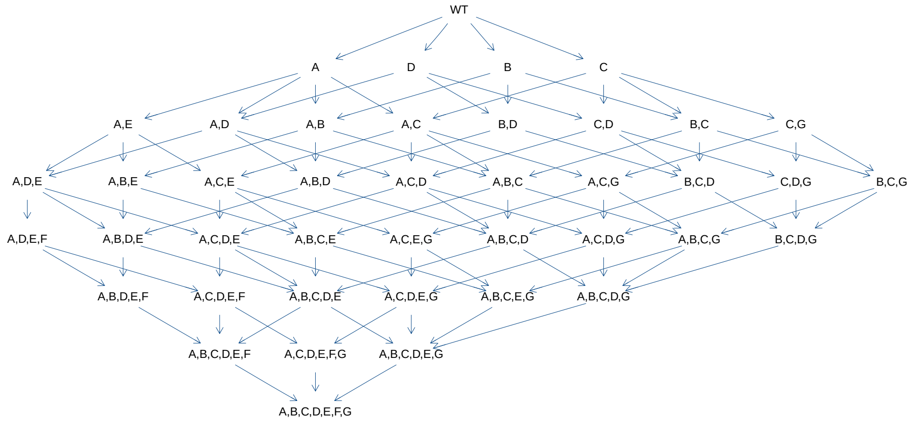

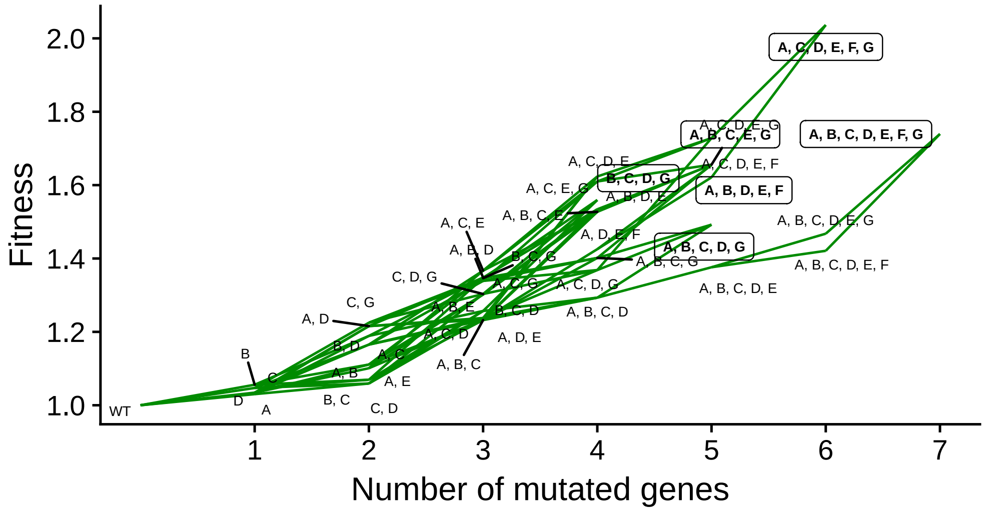

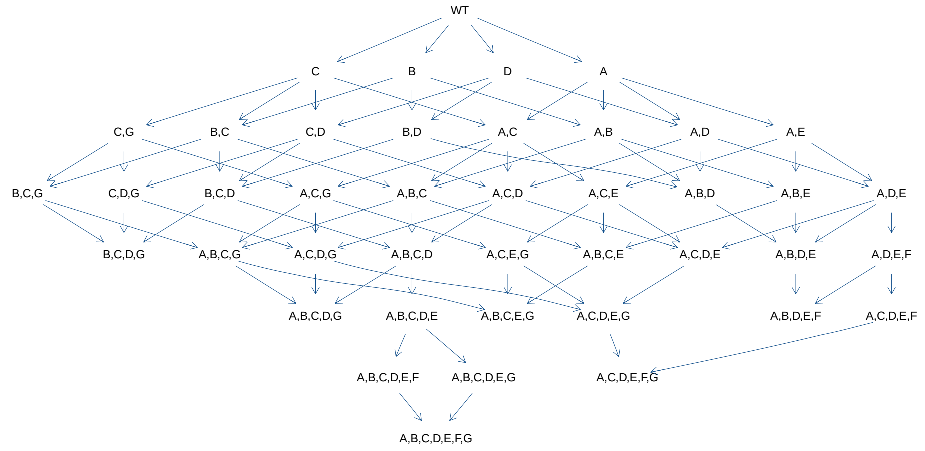

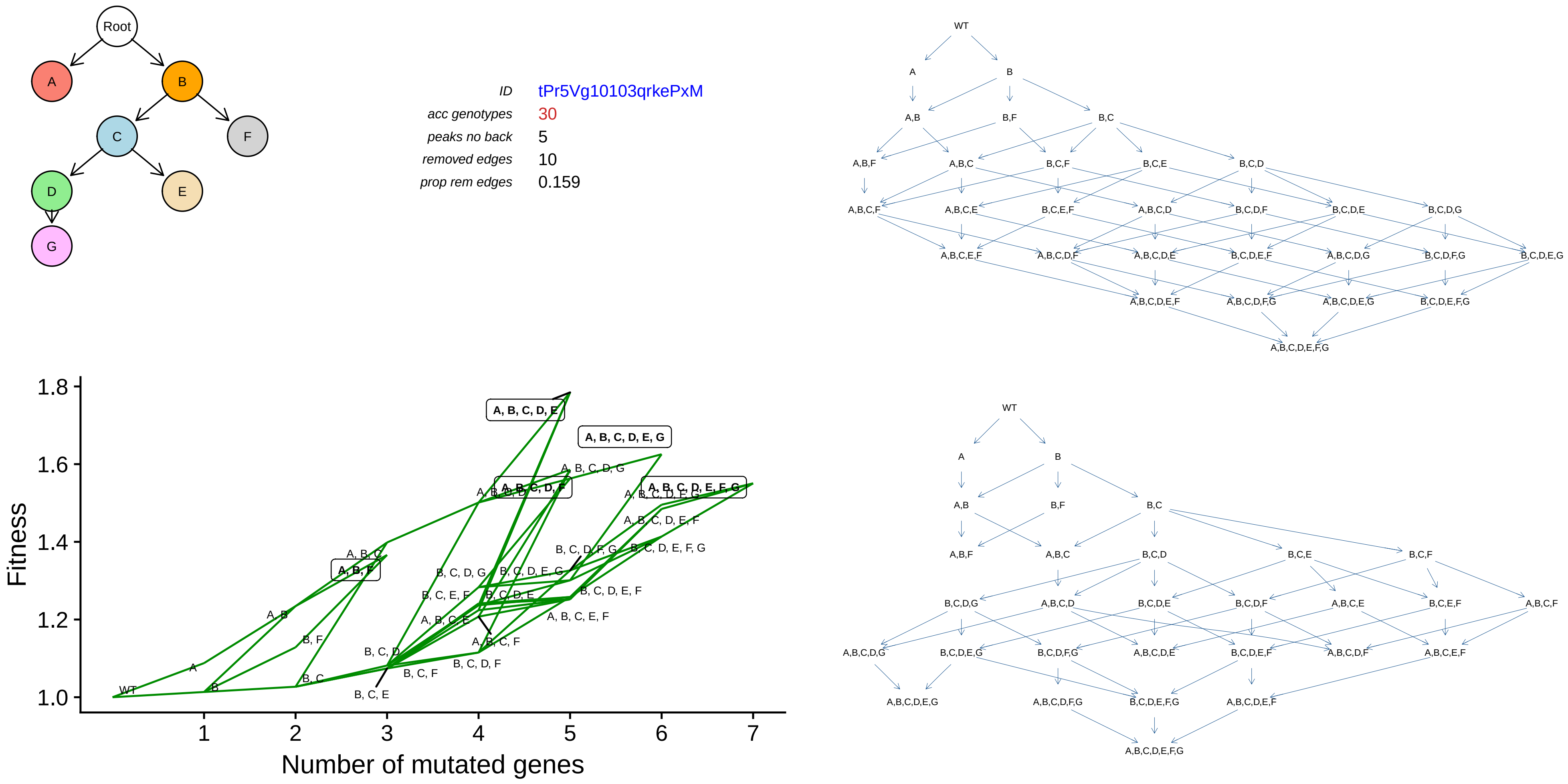
Root
A
B
C
F
D
E
G
ID tPr5Vg10103qrkePxM
acc genotypes 30
peaks no back 5
removed edges 10
prop rem edges 0.159
Fitness
Number of mutated genes
A, B, C, D, E
A, B, C, D, E, G
A, B, C, D, F
A, B, C, D, E, F, G
A, B, F

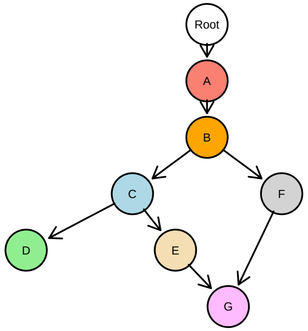

ID tZ722GsITVDN5PNF
acc genotypes 14
peaks no back 4
removed edges 4
prop rem edges 0.2

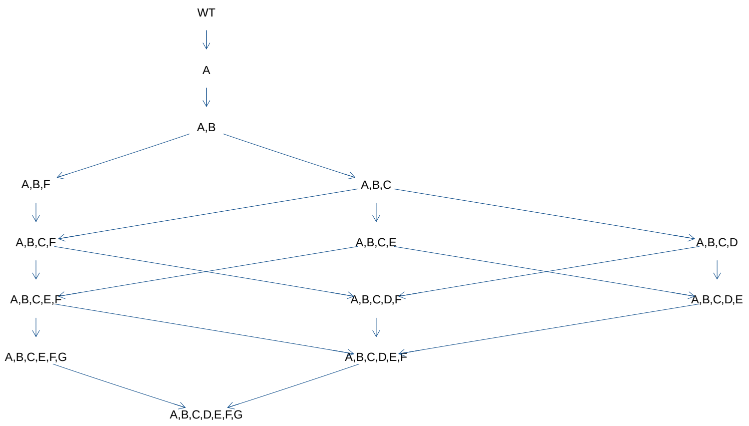

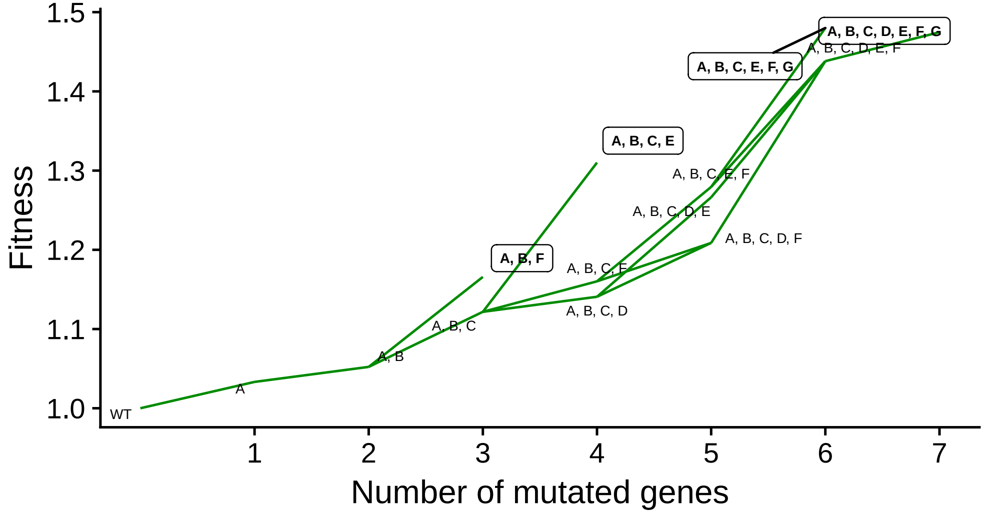

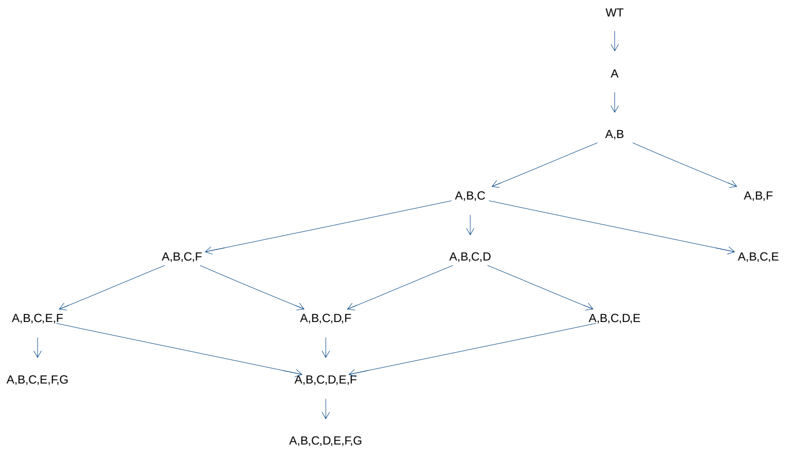

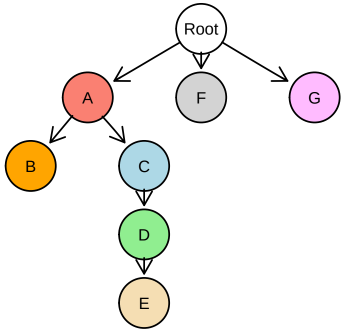

ID U3F9k2e17pCATfZA
acc genotypes 36
peaks no back 6
removed edges 9
prop rem edges 0.112

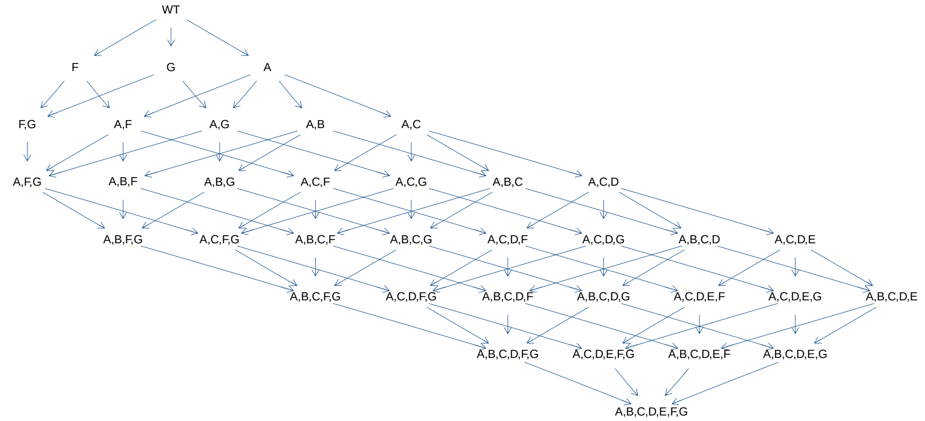

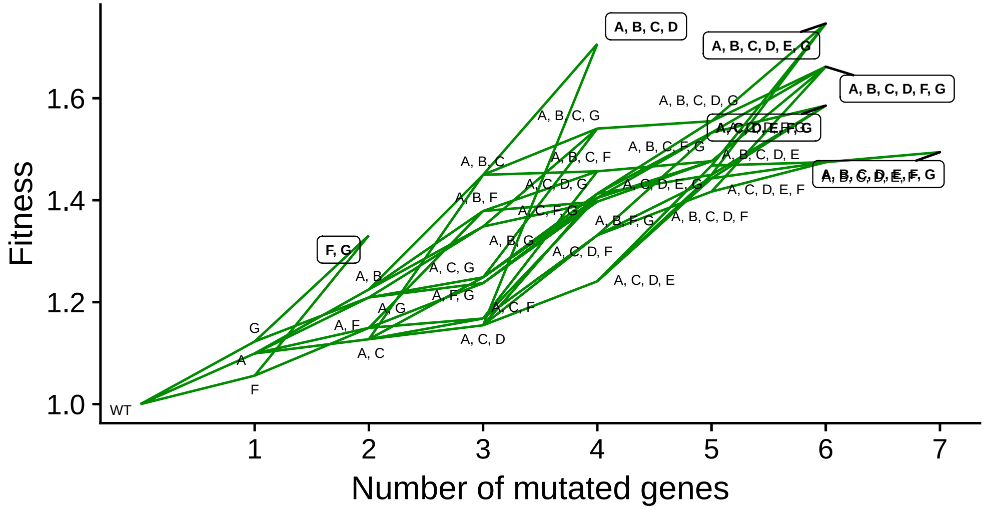

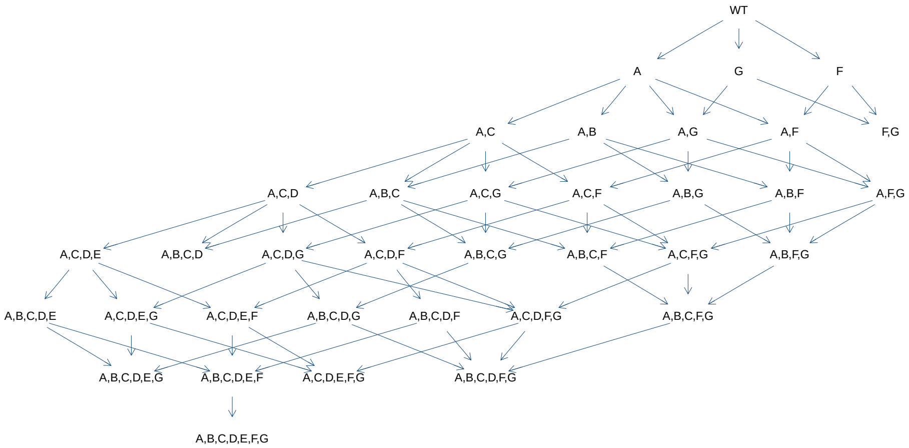

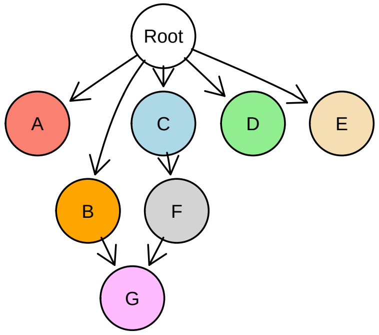

ID	u5c4a1CX10bL5RtgV
acc genotypes	56
peaks no back	7
removed edges	27
prop rem edges	0.182

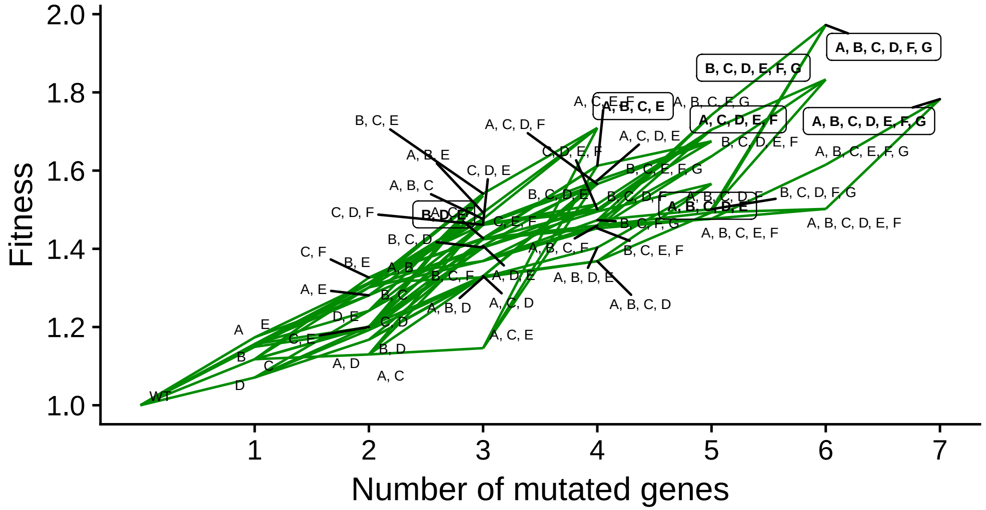

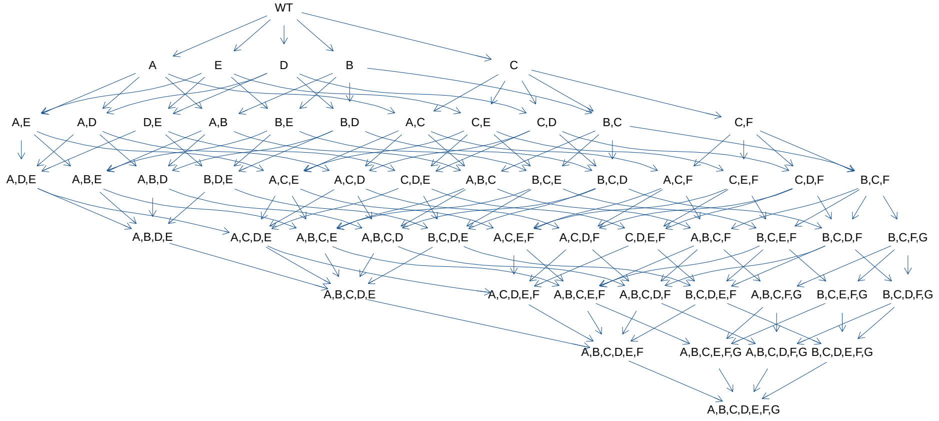

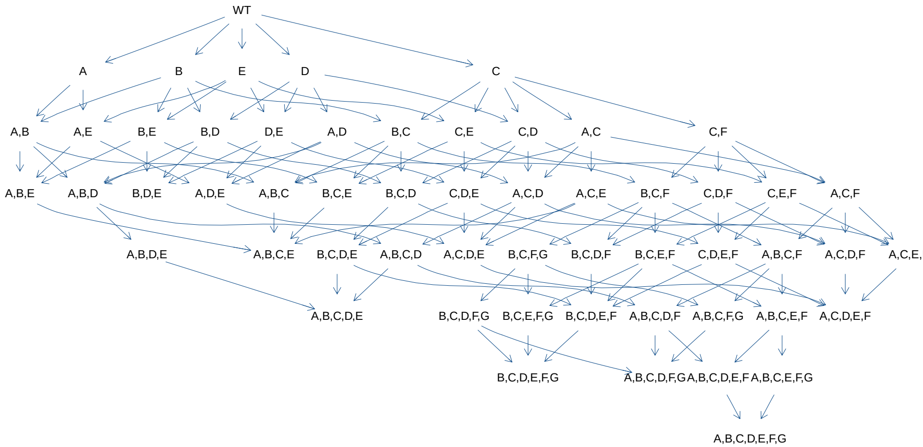

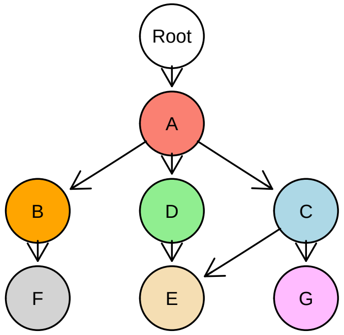

ID U7zMFhVmpGvNZWah
acc genotypes 25
peaks no back 6
removed edges 9
prop rem edges 0.191

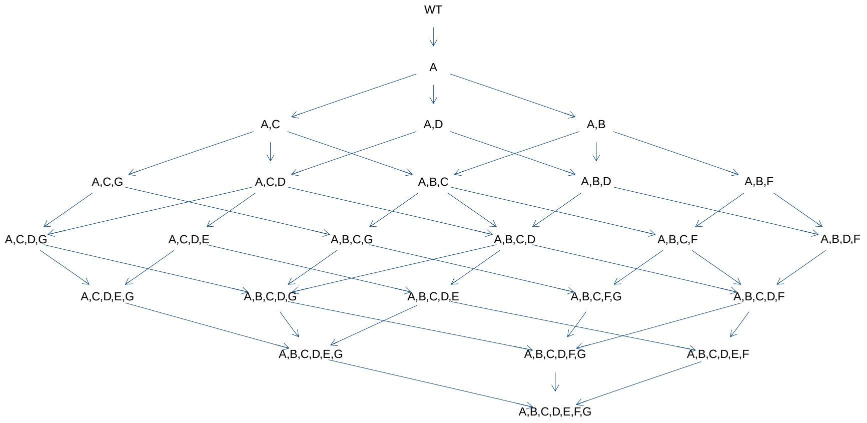

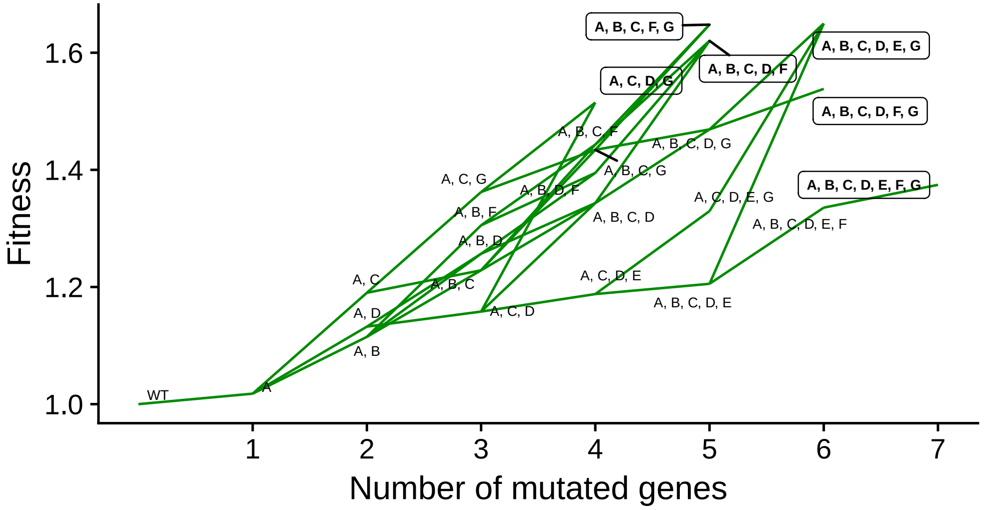

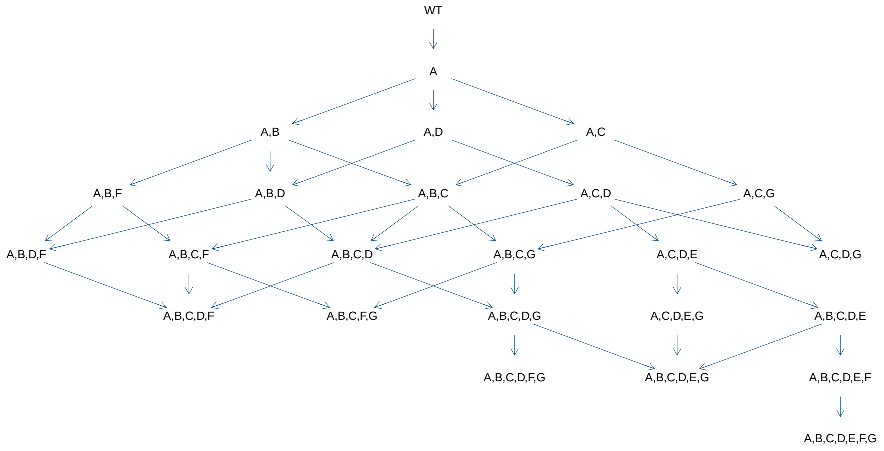

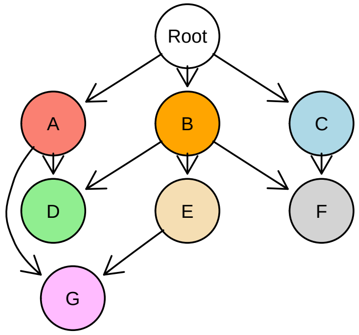

ID UlCZ6OQWroBIY8ER
acc genotypes 28
peaks no back 3
removed edges 4
prop rem edges 0.074

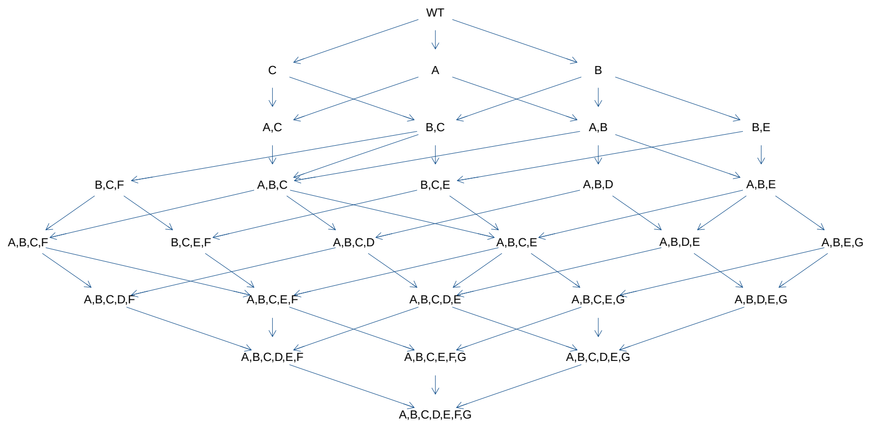

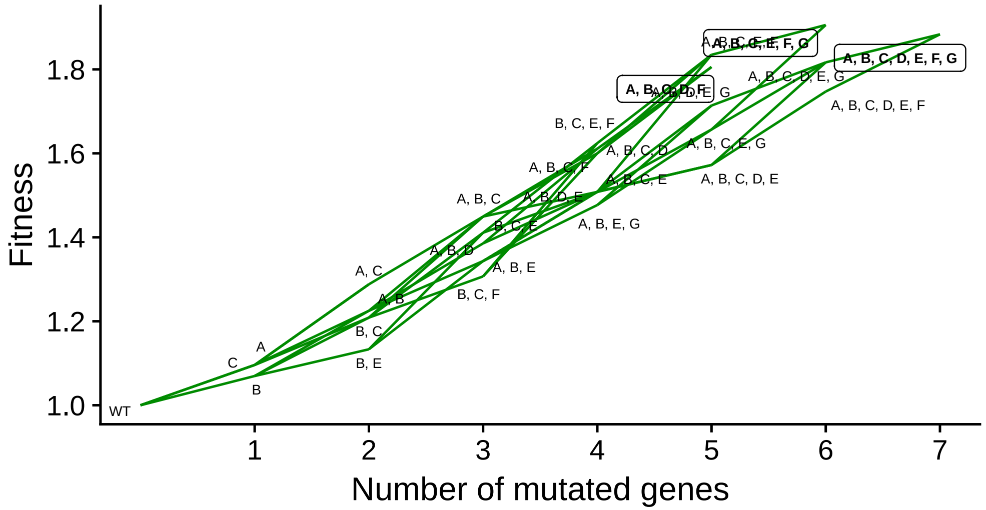

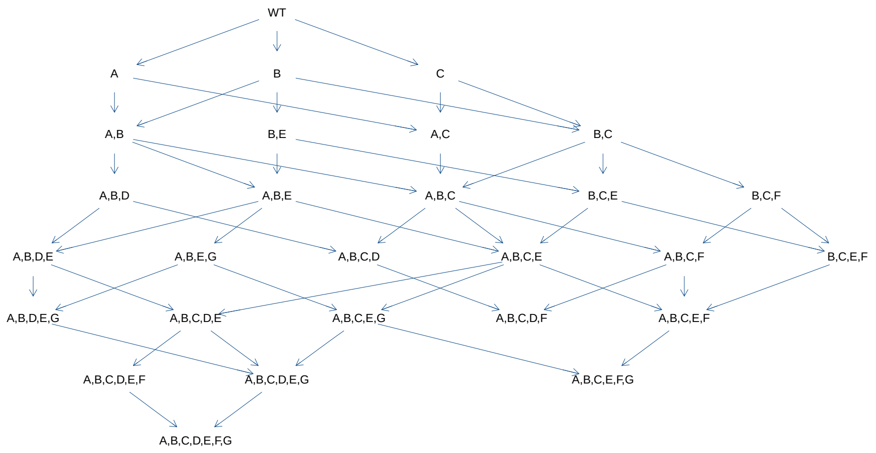

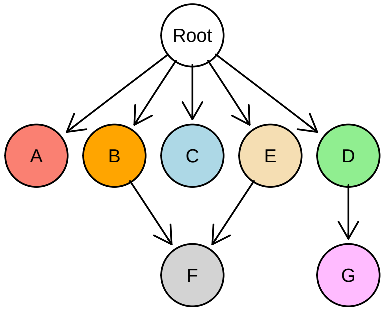

ID	unh1zVBv7V9gaHC5
acc genotypes	60
peaks no back	7
removed edges	29
prop rem edges	0.181

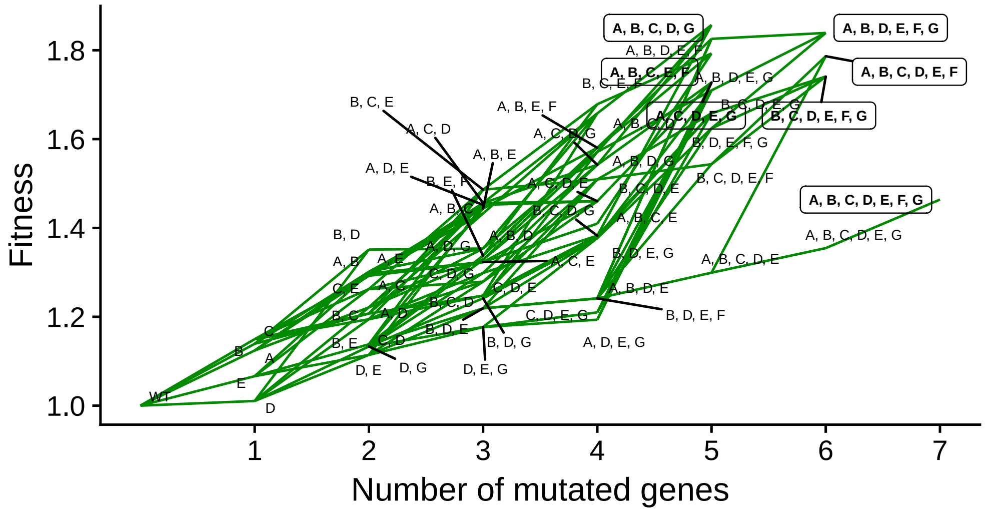

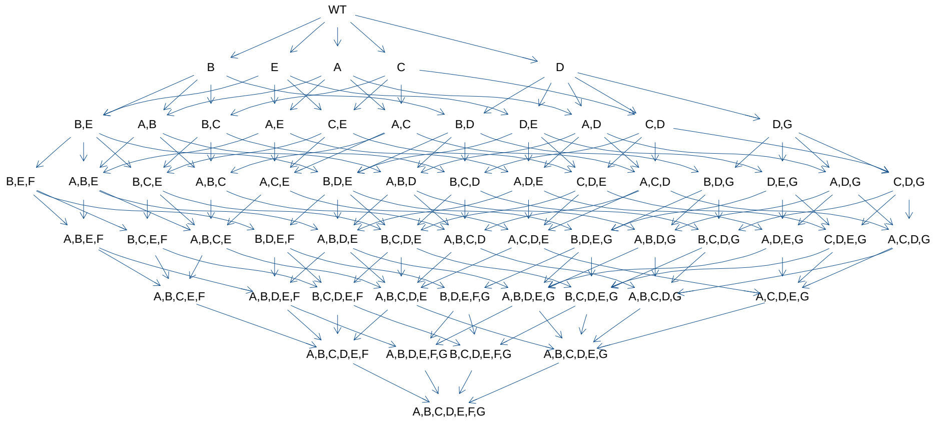

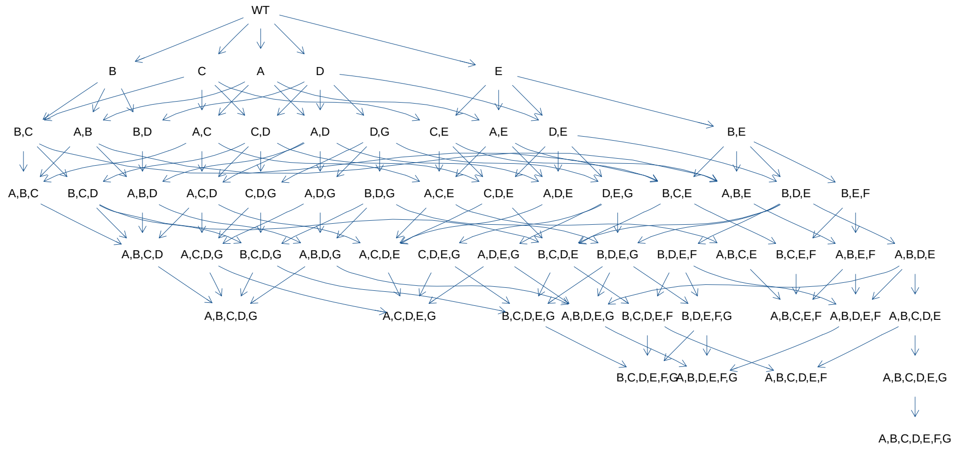

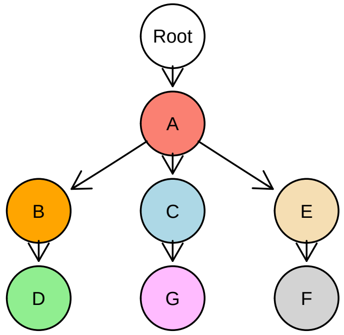

ID	uOql5ivL2U4HWUKS
acc genotypes	28
peaks no back	4
removed edges	4
prop rem edges	0.073

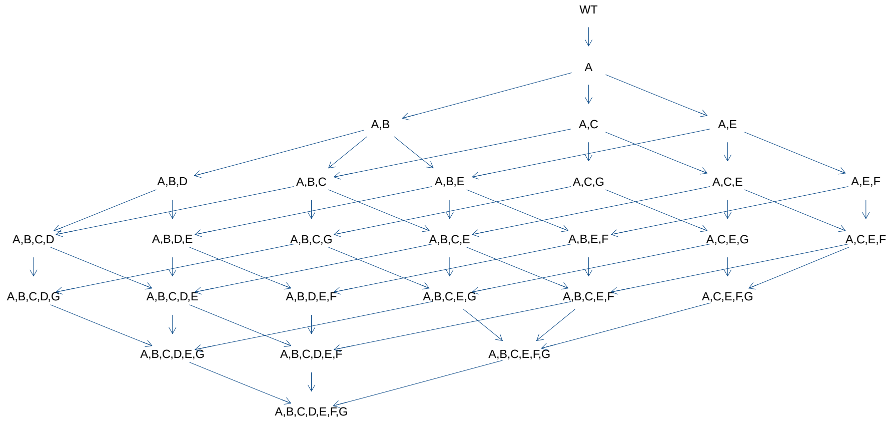

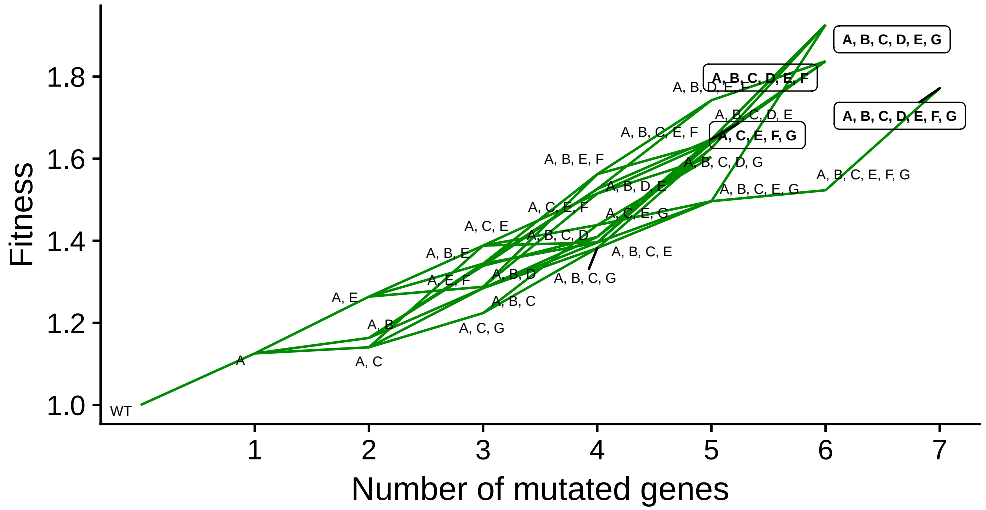

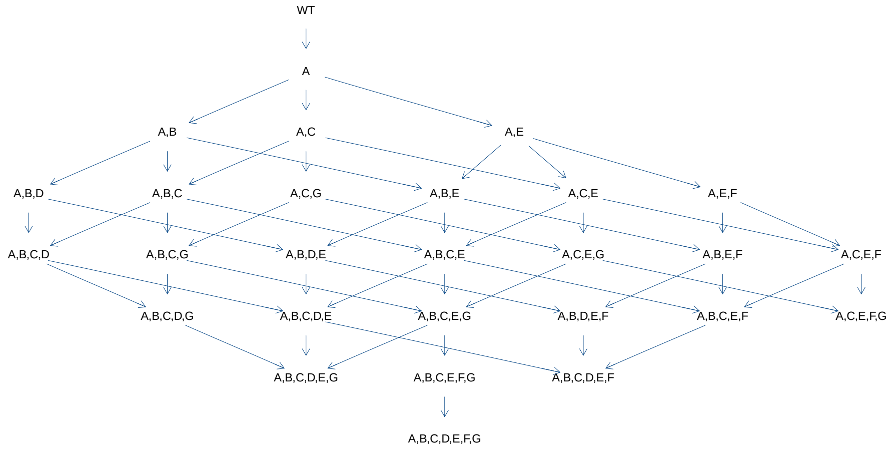

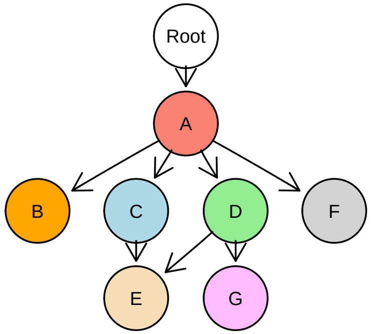

ID	upEZISo6oFVQcSEK
acc genotypes	33
peaks no back	6
removed edges	10
prop rem edges	0.137

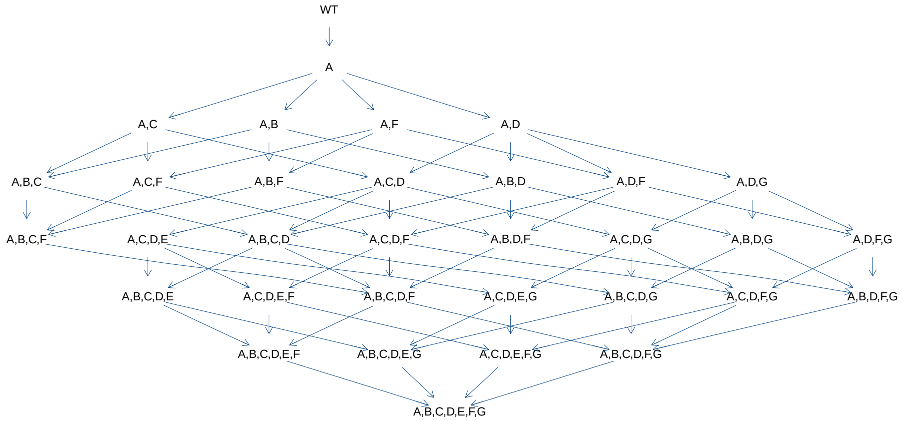

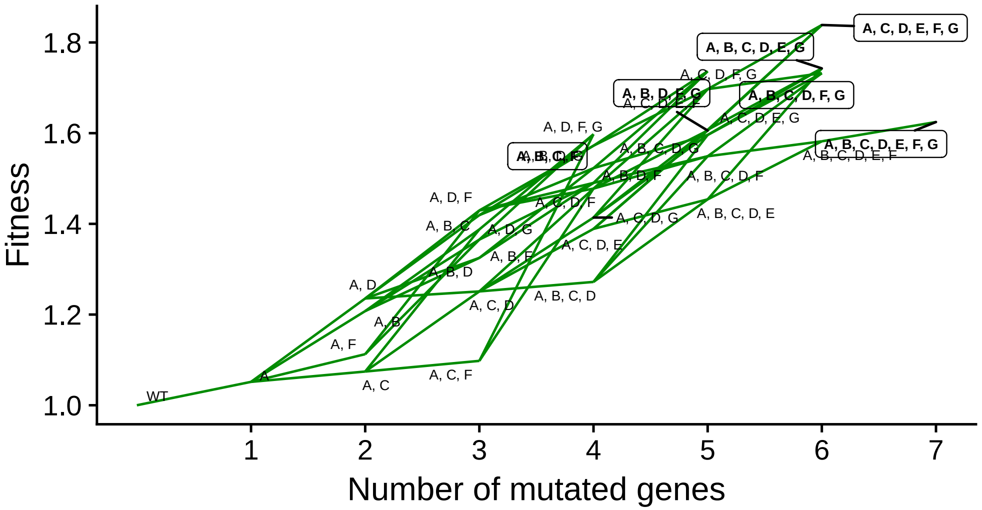

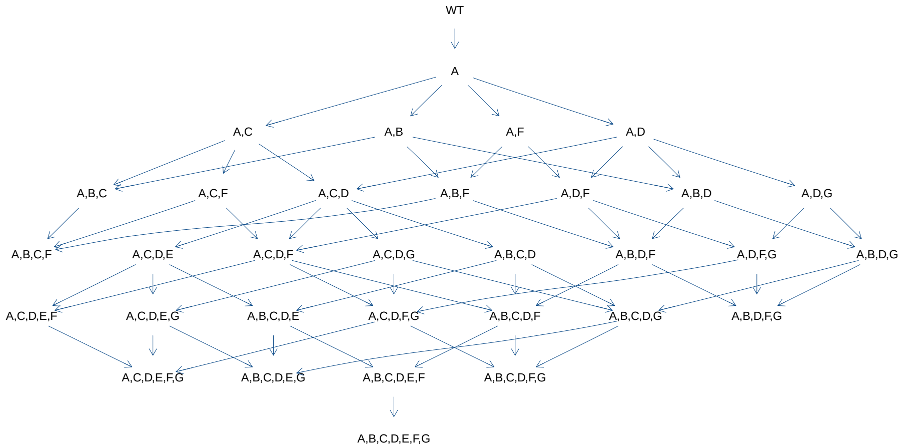

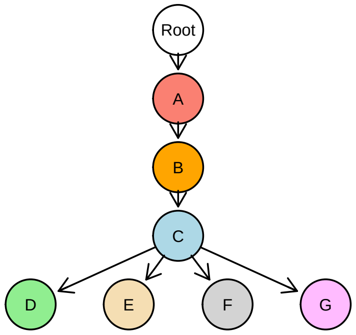

ID	UQu5n10aPzWHnGf59
acc genotypes	19
peaks no back	5
removed edges	6
prop rem edges	0.171

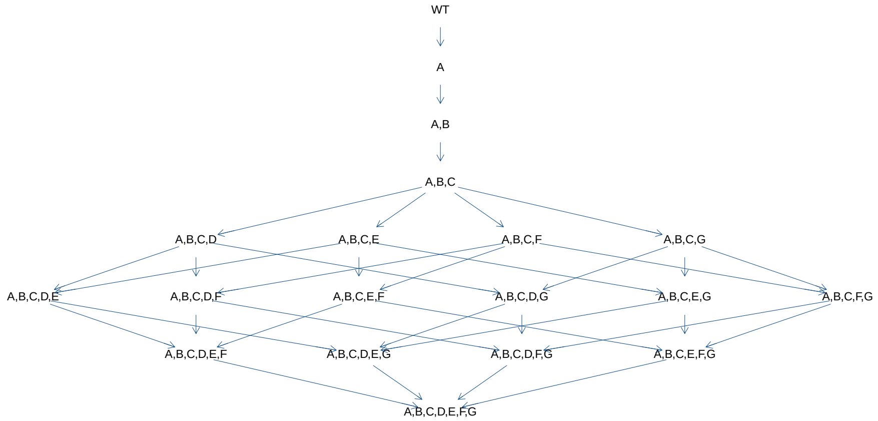

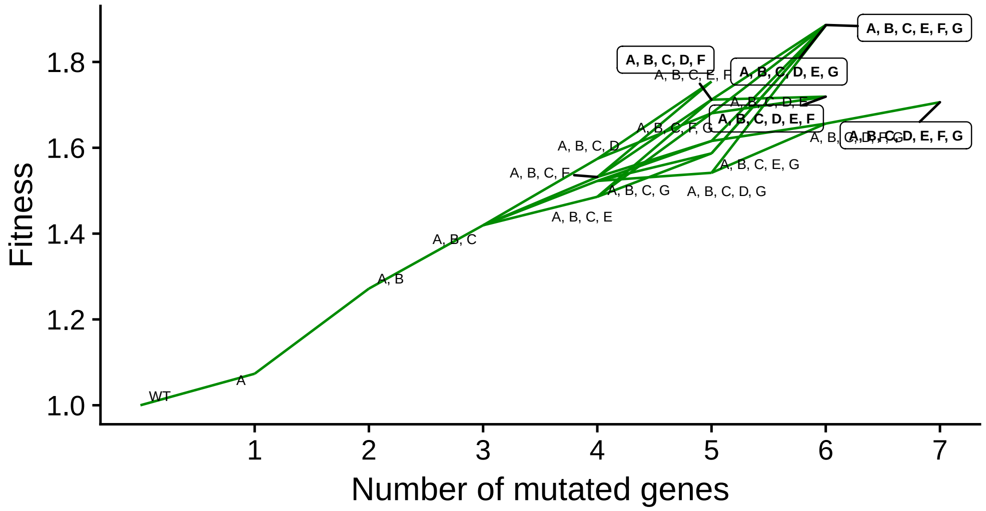

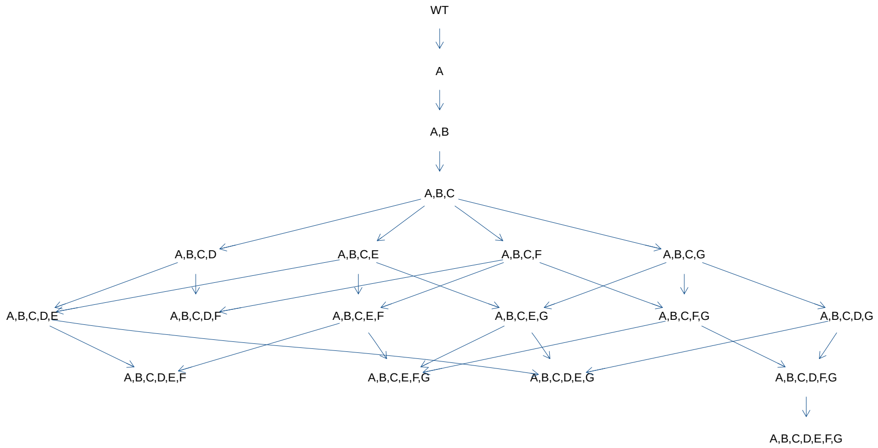

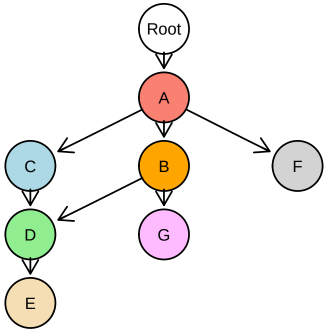

ID	uw7YHXhohAPISHwZ
acc genotypes	21
peaks no back	2
removed edges	8
prop rem edges	0.216

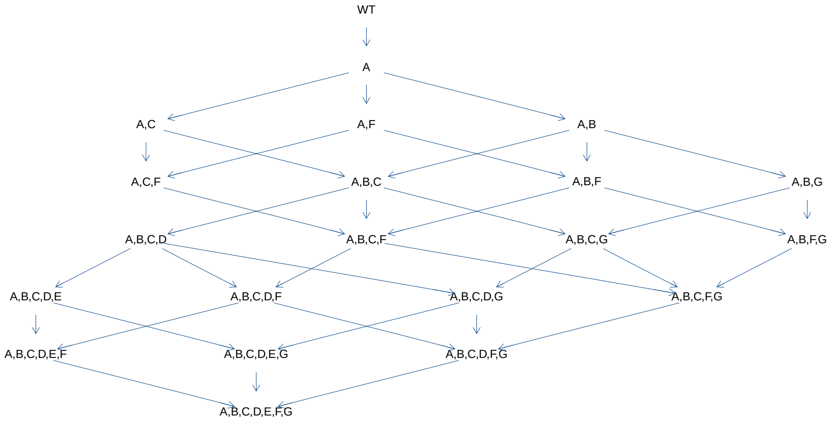

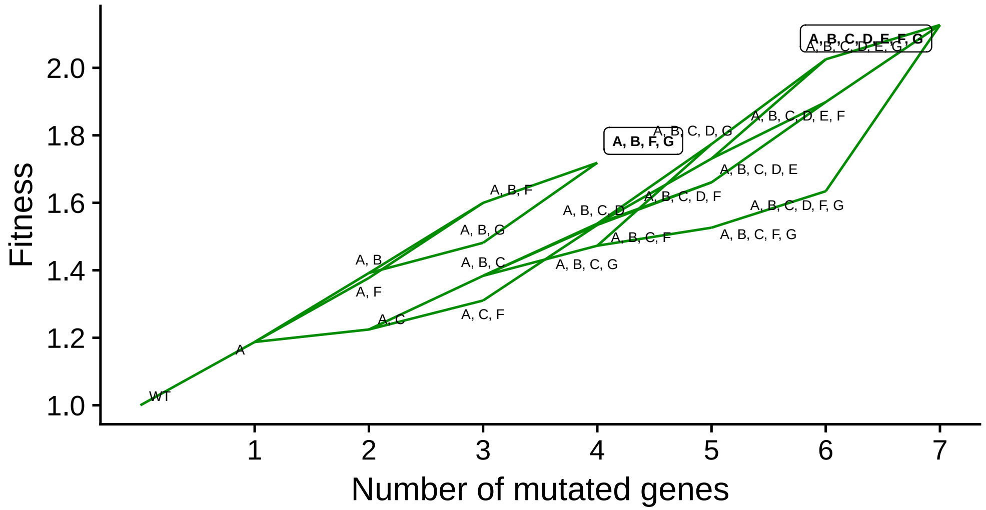

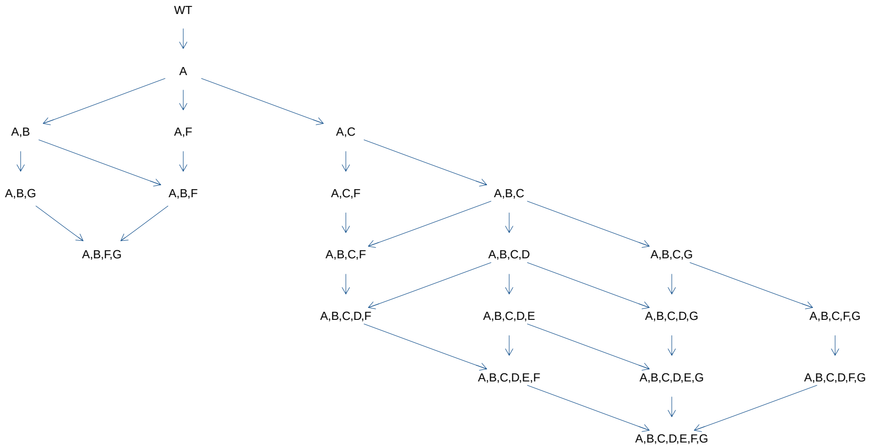

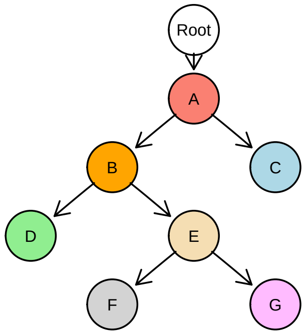

ID V4JKndnKWM310nITX
acc genotypes 23
peaks no back 4
removed edges 5
prop rem edges 0.114

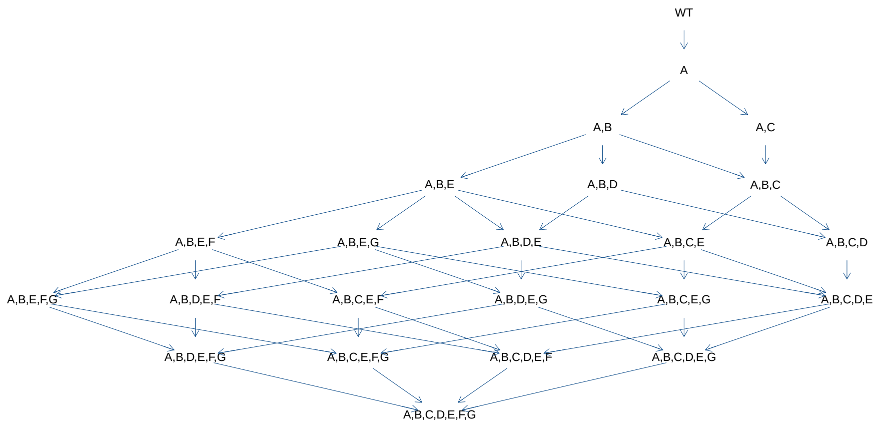

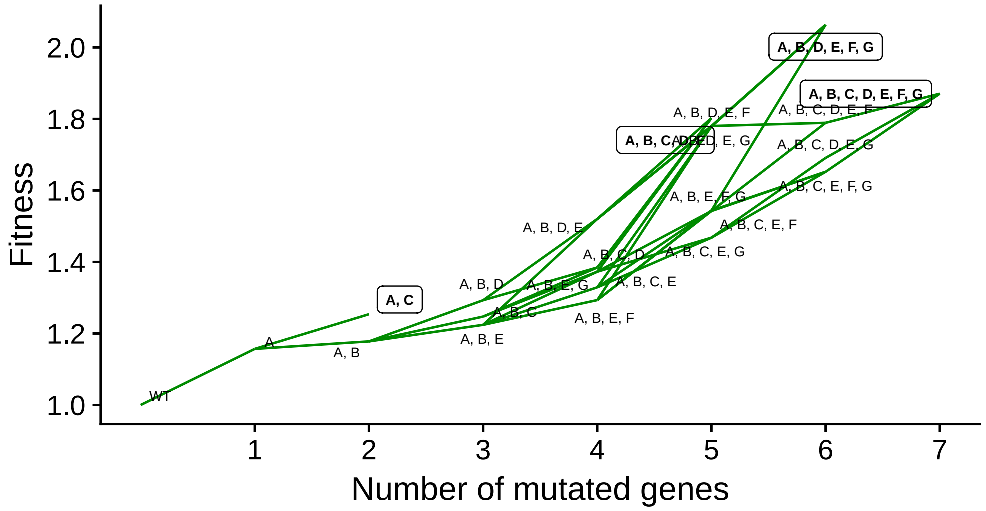

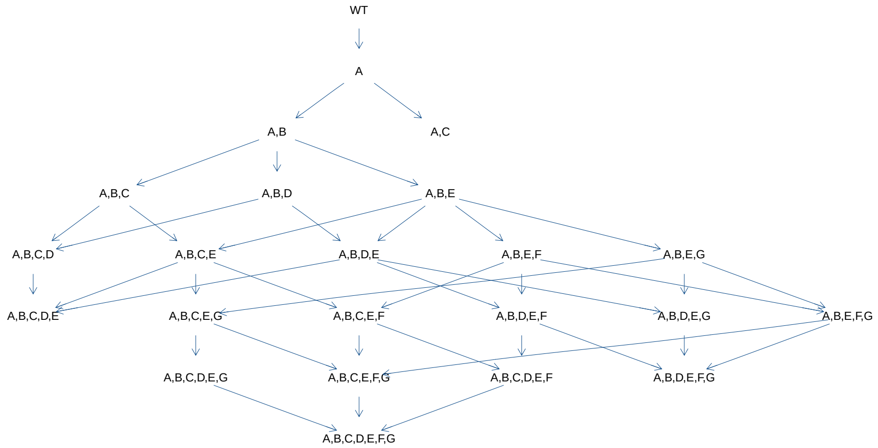

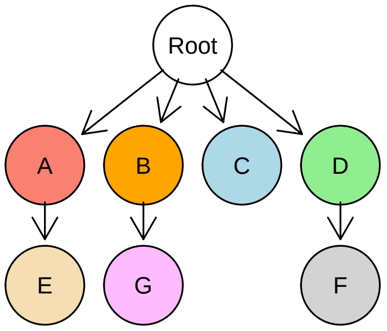

ID	V4TR5vUVudRPVWmM
acc genotypes	54
peaks no back	8
removed edges	24
prop rem edges	0.178

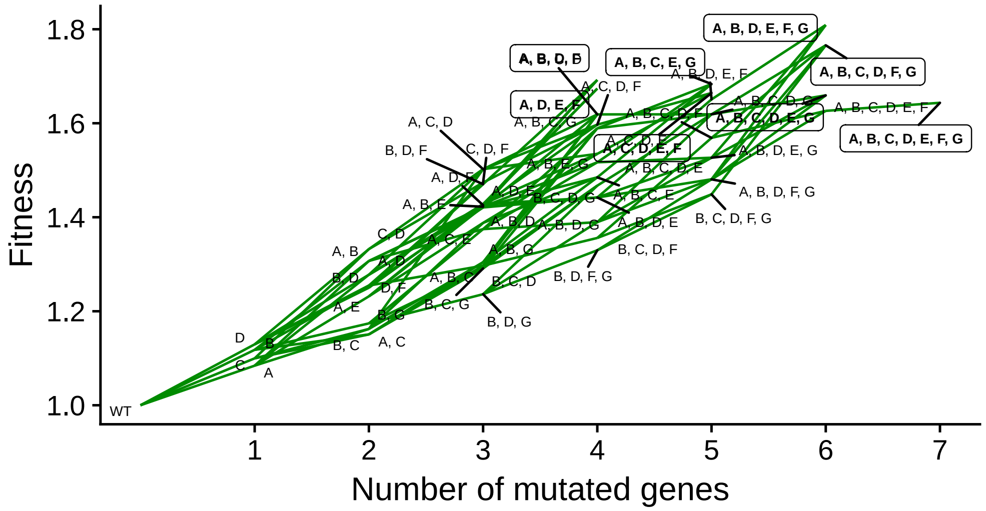

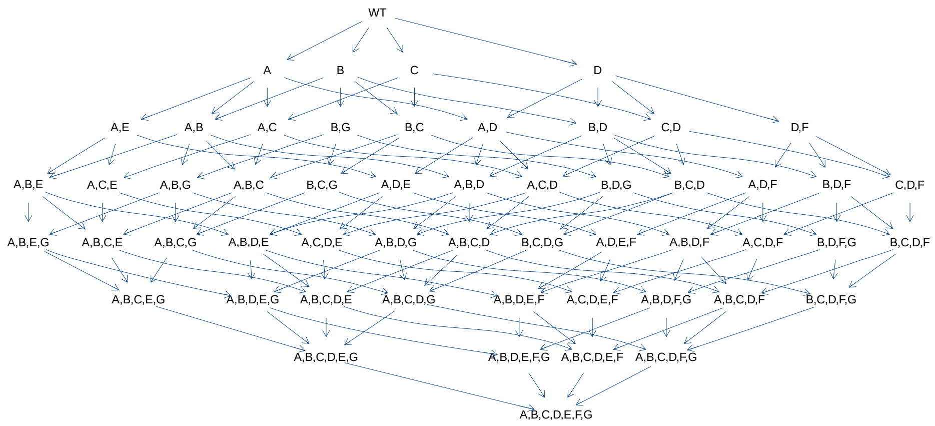

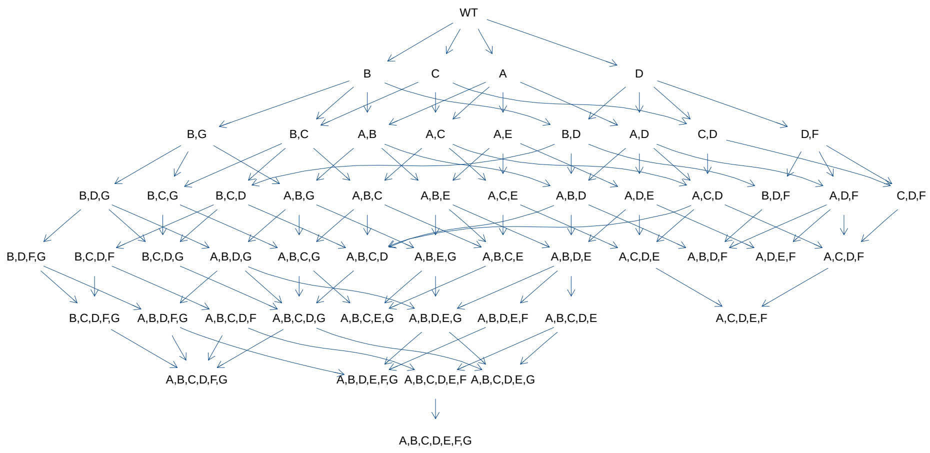

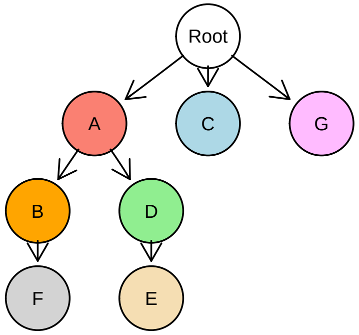

ID	vDqUv4dtGIoB94NH
acc genotypes	40
peaks no back	8
removed edges	13
prop rem edges	0.141

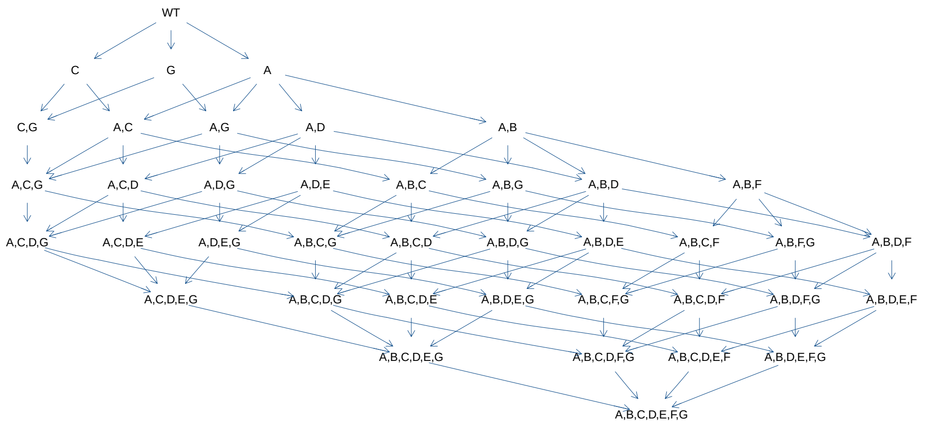

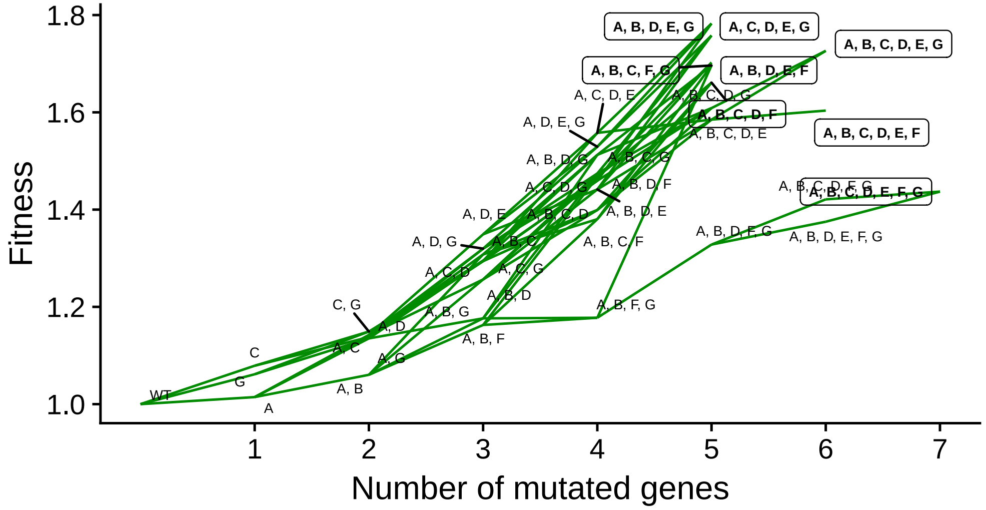

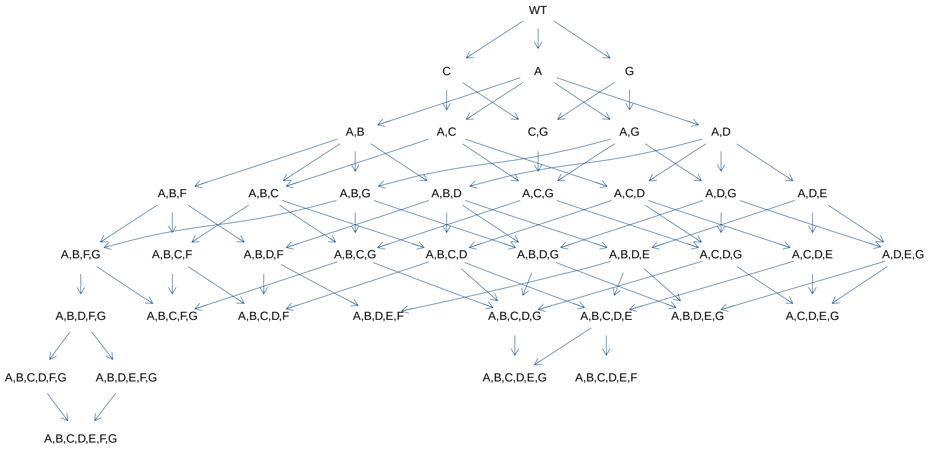

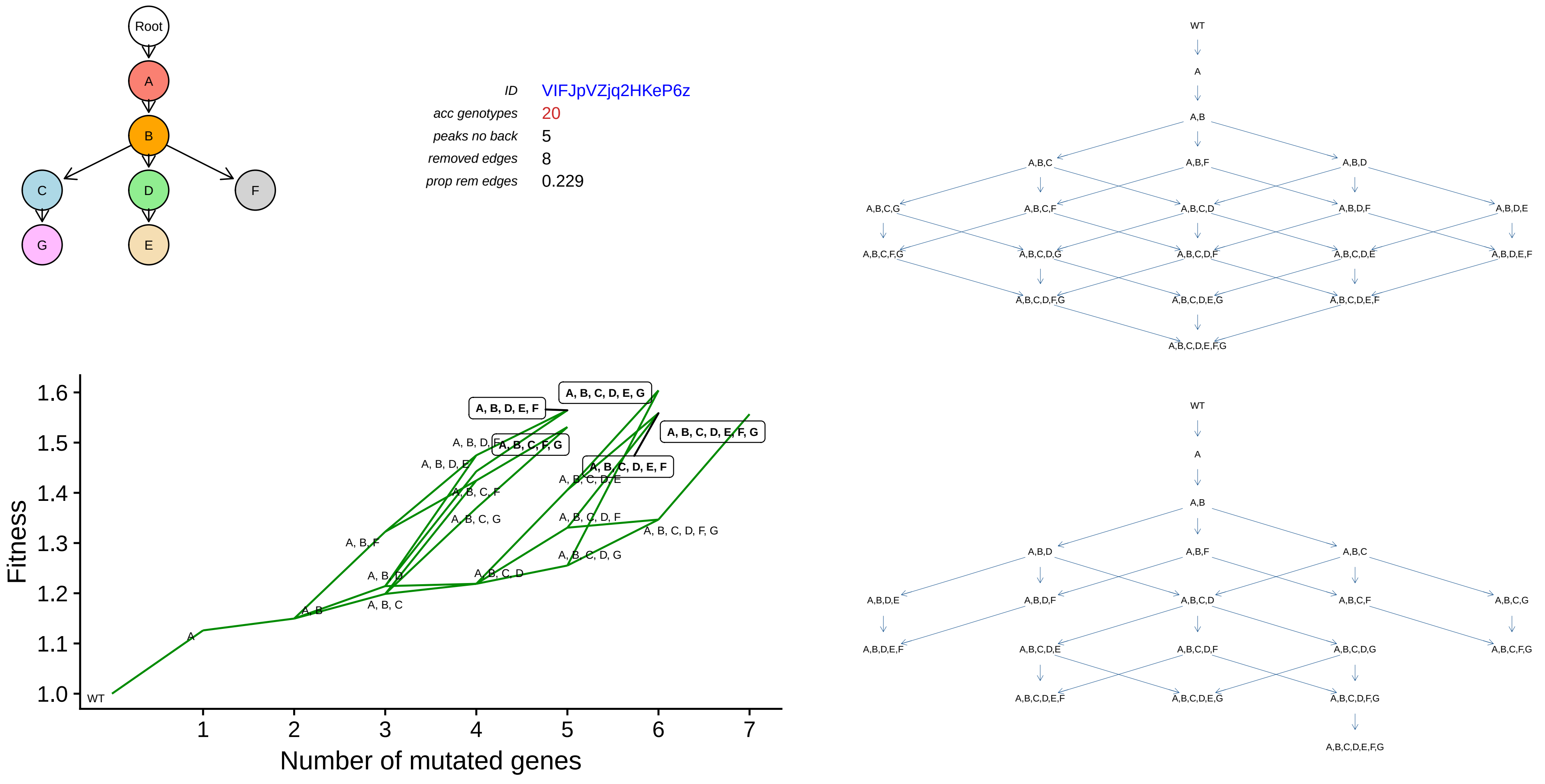
Root
A
B
C
D
F
G
E
ID VIFJpVZjq2HKeP6z
acc genotypes 20
peaks no back 5
removed edges 8
prop rem edges 0.229
Fitness
Number of mutated genes
A, B, D, E, F
A, B, C, D, E, G
A, B, C, F, G
A, B, C, D, E, F, G
A, B, C, D, E, F
WT
A
A, B
A, B, C
A, B, D
A, B, F
A, B, C, D
A, B, C, F
A, B, C, G
A, B, D, E
A, B, D, F
A, B, C, D, E
A, B, C, D, F
A, B, C, D, G
A, B, C, D, F, G

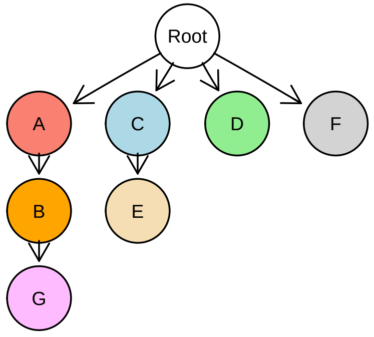

ID	vK7dEdwIvdYYs5rB
acc genotypes	48
peaks no back	10
removed edges	24
prop rem edges	0.207

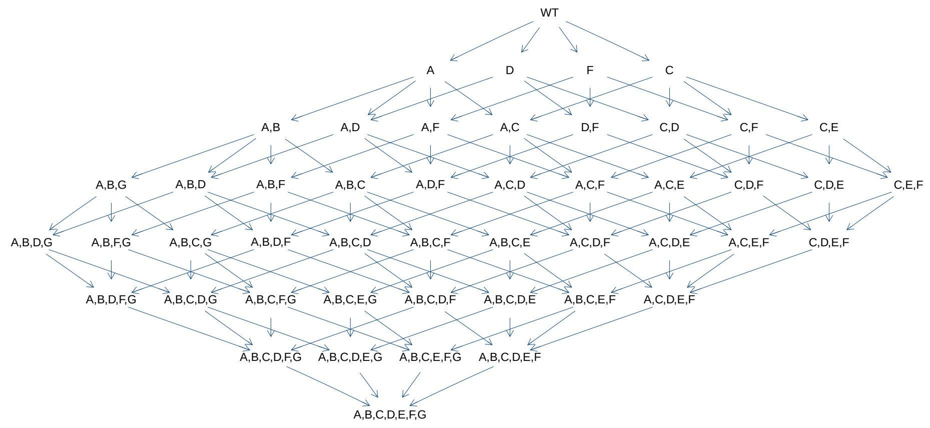

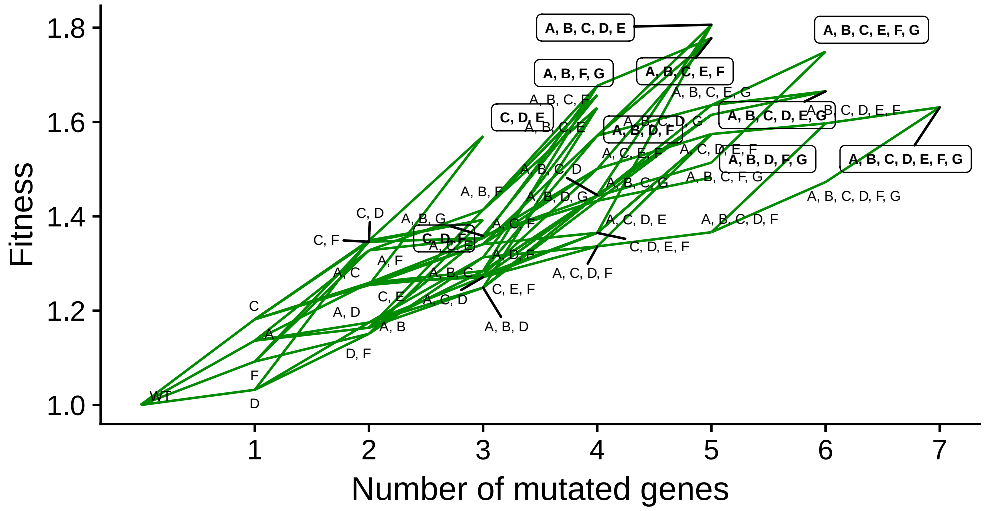

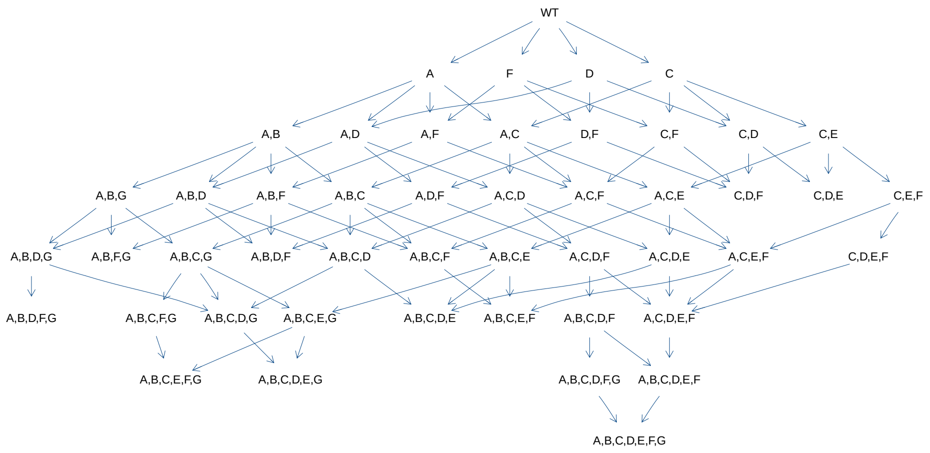

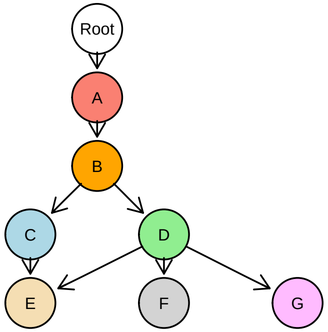

ID	VqVZDfELXTXwJzx10
acc genotypes	16
peaks no back	3
removed edges	2
prop rem edges	0.08

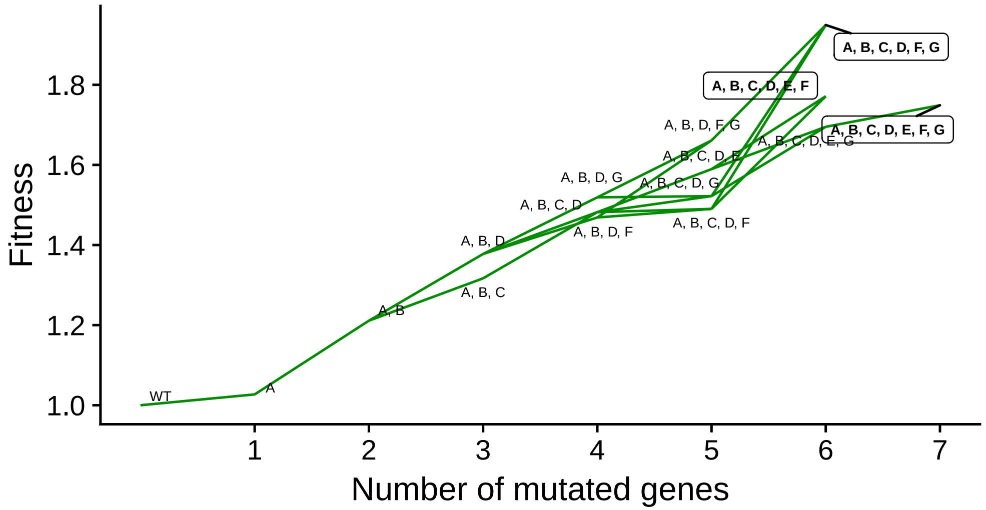

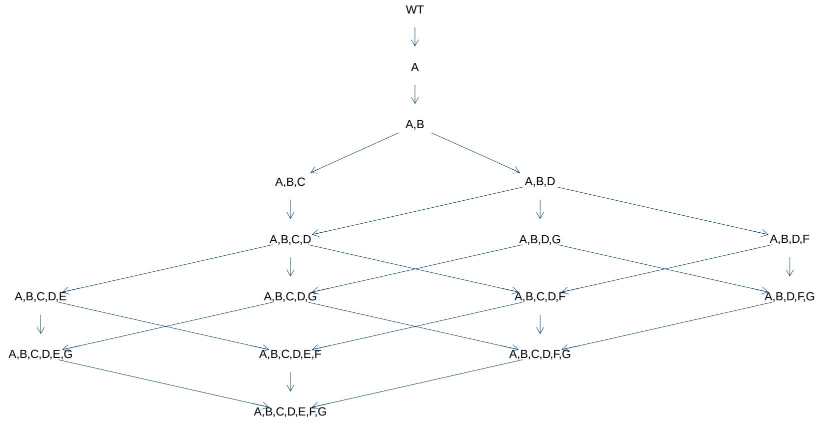

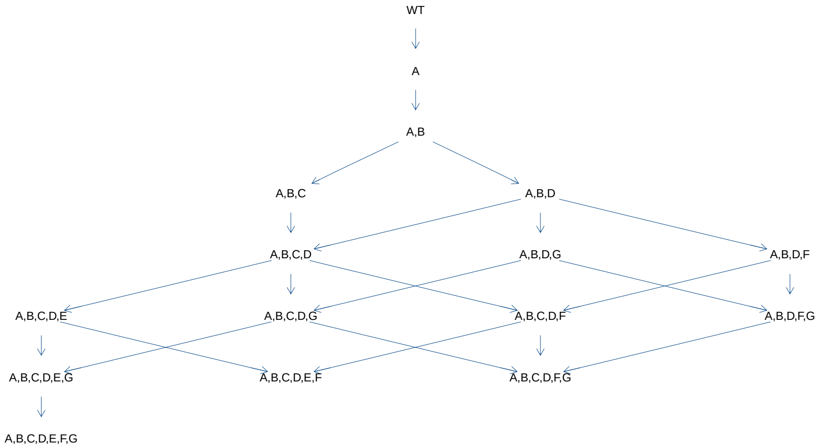

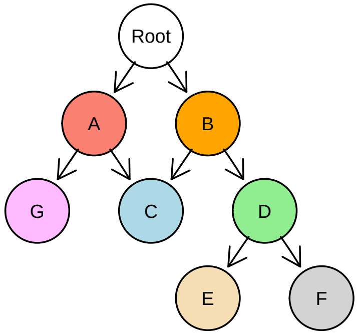

ID	vviAaqqVbYrKTfxK
acc genotypes	28
peaks no back	7
removed edges	12
prop rem edges	0.218

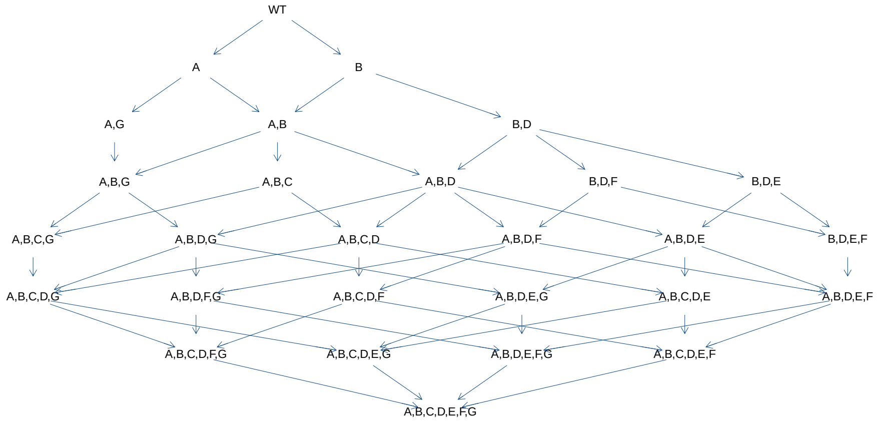

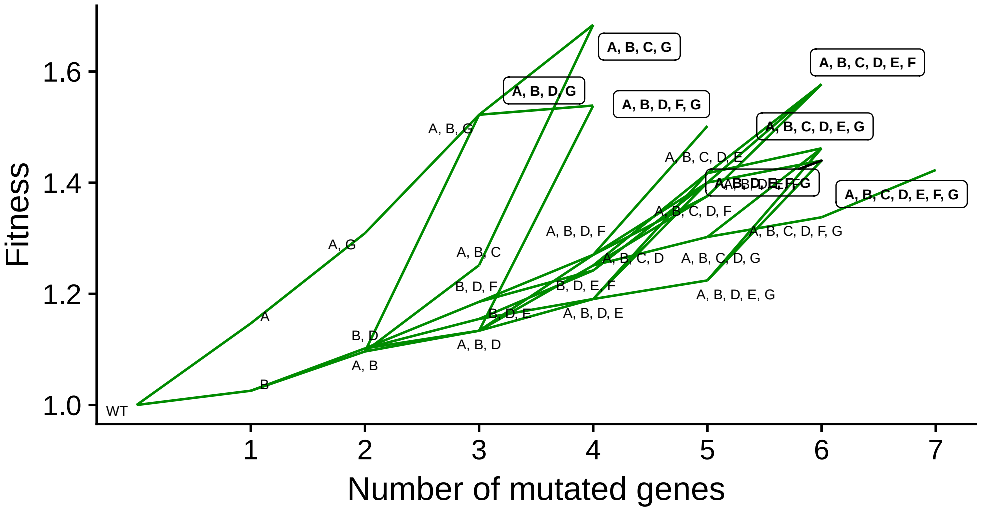

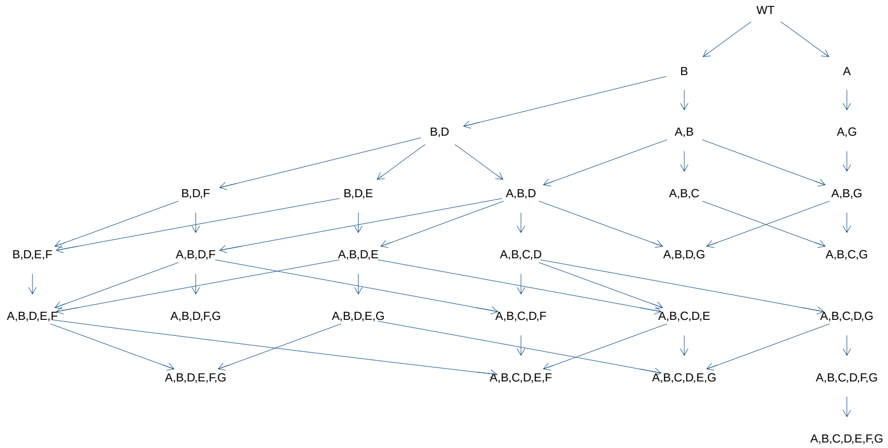

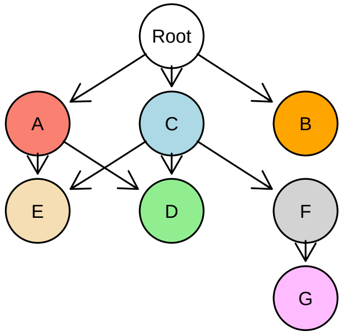

ID VYKTwc6PGOfo5RH5
acc genotypes 34
peaks no back 5
removed edges 12
prop rem edges 0.164

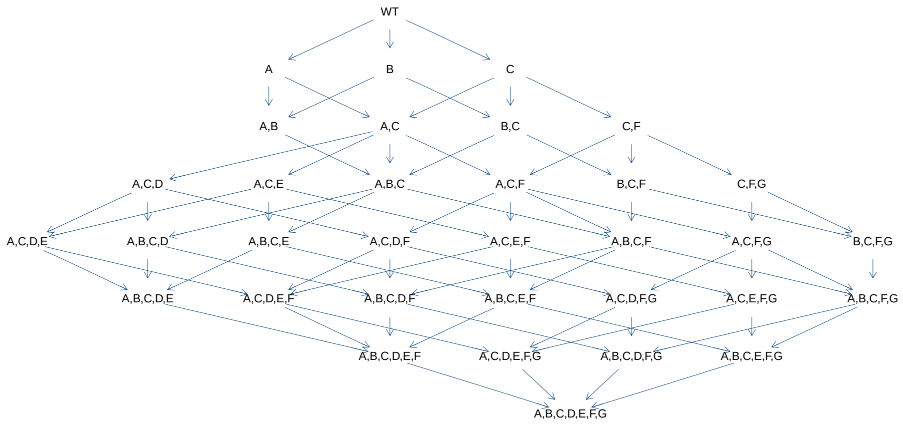

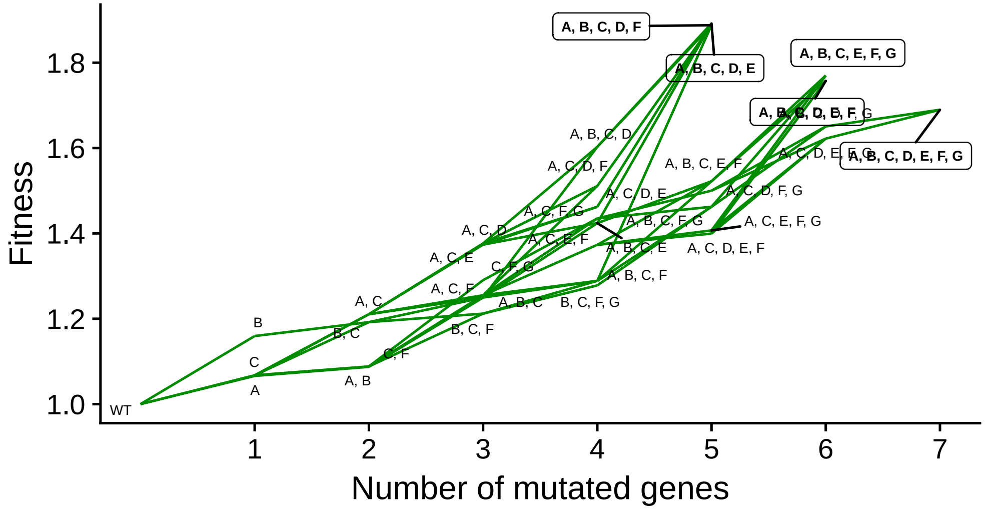

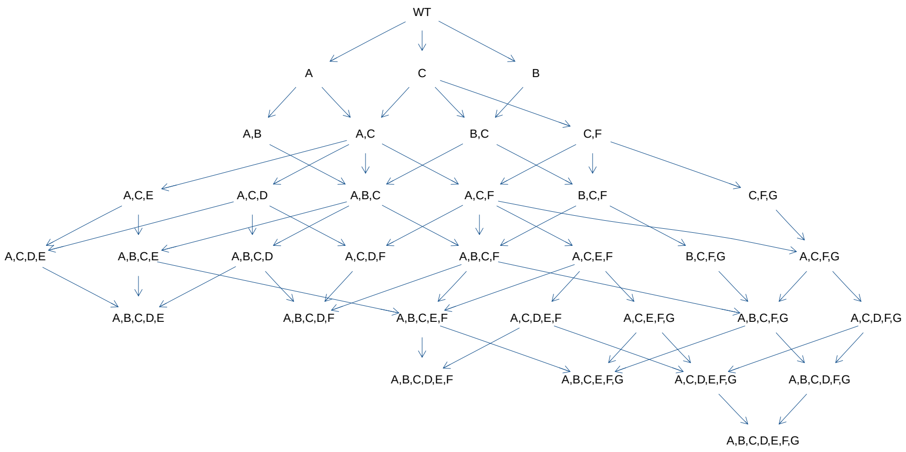

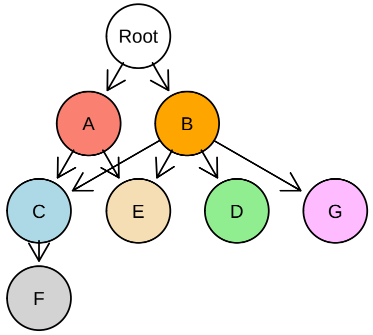

ID vz7pdw2T4KJ7Ey10T
acc genotypes 30
peaks no back 6
removed edges 14
prop rem edges 0.222

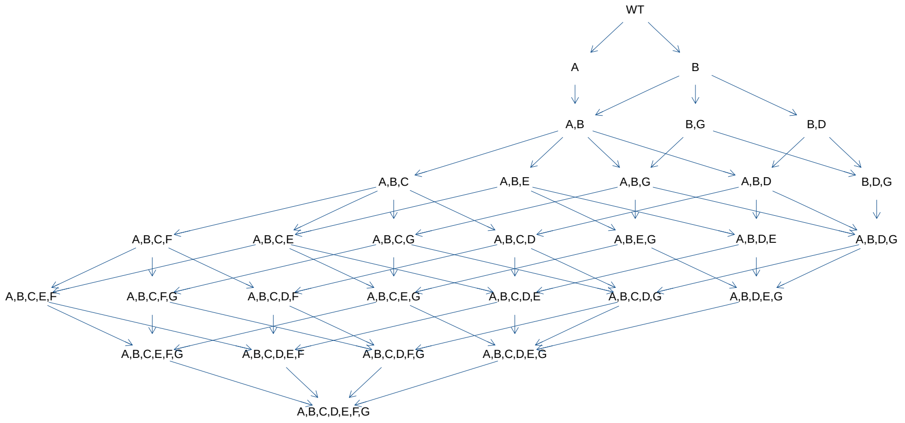

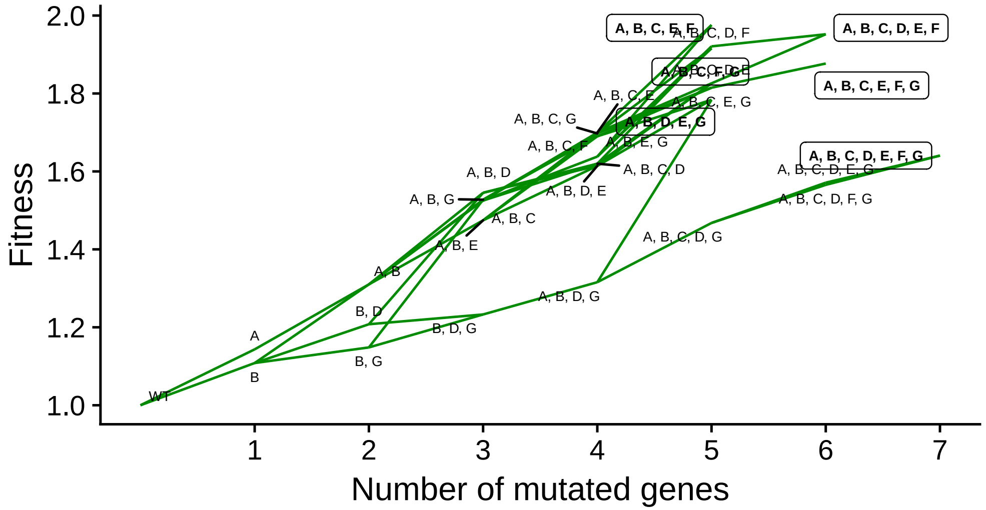

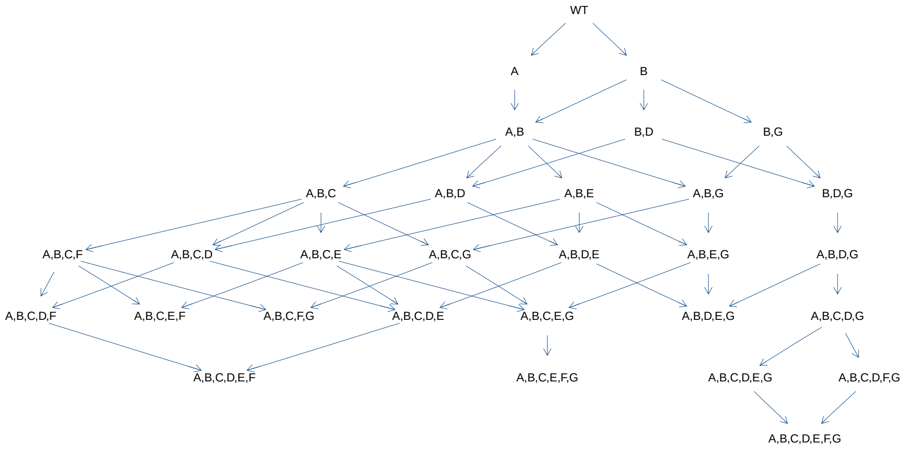

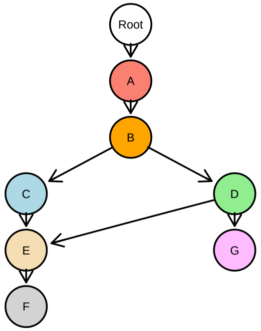

ID	WCthf7pnLrf65210C
acc genotypes	12
peaks no back	2
removed edges	1
prop rem edges	0.067

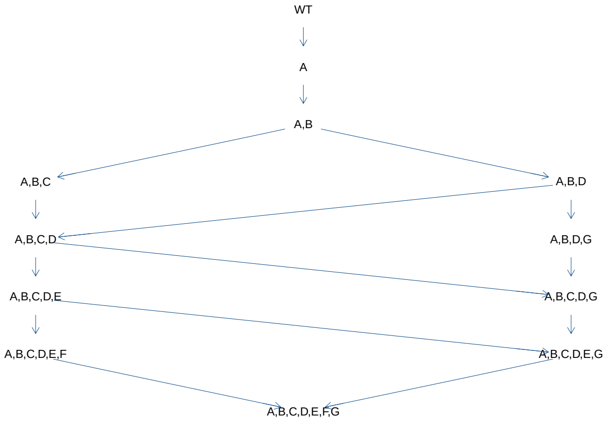

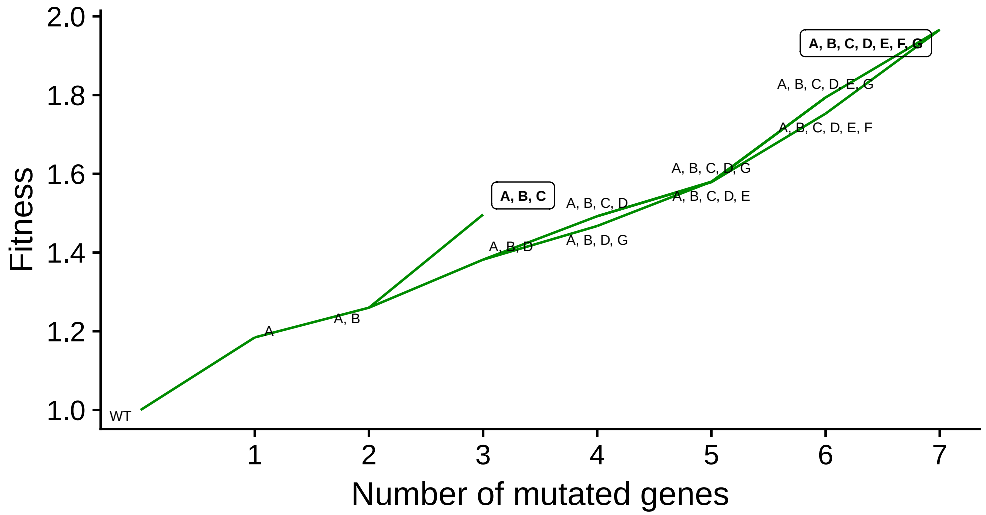

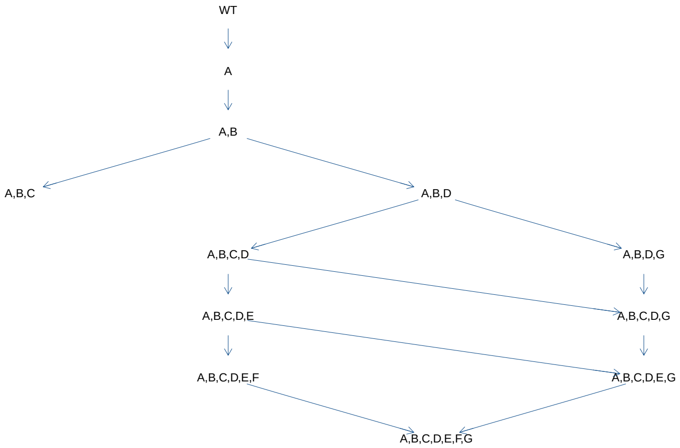

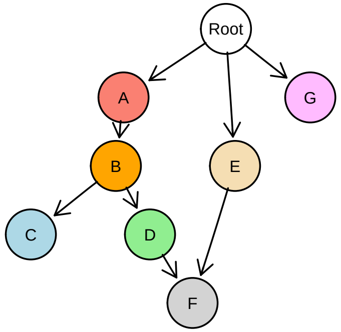

ID	wGM2GvMSYVAE2KwT
acc genotypes	28
peaks no back	6
removed edges	13
prop rem edges	0.232

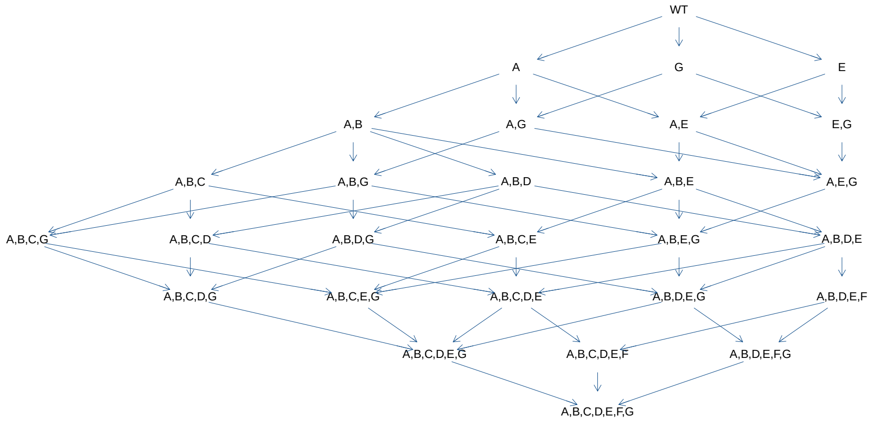

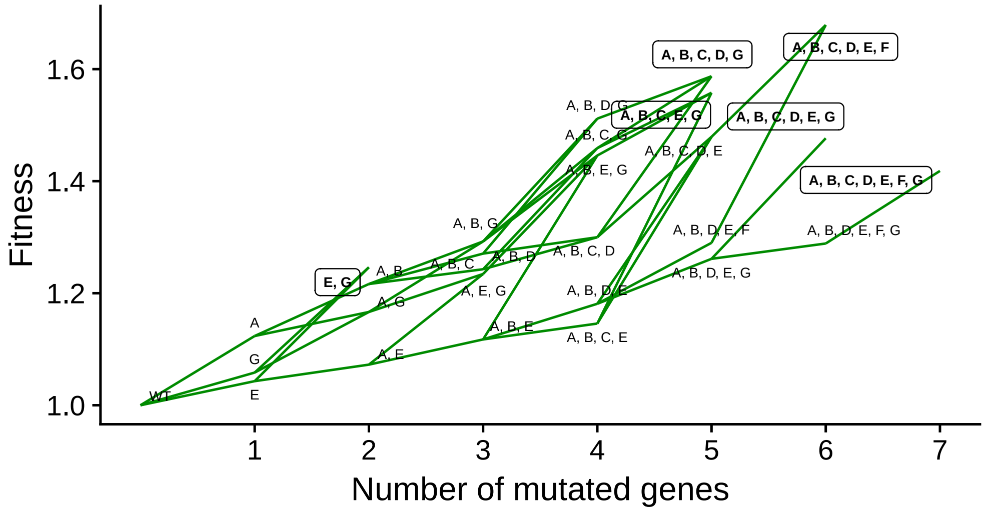

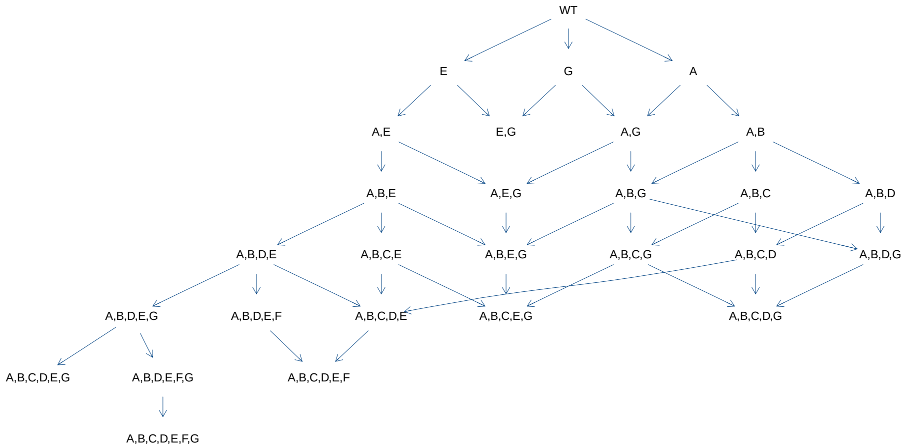

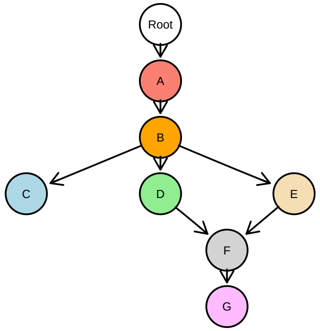

ID	WpF105HbEDoECa8vs
acc genotypes	14
peaks no back	2
removed edges	1
prop rem edges	0.05

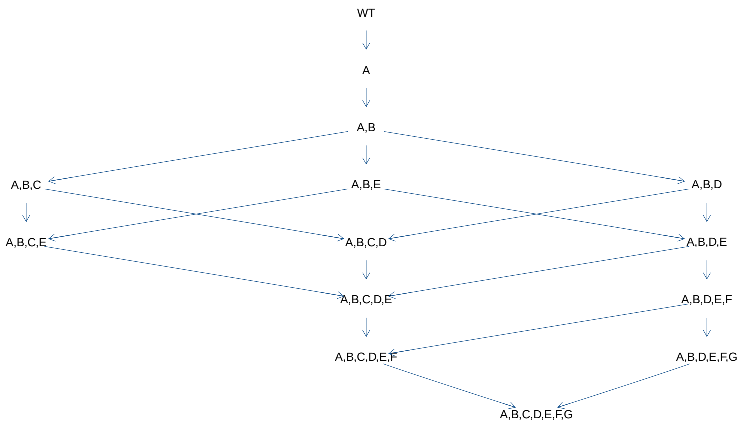

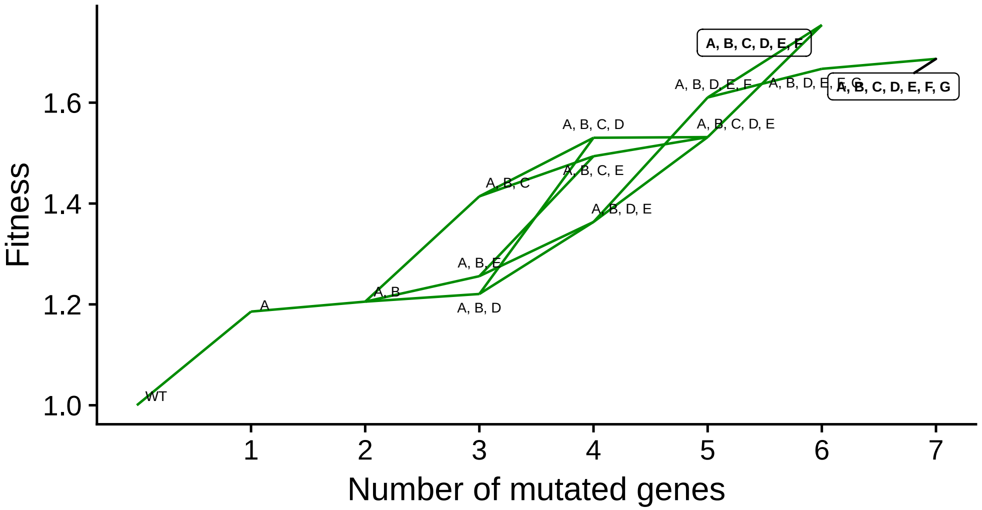

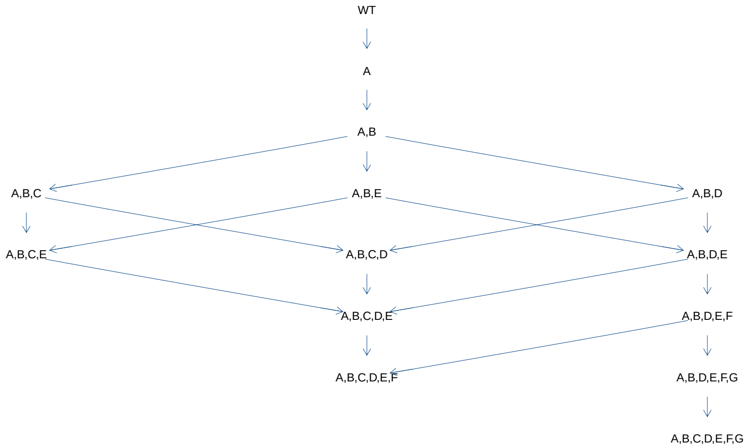

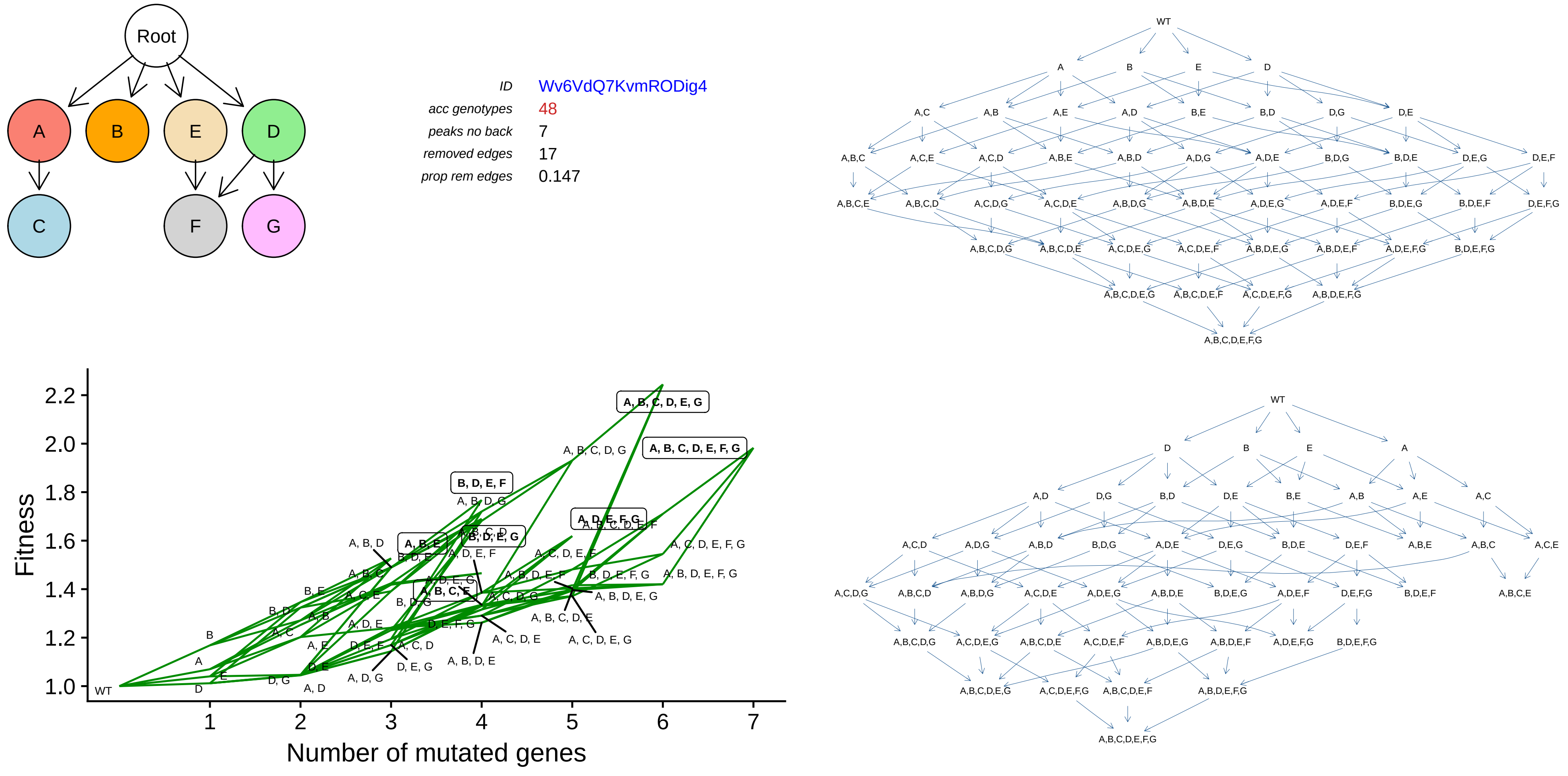
Root
A
B
E
D
C
F
G
ID
Wv6VdQ7KvmRODig4
acc genotypes
48
peaks no back
7
removed edges
17
prop rem edges
0.147
Fitness
Number of mutated genes
A, B, C, D, E, G
A, B, C, D, E, F, G
B, D, E, F
A, D, E, F, G
B, D, E, G
A, B, E
A, B, C, E
WT

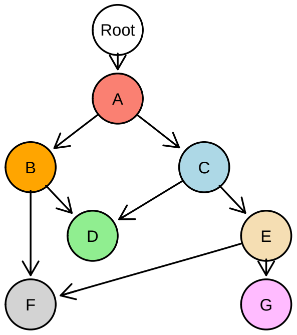

ID wXZP1BcX68smcT7W
acc genotypes 16
peaks no back 4
removed edges 4
prop rem edges 0.167

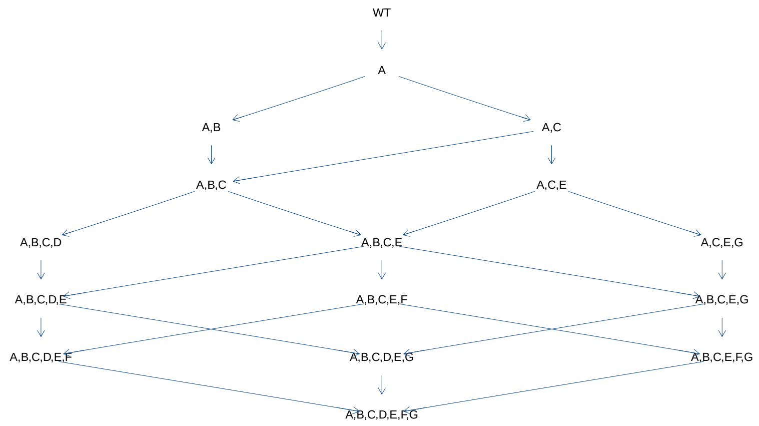

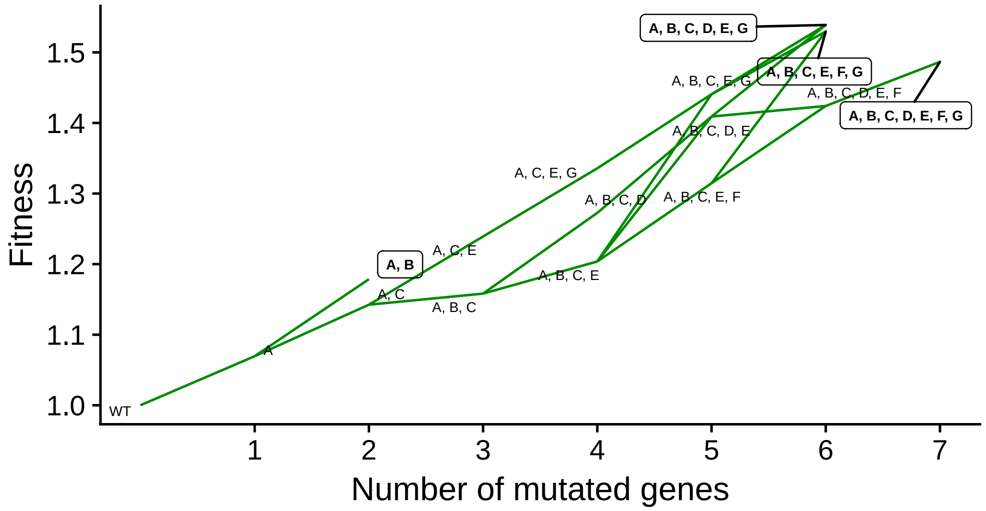

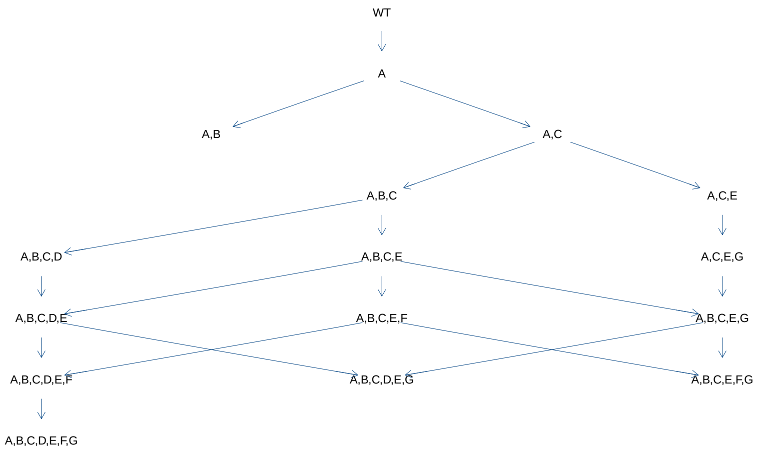

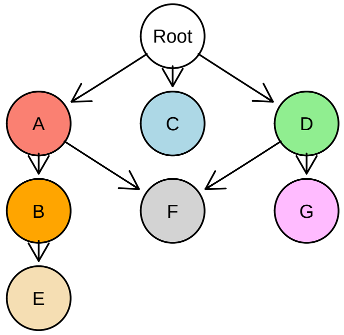

ID WzbE1eRFpaclNCxU

acc genotypes 36

peaks no back 6

removed edges 14

prop rem edges 0.179

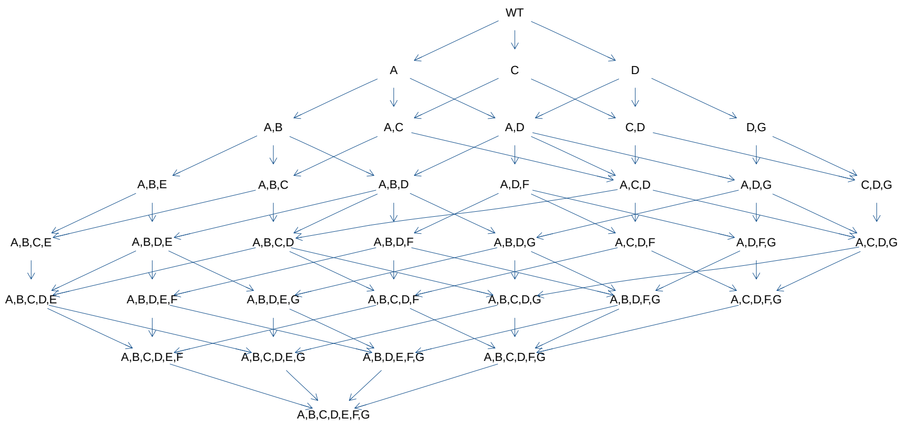

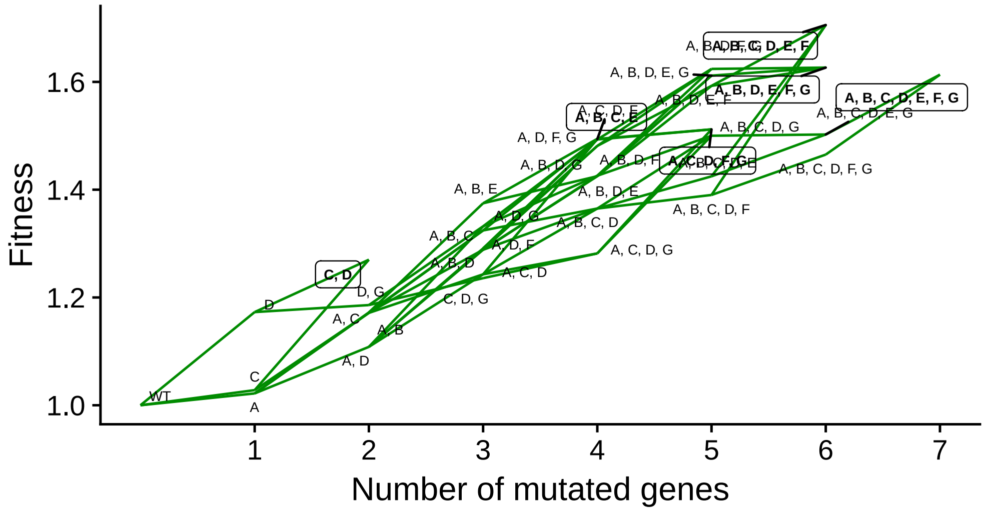

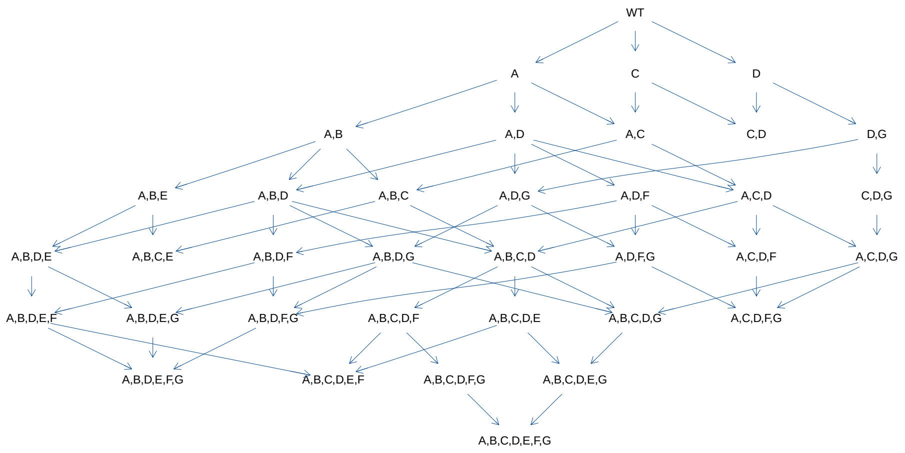

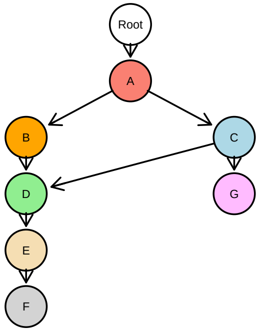

ID	wzBFbd7oN5KHrhdR
acc genotypes	13
peaks no back	2
removed edges	1
prop rem edges	0.059

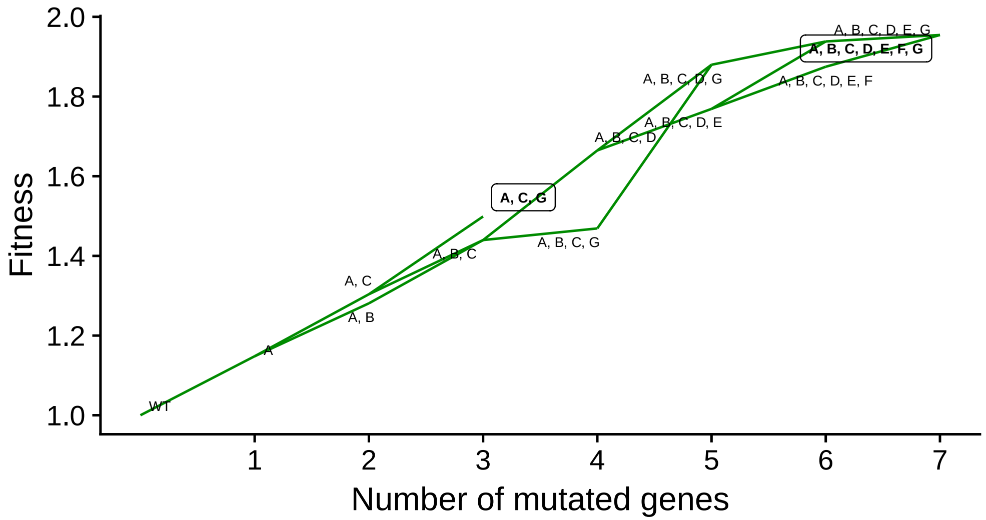

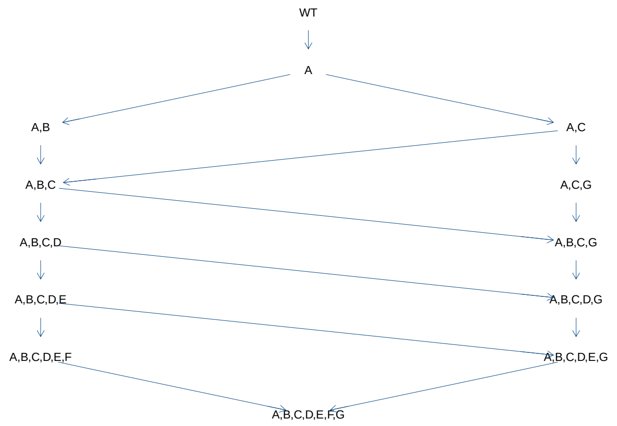

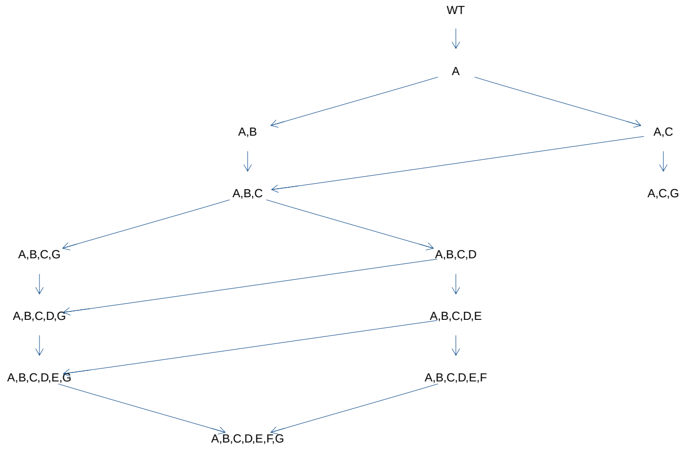

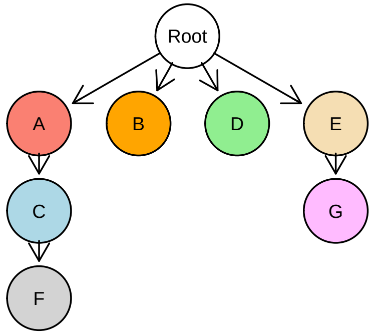

ID	X10HPXEjtf2hUa7l1
acc genotypes	48
peaks no back	9
removed edges	27
prop rem edges	0.233

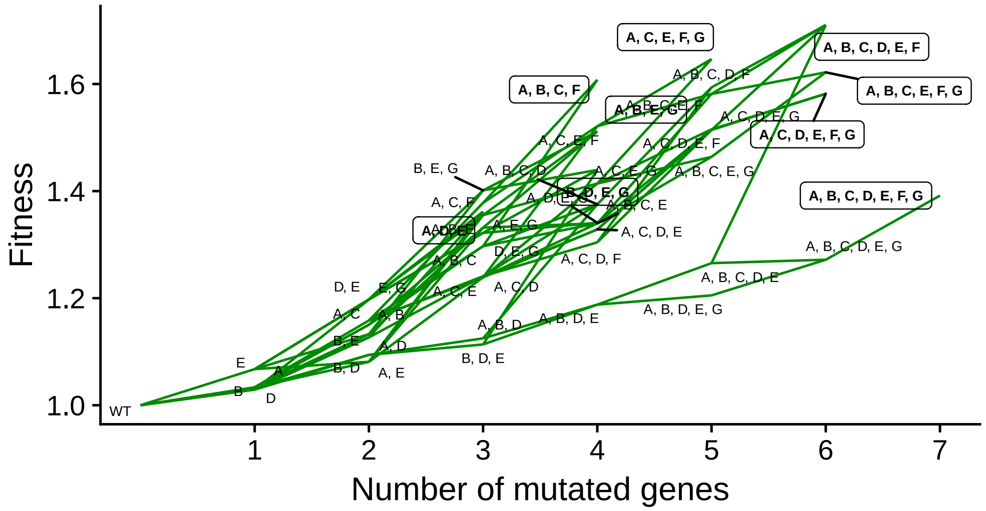

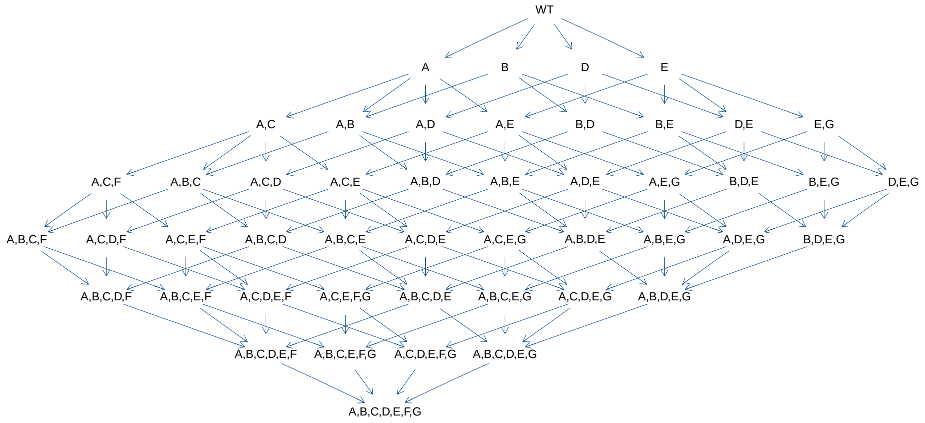

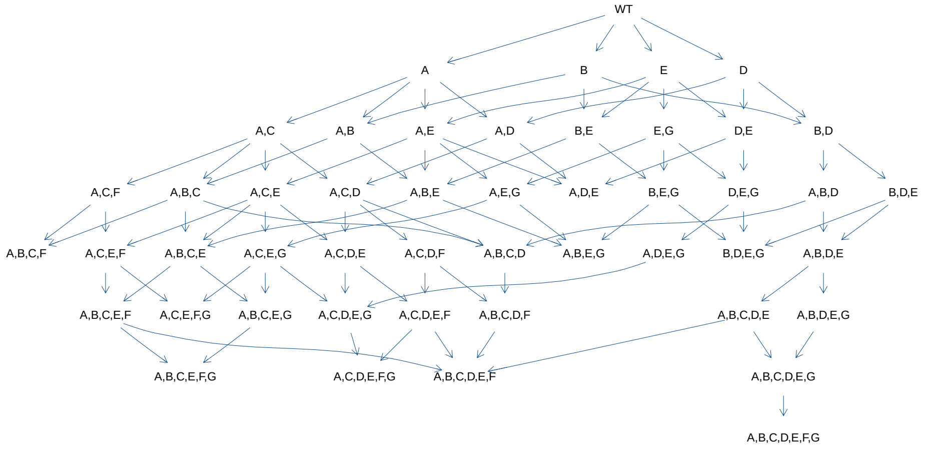

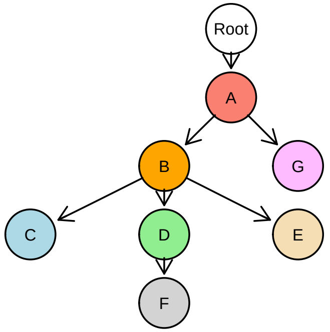

ID	x2Dh5bR5ksqmvSQp
acc genotypes	27
peaks no back	5
removed edges	14
prop rem edges	0.25

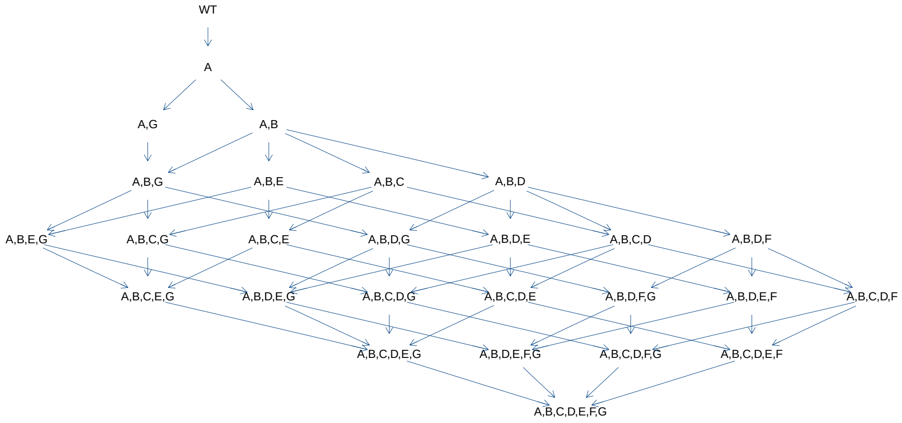

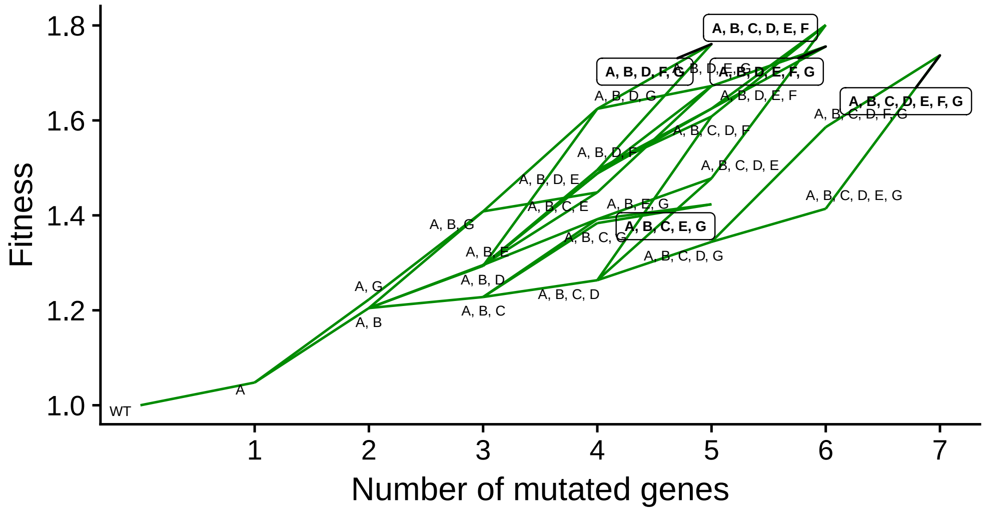

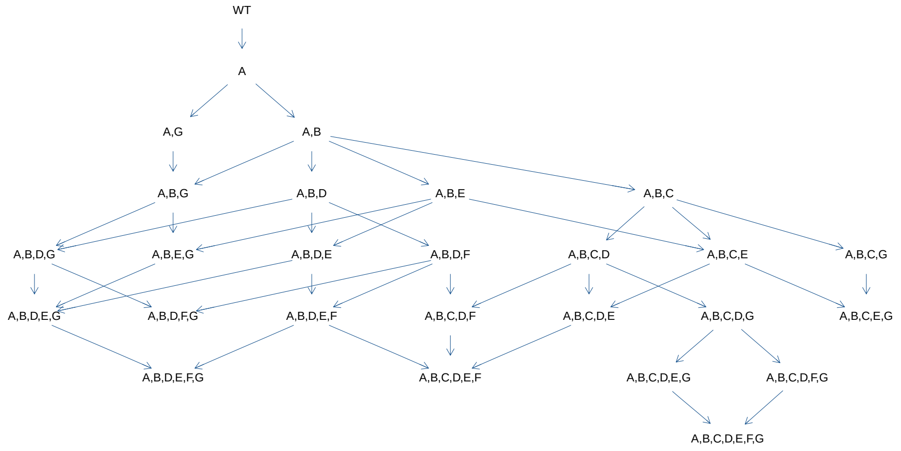

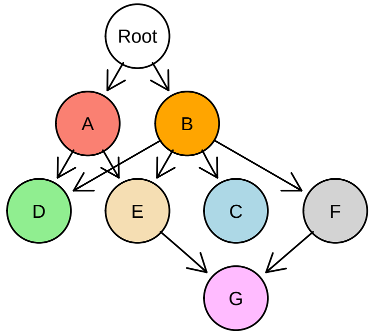

ID	XkAnRfxPG4FT103ju
acc genotypes	26
peaks no back	6
removed edges	12
prop rem edges	0.235

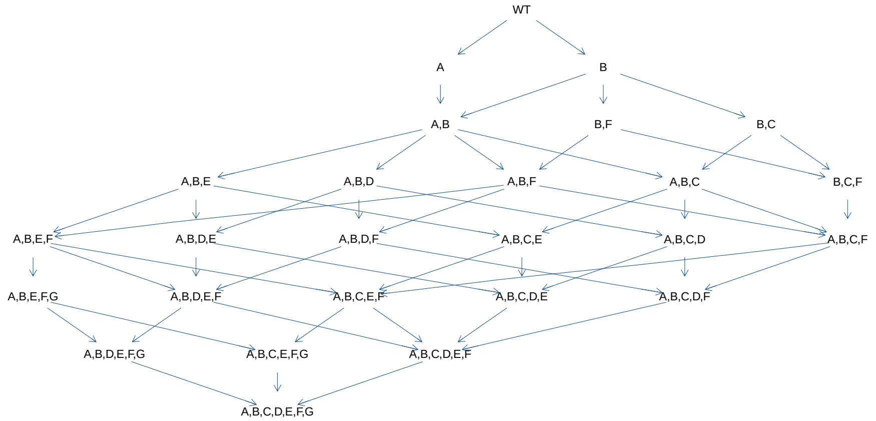

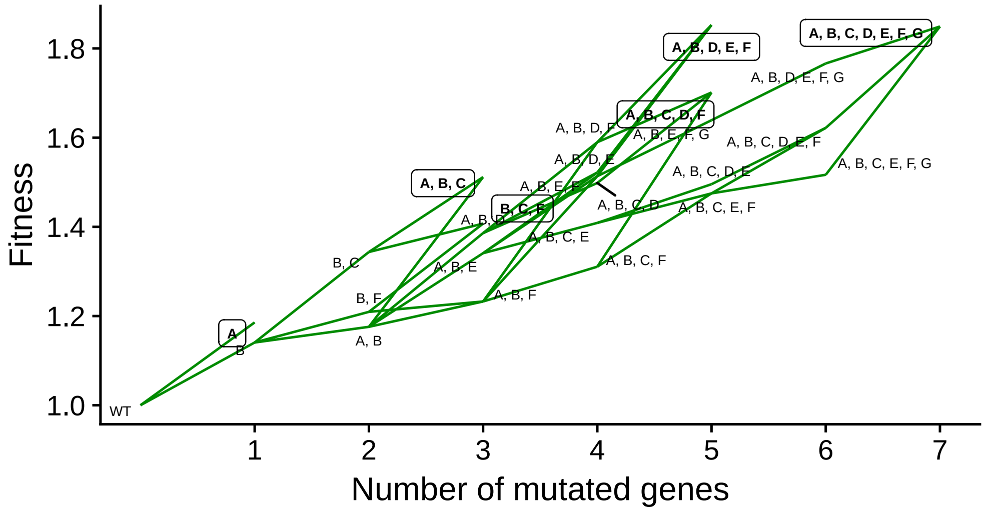

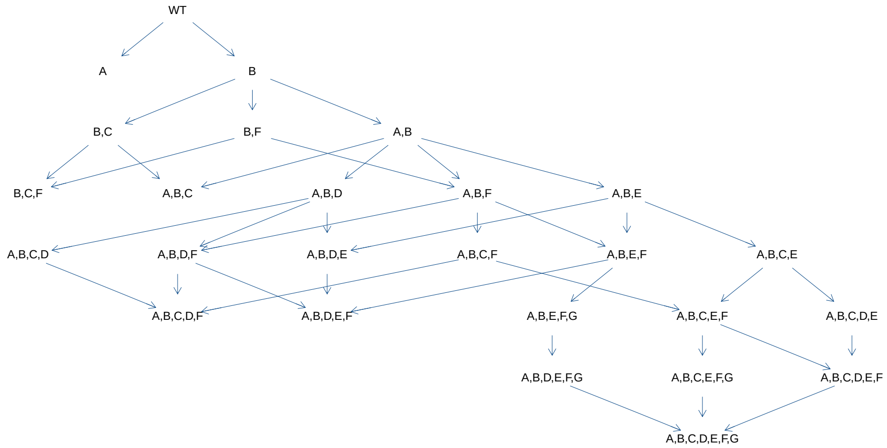

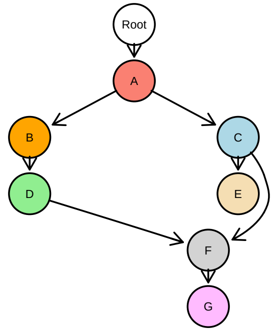

ID	XMtNwZBJ7EWu8yH7
acc genotypes	14
peaks no back	2
removed edges	2
prop rem edges	0.105

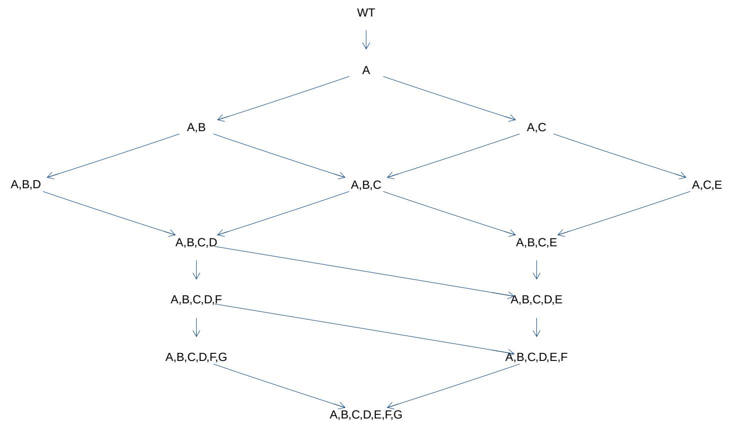

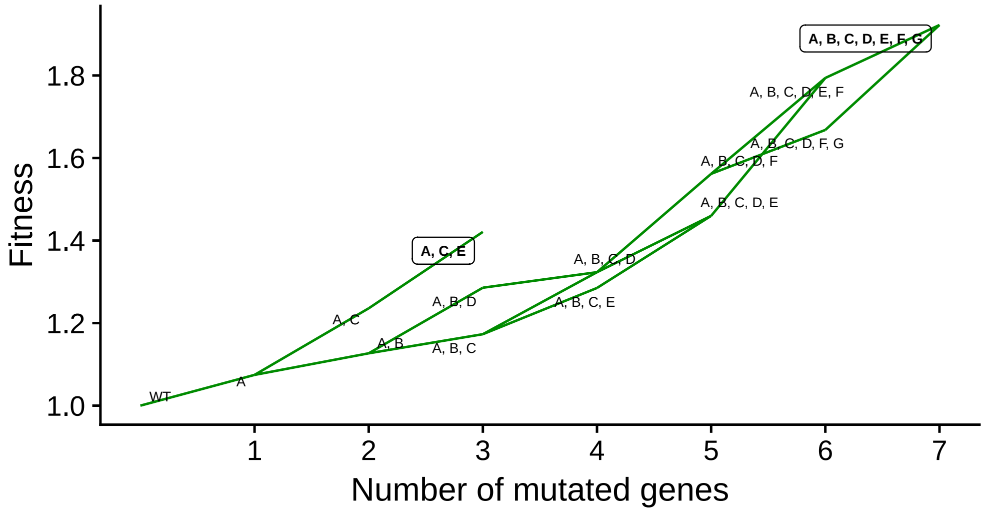

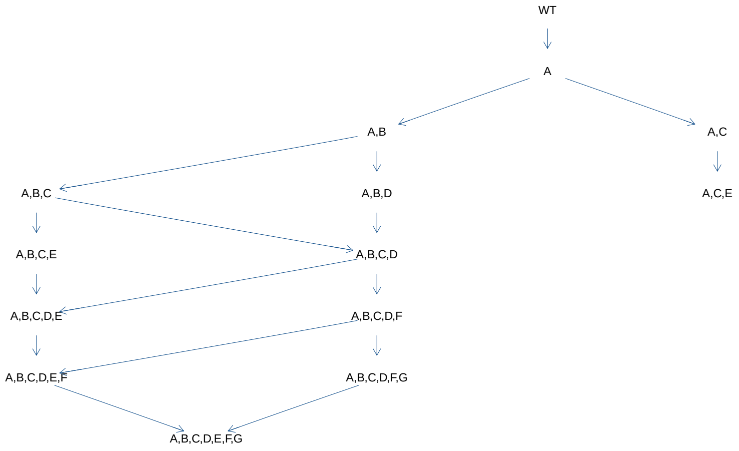

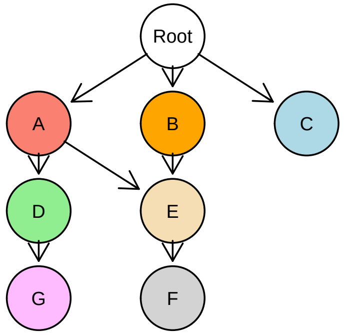

ID xN6jmjSdlYDl6ALk
acc genotypes 28
peaks no back 3
removed edges 5
prop rem edges 0.093

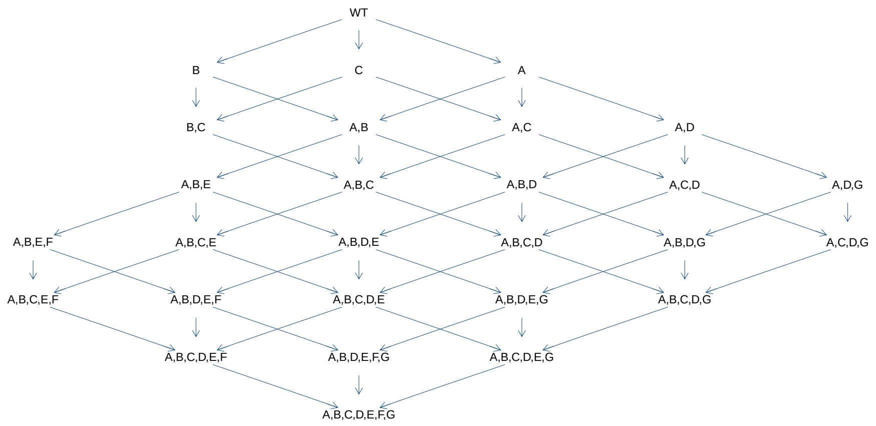

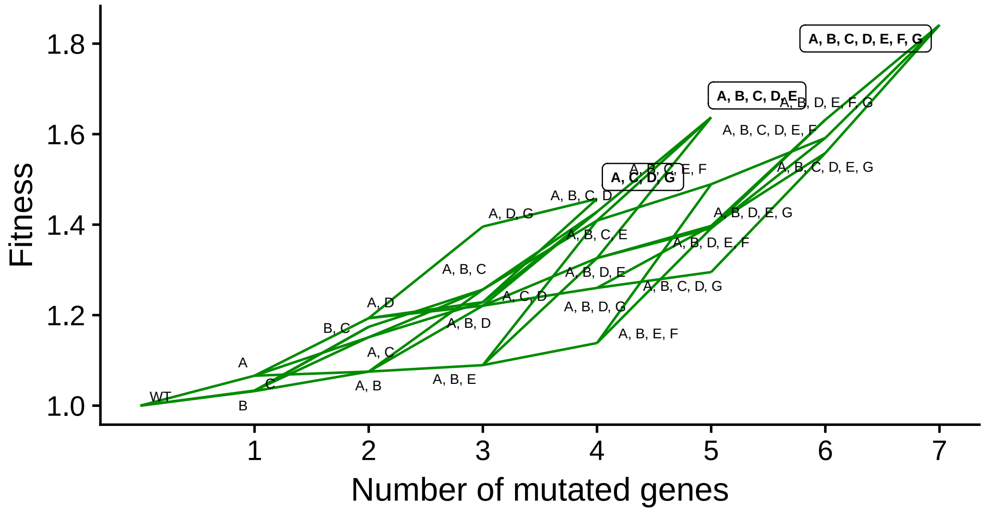

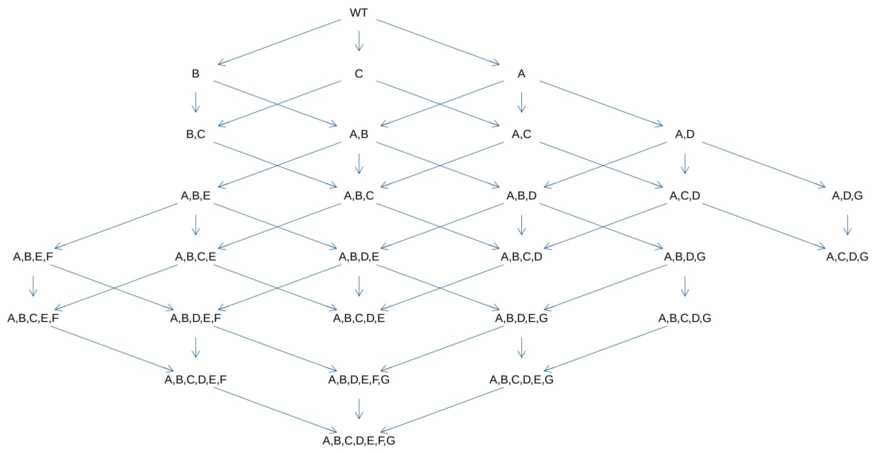

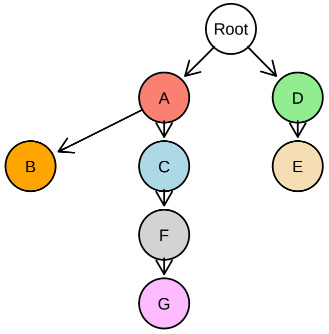

ID	xQCUt6eox10t8NYNj
acc genotypes	27
peaks no back	5
removed edges	7
prop rem edges	0.137

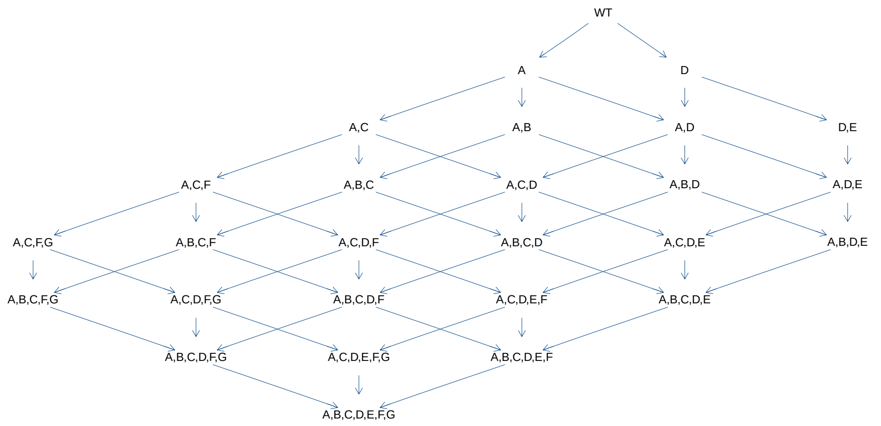

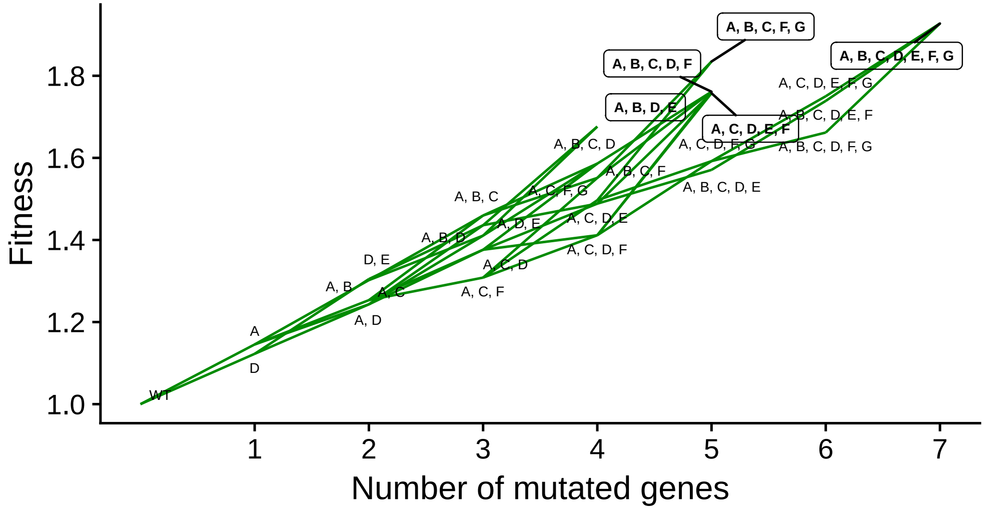

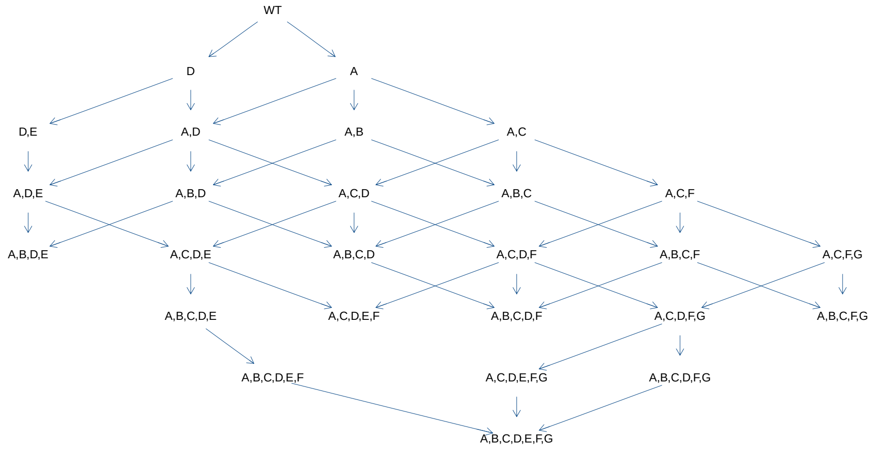

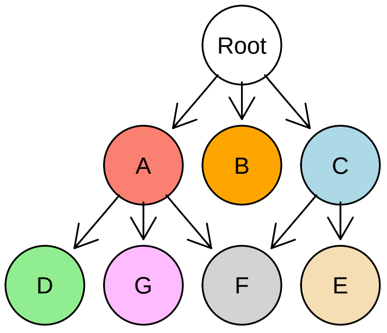

ID	xsLEpm8mUqbWuEtX
acc genotypes	46
peaks no back	6
removed edges	22
prop rem edges	0.195

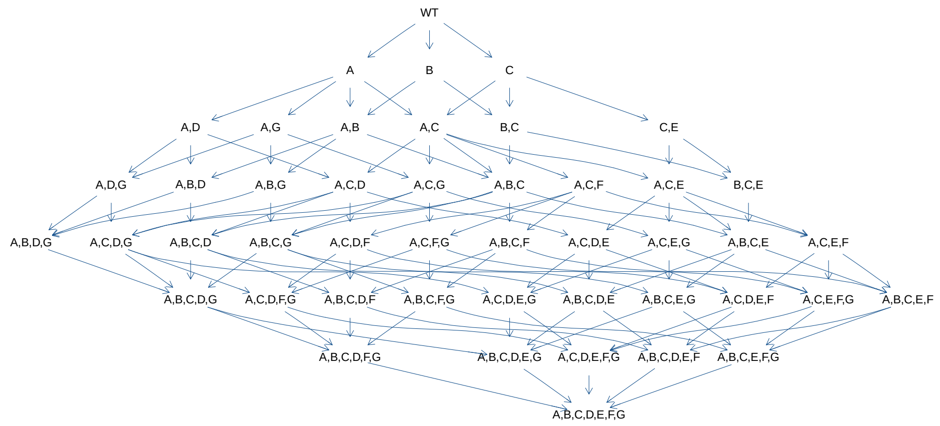

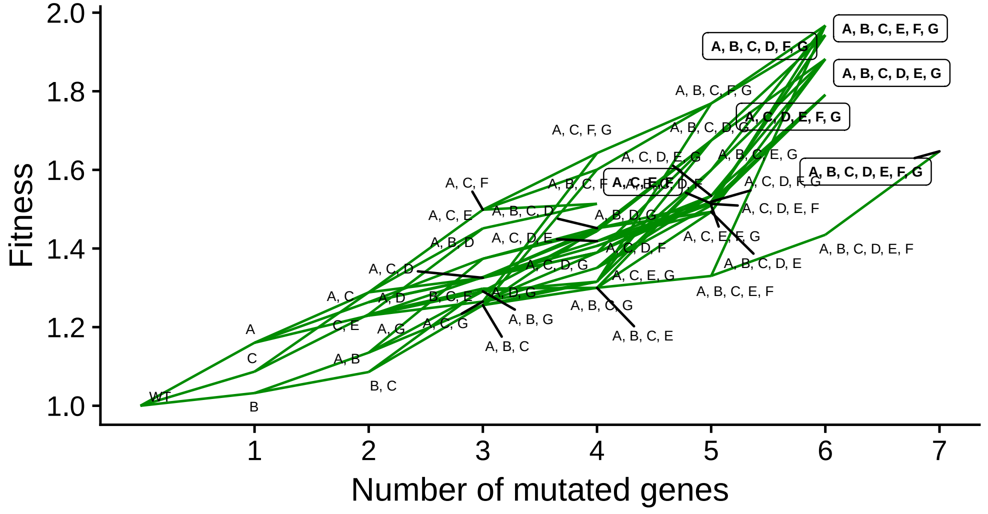

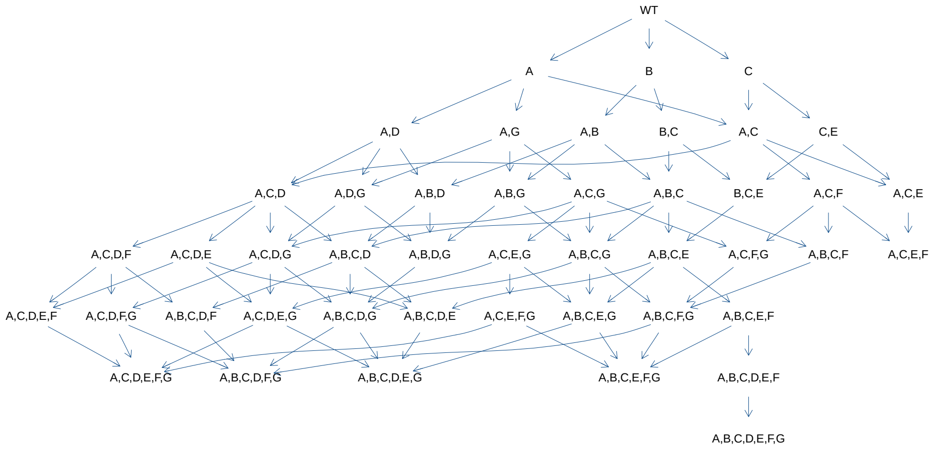

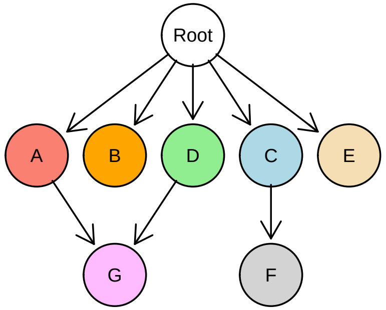

ID	XUvwmwc1Jrh7g5gR
acc genotypes	60
peaks no back	7
removed edges	26
prop rem edges	0.162

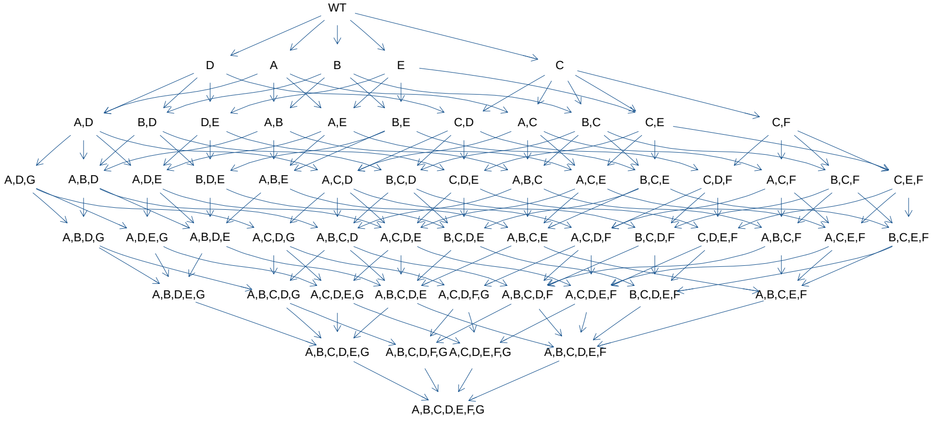

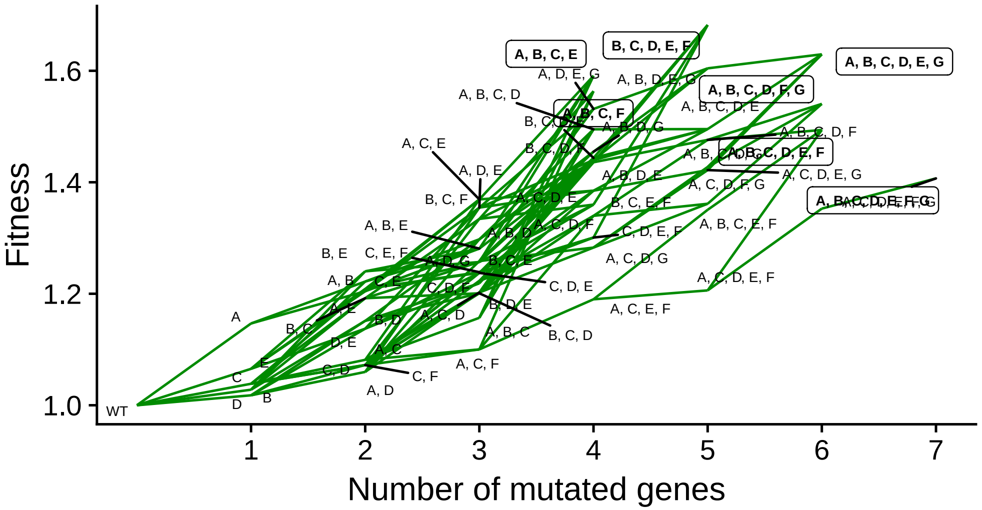

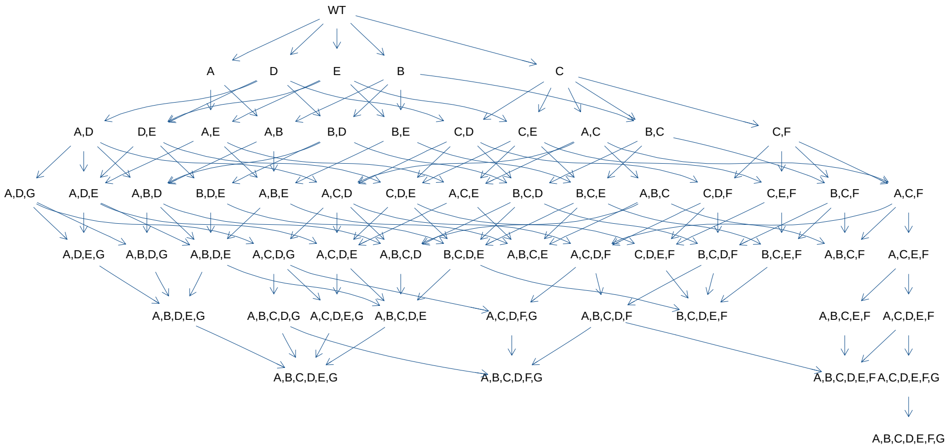

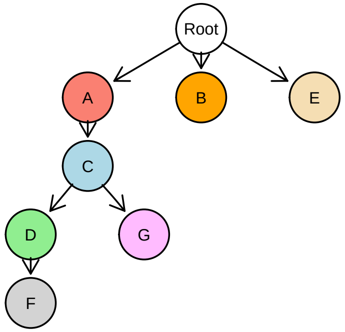

ID XvPBLPU6GY62cXv3
acc genotypes 32
peaks no back 6
removed edges 11
prop rem edges 0.162

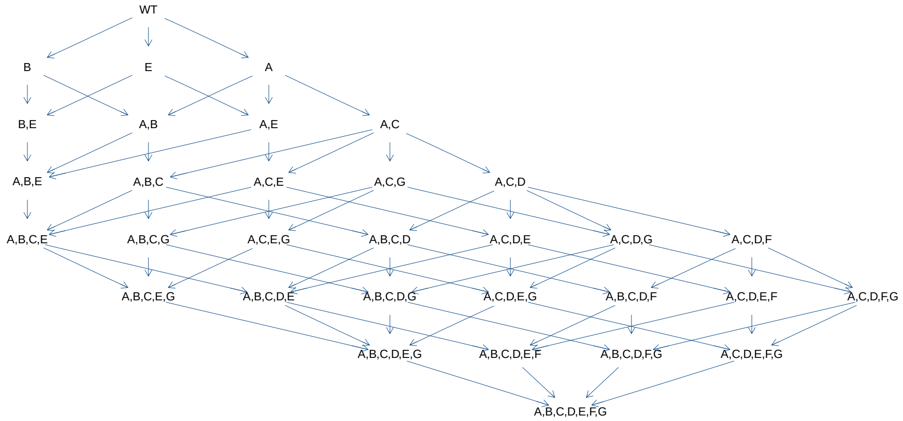

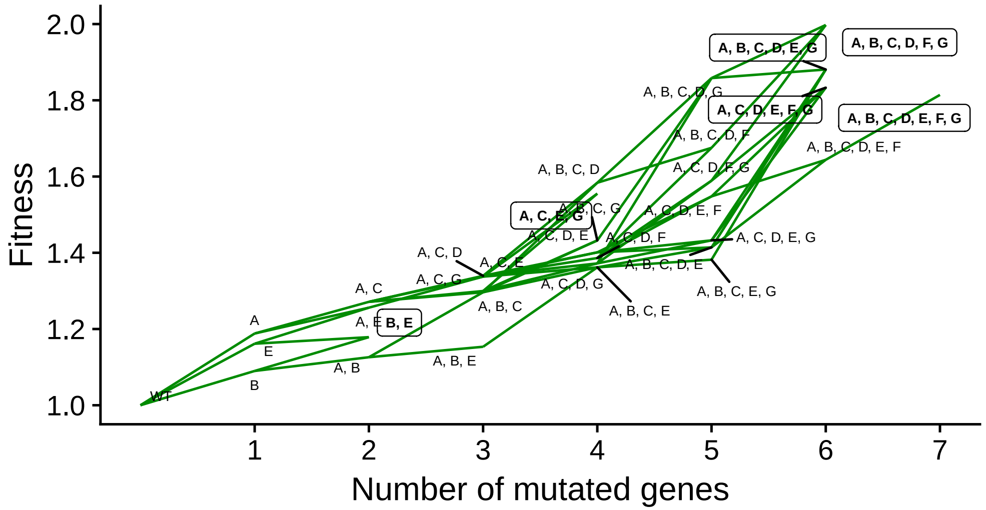

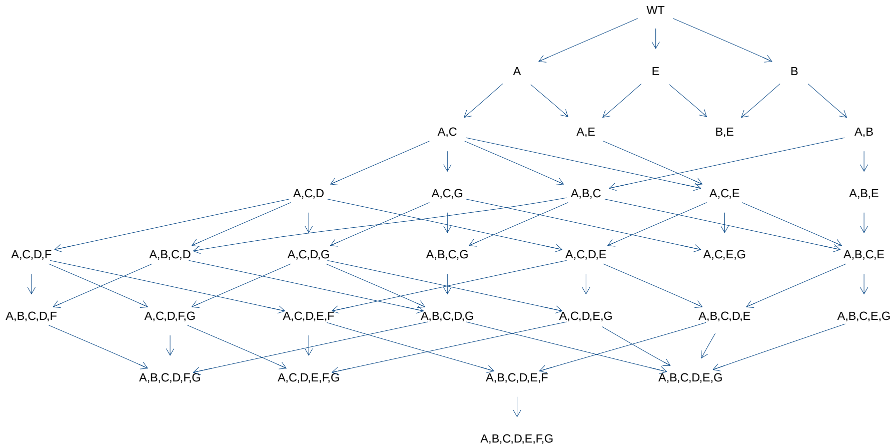

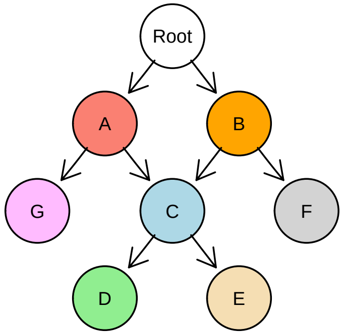

ID	yBXLyZECBuzOCwPP
acc genotypes	25
peaks no back	2
removed edges	4
prop rem edges	0.083

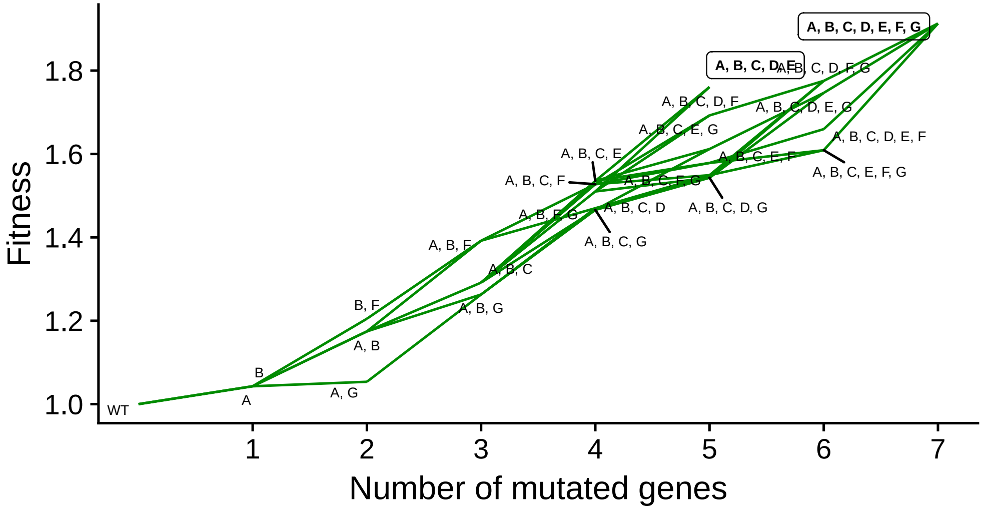

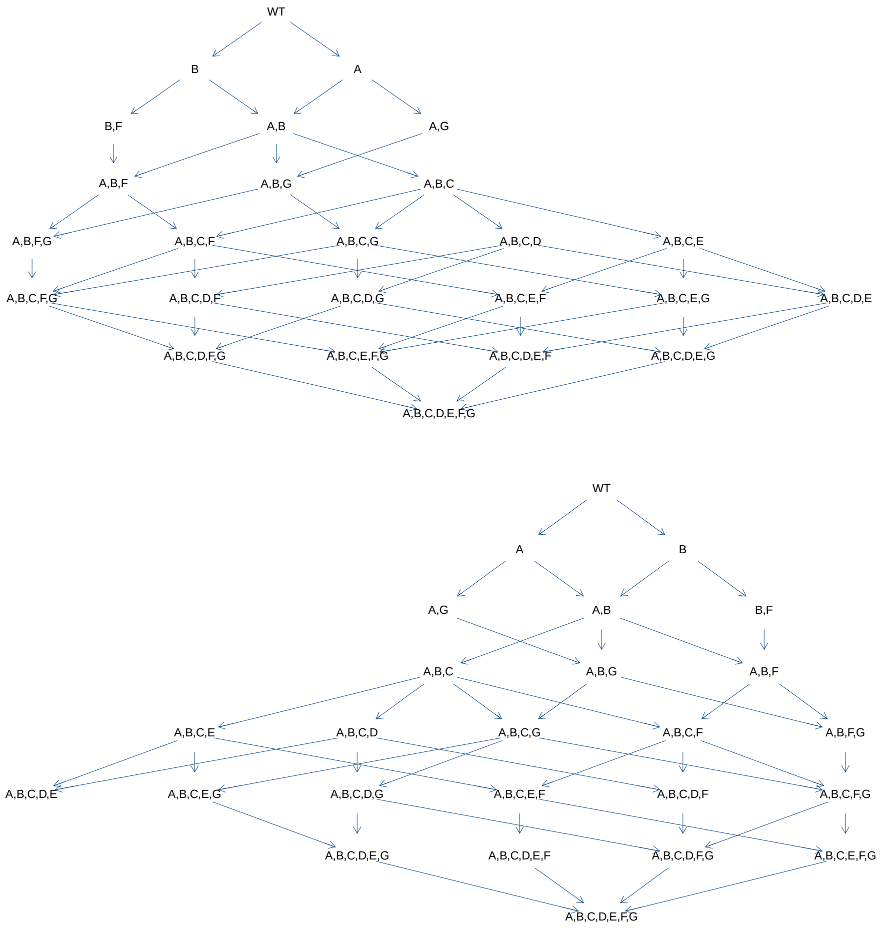

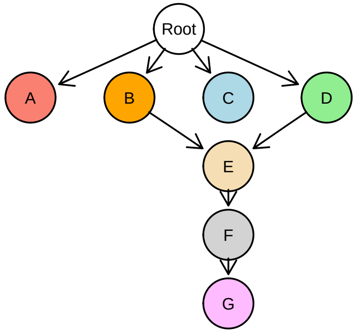

ID	ycfMUnx16qqaBjIW
acc genotypes	28
peaks no back	6
removed edges	11
prop rem edges	0.196

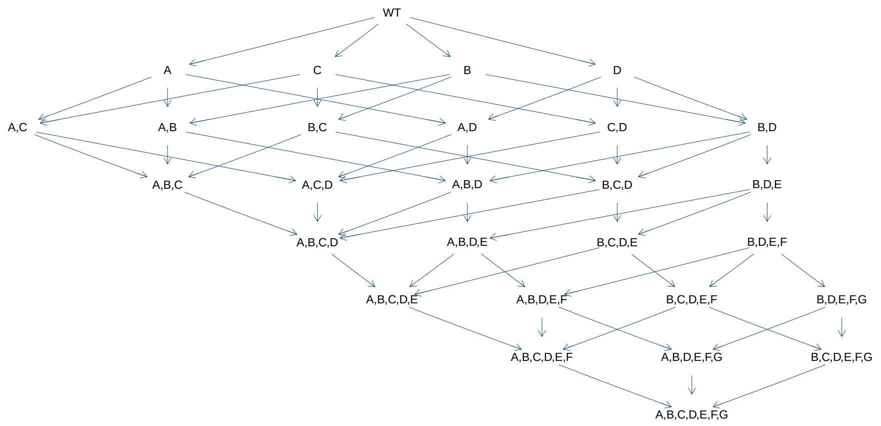

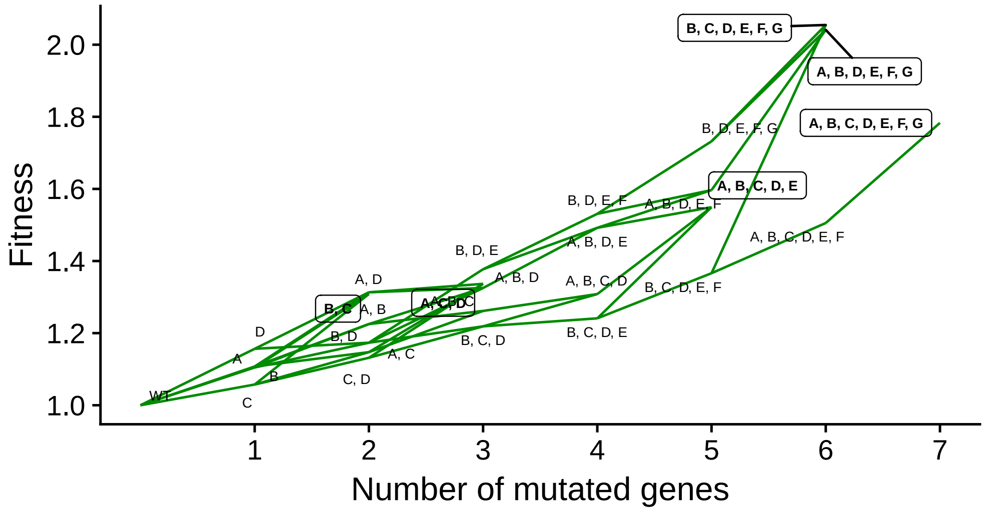

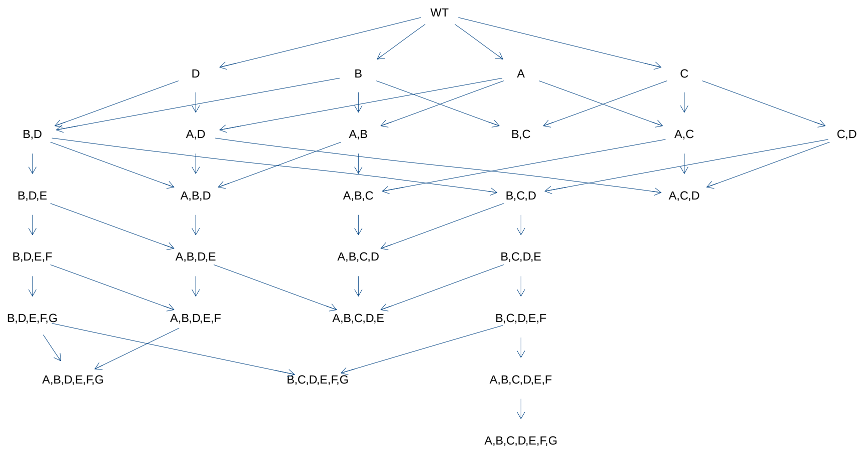

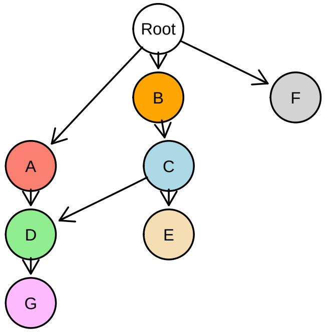

ID	yCxbC75Zji2W7wiD
acc genotypes	24
peaks no back	7
removed edges	11
prop rem edges	0.25

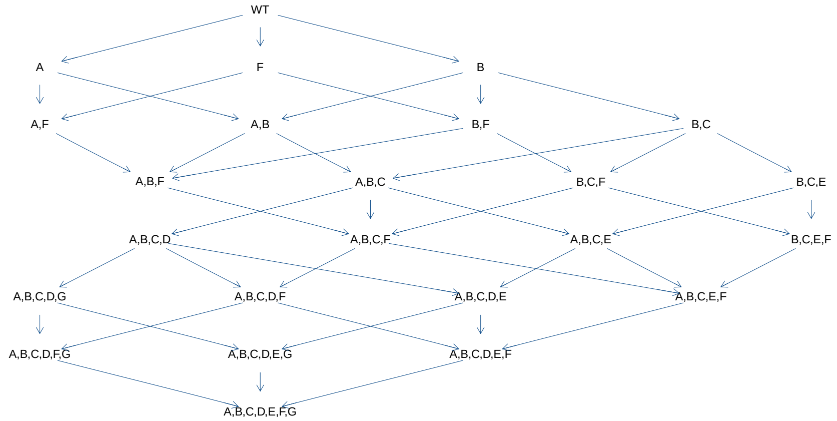

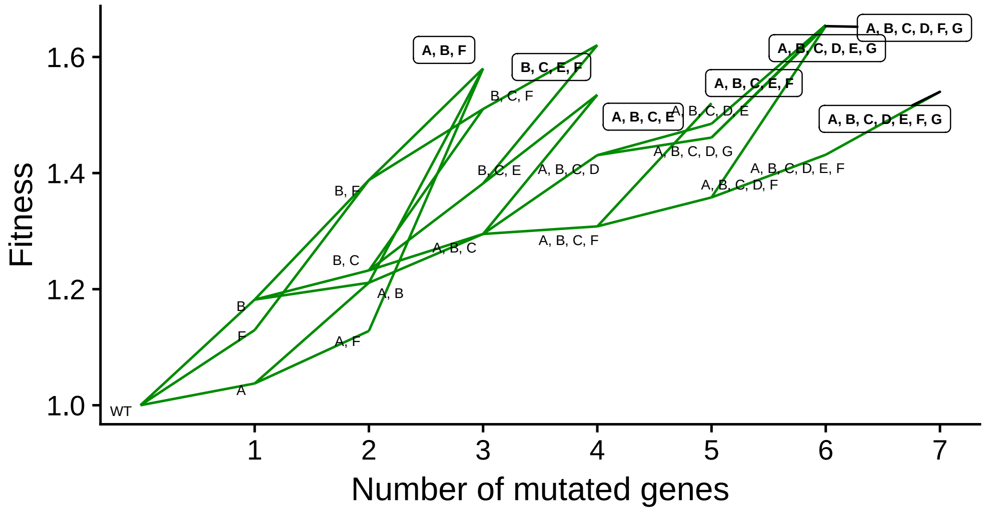

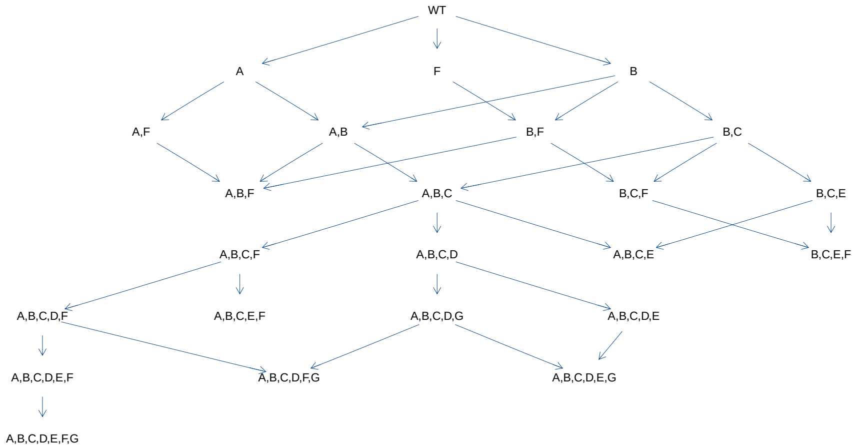

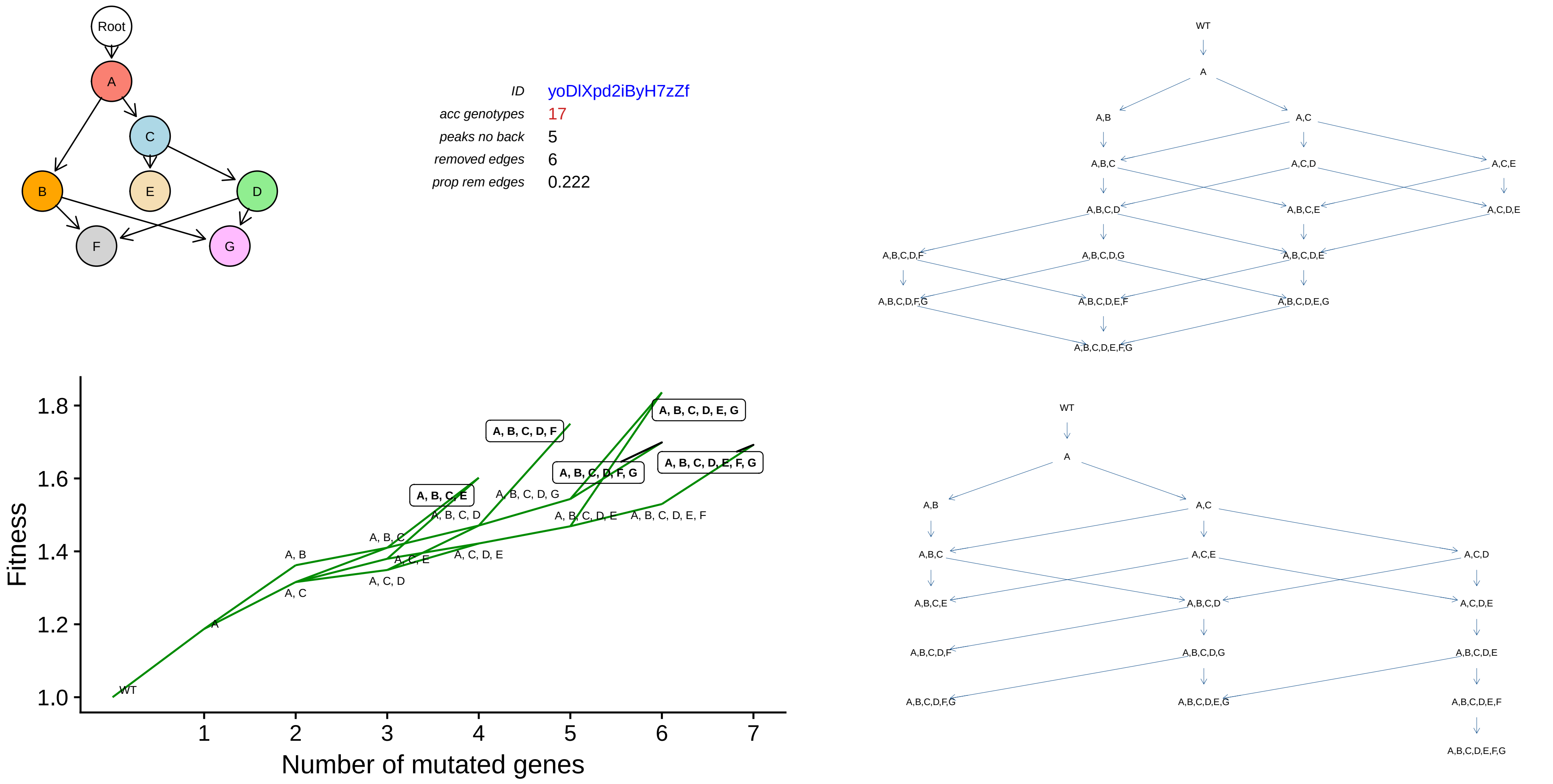

Root
A
B
C
D
E
F
G
ID yoDlXpd2iByH7zZf
acc genotypes 17
peaks no back 5
removed edges 6
prop rem edges 0.222
Fitness
Number of mutated genes
A, B, C, D, F
A, B, C, D, E, G
A, B, C, D, F, G
A, B, C, D, E, F, G
A, B, C, E
A, B, C, D, G
A, B, C, D
A, B, C, D, E
A, B, C, D, E, F
A, B, C
A, C, E
A, C, D, E
A, B
A, C, D
A, C
A
WT
WT
A
A,B
A,C
A,B,C
A,C,D
A,C,E
A,B,C,D
A,B,C,E
A,C,D,E
A,B,C,D,F
A,B,C,D,G
A,B,C,D,E
A,B,C,D,F,G
A,B,C,D,E,F
A,B,C,D,E,G
A,B,C,D,E,F,G

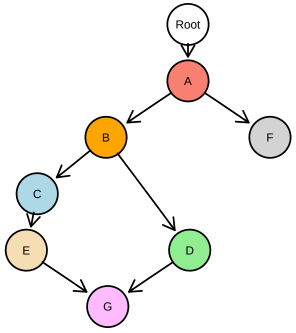

ID	YqJMzkvAccJ4gHYm
acc genotypes	17
peaks no back	3
removed edges	3
prop rem edges	0.111

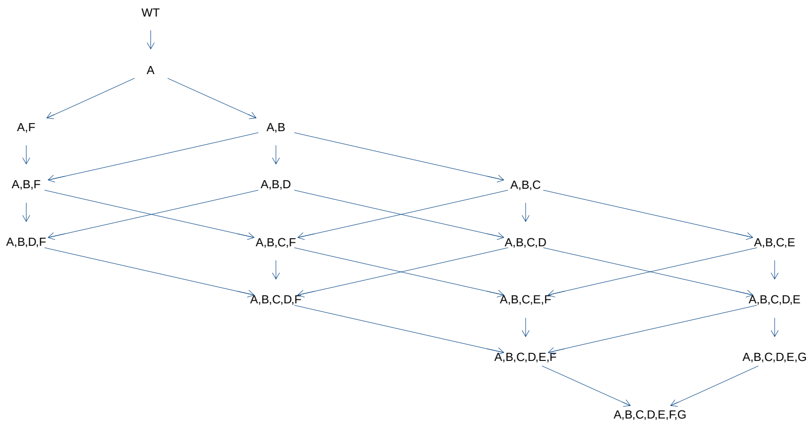

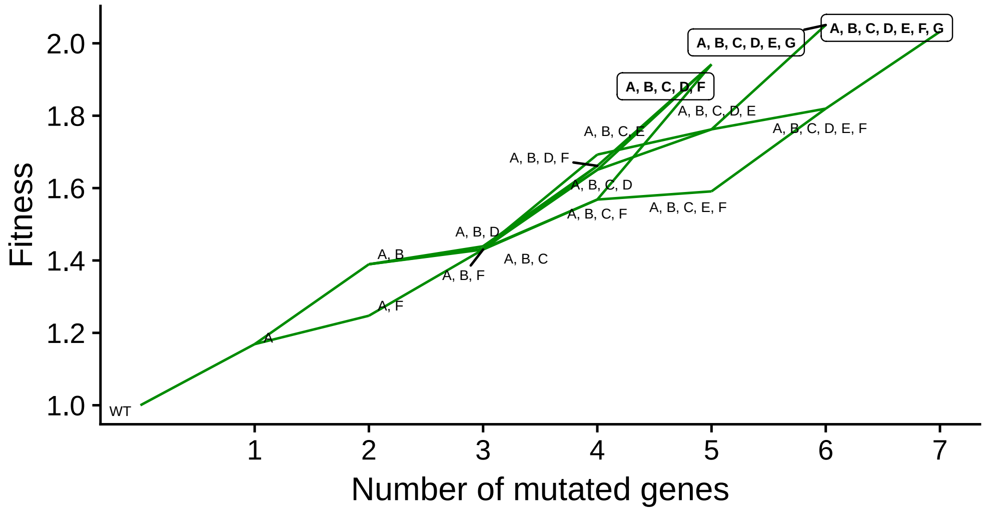

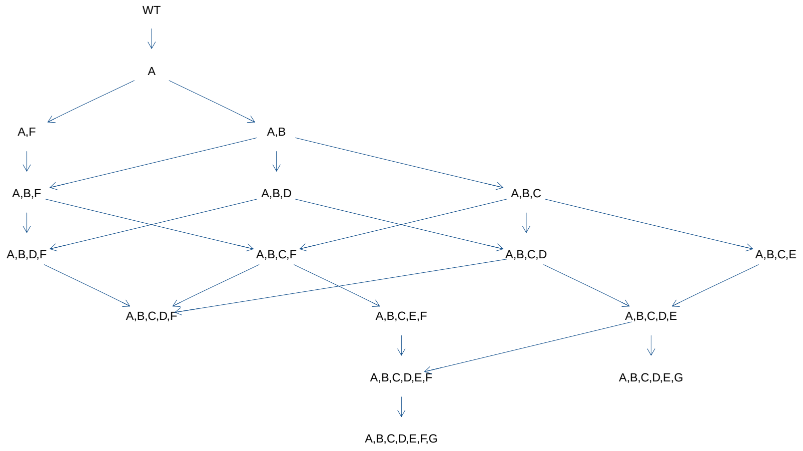

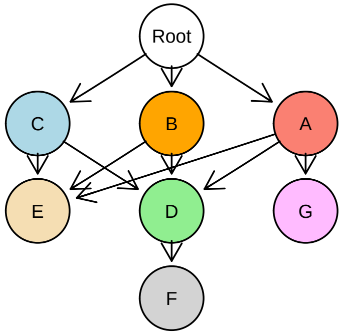

ID	yX5bopH3PiXCr10r7
acc genotypes	22
peaks no back	4
removed edges	8
prop rem edges	0.205

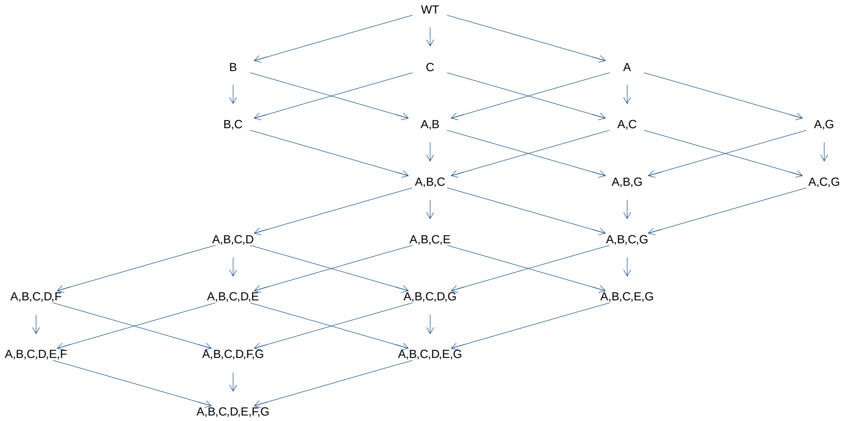

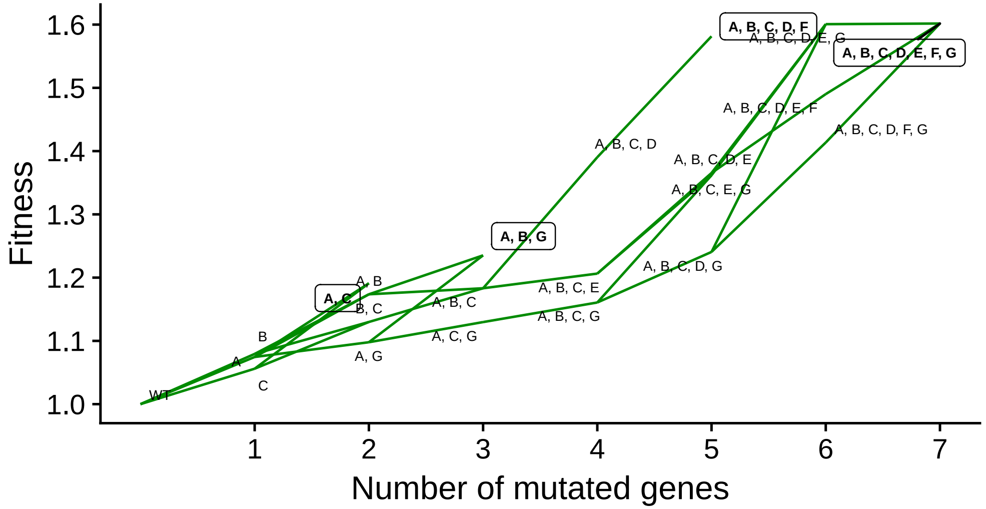

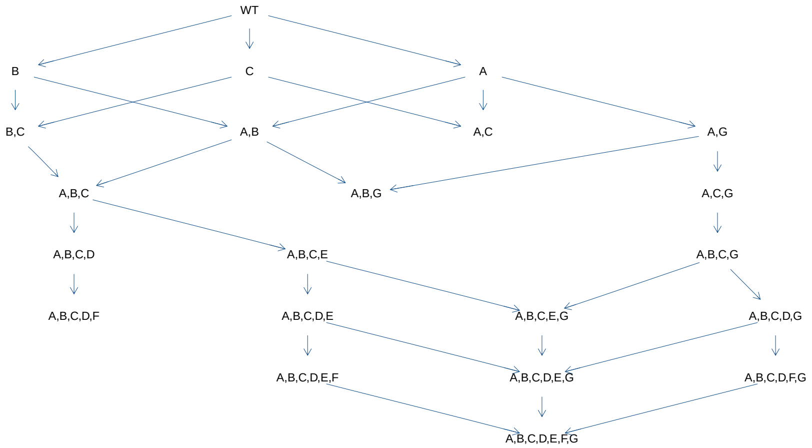

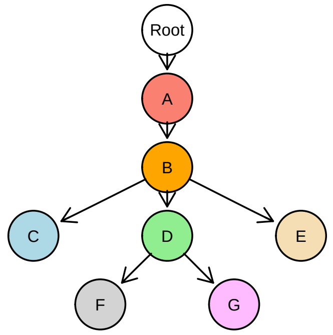

ID yy6dYwpU1ktFm49a
acc genotypes 22
peaks no back 6
removed edges 10
prop rem edges 0.238

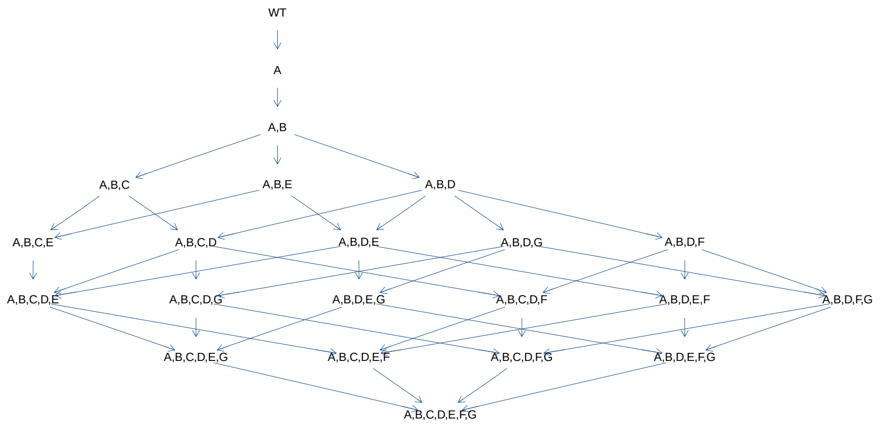

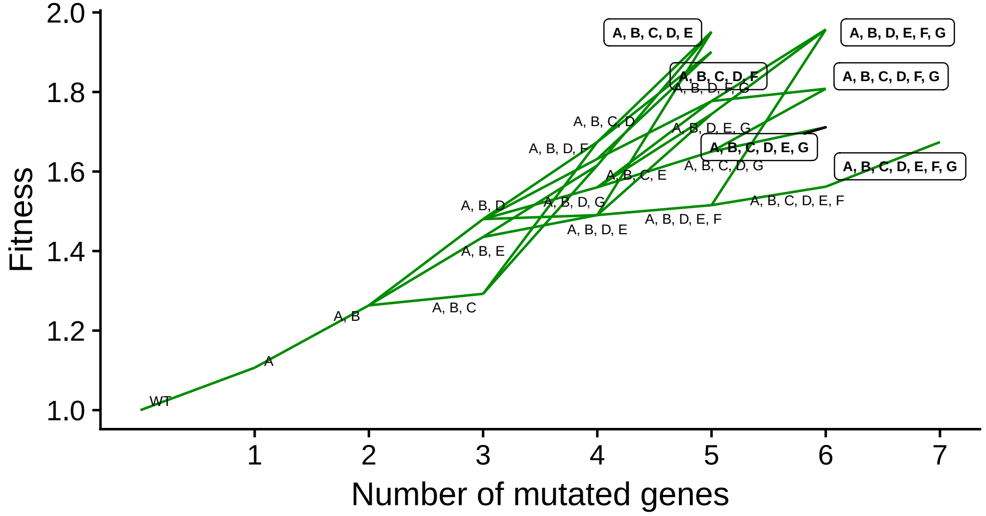

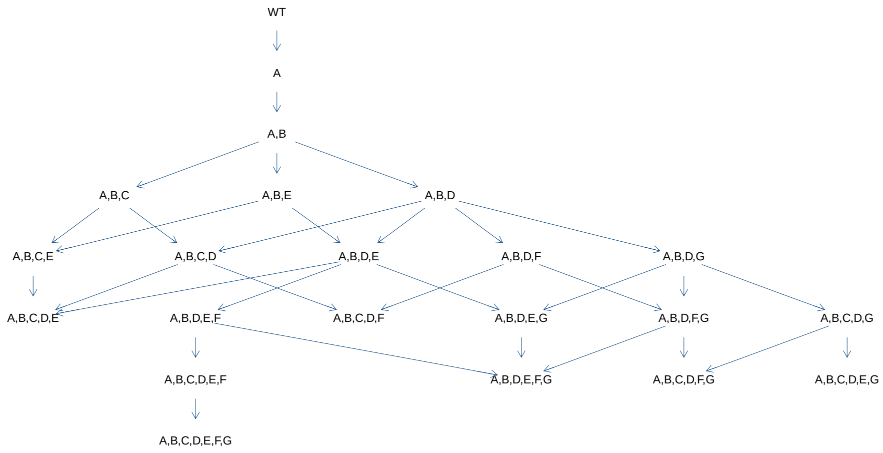

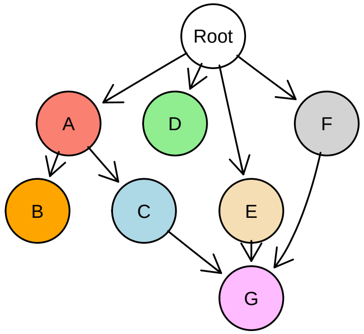

ID	zE6cfkQmrWiDYn5D
acc genotypes	44
peaks no back	5
removed edges	18
prop rem edges	0.167

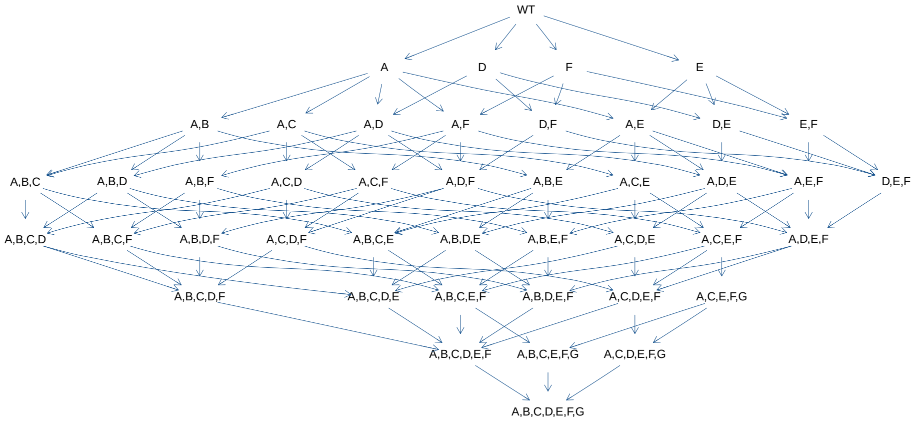

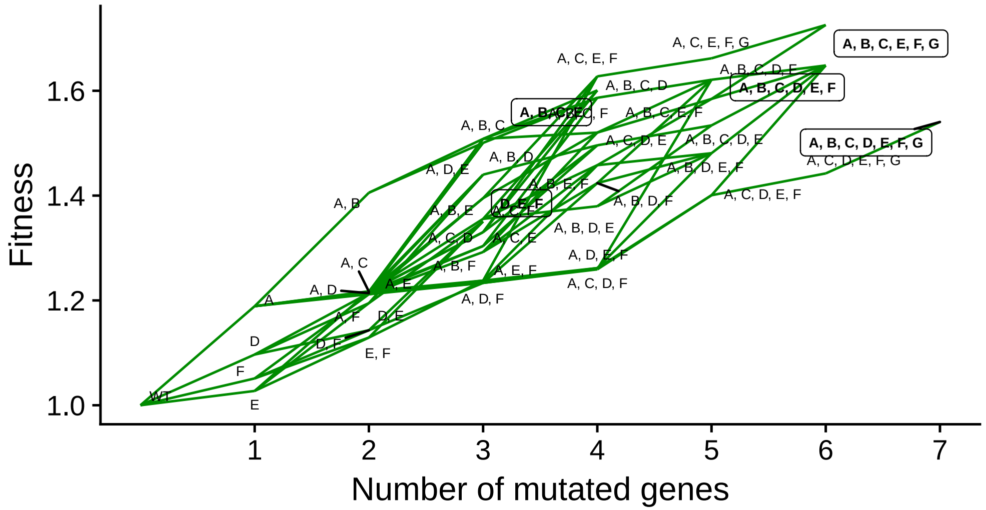

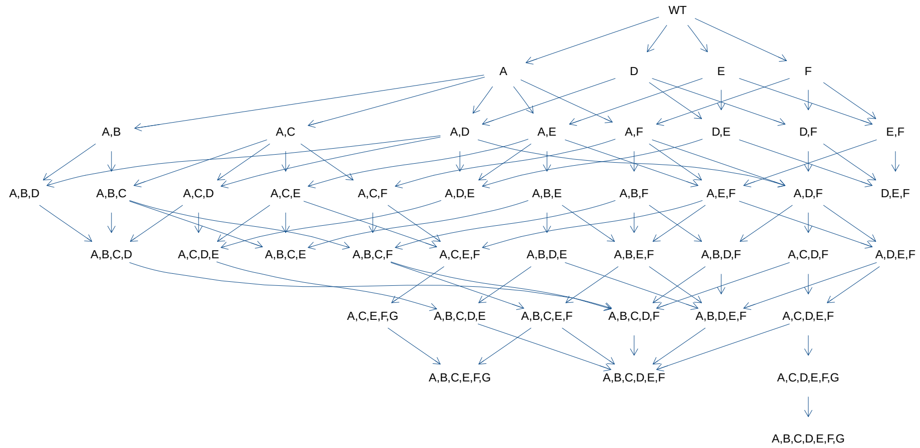

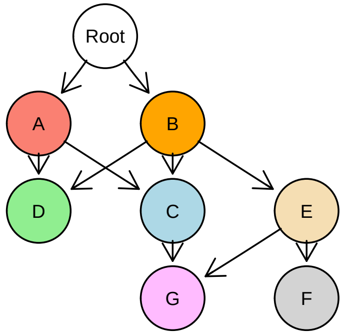

ID zHGCza3O5qL97M76
acc genotypes 21
peaks no back 2
removed edges 3
prop rem edges 0.083

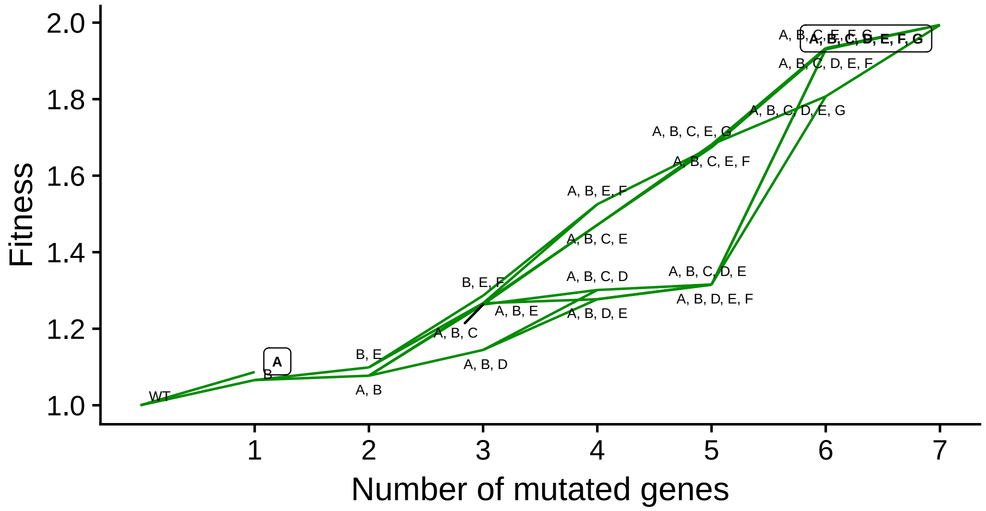

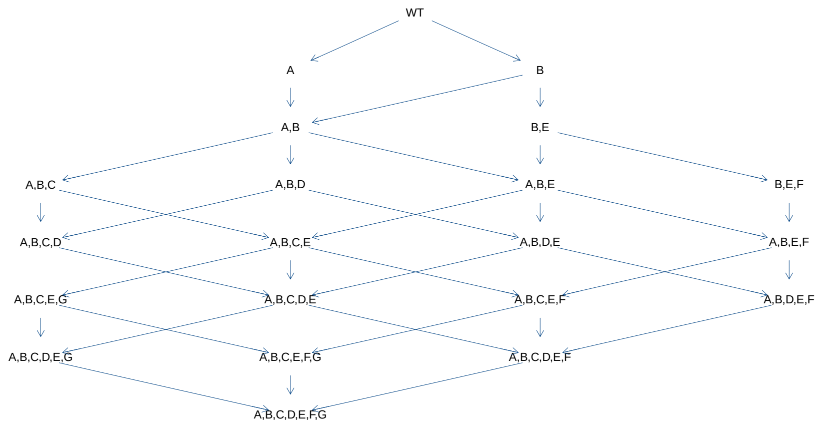

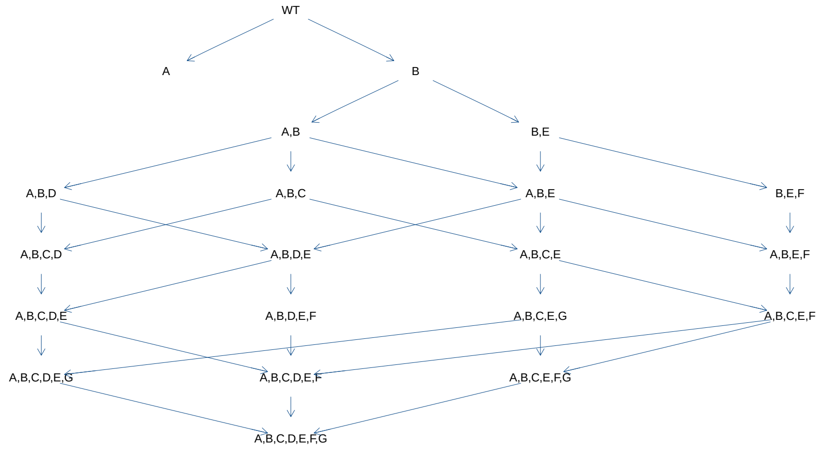

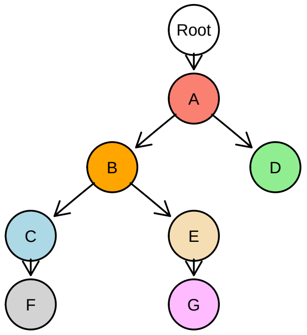

ID	ZkjYaBc10QMnrUp8W
acc genotypes	21
peaks no back	3
removed edges	2
prop rem edges	0.054

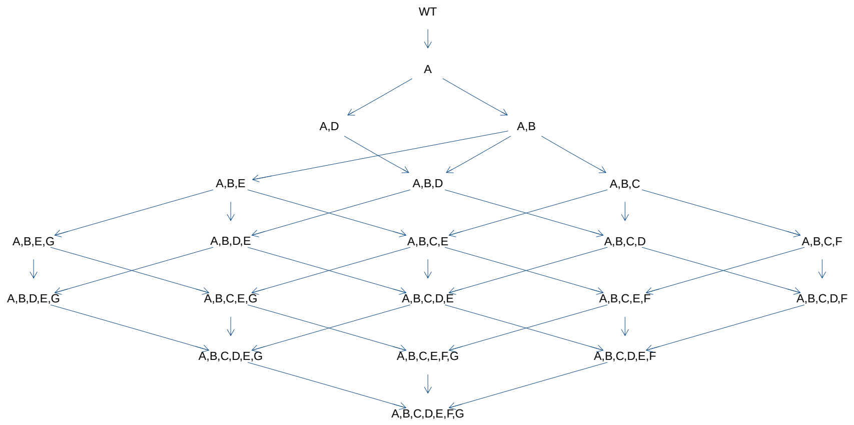

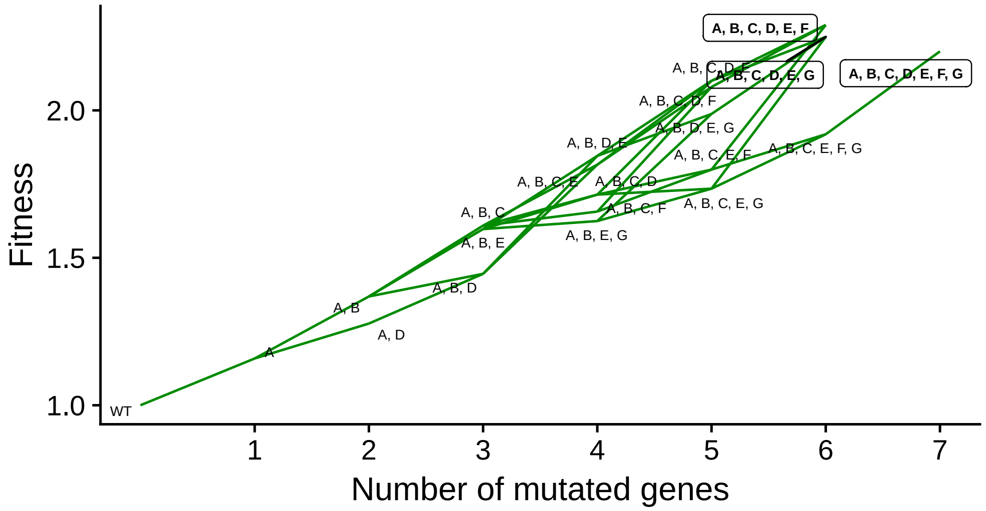

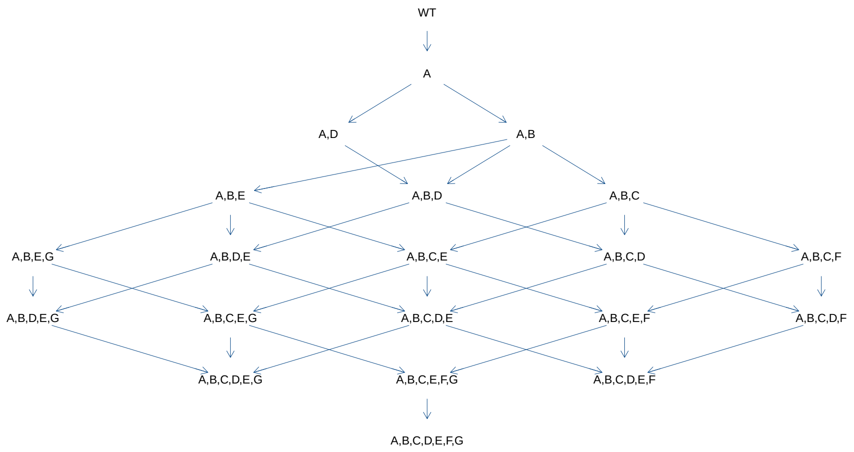

ID	10bxK1gc1IxCjpaJM
acc genotypes	24
peaks no back	9
removed edges	NA
prop rem edges	NA

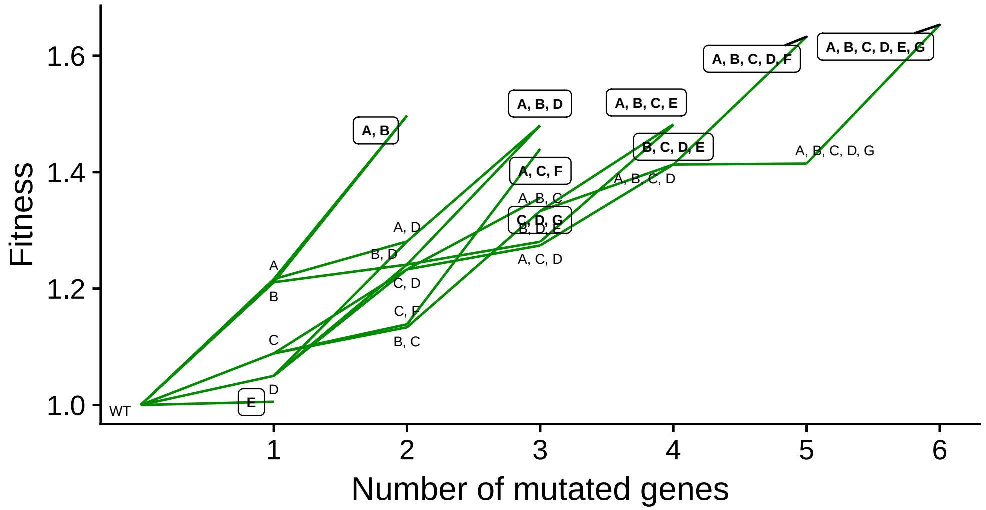

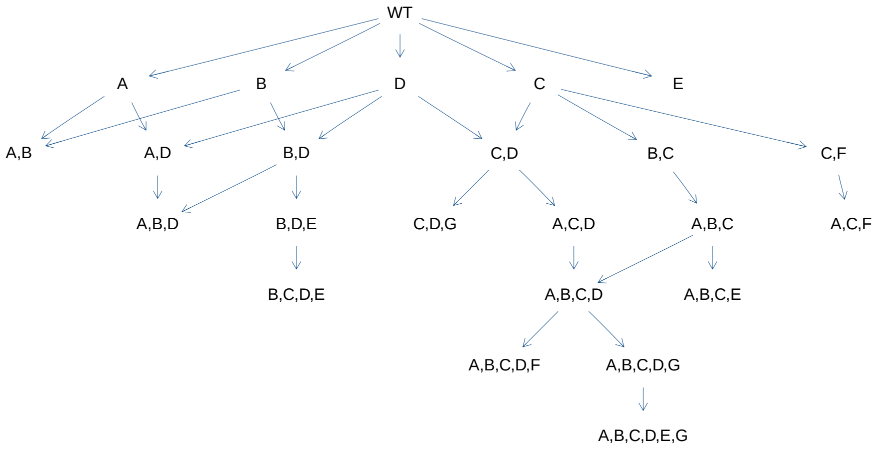

ID	10fs6bc54hwzLal8K
acc genotypes	99
peaks no back	14
removed edges	NA
prop rem edges	NA

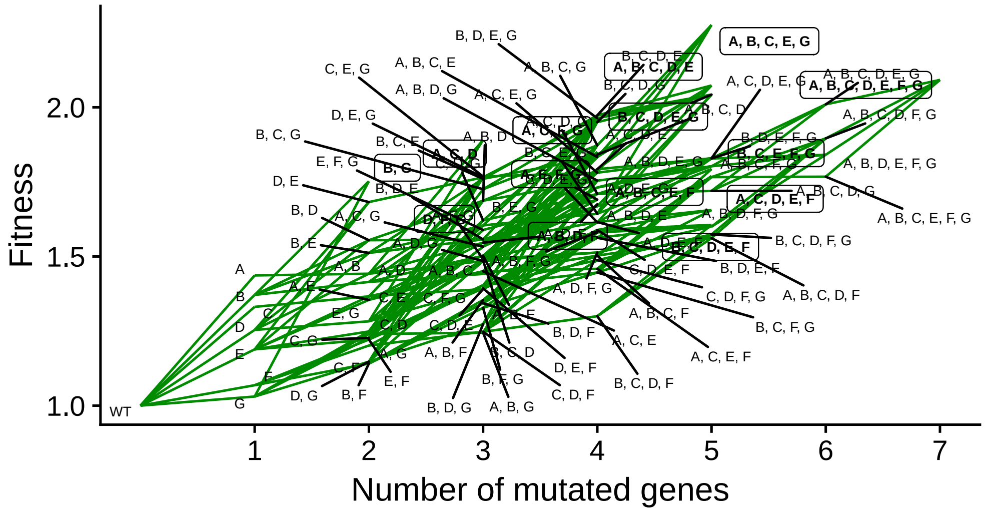

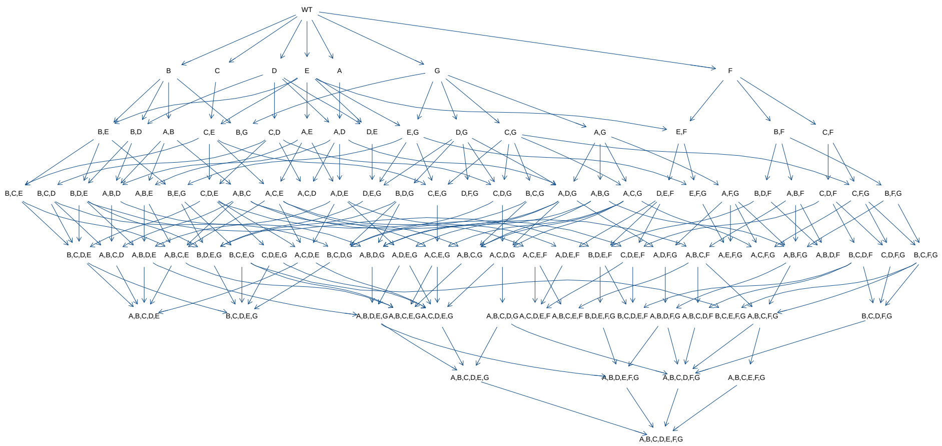

ID	10H8qiQAOoVrnY9T3
acc genotypes	42
peaks no back	15
removed edges	NA
prop rem edges	NA

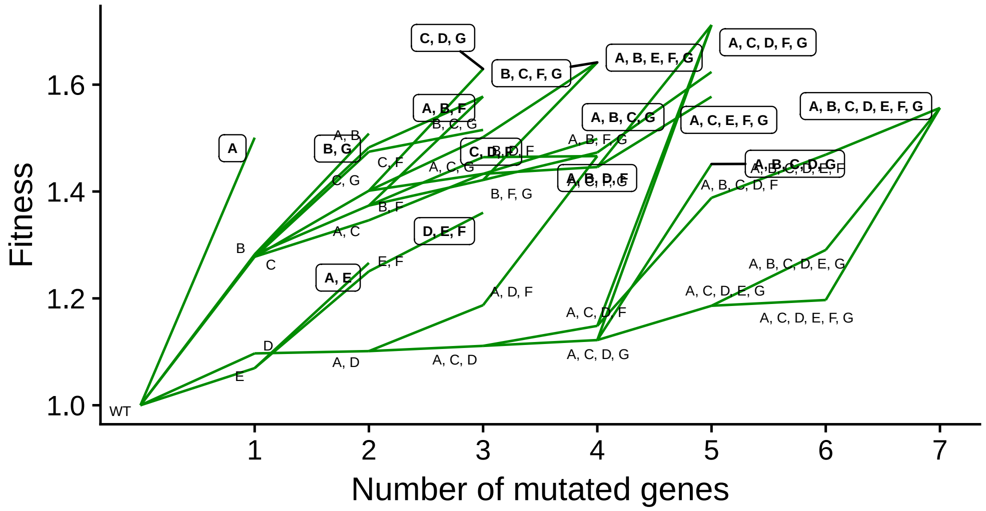

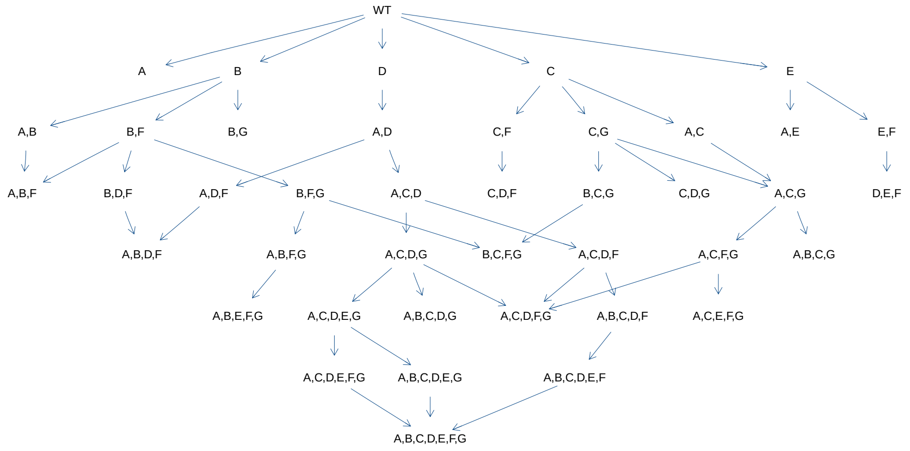

ID	10rLLqPV6rJMo5c6I
acc genotypes	28
peaks no back	9
removed edges	NA
prop rem edges	NA

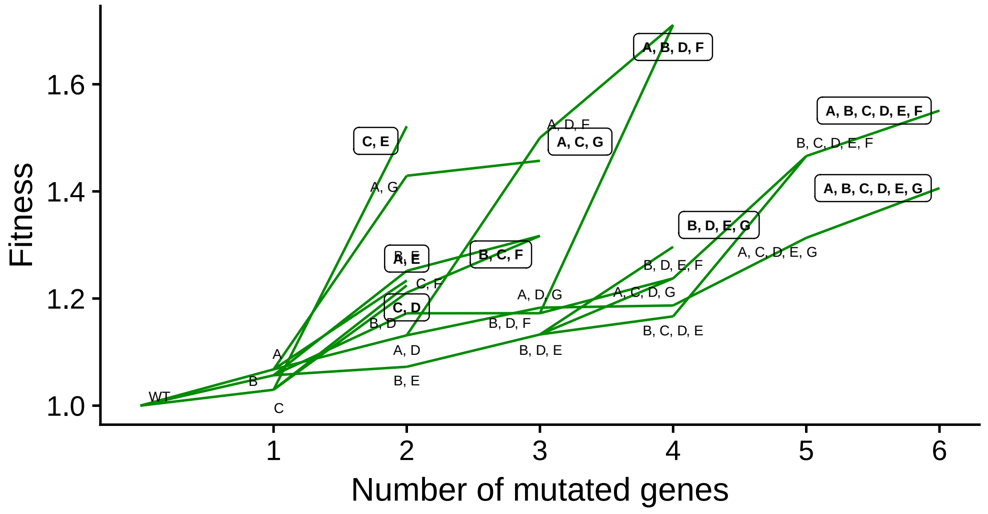

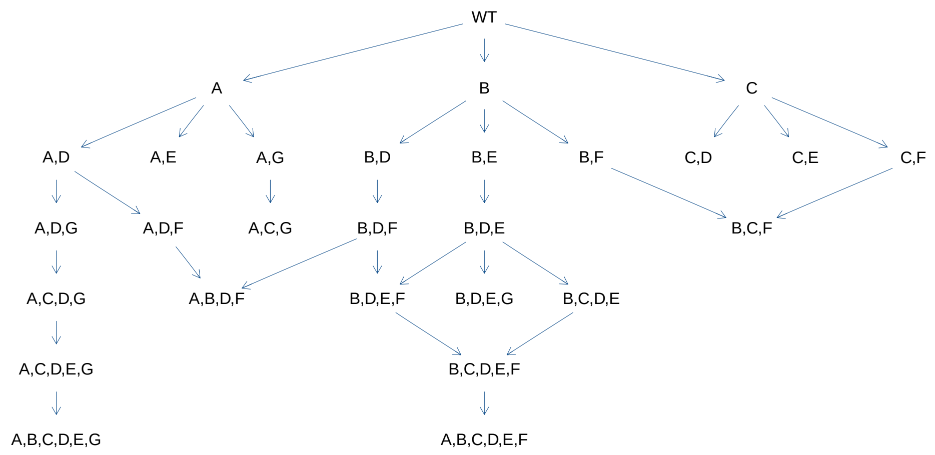

ID 10uENjADj4VYkG10Z6
acc genotypes 58
peaks no back 26
removed edges NA
prop rem edges NA

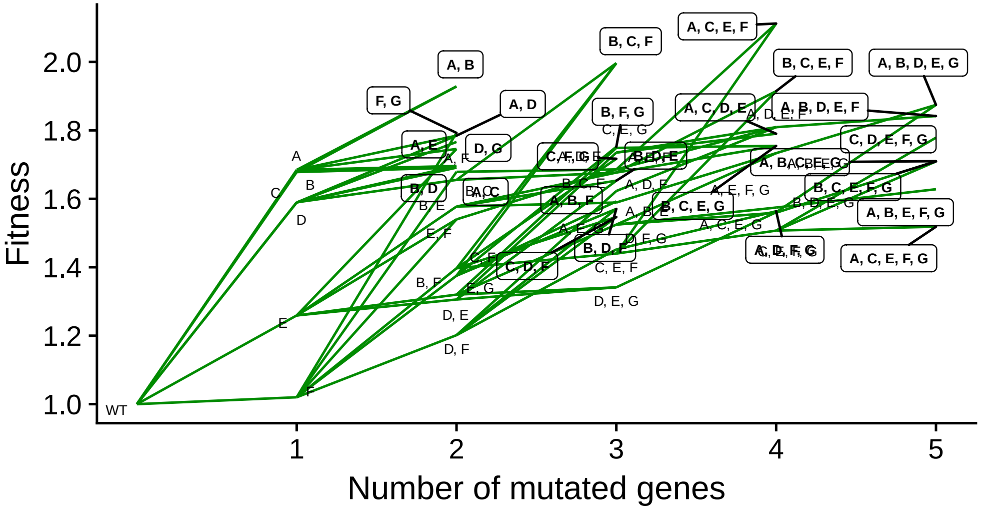

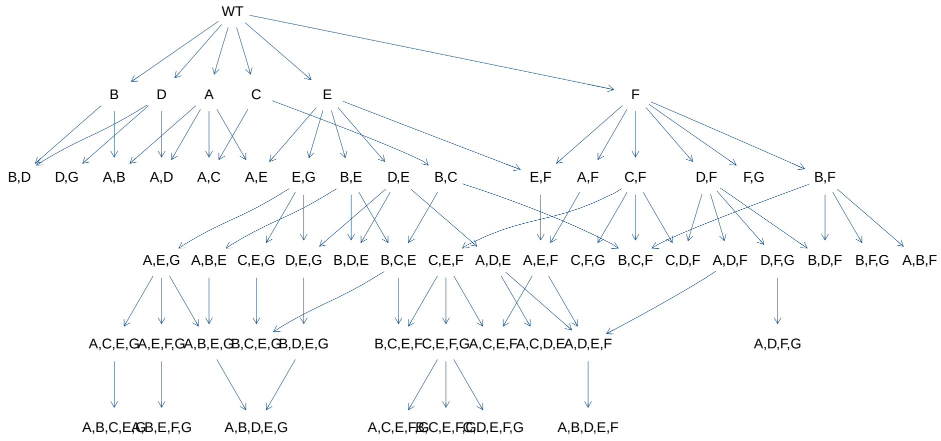

ID	16H2tTfJLrFoSFFU
acc genotypes	23
peaks no back	7
removed edges	NA
prop rem edges	NA

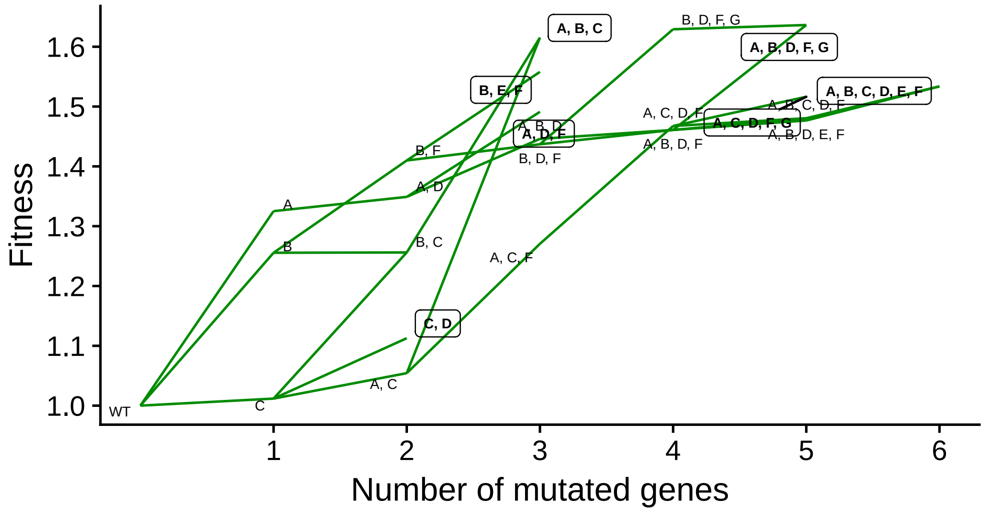

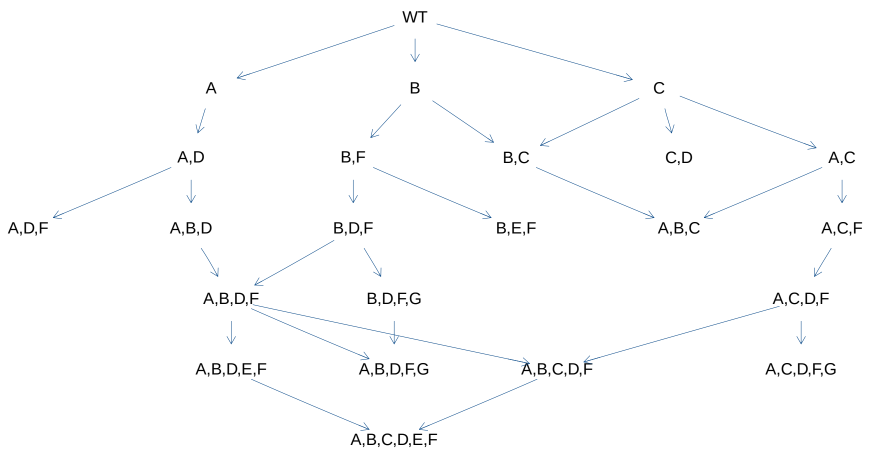

ID	18VIJjttIjPc2A62
acc genotypes	24
peaks no back	10
removed edges	NA
prop rem edges	NA

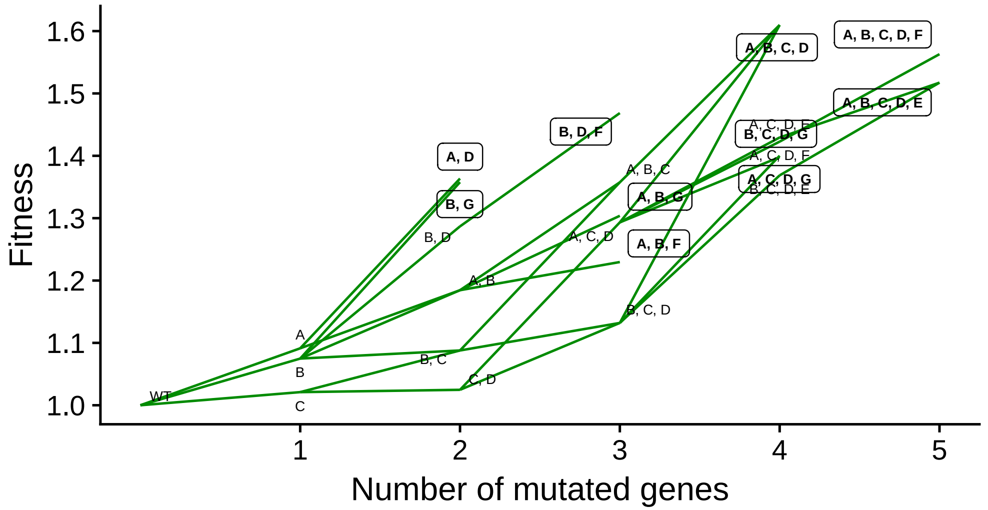

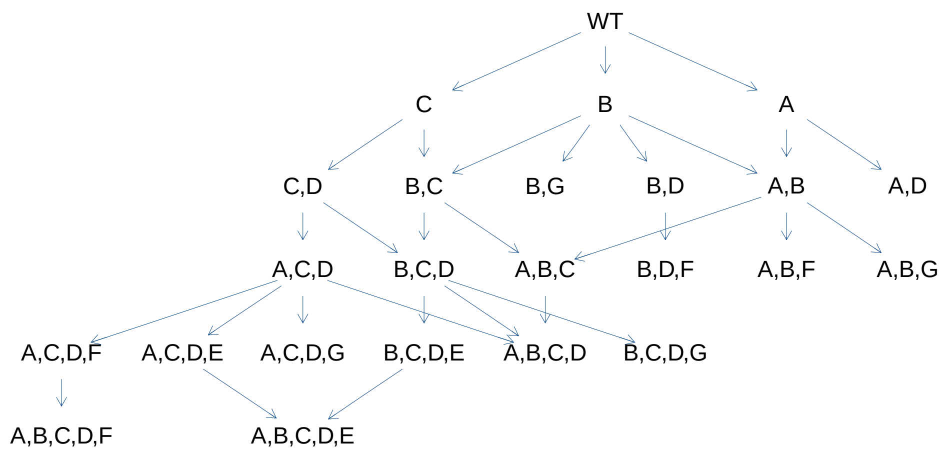

ID	1ylOxuiyzdWquD2u
acc genotypes	27
peaks no back	12
removed edges	NA
prop rem edges	NA

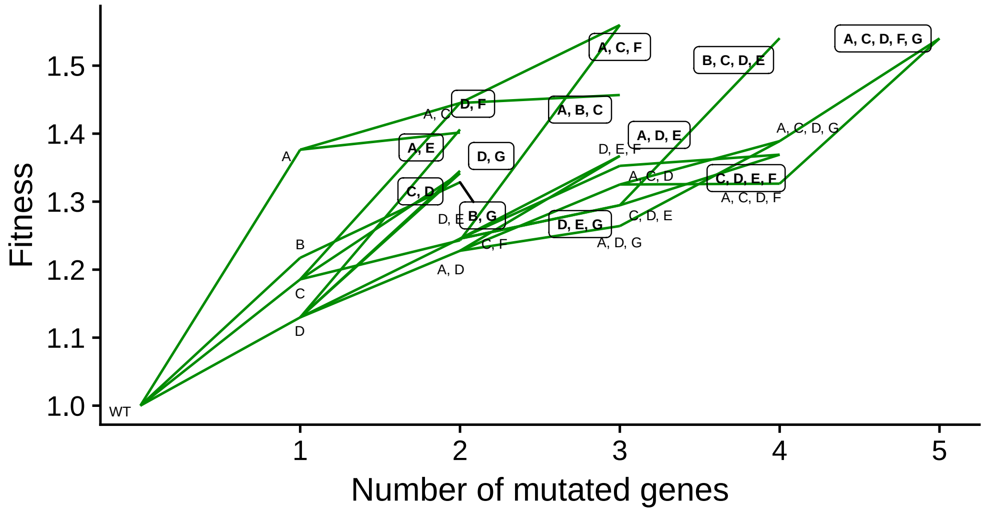

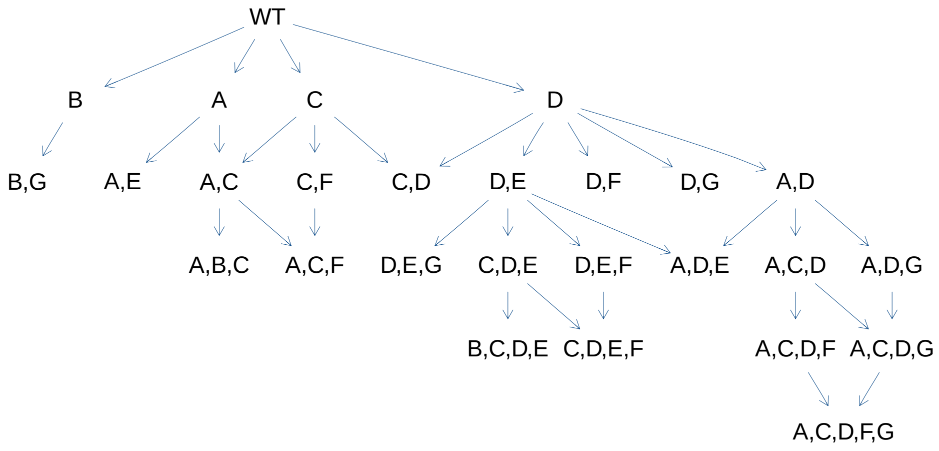

ID	28U7vxmKq8g5SSwV
acc genotypes	26
peaks no back	11
removed edges	NA
prop rem edges	NA

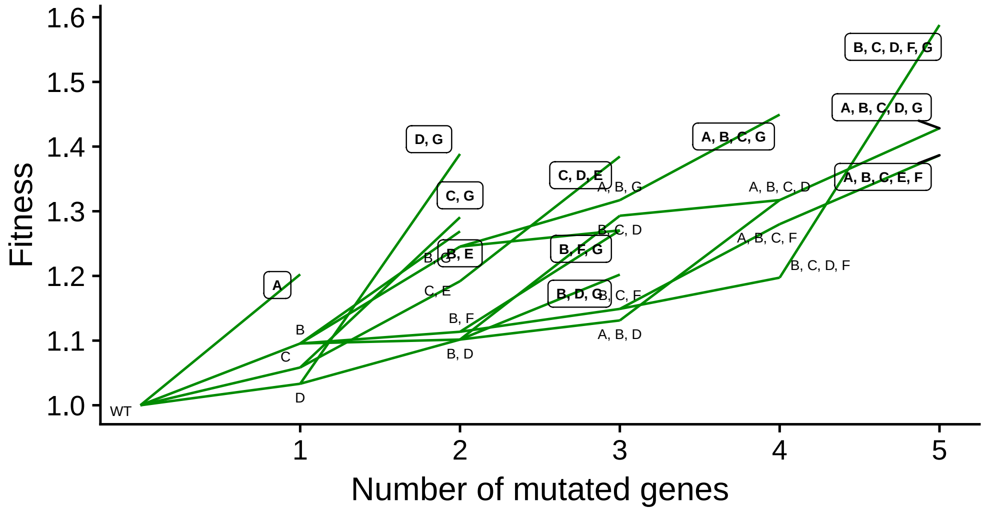

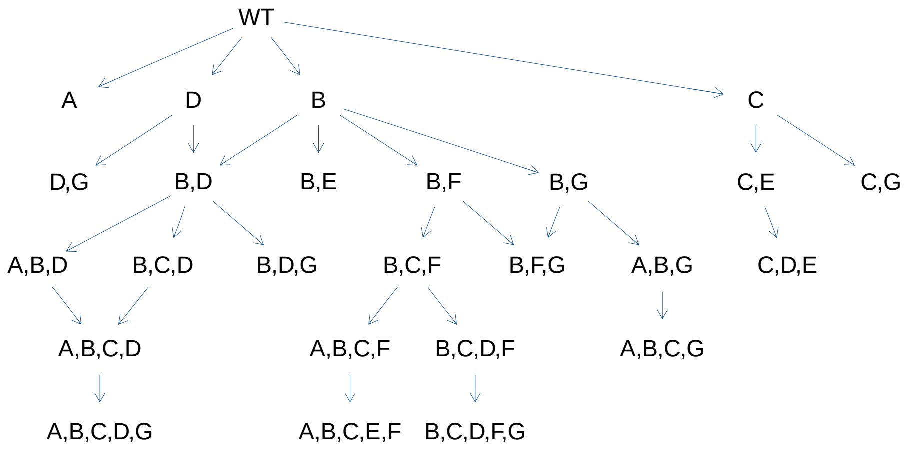

ID	2PO6NSrcnBJAxivm
acc genotypes	12
peaks no back	5
removed edges	NA
prop rem edges	NA

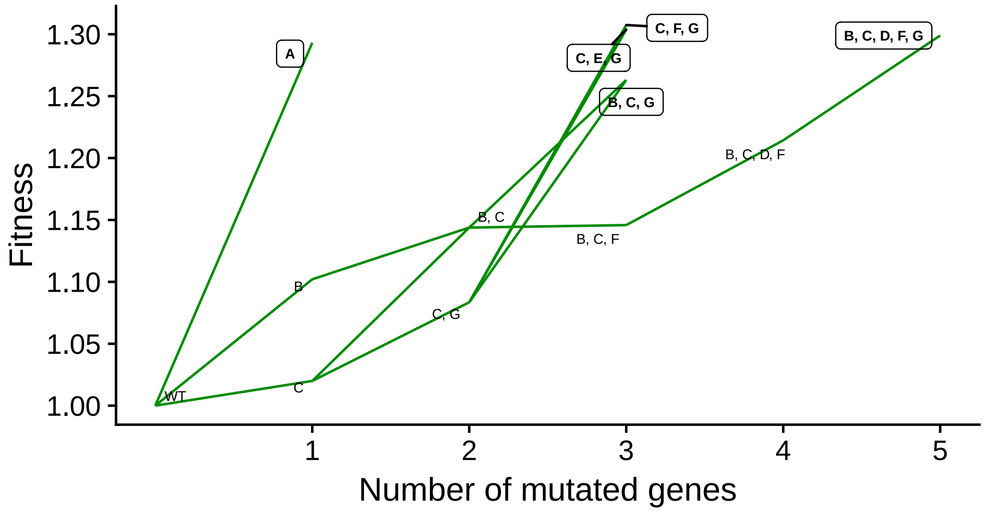

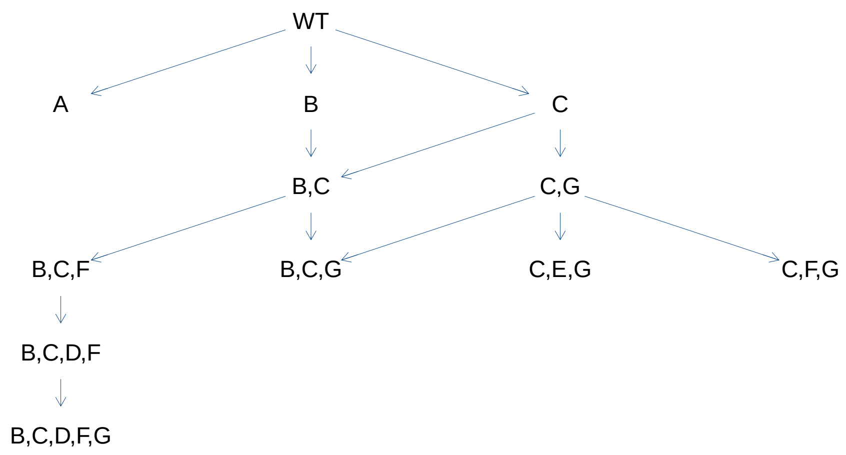

ID	344NmuCSF610HIunl
acc genotypes	44
peaks no back	17
removed edges	NA
prop rem edges	NA

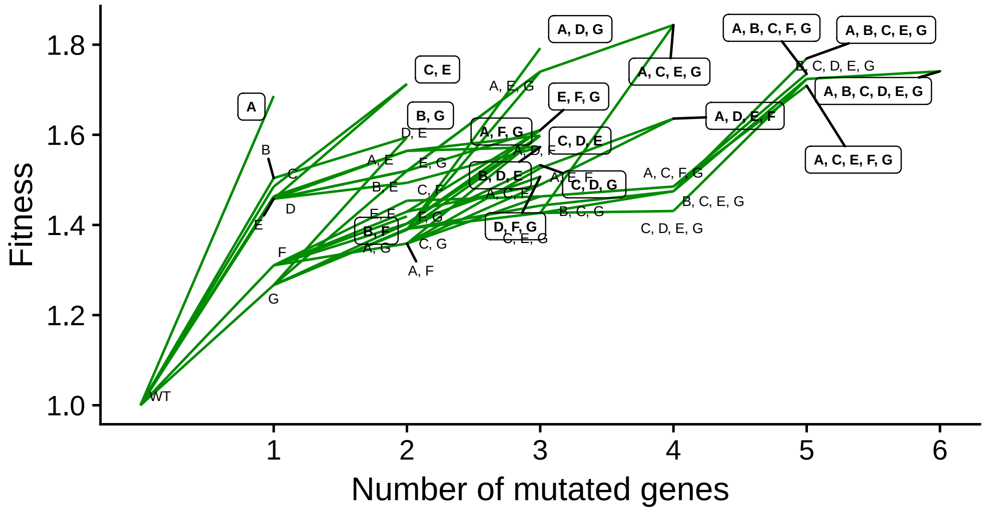

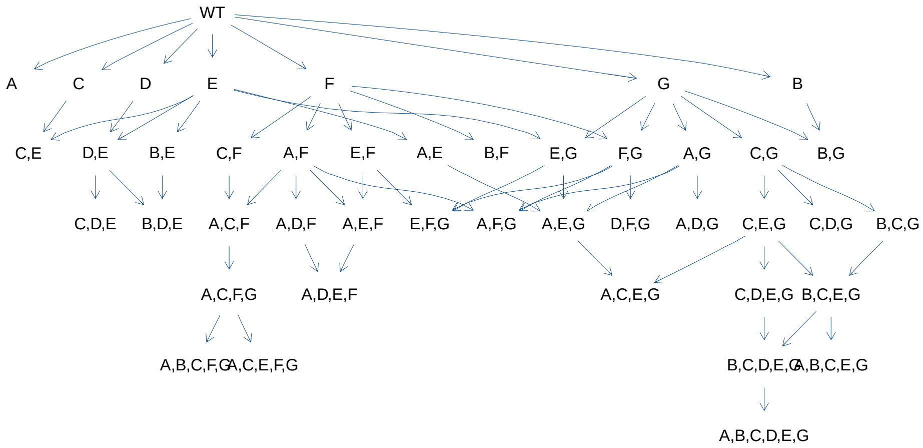

ID	3JtCUkHFLmBXxzdb
acc genotypes	25
peaks no back	13
removed edges	NA
prop rem edges	NA

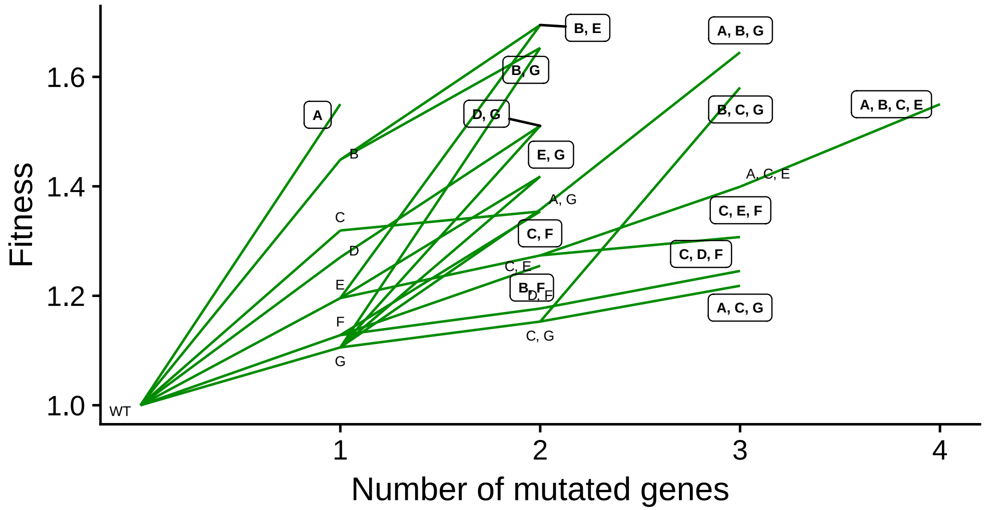

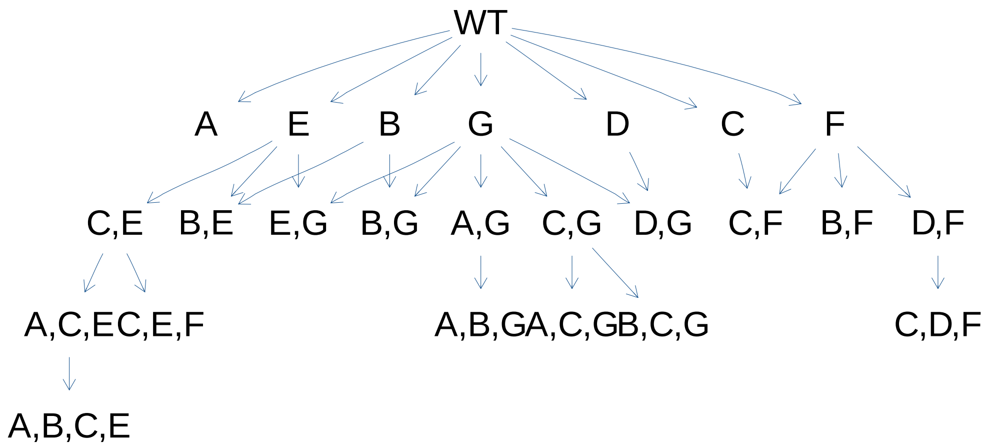

ID	3LxjVpequ1QHM105O
acc genotypes	26
peaks no back	13
removed edges	NA
prop rem edges	NA

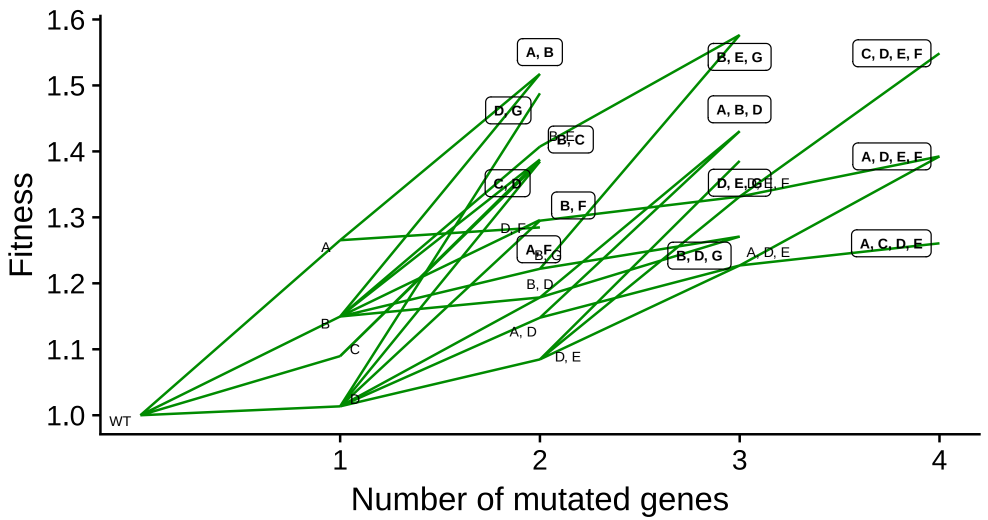

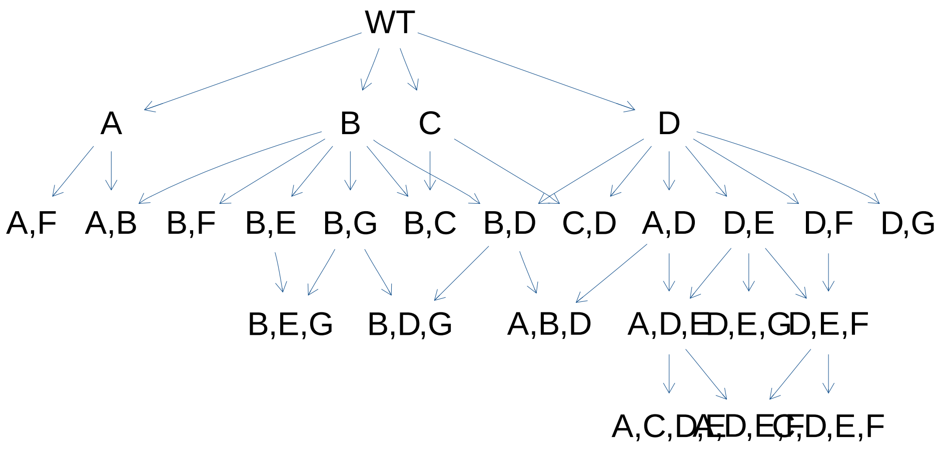

ID	3NwMO8WF7CzUnn4q
acc genotypes	57
peaks no back	15
removed edges	NA
prop rem edges	NA

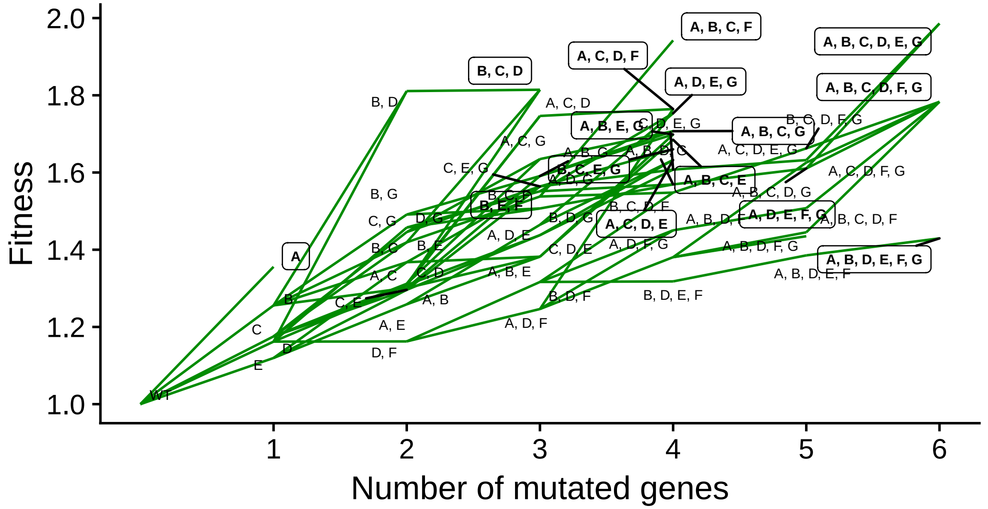

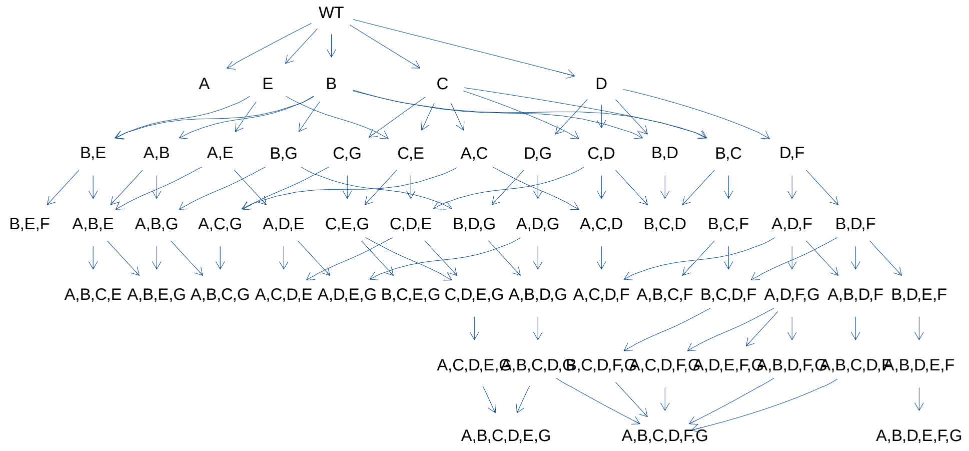

ID 4b99AWrdV4410hdsO
acc genotypes 78
peaks no back 21
removed edges NA
prop rem edges NA

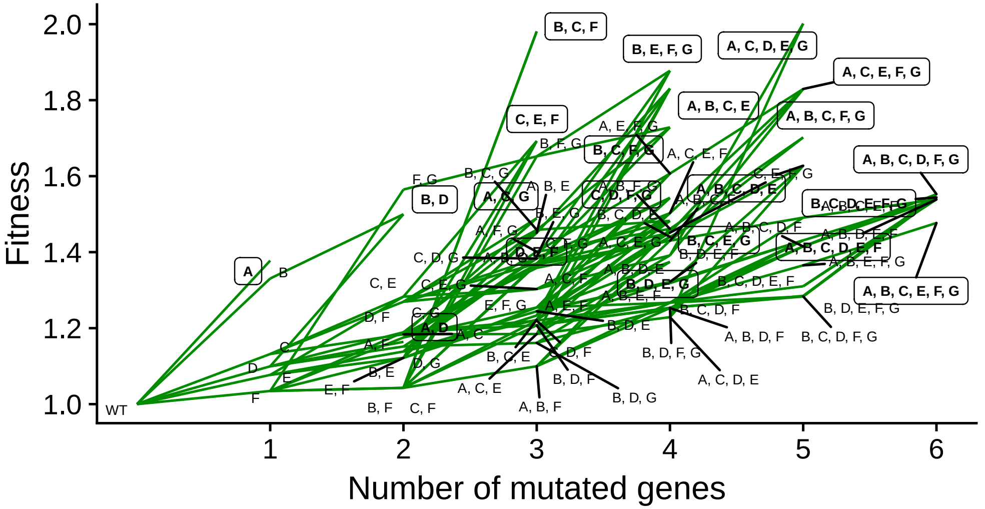

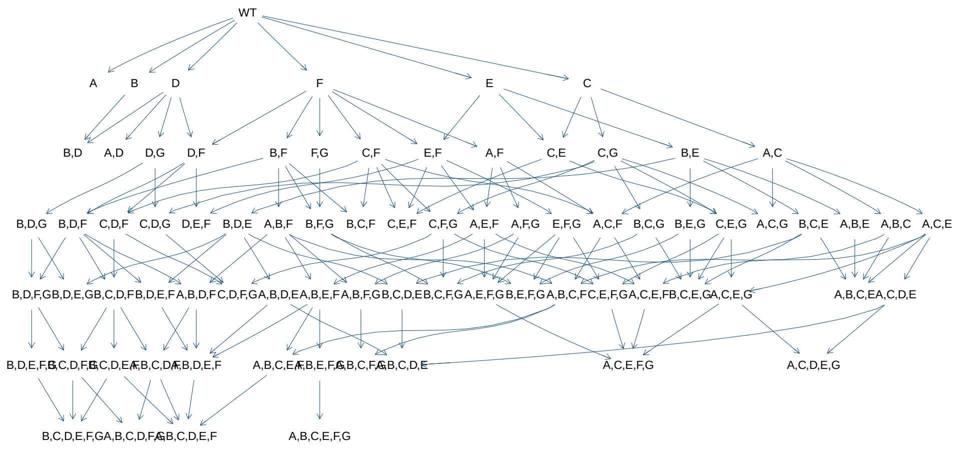

ID	4cDYPNJ8oYbtJWIe
acc genotypes	50
peaks no back	13
removed edges	NA
prop rem edges	NA

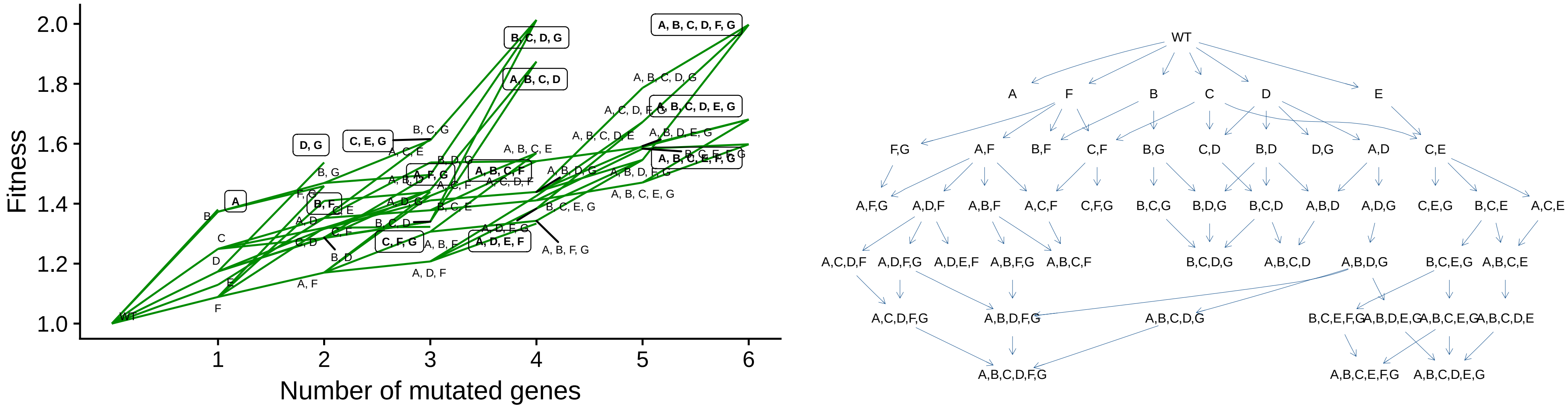

ID	4oM8TrevISckCyjy
acc genotypes	17
peaks no back	11
removed edges	NA
prop rem edges	NA

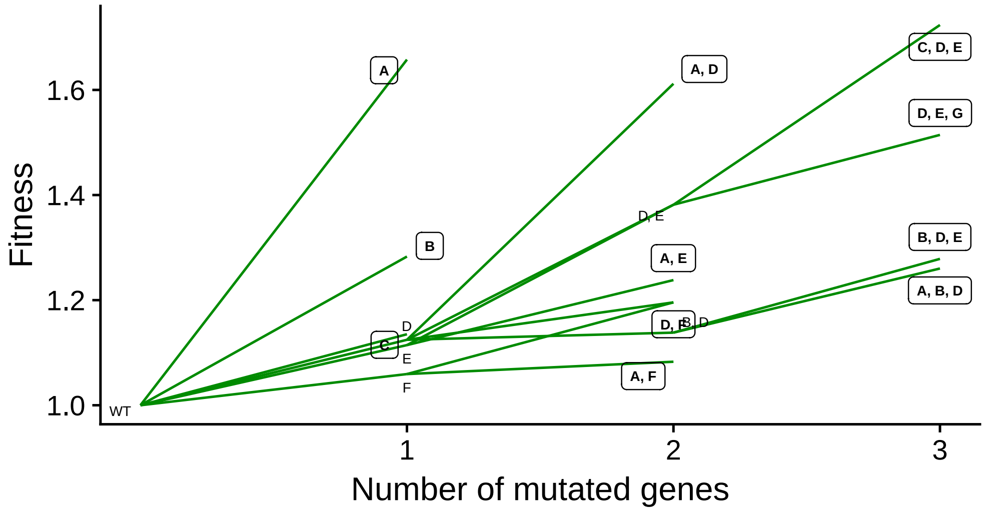

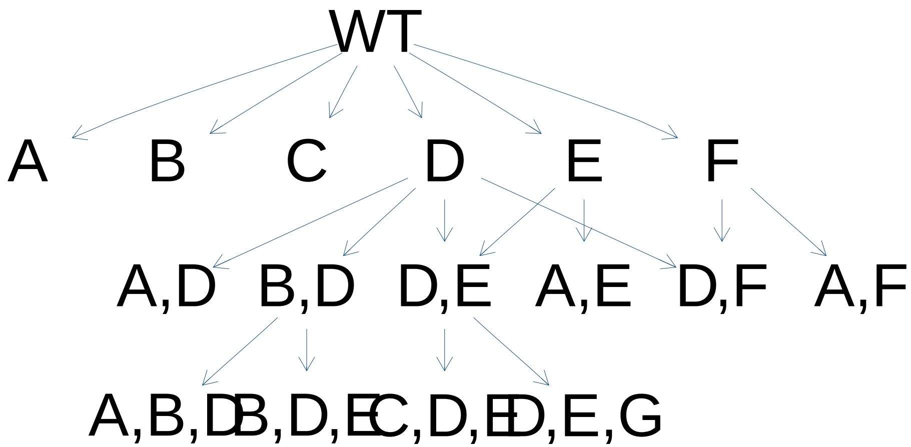

ID	4oYbhN10xm4ltLDHm
acc genotypes	115
peaks no back	12
removed edges	NA
prop rem edges	NA

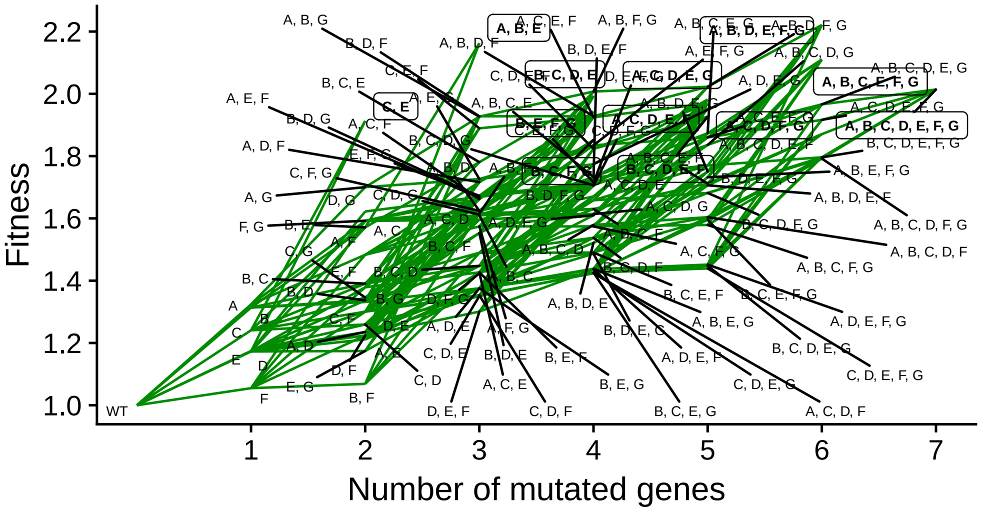

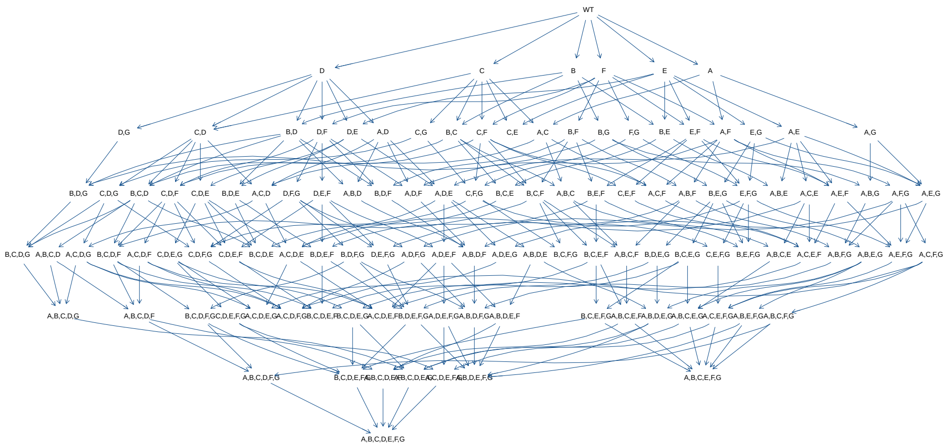

ID	4UvCBrp2StvG6sde
acc genotypes	68
peaks no back	27
removed edges	NA
prop rem edges	NA

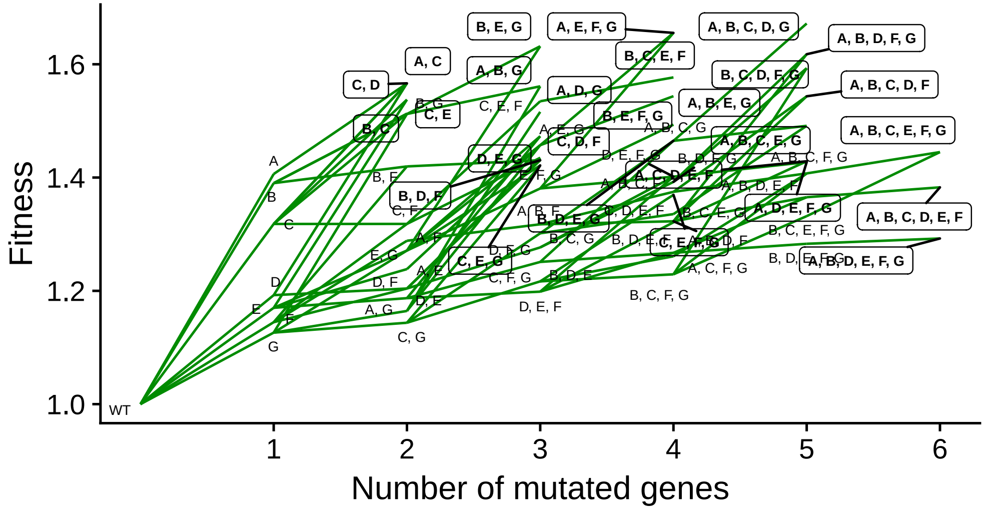

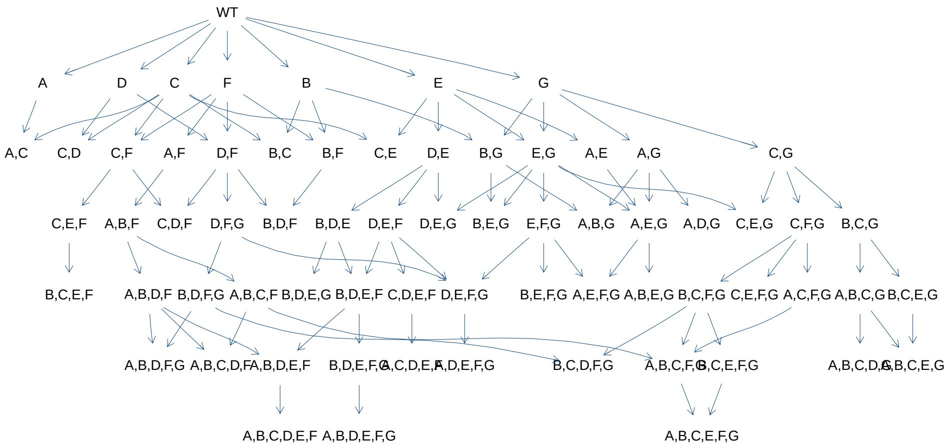

ID	4xoT2TA10PlN1Y8f9
acc genotypes	43
peaks no back	17
removed edges	NA
prop rem edges	NA

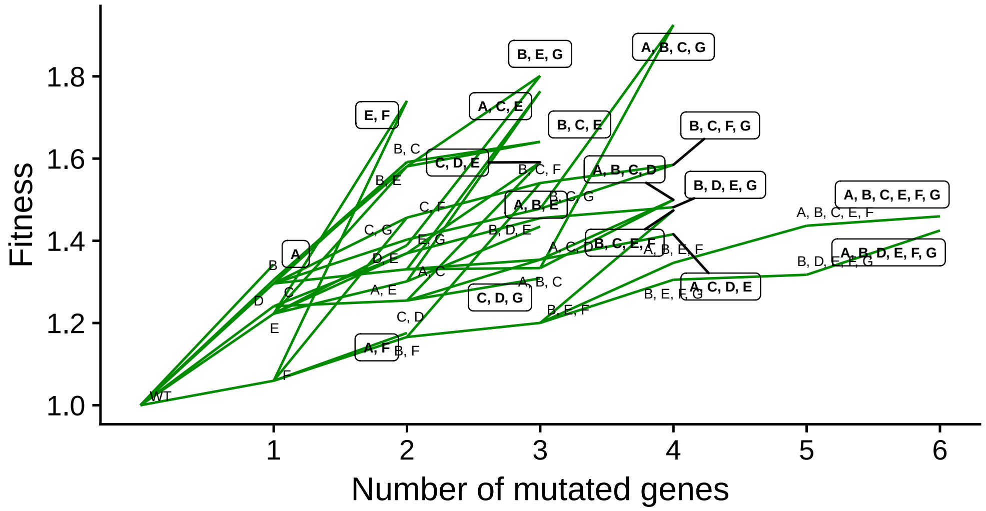

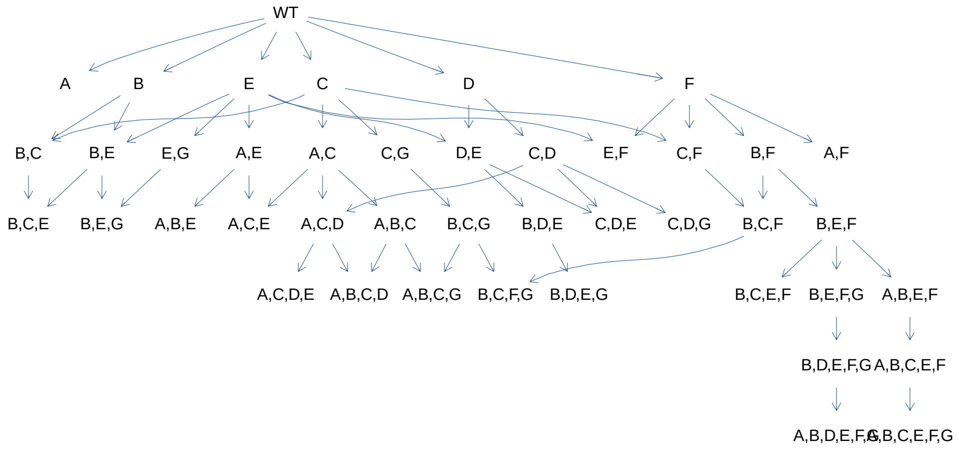

ID	5cropZmgpoQ1ehPe
acc genotypes	33
peaks no back	13
removed edges	NA
prop rem edges	NA

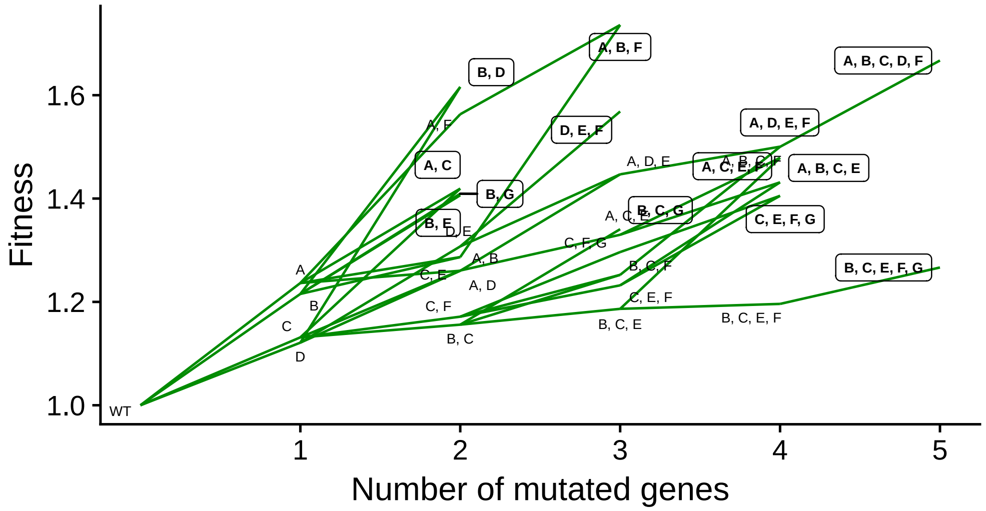

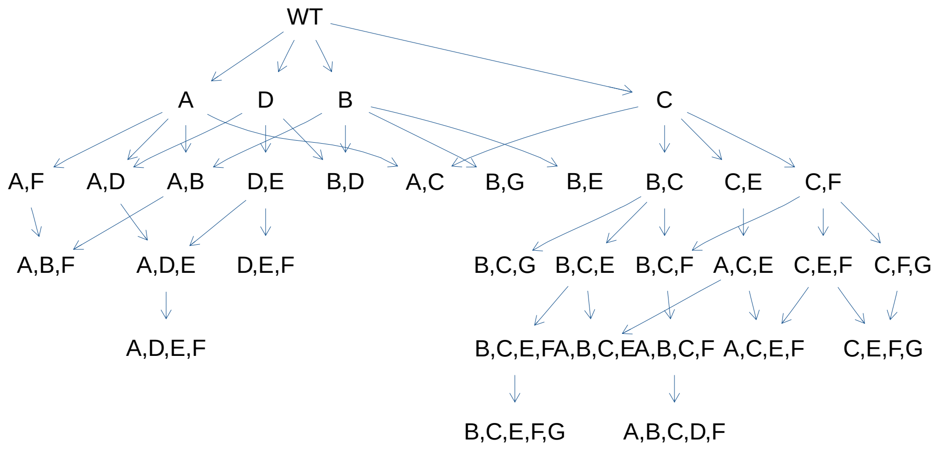

ID	5epf10NPUUcYzNrU9
acc genotypes	46
peaks no back	7
removed edges	NA
prop rem edges	NA

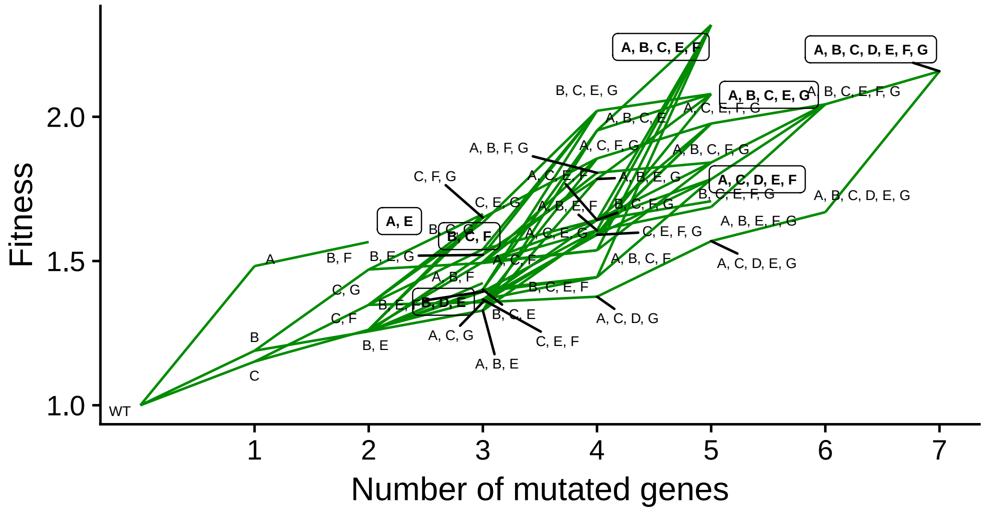

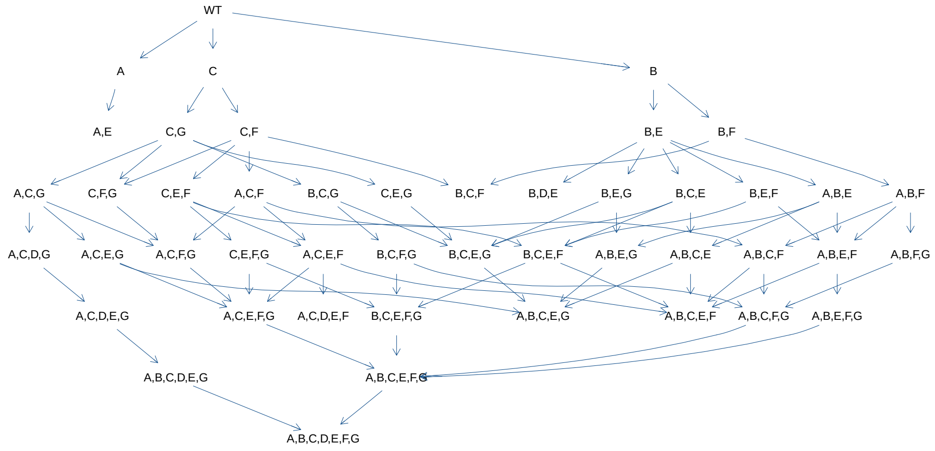

ID	5J10fJoXgI3k5FMVe
acc genotypes	81
peaks no back	18
removed edges	NA
prop rem edges	NA

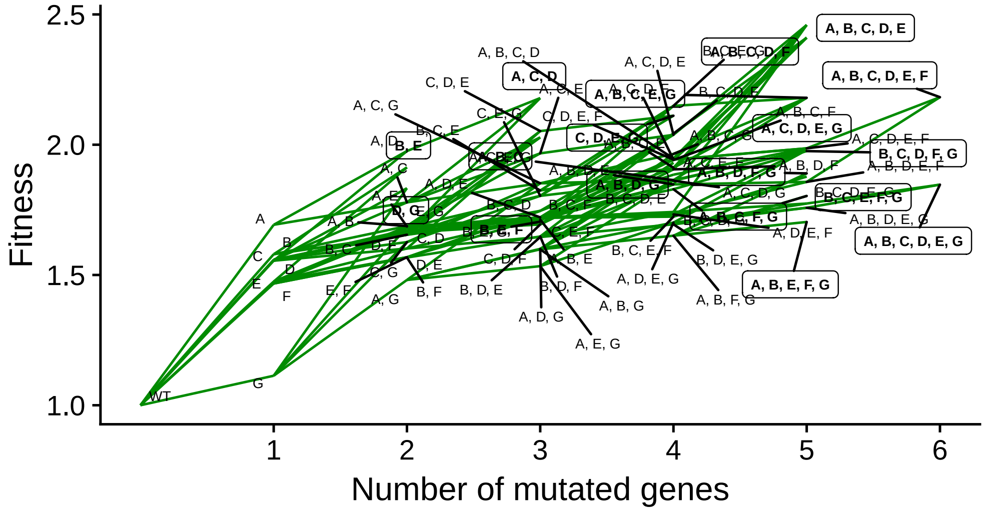

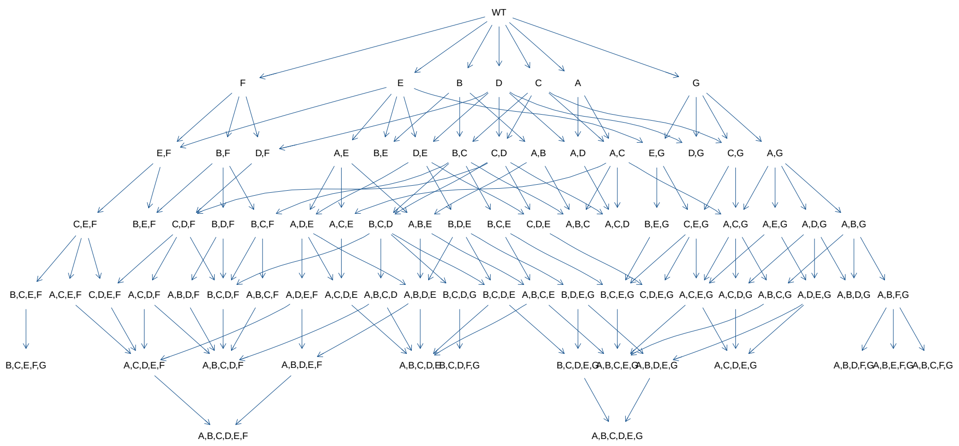

ID	5J6VWoMy7ROl8aOu
acc genotypes	72
peaks no back	23
removed edges	NA
prop rem edges	NA

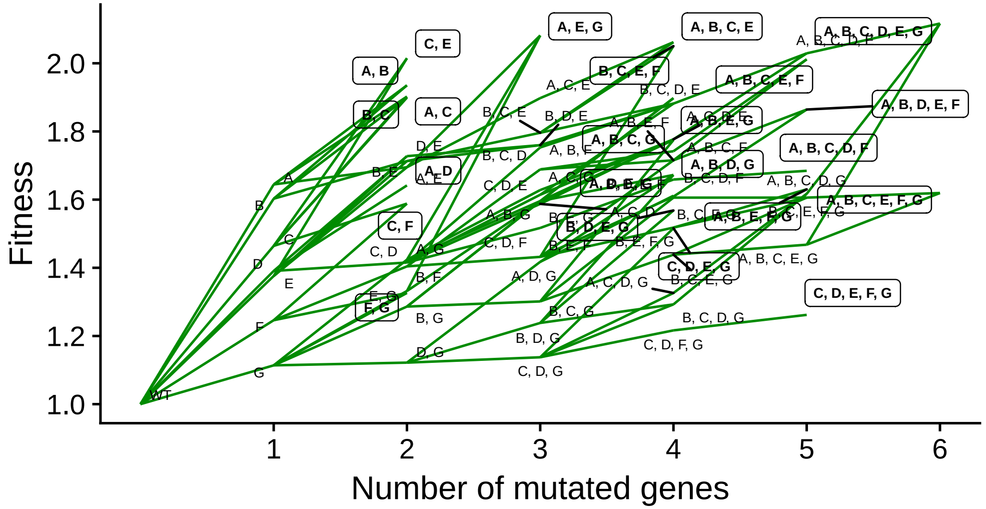

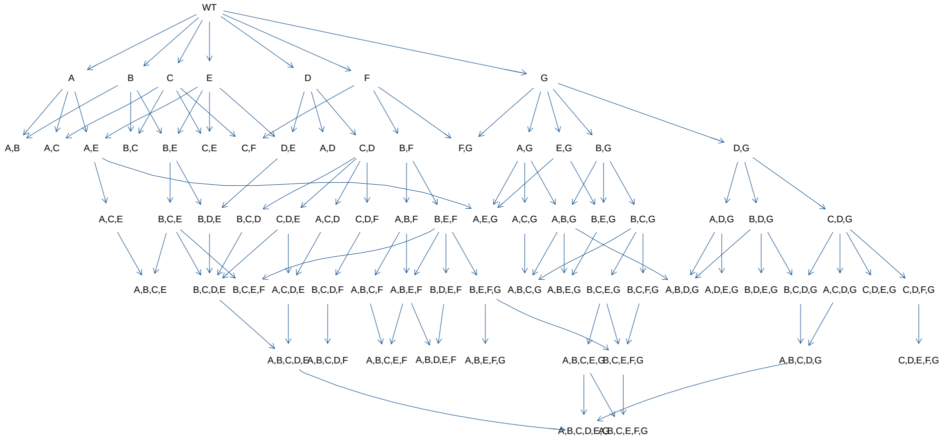

ID 5MozgxpY4MNYCtSZ

acc genotypes 80

peaks no back 28

removed edges NA

prop rem edges NA

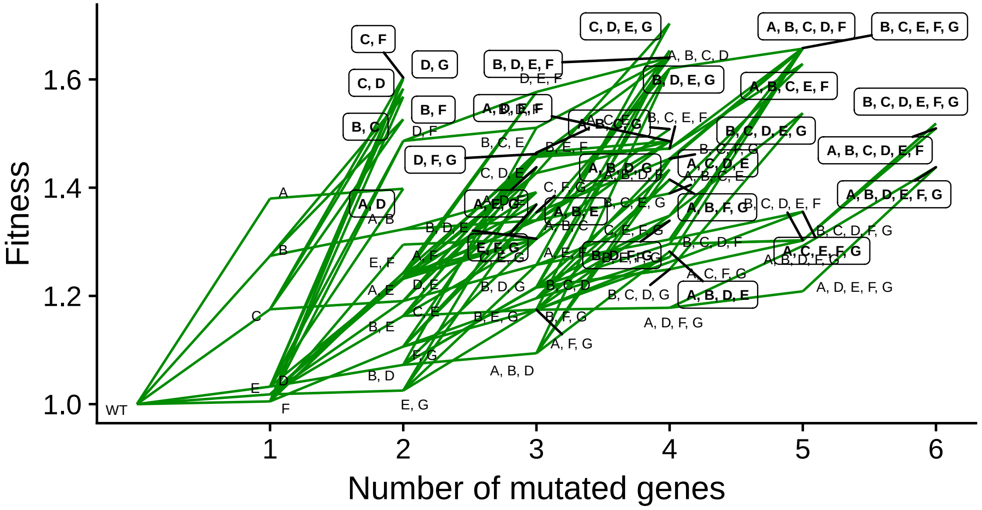

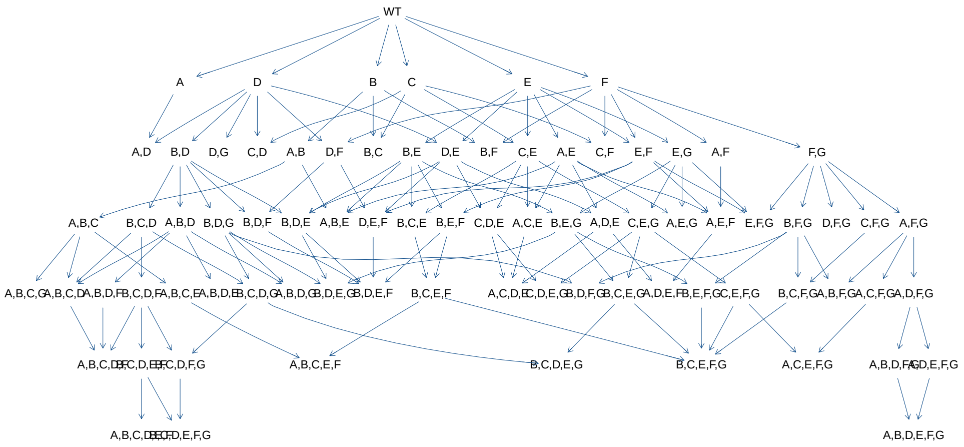

ID	6ARgJvilfN23NjA4
acc genotypes	81
peaks no back	22
removed edges	NA
prop rem edges	NA

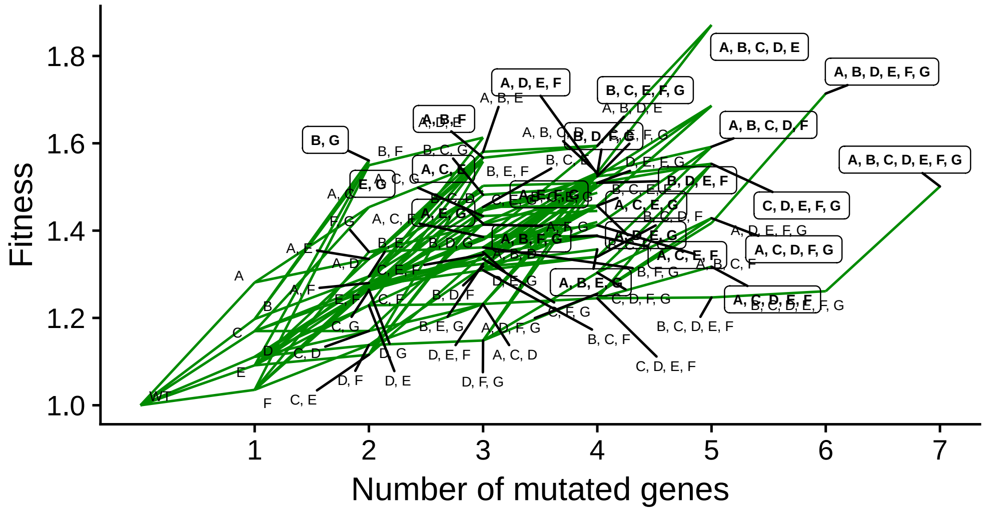

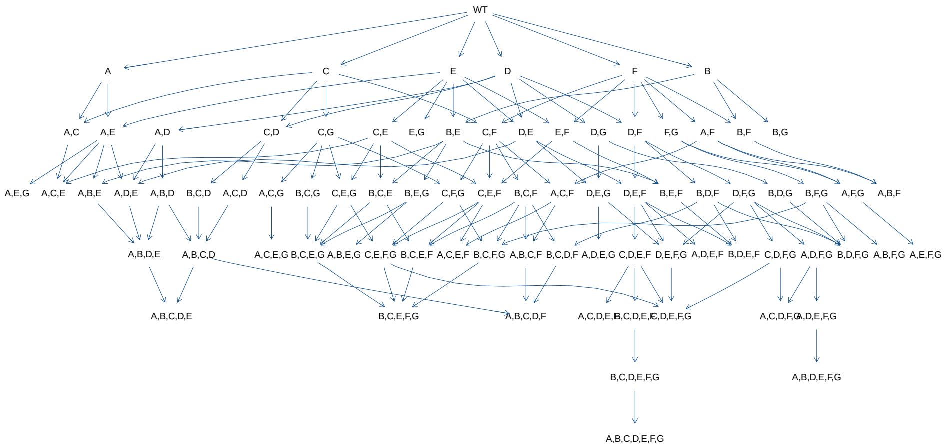

ID	6grkdzP10lm6kQ42g
acc genotypes	81
peaks no back	12
removed edges	NA
prop rem edges	NA

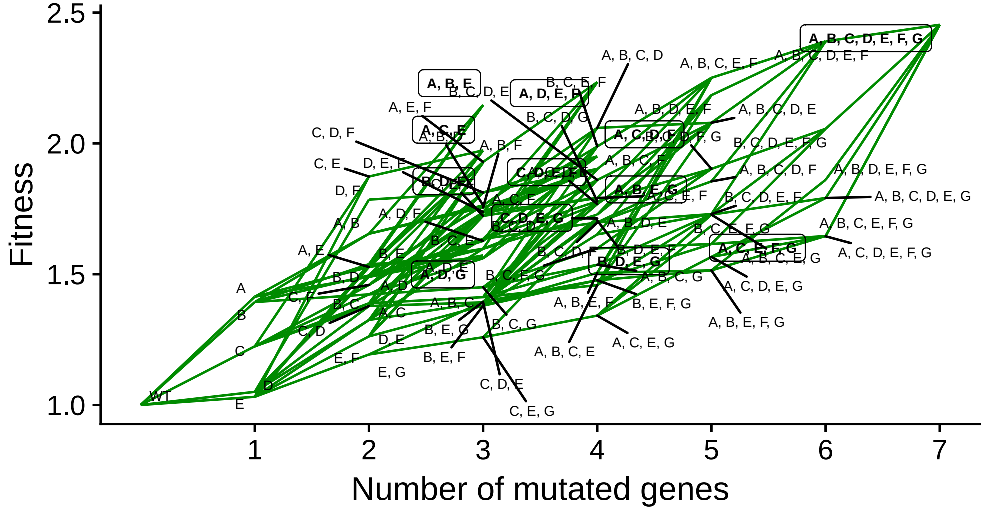

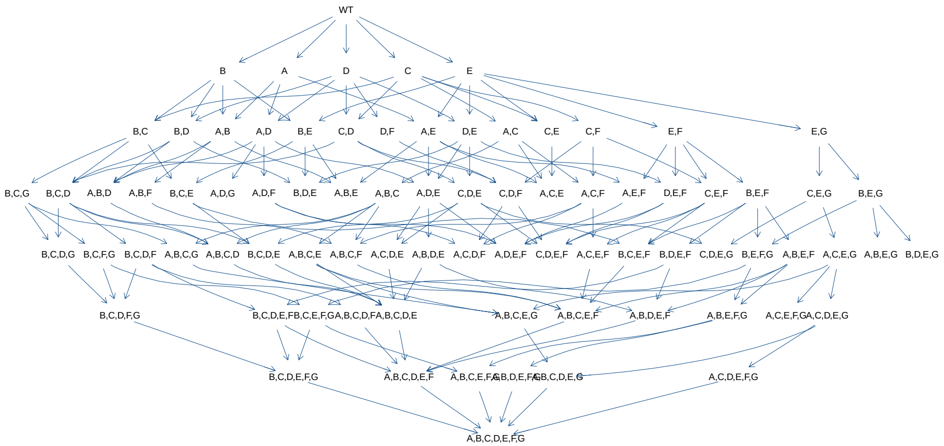

ID	75PXrtvV107d4XbmV
acc genotypes	70
peaks no back	19
removed edges	NA
prop rem edges	NA

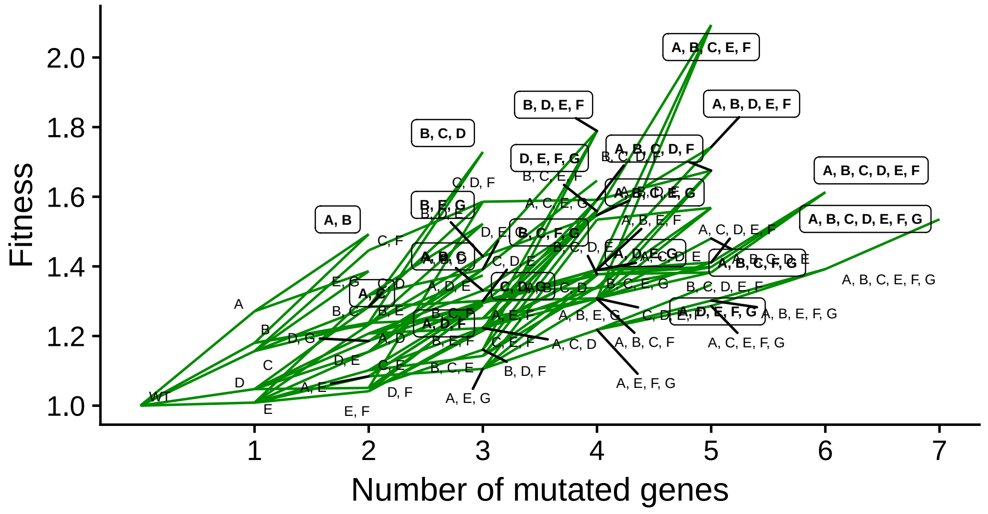

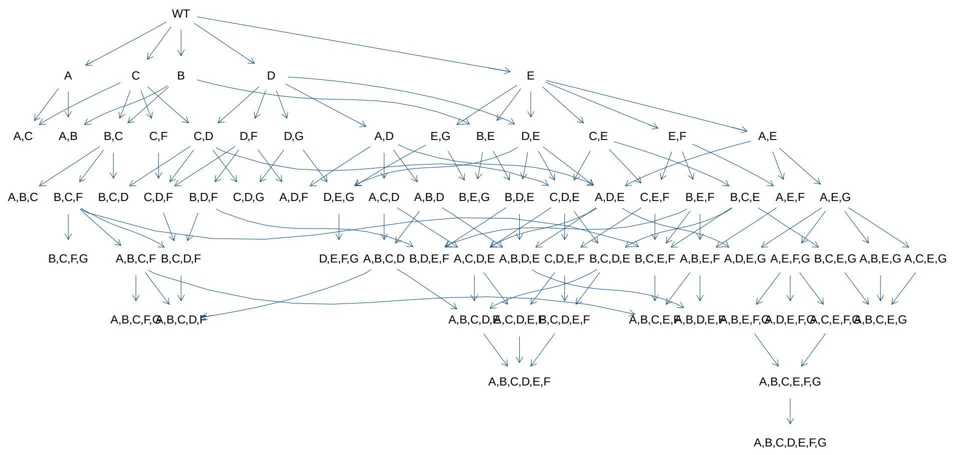

ID	84eRNCZIduWDrCW4
acc genotypes	39
peaks no back	15
removed edges	NA
prop rem edges	NA

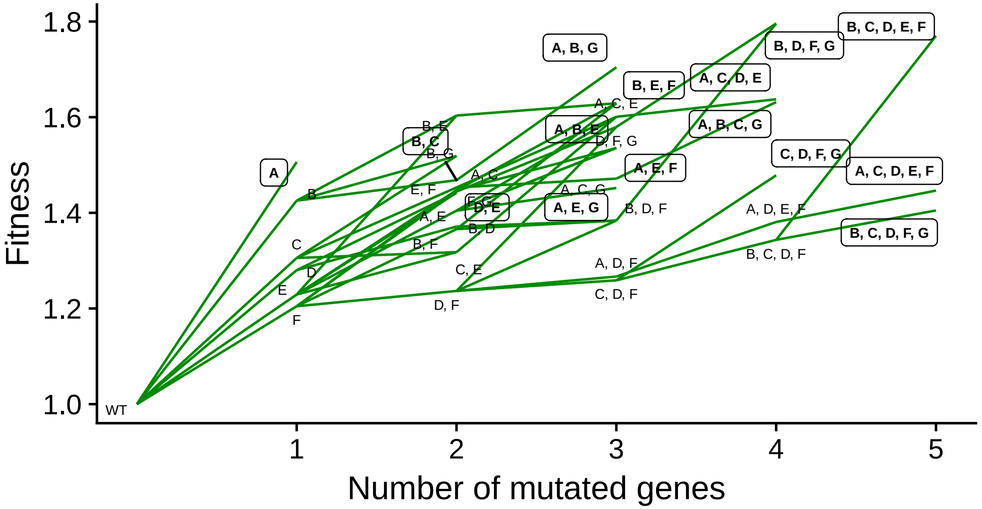

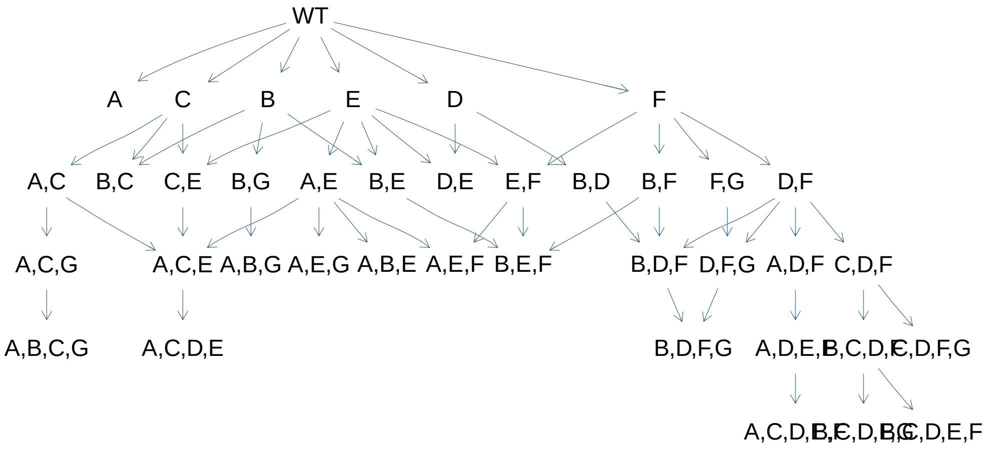

ID	84uIeheQaYaVNux8
acc genotypes	18
peaks no back	6
removed edges	NA
prop rem edges	NA

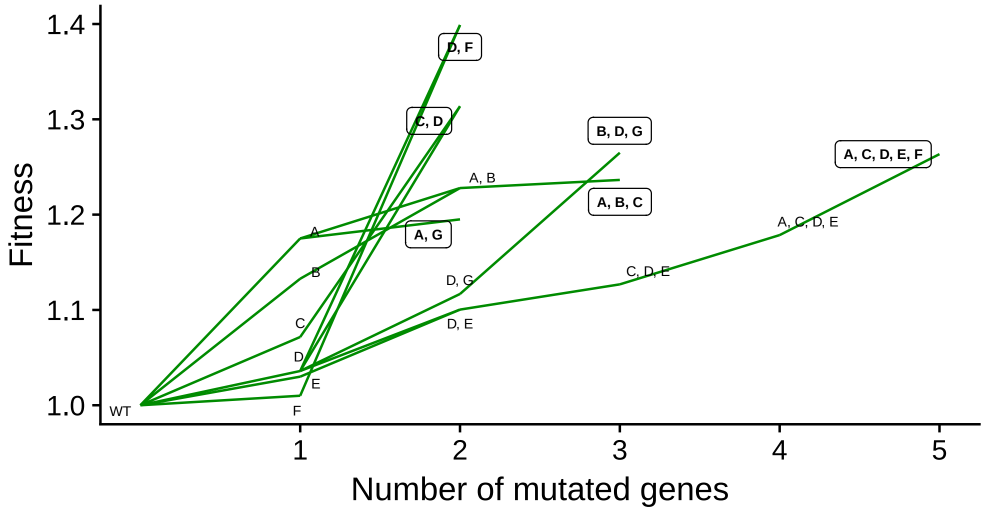

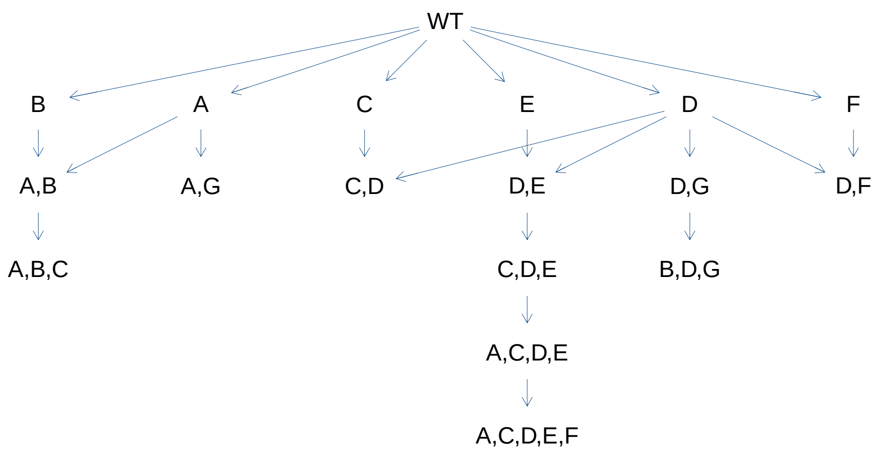

ID	8a9vNJyhKyFzgtGl
acc genotypes	29
peaks no back	6
removed edges	NA
prop rem edges	NA

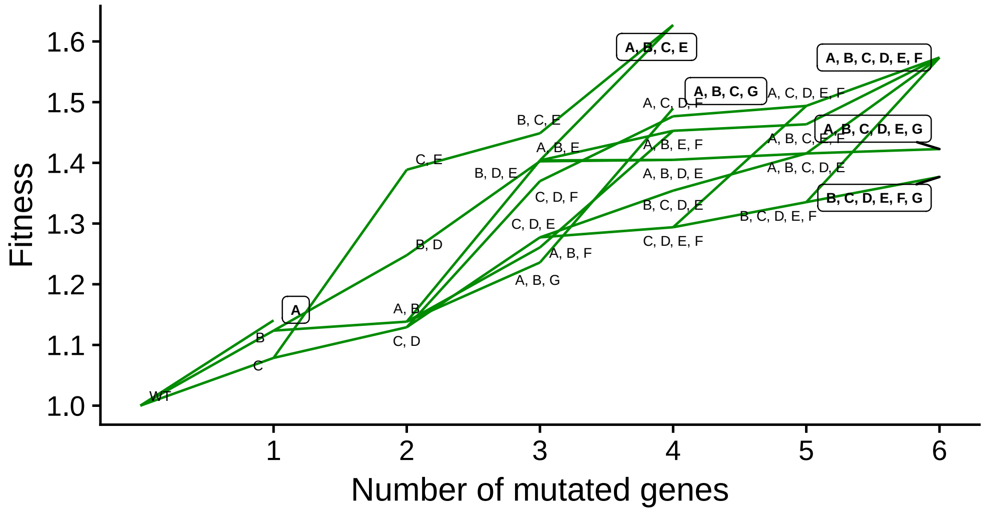

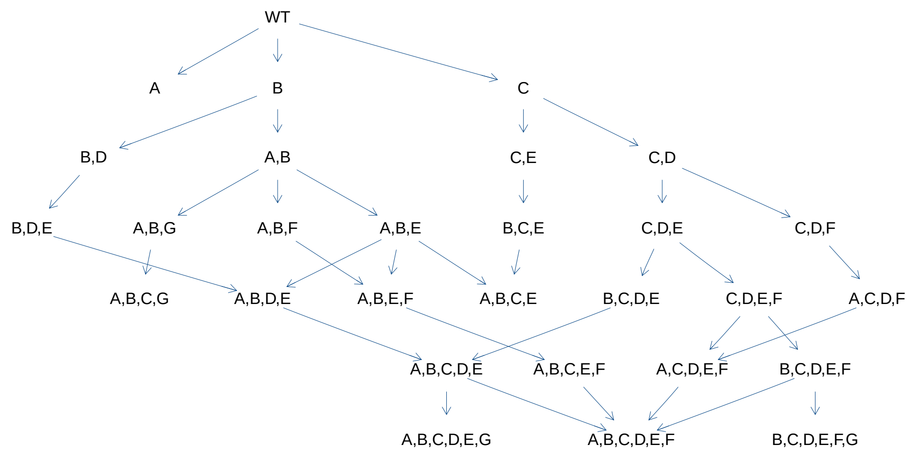

ID	8UysAOd1WpNJ6JEA
acc genotypes	67
peaks no back	15
removed edges	NA
prop rem edges	NA

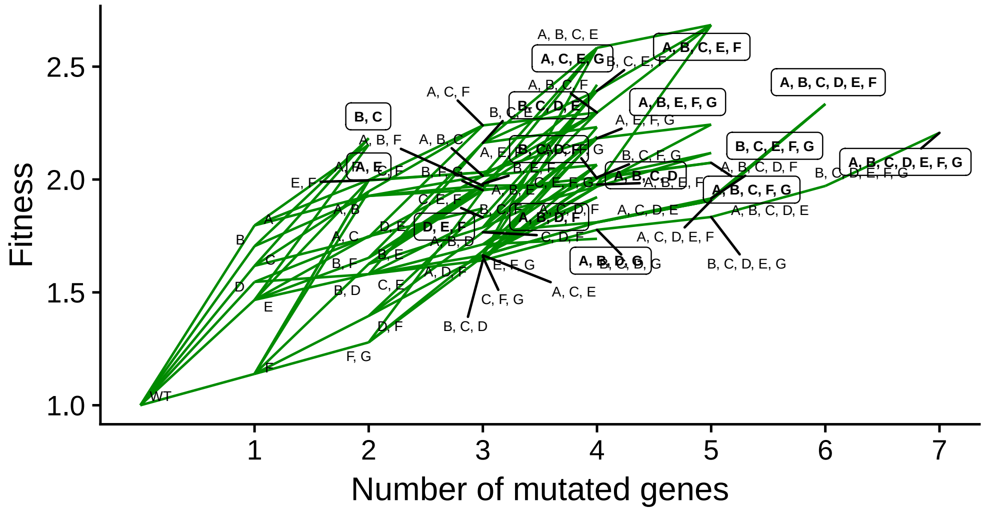

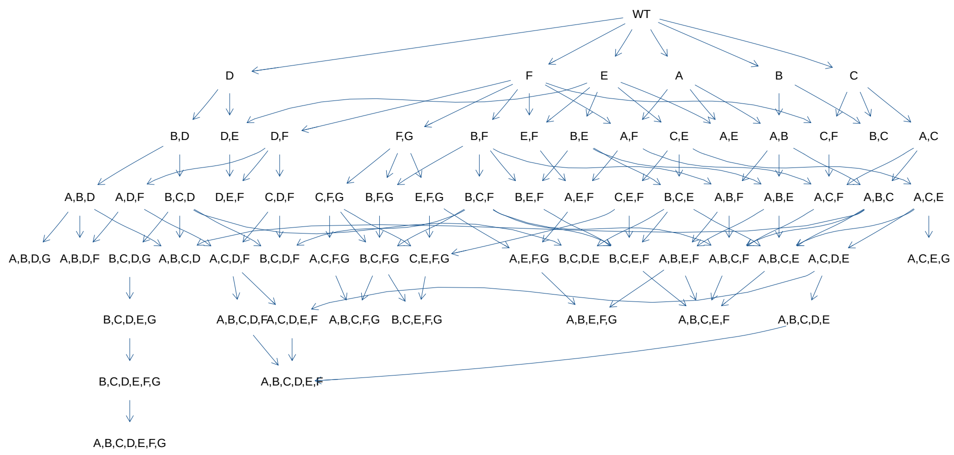

ID	8yQFlKWNFgRltIOW
acc genotypes	40
peaks no back	18
removed edges	NA
prop rem edges	NA

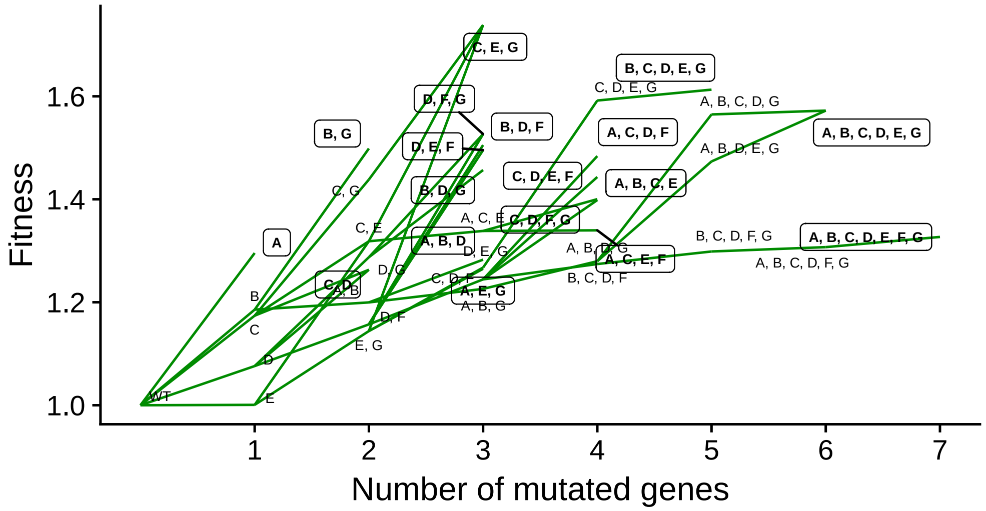

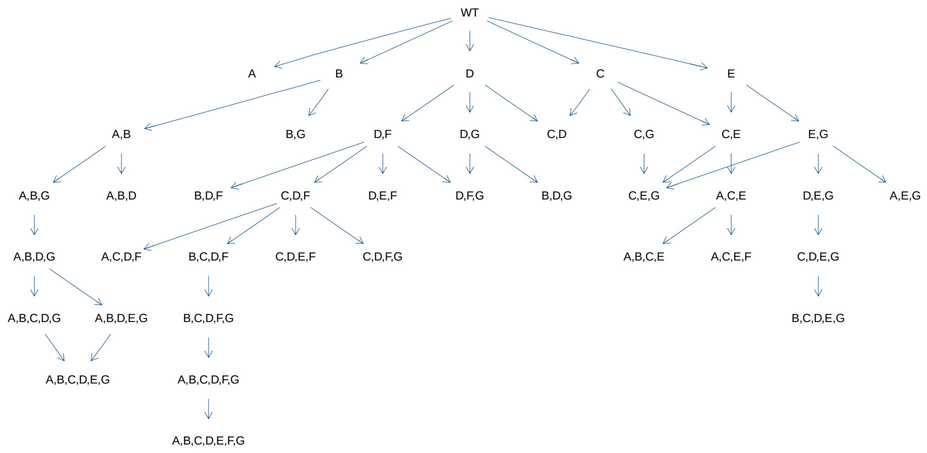

ID	98Ltpa3lXTnc4zbC
acc genotypes	29
peaks no back	12
removed edges	NA
prop rem edges	NA

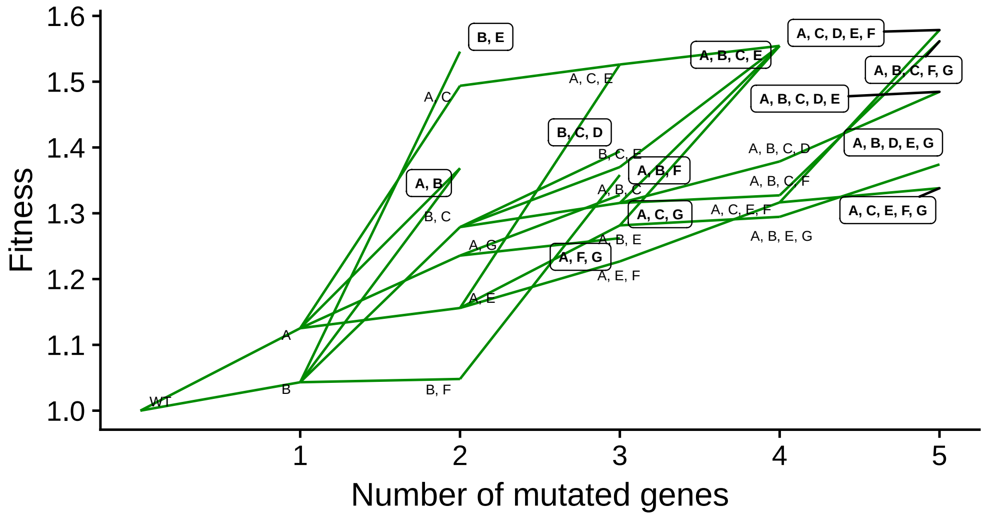

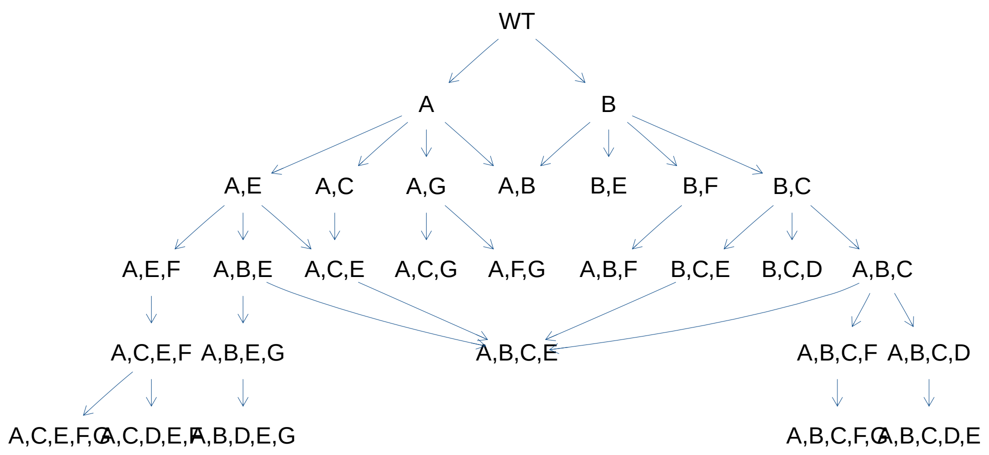

ID	9KrYrcUaMBo5BoOd
acc genotypes	89
peaks no back	13
removed edges	NA
prop rem edges	NA

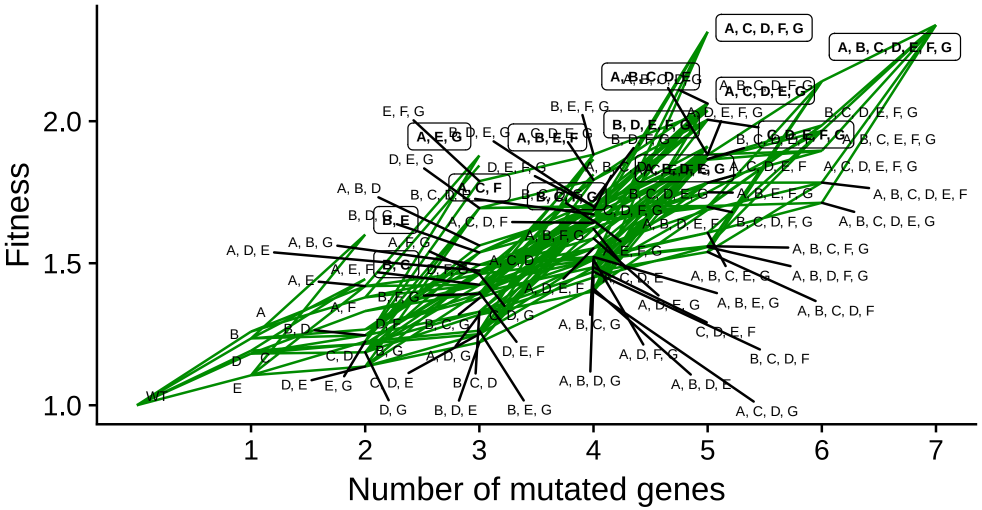

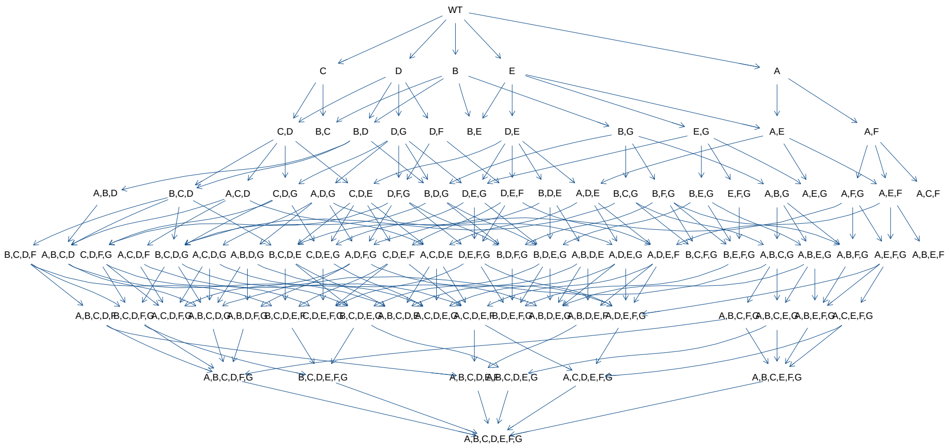

ID	9MBO3qg10BvSTzUqM
acc genotypes	52
peaks no back	15
removed edges	NA
prop rem edges	NA

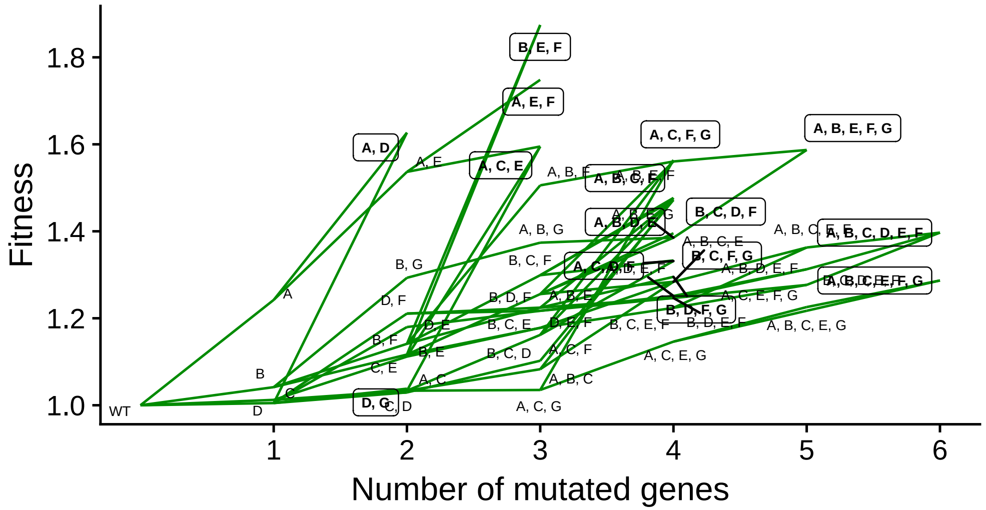

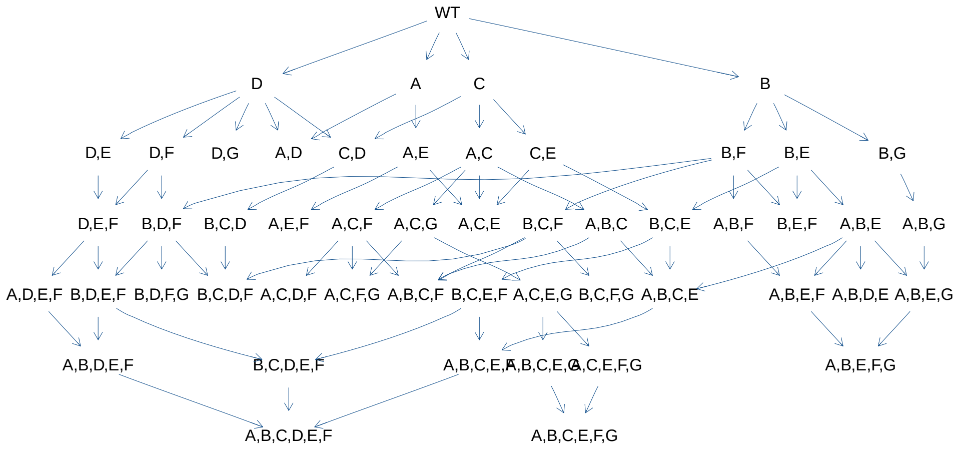

ID	9rebvjtcu7f5fc6O
acc genotypes	38
peaks no back	12
removed edges	NA
prop rem edges	NA

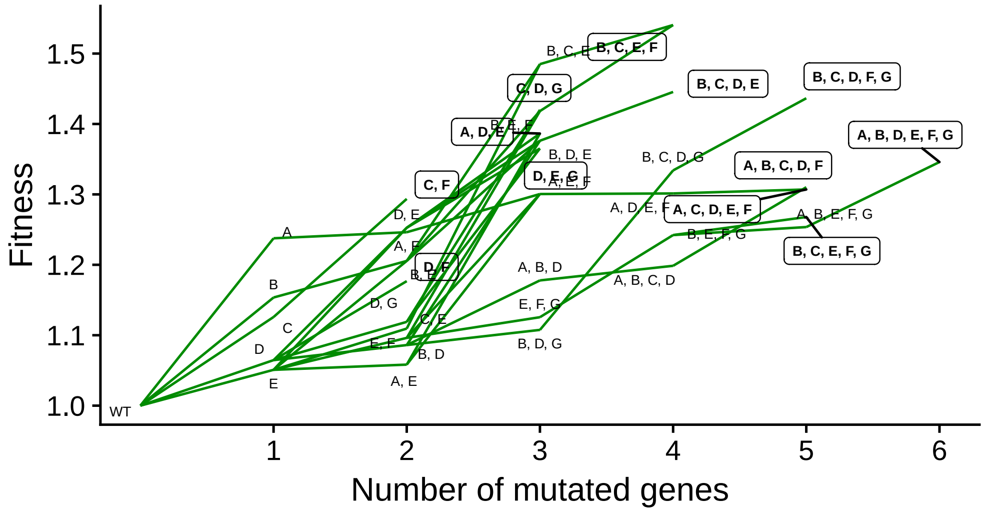

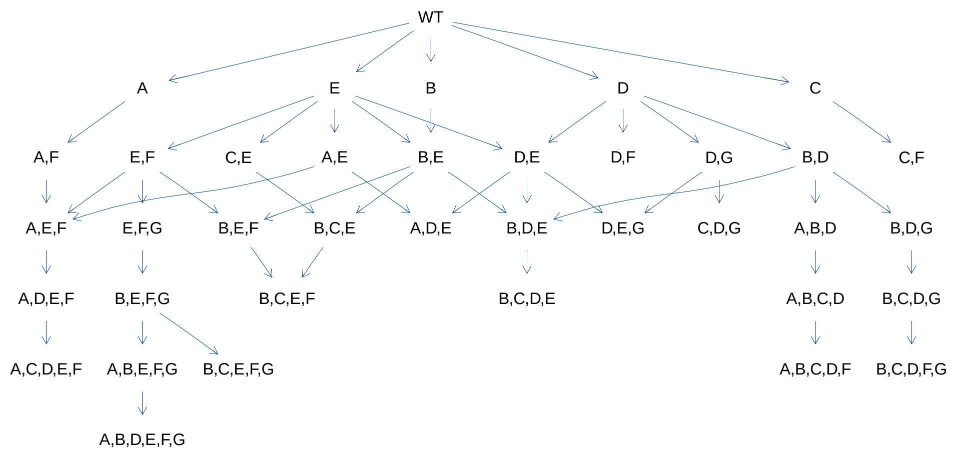

ID	A3LrldXjUIno4iNV
acc genotypes	77
peaks no back	19
removed edges	NA
prop rem edges	NA

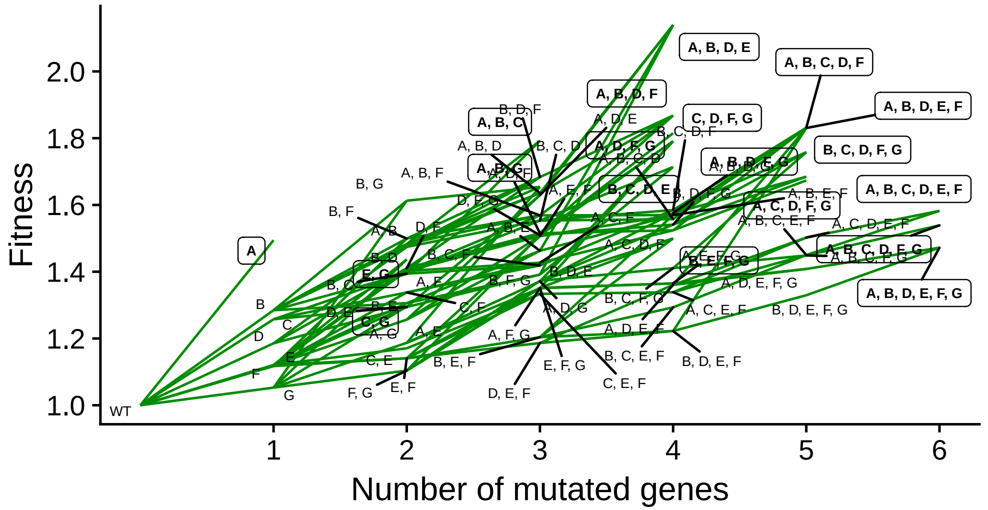

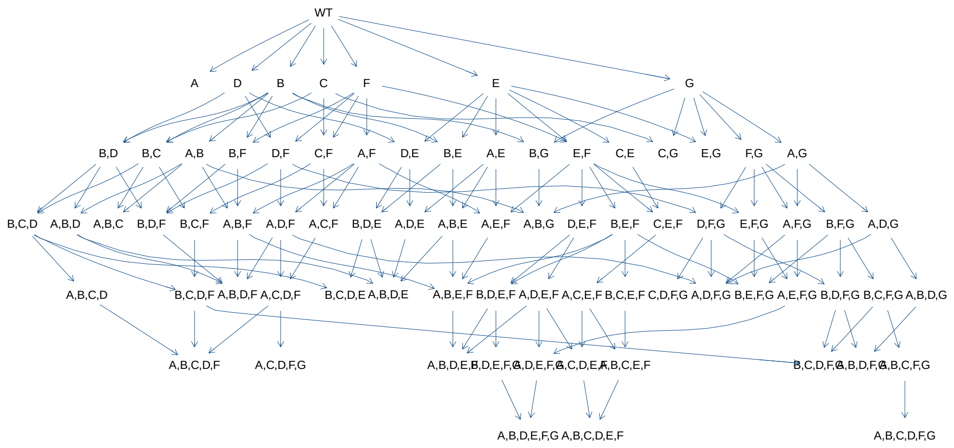

ID	aA3KFQazfmWZiKVN
acc genotypes	54
peaks no back	20
removed edges	NA
prop rem edges	NA

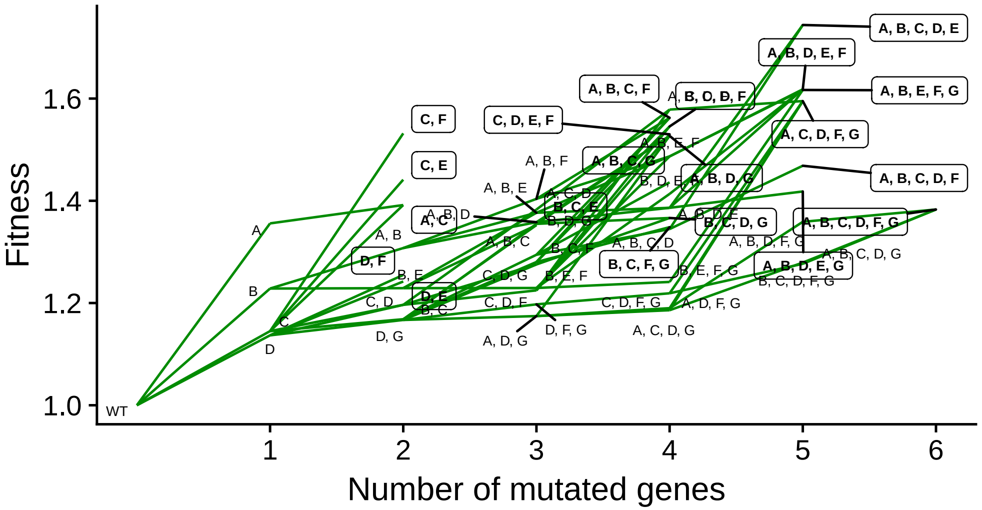

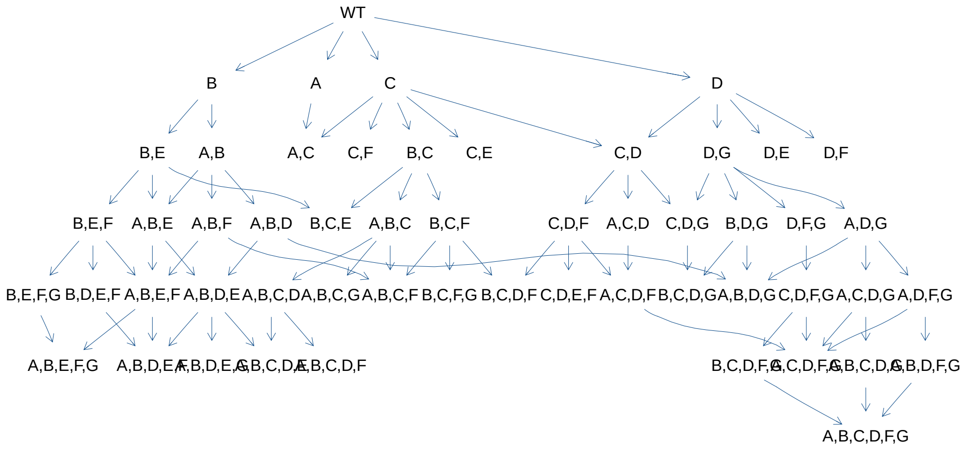

ID ACGaLODBO3L7xXPK
acc genotypes 70
peaks no back 19
removed edges NA
prop rem edges NA

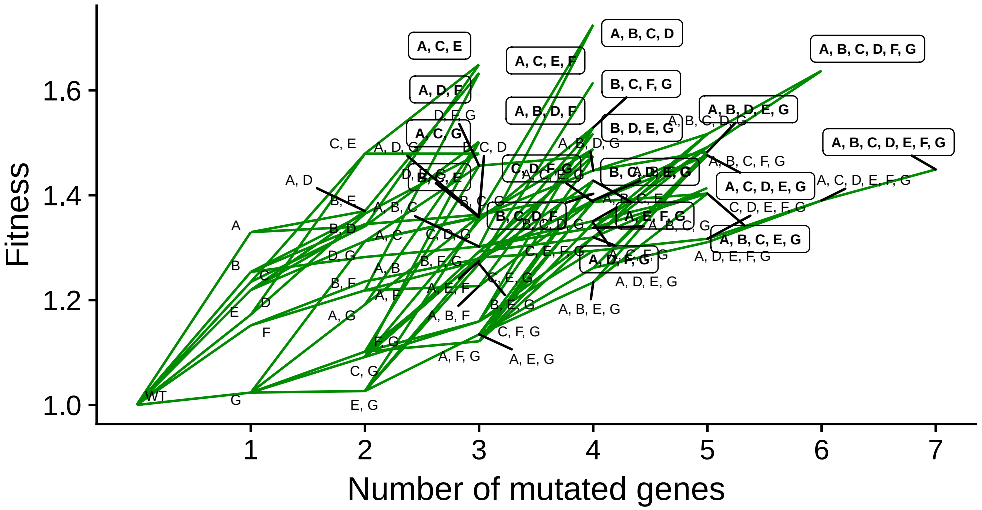

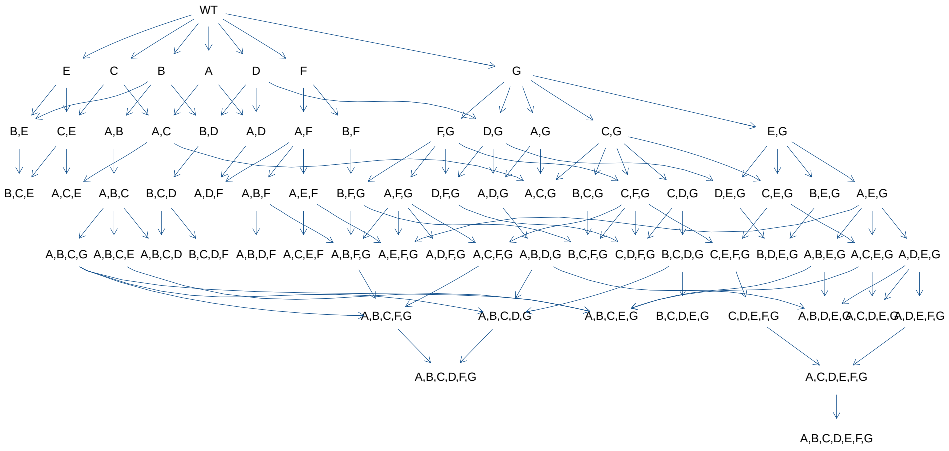

ID	AdlPqP6mmr9aElyf
acc genotypes	19
peaks no back	8
removed edges	NA
prop rem edges	NA

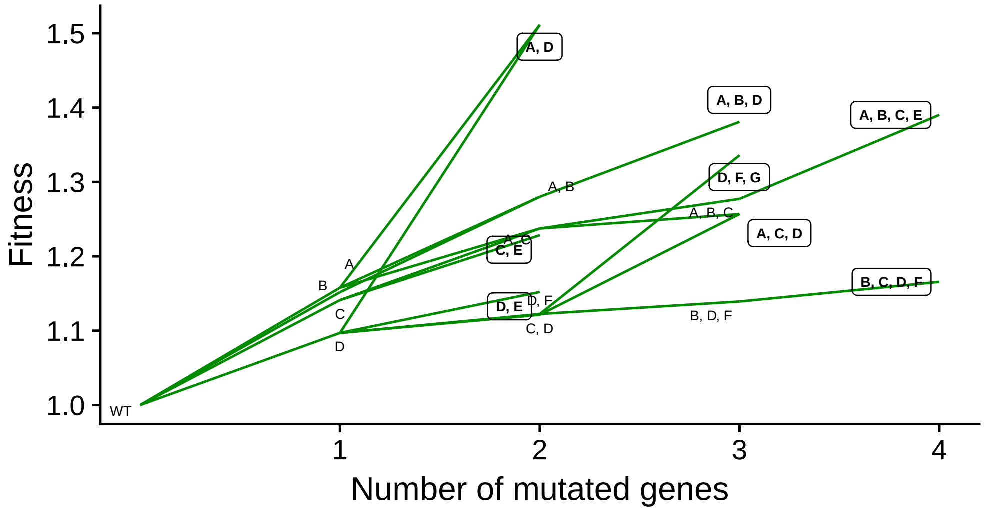

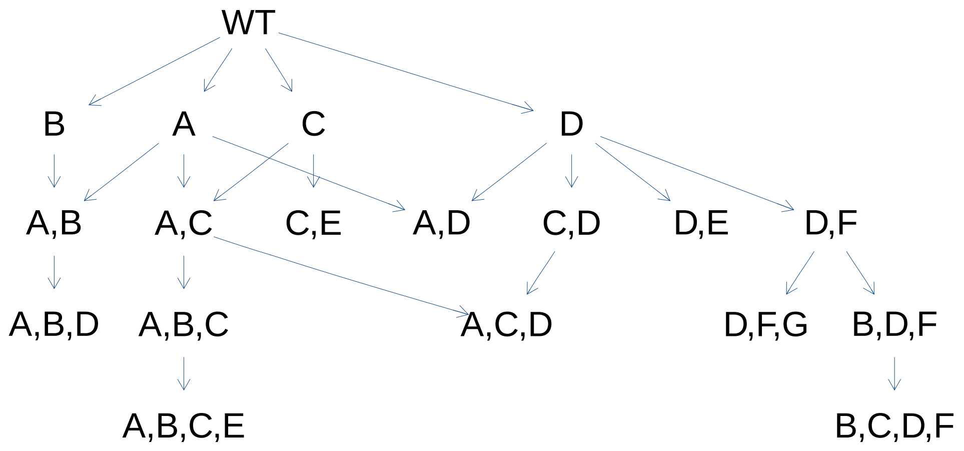

ID	aeQvEQMAOMlDD5Ee
acc genotypes	49
peaks no back	20
removed edges	NA
prop rem edges	NA

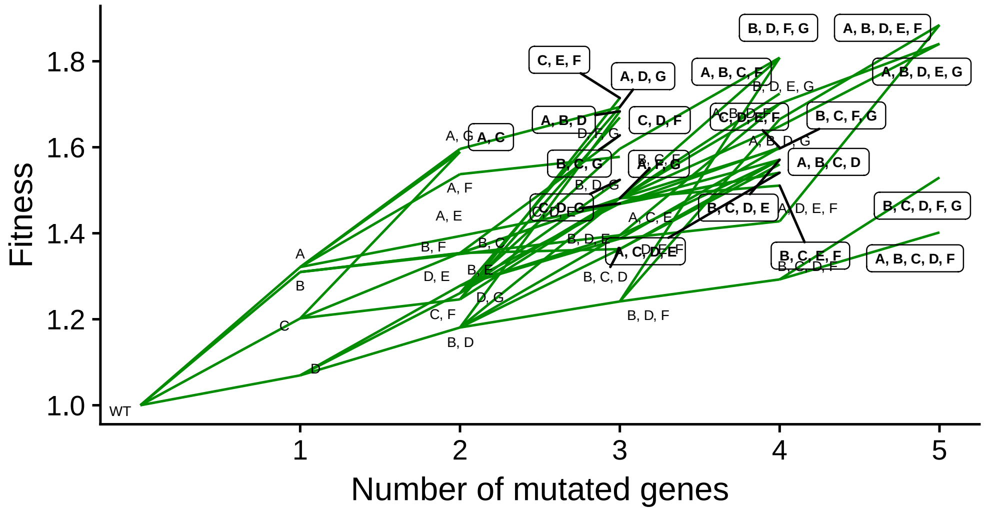

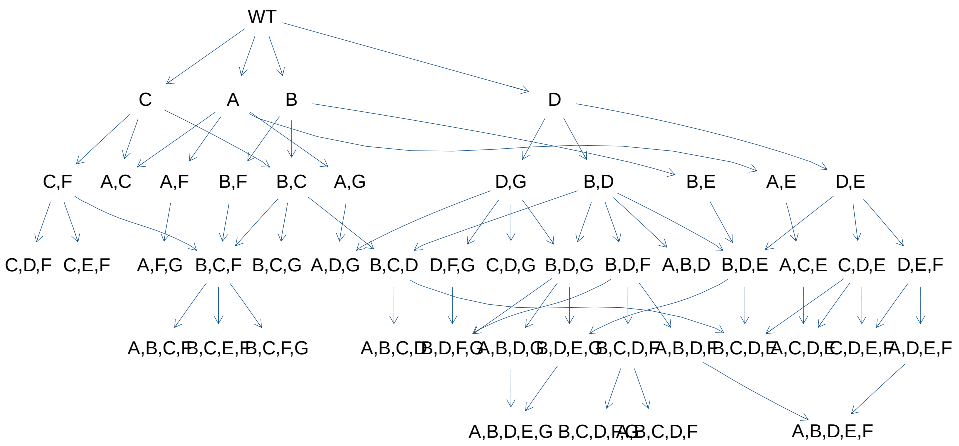

ID	AqnbiWCAFm6eogfg
acc genotypes	17
peaks no back	6
removed edges	NA
prop rem edges	NA

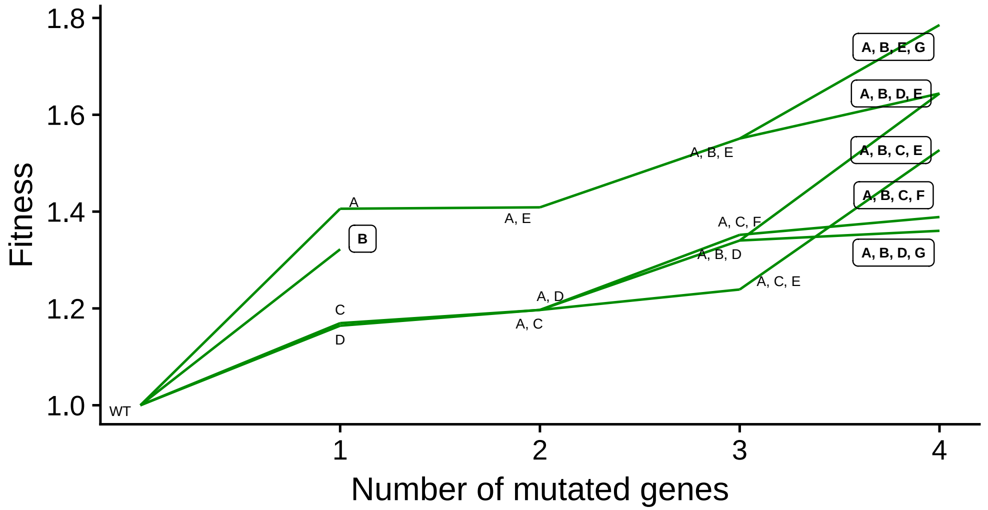

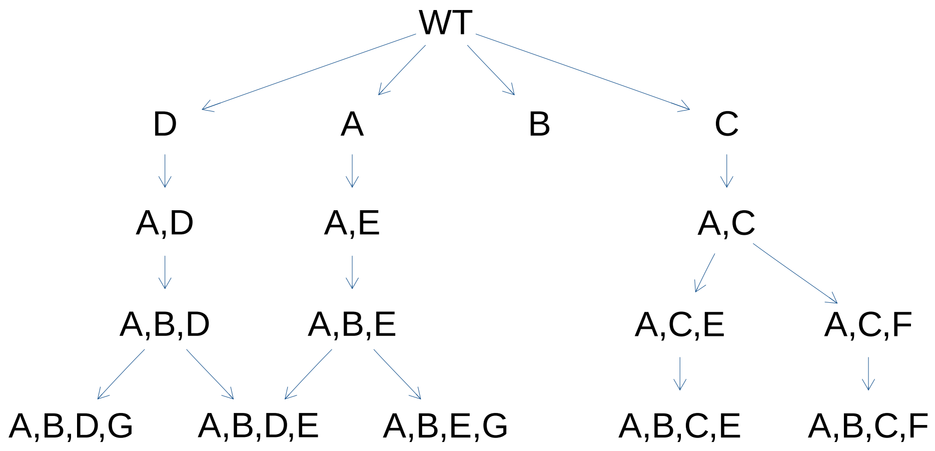

ID	aVdtnwMxlBa9JGAI
acc genotypes	48
peaks no back	14
removed edges	NA
prop rem edges	NA

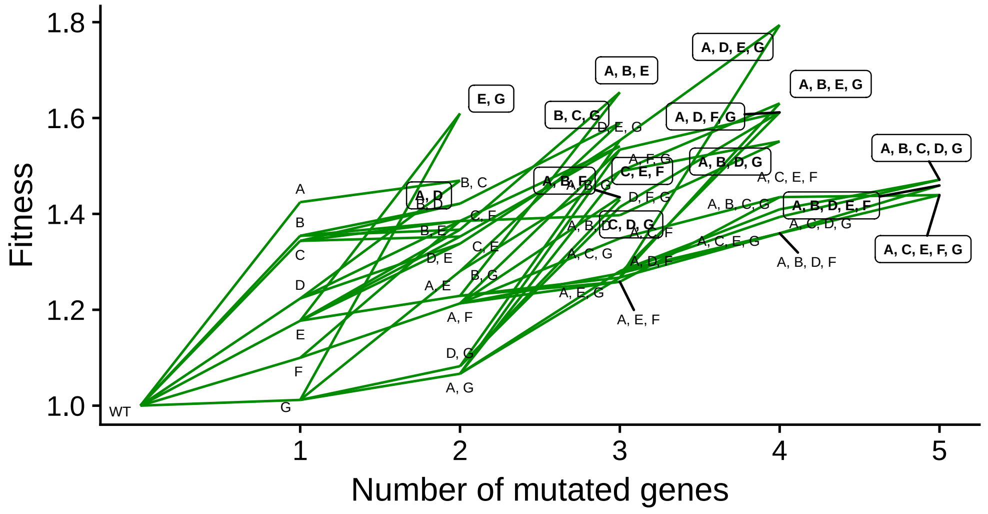

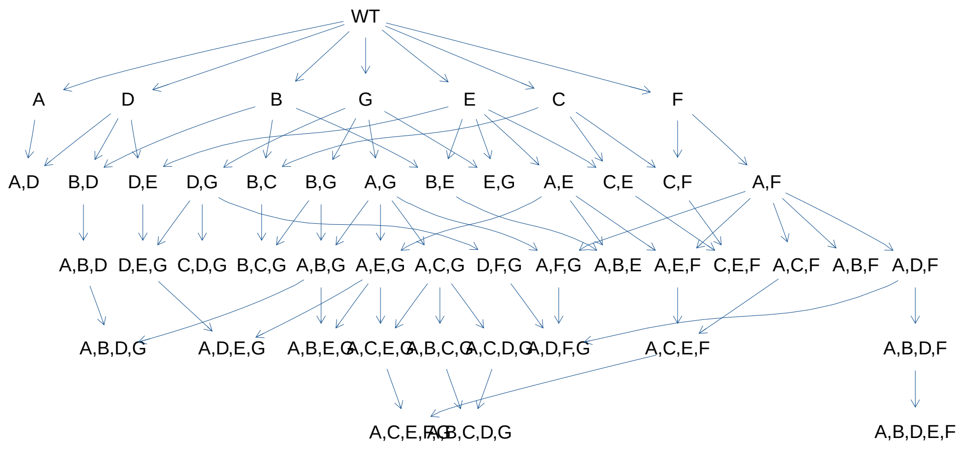

ID	Avmue778lBrop2vO
acc genotypes	44
peaks no back	11
removed edges	NA
prop rem edges	NA

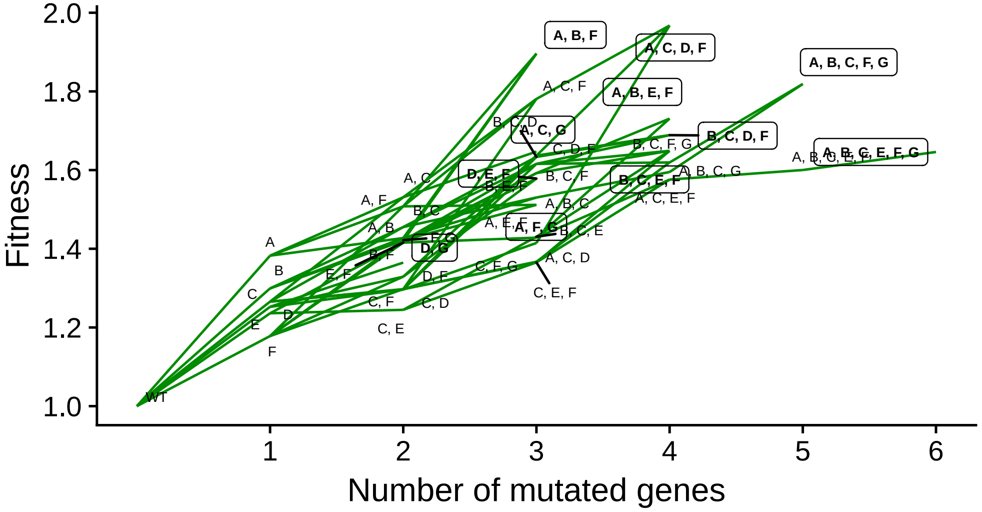

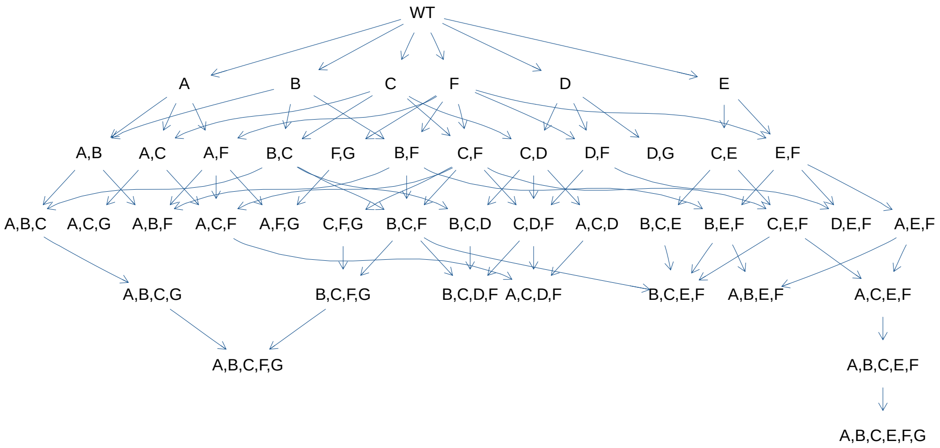

ID	AyannCS2nwfQ2TDc
acc genotypes	25
peaks no back	9
removed edges	NA
prop rem edges	NA

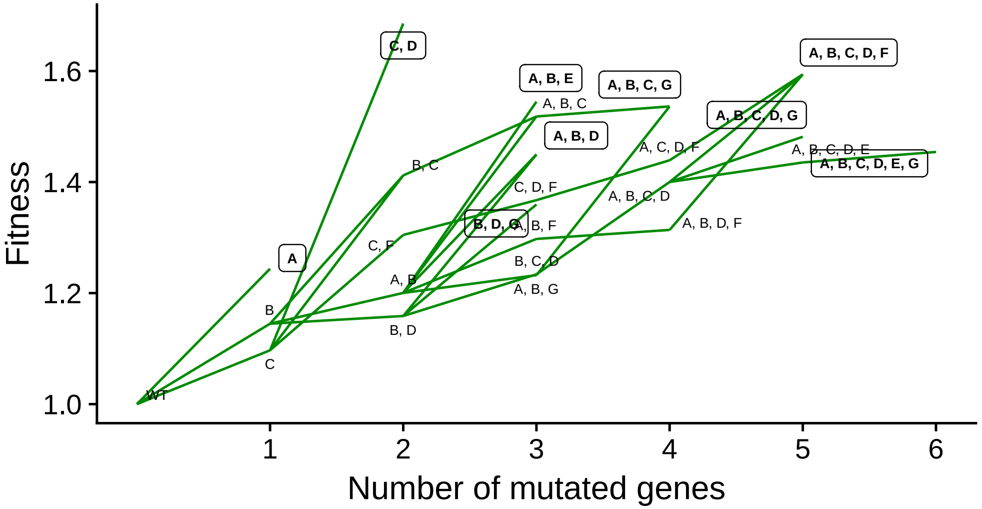

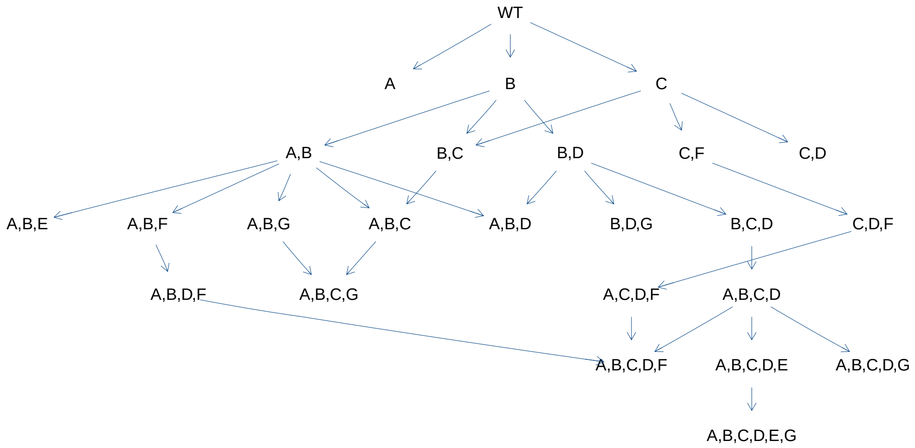

ID	aZQI57105TWpukXl1
acc genotypes	22
peaks no back	10
removed edges	NA
prop rem edges	NA

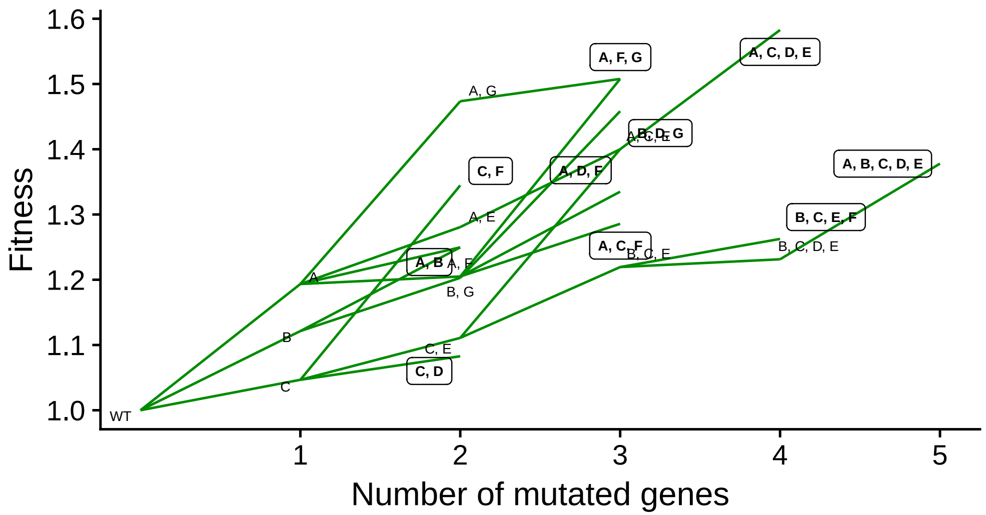

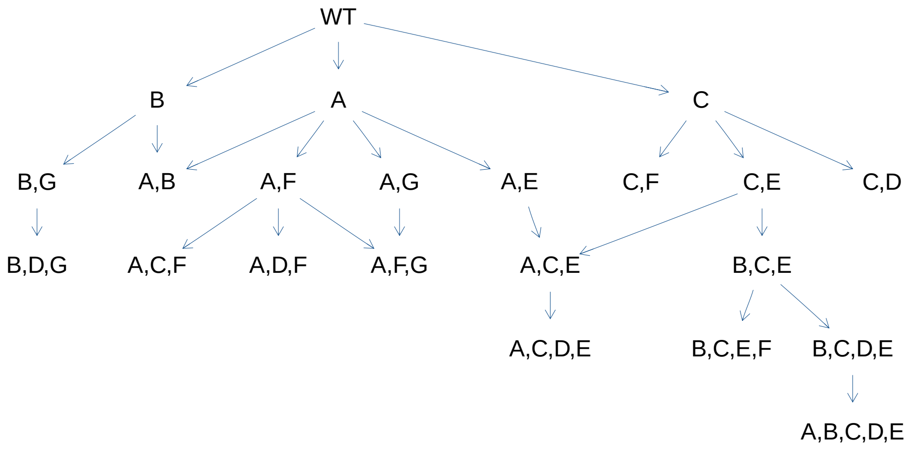

ID	BD3duuYOmGuZZNVr
acc genotypes	14
peaks no back	6
removed edges	NA
prop rem edges	NA

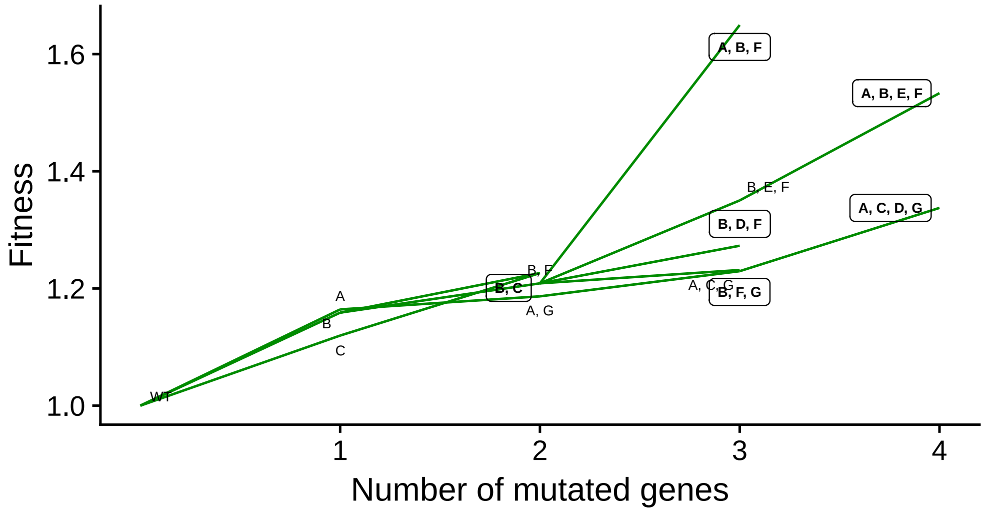

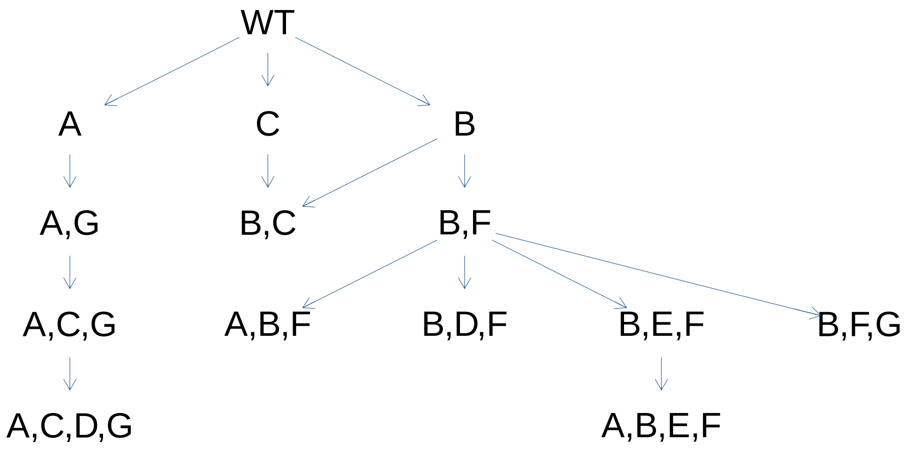

ID	BfGQkzq8zBGWdITj
acc genotypes	18
peaks no back	7
removed edges	NA
prop rem edges	NA

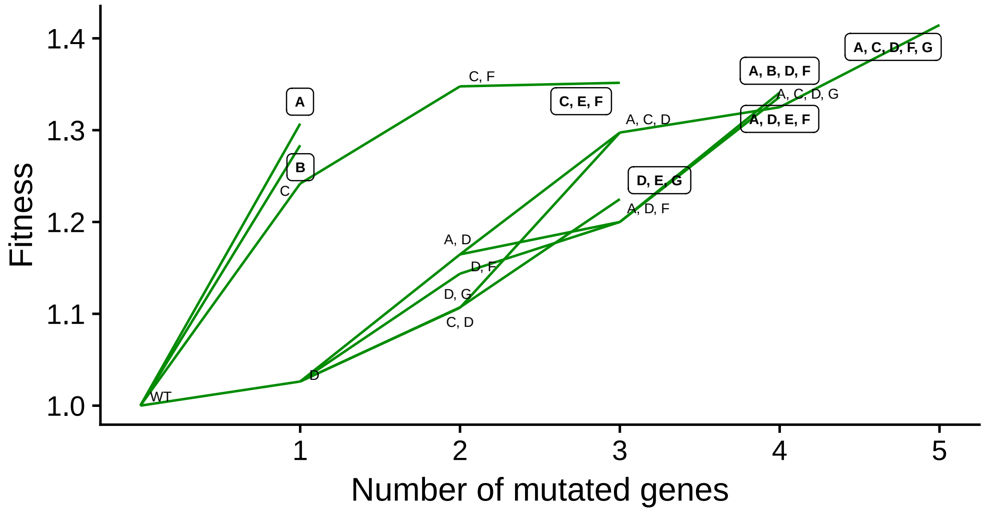

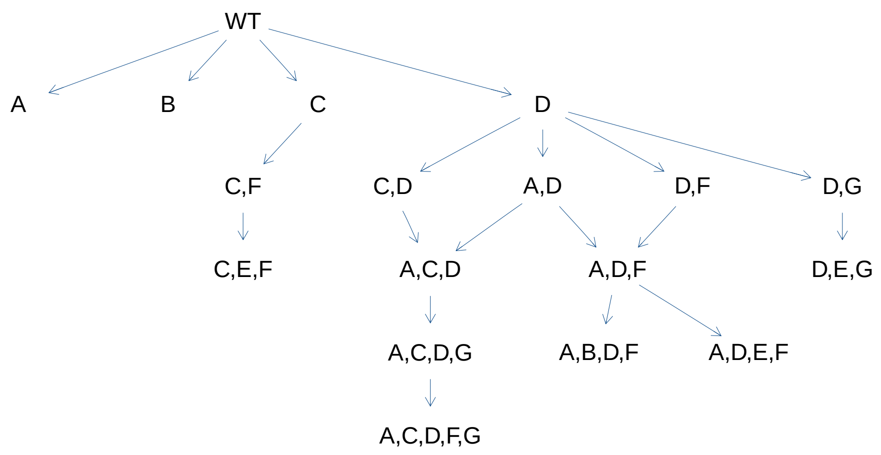

ID	bIGKwHhjkye10mnvq
acc genotypes	85
peaks no back	10
removed edges	NA
prop rem edges	NA

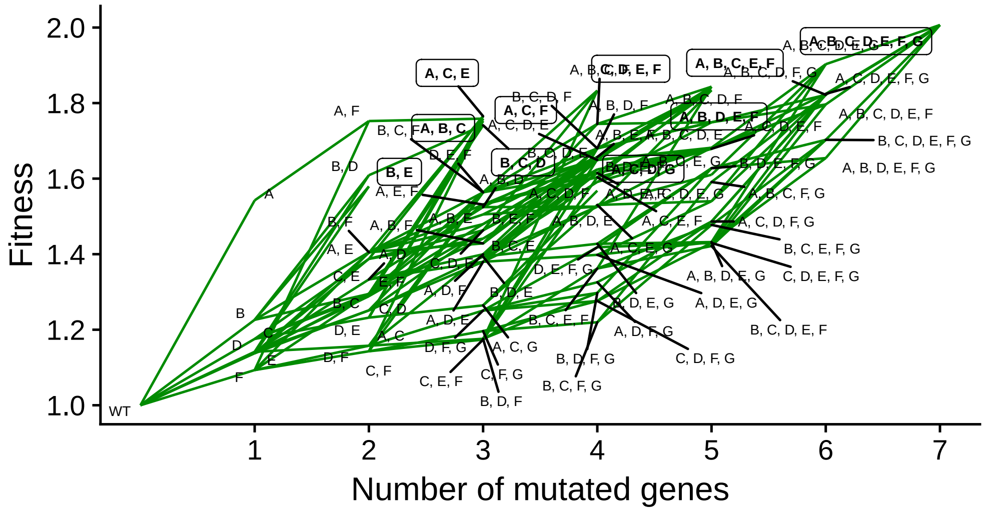

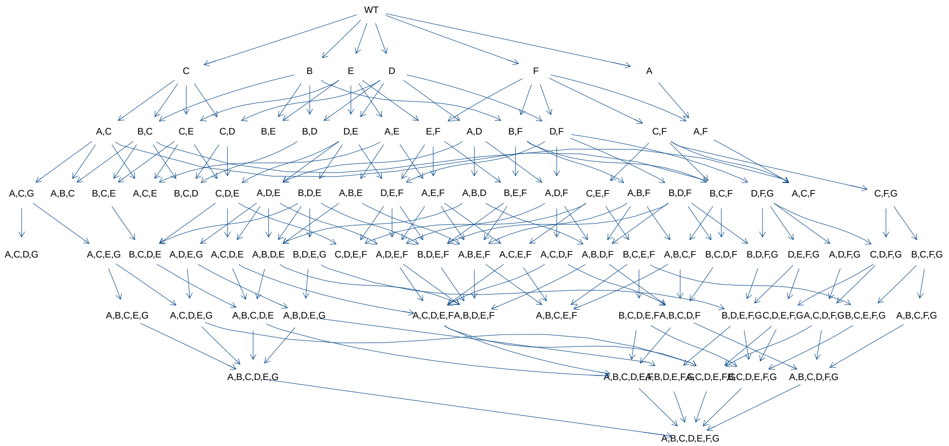

ID	bpM86wHSqemVZQ1G
acc genotypes	57
peaks no back	18
removed edges	NA
prop rem edges	NA

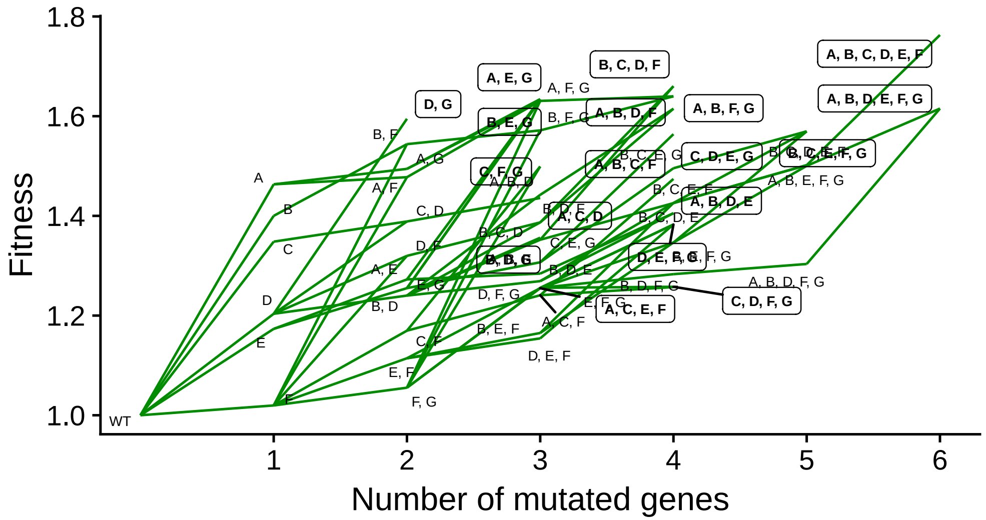

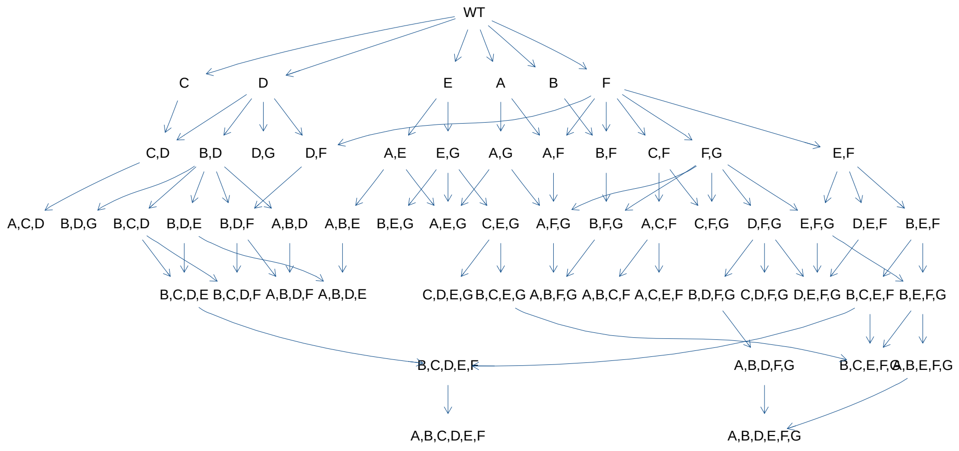

ID	BTJyOApuNPFWEPsU
acc genotypes	17
peaks no back	11
removed edges	NA
prop rem edges	NA

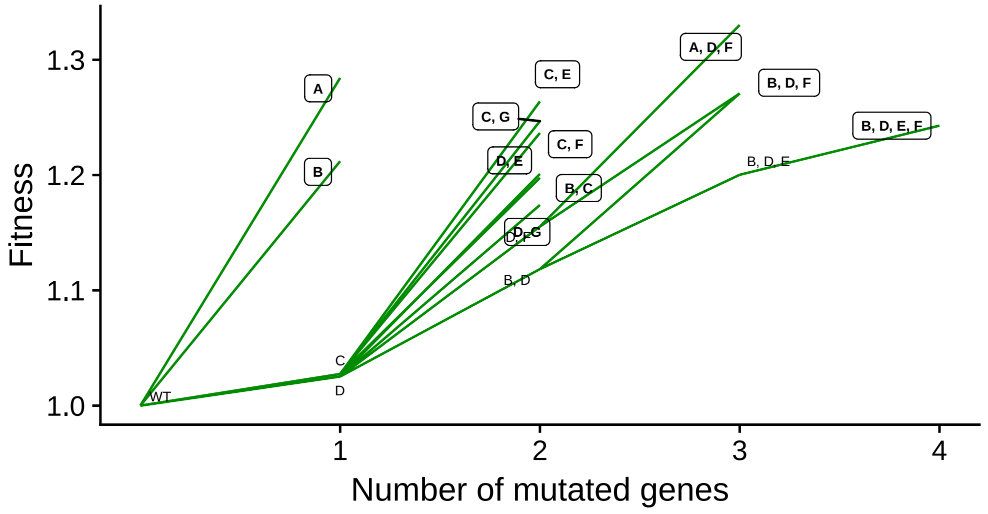

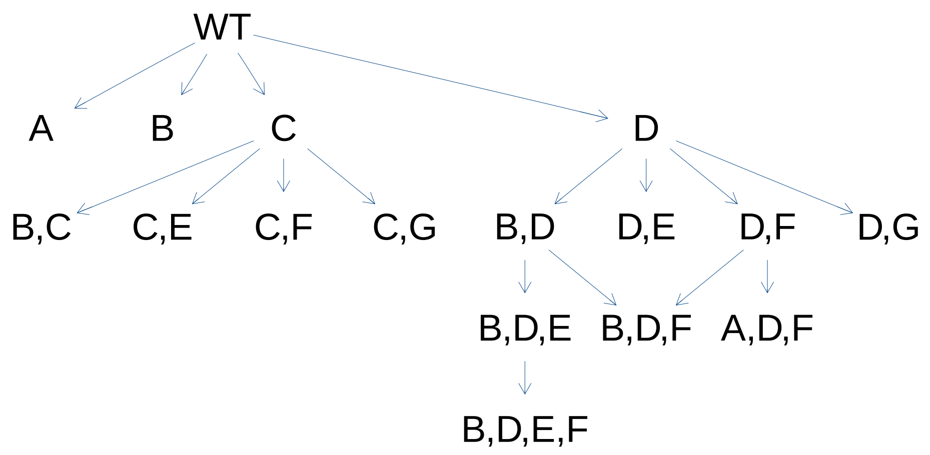

ID	BxBVJz10wDpOmersD
acc genotypes	90
peaks no back	21
removed edges	NA
prop rem edges	NA

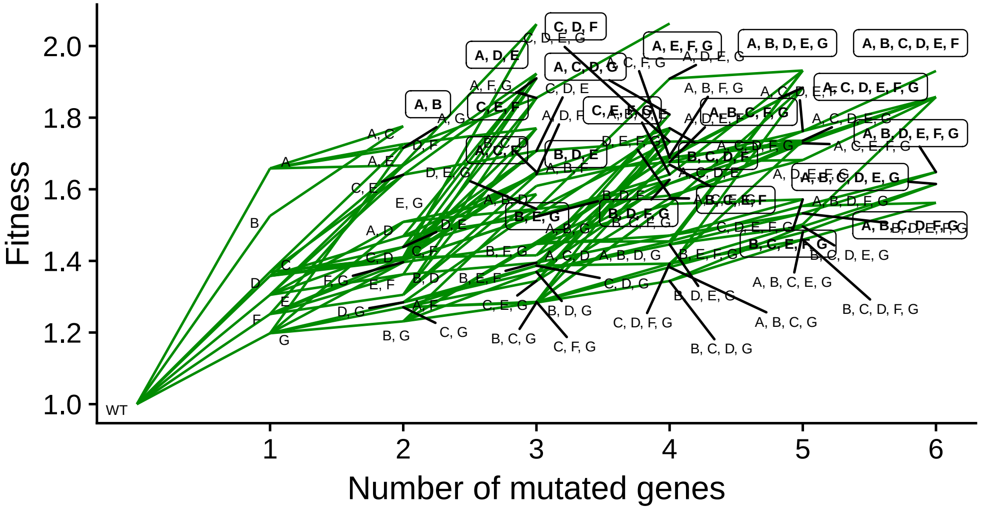

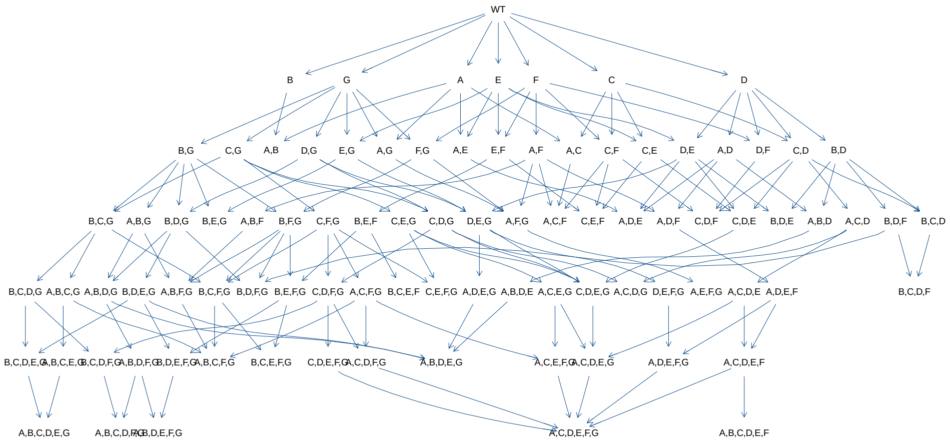

ID	c42Quu4ADM6baevo
acc genotypes	60
peaks no back	14
removed edges	NA
prop rem edges	NA

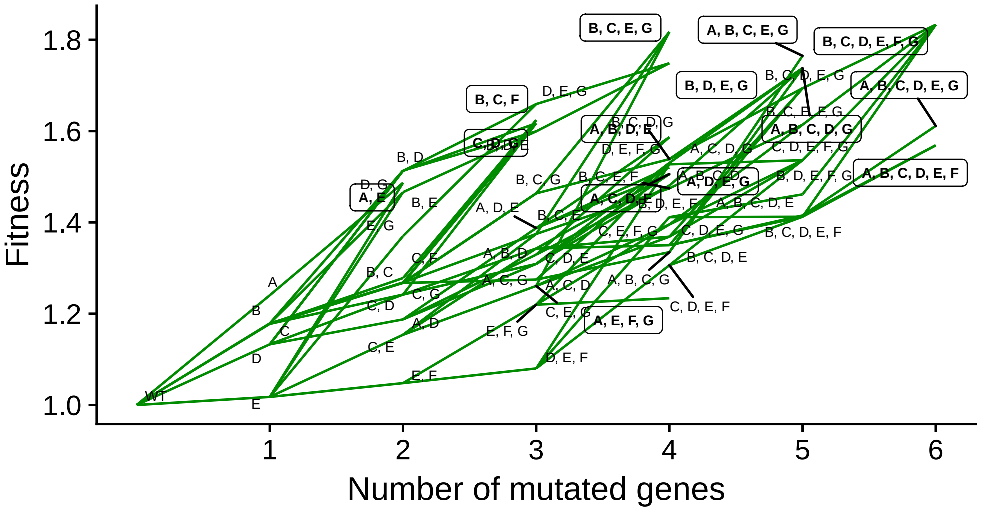

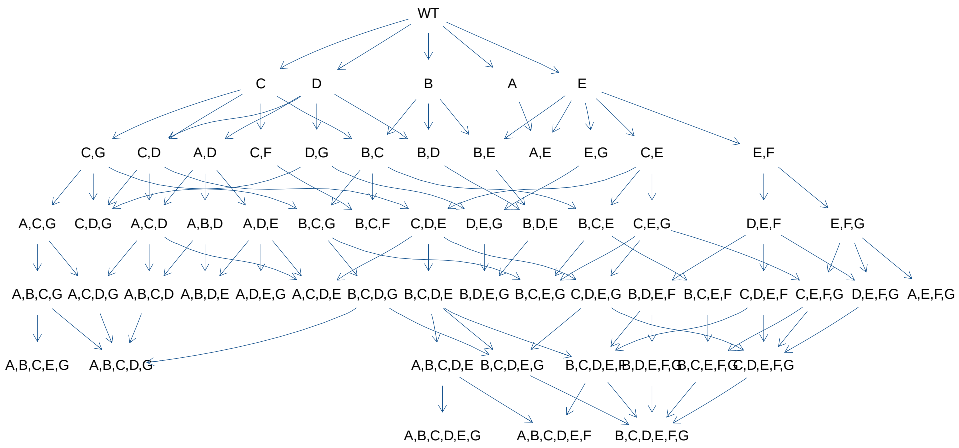

ID	c534FhXAH8kOwR3J
acc genotypes	53
peaks no back	24
removed edges	NA
prop rem edges	NA

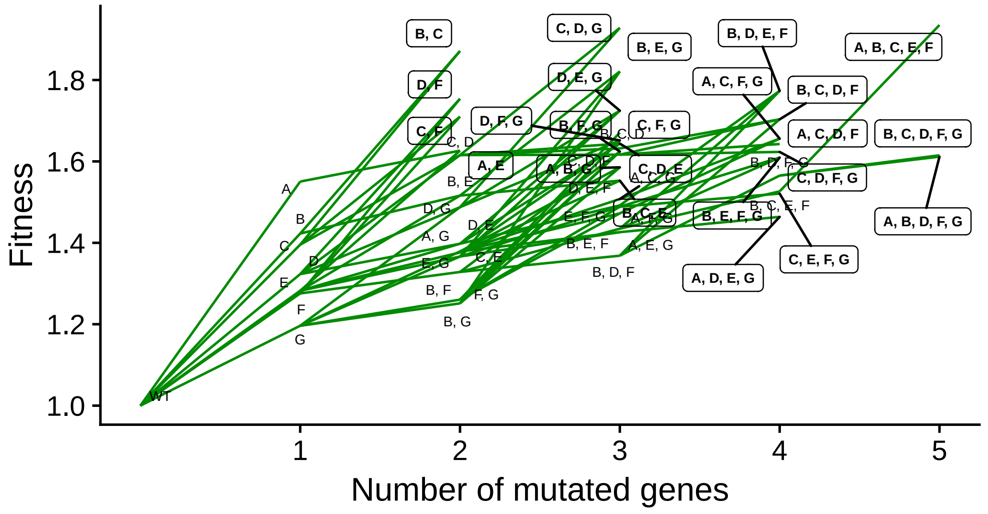

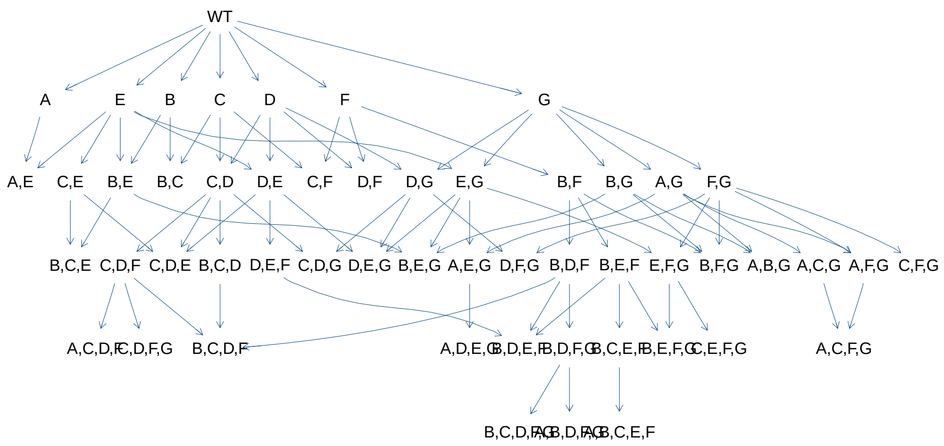

ID	CfrC3ZAnJWi3s9E3
acc genotypes	38
peaks no back	15
removed edges	NA
prop rem edges	NA

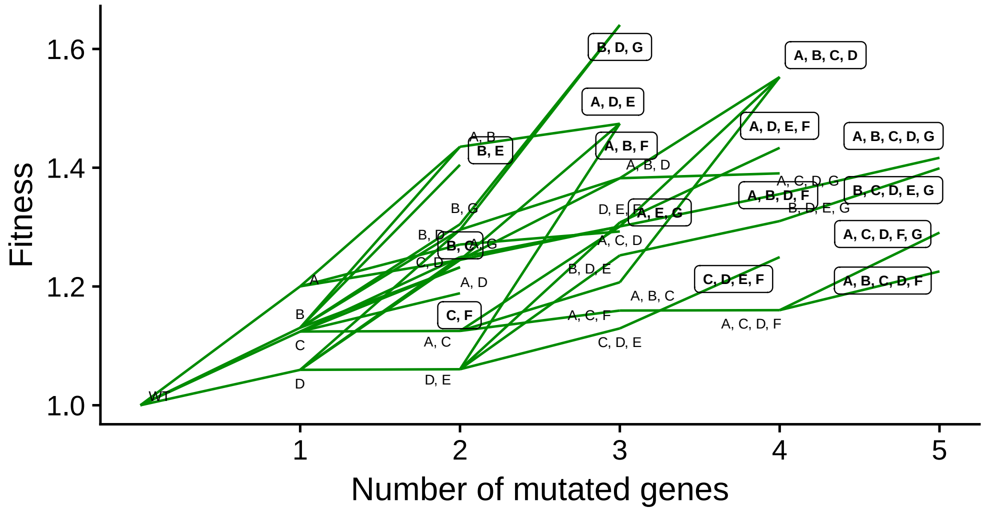

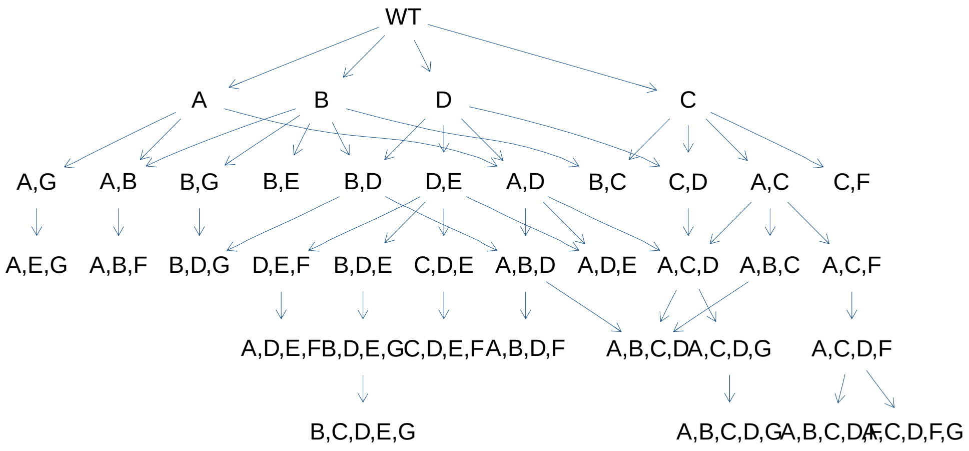

ID	CHYQvcfXo103swhhd
acc genotypes	26
peaks no back	9
removed edges	NA
prop rem edges	NA

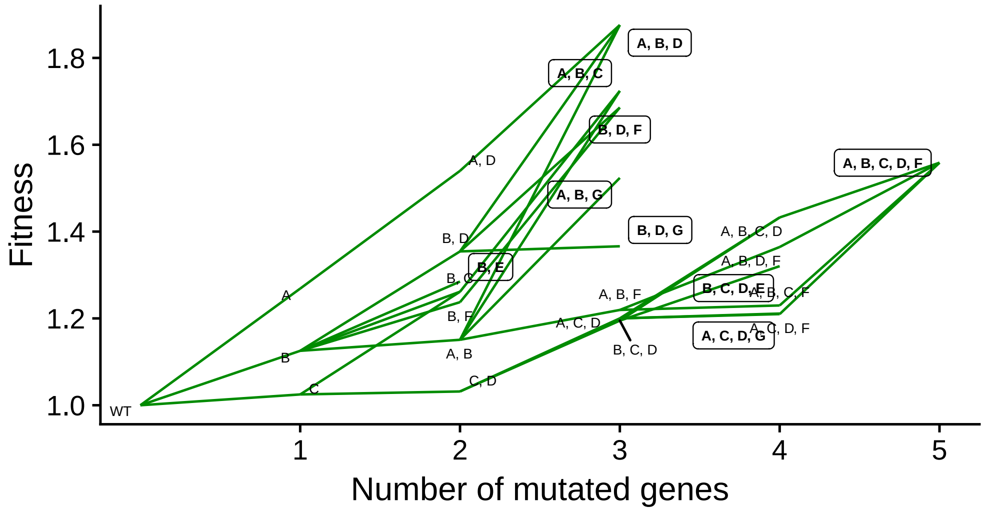

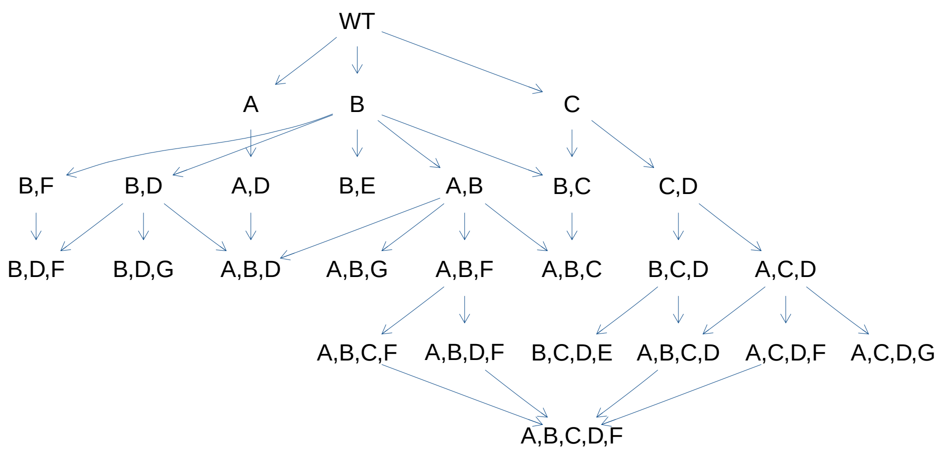

ID cI8oWQEqqPY7cX2o
acc genotypes 22
peaks no back 13
removed edges NA
prop rem edges NA

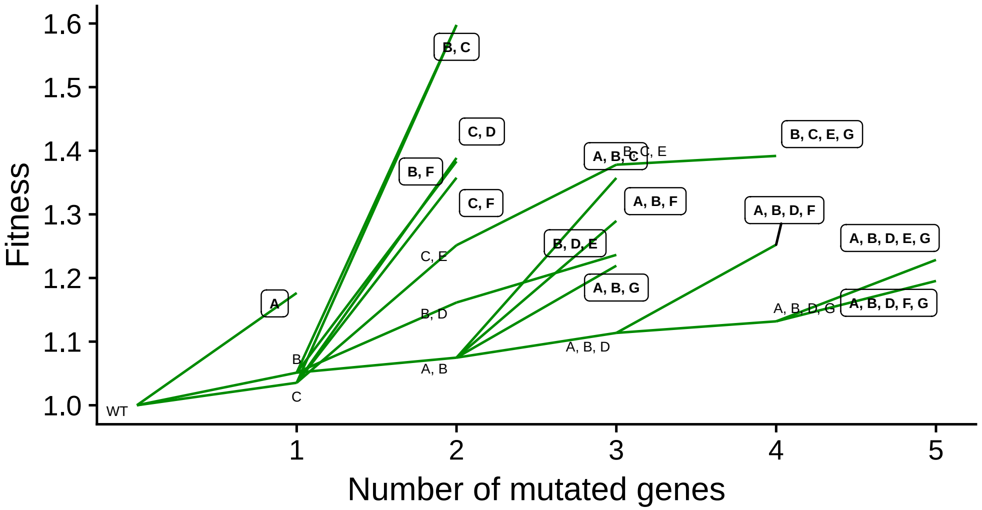

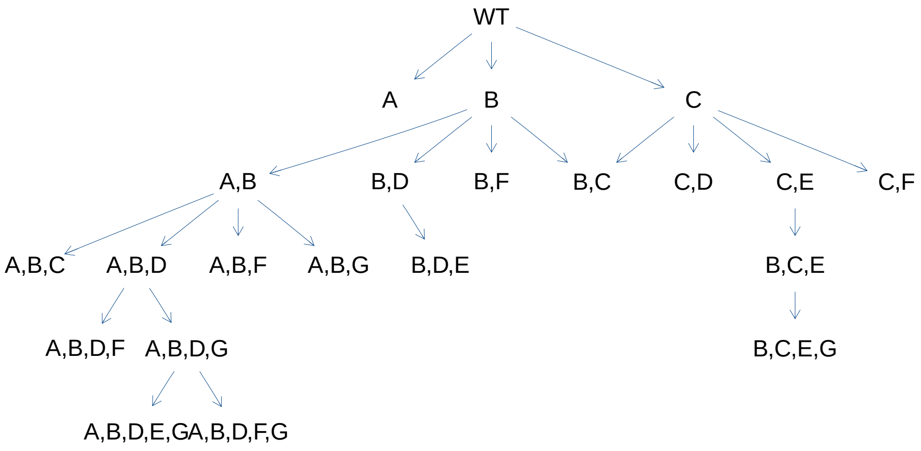

ID	CjzXJbJTcI5BNmE1
acc genotypes	74
peaks no back	21
removed edges	NA
prop rem edges	NA

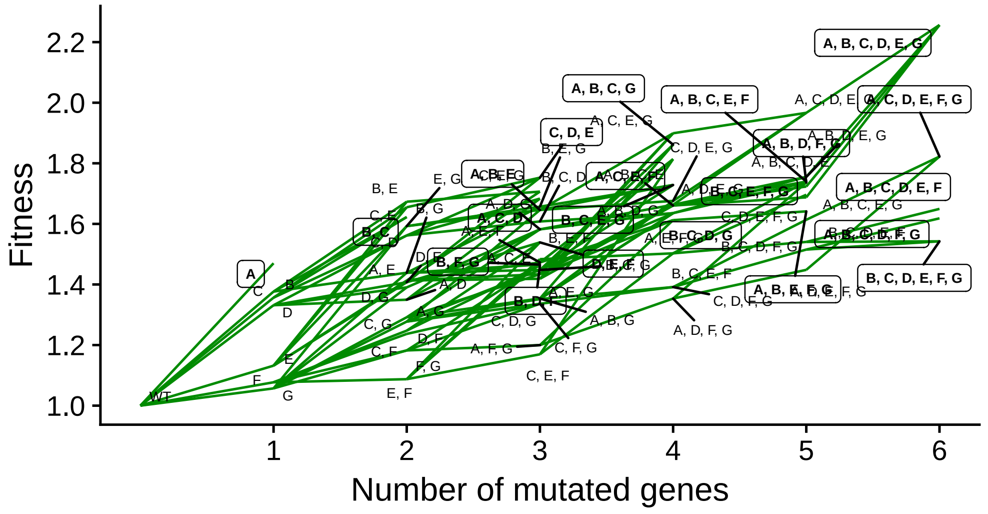

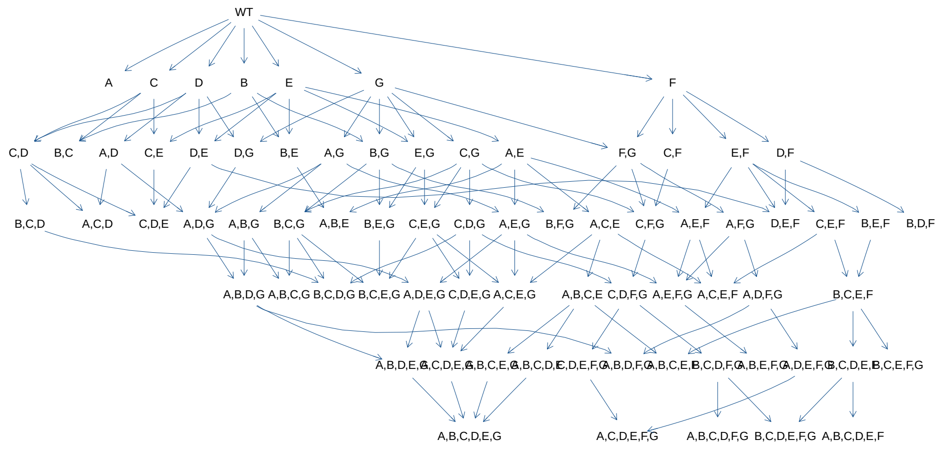

ID	Ck1zf9wAa7Dm9QHw
acc genotypes	24
peaks no back	9
removed edges	NA
prop rem edges	NA

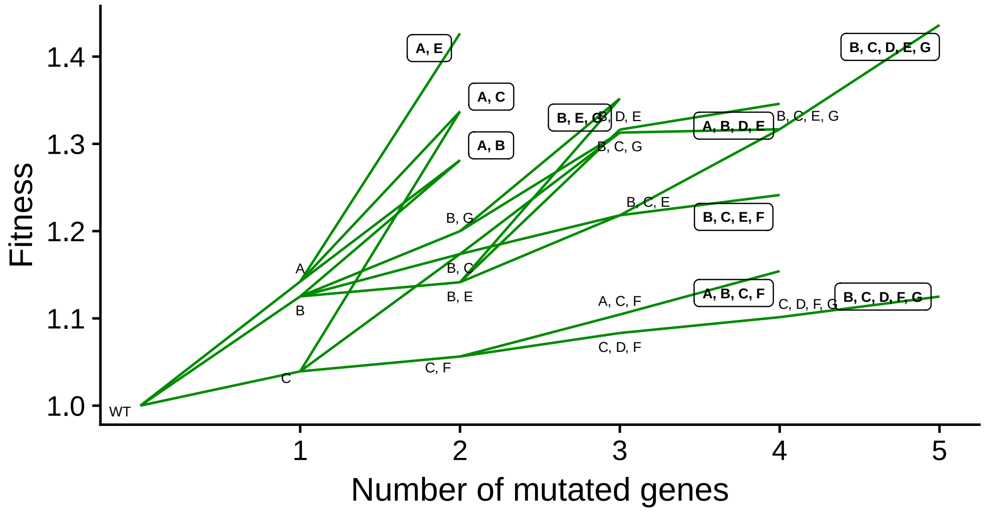

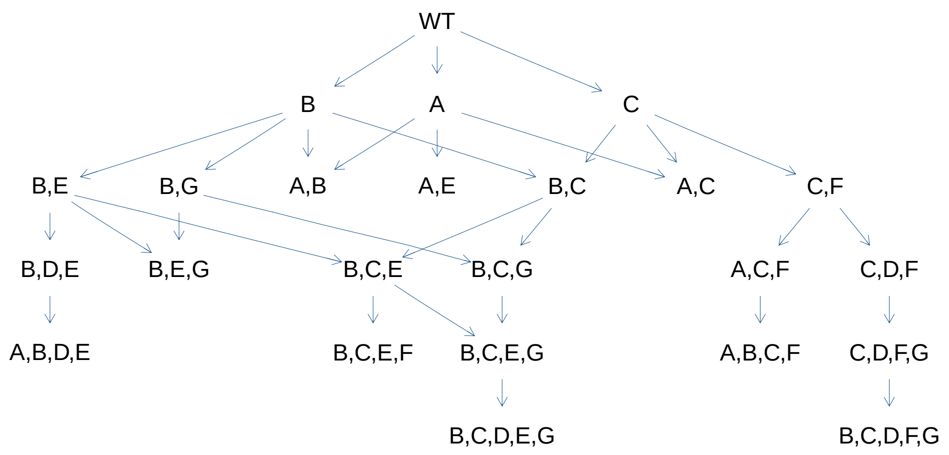

ID	CsQCk5oitWE3VWiq
acc genotypes	90
peaks no back	25
removed edges	NA
prop rem edges	NA

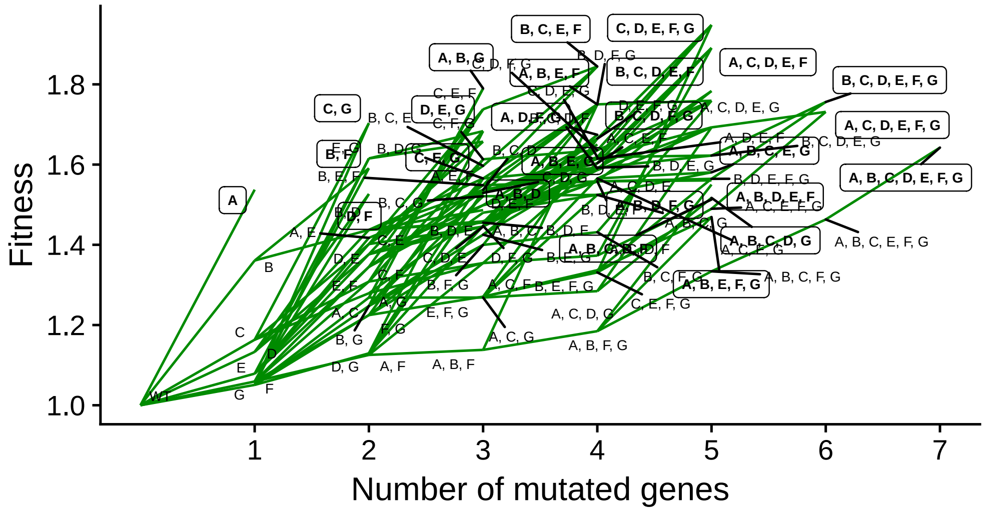

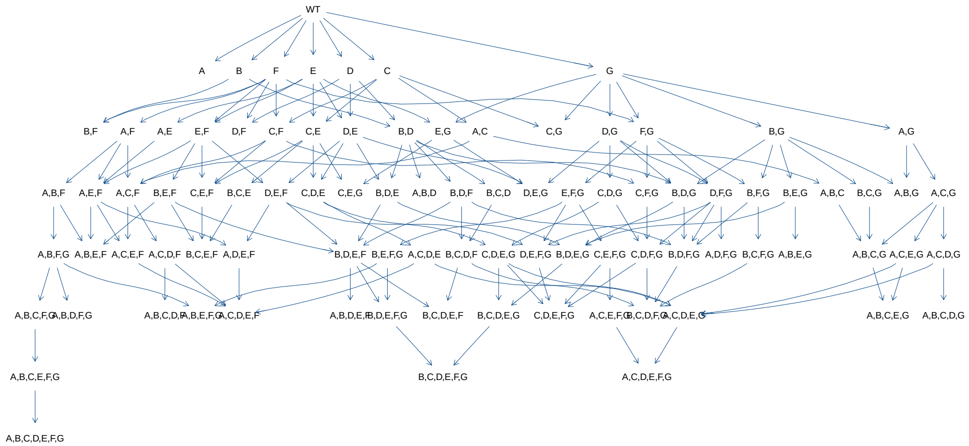

ID	cueZ258JKmvObVdj
acc genotypes	67
peaks no back	15
removed edges	NA
prop rem edges	NA

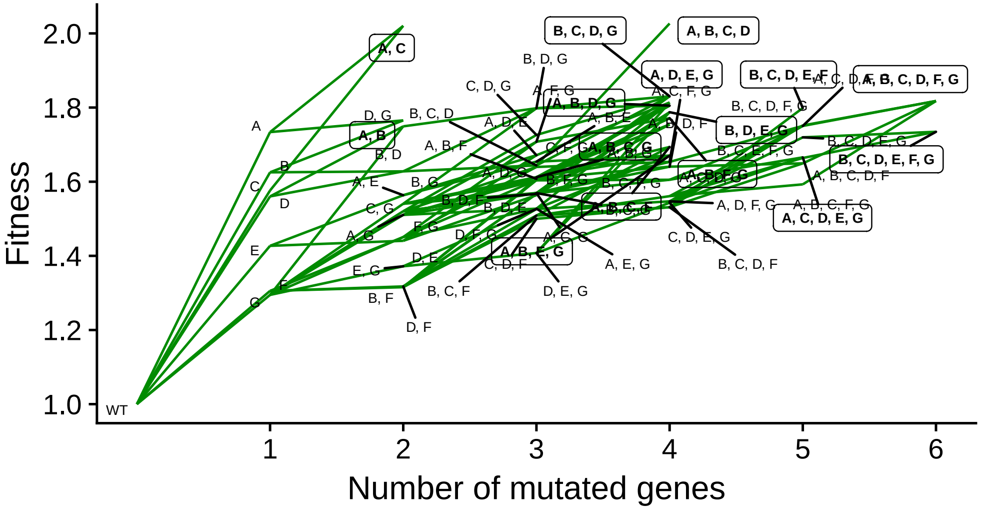

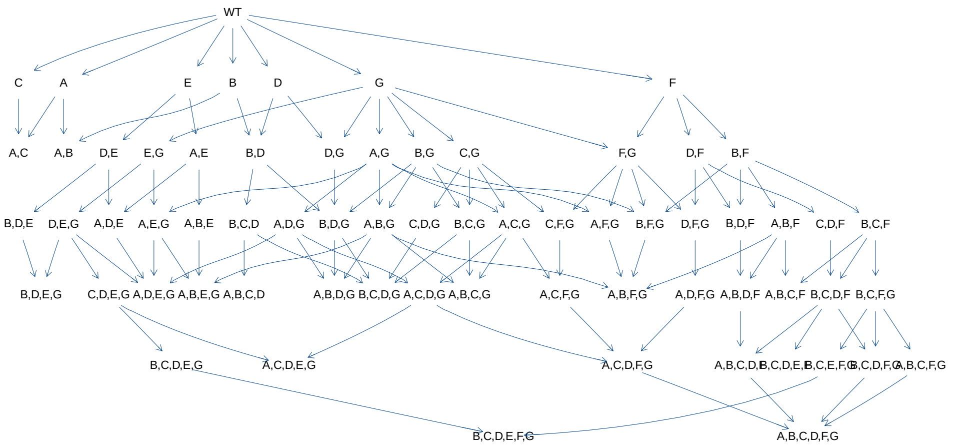

ID	CvnM3L5EYGnoEGpc
acc genotypes	44
peaks no back	19
removed edges	NA
prop rem edges	NA

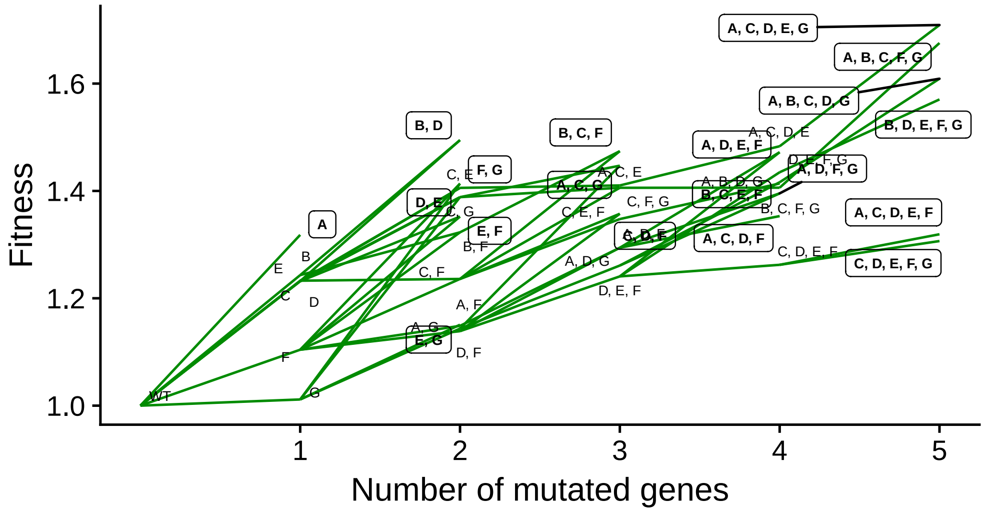

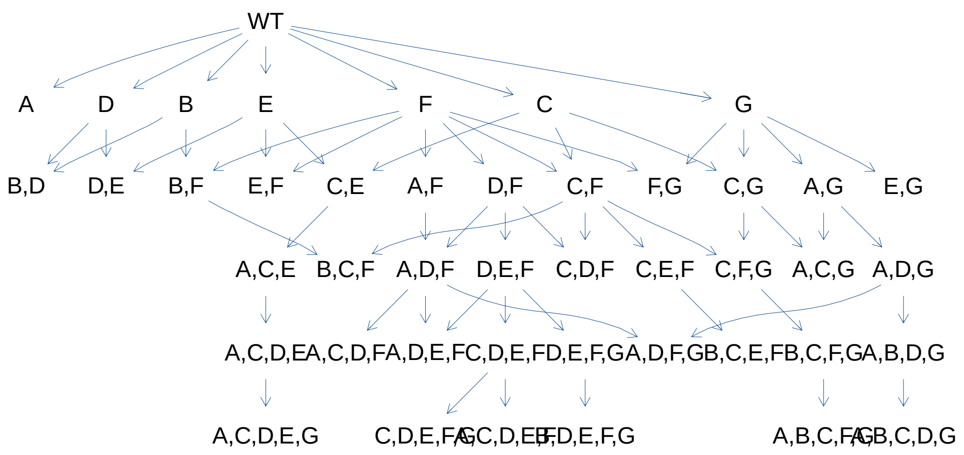

ID	D8vky9fRuaEmBWIV
acc genotypes	45
peaks no back	16
removed edges	NA
prop rem edges	NA

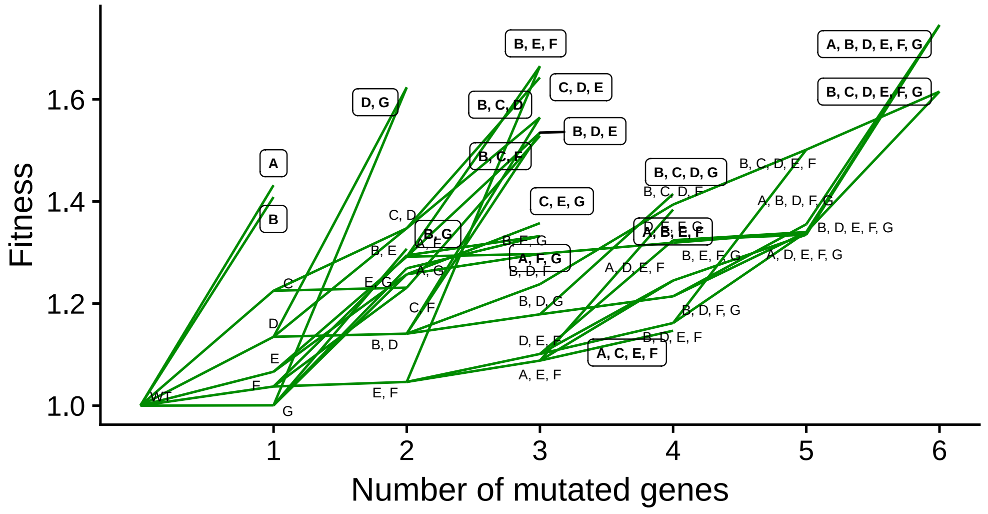

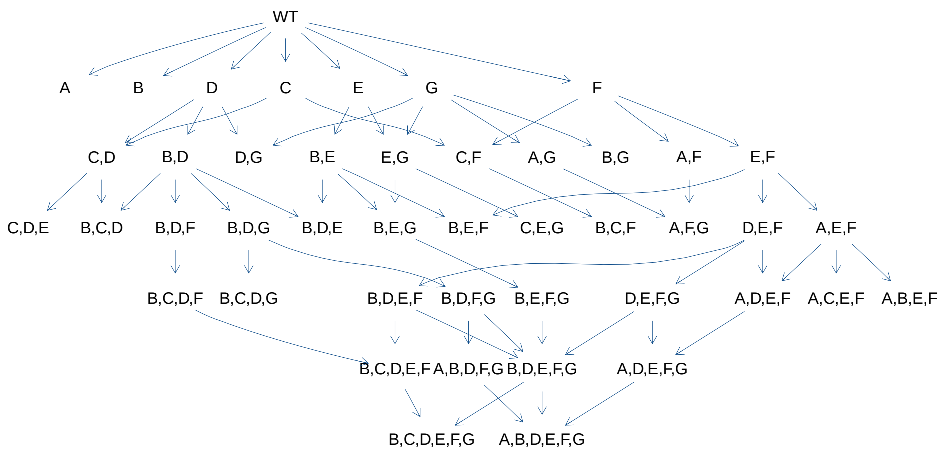

ID	Daq9vjIG10xrsjP23
acc genotypes	22
peaks no back	13
removed edges	NA
prop rem edges	NA

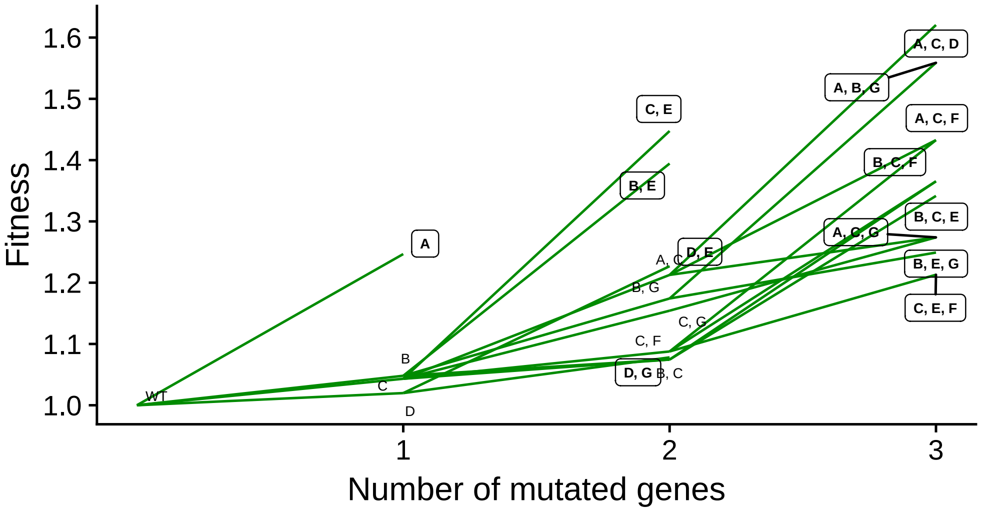

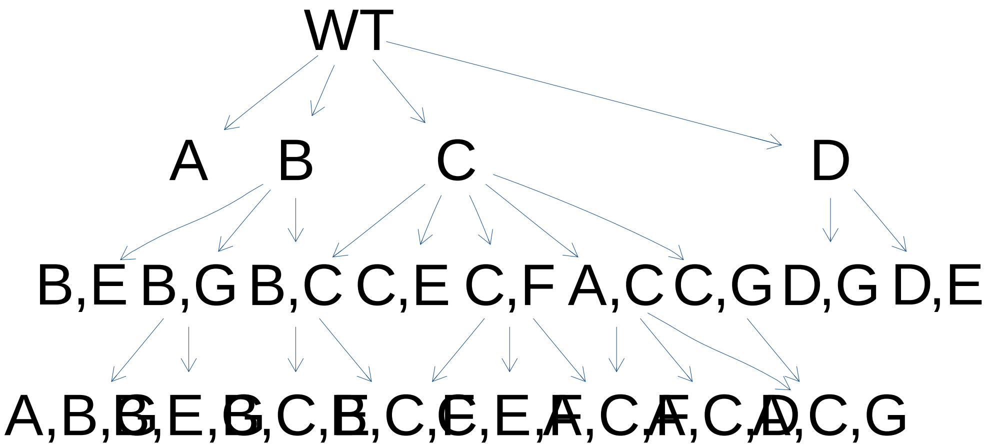

ID	deoj10TADCLDLOA5o
acc genotypes	30
peaks no back	8
removed edges	NA
prop rem edges	NA

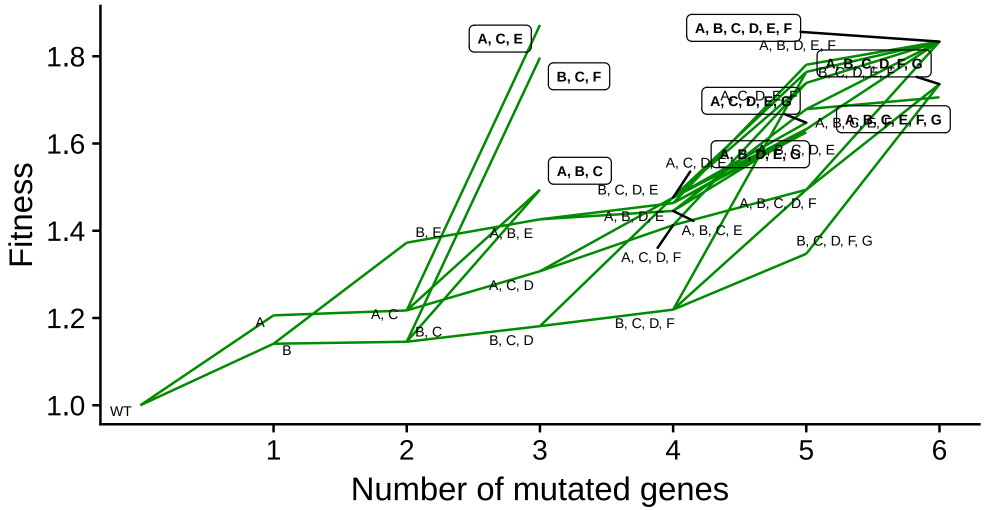

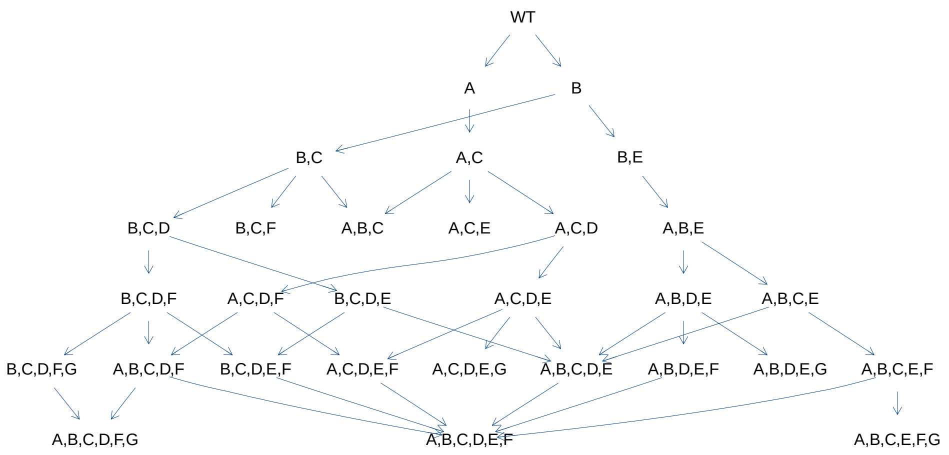

ID	dTwzjRHQb3XHk1Qo
acc genotypes	39
peaks no back	14
removed edges	NA
prop rem edges	NA

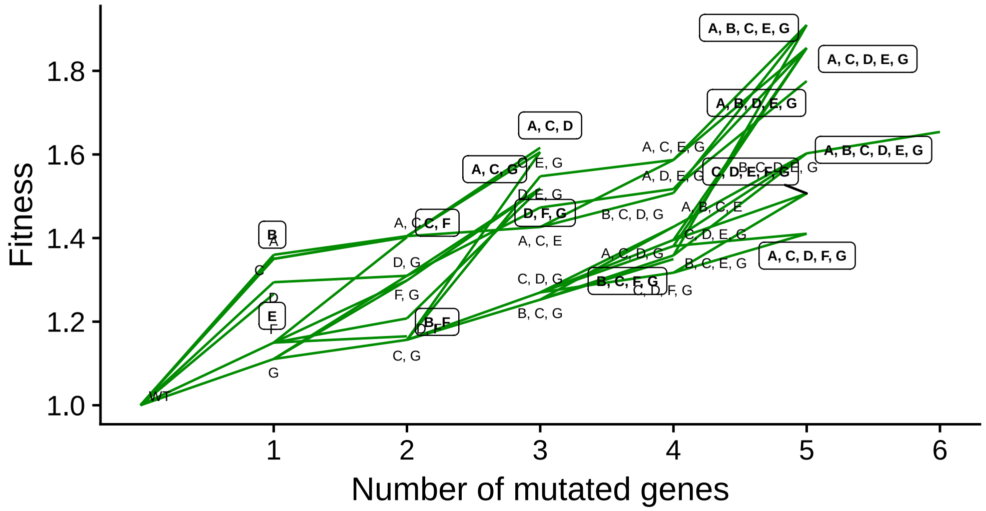

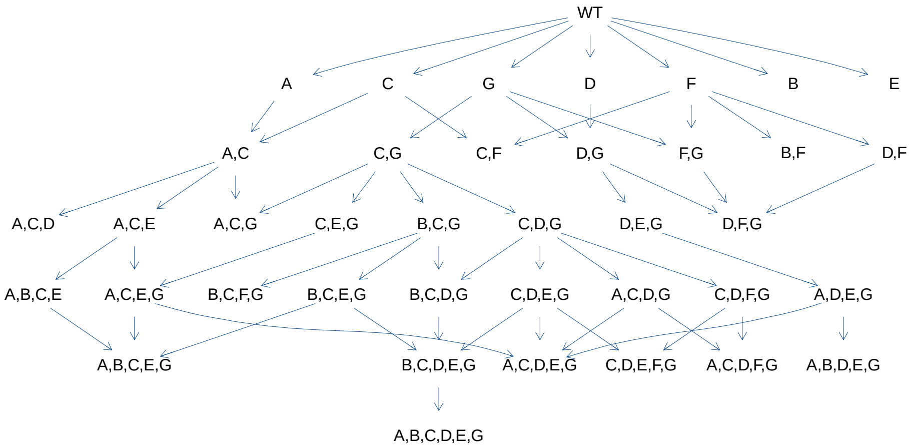

ID duoNzOQwAe8B75y5
acc genotypes 42
peaks no back 8
removed edges NA
prop rem edges NA

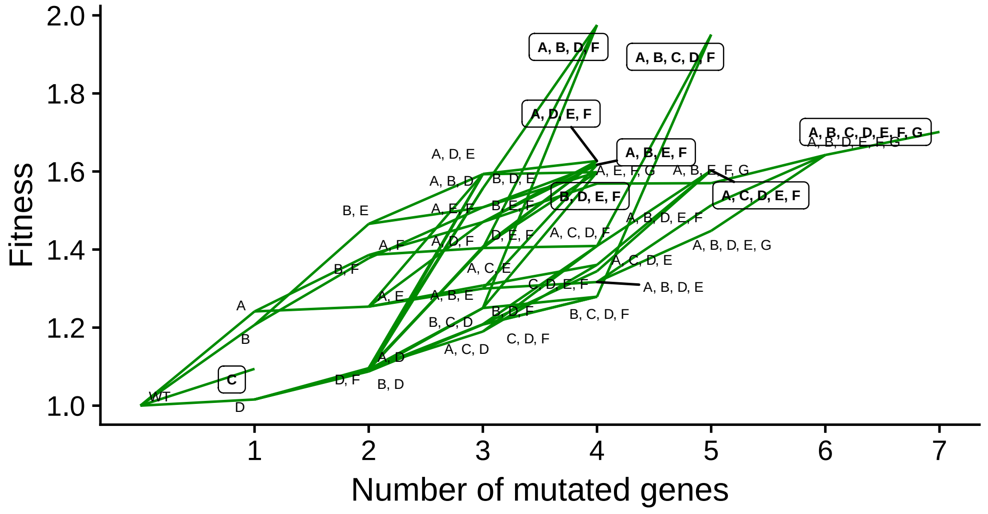

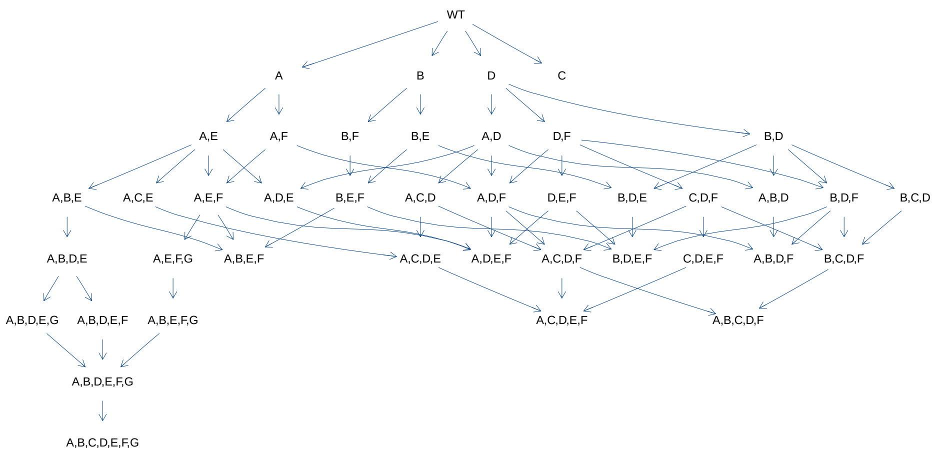

ID	Dv4AjSOStEZrnaox
acc genotypes	62
peaks no back	17
removed edges	NA
prop rem edges	NA

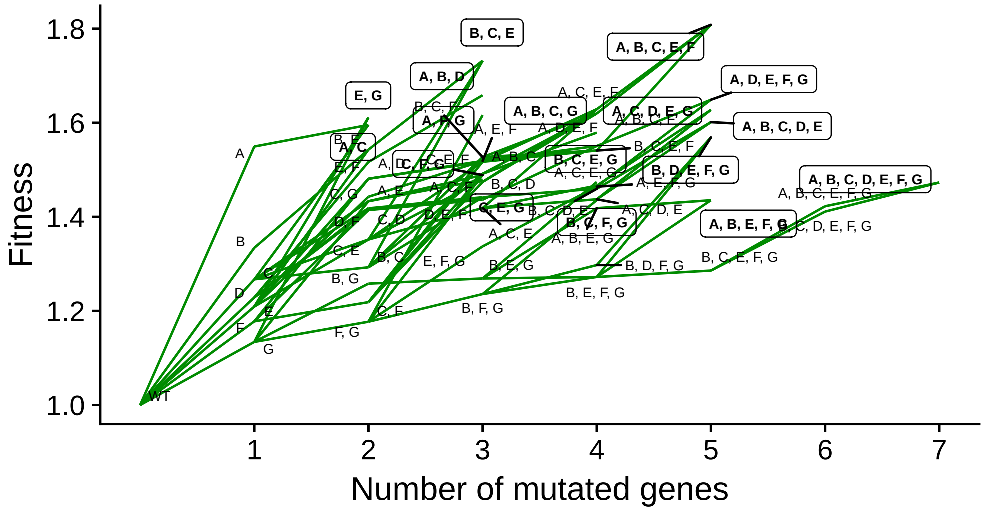

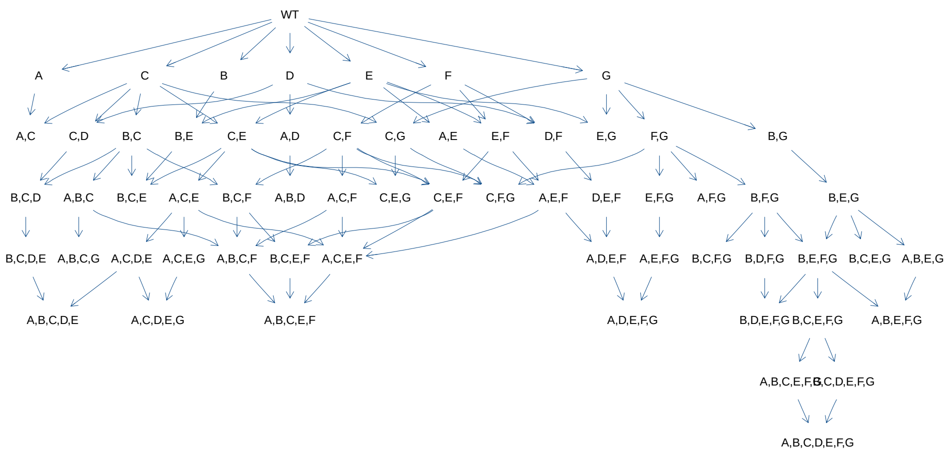

ID	E1088yM4b7OyoaM3C
acc genotypes	96
peaks no back	12
removed edges	NA
prop rem edges	NA

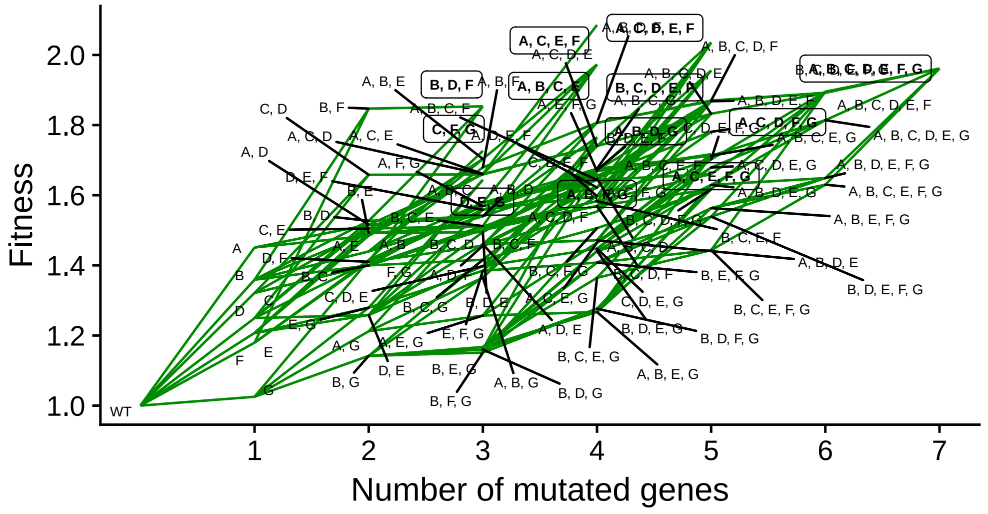

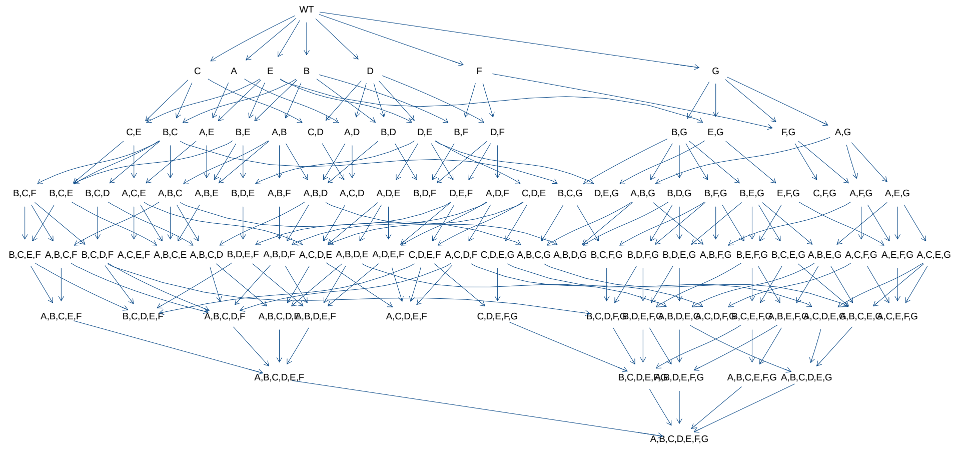

ID	EAI9G10BQGPjhOaTd
acc genotypes	20
peaks no back	9
removed edges	NA
prop rem edges	NA

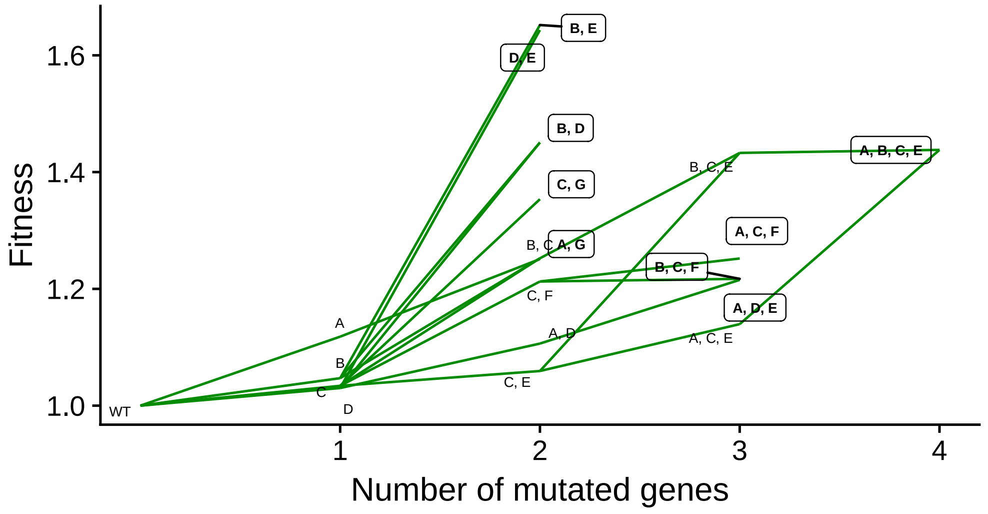

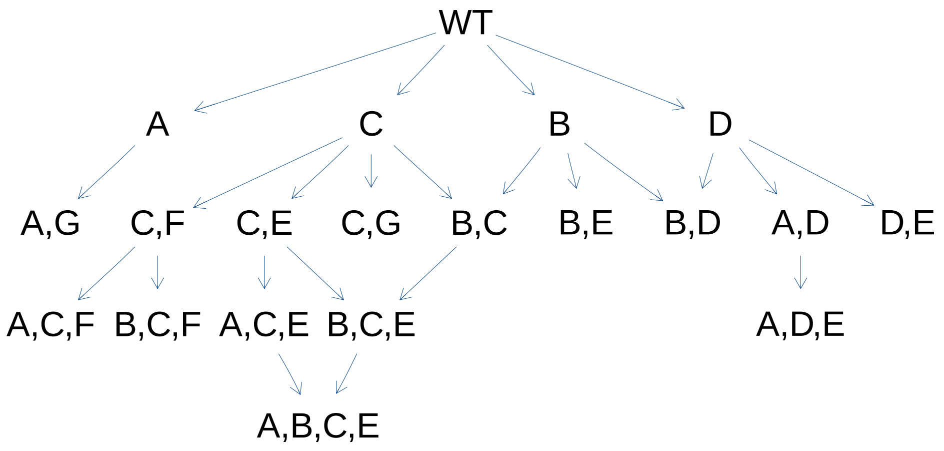

ID	eb4moeiVT2ClnVLV
acc genotypes	74
peaks no back	28
removed edges	NA
prop rem edges	NA

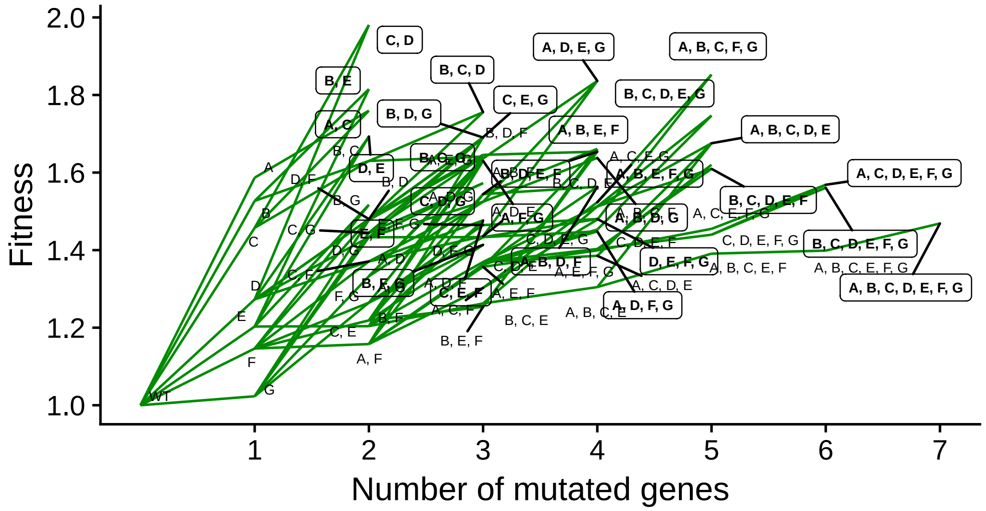

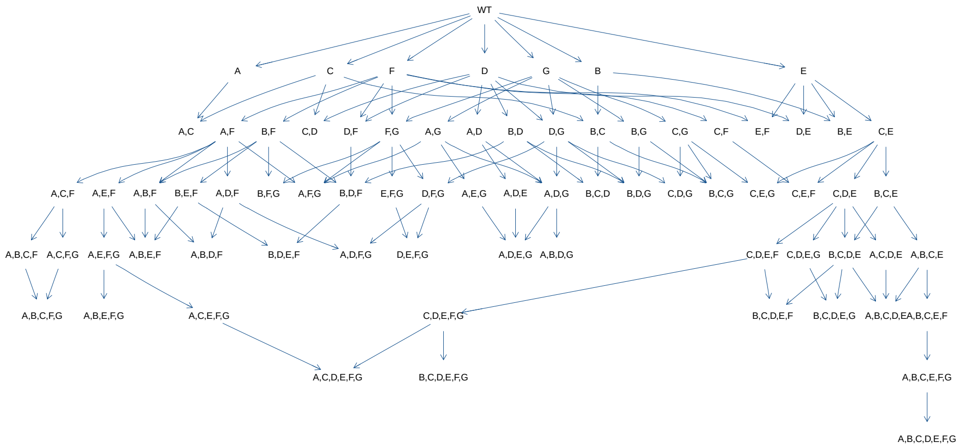

ID	EdoyPlJWzxCpBrys
acc genotypes	31
peaks no back	14
removed edges	NA
prop rem edges	NA

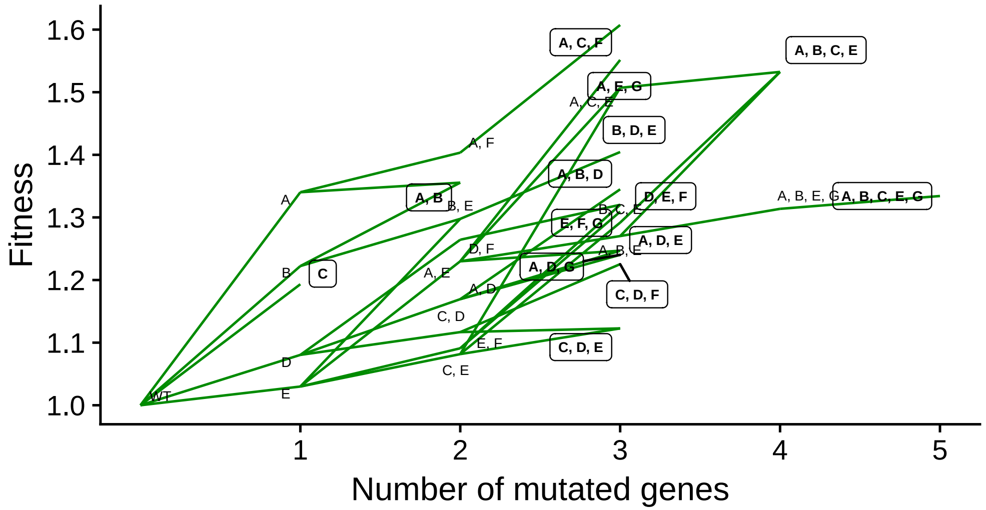

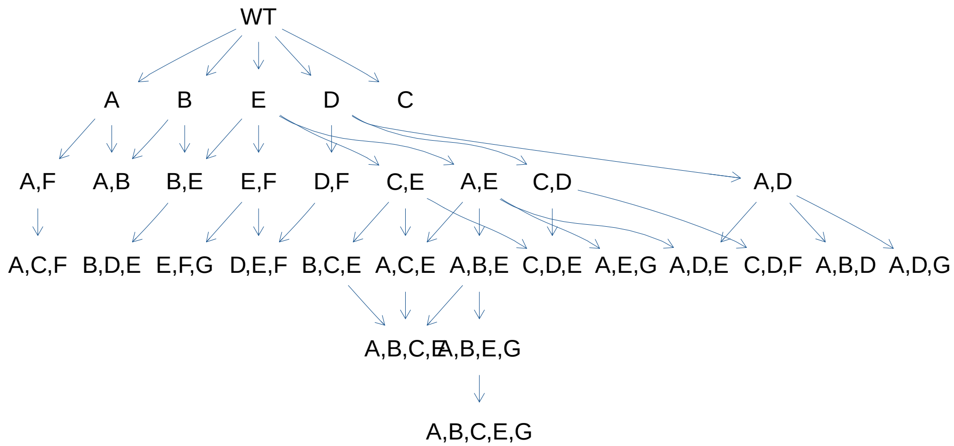

ID	EKkcYkyuQzPjnUDy
acc genotypes	54
peaks no back	16
removed edges	NA
prop rem edges	NA

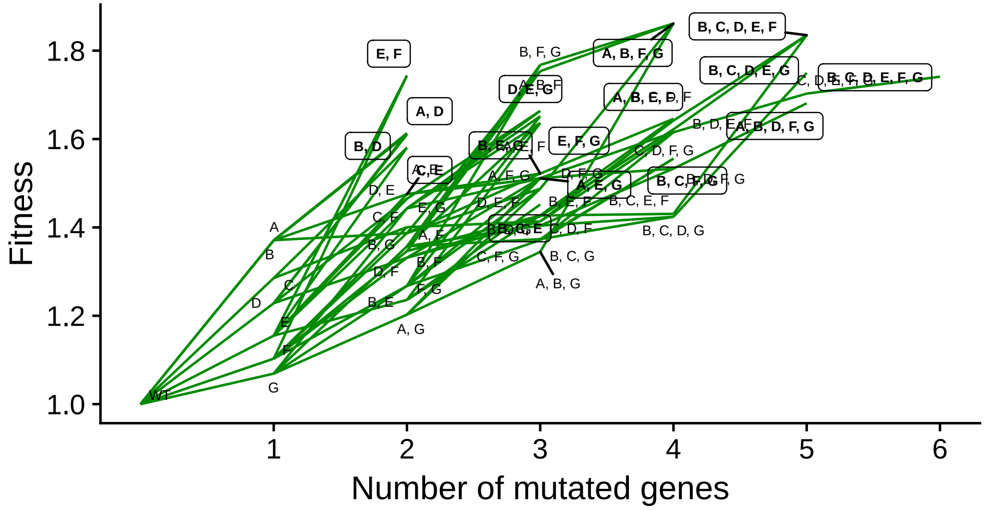

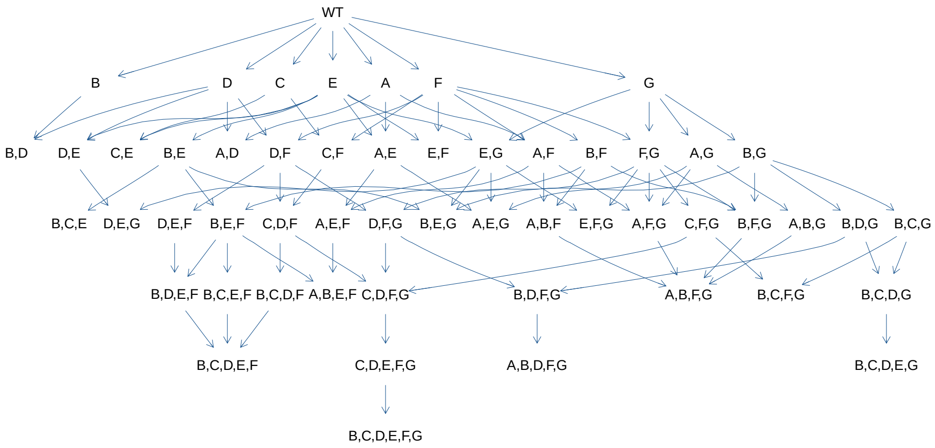

ID	EM68B9s53CXWI9KL
acc genotypes	41
peaks no back	16
removed edges	NA
prop rem edges	NA

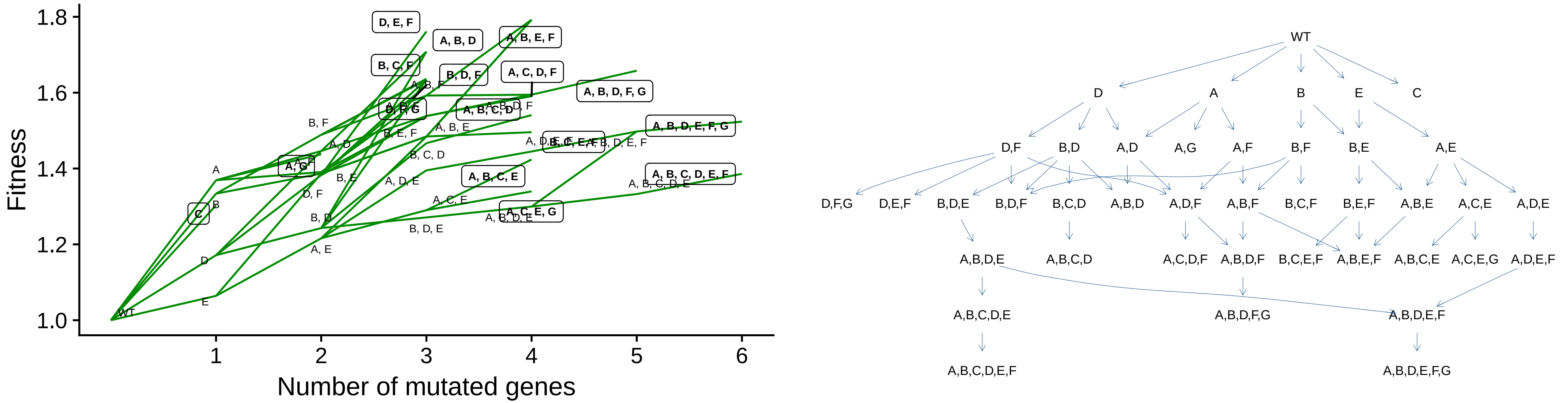

ID	eQOmKNmXnLoUPYw5
acc genotypes	30
peaks no back	14
removed edges	NA
prop rem edges	NA

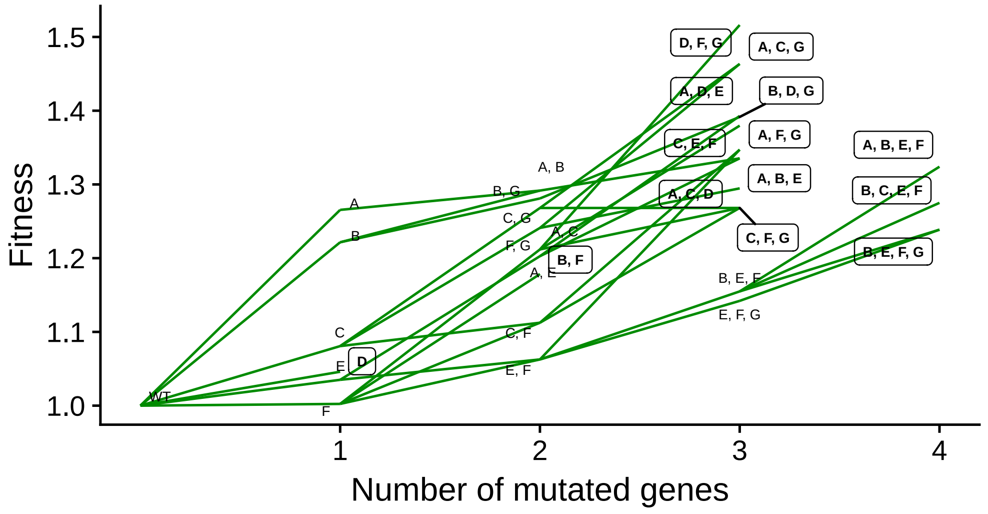

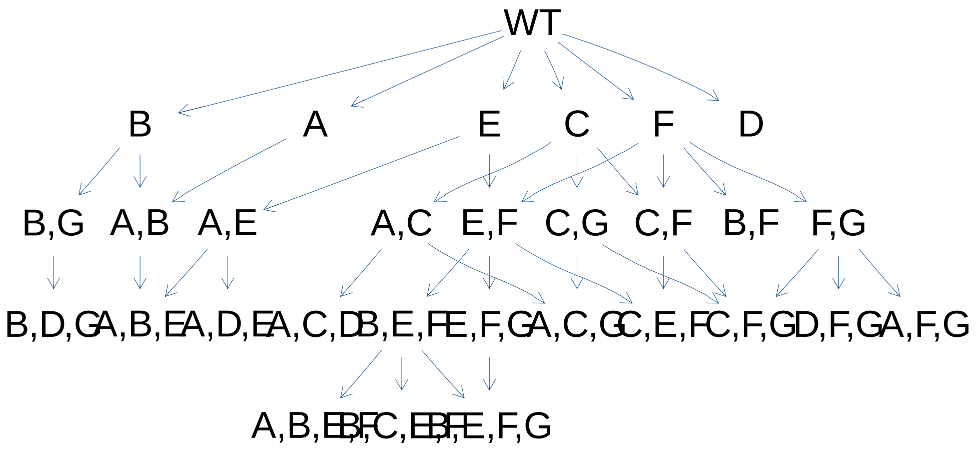

ID	EsTjWIE7vDhzVqNd
acc genotypes	69
peaks no back	20
removed edges	NA
prop rem edges	NA

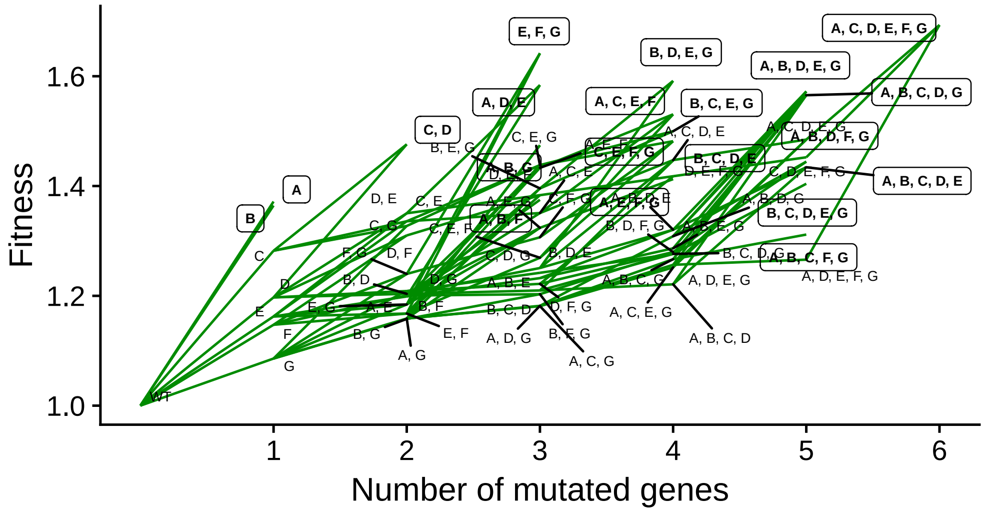

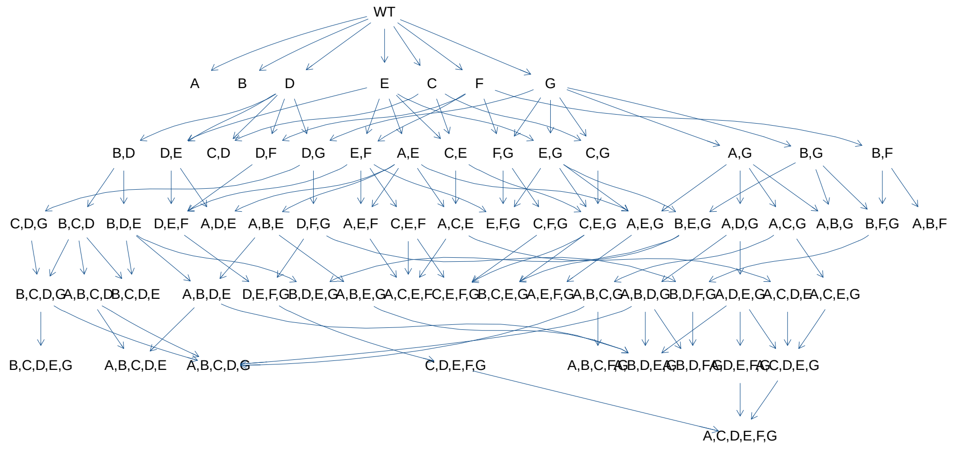

ID	ETH7102TvoUx6htCa
acc genotypes	21
peaks no back	9
removed edges	NA
prop rem edges	NA

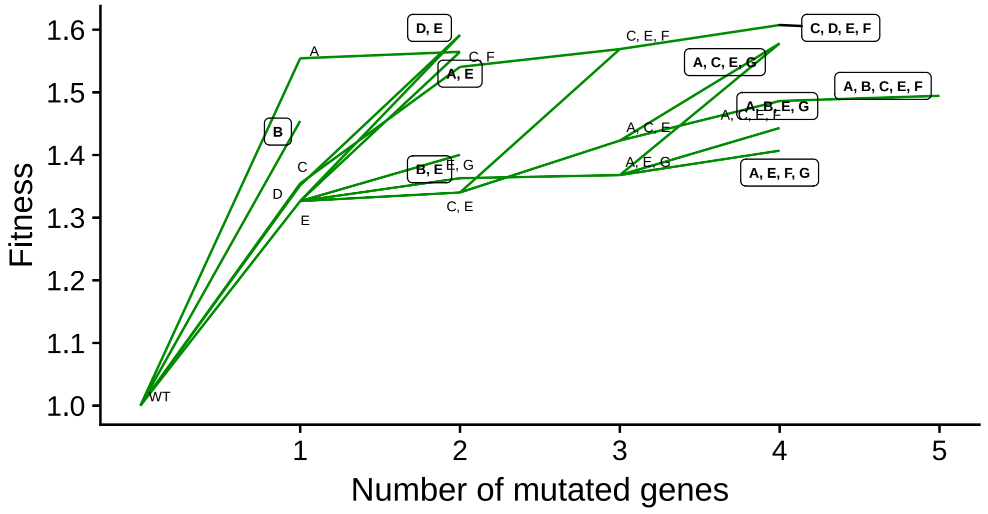

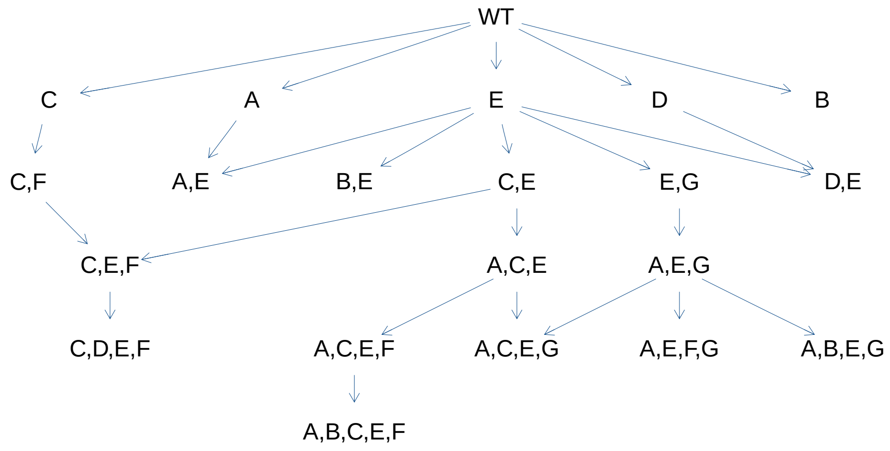

ID	eUzrnqLvhVknhTTj
acc genotypes	40
peaks no back	11
removed edges	NA
prop rem edges	NA

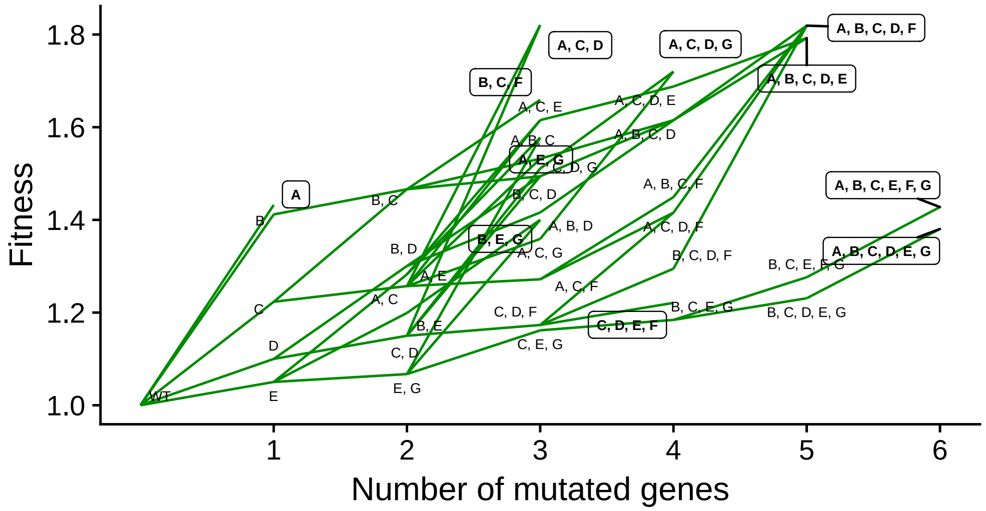

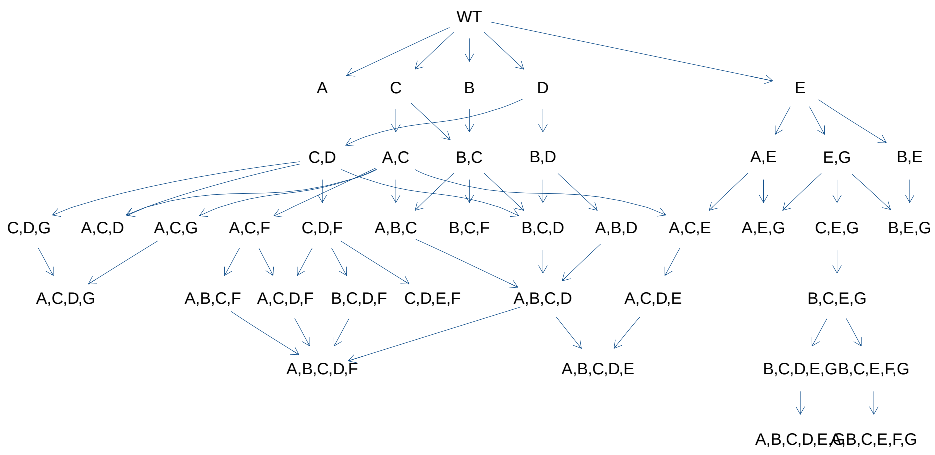

ID	fdZVeDlLcm54cJxM
acc genotypes	41
peaks no back	17
removed edges	NA
prop rem edges	NA

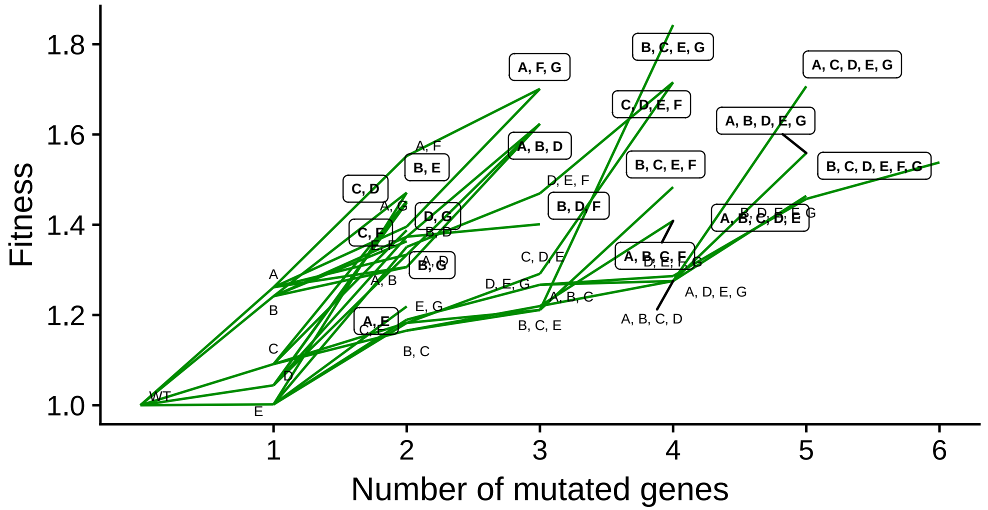

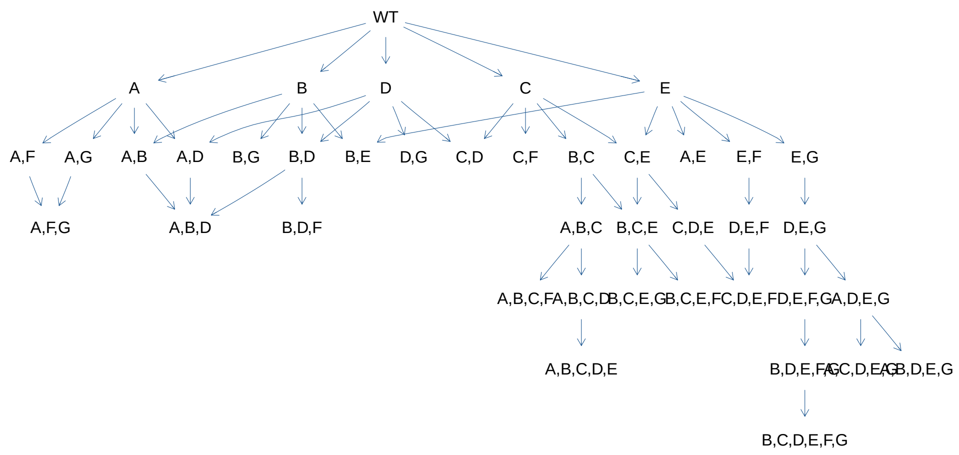

ID	fE3LkSfz106ZntdBy
acc genotypes	48
peaks no back	13
removed edges	NA
prop rem edges	NA

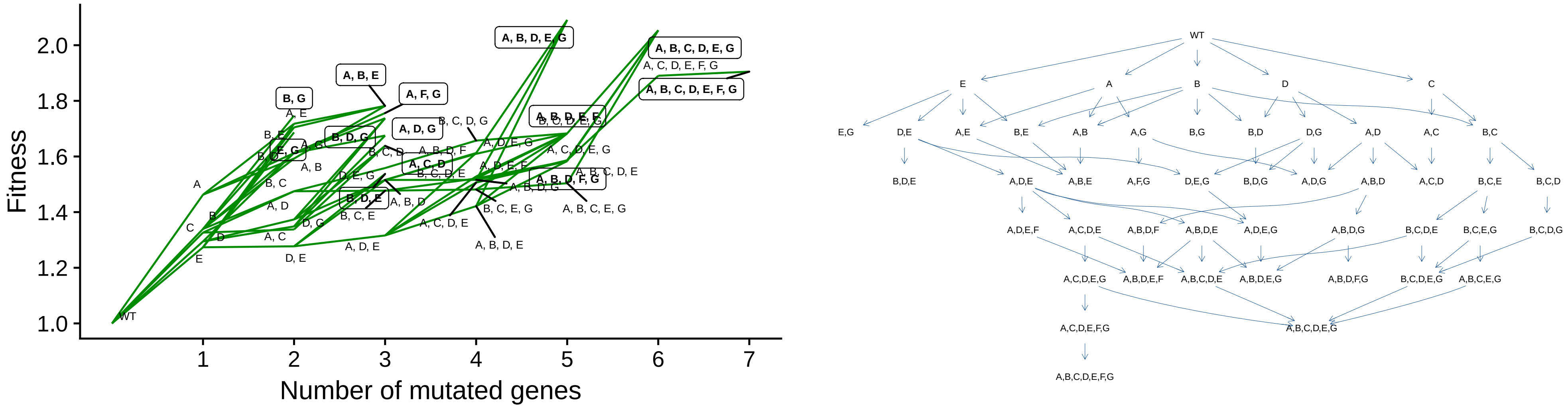

ID	fEwT1uohy1ShL5nH
acc genotypes	38
peaks no back	17
removed edges	NA
prop rem edges	NA

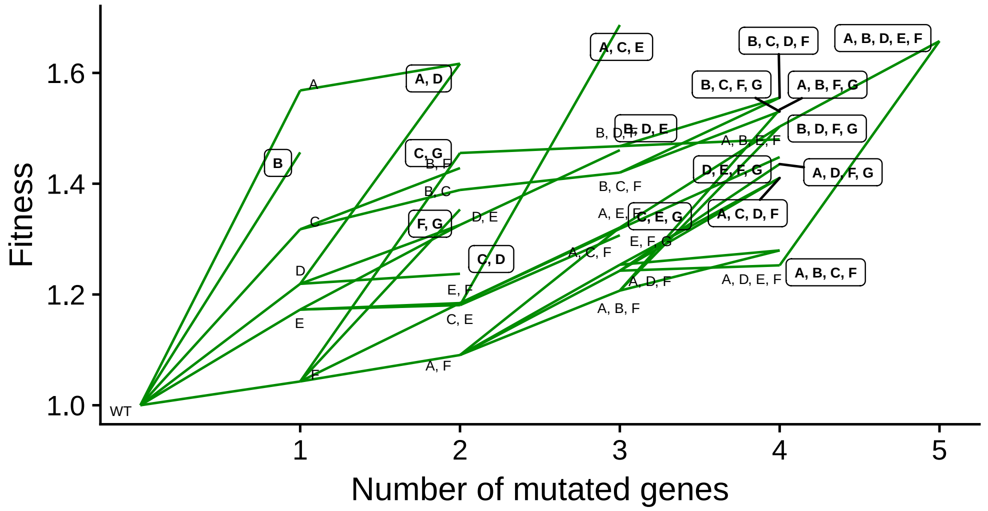

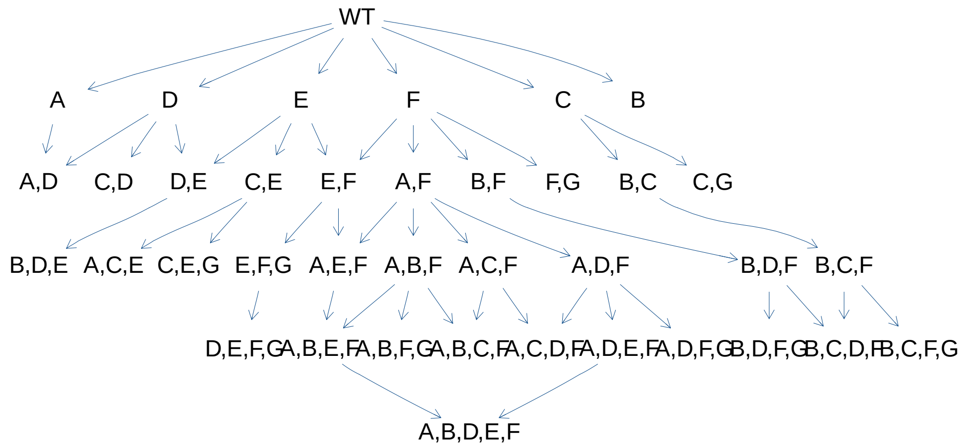

ID	fS10Ju2gMFkEFOANk
acc genotypes	20
peaks no back	8
removed edges	NA
prop rem edges	NA

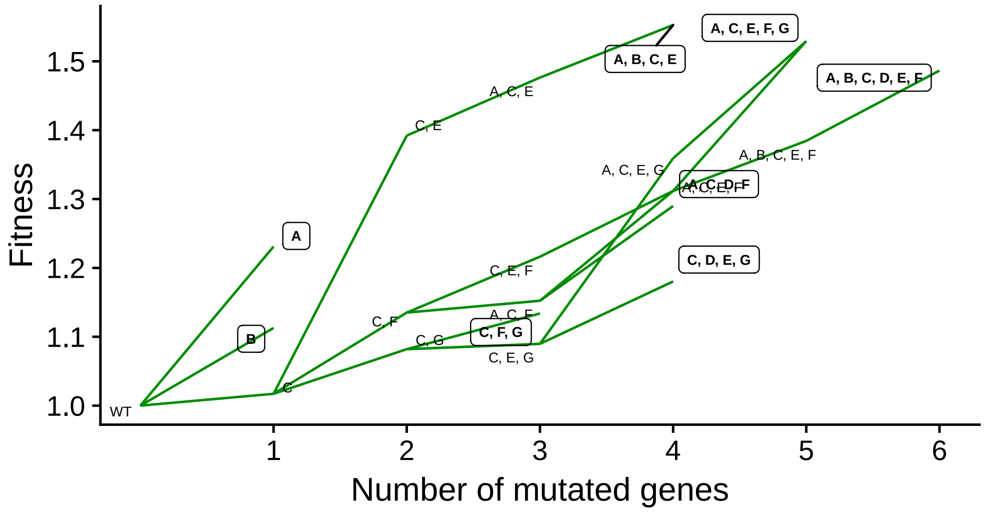

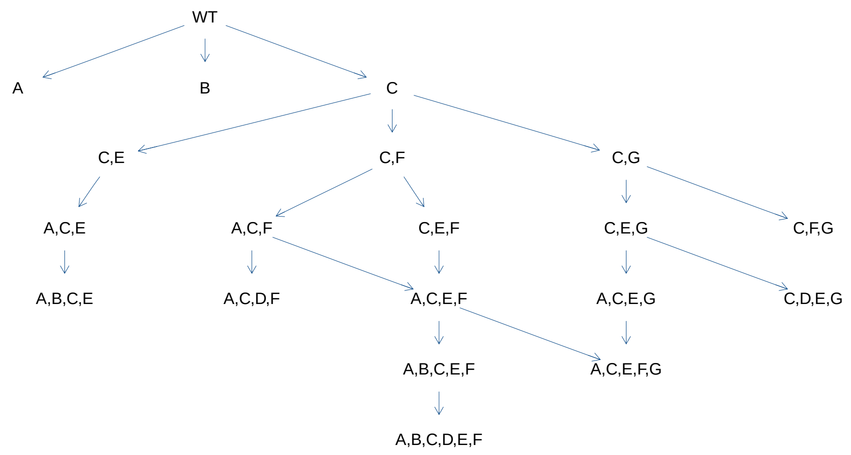

ID	Fwg3jWZQftuLfTc8
acc genotypes	43
peaks no back	13
removed edges	NA
prop rem edges	NA

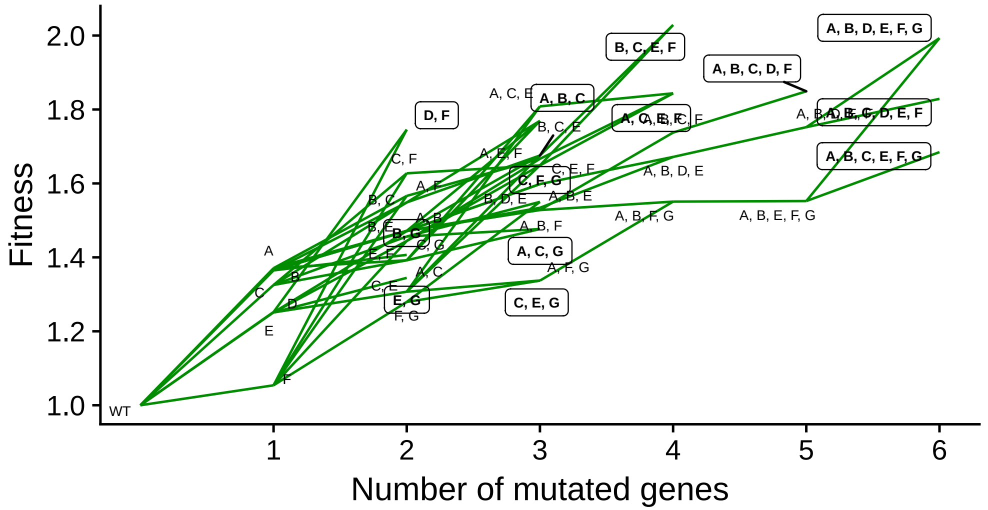

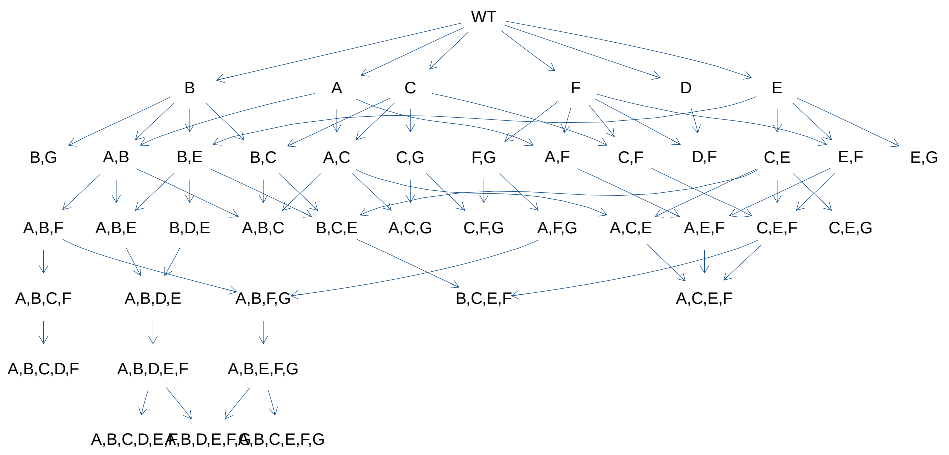

ID	fzwb5MOAwOWpF4B9
acc genotypes	62
peaks no back	18
removed edges	NA
prop rem edges	NA

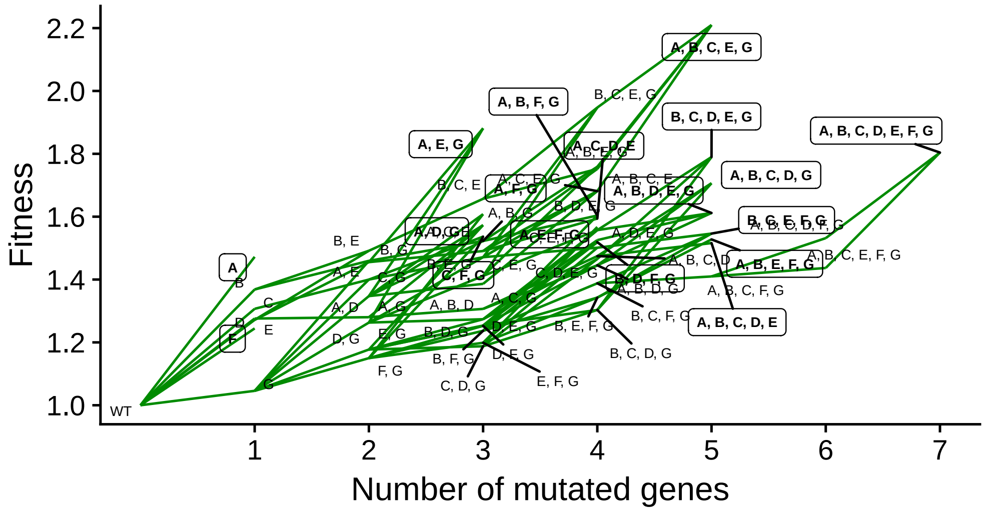

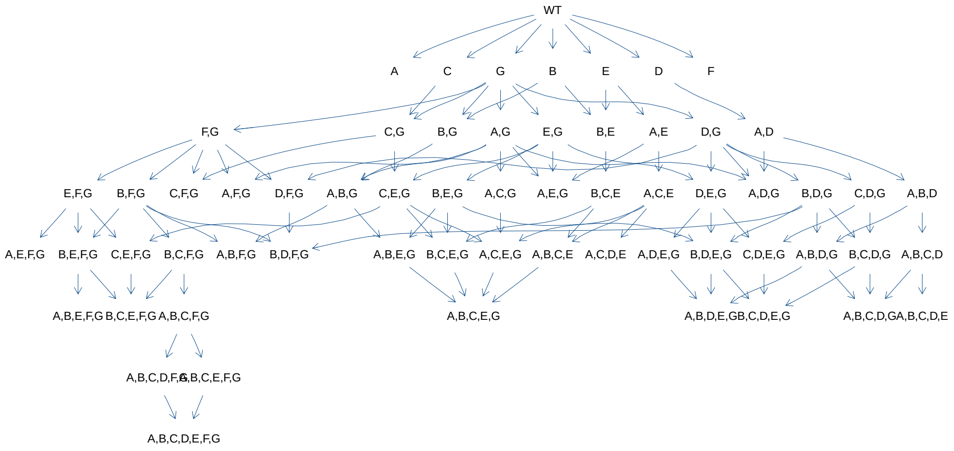

ID GDVo4gmjaDr6lo1c
acc genotypes 51
peaks no back 16
removed edges NA
prop rem edges NA

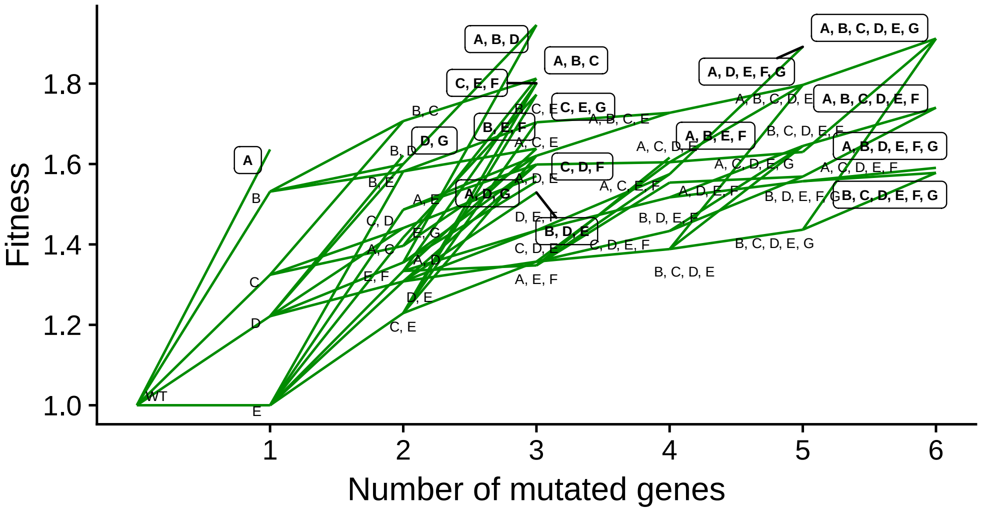

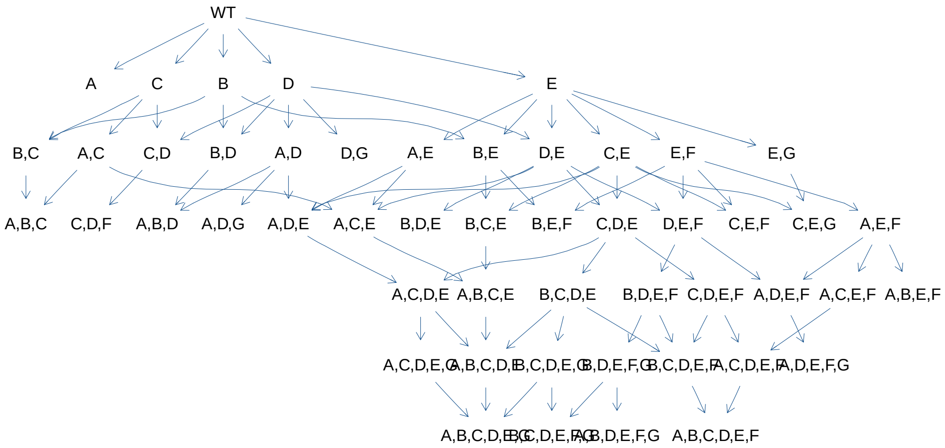

ID	gHFY6JsOn10PnBYf3
acc genotypes	40
peaks no back	13
removed edges	NA
prop rem edges	NA

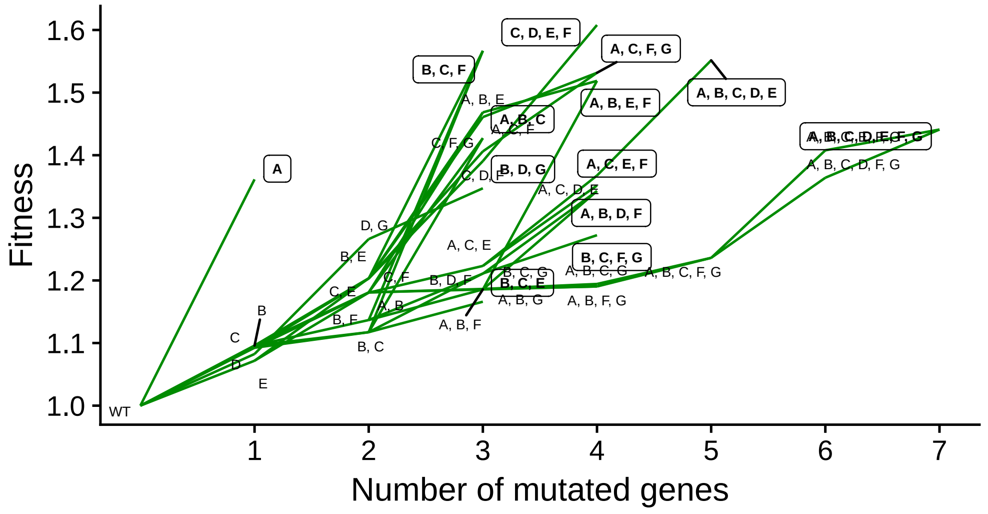

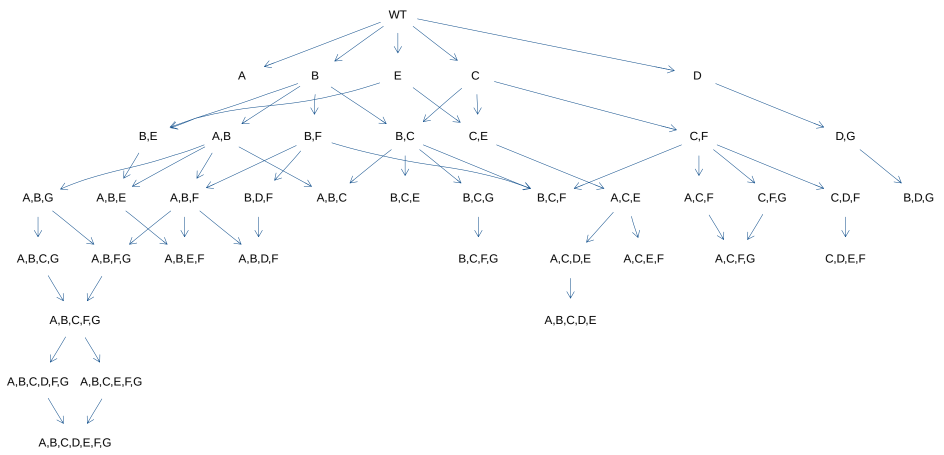

ID	GJH85dNm2AtrzoUI
acc genotypes	30
peaks no back	13
removed edges	NA
prop rem edges	NA

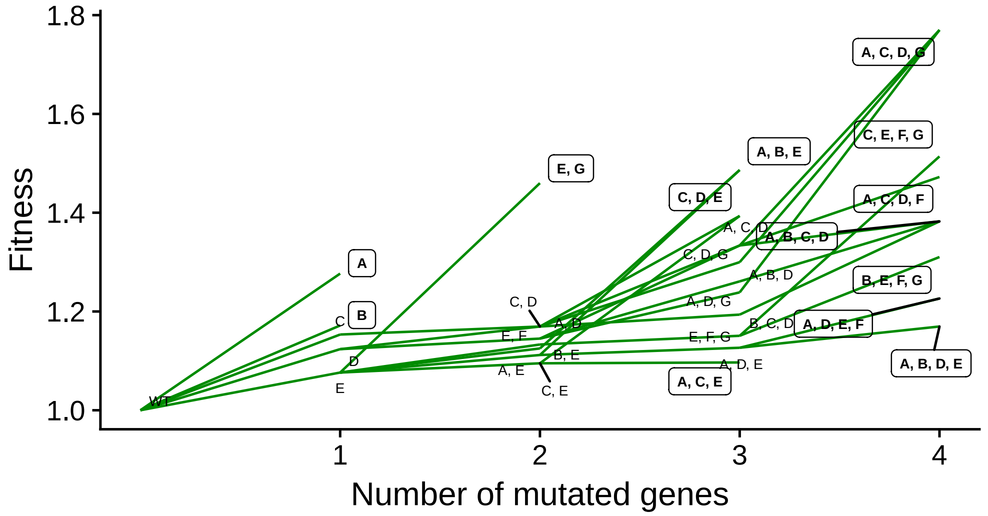

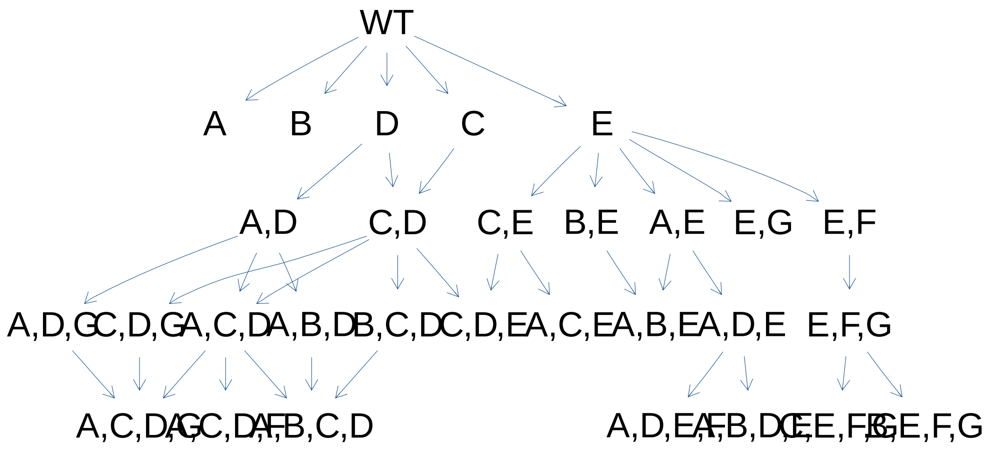

ID	GrnJzITUvPUix6BN
acc genotypes	51
peaks no back	19
removed edges	NA
prop rem edges	NA

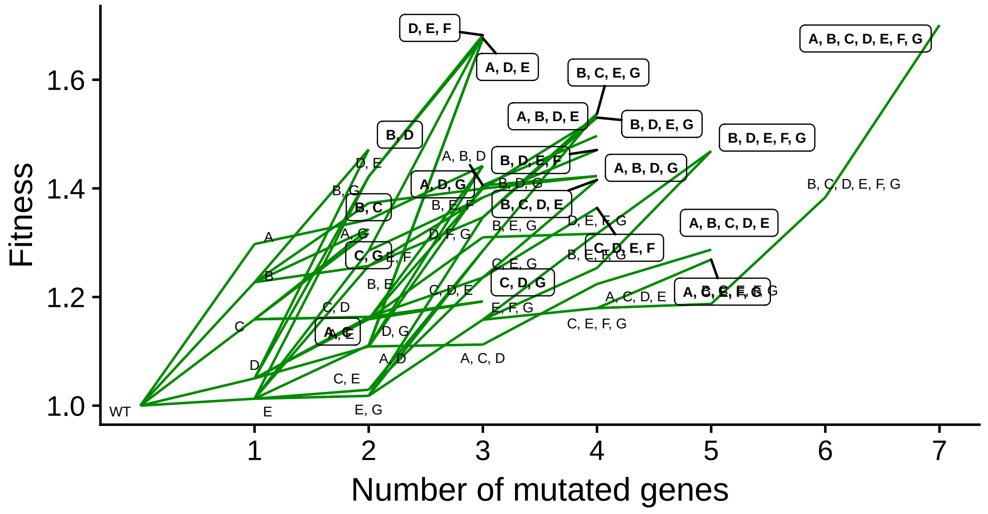

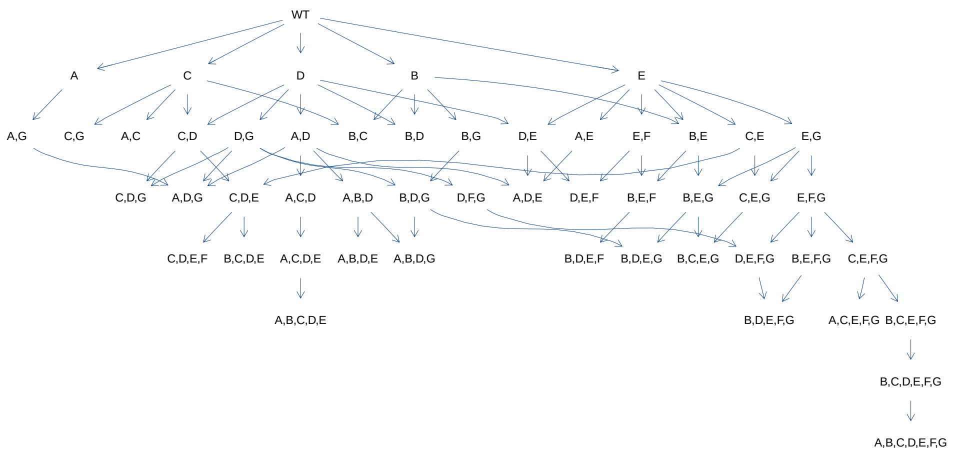

ID	Hm3E9vk10sP85Ykfb
acc genotypes	14
peaks no back	6
removed edges	NA
prop rem edges	NA

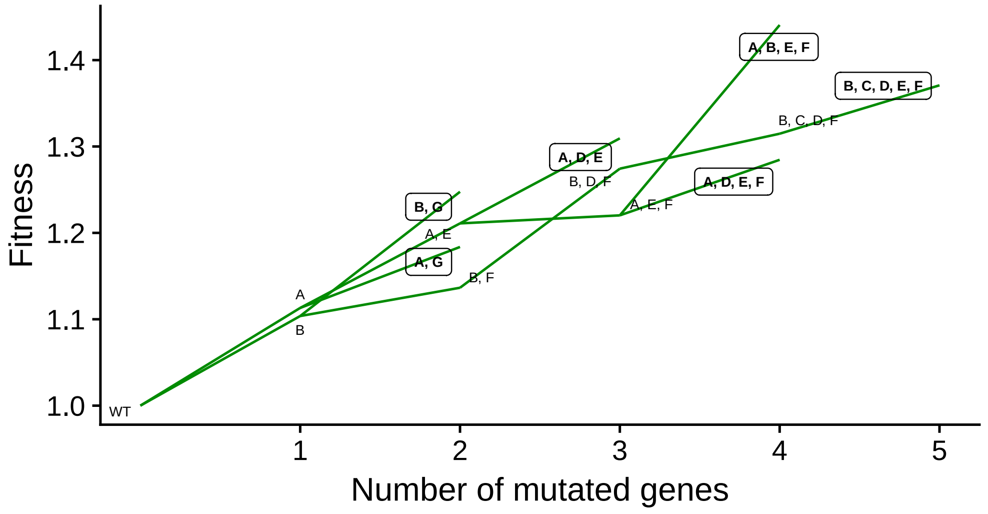

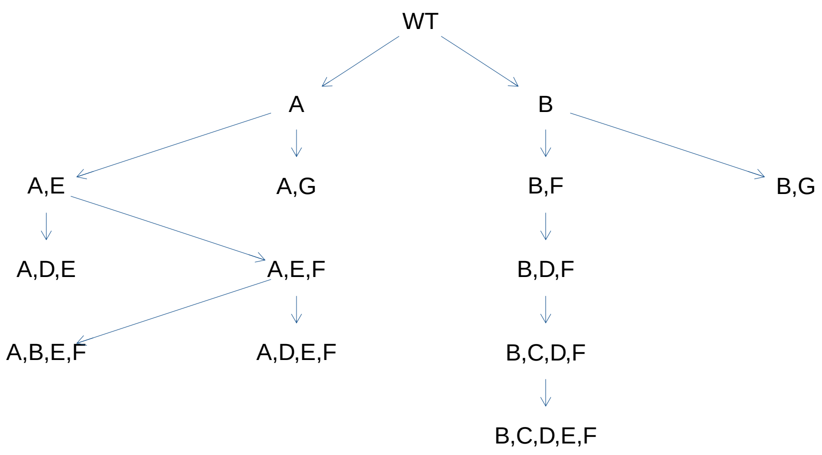

ID	hnKNIikQ9aKghXCm
acc genotypes	36
peaks no back	14
removed edges	NA
prop rem edges	NA

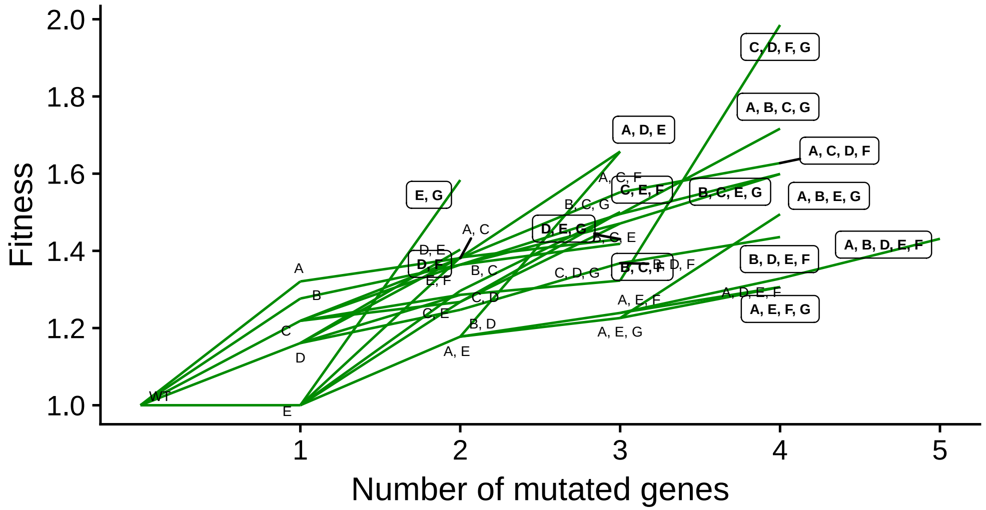

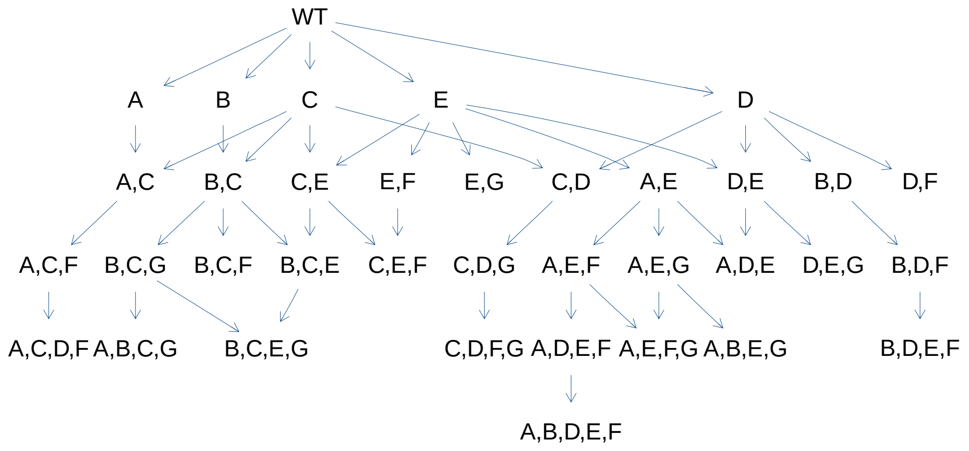

ID	HUGD4mPfT3Y9PL8i
acc genotypes	18
peaks no back	4
removed edges	NA
prop rem edges	NA

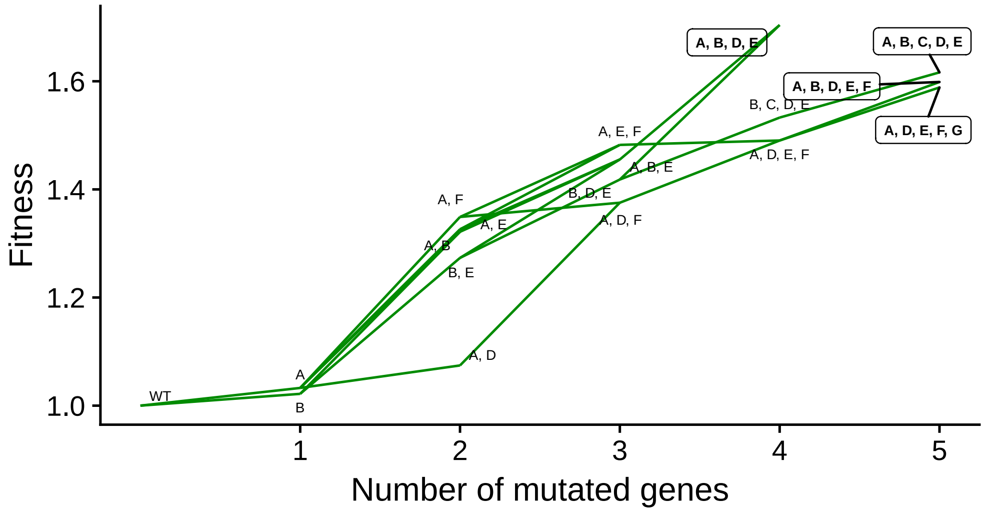

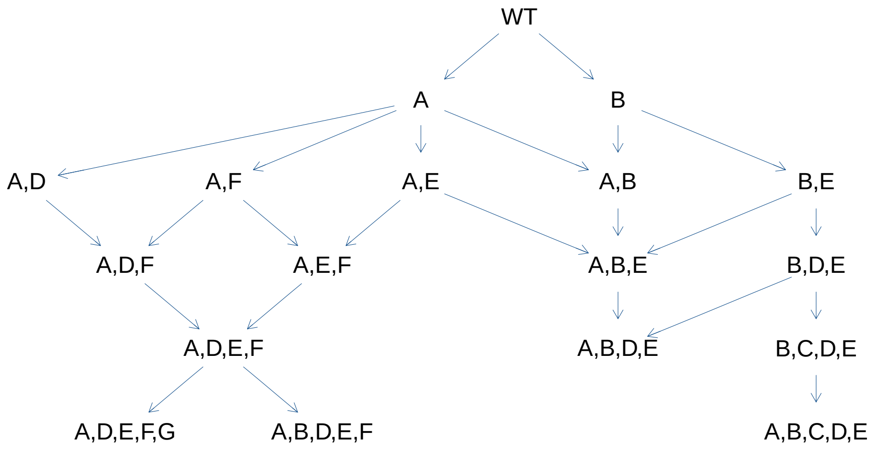

ID	HVUm1KRpYXeSvmPO
acc genotypes	28
peaks no back	13
removed edges	NA
prop rem edges	NA

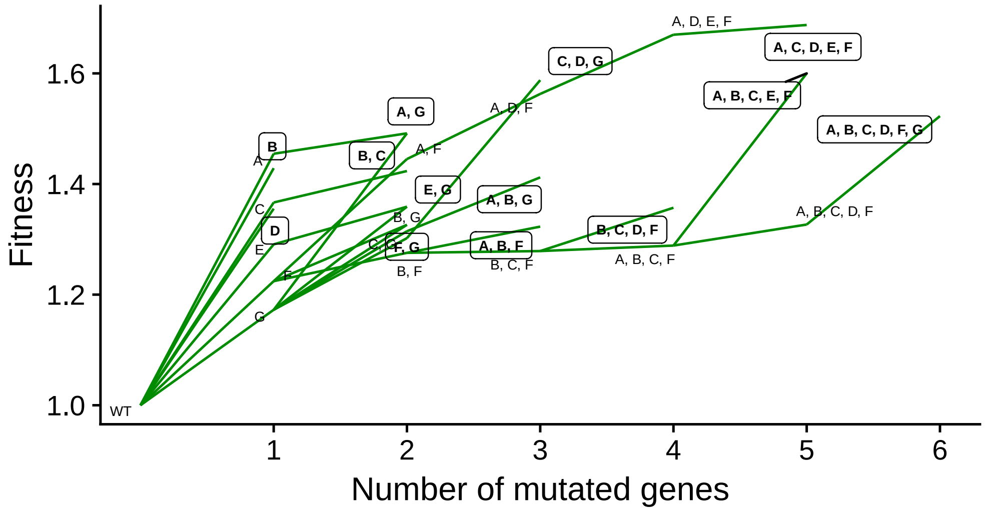

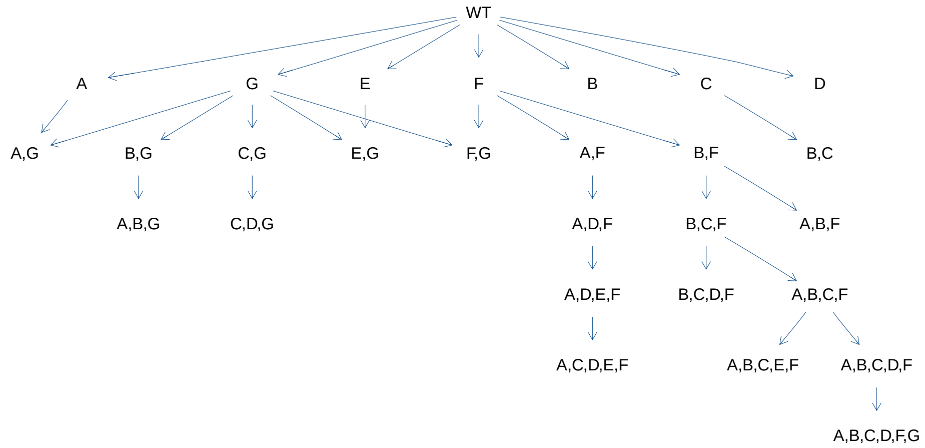

ID	hwWOucBKvmrZN9ue
acc genotypes	55
peaks no back	15
removed edges	NA
prop rem edges	NA

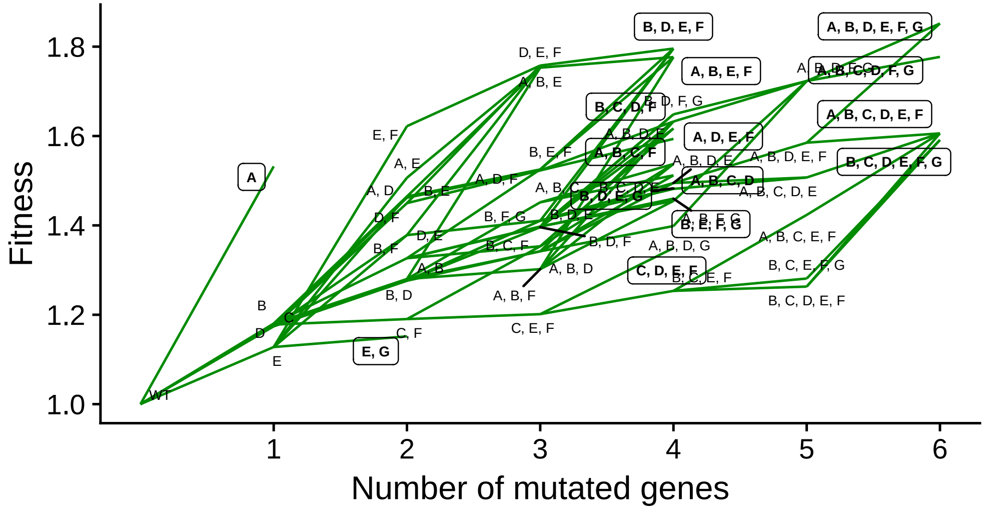

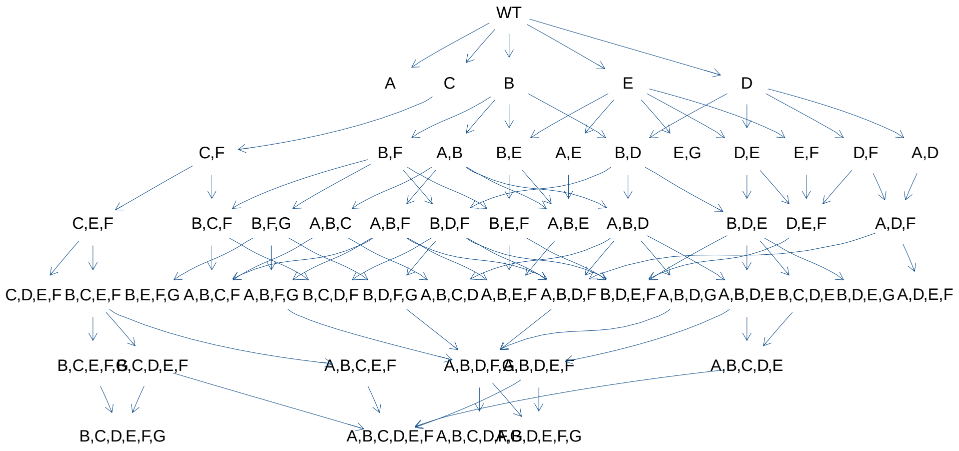

ID	hXnNgnV34hLXDvEz
acc genotypes	49
peaks no back	16
removed edges	NA
prop rem edges	NA

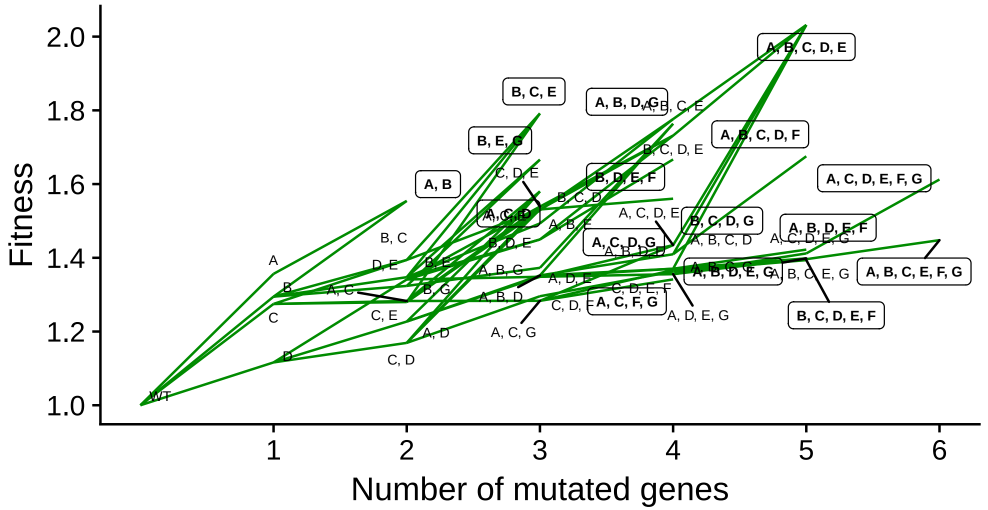

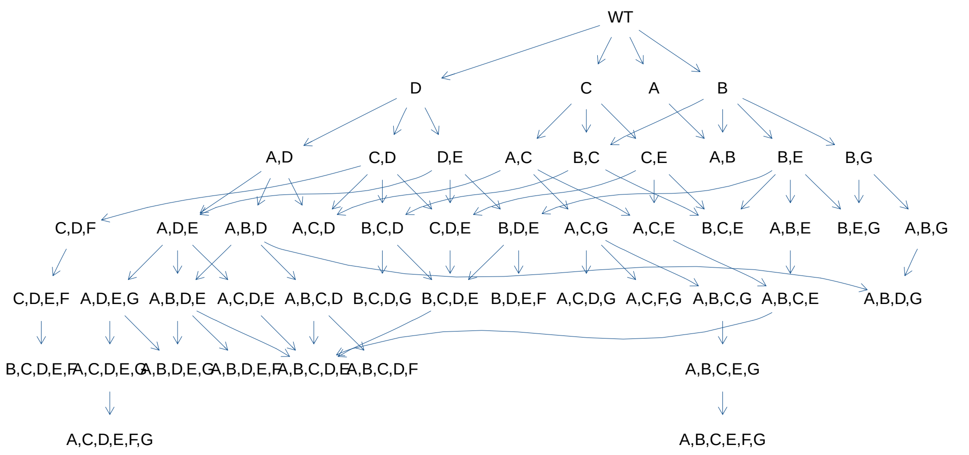

ID	hz9XzL5TRKBXl8Xk
acc genotypes	45
peaks no back	21
removed edges	NA
prop rem edges	NA

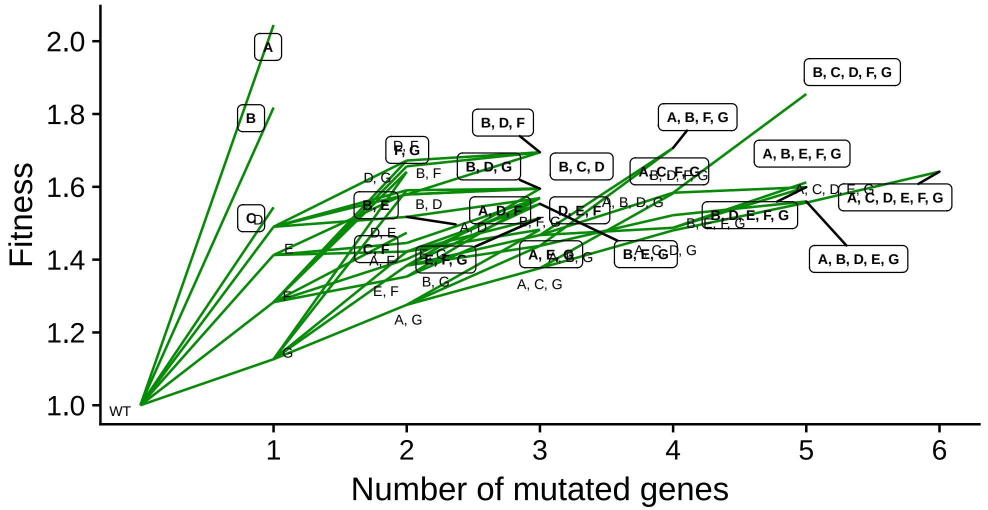

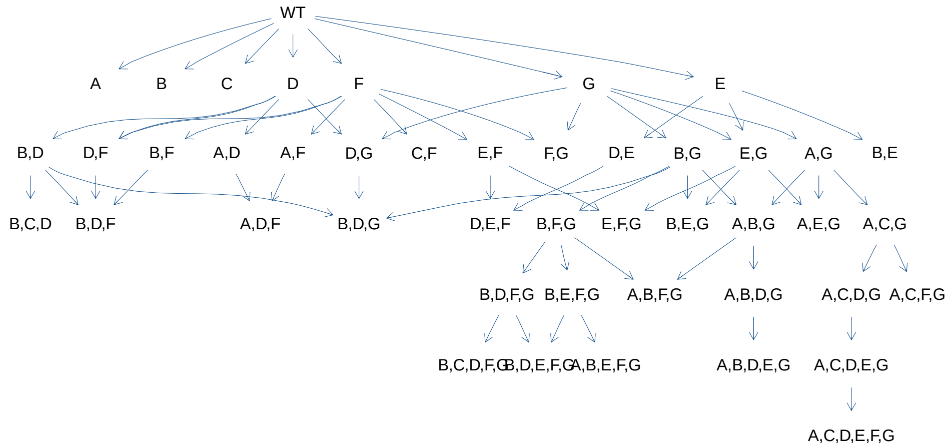

ID	i102joVz69wXudIlf
acc genotypes	26
peaks no back	11
removed edges	NA
prop rem edges	NA

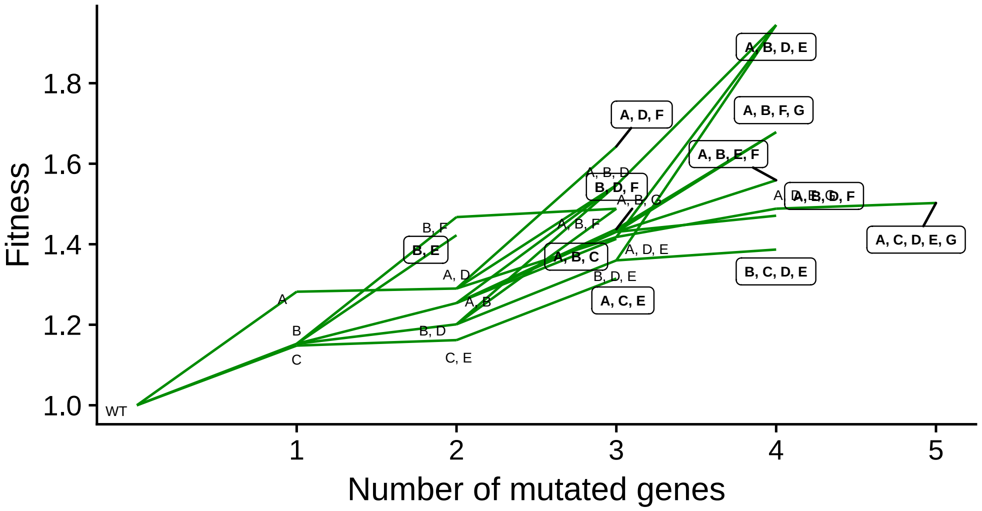

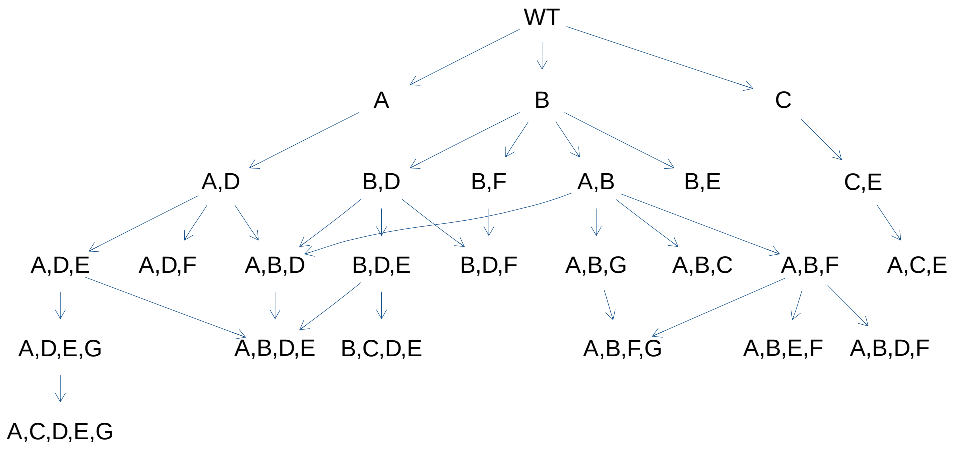

ID	I4wGMzShjuo8XyuN
acc genotypes	43
peaks no back	18
removed edges	NA
prop rem edges	NA

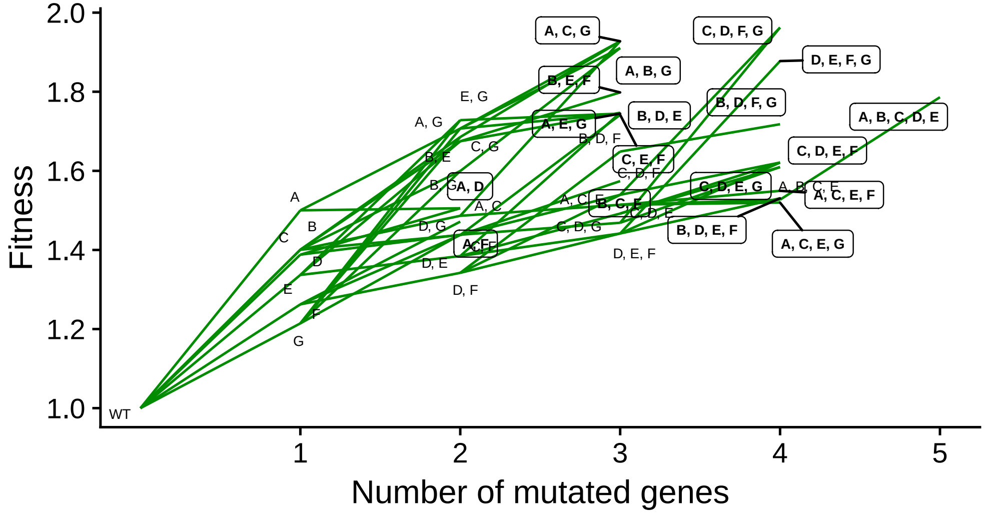

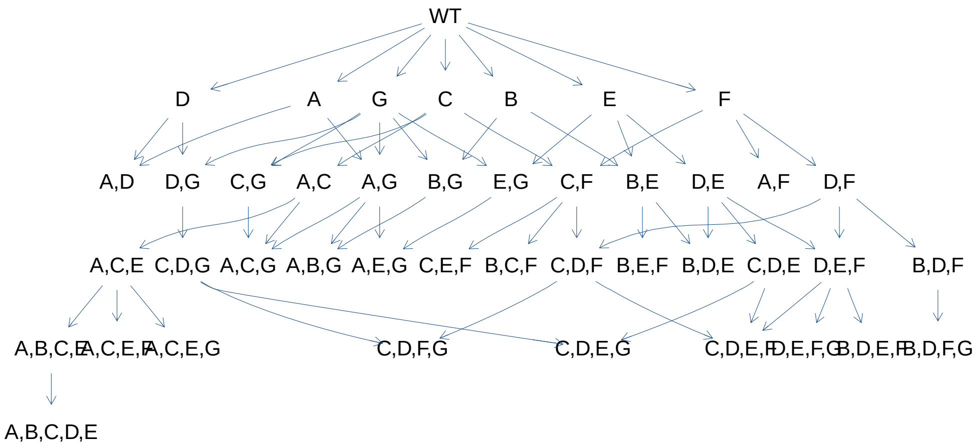

ID	IDLT5qGFT3uM1rEn
acc genotypes	50
peaks no back	19
removed edges	NA
prop rem edges	NA

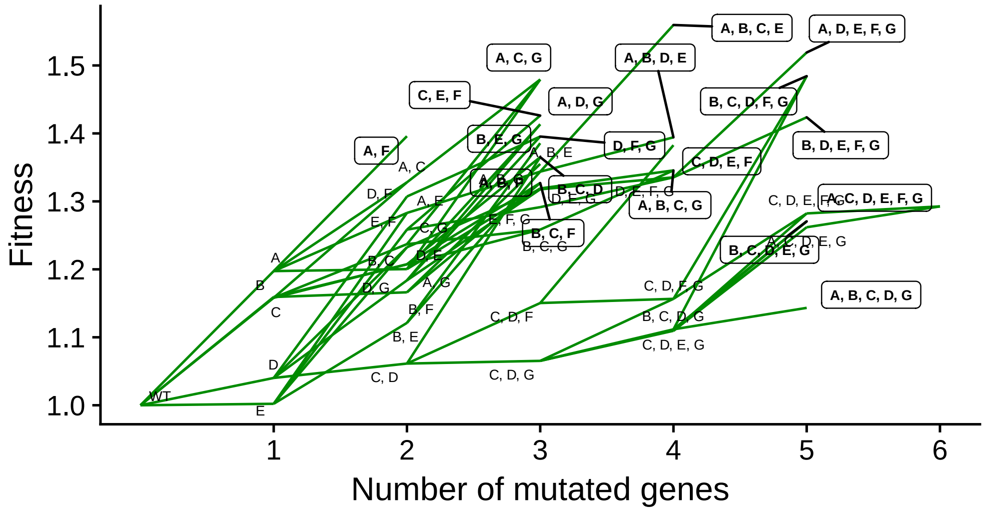

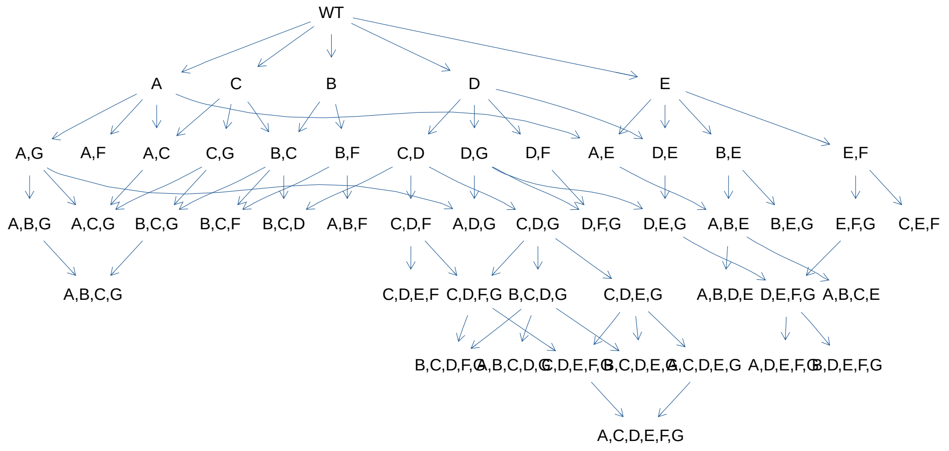

ID IJl10hVtdsgYgvoV7

acc genotypes 46

peaks no back 10

removed edges NA

prop rem edges NA

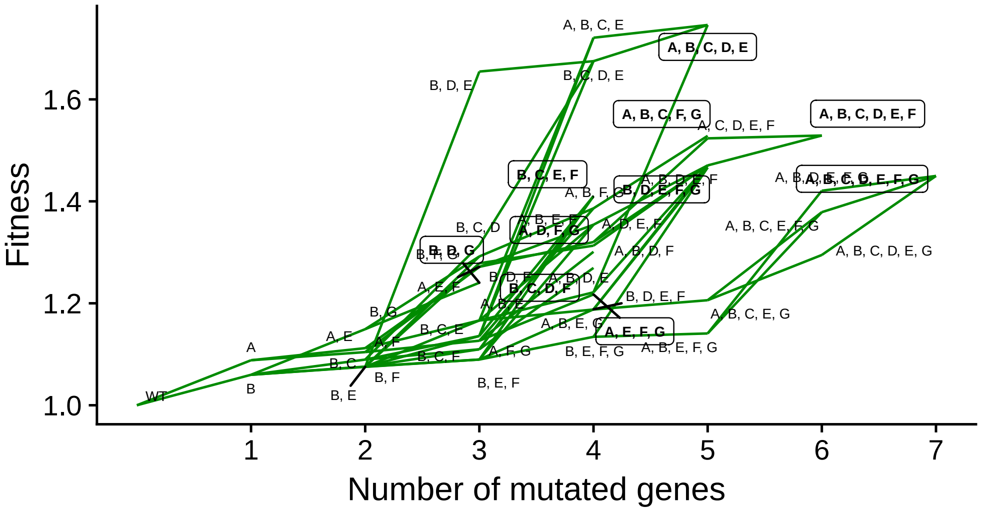

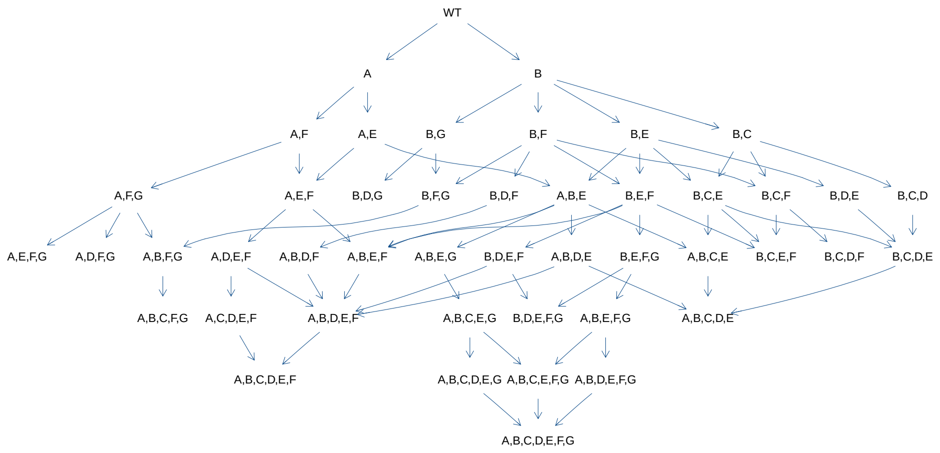

ID	iuizHj29wSFmqntf
acc genotypes	39
peaks no back	17
removed edges	NA
prop rem edges	NA

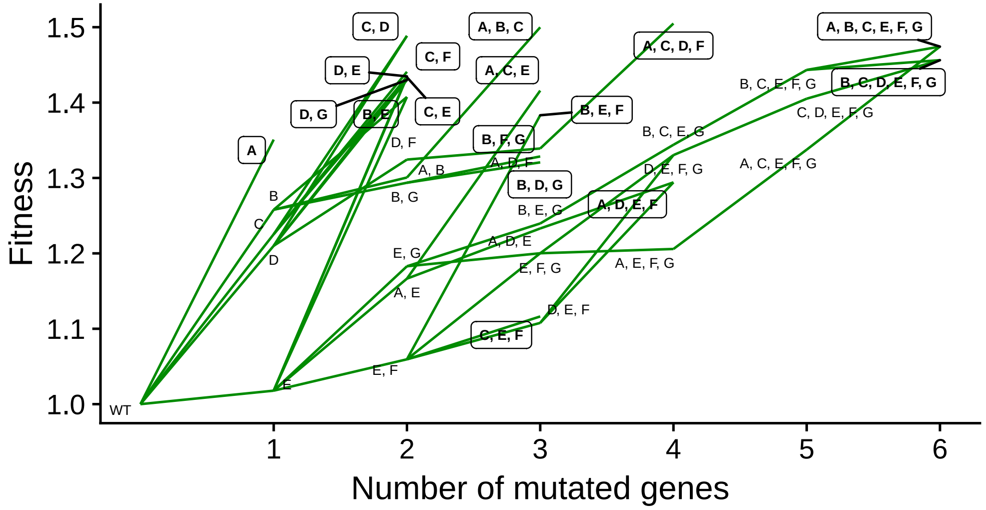

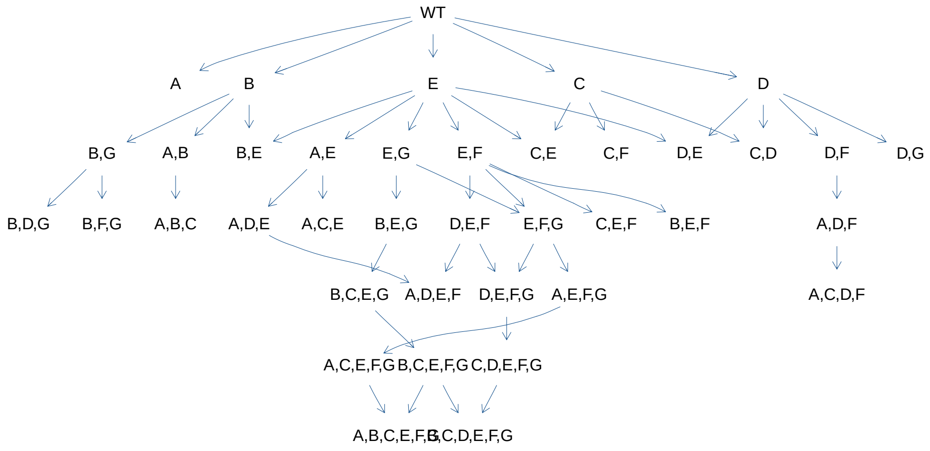

ID	jARDfKjvS5xgIbjy
acc genotypes	32
peaks no back	12
removed edges	NA
prop rem edges	NA

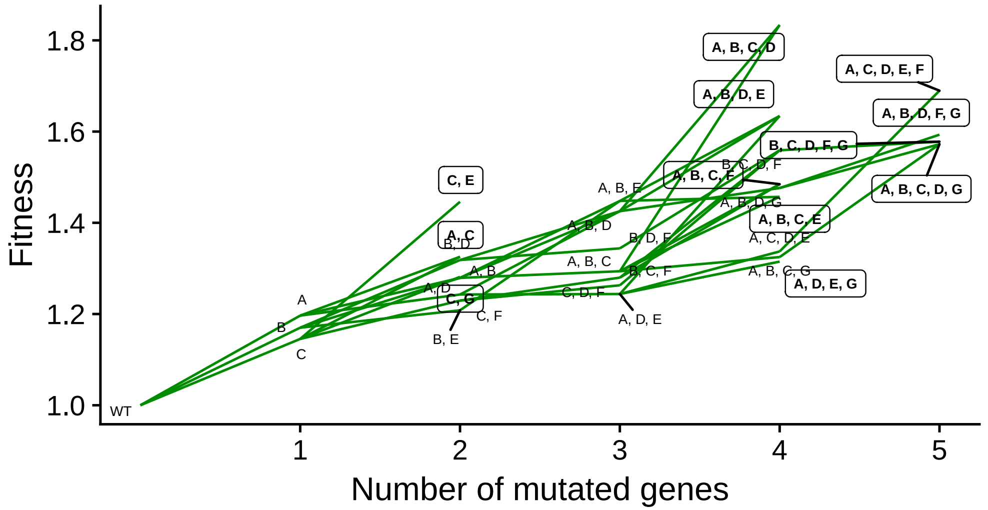

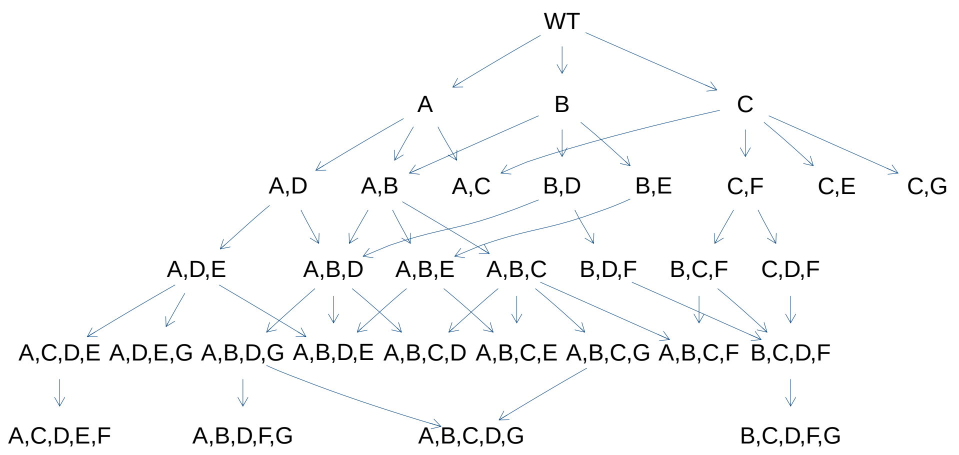

ID	JC7USXL8yt3B9M10N
acc genotypes	36
peaks no back	15
removed edges	NA
prop rem edges	NA

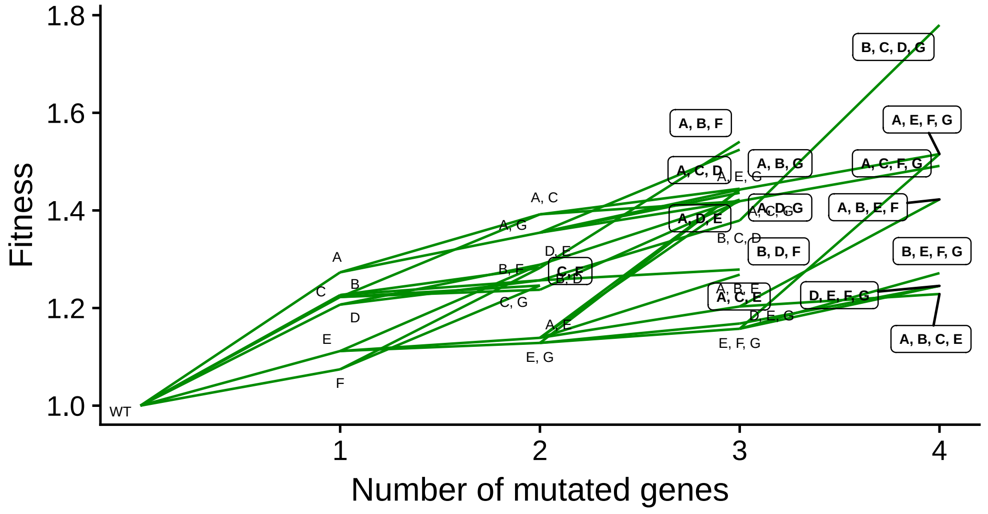

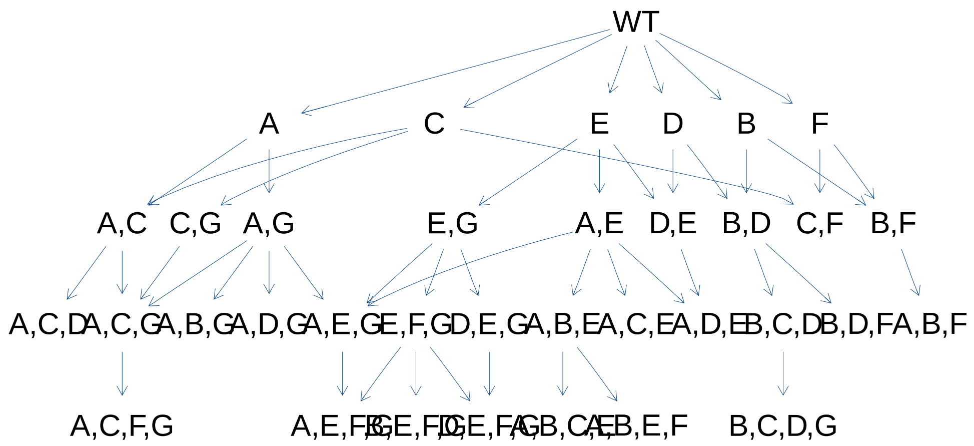

ID	JdIeKg10Z6a5TlQp5
acc genotypes	21
peaks no back	7
removed edges	NA
prop rem edges	NA

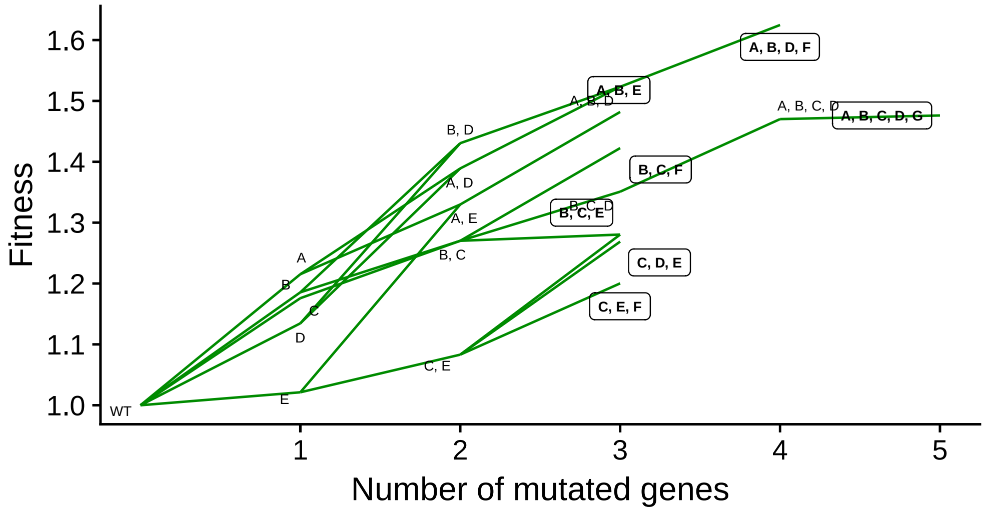

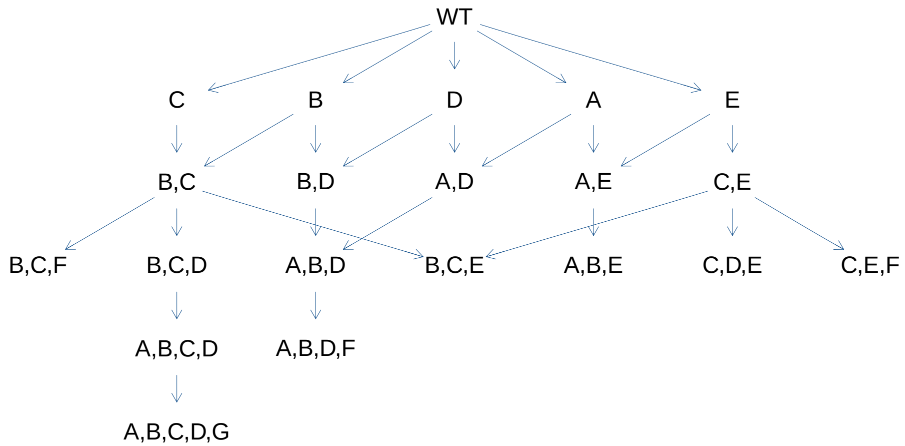

ID	jJkQq5oUGT4cTfIw
acc genotypes	11
peaks no back	5
removed edges	NA
prop rem edges	NA

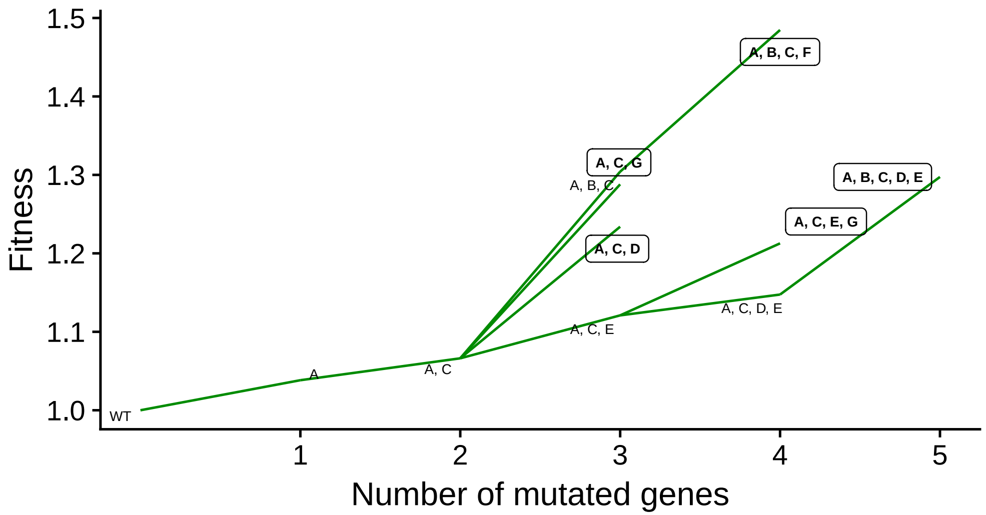

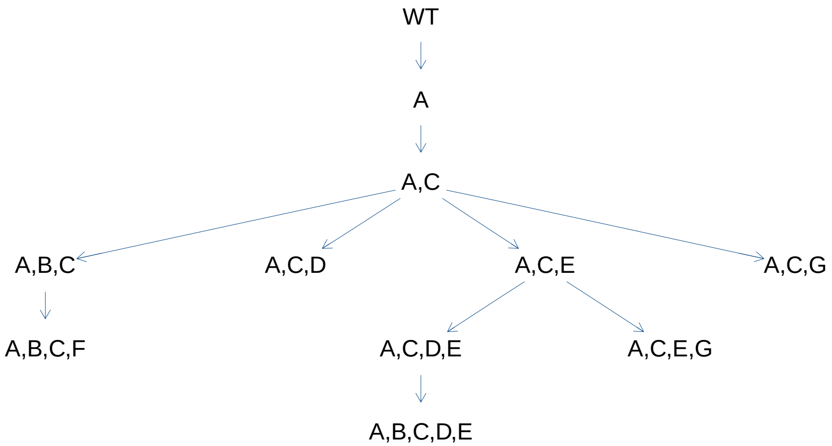

ID	JqytlTKKThh5QBbf
acc genotypes	11
peaks no back	5
removed edges	NA
prop rem edges	NA

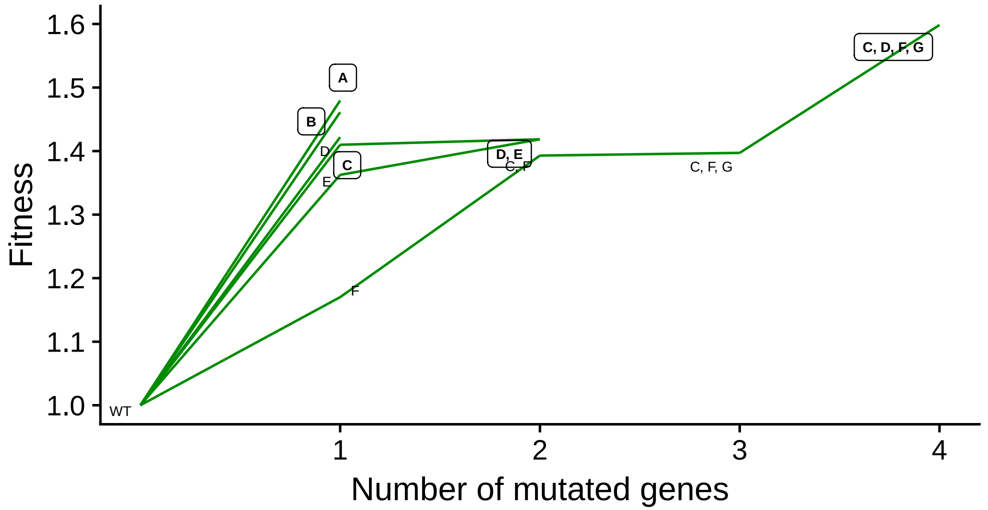

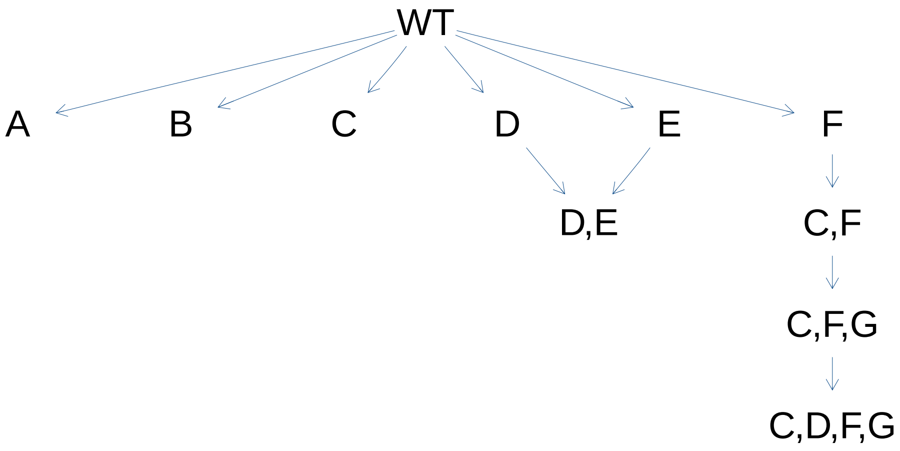

ID	jvGYmalfWVHjDSsr
acc genotypes	39
peaks no back	15
removed edges	NA
prop rem edges	NA

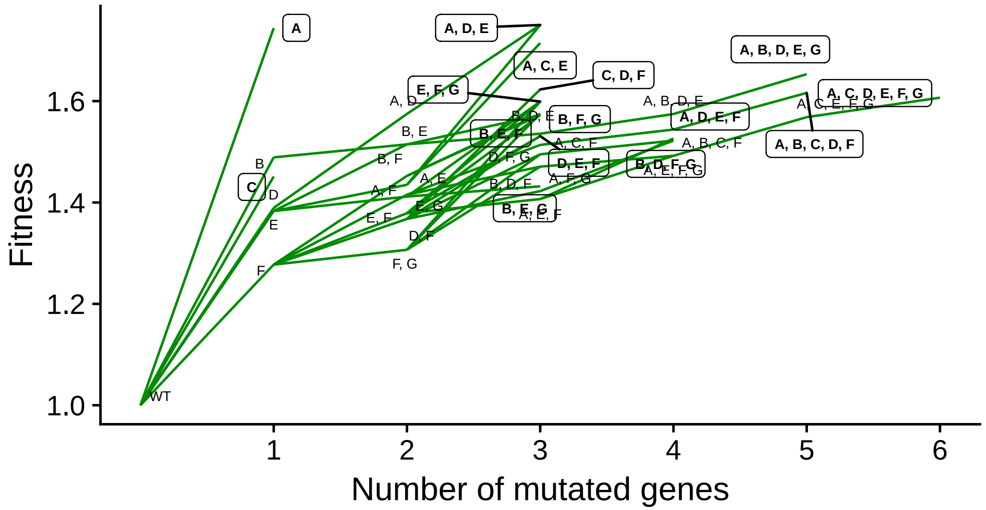

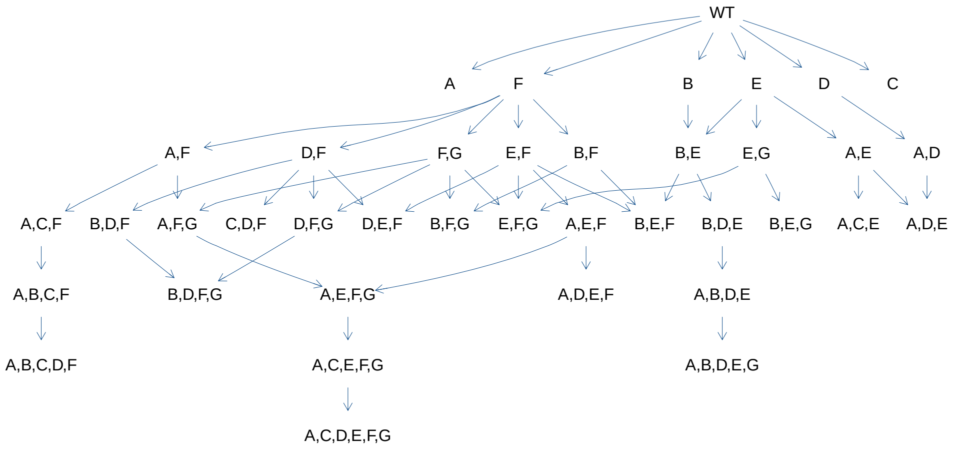

ID	jvTSHGXJvbnFtVLt
acc genotypes	30
peaks no back	9
removed edges	NA
prop rem edges	NA

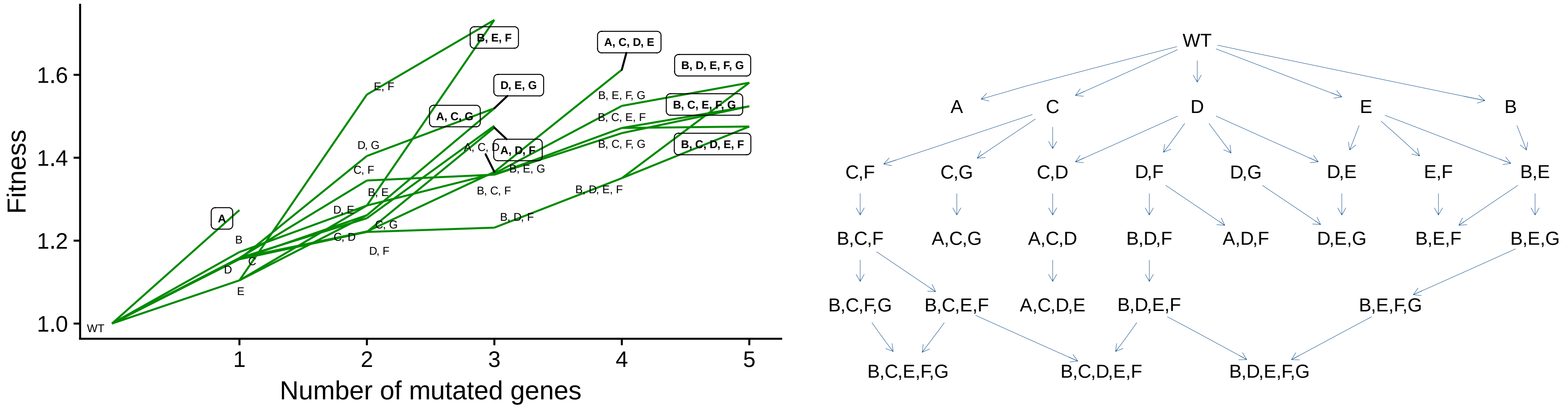

ID	k4wHgtkweyDrnCn8
acc genotypes	80
peaks no back	17
removed edges	NA
prop rem edges	NA

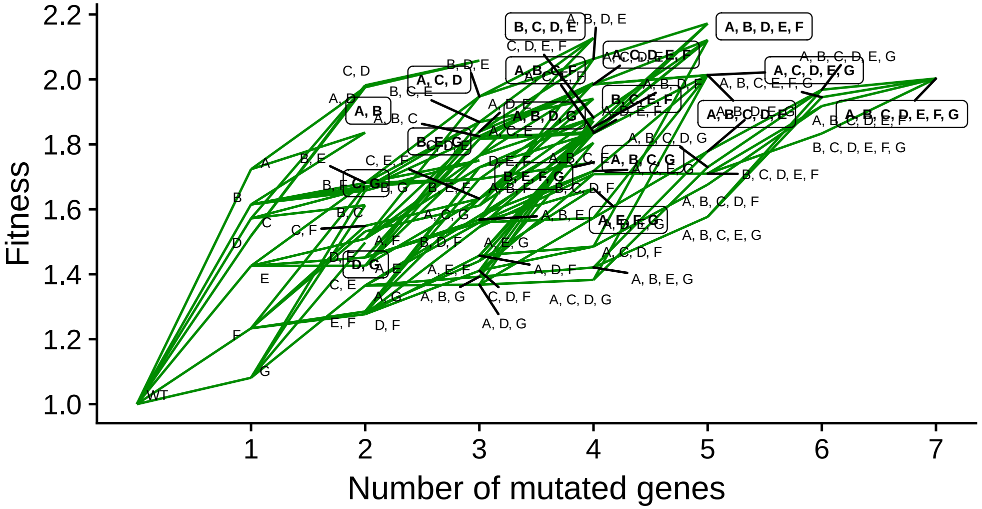

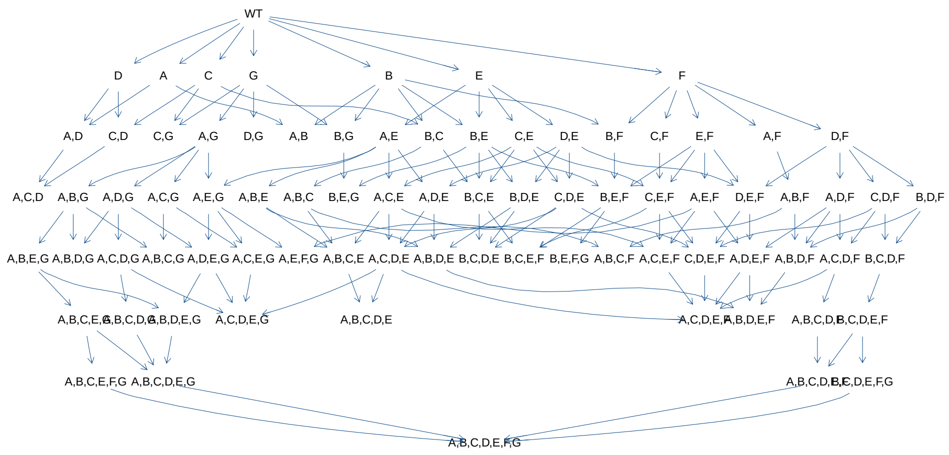

ID	kd10vK9jGcoOkaAMm
acc genotypes	75
peaks no back	7
removed edges	NA
prop rem edges	NA

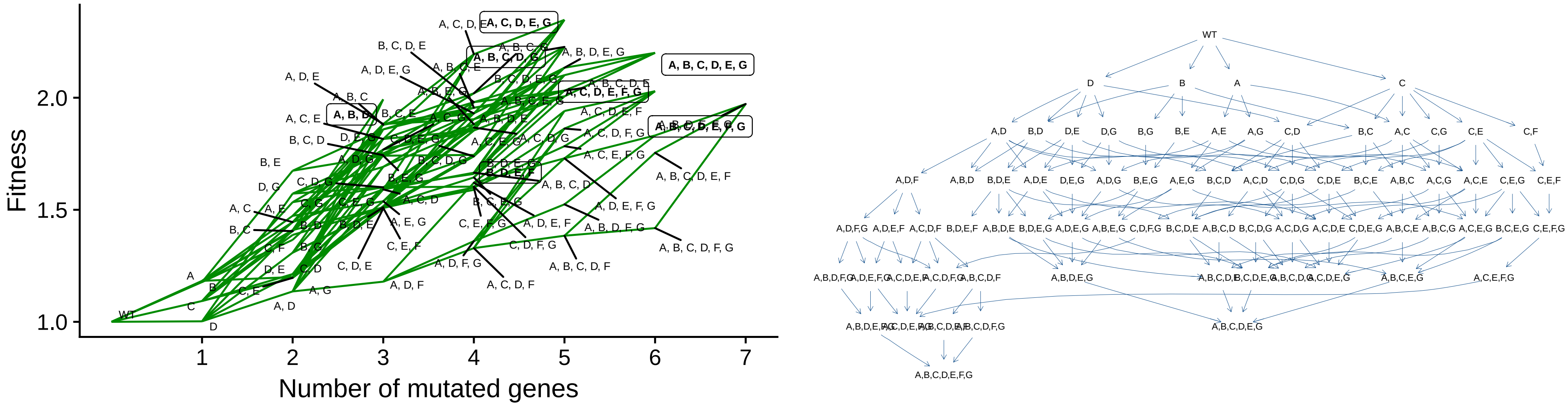

ID	kfB7NOk1J247FNsy
acc genotypes	18
peaks no back	8
removed edges	NA
prop rem edges	NA

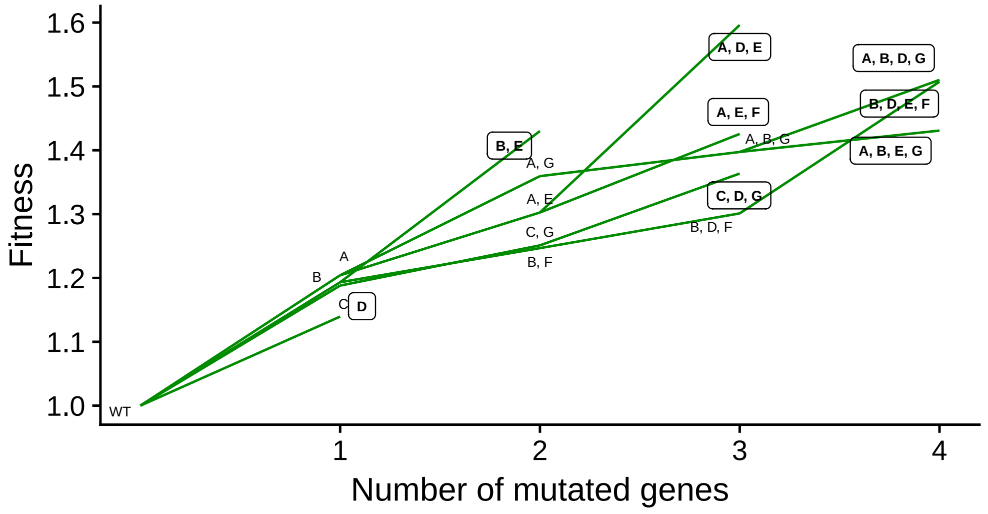

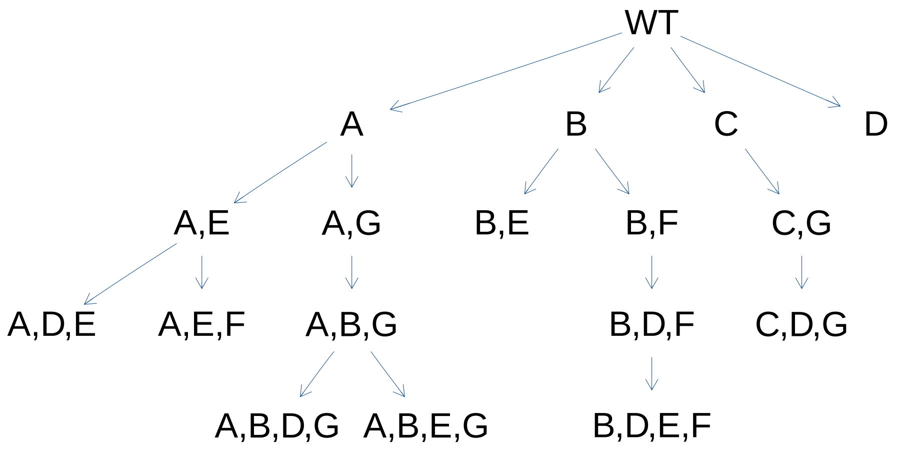

ID	kfC76EeHnueEMKzE
acc genotypes	70
peaks no back	19
removed edges	NA
prop rem edges	NA

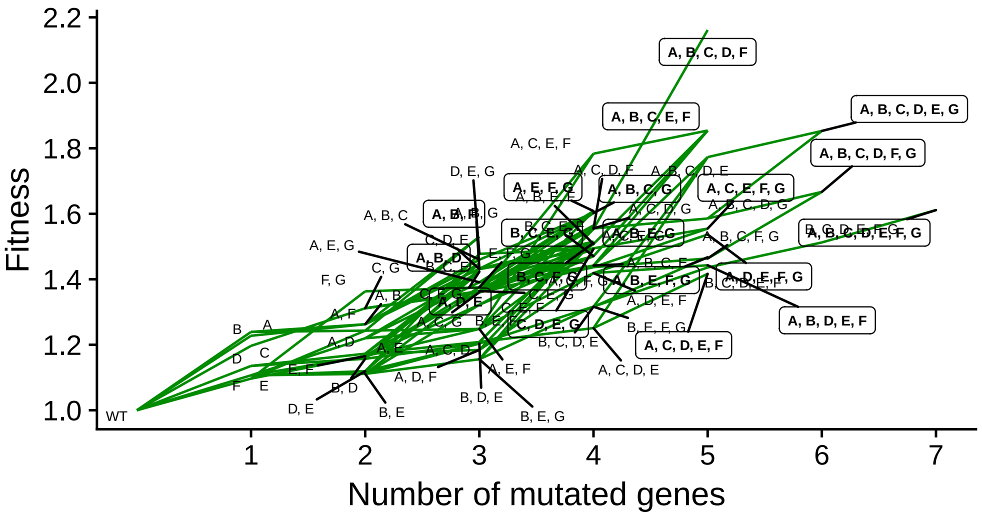

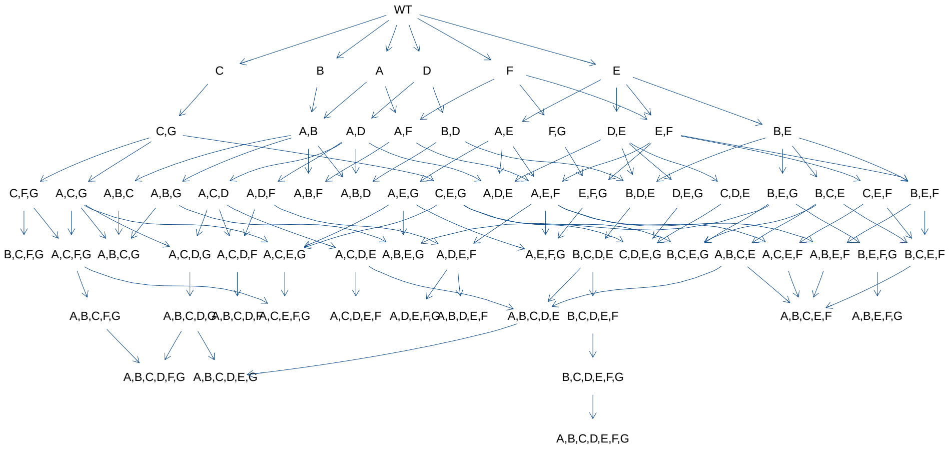

ID	knzBi4rUYms1ZmCY
acc genotypes	57
peaks no back	20
removed edges	NA
prop rem edges	NA

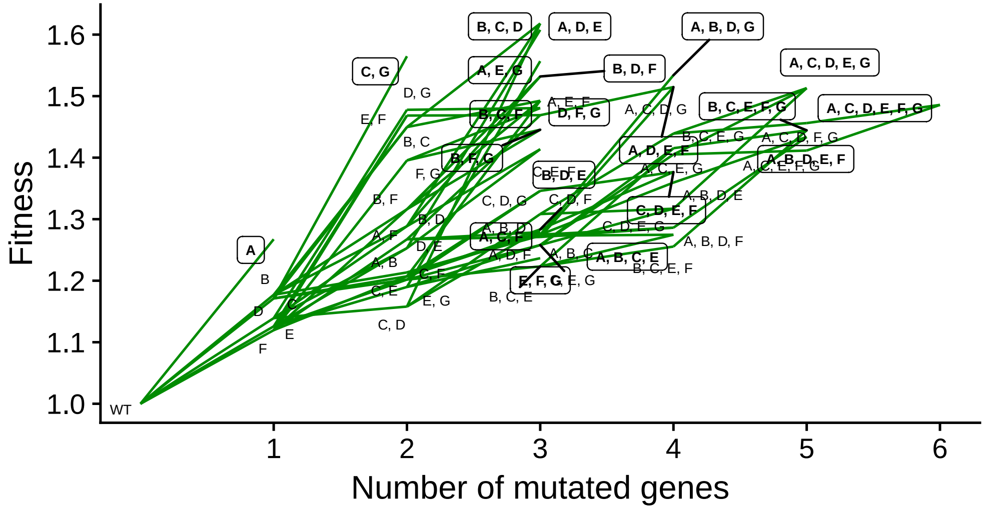

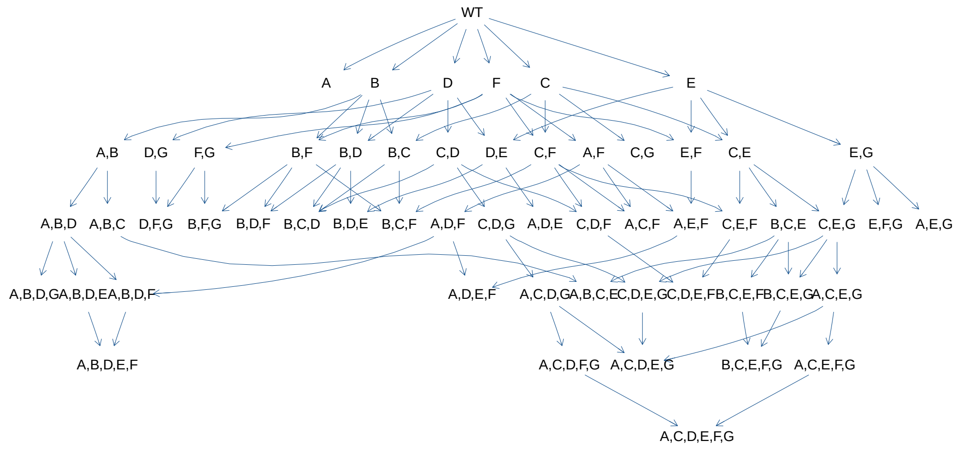

ID	KRi7SXjpurE910L5q
acc genotypes	37
peaks no back	13
removed edges	NA
prop rem edges	NA

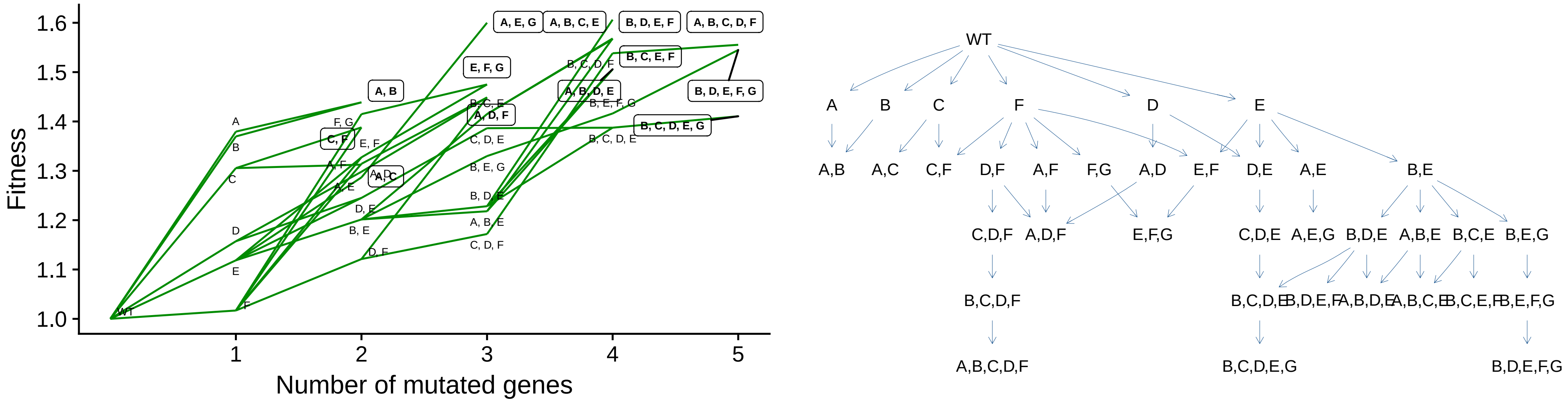

ID	KwdXNcrapMR7uPi7
acc genotypes	31
peaks no back	10
removed edges	NA
prop rem edges	NA

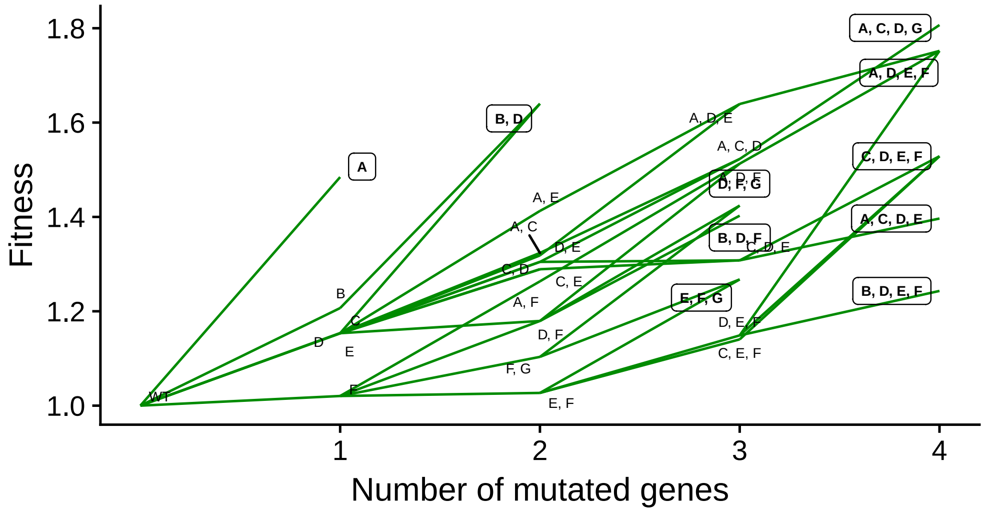

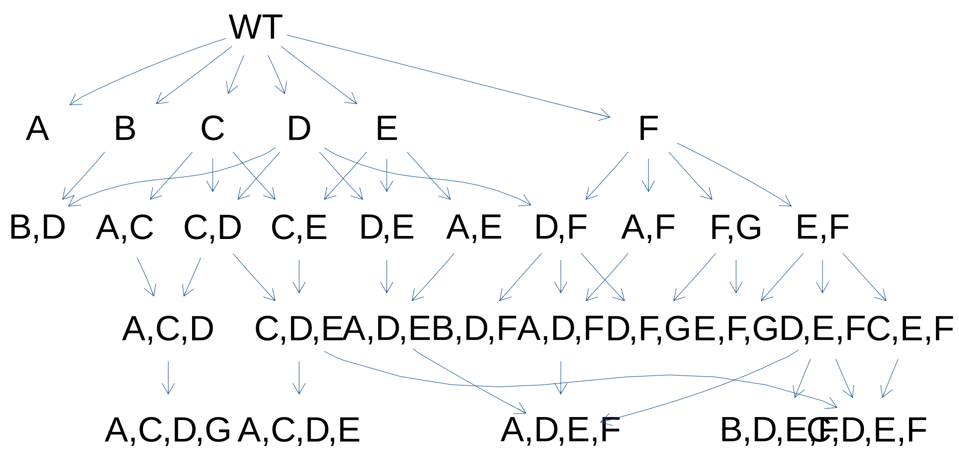

ID	L53nf1rd3BsJMUlj
acc genotypes	53
peaks no back	20
removed edges	NA
prop rem edges	NA

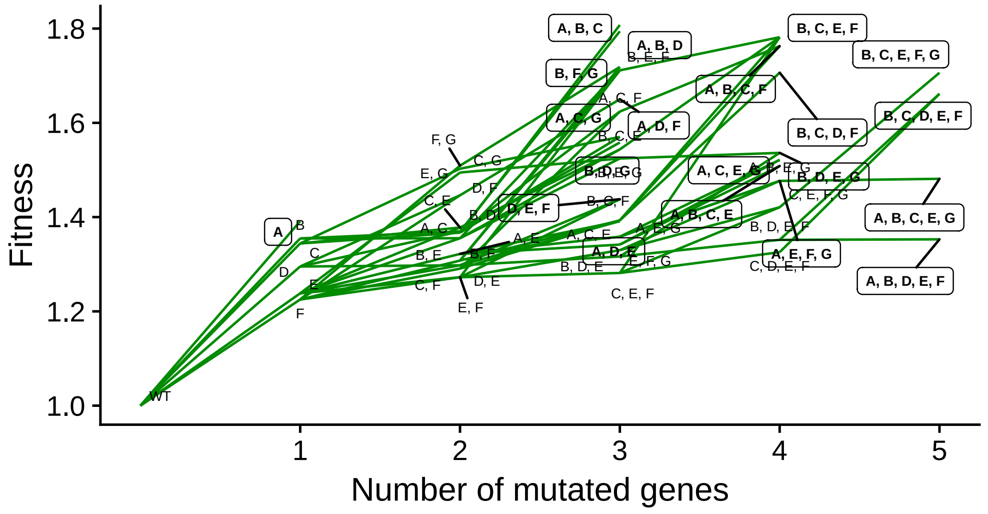

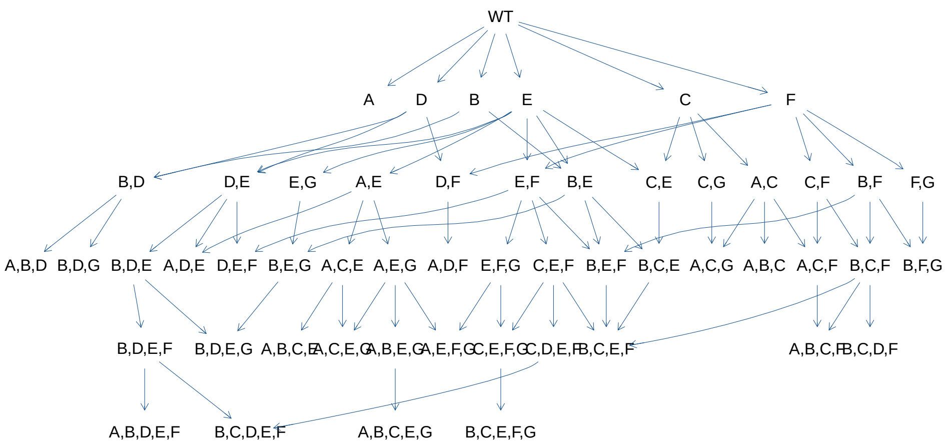

ID	L96VgnsLKl1UgcXs
acc genotypes	29
peaks no back	10
removed edges	NA
prop rem edges	NA

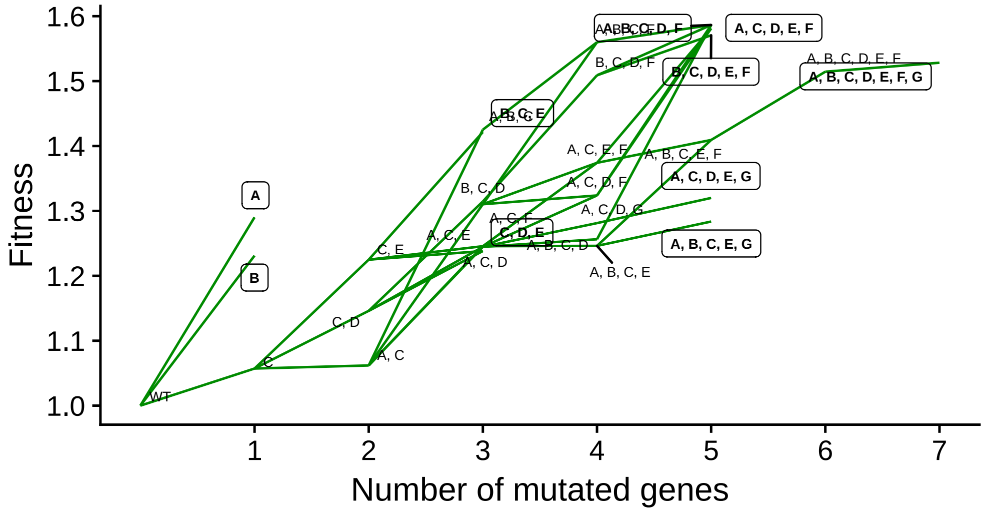

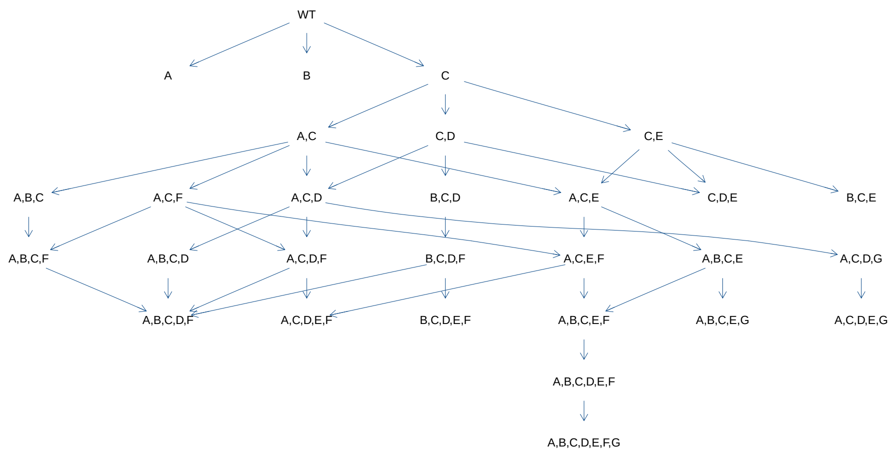

ID	lG7VsdckSOb10Utw8
acc genotypes	40
peaks no back	13
removed edges	NA
prop rem edges	NA

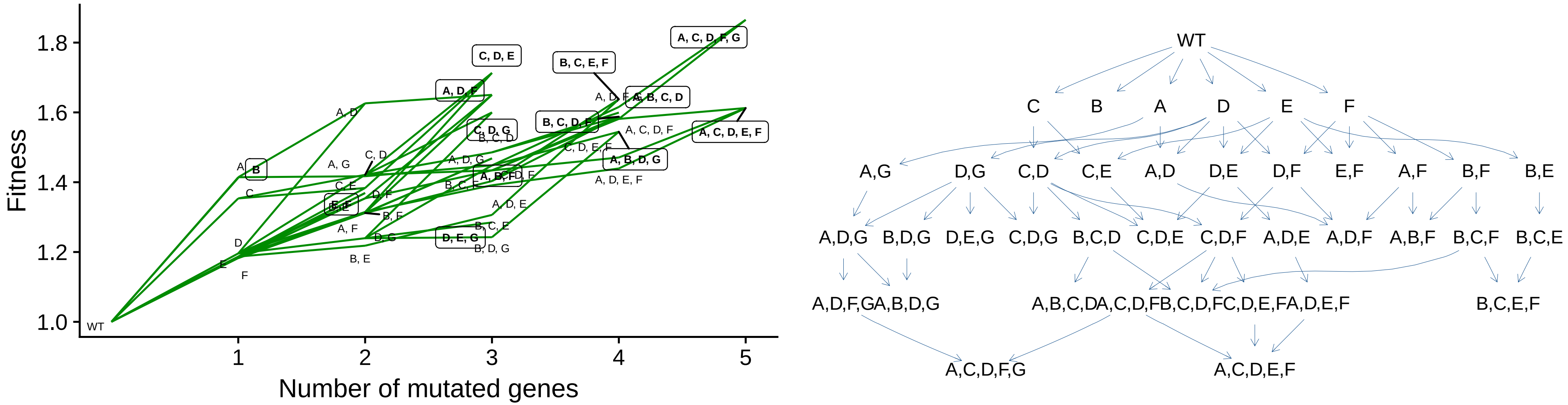

ID	LJ27jI8rSPxKS2A9
acc genotypes	52
peaks no back	19
removed edges	NA
prop rem edges	NA

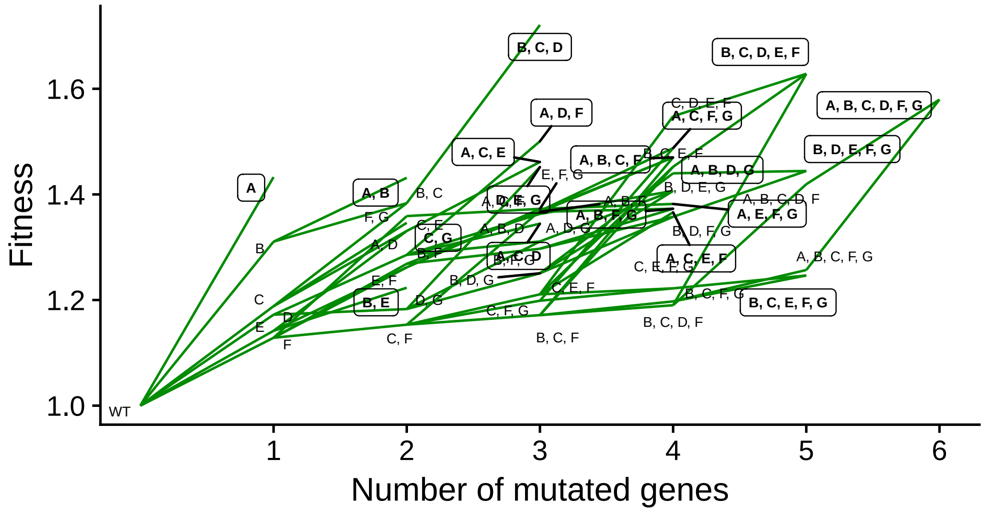

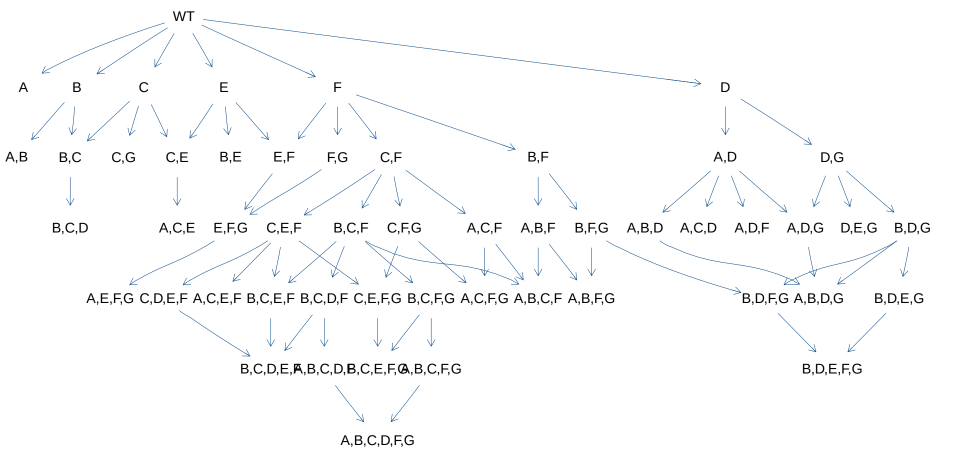

ID	M19kX109AWS4lET93
acc genotypes	21
peaks no back	10
removed edges	NA
prop rem edges	NA

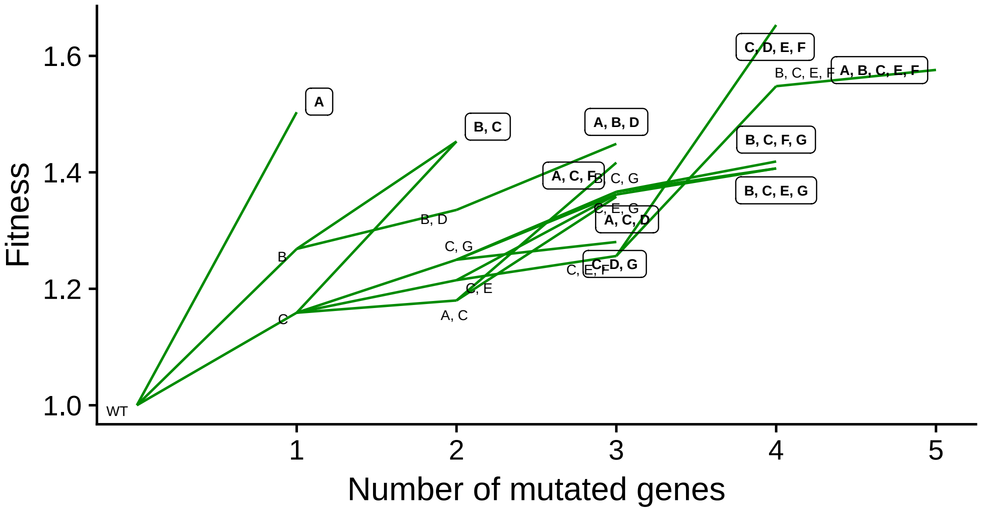

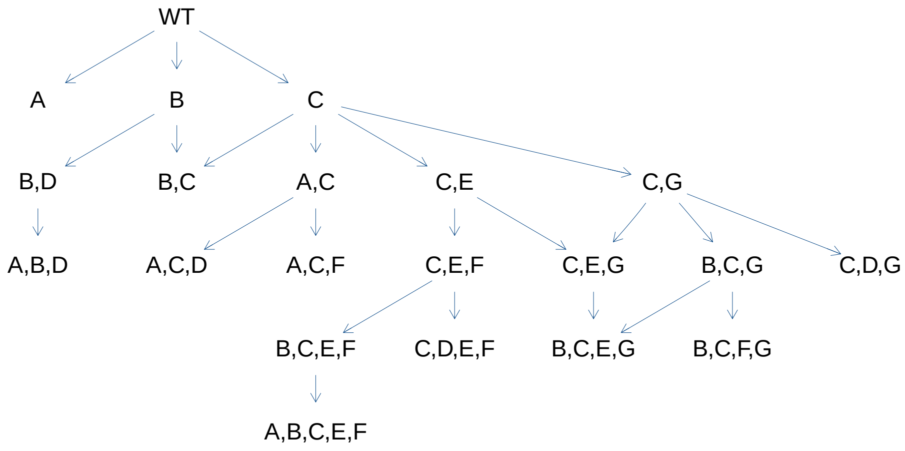

ID	mBMwivnTLA1F1fOQ
acc genotypes	26
peaks no back	11
removed edges	NA
prop rem edges	NA

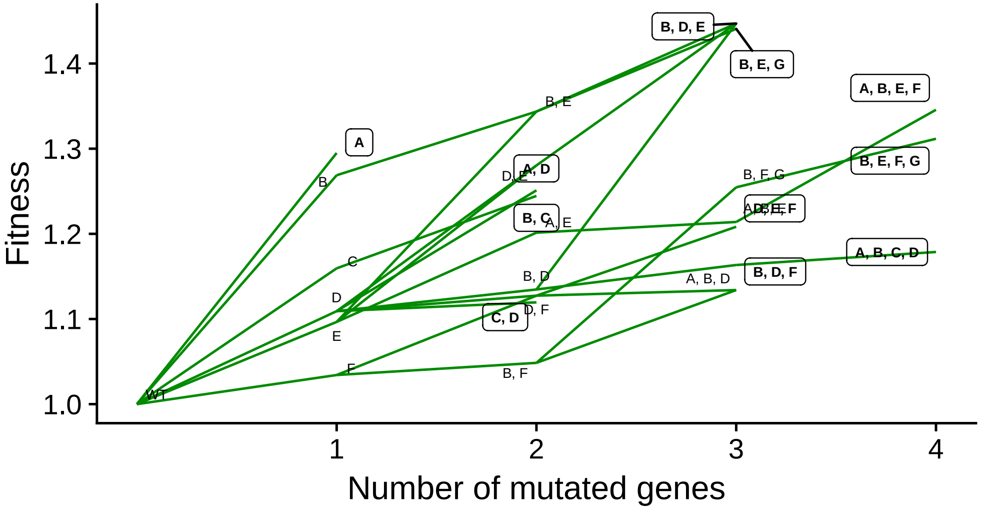

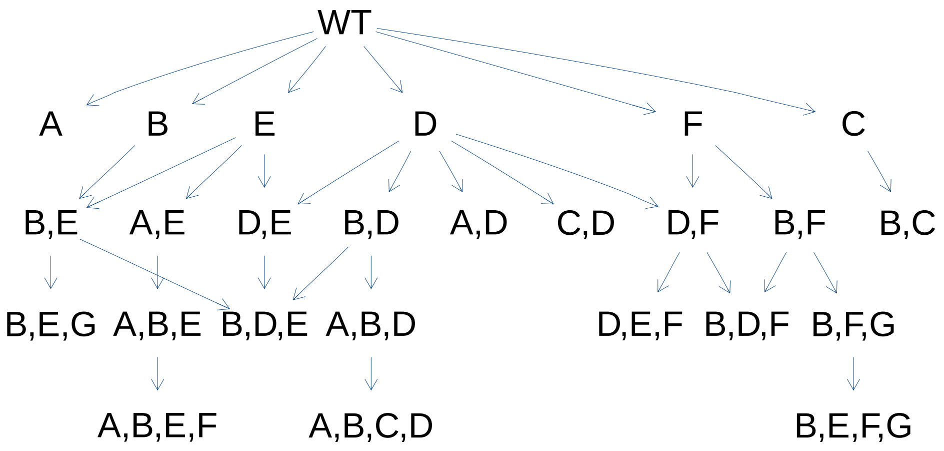

ID	MPJBgvRZOA1Xp4SQ
acc genotypes	53
peaks no back	19
removed edges	NA
prop rem edges	NA

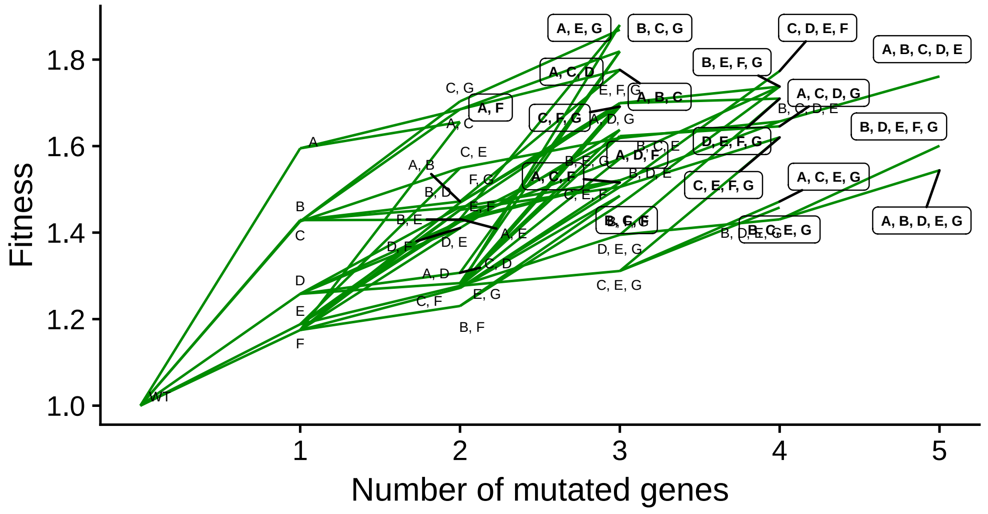

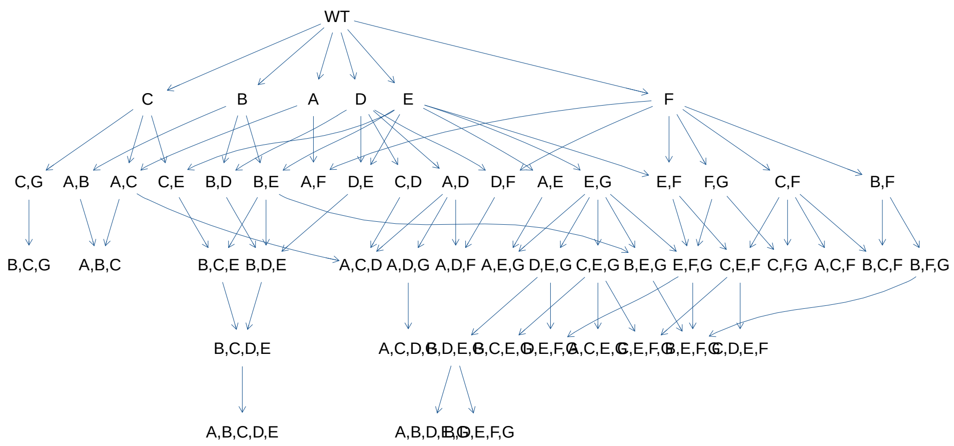

ID	Mq3VEO6q8h6j5Q2K
acc genotypes	43
peaks no back	17
removed edges	NA
prop rem edges	NA

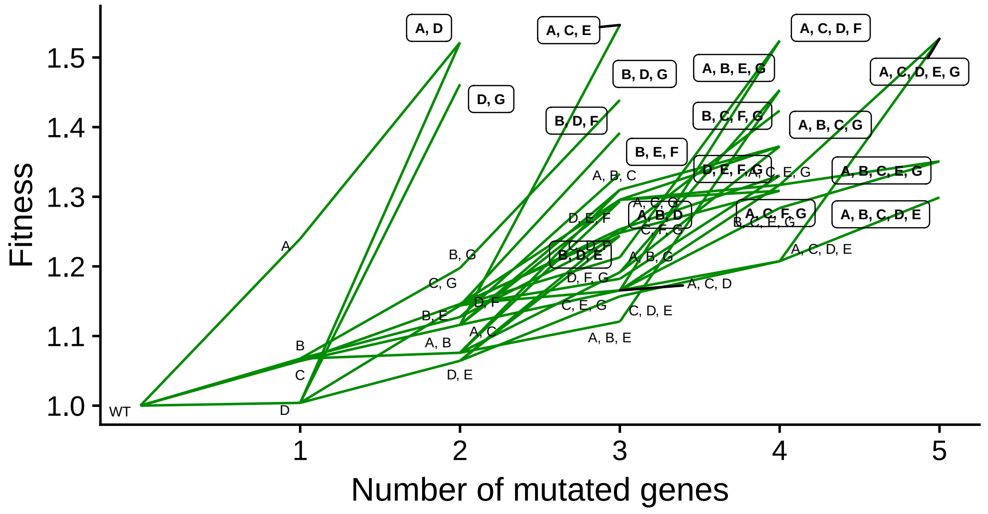

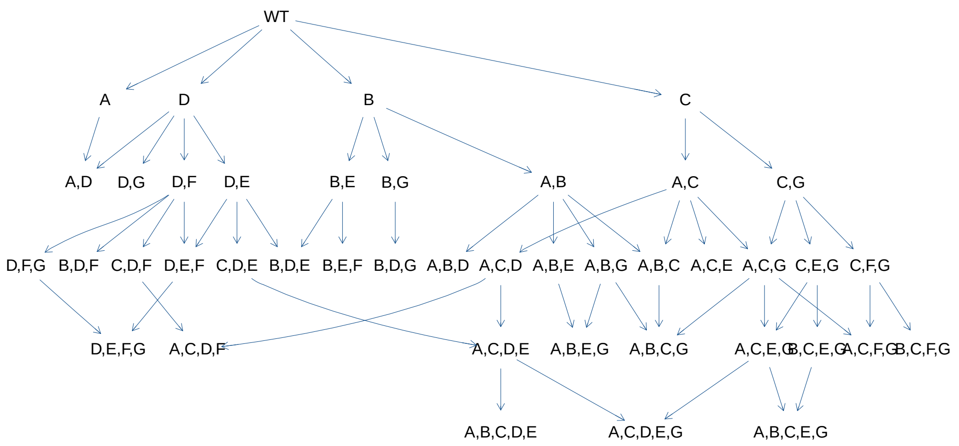

ID	mrmstEuzwuuKXnIi
acc genotypes	64
peaks no back	18
removed edges	NA
prop rem edges	NA

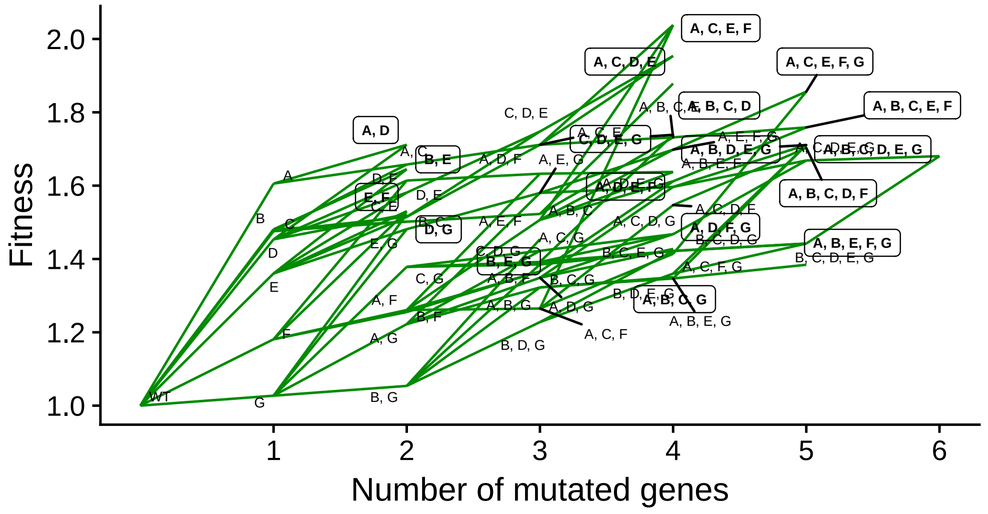

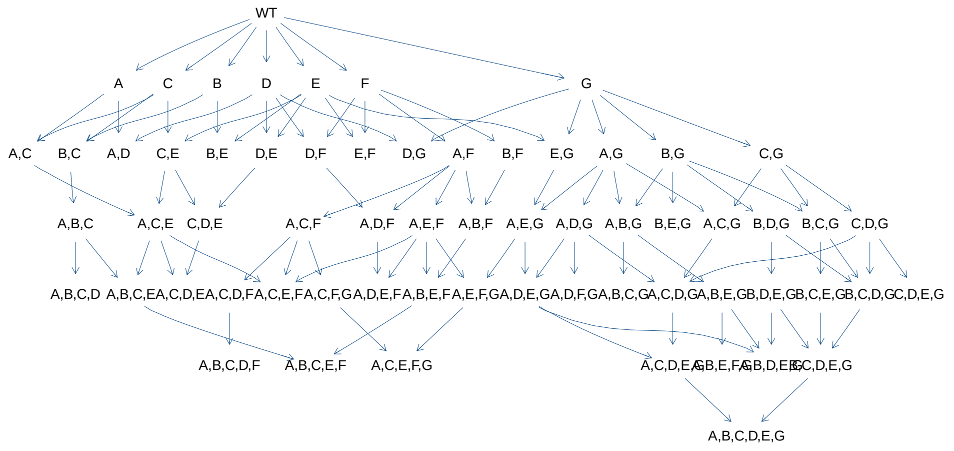

ID	mTWr1XzBqvHzZVjW
acc genotypes	63
peaks no back	22
removed edges	NA
prop rem edges	NA

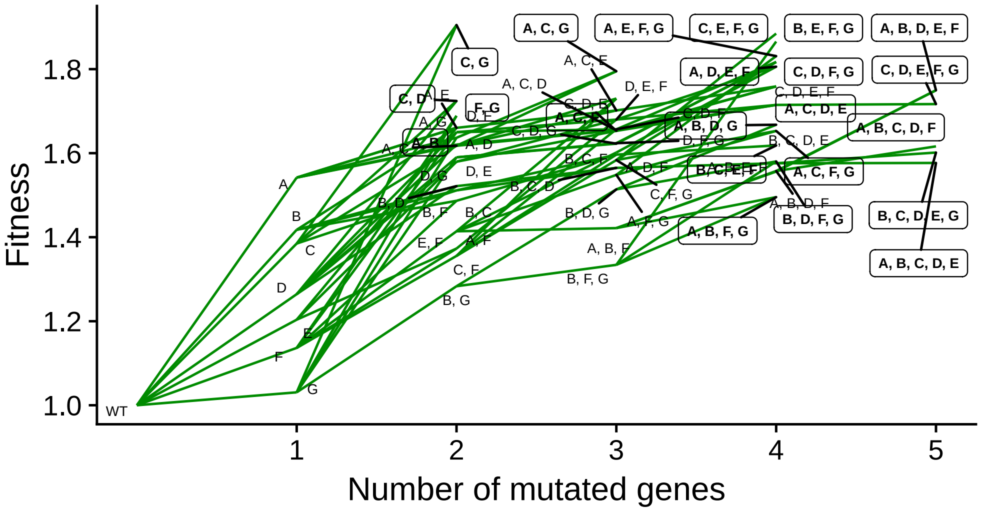

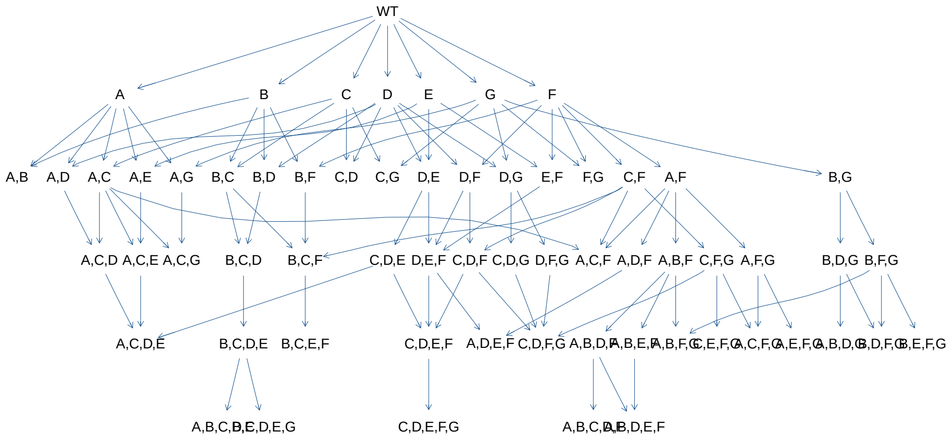

ID	mwHTaW8c10WKVxeLt
acc genotypes	71
peaks no back	22
removed edges	NA
prop rem edges	NA

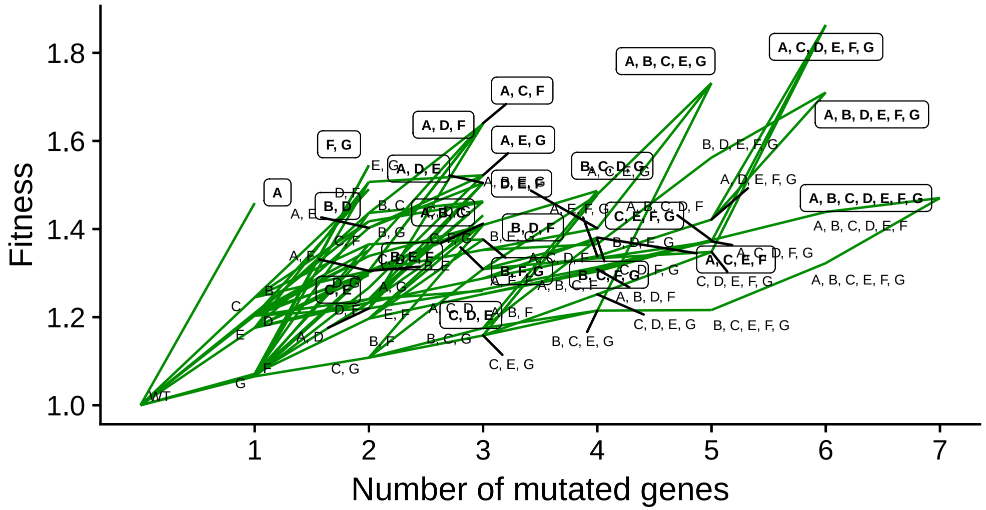

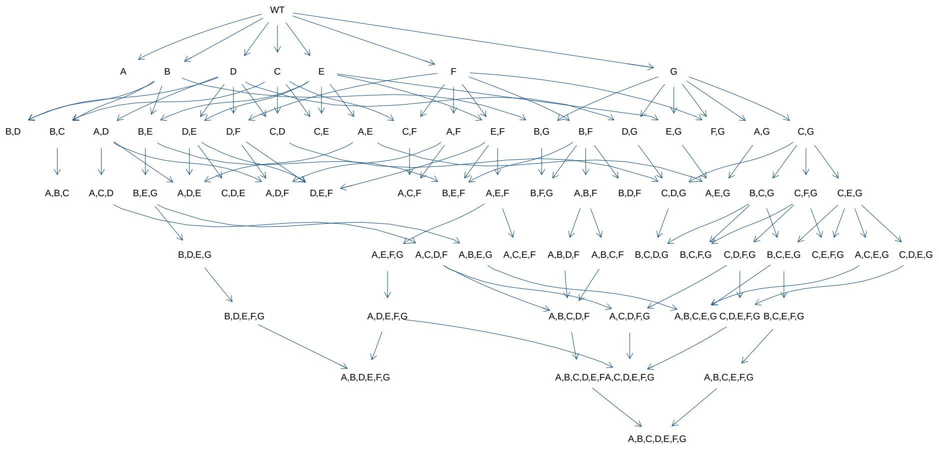

ID	n2WFR10n5VPYOSSSt
acc genotypes	61
peaks no back	17
removed edges	NA
prop rem edges	NA

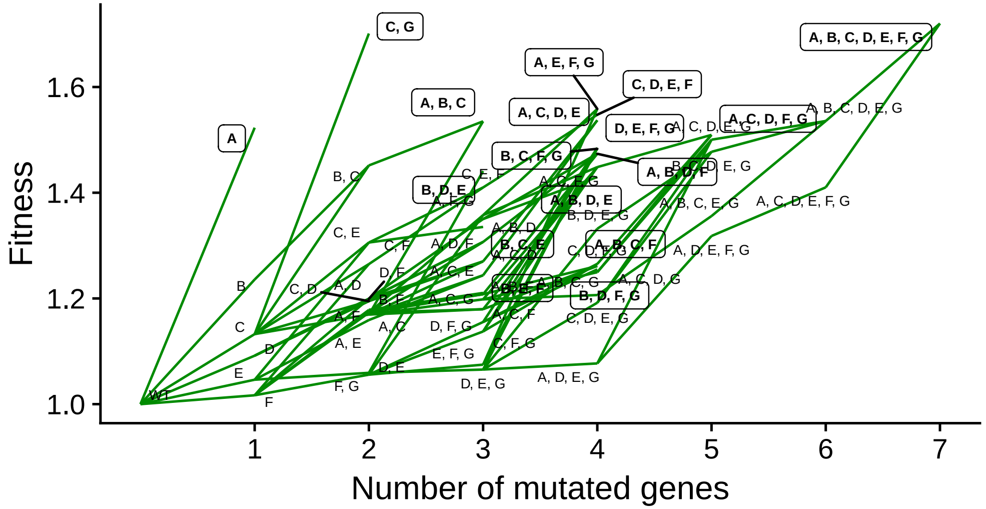

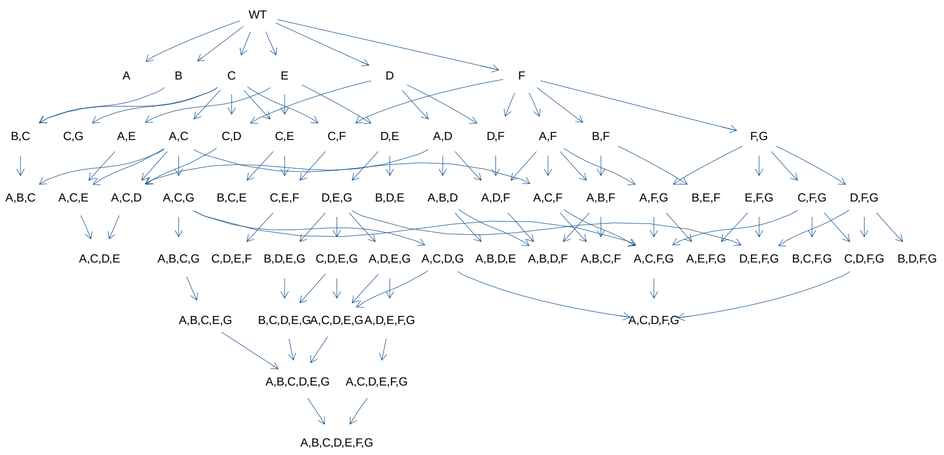

ID	NejgQ4lbqE6syfEk
acc genotypes	60
peaks no back	22
removed edges	NA
prop rem edges	NA

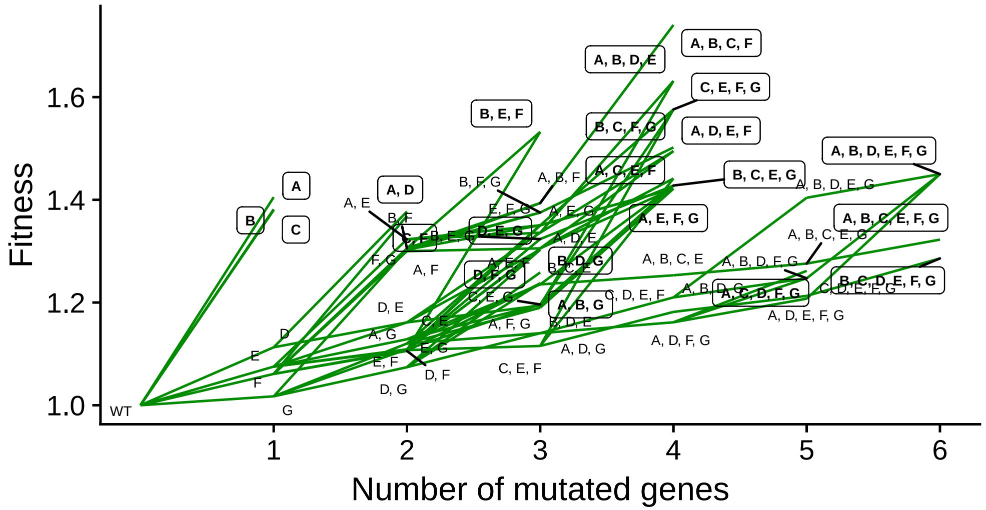

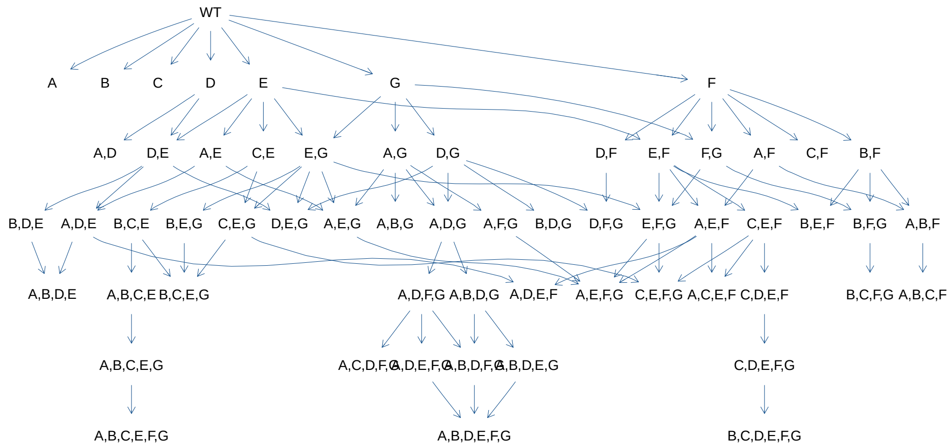

ID	nIkUt9Az1n9o6UtN
acc genotypes	40
peaks no back	17
removed edges	NA
prop rem edges	NA

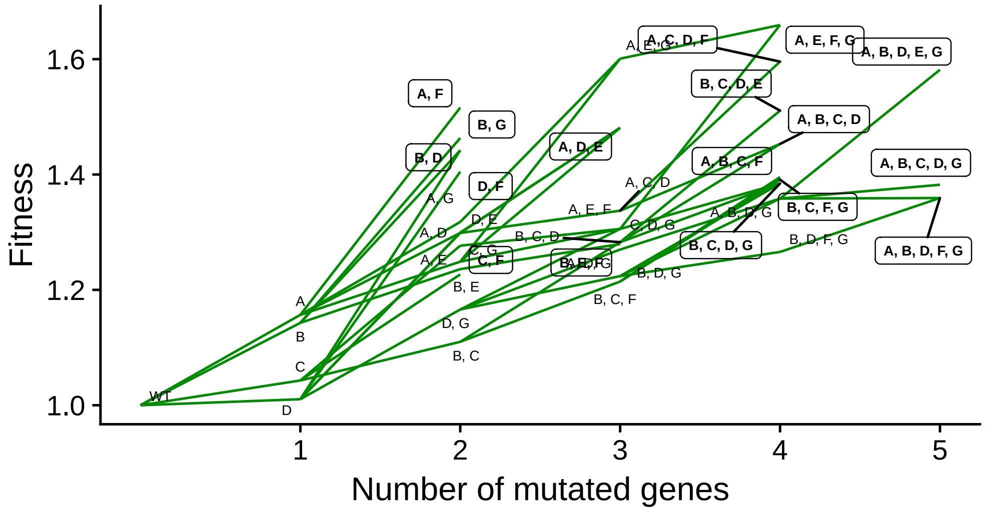

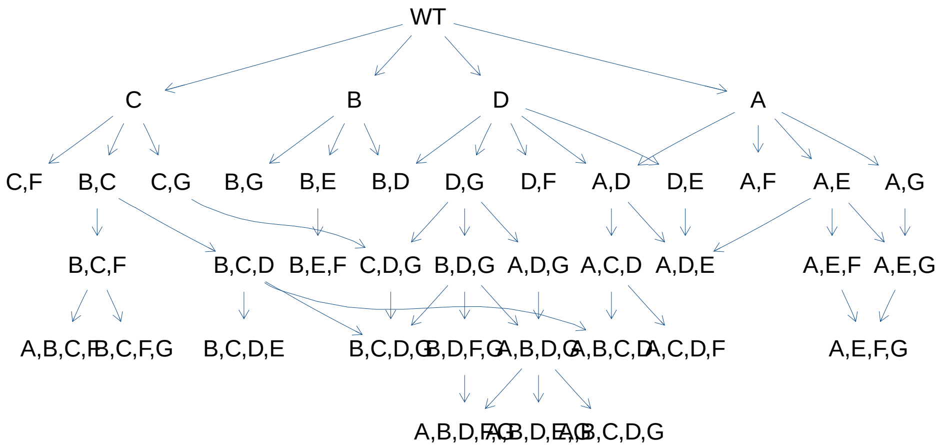

ID	NLcu10ZEMImemfBBs
acc genotypes	32
peaks no back	14
removed edges	NA
prop rem edges	NA

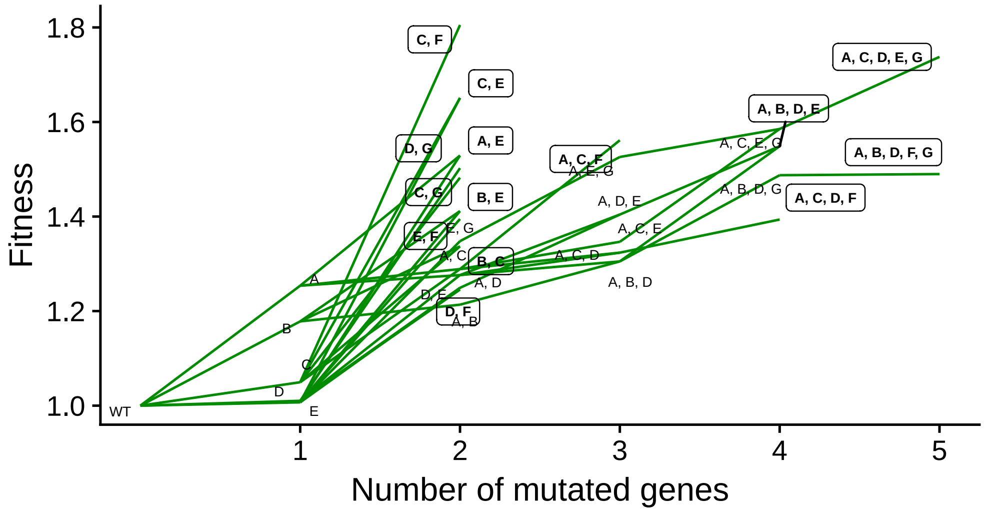

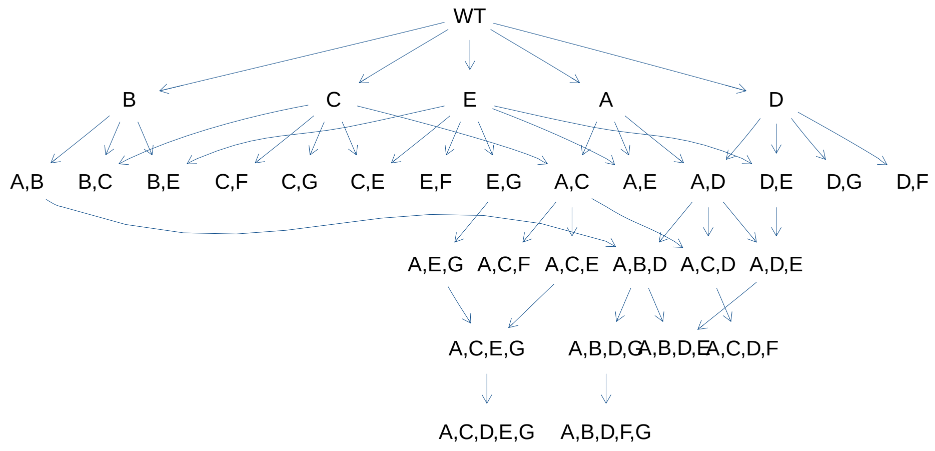

ID	nlx10k3xX8bswraYG
acc genotypes	19
peaks no back	5
removed edges	NA
prop rem edges	NA

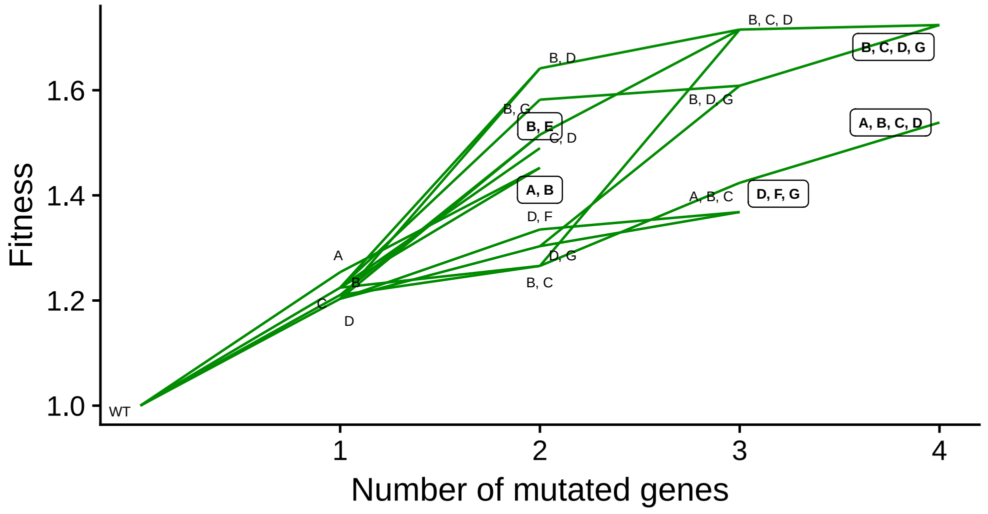

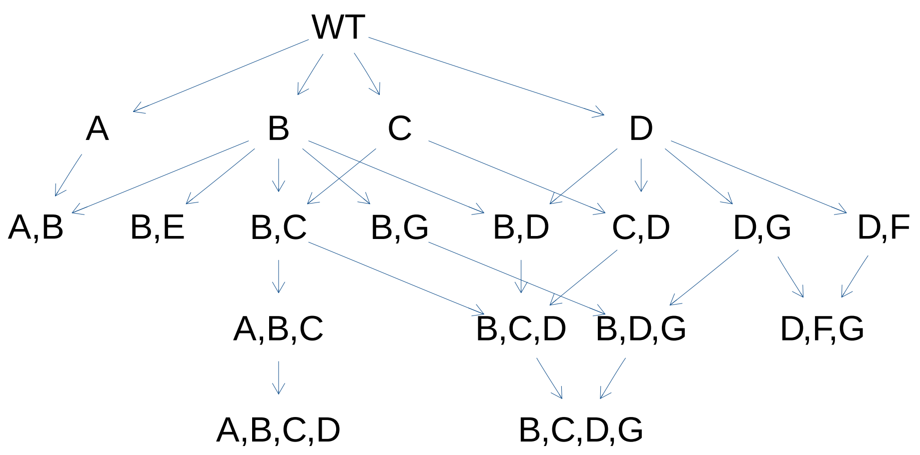

ID	Nq9C7m8MciTio6I4
acc genotypes	45
peaks no back	13
removed edges	NA
prop rem edges	NA

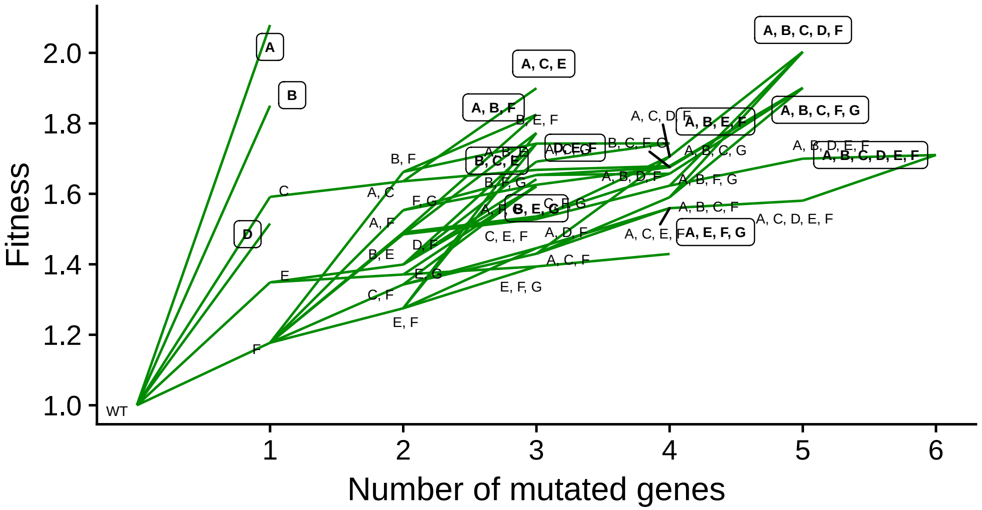

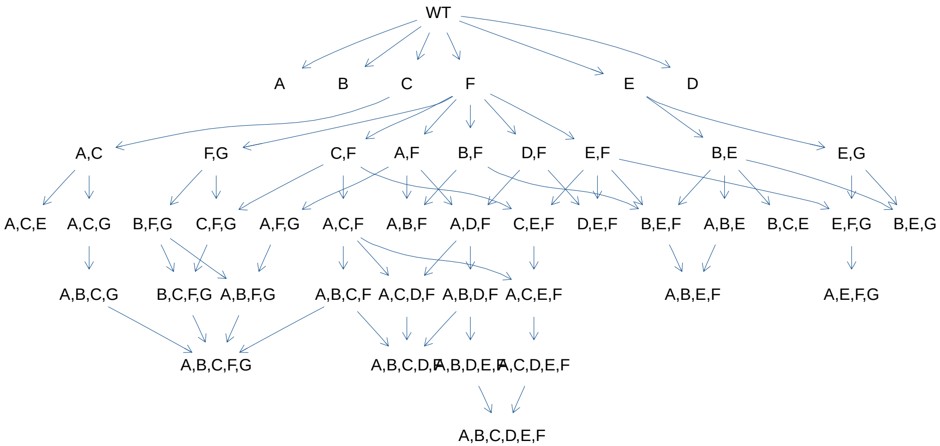

ID	NuYHUmW2Daw10xoxW
acc genotypes	61
peaks no back	15
removed edges	NA
prop rem edges	NA

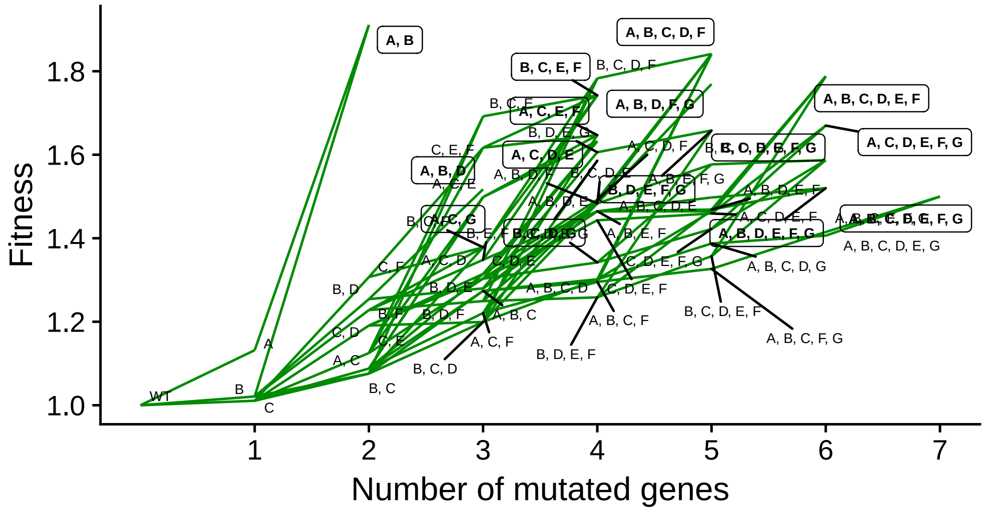

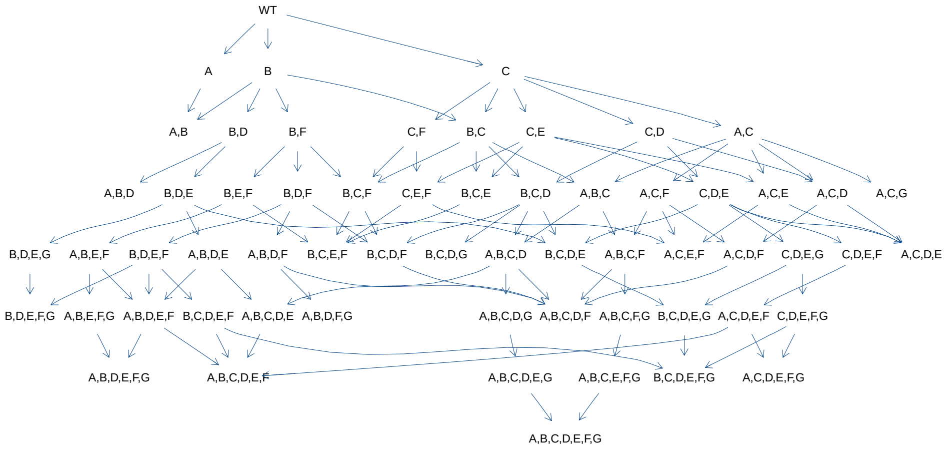

ID	nwLrcLgueac4e3RH
acc genotypes	57
peaks no back	16
removed edges	NA
prop rem edges	NA

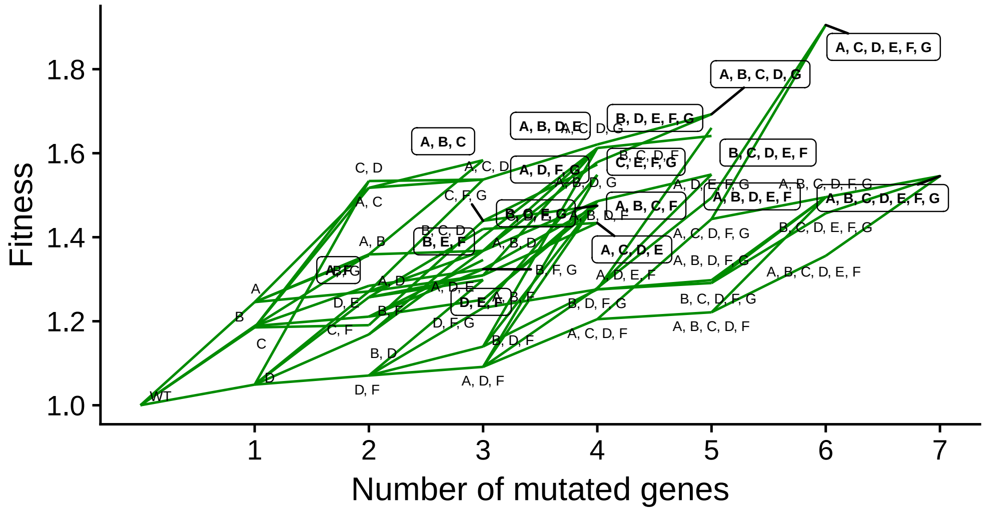

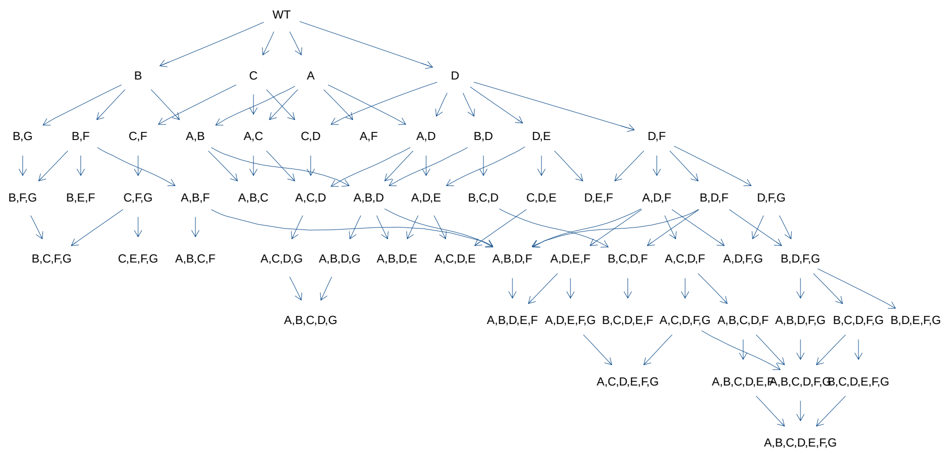

ID	om3Pj5IphjvaQhAk
acc genotypes	82
peaks no back	27
removed edges	NA
prop rem edges	NA

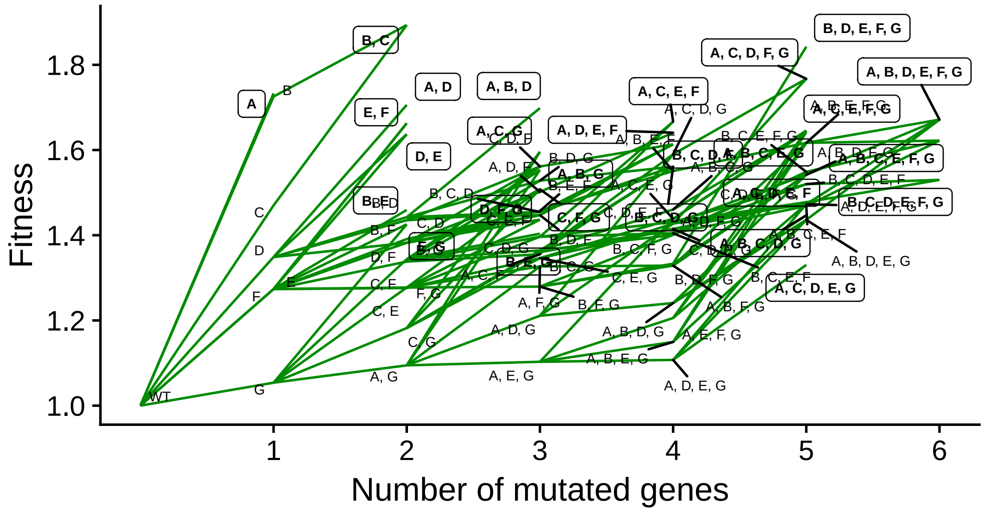

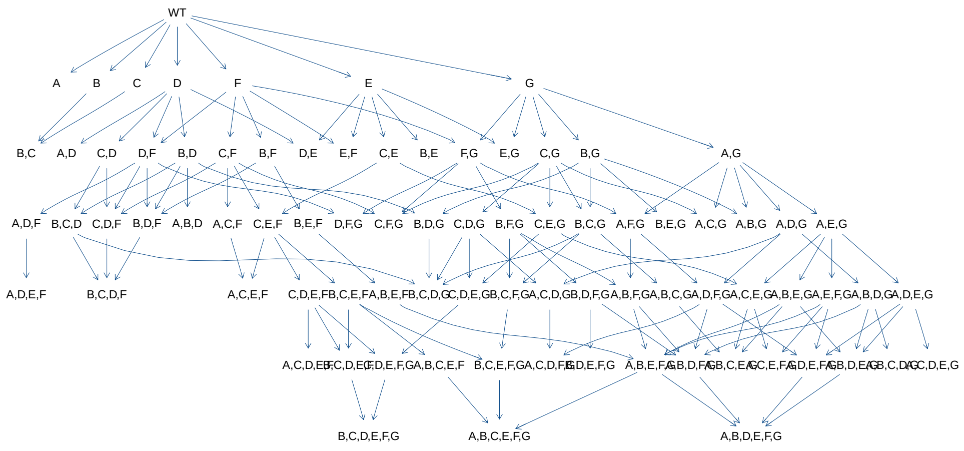

ID	OsDpSWcfqKrGoOGX
acc genotypes	31
peaks no back	10
removed edges	NA
prop rem edges	NA

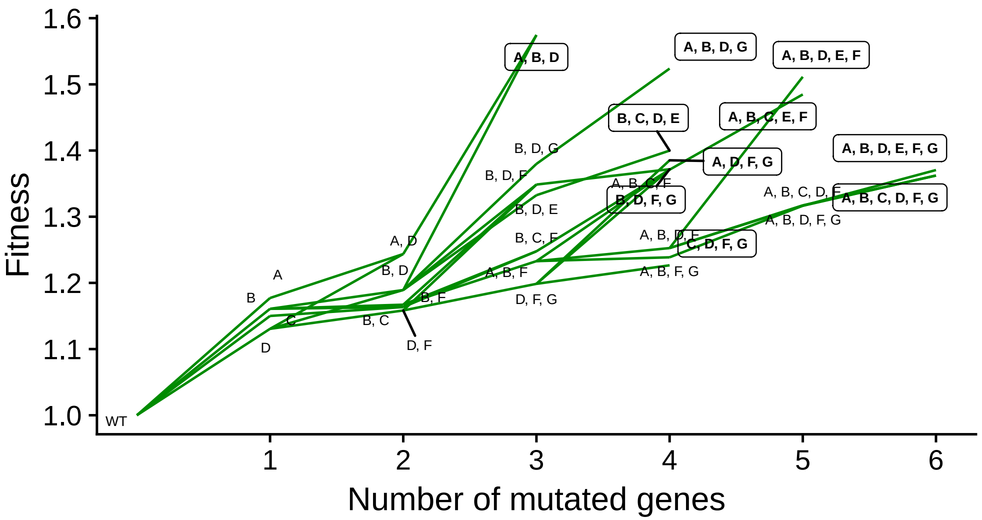

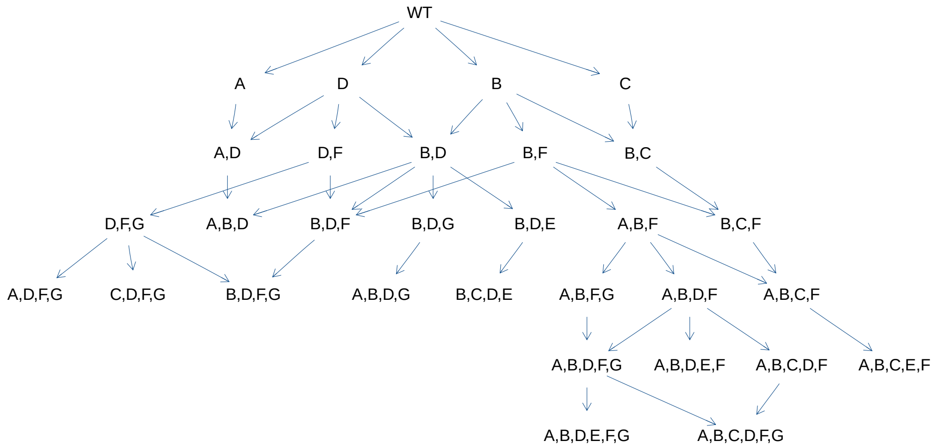

ID	ozA8bz3JzJBjMoZP
acc genotypes	38
peaks no back	11
removed edges	NA
prop rem edges	NA

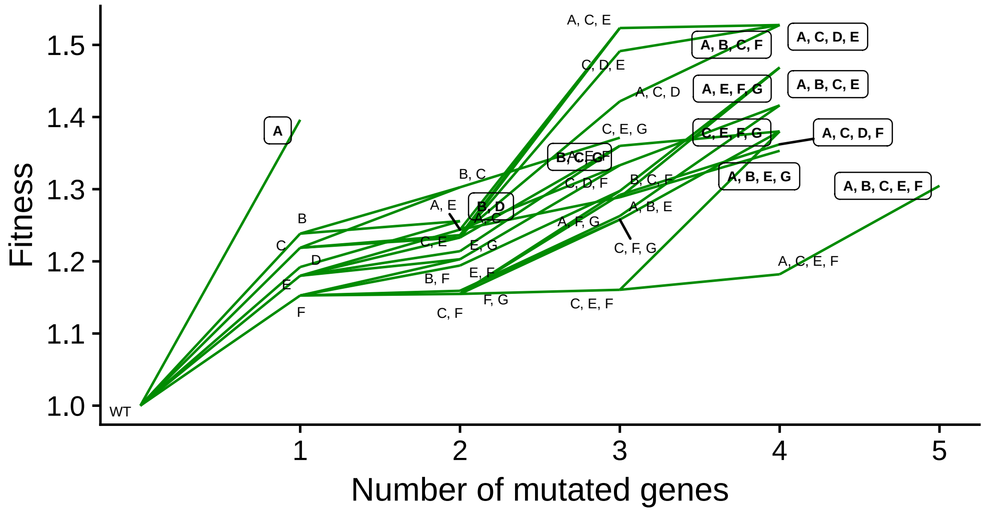

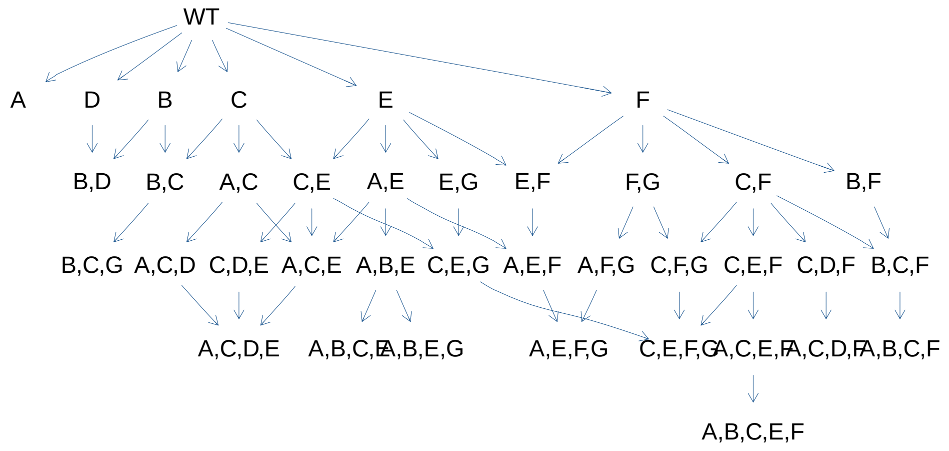

ID	p2rZTfzQSZaEzk7D
acc genotypes	42
peaks no back	11
removed edges	NA
prop rem edges	NA

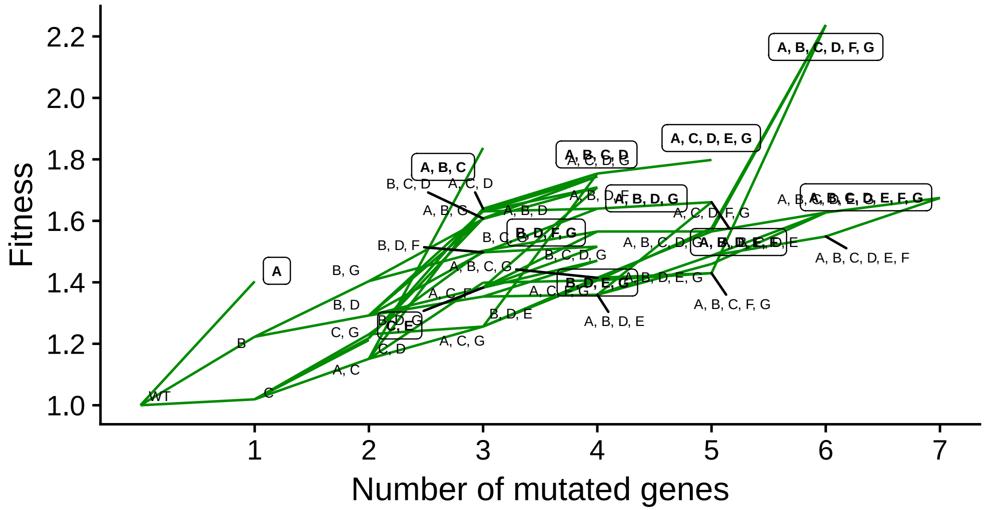

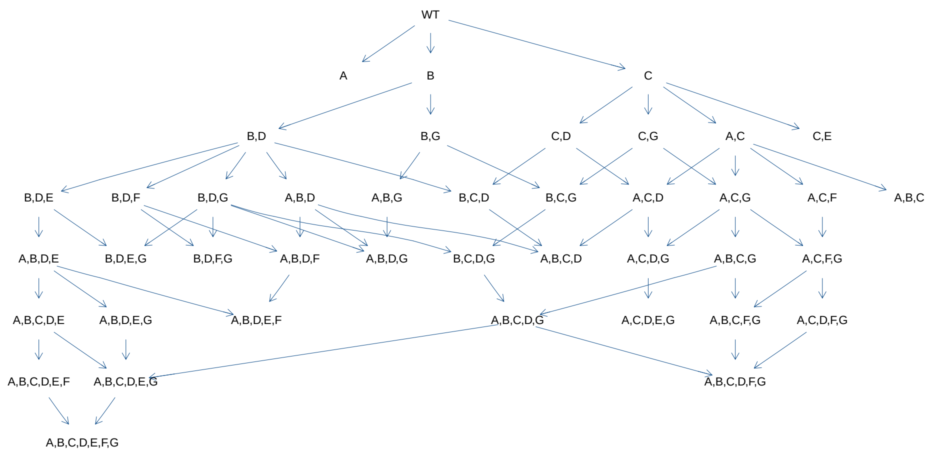

ID	PdBS51bgDdPXdV8W
acc genotypes	33
peaks no back	10
removed edges	NA
prop rem edges	NA

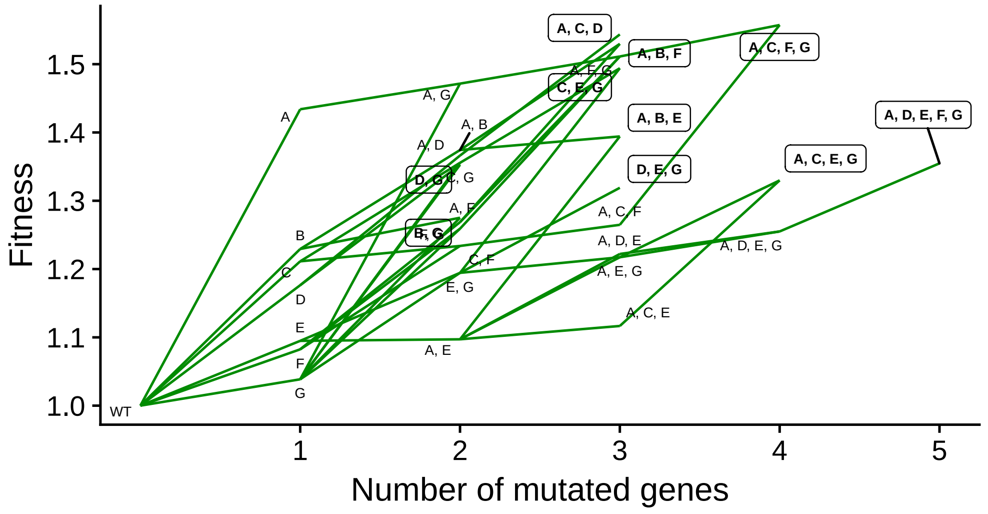

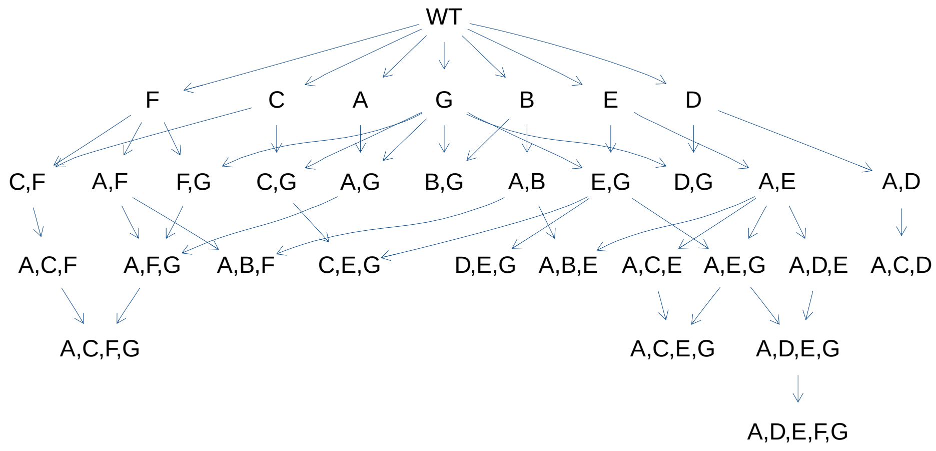

ID	PdEpnOBgly104Dp4A
acc genotypes	50
peaks no back	16
removed edges	NA
prop rem edges	NA

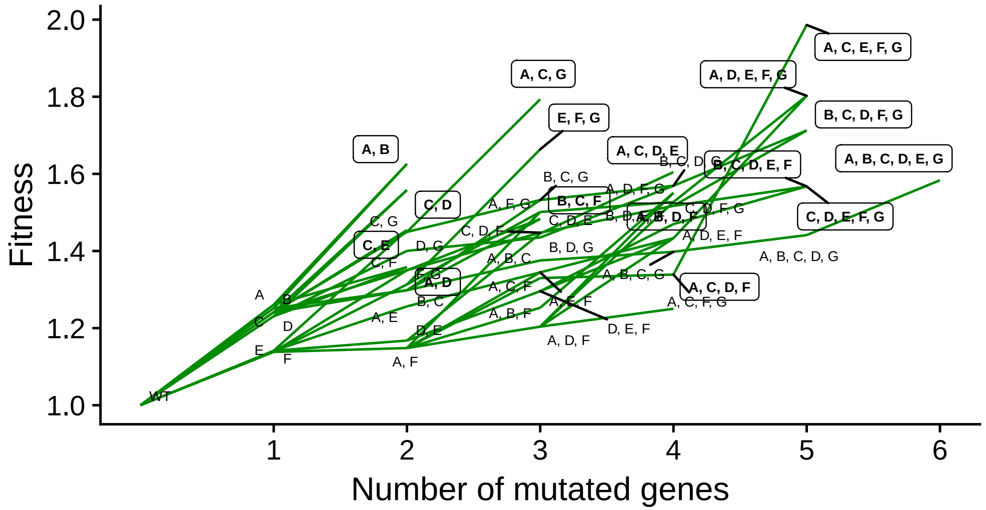

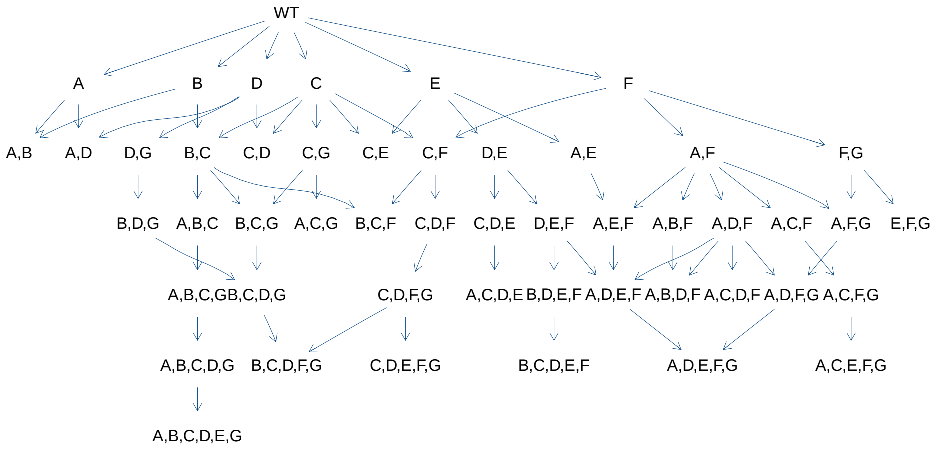

ID	pdLy5HbuIEcGMFw8
acc genotypes	39
peaks no back	12
removed edges	NA
prop rem edges	NA

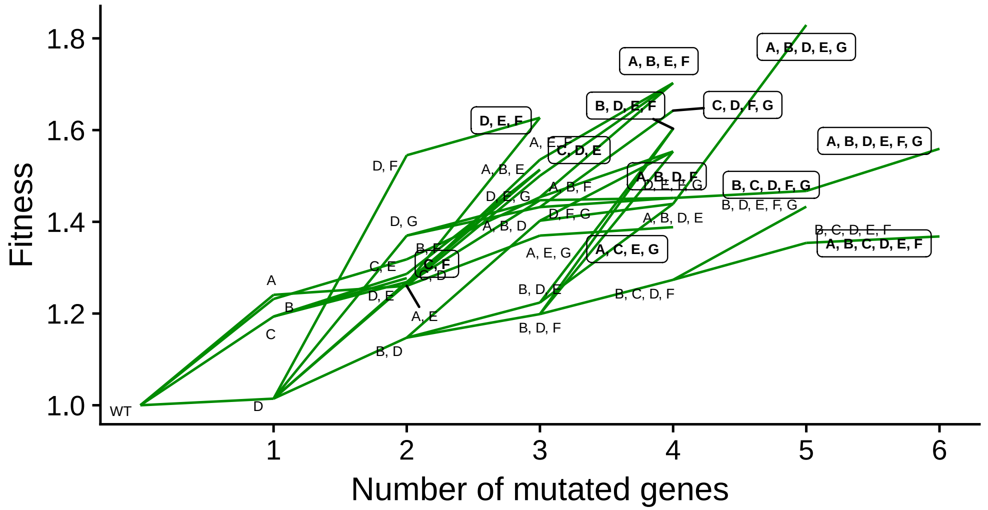

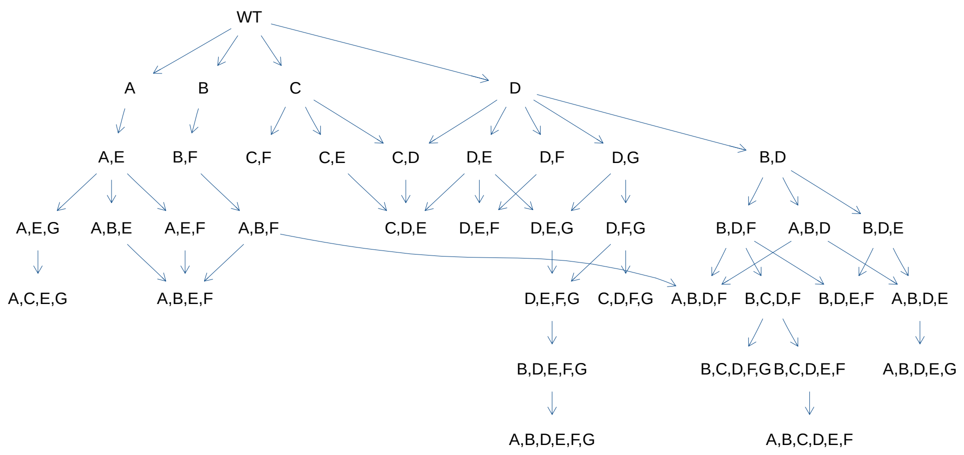

ID	PMnxoLN1P10vNrmKW
acc genotypes	22
peaks no back	8
removed edges	NA
prop rem edges	NA

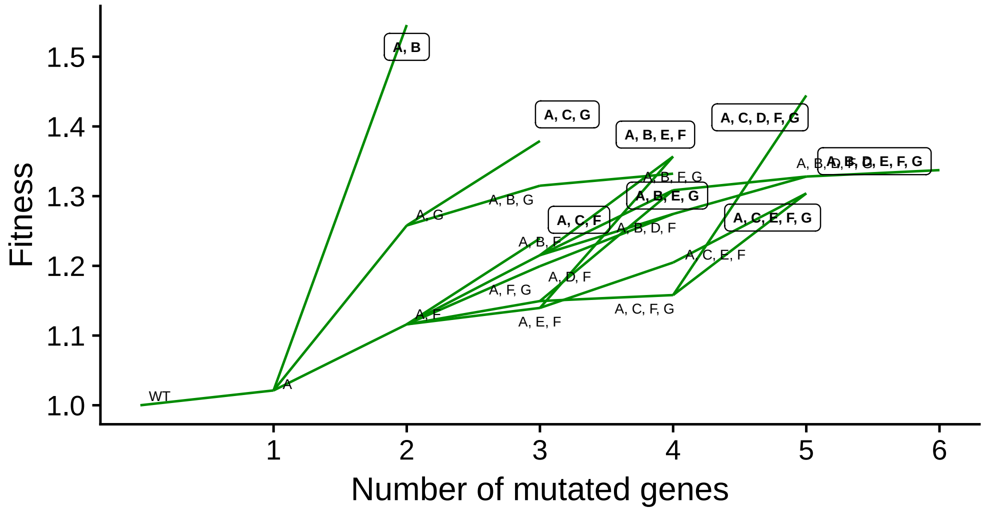

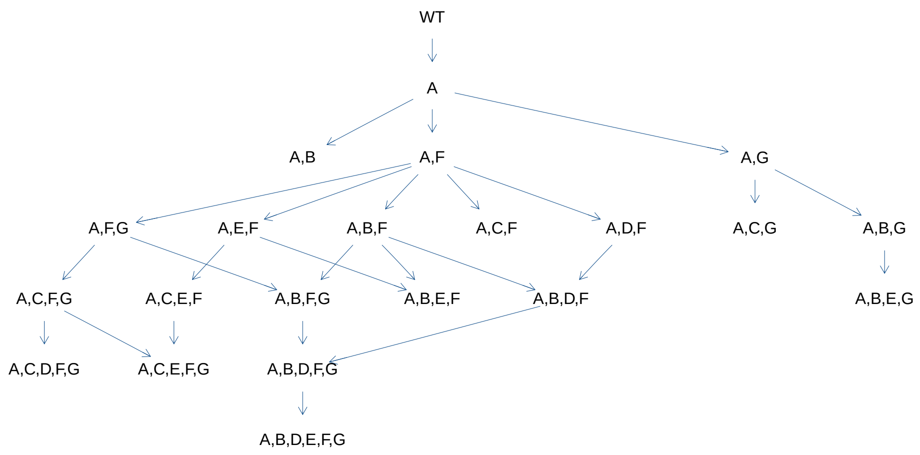

ID	poffFlI3VRWbB7qo
acc genotypes	49
peaks no back	17
removed edges	NA
prop rem edges	NA

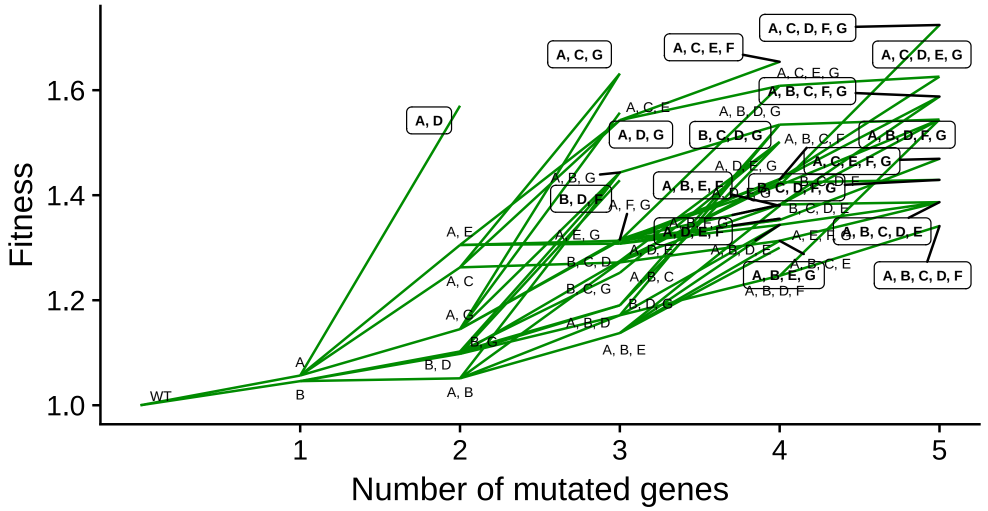

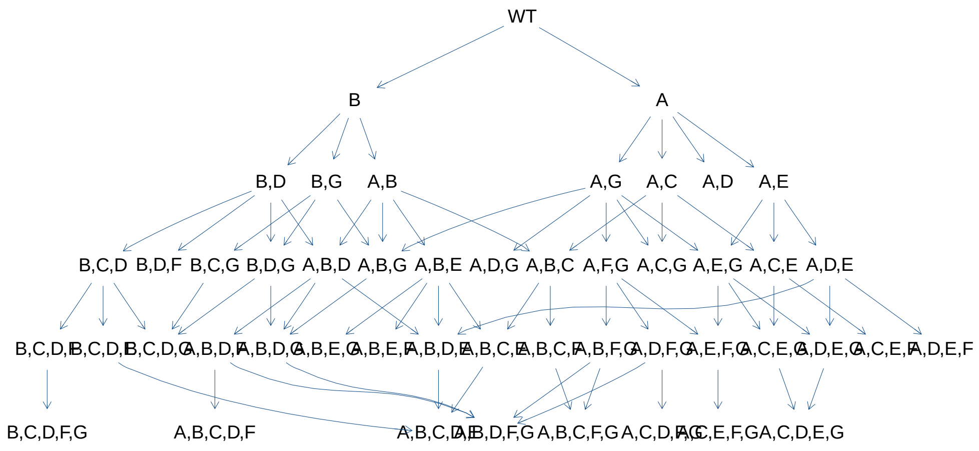

ID	pZAMLLsskT5d10Q10B
acc genotypes	41
peaks no back	16
removed edges	NA
prop rem edges	NA

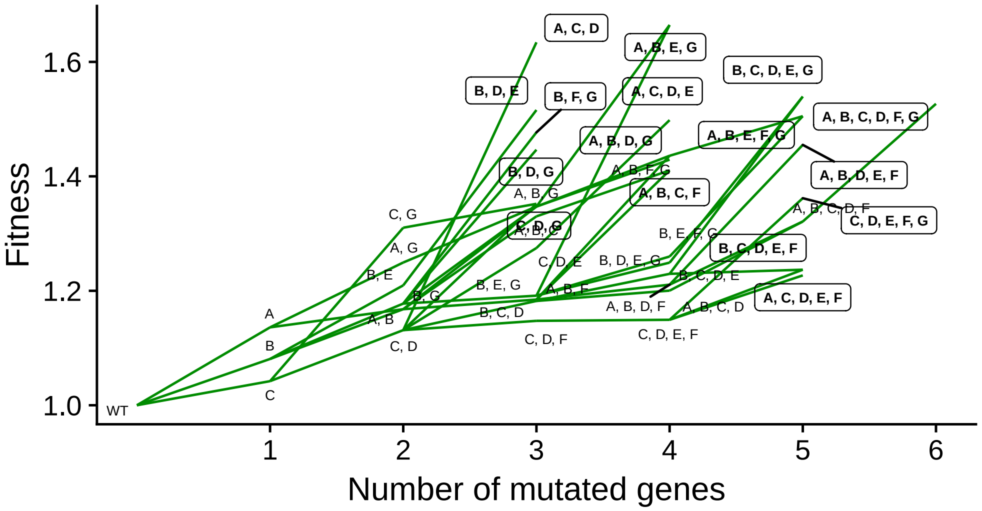

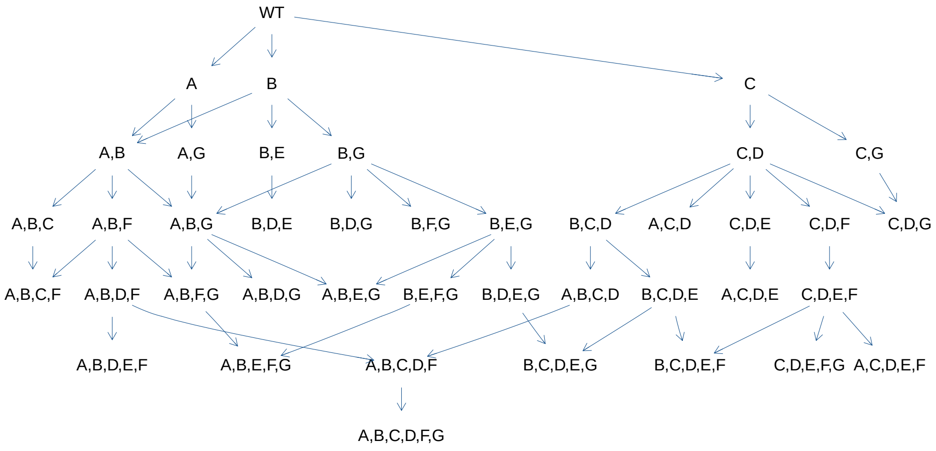

ID	QdO42eriCLzJlKPT
acc genotypes	56
peaks no back	22
removed edges	NA
prop rem edges	NA

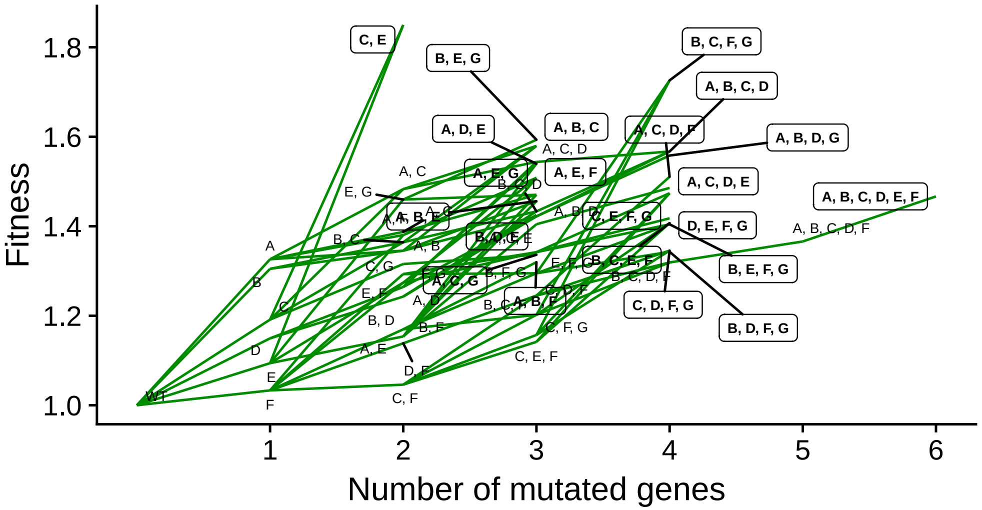

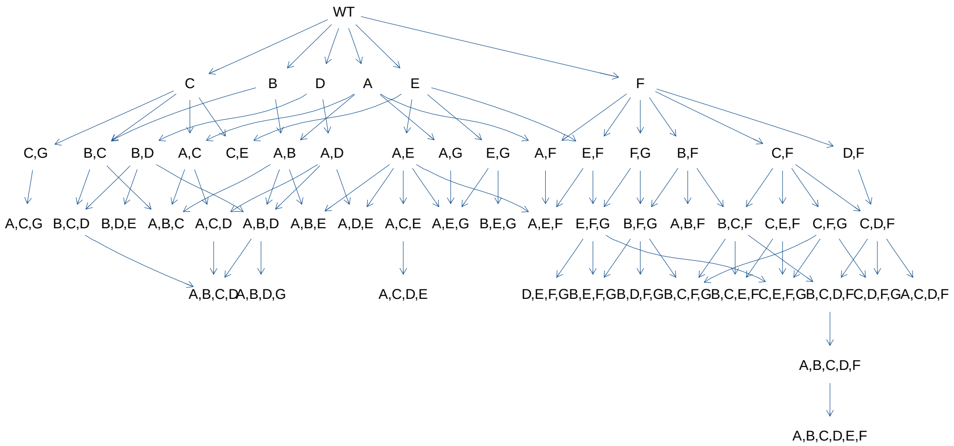

ID	QDPtbXAp8i6koPnT
acc genotypes	18
peaks no back	8
removed edges	NA
prop rem edges	NA

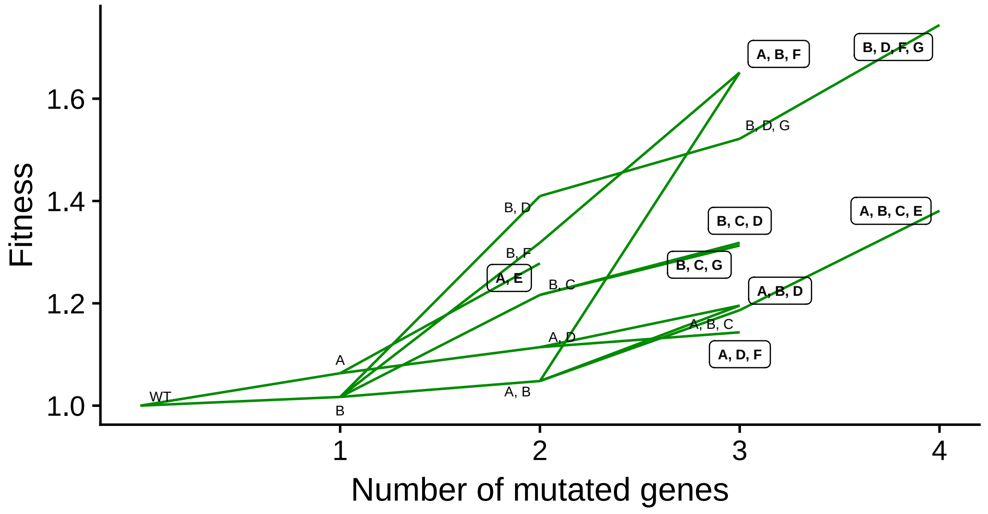

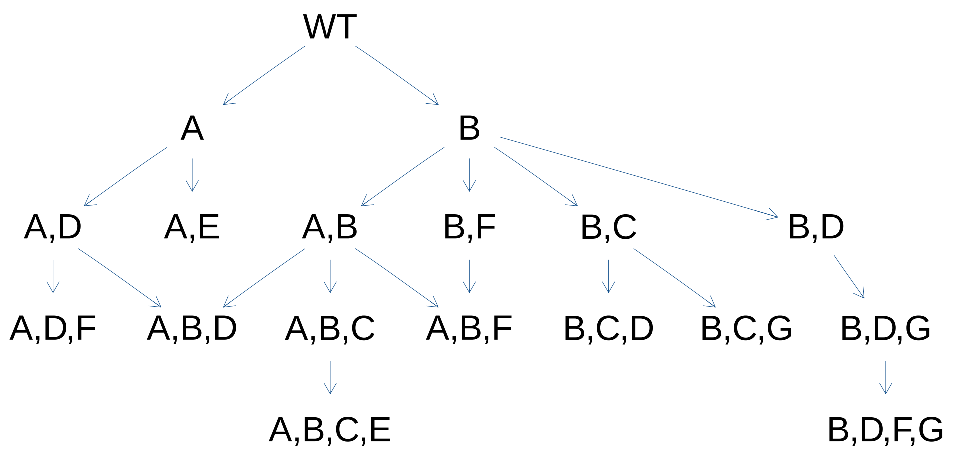

ID	QIQaxajtj6kjit1V
acc genotypes	27
peaks no back	10
removed edges	NA
prop rem edges	NA

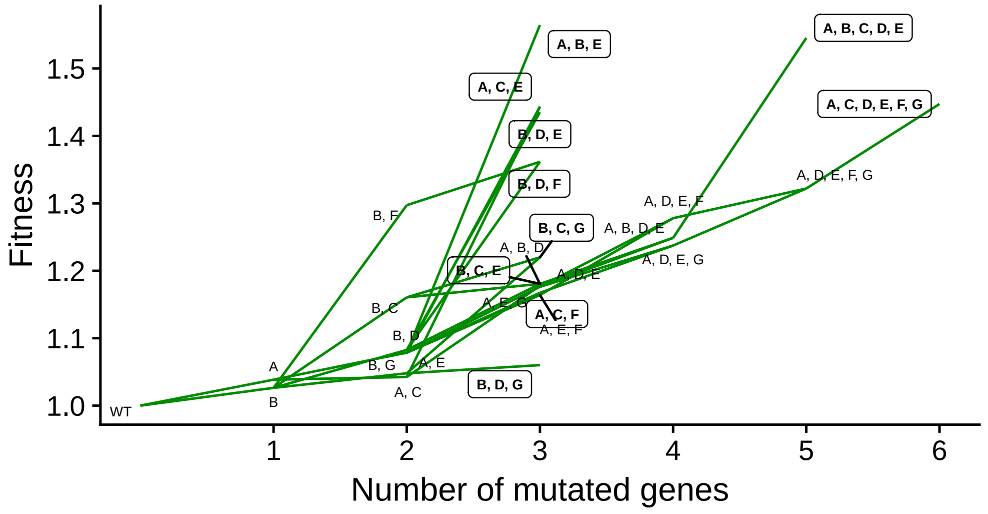

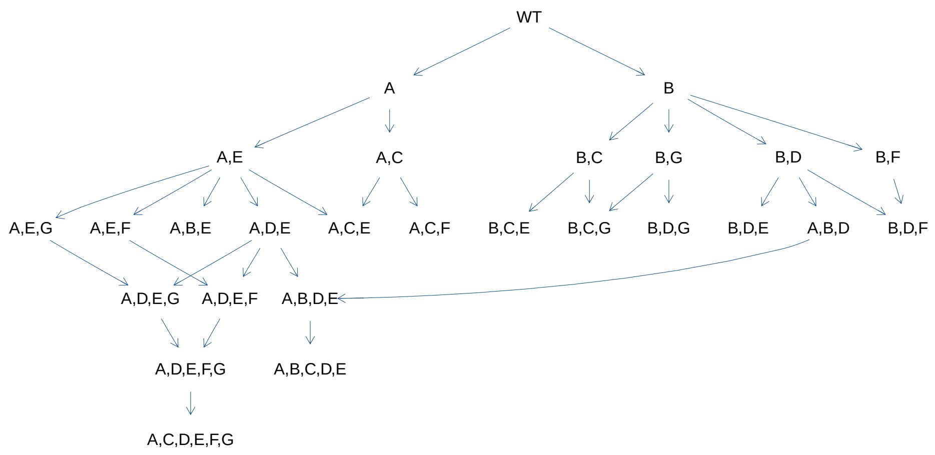

ID	QTFRtRudotyAGuqB
acc genotypes	55
peaks no back	18
removed edges	NA
prop rem edges	NA

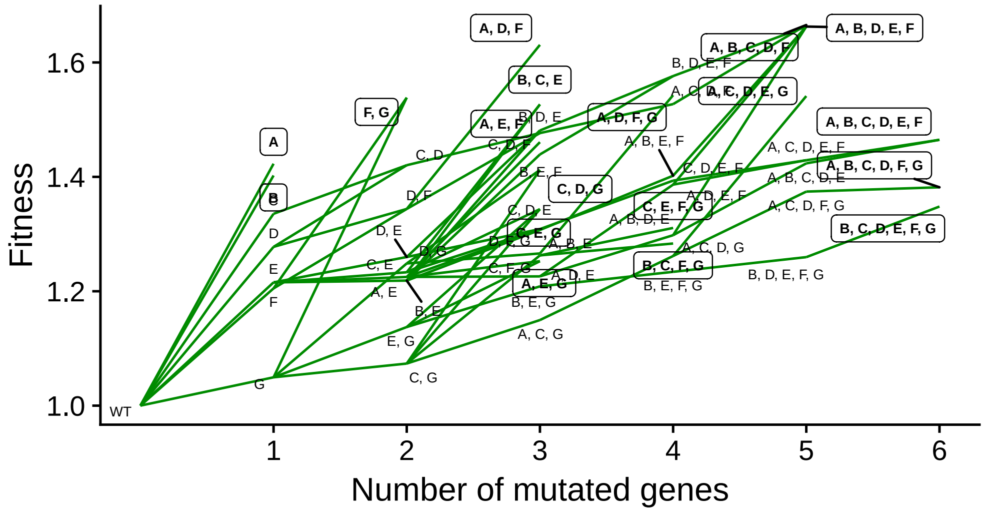

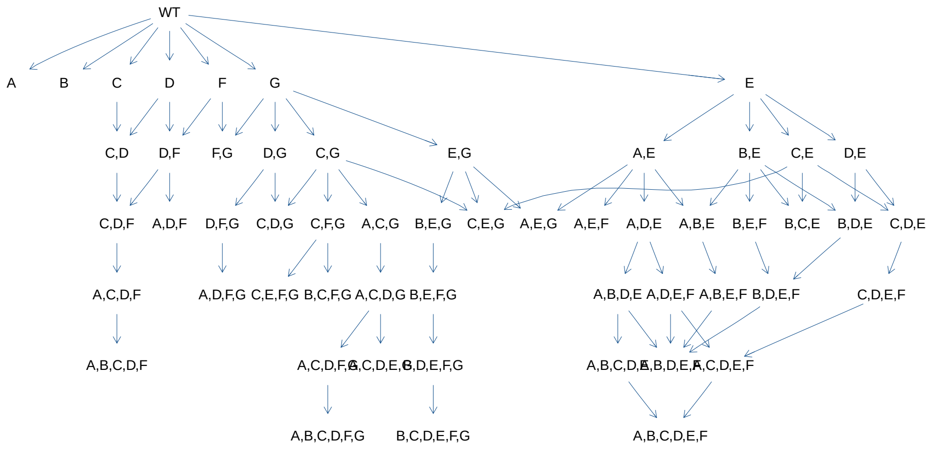

ID	qUmaRIOlDCO4dtyu
acc genotypes	44
peaks no back	17
removed edges	NA
prop rem edges	NA

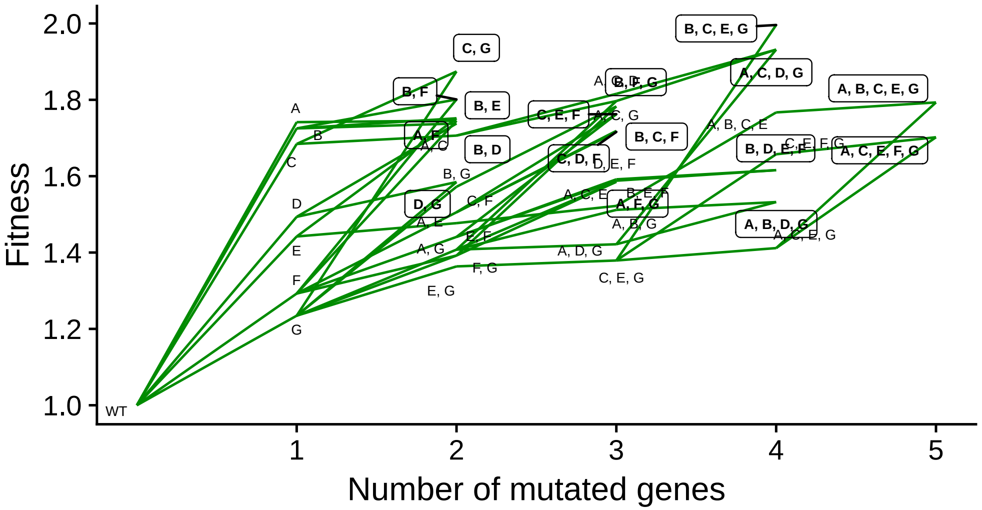

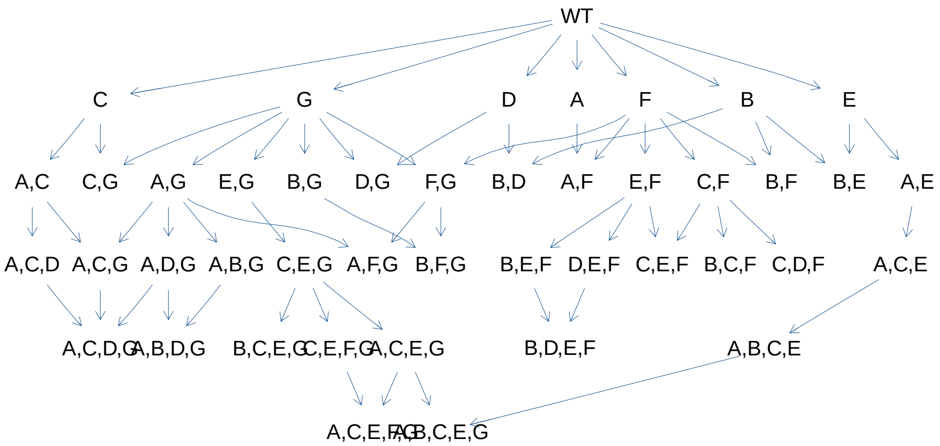

ID	r4JwqMTj8Y3k7yRZ
acc genotypes	52
peaks no back	15
removed edges	NA
prop rem edges	NA

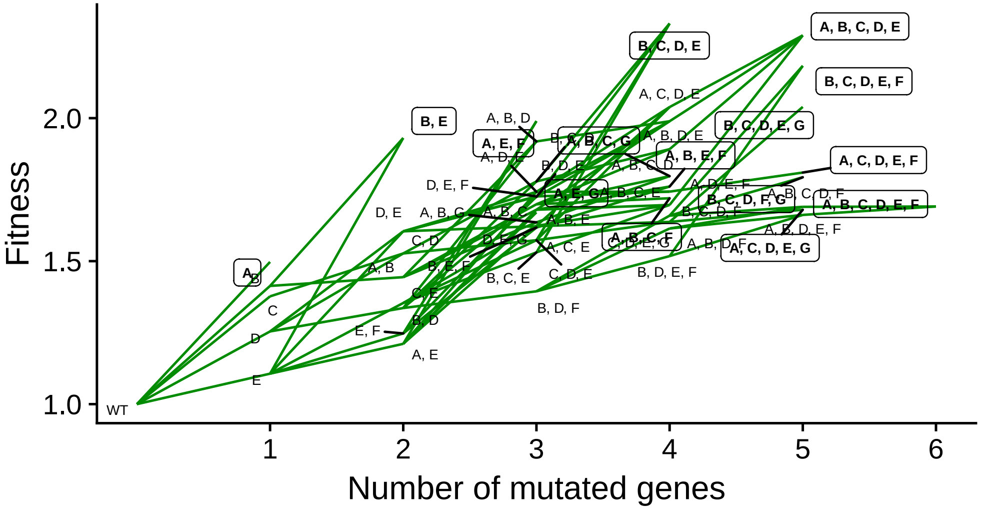

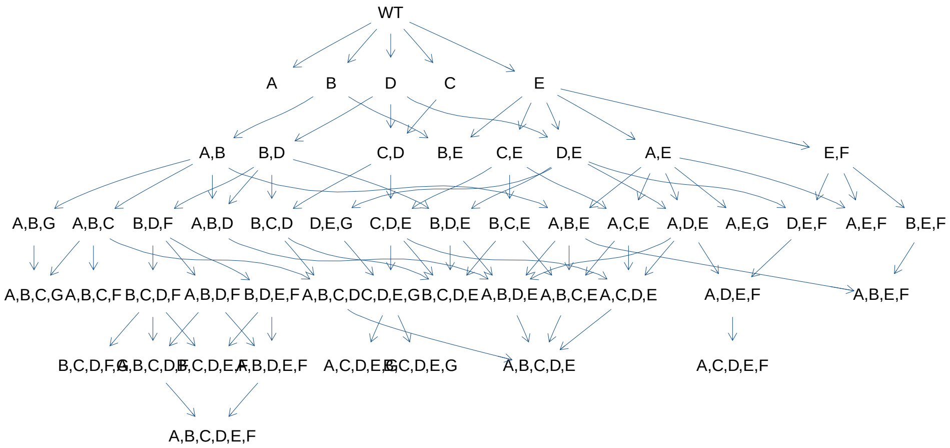

ID	rB10EATzKOO2QwOIv
acc genotypes	23
peaks no back	9
removed edges	NA
prop rem edges	NA

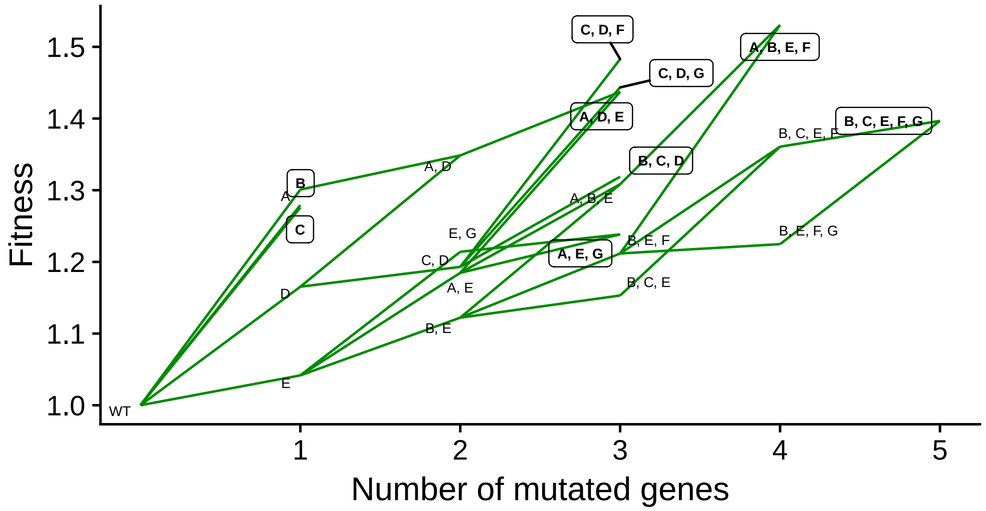

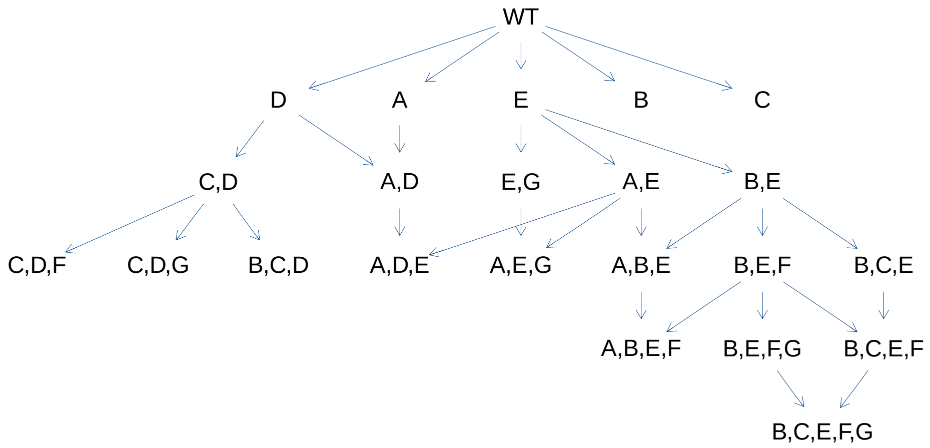

ID	rCDeBXmZNnImUHl1
acc genotypes	60
peaks no back	24
removed edges	NA
prop rem edges	NA

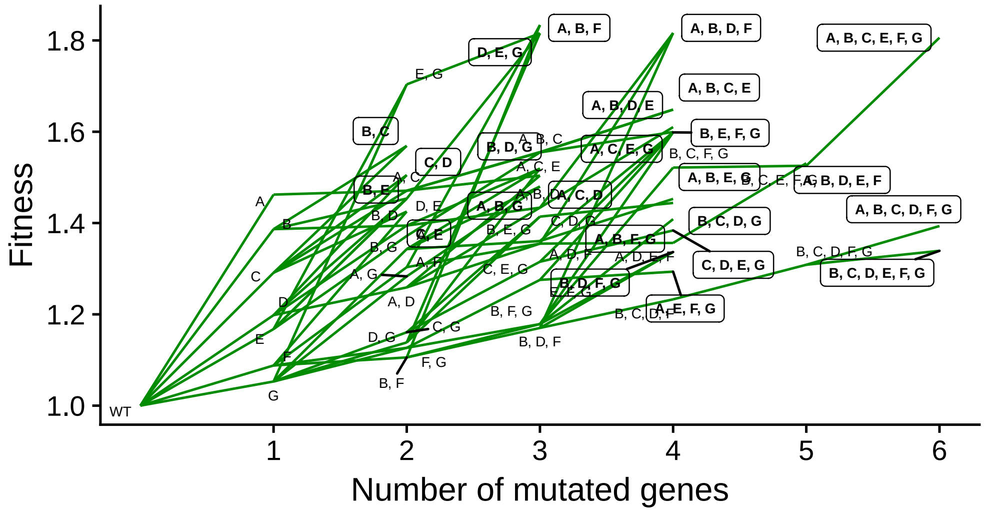

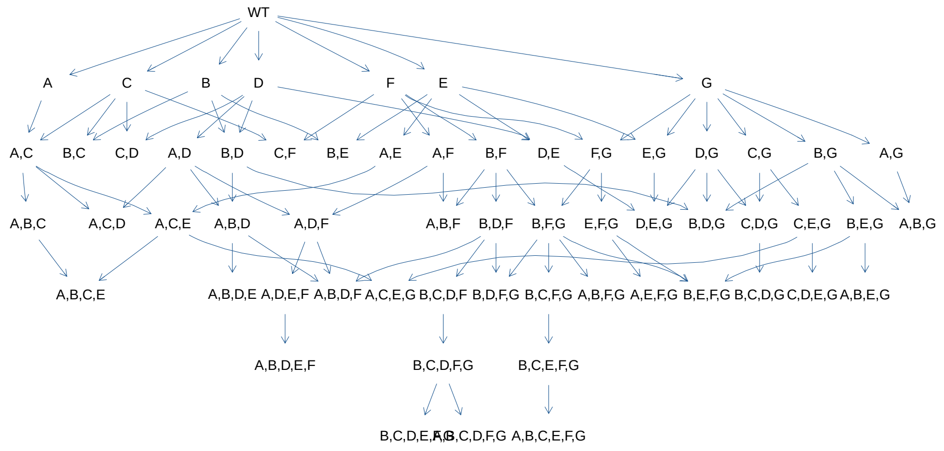

ID	ReBiTNyW6Sg6eJwN
acc genotypes	75
peaks no back	13
removed edges	NA
prop rem edges	NA

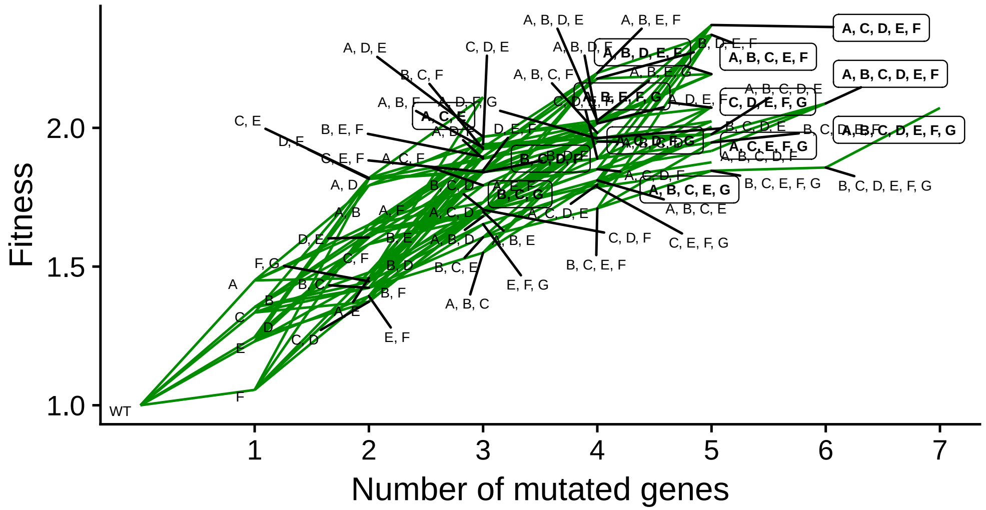

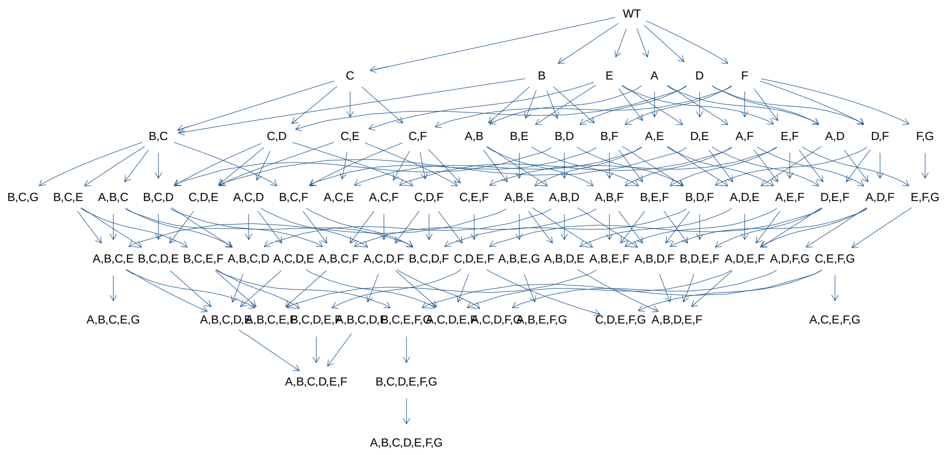

ID	rhc6UcRhymI47xh4
acc genotypes	12
peaks no back	6
removed edges	NA
prop rem edges	NA

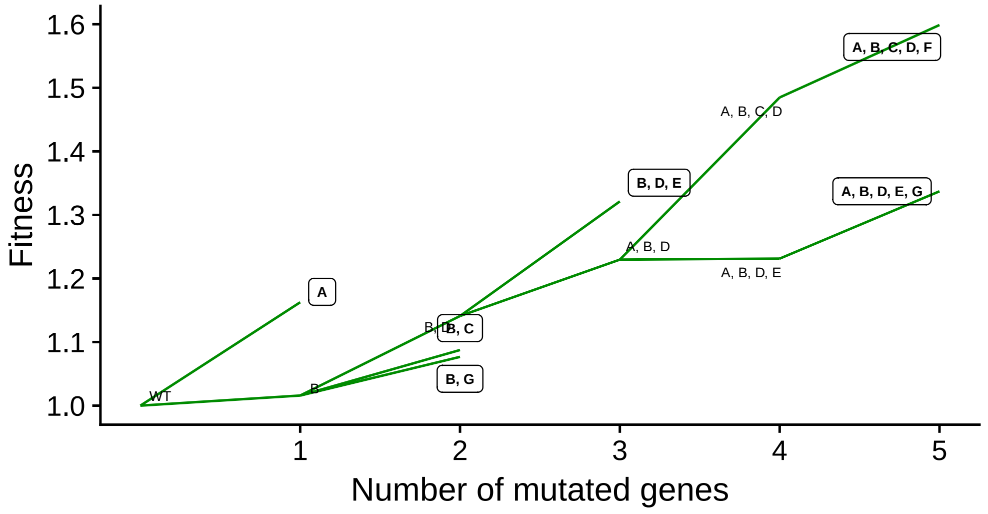

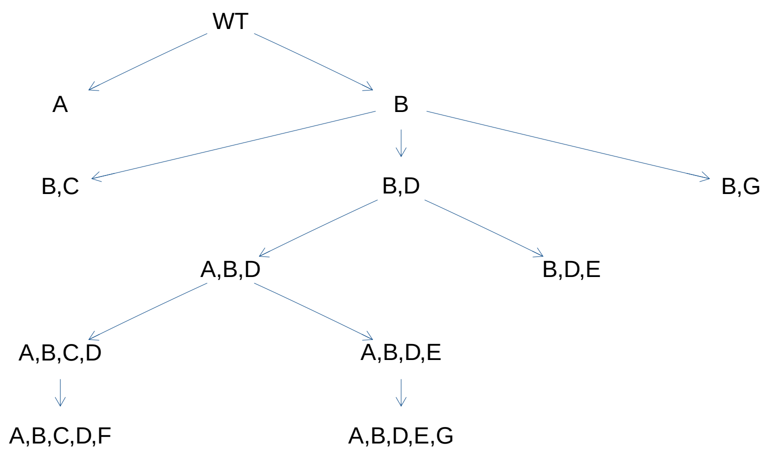

ID	RmugDTsuCjx1AI9r
acc genotypes	14
peaks no back	6
removed edges	NA
prop rem edges	NA

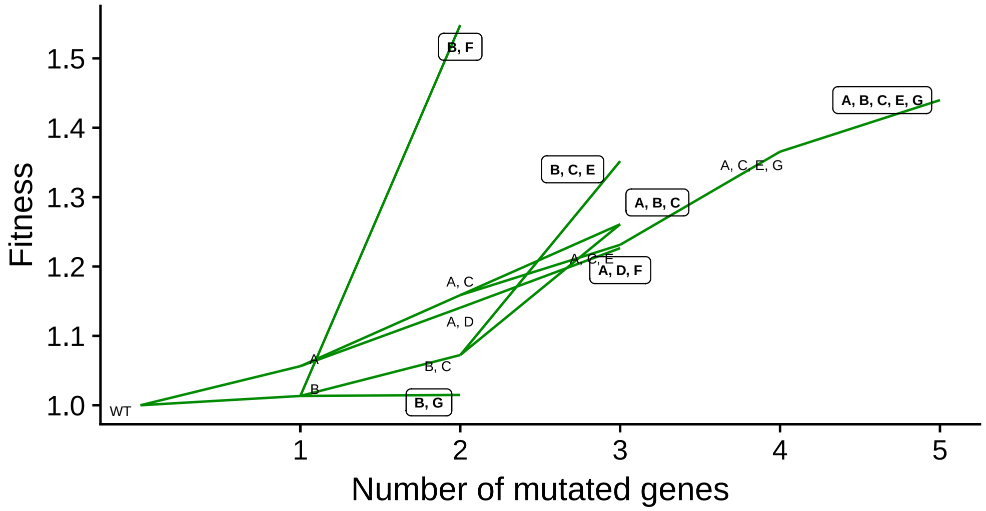

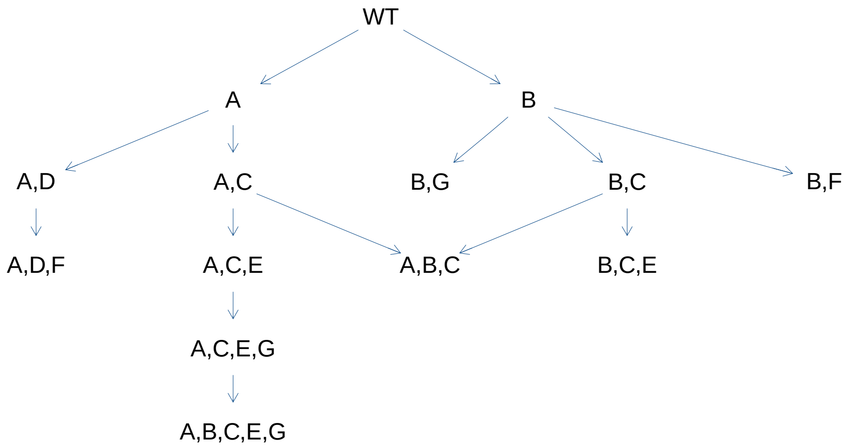

ID	RnHnxYc271fXVfMs
acc genotypes	57
peaks no back	15
removed edges	NA
prop rem edges	NA

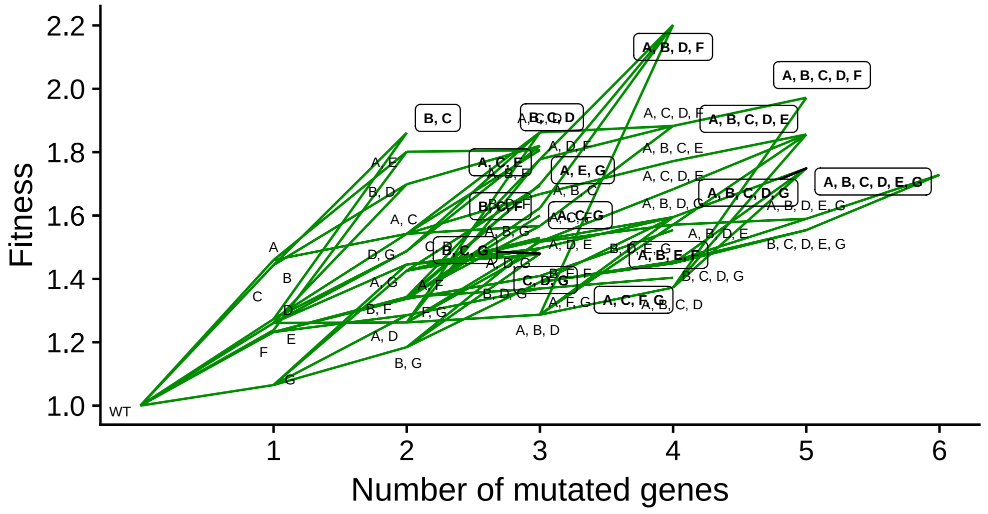

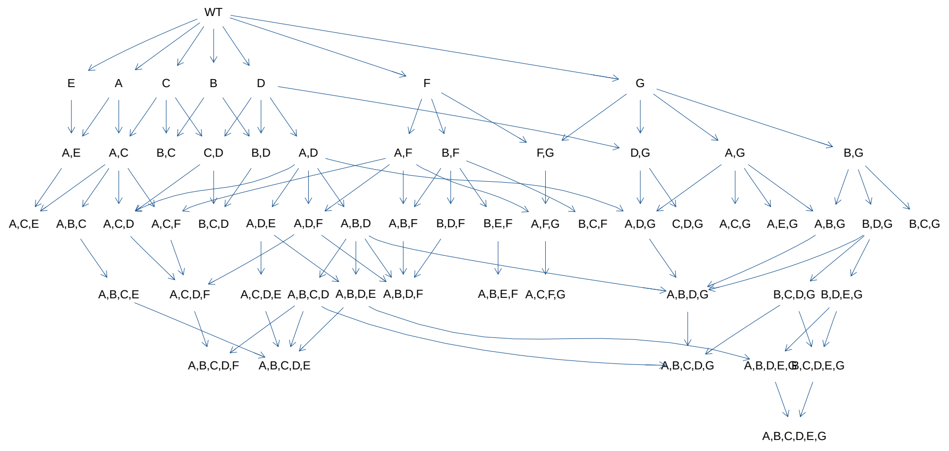

ID	rWquwMtuP4hFOLRB
acc genotypes	66
peaks no back	15
removed edges	NA
prop rem edges	NA

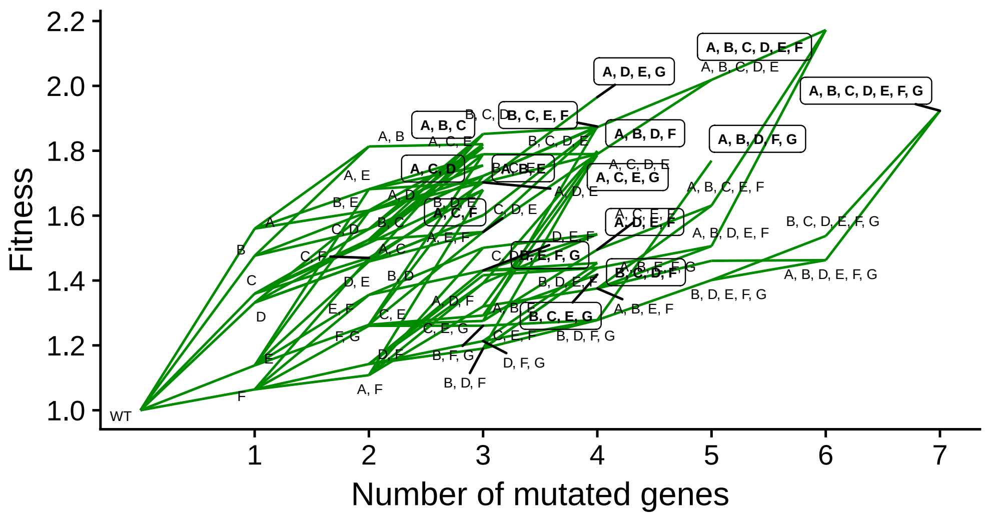

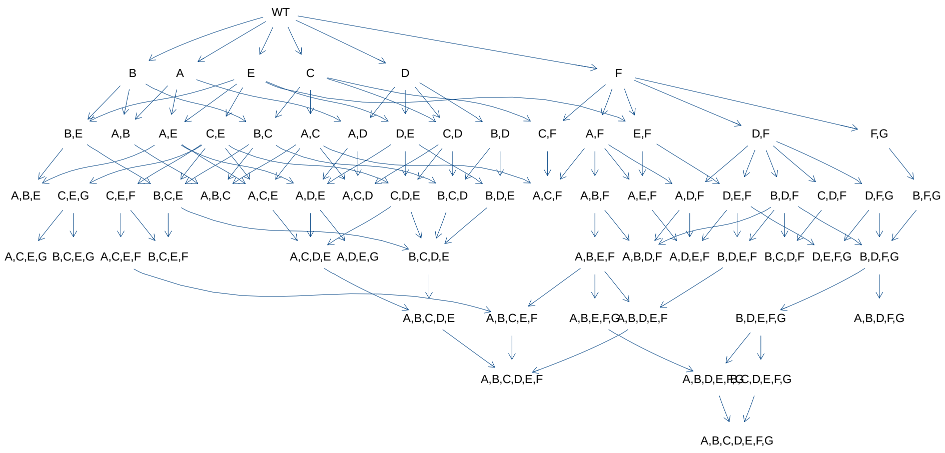

ID	rX57ibCOZcMSMm4z
acc genotypes	30
peaks no back	12
removed edges	NA
prop rem edges	NA

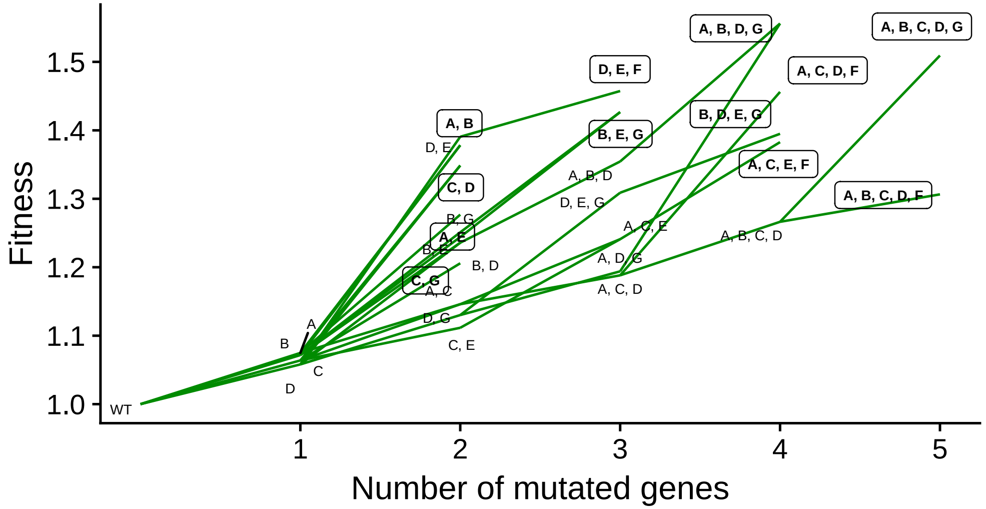

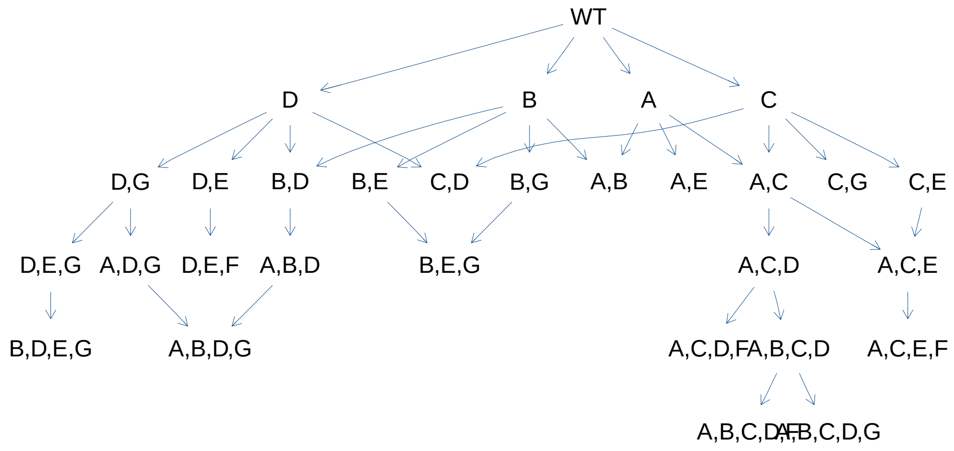

ID	s10ieWttHAueSTqaL
acc genotypes	74
peaks no back	25
removed edges	NA
prop rem edges	NA

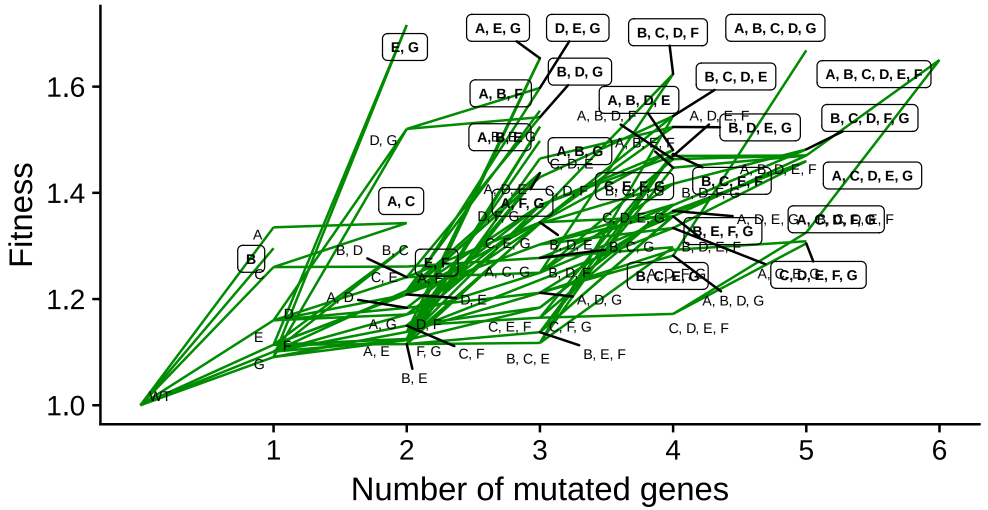

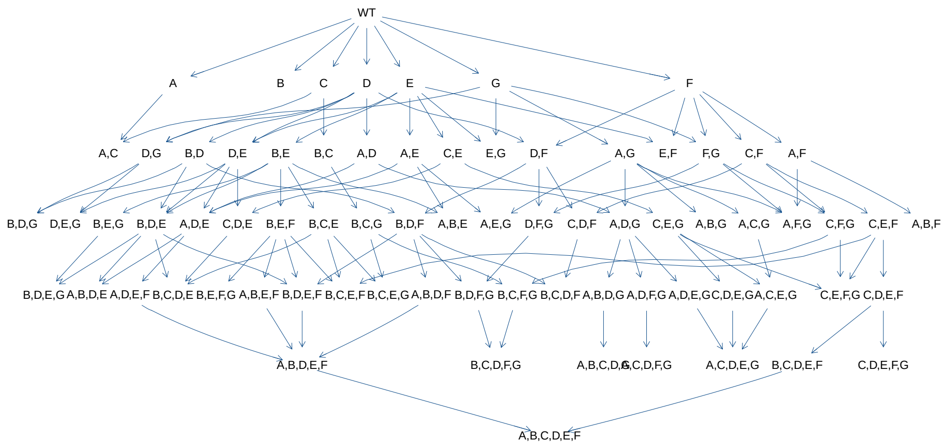

ID	s5lzMrcmZRB5b6Mo
acc genotypes	28
peaks no back	10
removed edges	NA
prop rem edges	NA

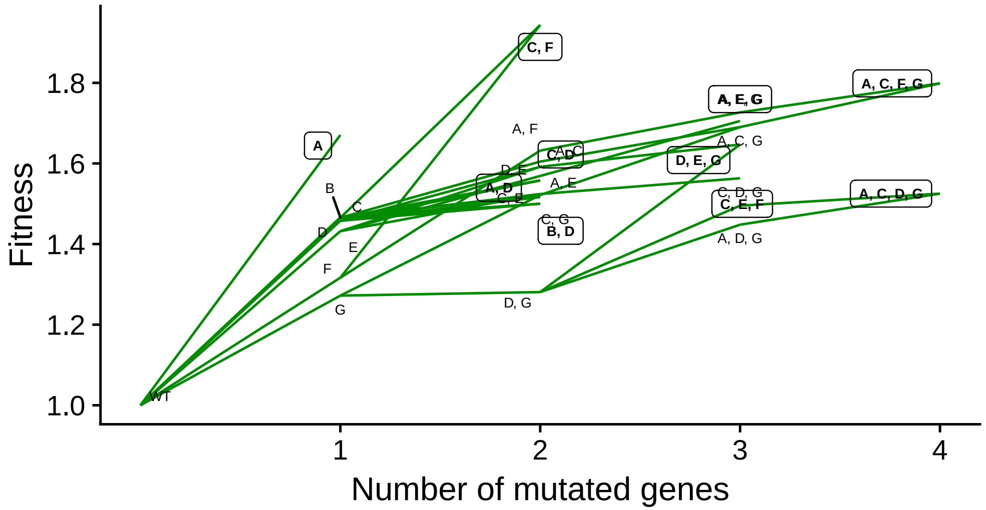

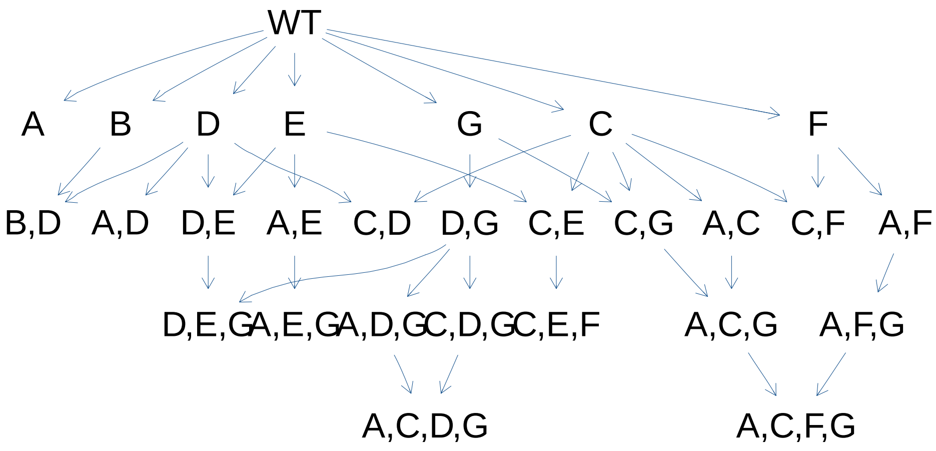

ID	sgP24AiGrN2flVGV
acc genotypes	43
peaks no back	10
removed edges	NA
prop rem edges	NA

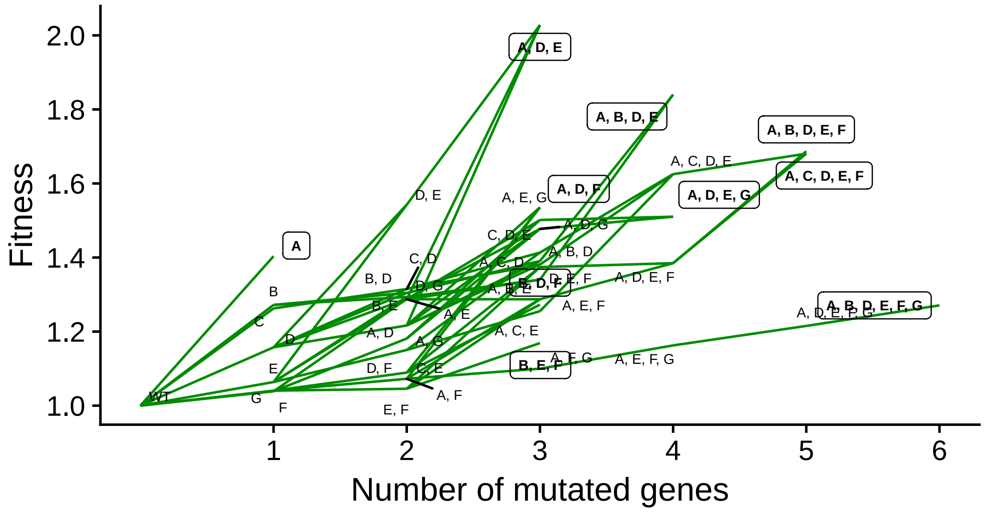

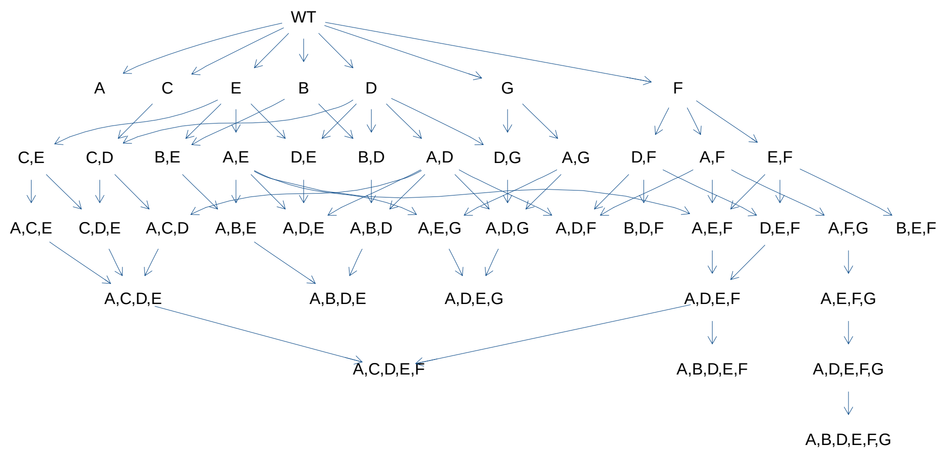

ID	sJhH8DjY2yo2ZQjo
acc genotypes	19
peaks no back	8
removed edges	NA
prop rem edges	NA

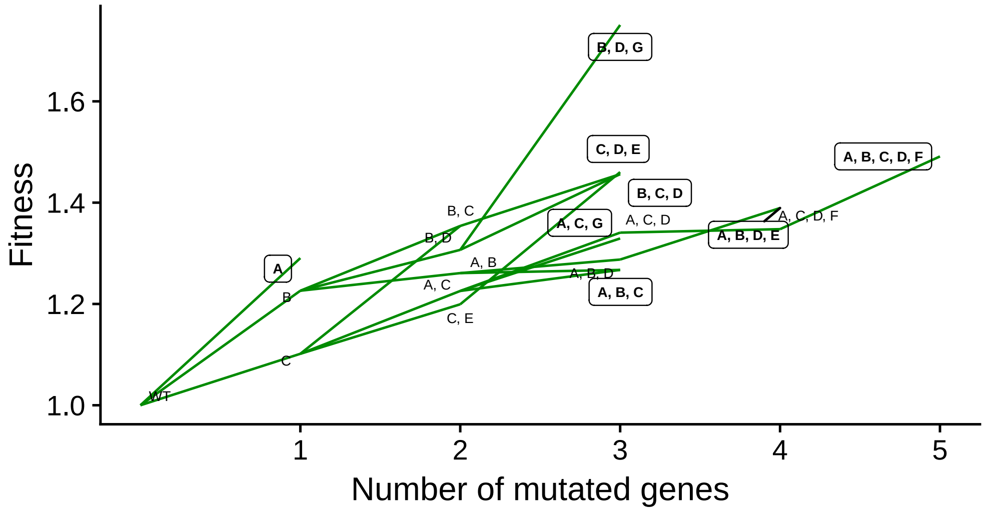

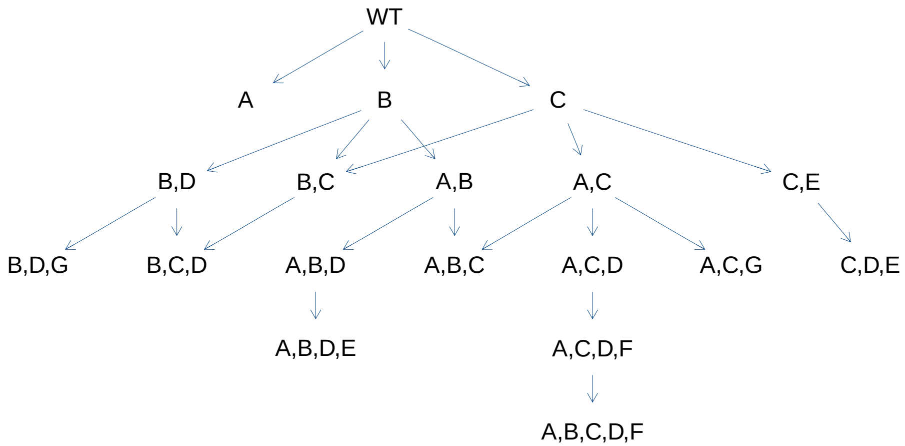

ID	SpvxDYG1010zub43g5
acc genotypes	25
peaks no back	12
removed edges	NA
prop rem edges	NA

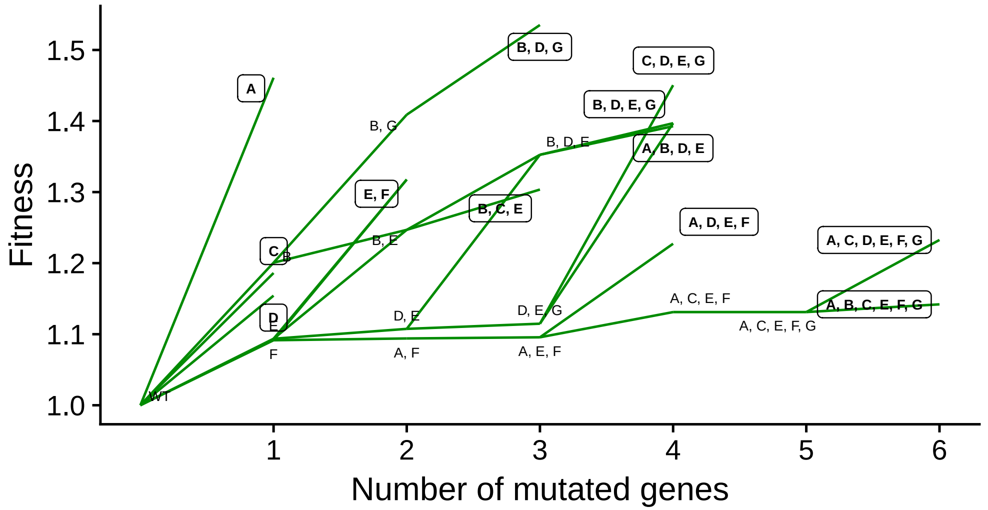

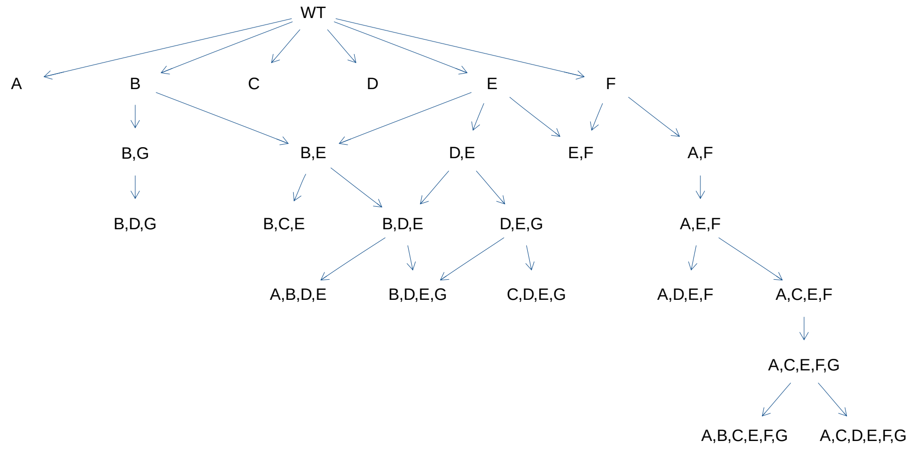

ID	SRBzzEiA3CEspfsm
acc genotypes	51
peaks no back	12
removed edges	NA
prop rem edges	NA

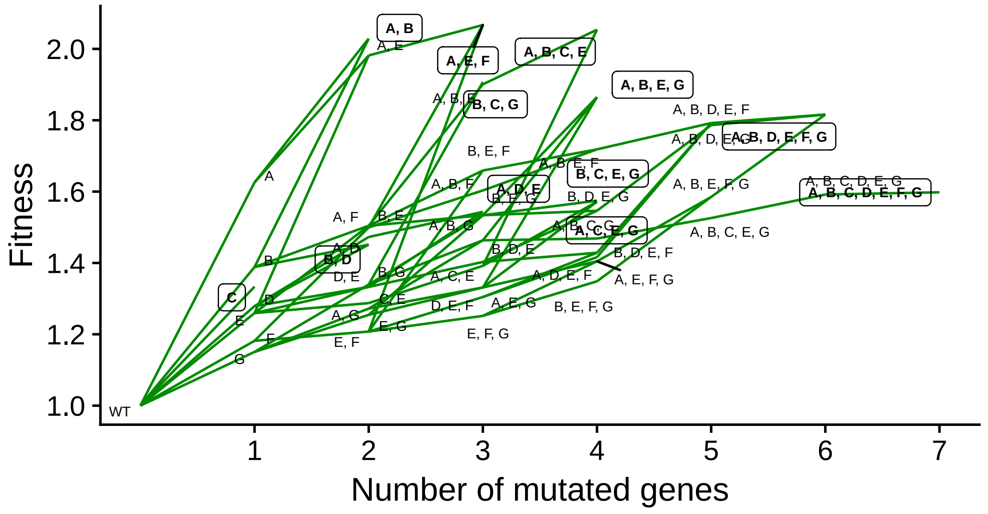

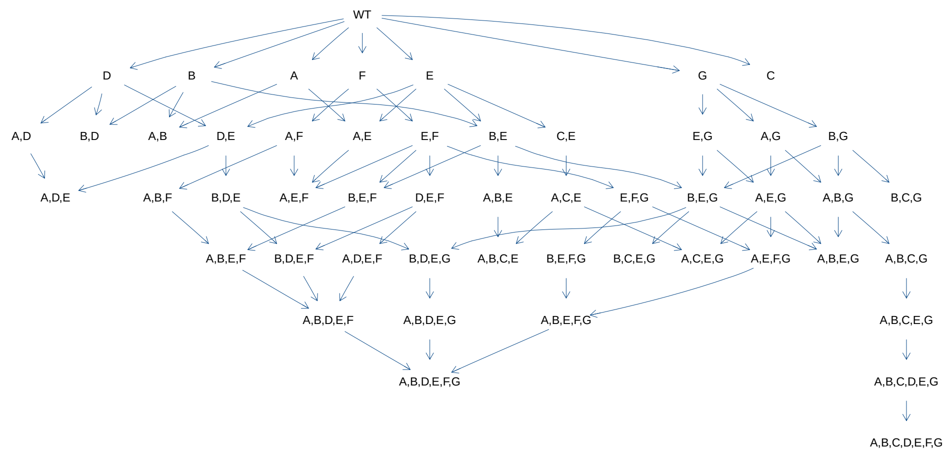

ID	SWE6nweARORL1yFW
acc genotypes	19
peaks no back	6
removed edges	NA
prop rem edges	NA

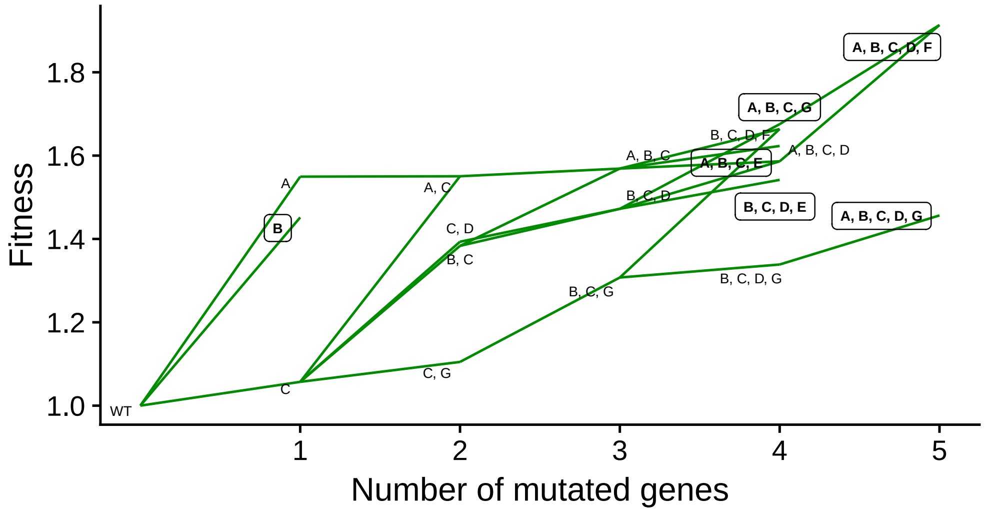

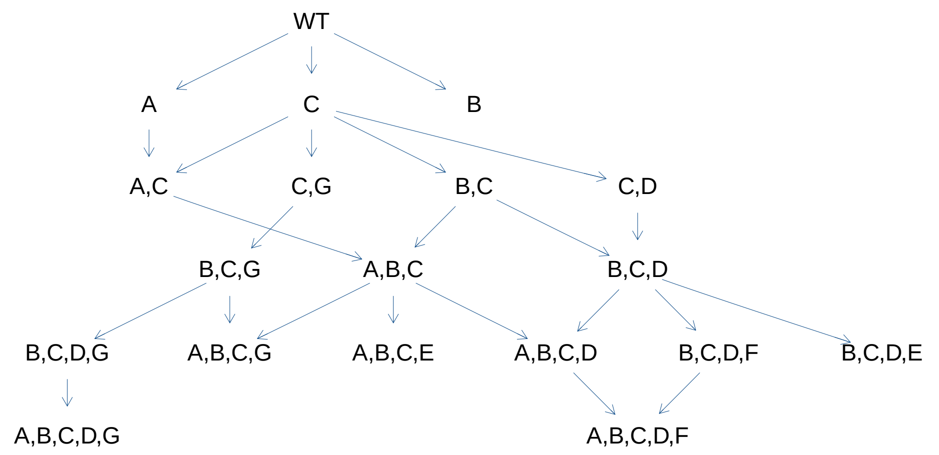

ID	t10CCmCuCK9esa9Rx
acc genotypes	55
peaks no back	20
removed edges	NA
prop rem edges	NA

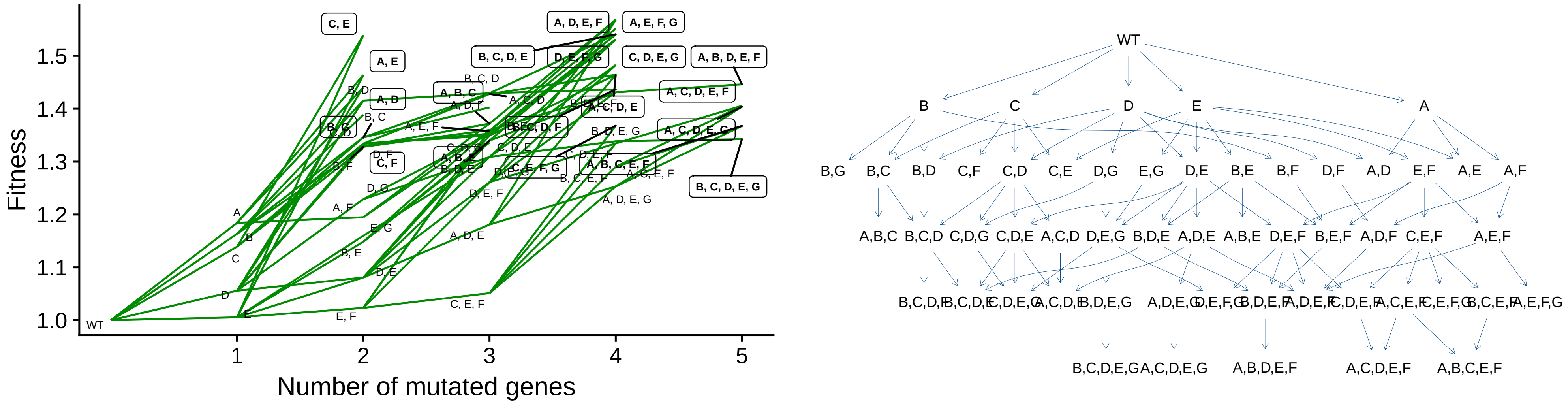

ID	tgqvbY64a2p366jT
acc genotypes	46
peaks no back	14
removed edges	NA
prop rem edges	NA

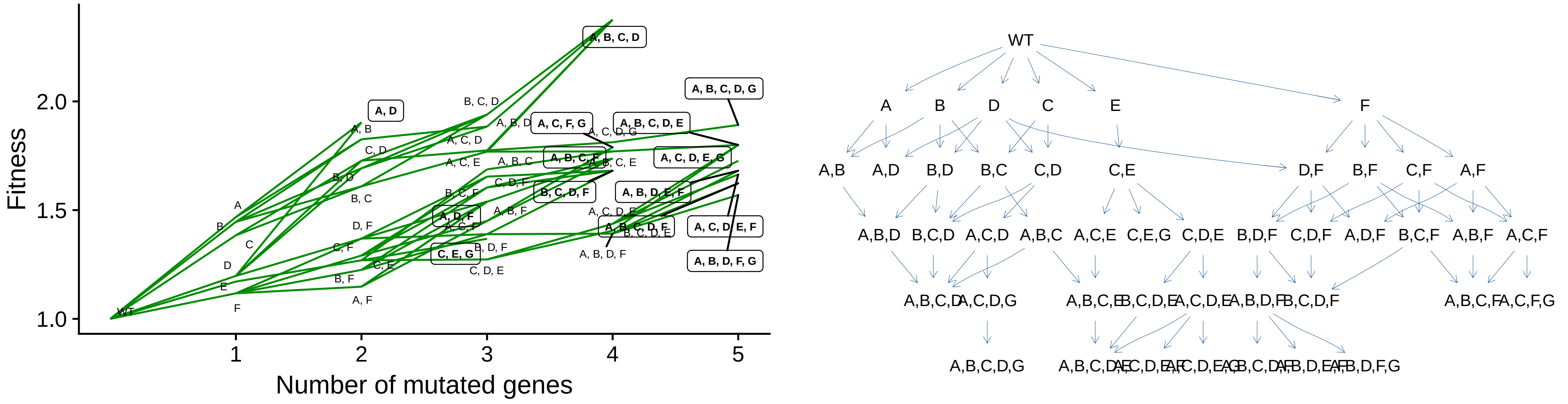

ID	TgUTHKueceu4QsBH
acc genotypes	18
peaks no back	9
removed edges	NA
prop rem edges	NA

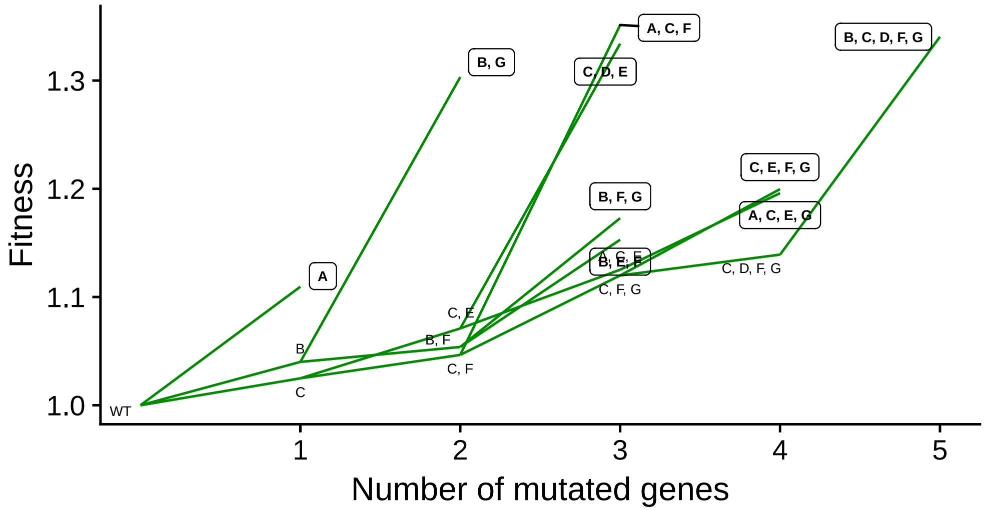

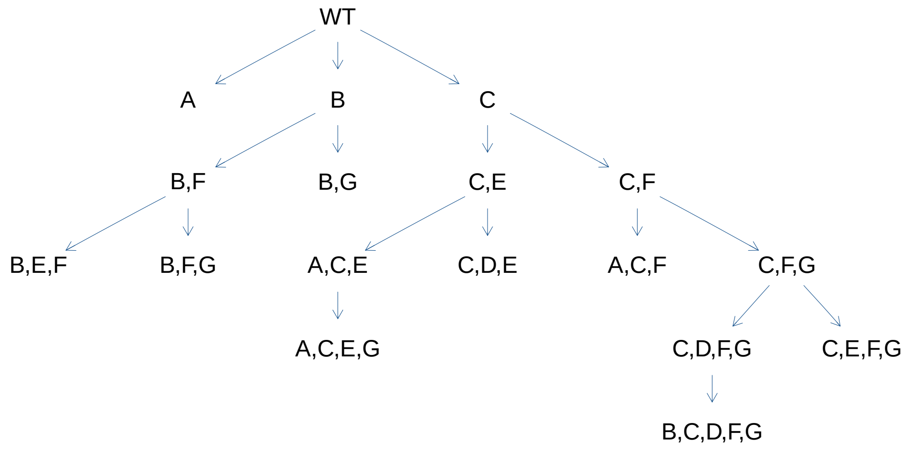

ID	TgvwxIvA3IzbdsYC
acc genotypes	38
peaks no back	14
removed edges	NA
prop rem edges	NA

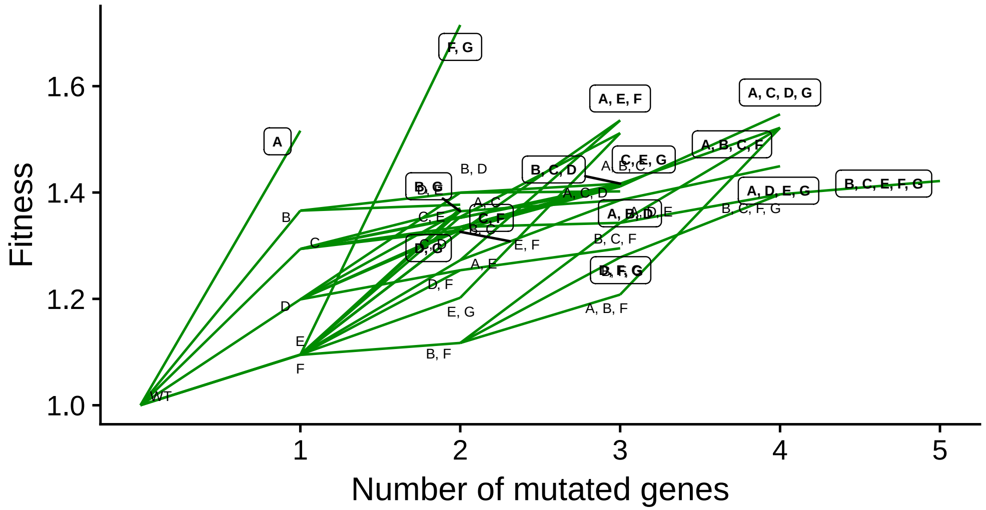

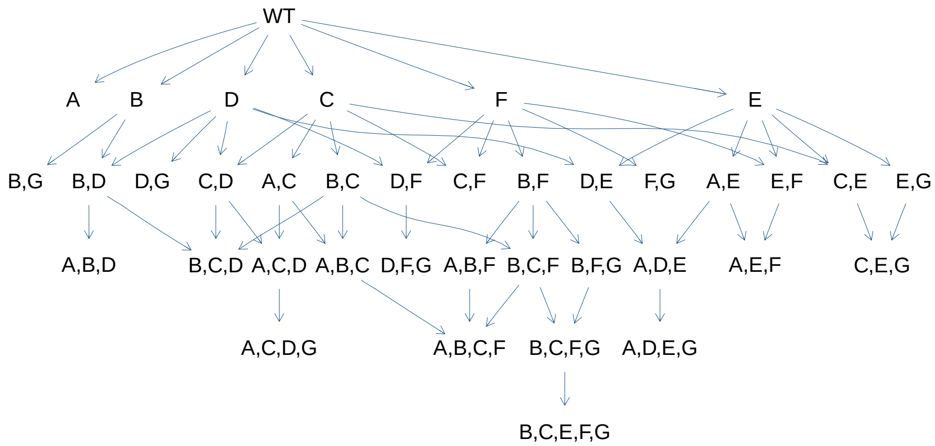

ID	tHfMAysqIiLPDZQ5
acc genotypes	23
peaks no back	7
removed edges	NA
prop rem edges	NA

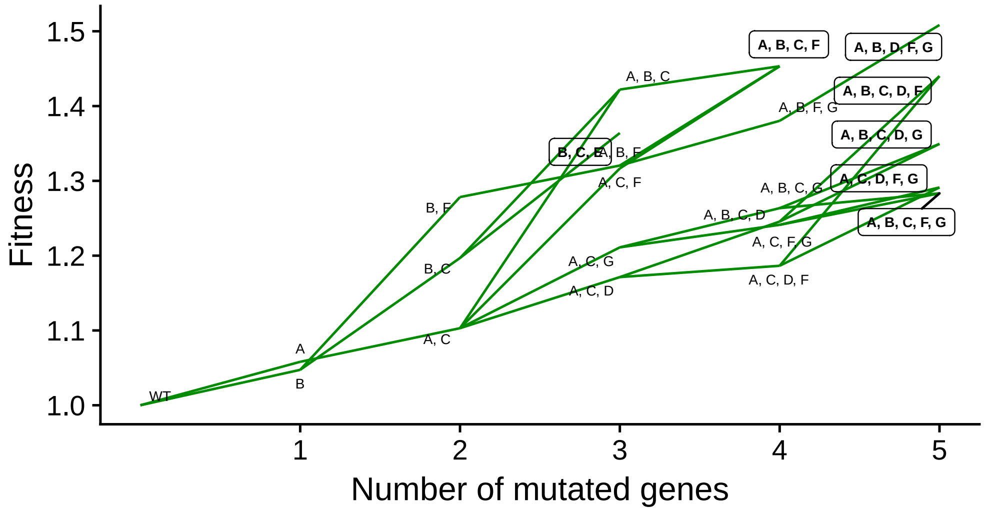

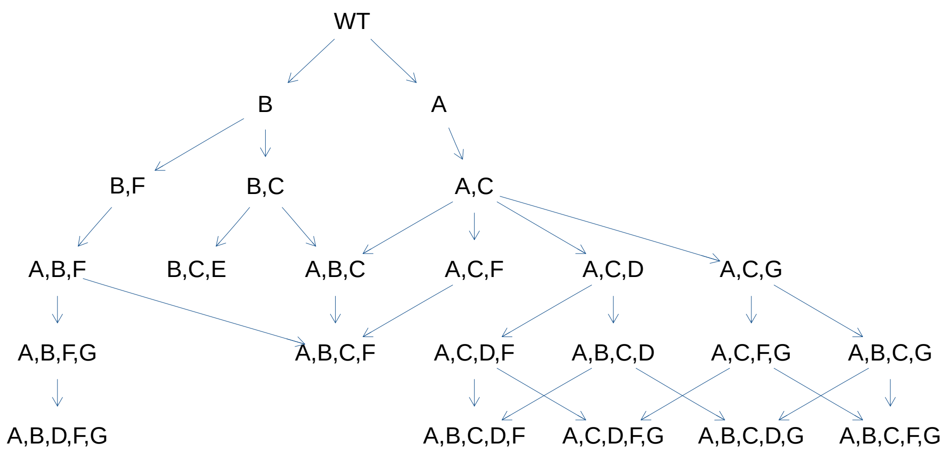

ID	TJaoNibPlMSgtl1f
acc genotypes	24
peaks no back	12
removed edges	NA
prop rem edges	NA

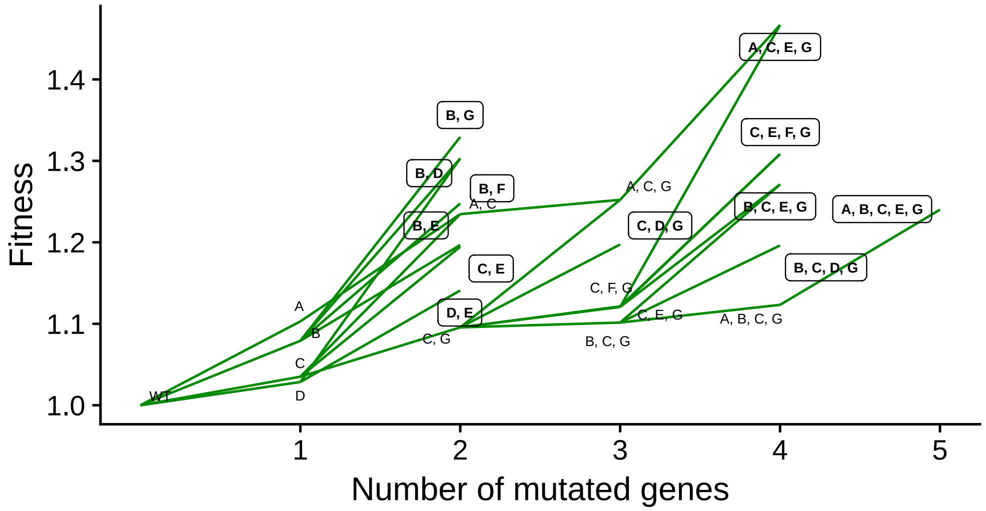

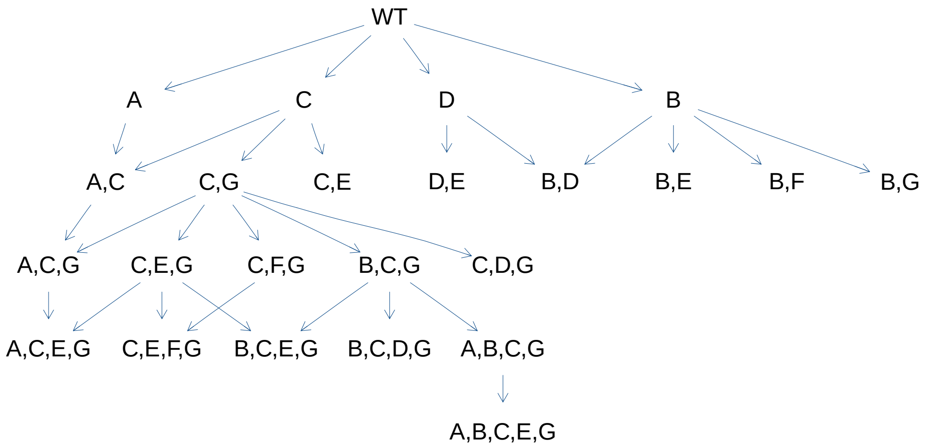

ID	Tl10ATX82Nhqew8T7
acc genotypes	80
peaks no back	15
removed edges	NA
prop rem edges	NA

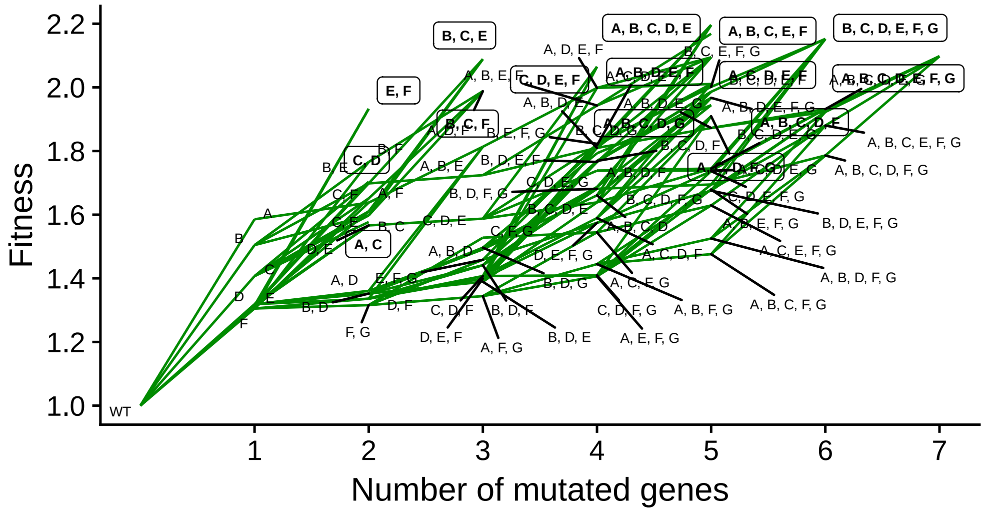

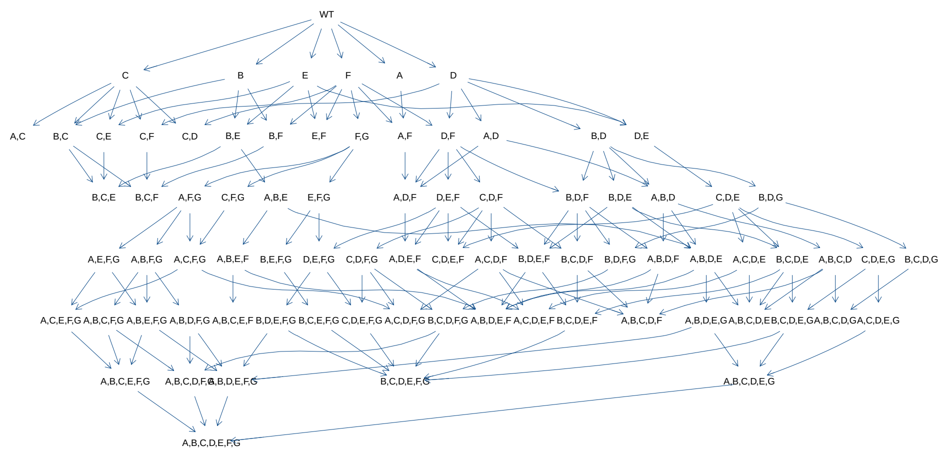

ID	tljFPA395CywDkbx
acc genotypes	17
peaks no back	8
removed edges	NA
prop rem edges	NA

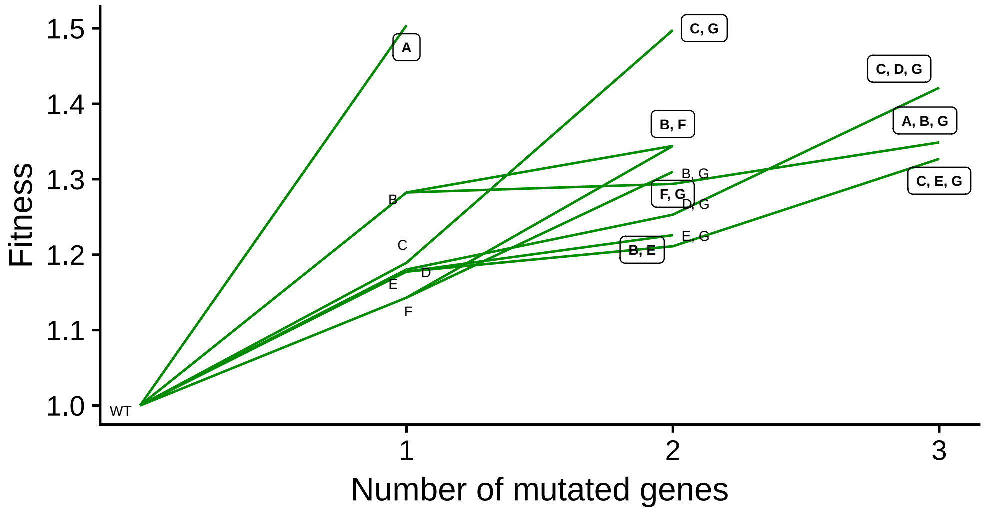

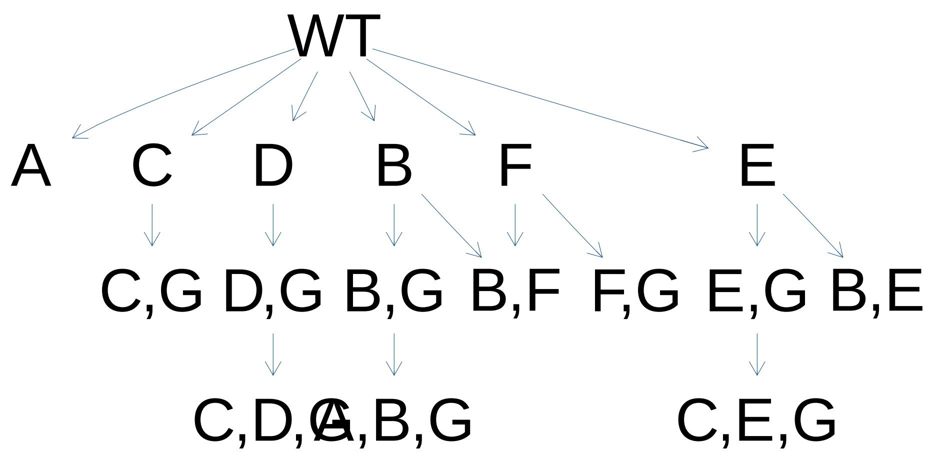

ID	TonSIXDkiuvbpNr6
acc genotypes	64
peaks no back	16
removed edges	NA
prop rem edges	NA

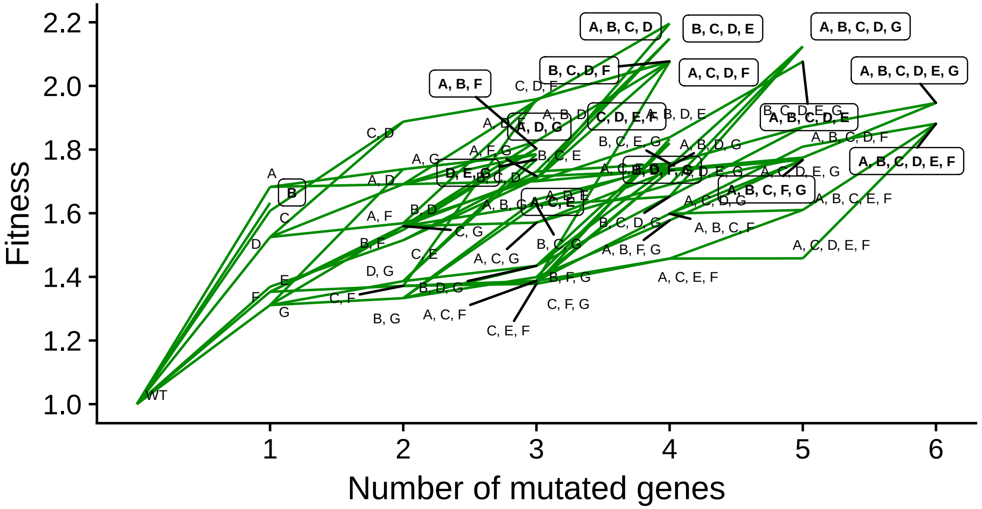

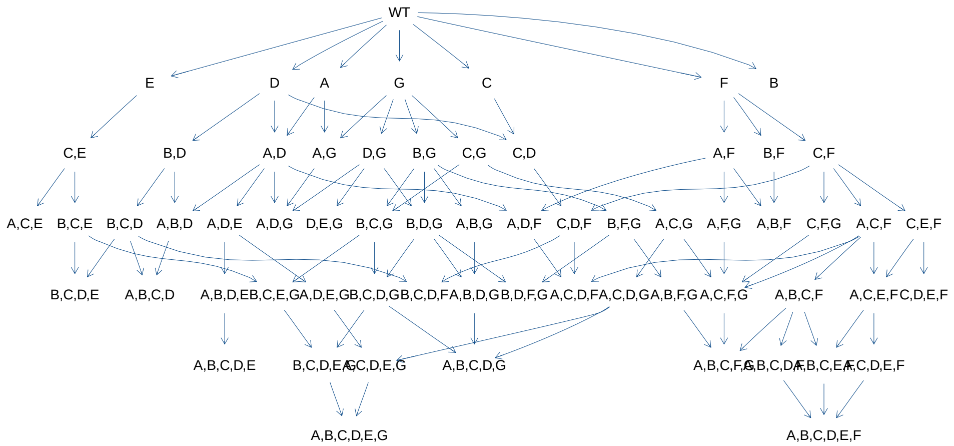

ID	TuigQ6ZYErsjqG8r
acc genotypes	19
peaks no back	9
removed edges	NA
prop rem edges	NA

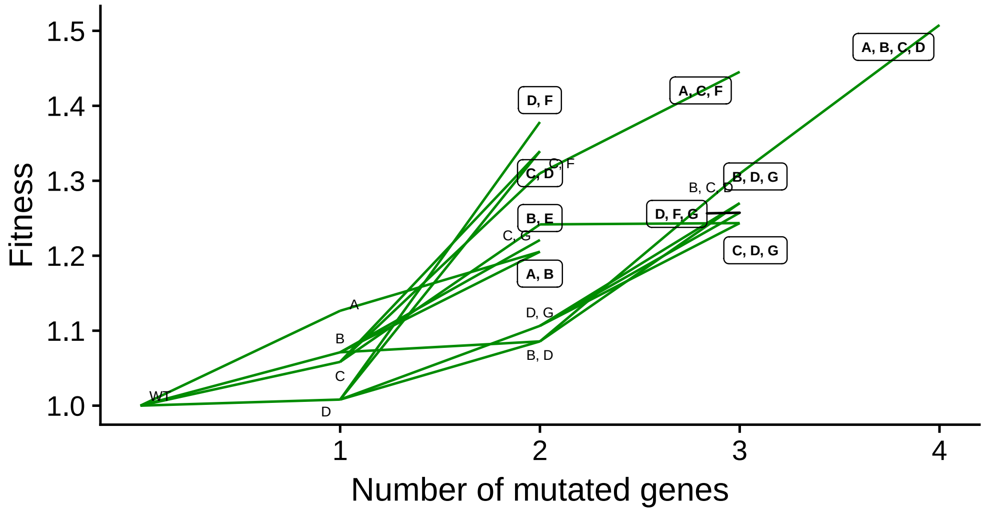

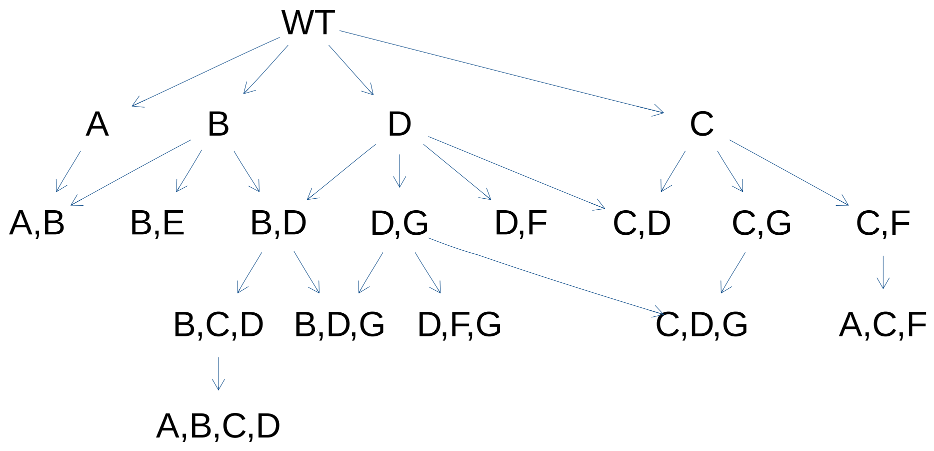

ID	U2qBdGfdtF10VB9Ww
acc genotypes	30
peaks no back	12
removed edges	NA
prop rem edges	NA

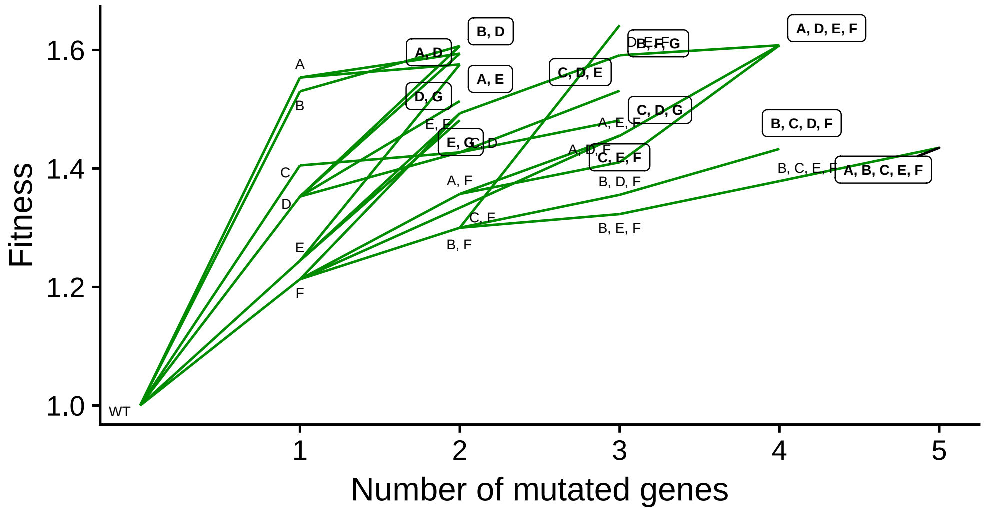

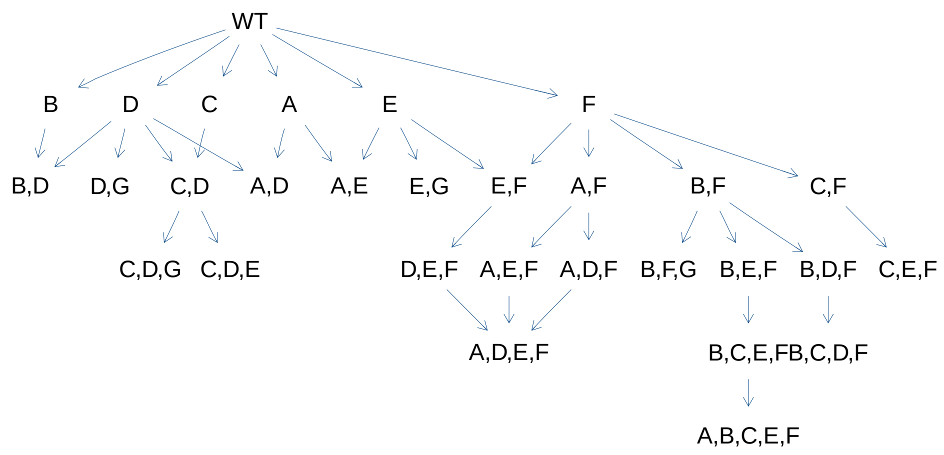

ID	U7R64nQkJqBxJIju
acc genotypes	43
peaks no back	14
removed edges	NA
prop rem edges	NA

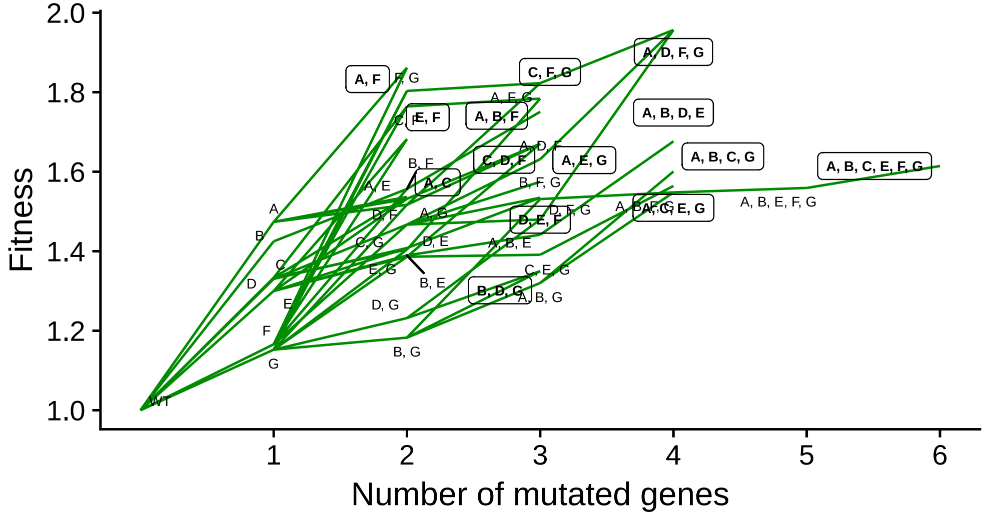

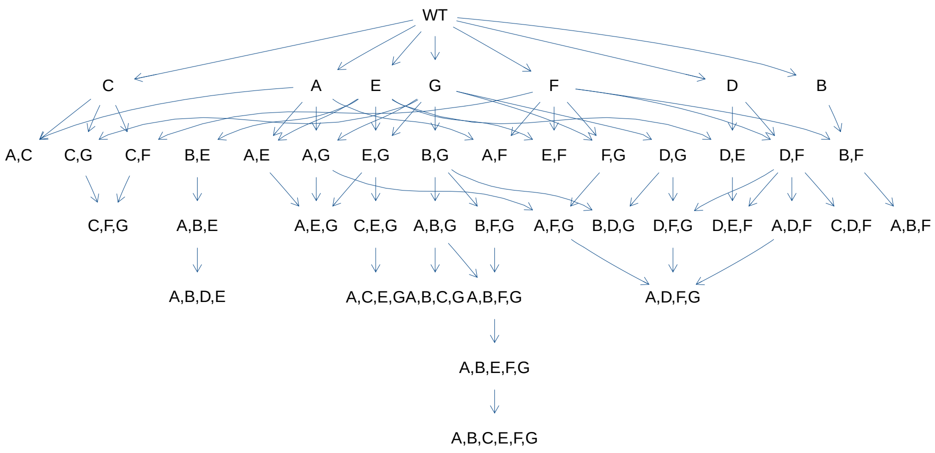

ID	usBeU10iBdpLaKISX
acc genotypes	14
peaks no back	7
removed edges	NA
prop rem edges	NA

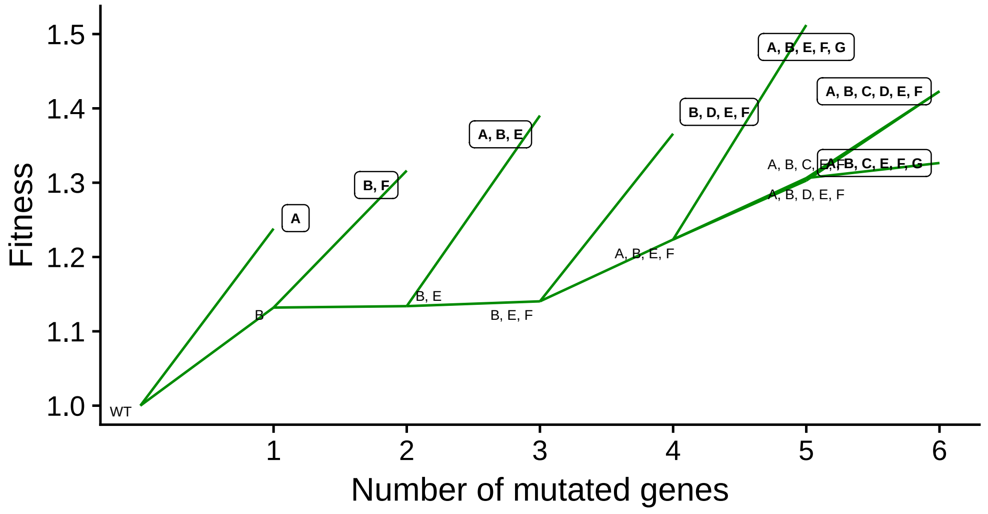

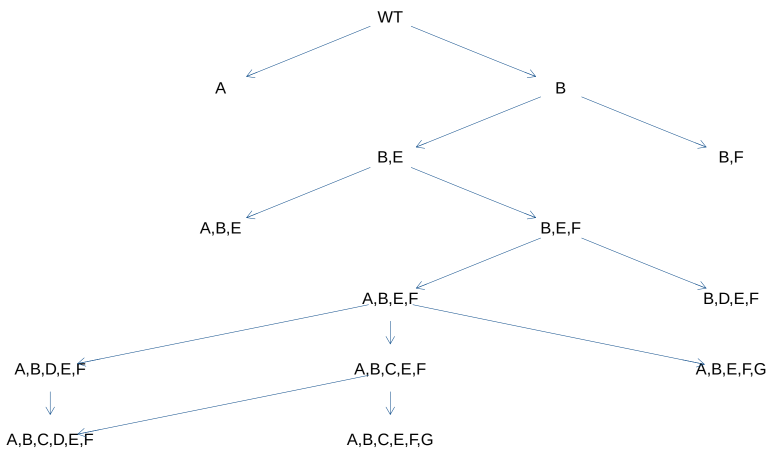

ID	uTuVU1GMy3Xg8Zye
acc genotypes	43
peaks no back	17
removed edges	NA
prop rem edges	NA

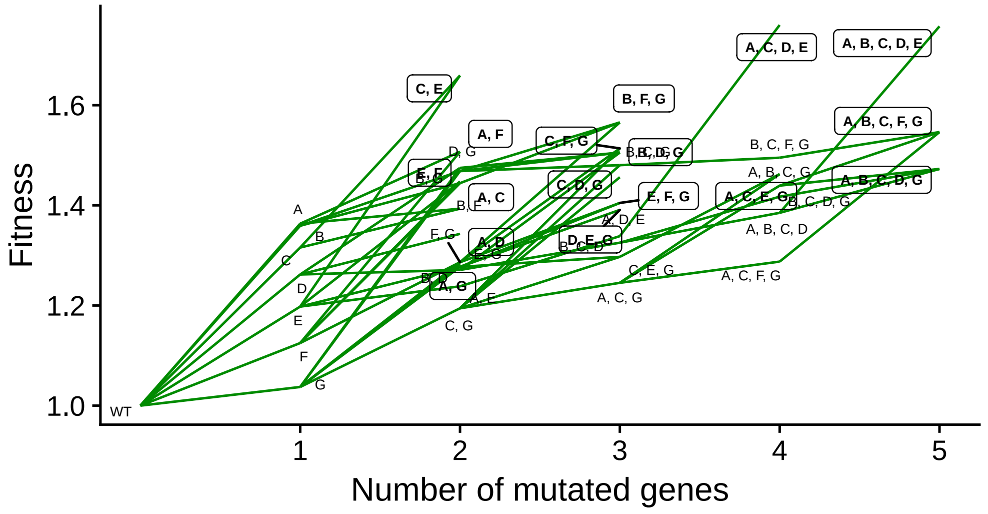

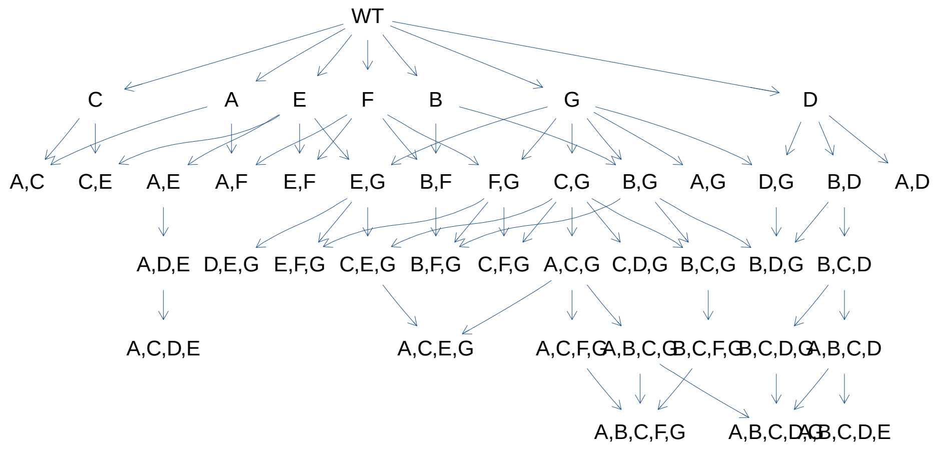

ID	uxdKQzl2TafUiD7b
acc genotypes	42
peaks no back	17
removed edges	NA
prop rem edges	NA

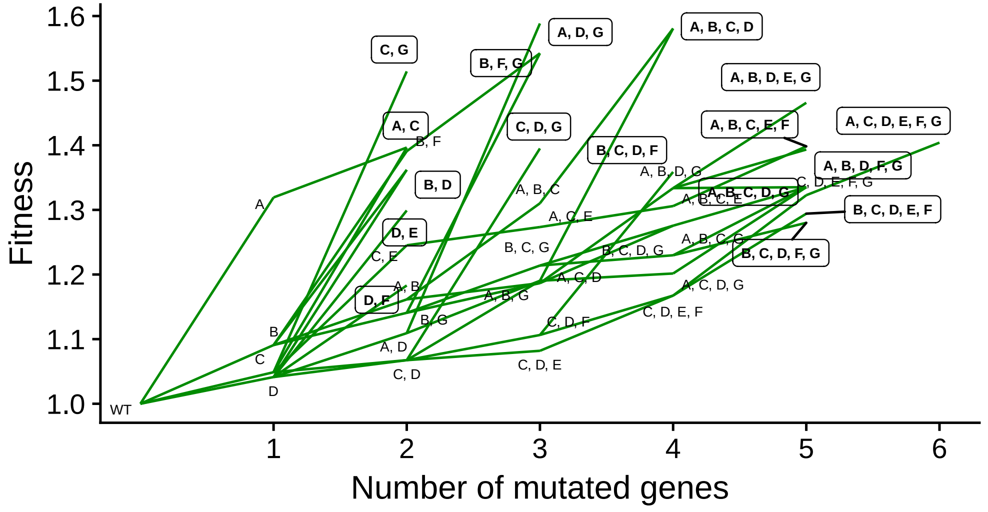

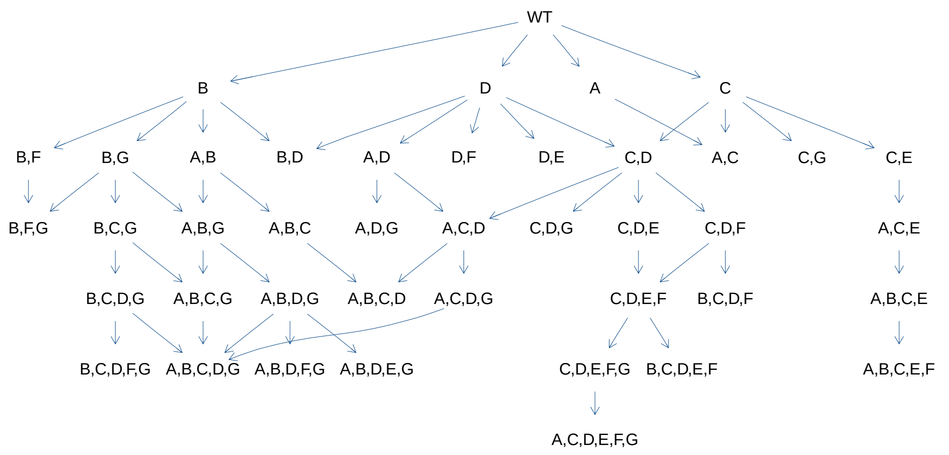

ID	vFhJDZxTJ3k4epfY
acc genotypes	49
peaks no back	19
removed edges	NA
prop rem edges	NA

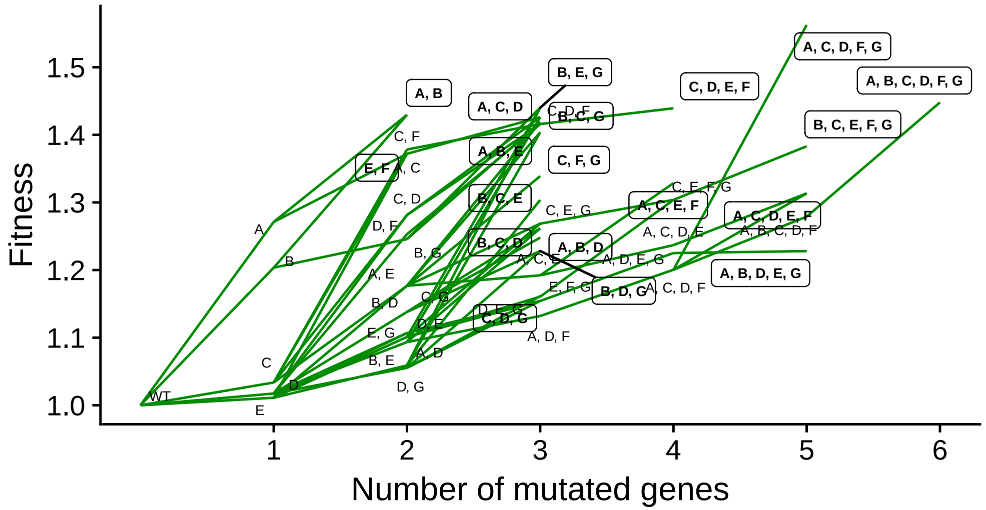

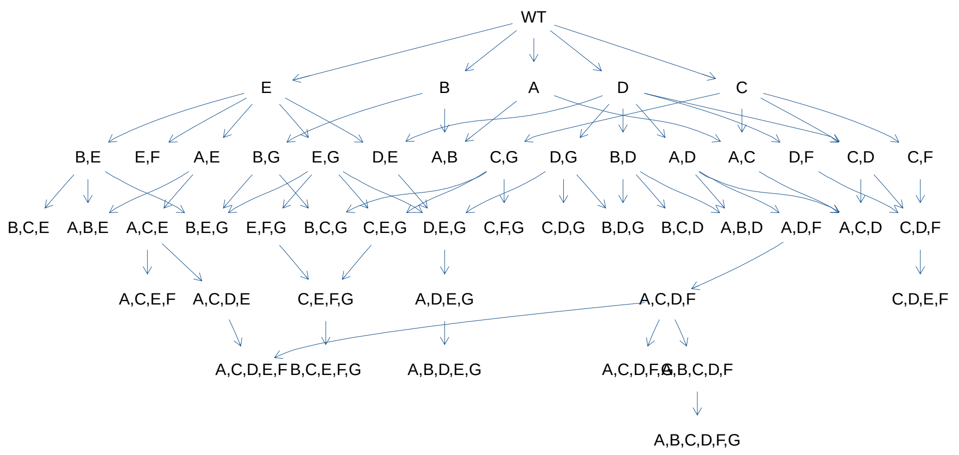

ID	vmQ3luxZBIZ10PMCg
acc genotypes	25
peaks no back	9
removed edges	NA
prop rem edges	NA

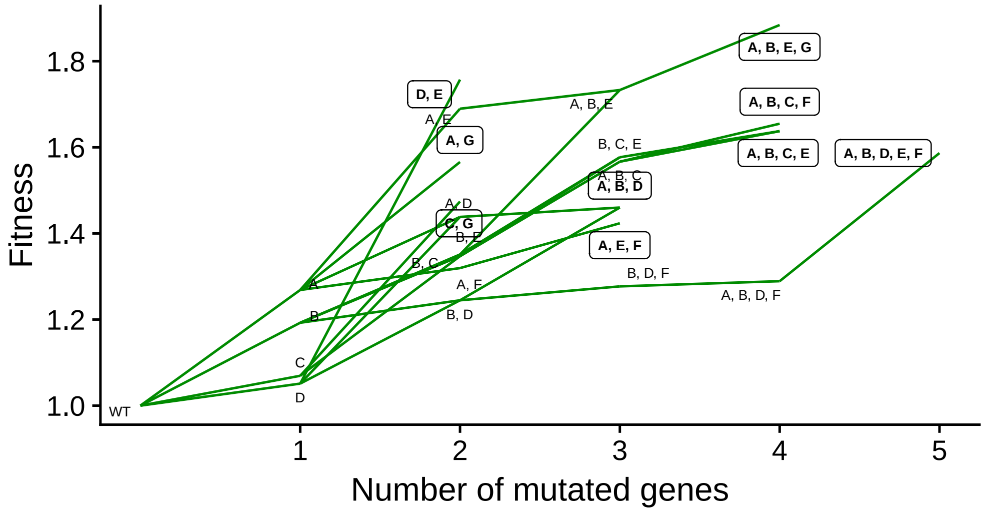

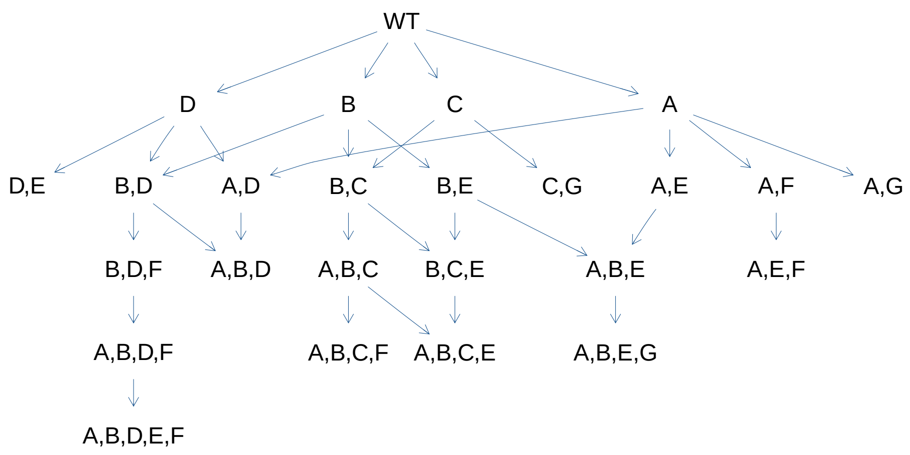

ID	vt56Tsvc76JVWmej
acc genotypes	20
peaks no back	5
removed edges	NA
prop rem edges	NA

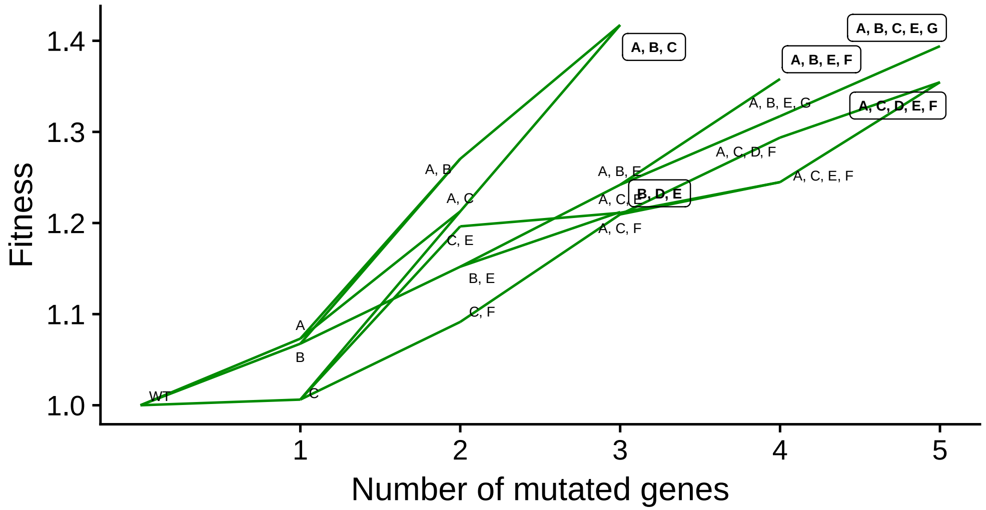

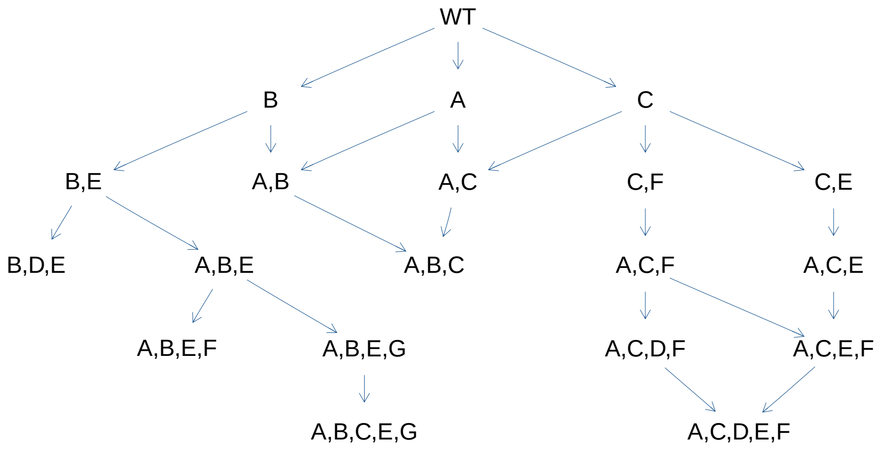

ID	vweucMX7s10v2ZeDw
acc genotypes	57
peaks no back	13
removed edges	NA
prop rem edges	NA

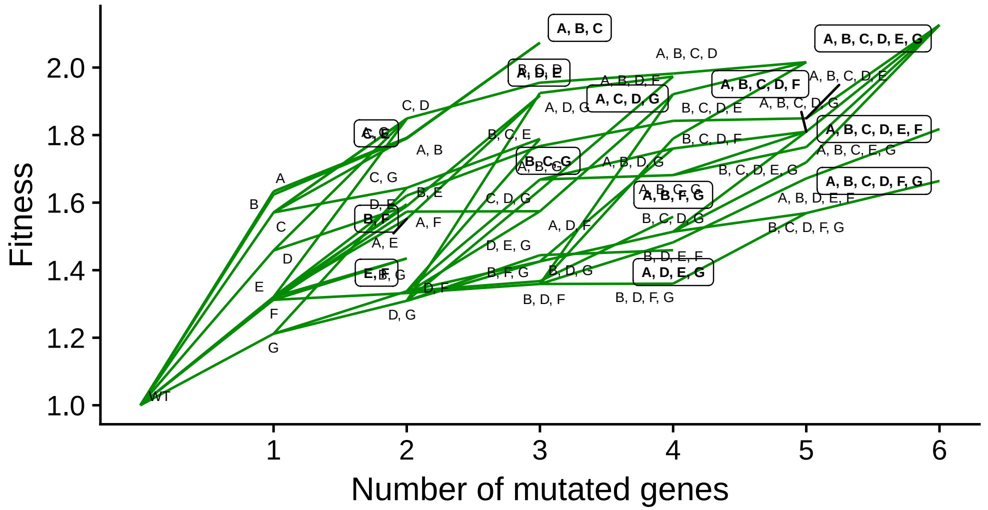

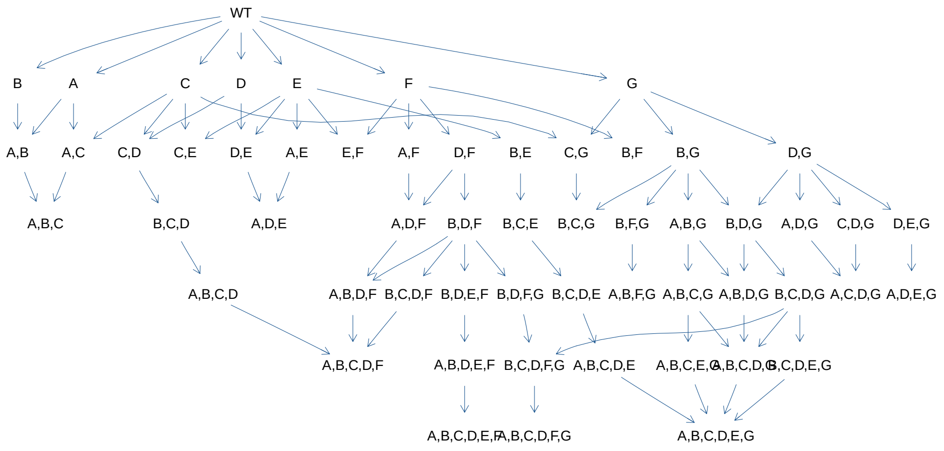

ID	w3y65IfSHEfh1Qrj
acc genotypes	45
peaks no back	15
removed edges	NA
prop rem edges	NA

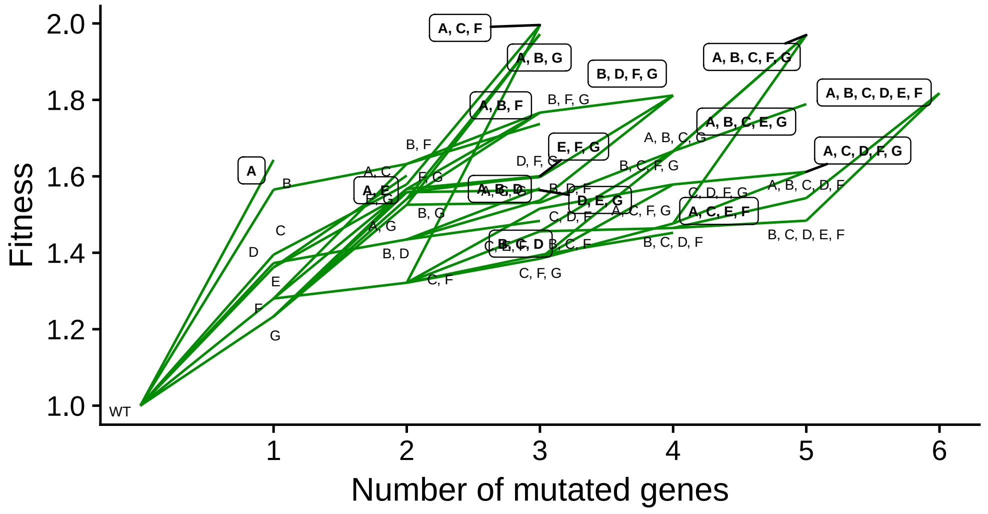

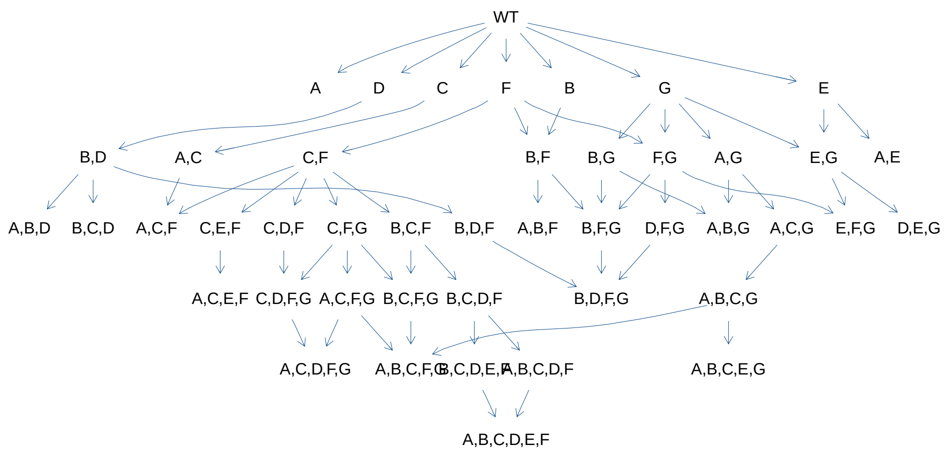

ID	W7VVpn96d4J5b6gp
acc genotypes	43
peaks no back	13
removed edges	NA
prop rem edges	NA

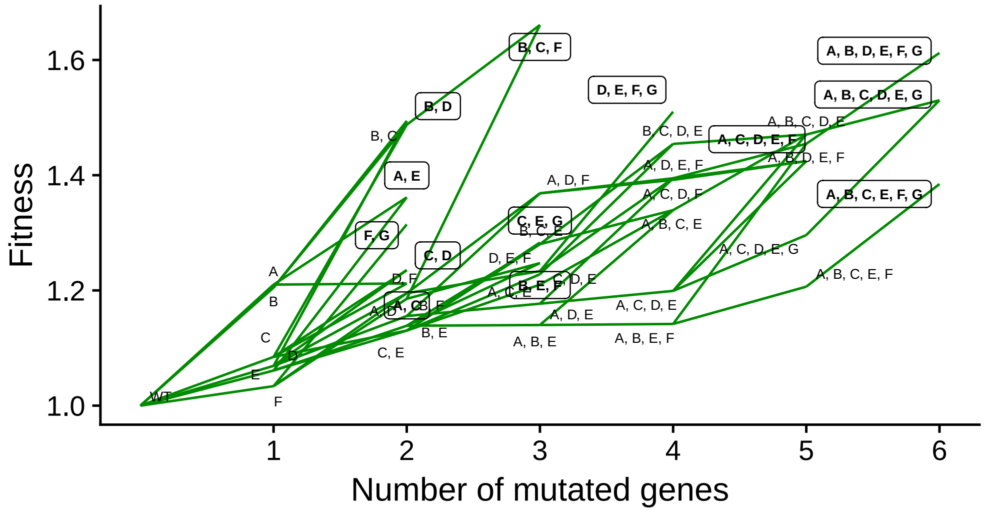

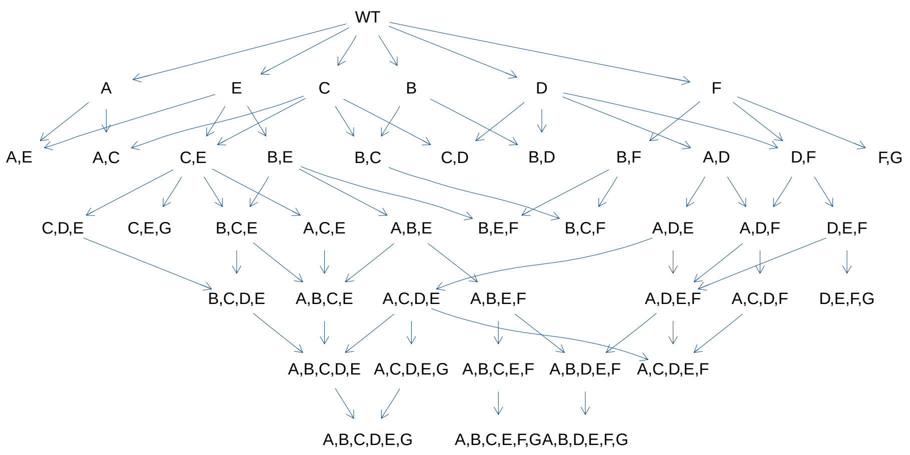

ID	w8IlHfJBXuIGpKP10
acc genotypes	57
peaks no back	18
removed edges	NA
prop rem edges	NA

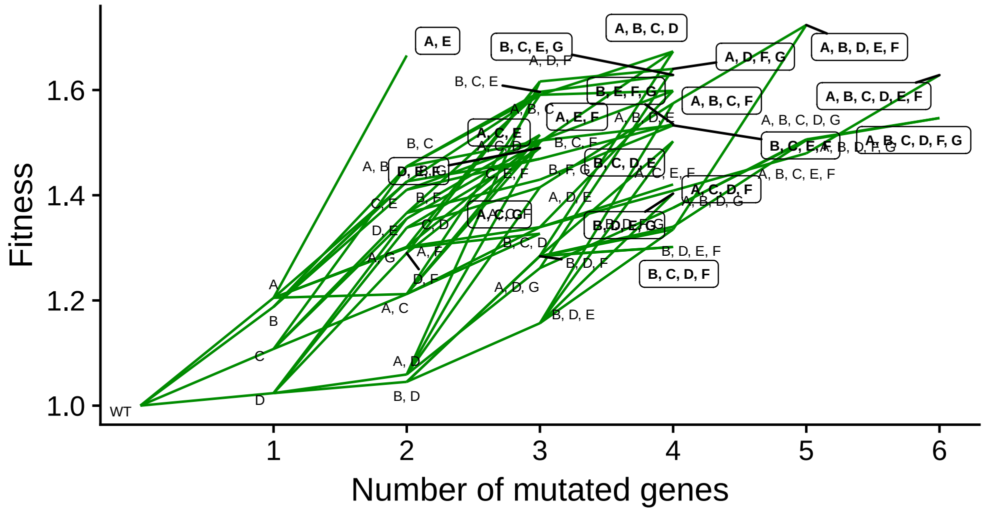

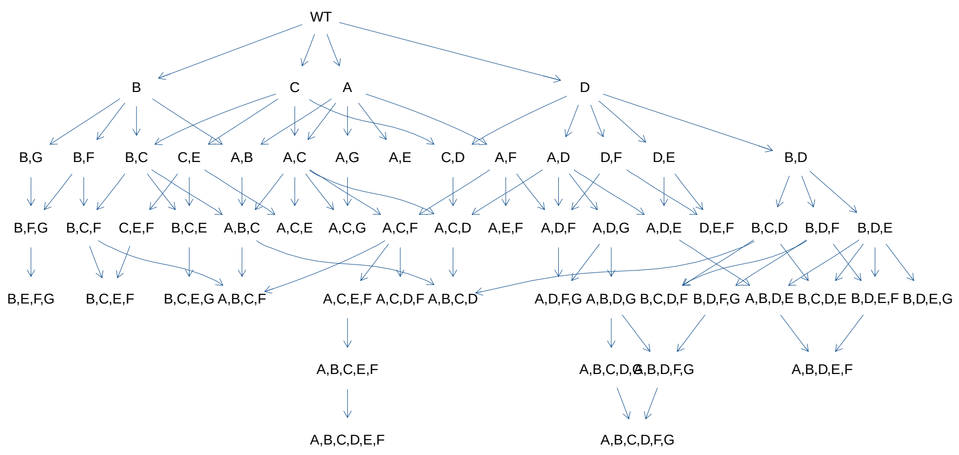

ID	WTbc1BmerHIsalgq
acc genotypes	38
peaks no back	15
removed edges	NA
prop rem edges	NA

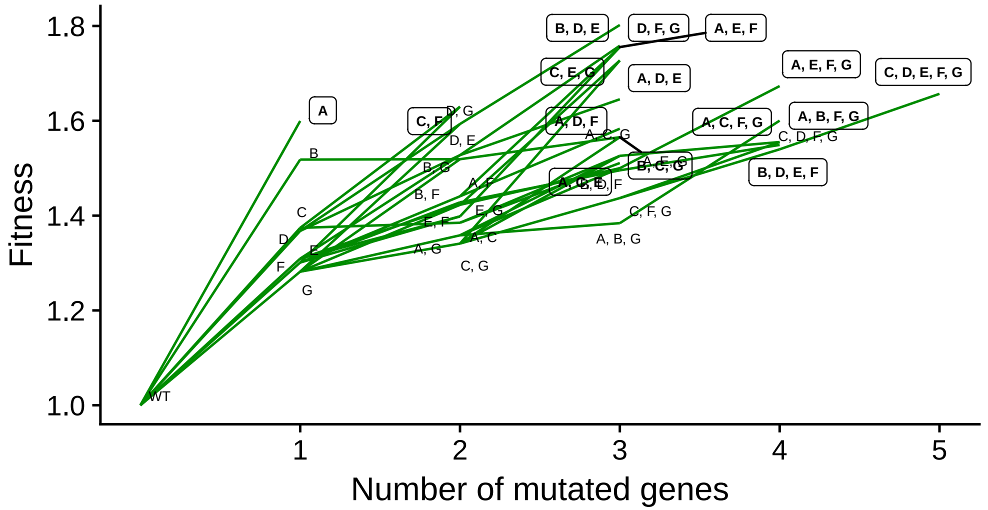

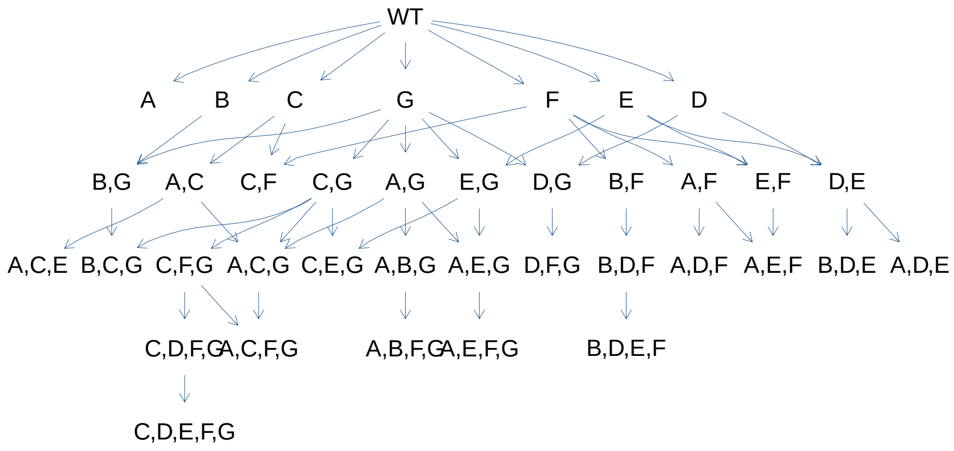

ID	WuYVFvHnmpxvVWQi
acc genotypes	72
peaks no back	20
removed edges	NA
prop rem edges	NA

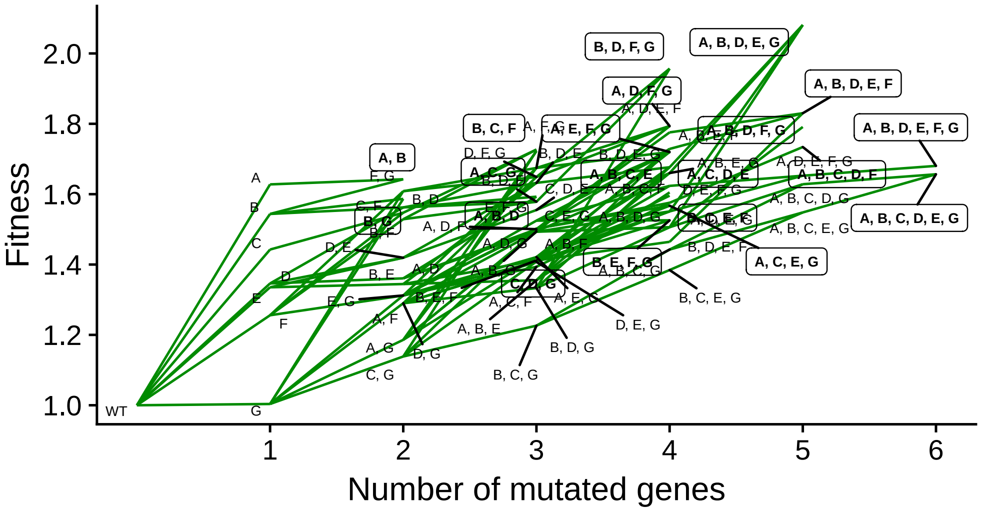

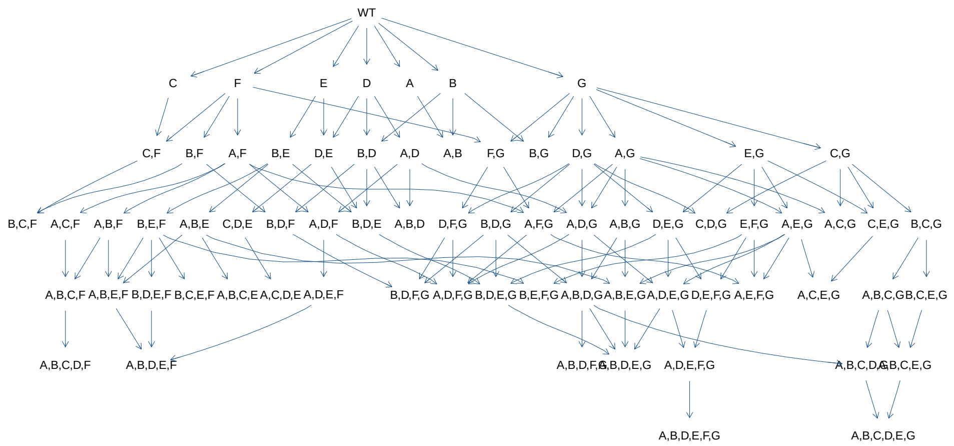

ID	WXvNSe1lPVS5wf7B
acc genotypes	46
peaks no back	14
removed edges	NA
prop rem edges	NA

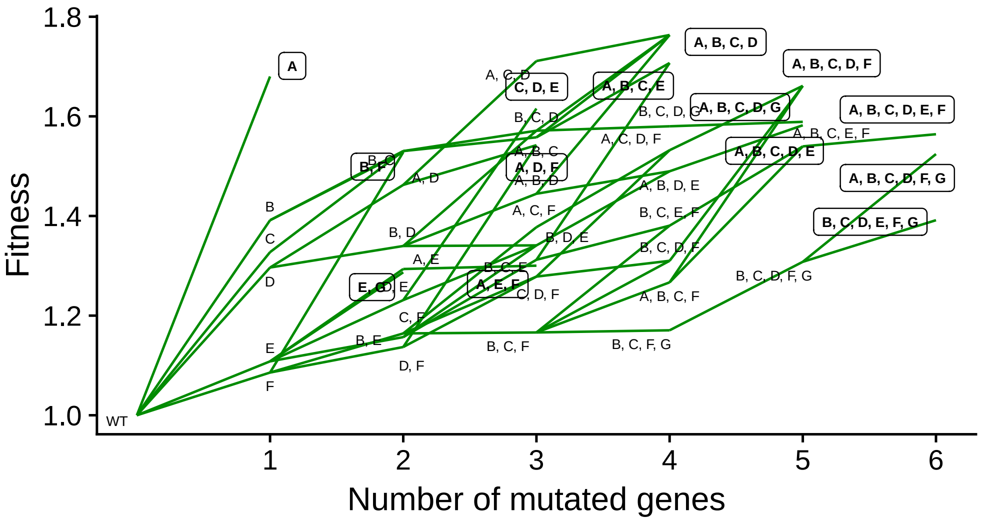

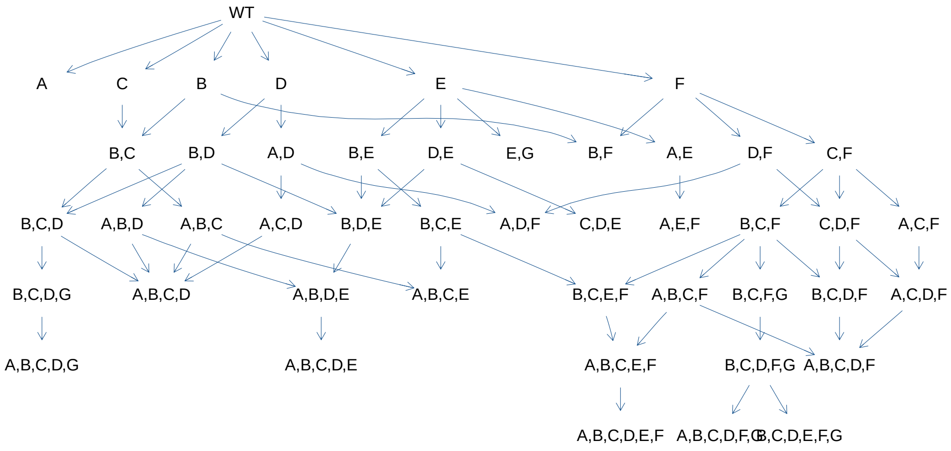

ID	x4ZFzf8D5q4Z4anR
acc genotypes	11
peaks no back	4
removed edges	NA
prop rem edges	NA

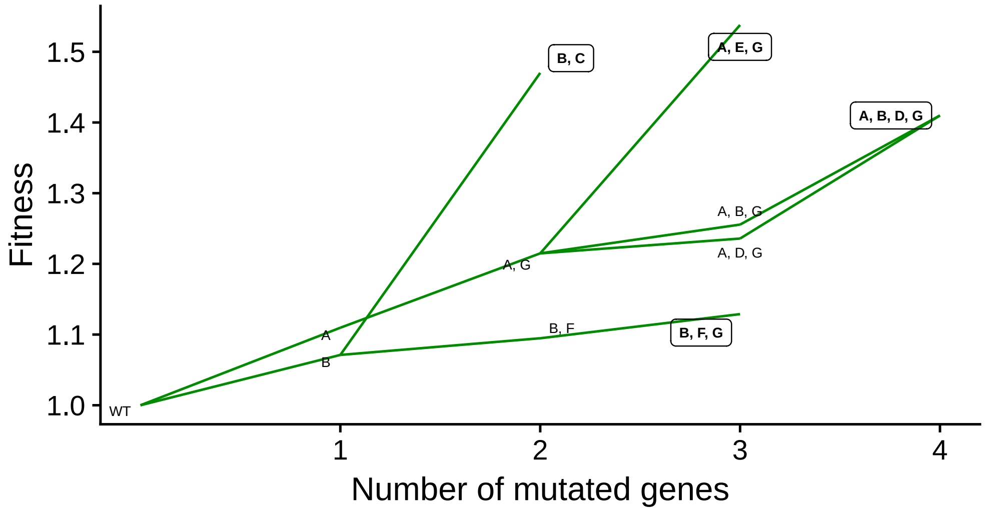

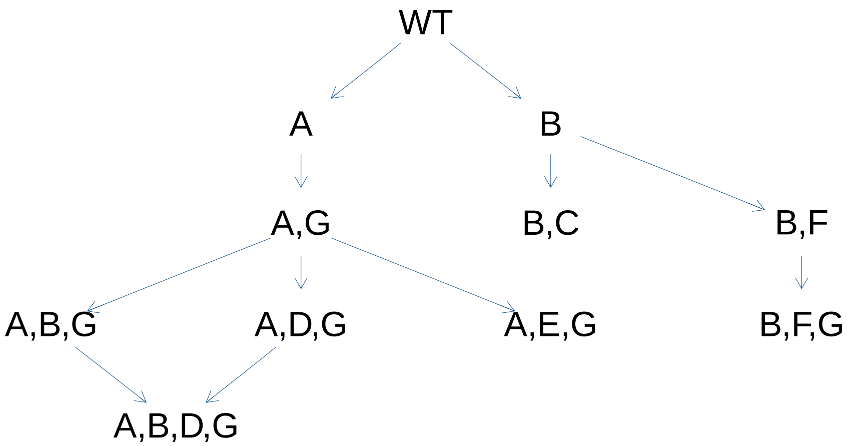

ID	x53kISylIWMWcjly
acc genotypes	22
peaks no back	6
removed edges	NA
prop rem edges	NA

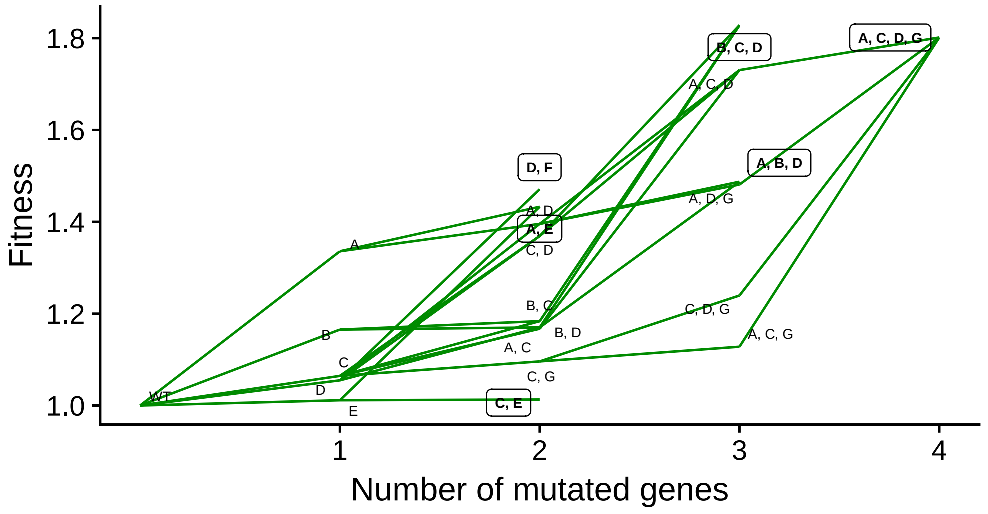

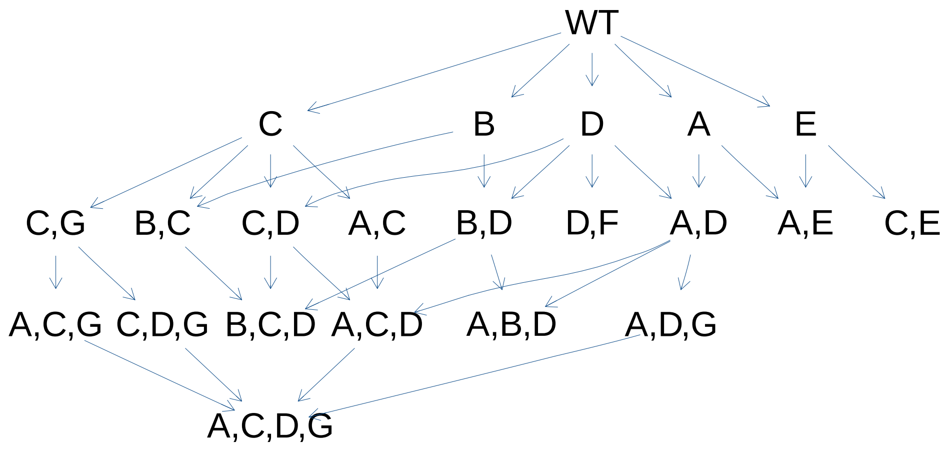

ID	x63vO10FZvDdTuarD
acc genotypes	20
peaks no back	9
removed edges	NA
prop rem edges	NA

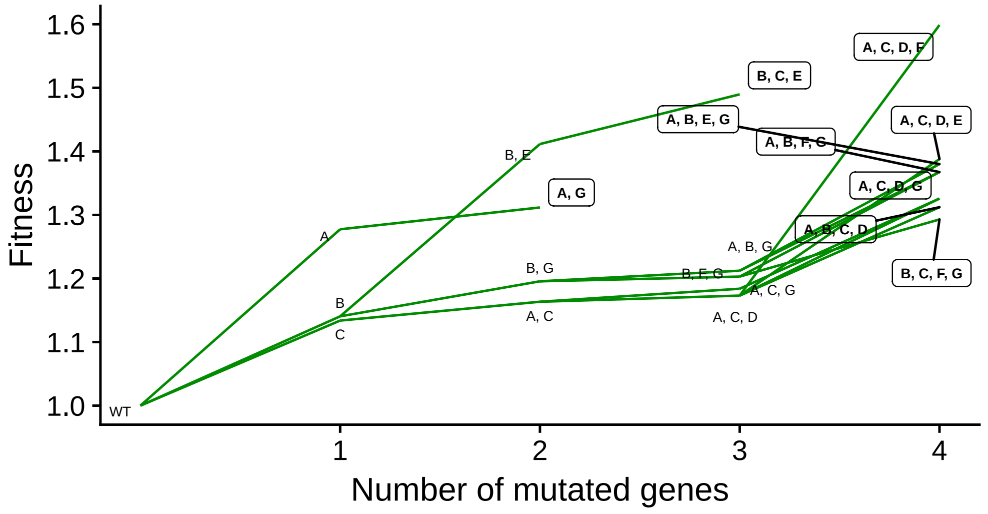

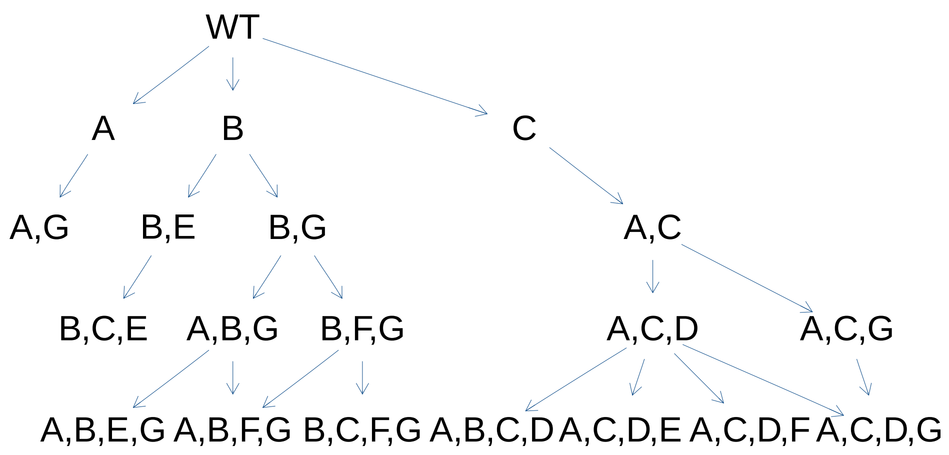

ID	XhJcAoB3WdJKttP7
acc genotypes	55
peaks no back	21
removed edges	NA
prop rem edges	NA

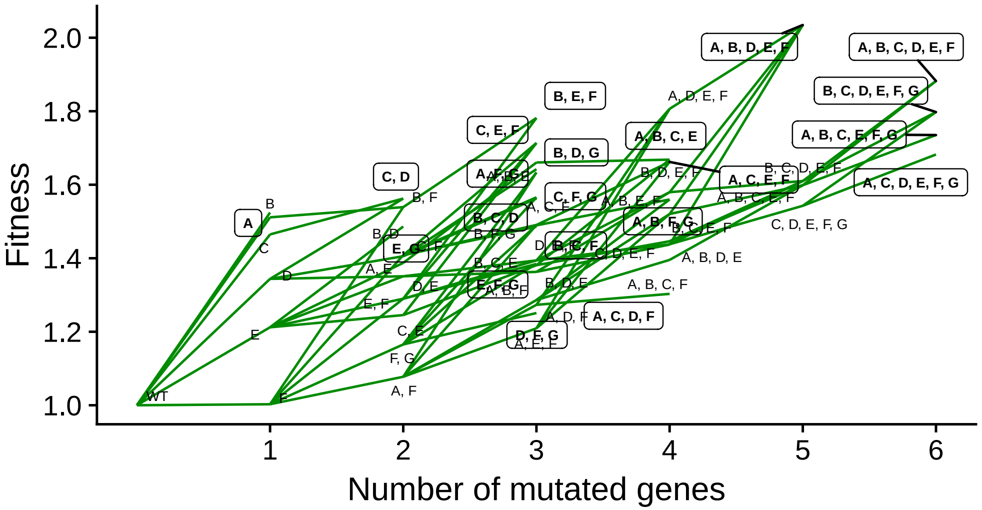

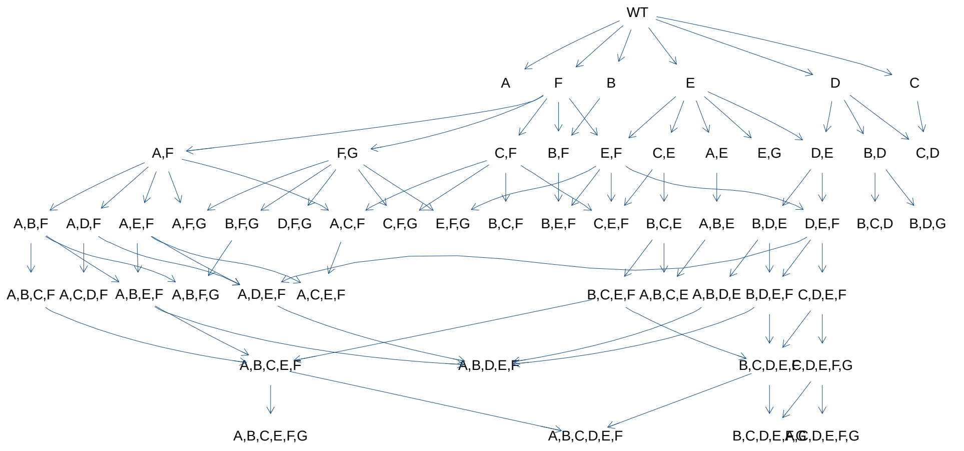

ID	xp6hoPCA8glPKMzG
acc genotypes	62
peaks no back	16
removed edges	NA
prop rem edges	NA

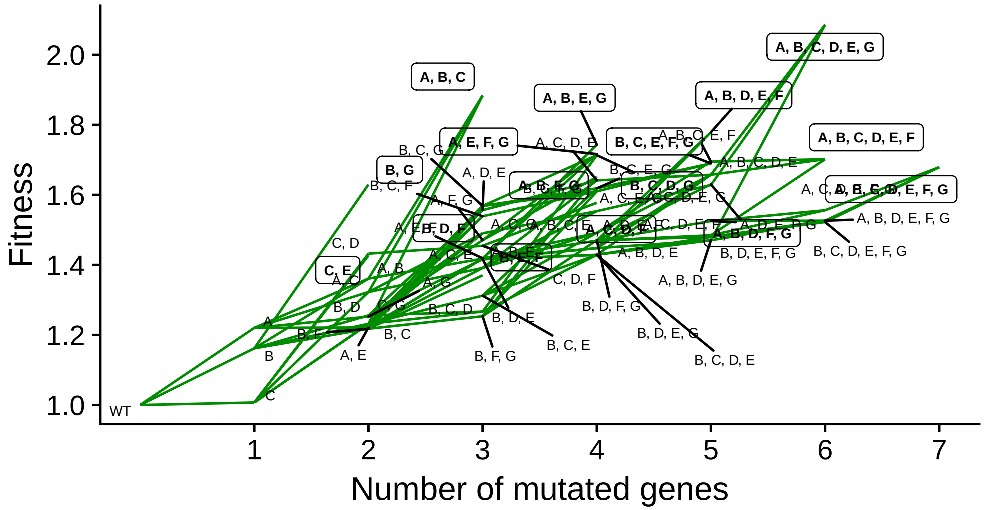

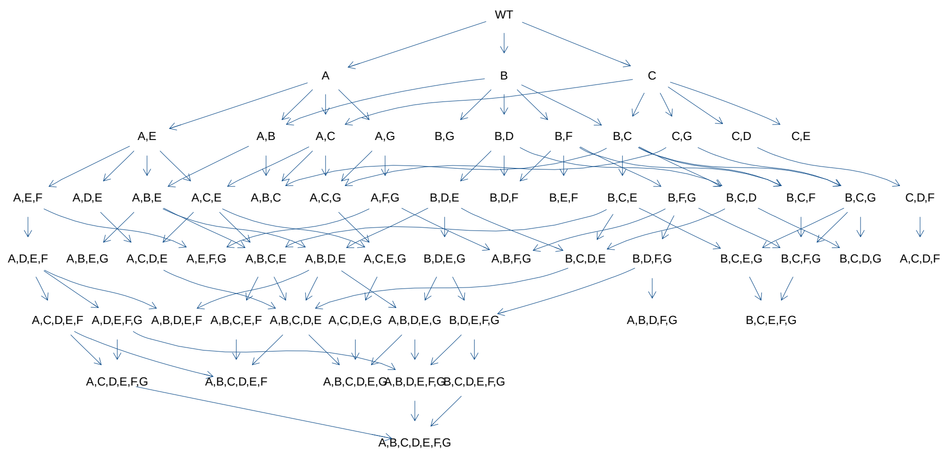

ID	xU2TICTIsth9hYb3
acc genotypes	23
peaks no back	7
removed edges	NA
prop rem edges	NA

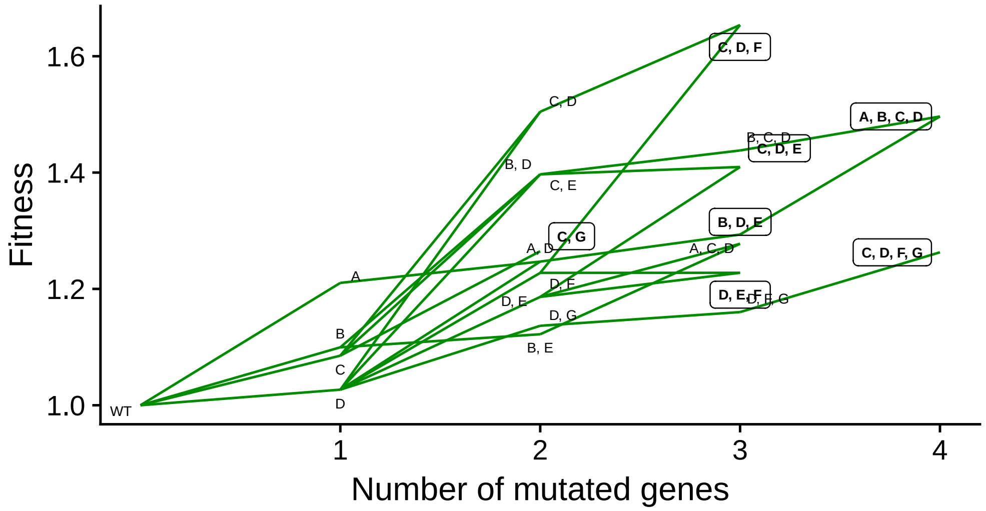

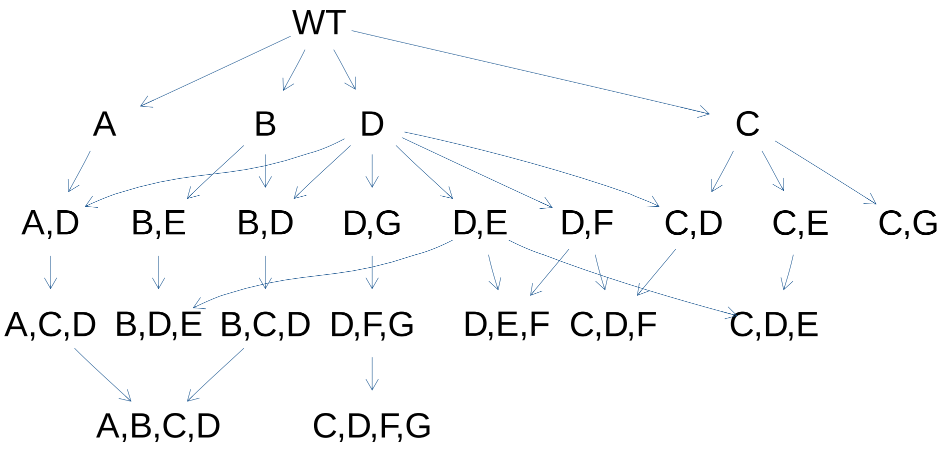

ID	xZGO107xRn107k9sKc
acc genotypes	37
peaks no back	12
removed edges	NA
prop rem edges	NA

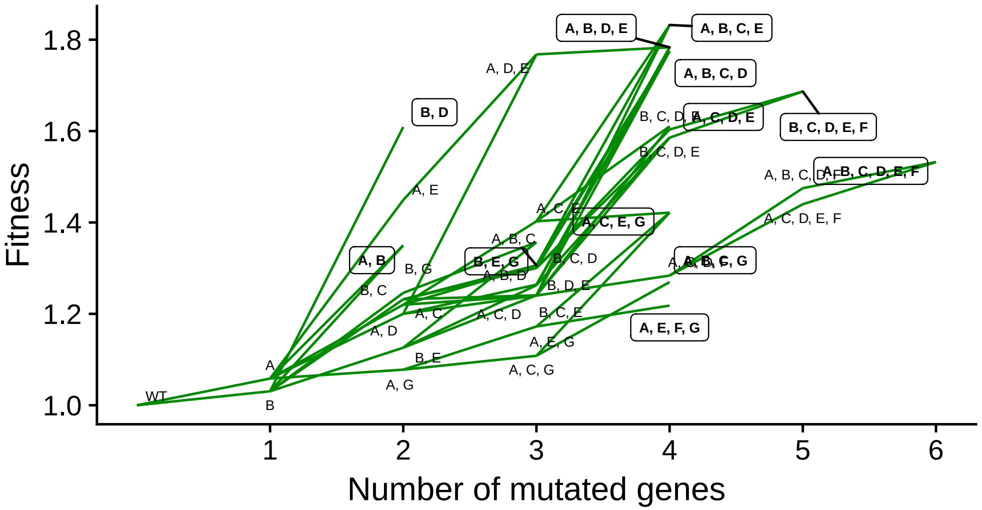

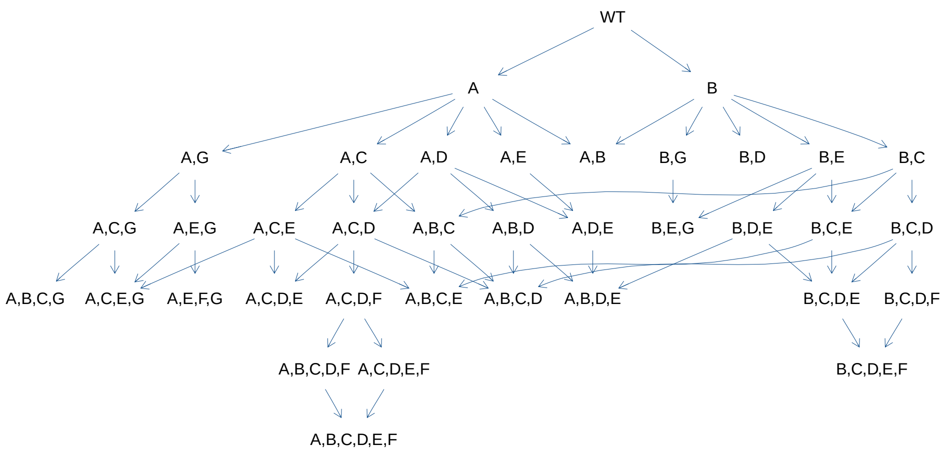

ID	Y8pEslk1p3jDN3PC
acc genotypes	16
peaks no back	7
removed edges	NA
prop rem edges	NA

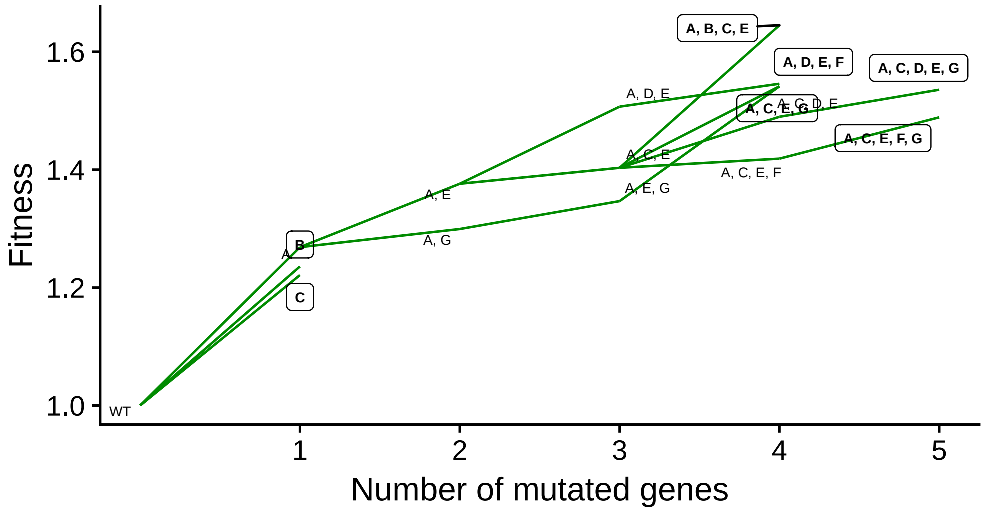

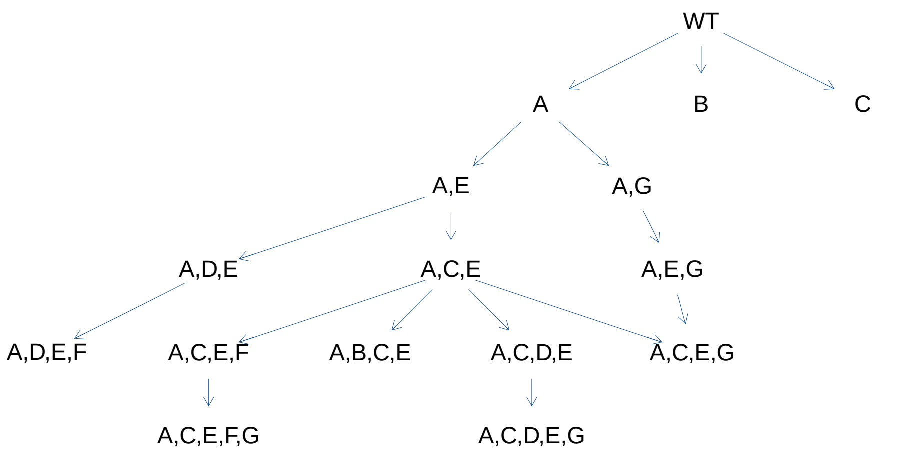

ID	yc851VadCH5z6cH10
acc genotypes	30
peaks no back	14
removed edges	NA
prop rem edges	NA

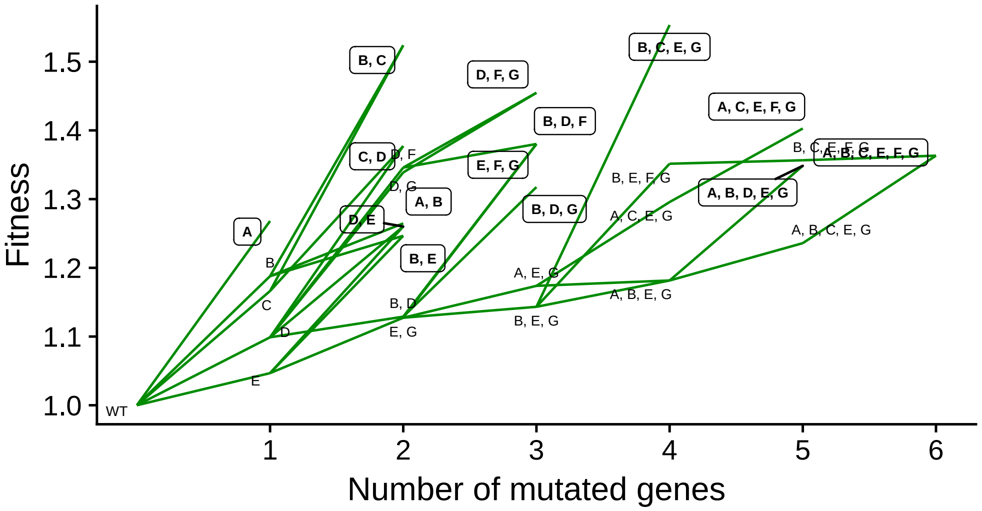

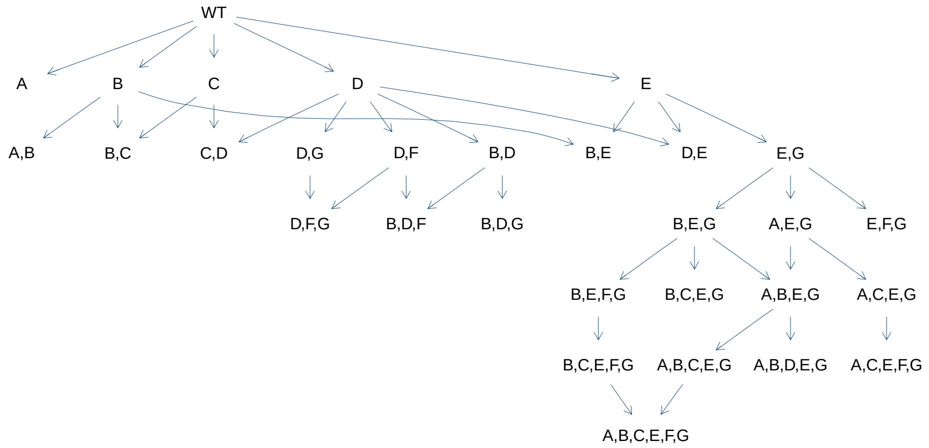

ID	yeCk19IGNHzq9Zia
acc genotypes	44
peaks no back	18
removed edges	NA
prop rem edges	NA

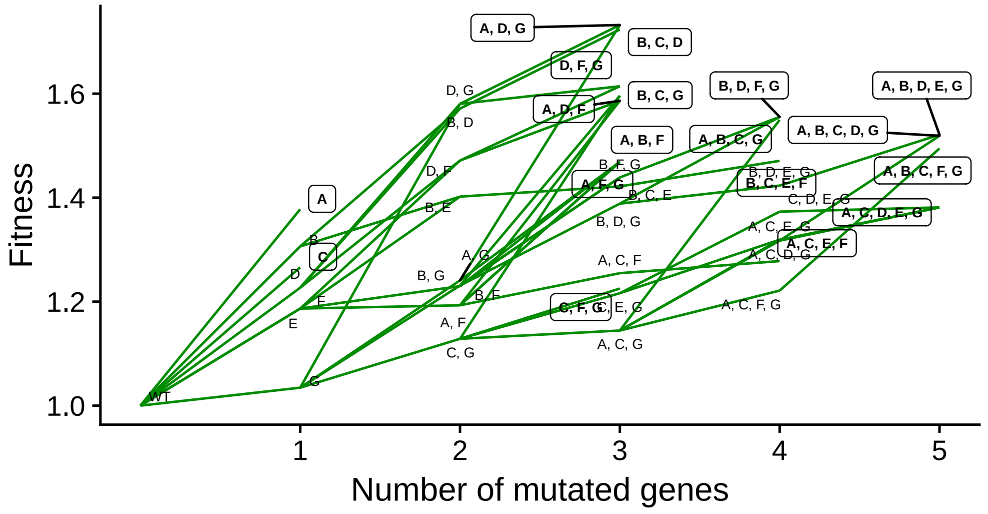

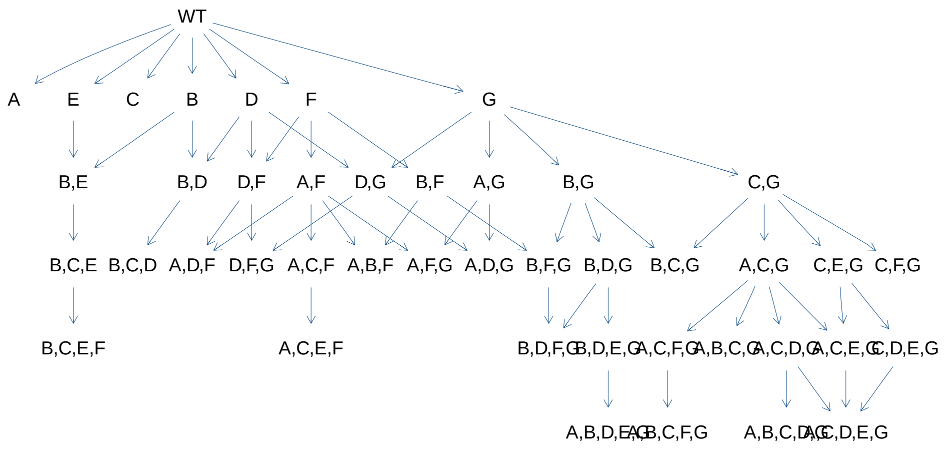

ID	YpVNspihDcE5kIsm
acc genotypes	48
peaks no back	12
removed edges	NA
prop rem edges	NA

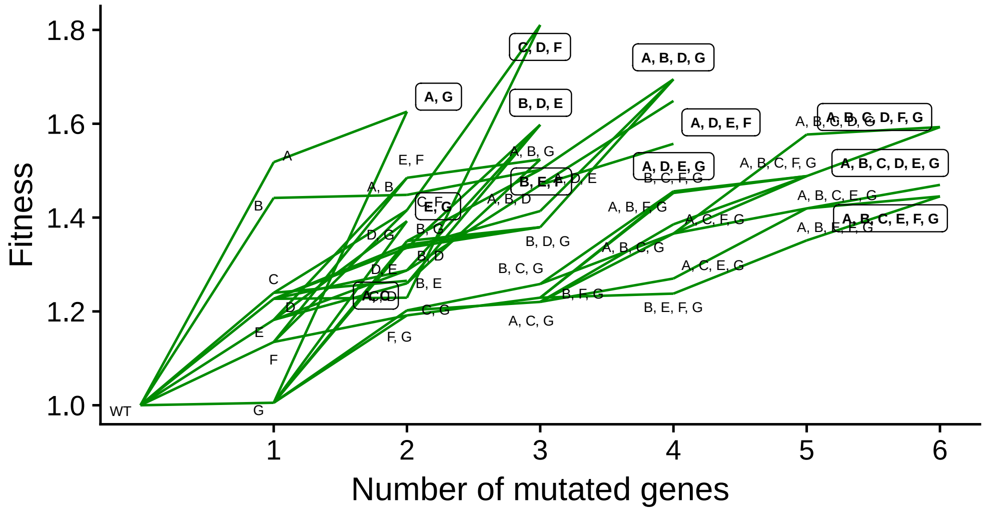

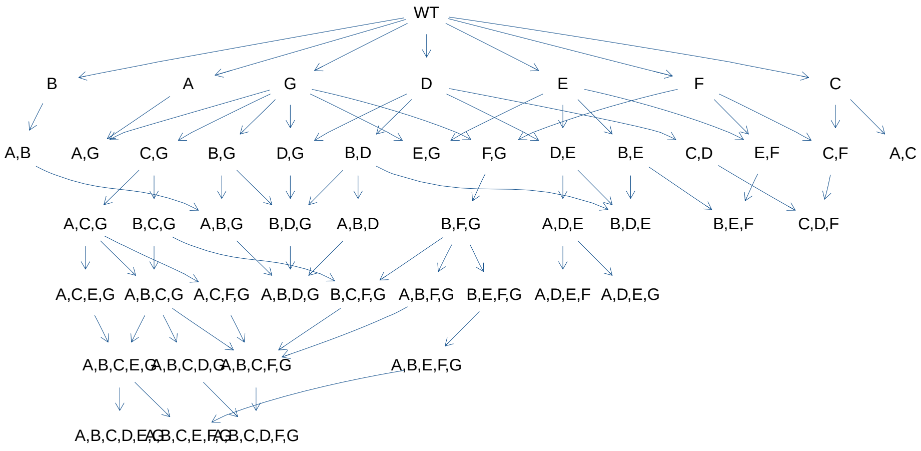

ID	YuMhOqw10Xkx33JOs
acc genotypes	61
peaks no back	17
removed edges	NA
prop rem edges	NA

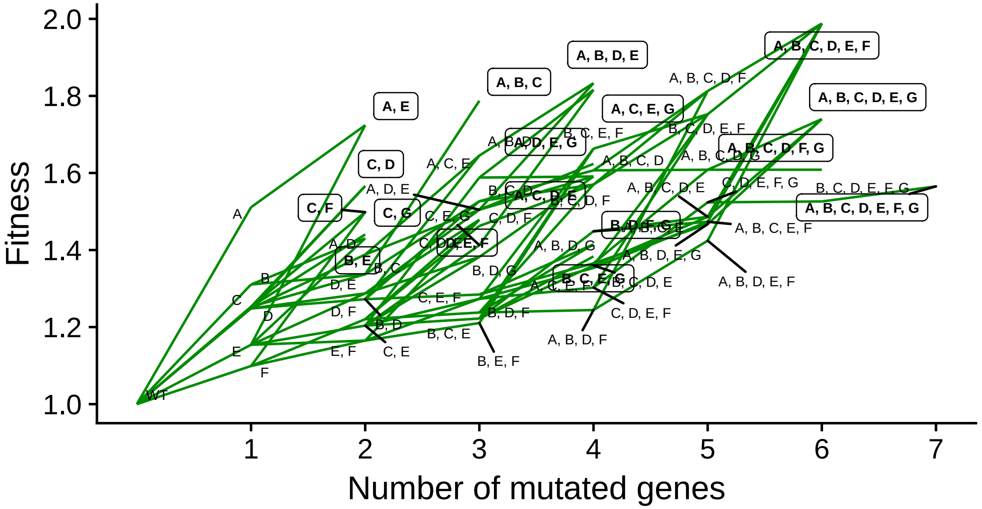

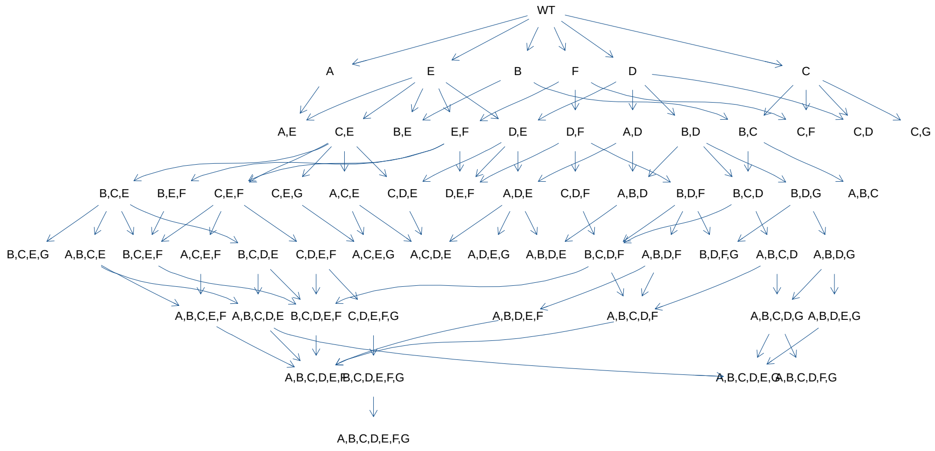

ID	yYOyPKhl3NOTcbHe
acc genotypes	50
peaks no back	18
removed edges	NA
prop rem edges	NA

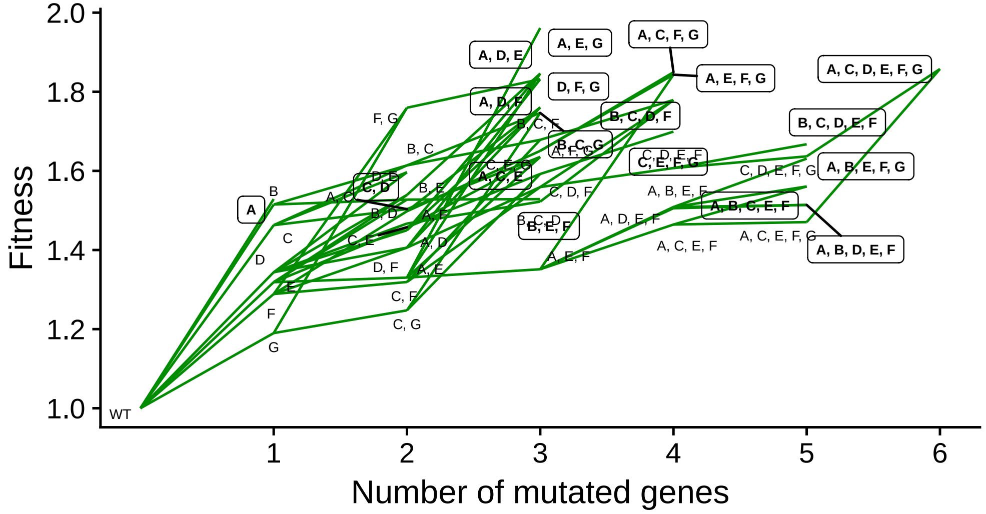

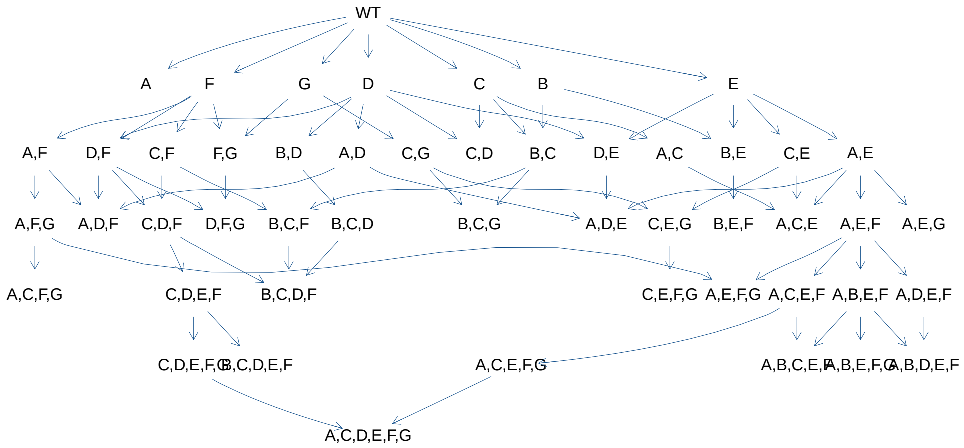

ID	Z10gb2ZT22WwOQVNk
acc genotypes	30
peaks no back	12
removed edges	NA
prop rem edges	NA

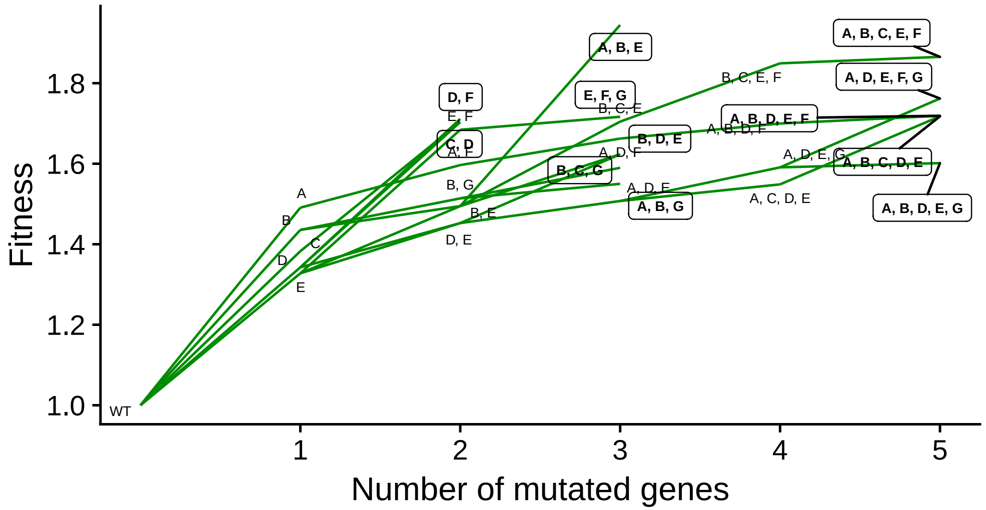

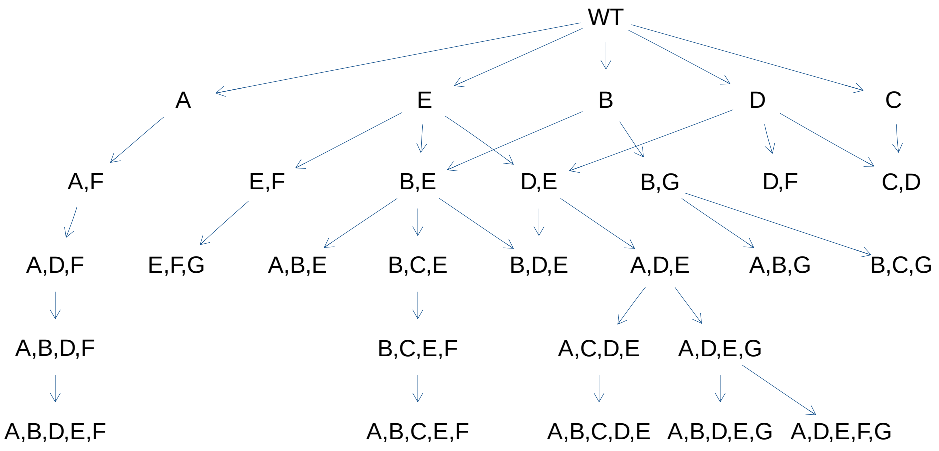

ID	Z7IpusjT8G9uuSKU
acc genotypes	59
peaks no back	18
removed edges	NA
prop rem edges	NA

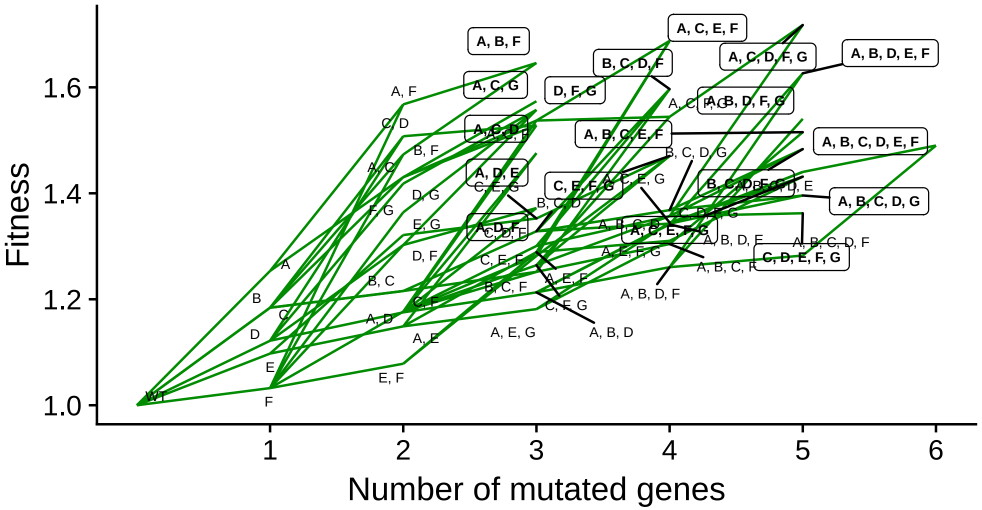

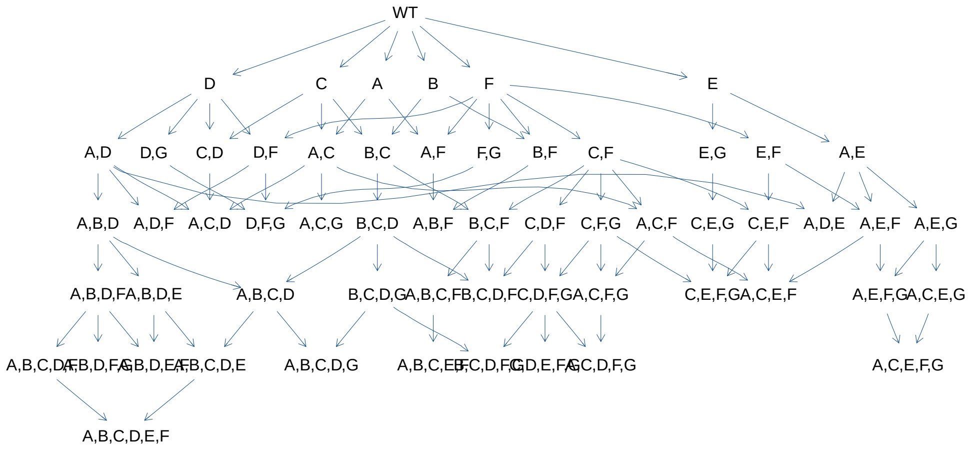

ID	ZGkYkR2uDqq44mge
acc genotypes	34
peaks no back	14
removed edges	NA
prop rem edges	NA

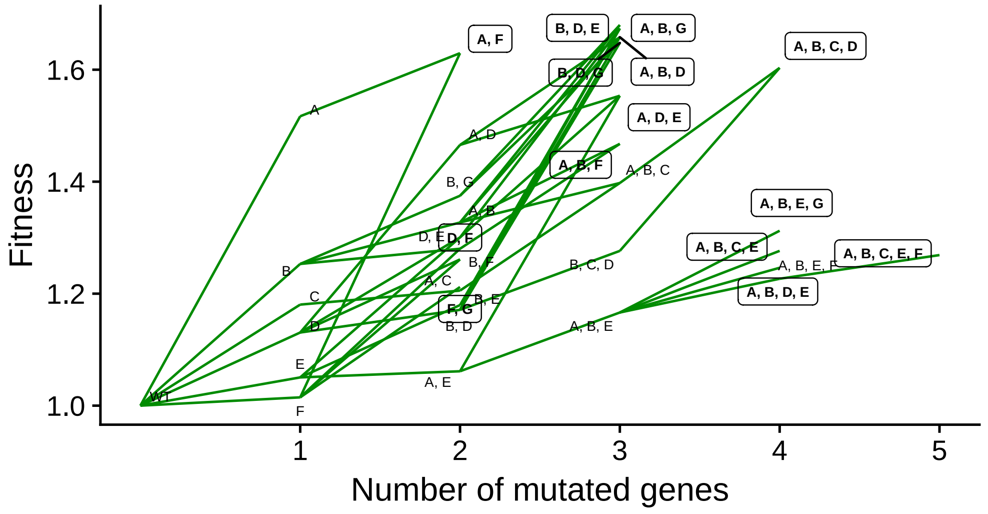

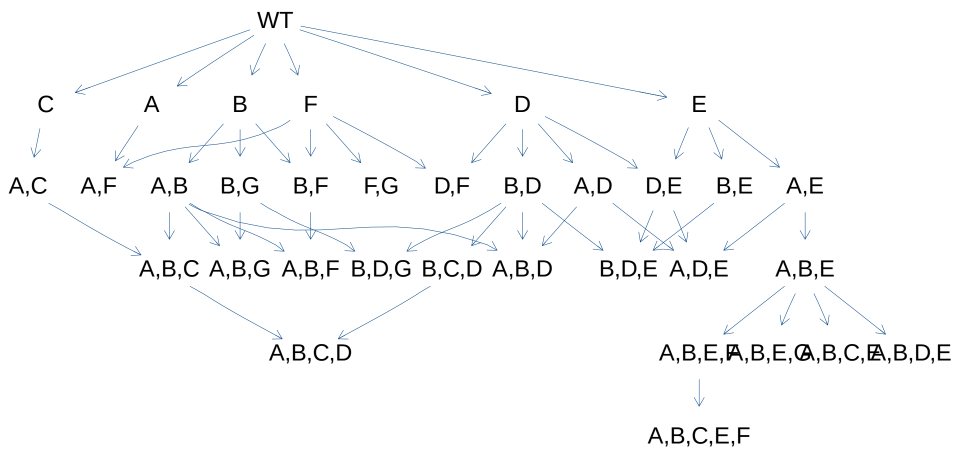

ID	ZjLMUPFuopk2WZUI
acc genotypes	22
peaks no back	7
removed edges	NA
prop rem edges	NA

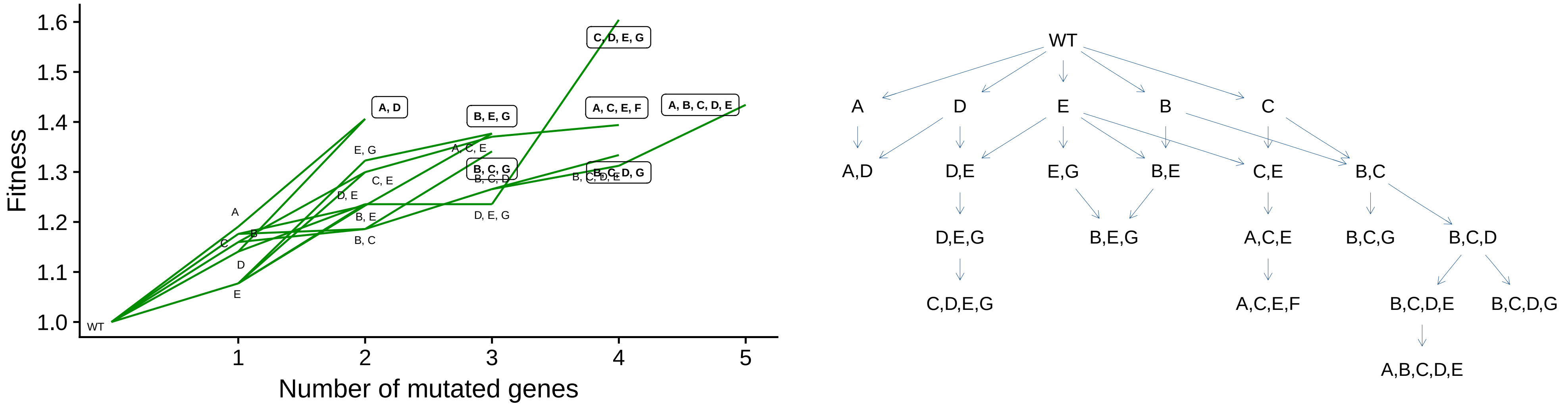

ID	ZVWECQo9V7rYLRNy
acc genotypes	72
peaks no back	10
removed edges	NA
prop rem edges	NA

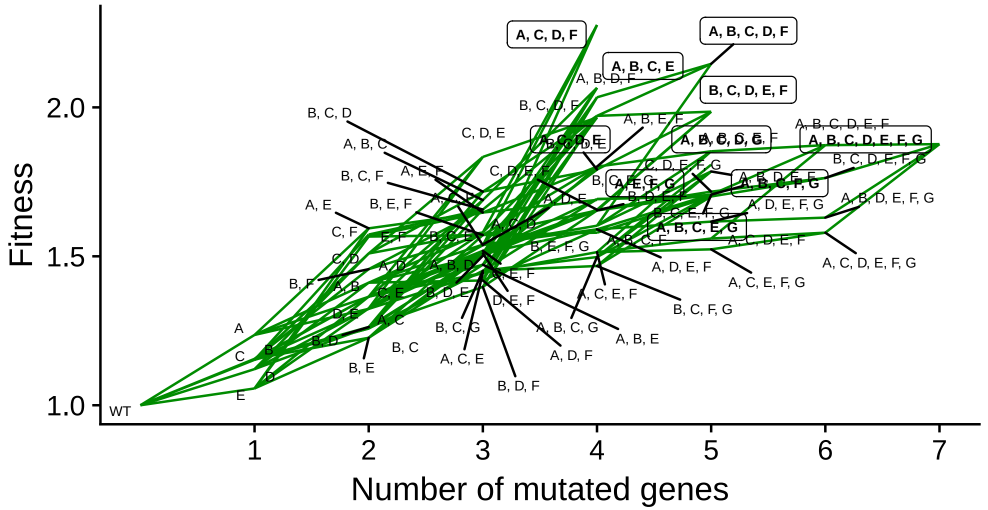

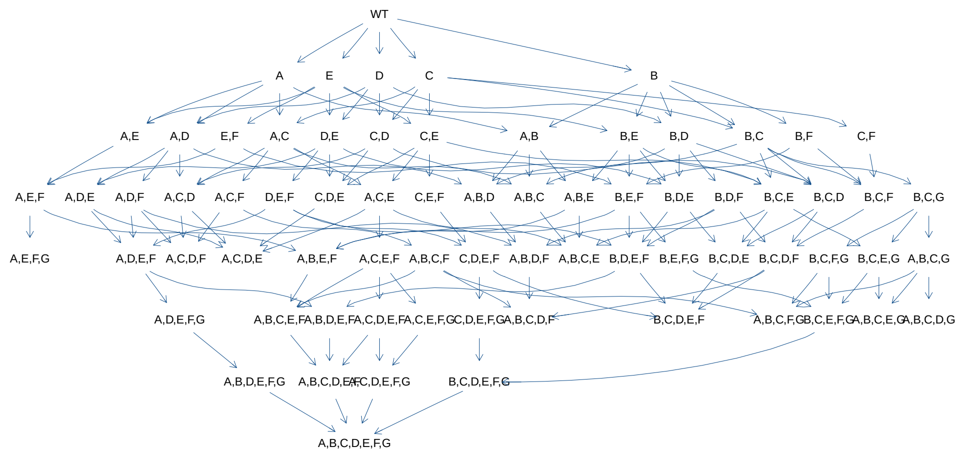

ID	ZWDP3y7TKpRZUzKJ
acc genotypes	46
peaks no back	15
removed edges	NA
prop rem edges	NA

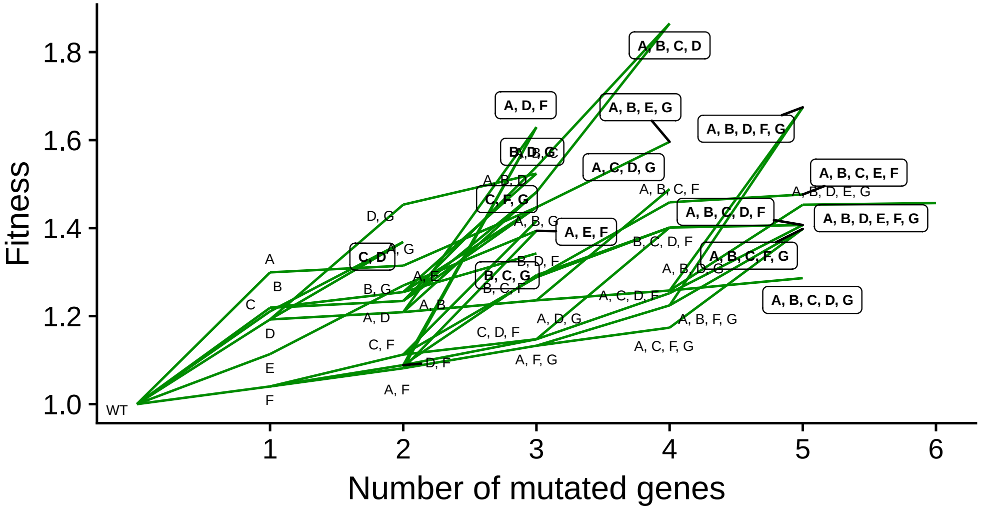

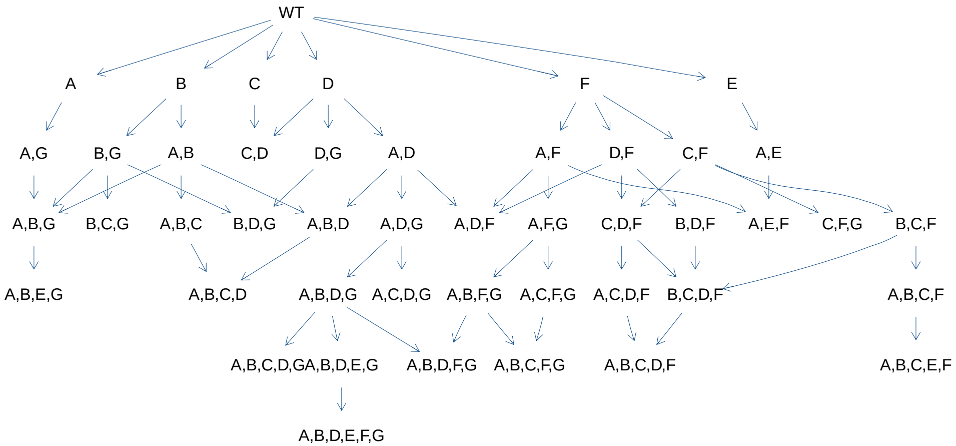

ID	ZXShHtwLyLLxewaU
acc genotypes	51
peaks no back	22
removed edges	NA
prop rem edges	NA

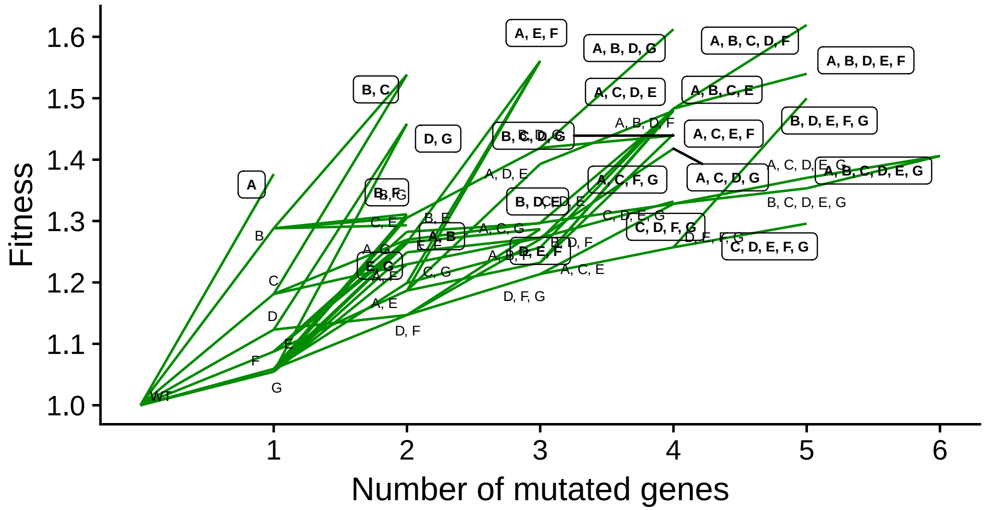

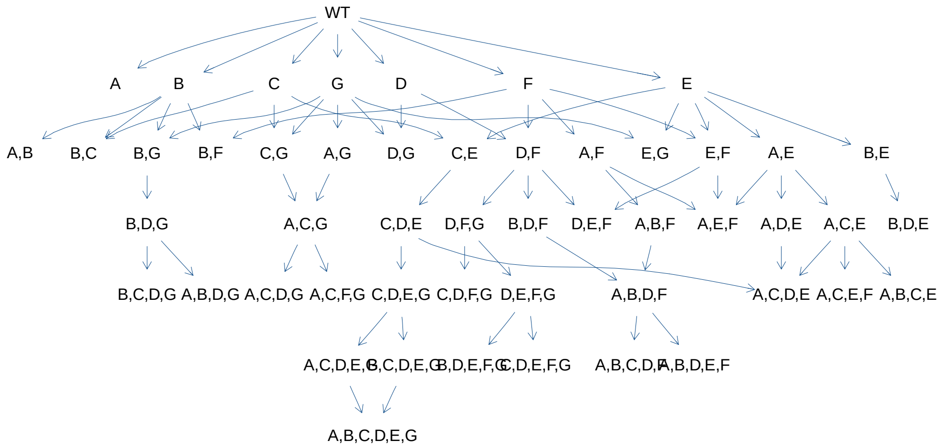

3. Ten genes

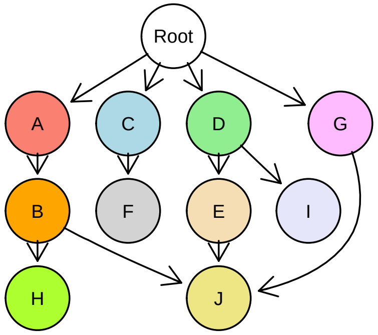

ID	10Ic5x6106RnZOmZ6Z
acc genotypes	132
peaks no back	1
removed edges	0
prop rem edges	0

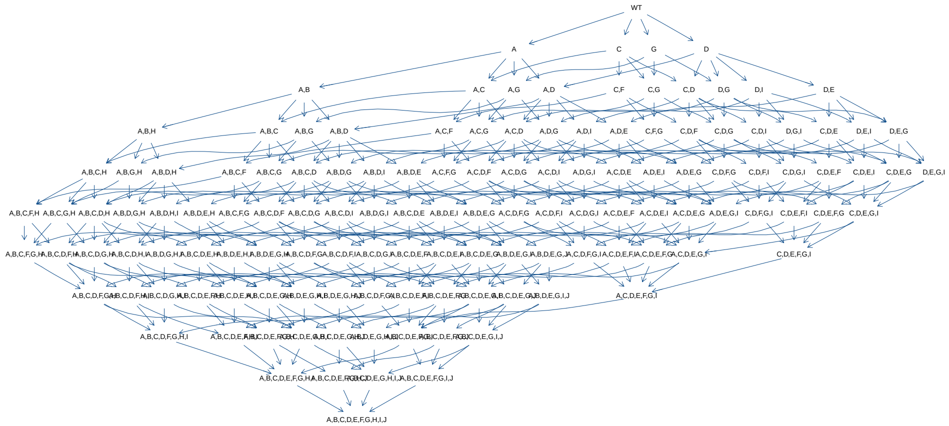

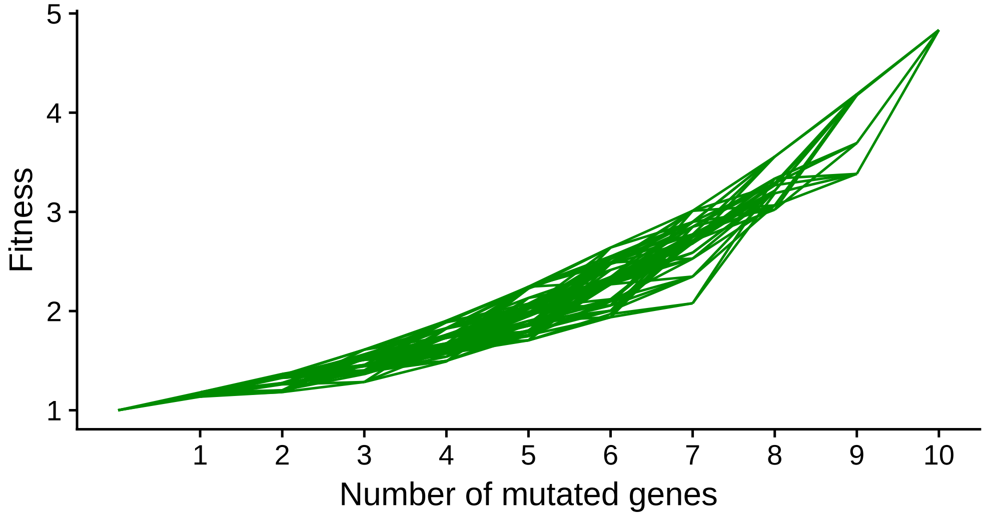

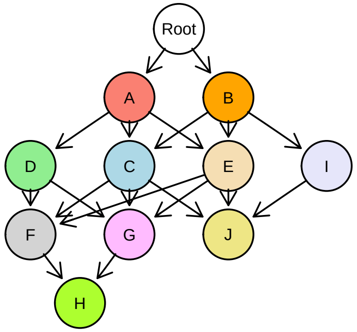

ID	10sA3fCTx7METho38
acc genotypes	35
peaks no back	1
removed edges	0
prop rem edges	0

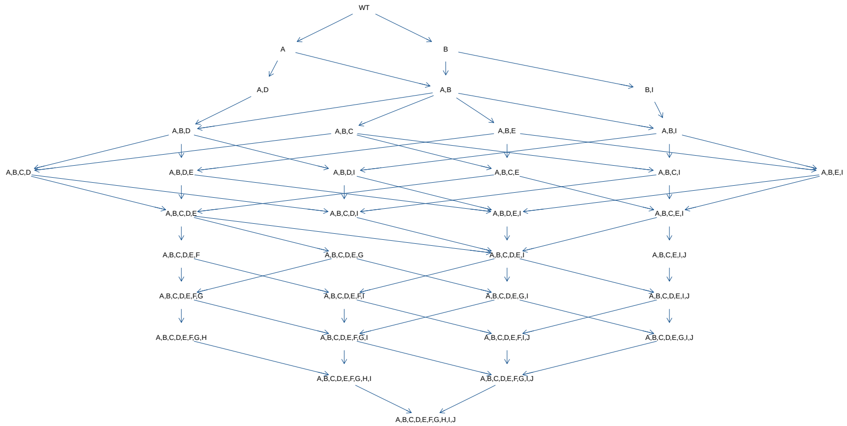

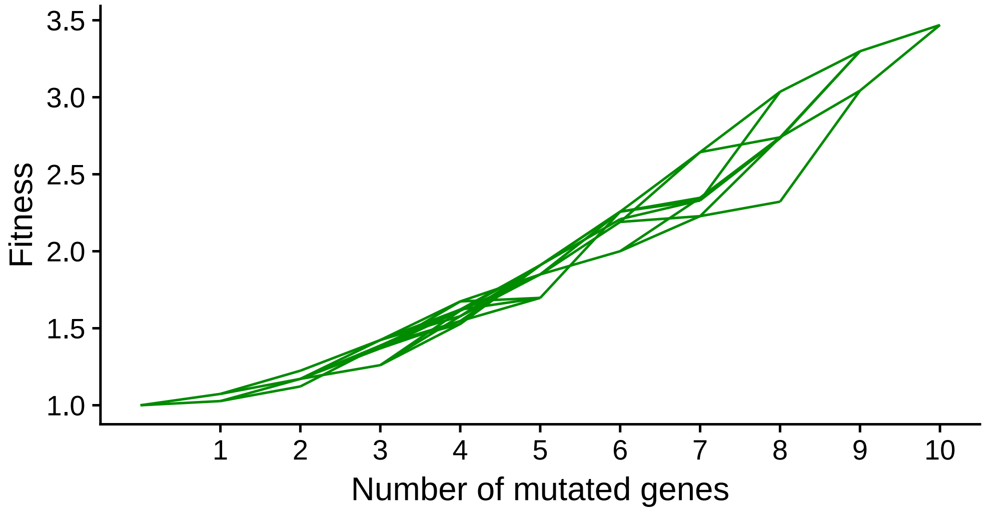

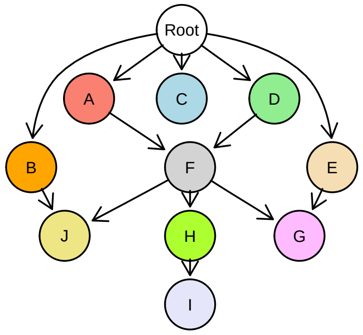

ID	1MaScdYnWRHseOZ7
acc genotypes	86
peaks no back	1
removed edges	0
prop rem edges	0

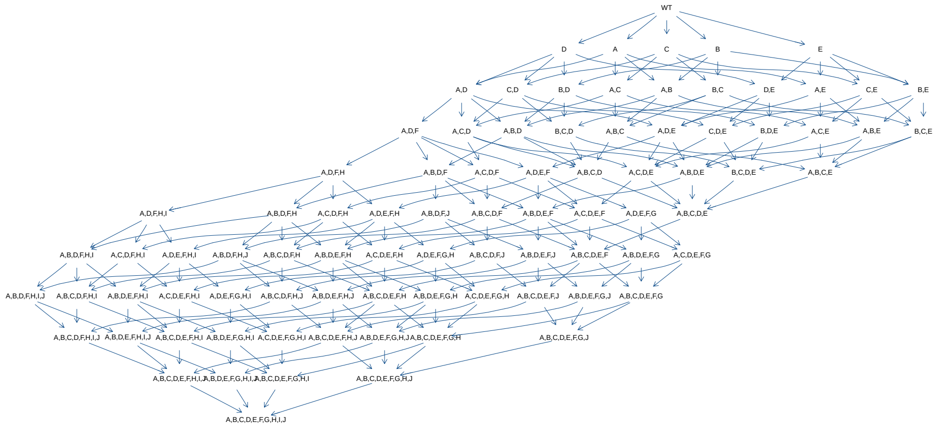

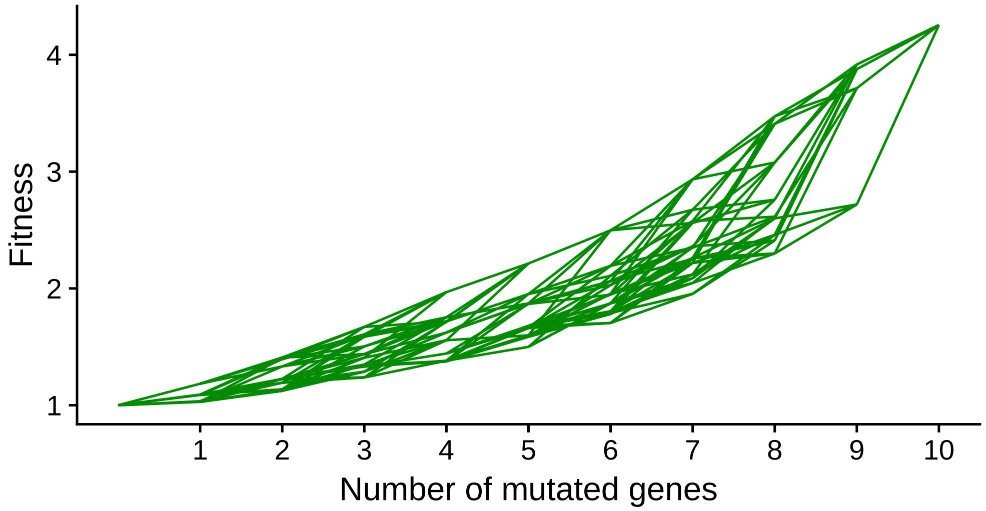

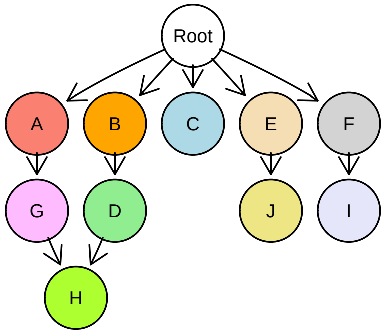

ID 1Nl1Lblu3EuEdbw8
acc genotypes 180
peaks no back 1
removed edges 0
prop rem edges 0

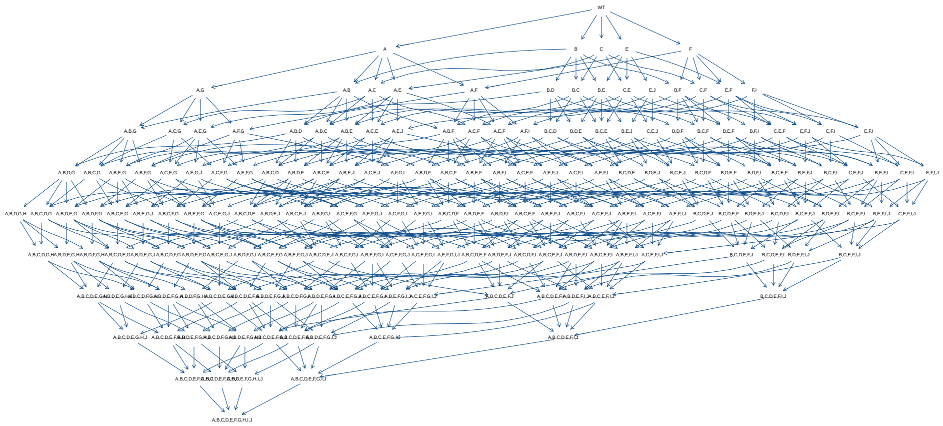

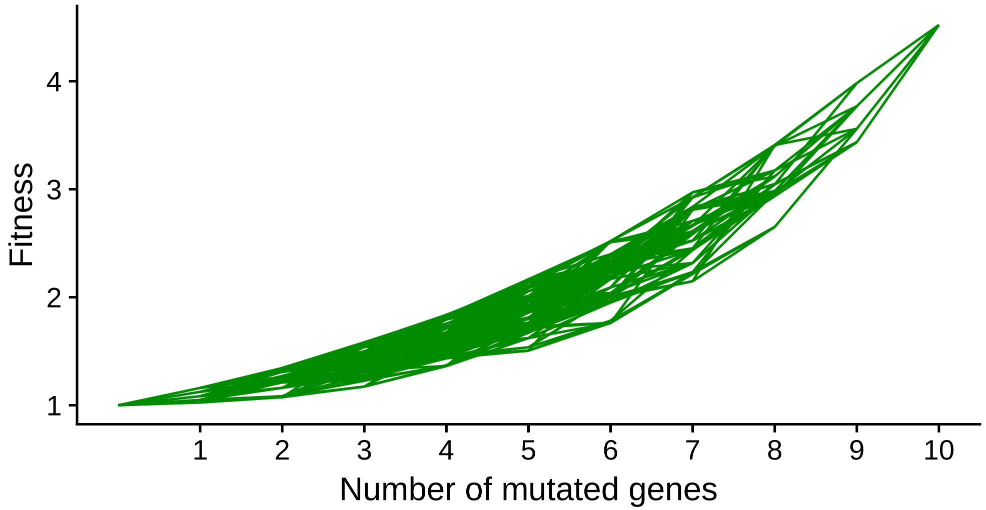

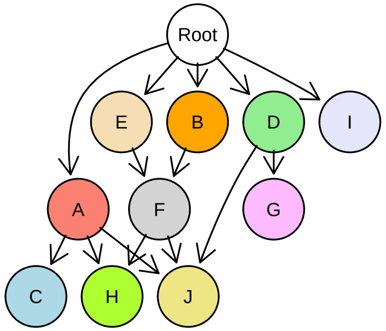

ID	2PhVbwXtsibgHoCR
acc genotypes	118
peaks no back	1
removed edges	0
prop rem edges	0

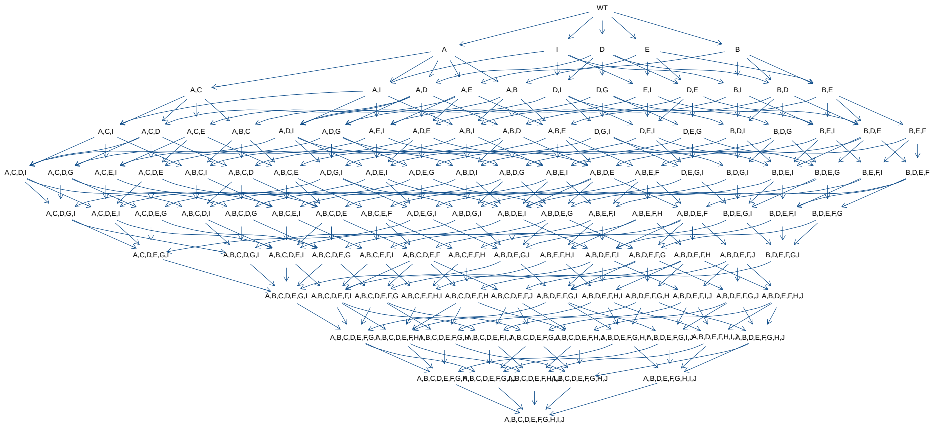

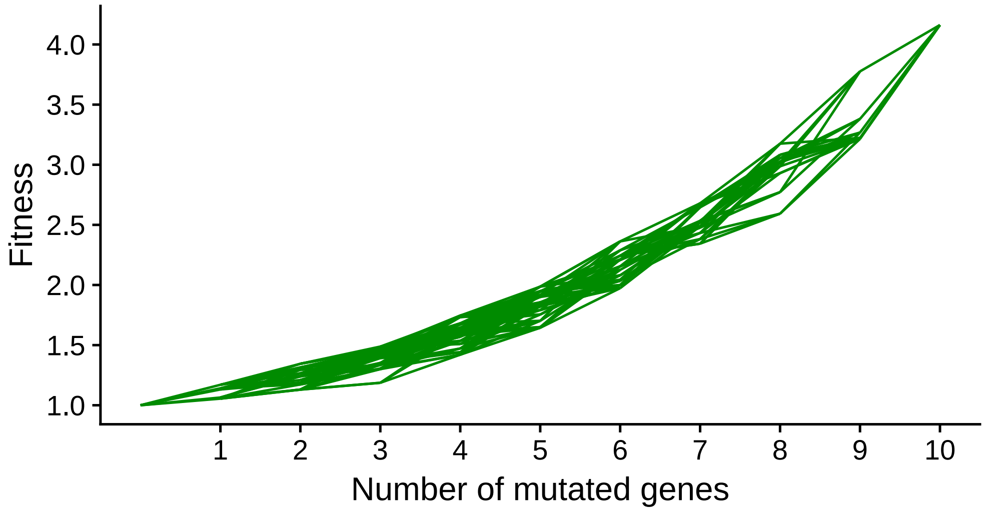

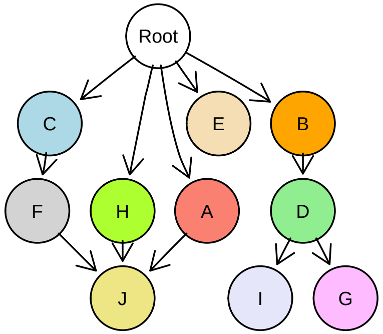

ID	2QFqp77NSVxKUuRw
acc genotypes	156
peaks no back	1
removed edges	0
prop rem edges	0

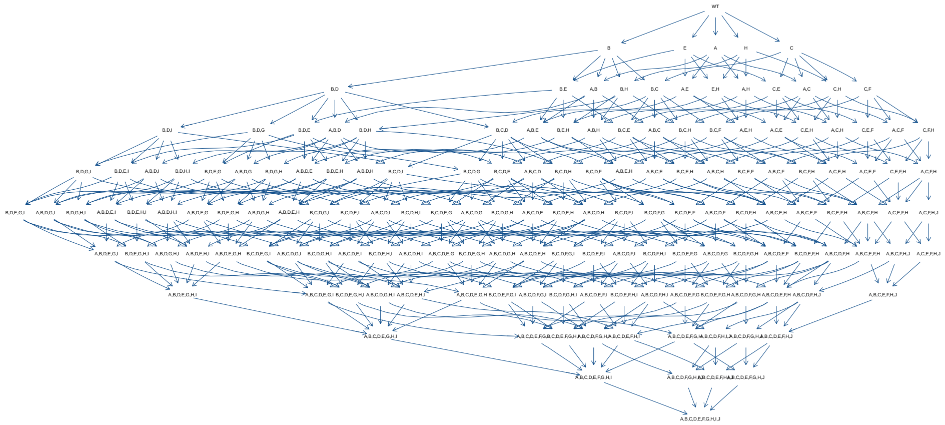

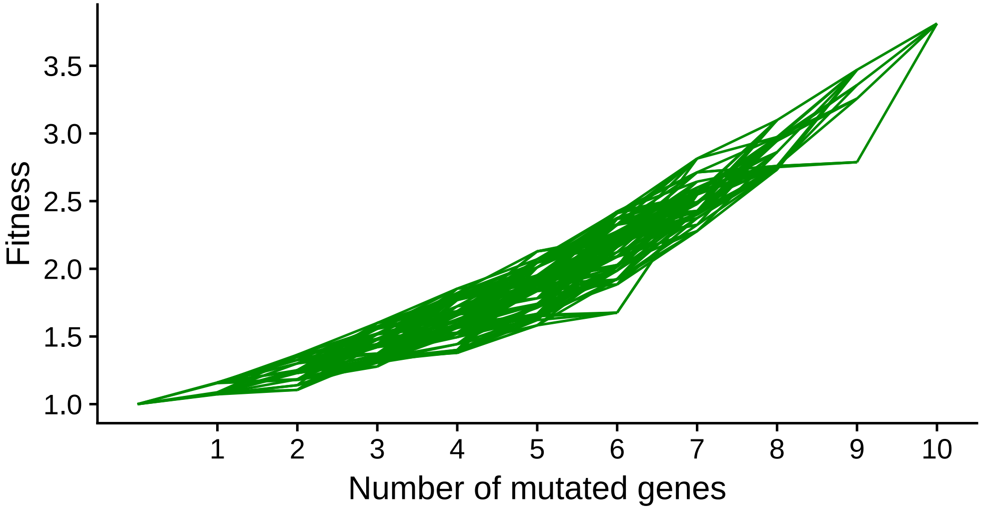

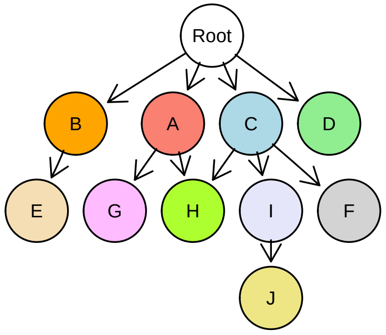

ID	3LaNuAy8eWU4GMwm
acc genotypes	198
peaks no back	1
removed edges	0
prop rem edges	0

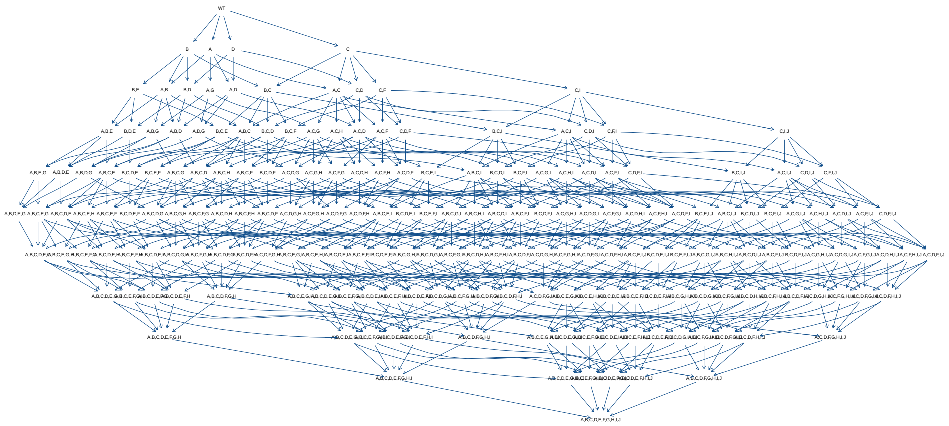

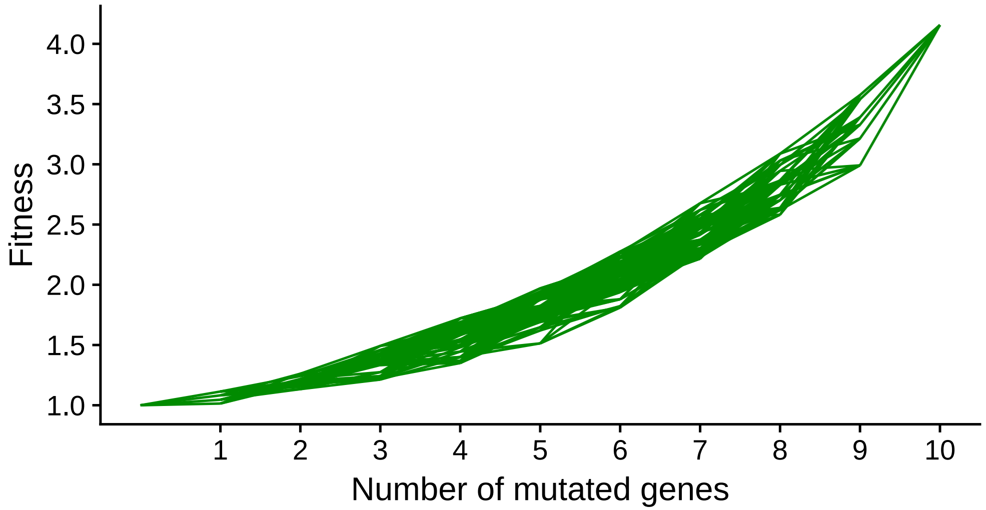

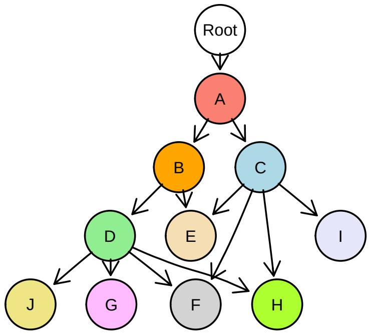

ID 3qwClNpvS1TW6yT3
acc genotypes 77
peaks no back 1
removed edges 0
prop rem edges 0

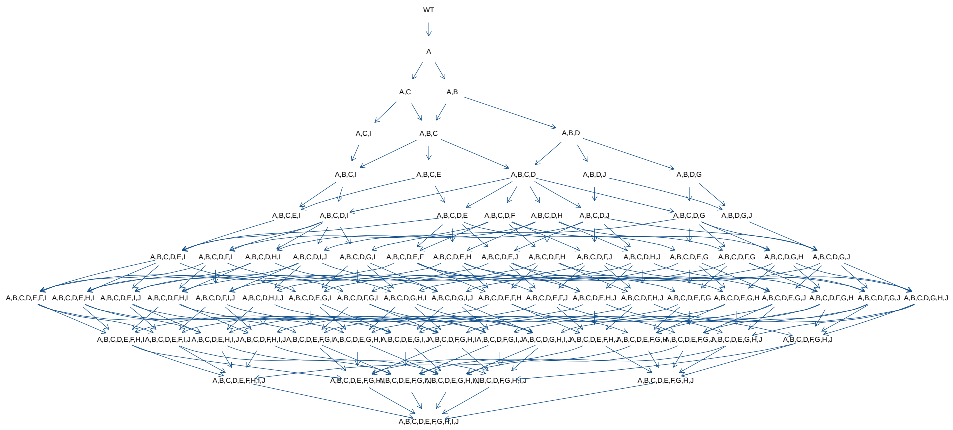

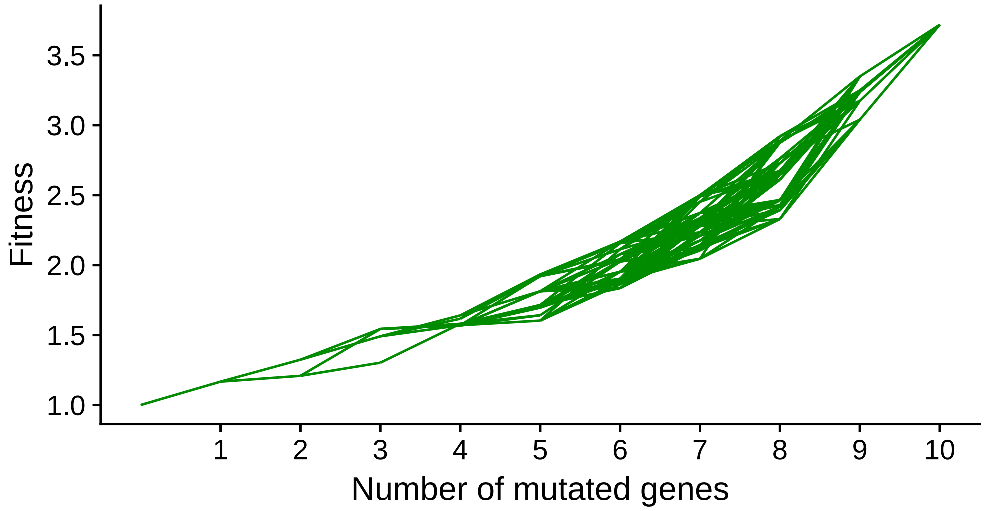

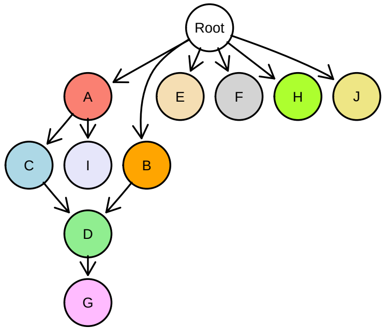

ID	3wRHh10MSegWf4SVc
acc genotypes	224
peaks no back	1
removed edges	0
prop rem edges	0

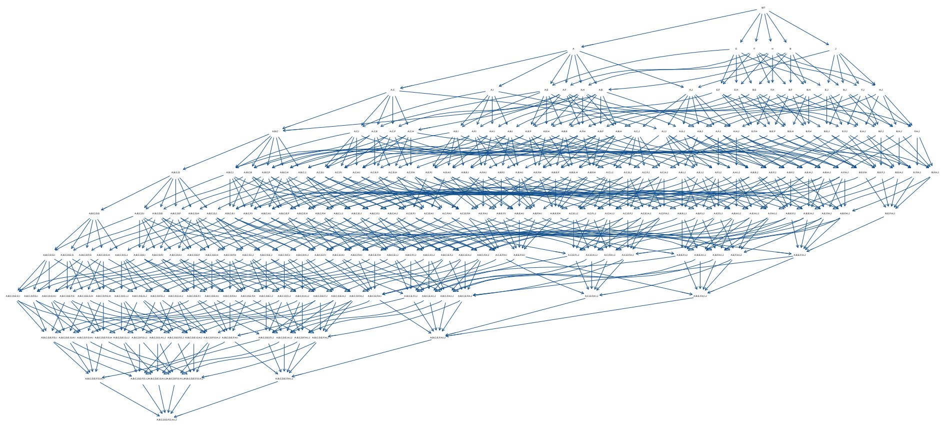

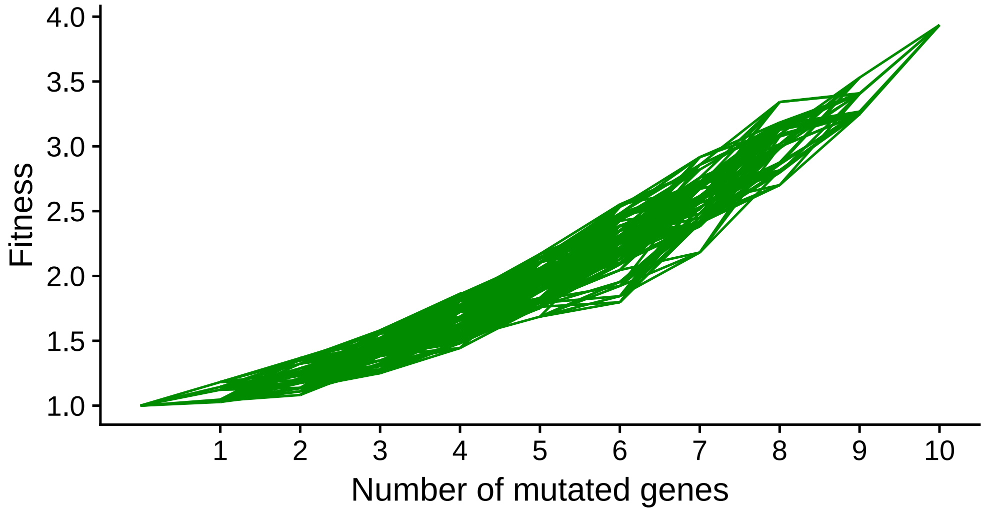

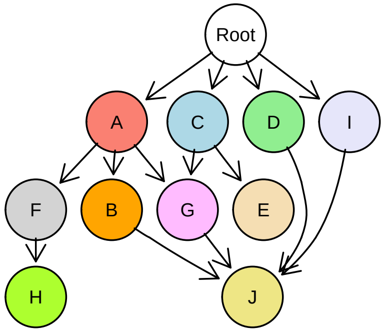

ID	3xUzonJpTvNpS9cY
acc genotypes	138
peaks no back	1
removed edges	0
prop rem edges	0

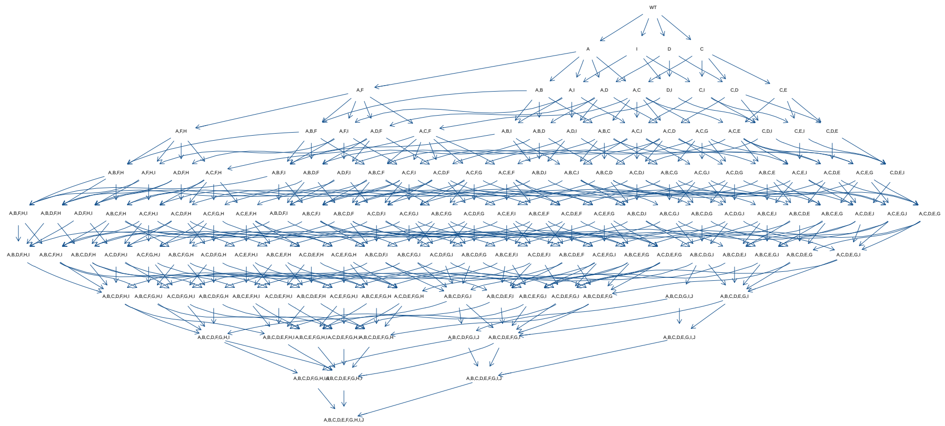

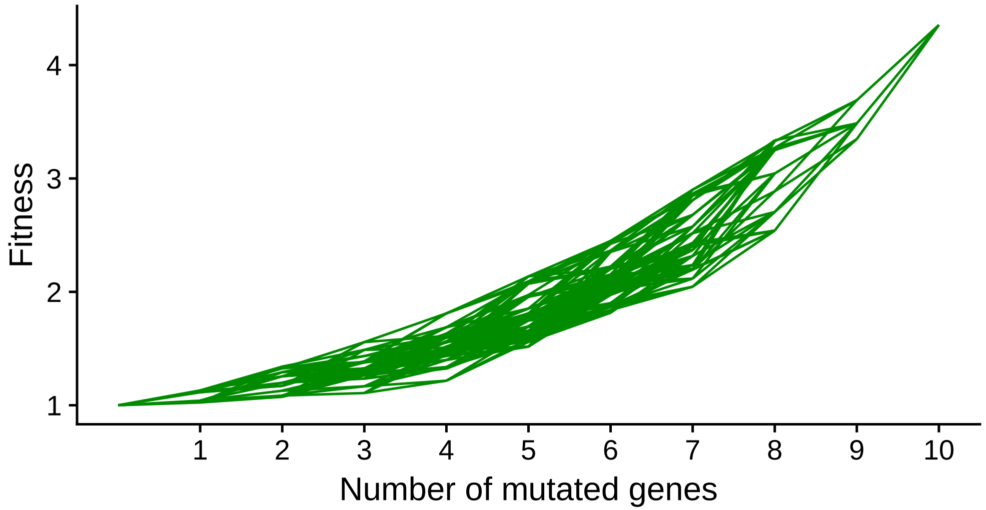

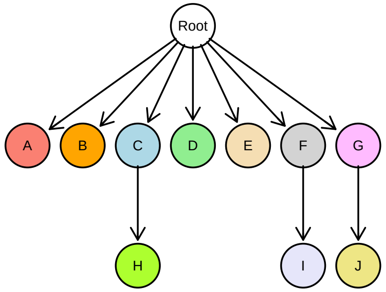

ID	41wj4xc10qjq6tByv
acc genotypes	432
peaks no back	1
removed edges	0
prop rem edges	0

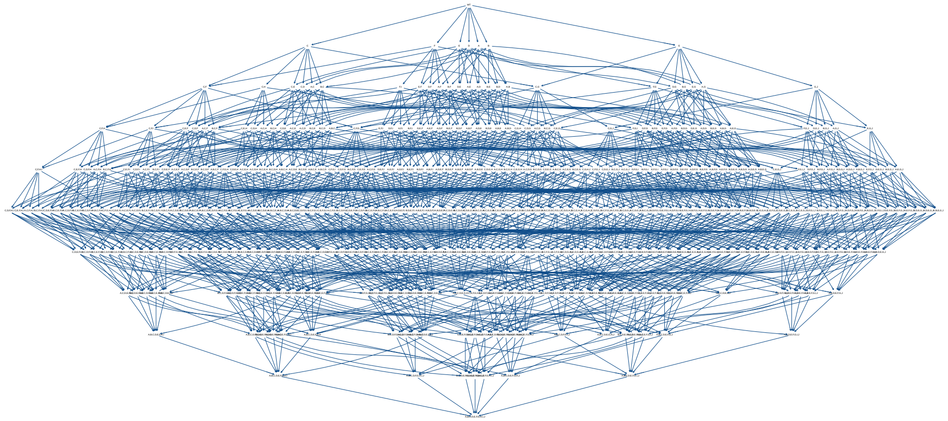

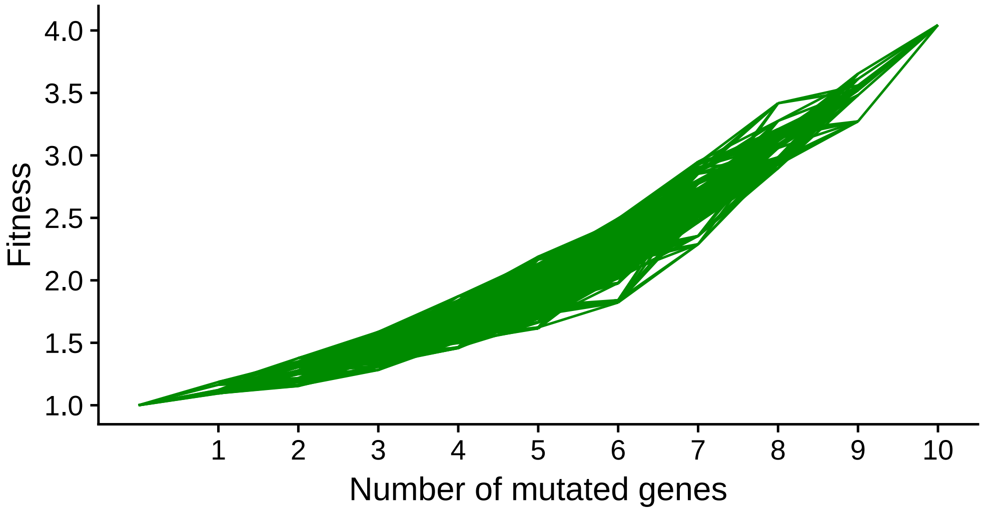

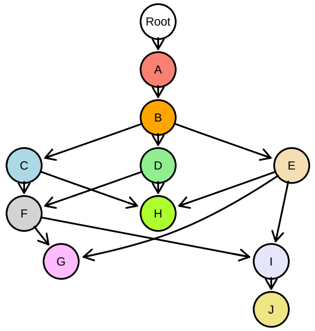

ID 43e2wOk4101CSHhe7

acc genotypes 24

peaks no back 1

removed edges 0

prop rem edges 0

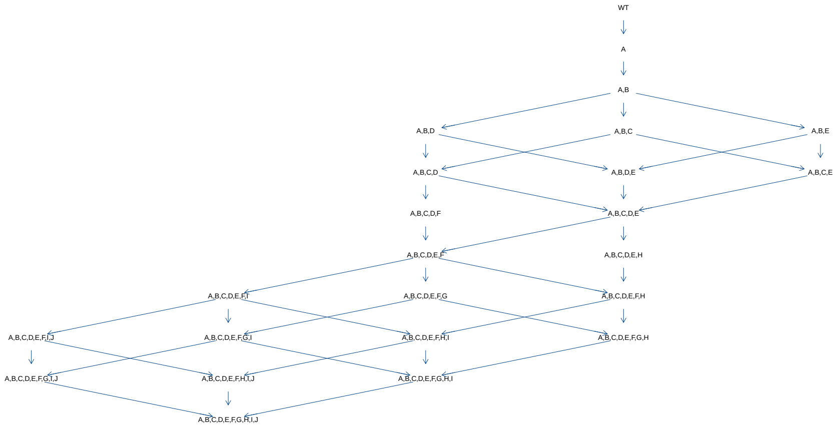

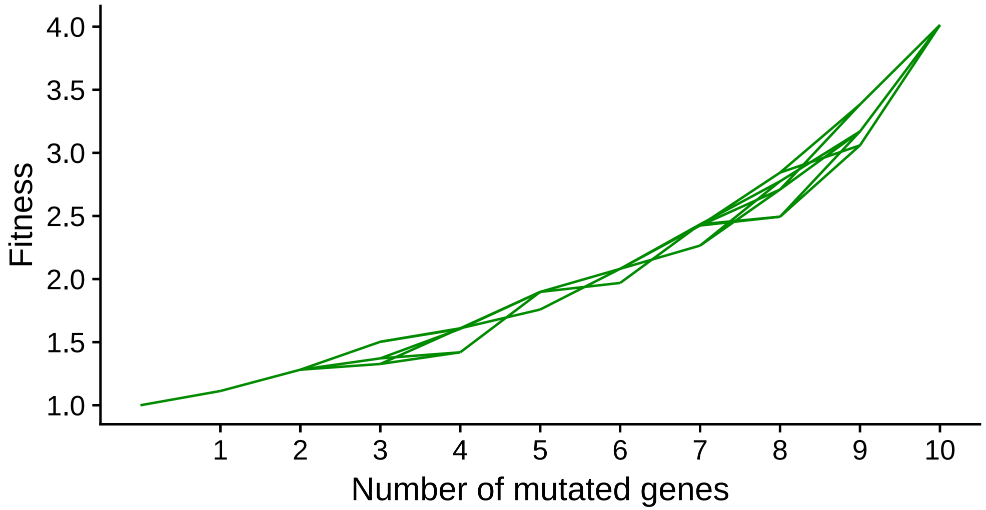

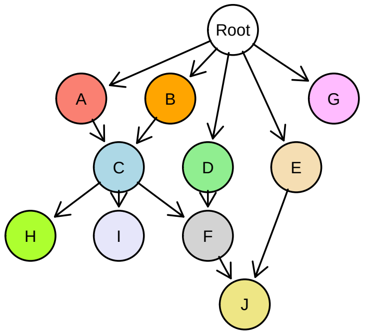

ID	45ZtV5kGH2IwAPAq
acc genotypes	88
peaks no back	1
removed edges	0
prop rem edges	0

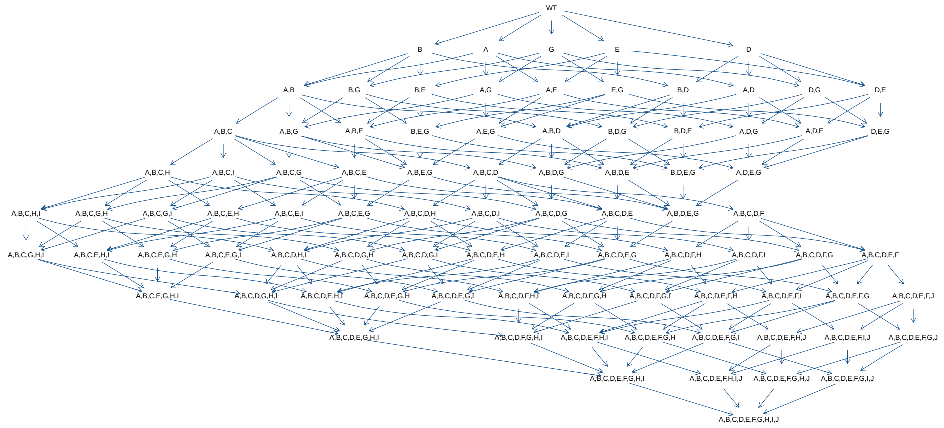

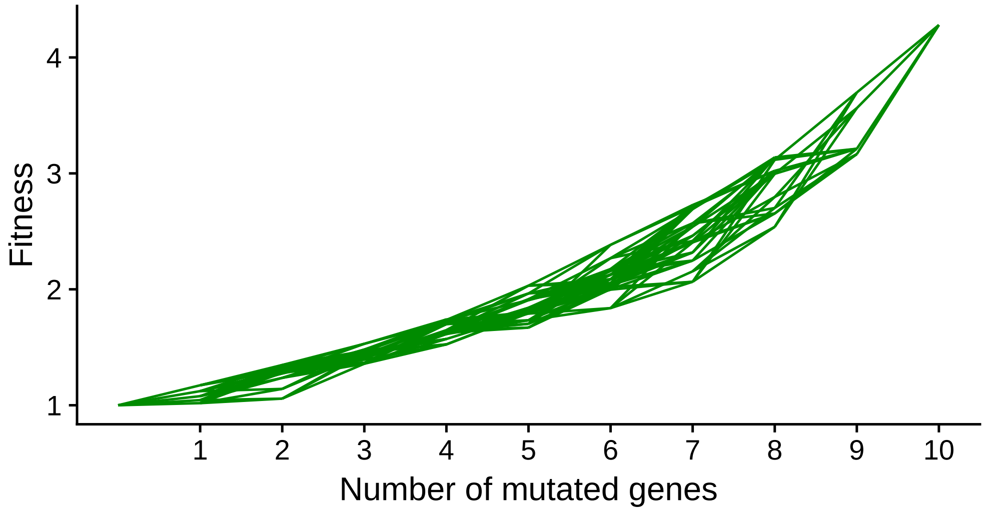

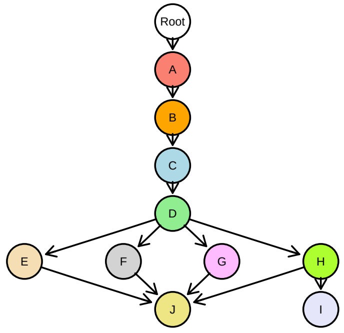

ID	510skoIlHNus2jjBB
acc genotypes	30
peaks no back	1
removed edges	0
prop rem edges	0

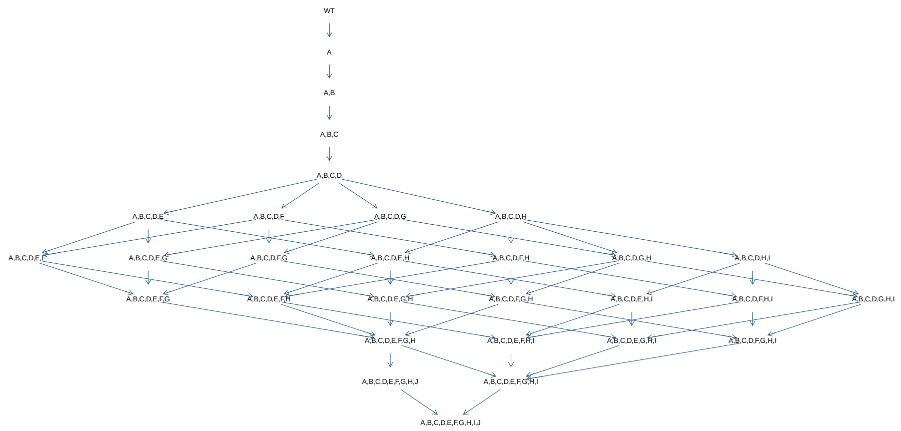

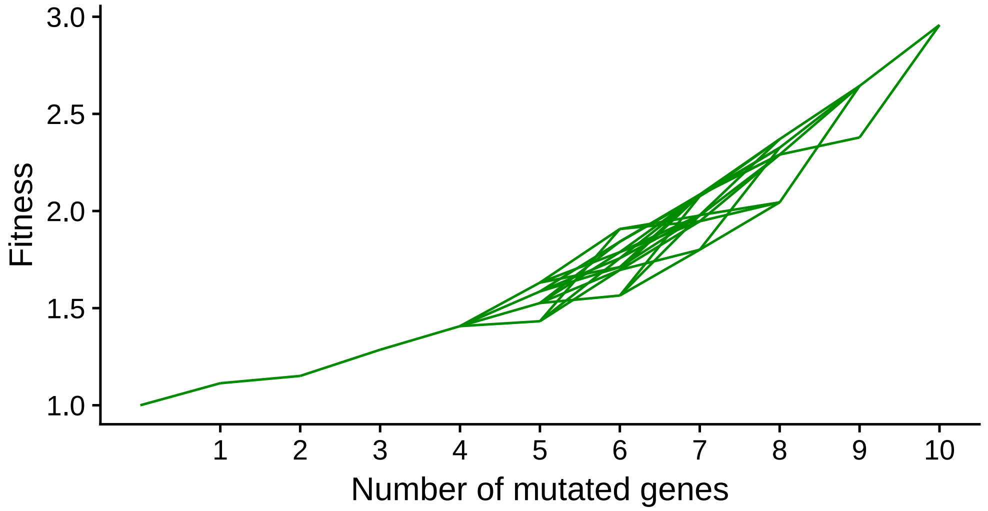

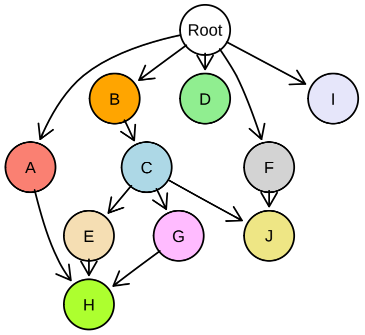

ID	56QJuQAd106HujMS5
acc genotypes	140
peaks no back	1
removed edges	0
prop rem edges	0

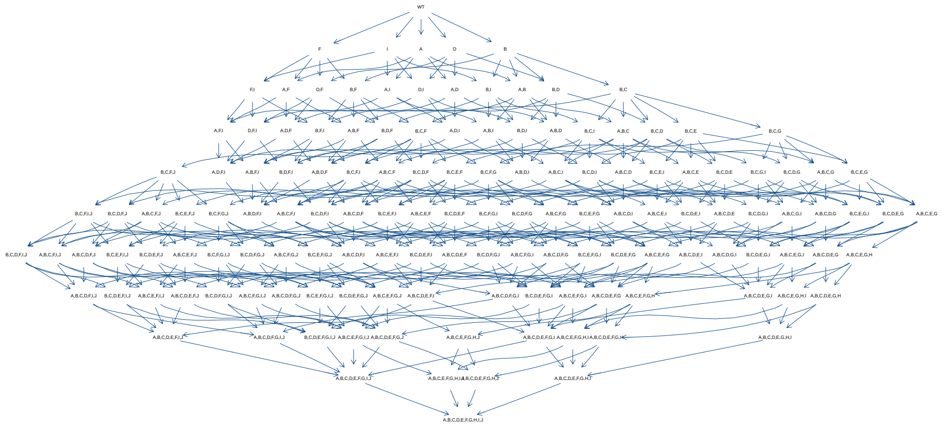

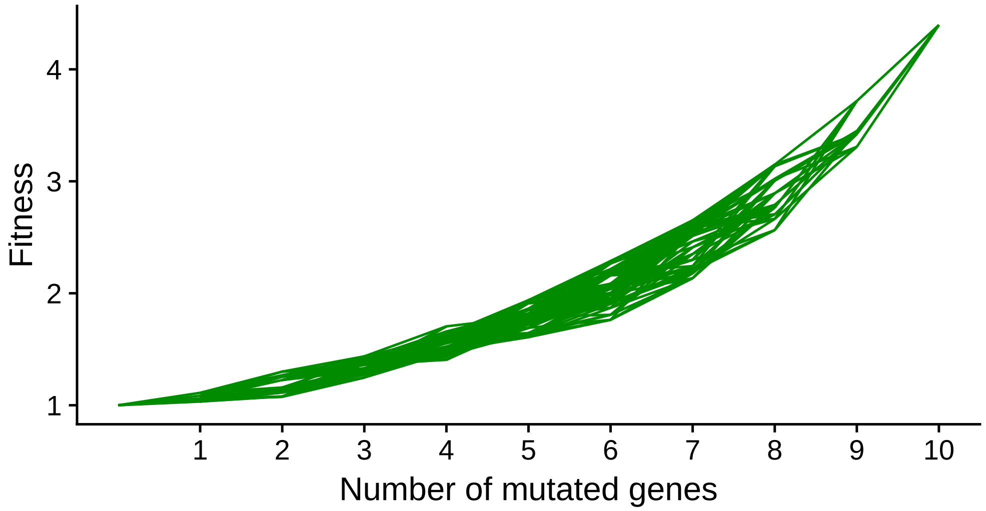

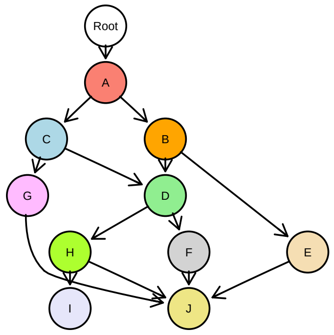

ID 59d4W3MnYvIGCNDg
acc genotypes 36
peaks no back 1
removed edges 0
prop rem edges 0

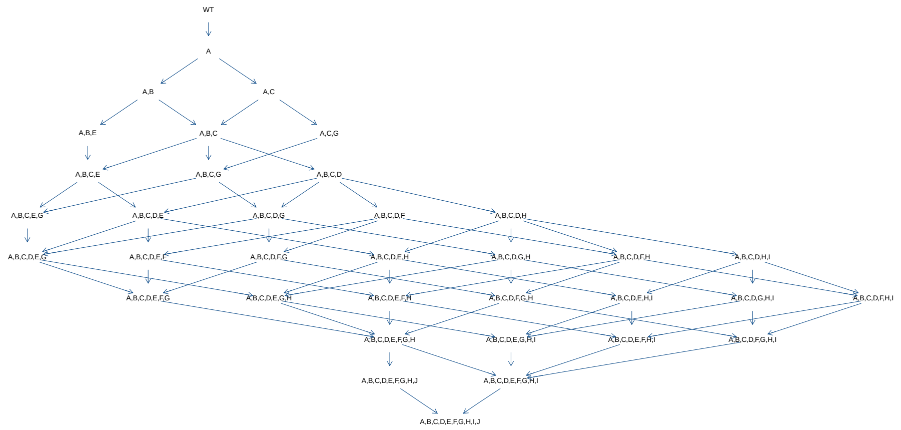

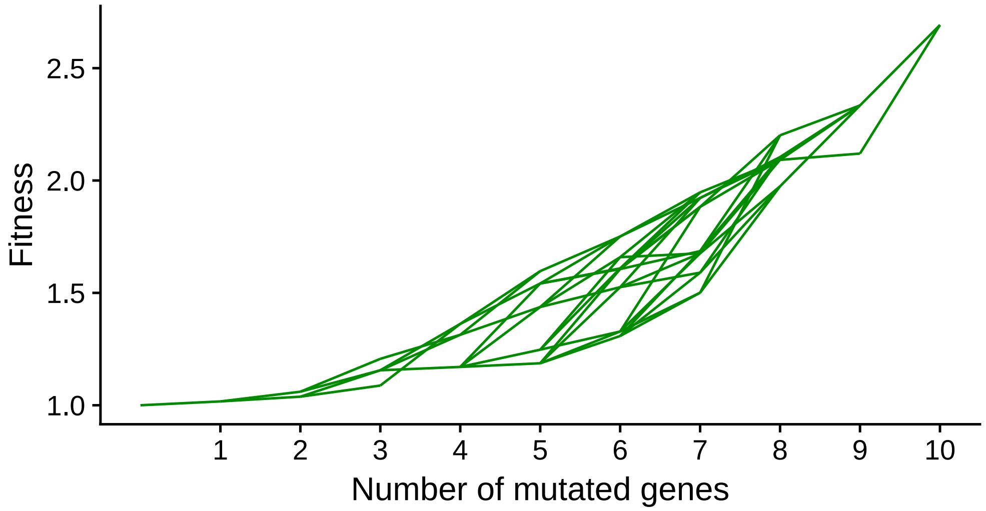

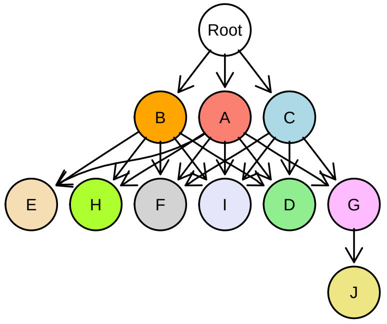

ID	5aGJFJcQGLZfPkJM
acc genotypes	108
peaks no back	1
removed edges	0
prop rem edges	0

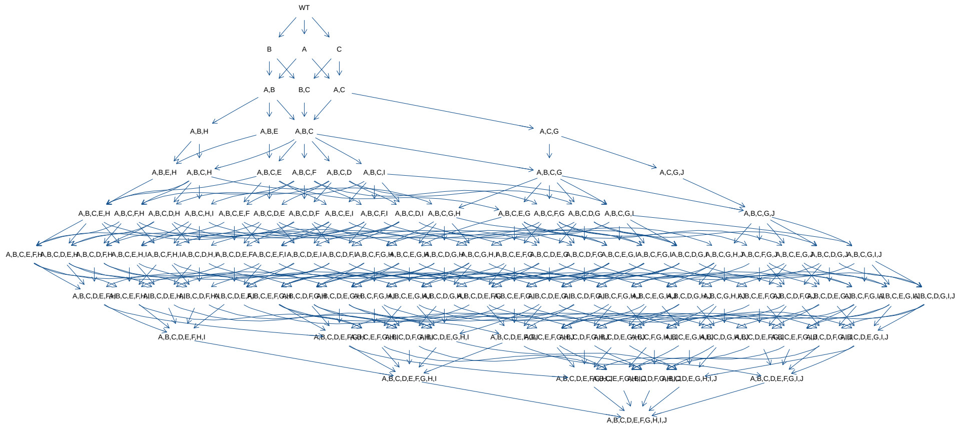

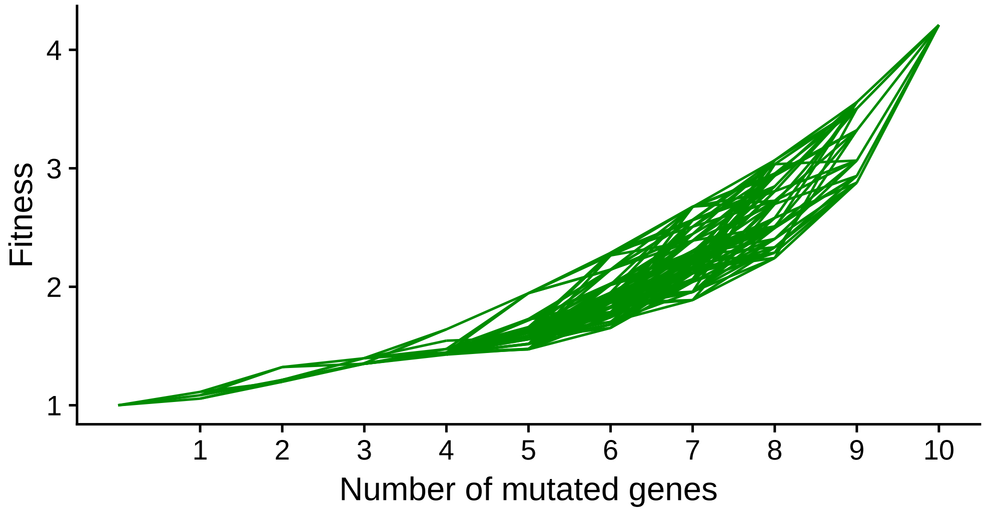

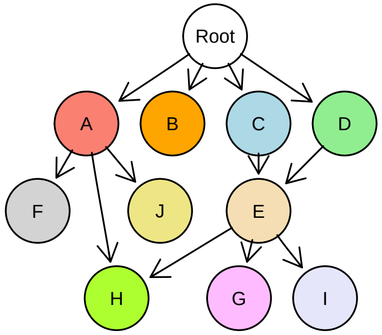

ID	5ism4PKhL1Vt6vub
acc genotypes	112
peaks no back	1
removed edges	0
prop rem edges	0

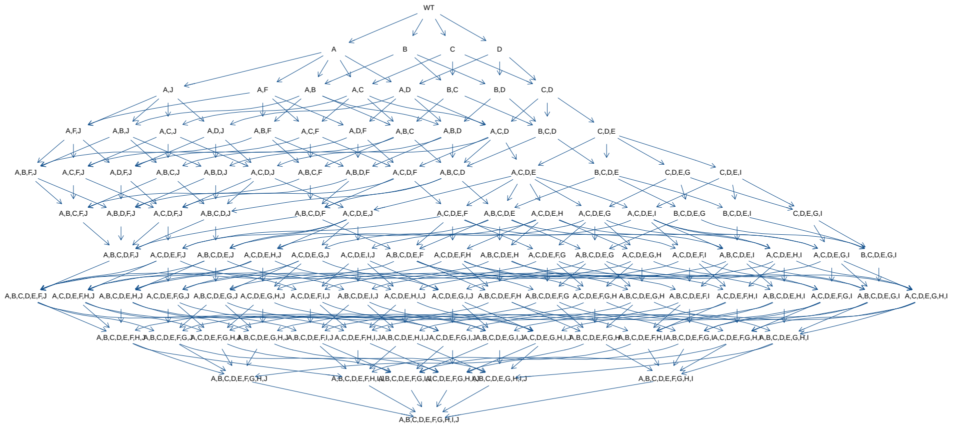

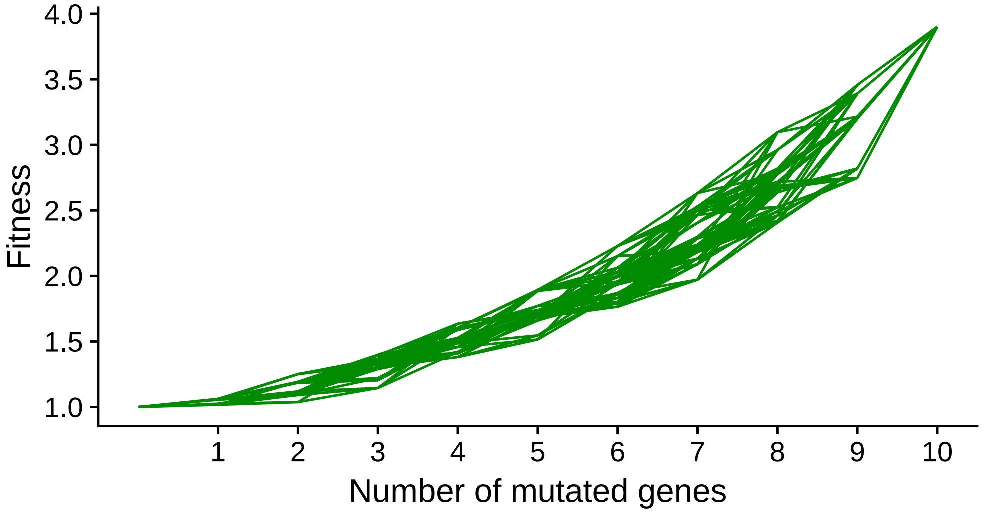

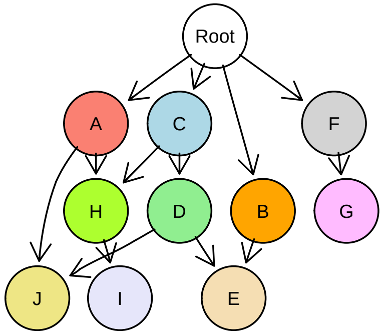

ID 5j8b10ekasUqQVF9H
acc genotypes 99
peaks no back 1
removed edges 0
prop rem edges 0

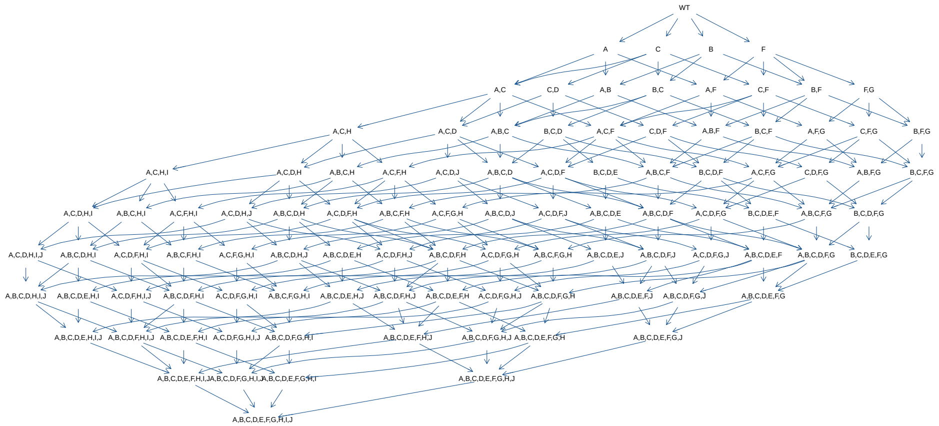

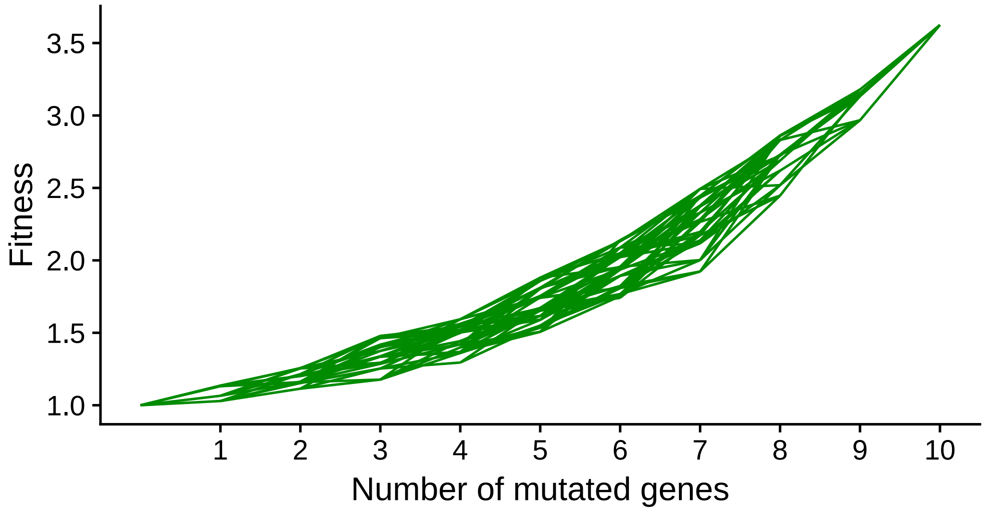

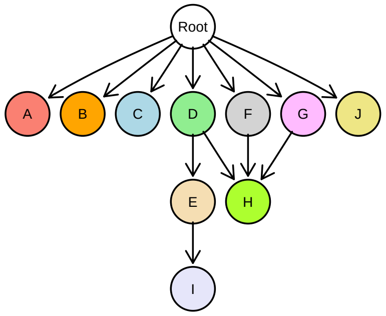

ID	5RbSm92BvC5zV10ss
acc genotypes	304
peaks no back	1
removed edges	0
prop rem edges	0

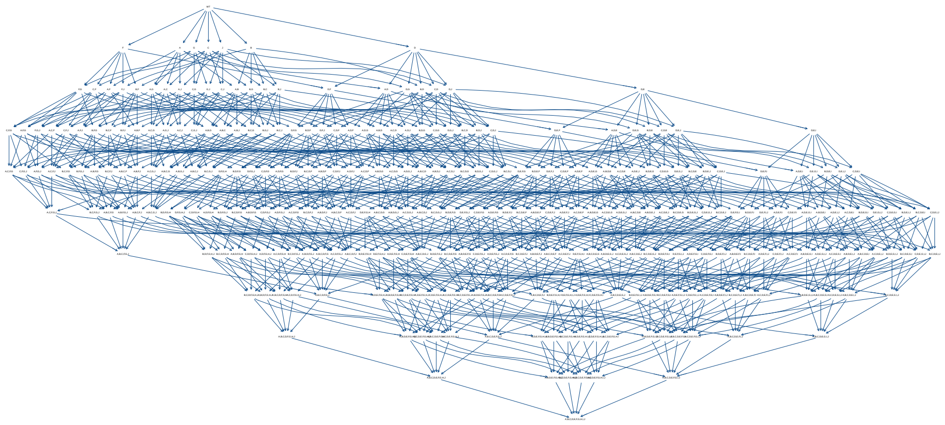

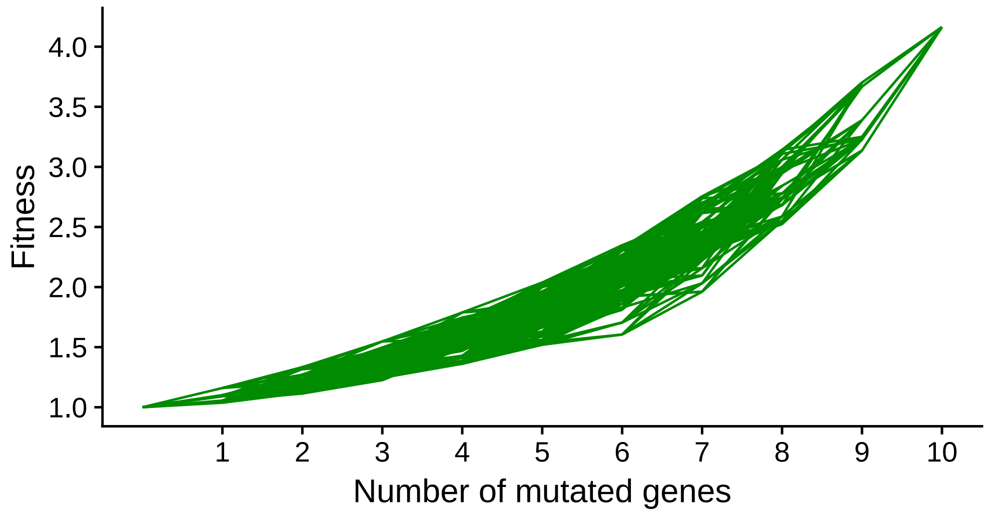

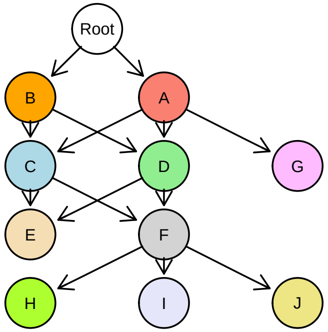

ID	6Lxt9hxPDEYs7HrO
acc genotypes	46
peaks no back	1
removed edges	0
prop rem edges	0

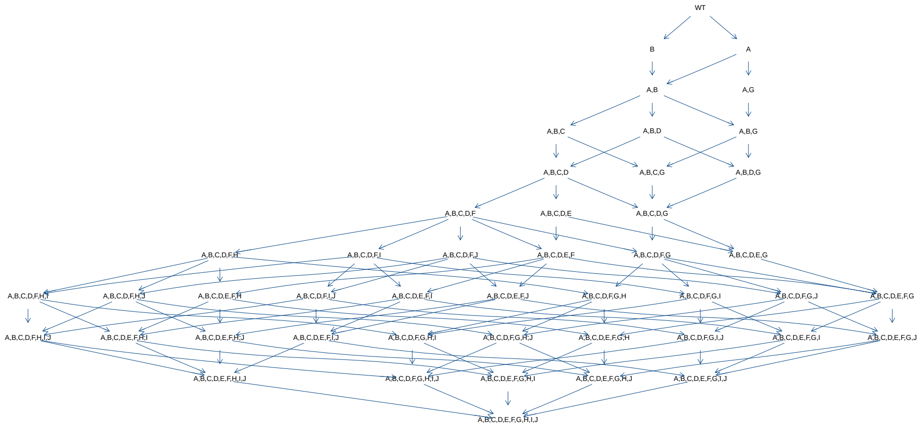

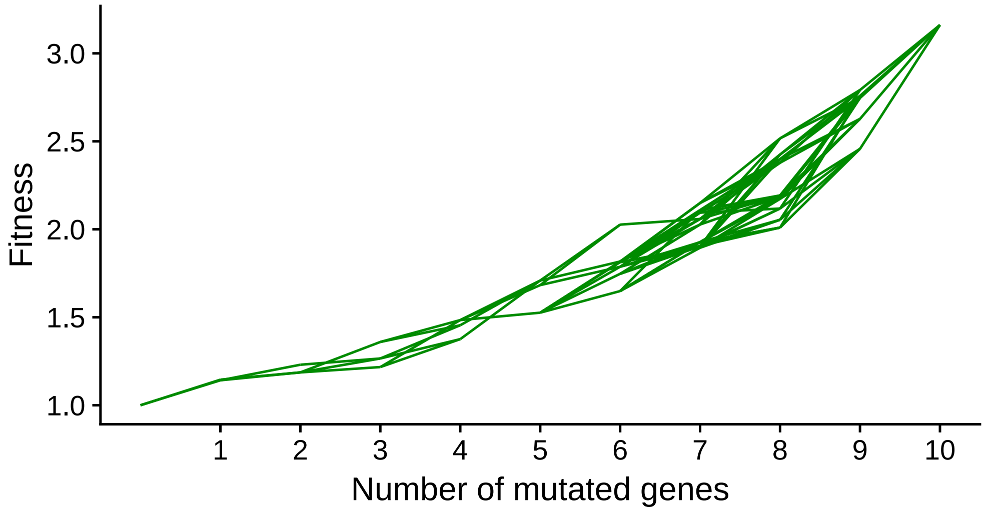

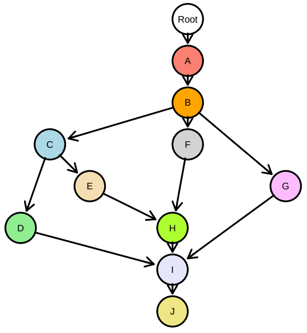

ID	7Fj7Va75pN1rV57O
acc genotypes	28
peaks no back	1
removed edges	0
prop rem edges	0

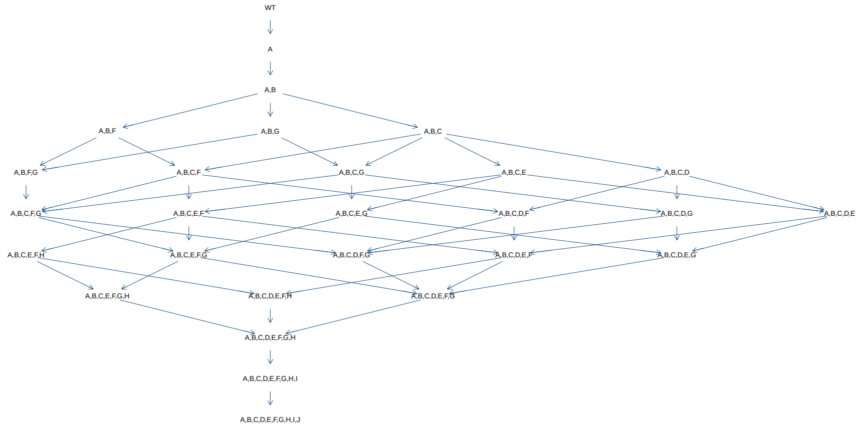

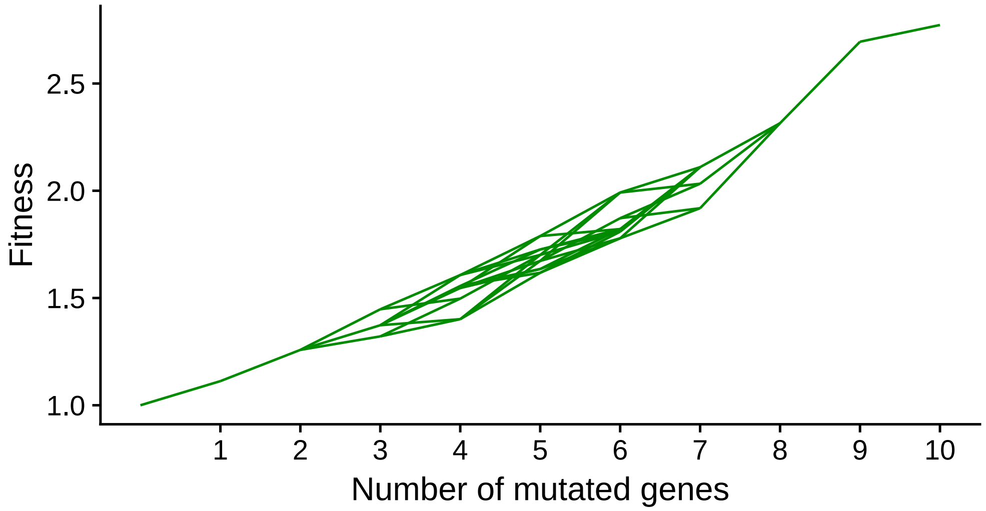

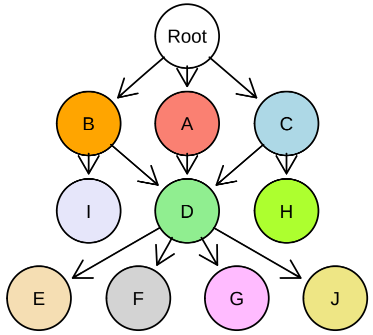

ID	7MYmvXyPDesfmMhs
acc genotypes	82
peaks no back	1
removed edges	0
prop rem edges	0

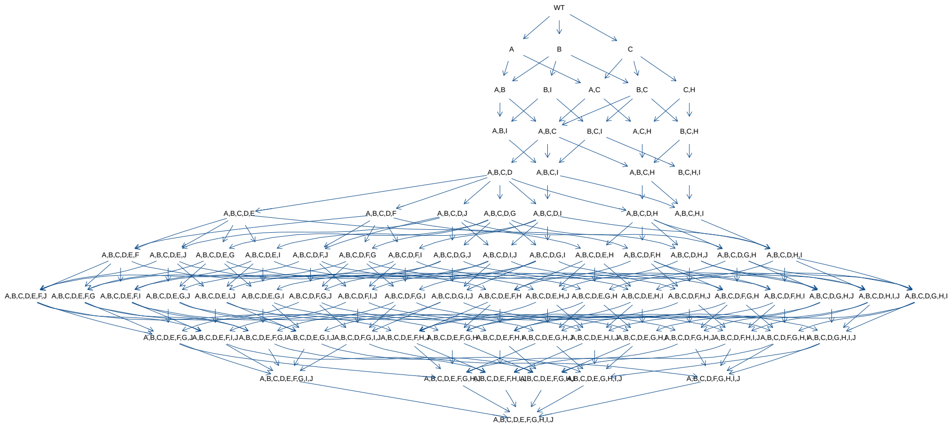

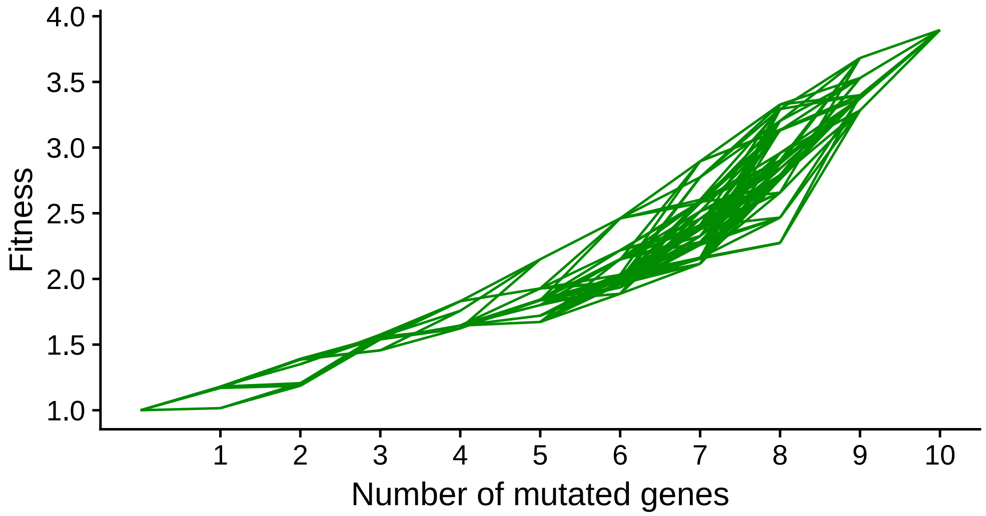

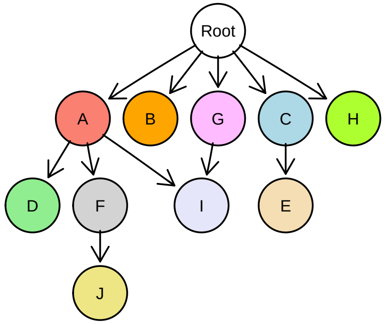

ID	7xha8OlAIqIZ4SXr
acc genotypes	240
peaks no back	1
removed edges	0
prop rem edges	0

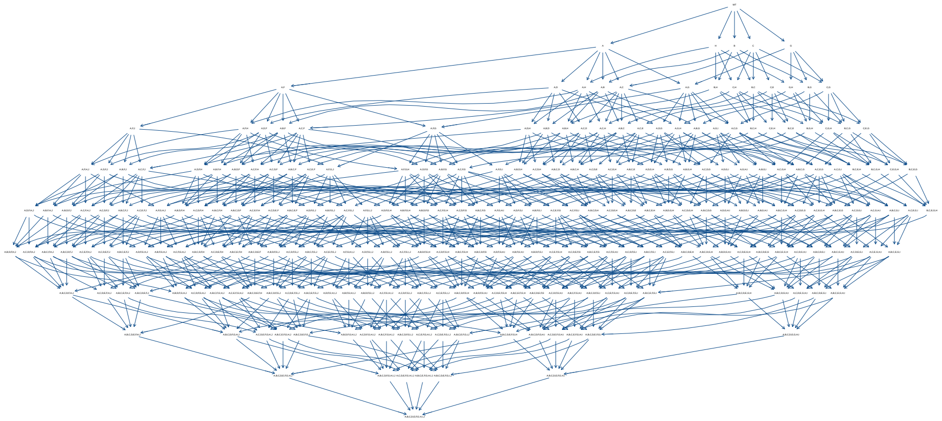

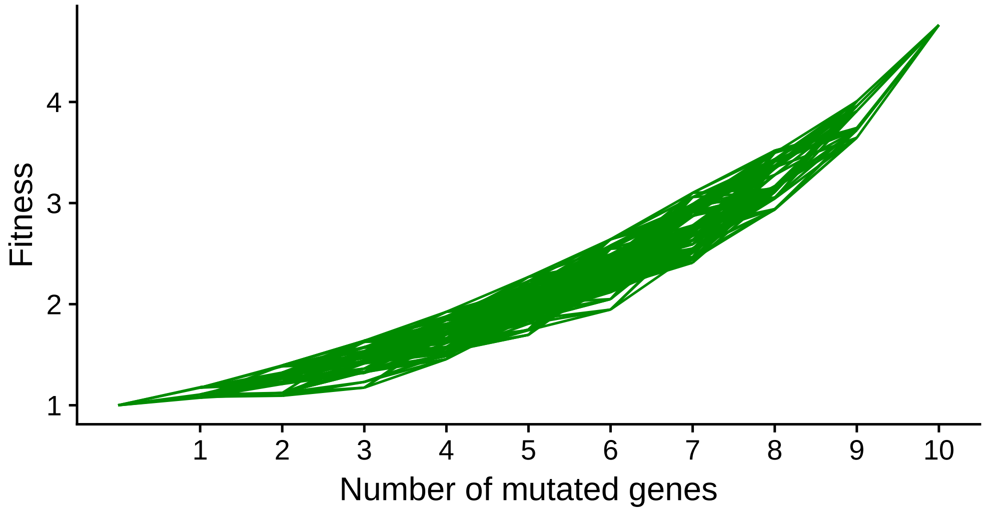

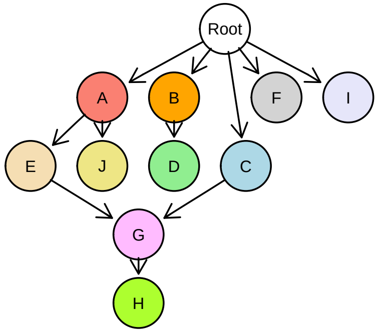

ID	84w3Yj1QSkf4zJx4
acc genotypes	168
peaks no back	1
removed edges	0
prop rem edges	0

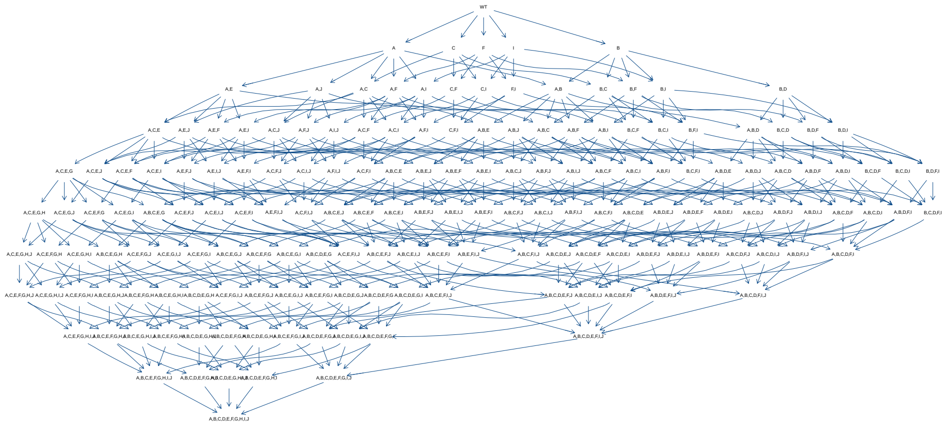

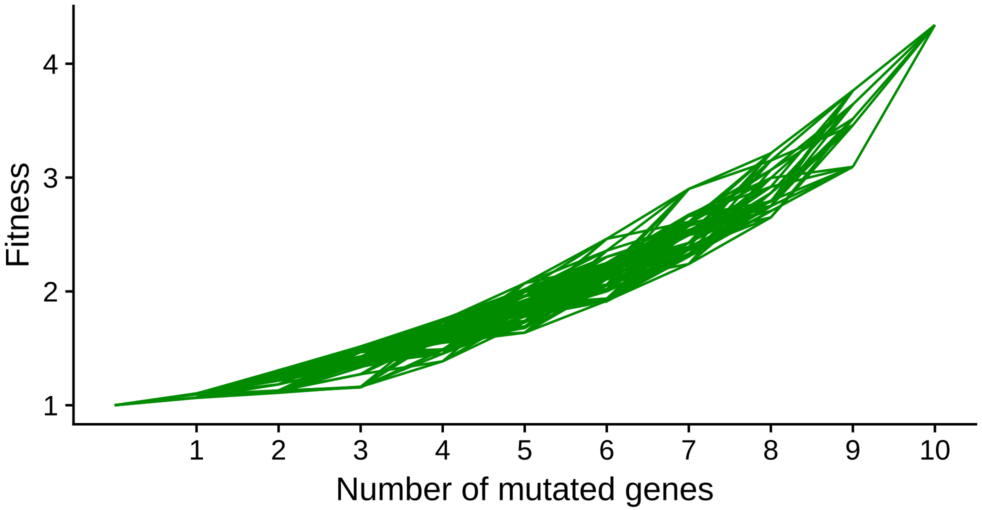

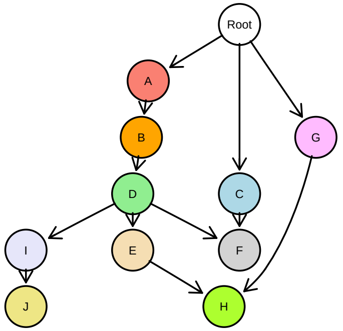

ID	8Libw9yeJgfs5Q95
acc genotypes	57
peaks no back	1
removed edges	0
prop rem edges	0

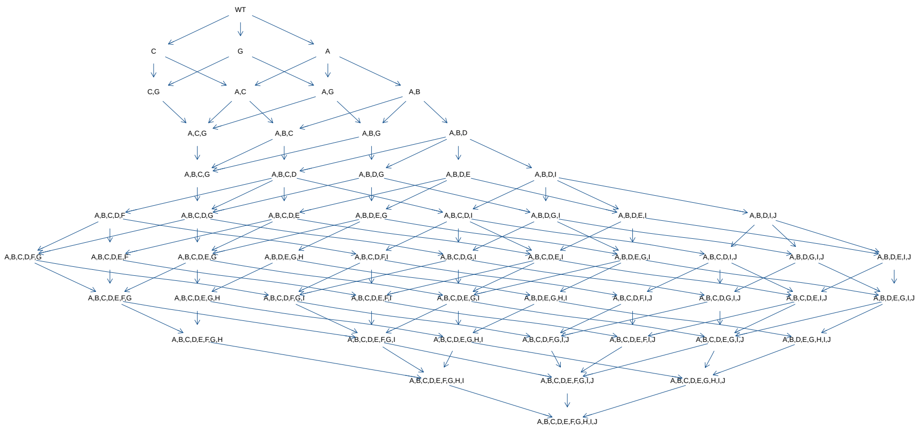

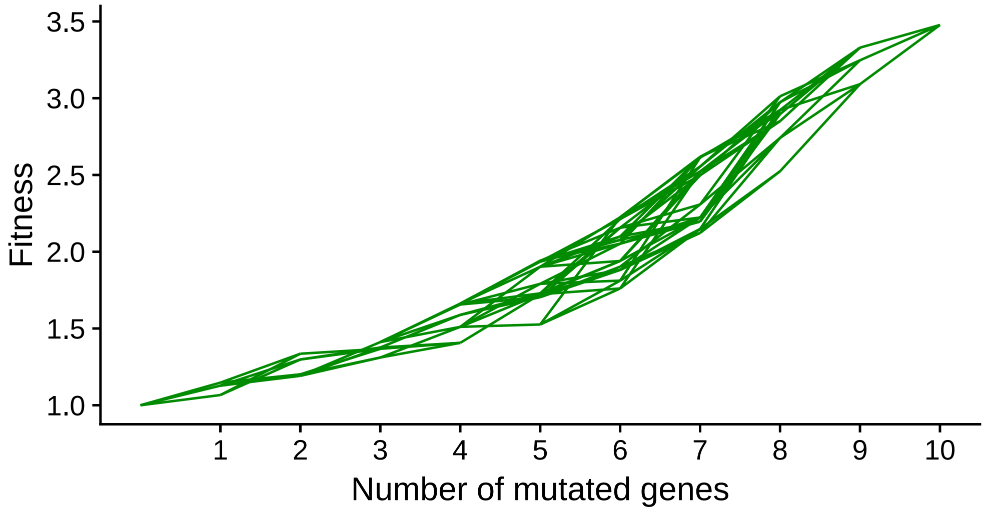

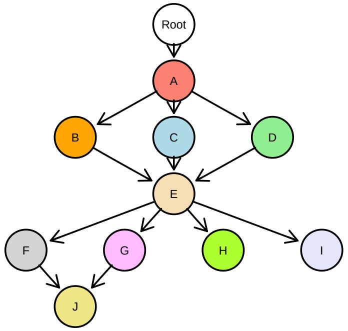

ID	9wKr6Qeu5I92VwmA
acc genotypes	29
peaks no back	1
removed edges	0
prop rem edges	0

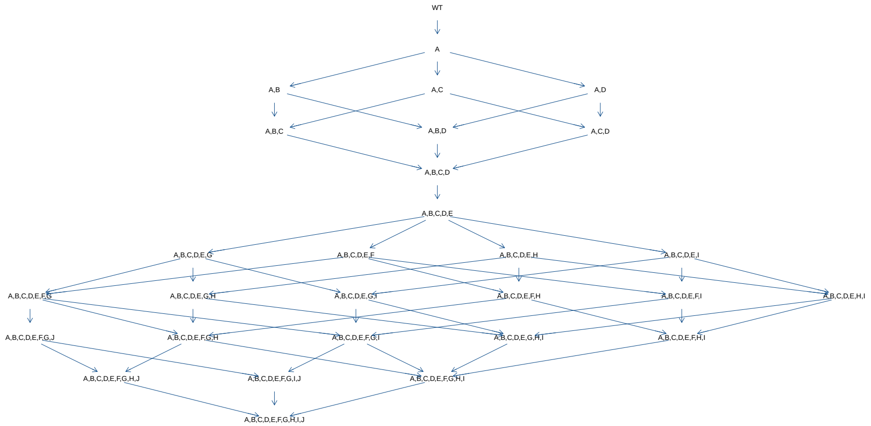

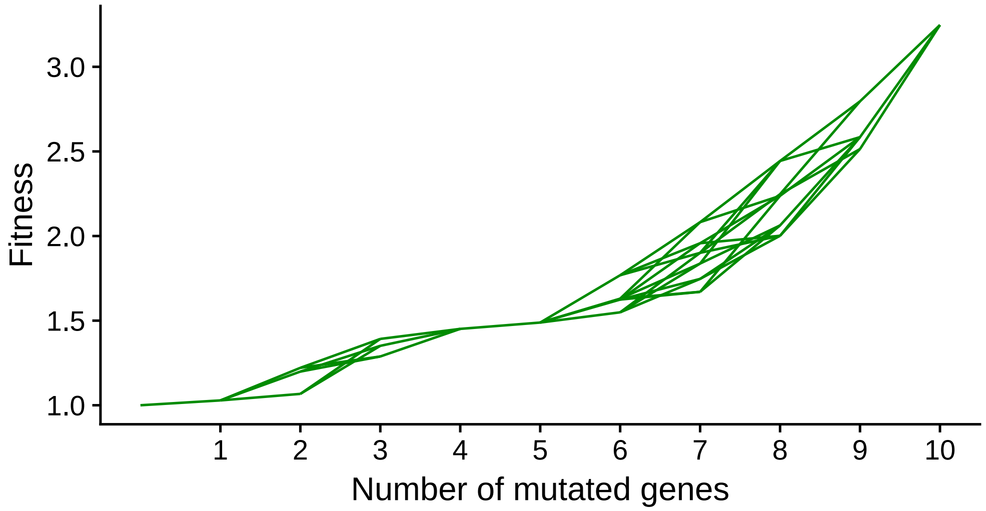

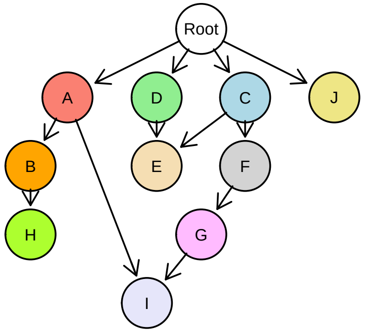

ID	ad9ndCIhE585lPSN
acc genotypes	106
peaks no back	1
removed edges	0
prop rem edges	0

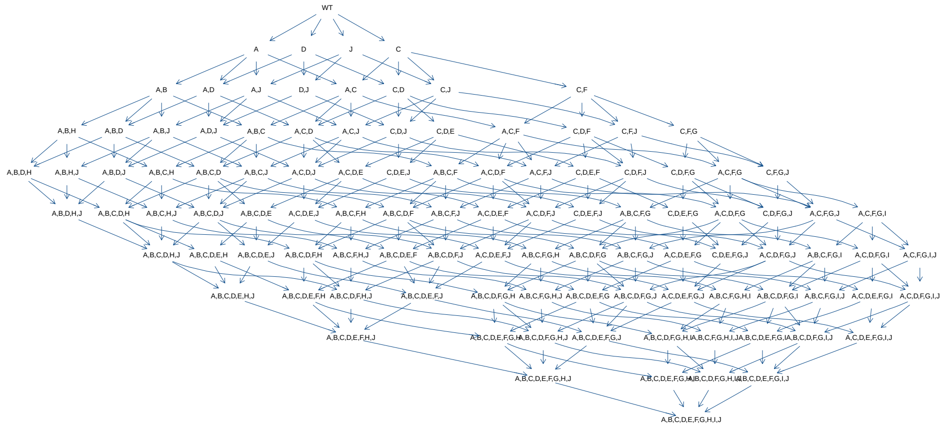

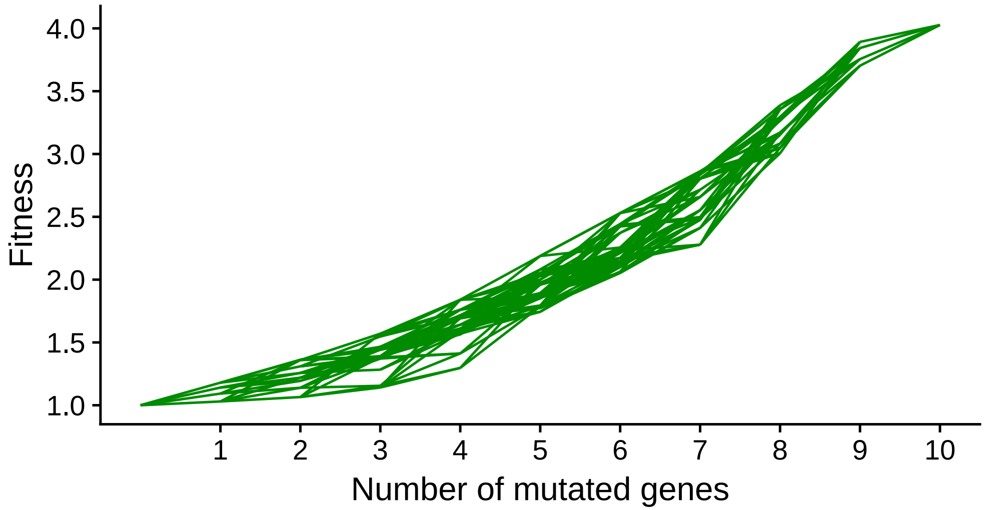

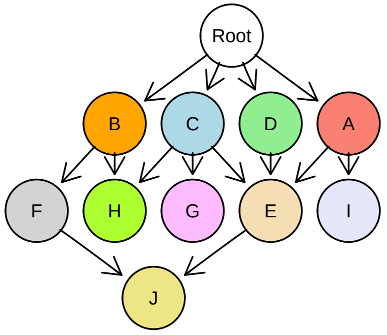

ID	AFZHDDg9eZ1wABOd
acc genotypes	106
peaks no back	1
removed edges	0
prop rem edges	0

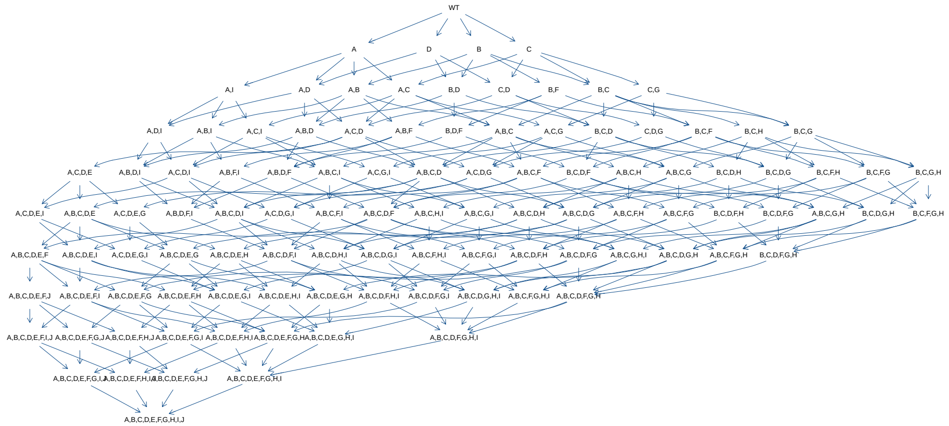

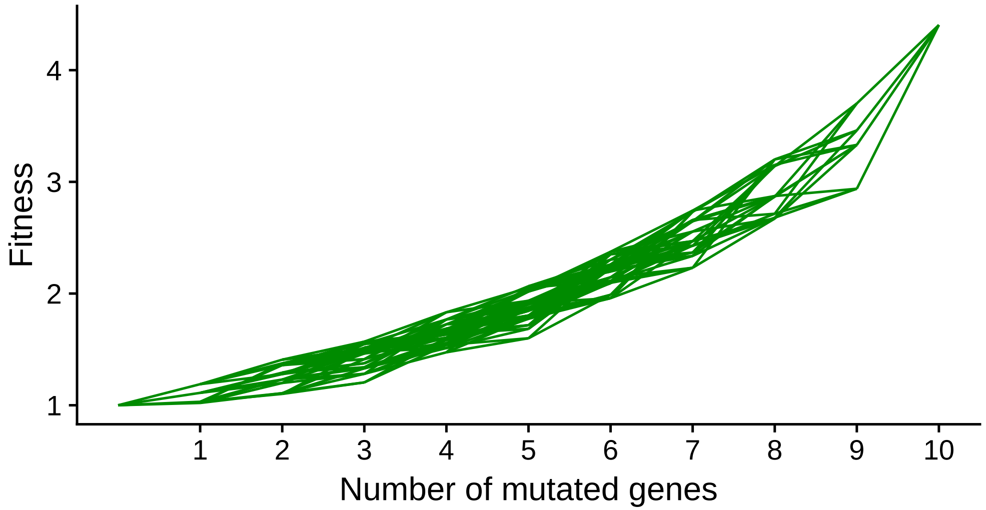

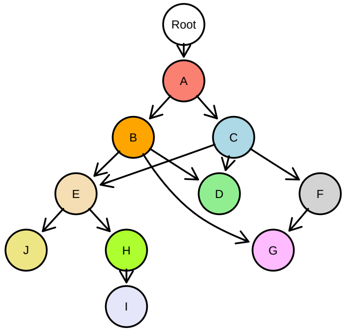

ID	ALSuQeDPTtbq8lNG
acc genotypes	47
peaks no back	1
removed edges	0
prop rem edges	0

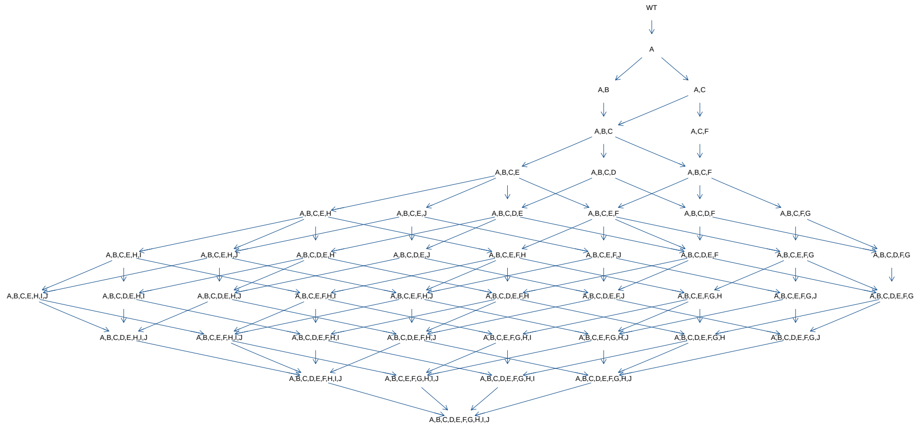

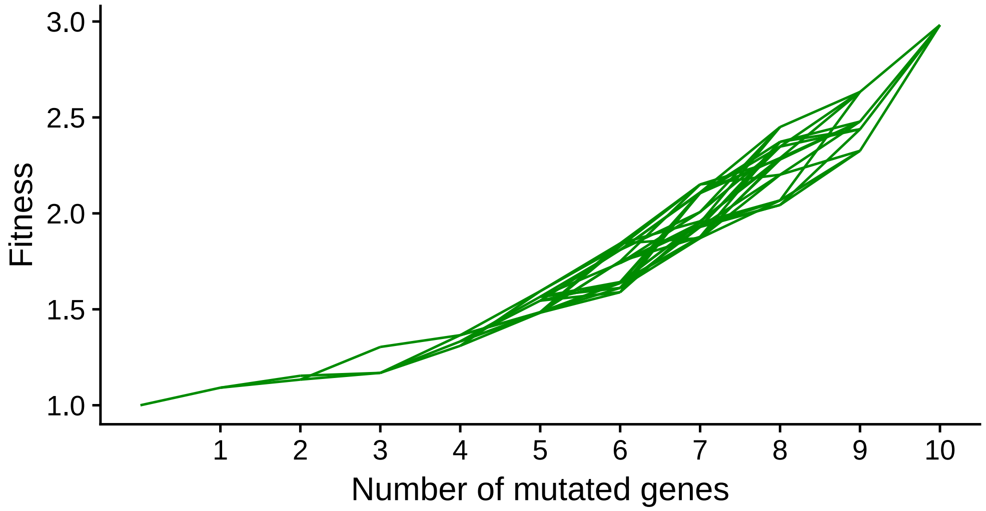

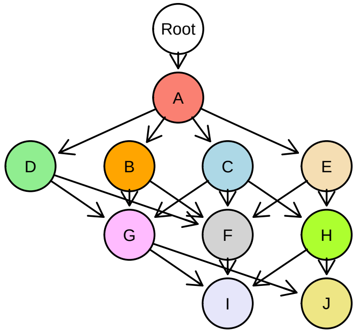

ID AV7D5TvJW78FIxWP
acc genotypes 32
peaks no back 1
removed edges 0
prop rem edges 0

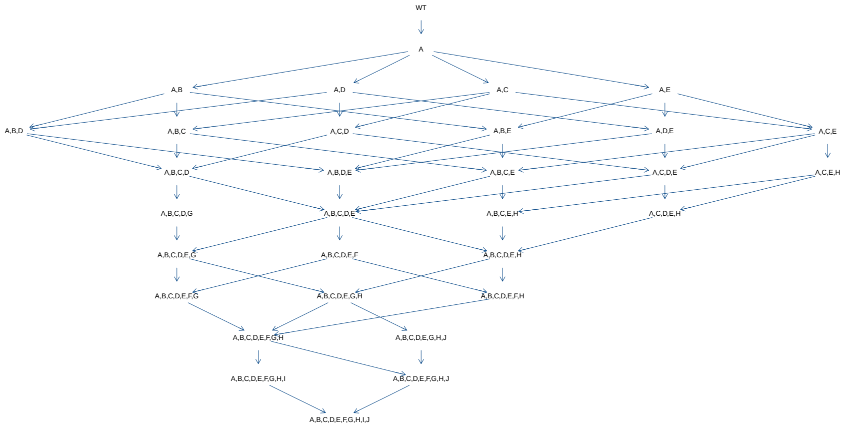

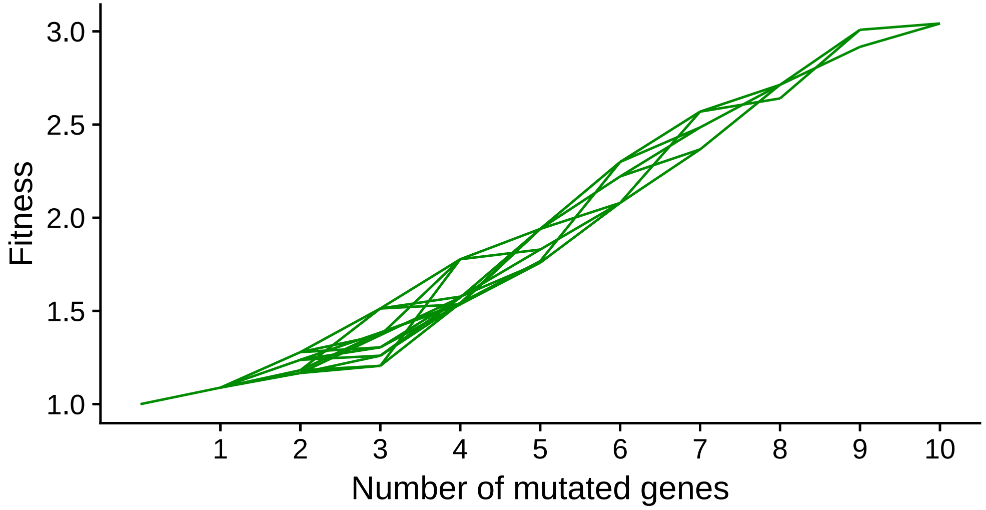

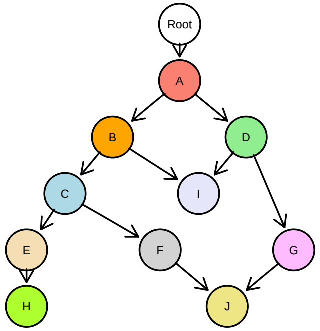

ID	AzcqFM9IjyJEevU2
acc genotypes	45
peaks no back	1
removed edges	0
prop rem edges	0

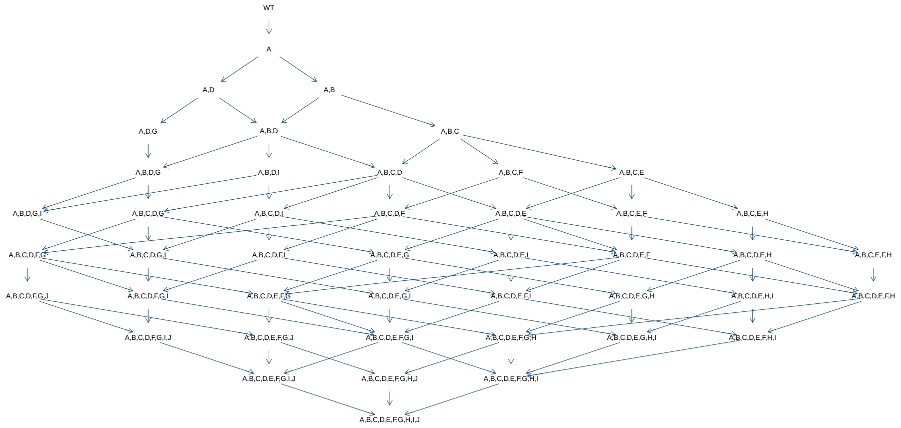

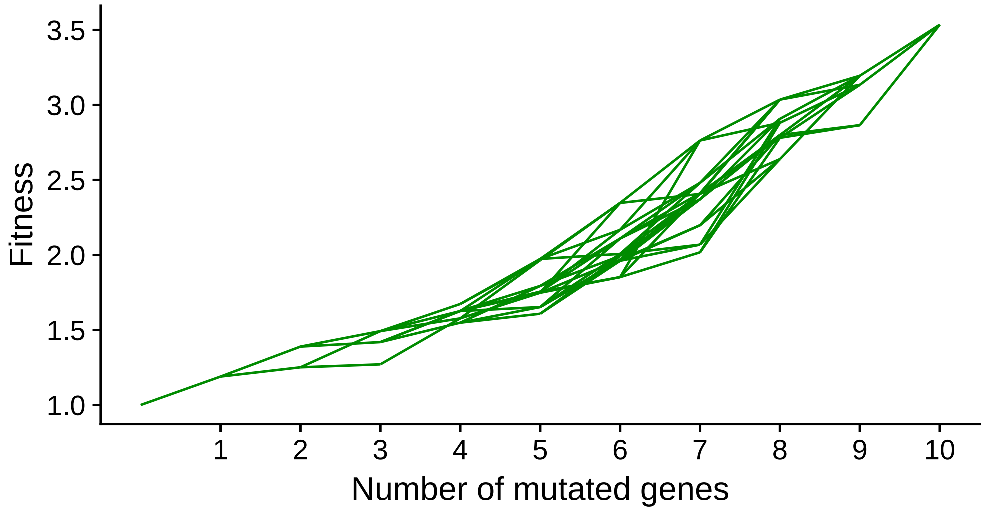

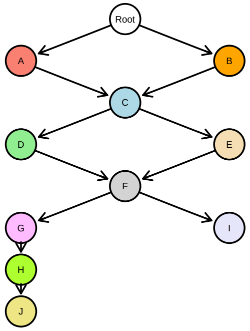

ID	B2Xyqnt5VTgzAZsS
acc genotypes	16
peaks no back	1
removed edges	0
prop rem edges	0

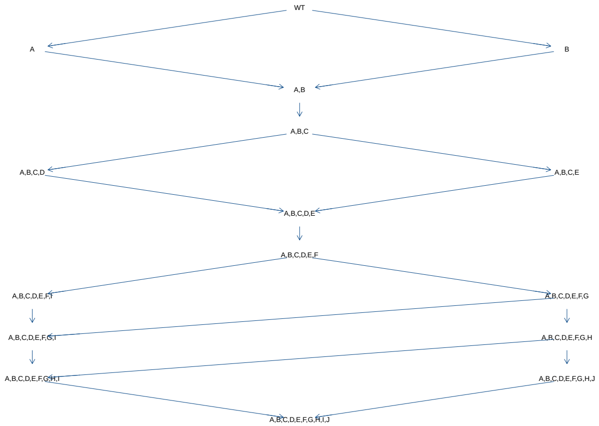

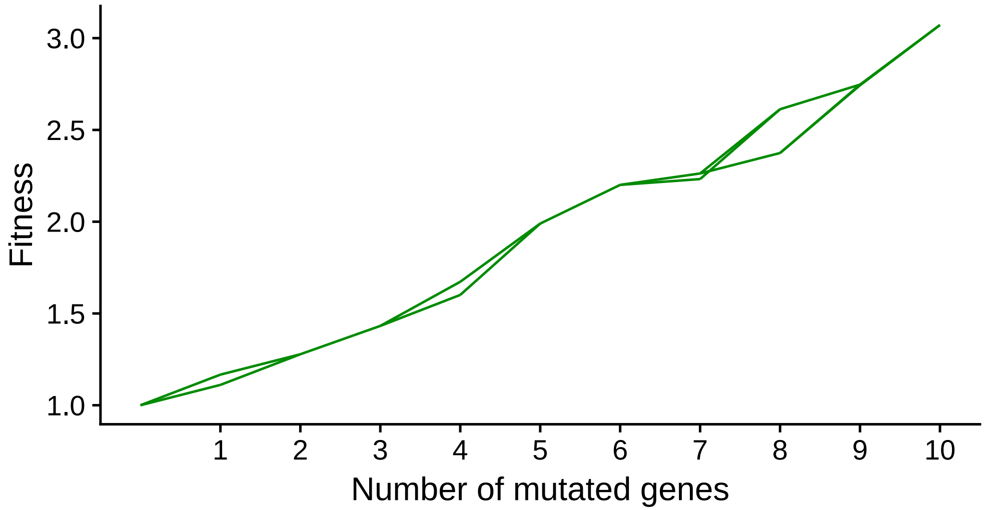

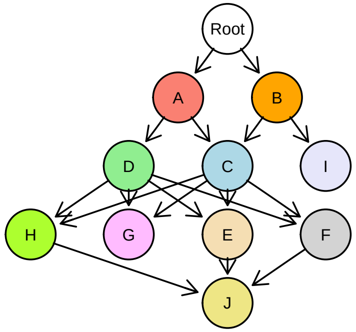

ID	b6IqOPrfURmxrTRJ
acc genotypes	47
peaks no back	1
removed edges	0
prop rem edges	0

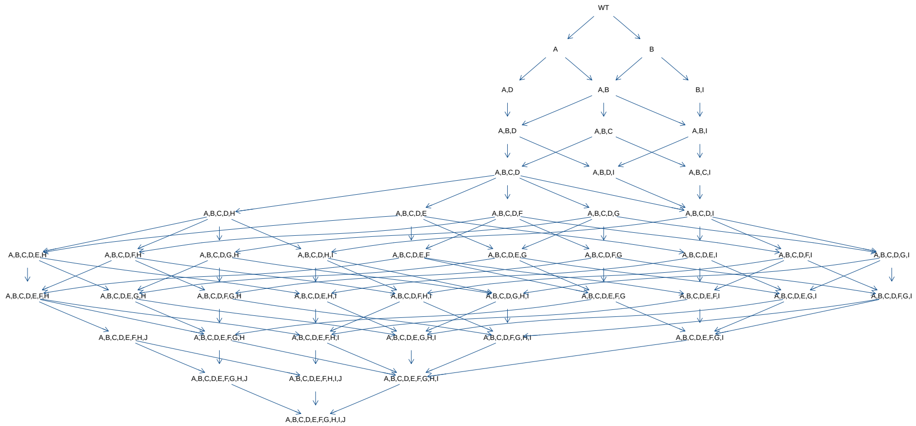

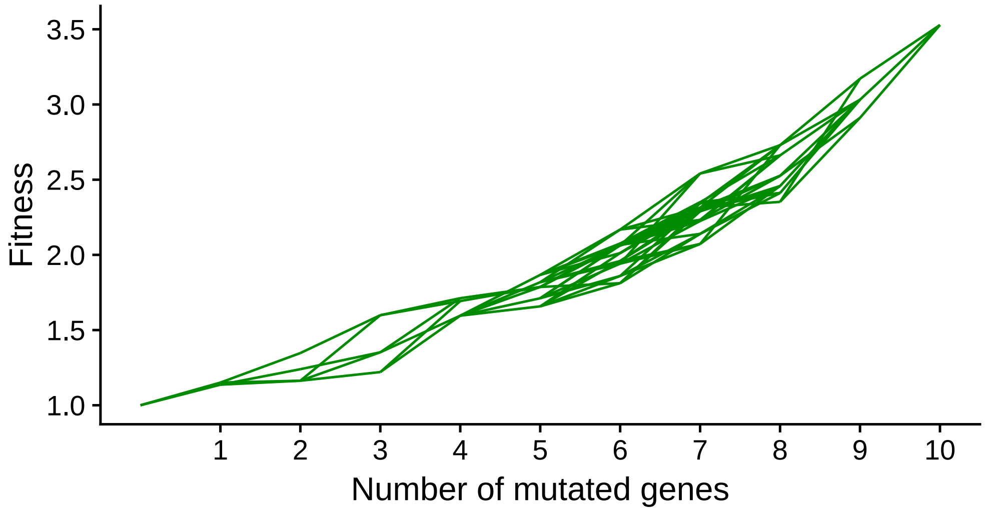

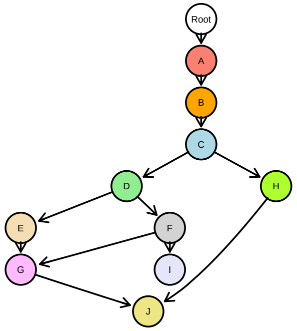

ID	BkSjd8cAWeN4eVyO
acc genotypes	23
peaks no back	1
removed edges	0
prop rem edges	0

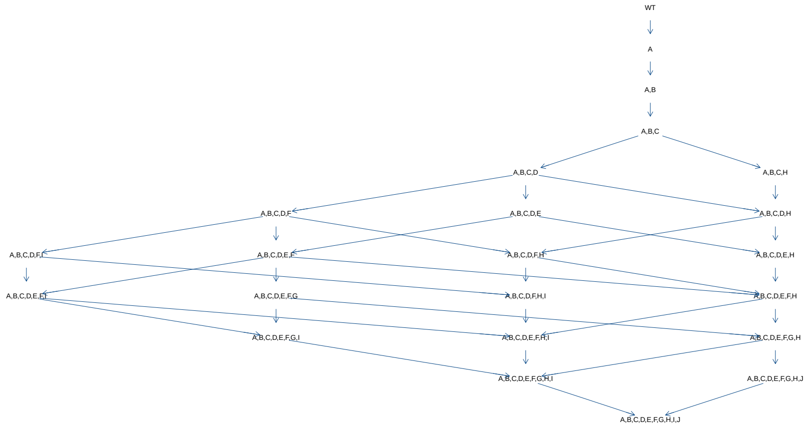

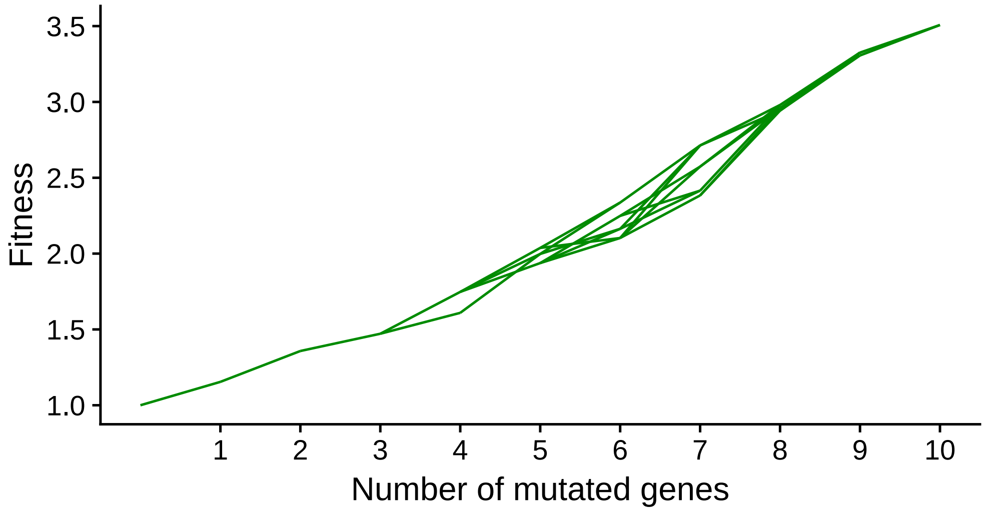

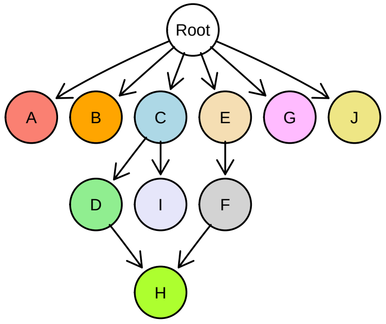

ID	BqTdapi2oHdGJNeQ
acc genotypes	272
peaks no back	1
removed edges	0
prop rem edges	0

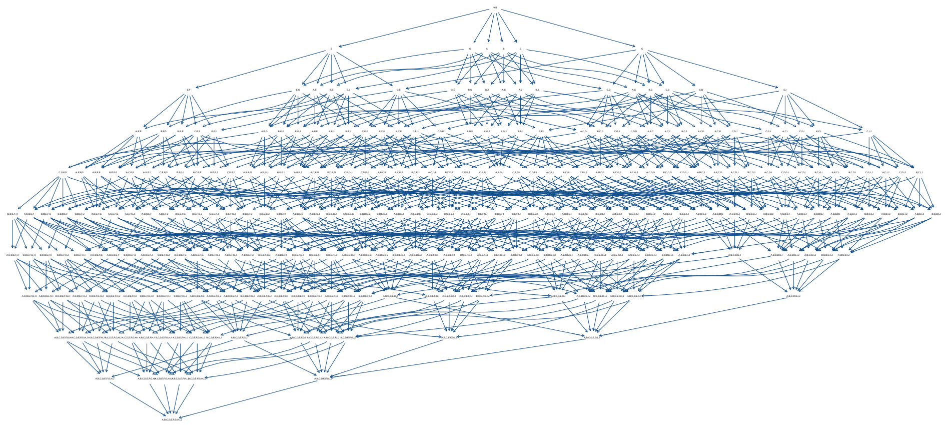

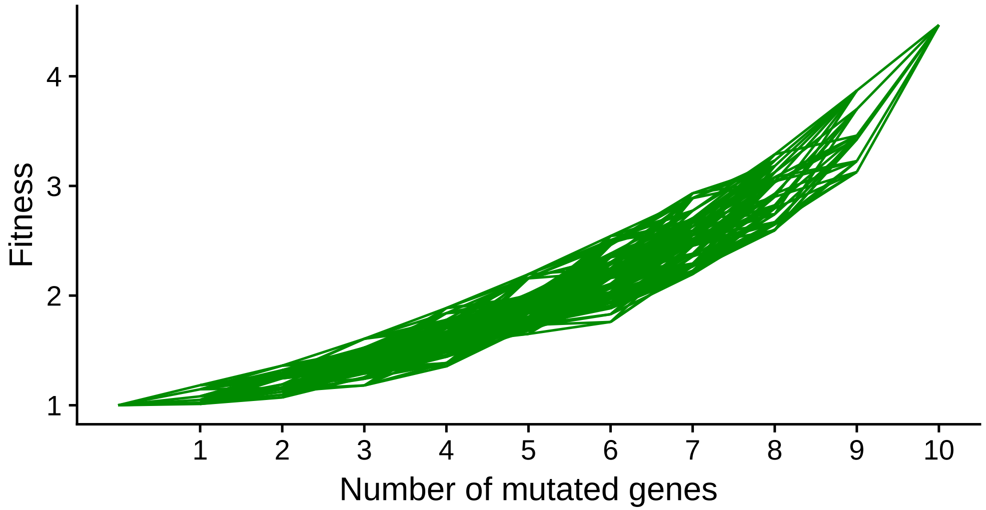

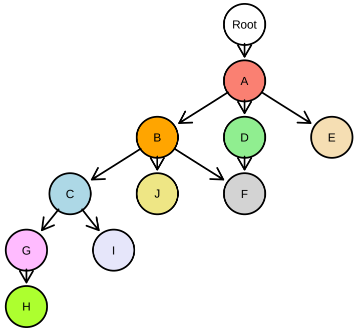

ID	bTi93VZQ3TjAOVaY
acc genotypes	89
peaks no back	1
removed edges	0
prop rem edges	0

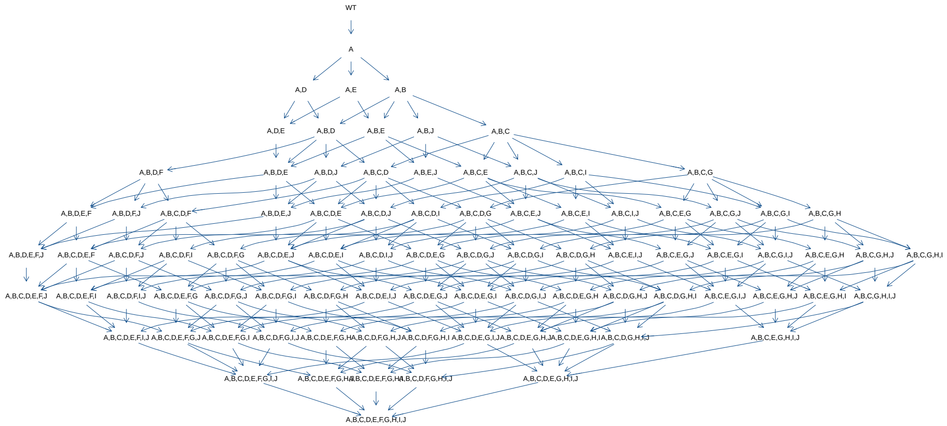

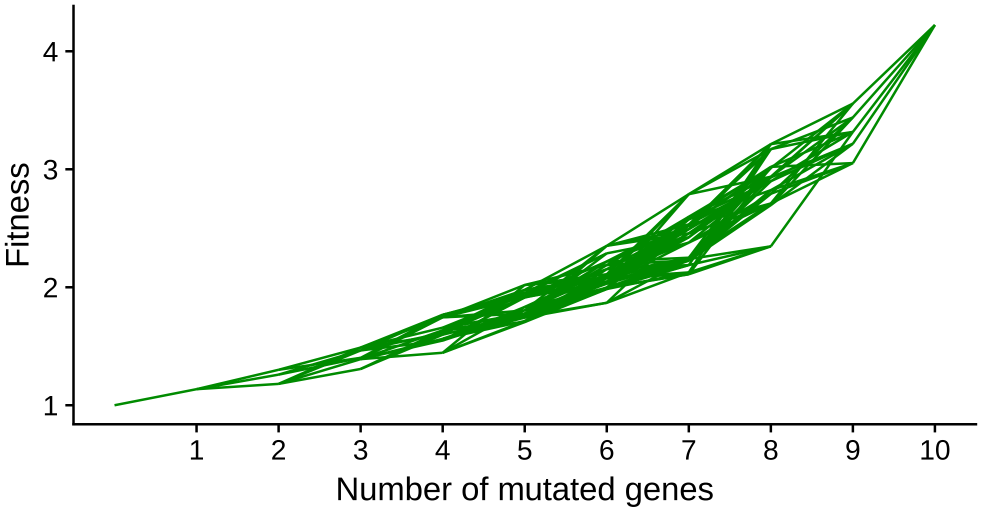

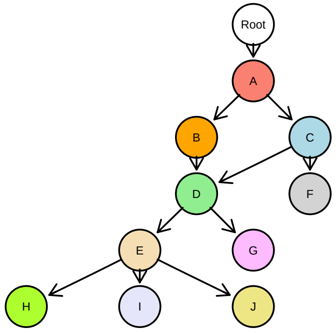

ID	BU2srhsWr1UElYvS
acc genotypes	43
peaks no back	1
removed edges	0
prop rem edges	0

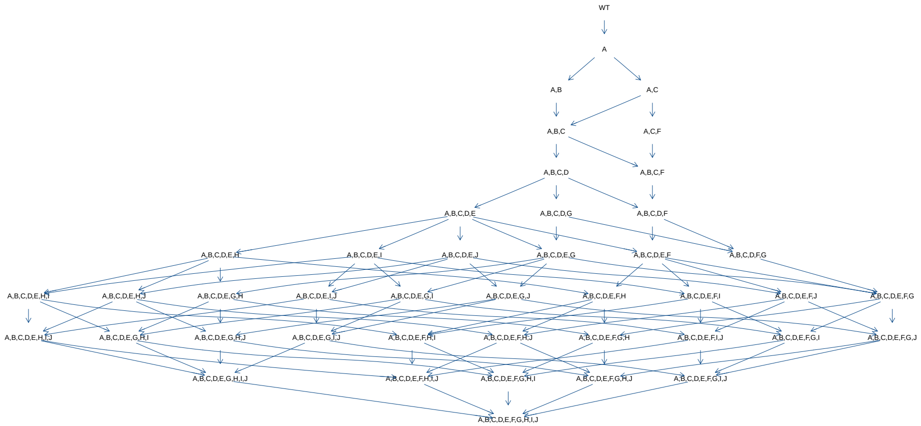

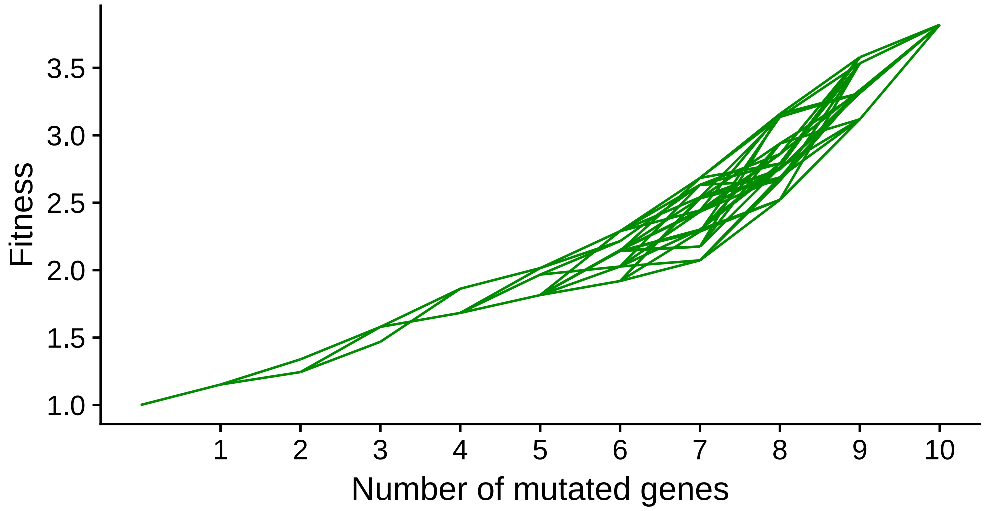

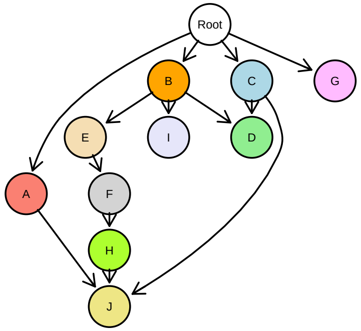

ID	bv105Mh6lDH10HPdOB
acc genotypes	112
peaks no back	1
removed edges	0
prop rem edges	0

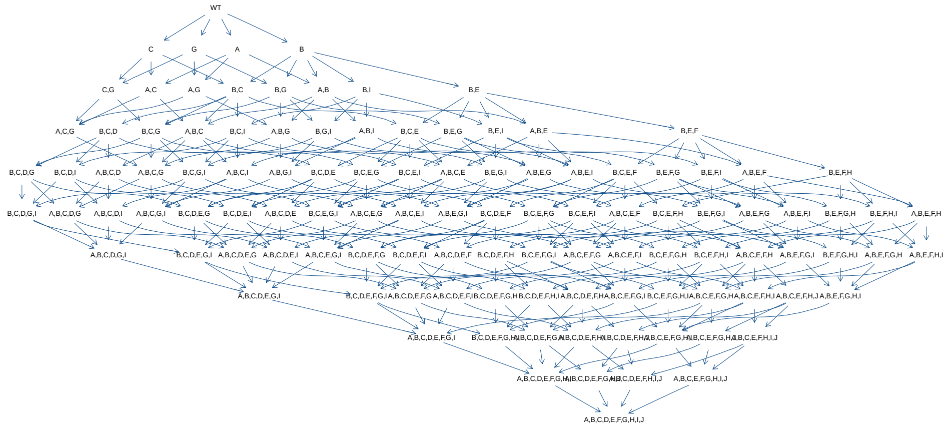

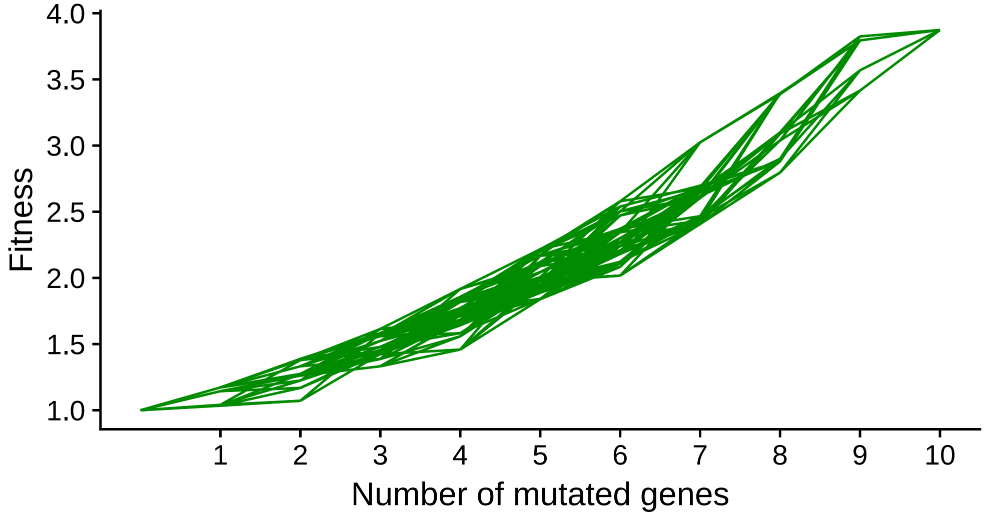

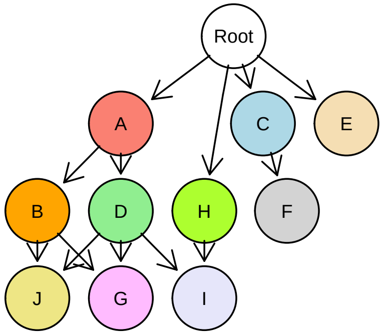

ID	BWkhGPzysoNbGoq1
acc genotypes	126
peaks no back	1
removed edges	0
prop rem edges	0

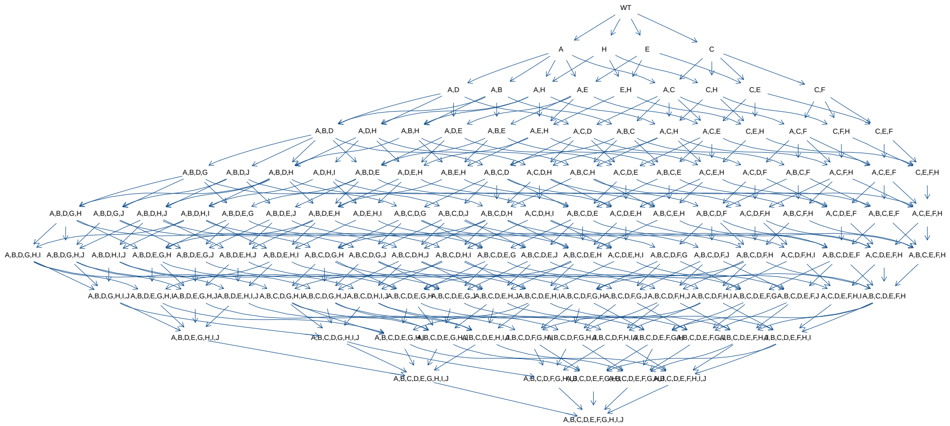

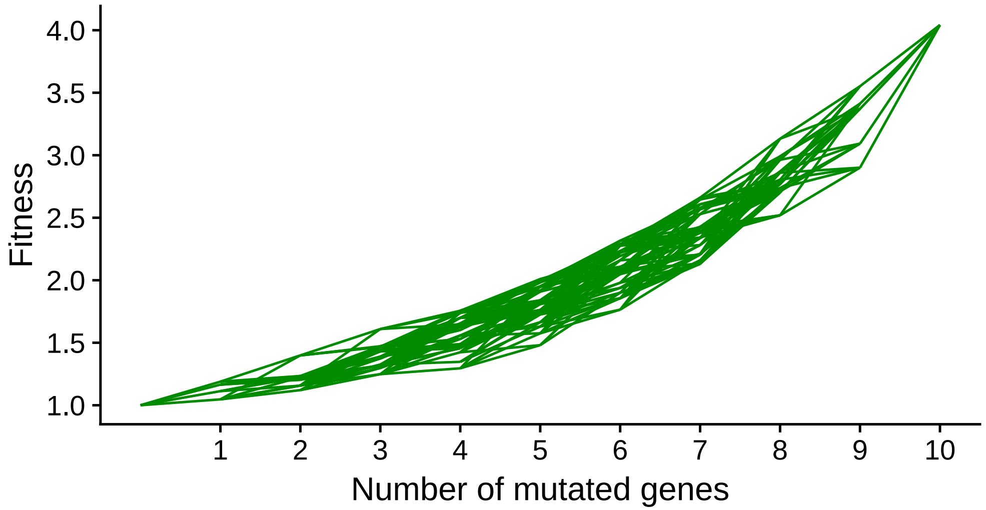

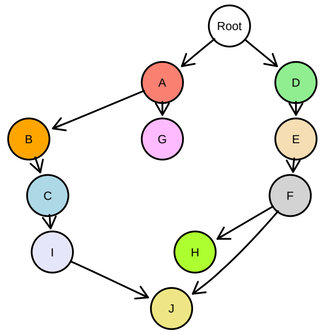

ID	bXvsBjdeGDgvk10nj
acc genotypes	49
peaks no back	1
removed edges	0
prop rem edges	0

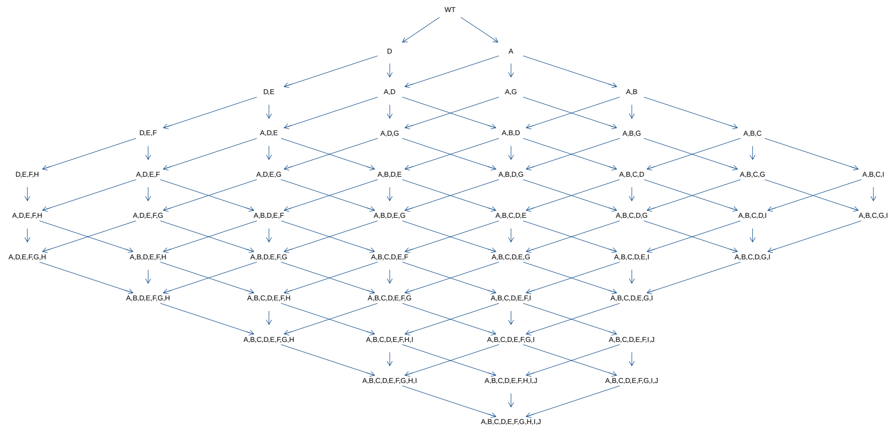

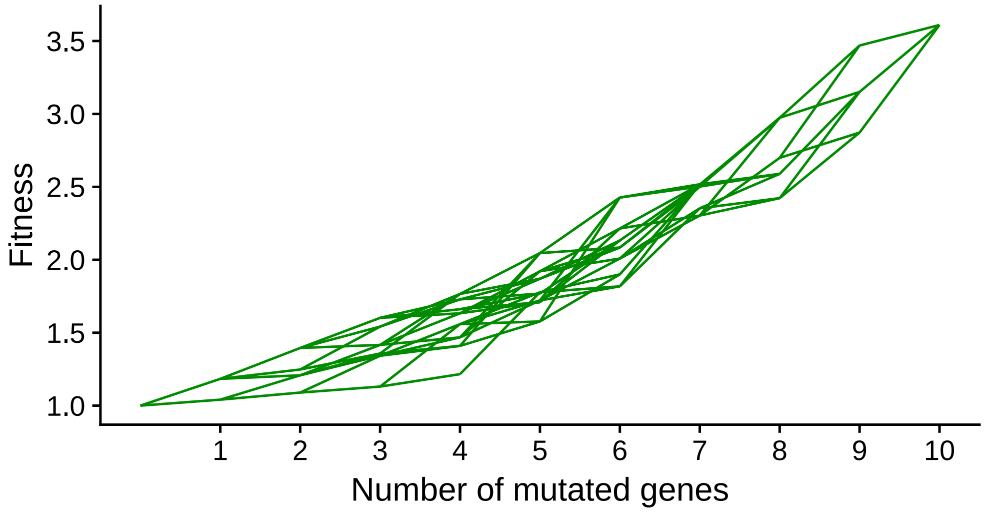

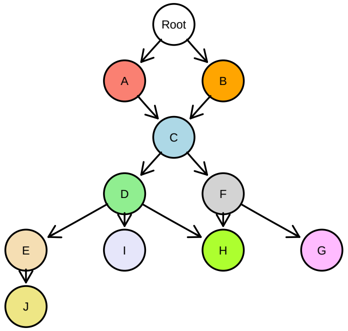

ID	c4ImcRlDBXQAiAza
acc genotypes	37
peaks no back	1
removed edges	0
prop rem edges	0

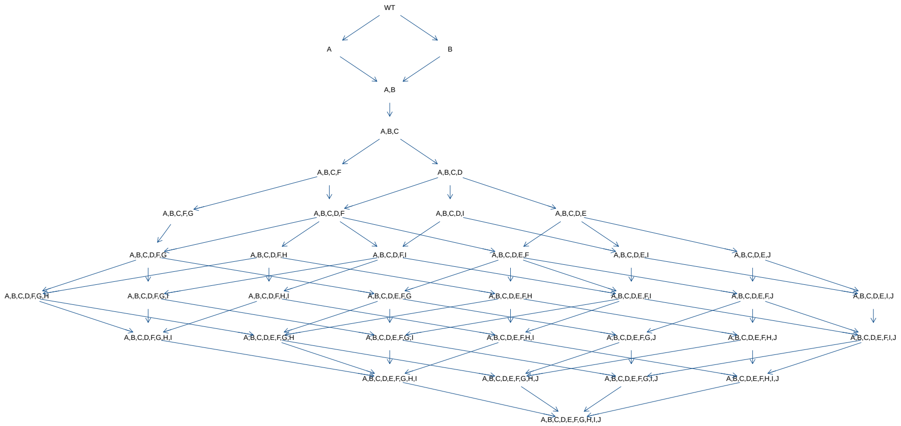

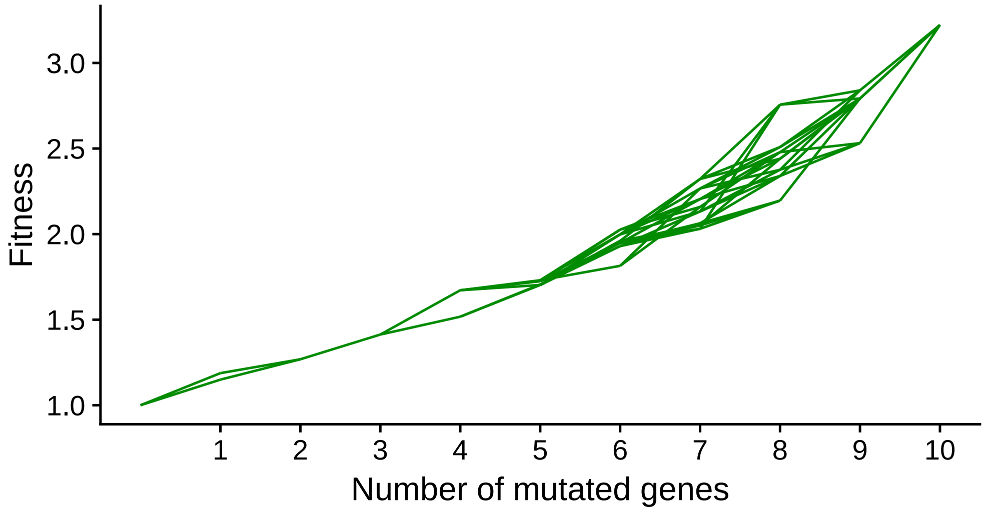

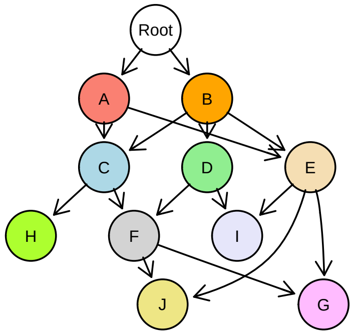

ID	cczLUqQeSfB6Czbr
acc genotypes	37
peaks no back	1
removed edges	0
prop rem edges	0

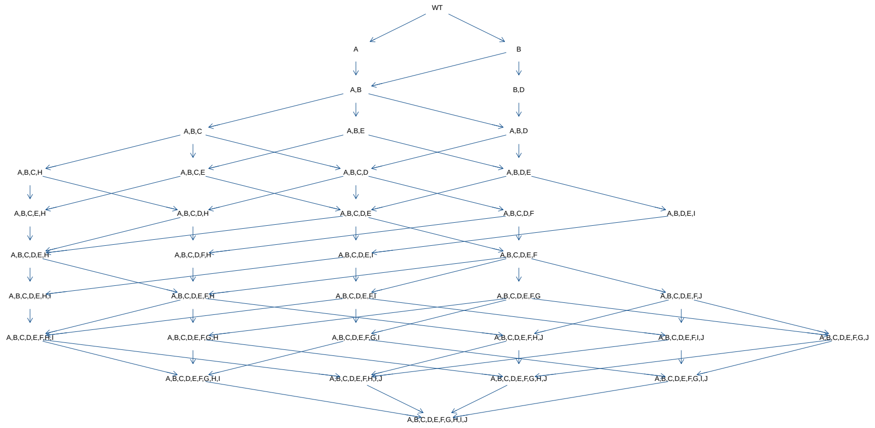

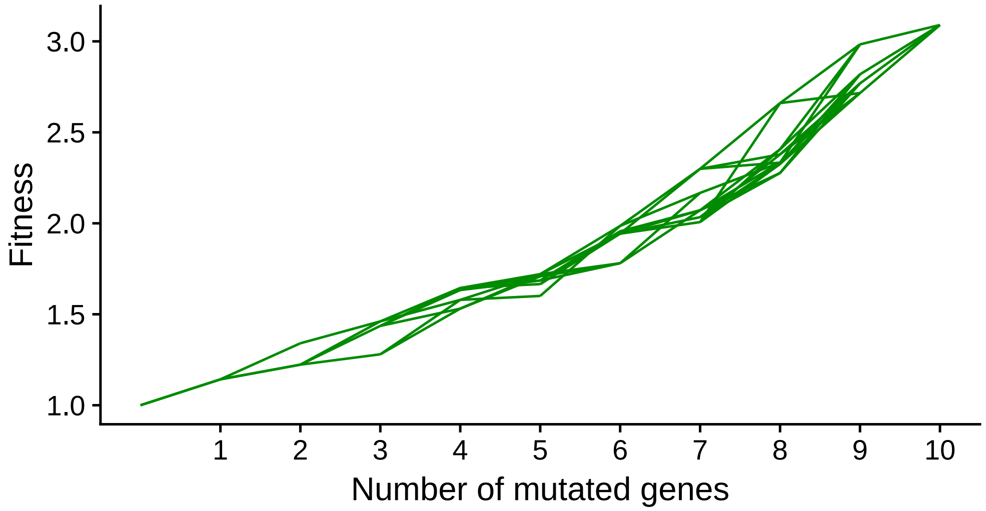

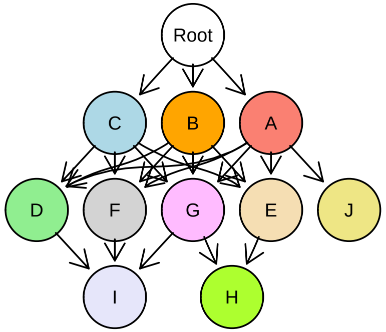

ID	CEKKz1011cQcW3hT7
acc genotypes	56
peaks no back	1
removed edges	0
prop rem edges	0

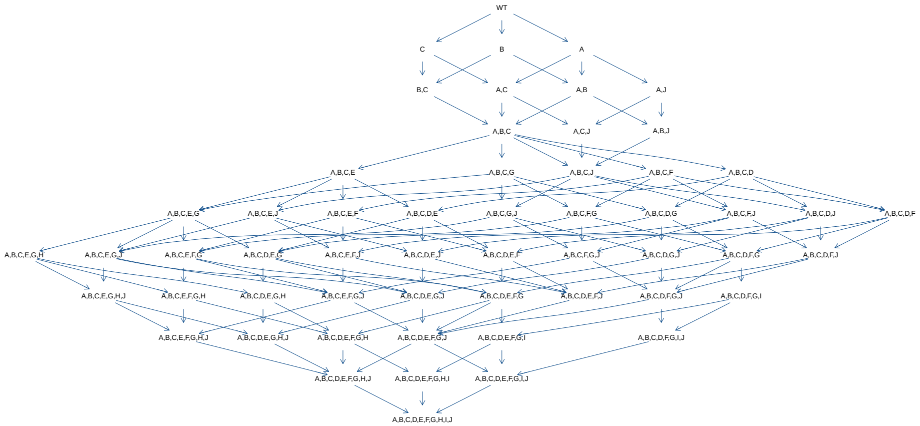

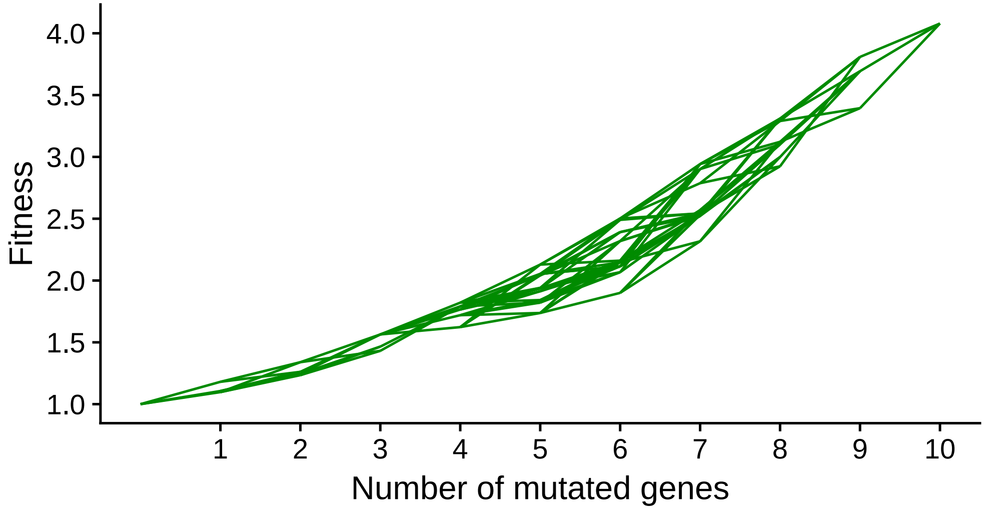

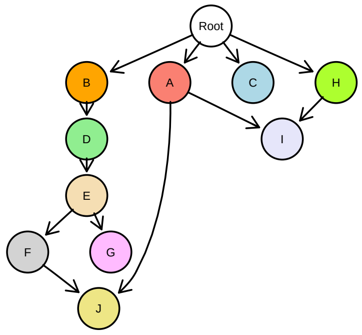

ID	COhbmk810lA2tC32X
acc genotypes	82
peaks no back	1
removed edges	0
prop rem edges	0

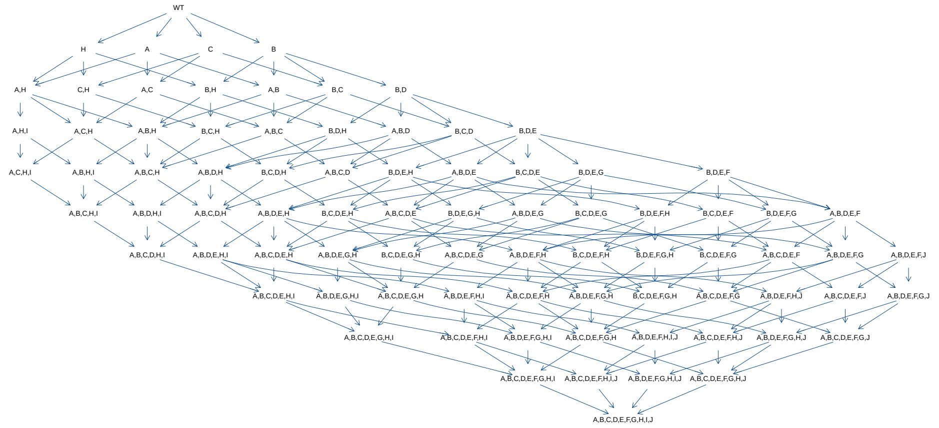

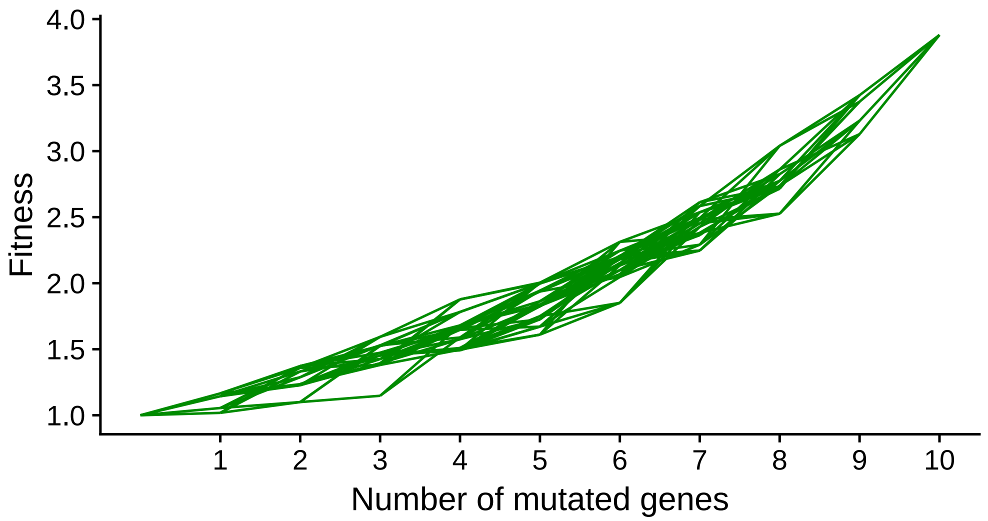

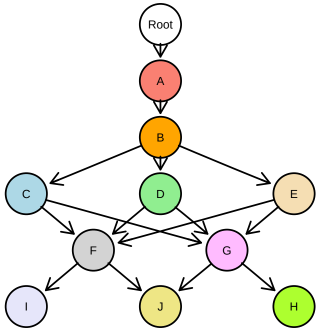

ID	CRSLV974aXfmgnX10
acc genotypes	22
peaks no back	1
removed edges	0
prop rem edges	0

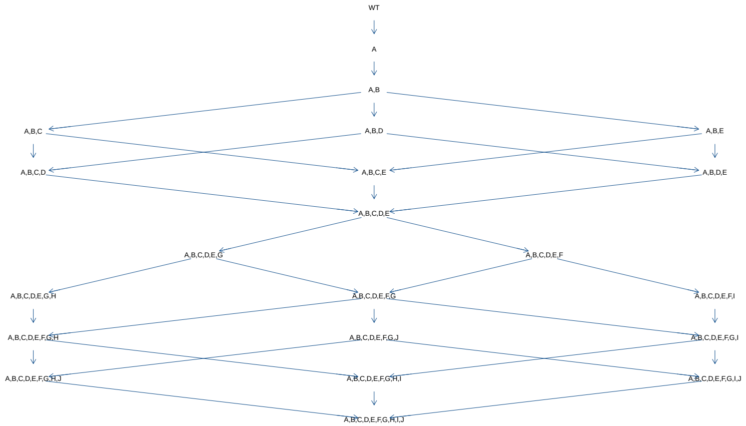

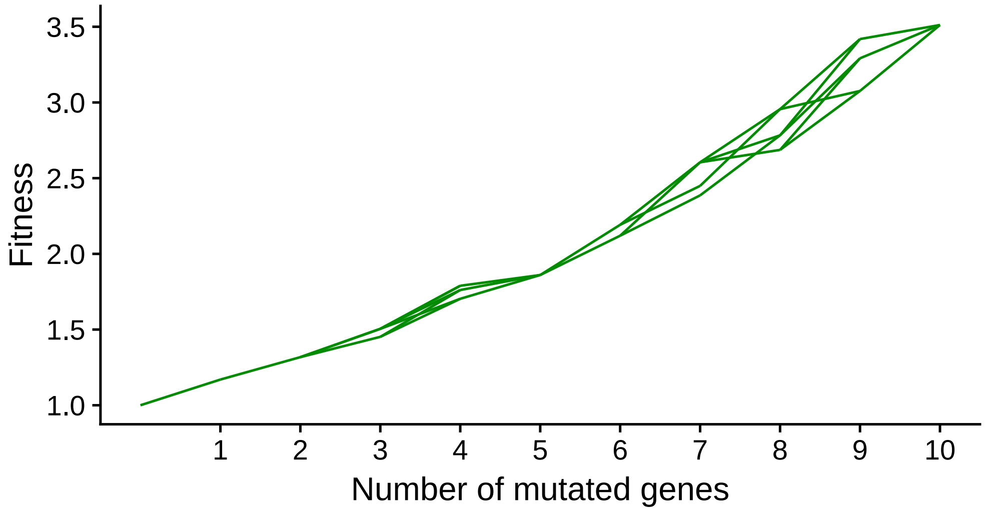

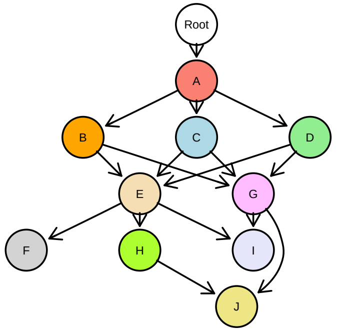

ID	cWla4xvRzx8wVebz
acc genotypes	26
peaks no back	1
removed edges	0
prop rem edges	0

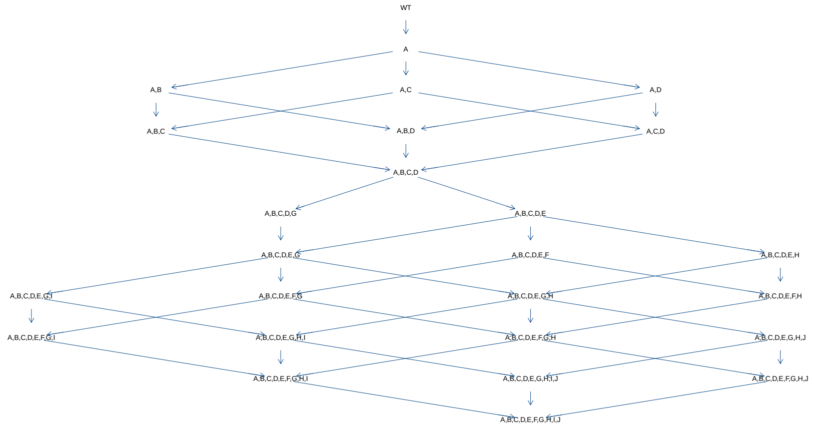

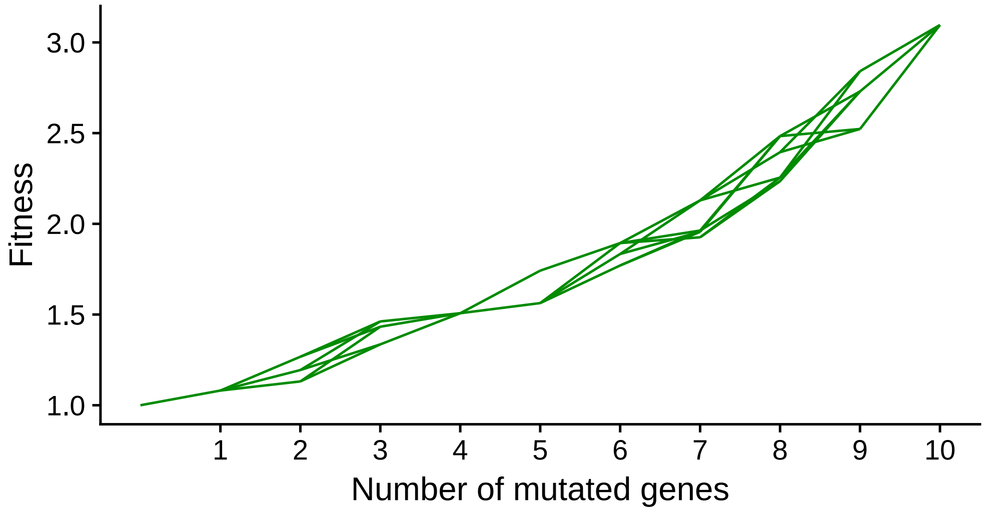

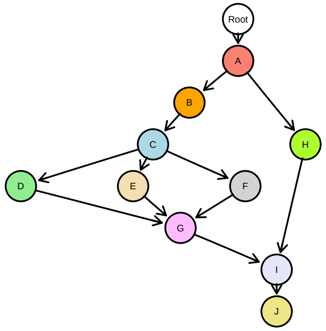

ID	d1i86X4sy10ttNPpW
acc genotypes	25
peaks no back	1
removed edges	0
prop rem edges	0

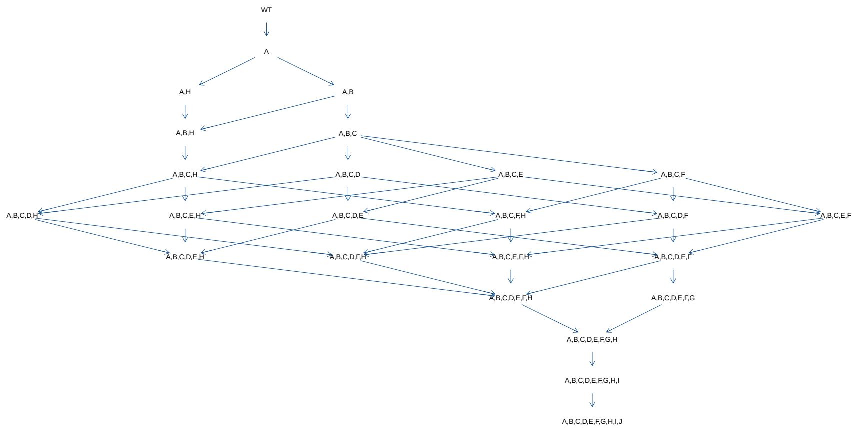

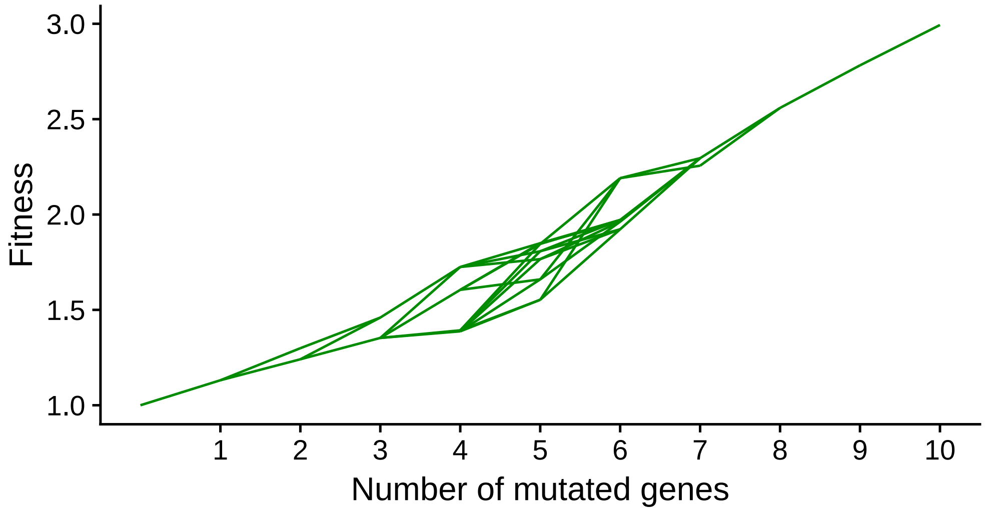

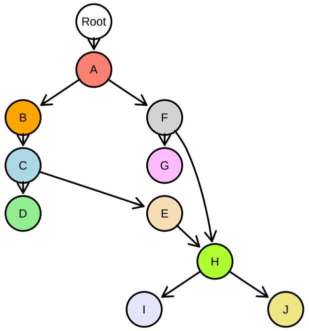

ID	D3yYb2Y5LZWHMlC9
acc genotypes	35
peaks no back	1
removed edges	0
prop rem edges	0

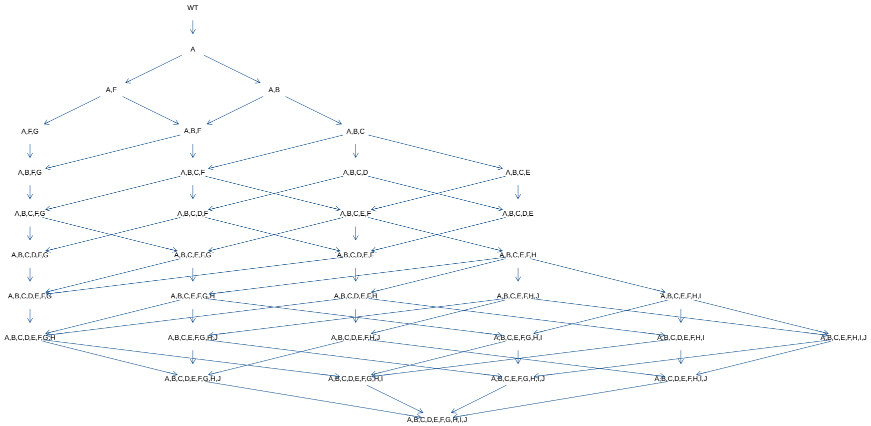

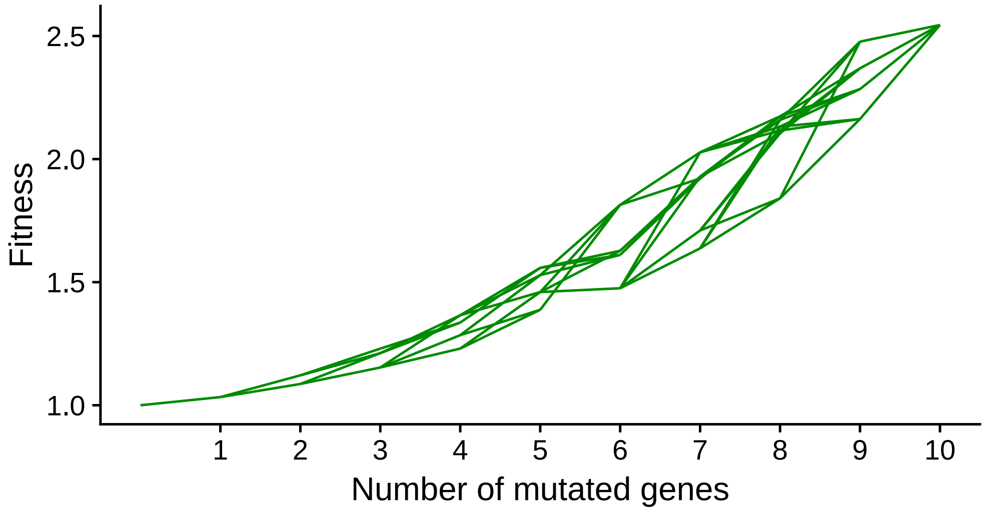

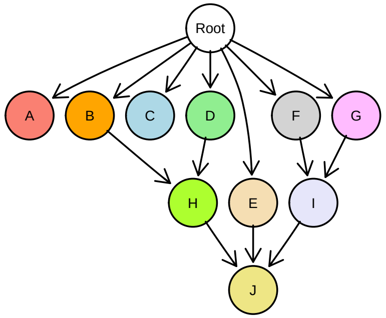

ID	DishuUtqtuc3gwcU
acc genotypes	204
peaks no back	1
removed edges	0
prop rem edges	0

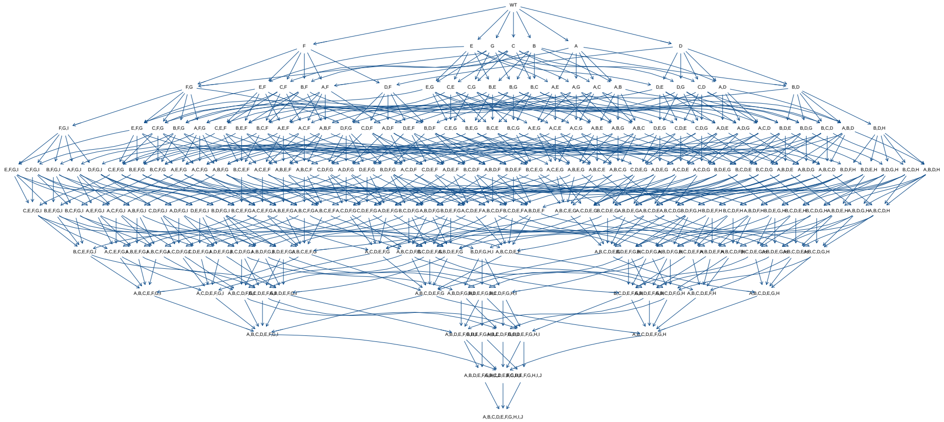

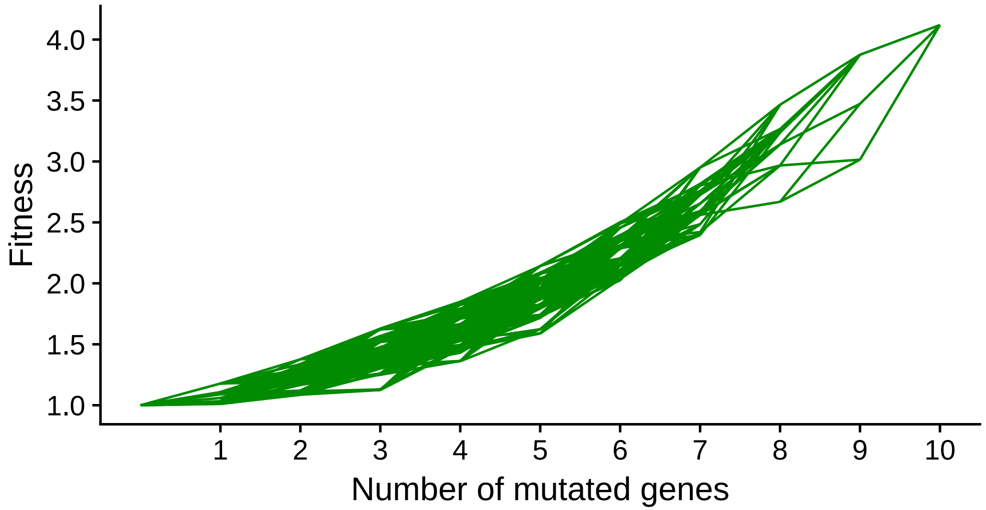

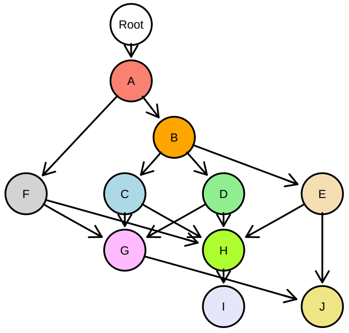

ID	DLJ64zEF6RPBgmZU
acc genotypes	28
peaks no back	1
removed edges	0
prop rem edges	0

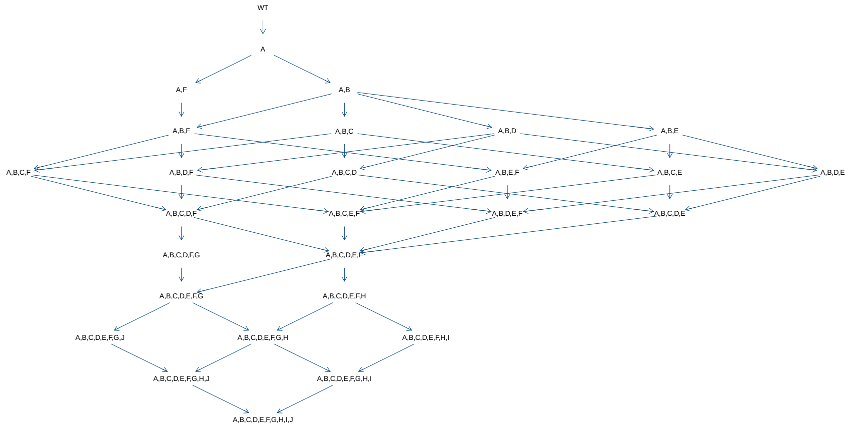

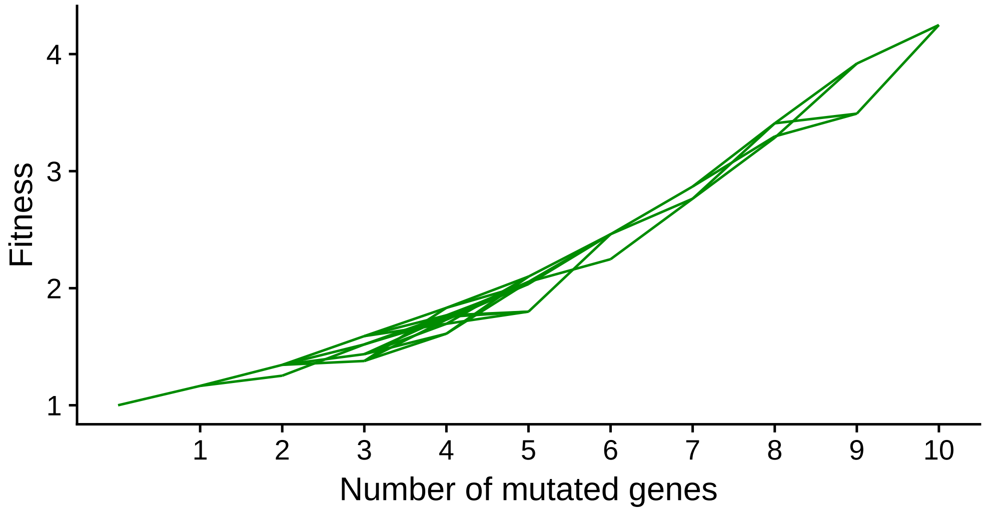

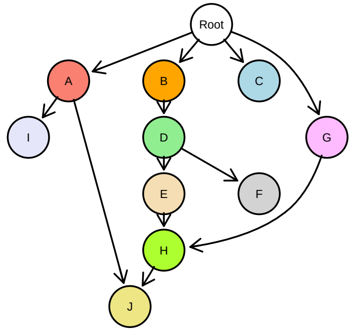

ID	dvBzM4I8nentNLyl
acc genotypes	92
peaks no back	1
removed edges	0
prop rem edges	0

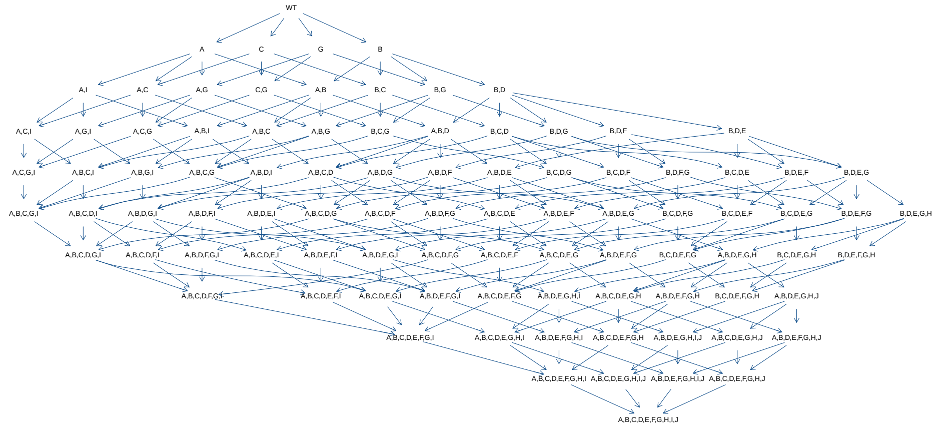

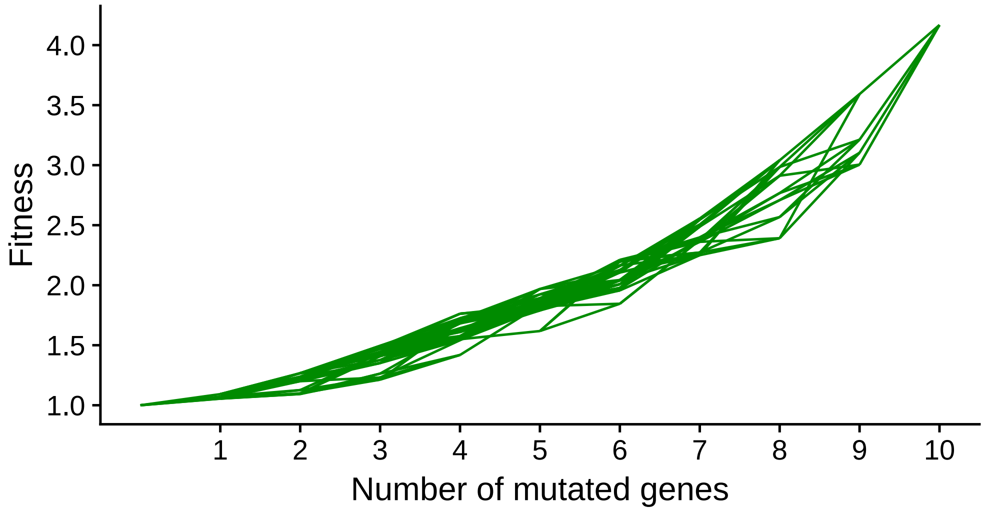

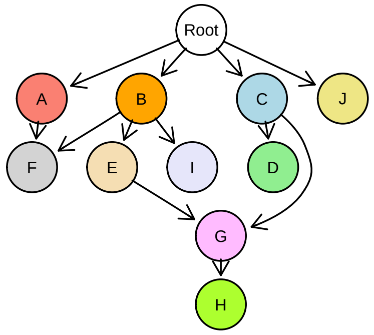

ID	dvQzUhETlllrU97u
acc genotypes	132
peaks no back	1
removed edges	0
prop rem edges	0

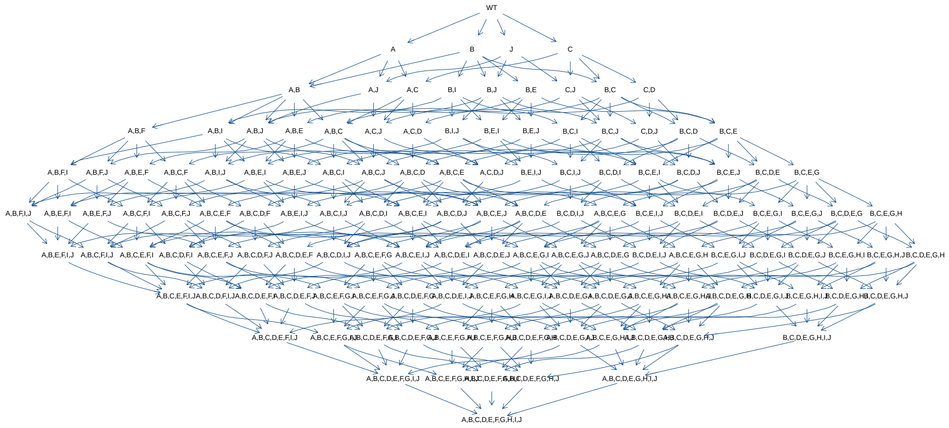

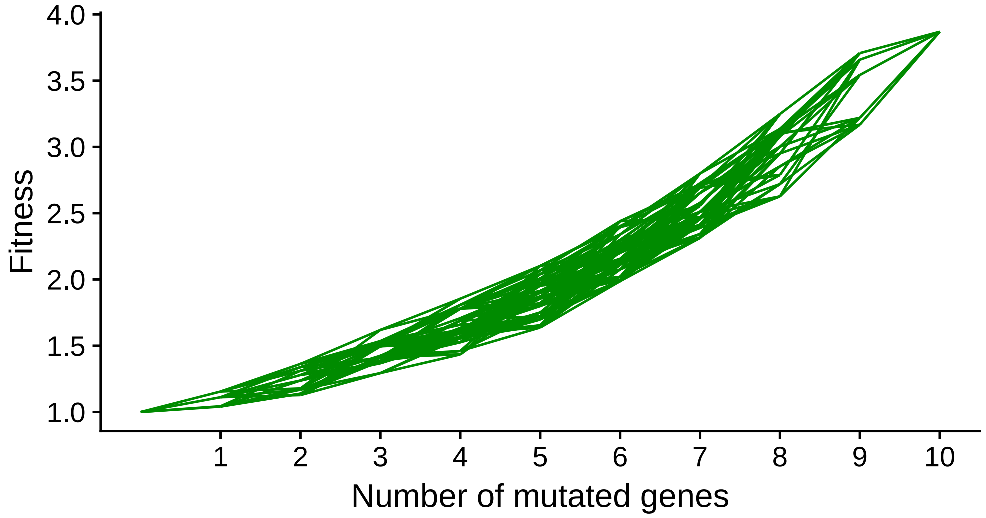

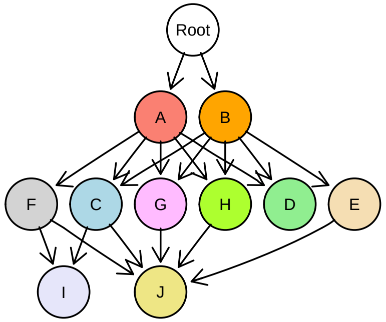

ID EBt810tAfY9D105FkY

acc genotypes 89

peaks no back 1

removed edges 0

prop rem edges 0

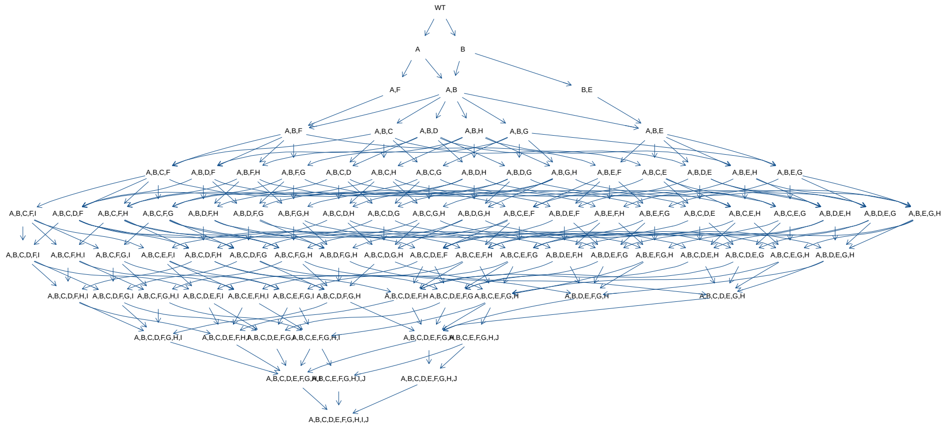

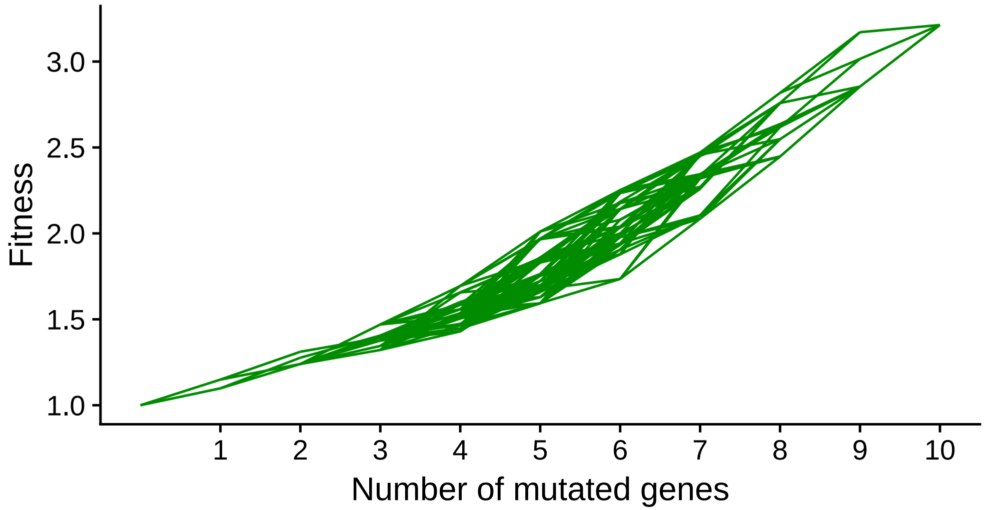

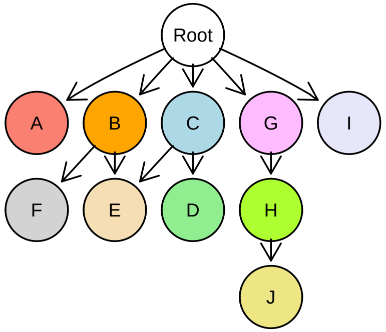

ID ekTHm442hqA10lVJm
acc genotypes 208
peaks no back 1
removed edges 0
prop rem edges 0

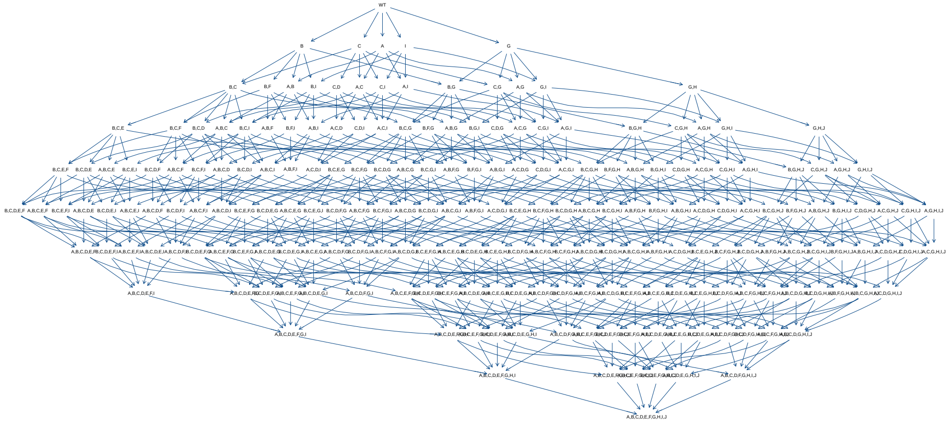

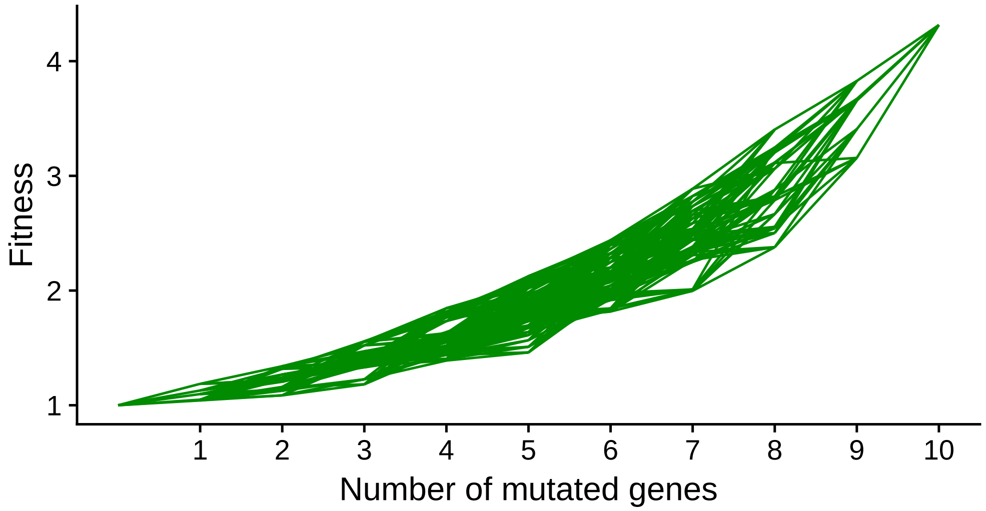

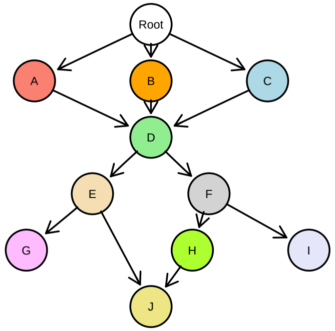

ID	EkuM7k8AJLiPR6hS
acc genotypes	27
peaks no back	1
removed edges	0
prop rem edges	0

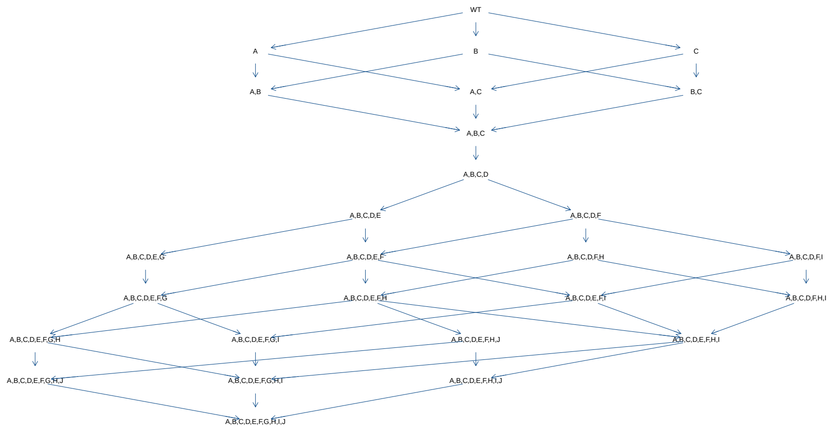

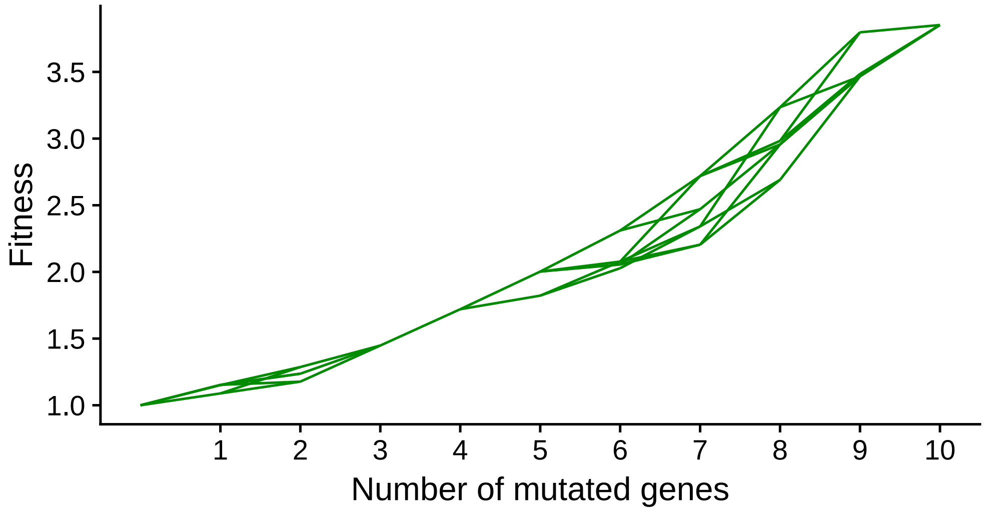

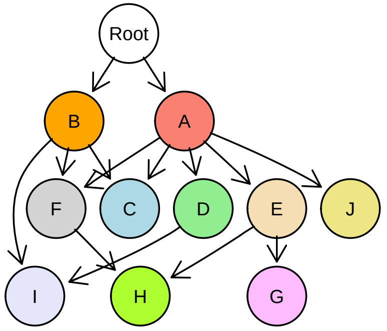

ID	eNPbGhByo2yD8VoG
acc genotypes	110
peaks no back	1
removed edges	0
prop rem edges	0

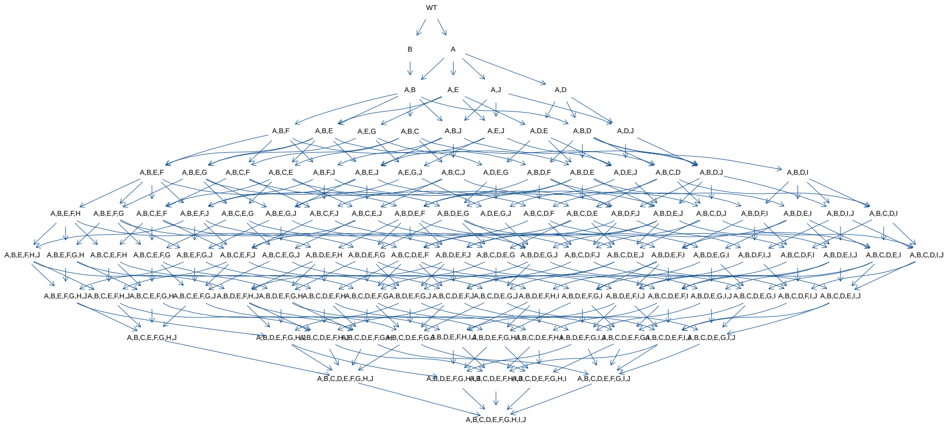

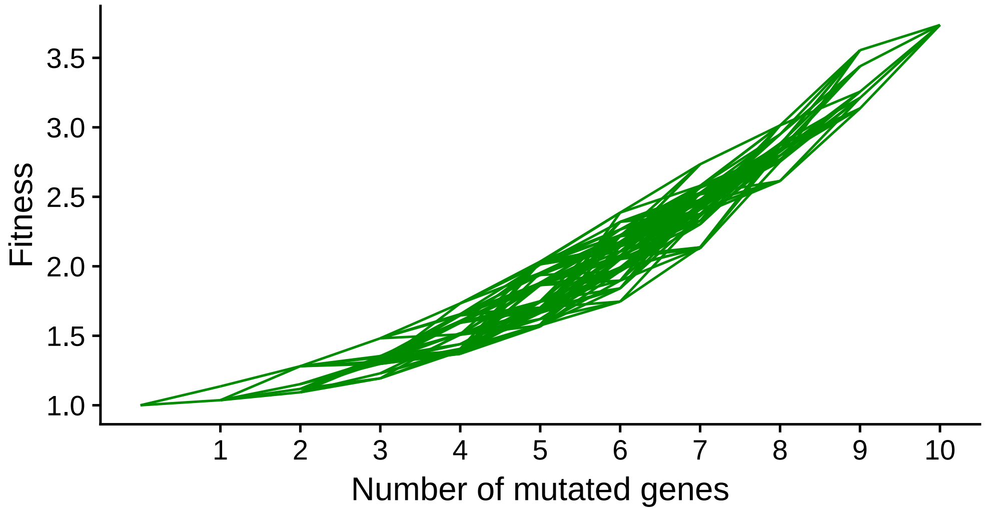

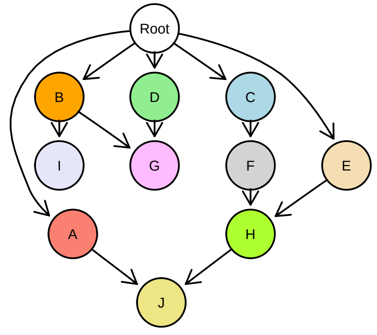

ID	EoCzYRJurCmFz168
acc genotypes	120
peaks no back	1
removed edges	0
prop rem edges	0

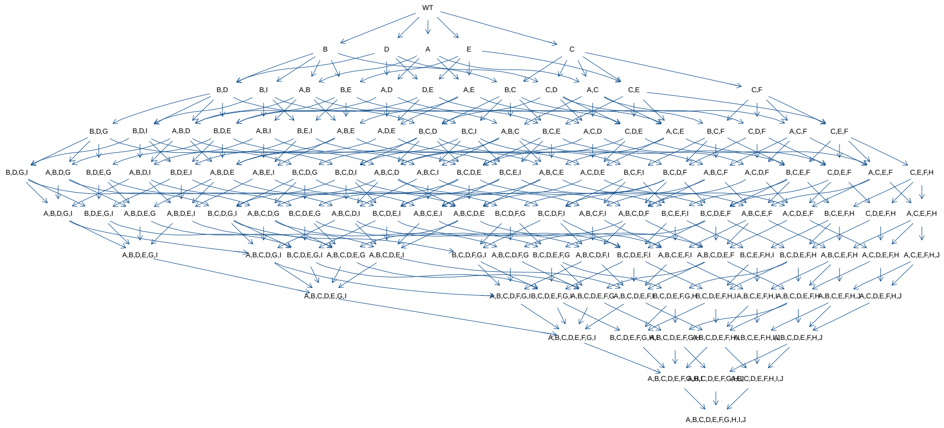

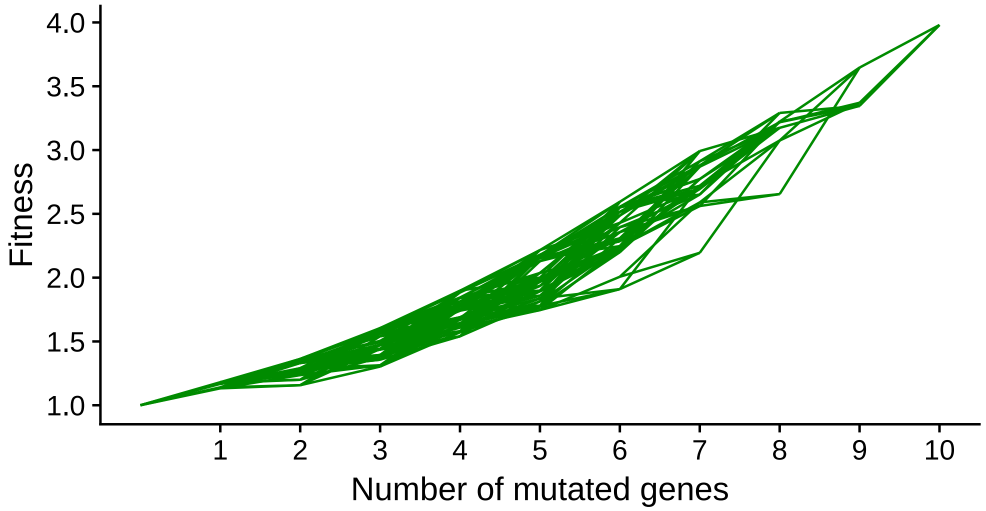

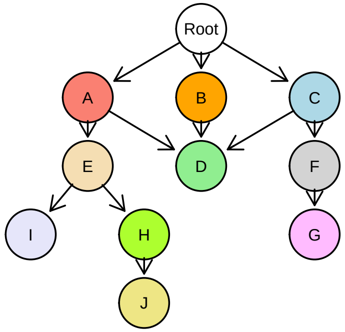

ID	eOGzHUxNXJg7737x
acc genotypes	85
peaks no back	1
removed edges	0
prop rem edges	0

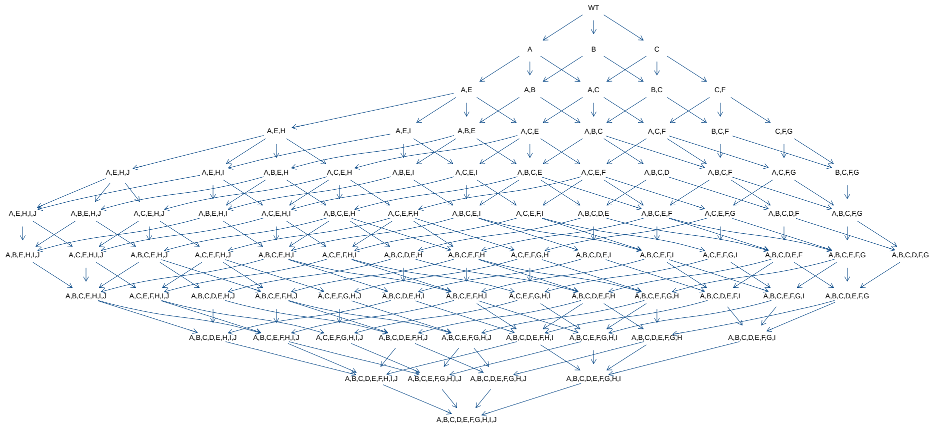

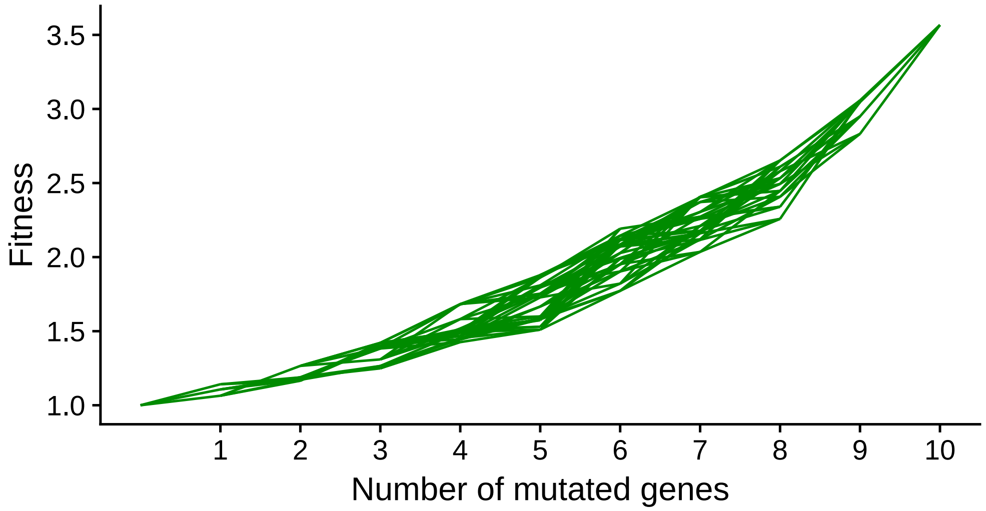

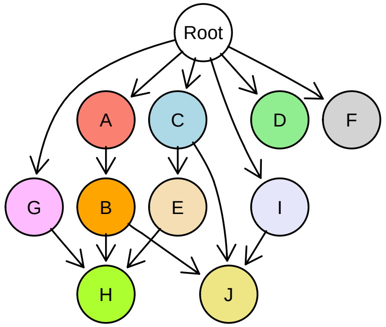

ID ESсCMp2k63ciS5e1
acc genotypes 172
peaks no back 1
removed edges 0
prop rem edges 0

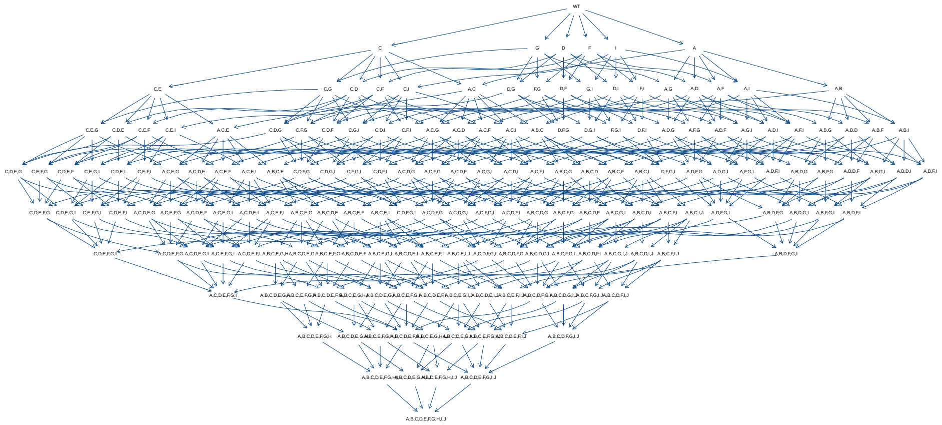

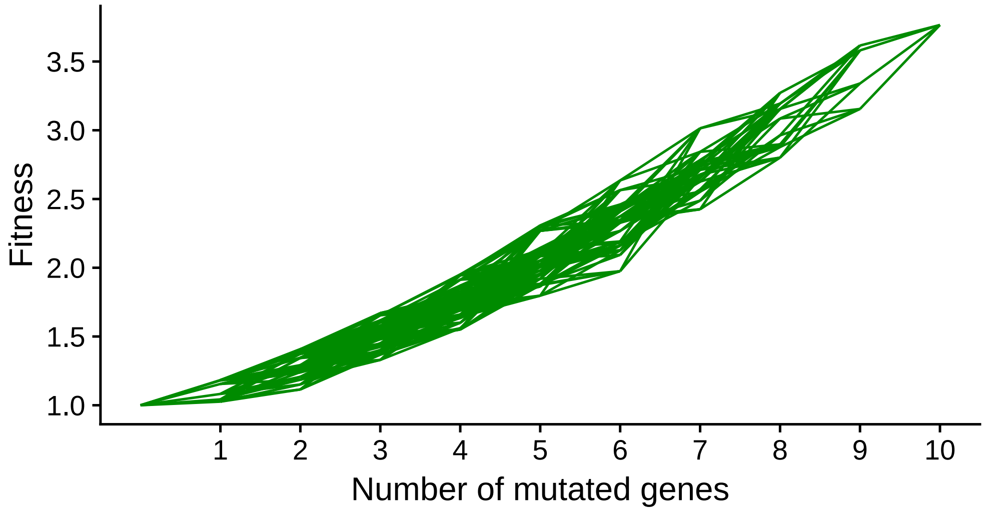

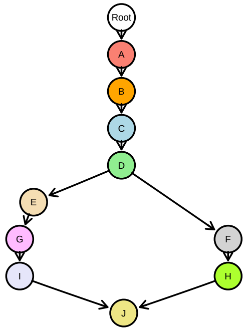

ID	EUl10dI8bbHyhh4nE
acc genotypes	17
peaks no back	1
removed edges	0
prop rem edges	0

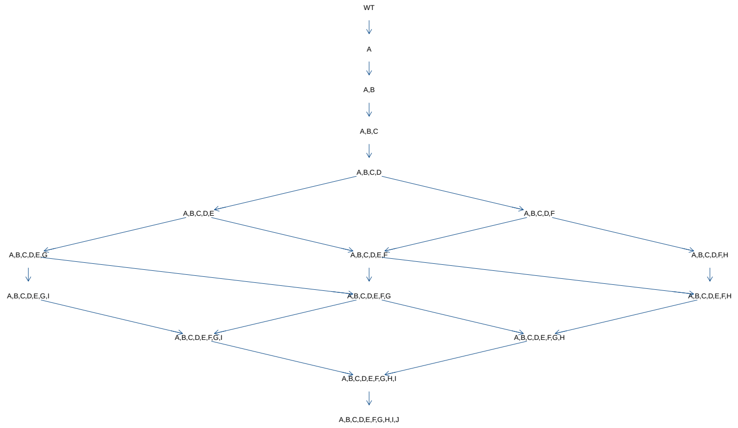

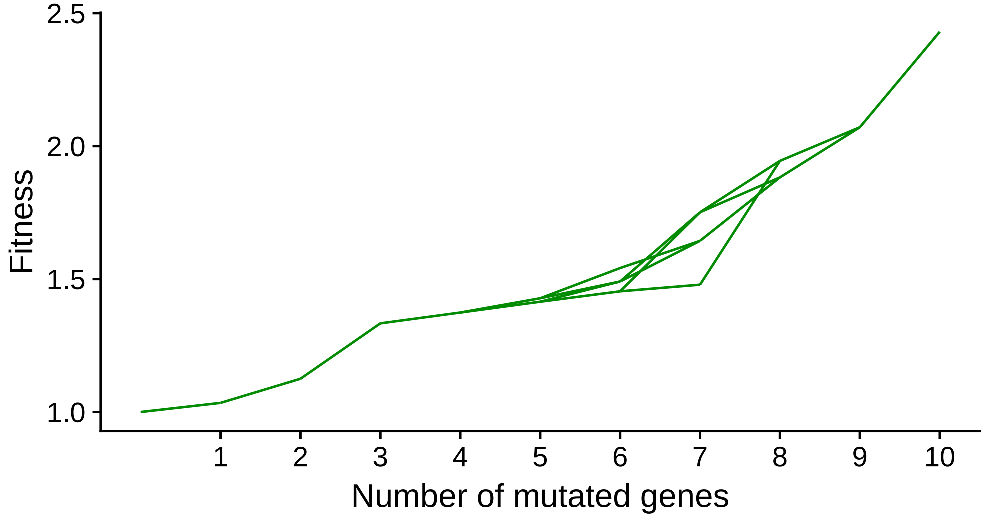

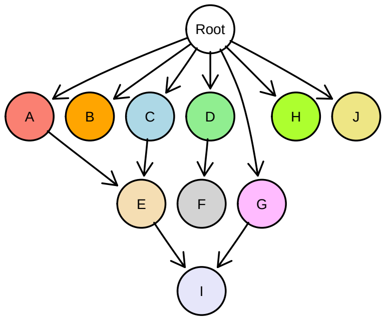

ID	ExaZNcadWsiZp1os
acc genotypes	264
peaks no back	1
removed edges	0
prop rem edges	0

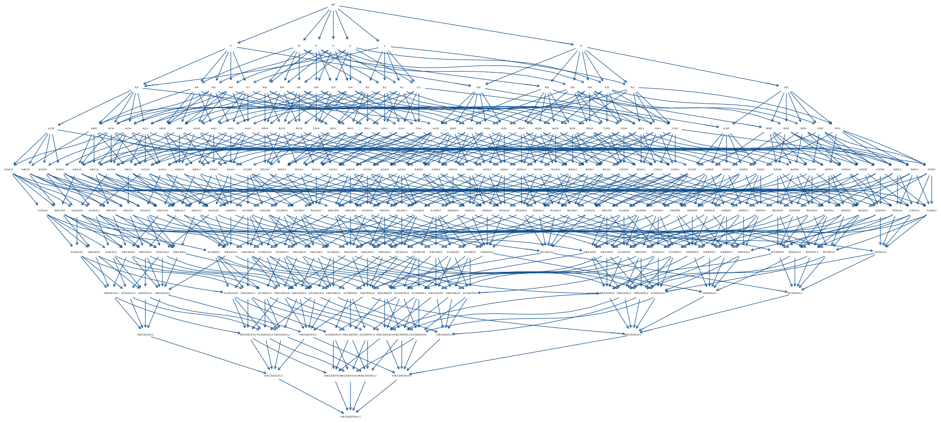

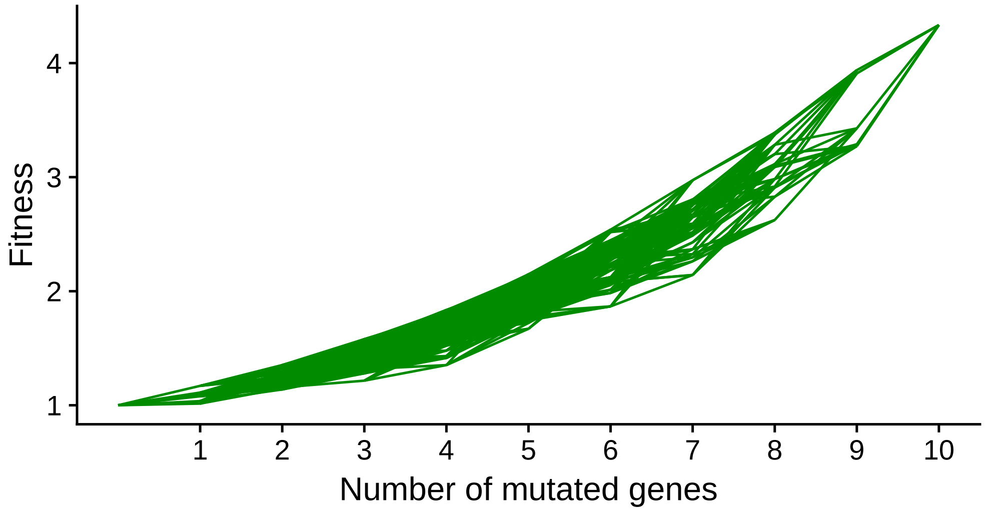

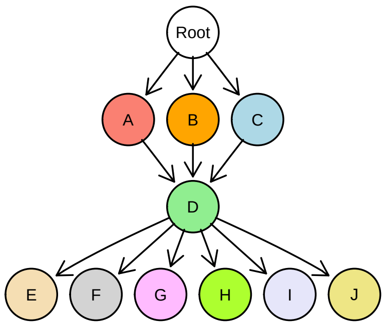

ID F74OrUlE2JUNgUl10
acc genotypes 72
peaks no back 1
removed edges 0
prop rem edges 0

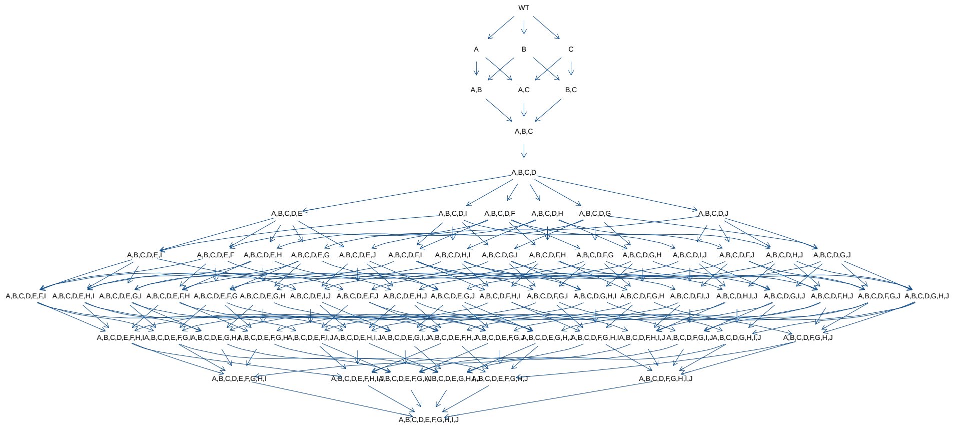

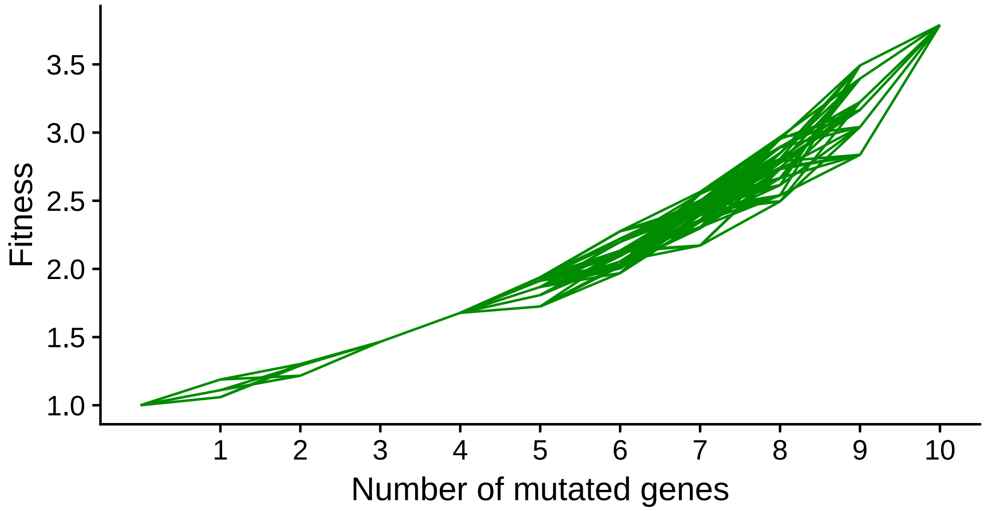

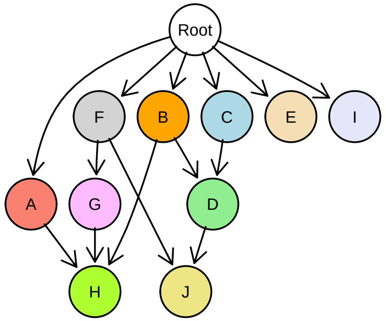

ID fDVyfwz5EixVTGkl
acc genotypes 152
peaks no back 1
removed edges 0
prop rem edges 0

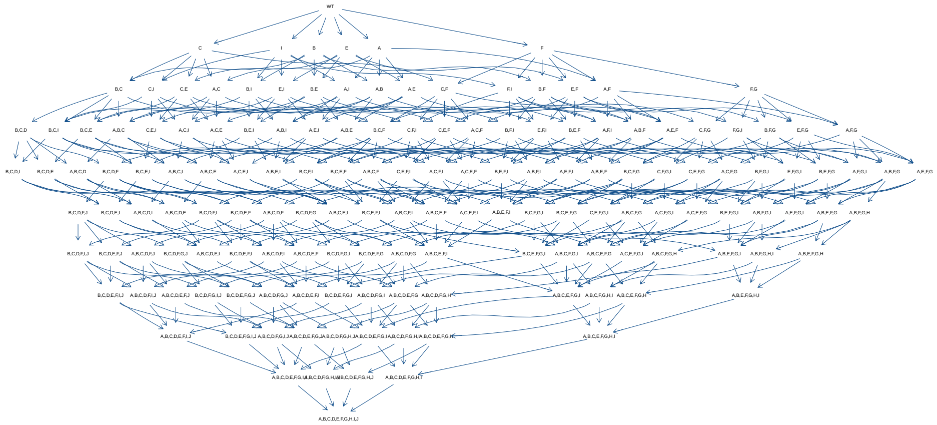

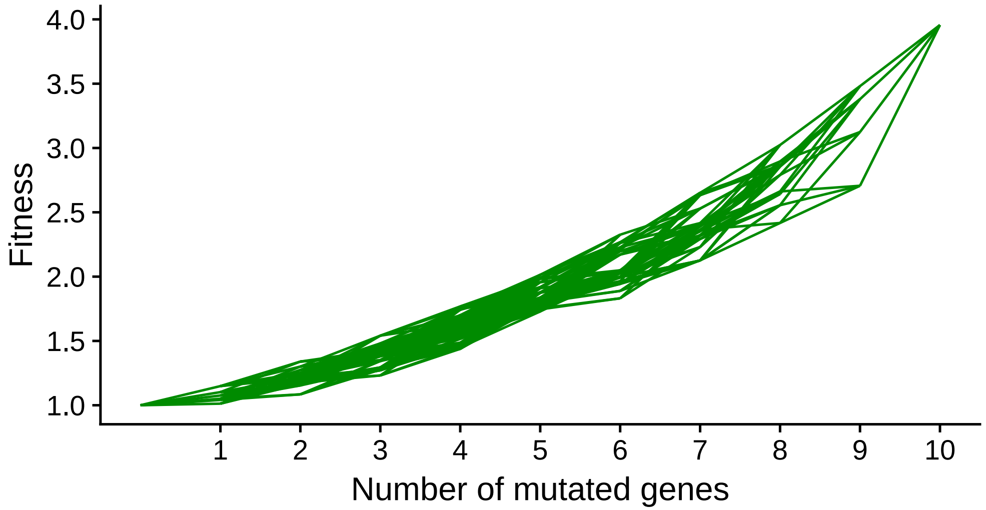

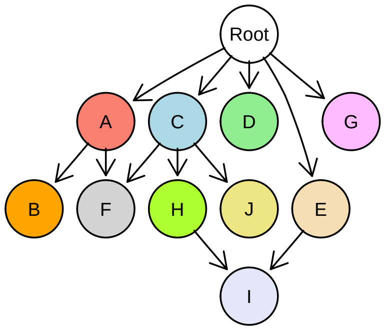

ID	FMa3on8bA8mNavrZ
acc genotypes	224
peaks no back	1
removed edges	0
prop rem edges	0

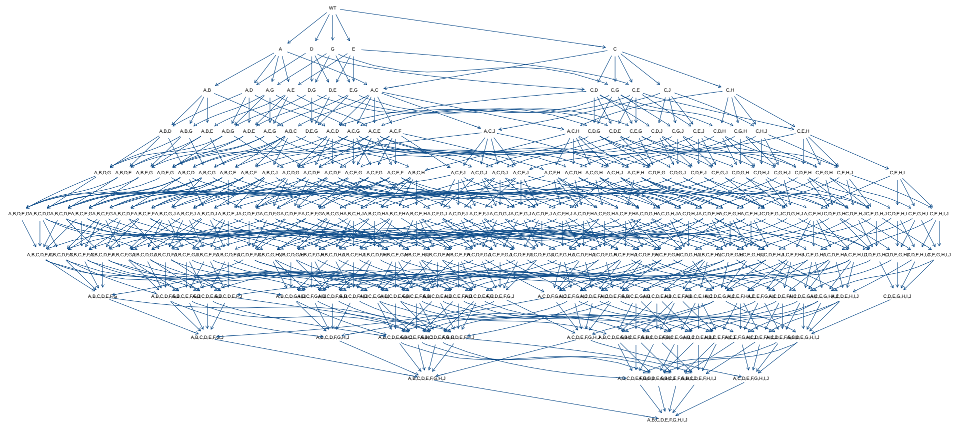

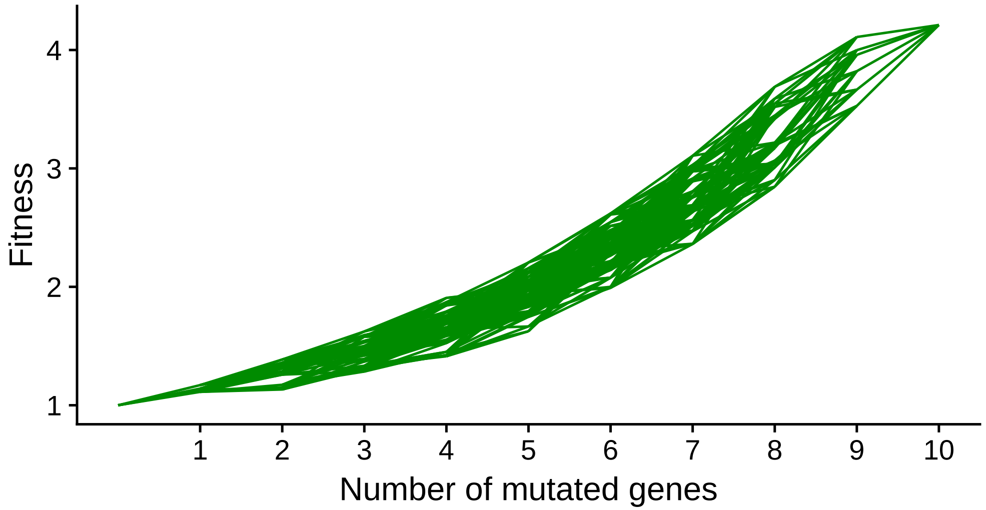

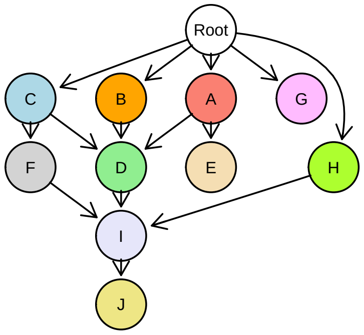

ID	Fsqw1LKsGEDExuVa
acc genotypes	96
peaks no back	1
removed edges	0
prop rem edges	0

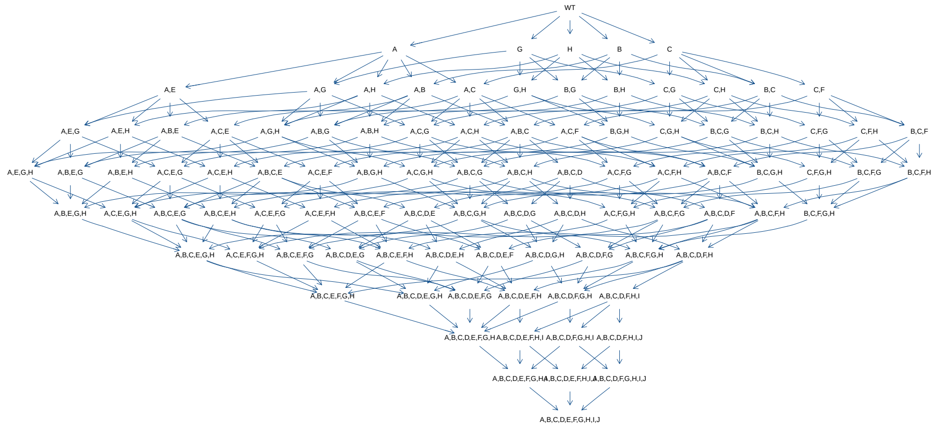

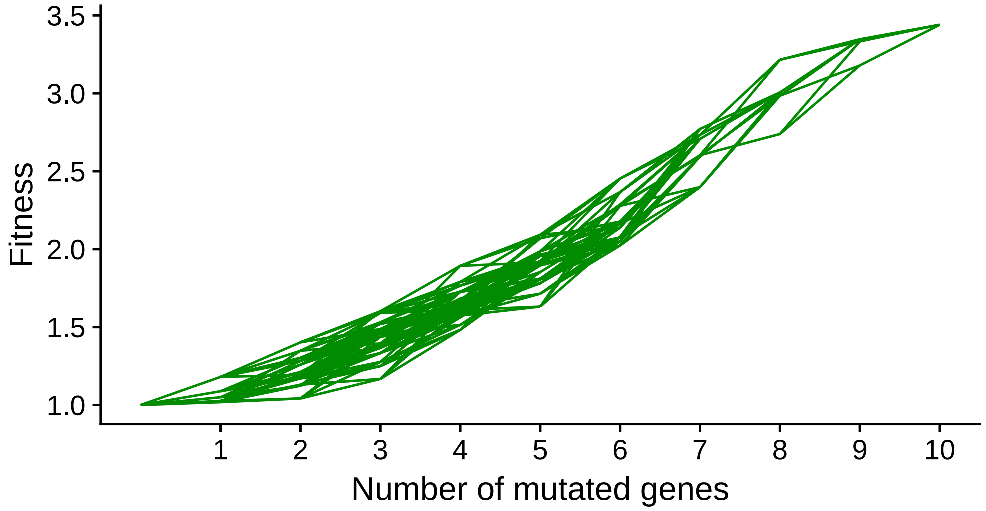

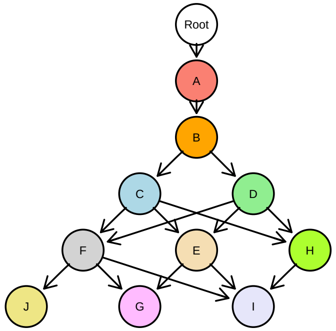

ID	FYcNi4WyweZ3YLZu
acc genotypes	25
peaks no back	1
removed edges	0
prop rem edges	0

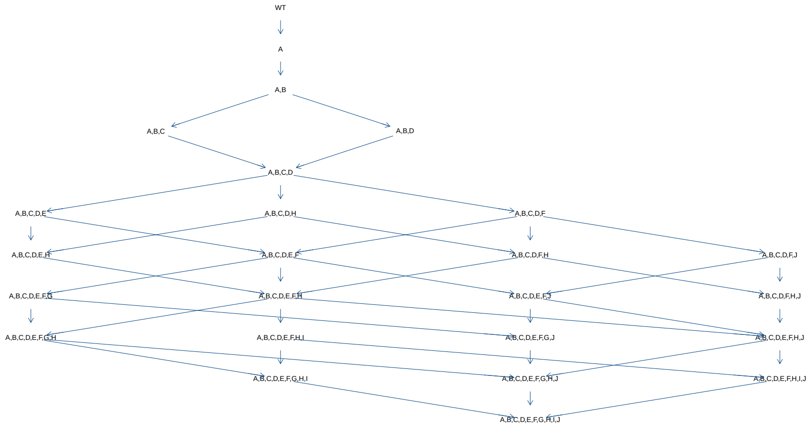

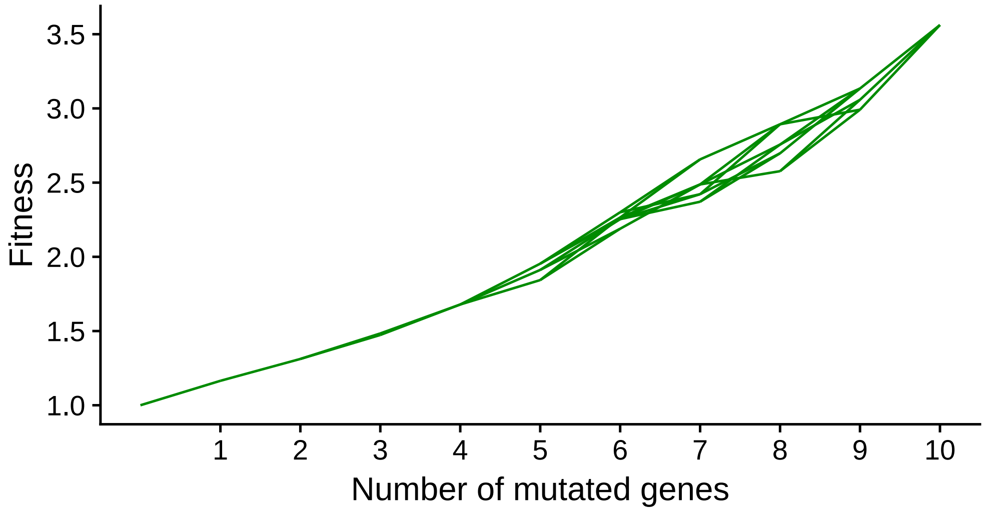

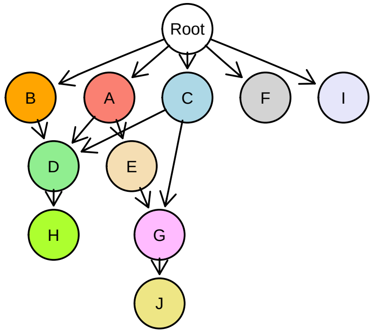

ID G3GJI10gesv99QKDi
acc genotypes 96
peaks no back 1
removed edges 0
prop rem edges 0

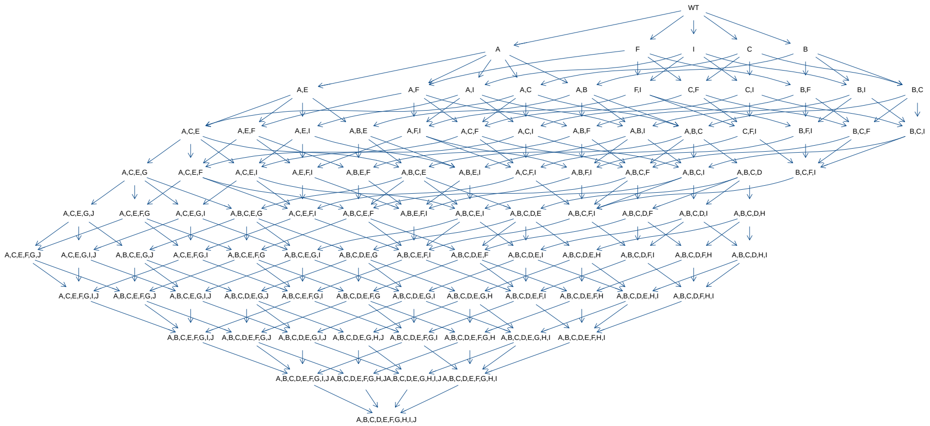

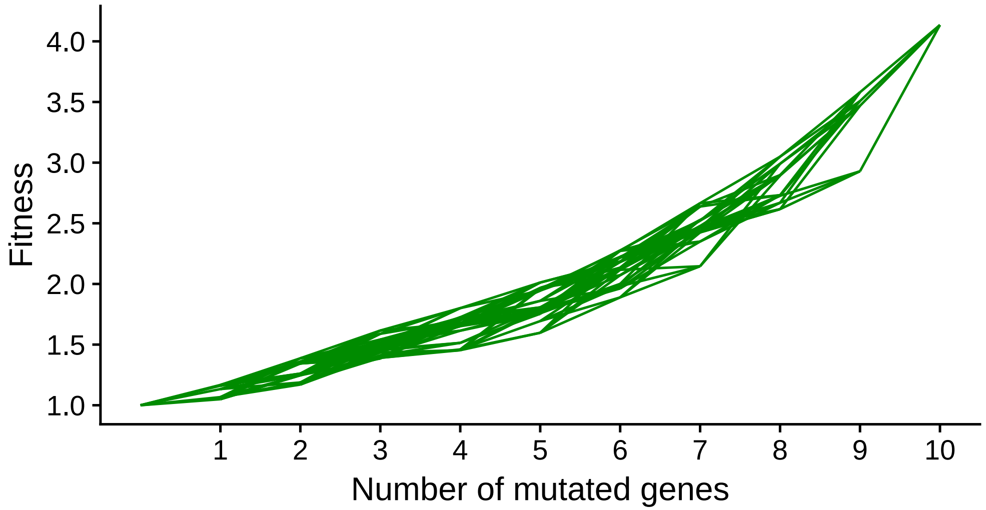

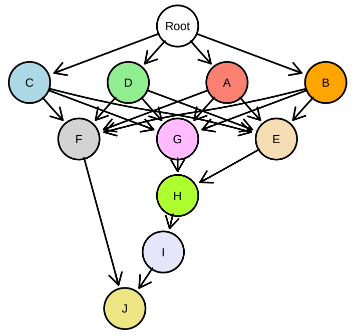

ID	gcBjSEP8ZTo7NX2w
acc genotypes	28
peaks no back	1
removed edges	0
prop rem edges	0

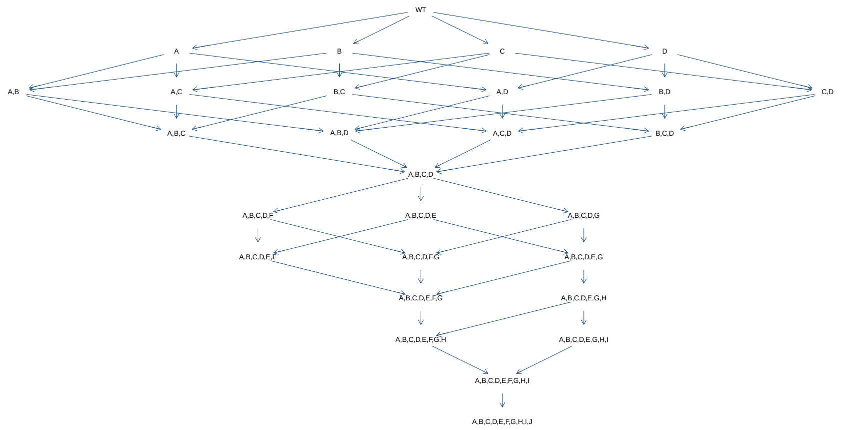

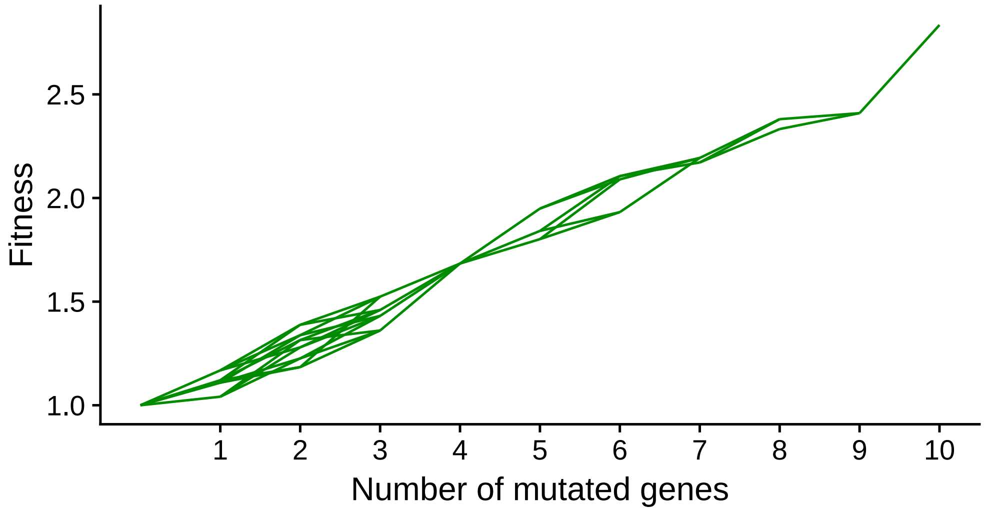

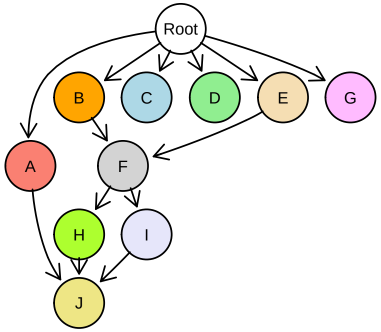

ID	GdKyxu7pbMBmXsha
acc genotypes	136
peaks no back	1
removed edges	0
prop rem edges	0

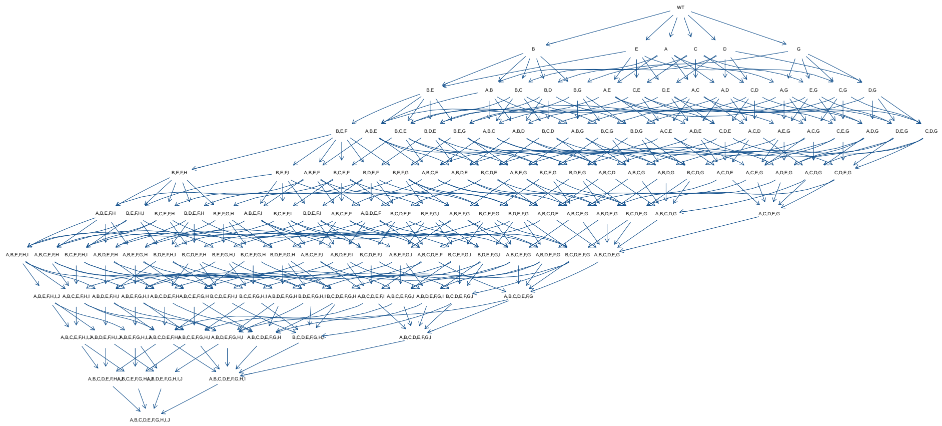

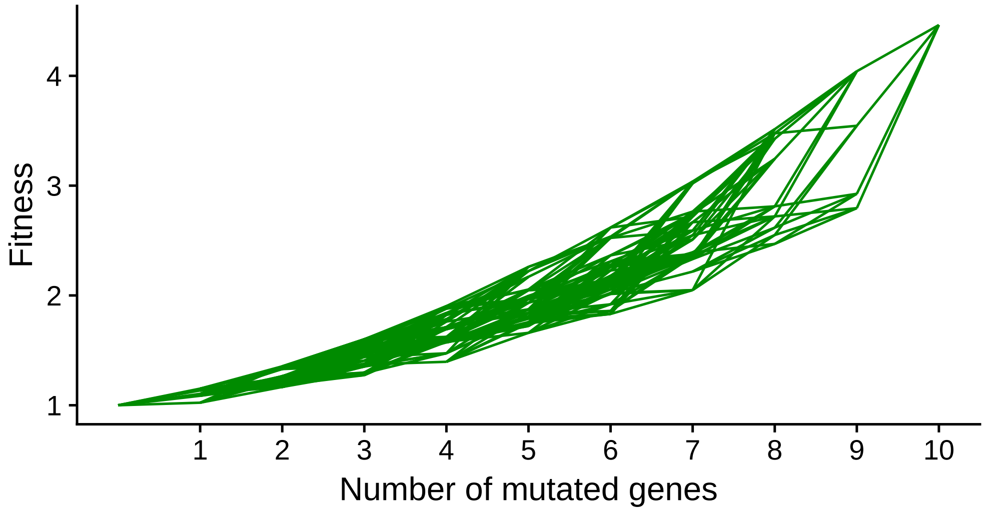

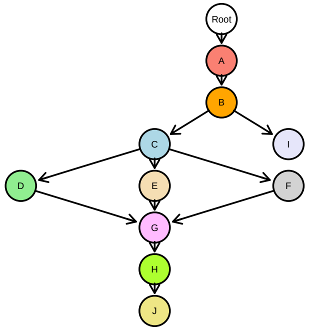

ID	GDWChzATMq5hpozJ
acc genotypes	26
peaks no back	1
removed edges	0
prop rem edges	0

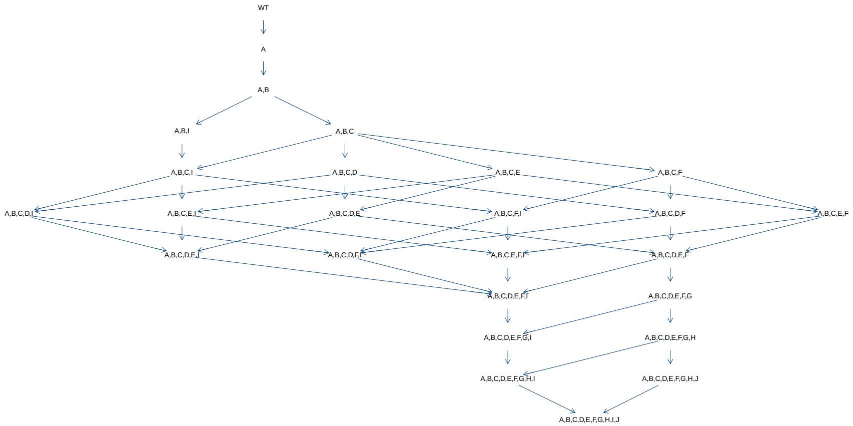

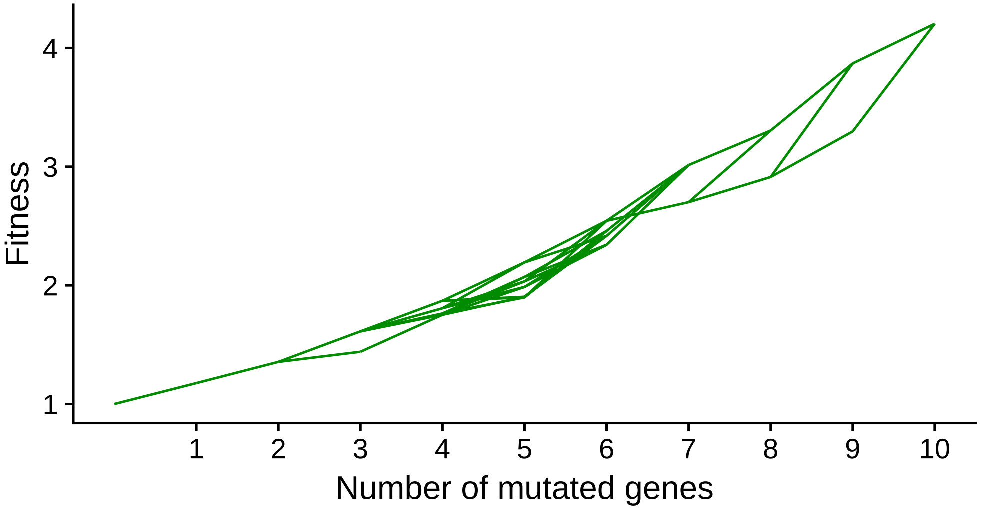

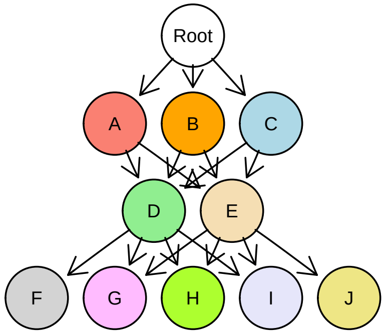

ID	gNDvvHs3jYcerc7l
acc genotypes	44
peaks no back	1
removed edges	0
prop rem edges	0

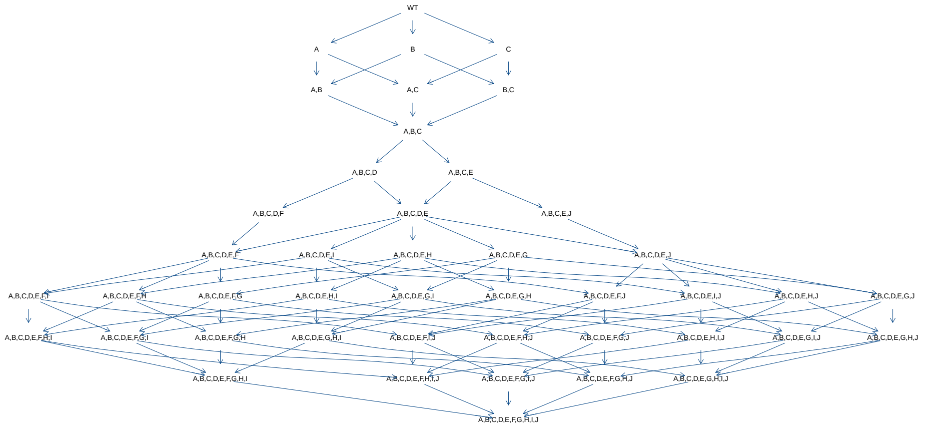

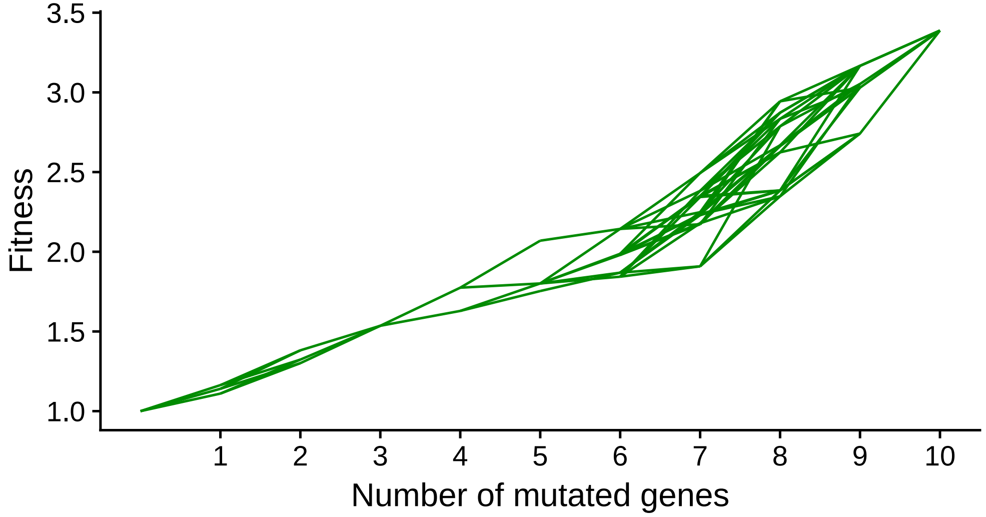

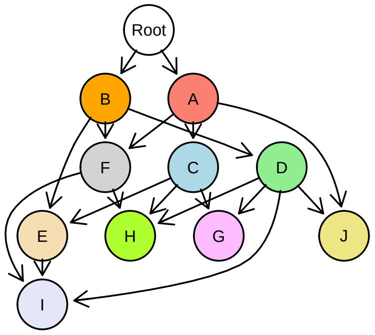

ID	gqmarqZNpxa6L2Zx
acc genotypes	47
peaks no back	1
removed edges	0
prop rem edges	0

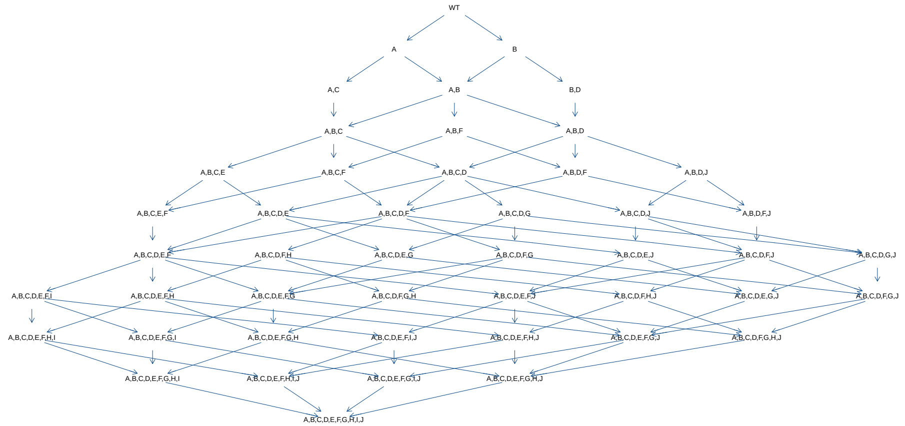

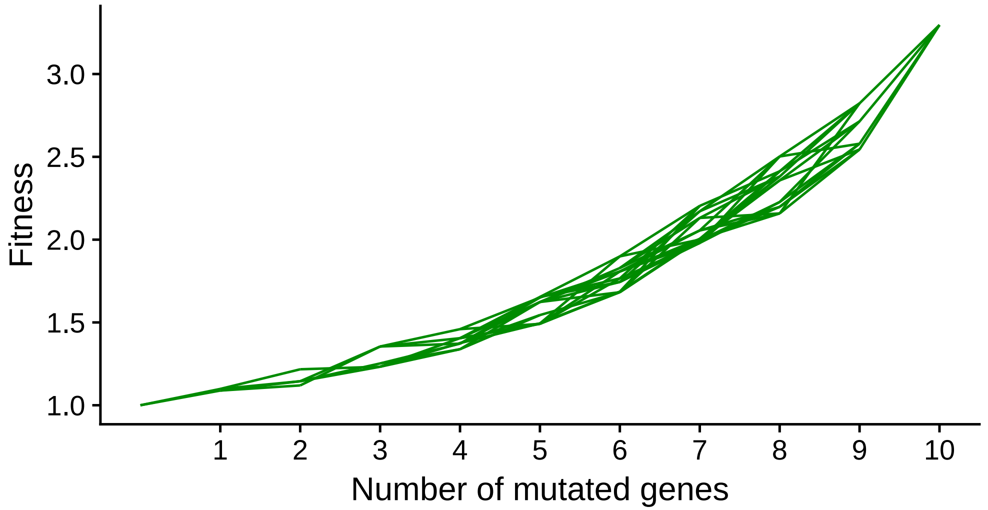

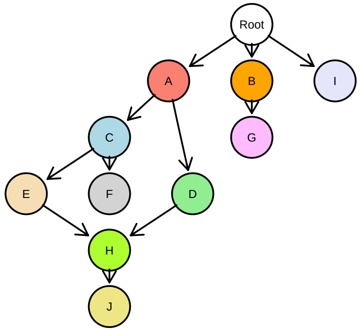

ID	gYZAxs6hKlMQf3Ca
acc genotypes	90
peaks no back	1
removed edges	0
prop rem edges	0

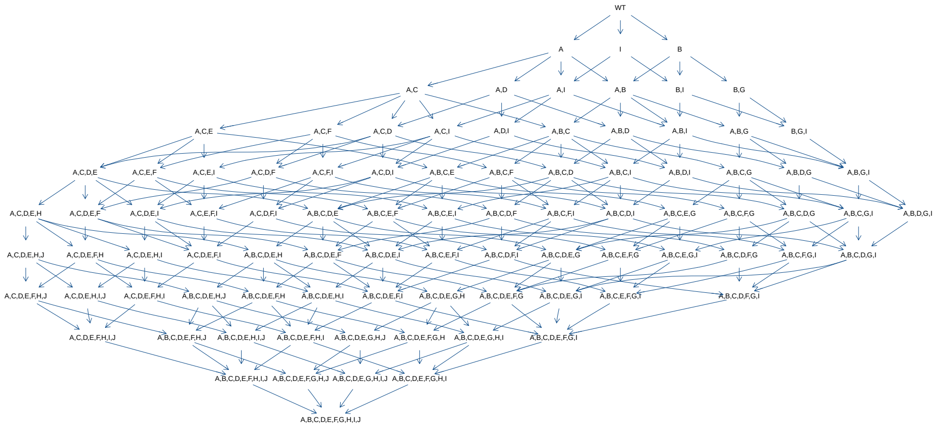

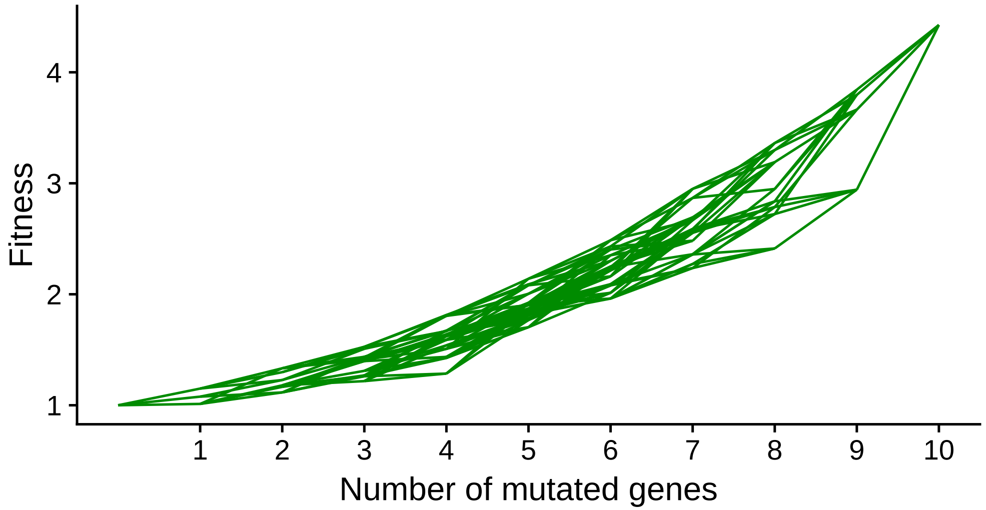

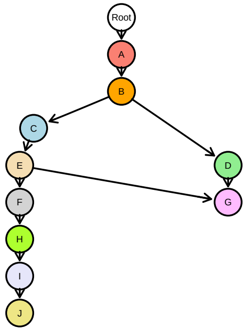

ID	H10RfX4lukGLEX1kv
acc genotypes	21
peaks no back	1
removed edges	0
prop rem edges	0

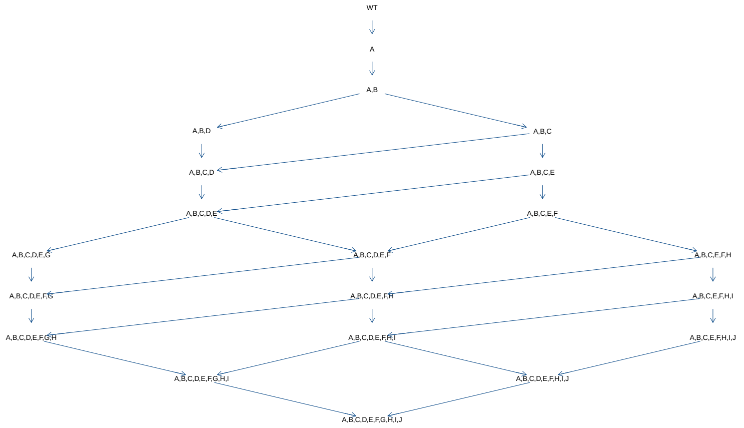

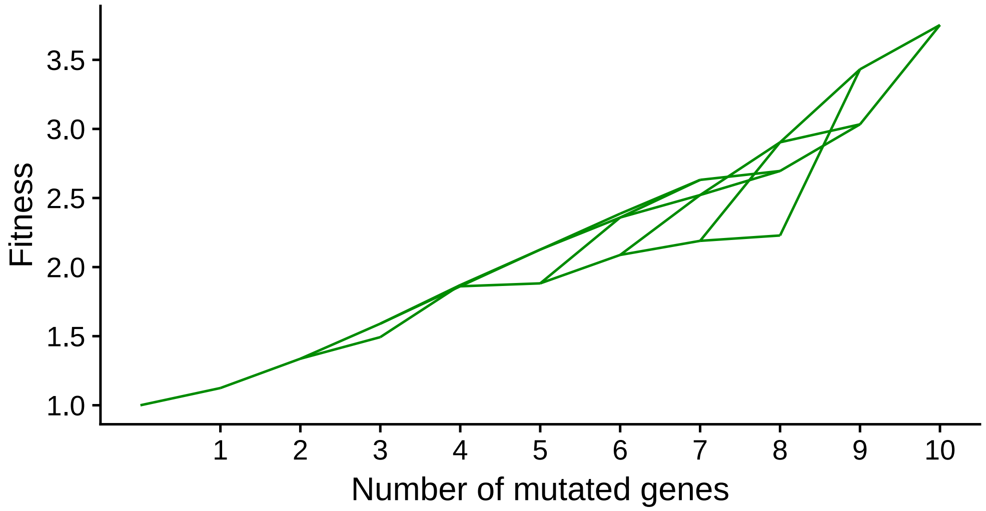

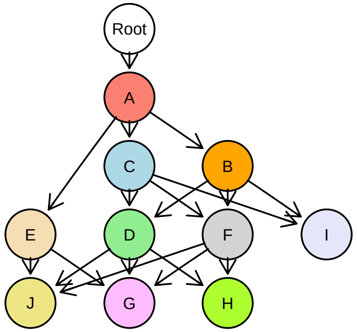

ID	HaVk9pjpjvDygF8e
acc genotypes	39
peaks no back	1
removed edges	0
prop rem edges	0

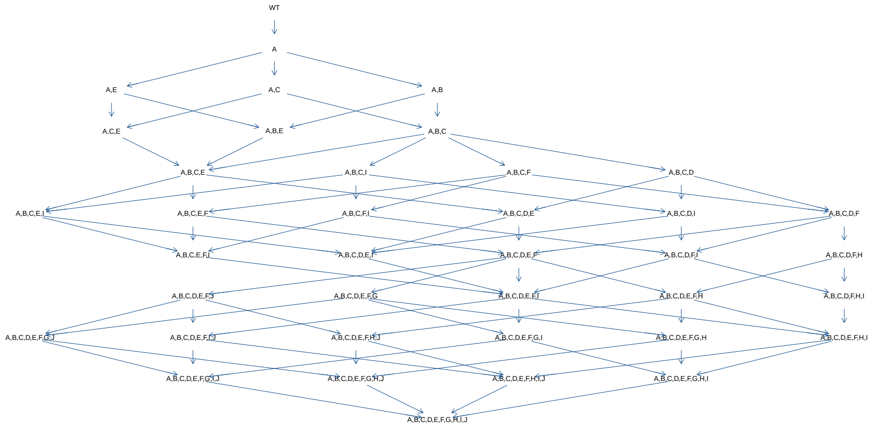

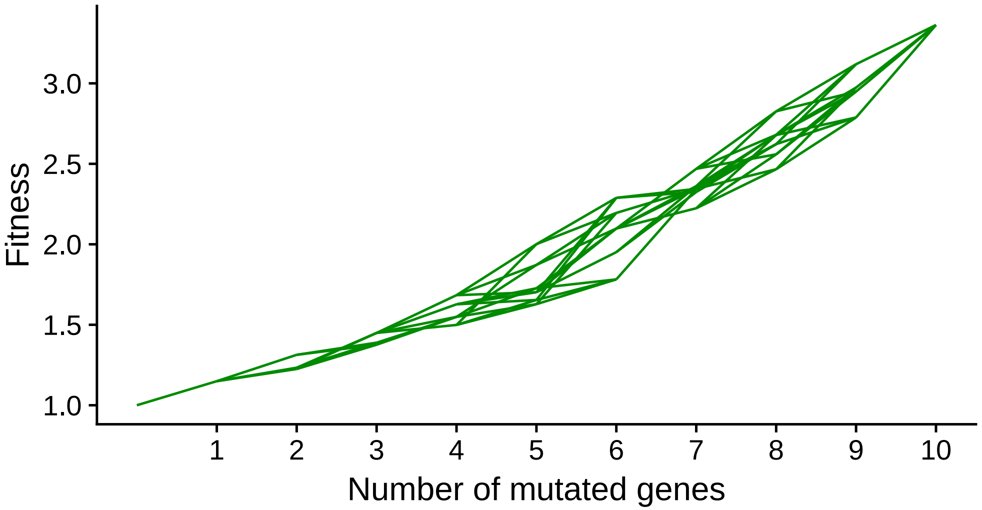

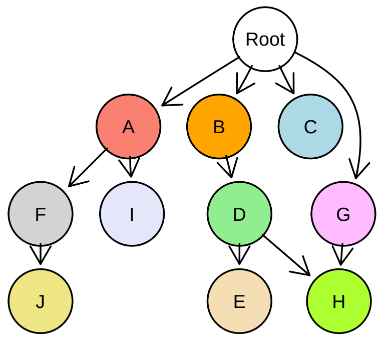

ID	hB10YoS6mVpFbv6aL
acc genotypes	140
peaks no back	1
removed edges	0
prop rem edges	0

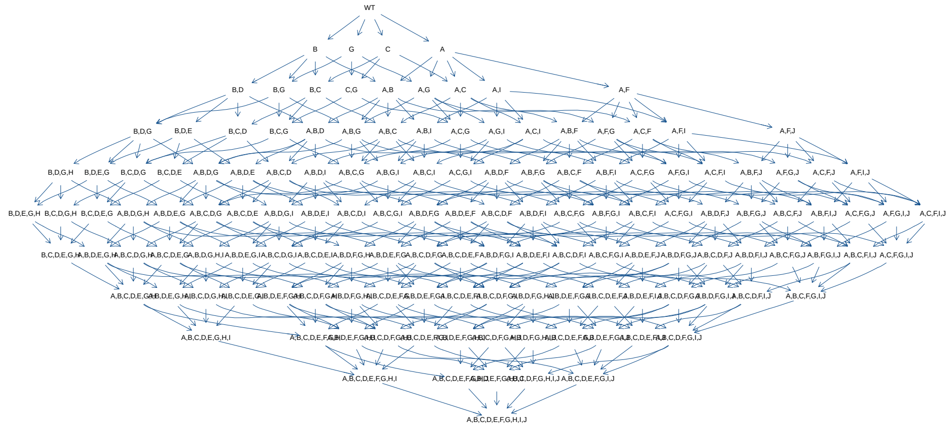

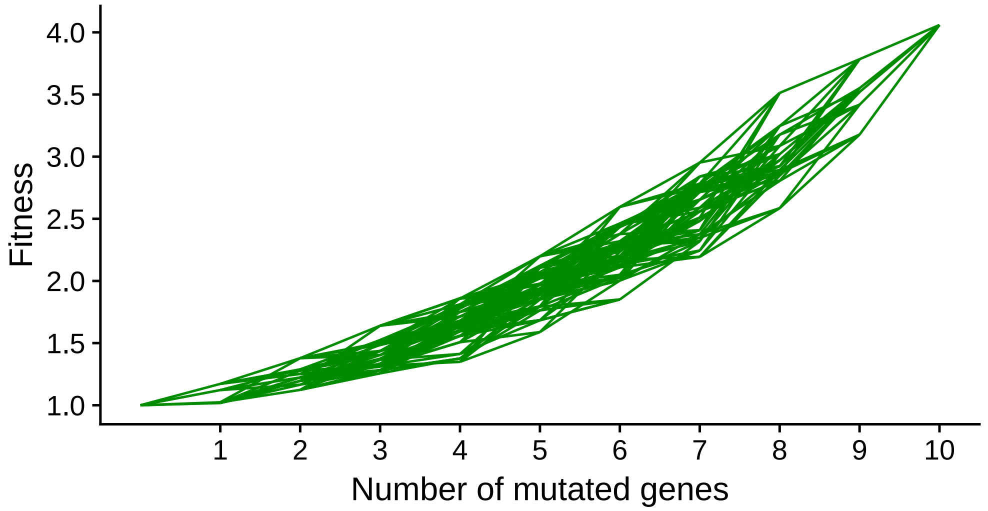

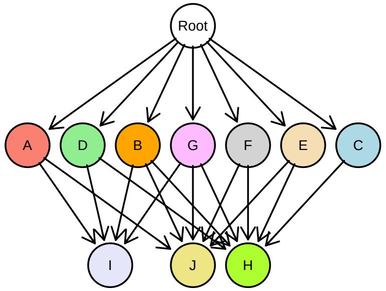

ID	hiImr3F710LYpQsGL
acc genotypes	147
peaks no back	1
removed edges	0
prop rem edges	0

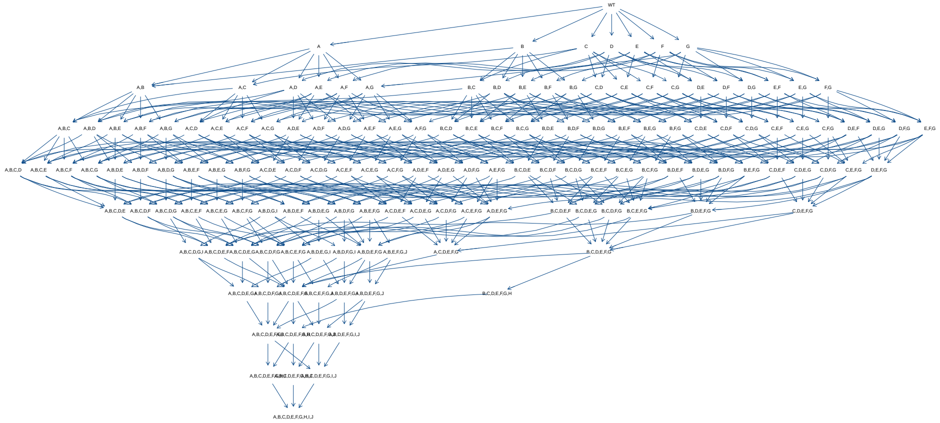

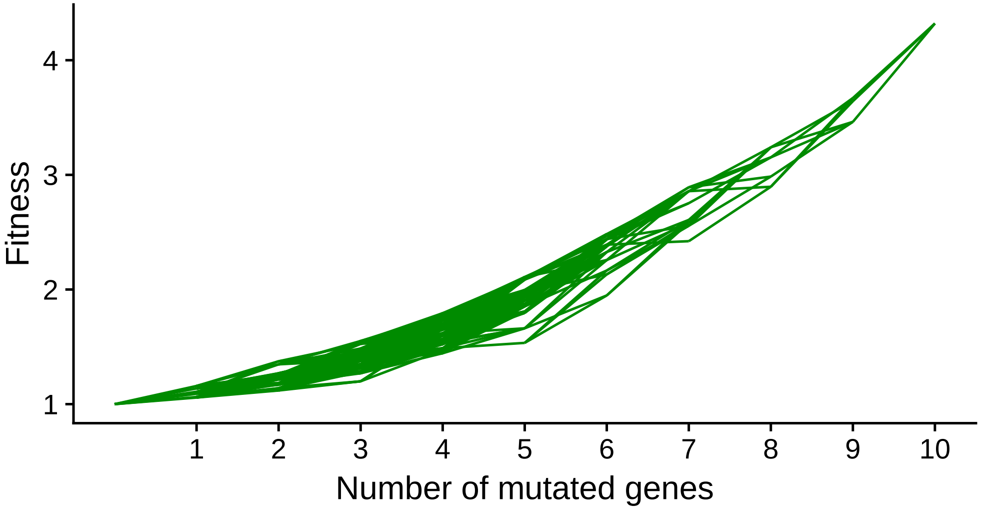

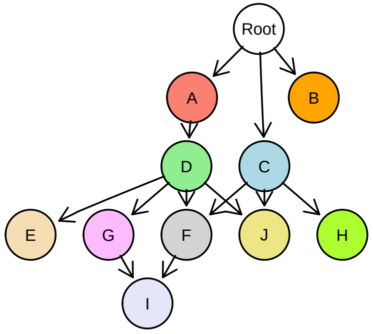

ID hNfoY8xLhb3Nf7ZZ
acc genotypes 100
peaks no back 1
removed edges 0
prop rem edges 0

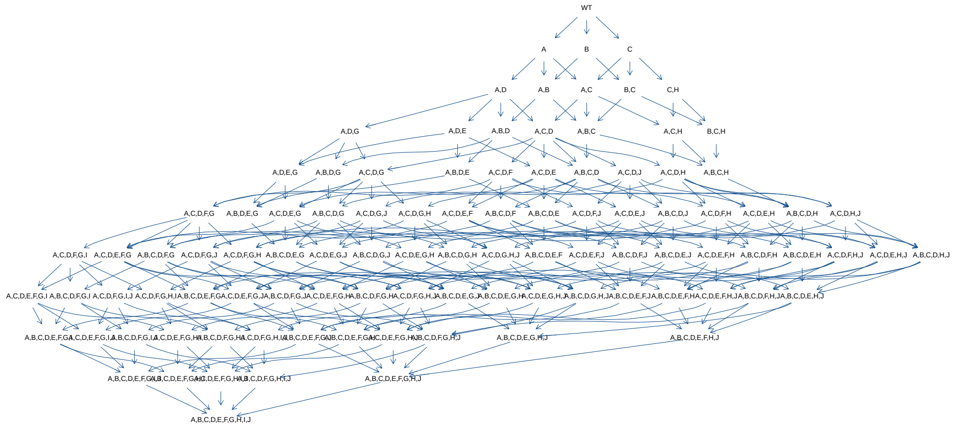

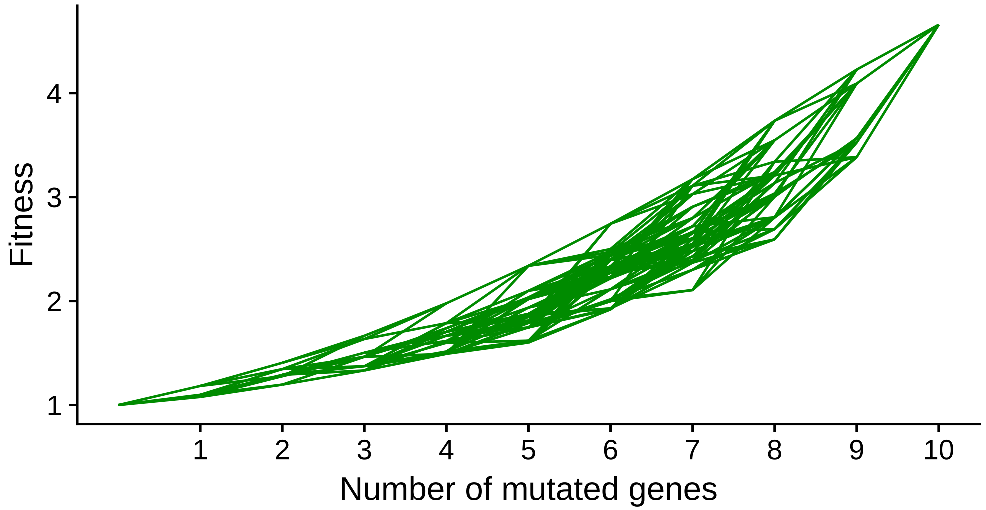

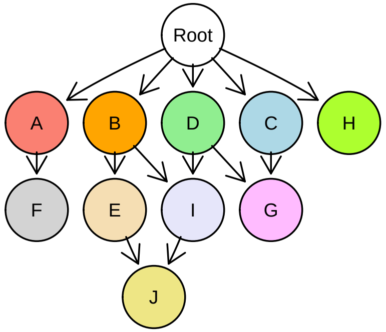

ID	HnZ10pnWSr3M1MXbv
acc genotypes	144
peaks no back	1
removed edges	0
prop rem edges	0

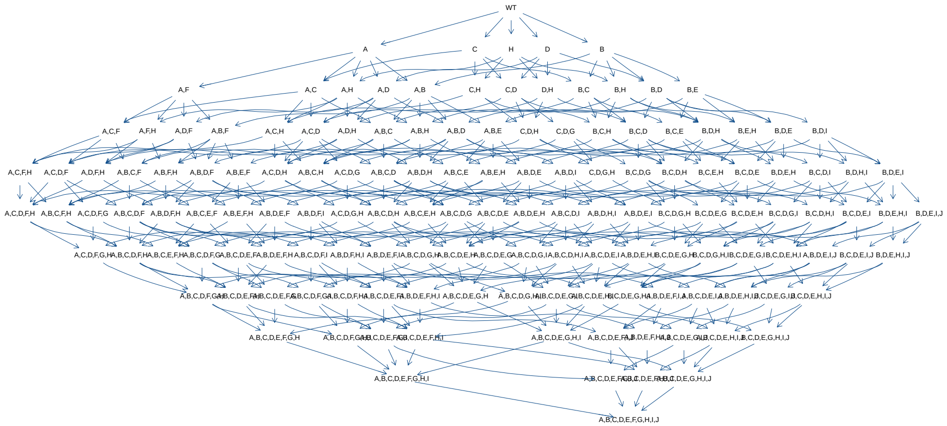

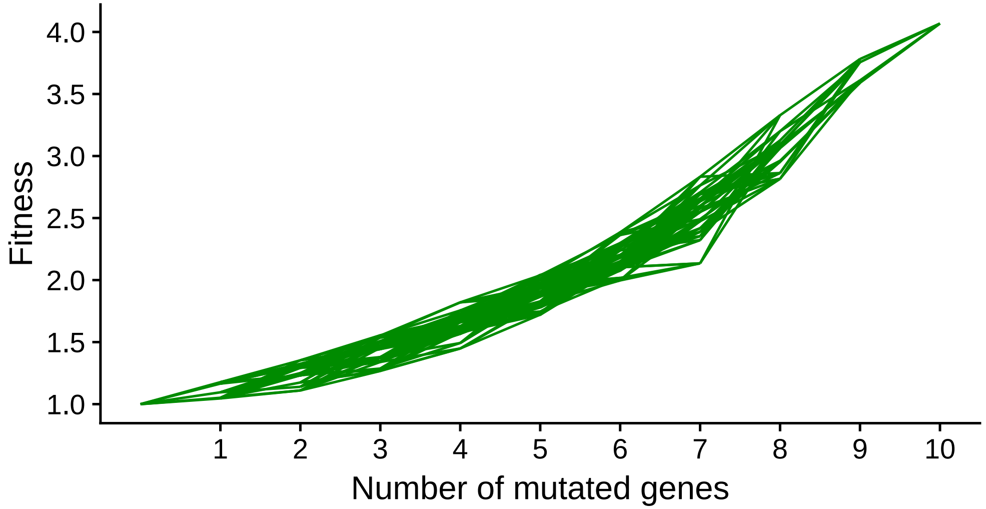

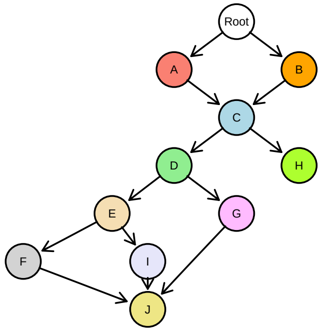

ID	Hon2Ud6RNbFJ826U
acc genotypes	28
peaks no back	1
removed edges	0
prop rem edges	0

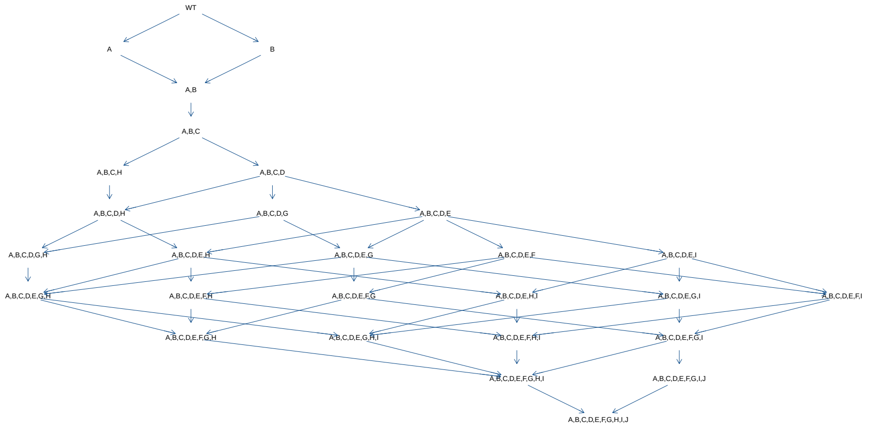

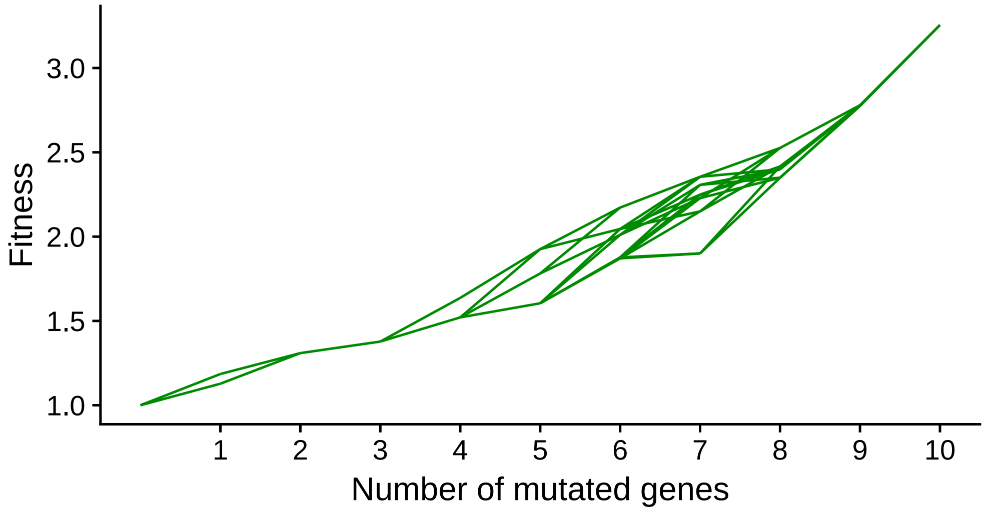

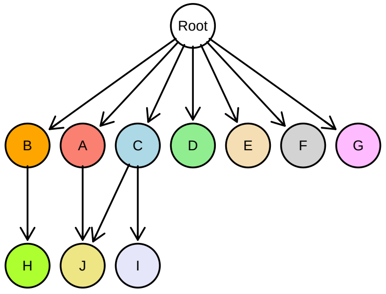

ID	hqhSp8AzeBqMadTe
acc genotypes	384
peaks no back	1
removed edges	0
prop rem edges	0

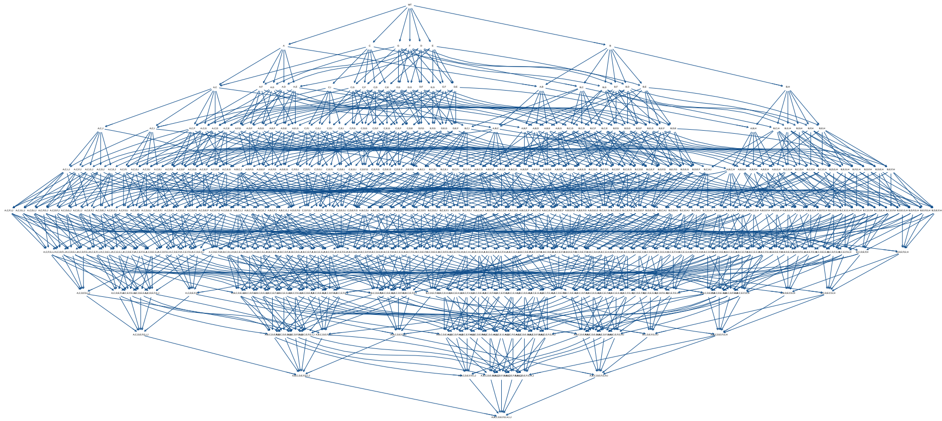

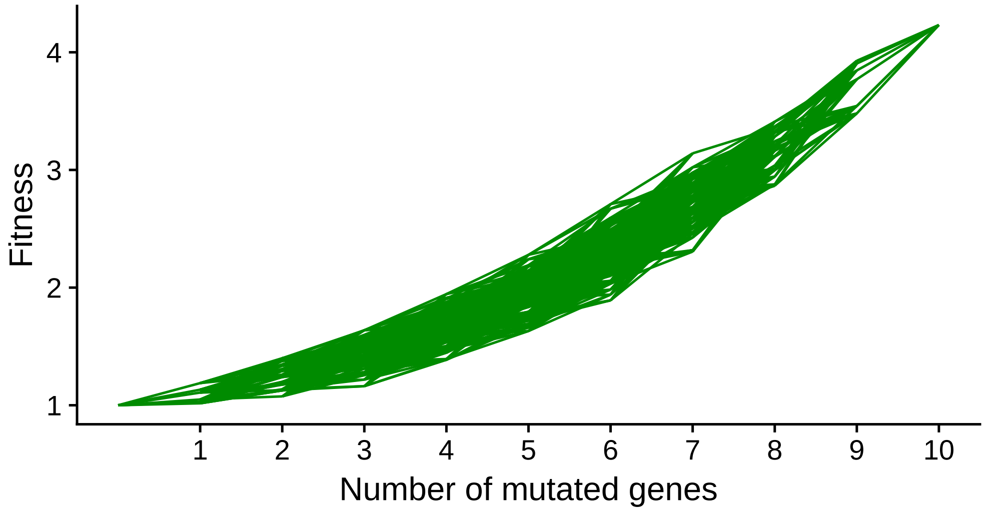

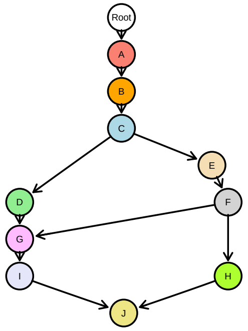

ID	hshAK10qbNh6UkHAB
acc genotypes	16
peaks no back	1
removed edges	0
prop rem edges	0

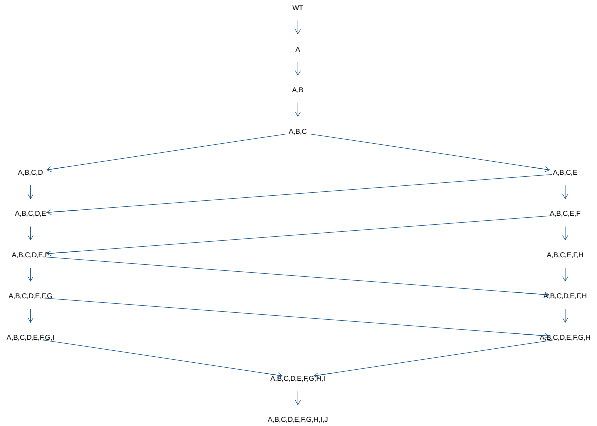

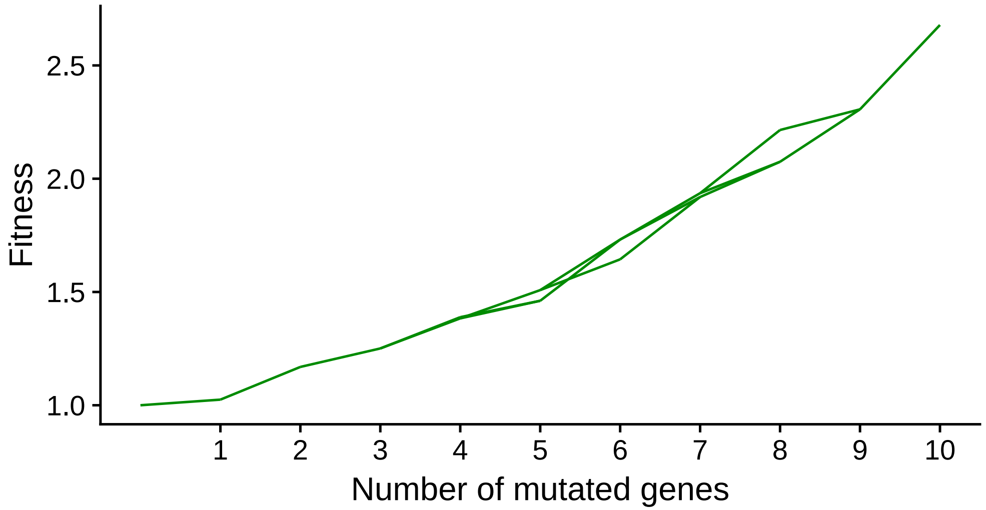

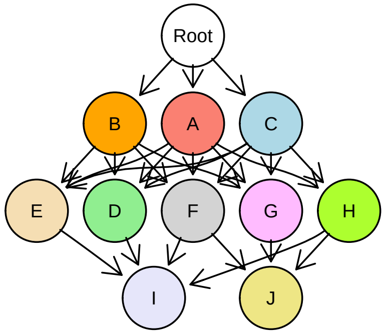

ID HXhpKGzcwUflLLeF
acc genotypes 47
peaks no back 1
removed edges 0
prop rem edges 0

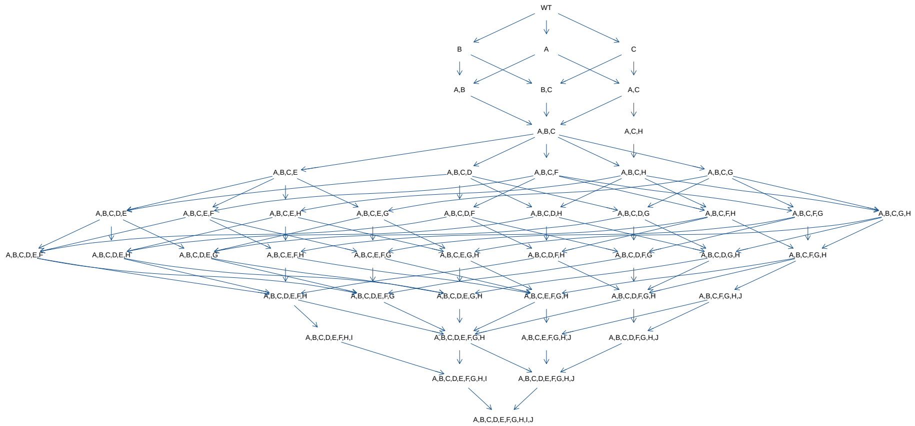

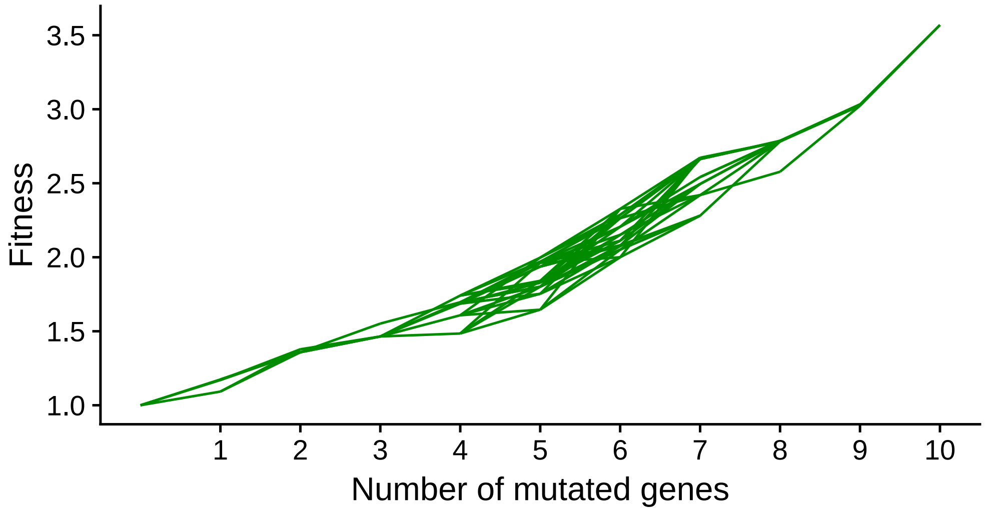

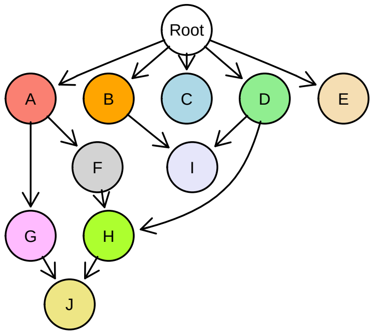

ID	HXibbT4wtp8elqYm
acc genotypes	136
peaks no back	1
removed edges	0
prop rem edges	0

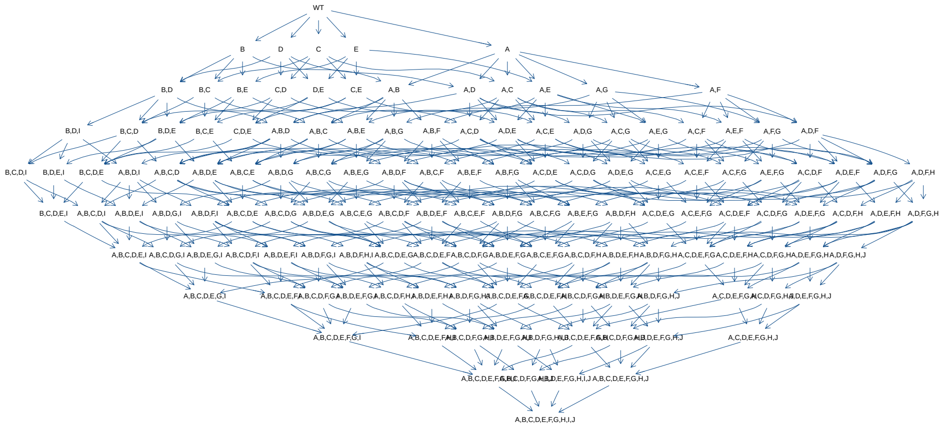

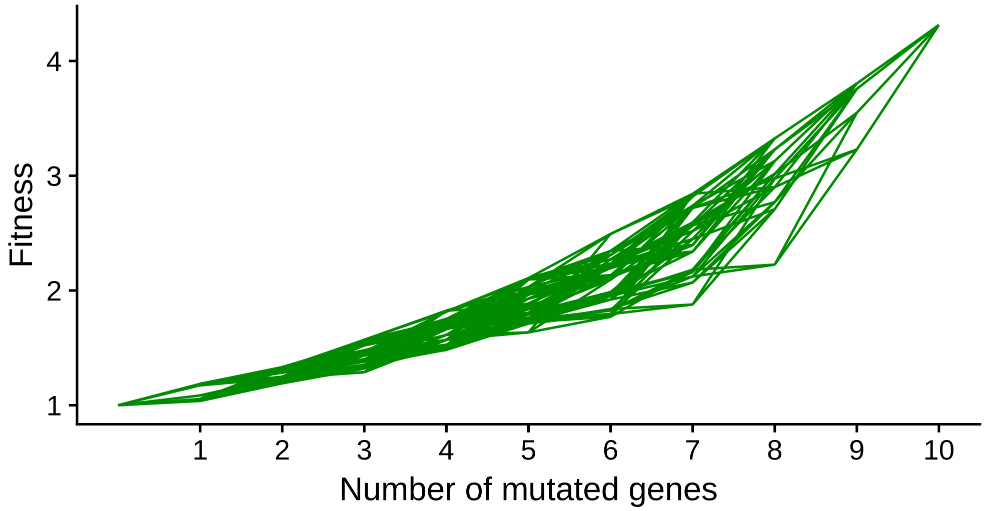

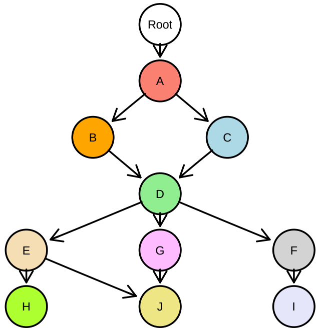

ID	IBfkuKvC5LnGkILI
acc genotypes	29
peaks no back	1
removed edges	0
prop rem edges	0

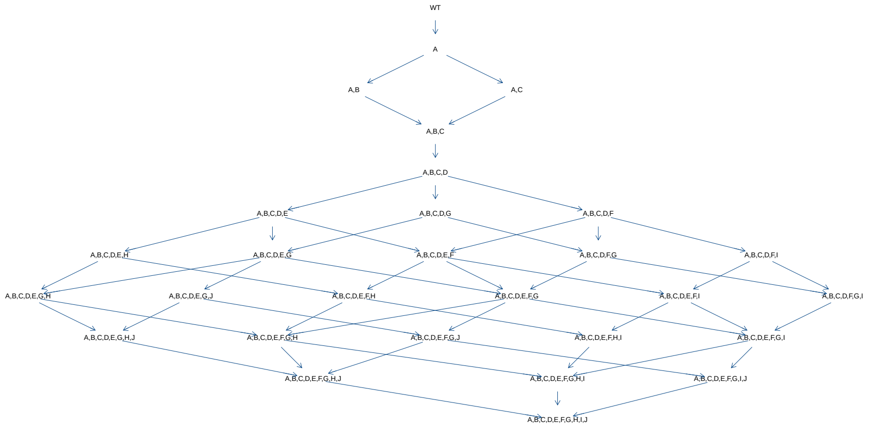

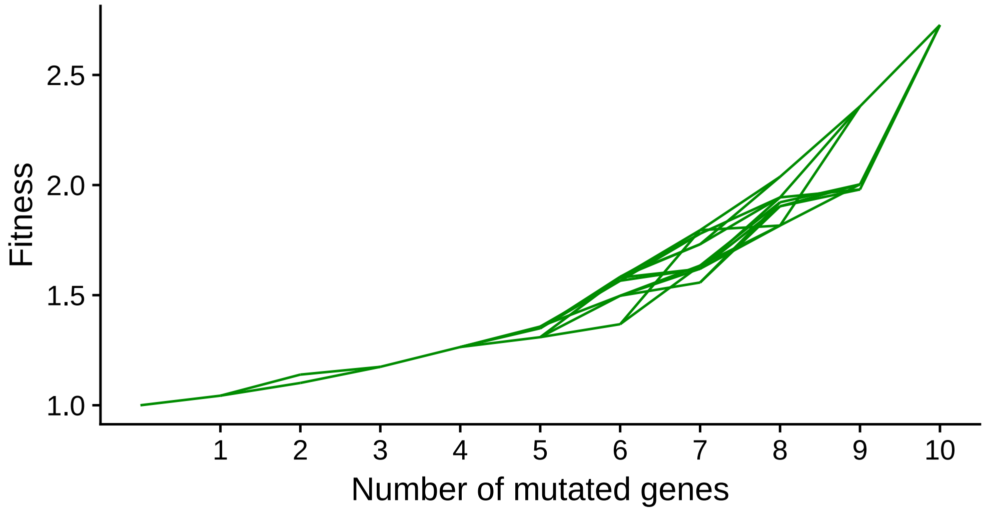

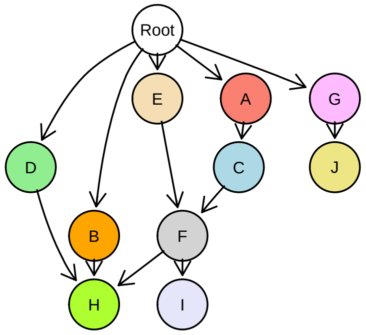

ID	ibqIgMnpLmhx8hJy
acc genotypes	102
peaks no back	1
removed edges	0
prop rem edges	0

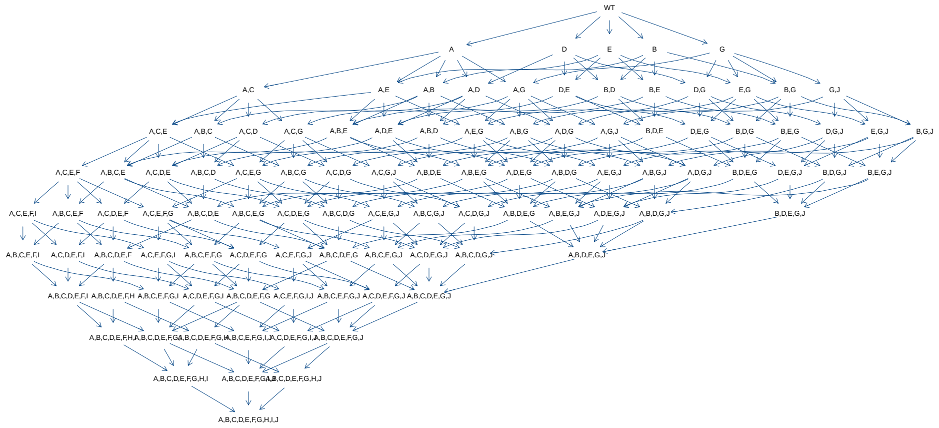

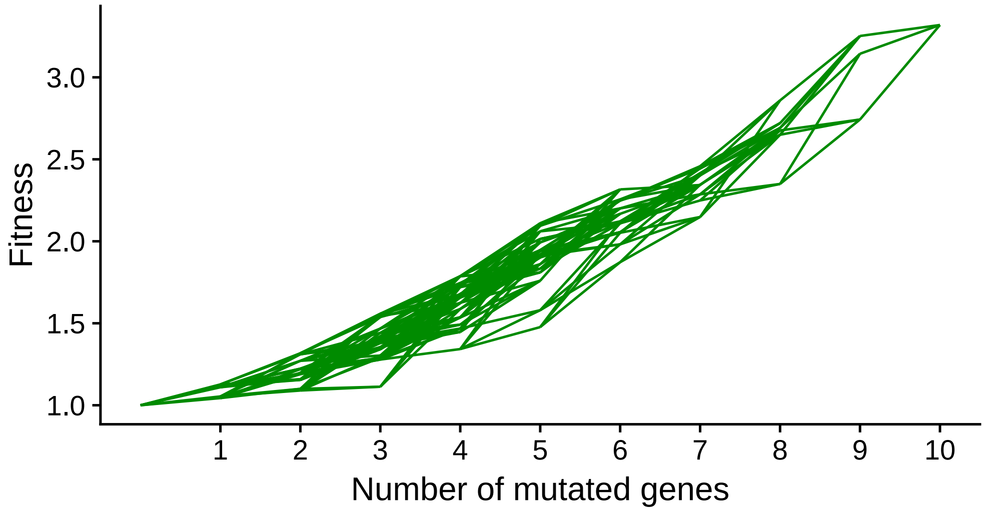

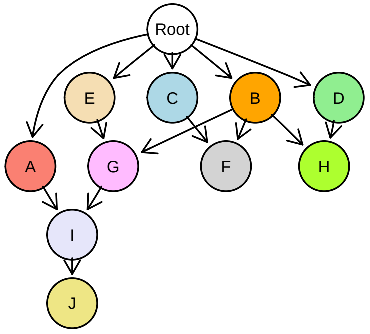

ID	IFzpx6g9rMSHRpvL
acc genotypes	88
peaks no back	1
removed edges	0
prop rem edges	0

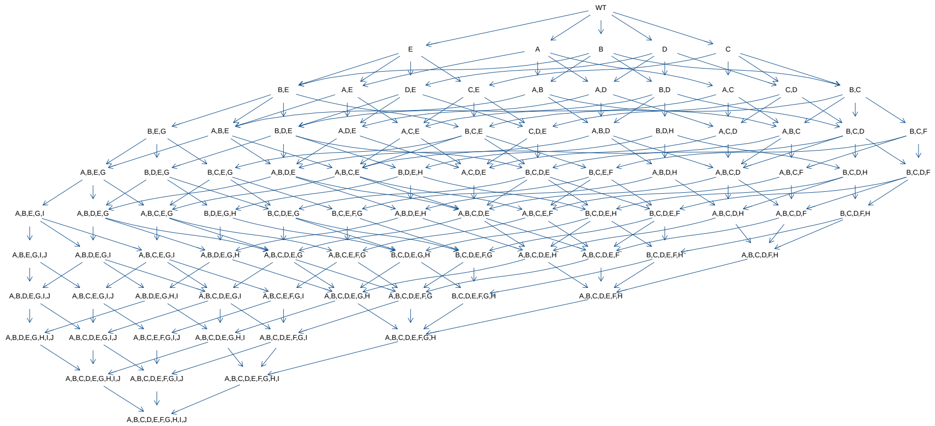

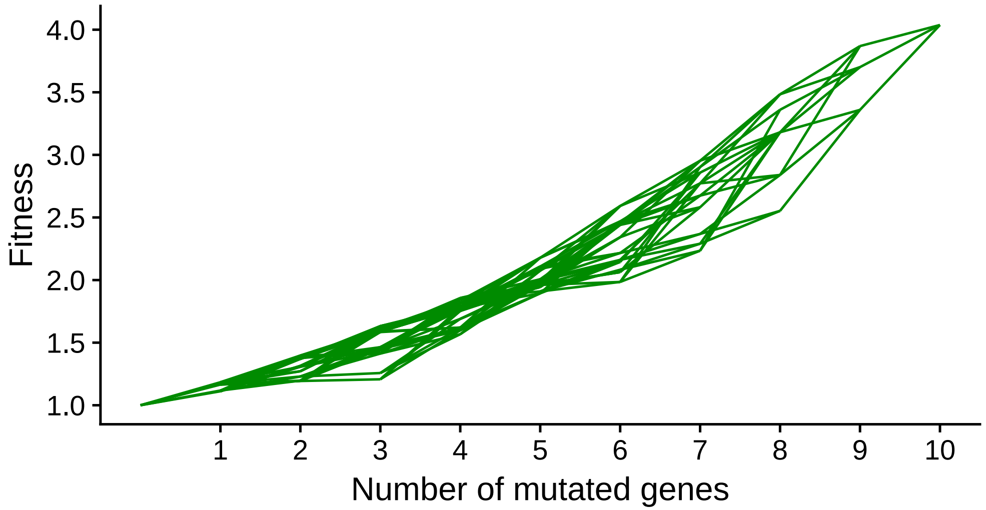

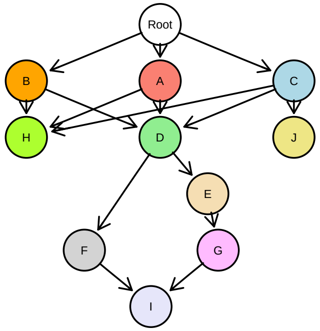

ID	IGNIamDyyL6pJkAH
acc genotypes	42
peaks no back	1
removed edges	0
prop rem edges	0

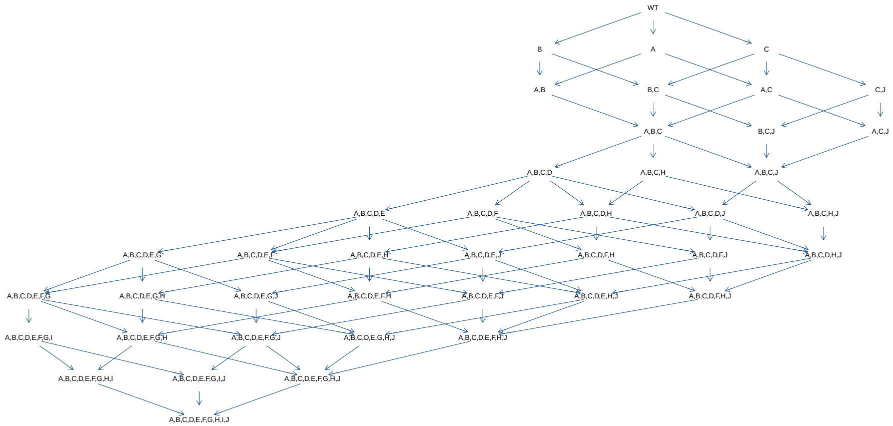

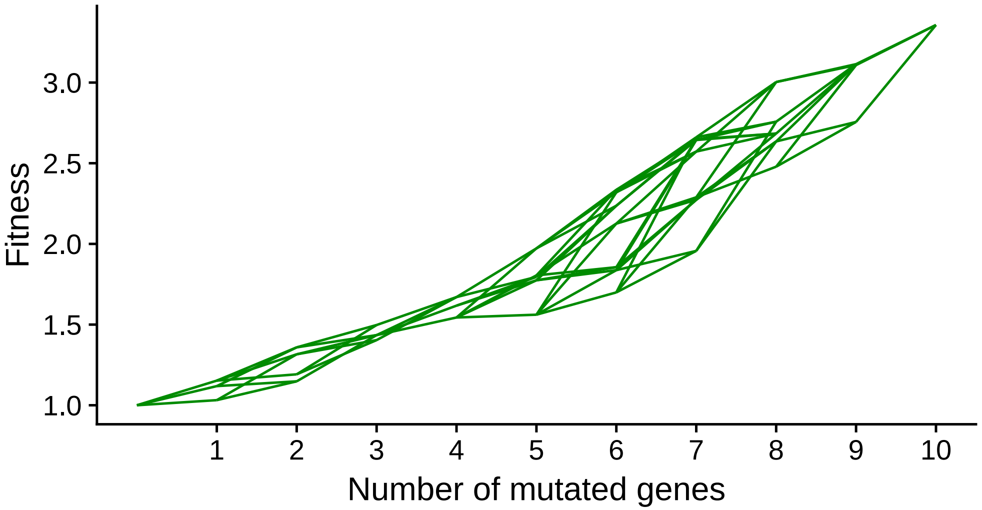

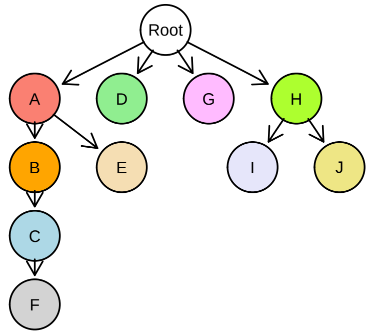

ID IIY6ewqbwnTGL76Q
acc genotypes 180
peaks no back 1
removed edges 0
prop rem edges 0

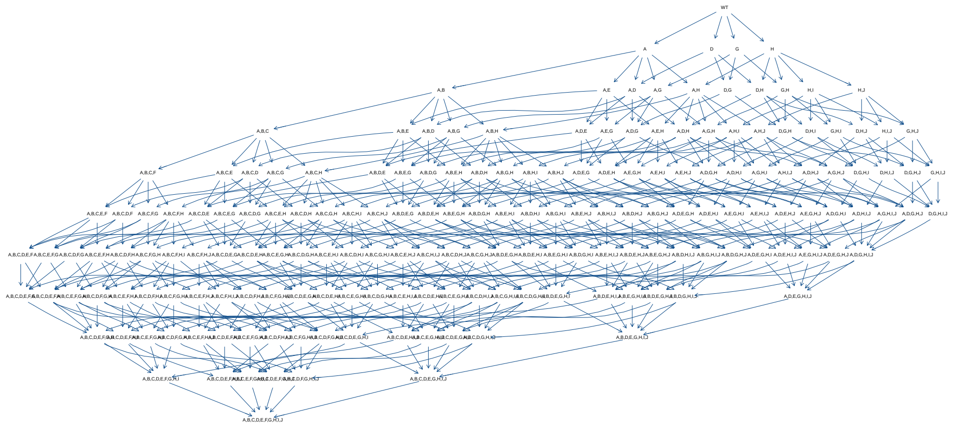

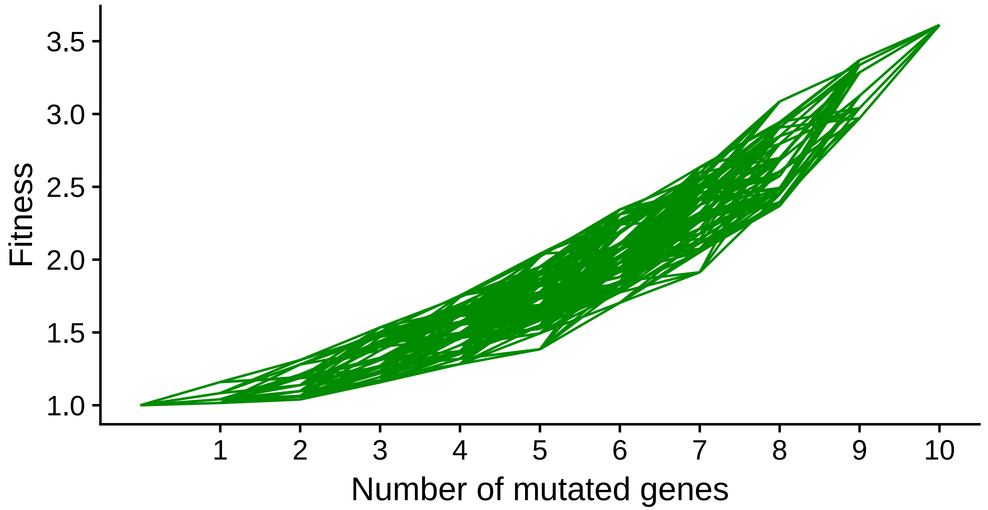

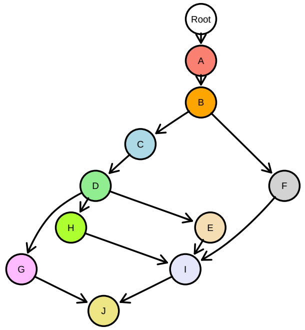

ID	IJ82SrhcD1ULypf8
acc genotypes	25
peaks no back	1
removed edges	0
prop rem edges	0

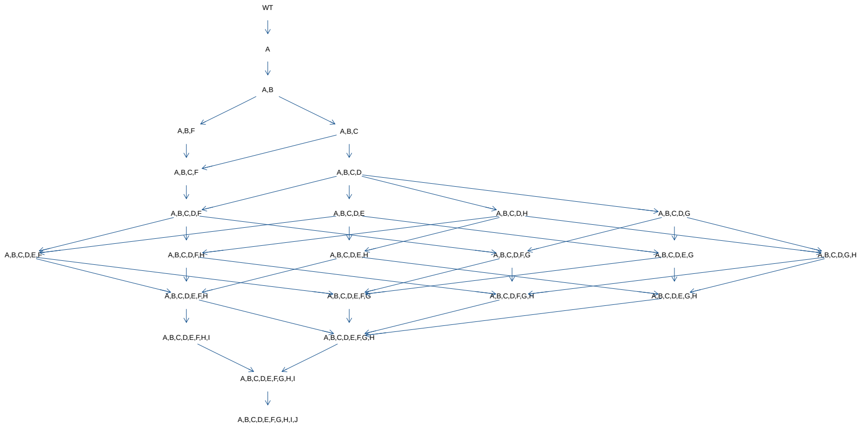

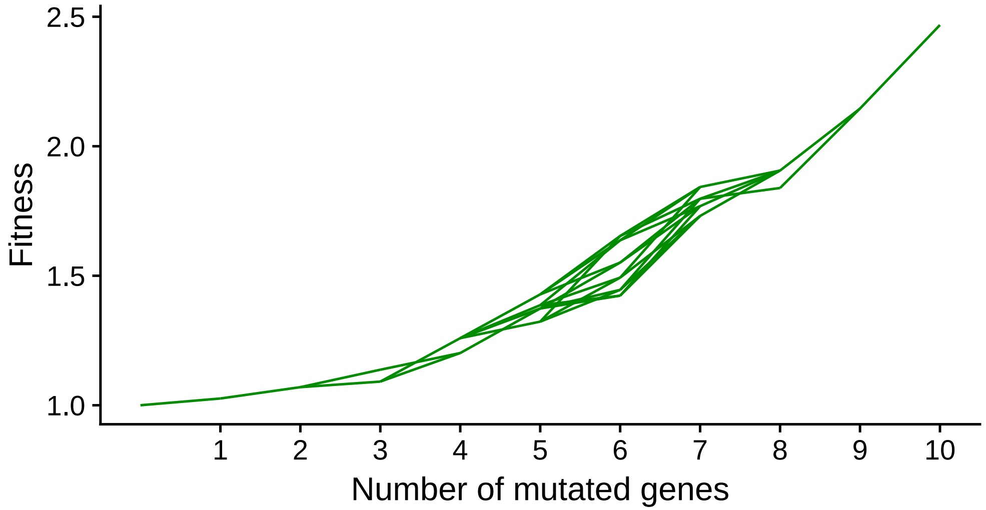

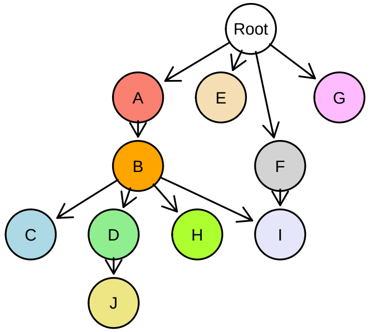

ID	IqyqZ4siy10VLgJ5s
acc genotypes	160
peaks no back	1
removed edges	0
prop rem edges	0

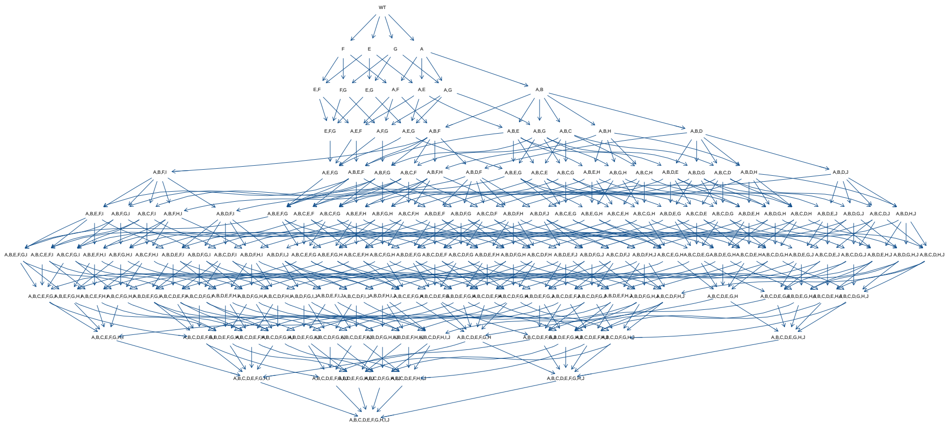

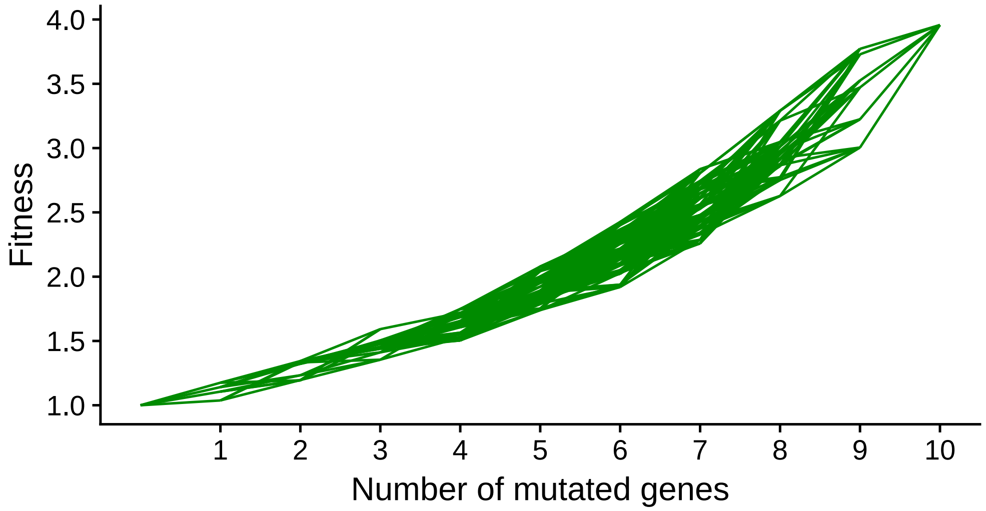

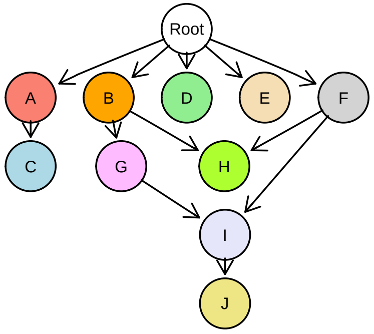

ID	ITJu10YflRKiTyZLt
acc genotypes	144
peaks no back	1
removed edges	0
prop rem edges	0

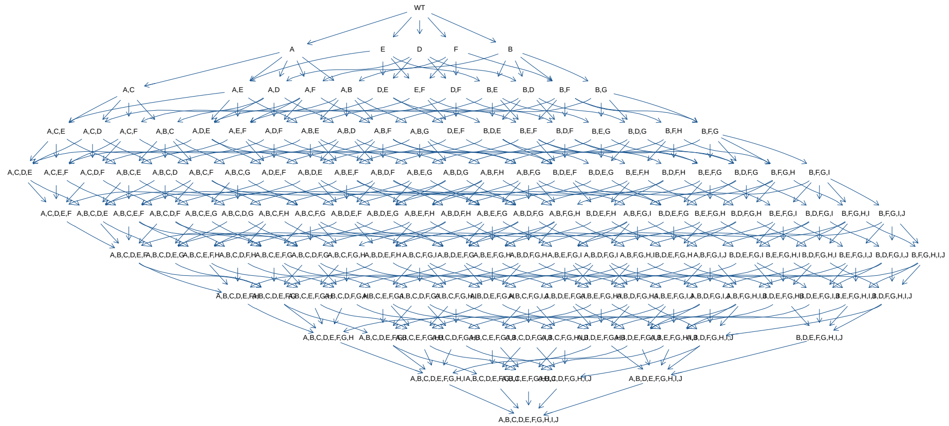

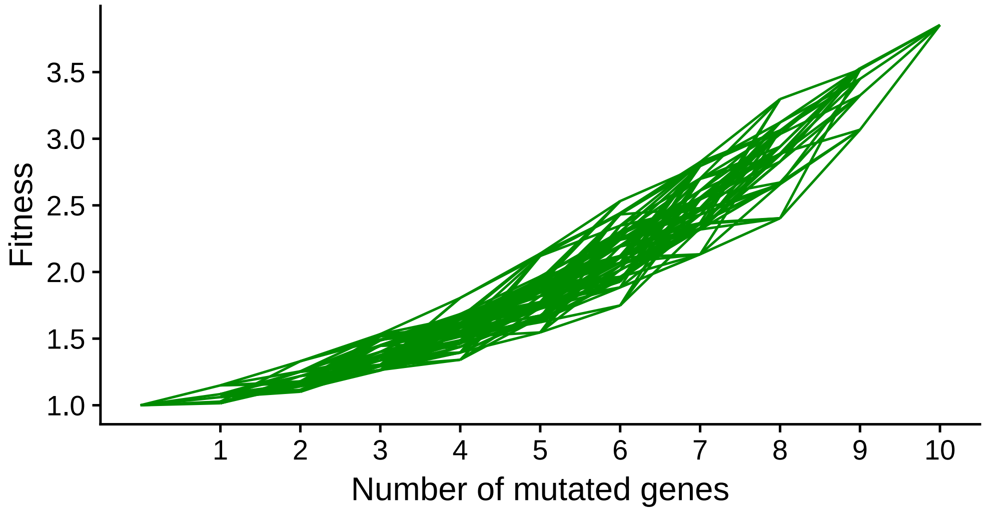

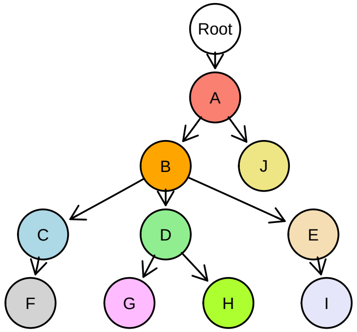

ID j5cMndnUwm51TTqp

acc genotypes 93

peaks no back 1

removed edges 0

prop rem edges 0

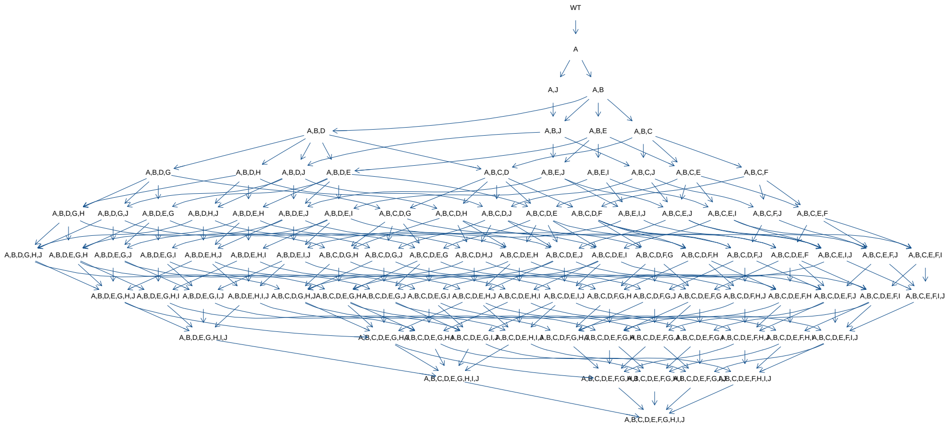

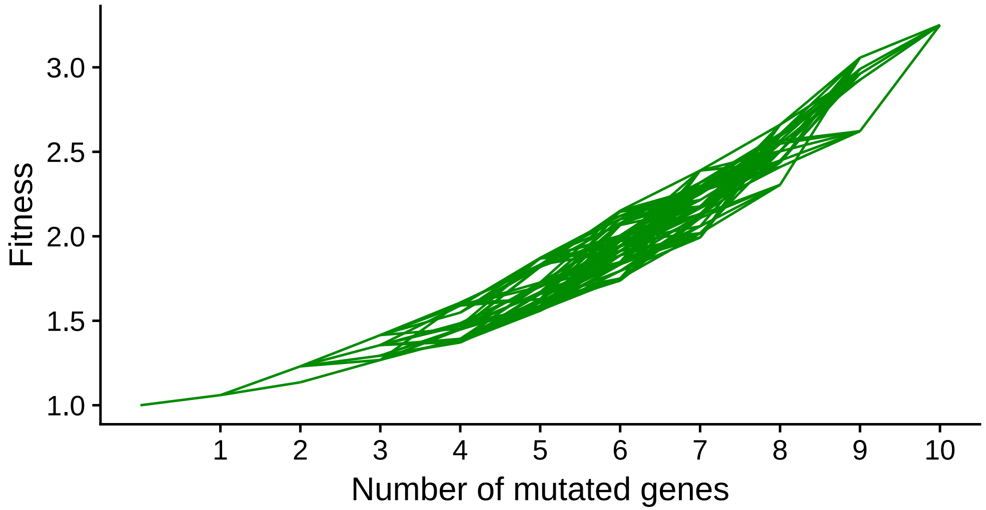

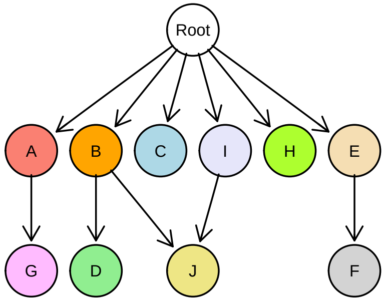

ID j6HW2RoAhJ2N68DN
acc genotypes 288
peaks no back 1
removed edges 0
prop rem edges 0

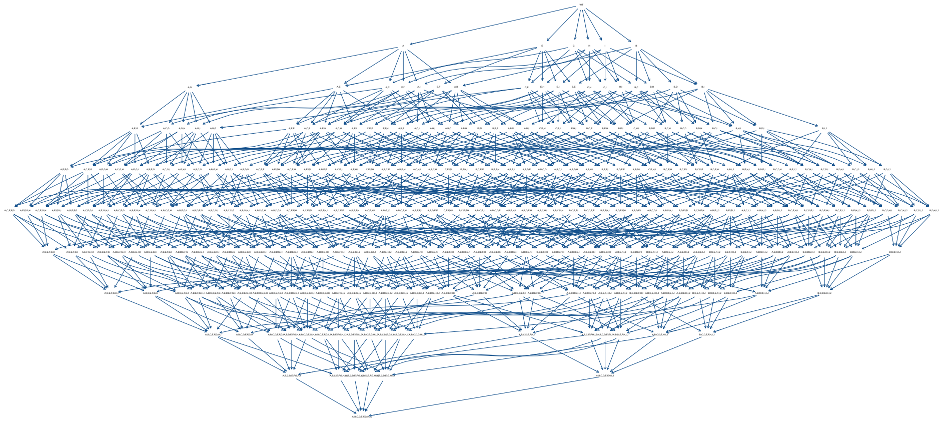

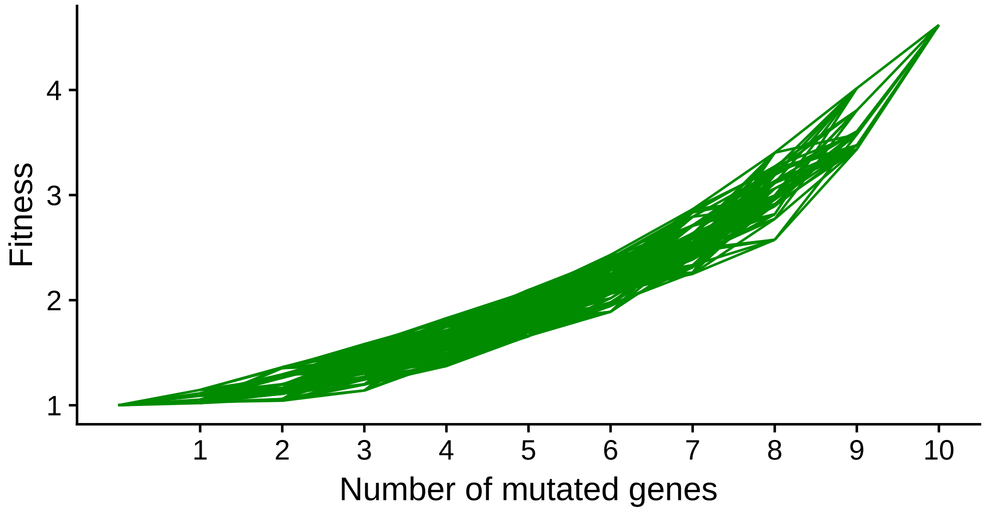

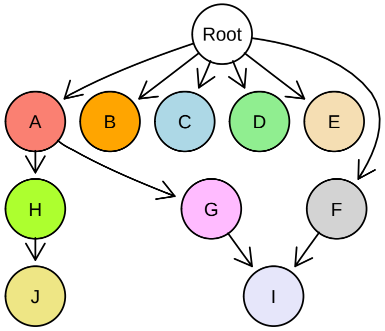

ID jAZ9fNKs3jEXePB4
acc genotypes 272
peaks no back 1
removed edges 0
prop rem edges 0

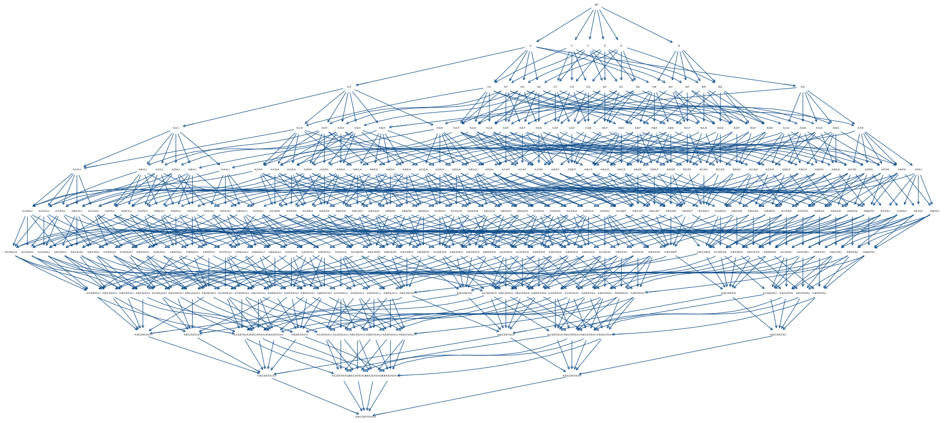

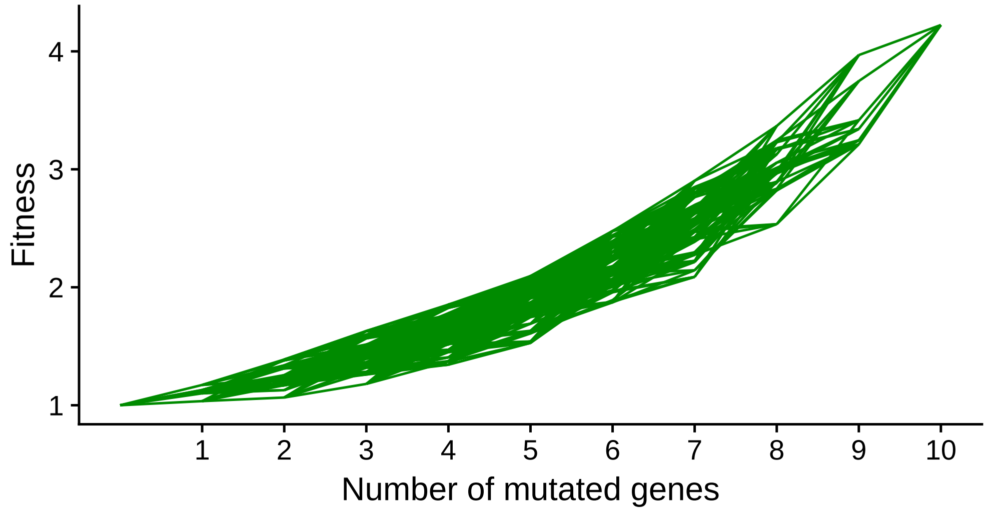

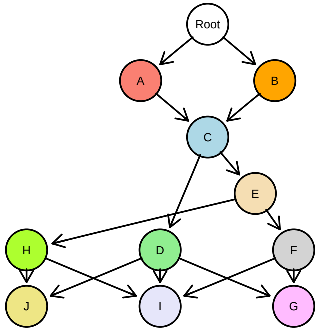

ID	jD4PcR3DEFwIK6qC
acc genotypes	23
peaks no back	1
removed edges	0
prop rem edges	0

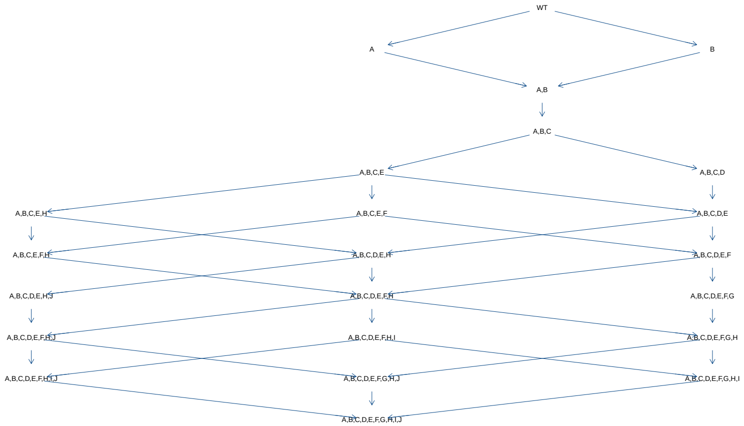

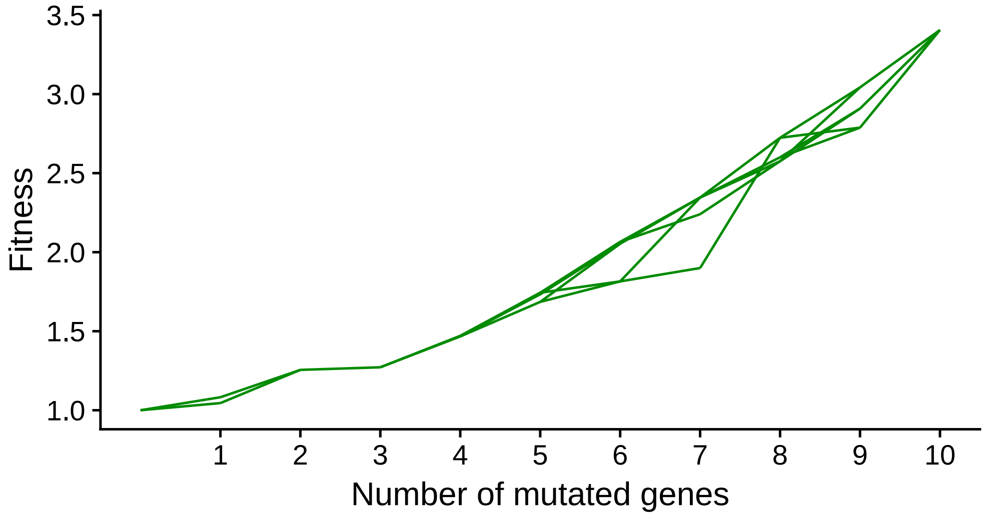

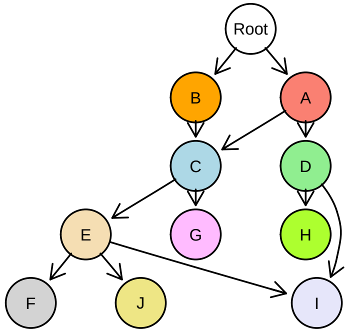

ID	Jh8glYdX10audNWJF
acc genotypes	54
peaks no back	1
removed edges	0
prop rem edges	0

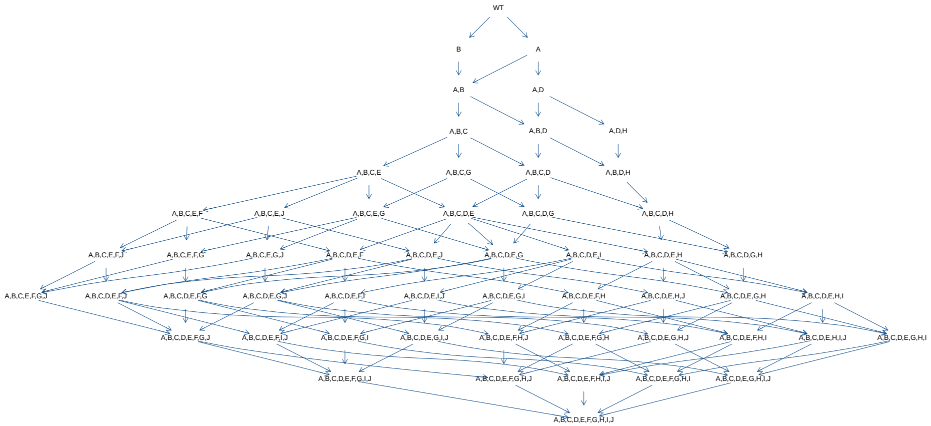

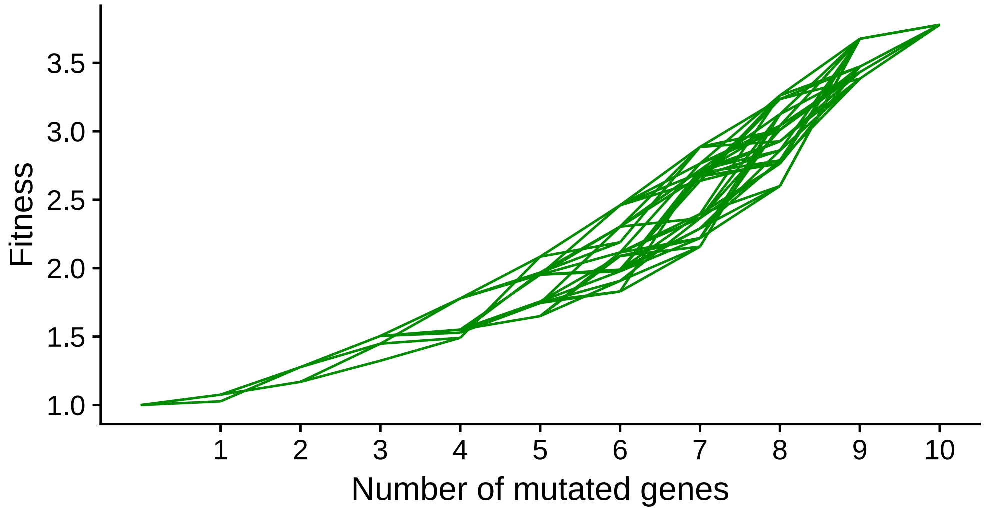

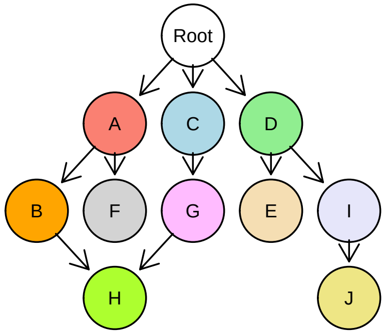

ID	JhPPnqoKAJCL8pvR
acc genotypes	119
peaks no back	1
removed edges	0
prop rem edges	0

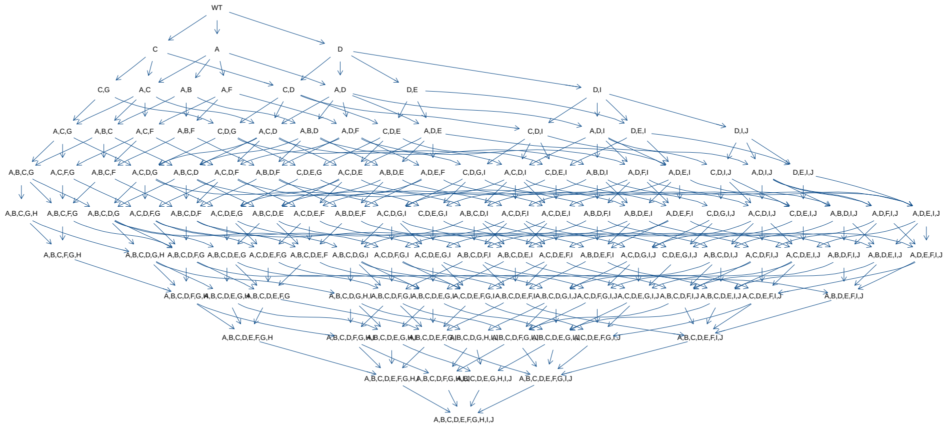

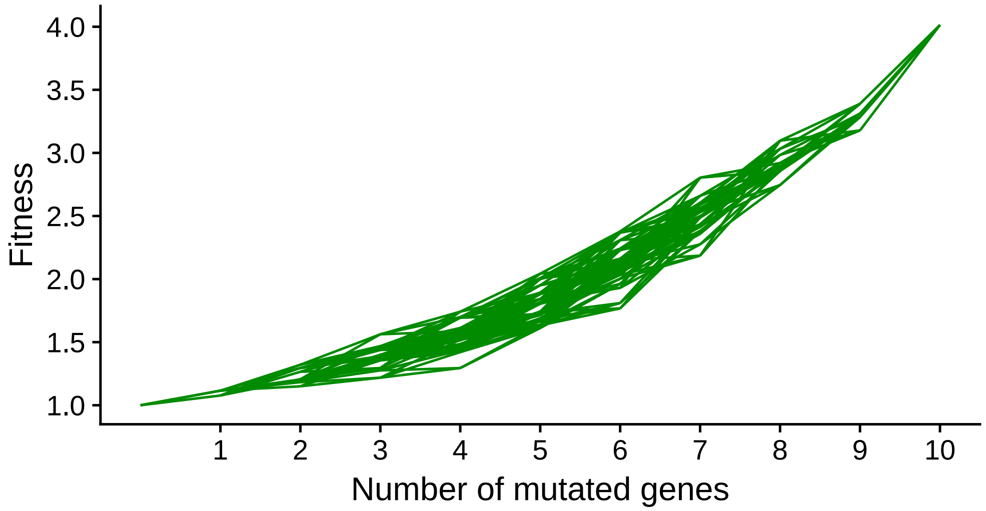

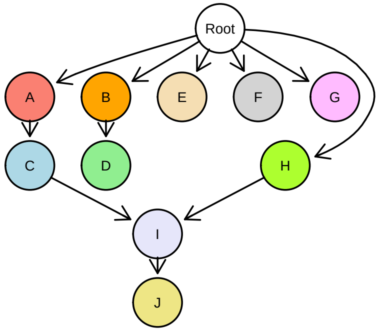

ID	JIKf7ckdhSo6ArQQ
acc genotypes	192
peaks no back	1
removed edges	0
prop rem edges	0

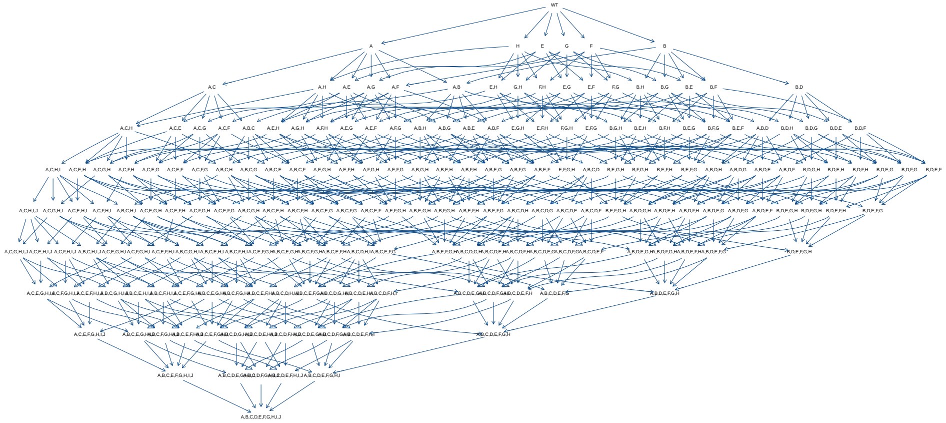

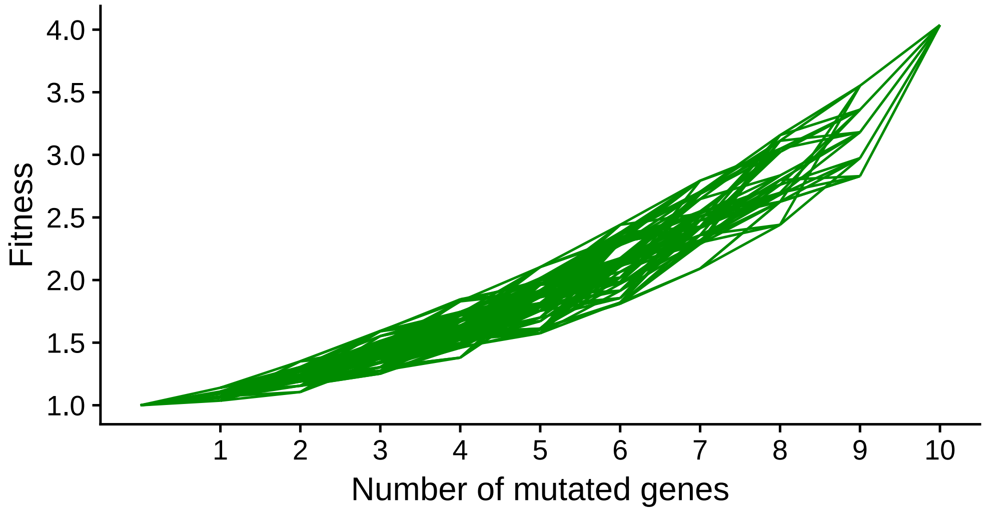

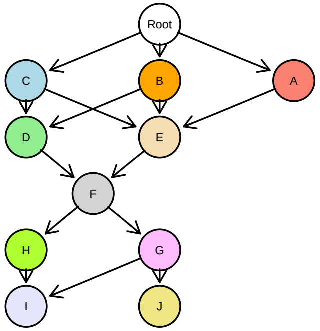

ID	JoiUrmmVqi67SyTm
acc genotypes	20
peaks no back	1
removed edges	0
prop rem edges	0

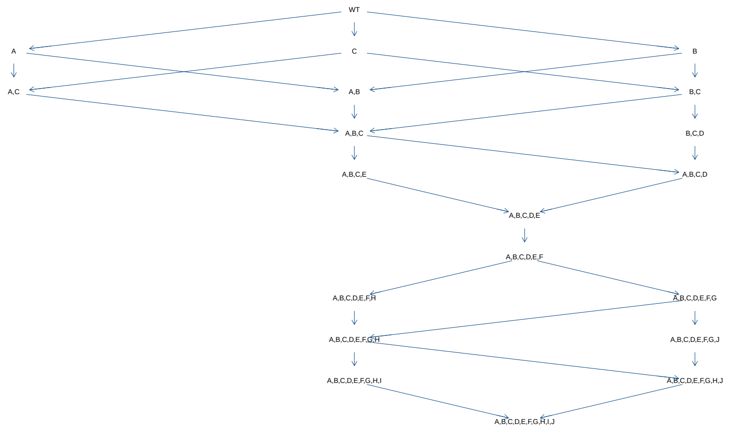

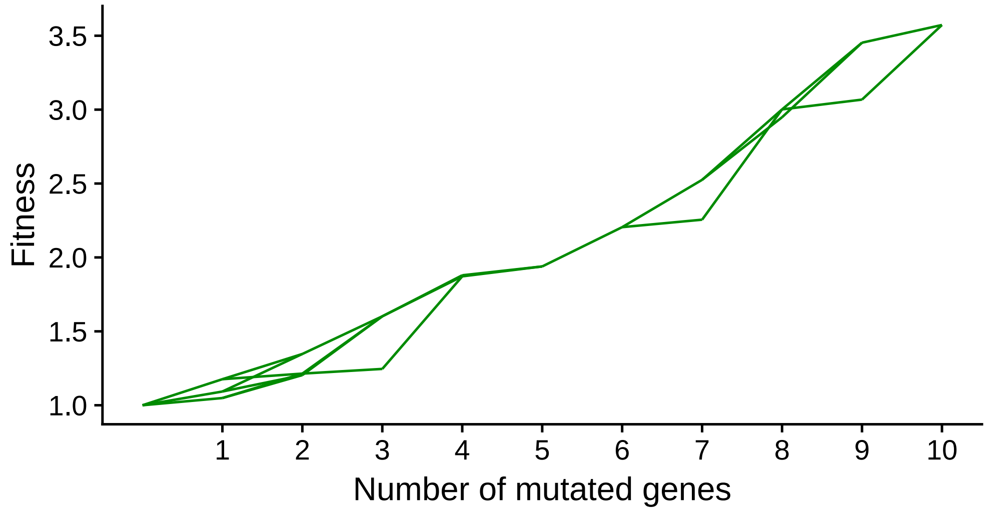

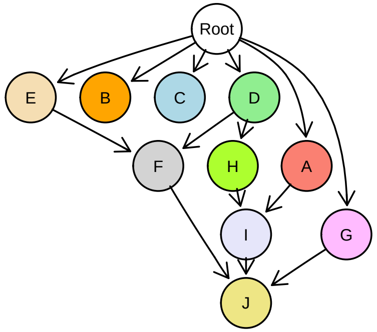

ID	jSQmuyLWDxqE106yj
acc genotypes	156
peaks no back	1
removed edges	0
prop rem edges	0

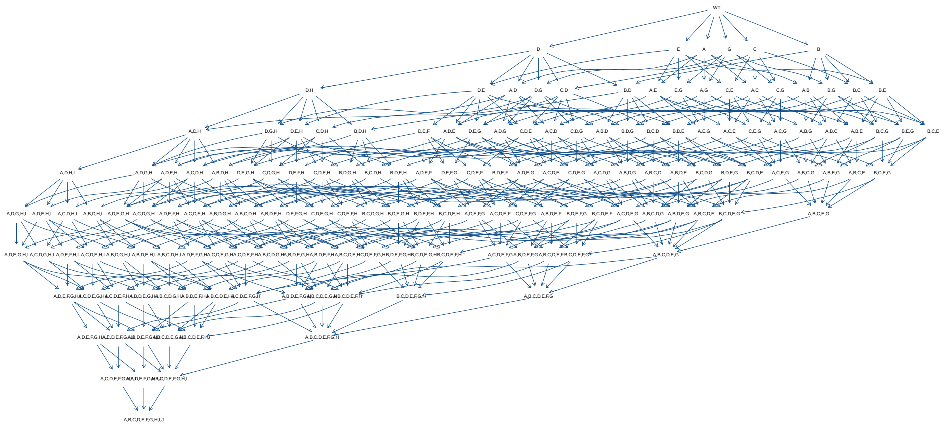

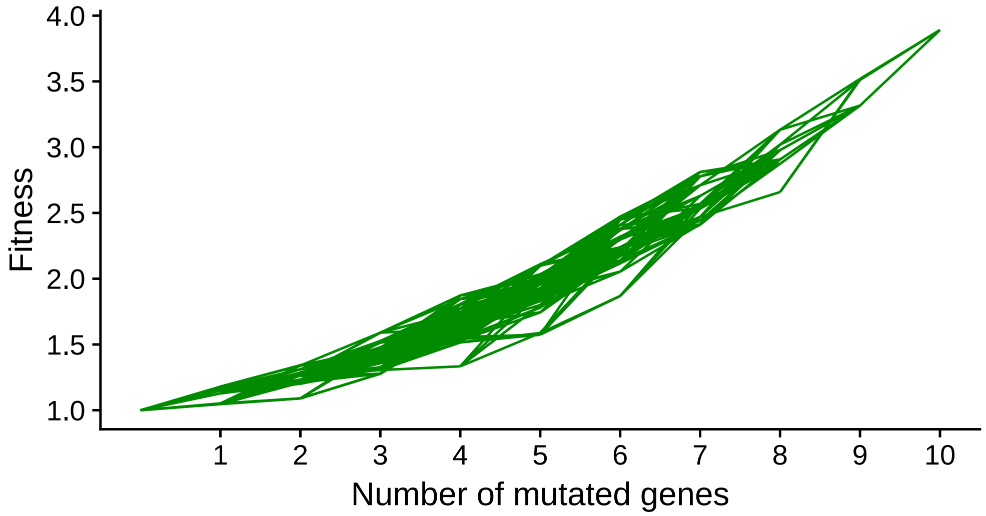

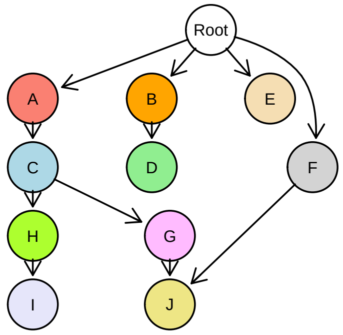

ID	jXDzA3wcvwQmXJvD
acc genotypes	114
peaks no back	1
removed edges	0
prop rem edges	0

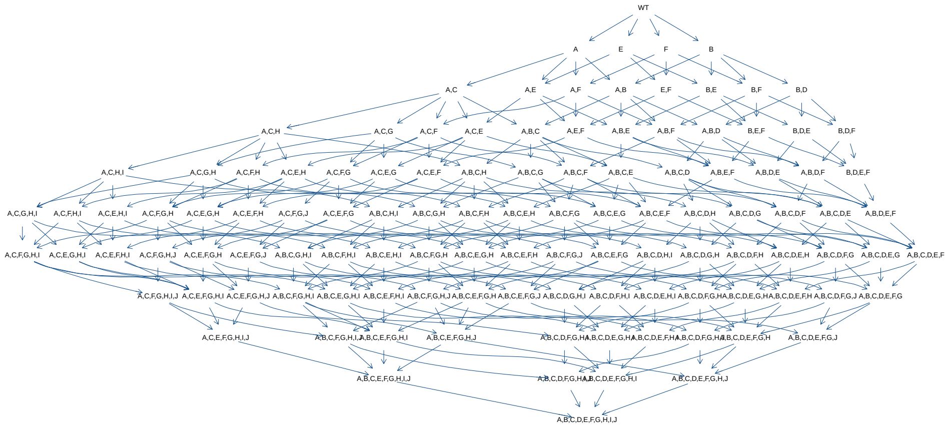

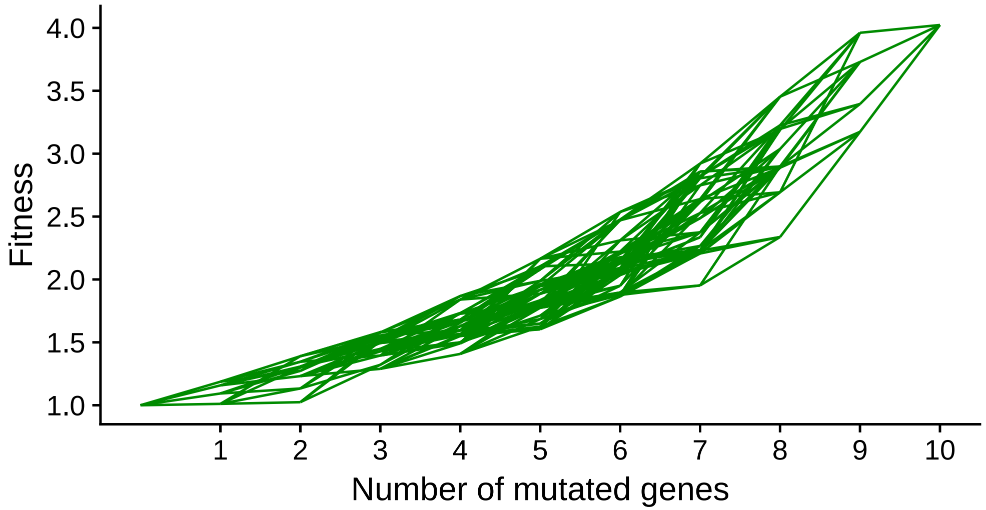

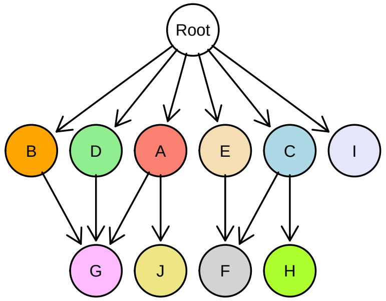

ID	K1jCHK7t4zCUPD6B
acc genotypes	224
peaks no back	1
removed edges	0
prop rem edges	0

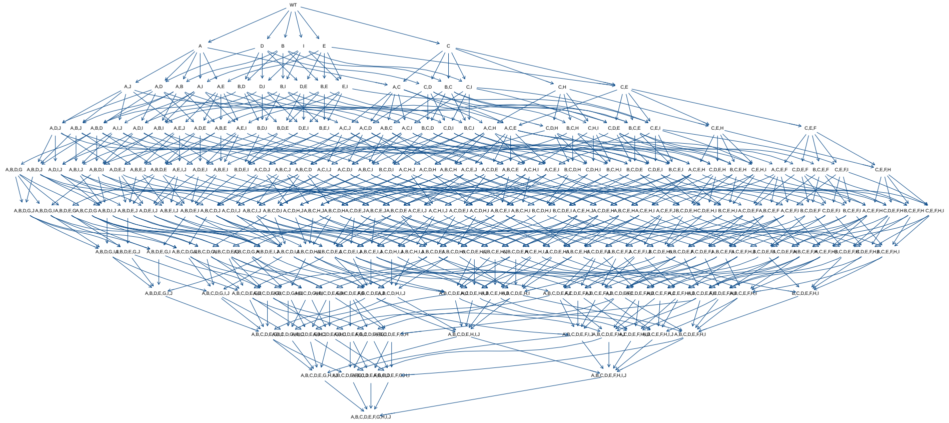

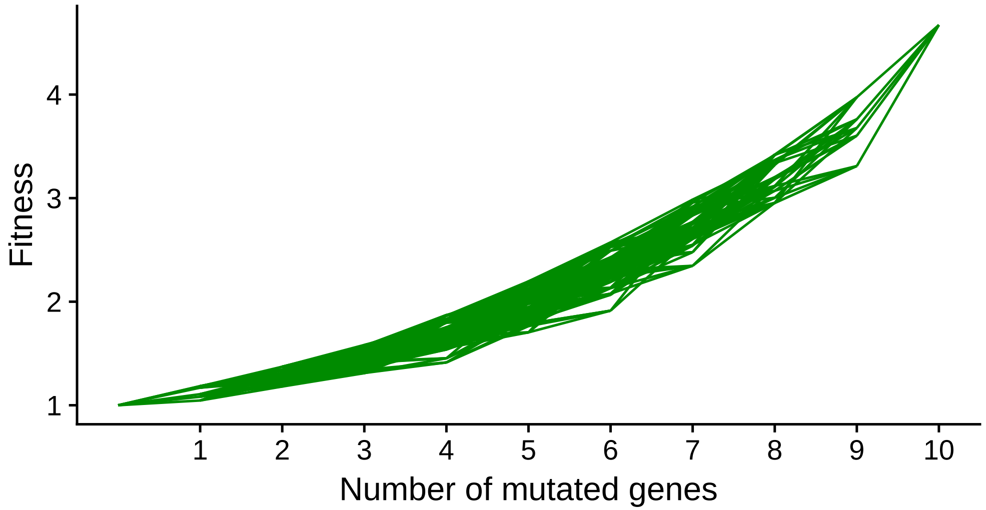

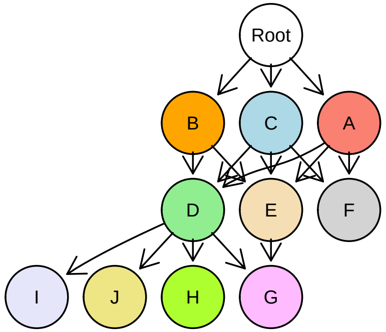

ID	KkQL102YepOlao9Bb
acc genotypes	60
peaks no back	1
removed edges	0
prop rem edges	0

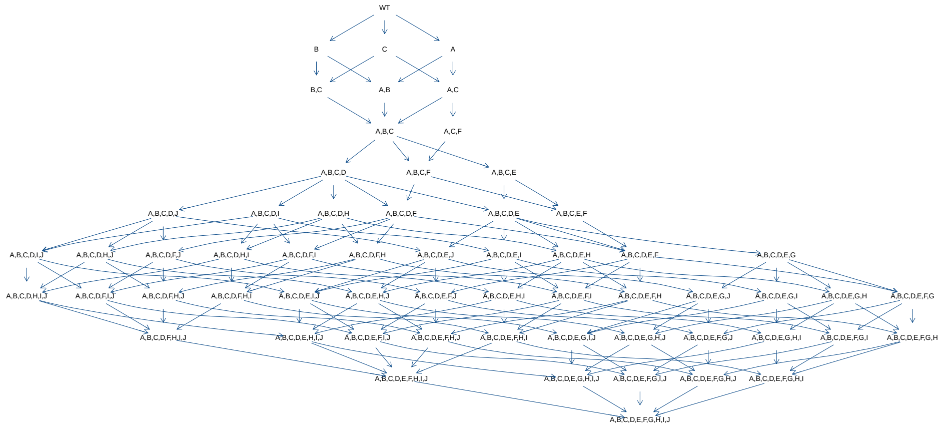

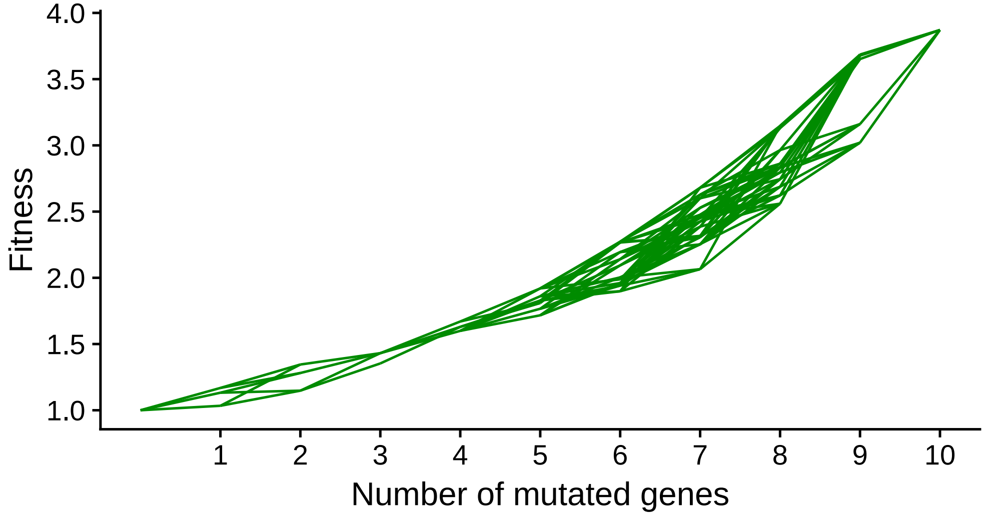

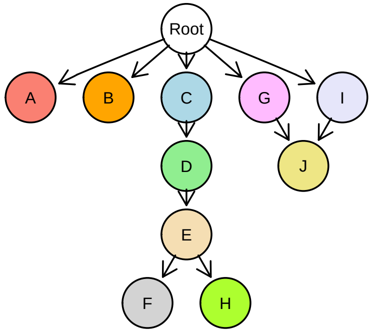

ID kpRFNq2Q10Ex8oSMG
acc genotypes 140
peaks no back 1
removed edges 0
prop rem edges 0

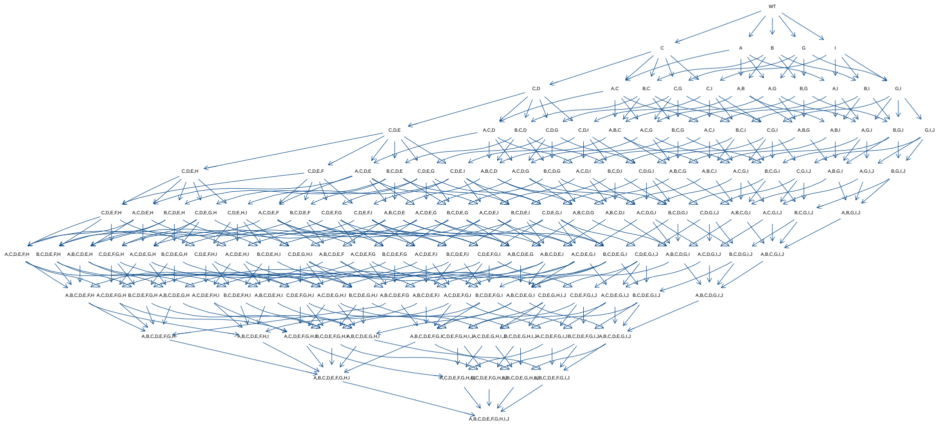

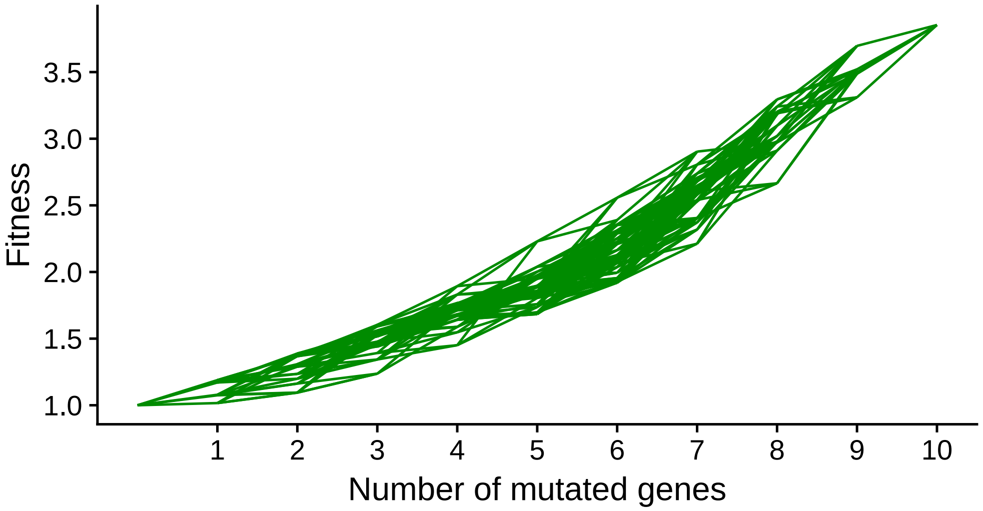

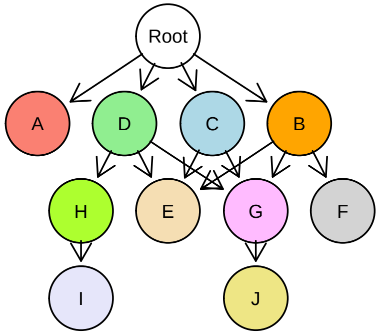

ID ktOHeXaq4zp10UQNO
acc genotypes 108
peaks no back 1
removed edges 0
prop rem edges 0

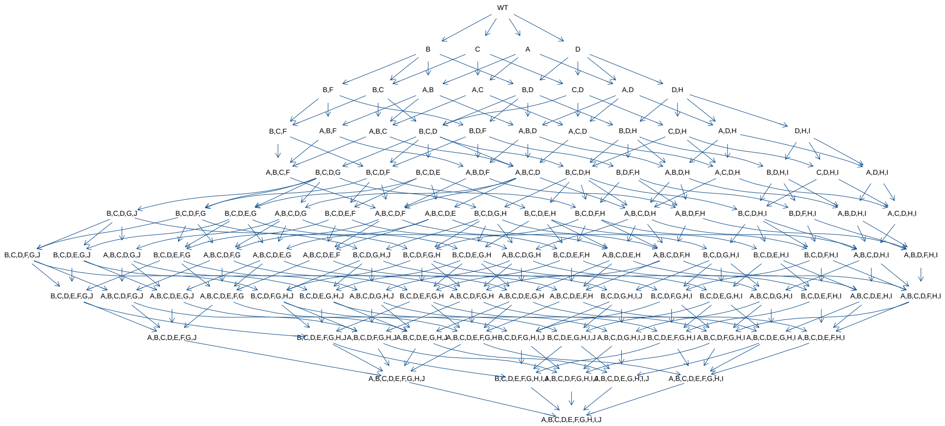

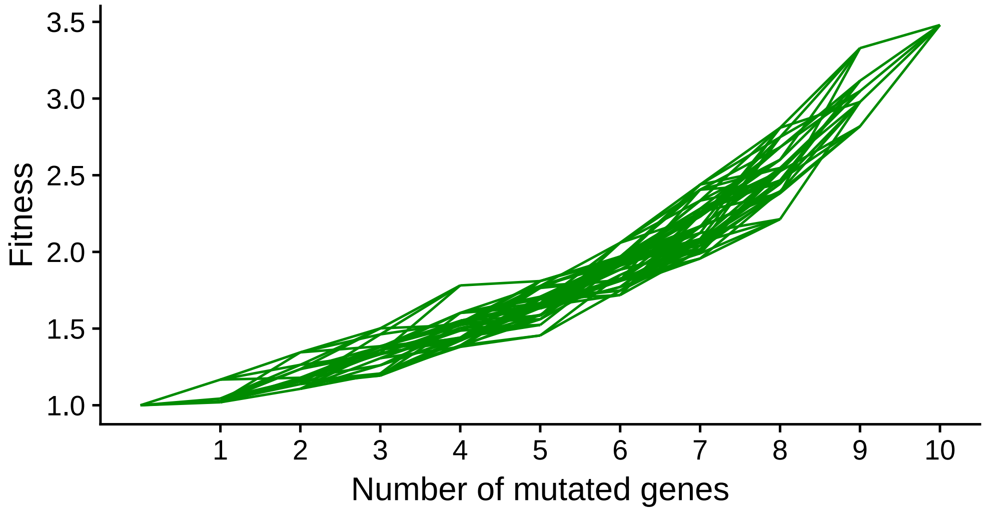

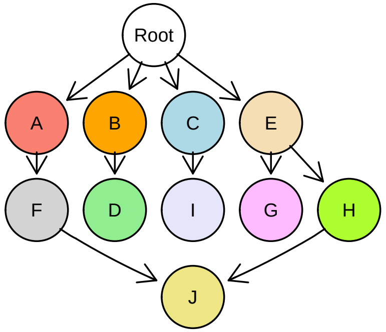

ID	kvDLqWZKOAz5w43U
acc genotypes	153
peaks no back	1
removed edges	0
prop rem edges	0

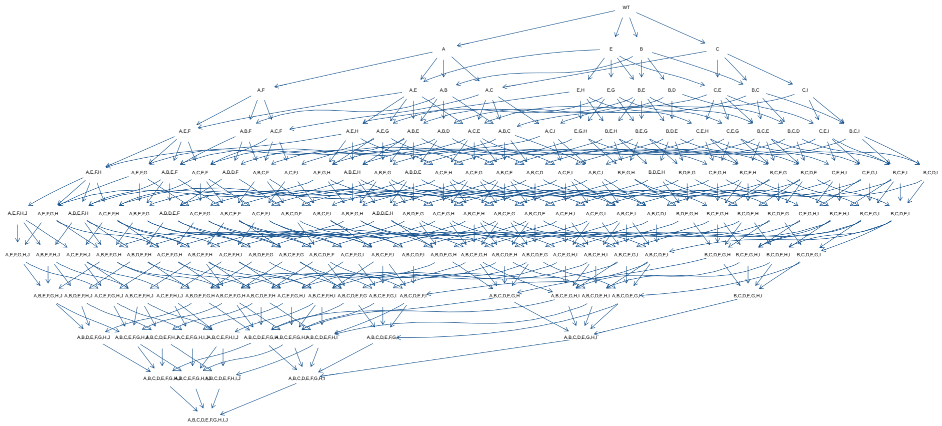

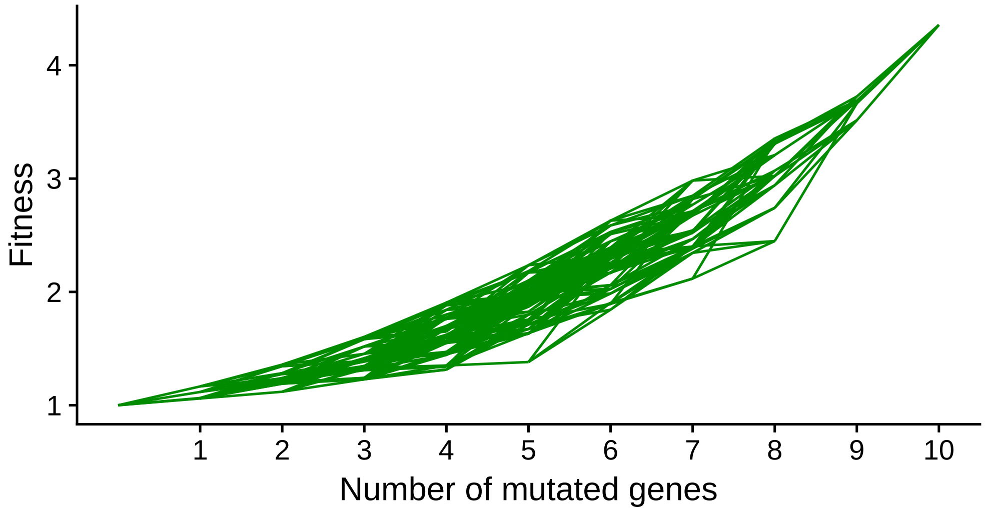

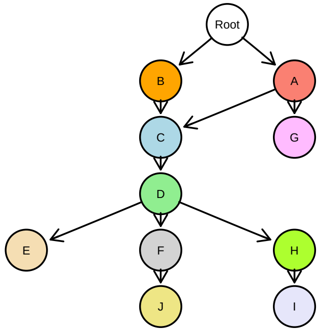

ID	LAiwaf4n74KiIOZv
acc genotypes	44
peaks no back	1
removed edges	0
prop rem edges	0

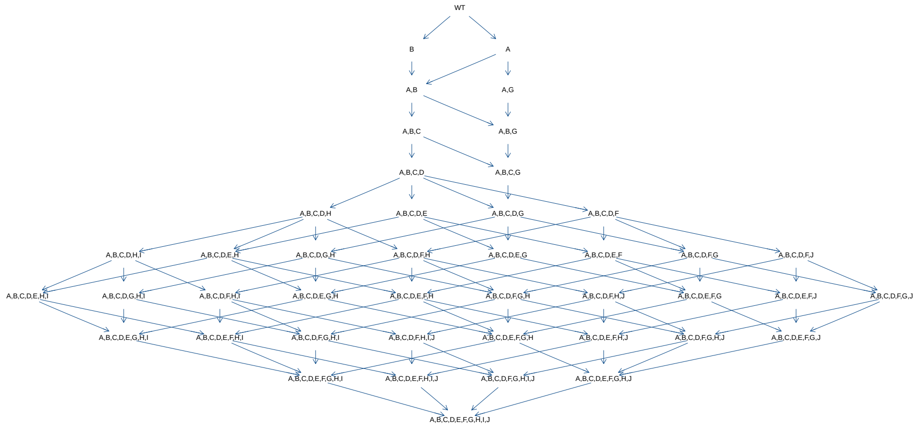

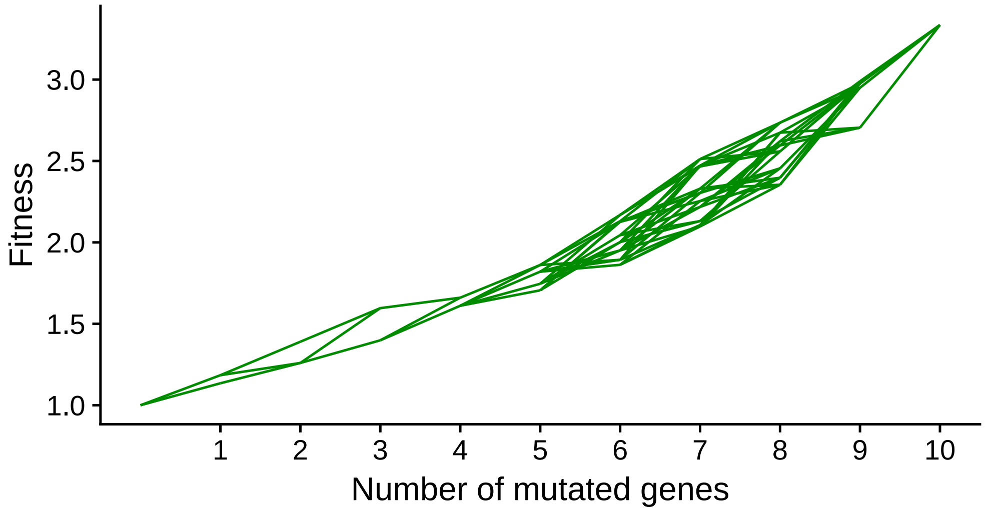

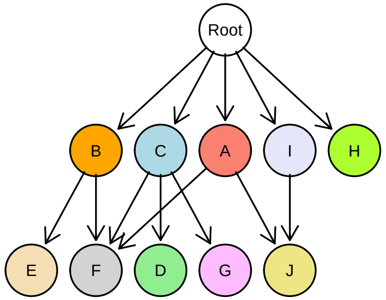

ID	LgHU63NzyxJ8cVAR
acc genotypes	198
peaks no back	1
removed edges	0
prop rem edges	0

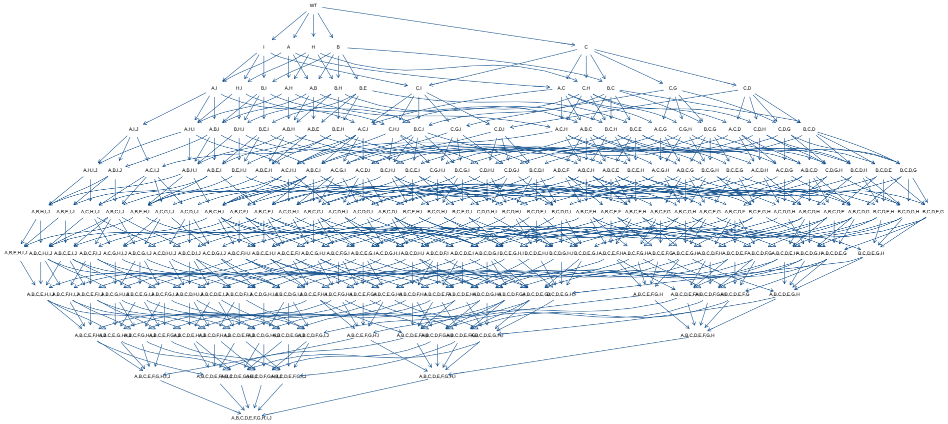

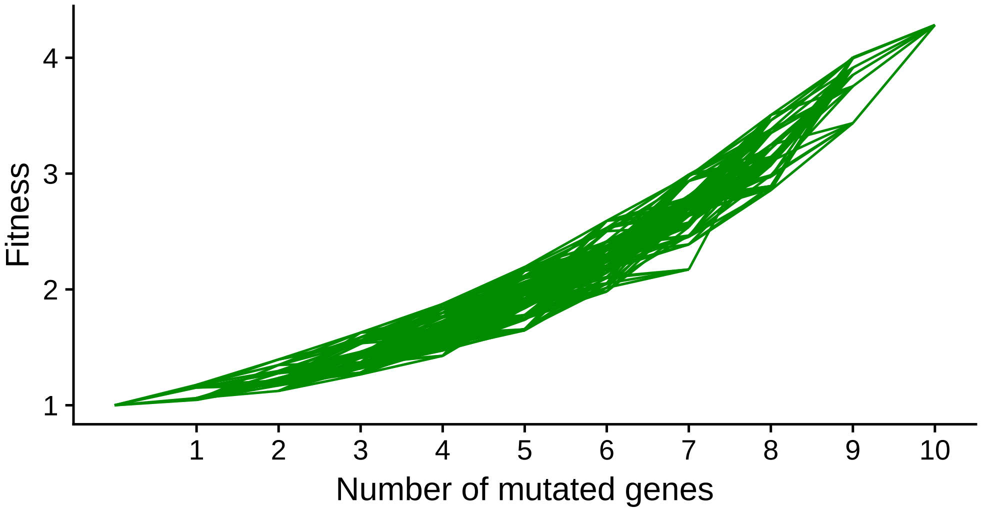

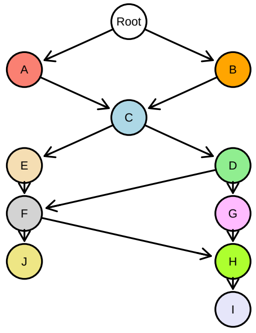

ID	lIOath10wN5BQcyee
acc genotypes	18
peaks no back	1
removed edges	0
prop rem edges	0

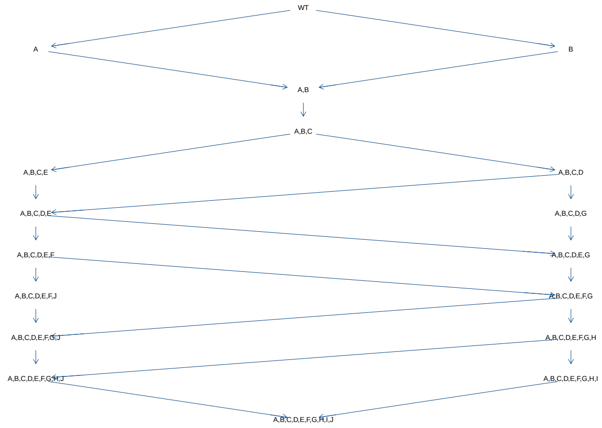

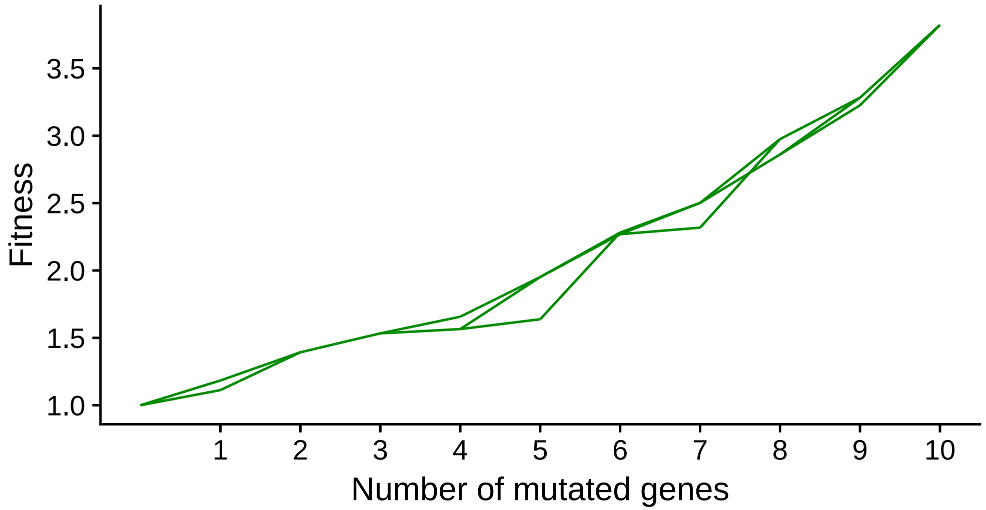

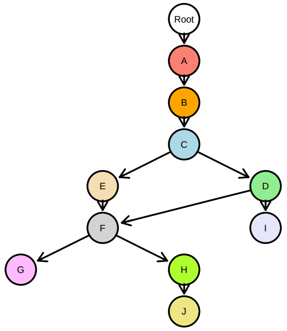

ID	LiqVyEk5185giJ87
acc genotypes	21
peaks no back	1
removed edges	0
prop rem edges	0

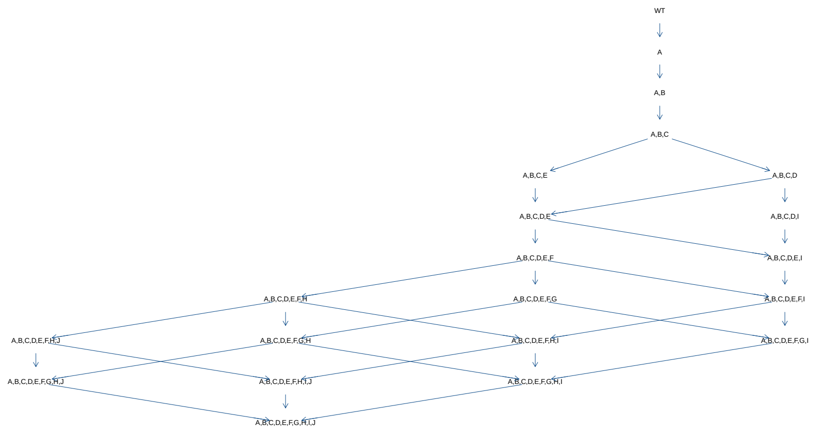

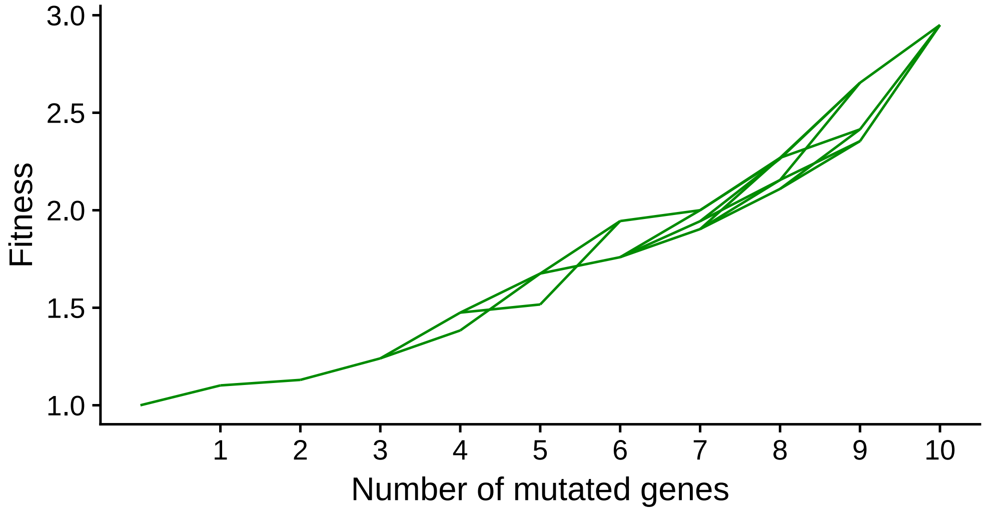

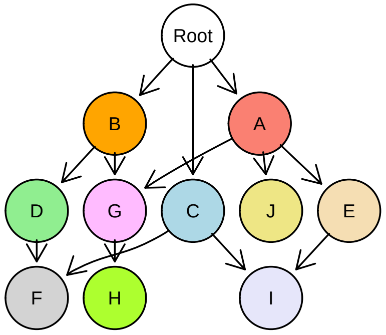

ID	IJ3M9zaA46iBzYO2
acc genotypes	95
peaks no back	1
removed edges	0
prop rem edges	0

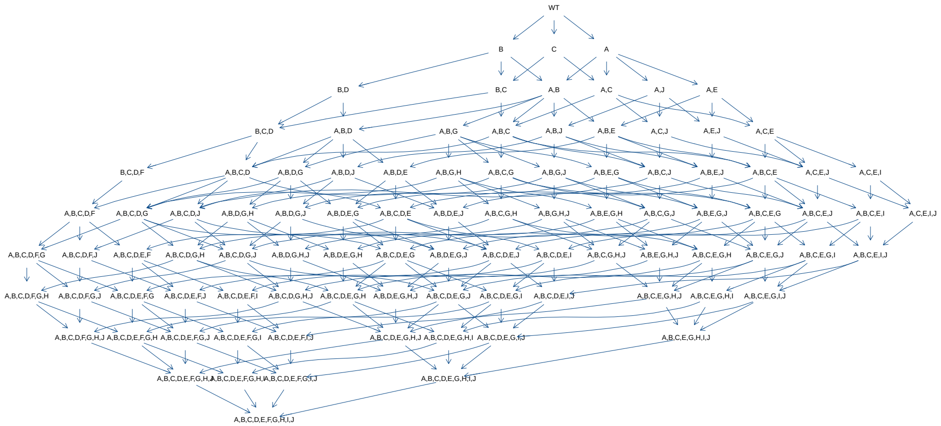

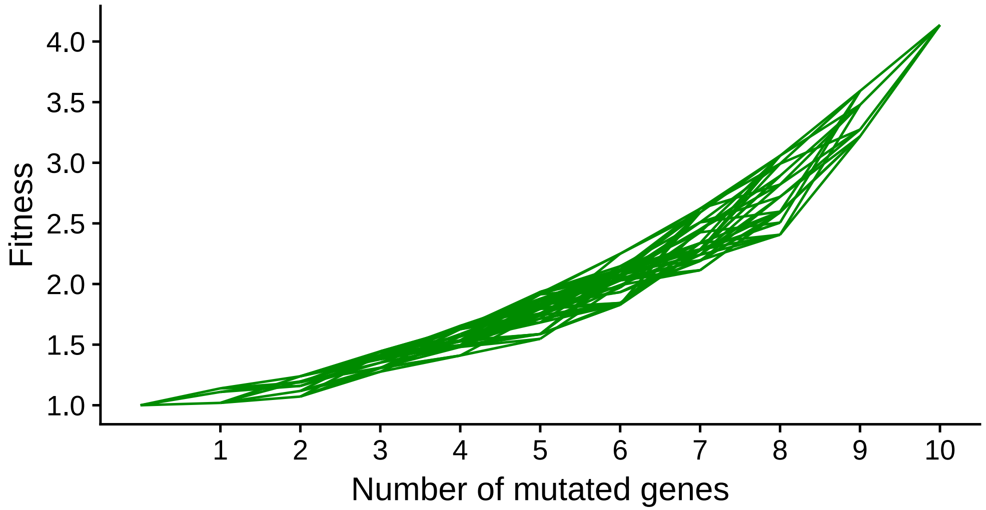

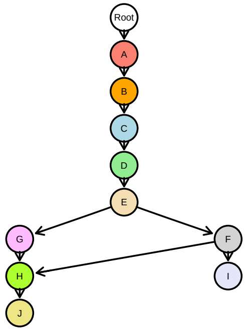

ID	LKzTjEtauR10FkWvI
acc genotypes	15
peaks no back	1
removed edges	0
prop rem edges	0

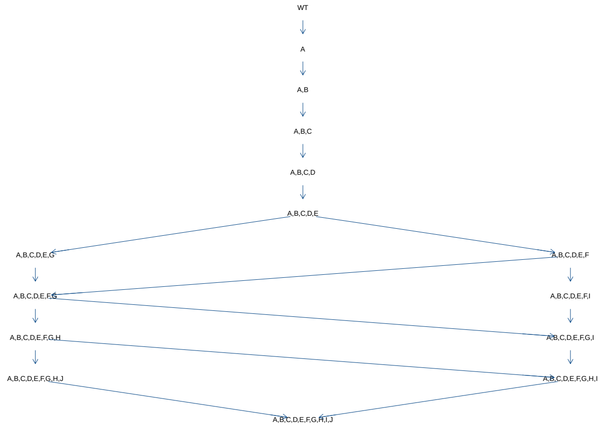

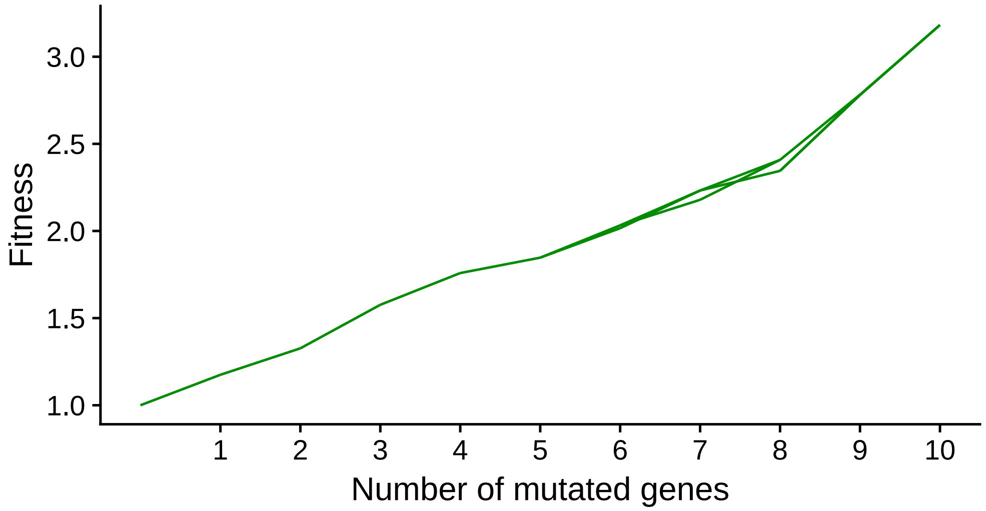

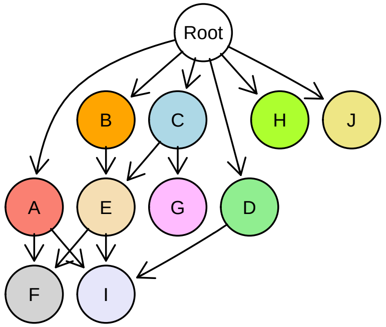

ID	LLARXmNmlvPm7fLH
acc genotypes	160
peaks no back	1
removed edges	0
prop rem edges	0

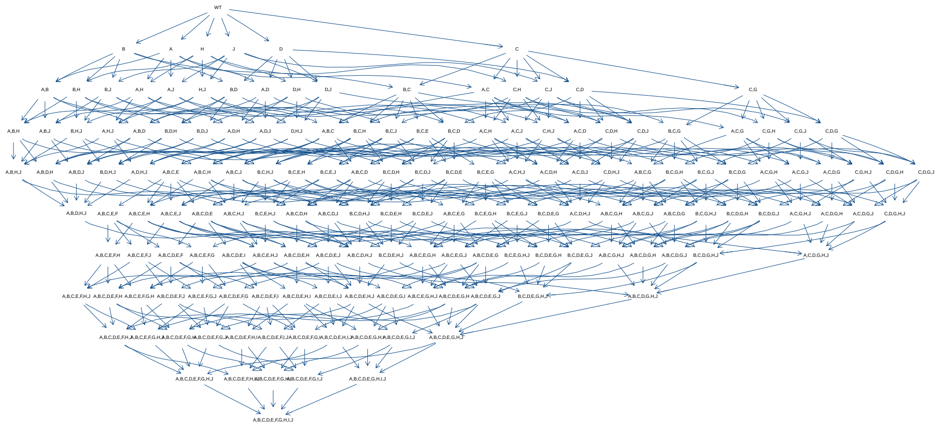

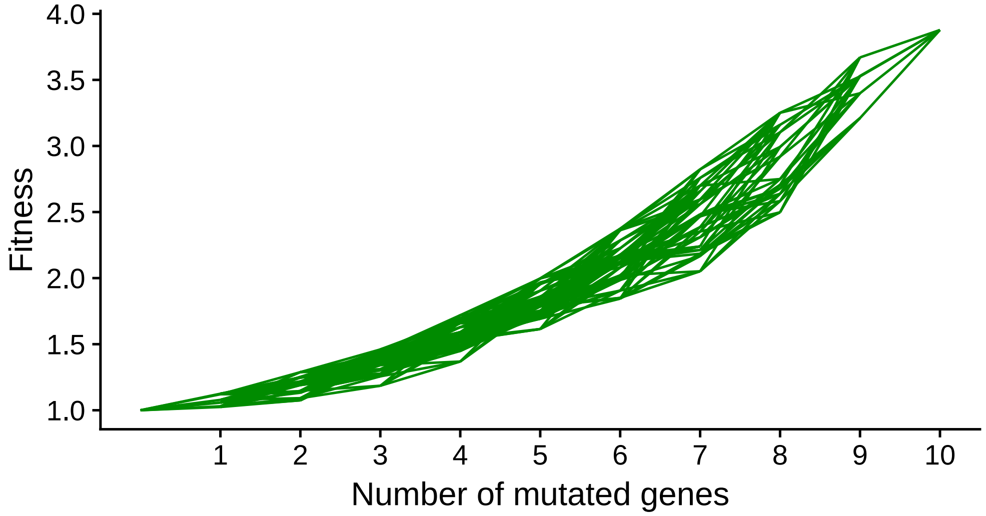

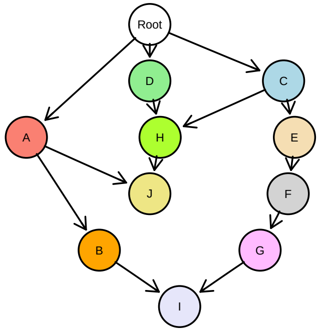

ID	LoVQjzyAttkUHwB8
acc genotypes	54
peaks no back	1
removed edges	0
prop rem edges	0

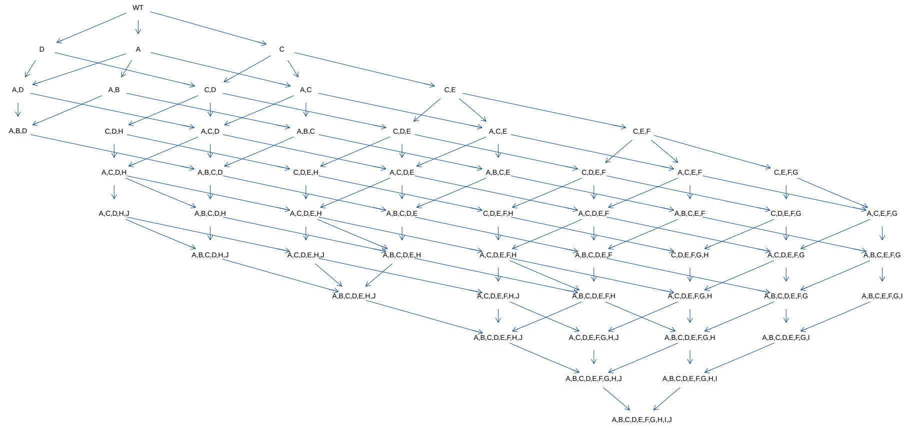

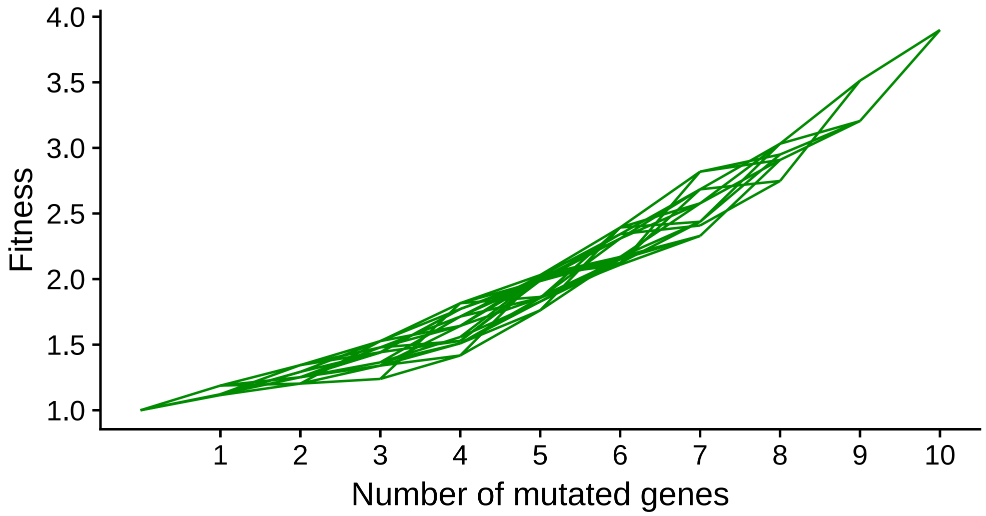

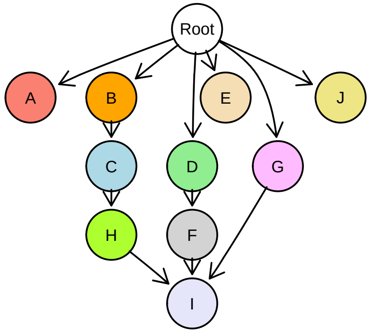

ID	m2LJE5DdVSyhA9R9
acc genotypes	200
peaks no back	1
removed edges	0
prop rem edges	0

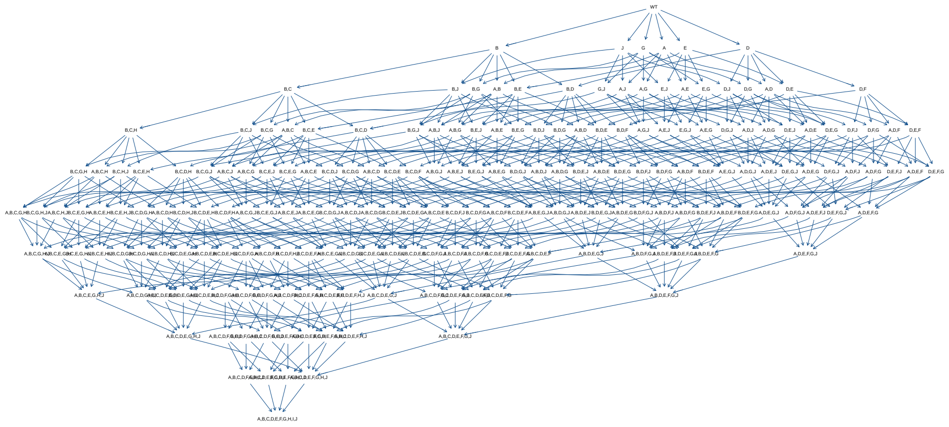

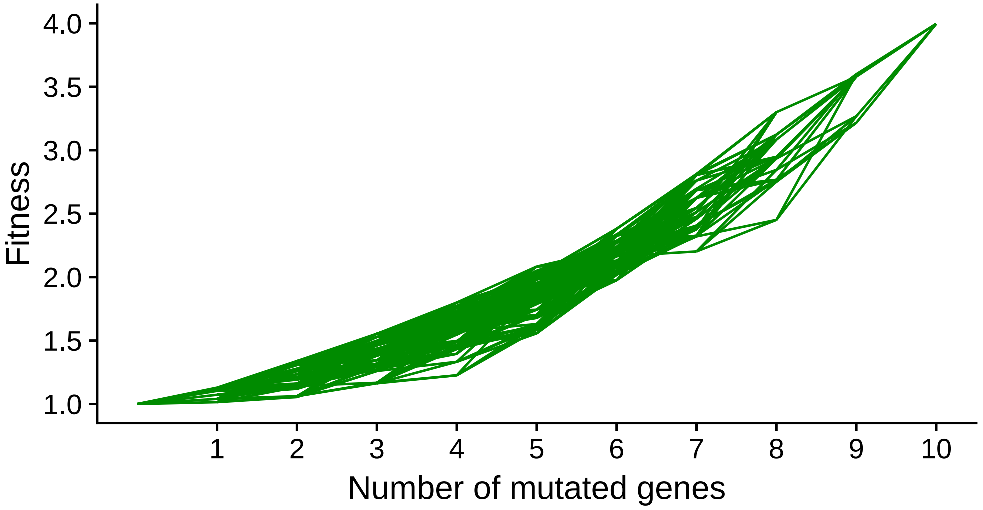

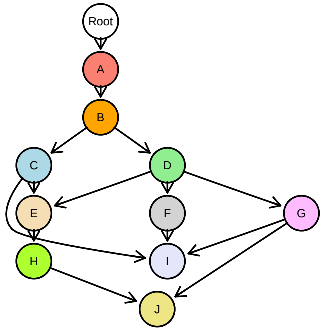

ID	M5NjxUHUHAkU7nQF
acc genotypes	26
peaks no back	1
removed edges	0
prop rem edges	0

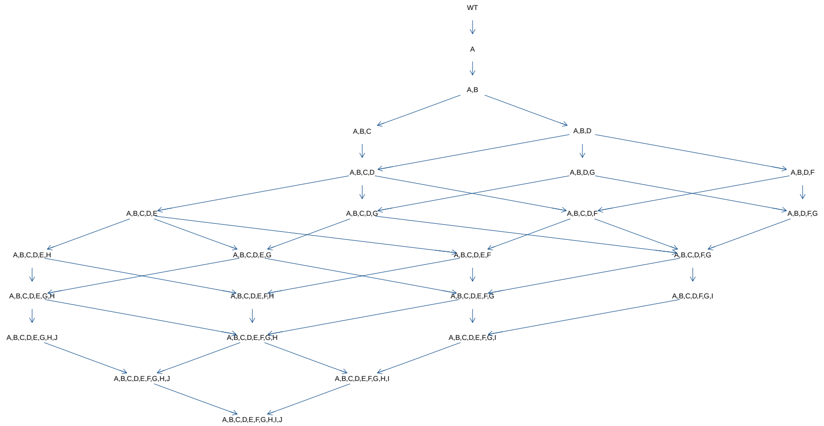

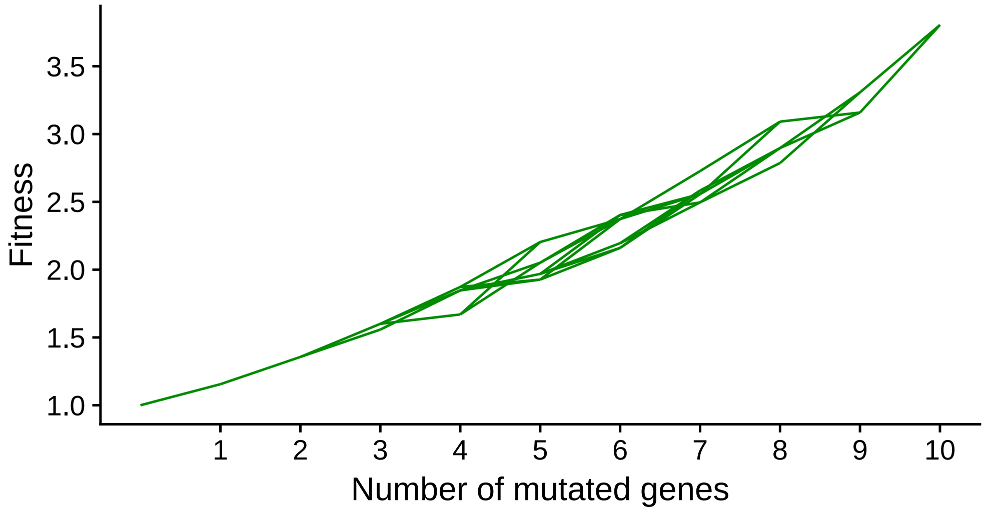

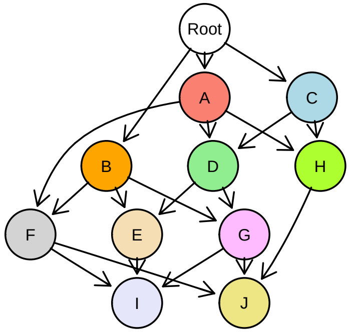

ID MJxO4QrzOUXVWzXM
acc genotypes 36
peaks no back 1
removed edges 0
prop rem edges 0

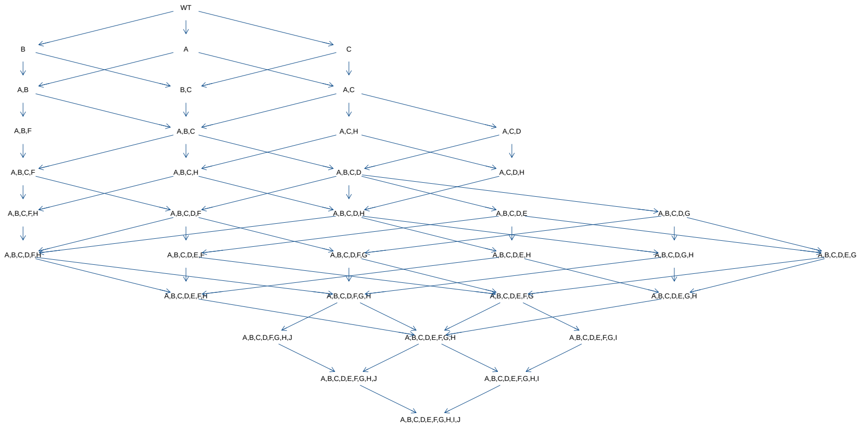

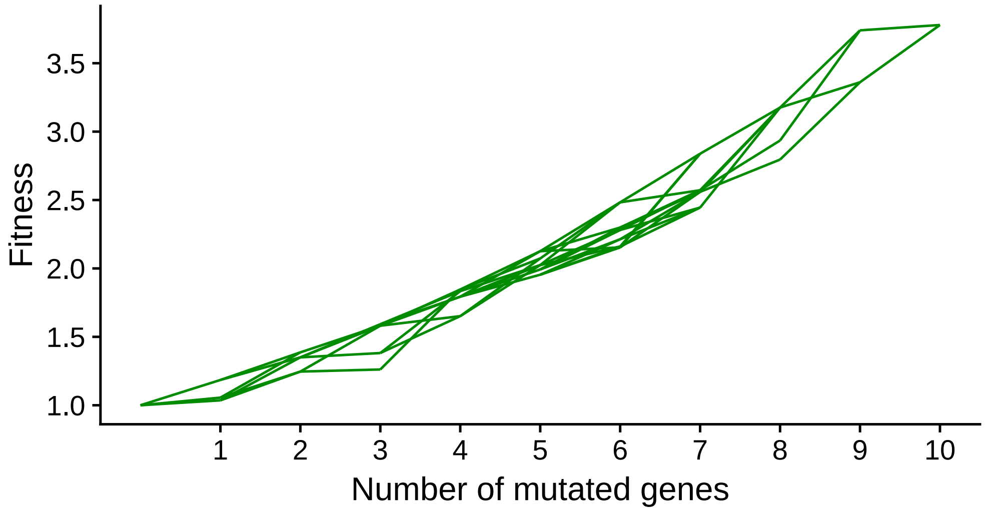

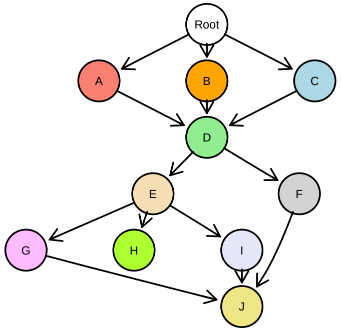

ID	MkJPmR2HWGDw34xG
acc genotypes	28
peaks no back	1
removed edges	0
prop rem edges	0

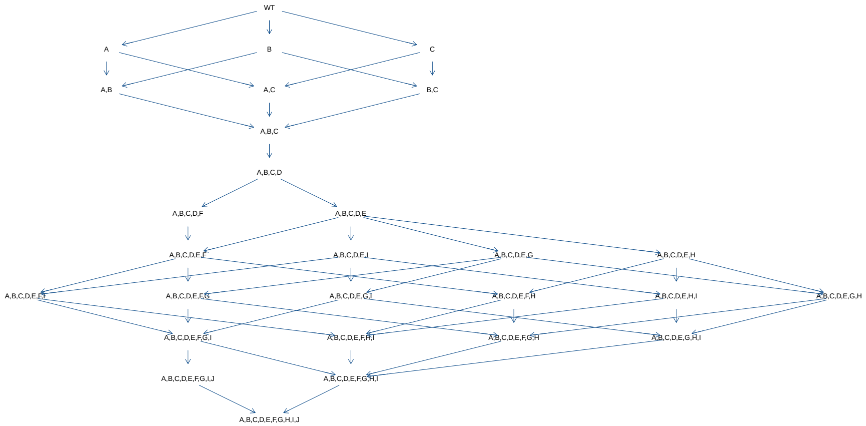

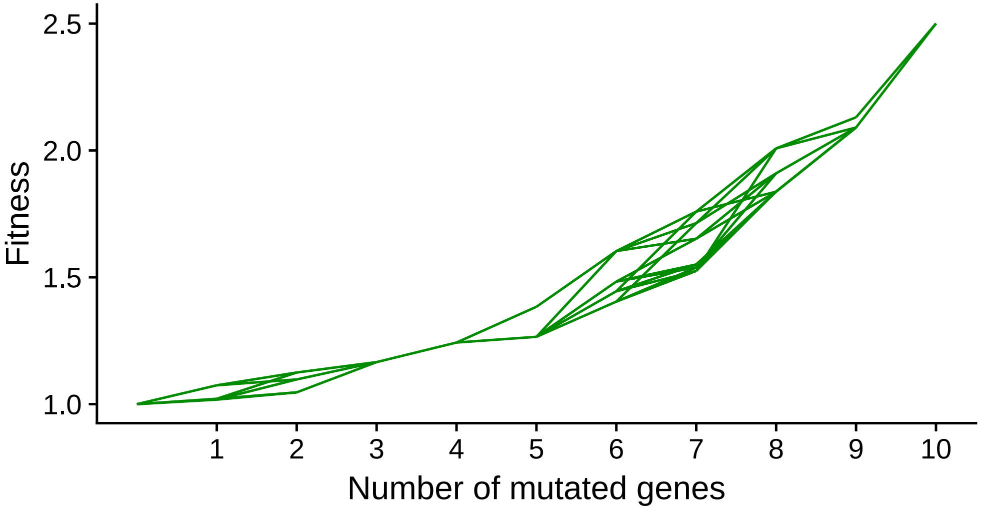

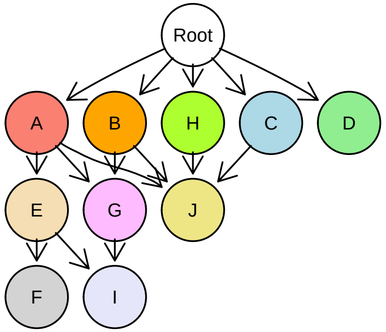

ID	MocksvQ5er102Pjpk
acc genotypes	120
peaks no back	1
removed edges	0
prop rem edges	0

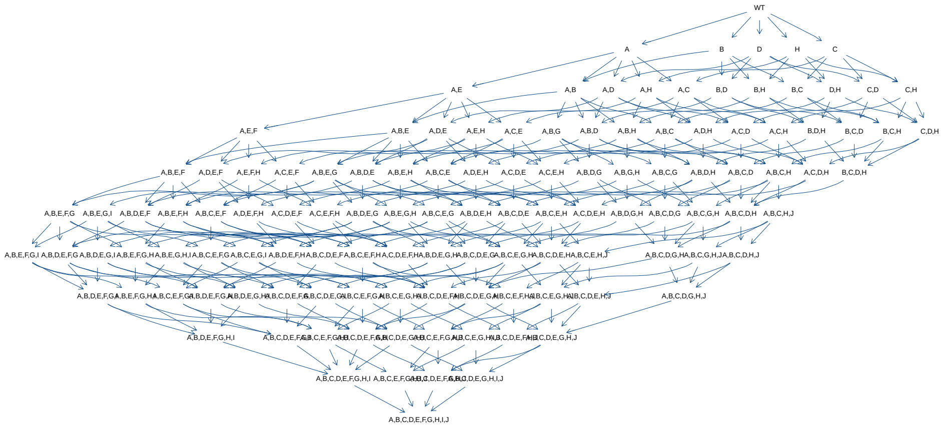

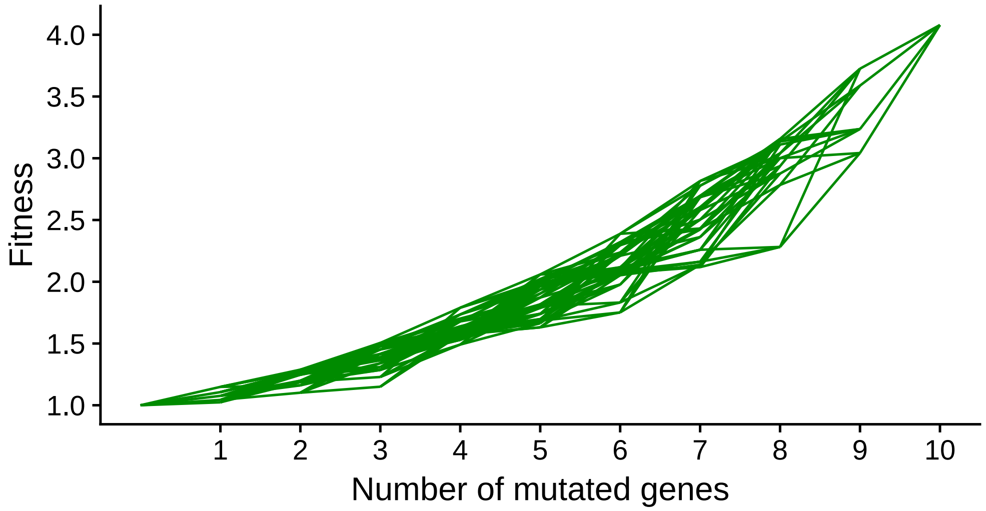

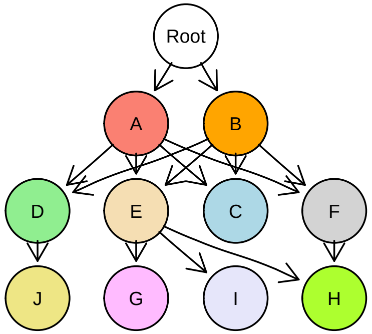

ID MPXnkjvgPoPYXZbV

acc genotypes 87

peaks no back 1

removed edges 0

prop rem edges 0

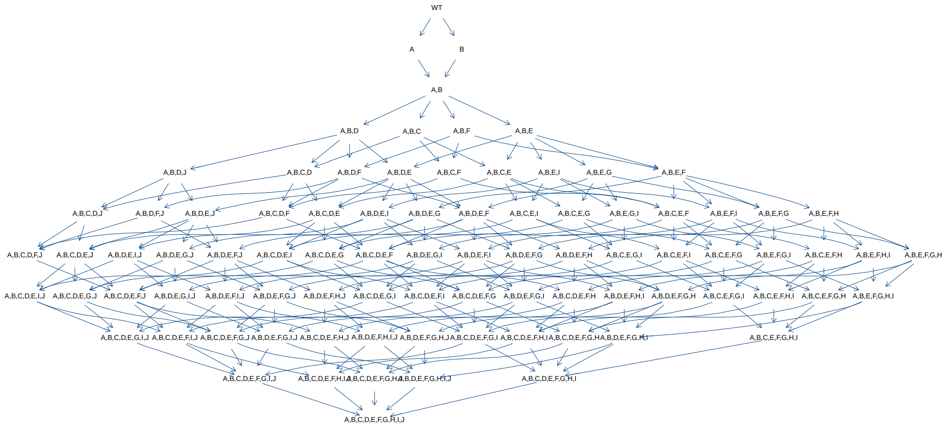

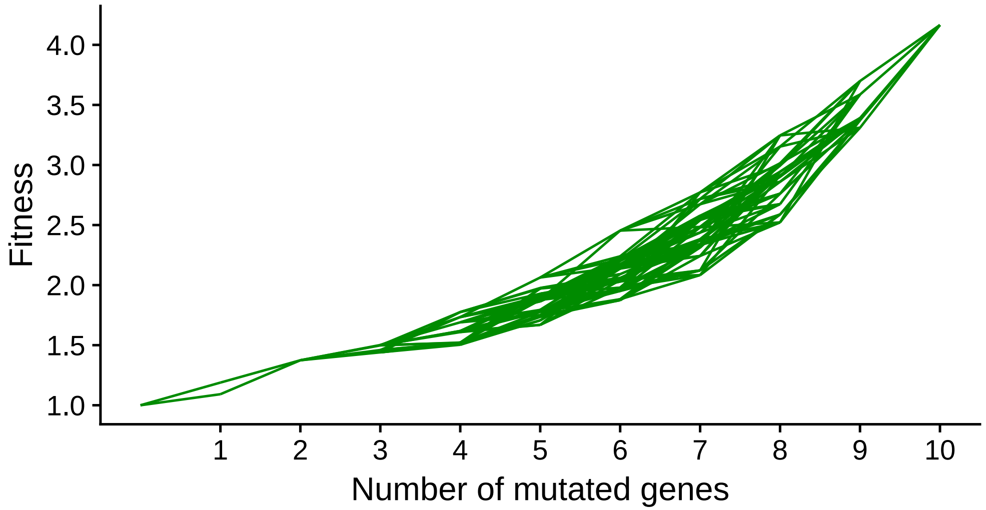

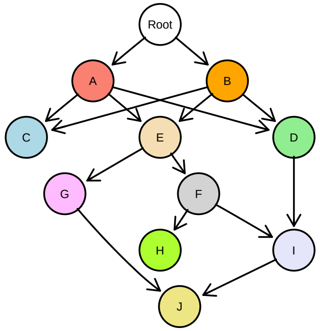

ID	msWBh10MLRgzEH10BX
acc genotypes	43
peaks no back	1
removed edges	0
prop rem edges	0

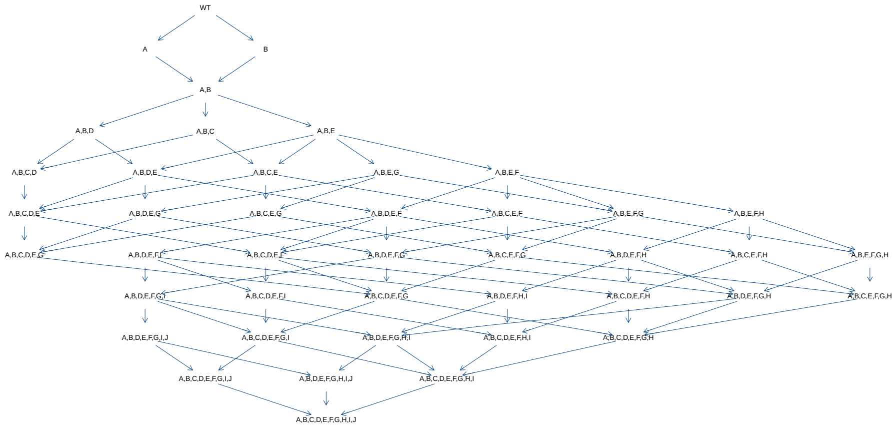

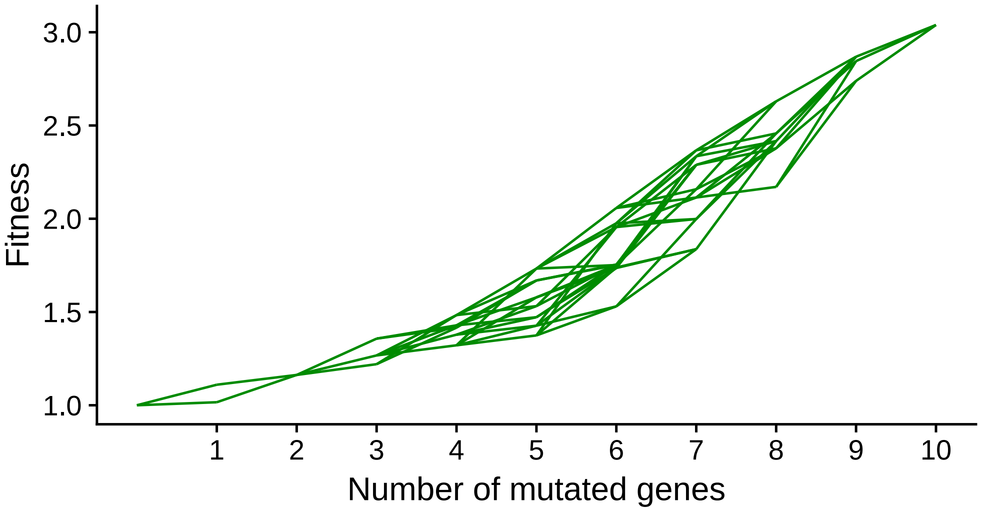

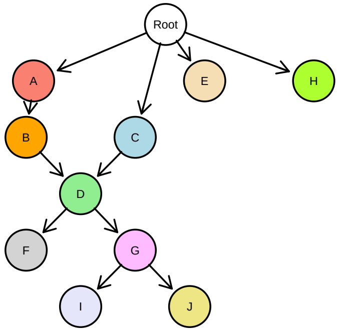

ID	MT7WzjxZkqx10snFO
acc genotypes	64
peaks no back	1
removed edges	0
prop rem edges	0

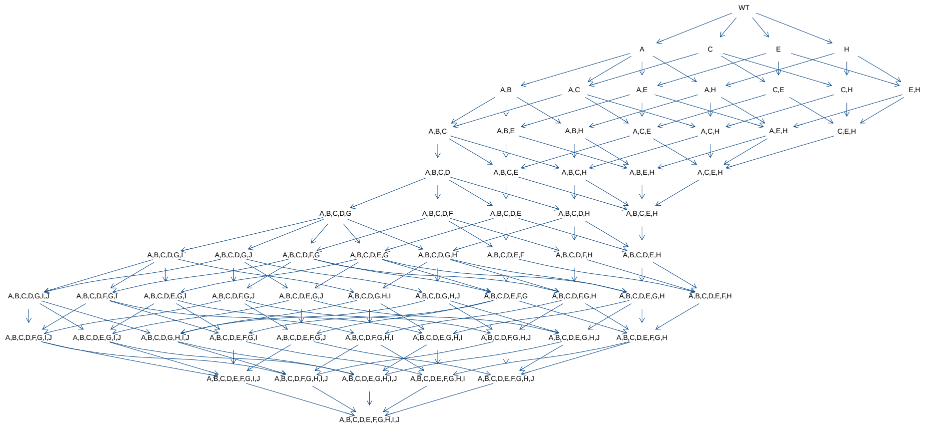

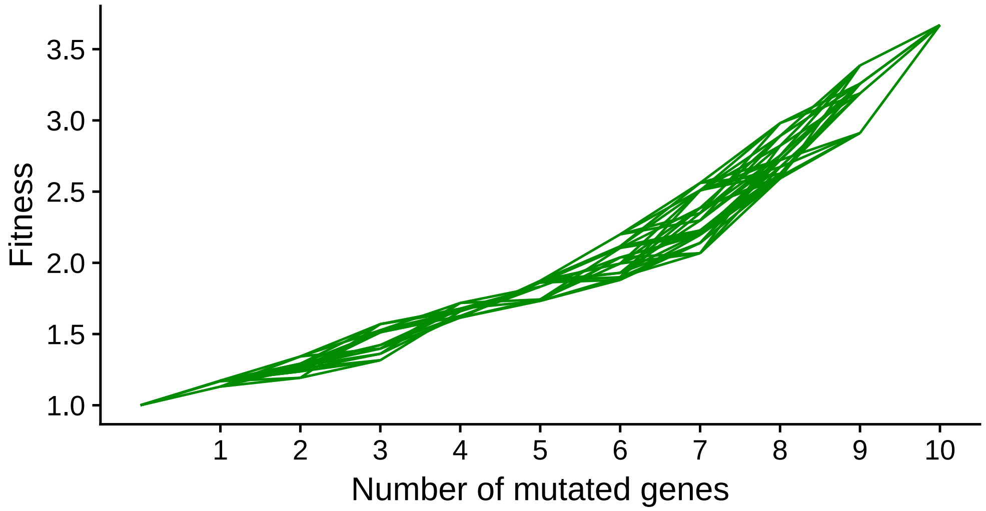

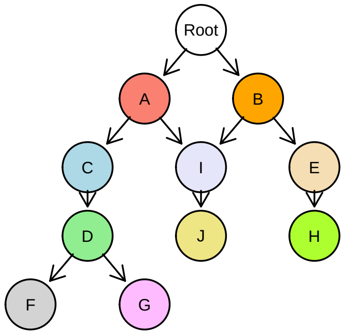

ID	n210BxEwpJXJV8VL6
acc genotypes	64
peaks no back	1
removed edges	0
prop rem edges	0

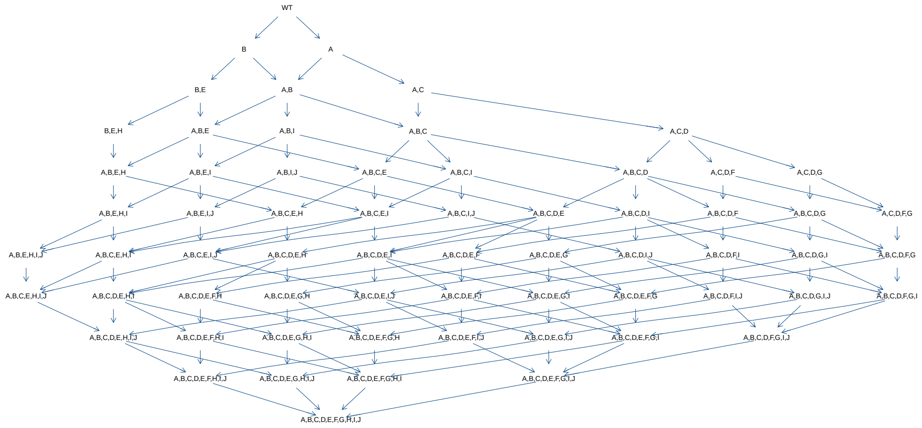

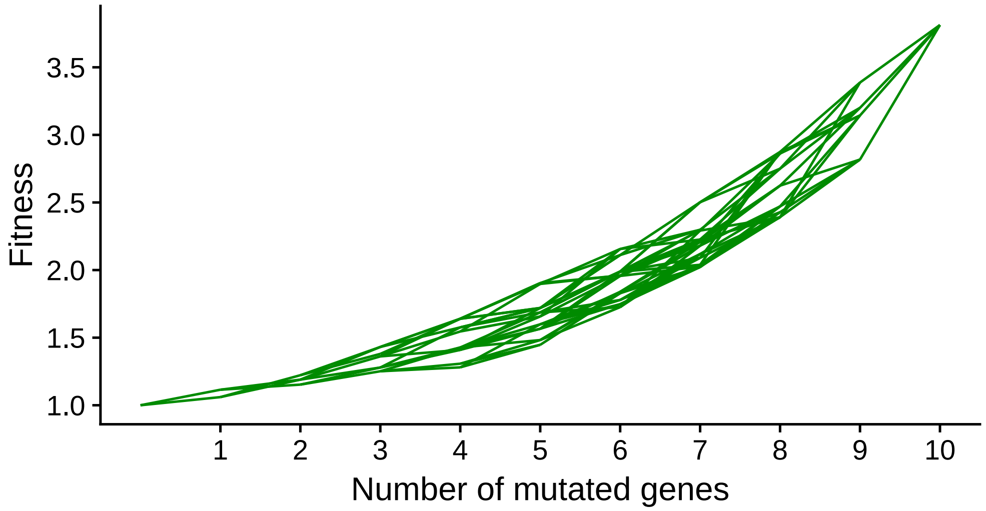

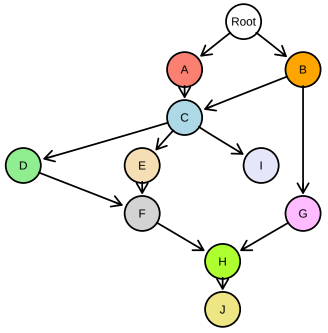

ID	Nd5tXtGonmFk3JtF
acc genotypes	30
peaks no back	1
removed edges	0
prop rem edges	0

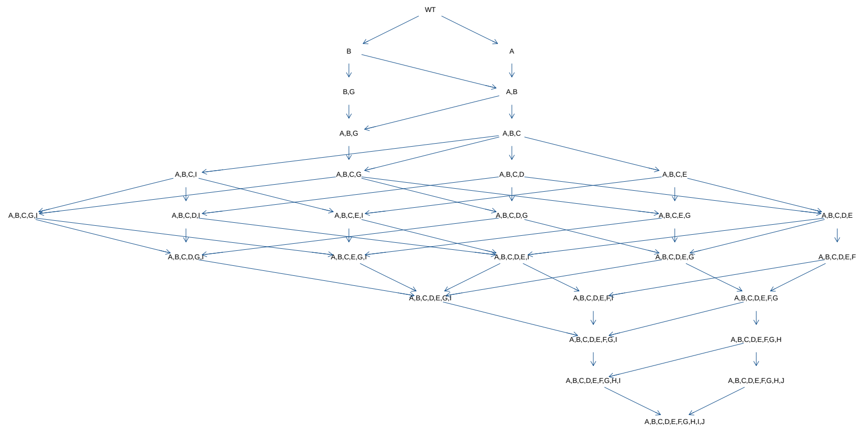

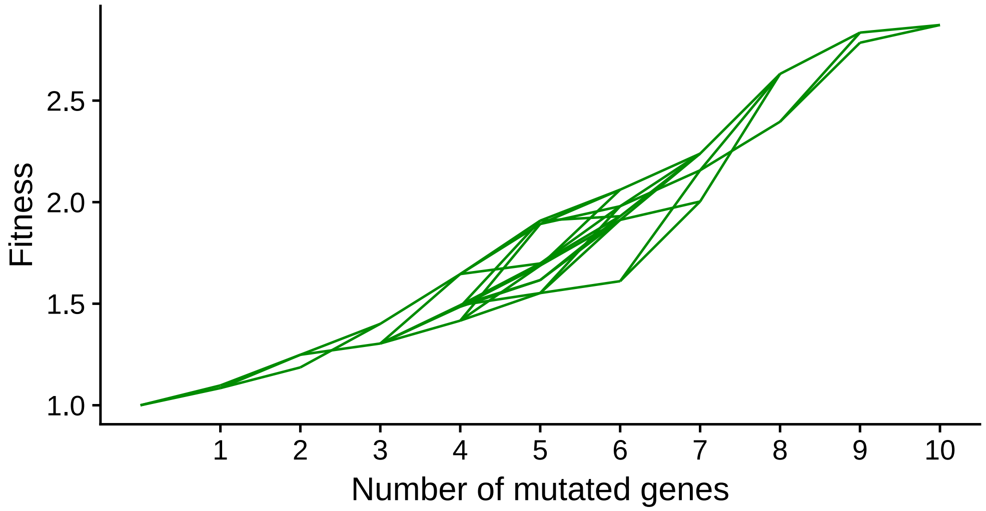

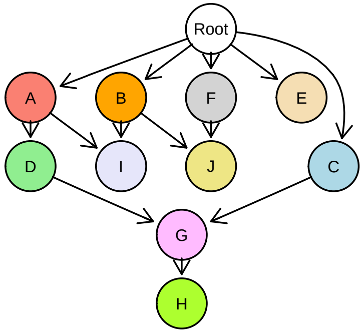

ID	nfjDxDAtFc4uTrco
acc genotypes	116
peaks no back	1
removed edges	0
prop rem edges	0

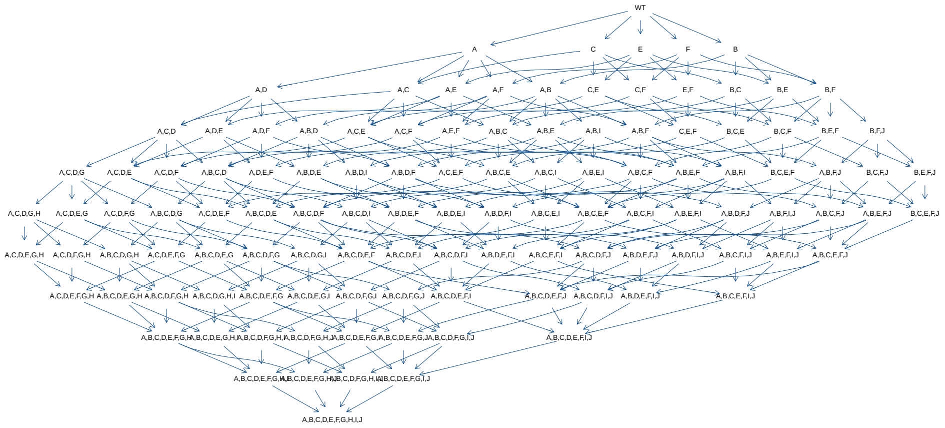

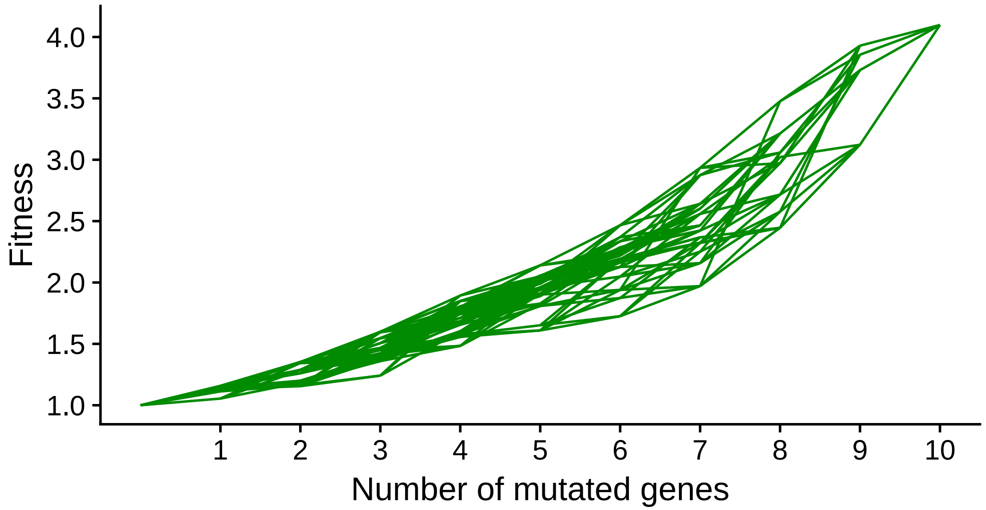

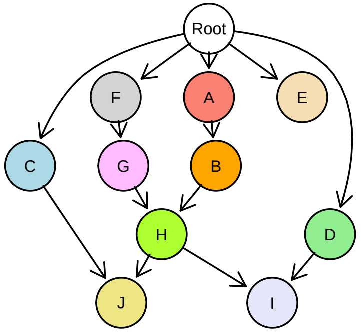

ID	nO5yu7D15ZhqKnN9
acc genotypes	90
peaks no back	1
removed edges	0
prop rem edges	0

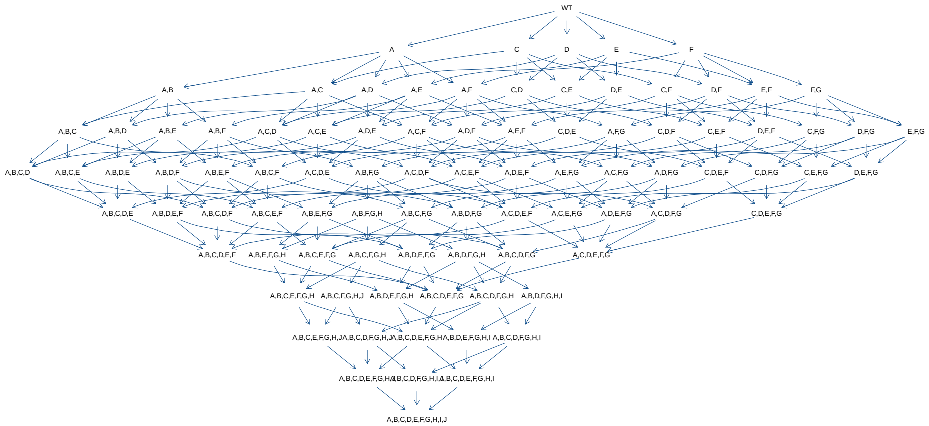

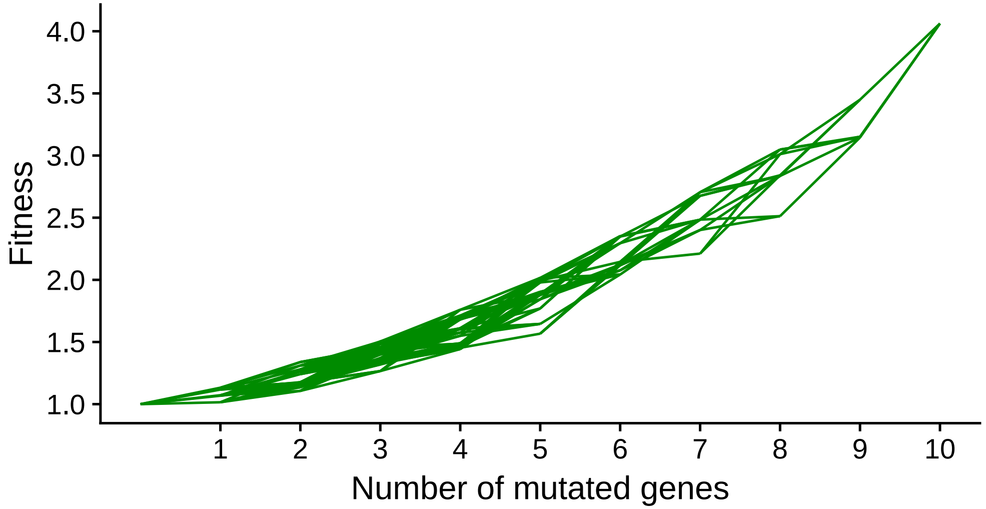

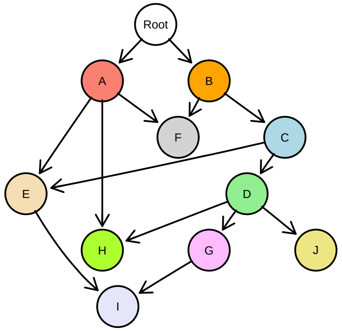

ID	NP7ssqpSWvIesNBx
acc genotypes	54
peaks no back	1
removed edges	0
prop rem edges	0

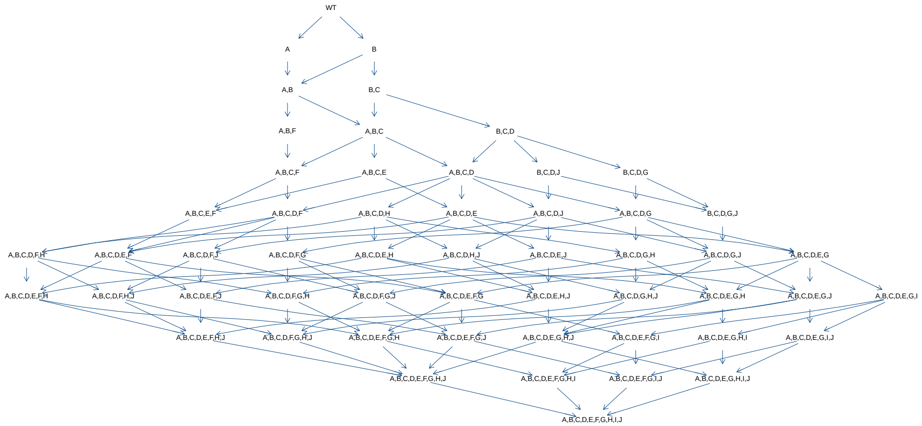

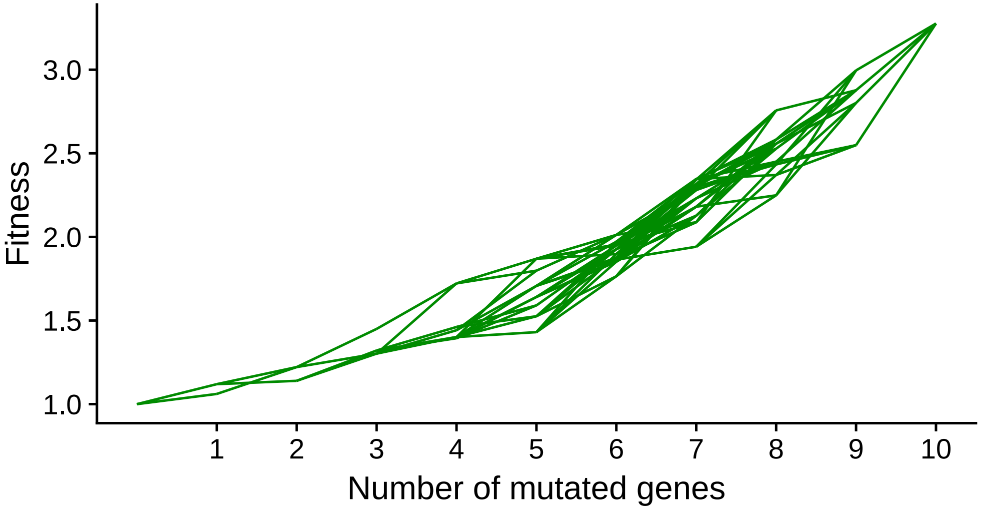

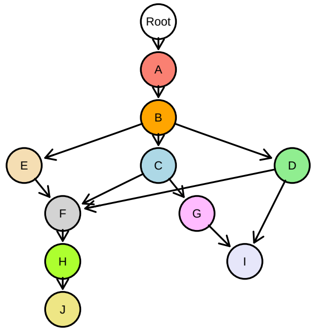

ID	NyMcE3XWsePNxuEs
acc genotypes	25
peaks no back	1
removed edges	0
prop rem edges	0

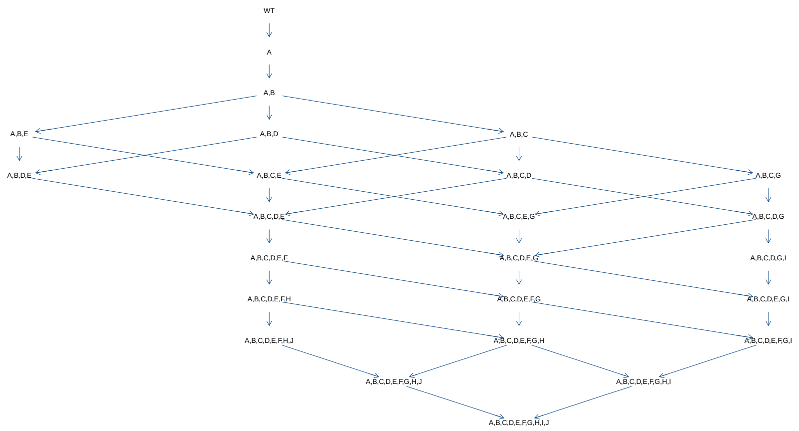

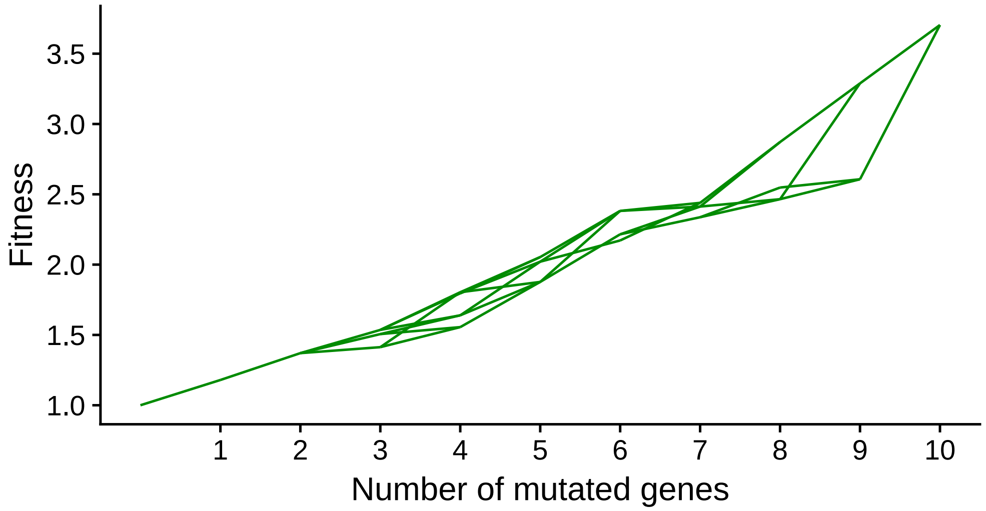

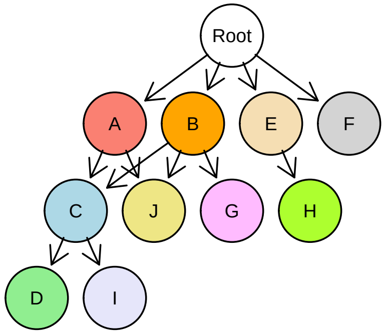

ID	o10BaslJKzTznq5sp
acc genotypes	144
peaks no back	1
removed edges	0
prop rem edges	0

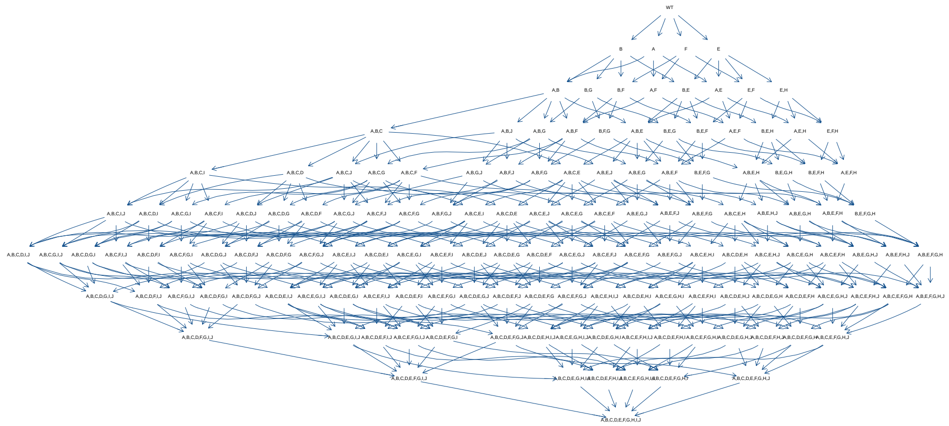

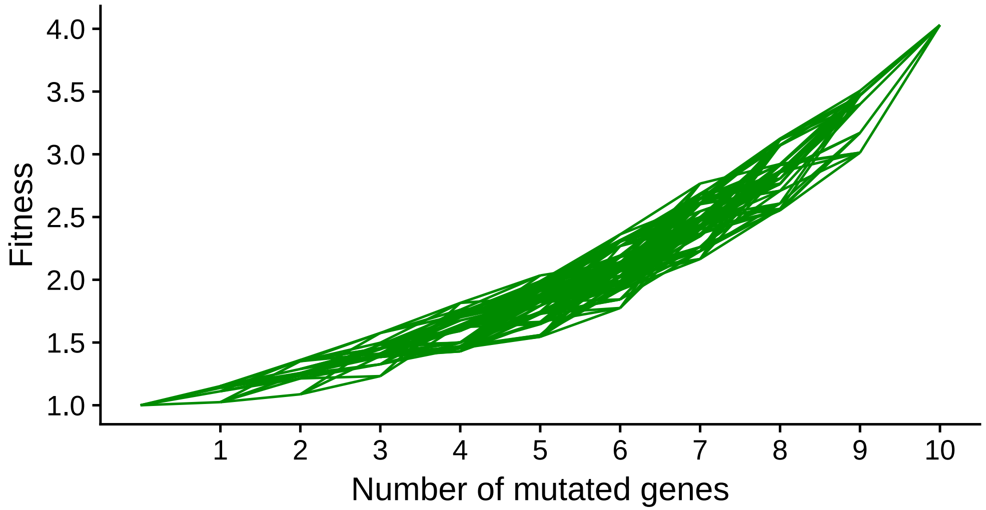

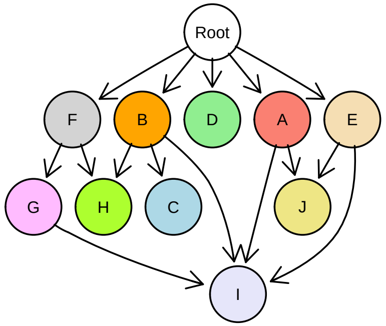

ID	o89L6ak94kWOZRNA
acc genotypes	146
peaks no back	1
removed edges	0
prop rem edges	0

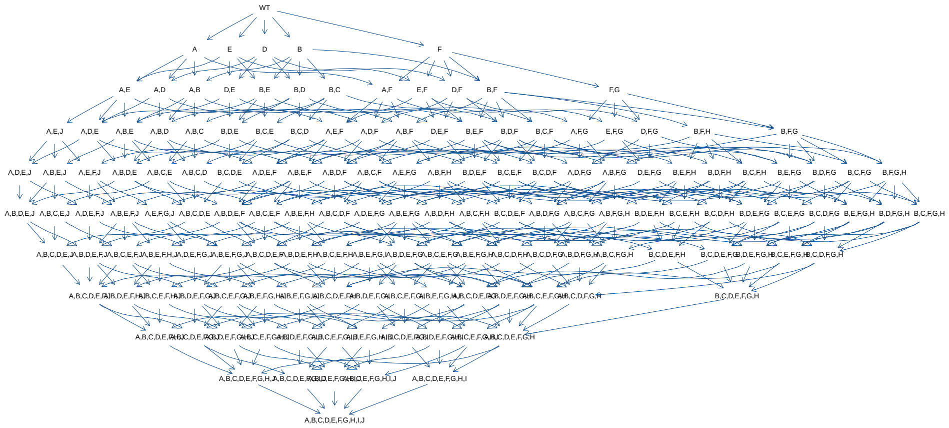

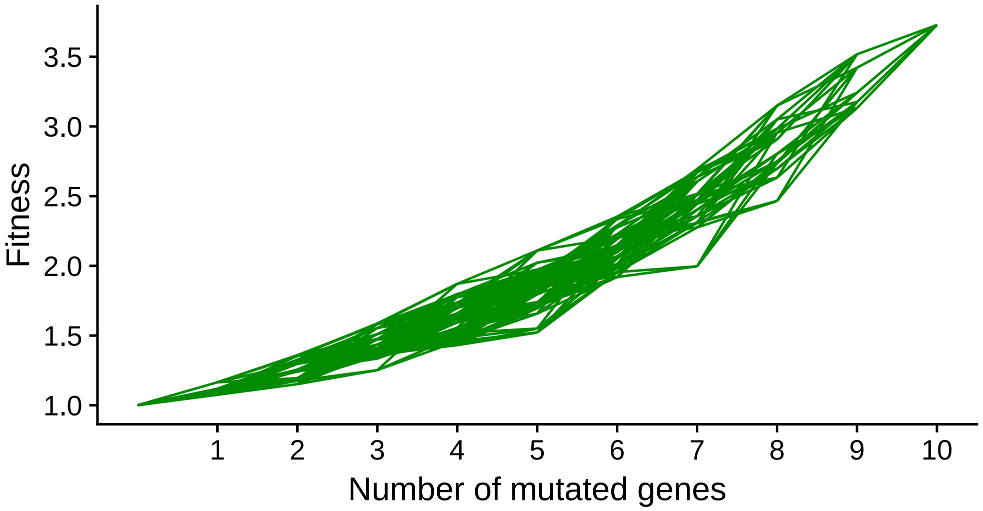

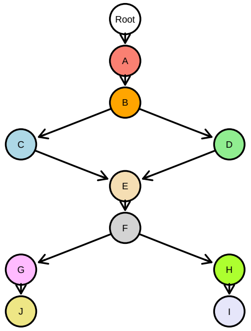

ID	ocx2Aw4wa94NsR64
acc genotypes	16
peaks no back	1
removed edges	0
prop rem edges	0

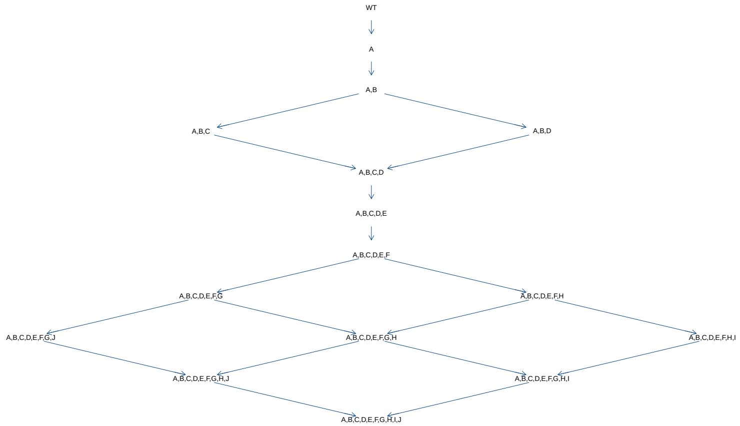

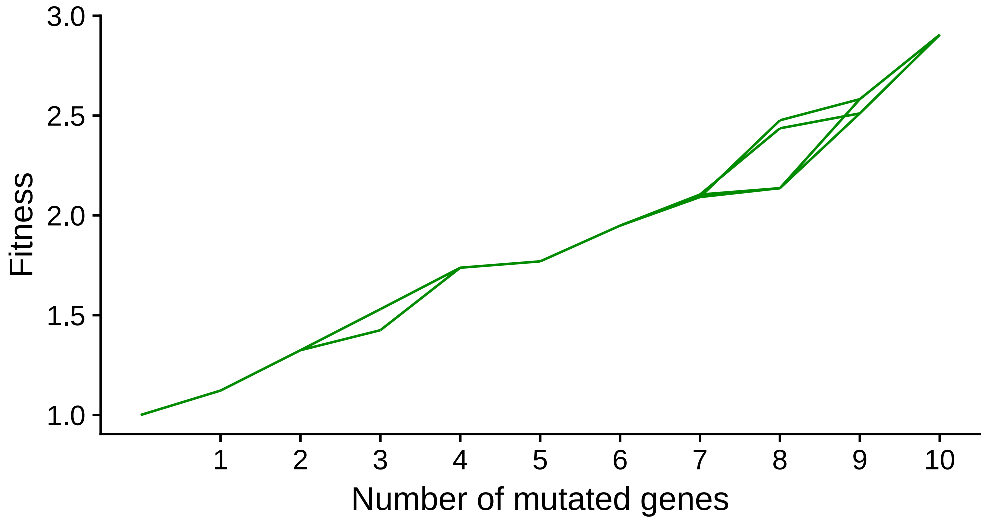

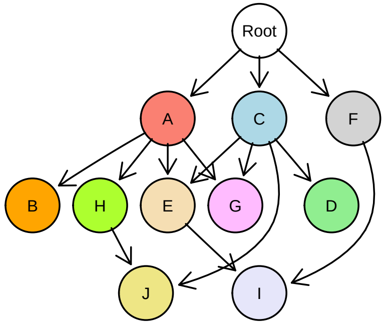

ID	OGeUcOzxp4J7ocOW
acc genotypes	134
peaks no back	1
removed edges	0
prop rem edges	0

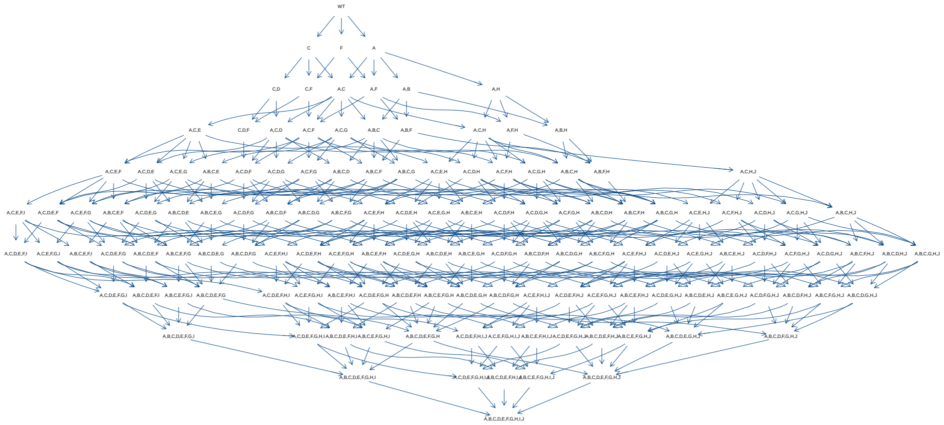

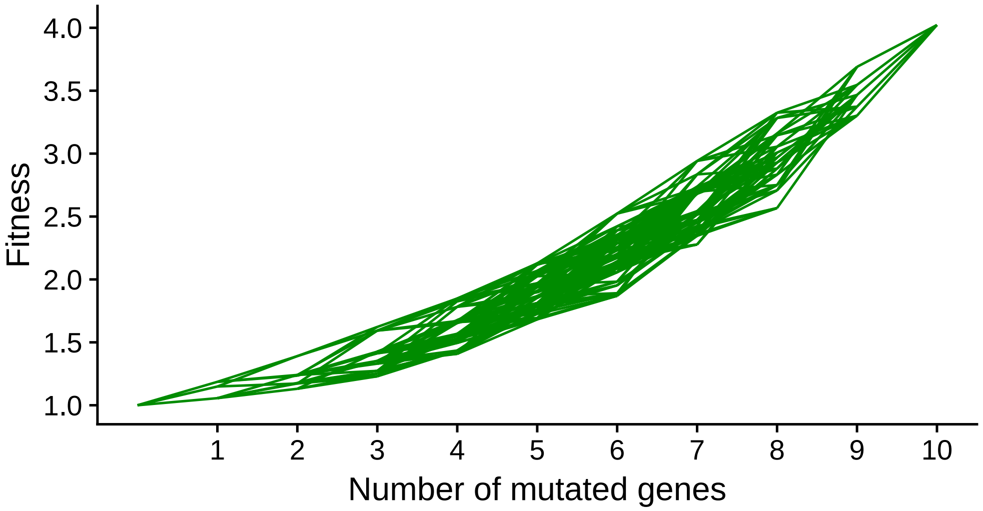

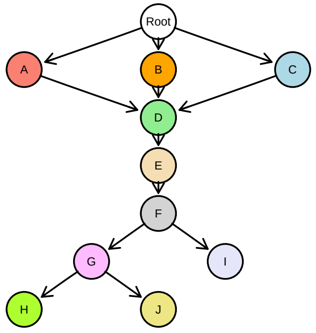

ID	oGhD99FEVEPwA8X8
acc genotypes	20
peaks no back	1
removed edges	0
prop rem edges	0

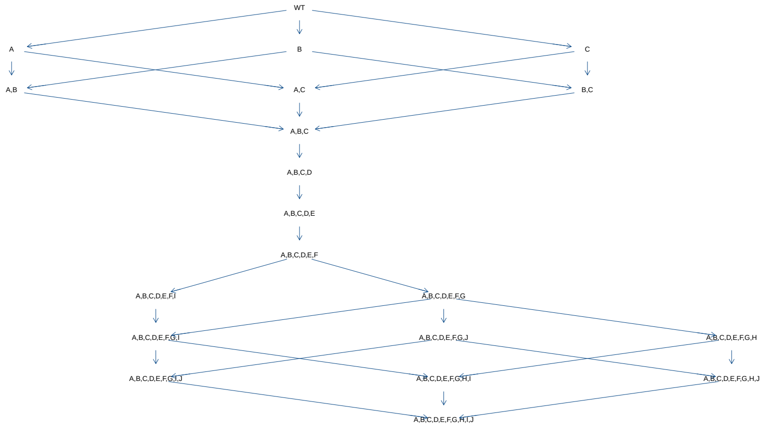

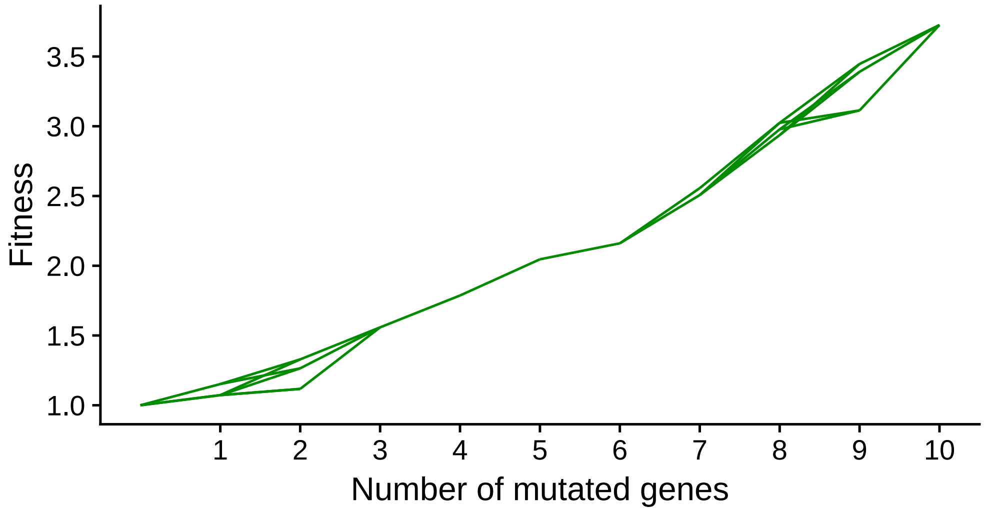

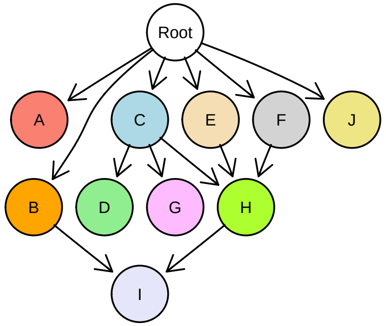

ID	ohfmWmj2mPmIjDEY
acc genotypes	208
peaks no back	1
removed edges	0
prop rem edges	0

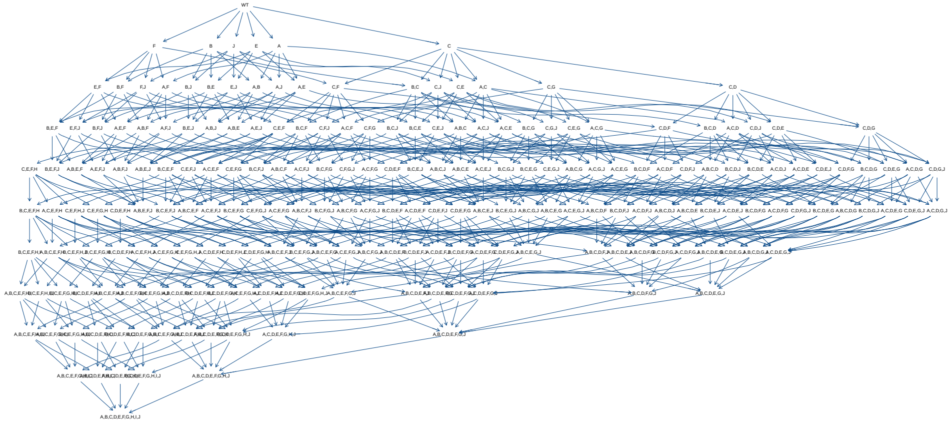

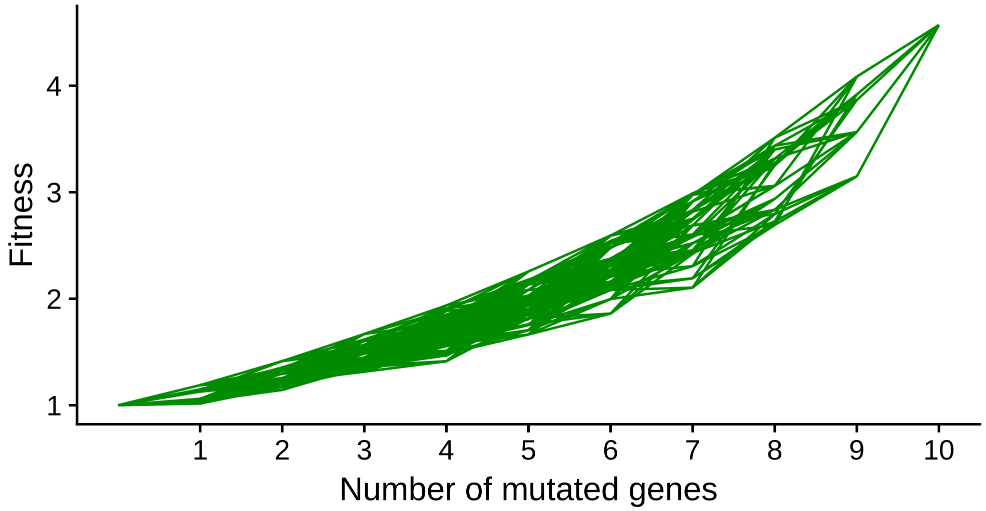

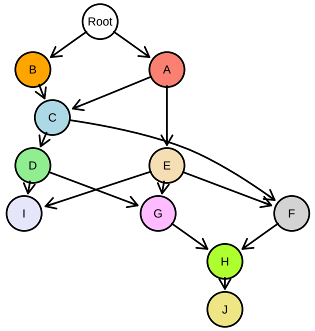

ID	oRJlh1e10mdF10GFUm
acc genotypes	22
peaks no back	1
removed edges	0
prop rem edges	0

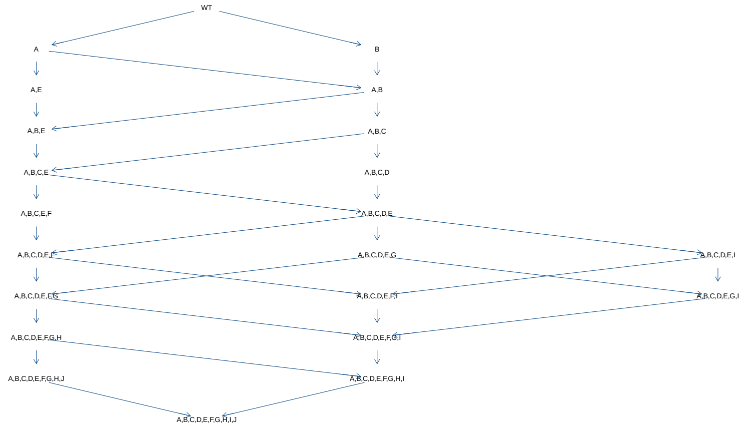

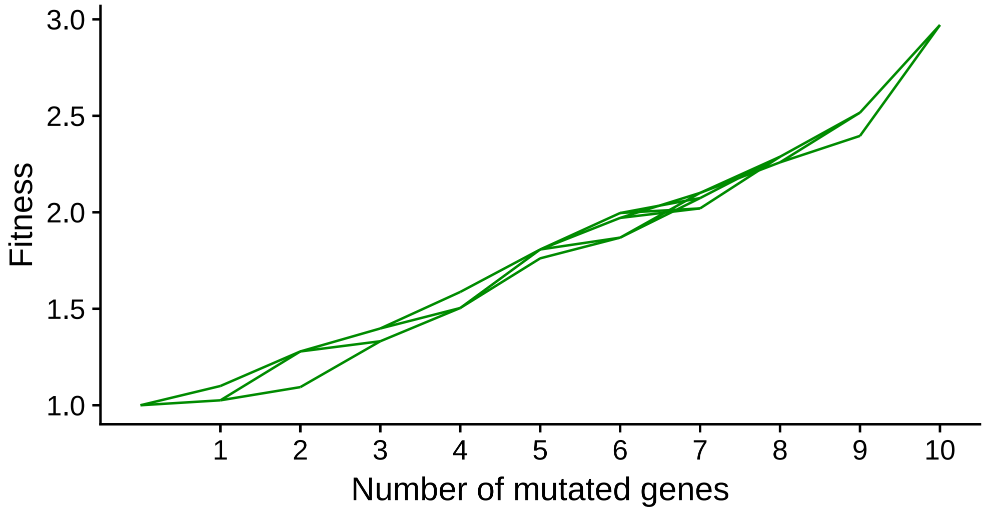

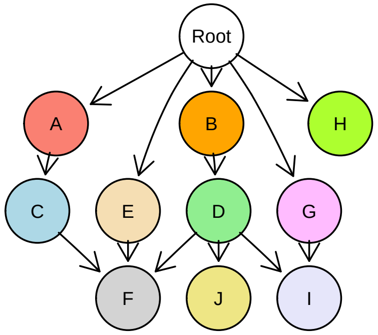

ID	PEUXKMJo1Ym7Cv4Z
acc genotypes	132
peaks no back	1
removed edges	0
prop rem edges	0

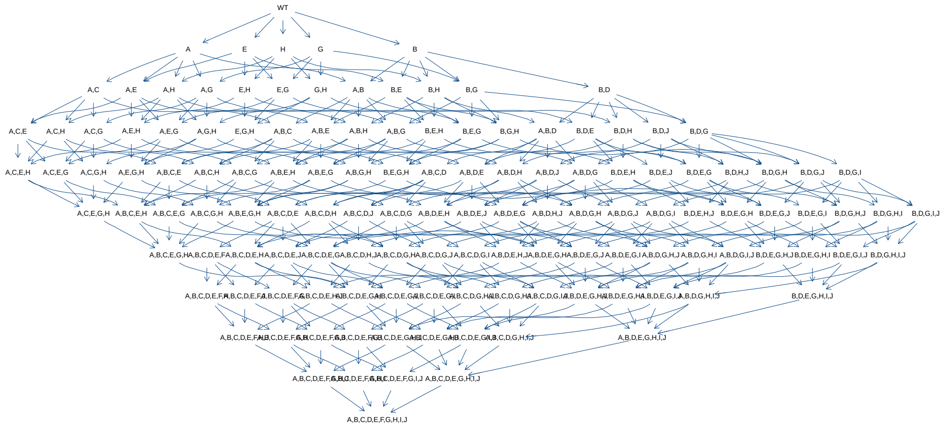

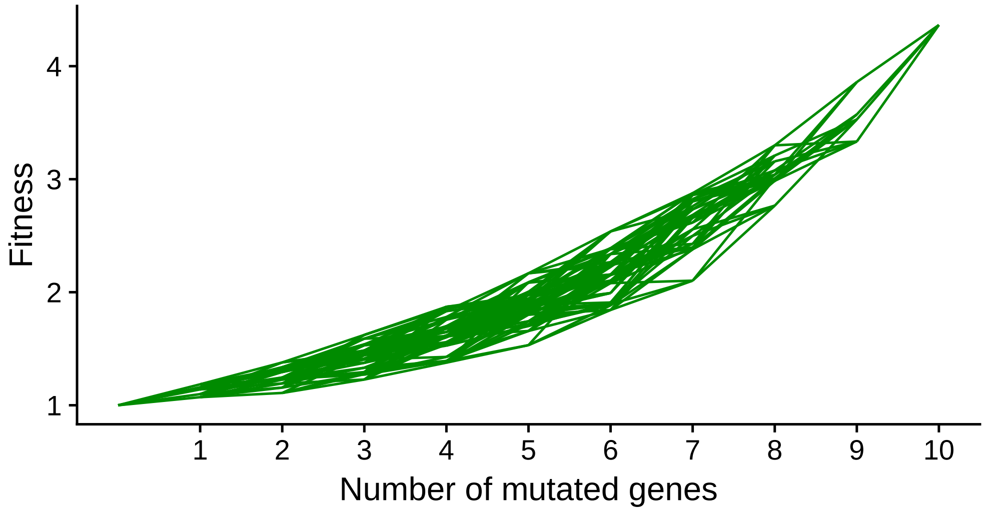

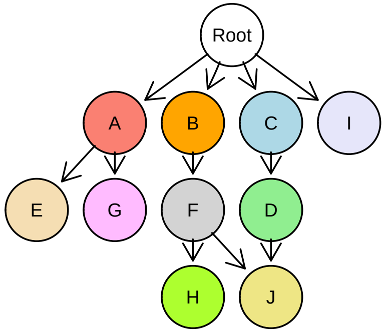

ID	PhaG1vE1MZA6aPS7
acc genotypes	140
peaks no back	1
removed edges	0
prop rem edges	0

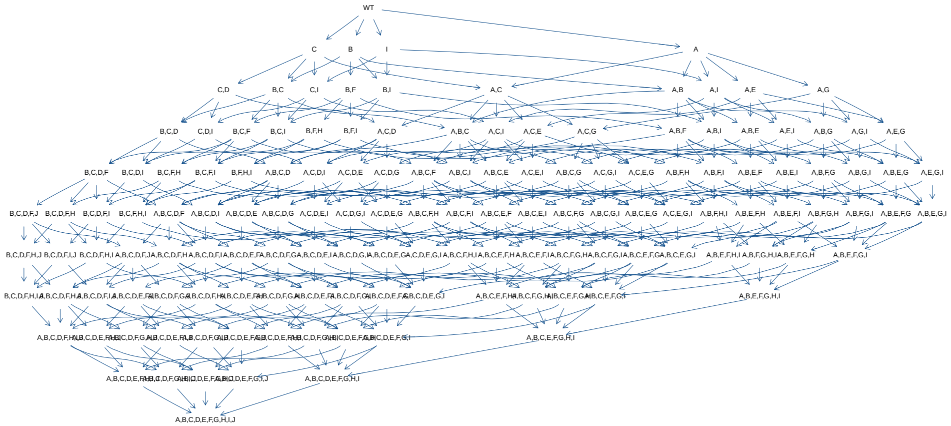

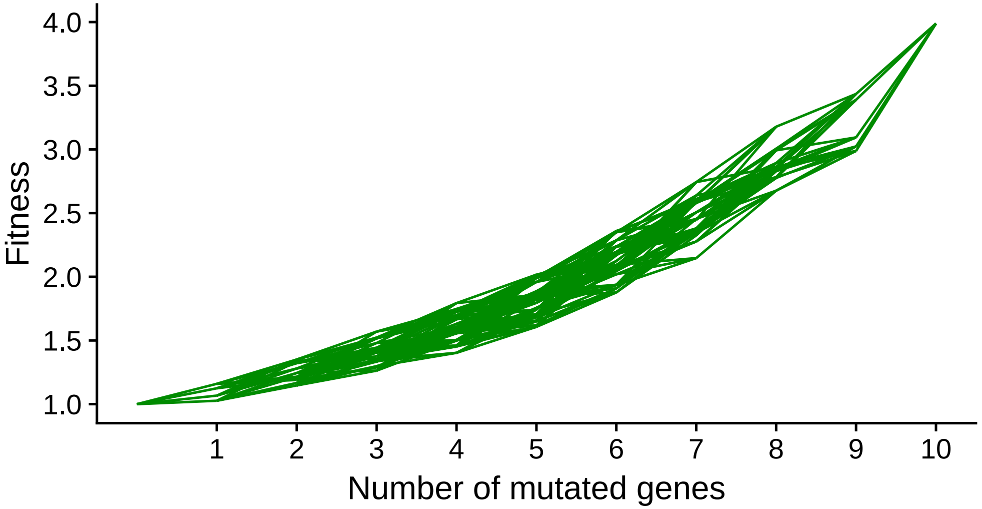

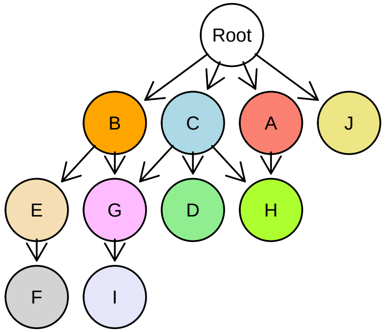

ID	Pl10ybVHbxKrShrRr
acc genotypes	136
peaks no back	1
removed edges	0
prop rem edges	0

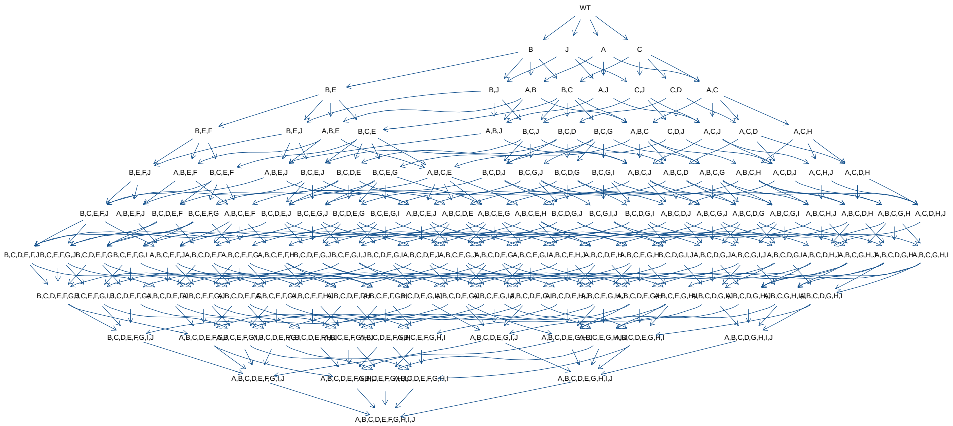

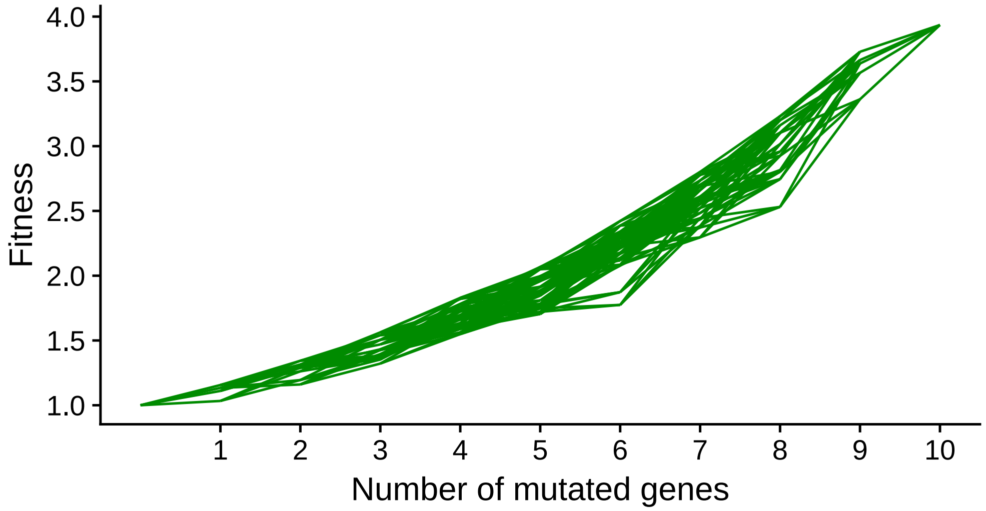

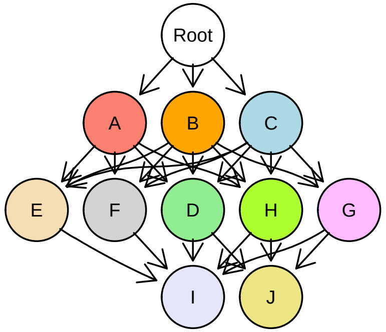

ID	pTNzMT9igvRrzlPi
acc genotypes	46
peaks no back	1
removed edges	0
prop rem edges	0

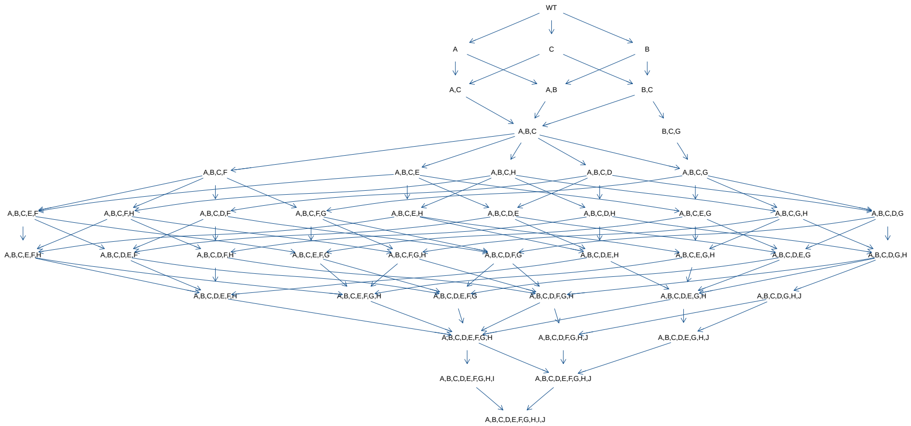

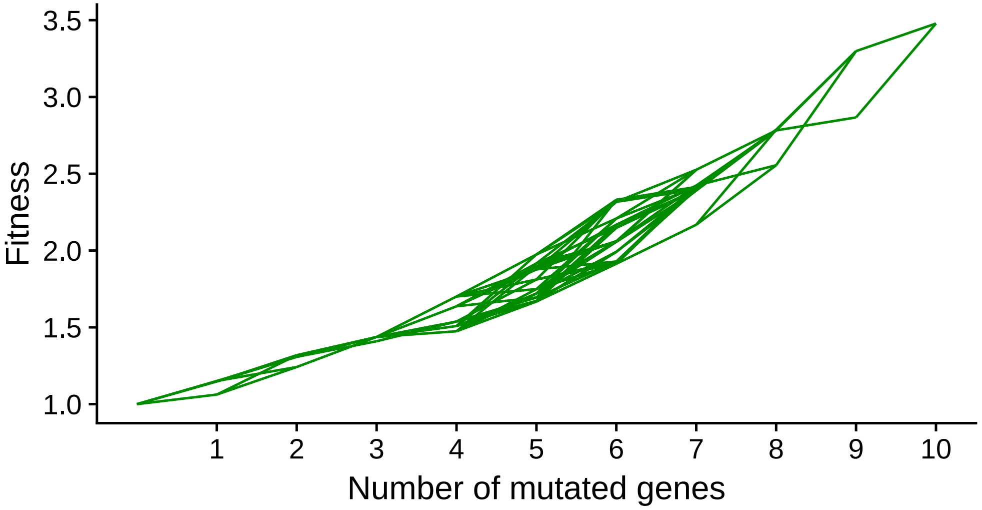

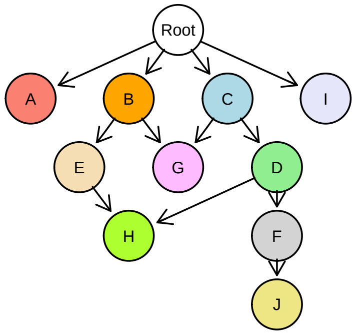

ID Pu5M3F61IUoT6zBD

acc genotypes 116

peaks no back 1

removed edges 0

prop rem edges 0

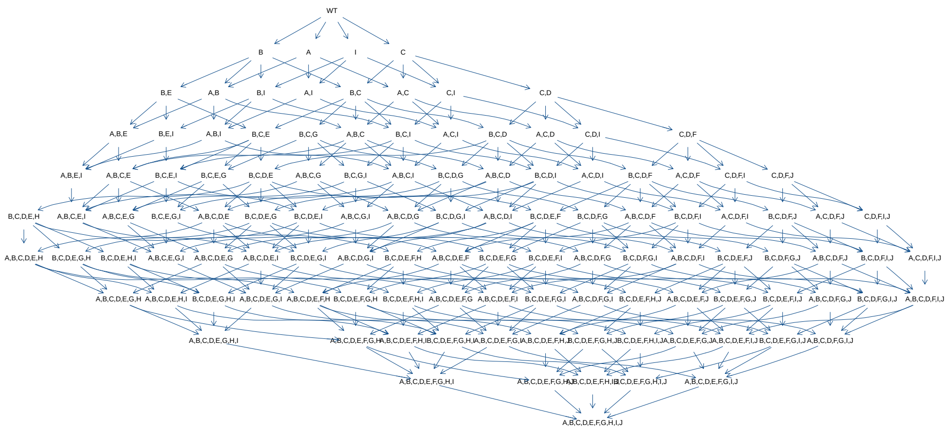

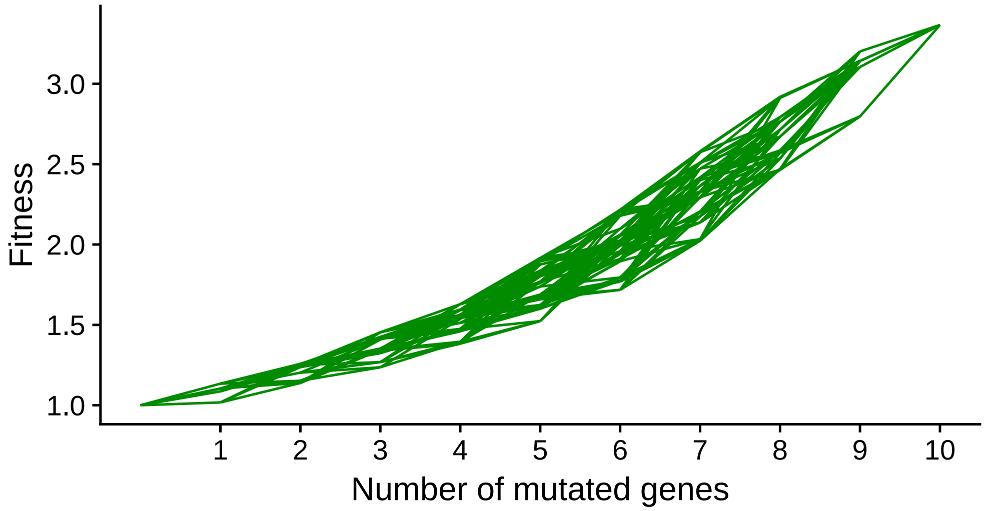

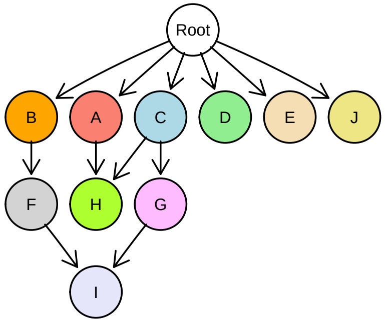

ID	PVfJTlNJUgHLfOjP
acc genotypes	216
peaks no back	1
removed edges	0
prop rem edges	0

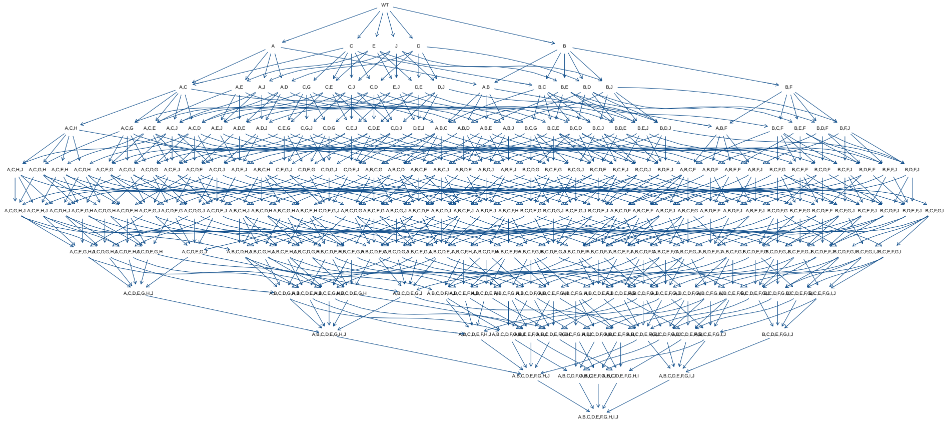

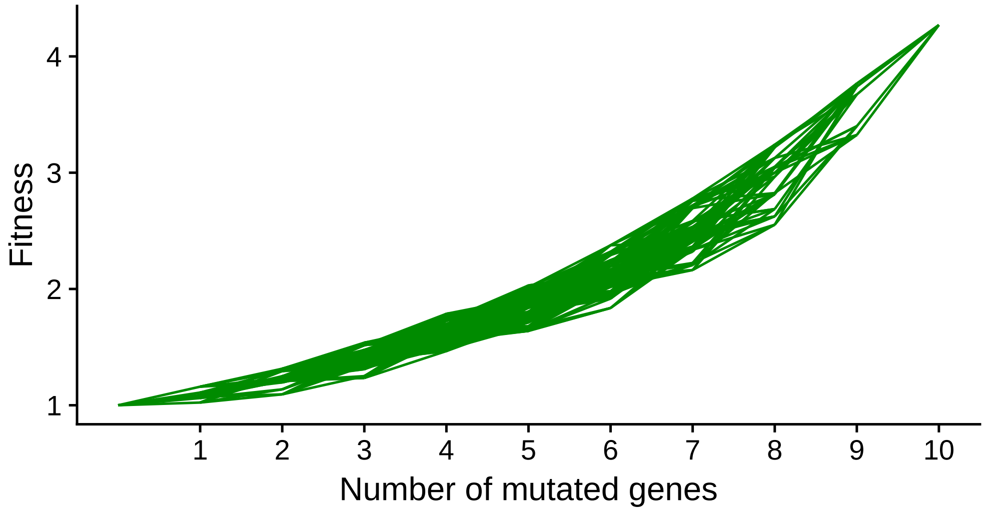

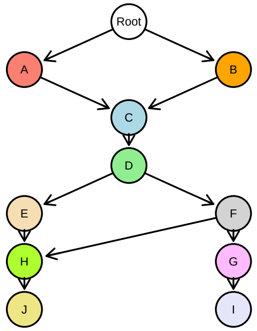

ID	pwyaau7tK7LZumPR
acc genotypes	19
peaks no back	1
removed edges	0
prop rem edges	0

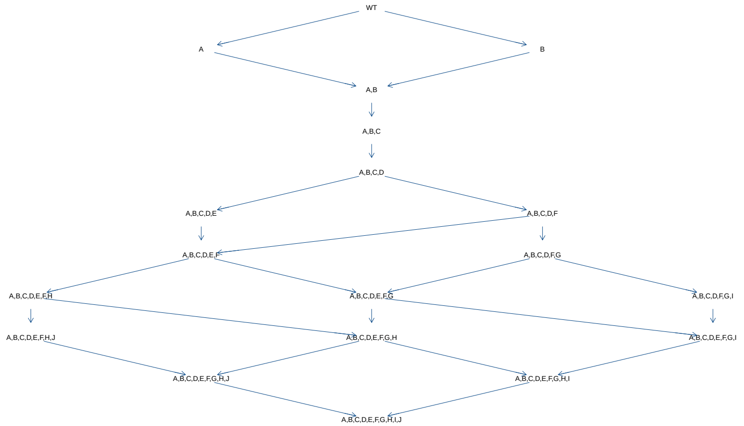

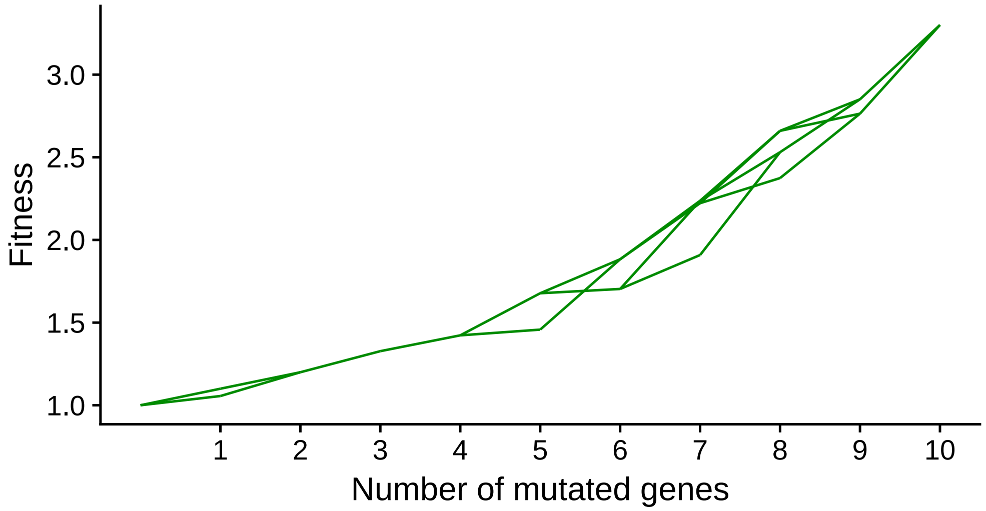

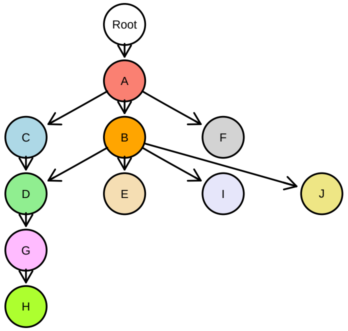

ID	QePxqp7BQd10uZ5Ko
acc genotypes	85
peaks no back	1
removed edges	0
prop rem edges	0

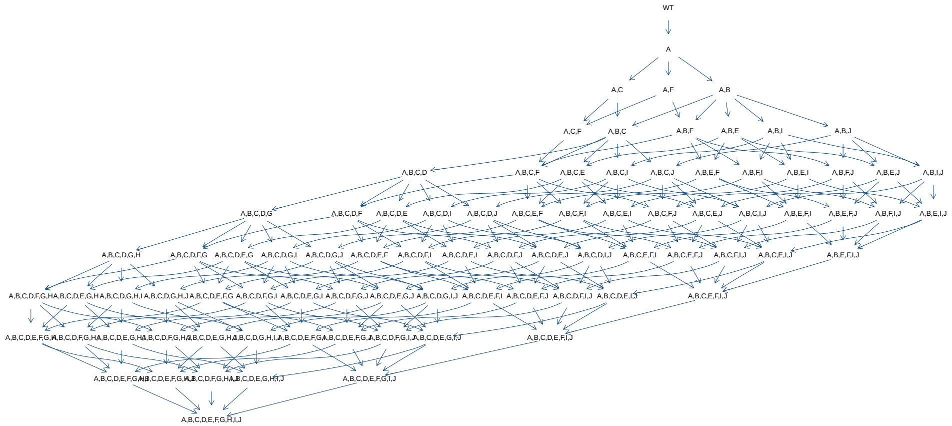

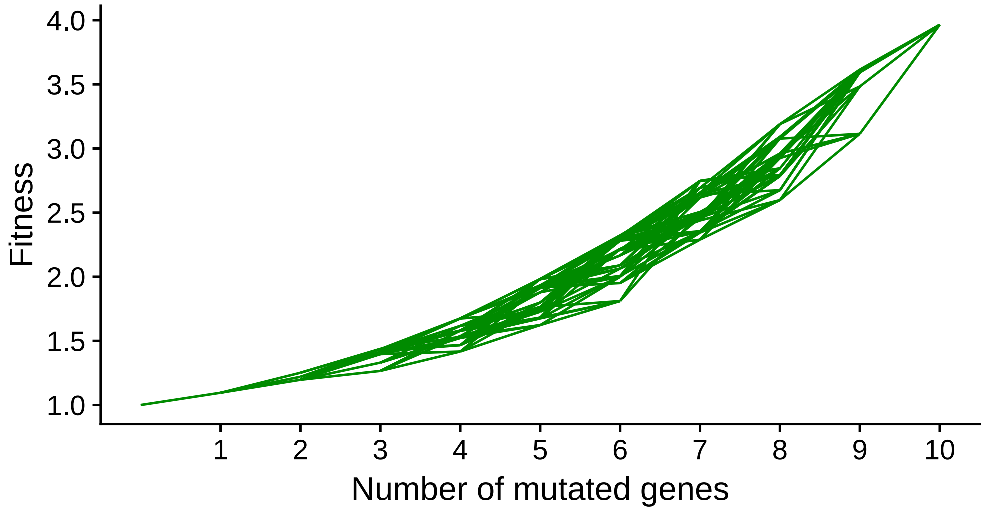

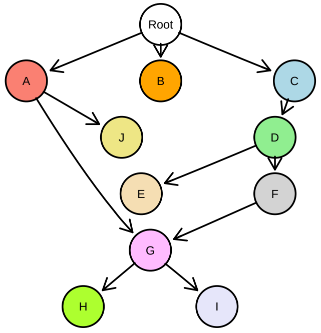

ID	QGRVlBiM3bkC395C
acc genotypes	68
peaks no back	1
removed edges	0
prop rem edges	0

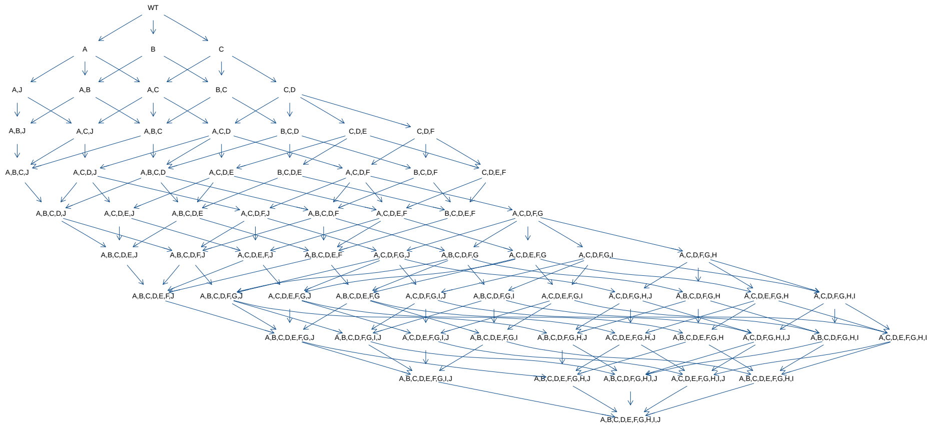

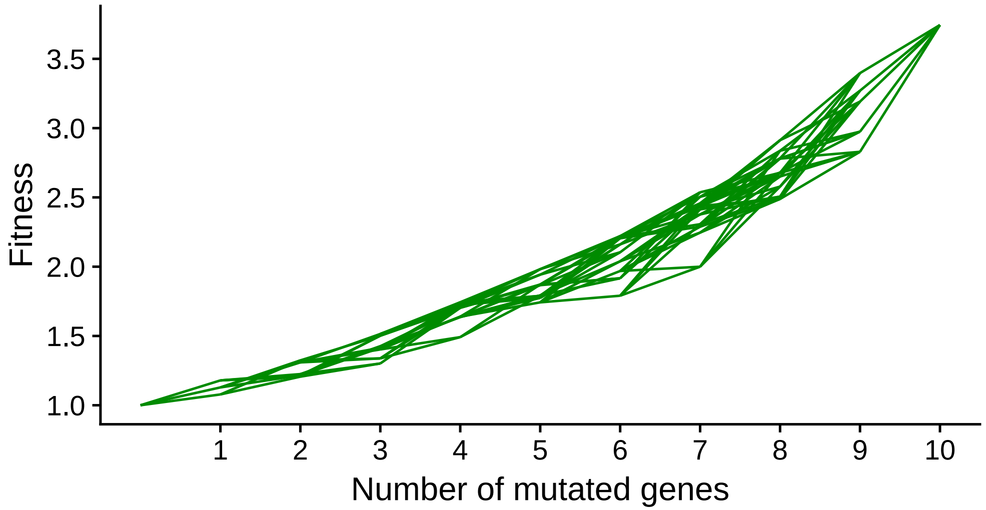

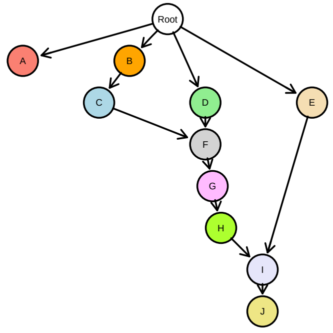

ID	Qt7h8PhrOcDVjbUJ
acc genotypes	40
peaks no back	1
removed edges	0
prop rem edges	0

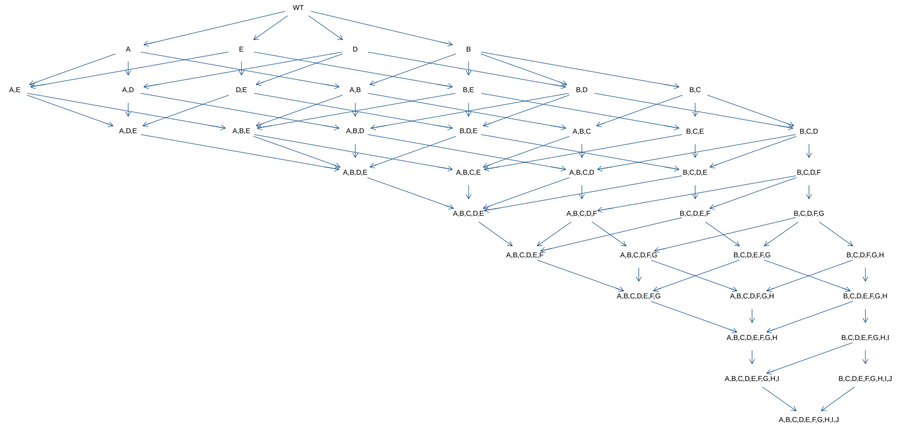

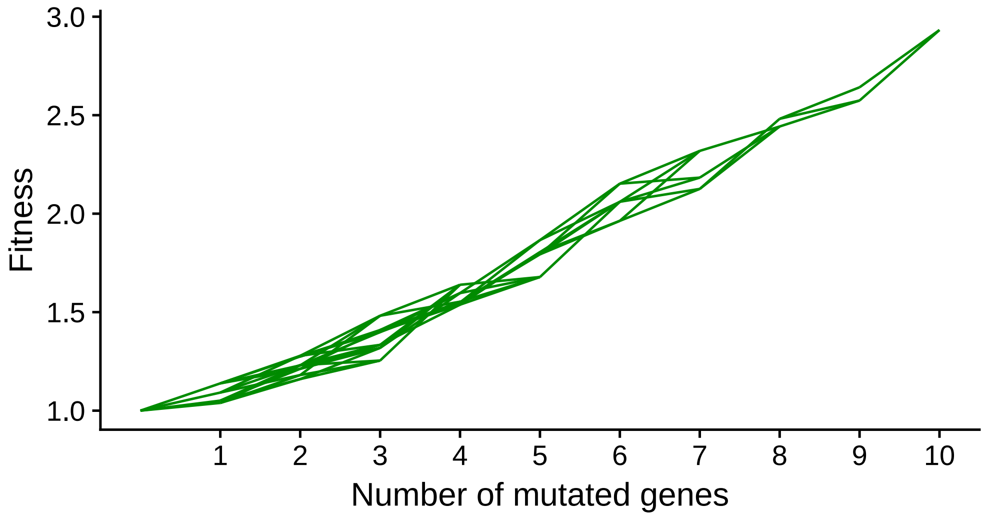

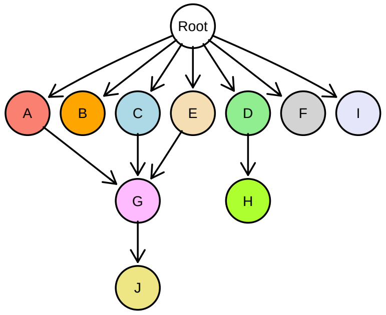

ID	Rgqi10zX3omumQ9oh
acc genotypes	240
peaks no back	1
removed edges	0
prop rem edges	0

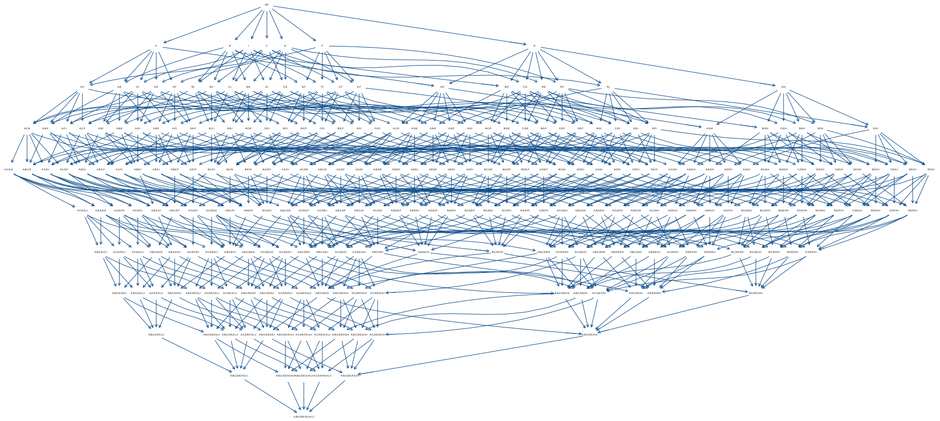

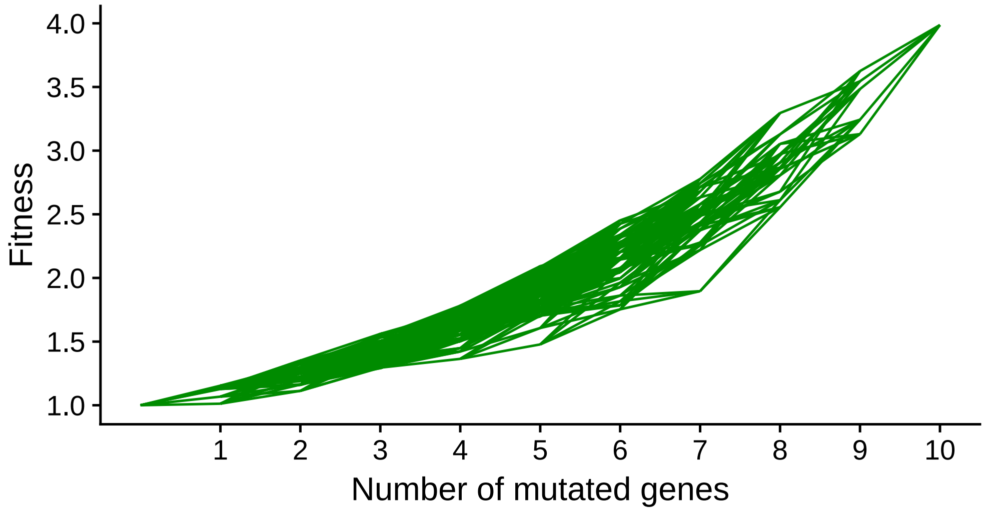

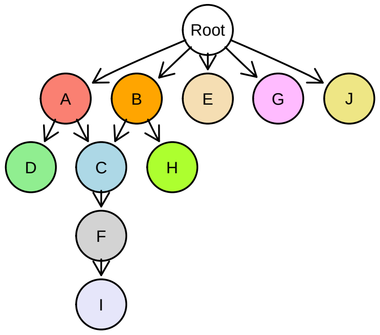

ID	RHdC3iuVYPR6IBD10
acc genotypes	168
peaks no back	1
removed edges	0
prop rem edges	0

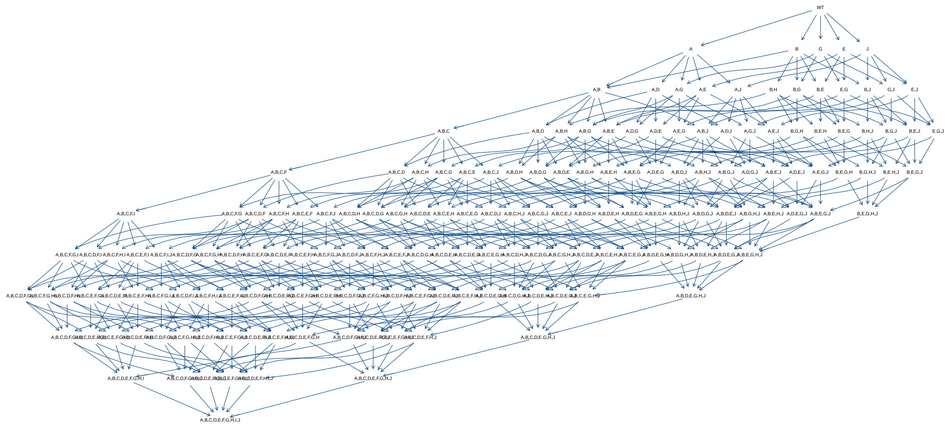

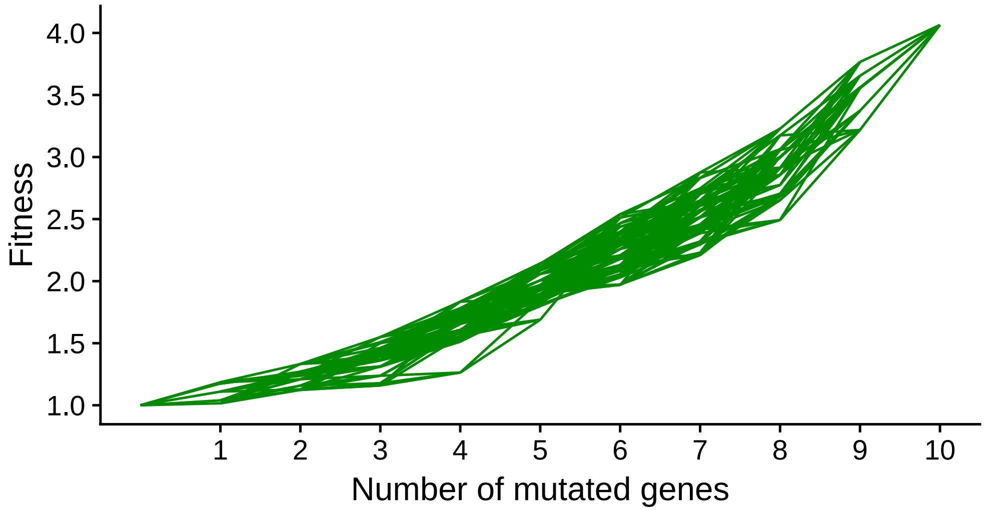

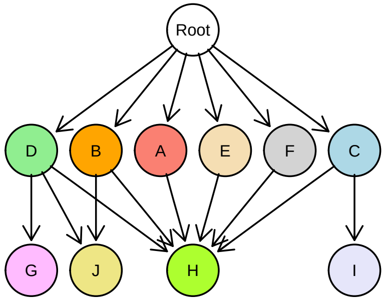

ID	RM2aZBkyRpQtZvKY
acc genotypes	200
peaks no back	1
removed edges	0
prop rem edges	0

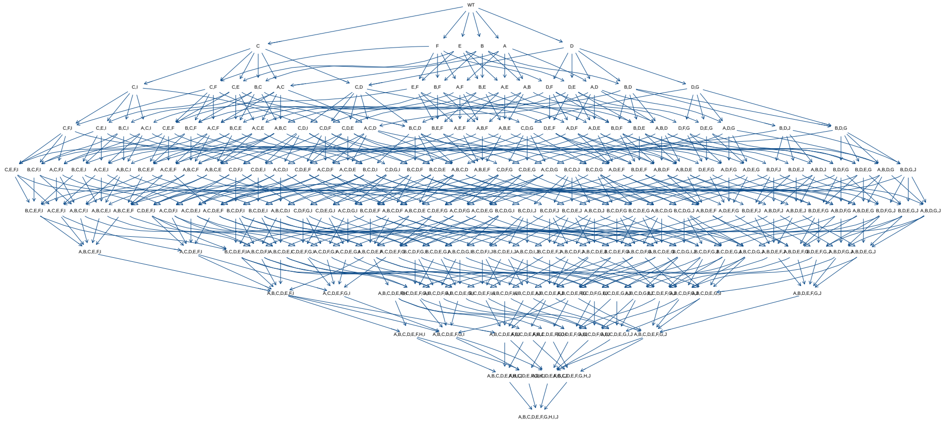

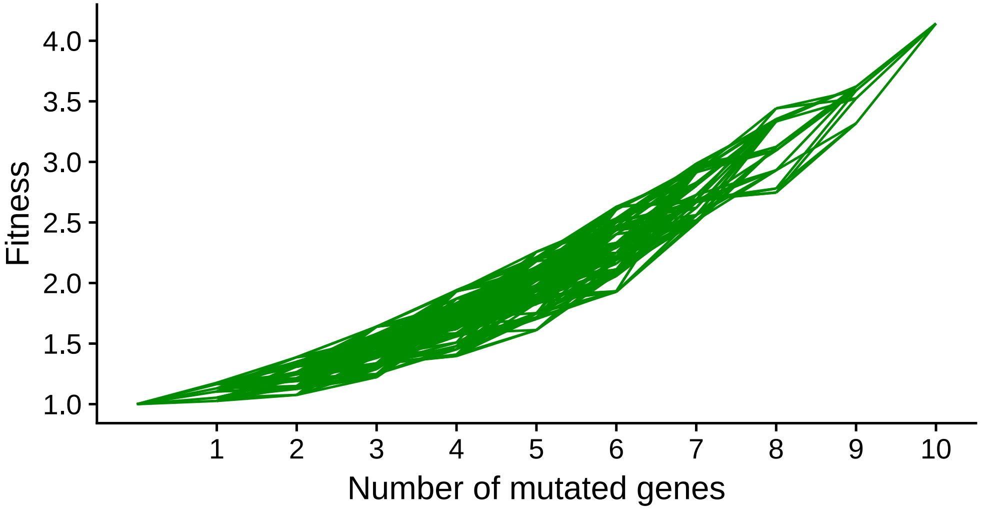

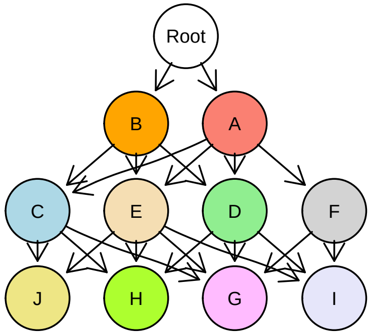

ID	RNqpQsaM6eeS7RWA
acc genotypes	41
peaks no back	1
removed edges	0
prop rem edges	0

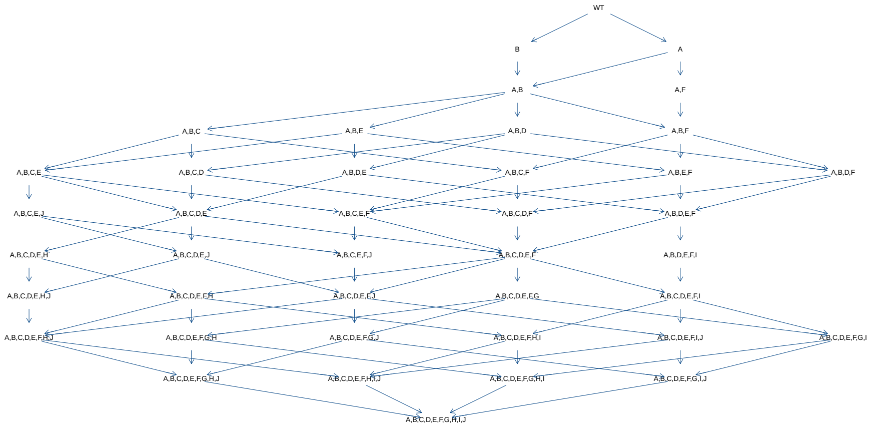

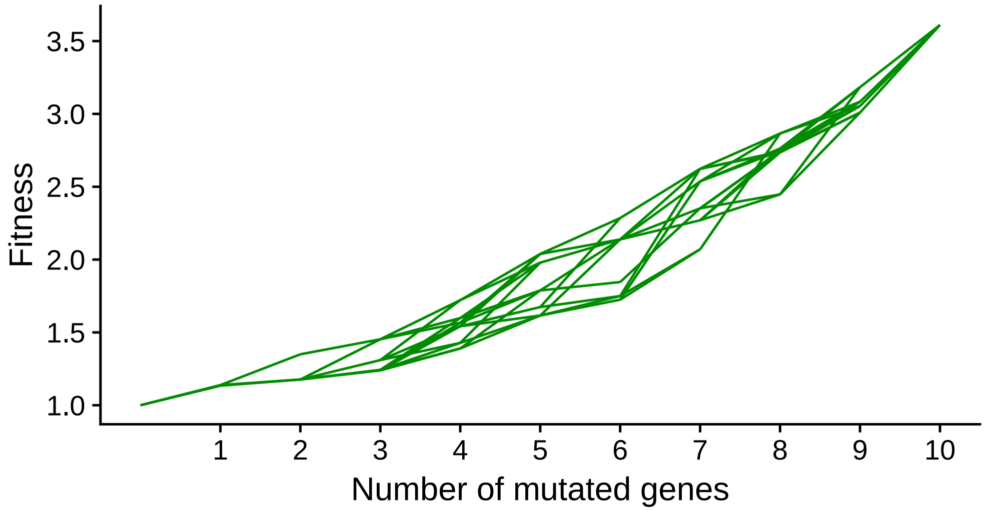

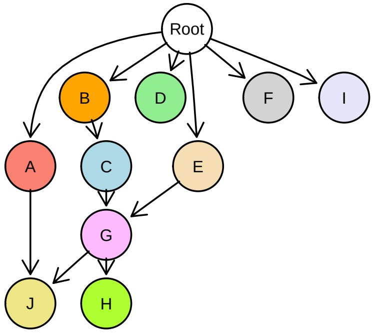

ID	rRVesv4UlXP10d31k
acc genotypes	144
peaks no back	1
removed edges	0
prop rem edges	0

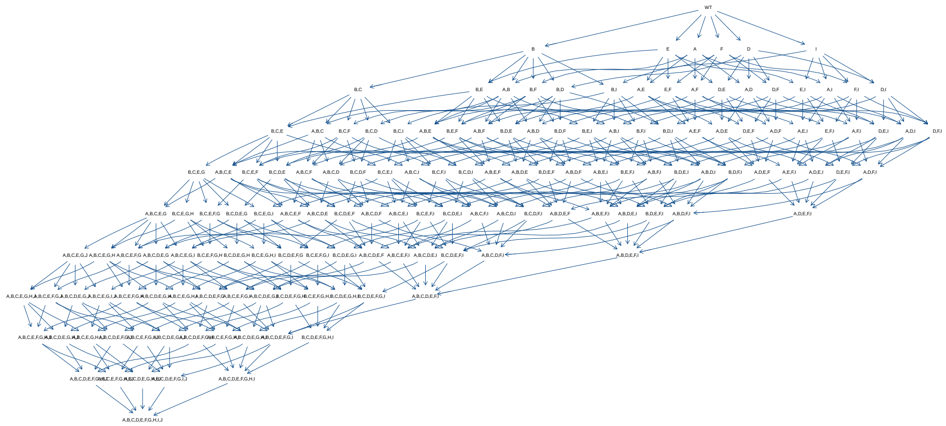

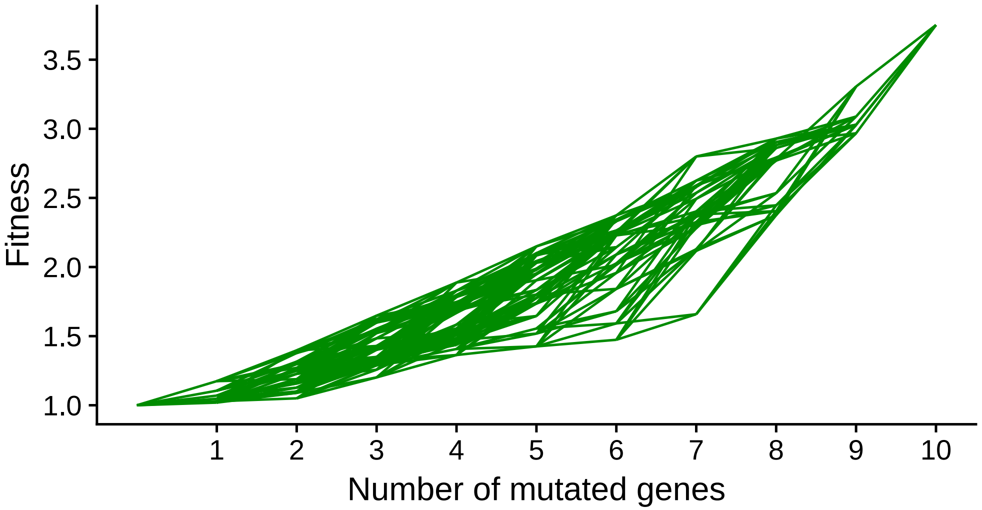

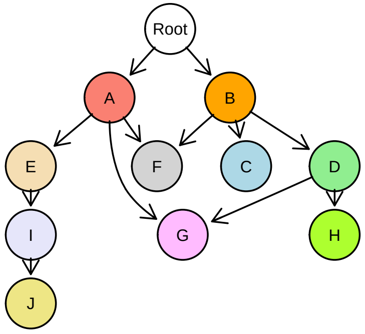

ID rsk7tIpjfEVKOLgb
acc genotypes 91
peaks no back 1
removed edges 0
prop rem edges 0

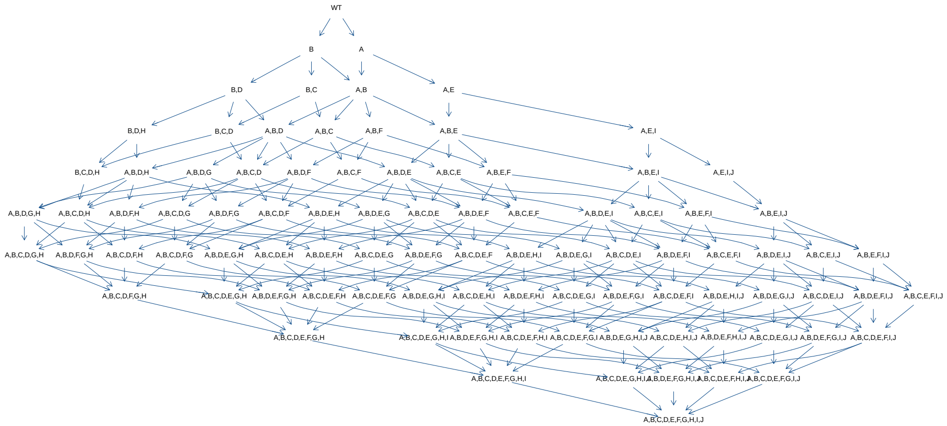

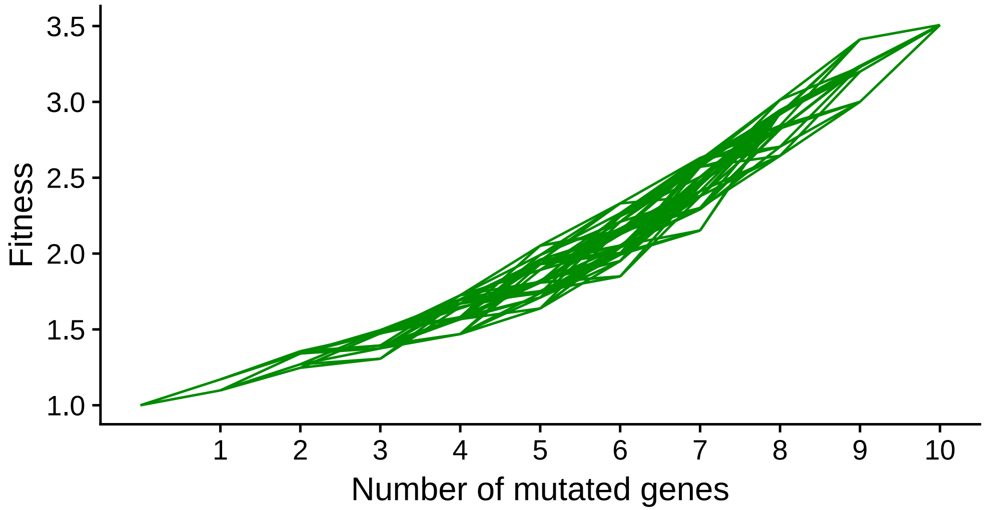

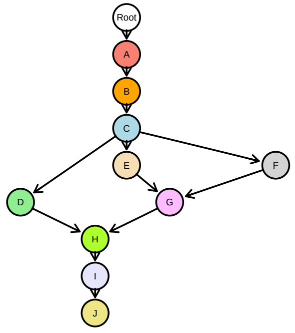

ID	rXEM4KMif4W386fC
acc genotypes	16
peaks no back	1
removed edges	0
prop rem edges	0

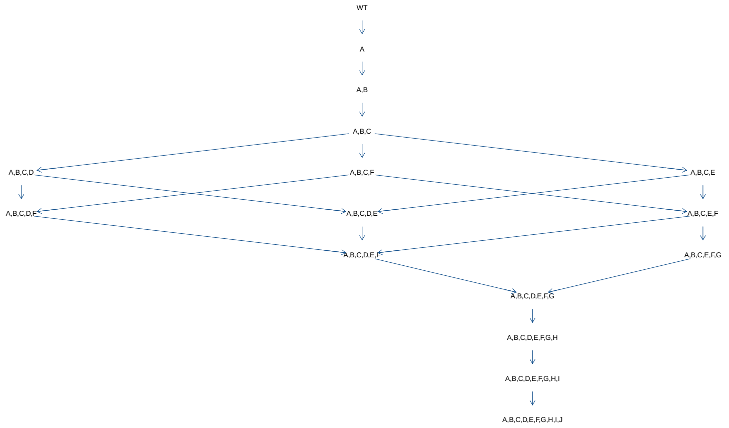

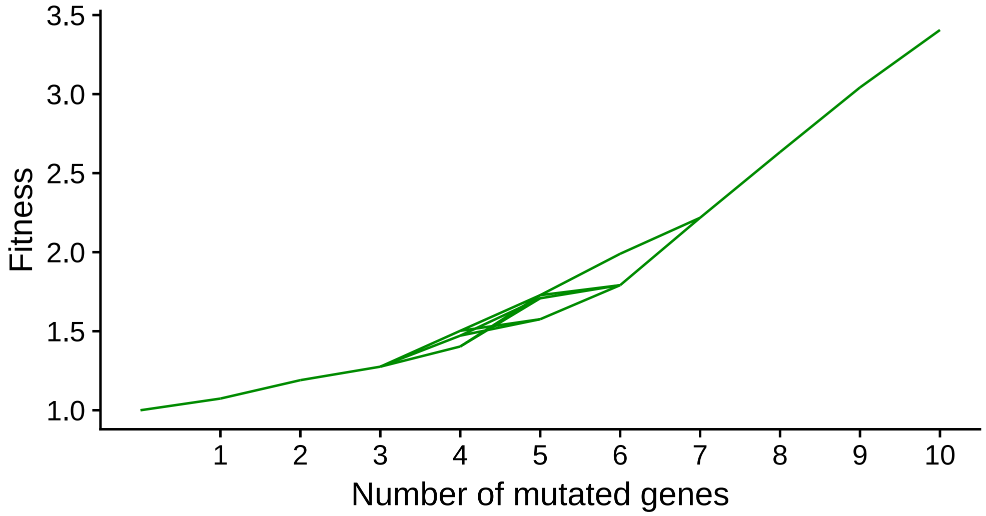

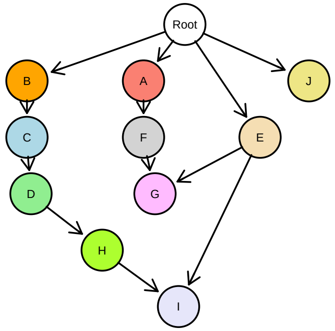

ID	rZYDGyZC7mmbrvG7
acc genotypes	78
peaks no back	1
removed edges	0
prop rem edges	0

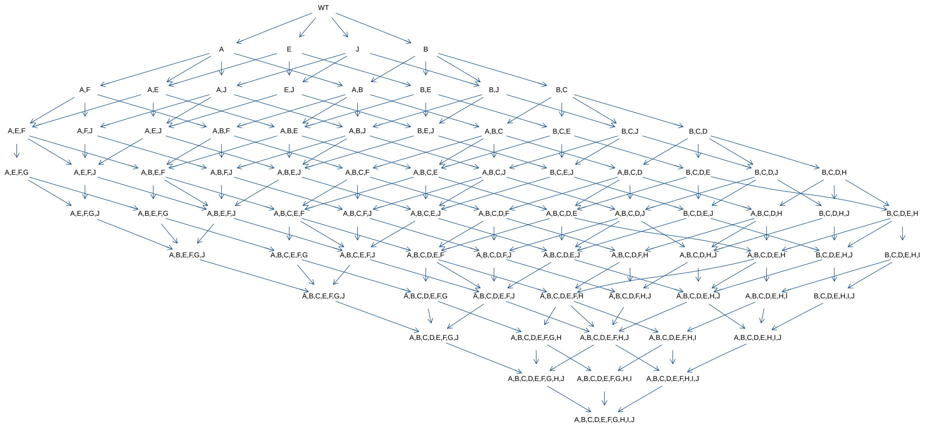

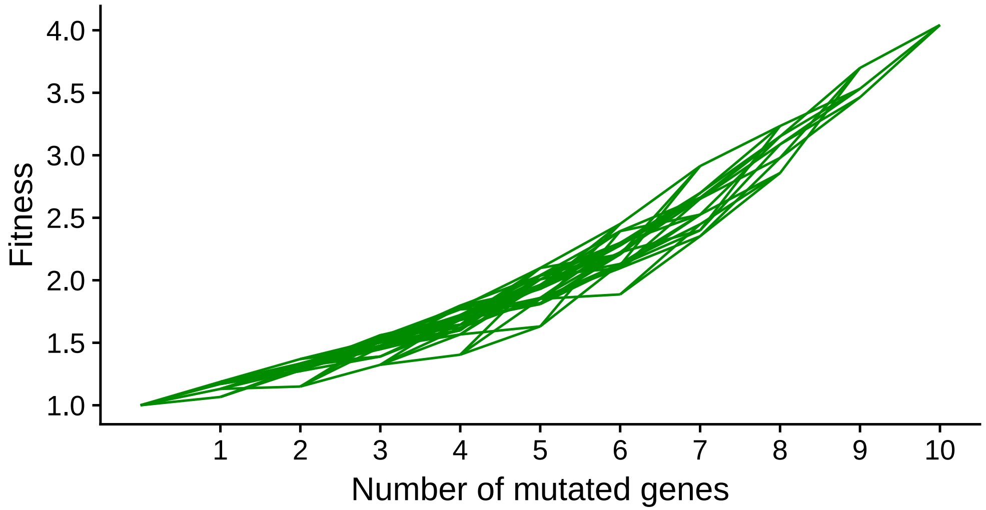

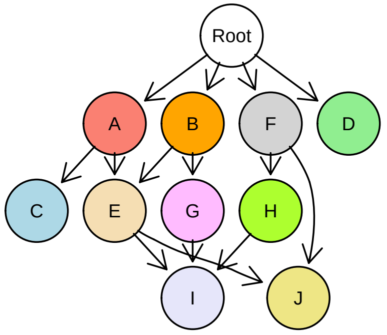

ID	sH934jpdql10yYeSf
acc genotypes	102
peaks no back	1
removed edges	0
prop rem edges	0

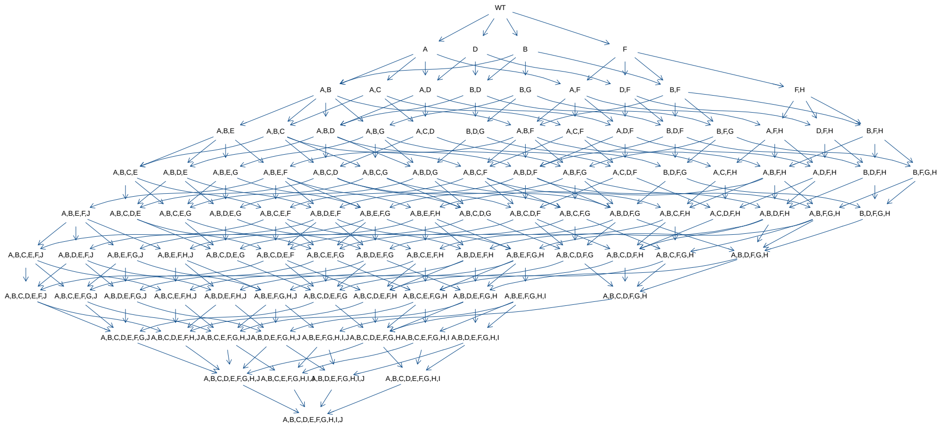

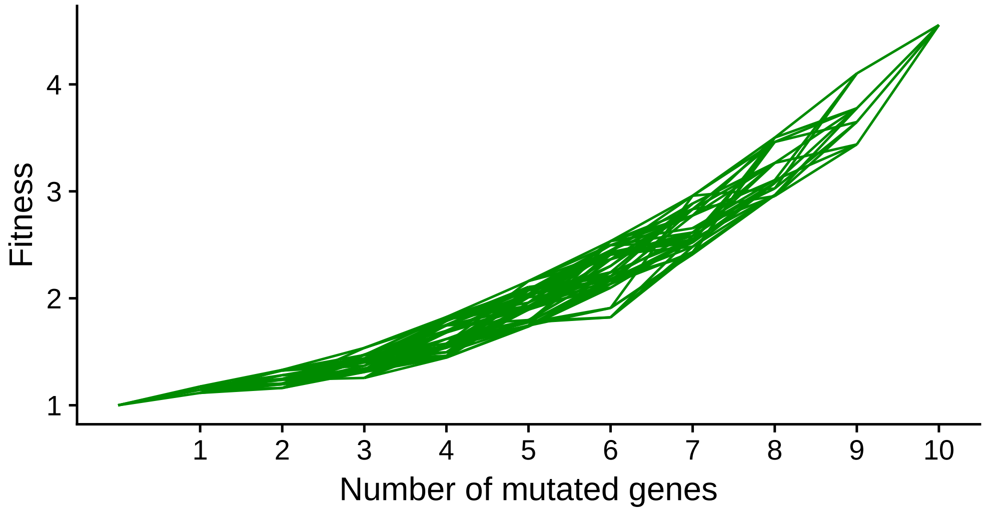

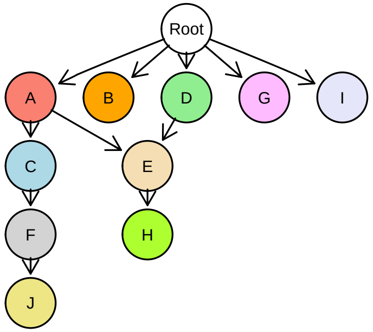

ID sisQewhG7QPpthsD
acc genotypes 144
peaks no back 1
removed edges 0
prop rem edges 0

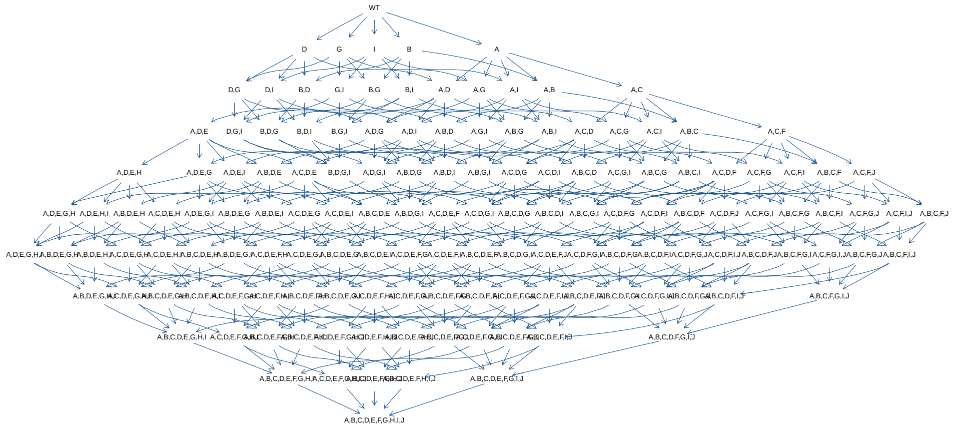

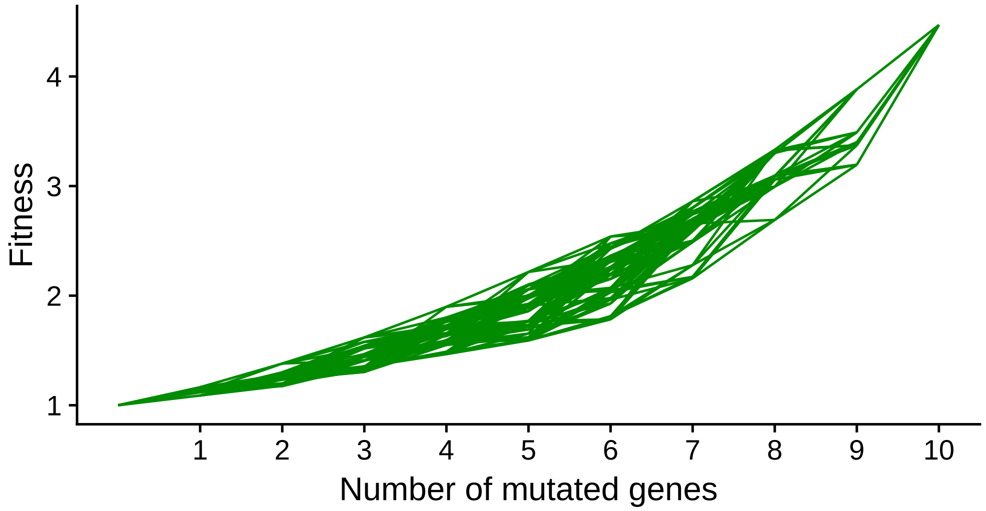

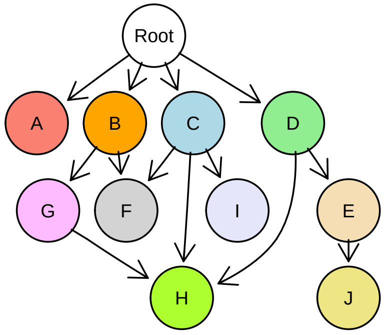

ID	sNgShm95dlSfotH4
acc genotypes	128
peaks no back	1
removed edges	0
prop rem edges	0

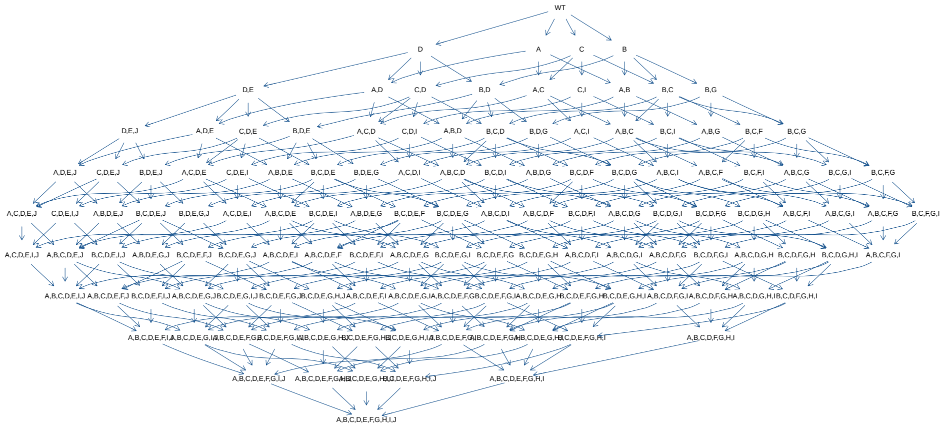

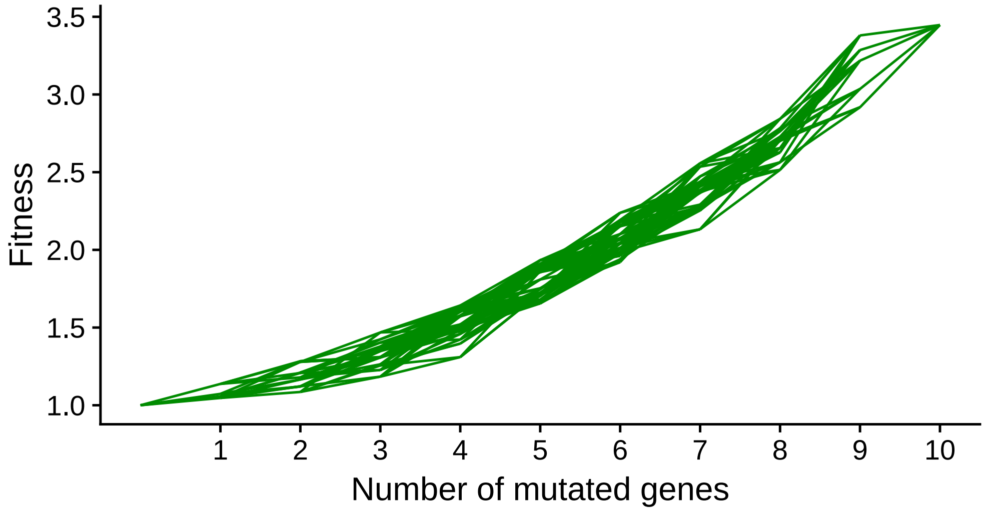

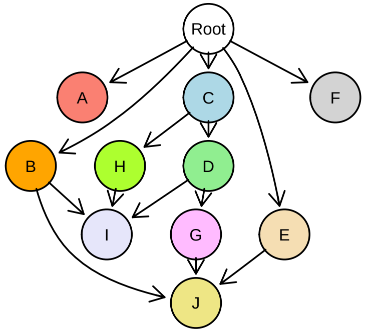

ID	sQiN7joZyxfMKsXn
acc genotypes	140
peaks no back	1
removed edges	0
prop rem edges	0

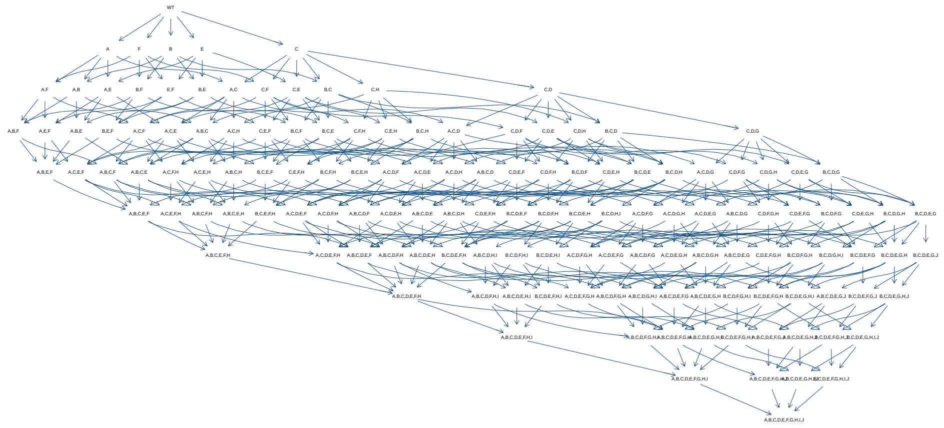

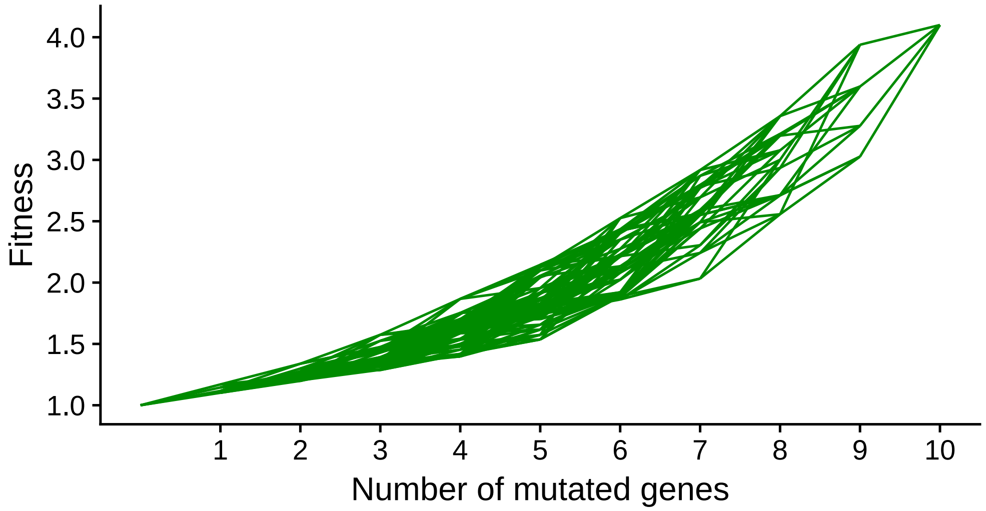

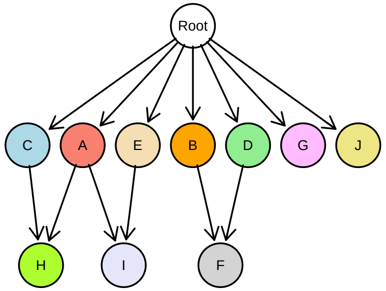

ID	sUfgZw6OOPt5SCir
acc genotypes	260
peaks no back	1
removed edges	0
prop rem edges	0

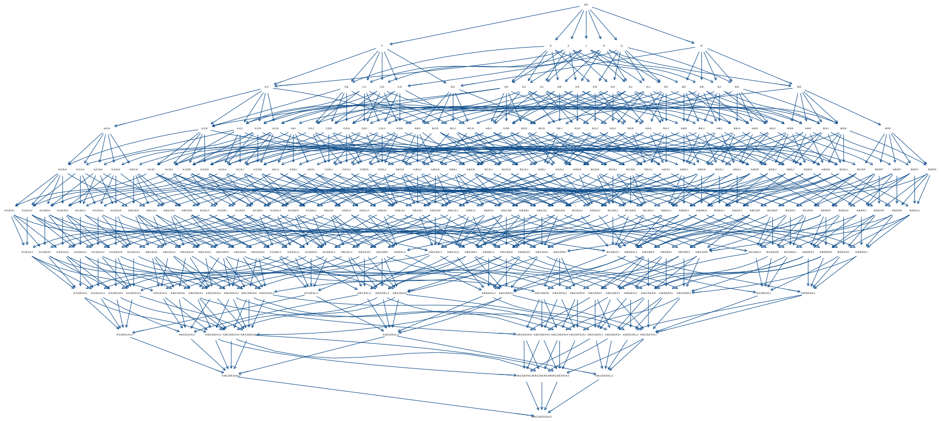

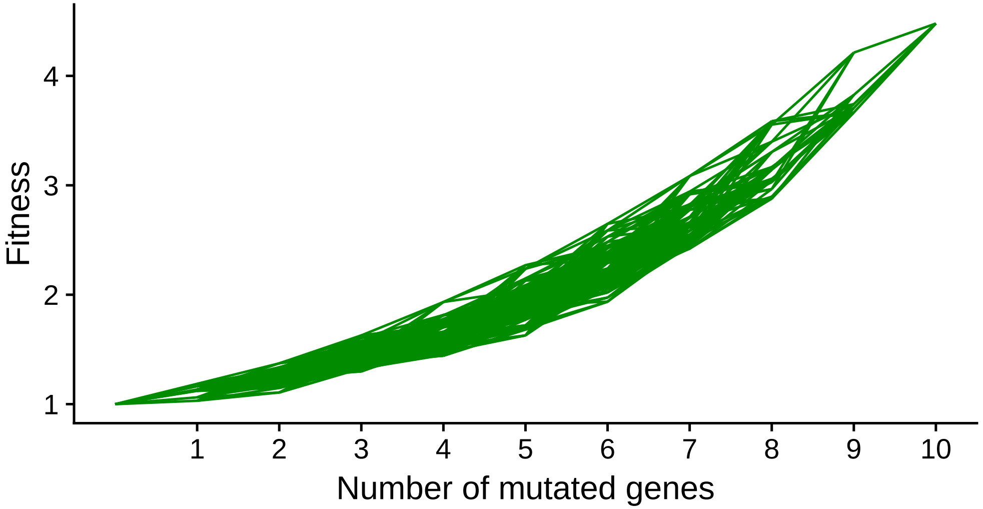

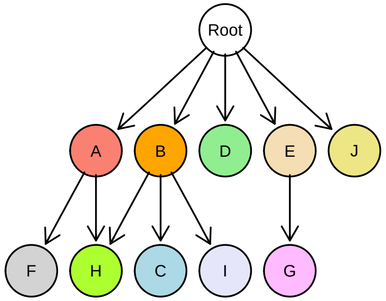

ID	svUNVWKM4yHG4ZG6
acc genotypes	276
peaks no back	1
removed edges	0
prop rem edges	0

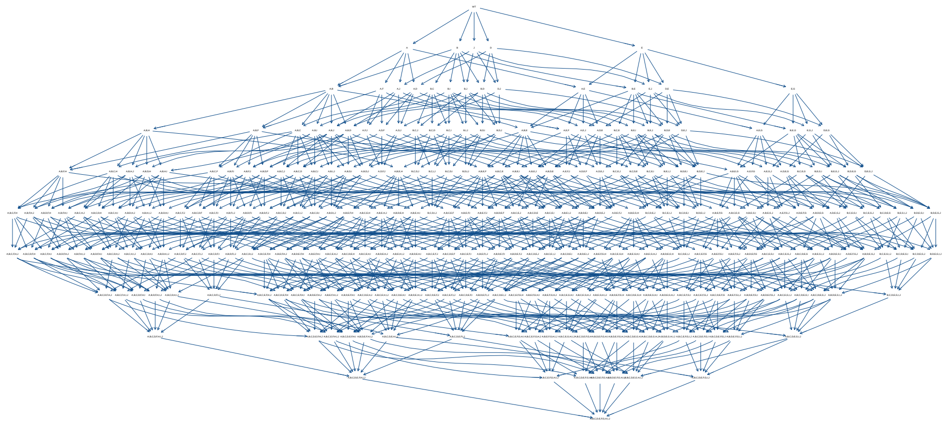

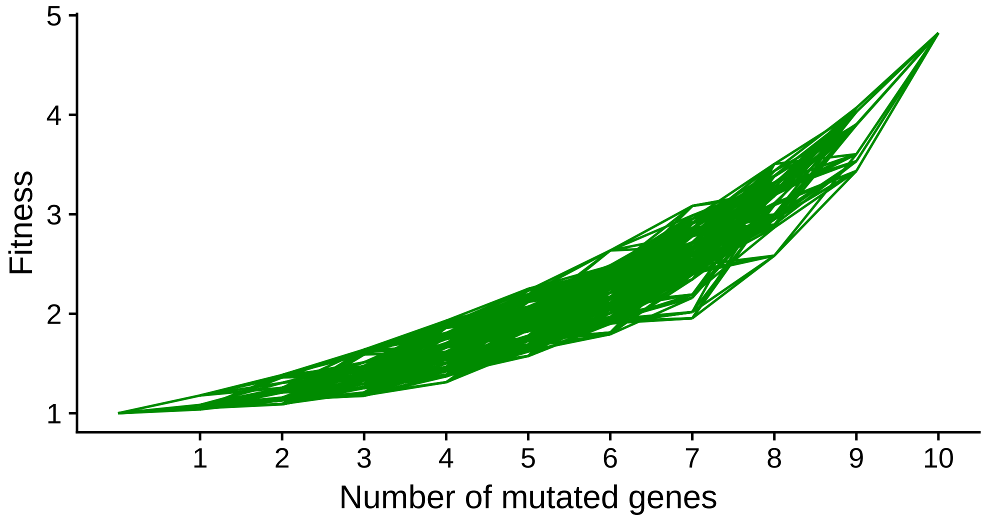

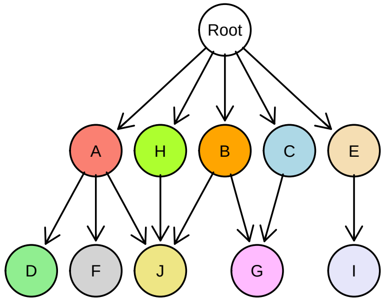

ID	tbhoAhVYmdTD8tmZ
acc genotypes	186
peaks no back	1
removed edges	0
prop rem edges	0

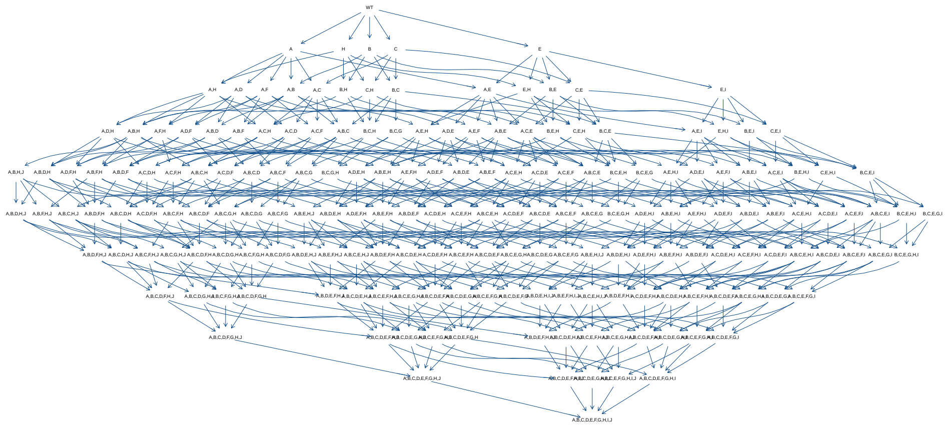

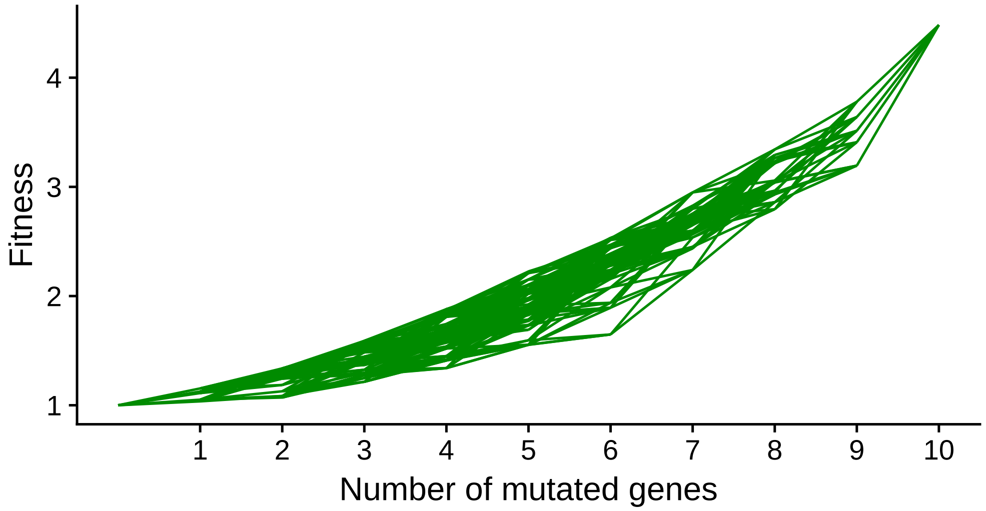

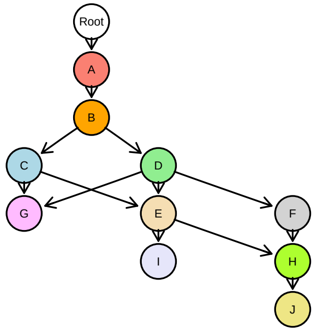

ID	TFxs2KPm5WO9Abi10
acc genotypes	26
peaks no back	1
removed edges	0
prop rem edges	0

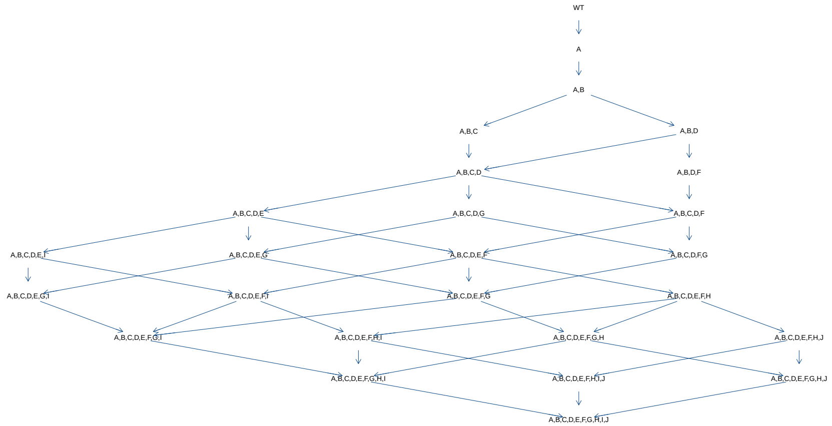

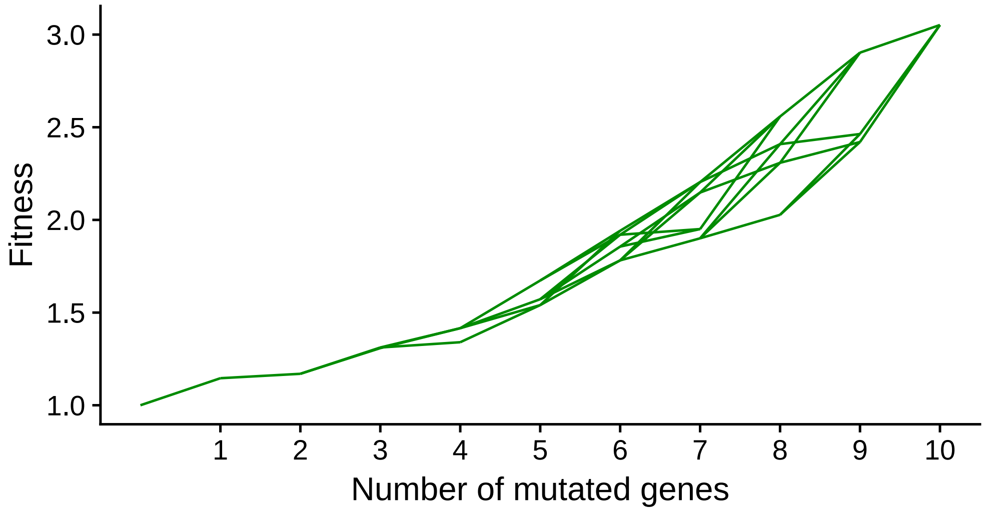

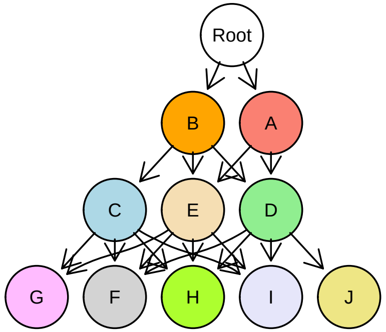

ID	TH4wayDxtpPQJ3zV
acc genotypes	47
peaks no back	1
removed edges	0
prop rem edges	0

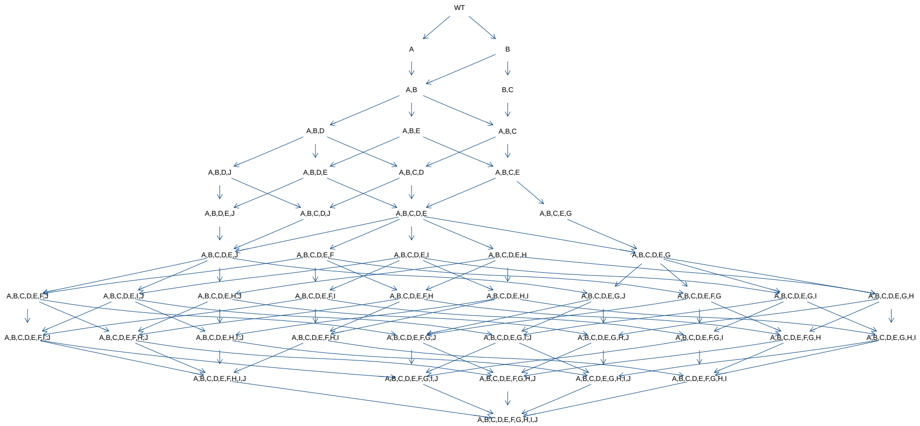

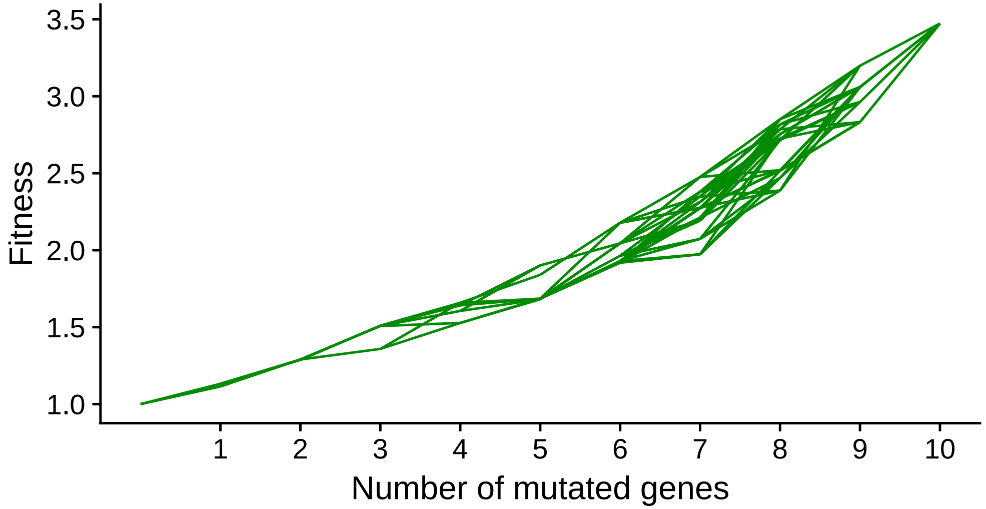

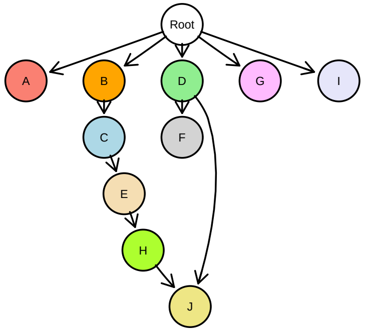

ID	tl7D6tClLmHkt9fG
acc genotypes	136
peaks no back	1
removed edges	0
prop rem edges	0

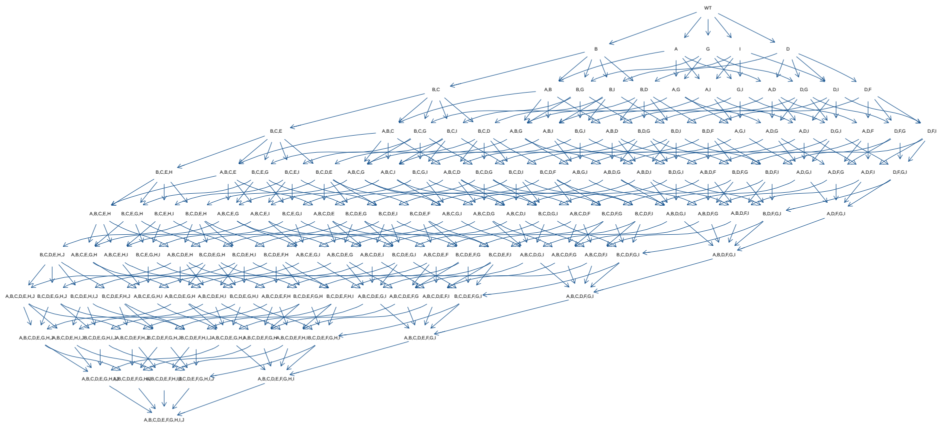

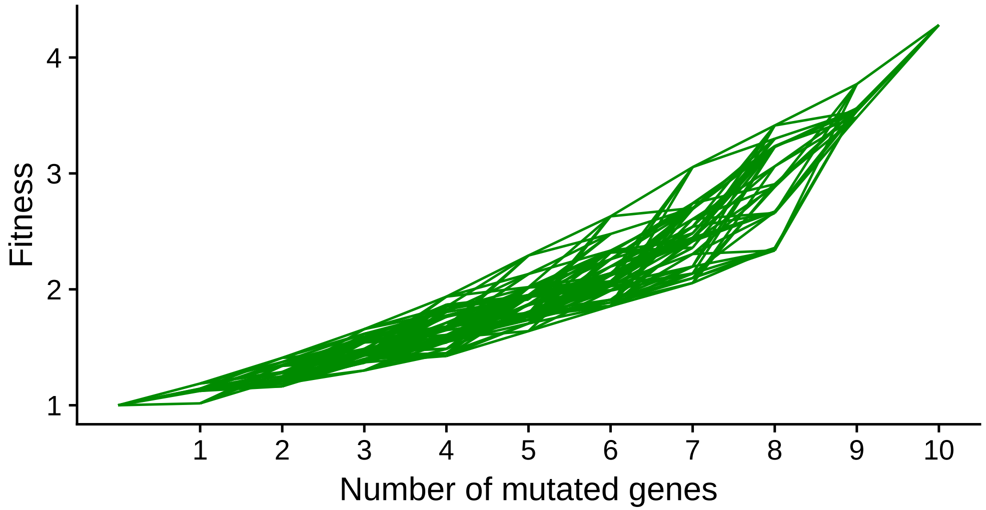

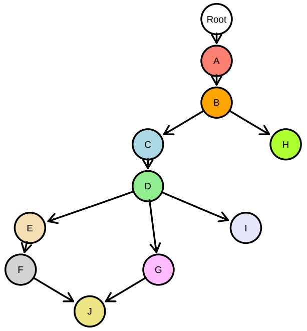

ID	tq7jgfG17AM4lqGW
acc genotypes	34
peaks no back	1
removed edges	0
prop rem edges	0

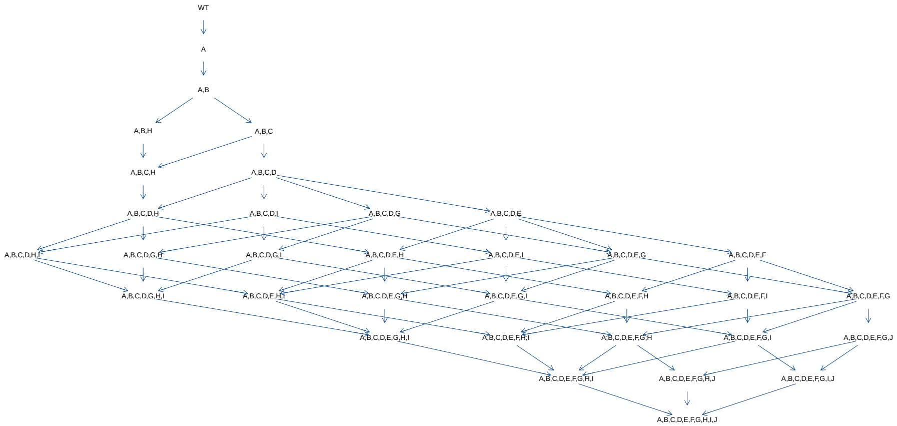

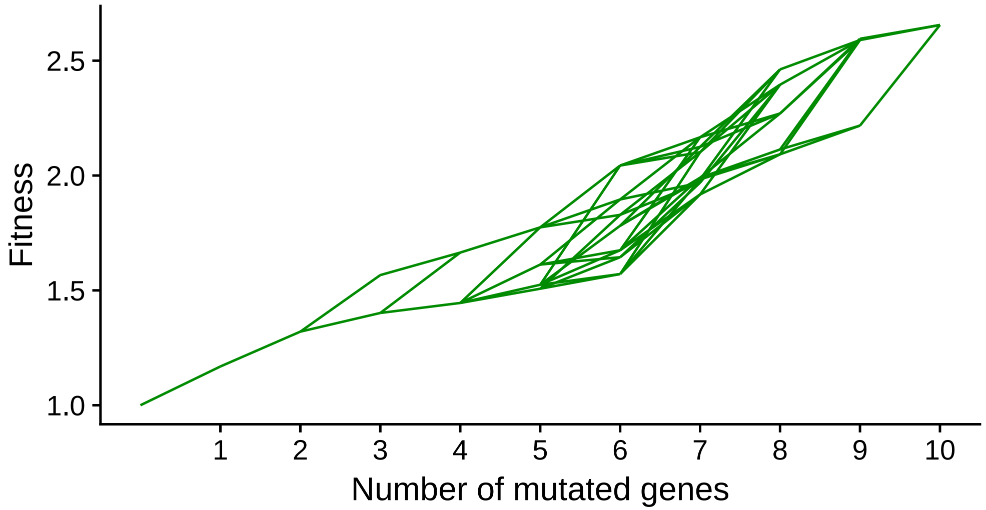

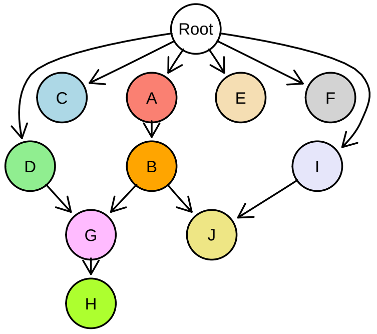

ID	TVJsMGLUm85XS67g
acc genotypes	160
peaks no back	1
removed edges	0
prop rem edges	0

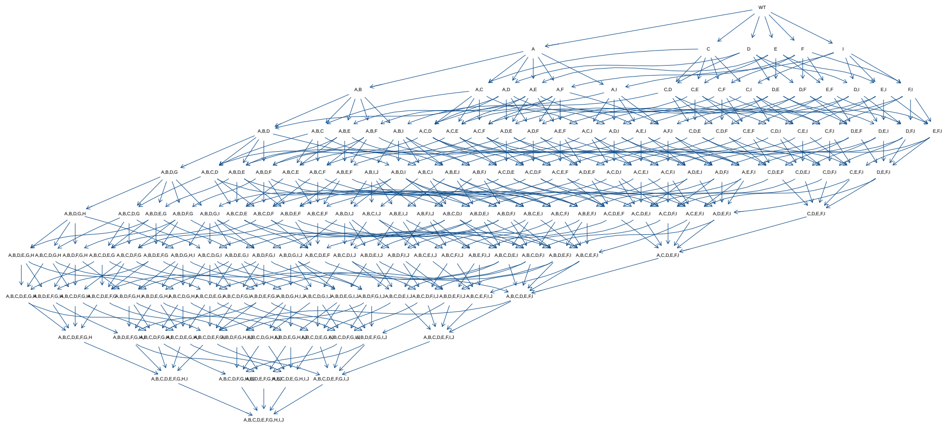

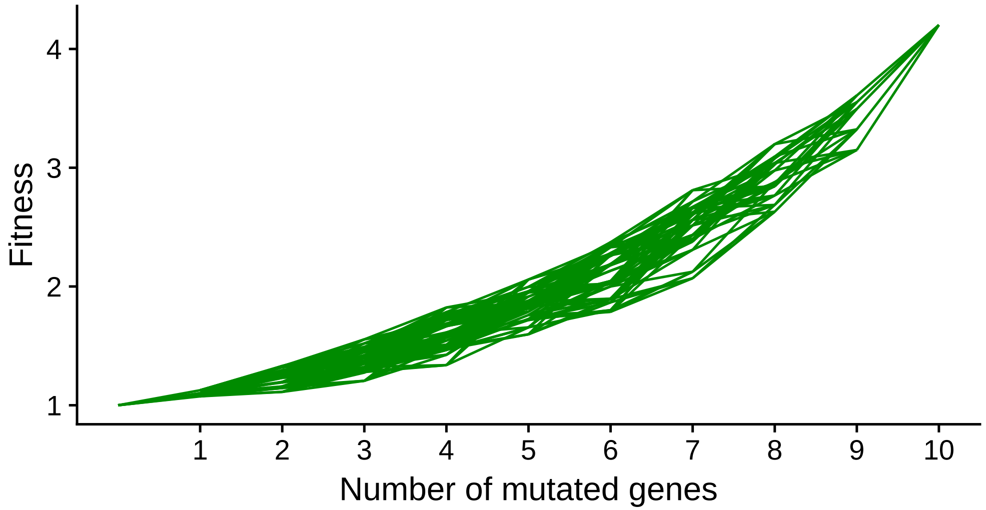

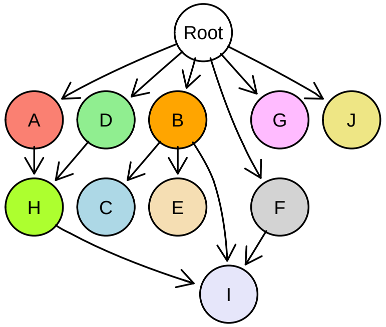

ID	TXSYGzI7tgHRqH7B
acc genotypes	216
peaks no back	1
removed edges	0
prop rem edges	0

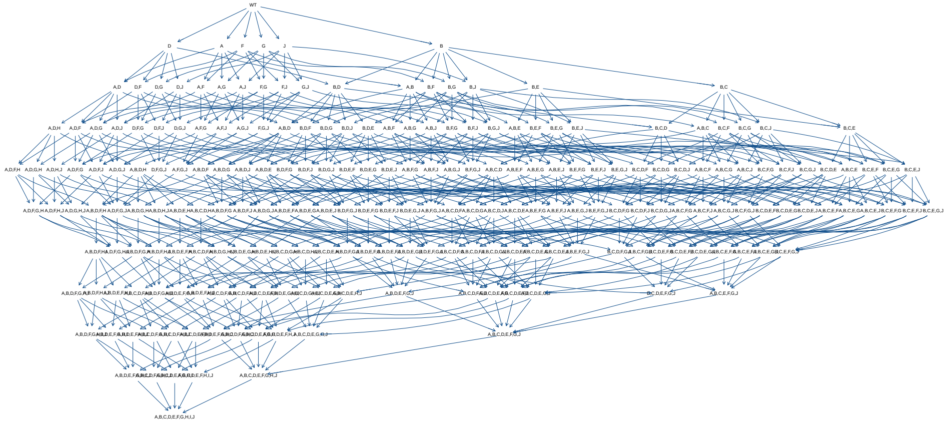

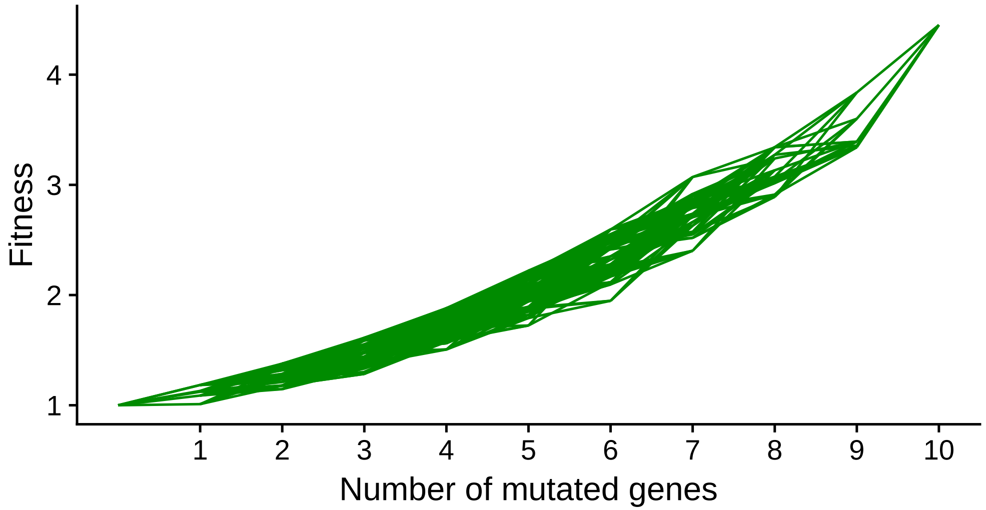

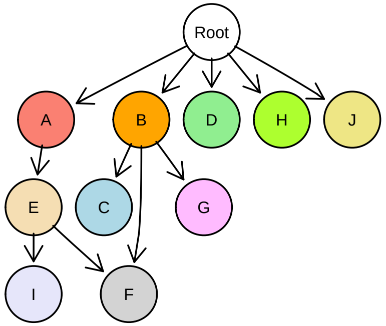

ID	u1PuIFhBeoVLcUwQ
acc genotypes	224
peaks no back	1
removed edges	0
prop rem edges	0

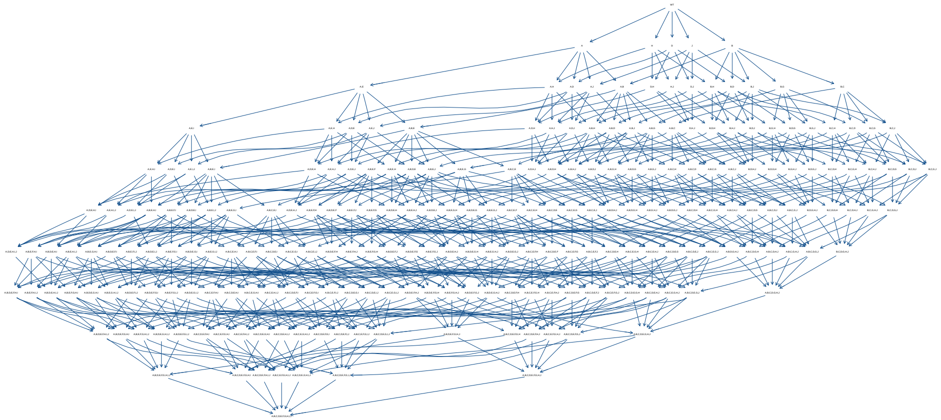

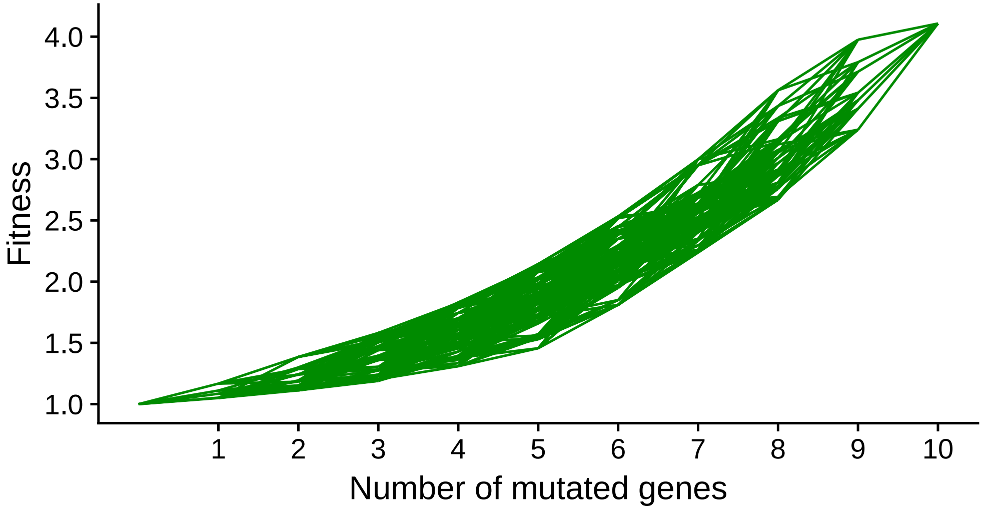

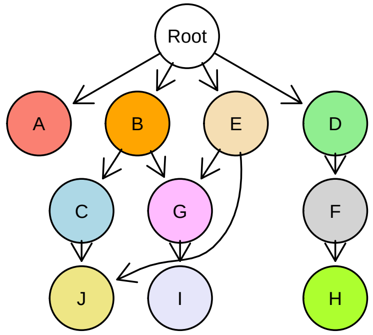

ID	u7NcTlp7H7vgrd6Z
acc genotypes	104
peaks no back	1
removed edges	0
prop rem edges	0

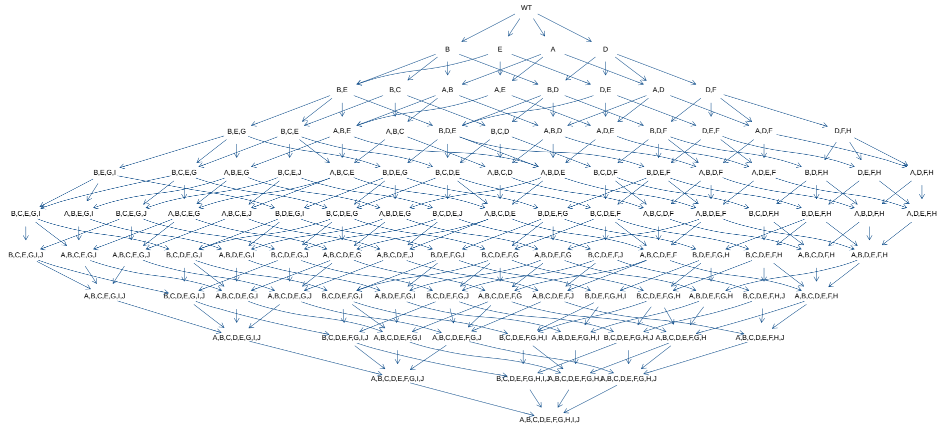

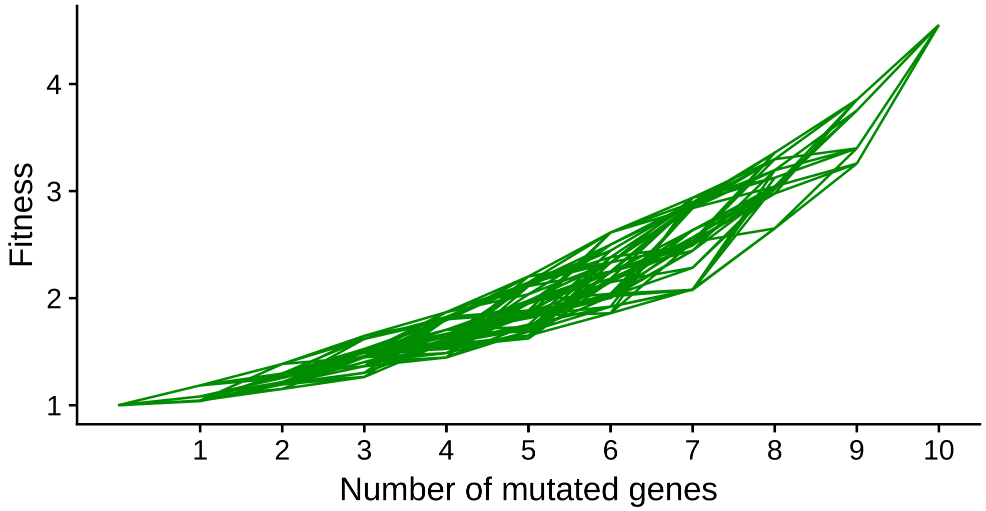

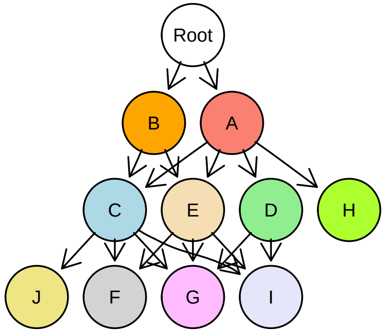

ID	u8LSkOke6emtVQOi
acc genotypes	62
peaks no back	1
removed edges	0
prop rem edges	0

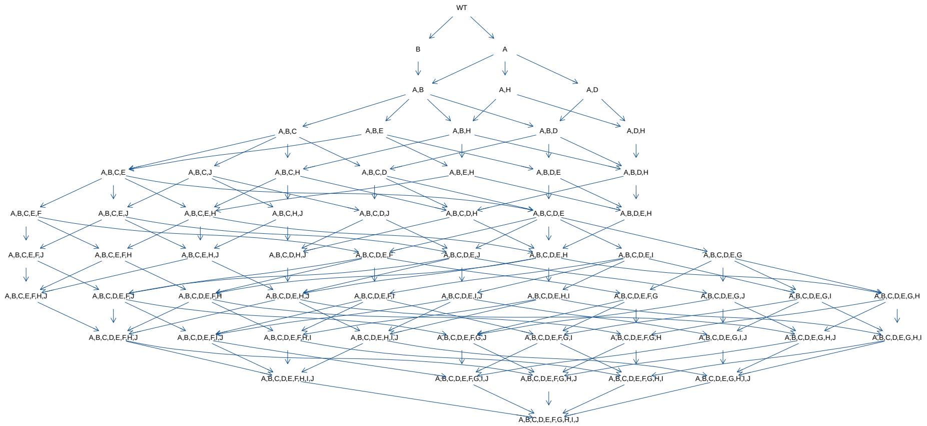

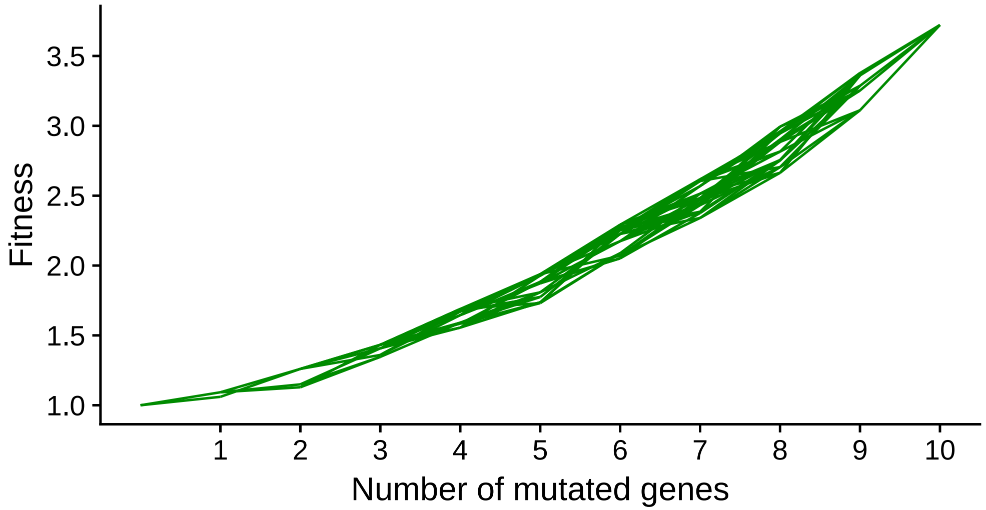

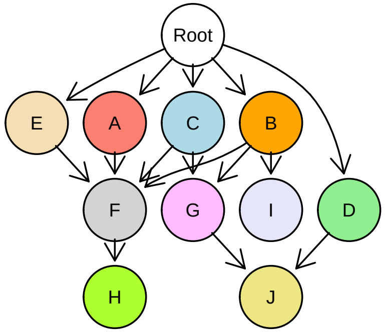

ID	UjXjH4wCYwemR9Ik
acc genotypes	92
peaks no back	1
removed edges	0
prop rem edges	0

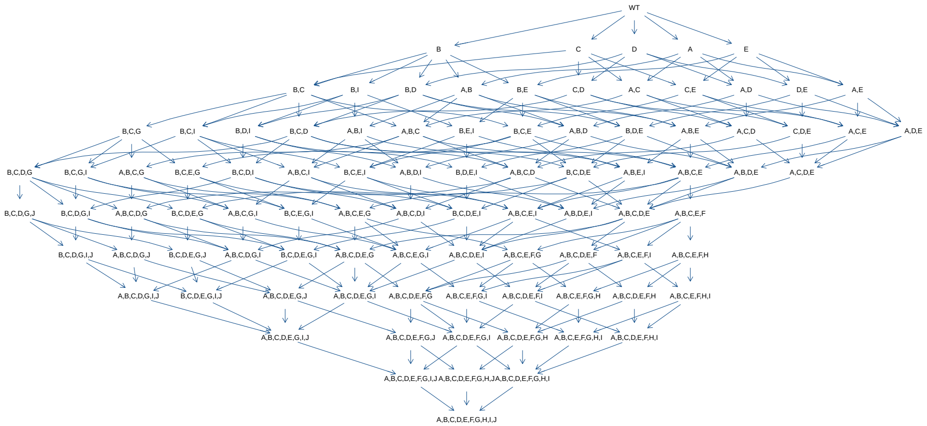

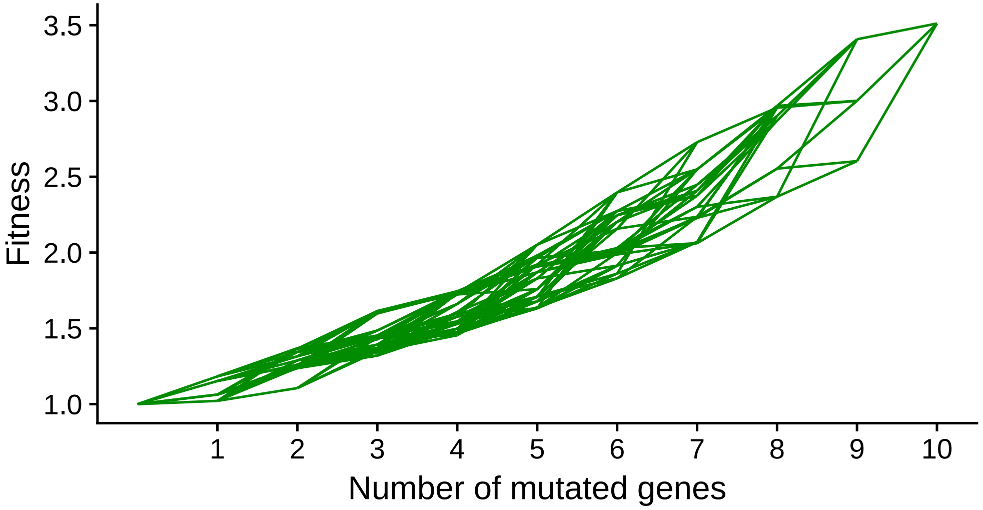

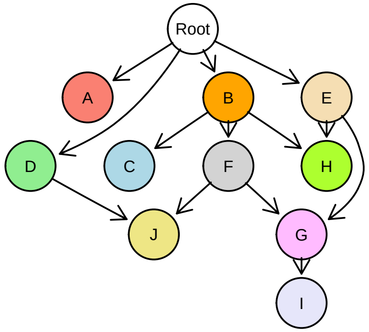

ID	UPacqPOLUgev2Qbz
acc genotypes	116
peaks no back	1
removed edges	0
prop rem edges	0

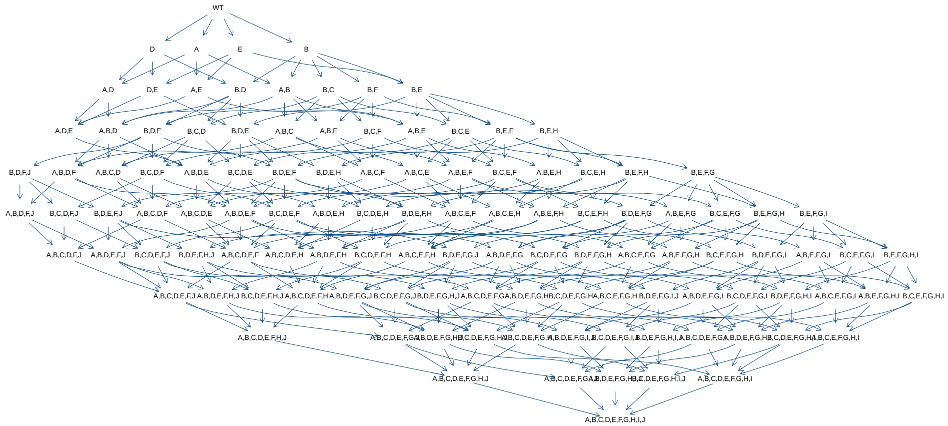

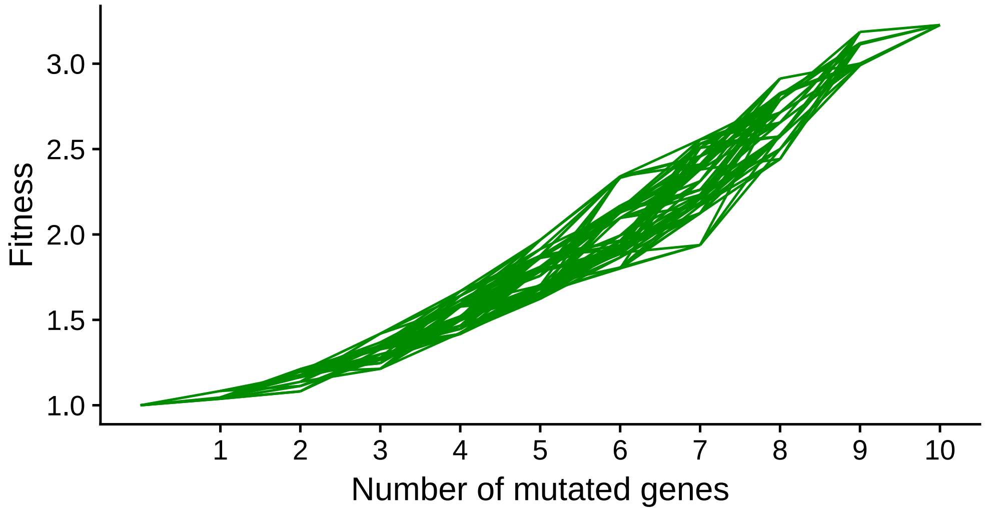

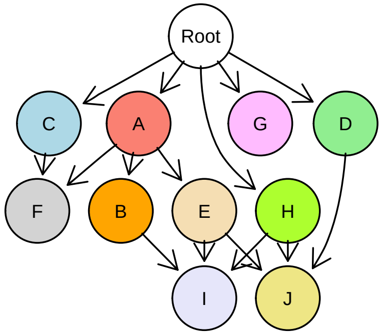

ID	UtTlZN4ssUPI1042T
acc genotypes	142
peaks no back	1
removed edges	0
prop rem edges	0

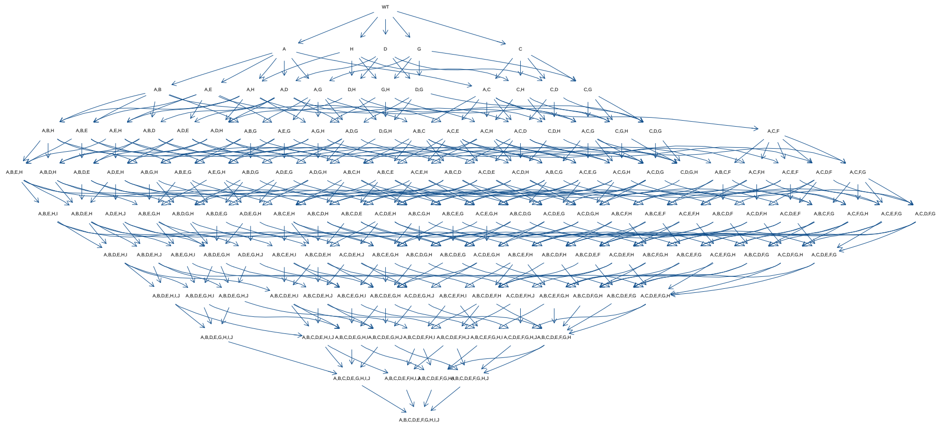

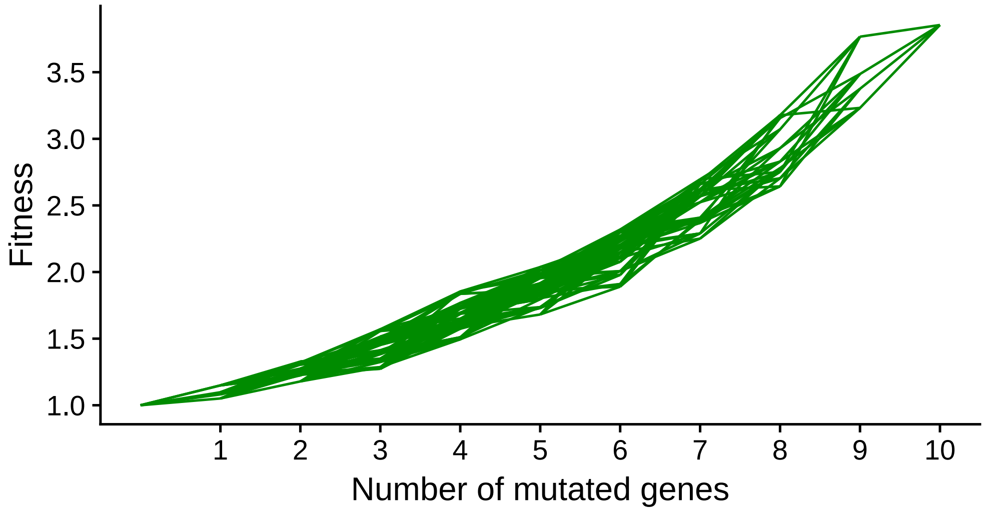

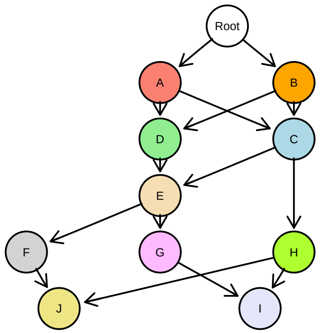

ID	uU3eWnOFxxJy7Vct
acc genotypes	22
peaks no back	1
removed edges	0
prop rem edges	0

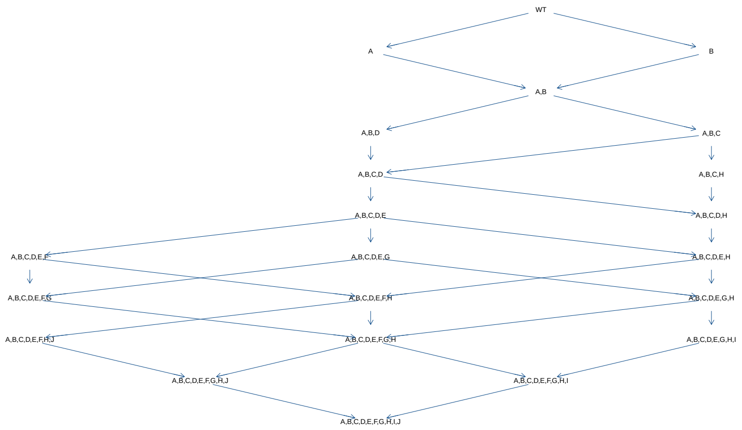

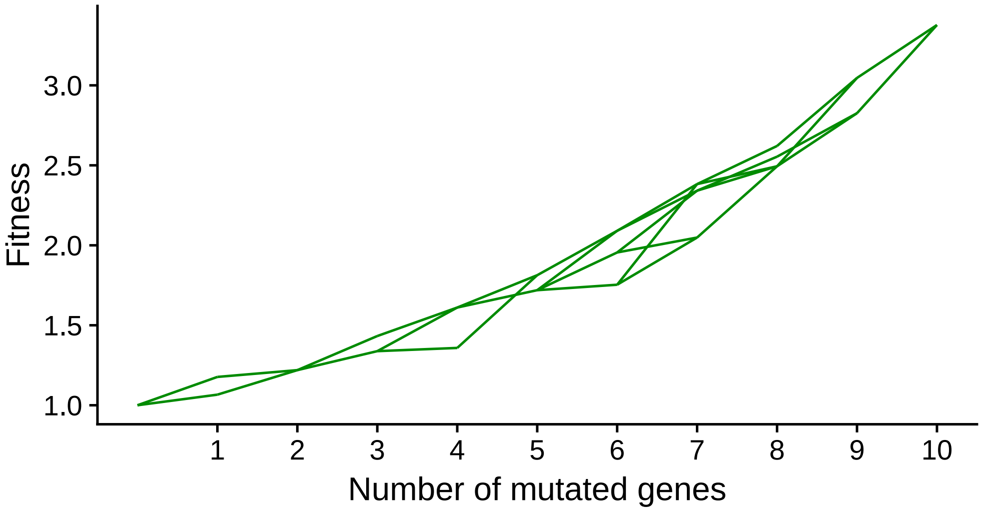

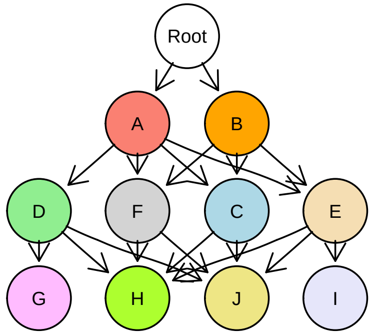

ID	UvbbY5R10xG3o3Fbv
acc genotypes	53
peaks no back	1
removed edges	0
prop rem edges	0

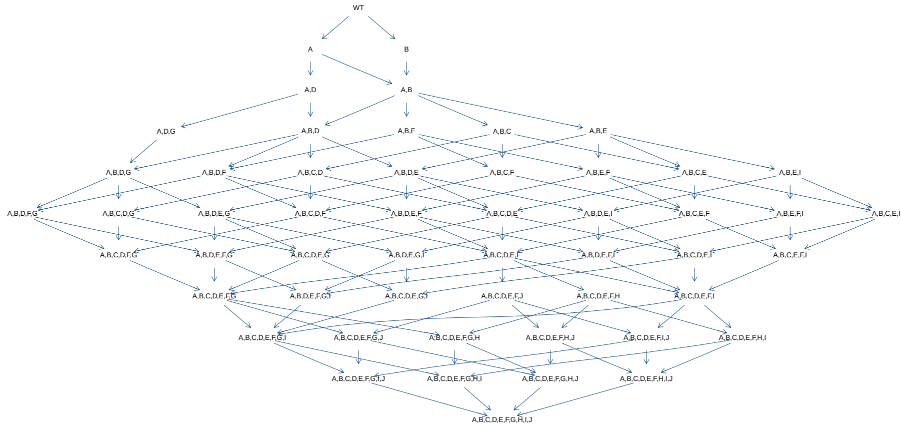

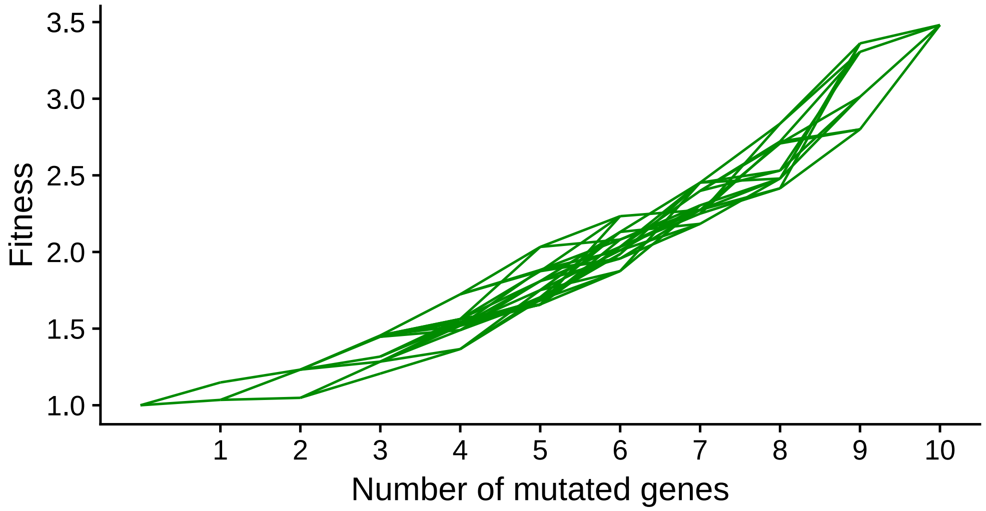

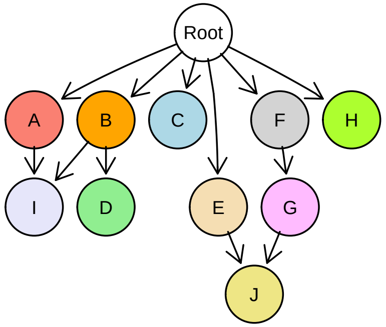

ID	uVPTmh9a12FoeCLG
acc genotypes	224
peaks no back	1
removed edges	0
prop rem edges	0

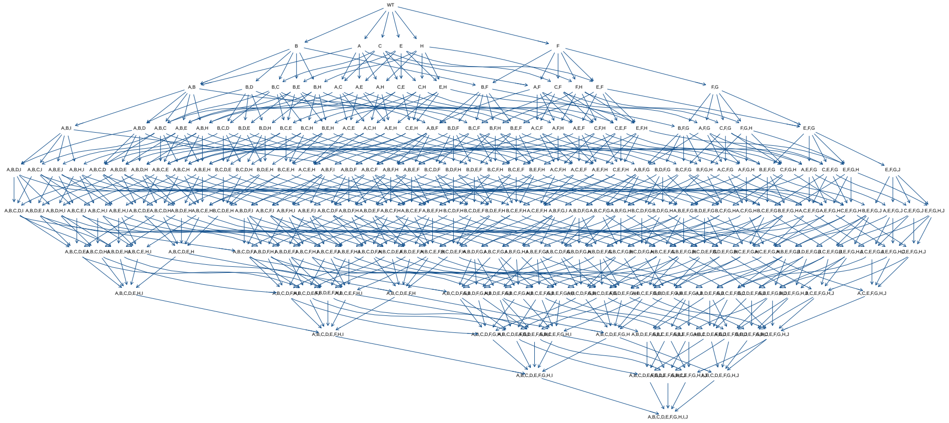

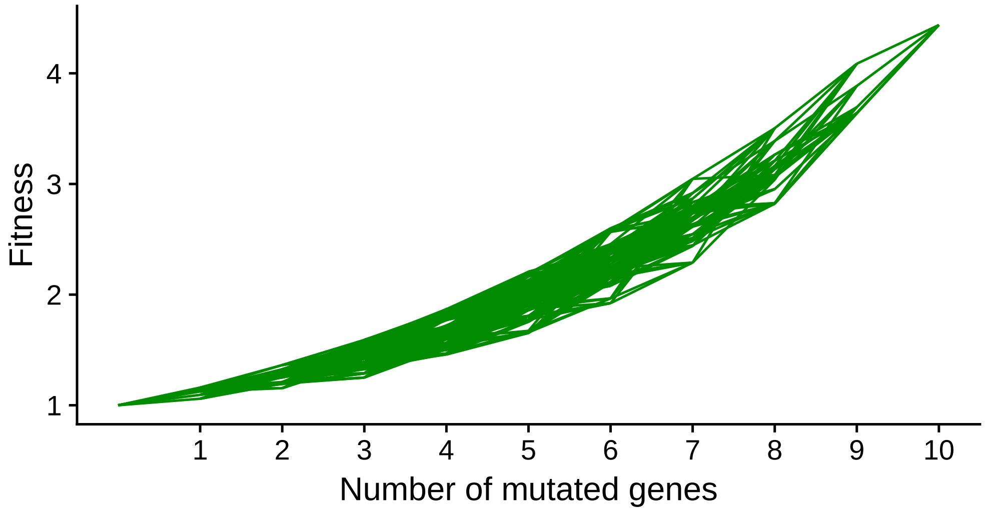

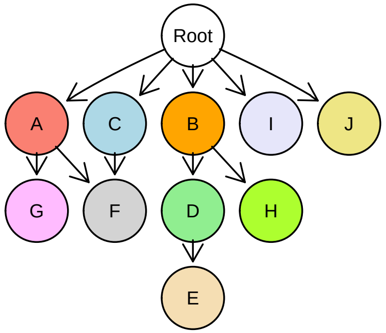

ID	uWAn38F57JFUrKmr
acc genotypes	224
peaks no back	1
removed edges	0
prop rem edges	0

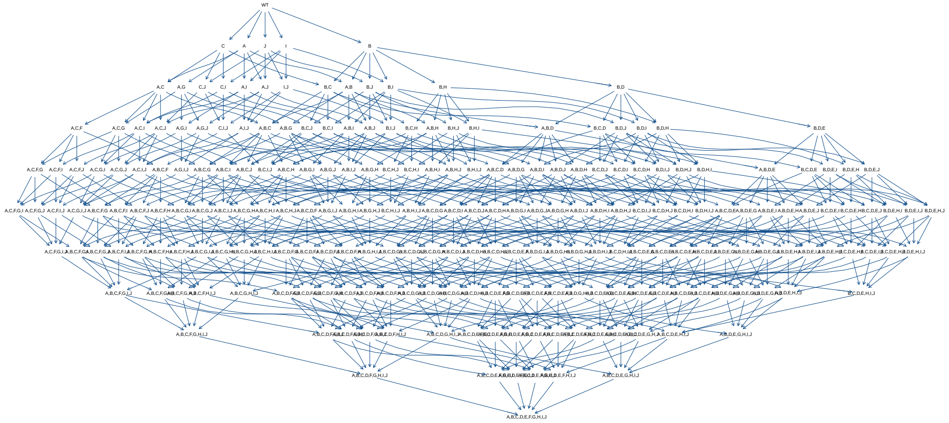

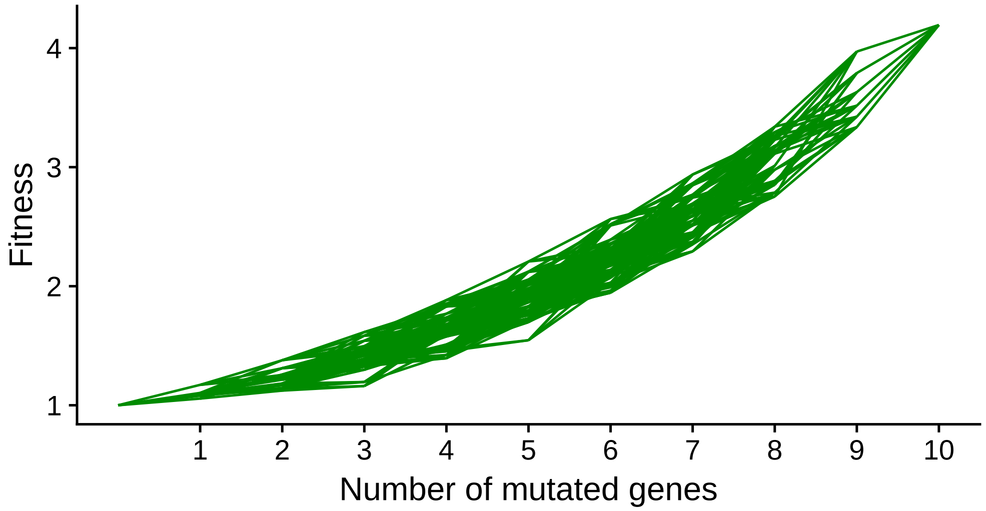

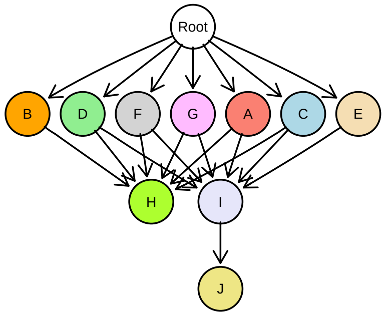

ID	UXtvbte61fs7Xr8g
acc genotypes	136
peaks no back	1
removed edges	0
prop rem edges	0

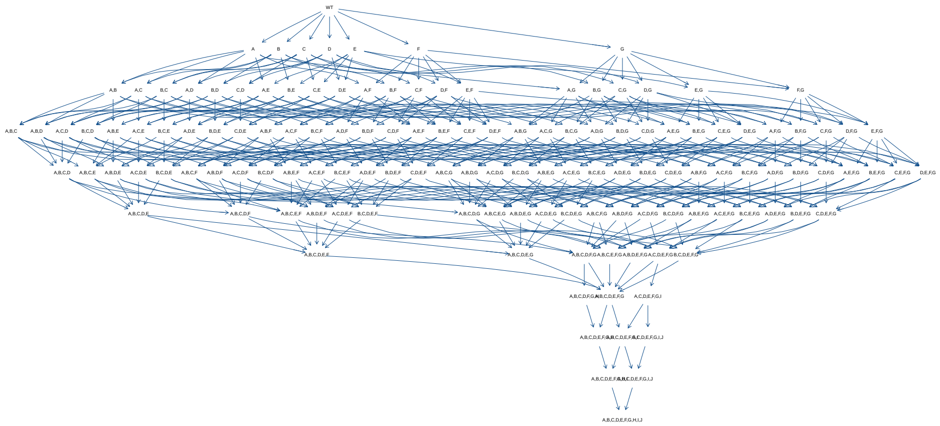

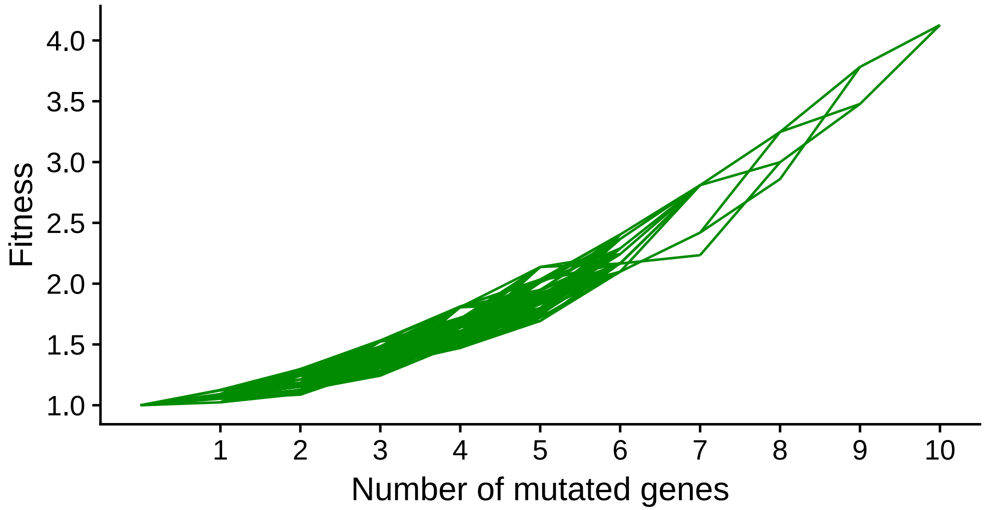

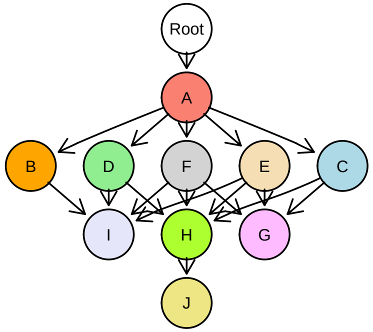

ID v7v1qJcKO4kxs78T
acc genotypes 52
peaks no back 1
removed edges 0
prop rem edges 0

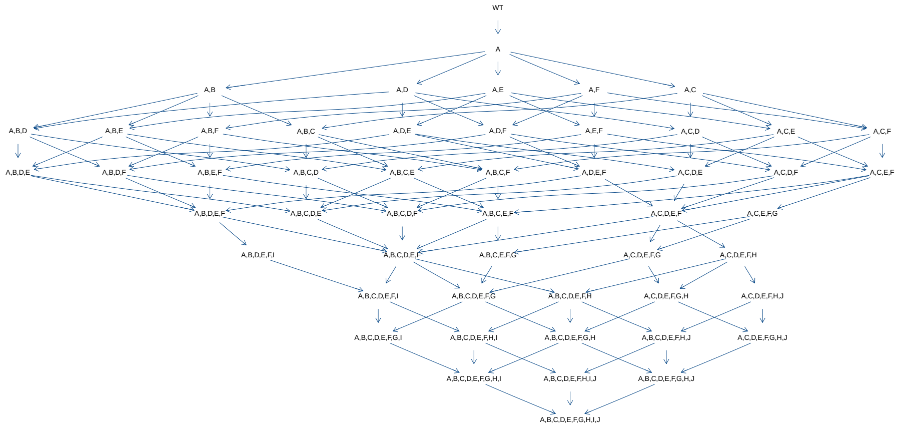

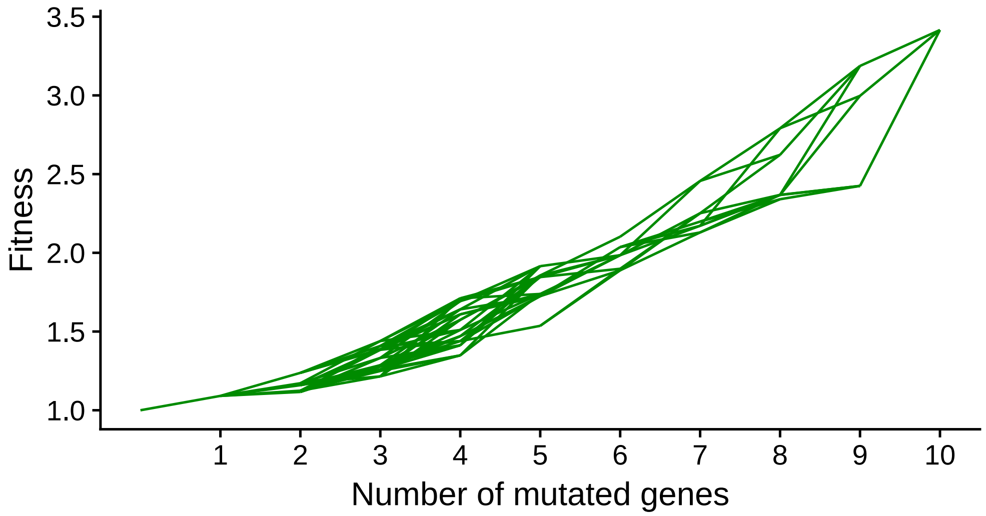

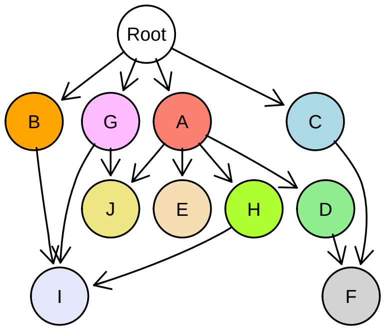

ID	VdRwaw10j2G10mqCxP
acc genotypes	148
peaks no back	1
removed edges	0
prop rem edges	0

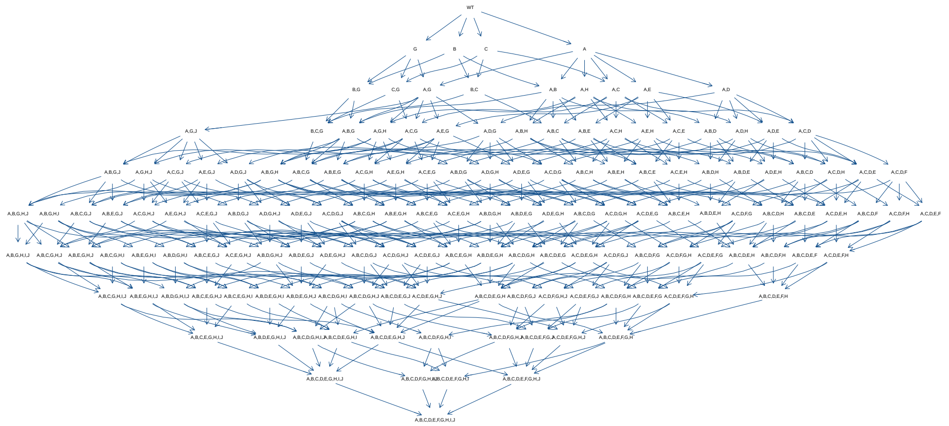

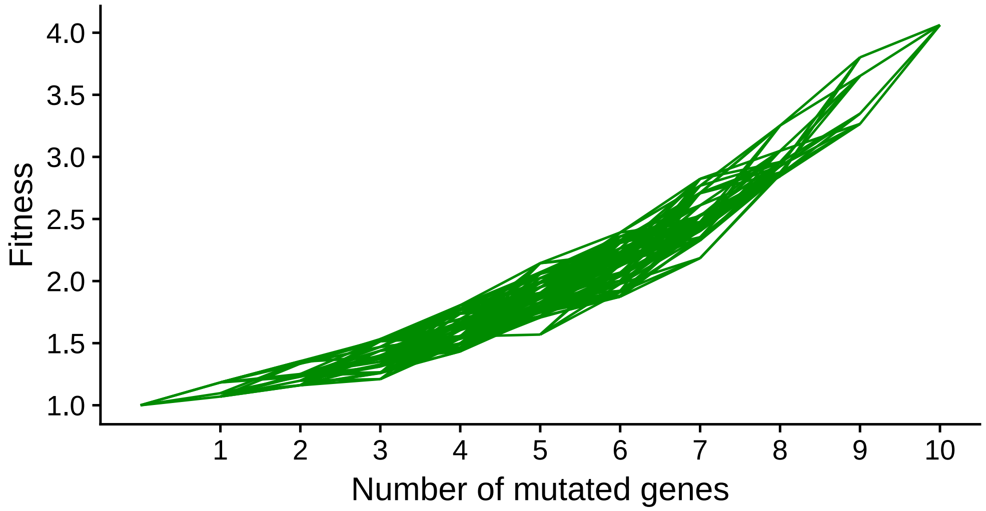

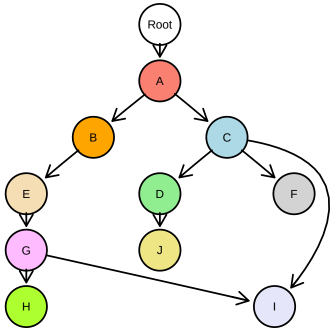

ID	VIlrIHndJDehvx8p
acc genotypes	48
peaks no back	1
removed edges	0
prop rem edges	0

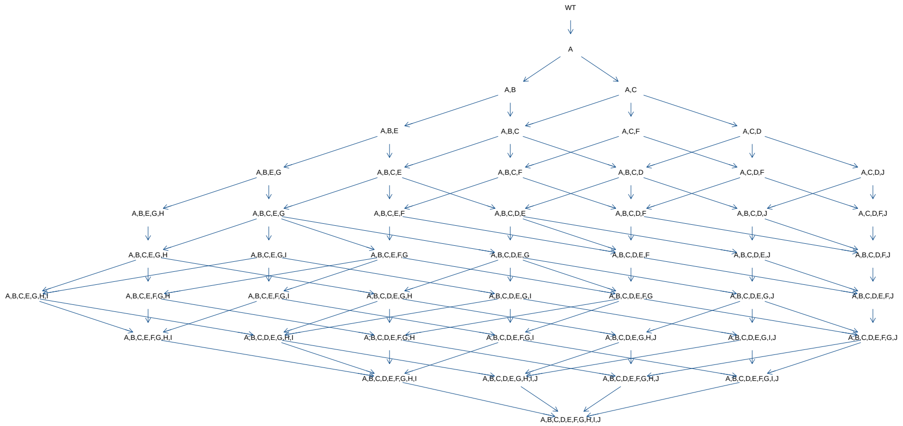

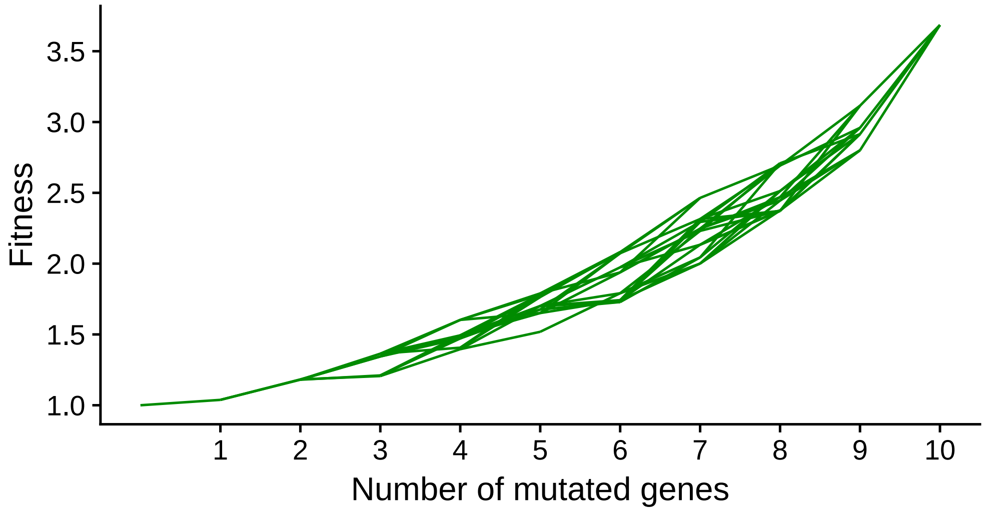

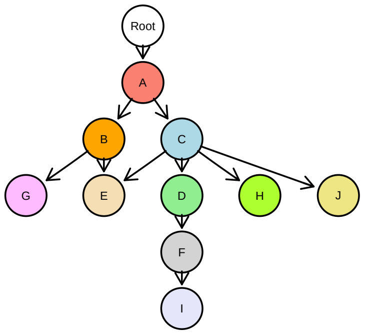

ID	vKqR1PxaeGDS6o2G
acc genotypes	84
peaks no back	1
removed edges	0
prop rem edges	0

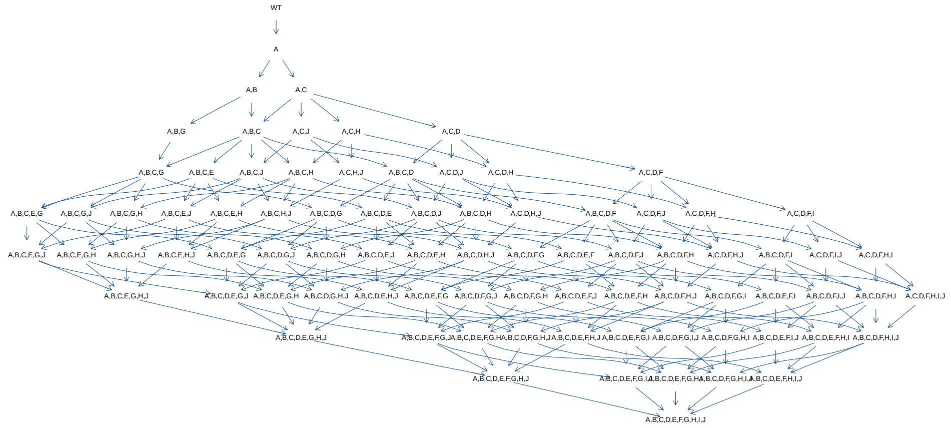

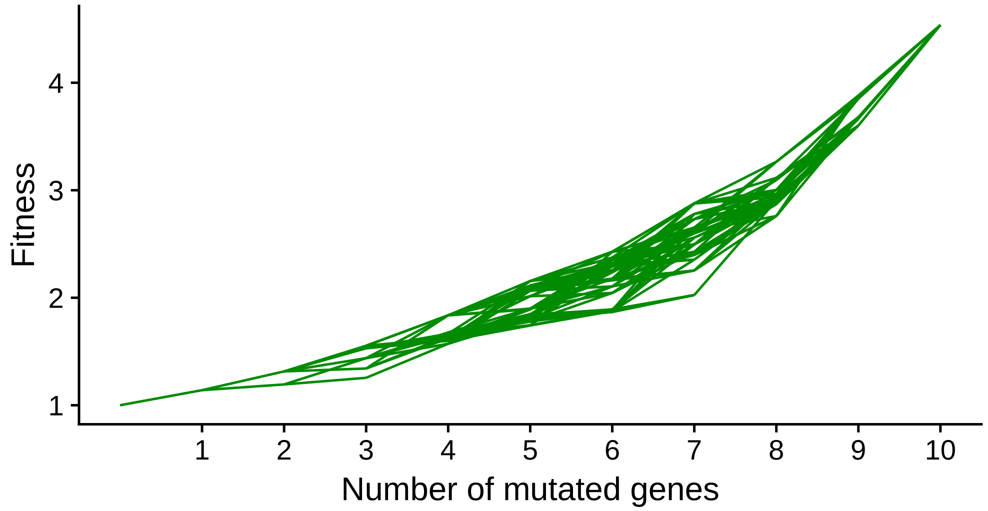

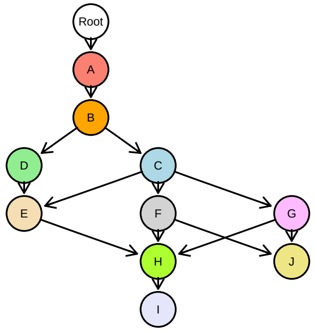

ID	vWa8dLmVr7VaMVrU
acc genotypes	23
peaks no back	1
removed edges	0
prop rem edges	0

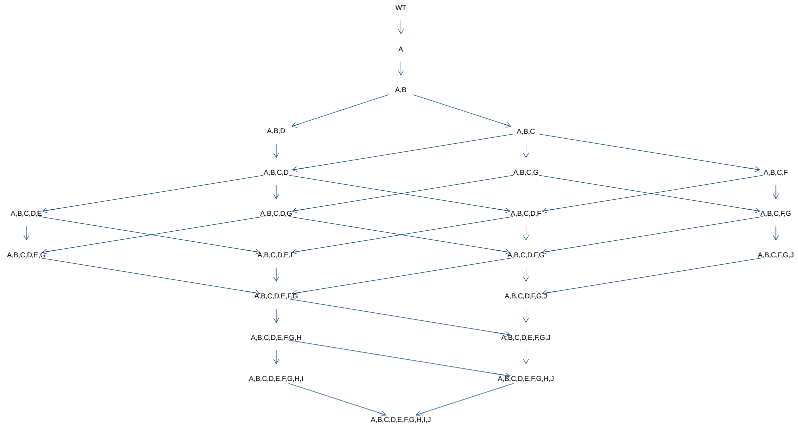

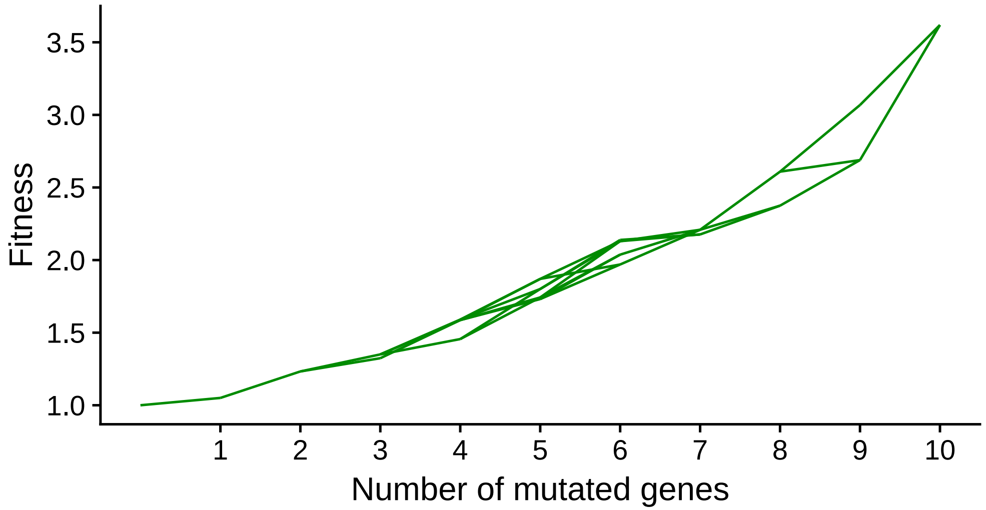

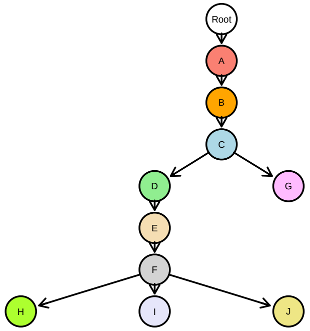

ID	W10RExqb1hvnBZhkz
acc genotypes	25
peaks no back	1
removed edges	0
prop rem edges	0

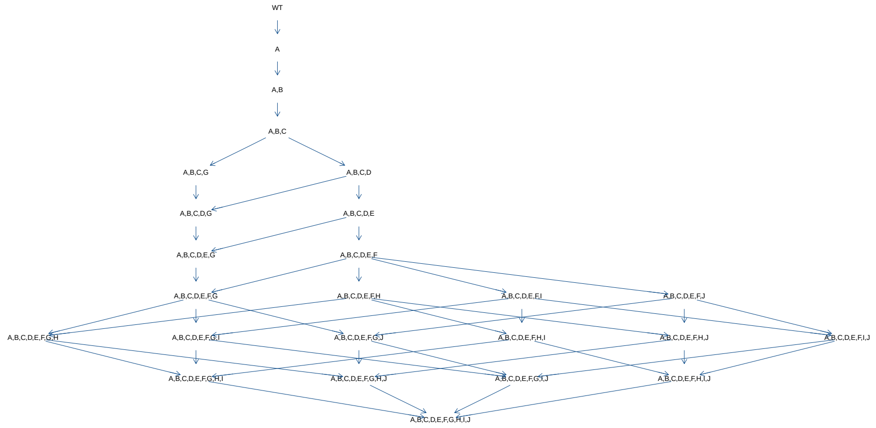

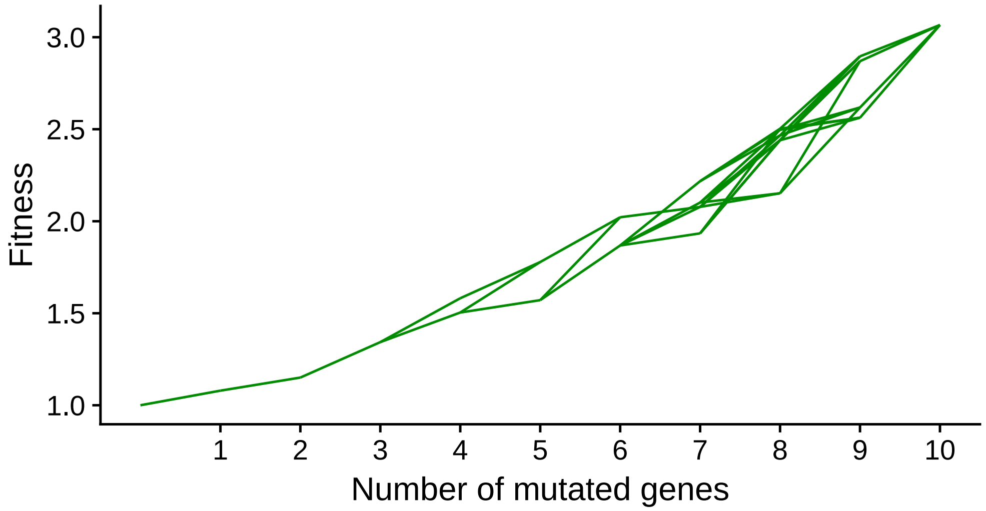

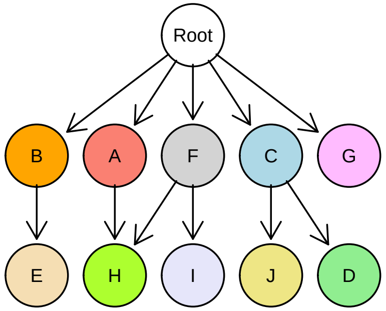

ID w2LGyrZeMILULslK
acc genotypes 240
peaks no back 1
removed edges 0
prop rem edges 0

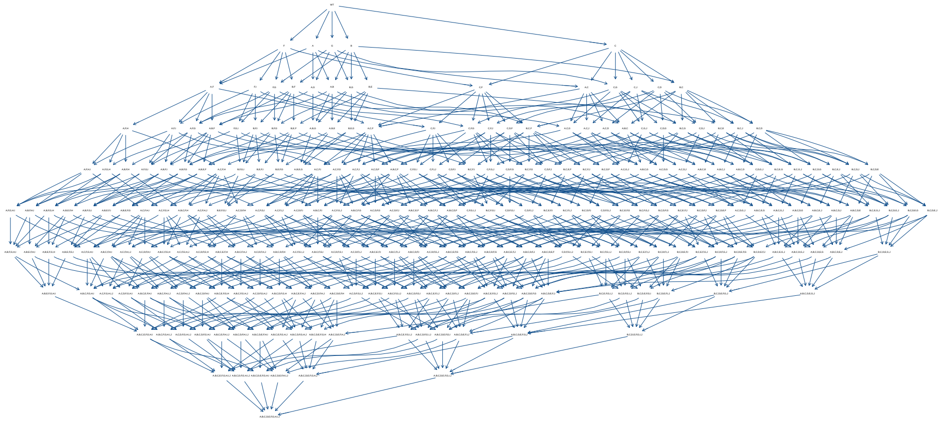

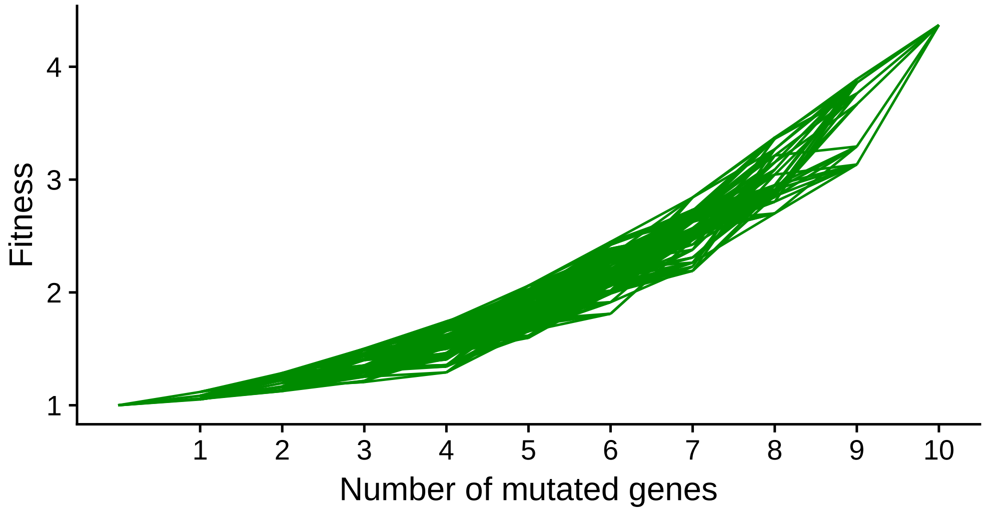

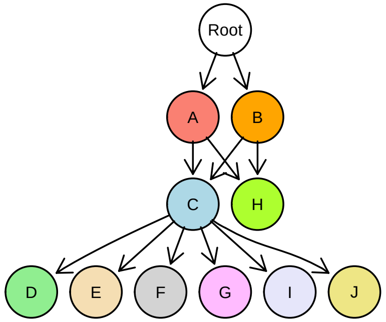

ID wOVnm2kwBXXY2Fm4
acc genotypes 133
peaks no back 1
removed edges 0
prop rem edges 0

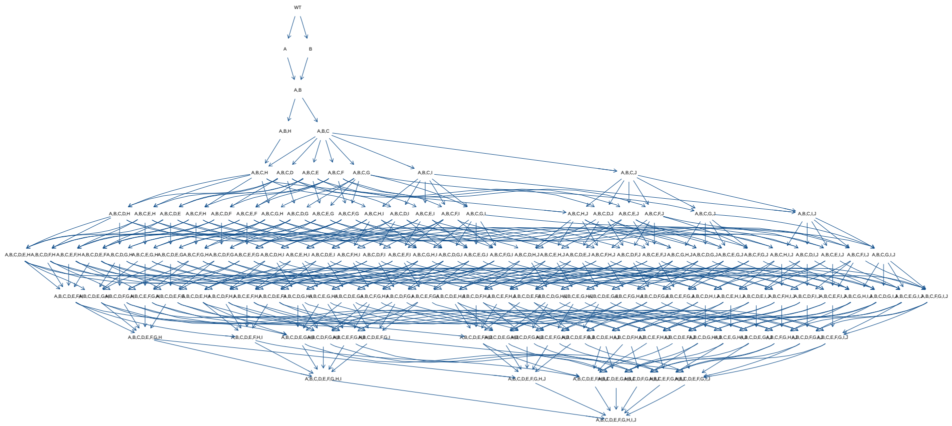

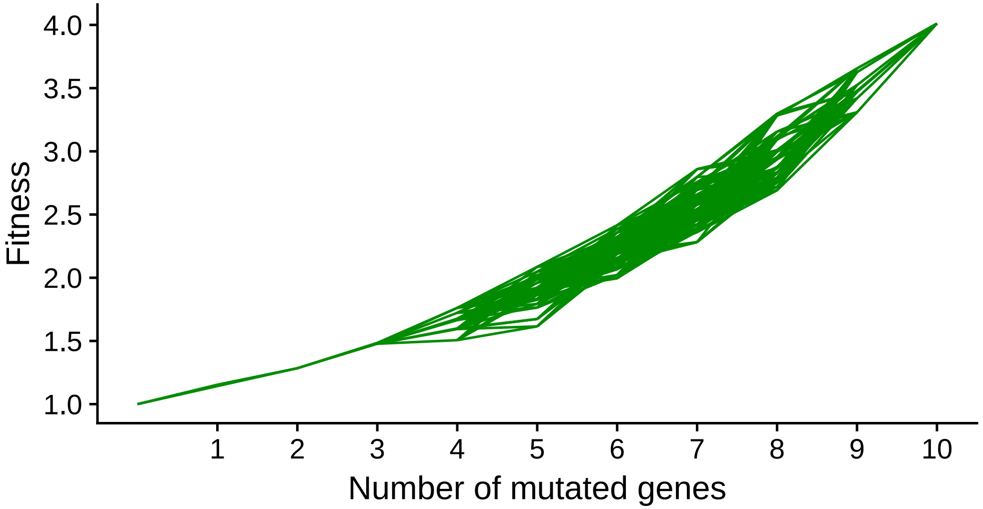

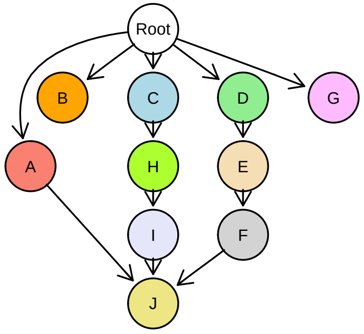

ID	WX2rq5CKiTz10Vbzs
acc genotypes	132
peaks no back	1
removed edges	0
prop rem edges	0

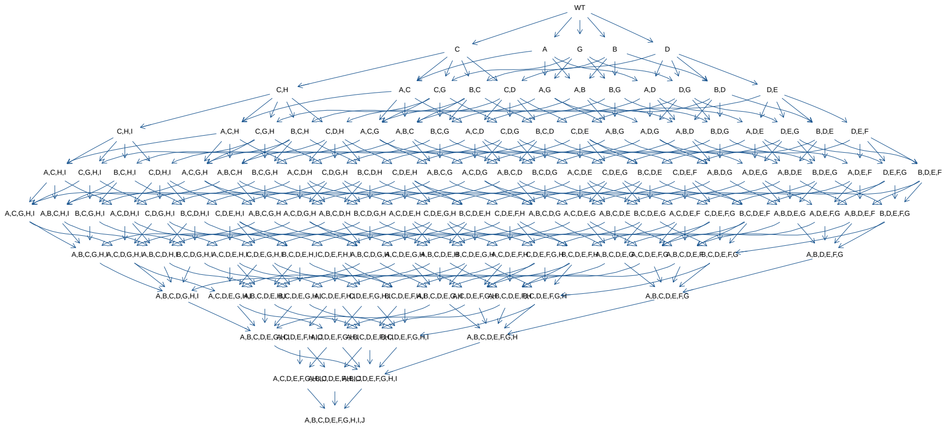

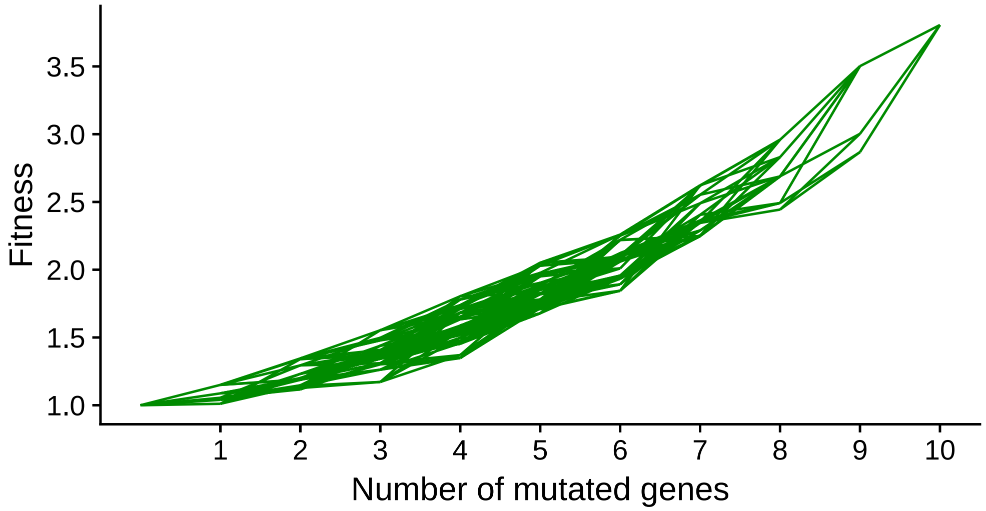

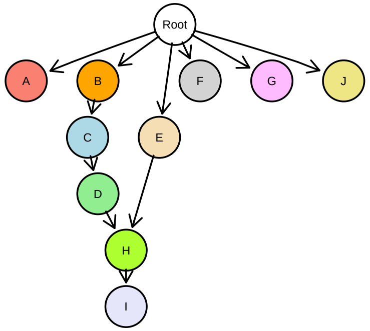

ID	X73CthbrahOJ65dM
acc genotypes	160
peaks no back	1
removed edges	0
prop rem edges	0

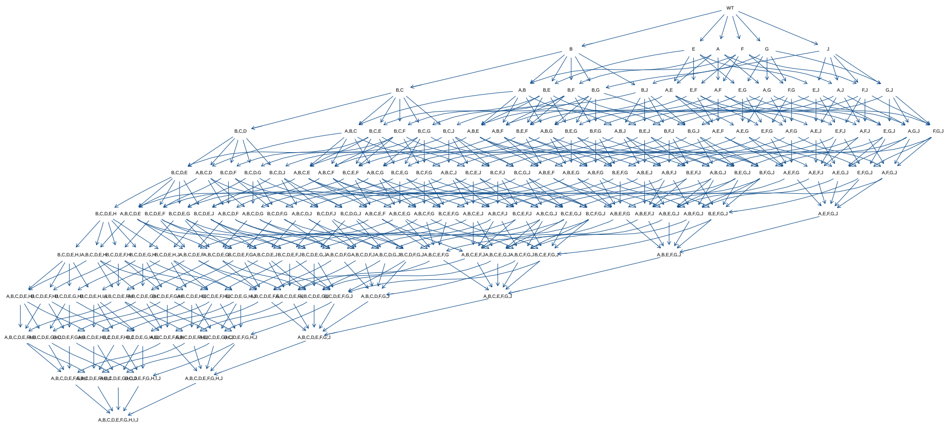

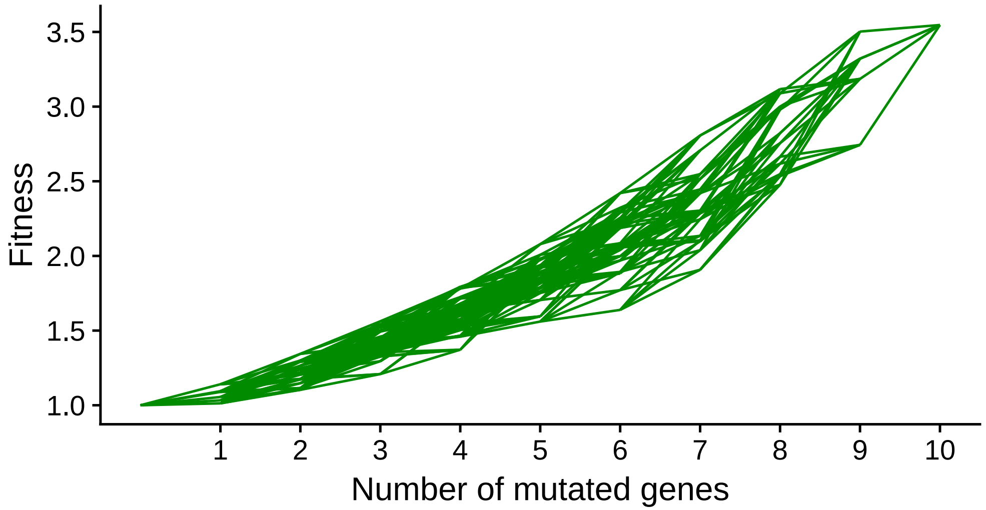

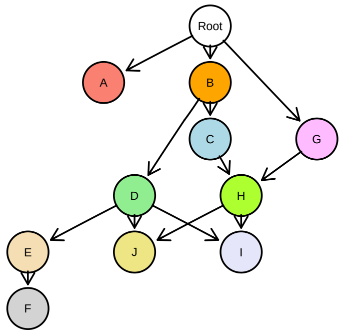

ID	xghzdPbC2Ste8yA4
acc genotypes	62
peaks no back	1
removed edges	0
prop rem edges	0

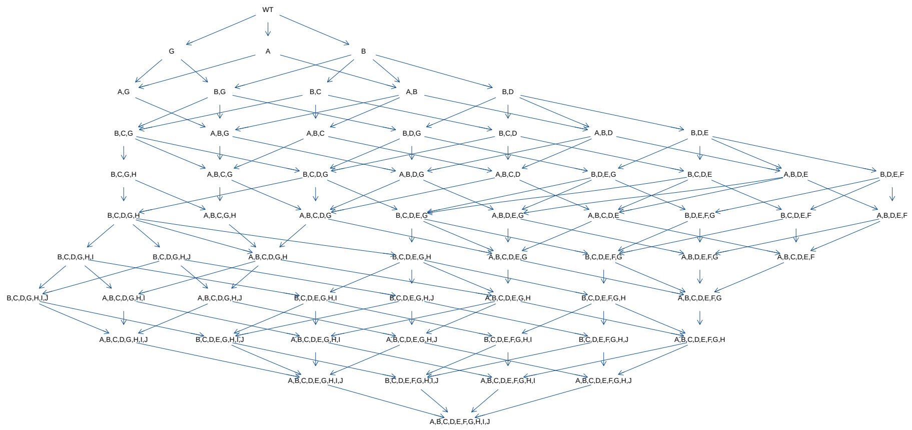

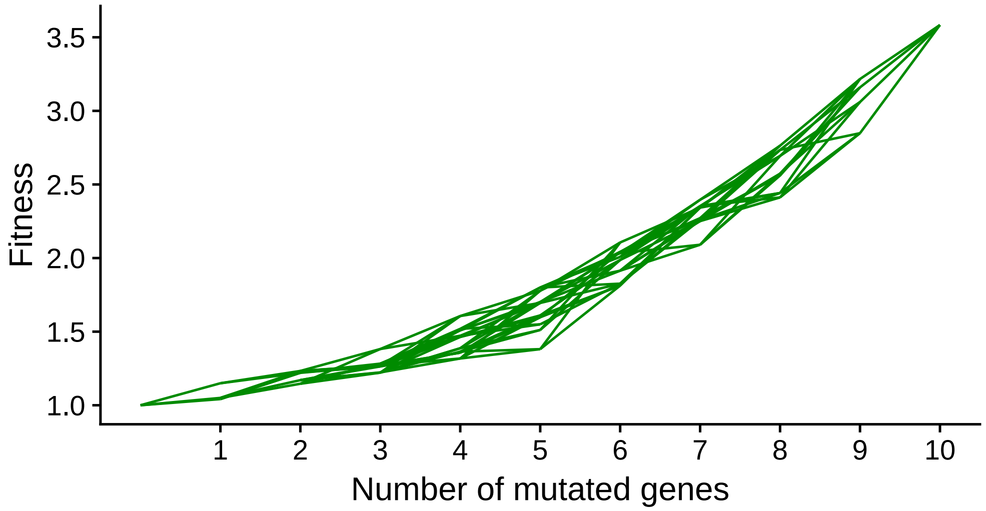

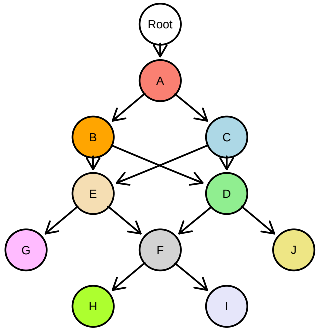

ID	xsBe1fvNRQrAYKEK
acc genotypes	29
peaks no back	1
removed edges	0
prop rem edges	0

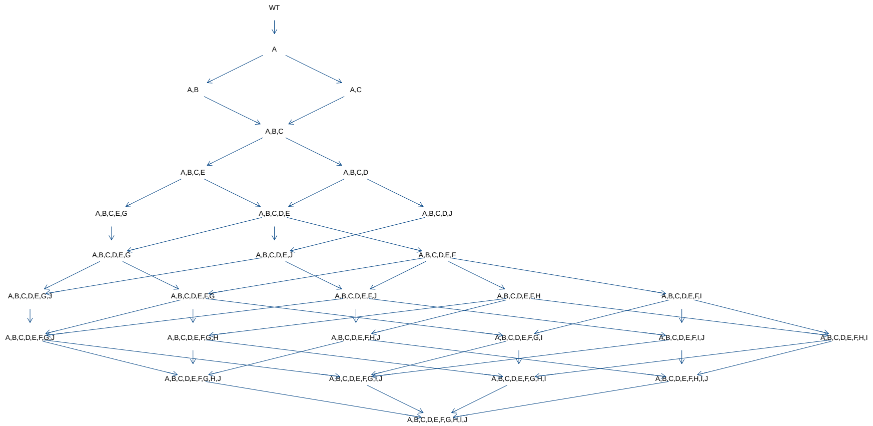

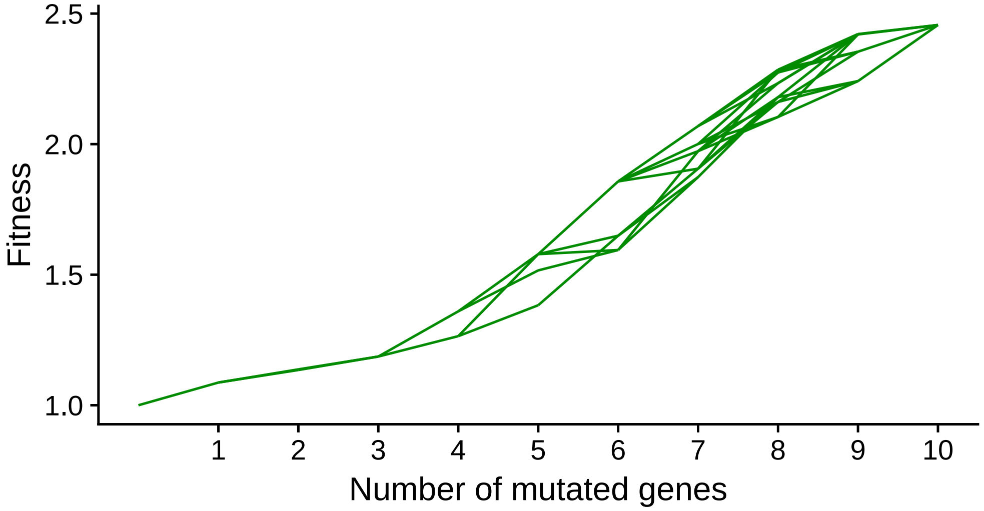

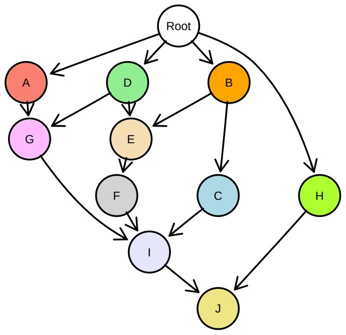

ID xTeo6GEUfqWOarjY
acc genotypes 57
peaks no back 1
removed edges 0
prop rem edges 0

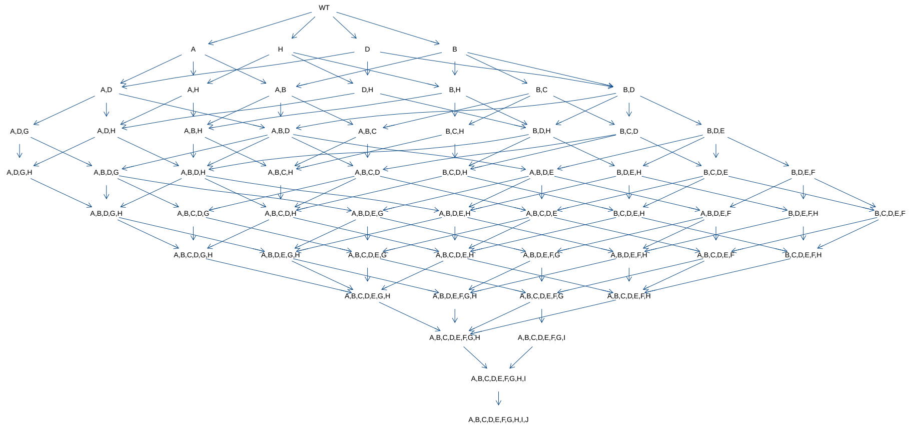

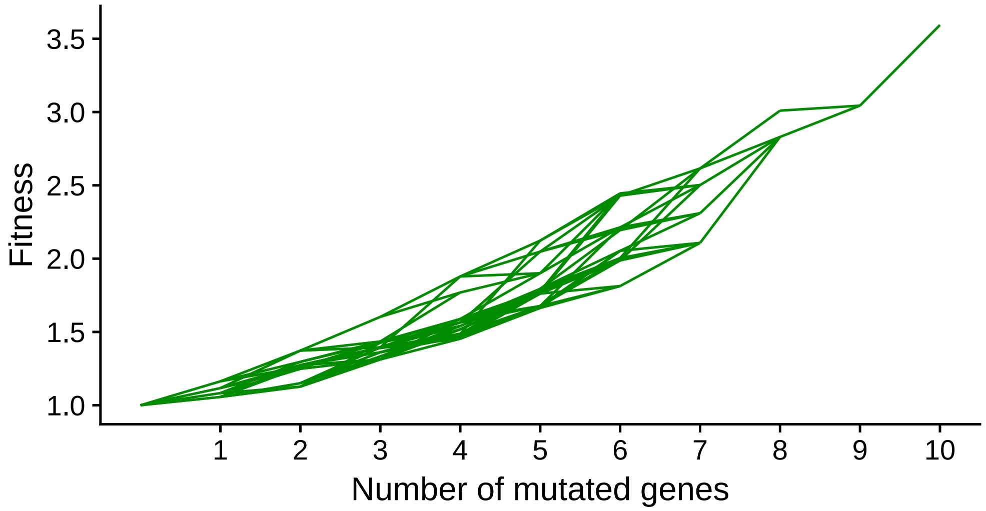

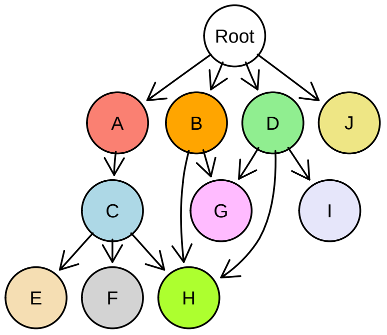

ID	xUB10YVej3P32RKIi
acc genotypes	128
peaks no back	1
removed edges	0
prop rem edges	0

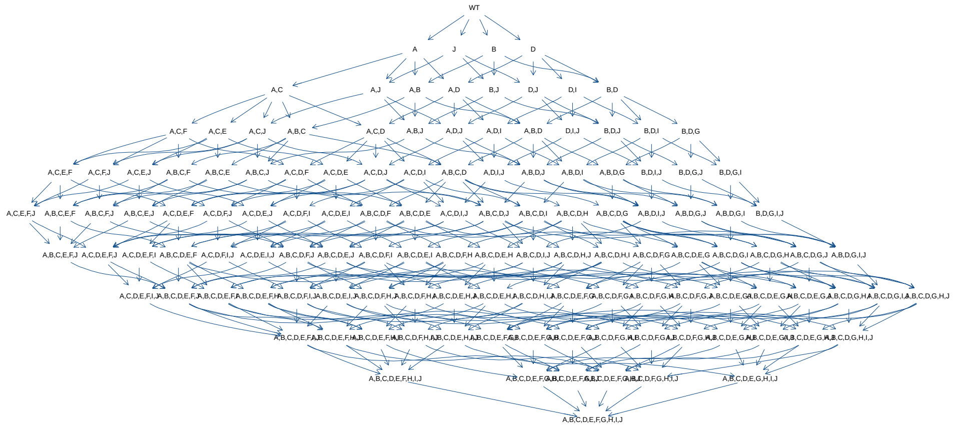

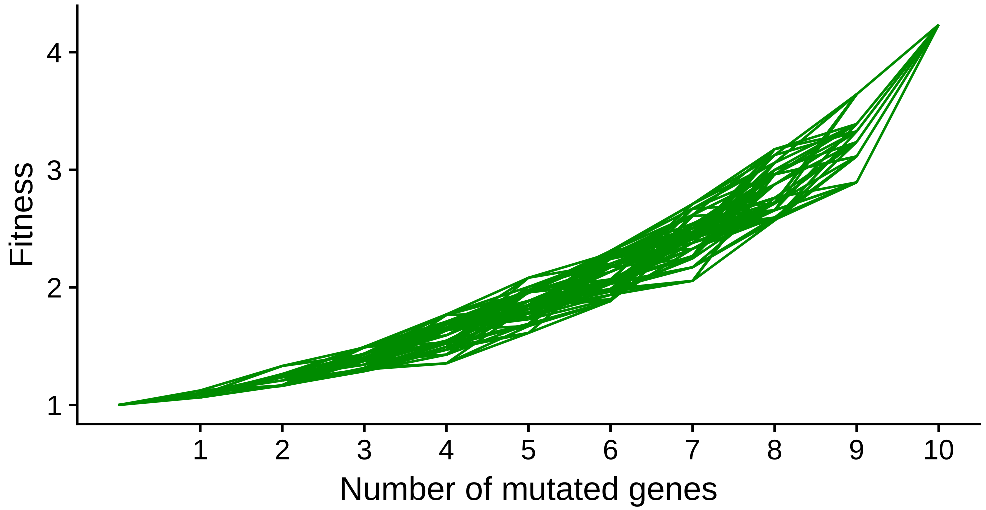

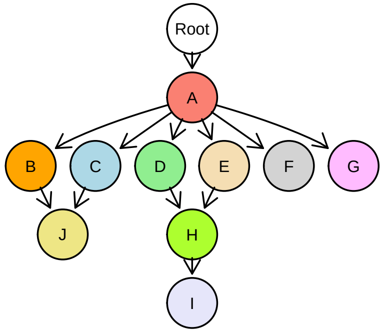

ID	xVYnsd10xBjzI3Lt2
acc genotypes	121
peaks no back	1
removed edges	0
prop rem edges	0

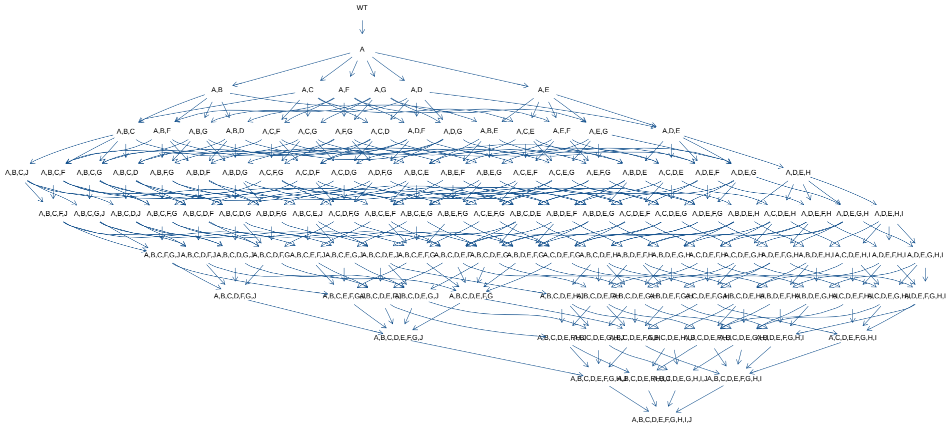

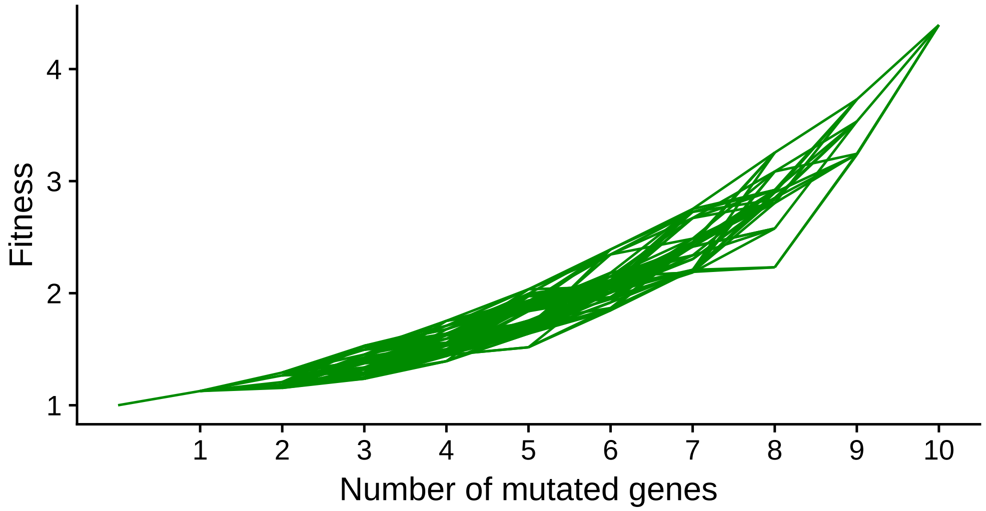

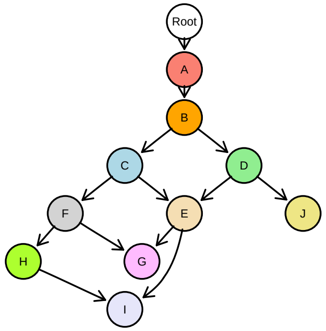

ID	xWR3MCuUy8RKrLHF
acc genotypes	28
peaks no back	1
removed edges	0
prop rem edges	0

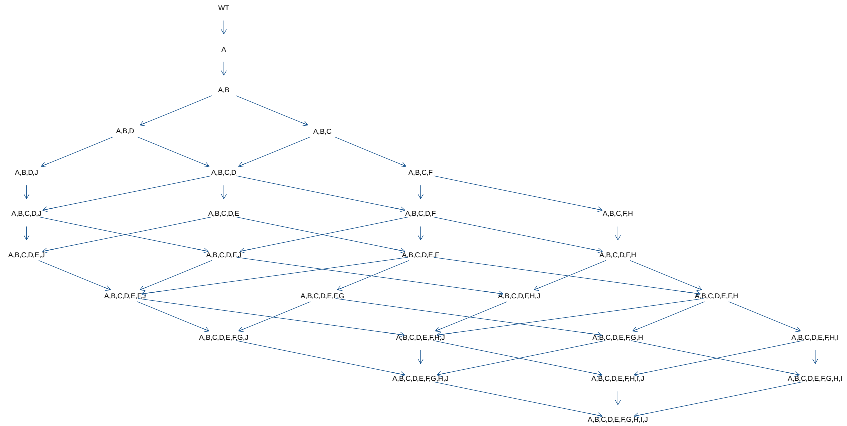

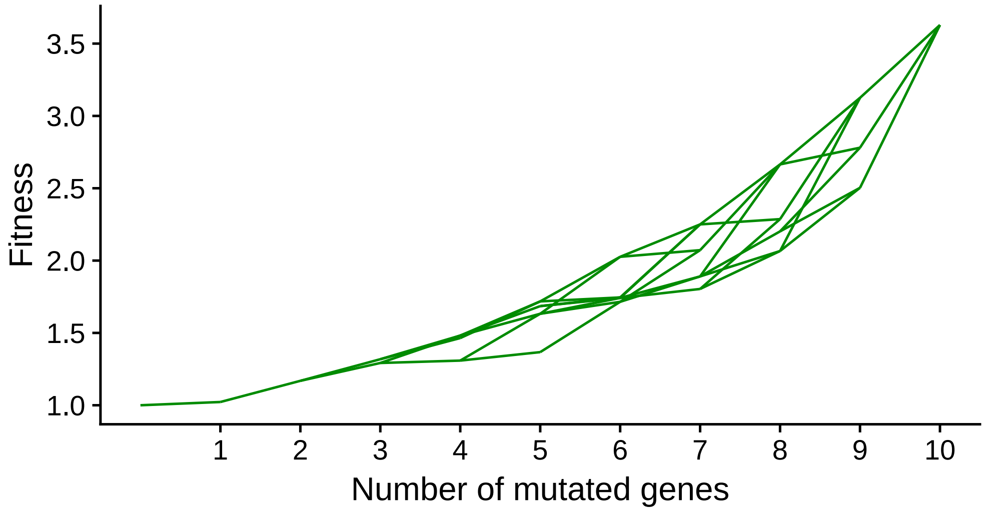

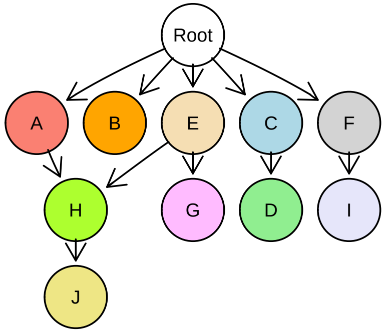

ID XYVDPlvrNBJywJzR
acc genotypes 180
peaks no back 1
removed edges 0
prop rem edges 0

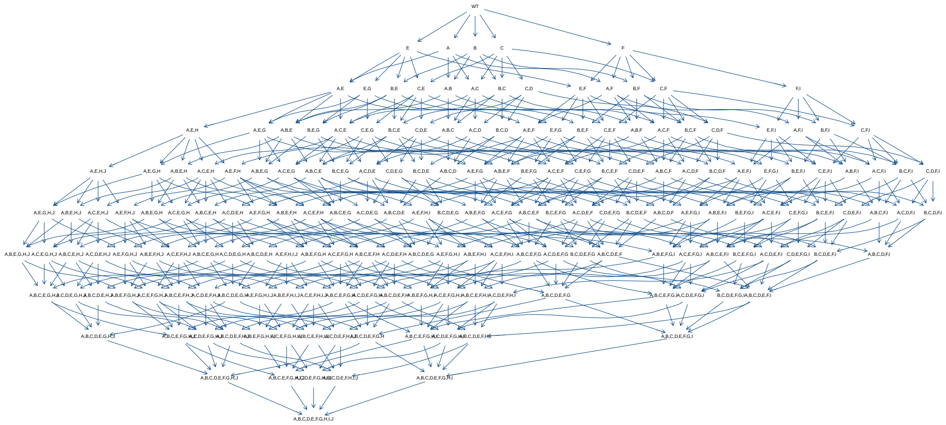

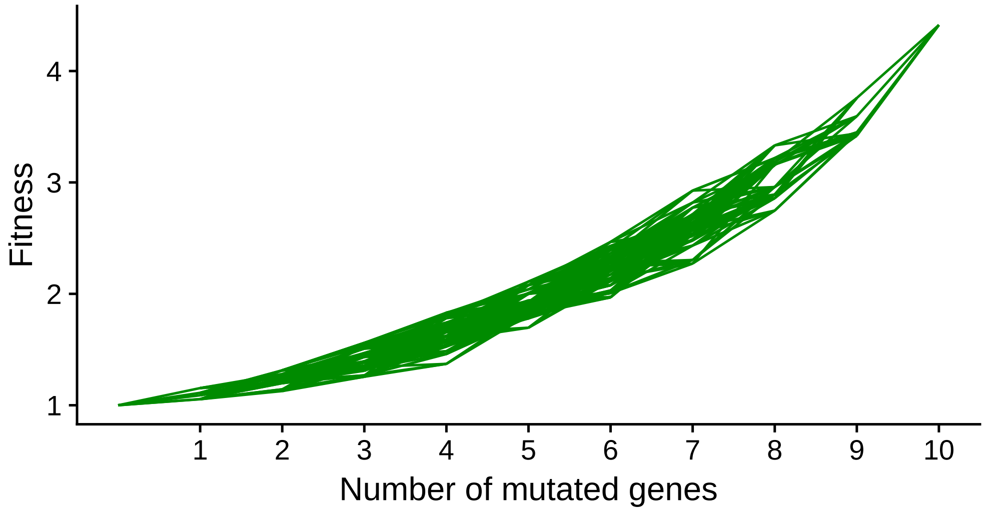

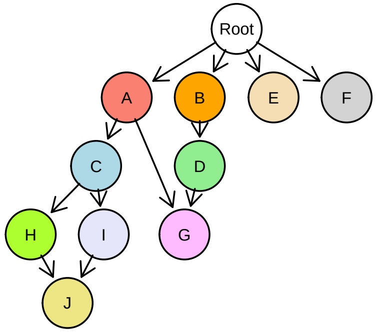

ID	y1DWAEMgCsc8ELl2
acc genotypes	108
peaks no back	1
removed edges	0
prop rem edges	0

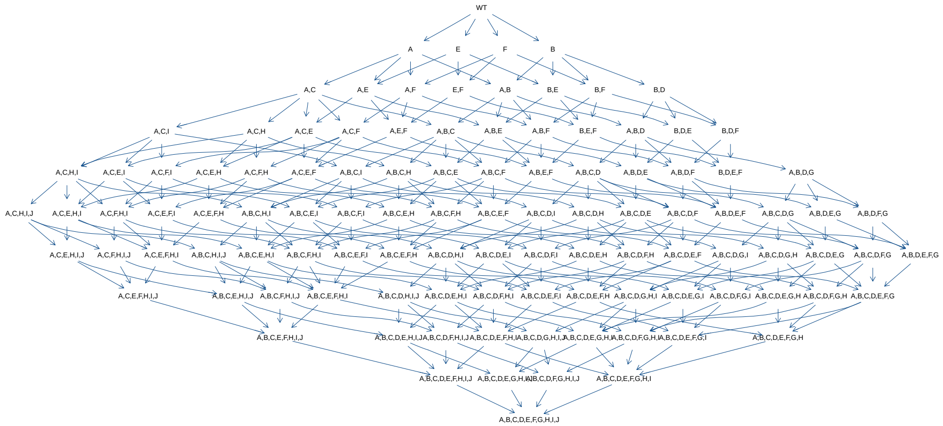

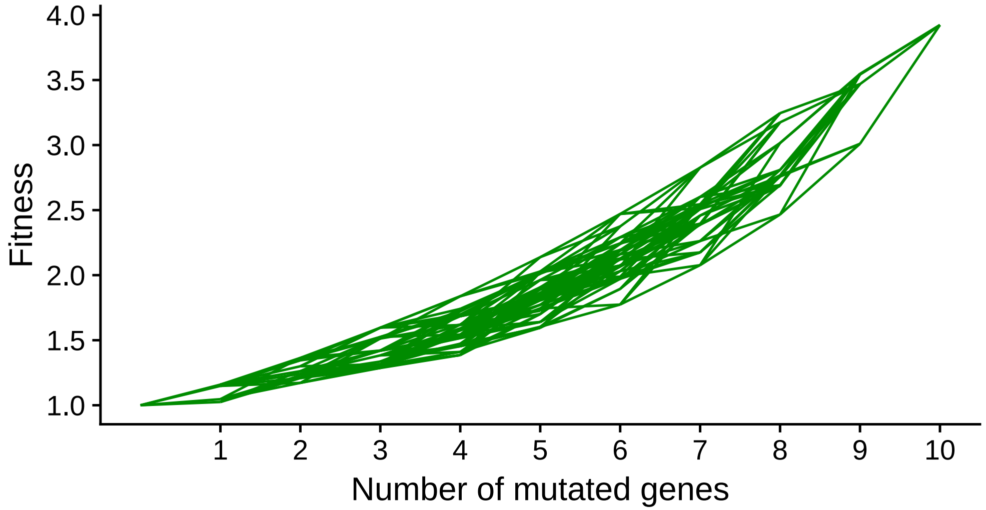

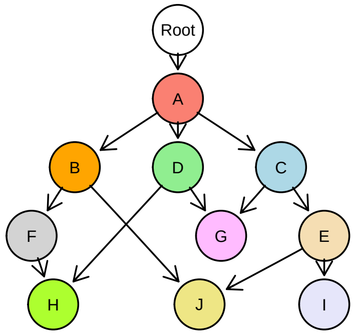

ID	y910g6HDlnpD8GSSB
acc genotypes	57
peaks no back	1
removed edges	0
prop rem edges	0

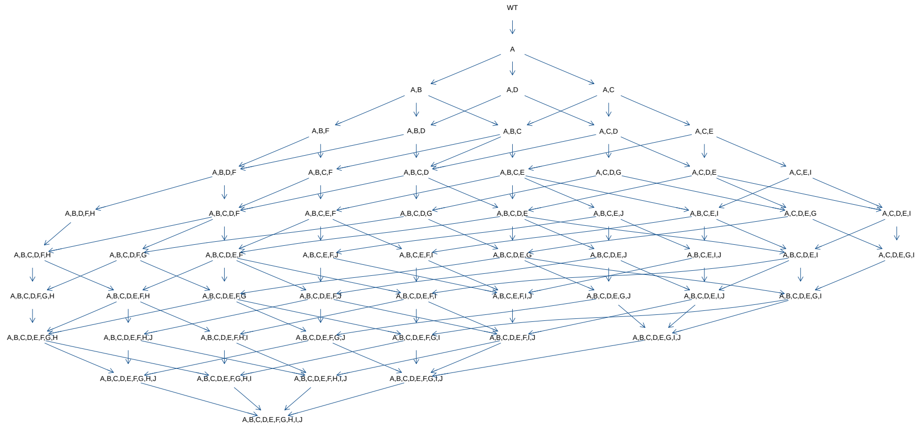

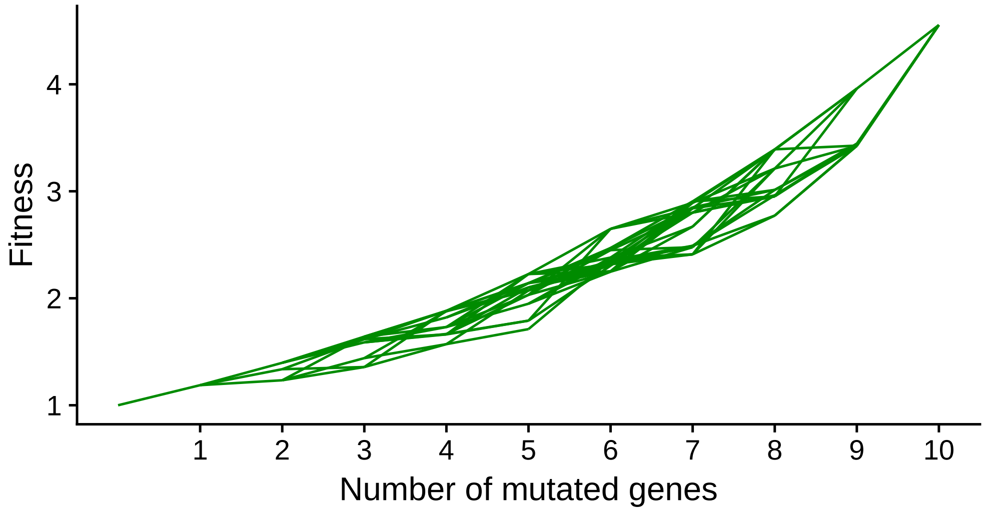

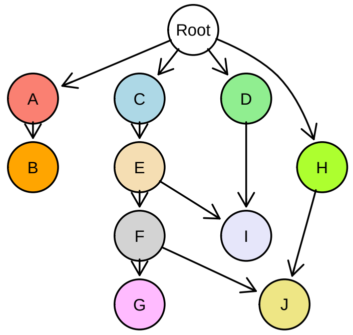

ID	YbPjwENMtPWo53FW
acc genotypes	96
peaks no back	1
removed edges	0
prop rem edges	0

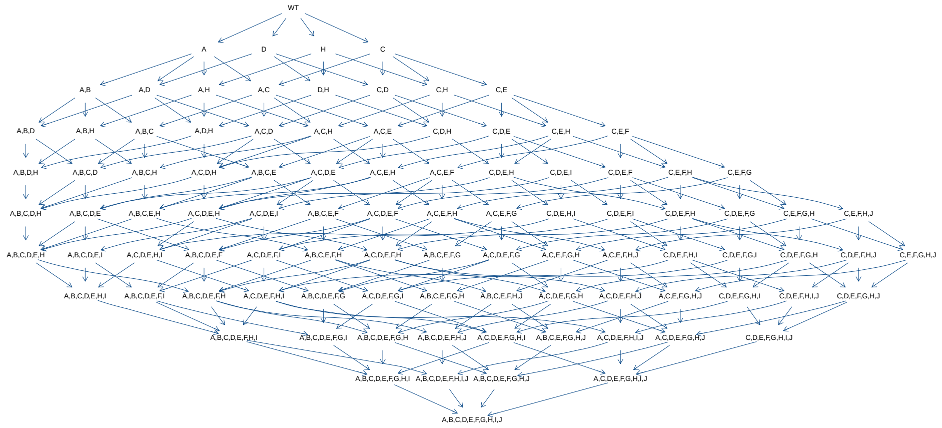

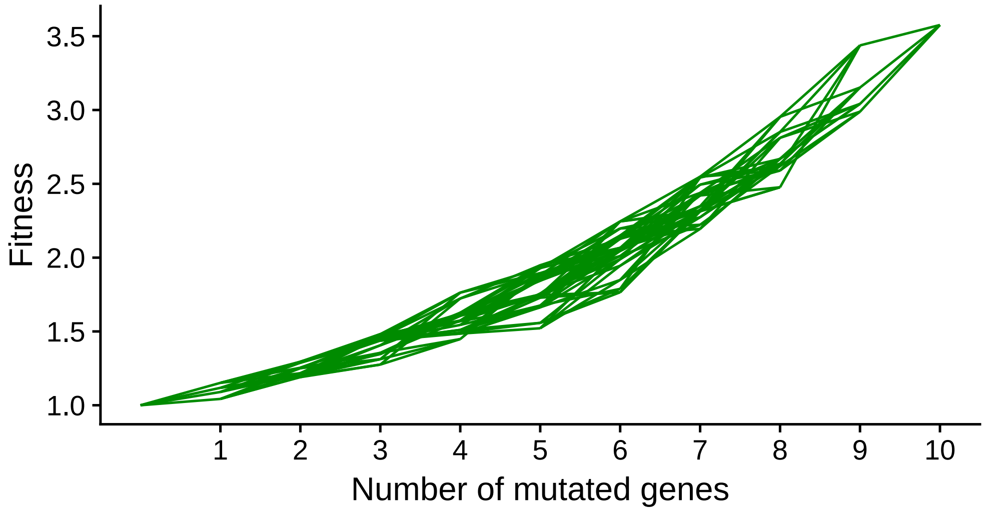

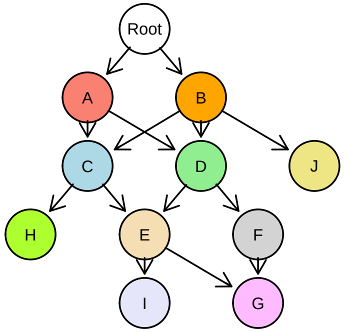

ID	YDOzLX10m8IHyrpAK
acc genotypes	46
peaks no back	1
removed edges	0
prop rem edges	0

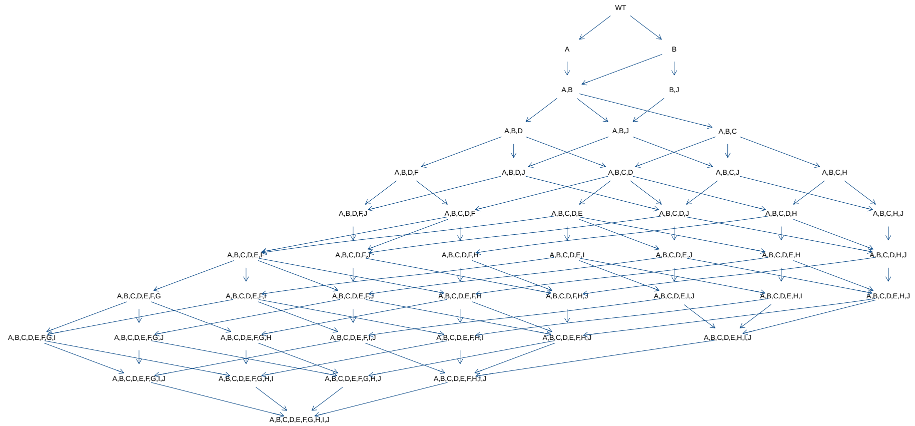

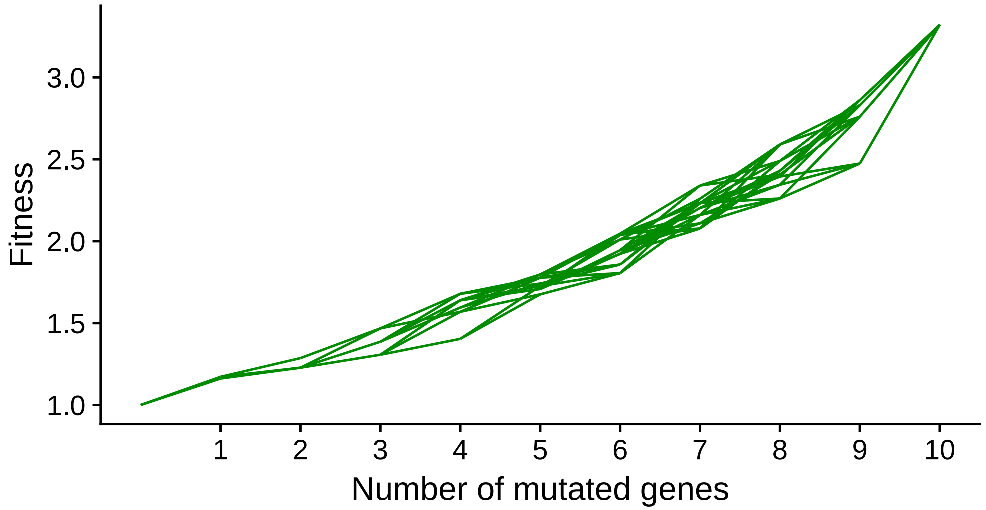

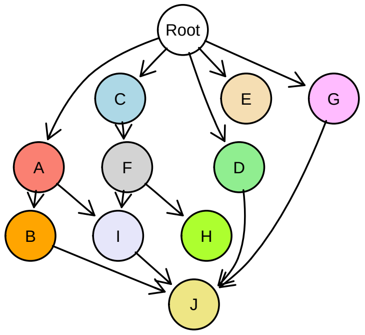

ID	yKg7RfKP7wBk10kV3
acc genotypes	132
peaks no back	1
removed edges	0
prop rem edges	0

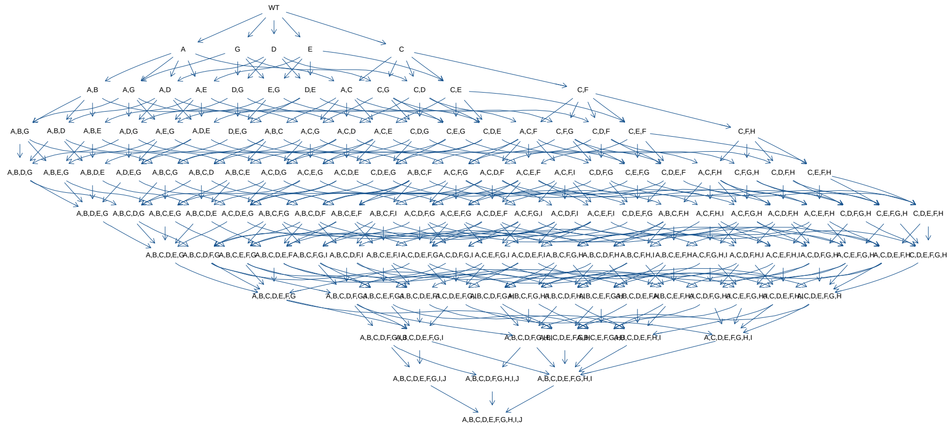

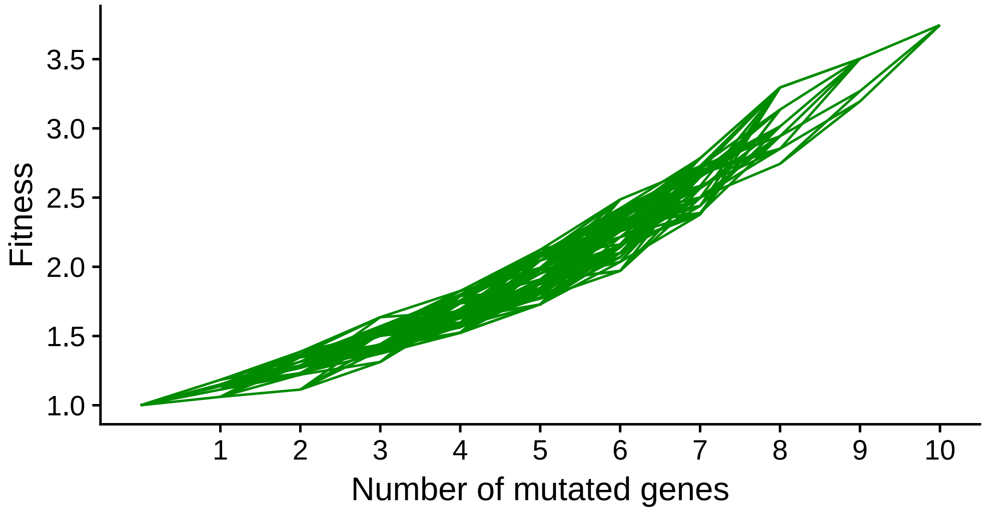

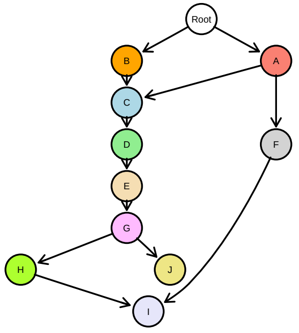

ID YlNcYnp363stUXfS
acc genotypes 22
peaks no back 1
removed edges 0
prop rem edges 0

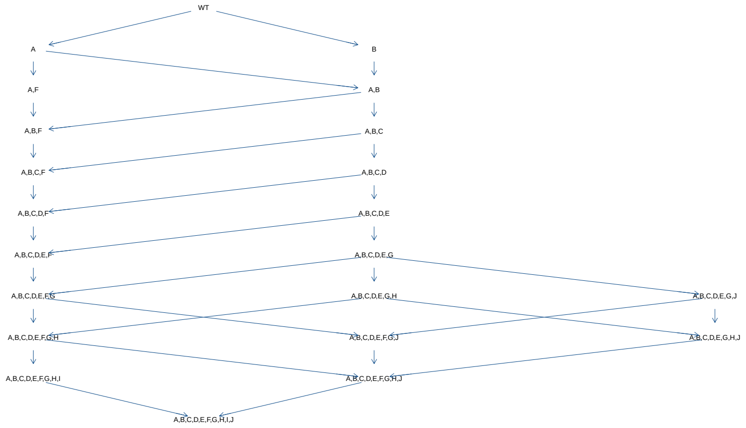

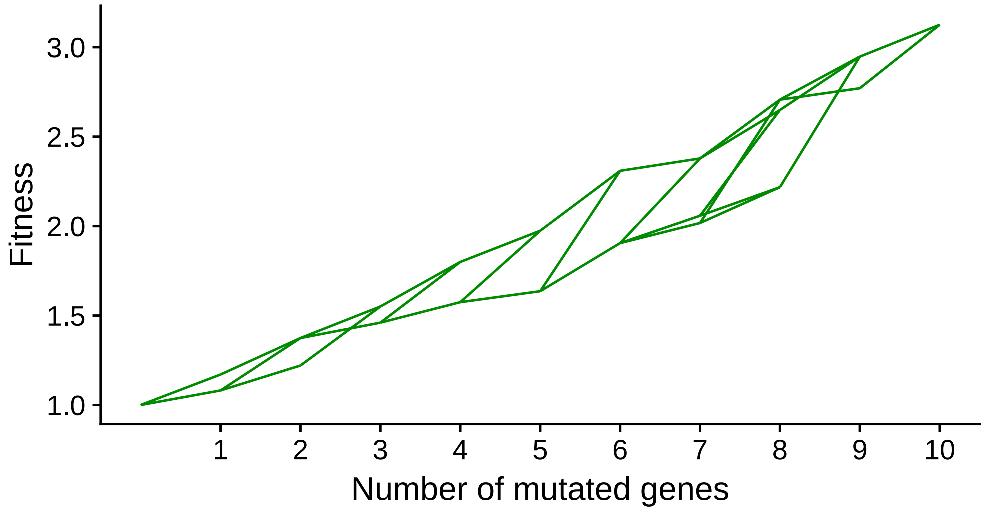

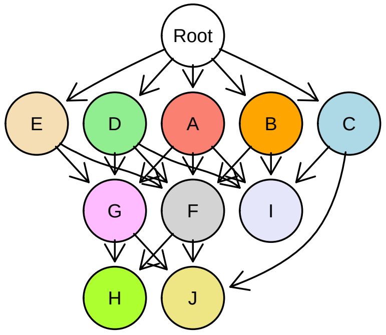

ID	YlnEDjC9bTAwZFRa
acc genotypes	52
peaks no back	1
removed edges	0
prop rem edges	0

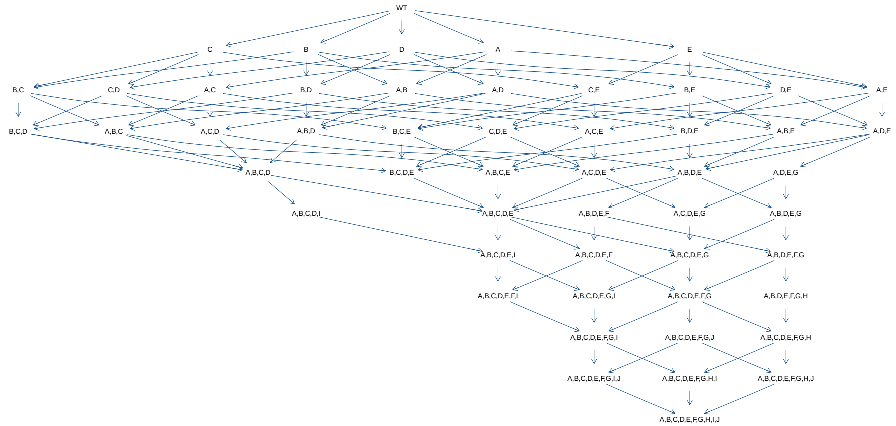

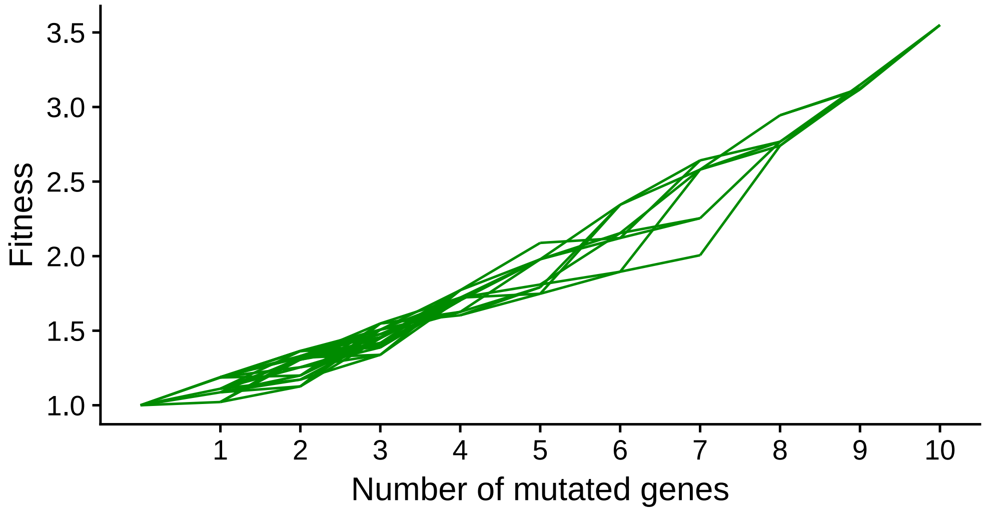

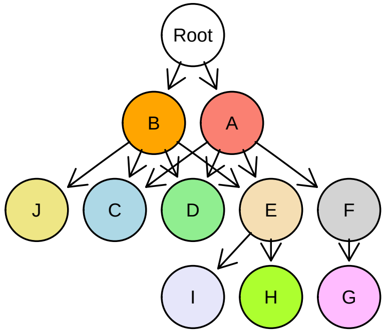

ID	YsJ3QBe72KOpkYgG
acc genotypes	126
peaks no back	1
removed edges	0
prop rem edges	0

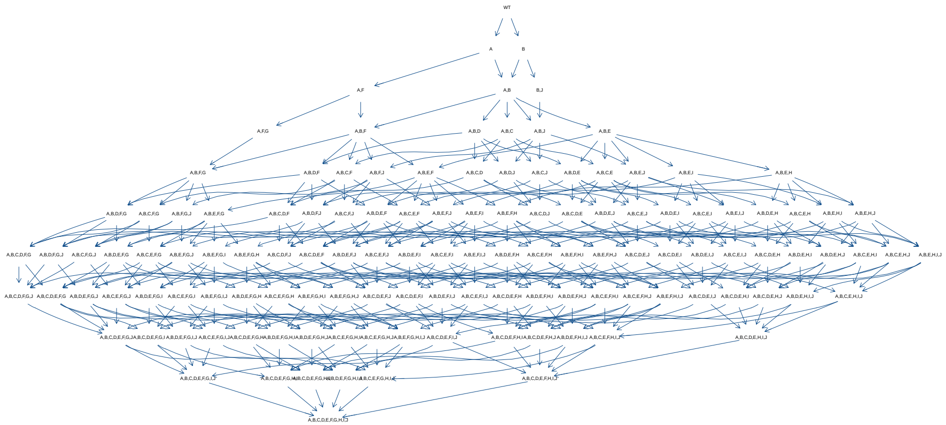

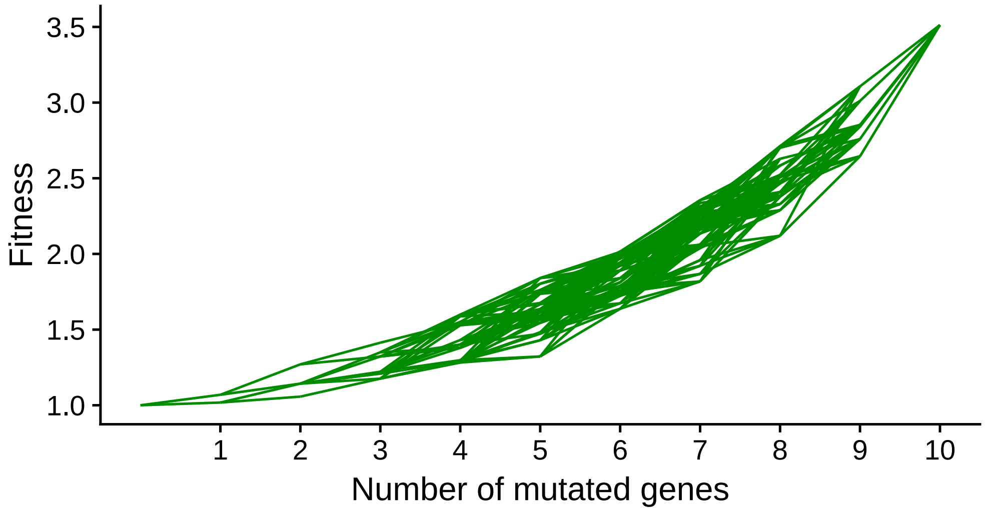

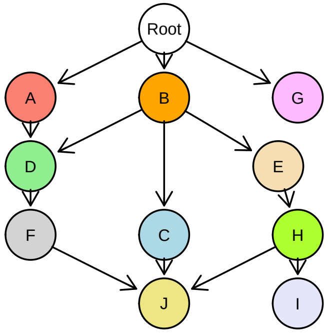

ID yUkCSfeekAS3UsYq
acc genotypes 72
peaks no back 1
removed edges 0
prop rem edges 0

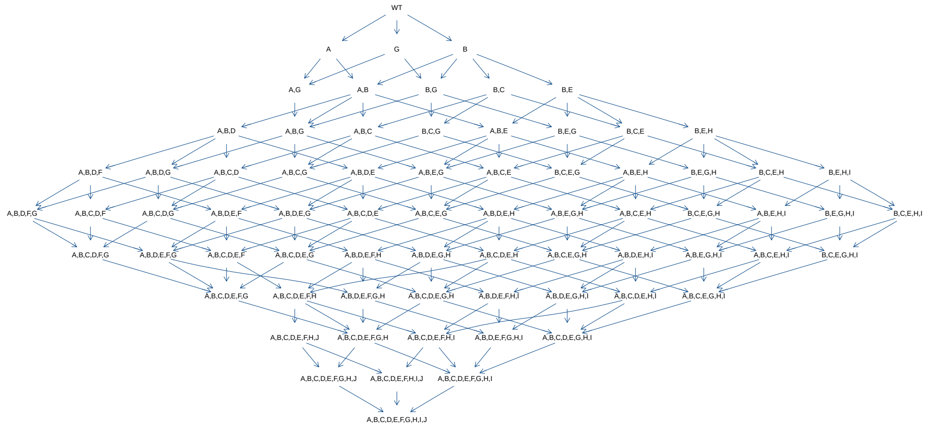

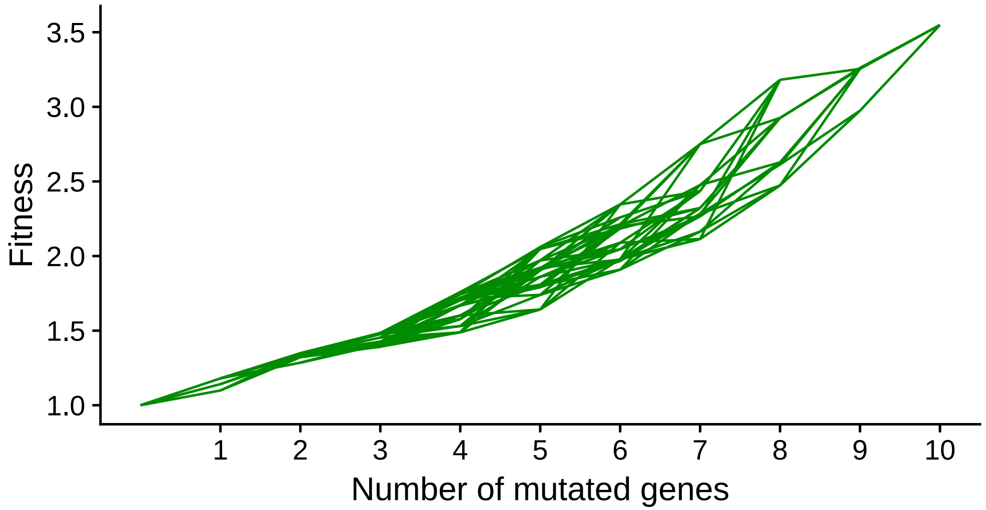

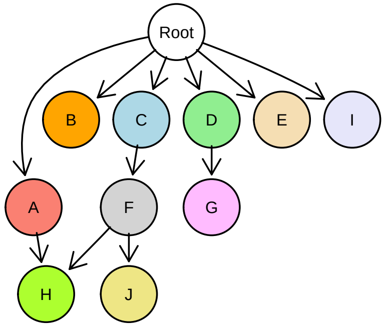

ID	z1gqmdhtodgm6biP
acc genotypes	240
peaks no back	1
removed edges	0
prop rem edges	0

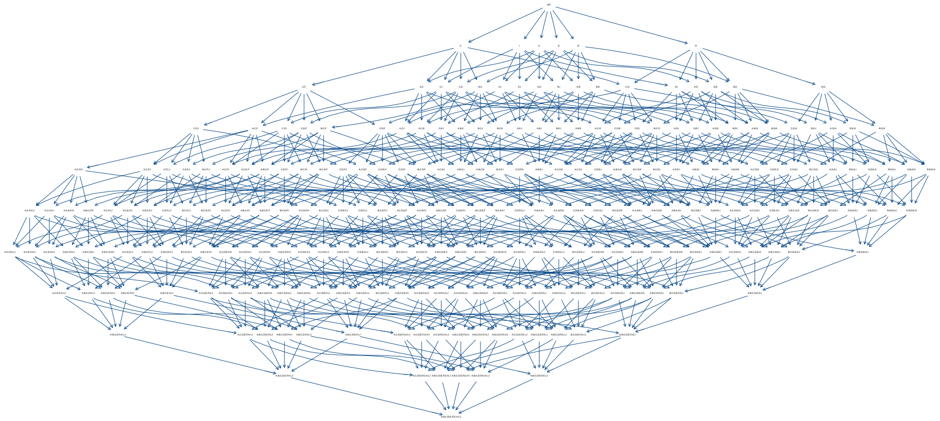

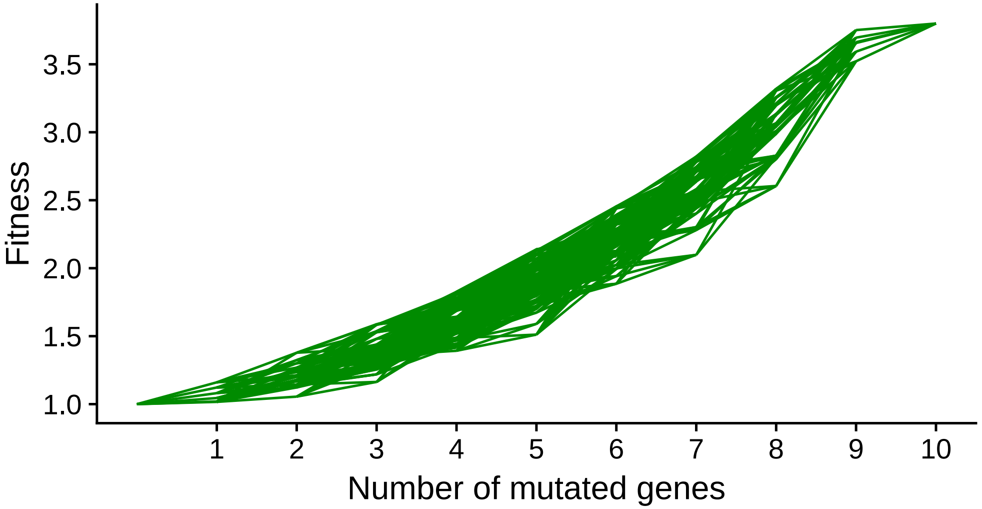

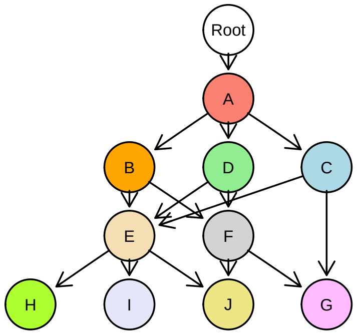

ID	z55GMs5PYH8kzvrX
acc genotypes	32
peaks no back	1
removed edges	0
prop rem edges	0

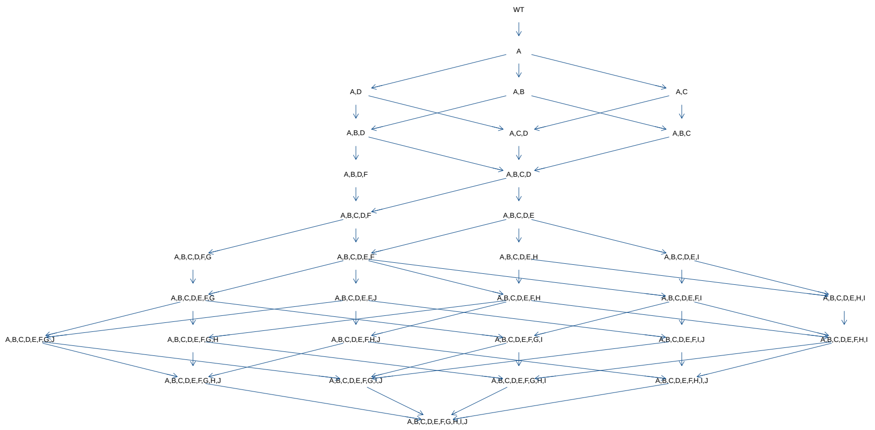

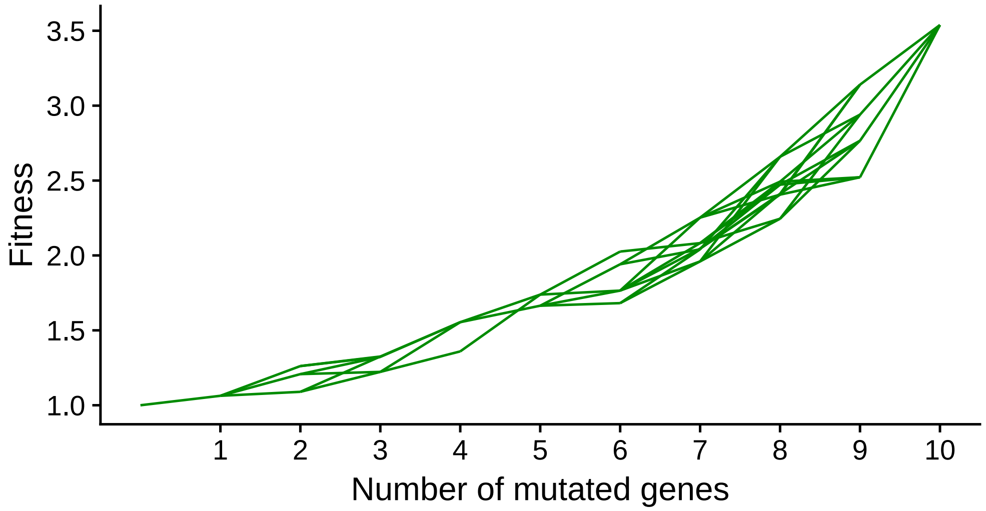

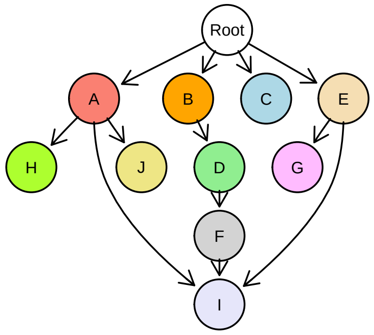

ID	ZEOZqZ8hAfpl29IT
acc genotypes	136
peaks no back	1
removed edges	0
prop rem edges	0

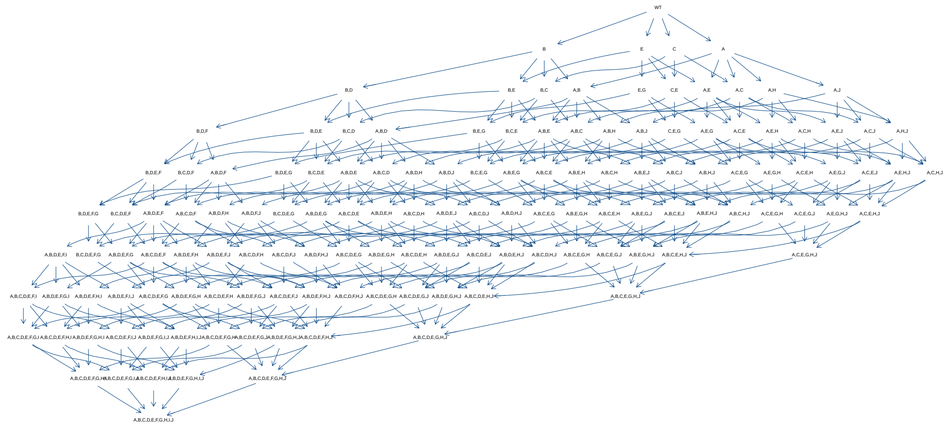

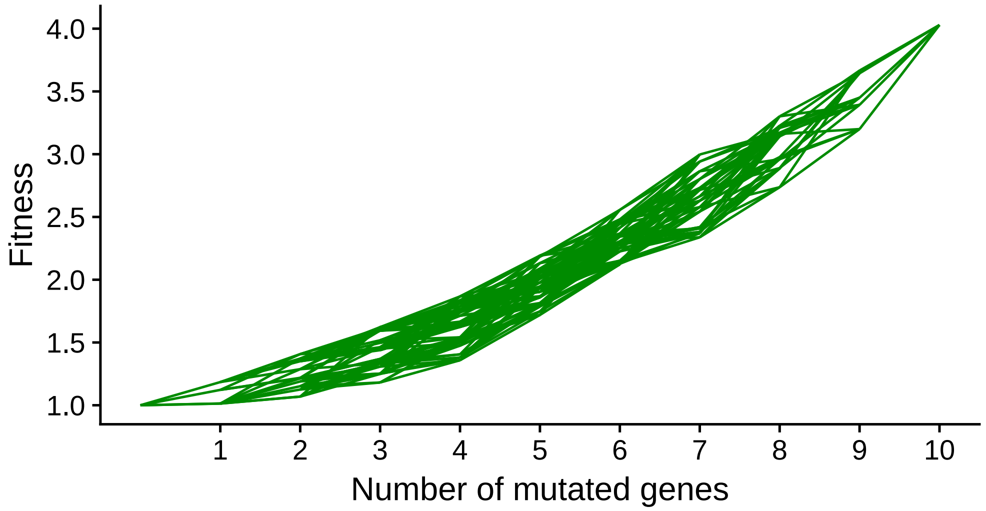

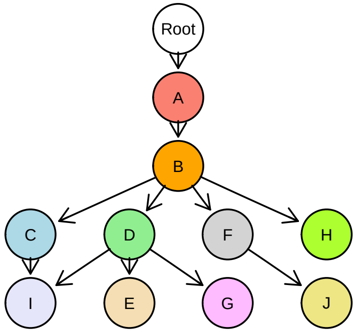

ID	zibjAEA10MPGwv10fw
acc genotypes	86
peaks no back	1
removed edges	0
prop rem edges	0

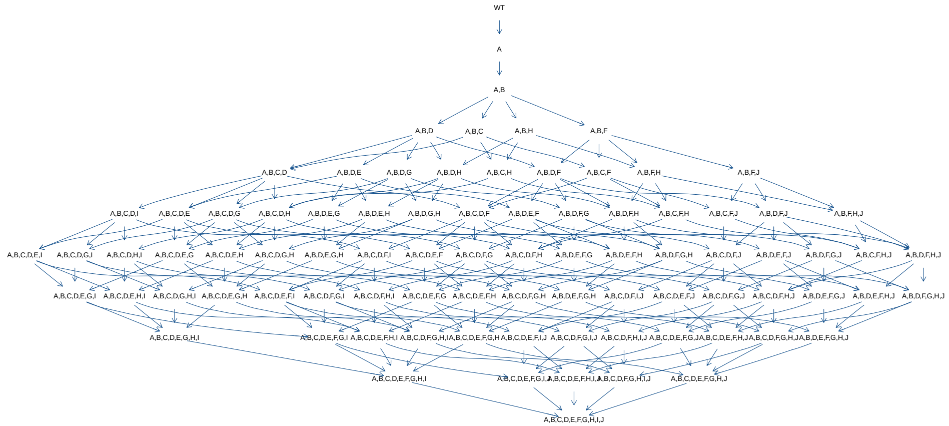

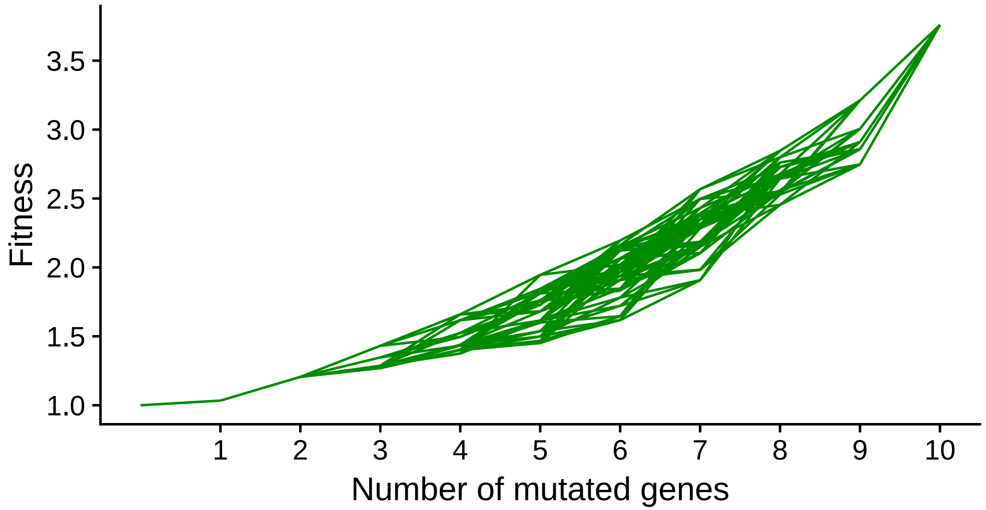

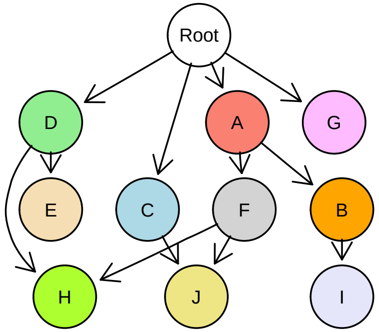

ID	zwBgTR5JXPWHnI1B
acc genotypes	138
peaks no back	1
removed edges	0
prop rem edges	0

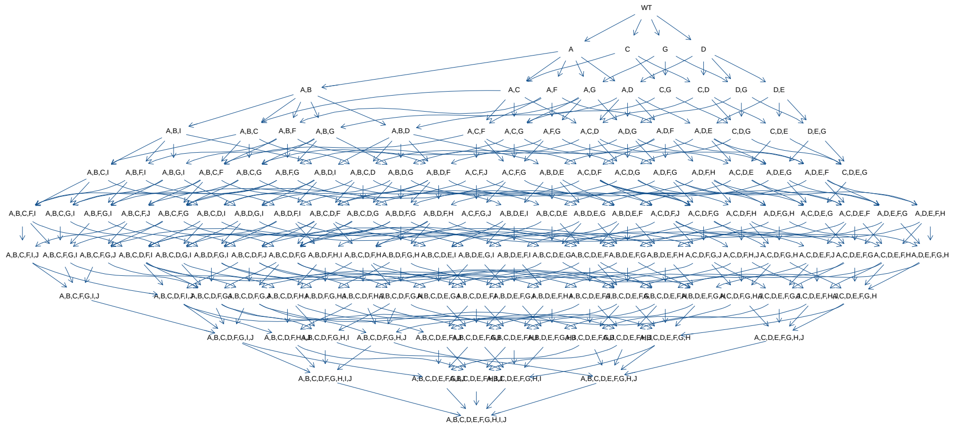

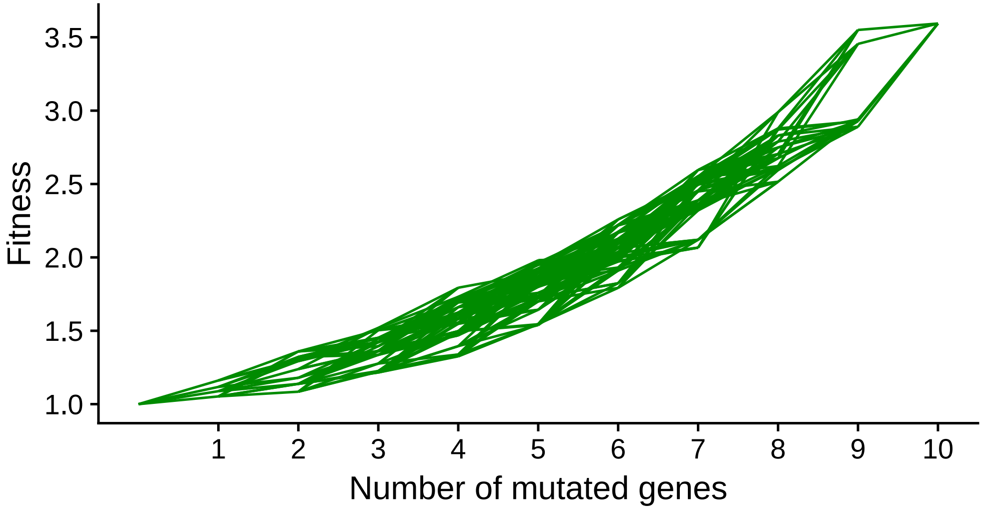

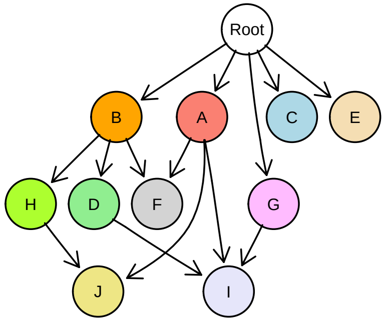

ID	10O7yB4hzcMcCLWhO
acc genotypes	168
peaks no back	15
removed edges	123
prop rem edges	0.228

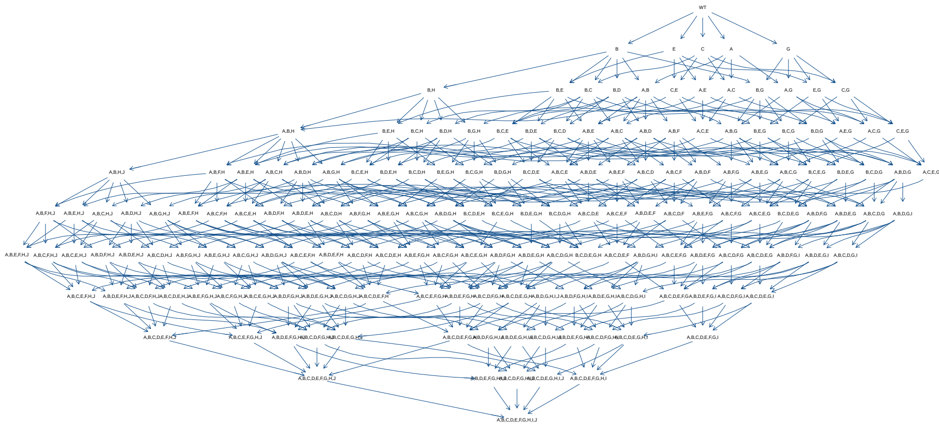

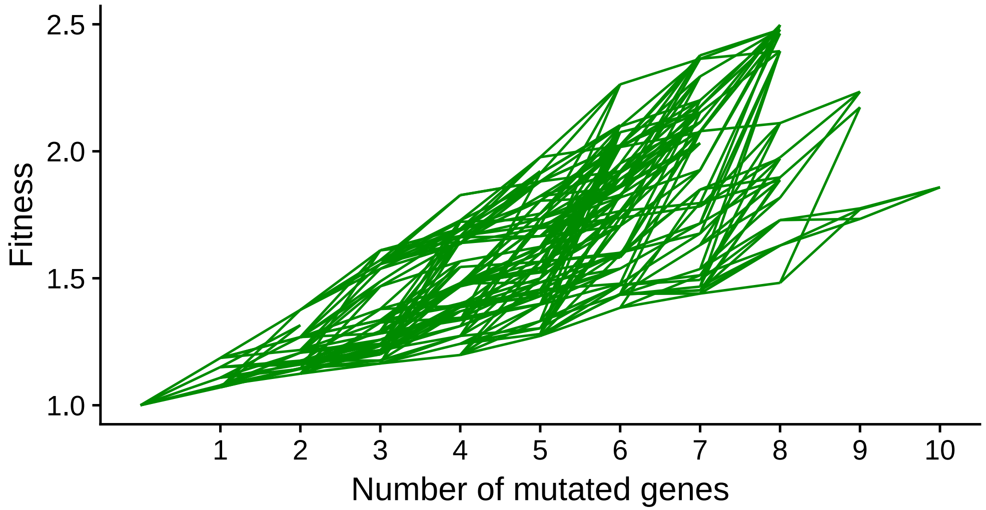

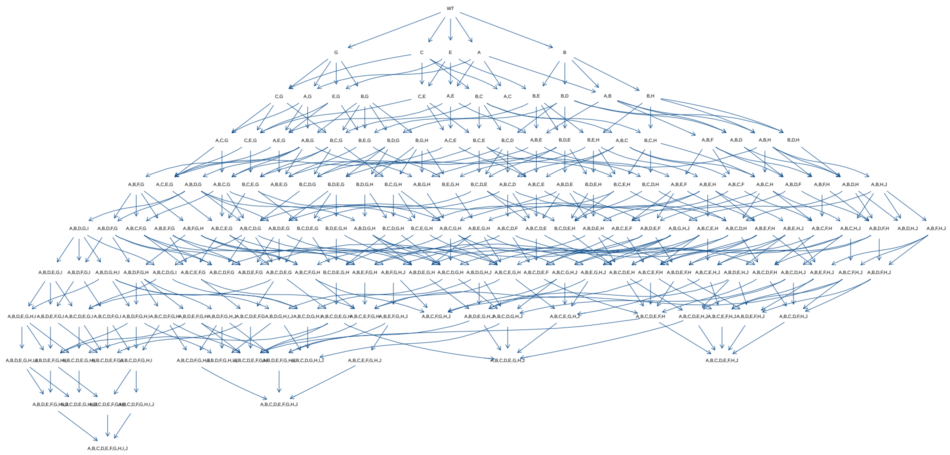

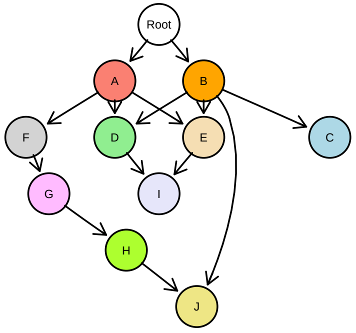

ID	1cfjEhrDN713Rg8k
acc genotypes	57
peaks no back	7
removed edges	19
prop rem edges	0.15

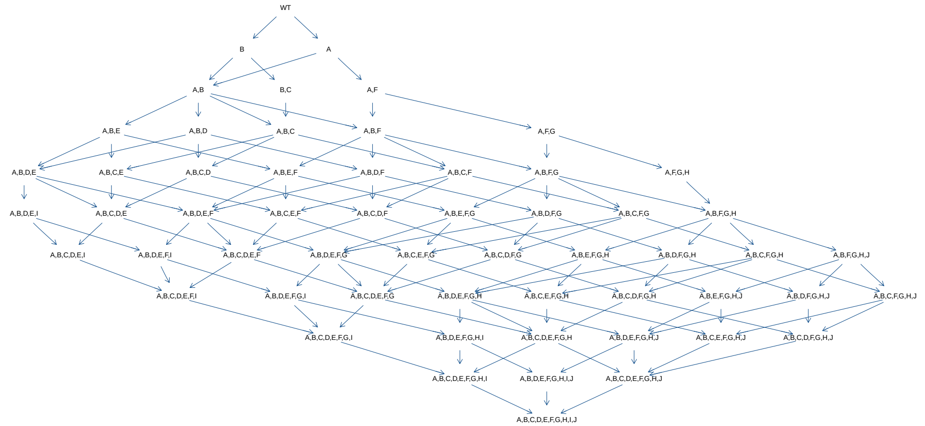

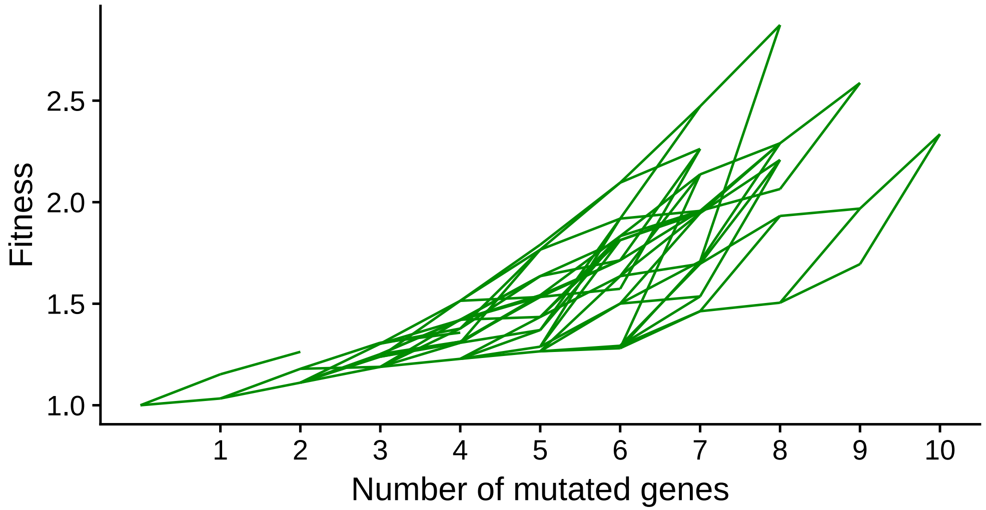

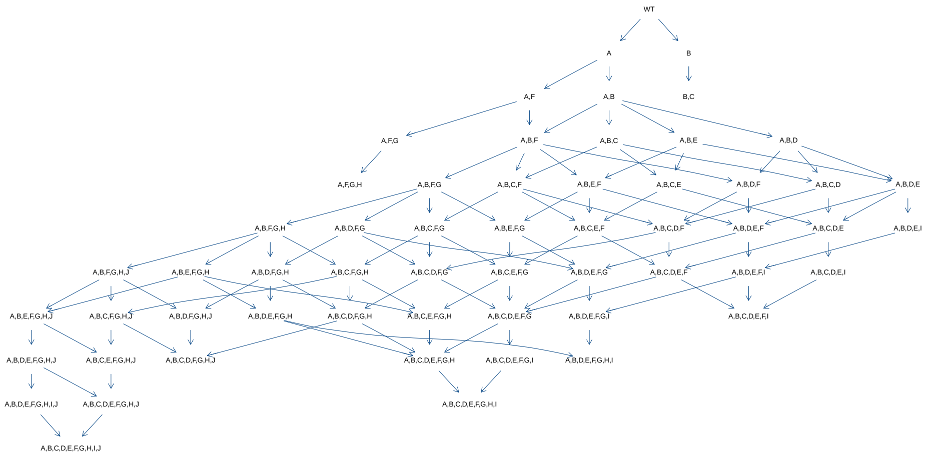

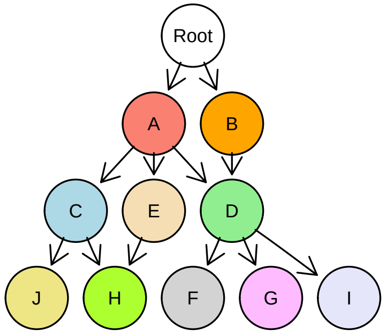

ID	1flJ9gvVIxHu10l4I
acc genotypes	82
peaks no back	10
removed edges	36
prop rem edges	0.167

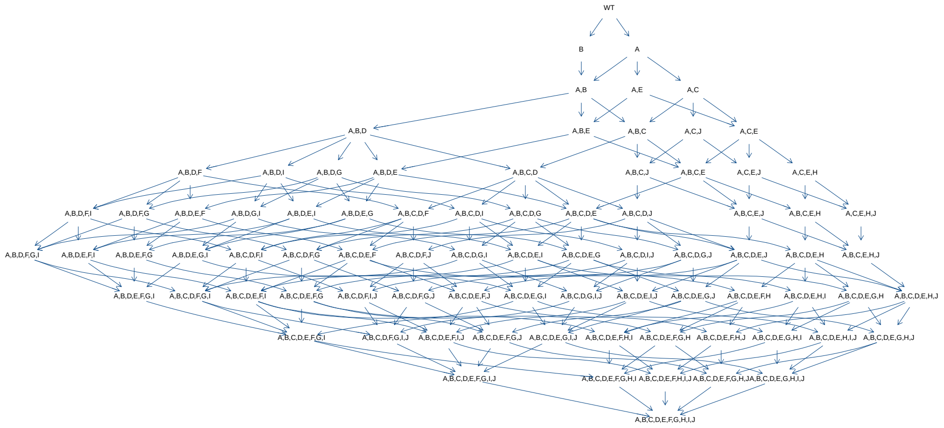

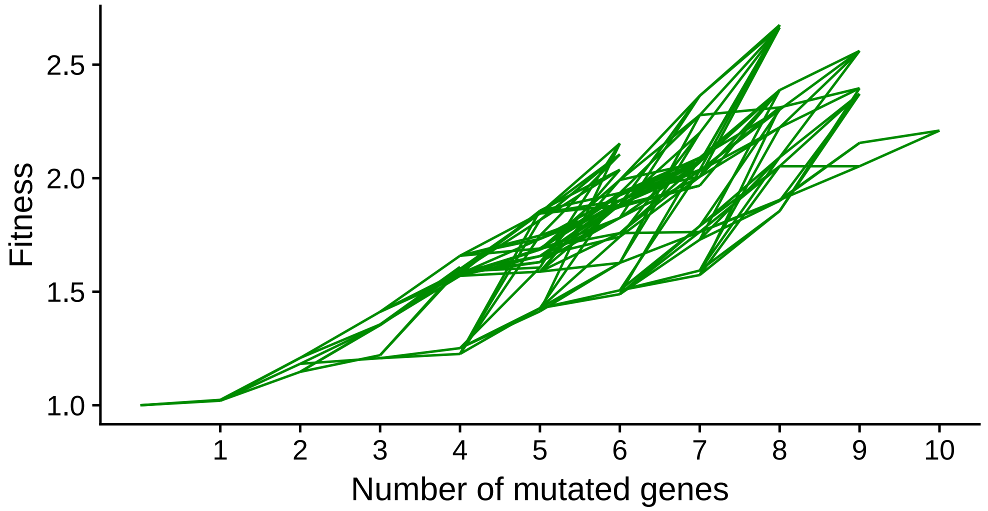

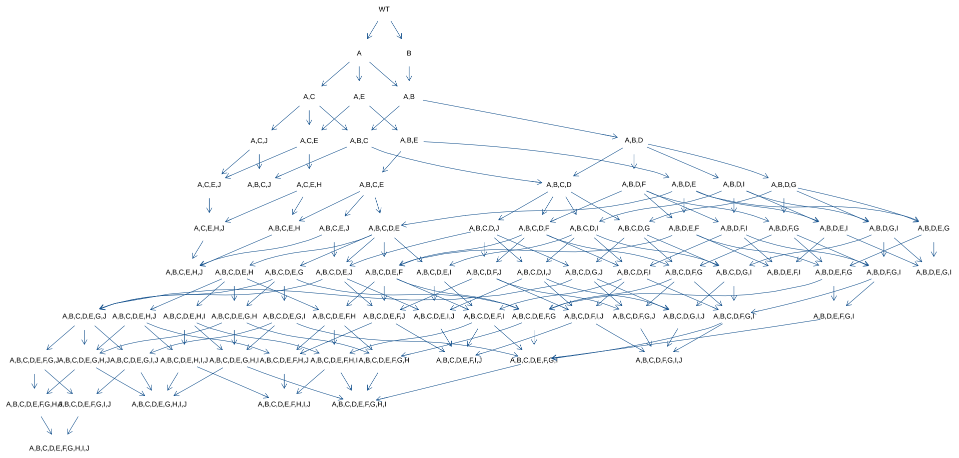

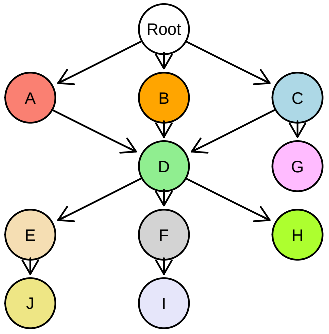

ID 1LRNl4YXQOtosQeO
acc genotypes 48
peaks no back 7
removed edges 20
prop rem edges 0.189

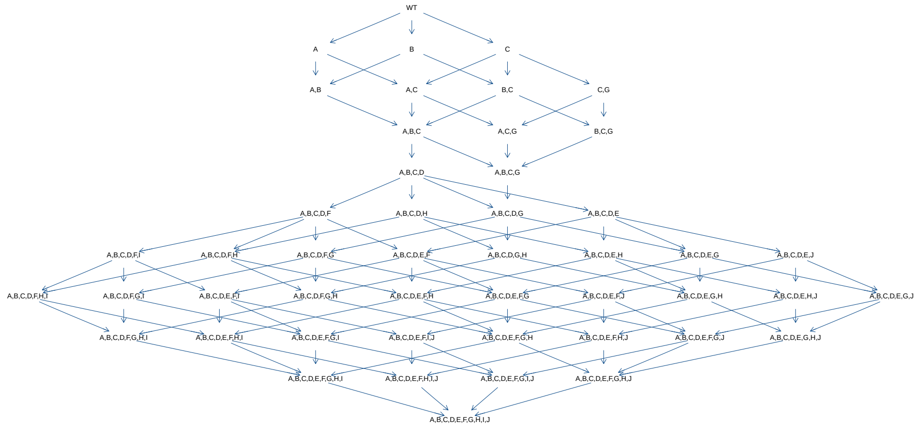

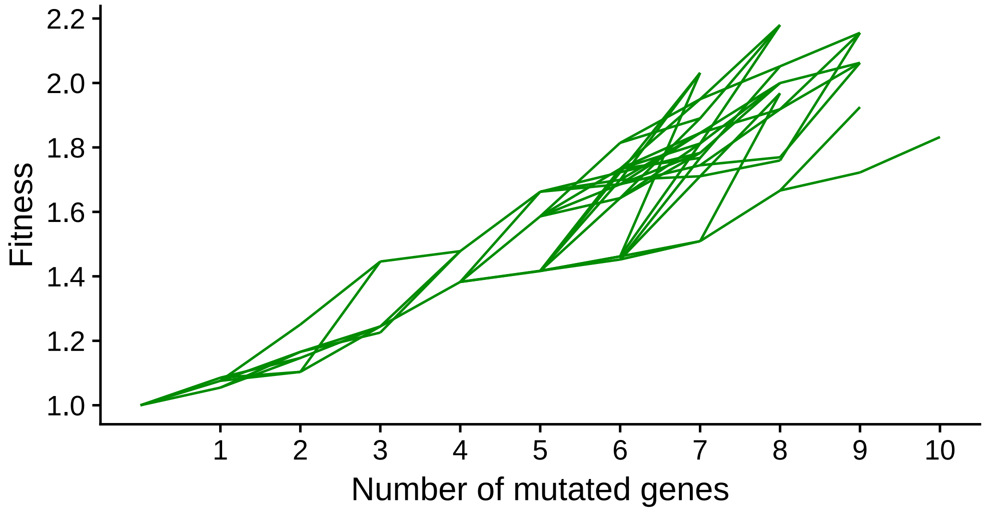

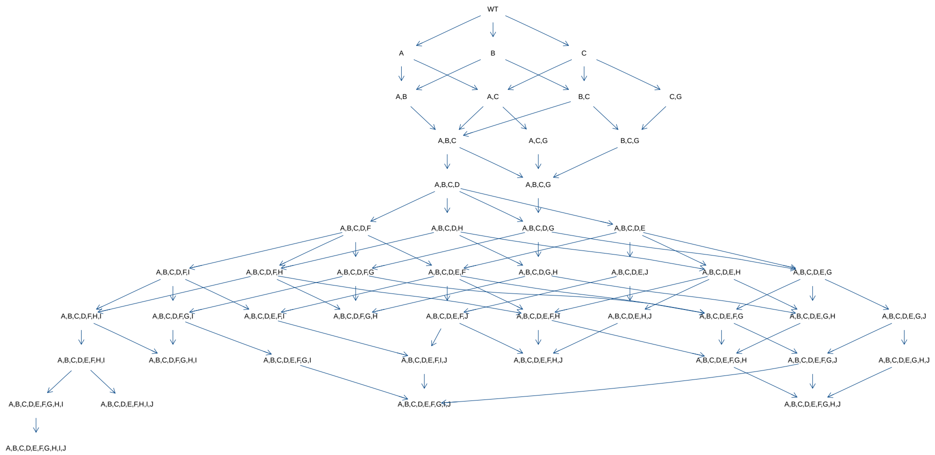

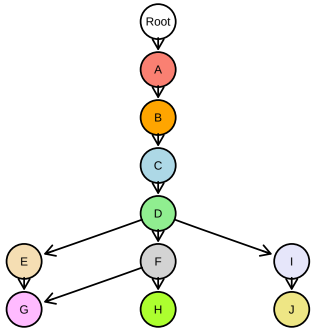

ID	24psYb2Z5uoeEIgL
acc genotypes	28
peaks no back	5
removed edges	9
prop rem edges	0.18

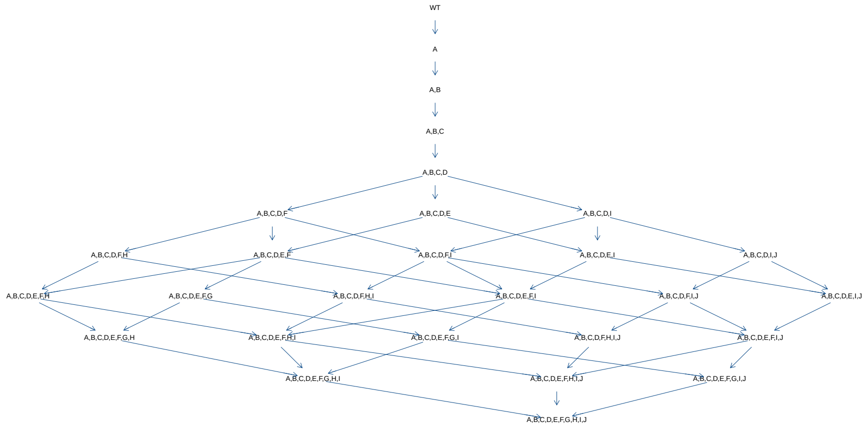

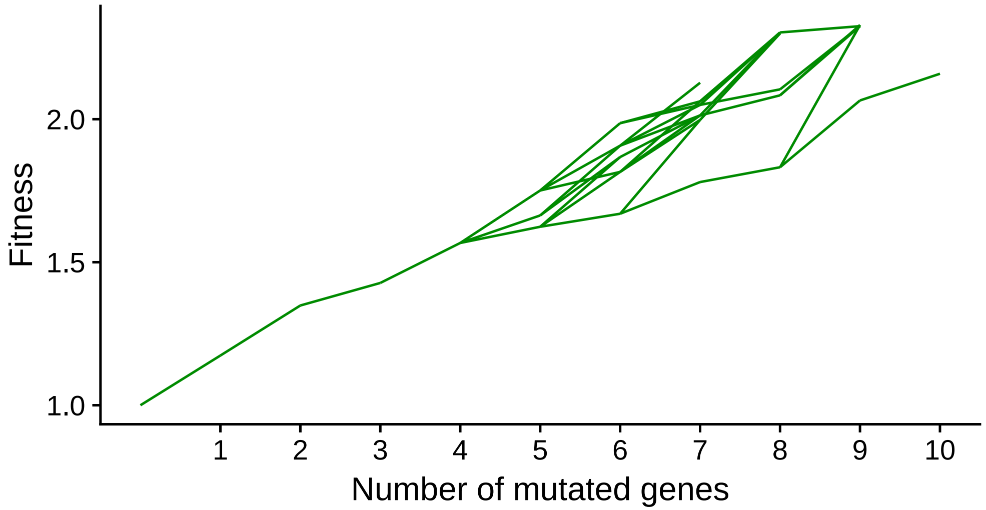

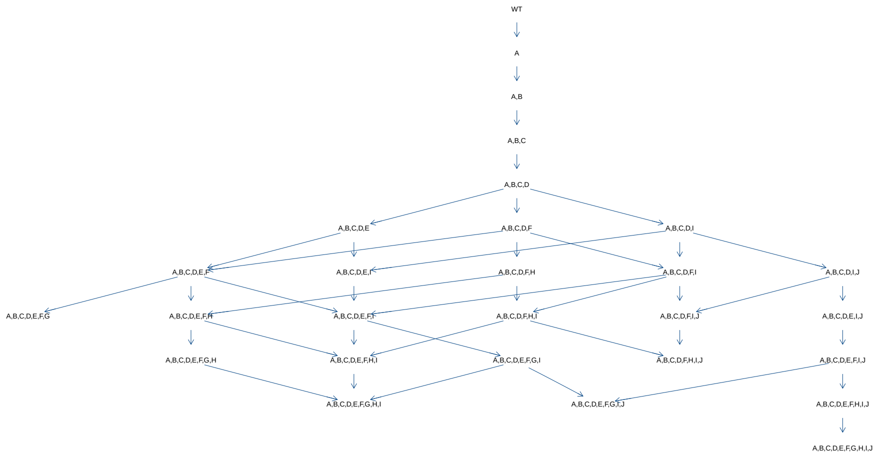

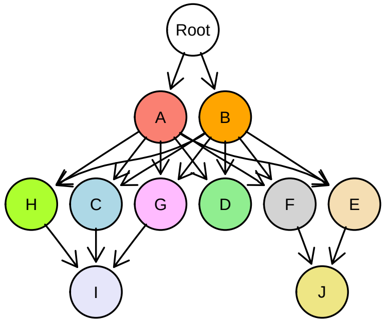

ID	26j4DR9Ab9swRweC
acc genotypes	93
peaks no back	10
removed edges	40
prop rem edges	0.149

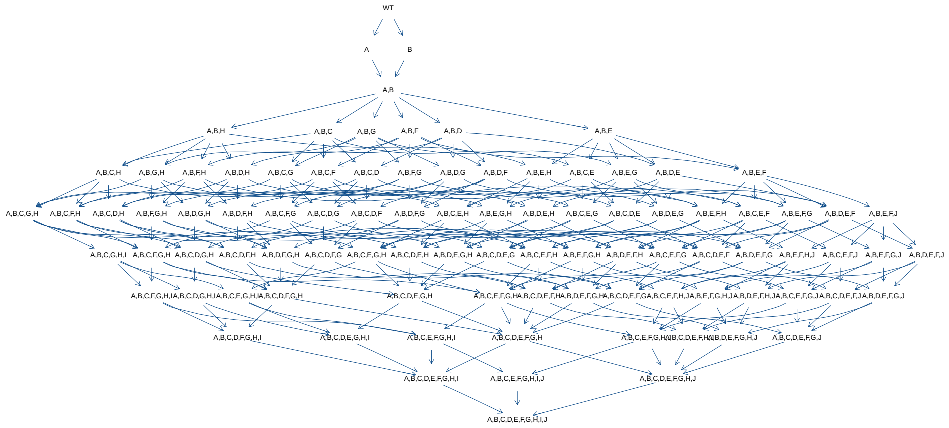

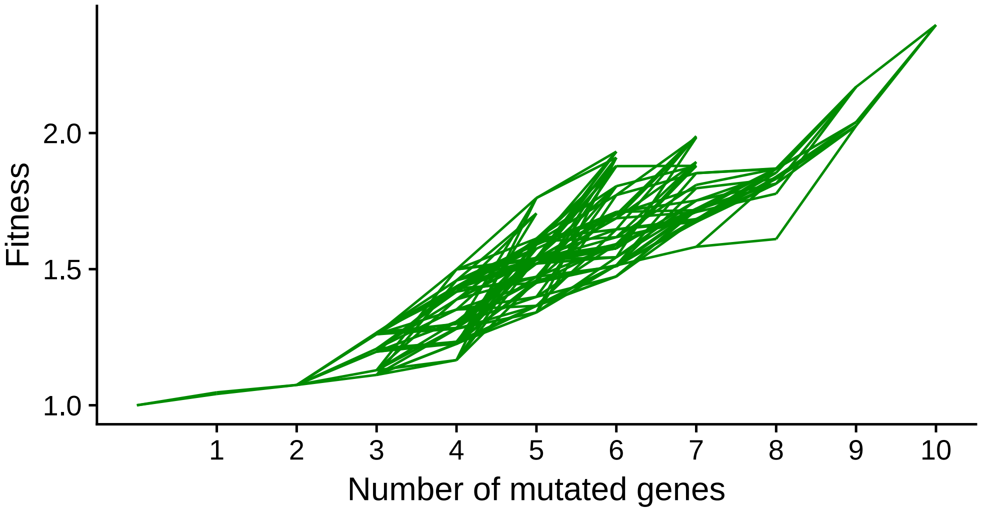

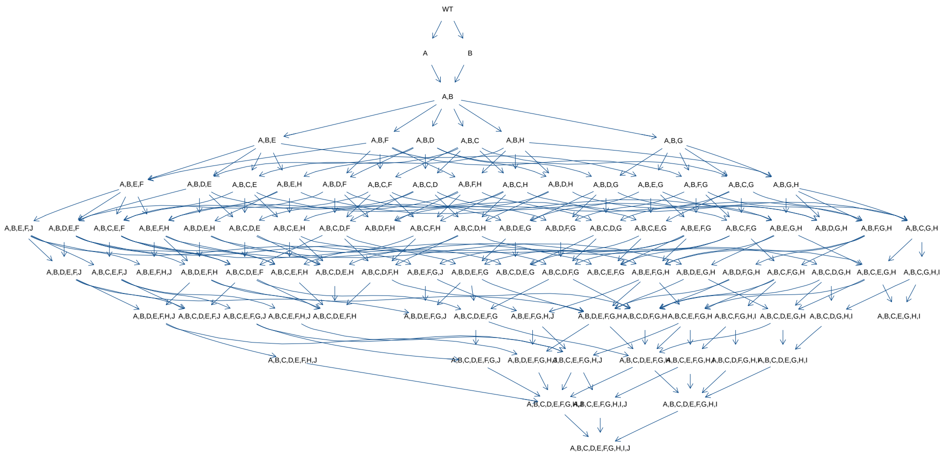

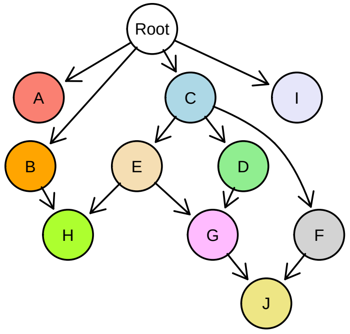

ID	29J8aMCesoGoSpvM
acc genotypes	124
peaks no back	10
removed edges	55
prop rem edges	0.149

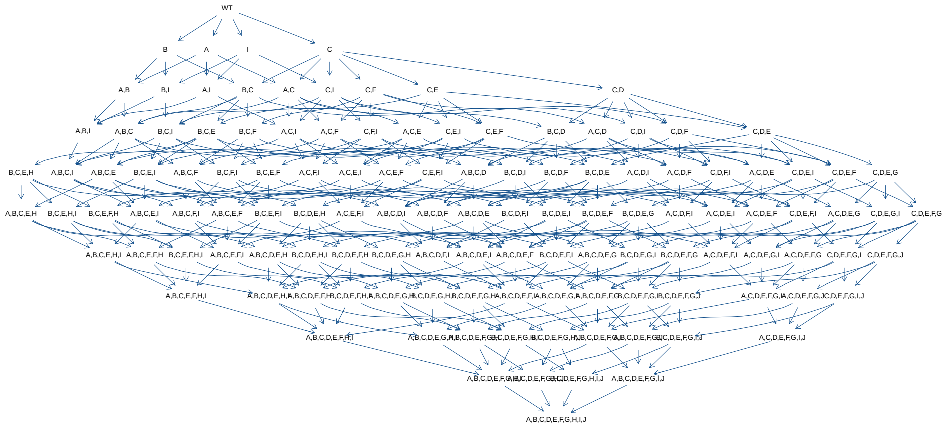

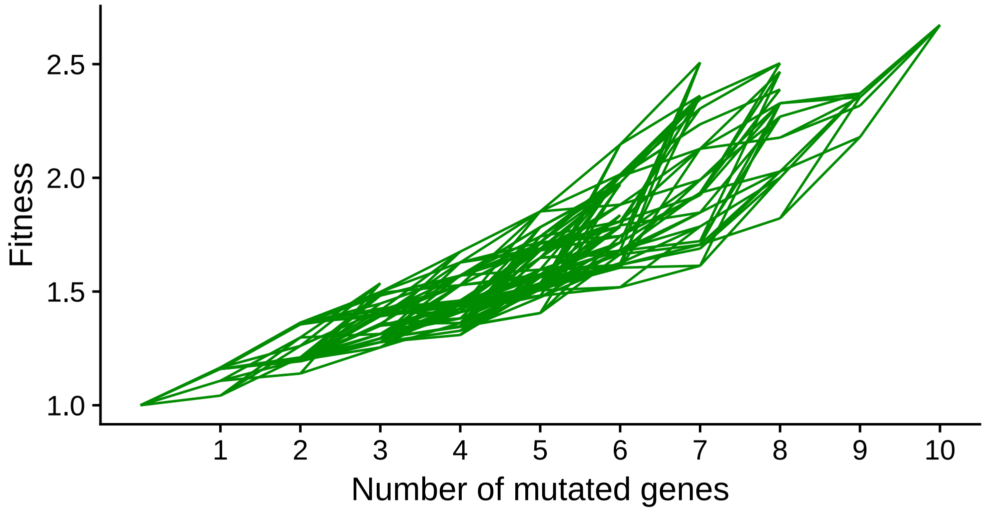

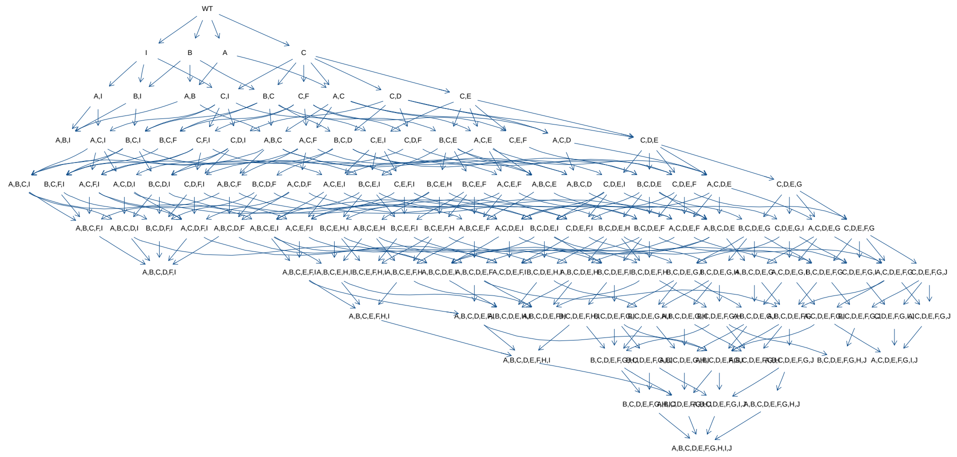

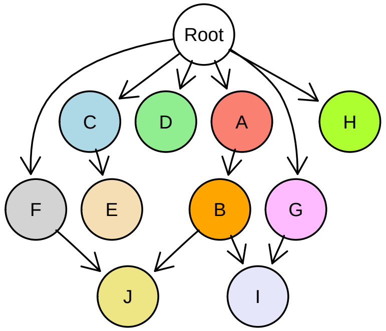

ID	2e8NWr3WSIIdSHqm
acc genotypes	204
peaks no back	16
removed edges	139
prop rem edges	0.206

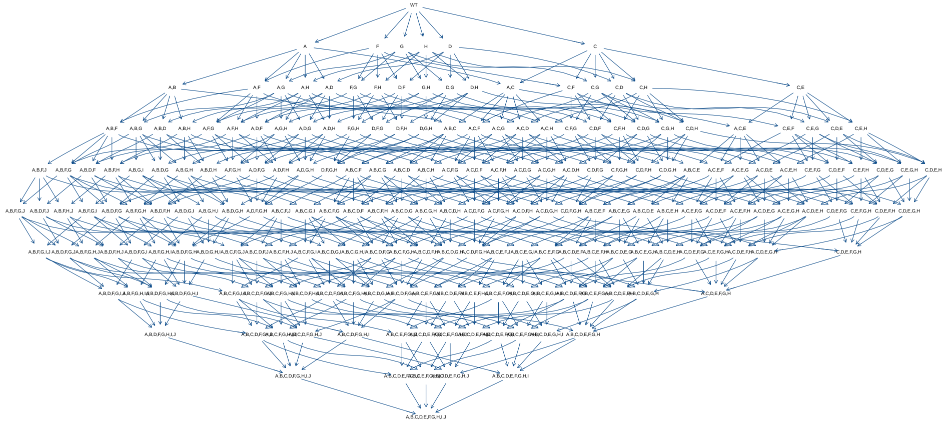

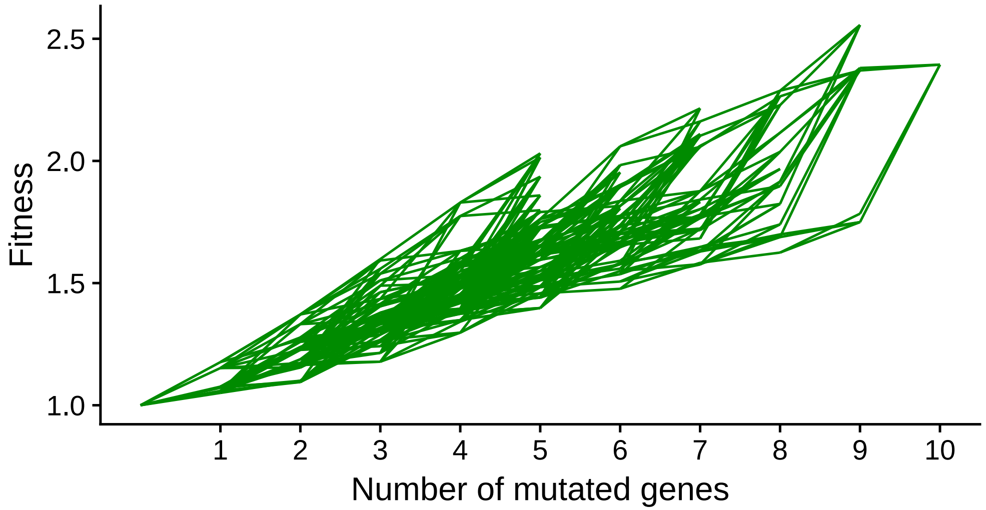

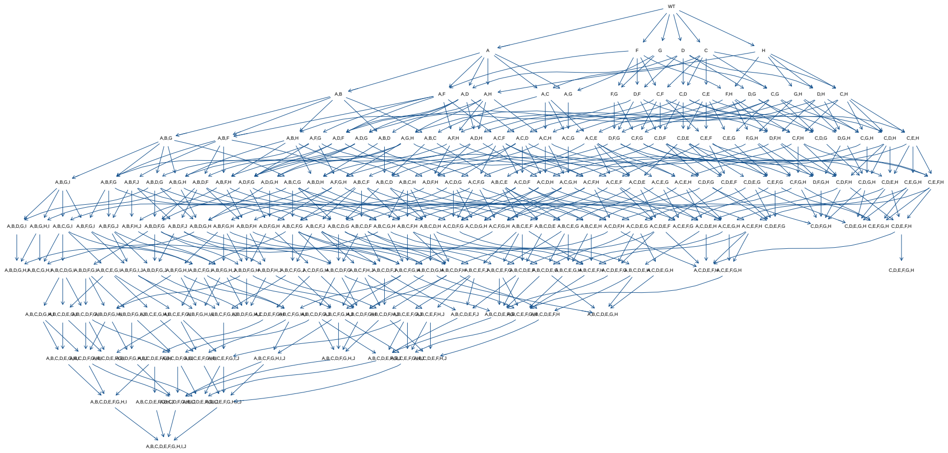

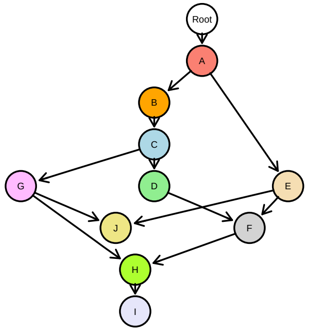

ID 2NKDNCMNshVULJ2E
acc genotypes 22
peaks no back 2
removed edges 3
prop rem edges 0.091

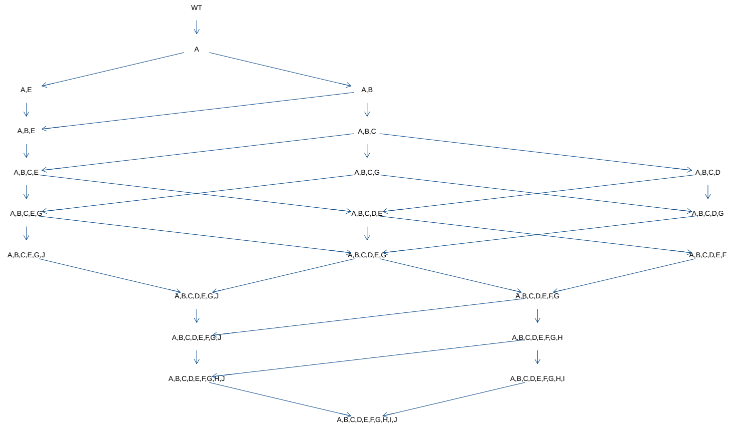

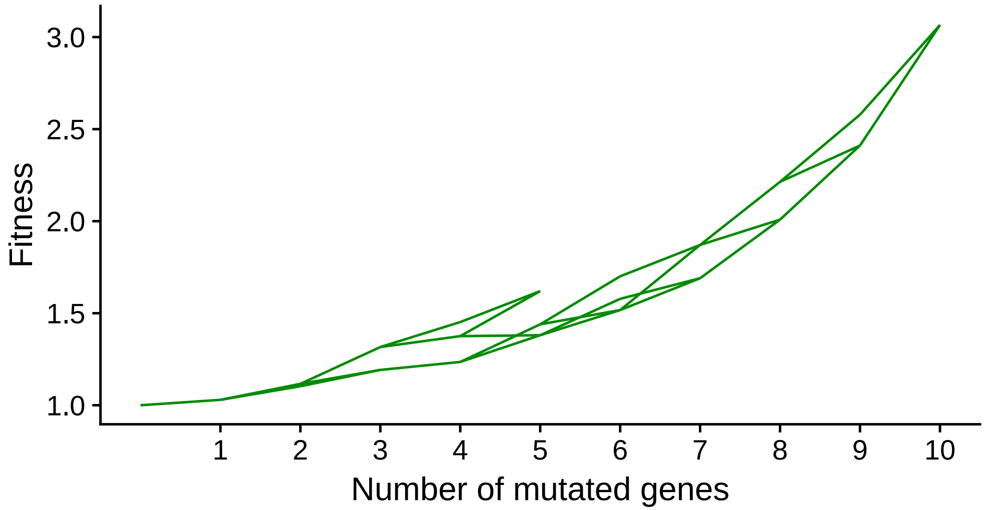

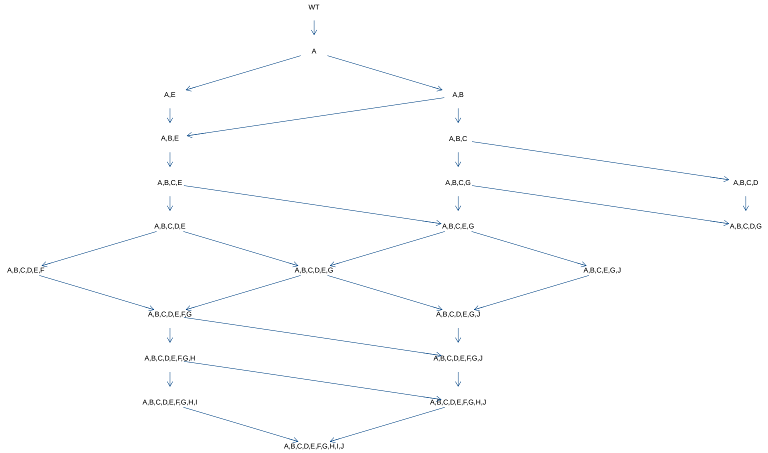

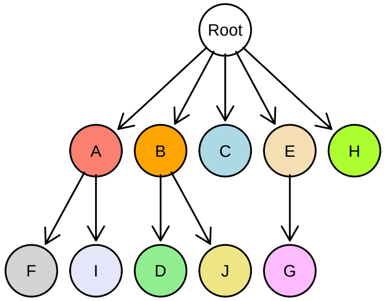

ID	2ti105XmgduWlCPHP
acc genotypes	300
peaks no back	24
removed edges	245
prop rem edges	0.223

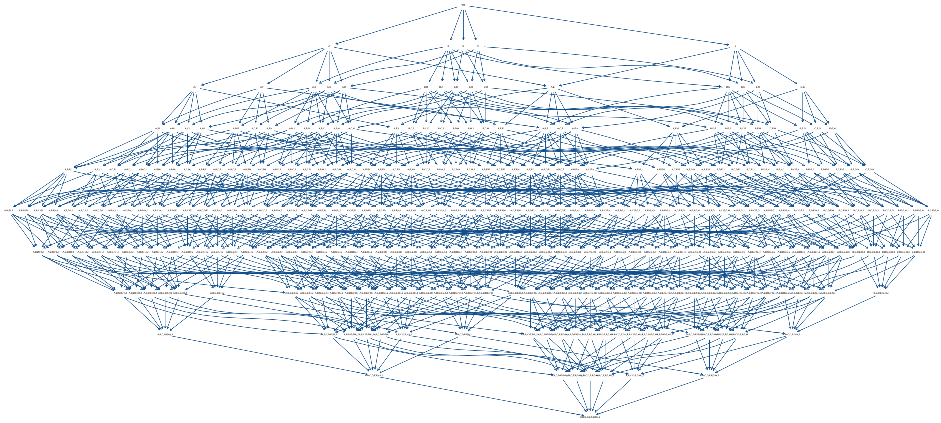

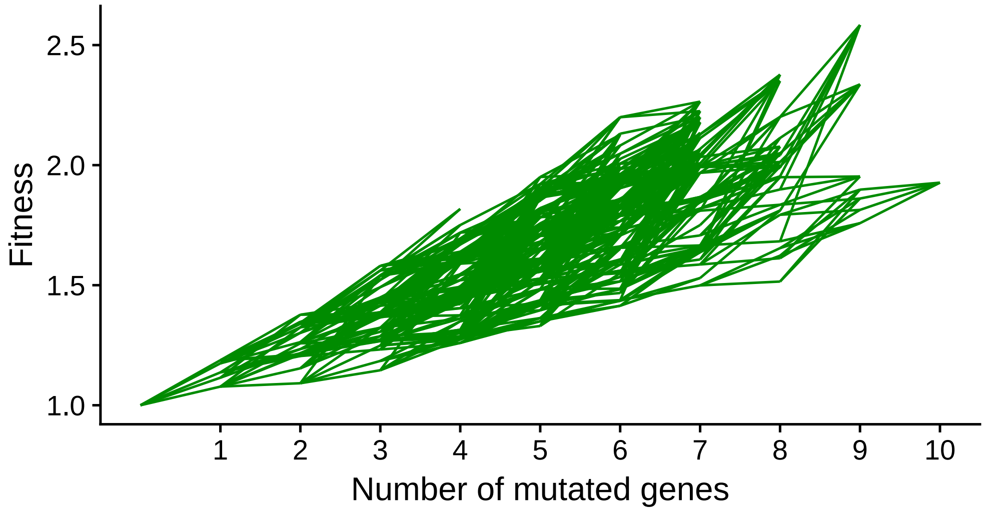

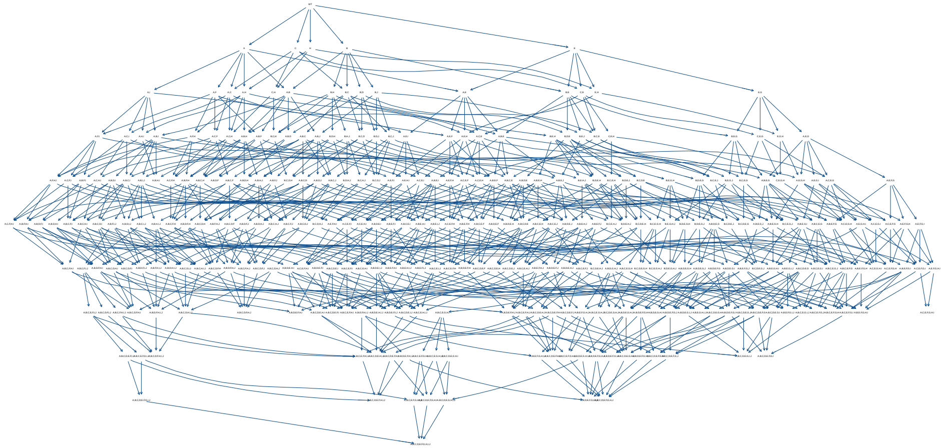

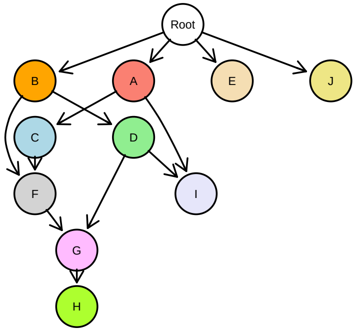

ID	3a7jMZPzBxEpCMra
acc genotypes	72
peaks no back	11
removed edges	42
prop rem edges	0.239

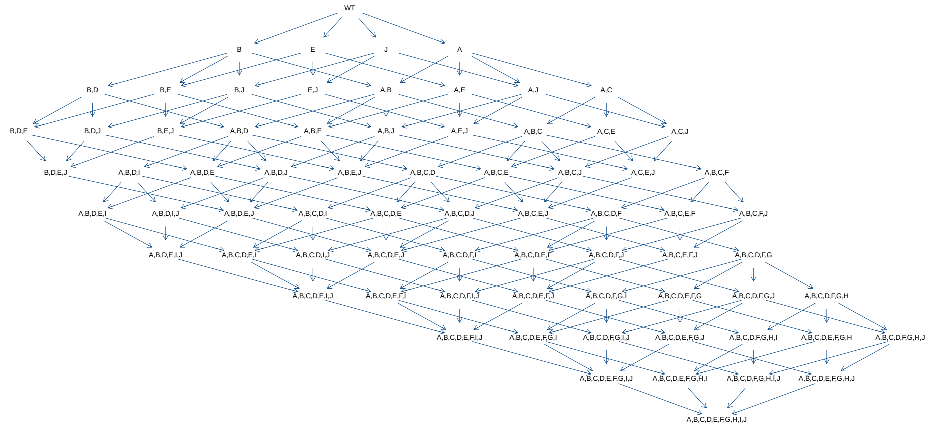

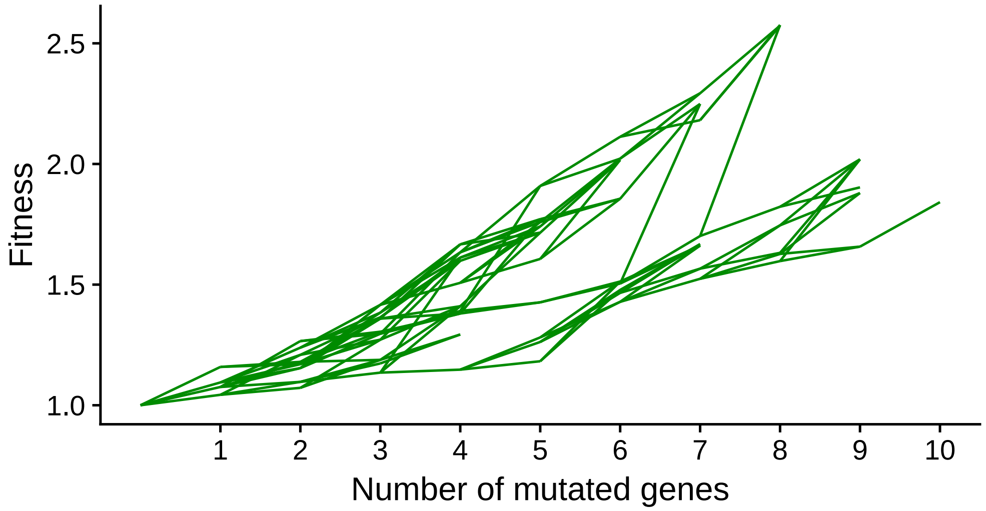

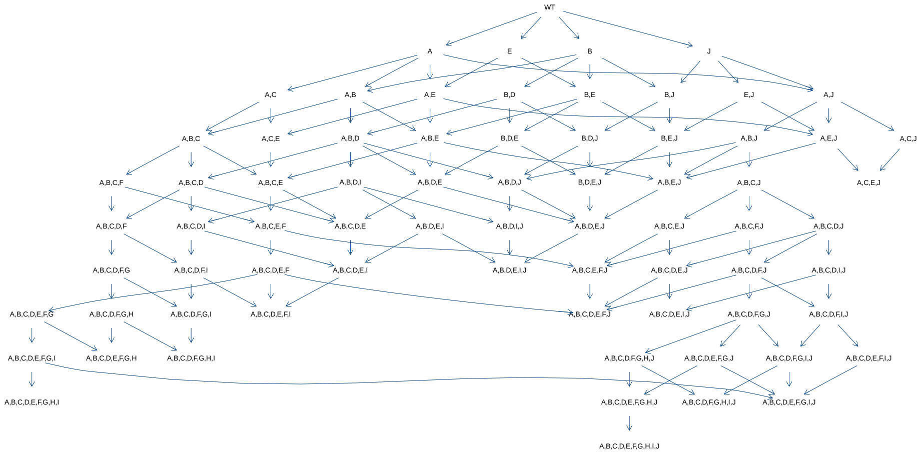

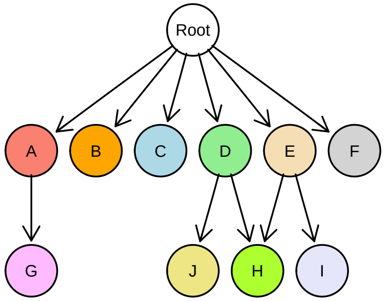

ID 46TSoH89nM3Ac4Jd
acc genotypes 312
peaks no back 23
removed edges 210
prop rem edges 0.182

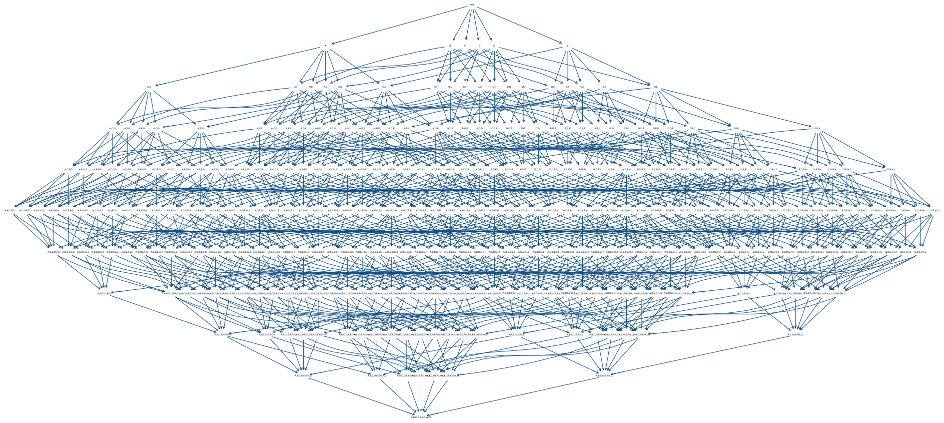

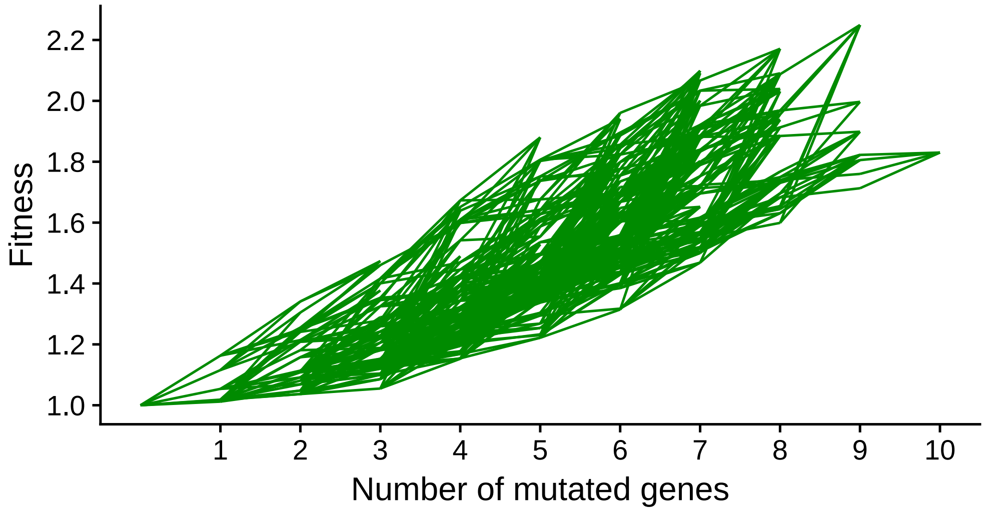

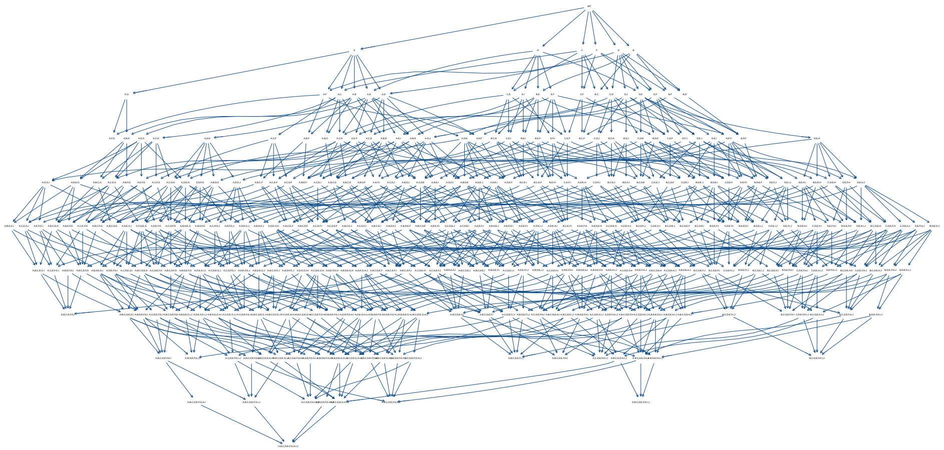

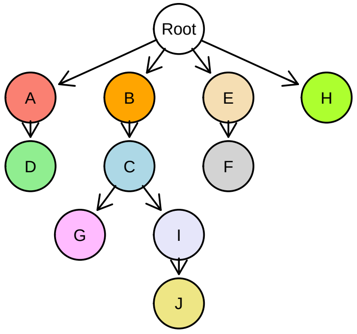

ID	4KWL9eg4fUjg1ndt
acc genotypes	144
peaks no back	15
removed edges	97
prop rem edges	0.228

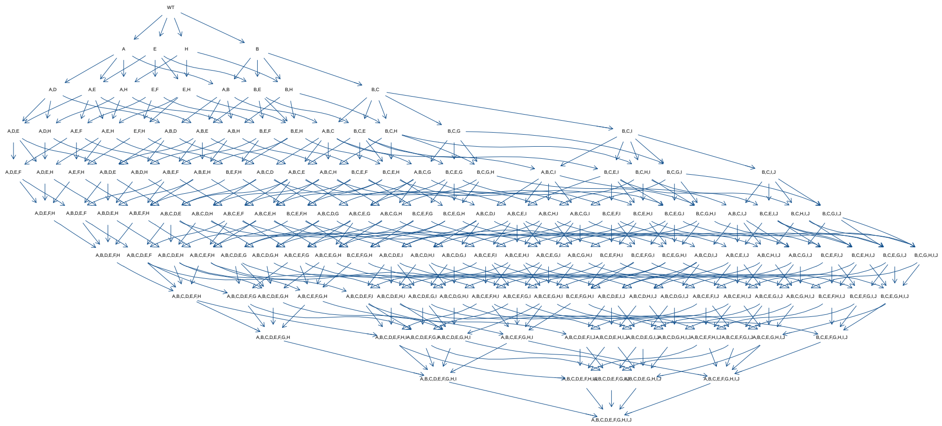

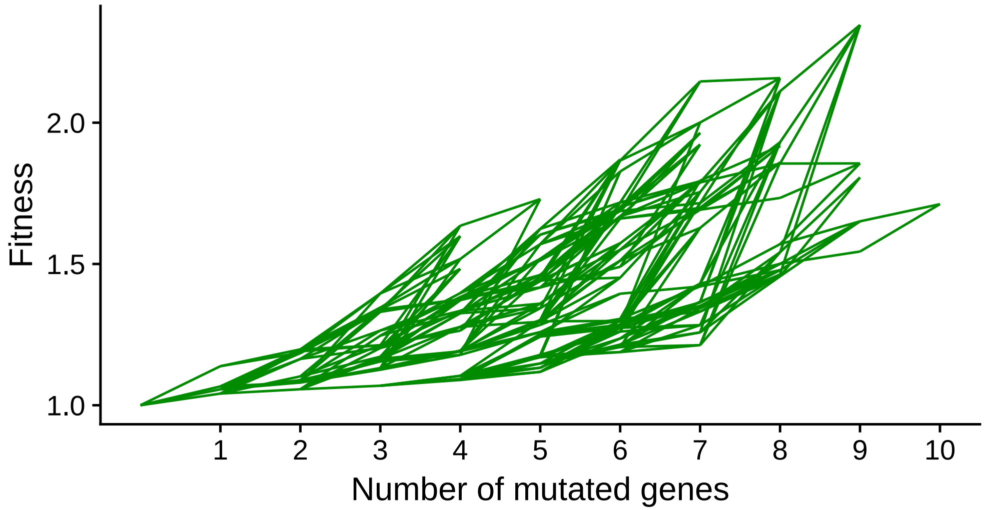

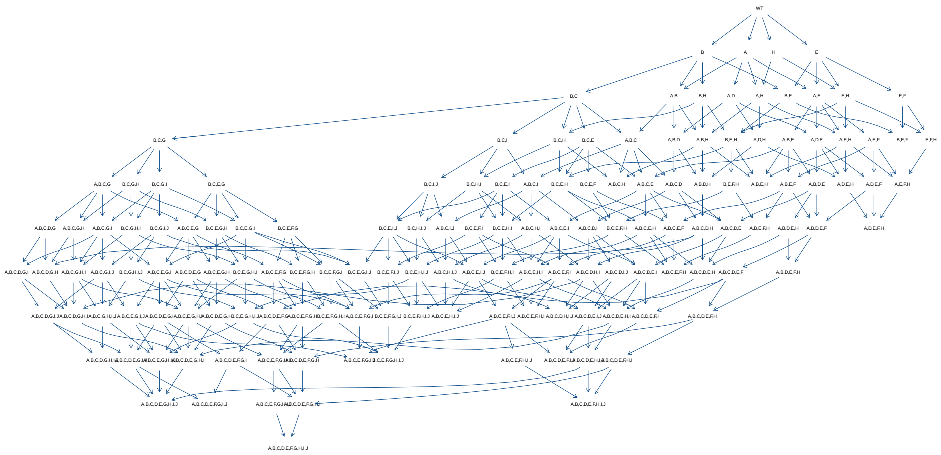

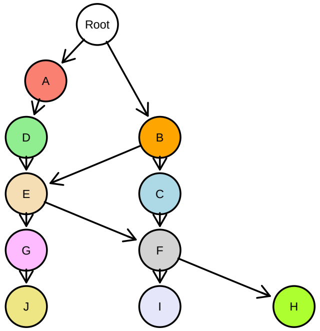

ID 4r7diBoGWUyNOWMY
acc genotypes 27
peaks no back 5
removed edges 6
prop rem edges 0.136

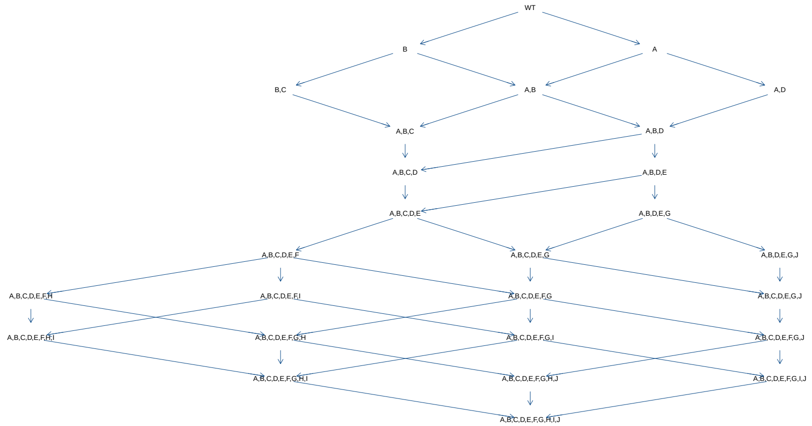

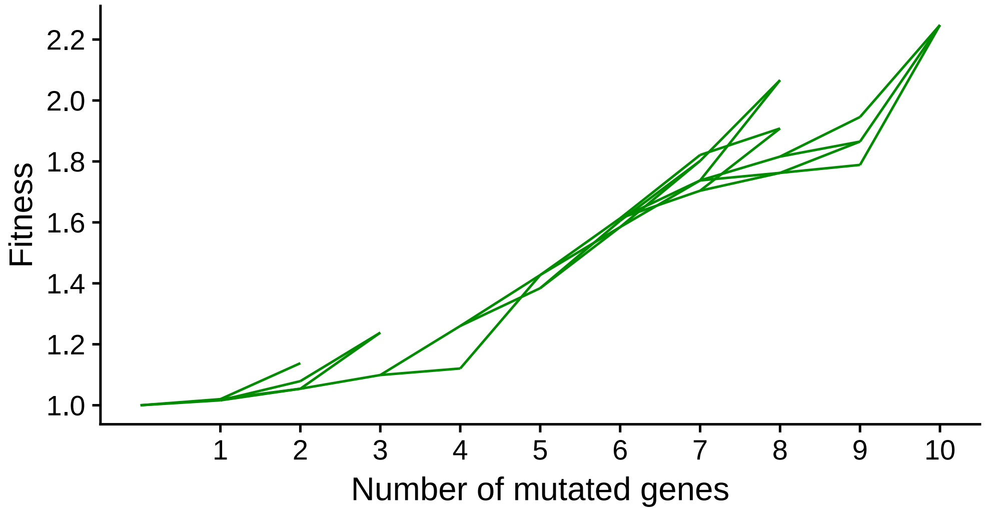

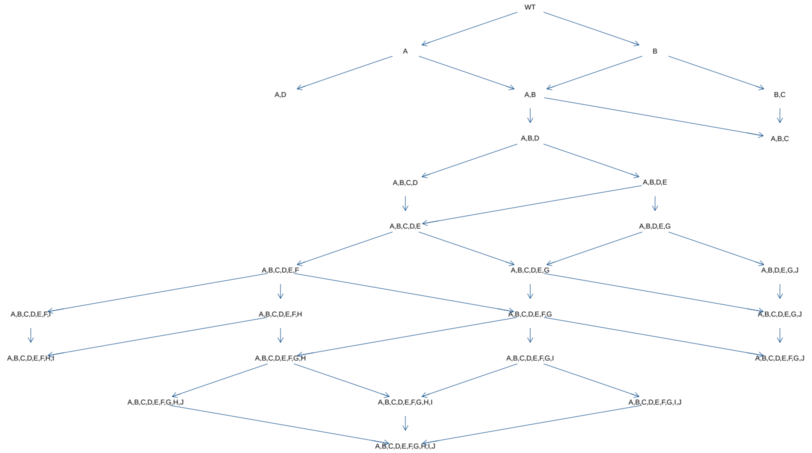

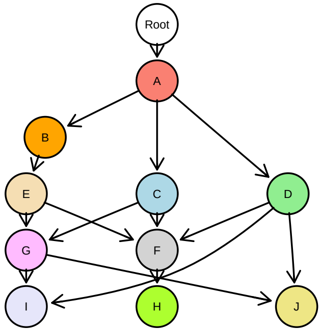

ID	56zMgtzUdnUxiwN9
acc genotypes	28
peaks no back	6
removed edges	8
prop rem edges	0.167

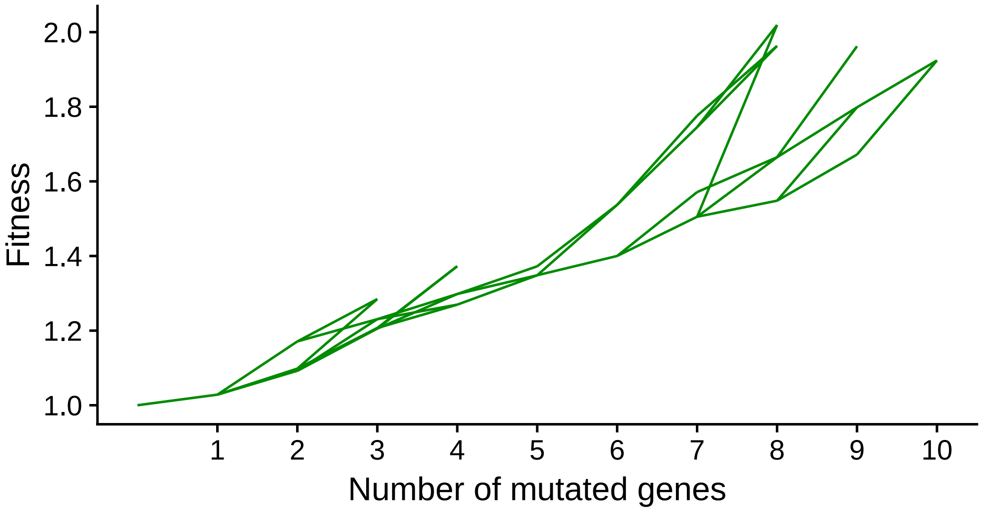

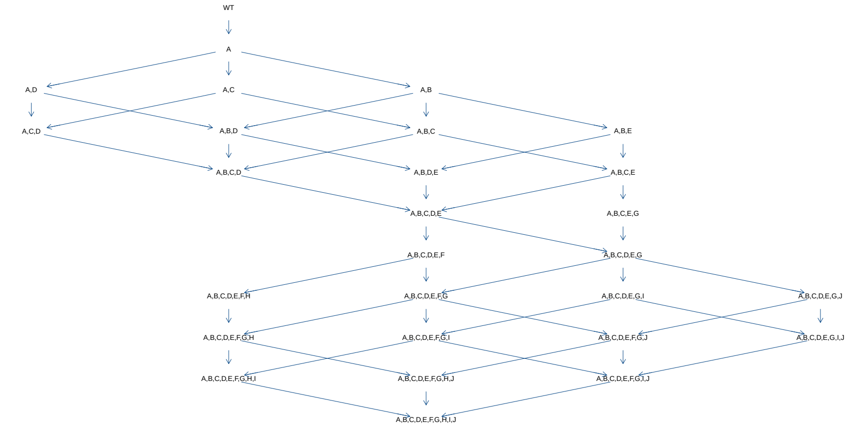

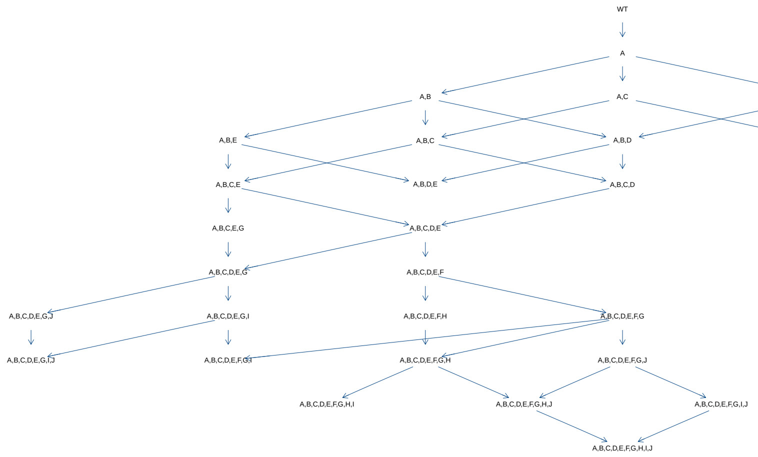

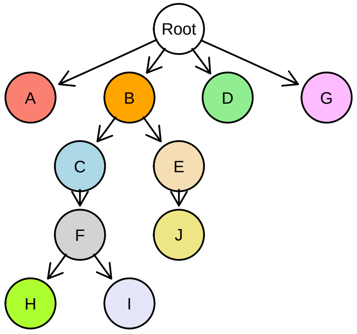

ID	5D4EKkcfeVoj4fHS
acc genotypes	152
peaks no back	14
removed edges	94
prop rem edges	0.197

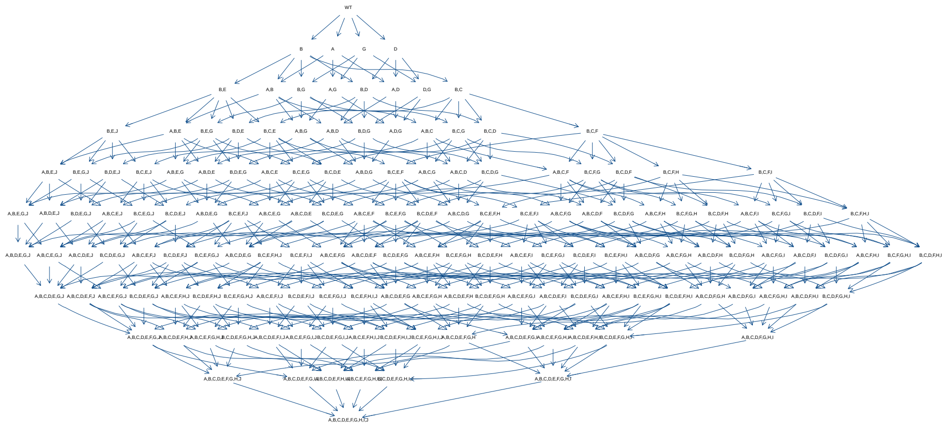

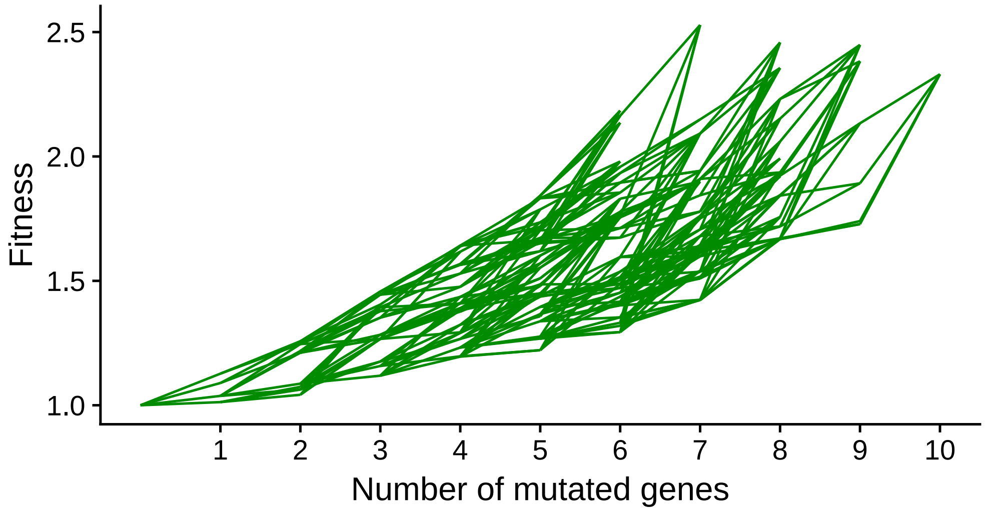

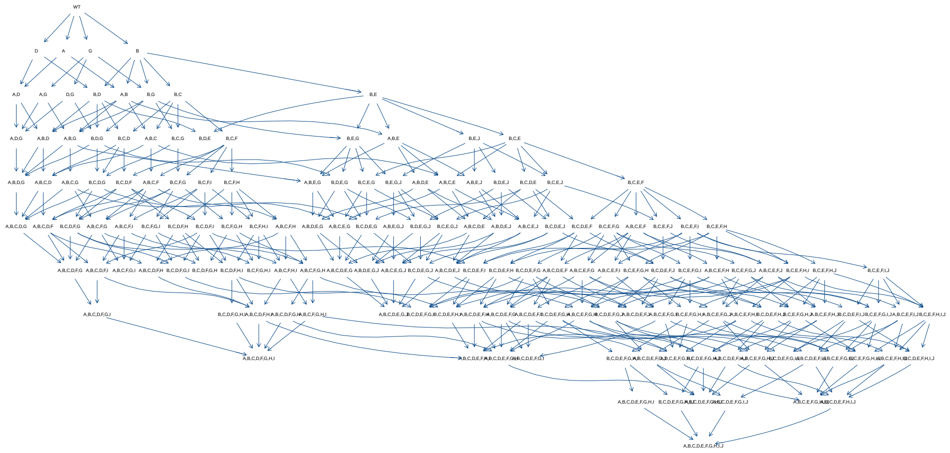

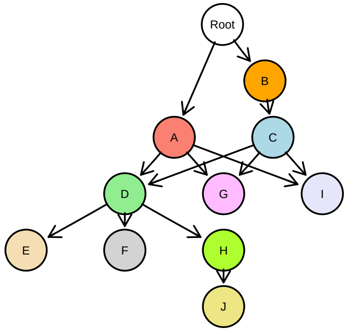

ID	5DSlnm8xyZVZ5wT9
acc genotypes	57
peaks no back	8
removed edges	20
prop rem edges	0.14

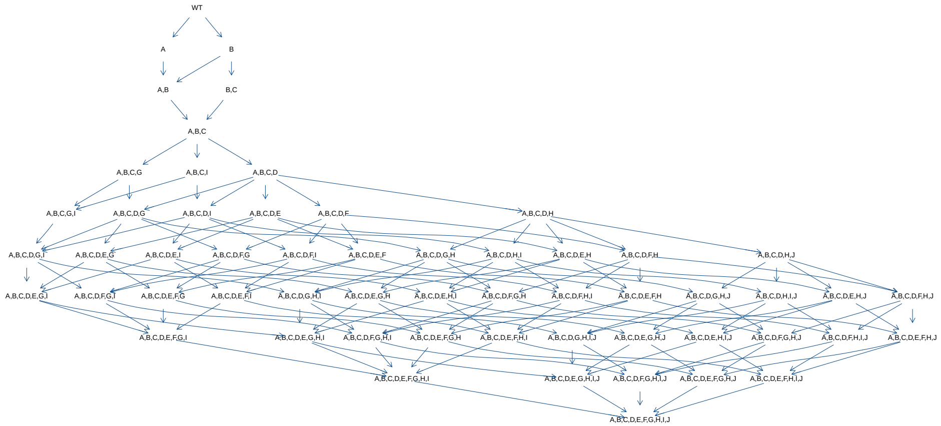

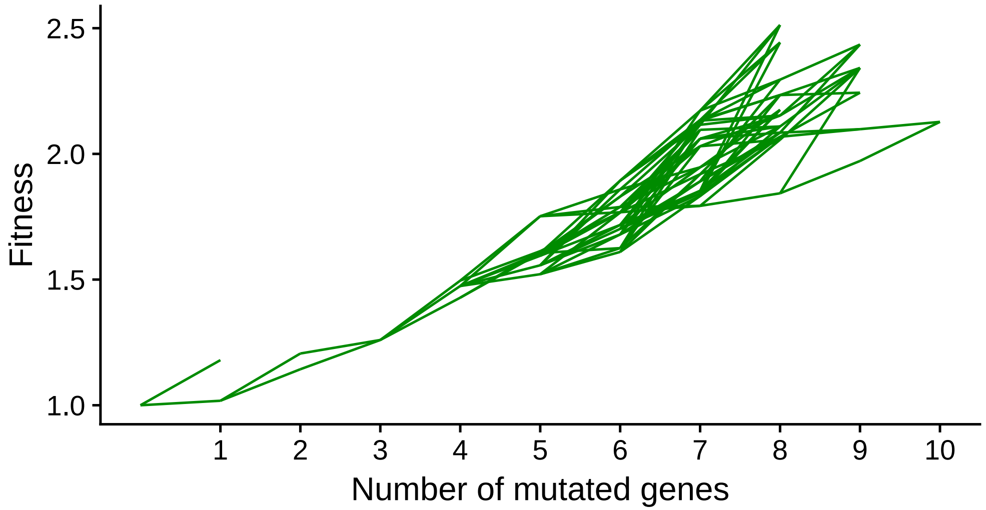

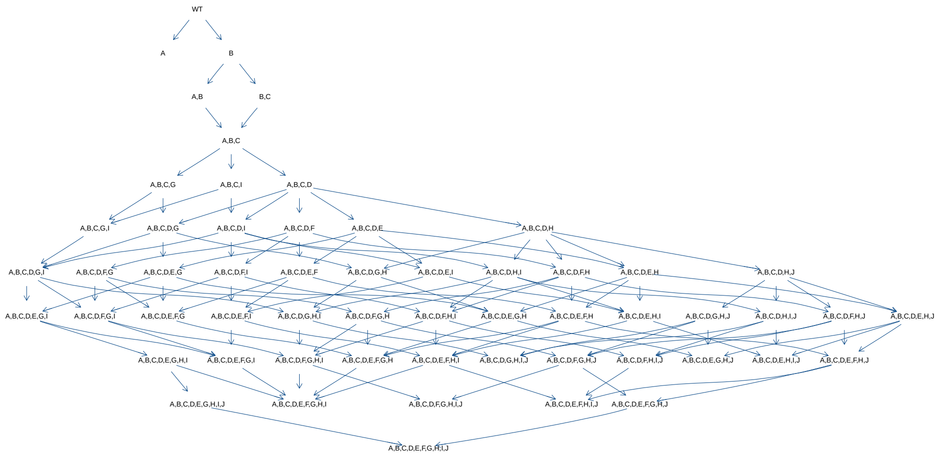

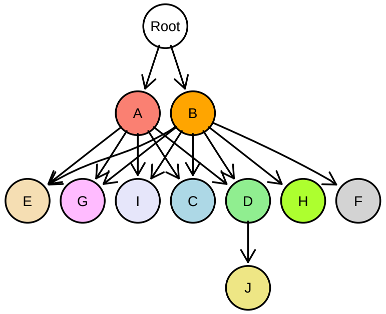

ID	5ervwavRLQyVUmM4
acc genotypes	198
peaks no back	19
removed edges	124
prop rem edges	0.173

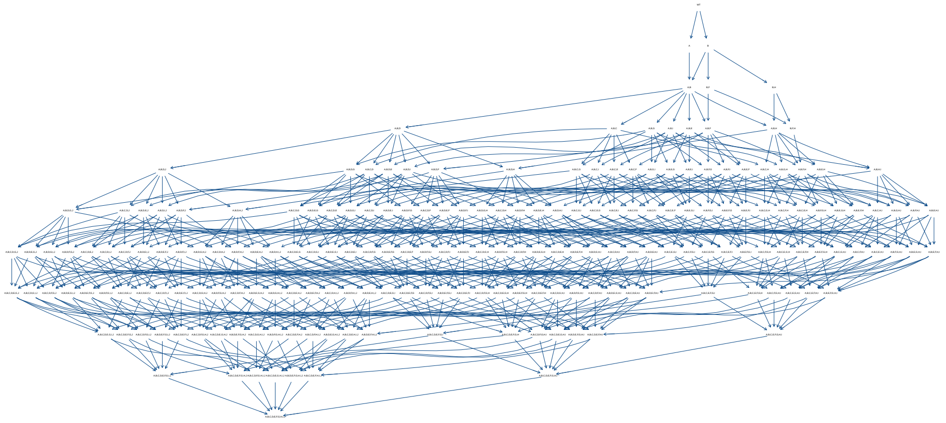

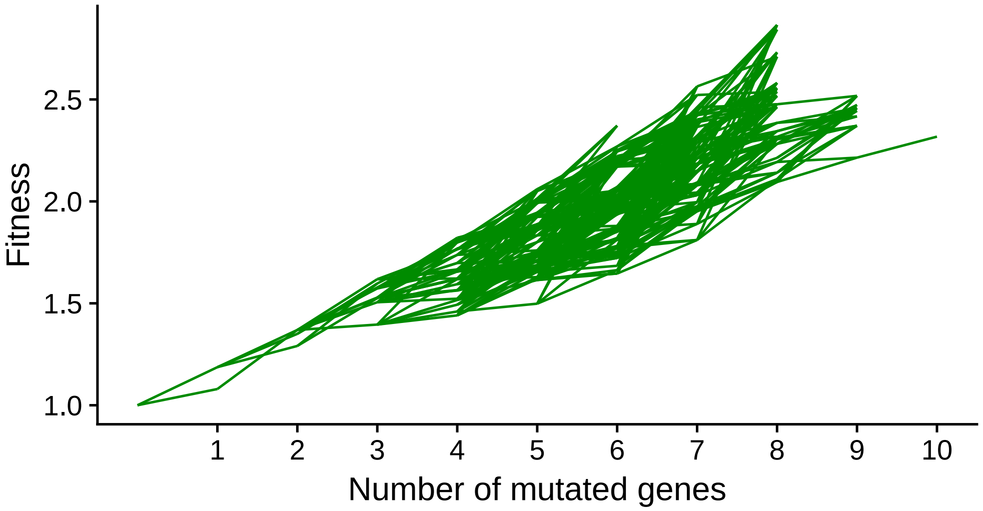

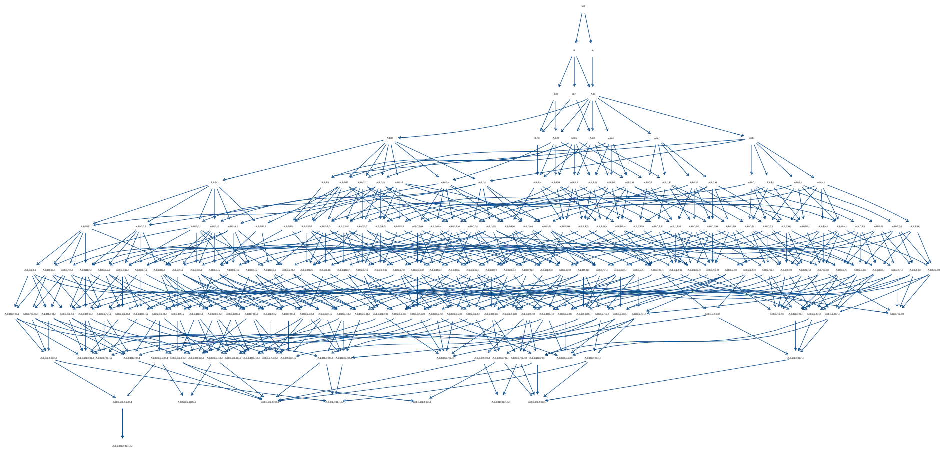

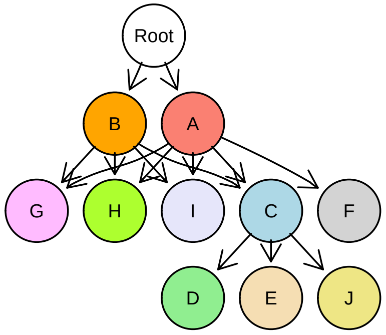

ID	5Kk3ENaa6v1KQQvo
acc genotypes	148
peaks no back	18
removed edges	100
prop rem edges	0.199

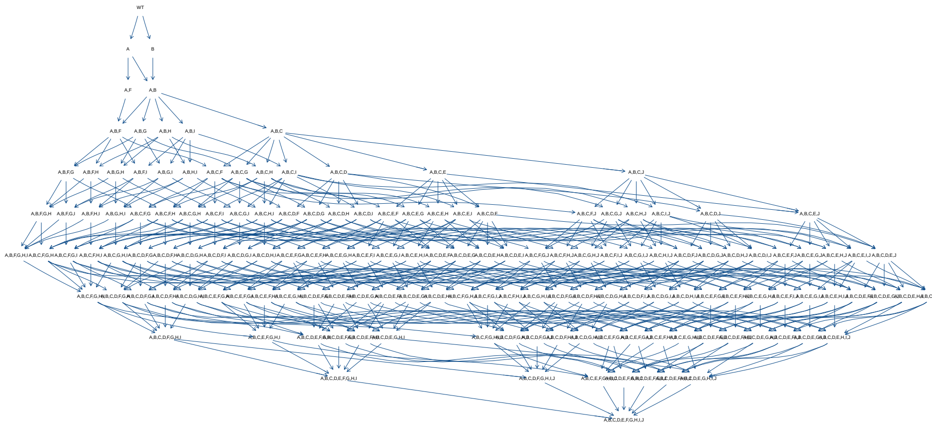

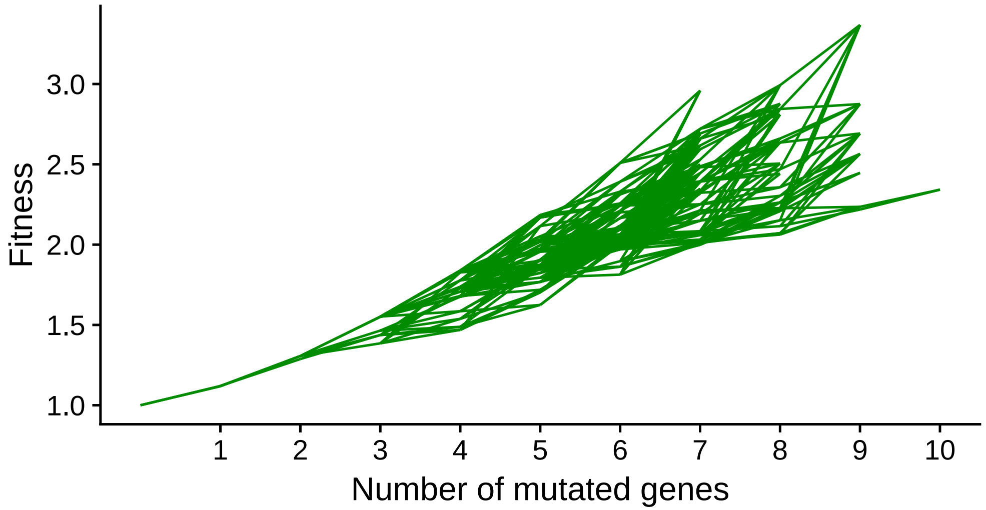

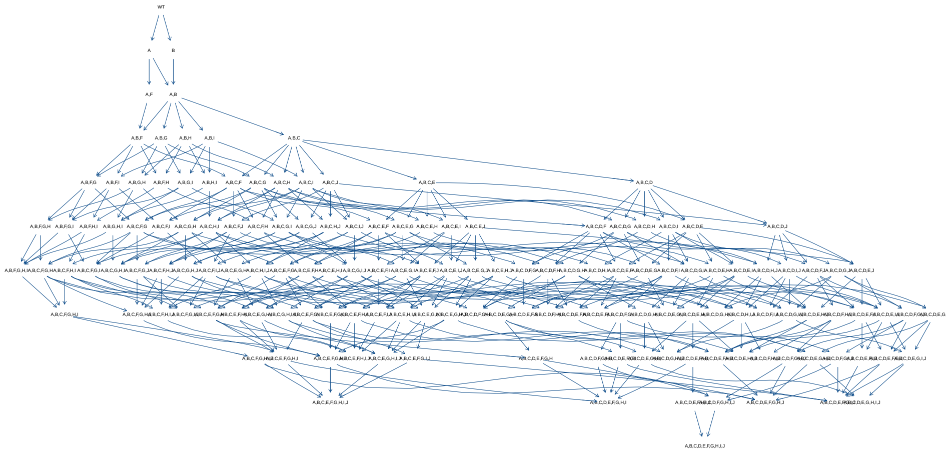

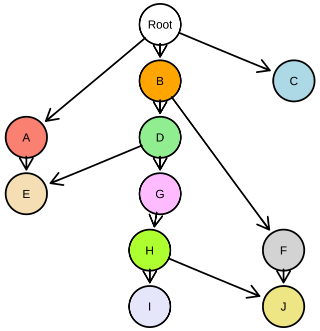

ID	5MCNYeerEvdy7pit
acc genotypes	72
peaks no back	8
removed edges	33
prop rem edges	0.188

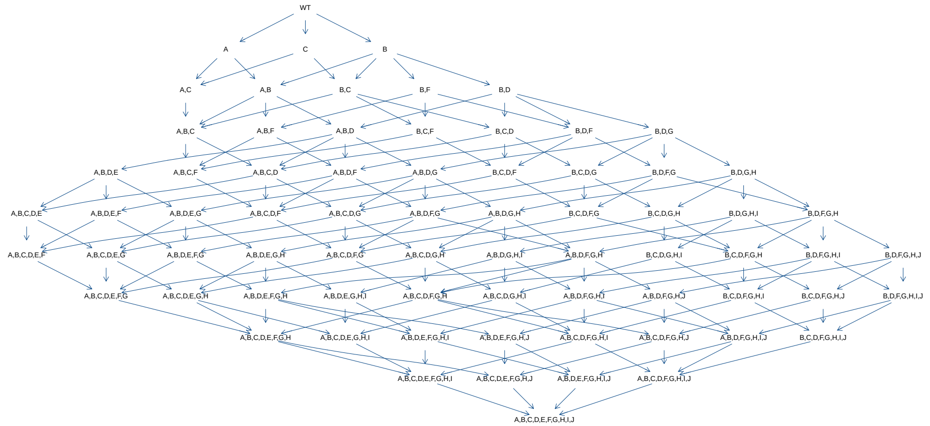

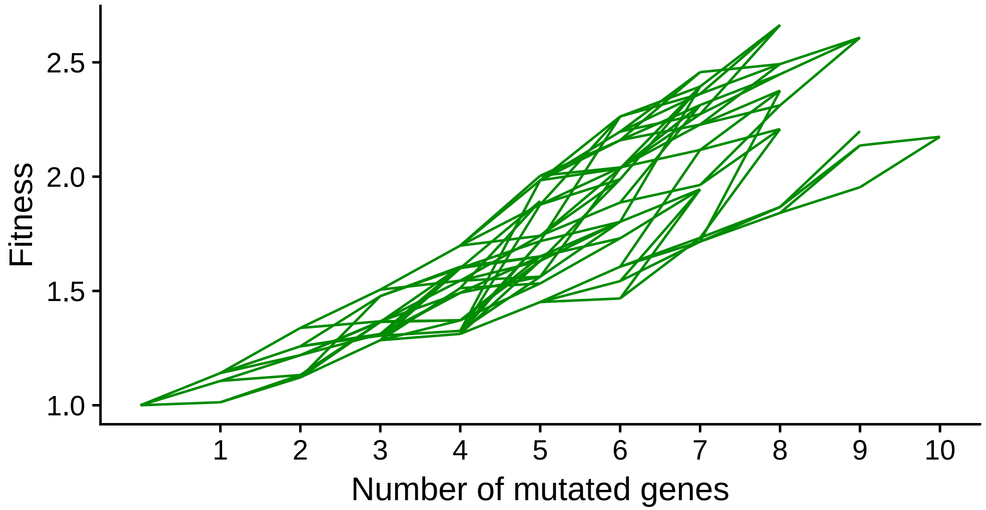

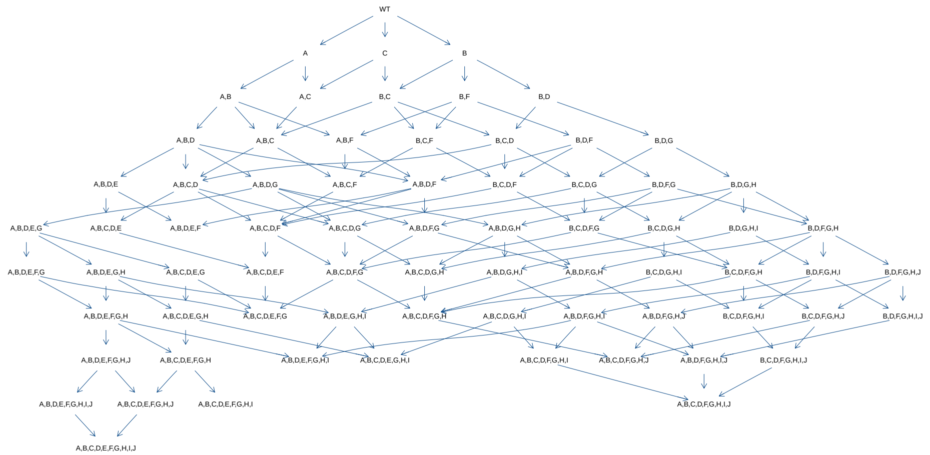

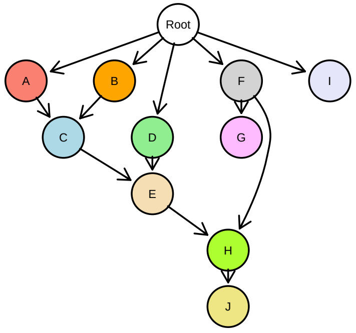

ID	5MxLARdmCMPqgUDW
acc genotypes	74
peaks no back	9
removed edges	35
prop rem edges	0.185

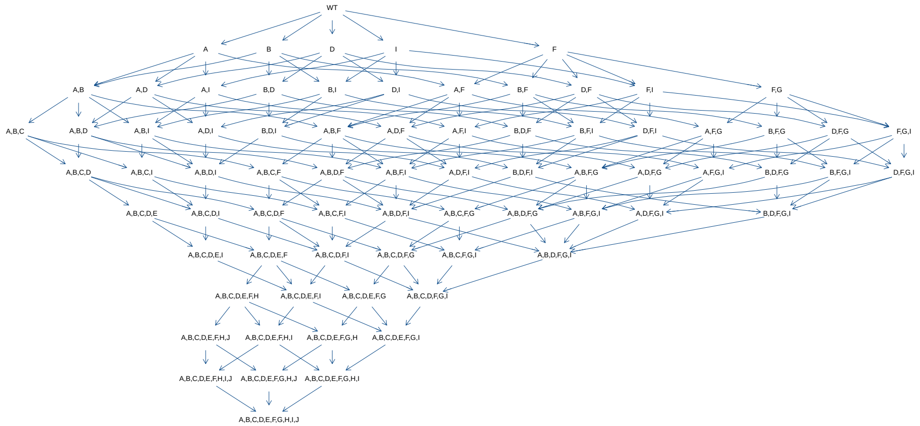

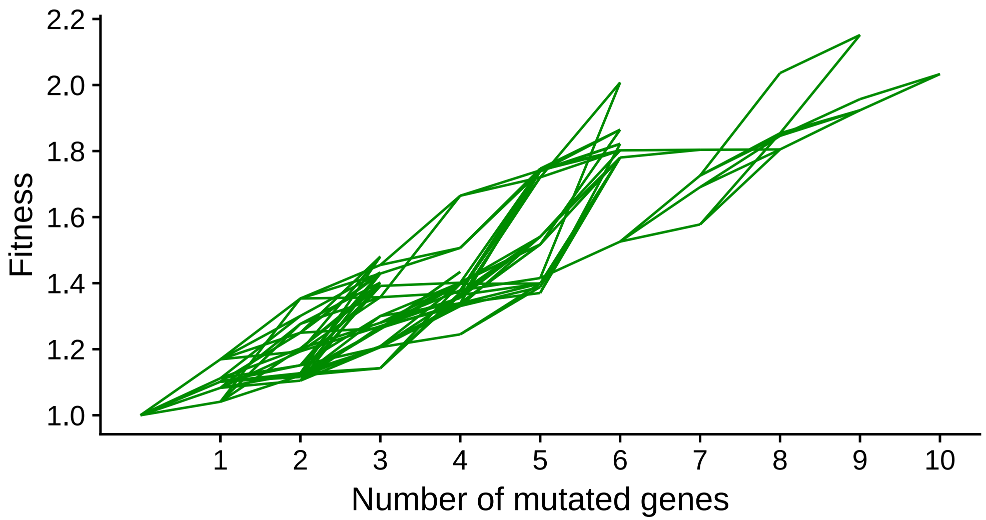

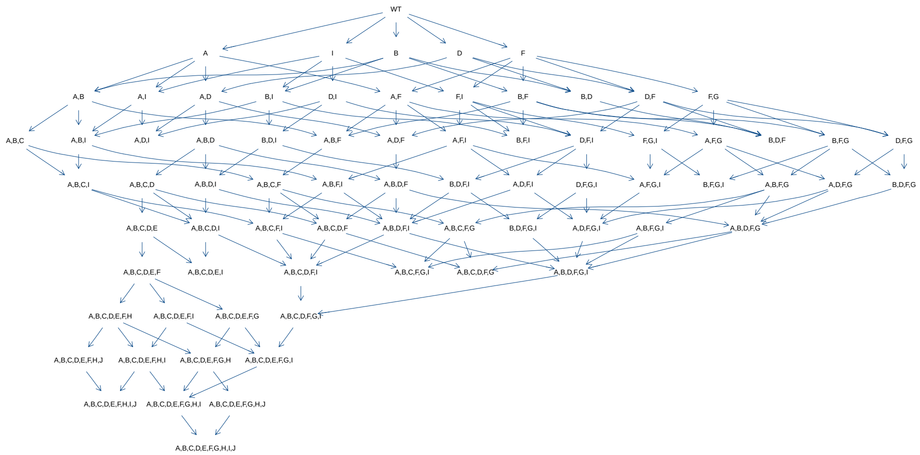

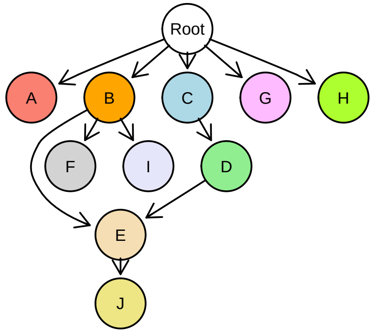

ID	5URt4ktIFolpEsp3
acc genotypes	184
peaks no back	19
removed edges	120
prop rem edges	0.199

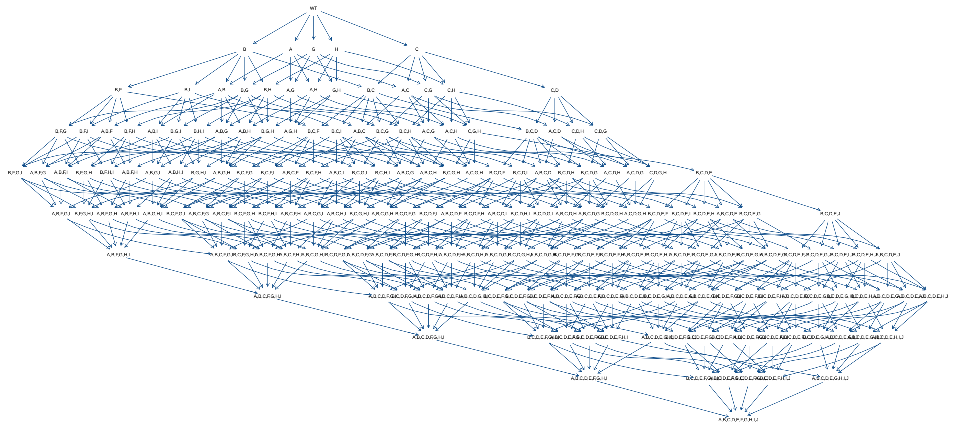

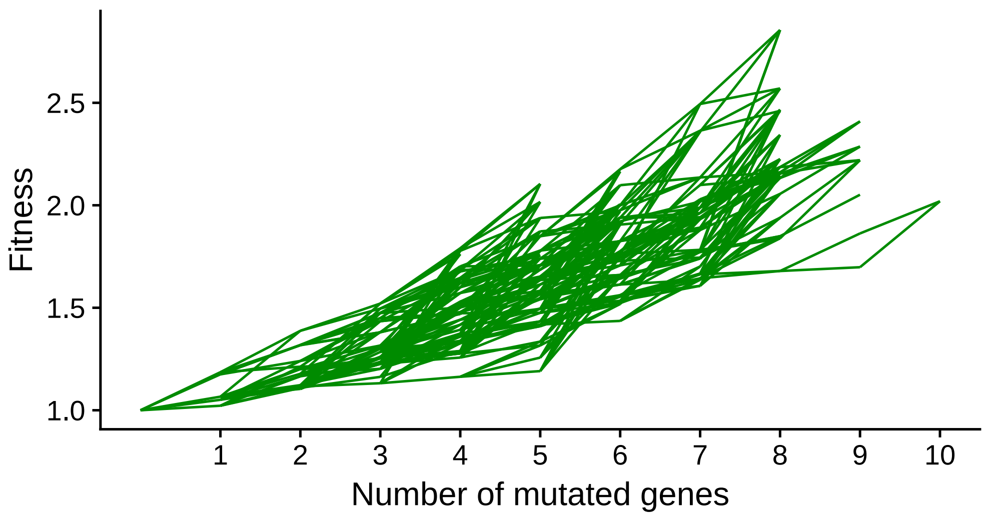

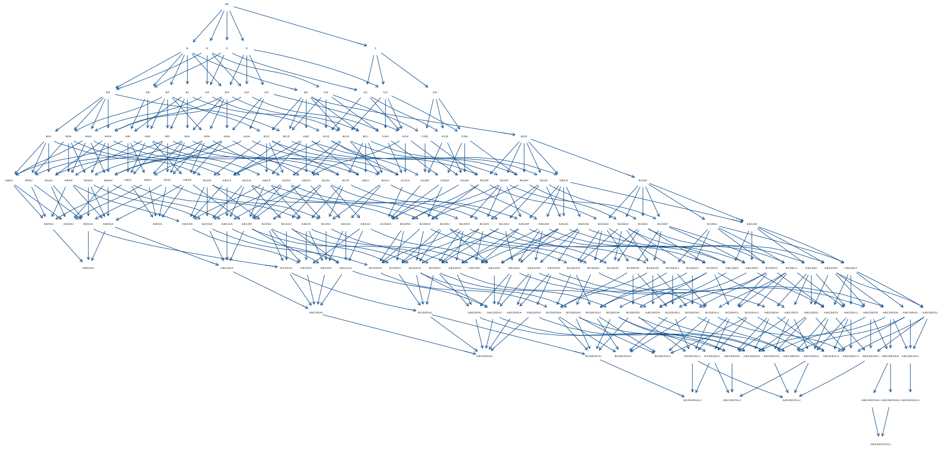

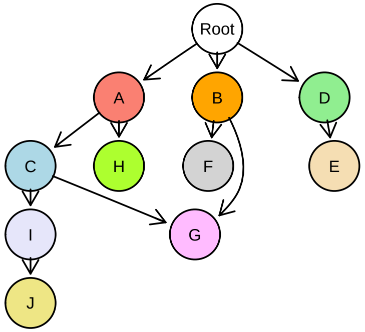

ID	64xXOCLCH1gfTB10v
acc genotypes	117
peaks no back	12
removed edges	78
prop rem edges	0.239

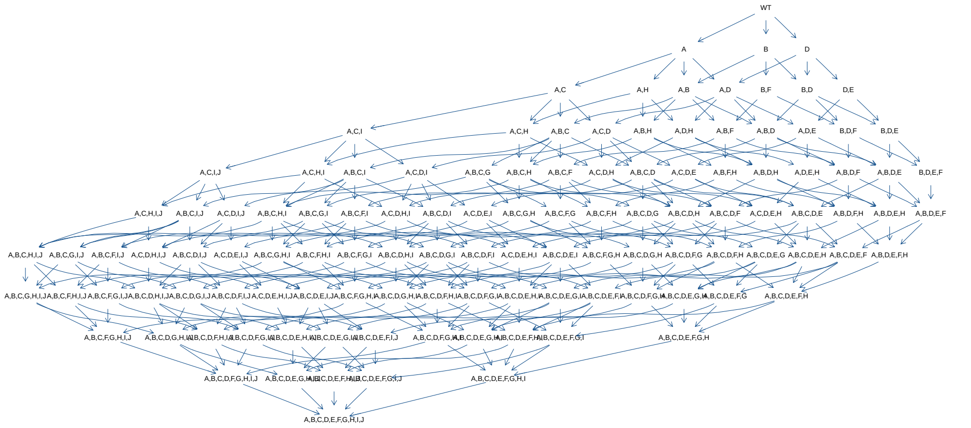

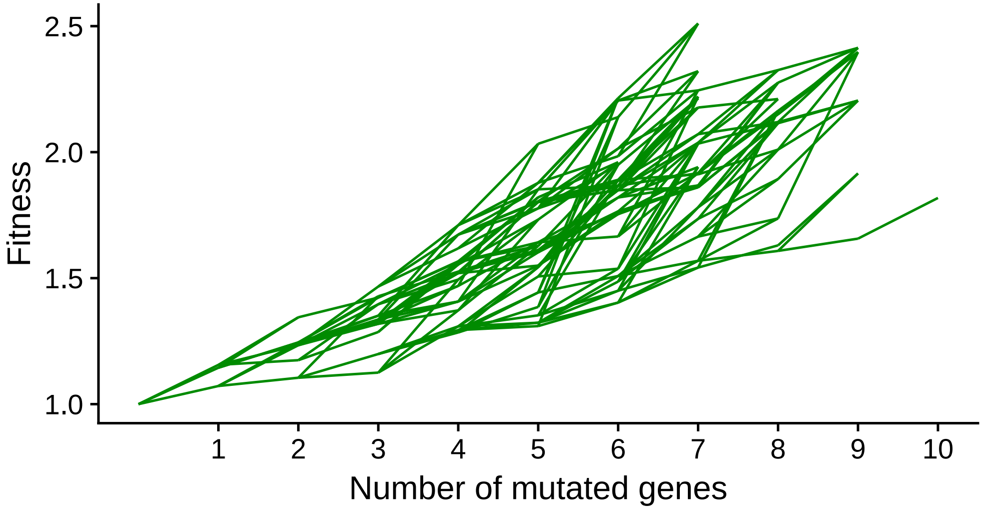

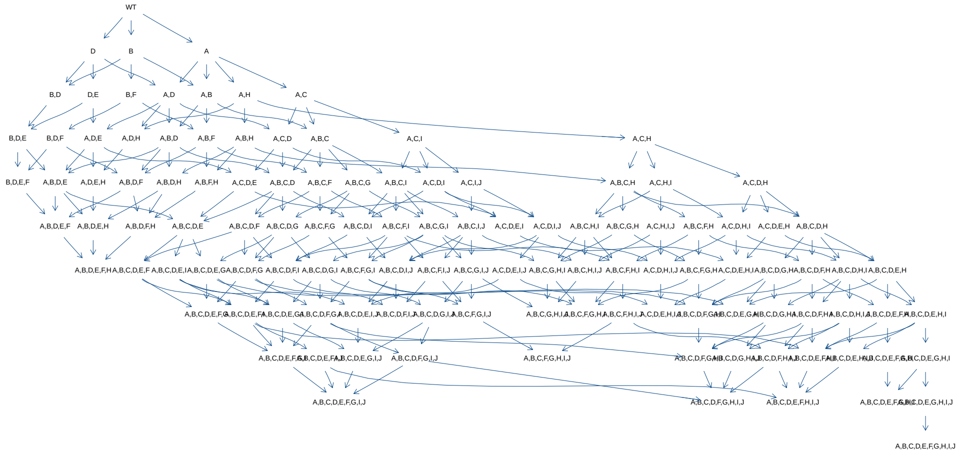

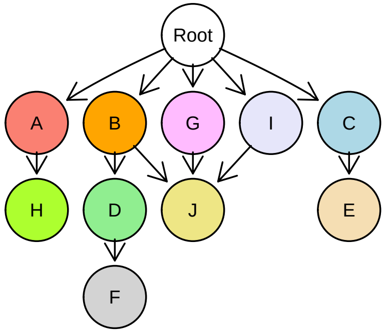

ID	69GLOdUeTxOq6MVW
acc genotypes	171
peaks no back	13
removed edges	102
prop rem edges	0.194

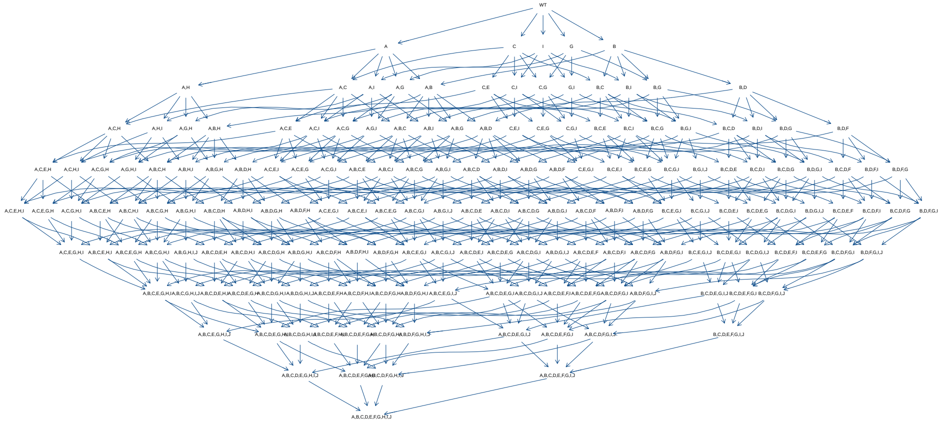

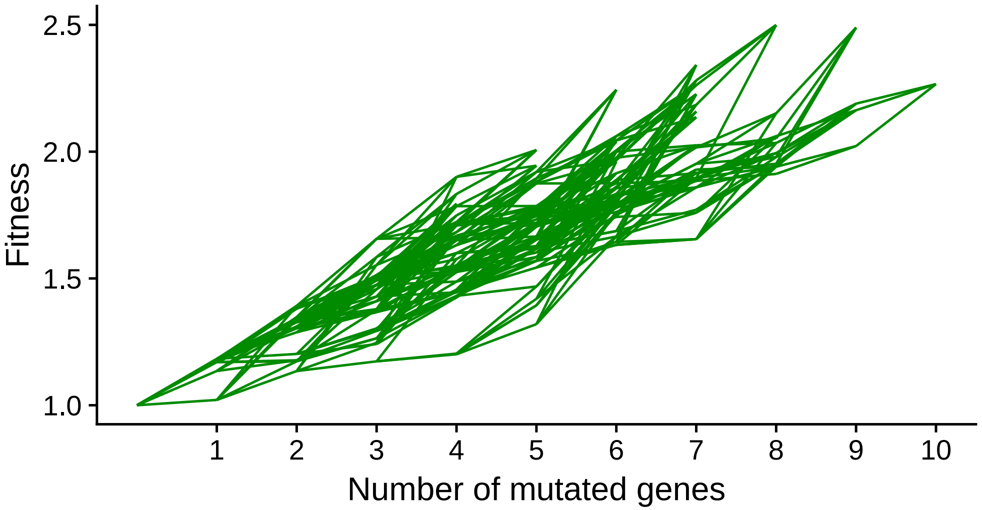

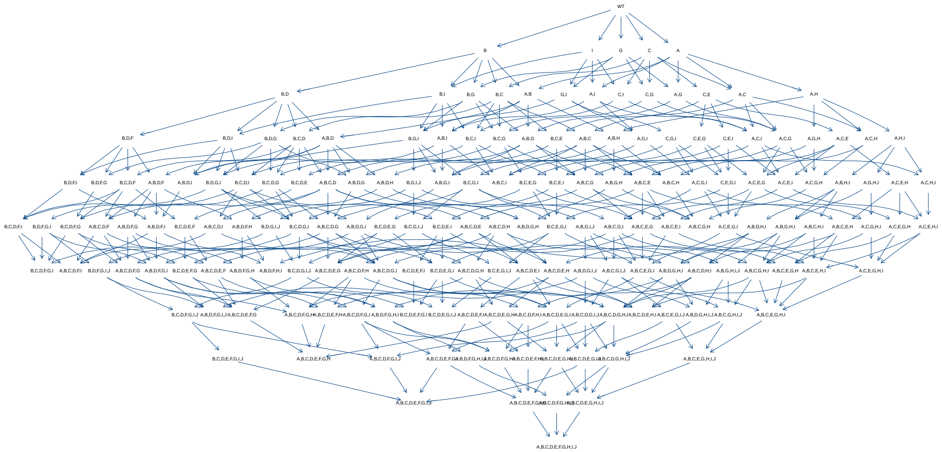

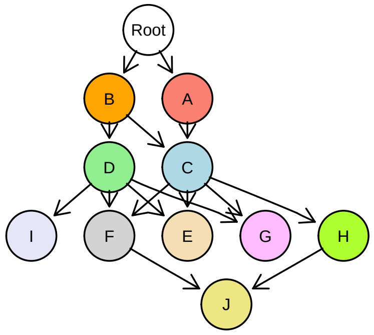

ID 6dNxcxBCKj6SRe7c
acc genotypes 50
peaks no back 7
removed edges 19
prop rem edges 0.164

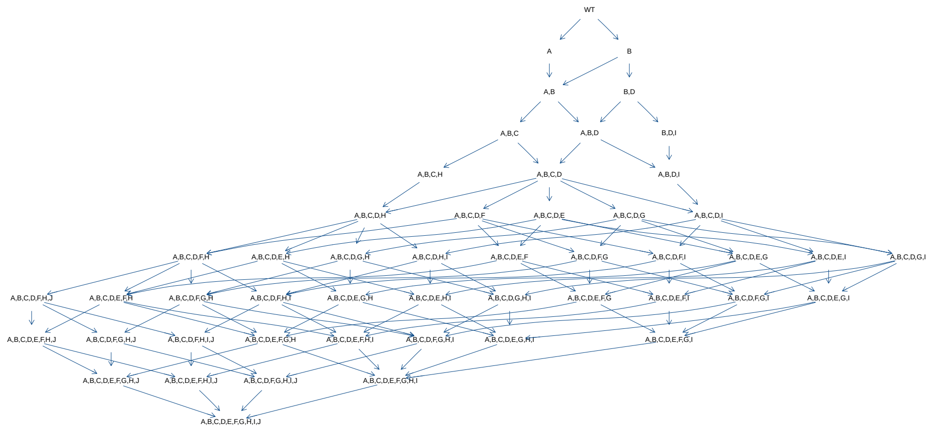

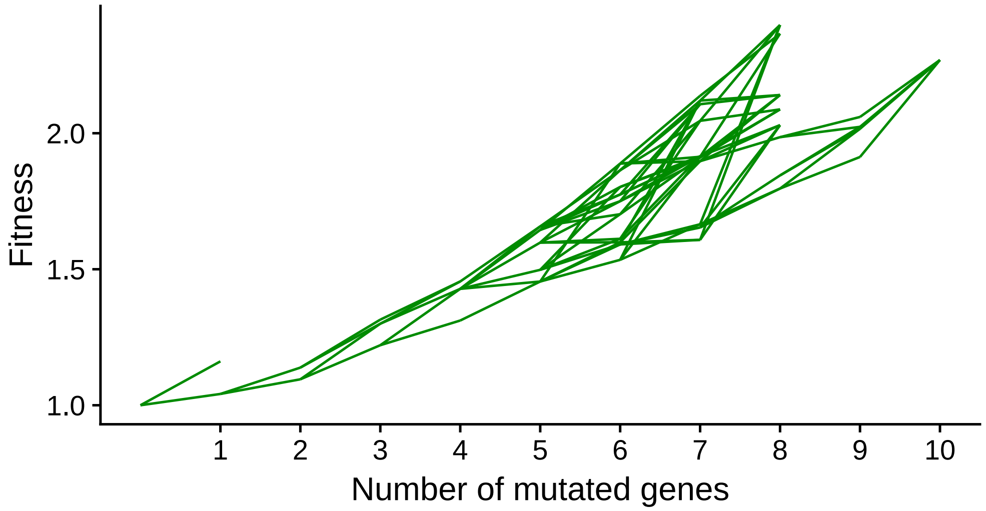

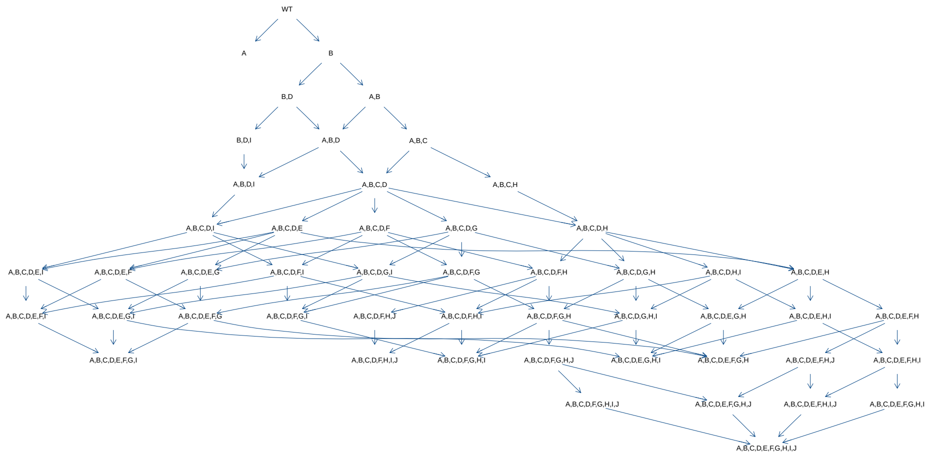

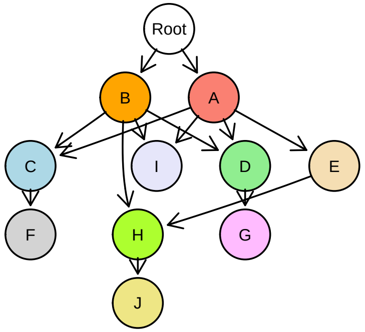

ID	6faIe6UPTkVnVhjC
acc genotypes	76
peaks no back	7
removed edges	24
prop rem edges	0.125

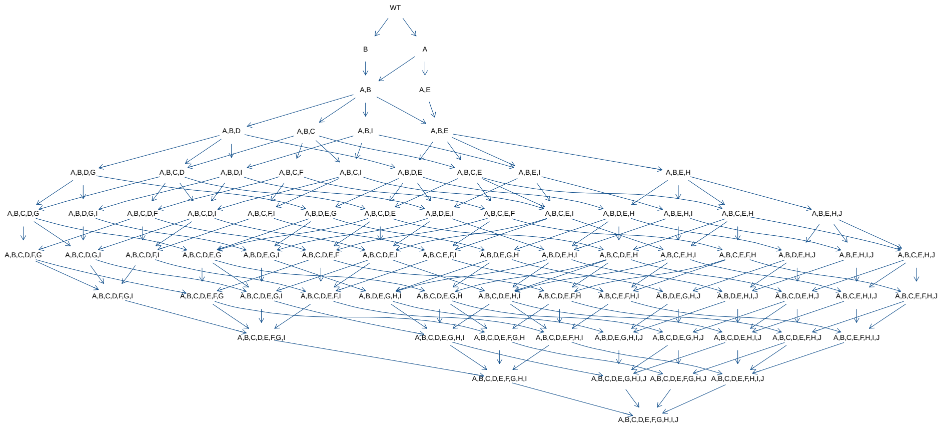

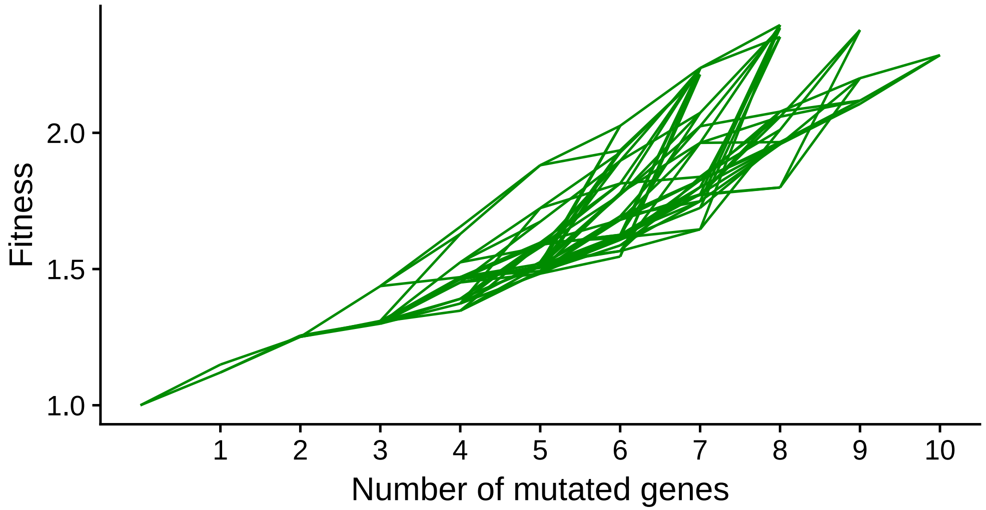

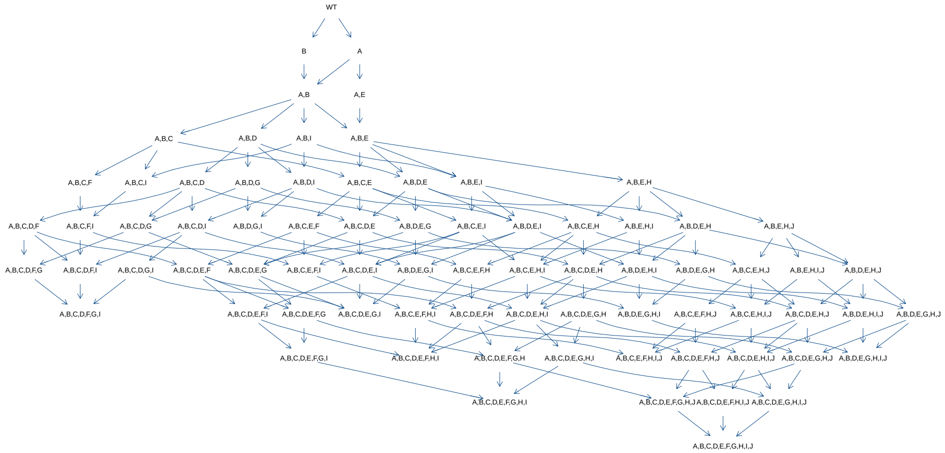

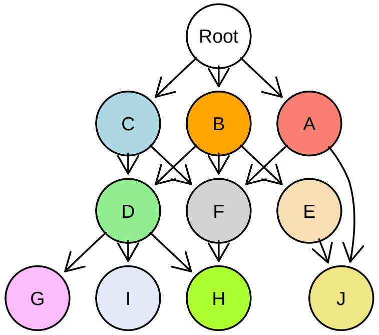

ID	6j2DyMAhErFeSJJe
acc genotypes	61
peaks no back	9
removed edges	26
prop rem edges	0.186

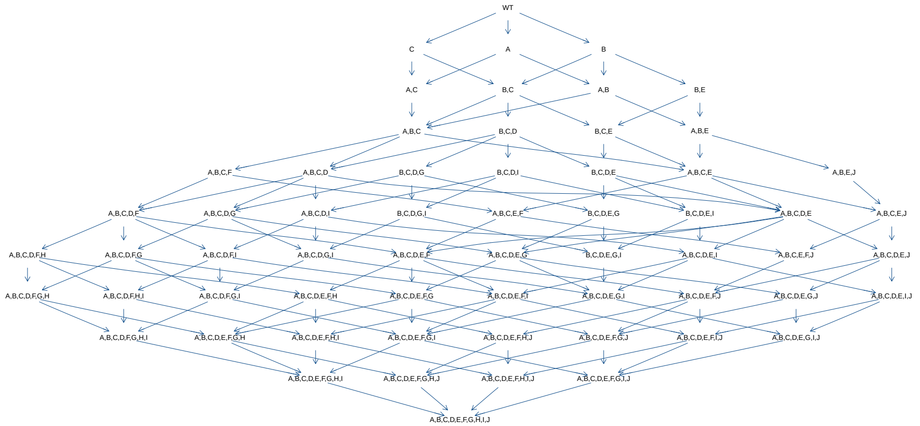

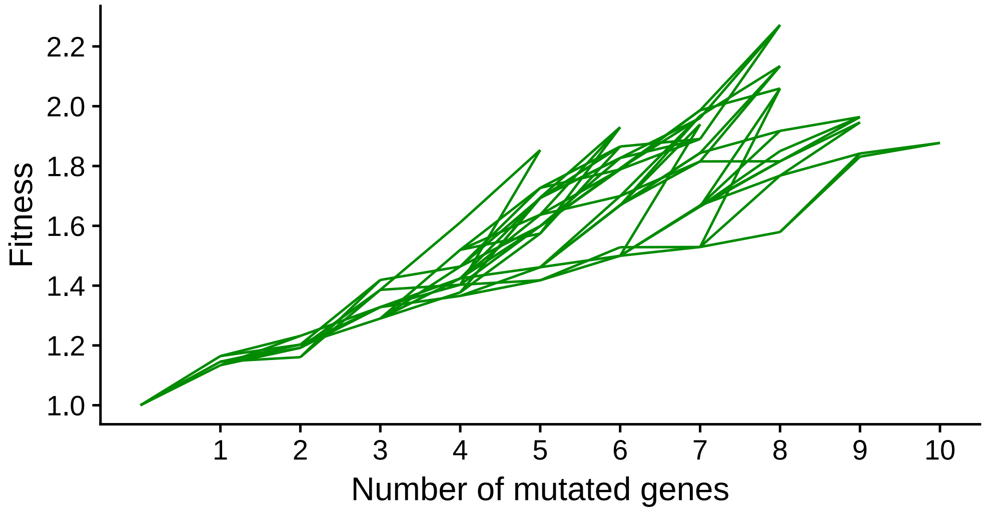

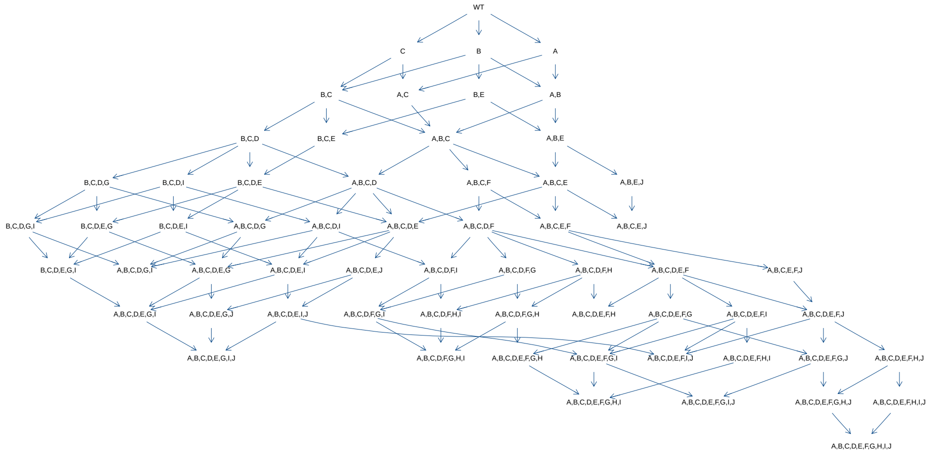

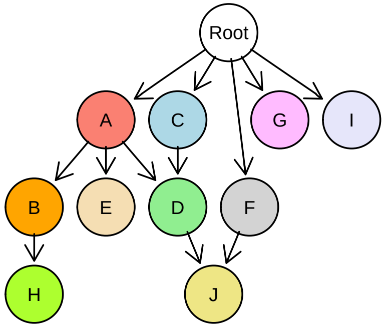

ID	7wUPQMRQPYFpPVSq
acc genotypes	184
peaks no back	17
removed edges	135
prop rem edges	0.224

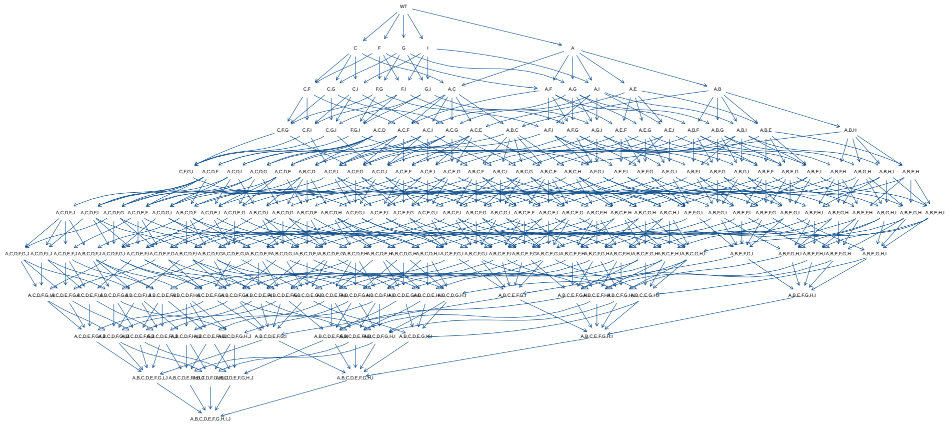

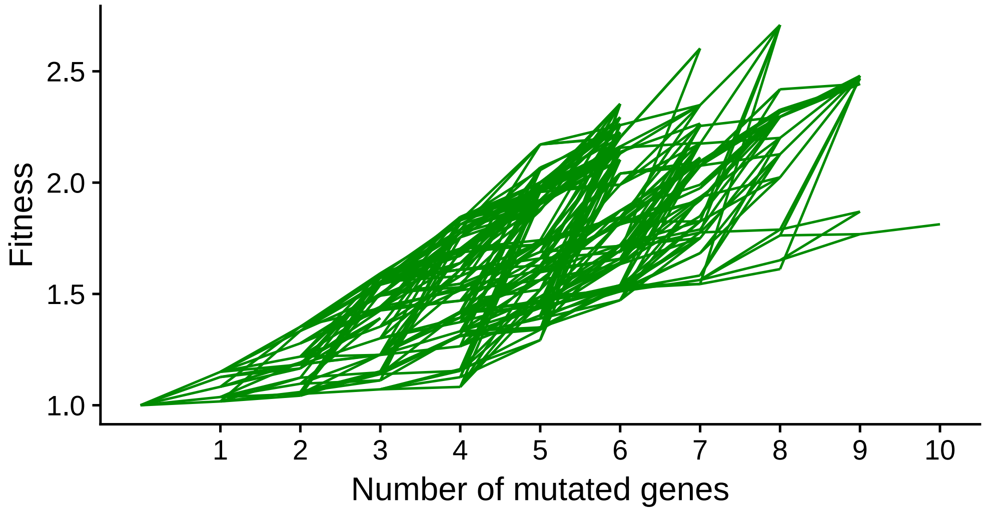

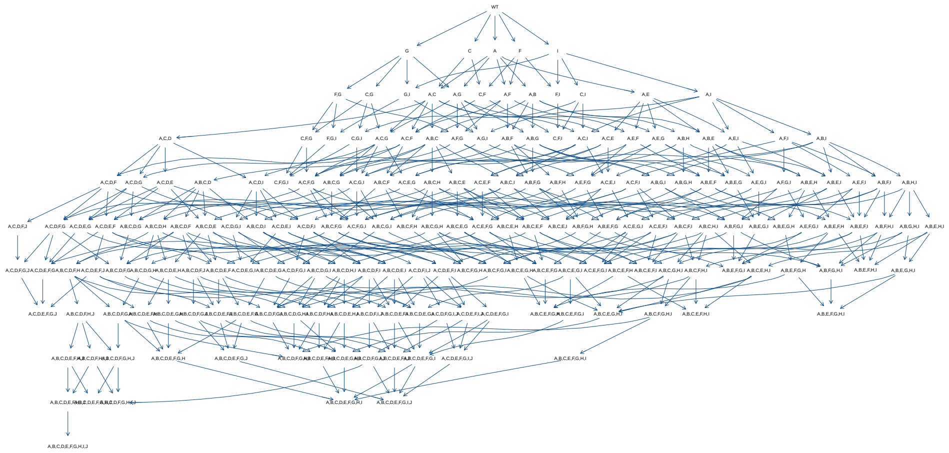

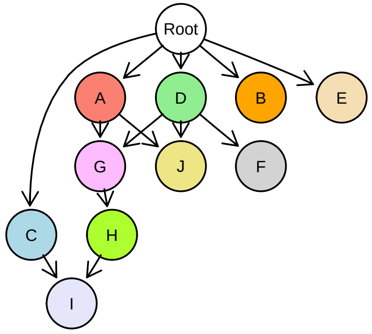

ID	87fcnp96NbP7zN7N
acc genotypes	144
peaks no back	13
removed edges	63
prop rem edges	0.141

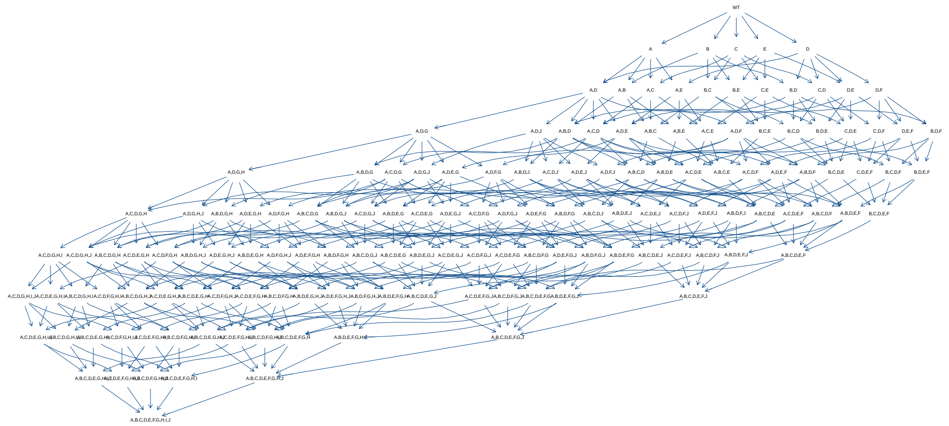

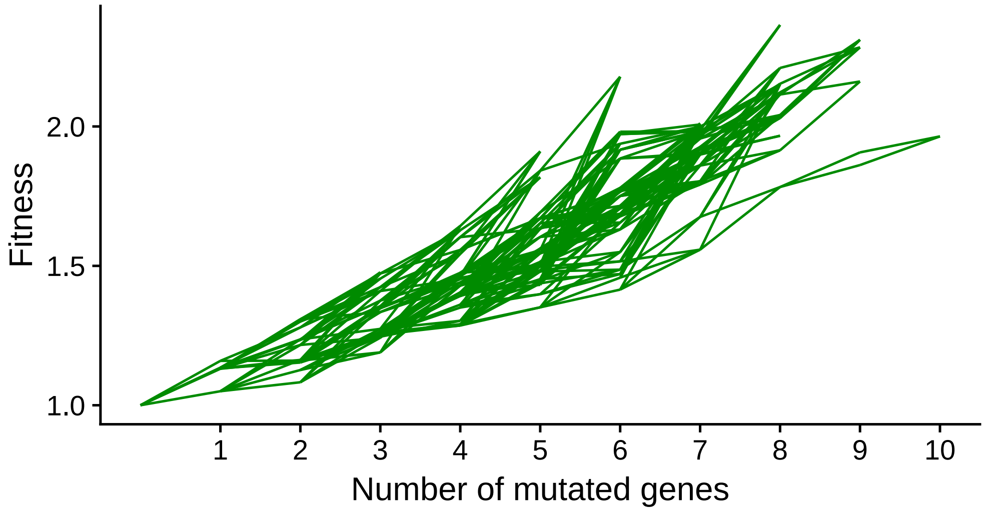

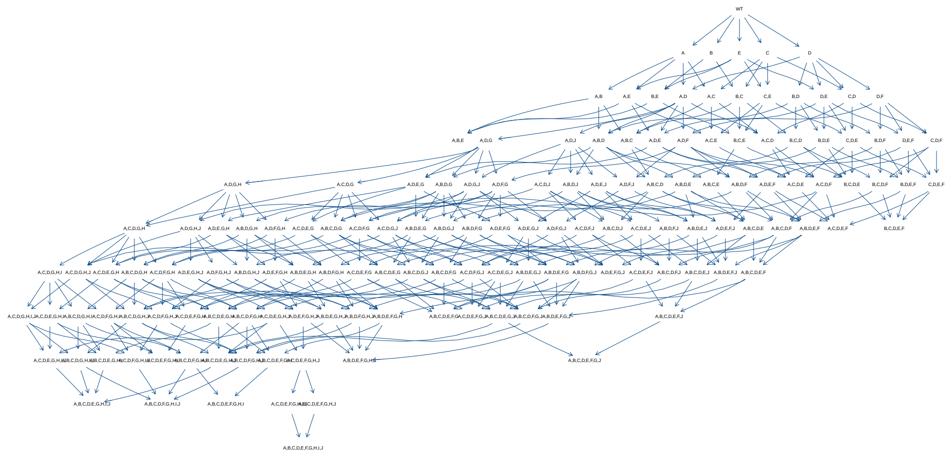

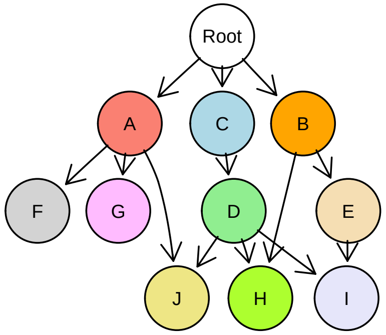

ID	8GlpgW8kSqHXEOja
acc genotypes	93
peaks no back	9
removed edges	40
prop rem edges	0.165

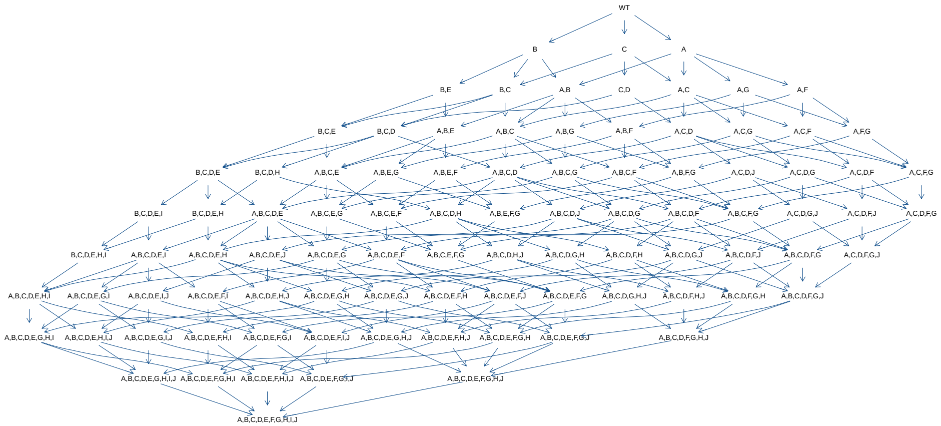

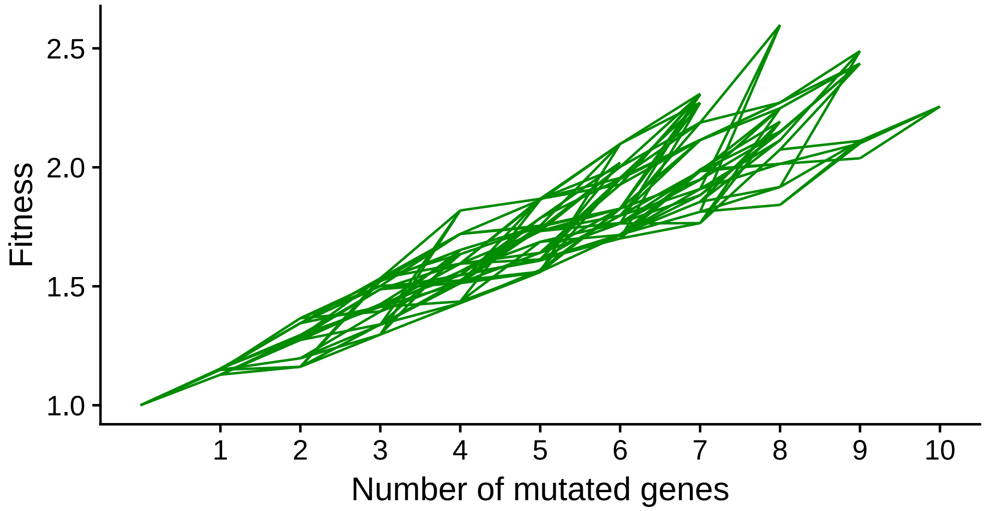

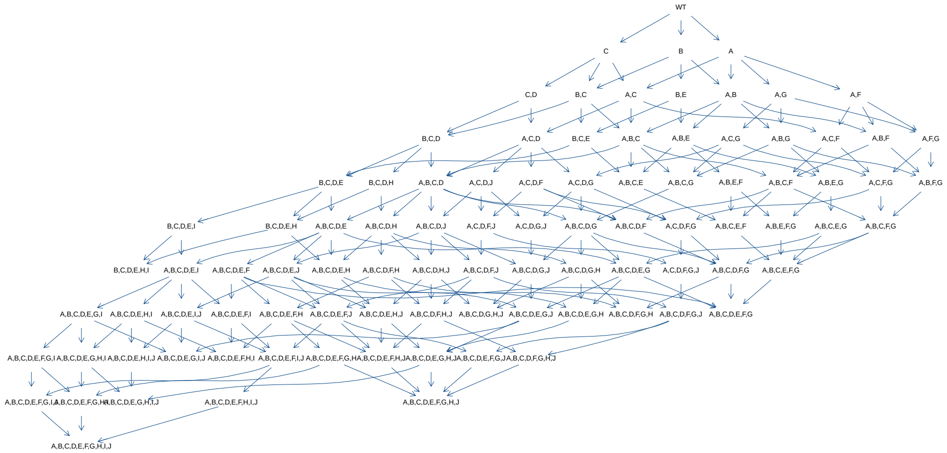

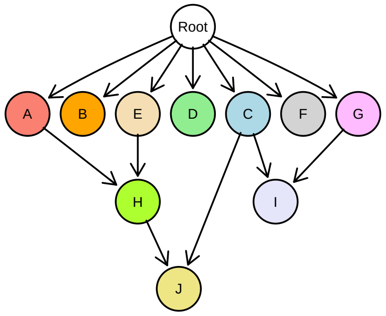

ID	8OAYTmnWfxxdHTRV
acc genotypes	224
peaks no back	18
removed edges	163
prop rem edges	0.21

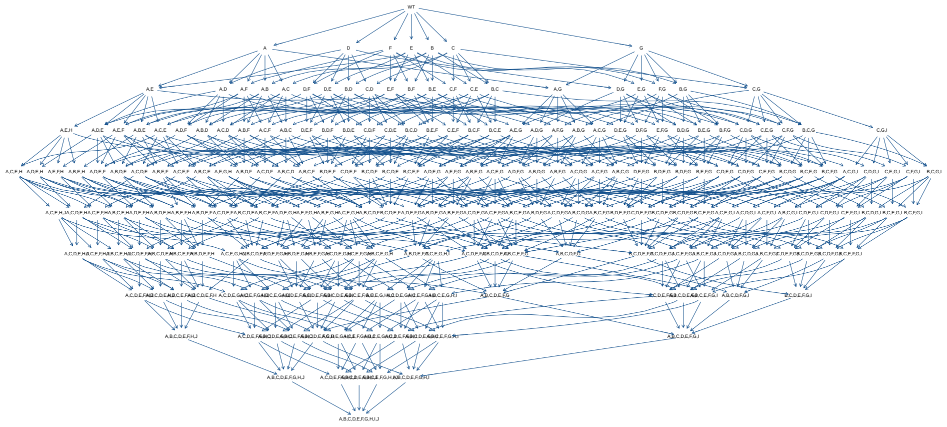

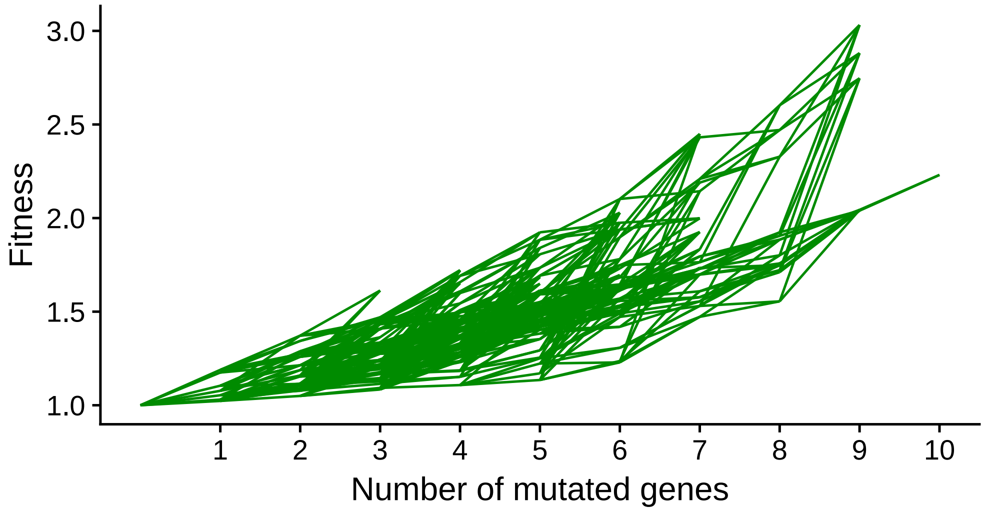

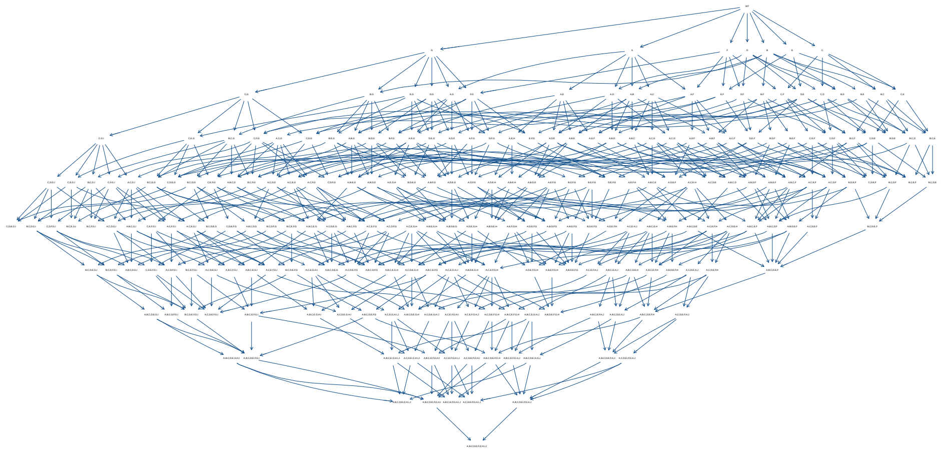

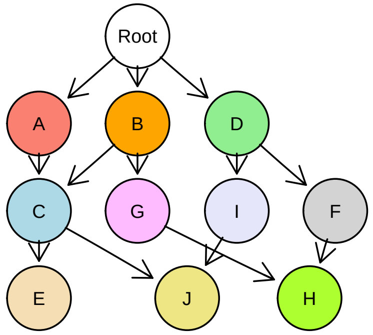

ID 8pBC5Dt22yGwrF10I
acc genotypes 68
peaks no back 2
removed edges 15
prop rem edges 0.095

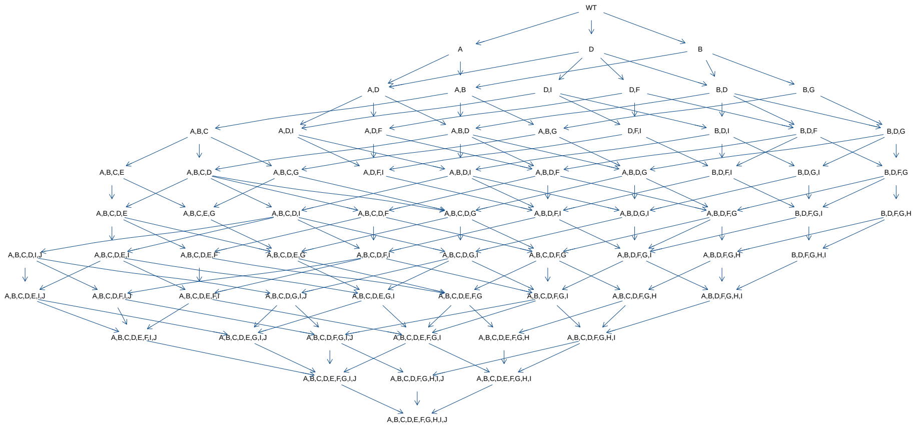

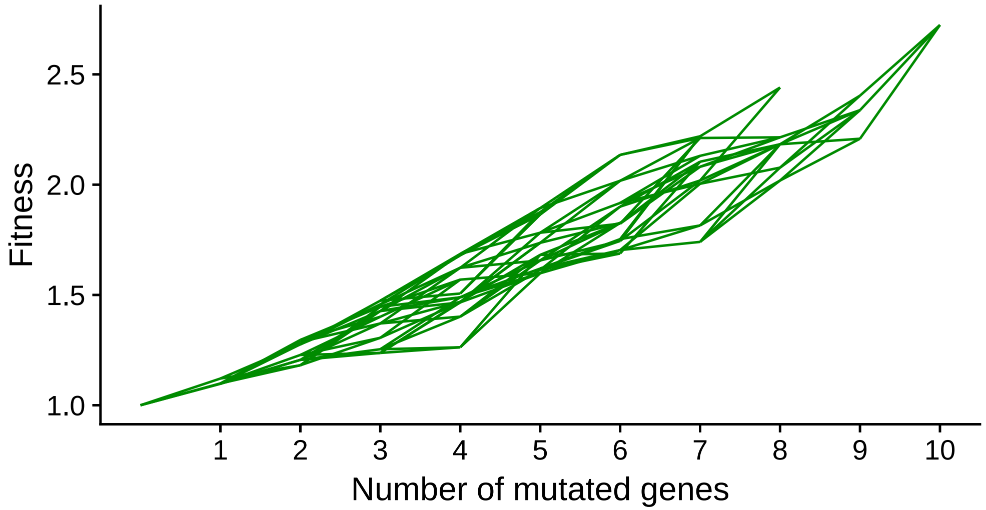

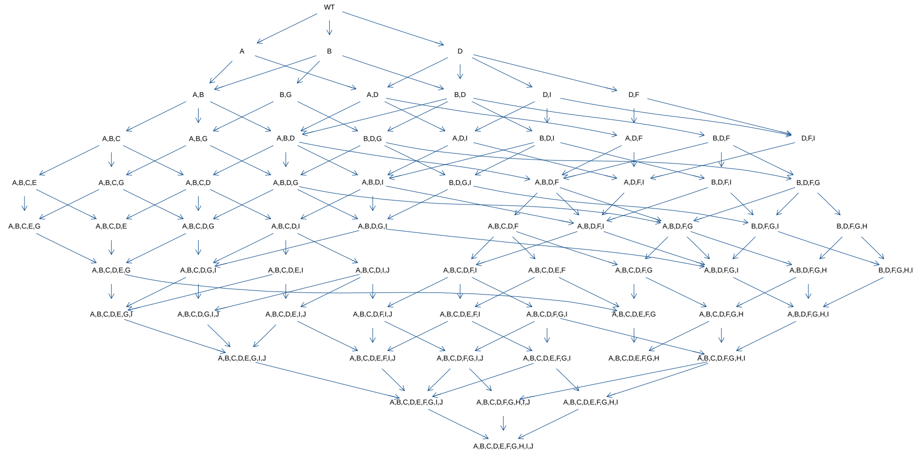

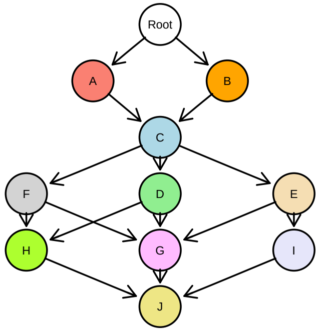

ID 8zRUVMJqJUMsBBkl
acc genotypes 24
peaks no back 4
removed edges 3
prop rem edges 0.077

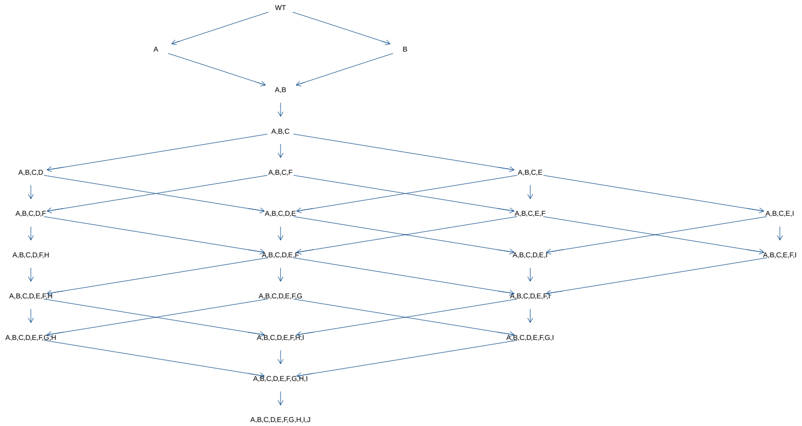

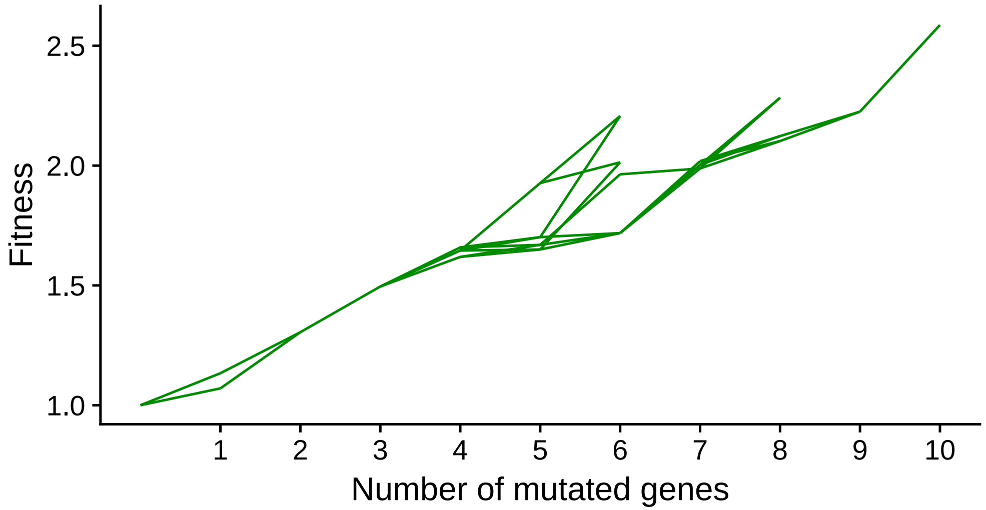

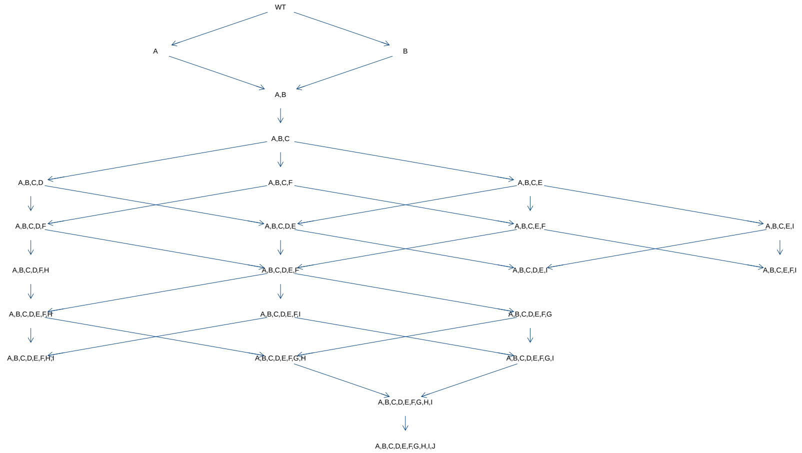

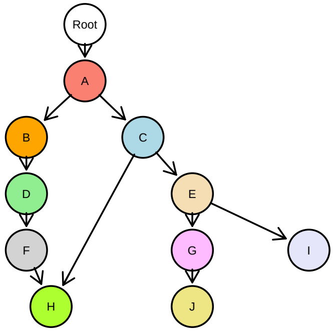

ID	8ZUcIGXESgYkqacc
acc genotypes	40
peaks no back	6
removed edges	13
prop rem edges	0.171

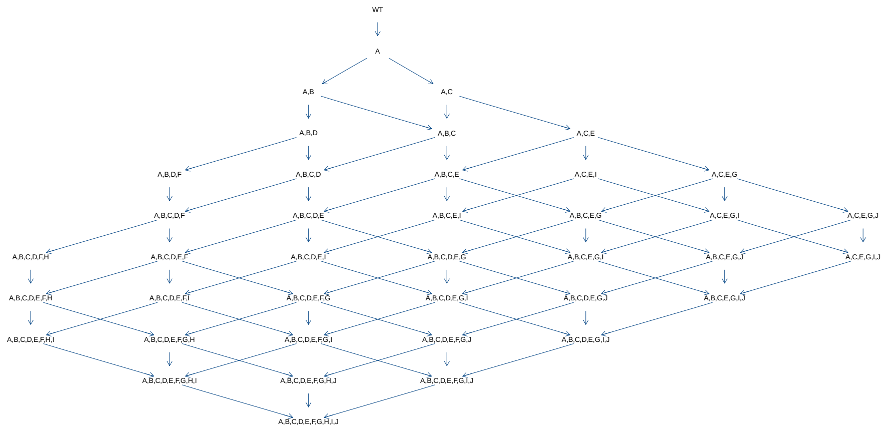

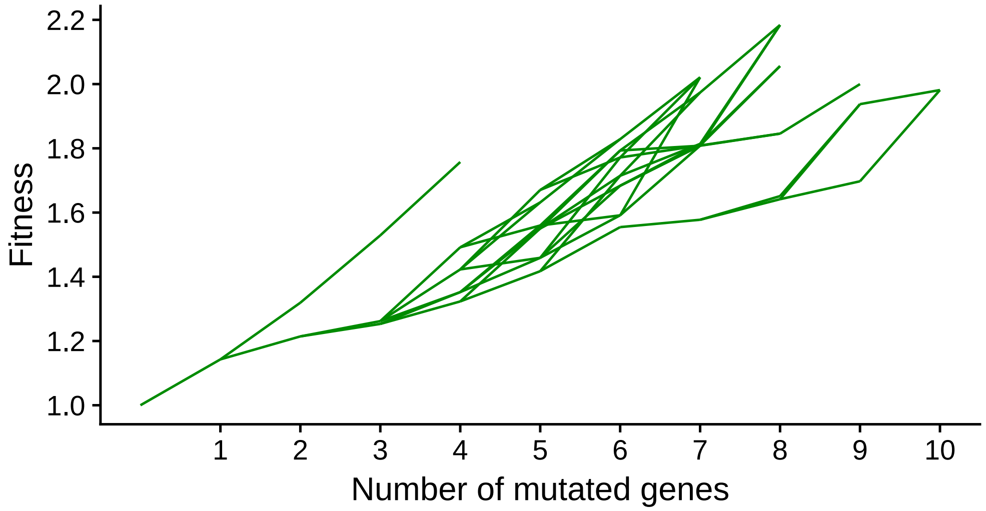

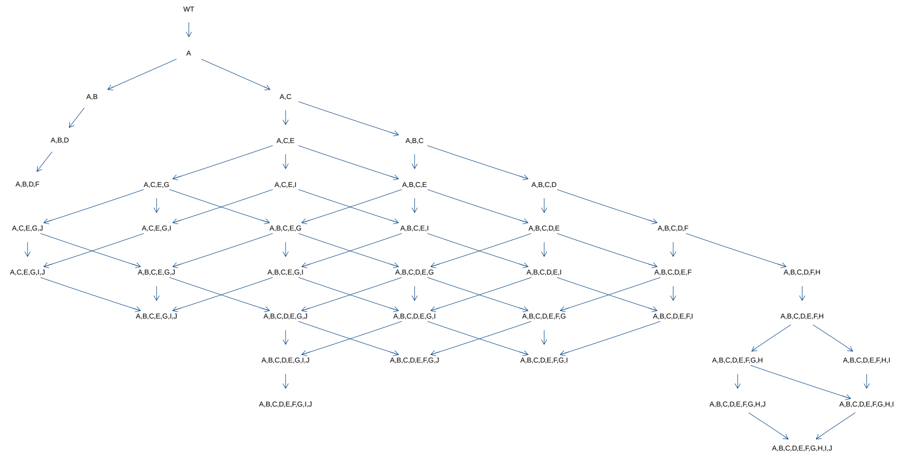

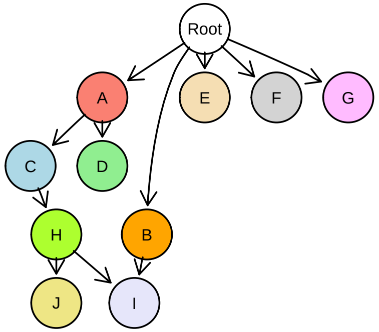

ID	92mZfe3axm10pyB10O
acc genotypes	176
peaks no back	15
removed edges	108
prop rem edges	0.188

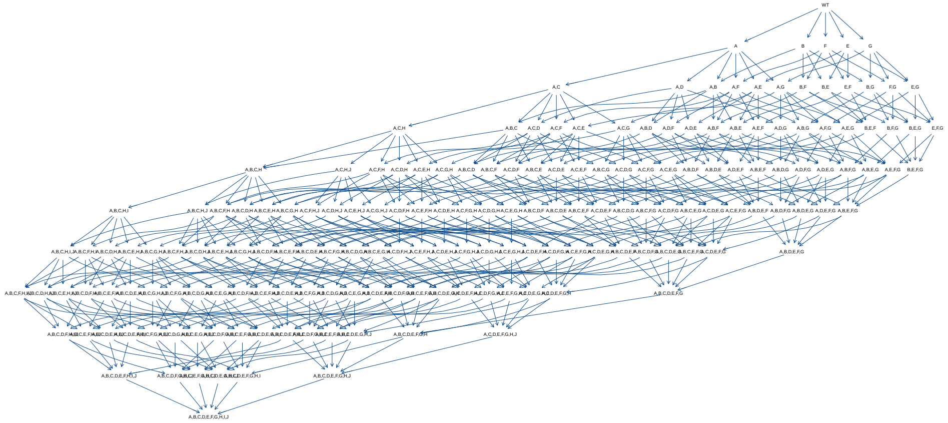

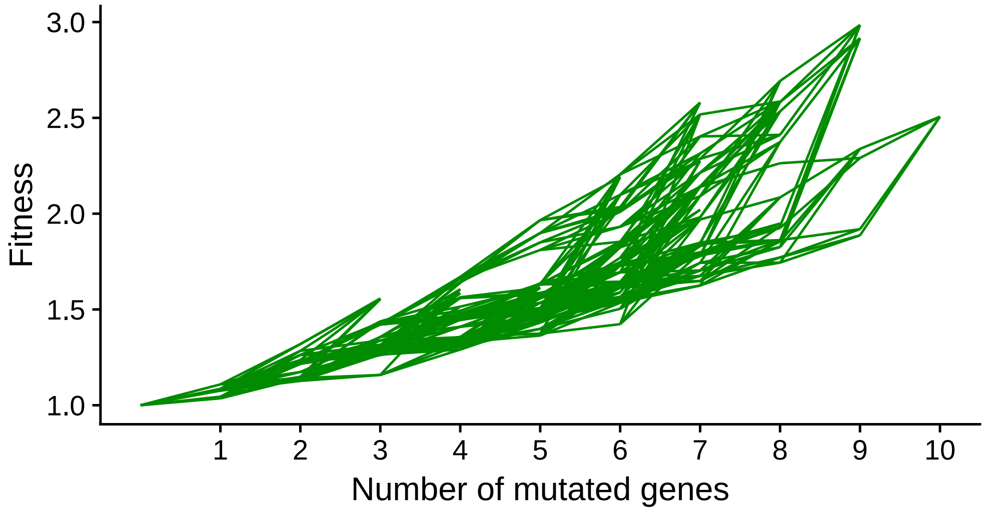

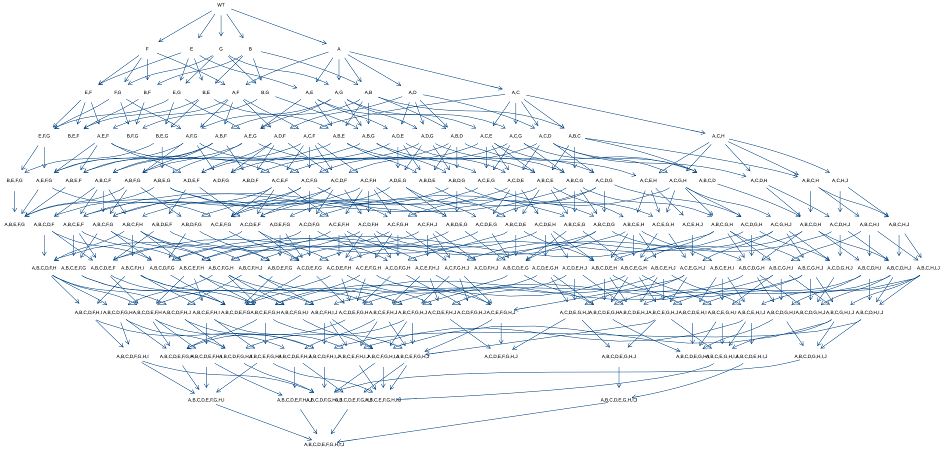

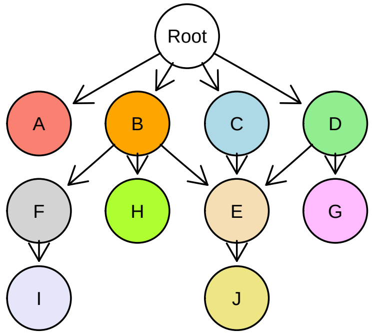

ID	9tzPJQwcPFgCvZpS
acc genotypes	132
peaks no back	12
removed edges	78
prop rem edges	0.201

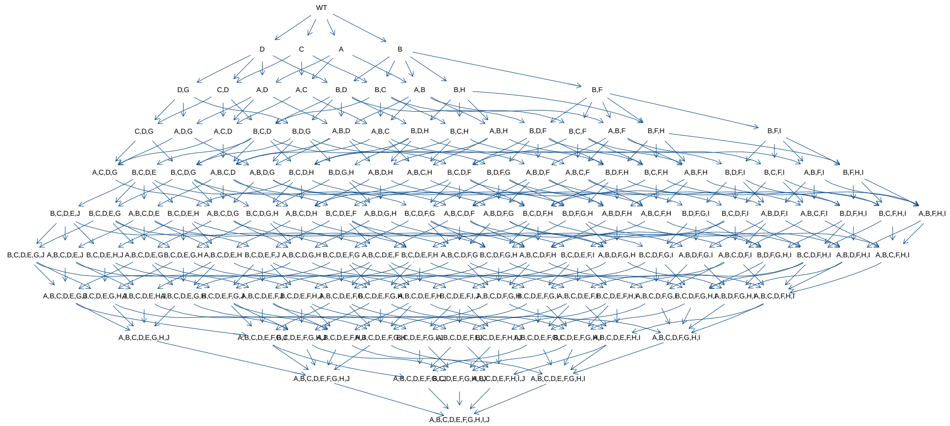

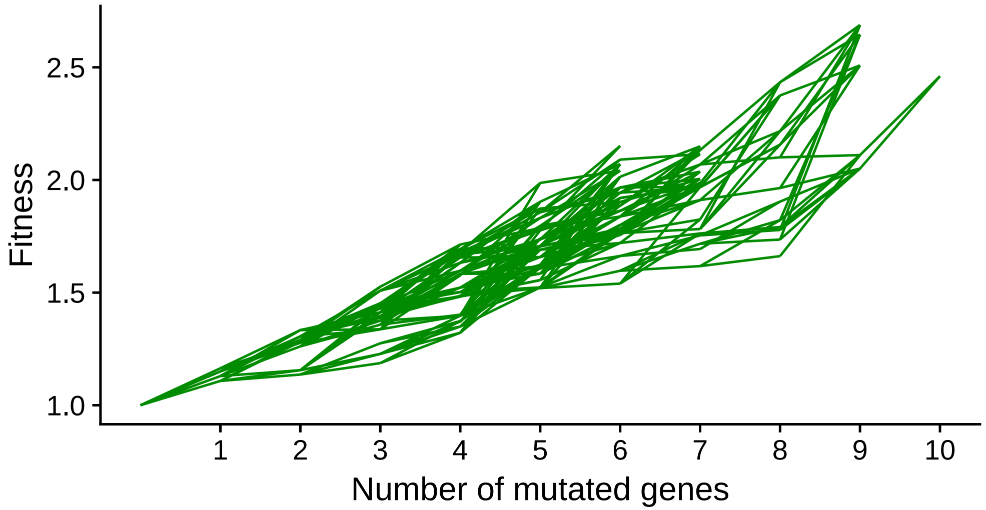

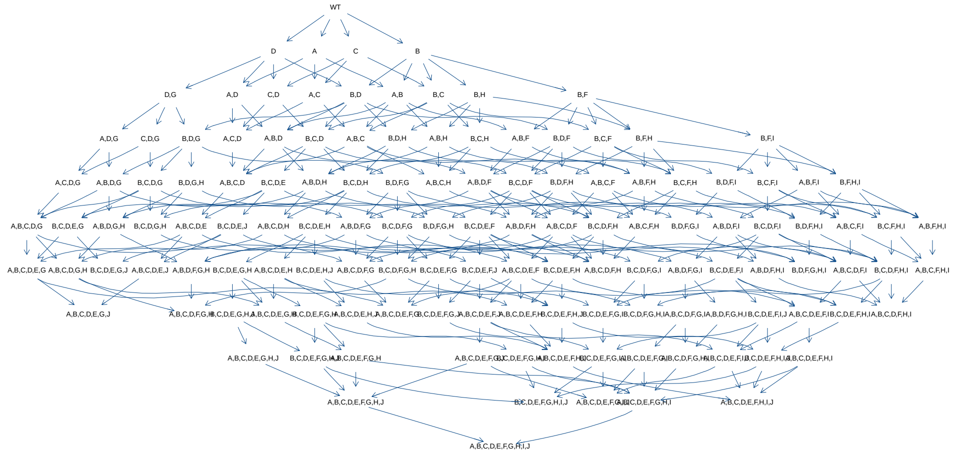

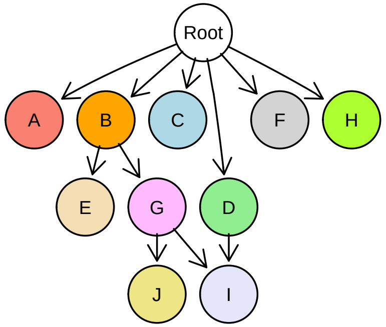

ID	9XhdPR4OnXtyF10ls
acc genotypes	288
peaks no back	21
removed edges	224
prop rem edges	0.209

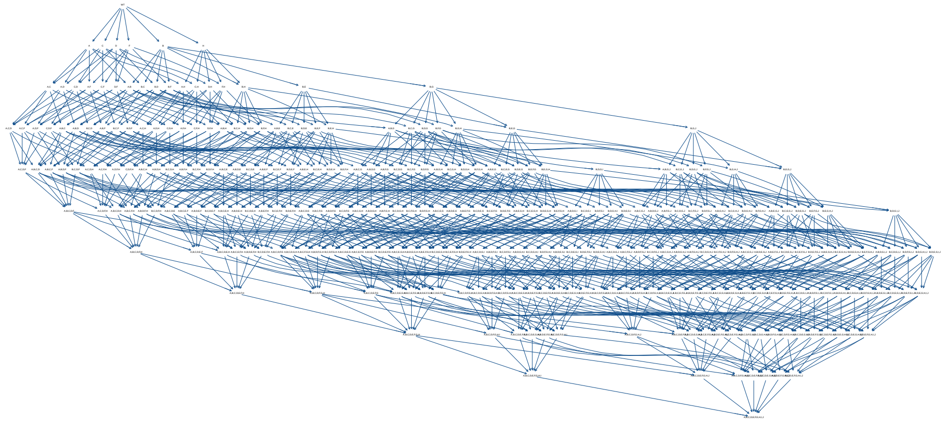

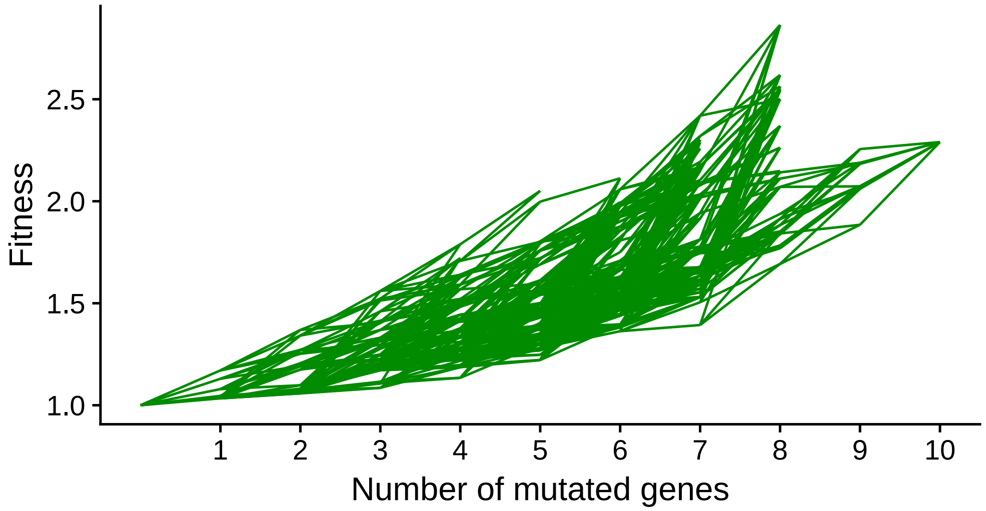

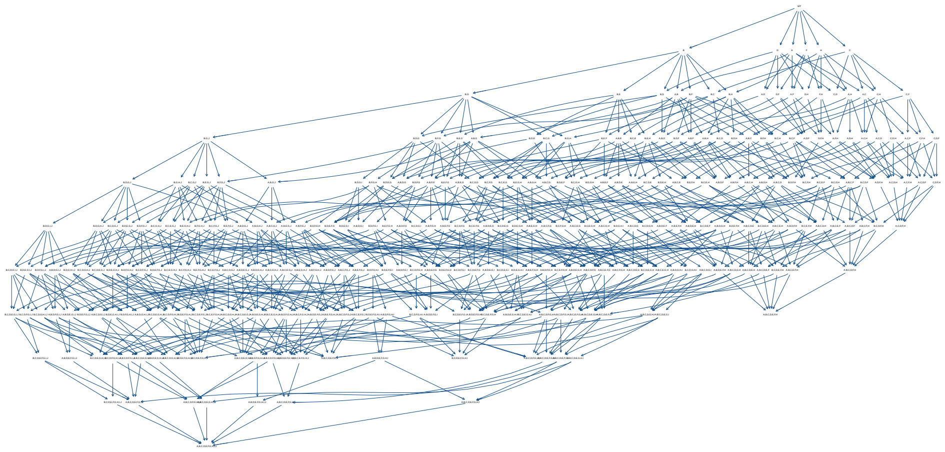

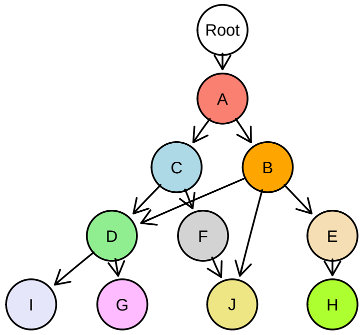

ID	AAvENuRd7EV7ymhh
acc genotypes	52
peaks no back	8
removed edges	21
prop rem edges	0.181

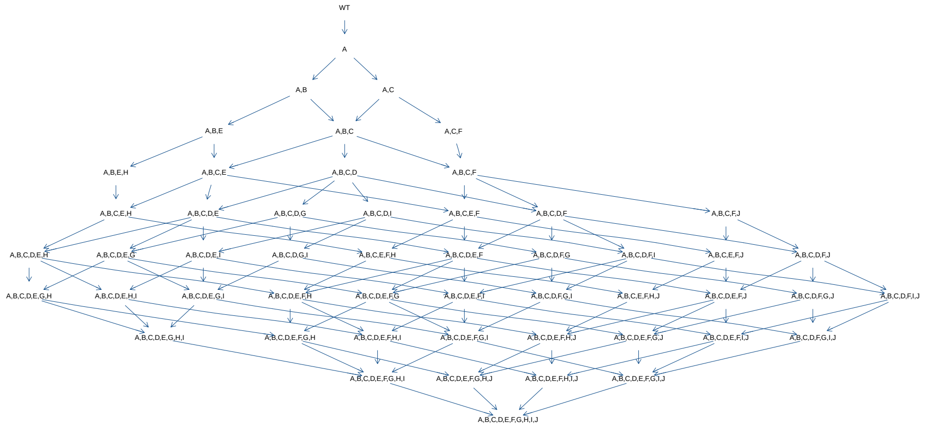

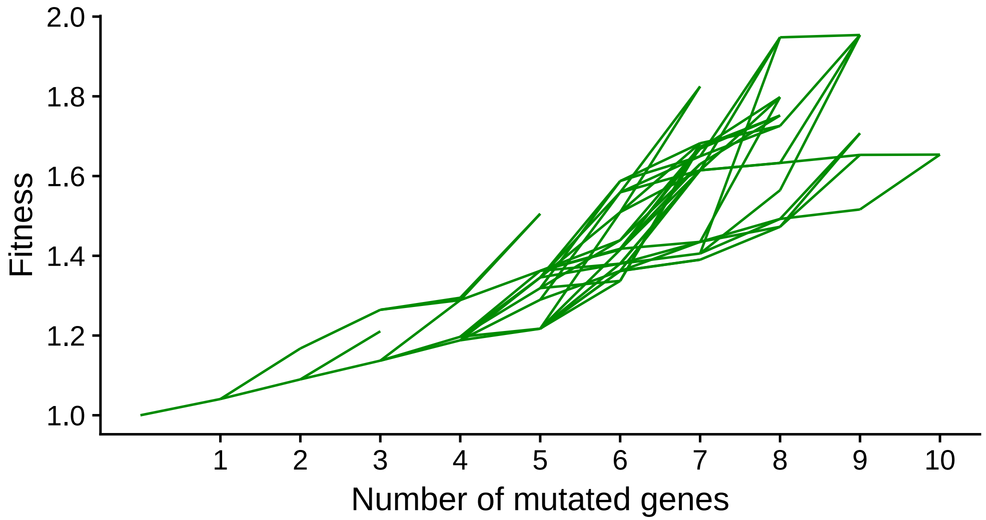

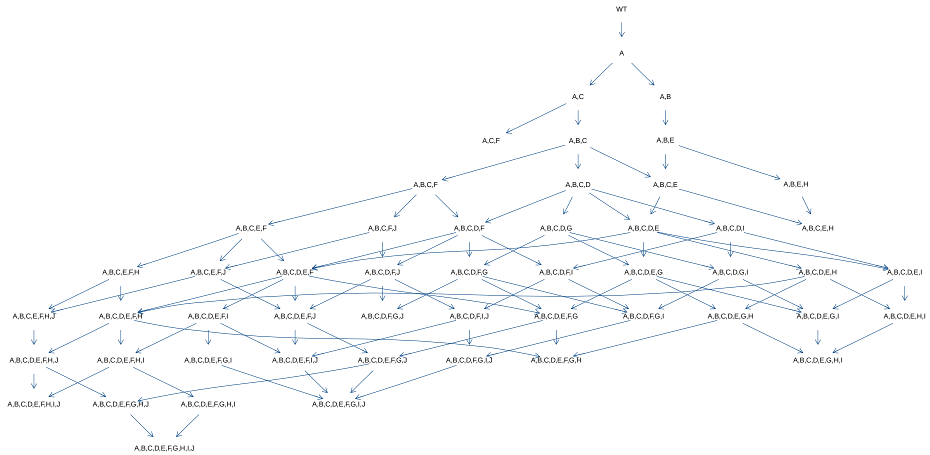

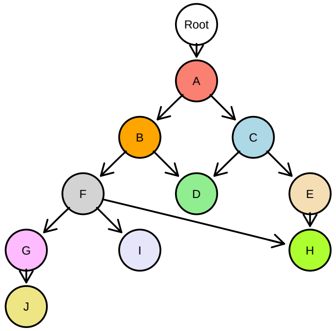

ID	aeSijOttBLlSw1Z1
acc genotypes	51
peaks no back	8
removed edges	21
prop rem edges	0.186

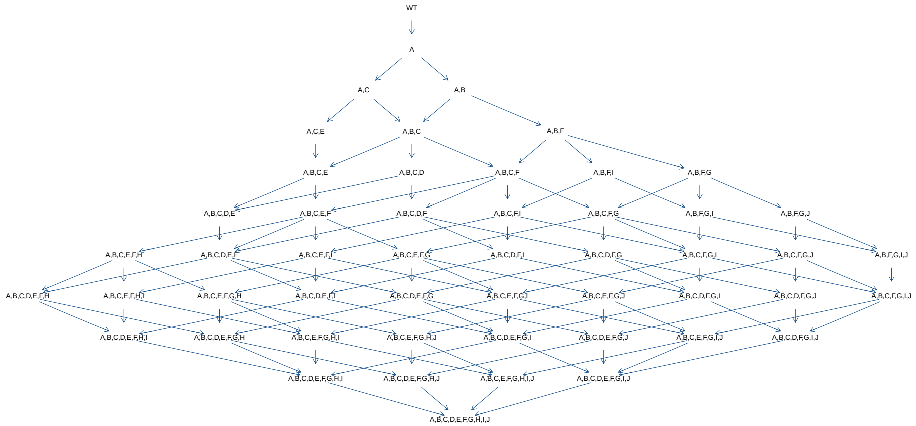

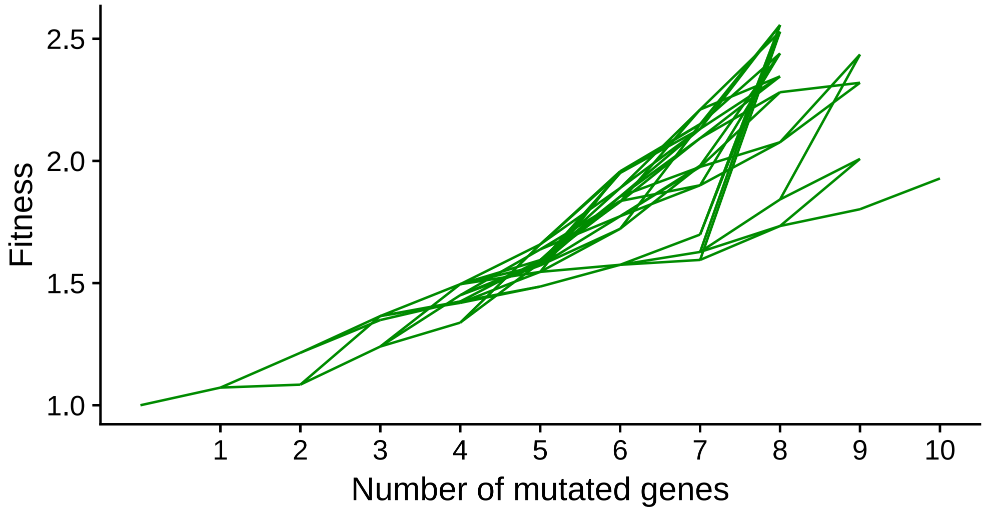

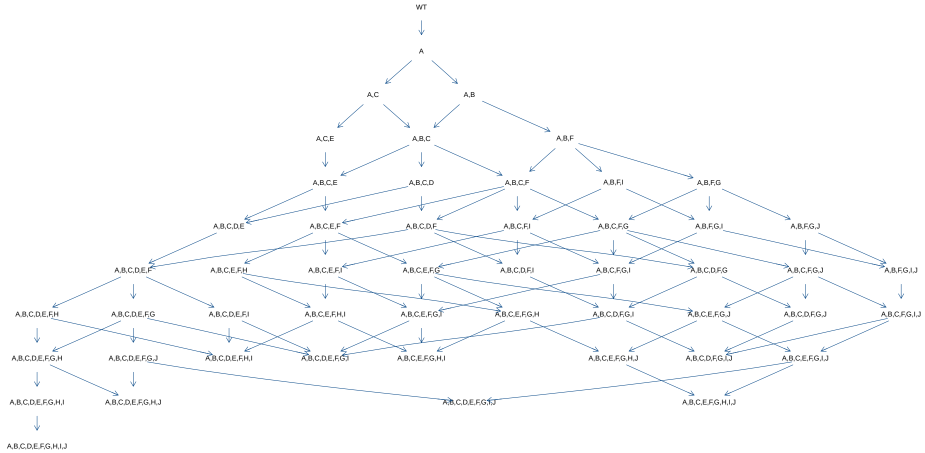

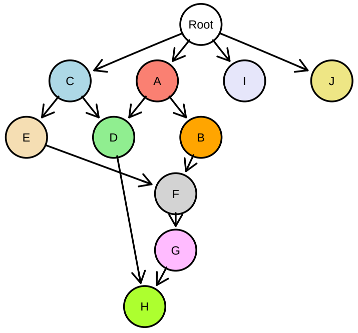

ID	aFSJF5G7y4DBZWSr
acc genotypes	72
peaks no back	8
removed edges	39
prop rem edges	0.217

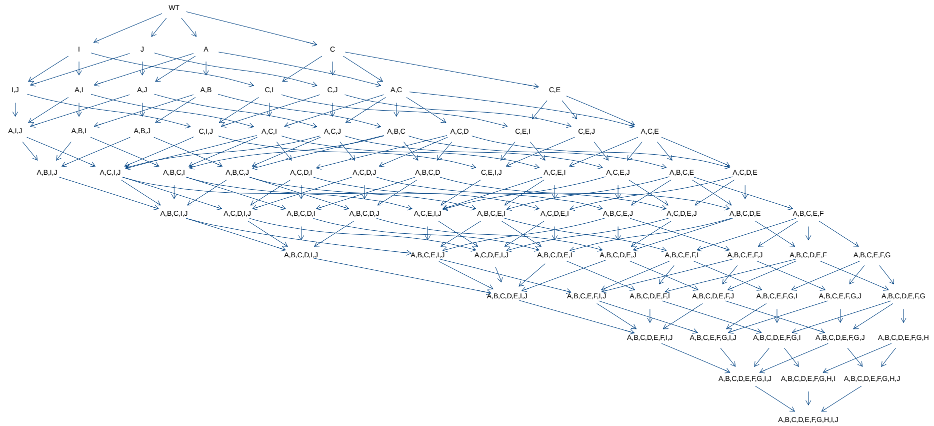

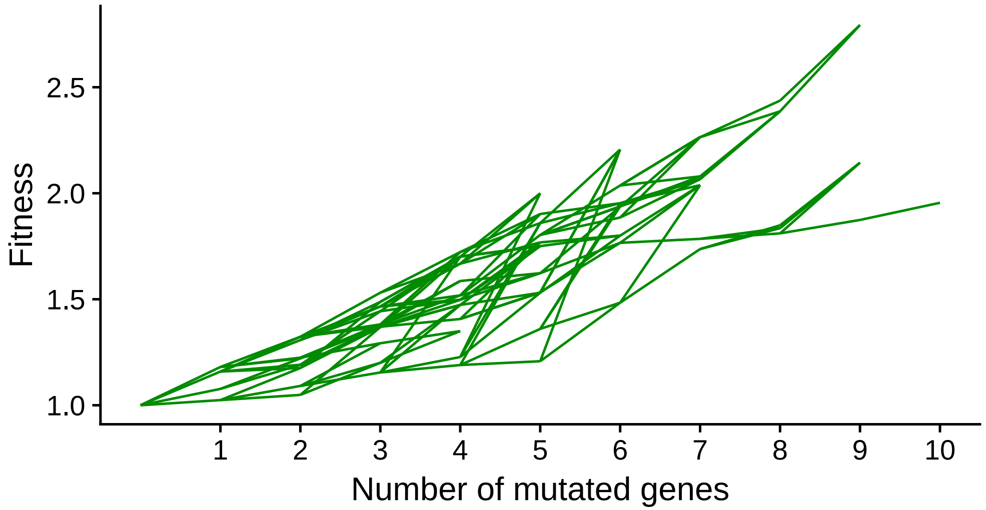

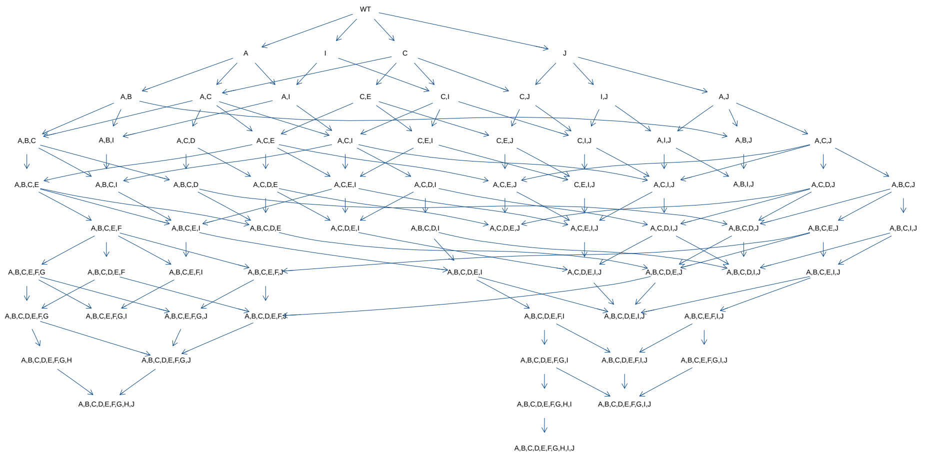

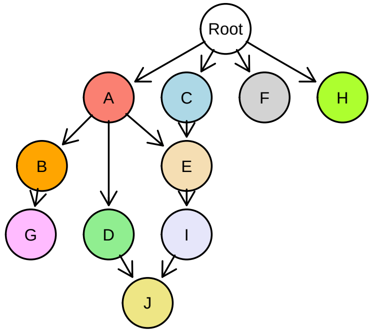

ID	AjFtq103DguBWTFLW
acc genotypes	116
peaks no back	10
removed edges	74
prop rem edges	0.223

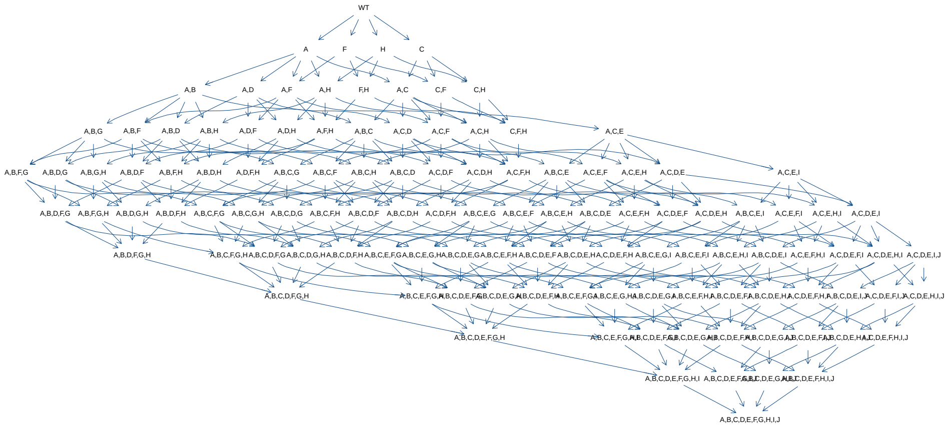

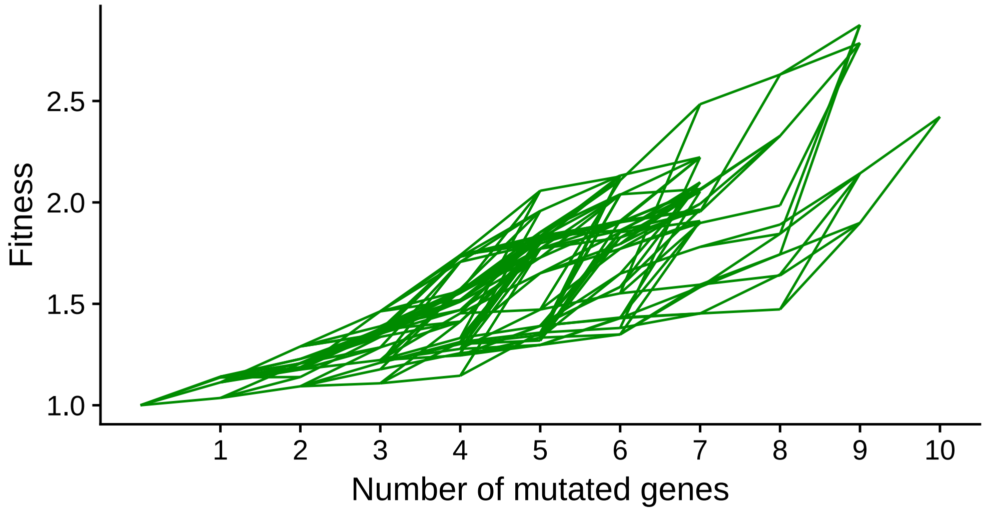

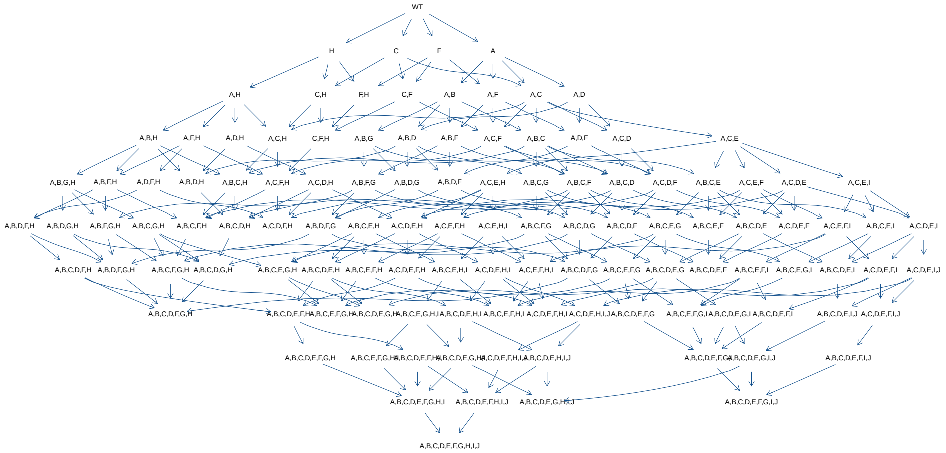

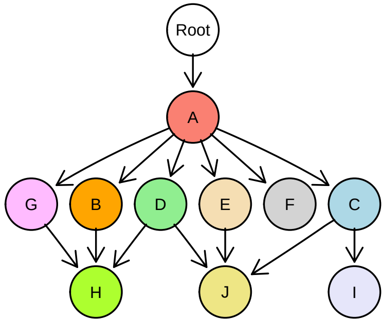

ID	B10D8cwK5cY9qVLNq
acc genotypes	129
peaks no back	13
removed edges	86
prop rem edges	0.217

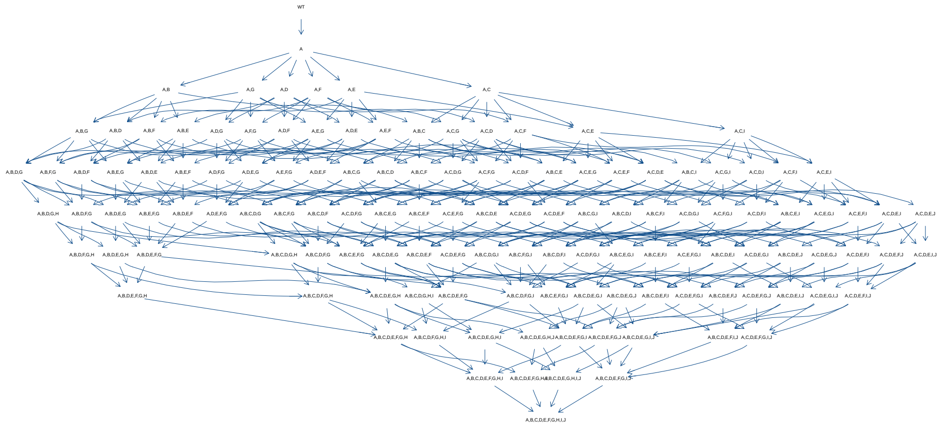

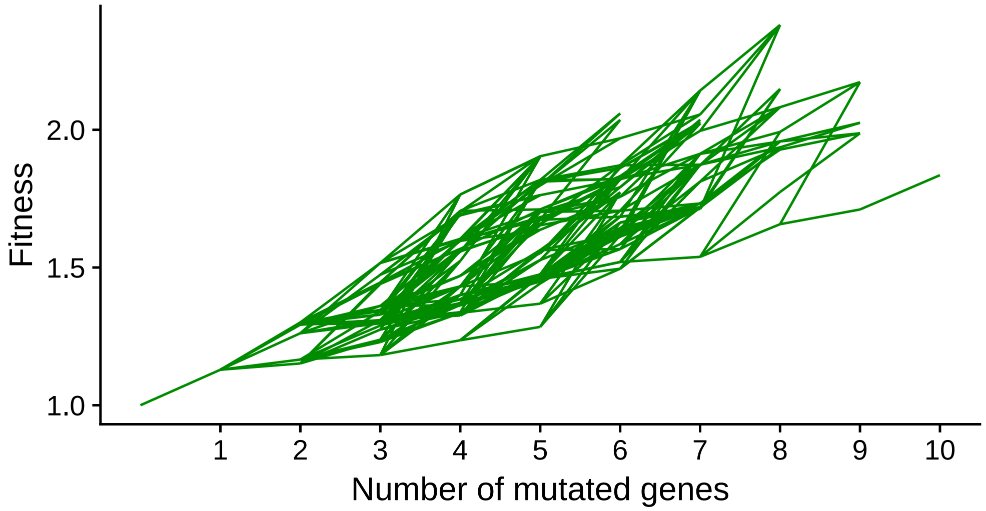

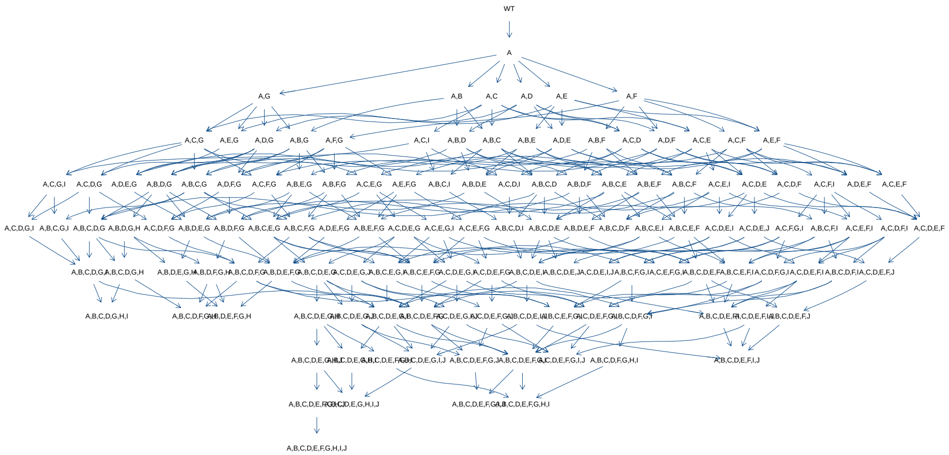

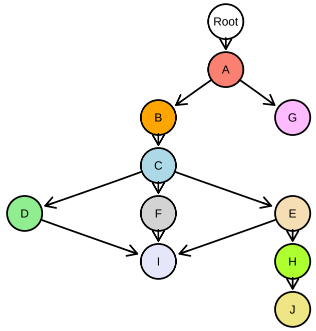

ID	b2dBTAqsSxgbkkTE
acc genotypes	43
peaks no back	7
removed edges	14
prop rem edges	0.152

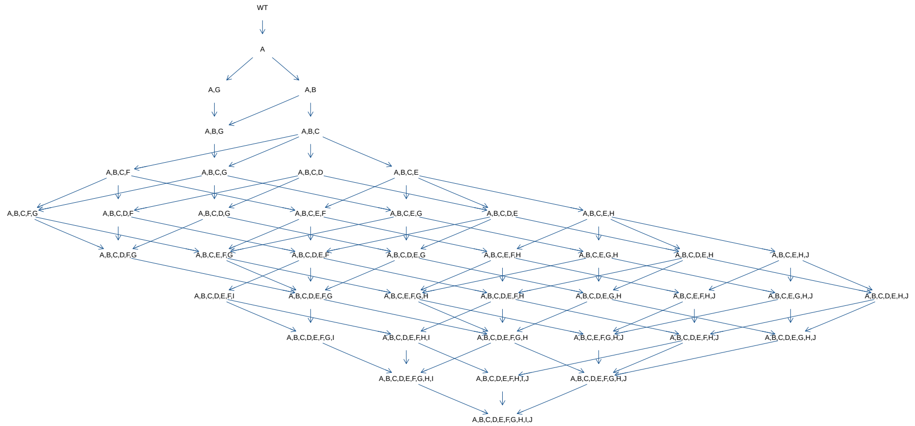

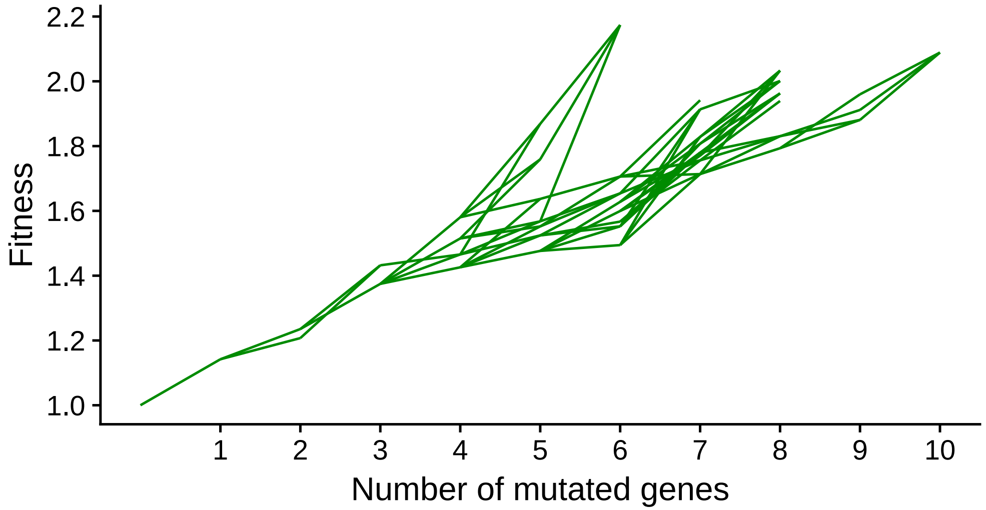

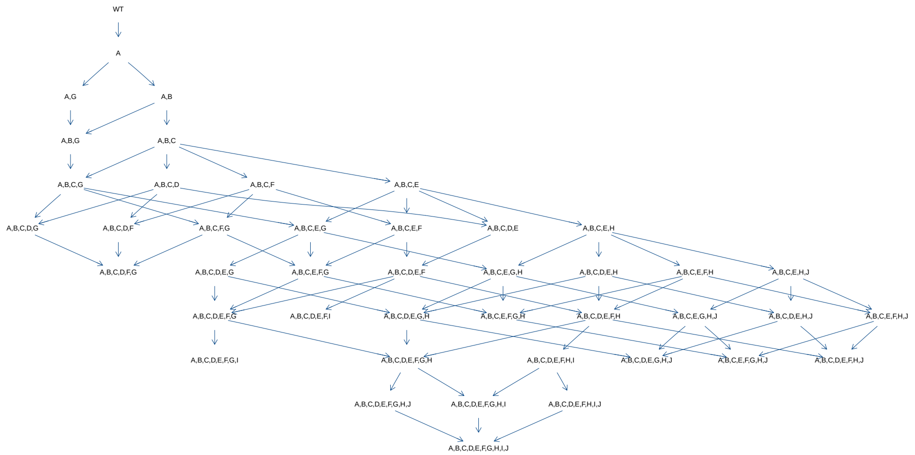

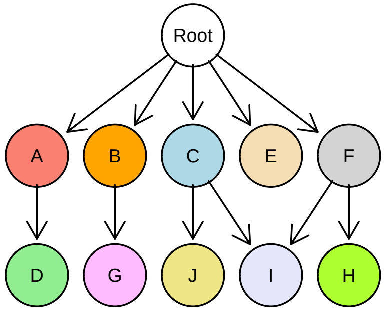

ID	B8LF4EJa7H2B21J3
acc genotypes	234
peaks no back	14
removed edges	130
prop rem edges	0.165

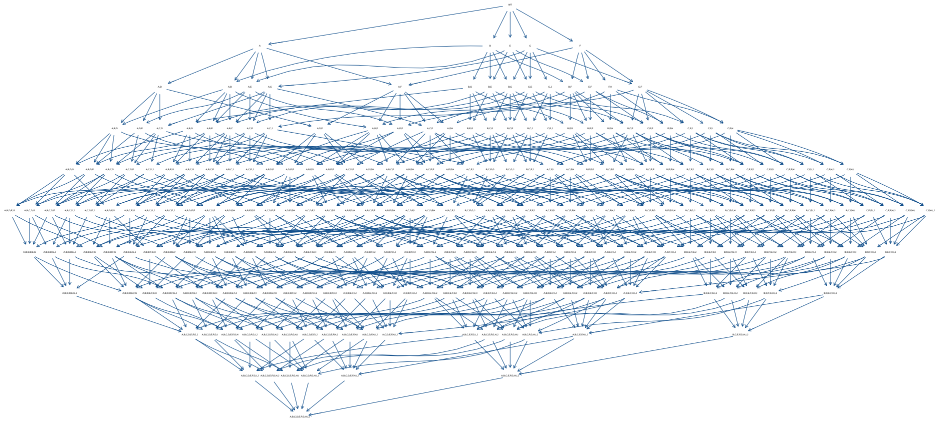

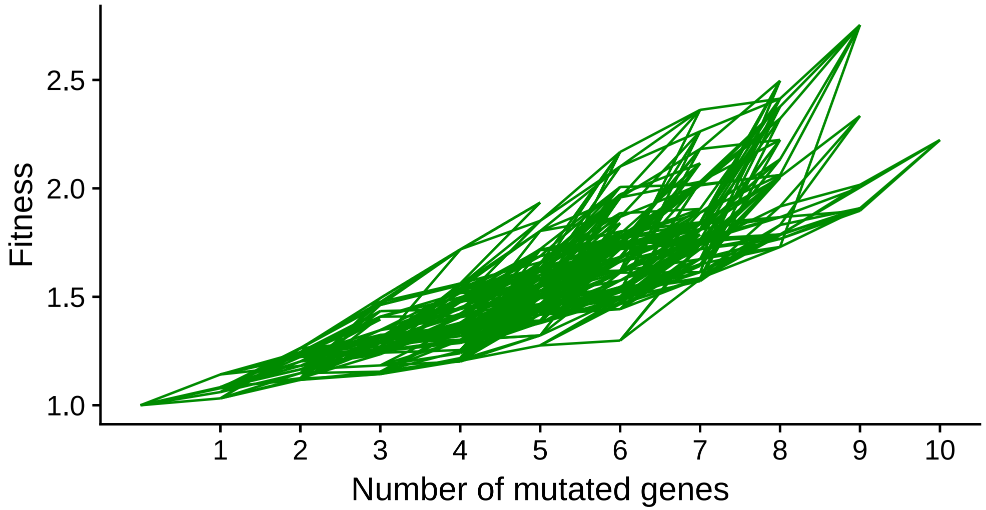

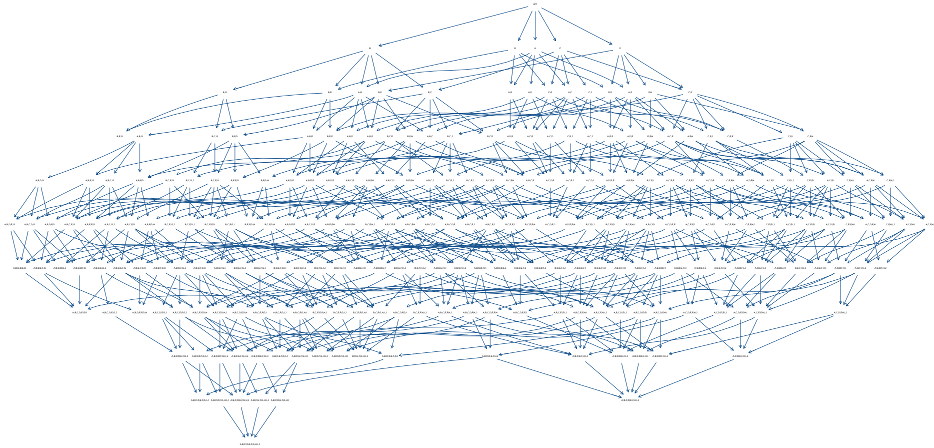

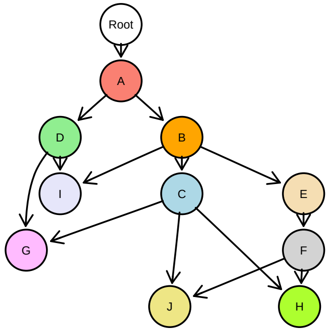

ID	BoCaqtWu6XR9fw10u
acc genotypes	42
peaks no back	9
removed edges	20
prop rem edges	0.235

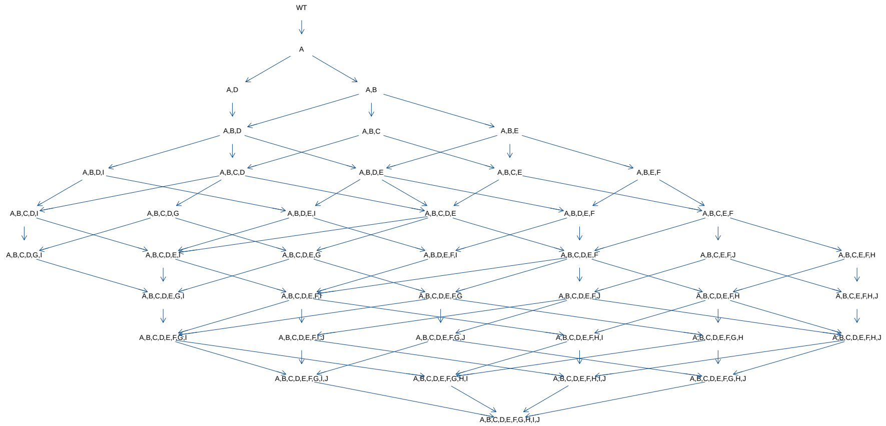

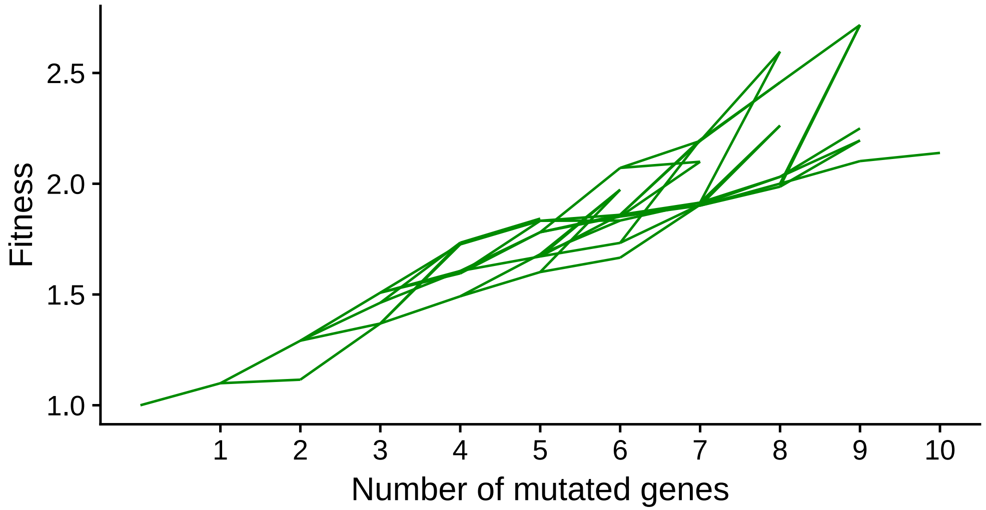

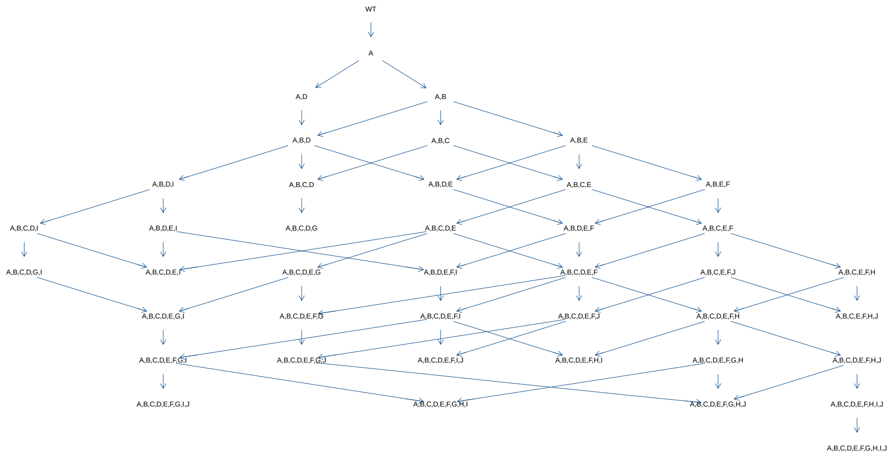

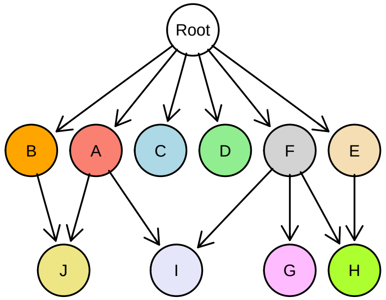

ID	BpzeoBLsEJ3xTD7v
acc genotypes	232
peaks no back	17
removed edges	155
prop rem edges	0.195

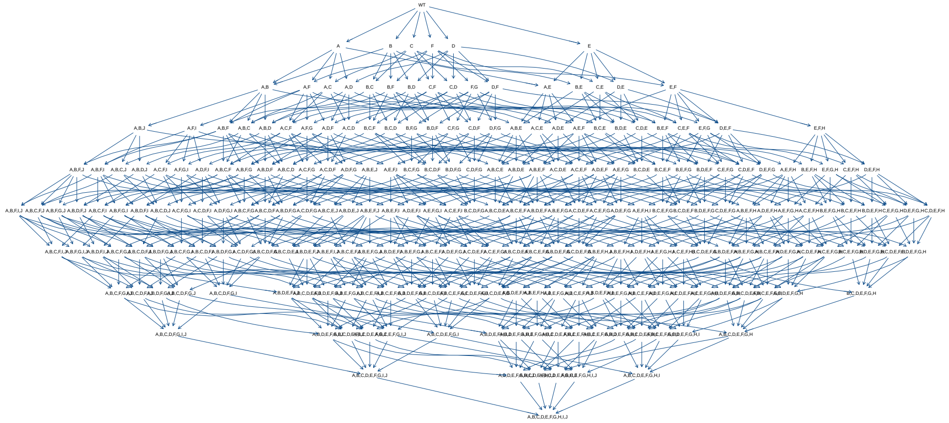

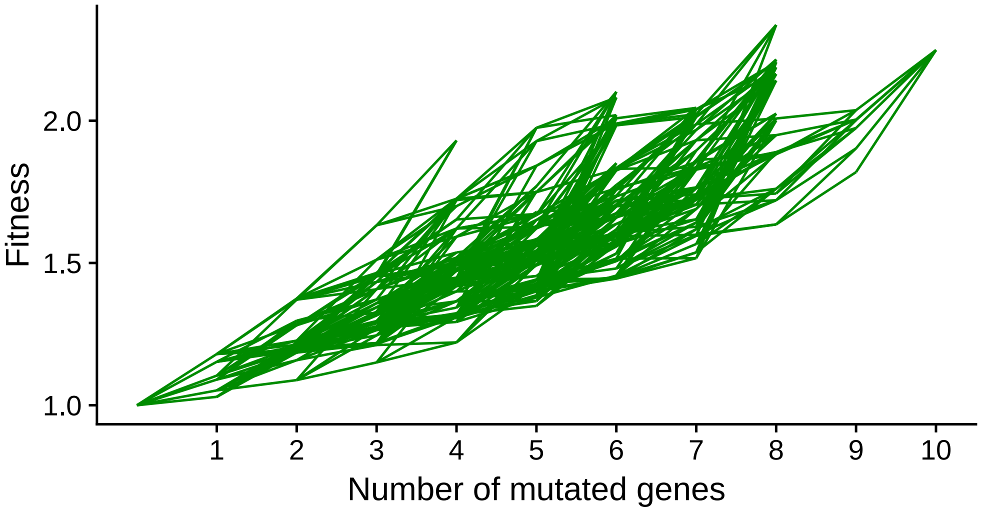

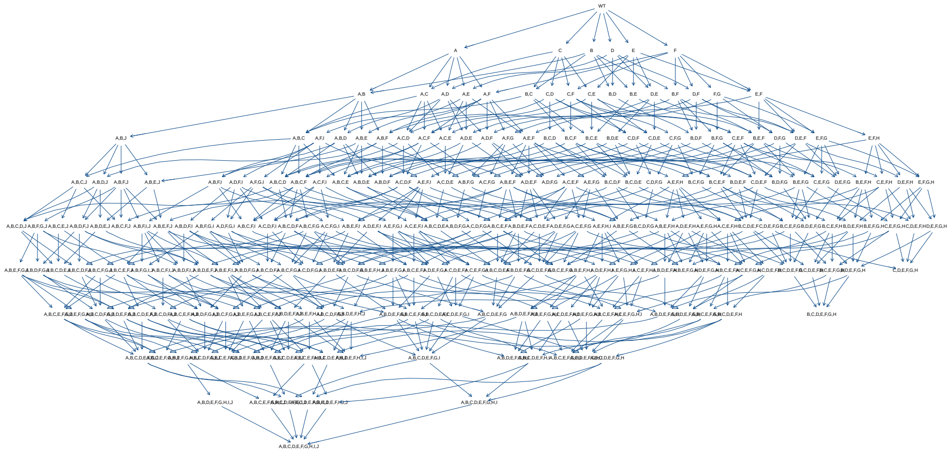

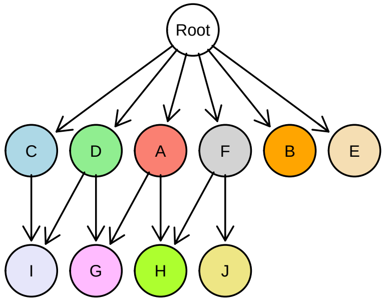

ID	Bqkt5YbsNE7niJz9
acc genotypes	220
peaks no back	19
removed edges	183
prop rem edges	0.247

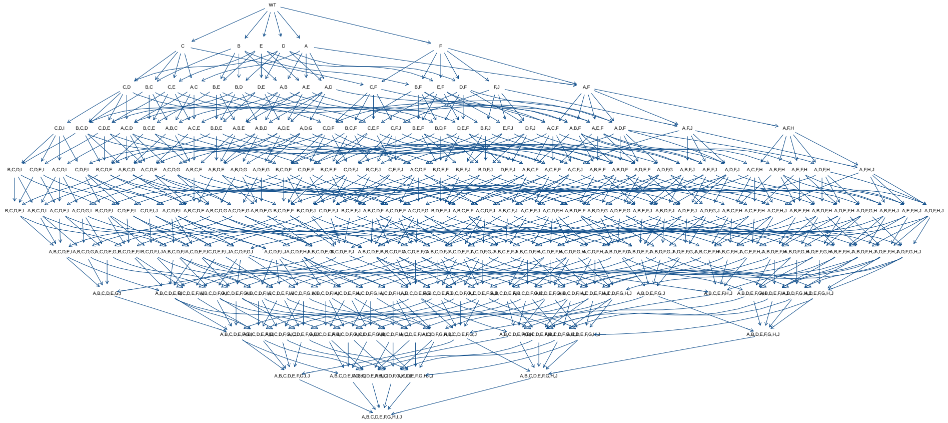

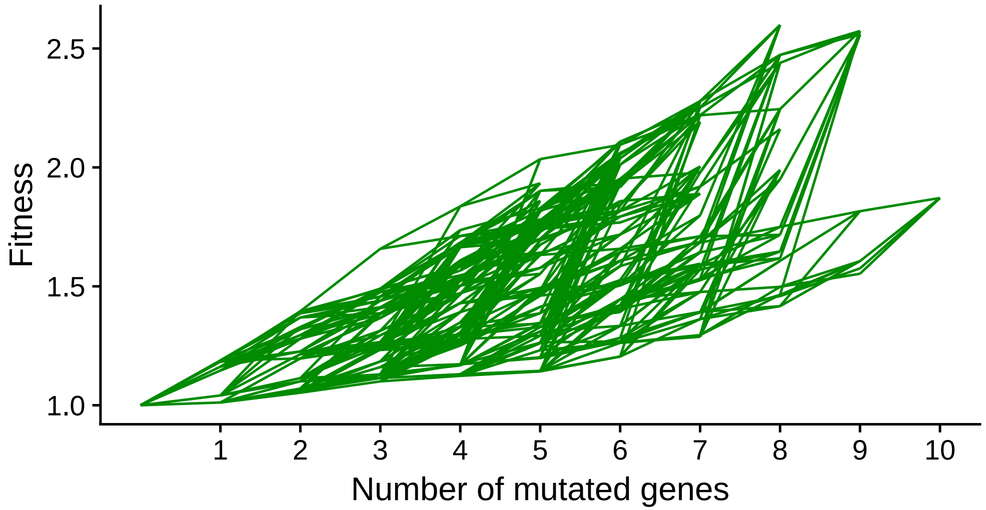

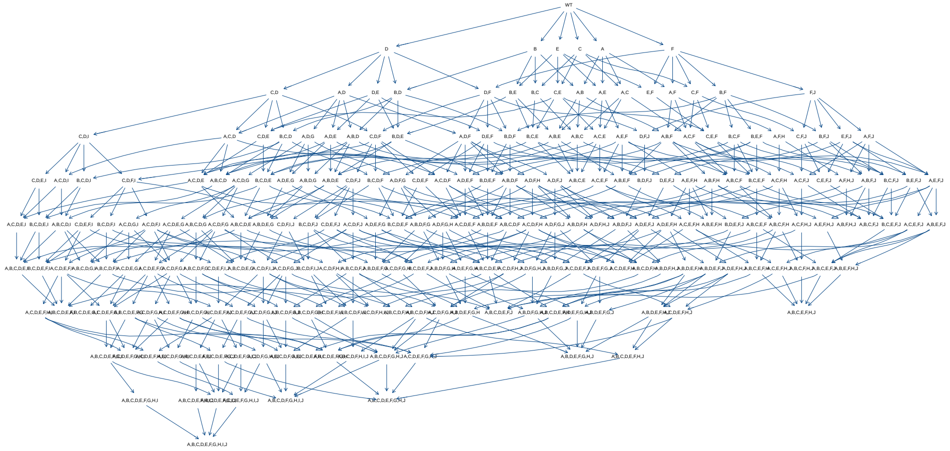

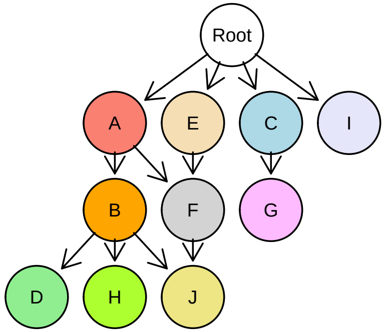

ID	c10jjy6210kvQdZBVg
acc genotypes	126
peaks no back	16
removed edges	70
prop rem edges	0.193

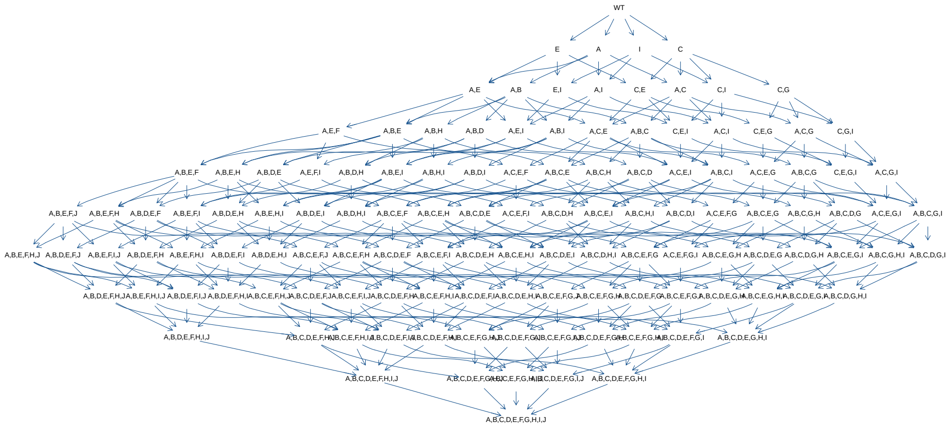

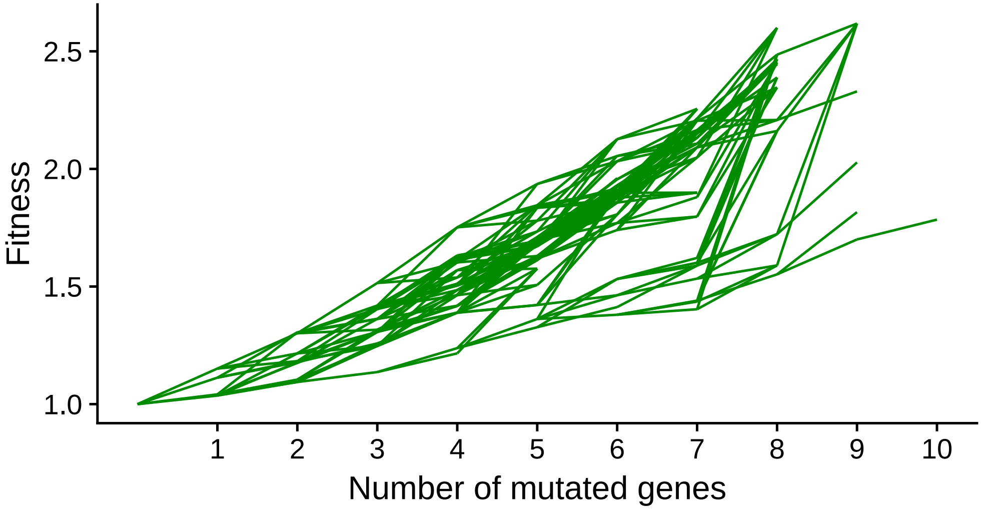

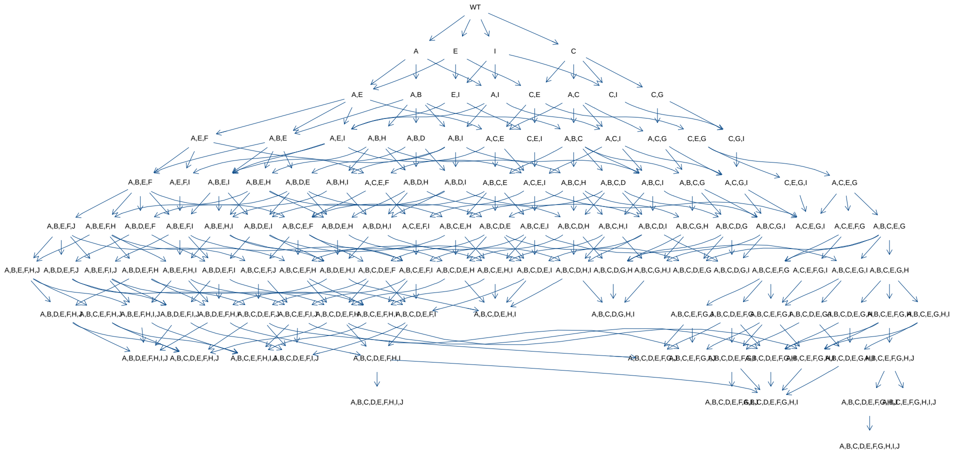

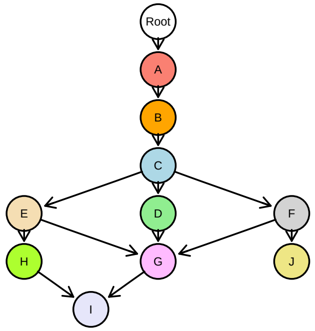

ID	C4drUyloTXoBU6JC
acc genotypes	27
peaks no back	4
removed edges	9
prop rem edges	0.191

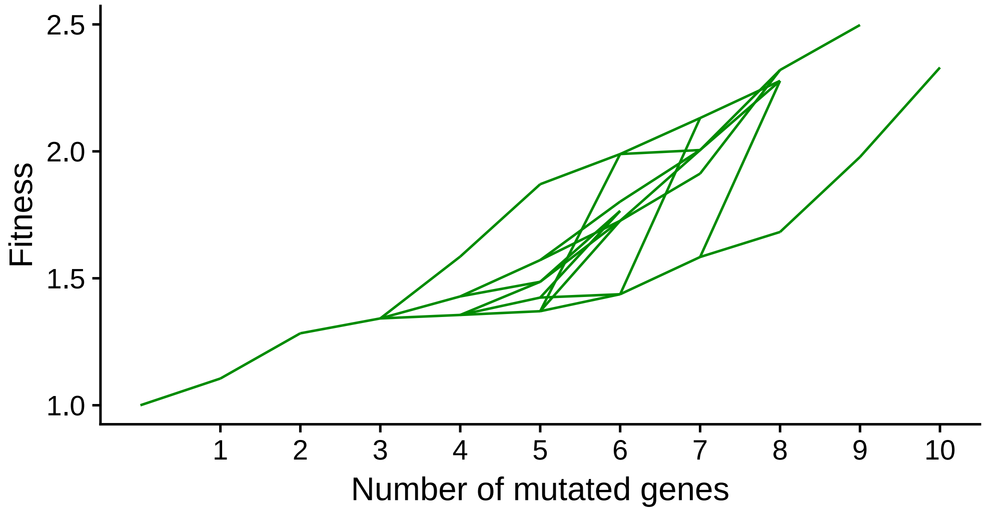

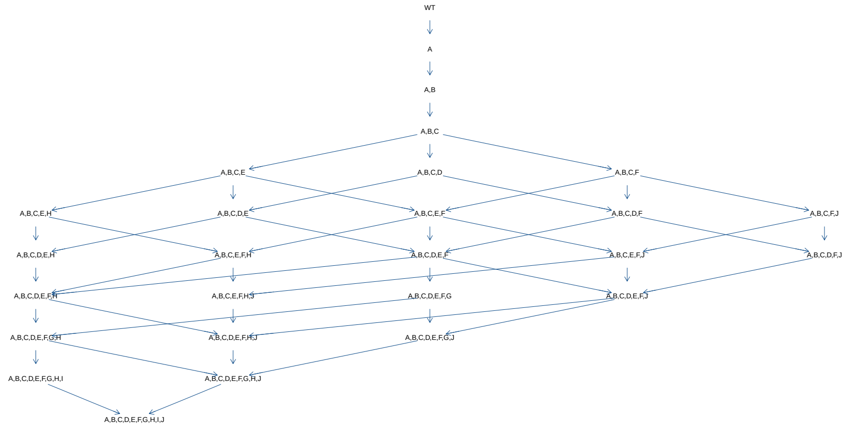

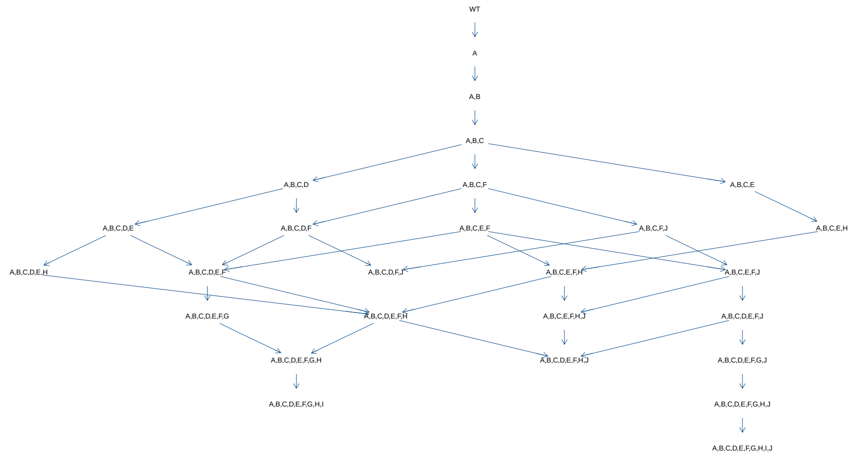

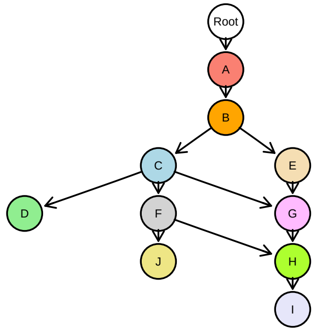

ID	CDprcI8u4ZcaFn7F
acc genotypes	30
peaks no back	4
removed edges	6
prop rem edges	0.111

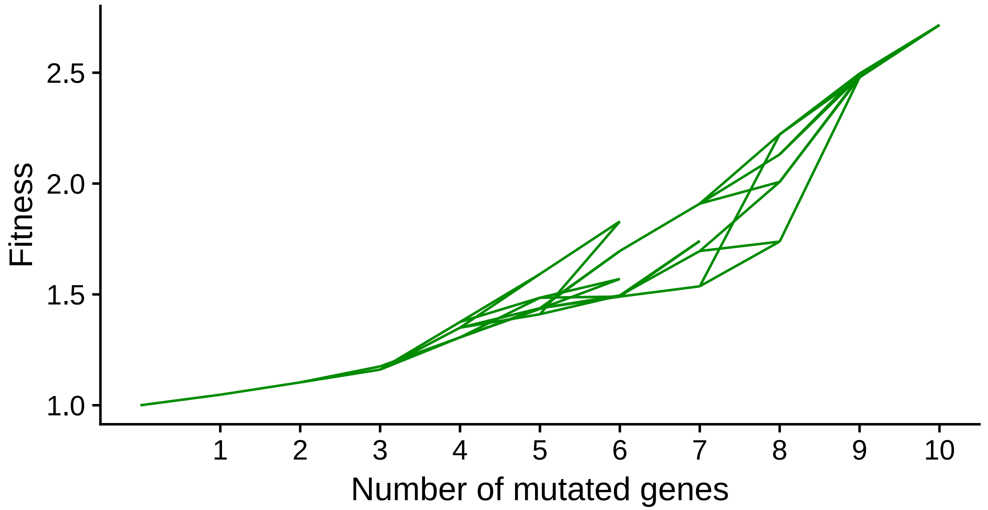

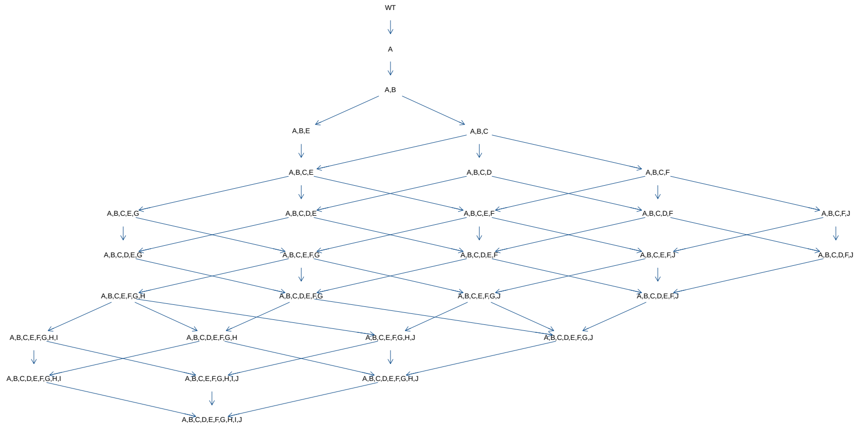

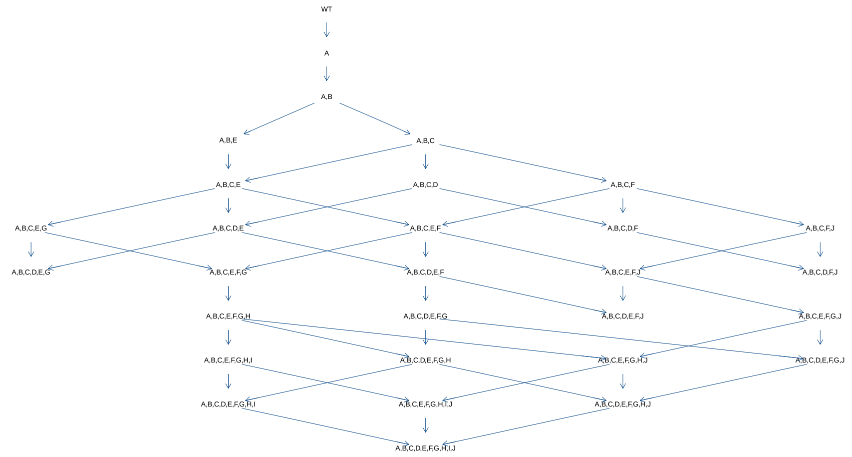

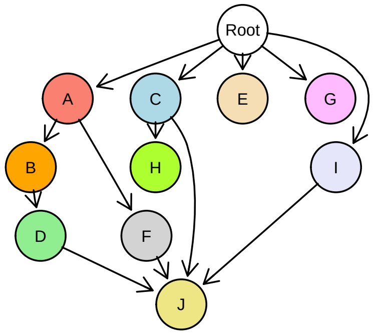

ID cGm7gRR3s42NqkyP
acc genotypes 176
peaks no back 16
removed edges 116
prop rem edges 0.201

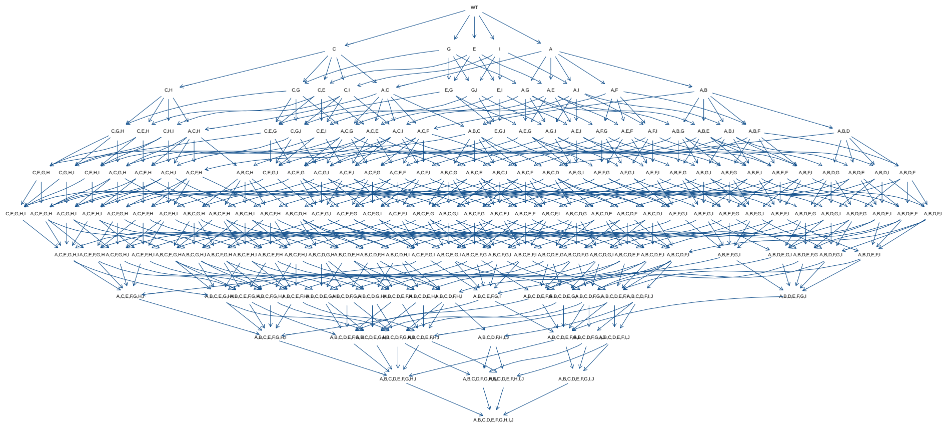

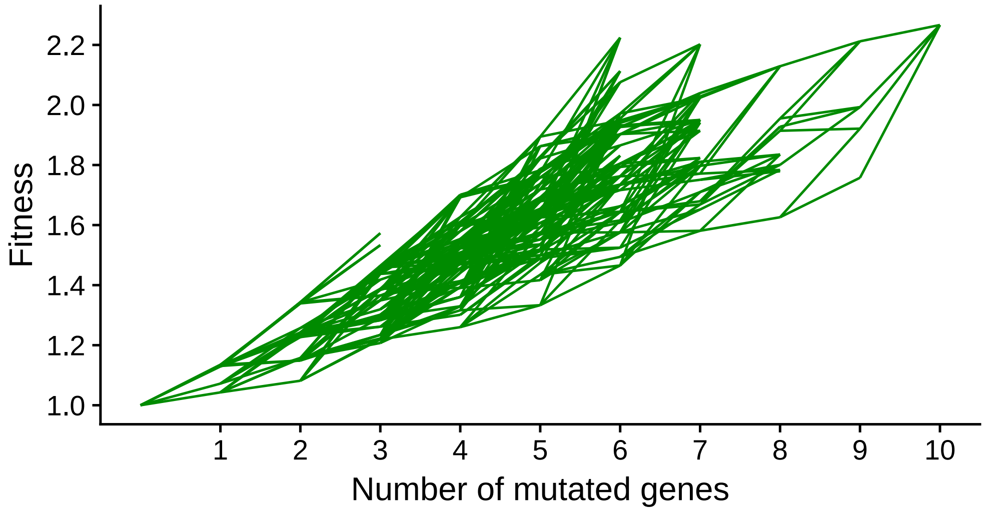

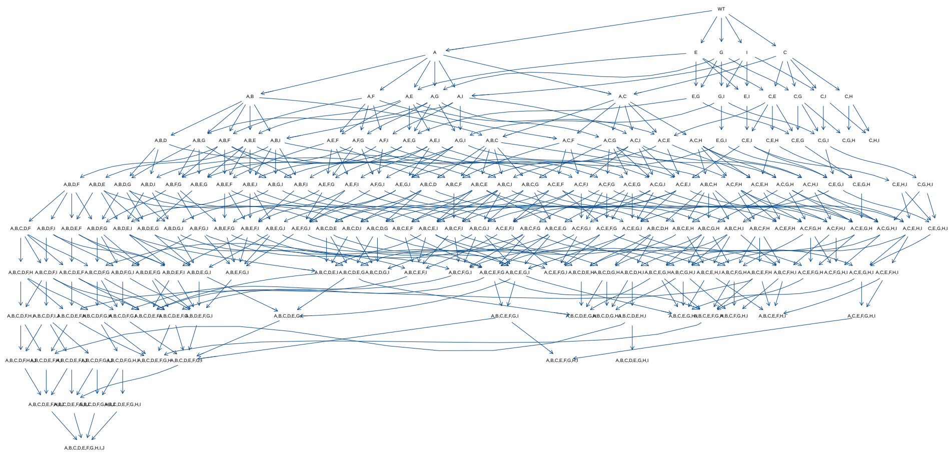

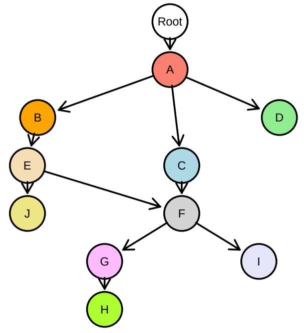

ID	CjDx5CPhar7wxbYH
acc genotypes	41
peaks no back	7
removed edges	22
prop rem edges	0.259

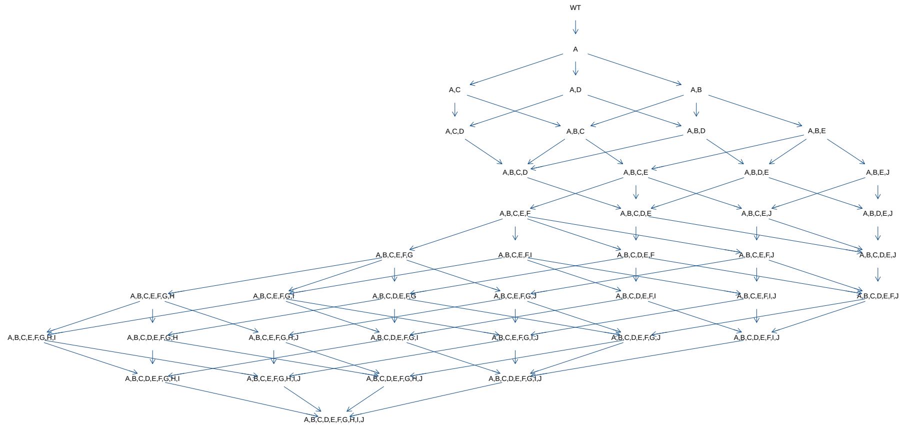

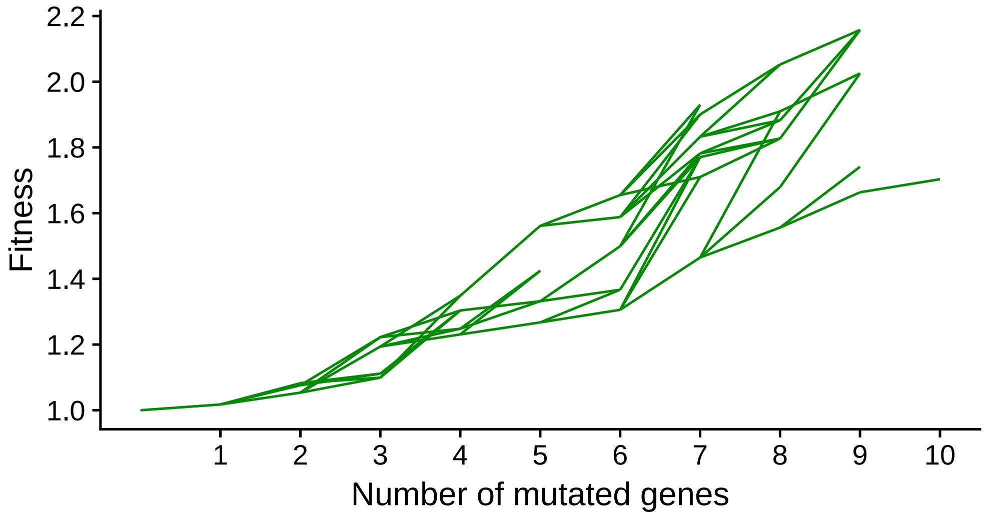

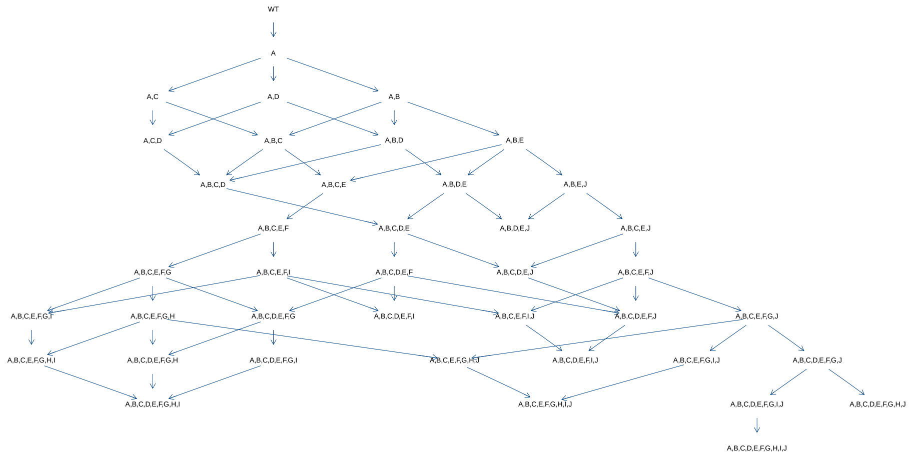

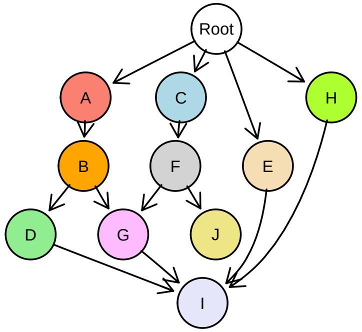

ID	dC6vMuFmbDjoadOo
acc genotypes	82
peaks no back	10
removed edges	39
prop rem edges	0.185

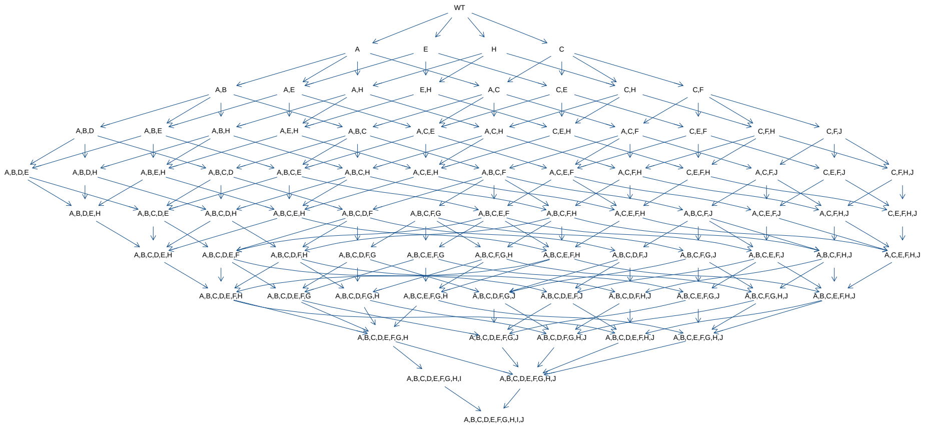

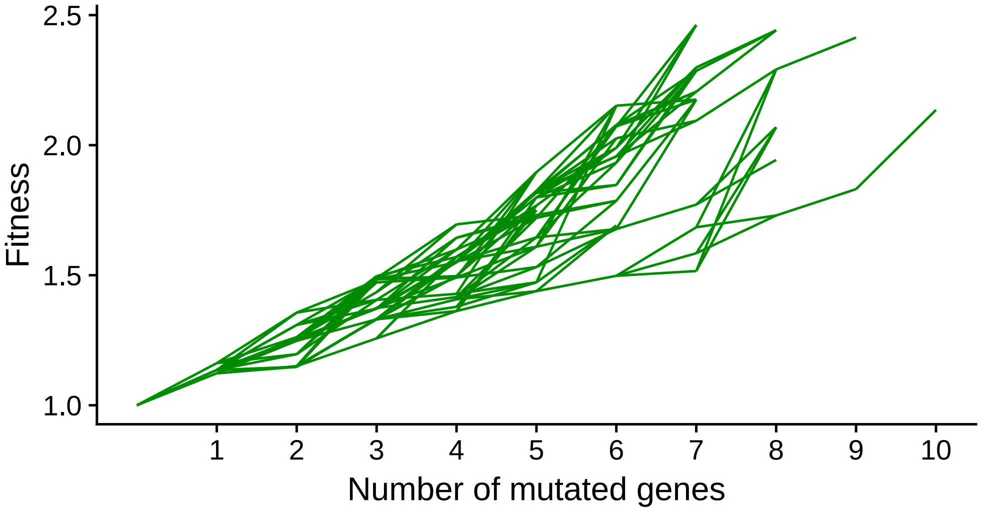

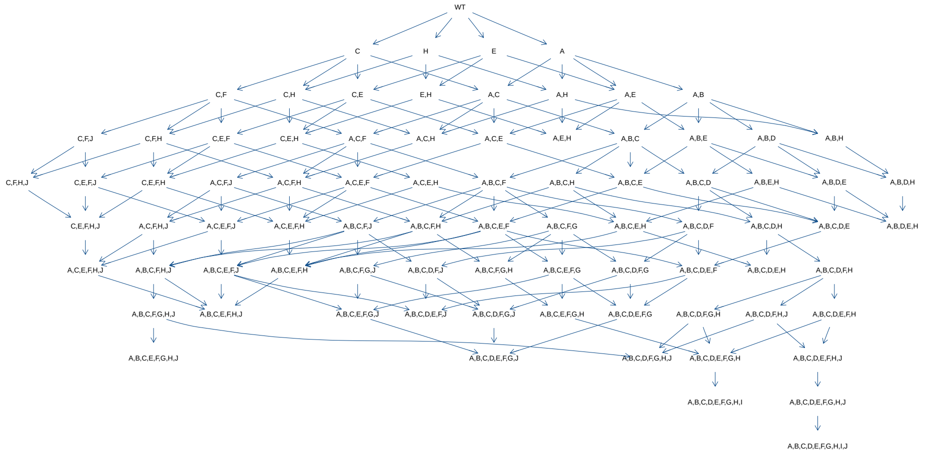

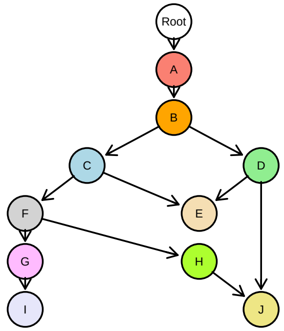

ID	DcAnAY4JLtfHe55I
acc genotypes	31
peaks no back	4
removed edges	8
prop rem edges	0.143

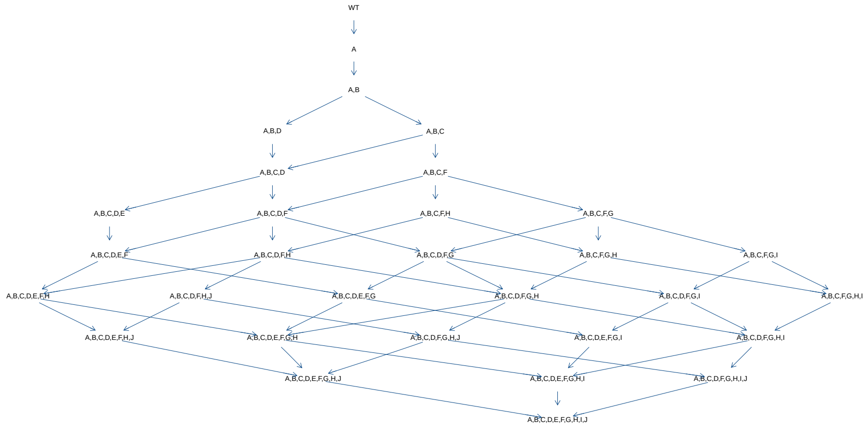

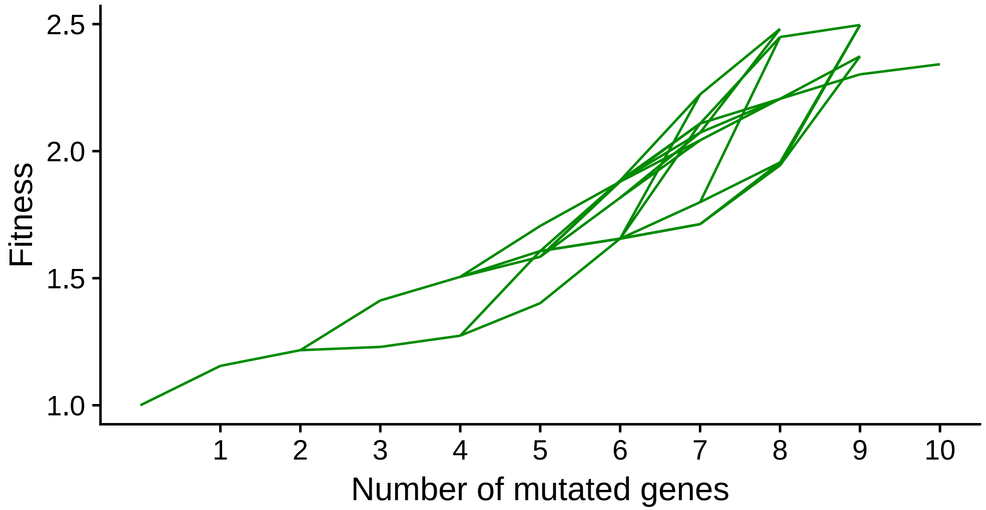

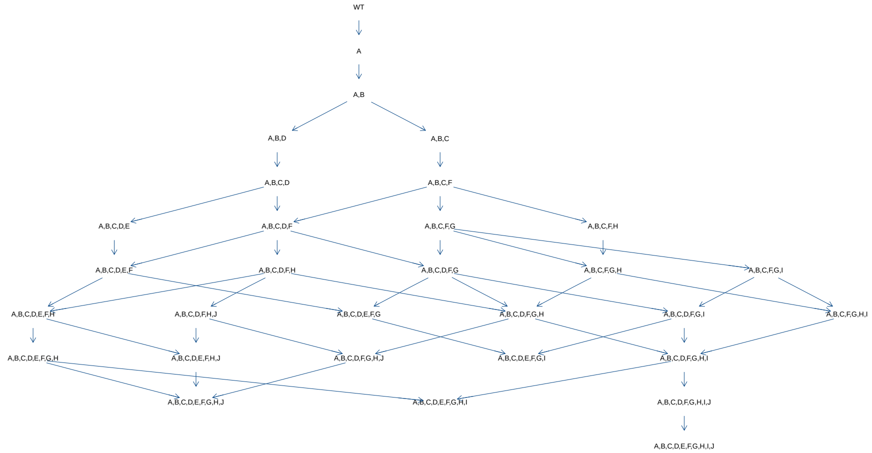

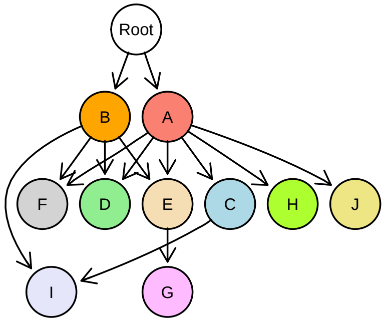

ID	dDZ4CvpAmrvxLFyS
acc genotypes	154
peaks no back	13
removed edges	111
prop rem edges	0.221

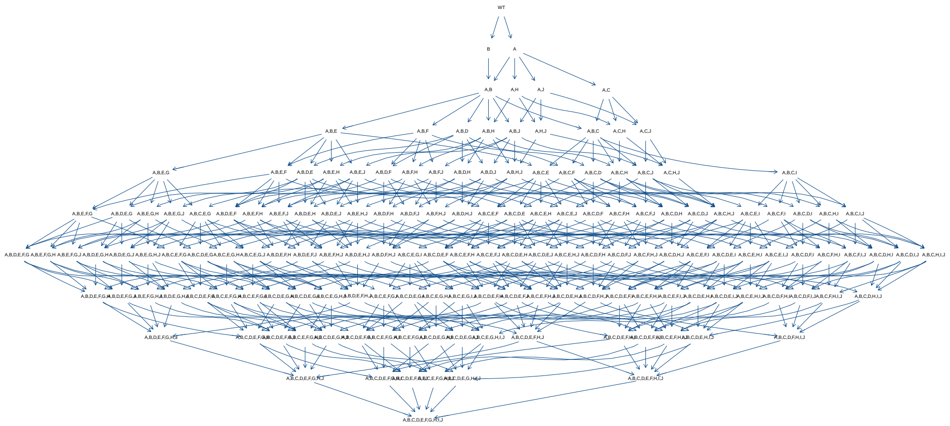

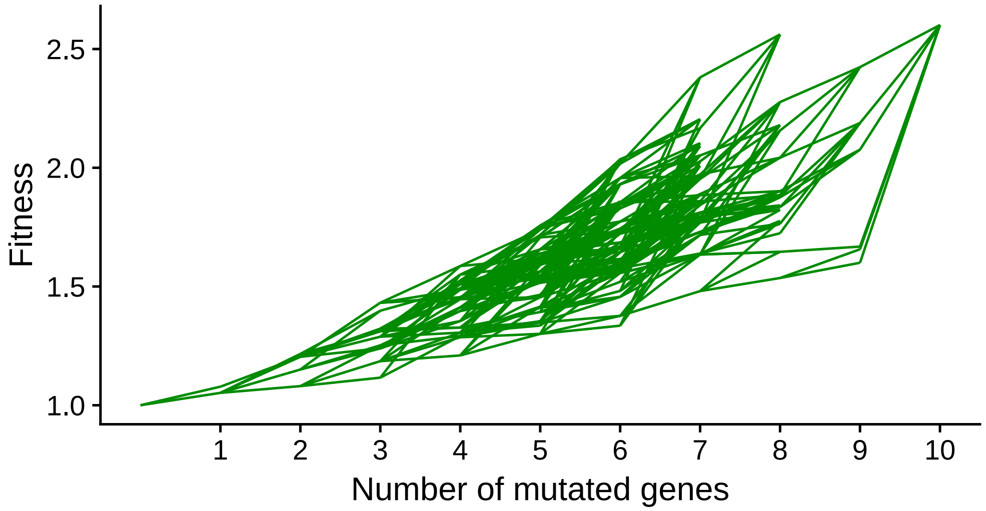

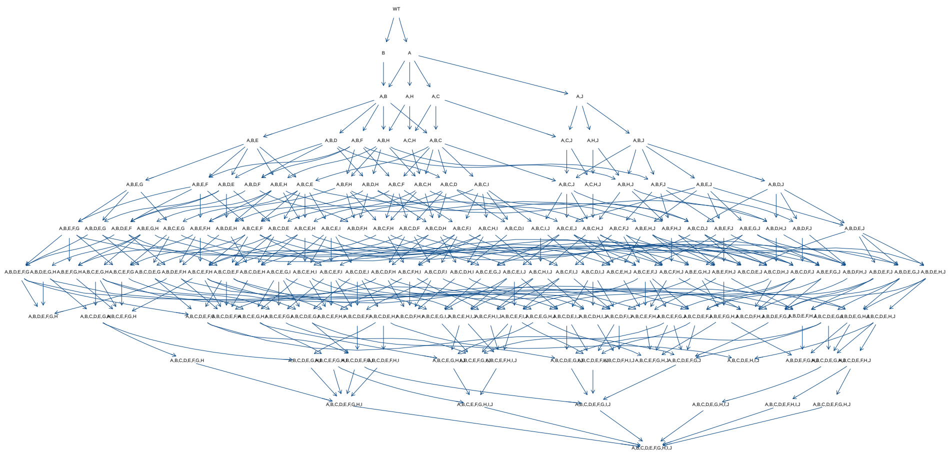

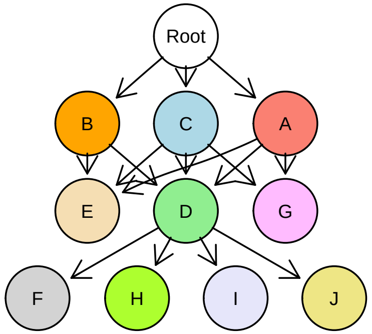

ID DFTK3ko7MlzoABxk
acc genotypes 76
peaks no back 9
removed edges 40
prop rem edges 0.187

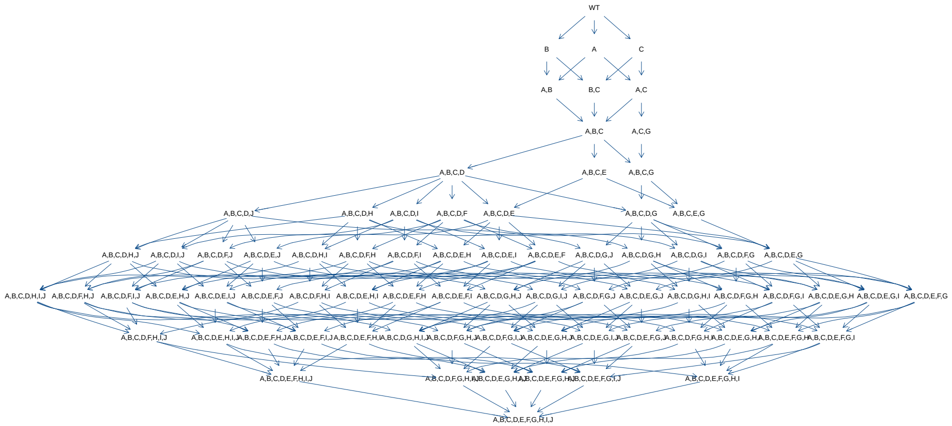

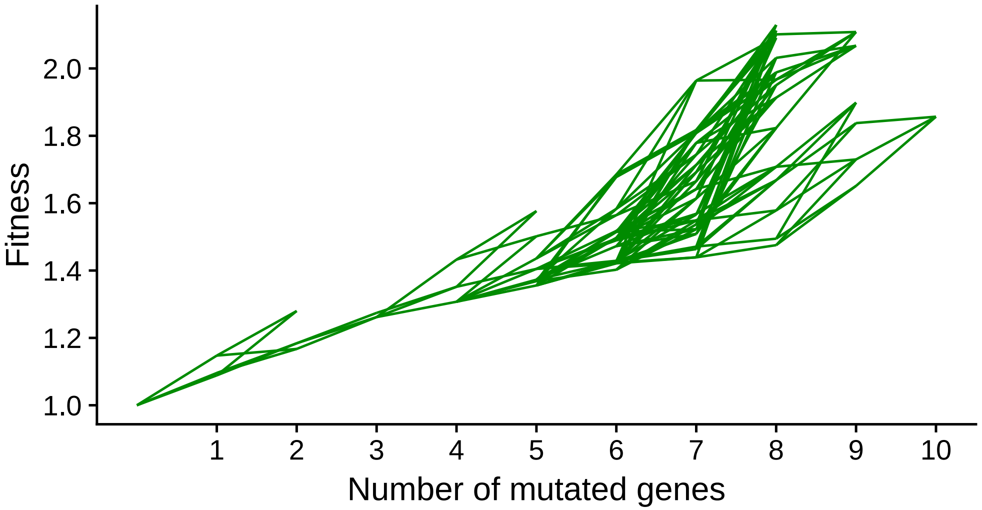

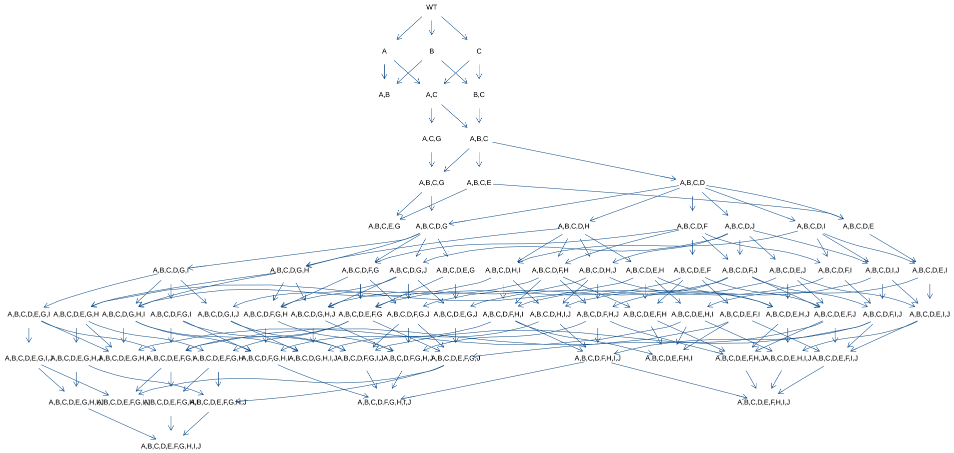

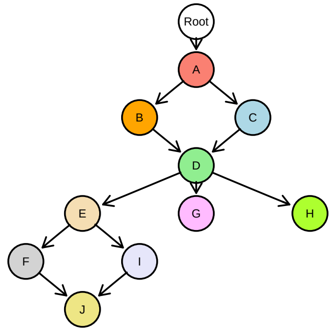

ID	DXTB5tpZMuV2IF2I
acc genotypes	29
peaks no back	5
removed edges	8
prop rem edges	0.148

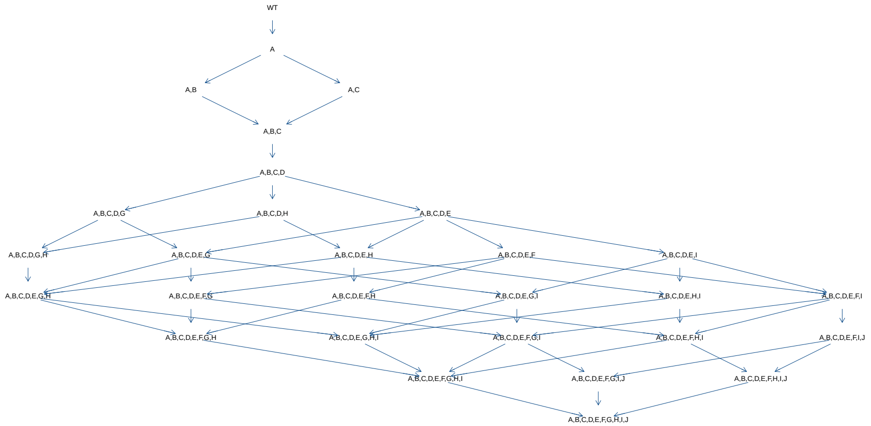

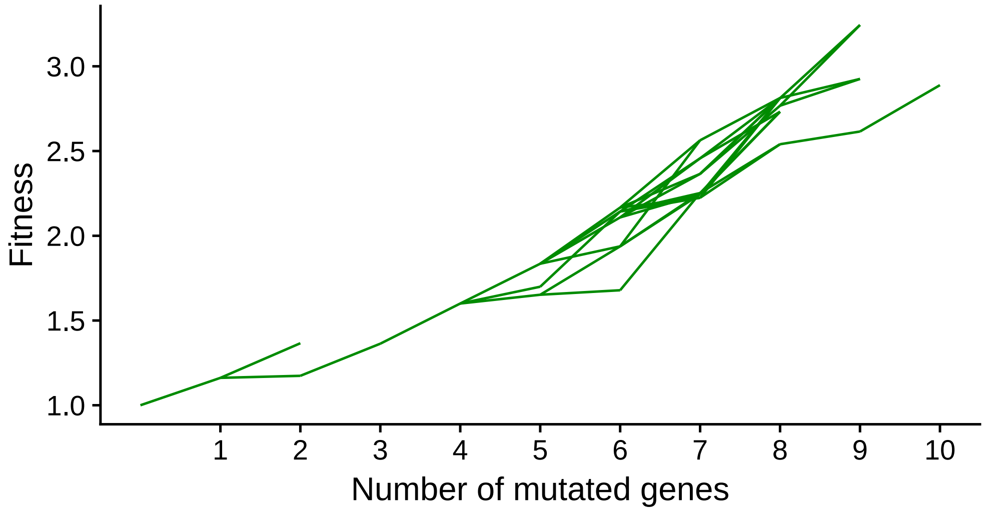

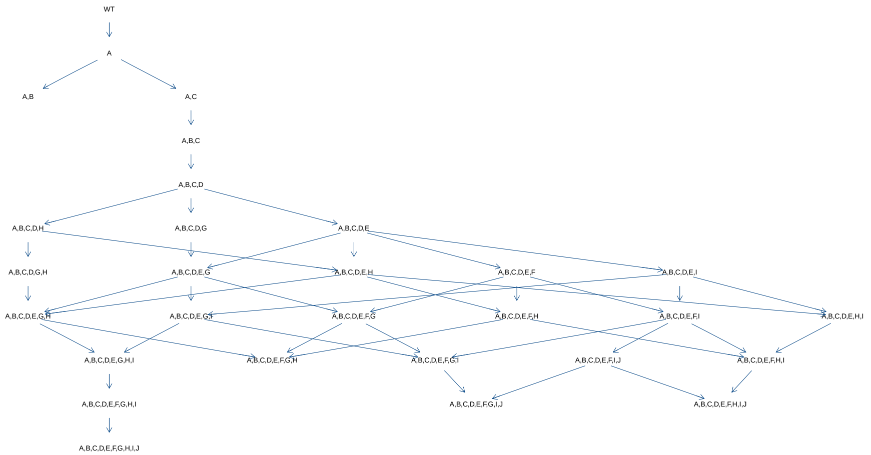

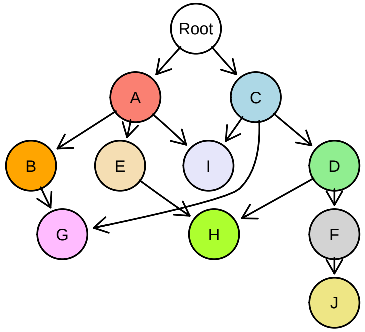

ID	dYeC4QsDciy4hwa3
acc genotypes	75
peaks no back	10
removed edges	32
prop rem edges	0.174

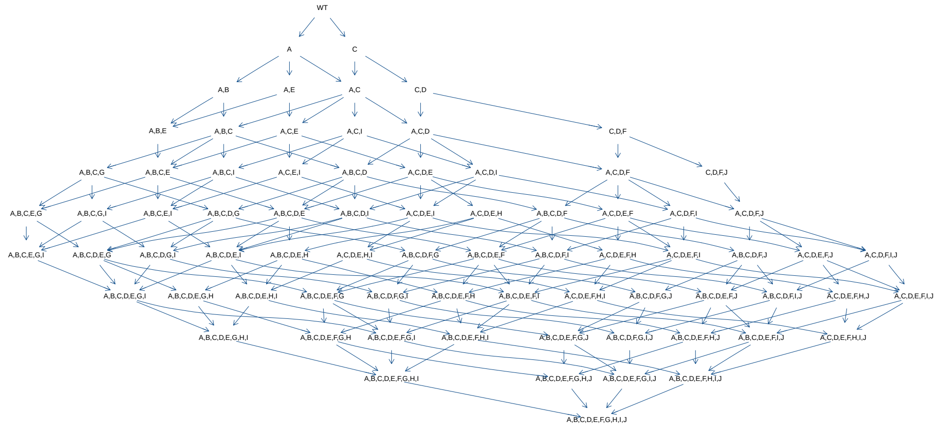

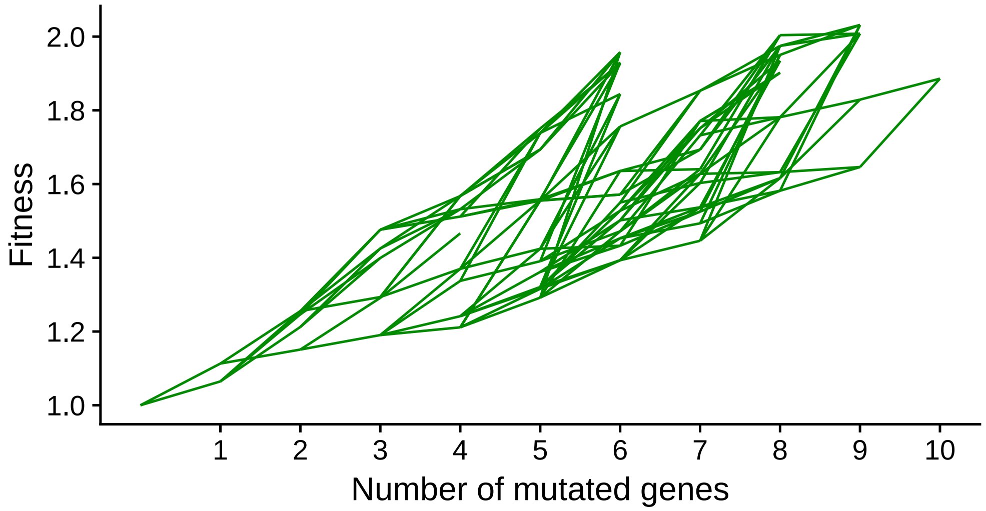

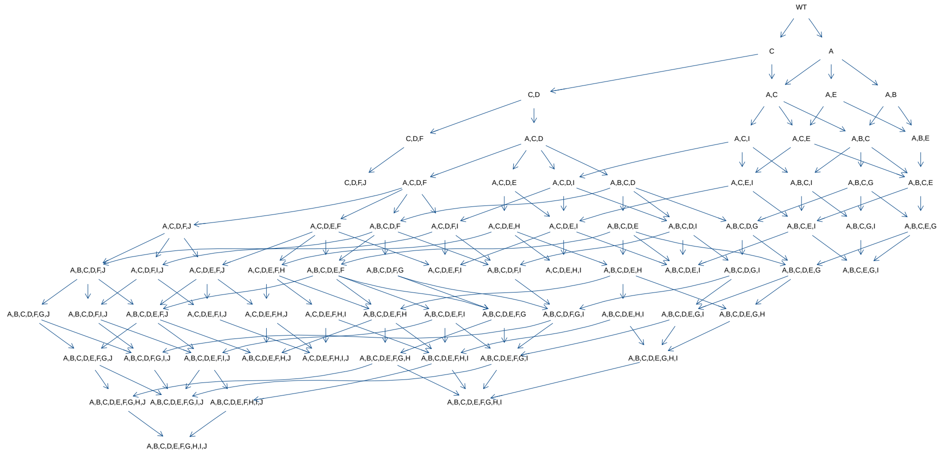

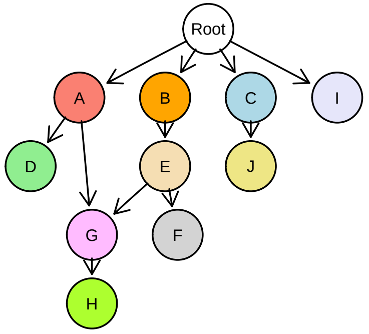

ID	E8phhmTg4KHAS8DL
acc genotypes	120
peaks no back	12
removed edges	61
prop rem edges	0.18

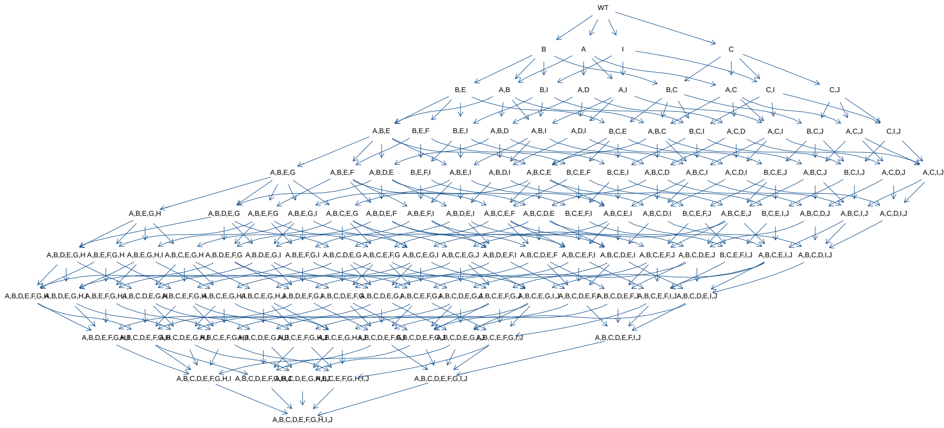

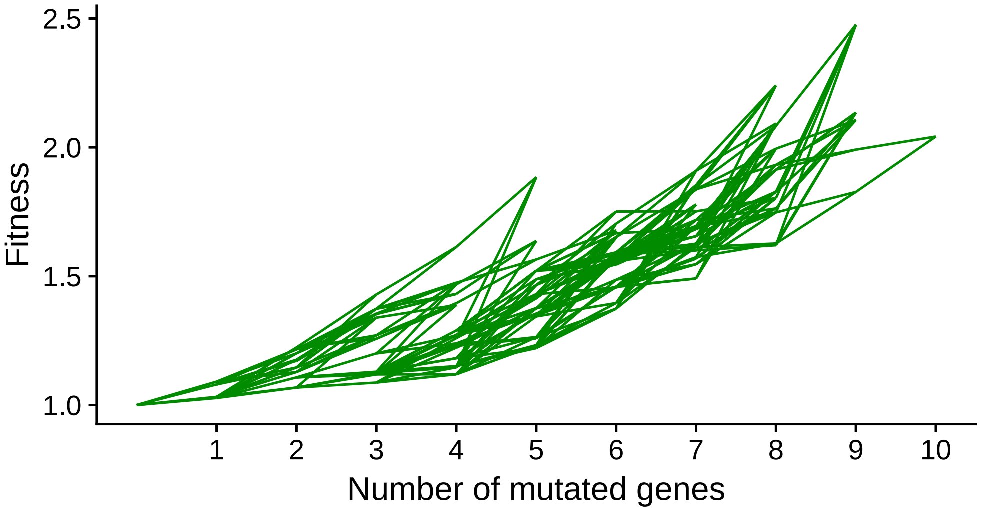

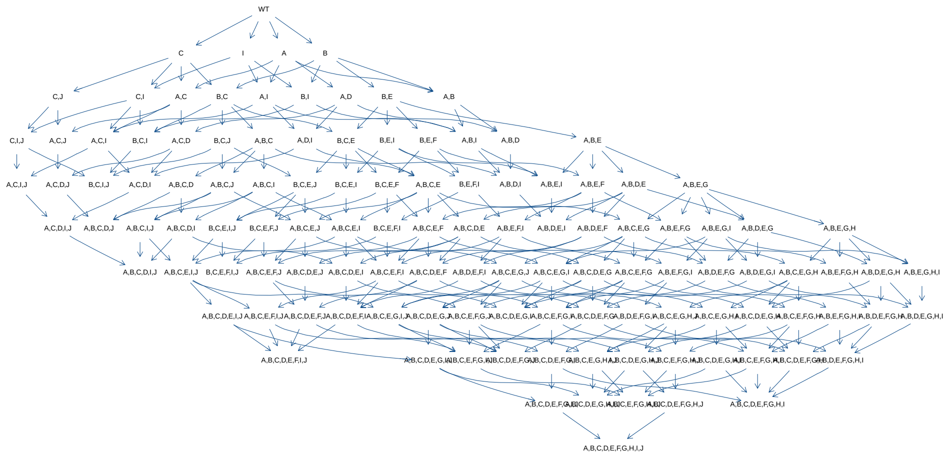

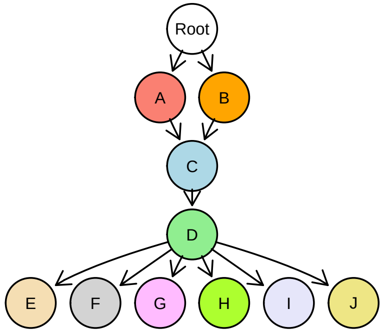

ID	eBeyysHajtLlNlLT
acc genotypes	69
peaks no back	8
removed edges	35
prop rem edges	0.177

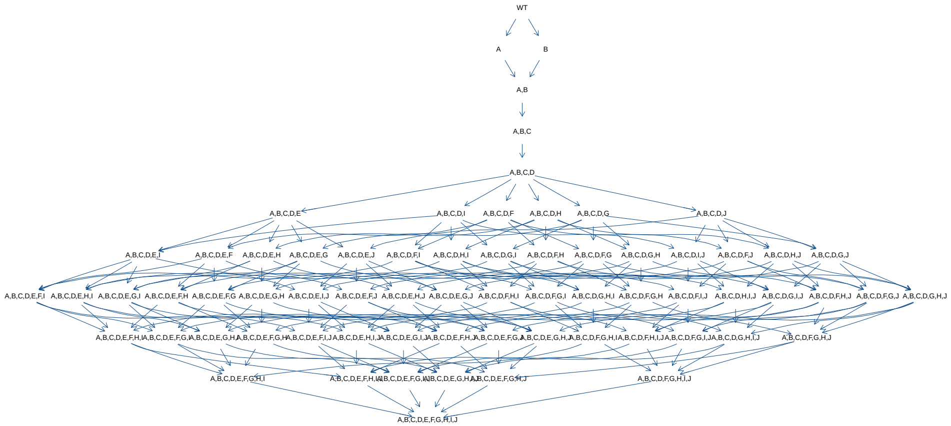

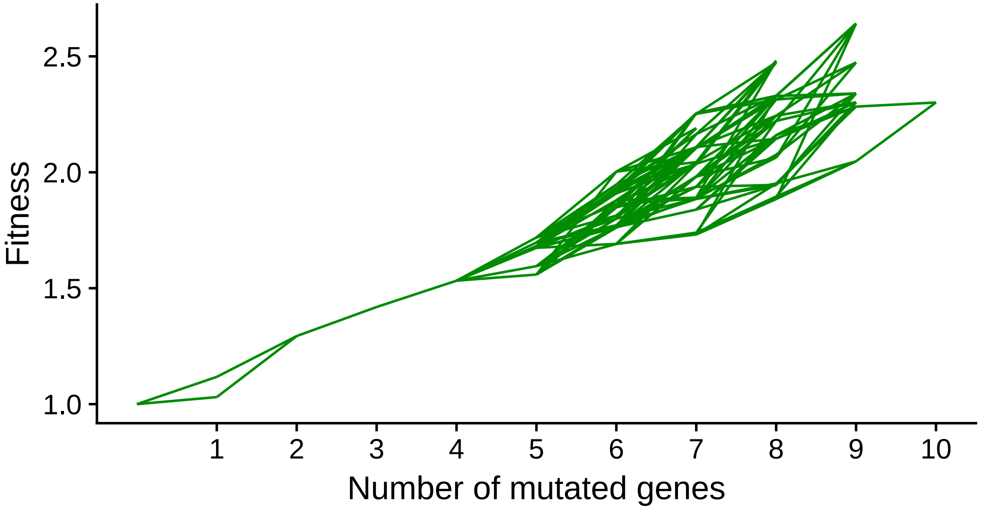

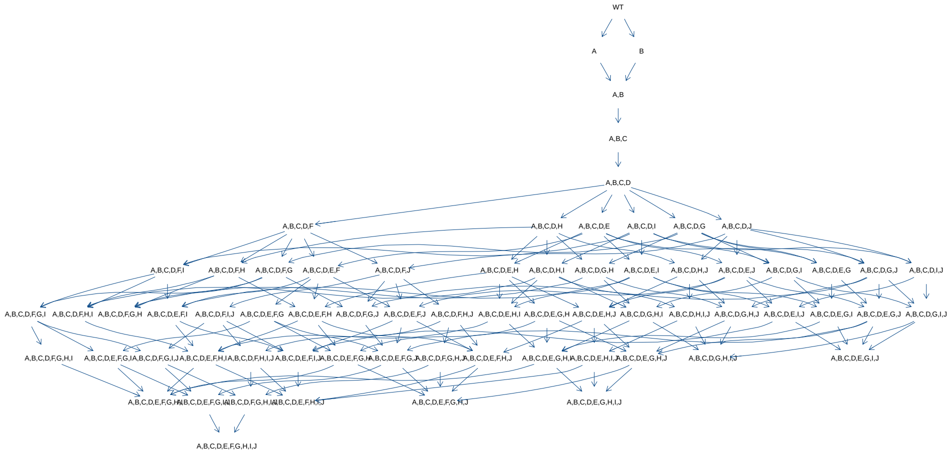

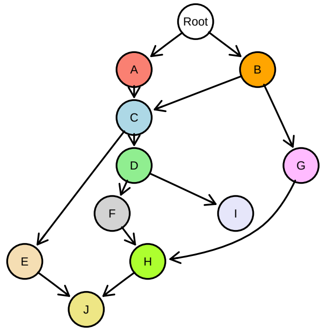

ID	EflrY474a8aE1bD2
acc genotypes	32
peaks no back	5
removed edges	8
prop rem edges	0.133

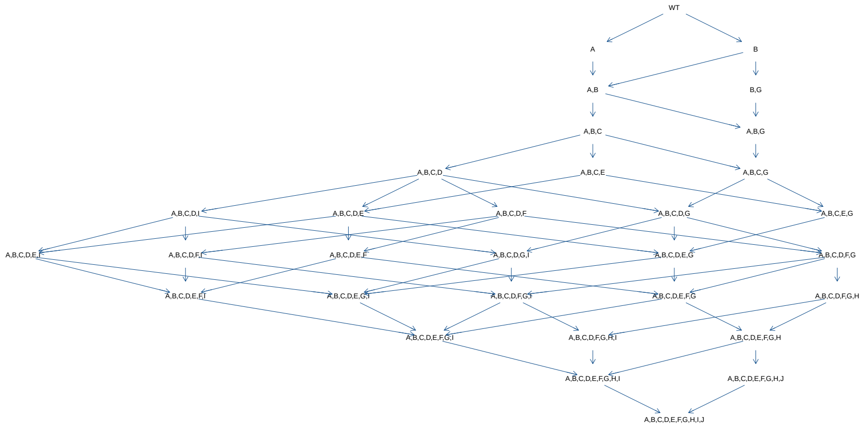

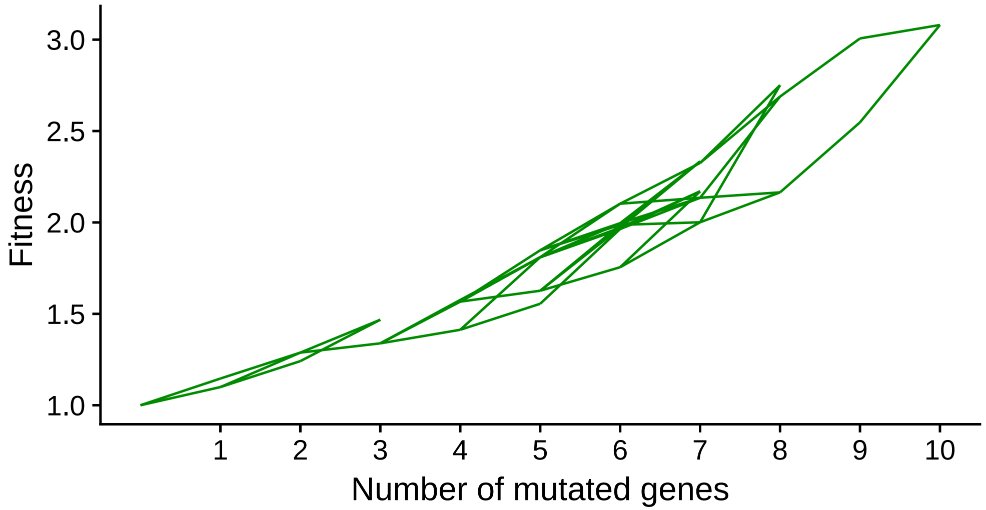

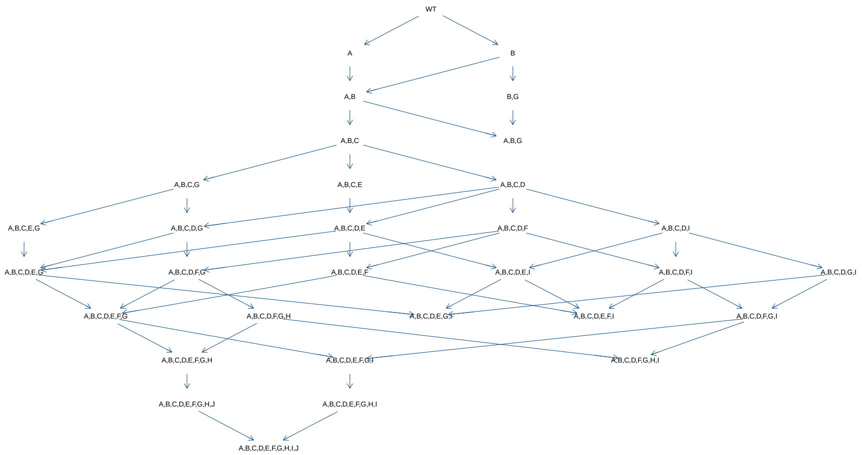

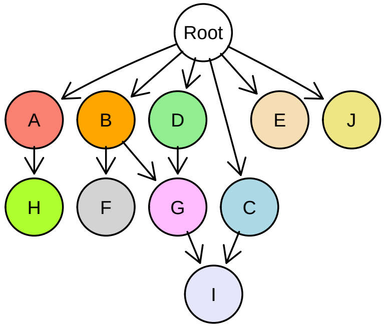

ID	EjeOkmz5yWV10GMyo
acc genotypes	216
peaks no back	14
removed edges	134
prop rem edges	0.183

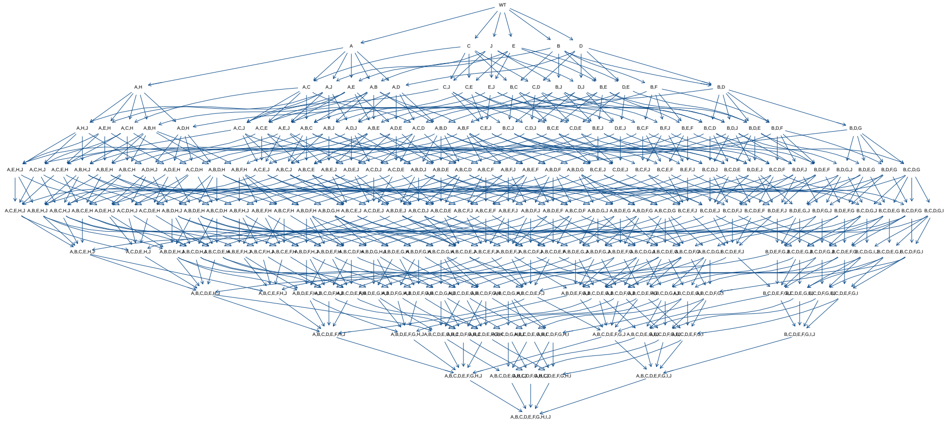

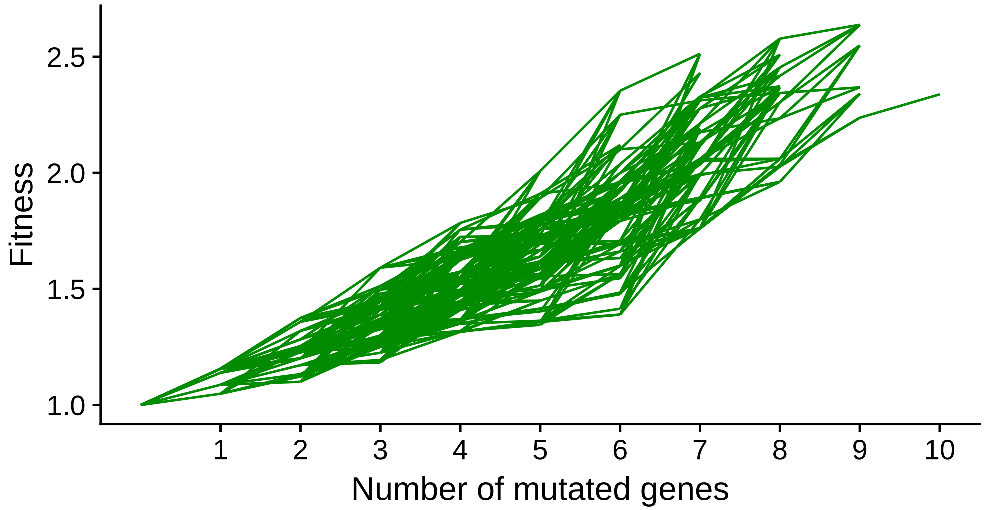

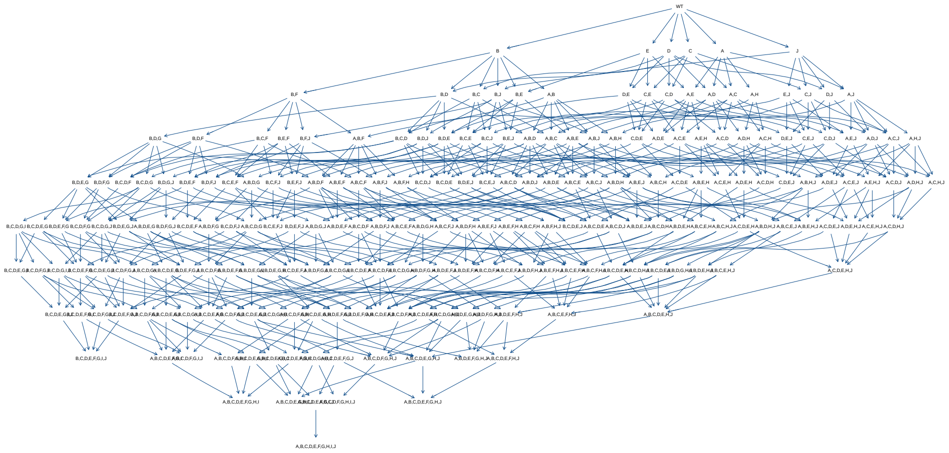

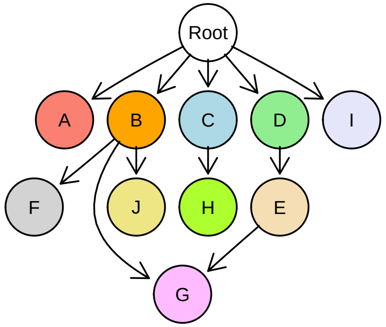

ID	EJVLcdkFsVSwbuKT
acc genotypes	228
peaks no back	18
removed edges	157
prop rem edges	0.202

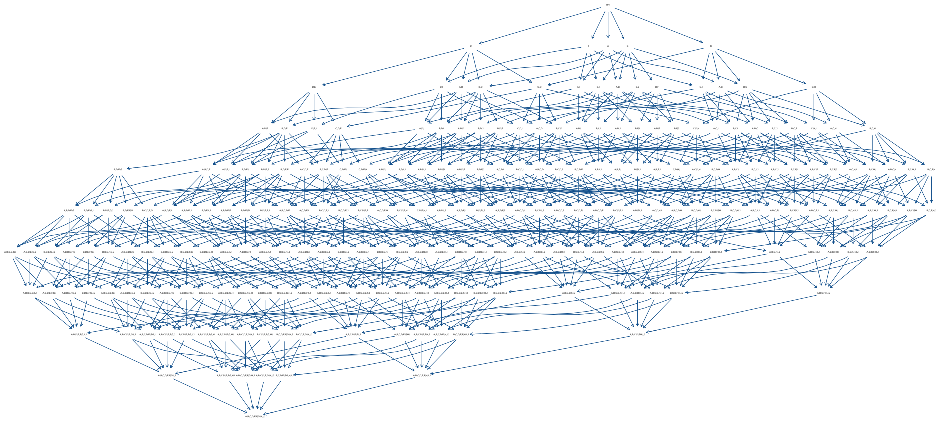

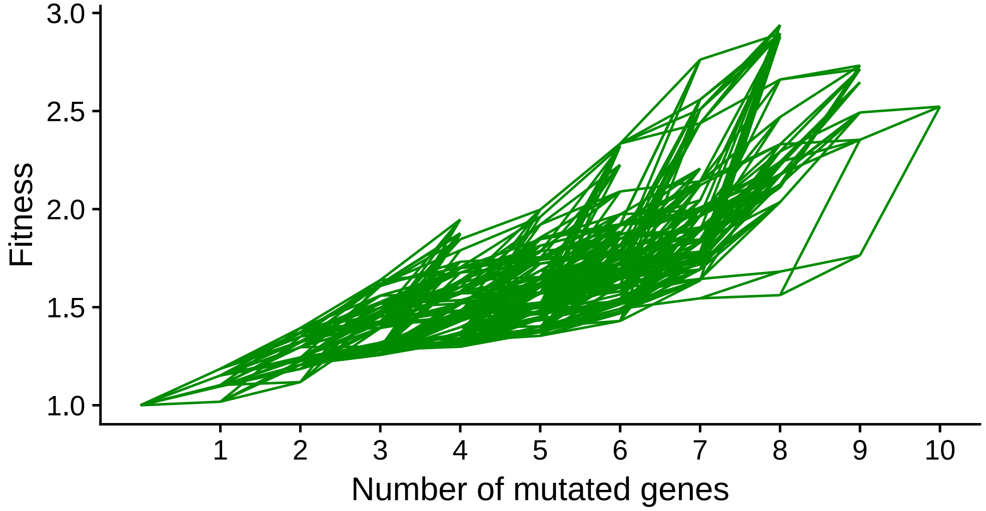

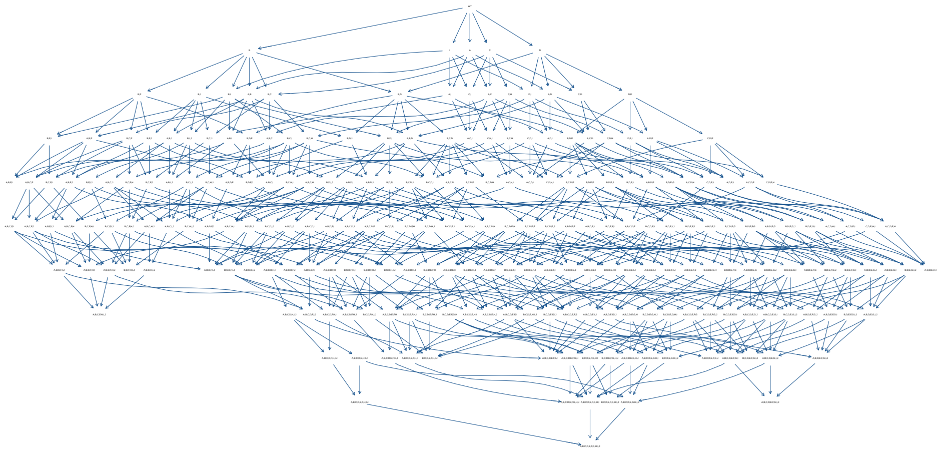

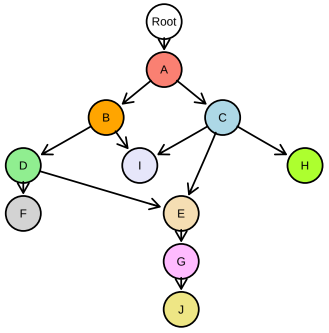

ID	EkGrTn9N4iF6FhfE
acc genotypes	43
peaks no back	5
removed edges	15
prop rem edges	0.165

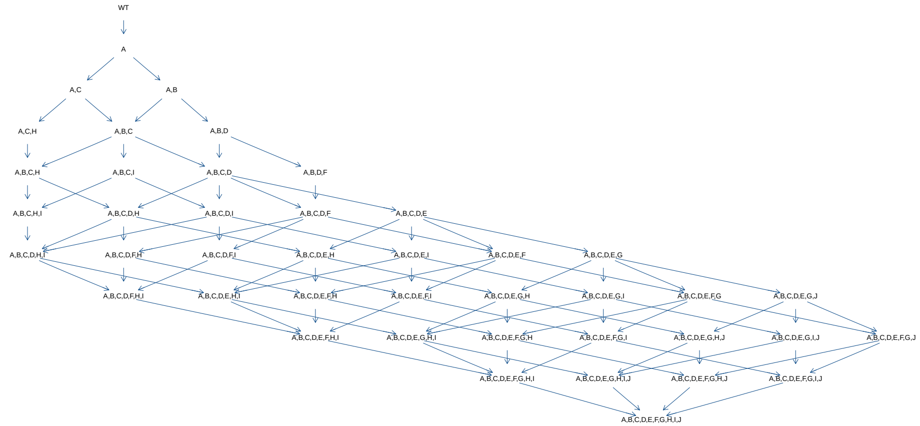

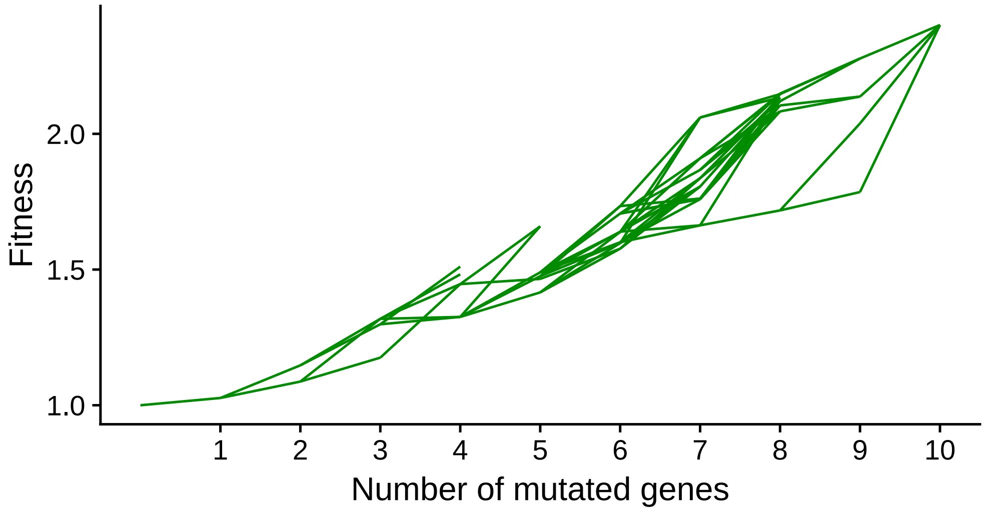

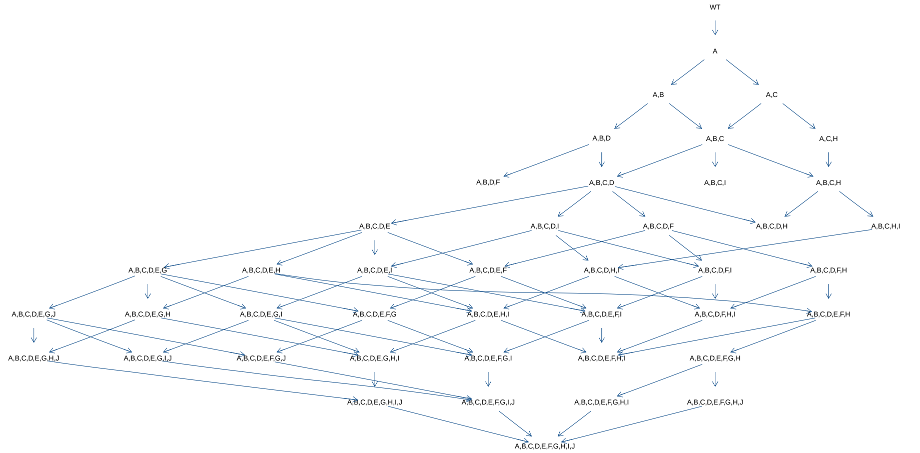

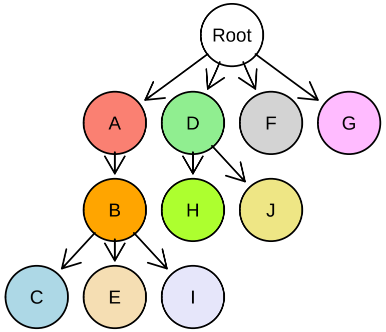

ID	EMu88ff7108oDY2Nd
acc genotypes	200
peaks no back	21
removed edges	136
prop rem edges	0.2

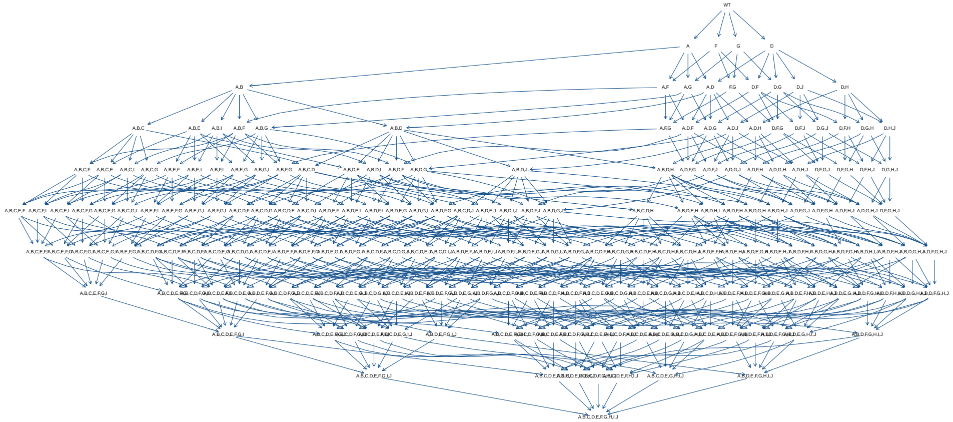

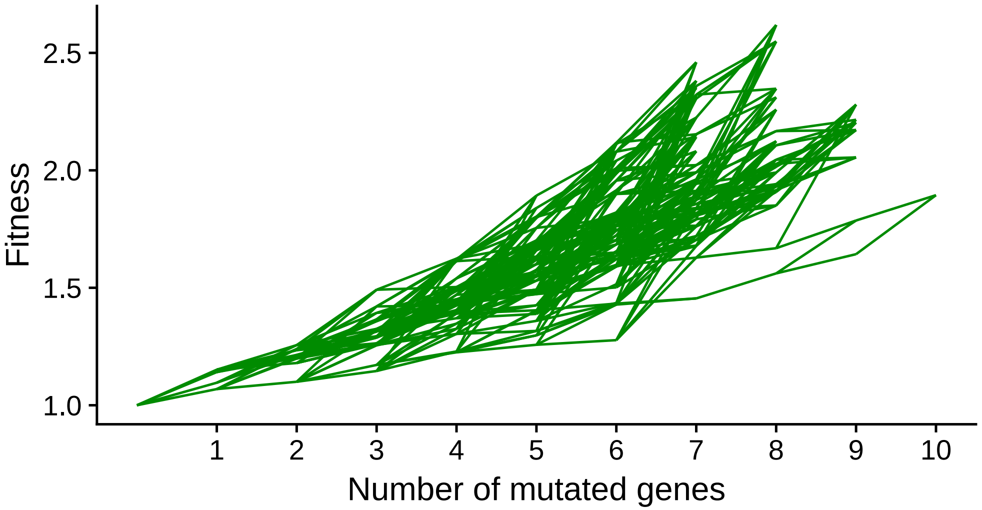

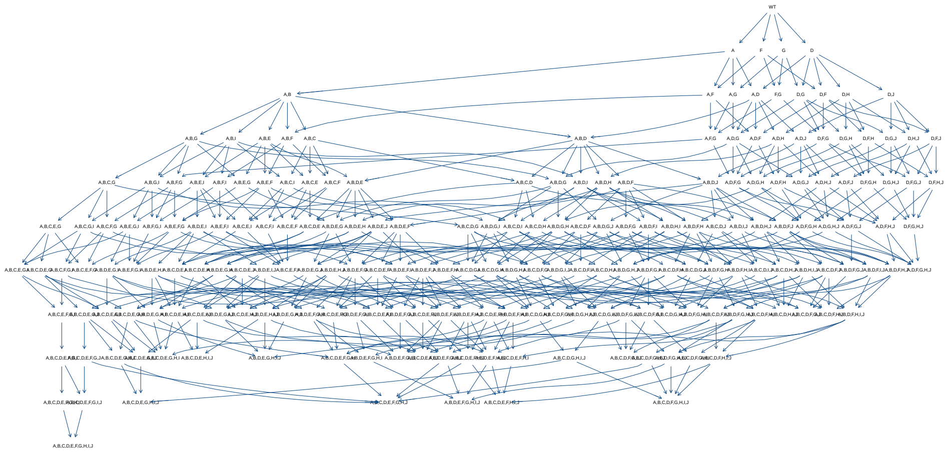

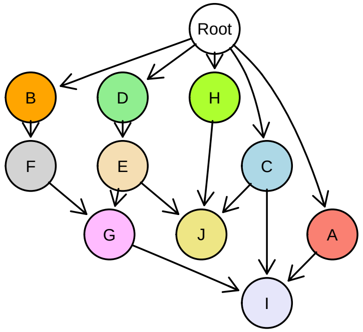

ID	EnDJ6doxjCtQ9zue
acc genotypes	91
peaks no back	11
removed edges	52
prop rem edges	0.211

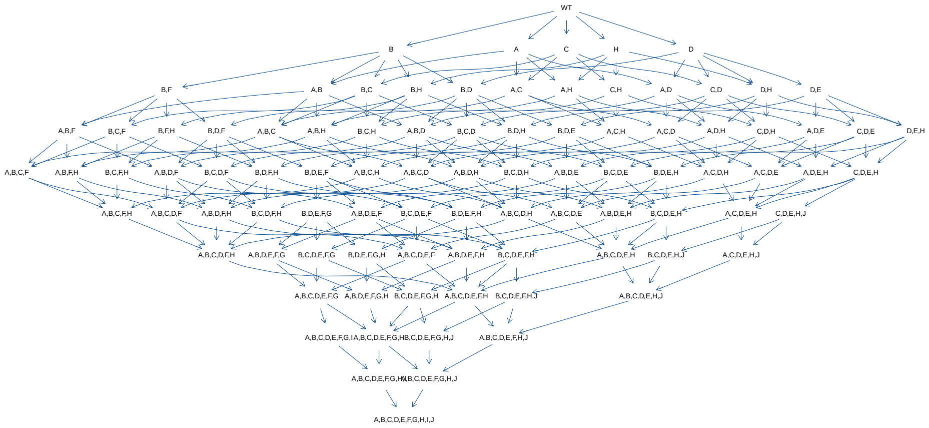

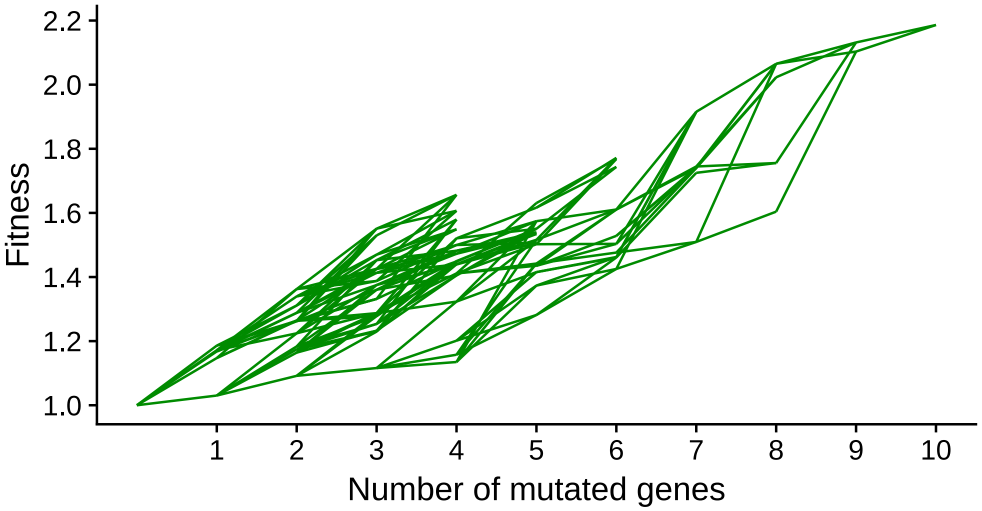

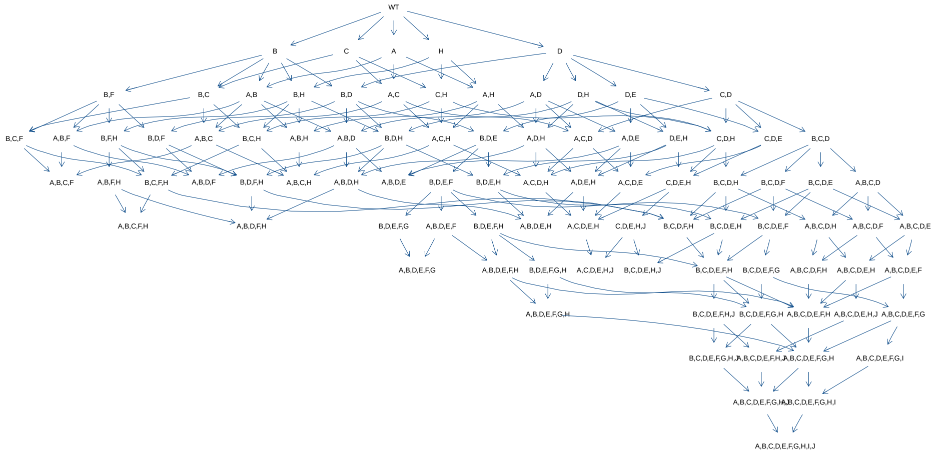

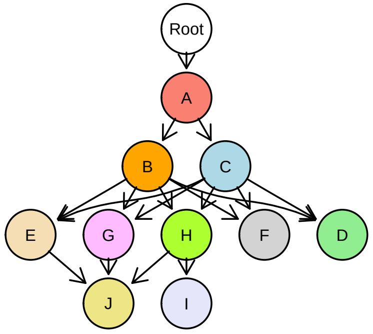

ID EPFiT9RotYTgEnMR
acc genotypes 60
peaks no back 7
removed edges 30
prop rem edges 0.196

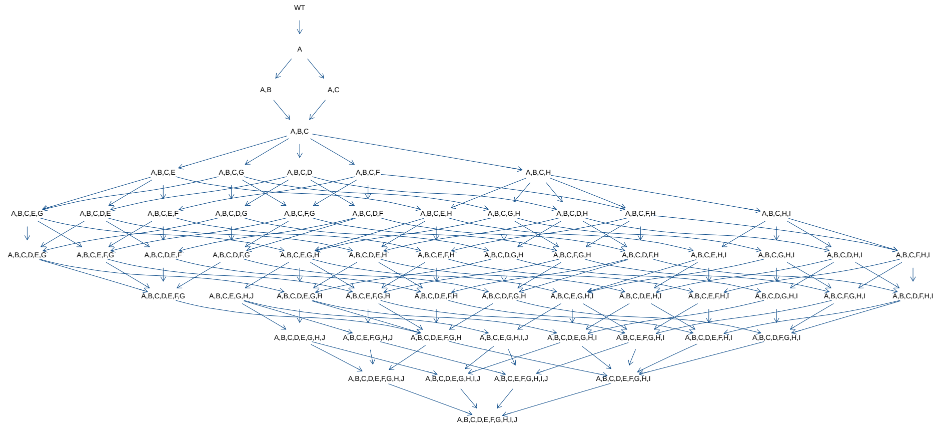

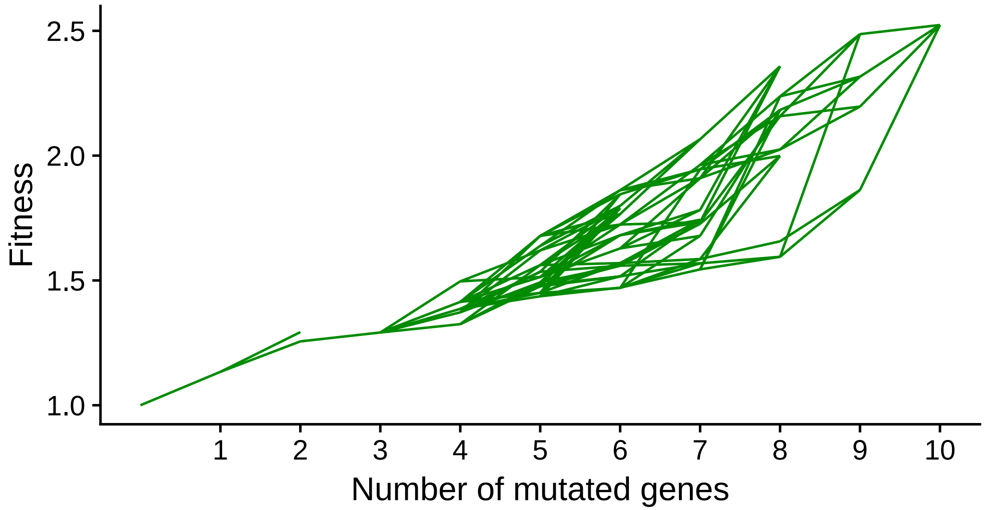

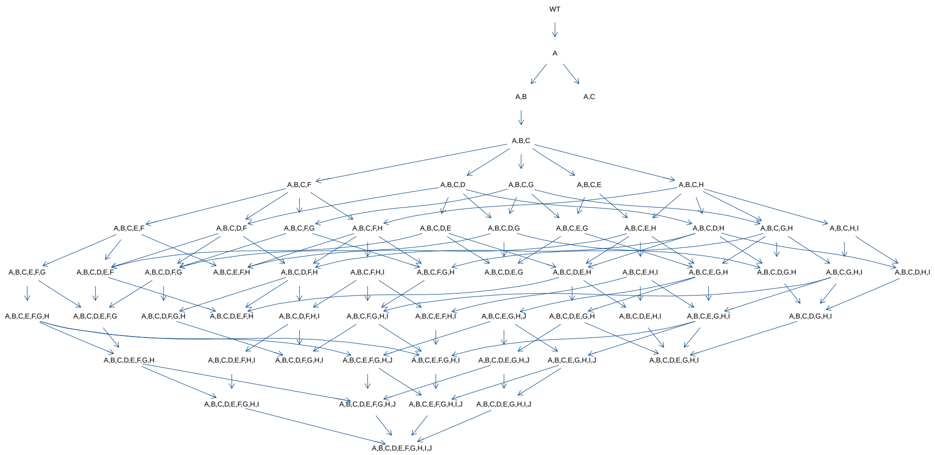

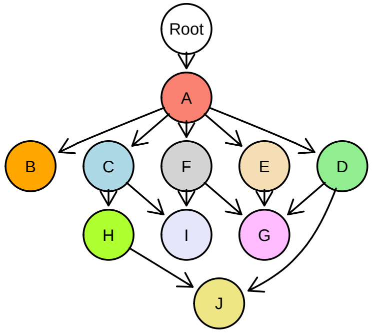

ID FcFcGhQtL910uSnWl
acc genotypes 91
peaks no back 10
removed edges 51
prop rem edges 0.207

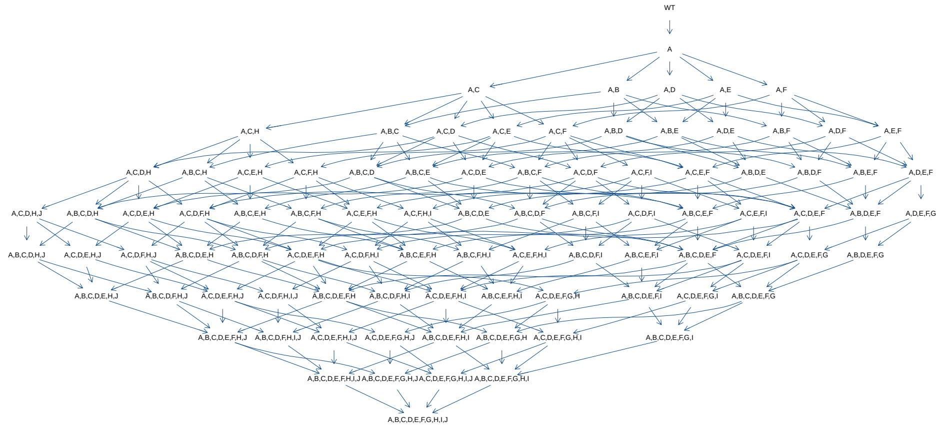

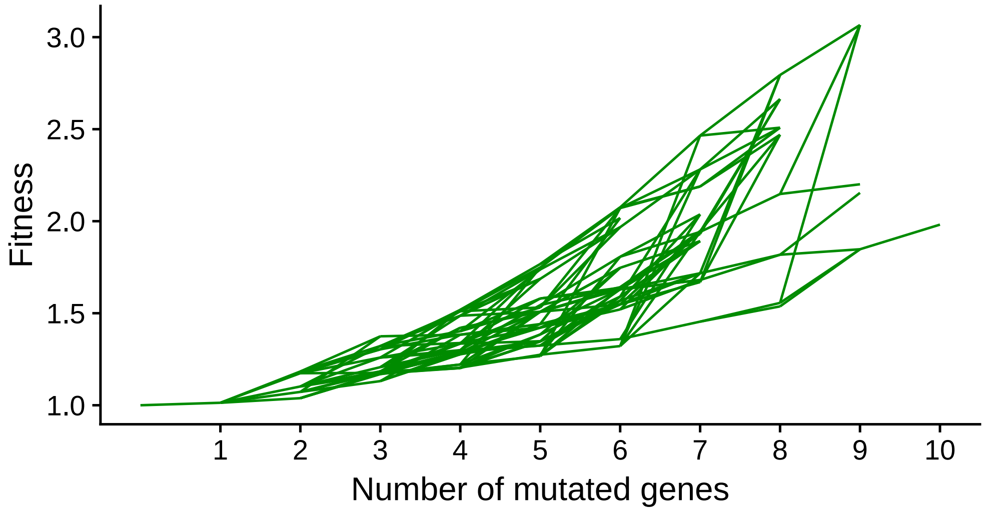

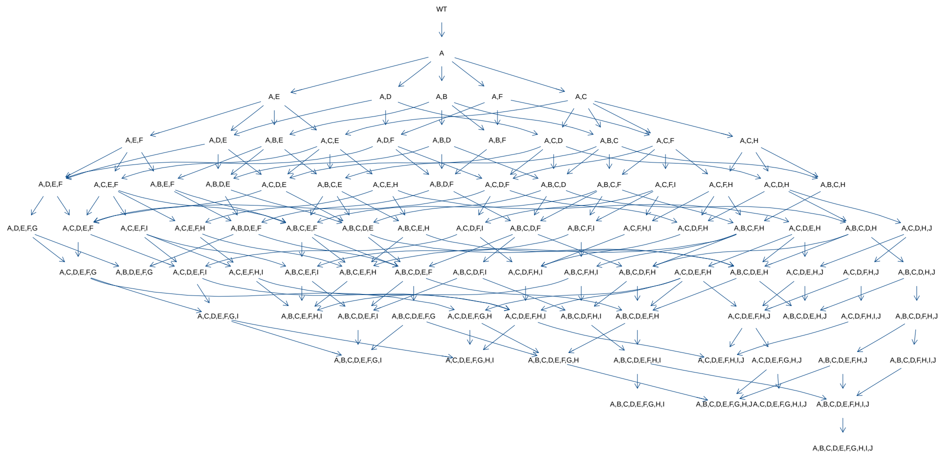

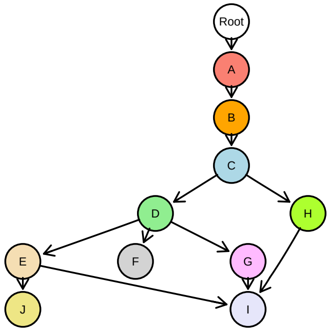

ID fCl5UMNFVGXtbOWb
acc genotypes 33
peaks no back 2
removed edges 4
prop rem edges 0.061

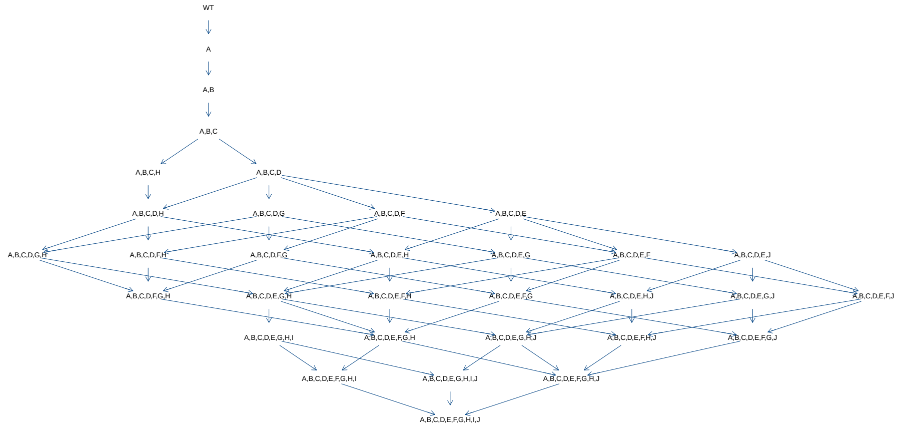

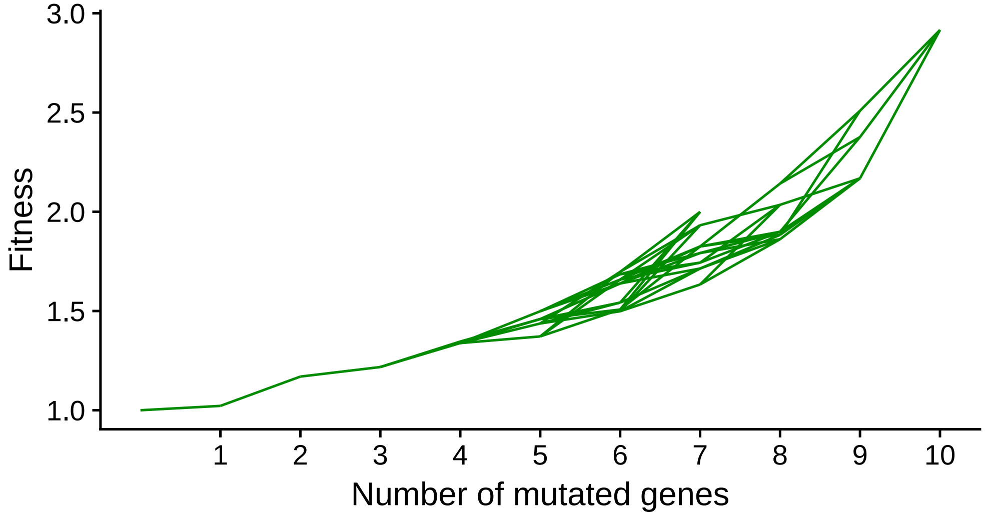

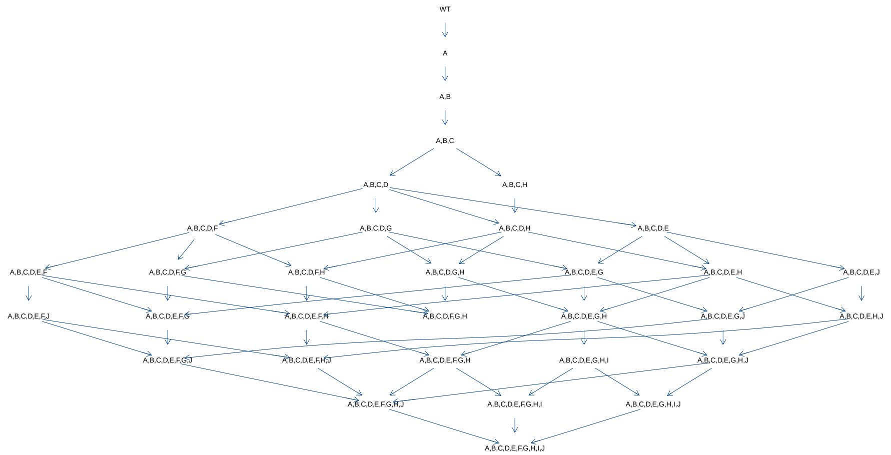

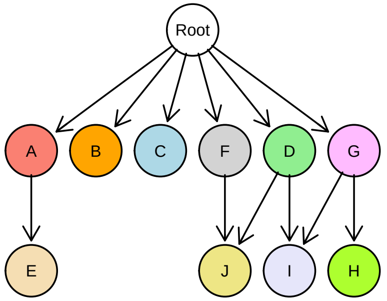

ID	fjYl5FajfI1l9TqE
acc genotypes	252
peaks no back	17
removed edges	166
prop rem edges	0.189

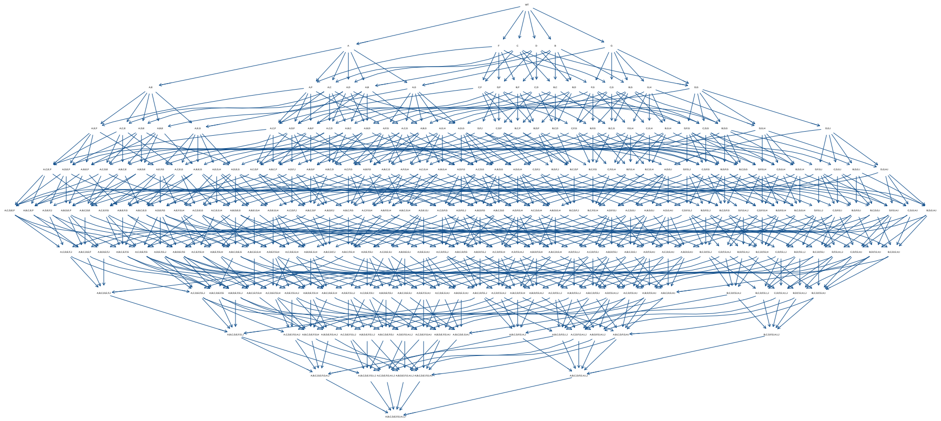

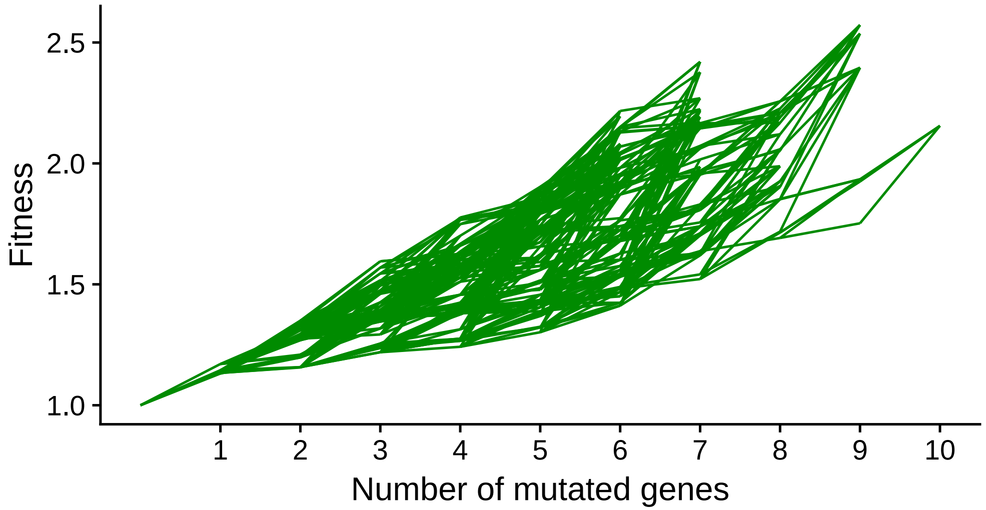

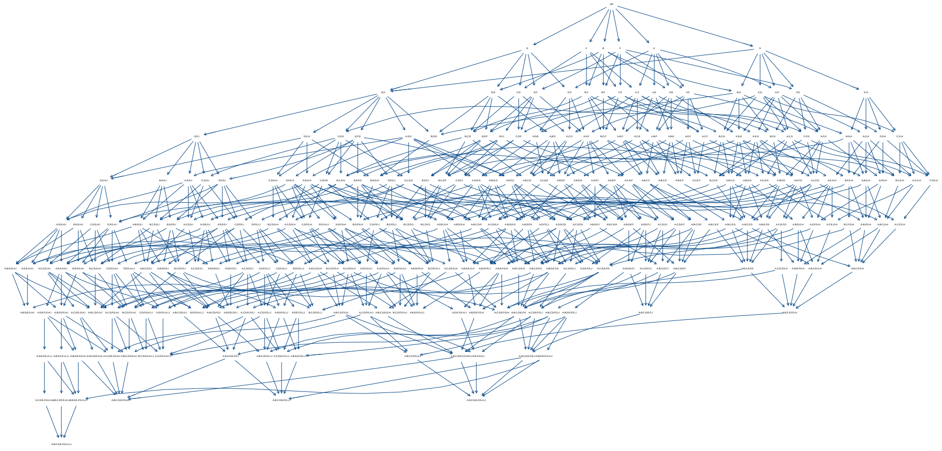

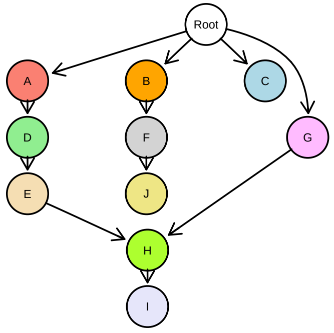

ID	FNDb716ELrbzr3xJ
acc genotypes	80
peaks no back	3
removed edges	22
prop rem edges	0.112

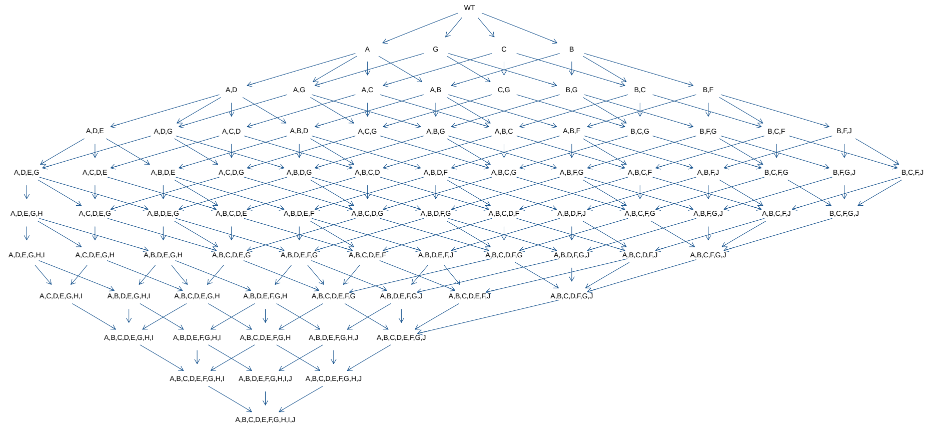

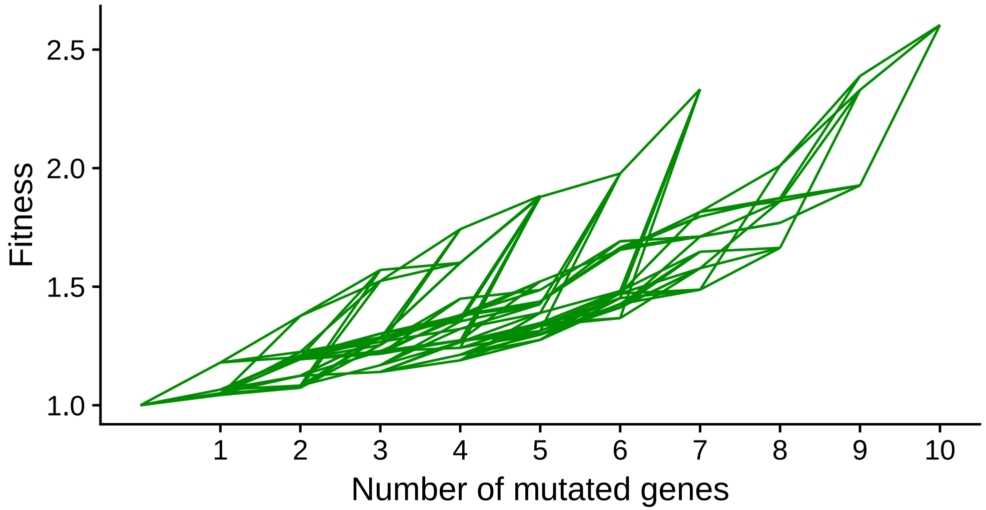

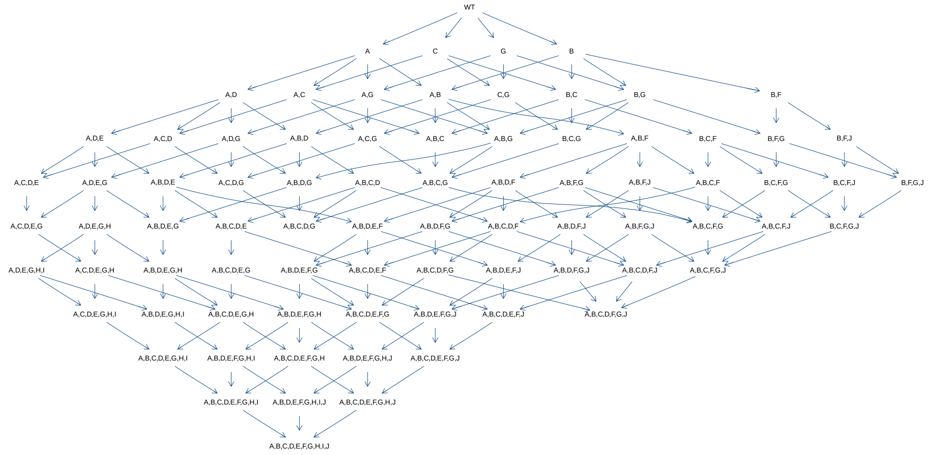

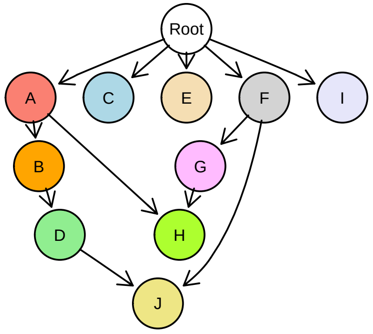

ID	Fp1fshyDCkcvIW97
acc genotypes	144
peaks no back	13
removed edges	95
prop rem edges	0.22

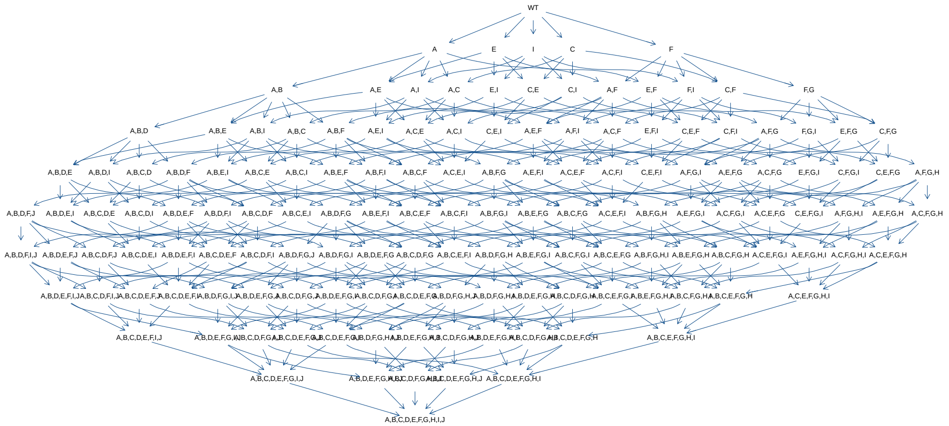

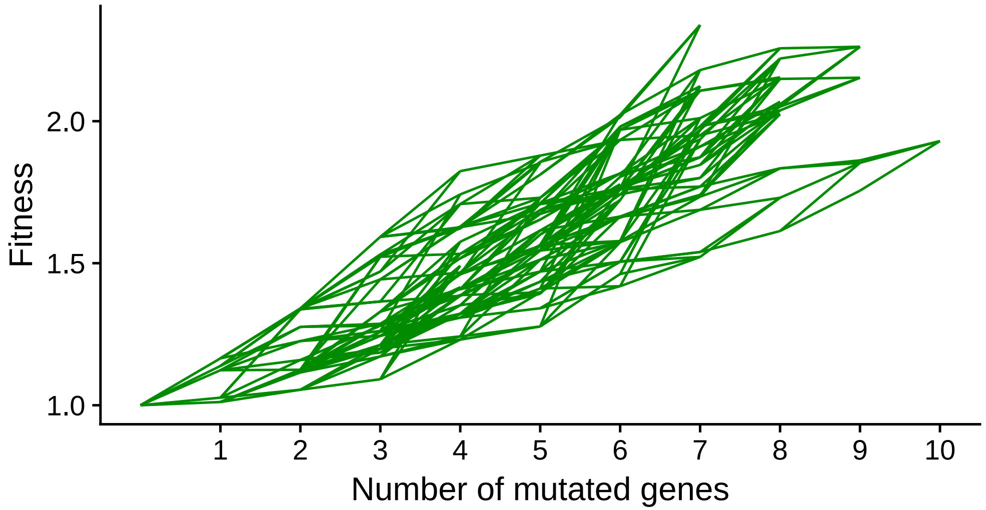

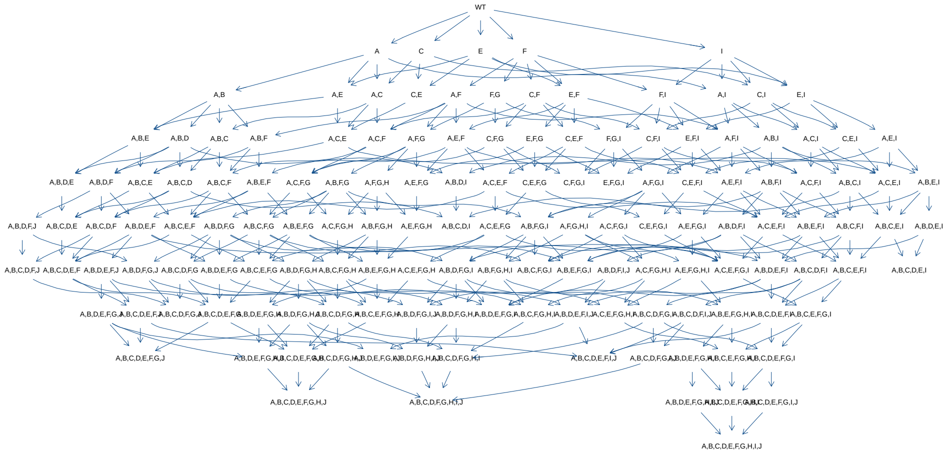

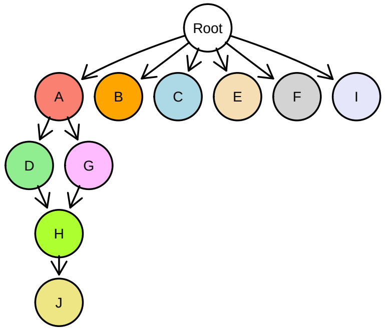

ID	ftHhRYTFn79eRdaP
acc genotypes	224
peaks no back	19
removed edges	143
prop rem edges	0.182

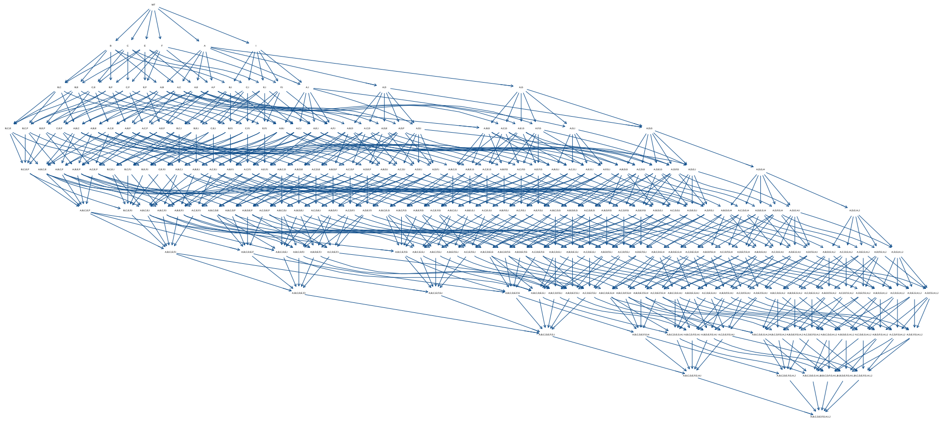

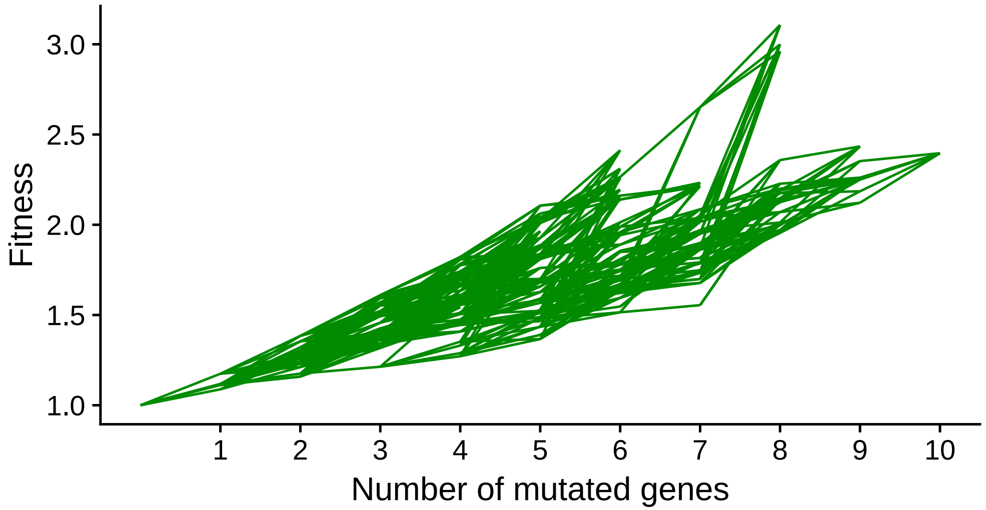

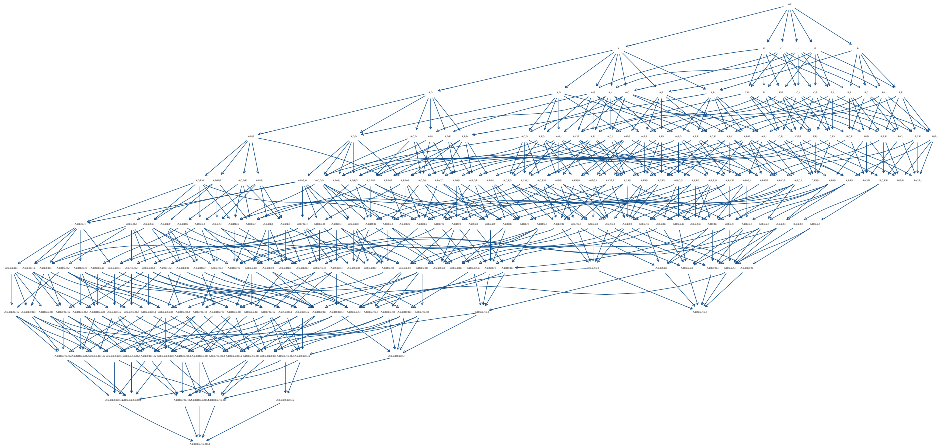

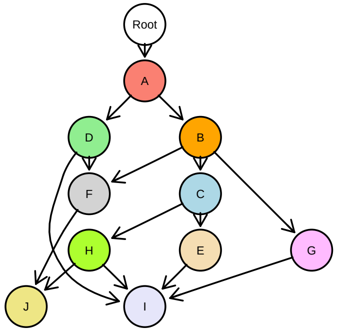

ID	fV68aMwNJcqHIZQX
acc genotypes	40
peaks no back	3
removed edges	11
prop rem edges	0.134

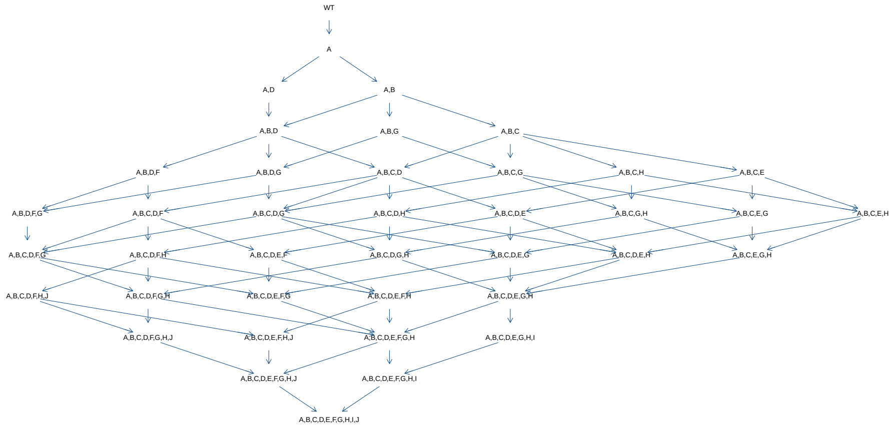

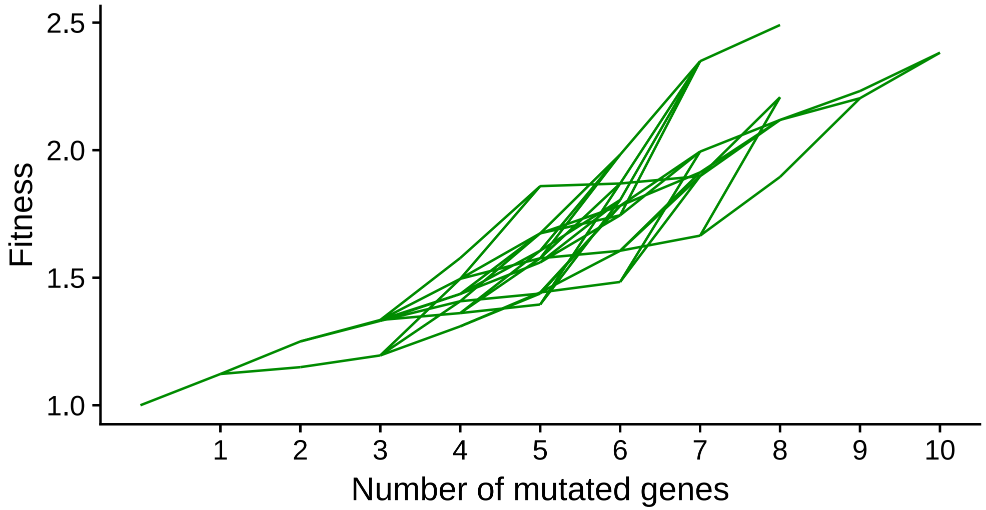

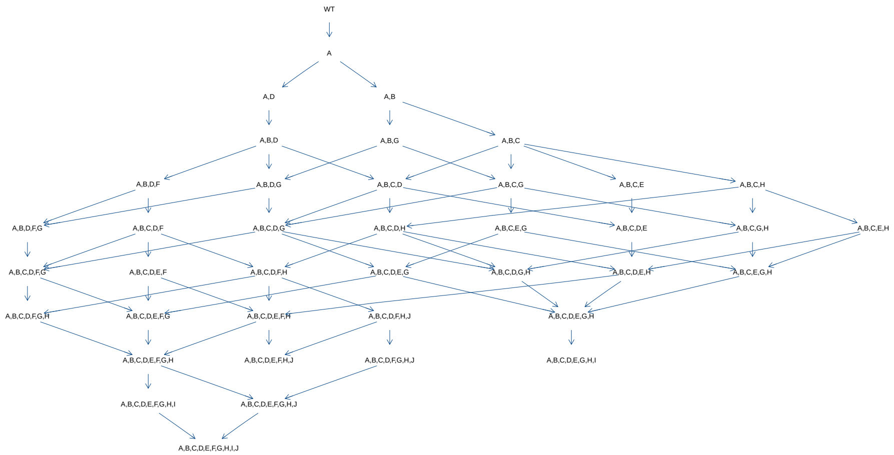

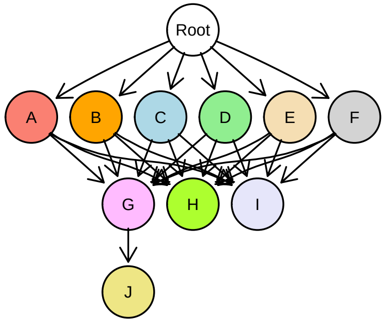

ID	g632uhVWzFmg59eK
acc genotypes	75
peaks no back	10
removed edges	31
prop rem edges	0.146

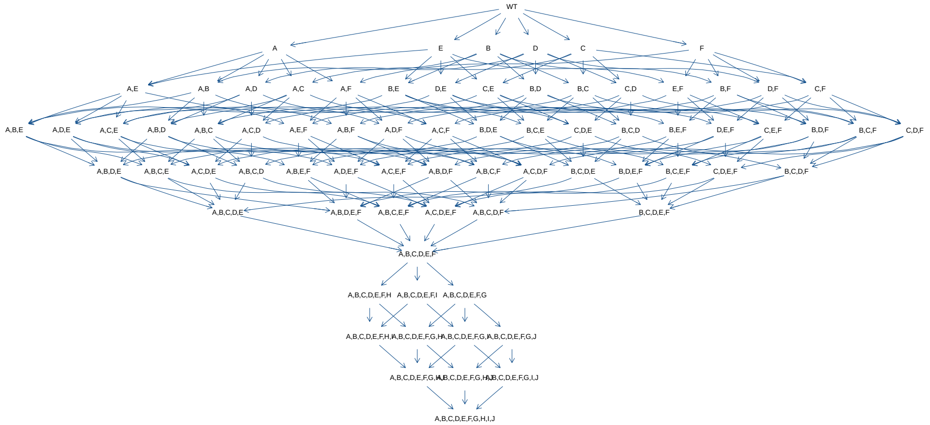

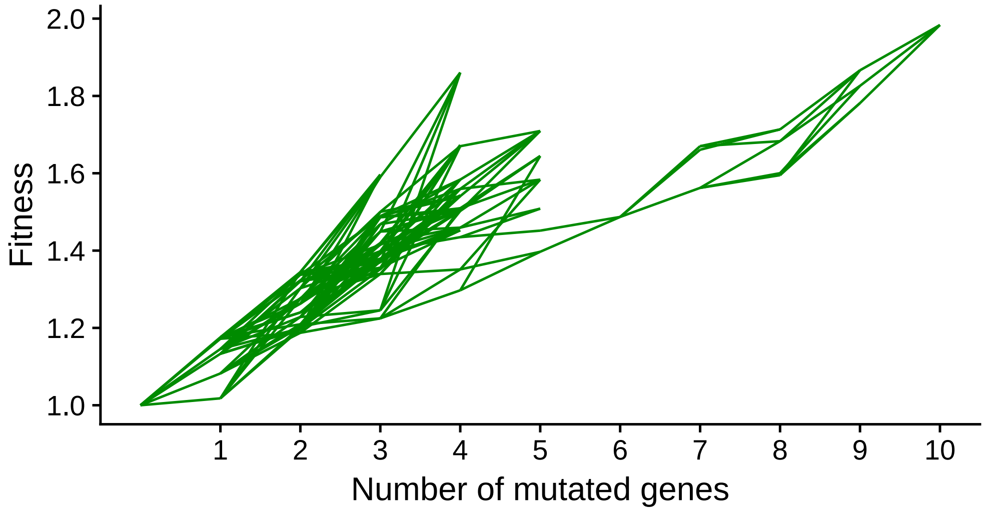

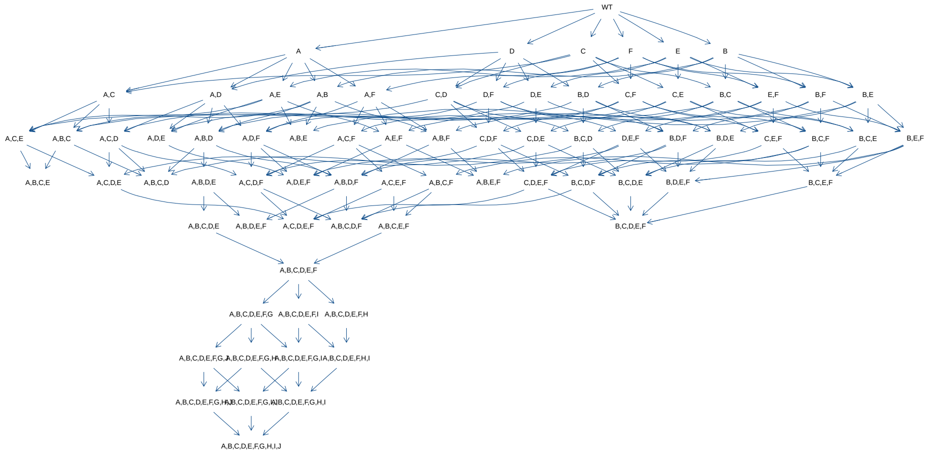

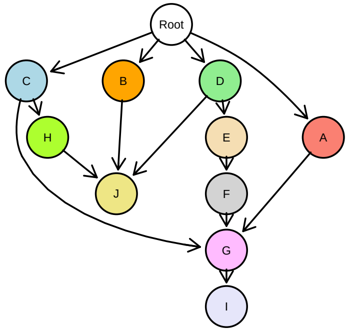

ID	G9yrL22aEcqd1Y23
acc genotypes	64
peaks no back	8
removed edges	28
prop rem edges	0.188

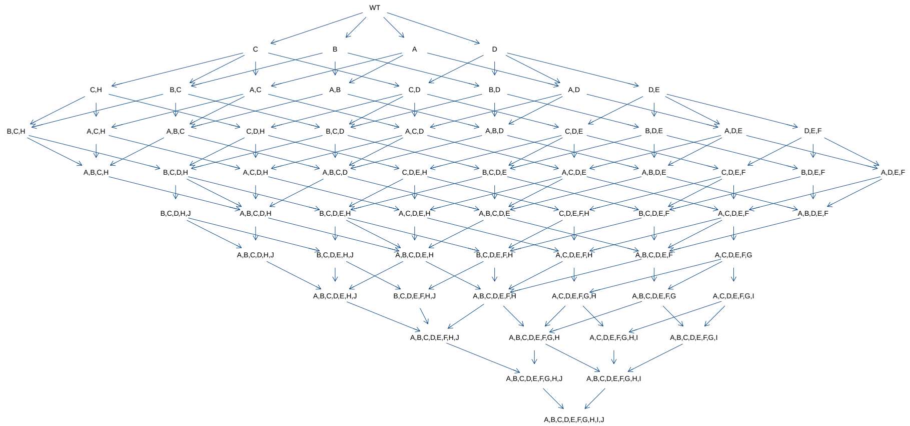

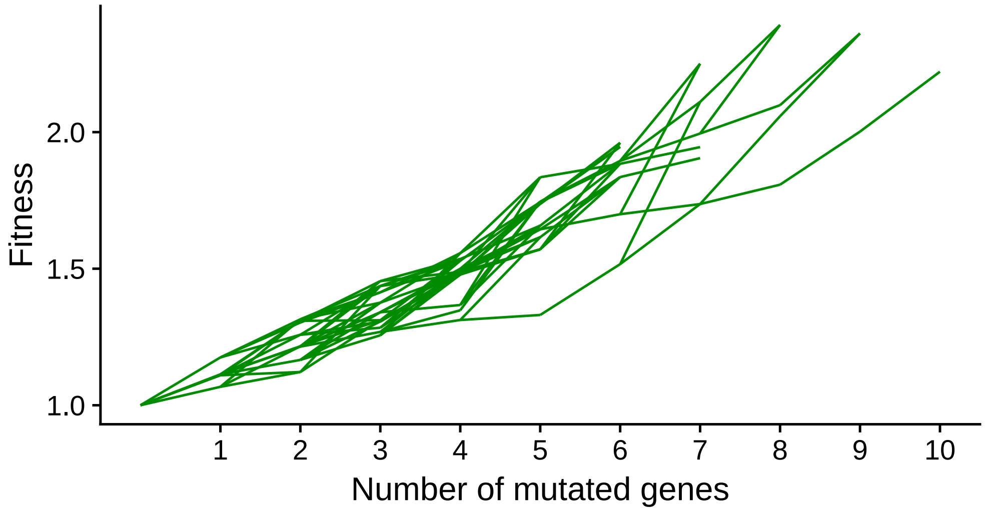

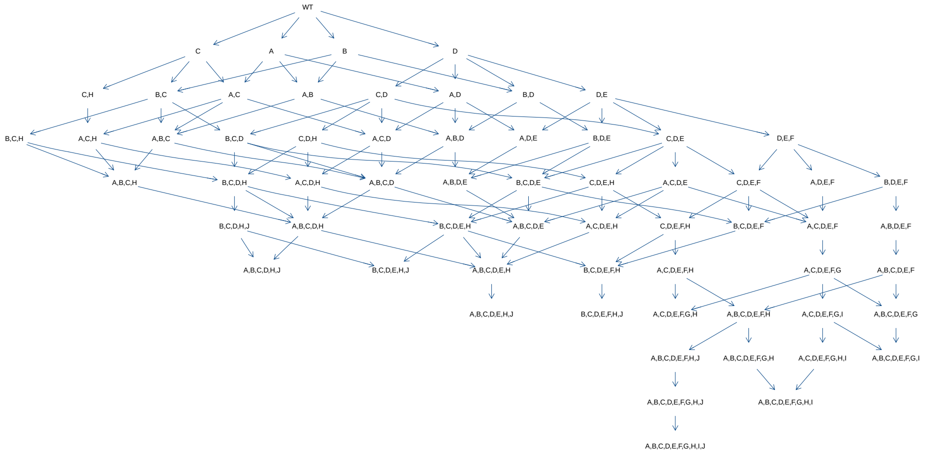

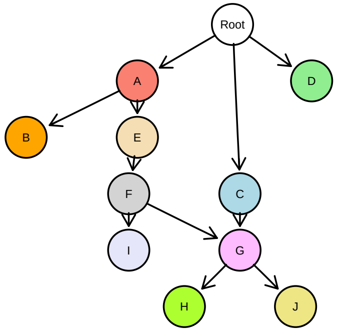

ID	gA76bffYxtXYMJa2
acc genotypes	68
peaks no back	11
removed edges	30
prop rem edges	0.179

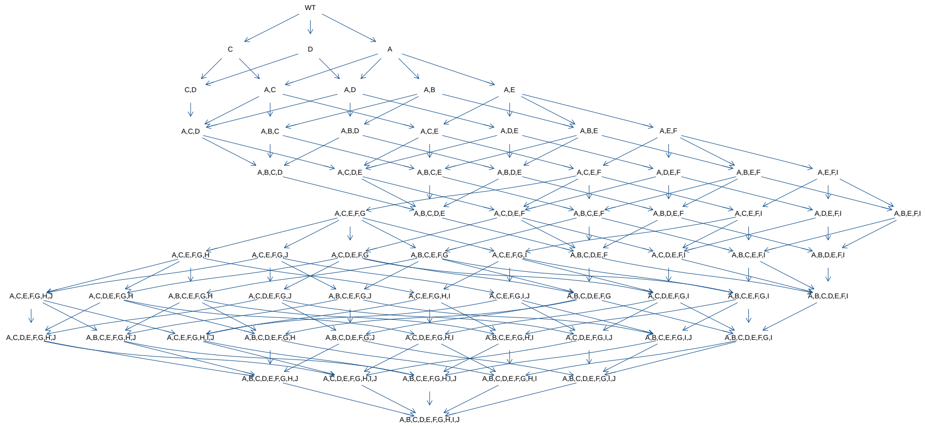

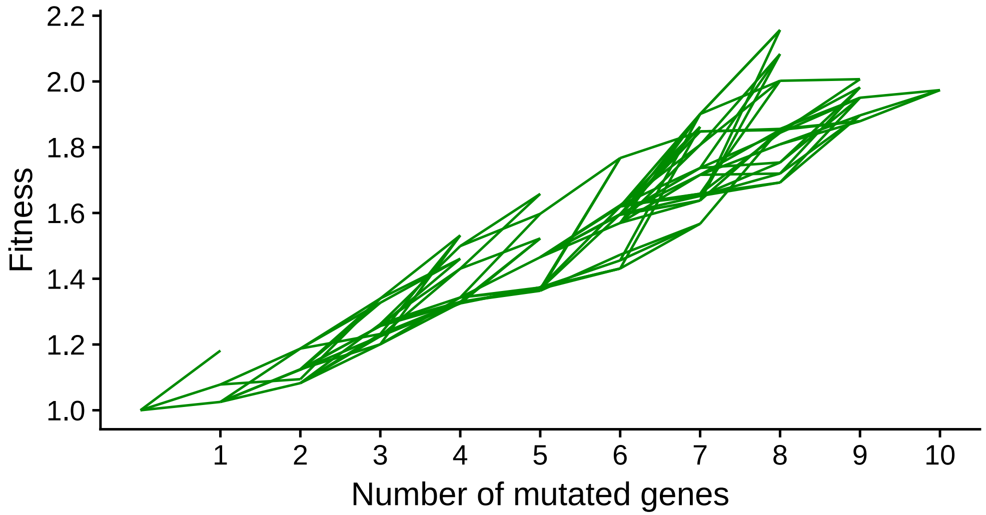

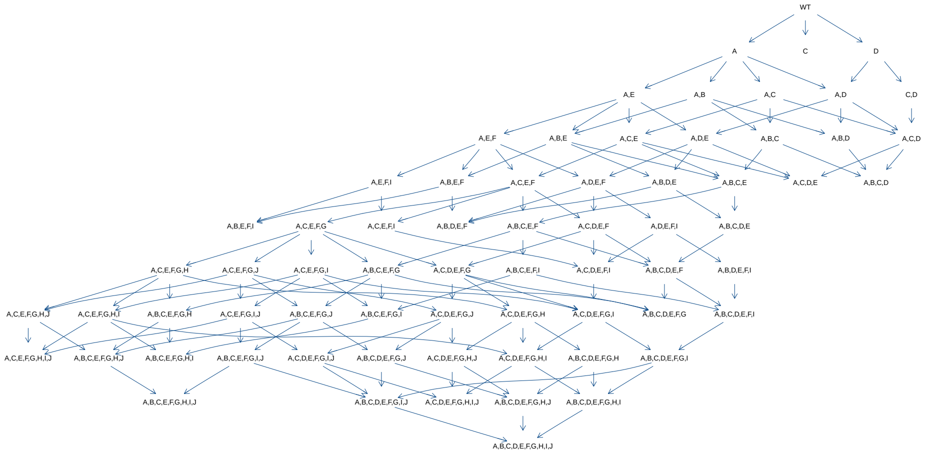

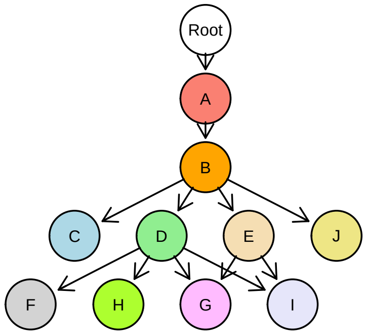

ID	GeE6ZiWGbvgLEf7V
acc genotypes	90
peaks no back	11
removed edges	56
prop rem edges	0.214

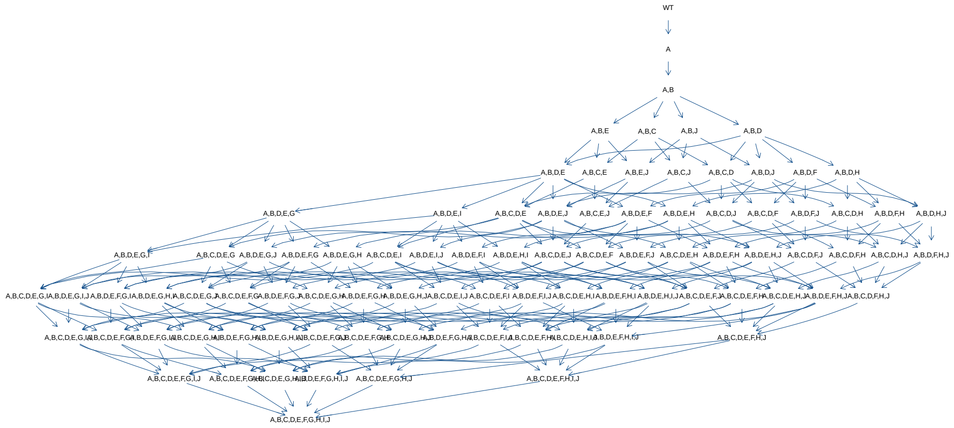

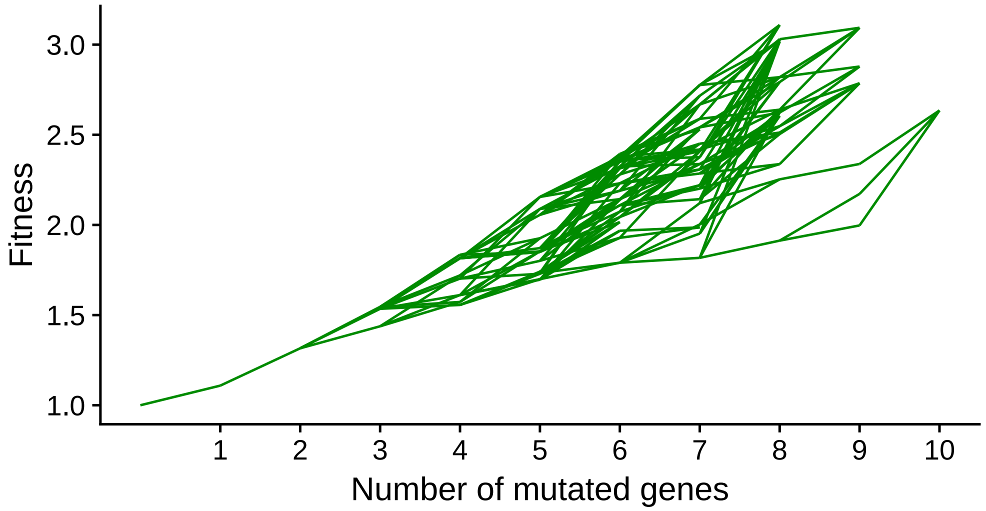

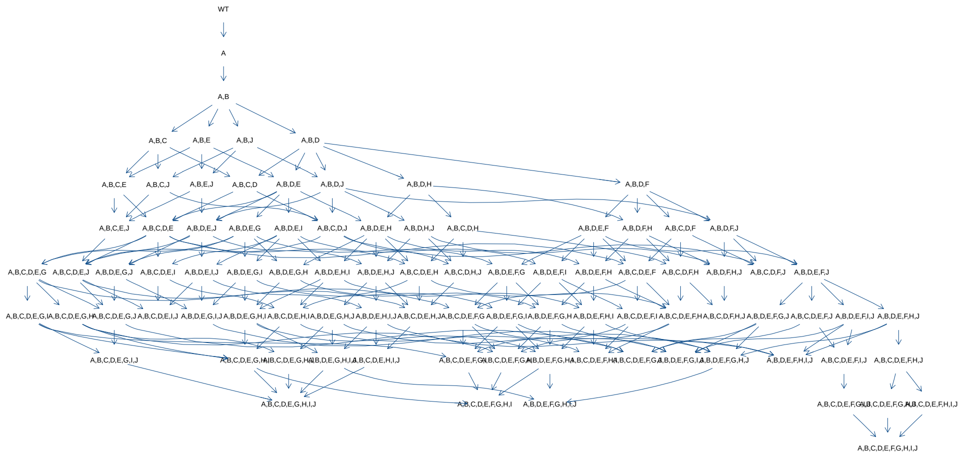

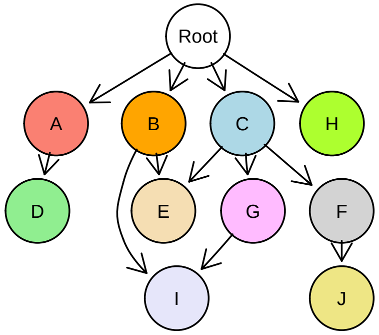

ID giZLyxZUhrKmiuIn
acc genotypes 156
peaks no back 15
removed edges 101
prop rem edges 0.212

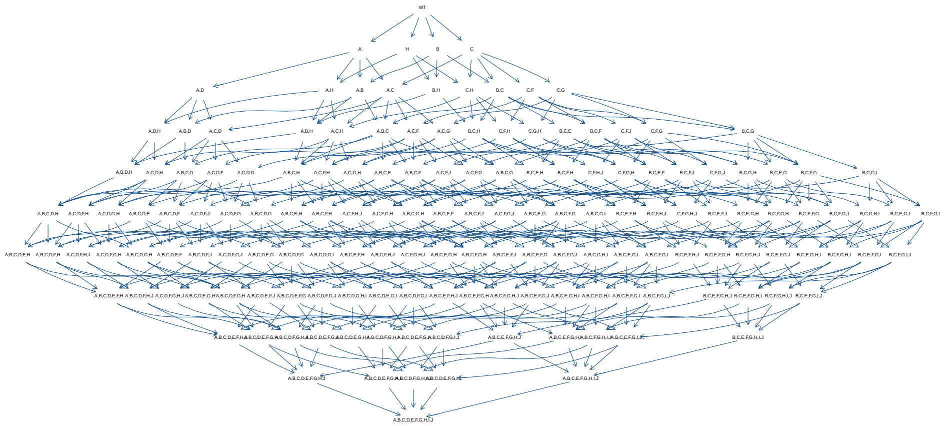

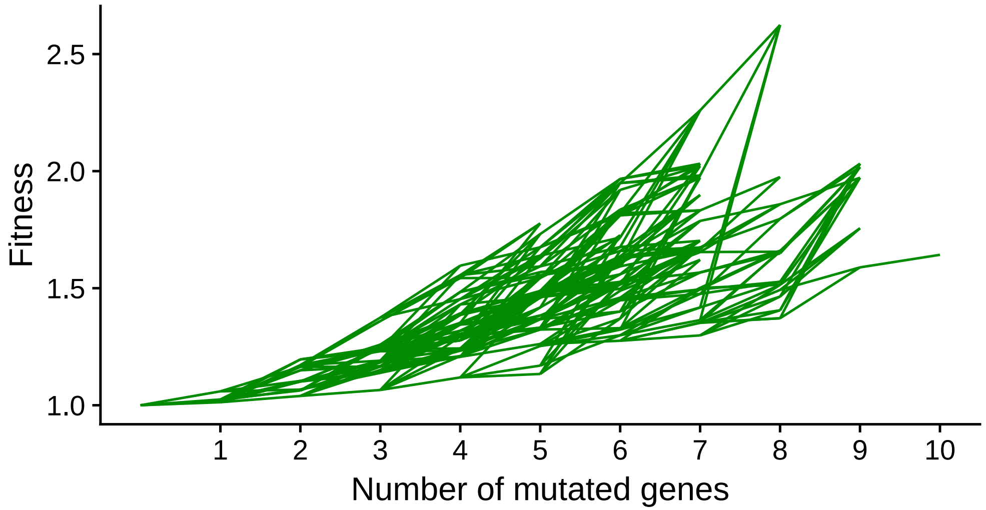

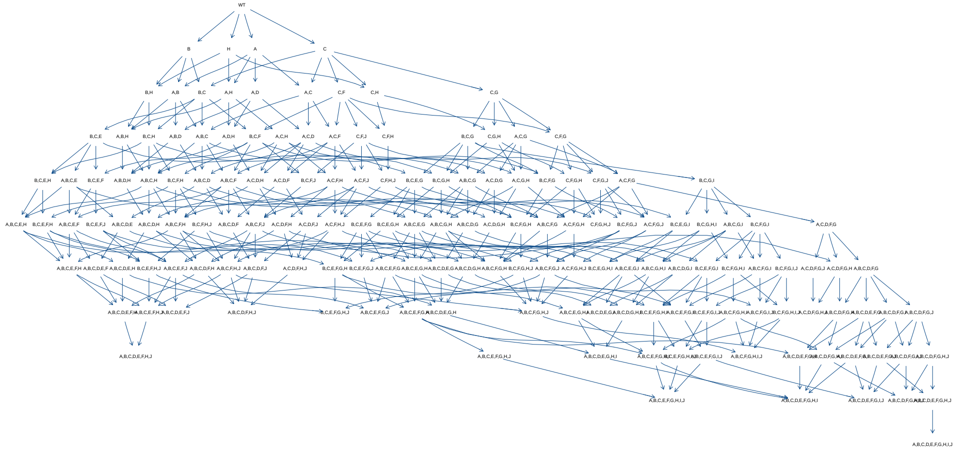

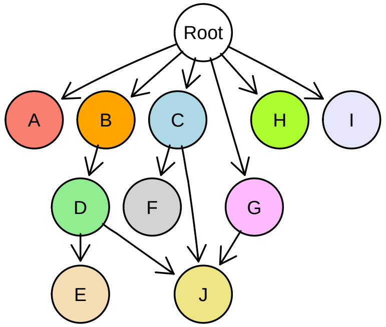

ID	glDXEi6qi95q3Iss
acc genotypes	224
peaks no back	18
removed edges	174
prop rem edges	0.227

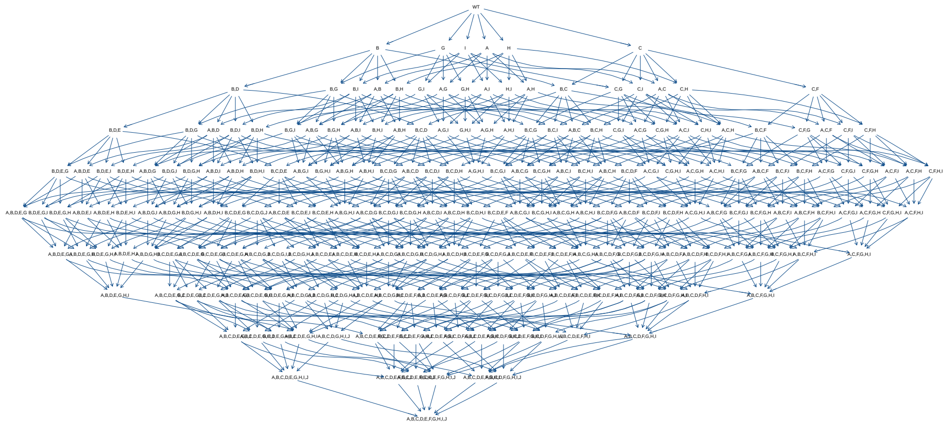

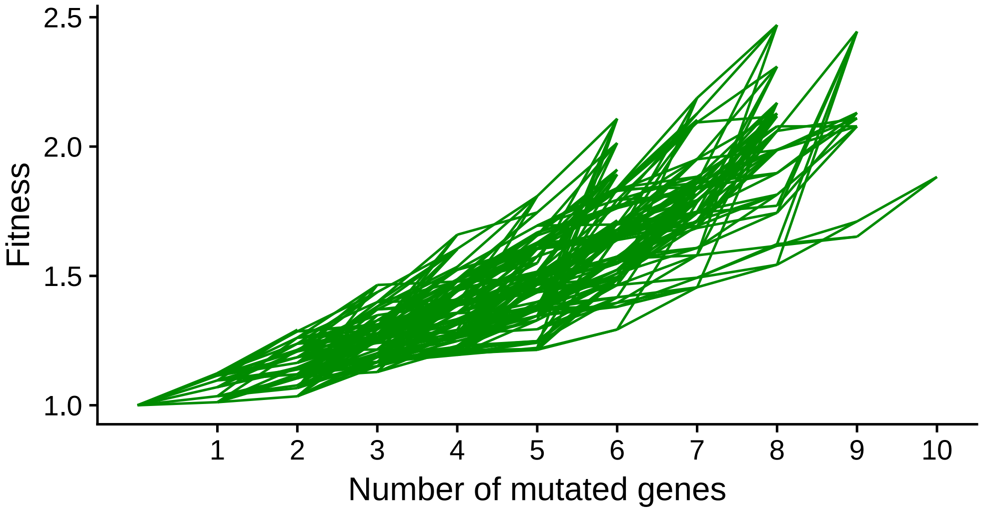

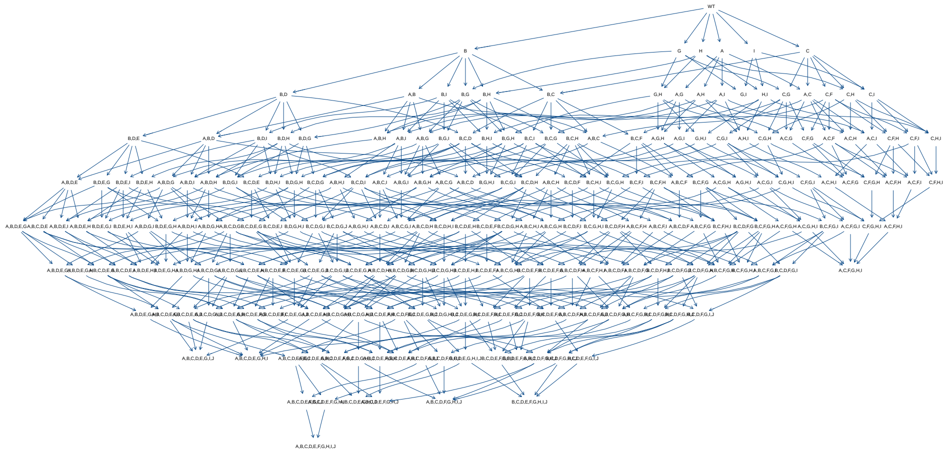

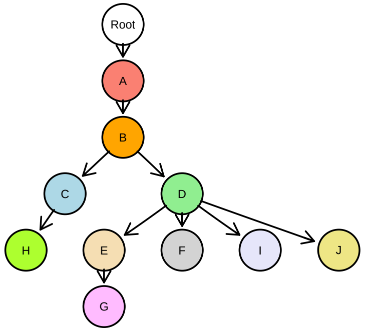

ID	gnxoxbzJ9TsWlPFr
acc genotypes	77
peaks no back	9
removed edges	45
prop rem edges	0.213

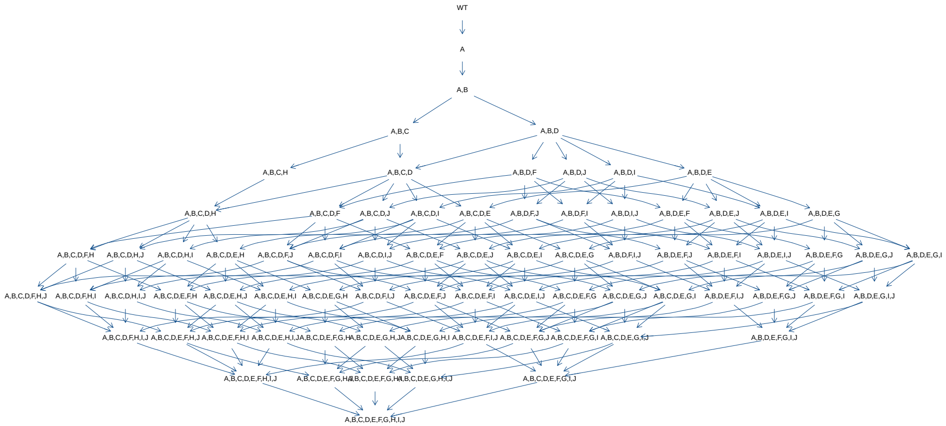

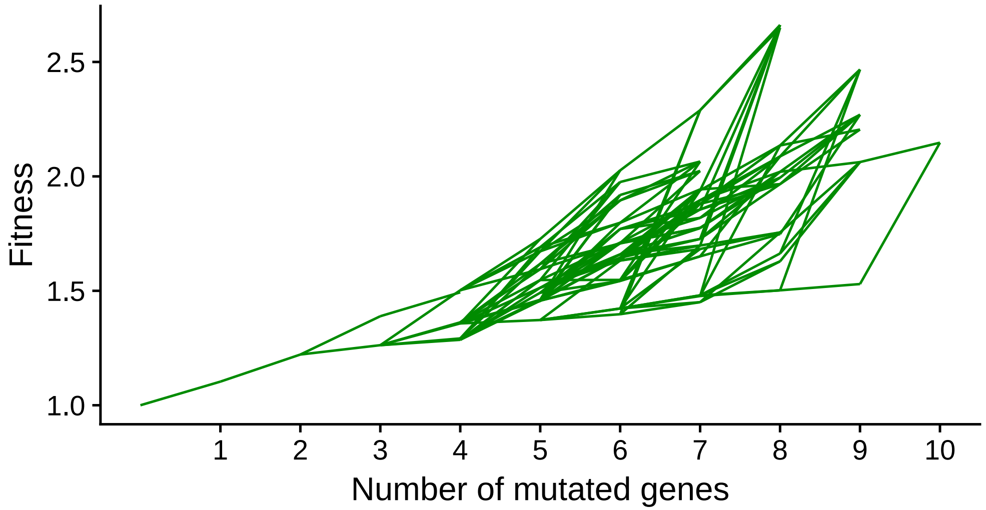

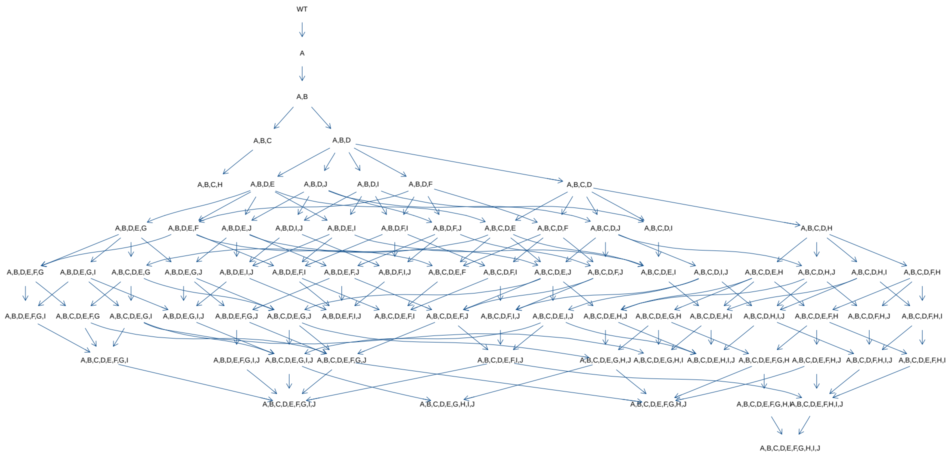

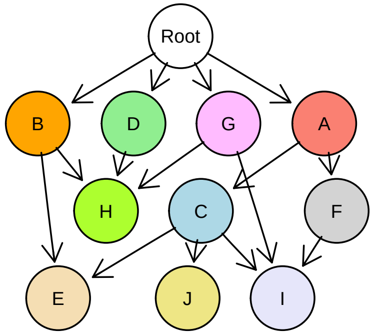

ID	Gw7Lw136jeS105BFu
acc genotypes	99
peaks no back	6
removed edges	44
prop rem edges	0.165

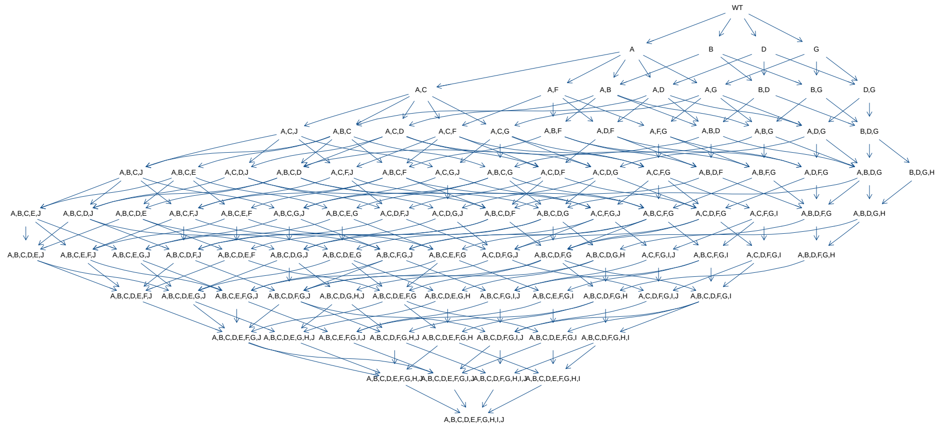

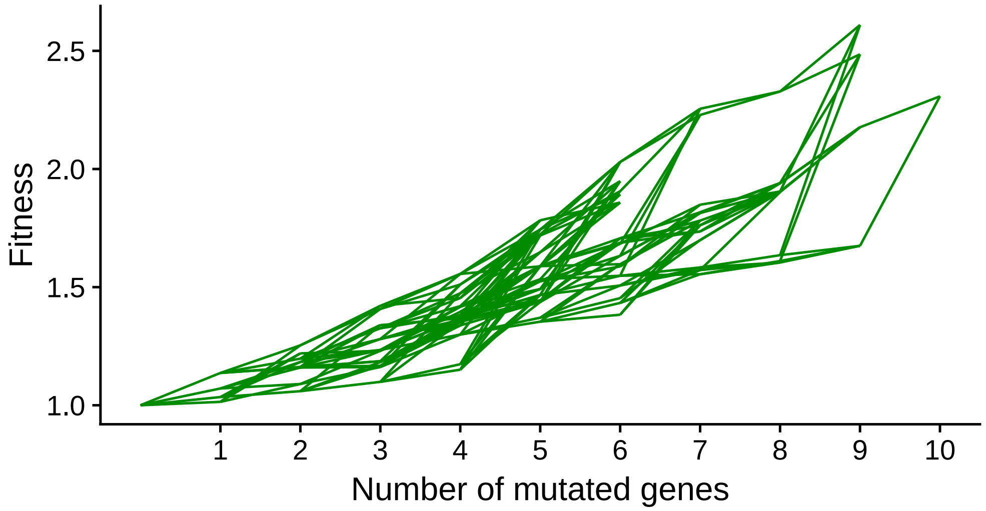

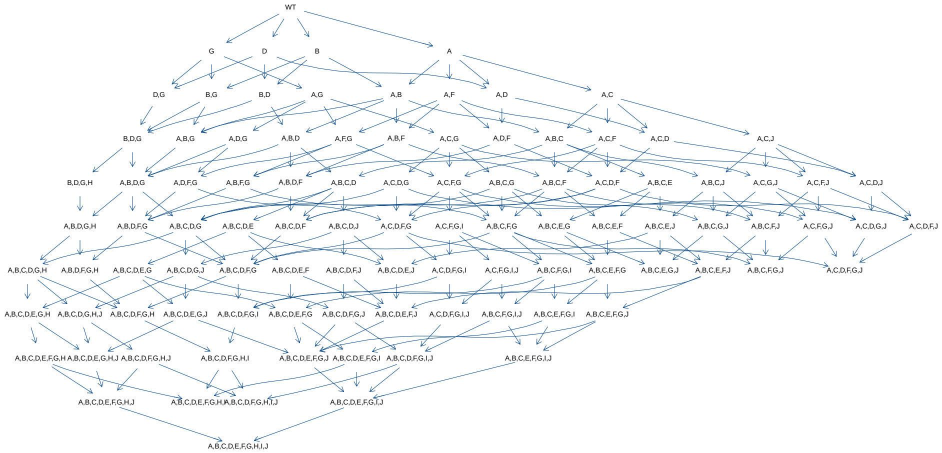

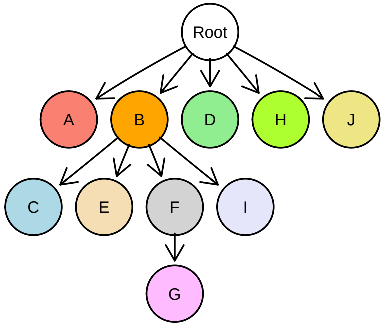

ID	hEMMzV7G9flteqwr
acc genotypes	400
peaks no back	24
removed edges	307
prop rem edges	0.186

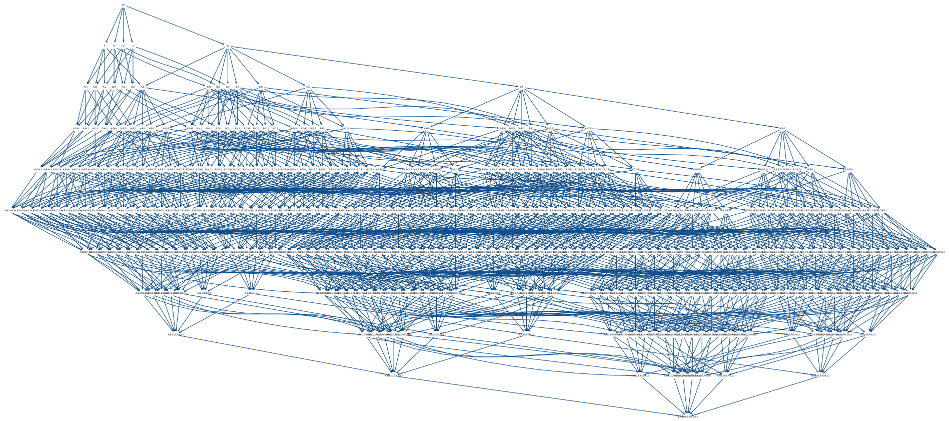

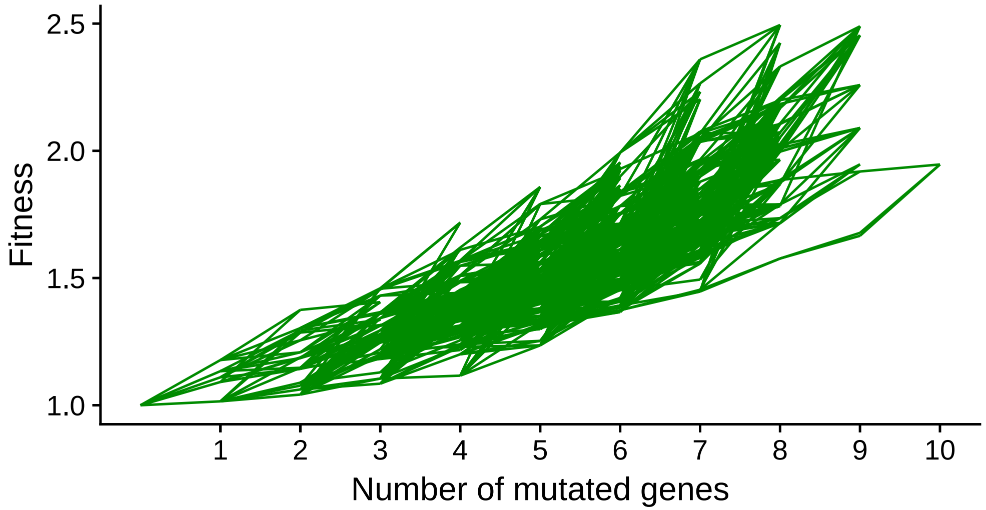

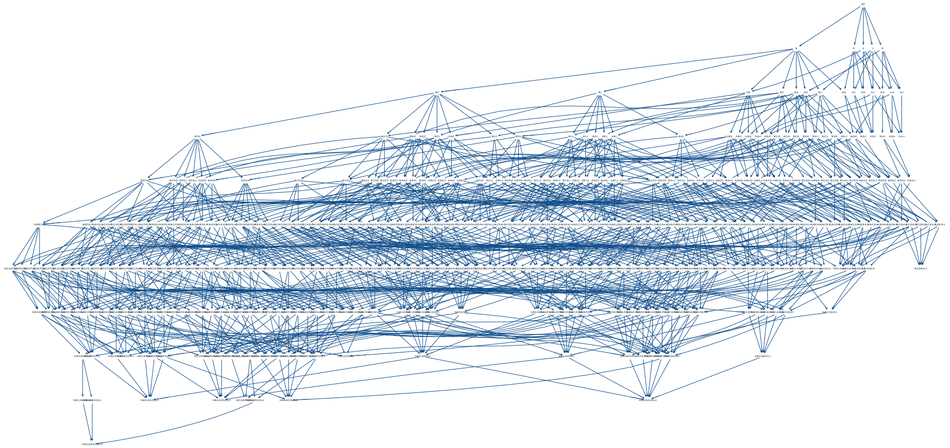

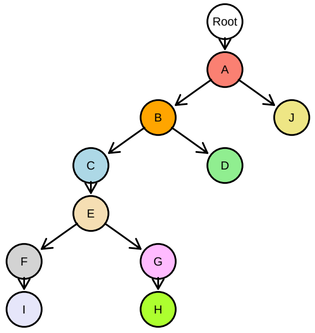

ID	HJf8BvCZrOXnhdpP
acc genotypes	47
peaks no back	5
removed edges	21
prop rem edges	0.202

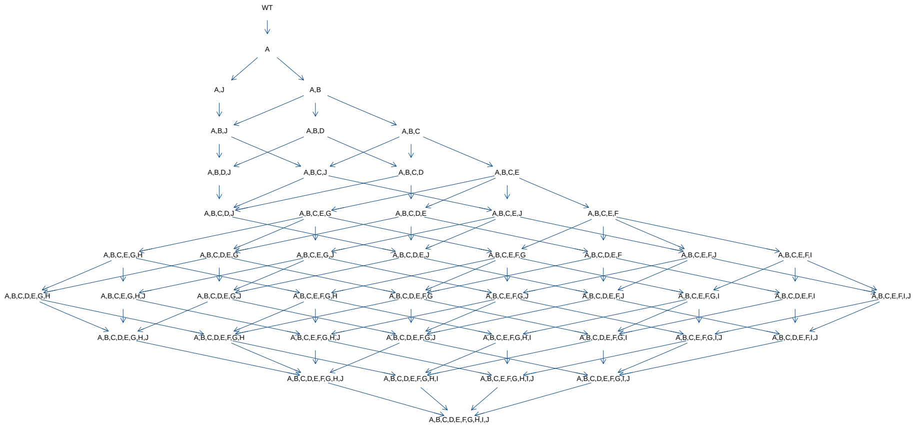

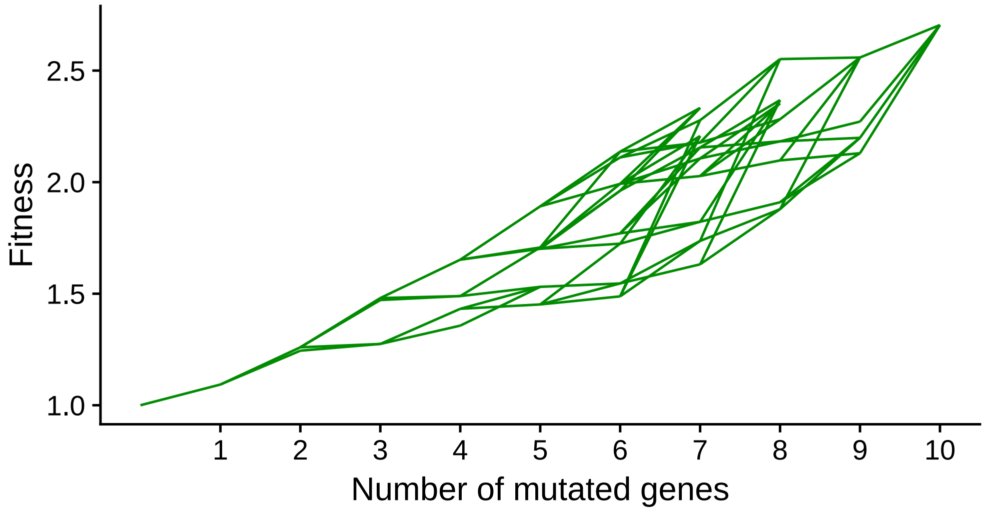

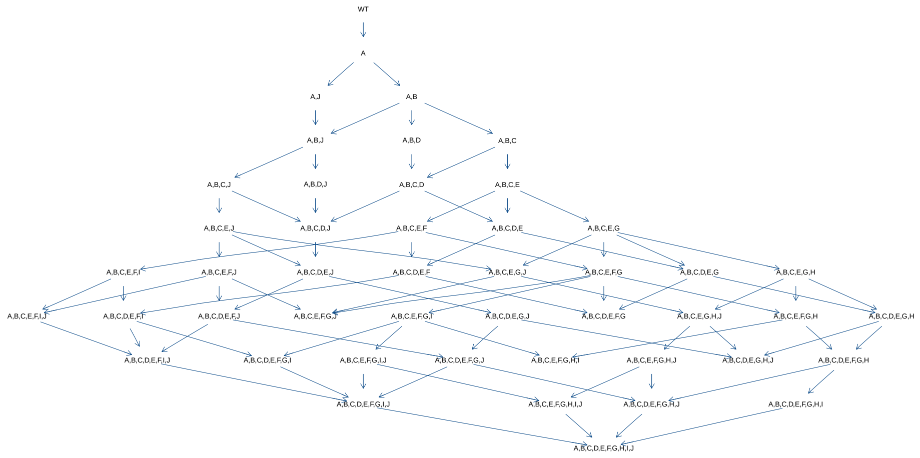

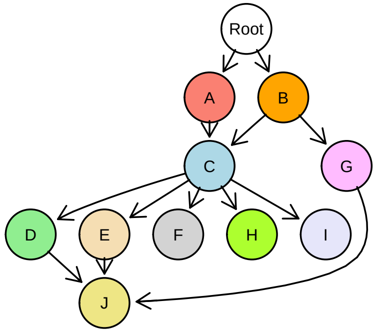

ID	hjGRWsJhOh106RXPY
acc genotypes	78
peaks no back	8
removed edges	48
prop rem edges	0.217

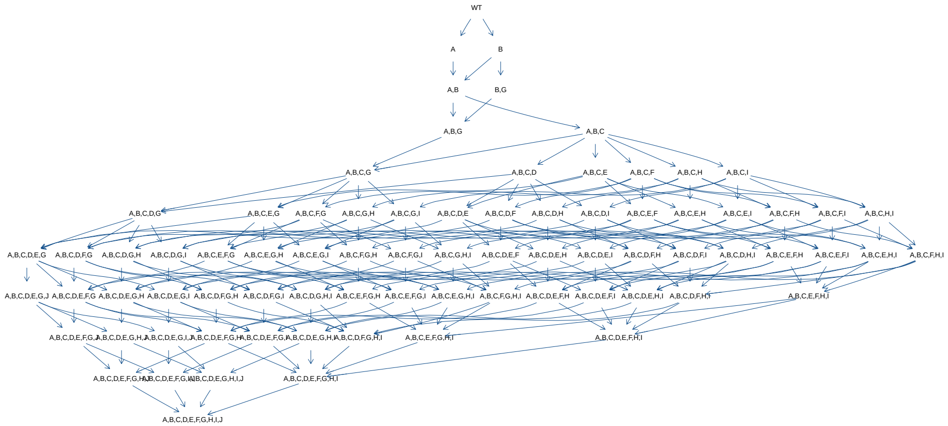

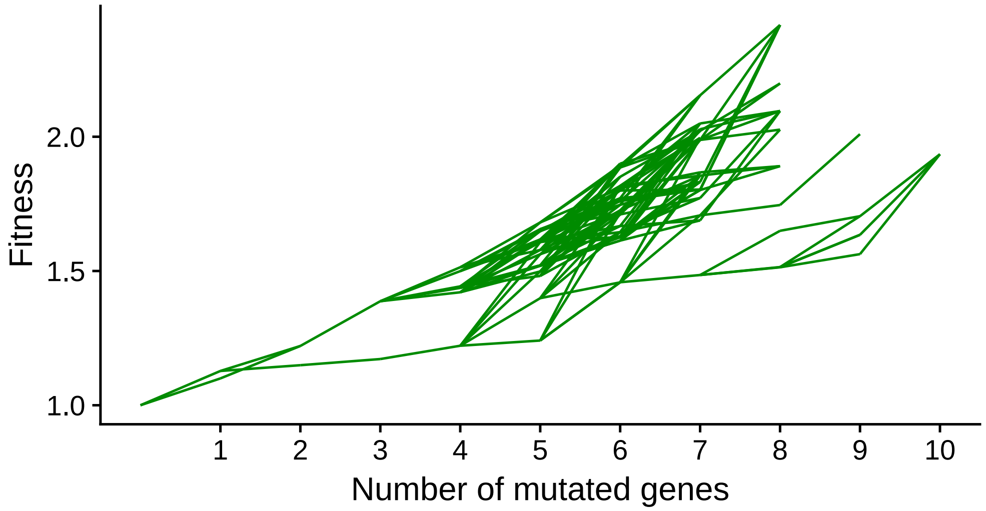

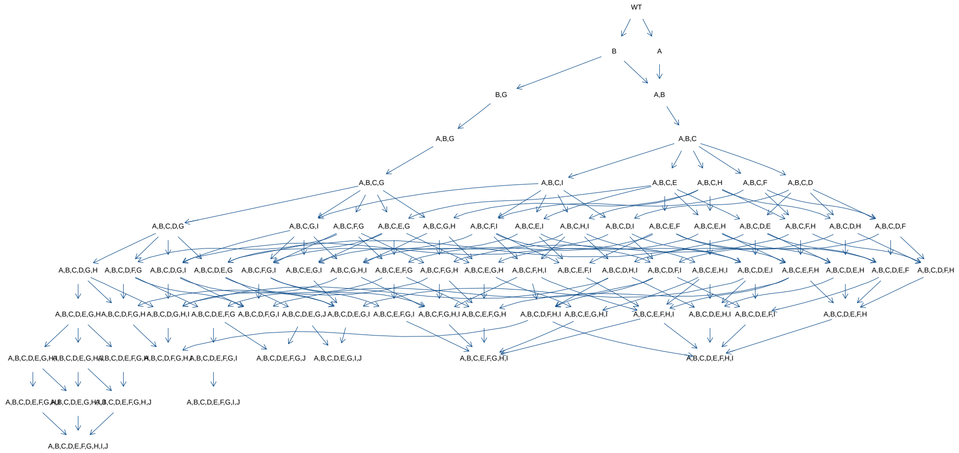

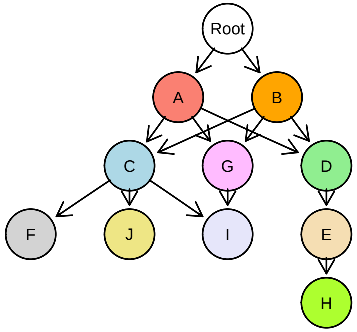

ID	HjmKaDaJUp3CbAMd
acc genotypes	59
peaks no back	9
removed edges	21
prop rem edges	0.152

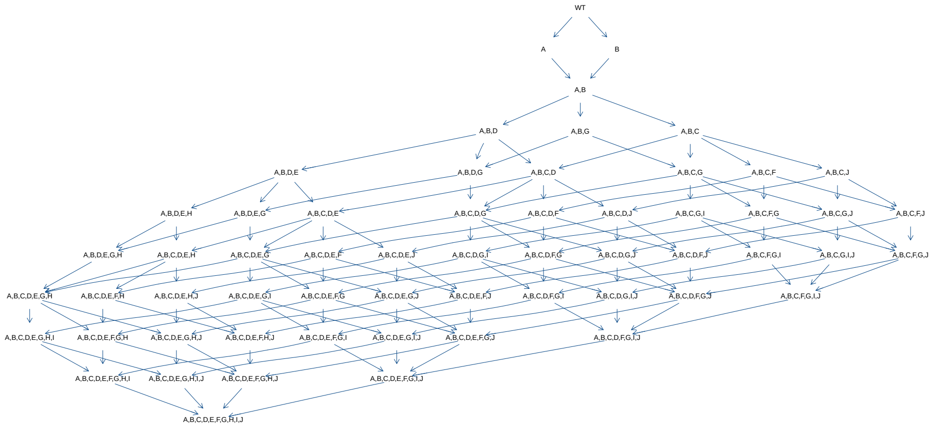

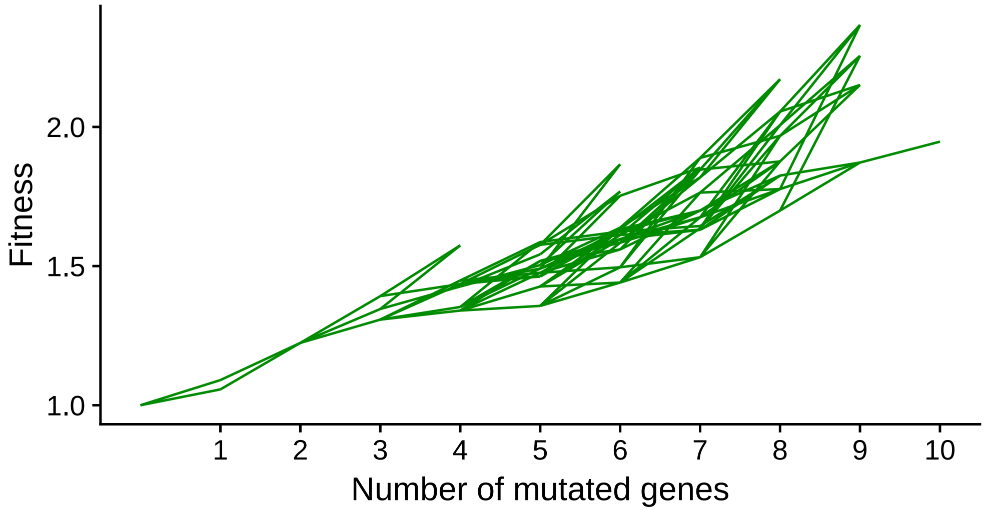

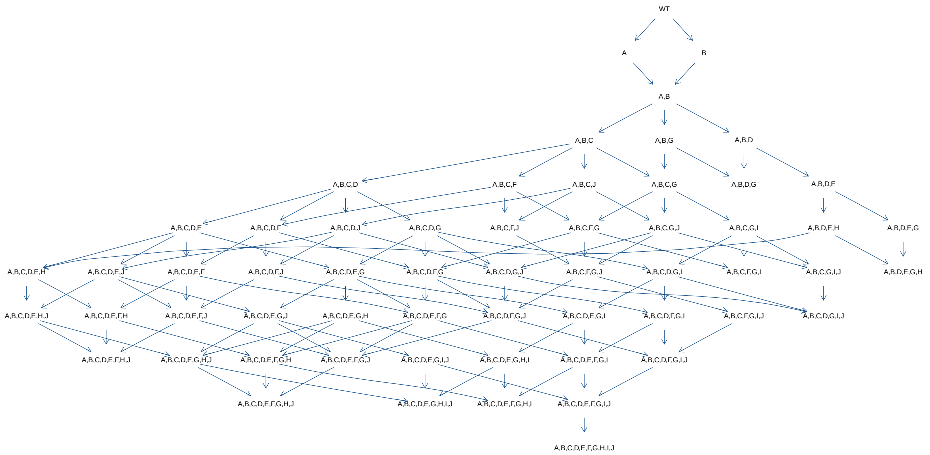

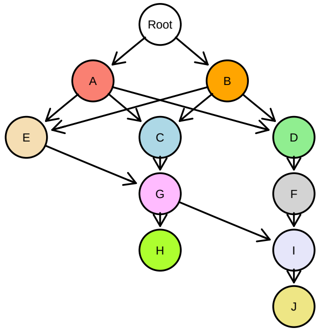

ID	hootgr4D2eT9wPQw
acc genotypes	25
peaks no back	2
removed edges	3
prop rem edges	0.075

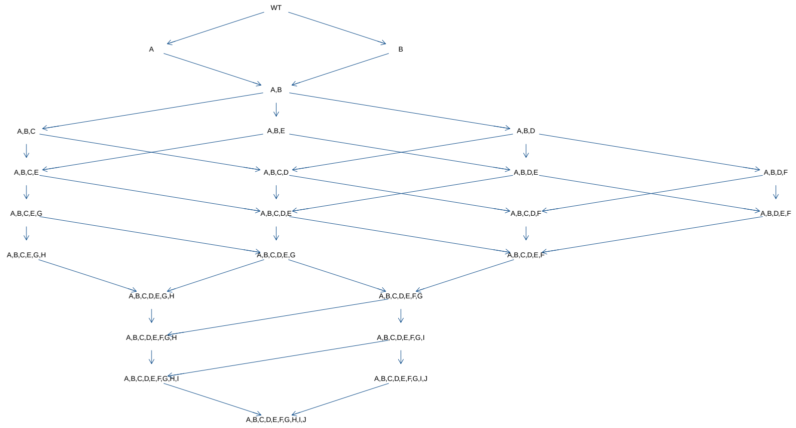

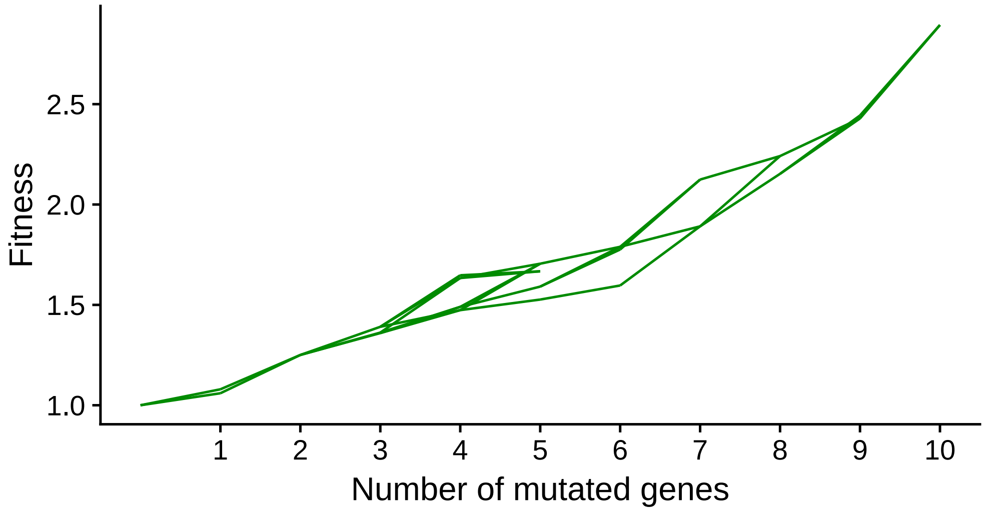

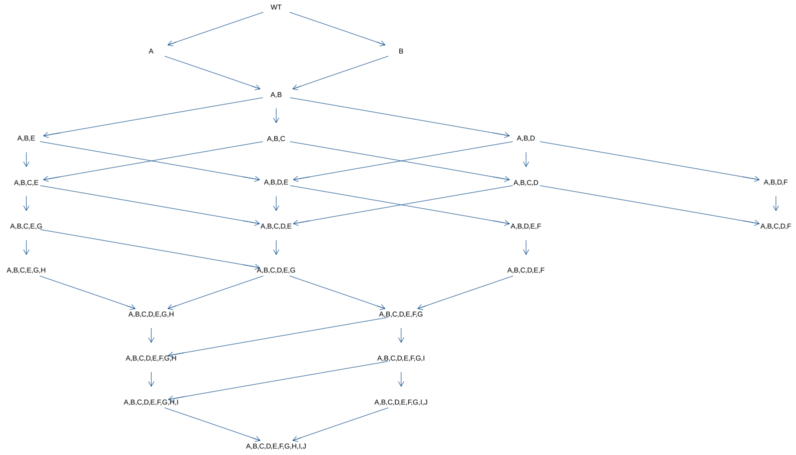

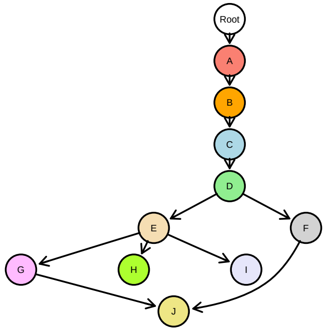

ID i210cnkWHYkaWwl3l
acc genotypes 26
peaks no back 4
removed edges 7
prop rem edges 0.149

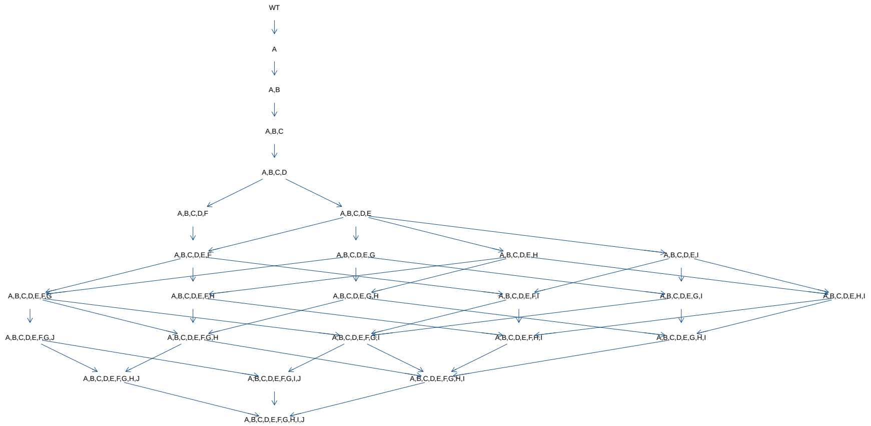

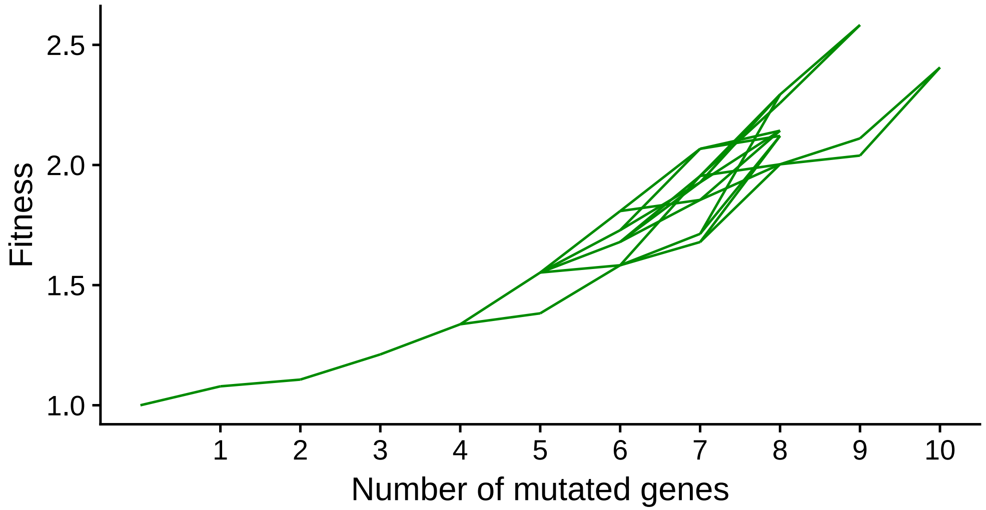

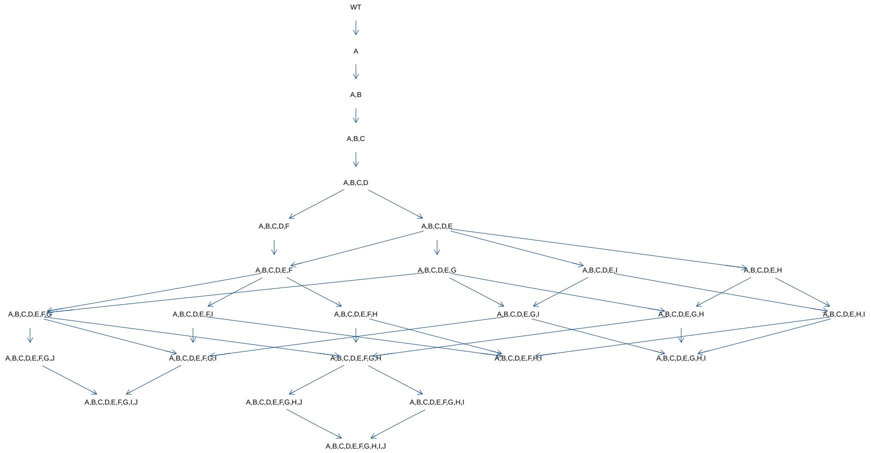

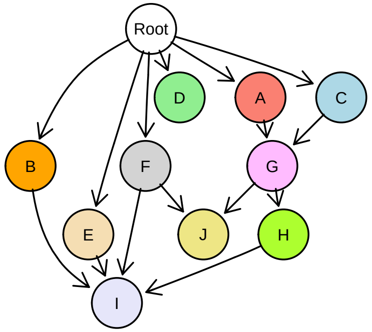

ID I9aZnfSBnGVPEGvO
acc genotypes 116
peaks no back 14
removed edges 89
prop rem edges 0.259

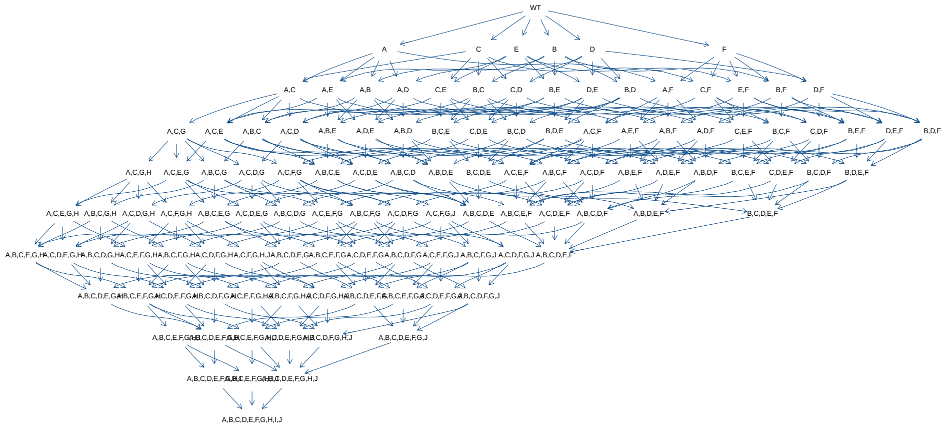

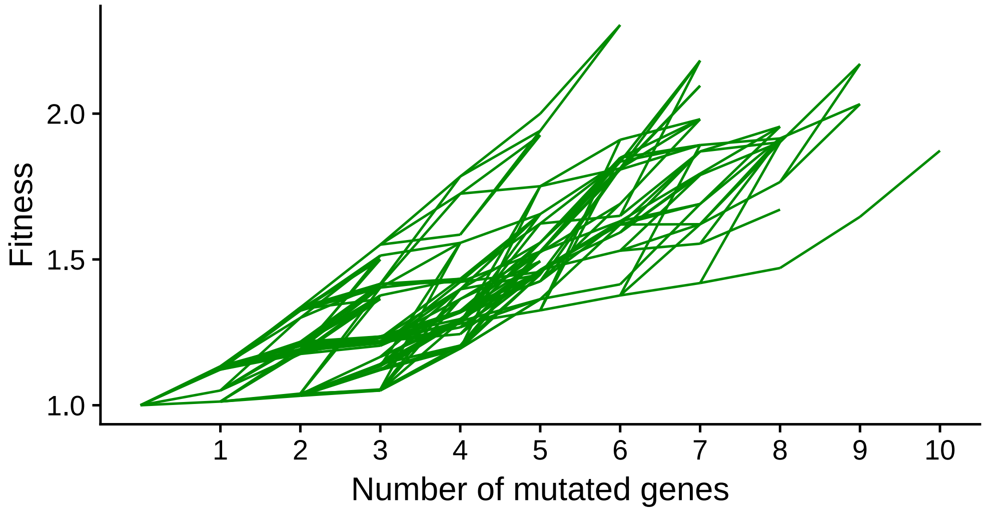

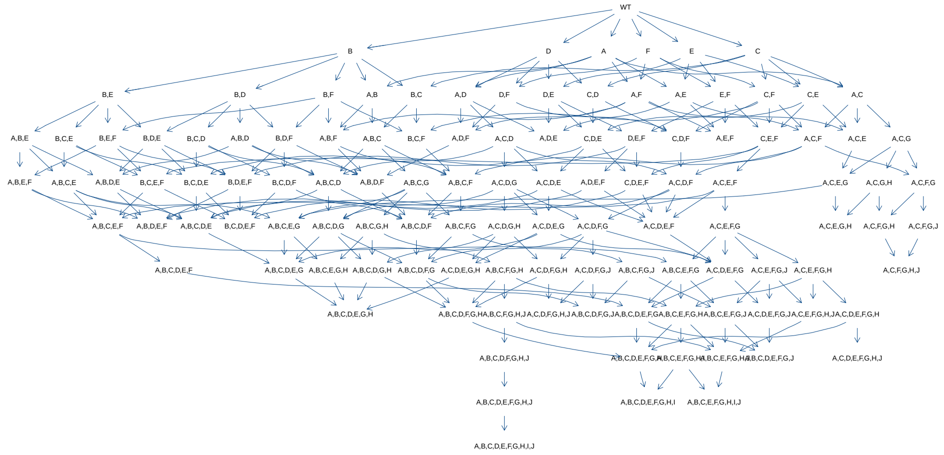

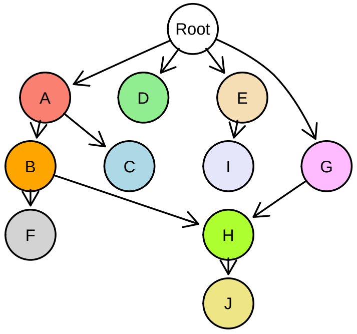

ID	ibcyCqVRdZgkjfmP
acc genotypes	132
peaks no back	13
removed edges	62
prop rem edges	0.16

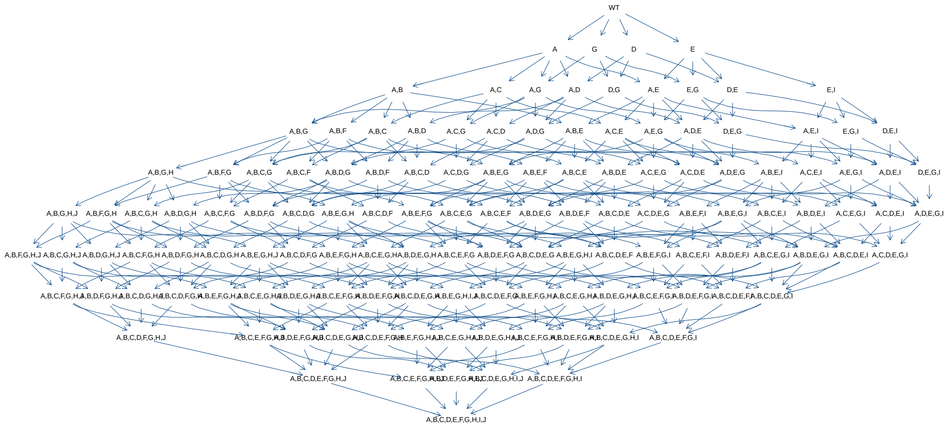

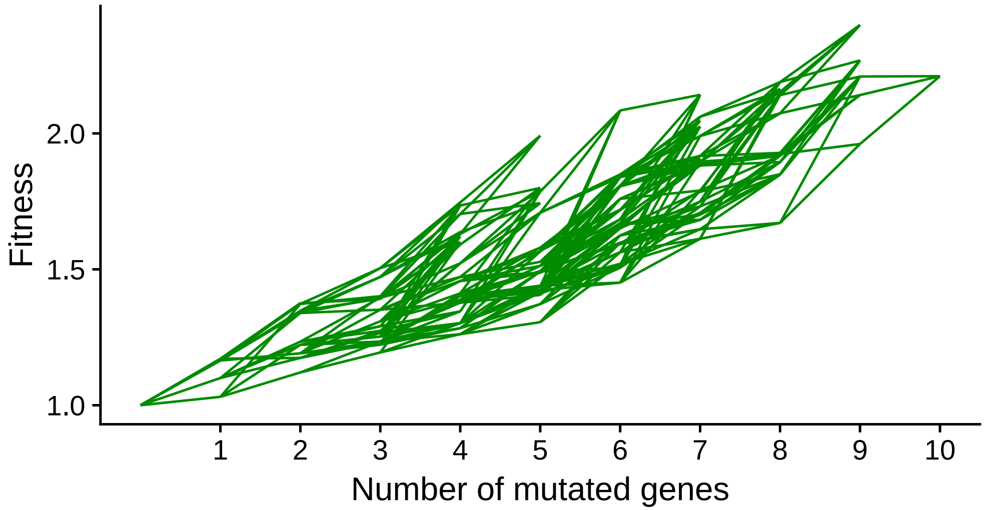

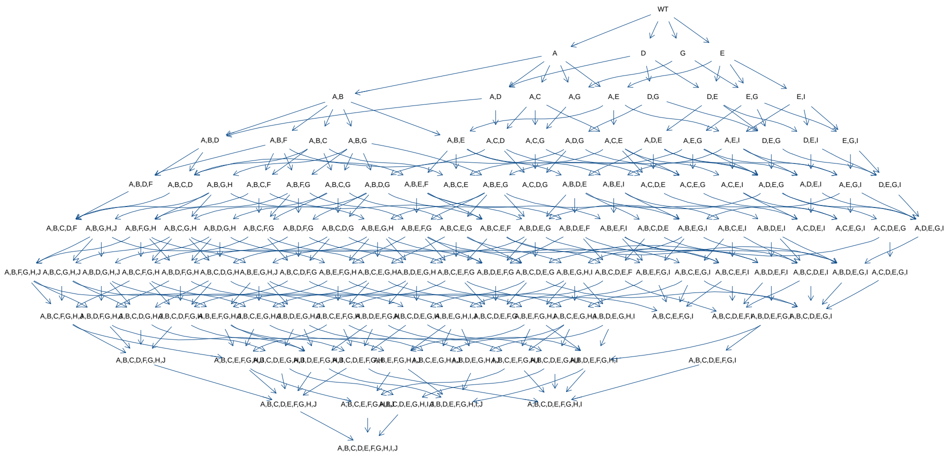

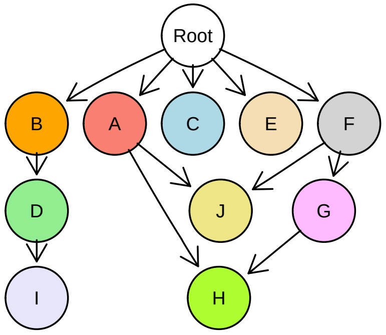

ID	Ihbe9R5g4X8LVErf
acc genotypes	160
peaks no back	12
removed edges	76
prop rem edges	0.156

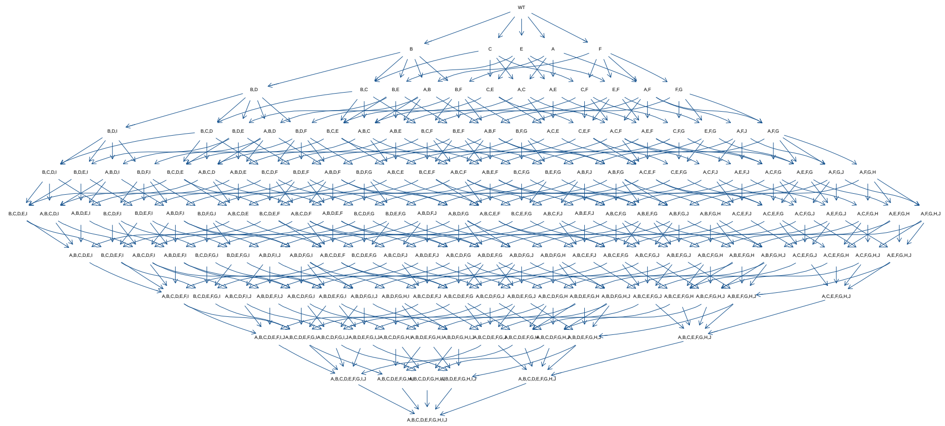

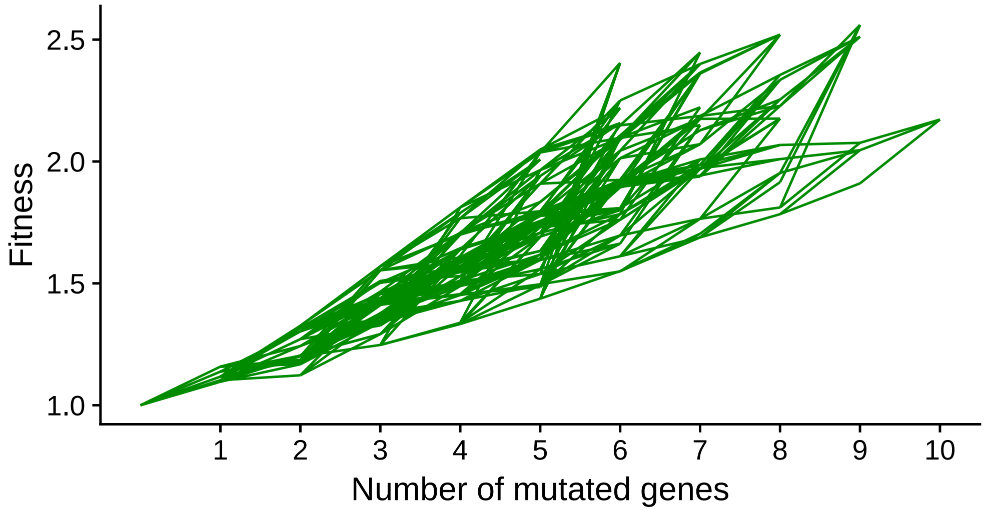

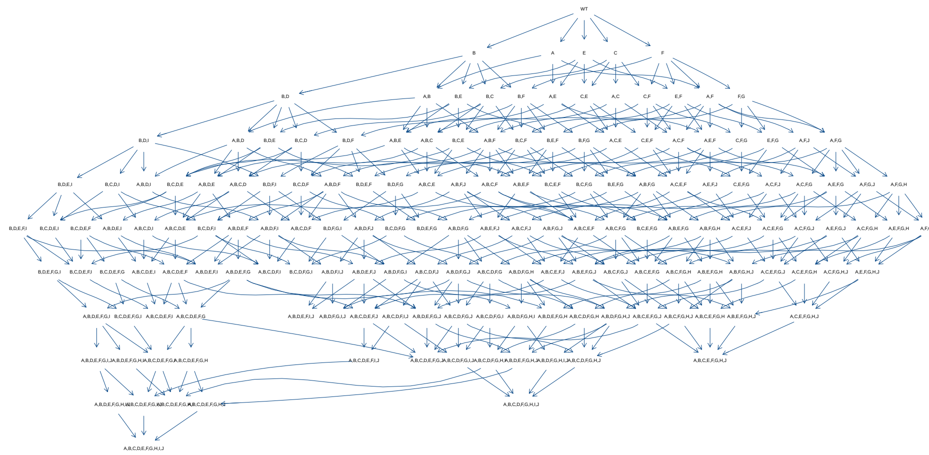

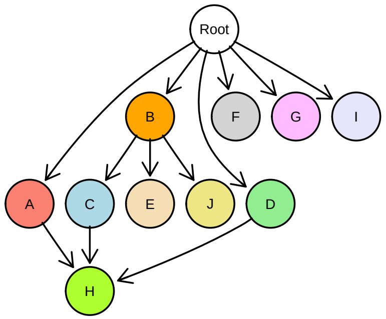

ID	IJHDmelG9vA10Rtnv
acc genotypes	320
peaks no back	21
removed edges	305
prop rem edges	0.244

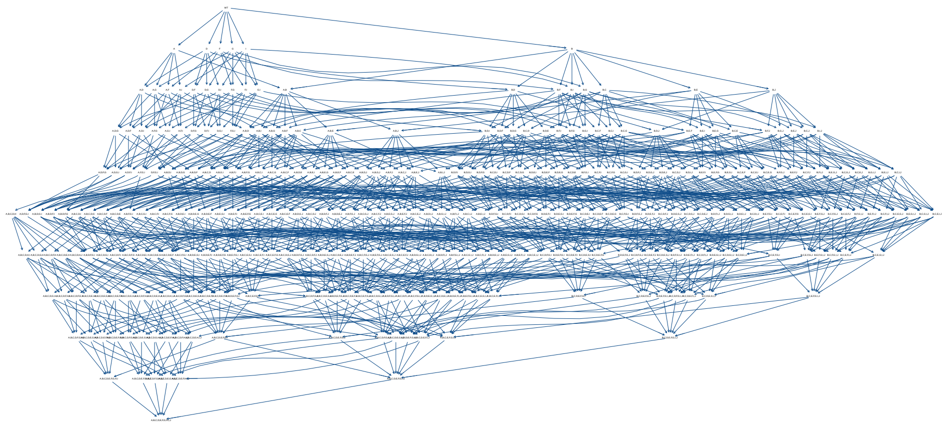

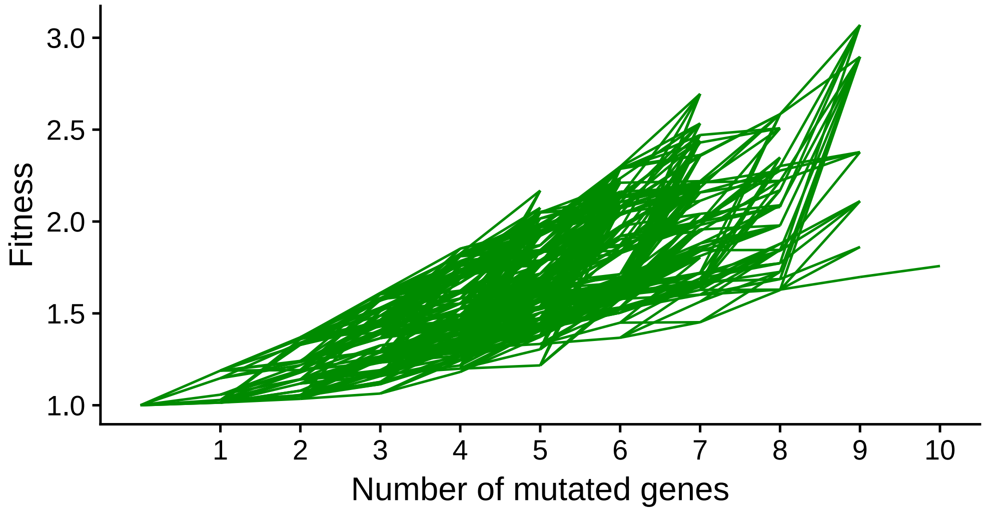

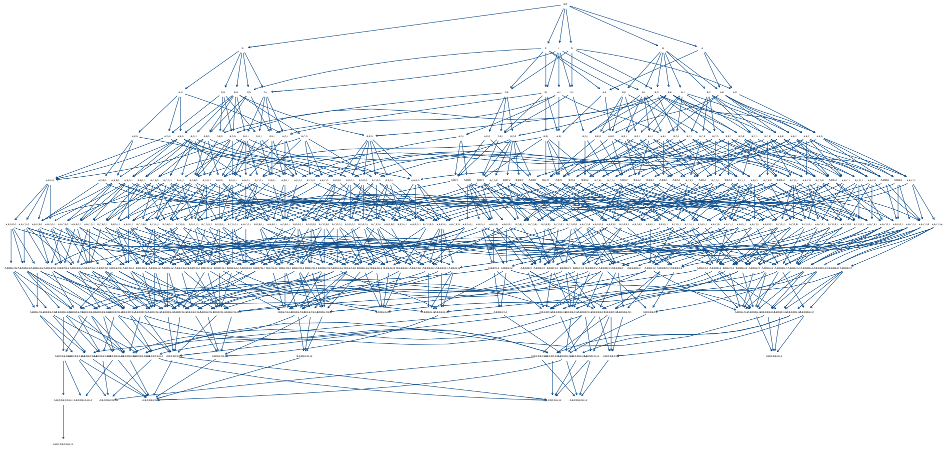

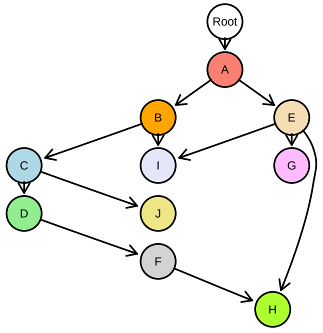

ID	InQKKevAQbH5g2SM
acc genotypes	47
peaks no back	7
removed edges	18
prop rem edges	0.178

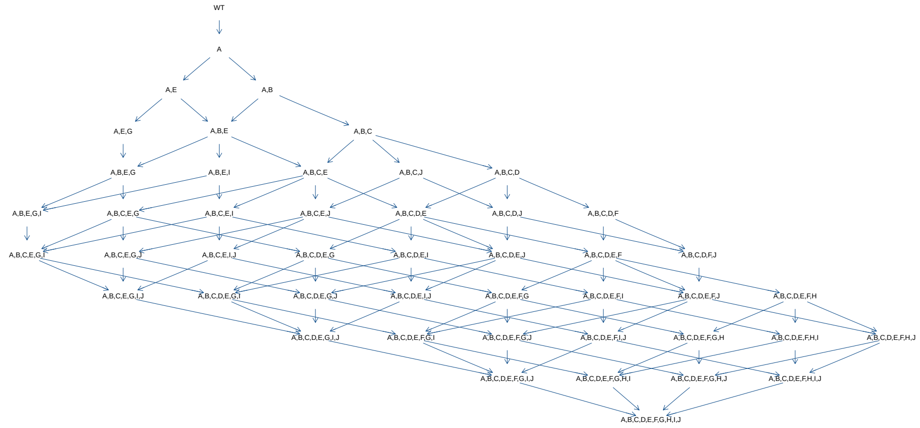

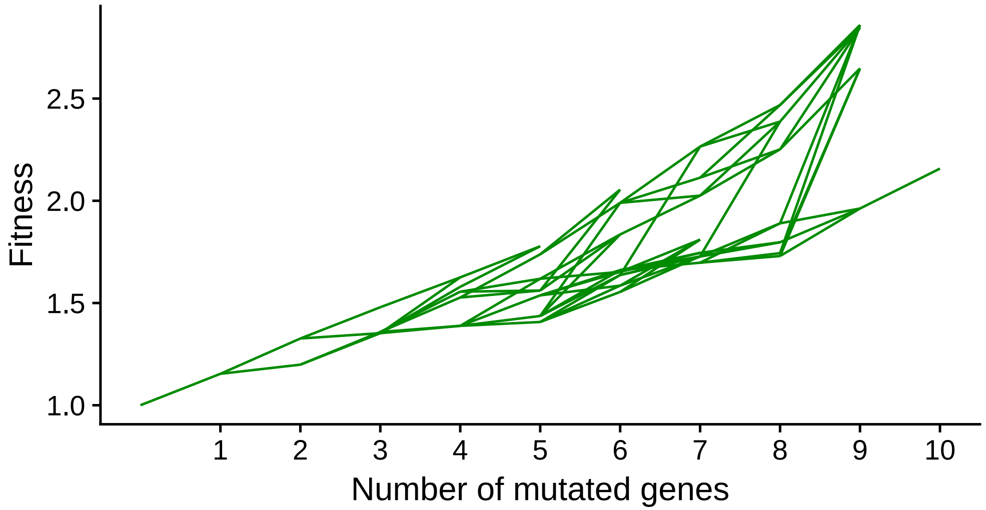

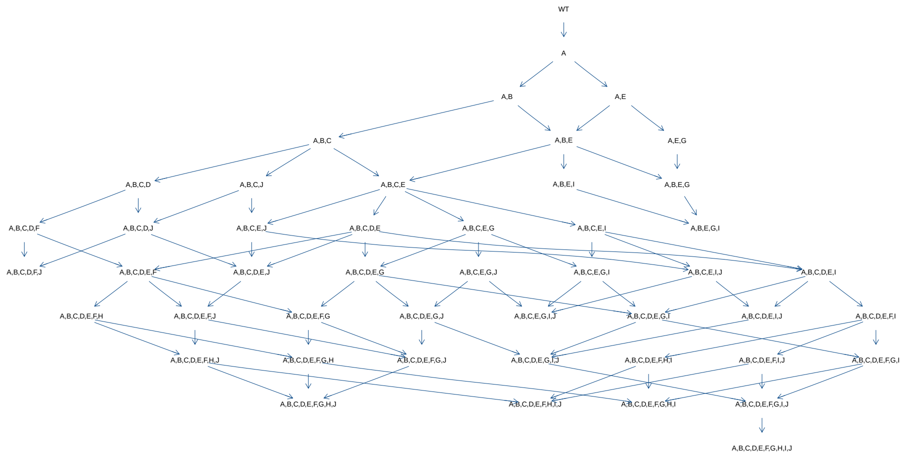

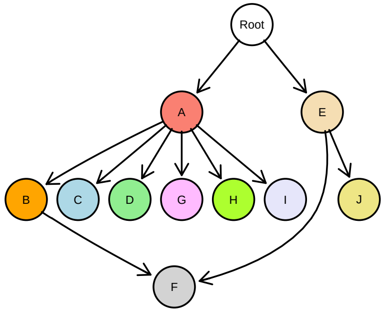

ID	iudi9ykQn8Kp10Gil
acc genotypes	259
peaks no back	19
removed edges	140
prop rem edges	0.145

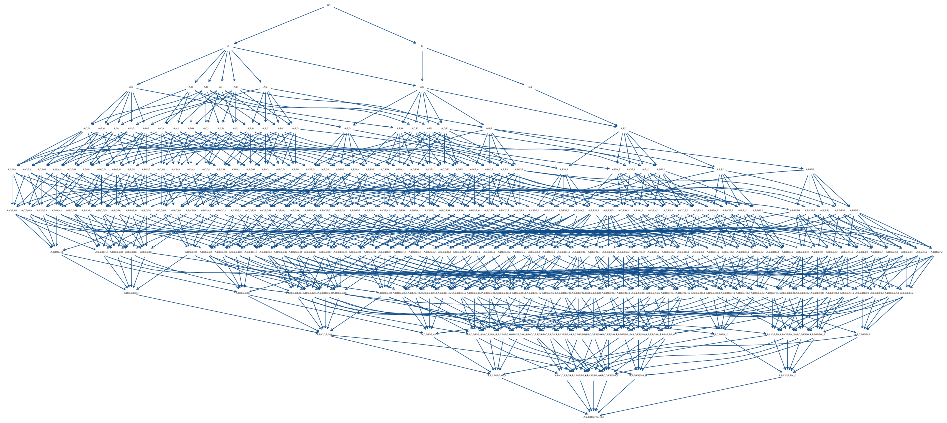

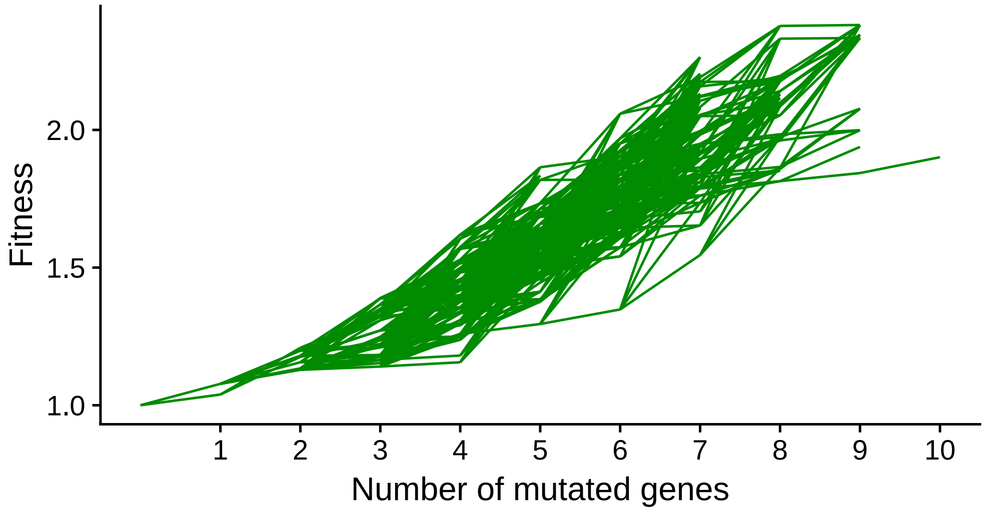

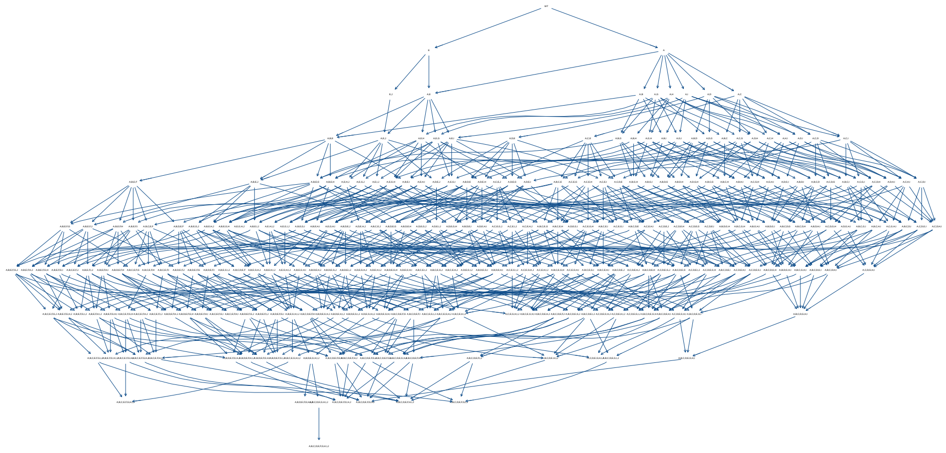

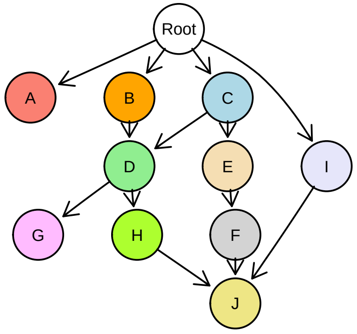

ID	iyeU83TTZkIieN106
acc genotypes	84
peaks no back	9
removed edges	37
prop rem edges	0.168

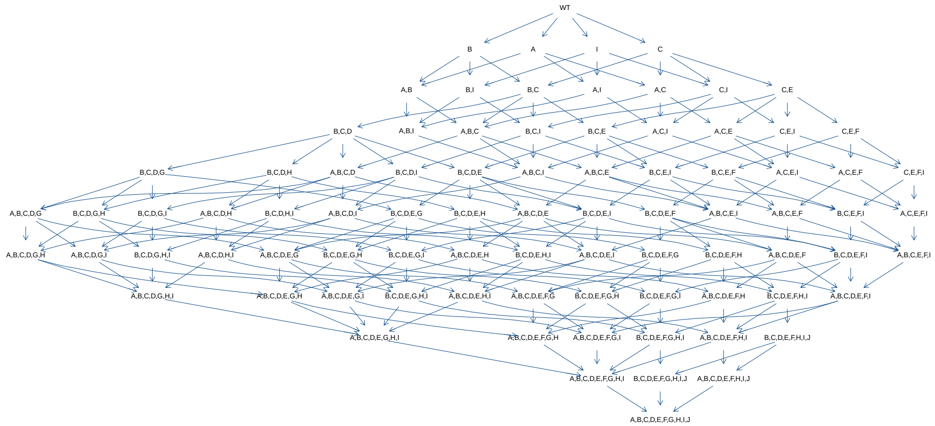

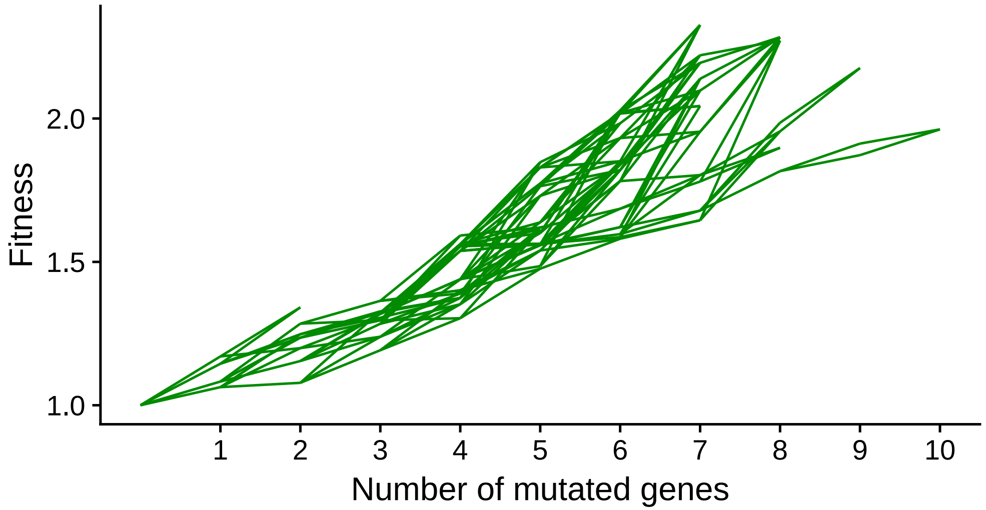

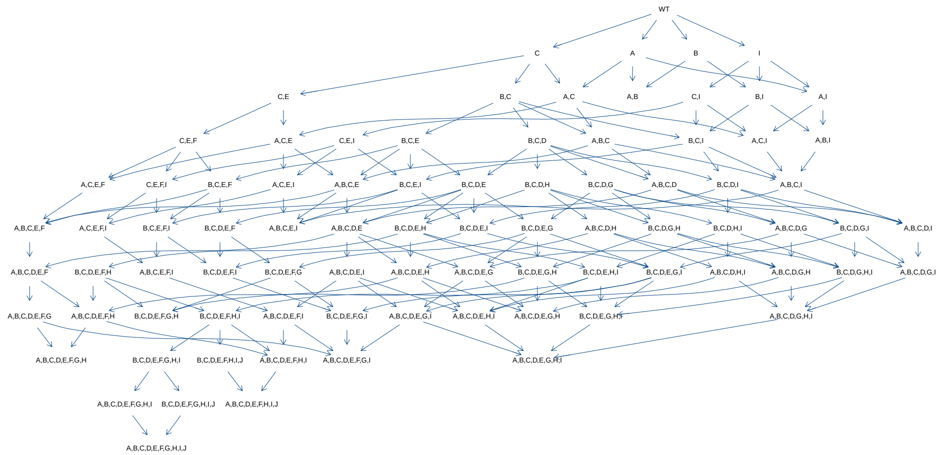

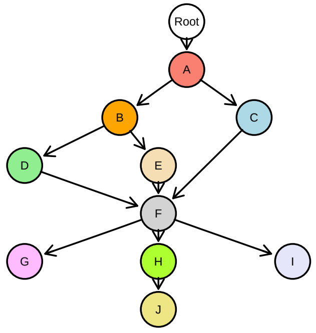

ID iyF6c65sIZnguySA
acc genotypes 23
peaks no back 2
removed edges 2
prop rem edges 0.054

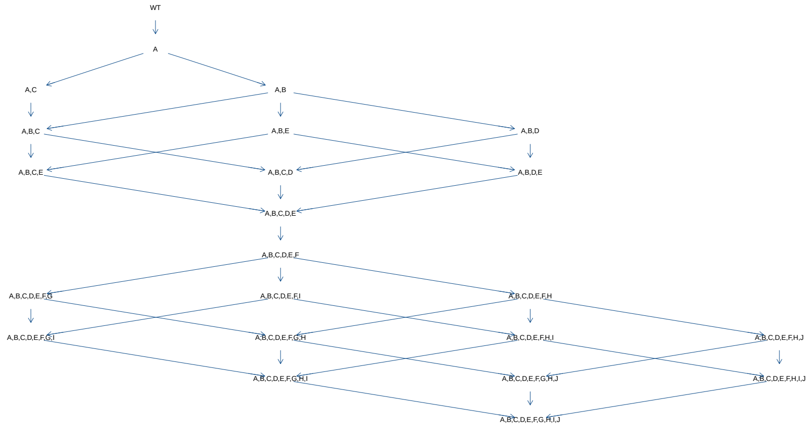

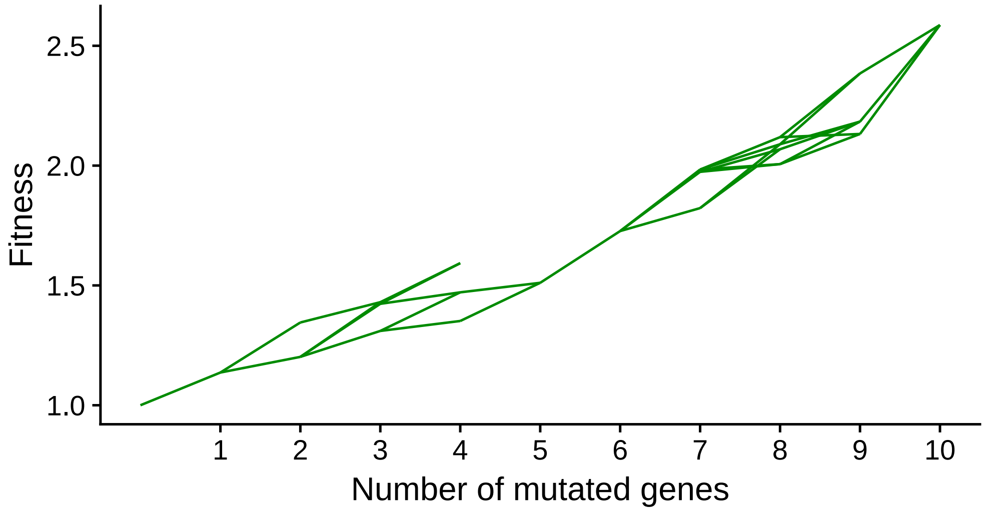

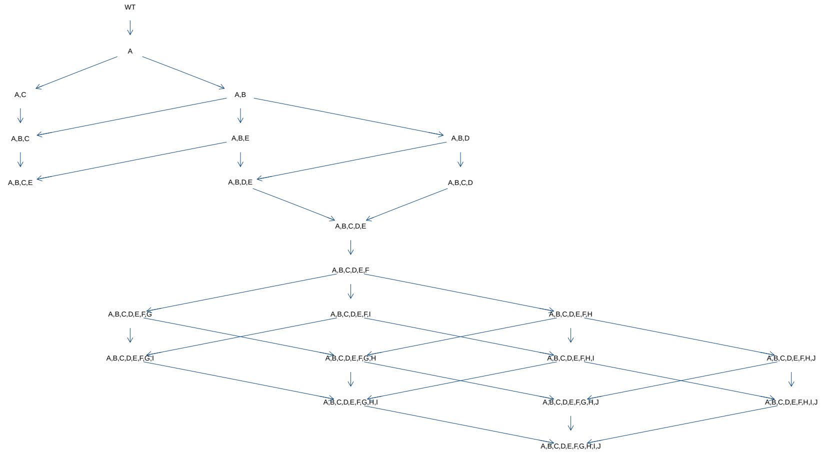

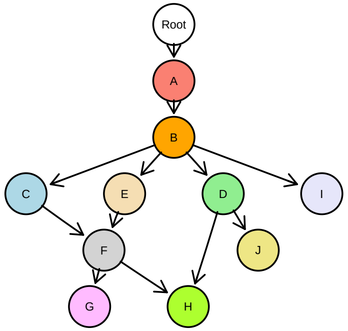

ID	iYOTCycsSTgt6LB1
acc genotypes	46
peaks no back	4
removed edges	12
prop rem edges	0.12

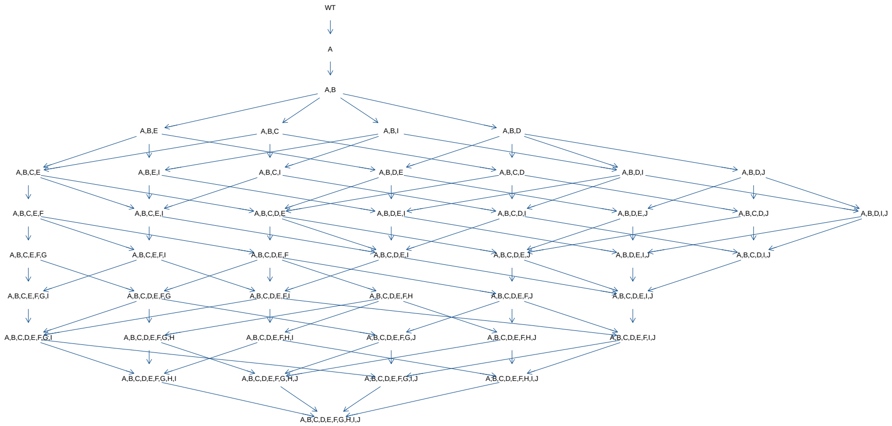

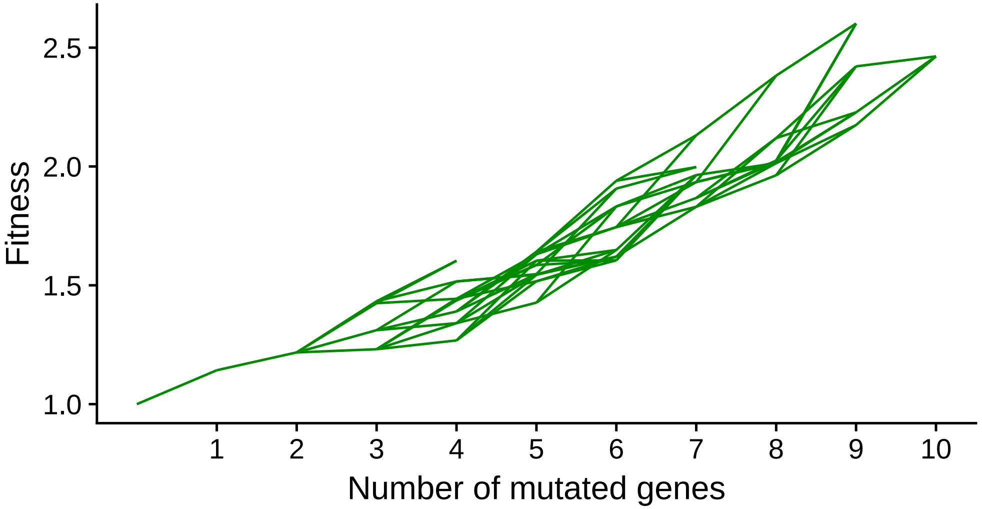

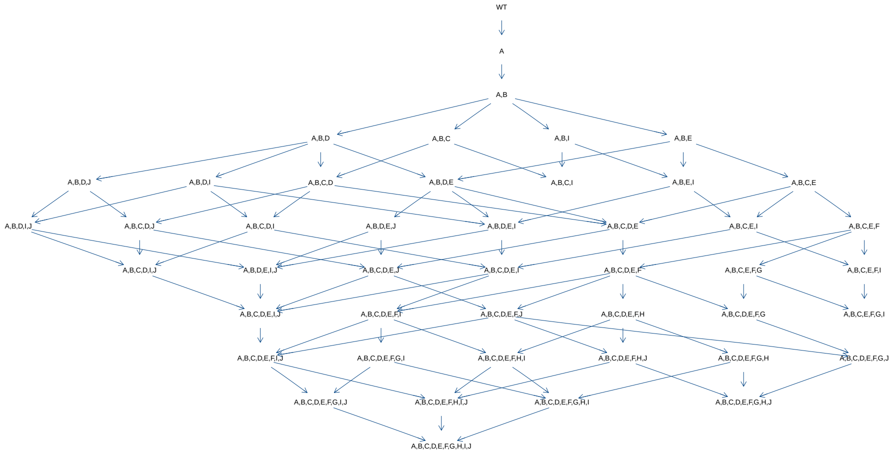

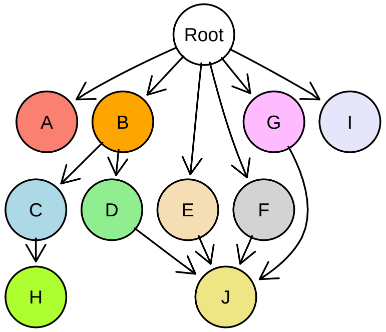

ID	ja5K99Pb6N9URztp
acc genotypes	236
peaks no back	17
removed edges	192
prop rem edges	0.226

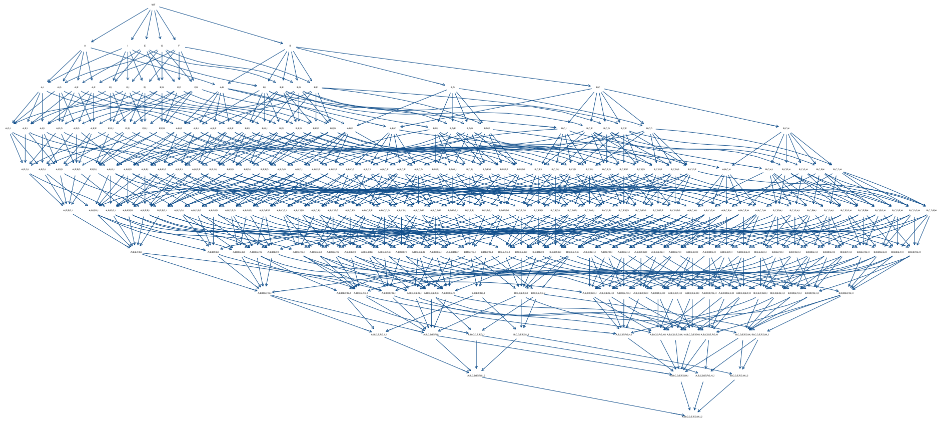

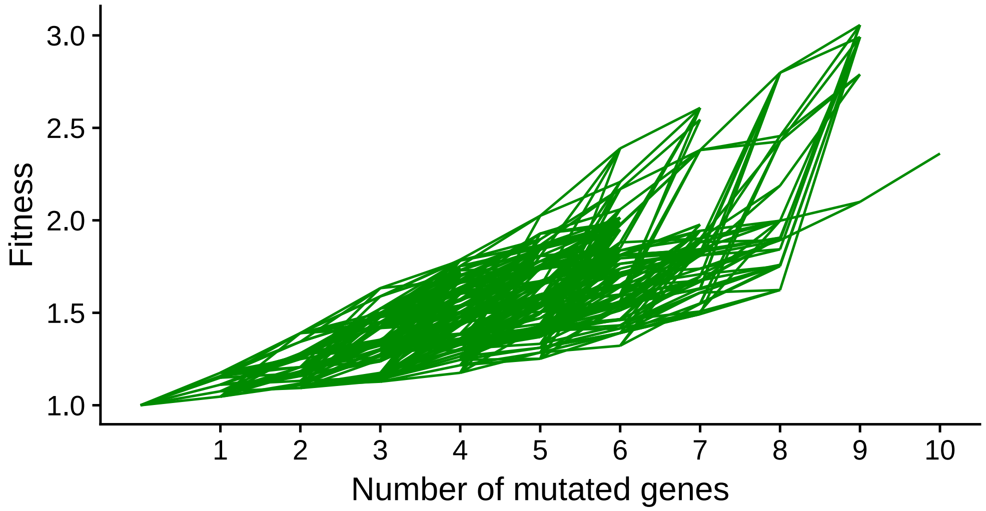

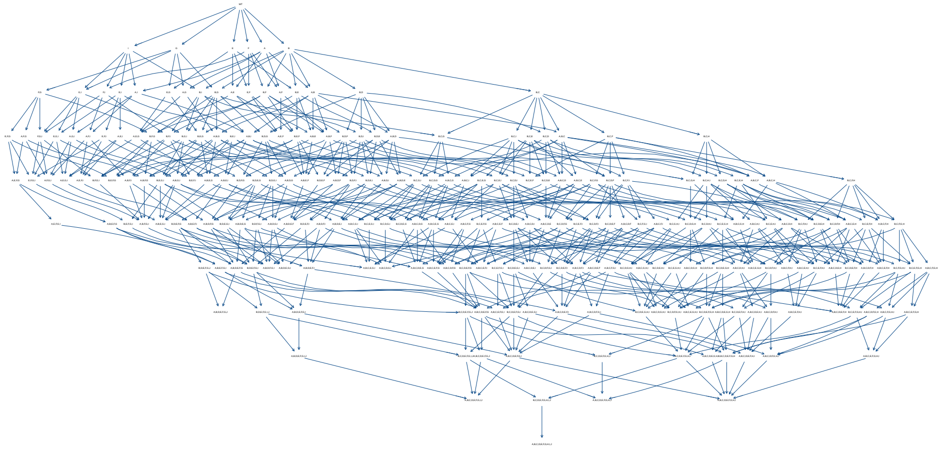

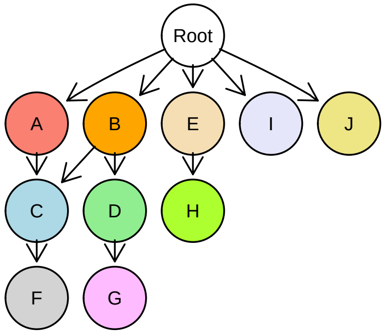

ID jBiKG5Scuo10UsyUf
acc genotypes 168
peaks no back 14
removed edges 111
prop rem edges 0.213

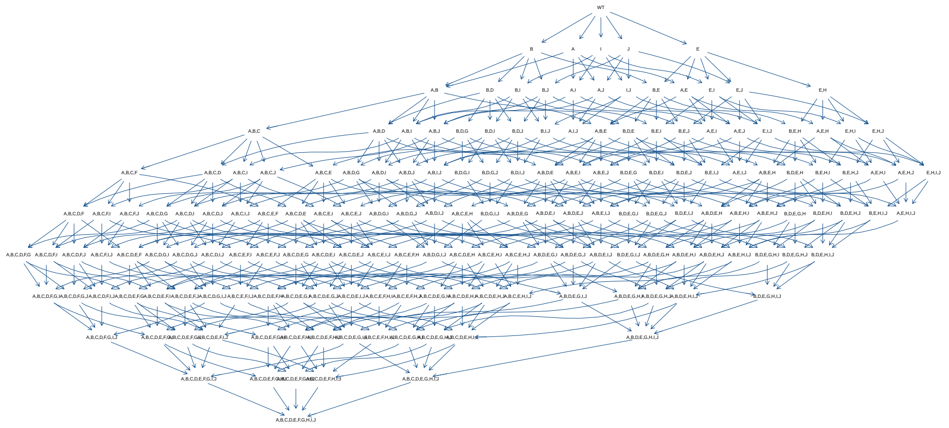

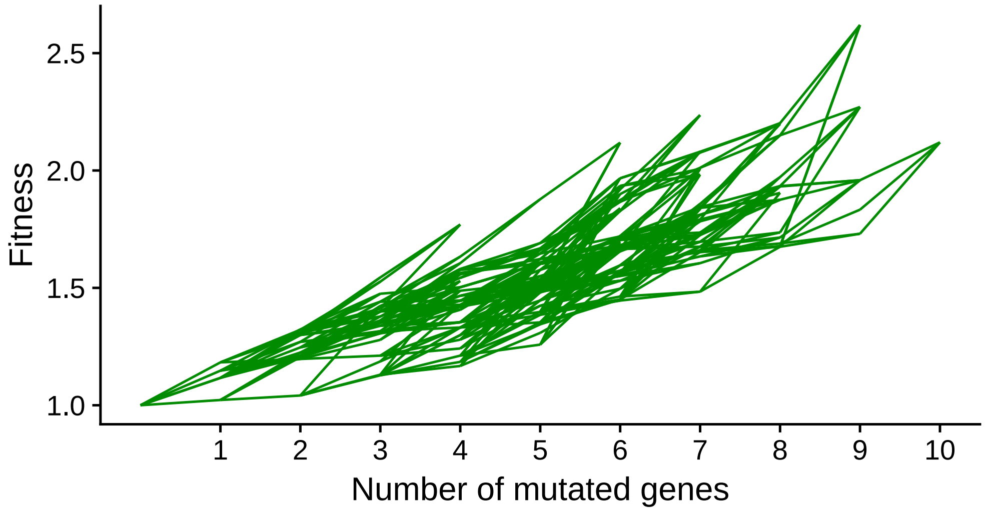

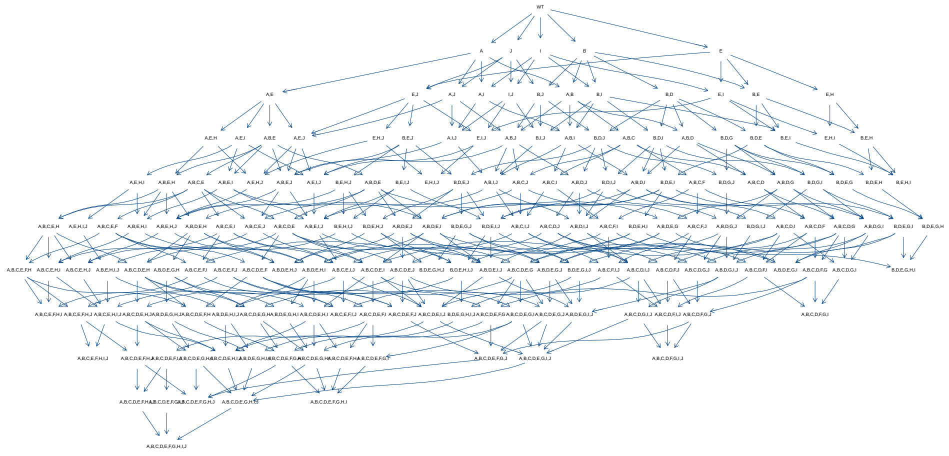

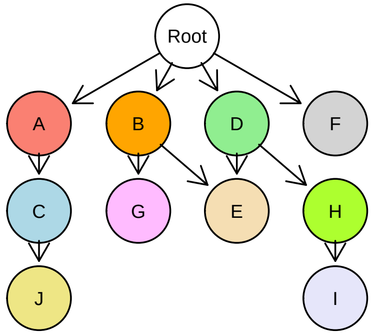

ID	jeIgNCENfpAlgfTL
acc genotypes	144
peaks no back	15
removed edges	89
prop rem edges	0.212

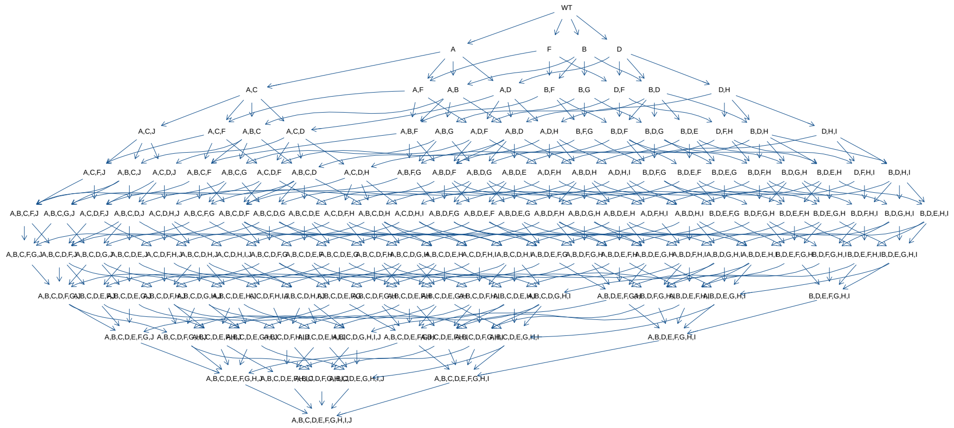

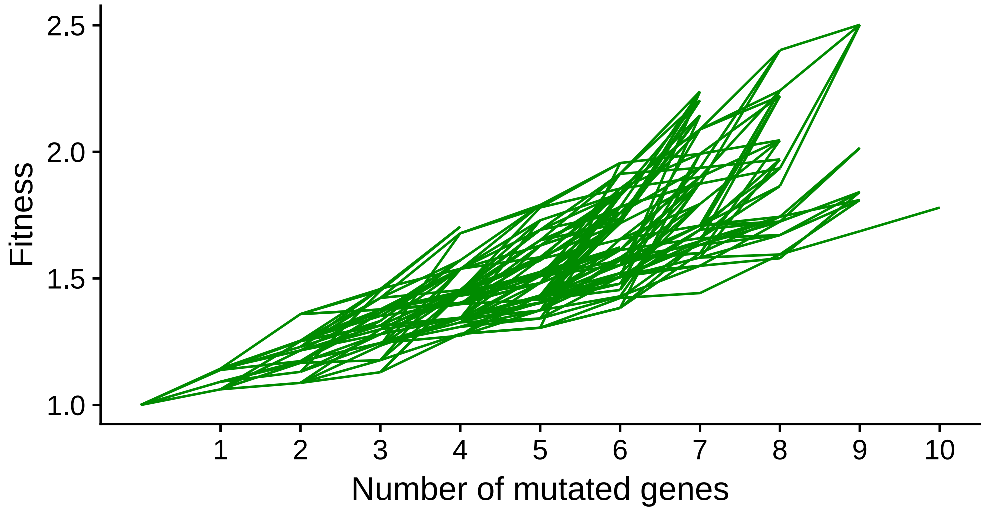

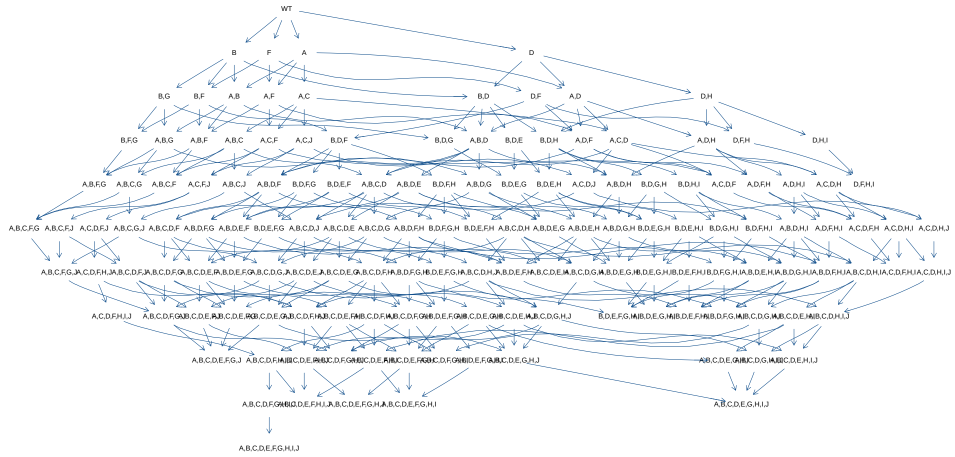

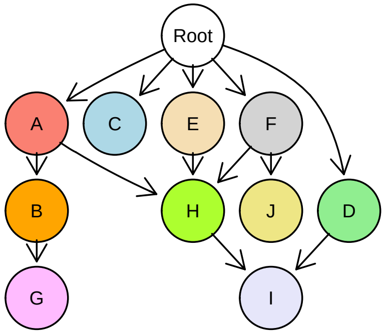

ID	k9iq8ysBwZDbv105Y
acc genotypes	132
peaks no back	12
removed edges	78
prop rem edges	0.201

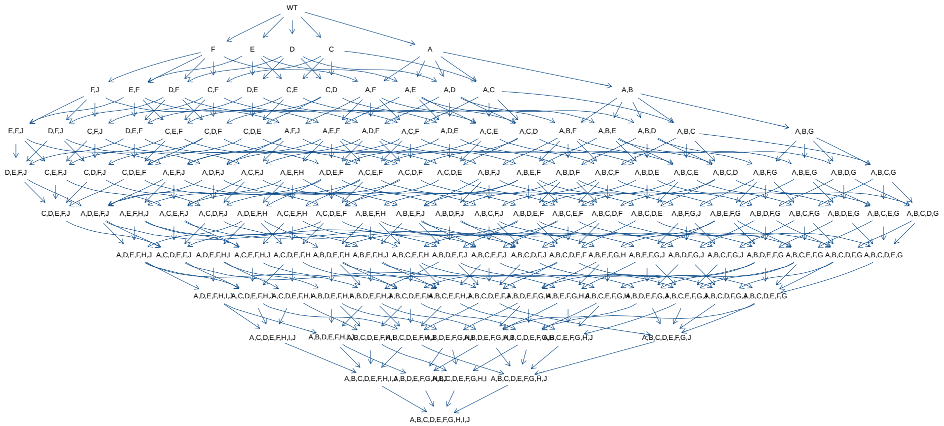

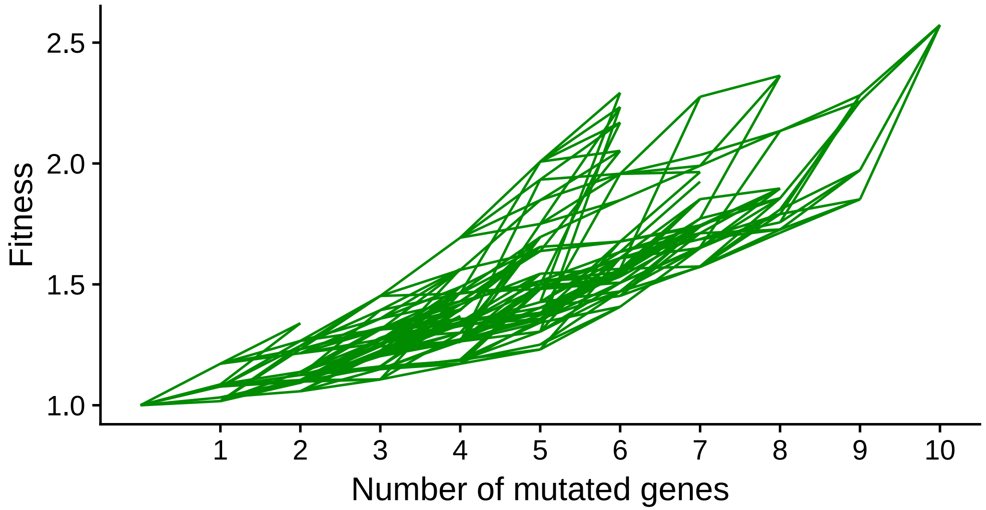

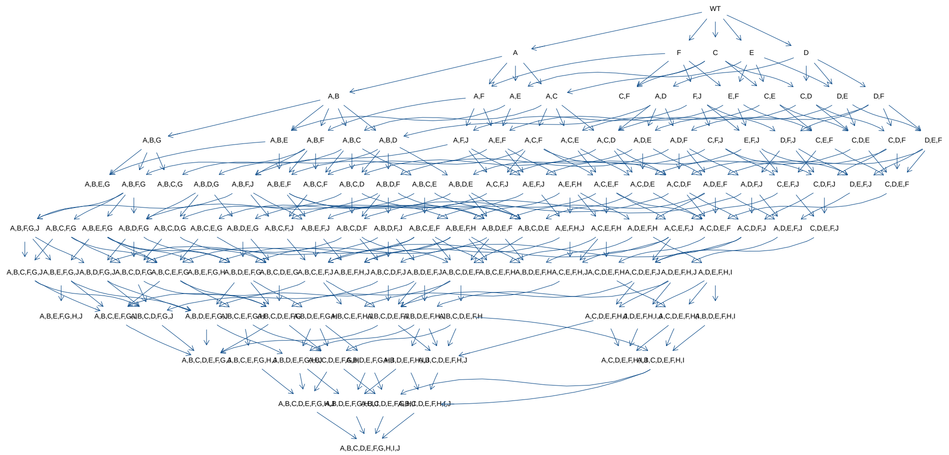

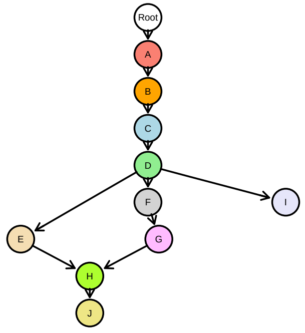

ID	KbbfhCf9UoSKy10IE
acc genotypes	20
peaks no back	2
removed edges	1
prop rem edges	0.033

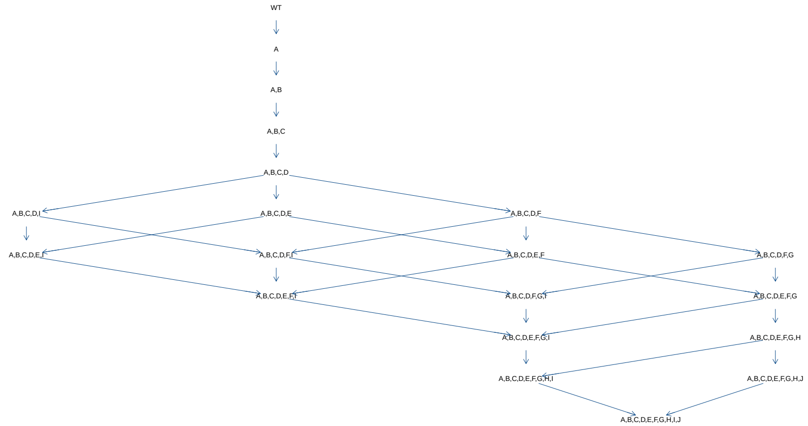

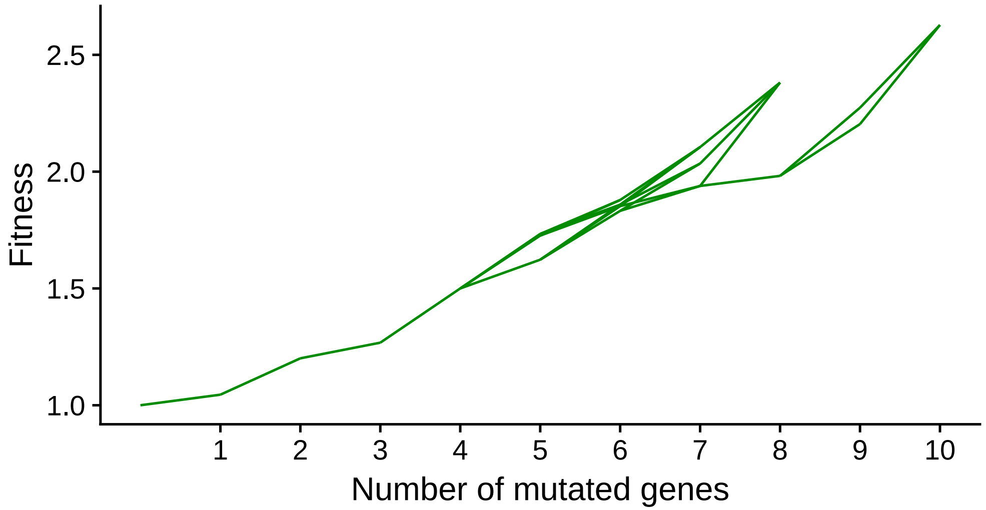

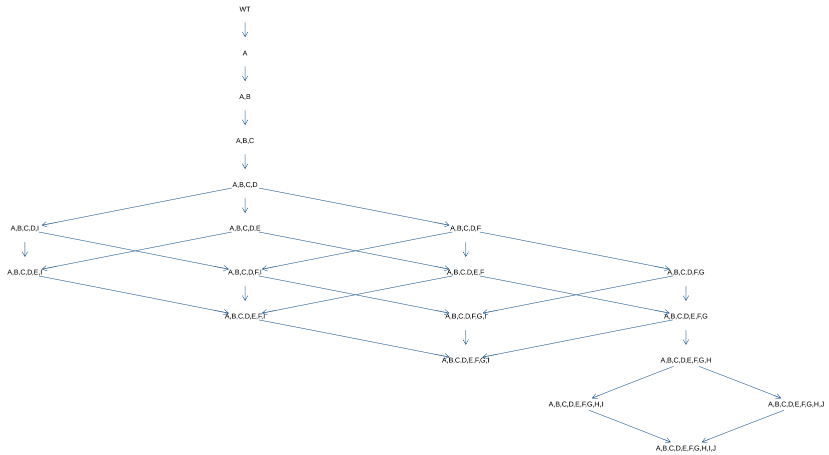

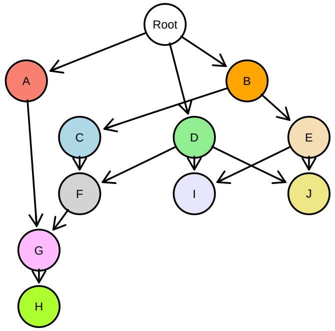

ID KEEVYiND4AYuzrNr
acc genotypes 52
peaks no back 7
removed edges 19
prop rem edges 0.168

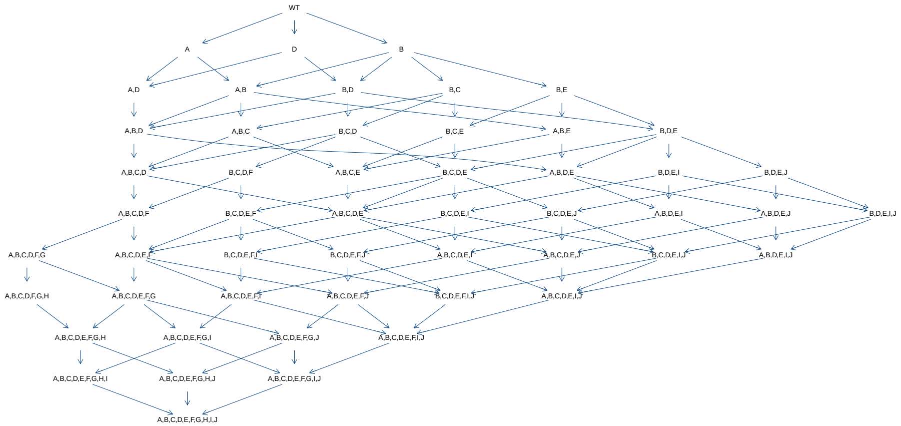

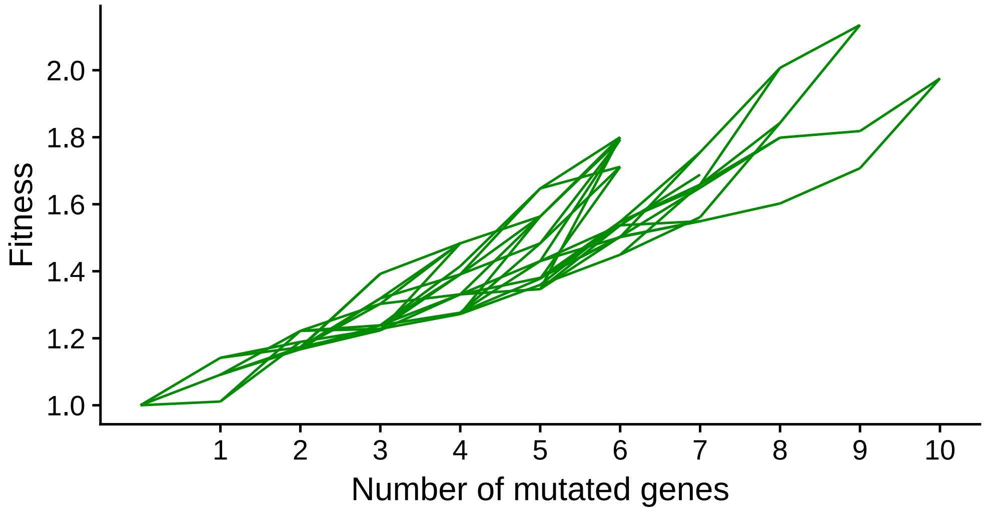

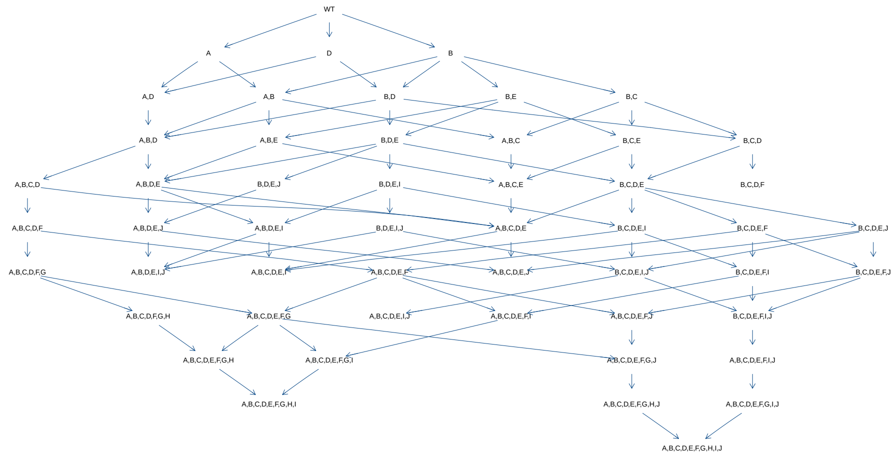

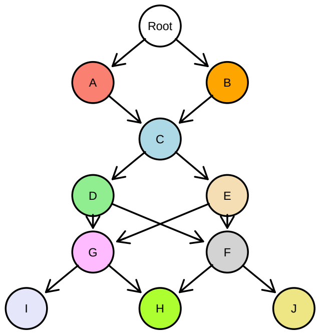

ID	KpASsdOSpsoQXAqF
acc genotypes	20
peaks no back	5
removed edges	5
prop rem edges	0.172

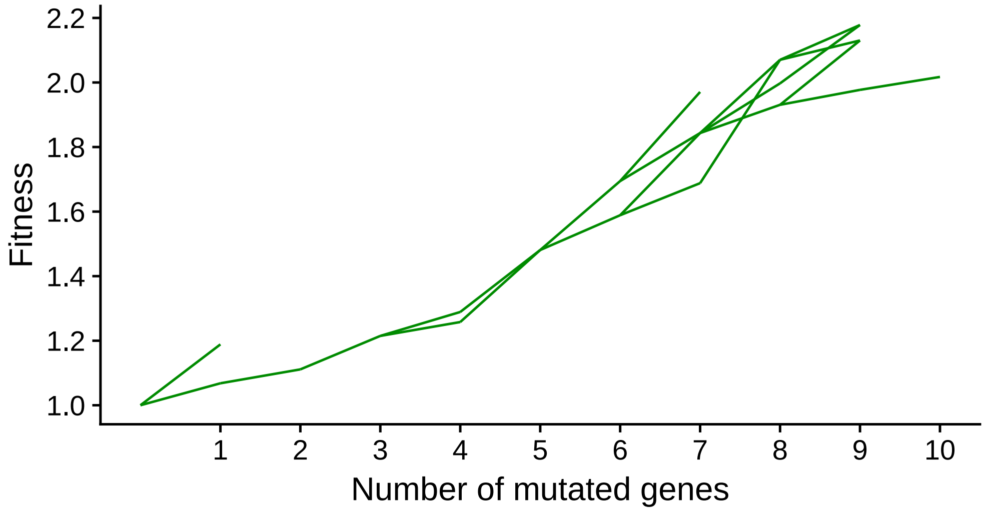

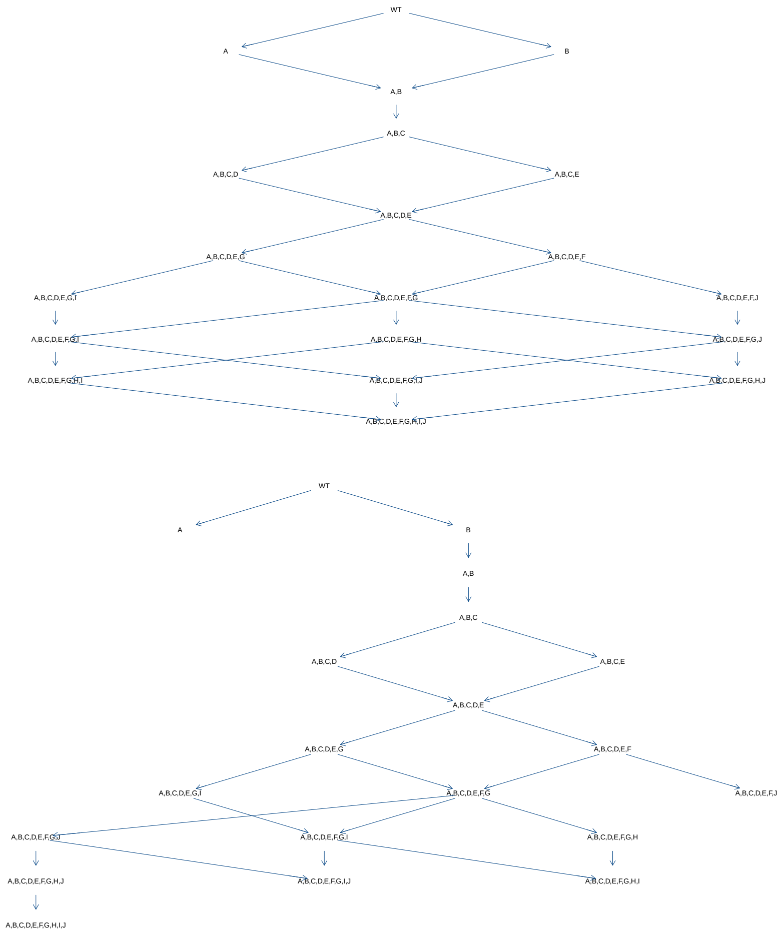

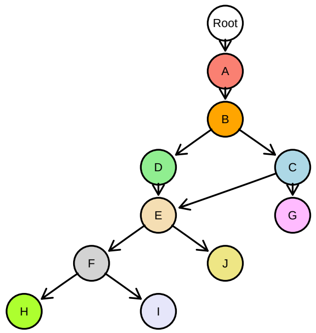

ID	KTPDesITE10I68VaZ
acc genotypes	28
peaks no back	5
removed edges	6
prop rem edges	0.118

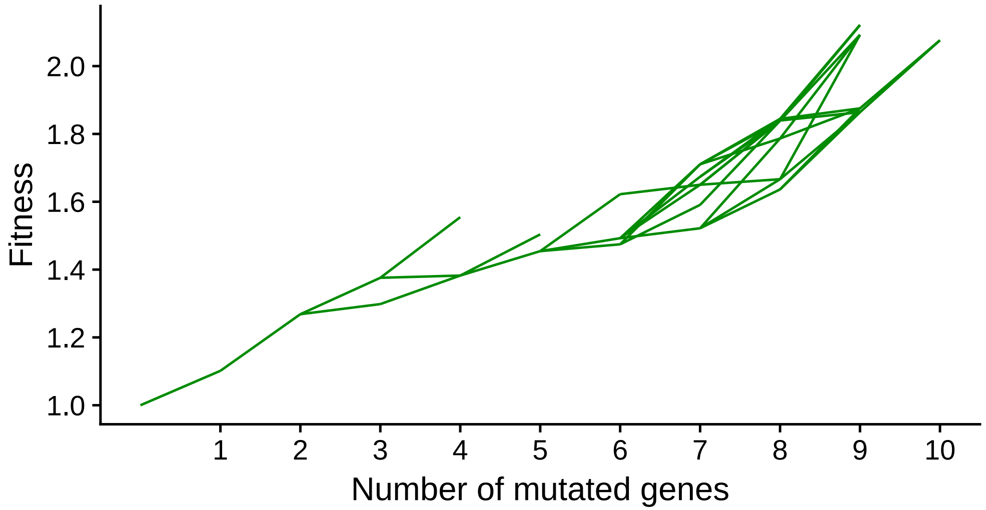

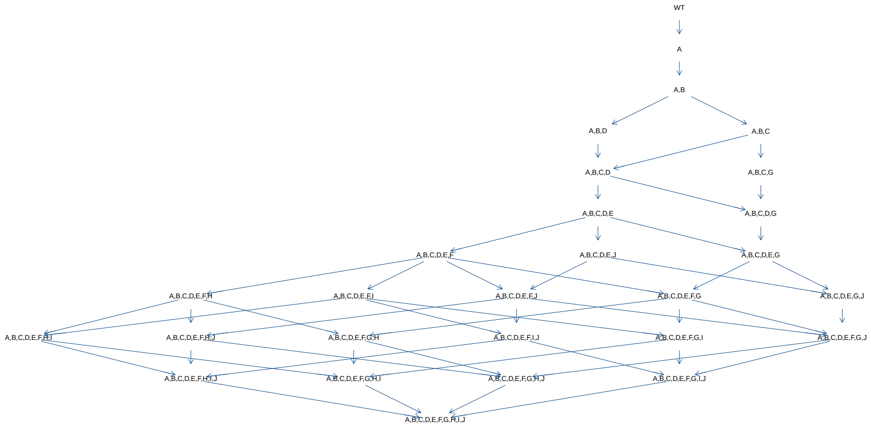

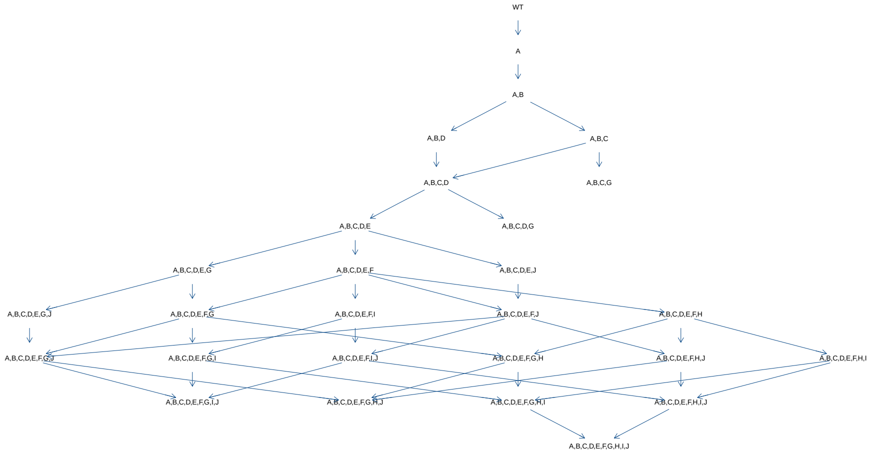

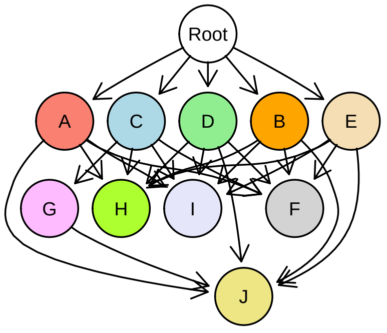

ID ku3OjGvU29I3Hlrg
acc genotypes 70
peaks no back 9
removed edges 32
prop rem edges 0.179

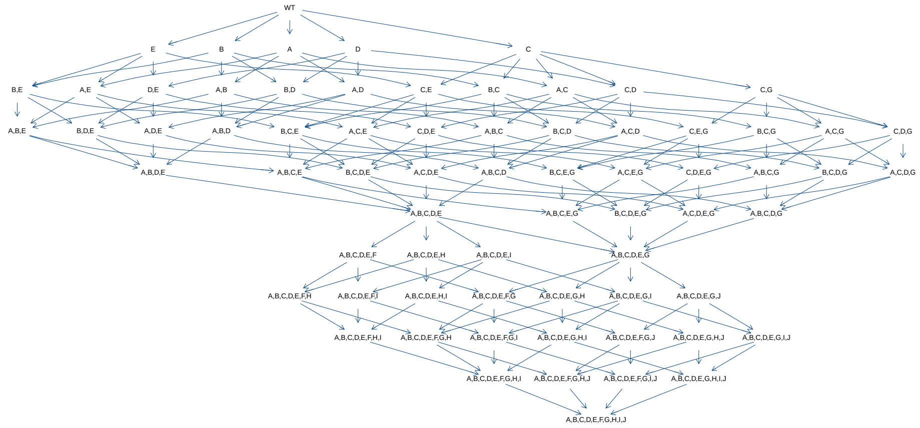

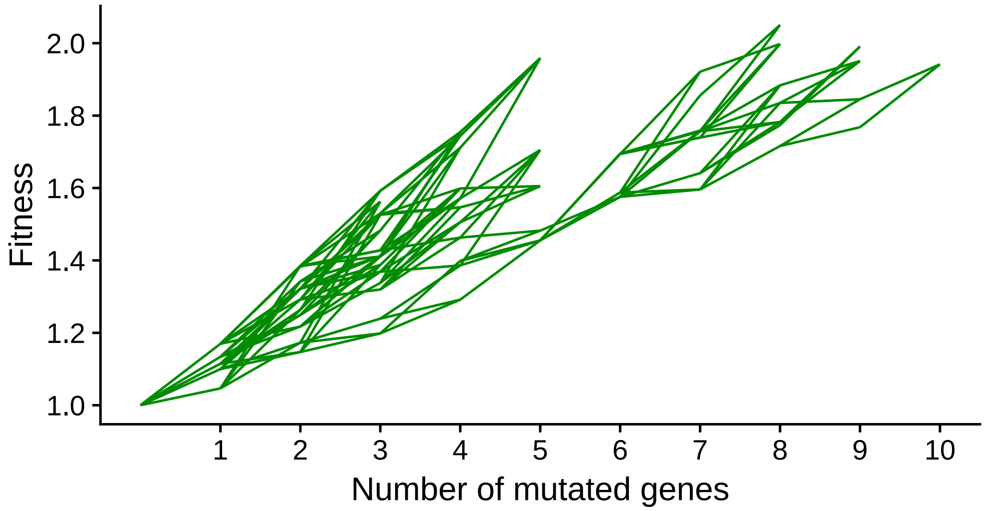

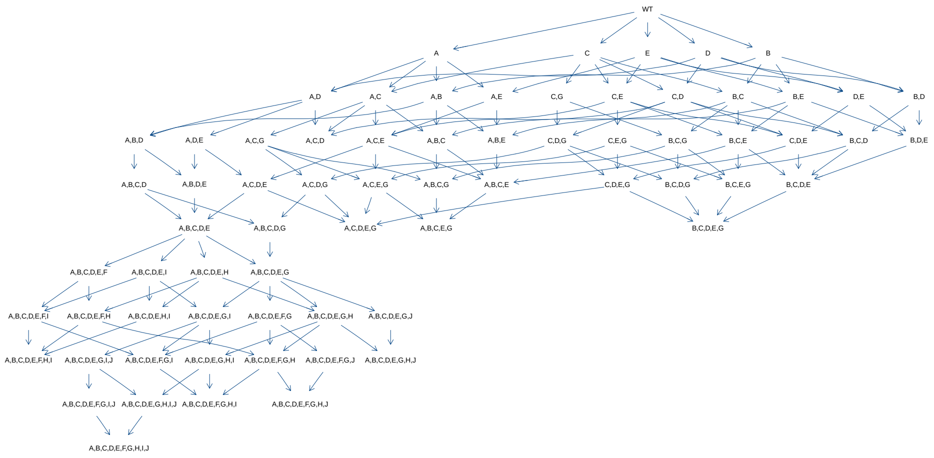

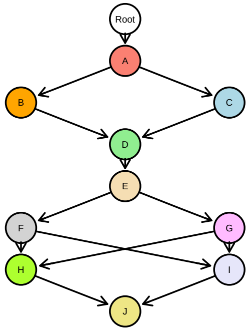

ID	kuiBBxD31RFF9NEj
acc genotypes	14
peaks no back	2
removed edges	1
prop rem edges	0.062

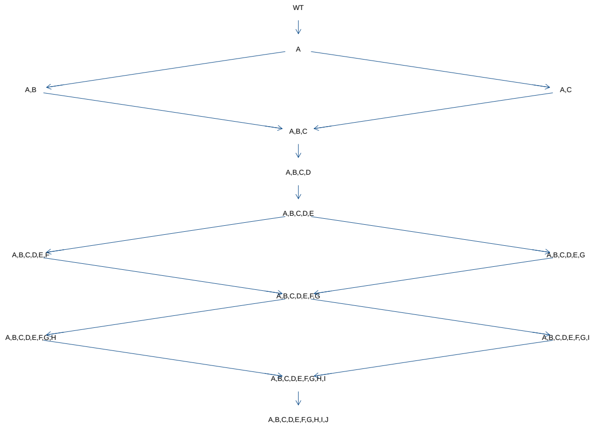

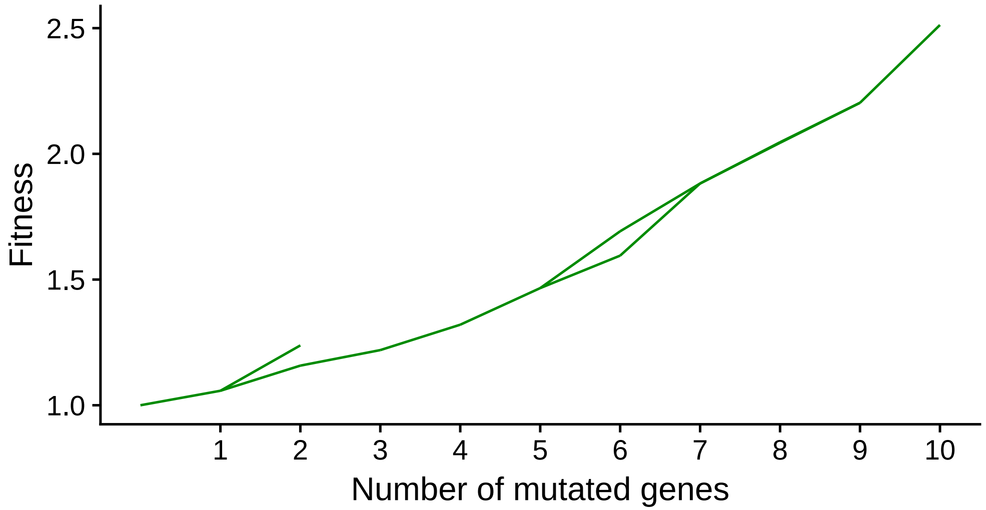

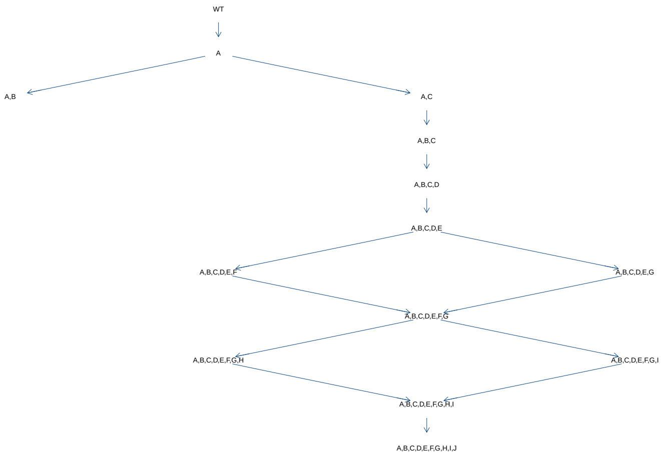

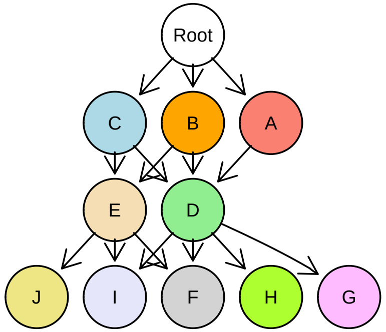

ID	kvFyL8XlKXwZvz1L
acc genotypes	48
peaks no back	9
removed edges	30
prop rem edges	0.275

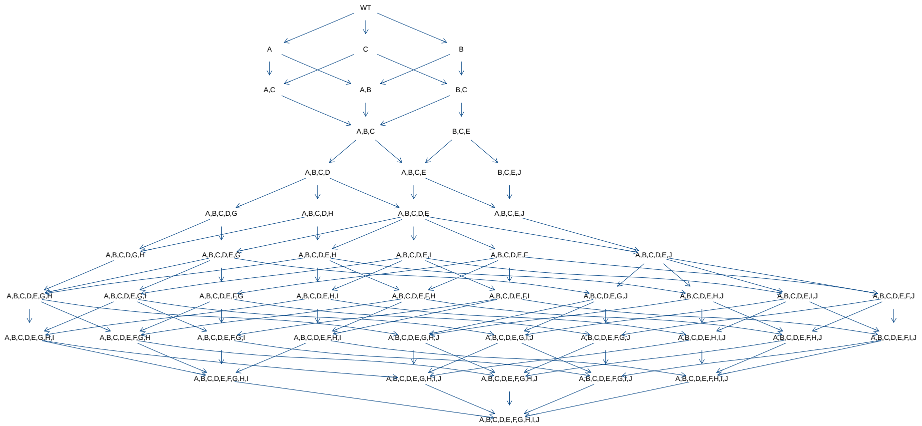

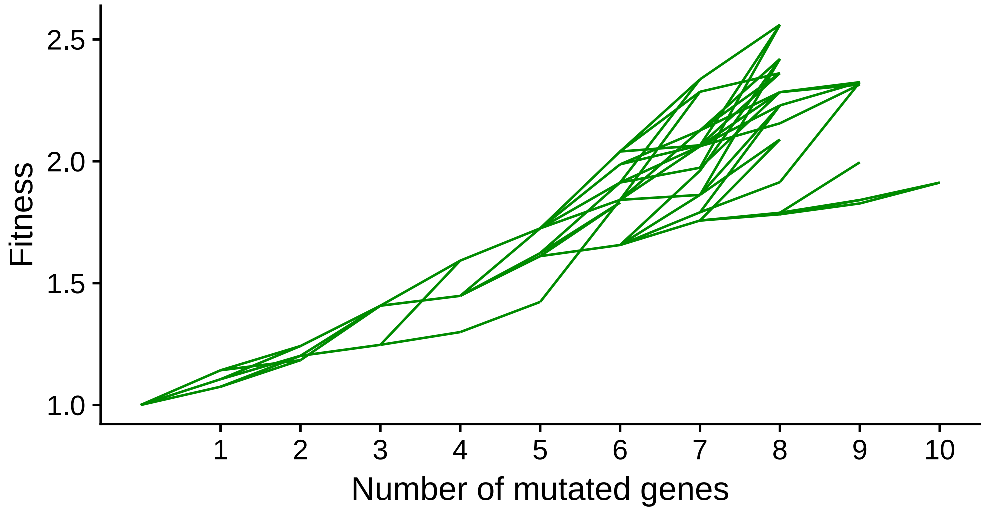

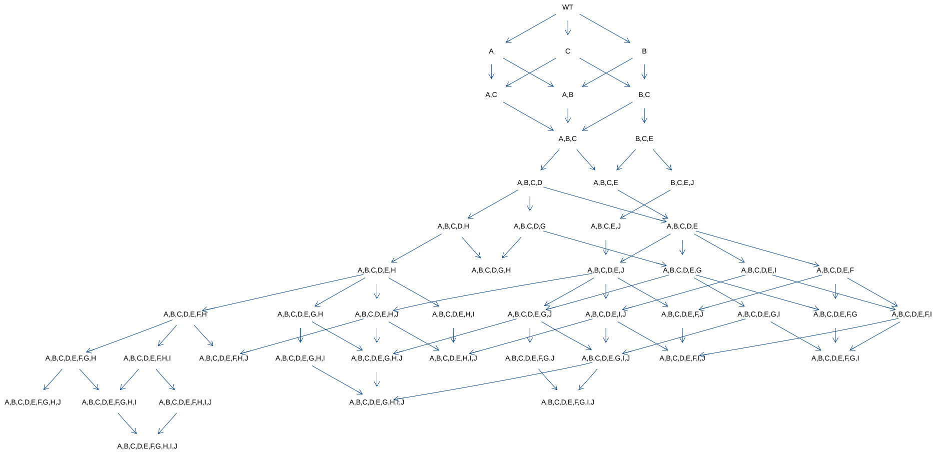

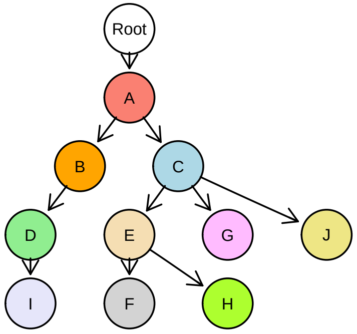

ID	l37sHoyzCWlv8at2
acc genotypes	85
peaks no back	10
removed edges	42
prop rem edges	0.184

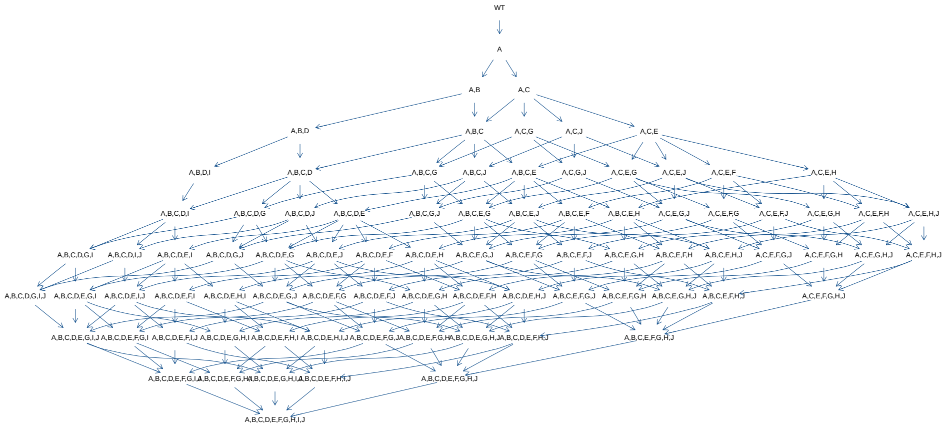

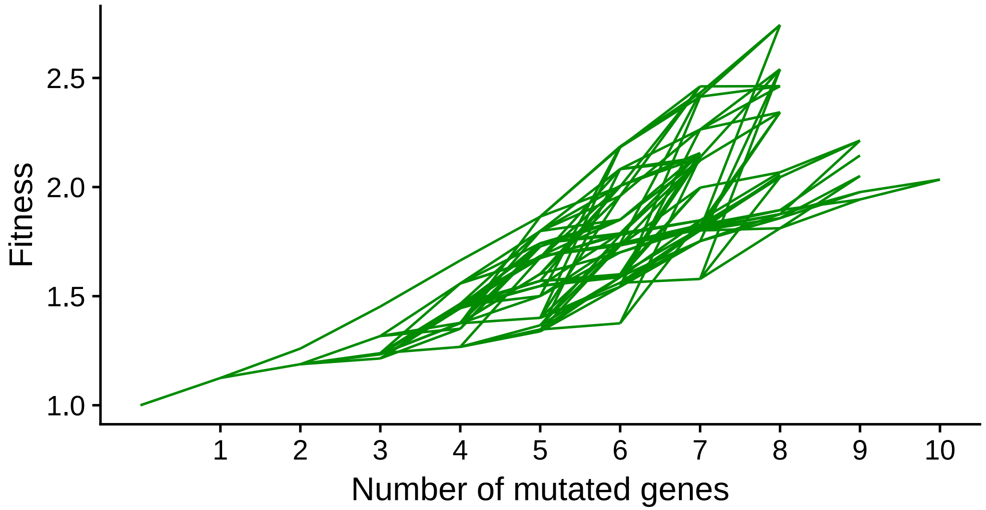

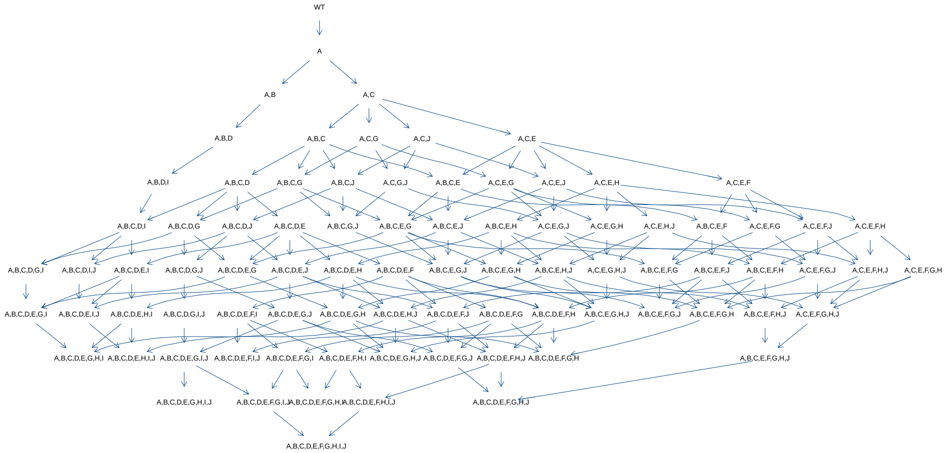

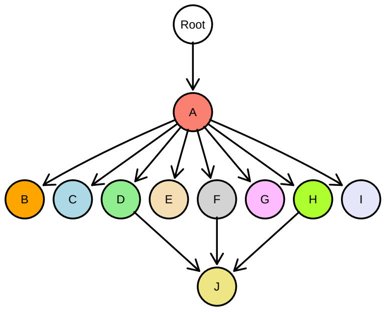

ID	lMrqdoPtFXSjearn
acc genotypes	289
peaks no back	21
removed edges	252
prop rem edges	0.222

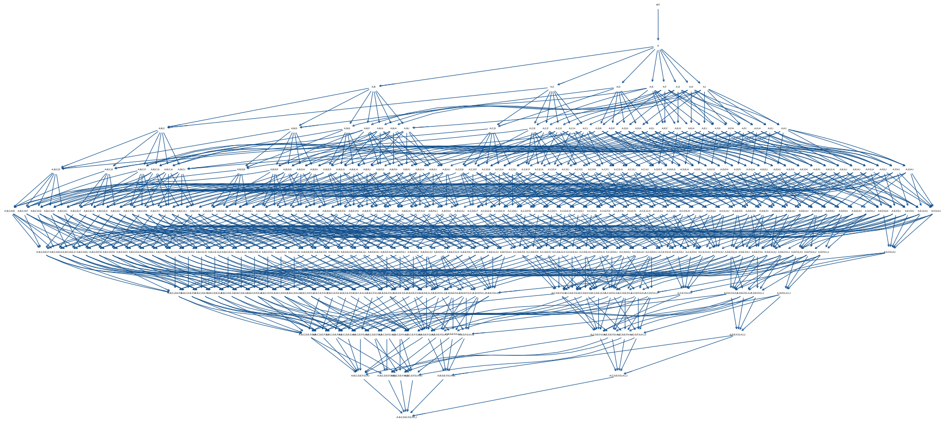

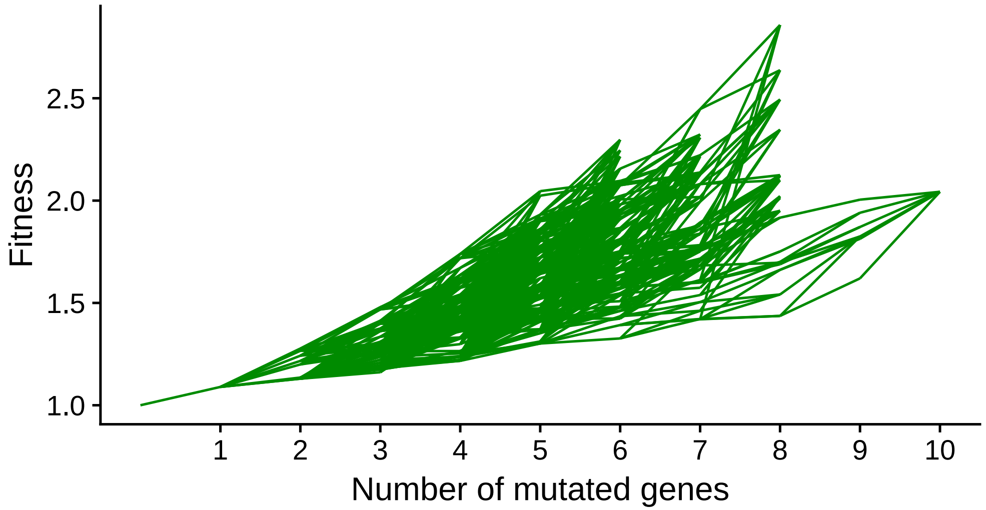

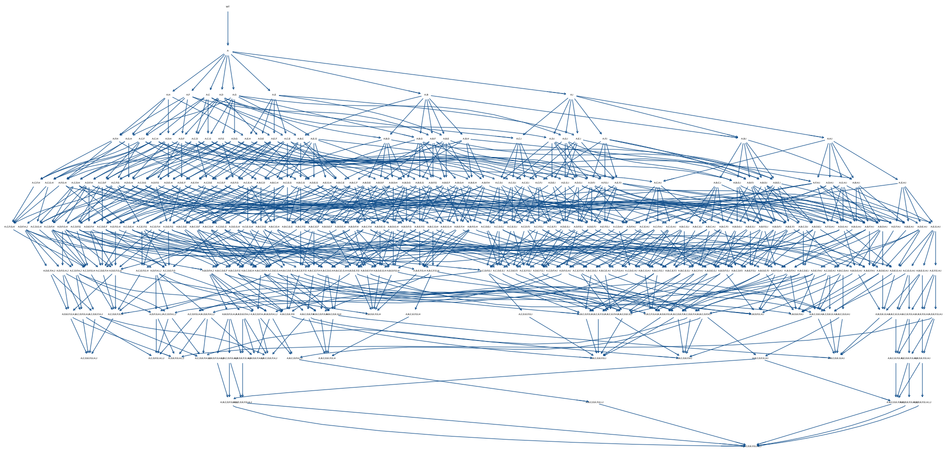

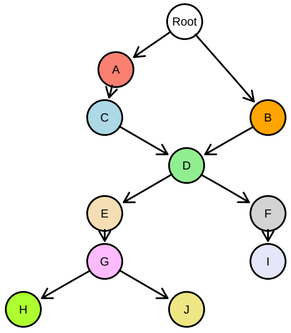

ID	LsTIGYZJWFqEXMCQ
acc genotypes	24
peaks no back	5
removed edges	6
prop rem edges	0.158

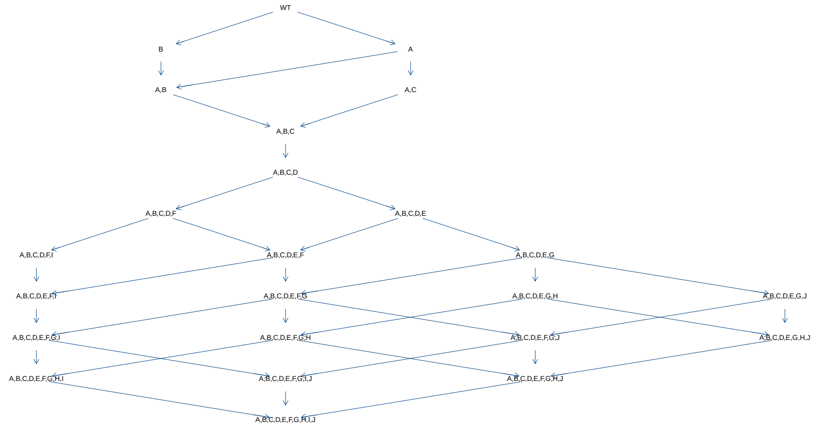

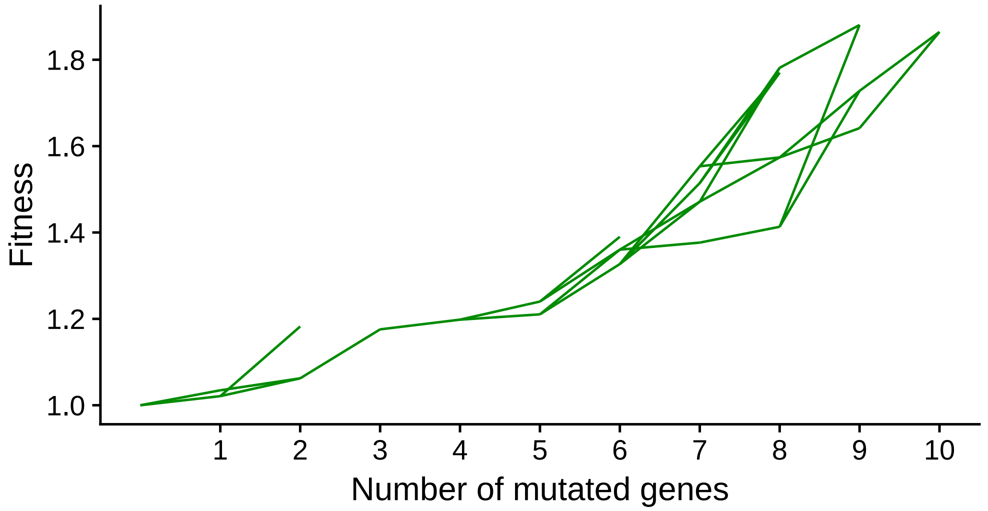

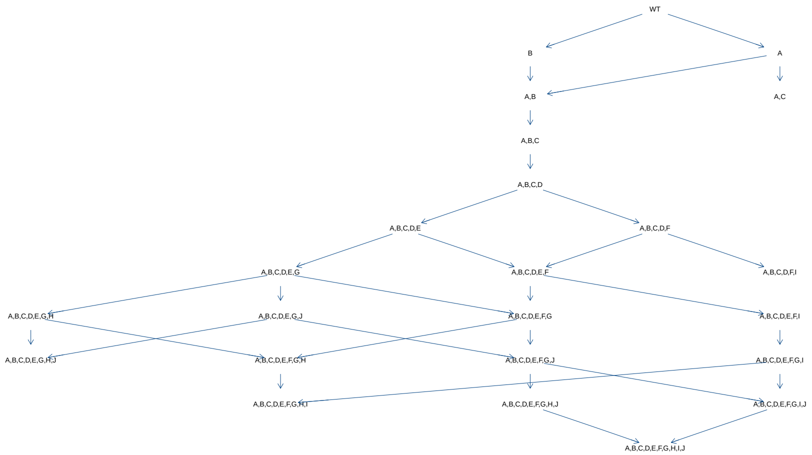

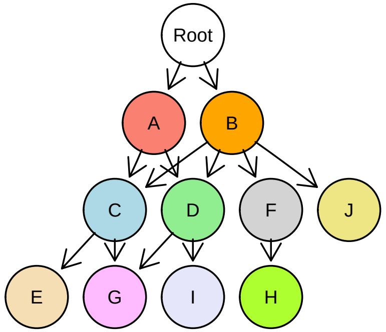

ID	LZ8ykaYM2CcUTFrI
acc genotypes	86
peaks no back	10
removed edges	44
prop rem edges	0.194

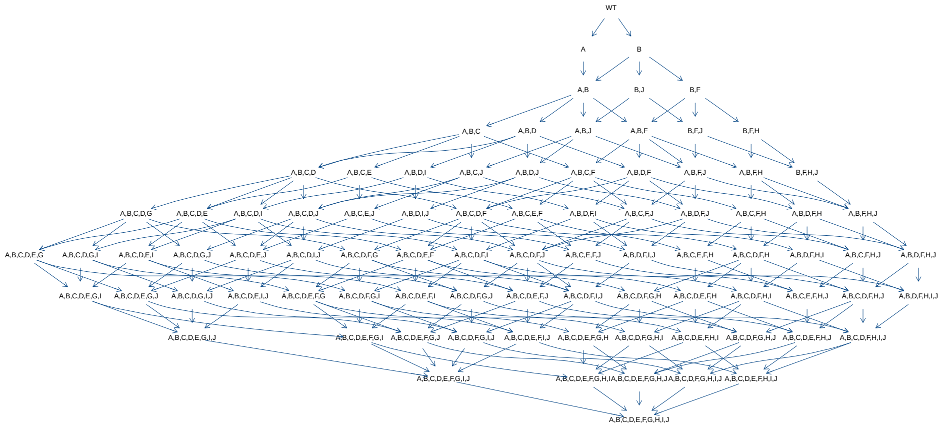

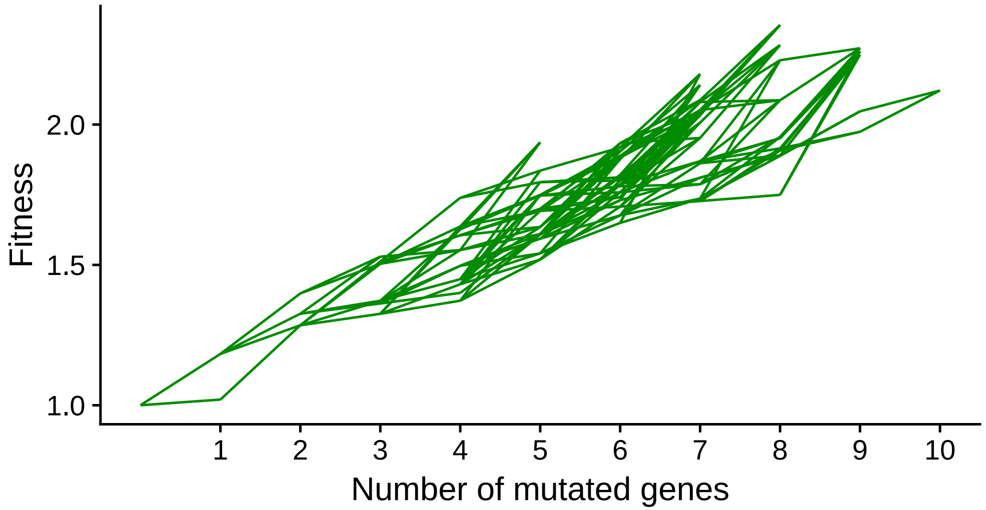

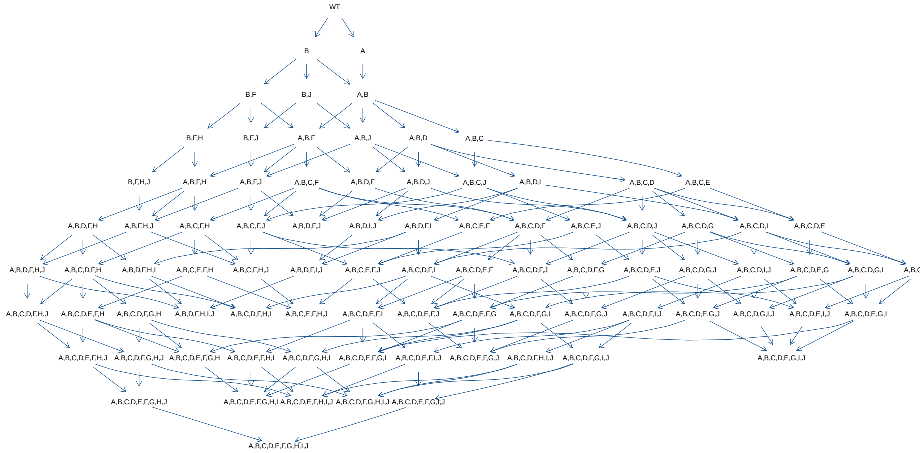

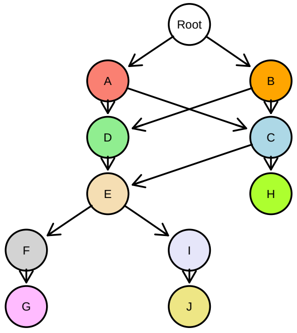

ID	m39LVsPQFf4Car10C
acc genotypes	27
peaks no back	4
removed edges	6
prop rem edges	0.13

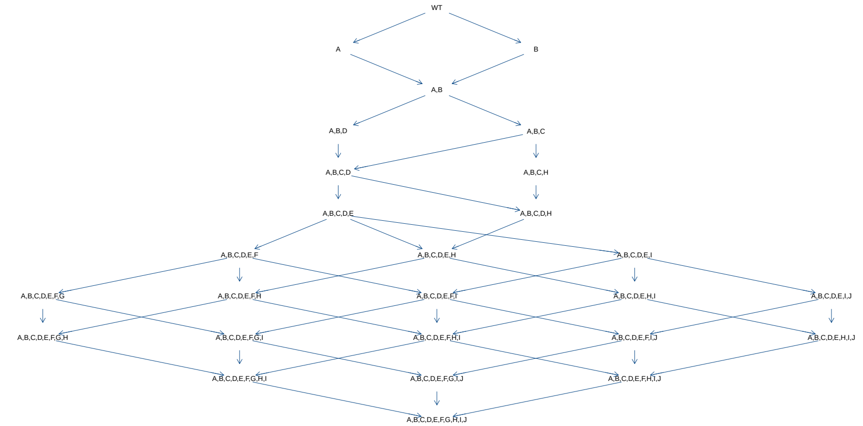

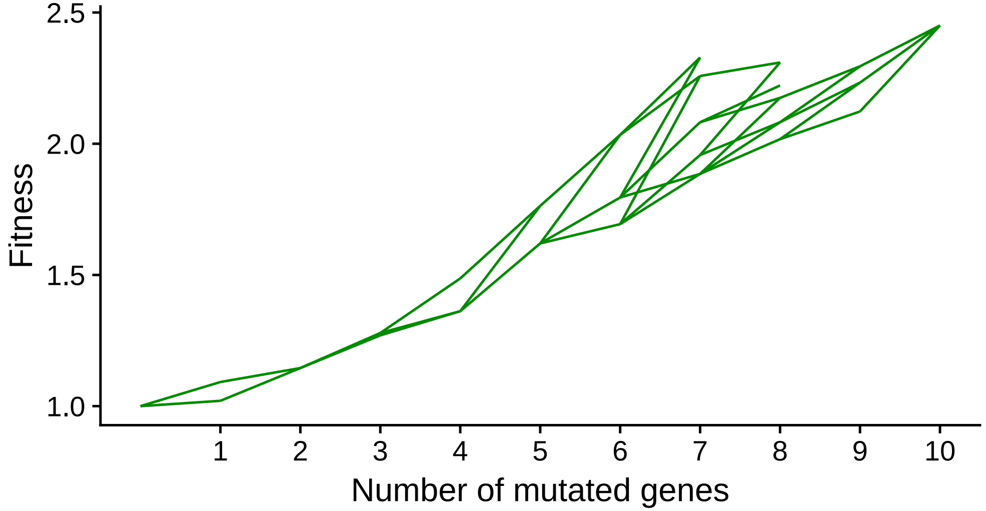

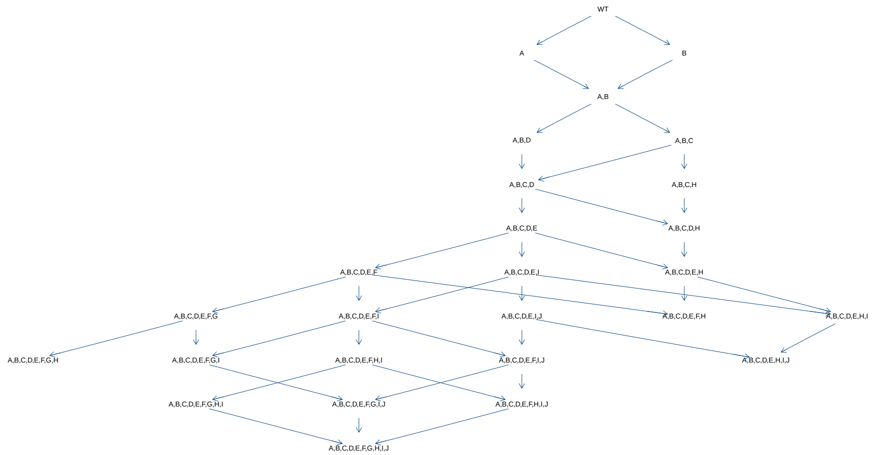

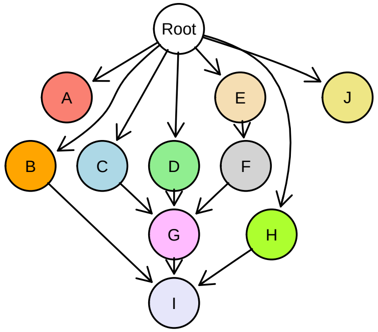

ID	M7TqwZkMb4FqiZxL
acc genotypes	212
peaks no back	18
removed edges	178
prop rem edges	0.234

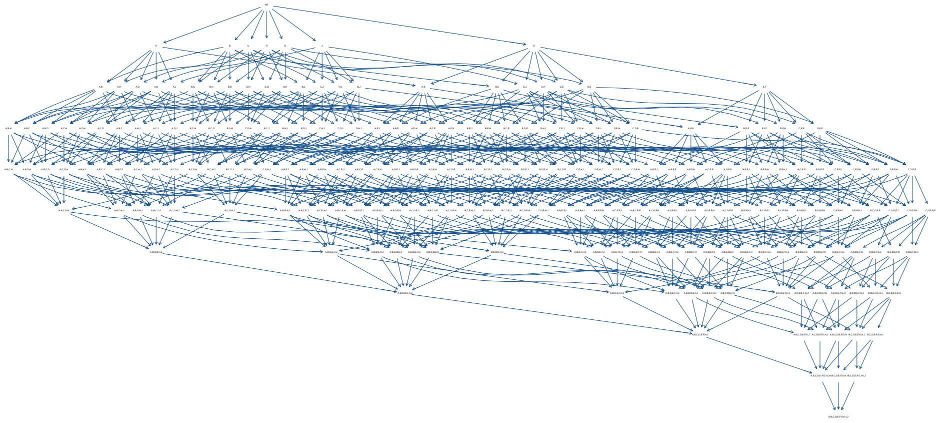

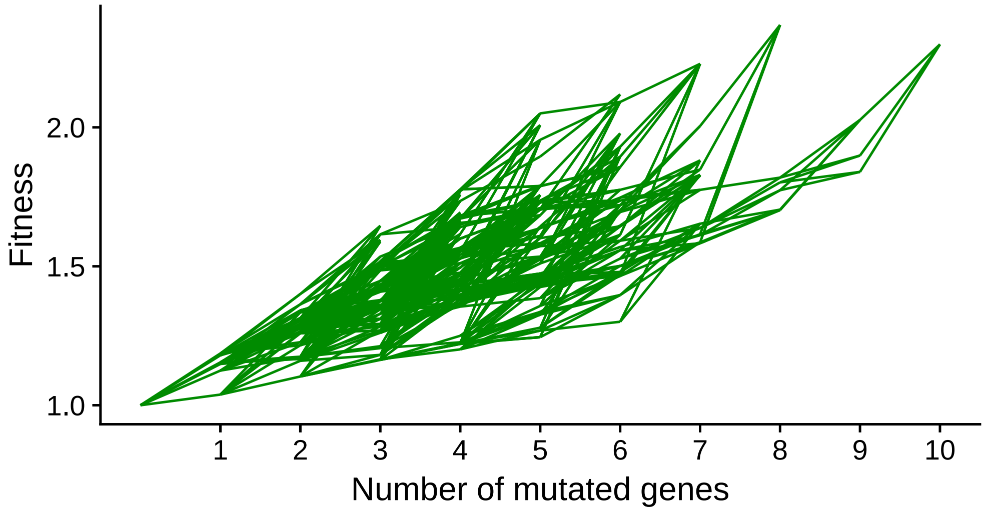

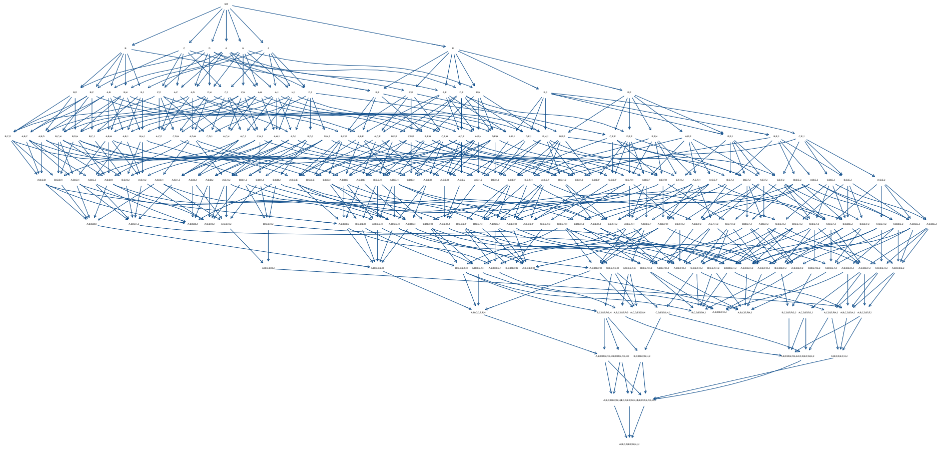

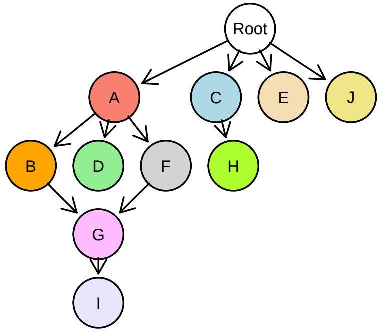

ID	MbPTZwyHjCzW5F3T
acc genotypes	156
peaks no back	12
removed edges	87
prop rem edges	0.178

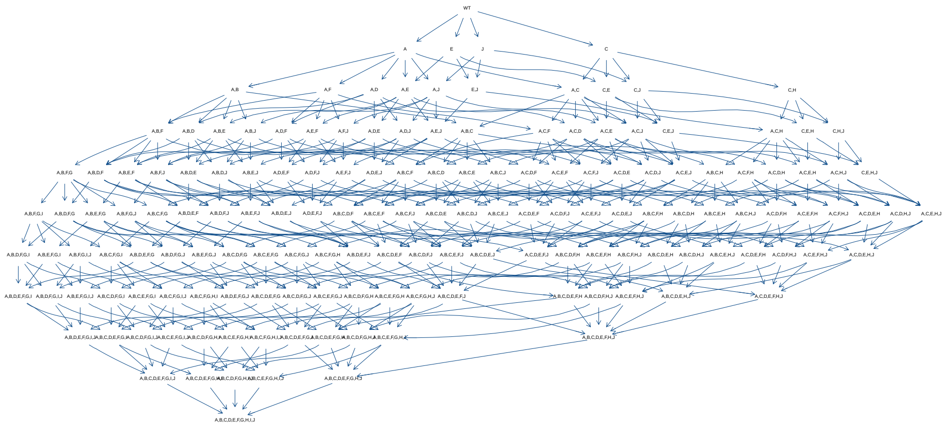

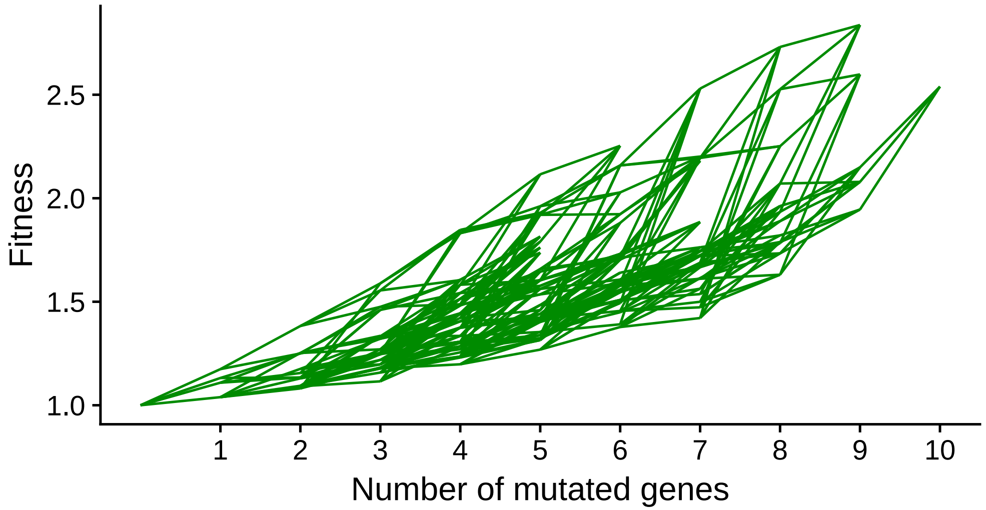

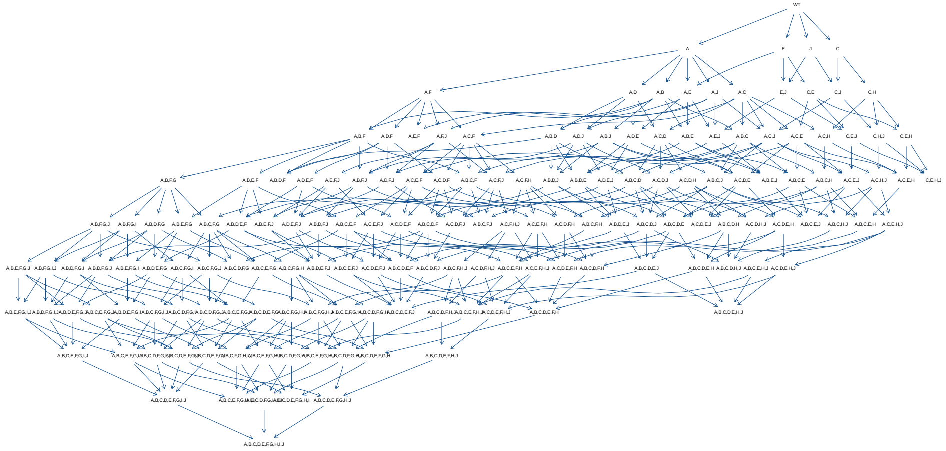

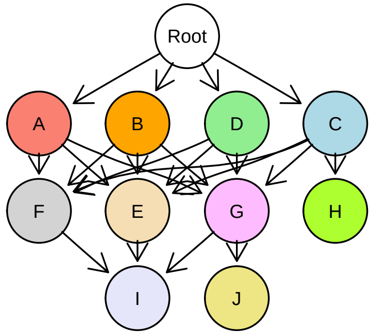

ID	MEBeweDPZbszojRQ
acc genotypes	50
peaks no back	9
removed edges	20
prop rem edges	0.18

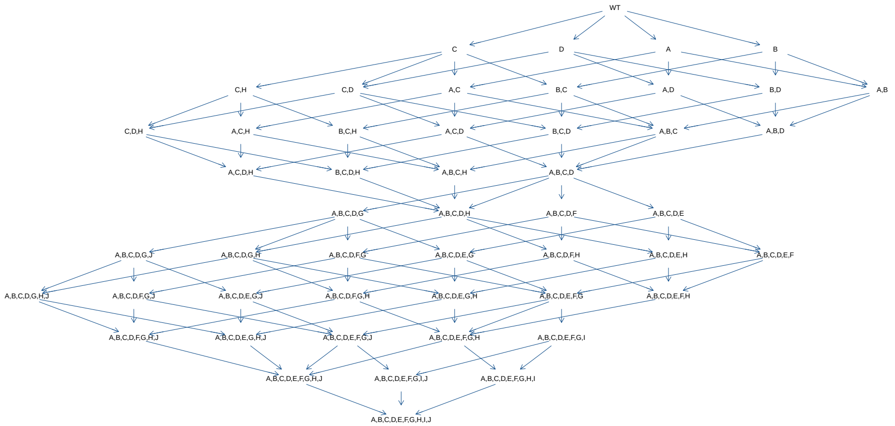

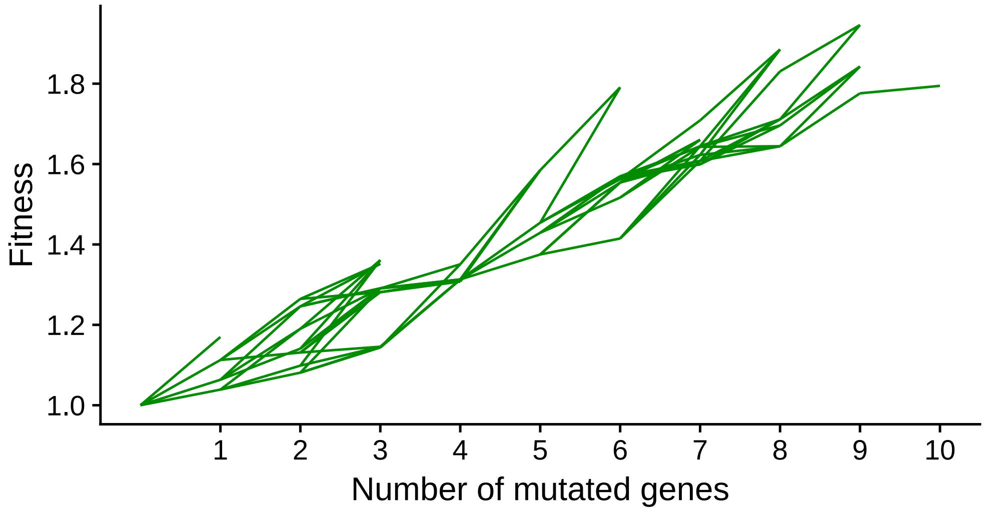

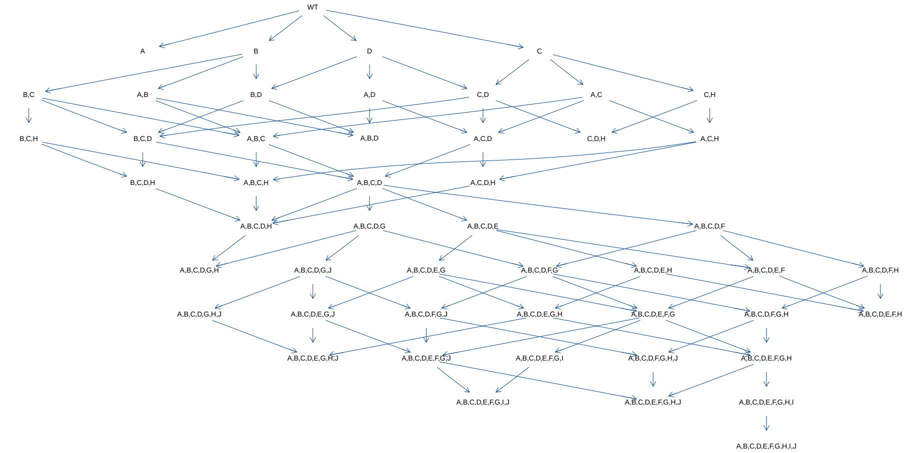

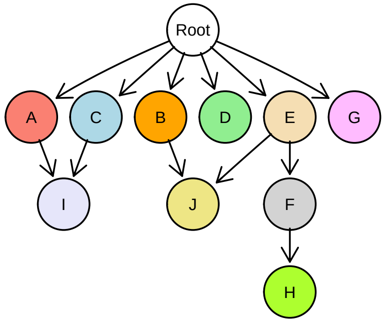

ID	MeQOZBLaWrCfm8Ns
acc genotypes	220
peaks no back	19
removed edges	135
prop rem edges	0.182

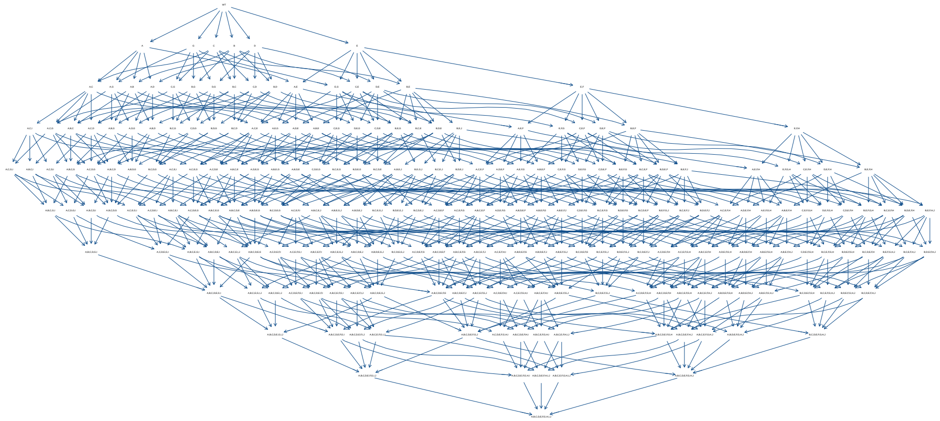

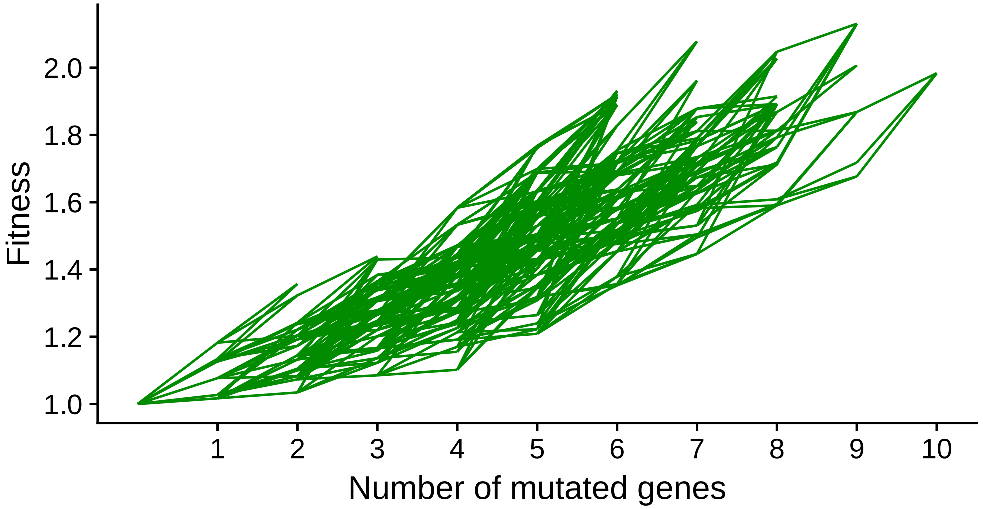

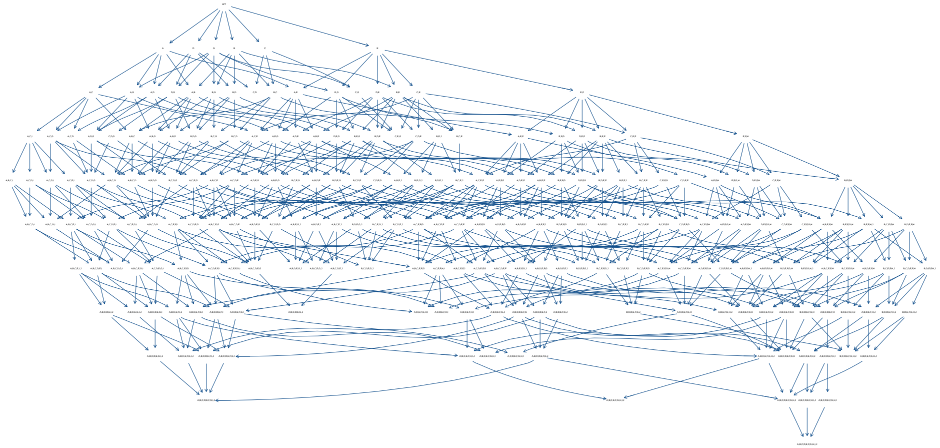

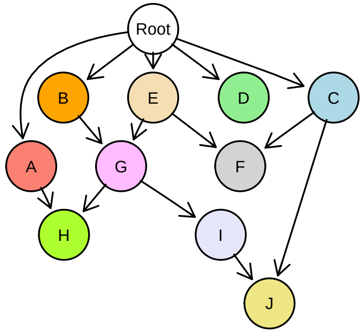

ID mHOgzGLqndvBGgxt

acc genotypes 88

peaks no back 10

removed edges 35

prop rem edges 0.154

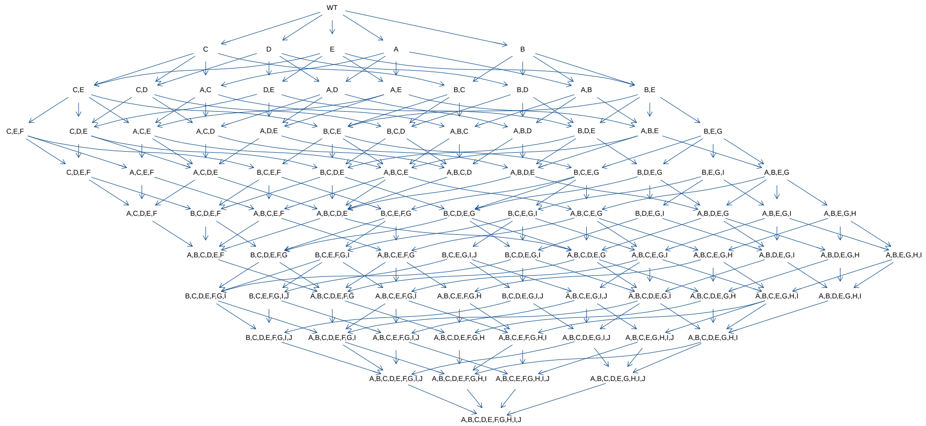

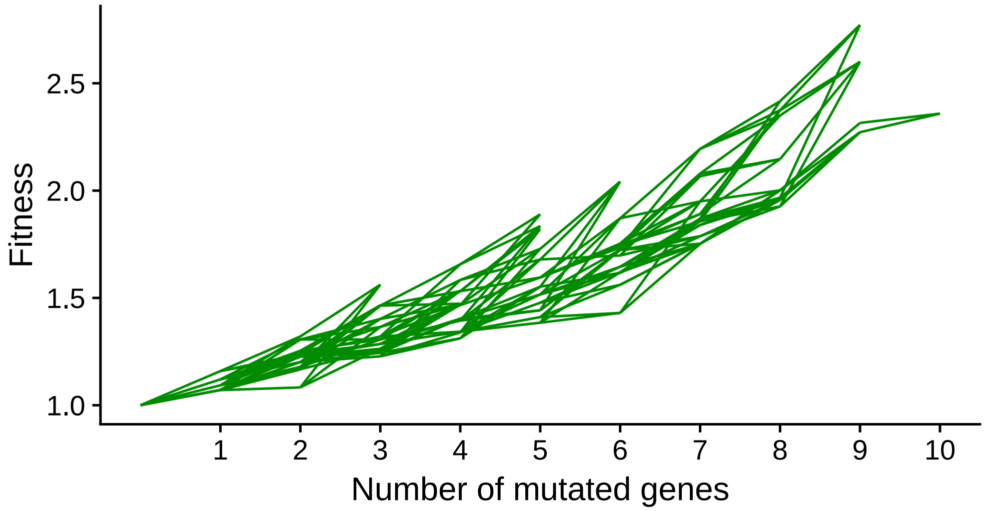

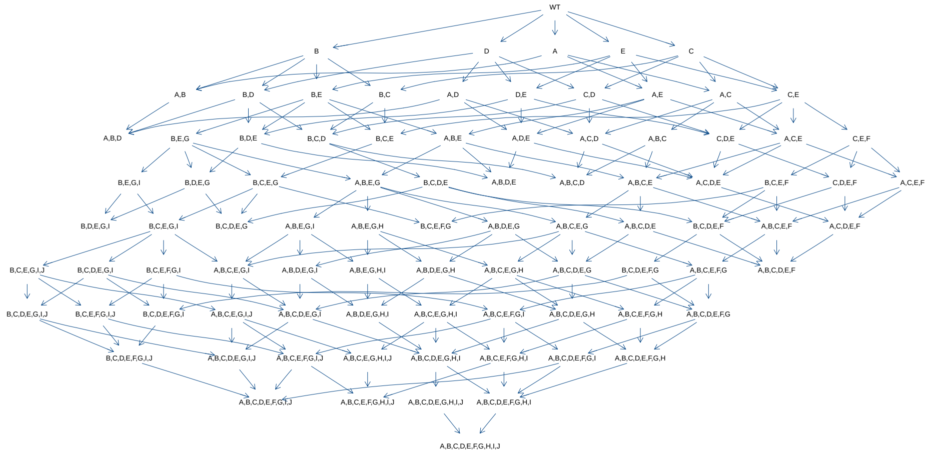

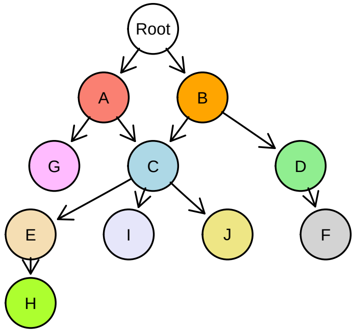

ID	mHXf10tSNigHlcwWO
acc genotypes	84
peaks no back	9
removed edges	44
prop rem edges	0.194

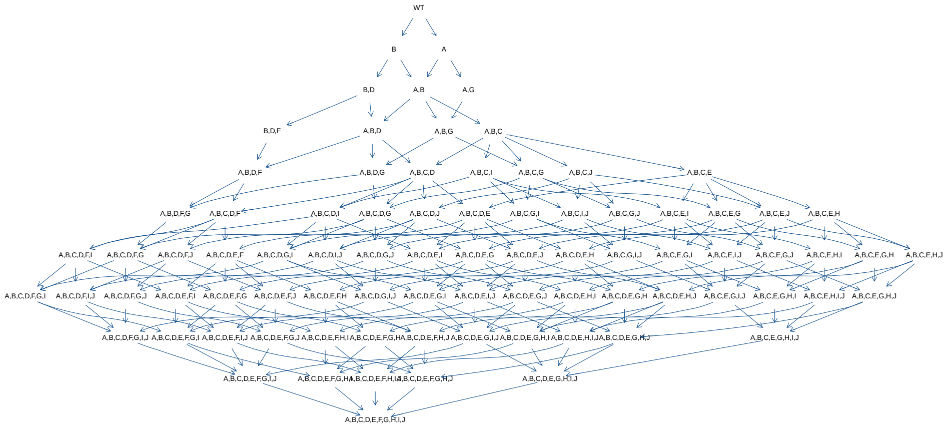

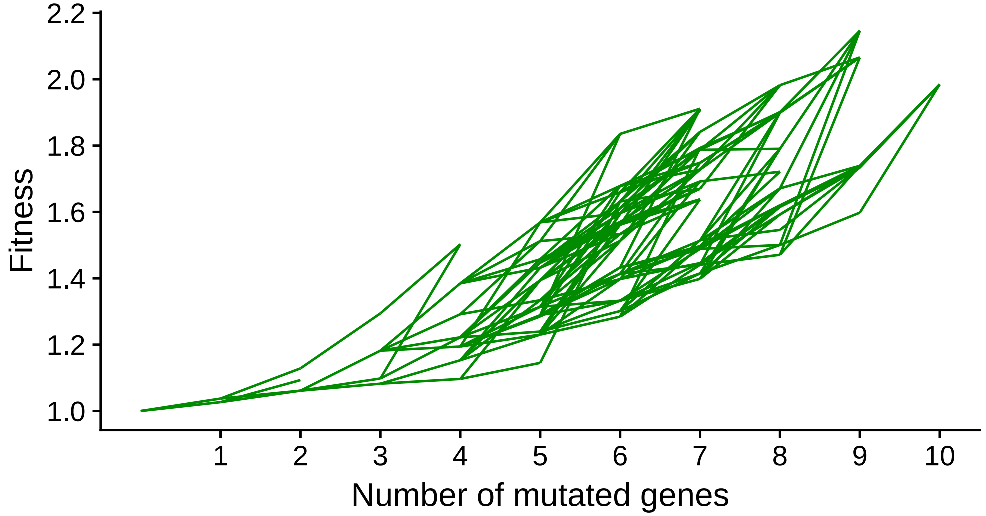

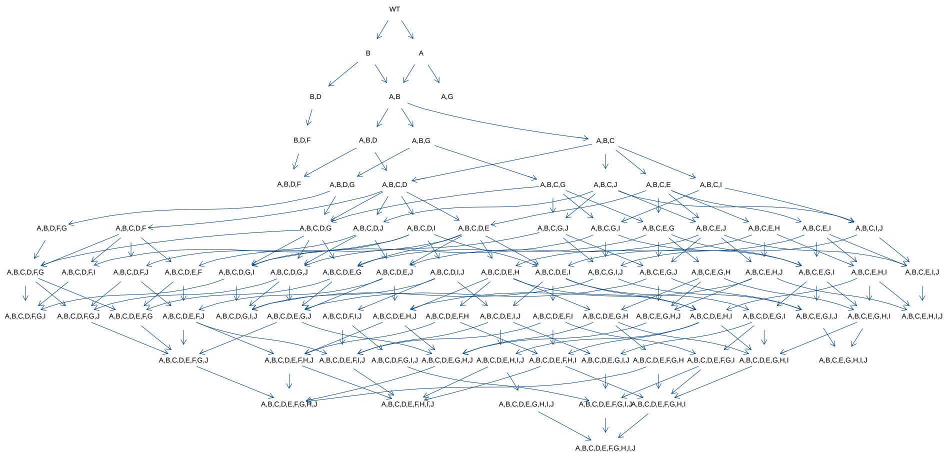

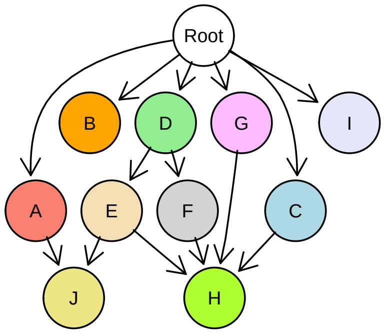

ID	MW1p5m710opywel6d
acc genotypes	204
peaks no back	15
removed edges	126
prop rem edges	0.179

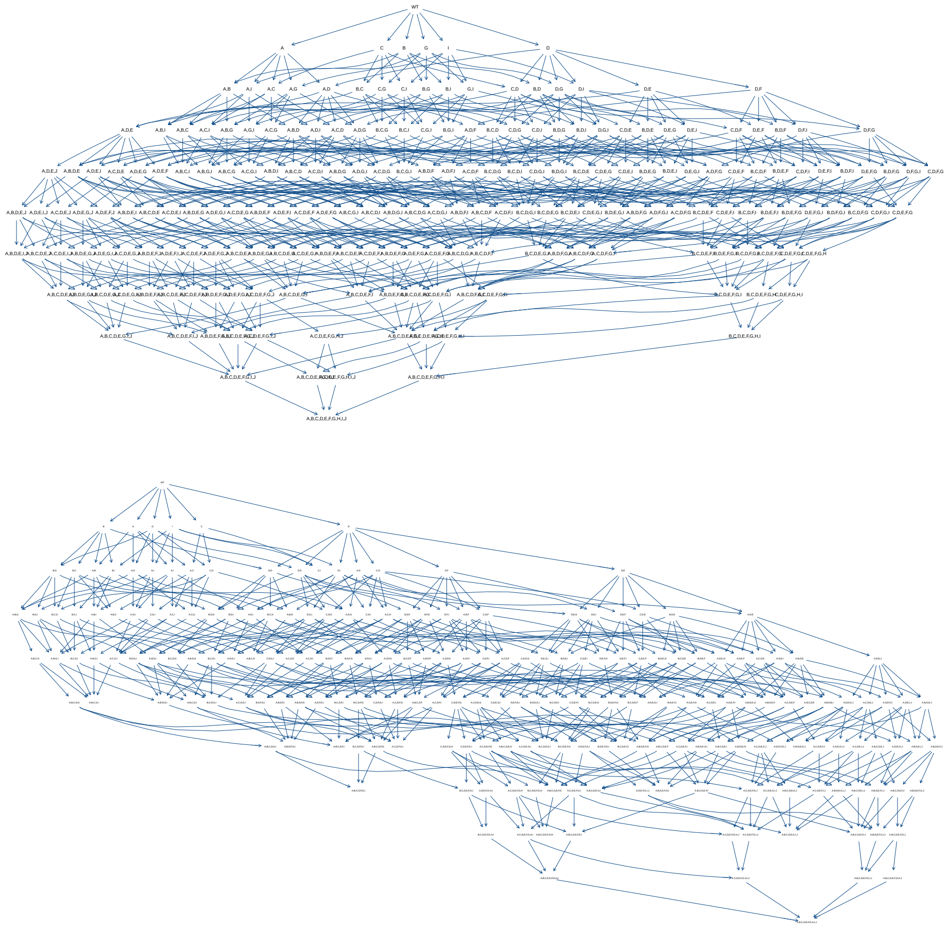

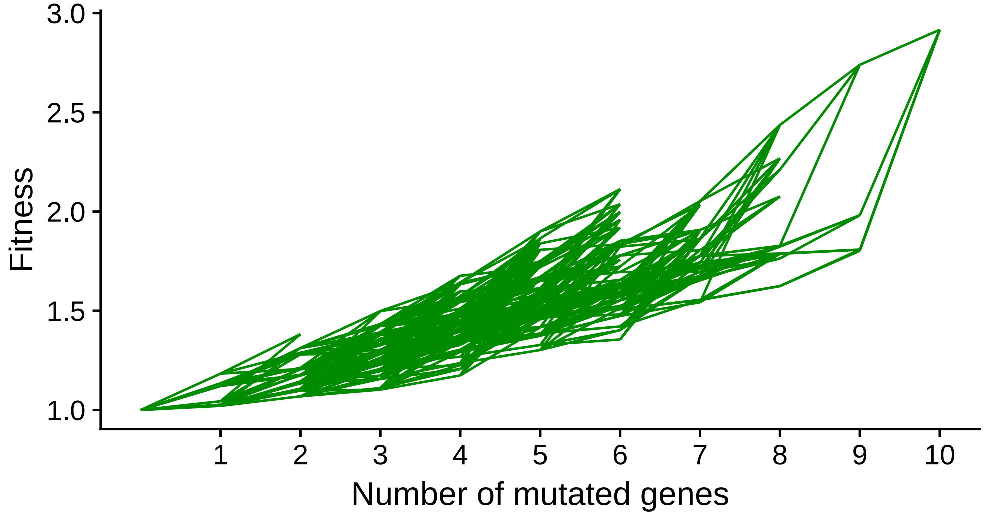

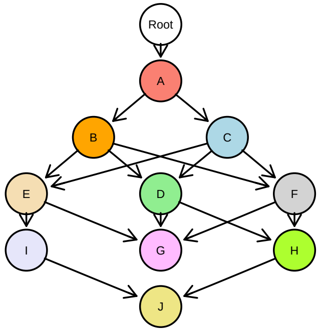

ID	N3AGZj5WtBq10w7rq
acc genotypes	25
peaks no back	5
removed edges	6
prop rem edges	0.146

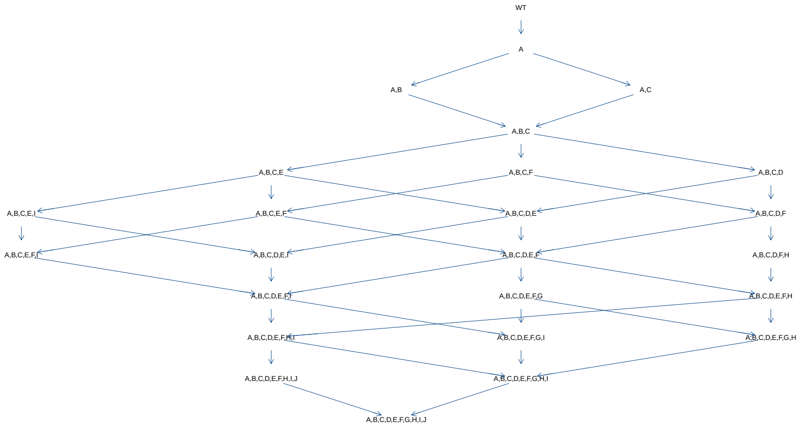

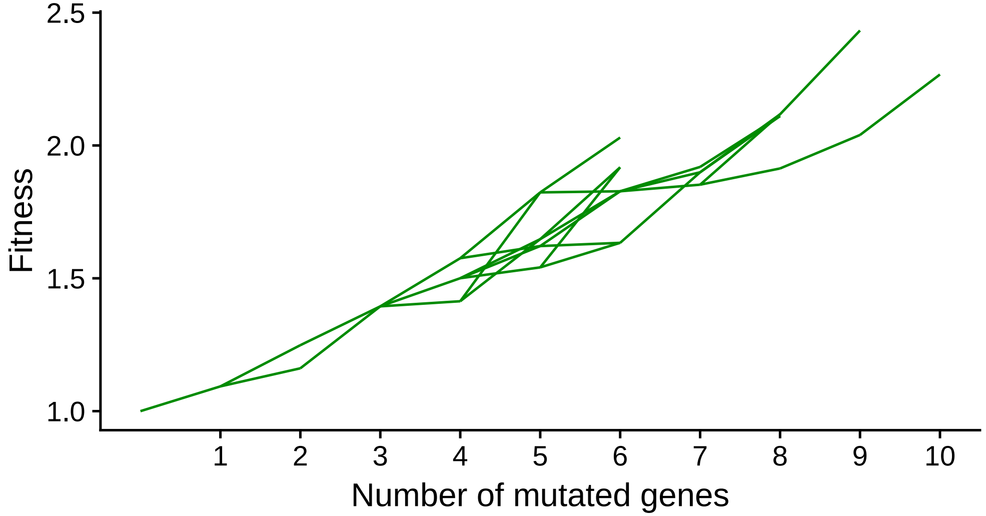

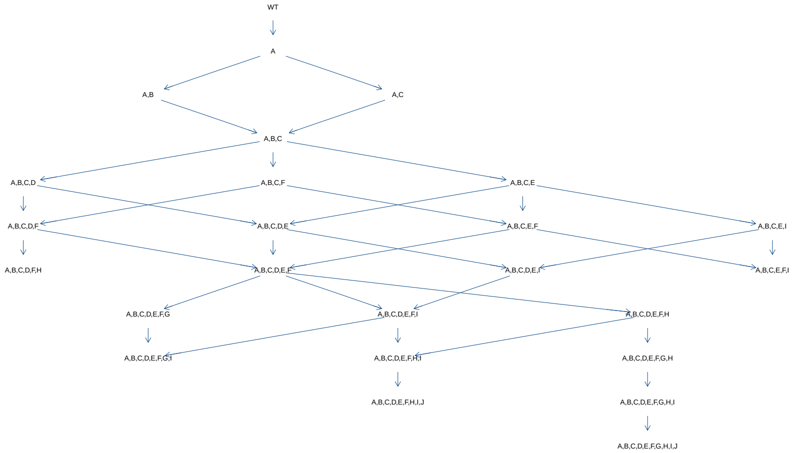

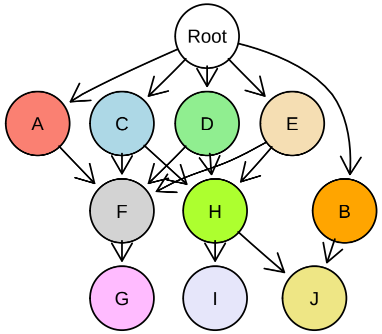

ID	n3mpftu4orkSLBLy
acc genotypes	60
peaks no back	10
removed edges	25
prop rem edges	0.179

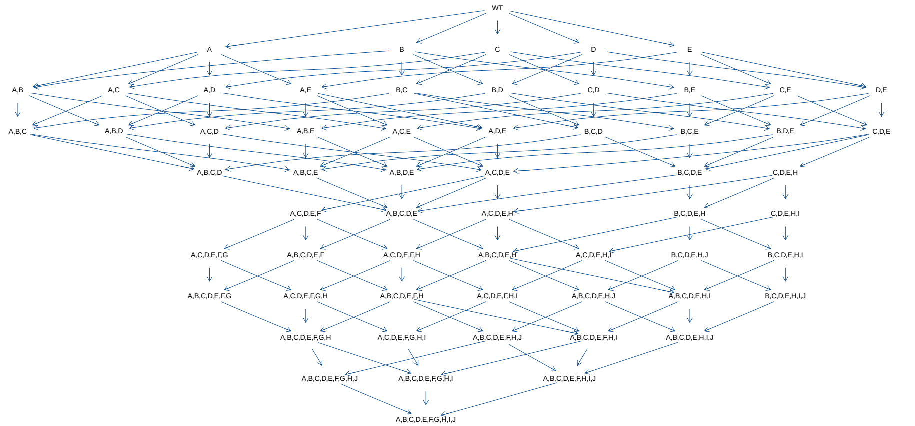

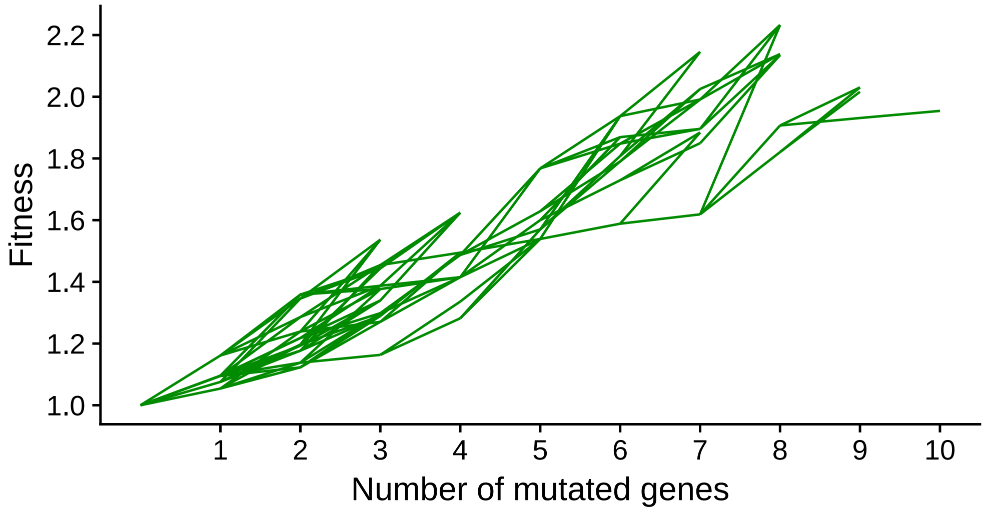

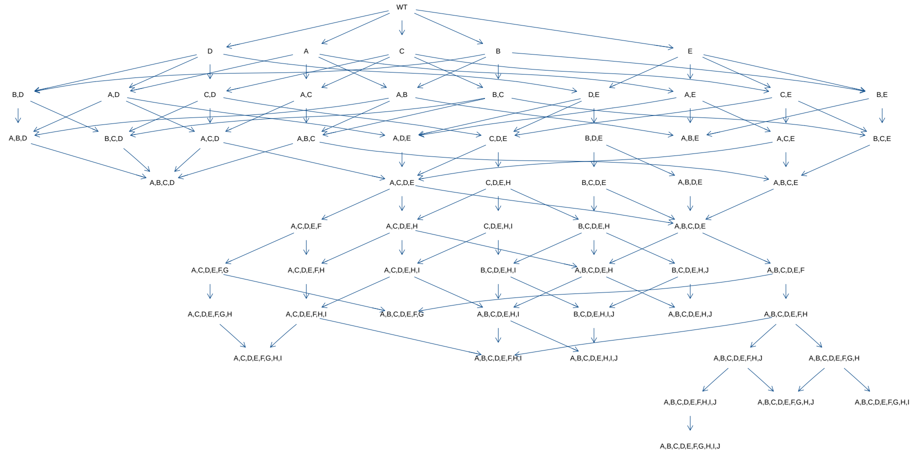

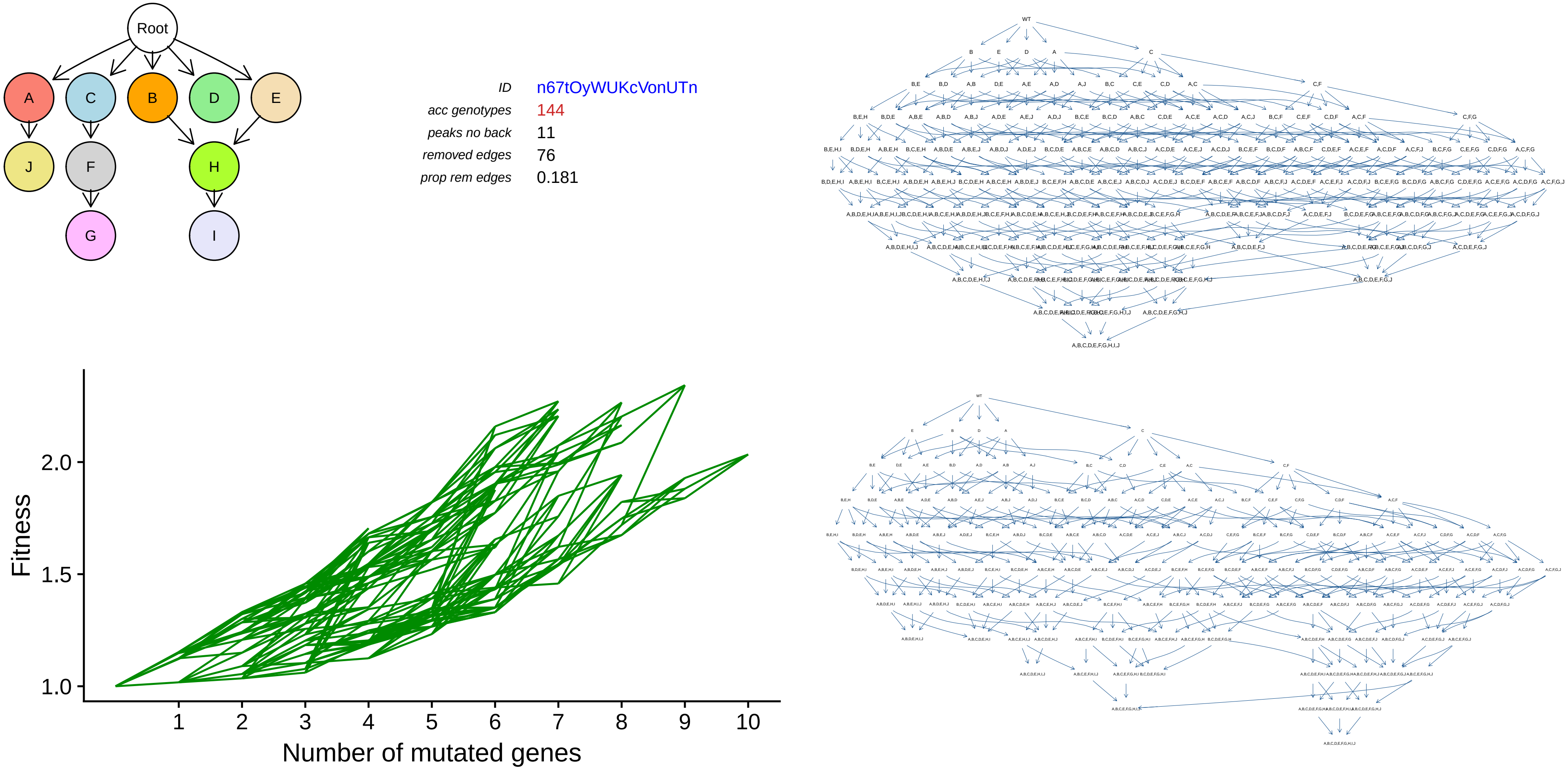
Root
A
C
B
D
E
J
F
H
G
I
ID n67tOyWUKcVonUTn
acc genotypes 144
peaks no back 11
removed edges 76
prop rem edges 0.181
Fitness
1.0
1.5
2.0
1
2
3
4
5
6
7
8
9
10
Number of mutated genes

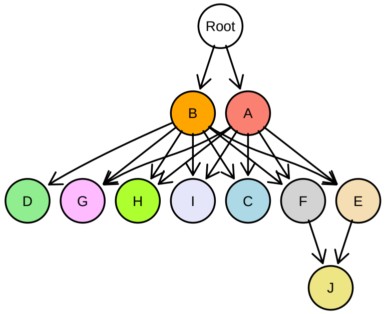

ID	n6N43lpBlanX2OCS
acc genotypes	164
peaks no back	15
removed edges	95
prop rem edges	0.168

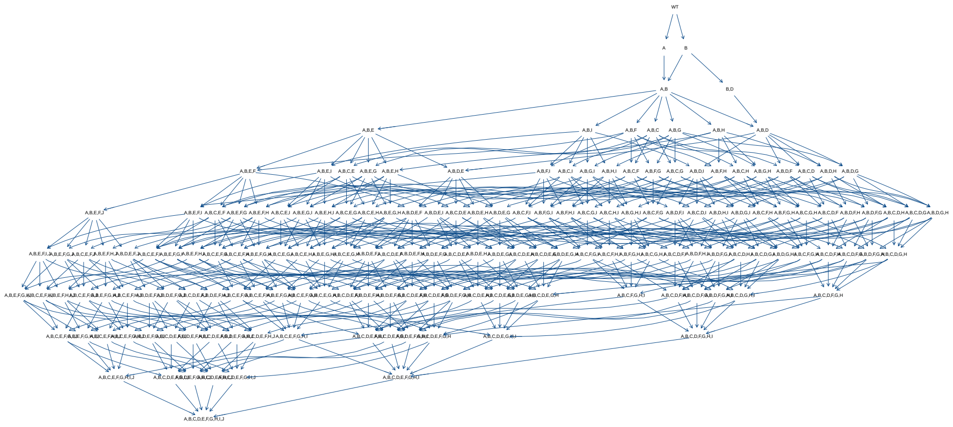

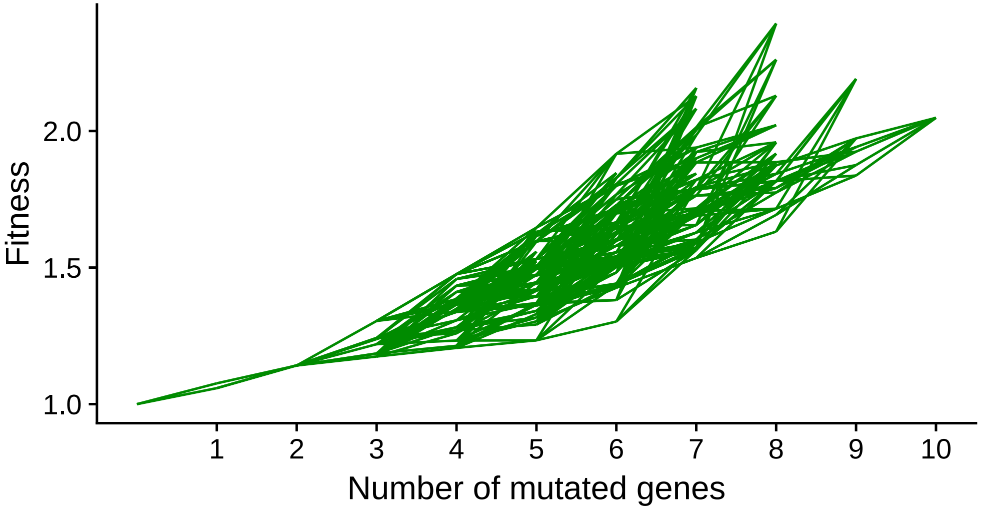

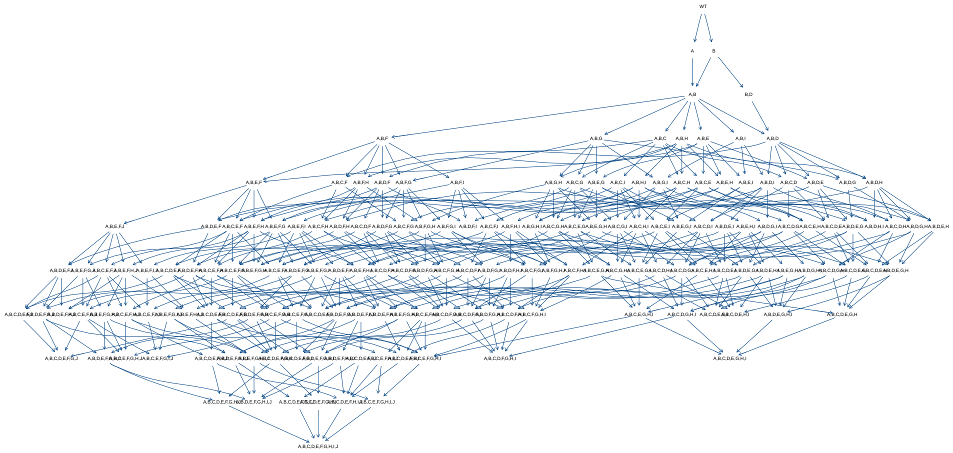

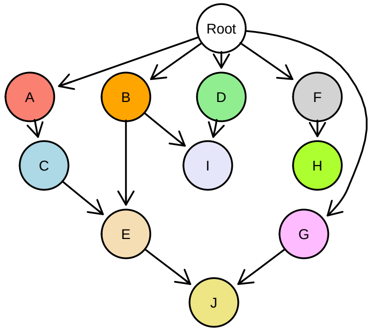

ID NHvOV5oluQFLza23
acc genotypes 117
peaks no back 11
removed edges 59
prop rem edges 0.18

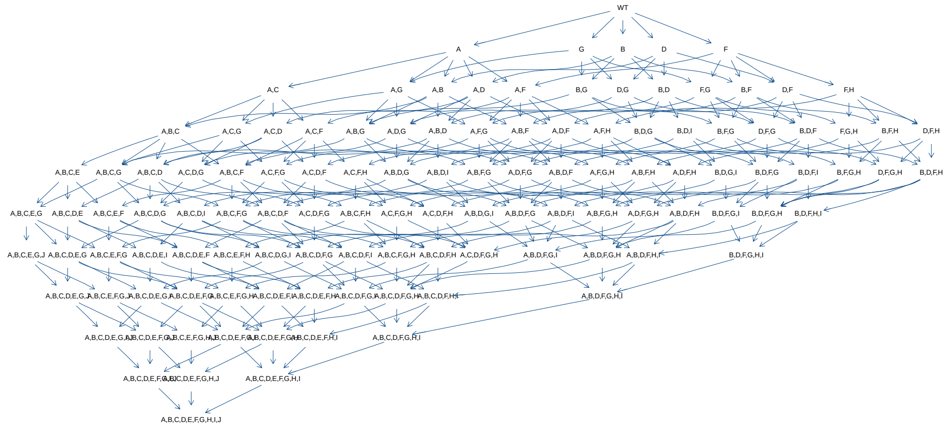

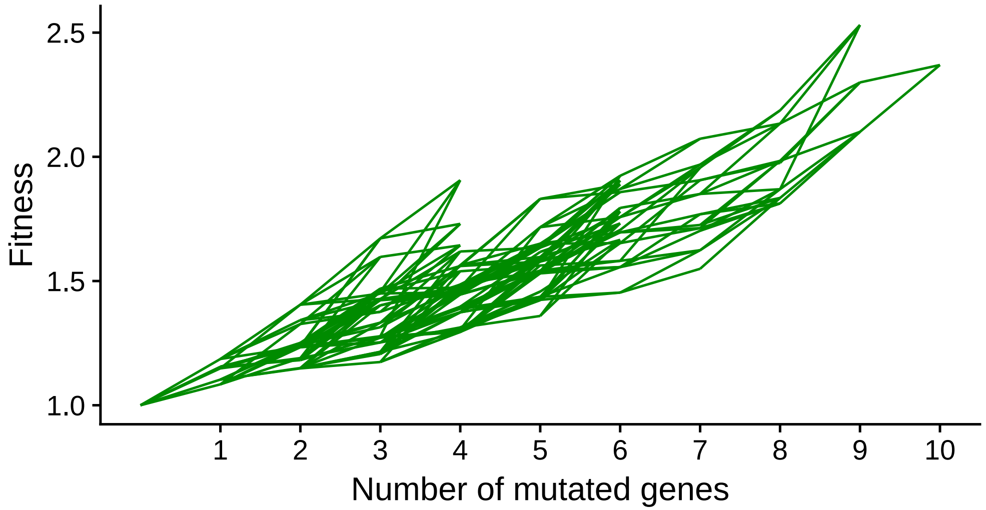

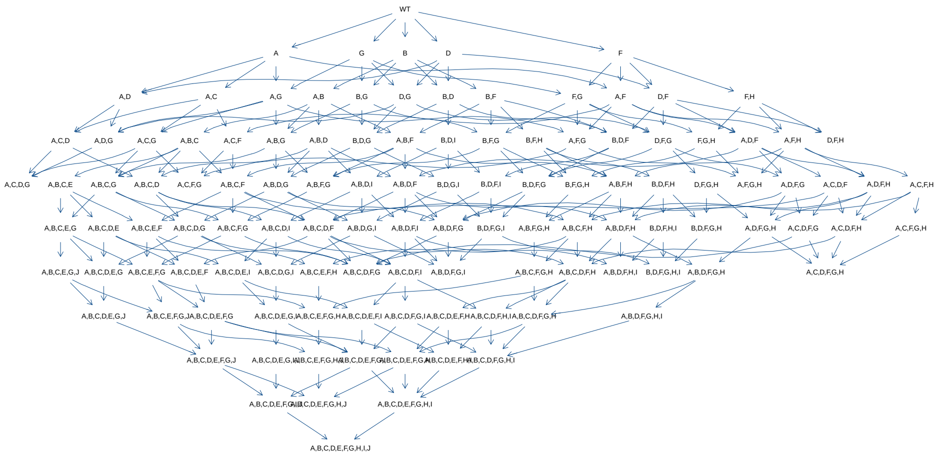

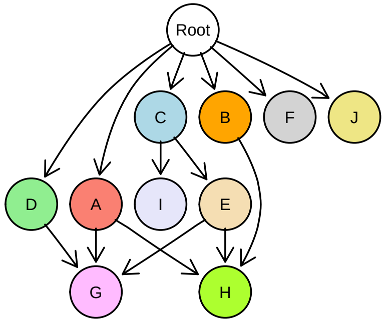

ID	nLayuUMxBrwIiw43
acc genotypes	200
peaks no back	17
removed edges	135
prop rem edges	0.197

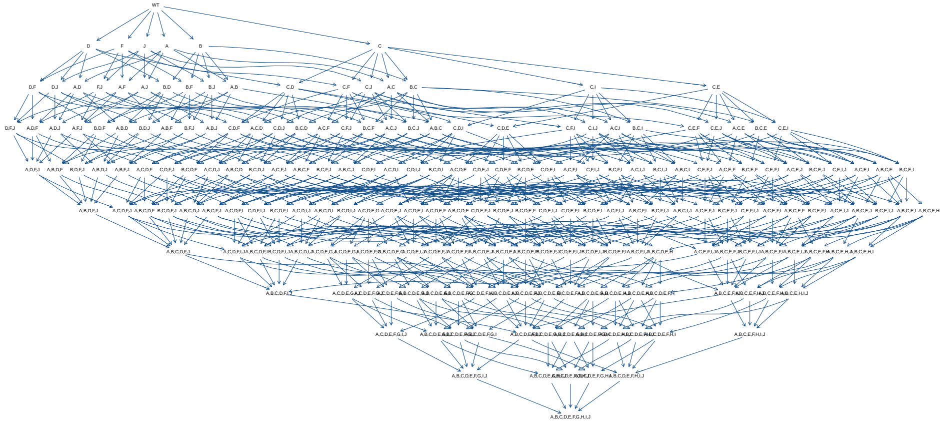

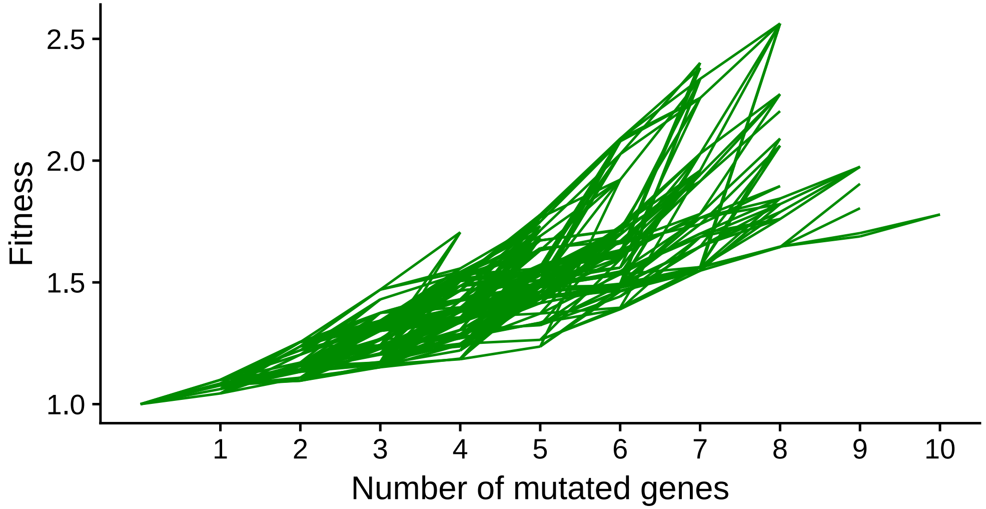

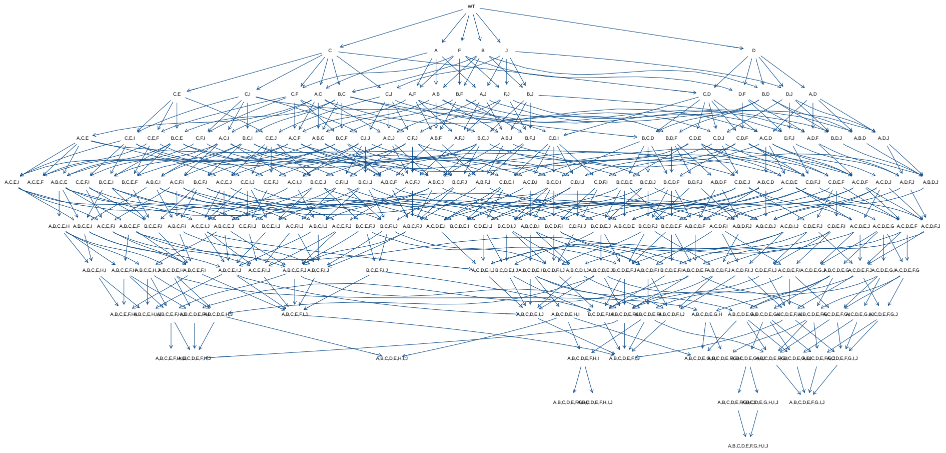

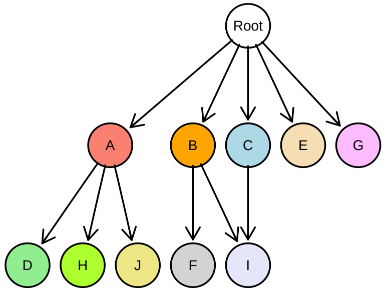

ID	nOAHFfiFZ4dc4UPV
acc genotypes	288
peaks no back	24
removed edges	255
prop rem edges	0.24

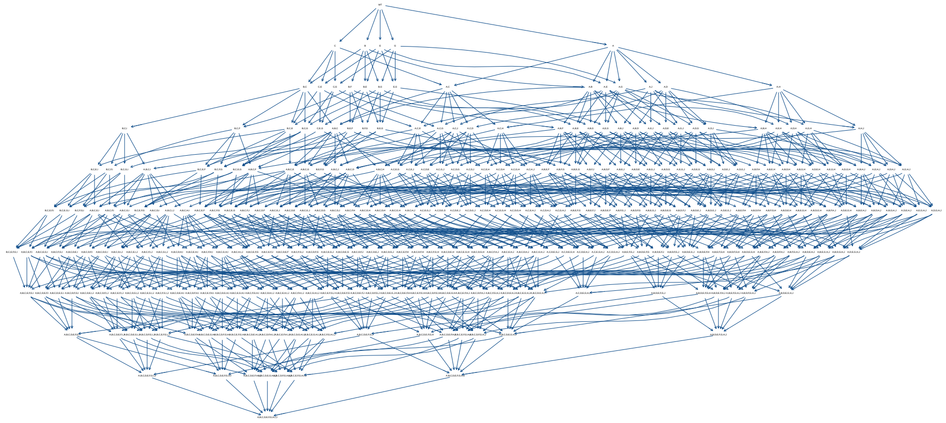

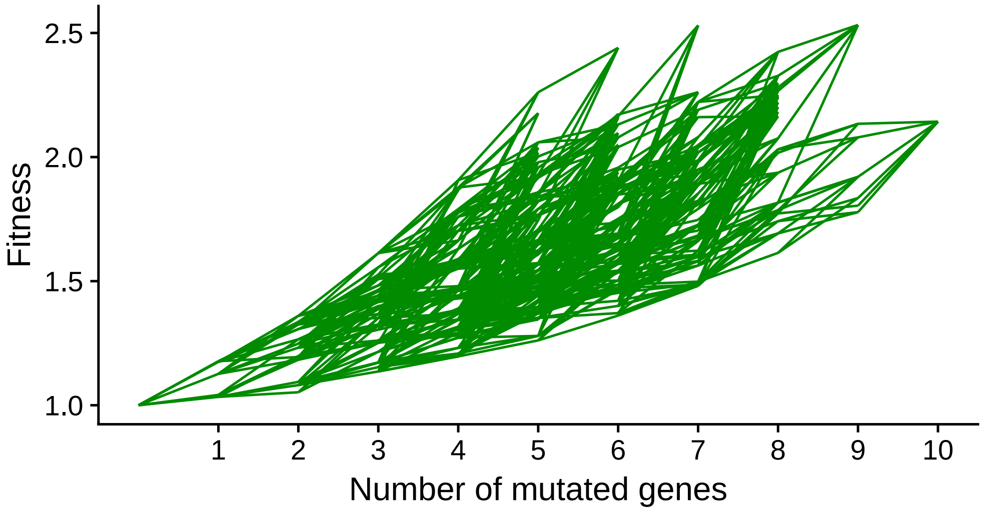

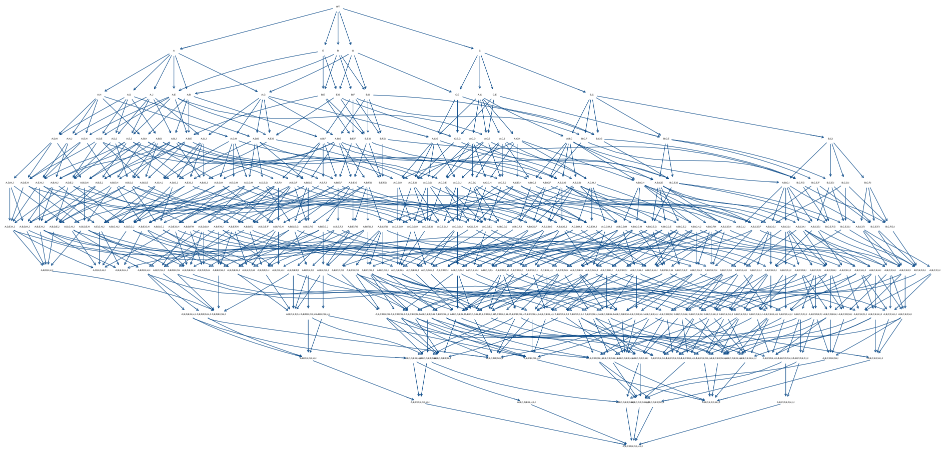

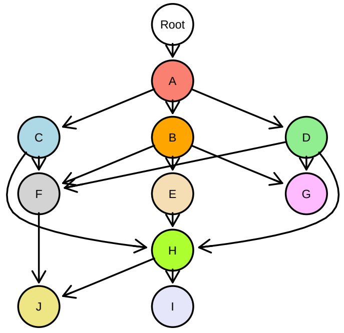

ID	noHAVtQI4KdH8hEq
acc genotypes	33
peaks no back	5
removed edges	12
prop rem edges	0.197

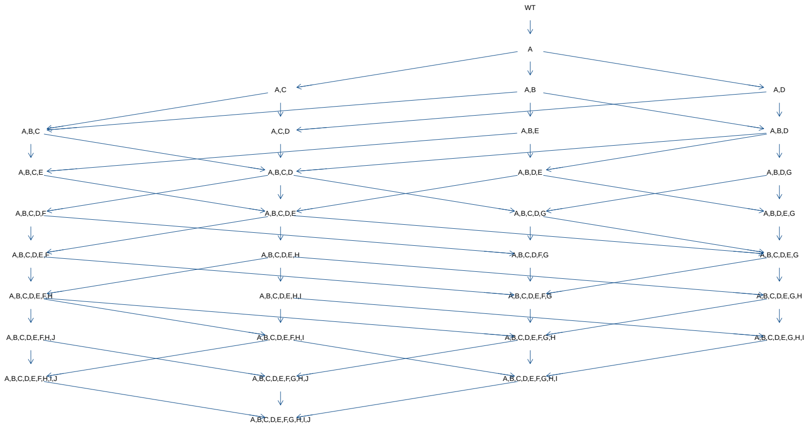

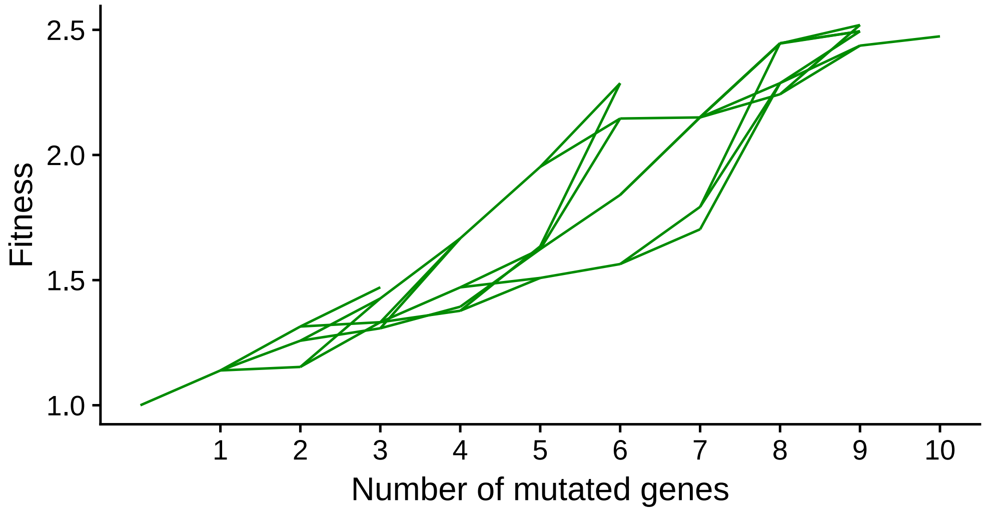

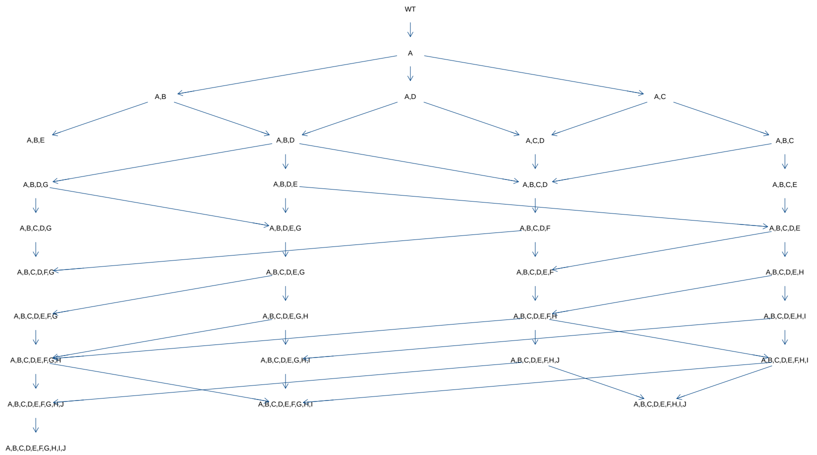

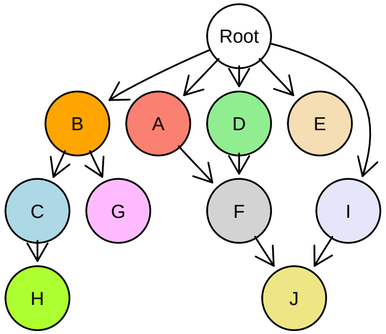

ID	NSqT8kGDJ9iU108hE
acc genotypes	154
peaks no back	11
removed edges	75
prop rem edges	0.157

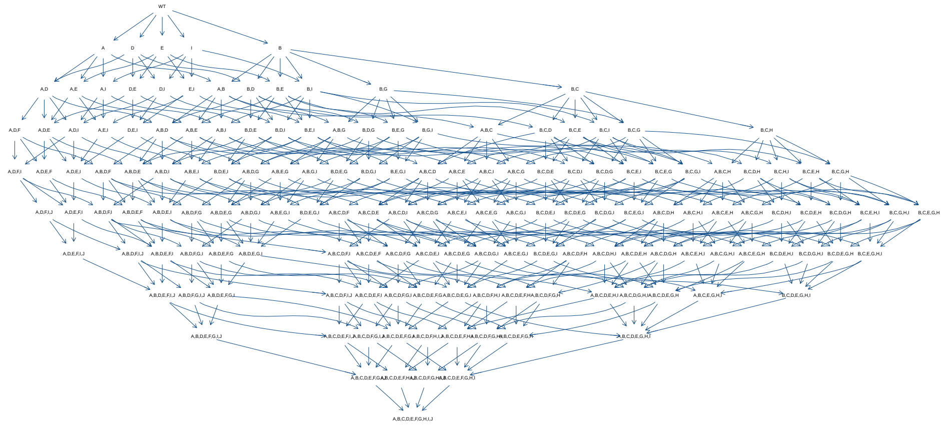

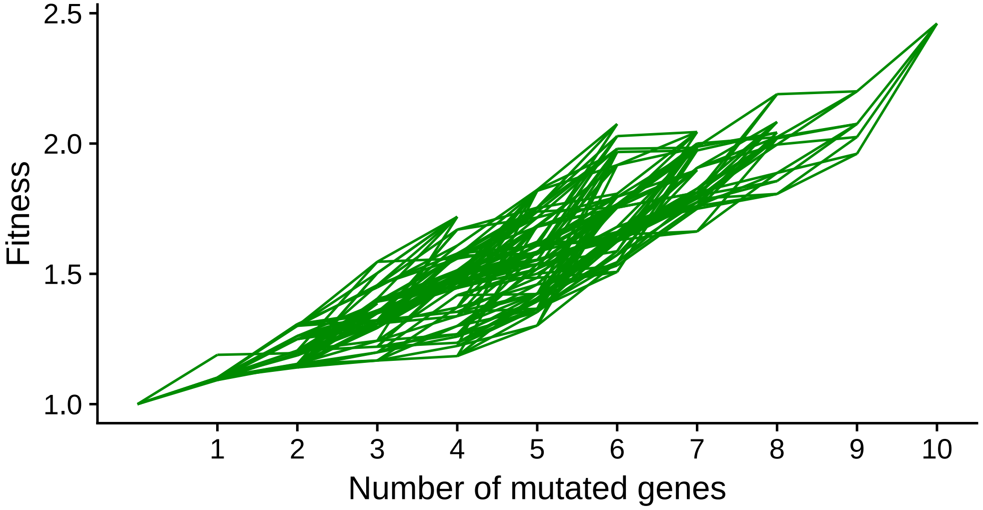

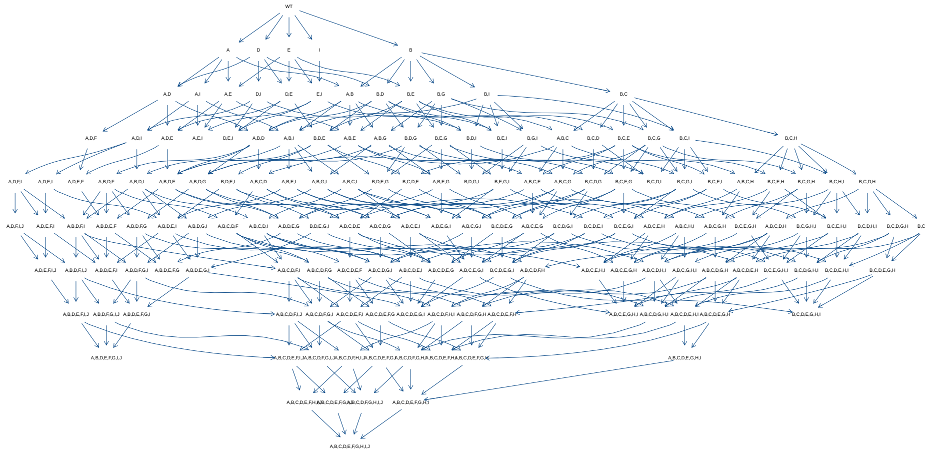

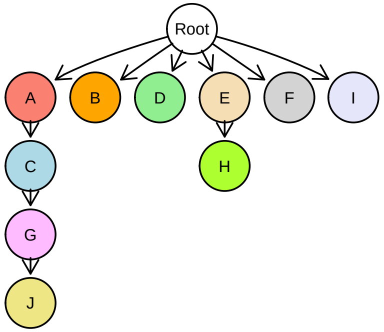

ID	nTjCF9qeG8JYDoN8
acc genotypes	240
peaks no back	19
removed edges	158
prop rem edges	0.19

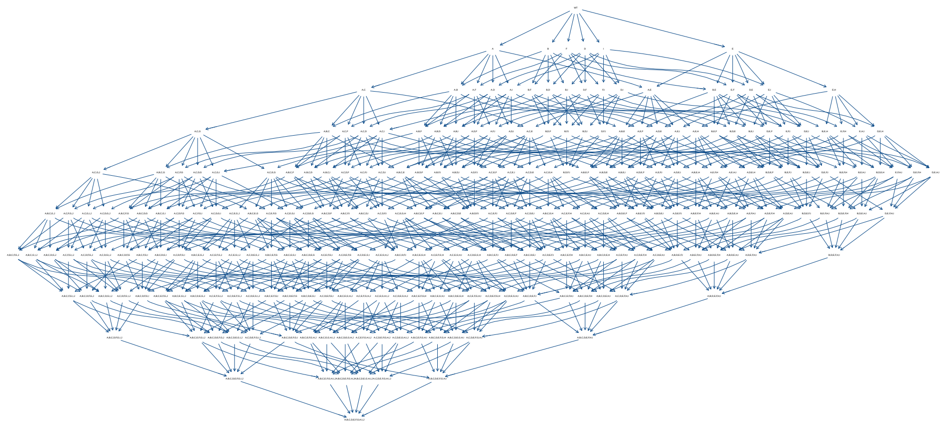

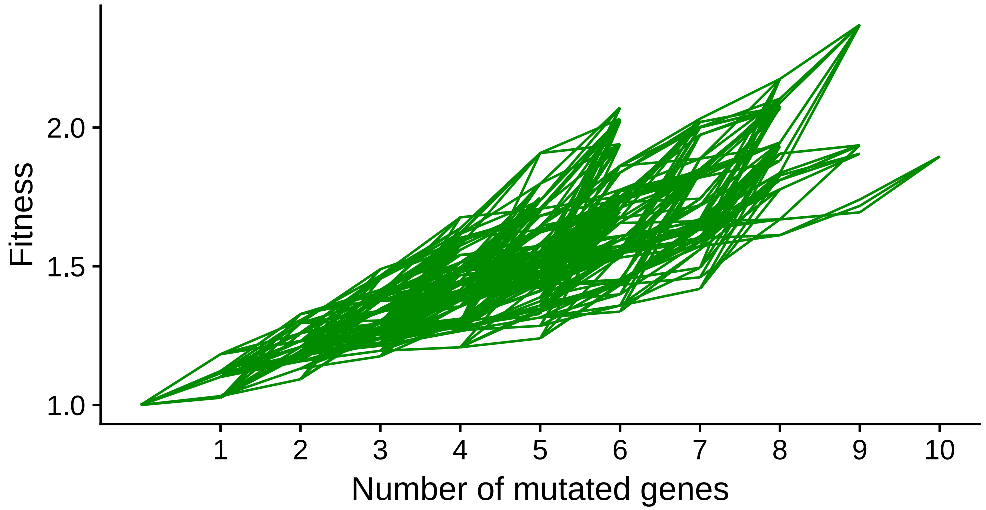

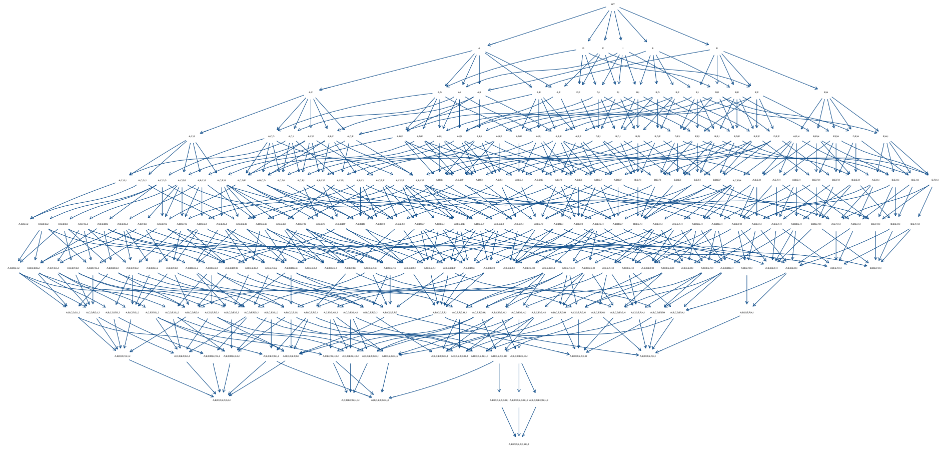

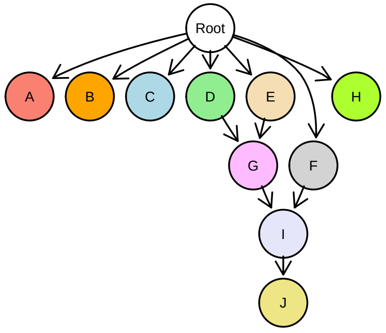

ID	nVi510c5OJbnQRcl5
acc genotypes	192
peaks no back	17
removed edges	126
prop rem edges	0.192

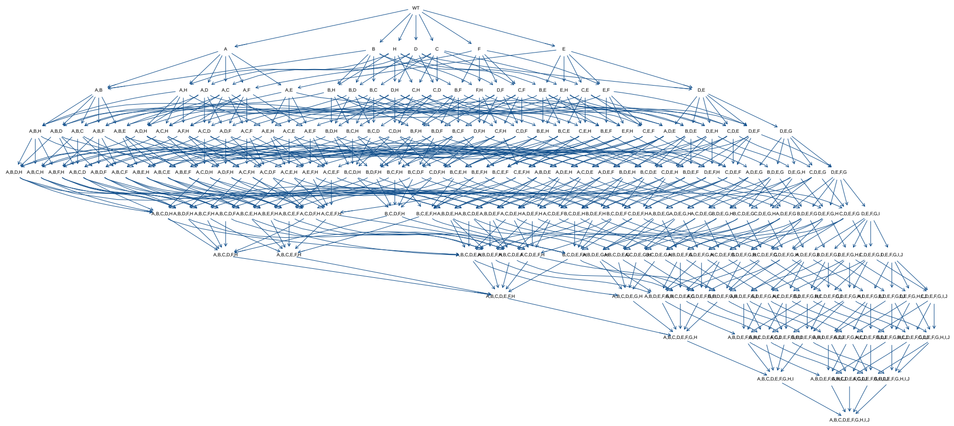

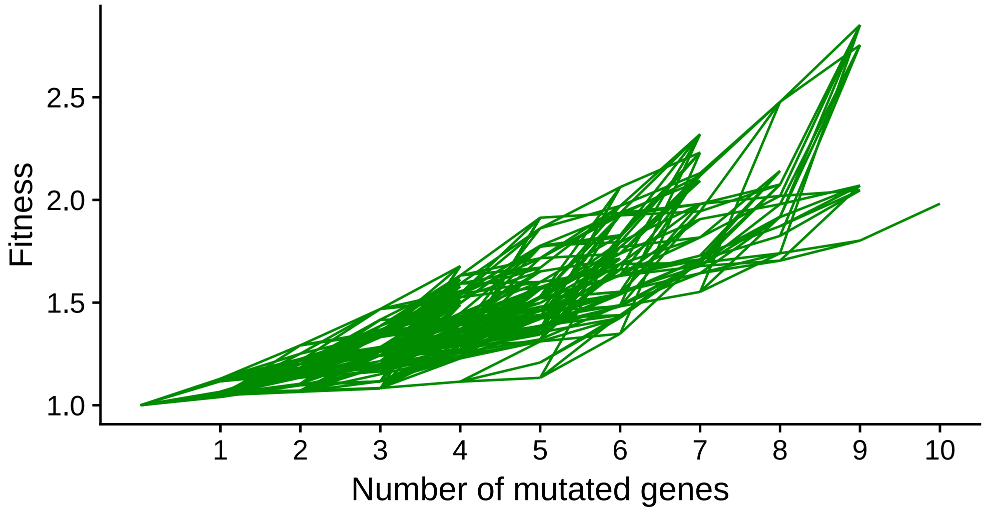

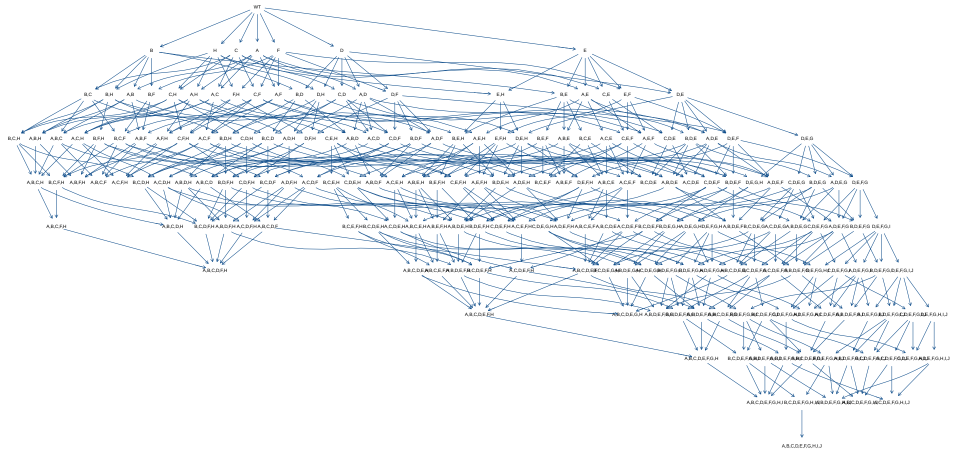

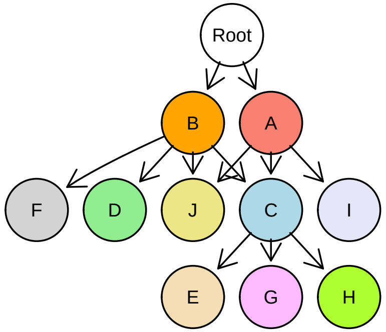

ID	NXDMDVPfzyoQDyuP
acc genotypes	151
peaks no back	11
removed edges	90
prop rem edges	0.177

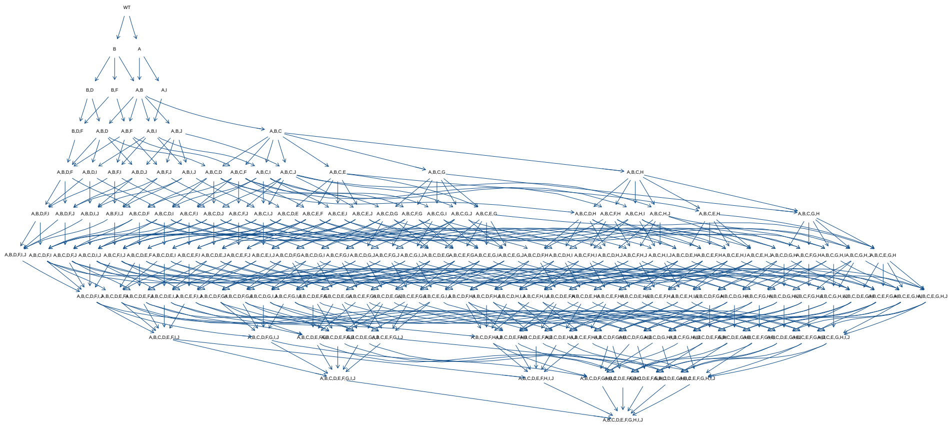

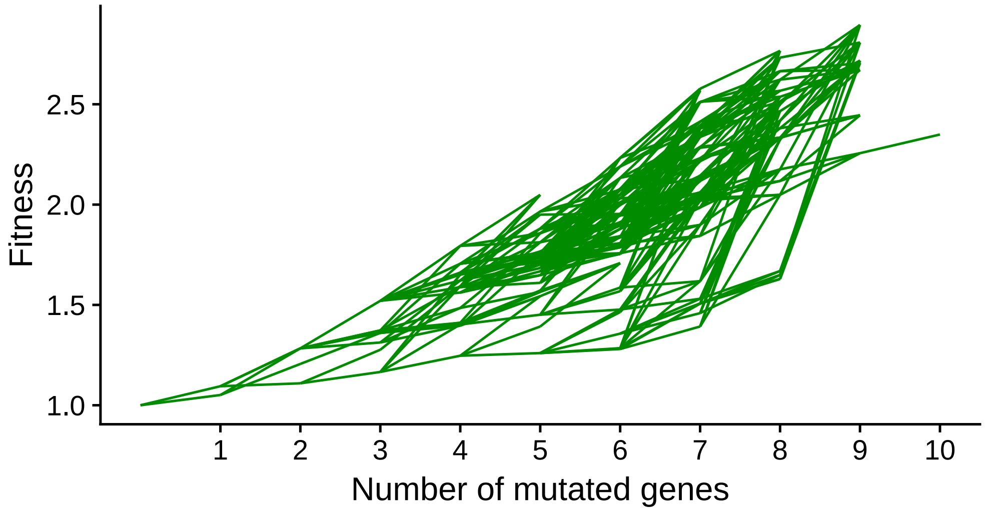

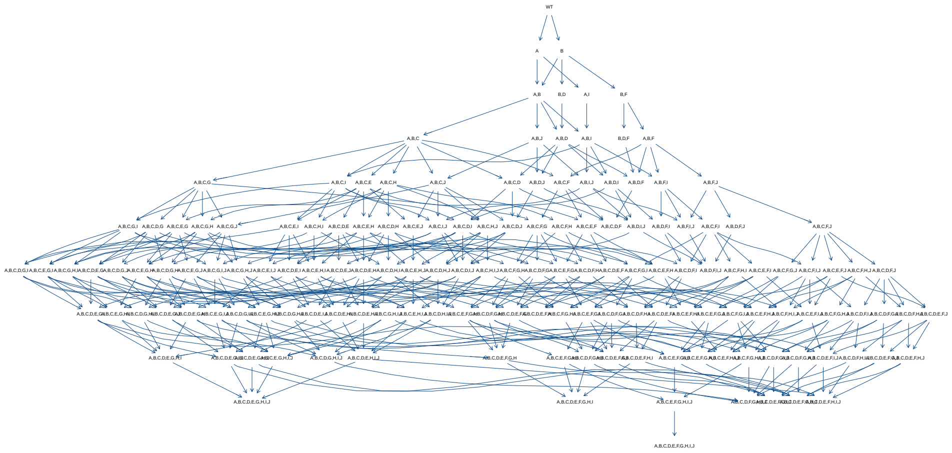

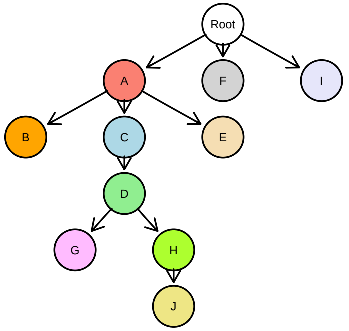

ID	o8OvpWIeTYvSAYxY
acc genotypes	132
peaks no back	13
removed edges	67
prop rem edges	0.164

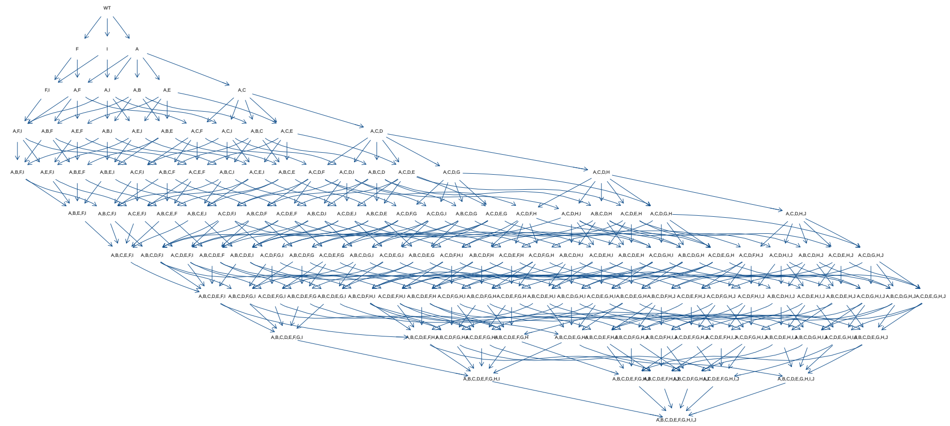

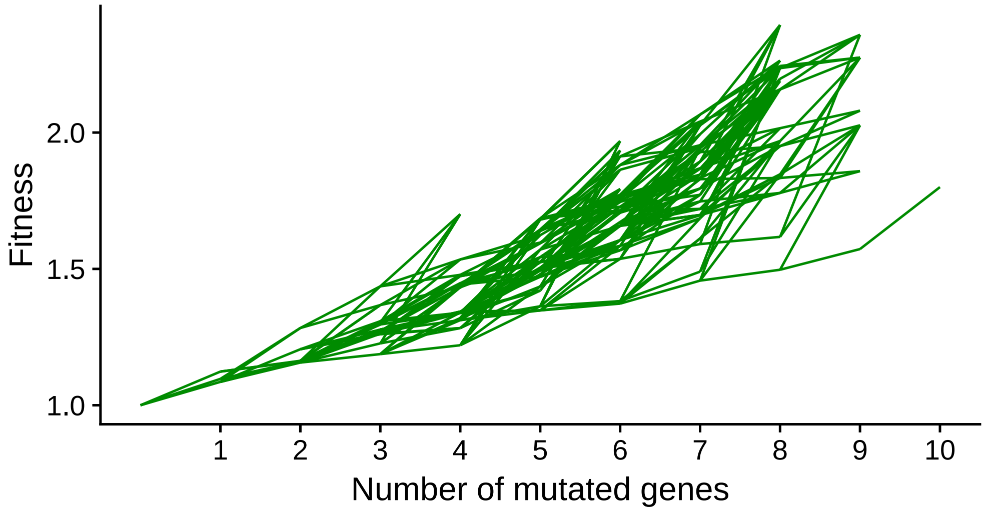

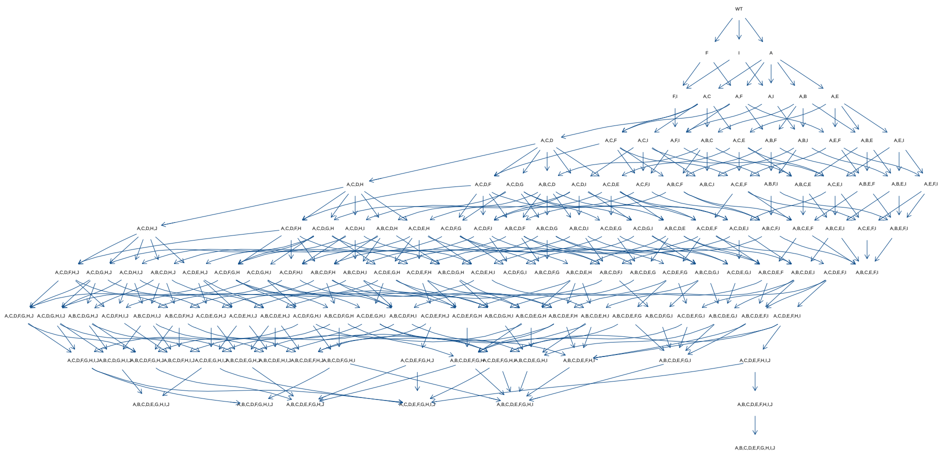

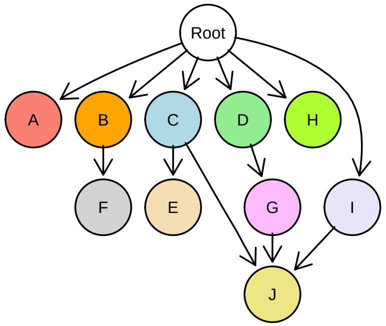

ID	ochEhkNsBx3ftyMD
acc genotypes	240
peaks no back	19
removed edges	173
prop rem edges	0.208

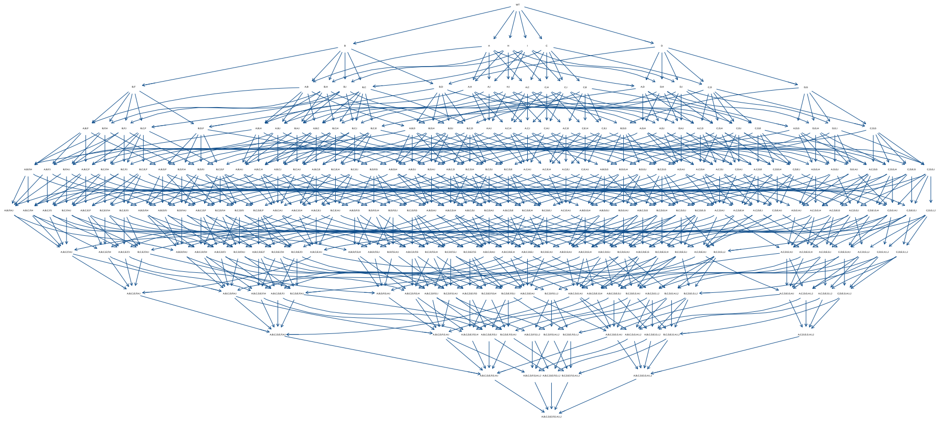

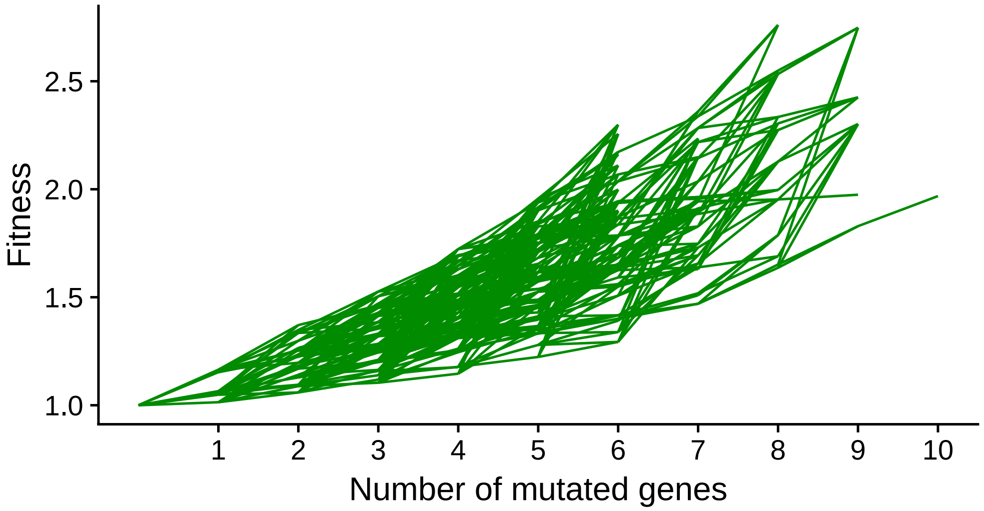

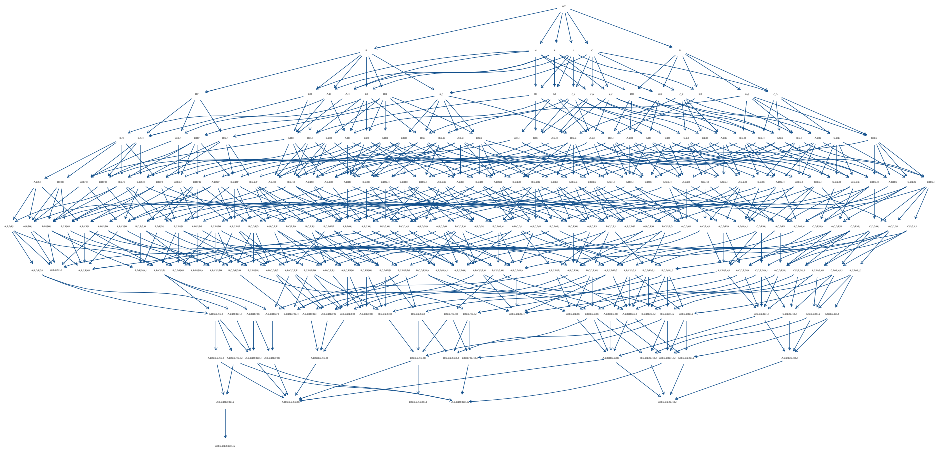

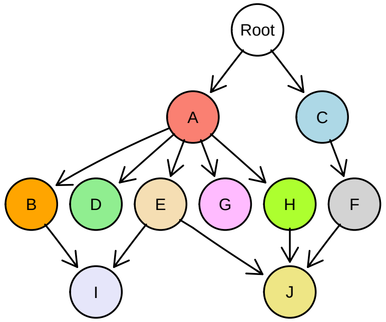

ID	OCRTvAy6n6Rv7KAn
acc genotypes	135
peaks no back	11
removed edges	80
prop rem edges	0.192

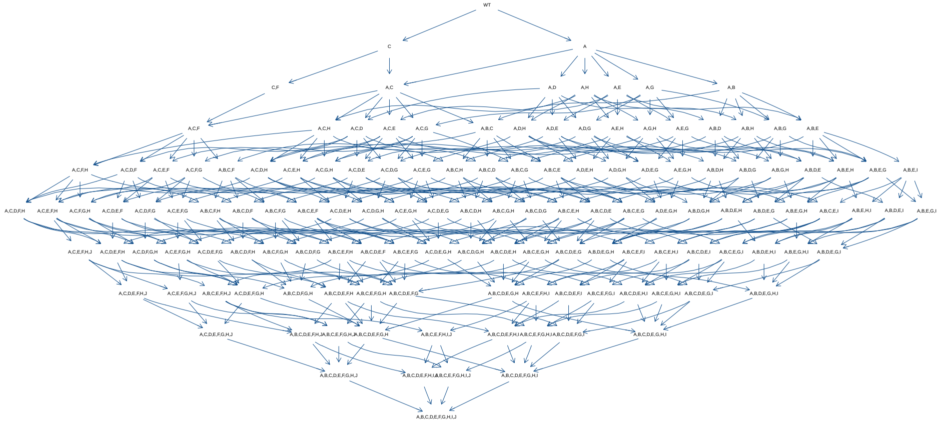

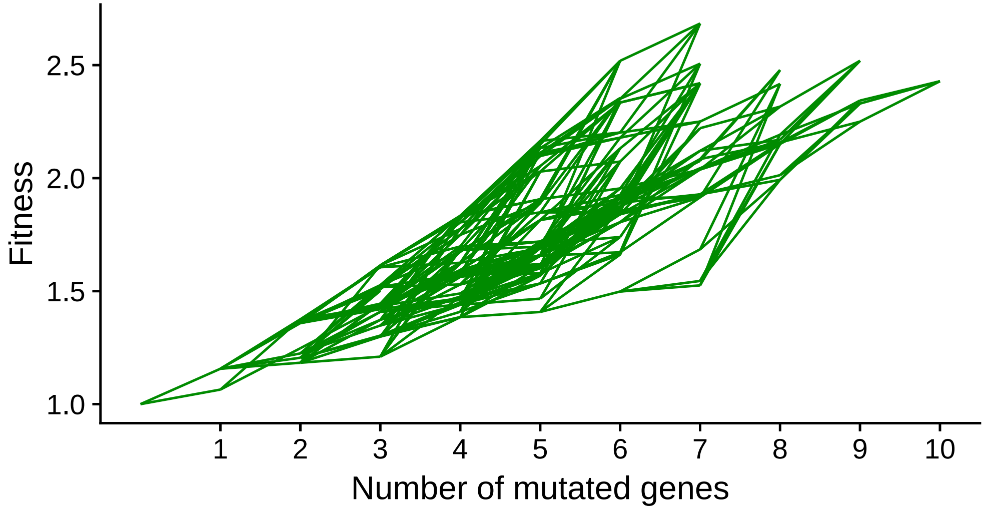

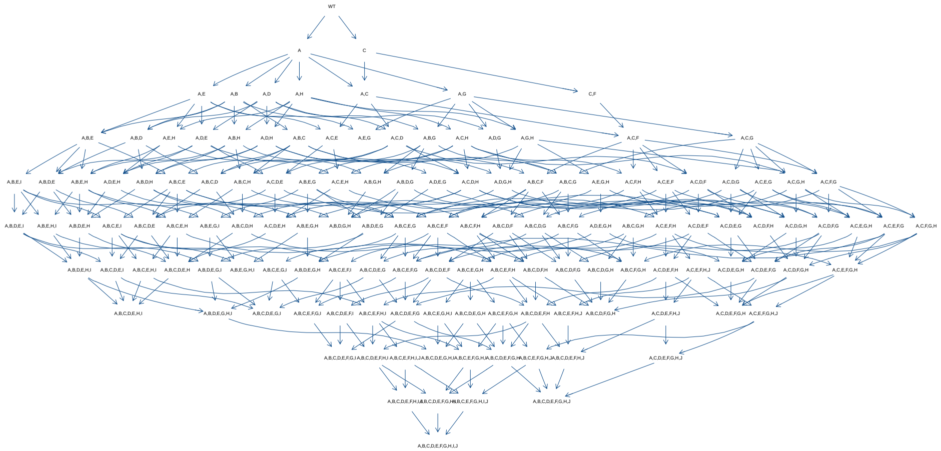

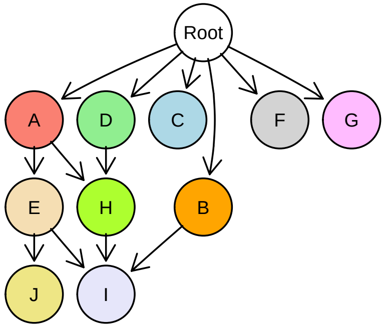

ID	Oo5hmM8b710XcCc1x
acc genotypes	192
peaks no back	15
removed edges	118
prop rem edges	0.184

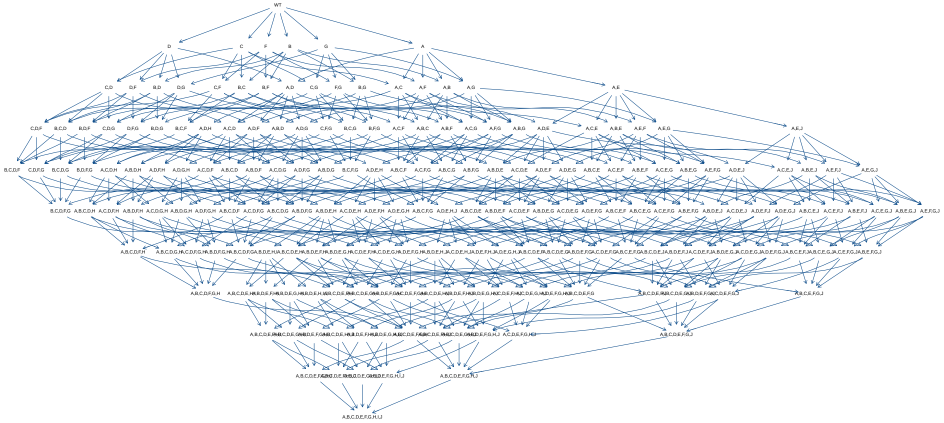

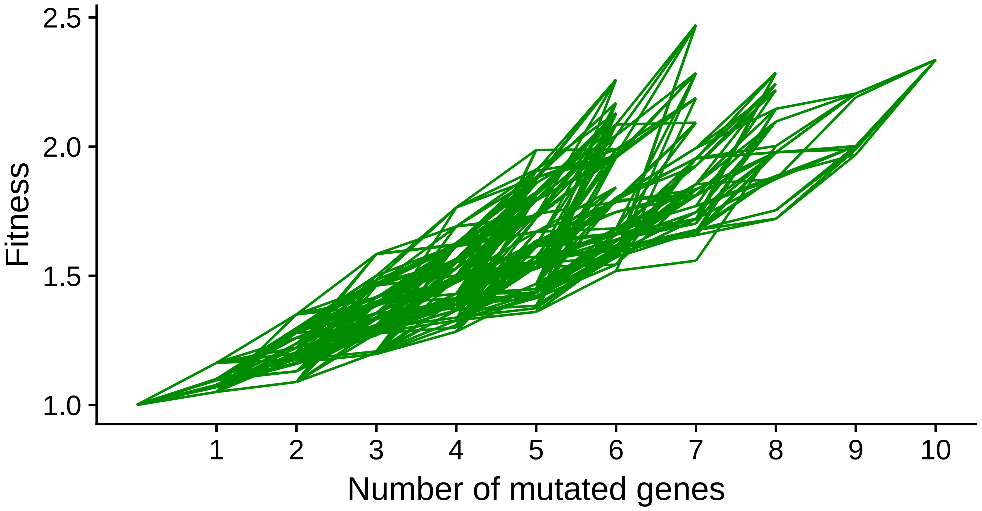

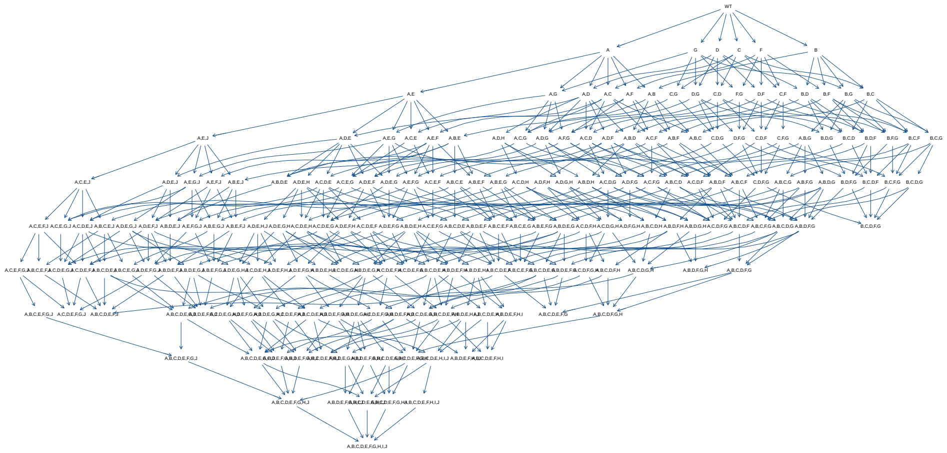

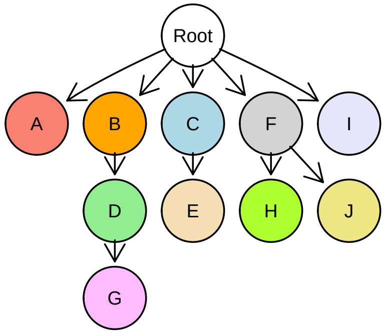

ID	OrLY4wrCbAM10e8mH
acc genotypes	240
peaks no back	16
removed edges	141
prop rem edges	0.172

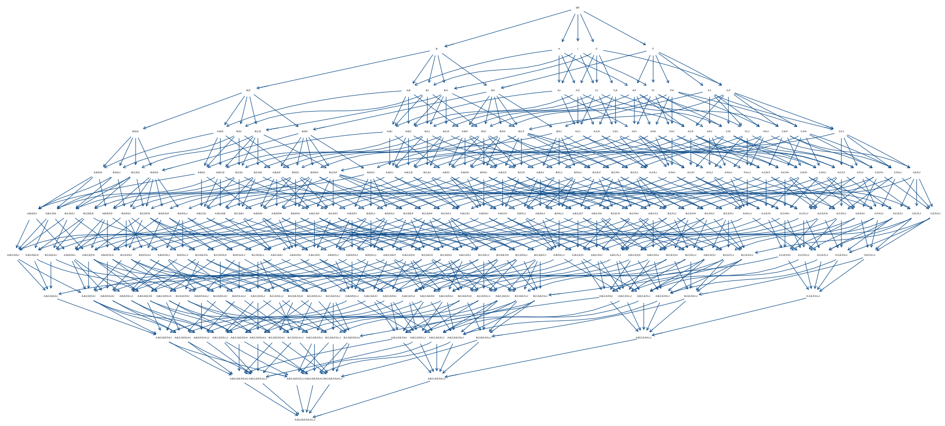

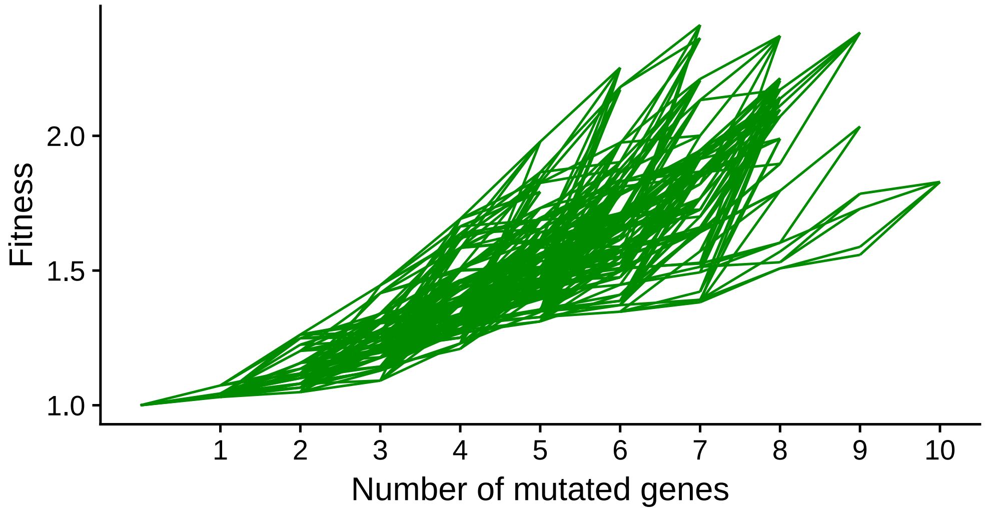

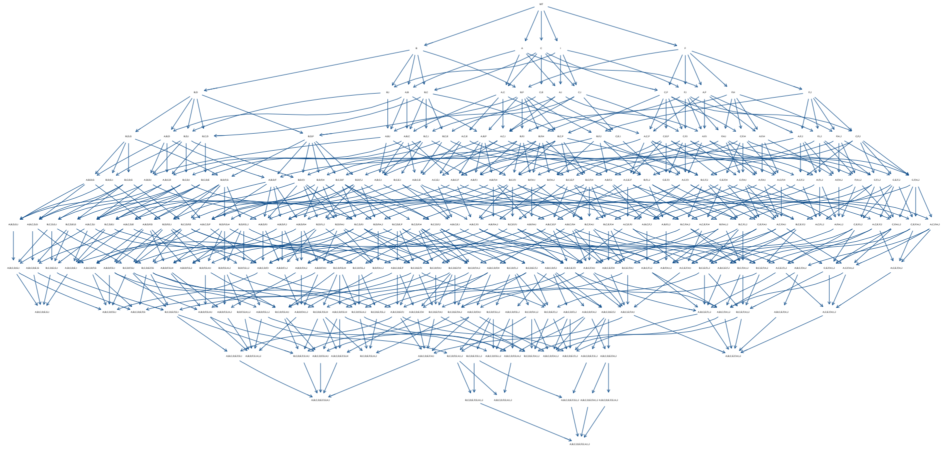

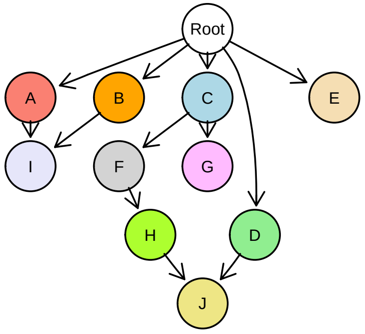

ID	OSiI7bIkeOUdWfpZ
acc genotypes	160
peaks no back	16
removed edges	100
prop rem edges	0.2

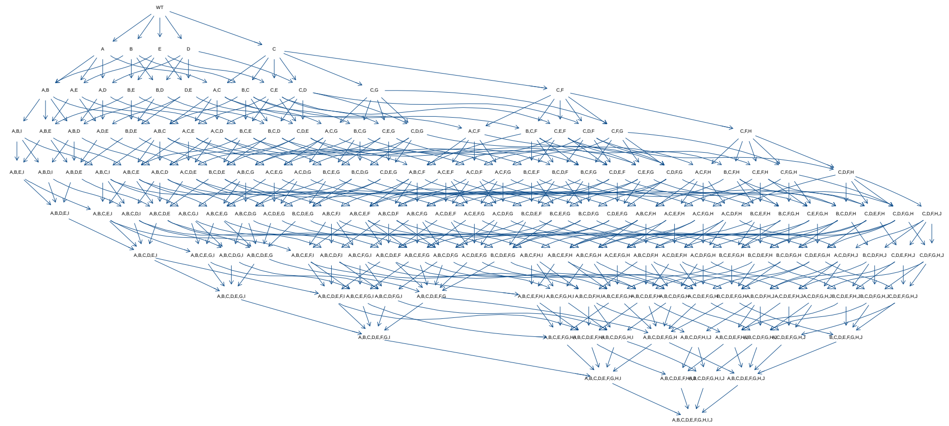

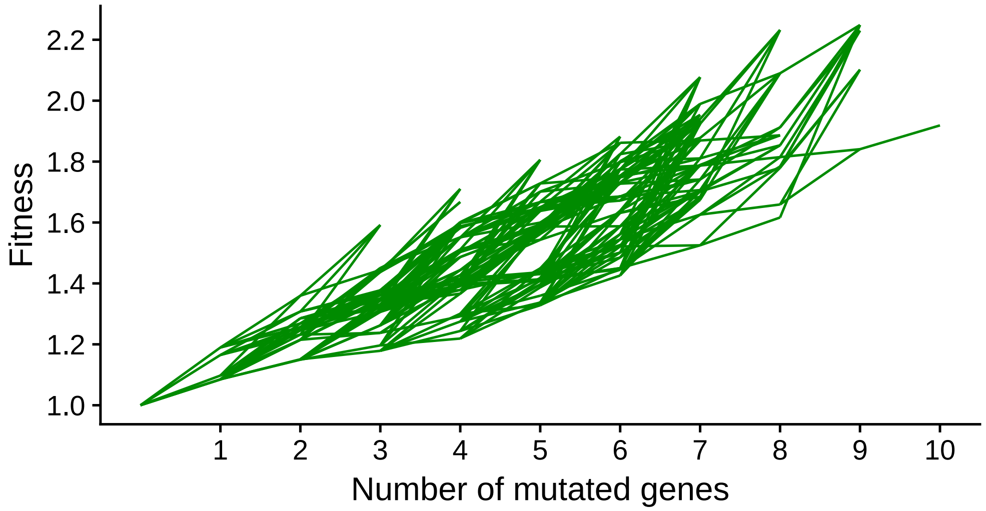

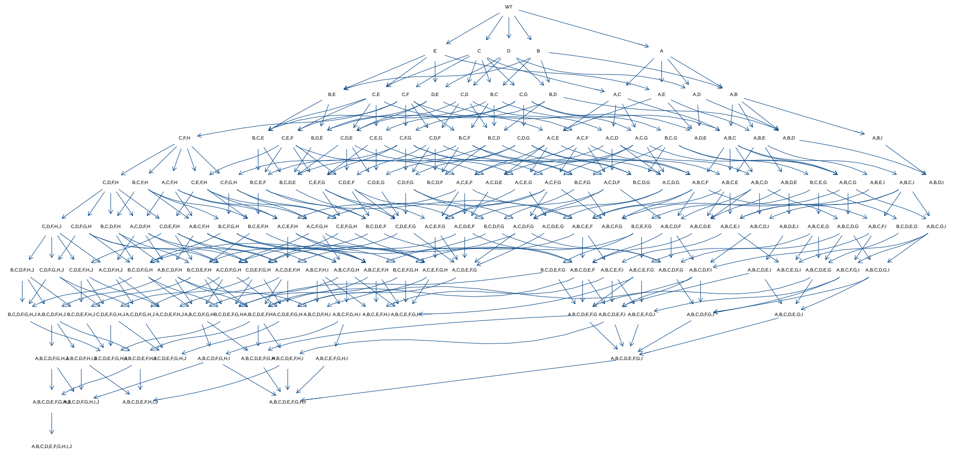

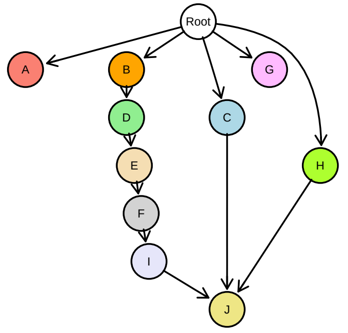

ID	OXbm8SnMAxb9VCsc
acc genotypes	100
peaks no back	10
removed edges	49
prop rem edges	0.175

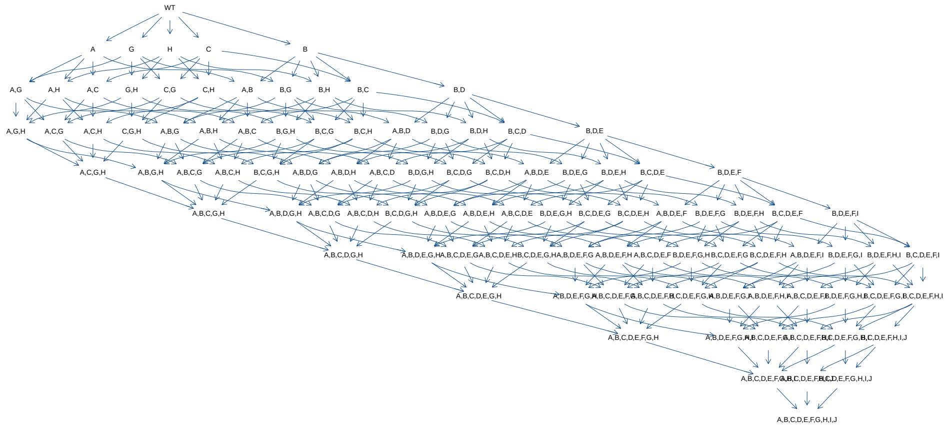

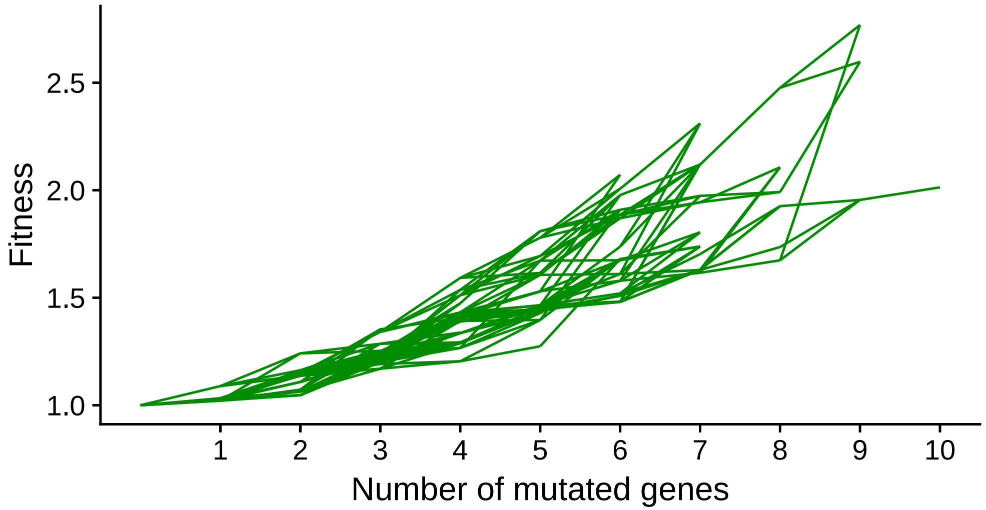

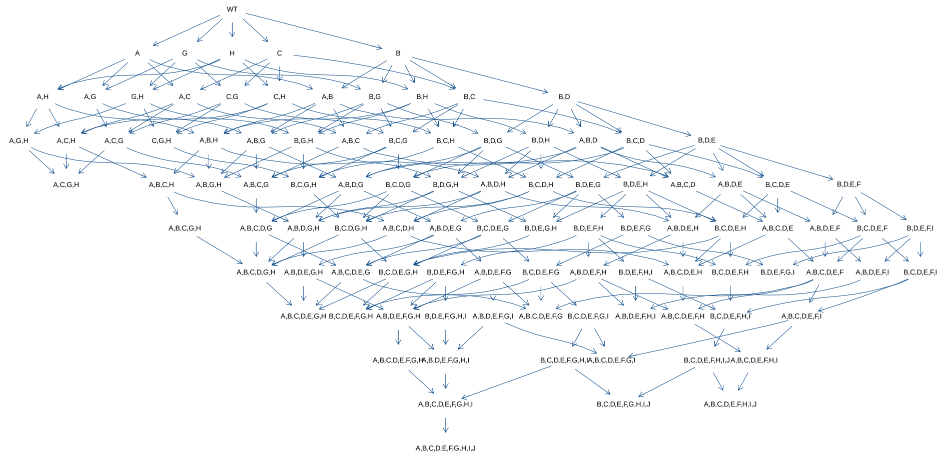

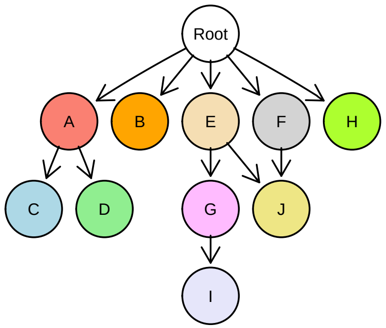

ID	PG8DkzMLvLzGnrF10
acc genotypes	220
peaks no back	18
removed edges	161
prop rem edges	0.218

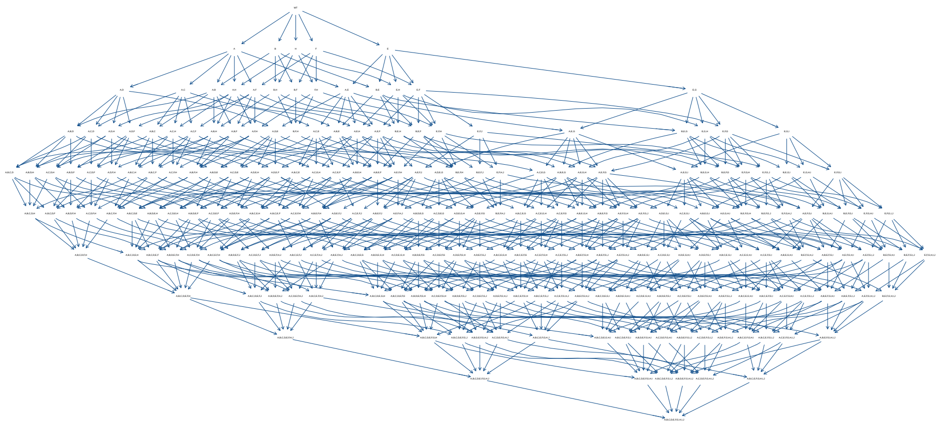

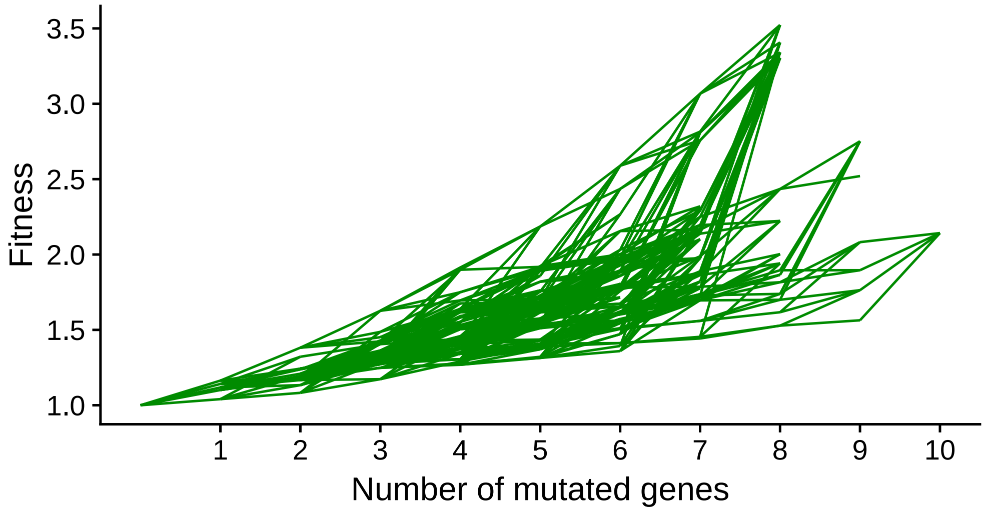

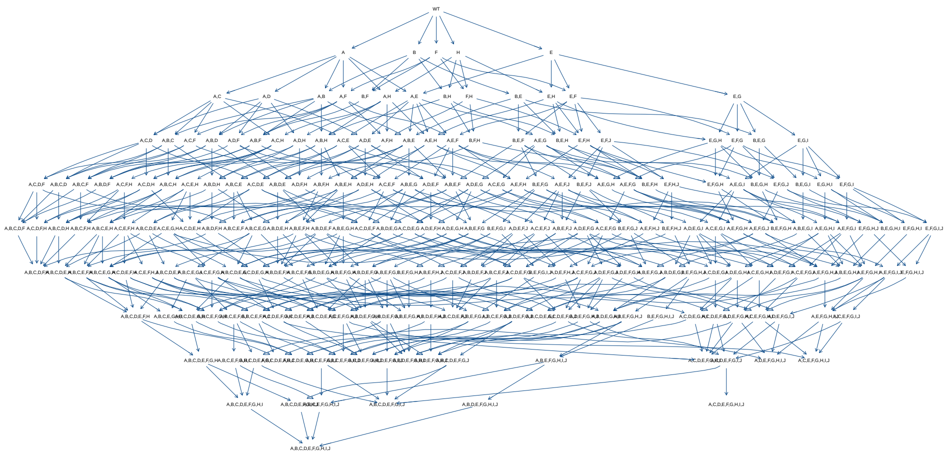

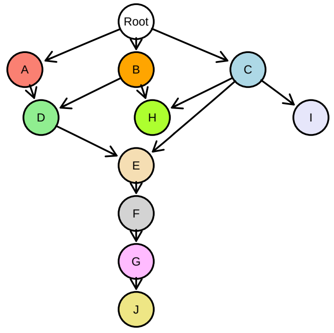

ID	pLto5Tz84T9OOoht
acc genotypes	37
peaks no back	6
removed edges	13
prop rem edges	0.186

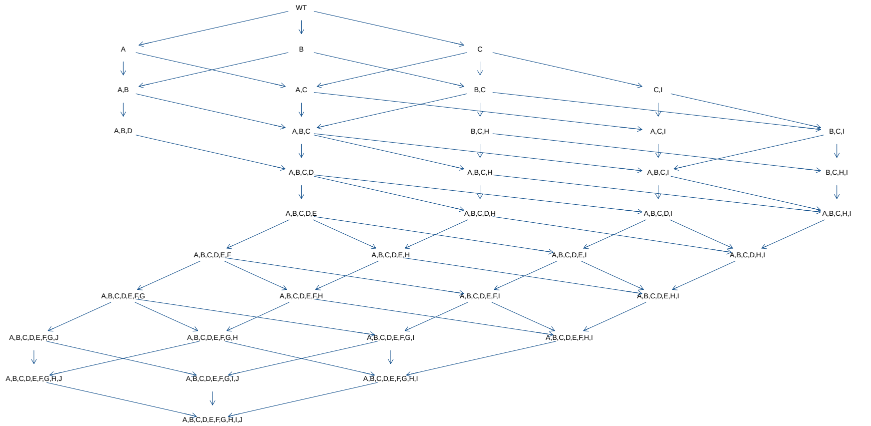

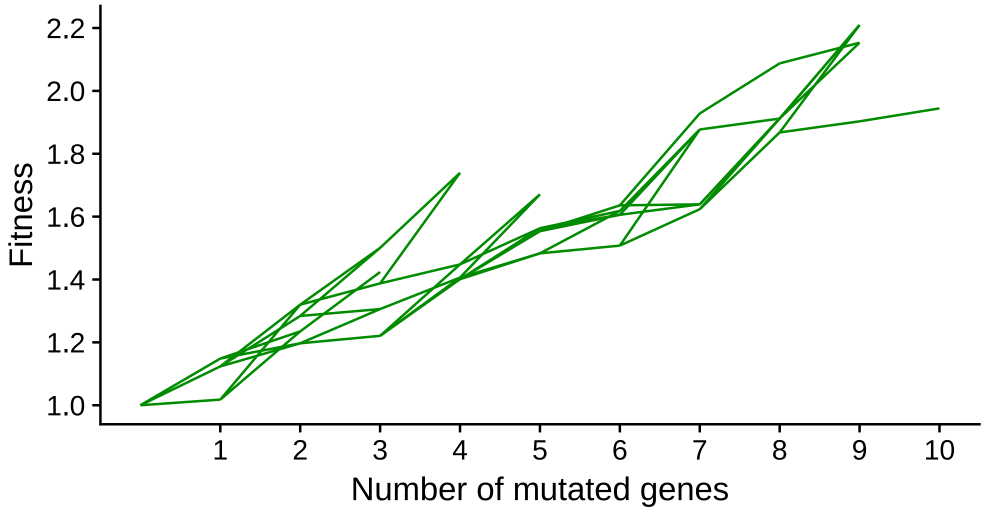

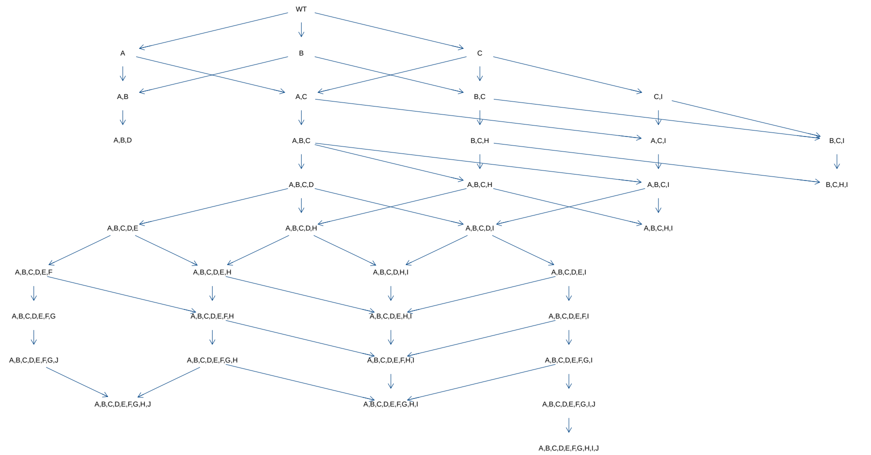

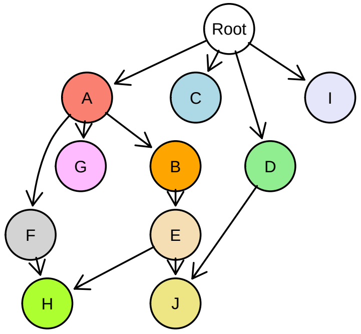

ID	PNEwdgl2i1dRkK3p
acc genotypes	144
peaks no back	15
removed edges	87
prop rem edges	0.194

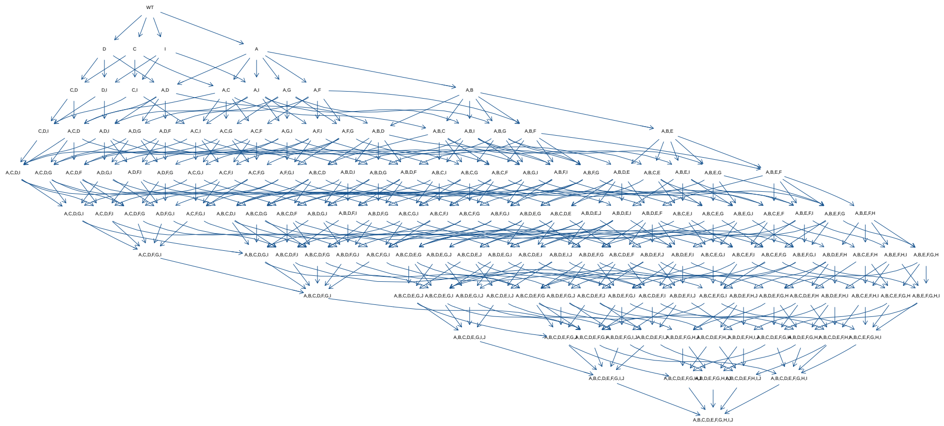

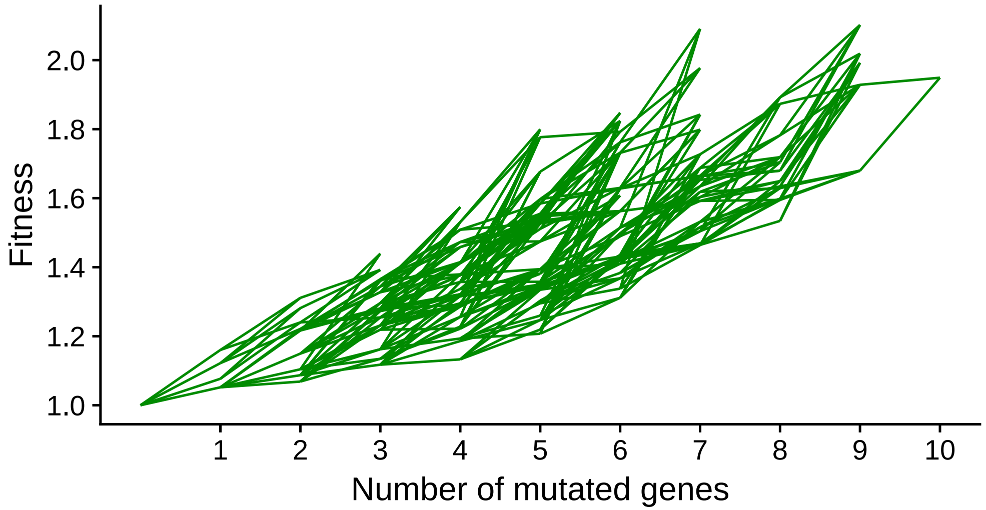

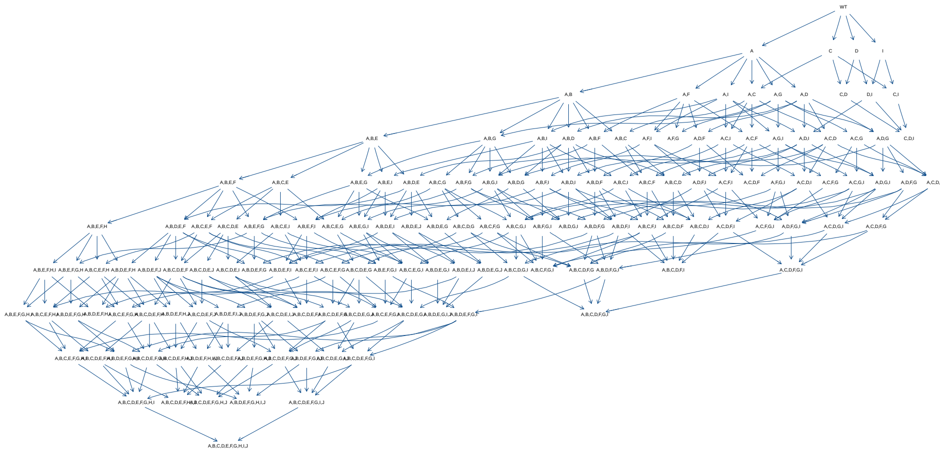

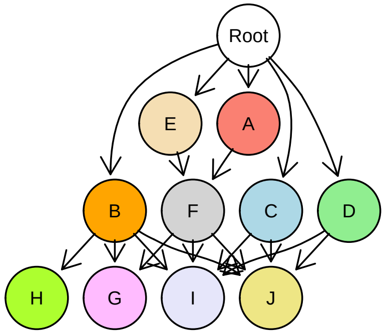

ID	PpAWVK9tbBJENITR
acc genotypes	80
peaks no back	12
removed edges	43
prop rem edges	0.207

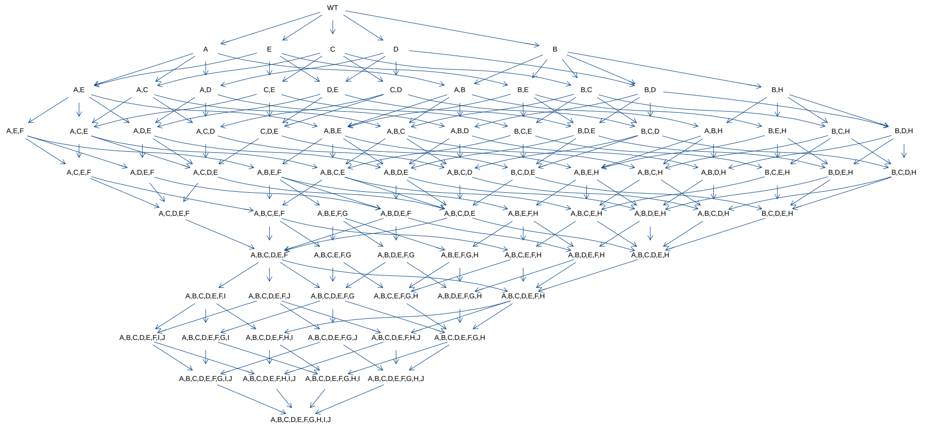

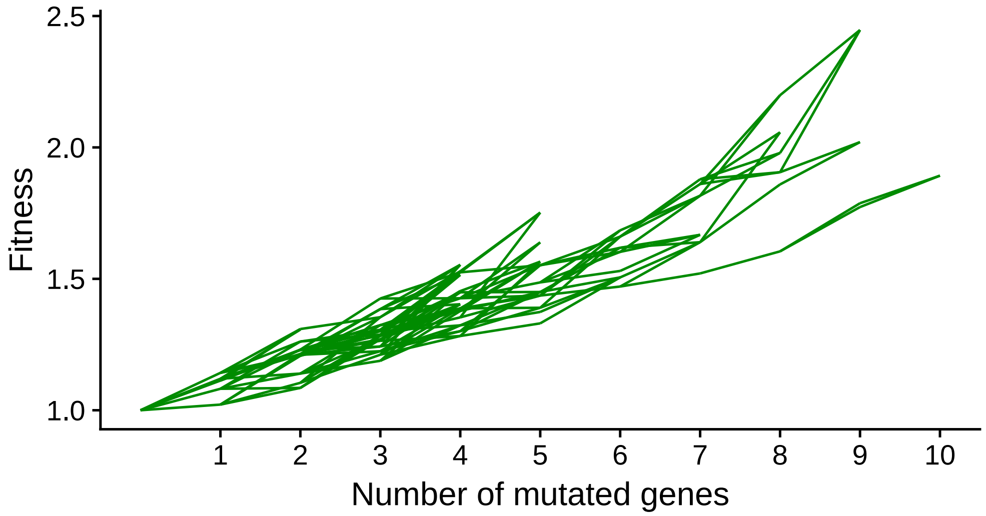

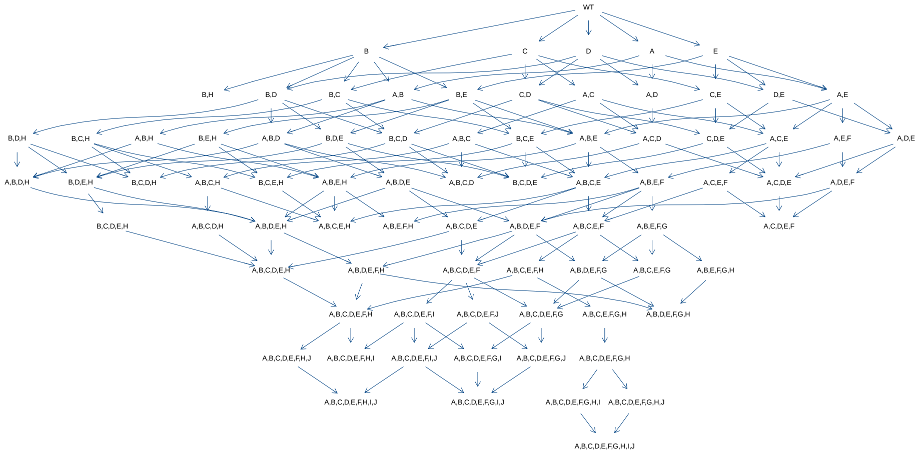

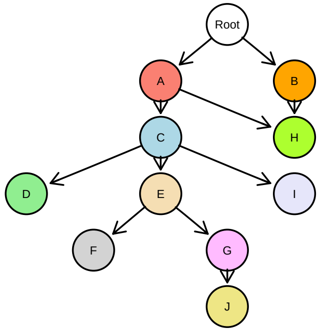

ID	Pqr5tjYF1SZIZUV10
acc genotypes	89
peaks no back	9
removed edges	46
prop rem edges	0.189

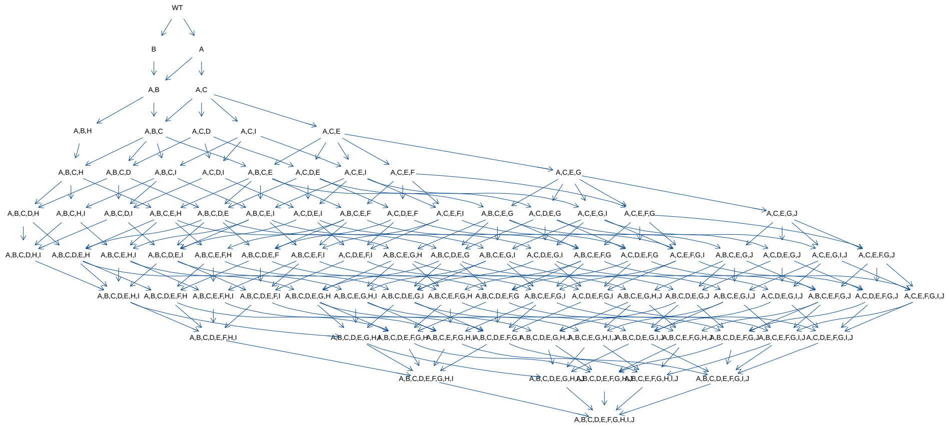

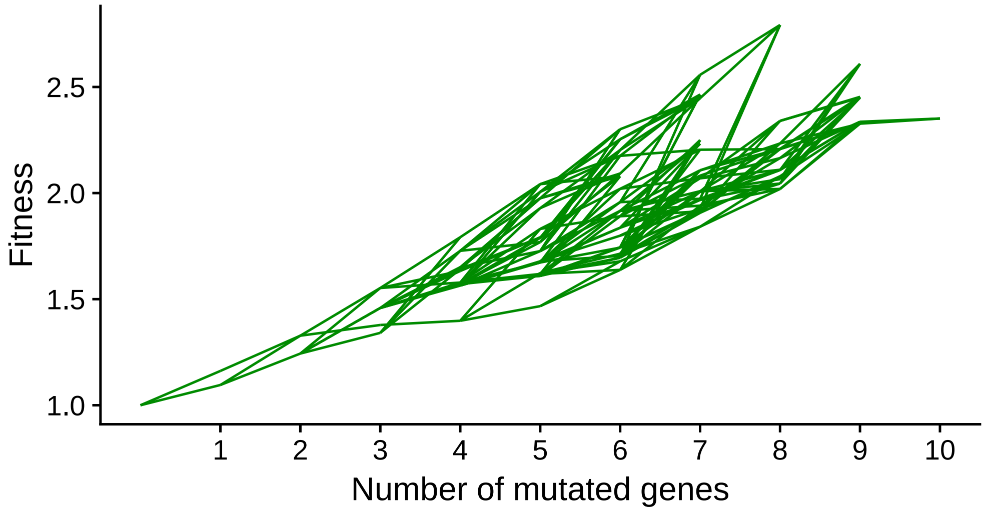

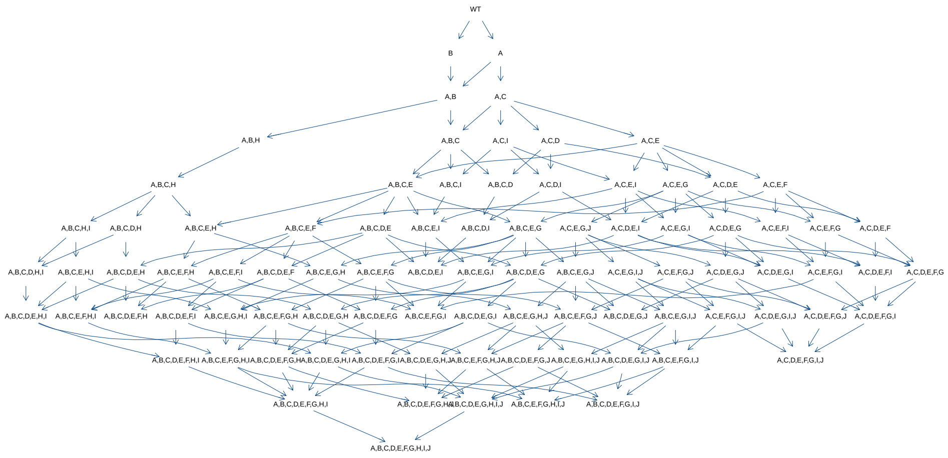

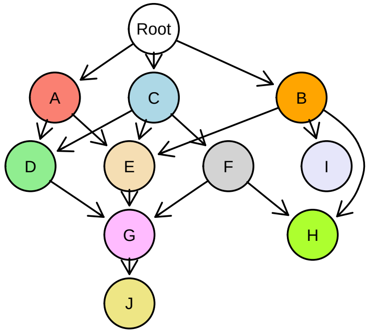

ID	q4xh5ljukpIhq9H9
acc genotypes	50
peaks no back	7
removed edges	20
prop rem edges	0.187

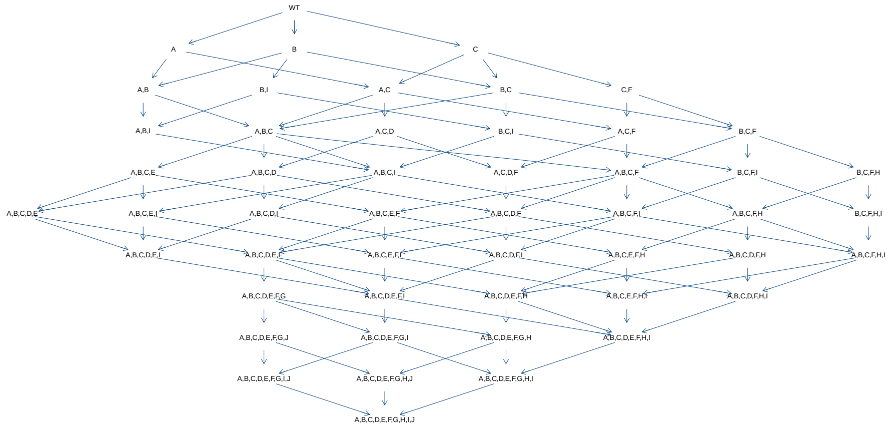

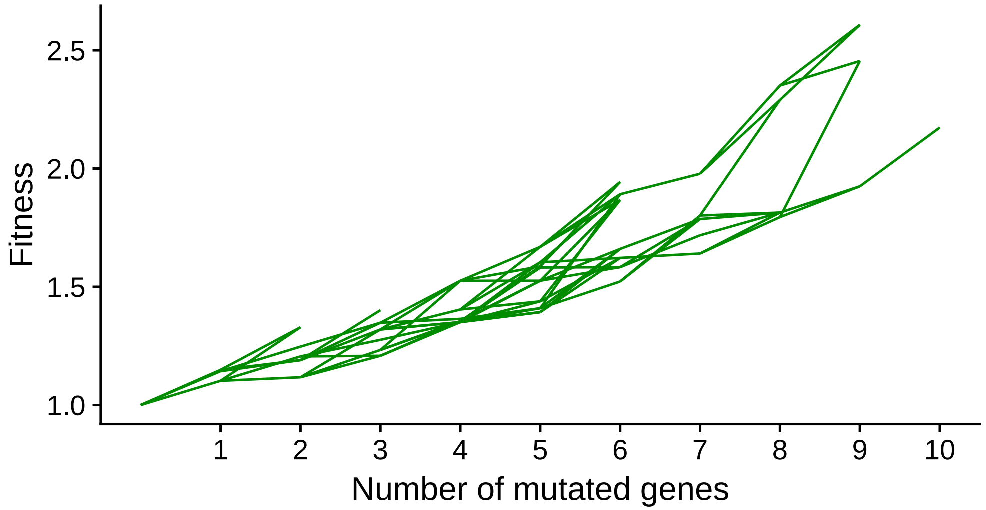

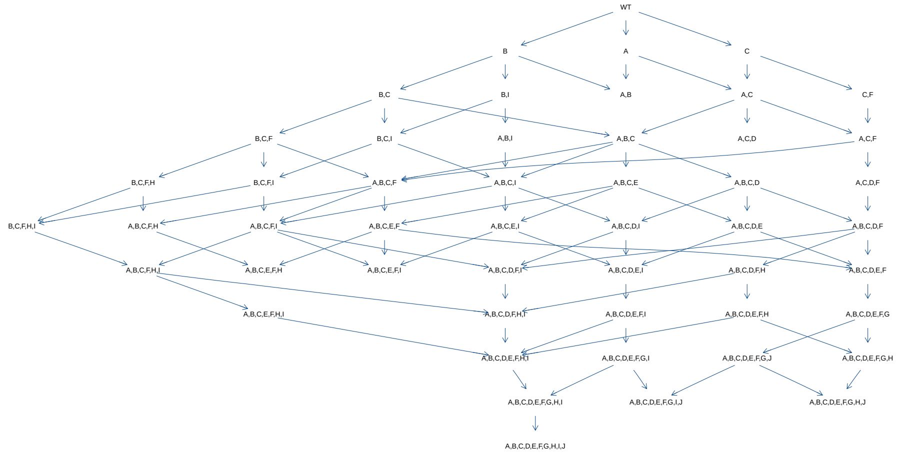

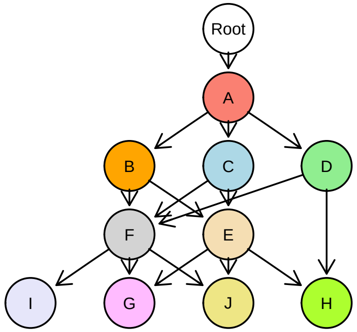

ID	q7yEvxoMttQHd9KY
acc genotypes	30
peaks no back	7
removed edges	10
prop rem edges	0.182

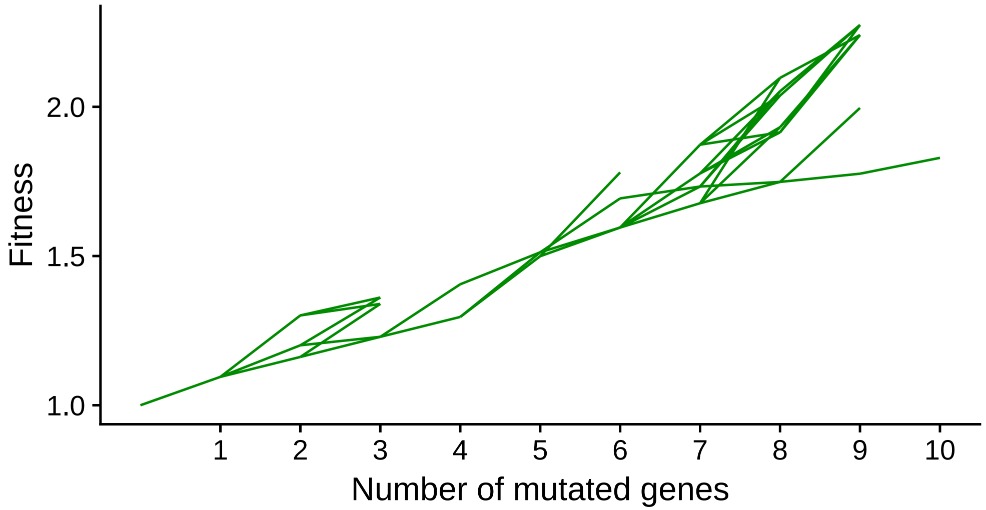

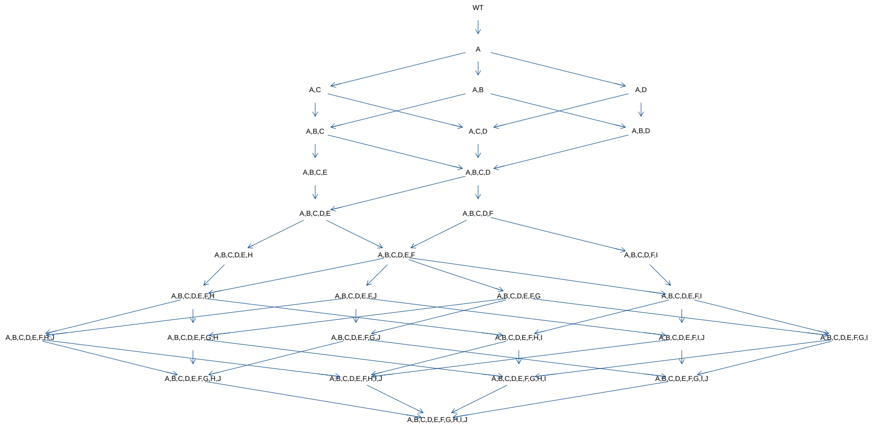

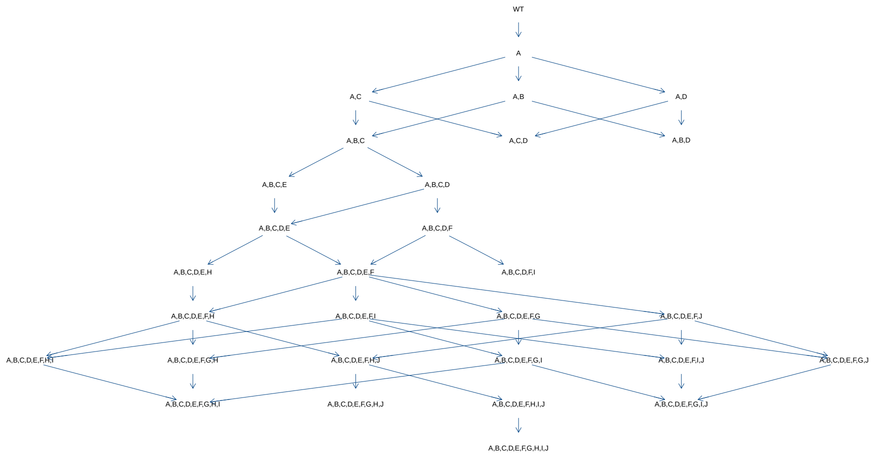

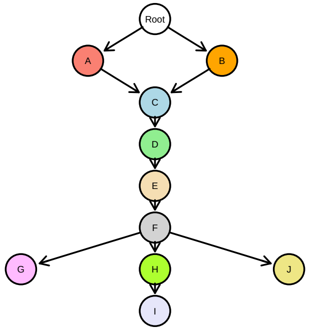

ID	qA5UjjBYFJWi5Ddc
acc genotypes	19
peaks no back	3
removed edges	2
prop rem edges	0.071

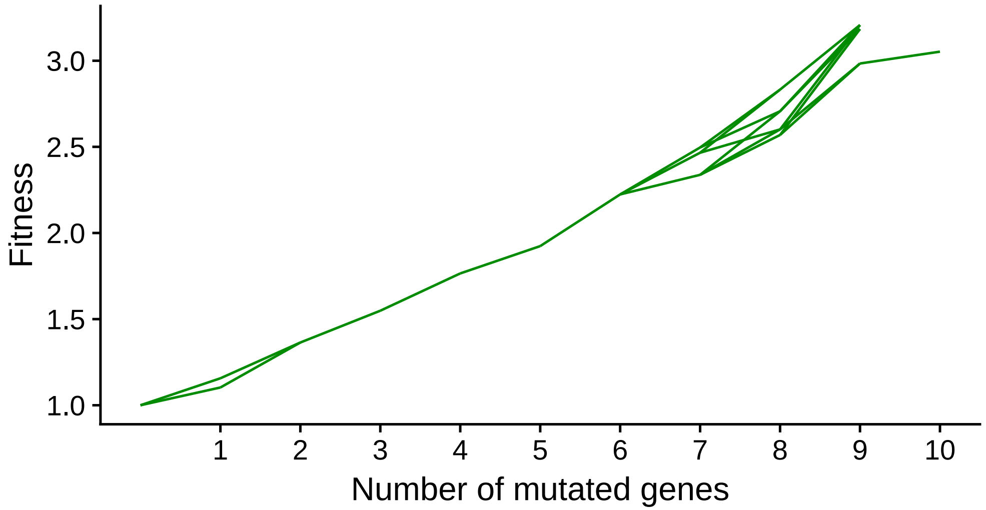

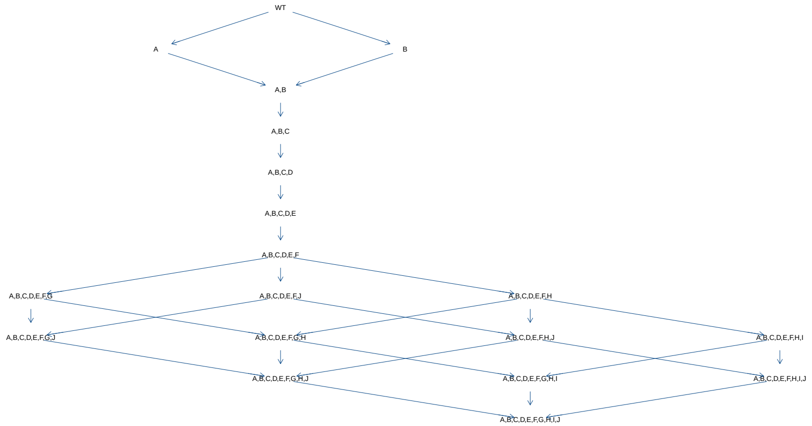

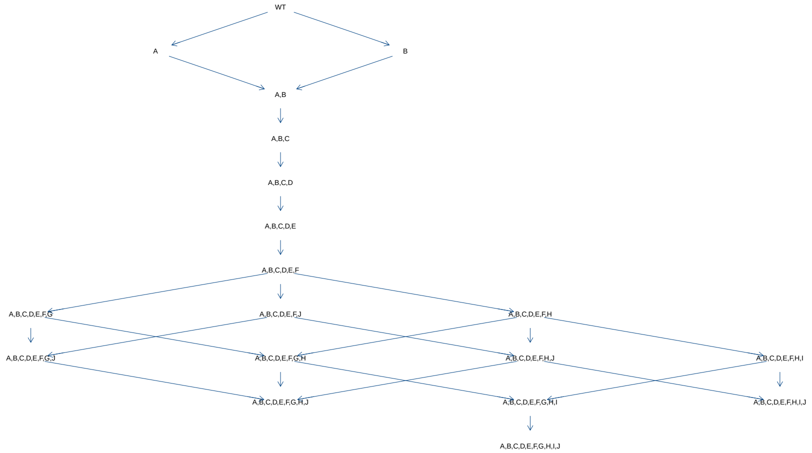

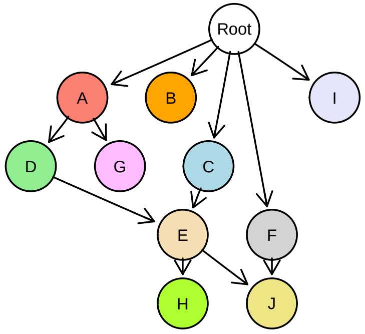

ID	QpSOnT7M3hBKpMNs
acc genotypes	128
peaks no back	13
removed edges	84
prop rem edges	0.219

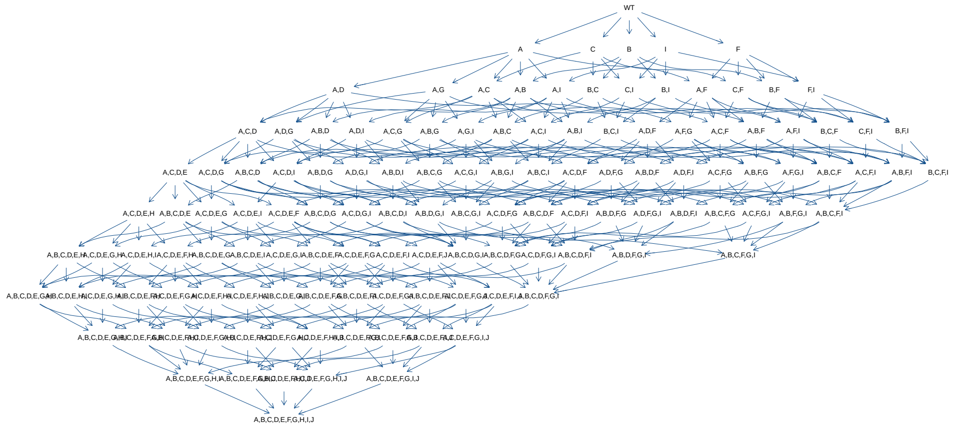

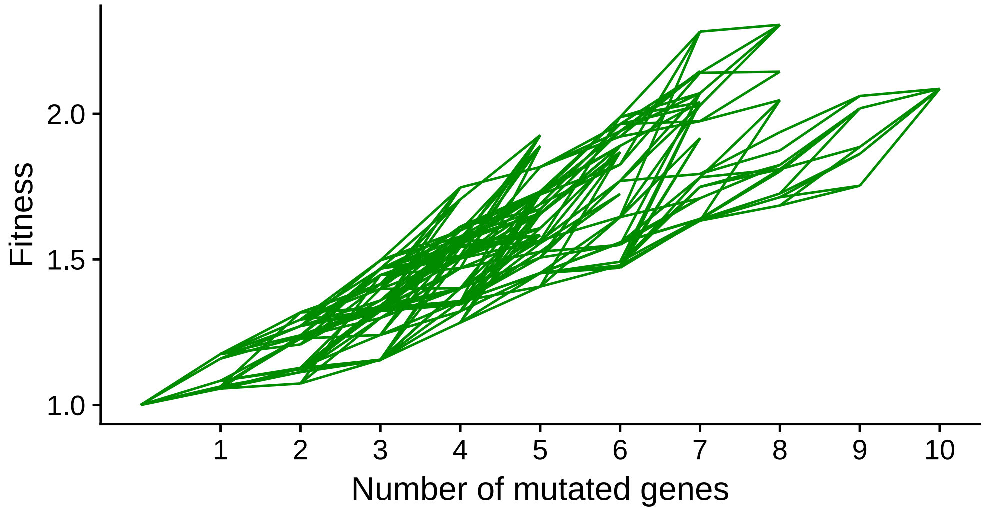

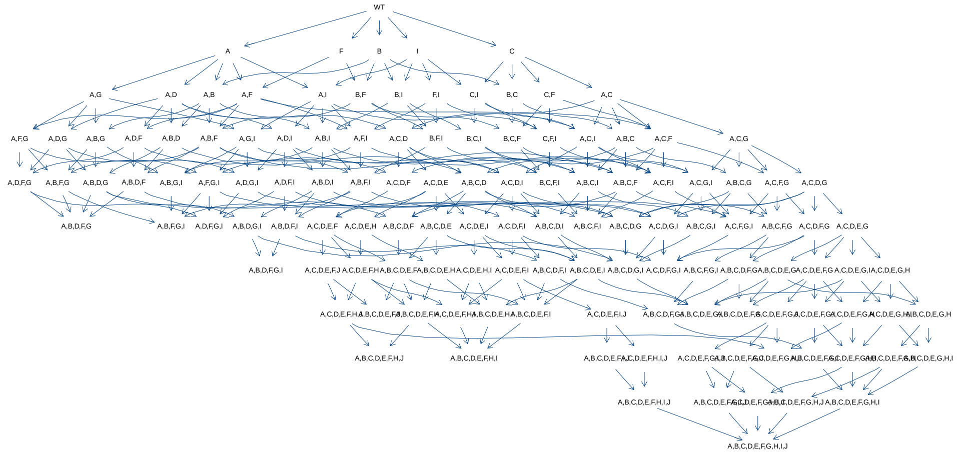

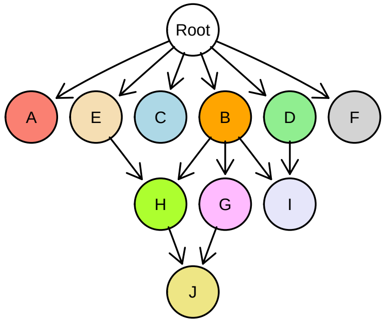

ID QTeBb3tzhv6dF9Iy
acc genotypes 200
peaks no back 16
removed edges 103
prop rem edges 0.154

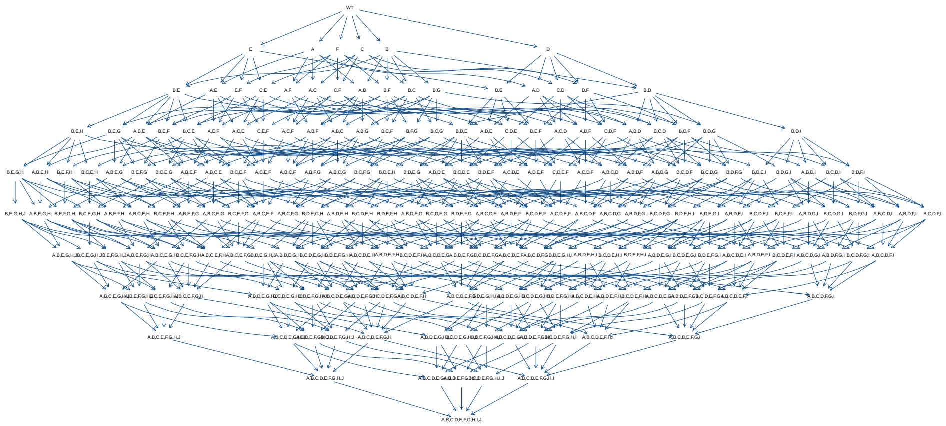

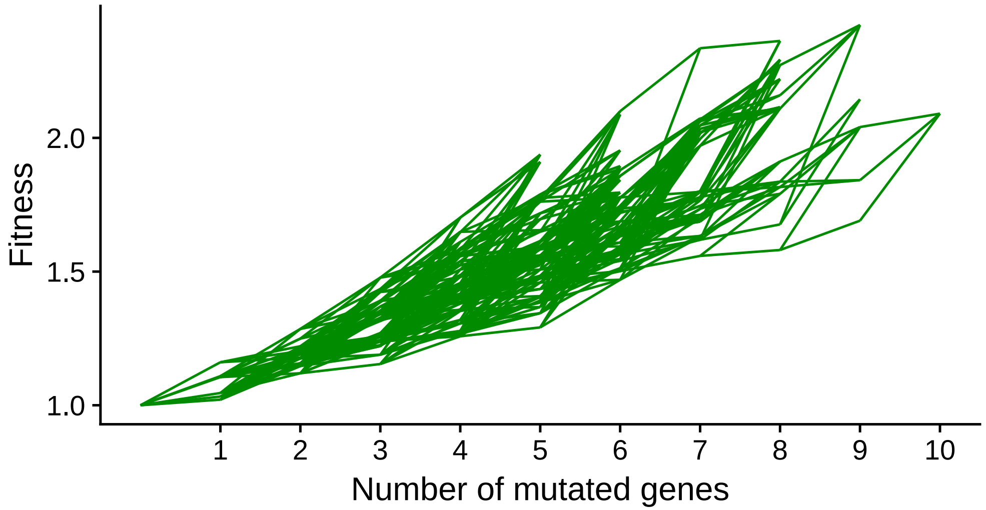

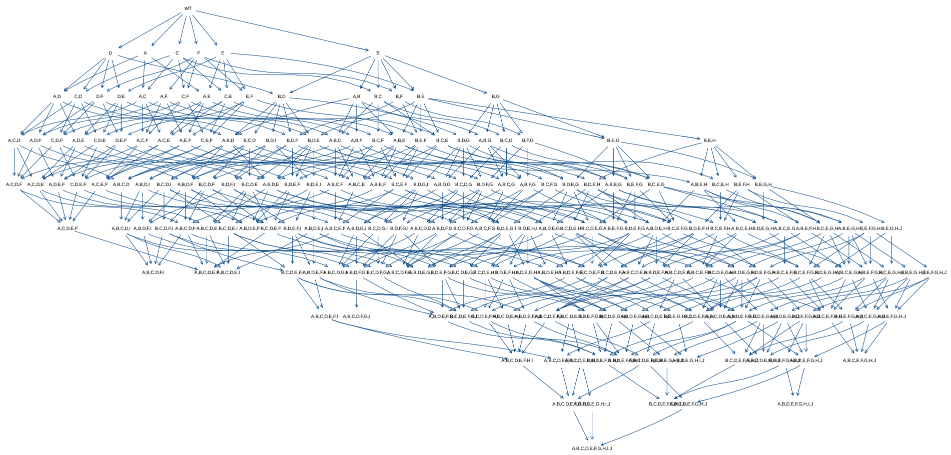

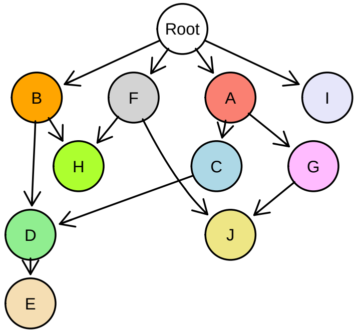

ID	QVEfHMuWmdeBZFrT
acc genotypes	94
peaks no back	11
removed edges	42
prop rem edges	0.171

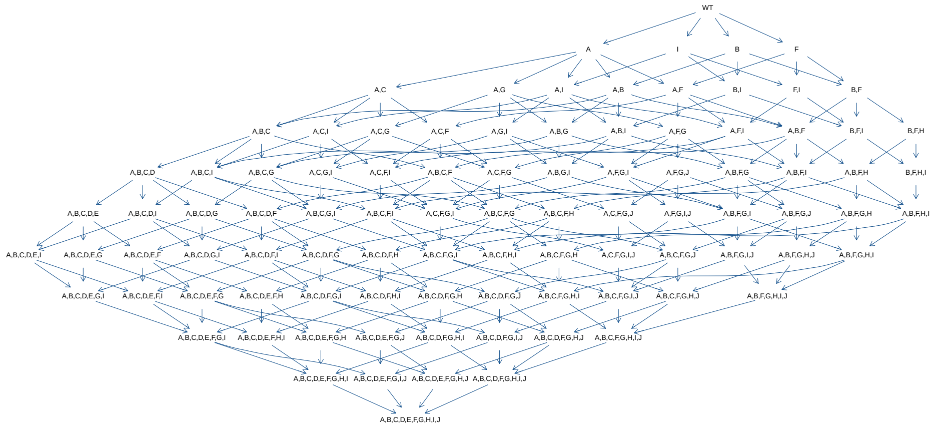

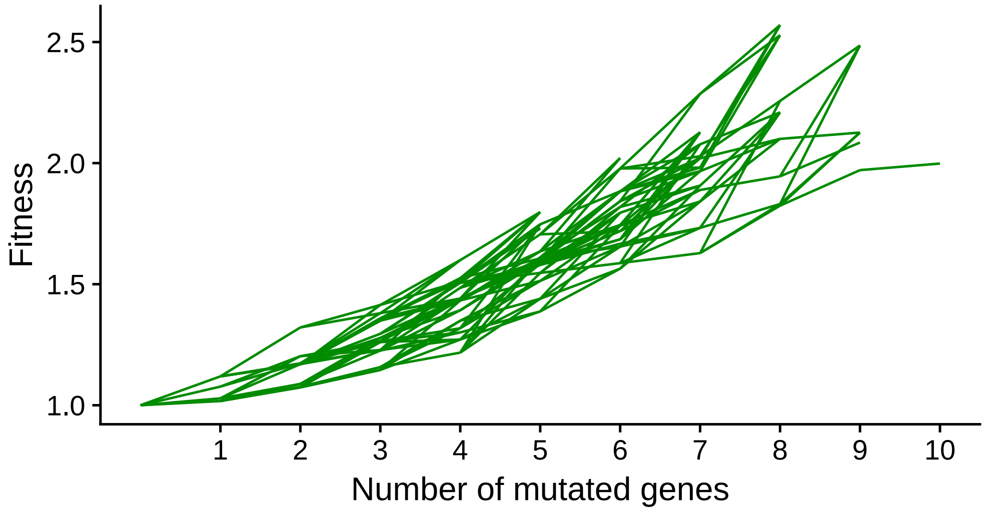

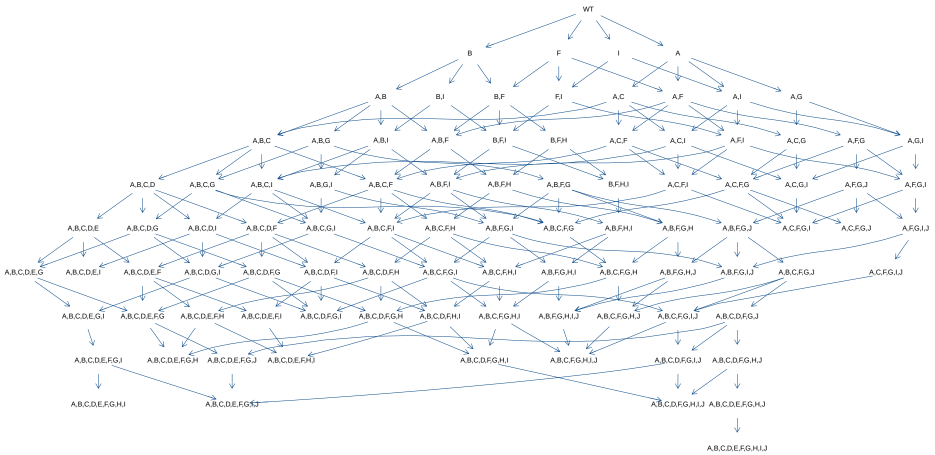

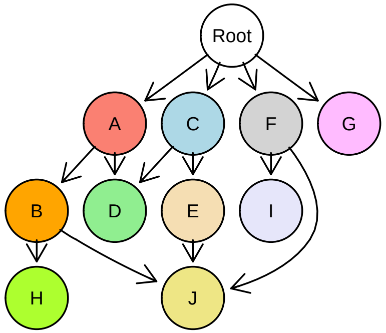

ID	qzPma2ONgy7W10OcP
acc genotypes	124
peaks no back	12
removed edges	68
prop rem edges	0.192

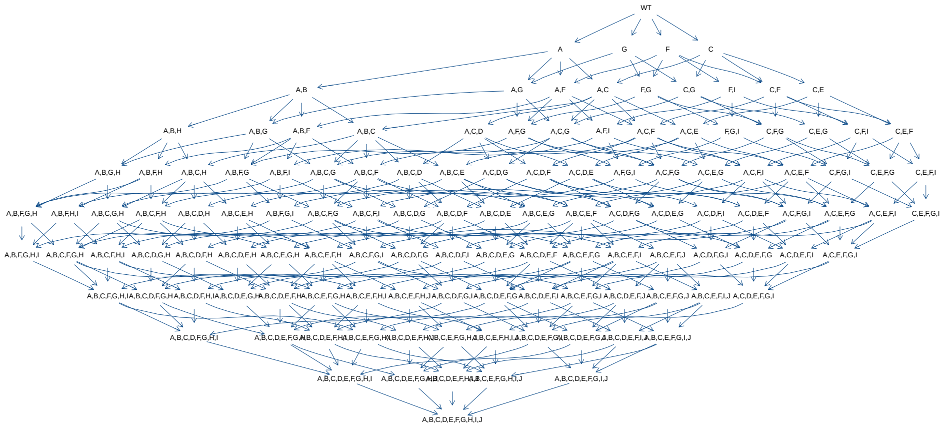

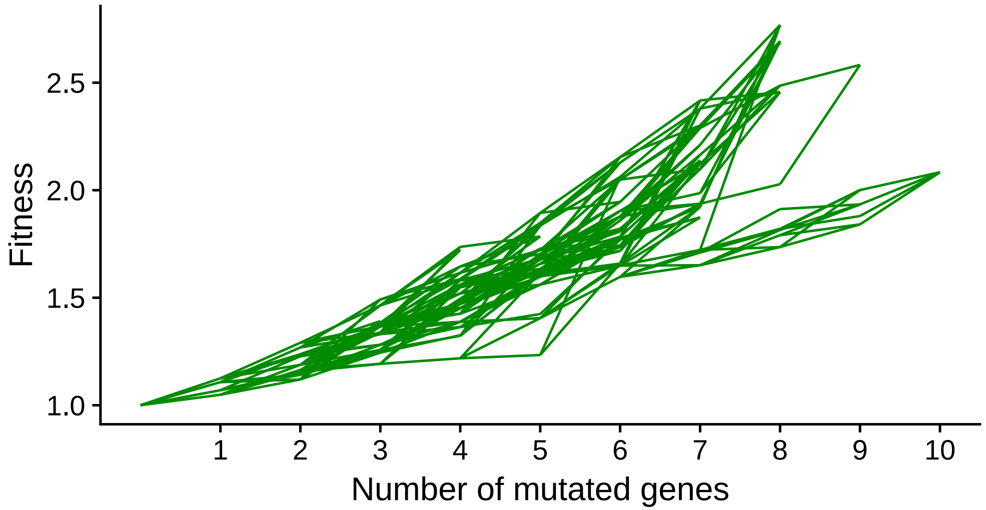

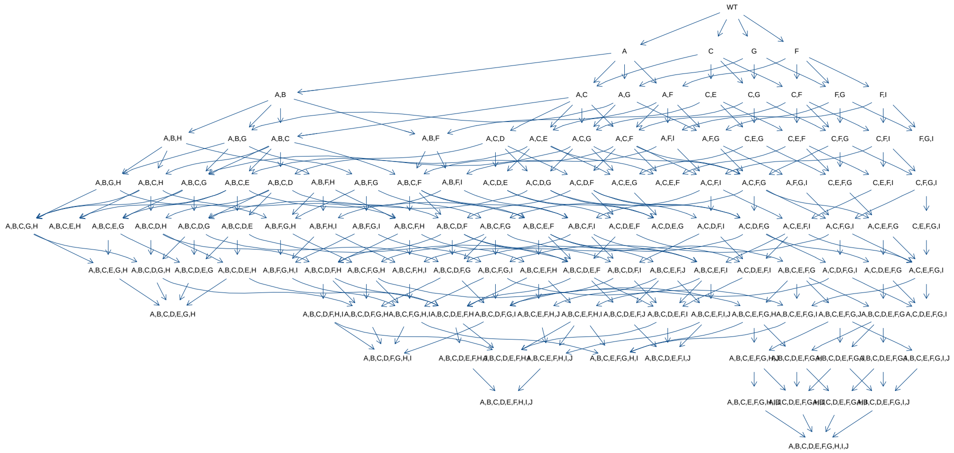

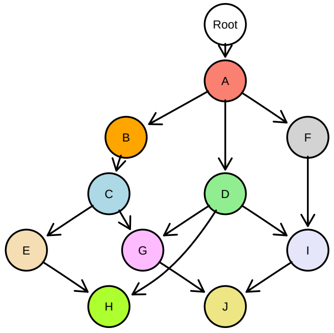

ID	r10ojqpU7Q5rU9ub3
acc genotypes	36
peaks no back	6
removed edges	12
prop rem edges	0.179

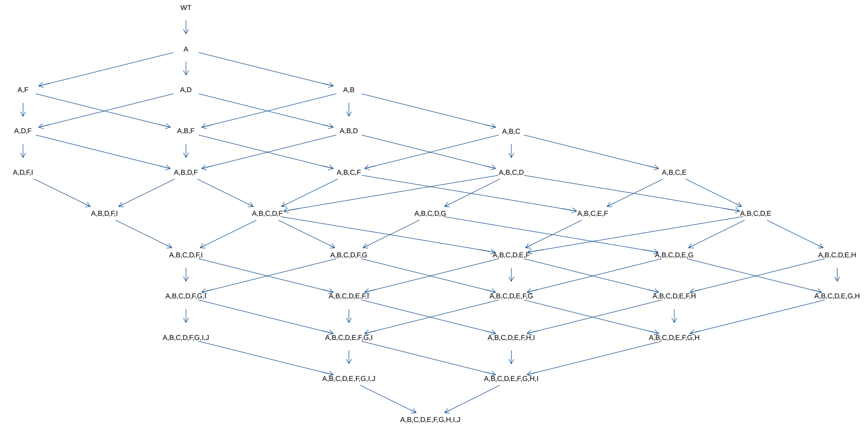

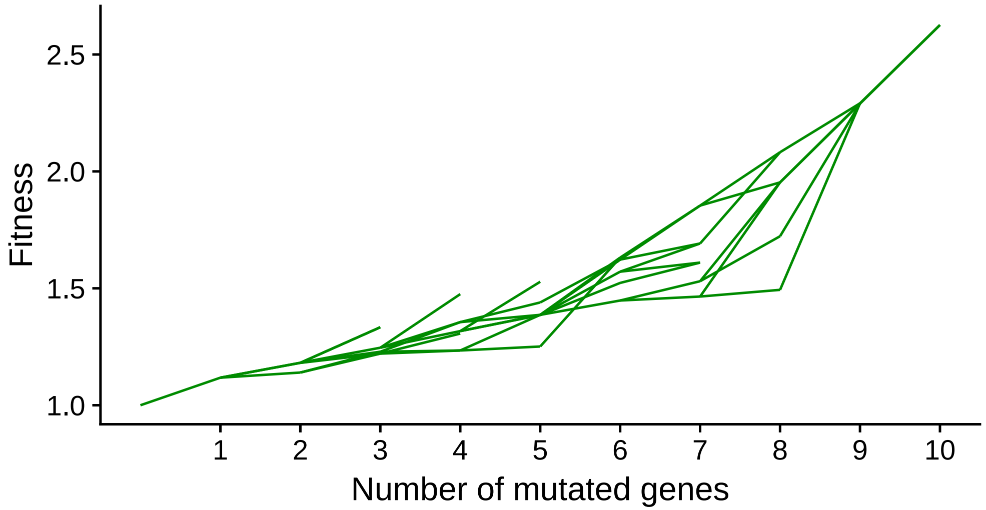

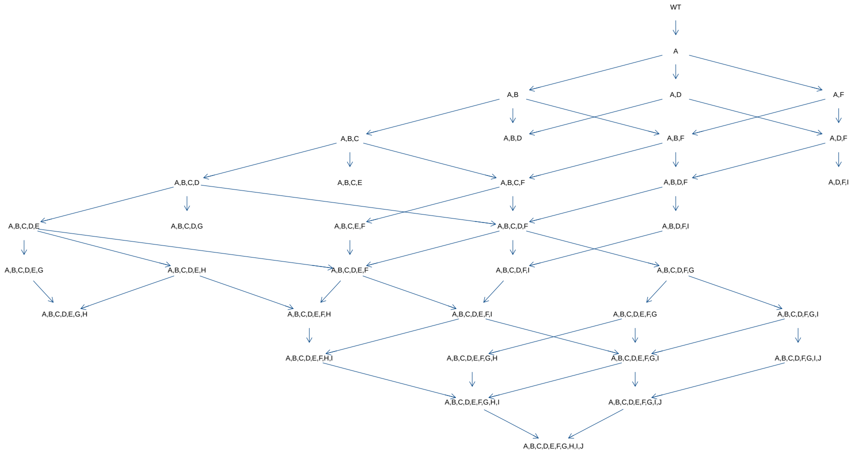

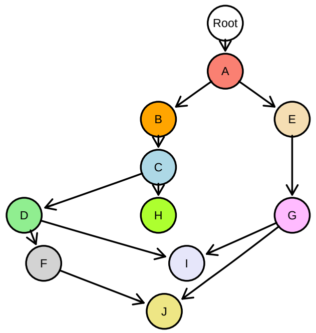

ID R4ZN7nnB4ujxYl9L

acc genotypes 33

peaks no back 5

removed edges 8

prop rem edges 0.133

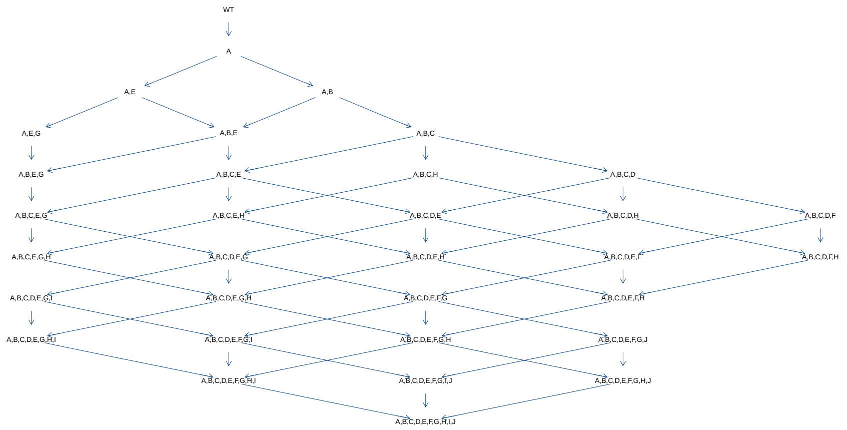

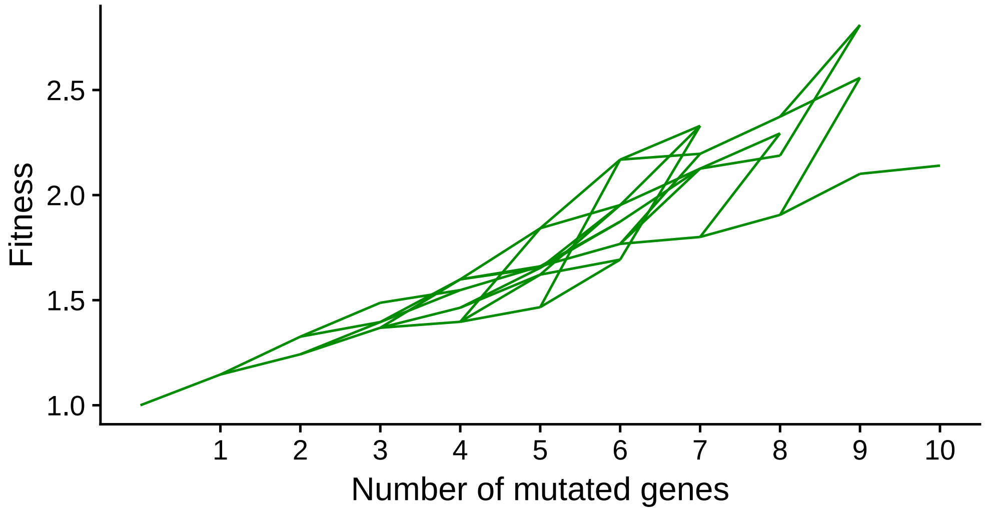

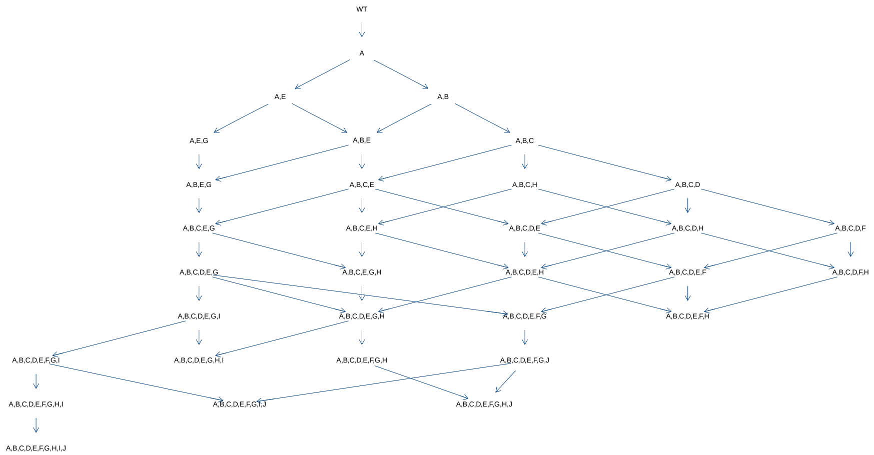

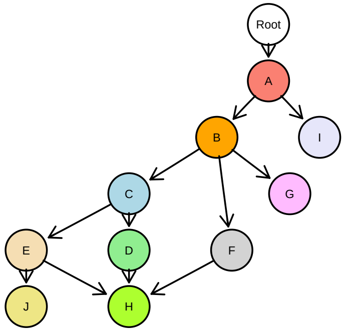

ID	R5UIg5w7GGy7xUcV
acc genotypes	67
peaks no back	8
removed edges	36
prop rem edges	0.209

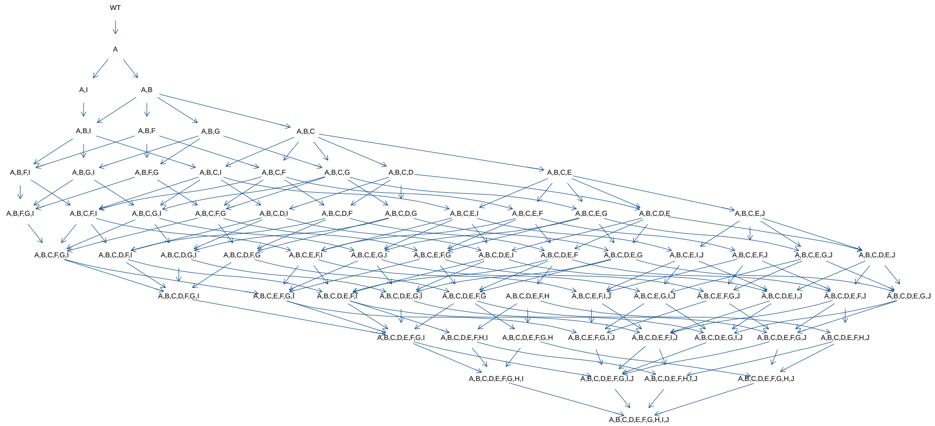

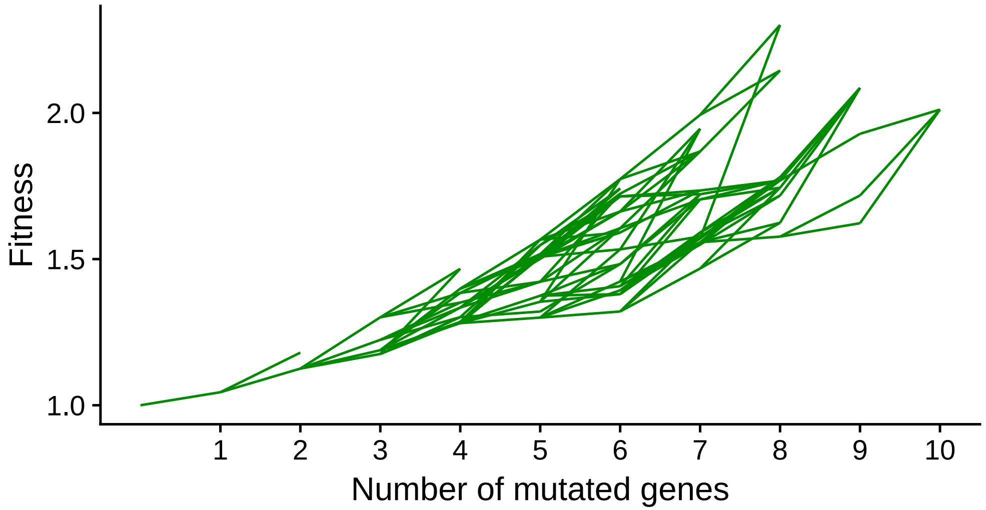

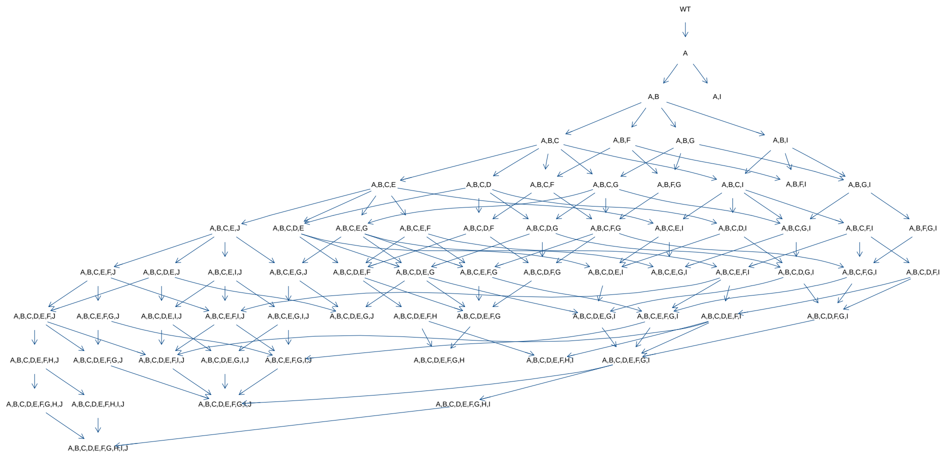

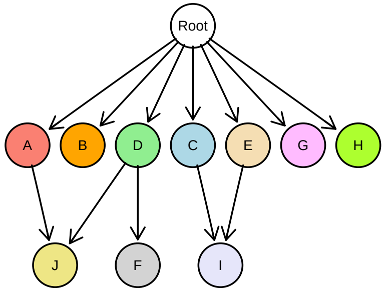

ID	RcLGnMwFAV7yHcPX
acc genotypes	320
peaks no back	19
removed edges	237
prop rem edges	0.198

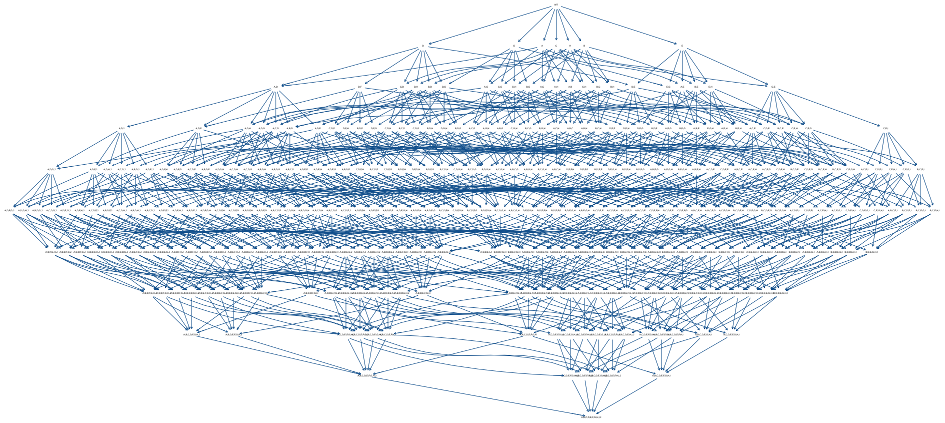

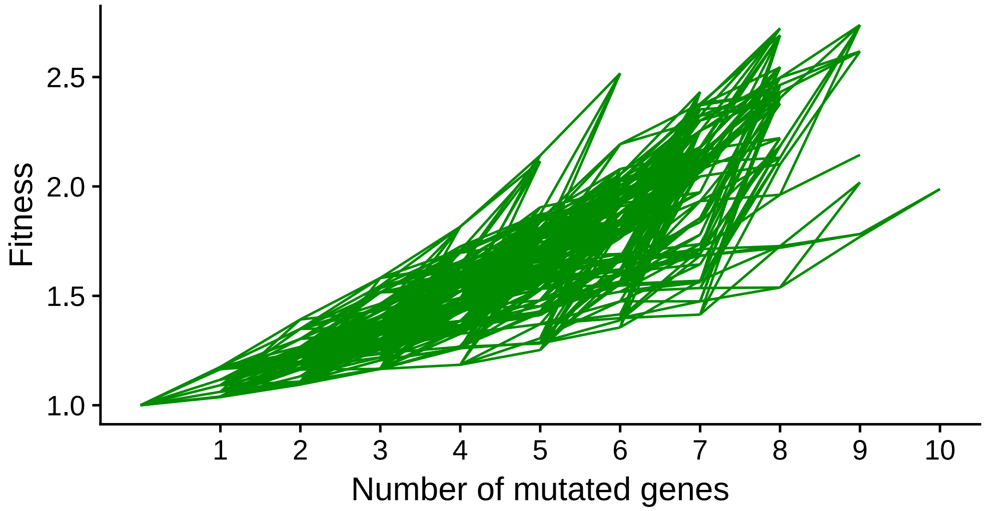

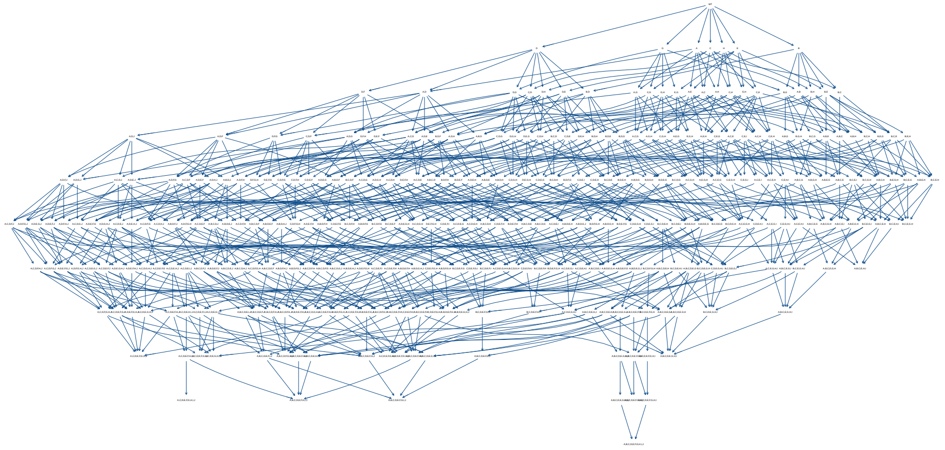

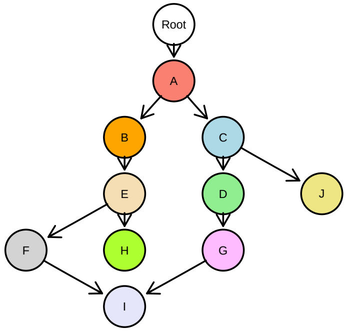

ID	RMPsjUicmruxvFMT
acc genotypes	47
peaks no back	8
removed edges	22
prop rem edges	0.222

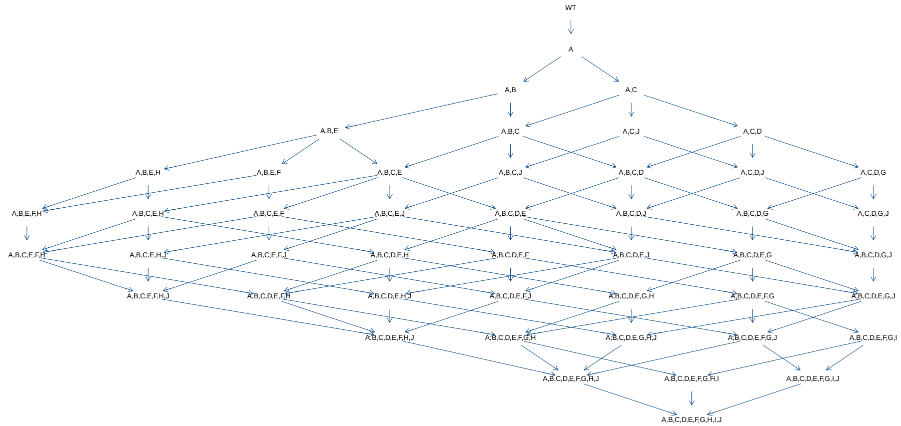

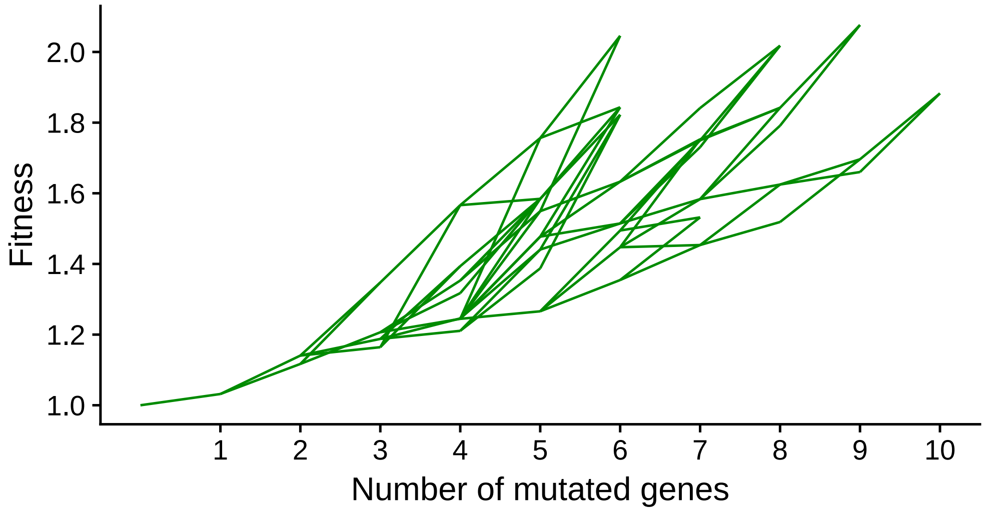

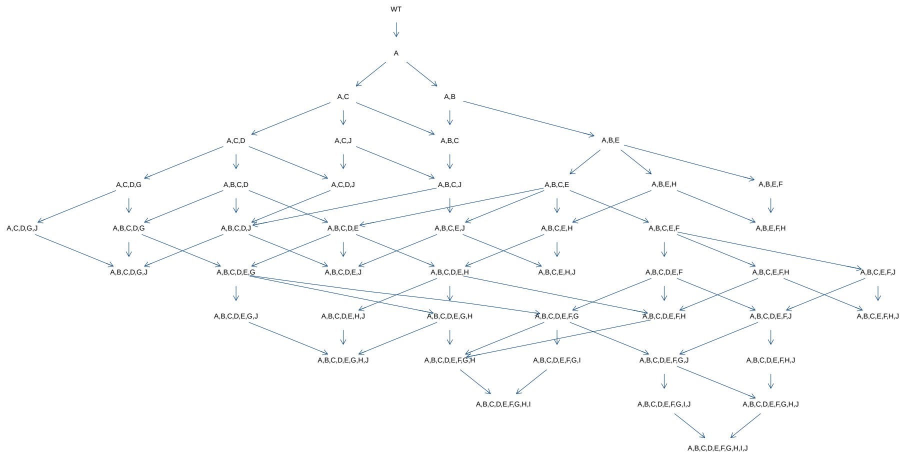

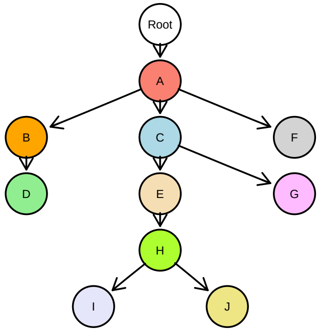

ID	rRQ3Bws3107fO7mHM
acc genotypes	79
peaks no back	11
removed edges	41
prop rem edges	0.199

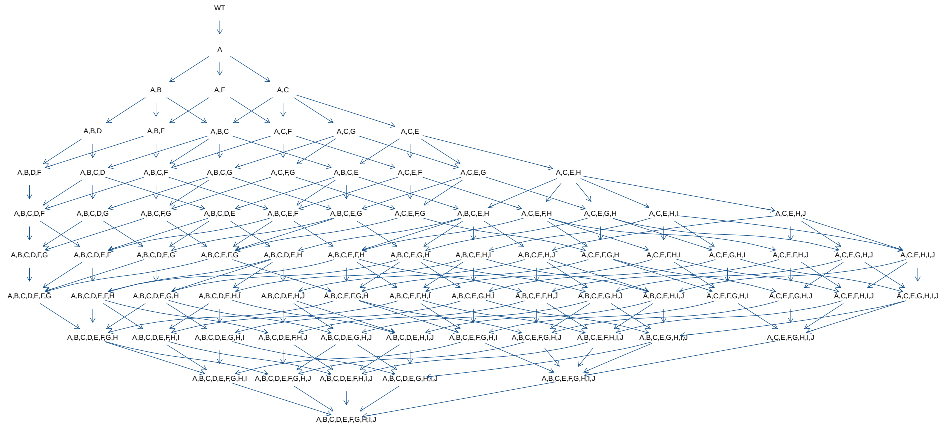

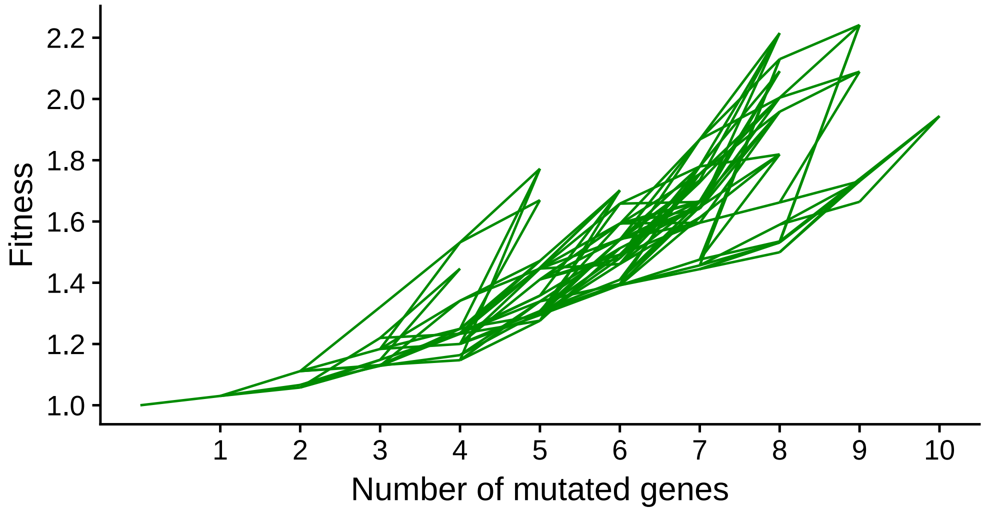

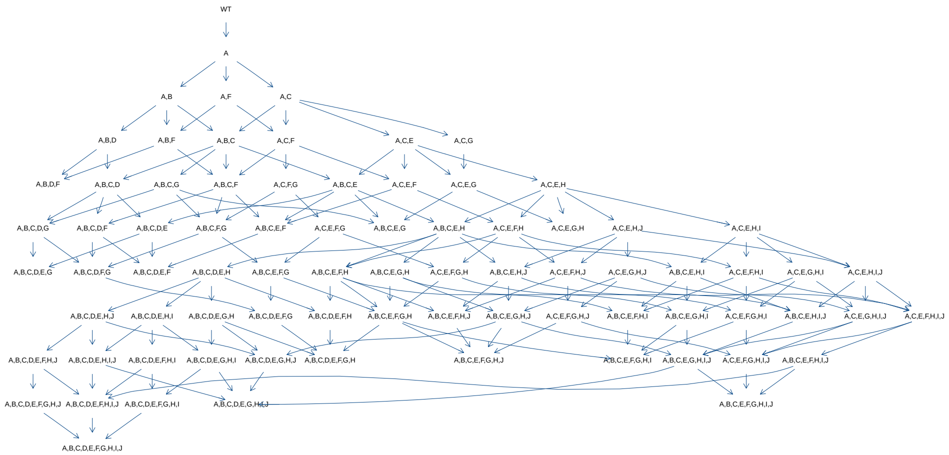

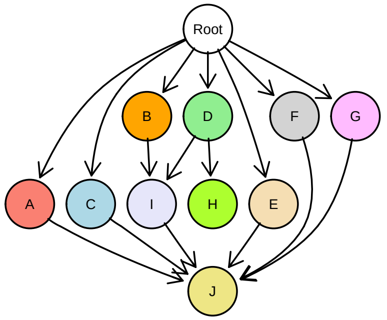

ID	SArTeHv6MV7tVCry
acc genotypes	258
peaks no back	16
removed edges	161
prop rem edges	0.167

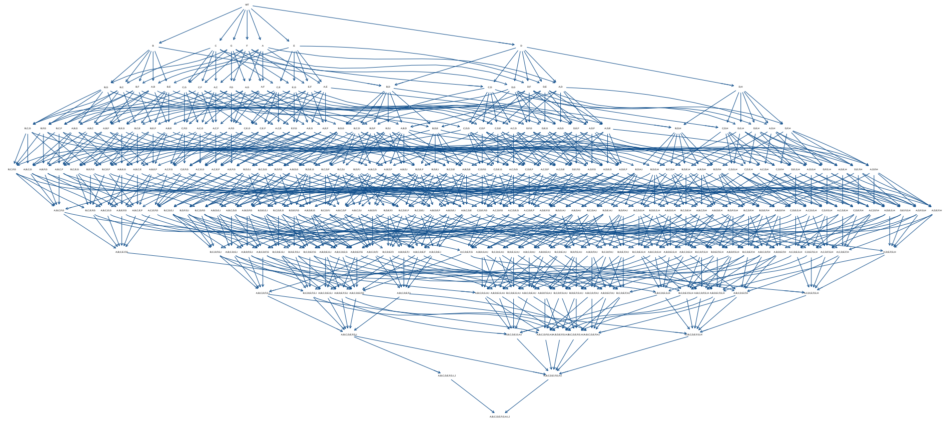

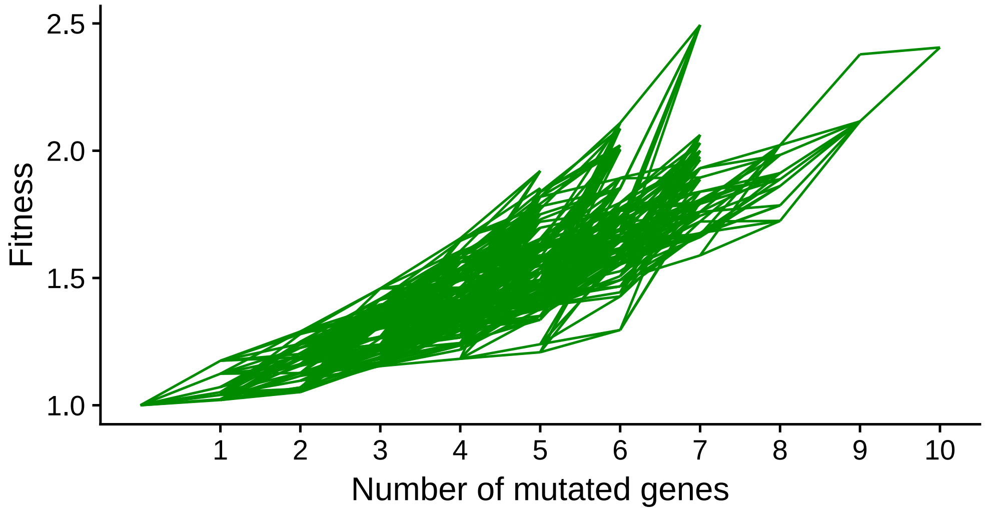

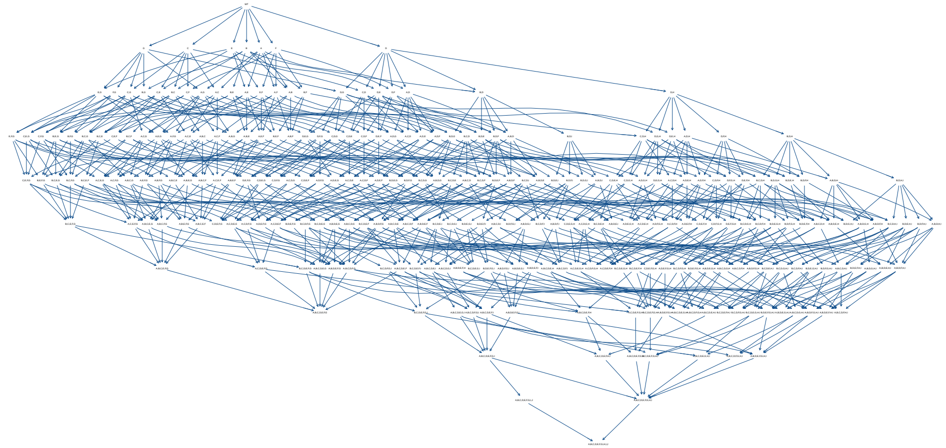

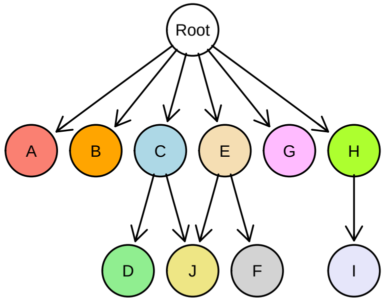

ID	SlYbZPVjDjsPd3OJ
acc genotypes	312
peaks no back	21
removed edges	259
prop rem edges	0.224

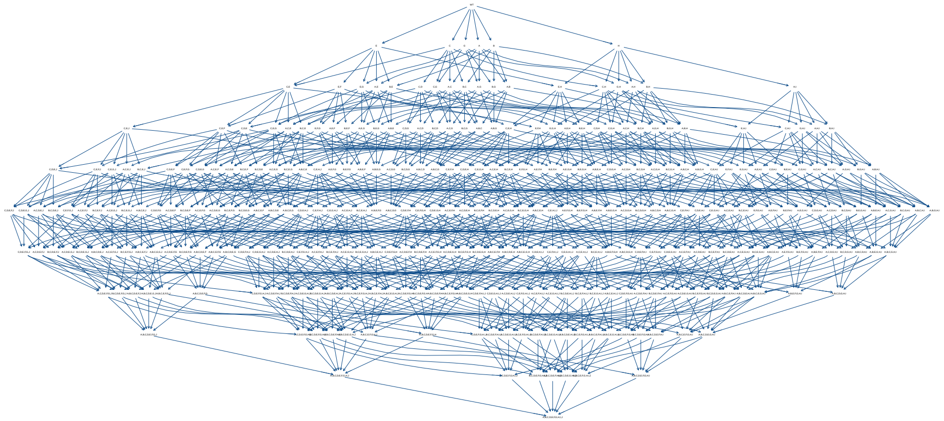

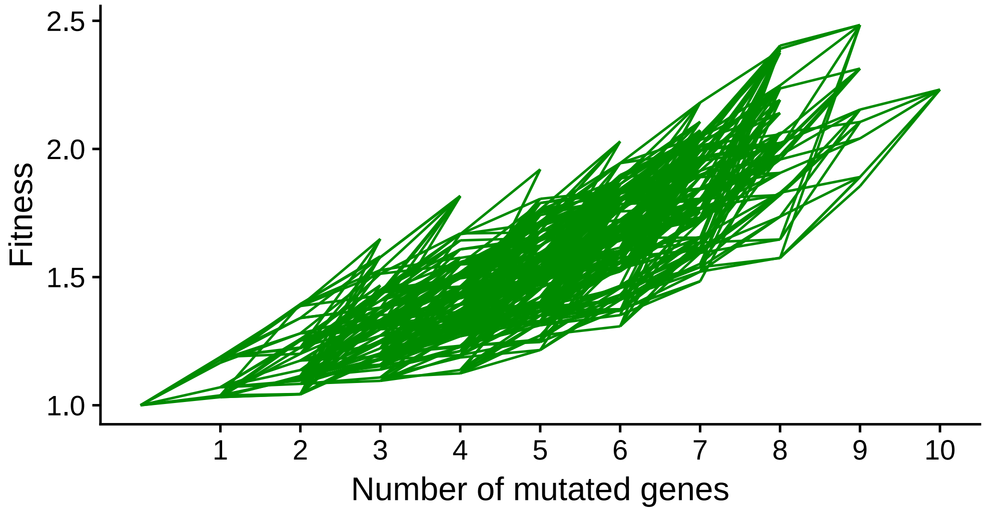

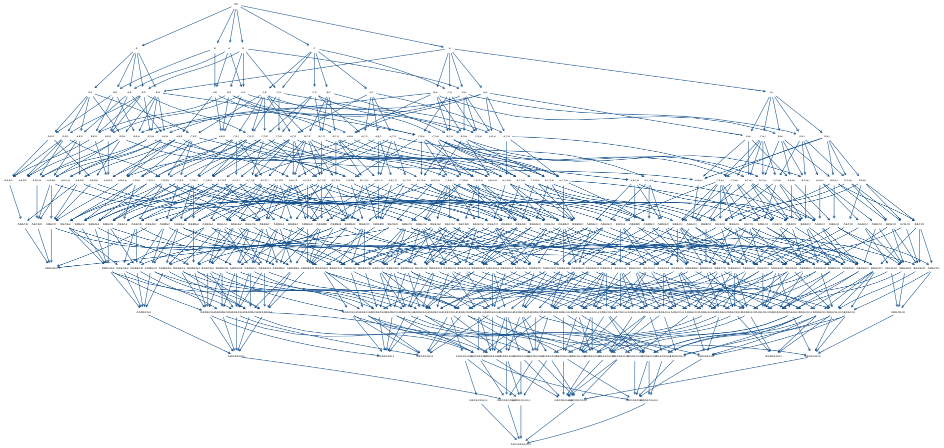

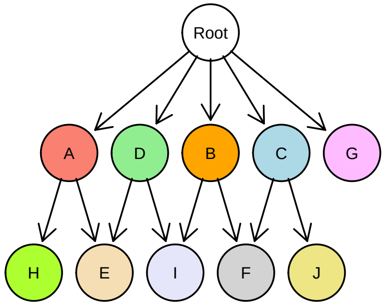

ID	smwY8F2IPkzSNFOb
acc genotypes	178
peaks no back	17
removed edges	101
prop rem edges	0.181

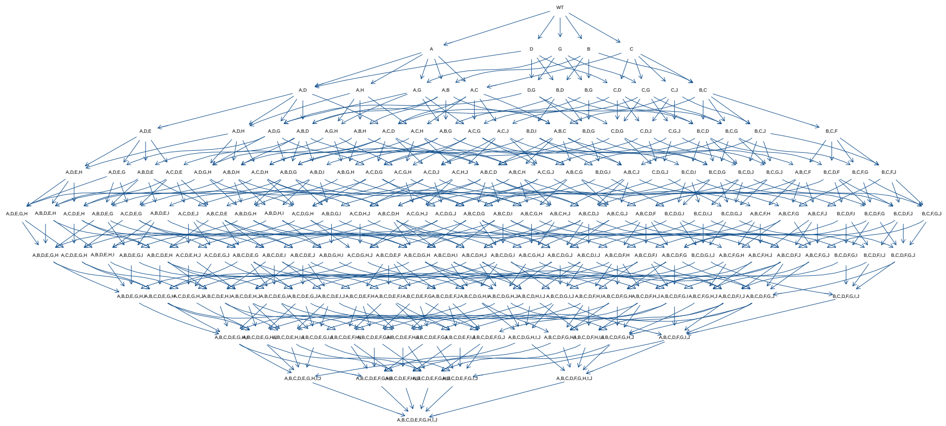

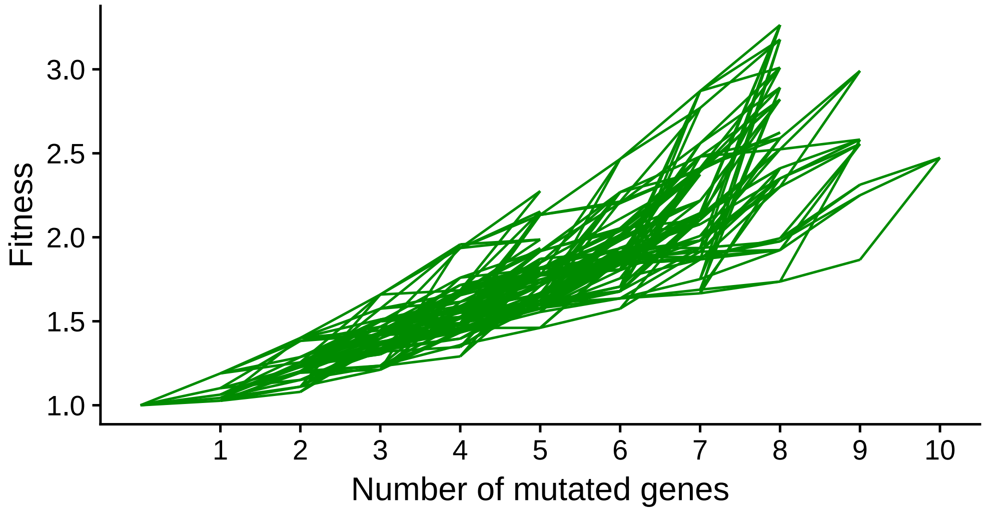

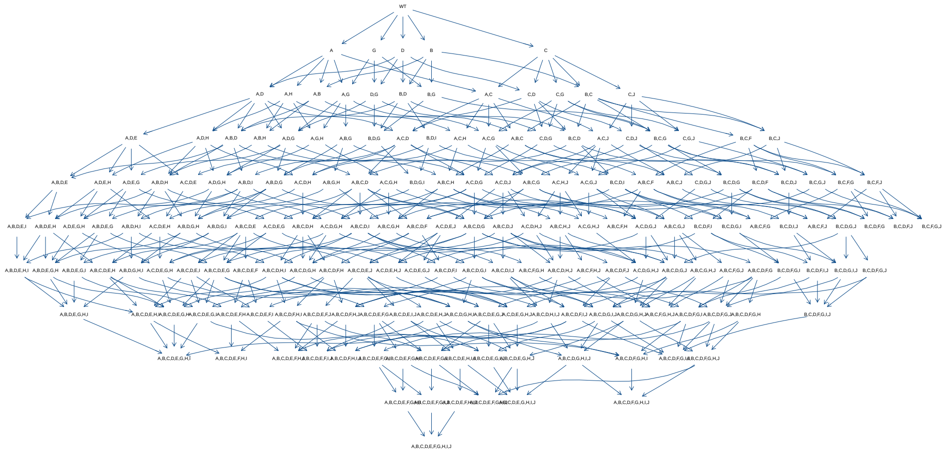

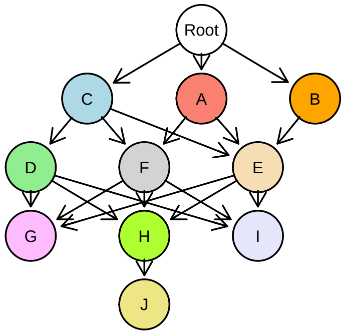

ID	sO7ye5yMrhvX10WZQ
acc genotypes	31
peaks no back	2
removed edges	2
prop rem edges	0.036

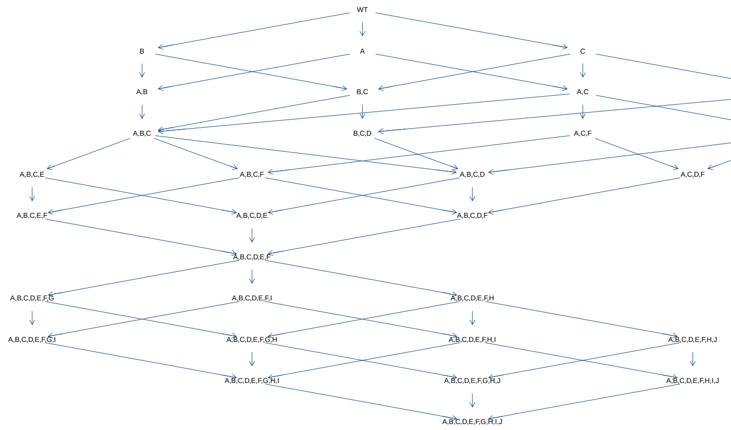

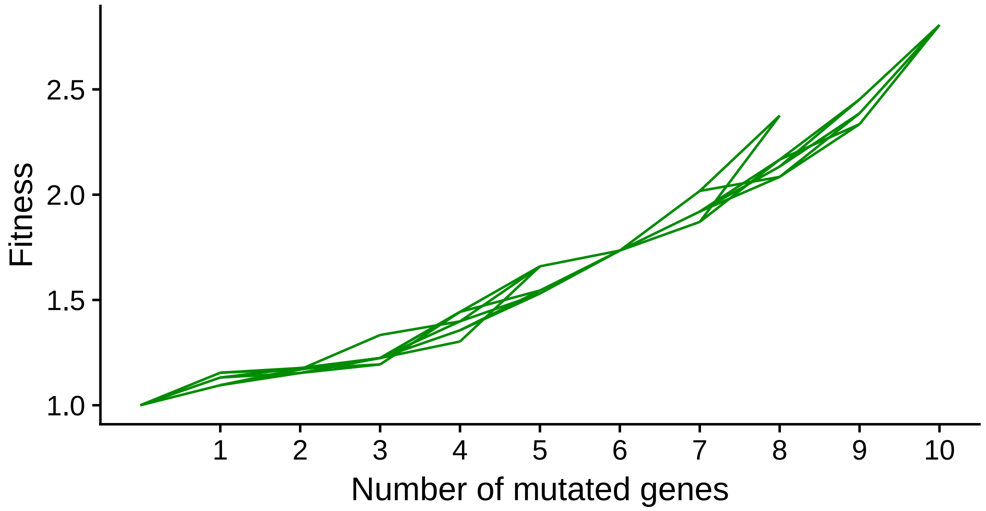

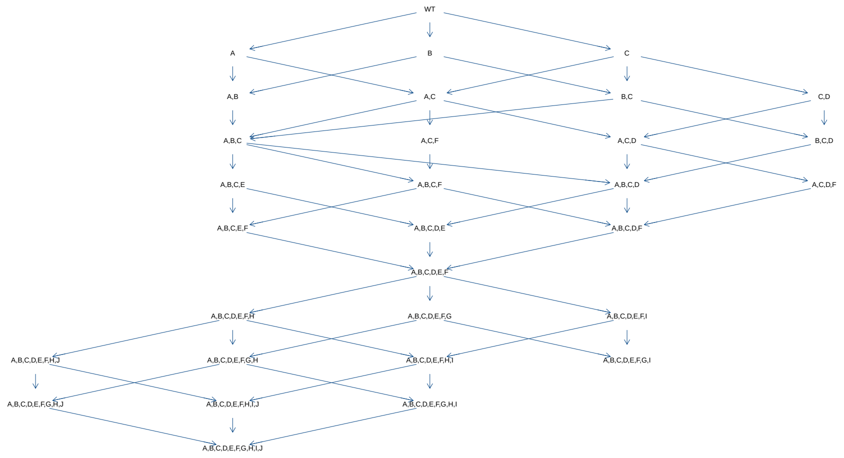

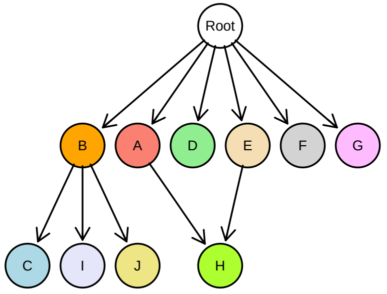

ID	sUe5FH9IUIMDiI10B
acc genotypes	360
peaks no back	23
removed edges	252
prop rem edges	0.177

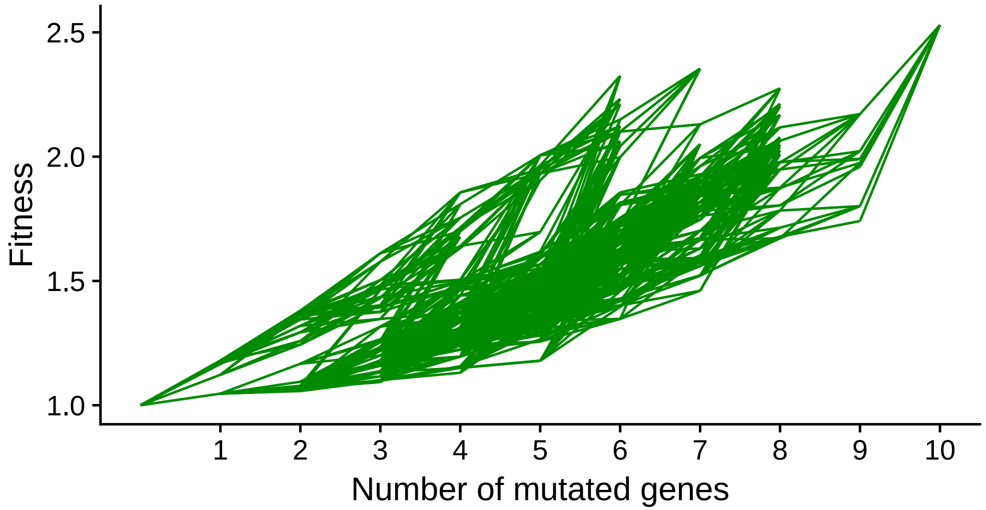

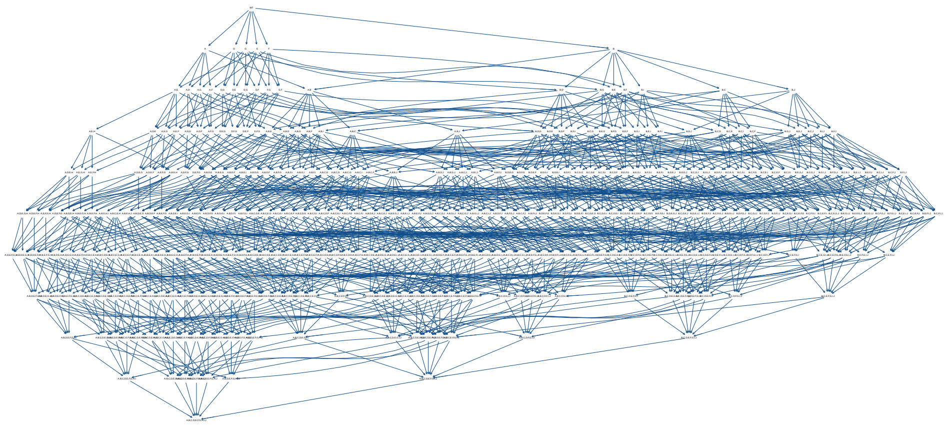

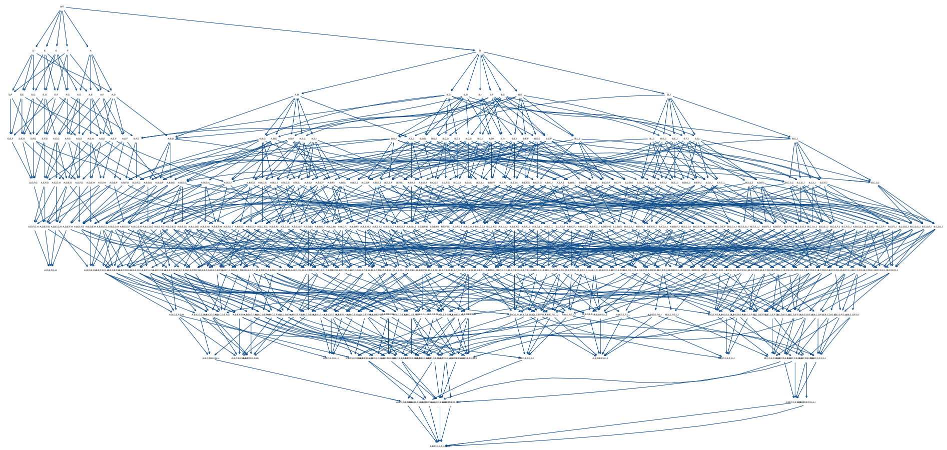

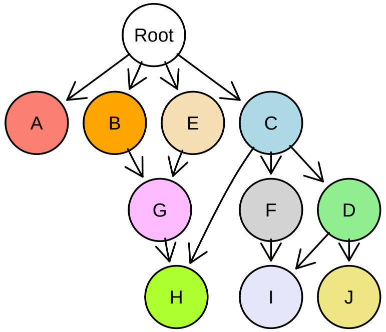

ID	SWgUDoSPxMcdnnQM
acc genotypes	106
peaks no back	12
removed edges	52
prop rem edges	0.18

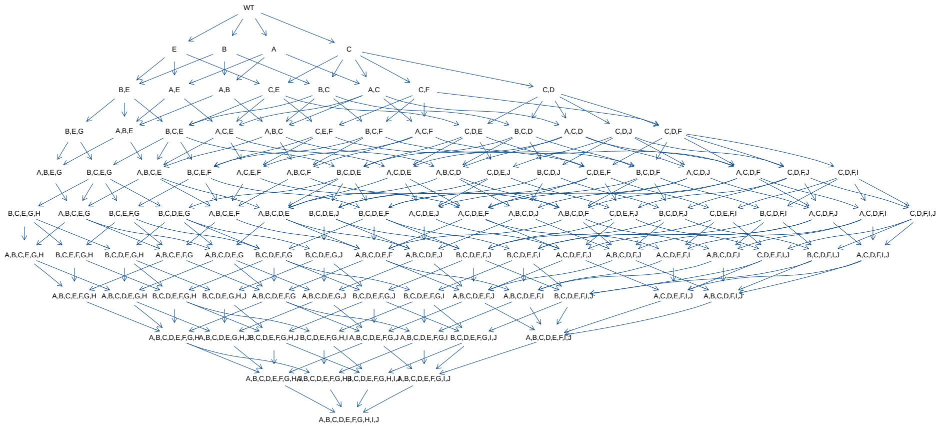

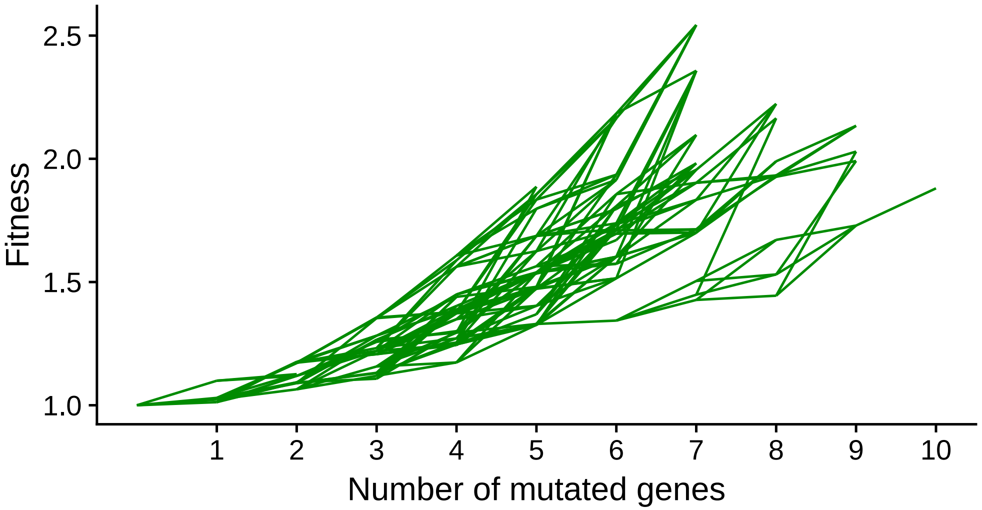

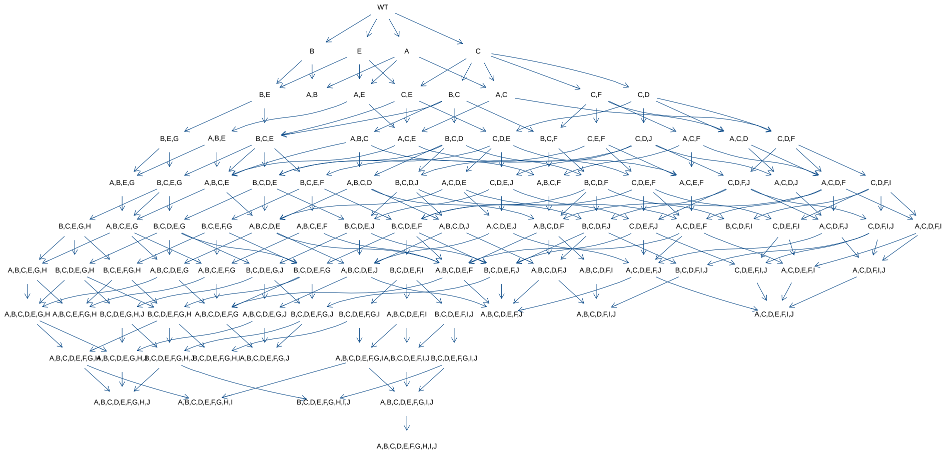

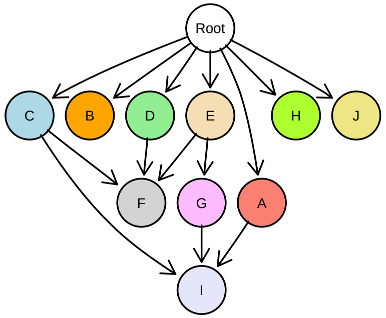

ID	sxE6qJHh512sjV9a
acc genotypes	248
peaks no back	16
removed edges	163
prop rem edges	0.183

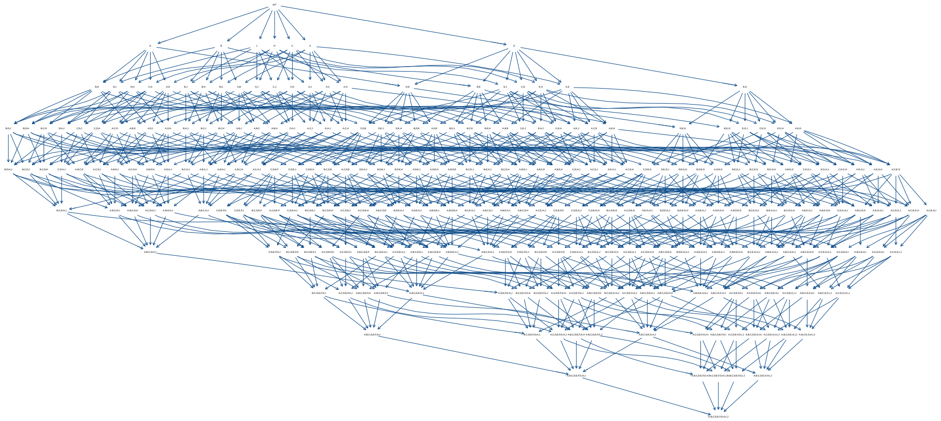

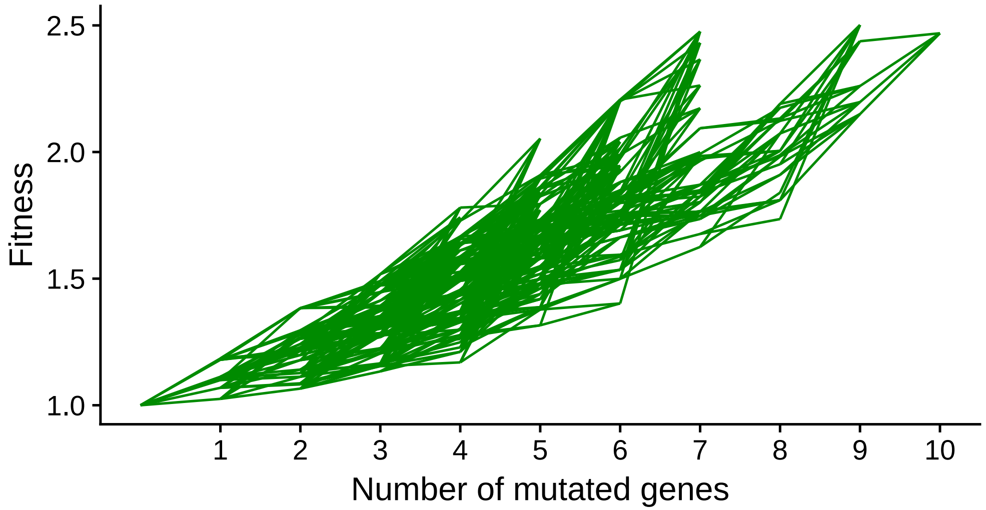

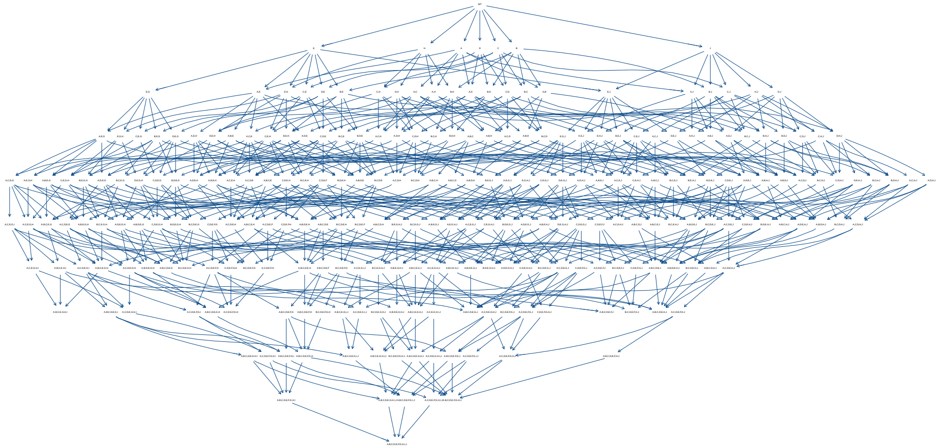

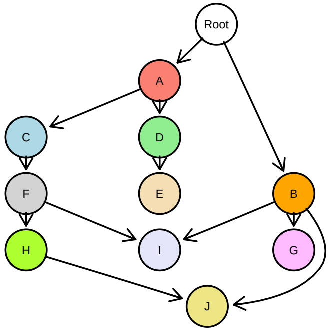

ID	t10jgW9R9zZrcAqjl
acc genotypes	63
peaks no back	12
removed edges	30
prop rem edges	0.208

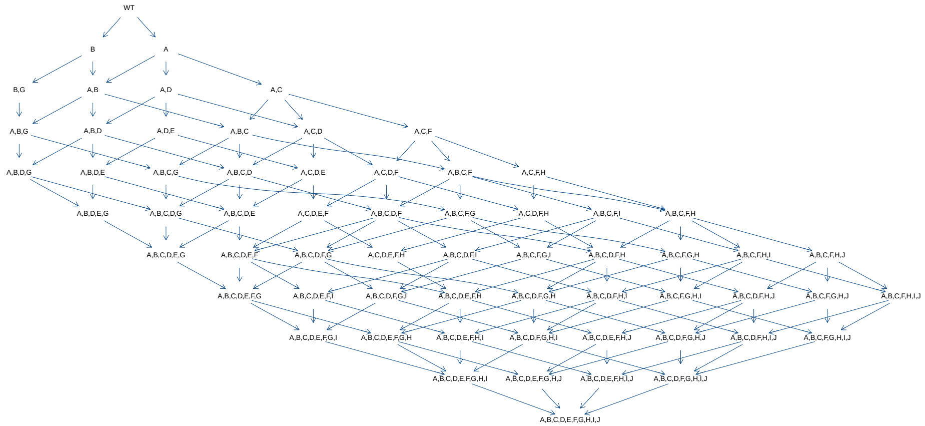

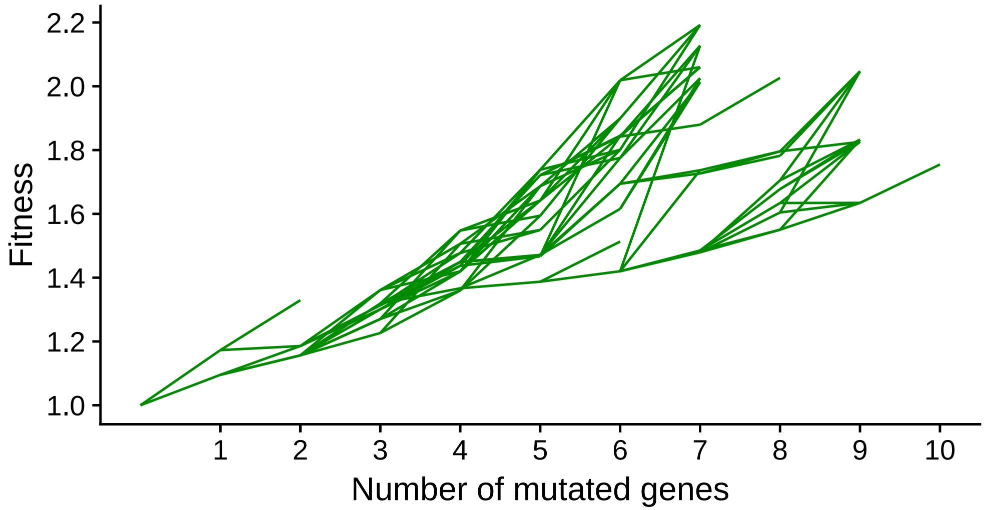

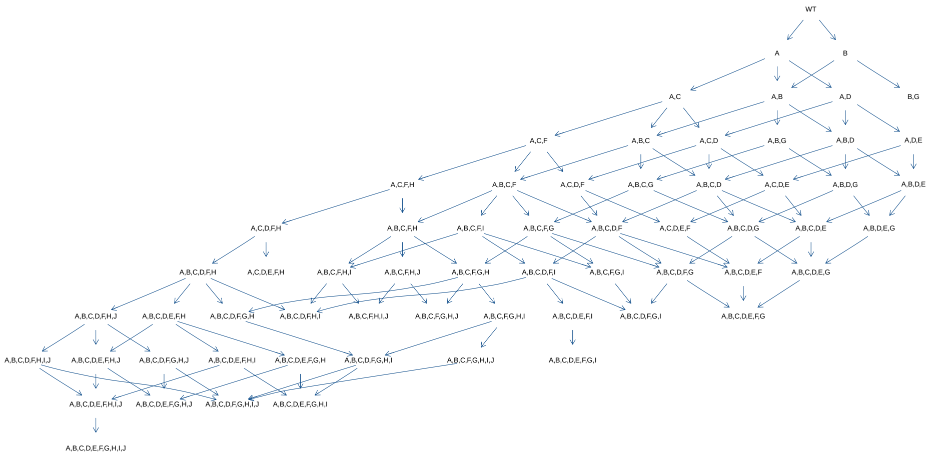

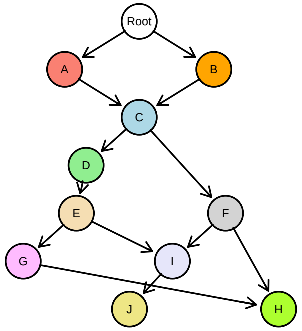

ID	t1yUXsv5fVuo10GRi
acc genotypes	19
peaks no back	2
removed edges	2
prop rem edges	0.077

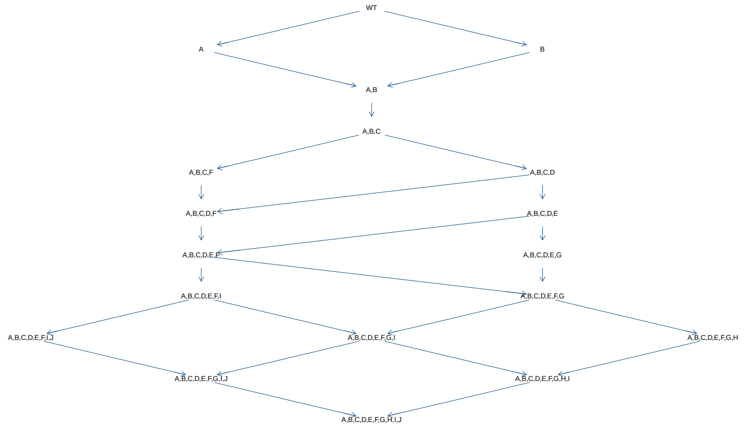

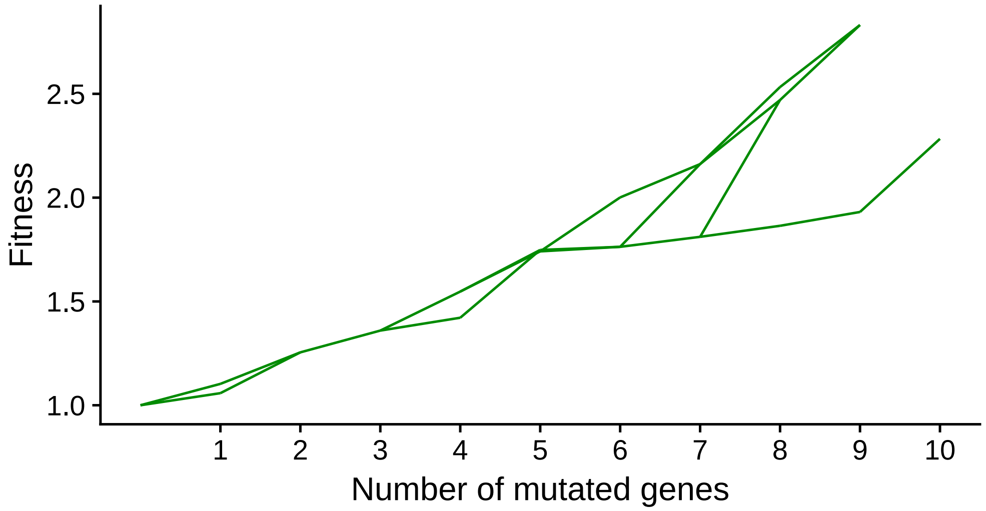

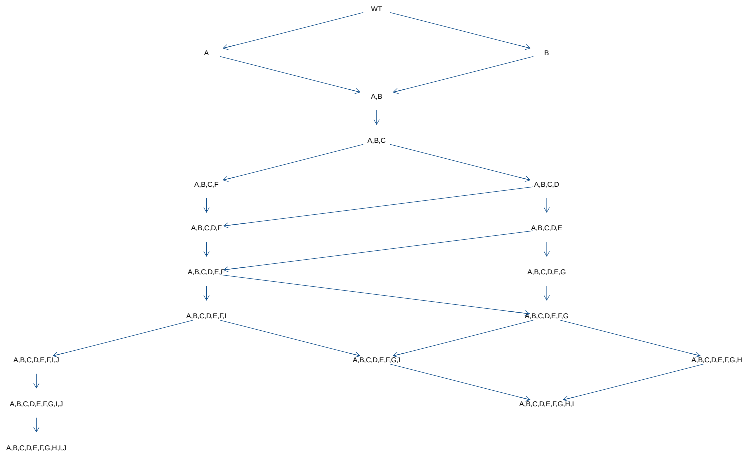

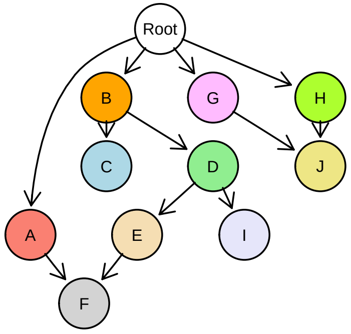

ID	tBuq3Z94BsQjawuL
acc genotypes	130
peaks no back	14
removed edges	92
prop rem edges	0.239

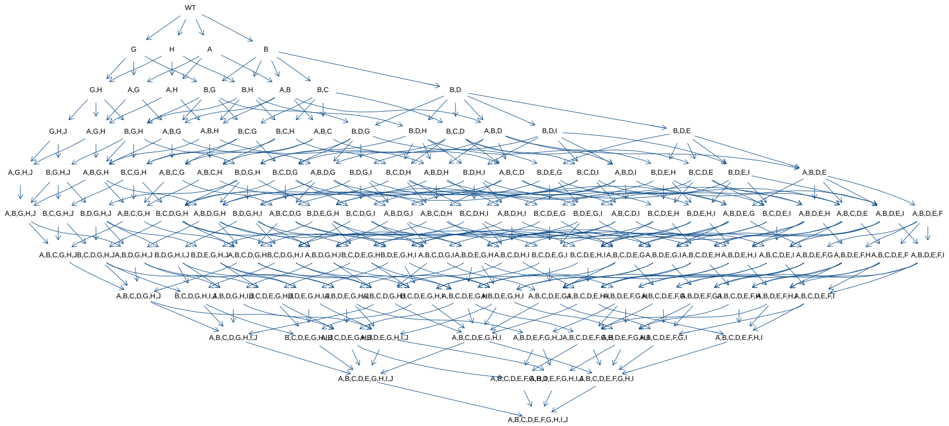

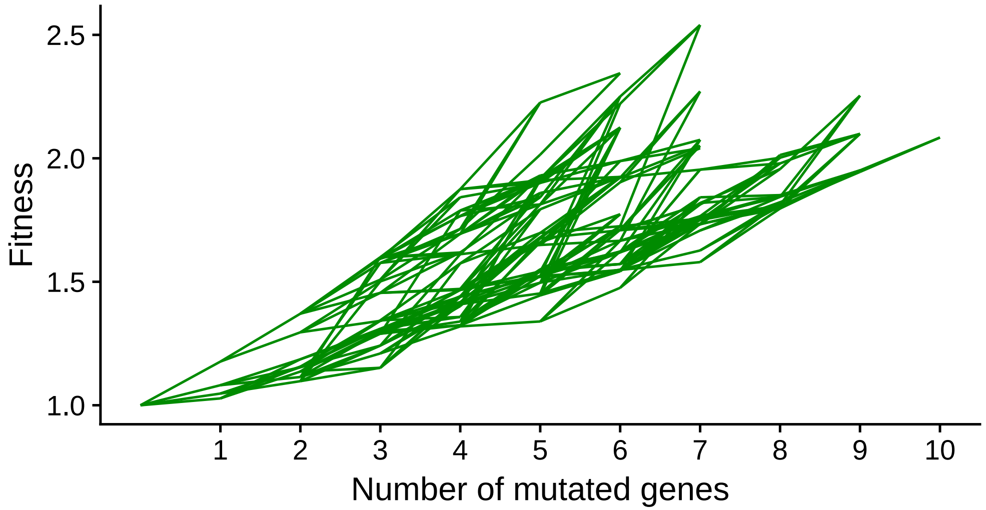

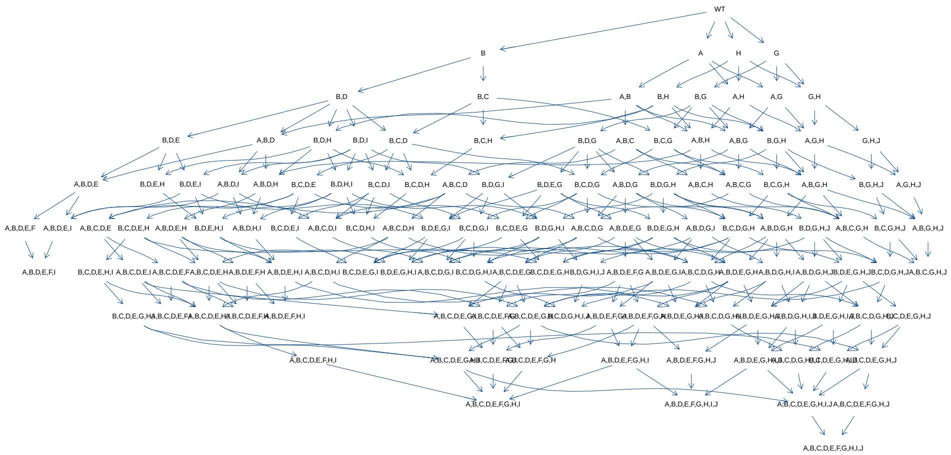

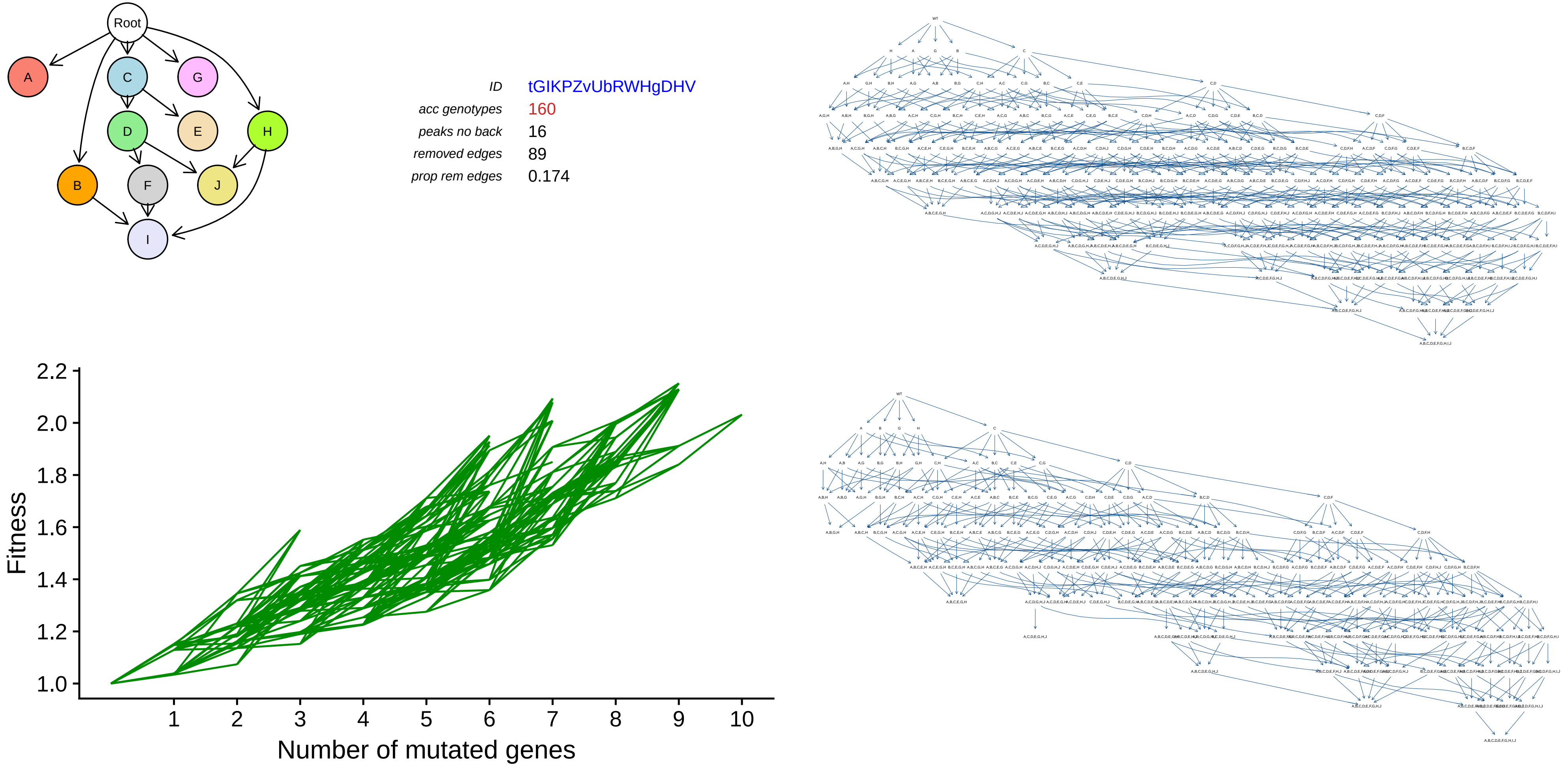
Root
A
B
C
D
E
F
G
H
I
J
ID
tGIKPZvUbRWHgDHV
acc genotypes
160
peaks no back
16
removed edges
89
prop rem edges
0.174
Fitness
Number of mutated genes
1.0
1.2
1.4
1.6
1.8
2.0
2.2
1
2
3
4
5
6
7
8
9
10

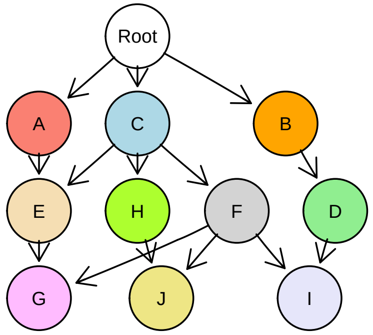

ID	TGzeGSf7DK4OTwG2
acc genotypes	72
peaks no back	8
removed edges	32
prop rem edges	0.19

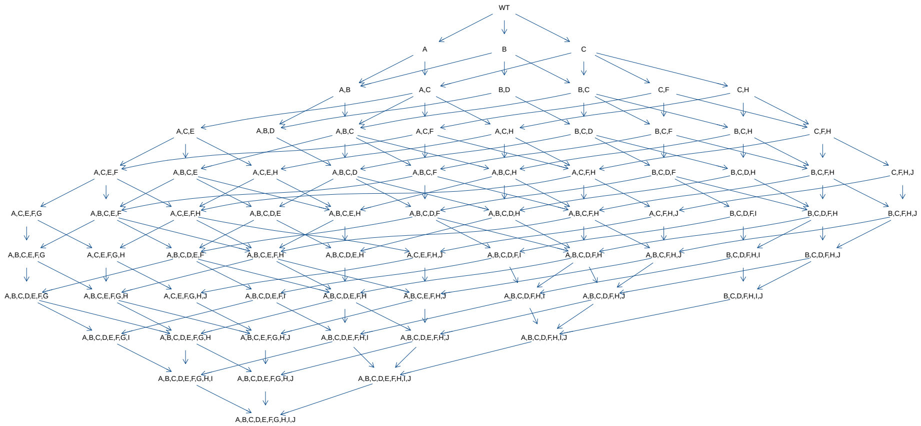

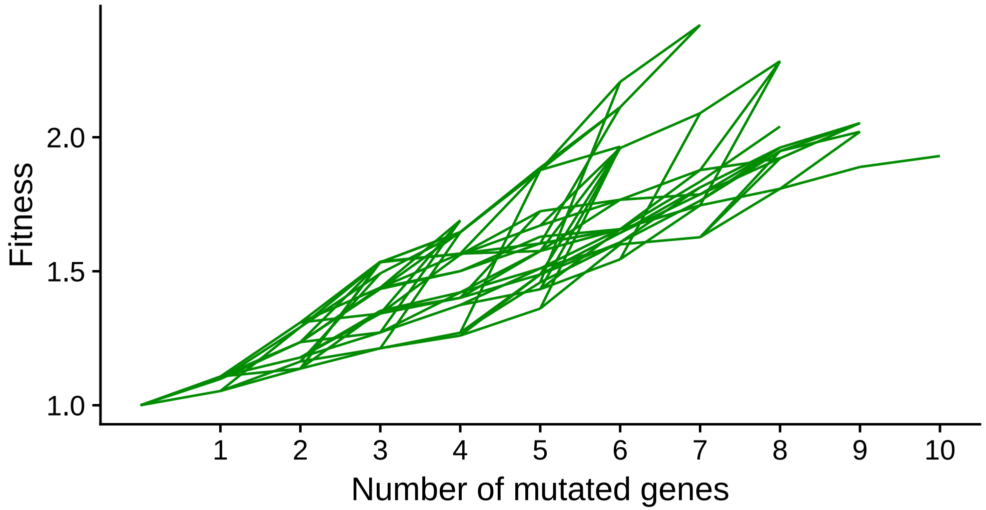

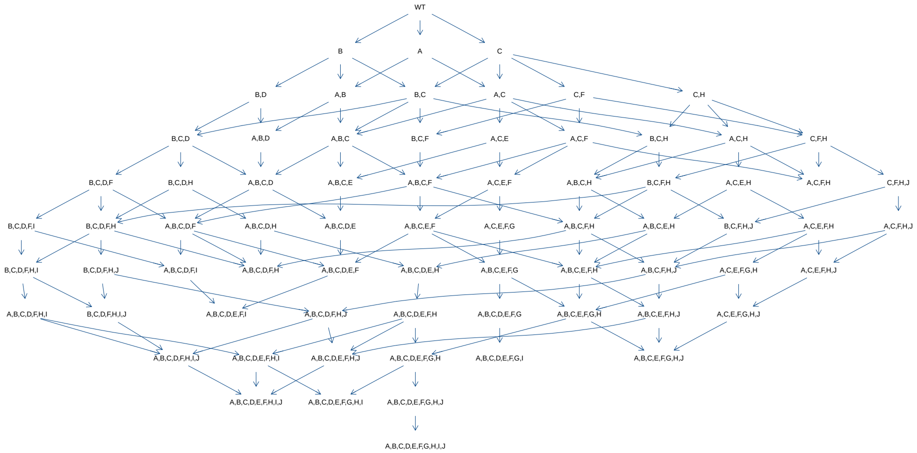

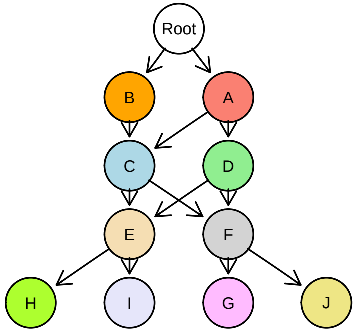

ID	tI8GpDHXTWuBAEB4
acc genotypes	32
peaks no back	6
removed edges	11
prop rem edges	0.183

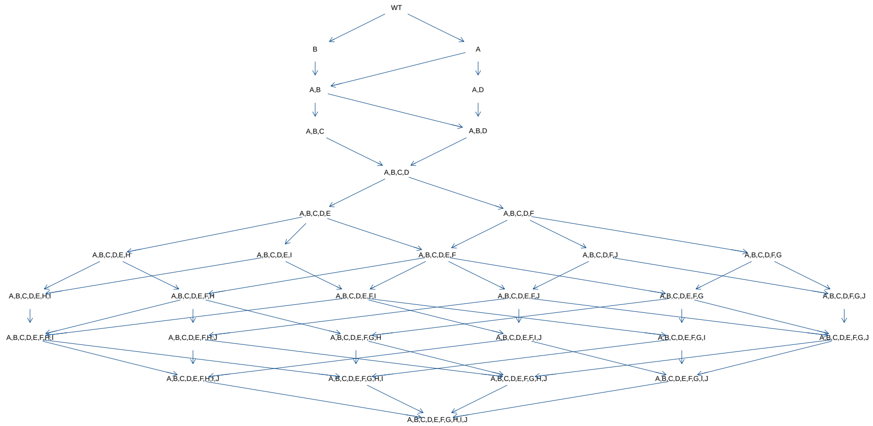

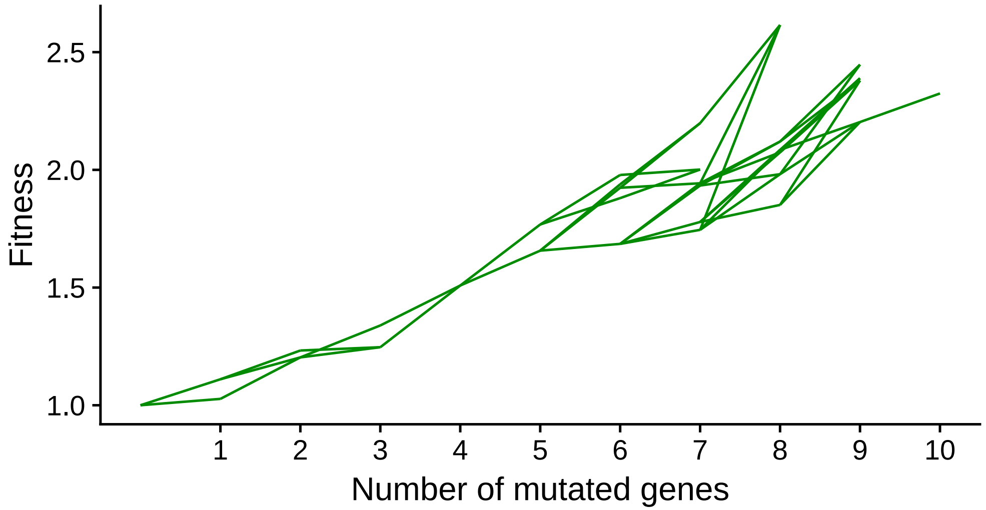

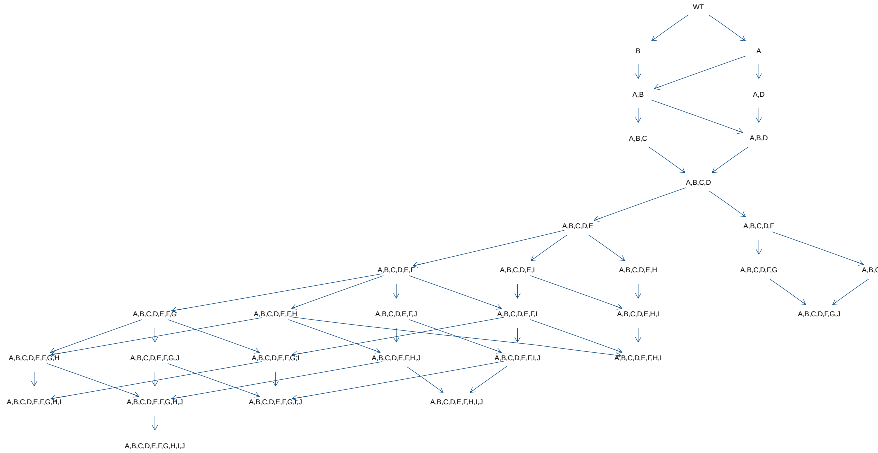

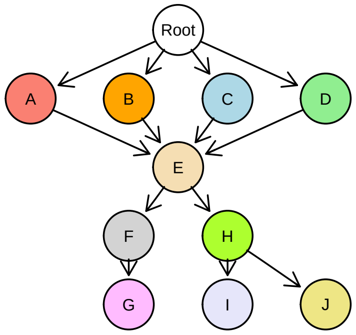

ID TnFE10L2B9qFy8hMT

acc genotypes 31

peaks no back 5

removed edges 8

prop rem edges 0.138

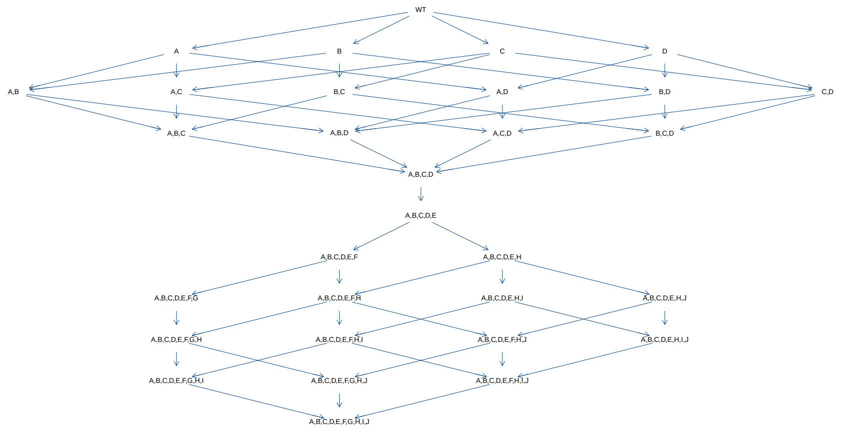

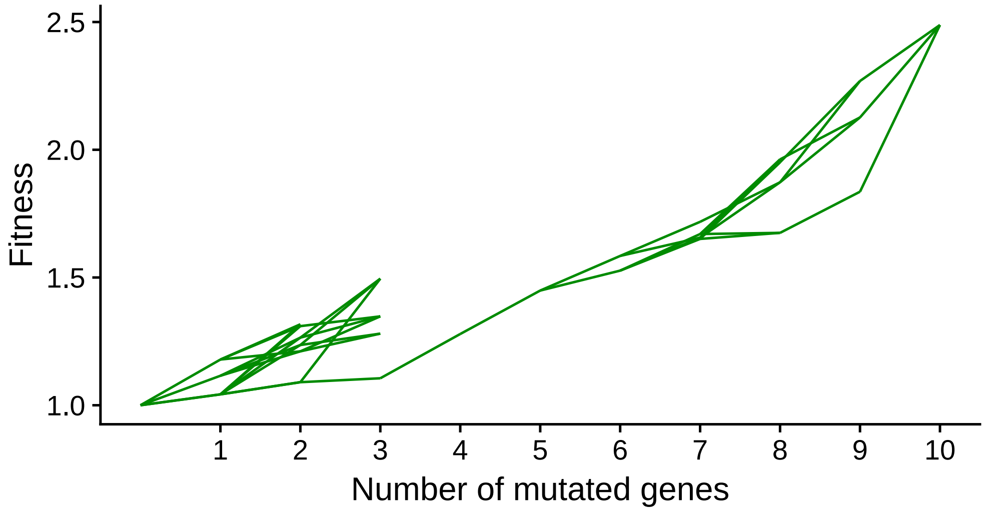

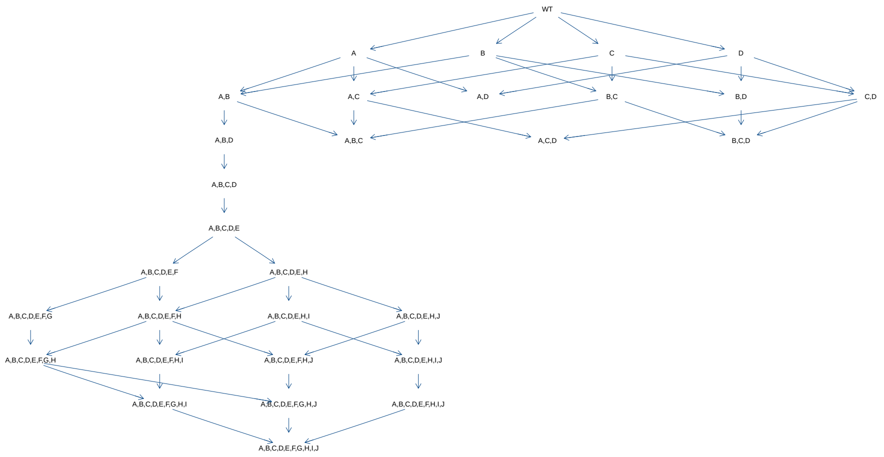

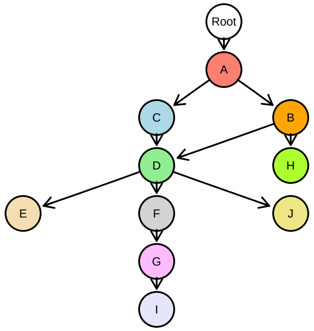

ID	ToRaV610sJqVtMv2b
acc genotypes	39
peaks no back	7
removed edges	14
prop rem edges	0.171

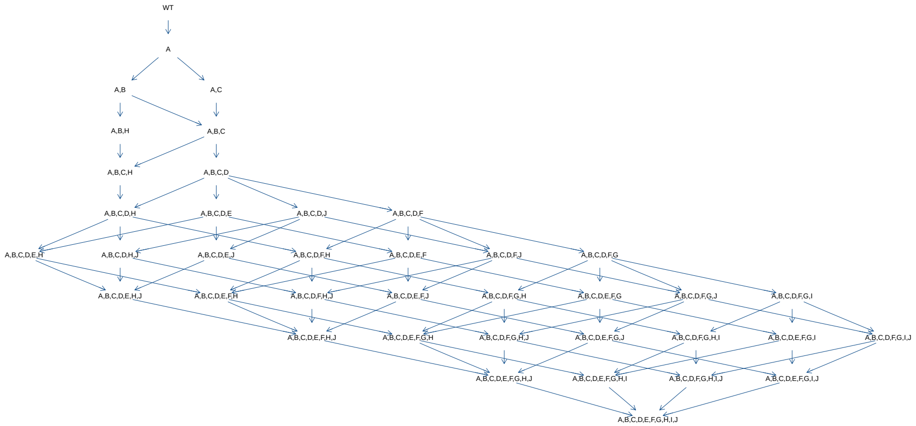

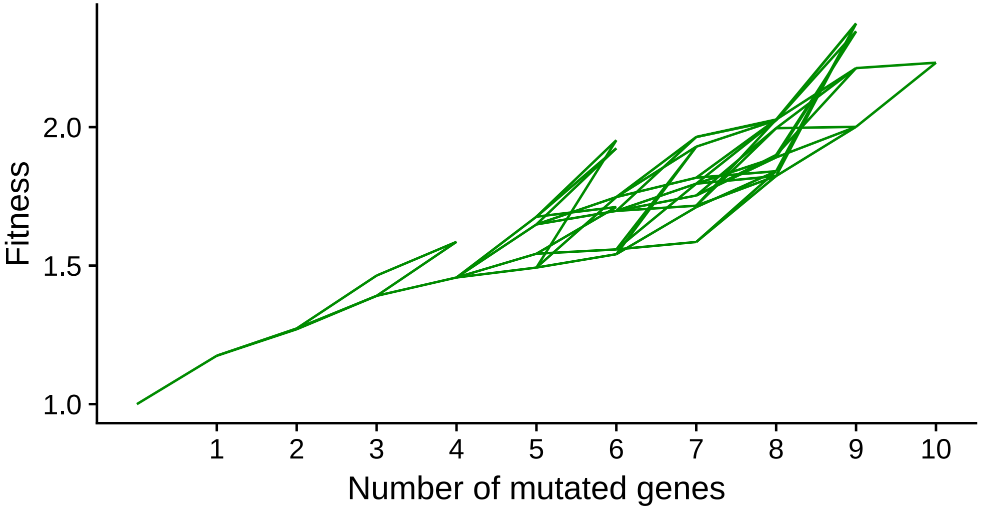

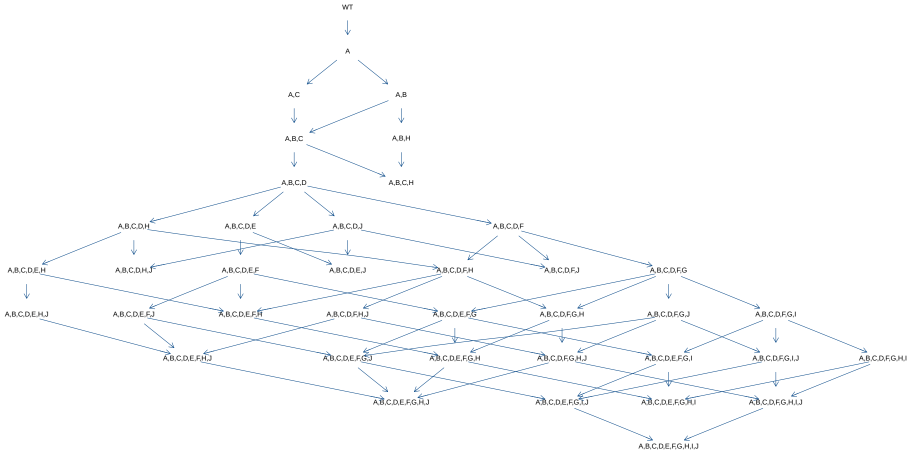

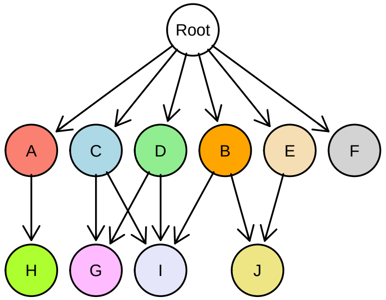

ID	TQnQiOlfxYRUpqrU
acc genotypes	186
peaks no back	13
removed edges	112
prop rem edges	0.188

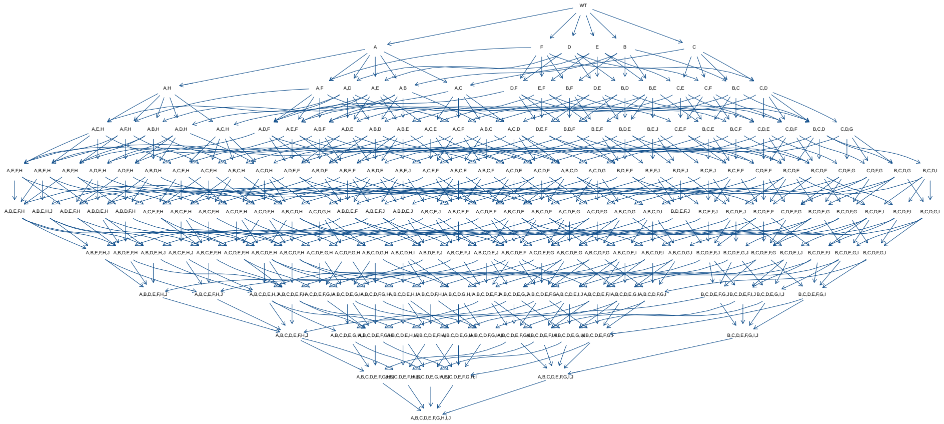

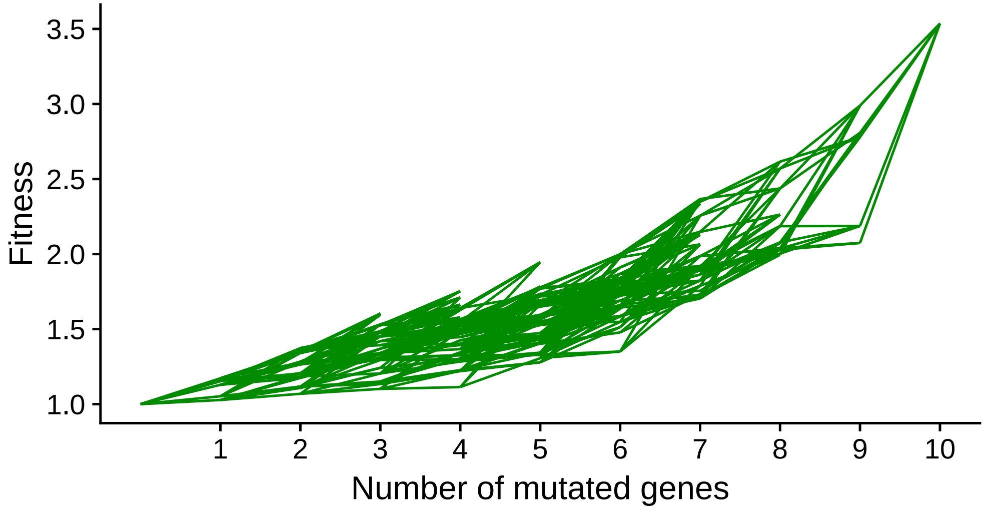

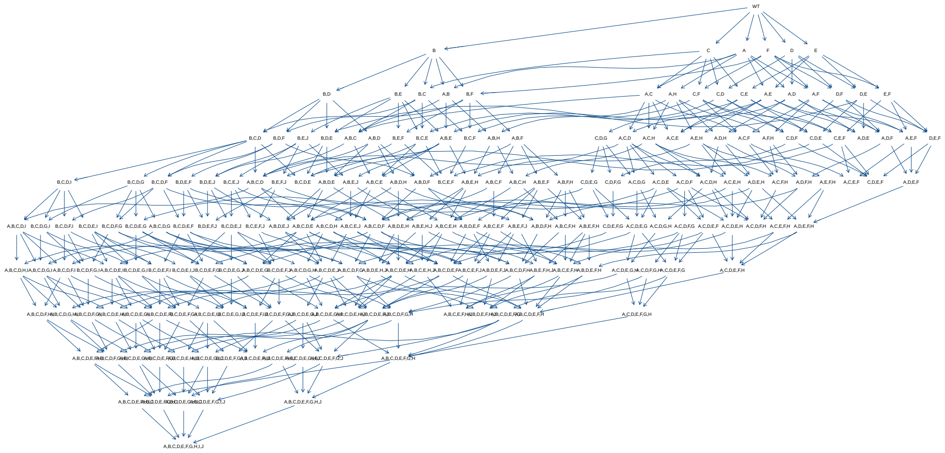

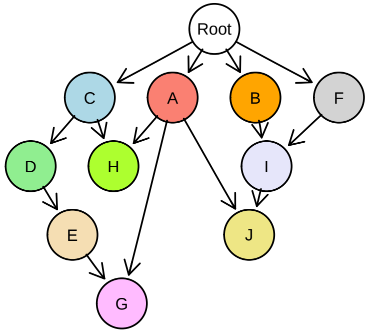

ID TVG98obaysdZVe7G
acc genotypes 74
peaks no back 8
removed edges 33
prop rem edges 0.189

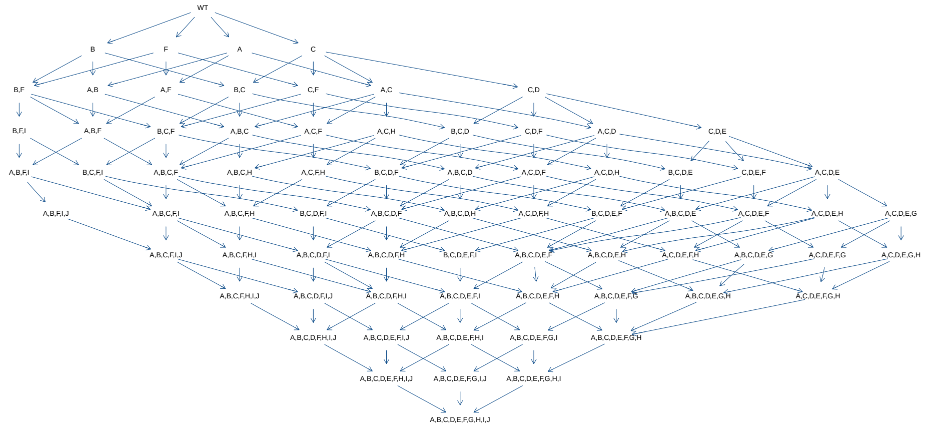

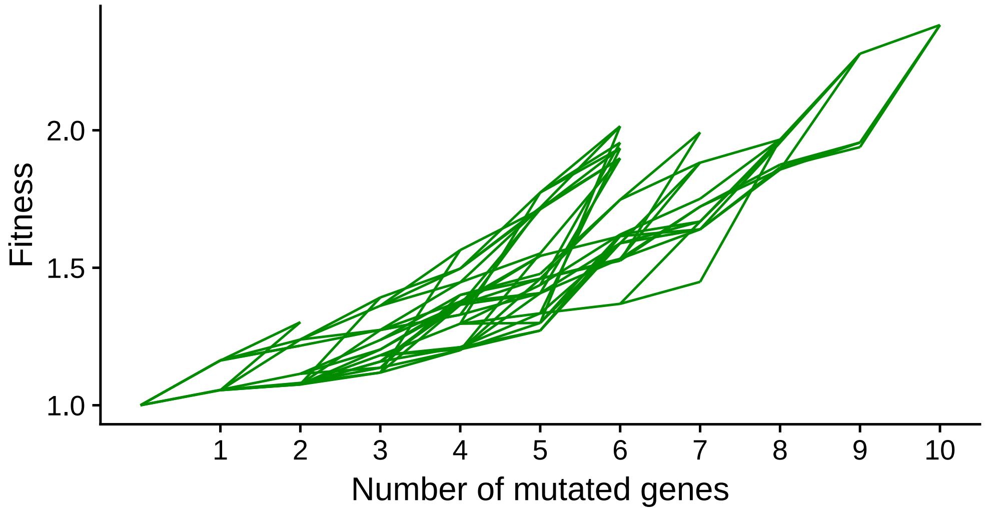

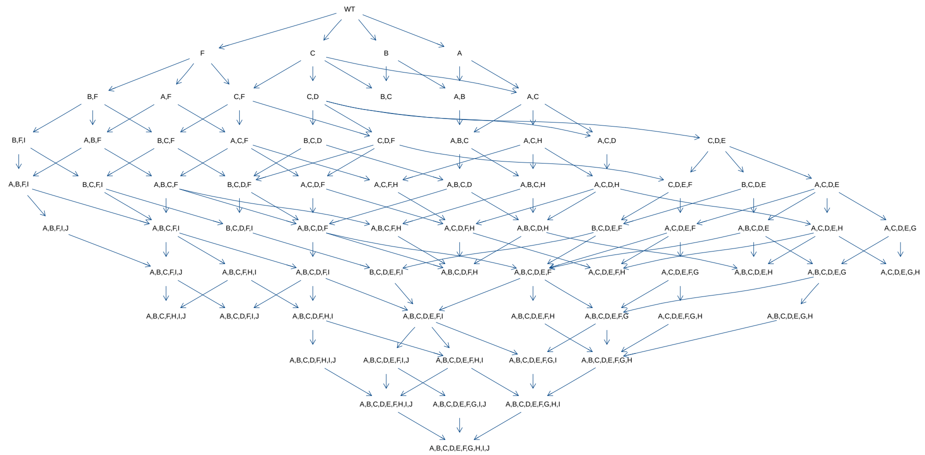

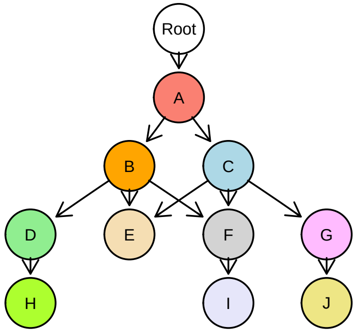

ID	tytbSnHIzi26I77A
acc genotypes	62
peaks no back	7
removed edges	18
prop rem edges	0.122

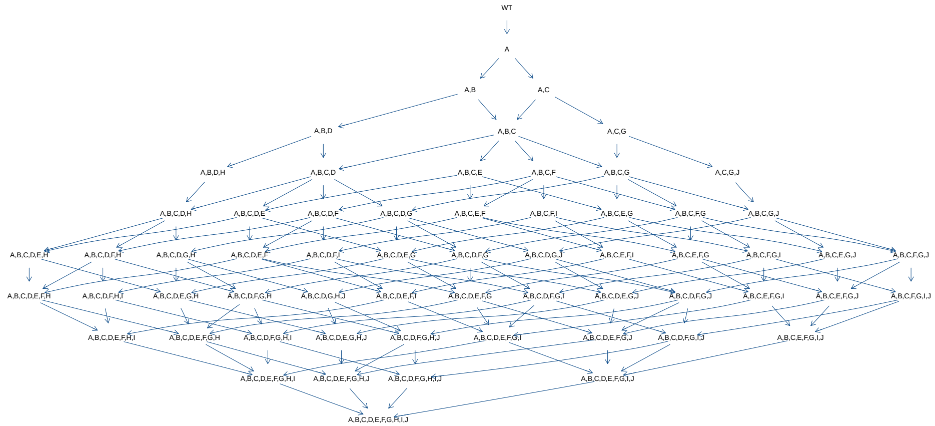

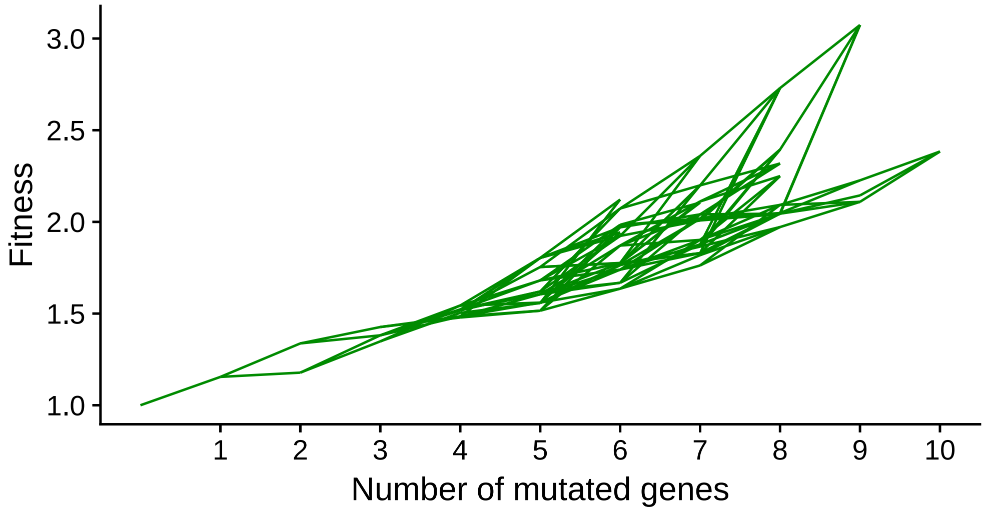

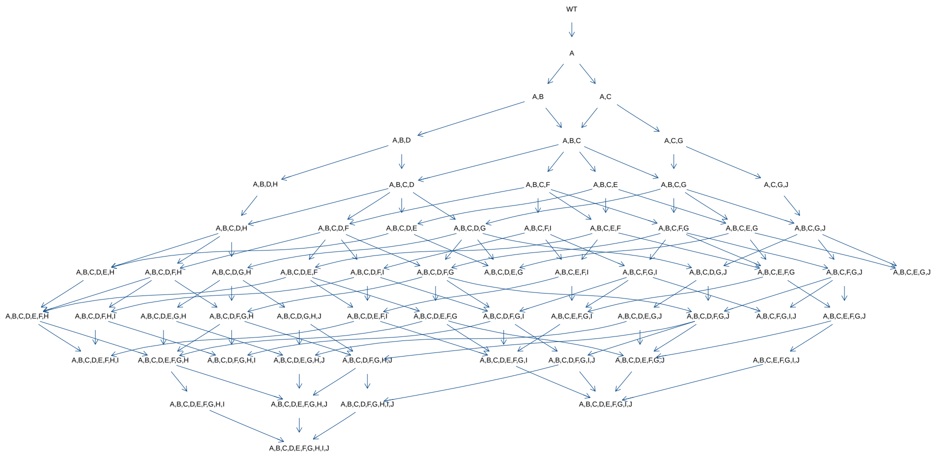

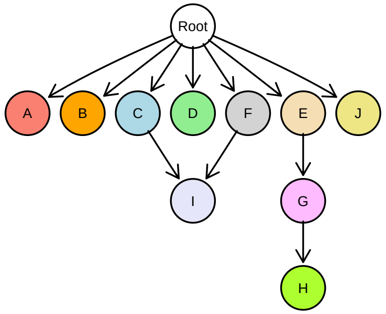

ID	TZFzcsBmlMqGXCih
acc genotypes	320
peaks no back	22
removed edges	231
prop rem edges	0.192

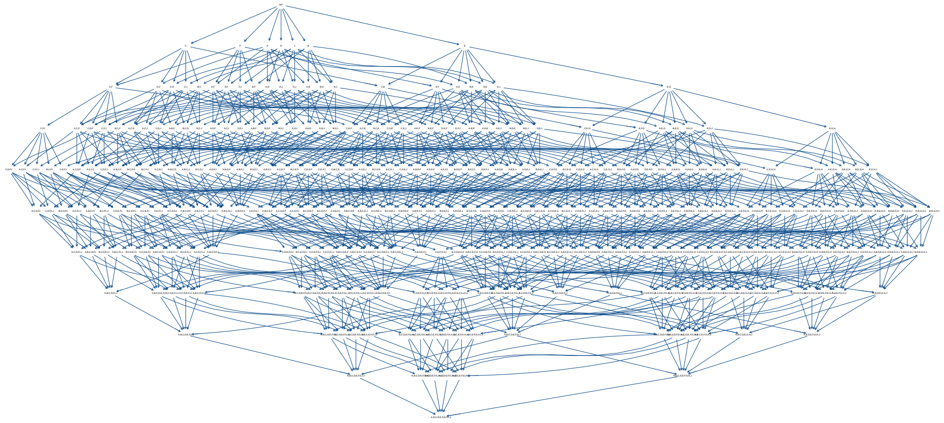

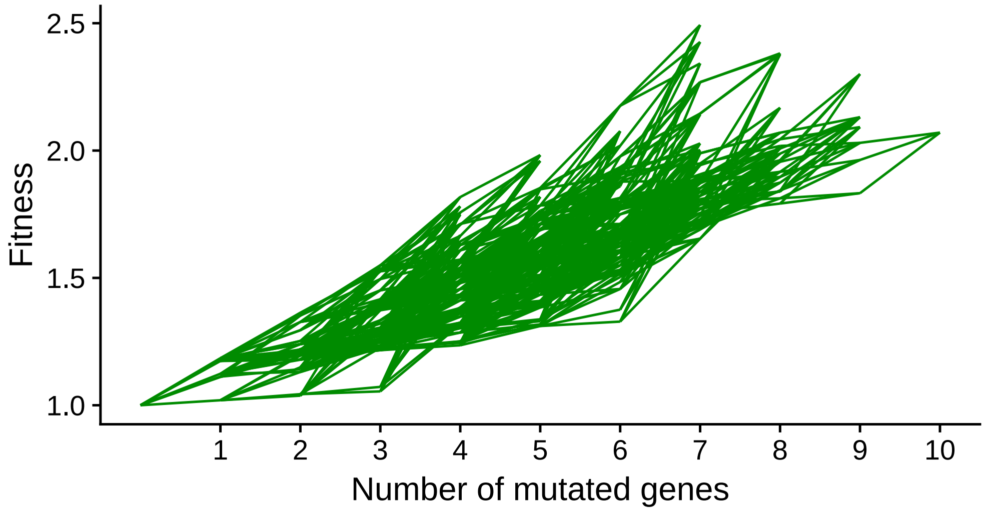

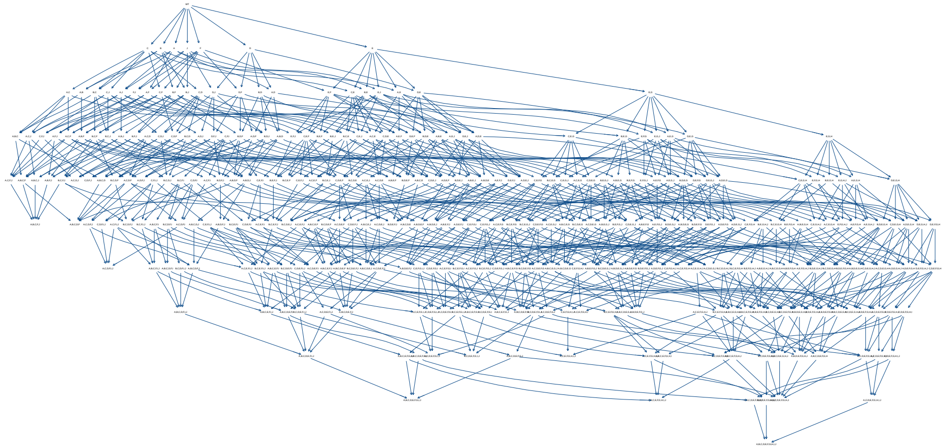

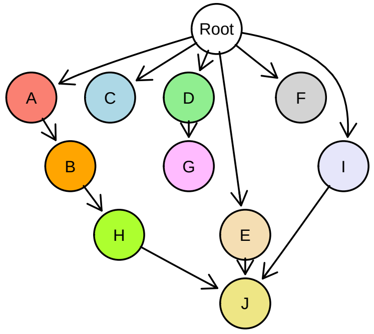

ID UBKJVjkElEDOKL7t
acc genotypes 204
peaks no back 14
removed edges 117
prop rem edges 0.17

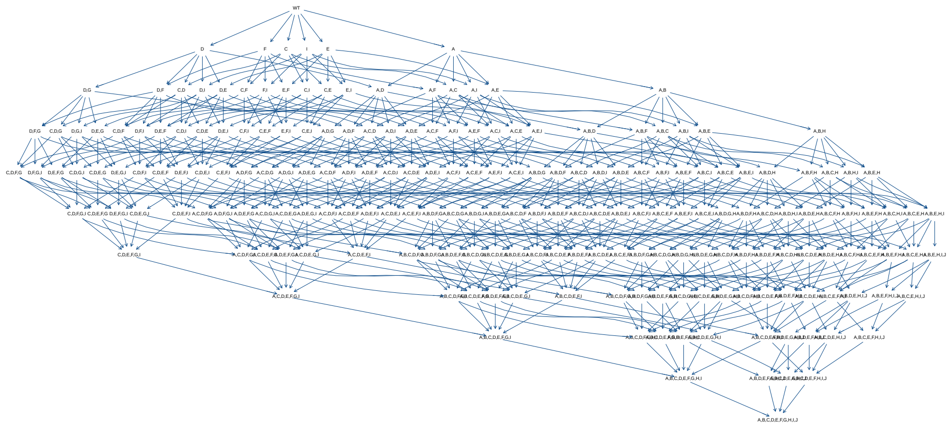

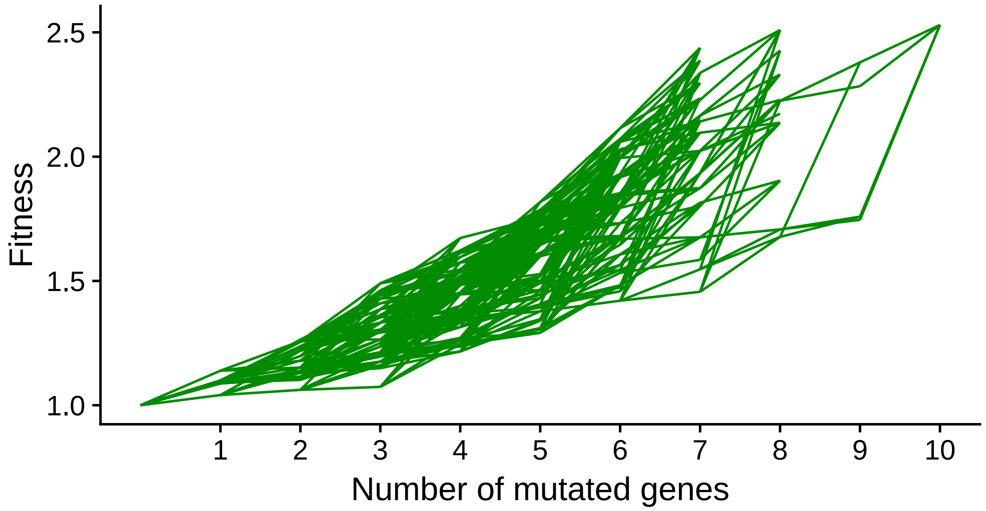

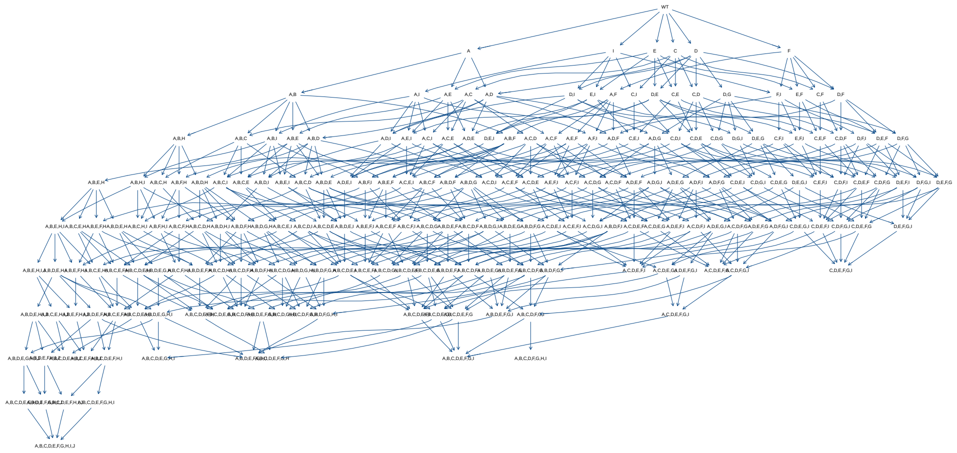

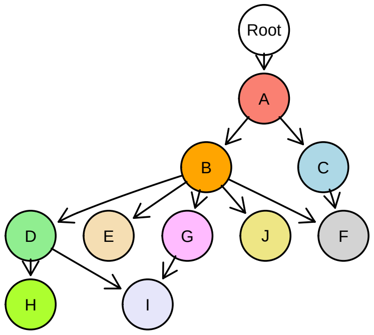

ID Ue10Y3AbLFXoQDQkX
acc genotypes 99
peaks no back 13
removed edges 62
prop rem edges 0.218

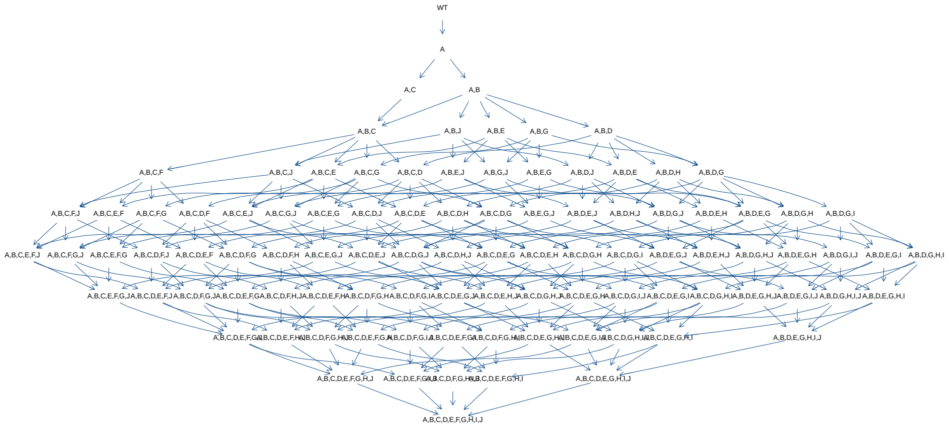

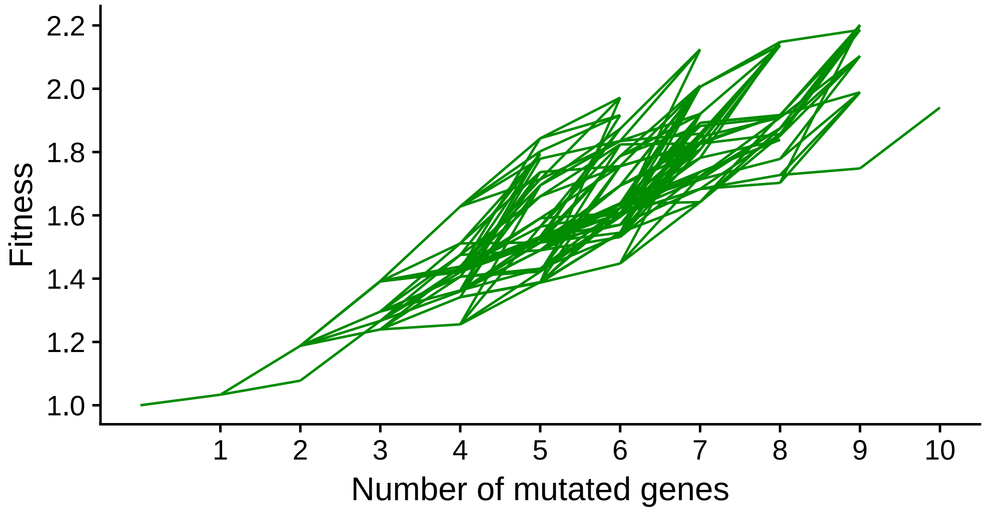

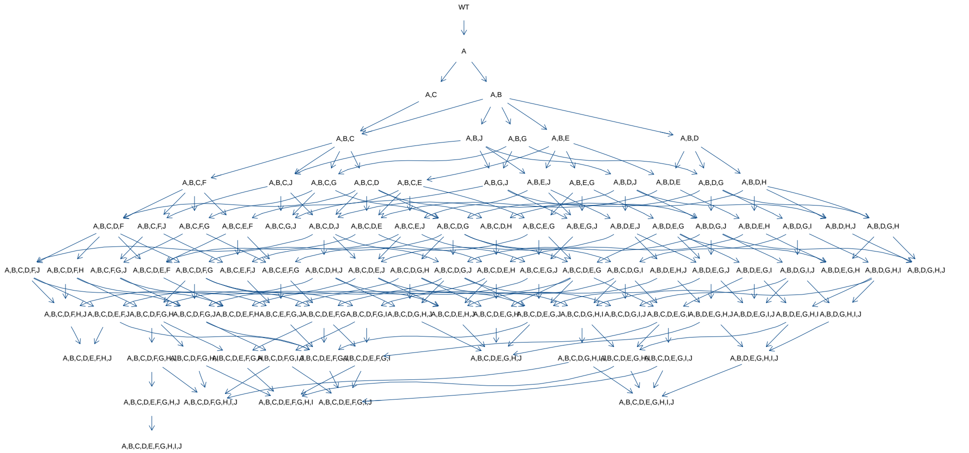

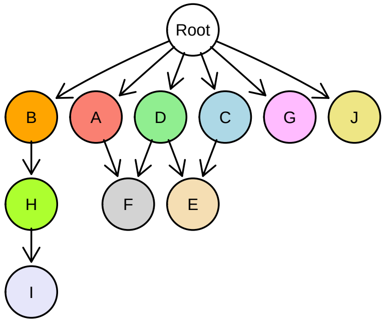

ID	UEcQih9YNrZ2uptX
acc genotypes	208
peaks no back	18
removed edges	127
prop rem edges	0.186

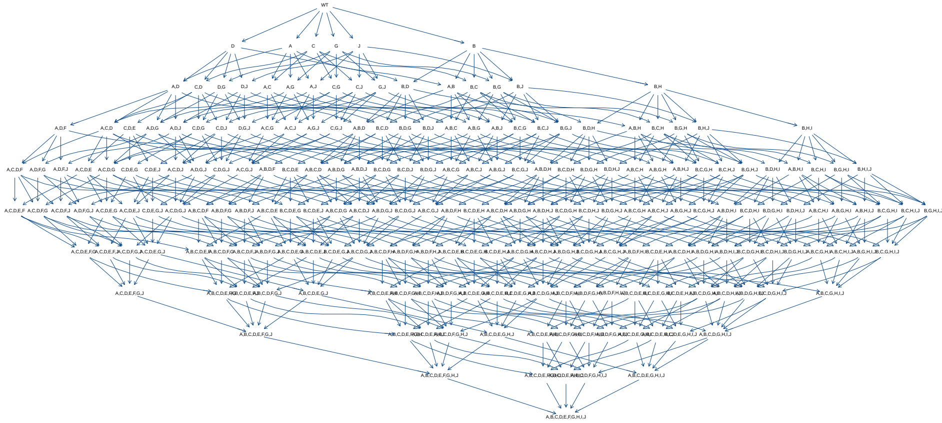

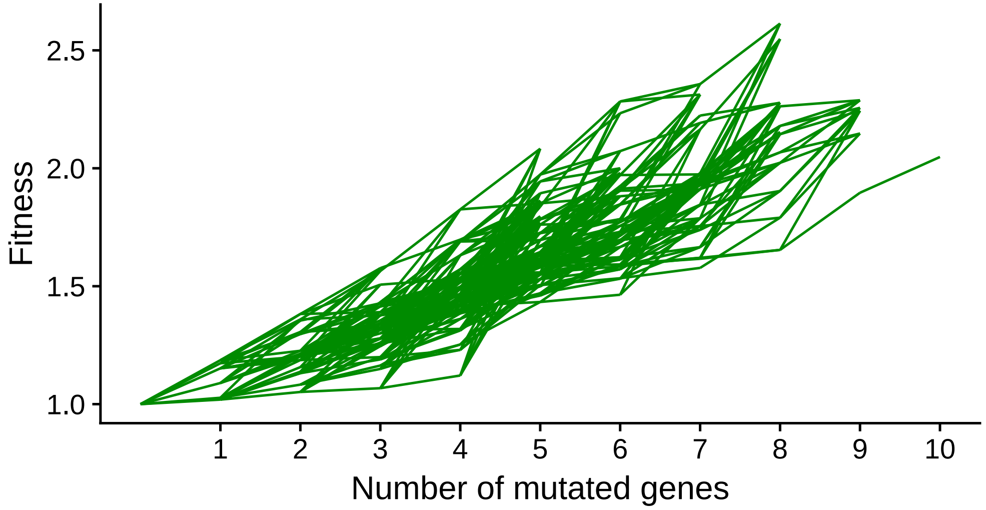

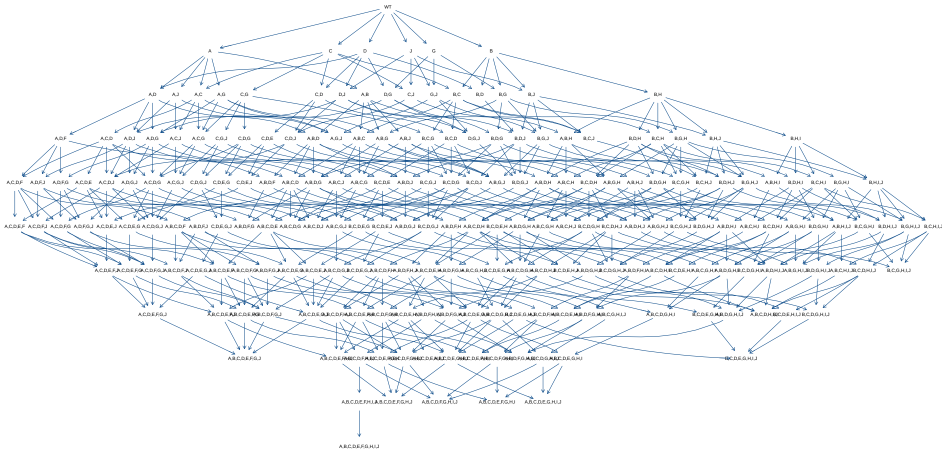

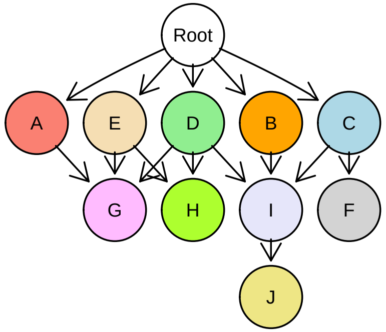

ID	UHMIcOOkS2hFJbTg
acc genotypes	104
peaks no back	10
removed edges	51
prop rem edges	0.182

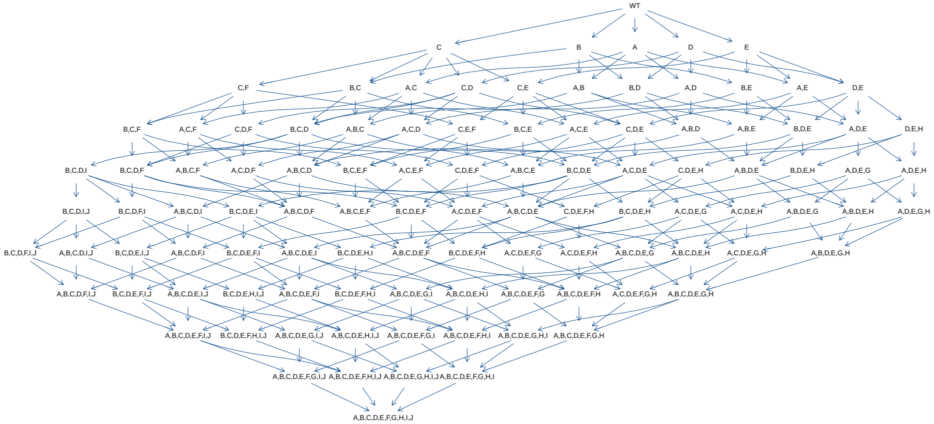

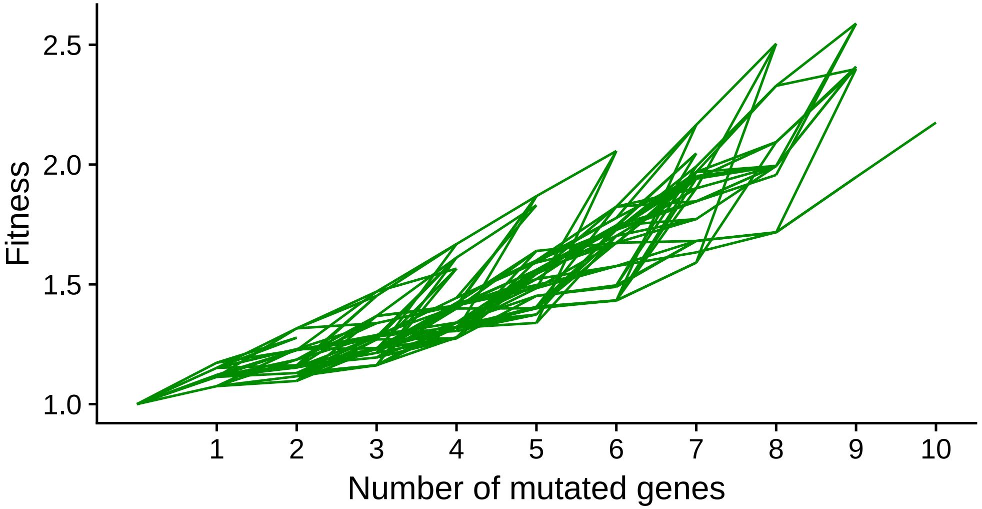

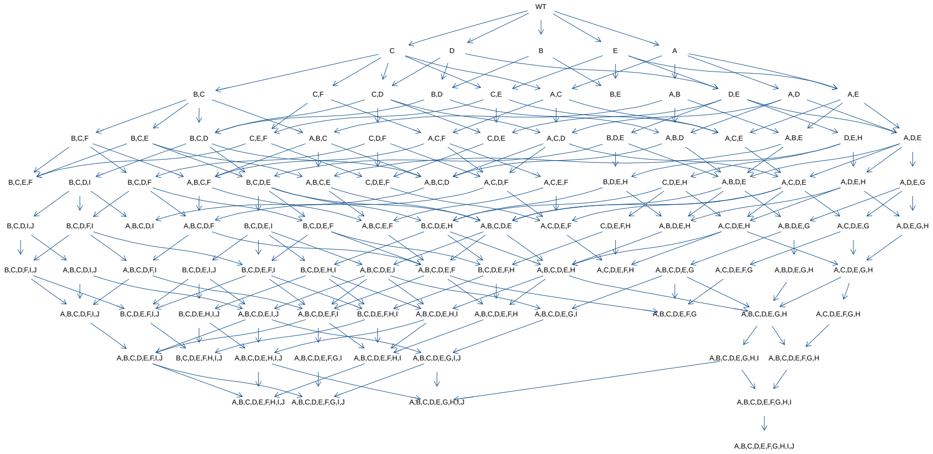

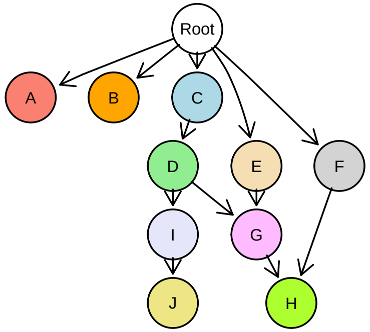

ID	uKmhMNcyKeLI3N10d
acc genotypes	116
peaks no back	12
removed edges	59
prop rem edges	0.178

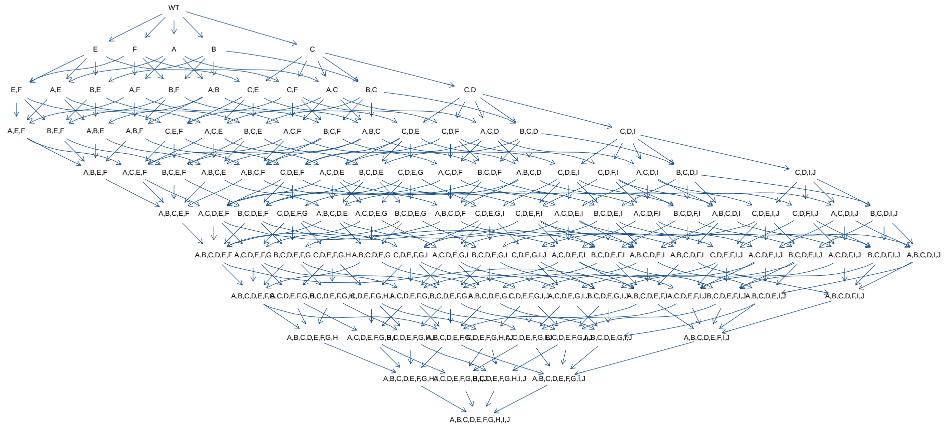

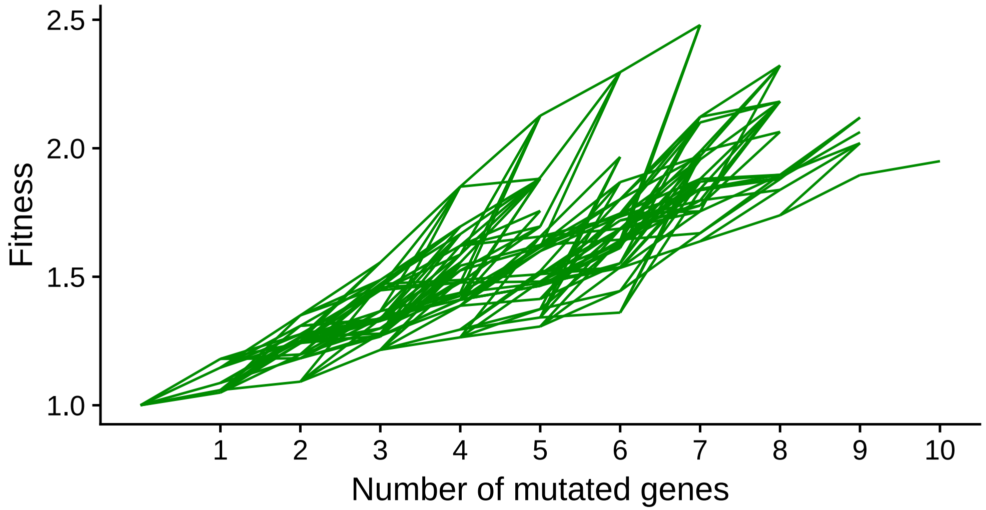

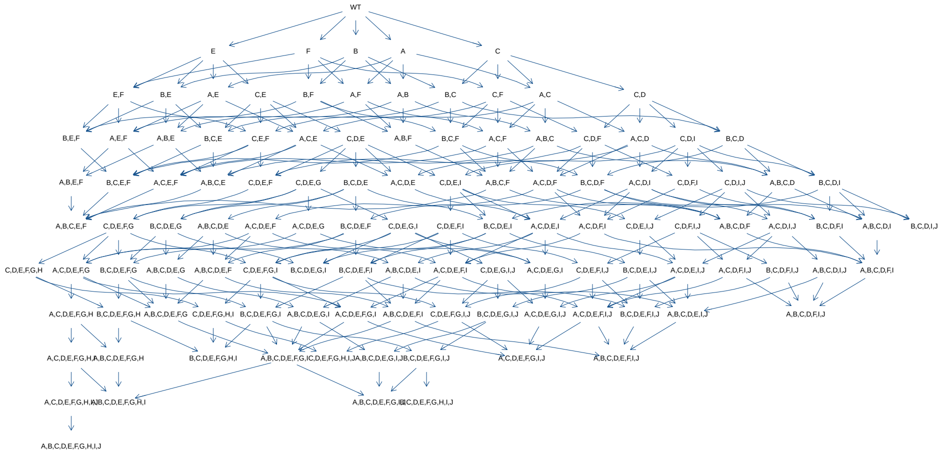

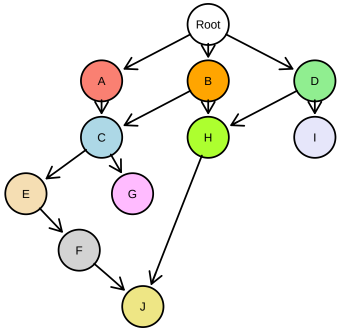

ID	UoHyw1GDeCOAnn4H
acc genotypes	50
peaks no back	6
removed edges	23
prop rem edges	0.217

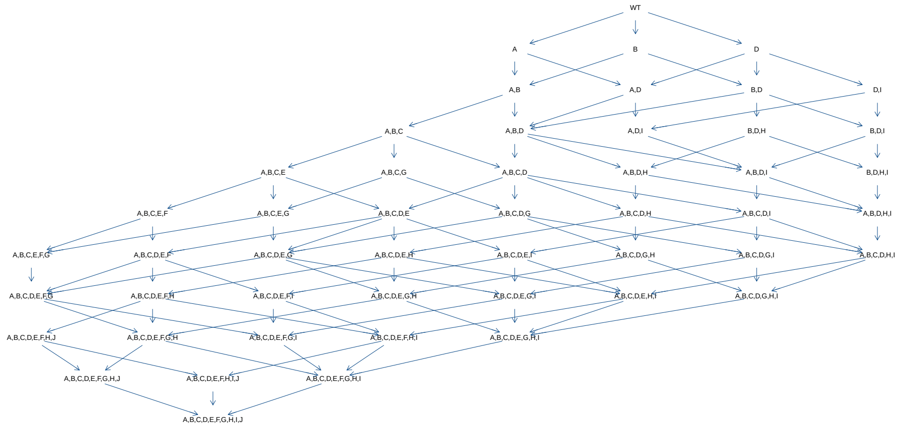

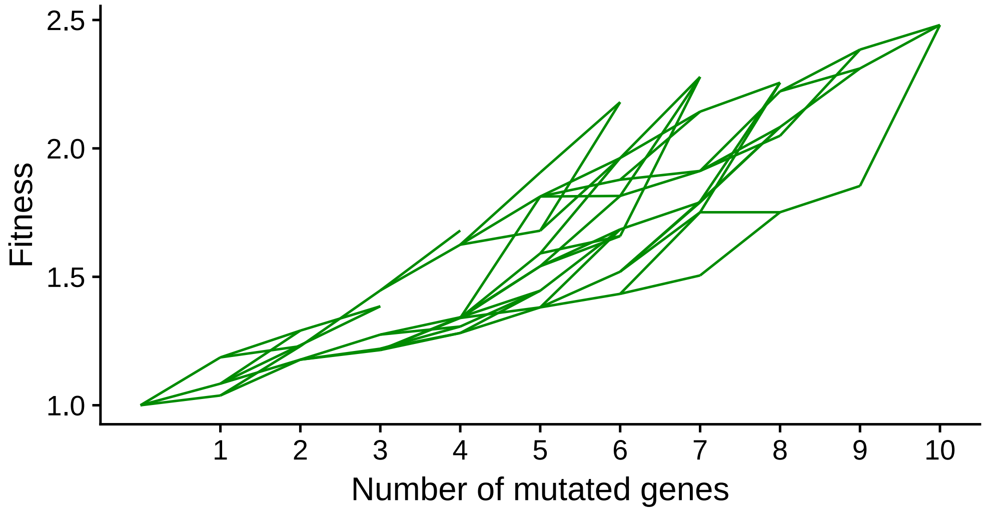

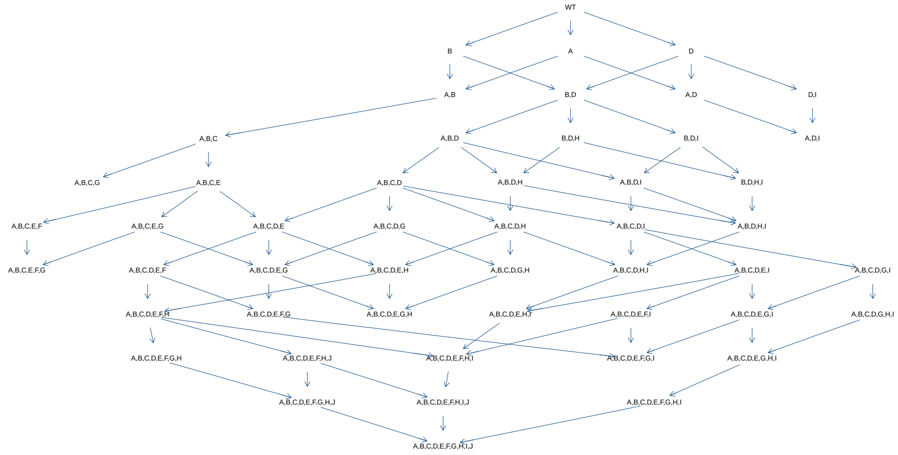

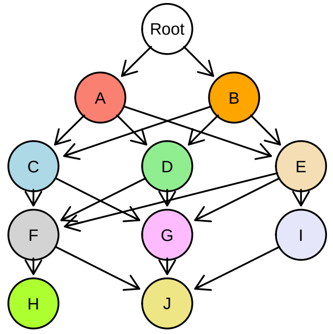

ID	utFV4f4ZwaTtF6F7
acc genotypes	27
peaks no back	5
removed edges	7
prop rem edges	0.152

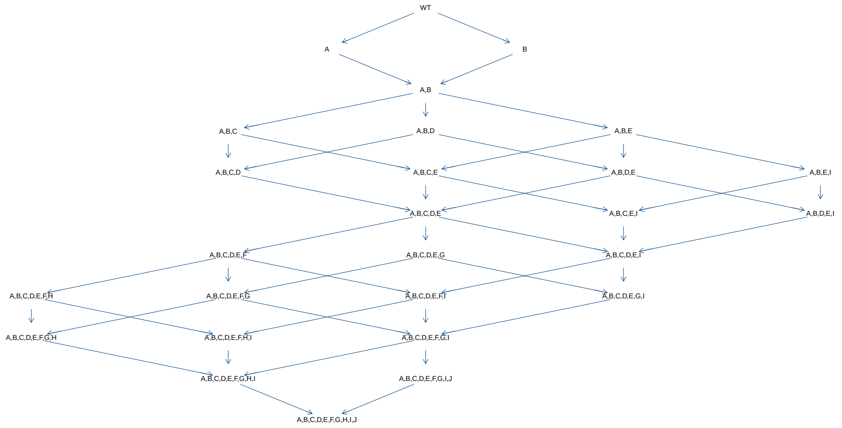

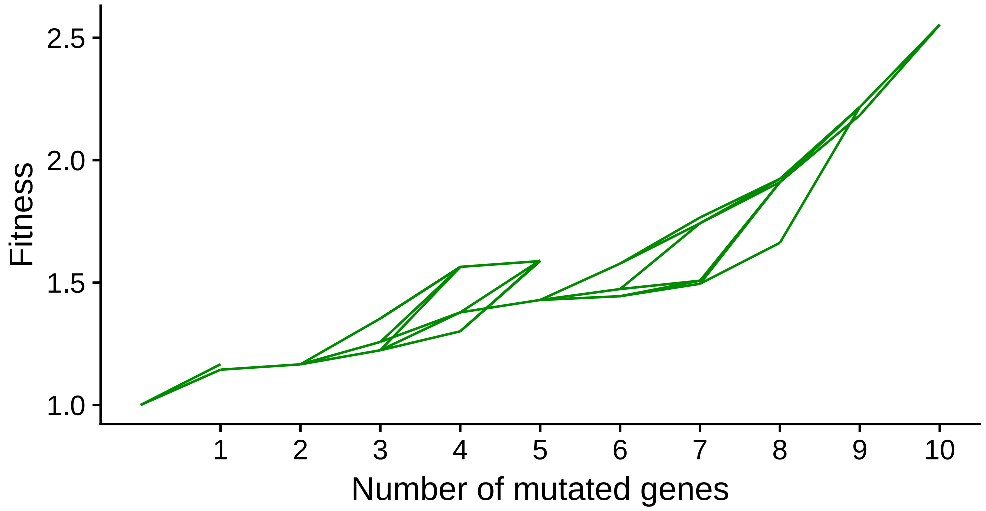

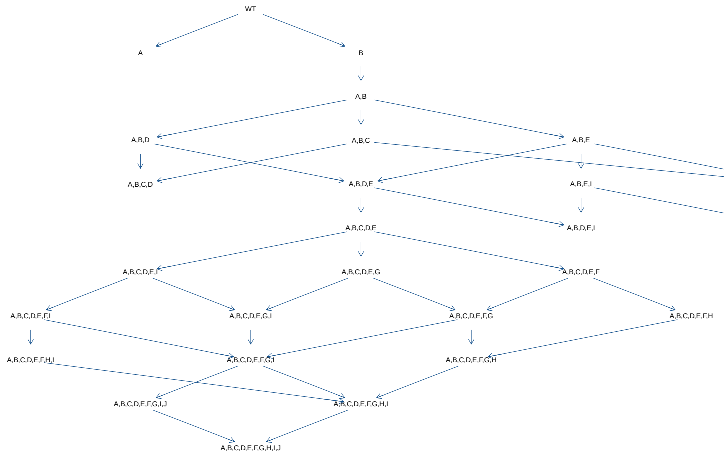

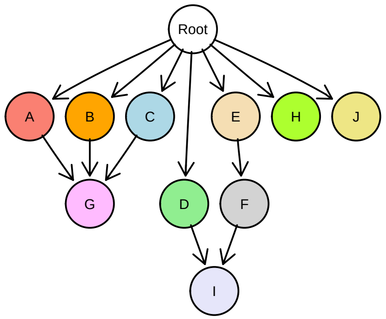

ID	uzycnOVWW3JNvHXq
acc genotypes	252
peaks no back	17
removed edges	162
prop rem edges	0.179

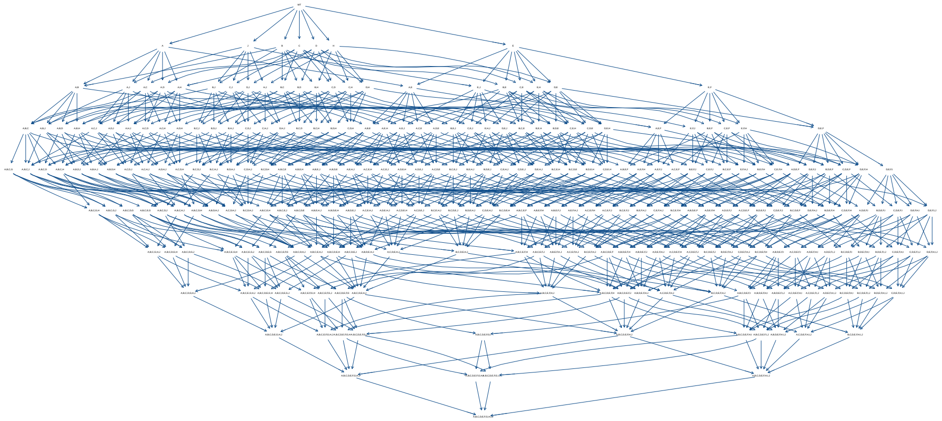

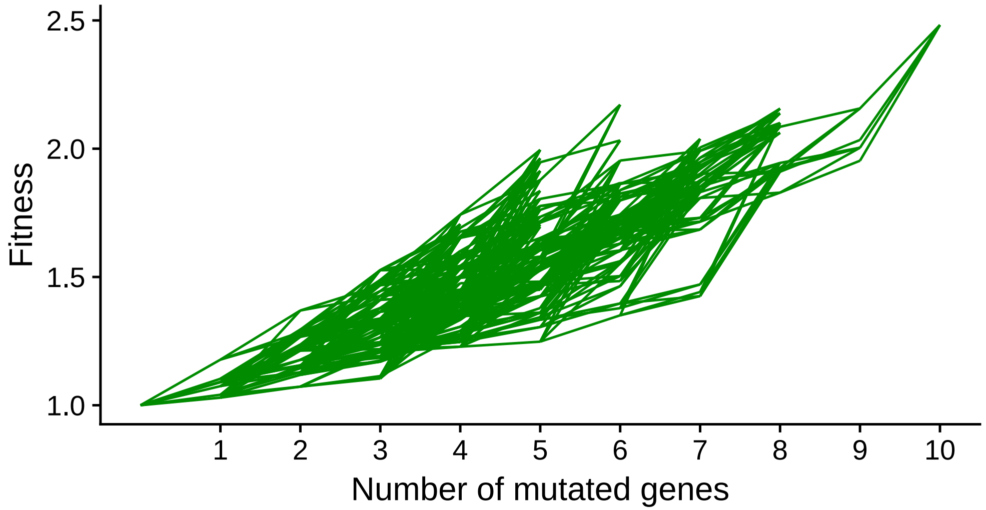

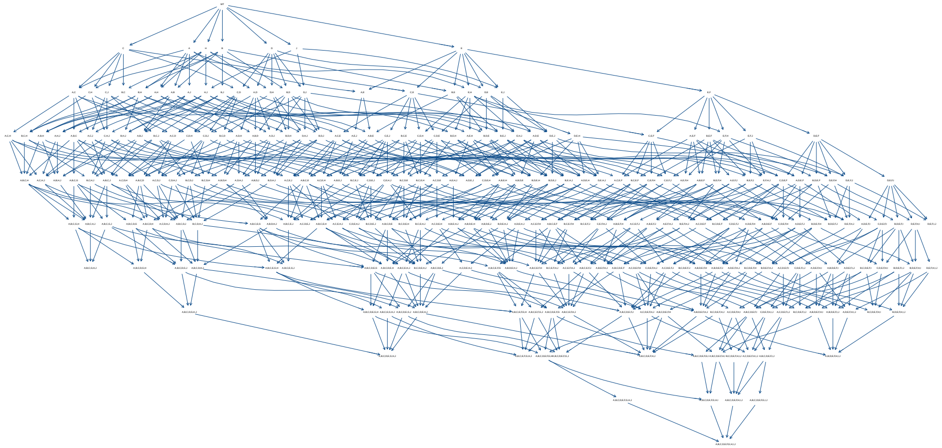

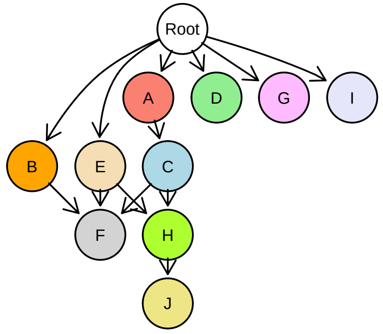

ID	V3Myq6Ap7yTWkyC1
acc genotypes	152
peaks no back	19
removed edges	116
prop rem edges	0.244

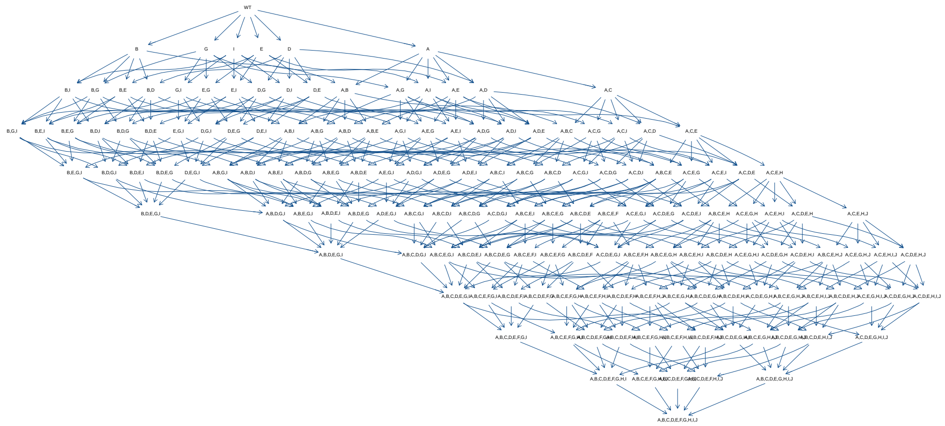

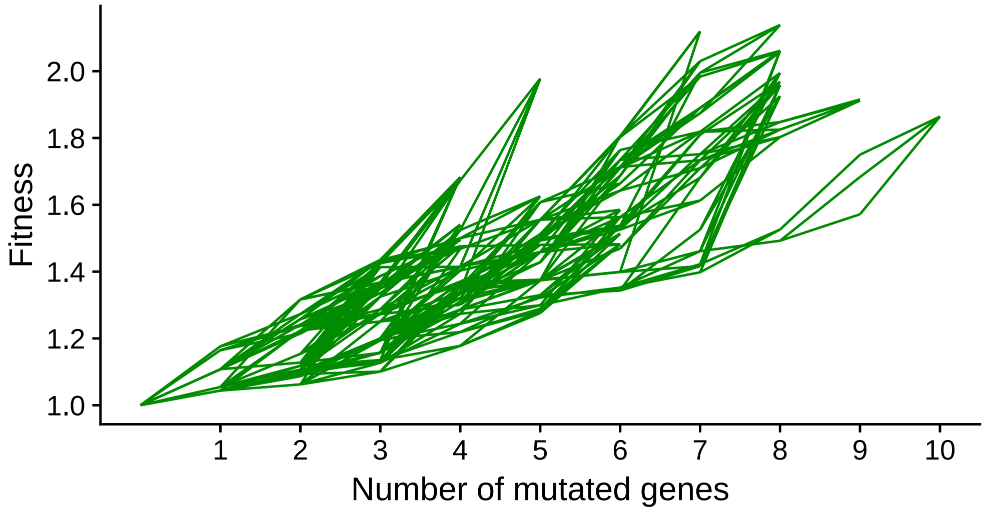

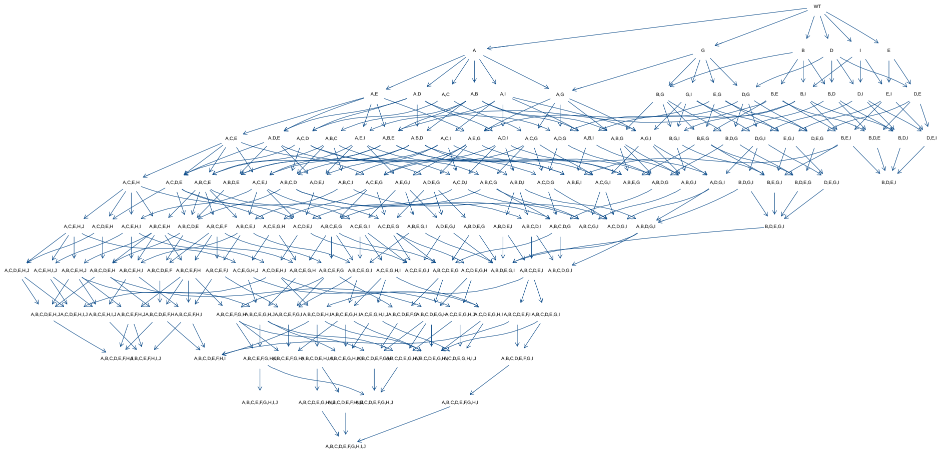

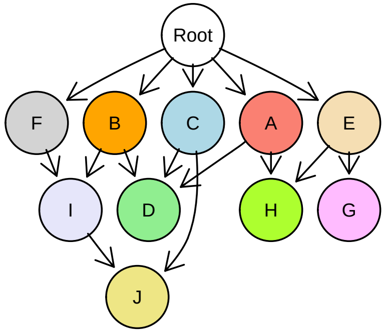

ID v3T1IG10jXOD5TPVs
acc genotypes 108
peaks no back 11
removed edges 62
prop rem edges 0.212

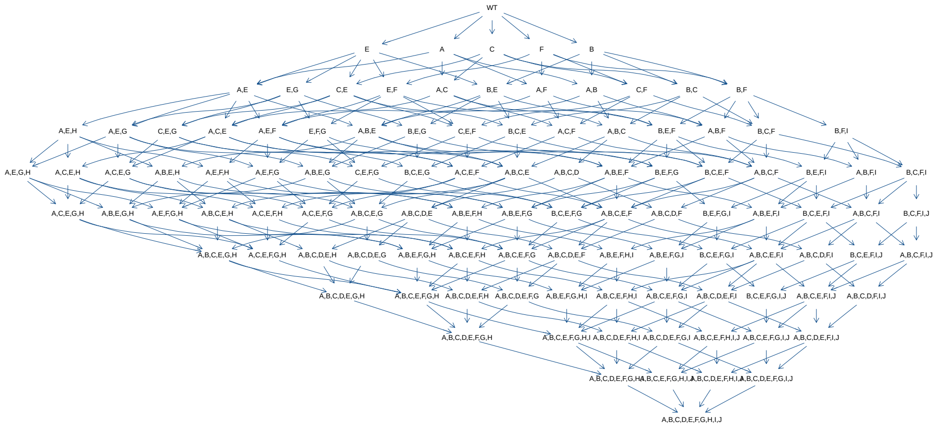

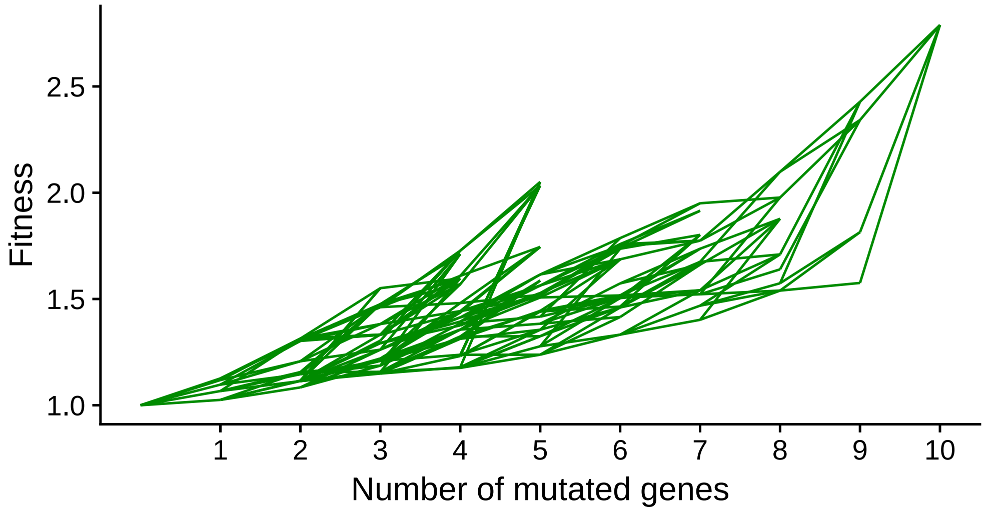

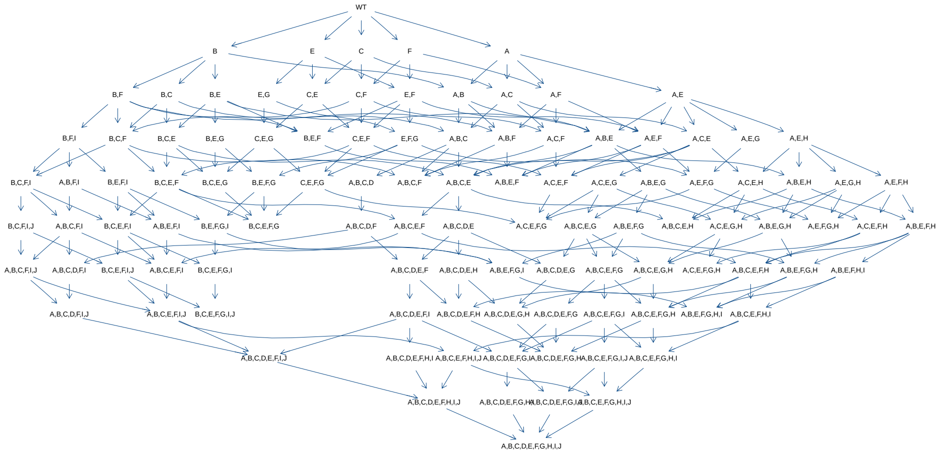

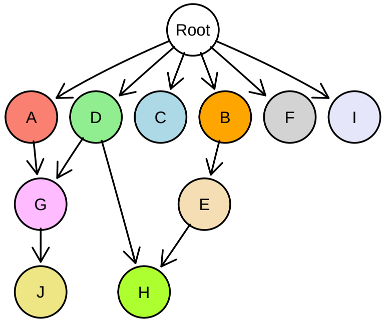

ID	vDscwX1MSzAXnUjn
acc genotypes	176
peaks no back	14
removed edges	95
prop rem edges	0.17

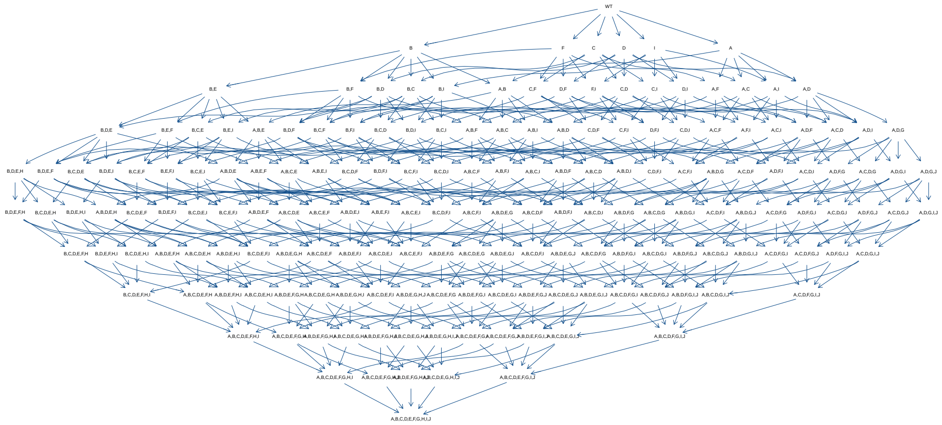

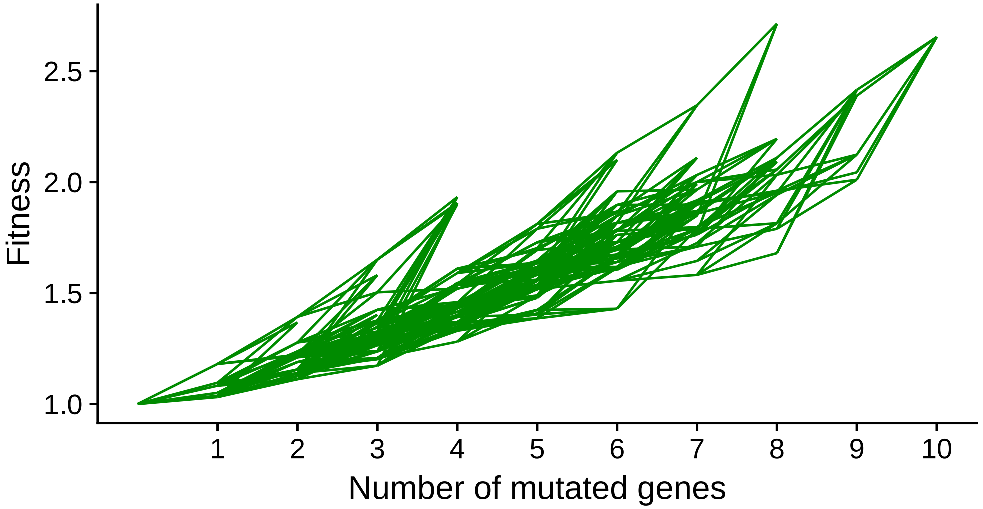

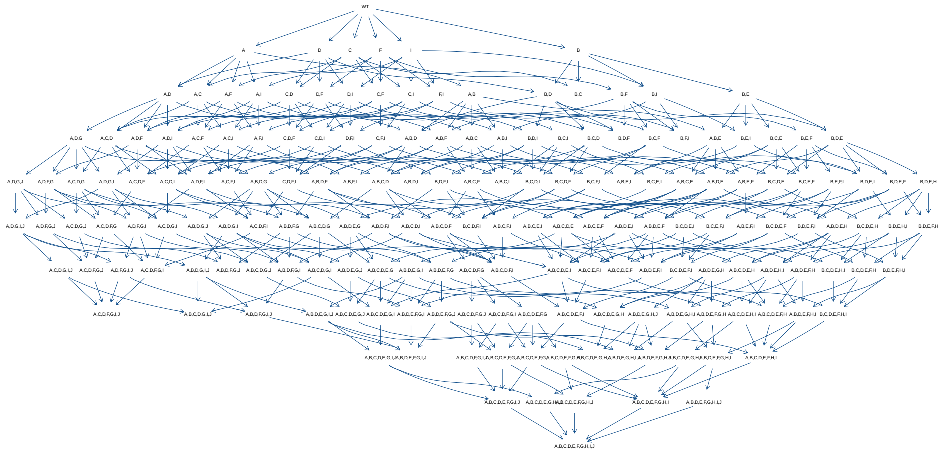

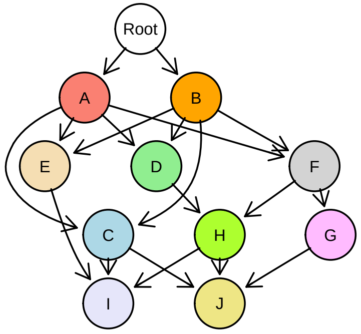

ID	veSOPRnIsirW3tXS
acc genotypes	40
peaks no back	7
removed edges	18
prop rem edges	0.214

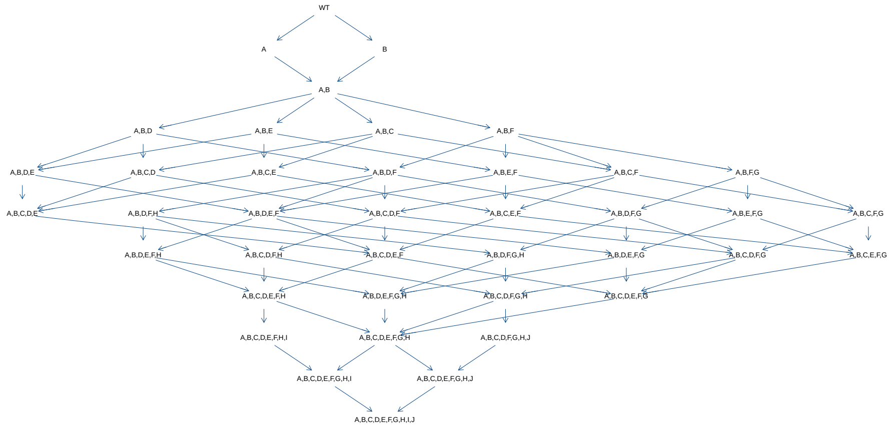

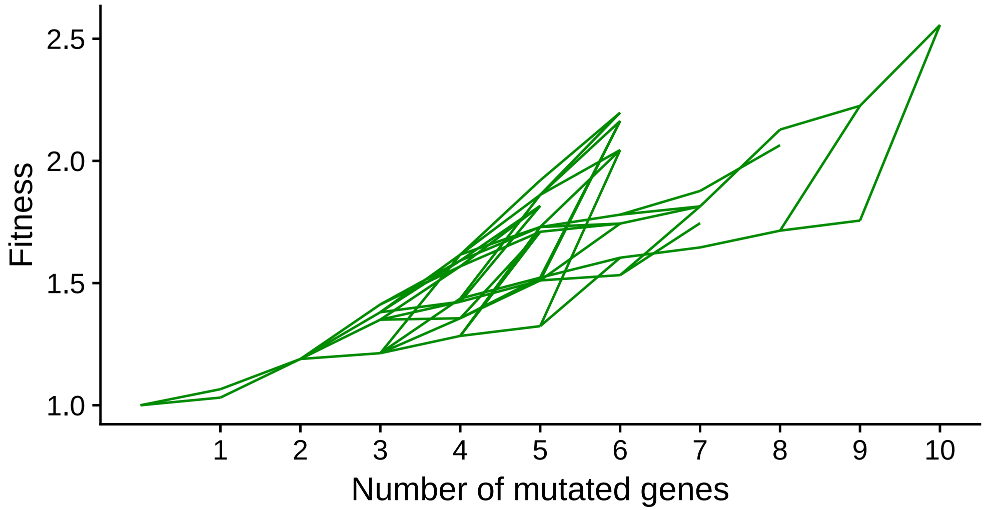

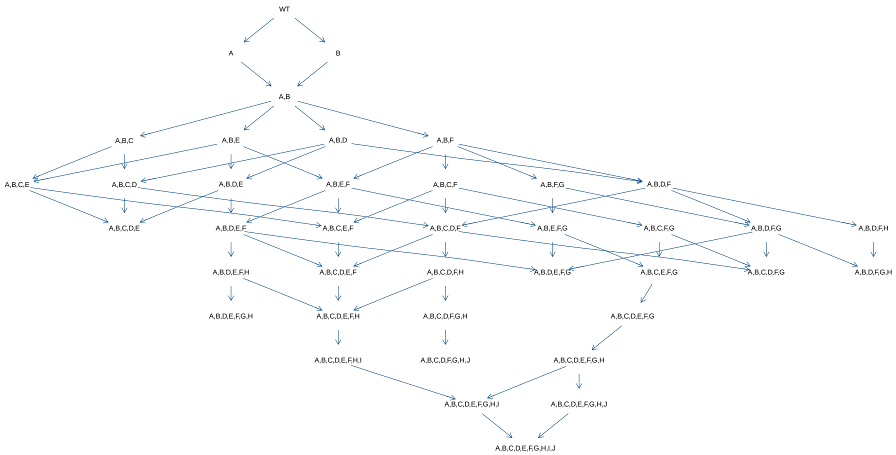

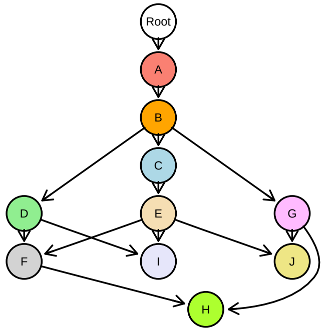

ID	vm585SzZroFfErOI
acc genotypes	29
peaks no back	2
removed edges	3
prop rem edges	0.059

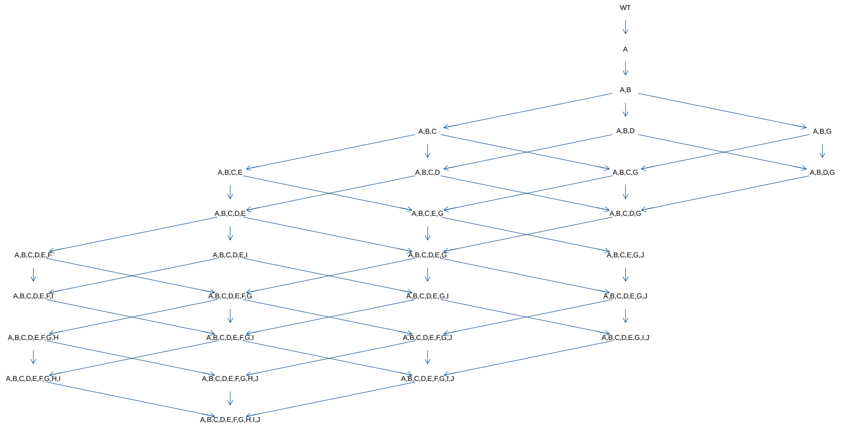

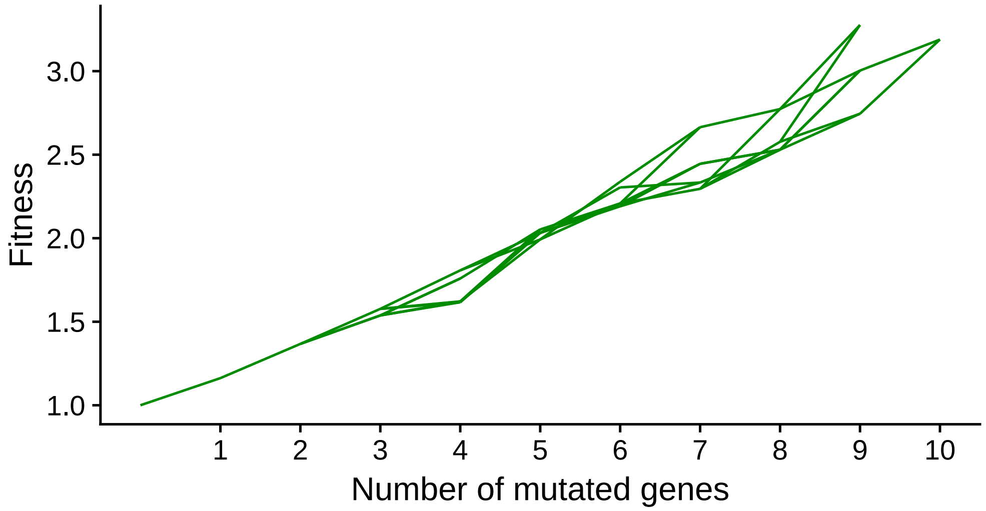

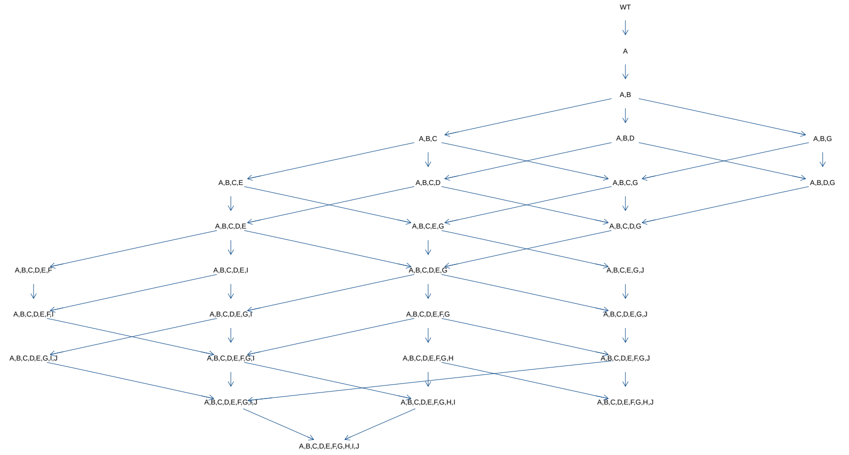

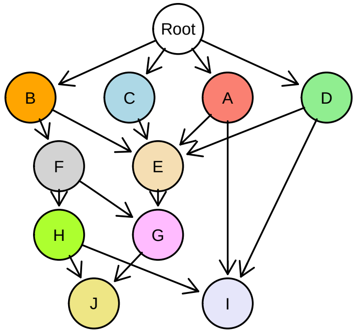

ID	VNq9x3A103vjj6H6C
acc genotypes	43
peaks no back	6
removed edges	18
prop rem edges	0.2

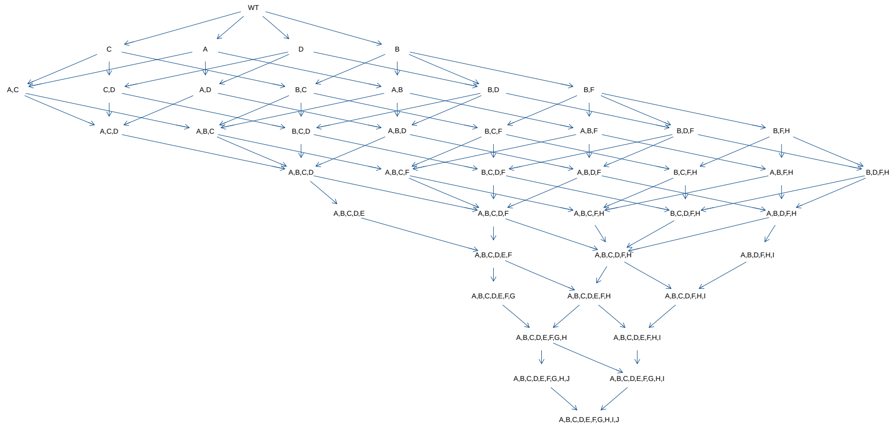

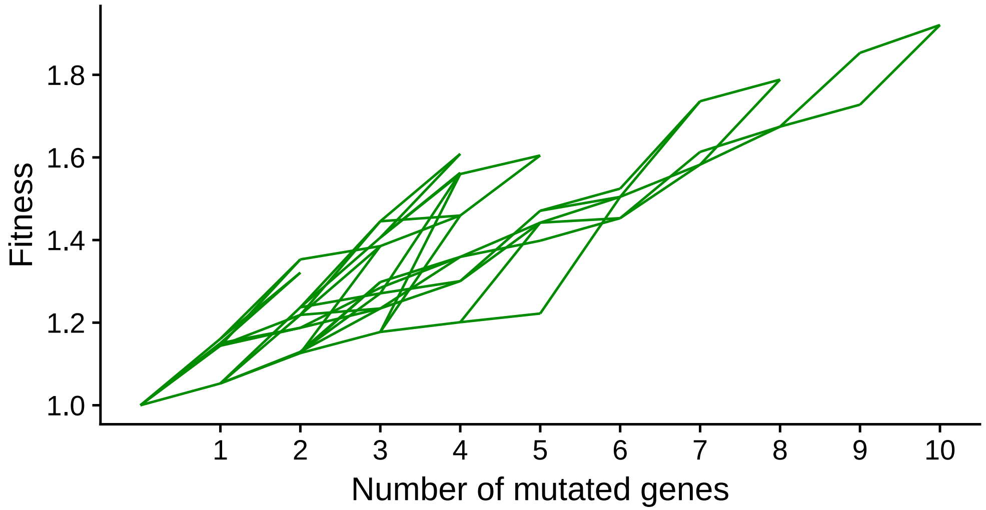

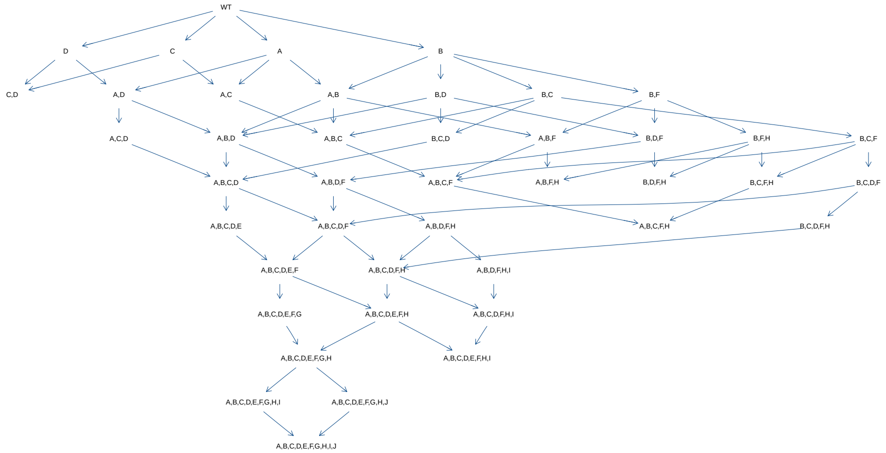

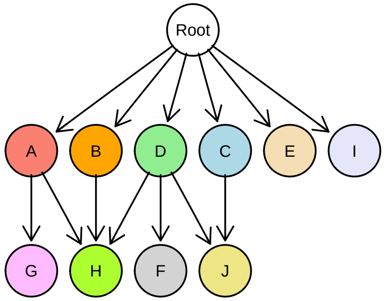

ID	w2gKASMJutkLe4I3
acc genotypes	240
peaks no back	20
removed edges	158
prop rem edges	0.19

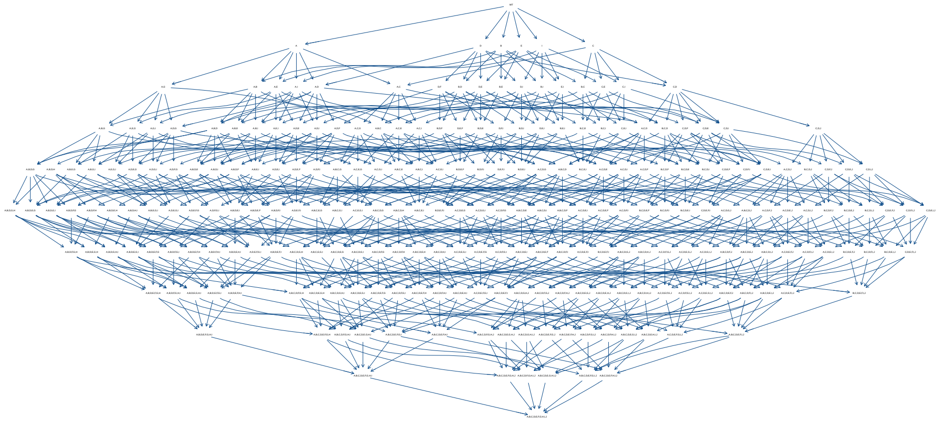

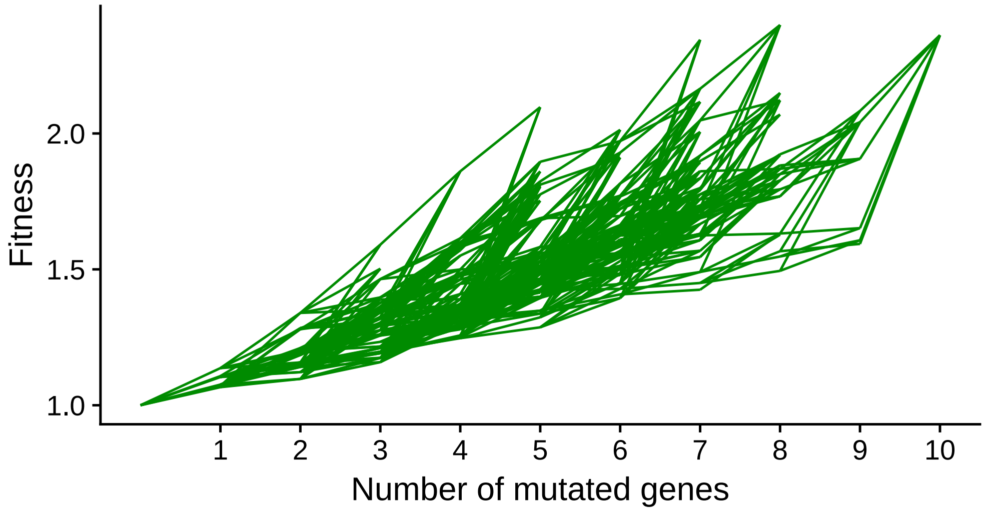

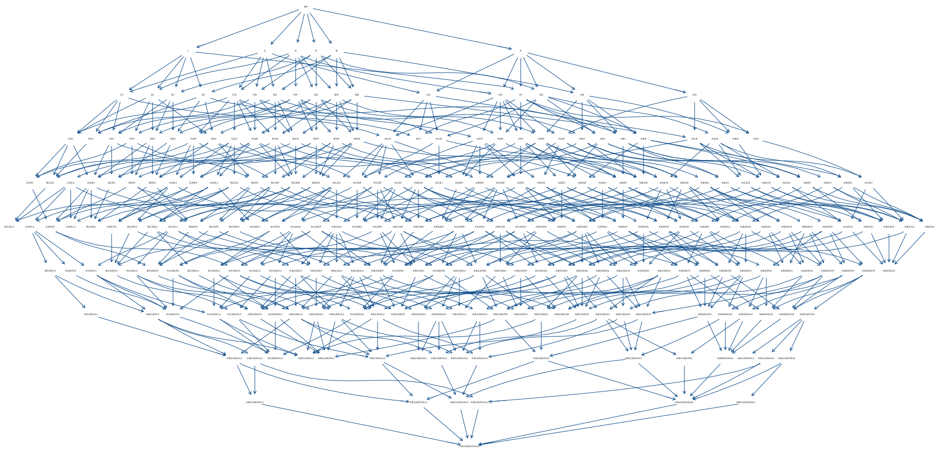

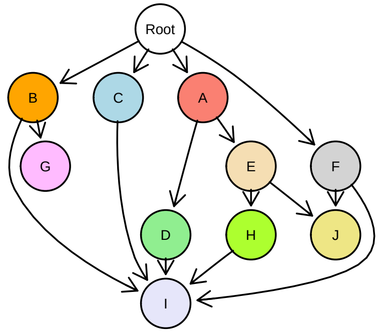

ID wavyEmnct10zXrBsJ

acc genotypes 112

peaks no back 11

removed edges 68

prop rem edges 0.212

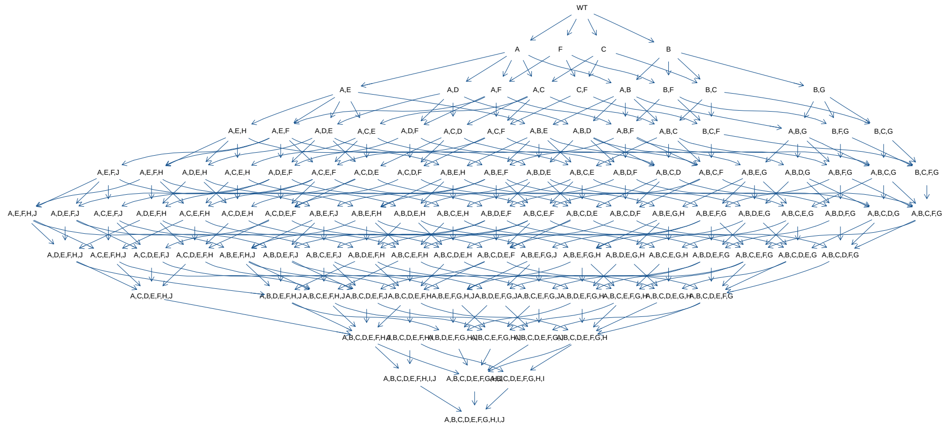

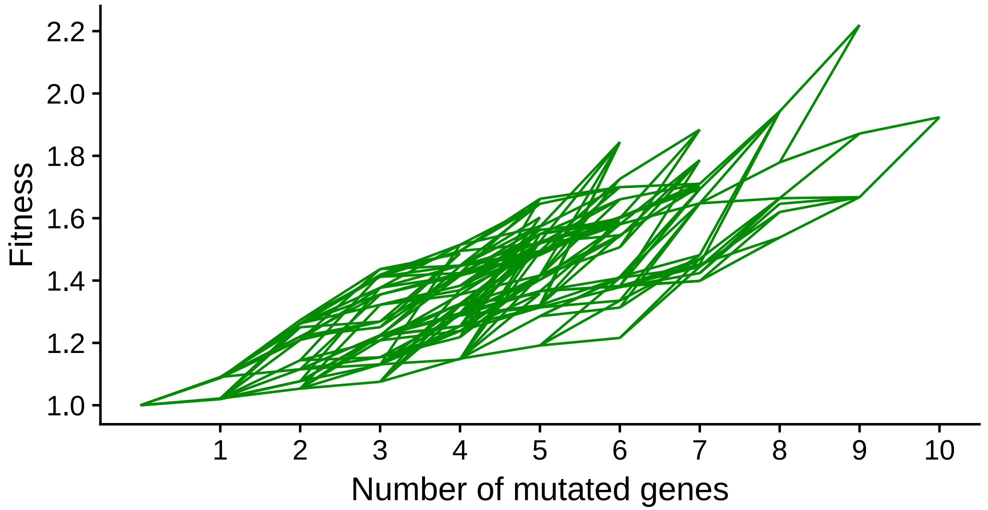

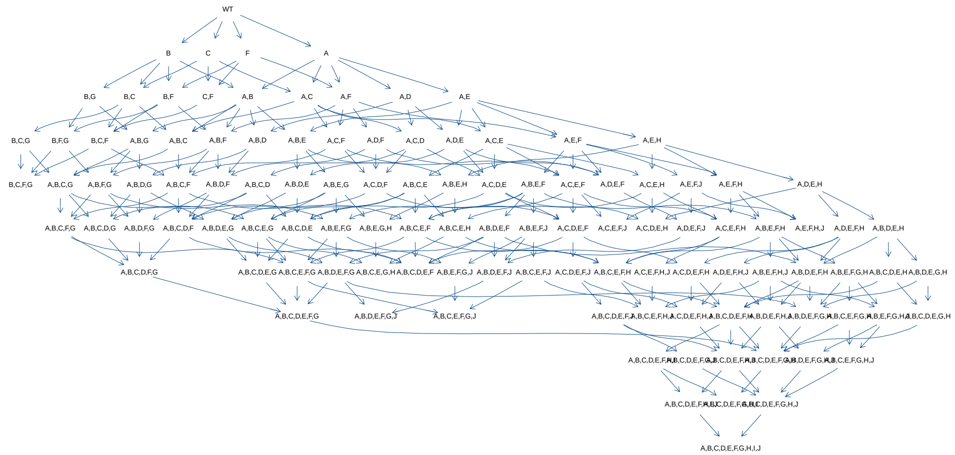

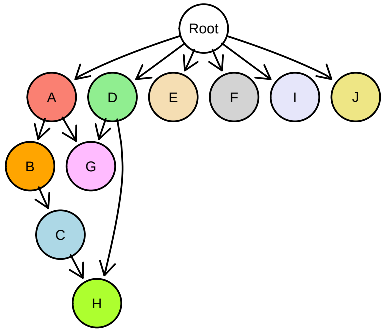

ID	wcH6gWqLOjM6KUvj
acc genotypes	208
peaks no back	21
removed edges	145
prop rem edges	0.206

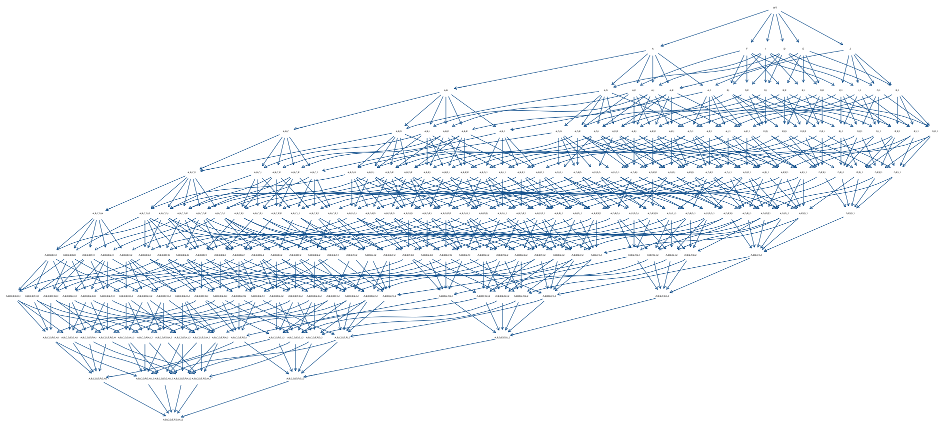

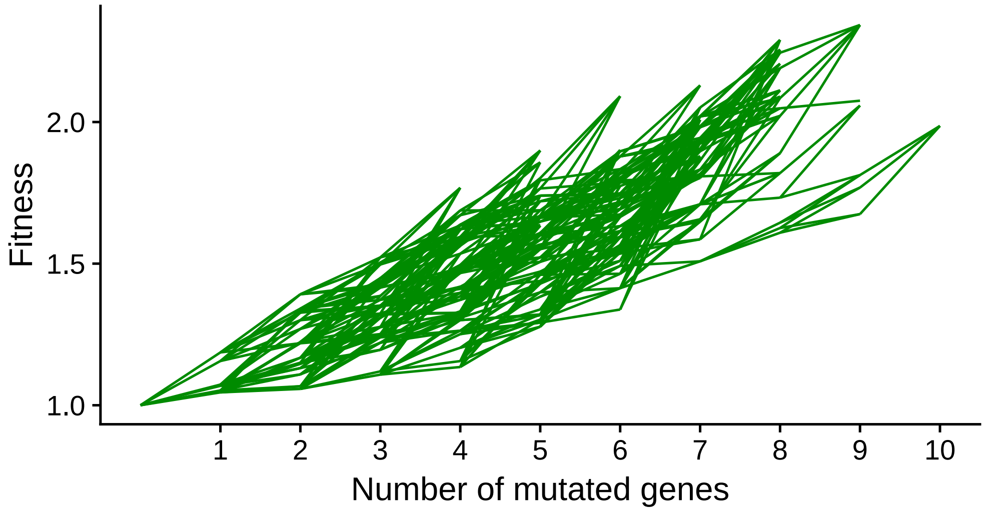

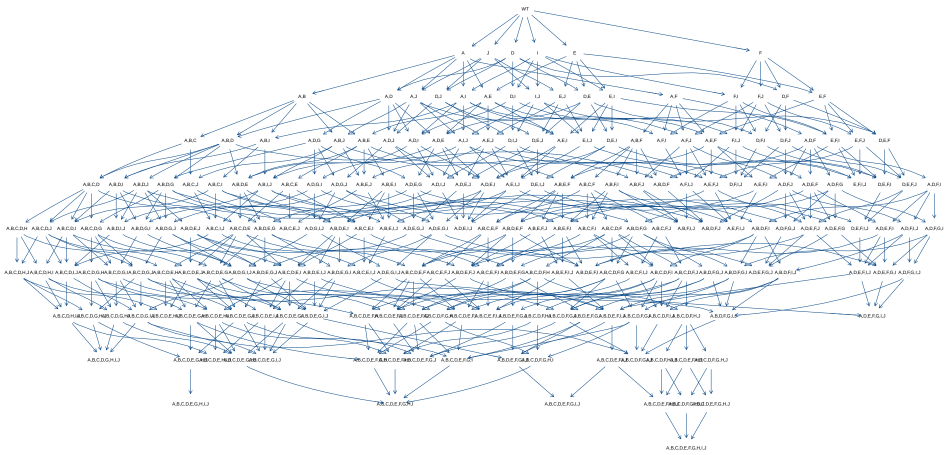

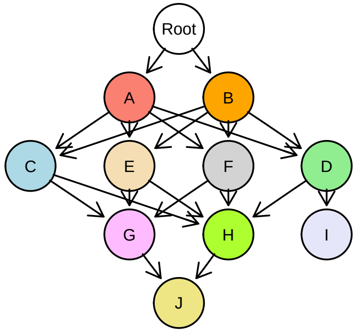

ID wCi29Nr110GfPJ9ih
acc genotypes 36
peaks no back 6
removed edges 10
prop rem edges 0.139

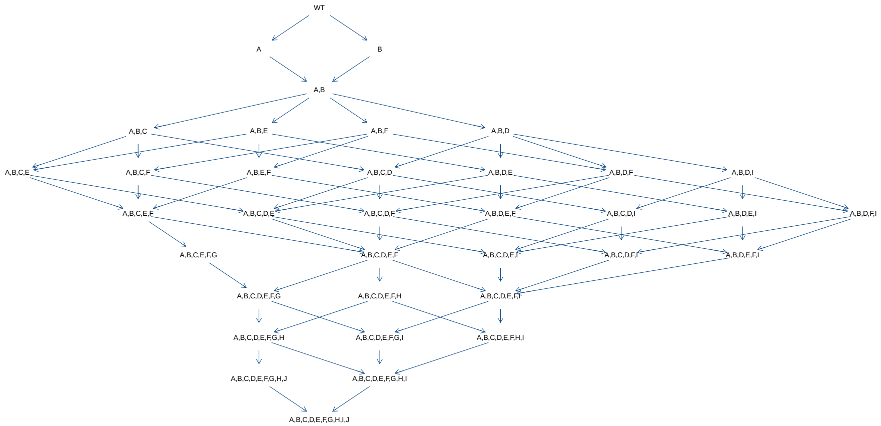

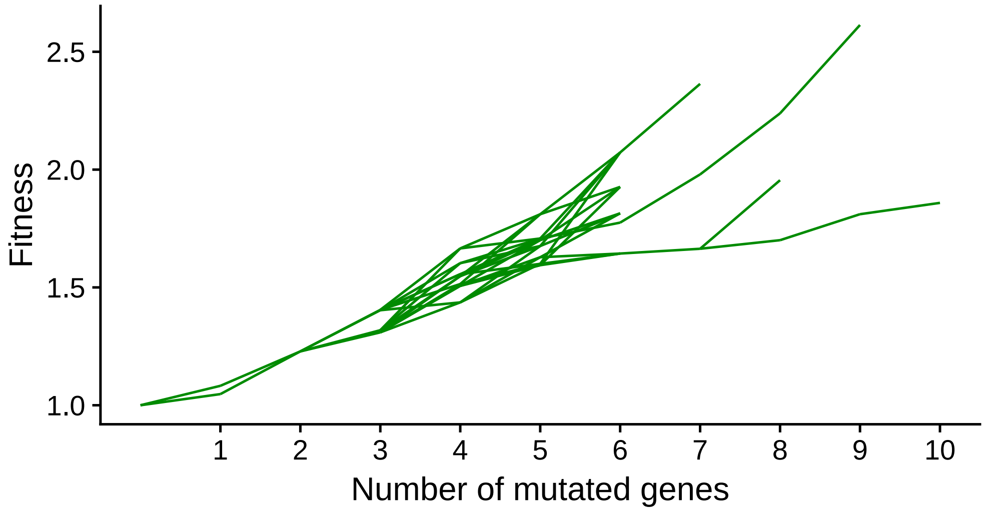

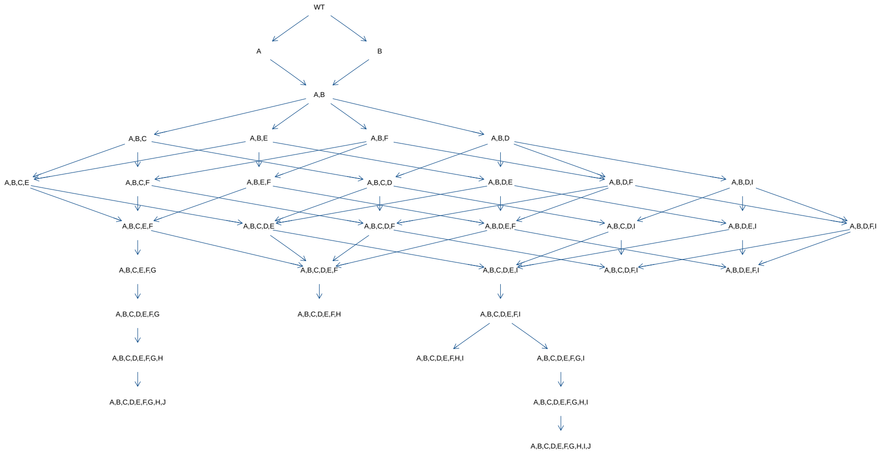

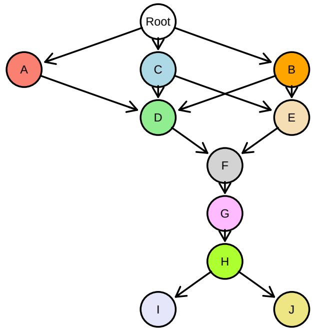

ID	WMUVgmnyiqxYFBg6
acc genotypes	18
peaks no back	2
removed edges	2
prop rem edges	0.08

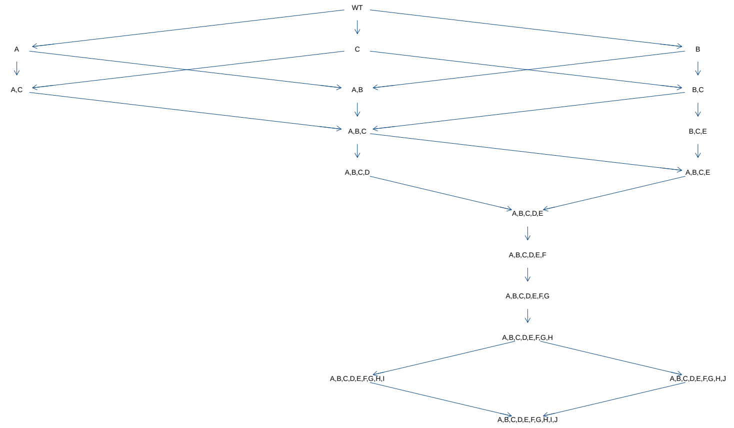

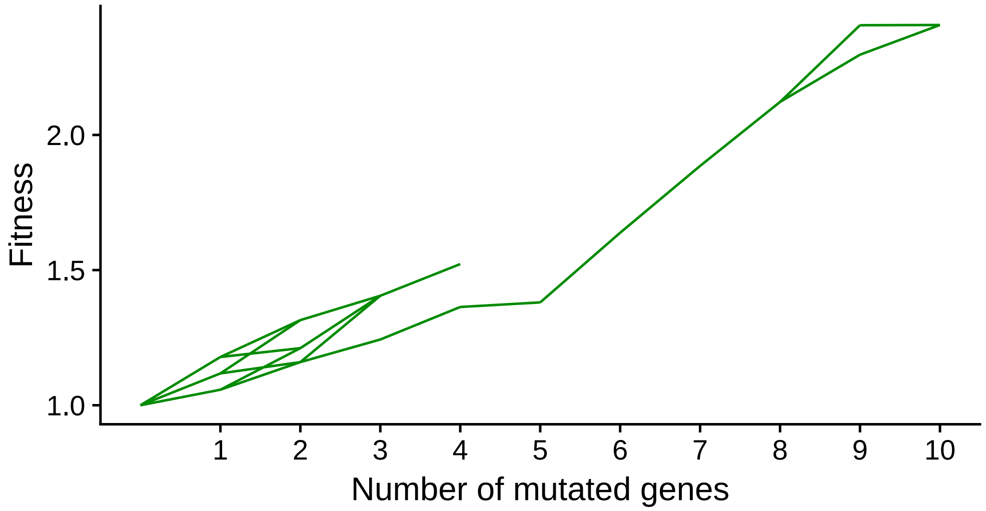

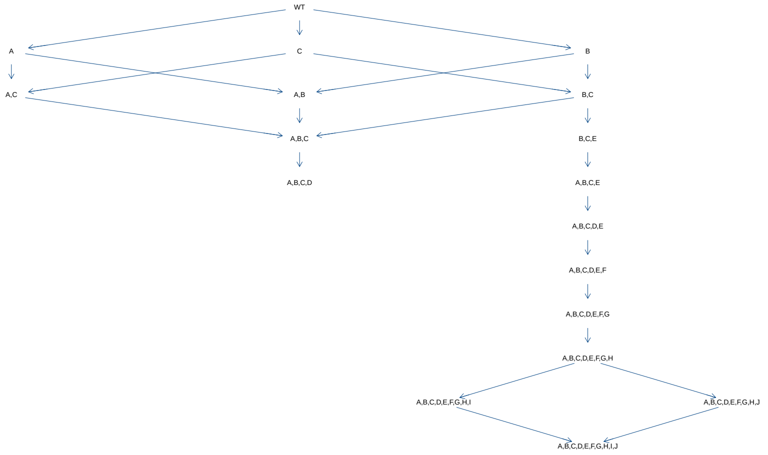

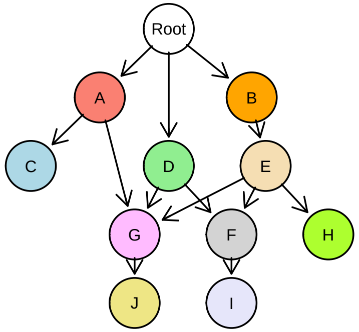

ID	WNDLFwNiHctCAAKK
acc genotypes	60
peaks no back	7
removed edges	24
prop rem edges	0.176

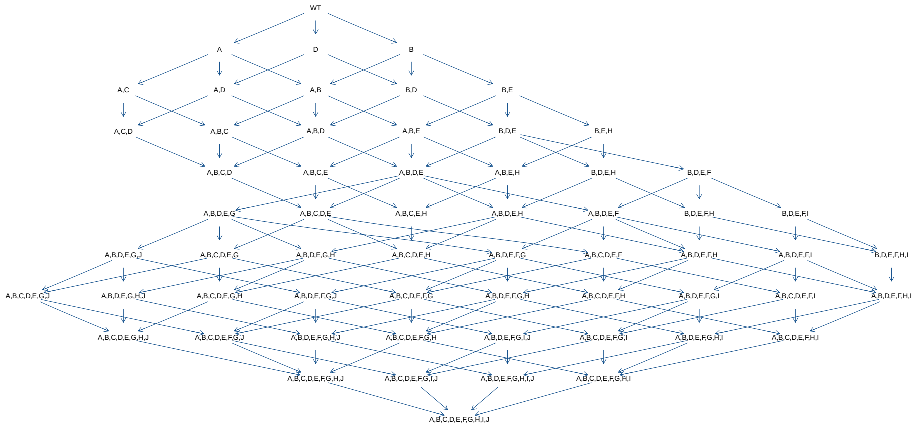

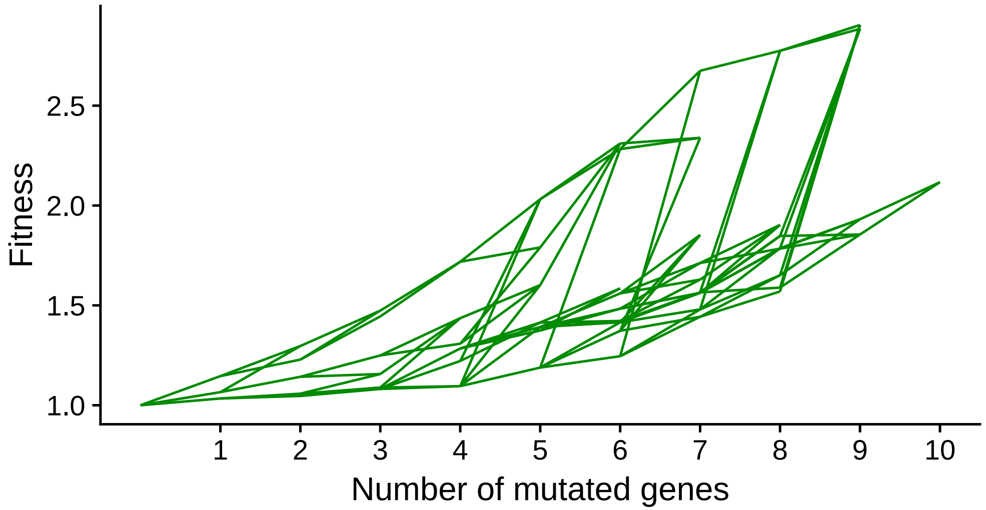

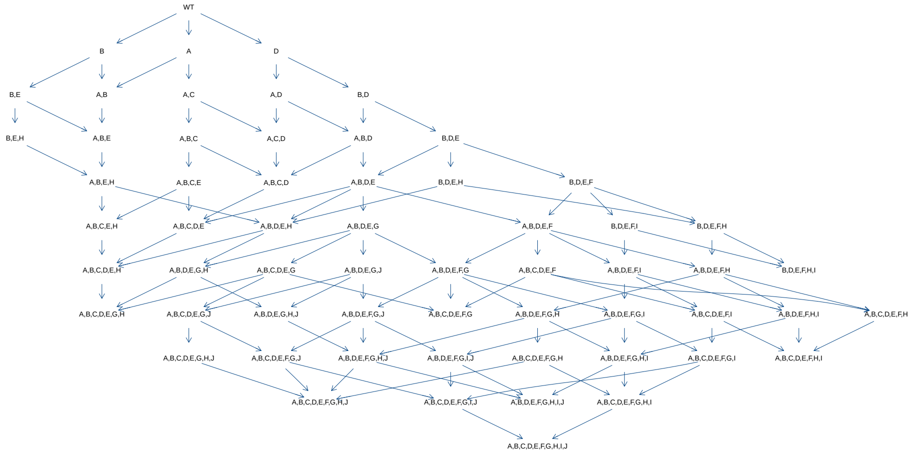

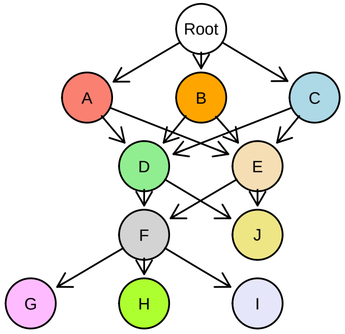

ID wrYzolpOzxMG9bWu
acc genotypes 28
peaks no back 4
removed edges 6
prop rem edges 0.118

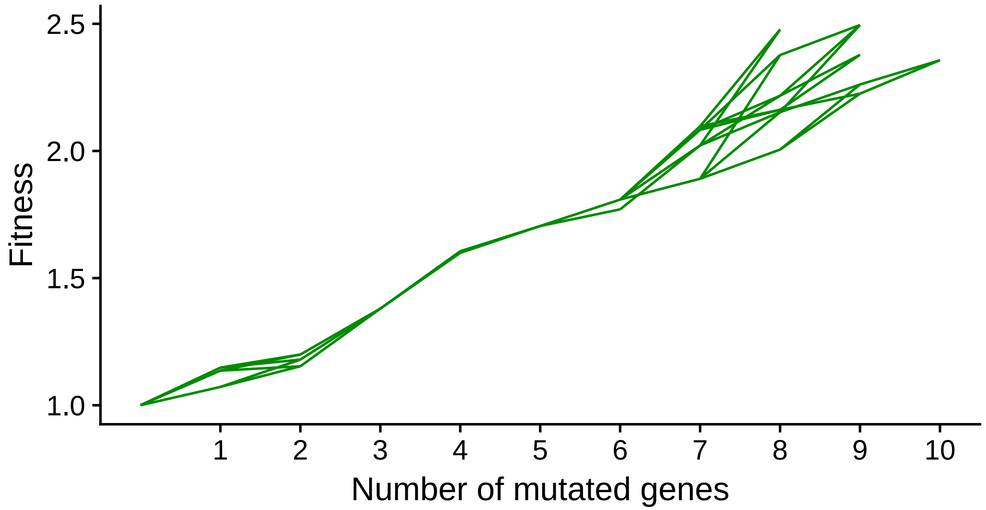

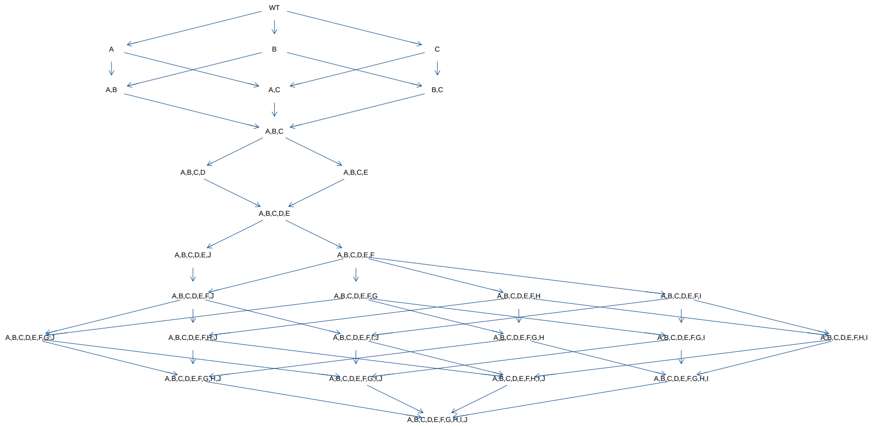

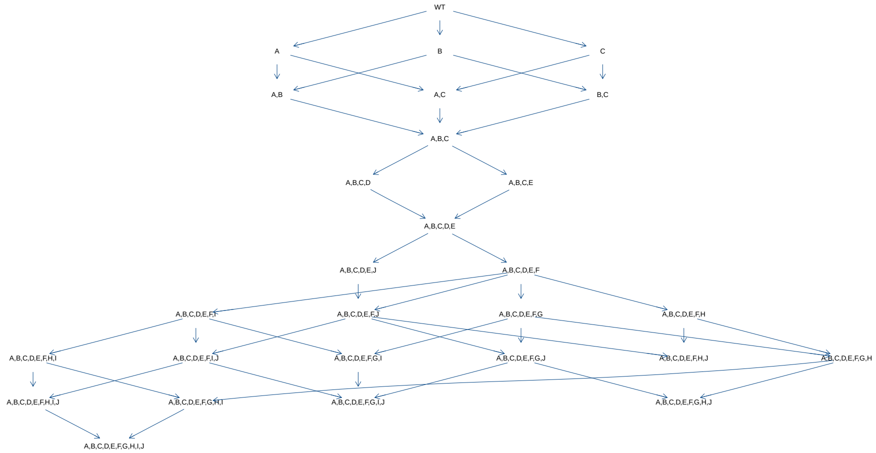

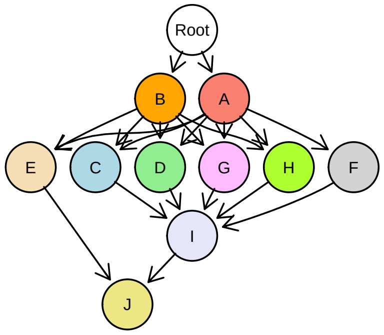

ID	ws10WbhFUWR9mXKoe
acc genotypes	71
peaks no back	8
removed edges	29
prop rem edges	0.144

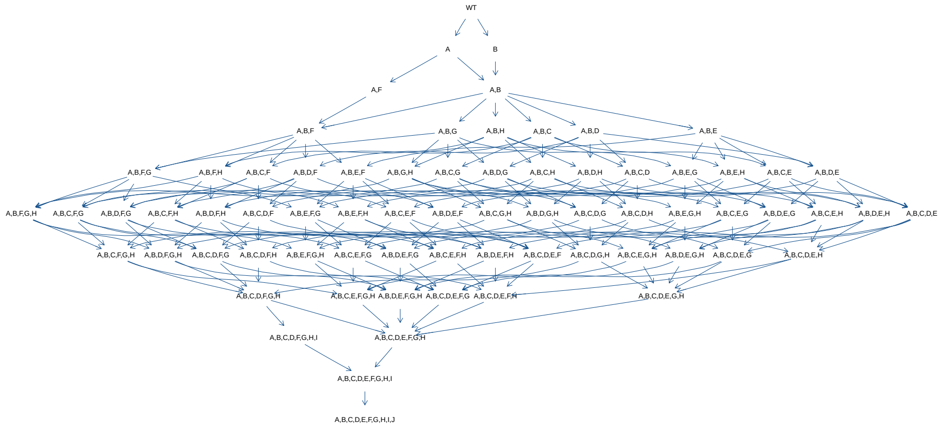

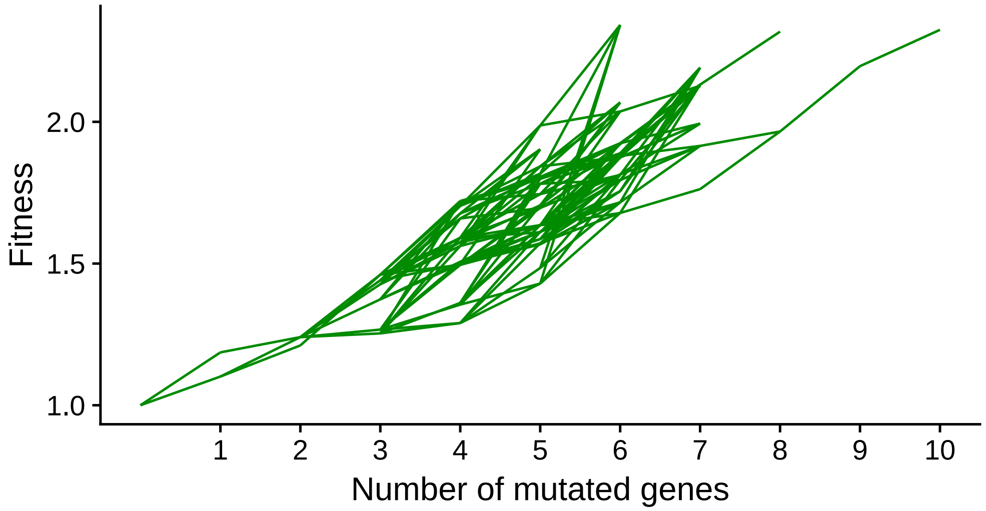

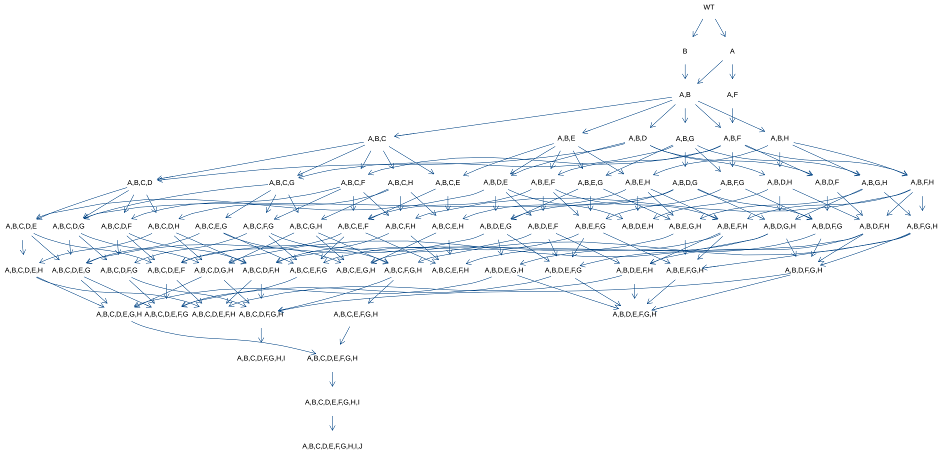

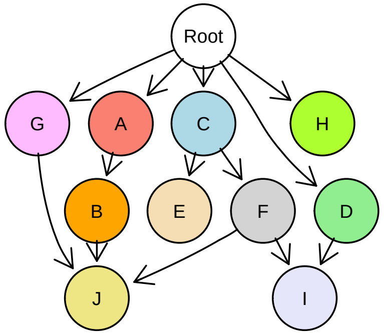

ID	wUaJscj5U6EXgDIC
acc genotypes	156
peaks no back	14
removed edges	87
prop rem edges	0.178

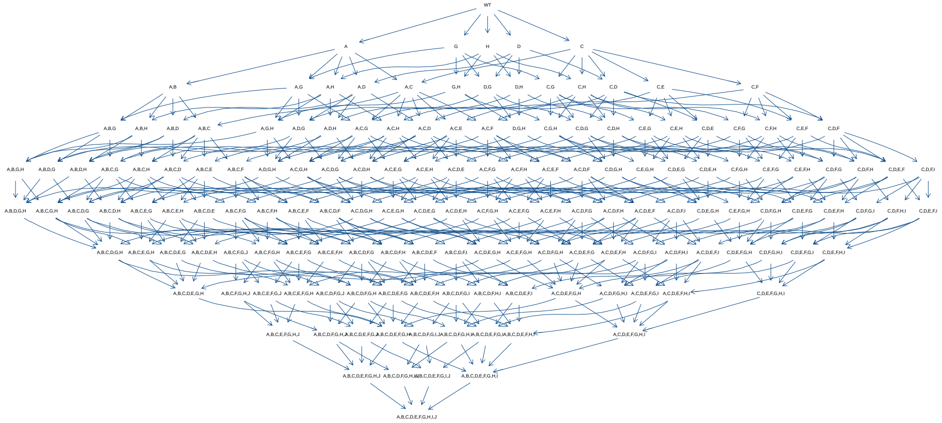

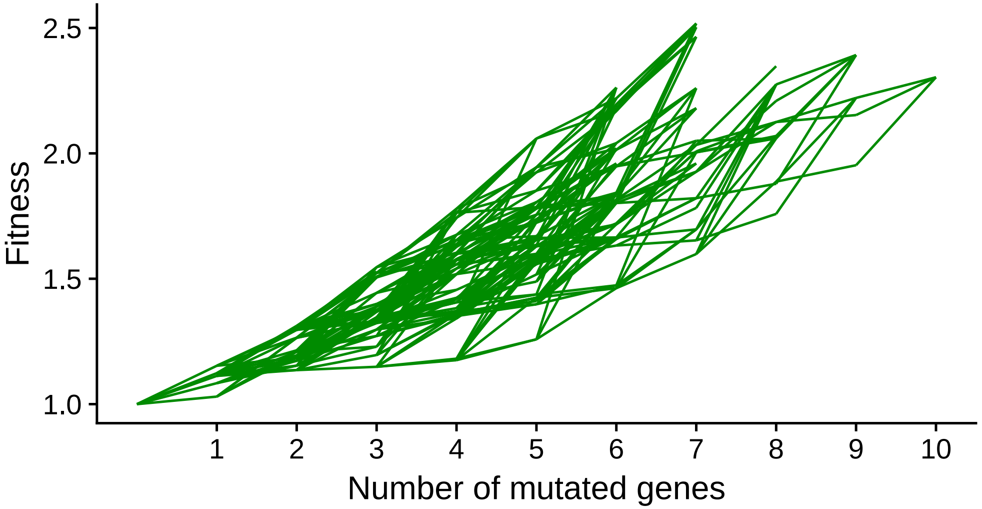

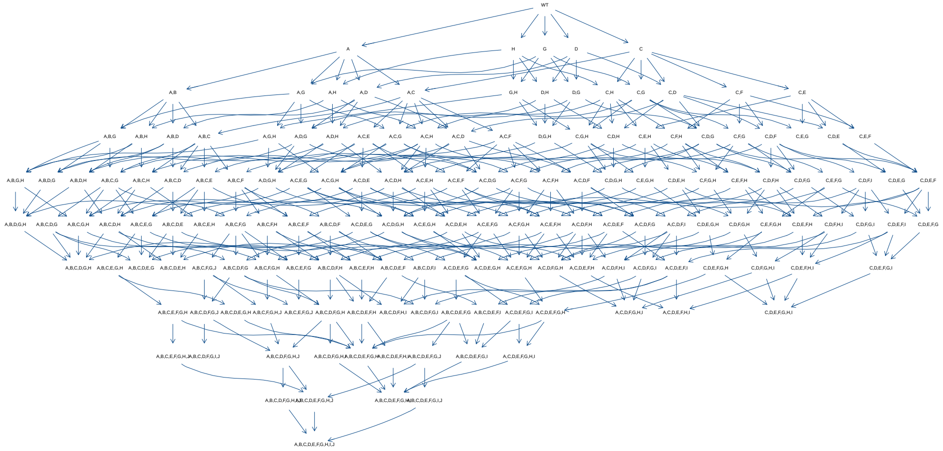

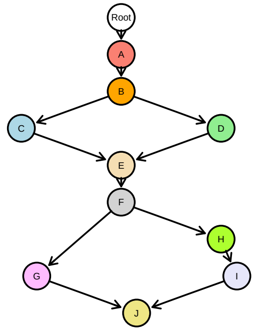

ID XajIk5zcAsLjK9cr
acc genotypes 14
peaks no back 3
removed edges 2
prop rem edges 0.125

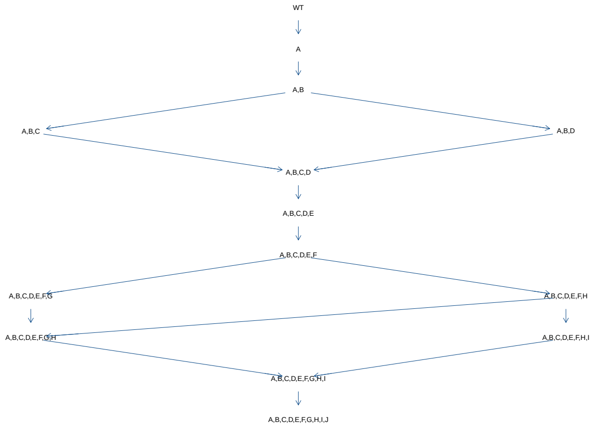

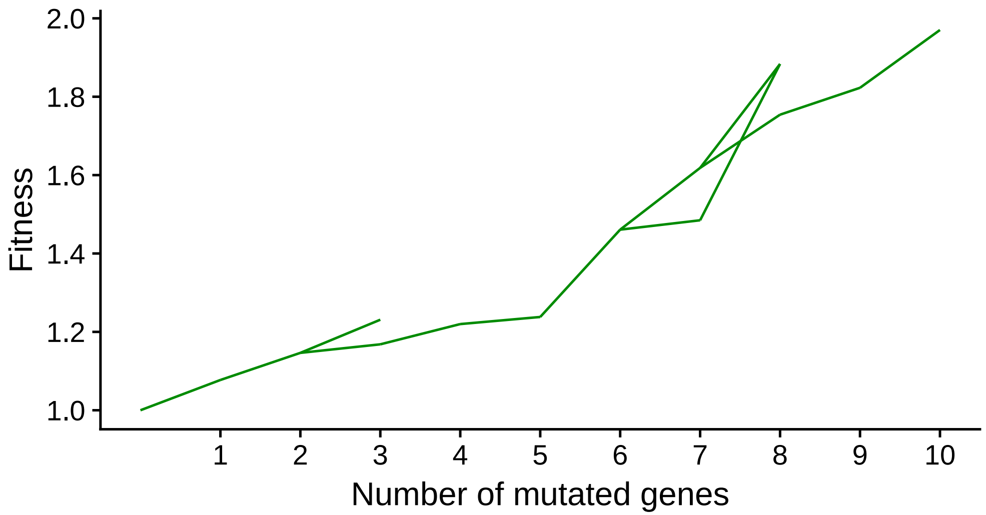

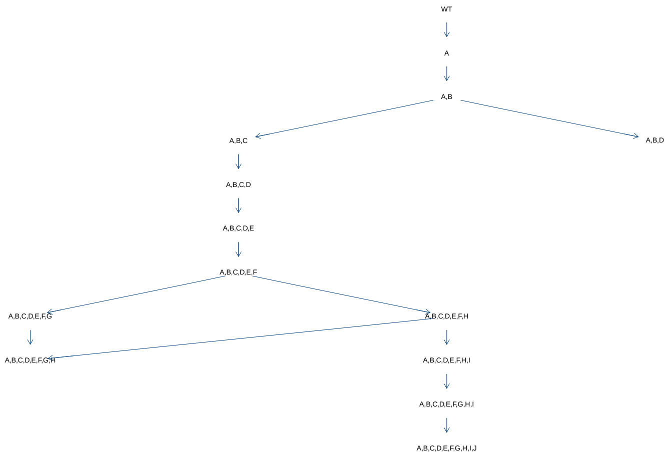

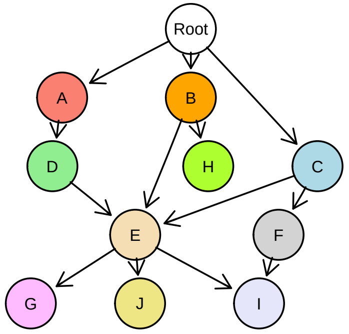

ID	xAYN10rolXAsrpbMd
acc genotypes	51
peaks no back	9
removed edges	16
prop rem edges	0.145

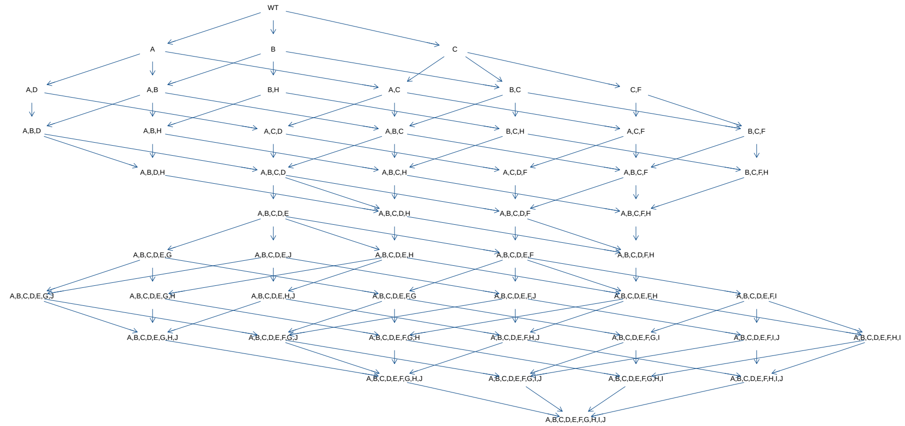

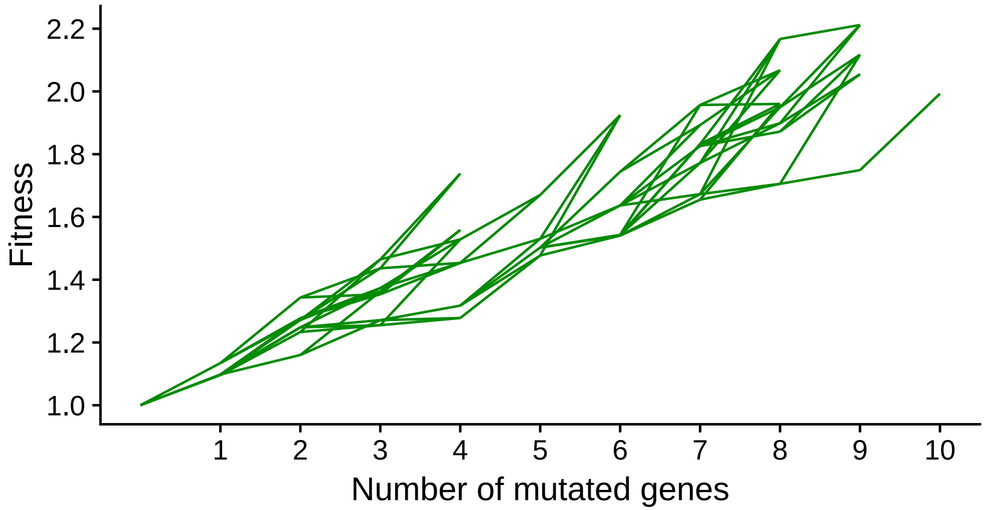

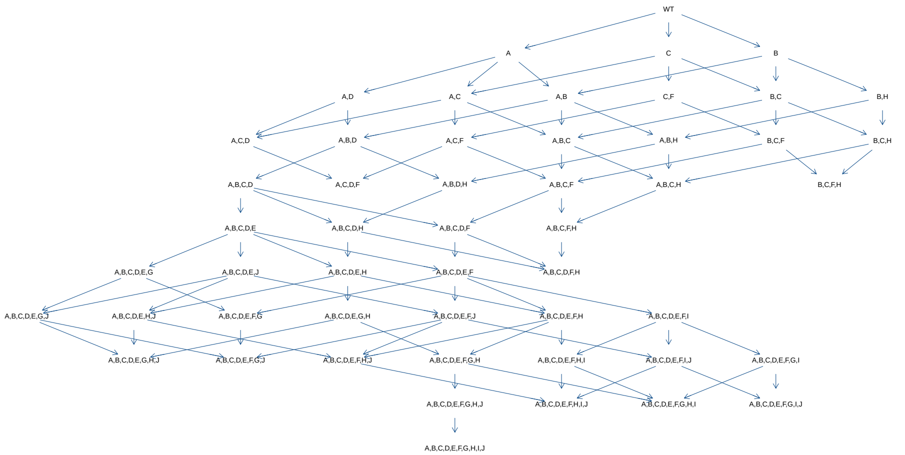

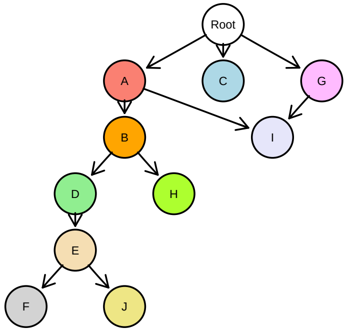

ID	XmnGrSjLNk1UkGT5
acc genotypes	82
peaks no back	12
removed edges	57
prop rem edges	0.268

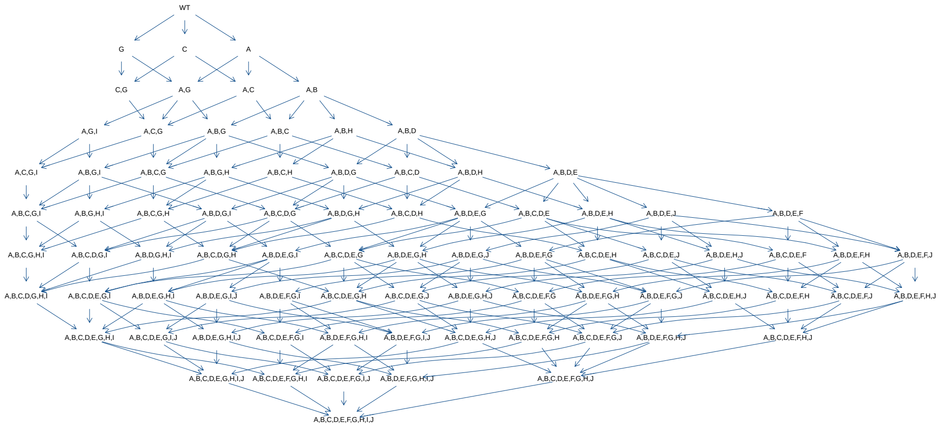

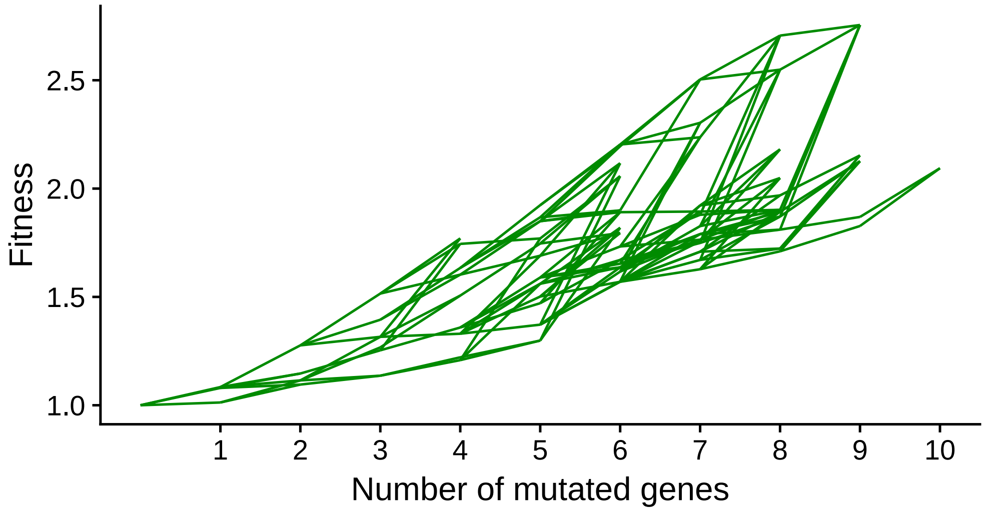

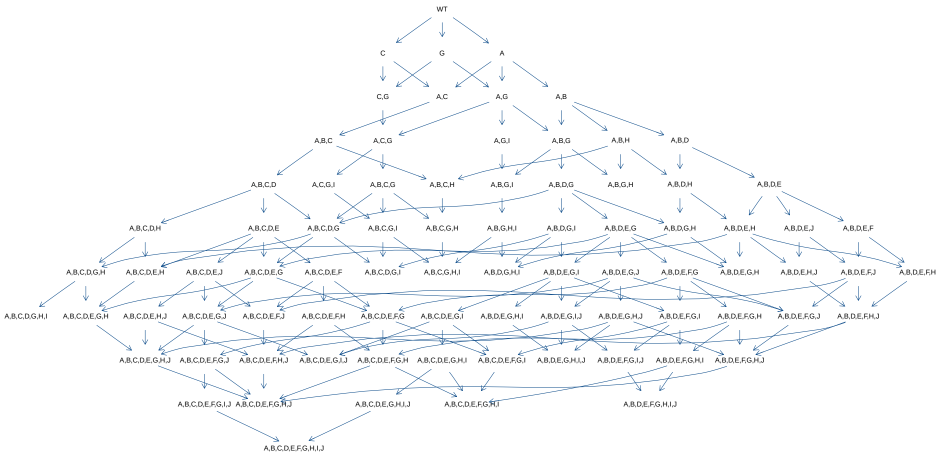

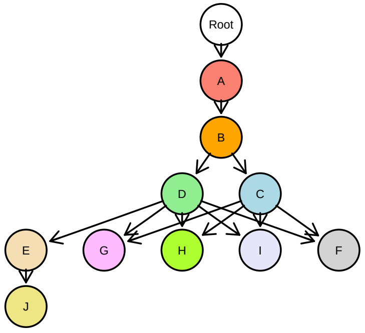

ID	y8uUVSfWKcZQiY4y
acc genotypes	55
peaks no back	8
removed edges	22
prop rem edges	0.159

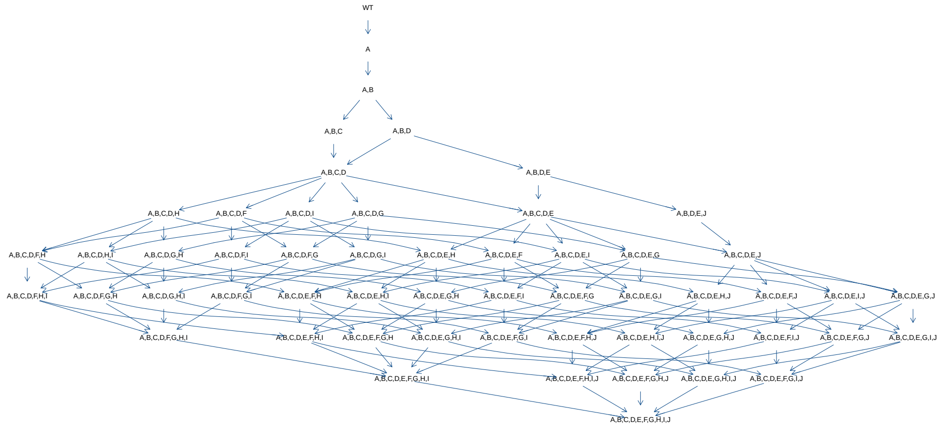

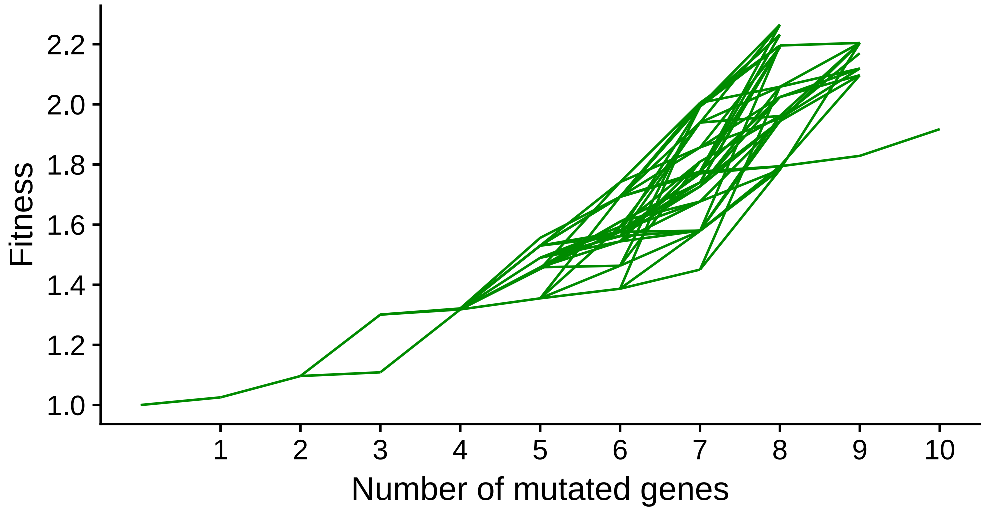

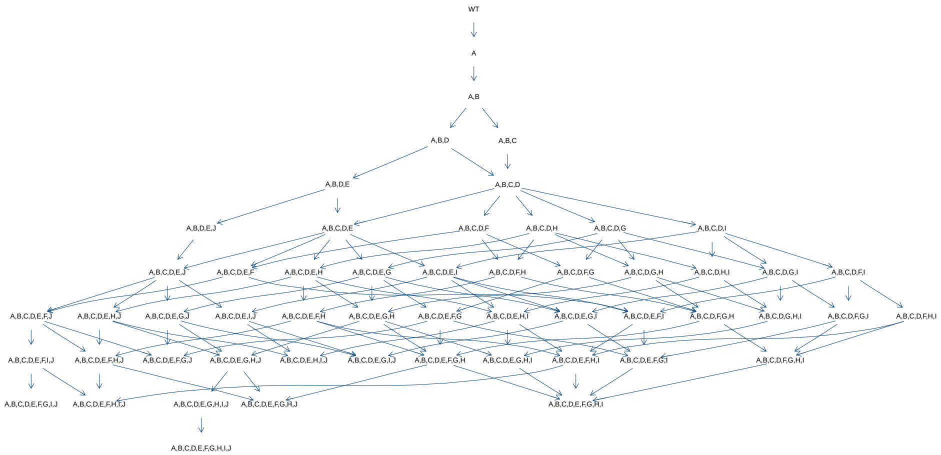

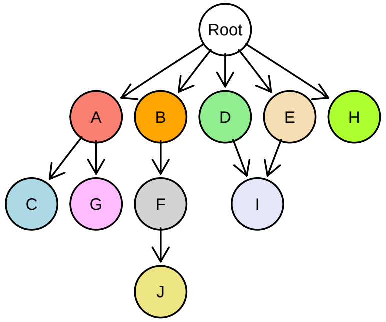

ID	ypRiC10Qof6jAjsVJ
acc genotypes	200
peaks no back	18
removed edges	112
prop rem edges	0.172

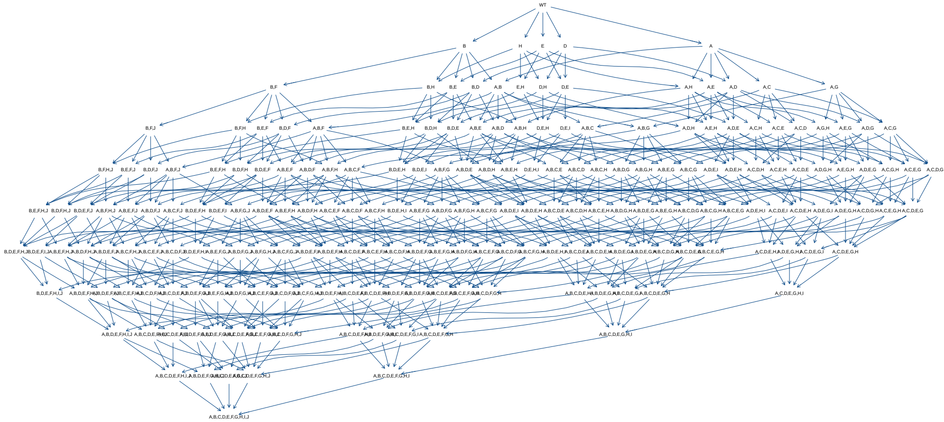

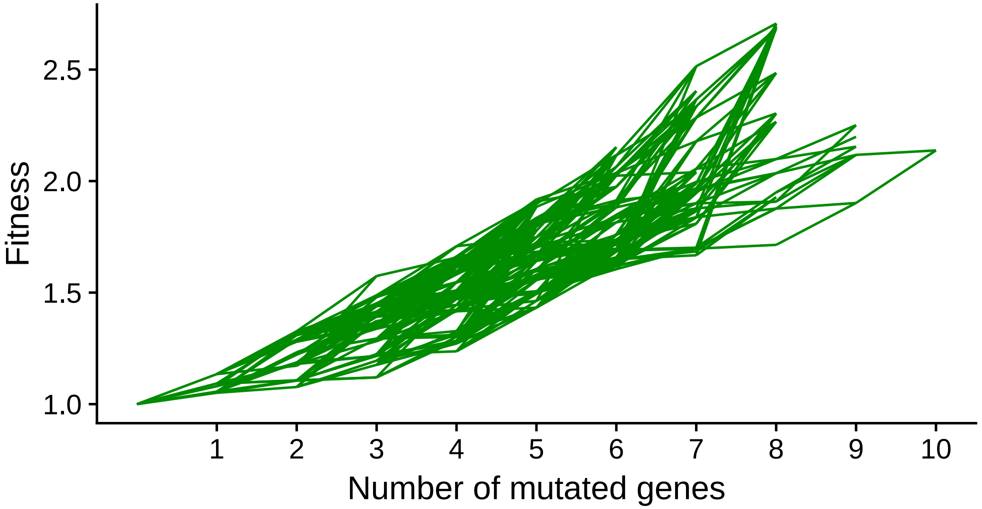

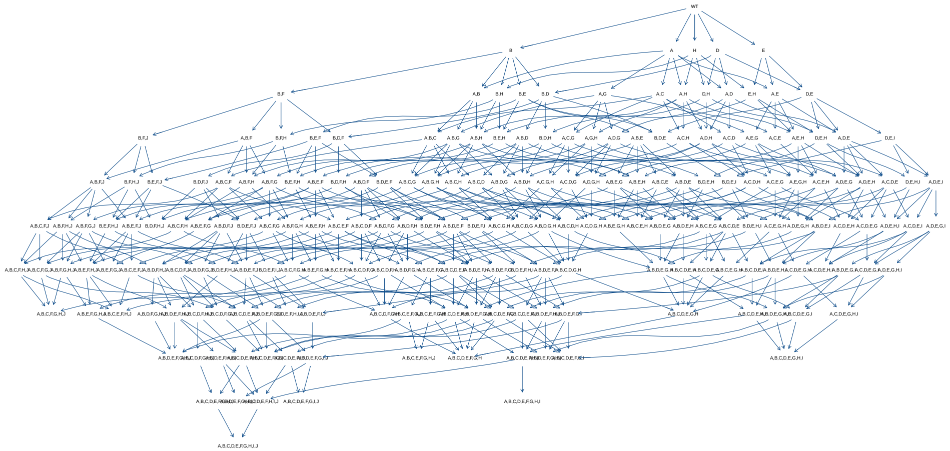

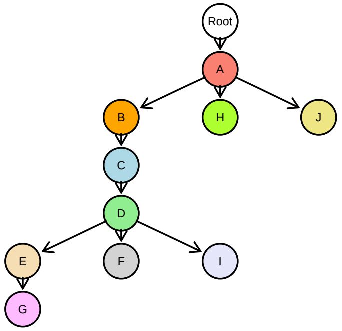

ID	YW7KQxwKMIe2i7ar
acc genotypes	61
peaks no back	9
removed edges	28
prop rem edges	0.183

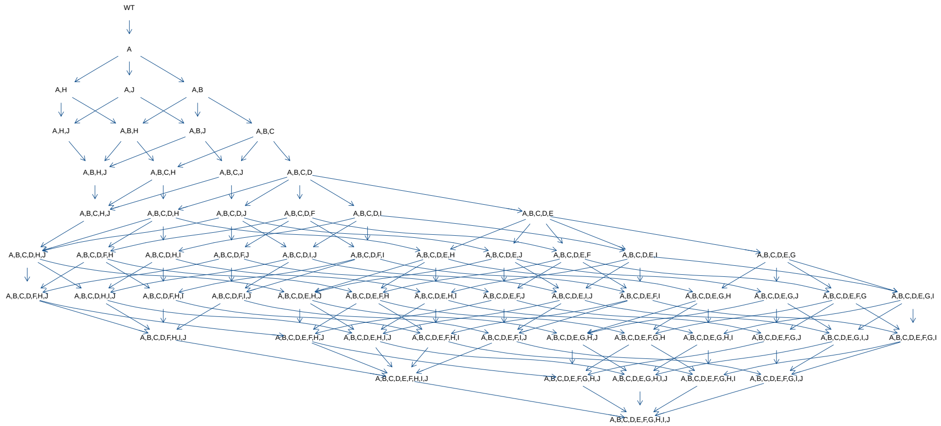

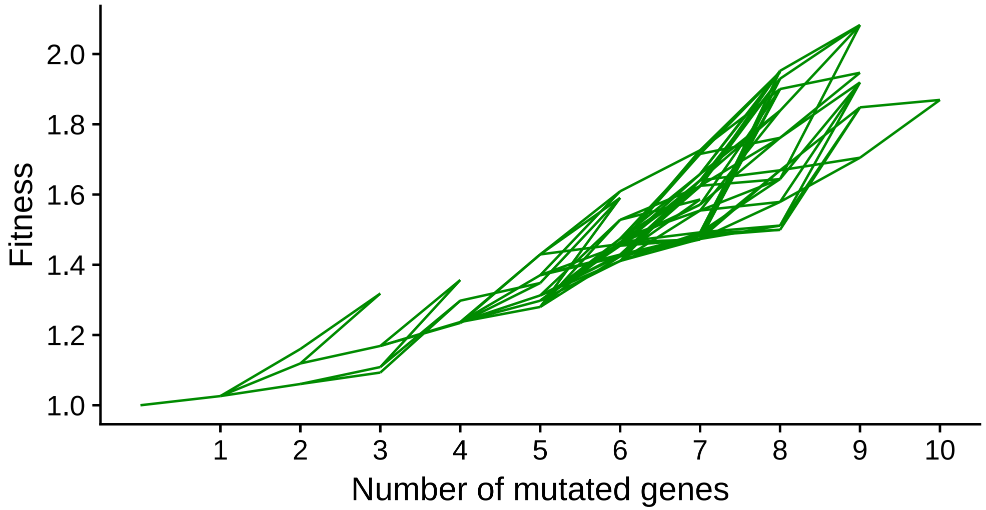

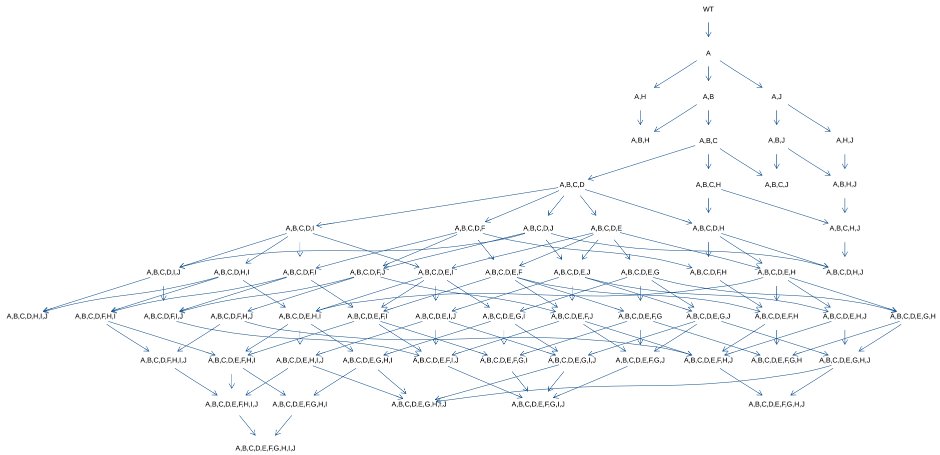

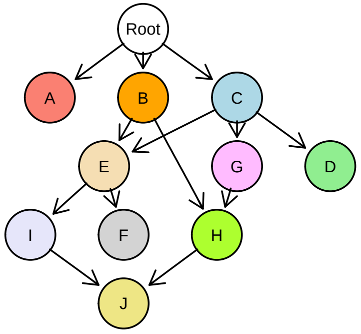

ID	z4RVEoFHLg5dwFWl
acc genotypes	80
peaks no back	9
removed edges	44
prop rem edges	0.212

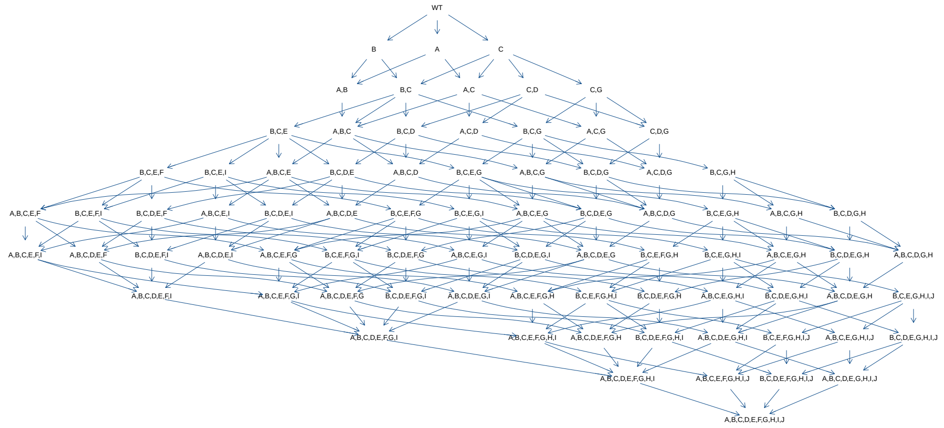

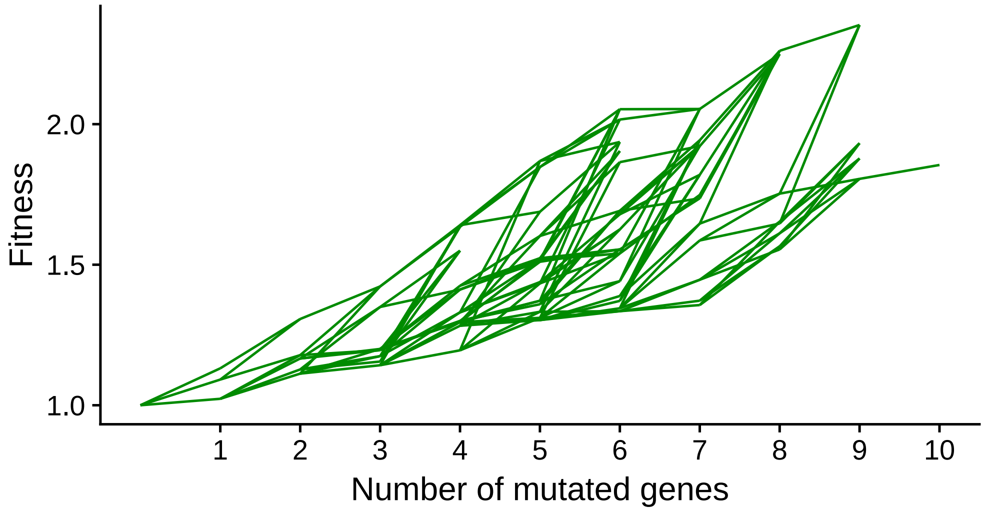

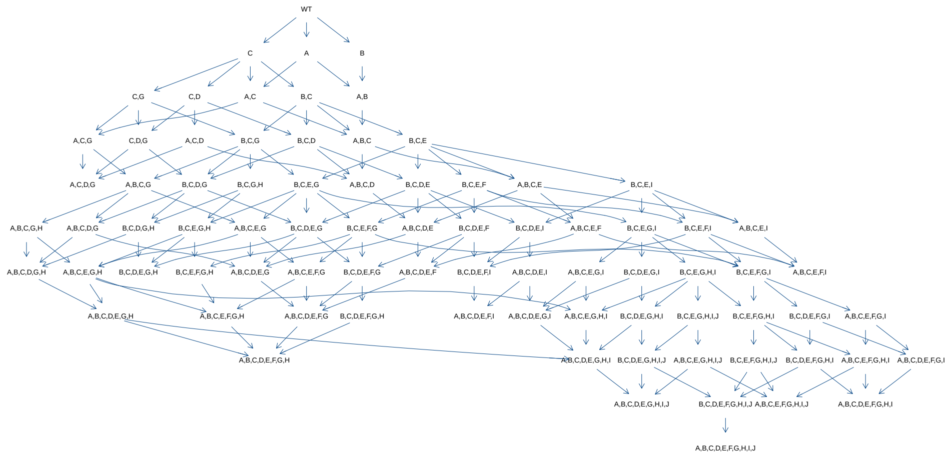

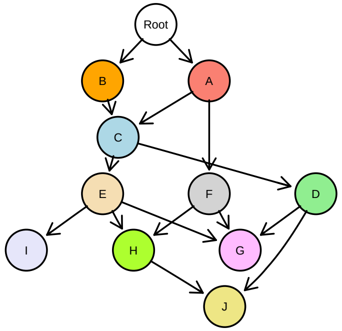

ID	zAMK2D5LDmq9X37r
acc genotypes	30
peaks no back	2
removed edges	3
prop rem edges	0.057

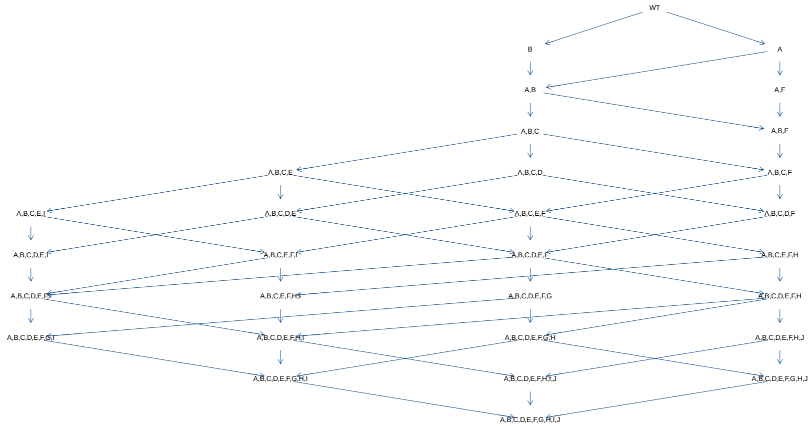

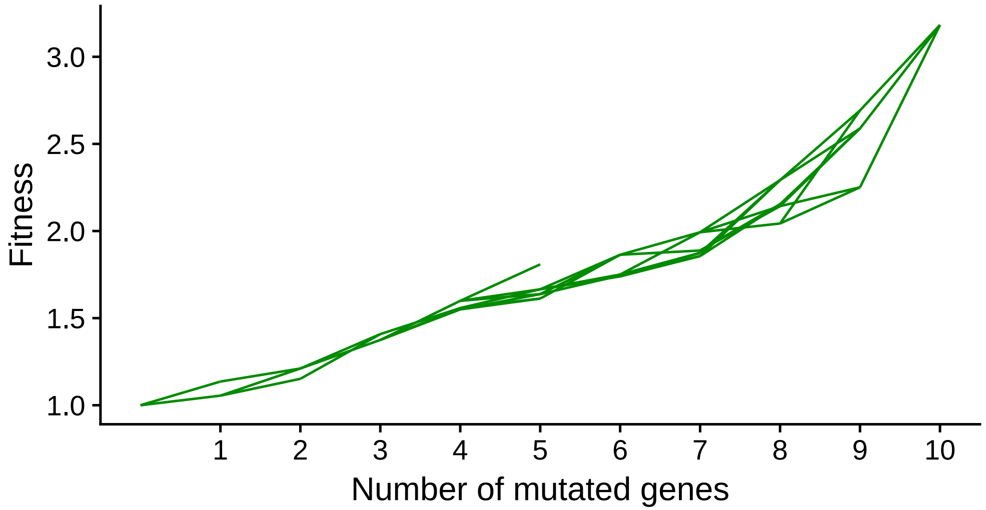

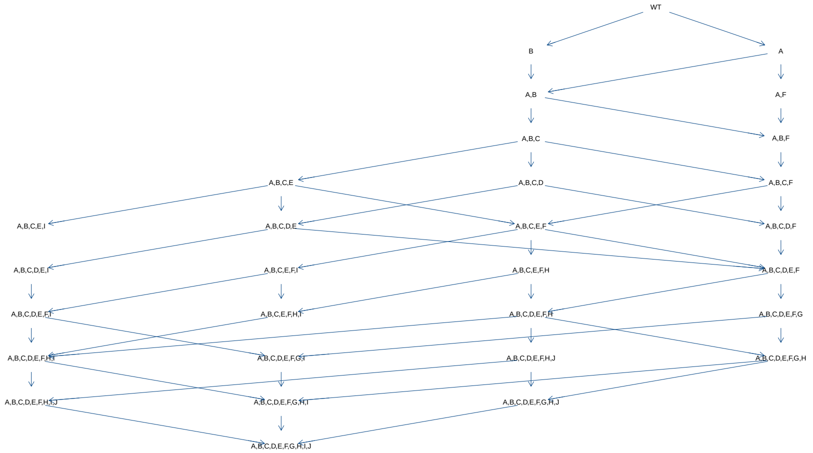

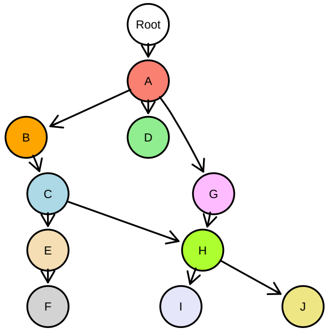

ID	ZIBdVWiOnxT3sPfI
acc genotypes	45
peaks no back	7
removed edges	11
prop rem edges	0.116

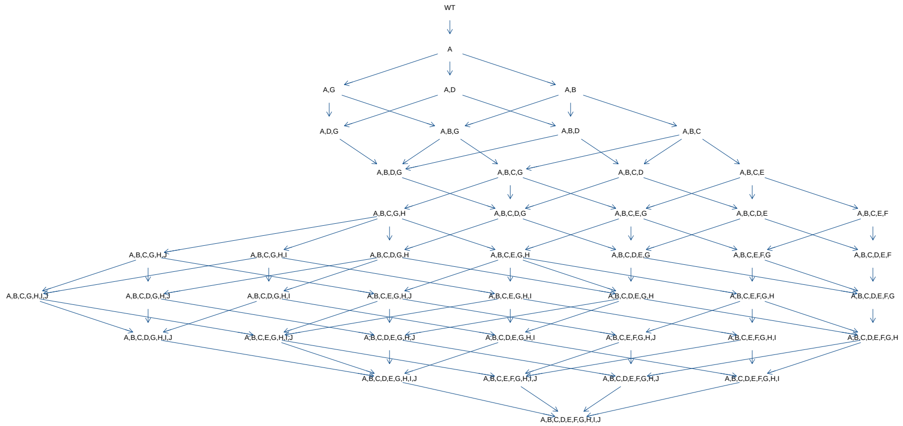

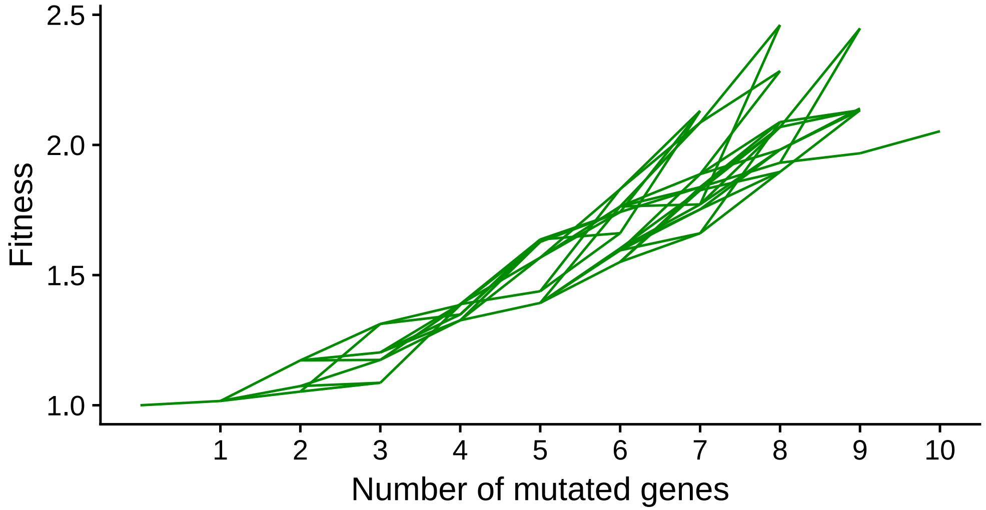

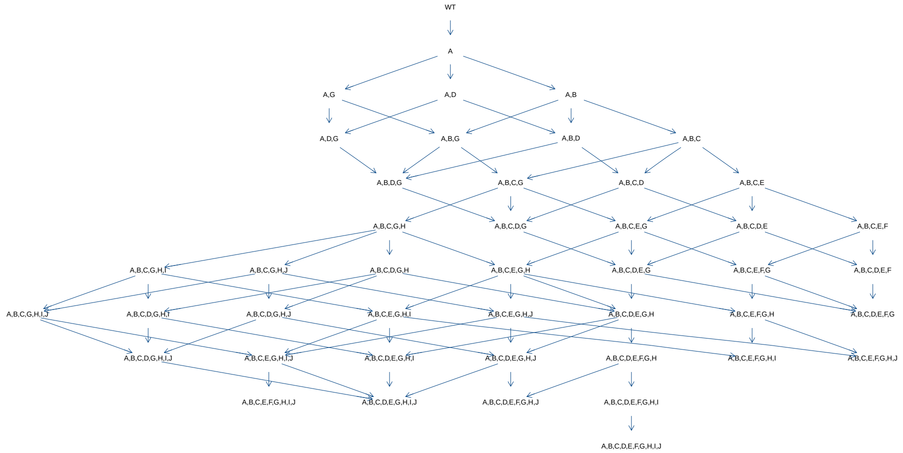

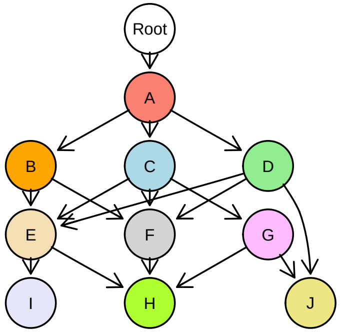

ID	zJtfsaG9cK5j5Hnm
acc genotypes	34
peaks no back	5
removed edges	12
prop rem edges	0.19

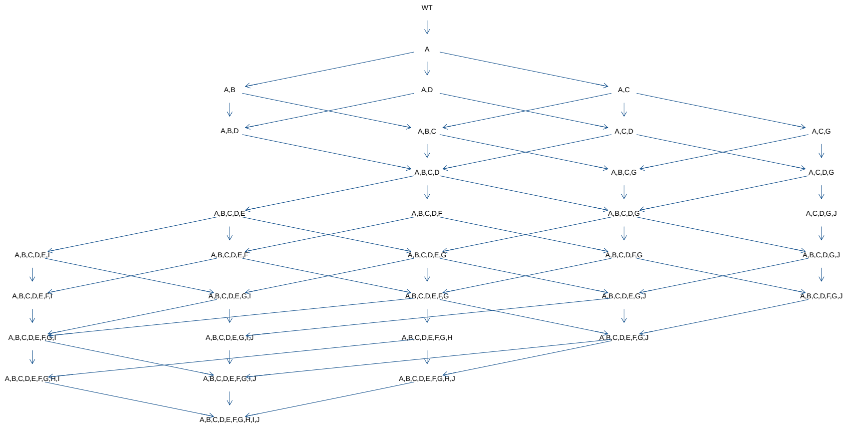

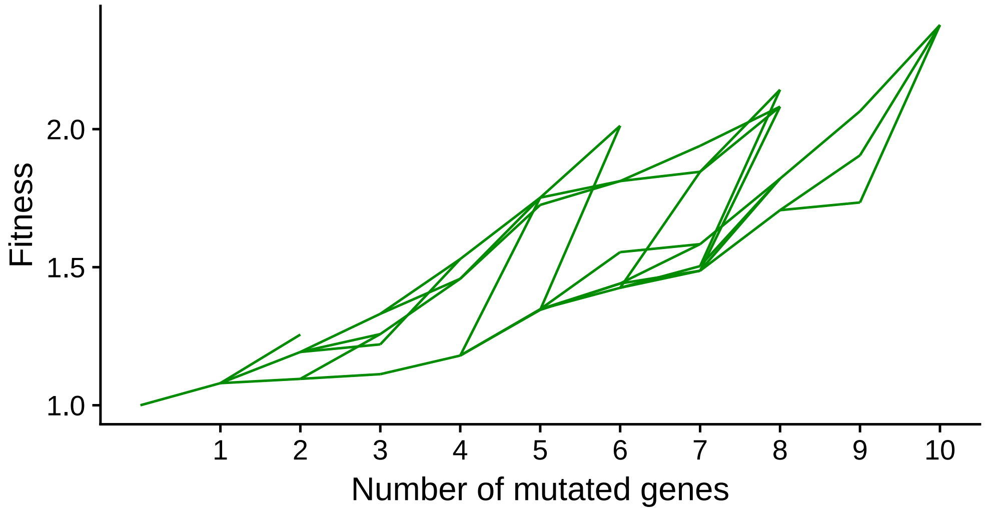

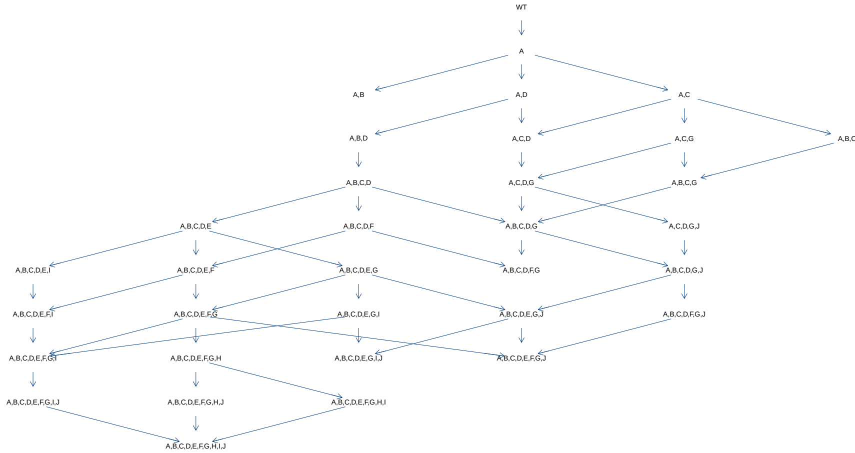

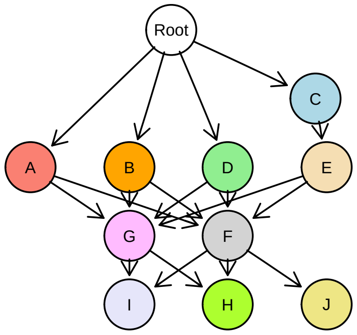

ID	ZKH10YvDZGVfsdHAY
acc genotypes	35
peaks no back	6
removed edges	9
prop rem edges	0.129

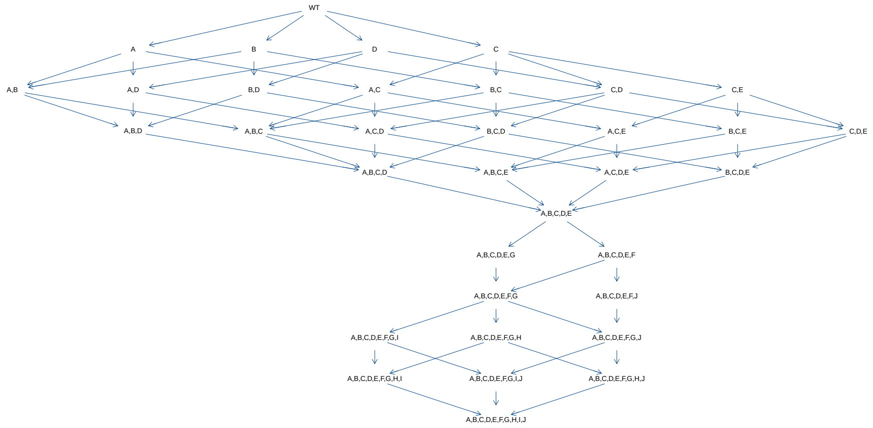

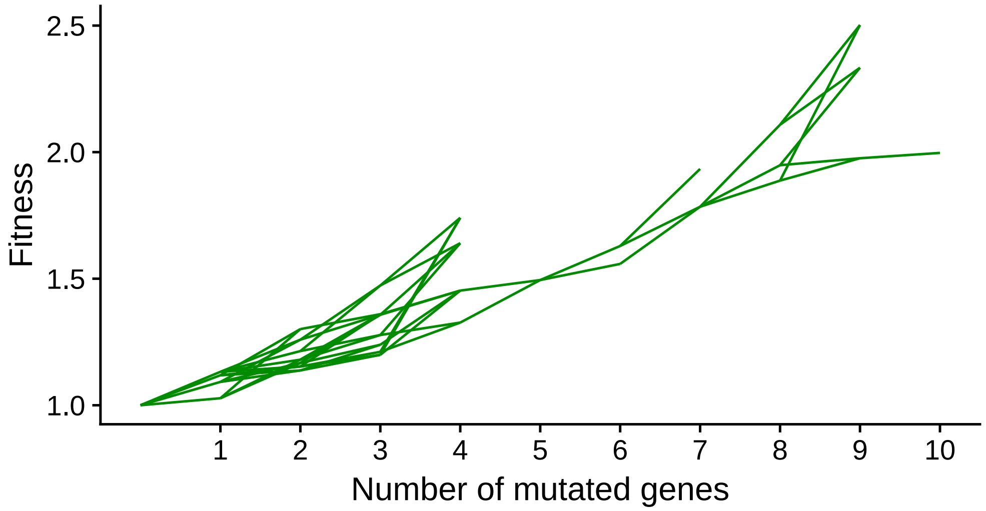

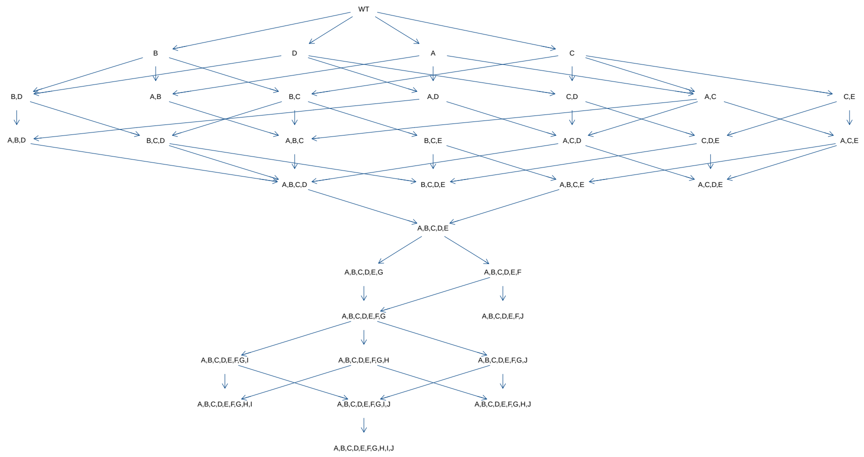

ID	103oxrJDcbfc2YpKa
acc genotypes	162
peaks no back	57
removed edges	NA
prop rem edges	NA

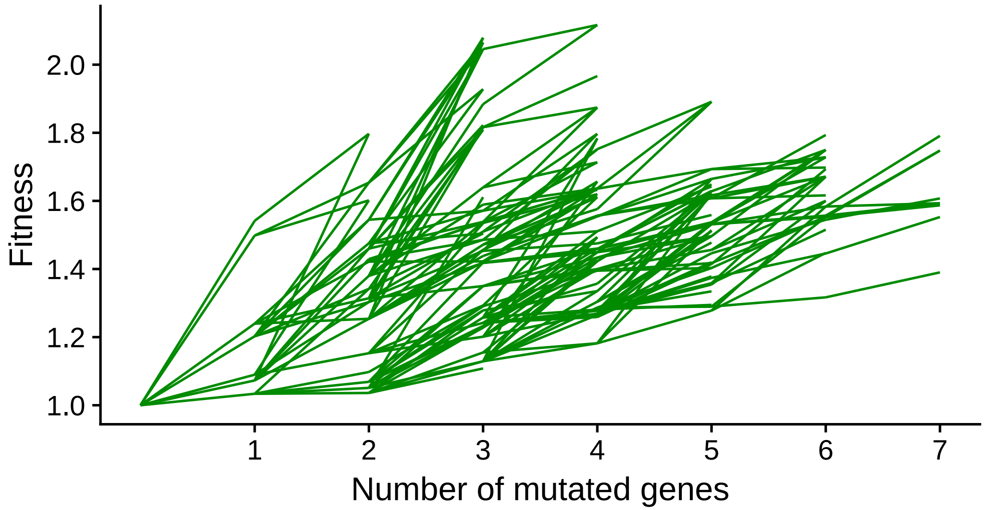

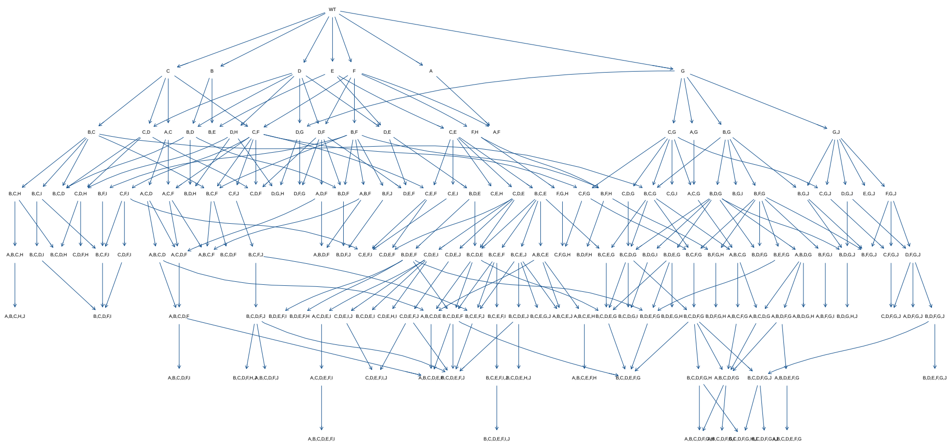

ID	10aJ10EAqKH7VA3ccp
acc genotypes	79
peaks no back	26
removed edges	NA
prop rem edges	NA

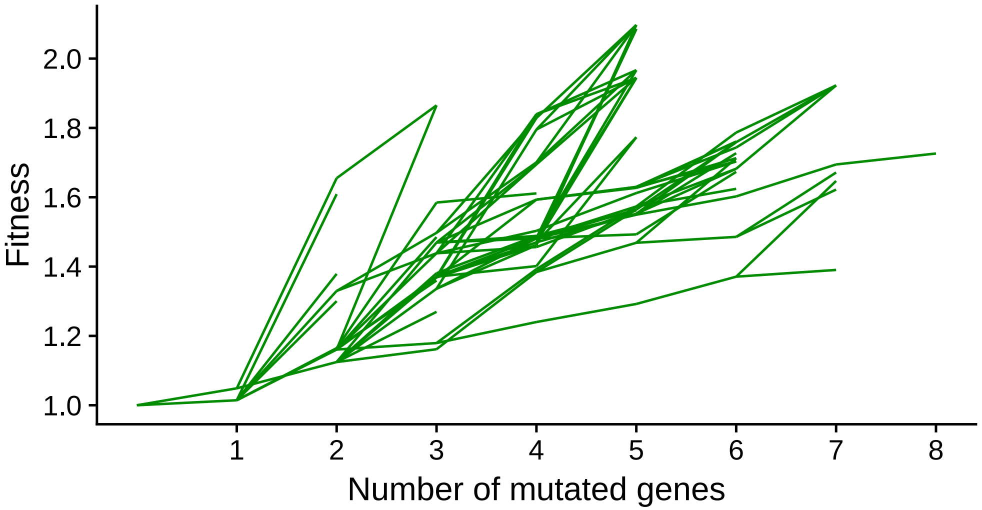

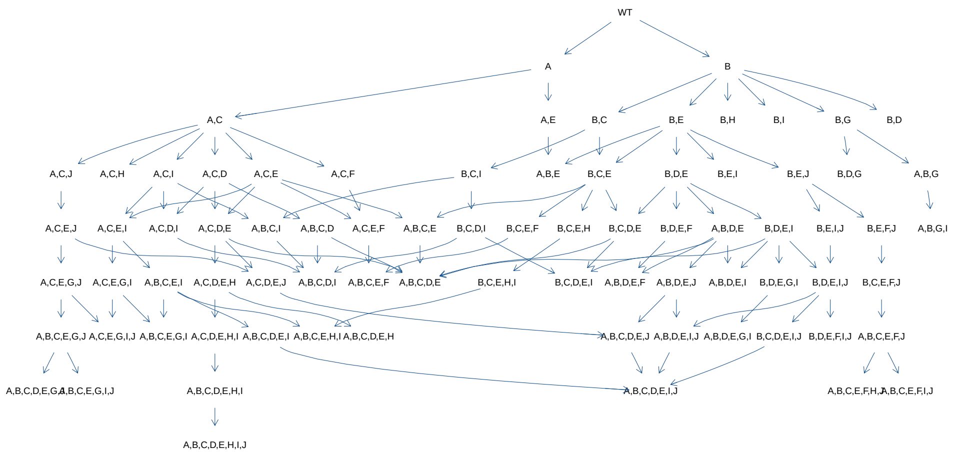

ID	10fyev4yPOAHqV3G4
acc genotypes	72
peaks no back	33
removed edges	NA
prop rem edges	NA

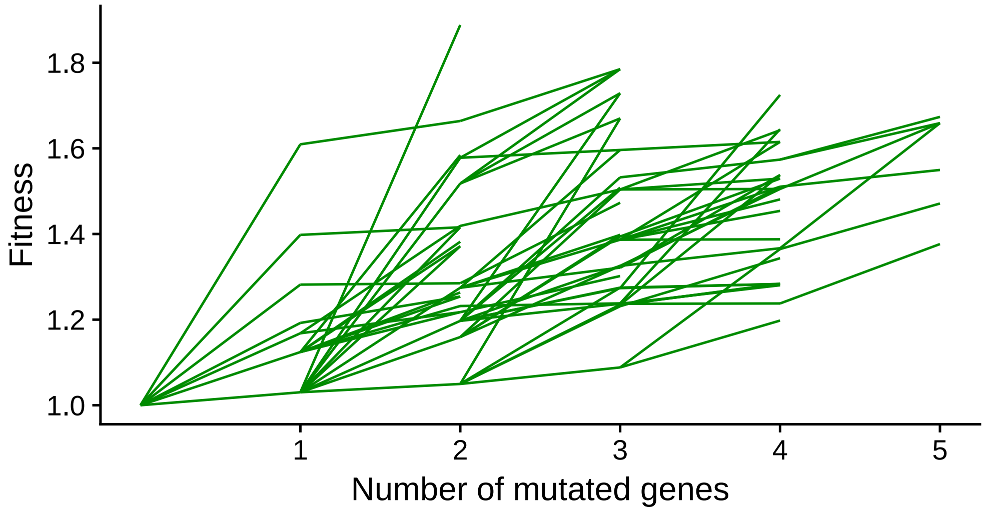

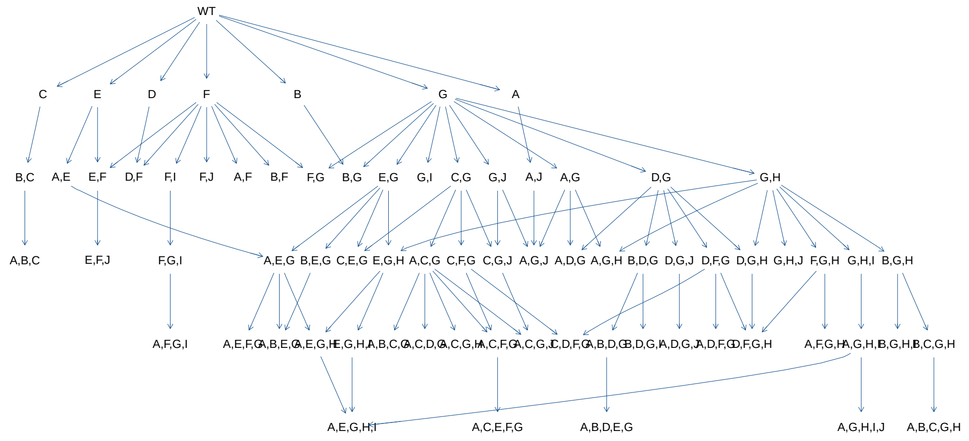

ID	10NjHwdZ4eQZuBASX
acc genotypes	326
peaks no back	116
removed edges	NA
prop rem edges	NA

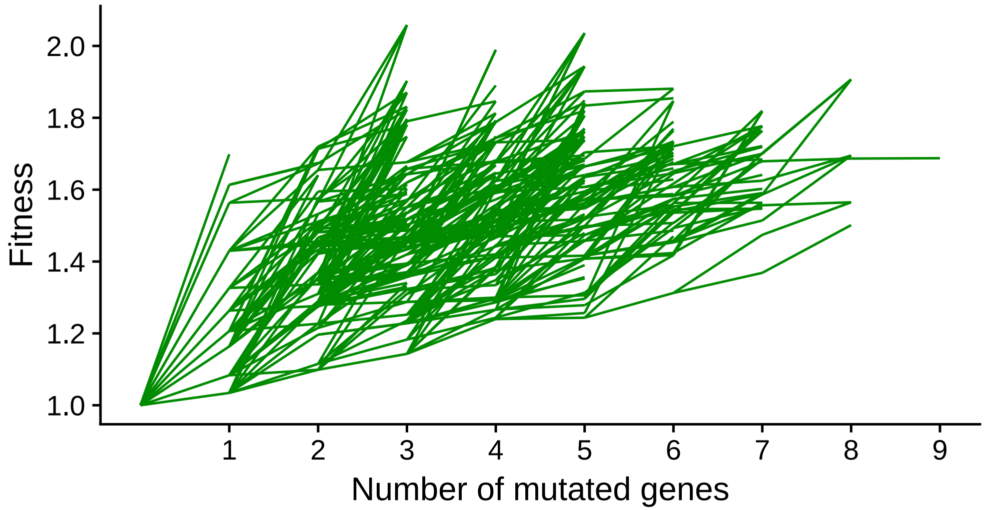

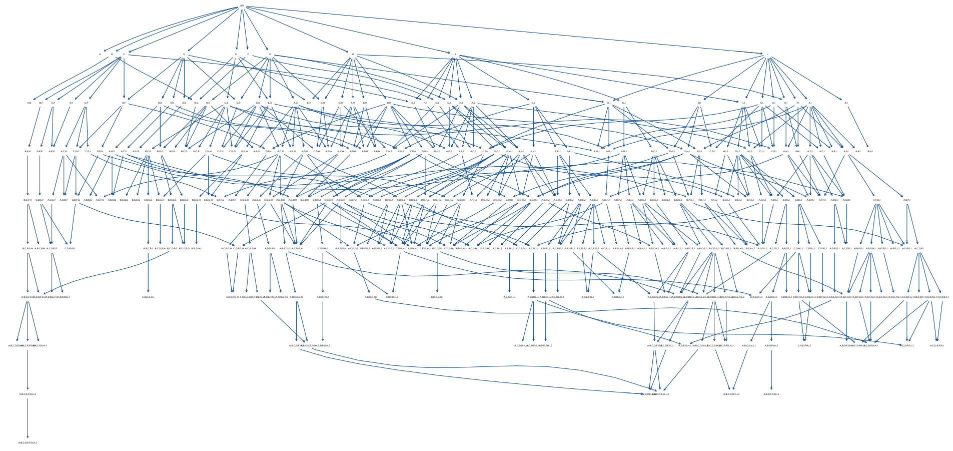

ID	10rm10ycggF6sUPqCV
acc genotypes	330
peaks no back	87
removed edges	NA
prop rem edges	NA

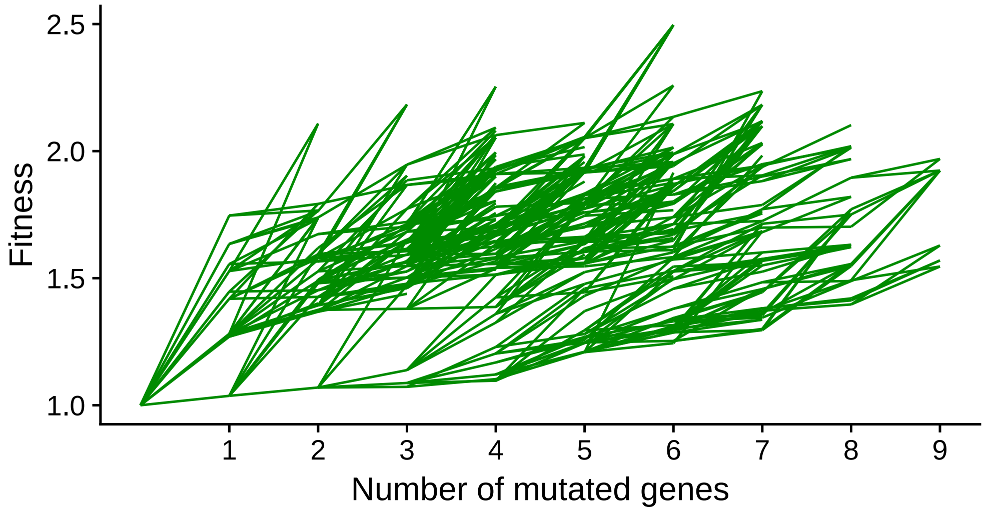

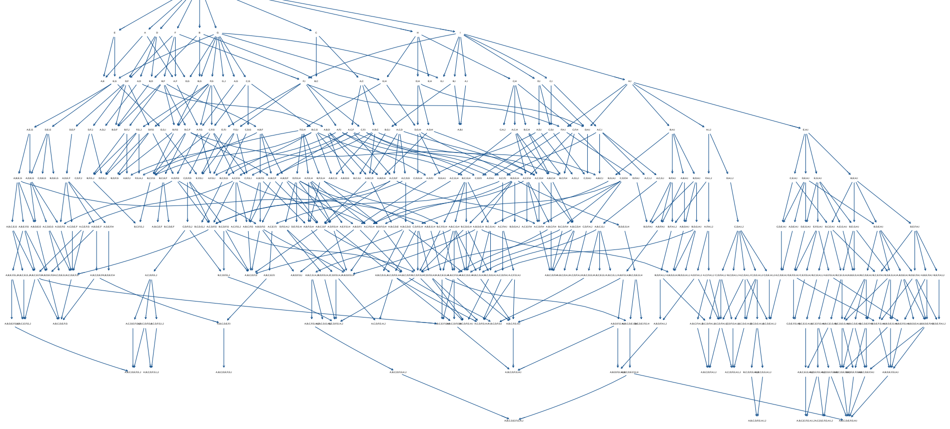

ID	10yJX7cQ5FF31Qv6f
acc genotypes	95
peaks no back	36
removed edges	NA
prop rem edges	NA

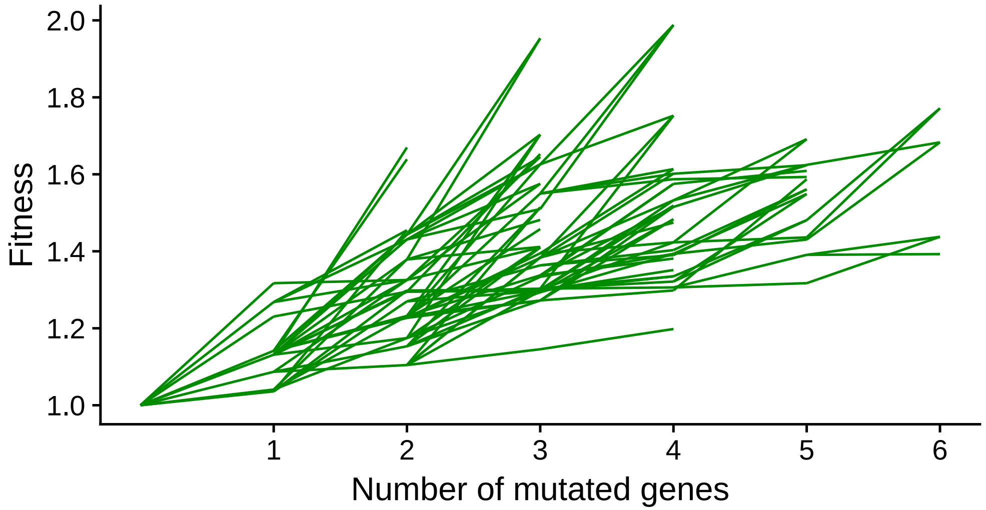

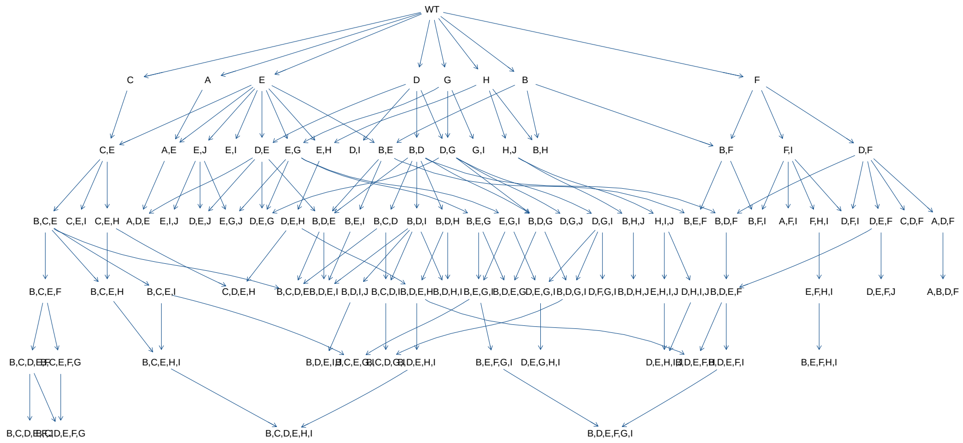

ID	1aUzp7cAshgfTlCQ
acc genotypes	44
peaks no back	16
removed edges	NA
prop rem edges	NA

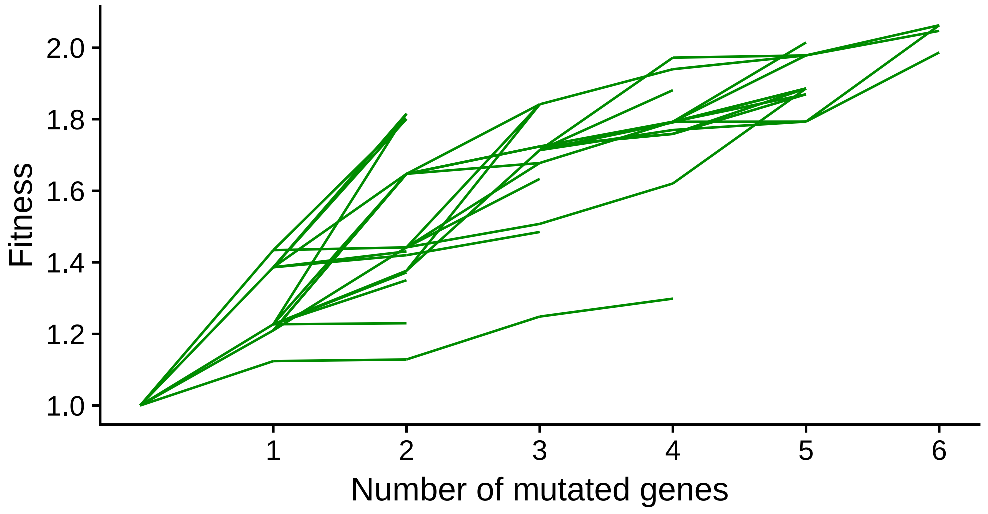

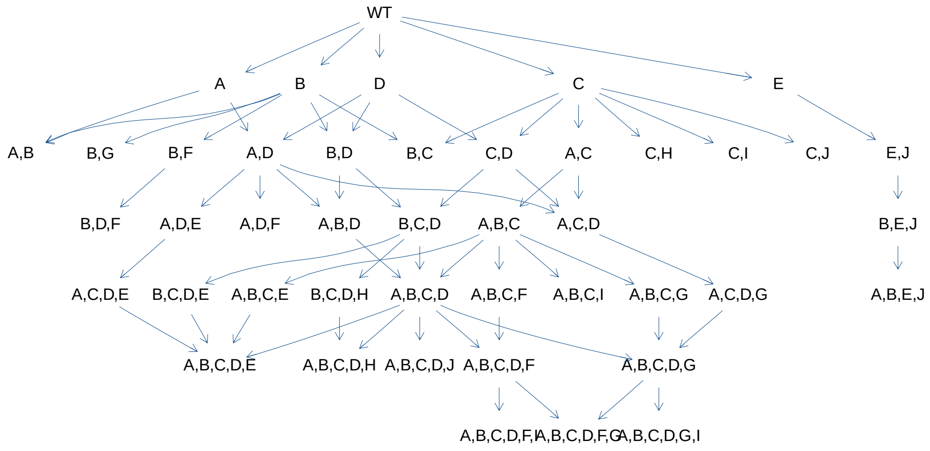

ID	1QFuLHW10HcmHRTX10
acc genotypes	217
peaks no back	81
removed edges	NA
prop rem edges	NA

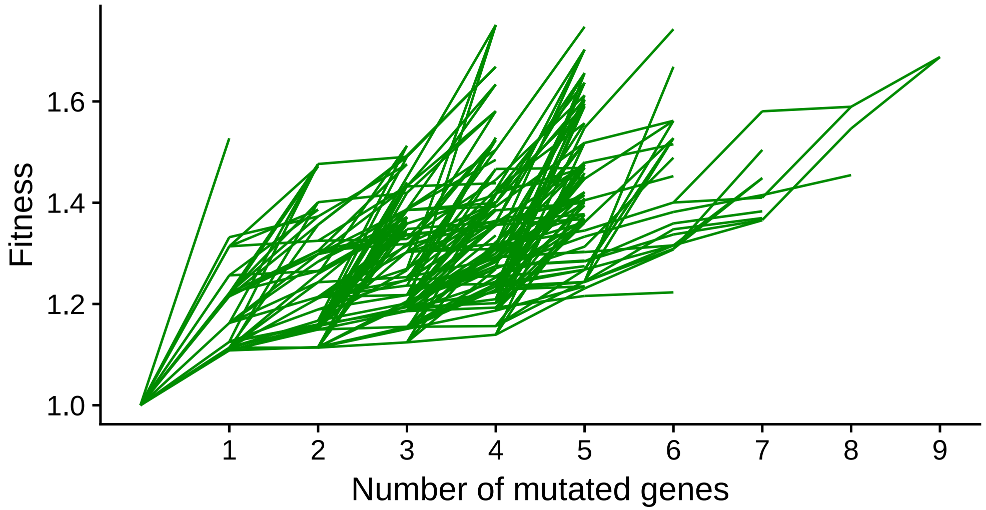

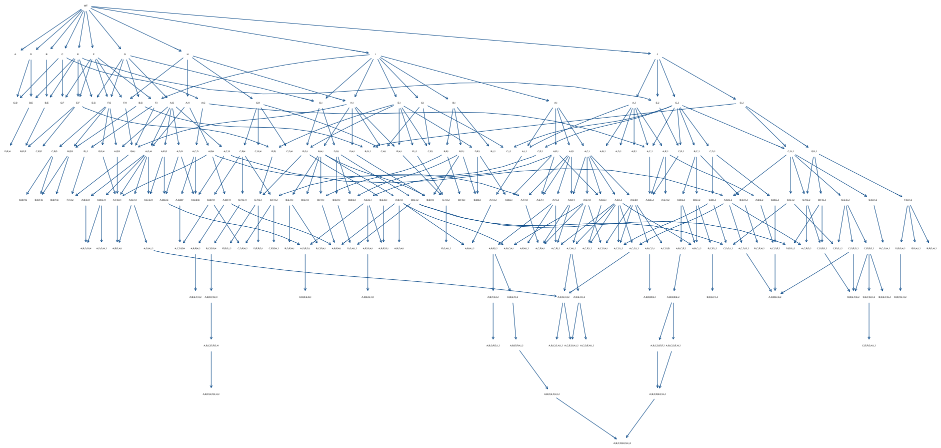

ID	2HoRF1oKBcGNaQYL
acc genotypes	597
peaks no back	113
removed edges	NA
prop rem edges	NA

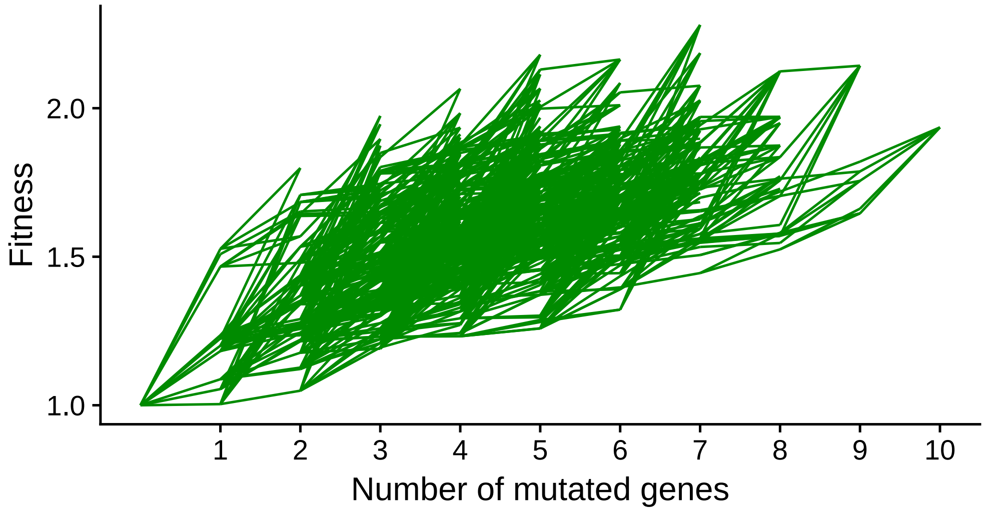

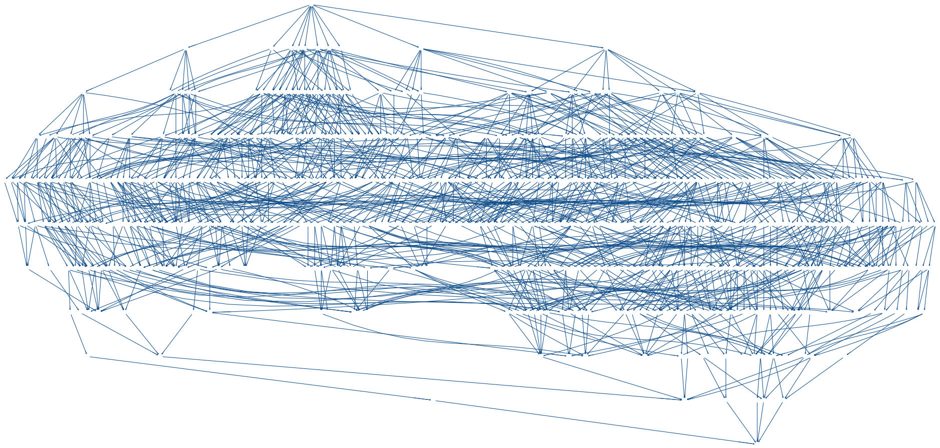

ID 2Ytm5OoPJh10HnzIe

acc genotypes 52

peaks no back 13

removed edges NA

prop rem edges NA

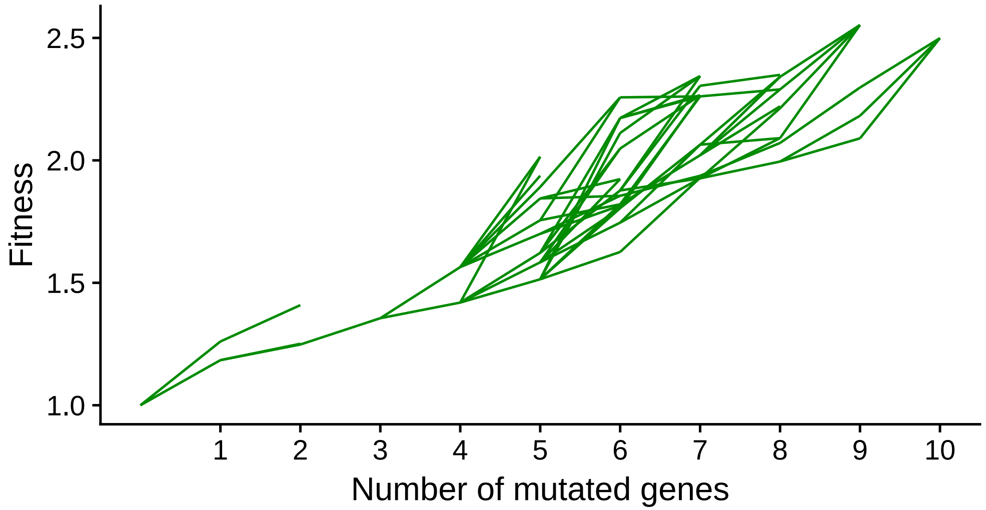

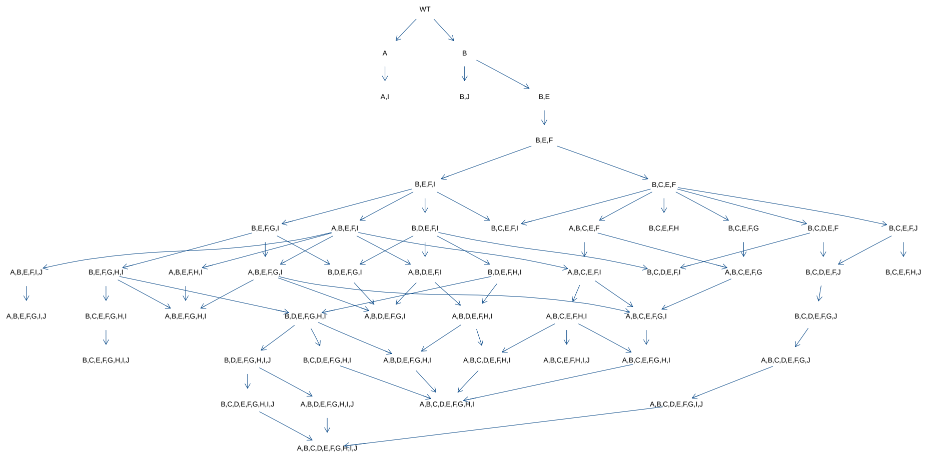

ID	2yuFuKxbV3XC3194
acc genotypes	282
peaks no back	63
removed edges	NA
prop rem edges	NA

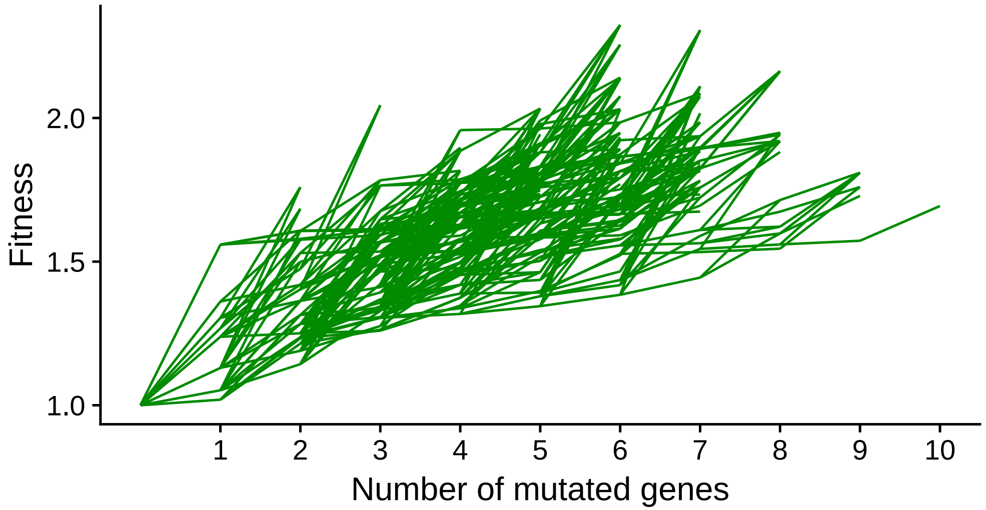

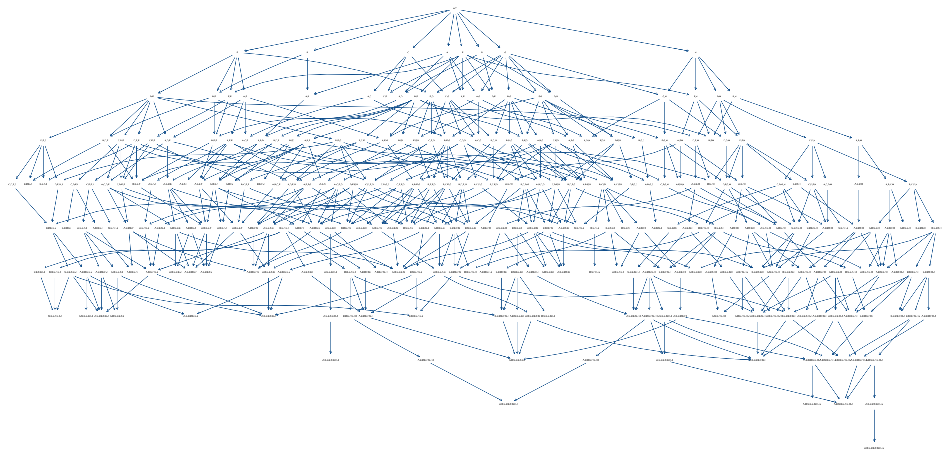

ID	341bQ6tMBQ3wbqIX
acc genotypes	204
peaks no back	68
removed edges	NA
prop rem edges	NA

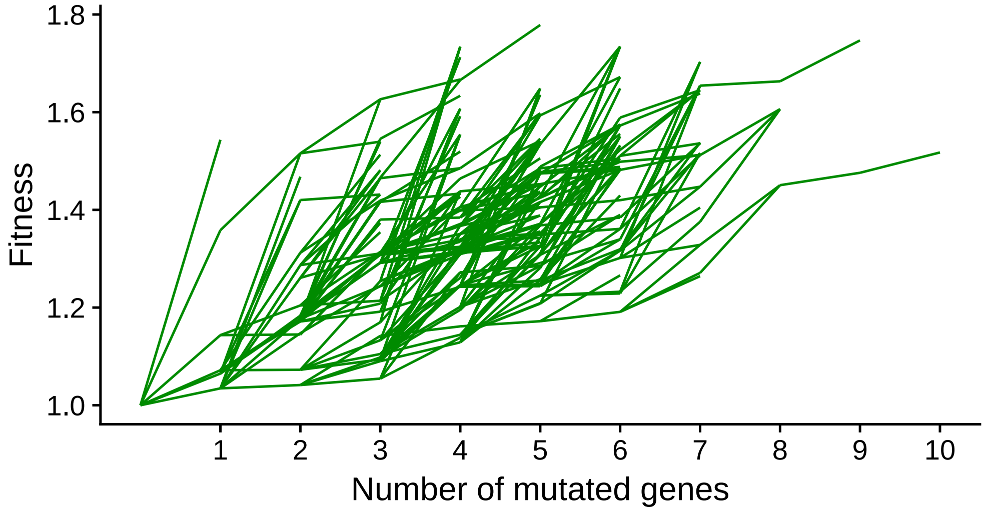

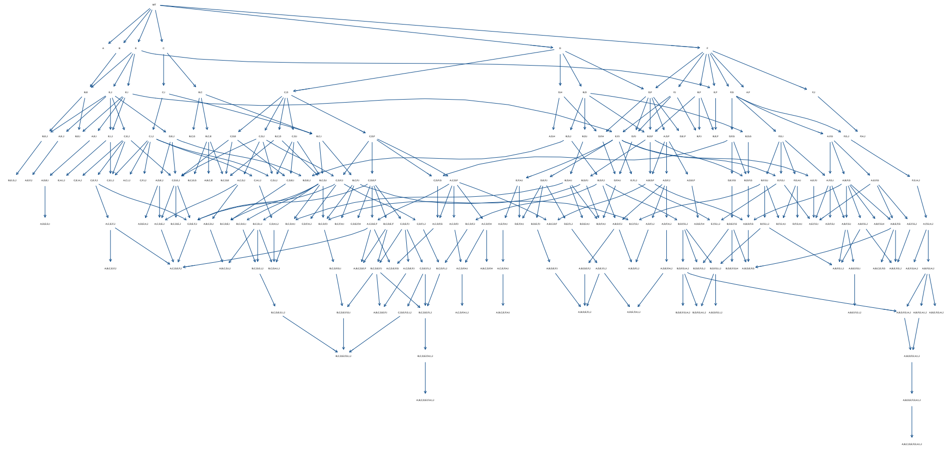

ID	3ageR5lDF1410rU5i
acc genotypes	51
peaks no back	25
removed edges	NA
prop rem edges	NA

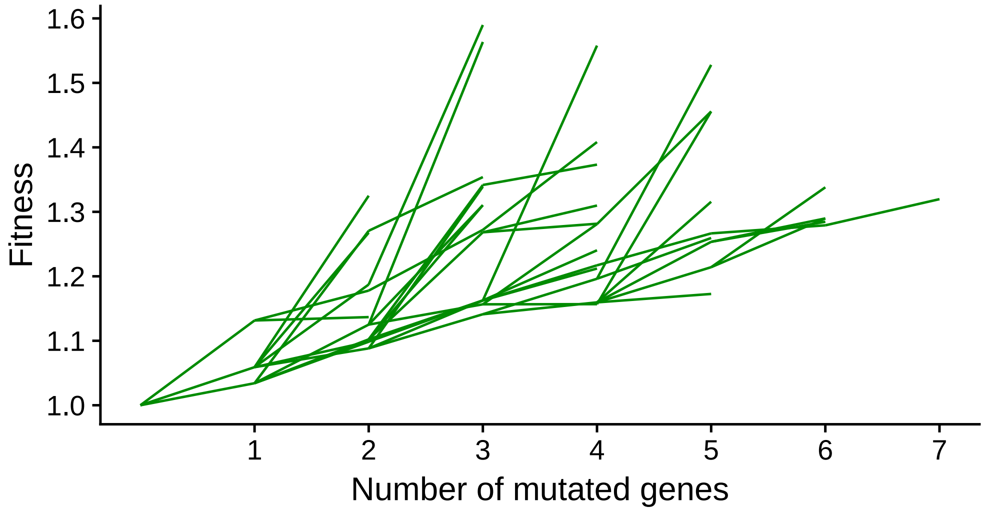

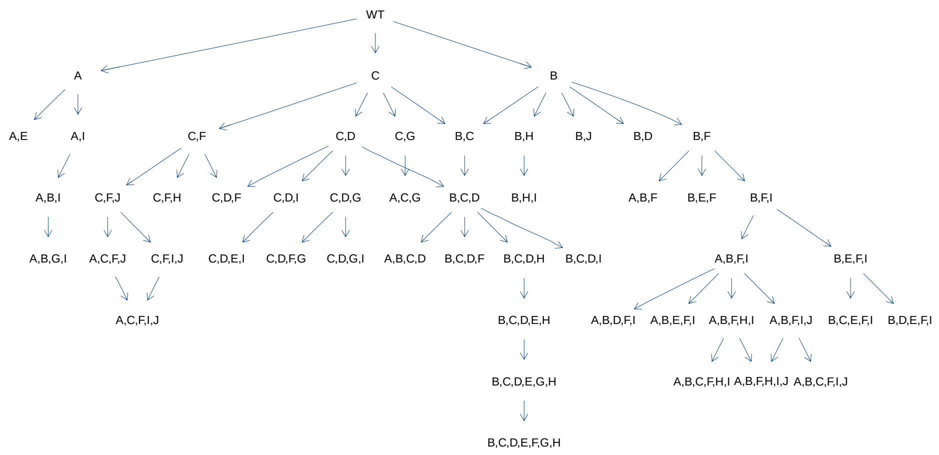

ID	3PJ410K7y10k7imair
acc genotypes	921
peaks no back	50
removed edges	NA
prop rem edges	NA

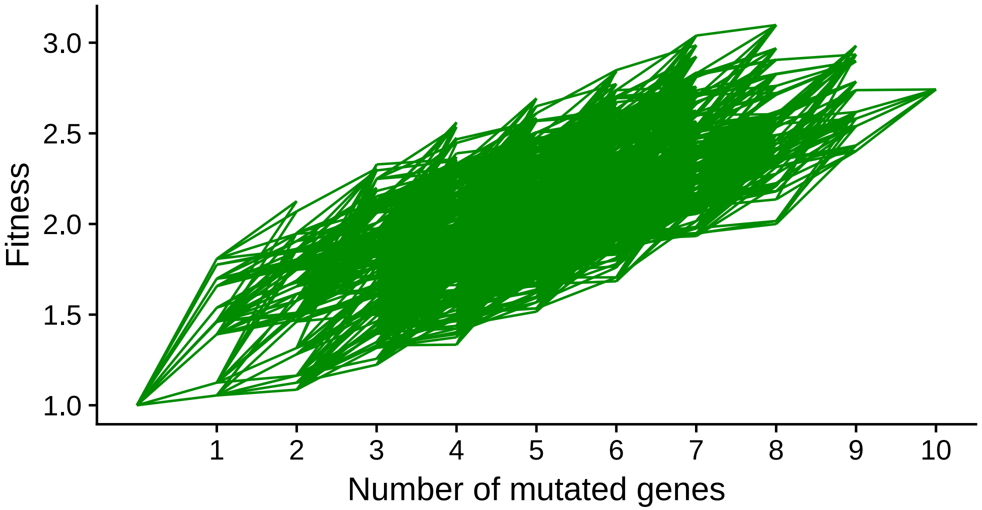

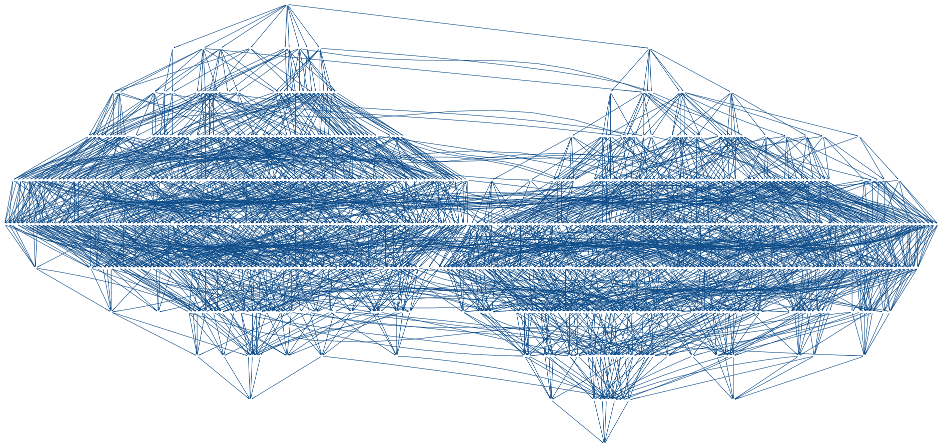

ID	3r101LFQ84LQ4QX2E
acc genotypes	66
peaks no back	29
removed edges	NA
prop rem edges	NA

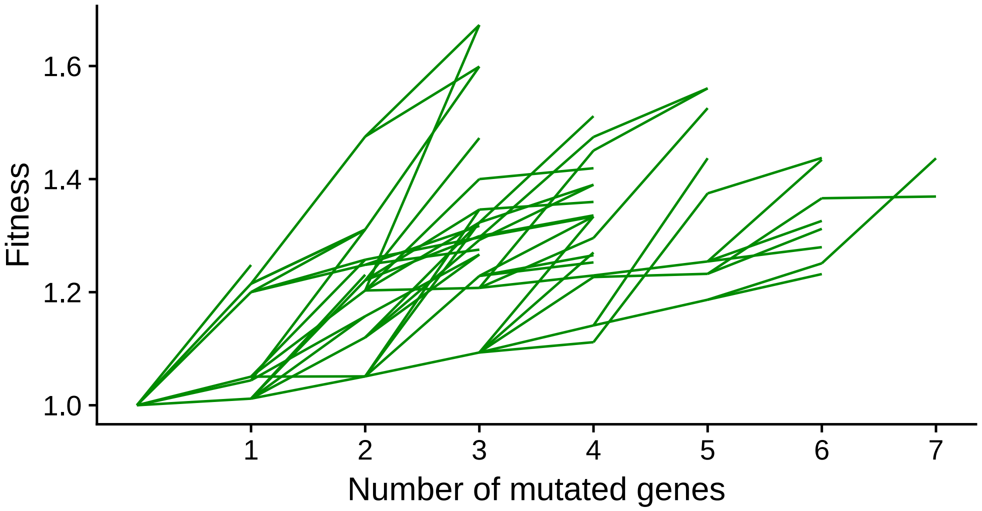

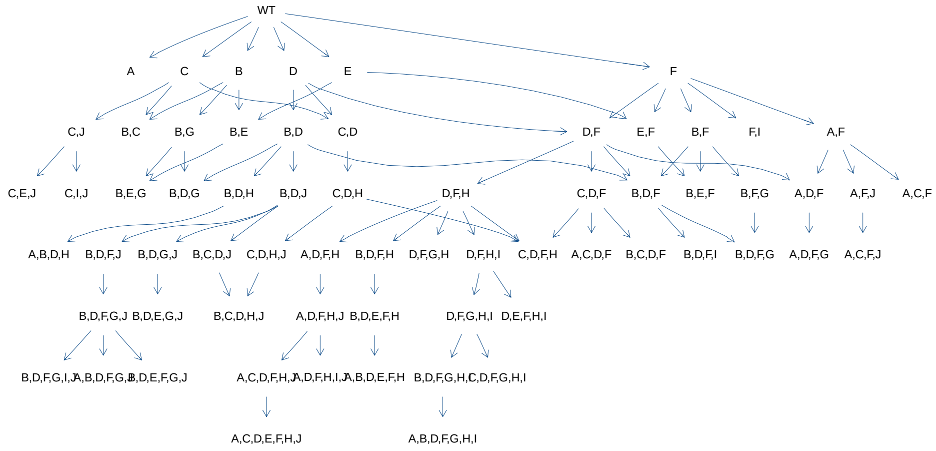

ID	3uTPGDsbMFbp45JR
acc genotypes	131
peaks no back	38
removed edges	NA
prop rem edges	NA

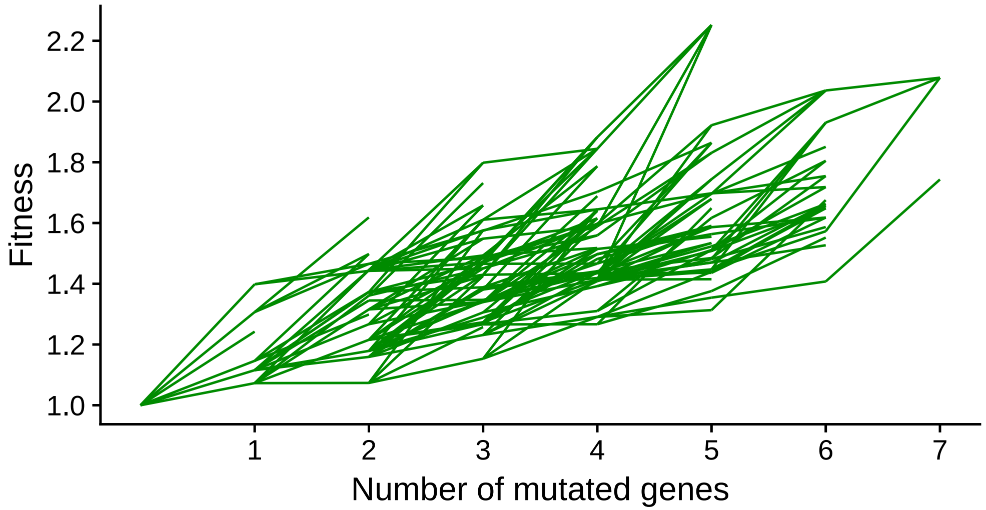

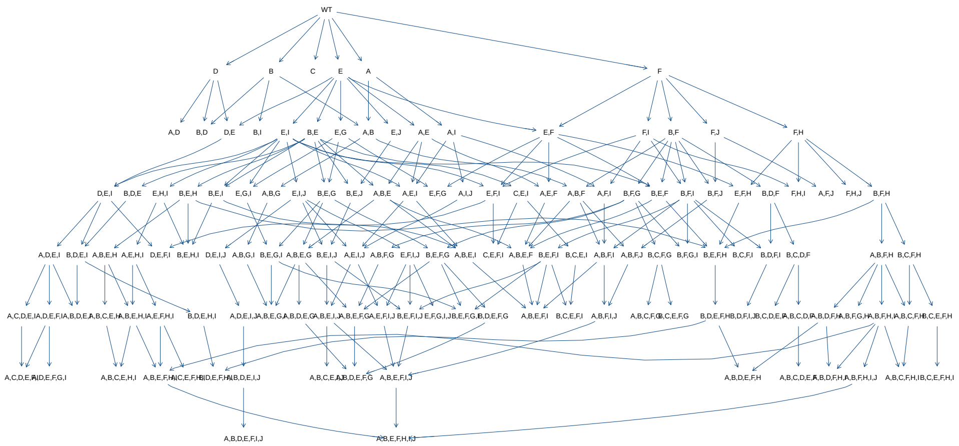

ID	3VdNxc10SG10x5vQBJ
acc genotypes	82
peaks no back	35
removed edges	NA
prop rem edges	NA

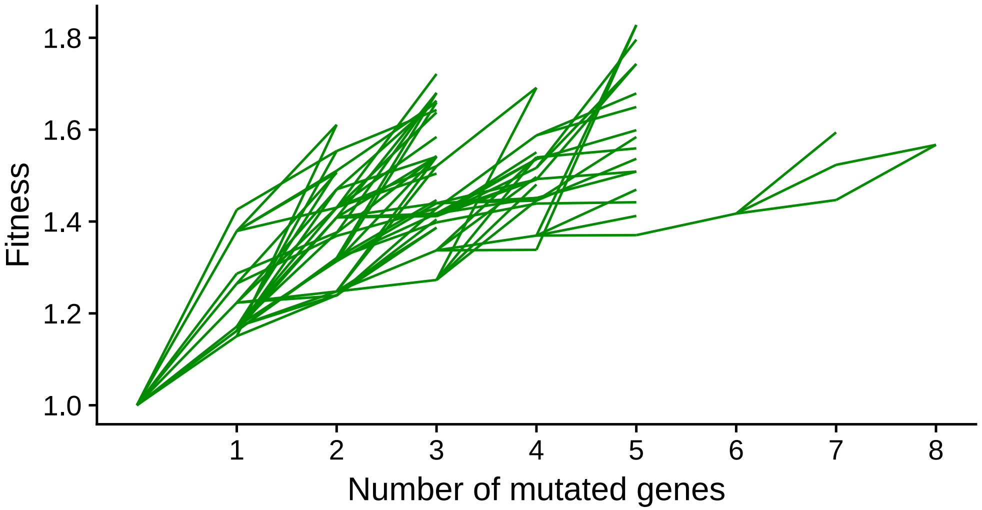

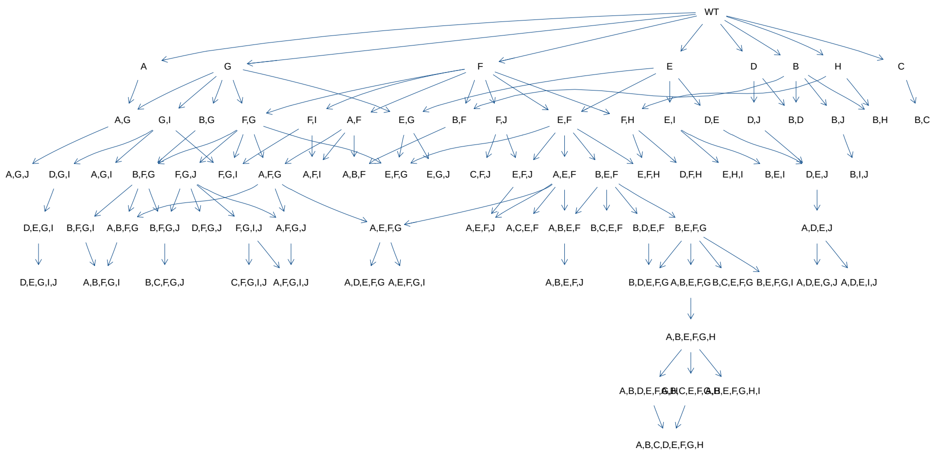

ID	4Ckjy1UeVlkV4sax
acc genotypes	191
peaks no back	38
removed edges	NA
prop rem edges	NA

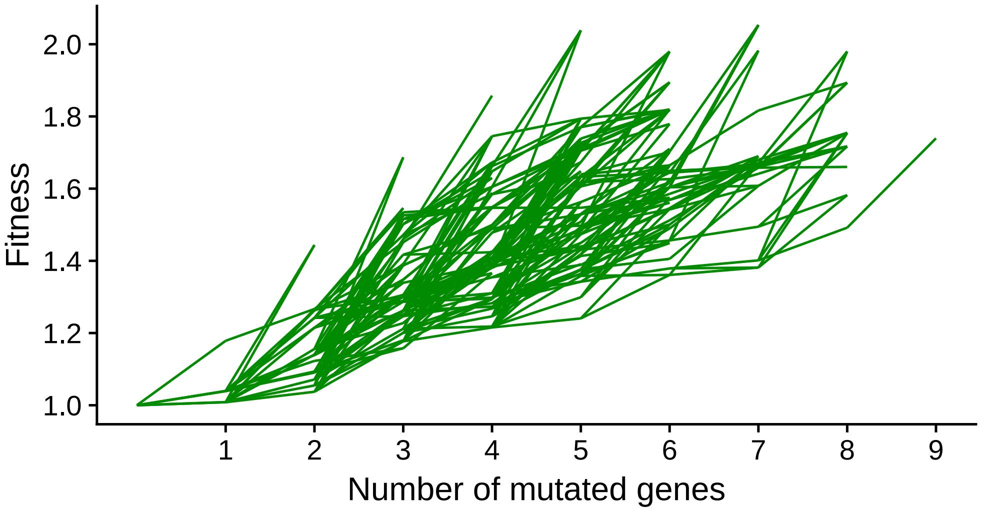

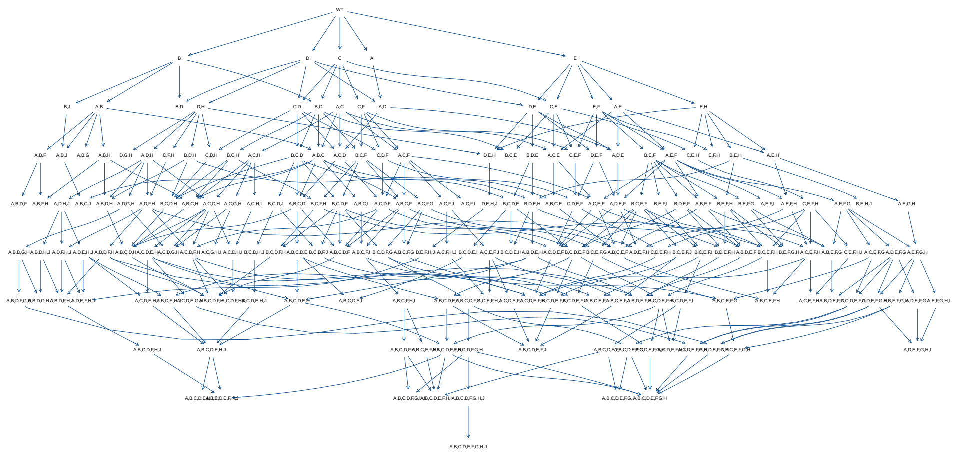

ID	4RVESRiVwzxmDTB9
acc genotypes	82
peaks no back	23
removed edges	NA
prop rem edges	NA

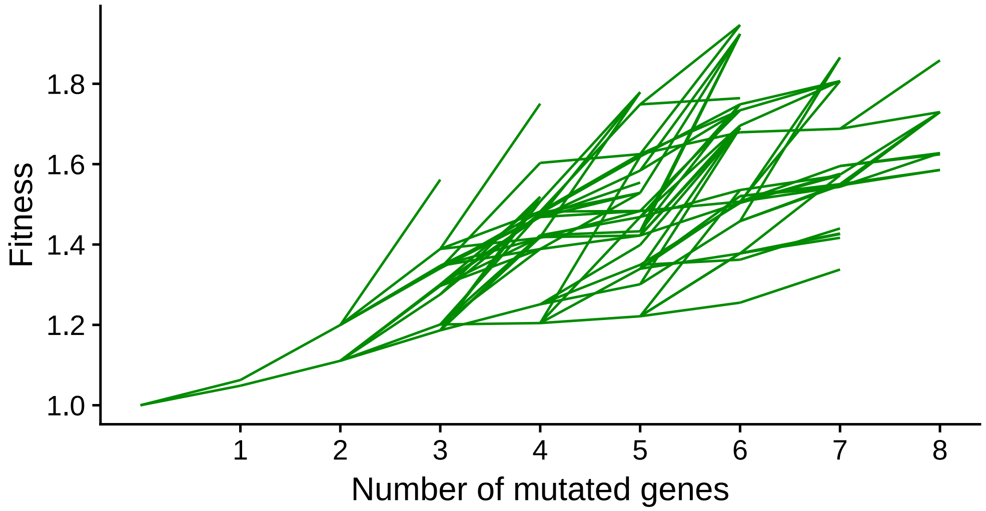

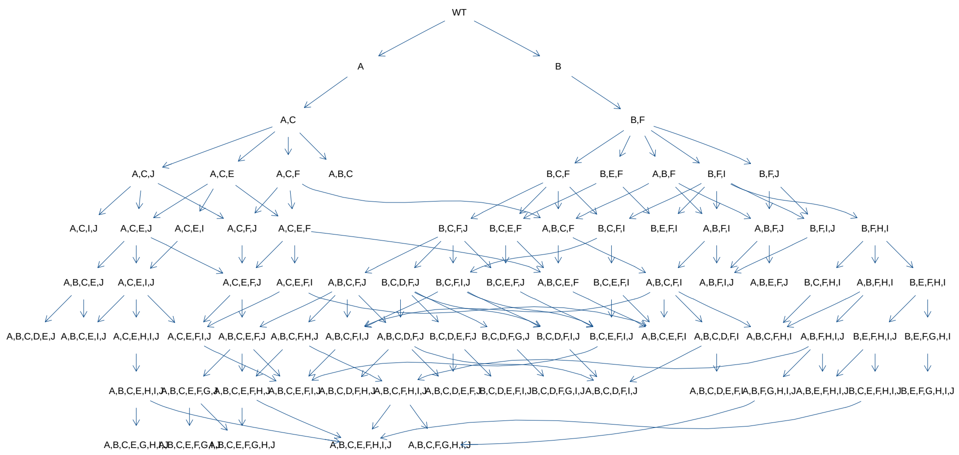

ID	5h10KTbvYD9O10OXnK
acc genotypes	59
peaks no back	28
removed edges	NA
prop rem edges	NA

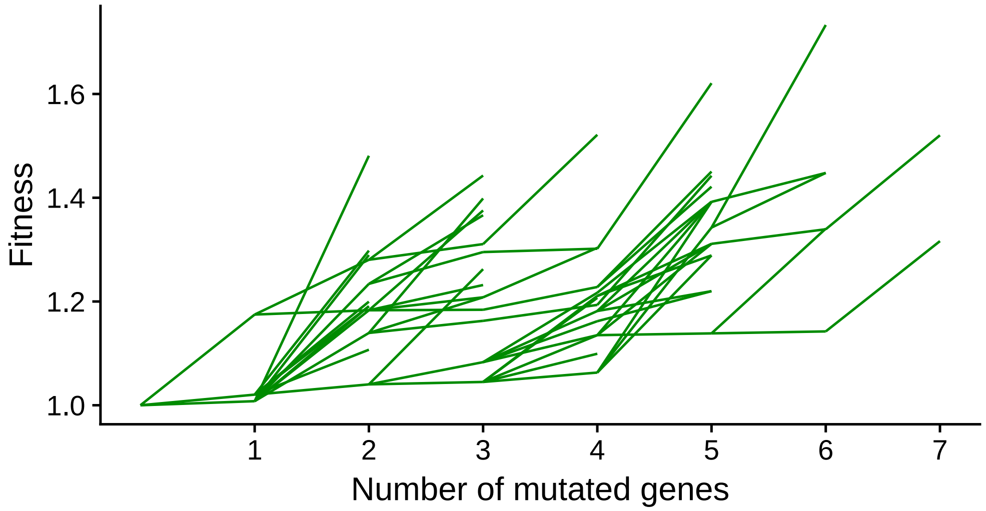

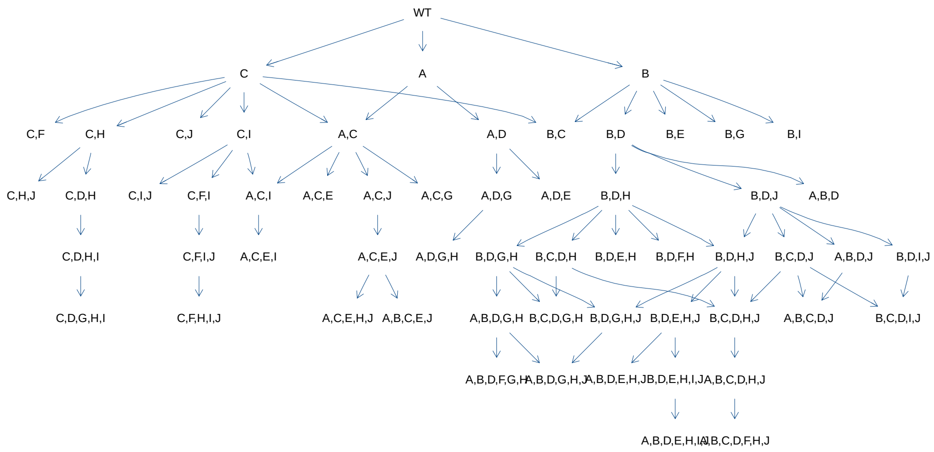

ID	5w3nMqdUbSKjUyuj
acc genotypes	336
peaks no back	69
removed edges	NA
prop rem edges	NA

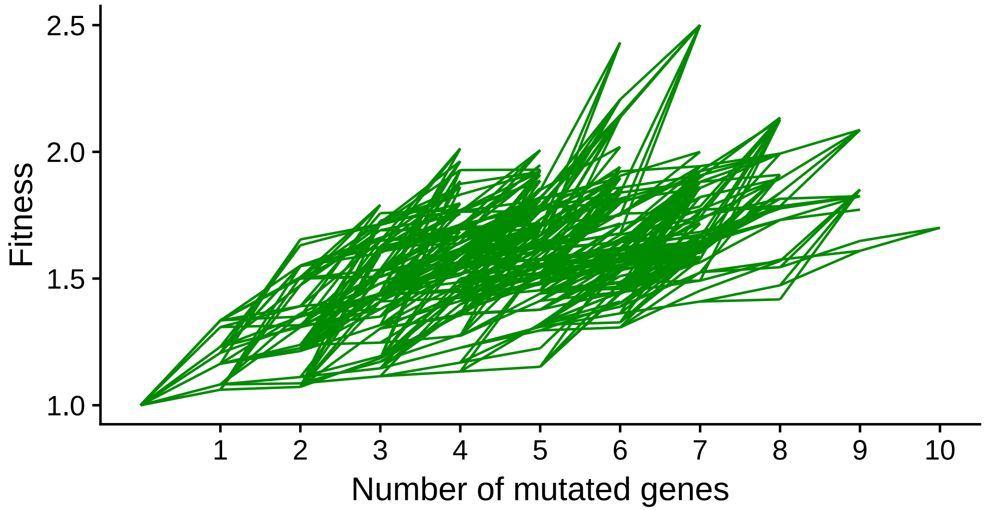

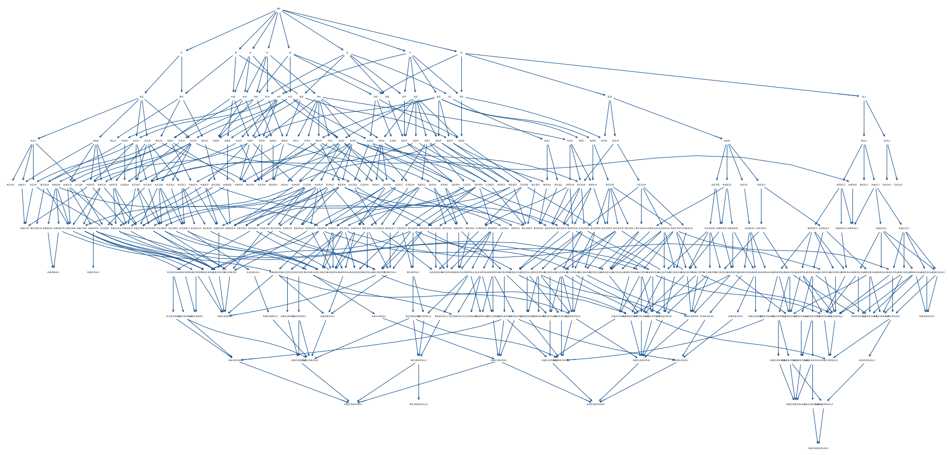

ID	5XTuzOyhioi24t2s
acc genotypes	48
peaks no back	21
removed edges	NA
prop rem edges	NA

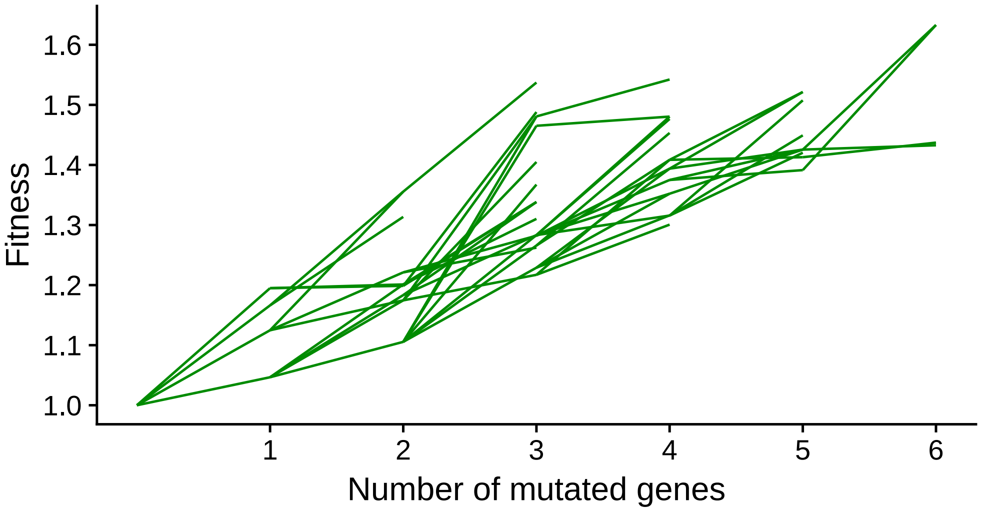

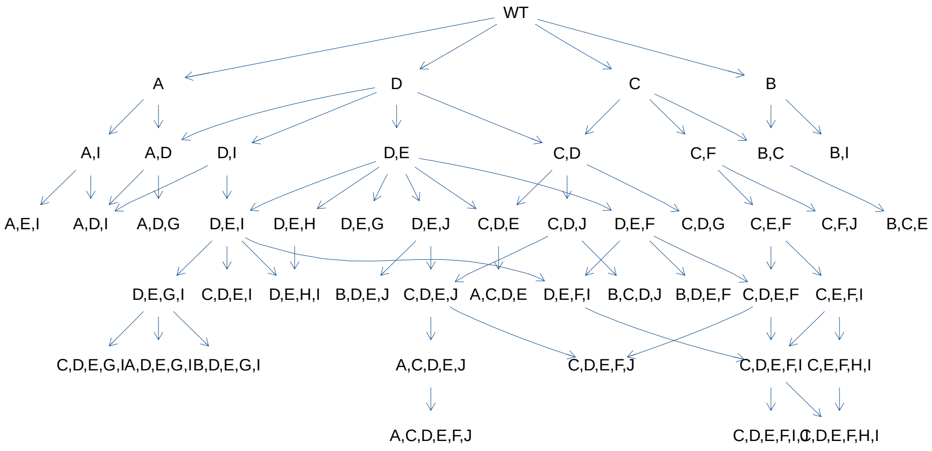

ID	75tsJre1YoxpOsyh
acc genotypes	187
peaks no back	70
removed edges	NA
prop rem edges	NA

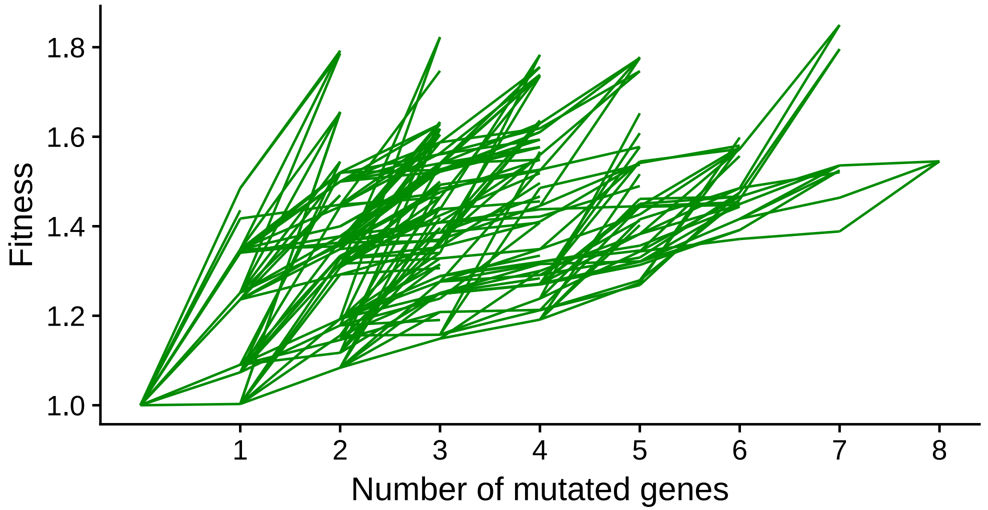

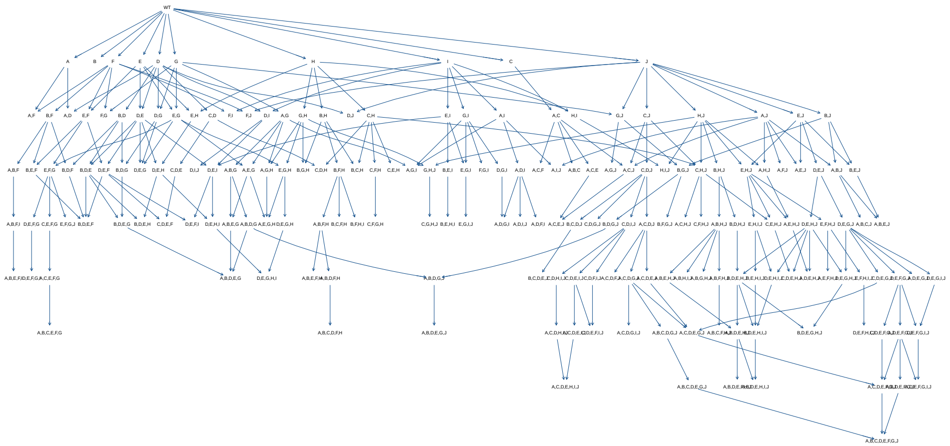

ID	7DaZyu10znssTnBfx
acc genotypes	674
peaks no back	56
removed edges	NA
prop rem edges	NA

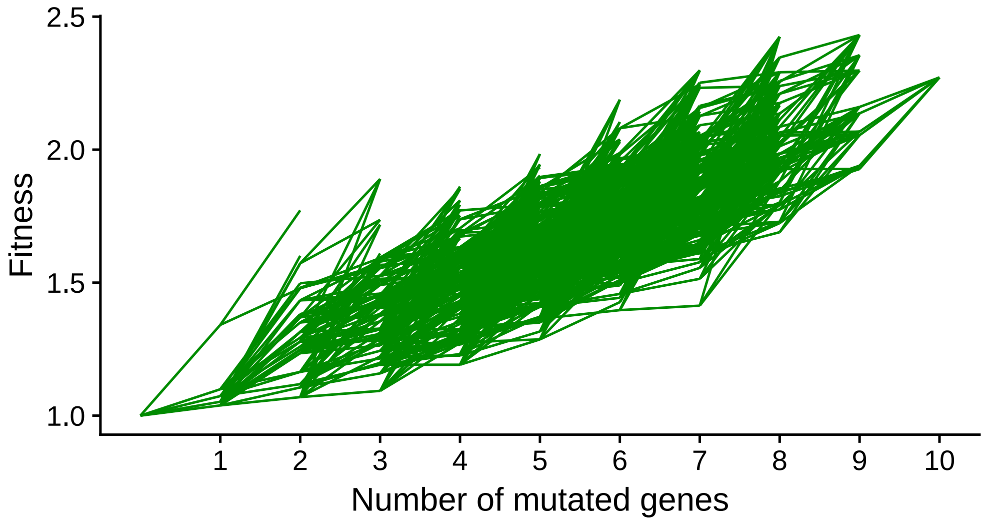

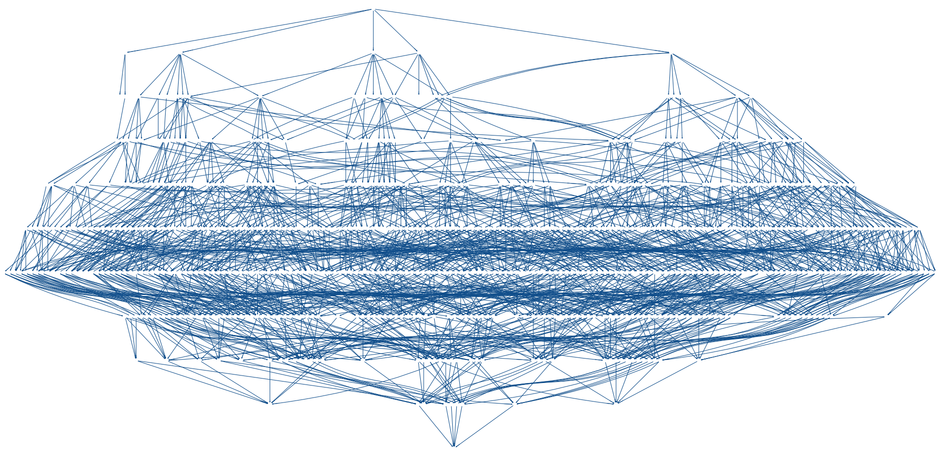

ID	7GapUNhPAnaZAI1M
acc genotypes	427
peaks no back	53
removed edges	NA
prop rem edges	NA

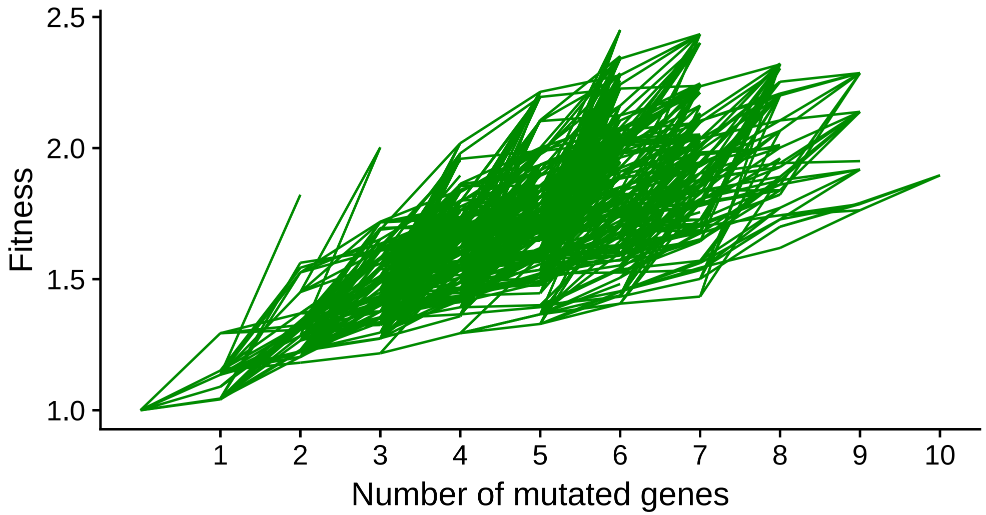

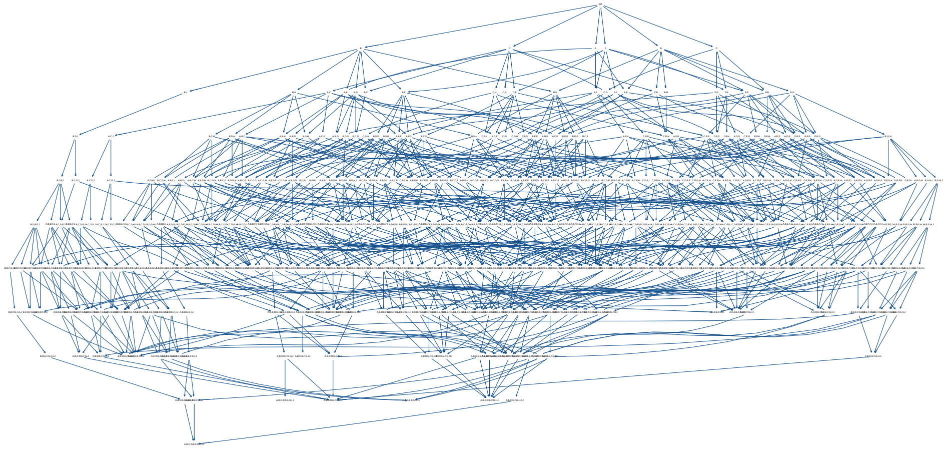

ID	7jxvBRIz6DAUbzBL
acc genotypes	24
peaks no back	12
removed edges	NA
prop rem edges	NA

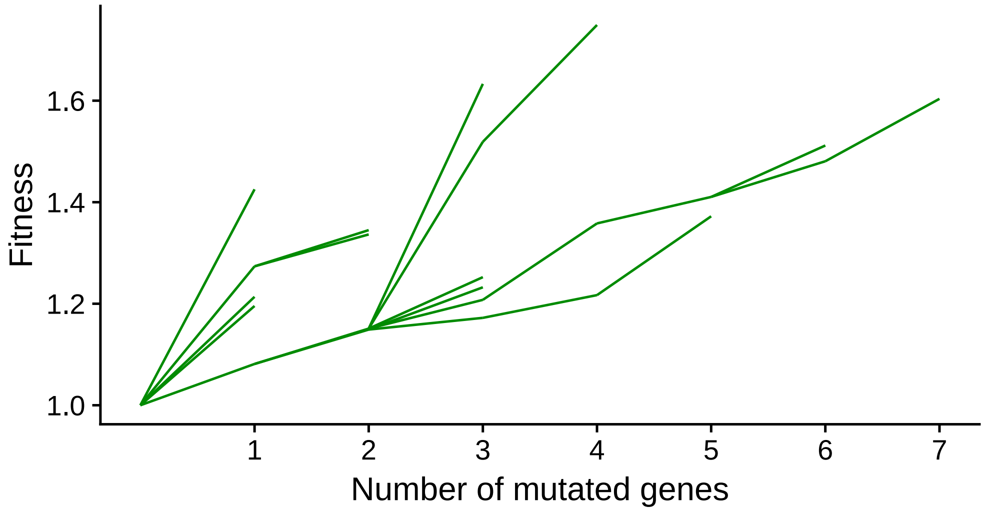

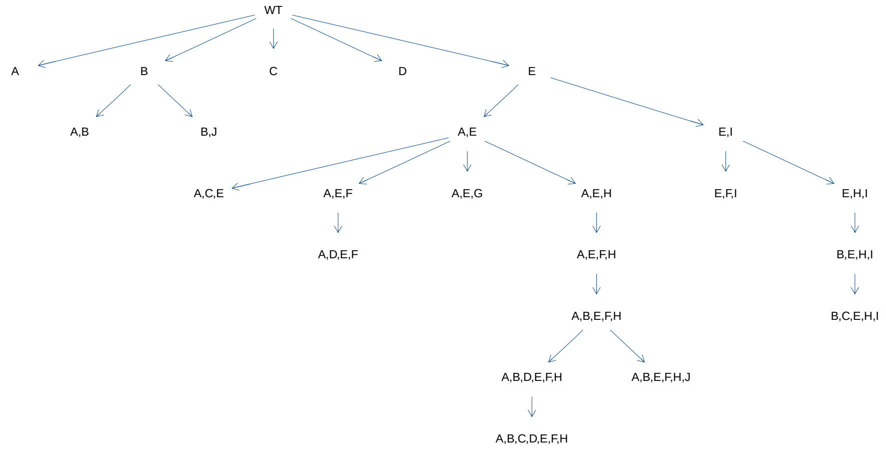

ID	7KQQodSTMHz6tzpM
acc genotypes	291
peaks no back	55
removed edges	NA
prop rem edges	NA

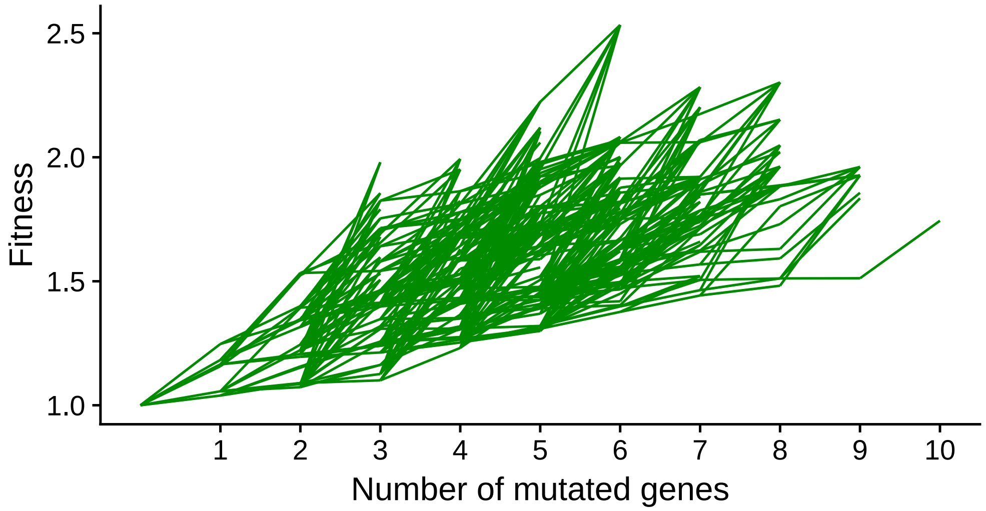

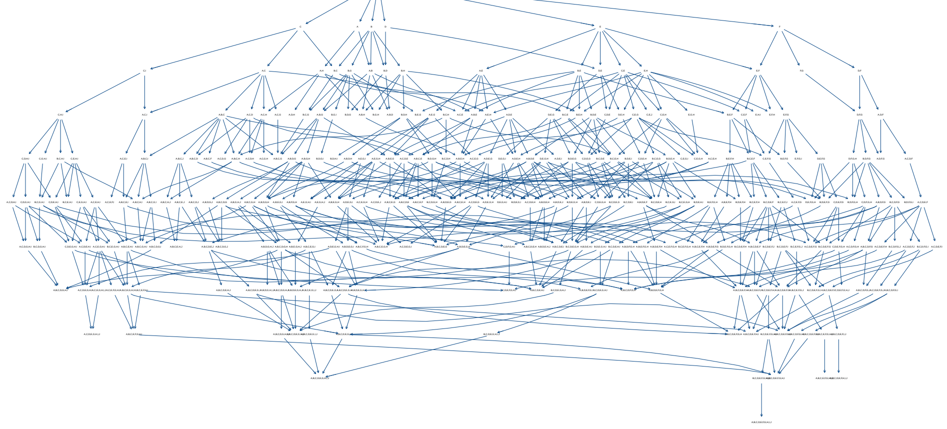

ID	7yMvs88xHRDYKybO
acc genotypes	182
peaks no back	62
removed edges	NA
prop rem edges	NA

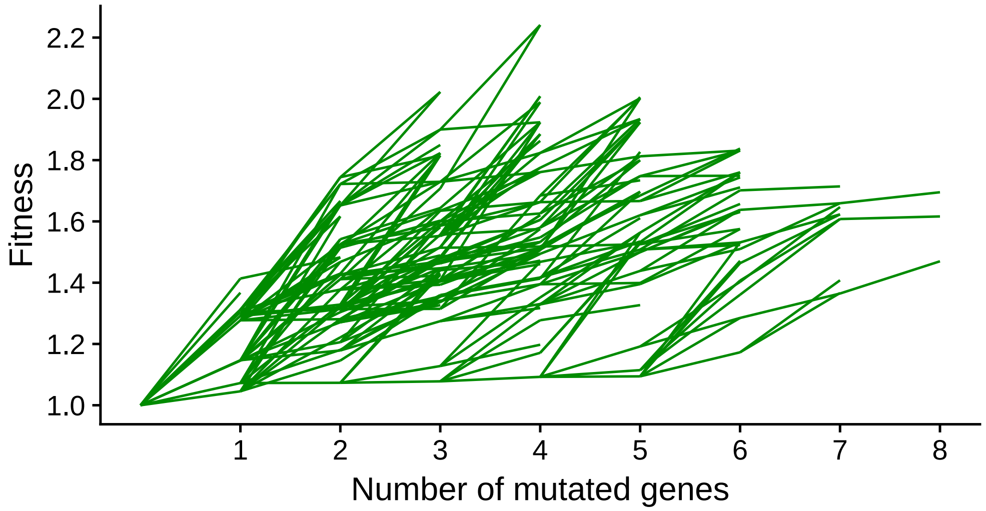

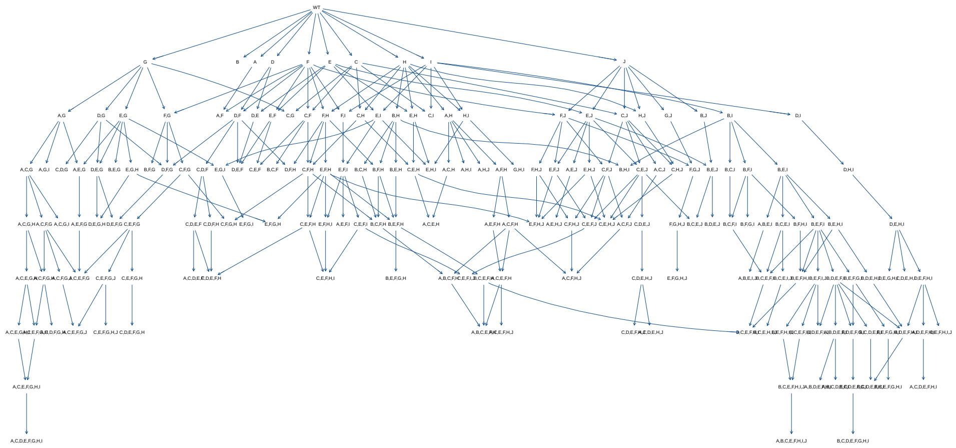

ID	7YYdBu6jr3vr7yKp
acc genotypes	197
peaks no back	75
removed edges	NA
prop rem edges	NA

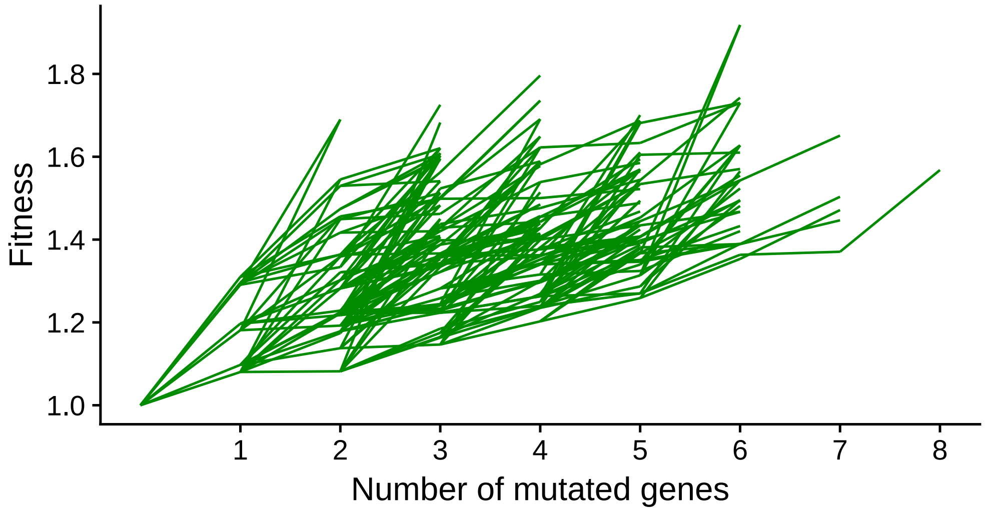

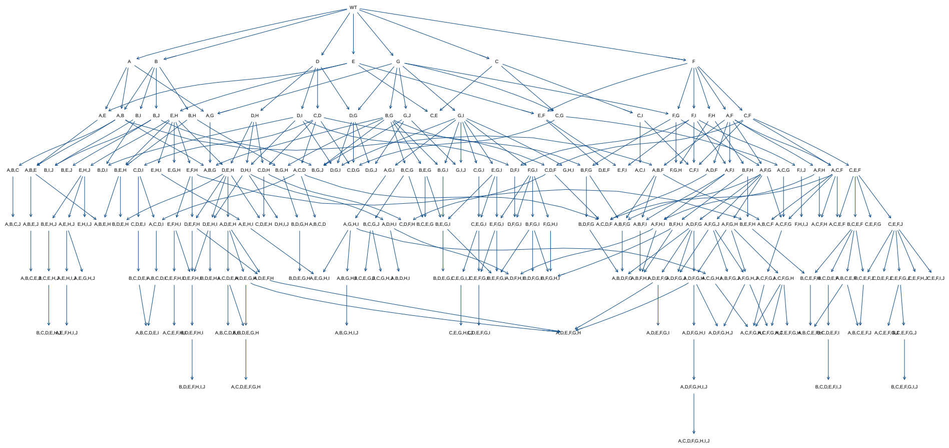

ID	8OiLROdLmPboaFyZ
acc genotypes	145
peaks no back	62
removed edges	NA
prop rem edges	NA

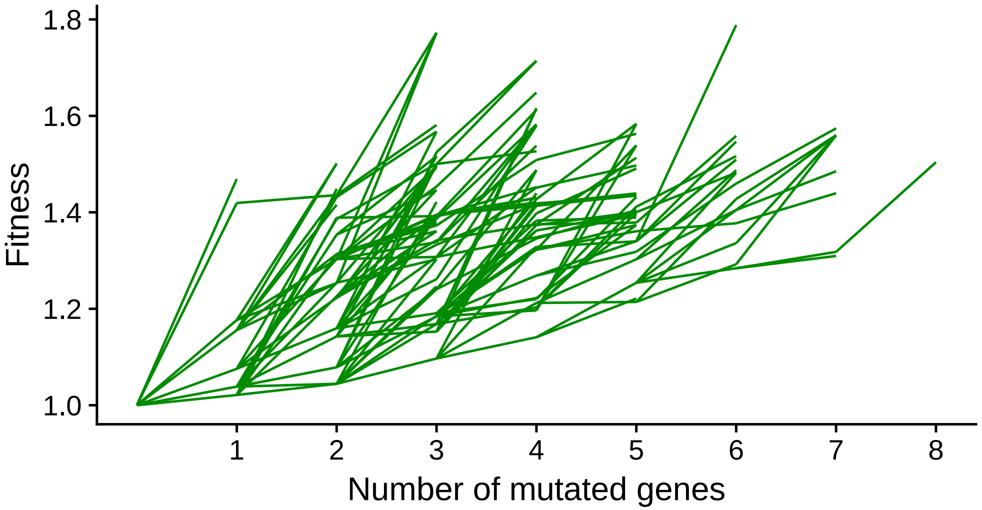

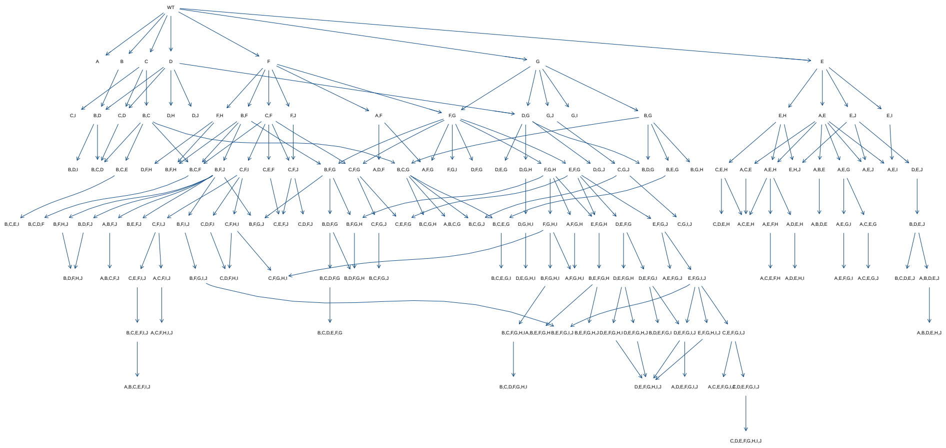

ID	8RIqFjyqv5Fe2F7q
acc genotypes	130
peaks no back	41
removed edges	NA
prop rem edges	NA

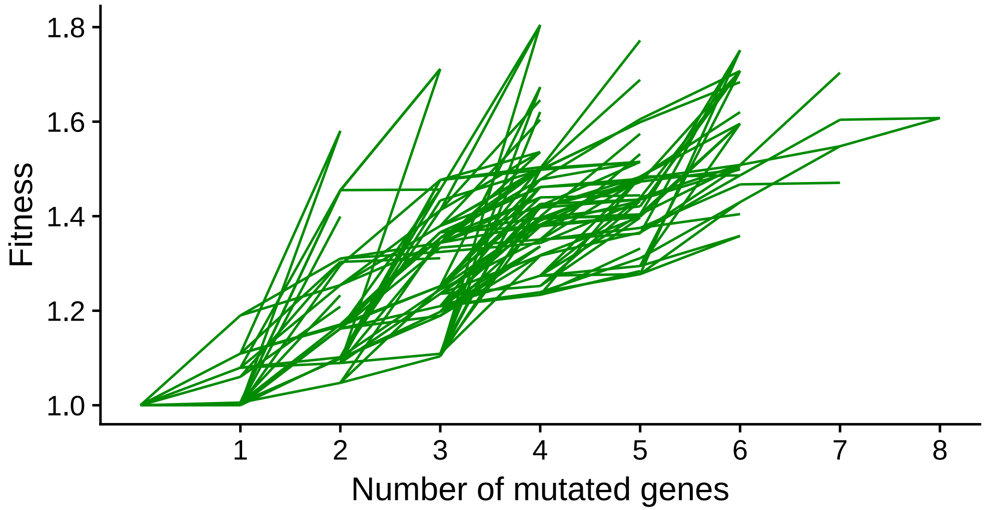

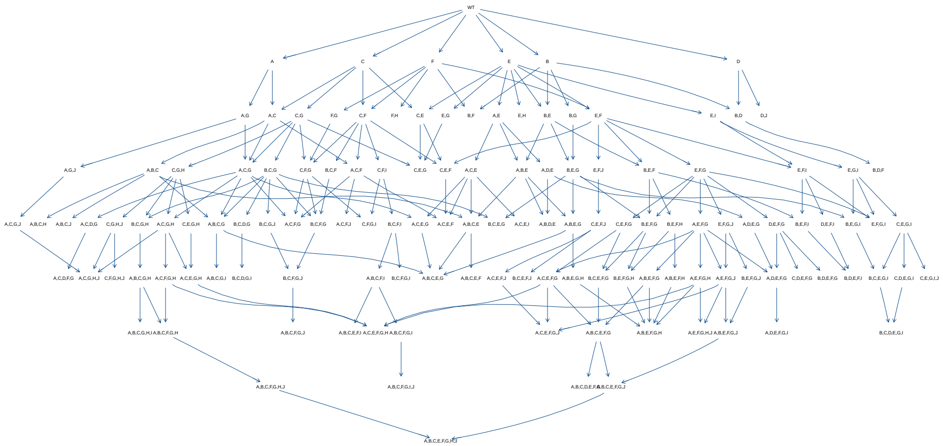

ID	8Wuwdsphcy5tFFeb
acc genotypes	117
peaks no back	46
removed edges	NA
prop rem edges	NA

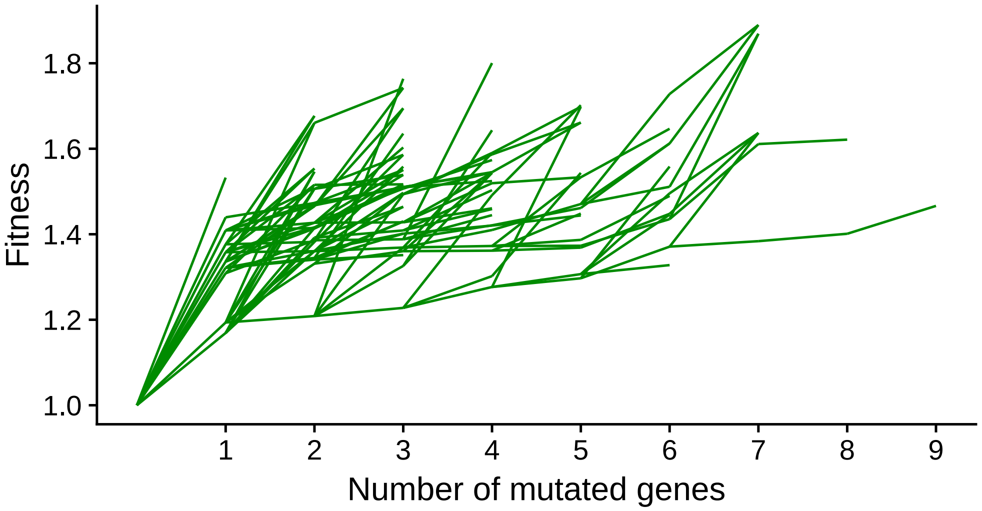

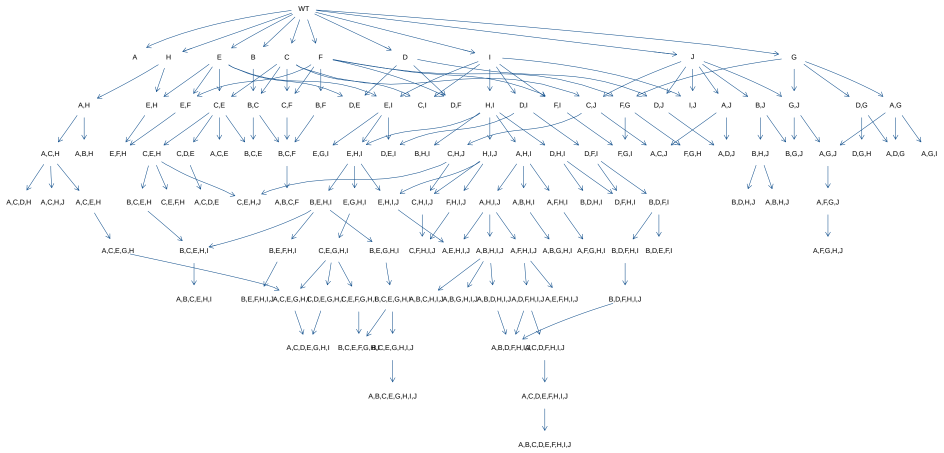

ID	913oZplLmQJl3z3r
acc genotypes	96
peaks no back	39
removed edges	NA
prop rem edges	NA

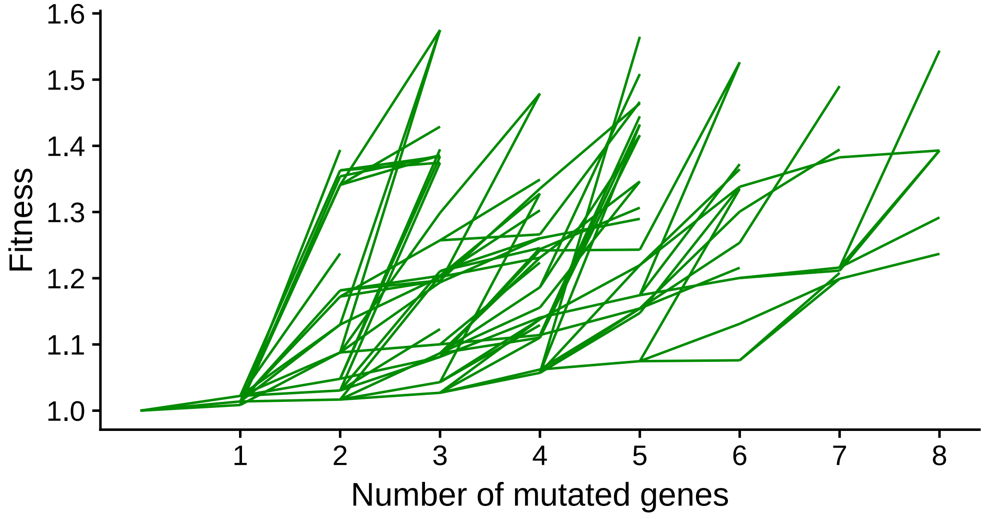

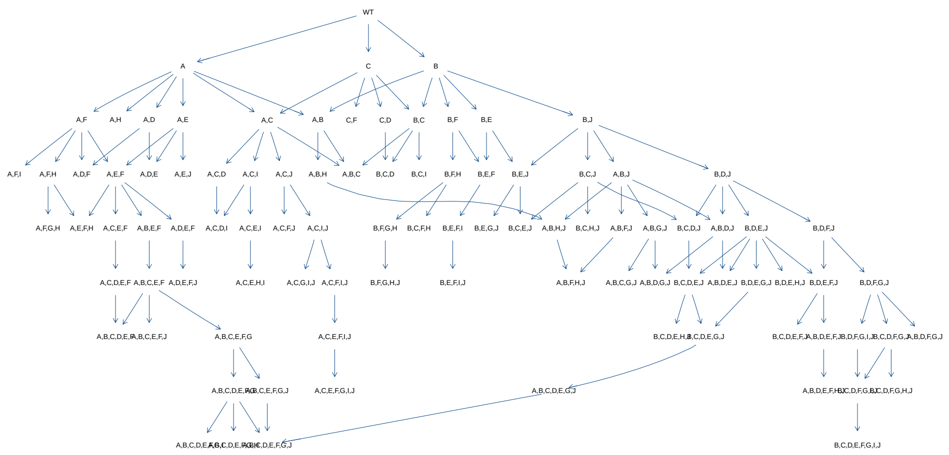

ID	9cep5DgsrTOPpWos
acc genotypes	163
peaks no back	43
removed edges	NA
prop rem edges	NA

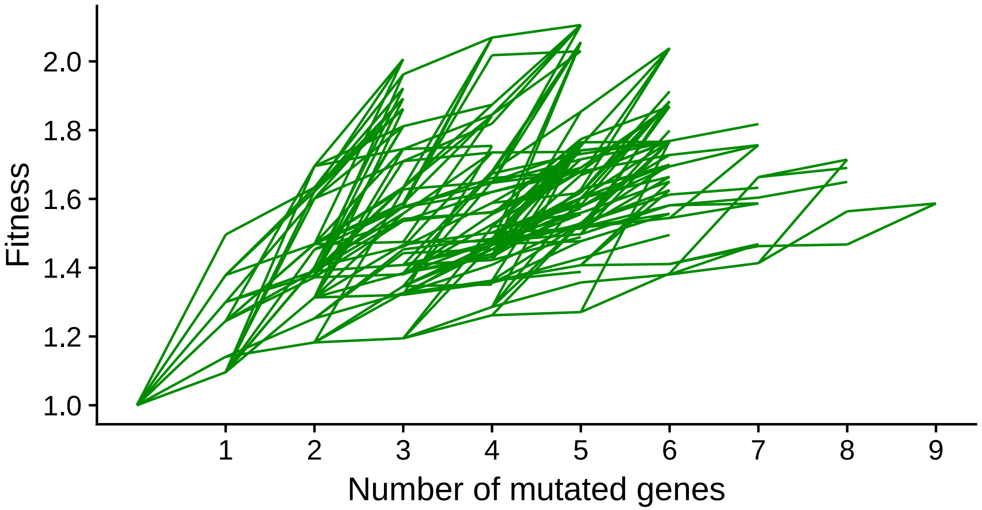

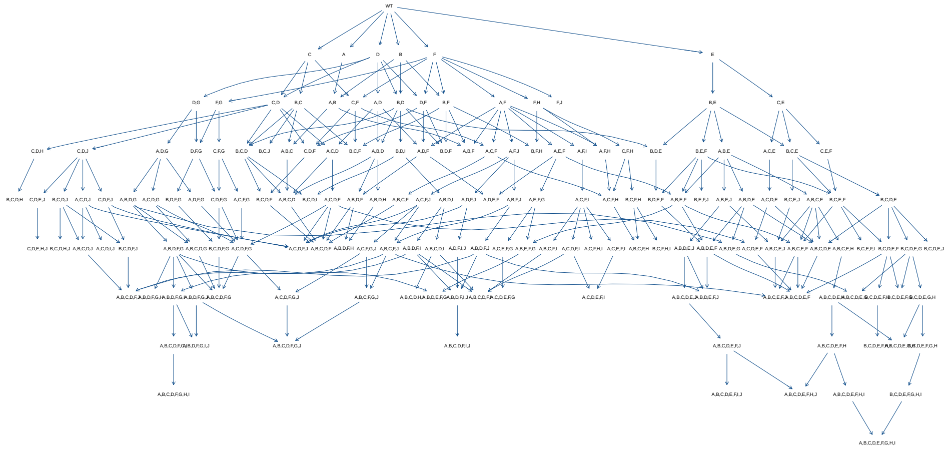

ID	9FoiB310FGDvgWcIE
acc genotypes	231
peaks no back	69
removed edges	NA
prop rem edges	NA

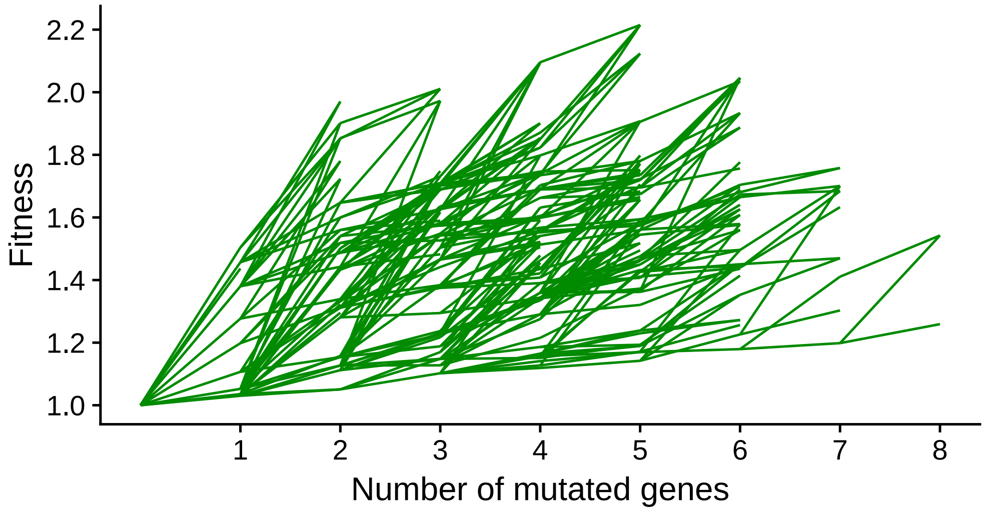

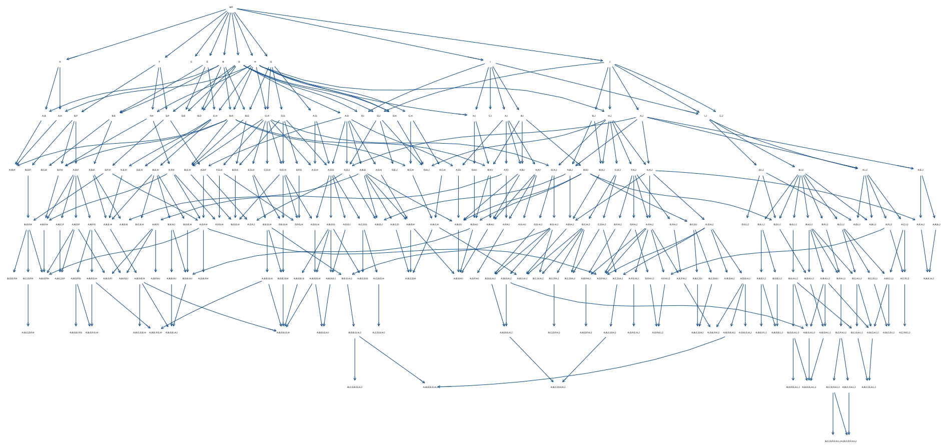

ID	A1JsKu9z21G7TWcc
acc genotypes	261
peaks no back	105
removed edges	NA
prop rem edges	NA

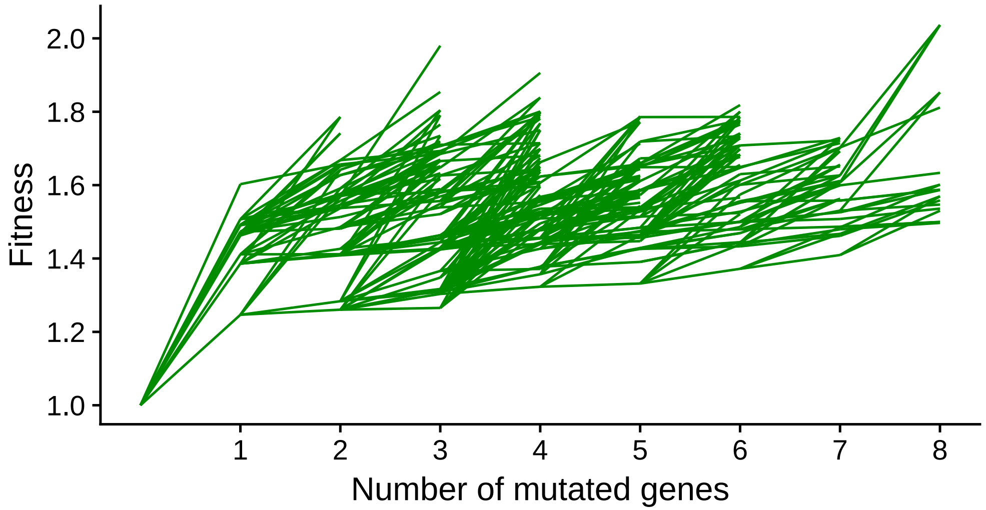

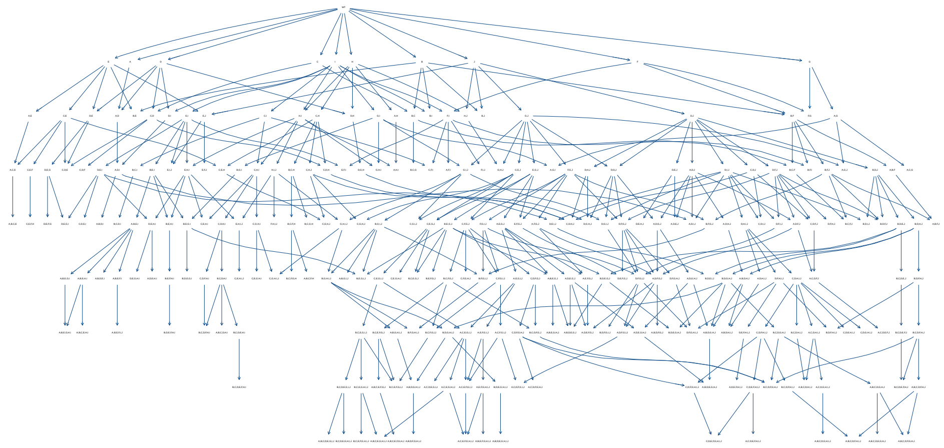

ID	a3mOmqYxncb9Ad9p
acc genotypes	284
peaks no back	99
removed edges	NA
prop rem edges	NA

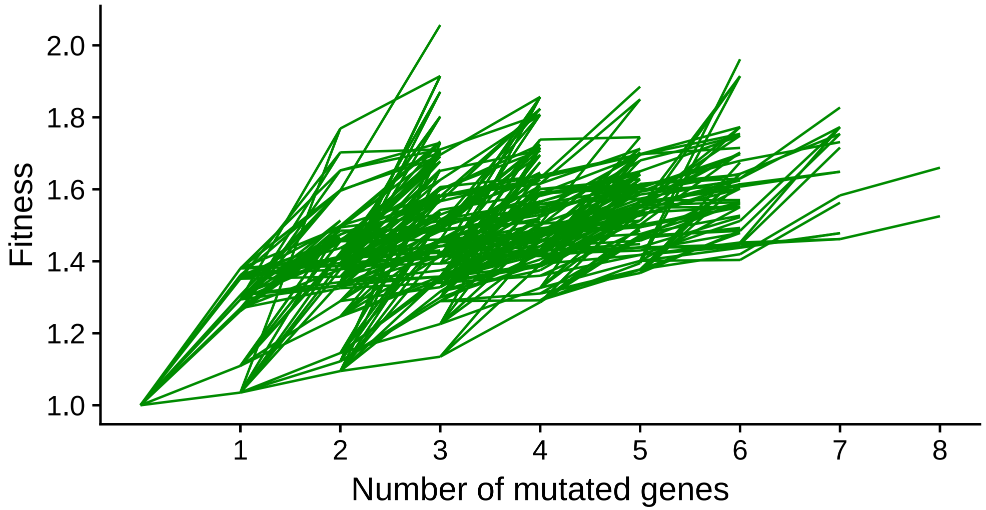

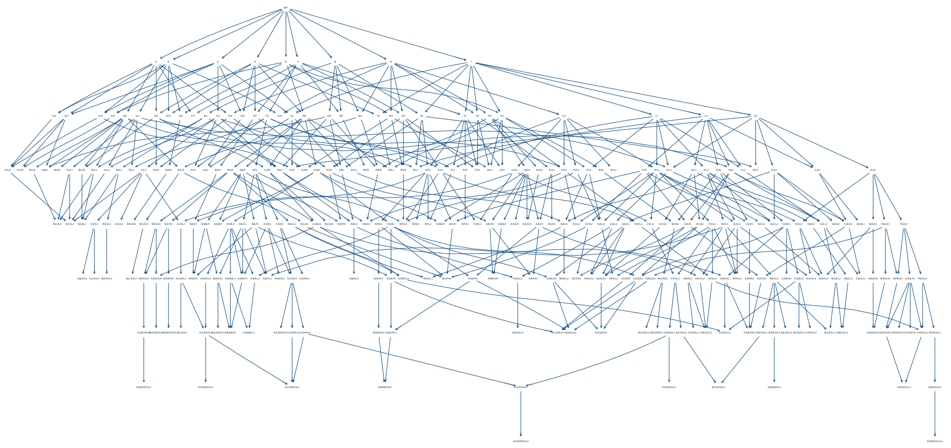

ID	AbVs2SaYTXrETNTU
acc genotypes	47
peaks no back	21
removed edges	NA
prop rem edges	NA

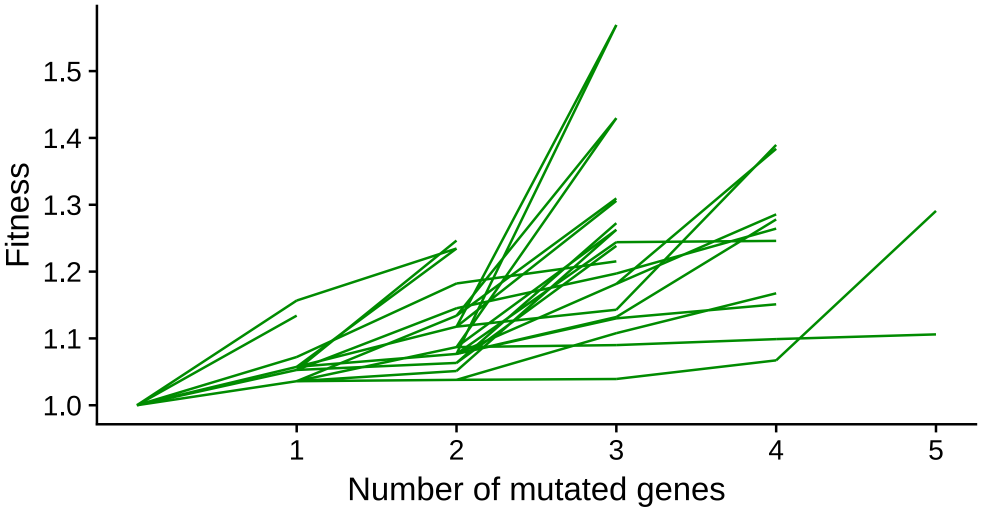

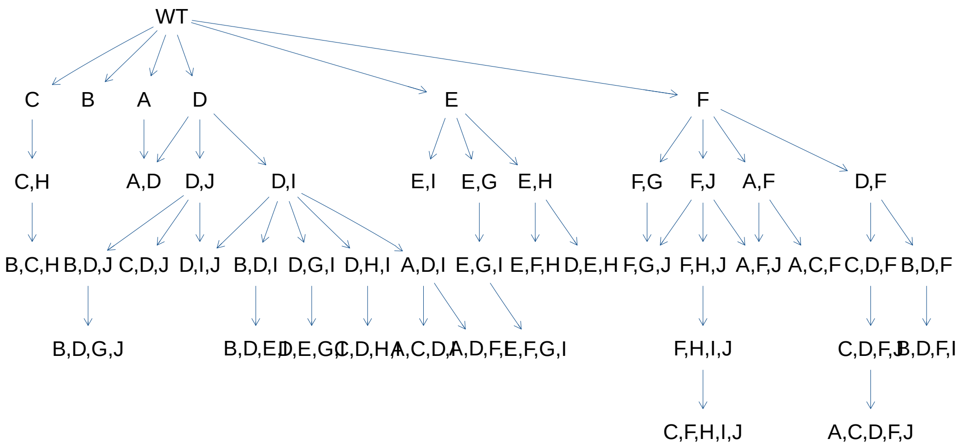

ID	aCr6IaKy6HuXLp5m
acc genotypes	180
peaks no back	53
removed edges	NA
prop rem edges	NA

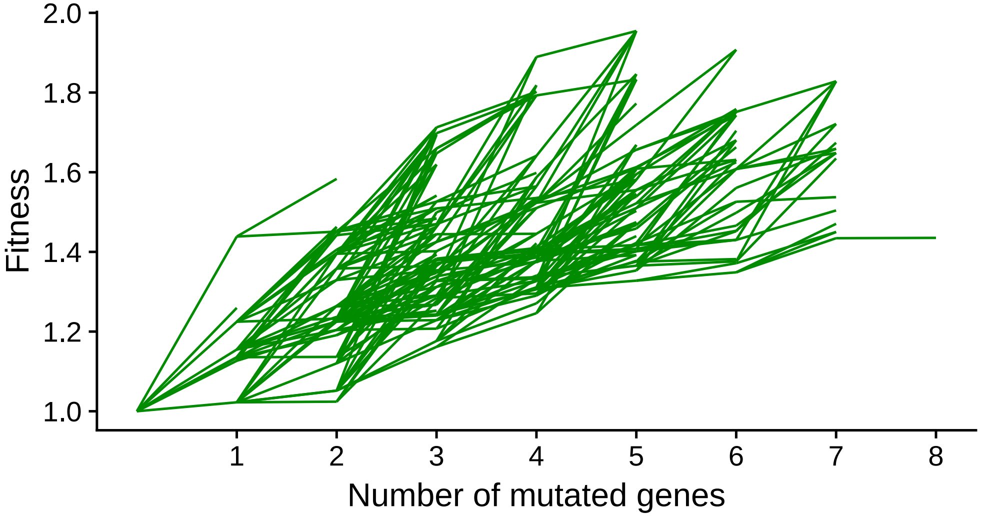

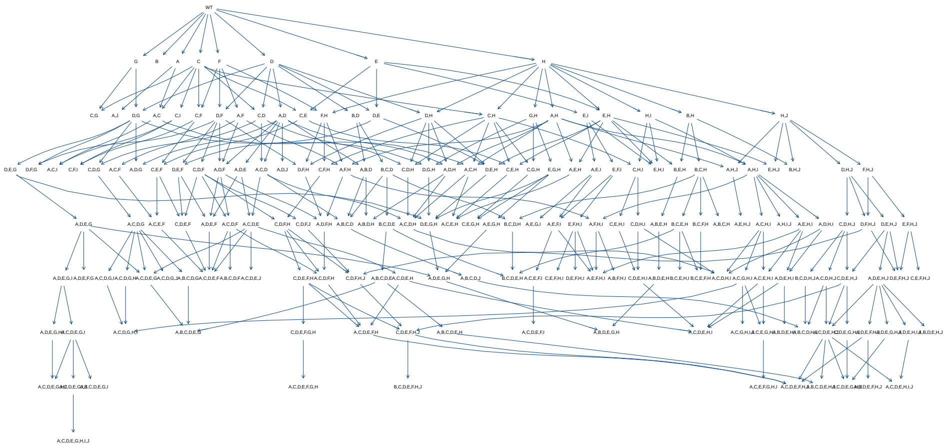

ID	aEeRemo3Lxmgcy8w
acc genotypes	311
peaks no back	94
removed edges	NA
prop rem edges	NA

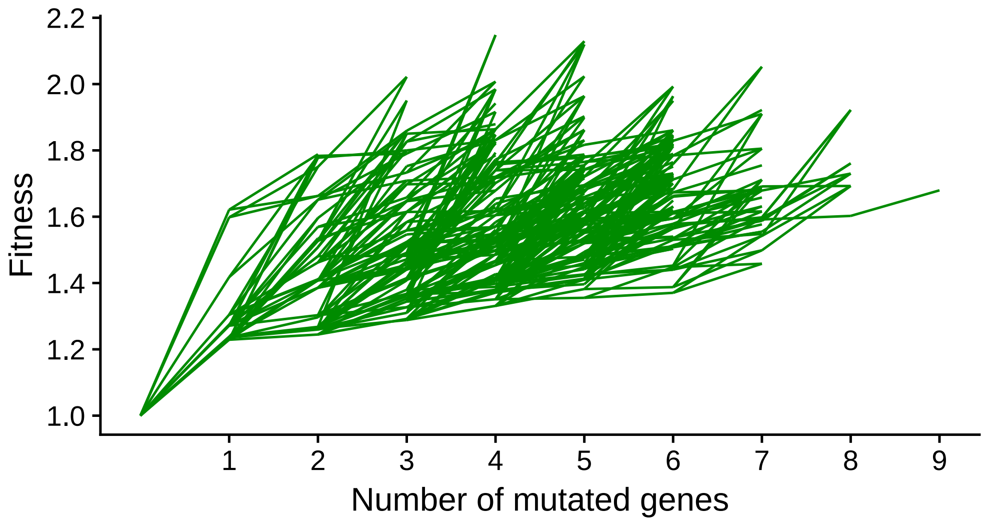

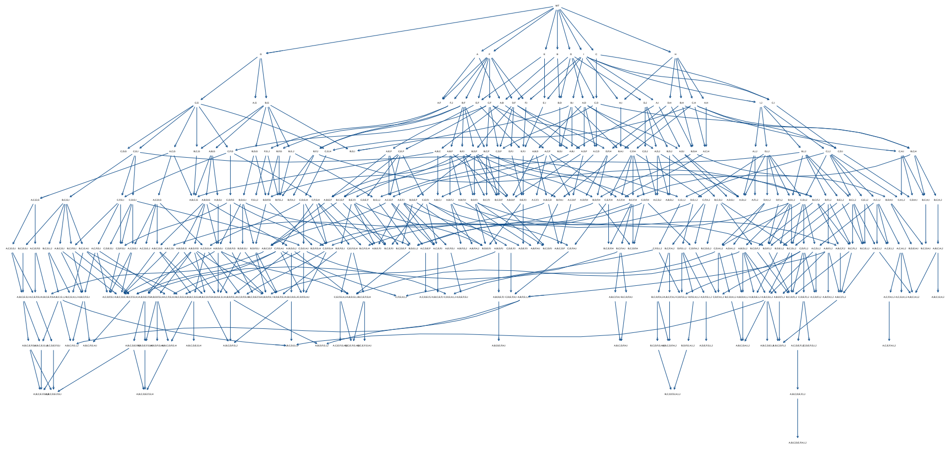

ID	Ag710LDVSvAEgCIJ3
acc genotypes	50
peaks no back	19
removed edges	NA
prop rem edges	NA

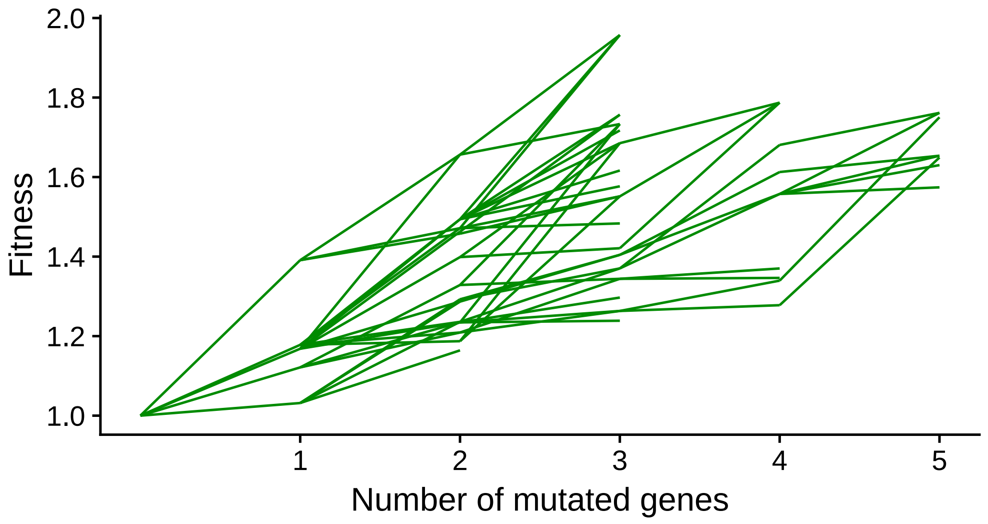

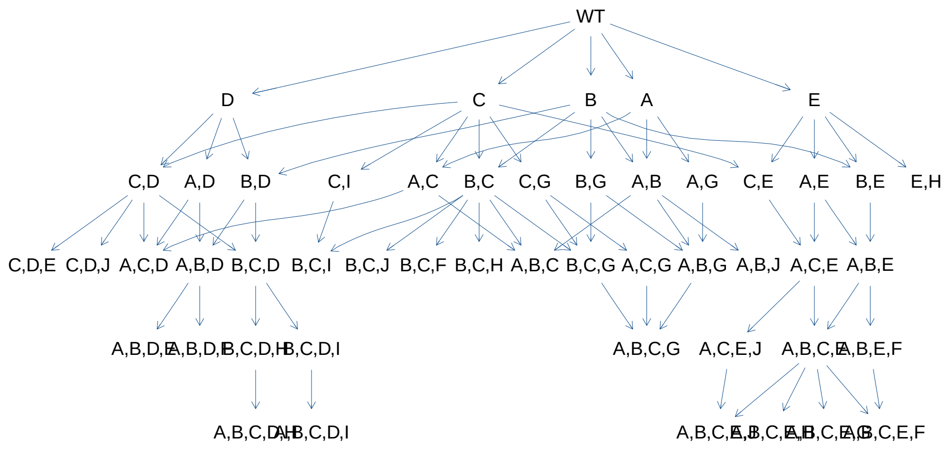

ID	ahVo16NivbfzL4Zy
acc genotypes	442
peaks no back	56
removed edges	NA
prop rem edges	NA

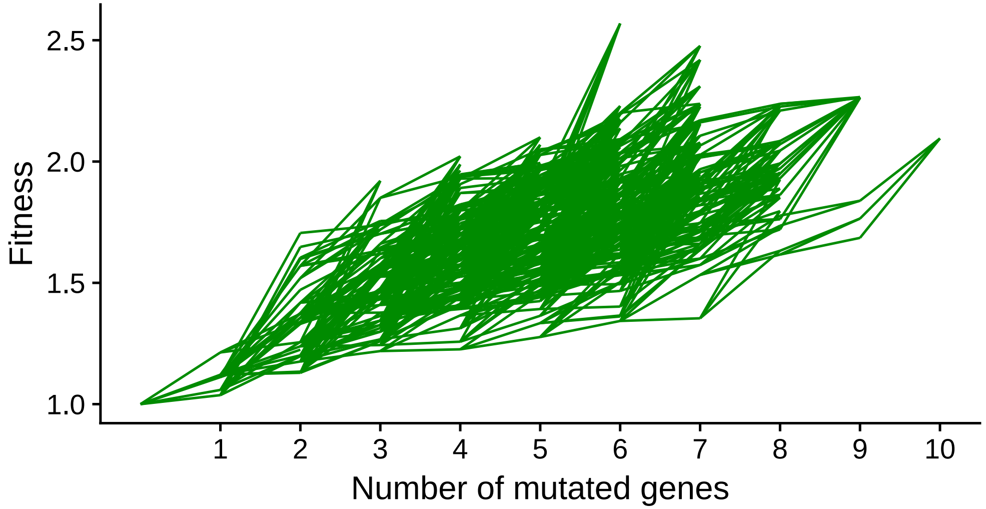

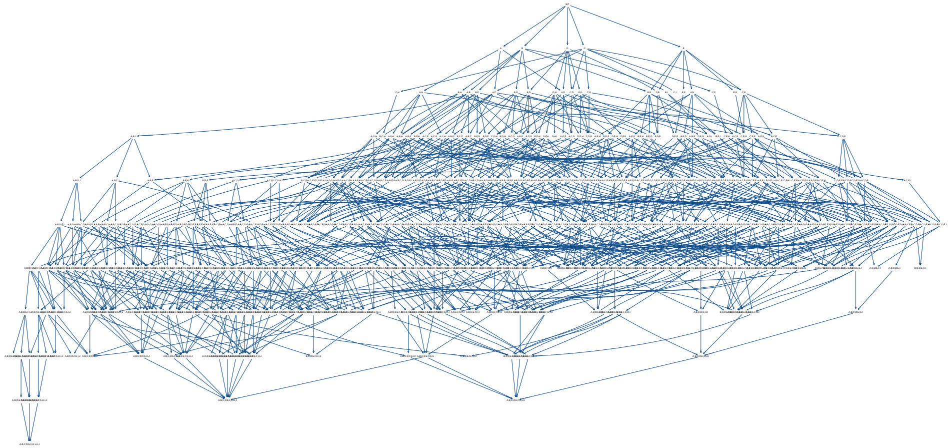

ID	AOgjTmG9Hs4vtlBq
acc genotypes	213
peaks no back	62
removed edges	NA
prop rem edges	NA

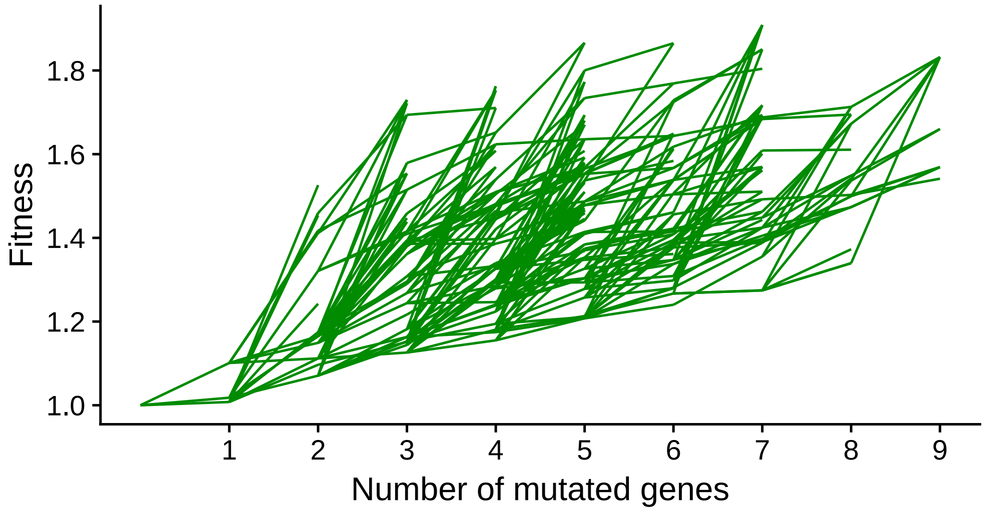

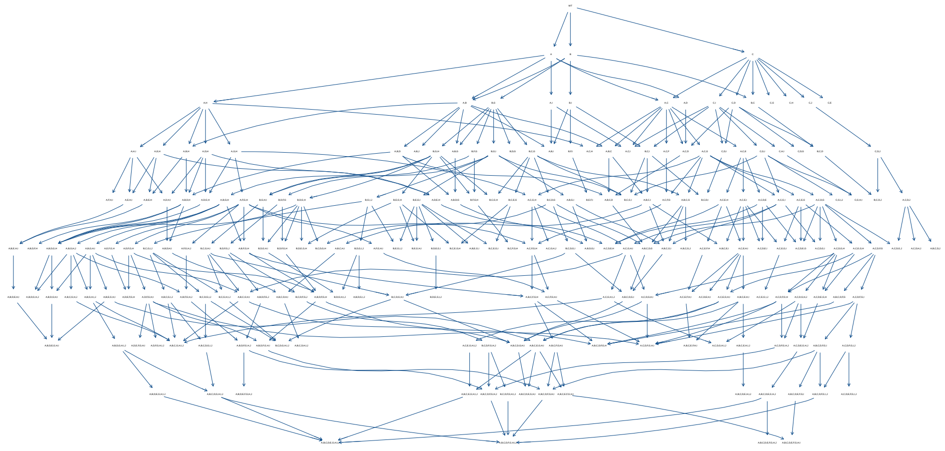

ID	AXZ6AsdBRokekeDx
acc genotypes	327
peaks no back	60
removed edges	NA
prop rem edges	NA

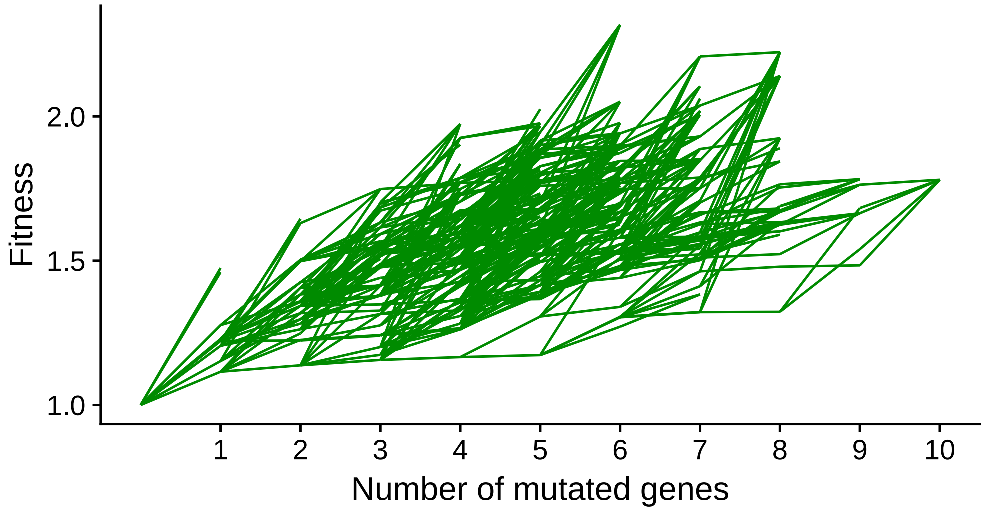

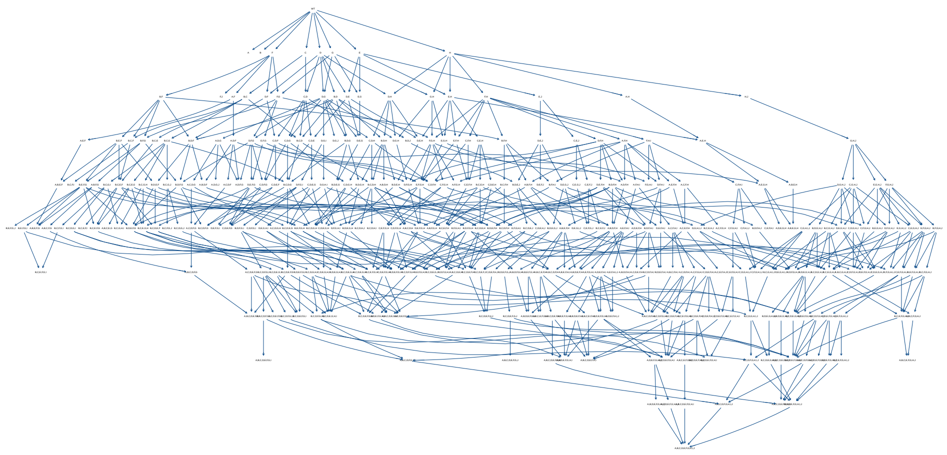

ID	bFdmuJ1681ocZTYL
acc genotypes	359
peaks no back	88
removed edges	NA
prop rem edges	NA

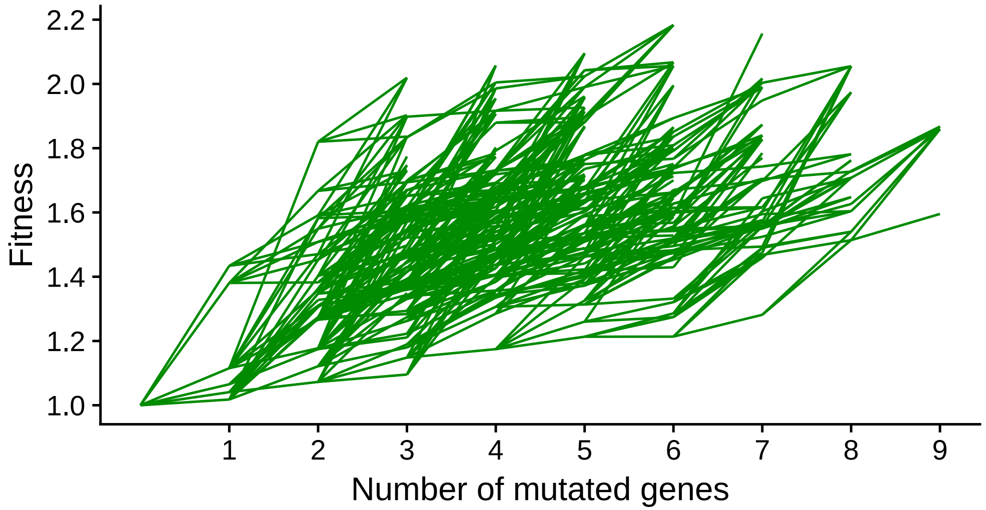

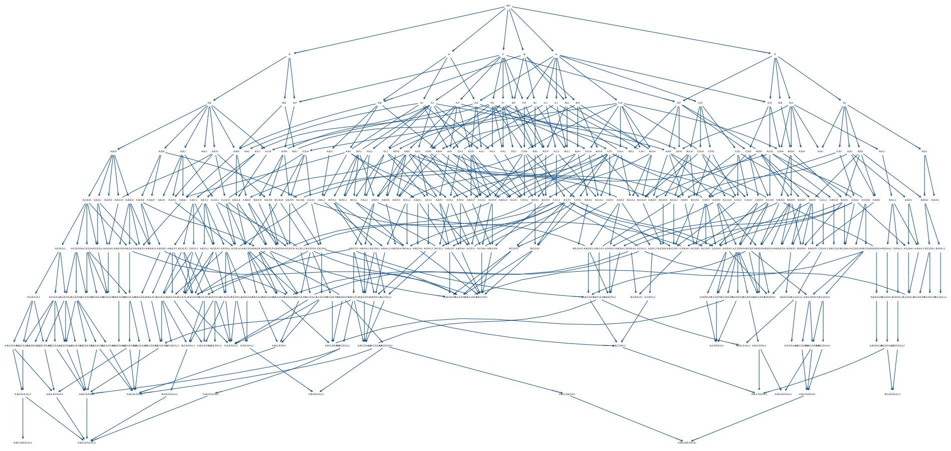

ID	bGijXeZCPrfEOBkI
acc genotypes	183
peaks no back	53
removed edges	NA
prop rem edges	NA

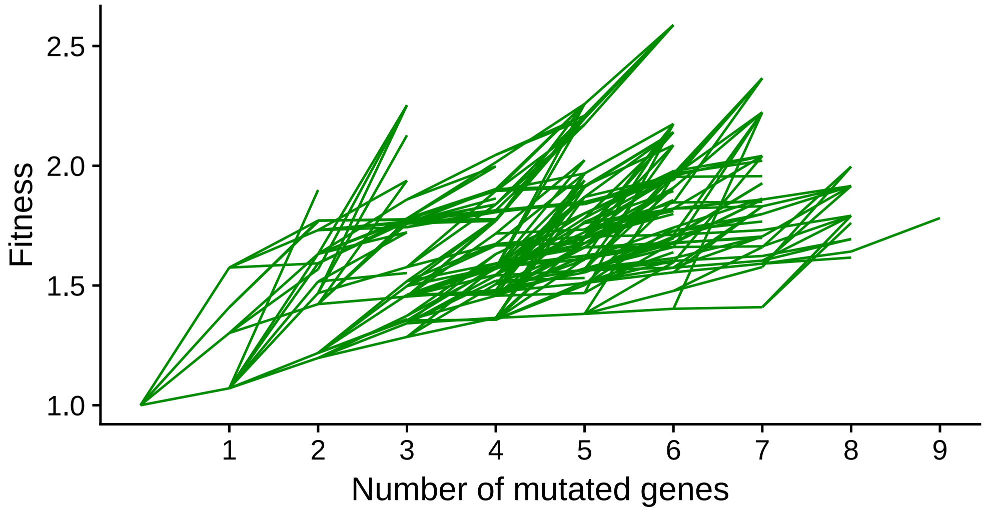

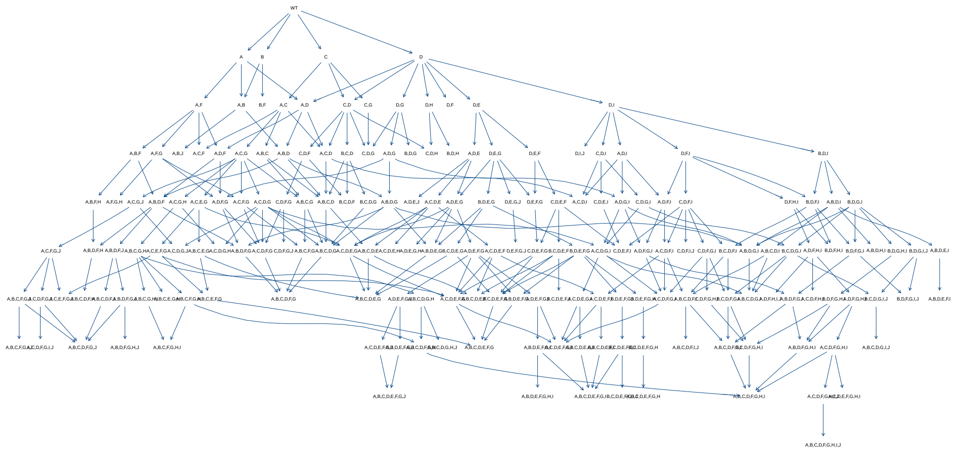

ID	BnCqeBo4erAk6AAn
acc genotypes	616
peaks no back	76
removed edges	NA
prop rem edges	NA

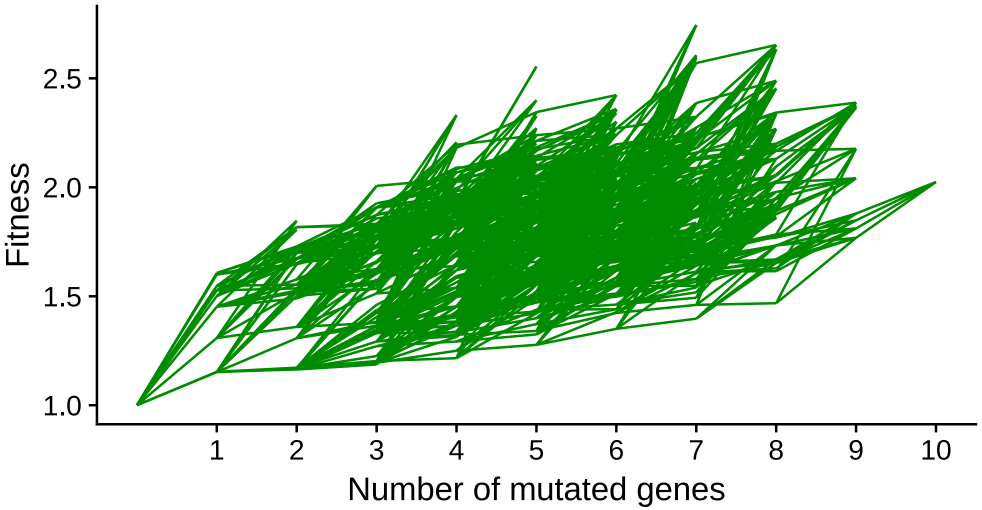

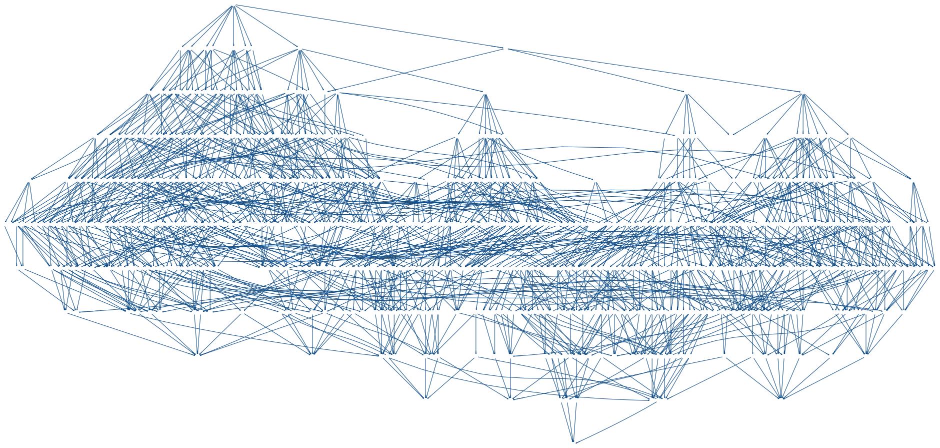

ID	bo1swth9Yj610ESJy
acc genotypes	133
peaks no back	40
removed edges	NA
prop rem edges	NA

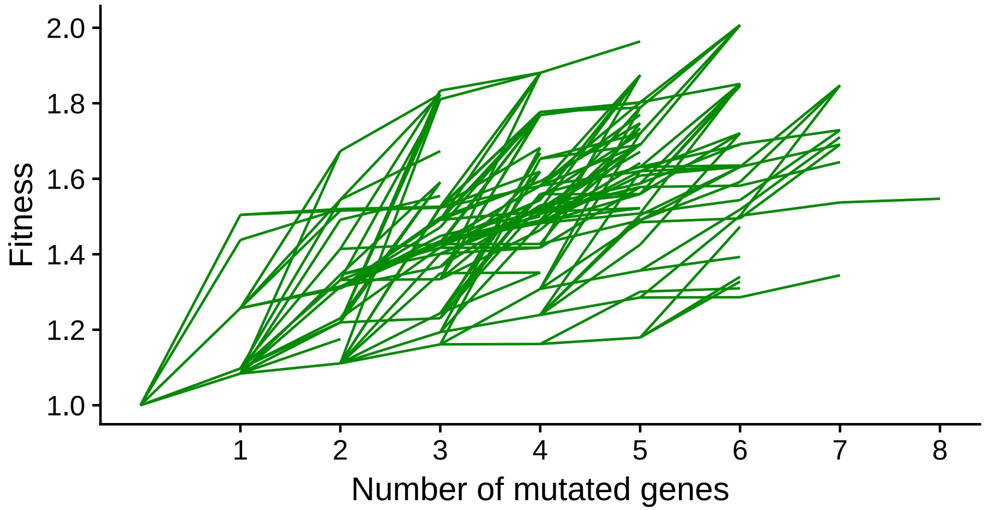

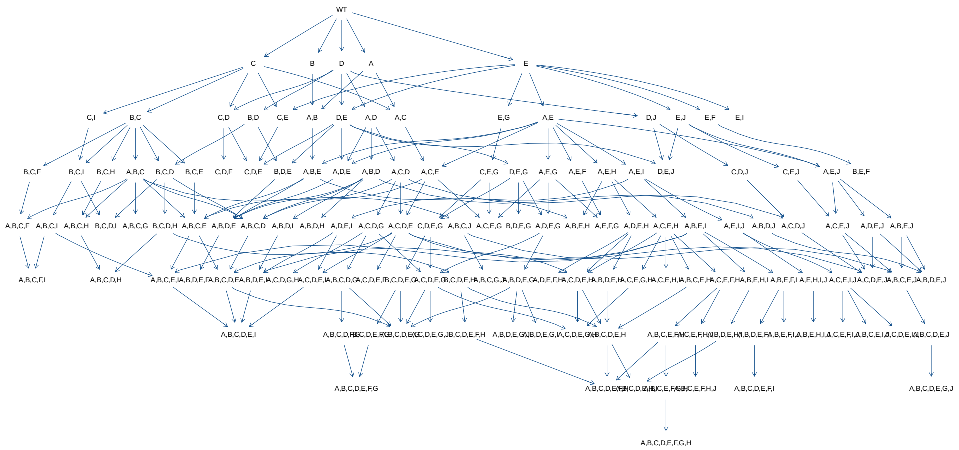

ID	bOw7SJCwZBwfZRmf
acc genotypes	187
peaks no back	71
removed edges	NA
prop rem edges	NA

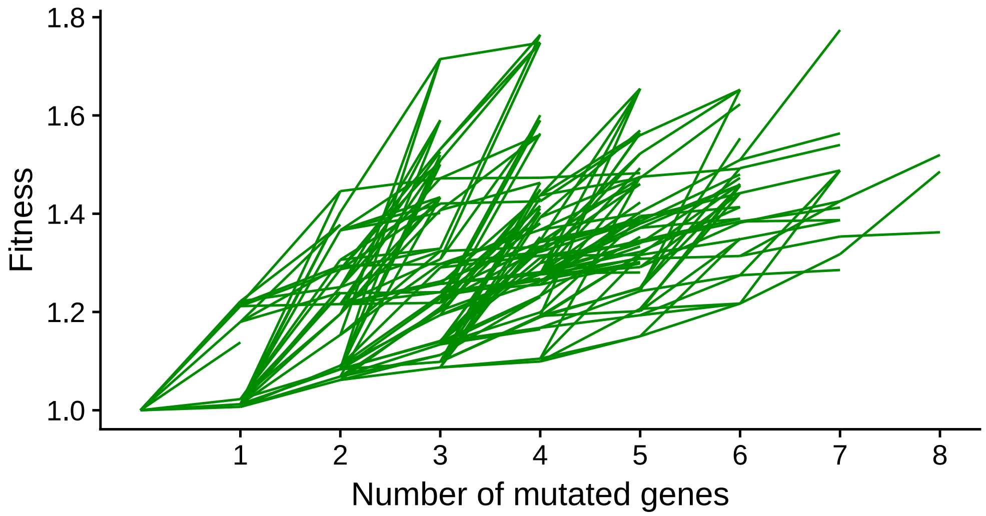

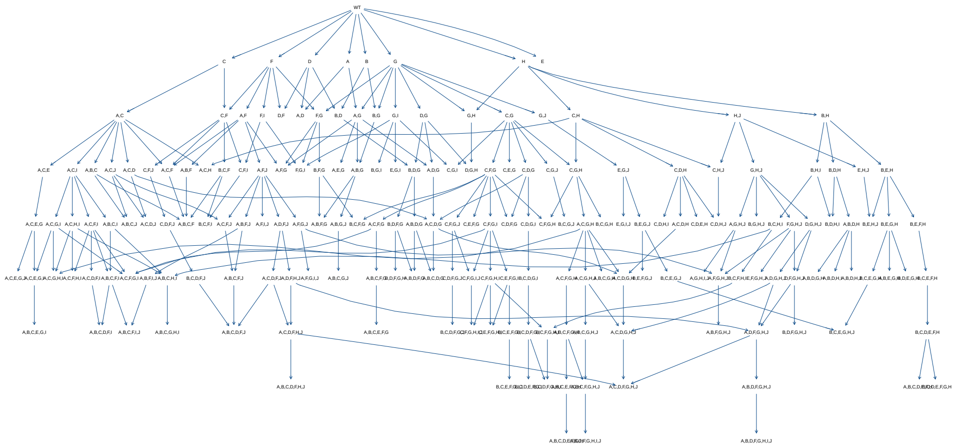

ID	BtsRtS3YmmkCe2Gg
acc genotypes	50
peaks no back	18
removed edges	NA
prop rem edges	NA

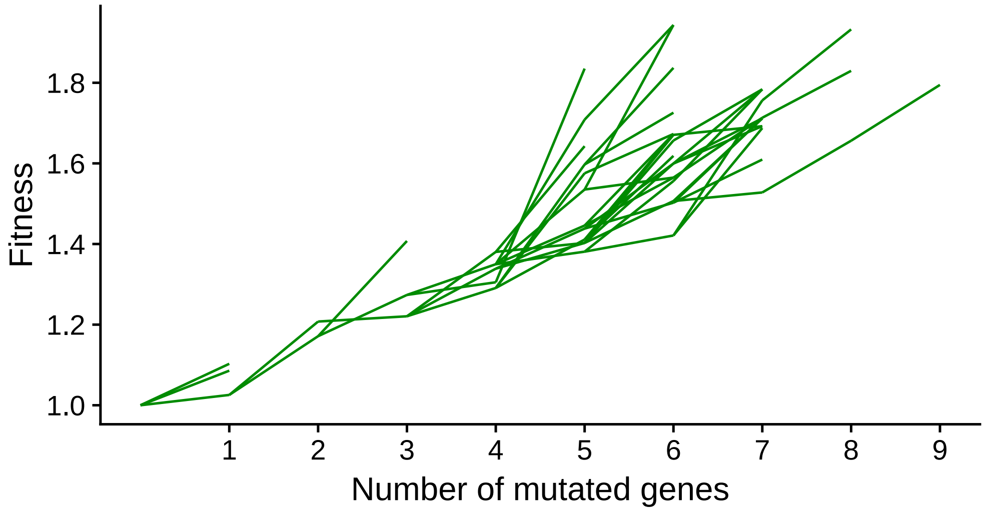

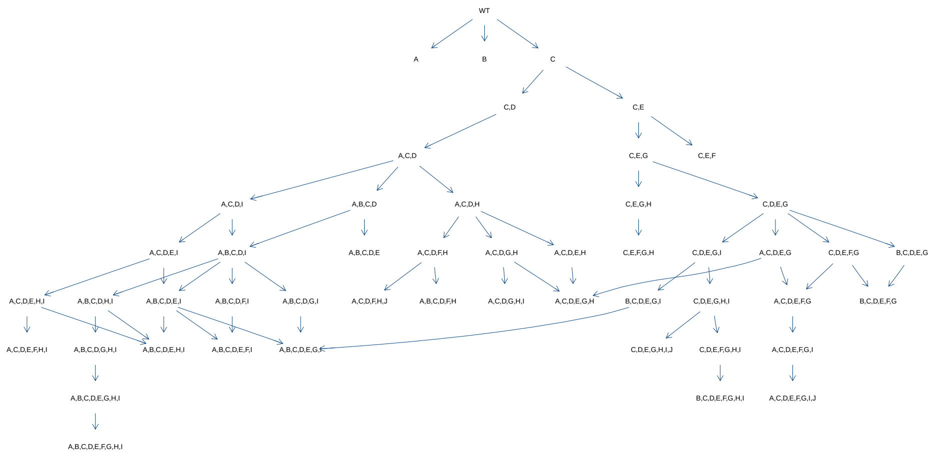

ID	BWbL4g3bDhB87PnG
acc genotypes	405
peaks no back	99
removed edges	NA
prop rem edges	NA

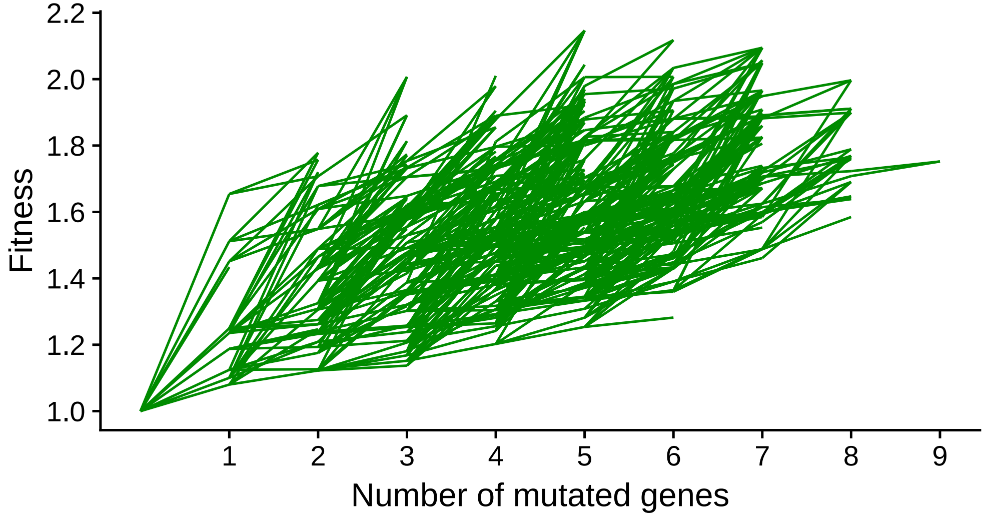

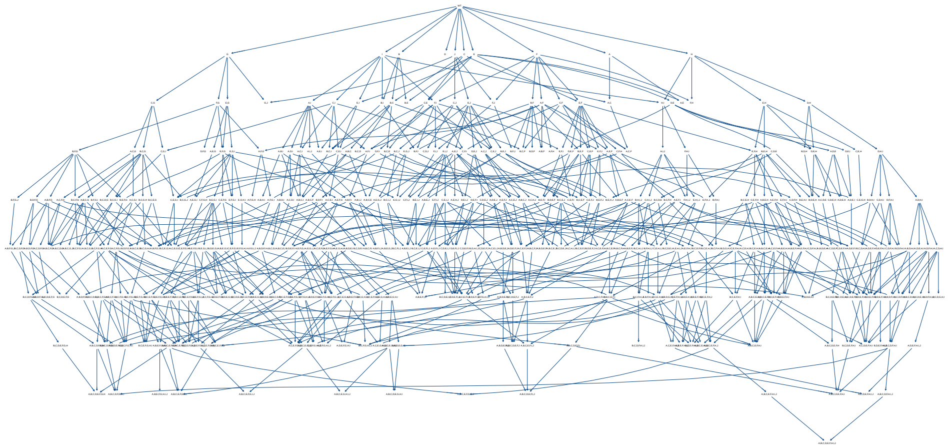

ID	C1zvP9CZFCg9avLi
acc genotypes	102
peaks no back	44
removed edges	NA
prop rem edges	NA

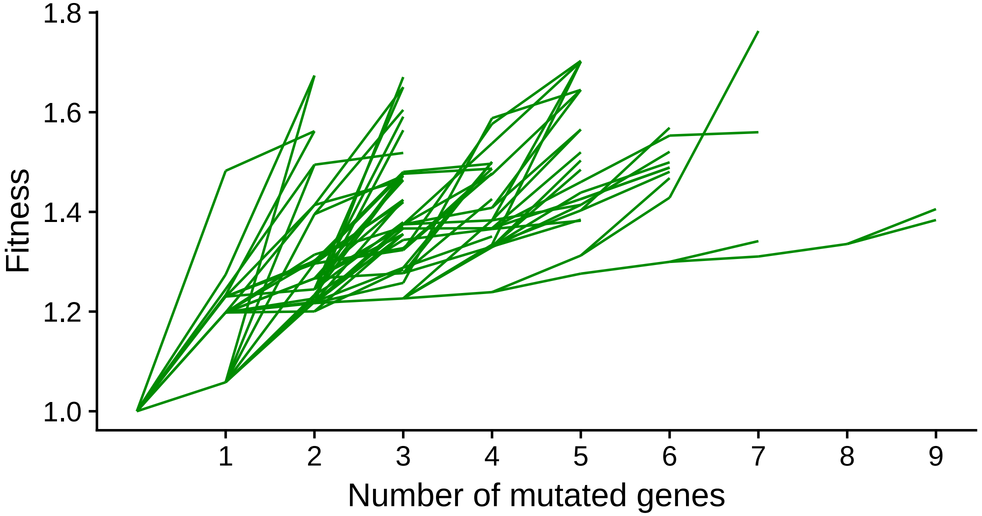

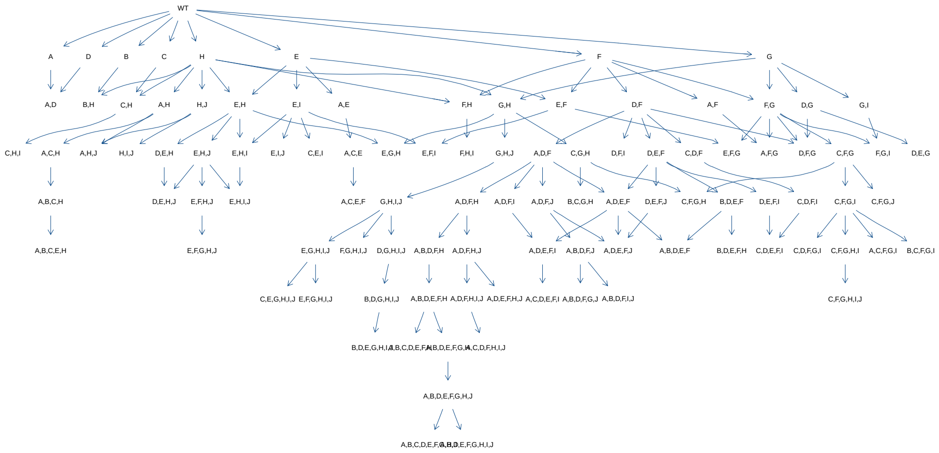

ID	c21LhpXlCZKWnkC5
acc genotypes	493
peaks no back	93
removed edges	NA
prop rem edges	NA

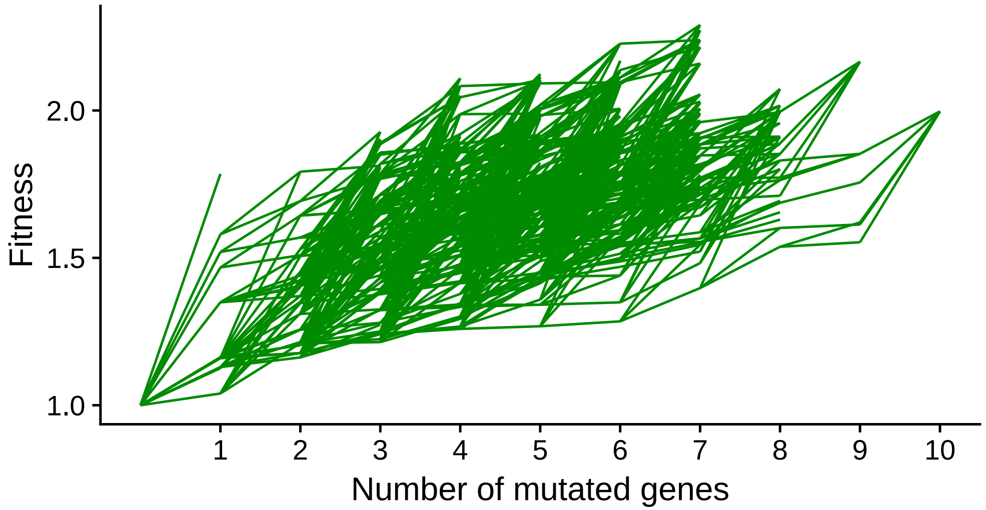

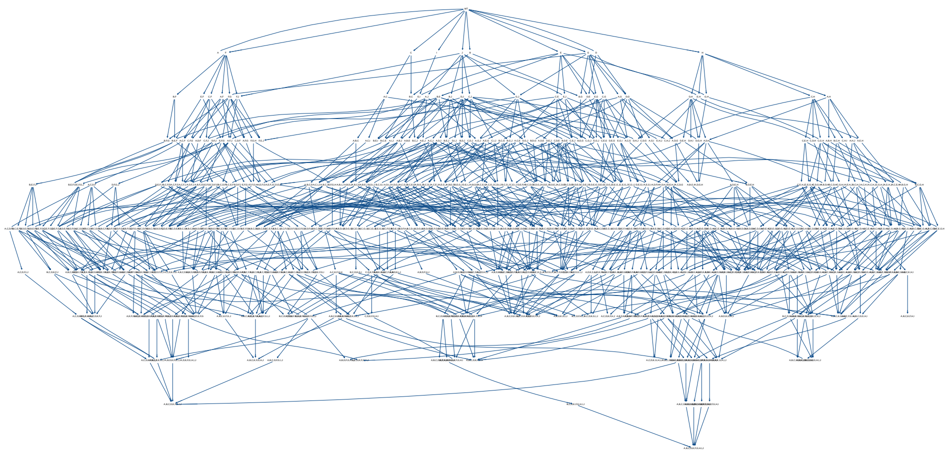

ID	C46MDiN7E3KDR5Fu
acc genotypes	143
peaks no back	61
removed edges	NA
prop rem edges	NA

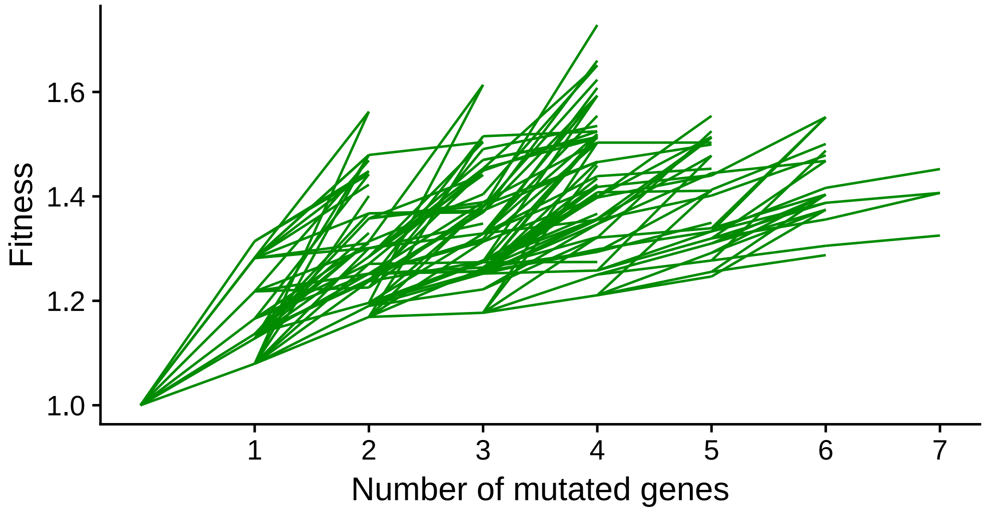

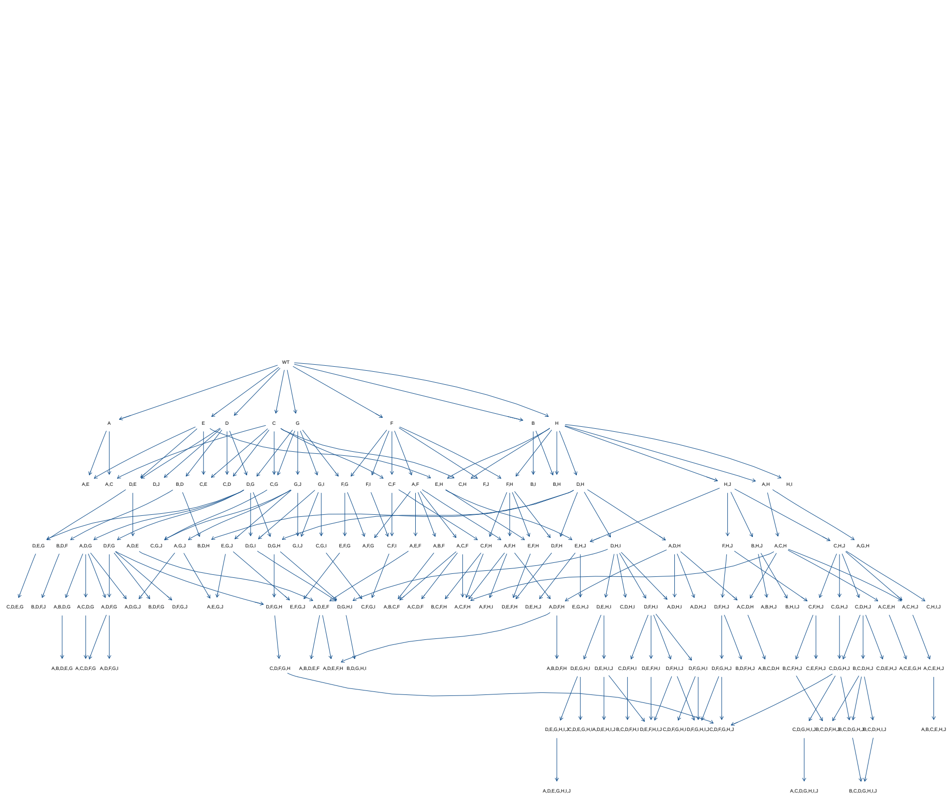

ID	COoTZ3S17BxN8HQx
acc genotypes	209
peaks no back	75
removed edges	NA
prop rem edges	NA

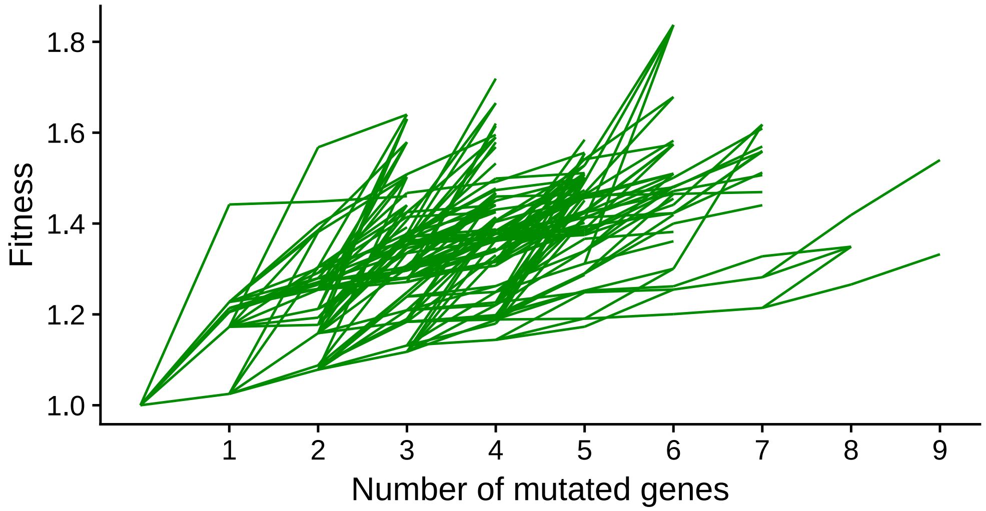

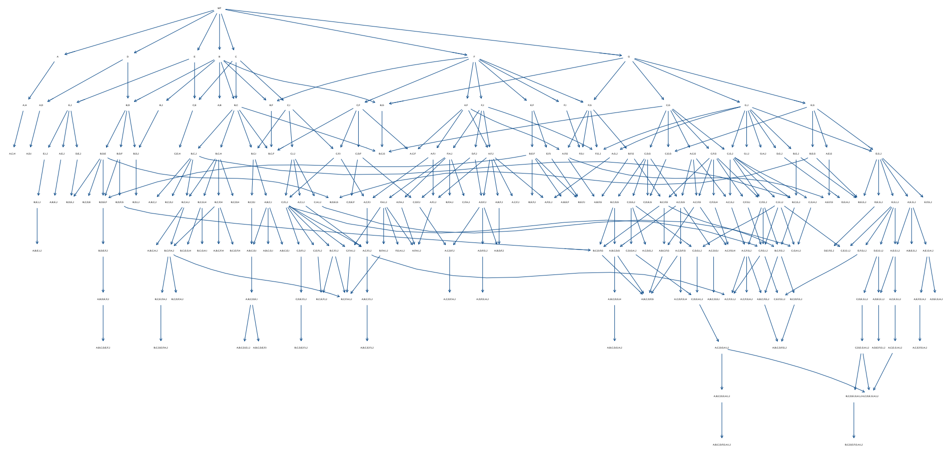

ID	CscphnR9HTju10jKi
acc genotypes	54
peaks no back	14
removed edges	NA
prop rem edges	NA

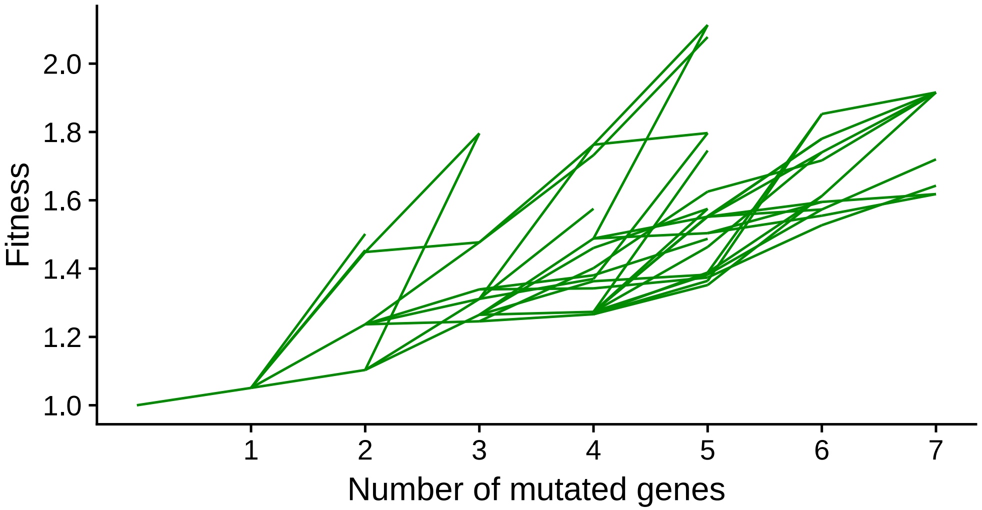

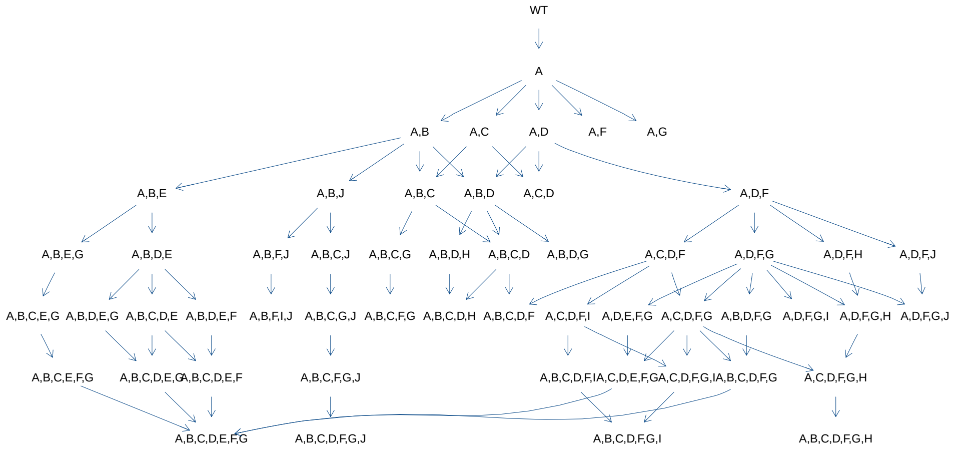

ID	CxyKl8ZkyGMlHEBy
acc genotypes	101
peaks no back	37
removed edges	NA
prop rem edges	NA

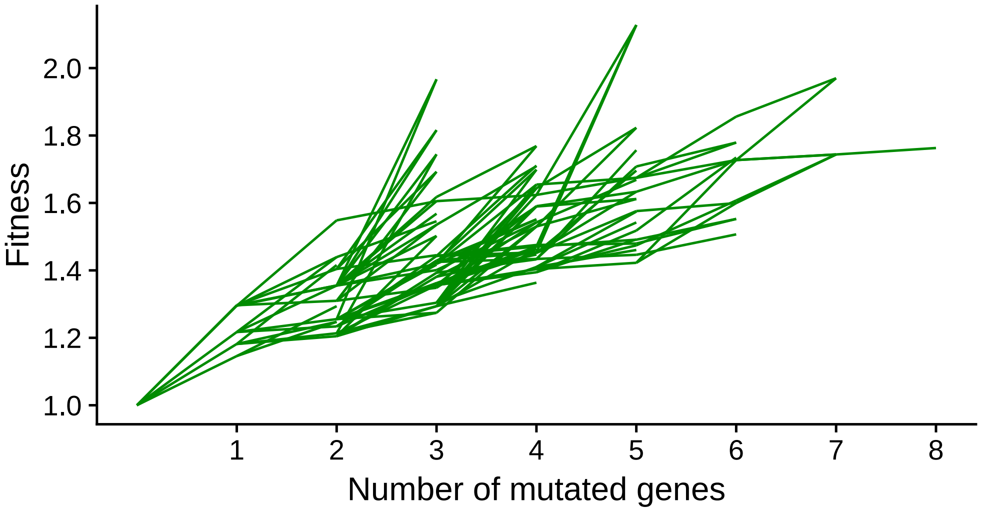

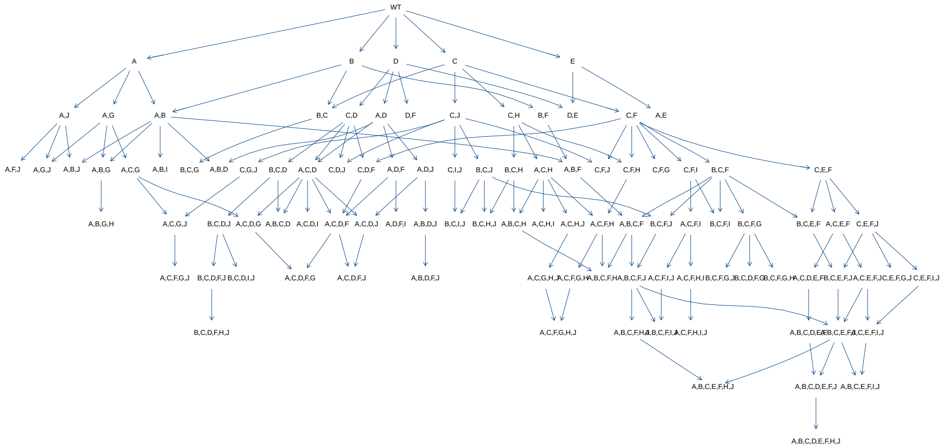

ID	d107ojrARayuzrHeN
acc genotypes	482
peaks no back	107
removed edges	NA
prop rem edges	NA

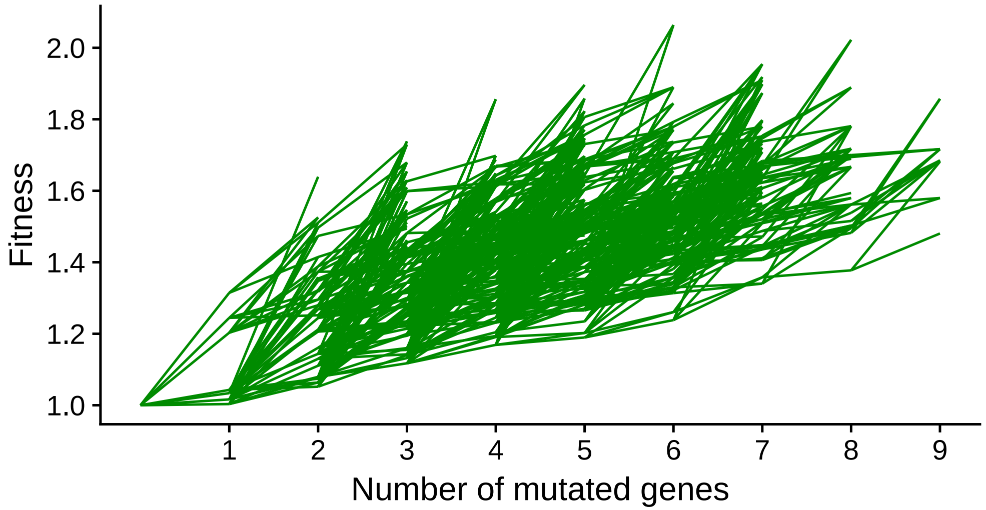

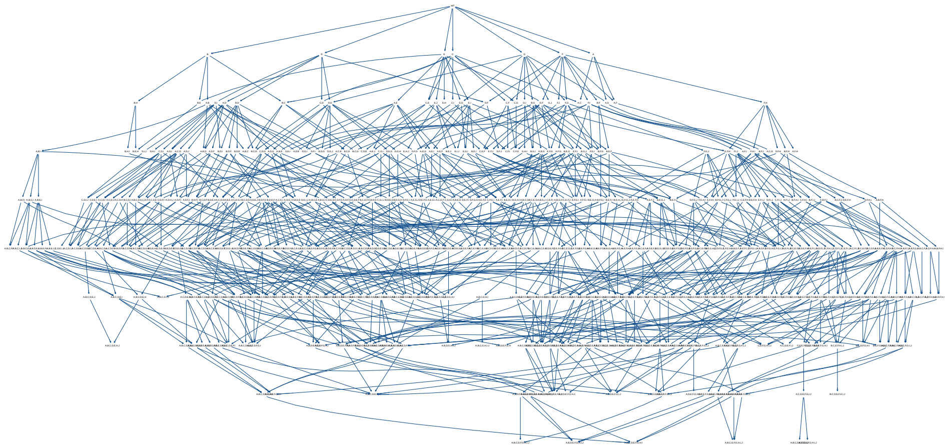

ID	DF1iteAjggjrOq9G
acc genotypes	240
peaks no back	84
removed edges	NA
prop rem edges	NA

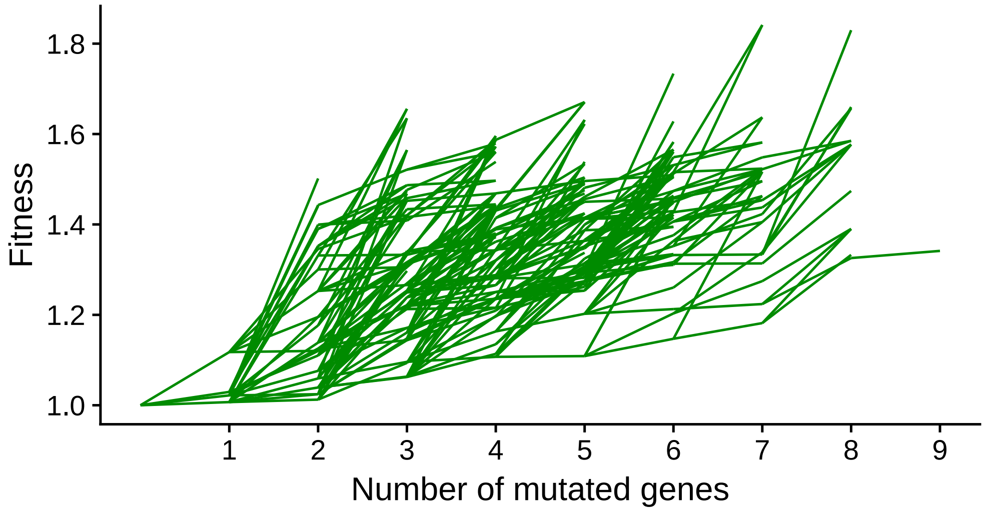

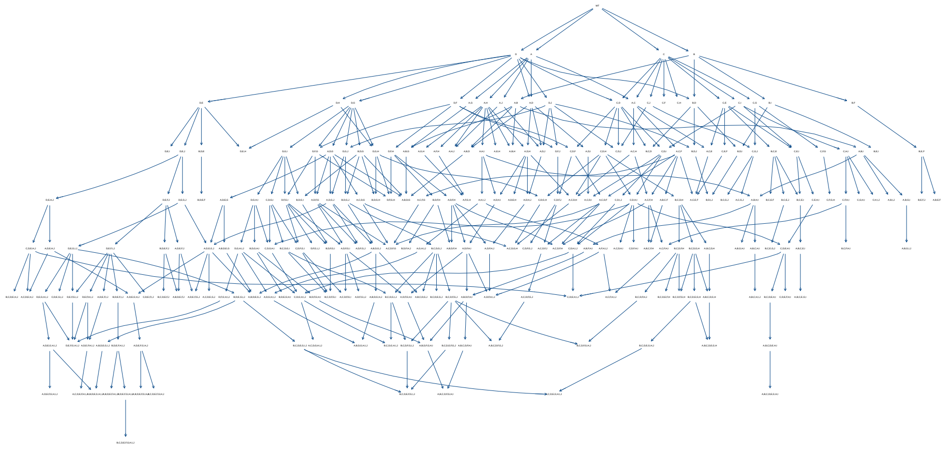

ID	DvGWOEzyGFaVOowT
acc genotypes	577
peaks no back	93
removed edges	NA
prop rem edges	NA

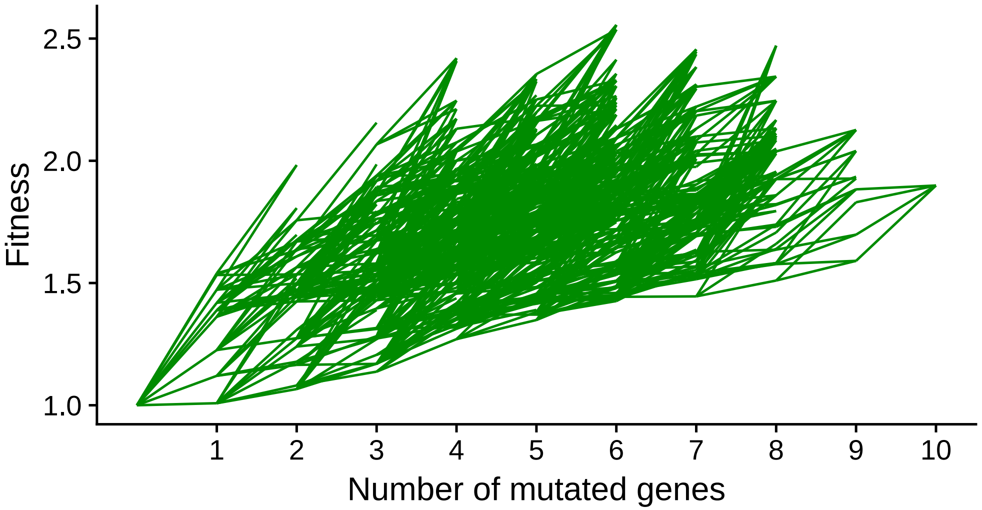

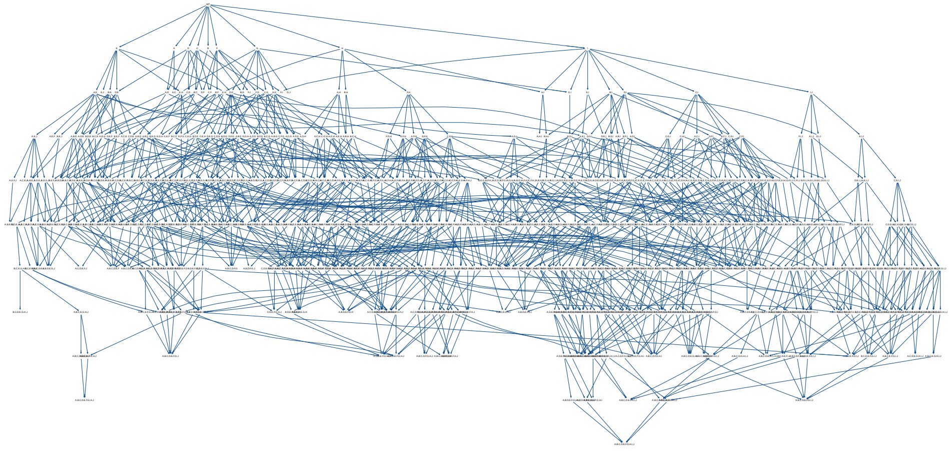

ID	dzb4OOvYv11WGeeP
acc genotypes	394
peaks no back	83
removed edges	NA
prop rem edges	NA

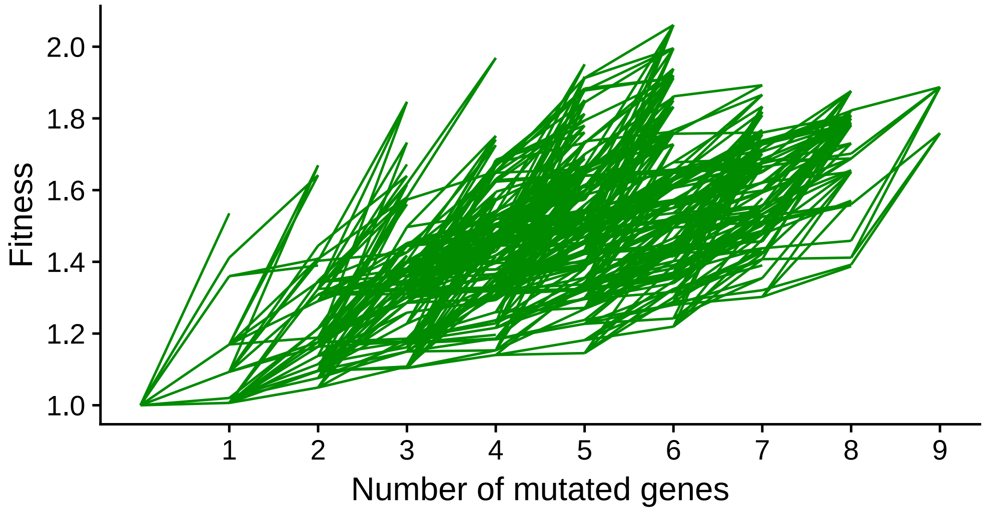

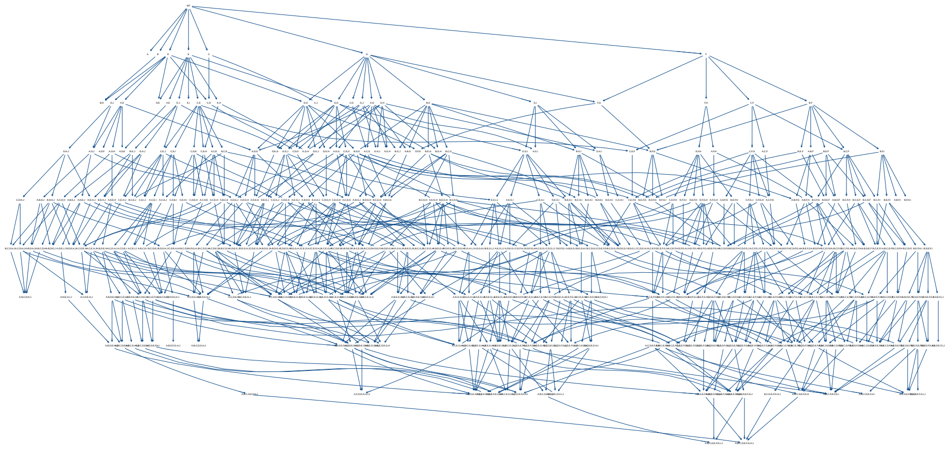

ID	EgkeUuvhLZsyQQ110
acc genotypes	283
peaks no back	50
removed edges	NA
prop rem edges	NA

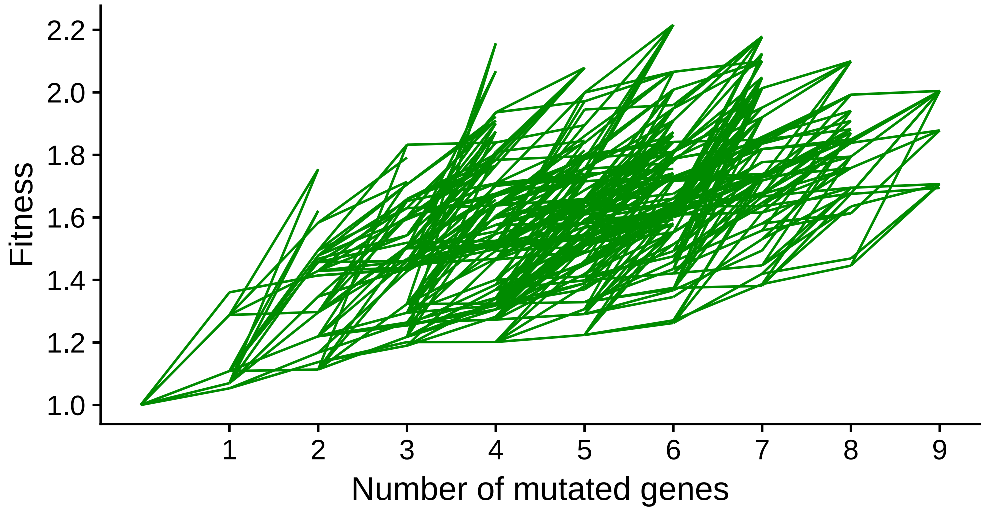

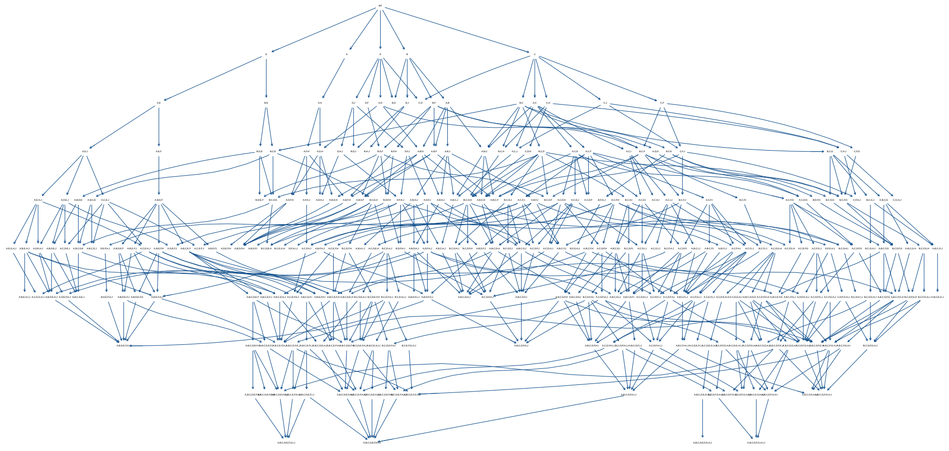

ID	eIl49J4HyU8o710iq
acc genotypes	576
peaks no back	83
removed edges	NA
prop rem edges	NA

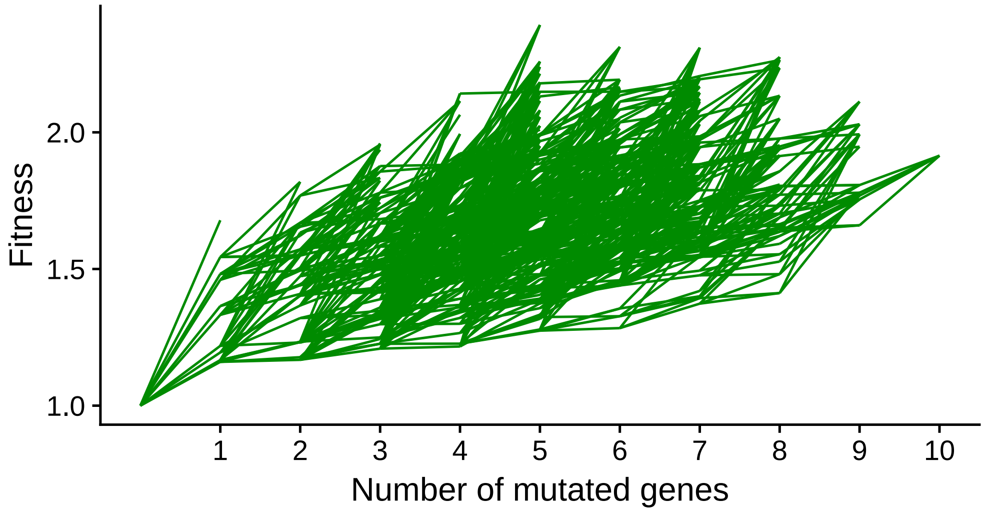

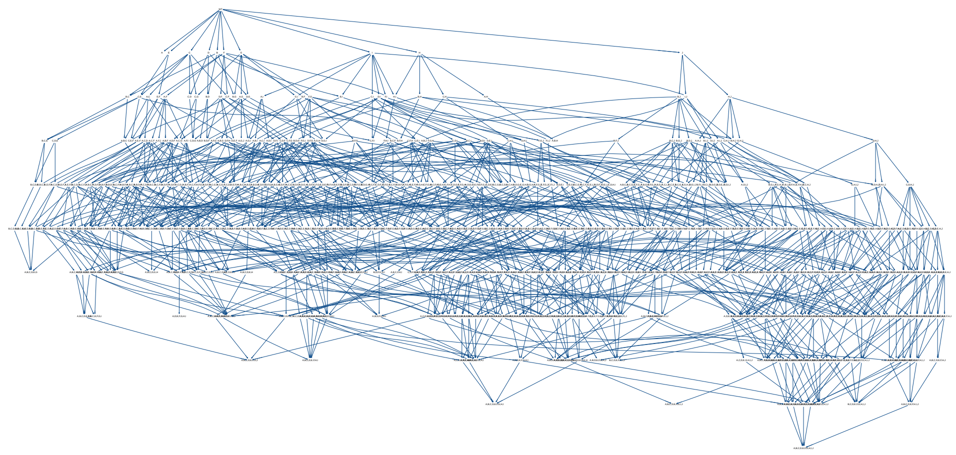

ID	EMbpnzl5vOYpYkCY
acc genotypes	262
peaks no back	64
removed edges	NA
prop rem edges	NA

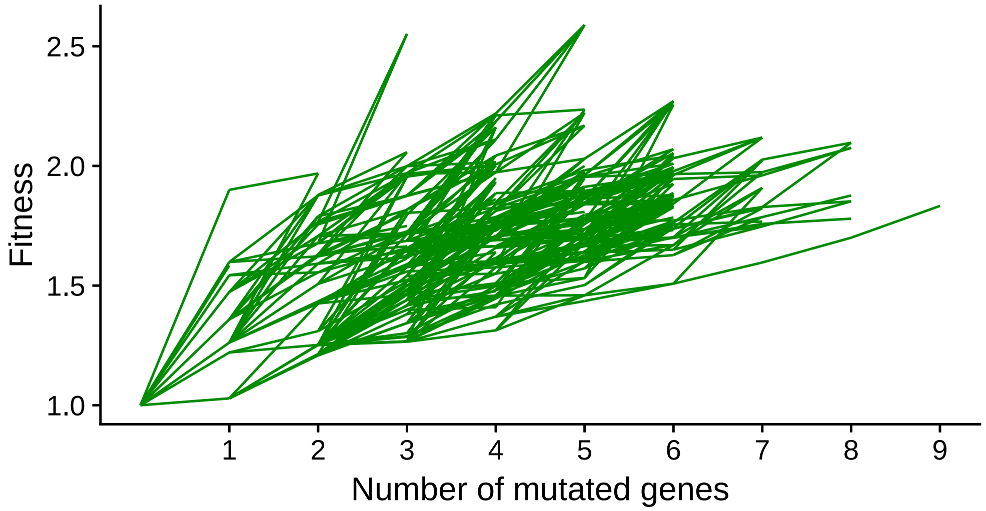

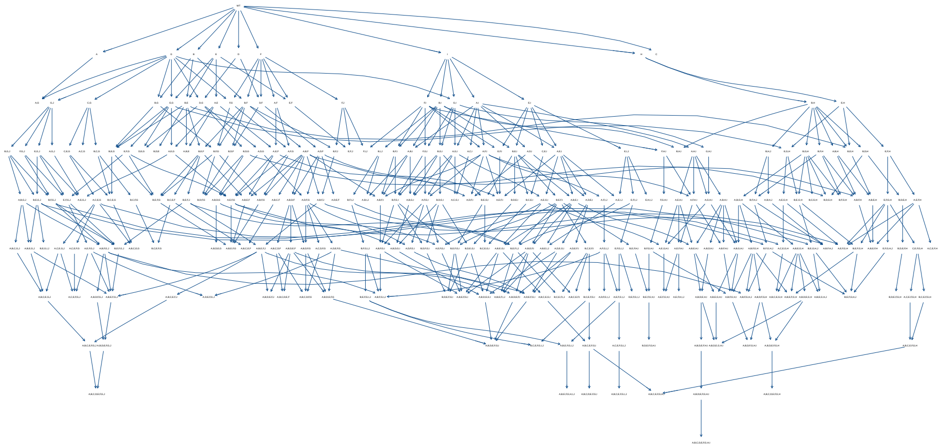

ID	ERn91KUoSwG1g1w9
acc genotypes	263
peaks no back	93
removed edges	NA
prop rem edges	NA

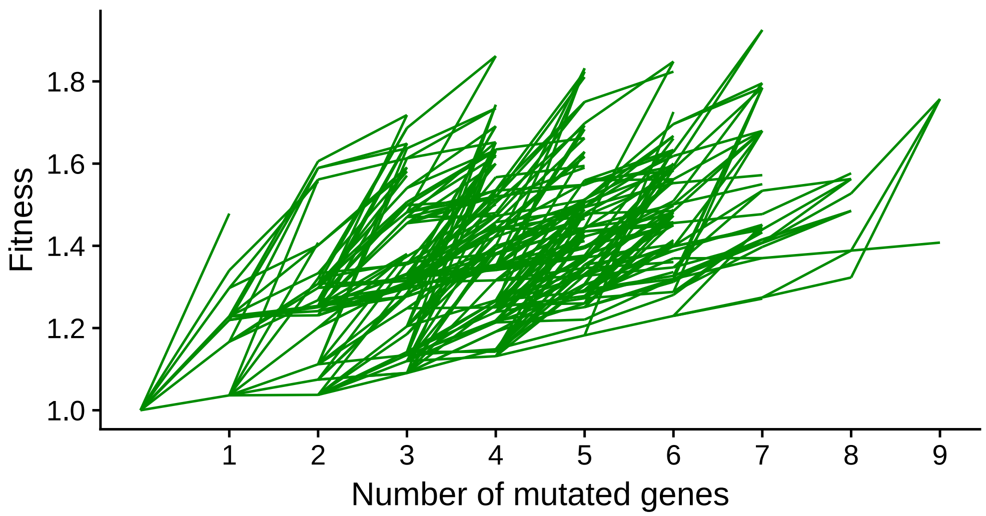

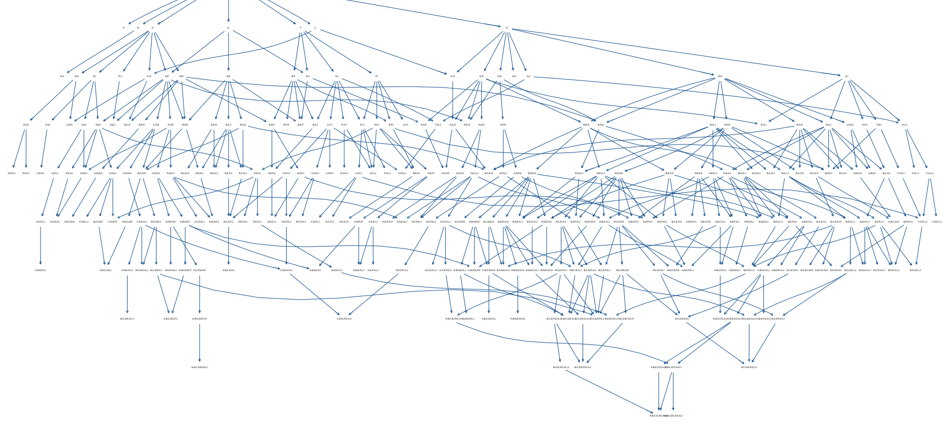

ID	erQFJpuljZzglgwp
acc genotypes	377
peaks no back	75
removed edges	NA
prop rem edges	NA

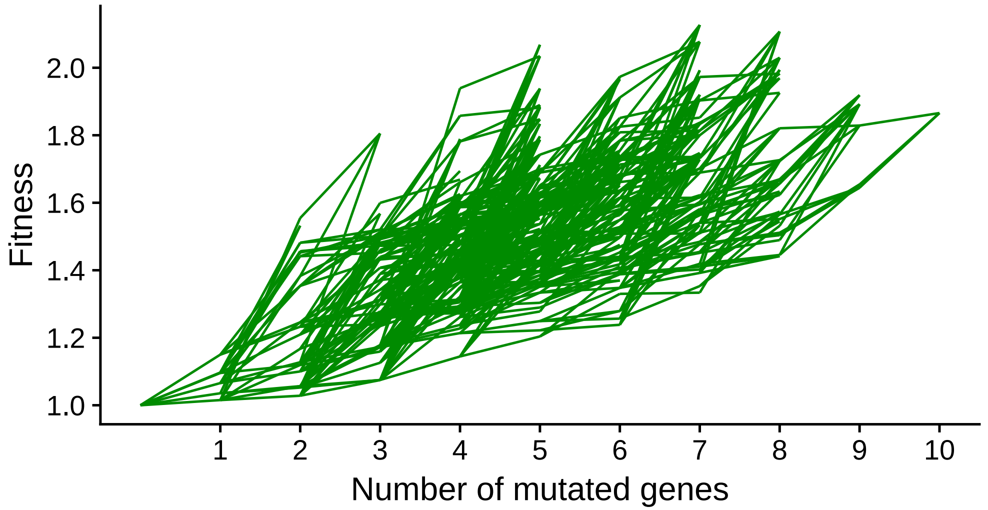

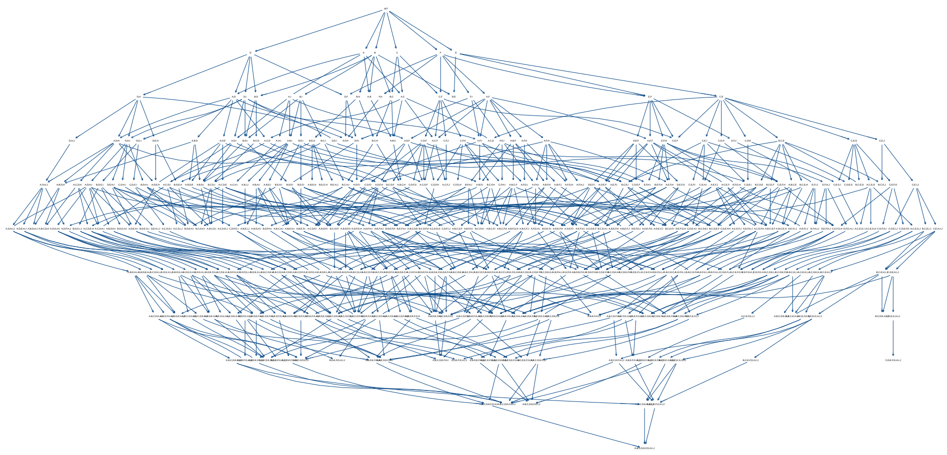

ID	EV5PzPVJ8yEpuh5C
acc genotypes	33
peaks no back	13
removed edges	NA
prop rem edges	NA

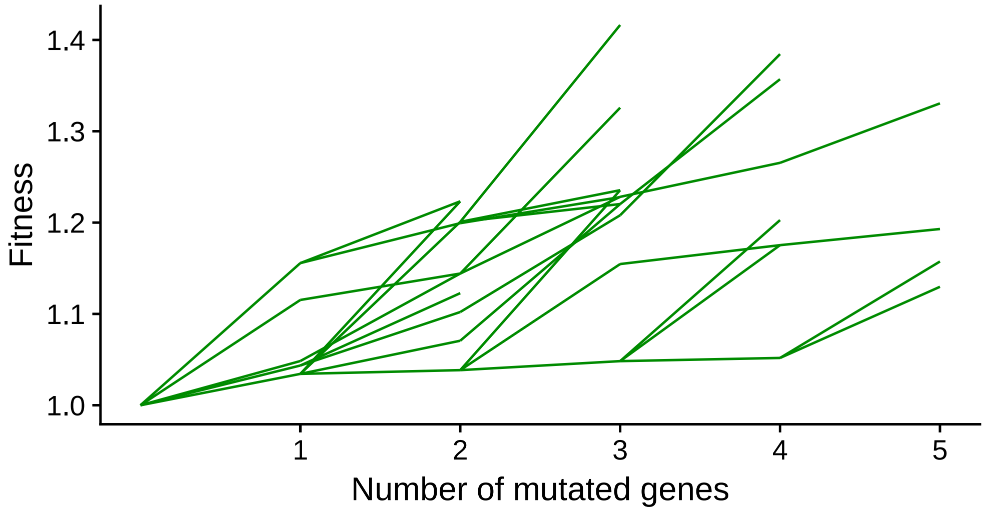

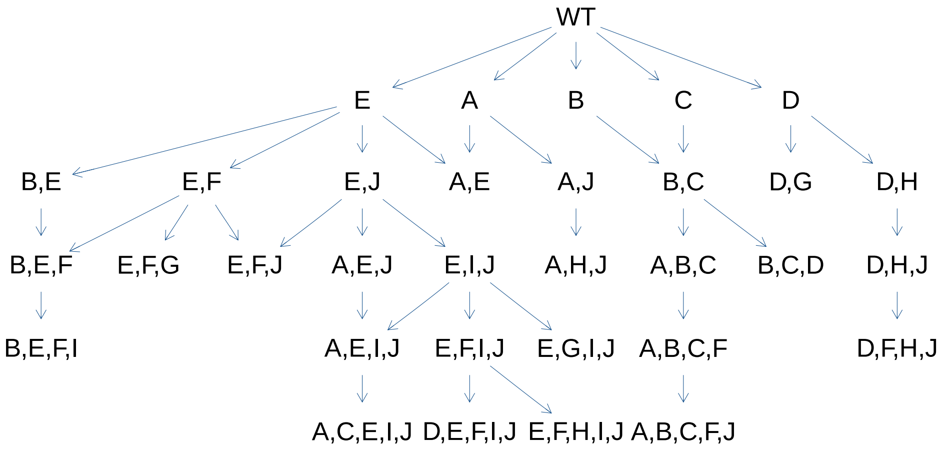

ID	f4lIMrTliuCuX6MS
acc genotypes	540
peaks no back	122
removed edges	NA
prop rem edges	NA

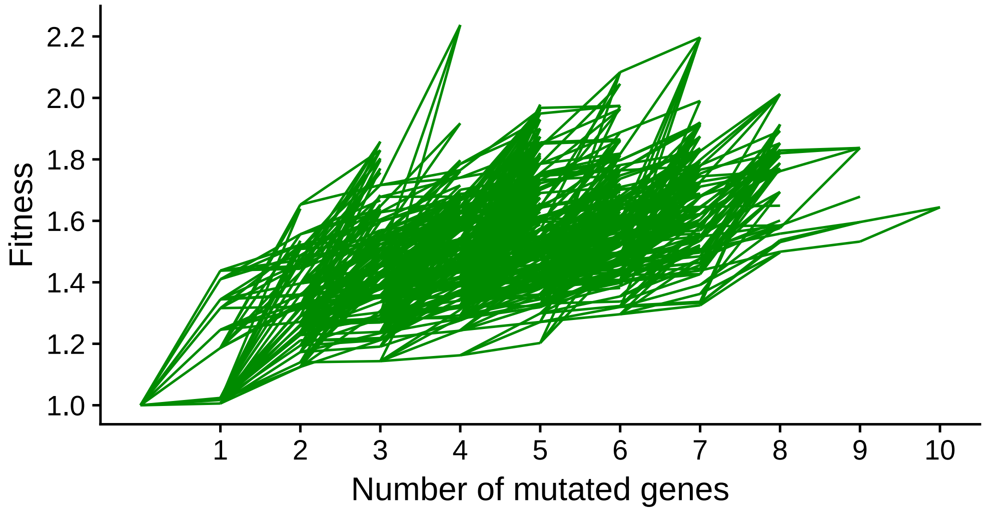

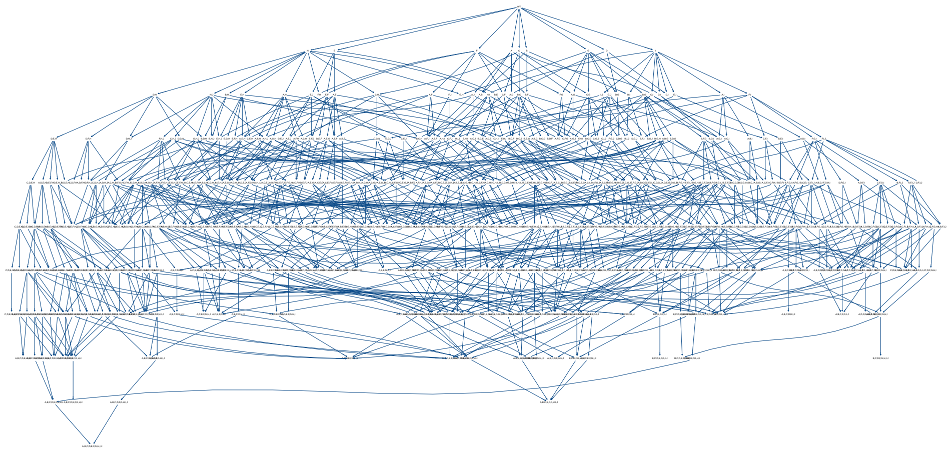

ID	FCcL7VfUNU6Igd6e
acc genotypes	400
peaks no back	62
removed edges	NA
prop rem edges	NA

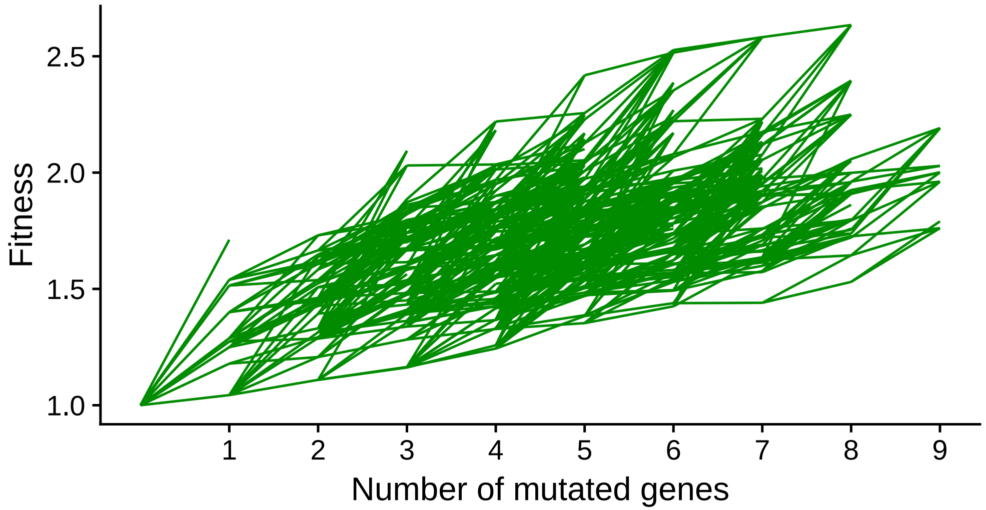

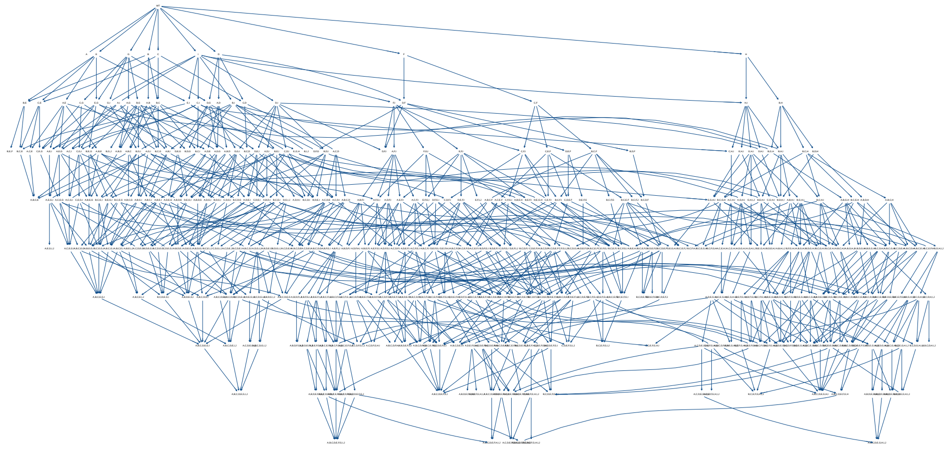

ID	fhP10WlHL7710GBMFE
acc genotypes	412
peaks no back	90
removed edges	NA
prop rem edges	NA

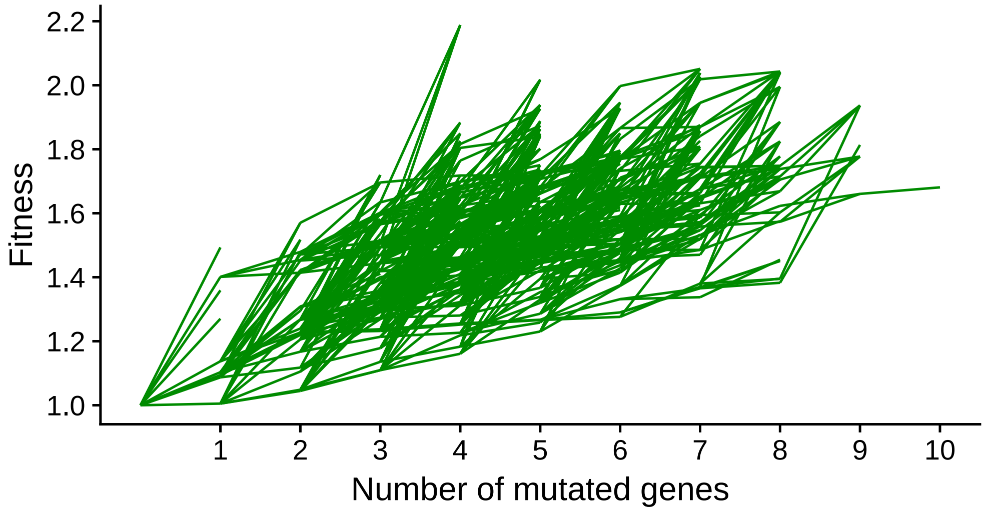

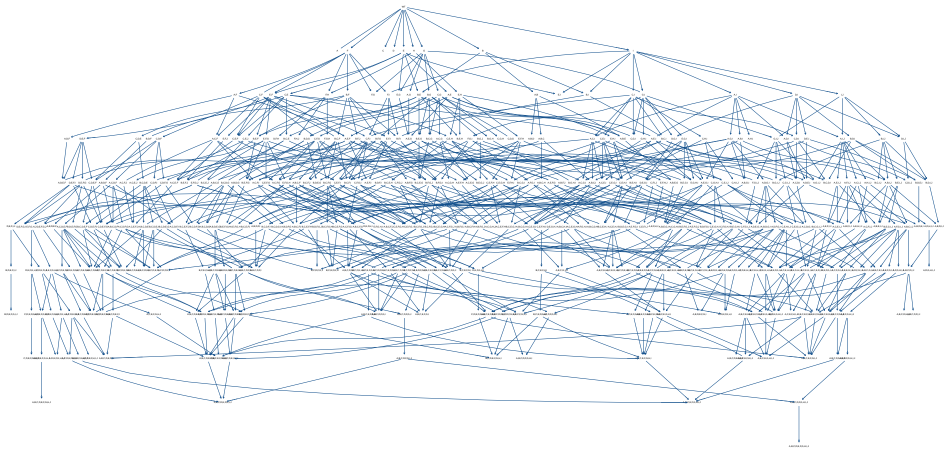

ID	fIL7P19LmiLt1csg
acc genotypes	338
peaks no back	63
removed edges	NA
prop rem edges	NA

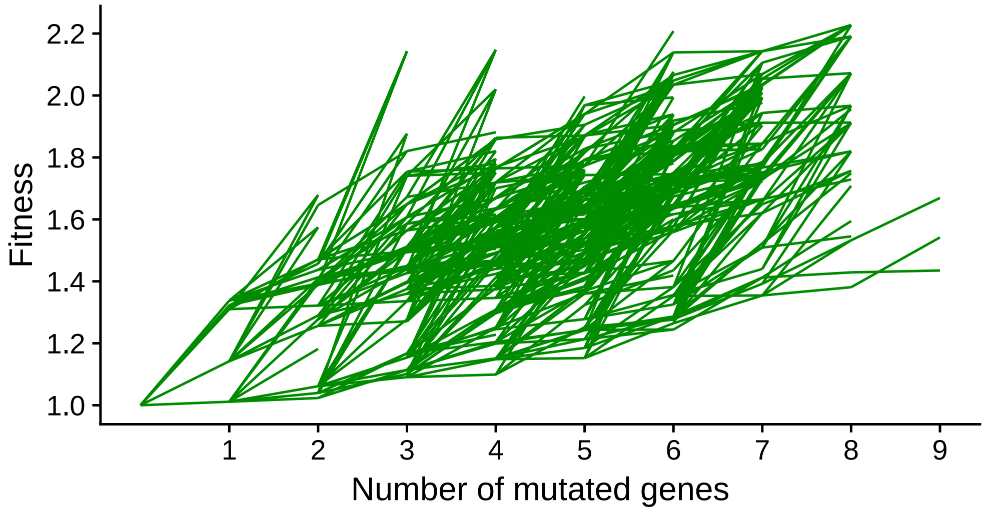

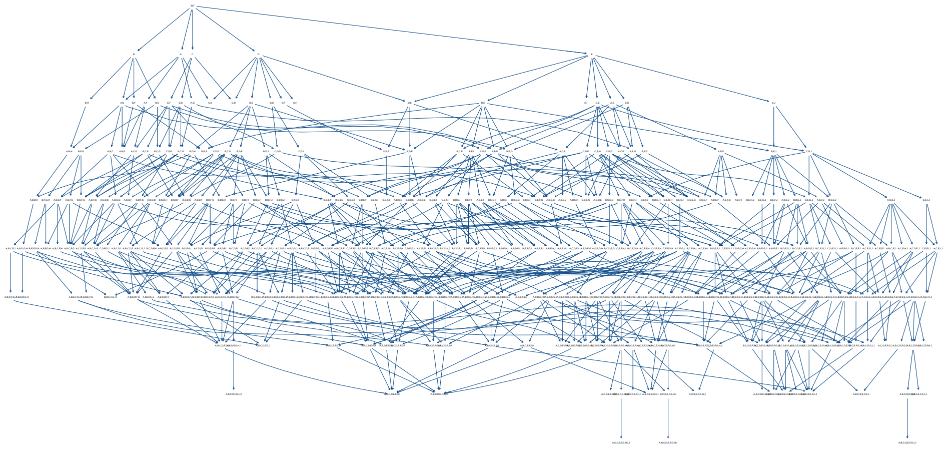

ID	FJPvUaTzze1FXsAz
acc genotypes	459
peaks no back	81
removed edges	NA
prop rem edges	NA

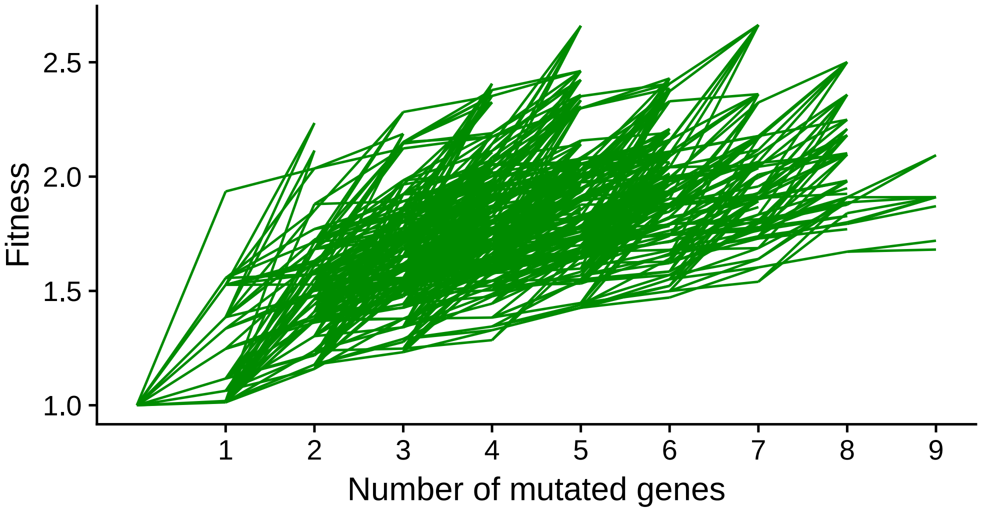

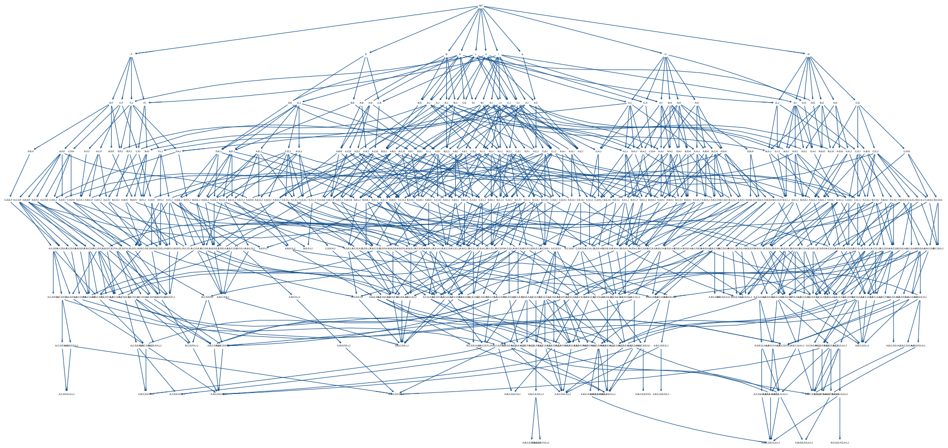

ID	fpSFxSYrkAEuTBdj
acc genotypes	355
peaks no back	123
removed edges	NA
prop rem edges	NA

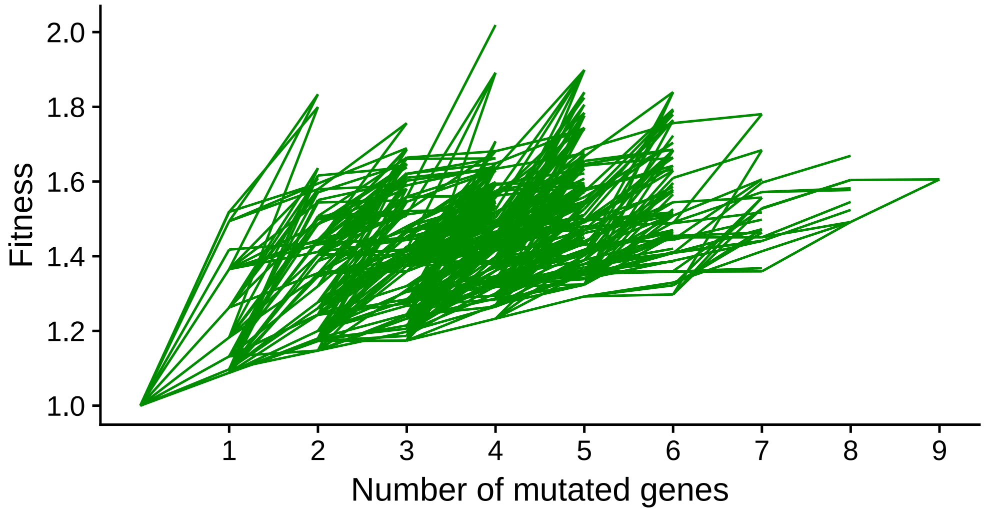

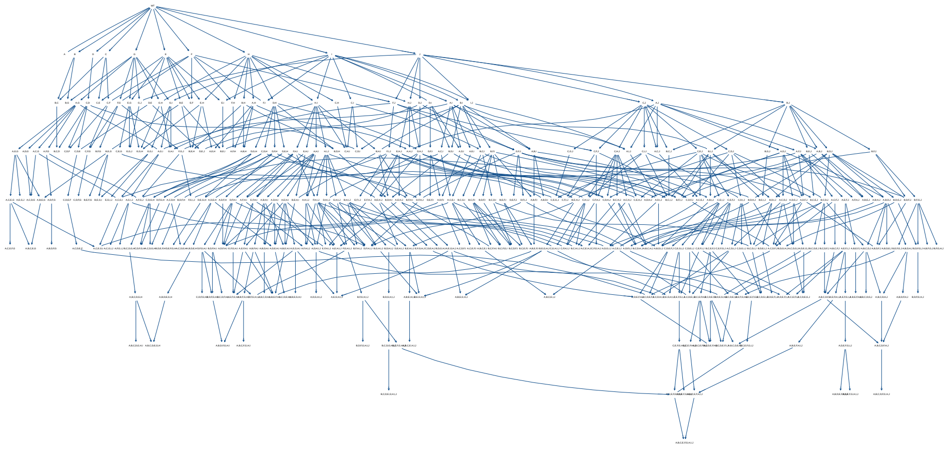

ID	FSw8uHKnSnOw6Xrh
acc genotypes	124
peaks no back	32
removed edges	NA
prop rem edges	NA

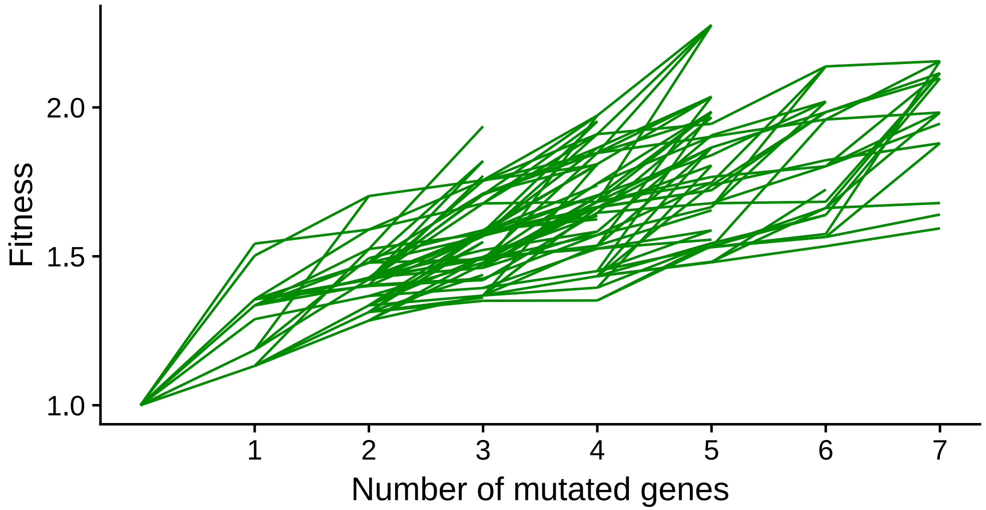

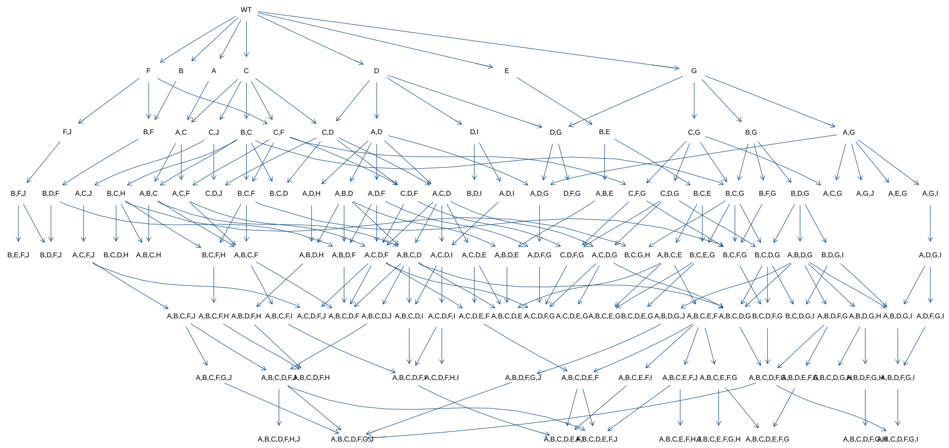

ID	fWEJplAFKvHJAViu
acc genotypes	208
peaks no back	52
removed edges	NA
prop rem edges	NA

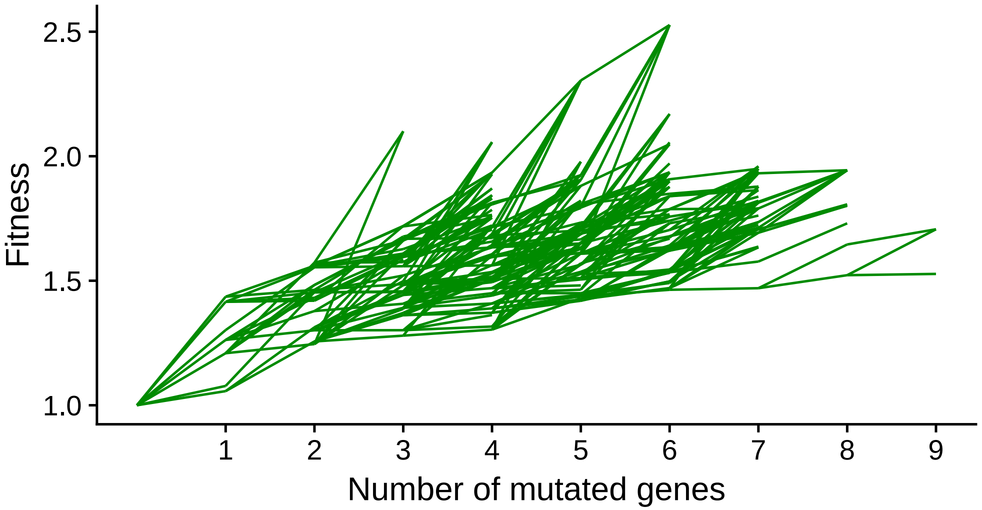

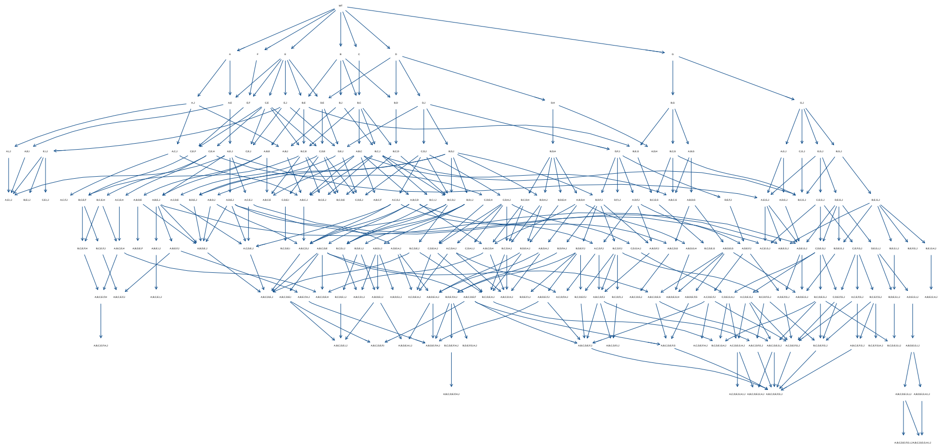

ID g4on7kMYrN7IvT9p
acc genotypes 106
peaks no back 48
removed edges NA
prop rem edges NA

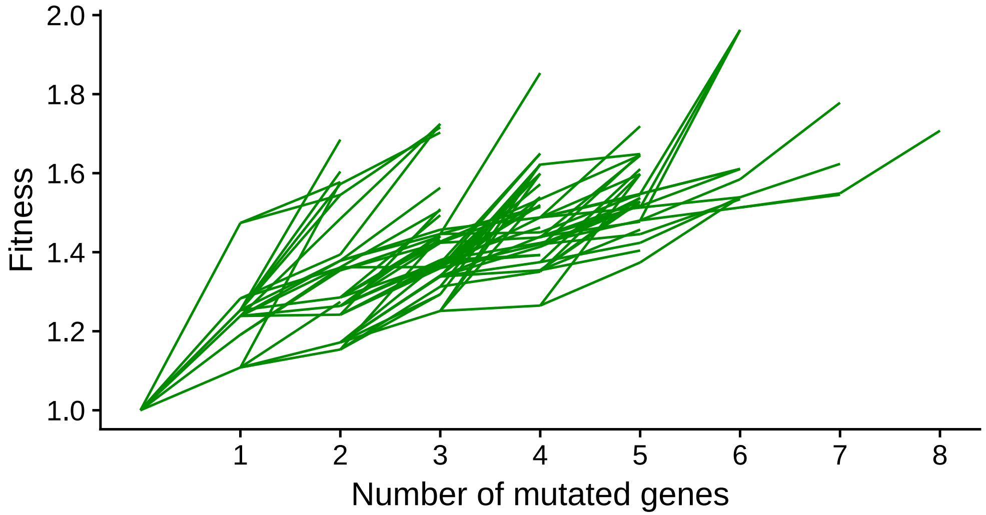

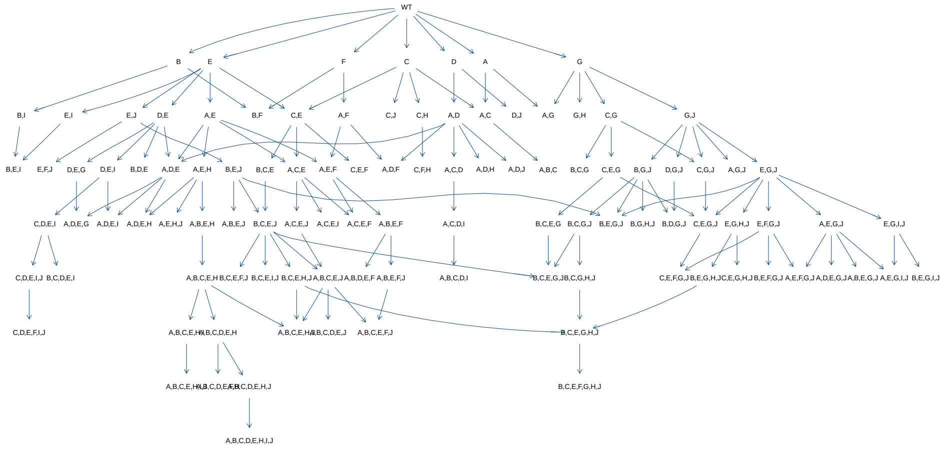

ID	g5rN9RqrUiS9arMn
acc genotypes	39
peaks no back	16
removed edges	NA
prop rem edges	NA

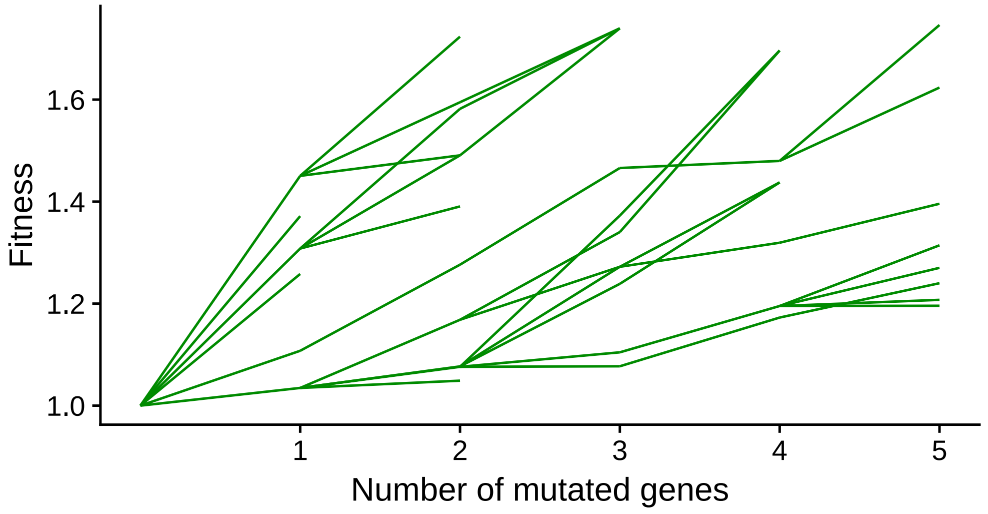

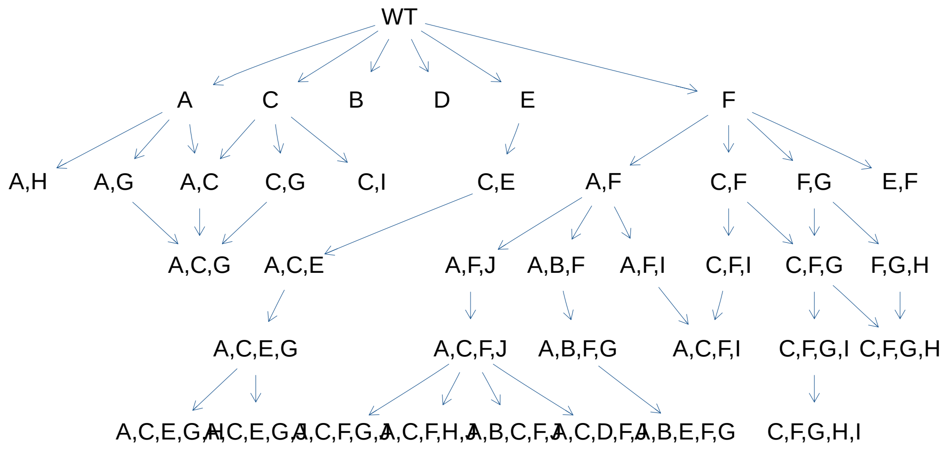

ID	g7EPSPvUEgZmblyv
acc genotypes	282
peaks no back	69
removed edges	NA
prop rem edges	NA

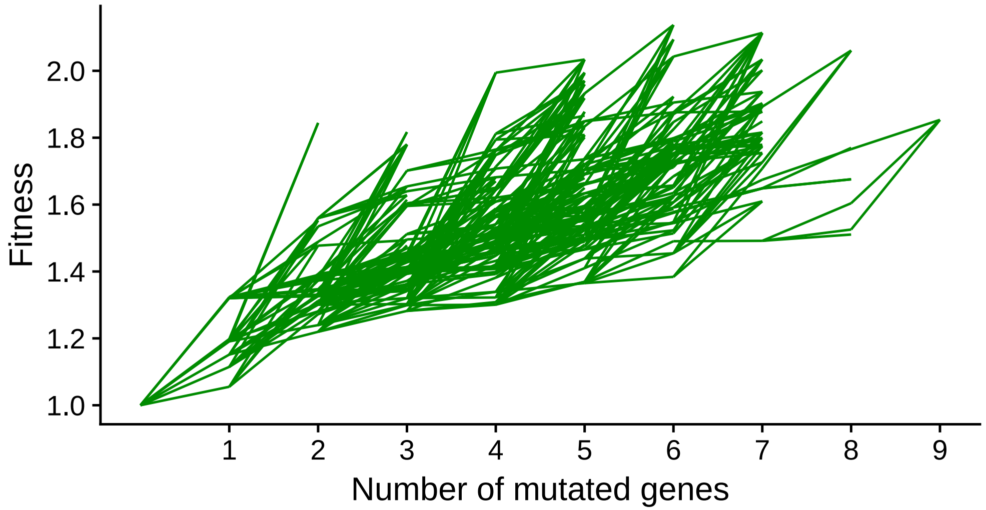

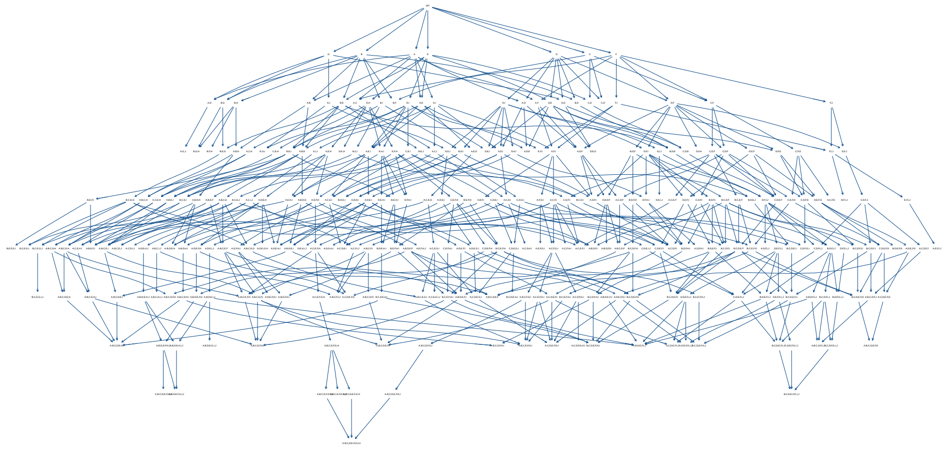

ID	GDPhLnRCnDMjG2D7
acc genotypes	185
peaks no back	64
removed edges	NA
prop rem edges	NA

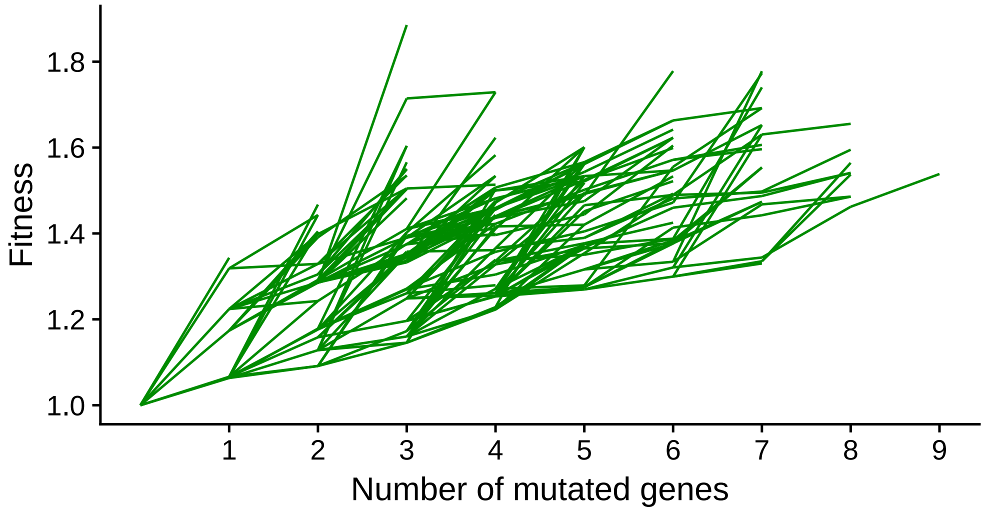

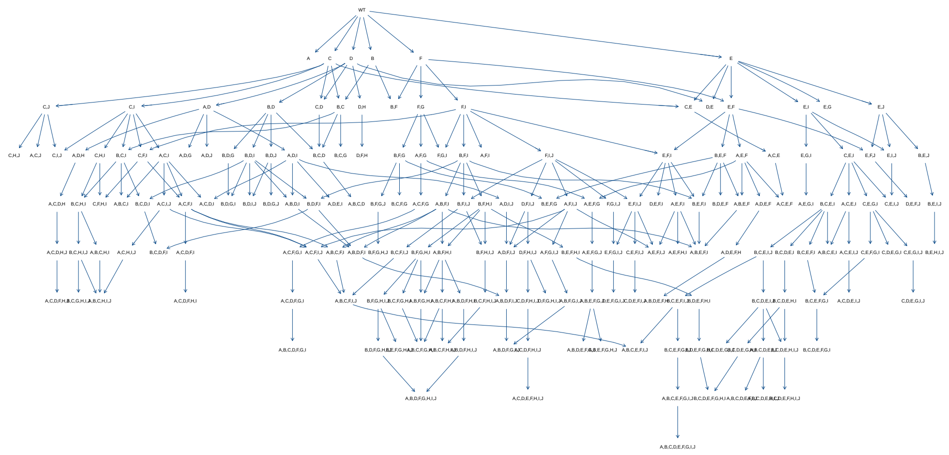

ID	Ggye5iq3jqoH3lvU
acc genotypes	314
peaks no back	112
removed edges	NA
prop rem edges	NA

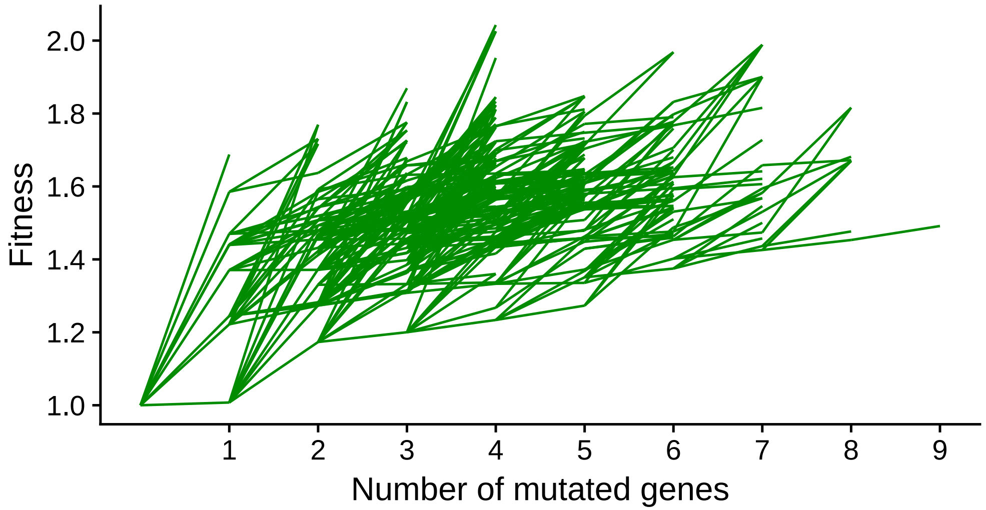

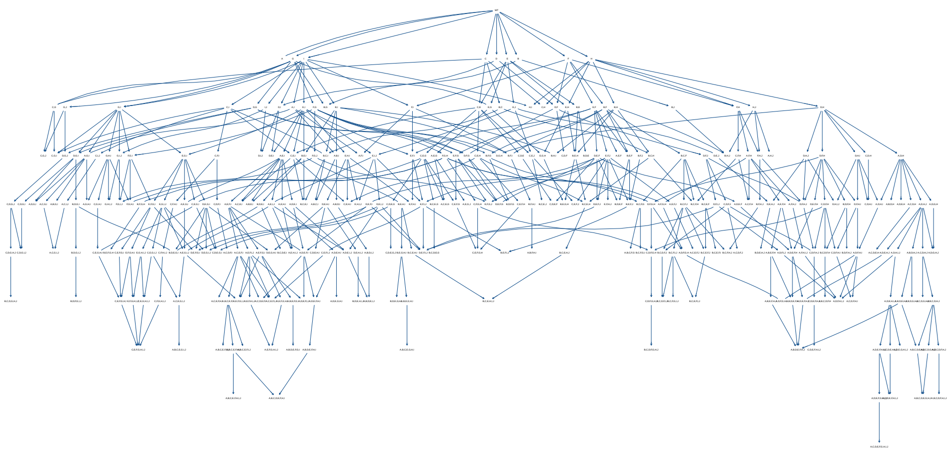

ID	gQ91ynxxLJpJfC10R
acc genotypes	53
peaks no back	23
removed edges	NA
prop rem edges	NA

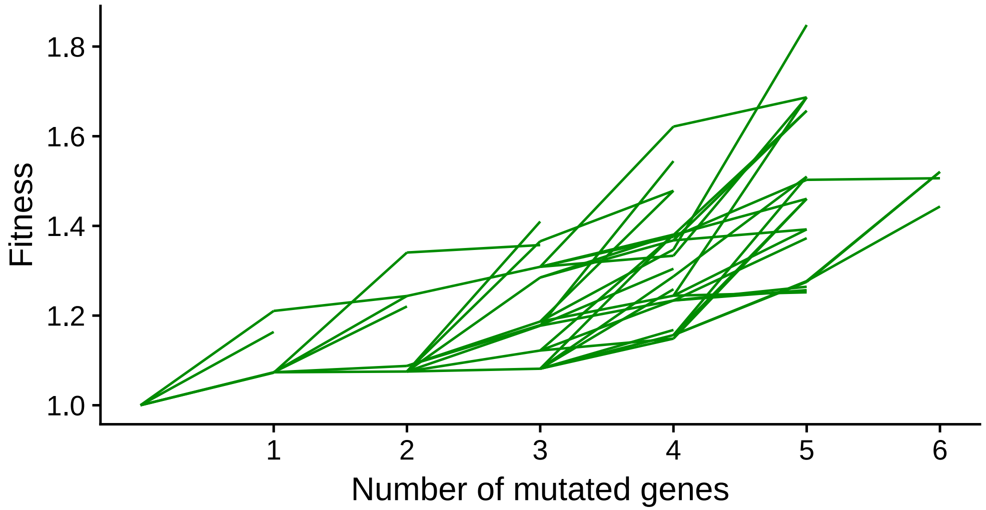

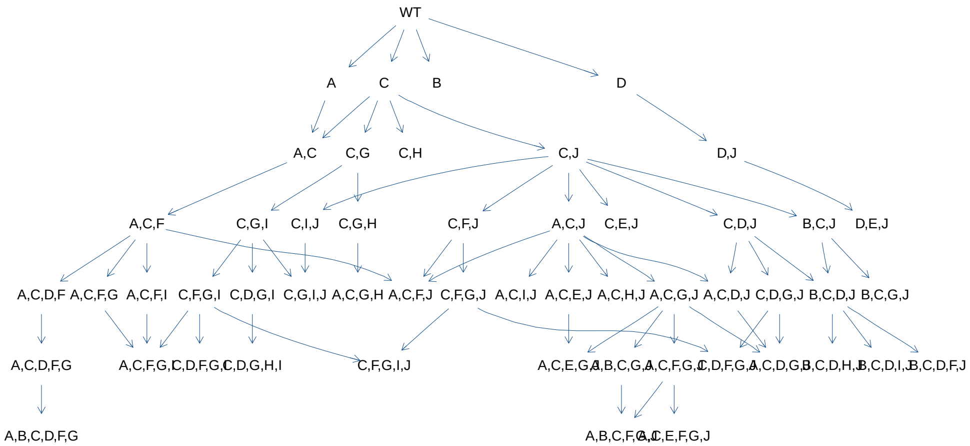

ID	GtsX2ANXEuqX4ryX
acc genotypes	48
peaks no back	21
removed edges	NA
prop rem edges	NA

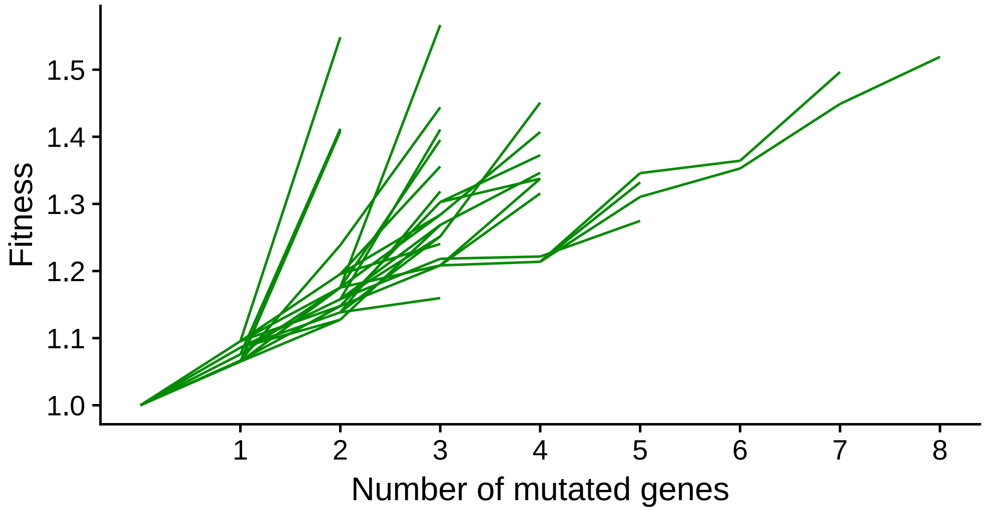

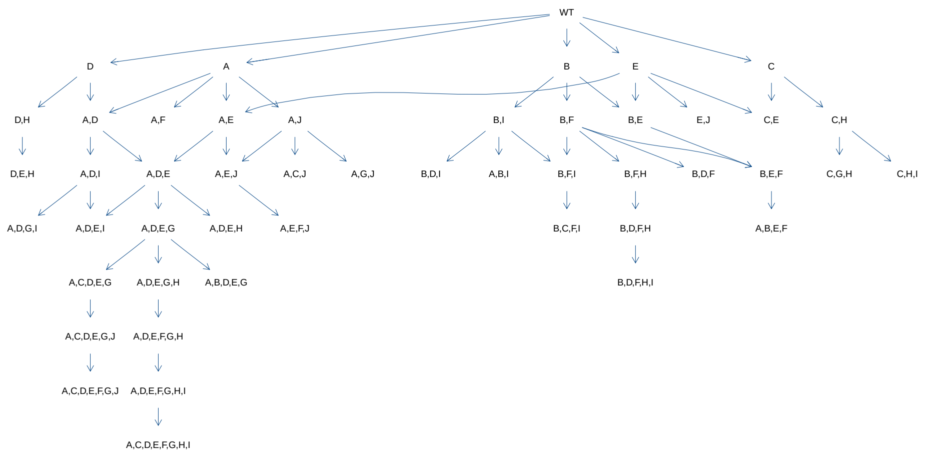

ID	gU6jF3rPQlacxvDT
acc genotypes	198
peaks no back	76
removed edges	NA
prop rem edges	NA

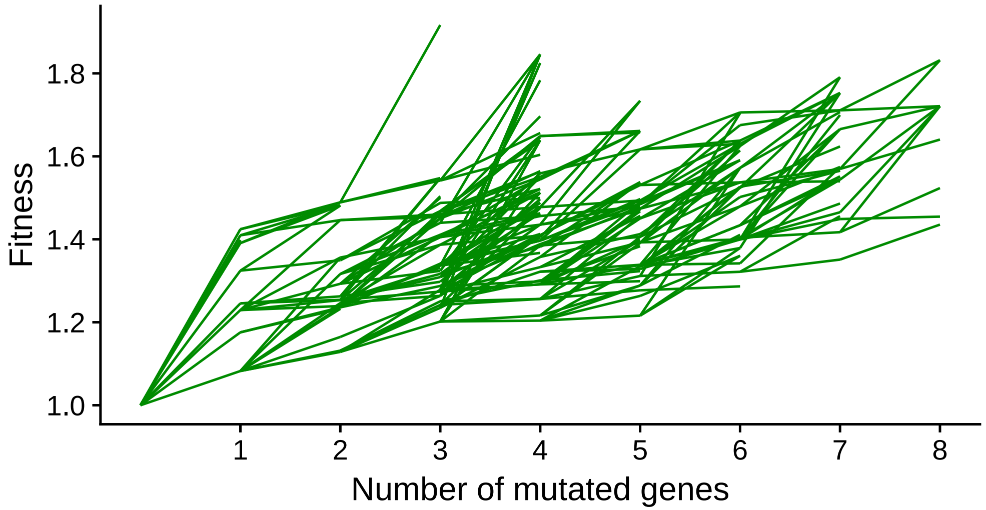

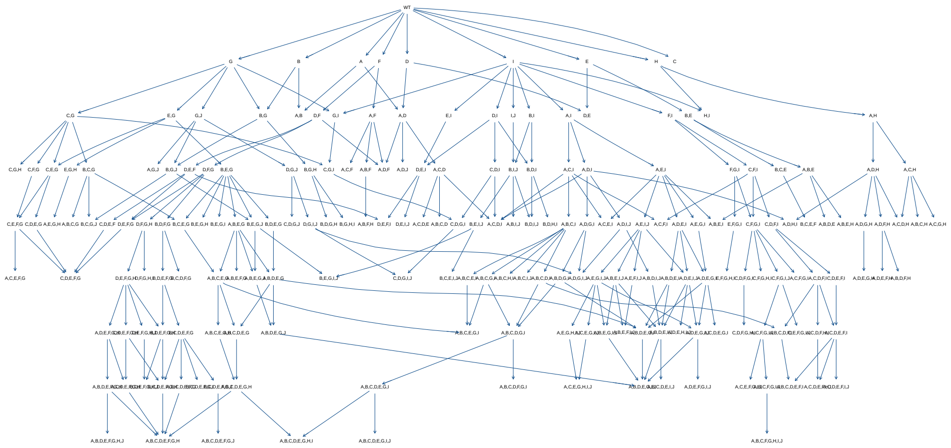

ID	h10Ra4qDED5CSQYCn
acc genotypes	472
peaks no back	79
removed edges	NA
prop rem edges	NA

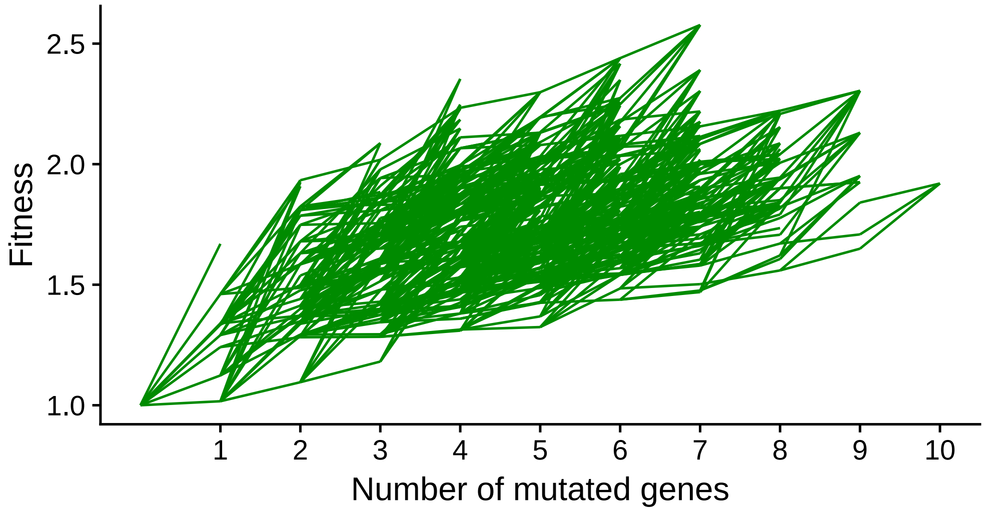

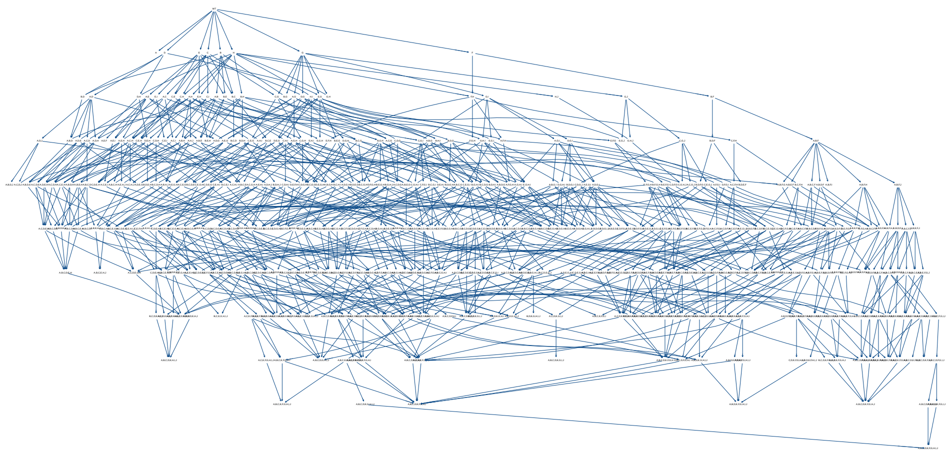

ID	H5F3U9TPZOWztRjG
acc genotypes	501
peaks no back	101
removed edges	NA
prop rem edges	NA

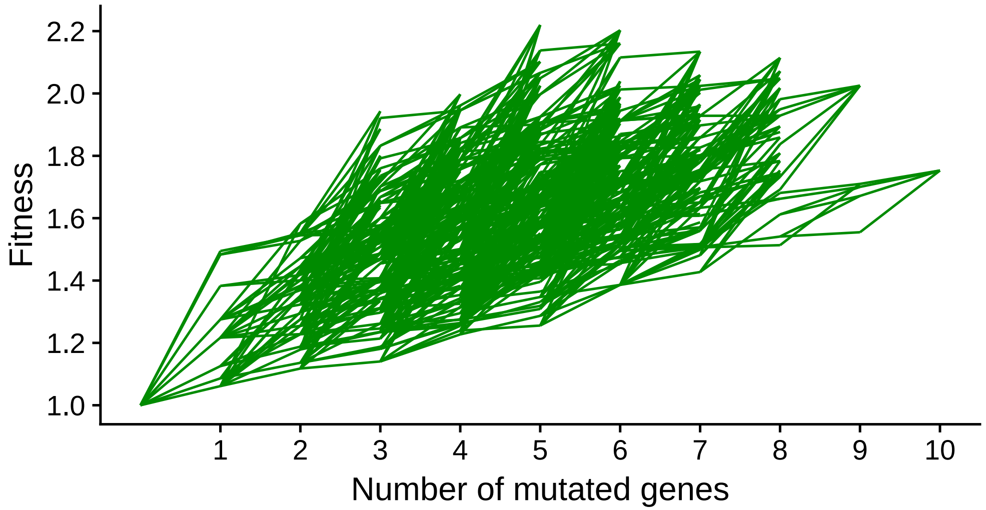

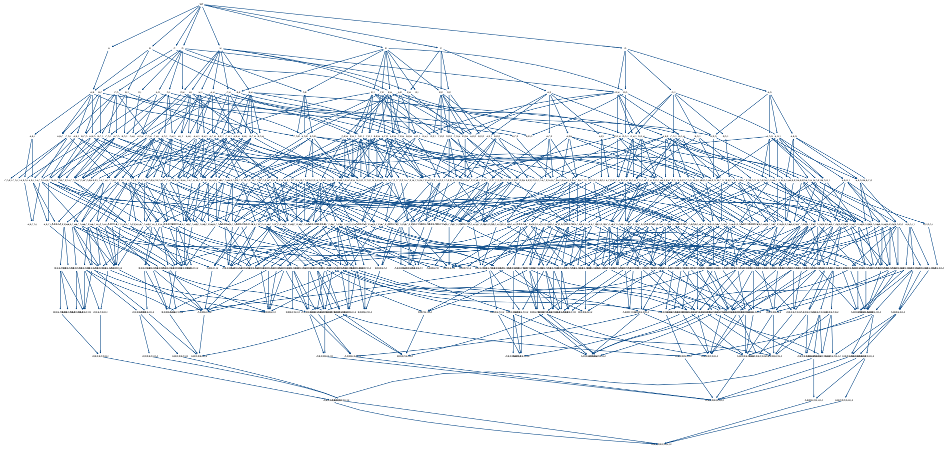

ID	HdswhOaHWIP1kOLZ
acc genotypes	389
peaks no back	95
removed edges	NA
prop rem edges	NA

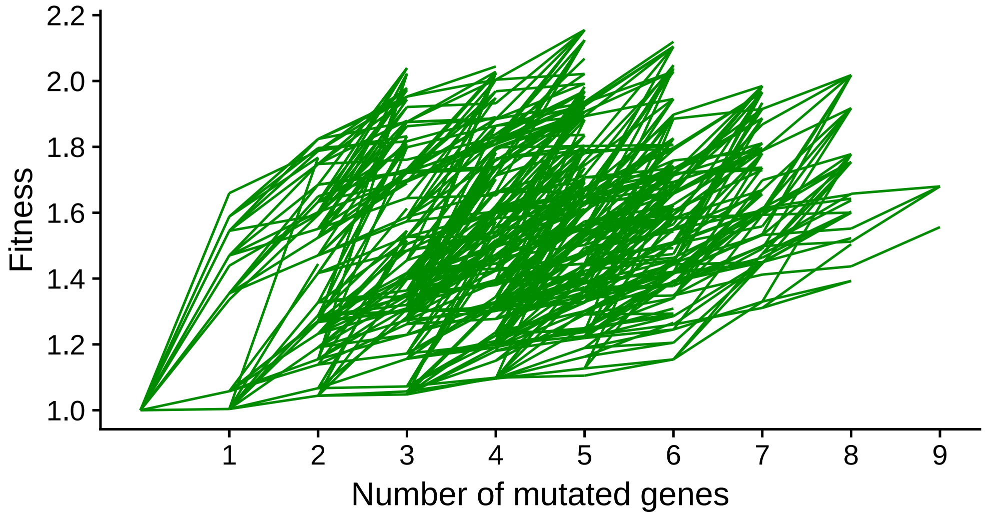

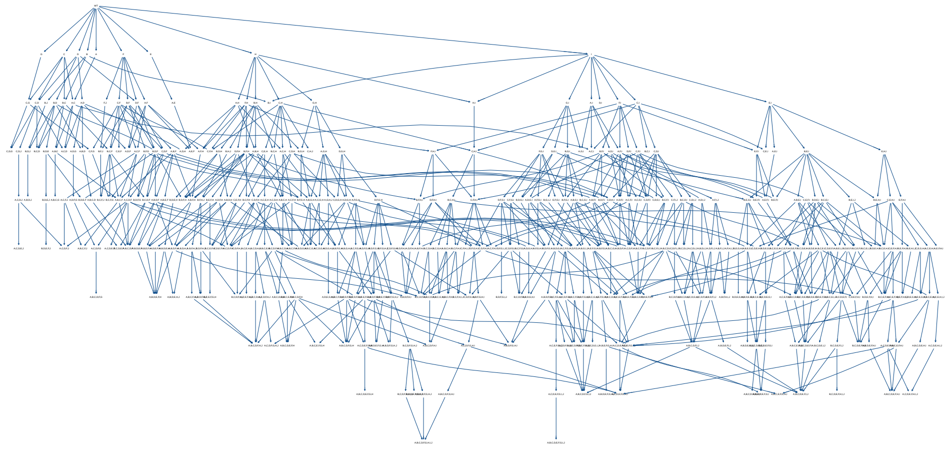

ID	heIHa8Gt8XkXkA6v
acc genotypes	19
peaks no back	8
removed edges	NA
prop rem edges	NA

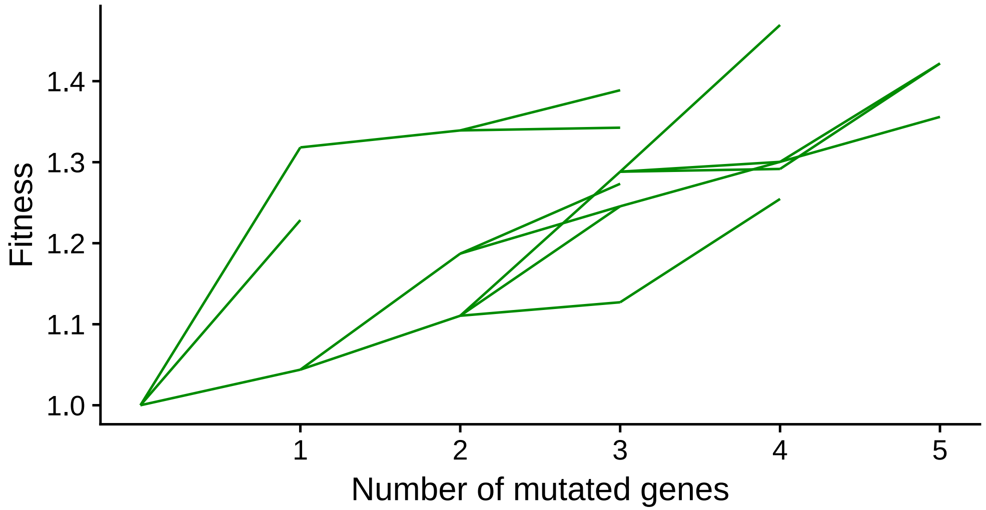

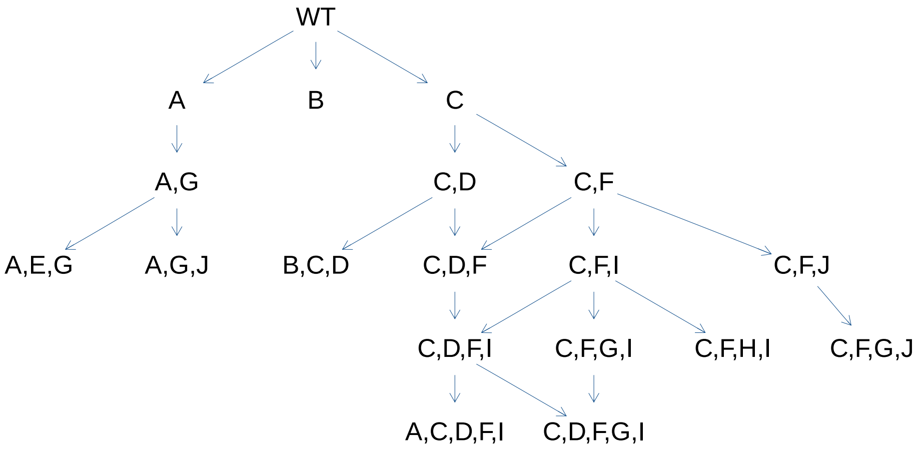

ID	hm10KP8sr2CYeQUji
acc genotypes	309
peaks no back	107
removed edges	NA
prop rem edges	NA

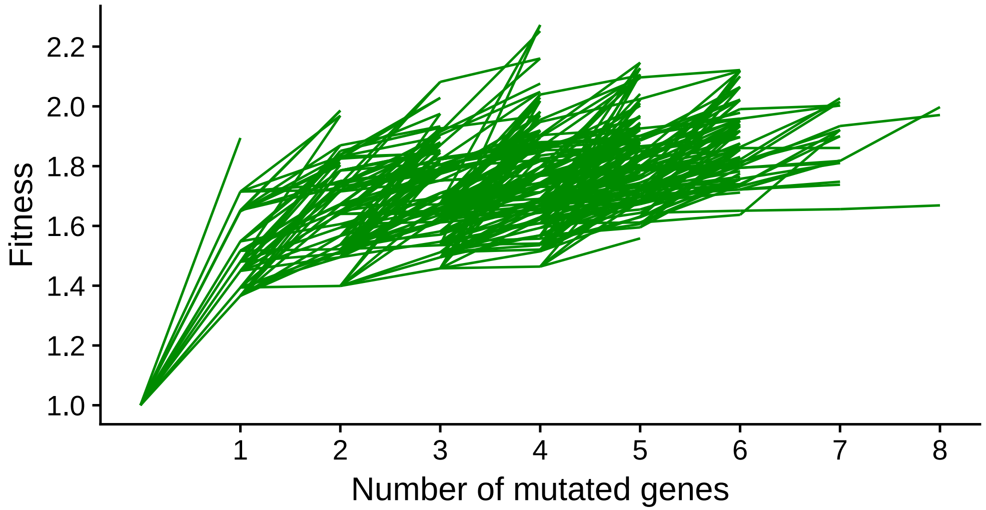

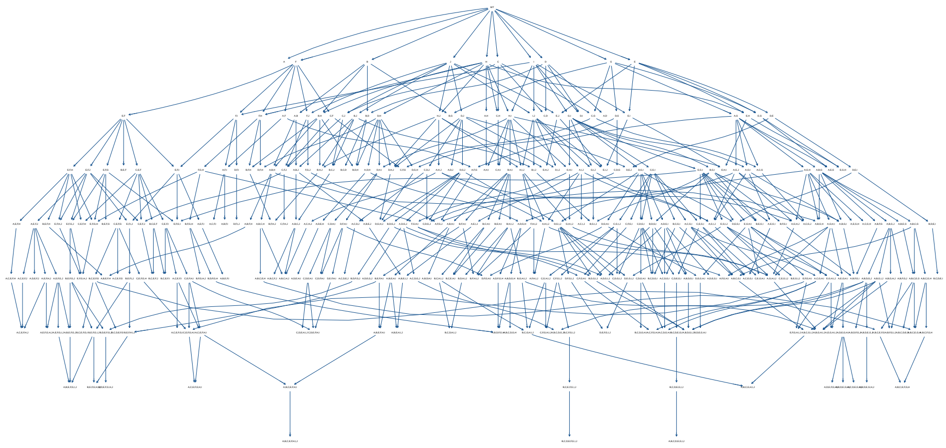

ID	hQhSt6SyCYjTd10y8
acc genotypes	593
peaks no back	84
removed edges	NA
prop rem edges	NA

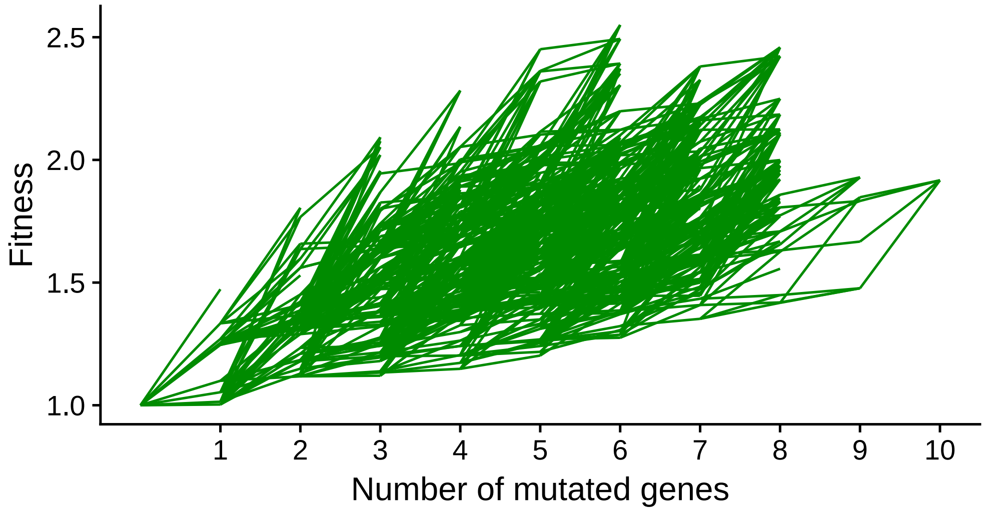

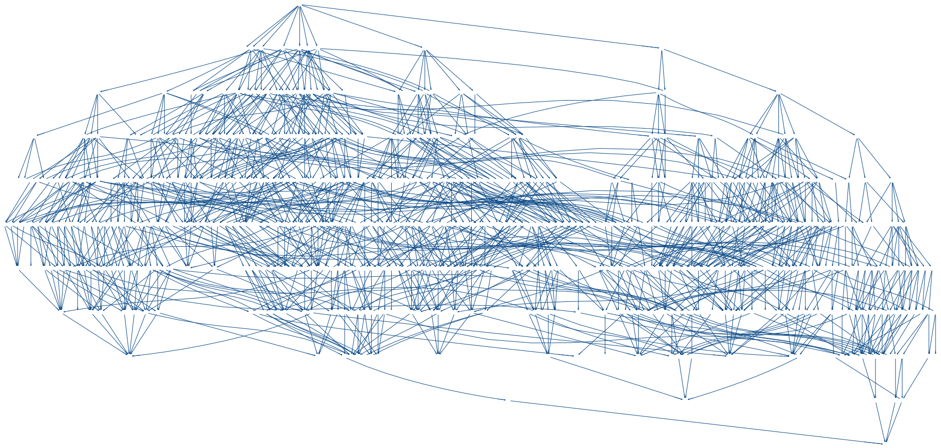

ID	hrKHhSGSCA4NDMJP
acc genotypes	157
peaks no back	43
removed edges	NA
prop rem edges	NA

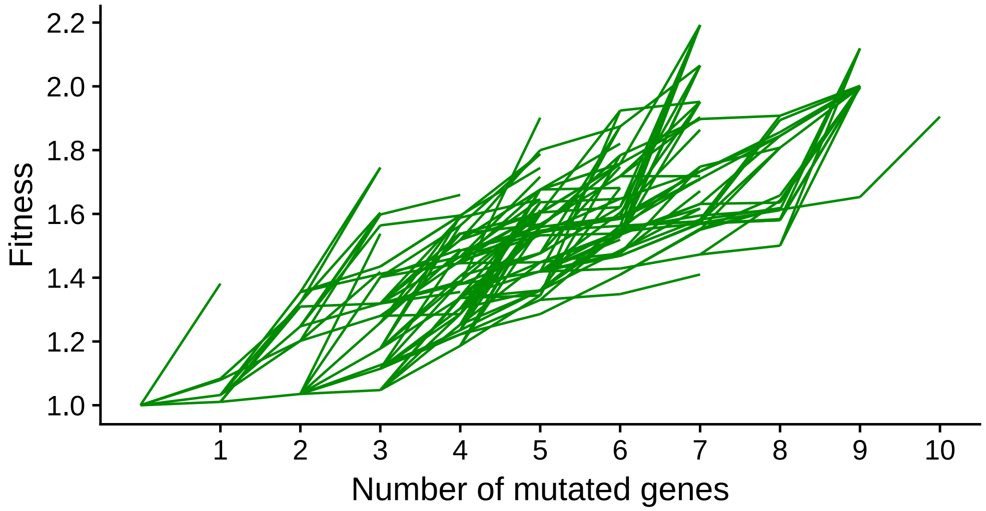

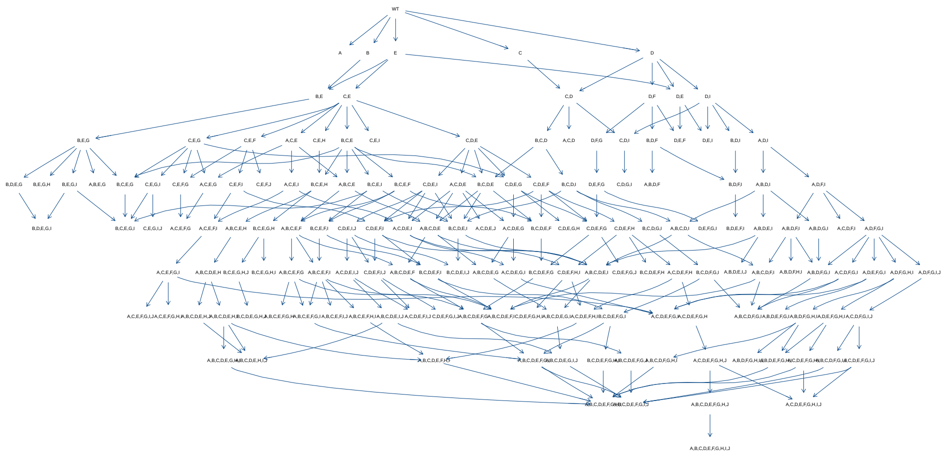

ID	I9q6GI3hisYql2on
acc genotypes	298
peaks no back	79
removed edges	NA
prop rem edges	NA

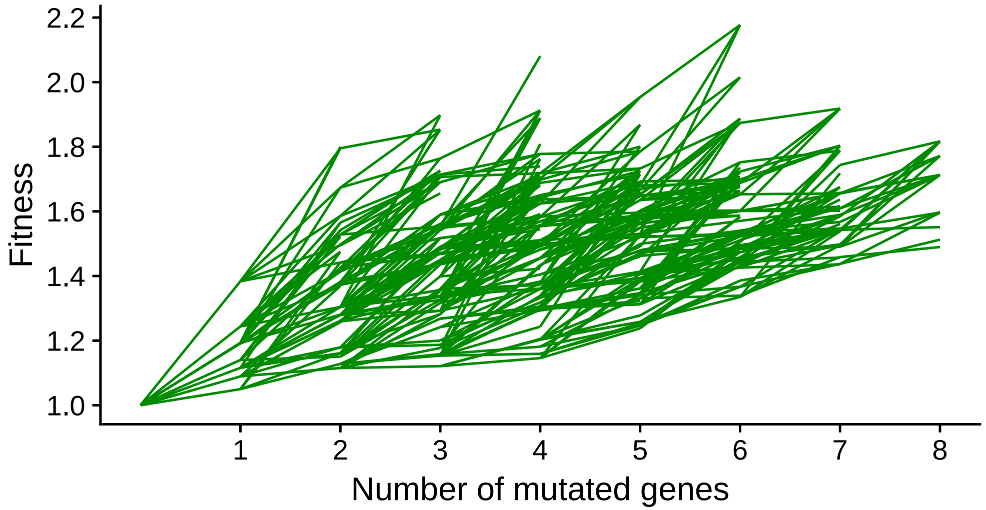

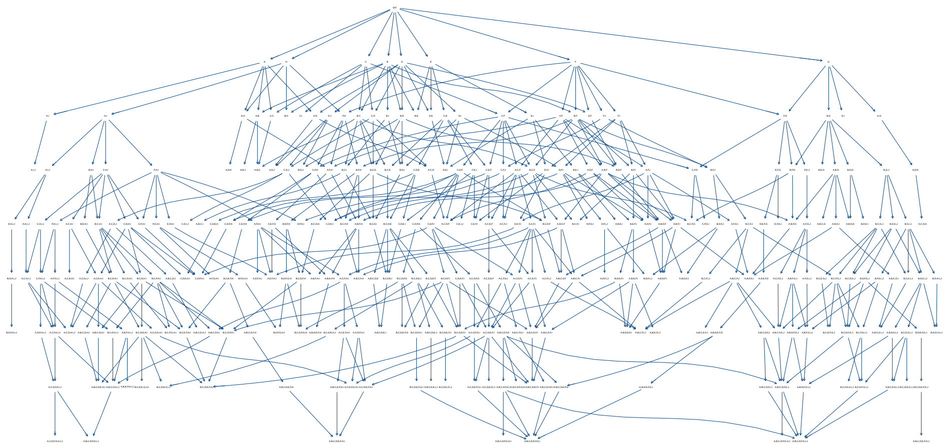

ID	ikuJV711Pr9P5gMR
acc genotypes	54
peaks no back	25
removed edges	NA
prop rem edges	NA

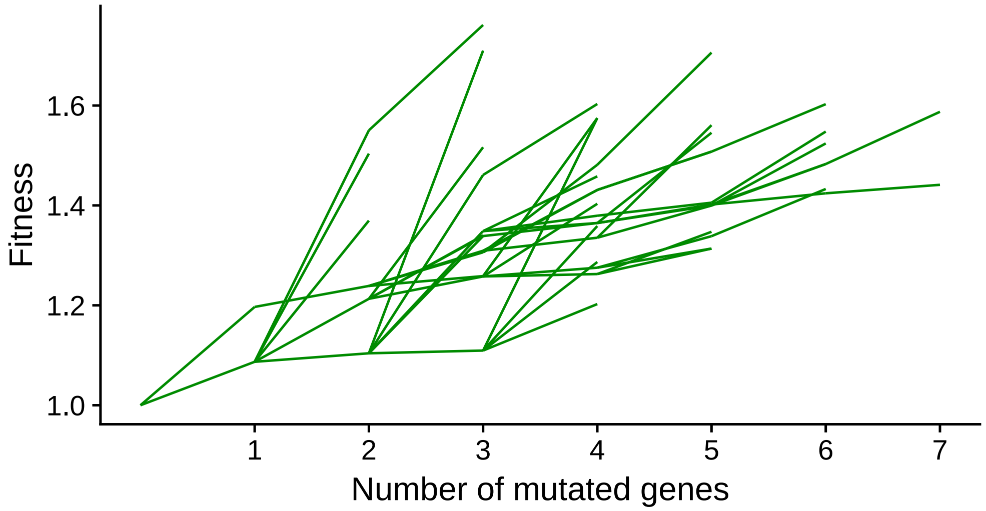

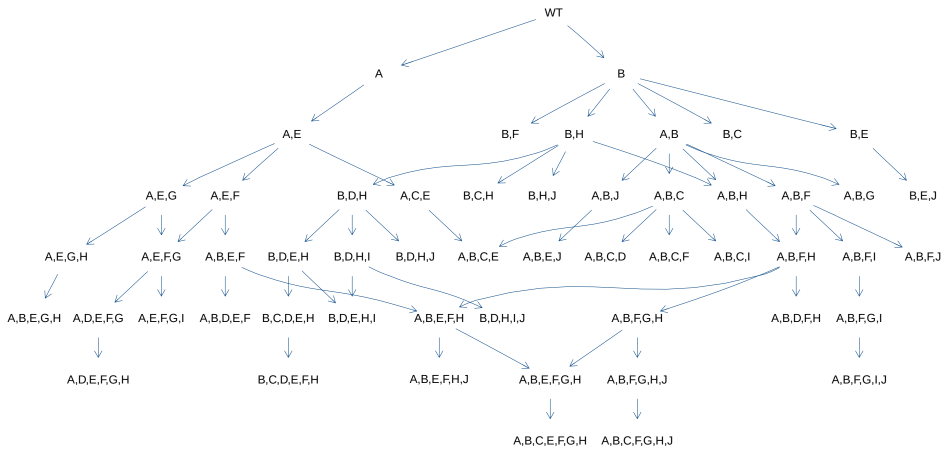

ID	irlmocvl1nEJmK32
acc genotypes	95
peaks no back	46
removed edges	NA
prop rem edges	NA

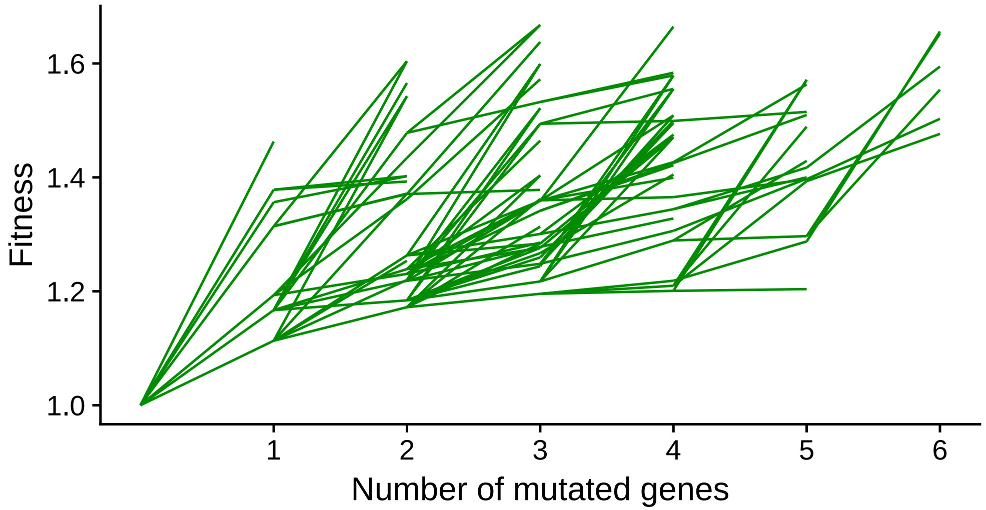

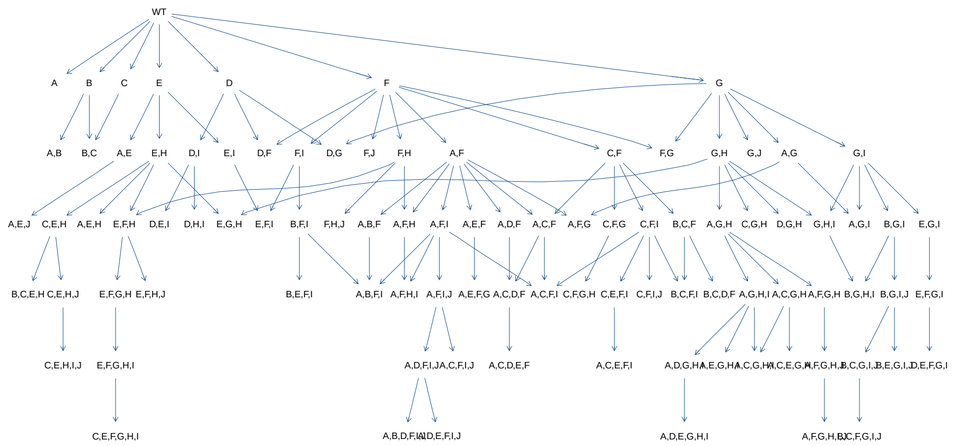

ID	itu7sgxZ42Rqmcbw
acc genotypes	140
peaks no back	36
removed edges	NA
prop rem edges	NA

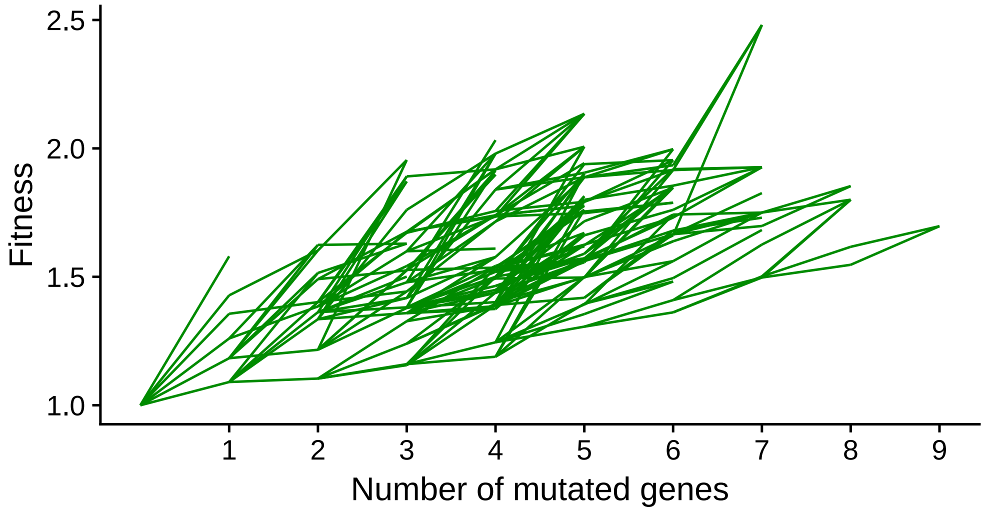

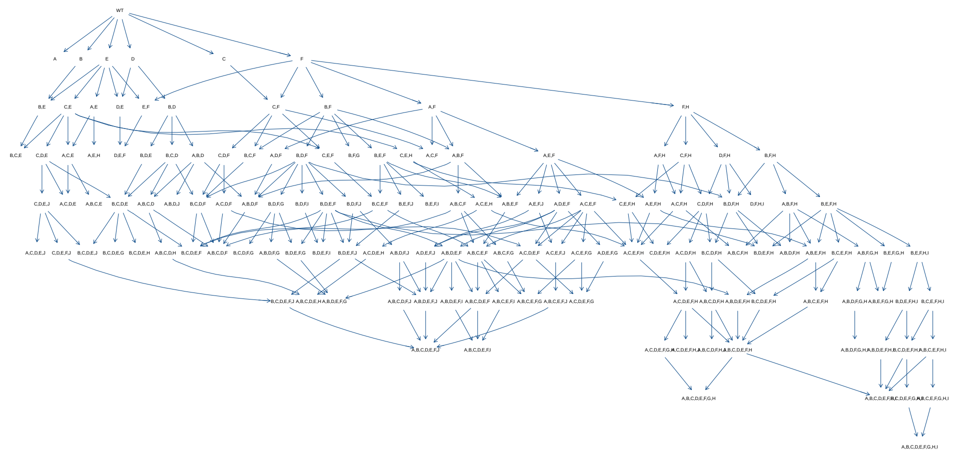

ID	IxTaa10yvF9m8psni
acc genotypes	188
peaks no back	61
removed edges	NA
prop rem edges	NA

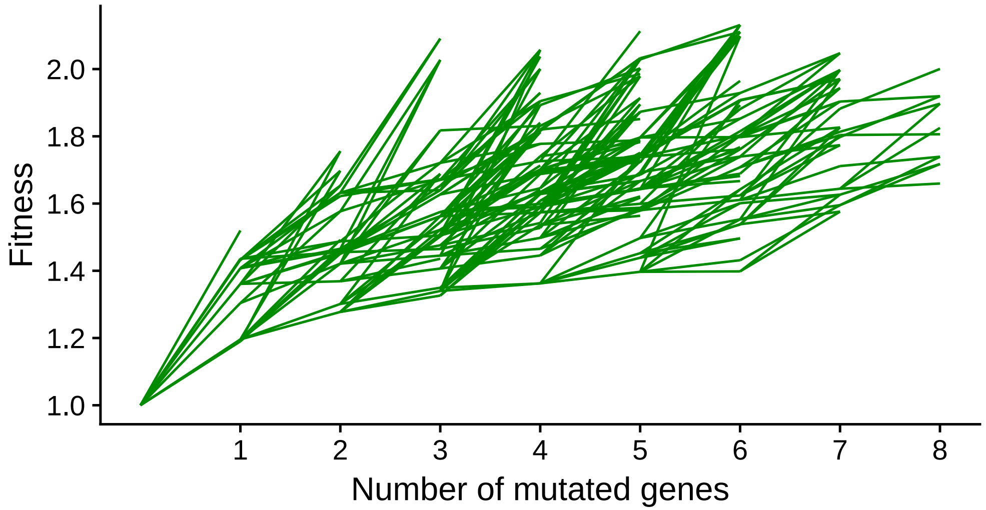

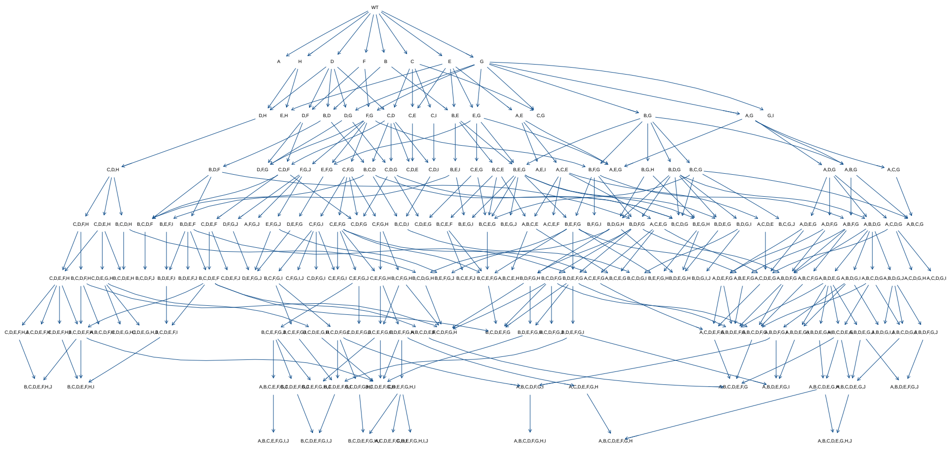

ID	j5Wlsy8xtbP8JPCd
acc genotypes	115
peaks no back	54
removed edges	NA
prop rem edges	NA

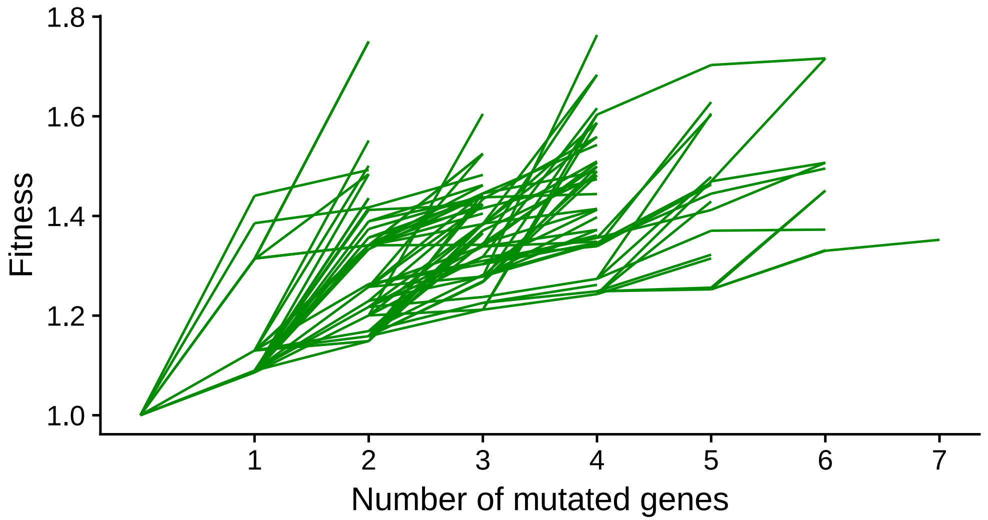

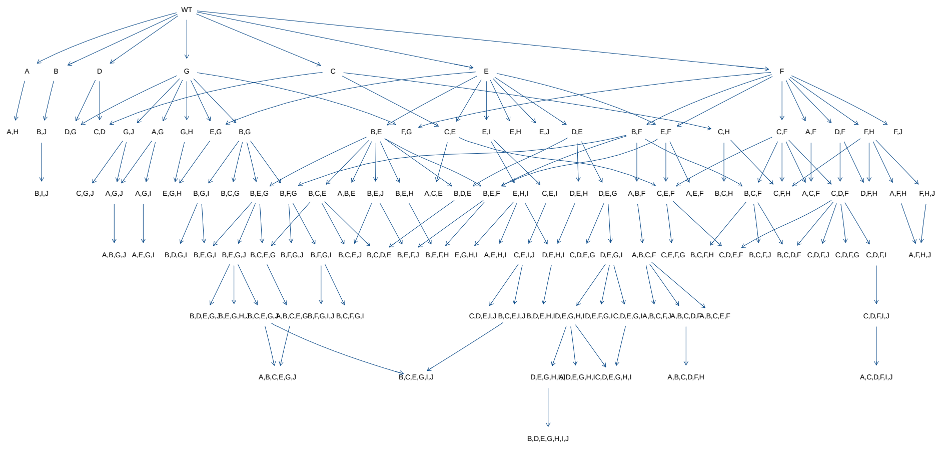

ID	J6cFc1nMWhianSvF
acc genotypes	189
peaks no back	73
removed edges	NA
prop rem edges	NA

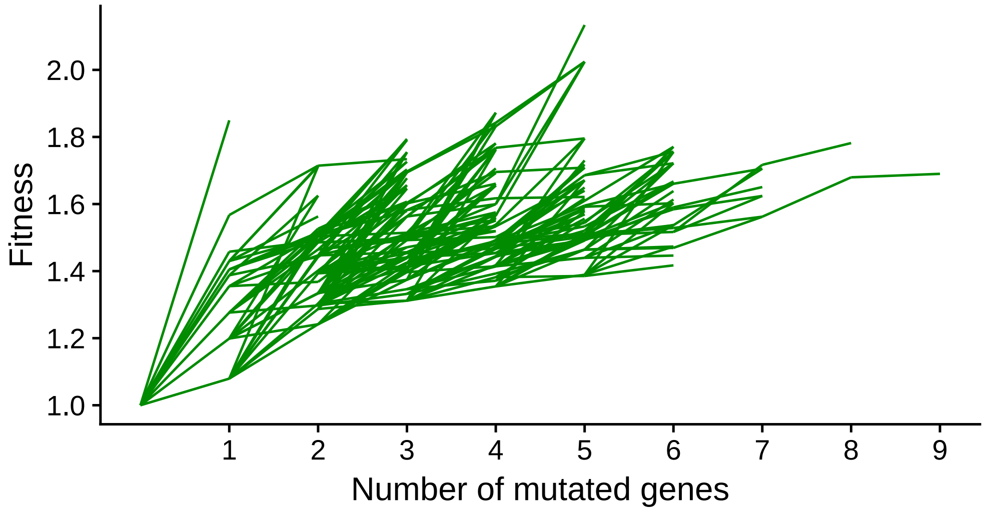

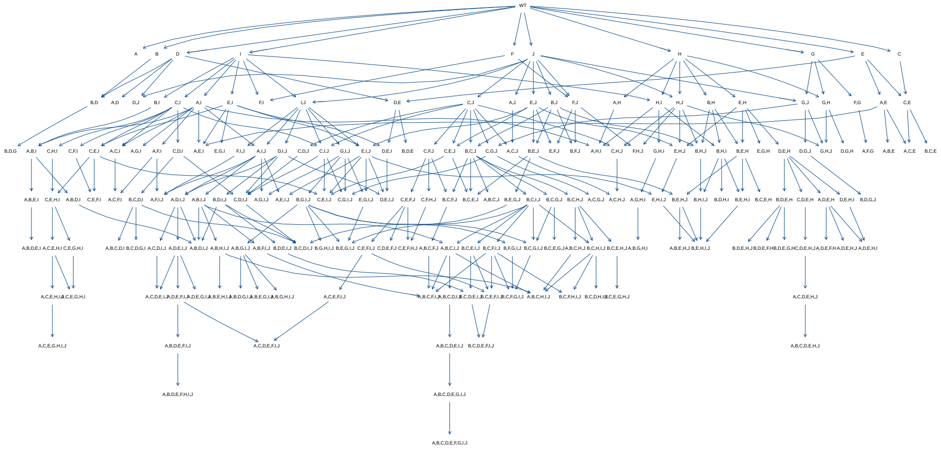

ID	jANbFMNMm7HJj8B10
acc genotypes	735
peaks no back	93
removed edges	NA
prop rem edges	NA

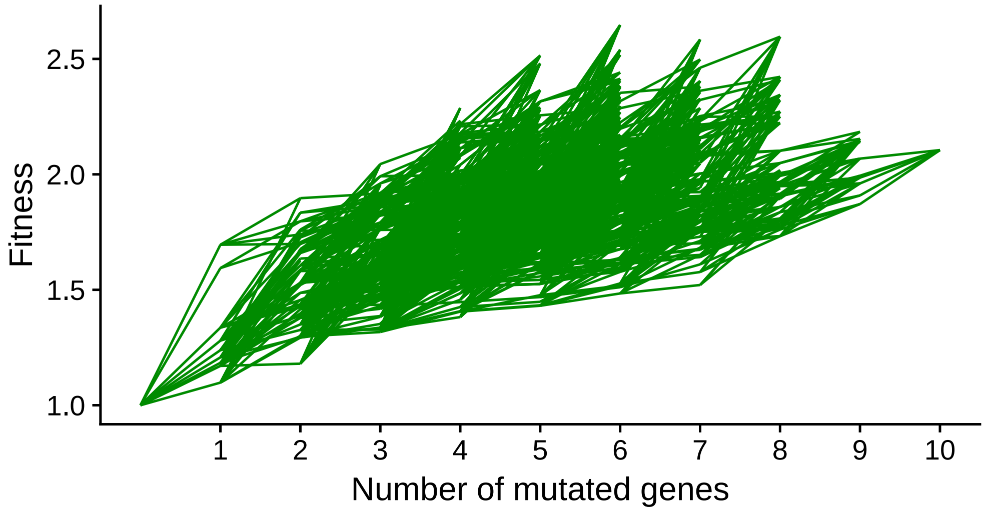

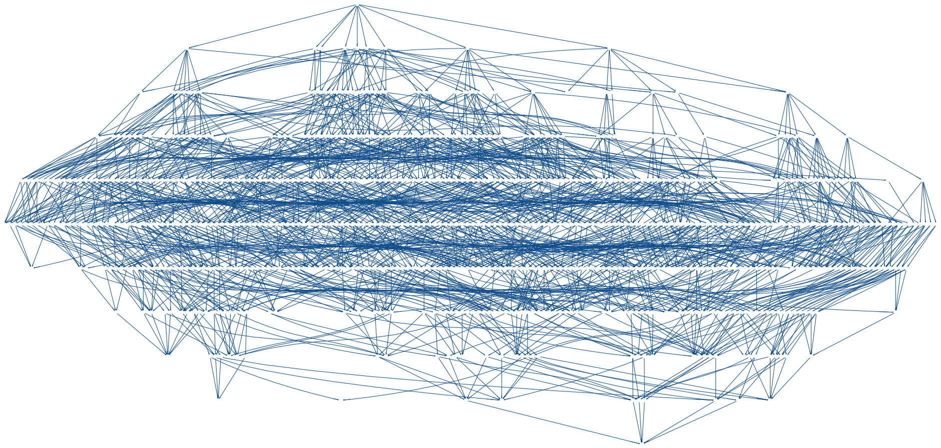

ID	jQBPZk8ZlI14NJWW
acc genotypes	88
peaks no back	31
removed edges	NA
prop rem edges	NA

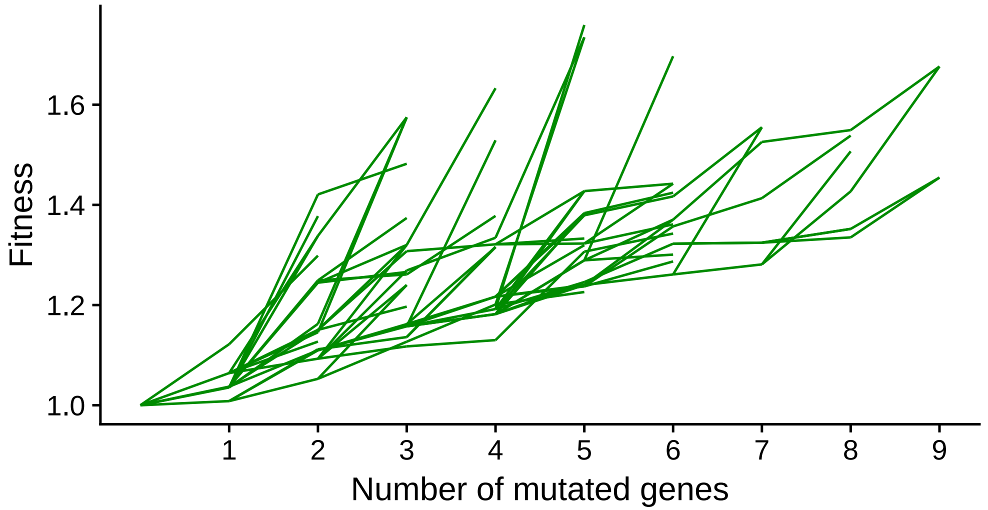

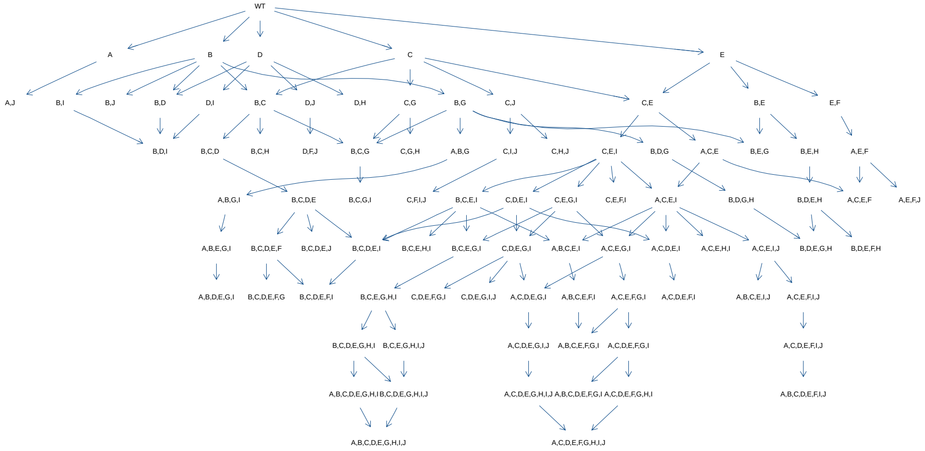

ID	K4fDyrolukDwi9Ma
acc genotypes	145
peaks no back	53
removed edges	NA
prop rem edges	NA

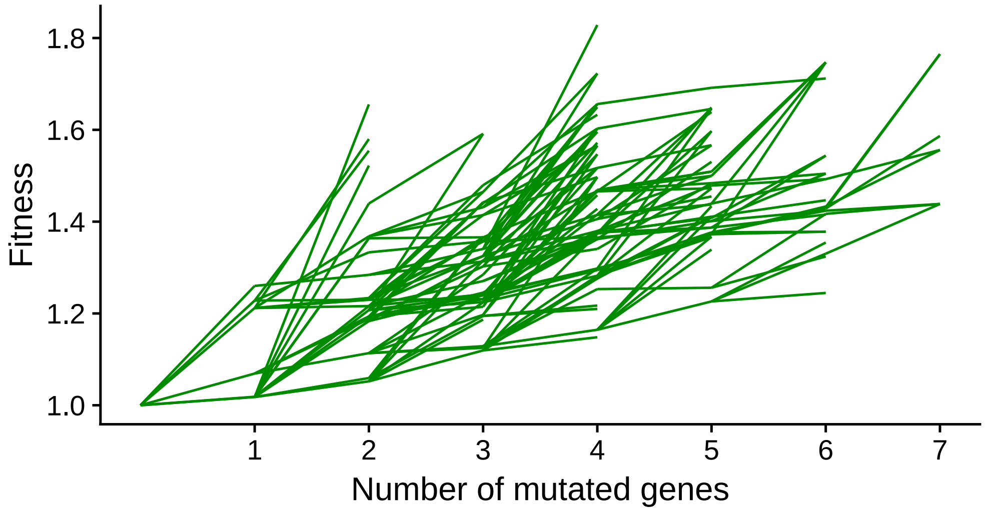

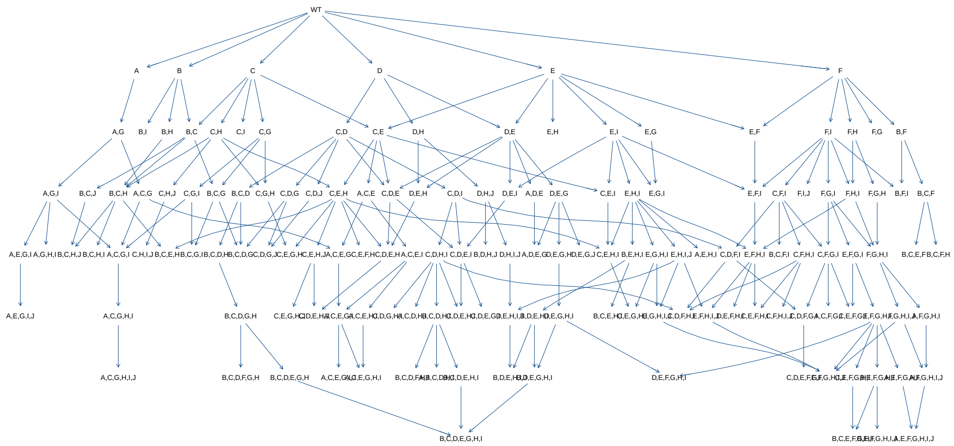

ID	k4Otptn3TVpfZN7m
acc genotypes	166
peaks no back	64
removed edges	NA
prop rem edges	NA

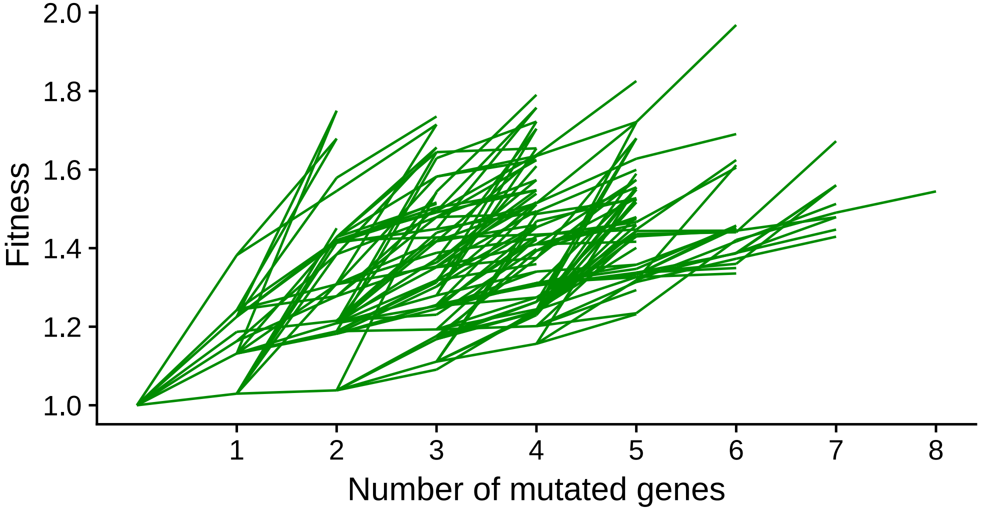

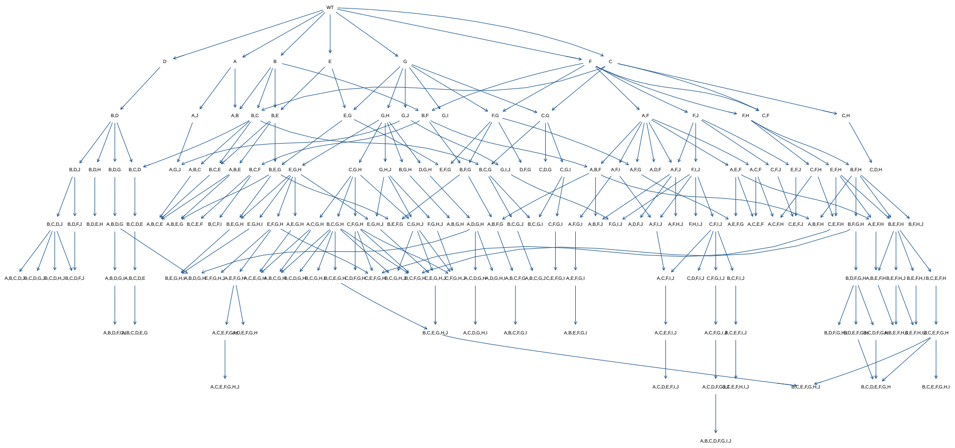

ID	k9EeYCy4KVgVd19I
acc genotypes	82
peaks no back	35
removed edges	NA
prop rem edges	NA

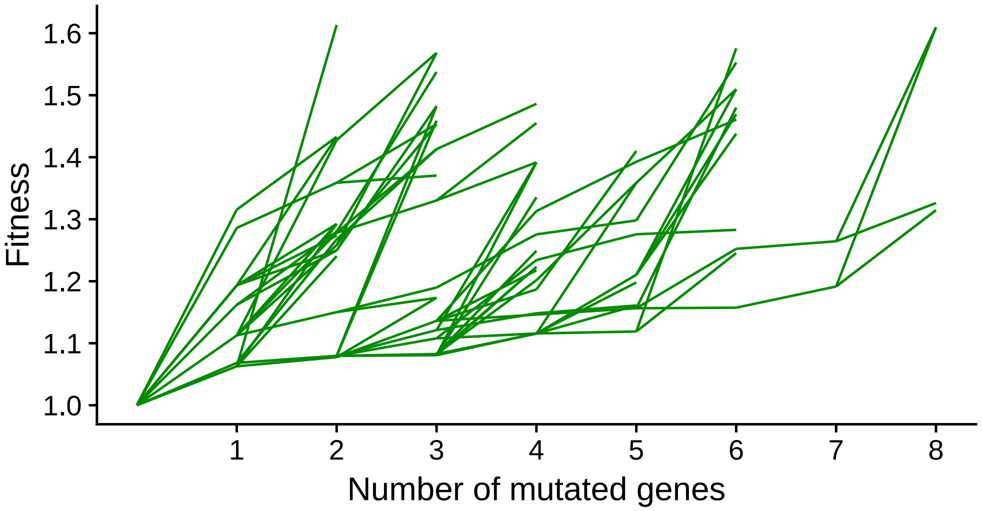

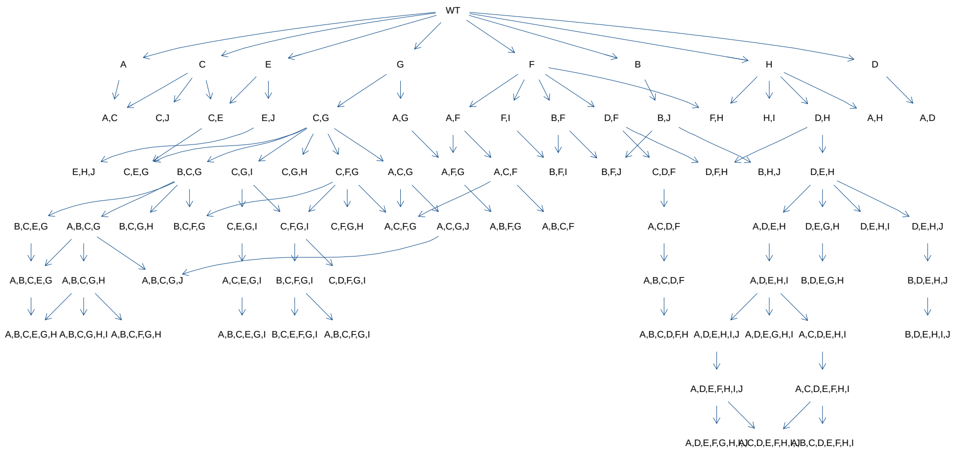

ID	KB10V10fR8eSnvo1Wn
acc genotypes	255
peaks no back	59
removed edges	NA
prop rem edges	NA

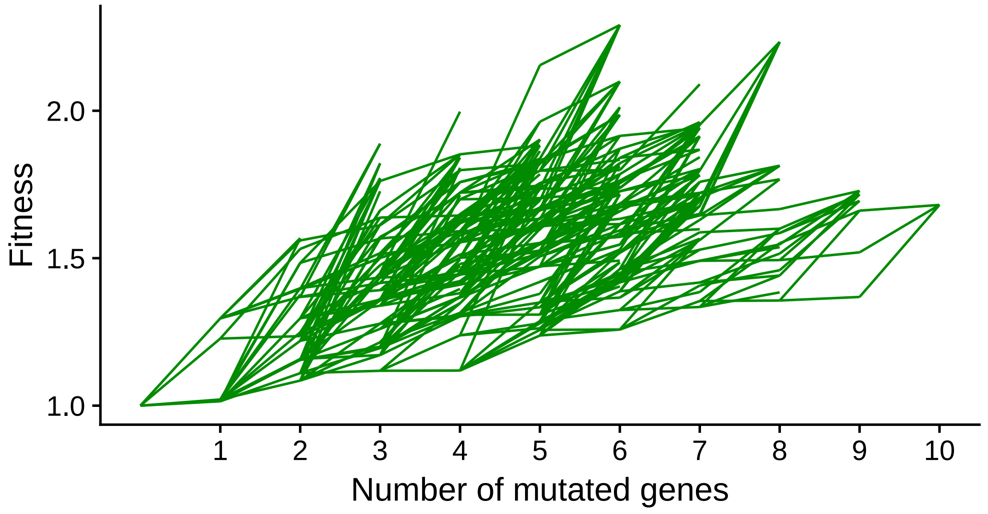

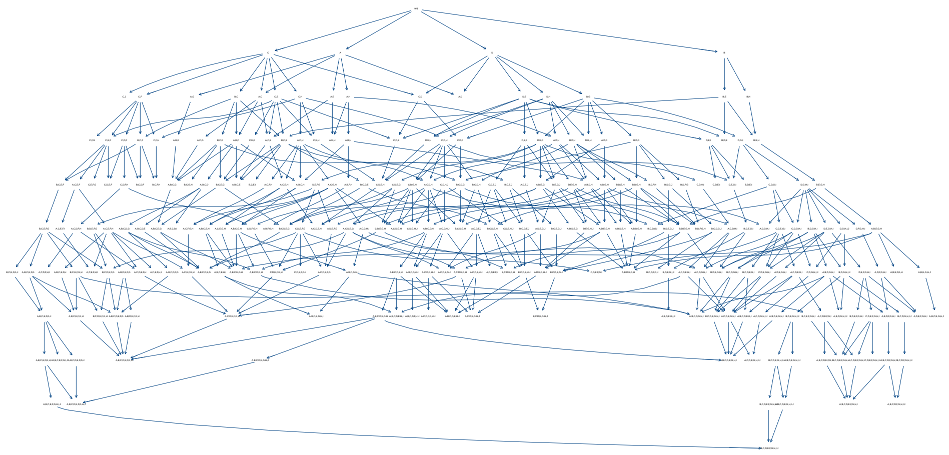

ID	kEEjEwfuinUR9Smg
acc genotypes	707
peaks no back	92
removed edges	NA
prop rem edges	NA

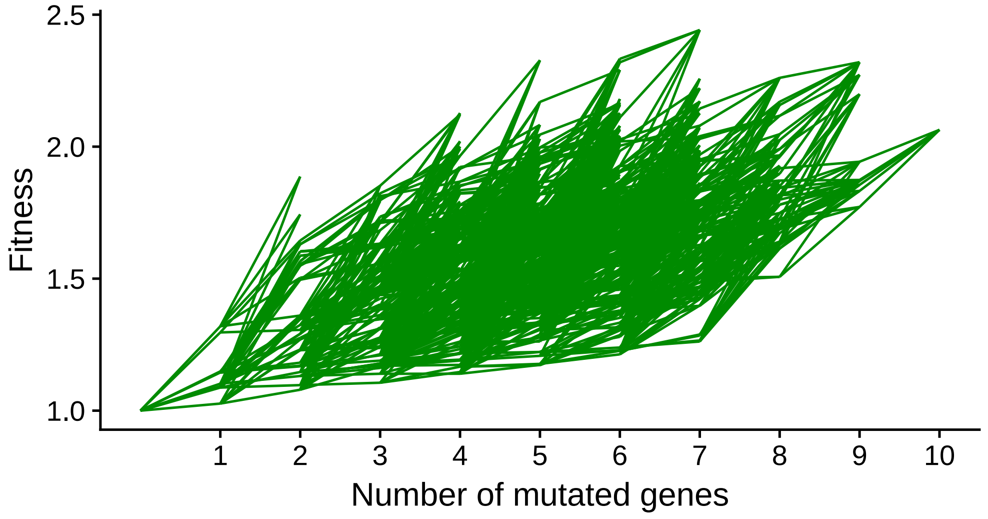

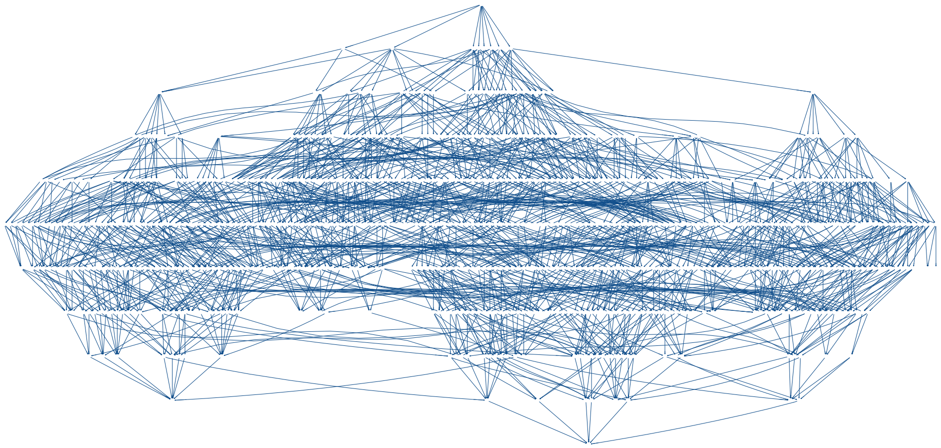

ID	kfHftKfTcY9lyTX3
acc genotypes	207
peaks no back	79
removed edges	NA
prop rem edges	NA

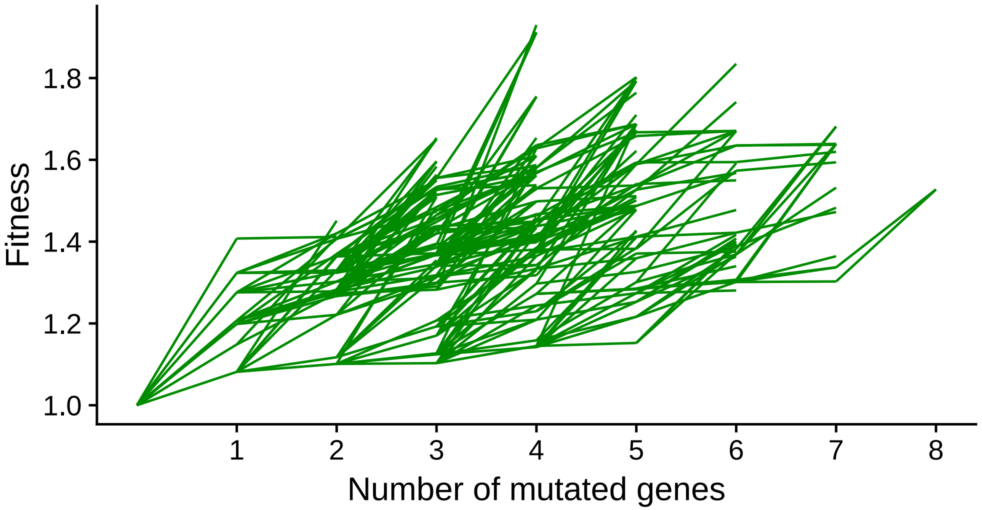

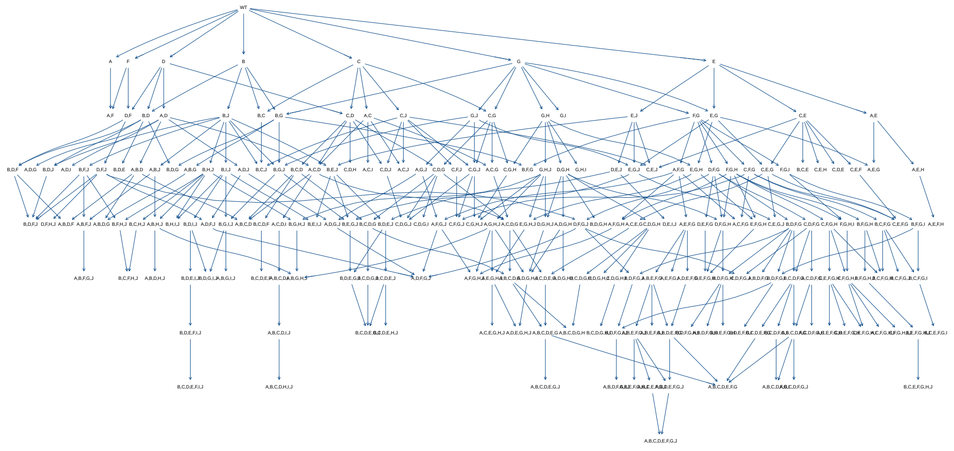

ID	KJpT1Yp64Tw10qzk10
acc genotypes	35
peaks no back	16
removed edges	NA
prop rem edges	NA

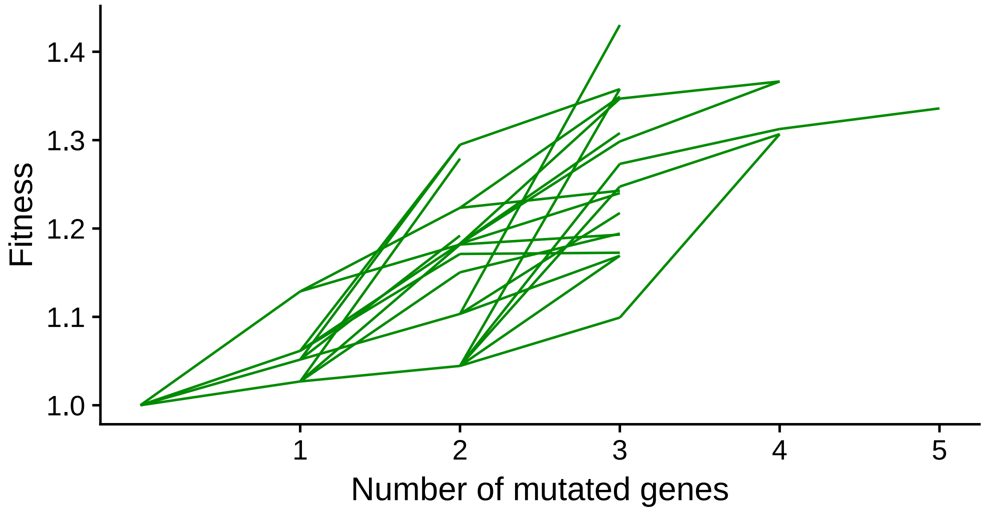

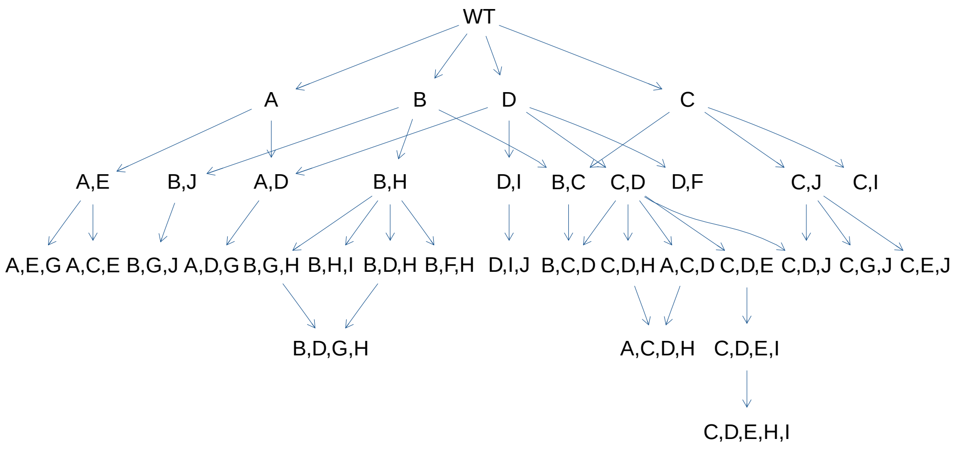

ID	KVDBUlaSExVhtPy5
acc genotypes	187
peaks no back	57
removed edges	NA
prop rem edges	NA

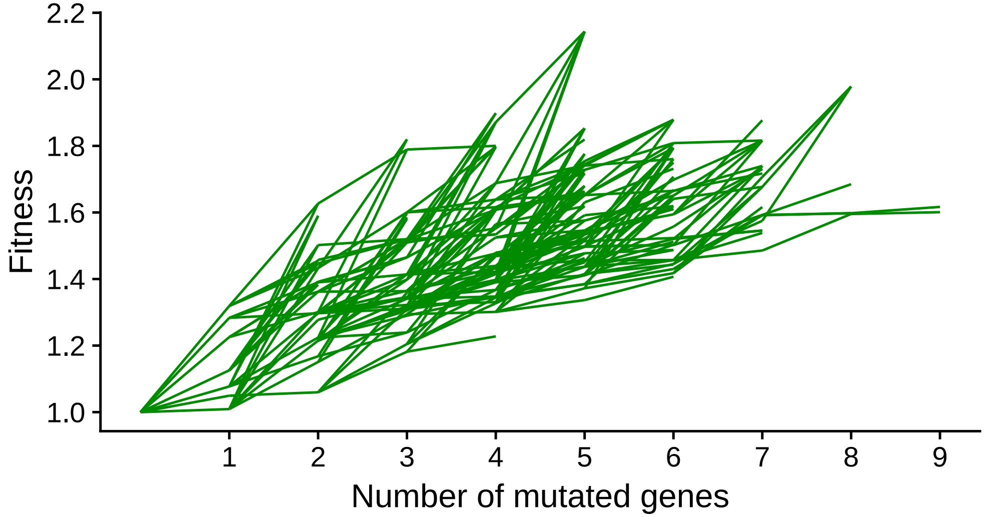

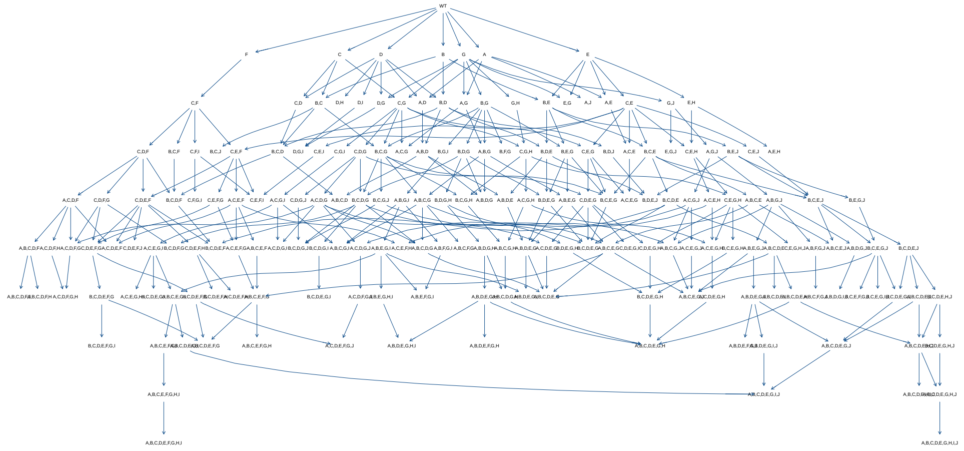

ID	l10y29Tq5MlxxOTsw
acc genotypes	465
peaks no back	98
removed edges	NA
prop rem edges	NA

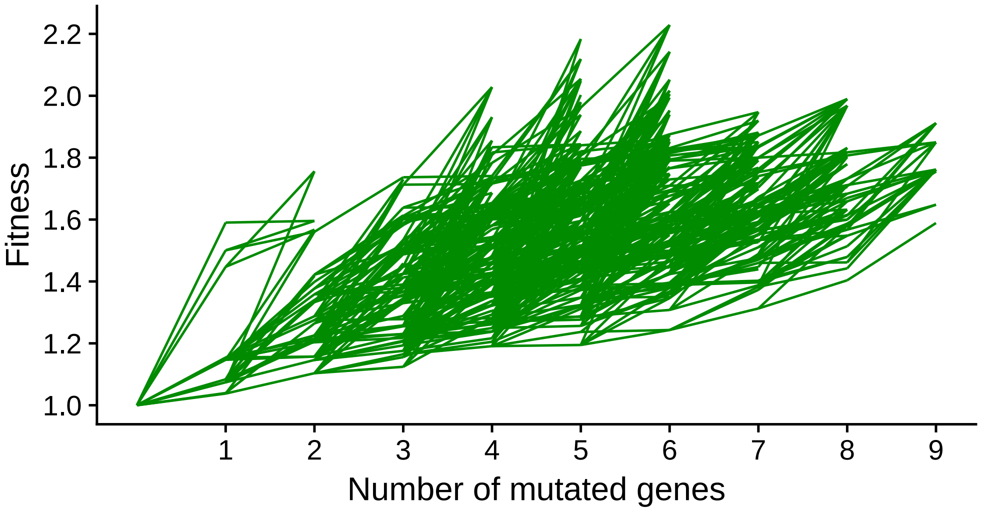

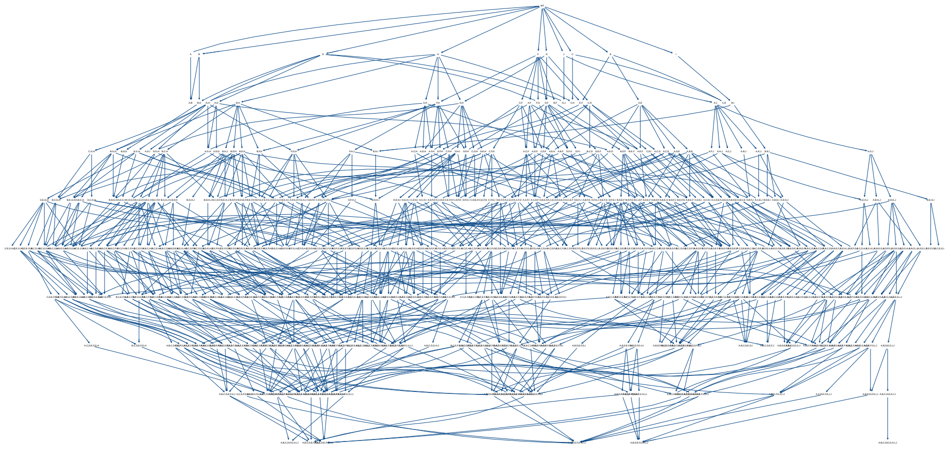

ID	La9fqNjWh3MIhyOc
acc genotypes	286
peaks no back	51
removed edges	NA
prop rem edges	NA

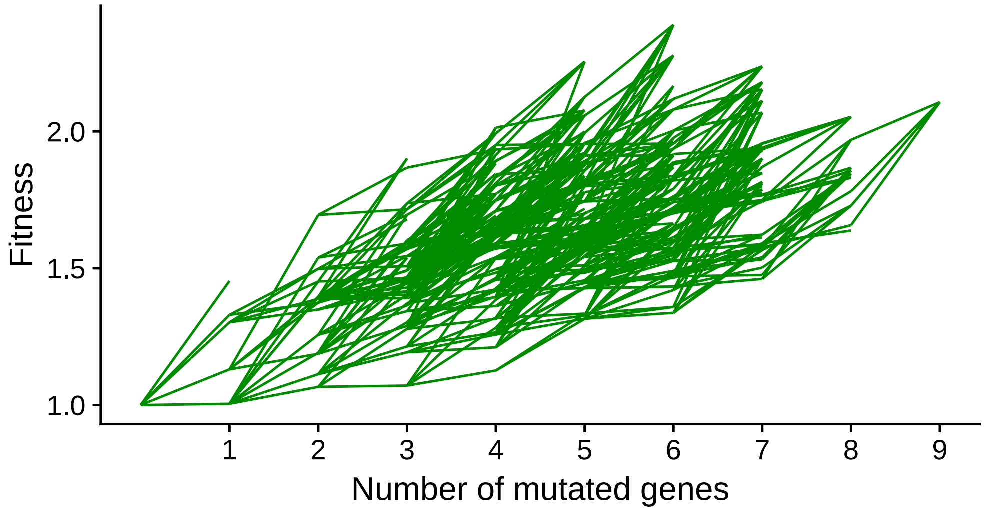

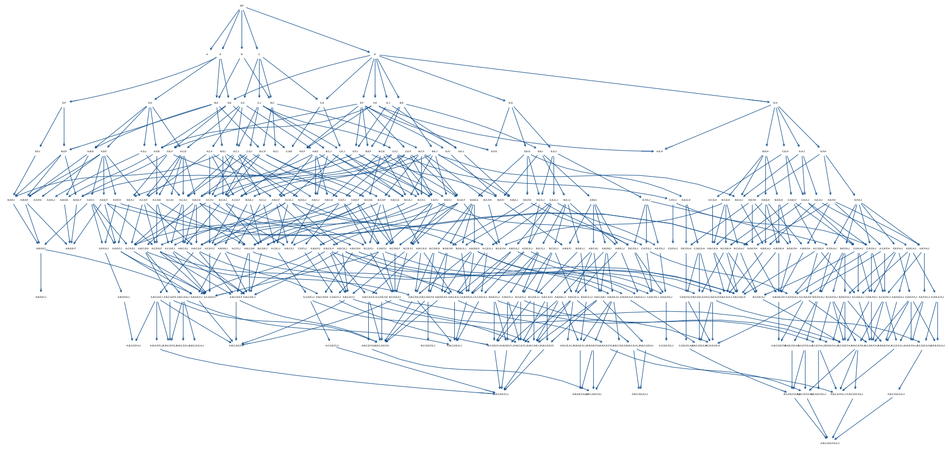

ID	LarH6pnhHub1WCoT
acc genotypes	119
peaks no back	34
removed edges	NA
prop rem edges	NA

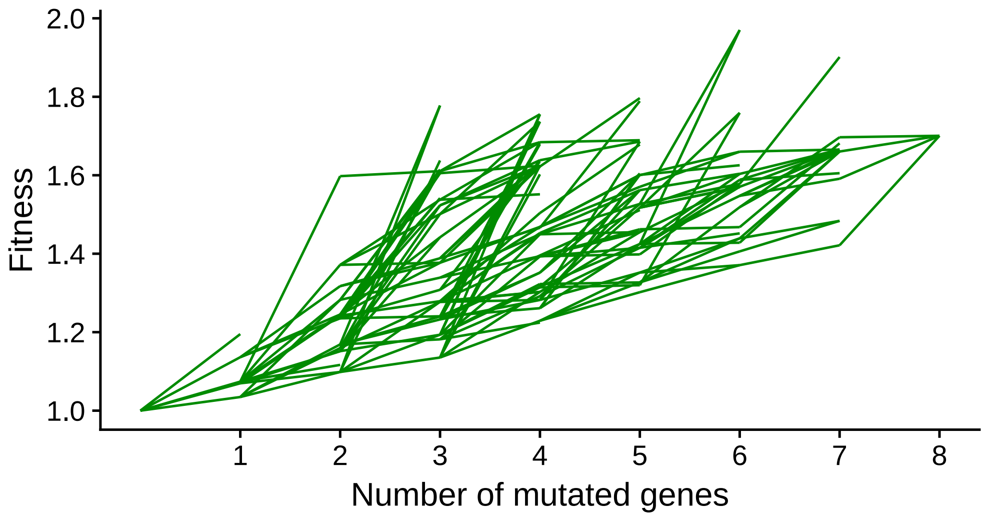

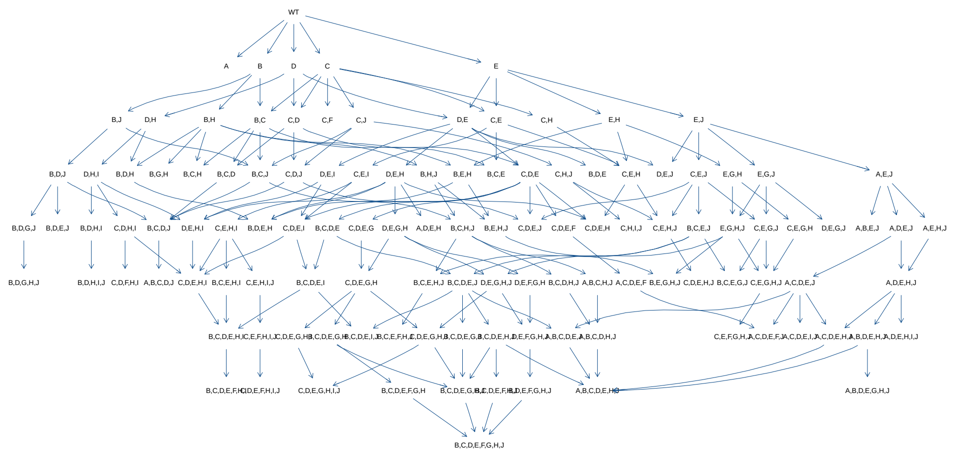

ID	ligIbsdWsdaVrYD4
acc genotypes	183
peaks no back	56
removed edges	NA
prop rem edges	NA

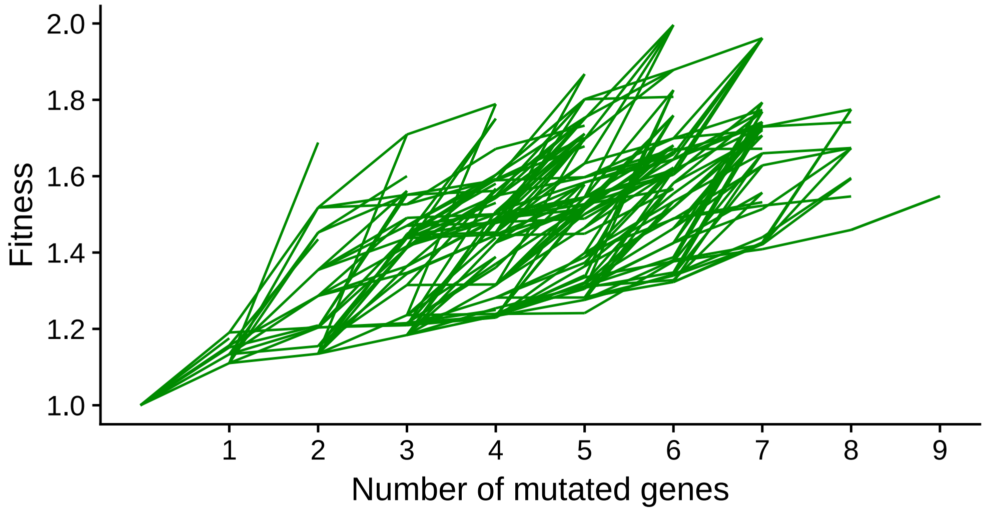

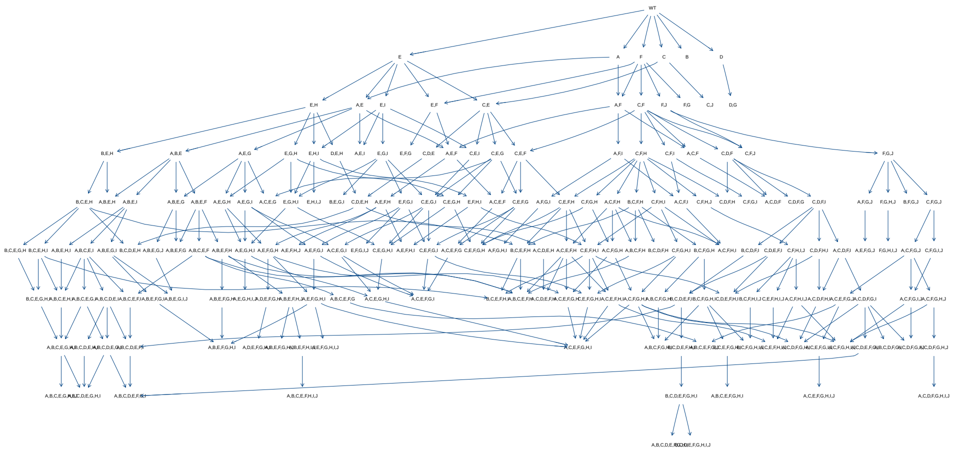

ID	LjALbpwaXmX67y1J
acc genotypes	200
peaks no back	60
removed edges	NA
prop rem edges	NA

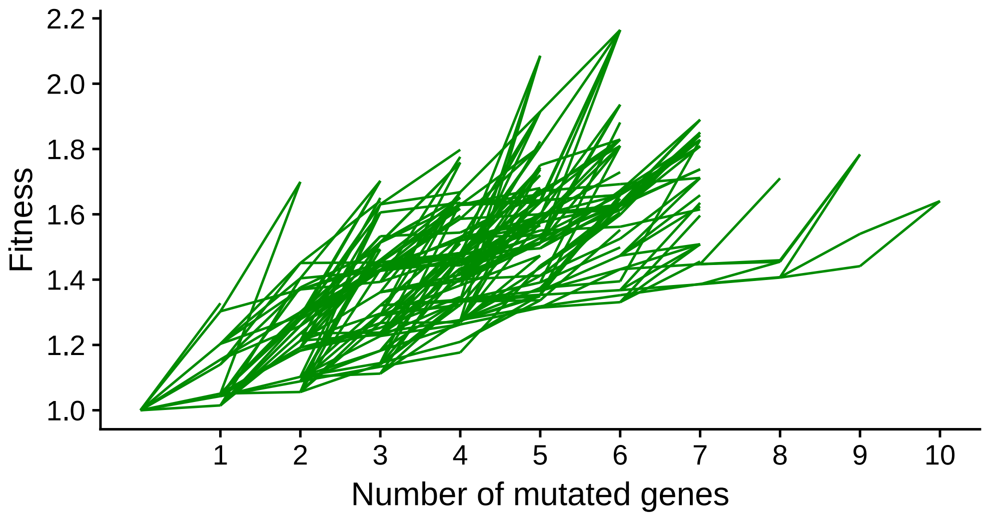

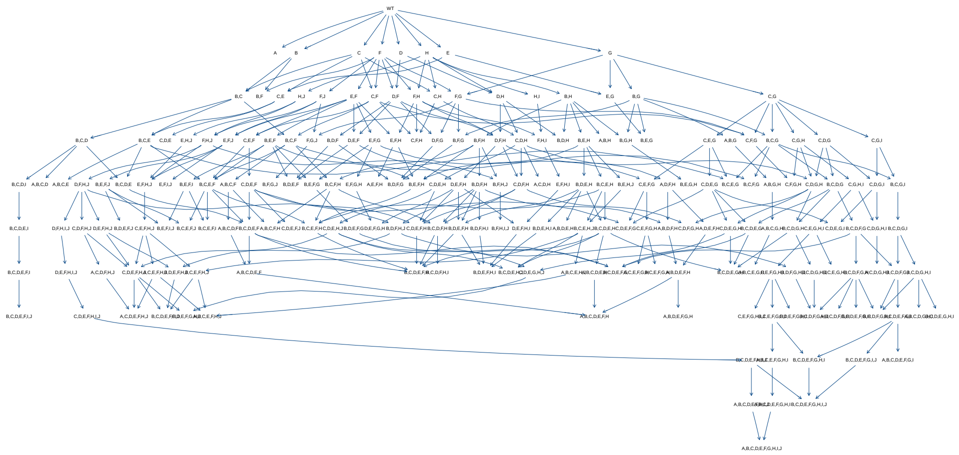

ID	LOsXXnHBlR9Q10wUS
acc genotypes	135
peaks no back	40
removed edges	NA
prop rem edges	NA

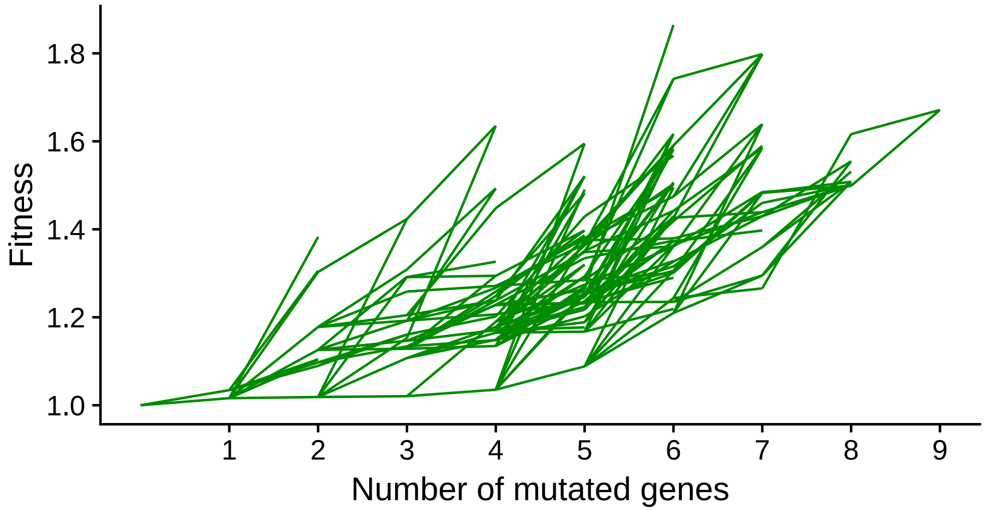

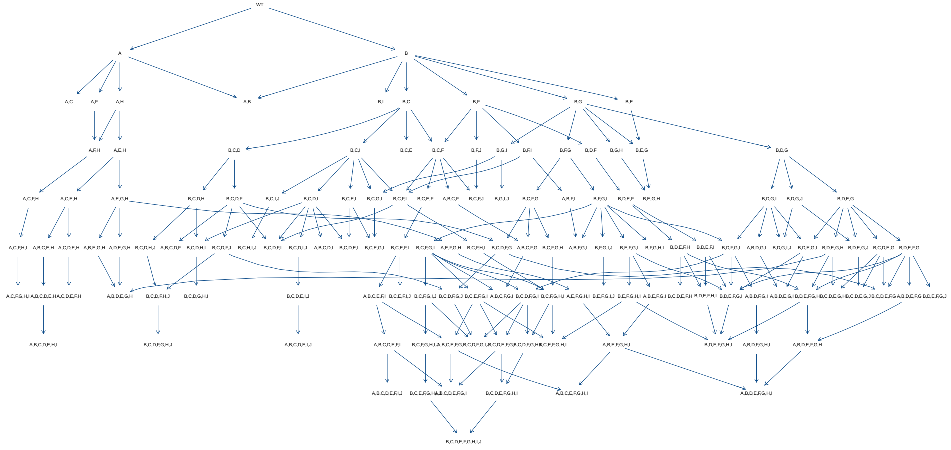

ID	LPrNa9xFzuJVcvTs
acc genotypes	132
peaks no back	51
removed edges	NA
prop rem edges	NA

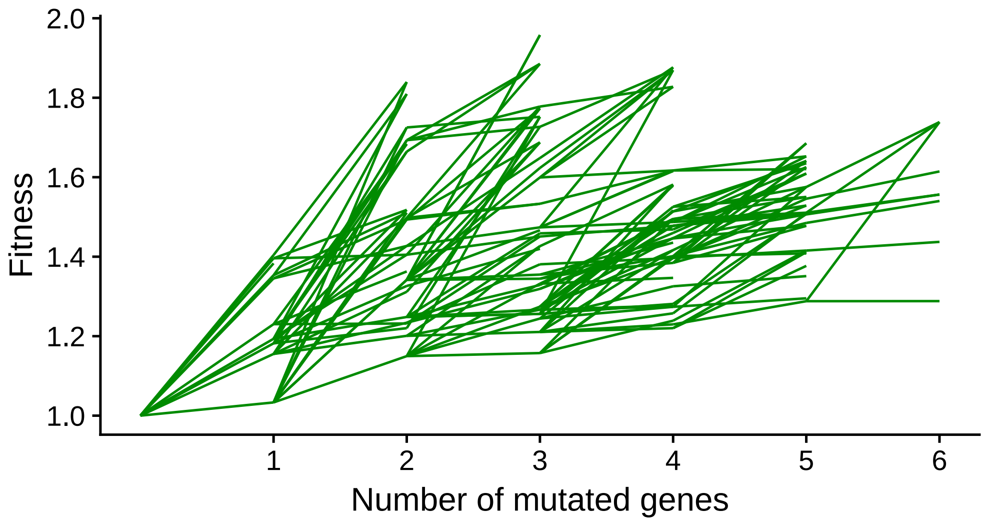

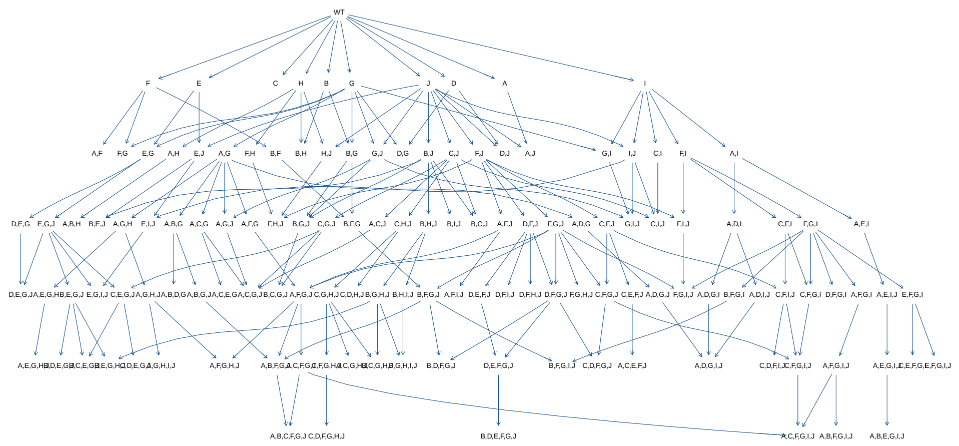

ID	ltssjb6NCBgdBABA
acc genotypes	232
peaks no back	86
removed edges	NA
prop rem edges	NA

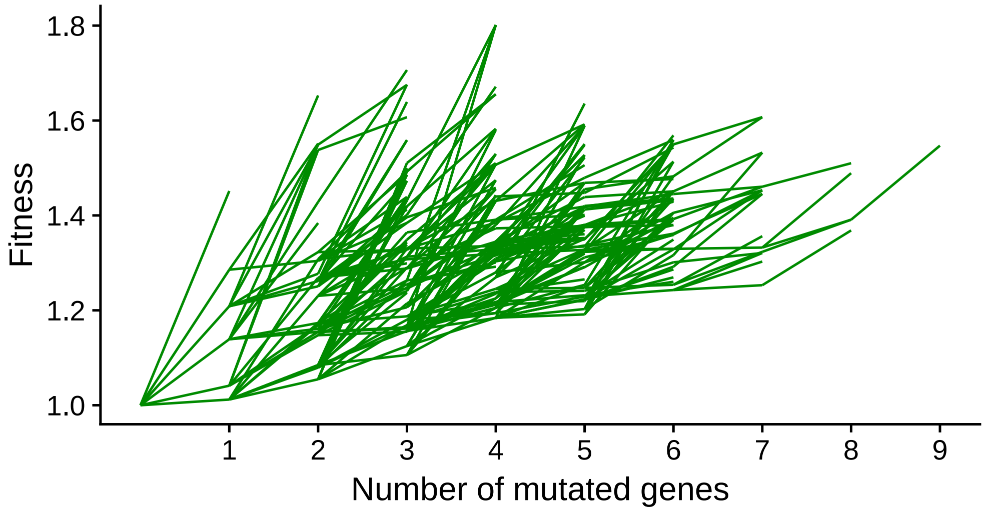

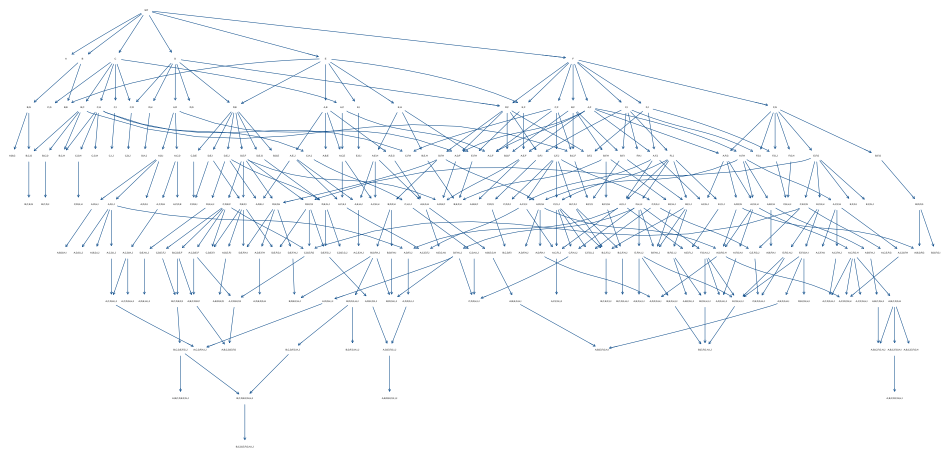

ID	LYJKKjl6A3xWjKwy
acc genotypes	289
peaks no back	75
removed edges	NA
prop rem edges	NA

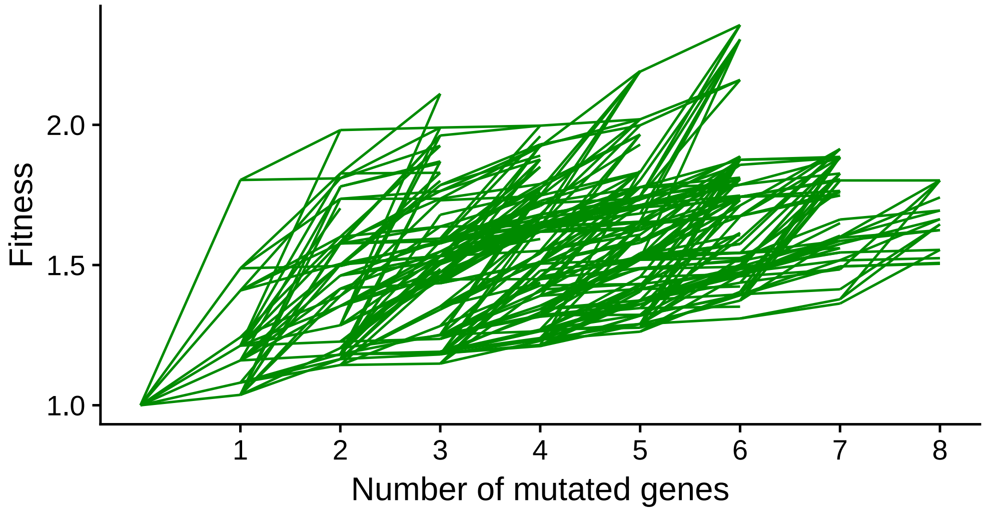

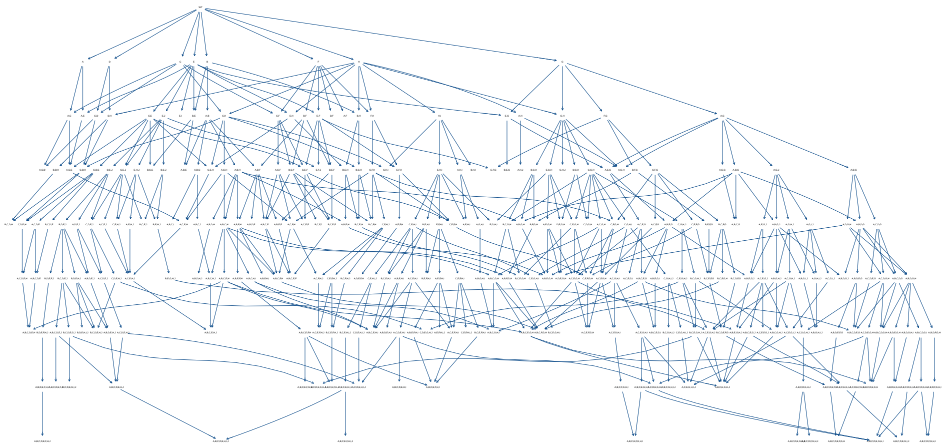

ID	MkpqetXw6W2muojH
acc genotypes	264
peaks no back	74
removed edges	NA
prop rem edges	NA

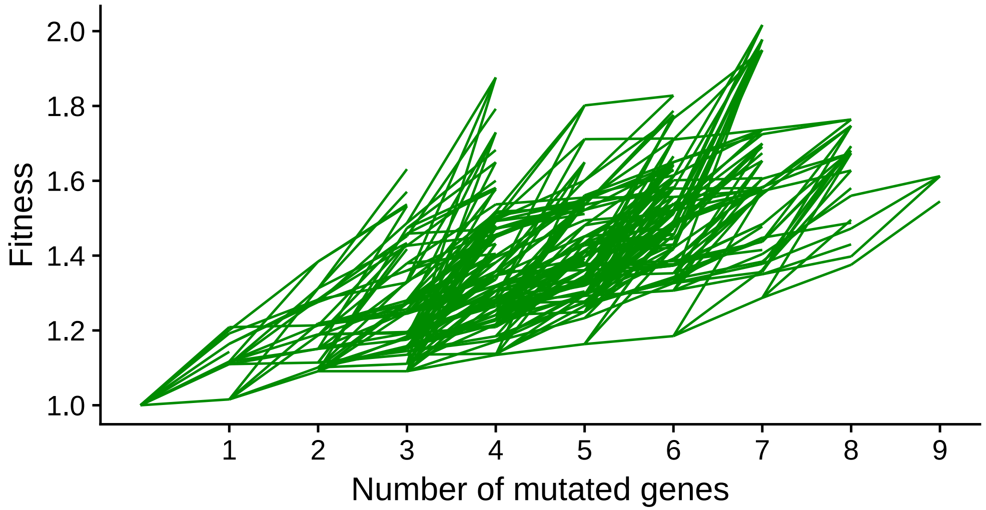

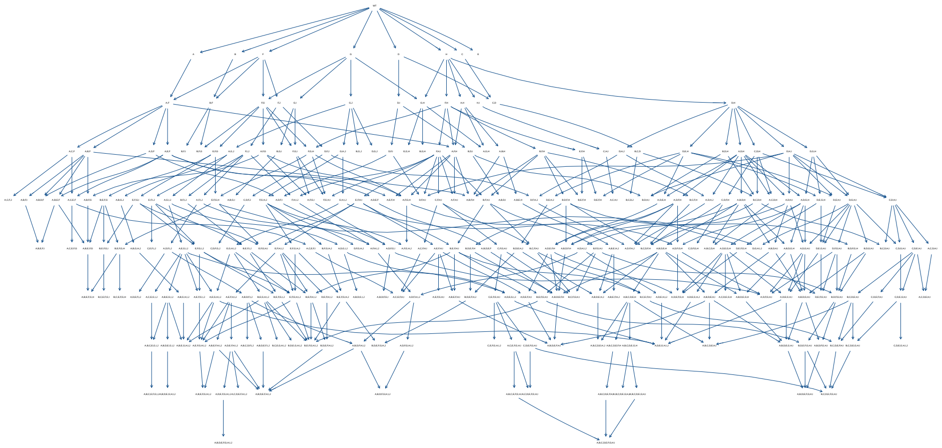

ID	mLM7OEw142104scrX
acc genotypes	107
peaks no back	42
removed edges	NA
prop rem edges	NA

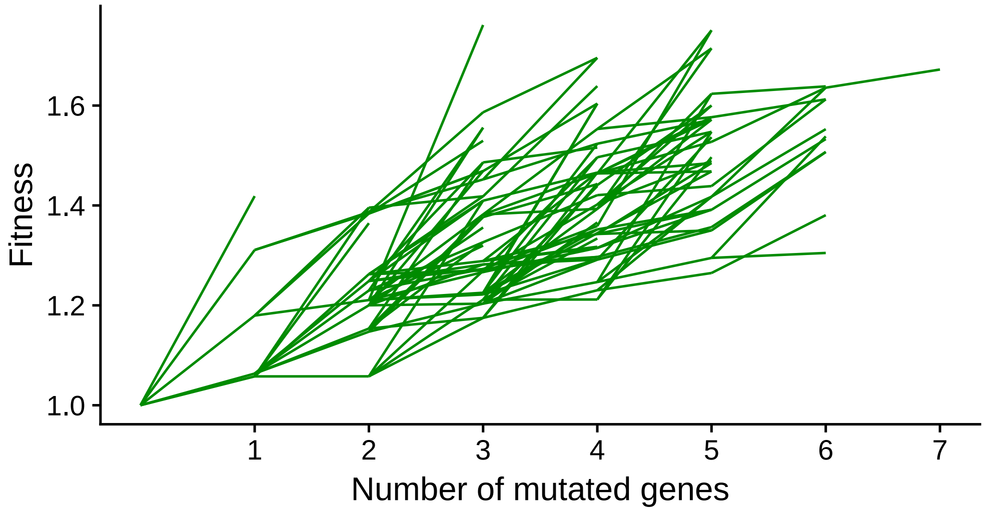

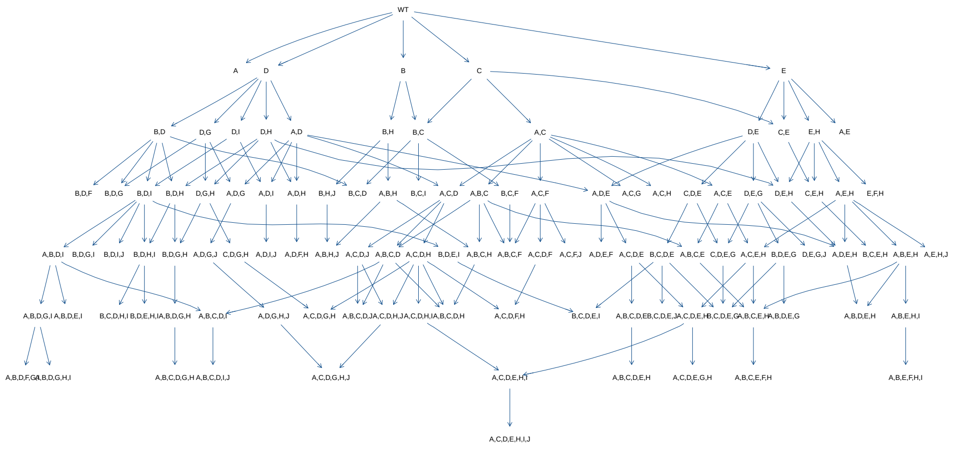

ID	mqC38cuoz4YoXqrC
acc genotypes	214
peaks no back	74
removed edges	NA
prop rem edges	NA

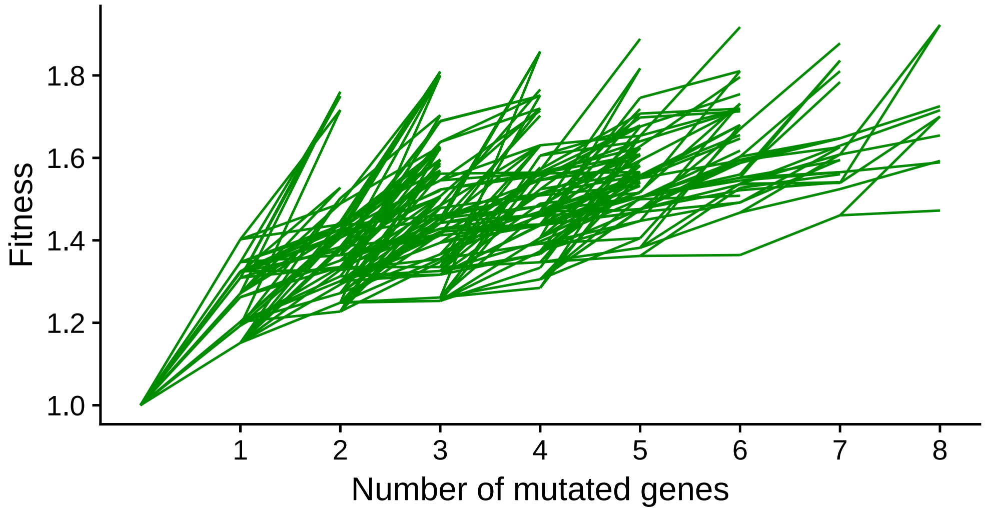

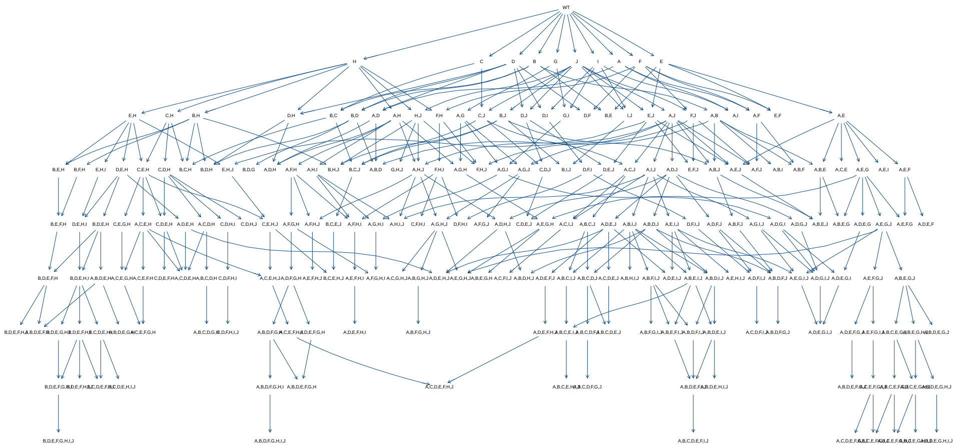

ID	MtpPxzteweNeL6dl
acc genotypes	295
peaks no back	63
removed edges	NA
prop rem edges	NA

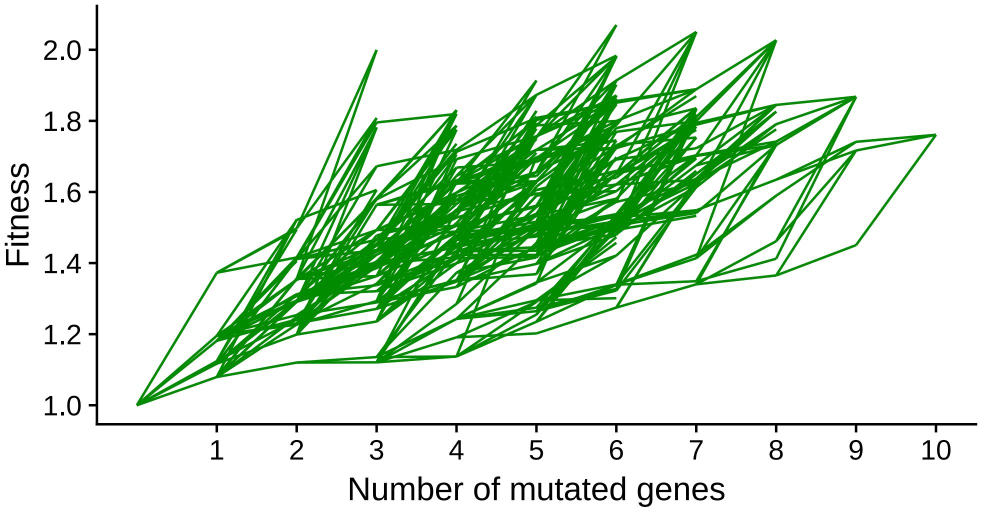

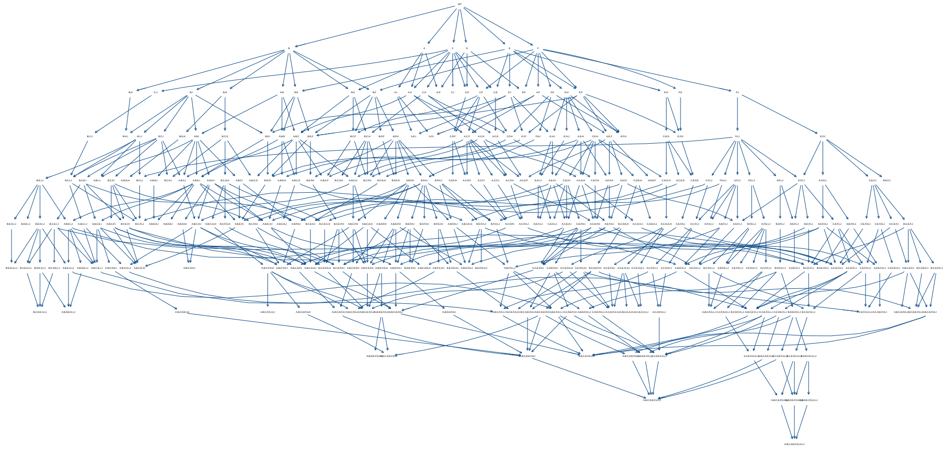

ID	mYuH5XdoLHUkC10ay
acc genotypes	440
peaks no back	88
removed edges	NA
prop rem edges	NA

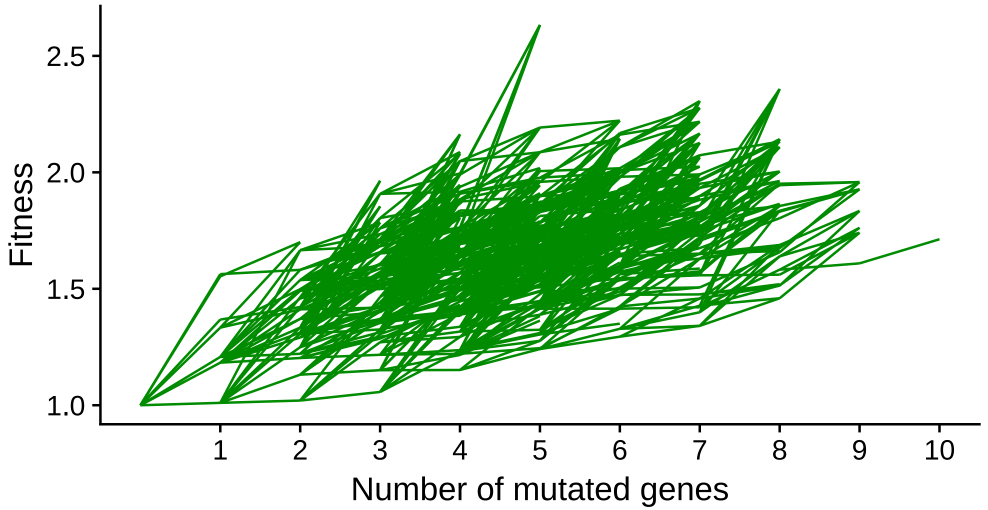

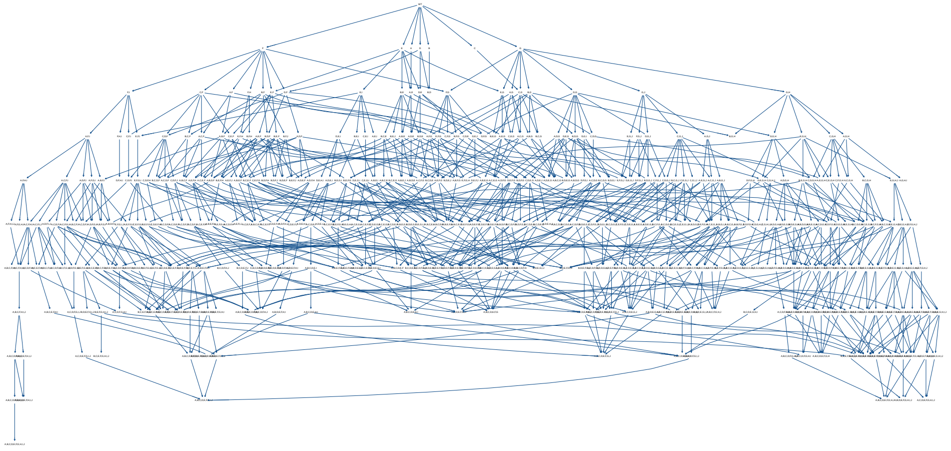

ID	n10oyDkkU10F10uiAs3
acc genotypes	228
peaks no back	73
removed edges	NA
prop rem edges	NA

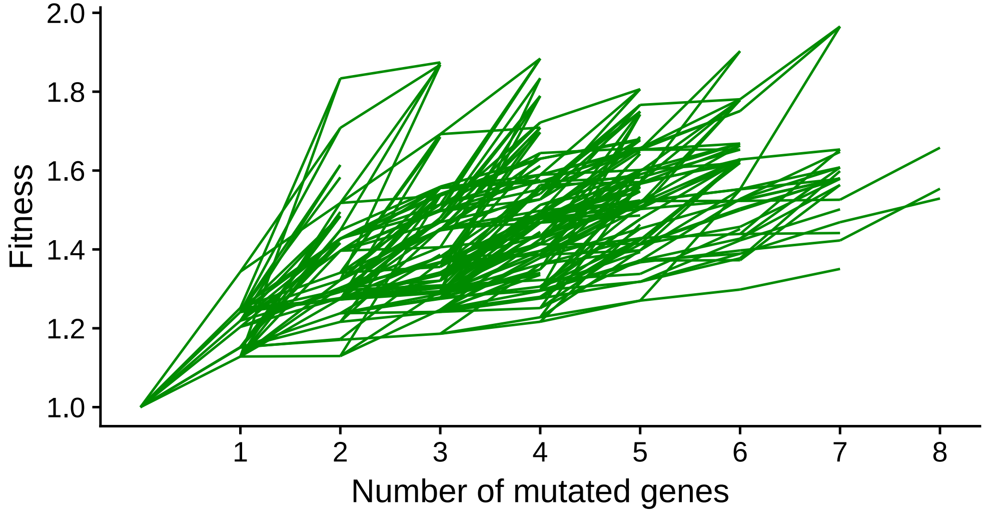

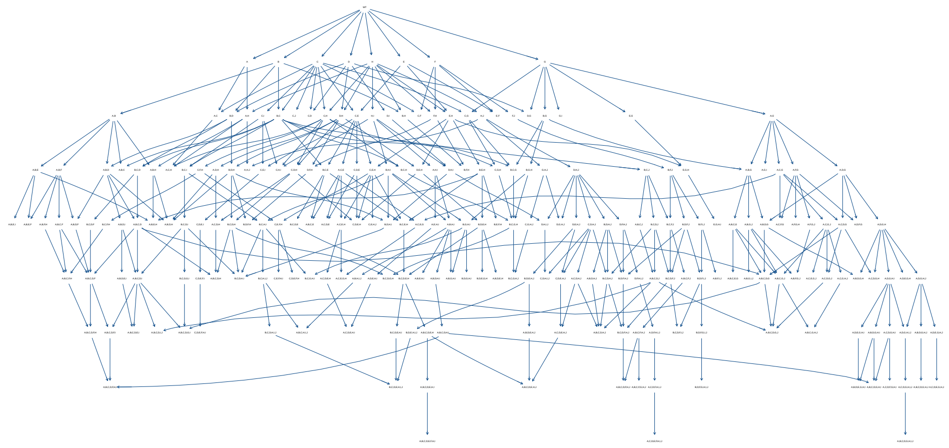

ID	Na1044PJp2RIHKSl6
acc genotypes	30
peaks no back	13
removed edges	NA
prop rem edges	NA

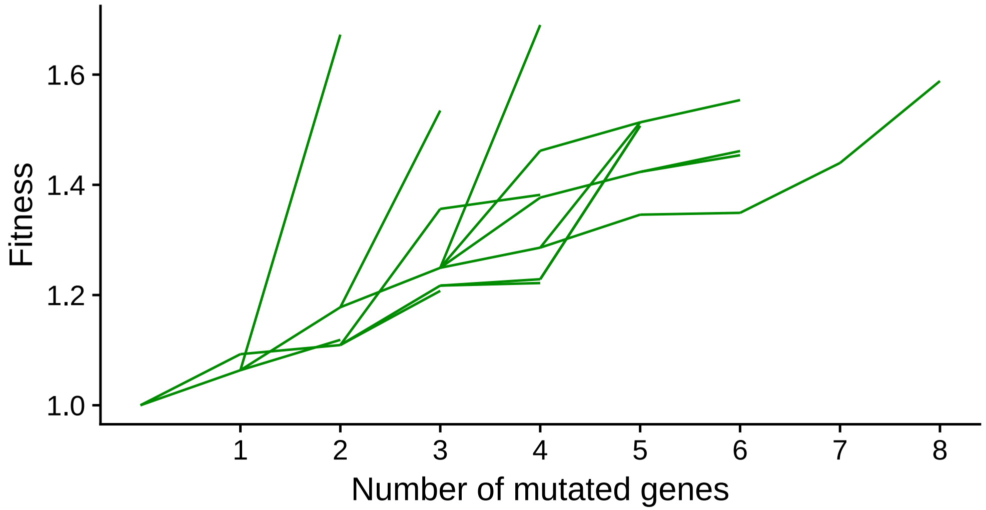

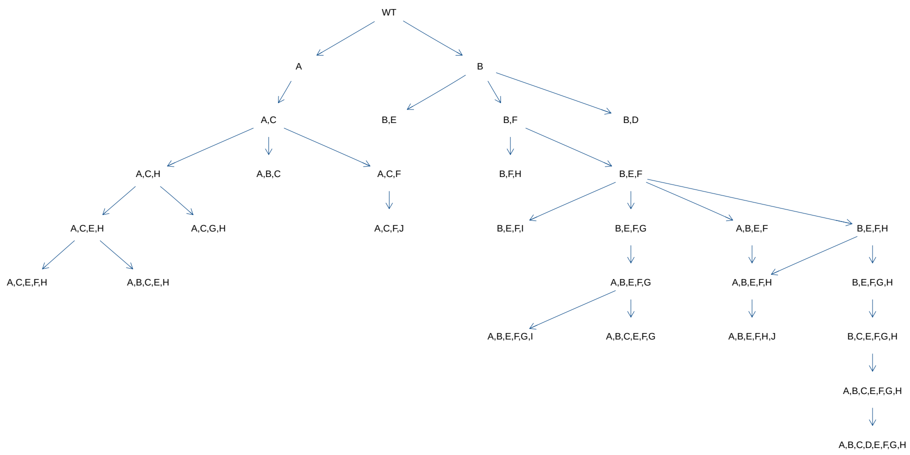

ID	Nckkjp8YxmOBJiLn
acc genotypes	176
peaks no back	45
removed edges	NA
prop rem edges	NA

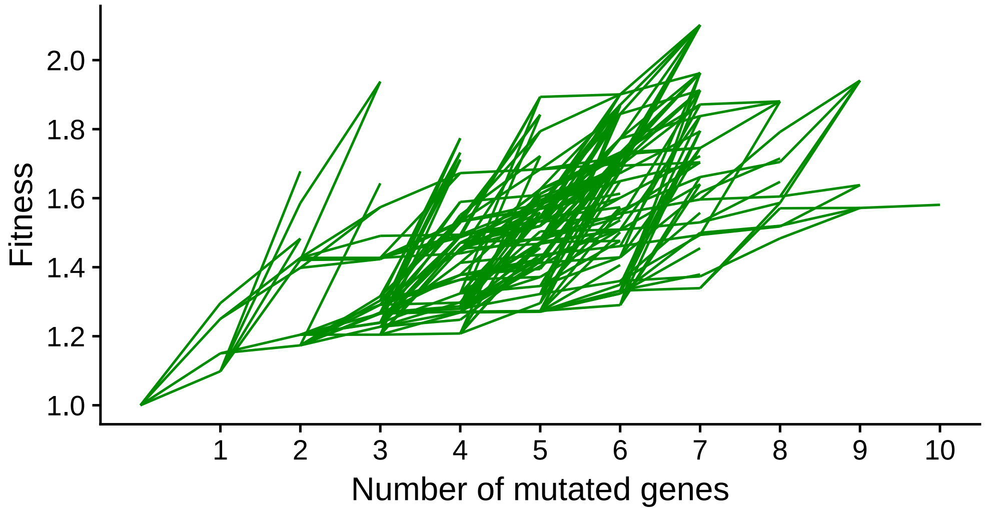

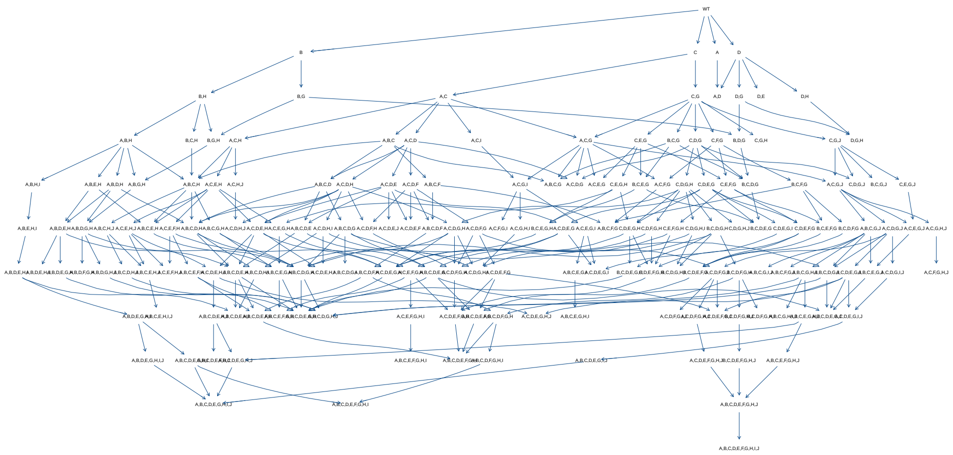

ID	nFFAJ57LcDAfooEJ
acc genotypes	537
peaks no back	73
removed edges	NA
prop rem edges	NA

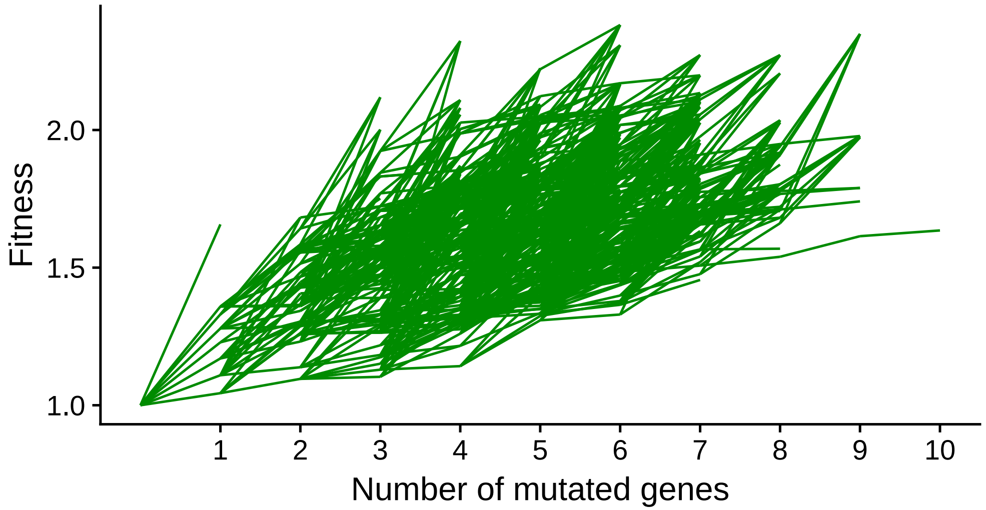

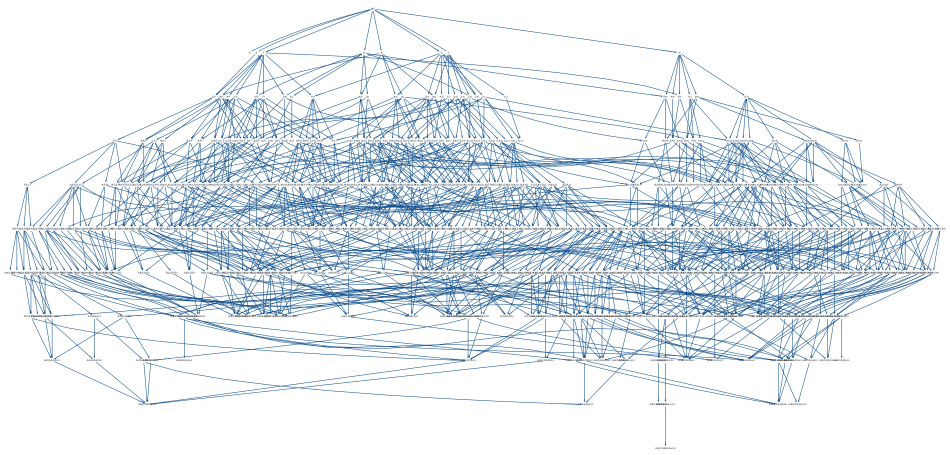

ID	nFGvQLr121eGqkyD
acc genotypes	138
peaks no back	51
removed edges	NA
prop rem edges	NA

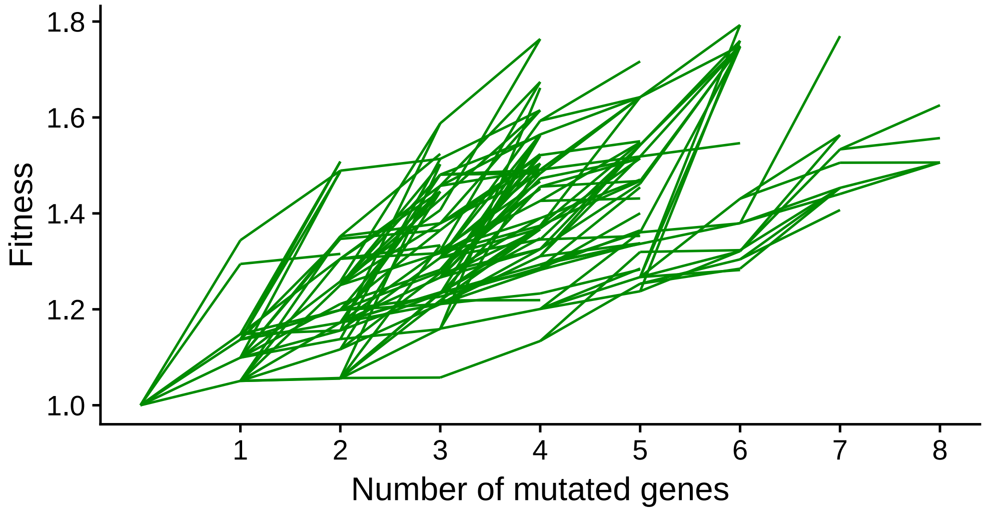

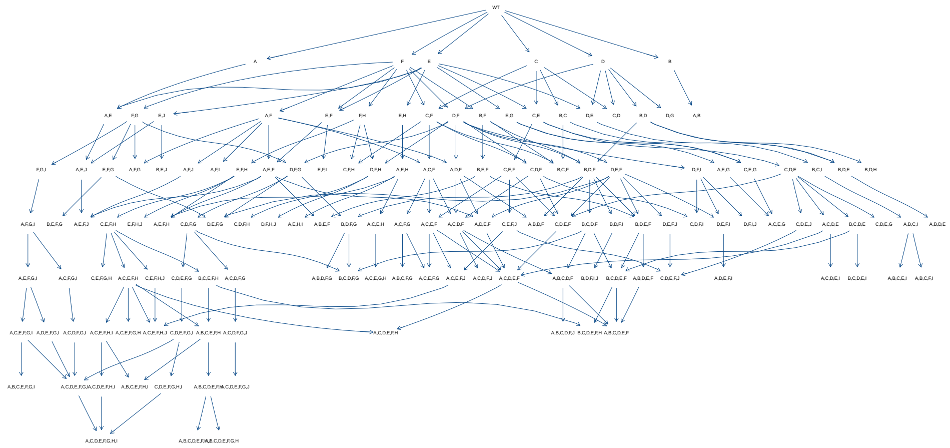

ID	OcPH3CcYp51kr5sW
acc genotypes	541
peaks no back	62
removed edges	NA
prop rem edges	NA

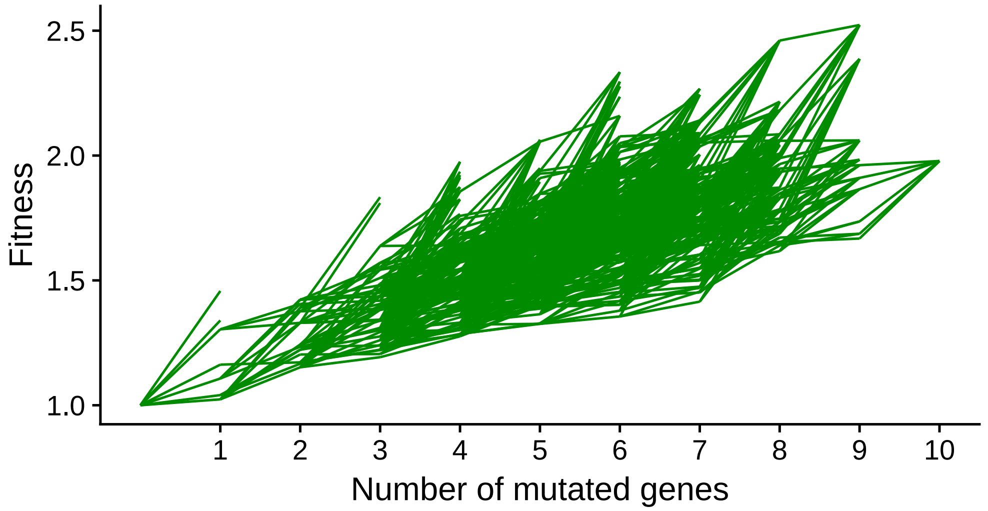

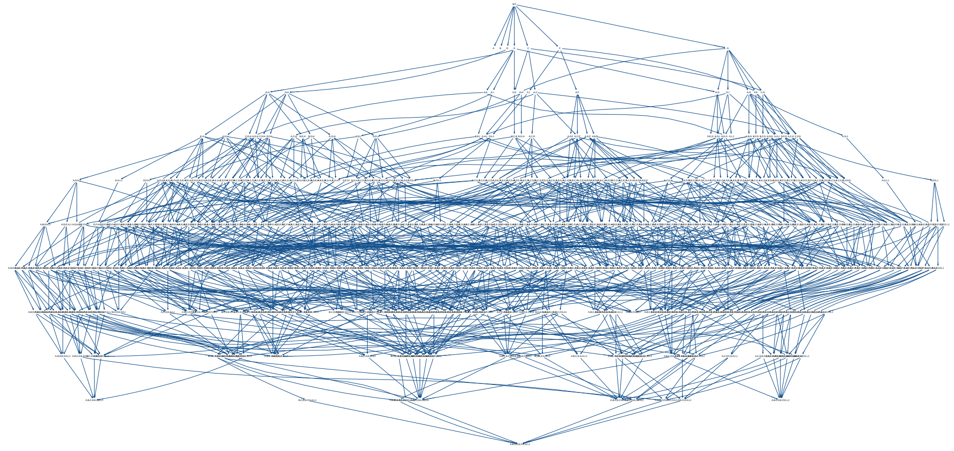

ID	OGxF8Oyv15NLRVHt
acc genotypes	147
peaks no back	63
removed edges	NA
prop rem edges	NA

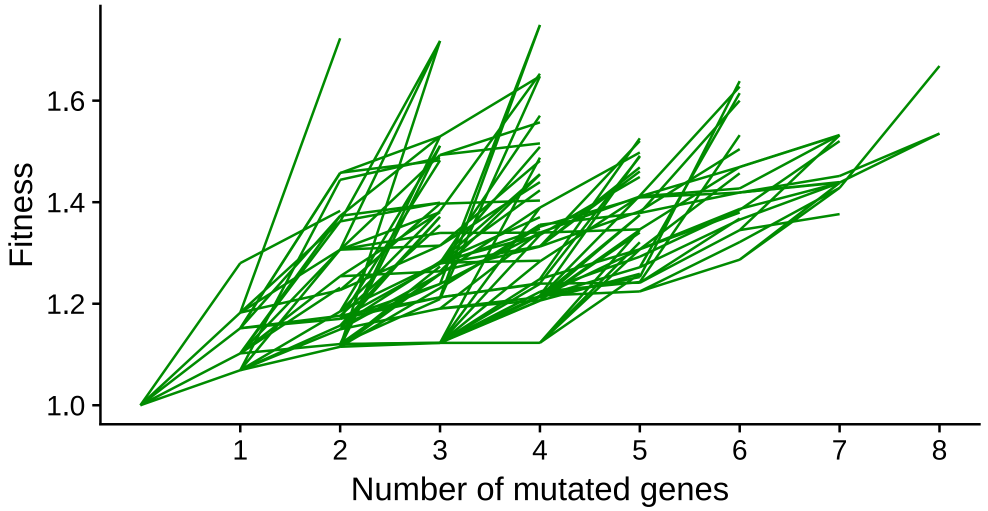

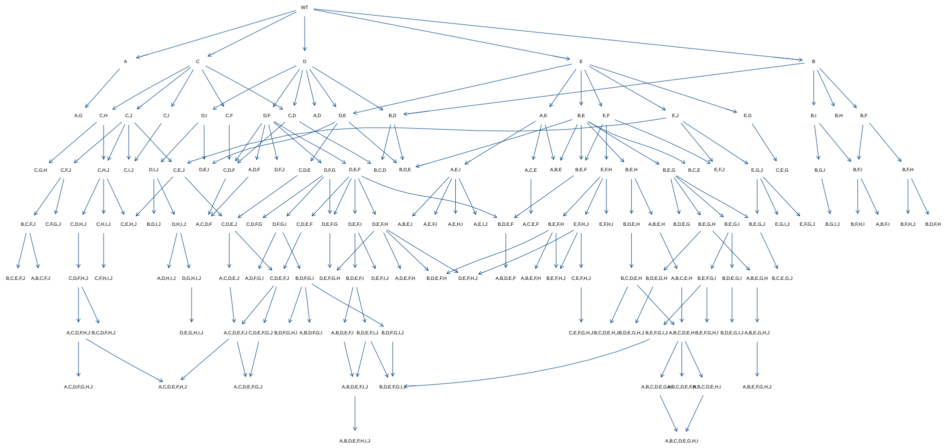

ID	Oh105Ra4cDXvsiqOA
acc genotypes	468
peaks no back	63
removed edges	NA
prop rem edges	NA

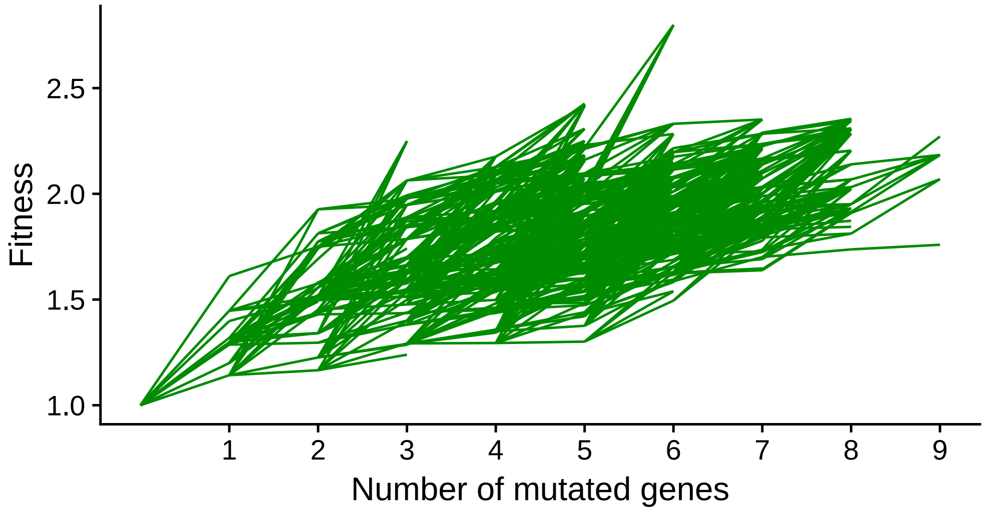

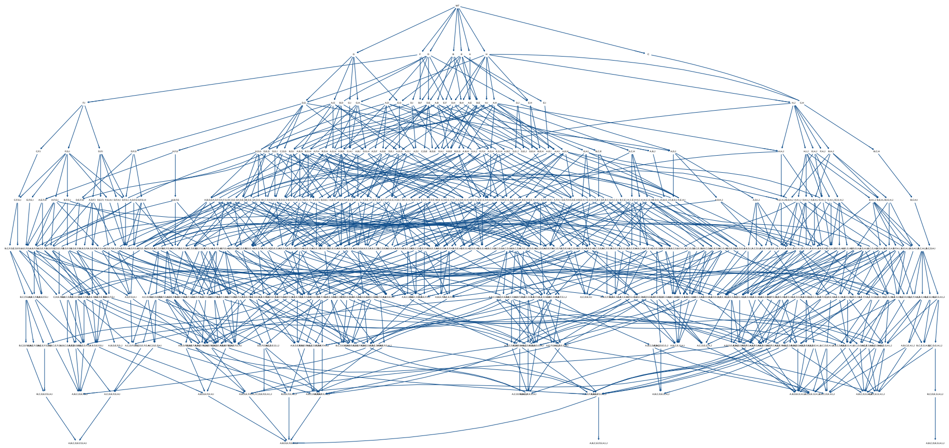

ID	Oj6oyBsD3bxROStD
acc genotypes	134
peaks no back	54
removed edges	NA
prop rem edges	NA

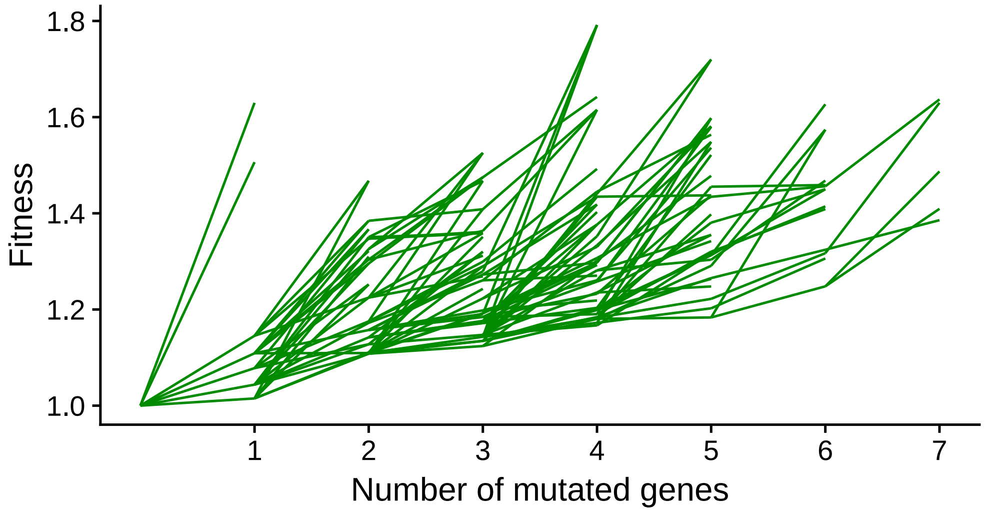

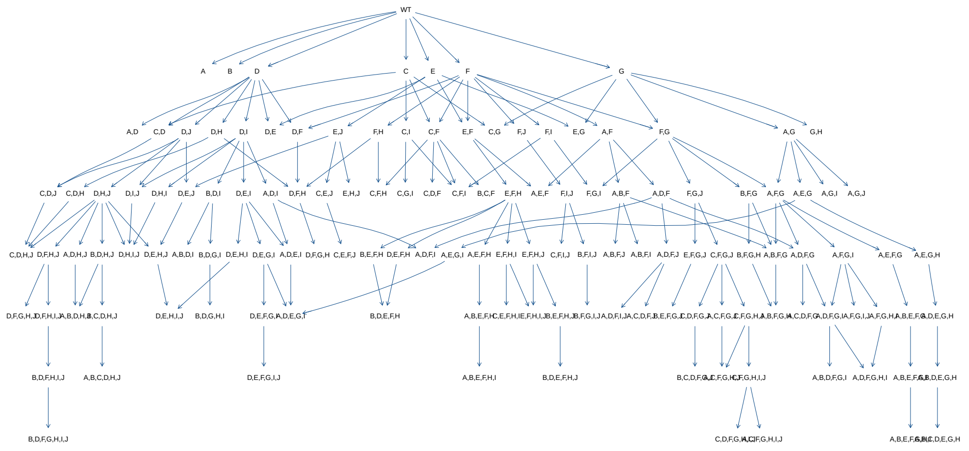

ID	Oyrh4S6yqbCq9BE1
acc genotypes	84
peaks no back	37
removed edges	NA
prop rem edges	NA

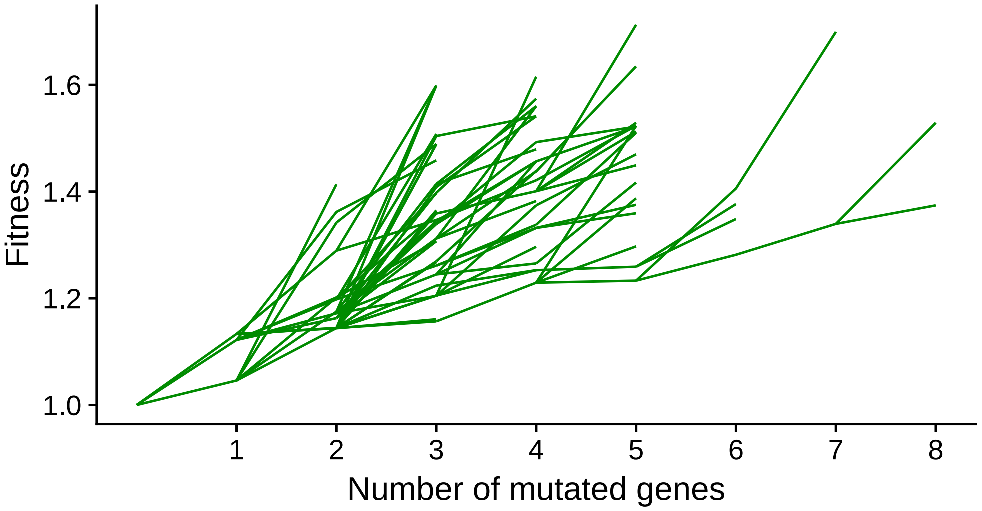

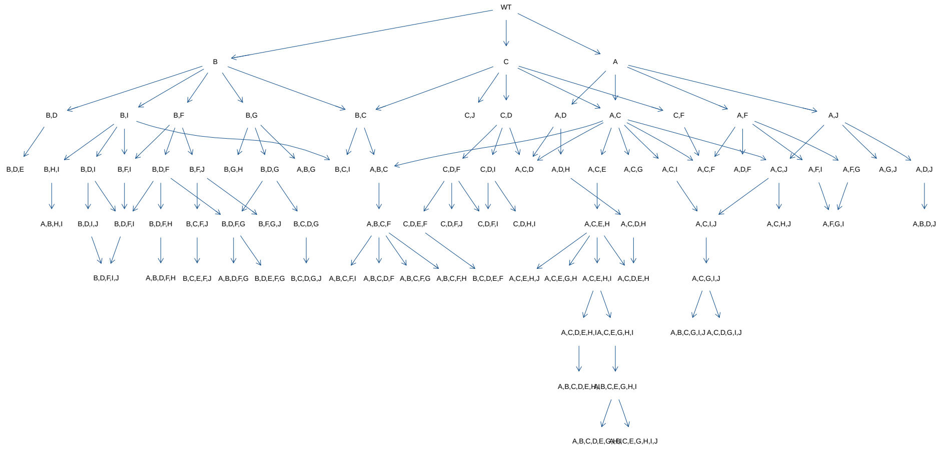

ID	p2Hy10RKJmoyhCKd10
acc genotypes	140
peaks no back	50
removed edges	NA
prop rem edges	NA

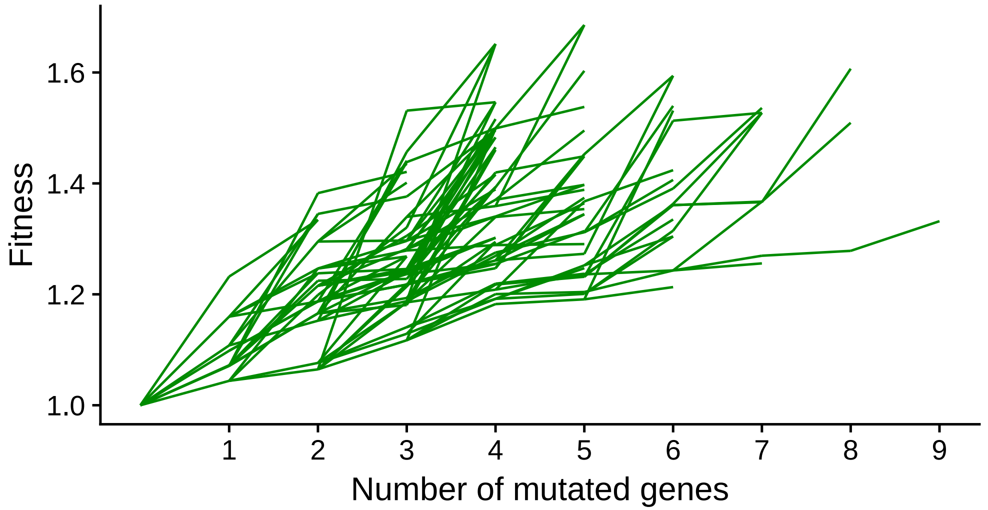

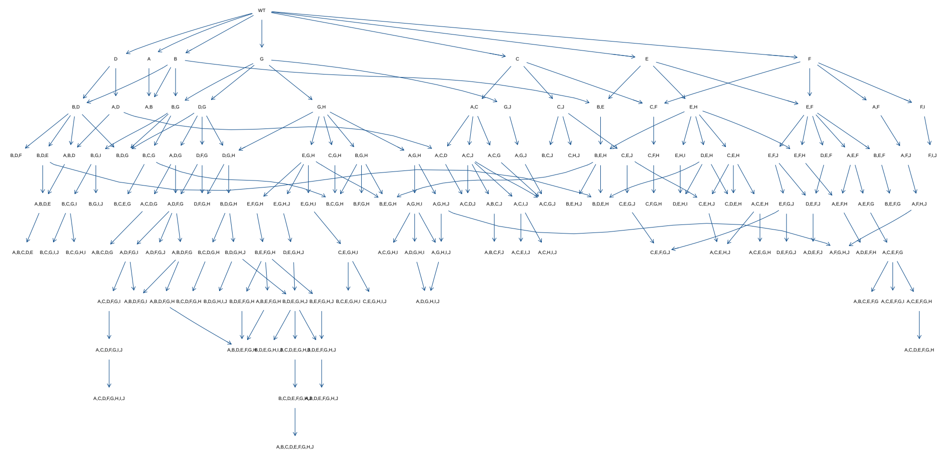

ID	pcFmFiSb1dmNWs7Q
acc genotypes	333
peaks no back	105
removed edges	NA
prop rem edges	NA

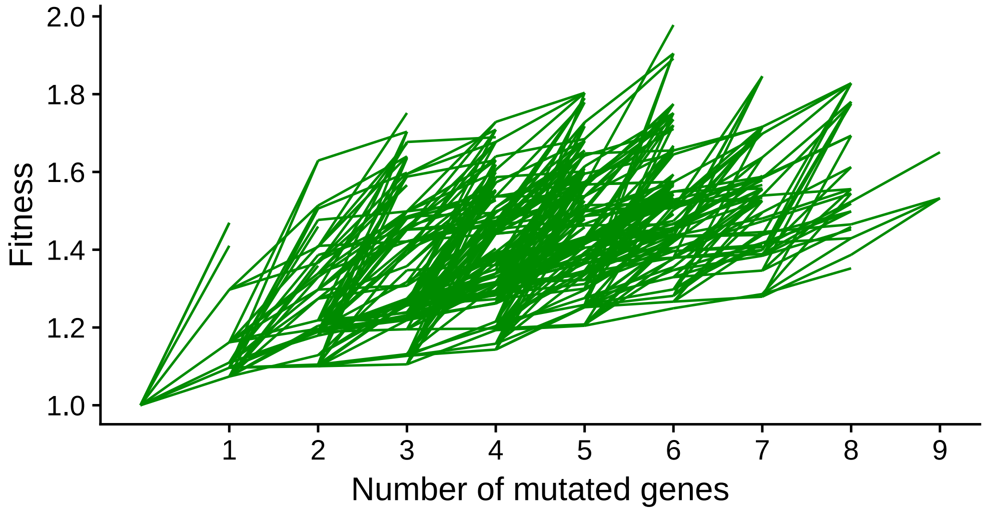

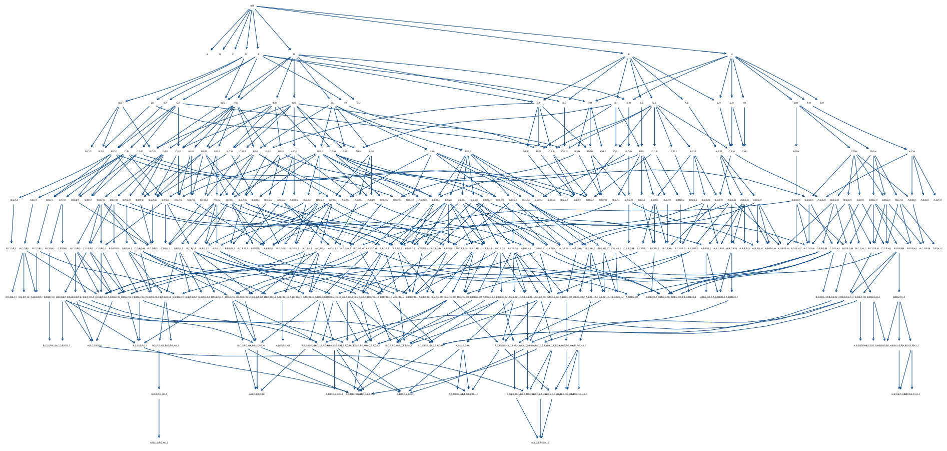

ID	pF515adN8ZsnzdgE
acc genotypes	276
peaks no back	100
removed edges	NA
prop rem edges	NA

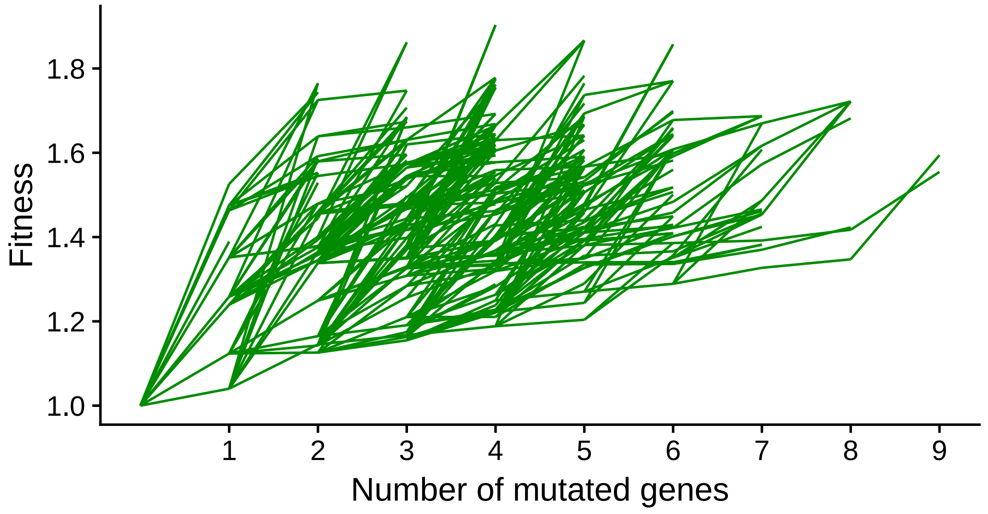

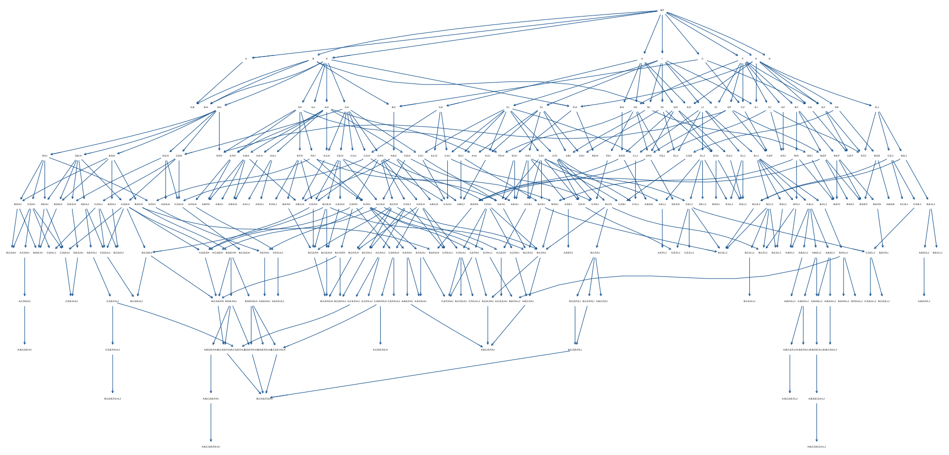

ID Pje92ruf6BDq1QiQ
acc genotypes 111
peaks no back 40
removed edges NA
prop rem edges NA

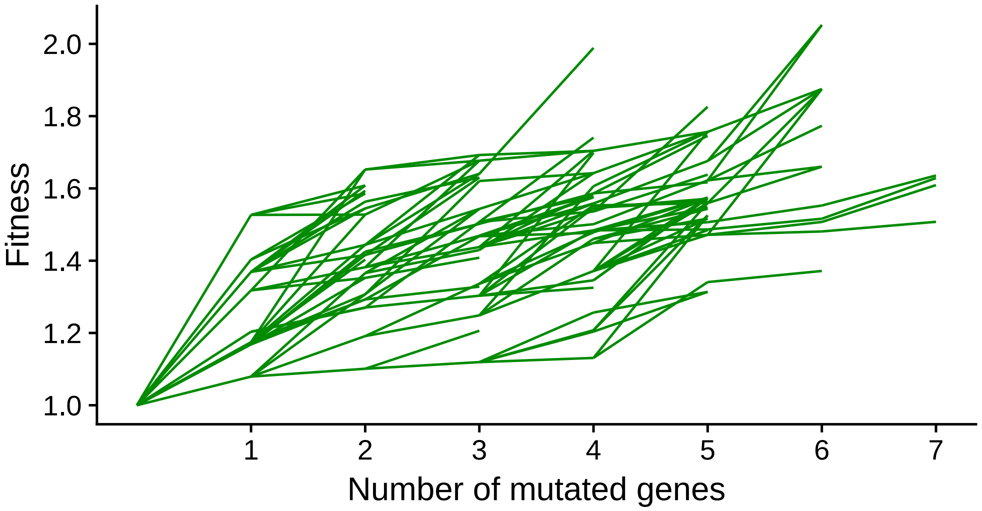

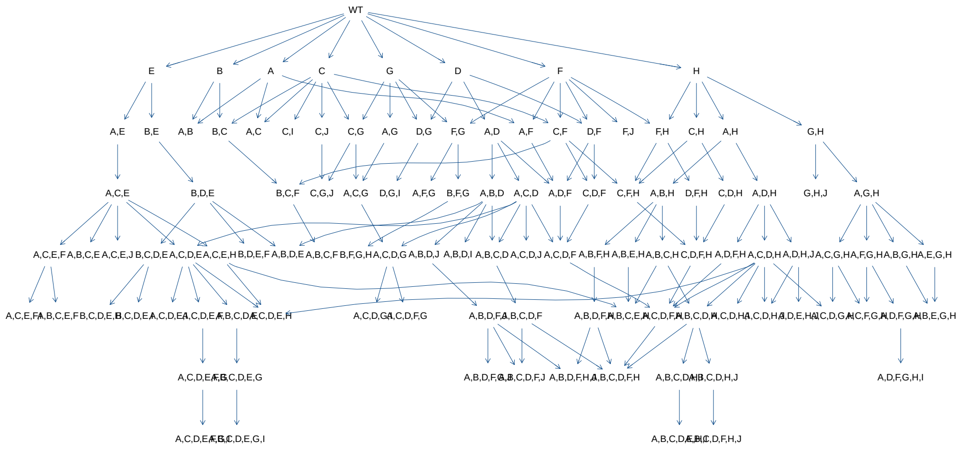

ID	PmQfeqFvnFM6z8Uz
acc genotypes	276
peaks no back	75
removed edges	NA
prop rem edges	NA

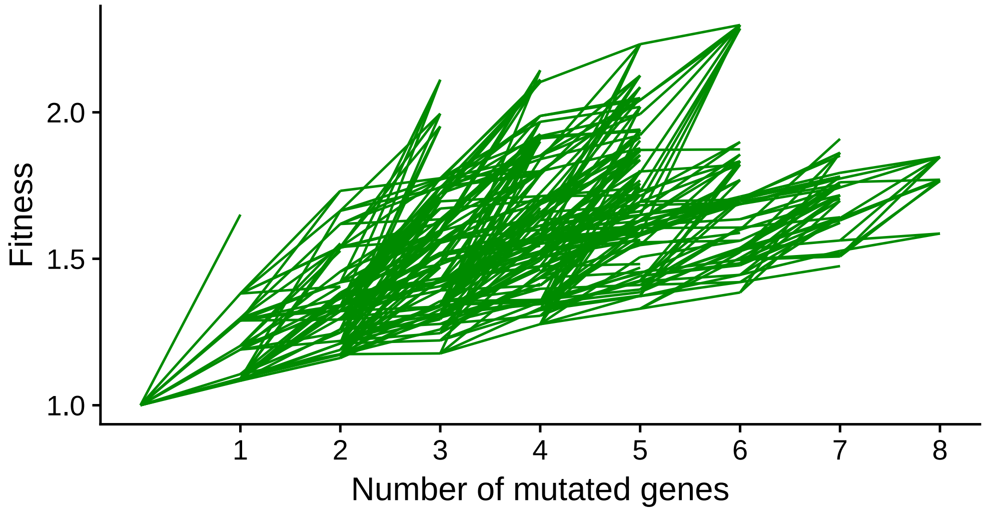

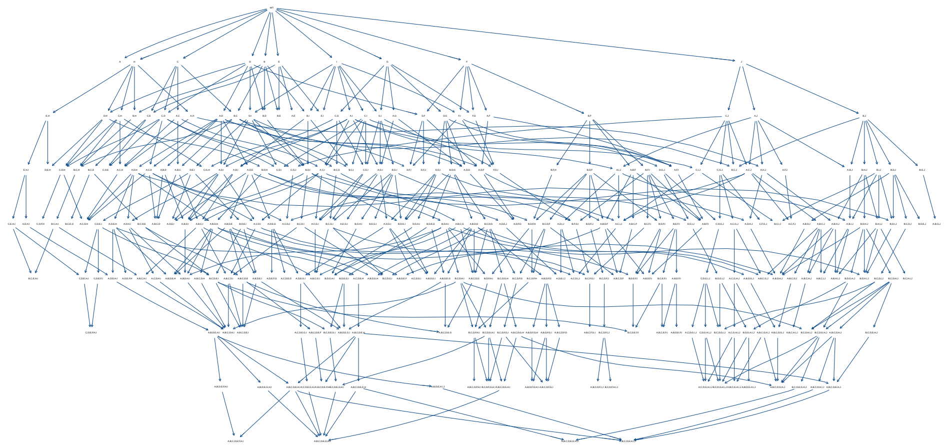

ID	ppgKWX3qfEYrvpoe
acc genotypes	98
peaks no back	22
removed edges	NA
prop rem edges	NA

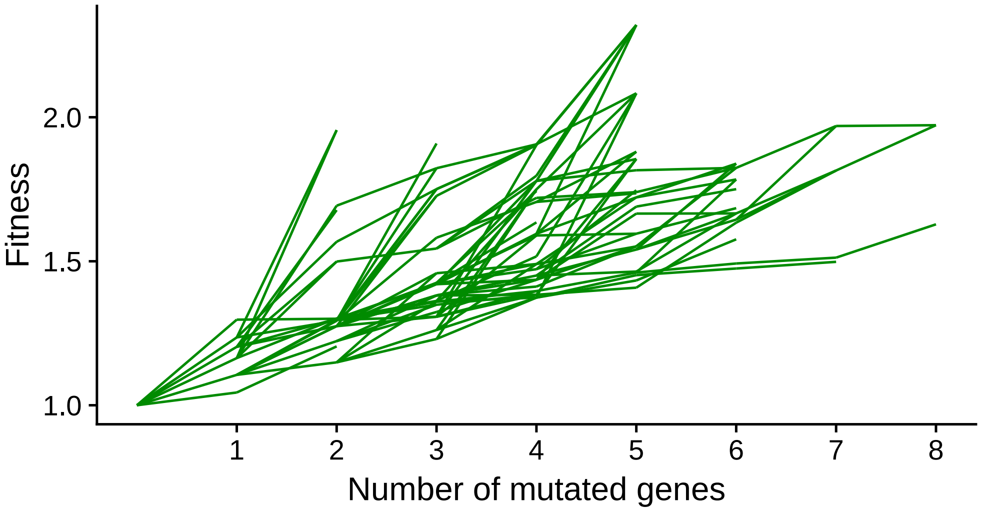

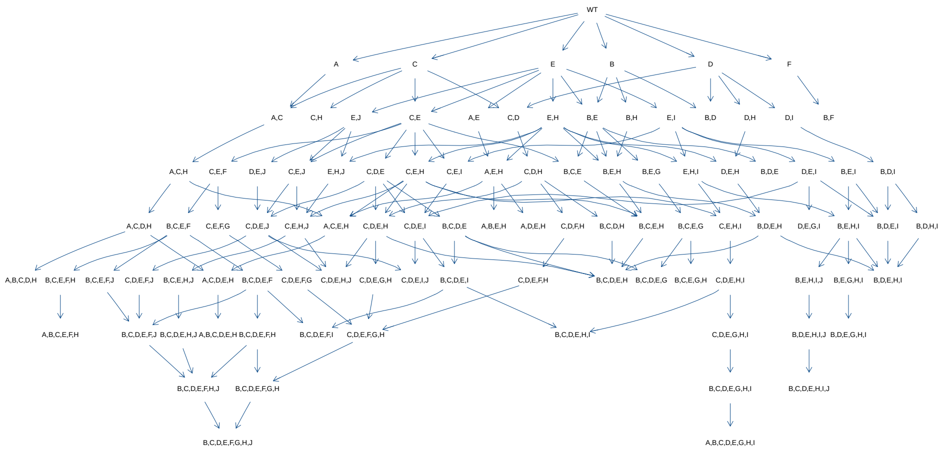

ID	q6ESfEnjdJLQJKzP
acc genotypes	110
peaks no back	41
removed edges	NA
prop rem edges	NA

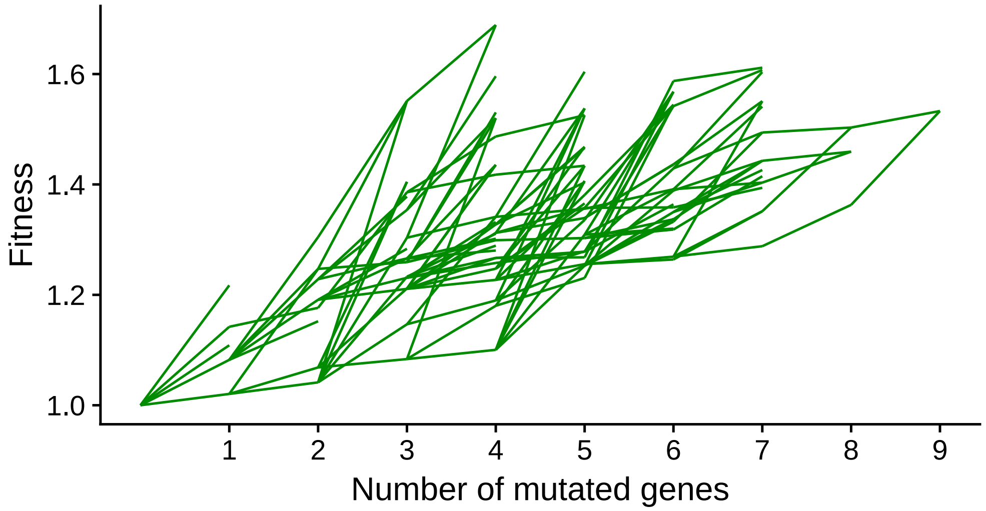

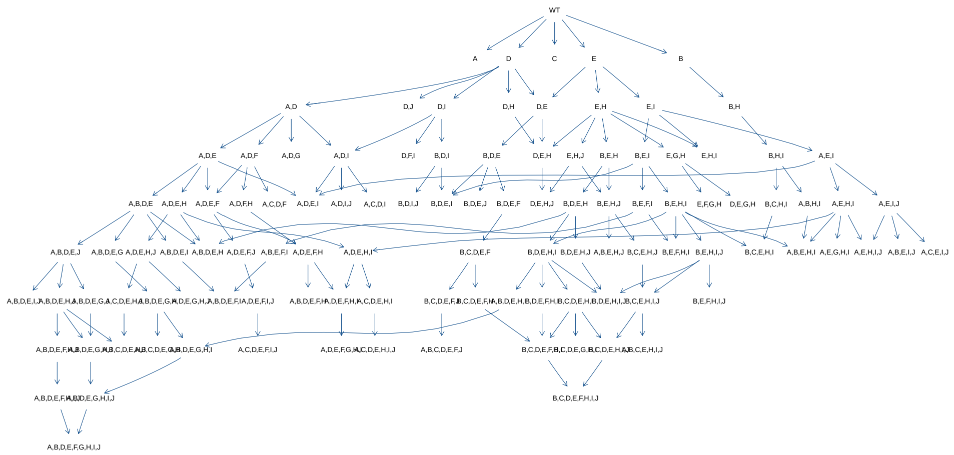

ID	QDlfwYRc6C9mw910Q
acc genotypes	251
peaks no back	69
removed edges	NA
prop rem edges	NA

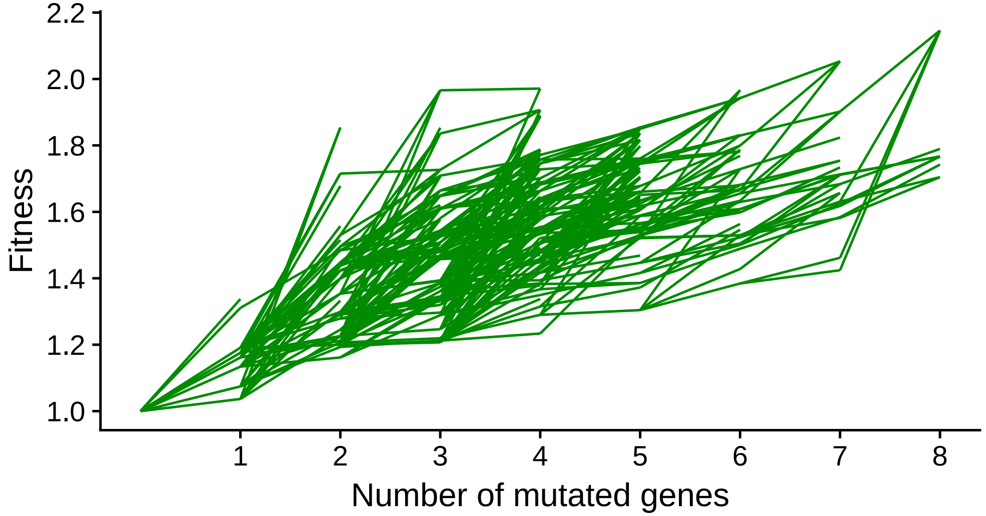

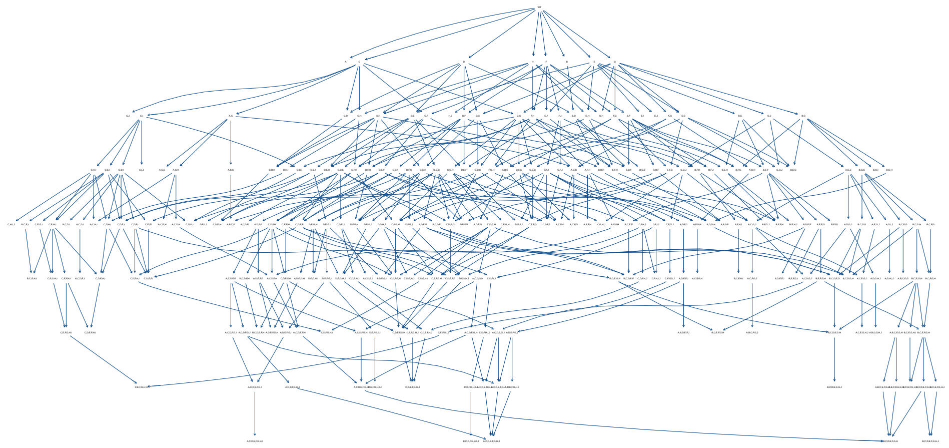

ID	QDnBQv95LeMoDZkz
acc genotypes	397
peaks no back	113
removed edges	NA
prop rem edges	NA

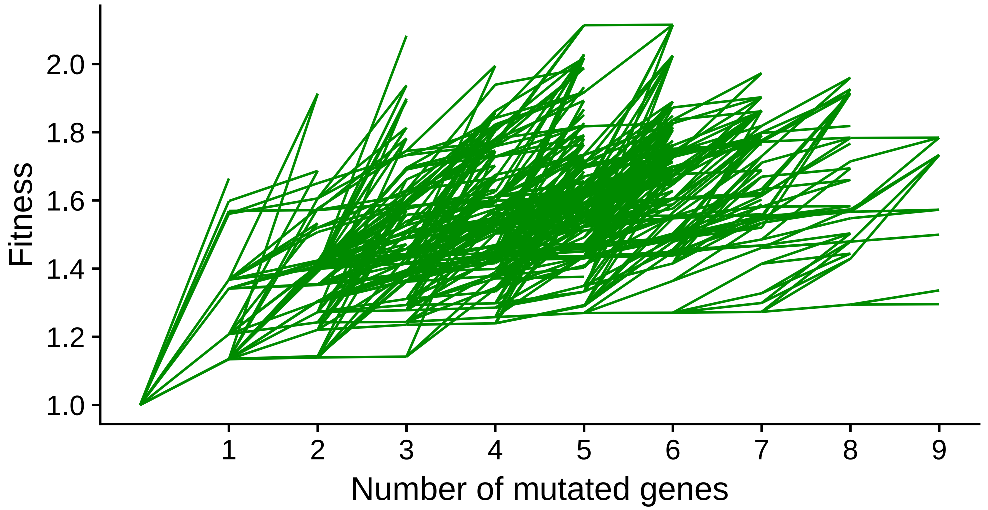

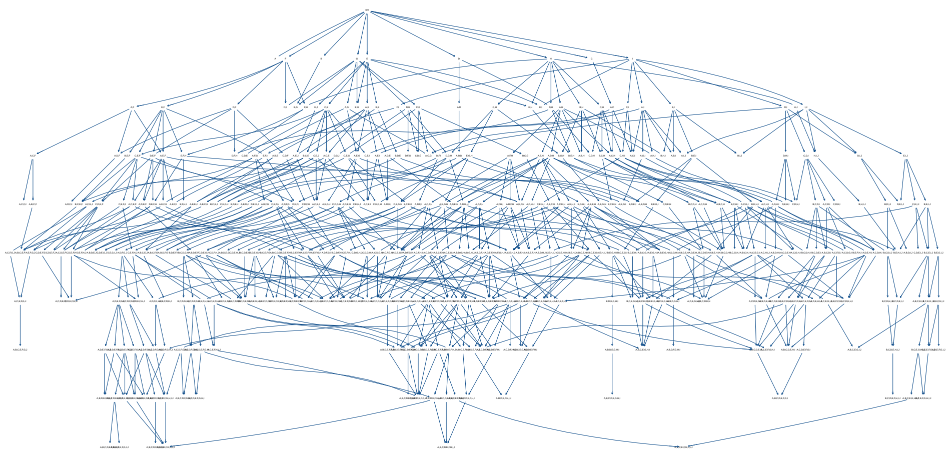

ID	qhoM9EMECJiKAEai
acc genotypes	301
peaks no back	43
removed edges	NA
prop rem edges	NA

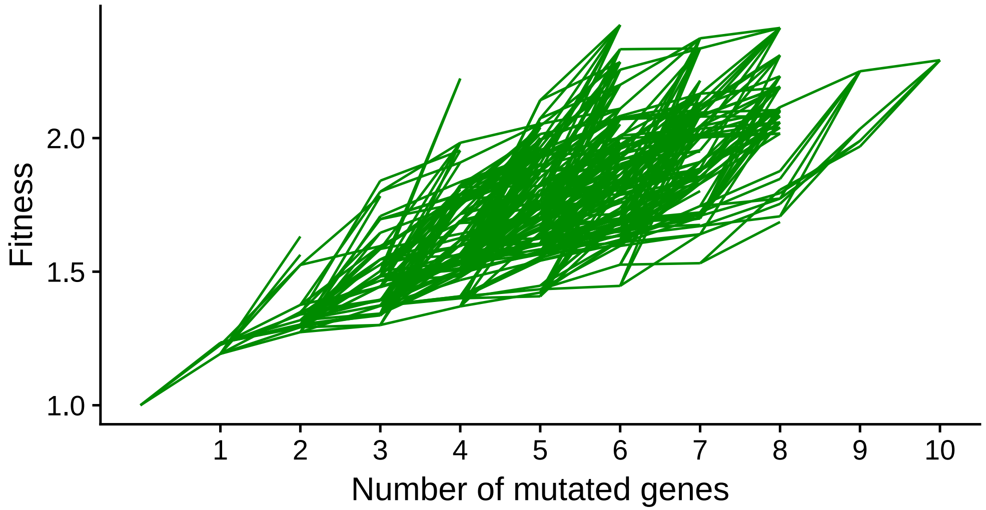

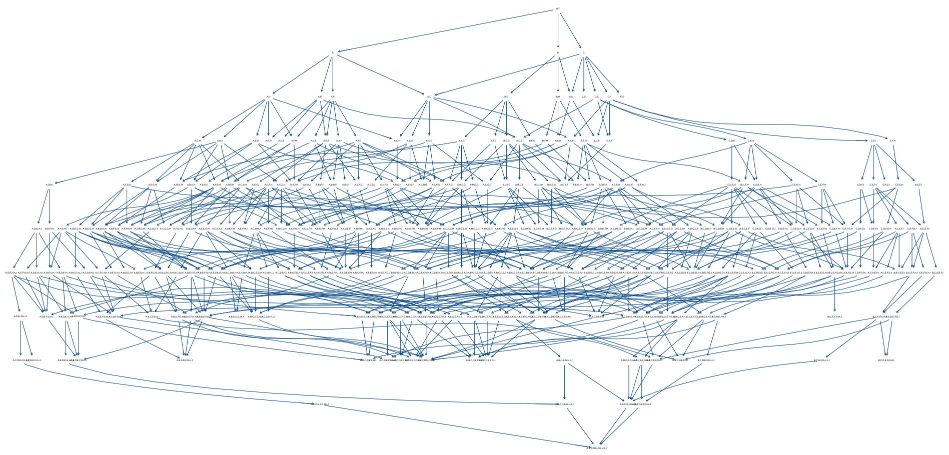

ID	qIG3omWesEf8erJY
acc genotypes	330
peaks no back	79
removed edges	NA
prop rem edges	NA

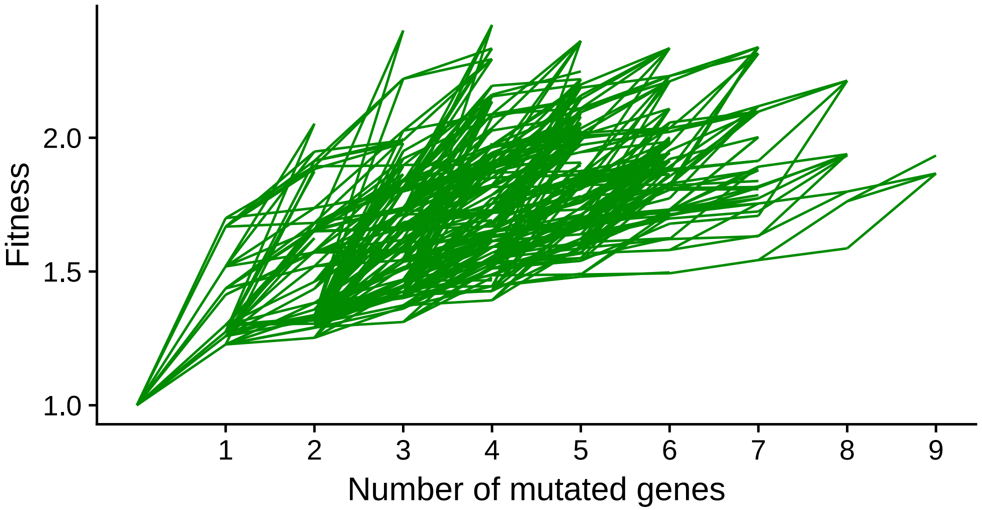

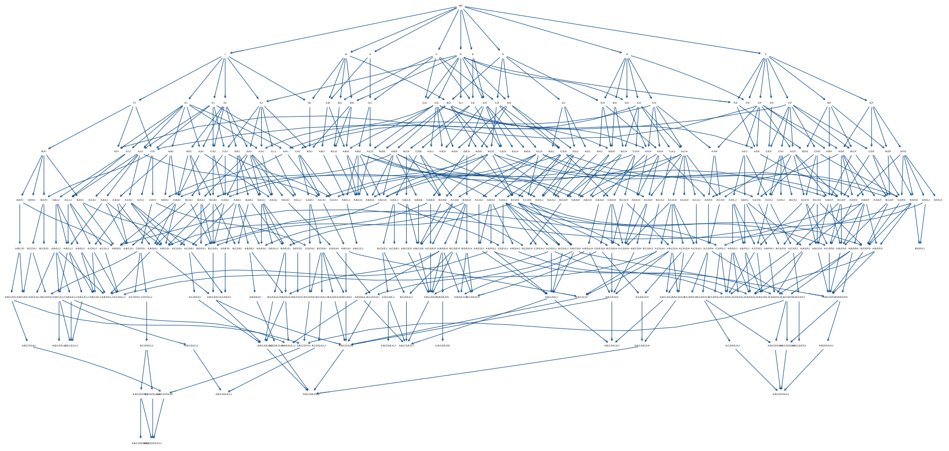

ID	qiw3nSX8uOEX3EE2
acc genotypes	123
peaks no back	37
removed edges	NA
prop rem edges	NA

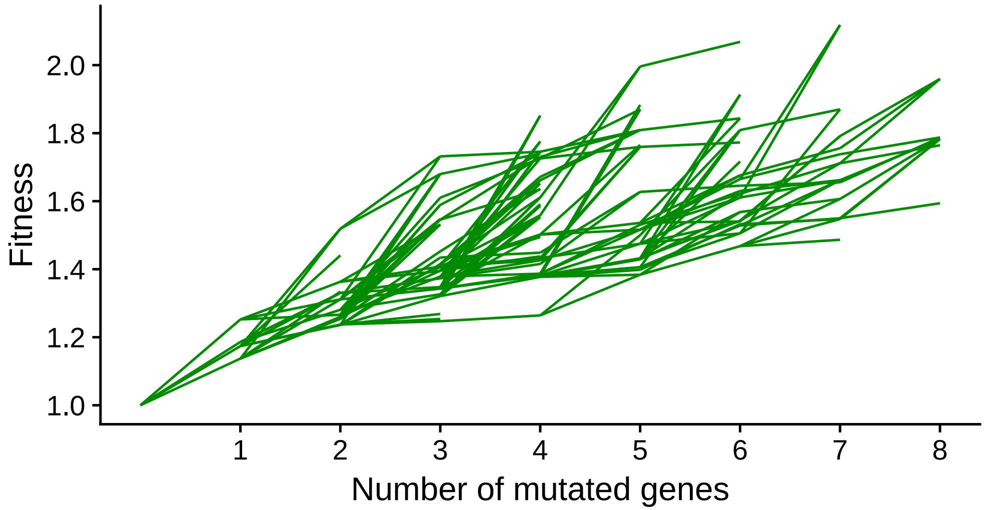

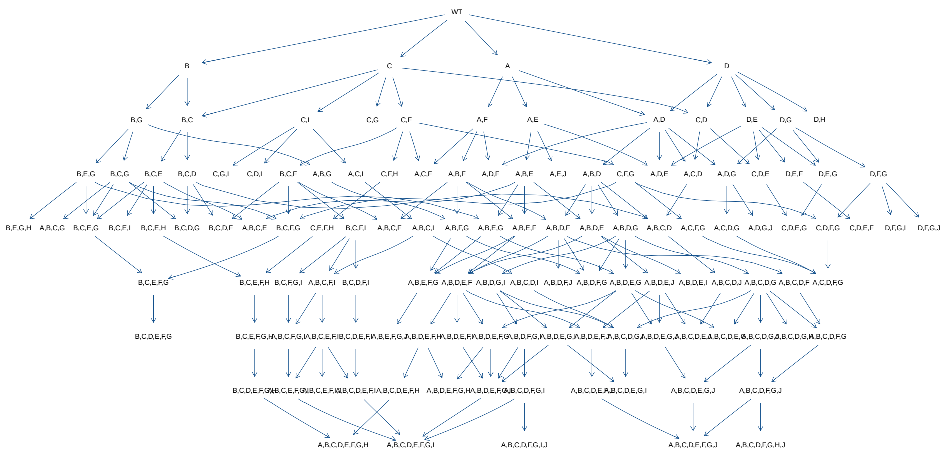

ID	QLvp54fgddHiWuDQ
acc genotypes	365
peaks no back	92
removed edges	NA
prop rem edges	NA

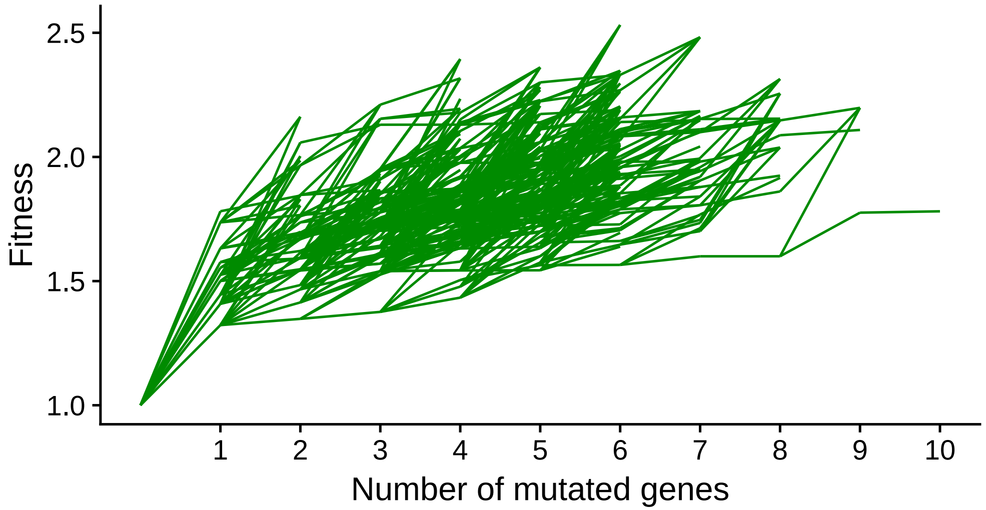

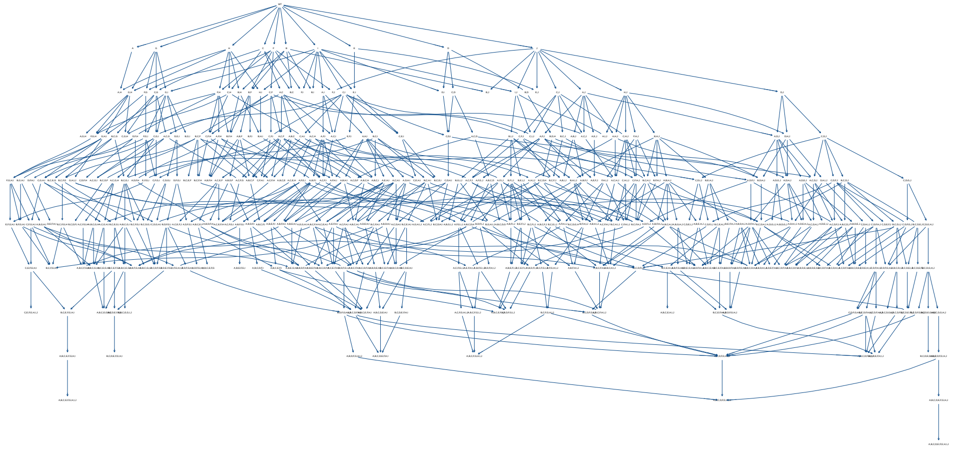

ID	R1rRFK7kvGTL7JOu
acc genotypes	95
peaks no back	39
removed edges	NA
prop rem edges	NA

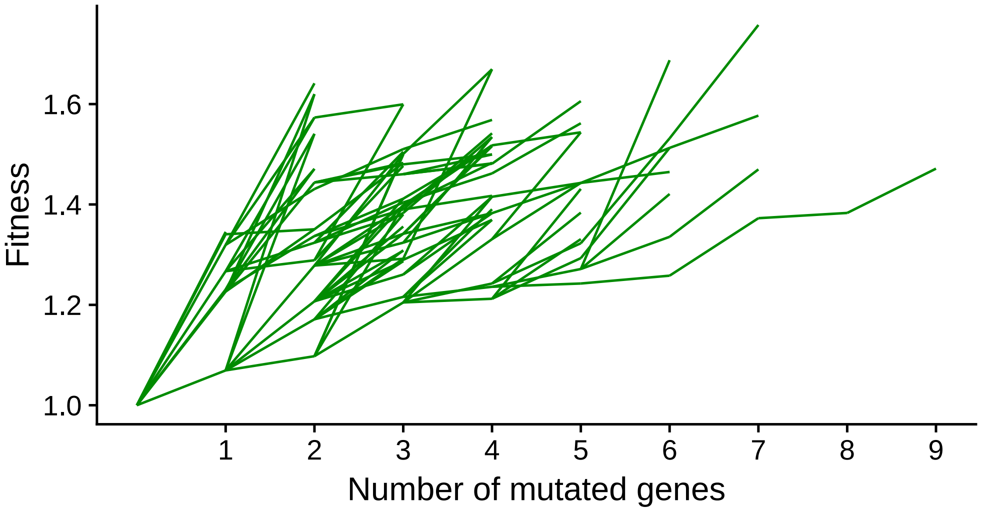

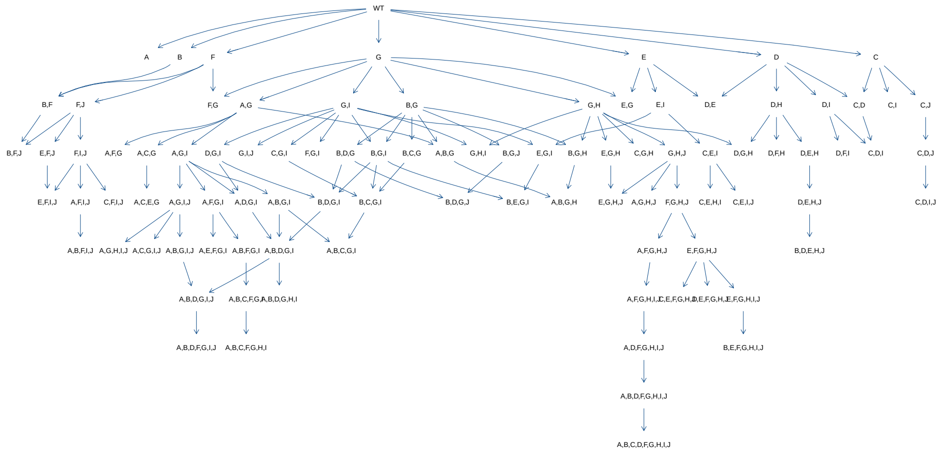

ID	r8ycAIoMMwveOtjh
acc genotypes	474
peaks no back	56
removed edges	NA
prop rem edges	NA

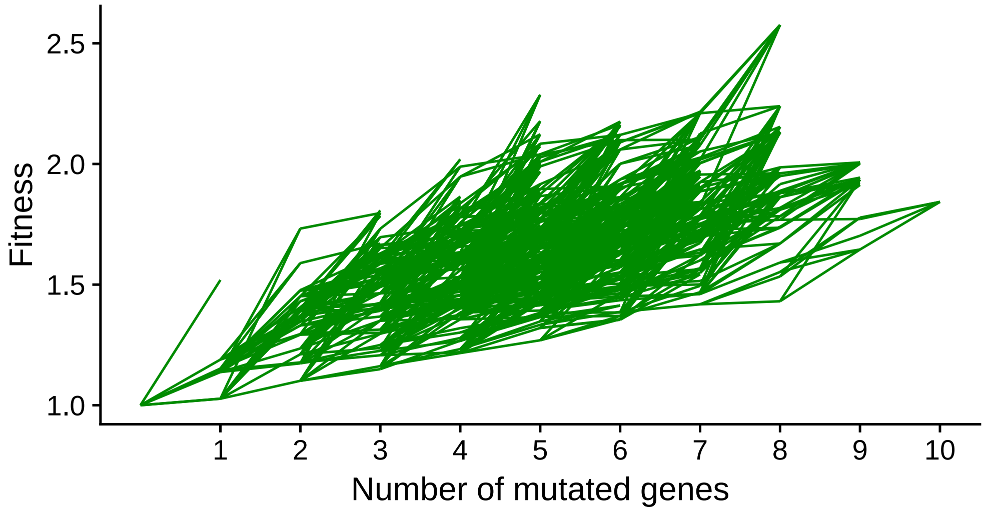

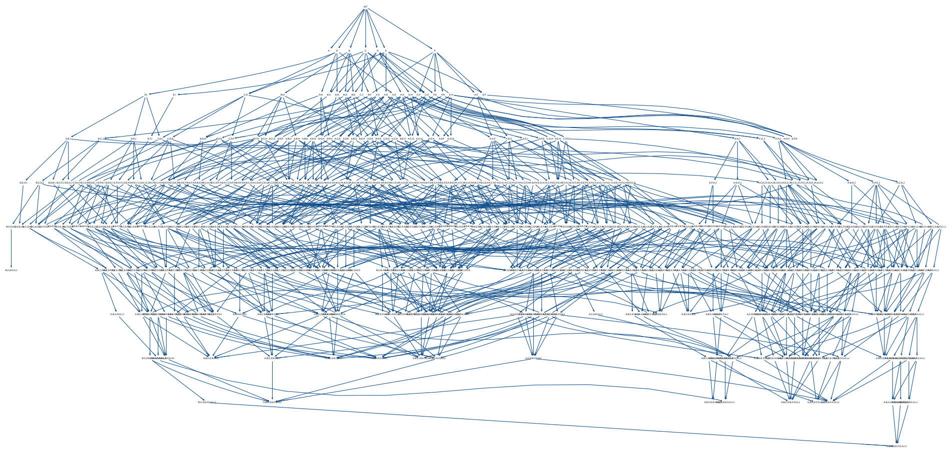

ID	rFXDokDyxb54xE8I
acc genotypes	267
peaks no back	95
removed edges	NA
prop rem edges	NA

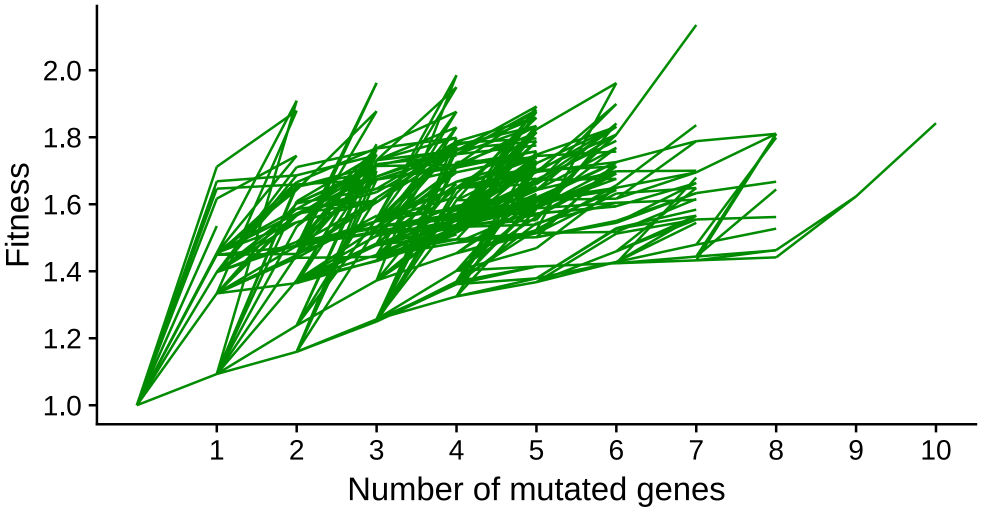

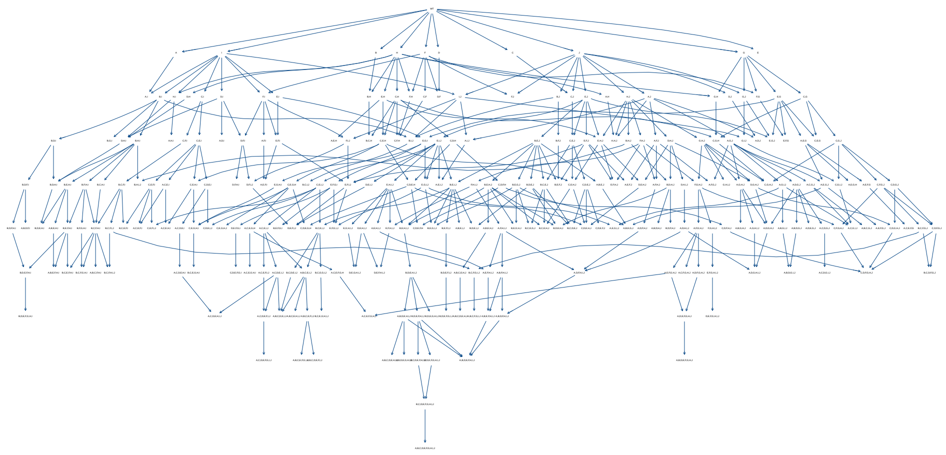

ID	Rgbd18KZtZQm310aF
acc genotypes	305
peaks no back	59
removed edges	NA
prop rem edges	NA

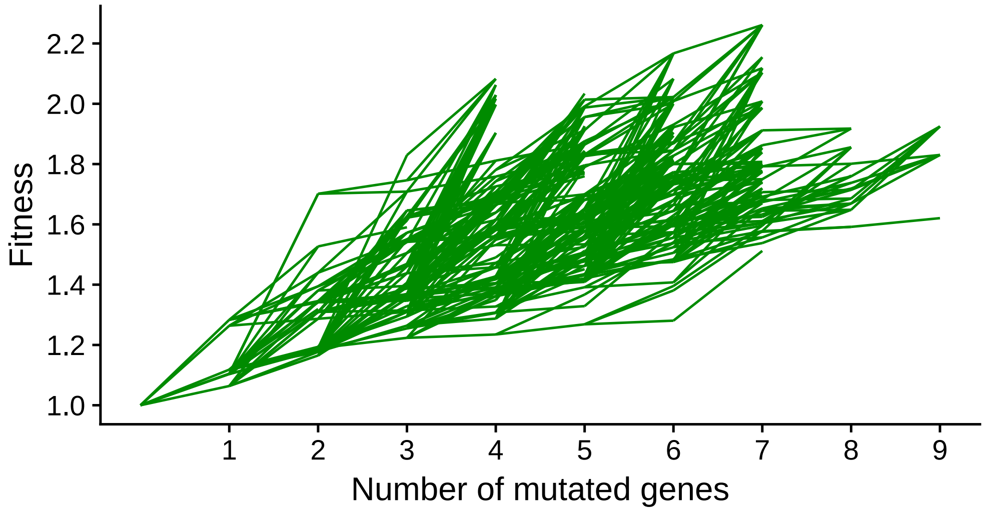

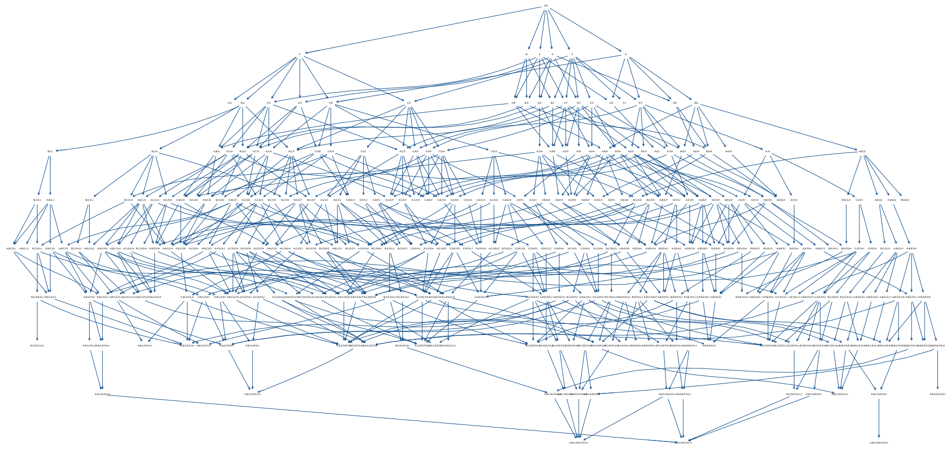

ID	RgQW10wScrRmd9eE8
acc genotypes	65
peaks no back	28
removed edges	NA
prop rem edges	NA

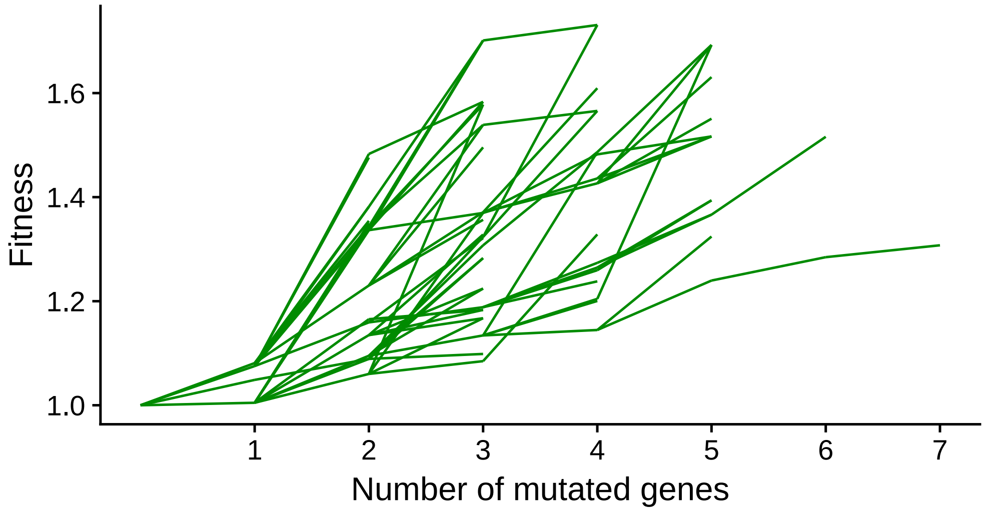

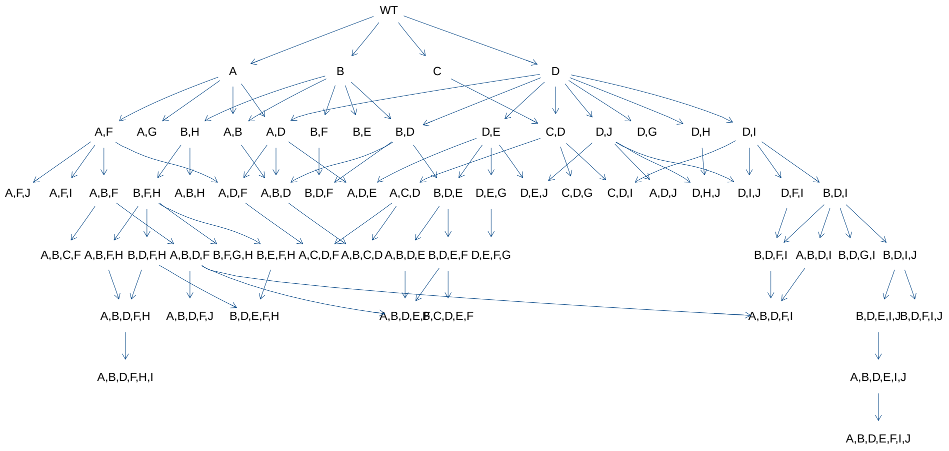

ID	RM1oRnvkQ74p10MYi
acc genotypes	211
peaks no back	85
removed edges	NA
prop rem edges	NA

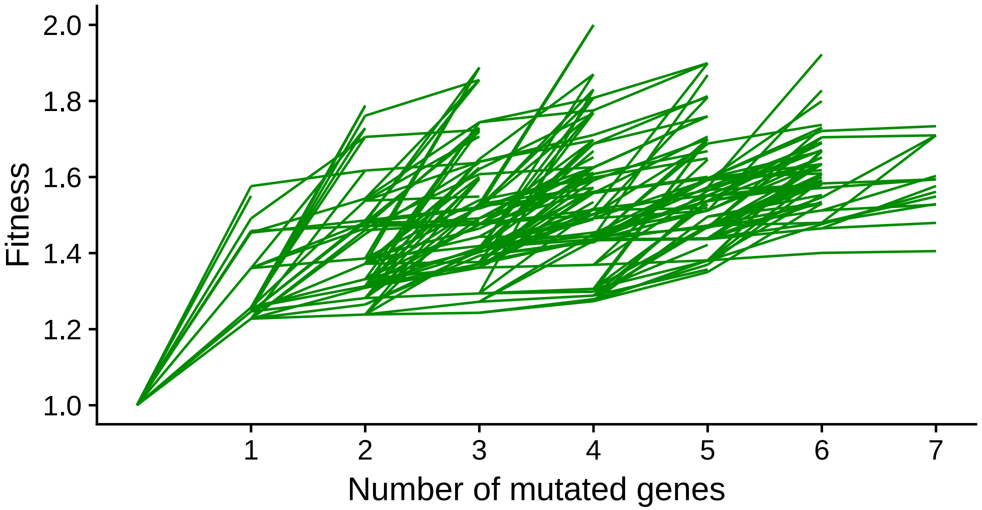

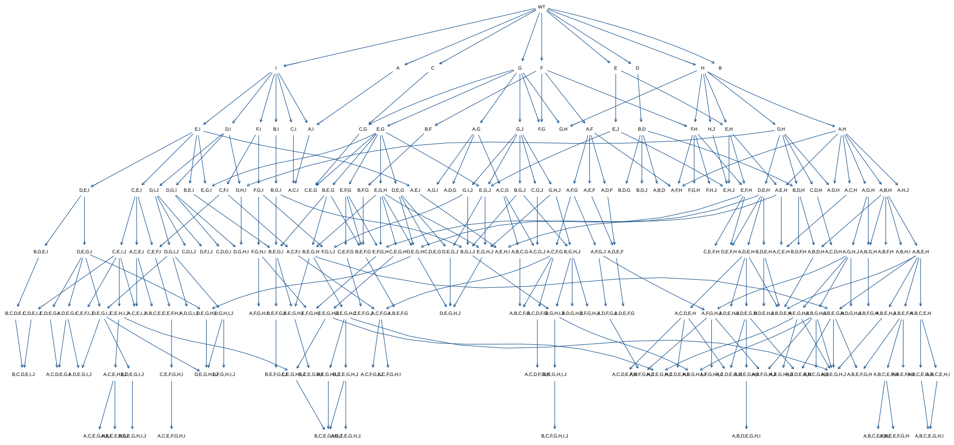

ID	rwEpMgO6p9XY5Fe3
acc genotypes	59
peaks no back	25
removed edges	NA
prop rem edges	NA

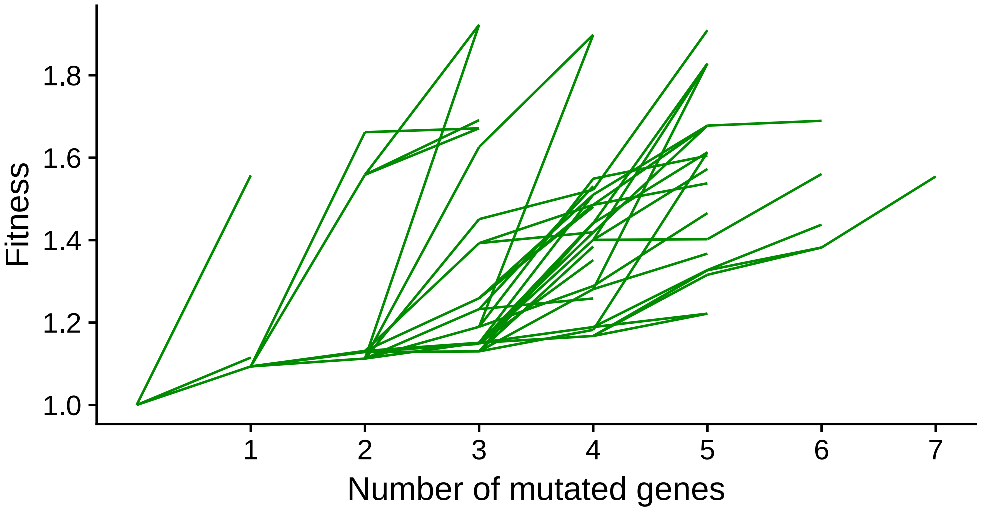

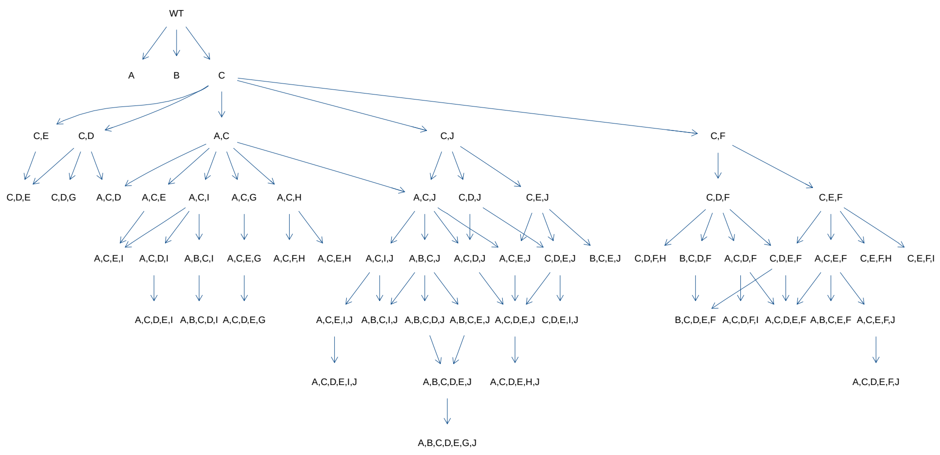

ID	siB10Dc3bBqHdWLsu
acc genotypes	152
peaks no back	59
removed edges	NA
prop rem edges	NA

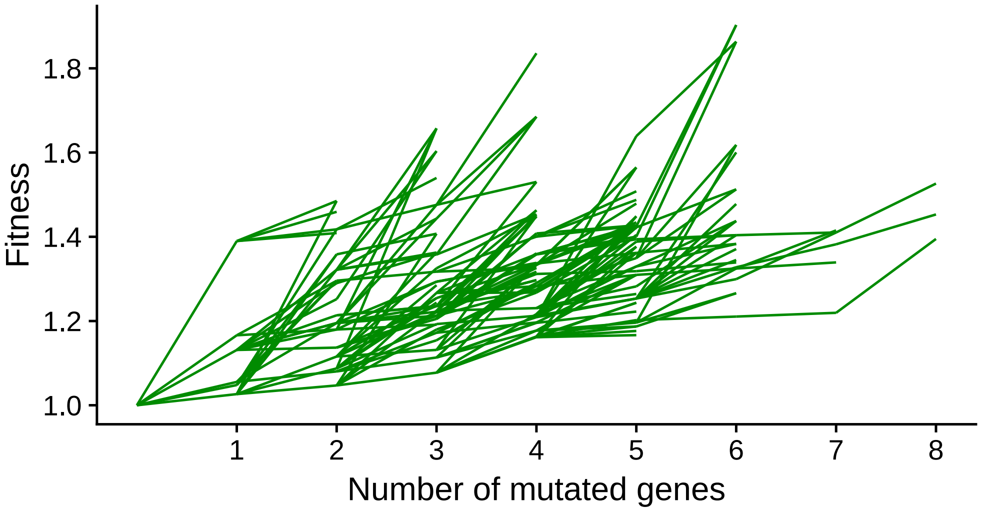

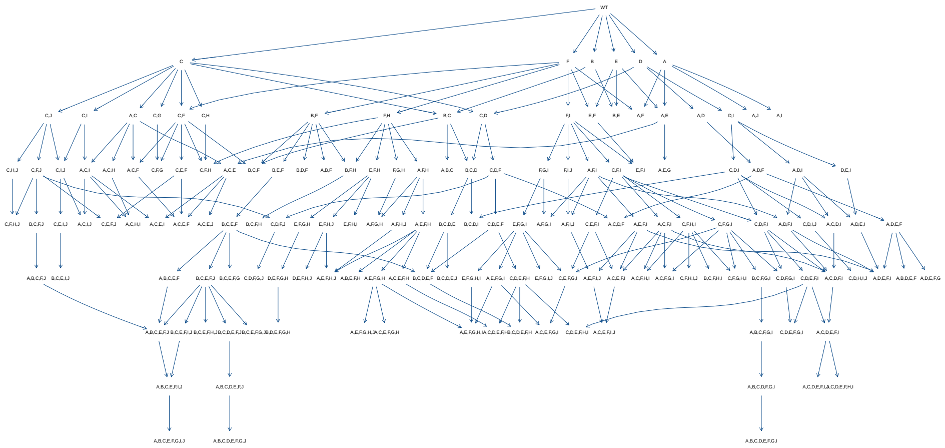

ID	SkrmpMeOx2A5XIjs
acc genotypes	41
peaks no back	14
removed edges	NA
prop rem edges	NA

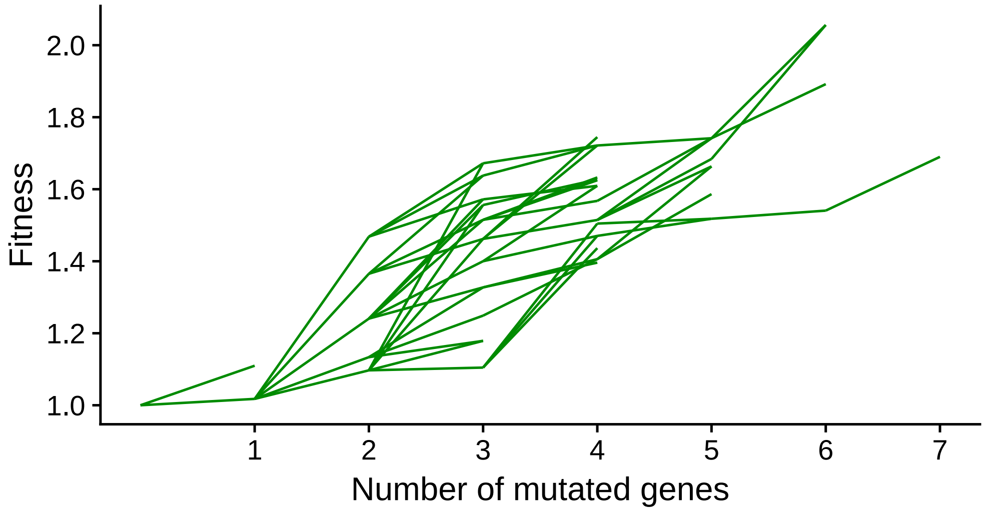

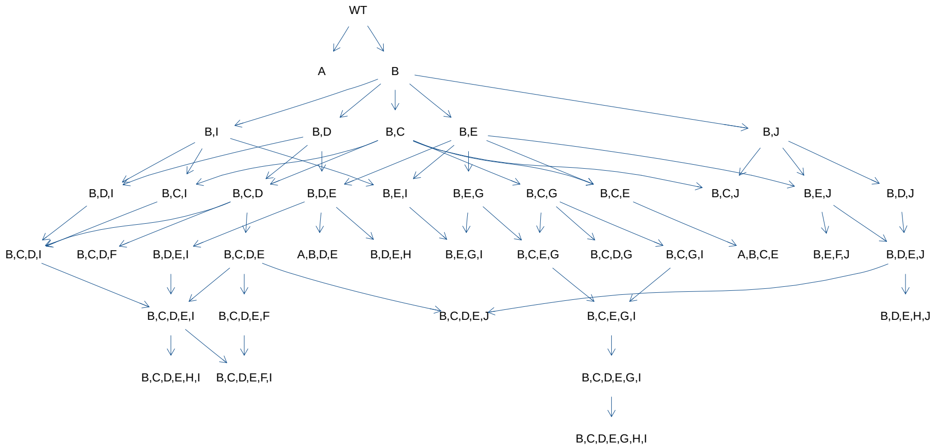

ID	sKV2JZzX6hoi6ZXJ
acc genotypes	302
peaks no back	68
removed edges	NA
prop rem edges	NA

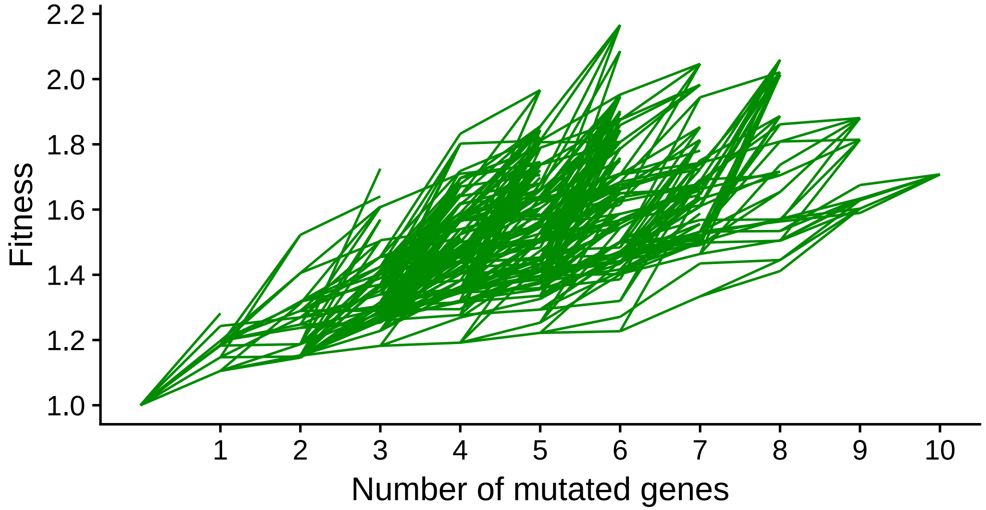

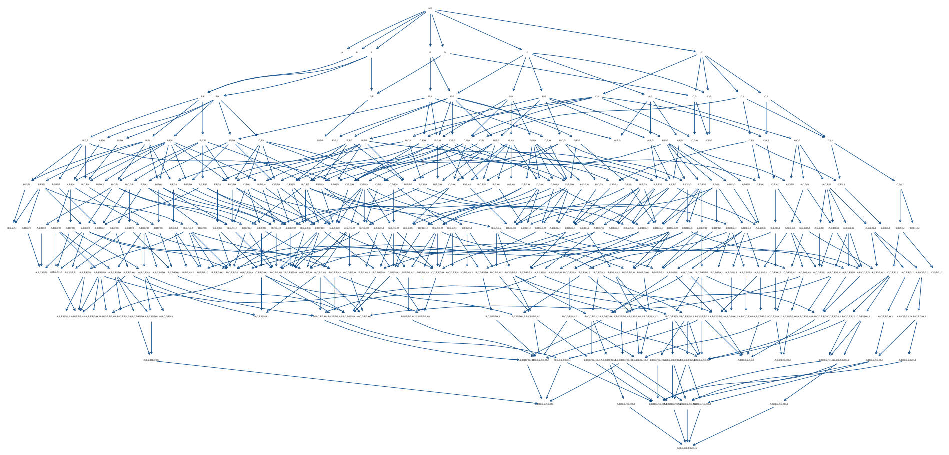

ID	SRQPFRnma8vPJxWK
acc genotypes	327
peaks no back	103
removed edges	NA
prop rem edges	NA

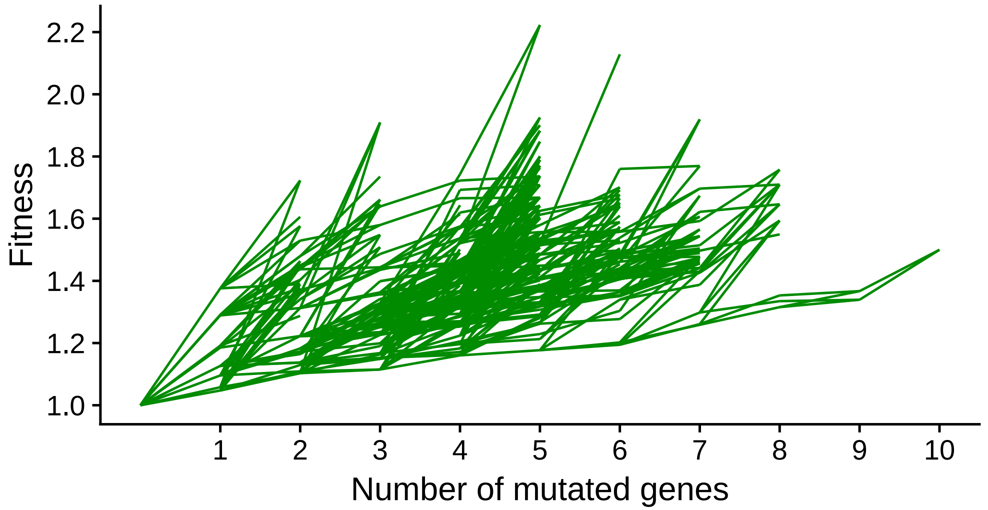

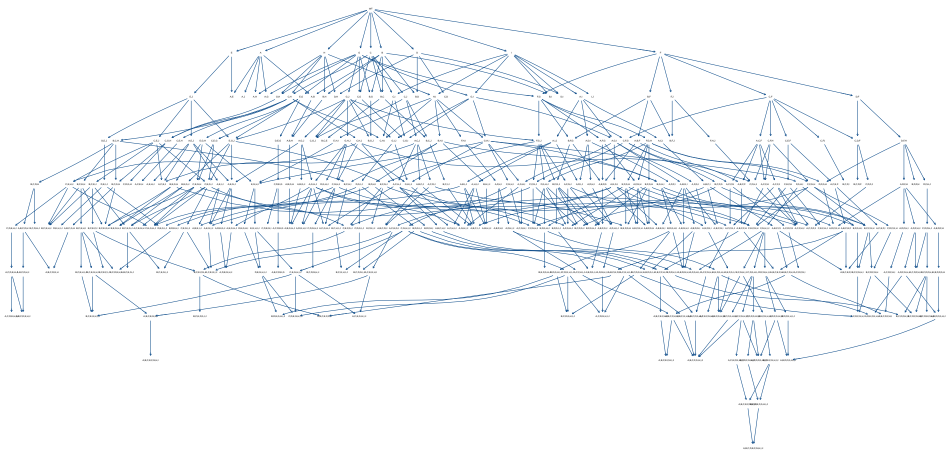

ID	suRv8SDkQ3Fspdke
acc genotypes	330
peaks no back	113
removed edges	NA
prop rem edges	NA

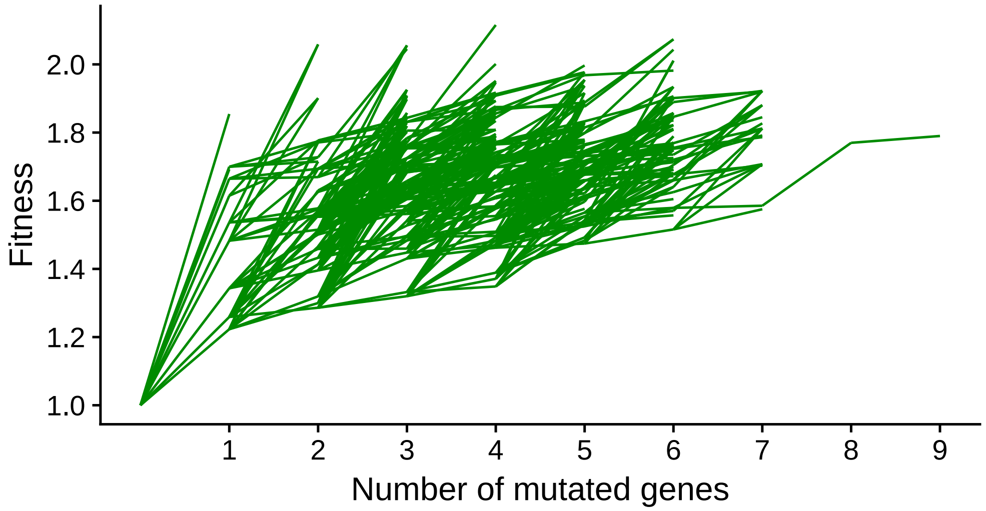

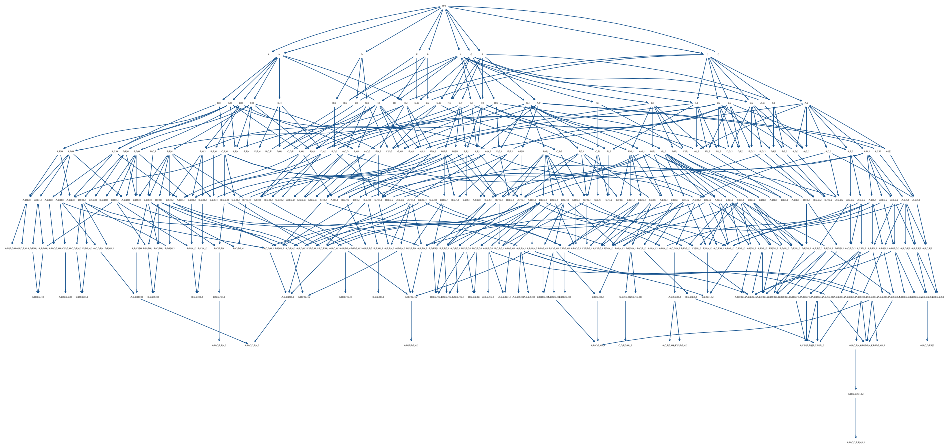

ID	SUX11JboEBbU7Sly
acc genotypes	159
peaks no back	49
removed edges	NA
prop rem edges	NA

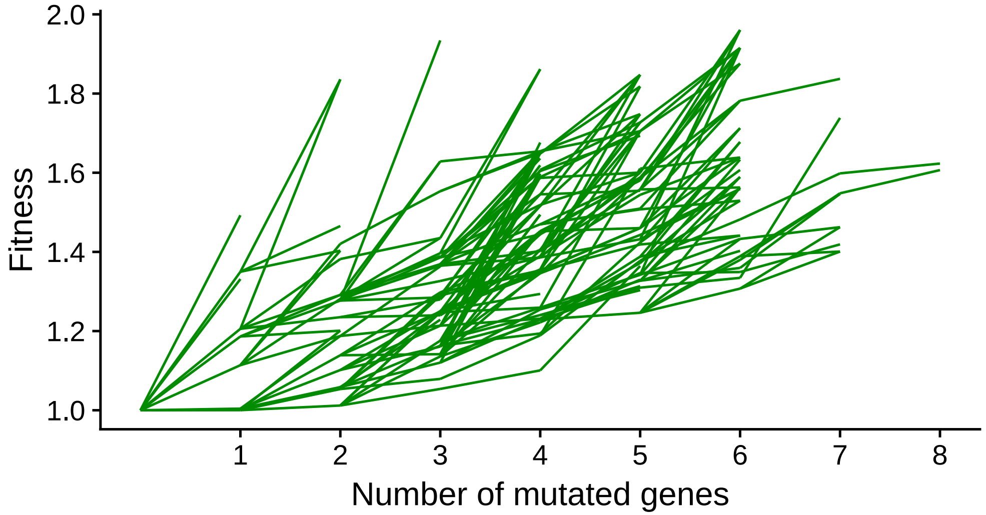

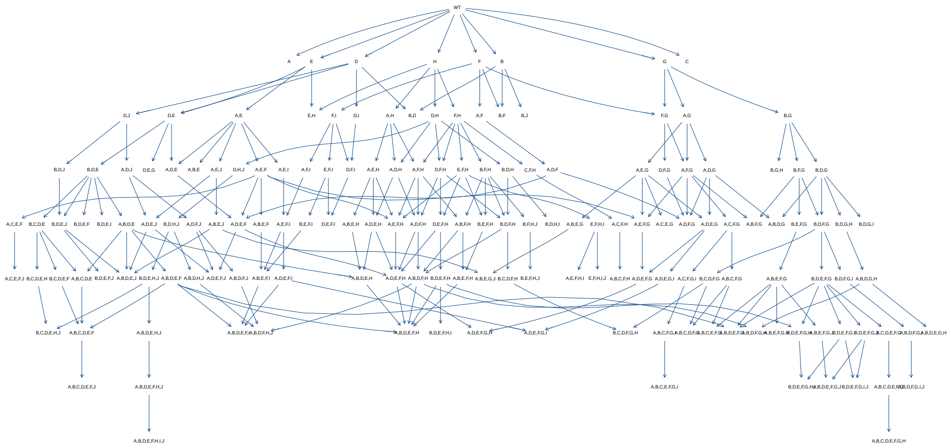

ID	T1lg10UC4ysvdG1vj
acc genotypes	75
peaks no back	31
removed edges	NA
prop rem edges	NA

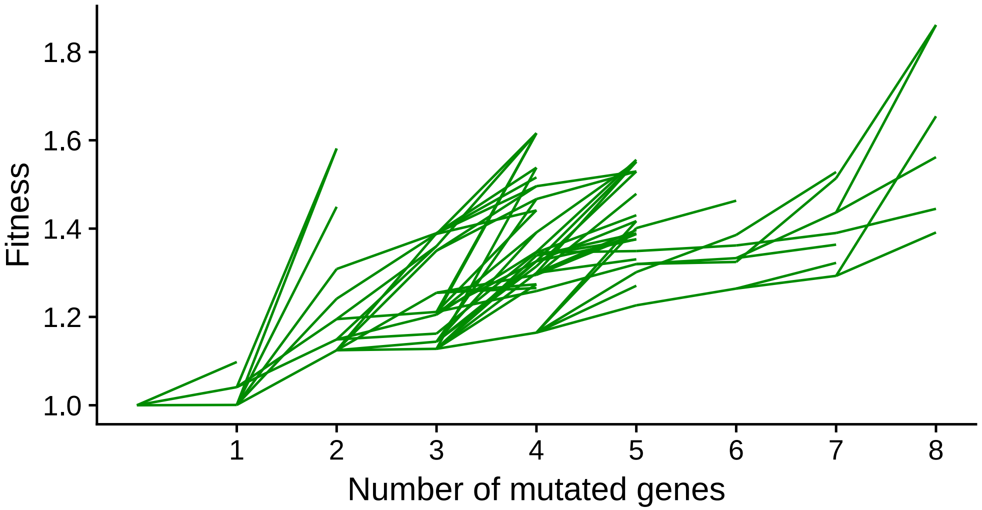

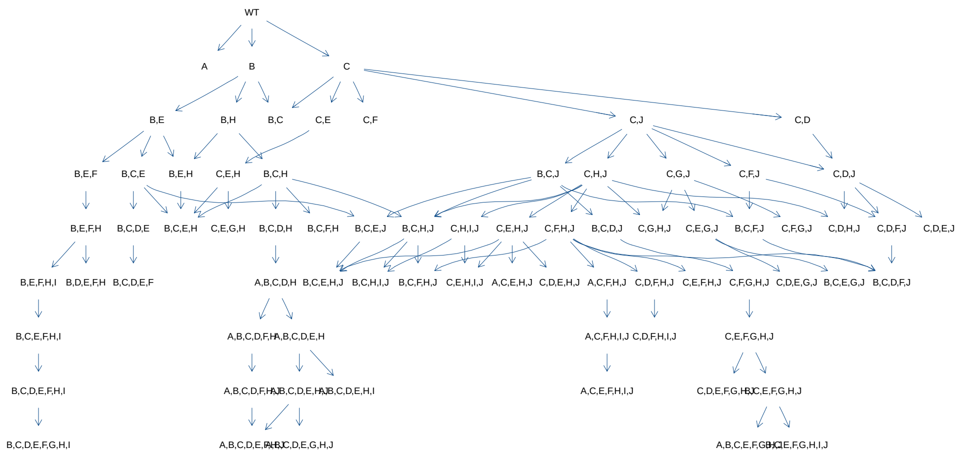

ID	T5oZEwzVo6JyuGLW
acc genotypes	125
peaks no back	52
removed edges	NA
prop rem edges	NA

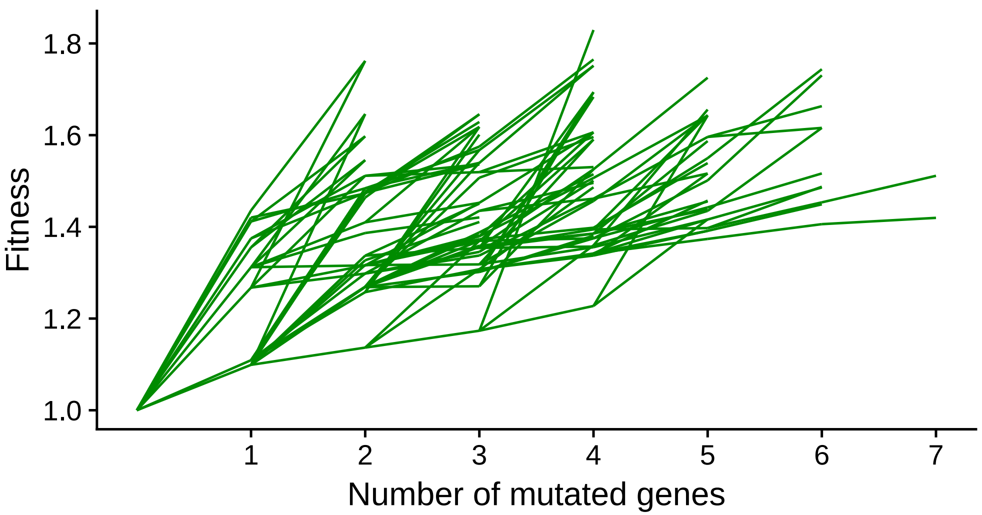

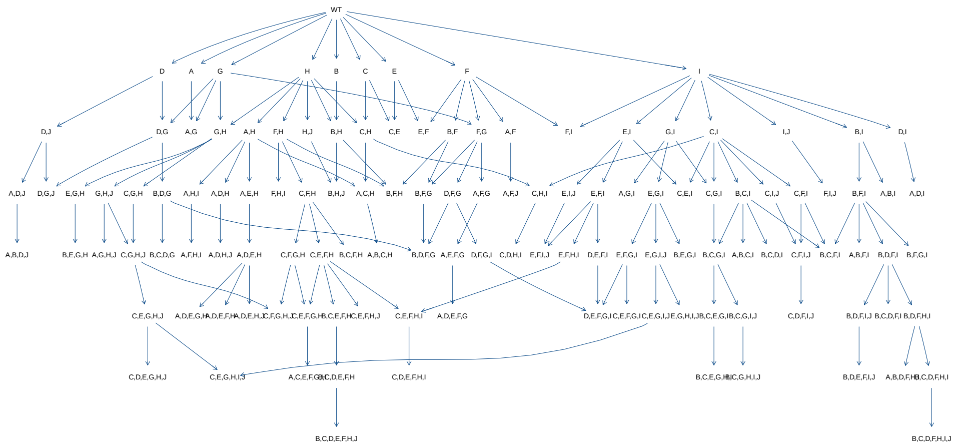

ID	Tklr7flyal9UtU86
acc genotypes	142
peaks no back	60
removed edges	NA
prop rem edges	NA

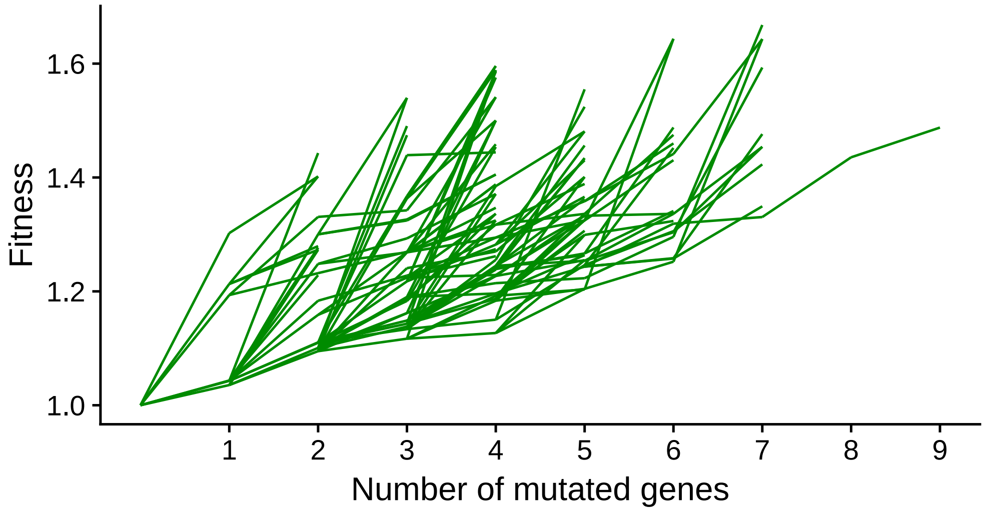

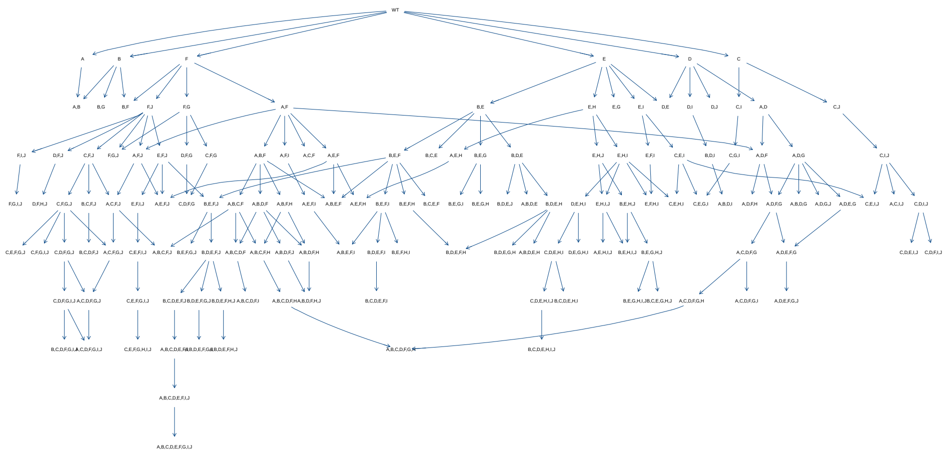

ID	tKkGKUAR1sBTETkR
acc genotypes	209
peaks no back	70
removed edges	NA
prop rem edges	NA

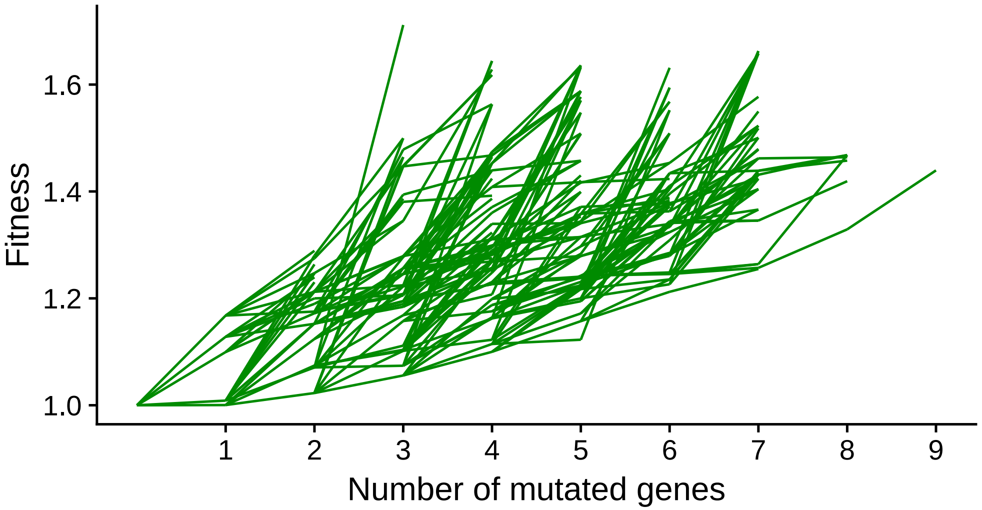

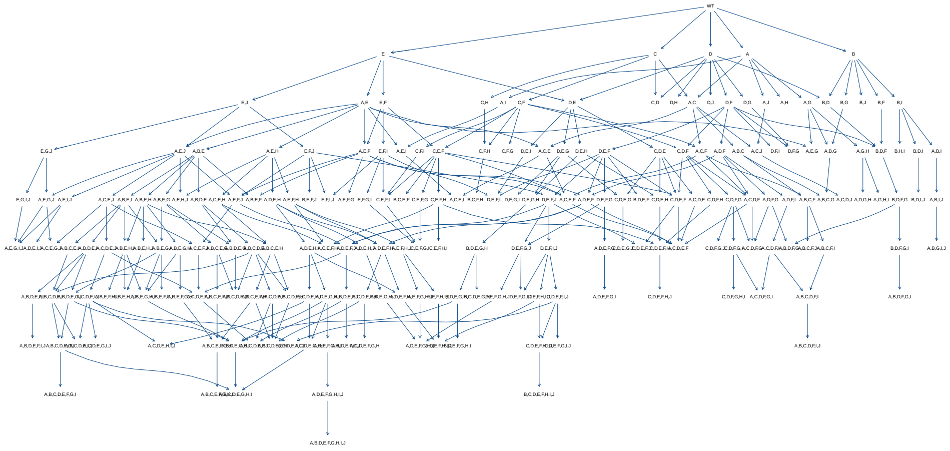

ID	tyHTZO351lxuXhpp
acc genotypes	173
peaks no back	40
removed edges	NA
prop rem edges	NA

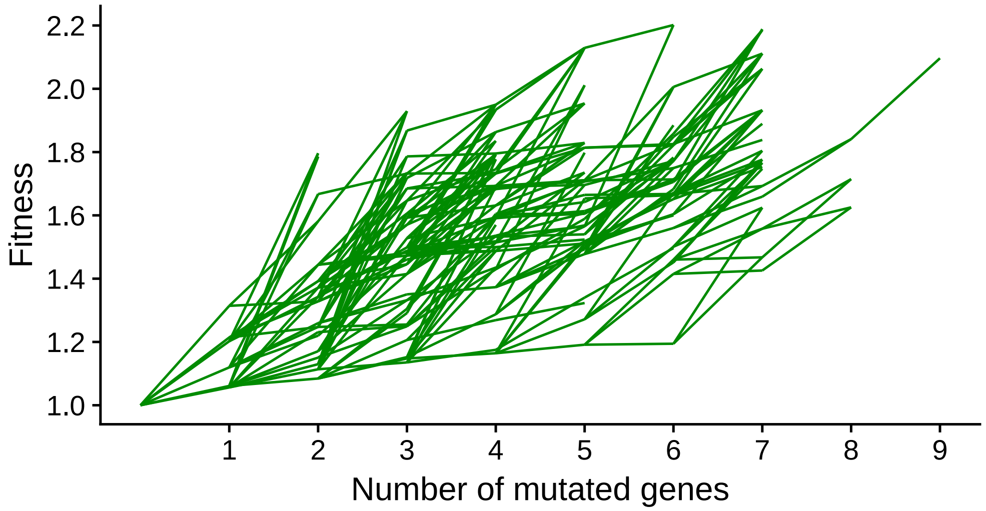

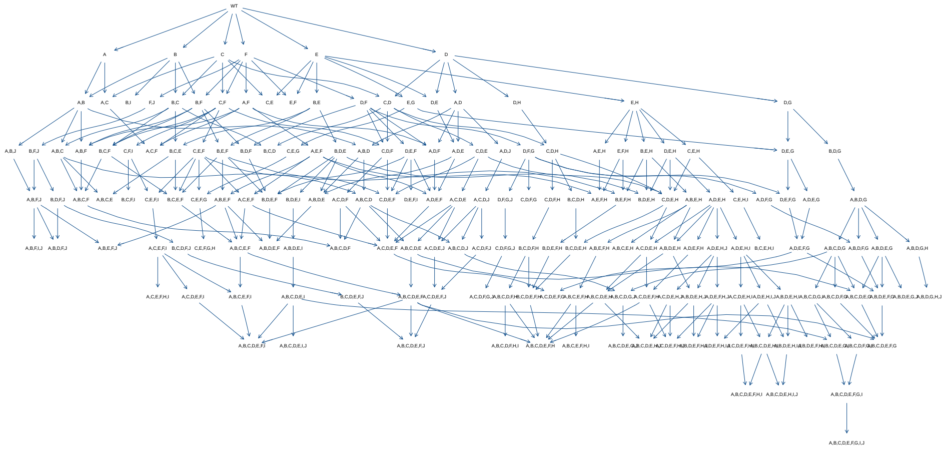

ID	u4QnEyMMh8u2Dmoq
acc genotypes	302
peaks no back	82
removed edges	NA
prop rem edges	NA

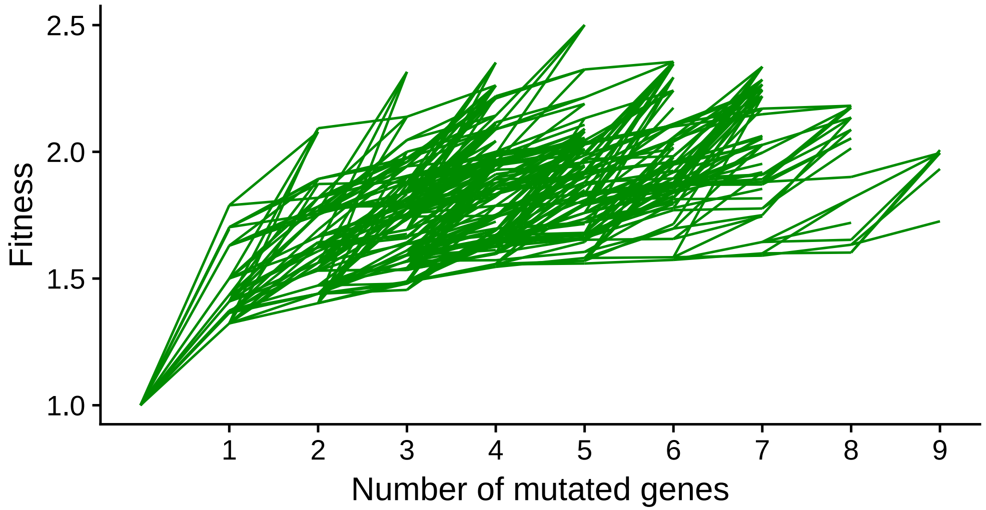

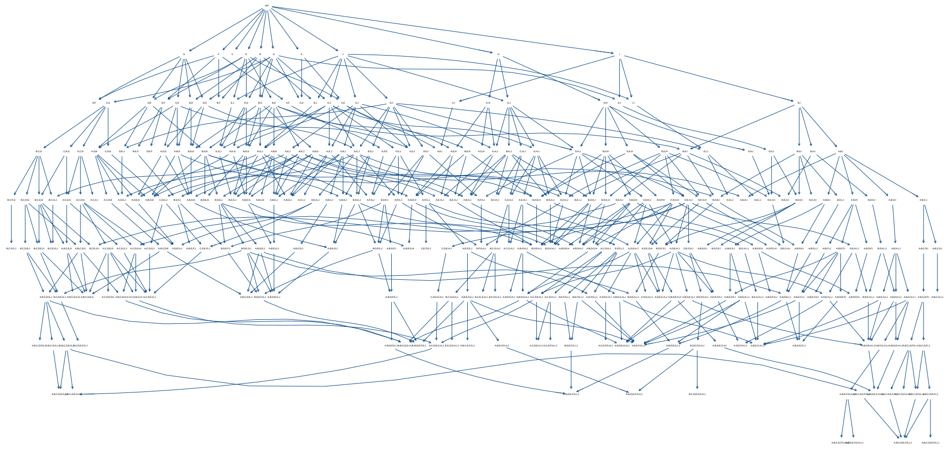

ID	u6D56PFLKLEytlrF
acc genotypes	243
peaks no back	53
removed edges	NA
prop rem edges	NA

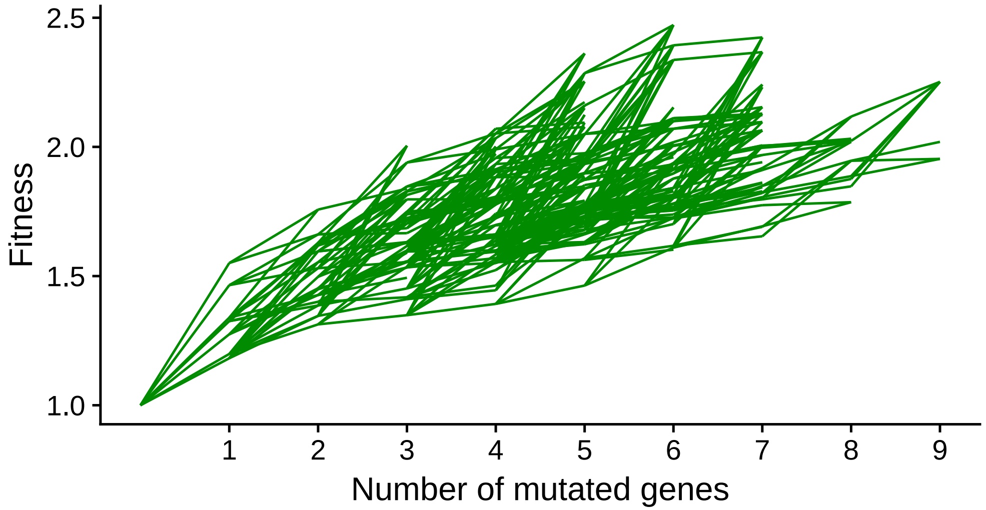

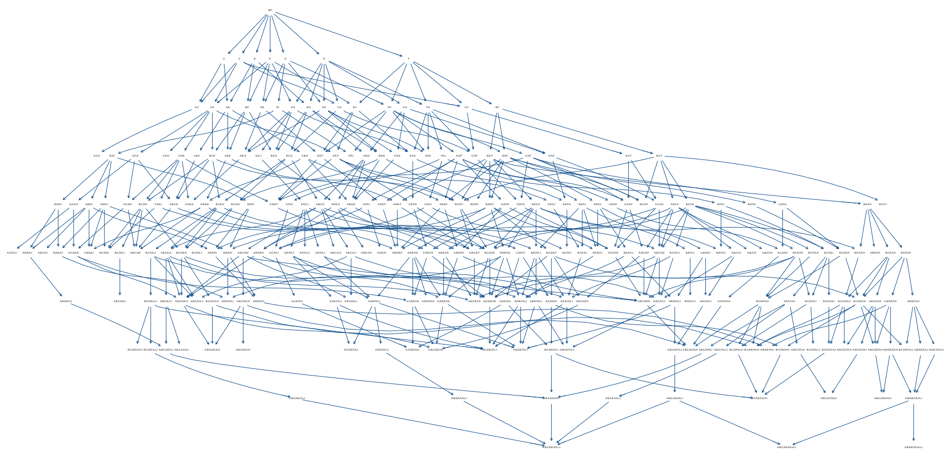

ID	u8aziQ10mxkwi2VPc
acc genotypes	57
peaks no back	19
removed edges	NA
prop rem edges	NA

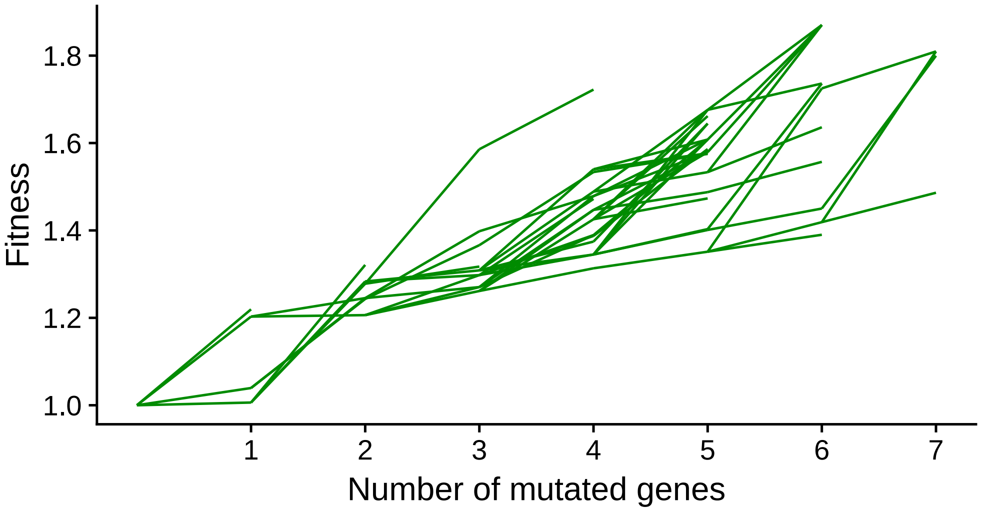

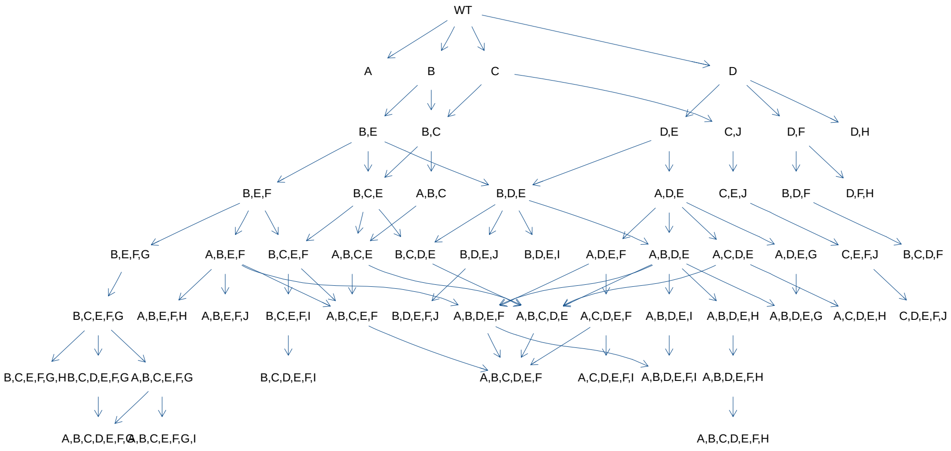

ID	U9tKbeEaFO915pcB
acc genotypes	151
peaks no back	66
removed edges	NA
prop rem edges	NA

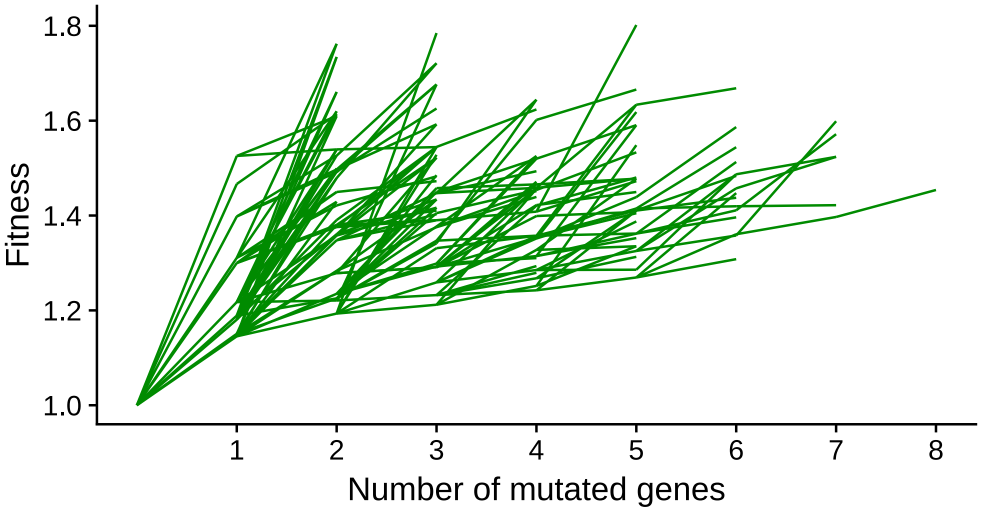

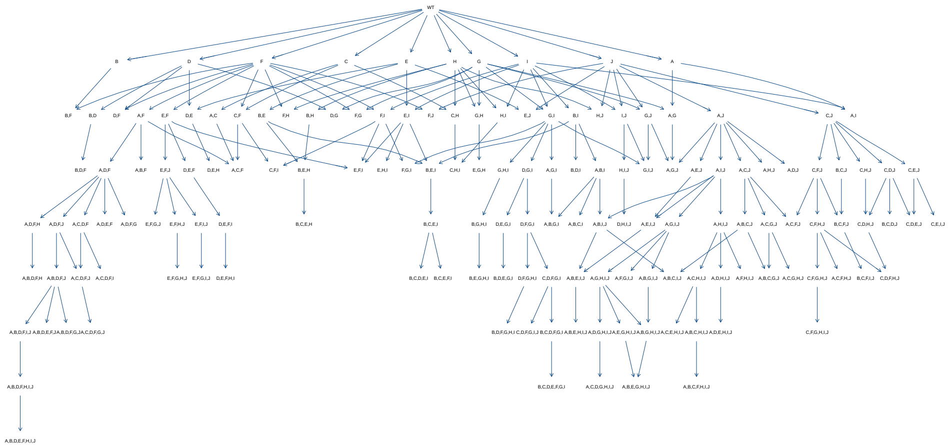

ID	uaHRvH1XC9BScLS5
acc genotypes	333
peaks no back	60
removed edges	NA
prop rem edges	NA

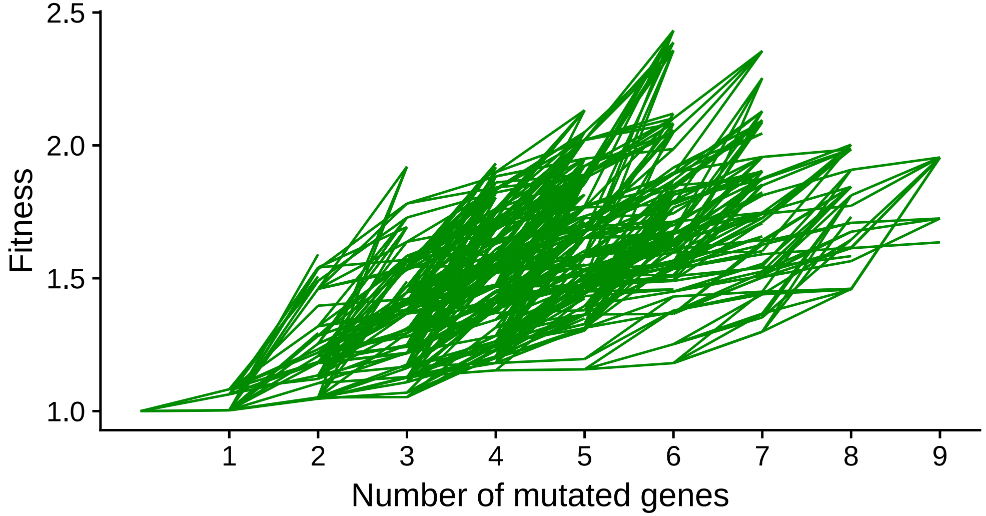

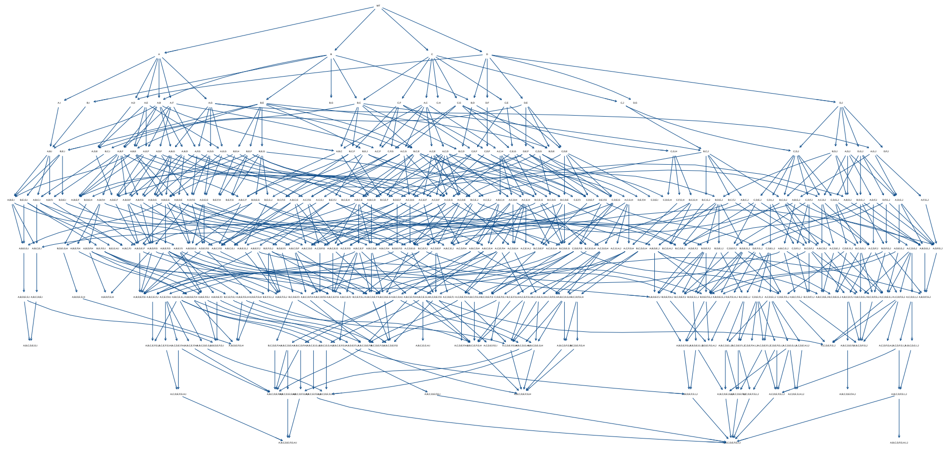

ID	UdIzVbWbljfForTa
acc genotypes	206
peaks no back	78
removed edges	NA
prop rem edges	NA

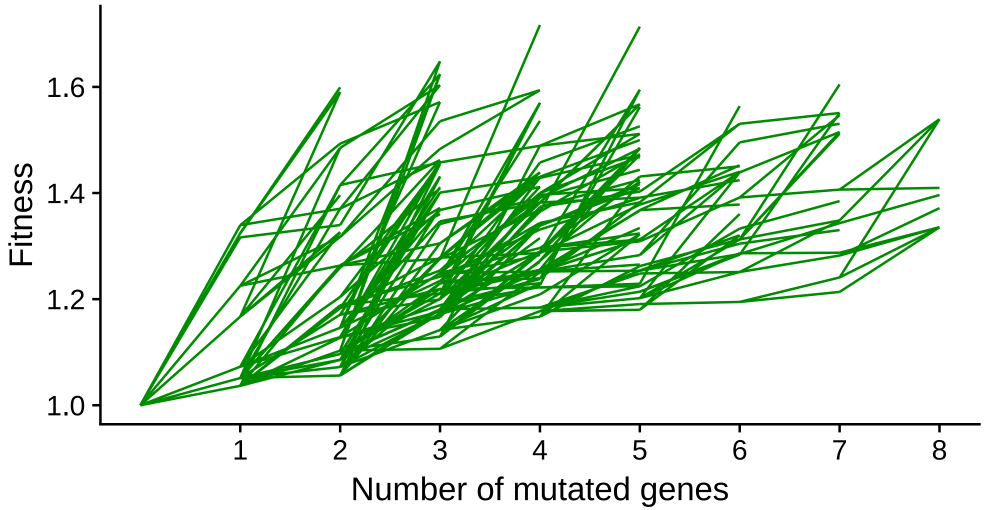

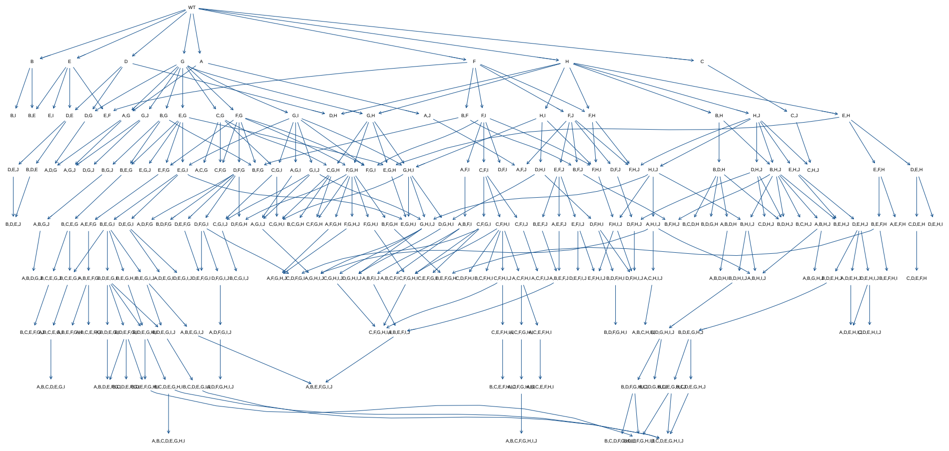

ID	Ug5ffs10lfz6uMbgO
acc genotypes	225
peaks no back	65
removed edges	NA
prop rem edges	NA

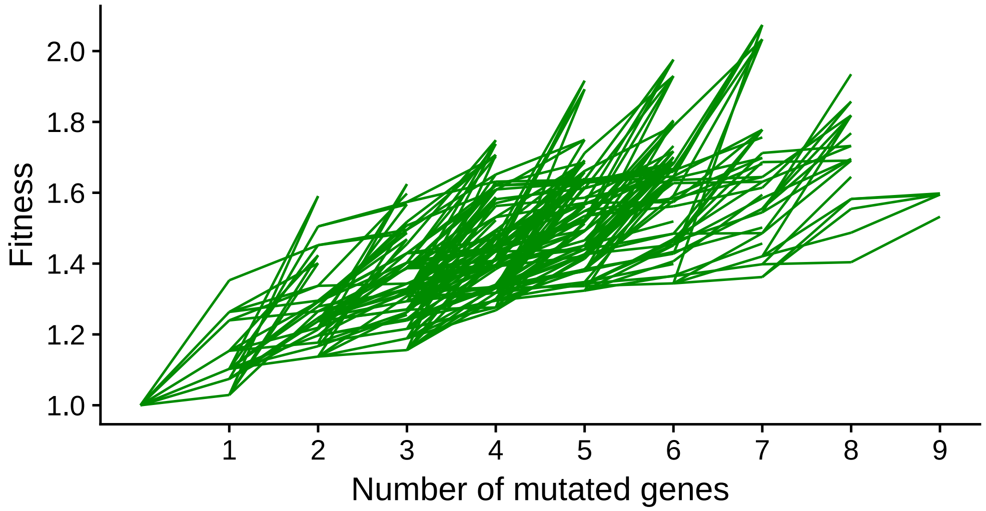

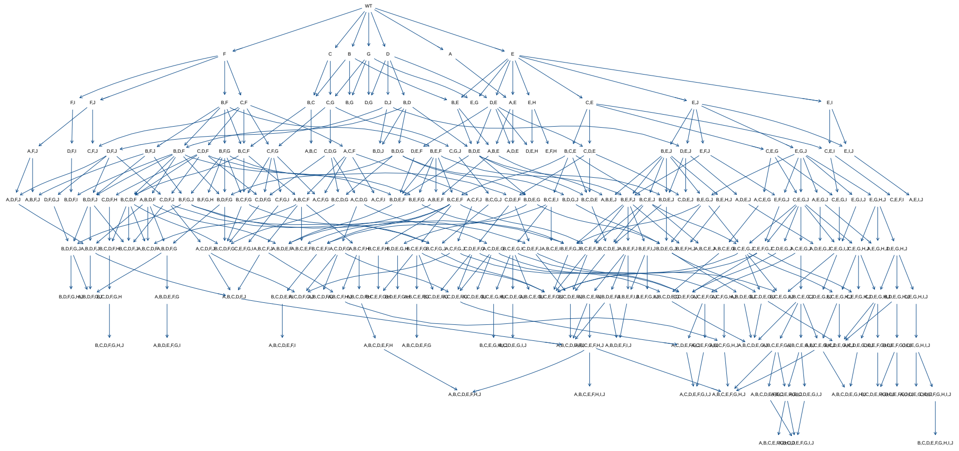

ID	UmunqSzUuUKy8o5I
acc genotypes	172
peaks no back	56
removed edges	NA
prop rem edges	NA

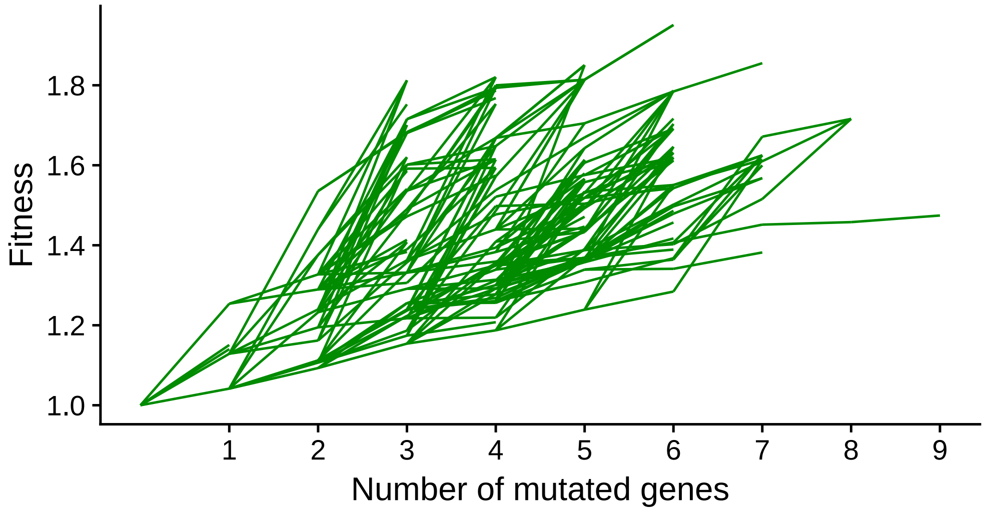

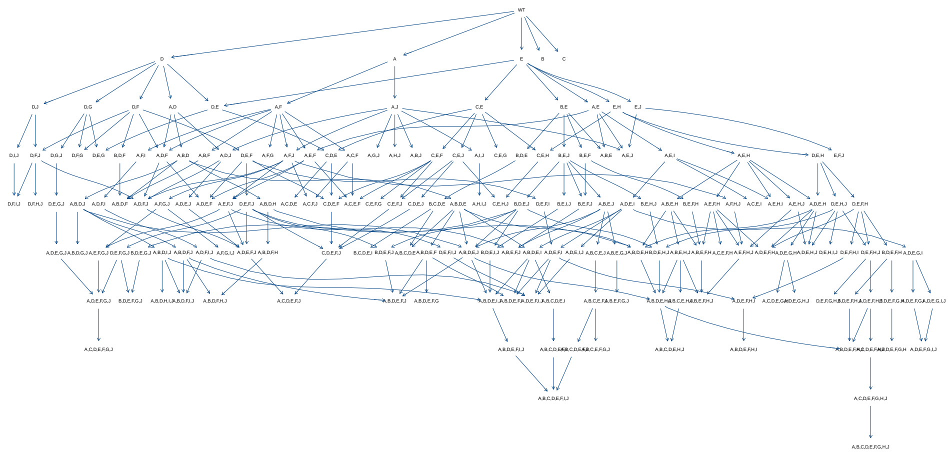

ID	UOqyH3eyYqb108SQa
acc genotypes	360
peaks no back	71
removed edges	NA
prop rem edges	NA

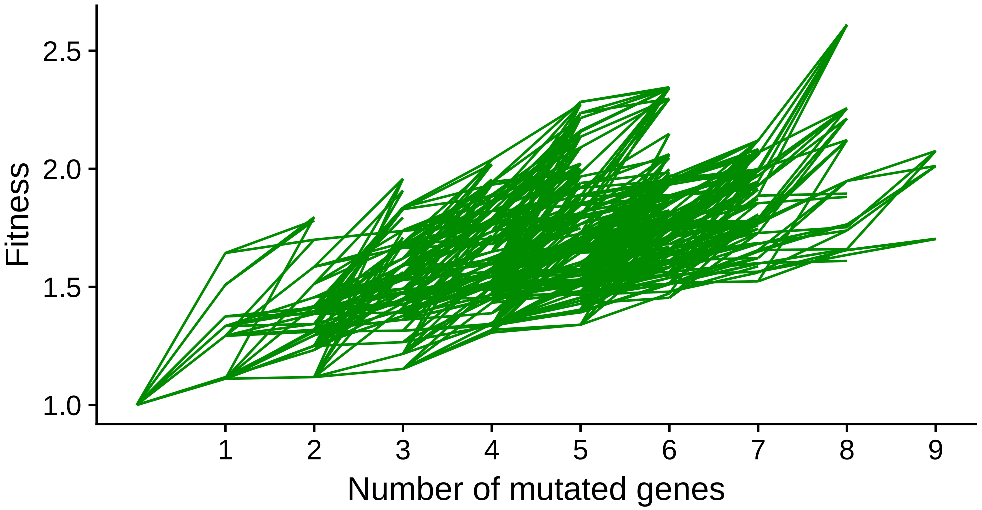

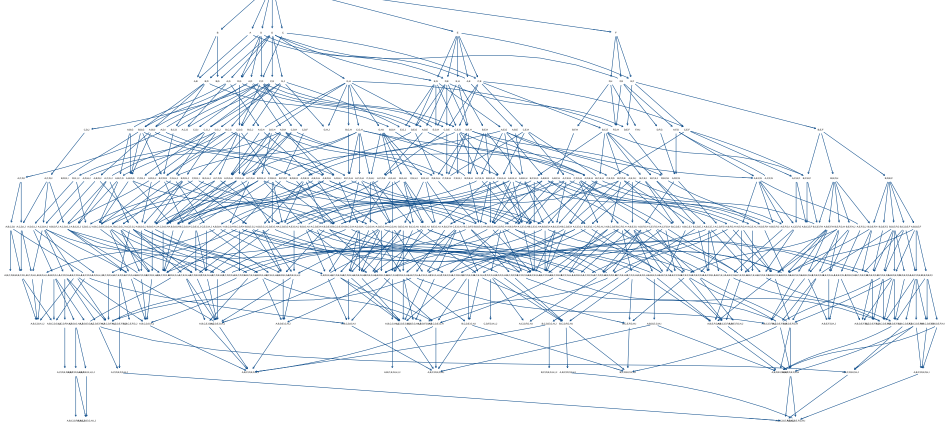

ID	UpYY82ecse62Y3kB
acc genotypes	102
peaks no back	28
removed edges	NA
prop rem edges	NA

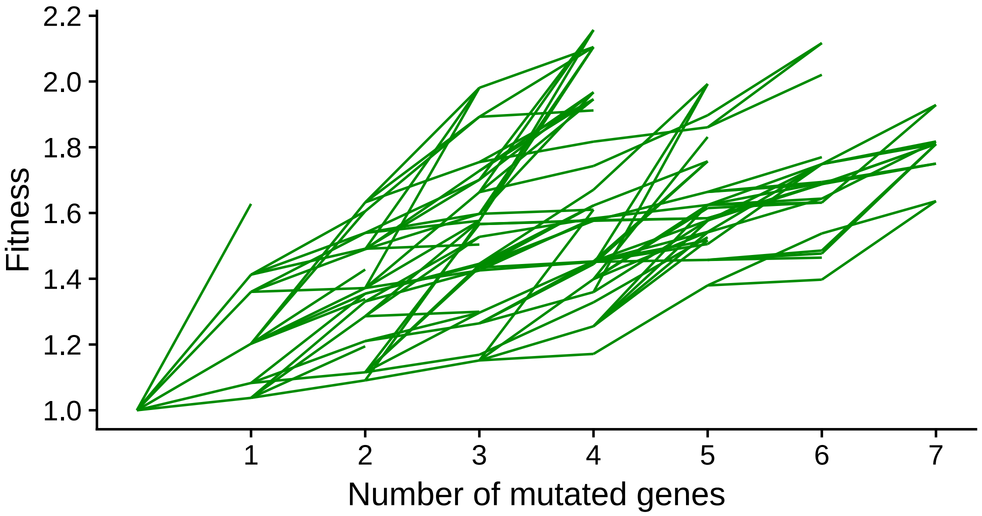

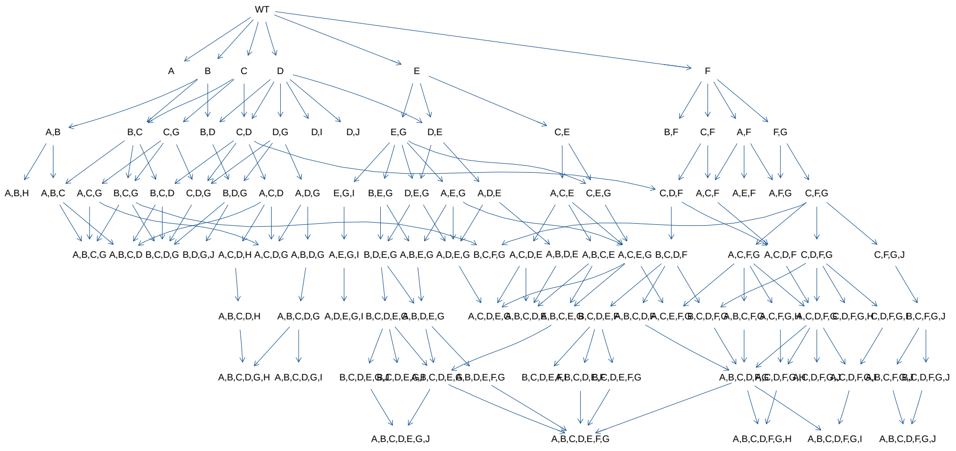

ID	UR8nbozIYY5kAyVm
acc genotypes	238
peaks no back	54
removed edges	NA
prop rem edges	NA

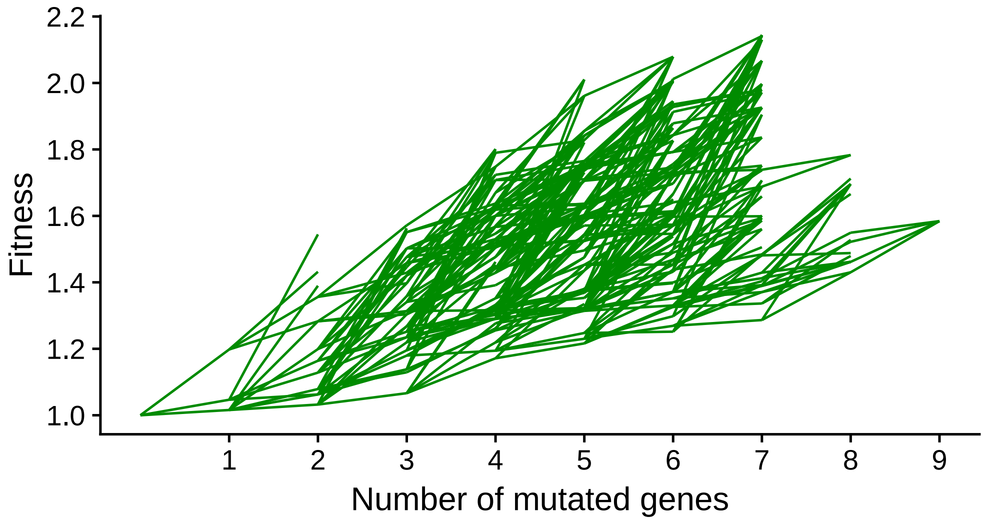

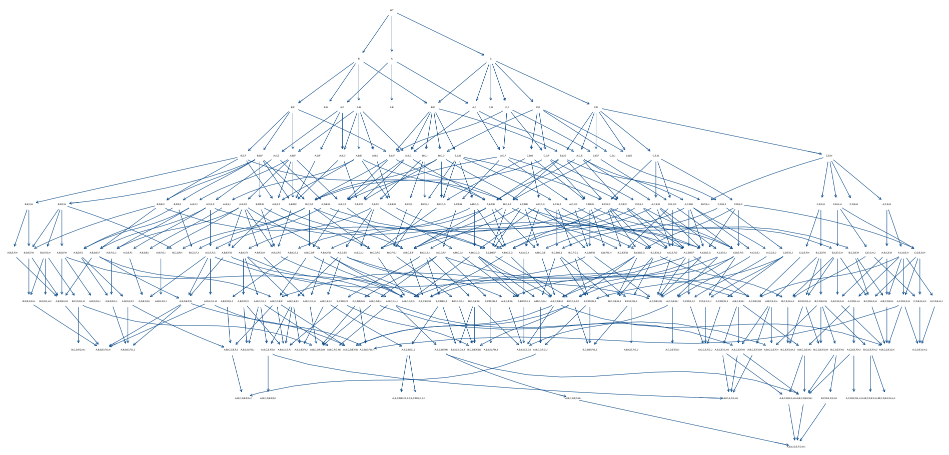

ID	vhfO10CYjbTCUd5Oc
acc genotypes	189
peaks no back	49
removed edges	NA
prop rem edges	NA

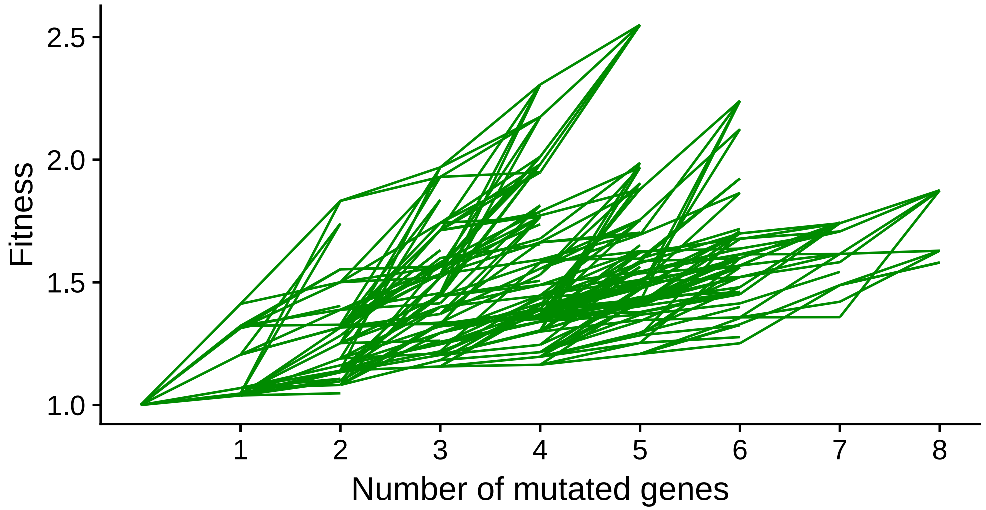

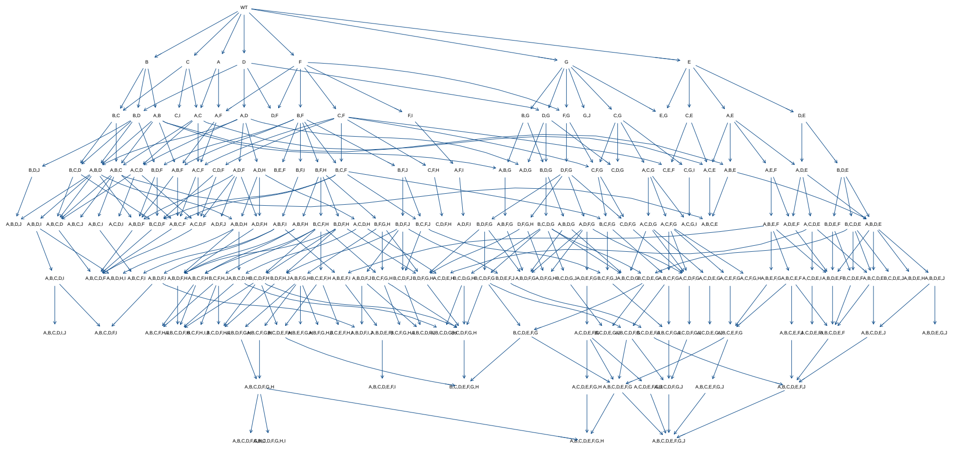

ID	VLbJrKnG7tDZJCX7
acc genotypes	738
peaks no back	105
removed edges	NA
prop rem edges	NA

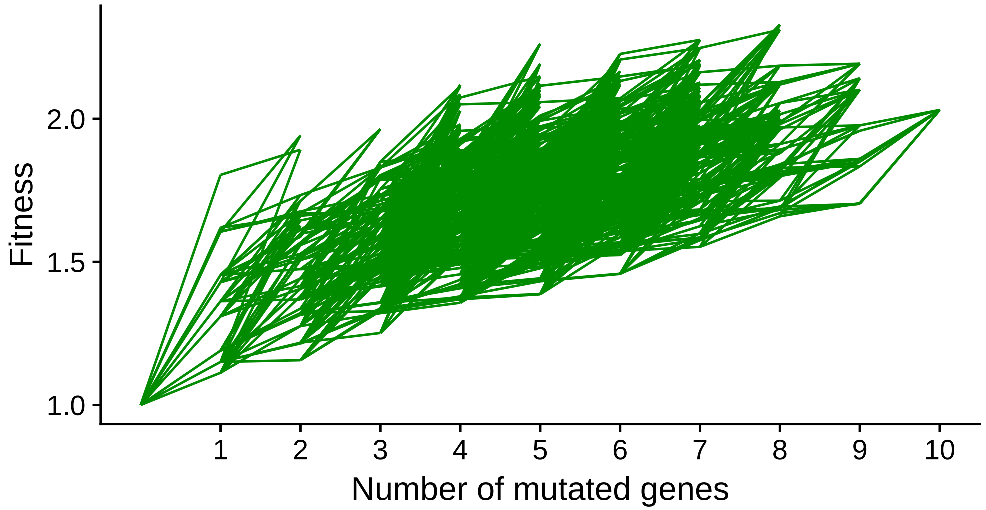

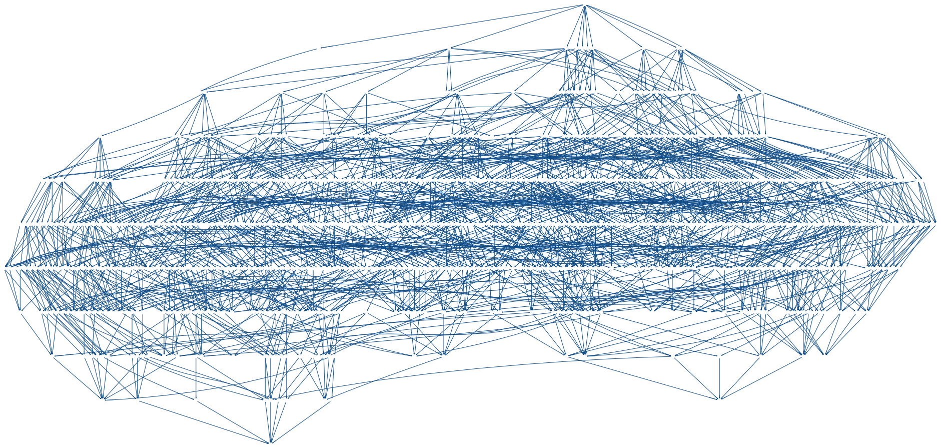

ID	vlOhJ52j3mkgx4bO
acc genotypes	179
peaks no back	73
removed edges	NA
prop rem edges	NA

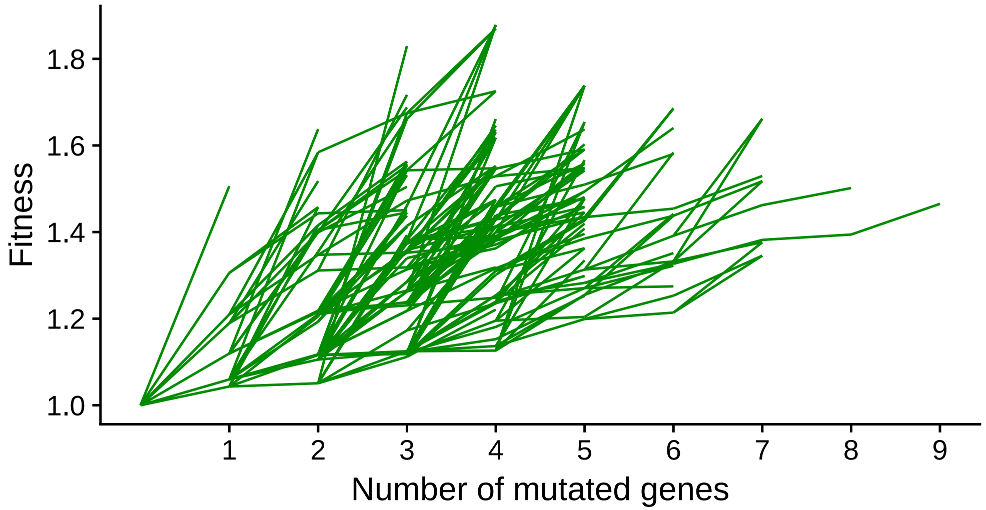

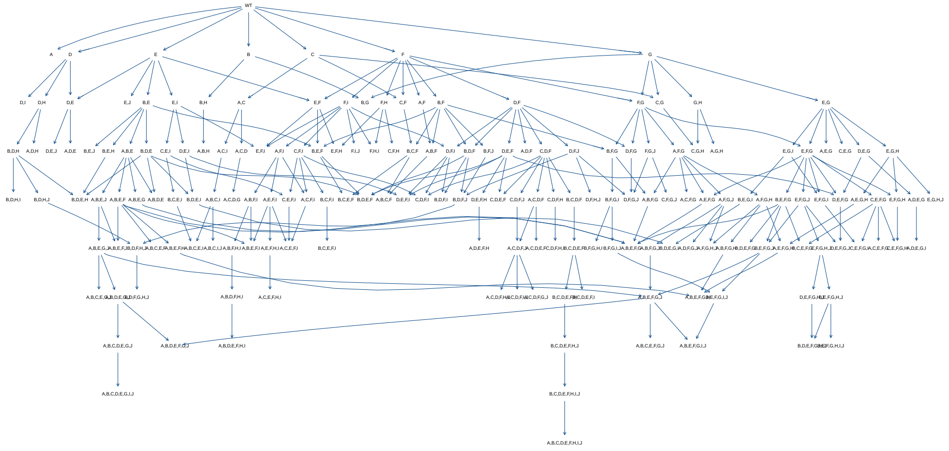

ID	W10IjZVnZ1043hQUxi
acc genotypes	388
peaks no back	70
removed edges	NA
prop rem edges	NA

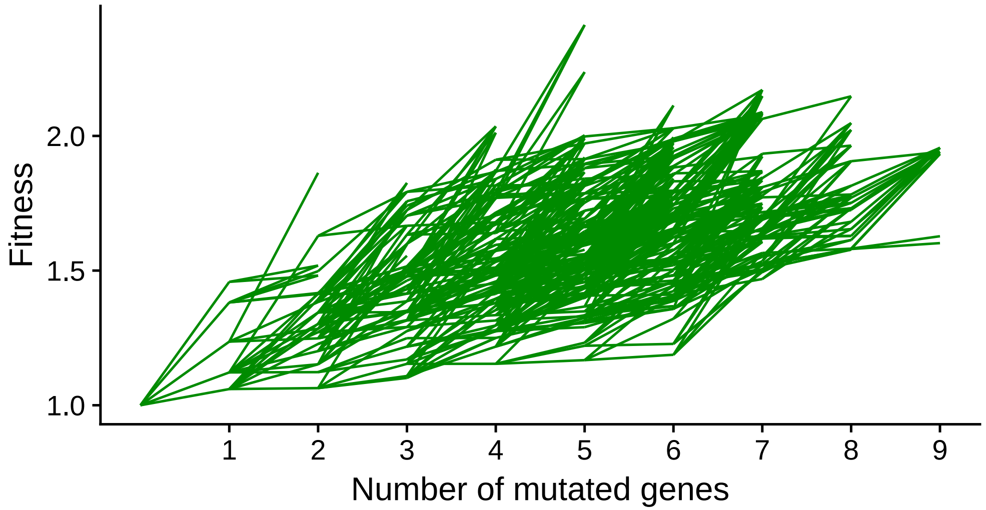

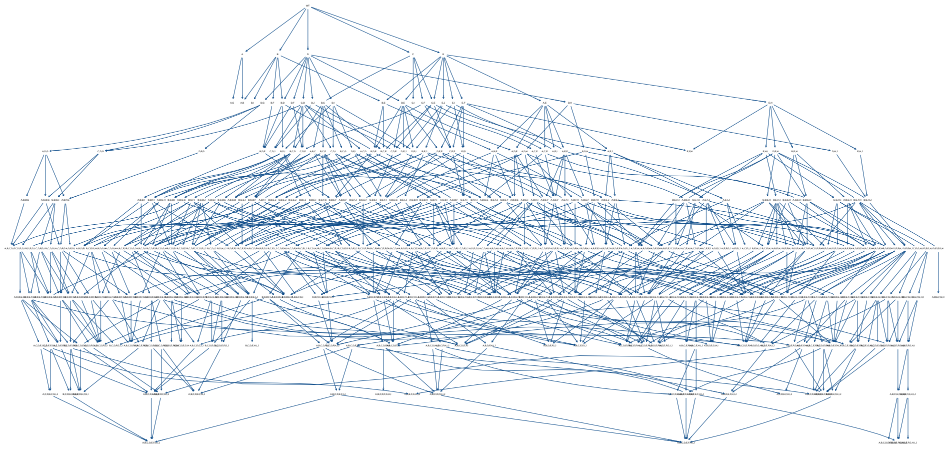

ID	wM4mouEZ5iUOoAtX
acc genotypes	143
peaks no back	52
removed edges	NA
prop rem edges	NA

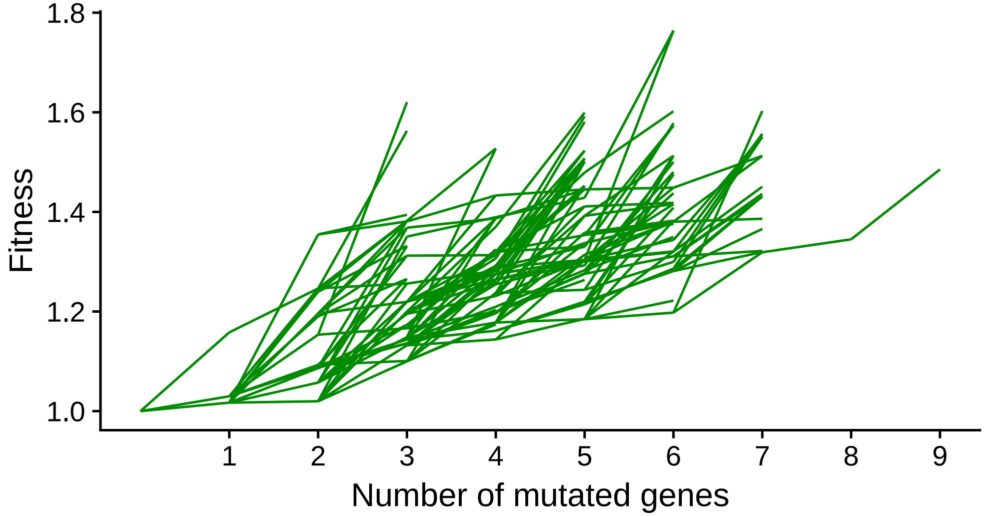

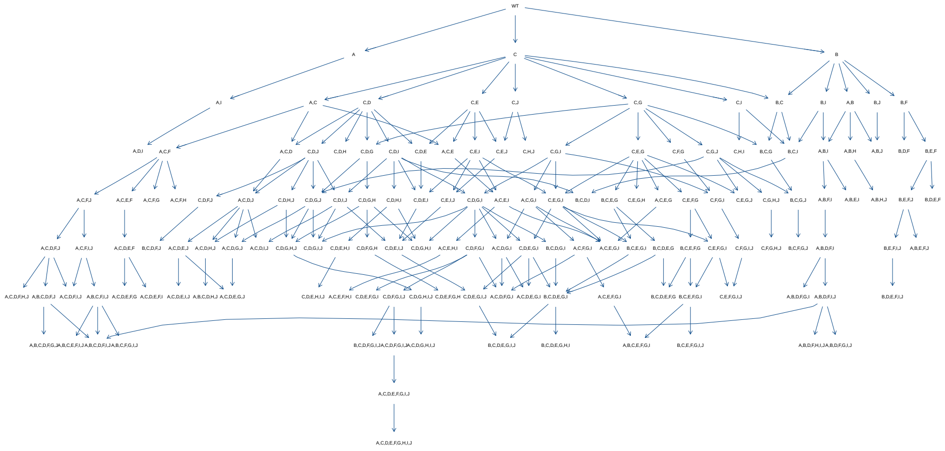

ID	wOuBIsYKAAzBpTkz
acc genotypes	191
peaks no back	69
removed edges	NA
prop rem edges	NA

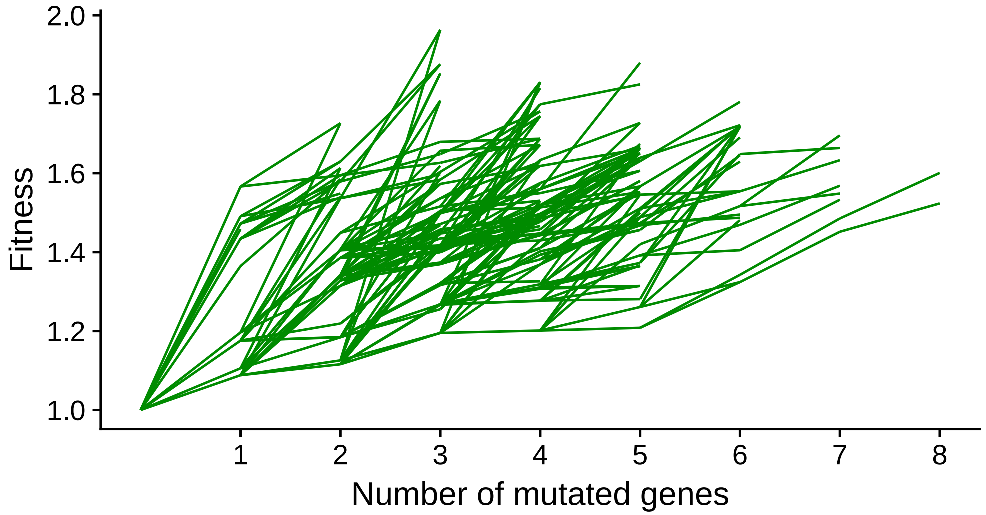

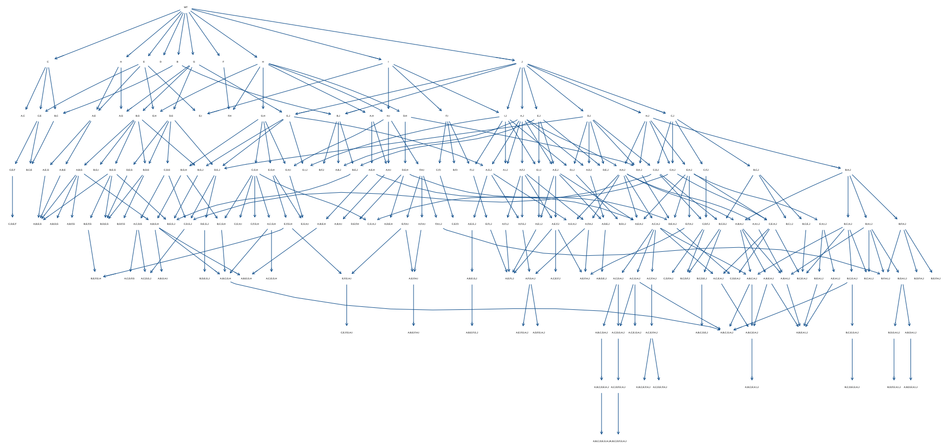

ID	wuF5q9kOxSCRE7RX
acc genotypes	728
peaks no back	82
removed edges	NA
prop rem edges	NA

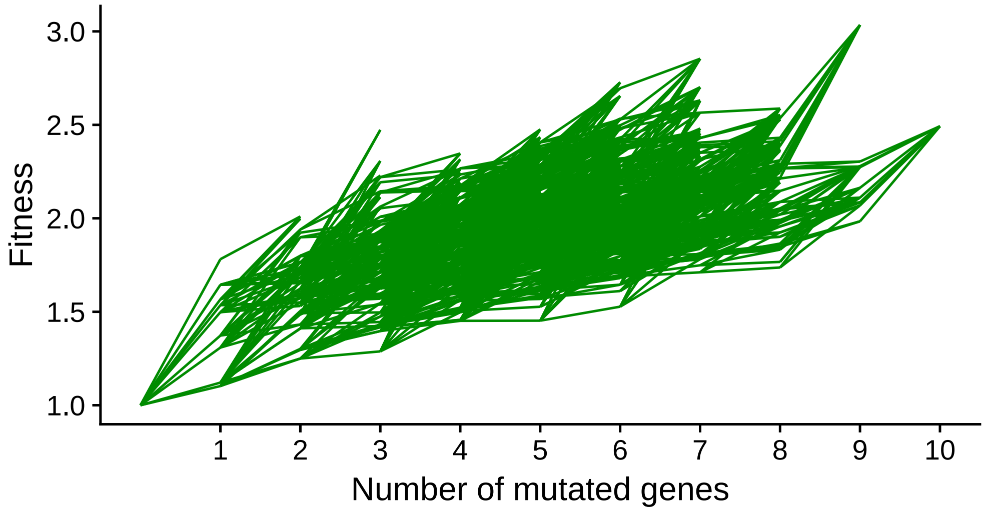

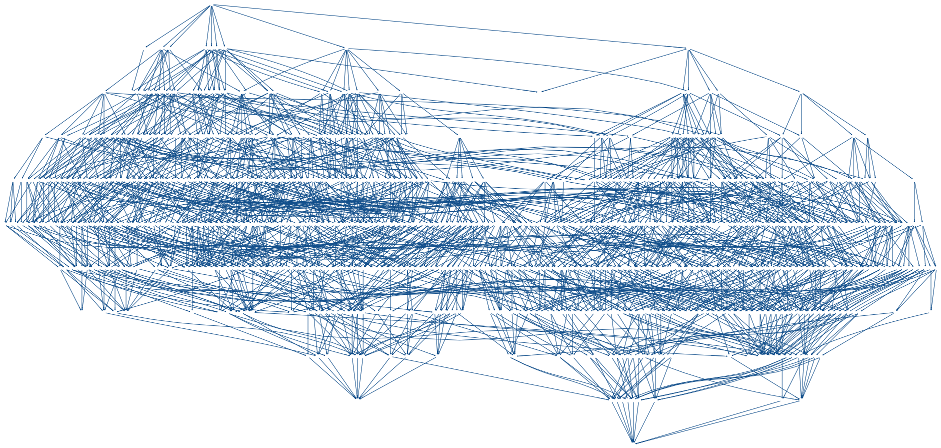

ID	wwBtyDIZSVYRmVqi
acc genotypes	222
peaks no back	83
removed edges	NA
prop rem edges	NA

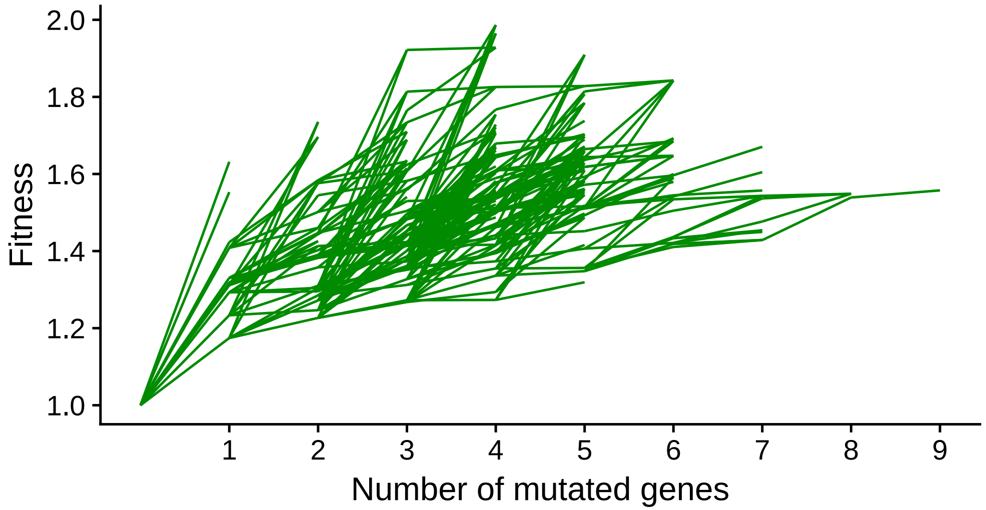

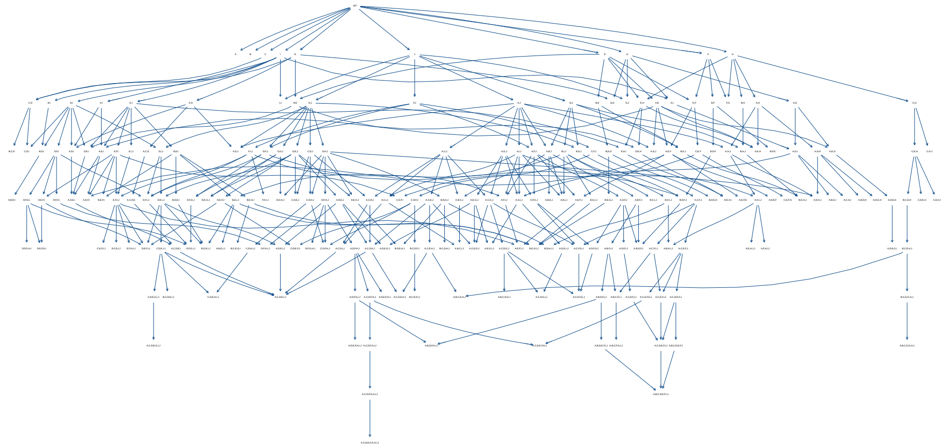

ID	wXZJGUyFMo10Nl4dV
acc genotypes	89
peaks no back	42
removed edges	NA
prop rem edges	NA

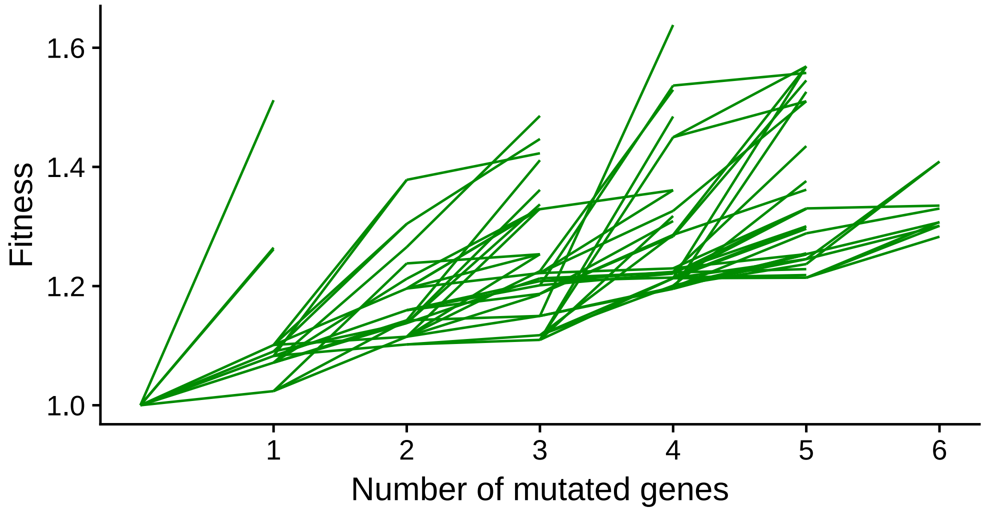

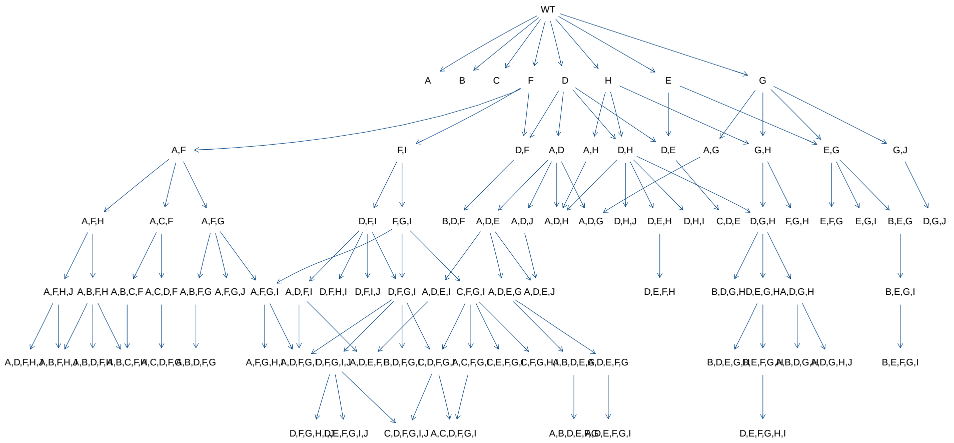

ID	WYBRgQ10JLk3RhGLo
acc genotypes	433
peaks no back	115
removed edges	NA
prop rem edges	NA

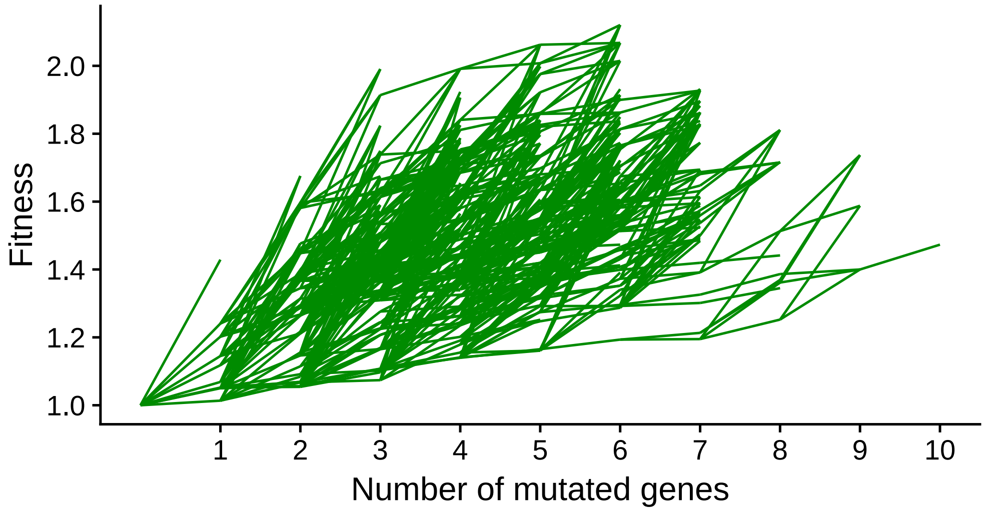

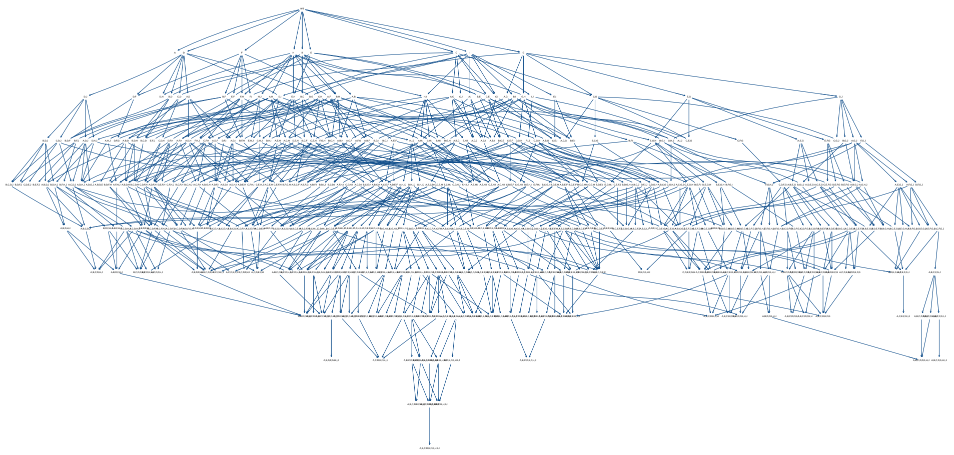

ID	WZ96wrbzeKdxcOJ3
acc genotypes	141
peaks no back	57
removed edges	NA
prop rem edges	NA

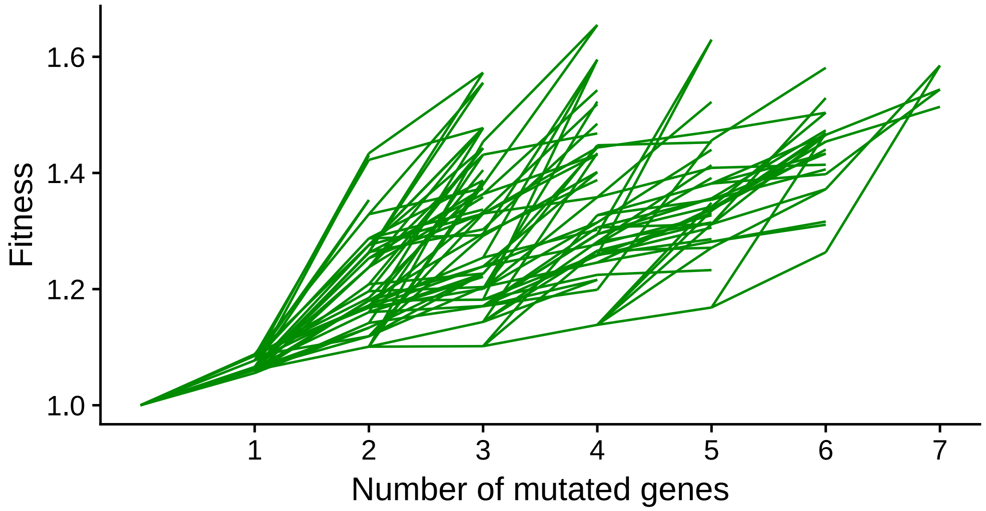

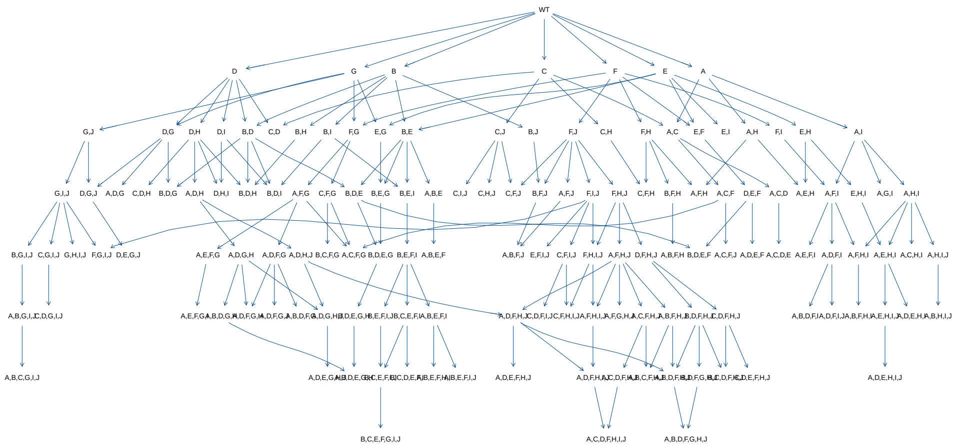

ID	X6Z5TgU29GZVilQy
acc genotypes	620
peaks no back	75
removed edges	NA
prop rem edges	NA

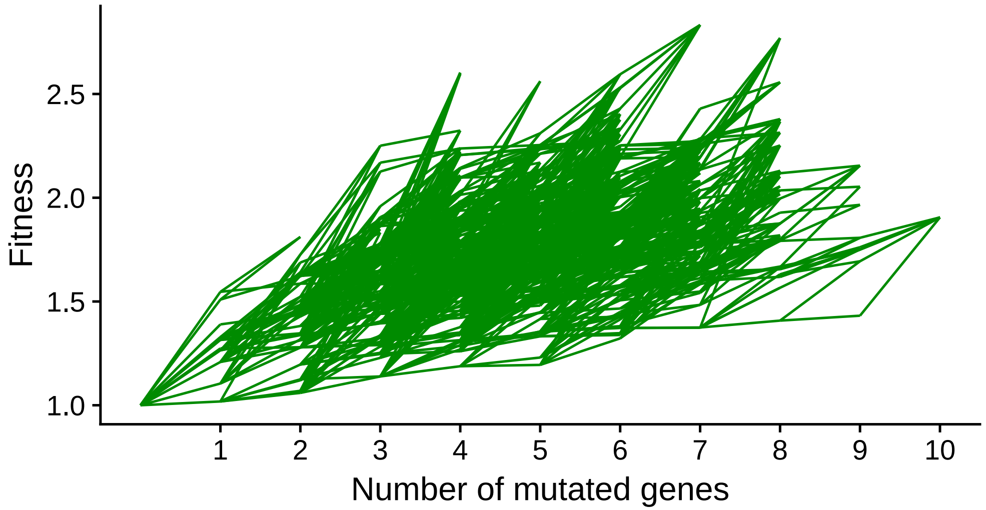

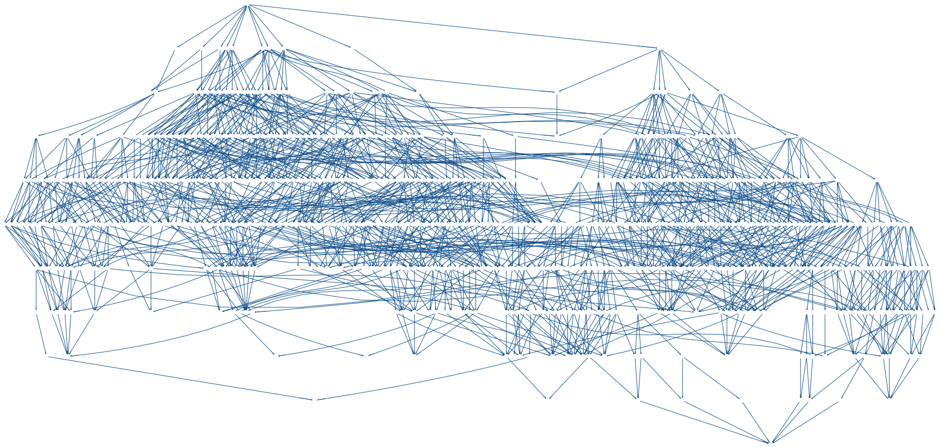

ID	x8IUOobt10WI39iG2
acc genotypes	209
peaks no back	81
removed edges	NA
prop rem edges	NA

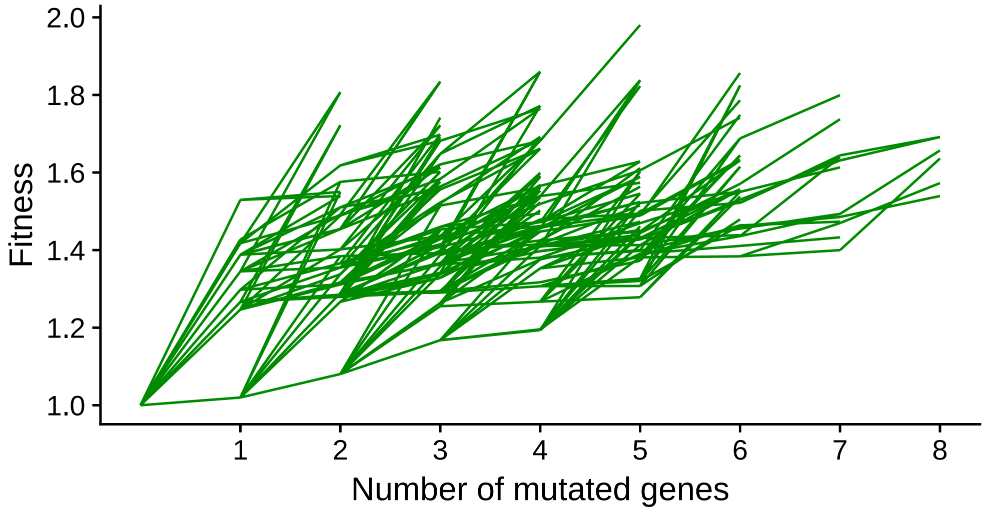

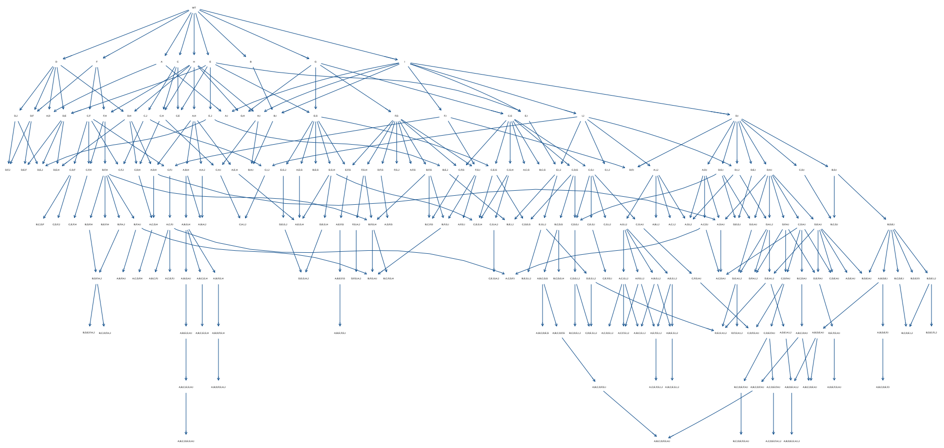

ID	xcdQXsmxI7kh5NDZ
acc genotypes	184
peaks no back	73
removed edges	NA
prop rem edges	NA

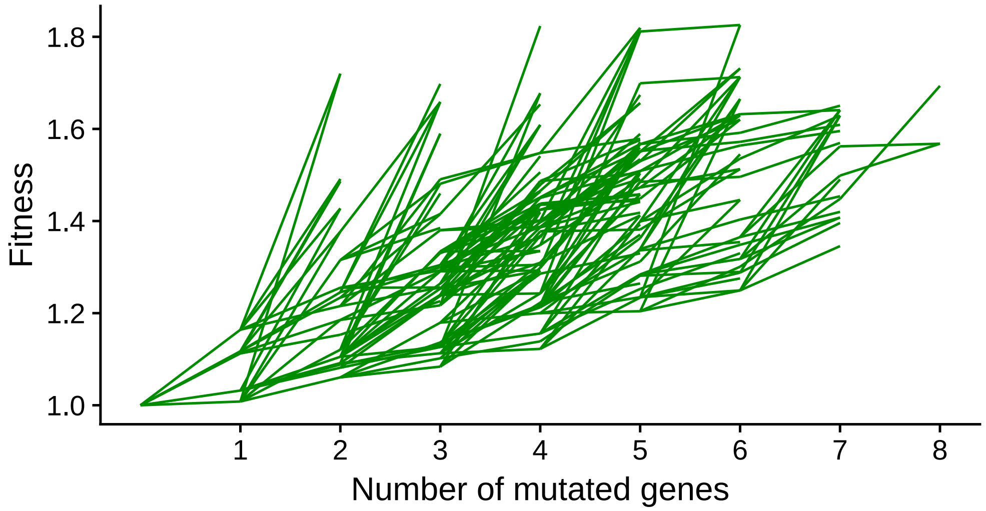

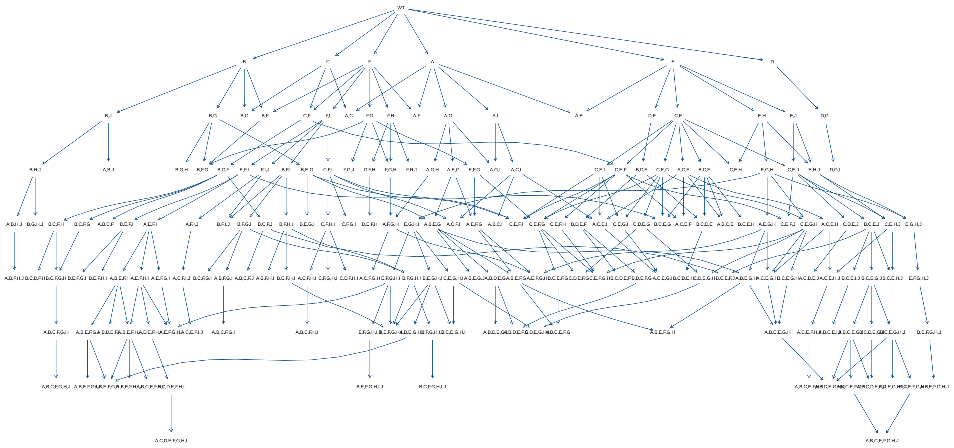

ID	Xh10HKozlpe9ybd7i
acc genotypes	200
peaks no back	89
removed edges	NA
prop rem edges	NA

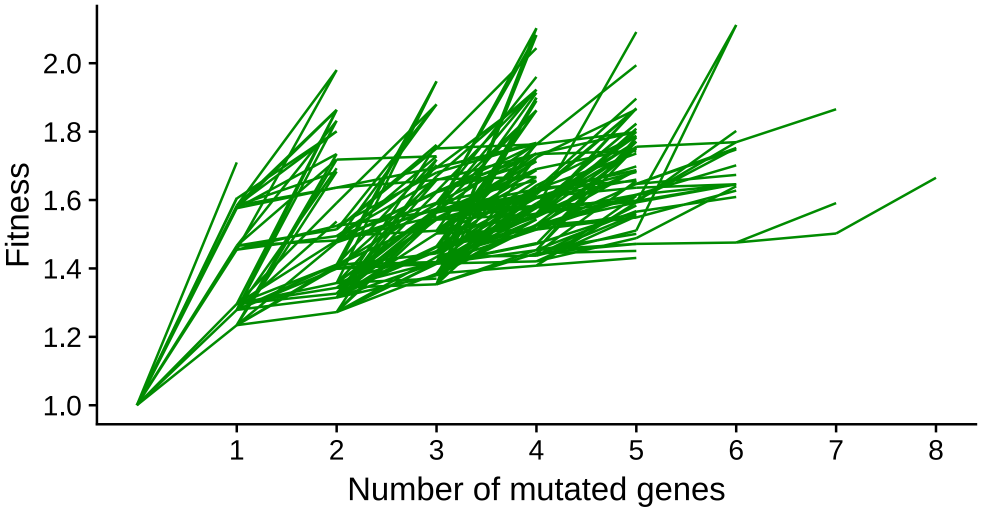

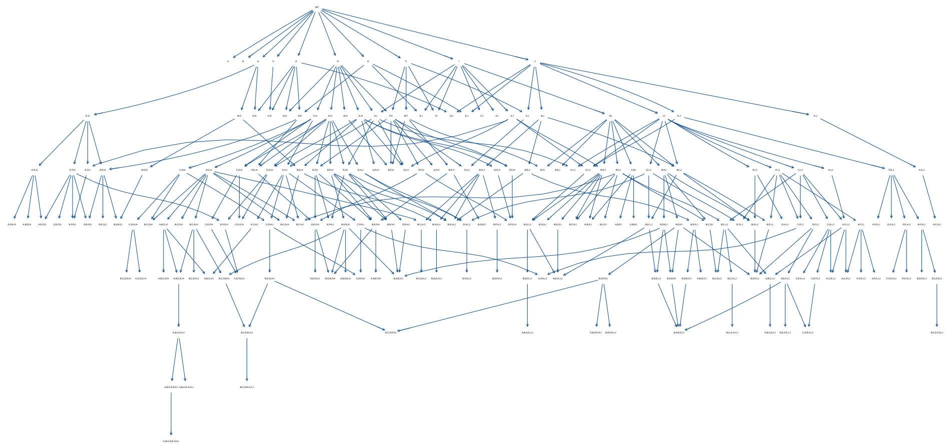

ID	XKTMaTNASYWPlHNl
acc genotypes	352
peaks no back	105
removed edges	NA
prop rem edges	NA

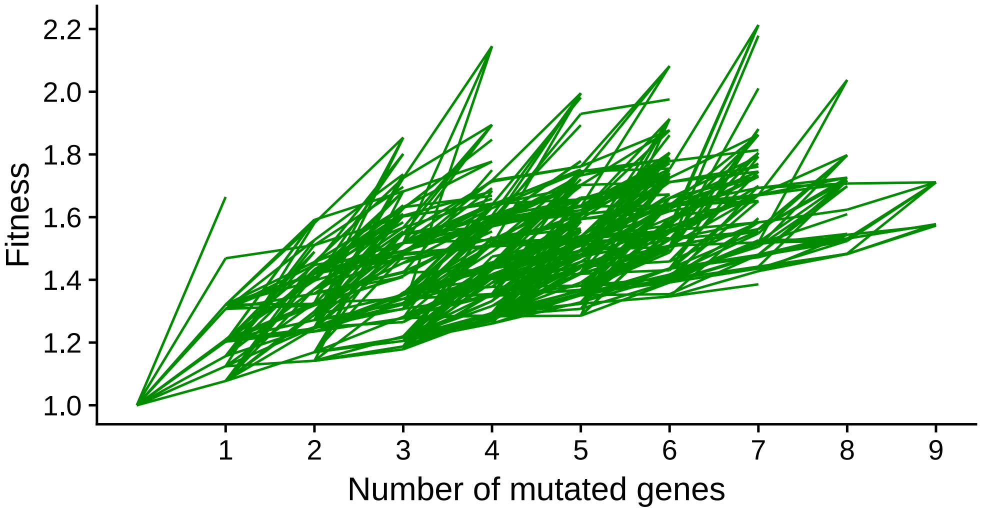

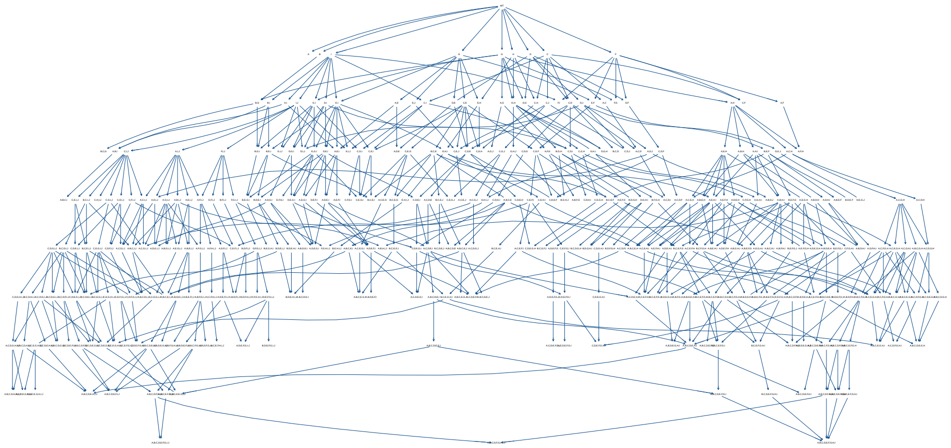

ID	XNRZEeV3l3oHG9g1
acc genotypes	424
peaks no back	121
removed edges	NA
prop rem edges	NA

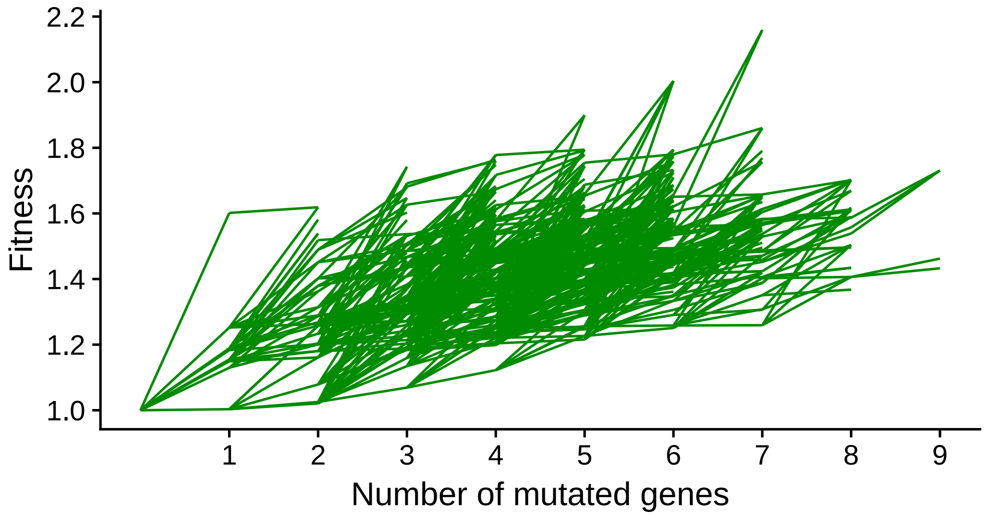

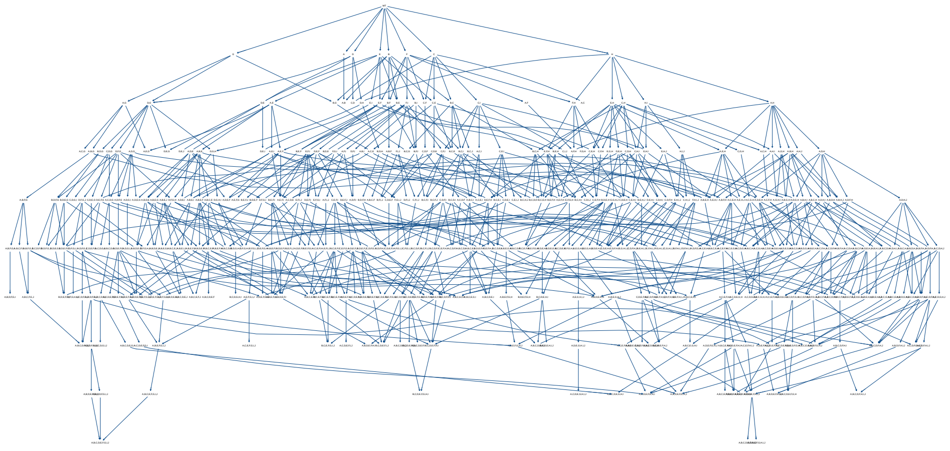

ID	XrjsetS7KL5noi92
acc genotypes	89
peaks no back	26
removed edges	NA
prop rem edges	NA

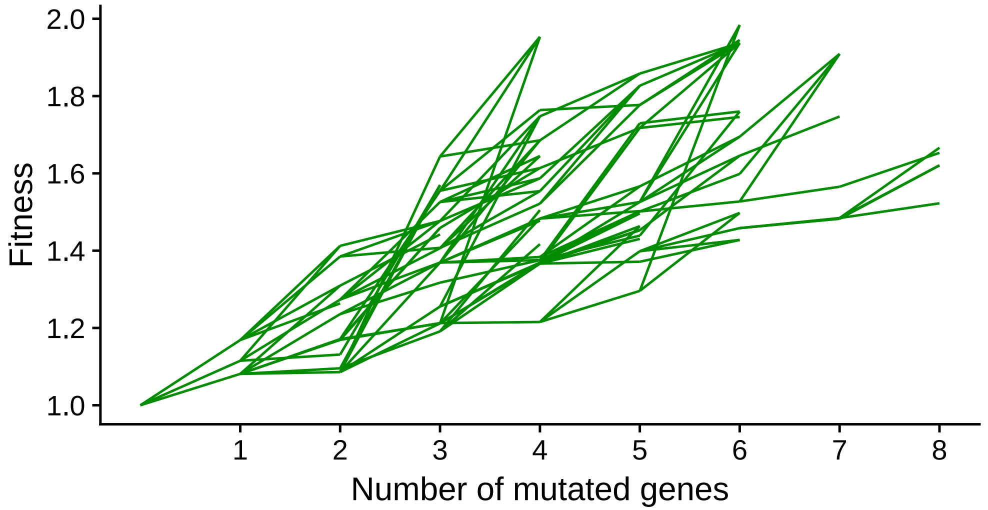

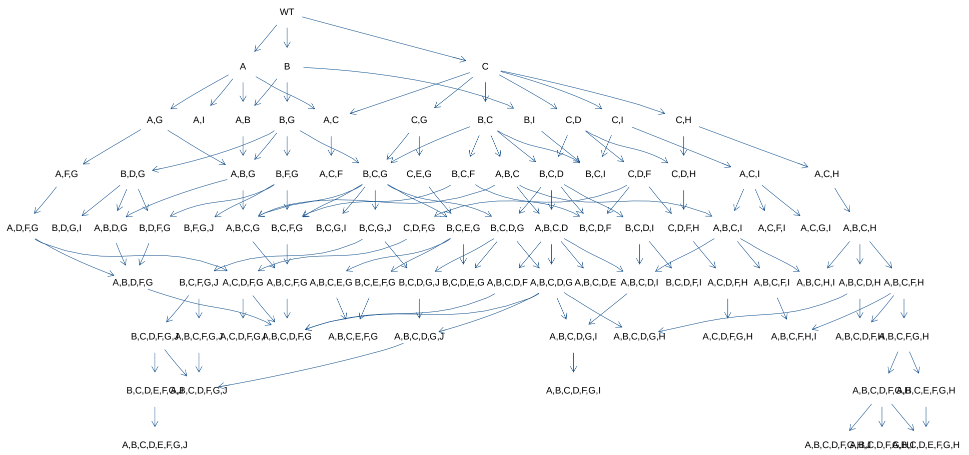

ID	xvHgdwCzOGa7Ev7e
acc genotypes	55
peaks no back	19
removed edges	NA
prop rem edges	NA

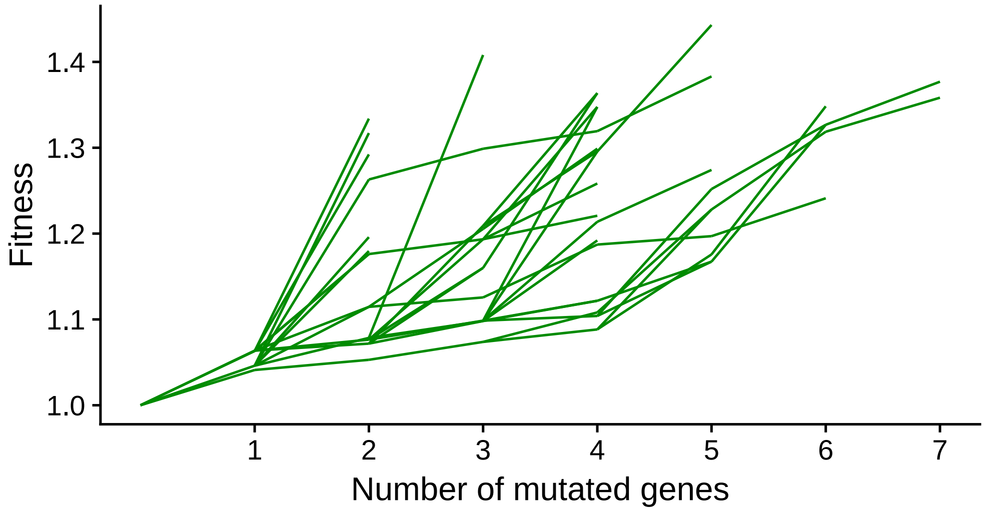

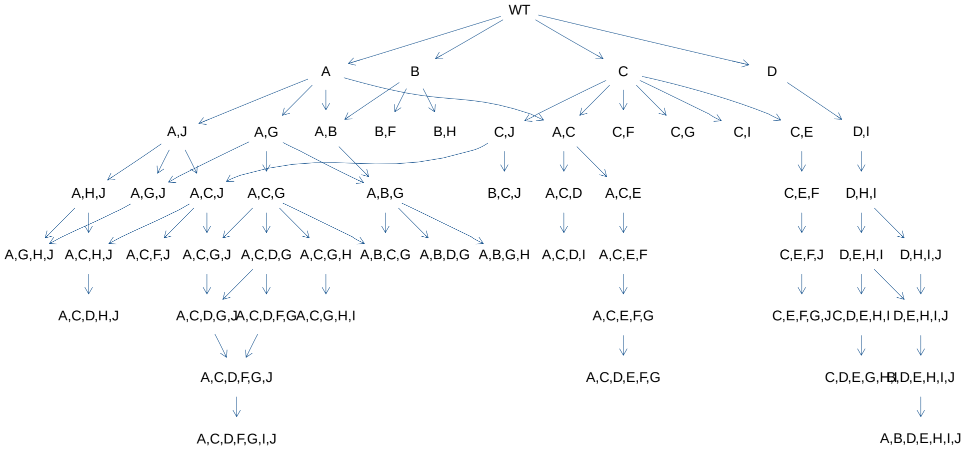

ID	xwEXGxQHVPacEqsW
acc genotypes	67
peaks no back	26
removed edges	NA
prop rem edges	NA

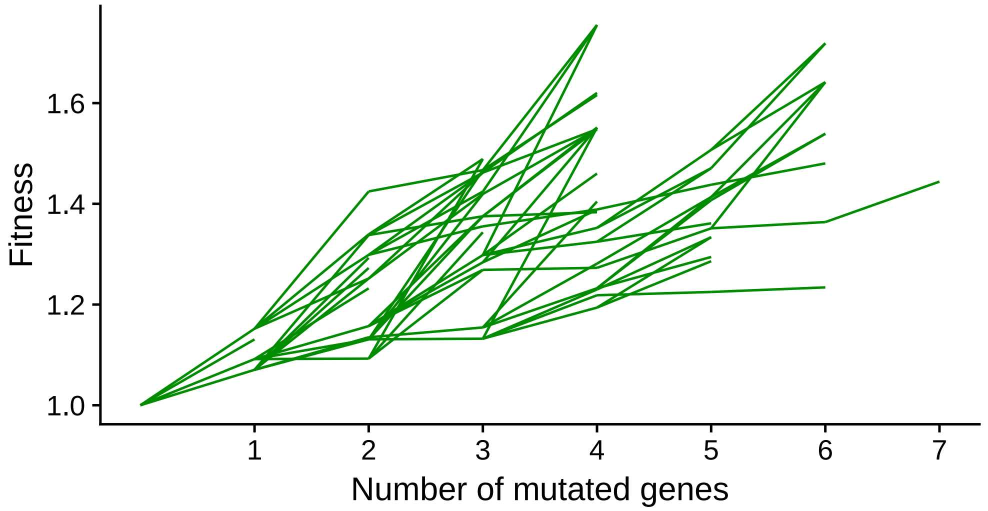

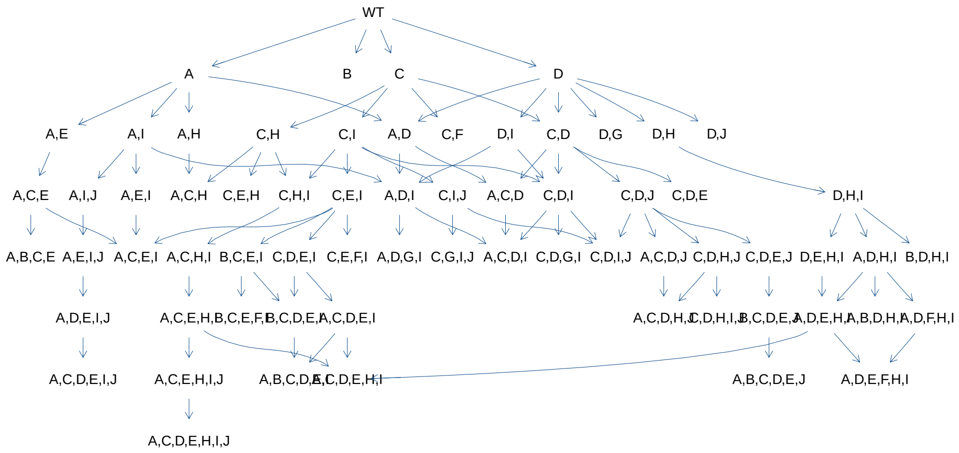

ID	xZ632I1FnE2fDF10q
acc genotypes	663
peaks no back	72
removed edges	NA
prop rem edges	NA

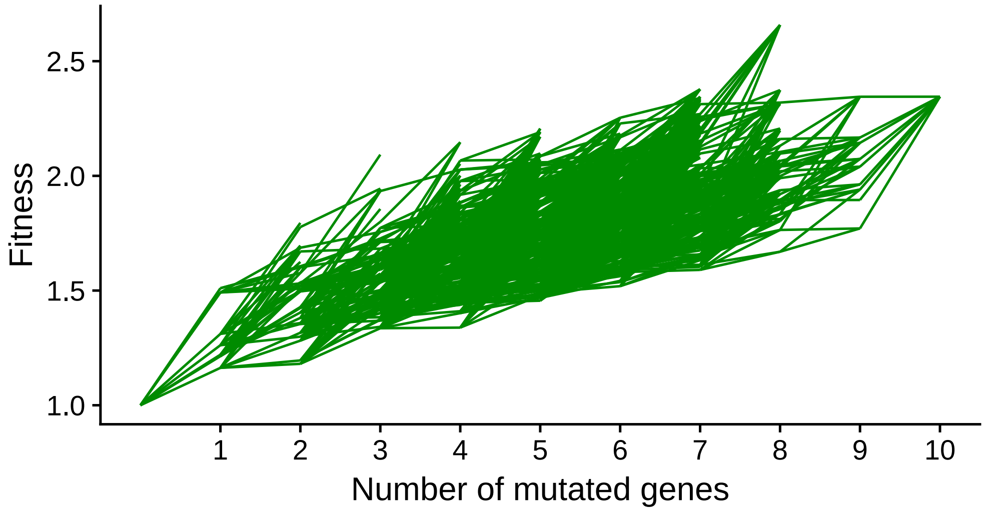

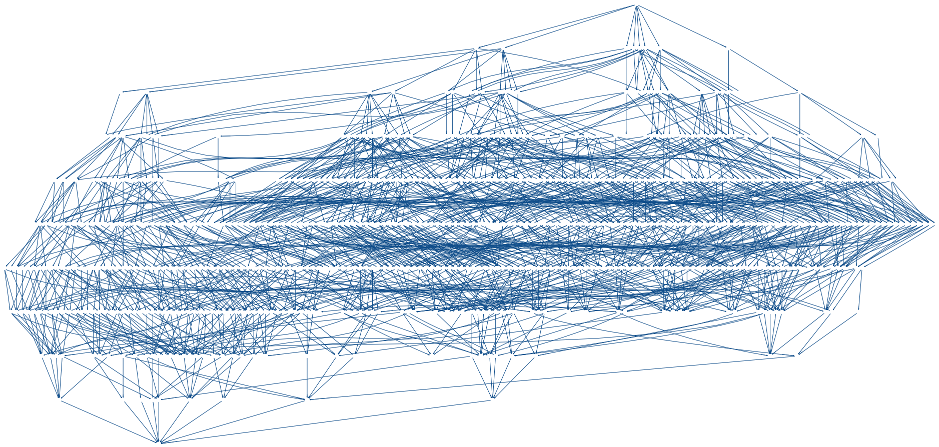

ID	y5oNzDs3V3q7y3ih
acc genotypes	297
peaks no back	91
removed edges	NA
prop rem edges	NA

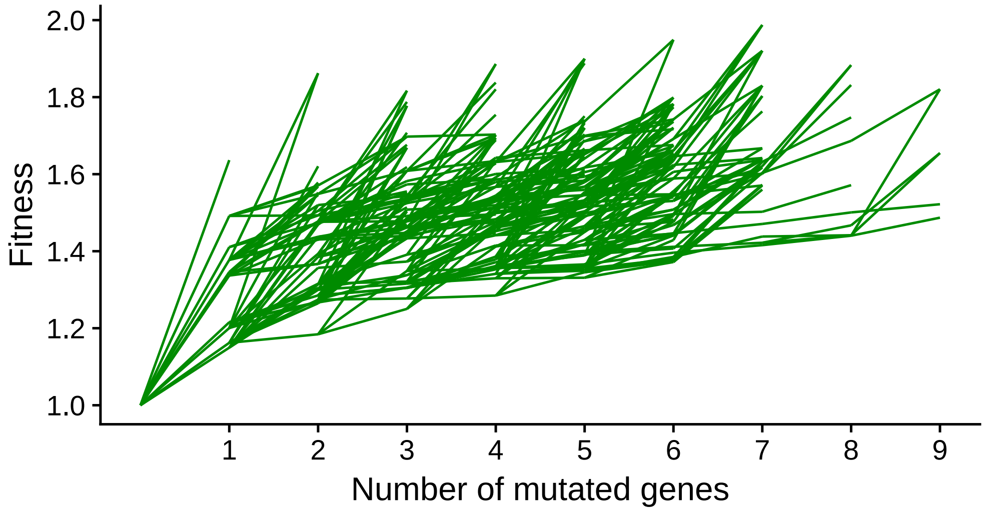

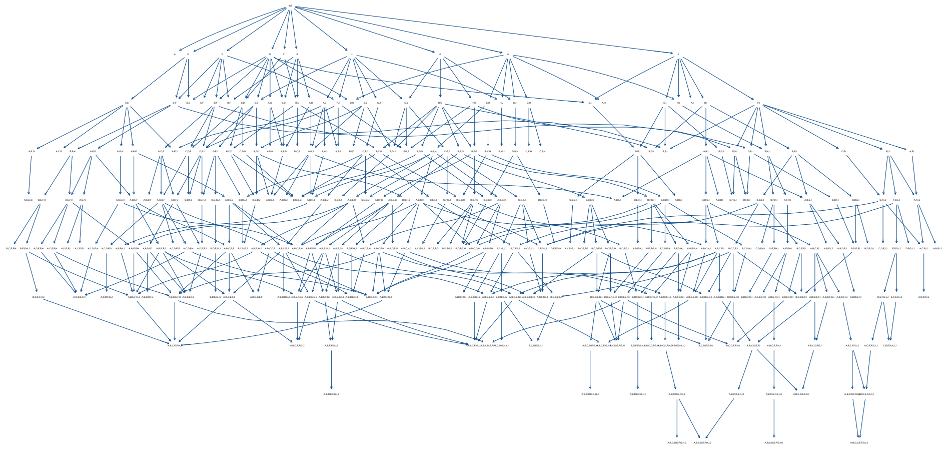

ID	YeUw4gB10IT8FRdhX
acc genotypes	110
peaks no back	46
removed edges	NA
prop rem edges	NA

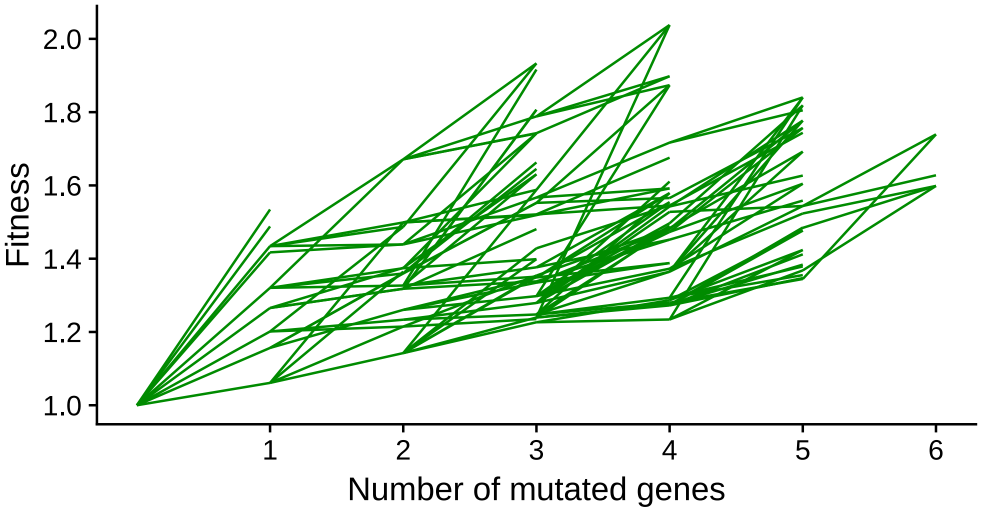

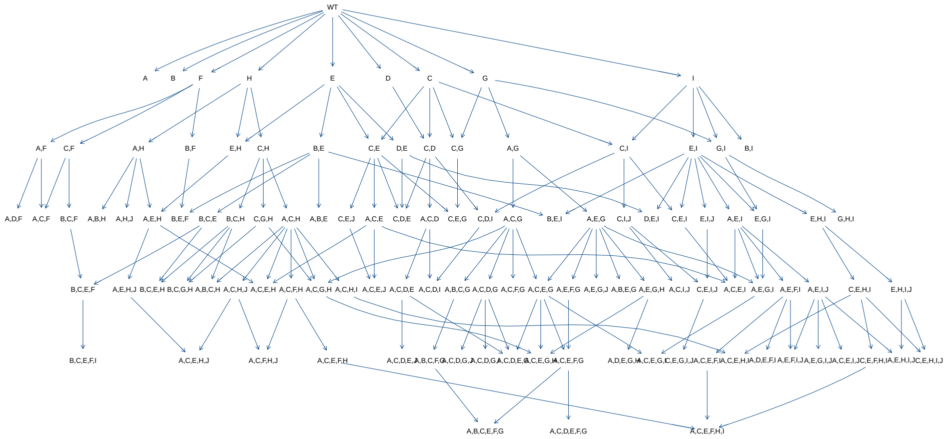

ID	Yg8zLevZ7wA4DC3B
acc genotypes	147
peaks no back	48
removed edges	NA
prop rem edges	NA

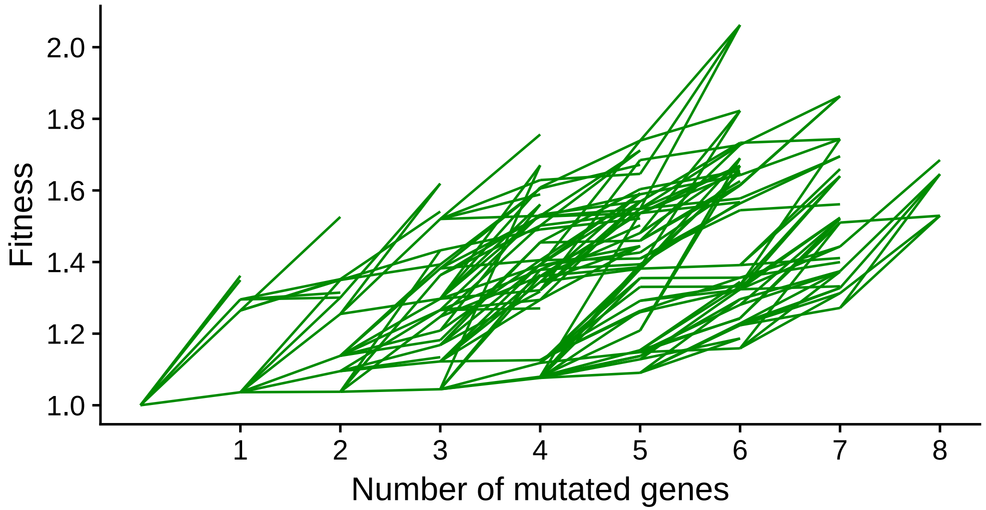

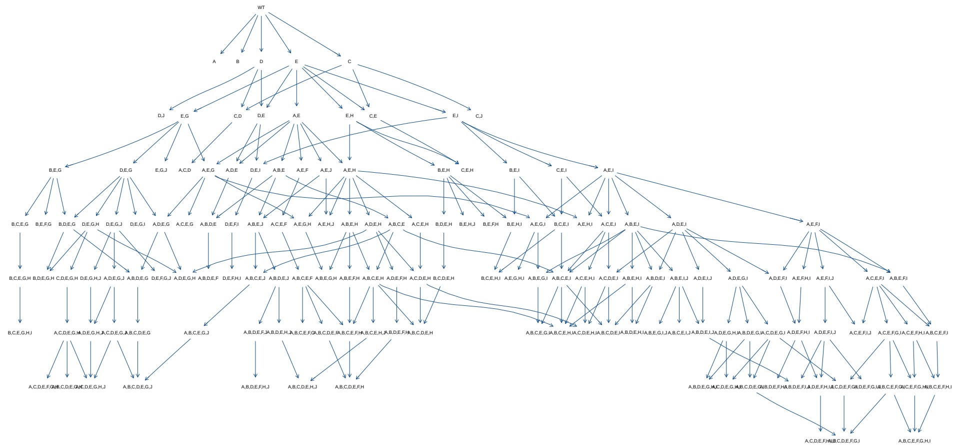

ID	YqlzMXE6SnRlTBq5
acc genotypes	73
peaks no back	37
removed edges	NA
prop rem edges	NA

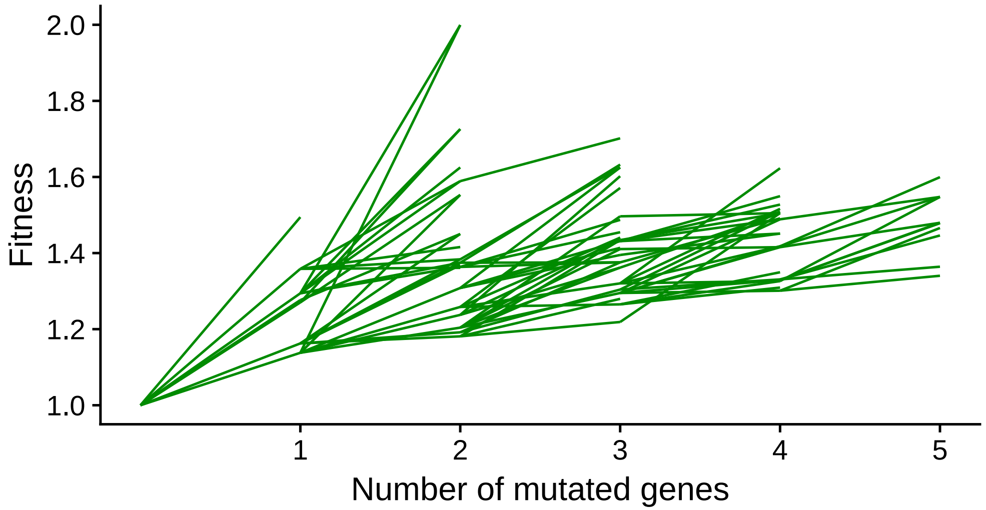

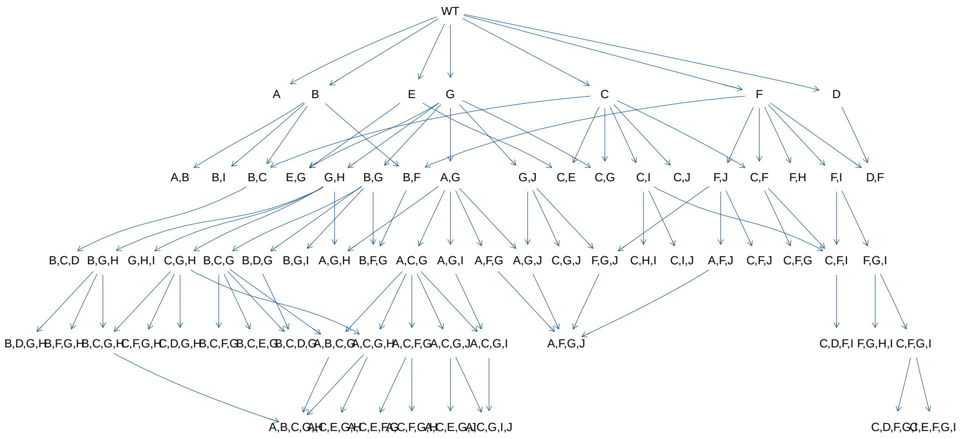

ID	YSwbWvqbUeR10s1p8
acc genotypes	279
peaks no back	73
removed edges	NA
prop rem edges	NA

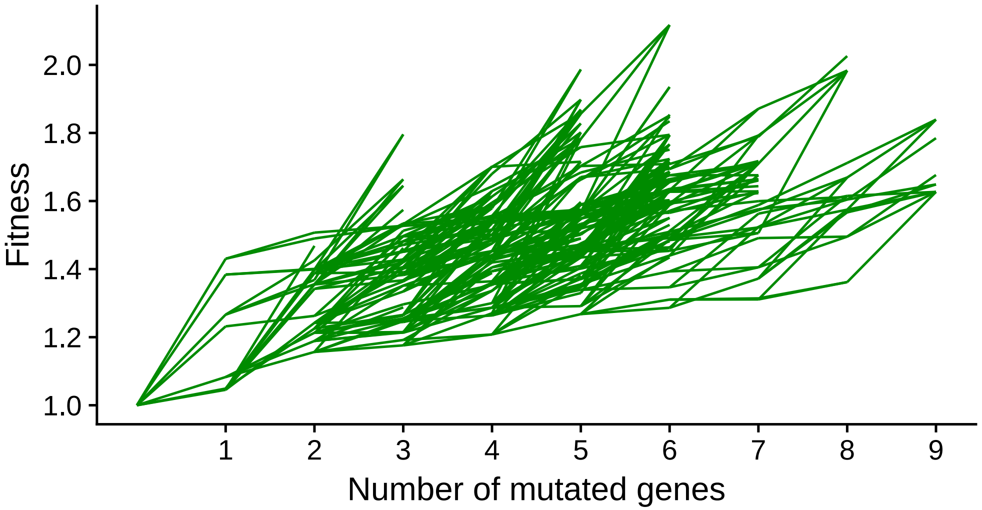

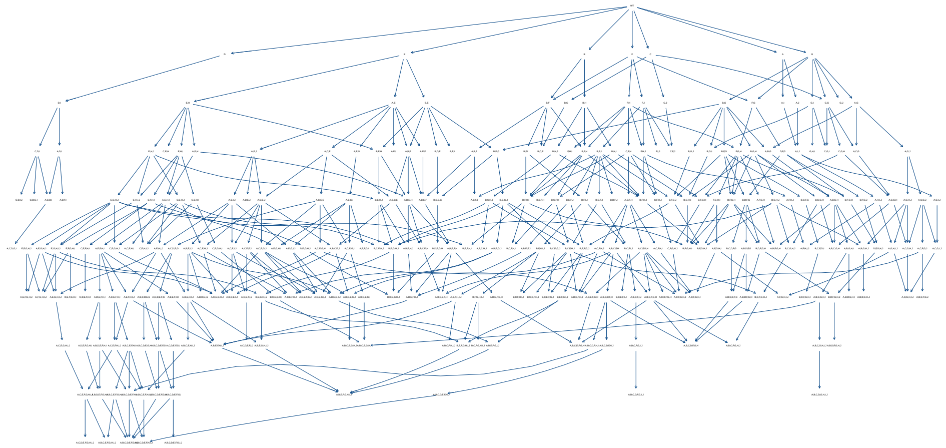

ID	zaGCXdhS1lAYBVFr
acc genotypes	242
peaks no back	91
removed edges	NA
prop rem edges	NA

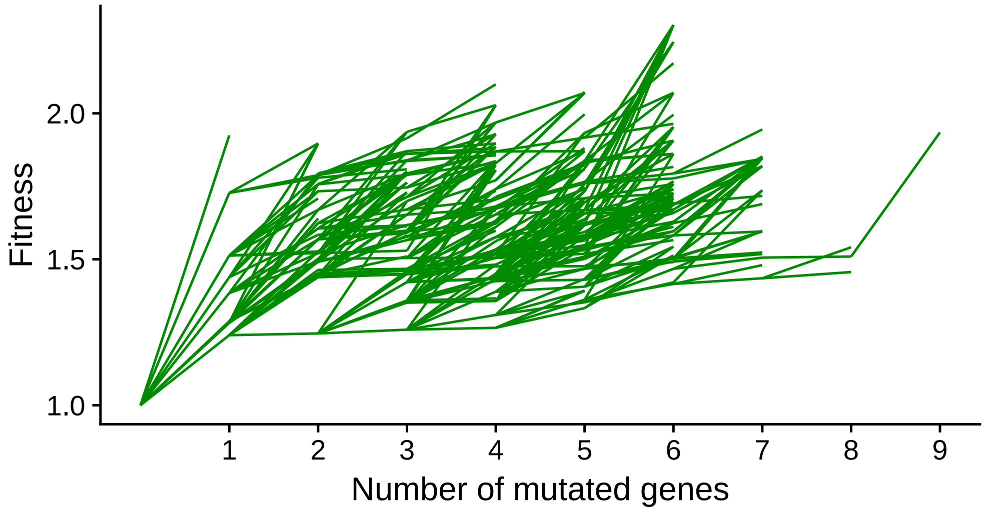

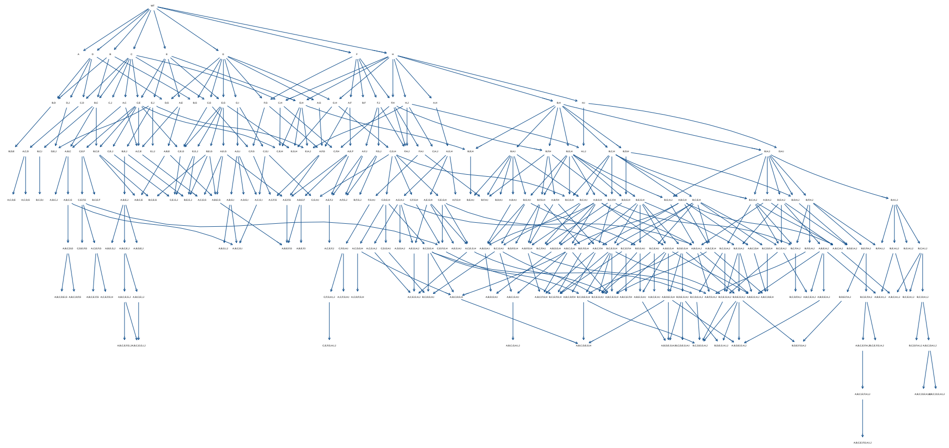

ID	ZARoxejcvdMx5gej
acc genotypes	335
peaks no back	87
removed edges	NA
prop rem edges	NA

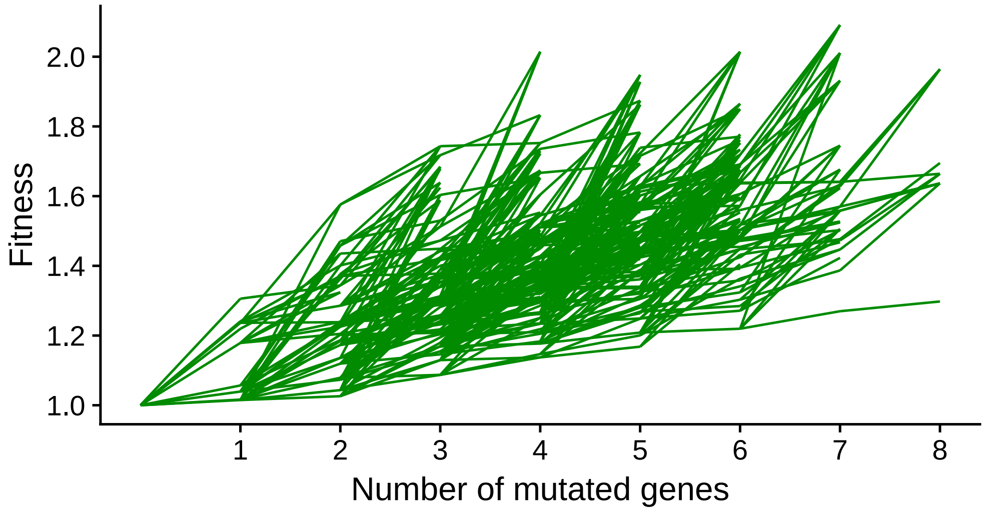

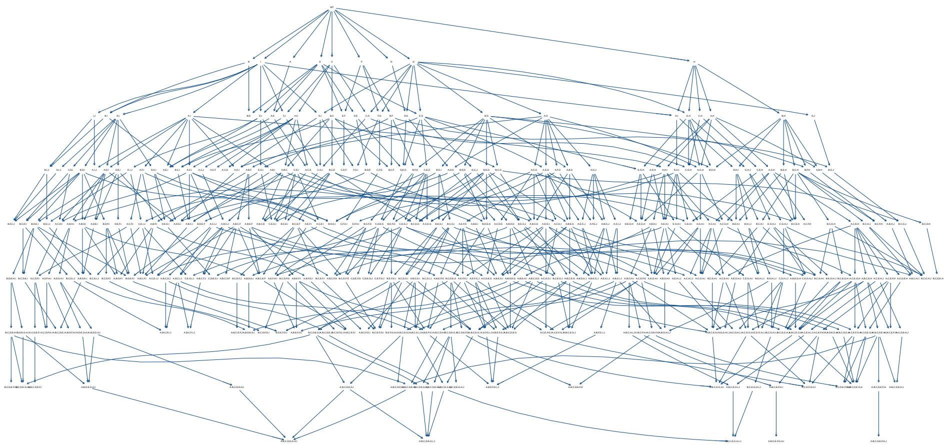

ID	zdCbK9OwSeu3jN2k
acc genotypes	26
peaks no back	12
removed edges	NA
prop rem edges	NA

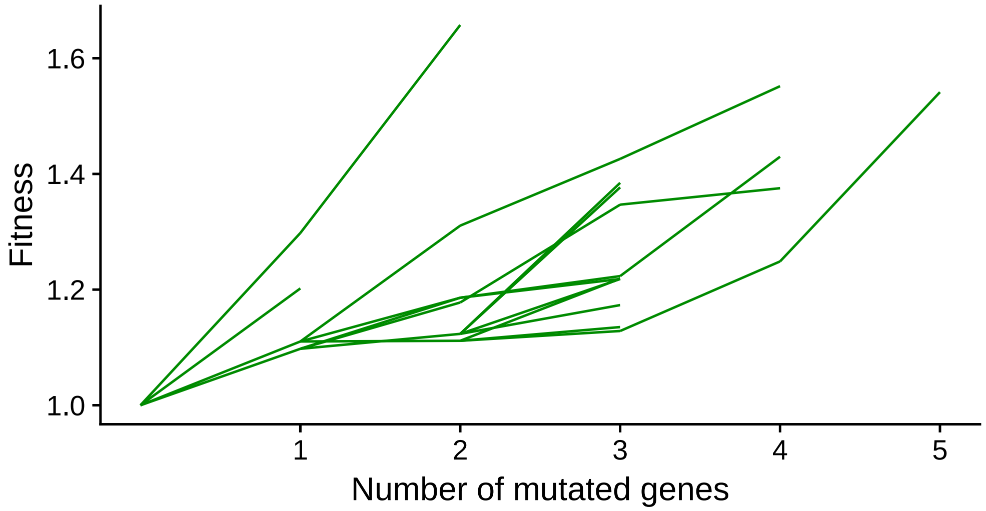

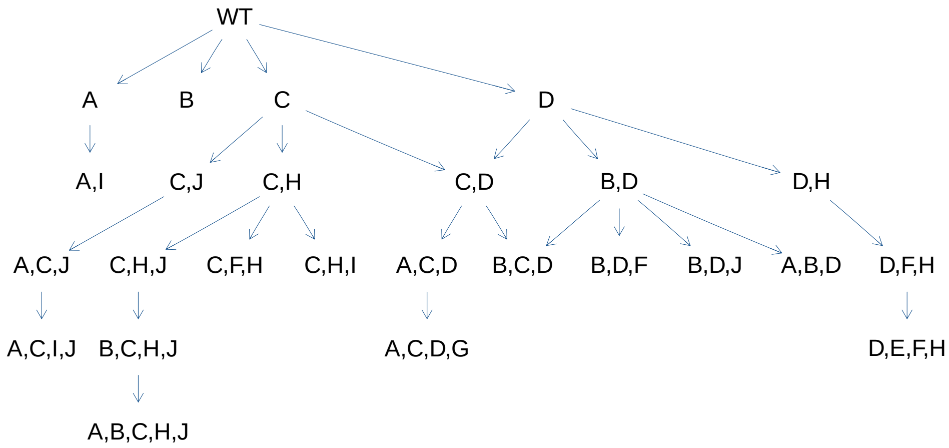

ID	Zi10rjNfw5yh1JEKK
acc genotypes	37
peaks no back	15
removed edges	NA
prop rem edges	NA

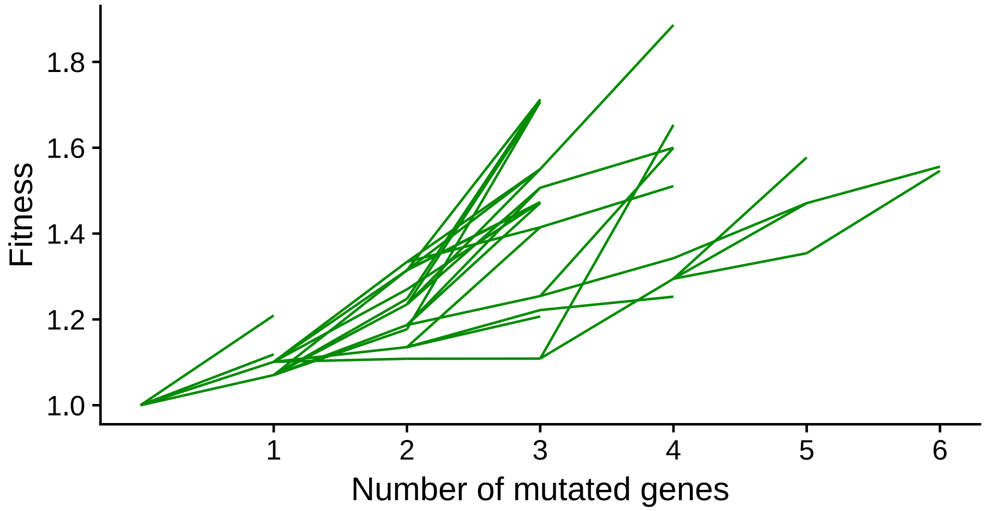

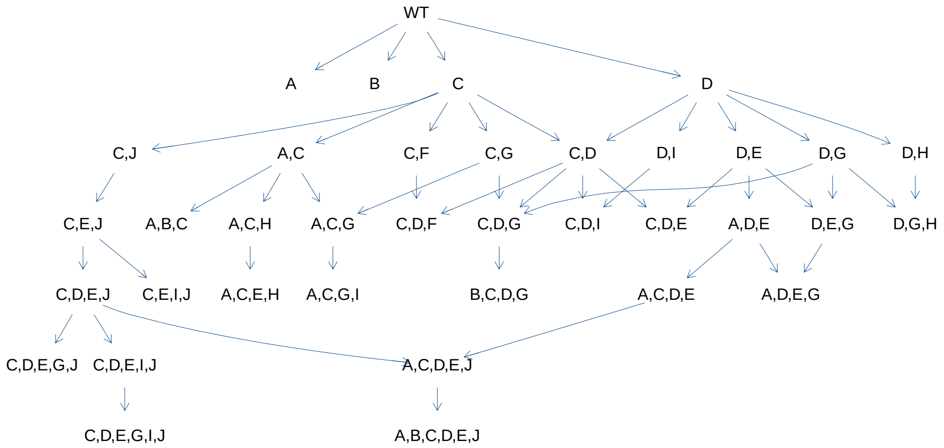

ID	ZIwKQjyTXgfkZAaS
acc genotypes	367
peaks no back	83
removed edges	NA
prop rem edges	NA

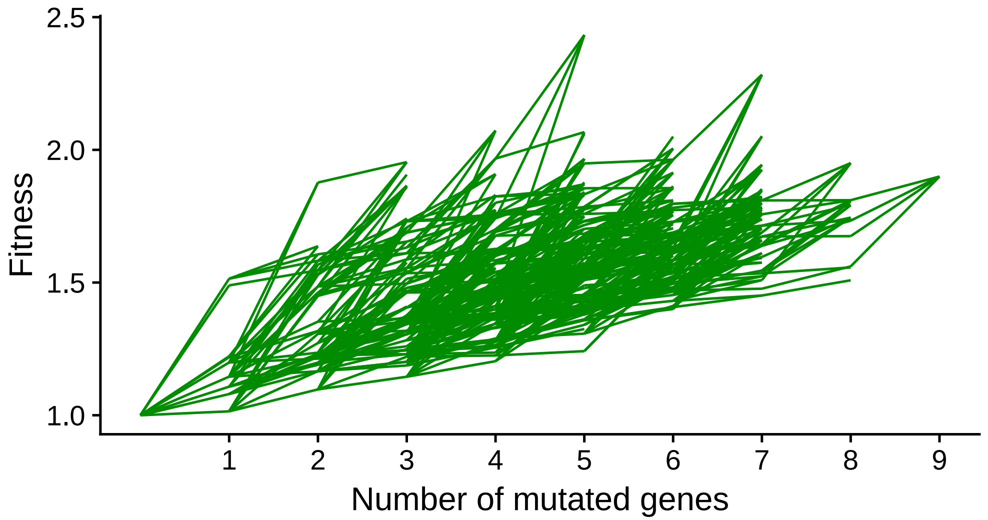

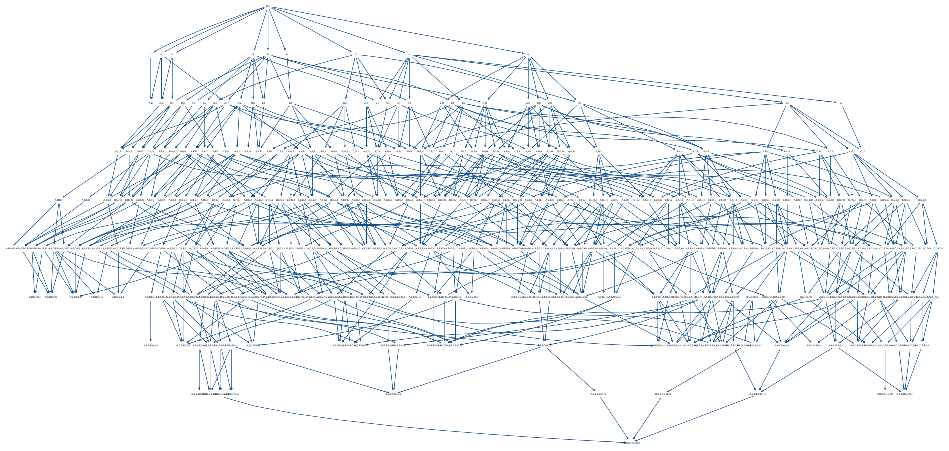

ID	zja2YeEj3wZTfoRu
acc genotypes	121
peaks no back	36
removed edges	NA
prop rem edges	NA

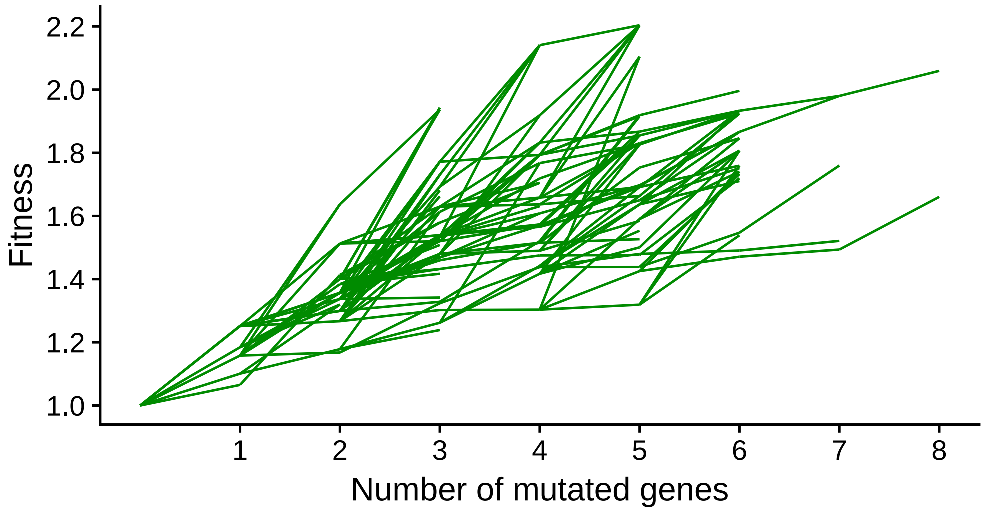

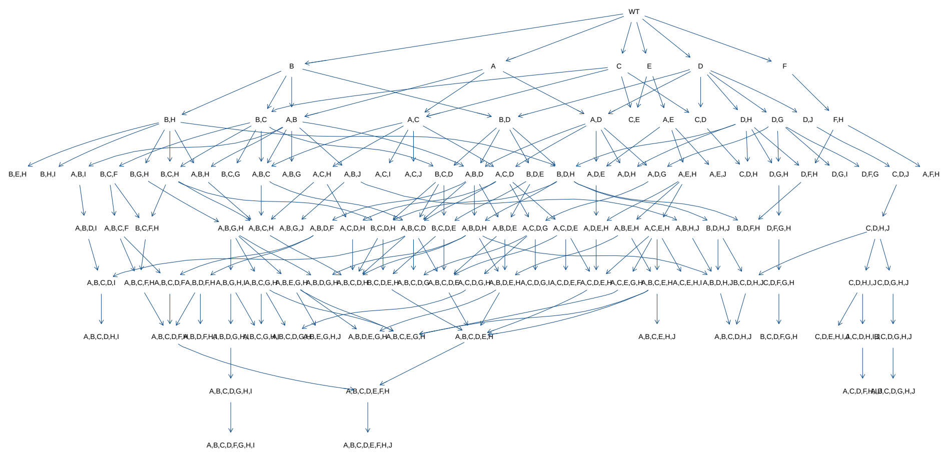

ID	zk5E1osYtIlv7Cob
acc genotypes	307
peaks no back	54
removed edges	NA
prop rem edges	NA

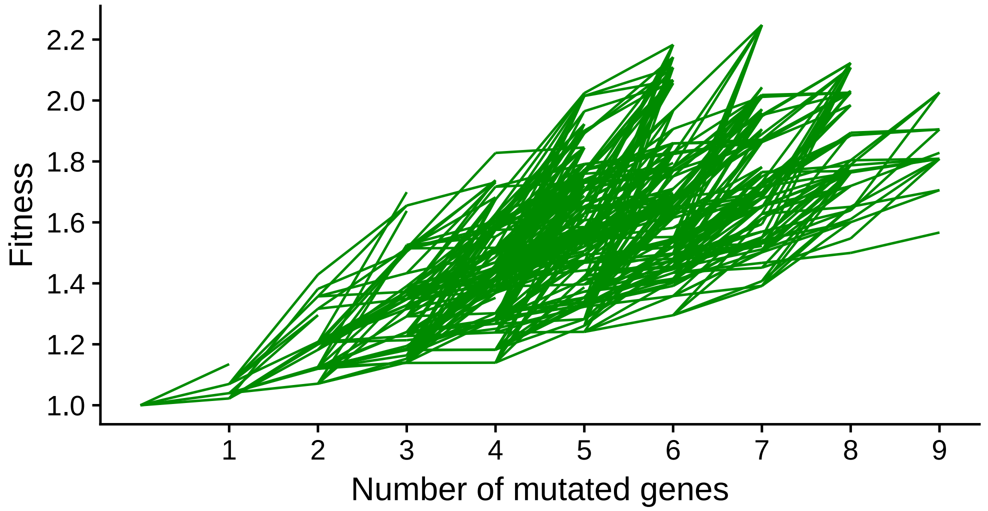

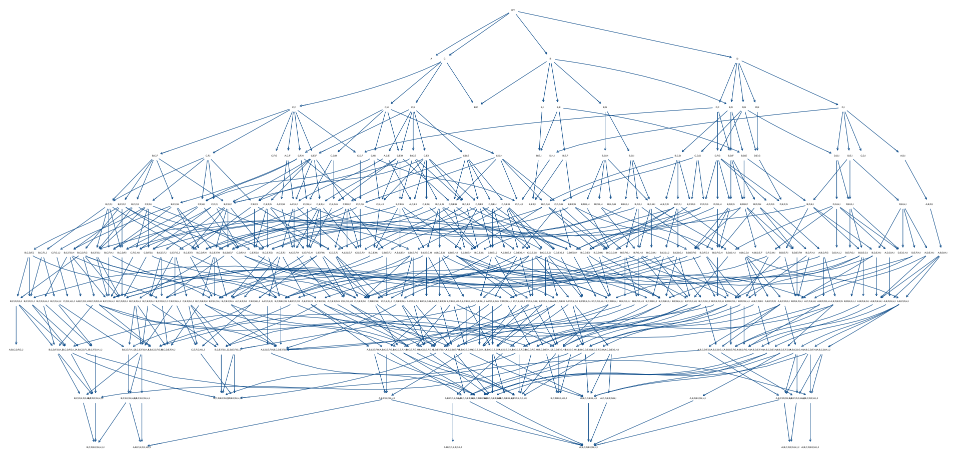

ID	zSLtW8y3ke10umntK
acc genotypes	98
peaks no back	26
removed edges	NA
prop rem edges	NA

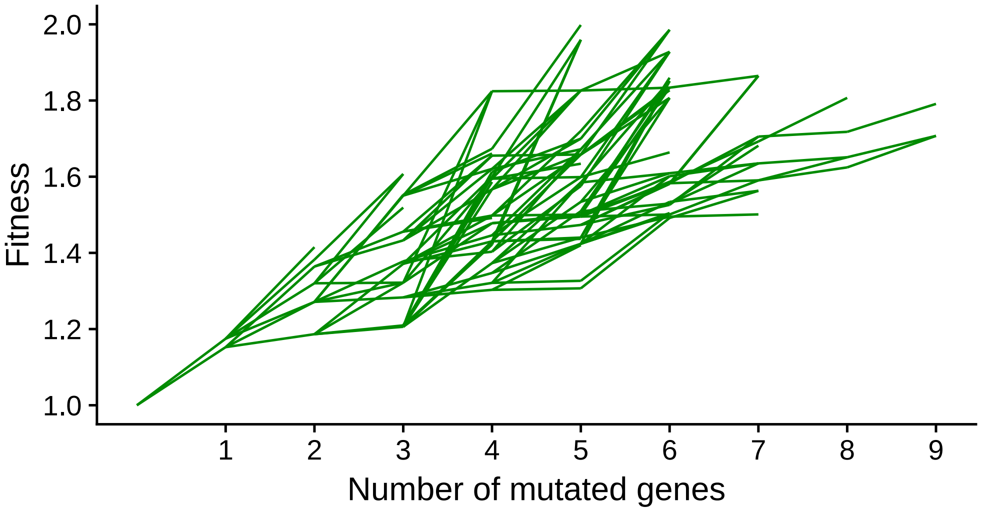

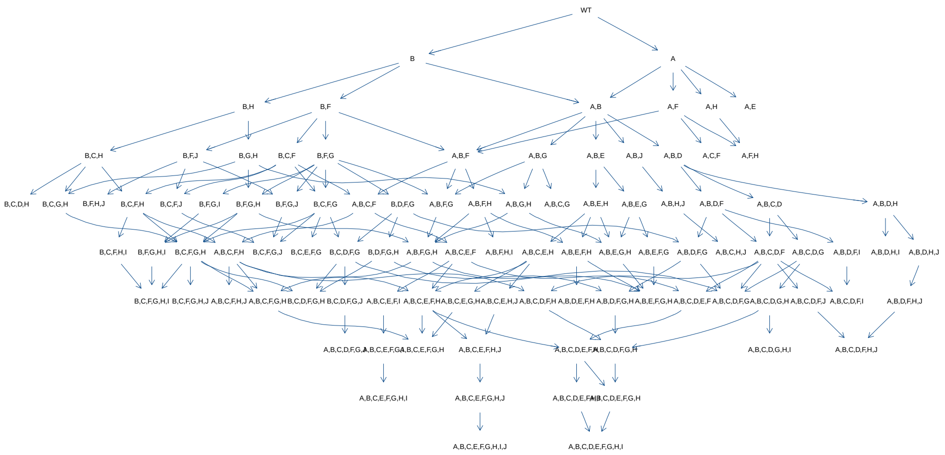

ID	ZTNMfNi10xGkfS7ED
acc genotypes	534
peaks no back	52
removed edges	NA
prop rem edges	NA

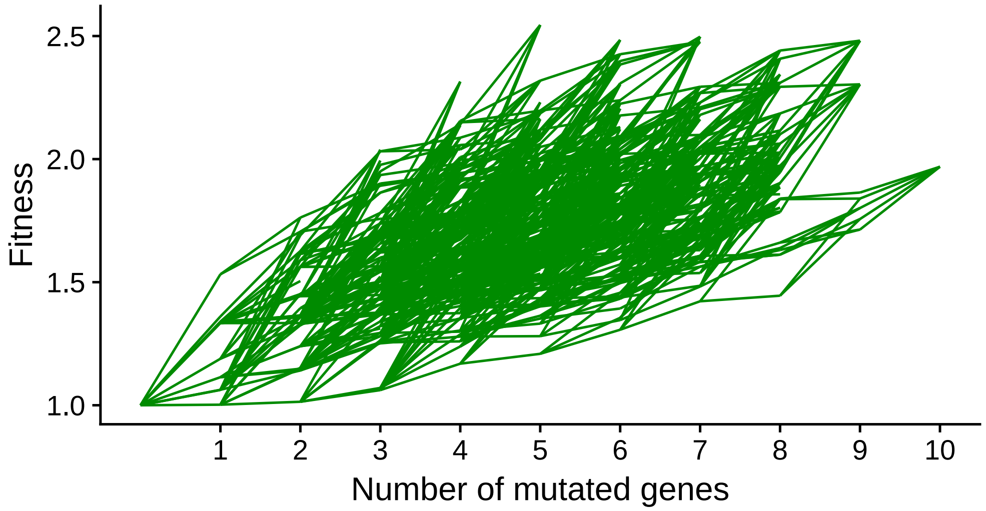

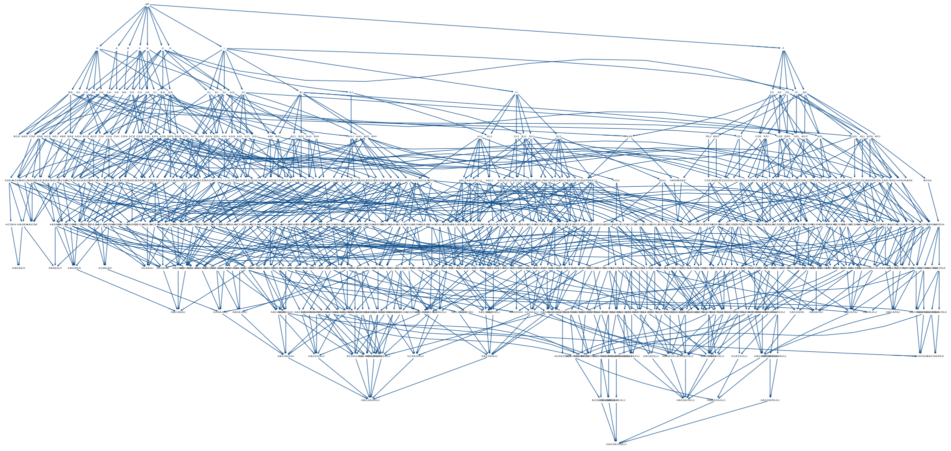

ID	ZVzsqZQydHbT4TYZ
acc genotypes	42
peaks no back	20
removed edges	NA
prop rem edges	NA

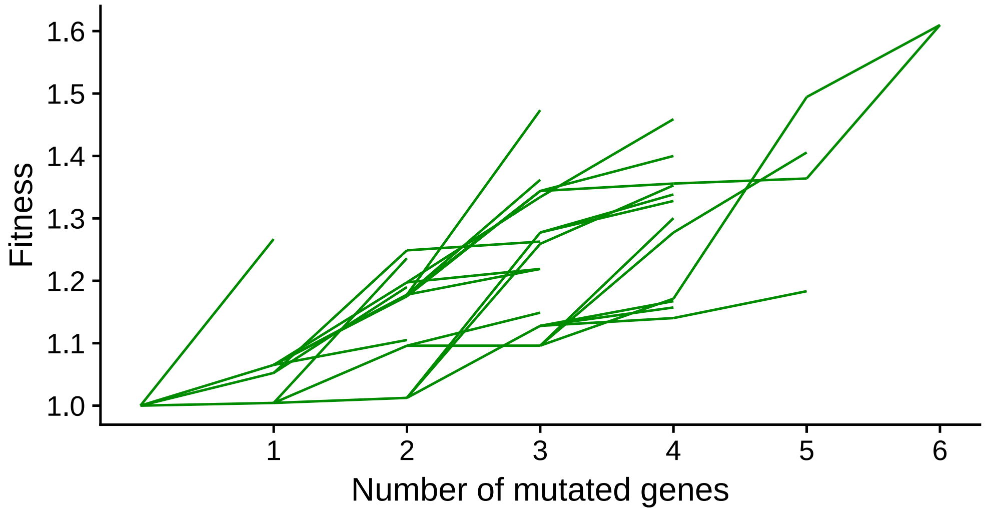

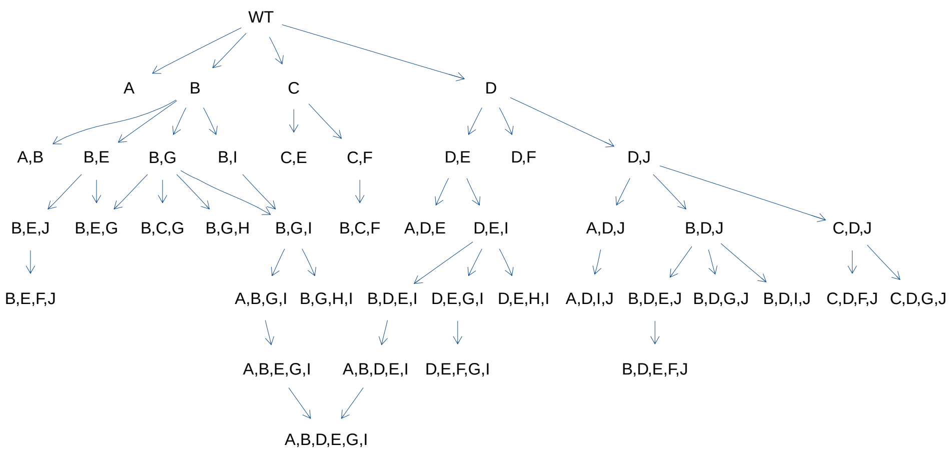

ID	zWjhPCHtGUy14jOB
acc genotypes	154
peaks no back	34
removed edges	NA
prop rem edges	NA

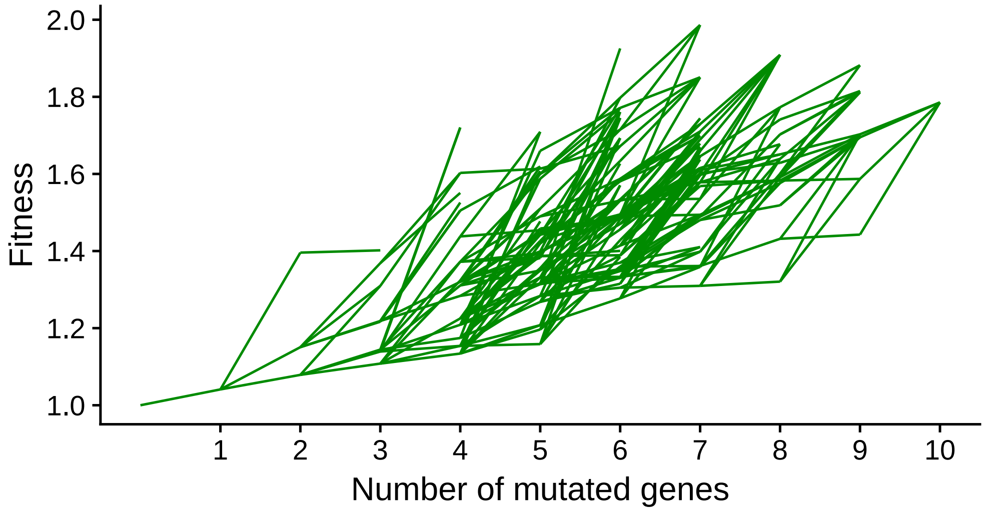

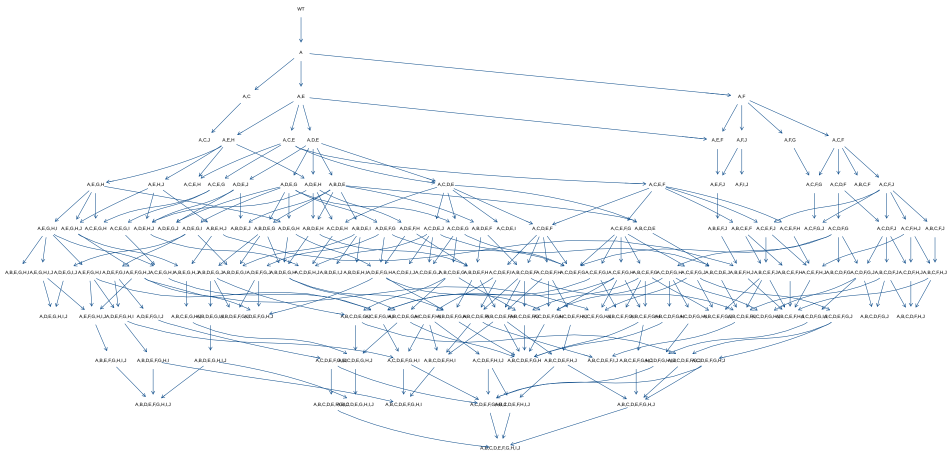

Supplement: S1 Fig — Plots of the 1260 fitness landscapes (and corresponding fitness graphs) used. (PDF) [file pcbi.1007246.s001.pdf]
