# Supplementary material for: Every which way? On predicting tumor evolution using cancer progression models
Source: PLoS Comput Biol. 2019 Aug 2;15(8):e1007246. doi: 10.1371/journal.pcbi.1007246 (PMC6693785; doi:10.1371/journal.pcbi.1007246)
Supplement: S2 Text — (PDF) [file pcbi.1007246.s004.pdf]

Supporting Information for "Every which way? On predicting tumor evolution using cancer progression models"

Ramon Diaz-Uriarte[1,2*], Claudia Vasallo[1,2]

1 Dept. Biochemistry, Universidad Autónoma de Madrid, Madrid, Spain
2 Instituto de Investigaciones Biomédicas "Alberto Sols" (UAM-CSIC), Madrid, Spain

* ramon.diaz@iib.uam.es

S2 Text. Generating random fitness landscapes

Contents

1 Representable fitness landscapes 1

2 Rough Mount Fuji 2

3 Local maxima without reciprocal sign epistasis? 2

4 Passenger mutations, deleterious mutations, and local maxima 3

5 References 4

---

1. Representable fitness landscapes

For the representable and local maxima fitness landscapes, we started by generating random DAGs. Since no agreed upon model exists for the distribution of DAGs in CPMs, we have used two different procedures, choosing each one randomly with the same probability. One of the procedures uses the function `simOGraph`, in the OncoSimulR package. To generate random DAGs with `simOGraph` for N genes, the genes were first randomly split in a number of levels, where the number of levels used was a randomly chosen integer between 3 and $N - 1$, both included. Then, each gene from each level i was randomly connected (as descendant) to randomly chosen genes (the ancestors) from levels j, where $j < i$; the number of incoming connections of each gene is a randomly chosen integer between 1 and $maxp$ (both included), where $maxp$ is a randomly chosen integer between 2 and $N - 2$ ($maxp$ is common for all genes in a DAG, but can vary between DAGs). The DAG we use is the transitive reduction of the above generated DAG. (Note that this procedure can occasionally result in star DAGs, i.e., DAGs without any dependencies; in such a case, the DAG was discarded and a new one obtained). The other procedure uses the function `random_poset` in package `mccbn` (https://github.com/cbg-ethz/MC-CBN); this function is undocumented, but it returns the transitive reduction of a randomly-filled adjacency matrix for a DAG where the initial number of non-zero connections is equal to the number of possible connections times a constant; we used the default value of that constant (0.15).

2. Rough Mount Fuji

In the Rough Mount Fuji fitness landscapes the reference genotype (i.e., the genotype with maximum fitness) was randomly chosen (setting `reference = 'random'` in the `rfitness` function in OncoSimulR). The standard deviation, *sd*, of the random normal variate was set to 0.2 and the decrease in fitness (strictly, birth rate) of a genotype per each unit increase in Hamming distance from the reference genotype, c, was chosen from a uniform $U(0, 0.2)$ distribution. This gives a wide variety of fitness landscapes that encompass from close to additive (large values of c) to House of Cards (c close to 0), with maximum fitness (birth rate) comparable to those of the representable and local maxima fitness landscapes.

The generated RMF fitness landscape was checked to ensure that all 7 or 10 genes were present in at least one accessible genotype; if they were not, a new fitness landscape was generated (with, possibly, different values of c and reference genotype). Function `rfitness` from the OncoSimulR package [1] was used.

3. Local maxima without reciprocal sign epistasis?

As explained in the paper, creating fitness landscapes with local maxima generally results in creating reciprocal sign epistasis and the number of local fitness maxima is associated with reciprocal sign epistasis —see Figures in section S2 Figure. There were, however, seven cases (out of 420) where introduction of local fitness maxima did not lead to introduction of reciprocal sign epistasis. Is this a contradiction of the statement that reciprocal sign epistasis is a necessary condition for local fitness maxima [2]? No: the key feature of all the models we consider is that back mutation is not allowed, and this explains why scenarios that have local fitness maxima (when no back mutation is allowed) might not have reciprocal sign epistasis.

We will look at one example in detail. The seven cases (which can be seen in S1 Figure) are landscapes with IDs "7E10pIyu7UguIUl8I", "8QlFQCUlUVfC10PZr", " bCsk2Qo5VMVm55fM", "GedZaWDeb1029Mf88", " hw8kQ4g44p4XAkDa", "WpF105HbEDoECa8vs", "t1yUXsv5fVuo10GRi". We will examine in detail fitness landscape "GedZaWDeb1029Mf88" (it is the smallest one — see page 299 in S1 Figure). The fitness of four of the relevant genotypes are

```
ABCDFG : 2.007
ABCDEFG: 1.8
ABCDF: 1.749
ABCDEF: 1.712
```

so there is no reciprocal sign epistasis (use, for example, the graphical criterion in [3] or [4]) and "ABCDFG" is a global maximum.

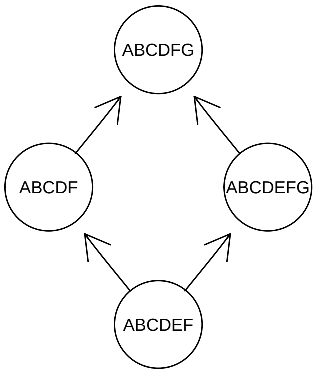

Under an evolutionary model that assumes no back mutations (as is the case of all models considered here), two of those transitions, those that involve loosing "E" (`ABCDEF -> ABCDF` and `ABCDEFG -> ABCDFG`) are not allowed, leading to two local fitness maxima (`ABCDFG, ABCDEFG`). To put it differently, under back mutations this fitness landscape

would have only a single maximum (ABCDFG); there is no reciprocal sign epistasis and there would be no local maxima. But once back mutation is not allowed, a local fitness maximum appears as some transitions are not allowed.

Note also that here, for that set of four genotypes, mutating gene E decreases fitness. But mutating E increases fitness in genotypes "ABC" or "ABCD". Thus, this fitness landscape does not fulfill either the assumption that a mutation never decreases the probability of acquiring other mutations (even if the fraction of pairs of genotypes with reciprocal sign epistasis is 0). Regardless, one can also simply focus on the fact that this fitness landscape contains local maxima (and is missing paths relative to the corresponding fitness graph from the DAG of restrictions).

4. Passenger mutations, deleterious mutations, and local maxima

CPMs model mutations in driver genes, and Figure 1 in the paper depicts several scenarios with respect to driver genes. If other genes can acquire mutations that are deleterious regardless of the genetic background (unconditionally deleterious —see below) then even under the CPM model there will be local fitness maxima if back mutation is not allowed.

The local maxima, in the absence of reciprocal sign epistasis, appear **because** back mutation is not allowed; with back mutation, reciprocal sign epistasis is a necessary condition for local fitness maxima (see [2]); this issue has also been illustrated above (section 3).

The following diagram shows the scenario with unconditionally deleterious mutations:

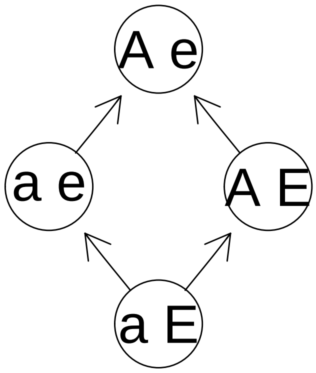

We use "A/a" for any of the drivers in the model (with its restrictions satisfied), where "A" means mutated and "a" not mutated. "E/e" denotes the genes with possible deleterious mutation(s), where "E" means mutated and "e" not mutated. If back mutations were possible, there would be a single maximum: "A e". Without back mutation, there are two maxima: "A e" and "A E" (without back mutation we cannot reach "A e" from "A E").

So for any specific genotype with respect to the CPM drivers in the model (i.e., for a given genetic background, specified as a configuration of CPM drivers), gaining any mutation in the deleterious genes will decrease fitness. And this is why we said above "mutations that are deleterious regardless of genetic background". Conversely, for any specific genotype of the deleterious loci, gaining a mutation in the drivers (with their restrictions satisfied) will increase fitness under the CPM model.

Thus, conditioning on a configuration of deleterious loci, there is a single fitness maximum, the genotype with all driver genes mutated. And regardless of the existence or not of these genes that are unconditionally deleterious when mutated, CPMs assume that the acquisition of a mutation in a driver gene, when all its possible dependencies on other genes are satisfied, does not decrease the probability of gaining a mutation in another driver gene: acquiring driver mutations (when their dependencies on other genes are satisfied) cannot decrease fitness.

It should be noted that this is a feature common to both strongly deleterious mutations and to mildly deleterious passenger mutations, including scenarios such as those in, e.g., [5, 6], where the accumulation of mildly deleterious mutations can lead to mutational meltdown.

We restricted this section to genes that are deleterious when mutated regardless of genetic background (i.e., unconditionally deleterious): these genes are not driver genes. What if some of the above deleterious mutations were beneficial under a given genetic background, one that includes one or more of the drivers considered by the CPM? Then, by definition, these would not be genes where a mutation is always, or unconditionally, deleterious but, rather, genes that should have been included in the CPM model as drivers: their mutation can be beneficial under certain genetic backgrounds and thus they show sign (and possibly reciprocal sign) epistasis relationships with the other genes in the model. In terms of Figure 1 in the paper, therefore, these are genes that should appear in the fitness landscapes depicted.

5. References

1. Diaz-Uriarte R. OncoSimulR: Genetic Simulation with Arbitrary Epistasis and Mutator Genes in Asexual Populations. Bioinformatics. 2017;33(12):1898–1899. doi:10.1093/bioinformatics/btx077.

2. Poelwijk FJ, Tănase-Nicola S, Kiviet DJ, Tans SJ. Reciprocal Sign Epistasis Is a Necessary Condition for Multi-Peaked Fitness Landscapes. Journal of Theoretical Biology. 2011;272(1):141–144. doi:10.1016/j.jtbi.2010.12.015.

3. Crona K, Greene D, Barlow M. The Peaks and Geometry of Fitness Landscapes. Journal of Theoretical Biology. 2013;317:1–10. doi:10.1016/j.jtbi.2012.09.028.

4. Ferretti L, Schmiegelt B, Weinreich D, Yamauchi A, Kobayashi Y, Tajima F, et al. Measuring Epistasis in Fitness Landscapes: The Correlation of Fitness Effects of Mutations. Journal of Theoretical Biology. 2016;396:132–143. doi:10.1016/j.jtbi.2016.01.037.

5. McFarland C, Mirny L, Korolev KS. A Tug-of-War between Driver and Passenger Mutations in Cancer and Other Adaptive Processes. Proc Natl Acad Sci U S A. 2014;111(42):15138–15143. doi:10.1101/003053.

6. McFarland CD, Yaglom JA, Wojtkowiak JW, Scott JG, Morse DL, Sherman MY, et al. The Damaging Effect of Passenger Mutations on Cancer Progression. Cancer Res. 2017;77(18):4763–4772. doi:10.1158/0008-5472.CAN-15-3283-T.
