# Supplementary material for: Every which way? On predicting tumor evolution using cancer progression models
Source: PLoS Comput Biol. 2019 Aug 2;15(8):e1007246. doi: 10.1371/journal.pcbi.1007246 (PMC6693785; doi:10.1371/journal.pcbi.1007246)
Supplement: S5 Text — (PDF) [file pcbi.1007246.s007.pdf]

Supporting Information for "Every which way? On predicting tumor evolution using cancer progression models"

Ramon Diaz-Uriarte[1,2*], Claudia Vasallo[1,2]

1 Dept. Biochemistry, Universidad Autónoma de Madrid, Madrid, Spain
2 Instituto de Investigaciones Biomédicas "Alberto Sols" (UAM-CSIC), Madrid, Spain

* ramon.diaz@iib.uam.es

S5 Text. Cancer data sets: sources, characteristics, additional results.

Contents

List of Figures **1**

1 Cancer data sets **2**
1.1 Cancer data sets: sources and characteristics 3
1.2 Bootstrapping on the cancer data sets 6

2 Cancer data sets: additional results, figures **7**
2.1 Cancer data sets: JS_{ob} and unpredictability for the bootstrap runs 8
2.1.1 Cancer data sets: distribution of number of mutations per subject 9
2.1.2 Cancer data sets: proportion of individuals in which a mutation is present 10
2.1.3 Cancer data sets: scatterplots of $JS_{o,b}$, S_c, and number of paths to the maximum 11

3 References **12**

List of Figures

A Results from the cancer data sets analyzed with H-CBN. Data sets have been ordered by increasing sample size, and the x-axis labels provide the acronym (shown in full in the inset legend). Below the data set acronym are the number of subjects and the total number of features, respectively. Analysis were run three times, limiting the number of features analyzed to the 7, 10, and 12 most common ones; the boxplots for each data set are shown in increasing order of number of features. For data sets such as, say, Pancreas genes (Pan_Ge), with 7 features, using 7, 10, or 12 maximum features makes no difference in the number of features analyzed; the three replicate runs show run-to-run variability. A) $JS_{o,b}$: JS statistic for the comparison of the distribution of paths from running H-CBN on the original data set against the distribution of paths from running H-CBN on each one of the bootstrap runs. B) Diamonds show the S_c from the full data, and boxplots the S_c from the bootstrap runs. Right axis labeled by number of equiprobable paths equivalent to the S_c. 8

B Cancer data sets: Histograms of number of mutations per subject in the data sets. 9

C Cancer data sets: Histograms of proportion of individuals in which each mutation is present. For example, in the PP data set, there are four mutations that are present in 80% to 90% of the individuals in the data set, 1 mutation present in 90% to 100% of the individuals, 1 mutation in between 0 and 10% of the individuals, and 1 in between 10% and 15%. 10
D Cancer data sets: scatterplots of the relationship between $JS_{o,b}$, S_c, and number of paths to the maximum, using the data labels, using the statistics from analyses with 12 features. 11

---

1. Cancer data sets

The cancer data sets used here are a representative example of data sets to which researchers have applied CPMs or data sets to which researchers might want to apply CPMs. All of these data sets (in at least one of their variants) have been used previously in studies with CPMs except for the BRCA data sets, that were obtained *de novo* for this paper.

These data sets vary in:

- sample size (27 to 594 samples);
- data types (nonsynonymous somatic mutations, and copy number aberrations, or both);
- levels of analysis: altered/non-altered pathways —e.g., Pan_pa, GBM_pa, Col_pa, all _pa—, functional modules —GBM_mo–, exclusivity groups [1] —Col_msi_co, Col_mss_co, ACML_co—, genes —e.g., BRCA_ba_s, BRCA_he_s, Pan_ge, GBM_ge, Col_ge, Ov, Lu—, and different types of gene-level events as insertion/deletions, missense point mutations, nonsense point mutations —ACML and ACML_co.
- different procedures for driver selection, from simple frequency-based selection of features (e.g., GBM_ge, Pan_ge, Col_ge) to state-of-the-art methods for the identification of significantly altered genes [2] (e.g., BRCA_he_ s, BRCA_ba_s, Col_msi, Col_mss, GBM_CNA);
- restriction of patient subtypes (with the purpose of achieving sample homogeneity —e.g., BRCA_ba_s, BRCA_he_s, Col_msi, Col_mss);

Thus, in several cases the same source data set has been processed in different ways to produce two different versions. For three of the data sets, two versions, one coded in terms of mutations of genes and one in terms of pathway alterations, were available (Col_ge and Col_pa, GBM_ge and GBM_pa, Pan_ge and Pan_pa). For three other data sets, we have analyzed both the original data (Col_msi, Col_mss, ACML), and the same data after accounting for so-called "exclusivity relations" (see 1; Col_msi_co, Col_mss_co, ACML_co). Another data set, GBM_CNA, was also analyzed in terms of "functional modules" (GBM_mo).

Other data sets have been obtained from a single source and split to increase subject homogeneity (e.g., BRCA_ba_s and BRCA_he_s; Col_msi, Col_mss).

1.1. Cancer data sets: sources and characteristics

Name	Source	Original source	Number of features	Number of subjects	Type of event	Abbreviation
All Pathways	[3]	(From sources for colon, glioblastoma, and pancreas genes data sets)	12	268	candidate mut	all_pa
Colon Genes	[3]	[4]	8	95	candidate mut	Col_ge
Colon Pathways	[3]	[4]	10	95	candidate mut	Col_pa
Glioblastoma Genes	[3]	[5]	8	78	candidate mut	GBM_ge
Glioblastoma Pathways	[3]	[5]	10	78	candidate mut	GBM_pa
Pancreas Genes	[3]	[6]	7	90	candidate mut	Pan_ge
Pancreas Pathways	[3]	[6]	7	90	candidate mut	Pan_pa
Lung	[7]	[8]	51	161	recurrent mut	Lu
Ovarian	[7]	[9]	192	326	recurrent mut	Ov
Ovarian driver	[7]	[9]	9	326	significant mut	Ov_drv
Colon MSI	[1]	[10]	30	27	significant mut and CNA	Col_msi
Colon MSS	[1]	[10]	34	152	significant mut and CNA	Col_mss
Colon MSI mutual exclusivity groups collapsed	[1]	[10]	20	27	significant mut and CNA	Col_msi_co
Colon MSS mutual exclusivity groups collapsed	[1]	[10]	13	152	significant mut and CNA	Col_mss_co
ACML	[11, 12]	[13]	16	64	recurrent mut	ACML
ACML mutual exclusivity groups collapsed	[11, 12]	[13]	11	64	recurrent mut	ACML_co
GBM CNA	[14, 15]	[16]	48	563	significant CNA	GBM_CNA
GBM CNA modules	[14, 15]	[16]	9	563	significant CNA	GBM_mo
GBM co-occurrent	[17]	[18, 19]	3	594	significant co-occurrent CNA	GBM_coo
Ovarian CNV	[20]	[21]	7	87	recurrent arm-level CNA	Ov_CNV
BRCA HER2, subtypes	[14, 15]	[22]	4	57	significant mut	BRCA_he_s
BRCA basal-like, subtypes	[14, 15]	[22]	6	81	significant mut	BRCA_ba_s

Table A: Cancer data sets used. Source refers to where the data have been obtained from, generally also the first reference where data set has been used with CPMs. Data sets BRCA_he_s and BRCA_ba_s have been obtained from original sources for this paper. A data set very similar to GBM_CNA was used in [23], but we obtained it from [14, 15], as explained in the text. Type of event: mut: nonsynonymous somatic mutations; CNA: copy number alterations; candidate mut: nonsynonymous mutations on candidate genes [2, 4, 24]; significant mut: nonsynonymous mutations on significant mutated genes, as defined by state-of-the-art algorithms [2] MuSiC [25] or MutSigCV [26]; significant CNA: significant copy number alterations, as defined by GISTIC2.0 [27].

These are further details about how the data were obtained and the rationale for the data processing:

All Pathways and Colon, Glioblastoma, Pancreas pathways Data sets Colon Genes, Glioblastoma genes, and Pancreas genes are from [3], with original sources [4], [5], and [6], respectively.

For the corresponding data sets in terms of pathways, the mapping from genes to pathways was done by [3], from the original papers with data sets. Our scripts to reproduce the analysis are provided with the code. Note that for Pancreas pathways we eliminate the four pathways that were present in all subjects (see also [3] and notes in the code for details). For Glioblastoma pathways, two pathways had identical patterns (Apoptosis and Small GTPase-dependent signaling (other than KRAS)) and only one was used.

What we call "All Pathways" here, for brevity, is called "All cancer types" in [3].

Lung Original data from [8]. They were obtained from text file `Lung_SM4` from the supplementary material of [7] (file "BMLv1.tar.gz").

Ovarian Original data from [9]. They were obtained from text file `OV_SM5` from the supplementary material of [7] (file "BMLv1.tar.gz").

Ovarian driver Data come from data set Ovarian, restricting events to 9 significantly mutated genes described in Table 2 of source paper [9].

Colon MSI Colorectal cancer, microsatellite unstable tumors. The original data (as well as Colon MSS) come from COADREAD [10]; we obtained them from [1], where the original data were split by tumor subtype into MSI and MSS (see also comments about patient stratification under "BRCA basal-like, subtypes, BRCA HER2, subtypes").

We used GIMP to open the PDF file page (Figure 3 on page 6 of [1]) where the figure was and cropped the grid of the figure and exported it as JPEG with high resolution. Then we imported it in ImageJ(Fiji) (https://fiji.sc/), converted it to 8-bit, applied threshold option and set it to B/W, then exported it as text image (matrix as txt). Then we imported the text image in R and used the code in `fig_to_matrix_capri_pnas.R` to convert the text image into a matrix of genotypes. The data were checked against the original figures.

Colon MSS Colorectal cancer, microsatellite stable tumors. The original data come from COADREAD [10] and we obtained them from [1], where the original data were split by tumor subtype into MSI and MSS (see above).

From [1]. Same process as for Colon MSI; the figure is Figure S5 from page 16 of the supplementary material to [1]. The authors explain that "Events selected for reconstruction are those involving genes altered in at least 5% of the cases, or part of group of alterations showing an exclusivity trend (see Figure S4)."

Colon MSI mutual exclusivity groups collapsed, Colon MSS mutual exclusivity groups collapsed Data sets were obtained from data sets **Colon MSI** and **Colon MSS**, respectively, processed so events showing mutual exclusivity patterns described in [1] were collapsed in a single event representing an exclusivity group.

Mutual exclusivity patterns, as explained in [1], could decrease the performance of CPMs (as CPMs assume no events show exclusivity or otherwise reduce the probability of another event occurring [7]). How to deal with these exclusivity patterns with CPMs such as CBN, OT, CAPRESE, or CAPRI without additional formulas, is not clear, however. For the Colon MSI and Colon MSS data sets, the exclusivity groups identified in Caravagna *et al.* (2016), [1], are supposed to represent fitness-equivalent exclusive sets of alterations. Some of these exclusivity groups share events, some represent "hard" exclusivity relations whereas others represent "soft" exclusivity relations [1]. What we have

done is consider each one of the exclusivity groups as analogous to a pathway in Gerstung *et al.* (2011) [3] or a module in Cheng *et al.* (2012) [23] (where the same gene can be part of different pathways/modules or, in this case, different exclusivity groups). This amounts to considering each exclusivity group as a "fitness equivalent" (*sensu* [1]) set of alterations for some "phenotype" shared by the exclusivity group, similar to what [1] did, and should not introduce any additional difficulties for the inference of downstream dependencies.

We removed from the data set any alteration that was a member of one or more exclusivity groups as an individual alteration. Thus, the data sets with exclusivity groups differ from the original ones by adding exclusivity groups and removing alterations that belong to those exclusivity groups. We used the Table S3 of the Supplementary Material of [1] as the canonical source of exclusivity groups. However, notice that there must be the following mistakes in Table S3: in row 6 it says ACVR1B:a, but there is no amplification event that affects ACVR1B in data set **Colon MSI**, according to Figure 3 in the paper (and Figure 5 and Figure S11), but mutation and deletion; in row 7 it says NRAS:a but there is no amplification event that affects NRAS in data set **Colon MSI**, according to Figure 3 in the paper (and Figure 5 and Figure S11), but mutation and deletion; in row 15 it says NRAS:d but there is no deletion event that affects NRAS in data set **Colon MSS**, according to Figure S5 (and Figure S4 and Figure S10), but mutation and amplification. So we assumed it should say ACVR1B:d in row 6, NRAS:d in row 7 and NRAS:a in row 15. Additionally, when a mutual exclusivity group in Table S3 was contained in another (for instance, group in row 5 is part of group of row 1) we used only the bigger one. This procedure results, for **Colon MSI**, in a new data set of 27 subjects and 20 columns (five from the exclusivity groups, and 15 from the 30 alterations in the original data set minus the 15 removed as they are in one or more exclusivity groups). For **Colon MSS**, this results in a new data set of 152 subjects and 13 columns (11 from the exclusivity groups, and 2 from the 34 alterations in the original data set minus the 32 removed as they are in one or more exclusivity groups).

ACML Data are originally from [13], and were obtained from the `aCML` data set in R package "TRONCO" [12] and processed to keep the 16 events used in [11]. The data includes alterations with a frequency above 5 % in original data set from [13] and additional selected alterations hypothesized to be part of a functional ACML progression path in the literature and are shown in Figure 5 of [11]. As explained in [11], events are categorized as insertion/deletions, missense point mutations, and nonsense point mutations. This data set shows mutual exclusivity patterns described in Section 4.2 of [11].

ACML mutual exclusivity groups collapsed Data come from data set **ACML**, processed so events showing mutual exclusivity patterns described in [11] were collapsed in a single event. As with data sets **Colon MSI** and **Colon MSS**, here we dealt with mutual exclusivity patterns in the data by collapsing individual events in exclusivity groups considered as "fitness equivalent" groups. The two exclusivity groups were obtained from Section 4.2 of [11]: one involves all types of alterations of genes ASXL1 and SF3B1 (ASXL1 nonsense point, ASXL1 ins/del, SF3B1 missense point) and the other involves all types of alterations of genes TET2 and IDH2 (TET2 nonsense point, TET2 missense point, TET2 ins/del, IDH2 missense point); thus, there are 11 columns, 2 from the exclusivity groups, and 9 from the 16 alterations minus the 7 removed as they belong into exclusivity groups.

GBM CNA Data come from TCGA GBM PUB CNA data set [16] and were obtained from cBioPortal [14, 15] using the R package "cgdsr" [28], selecting only CNA data from 51 driver genes used by Cheng *et al.* (2012) and detailed in Table 1 of [23], with a GISTIC score of 2 or -2. By doing so, we intended to follow the author's indications to obtain the same data set Cheng *et al.* (2012), [23], used to infer a cancer progression model. Although [23] cite [19] as the source of their data, we understand that the original data set used

in [23] should have been the "Provisional" TCGA data set at that time since they got more patients (462) than the total number of patients in the only published TCGA glioblastoma study at the date [19] (206). So, we used the data from the TCGA glioblastoma study published in 2013 [16] on the belief that this is the closest we can get to reproduce the data set used in [23] (the study of 2013, [16], contains 198 patients in common with the study of 2008, [19]). Note that 3 of the 51 genes were not altered in any subject and then were removed from the data set. Also note that, contrary to [23], to avoid mutual exclusivity patterns we only analyze one level of gain/loss per gene; so, here we used high-level amplification (GISTIC score of 2) and homozygous deletion (GISTIC score of - 2).

GBM CNA modules Data come from **GBM CNA**, processed so individual alterations were grouped in modules at phenotype level of cancer-related pathways.

We reproduced the procedure used in Cheng *et al.* (2012) [23] to map alterations to functional modules of positive or negative effects within different cancer-related pathways as originally described in [29] and as detailed in Table 1 of [23].

GBM co-occurrent Data come originally from [19] and [18] and were obtained from Supplementary Material file `ST01.xls` (GBM_copy_number tab) of Attollini *et al.* (2010) and processed to keep only three highly correlated events described in [17], namely PTEN homozygous deletion, P16 homozygous deletion and EFGR low-level amplification.

Ovarian CNV Obtained from data set `ov.cgh` in the R package "Oncotree" [20]. Data are originally from [21]; `ov.cgh` from the "Oncotree" R package has also been used in [30].

BRCA basal-like, subtypes, BRCA HER2, subtypes Original data come from TCGA BRCA PUB mutation data set [22] and were obtained from cBioPortal [14, 15] using the R package "cgdsr" [28], restricting the data to subtype-specific significantly mutated genes within patients subtypes.

The data were then split in two, restricting the subjects to those classified as basal-like and HER2-enriched subtypes, respectively. To split the data set according to cancer subtypes we used the patient's classification by the gene expression-based PAM50 technique as detailed in Supplementary Table 1 of [22]. Then, for each subtype, we restricted the features to subtype-specific significantly mutated genes identified by MuSiC algorithm [25] and detailed in Supplementary Table 2 of [22] (Supplementary Tables 1-4.xls file in supplementary file nature11412-s2.zip).

The reason for splitting the data into two subsets is that cancer subtypes are believed to follow distinct evolutionary trajectories, and hence rely on at least some different drivers and/or pathways, and show differences in the chronology of accumulation of alterations [23, 31, 32]. Thus, sample heterogeneity in terms of different cancer subtypes (intertumor heterogeneity) can be confounding and hamper the identification of existing relations in the data. Sample stratification can alleviate this to some extent and should allow to focus on relevant events for specific subsets of subjects [1].

1.2. Bootstrapping on the cancer data sets

If the bootstrapping process resulted in a feature becoming absent from the data, or two or more features having identical patterns (i.e., one feature being identical to another) we discarded the bootstrap sample and obtained a new one; this is done to ensure that all bootstrapped data sets have paths of identical length (see also section "Preprocessing of data for CPMs" in S4 Text). This, therefore, leads to JS values that are more optimistic (smaller).

2. Cancer data sets: additional results, figures

2.1. Cancer data sets: JS_{ob} and unpredictability for the bootstrap runs

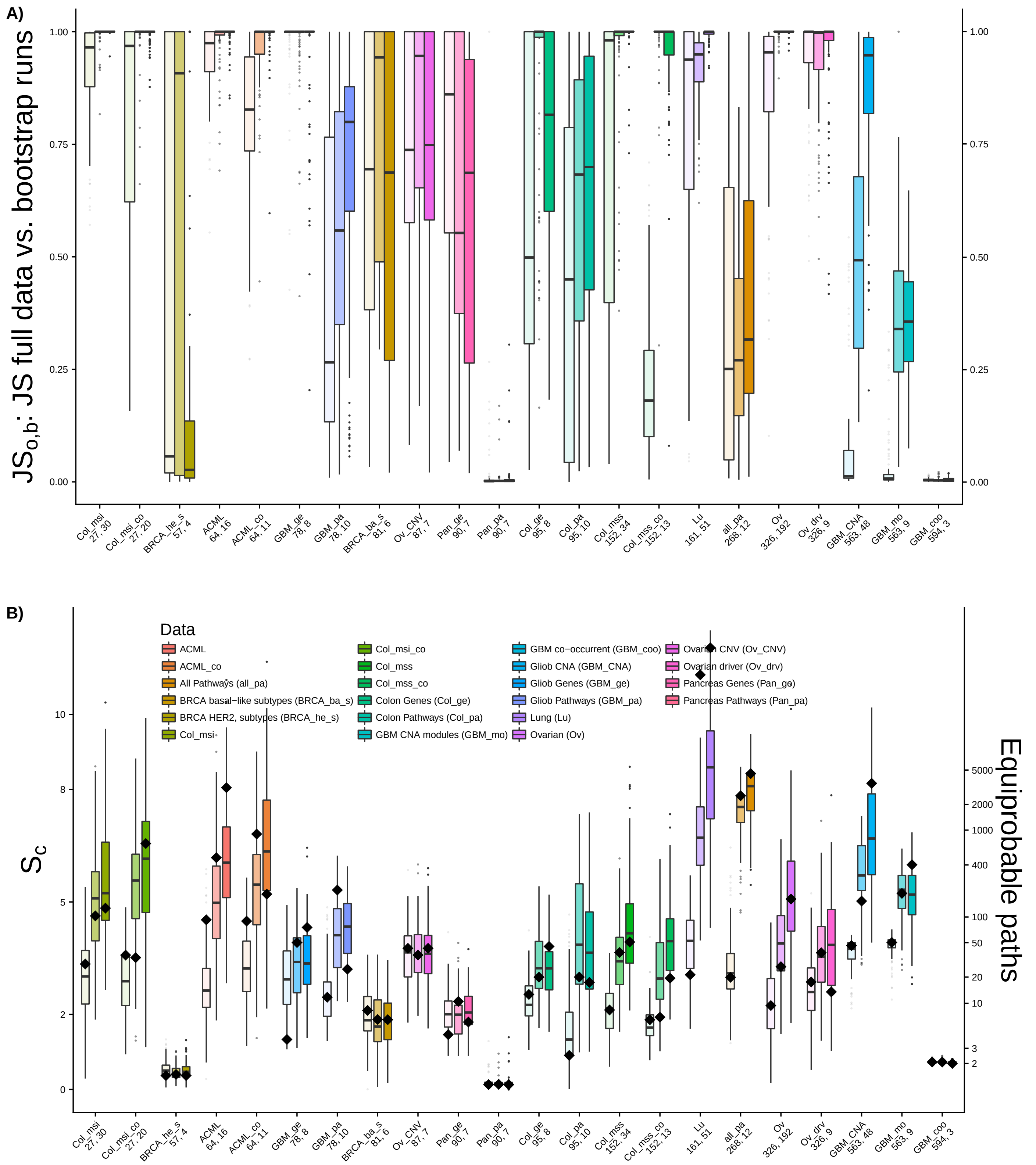

Figure A: Results from the cancer data sets analyzed with H-CBN. Data sets have been ordered by increasing sample size, and the x-axis labels provide the acronym (shown in full in the inset legend). Below the data set acronym are the number of subjects and the total number of features, respectively. Analysis were run three times, limiting the number of features analyzed to the 7, 10, and 12 most common ones; the boxplots for each data set are shown in increasing order of number of features. For data sets such as, say, Pancreas genes (Pan_Ge), with 7 features, using 7, 10, or 12 maximum features makes no difference in the number of features analyzed; the three replicate runs show run-to-run variability. A) $JS_{o,b}$: JS statistic for the comparison of the distribution of paths from running H-CBN on the original data set against the distribution of paths from running H-CBN on each one of the bootstrap runs. B) Diamonds show the S_c from the full data, and boxplots the S_c from the bootstrap runs. Right axis labeled by number of equiprobable paths equivalent to the S_c.

2.1.1. Cancer data sets: distribution of number of mutations per subject

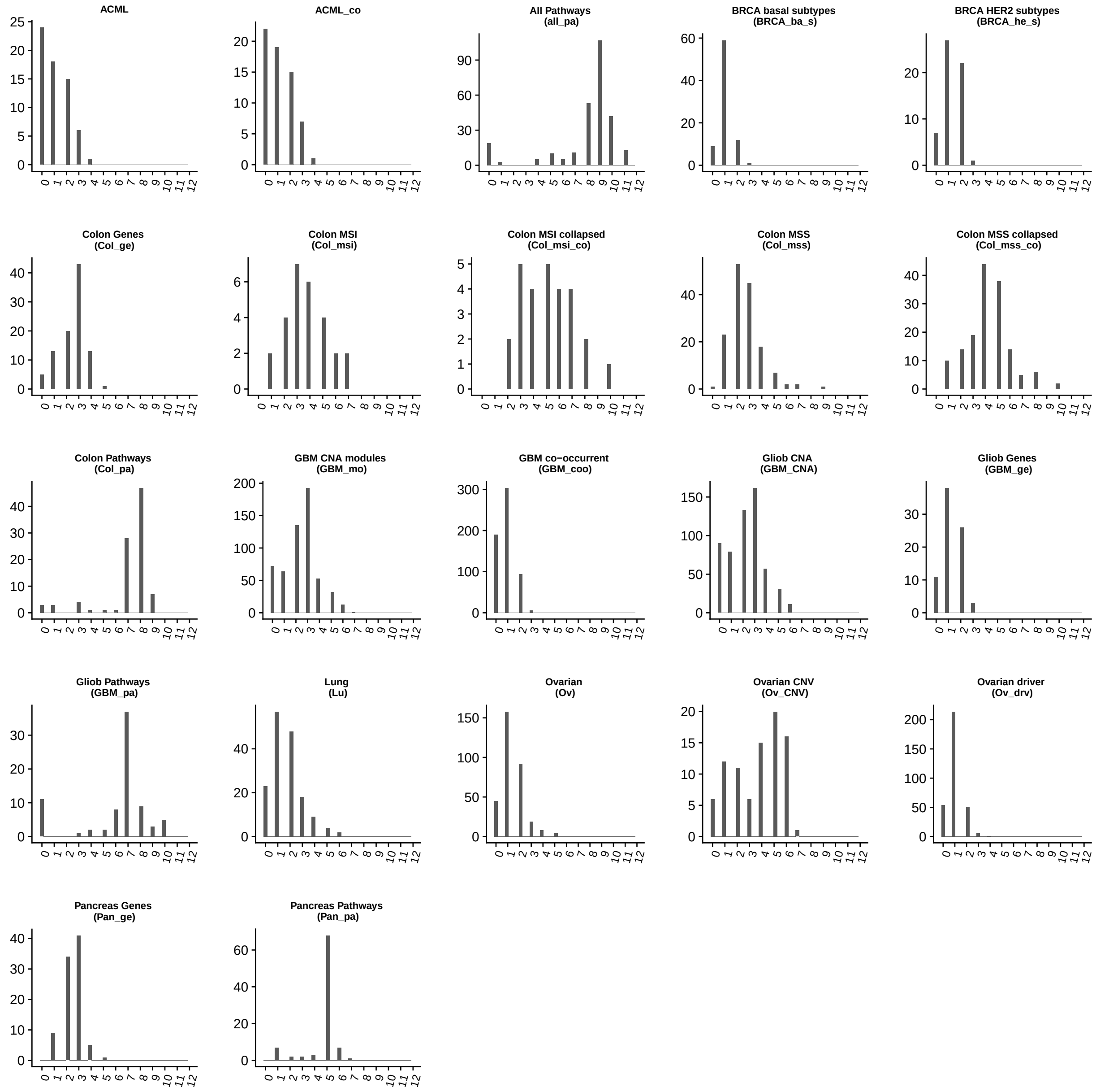

Figure B: Cancer data sets: Histograms of number of mutations per subject in the data sets.

2.1.2. Cancer data sets: proportion of individuals in which a mutation is present

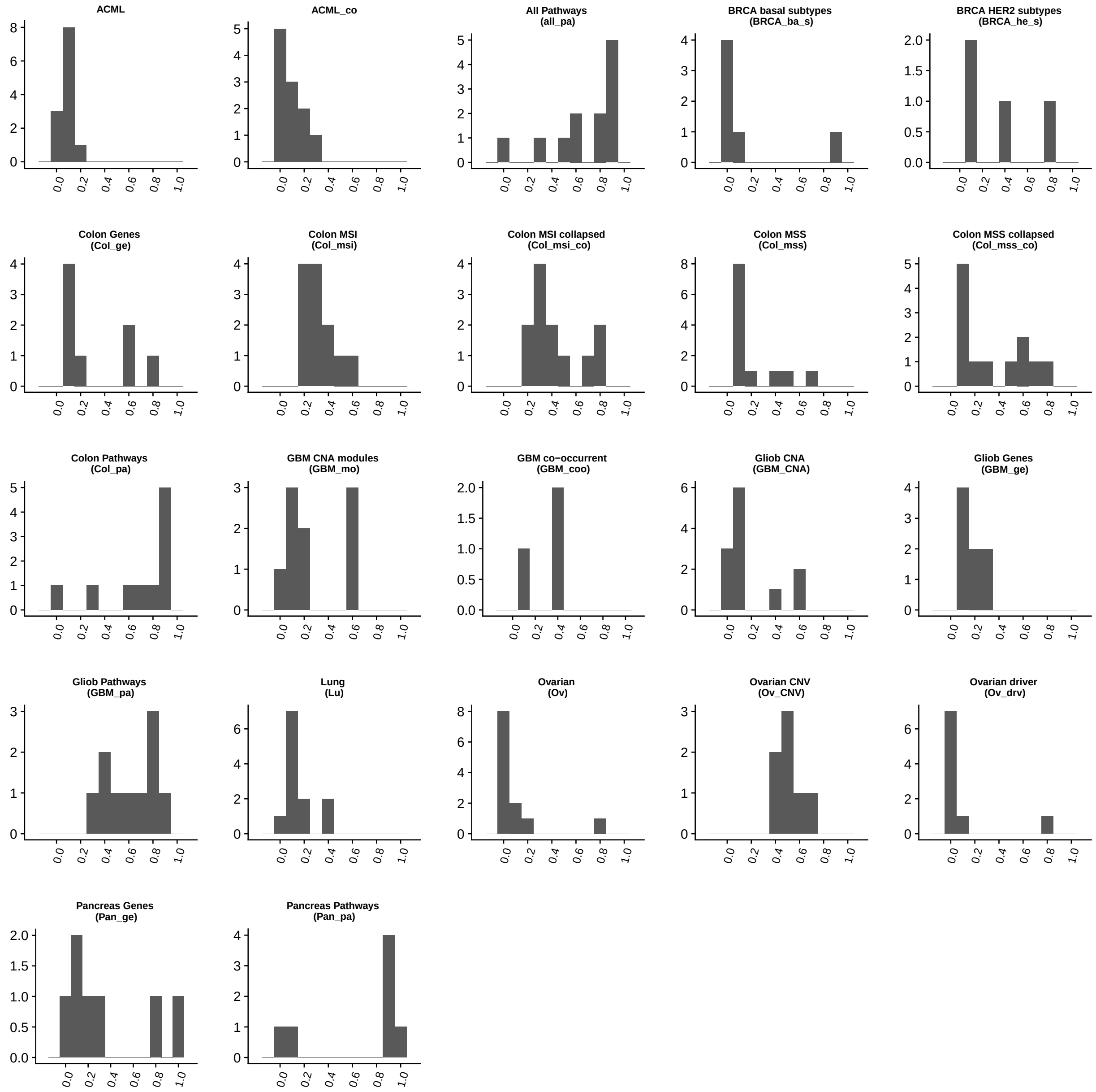

Figure C: Cancer data sets: Histograms of proportion of individuals in which each mutation is present. For example, in the PP data set, there are four mutations that are present in 80% to 90% of the individuals in the data set, 1 mutation present in 90% to 100% of the individuals, 1 mutation in between 0 and 10% of the individuals, and 1 in between 10% and 15%.

2.1.3. Cancer data sets: scatterplots of $JS_{o,b}$, S_c, and number of paths to the maximum

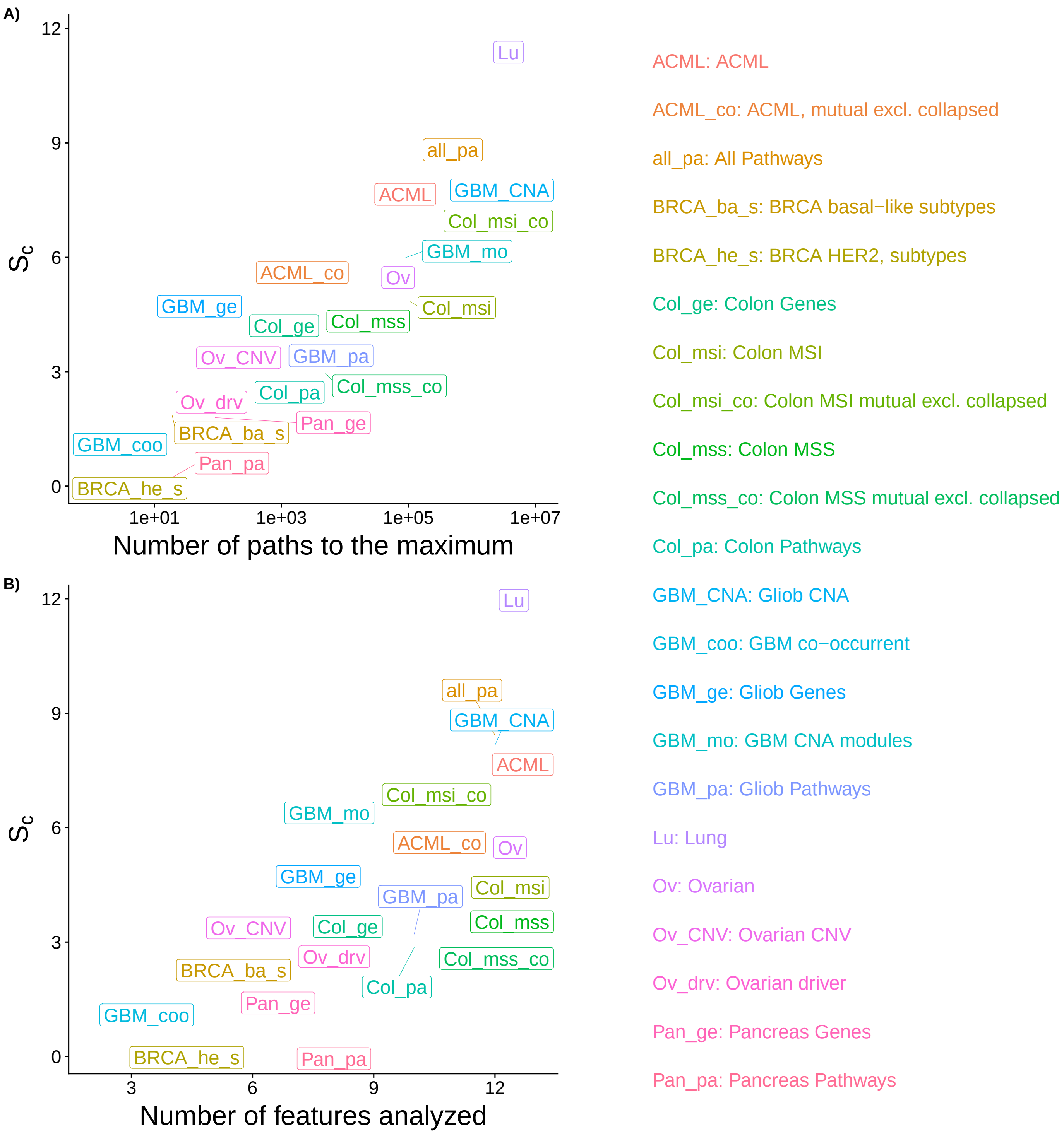

Figure D: Cancer data sets: scatterplots of the relationship between $JS_{o,b}$, S_c, and number of paths to the maximum, using the data labels, using the statistics from analyses with 12 features.

3. References

1. Caravagna G, Graudenzi A, Ramazzotti D, Sanz-Pamplona R, Sano LD, Mauri G, et al. Algorithmic Methods to Infer the Evolutionary Trajectories in Cancer Progression. PNAS. 2016;113(28):E4025–E4034. doi:10.1073/pnas.1520213113.

2. Tokheim CJ, Papadopoulos N, Kinzler KW, Vogelstein B, Karchin R. Evaluating the Evaluation of Cancer Driver Genes. PNAS. 2016;113(50):14330–14335. doi:10.1073/pnas.1616440113.

3. Gerstung M, Eriksson N, Lin J, Vogelstein B, Beerenwinkel N. The Temporal Order of Genetic and Pathway Alterations in Tumorigenesis. PLoS ONE. 2011;6(11):e27136. doi:10.1371/journal.pone.0027136.

4. Wood LD, Parsons DW, Jones S, Lin J, Sjoblom T, Leary RJ, et al. The Genomic Landscapes of Human Breast and Colorectal Cancers. Science. 2007;318(5853):1108–1113. doi:10.1126/science.1145720.

5. Parsons DW, Jones S, Zhang X, Lin JCH, Leary RJ, Angenendt P, et al. An Integrated Genomic Analysis of Human Glioblastoma Multiforme. Science. 2008;321(5897):1807–1812. doi:10.1126/science.1164382.

6. Jones S, Zhang X, Parsons DW, Lin JCH, Leary RJ, Angenendt P, et al. Core Signaling Pathways in Human Pancreatic Cancers Revealed by Global Genomic Analyses. Science (New York, NY). 2008;321(5897):1801–6. doi:10.1126/science.1164368.

7. Misra N, Szczurek E, Vingron M. Inferring the Paths of Somatic Evolution in Cancer. Bioinformatics (Oxford, England). 2014;30(17):2456–2463. doi:10.1093/bioinformatics/btu319.

8. Ding L, Getz G, Wheeler DA, Mardis ER, McLellan MD, Cibulskis K, et al. Somatic Mutations Affect Key Pathways in Lung Adenocarcinoma. Nature. 2008;455(7216):1069–1075. doi:10.1038/nature07423.

9. Cancer Genome Atlas Research Network. Integrated Genomic Analyses of Ovarian Carcinoma. Nature. 2011;474(7353):609–615. doi:10.1038/nature10166.

10. Cancer Genome Atlas Research Network. Comprehensive Molecular Characterization of Human Colon and Rectal Cancer. Nature. 2012;487(7407):330–337. doi:10.1038/nature11252.

11. Ramazzotti D, Caravagna G, Olde Loohuis L, Graudenzi A, Korsunsky I, Mauri G, et al. CAPRI: Efficient Inference of Cancer Progression Models from Cross-Sectional Data. Bioinformatics. 2015;31(18):3016–3026. doi:10.1093/bioinformatics/btv296.

12. De Sano L, Caravagna G, Ramazzotti D, Graudenzi A, Mauri G, Mishra B, et al. TRONCO: An R Package for the Inference of Cancer Progression Models from Heterogeneous Genomic Data. Bioinformatics. 2016;32(12):1911–1913. doi:10.1093/bioinformatics/btw035.

13. Piazza R, Valletta S, Winkelmann N, Redaelli S, Spinelli R, Pirola A, et al. Recurrent SETBP1 Mutations in Atypical Chronic Myeloid Leukemia. Nature Genetics. 2013;45(1):18–24.

14. Cerami E, Gao J, Dogrusoz U, Gross BE, Sumer SO, Aksoy BA, et al. The cBio Cancer Genomics Portal: An Open Platform for Exploring Multidimensional Cancer Genomics Data: Figure 1. Cancer Discovery. 2012;2(5):401–404.

15. Gao J, Aksoy BA, Dogrusoz U, Dresdner G, Gross B, Sumer SO, et al. Integrative Analysis of Complex Cancer Genomics and Clinical Profiles Using the cBioPortal. Science Signaling. 2013;6(269):pl1. doi:10.1126/scisignal.2004088.

16. Brennan CW, Verhaak RGW, McKenna A, Campos B, Noushmehr H, Salama SR, et al. The Somatic Genomic Landscape of Glioblastoma. Cell. 2013;155(2):462–477.

17. Attolini C, Cheng Y, Beroukhim R, Getz G, Abdel-Wahab O, Levine RL, et al. A Mathematical Framework to Determine the Temporal Sequence of Somatic Genetic Events in Cancer. Proceedings of the National Academy of Sciences. 2010;107(41):17604–17609. doi:10.1073/pnas.1009117107/-/DCSupplemental.www.pnas.org/cgi/doi/10.1073/pnas.1009117107.

18. Bamford S, Dawson E, Forbes S, Clements J, Pettett R, Dogan A, et al. The COSMIC (Catalogue of Somatic Mutations in Cancer) Database and Website. Br J Cancer. 2004;91(2):355–358. doi:10.1038/sj.bjc.6601894.

19. Cancer Genome Atlas Research Network. Comprehensive Genomic Characterization Defines Human Glioblastoma Genes and Core Pathways. Nature. 2008;455(7216):1061–1068.

20. Szabo A, Pappas L. Oncotree: Estimating Oncogenetic Trees. R Package Version 0.3.3.; 2013. Available from: http://cran.r-project.org/package=Oncotree.

21. Knutsen T, Gobu V, Knaus R, Padilla-Nash H, Augustud M, Strausberg RL, et al. The Interactive Online SKY/M-FISH & CGH Database and the Entrez Cancer Chromosomes Search Database: Linkage of Chromosomal Aberrations with the Genome Sequence. Genes, Chromosomes and Cancer. 2005;44(1):52–64.

22. Cancer Genome Atlas Research Network. Comprehensive Molecular Portraits of Human Breast Tumours. Nature. 2012;490(7418):61–70.

23. Cheng YK, Beroukhim R, Levine RL, Mellinghoff IK, Holland EC, Michor F. A Mathematical Methodology for Determining the Temporal Order of Pathway Alterations Arising during Gliomagenesis. PLoS computational biology. 2012;8(1):e1002337. doi:10.1371/journal.pcbi.1002337.

24. Sjoblom T, Jones S, Wood LD, Parsons DW, Lin J, Barber TD, et al. The Consensus Coding Sequences of Human Breast and Colorectal Cancers. Science. 2006;314(5797):268–274. doi:10.1126/science.1133427.

25. Dees ND, Zhang Q, Kandoth C, Wendl MC, Schierding W, Koboldt DC, et al. MuSiC: Identifying Mutational Significance in Cancer Genomes. Genome Research. 2012;22(8):1589–1598.

26. Lawrence M, Huber W, Pagès H, Aboyoun P, Carlson M, Gentleman R, et al. Software for Computing and Annotating Genomic Ranges. PLoS computational biology. 2013;9(8):e1003118. doi:10.1371/journal.pcbi.1003118.

27. Mermel CH, Schumacher SE, Hill B, Meyerson ML, Beroukhim R, Getz G. GISTIC2.0 Facilitates Sensitive and Confident Localization of the Targets of Focal Somatic Copy-Number Alteration in Human Cancers. Genome Biology. 2011;12(4):R41.

28. Jacobsen A, Questions c. Cgdsr: R-Based API for Accessing the MSKCC Cancer Genomics Data Server (CGDS); 2018. Available from: https://CRAN.R-project.org/package=cgdsr.

29. Cerami E, Demir E, Schultz N, Taylor BS, Sander C. Automated Network Analysis Identifies Core Pathways in Glioblastoma. PLoS ONE. 2010;5(2):e8918.

30. Olde Loohuis L, Caravagna G, Graudenzi A, Ramazzotti D, Mauri G, Antoniotti M, et al. Inferring Tree Causal Models of Cancer Progression with Probability Raising. PLOS ONE. 2014;9(10):e108358. doi:10.1371/journal.pone.0108358.

31. a Burrell R, McGranahan N, Bartek J, Swanton C. The Causes and Consequences of Genetic Heterogeneity in Cancer Evolution. Nature. 2013;501(7467):338–345. doi:10.1038/nature12625.

32. Anderson WF, Rosenberg PS, Prat A, Perou CM, Sherman ME. How Many Etiological Subtypes of Breast Cancer:Two,Three, Four, or More? JNCI: Journal of the National Cancer Institute. 2014;106(8):1–11.
