# Supplementary material for: Every which way? On predicting tumor evolution using cancer progression models
Source: PLoS Comput Biol. 2019 Aug 2;15(8):e1007246. doi: 10.1371/journal.pcbi.1007246 (PMC6693785; doi:10.1371/journal.pcbi.1007246)
Supplement: S6 Text — (PDF) [file pcbi.1007246.s008.pdf]

Supporting Information for “Every which way? On predicting tumor evolution using cancer progression models”

Ramon Diaz-Uriarte[1,2*], Claudia Vasallo[1,2]

1 Dept. Biochemistry, Universidad Autónoma de Madrid, Madrid, Spain
2 Instituto de Investigaciones Biomédicas “Alberto Sols” (UAM-CSIC), Madrid, Spain

* ramon.diaz@iib.uam.es

S6 Text. Additional results.

Contents

List of Figures 2

1 Overall patterns for the six CPM procedures 3

2 Probability of recovering the most common LOD 4

3 OT and H-CBN, JS, weighted vs. unweighted 6

4 CAPRI and H-CBN, 1-precision, unweighted 7

5 CAPRESE and OT, 1-precision, unweighted 8

6 Coefficient of variation of JS 9

7 Number of paths inferred 10

8 Slopes of regressions of 1-recall and 1-precision on LOD diversity, S_p 11

9 Coefficient of variation of S_c 12

10 Estimated S_c by H-CBN 13

11 Analysis of deviance tables for fitted models 14
11.1 Models fitted to the complete data set 15
11.1.1 Two-way interactions 15
11.1.2 Three-way interactions 16
11.1.3 Four-way interactions 17
11.2 Models fitted to each combination of fitness landscape by CPM 19
11.2.1 Main effects 19
11.2.2 Two-way interactions 21
11.2.3 Four-way interactions 24

12 Number of local maxima, mutations of local maxima, reciprocal sign epistasis and performance 29

13 LOD and CPM diversity: ratios and slopes 30

14 Example of good and moderate performance 30

15 References 31

List of Figures

A Summary performance measures for all six CPM procedures for all combinations of sample size, type of landscape, detection regime, and number of genes. For all measures, smaller is better. For OT, H-CBN, and MCCBN, Jensen-Shannon (JS) entropy and 1-precision use probability-weighted predicted paths (see text). Each point represented is the average of 210 points (35 replicates of each one of the six combinations of 3 initial size by 2 mutation rate regimes; we are thus marginalizing over initial size by mutation rate; each one of the 210 points is, itself, the average of five runs on different partitions of the simulated data. 3

B Probability of recovering the most common LOD: probability that the most common observed path to the maximum is among the paths allowed by the CPMs. . 4

C Probability of recovering the most common LOD and 1-recall: relationship. . . . 5

D Comparison of the performance of OT and H-CBN using weighted and unweighted probabilities of paths to the maximum. 6

E Comparison of the performance of CAPRI with H-CBN using weighted and unweighted probabilities of paths to the maximum. 7

F Comparison of the performance of CAPRESE with OT using weighted and unweighted probabilities of paths to the maximum. 8

G Coefficient of variation (standard deviation/mean) of JS for each combination of model and type of fitness landscape. The bottom figure represents the same information as the top one but only for the three models shown in the paper. The coefficient of variation has been computed from the five runs for each landscapes on each combination of sample size and detection regime. 9

H Number of paths to the maximum according to the CPMs. 10

I Slopes of regressions of 1-recall and 1-precision on LOD diversity, S_p 11

J Coefficient of variation (standard deviation/mean) of S_c for each combination of model and type of fitness landscape. The coefficient of variation has been computed from the five runs for each landscapes on each combination of sample size and detection regime. For OT and H-CBN, it is computed using the probability-weighted predicted paths (see text). Each point plotted is the average of 210 points. 12

K Estimated S_c by H-CBN for all combinations of sample size by type of landscape by detection regime by number of genes. Each box plot shows 1050 points. 13

L Slopes of regression of JS and 1-recall on mean number of mutations of local maxima. 29

1. Overall patterns for the six CPM procedures

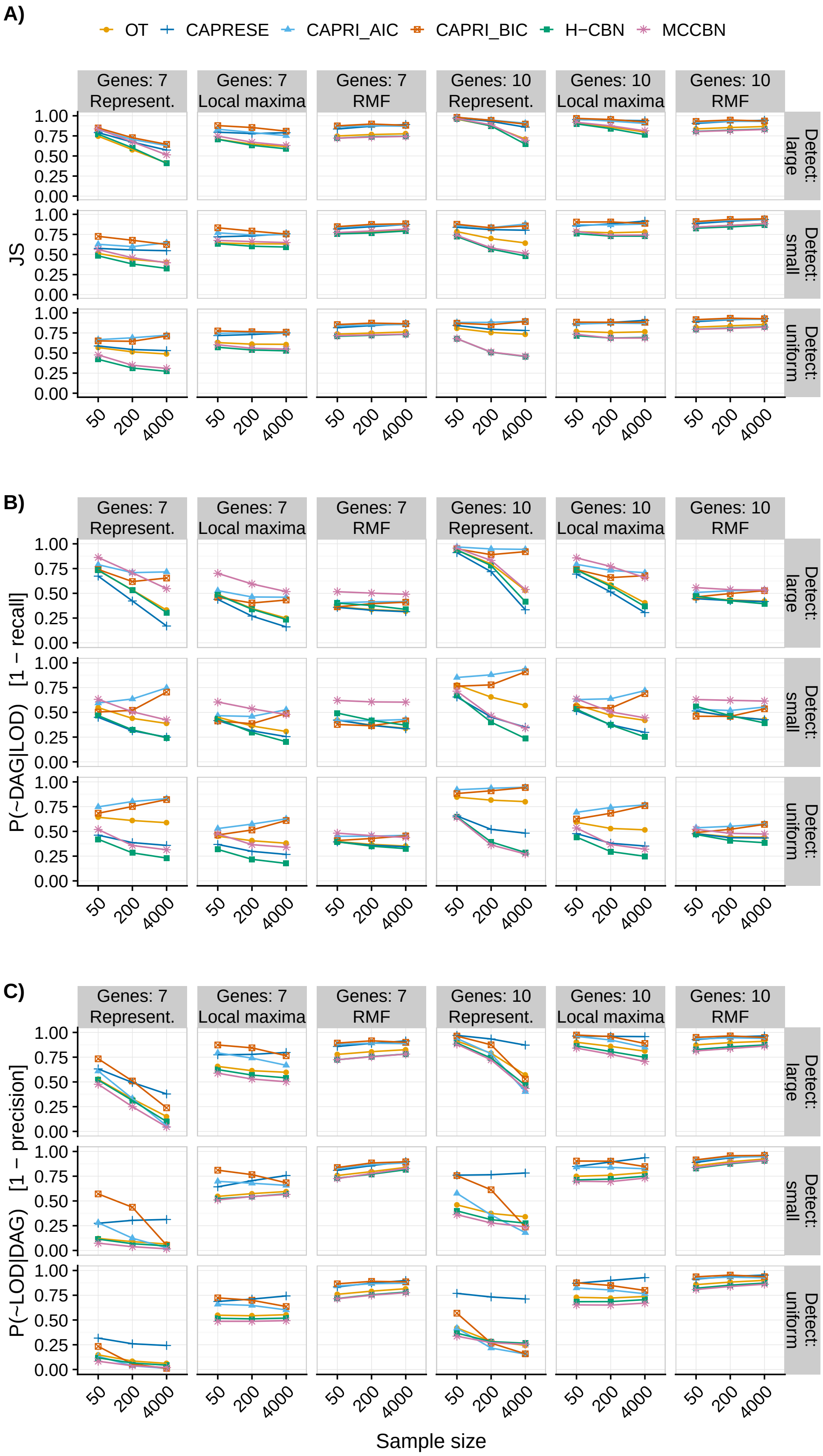

Figure A: Summary performance measures for all six CPM procedures for all combinations of sample size, type of landscape, detection regime, and number of genes. For all measures, smaller is better. For OT, H-CBN, and MCCBN, Jensen-Shannon (JS) entropy and 1-precision use probability-weighted predicted paths (see text). Each point represented is the average of 210 points (35 replicates of each one of the six combinations of 3 initial size by 2 mutation rate regimes; we are thus marginalizing over initial size by mutation rate; each one of the 210 points is, itself, the average of five runs on different partitions of the simulated data.

2. Probability of recovering the most common LOD

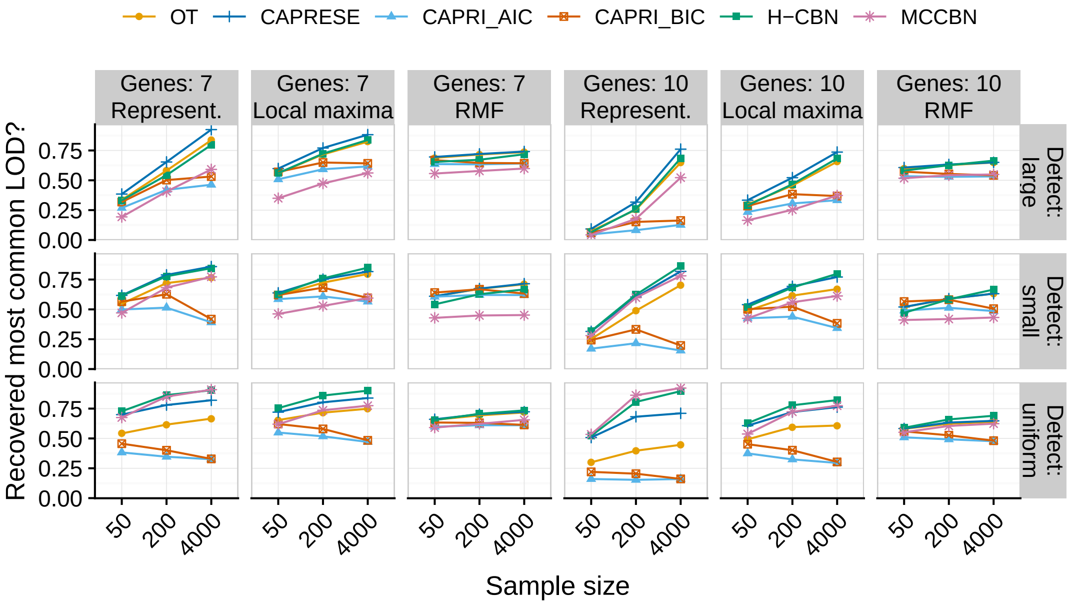

Figure B: Probability of recovering the most common LOD: probability that the most common observed path to the maximum is among the paths allowed by the CPMs.

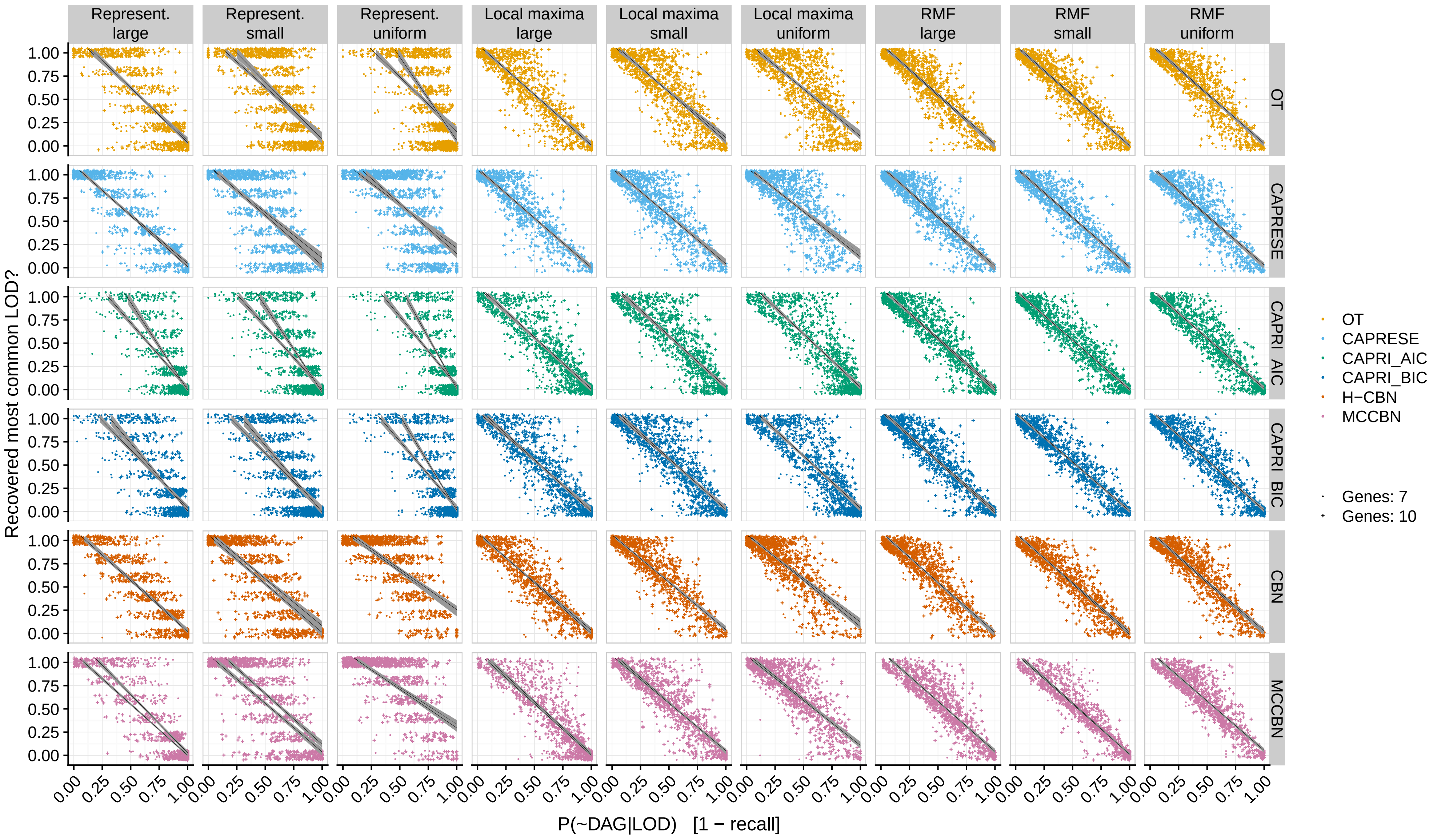

Figure C: Probability of recovering the most common LOD and 1-recall: relationship.

3. OT and H-CBN, JS, weighted vs. unweighted

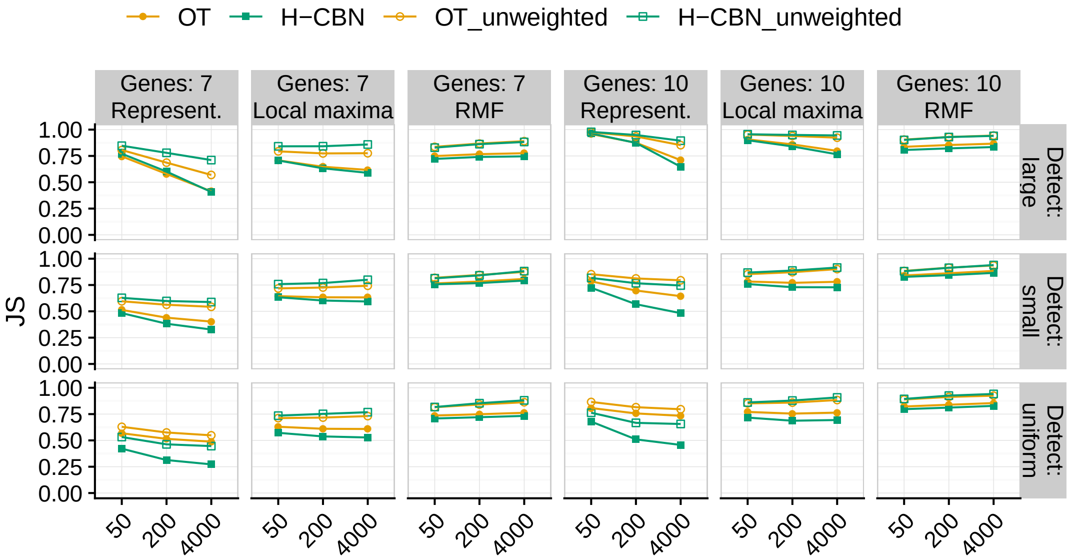

Figure D: Comparison of the performance of OT and H-CBN using weighted and unweighted probabilities of paths to the maximum.

4. CAPRI and H-CBN, 1-precision, unweighted

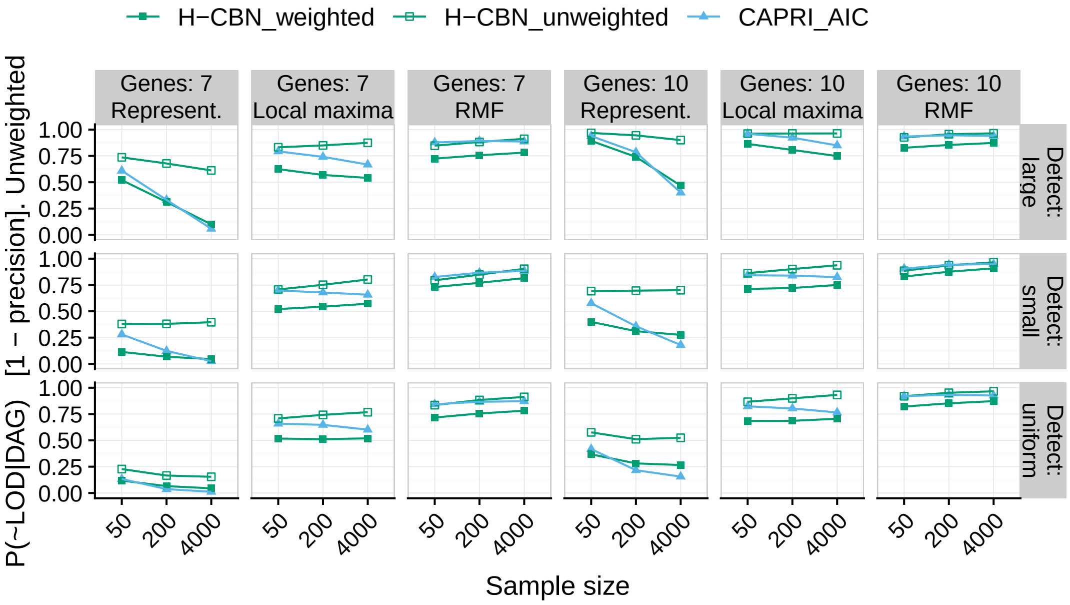

Figure E: Comparison of the performance of CAPRI with H-CBN using weighted and unweighted probabilities of paths to the maximum.

5. CAPRESE and OT, 1-precision, unweighted

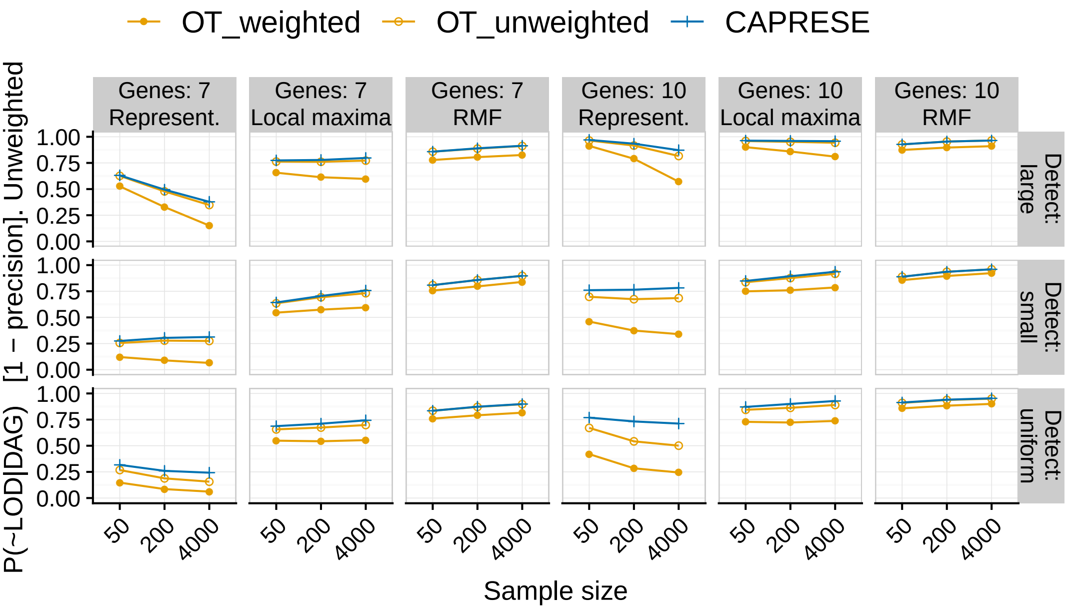

Figure F: Comparison of the performance of CAPRESE with OT using weighted and unweighted probabilities of paths to the maximum.

The results of OT here are remarkable because OT can only build trees, and therefore cannot reflect the dependency of a mutation on two or more upstream mutations so it is prone to allow more paths to the maximum. The results of OT contrasts with those of CAPRESE, the other model that only builds trees. CAPRESE is building DAGs of restrictions that have too few restrictions and, therefore, allow for too many paths to the maximum. One notable difference between the two models is that with OT it is relatively simple to use a measure of 1-precision that weights by the probability of each path. The performance of OT, even if we use unweighted probabilities of paths, is much better than that of CAPRESE but improves even further when using weighted paths, again highlighting the usefulness of weighting paths to obtain more accurate predictions.

6. Coefficient of variation of JS

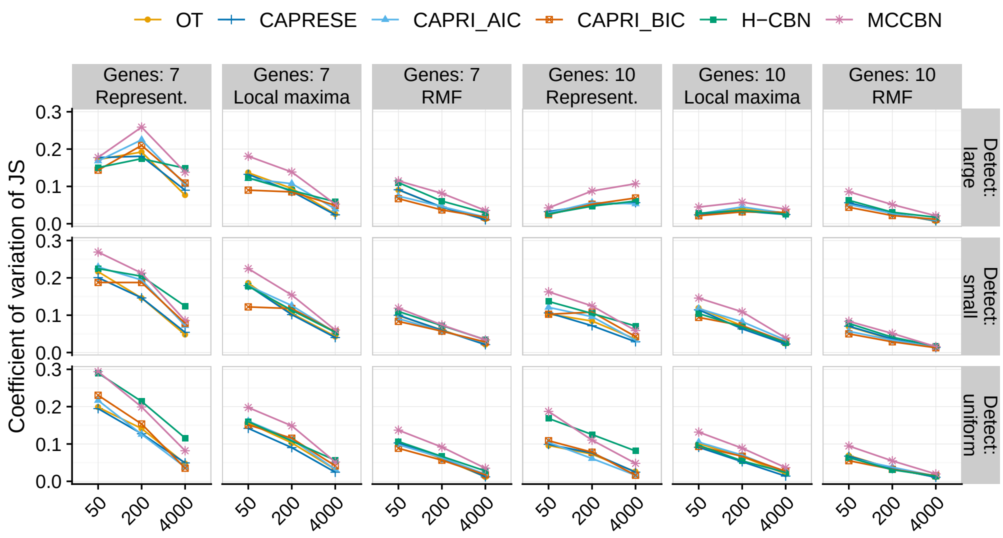

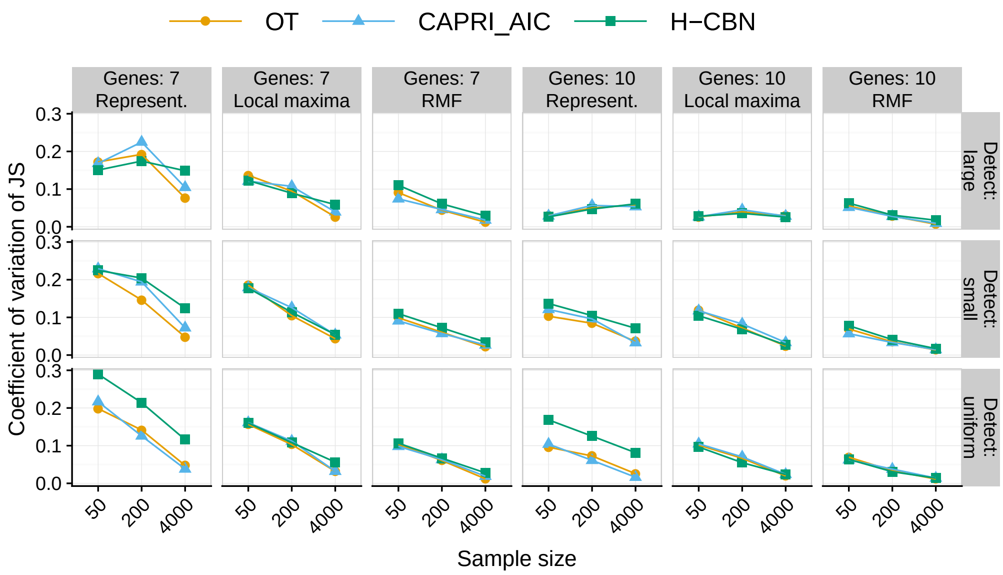

Figure G: Coefficient of variation (standard deviation/mean) of JS for each combination of model and type of fitness landscape. The bottom figure represents the same information as the top one but only for the three models shown in the paper. The coefficient of variation has been computed from the five runs for each landscapes on each combination of sample size and detection regime.

7. Number of paths inferred

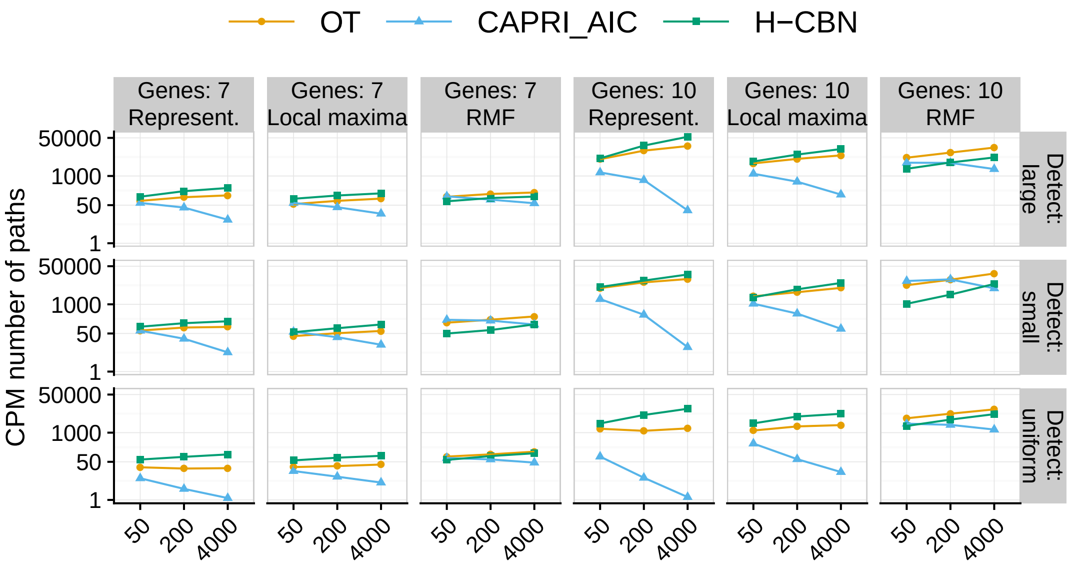

Figure H: Number of paths to the maximum according to the CPMs.

8. Slopes of regressions of 1-recall and 1-precision on LOD diversity, S_p

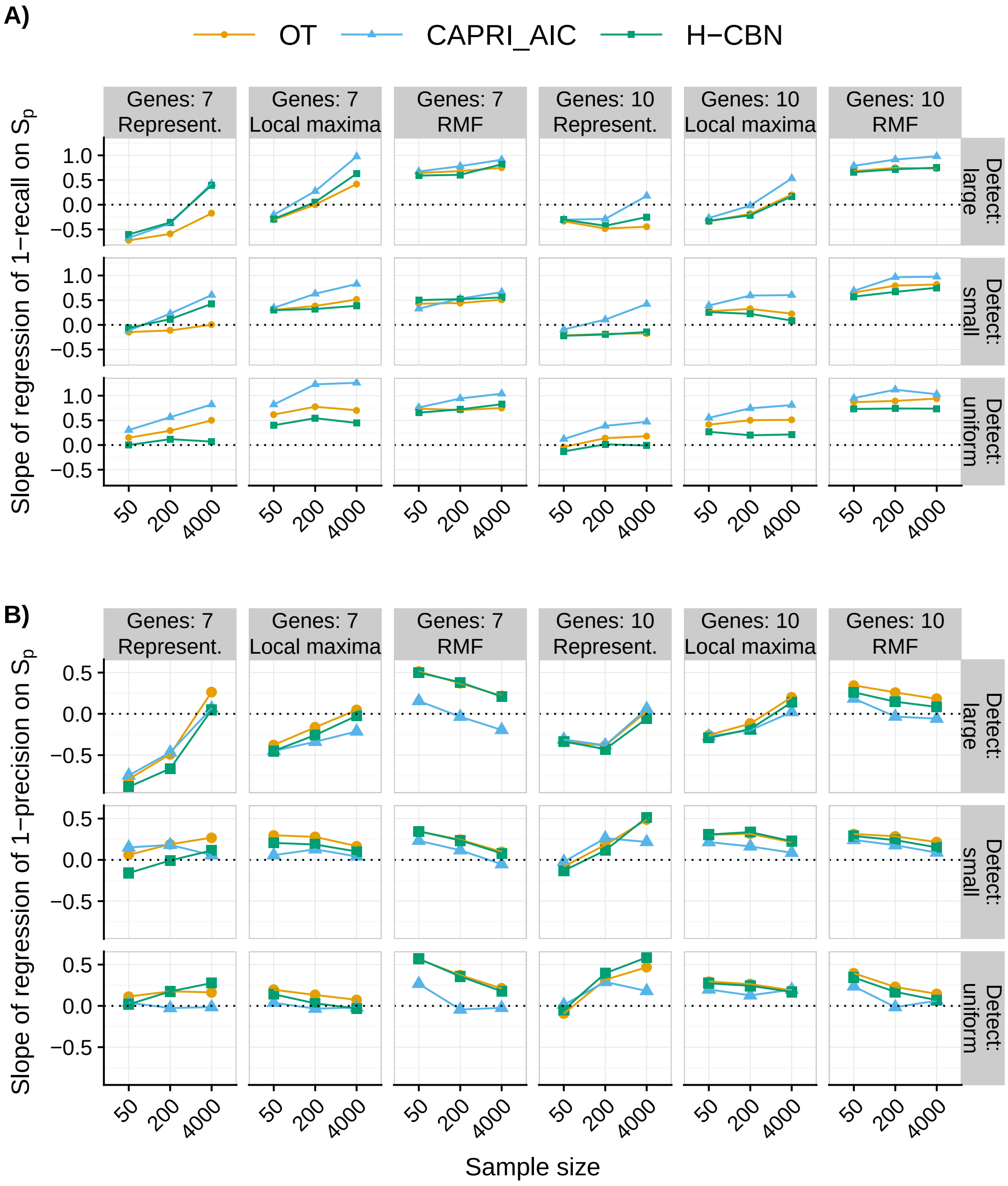

Figure I: Slopes of regressions of 1-recall and 1-precision on LOD diversity, S_p

9. Coefficient of variation of S_c

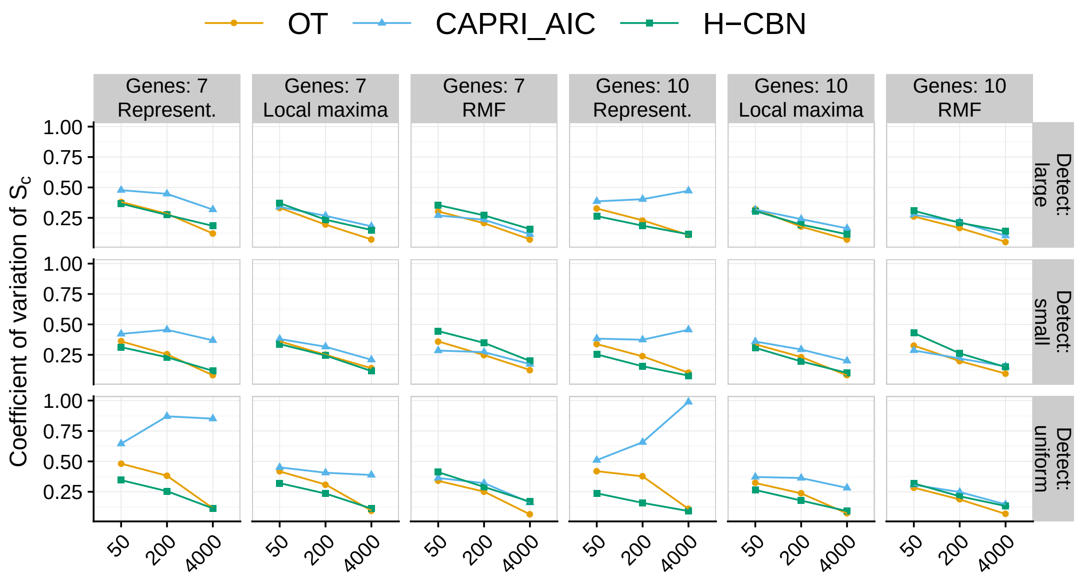

Figure J: Coefficient of variation (standard deviation/mean) of S_c for each combination of model and type of fitness landscape. The coefficient of variation has been computed from the five runs for each landscapes on each combination of sample size and detection regime. For OT and H-CBN, it is computed using the probability-weighted predicted paths (see text). Each point plotted is the average of 210 points.

10. Estimated S_c by H-CBN

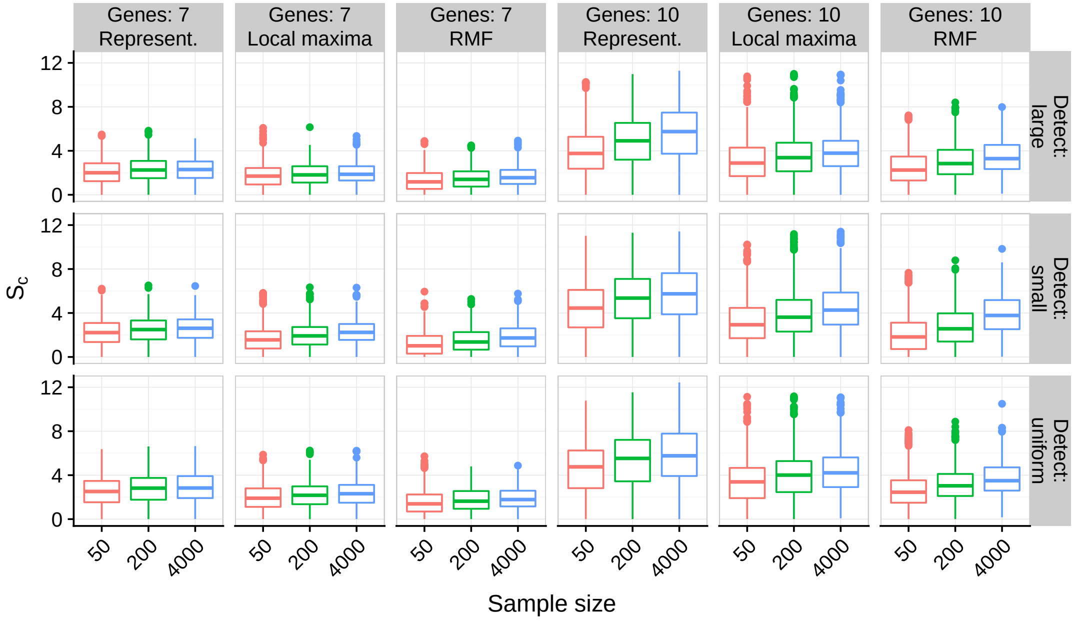

Figure K: Estimated S_c by H-CBN for all combinations of sample size by type of landscape by detection regime by number of genes. Each box plot shows 1050 points.

11. Analysis of deviance tables for fitted models

The tables below show the analysis of deviance tables for the generalized (beta regressions) linear mixed effects models. Models where fitted using the R package glmmTMB [1]. Analysis of deviance tables are from package car [2]. All analysis of deviance tables use Type II Wald chi-square tests.

Analysis have been run on the complete data set (section 11.1), and after splitting for the different combinations of CPM and fitness landscape type (section 11.2).

11.1. Models fitted to the complete data set

(Notice the strong evidence we see for three and four and even five way interactions.)

11.1.1. Two-way interactions

	Chisq	Df	Pr(>Chisq)
Num. Genes	223.34	1.00	$< .0001$
CPM	9459.99	2.00	$< .0001$
Landscape	322.37	2.00	$< .0001$
Detection	4768.56	2.00	$< .0001$
Sample Size	1599.17	2.00	$< .0001$
LOD diversity	139.98	1.00	$< .0001$
Num. Genes:CPM	22.98	2.00	$< .0001$
Num. Genes:Landscape	91.35	2.00	$< .0001$
Num. Genes:Detection	2318.60	2.00	$< .0001$
Num. Genes:Sample Size	381.80	2.00	$< .0001$
Num. Genes:LOD diversity	8.72	1.00	0.0032
CPM:Landscape	192.88	4.00	$< .0001$
CPM:Detection	678.41	4.00	$< .0001$
CPM:Sample Size	818.96	4.00	$< .0001$
CPM:LOD diversity	170.20	2.00	$< .0001$
Landscape:Detection	6534.14	4.00	$< .0001$
Landscape:Sample Size	3110.94	4.00	$< .0001$
Landscape:LOD diversity	78.21	2.00	$< .0001$
Detection:Sample Size	2420.56	4.00	$< .0001$
Detection:LOD diversity	3303.42	2.00	$< .0001$
Sample Size:LOD diversity	939.31	2.00	$< .0001$

Table A: Full statistical model (all CPMs), 2-way interactions

11.1.2. Three-way interactions

	Chisq	Df	Pr(>Chisq)
Num. Genes	197.19	1.00	< .0001
CPM	11331.58	2.00	< .0001
Landscape	411.00	2.00	< .0001
Detection	4217.33	2.00	< .0001
Sample Size	1536.56	2.00	< .0001
LOD diversity	146.46	1.00	< .0001
Num. Genes:CPM	52.74	2.00	< .0001
Num. Genes:Landscape	80.98	2.00	< .0001
Num. Genes:Detection	2394.91	2.00	< .0001
Num. Genes:Sample Size	354.72	2.00	< .0001
Num. Genes:LOD diversity	8.15	1.00	0.0043
CPM:Landscape	285.47	4.00	< .0001
CPM:Detection	707.50	4.00	< .0001
CPM:Sample Size	842.82	4.00	< .0001
CPM:LOD diversity	117.61	2.00	< .0001
Landscape:Detection	6924.74	4.00	< .0001
Landscape:Sample Size	3407.98	4.00	< .0001
Landscape:LOD diversity	77.13	2.00	< .0001
Detection:Sample Size	2507.80	4.00	< .0001
Detection:LOD diversity	4229.09	2.00	< .0001
Sample Size:LOD diversity	1120.46	2.00	< .0001
Num. Genes:CPM:Landscape	90.47	4.00	< .0001
Num. Genes:CPM:Detection	36.64	4.00	< .0001
Num. Genes:CPM:Sample Size	10.43	4.00	0.0338
Num. Genes:CPM:LOD diversity	41.35	2.00	< .0001
Num. Genes:Landscape:Detection	1302.06	4.00	< .0001
Num. Genes:Landscape:Sample Size	347.91	4.00	< .0001
Num. Genes:Landscape:LOD diversity	3.70	2.00	0.1572
Num. Genes:Detection:Sample Size	553.21	4.00	< .0001
Num. Genes:Detection:LOD diversity	3.91	2.00	0.1417
Num. Genes:Sample Size:LOD diversity	56.88	2.00	< .0001
CPM:Landscape:Detection	250.27	8.00	< .0001
CPM:Landscape:Sample Size	192.75	8.00	< .0001
CPM:Landscape:LOD diversity	605.91	4.00	< .0001
CPM:Detection:Sample Size	19.44	8.00	0.0127
CPM:Detection:LOD diversity	126.85	4.00	< .0001
CPM:Sample Size:LOD diversity	117.71	4.00	< .0001
Landscape:Detection:Sample Size	2084.94	8.00	< .0001
Landscape:Detection:LOD diversity	867.54	4.00	< .0001
Landscape:Sample Size:LOD diversity	1163.94	4.00	< .0001
Detection:Sample Size:LOD diversity	736.44	4.00	< .0001

Table B: Full statistical model (all CPMs), 3-way interactions

11.1.3. Four-way interactions

	Chisq	Df	Pr(>Chisq)
Num. Genes	193.68	1.00	$< .0001$
CPM	11619.48	2.00	$< .0001$
Landscape	426.49	2.00	$< .0001$
Detection	4059.78	2.00	$< .0001$
Sample Size	1500.59	2.00	$< .0001$
LOD diversity	150.27	1.00	$< .0001$
Num. Genes:CPM	54.25	2.00	$< .0001$
Num. Genes:Landscape	77.19	2.00	$< .0001$
Num. Genes:Detection	2370.70	2.00	$< .0001$
Num. Genes:Sample Size	357.14	2.00	$< .0001$
Num. Genes:LOD diversity	8.47	1.00	0.0036
CPM:Landscape	298.90	4.00	$< .0001$
CPM:Detection	716.32	4.00	$< .0001$
CPM:Sample Size	841.73	4.00	$< .0001$
CPM:LOD diversity	119.30	2.00	$< .0001$
Landscape:Detection	6697.76	4.00	$< .0001$
Landscape:Sample Size	3459.44	4.00	$< .0001$
Landscape:LOD diversity	77.85	2.00	$< .0001$
Detection:Sample Size	2466.93	4.00	$< .0001$
Detection:LOD diversity	4132.54	2.00	$< .0001$
Sample Size:LOD diversity	1103.02	2.00	$< .0001$
Num. Genes:CPM:Landscape	101.44	4.00	$< .0001$
Num. Genes:CPM:Detection	35.22	4.00	$< .0001$
Num. Genes:CPM:Sample Size	11.99	4.00	0.0174
Num. Genes:CPM:LOD diversity	43.71	2.00	$< .0001$
Num. Genes:Landscape:Detection	1262.35	4.00	$< .0001$
Num. Genes:Landscape:Sample Size	350.64	4.00	$< .0001$
Num. Genes:Landscape:LOD diversity	3.55	2.00	0.1699
Num. Genes:Detection:Sample Size	532.95	4.00	$< .0001$
Num. Genes:Detection:LOD diversity	2.93	2.00	0.231
Num. Genes:Sample Size:LOD diversity	50.25	2.00	$< .0001$
CPM:Landscape:Detection	229.42	8.00	$< .0001$
CPM:Landscape:Sample Size	215.46	8.00	$< .0001$
CPM:Landscape:LOD diversity	627.29	4.00	$< .0001$
CPM:Detection:Sample Size	21.27	8.00	0.0065
CPM:Detection:LOD diversity	131.95	4.00	$< .0001$
CPM:Sample Size:LOD diversity	88.97	4.00	$< .0001$
Landscape:Detection:Sample Size	2216.86	8.00	$< .0001$
Landscape:Detection:LOD diversity	867.13	4.00	$< .0001$
Landscape:Sample Size:LOD diversity	1175.97	4.00	$< .0001$
Detection:Sample Size:LOD diversity	819.51	4.00	$< .0001$
Num. Genes:CPM:Landscape:Detection	12.73	8.00	0.1214
Num. Genes:CPM:Landscape:Sample Size	7.44	8.00	0.4898
Num. Genes:CPM:Landscape:LOD diversity	14.84	4.00	0.0051
Num. Genes:CPM:Detection:Sample Size	25.87	8.00	0.0011
Num. Genes:CPM:Detection:LOD diversity	53.99	4.00	$< .0001$
Num. Genes:CPM:Sample Size:LOD diversity	2.51	4.00	0.6425
Num. Genes:Landscape:Detection:Sample Size	321.87	8.00	$< .0001$
Num. Genes:Landscape:Detection:LOD diversity	74.72	4.00	$< .0001$
Num. Genes:Landscape:Sample Size:LOD diversity	101.05	4.00	$< .0001$

Num. Genes:Detection:Sample Size:LOD diversity	115.78	4.00	$< .0001$
CPM:Landscape:Detection:Sample Size	24.77	16.00	0.0739
CPM:Landscape:Detection:LOD diversity	19.36	8.00	0.013
CPM:Landscape:Sample Size:LOD diversity	23.93	8.00	0.0024
CPM:Detection:Sample Size:LOD diversity	10.02	8.00	0.2634
Landscape:Detection:Sample Size:LOD diversity	237.96	8.00	$< .0001$

Table C: Full statistical model (all CPMs), 4-way interactions

11.2. Models fitted to each combination of fitness landscape by CPM

Remember each model uses 3780 observations: 35 replicates, 3 mutation rates, 2 variance settings, 2 number of genes, 3 detection regimes, 3 sample sizes. These correspond to 420 different fitness landscapes: 35 by 3 by 2 by 2. Each observation is itself the average of five different splits of the set of 20000 simulations.

11.2.1. Main effects

	Chisq	Df	Pr(>Chisq)
Num. Genes	325.32	1.00	$< .0001$
Sample Size	797.56	2.00	$< .0001$
Detection	1101.14	2.00	$< .0001$
LOD diversity	2.41	1.00	0.1206

Table D: Represent..OT

	Chisq	Df	Pr(>Chisq)
Num. Genes	84.38	1.00	$< .0001$
Sample Size	179.66	2.00	$< .0001$
Detection	470.52	2.00	$< .0001$
LOD diversity	63.71	1.00	$< .0001$

Table E: Local Peaks.OT

	Chisq	Df	Pr(>Chisq)
Num. Genes	9.65	1.00	0.0019
Sample Size	199.97	2.00	$< .0001$
Detection	206.52	2.00	$< .0001$
LOD diversity	162.60	1.00	$< .0001$

Table F: RMF.OT

	Chisq	Df	Pr(>Chisq)
Num. Genes	206.70	1.00	$< .0001$
Sample Size	62.82	2.00	$< .0001$
Detection	692.92	2.00	$< .0001$
LOD diversity	79.61	1.00	$< .0001$

Table G: Represent..CAPRI_AIC

	Chisq	Df	Pr(>Chisq)
Num. Genes	110.80	1.00	$< .0001$
Sample Size	48.42	2.00	$< .0001$
Detection	383.72	2.00	$< .0001$
LOD diversity	81.86	1.00	$< .0001$

Table H: Local Peaks.CAPRI_AIC

	Chisq	Df	Pr(>Chisq)
Num. Genes	35.67	1.00	< .0001
Sample Size	144.12	2.00	< .0001
Detection	48.52	2.00	< .0001
LOD diversity	70.86	1.00	< .0001

Table I: RMF.CAPRI_AIC

	Chisq	Df	Pr(>Chisq)
Num. Genes	157.75	1.00	< .0001
Sample Size	1331.50	2.00	< .0001
Detection	2493.82	2.00	< .0001
LOD diversity	0.35	1.00	0.5538

Table J: Represent..H-CBN

	Chisq	Df	Pr(>Chisq)
Num. Genes	71.83	1.00	< .0001
Sample Size	315.26	2.00	< .0001
Detection	823.90	2.00	< .0001
LOD diversity	64.31	1.00	< .0001

Table K: Local Peaks.H-CBN

	Chisq	Df	Pr(>Chisq)
Num. Genes	3.82	1.00	0.0507
Sample Size	100.38	2.00	< .0001
Detection	320.43	2.00	< .0001
LOD diversity	151.14	1.00	< .0001

Table L: RMF.H-CBN

11.2.2. Two-way interactions

	Chisq	Df	Pr(>Chisq)
Num. Genes	229.87	1.00	< .0001
Sample Size	1084.79	2.00	< .0001
Detection	1145.03	2.00	< .0001
LOD diversity	0.02	1.00	0.8958
Num. Genes:Sample Size	168.67	2.00	< .0001
Num. Genes:Detection	705.00	2.00	< .0001
Num. Genes:LOD diversity	4.46	1.00	0.0347
Sample Size:Detection	726.35	4.00	< .0001
Sample Size:LOD diversity	308.31	2.00	< .0001
Detection:LOD diversity	1019.12	2.00	< .0001

Table M: Represent..OT

	Chisq	Df	Pr(>Chisq)
Num. Genes	66.47	1.00	< .0001
Sample Size	163.41	2.00	< .0001
Detection	449.46	2.00	< .0001
LOD diversity	74.54	1.00	< .0001
Num. Genes:Sample Size	34.28	2.00	< .0001
Num. Genes:Detection	443.26	2.00	< .0001
Num. Genes:LOD diversity	0.02	1.00	0.895
Sample Size:Detection	307.15	4.00	< .0001
Sample Size:LOD diversity	21.38	2.00	< .0001
Detection:LOD diversity	403.27	2.00	< .0001

Table N: Local Peaks.OT

	Chisq	Df	Pr(>Chisq)
Num. Genes	9.65	1.00	0.0019
Sample Size	213.84	2.00	< .0001
Detection	215.42	2.00	< .0001
LOD diversity	155.94	1.00	< .0001
Num. Genes:Sample Size	31.75	2.00	< .0001
Num. Genes:Detection	5.39	2.00	0.0676
Num. Genes:LOD diversity	0.05	1.00	0.8191
Sample Size:Detection	7.68	4.00	0.1041
Sample Size:LOD diversity	190.09	2.00	< .0001
Detection:LOD diversity	9.29	2.00	0.0096

Table O: RMF.OT

	Chisq	Df	Pr(>Chisq)
Num. Genes	144.41	1.00	< .0001
Sample Size	70.06	2.00	< .0001
Detection	720.04	2.00	< .0001
LOD diversity	89.03	1.00	< .0001
Num. Genes:Sample Size	154.58	2.00	< .0001
Num. Genes:Detection	574.17	2.00	< .0001
Num. Genes:LOD diversity	5.16	1.00	0.0231
Sample Size:Detection	691.60	4.00	< .0001
Sample Size:LOD diversity	734.28	2.00	< .0001
Detection:LOD diversity	958.49	2.00	< .0001

Table P: Represent..CAPRI_AIC

	Chisq	Df	Pr(>Chisq)
Num. Genes	90.86	1.00	< .0001
Sample Size	36.29	2.00	< .0001
Detection	351.07	2.00	< .0001
LOD diversity	89.21	1.00	< .0001
Num. Genes:Sample Size	8.98	2.00	0.0112
Num. Genes:Detection	309.60	2.00	< .0001
Num. Genes:LOD diversity	0.01	1.00	0.91
Sample Size:Detection	262.51	4.00	< .0001
Sample Size:LOD diversity	80.39	2.00	< .0001
Detection:LOD diversity	429.26	2.00	< .0001

Table Q: Local Peaks.CAPRI_AIC

	Chisq	Df	Pr(>Chisq)
Num. Genes	33.69	1.00	< .0001
Sample Size	164.54	2.00	< .0001
Detection	50.28	2.00	< .0001
LOD diversity	65.36	1.00	< .0001
Num. Genes:Sample Size	34.37	2.00	< .0001
Num. Genes:Detection	2.23	2.00	0.3273
Num. Genes:LOD diversity	1.24	1.00	0.2663
Sample Size:Detection	15.51	4.00	0.0037
Sample Size:LOD diversity	154.86	2.00	< .0001
Detection:LOD diversity	40.59	2.00	< .0001

Table R: RMF.CAPRI_AIC

	Chisq	Df	Pr(>Chisq)
Num. Genes	111.35	1.00	< .0001
Sample Size	1727.19	2.00	< .0001
Detection	2829.04	2.00	< .0001
LOD diversity	2.47	1.00	0.116
Num. Genes:Sample Size	252.55	2.00	< .0001
Num. Genes:Detection	583.85	2.00	< .0001
Num. Genes:LOD diversity	11.81	1.00	6e-04
Sample Size:Detection	583.96	4.00	< .0001
Sample Size:LOD diversity	367.88	2.00	< .0001
Detection:LOD diversity	634.67	2.00	< .0001

Table S: Represent..H-CBN

	Chisq	Df	Pr(>Chisq)
Num. Genes	56.06	1.00	$< .0001$
Sample Size	313.03	2.00	$< .0001$
Detection	836.24	2.00	$< .0001$
LOD diversity	76.98	1.00	$< .0001$
Num. Genes:Sample Size	35.78	2.00	$< .0001$
Num. Genes:Detection	413.32	2.00	$< .0001$
Num. Genes:LOD diversity	0.30	1.00	0.5868
Sample Size:Detection	296.90	4.00	$< .0001$
Sample Size:LOD diversity	19.84	2.00	$< .0001$
Detection:LOD diversity	292.03	2.00	$< .0001$

Table T: Local Peaks.H-CBN

	Chisq	Df	Pr(>Chisq)
Num. Genes	3.82	1.00	0.0506
Sample Size	107.26	2.00	$< .0001$
Detection	331.99	2.00	$< .0001$
LOD diversity	144.95	1.00	$< .0001$
Num. Genes:Sample Size	21.97	2.00	$< .0001$
Num. Genes:Detection	4.24	2.00	0.12
Num. Genes:LOD diversity	1.16	1.00	0.2823
Sample Size:Detection	4.15	4.00	0.3856
Sample Size:LOD diversity	181.70	2.00	$< .0001$
Detection:LOD diversity	1.36	2.00	0.5071

Table U: RMF.H-CBN

11.2.3. Four-way interactions

	Chisq	Df	Pr(>Chisq)
Num. Genes	236.38	1.00	< .0001
Sample Size	1105.42	2.00	< .0001
Detection	1046.08	2.00	< .0001
LOD diversity	0.84	1.00	0.3593
Num. Genes:Sample Size	193.98	2.00	< .0001
Num. Genes:Detection	778.62	2.00	< .0001
Num. Genes:LOD diversity	6.38	1.00	0.0116
Sample Size:Detection	722.33	4.00	< .0001
Sample Size:LOD diversity	322.04	2.00	< .0001
Detection:LOD diversity	1027.32	2.00	< .0001
Num. Genes:Sample Size:Detection	96.81	4.00	< .0001
Num. Genes:Sample Size:LOD diversity	28.18	2.00	< .0001
Num. Genes:Detection:LOD diversity	26.39	2.00	< .0001
Sample Size:Detection:LOD diversity	116.00	4.00	< .0001
Num. Genes:Sample Size:Detection:LOD diversity	76.54	4.00	< .0001

Table V: Represent..OT

	Chisq	Df	Pr(>Chisq)
Num. Genes	57.63	1.00	< .0001
Sample Size	166.26	2.00	< .0001
Detection	380.88	2.00	< .0001
LOD diversity	72.98	1.00	< .0001
Num. Genes:Sample Size	35.33	2.00	< .0001
Num. Genes:Detection	409.75	2.00	< .0001
Num. Genes:LOD diversity	0.04	1.00	0.8394
Sample Size:Detection	326.02	4.00	< .0001
Sample Size:LOD diversity	24.07	2.00	< .0001
Detection:LOD diversity	412.53	2.00	< .0001
Num. Genes:Sample Size:Detection	198.32	4.00	< .0001
Num. Genes:Sample Size:LOD diversity	1.85	2.00	0.3967
Num. Genes:Detection:LOD diversity	5.28	2.00	0.0715
Sample Size:Detection:LOD diversity	219.97	4.00	< .0001
Num. Genes:Sample Size:Detection:LOD diversity	8.77	4.00	0.0672

Table W: Local Peaks.OT

	Chisq	Df	Pr(>Chisq)
Num. Genes	9.69	1.00	0.0019
Sample Size	215.06	2.00	$< .0001$
Detection	215.20	2.00	$< .0001$
LOD diversity	155.03	1.00	$< .0001$
Num. Genes:Sample Size	32.40	2.00	$< .0001$
Num. Genes:Detection	5.40	2.00	0.0673
Num. Genes:LOD diversity	0.05	1.00	0.8191
Sample Size:Detection	7.55	4.00	0.1096
Sample Size:LOD diversity	191.25	2.00	$< .0001$
Detection:LOD diversity	9.34	2.00	0.0094
Num. Genes:Sample Size:Detection	0.26	4.00	0.9925
Num. Genes:Sample Size:LOD diversity	1.73	2.00	0.4219
Num. Genes:Detection:LOD diversity	32.05	2.00	$< .0001$
Sample Size:Detection:LOD diversity	1.65	4.00	0.799
Num. Genes:Sample Size:Detection:LOD diversity	1.63	4.00	0.8035

Table X: RMF.OT

	Chisq	Df	Pr(>Chisq)
Num. Genes	163.81	1.00	$< .0001$
Sample Size	53.20	2.00	$< .0001$
Detection	661.33	2.00	$< .0001$
LOD diversity	100.78	1.00	$< .0001$
Num. Genes:Sample Size	170.47	2.00	$< .0001$
Num. Genes:Detection	654.22	2.00	$< .0001$
Num. Genes:LOD diversity	5.37	1.00	0.0205
Sample Size:Detection	691.21	4.00	$< .0001$
Sample Size:LOD diversity	750.44	2.00	$< .0001$
Detection:LOD diversity	972.35	2.00	$< .0001$
Num. Genes:Sample Size:Detection	48.16	4.00	$< .0001$
Num. Genes:Sample Size:LOD diversity	24.57	2.00	$< .0001$
Num. Genes:Detection:LOD diversity	42.41	2.00	$< .0001$
Sample Size:Detection:LOD diversity	116.19	4.00	$< .0001$
Num. Genes:Sample Size:Detection:LOD diversity	53.68	4.00	$< .0001$

Table Y: Represent..CAPRI_AIC

	Chisq	Df	Pr(>Chisq)
Num. Genes	87.57	1.00	< .0001
Sample Size	35.06	2.00	< .0001
Detection	329.14	2.00	< .0001
LOD diversity	88.97	1.00	< .0001
Num. Genes:Sample Size	9.80	2.00	0.0074
Num. Genes:Detection	309.31	2.00	< .0001
Num. Genes:LOD diversity	0.03	1.00	0.8725
Sample Size:Detection	272.21	4.00	< .0001
Sample Size:LOD diversity	87.34	2.00	< .0001
Detection:LOD diversity	437.56	2.00	< .0001
Num. Genes:Sample Size:Detection	63.36	4.00	< .0001
Num. Genes:Sample Size:LOD diversity	1.44	2.00	0.4879
Num. Genes:Detection:LOD diversity	4.16	2.00	0.1248
Sample Size:Detection:LOD diversity	73.43	4.00	< .0001
Num. Genes:Sample Size:Detection:LOD diversity	2.63	4.00	0.6213

Table Z: Local Peaks.CAPRI_AIC

	Chisq	Df	Pr(>Chisq)
Num. Genes	33.91	1.00	< .0001
Sample Size	165.45	2.00	< .0001
Detection	50.85	2.00	< .0001
LOD diversity	65.50	1.00	< .0001
Num. Genes:Sample Size	34.42	2.00	< .0001
Num. Genes:Detection	2.30	2.00	0.3163
Num. Genes:LOD diversity	1.25	1.00	0.2641
Sample Size:Detection	15.66	4.00	0.0035
Sample Size:LOD diversity	155.70	2.00	< .0001
Detection:LOD diversity	41.21	2.00	< .0001
Num. Genes:Sample Size:Detection	1.76	4.00	0.7805
Num. Genes:Sample Size:LOD diversity	9.35	2.00	0.0093
Num. Genes:Detection:LOD diversity	3.06	2.00	0.2169
Sample Size:Detection:LOD diversity	2.52	4.00	0.6414
Num. Genes:Sample Size:Detection:LOD diversity	0.83	4.00	0.9342

Table AA: RMF.CAPRI_AIC

	Chisq	Df	Pr(>Chisq)
Num. Genes	109.99	1.00	< .0001
Sample Size	1798.63	2.00	< .0001
Detection	2781.42	2.00	< .0001
LOD diversity	4.29	1.00	0.0384
Num. Genes:Sample Size	292.35	2.00	< .0001
Num. Genes:Detection	633.77	2.00	< .0001
Num. Genes:LOD diversity	13.30	1.00	3e-04
Sample Size:Detection	577.47	4.00	< .0001
Sample Size:LOD diversity	383.39	2.00	< .0001
Detection:LOD diversity	647.75	2.00	< .0001
Num. Genes:Sample Size:Detection	91.32	4.00	< .0001
Num. Genes:Sample Size:LOD diversity	48.42	2.00	< .0001
Num. Genes:Detection:LOD diversity	16.31	2.00	3e-04
Sample Size:Detection:LOD diversity	97.29	4.00	< .0001
Num. Genes:Sample Size:Detection:LOD diversity	98.90	4.00	< .0001

Table AB: Represent..H-CBN

	Chisq	Df	Pr(>Chisq)
Num. Genes	46.47	1.00	< .0001
Sample Size	326.11	2.00	< .0001
Detection	762.69	2.00	< .0001
LOD diversity	74.96	1.00	< .0001
Num. Genes:Sample Size	35.59	2.00	< .0001
Num. Genes:Detection	377.15	2.00	< .0001
Num. Genes:LOD diversity	0.43	1.00	0.5135
Sample Size:Detection	315.03	4.00	< .0001
Sample Size:LOD diversity	21.87	2.00	< .0001
Detection:LOD diversity	296.12	2.00	< .0001
Num. Genes:Sample Size:Detection	213.53	4.00	< .0001
Num. Genes:Sample Size:LOD diversity	2.25	2.00	0.3248
Num. Genes:Detection:LOD diversity	20.79	2.00	< .0001
Sample Size:Detection:LOD diversity	233.91	4.00	< .0001
Num. Genes:Sample Size:Detection:LOD diversity	4.47	4.00	0.3461

Table AC: Local Peaks.H-CBN

	Chisq	Df	Pr(>Chisq)
Num. Genes	3.85	1.00	0.0499
Sample Size	107.17	2.00	$< .0001$
Detection	332.57	2.00	$< .0001$
LOD diversity	143.99	1.00	$< .0001$
Num. Genes:Sample Size	22.27	2.00	$< .0001$
Num. Genes:Detection	4.25	2.00	0.1195
Num. Genes:LOD diversity	1.16	1.00	0.2811
Sample Size:Detection	4.19	4.00	0.3803
Sample Size:LOD diversity	182.19	2.00	$< .0001$
Detection:LOD diversity	1.29	2.00	0.5253
Num. Genes:Sample Size:Detection	0.42	4.00	0.9807
Num. Genes:Sample Size:LOD diversity	0.44	2.00	0.8014
Num. Genes:Detection:LOD diversity	24.00	2.00	$< .0001$
Sample Size:Detection:LOD diversity	8.52	4.00	0.0744
Num. Genes:Sample Size:Detection:LOD diversity	1.49	4.00	0.8284

Table AD: RMF.H-CBN

12. Number of local maxima, mutations of local maxima, reciprocal sign epistasis and performance

(When interpreting these figures, you should focus on sign of slope and comparisons between models within panel. Comparisons between panels in terms of magnitudes of slopes are complicated because the range of the independent variable can vary a lot between panels —e.g., the range of mean number of mutations in local fitness maxima in the local maxima vs. RMF fitness landscapes.)

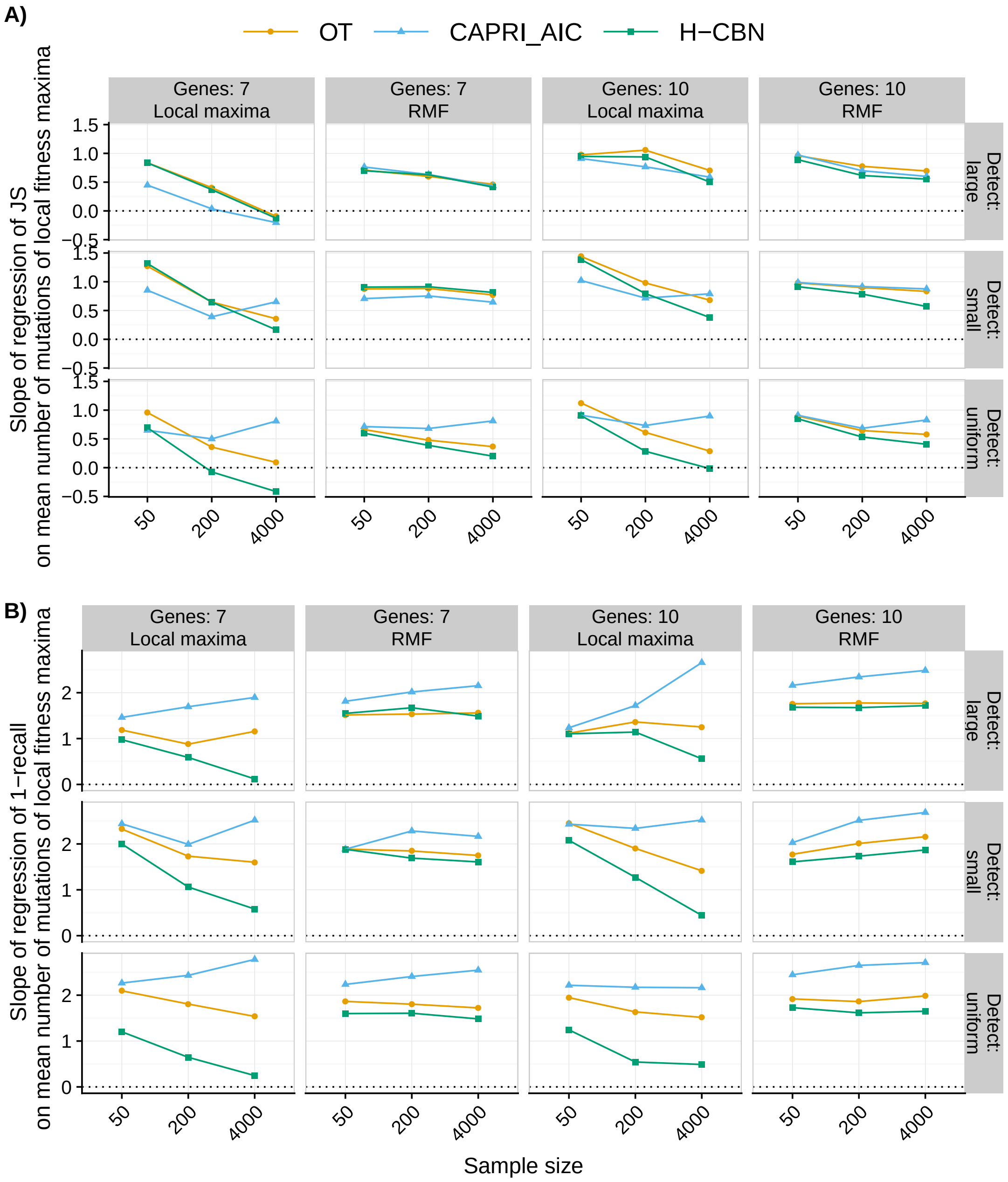

Figure L: Slopes of regression of JS and 1-recall on mean number of mutations of local maxima.

13. LOD and CPM diversity: ratios and slopes

The following R code will, via a simple example, show that it is easy to have data where the average of the ratios is larger than one whereas the slope of the regression is negative:

```
a <- 10
n <- 100
sd <- 0.5
x <- runif(n, min = 1, max = 5)
y <- -1 * x + a + rnorm(n, mean = 0, sd = sd)
plot(y ~ x)
summary(lm(y ~ x))
mean(y/x)
```

14. Example of good and moderate performance

To give a simple, intuitive view of the kind of the kinds of results obtained, we provide below the output for the frequency of LODs and the estimated probabilities of paths to the maximum from H-CBN with sample size 200 and uniform sampling for two replicate runs from fitness landscape "uW1bRozHBgg4IHvp", a relatively simple case with only six different paths to the maximum.

Frequencies of lines of descent (LODs)

```
                                                                                                       LOD     Freq
1 WT -> A -> A, B -> A, B, C -> A, B, C, D -> A, B, C, D, E -> A, B, C, D, E, F -> A, B, C, D, E, F, G   0.01400
2 WT -> A -> A, B -> A, B, C -> A, B, C, D -> A, B, C, D, F -> A, B, C, D, E, F -> A, B, C, D, E, F, G   0.11390
3 WT -> A -> A, B -> A, B, C -> A, B, C, F -> A, B, C, D, F -> A, B, C, D, E, F -> A, B, C, D, E, F, G   0.31340
4 WT -> B -> A, B -> A, B, C -> A, B, C, D -> A, B, C, D, E -> A, B, C, D, E, F -> A, B, C, D, E, F, G   0.01885
5 WT -> B -> A, B -> A, B, C -> A, B, C, D -> A, B, C, D, F -> A, B, C, D, E, F -> A, B, C, D, E, F, G   0.14235
6 WT -> B -> A, B -> A, B, C -> A, B, C, F -> A, B, C, D, F -> A, B, C, D, E, F -> A, B, C, D, E, F, G   0.39750
```

A model with a JS divergence of 0.16:

```
                path                                                                                          Prob
1 WT -> A -> A, B -> A, B, C -> A, B, C, F -> A, B, C, D, F -> A, B, C, D, E, F -> A, B, C, D, E, F, G     0.4547
2 WT -> B -> A, B -> A, B, C -> A, B, C, F -> A, B, C, D, F -> A, B, C, D, E, F -> A, B, C, D, E, F, G     0.5453
```

Notice how this model only predicts two paths to the maximum, but those are the paths that correspond to the LODs with larger frequencies.

A model with a JS divergence of 0.35:

```
                path                                                                                       Prob
1 WT -> A -> A, B -> A, B, C -> A, B, C, F -> A, B, C, D, F -> A, B, C, D, E, F -> A, B, C, D, E, F, G  0.6305357
2 WT -> B -> A, B -> A, B, C -> A, B, C, F -> A, B, C, D, F -> A, B, C, D, E, F -> A, B, C, D, E, F, G  0.1372609
3 WT -> B -> B, C -> A, B, C -> A, B, C, F -> A, B, C, D, F -> A, B, C, D, E, F -> A, B, C, D, E, F, G  0.1739520
4 WT -> B -> B, C -> B, C, F -> A, B, C, F -> A, B, C, D, F -> A, B, C, D, E, F -> A, B, C, D, E, F, G  0.0153354
5 WT -> B -> B, C -> B, C, F -> B, C, D, F -> A, B, C, D, F -> A, B, C, D, E, F -> A, B, C, D, E, F, G  0.0356487
6 WT -> B -> B, C -> B, C, F -> B, C, D, F -> B, C, D, E, F -> A, B, C, D, E, F -> A, B, C, D, E, F, G  0.0067255
7 WT -> B -> B, C -> B, C, F -> B, C, D, F -> B, C, D, E, F -> B, C, D, E, F, G -> A, B, C, D, E, F, G  0.0005419
```

This model predicts seven paths to the maximum and in fact only the first two correspond to possible paths to the maximum.

15. References

1. Brooks ME, Kristensen K, van Benthem KJ, Magnusson A, Berg CW, Nielsen A, et al. glmmTMB Balances Speed and Flexibility Among Packages for Zero-Inflated Generalized Linear Mixed Modeling. The R Journal. 2017;9(2):378–400.

2. Fox J, Weisberg S. An R Companion to Applied Regression, 2nd Ed. Thousand Oaks, CA: Sage; 2011.
