# Supplementary material for: Every which way? On predicting tumor evolution using cancer progression models
Source: PLoS Comput Biol. 2019 Aug 2;15(8):e1007246. doi: 10.1371/journal.pcbi.1007246 (PMC6693785; doi:10.1371/journal.pcbi.1007246)
Supplement: S2 Fig — (PDF) [file pcbi.1007246.s002.pdf]

# Supporting Information for "Every which way? On predicting tumor evolution using cancer progression models"

Ramon Diaz-Uriarte[1,2*], Claudia Vasallo[1,2]

**1** Dept. Biochemistry, Universidad Autónoma de Madrid, Madrid, Spain
**2** Instituto de Investigaciones Biomédicas "Alberto Sols" (UAM-CSIC), Madrid, Spain

* ramon.diaz@iib.uam.es

# S2 Figure. Simulated fitness landscapes and fitness graphs: characteristics, evolutionary unpredictability, clonal interference, and sampled genotypes

---

## Contents

**List of Figures** 1

**1 Description** 2

1.1 Terminology . . . . . . . . . . 2

## List of Figures

A Simulated fitness landscapes: Number of accessible genotypes . . . . . 3
B Simulated fitness landscapes: Number of local fitness maxima . . . . . 4
C Simulated fitness landscapes: reciprocal sign epistasis . . . . . 5
D Simulated fitness landscapes: clonal interference (frequency of most frequent genotype) . . . . . 6
E Simulated fitness landscapes: clonal interference (average number of clones with frequency $> 5\%$) . . . . . 7
F Simulated fitness landscapes: LOD . . . . . 8
G Simulated fitness landscapes: $S_p$ . . . . . 9
H Simulated fitness landscapes: number of observed local fitness maxima . . . . . 10
I Simulated fitness landscapes: diversity of observed fitness maxima . . . . . 11
J Simulated fitness landscapes: $S_p$ vs. accessible genotypes . . . . . 12
K Simulated fitness landscapes: number of mutations of fitness maxima . . . . . 13
L Simulated samples' characteristics: number of genotypes . . . . . 14
M Simulated samples' characteristics: diversity of genotypes . . . . . 15
N Simulated samples' characteristics: mean number of mutations in genotypes . . . 16
O Simulated samples' characteristics: median number of mutations in genotypes . 17
P Simulated samples' characteristics: standard deviation number of mutations in genotypes . . . . . 18
Q Simulated samples' characteristics: coefficient of variation in number of mutations in genotypes . . . . . 19

---

# 1. Description

The following figures show the main fitness landscape characteristics and the resulting variation in evolutionary predictability, clonal interference, and sampling characteristics, between types of fitness landscapes and simulation conditions (initial population size and mutation rate). Note that the "static fitness landscape" characteristics do not depend on the simulations.

## 1.1. Terminology

The **number of local (fitness) maxima** is a static feature of the landscape (number of genotypes such that all genotypes within a distance of one mutation have lower fitness). The number of **observed local (fitness) maxima** can be smaller, since some peaks (local maxima) might never be visited. For representable fitness landscapes, both numbers are 1. For the other two landscapes, those numbers were $\geq 2$.

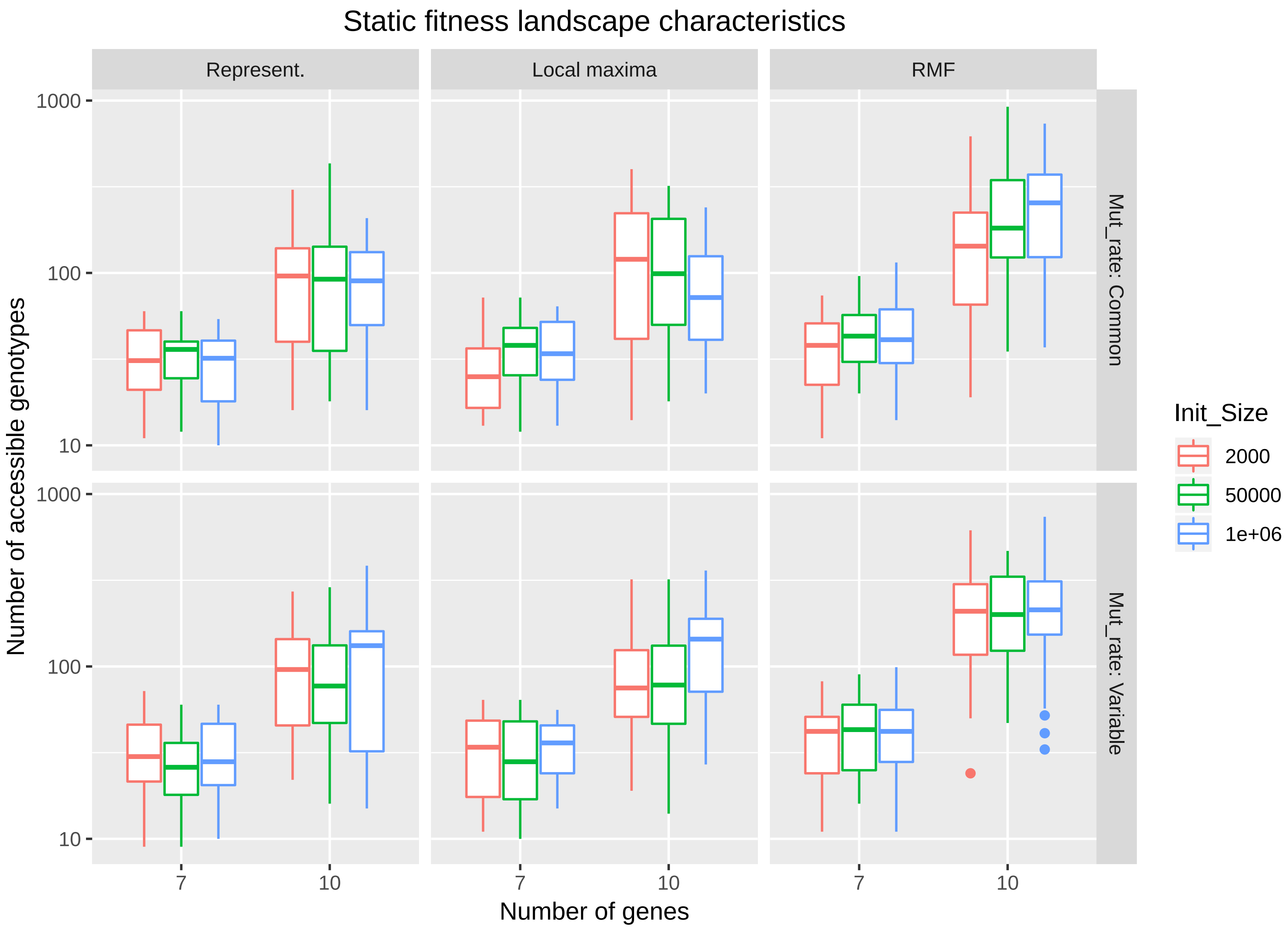


Figure A: Simulated fitness landscapes: Number of accessible genotypes

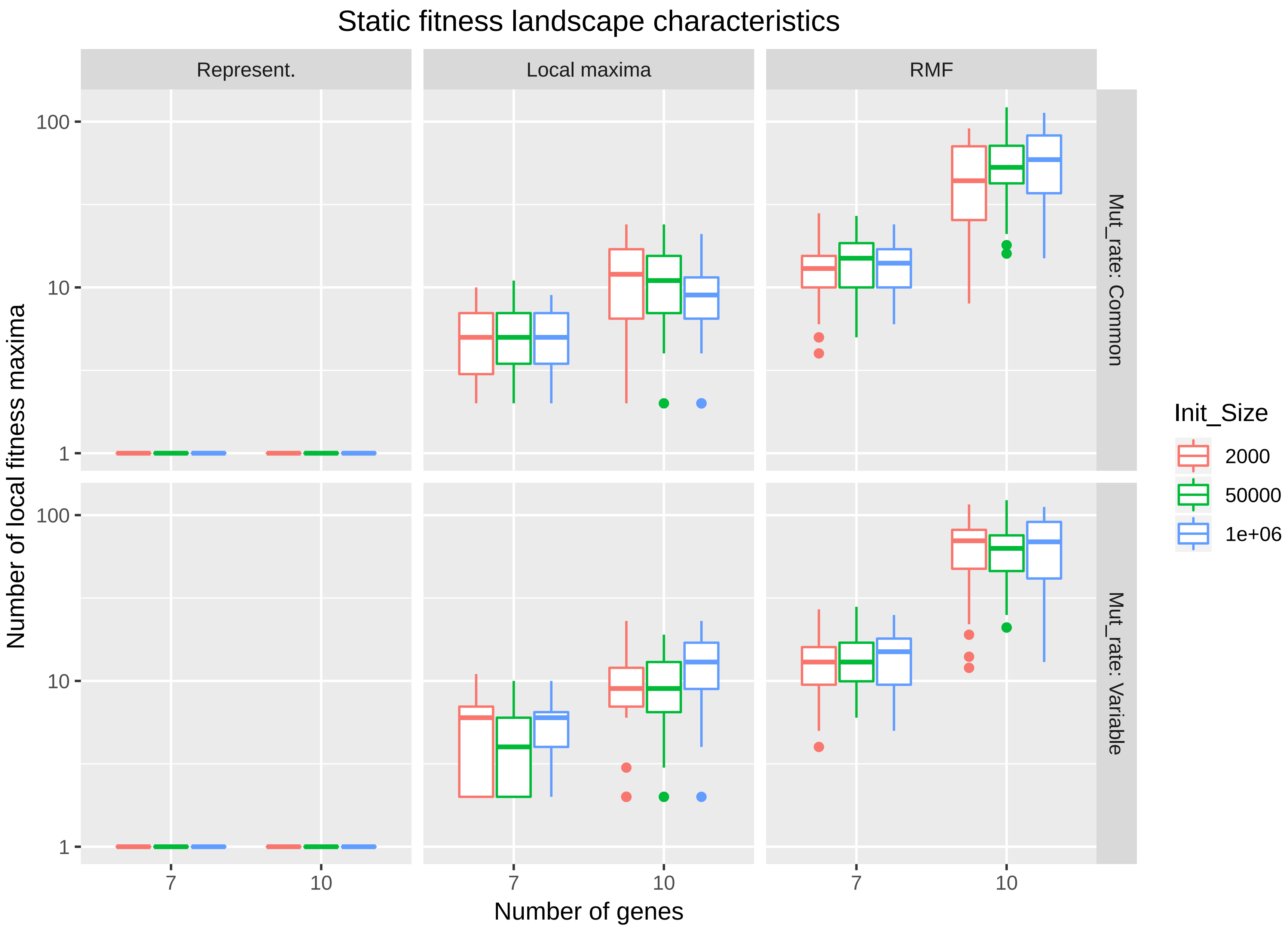


Figure B: Simulated fitness landscapes: Number of local fitness maxima

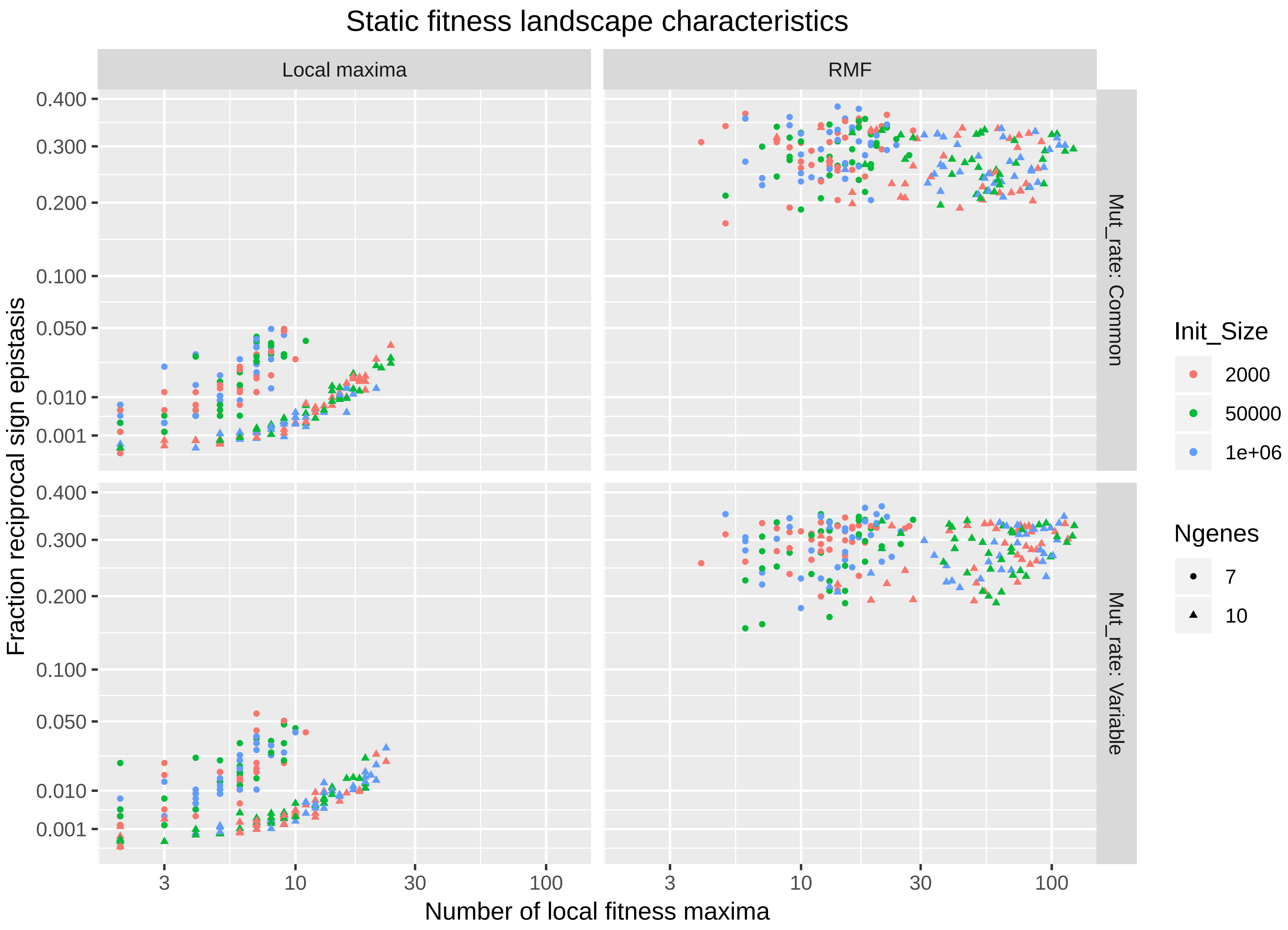


Figure C: Simulated fitness landscapes: reciprocal sign epistasis

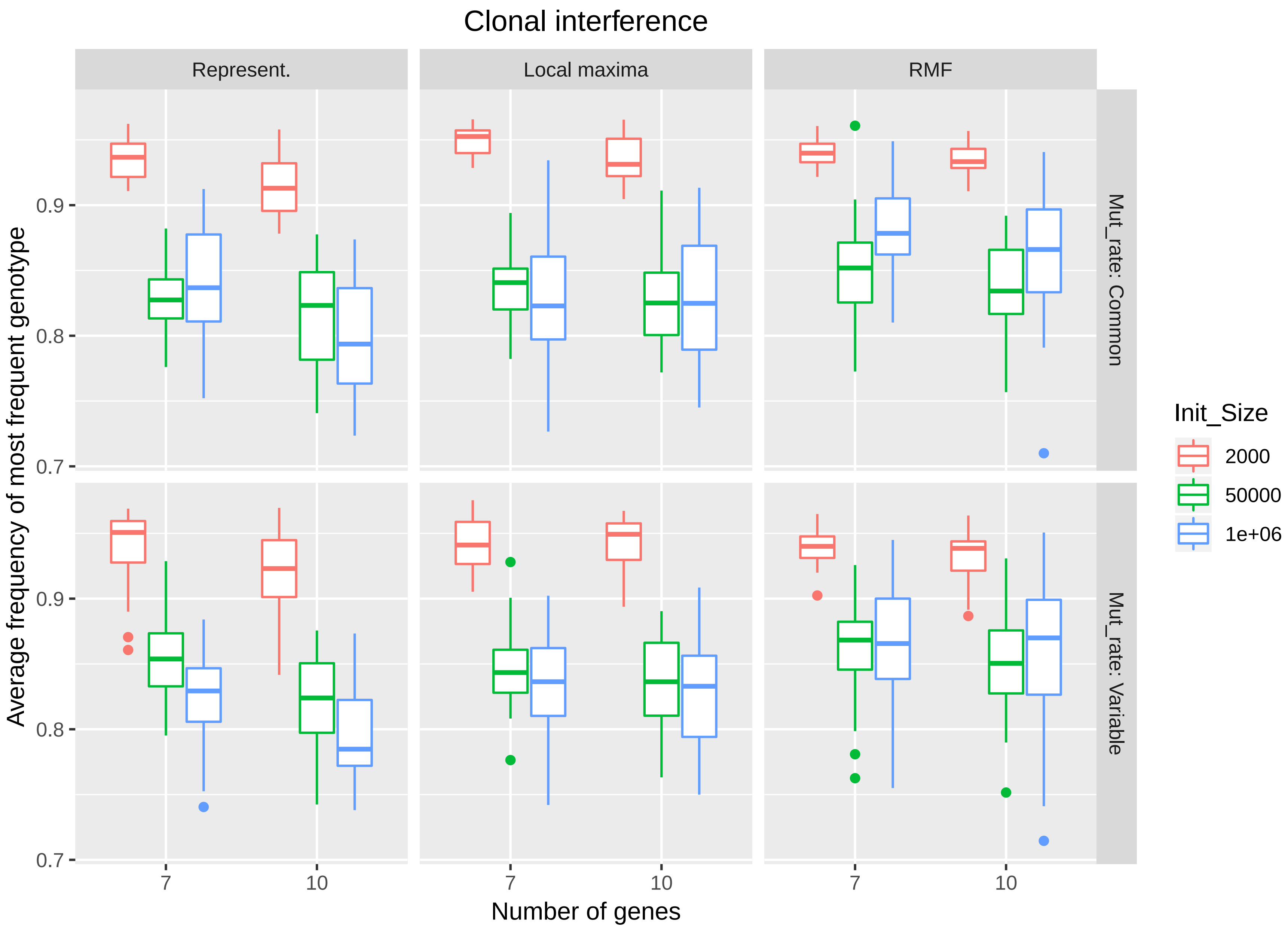


Figure D: Simulated fitness landscapes: clonal interference (frequency of most frequent genotype)

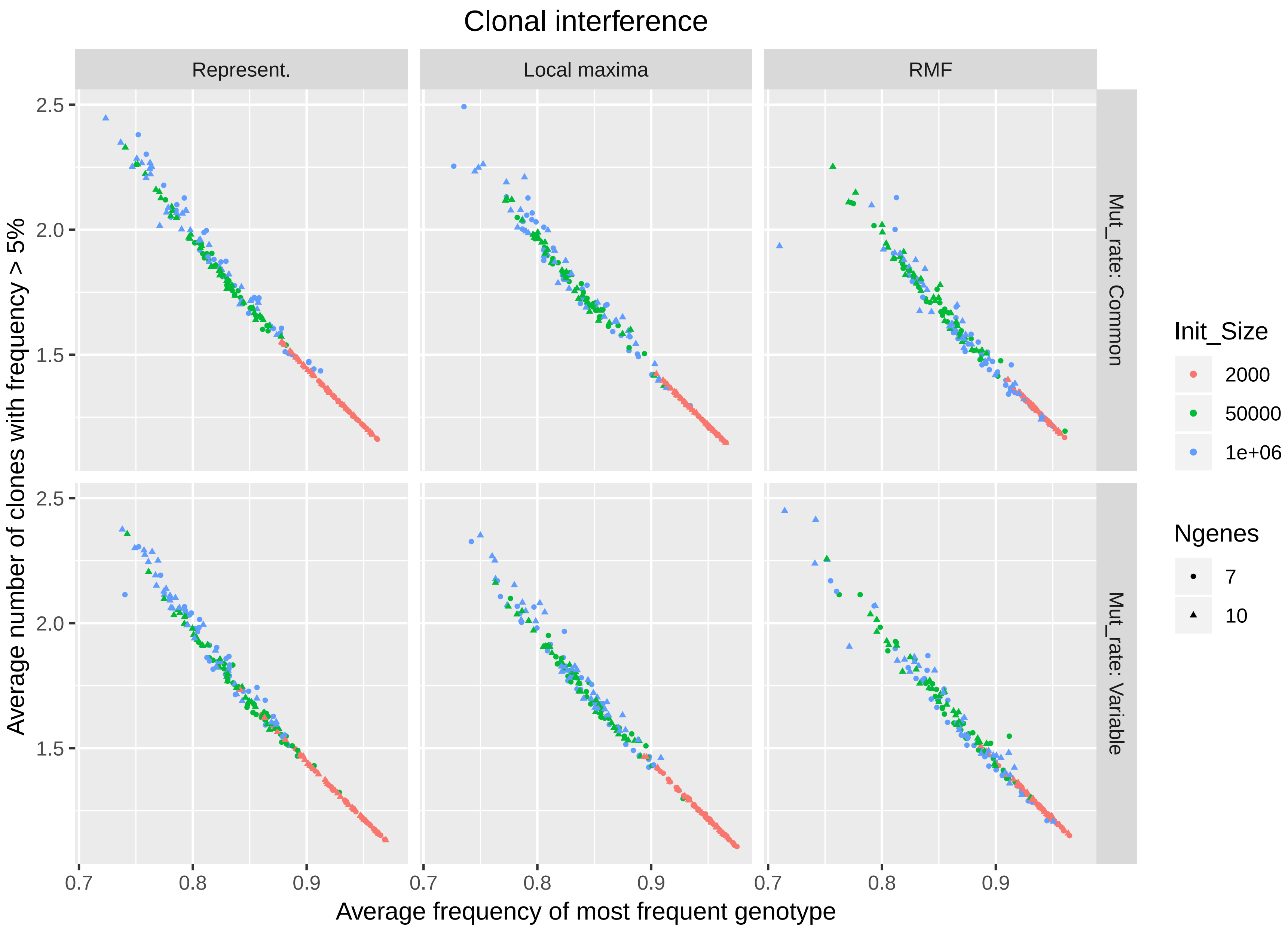


Figure E: Simulated fitness landscapes: clonal interference (average number of clones with frequency $> 5\%$)

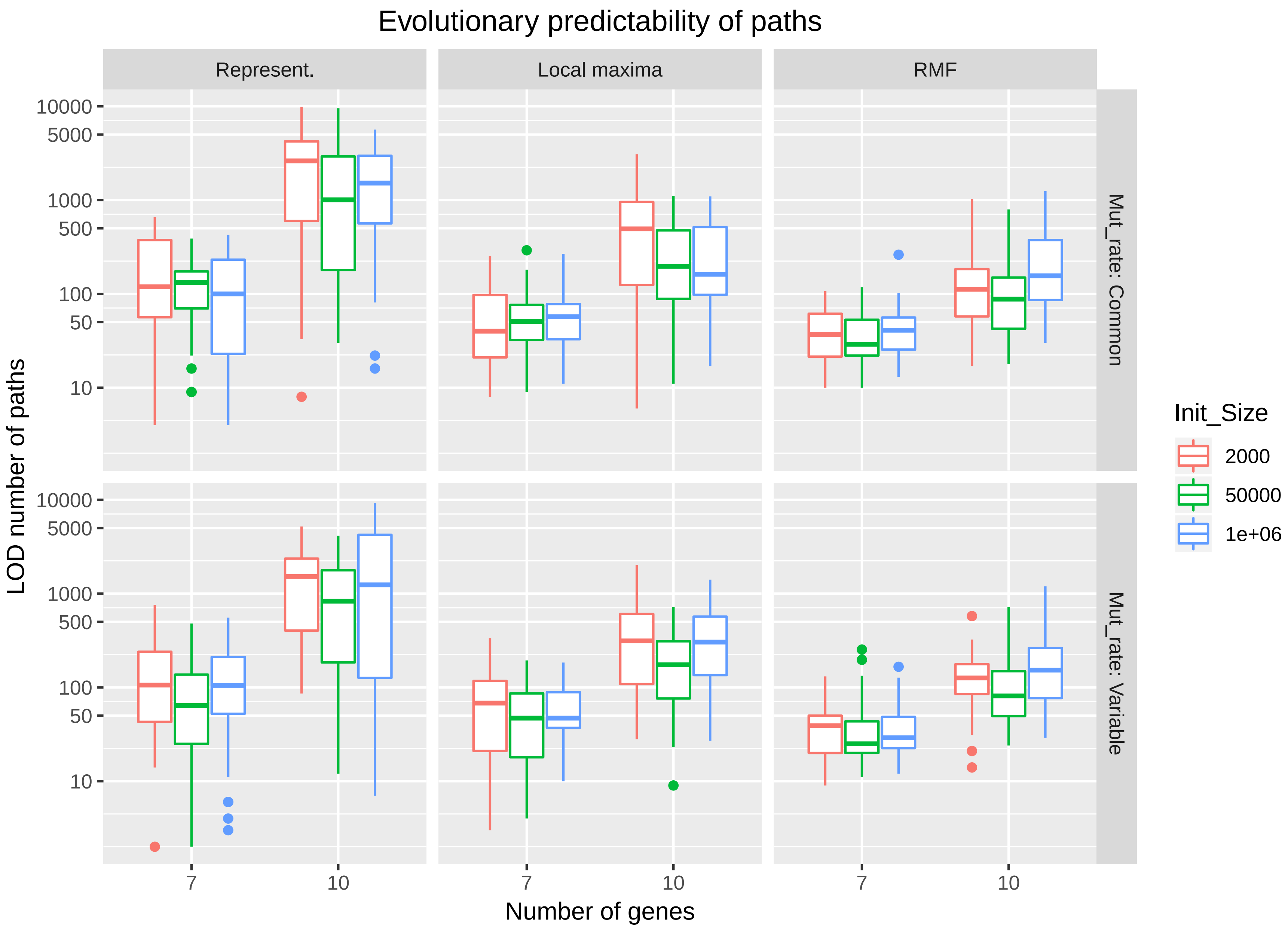


Figure F: Simulated fitness landscapes: LOD

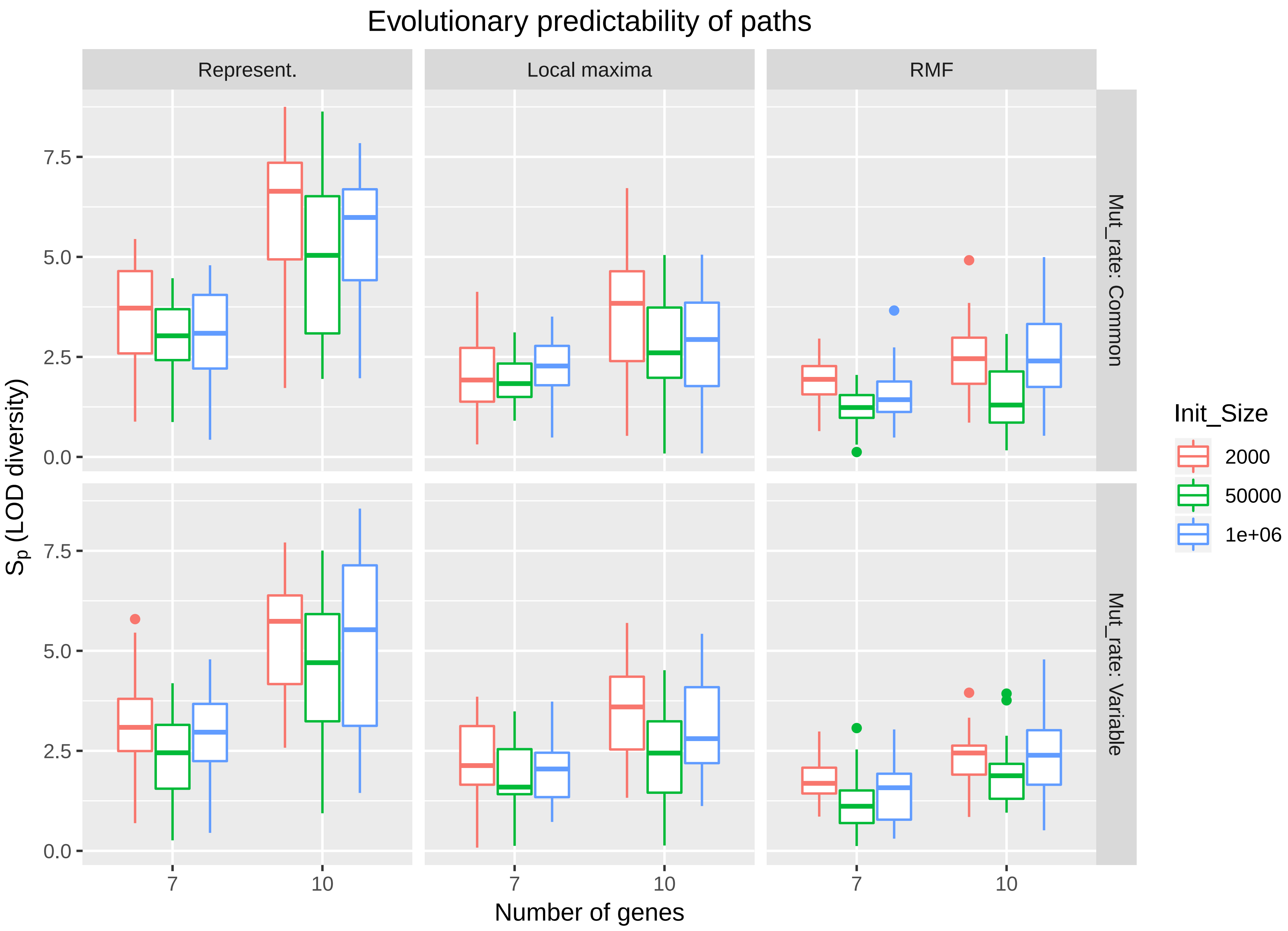


Figure G: Simulated fitness landscapes: $S_p$

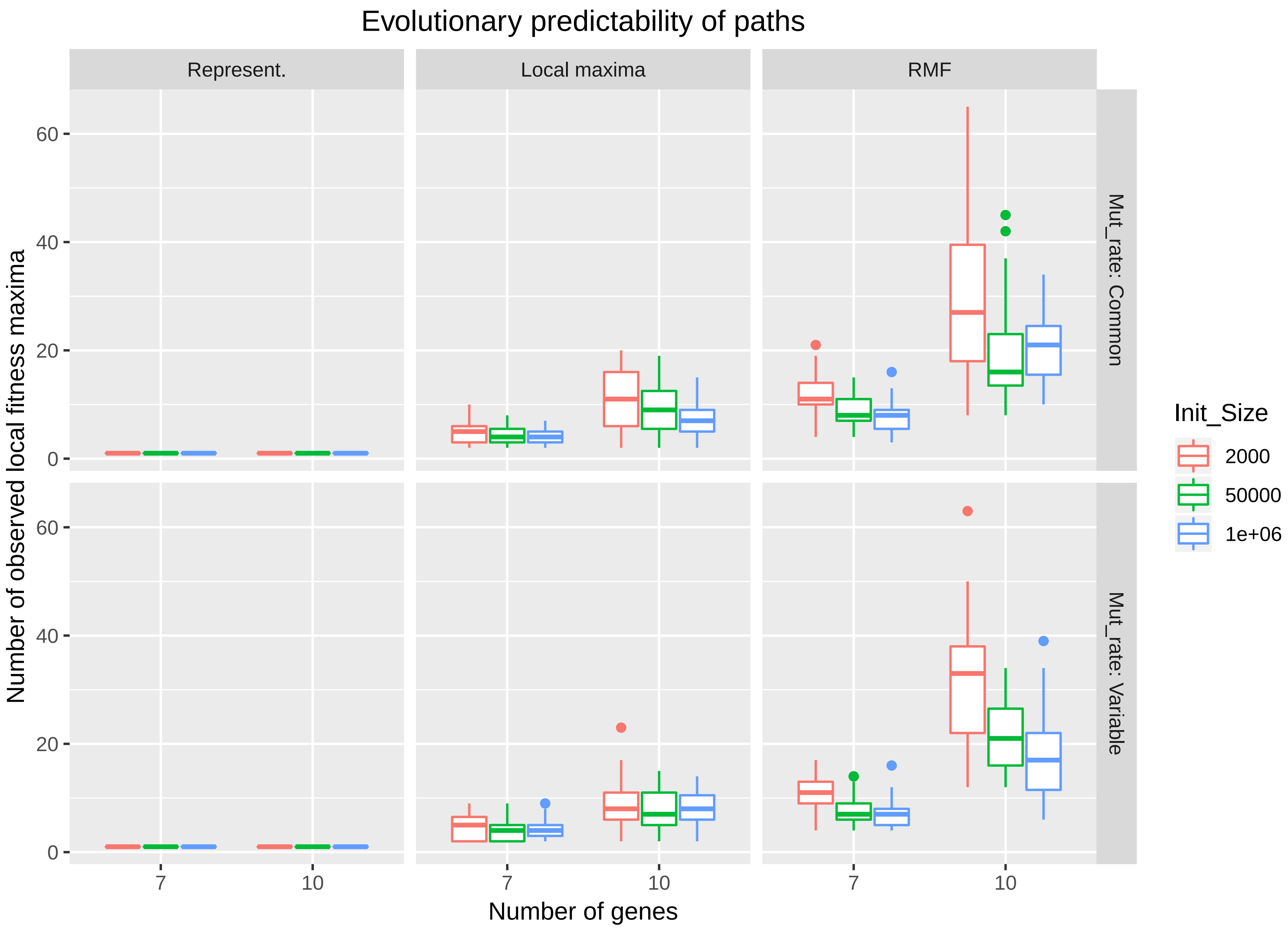


Figure H: Simulated fitness landscapes: number of observed local fitness maxima

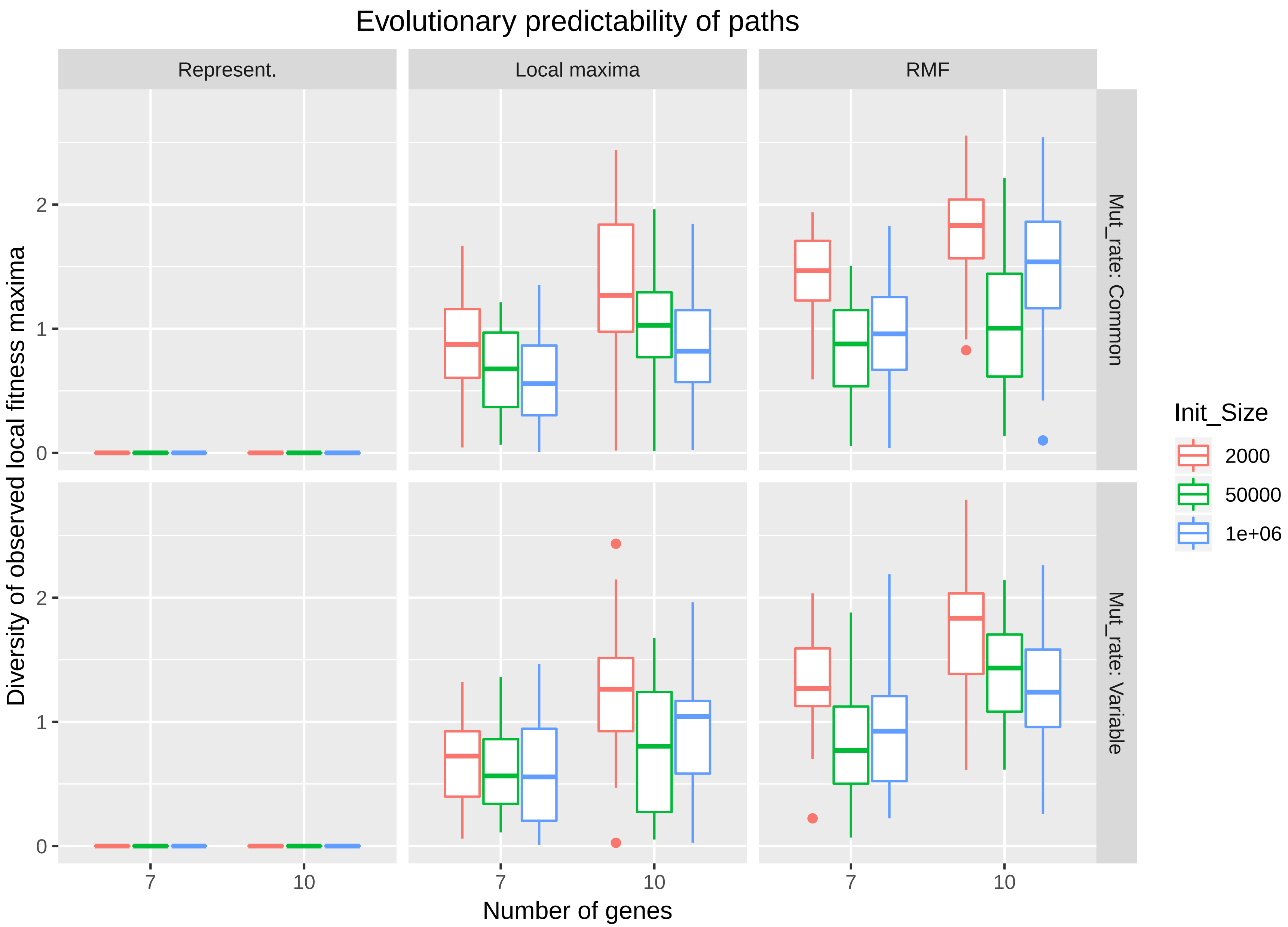


Figure I: Simulated fitness landscapes: diversity of observed fitness maxima

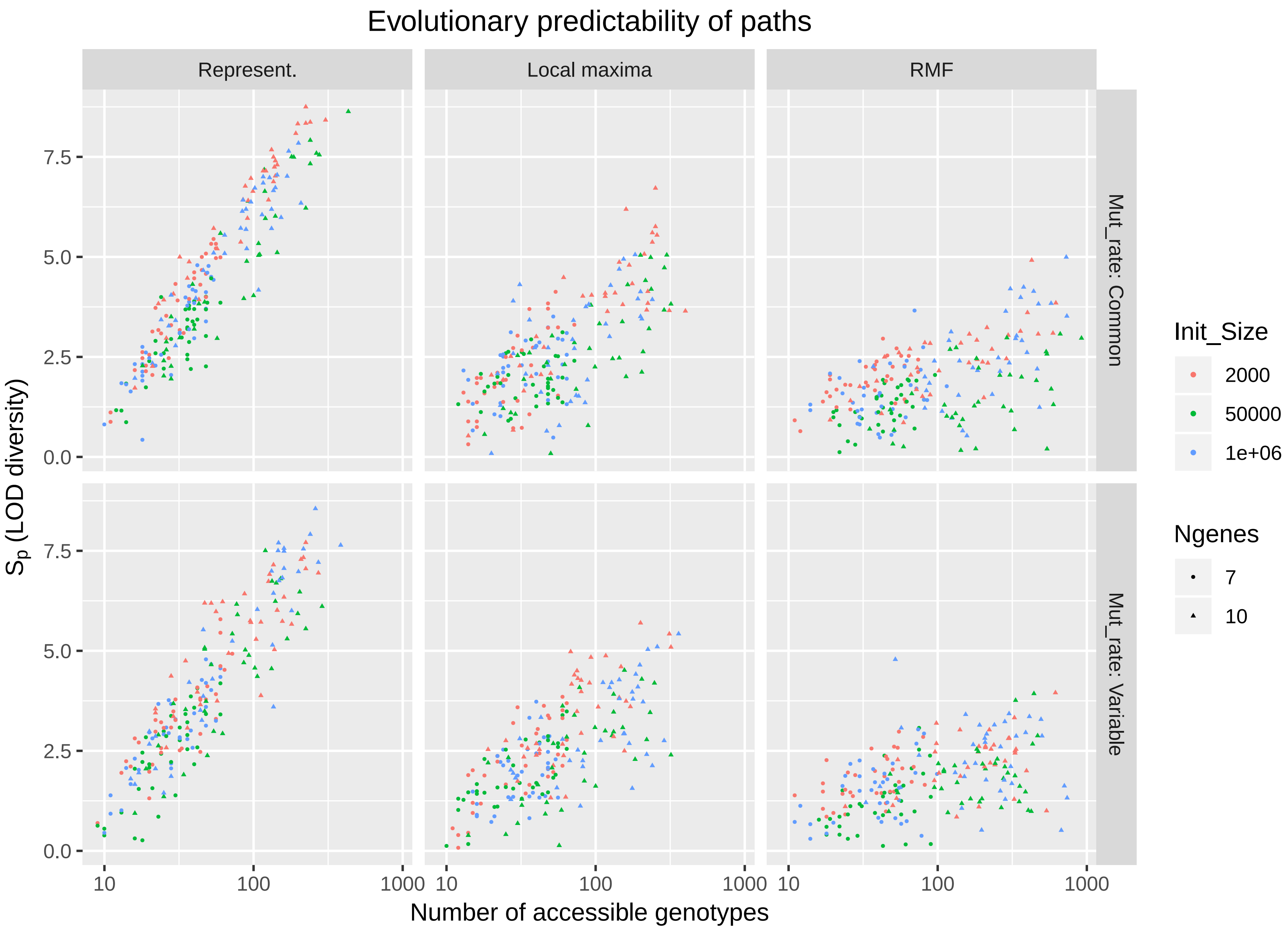


Figure J: Simulated fitness landscapes: $S_p$ vs. accessible genotypes

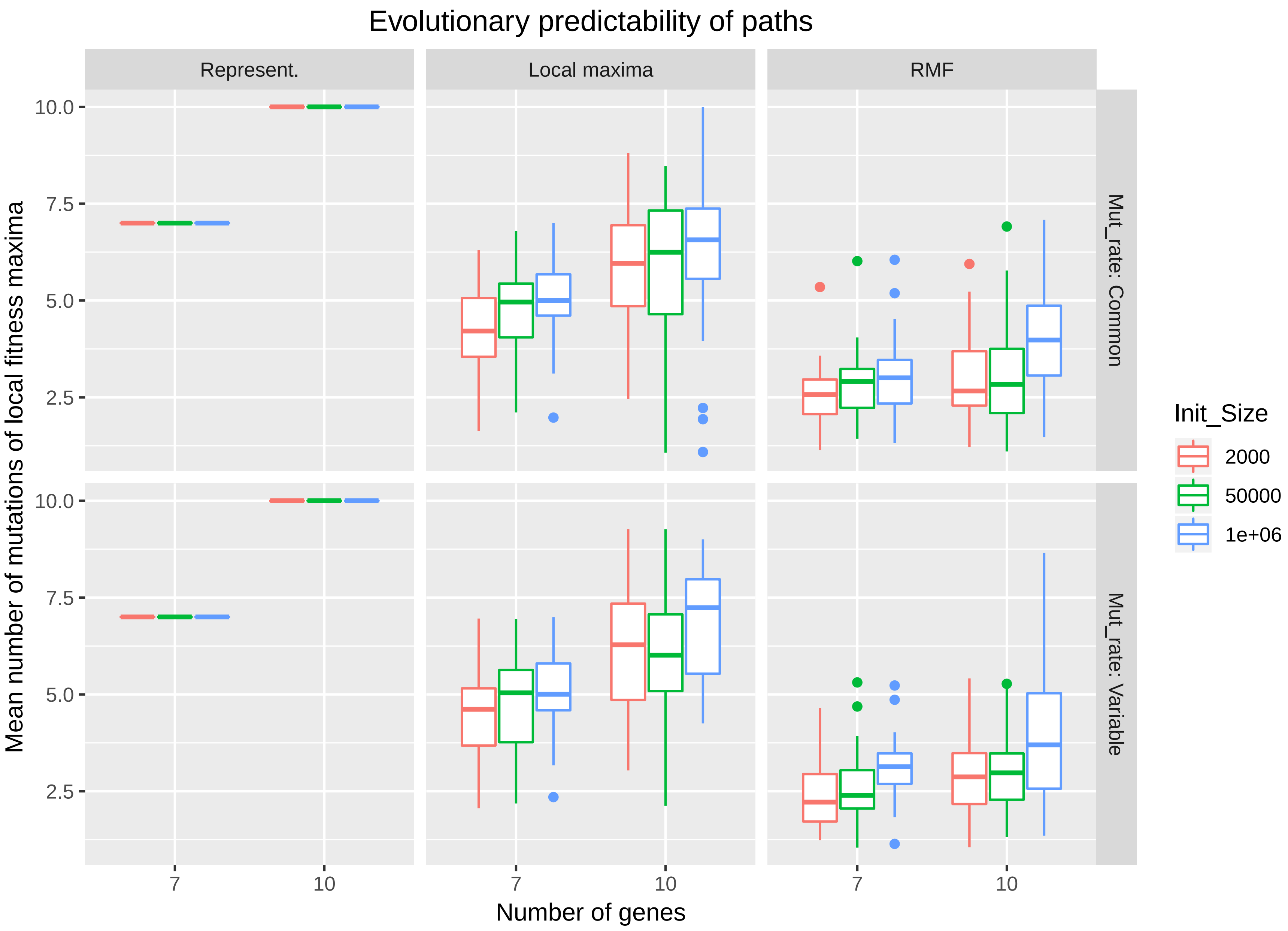


Figure K: Simulated fitness landscapes: number of mutations of fitness maxima

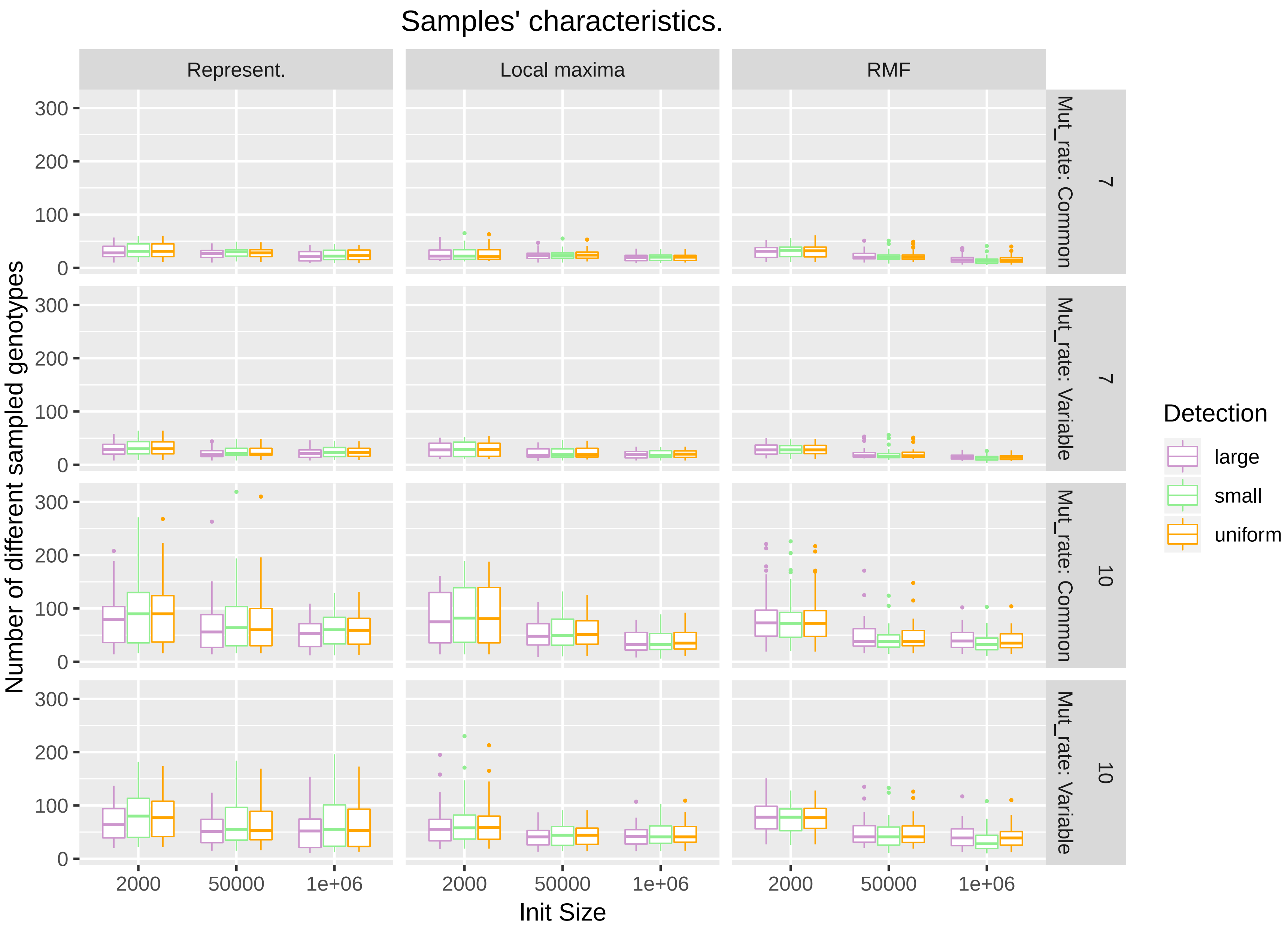


Figure L: Simulated samples' characteristics: number of genotypes

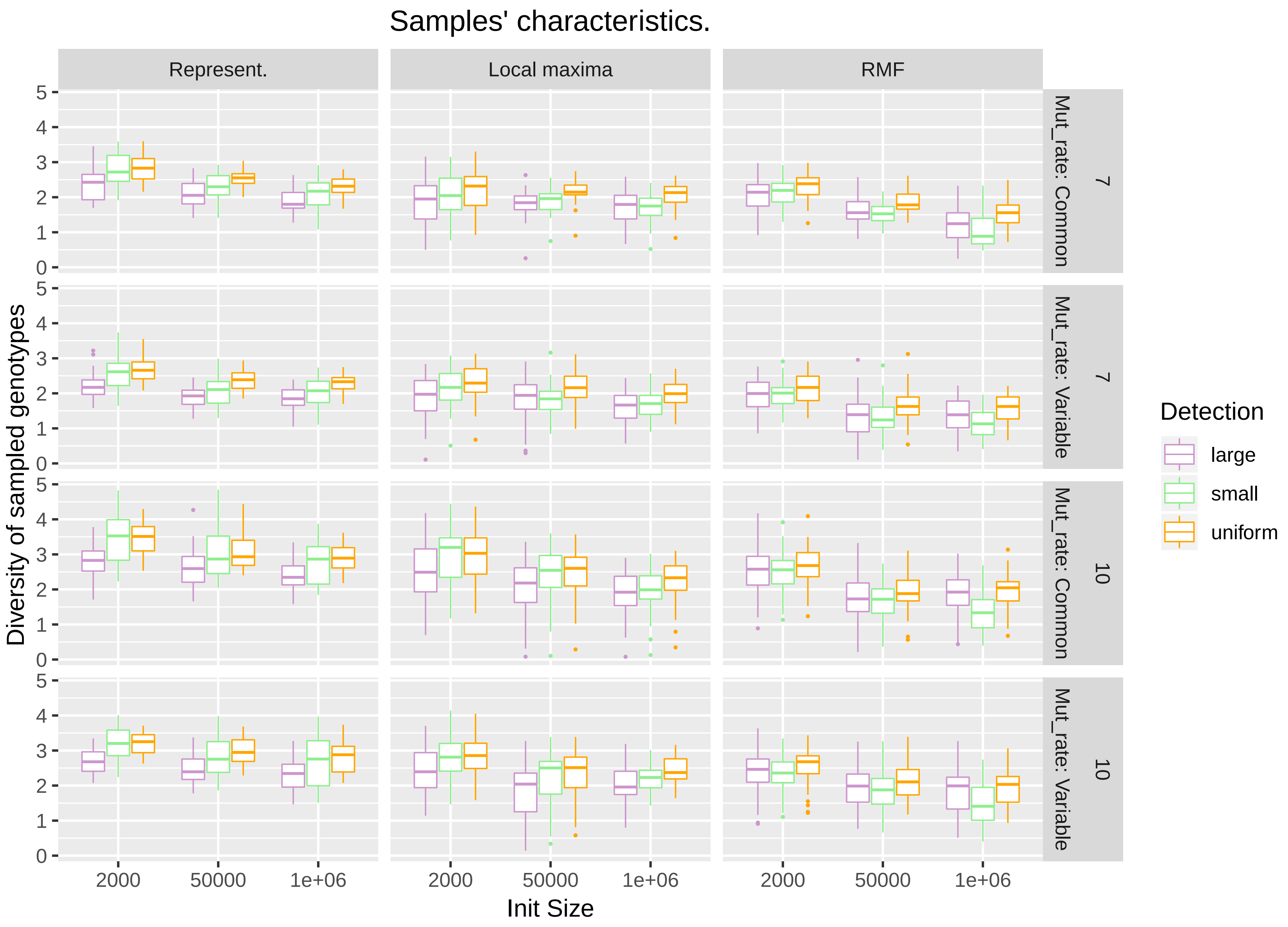


Figure M: Simulated samples' characteristics: diversity of genotypes

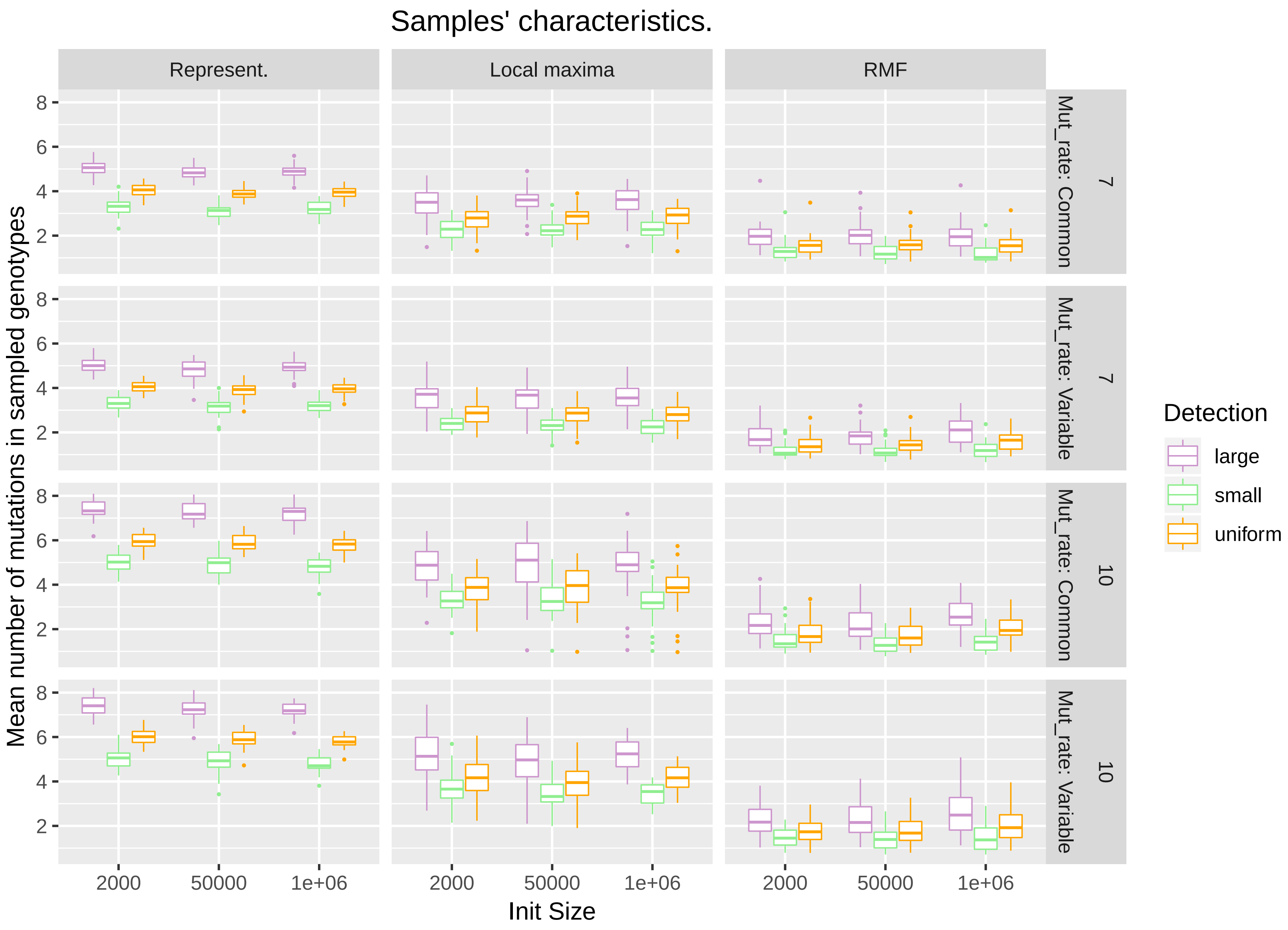


Figure N: Simulated samples' characteristics: mean number of mutations in genotypes

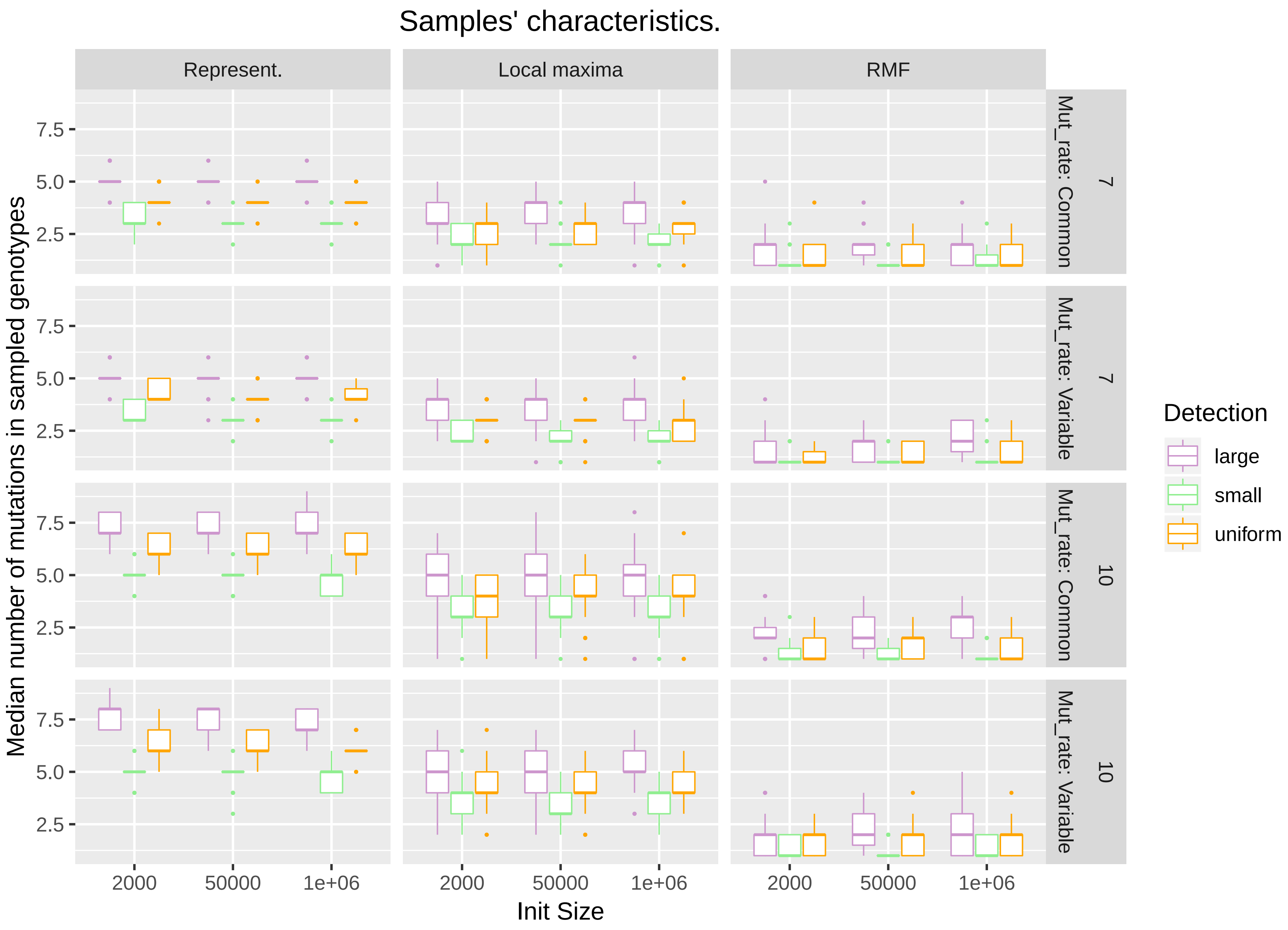


Figure O: Simulated samples' characteristics: median number of mutations in genotypes

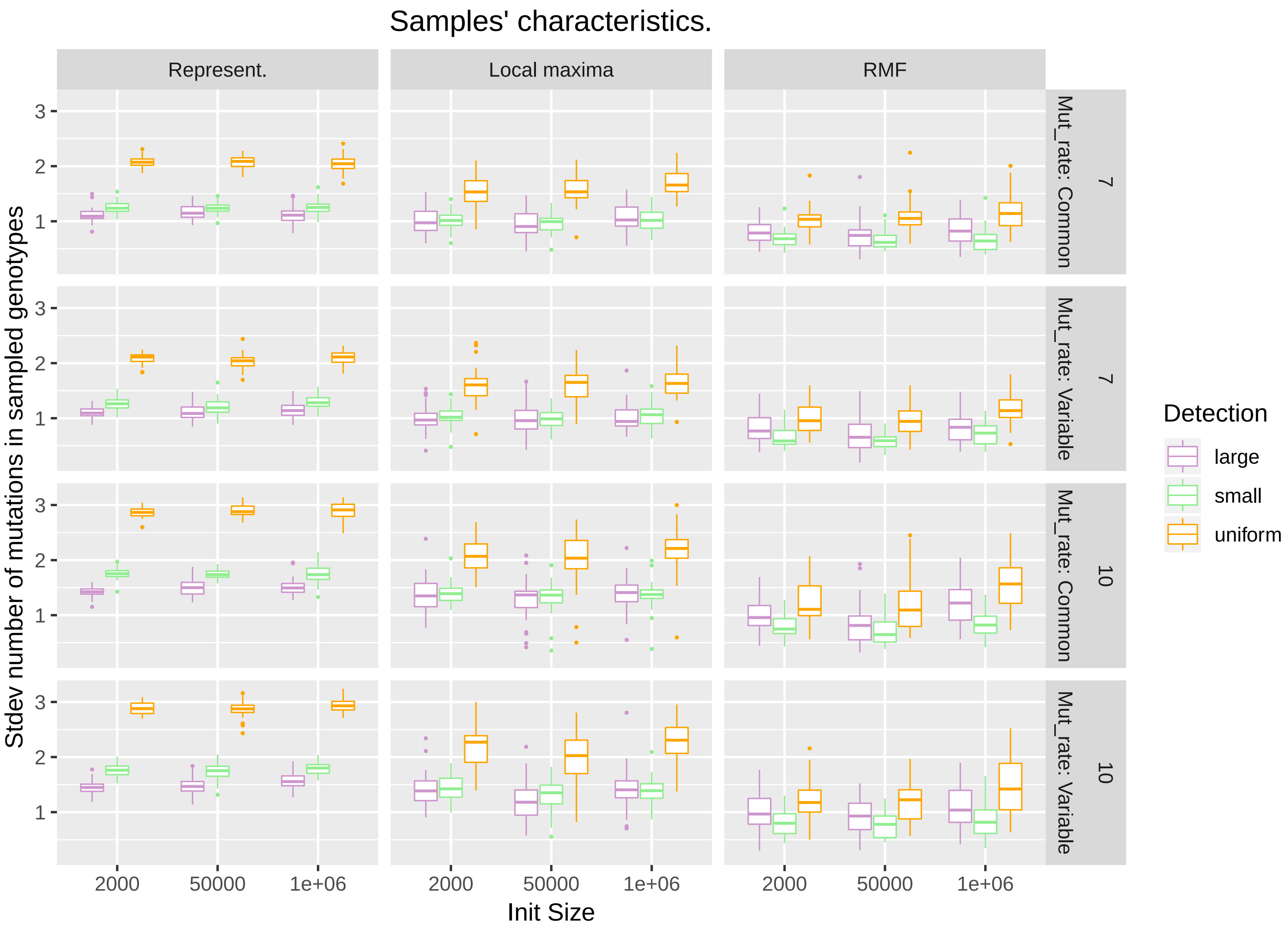


Figure P: Simulated samples' characteristics: standard deviation number of mutations in genotypes

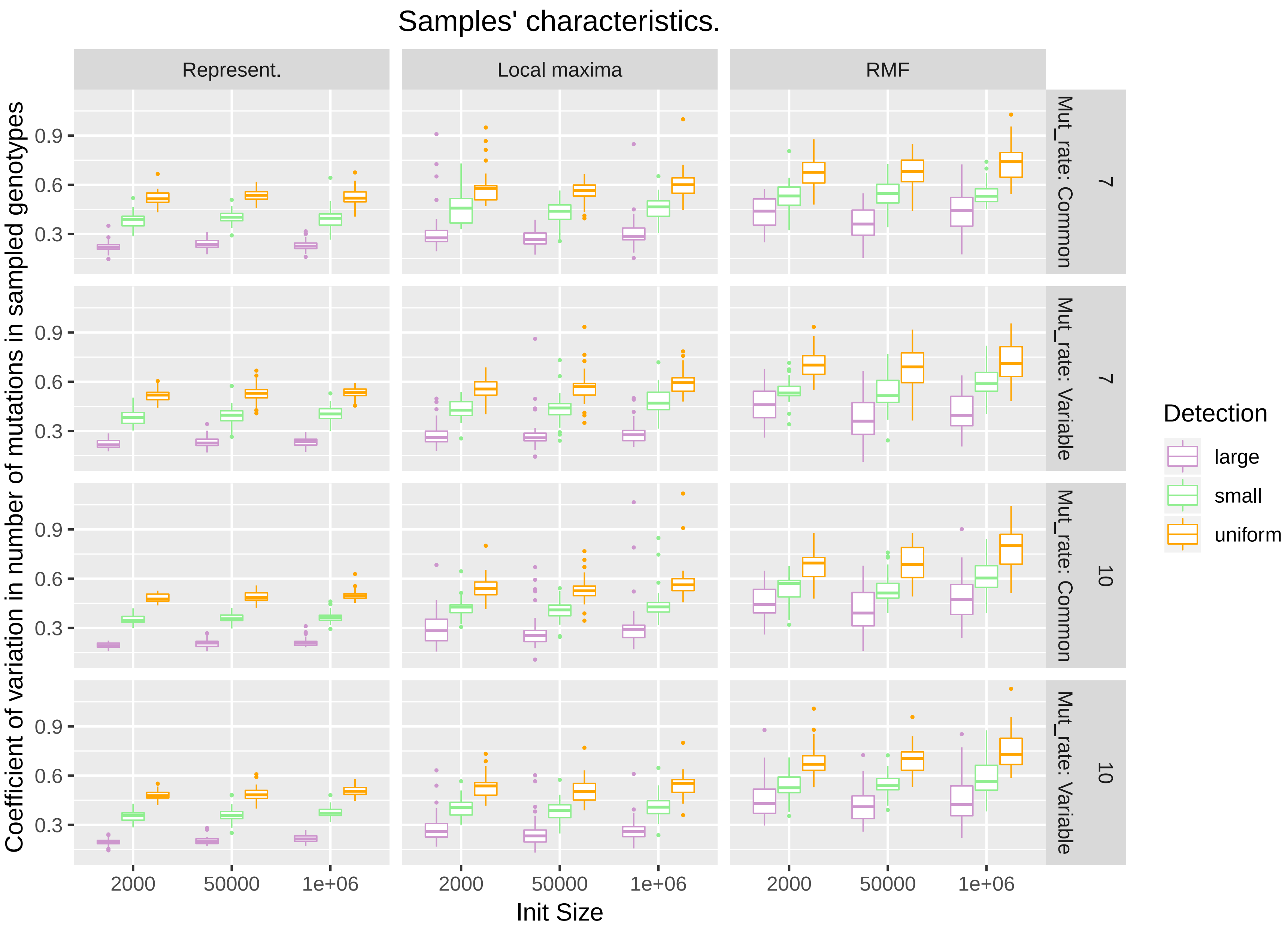


Figure Q: Simulated samples' characteristics: coefficient of variation in number of mutations in genotypes
